# AI-driven virtual screening platform identifies novel NSUN2 inhibitor candidates for targeted cancer therapy: a computational drug discovery approach

**DOI:** 10.1038/s41698-026-01296-2

**Published:** 2026-01-30

**Authors:** Shuangqi Yu, Qiao Peng, Wei Wei, Xiang Li, Shengrong Long

**Affiliations:** 1 Department of Thoracic Surgery, Tongji Hospital, Tongji Medical College, Huazhong University of Science and Technology, Wuhan, China; 2 Brain Research Center, Zhongnan Hospital of Wuhan University, Wuhan, China; 3 Department of Neurosurgery, Zhongnan Hospital of Wuhan University, Wuhan, China; 4 Frontier Science Center for Immunology and Metabolism, Wuhan University, Wuhan, China; 5 Medical Research Institute, Wuhan University, Wuhan, China; 6 Sino-Italian Ascula Brain Science Joint Laboratory, Zhongnan Hospital of Wuhan University, Wuhan, China

**Keywords:** Cancer, Computational biology and bioinformatics, Drug discovery

## Abstract

The RNA cytosine-5 methyltransferase NSUN2 is an emerging therapeutic target in precision oncology, with aberrant overexpression driving tumor progression, metastasis, and therapy resistance across multiple malignancies. Despite its critical role in cancer biology, selective small-molecule inhibitors remain limited. We employed an AI-accelerated workflow to screen approximately 101 million compounds from the ZINC database using structure-based virtual screening. The AlphaFold2-predicted human NSUN2 structure was aligned with the experimentally determined M. jannaschii TRM4 homolog (PDB: 3A4T, 34.2% sequence identity, 1.82 Å RMSD). A CatBoost ensemble classifier trained on Morgan fingerprint descriptors with AutoDock Vina-derived labels achieved robust performance (training: recall 0.87, ROC-AUC 0.89; test: recall 0.71, ROC-AUC 0.85), with low test precision reflecting extreme class imbalance inherent to virtual screening. Multi-stage filtering identified 12,000 high-scoring compounds with binding affinities of −9.933 to −8.375 kcal/mol. ADMET profiling yielded 34 drug-like candidates with favorable pharmacokinetic and toxicological profiles. Molecular dynamics simulations over 50 nanoseconds validated binding stability of lead compounds ZINC-1000507789 and ZINC-1000507824. These structurally diverse non-covalent reversible inhibitors targeting the SAM cofactor binding pocket warrant experimental validation through biochemical assays and cellular studies to overcome therapeutic resistance in NSUN2-driven malignancies.

## Introduction

The RNA cytosine-5 methyltransferase NSUN2 (NOP2/Sun RNA Methyltransferase 2) catalyzes the formation of 5-methylcytosine (m5C) modifications in various RNA species, including transfer RNAs (tRNAs), messenger RNAs (mRNAs), and long non-coding RNAs (lncRNAs). This epitranscriptomic modification plays crucial roles in regulating RNA stability, translation efficiency, and nucleocytoplasmic export, thereby influencing fundamental cellular processes including gene expression, protein synthesis, and cellular differentiation^1,2^. As one of the most extensively characterized members of the NSUN family of RNA methyltransferases, NSUN2 has emerged as a pivotal regulator in diverse biological contexts, with its dysregulation increasingly implicated in human disease pathogenesis^3^.

The pathological significance of NSUN2 is particularly evident in oncology, where aberrant expression of this methyltransferase has been documented across multiple cancer types. Elevated NSUN2 expression has been consistently associated with enhanced tumor cell proliferation, migration, invasion, and poor prognosis in diverse malignancies including non-small cell lung cancer, gastric cancer, pancreatic cancer, breast cancer, and esophageal squamous cell carcinoma^4,5^. Mechanistically, NSUN2-mediated m5C modifications stabilize oncogenic mRNAs and activate critical signaling pathways such as PI3K/AKT and ERK/MAPK, thereby promoting tumorigenesis and cancer progression^6,7^. Furthermore, NSUN2 has been implicated in mediating resistance to targeted therapies, including EGFR tyrosine kinase inhibitors in lung cancer^8^, highlighting its multifaceted role in cancer biology.

Beyond oncology, NSUN2 plays essential roles in neurological function, with loss-of-function mutations causing intellectual disability and neurodegenerative phenotypes^9–12^. While NSUN2 loss-of-function impairs neuronal homeostasis, the therapeutic application of NSUN2 inhibitors targets cancer cells with aberrant overexpression. This dual biology underscores the importance of developing highly selective inhibitors with minimal off-target effects and appropriate therapeutic windows.

Despite the mounting evidence implicating NSUN2 in cancer progression, the development of selective small-molecule inhibitors targeting this enzyme has been significantly limited. While NSUN2 represents an attractive therapeutic target given its overexpression in multiple cancers and its role in disease progression, the lack of specific inhibitors has hindered both mechanistic studies and clinical translation^13^. Recent breakthrough studies have reported the discovery of covalent azetidine acrylamide compounds that stereo selectively target the catalytic cysteine residue (C271) of NSUN2, demonstrating high isotype selectivity, minimal cross-reactivity and cell-based activity^14^. These inhibitors function through covalent, irreversible modification of the catalytic cysteine, providing proof-of-concept for NSUN2 druggability. However, these initial compounds require further optimization to achieve suitable pharmacokinetic properties and therapeutic efficacy. The scarcity of diverse chemical scaffolds targeting NSUN2, particularly non-covalent reversible inhibitors that may offer complementary advantages including different pharmacokinetic profiles and therapeutic efficacy, emphasizes the urgent need for a better theoretical framework to identify novel lead compounds with improved drug-like properties.

Virtual screening has emerged as a powerful and cost-effective strategy in modern drug discovery, enabling the rapid evaluation of vast chemical libraries to identify potential therapeutic candidates. Structure-based virtual screening employs molecular docking techniques to predict binding affinities and poses of small molecules within protein binding sites, substantially reducing the time and cost associated with experimental high-throughput screening^15–17^. Recent advances in artificial intelligence and machine learning have fundamentally transformed virtual screening capabilities, addressing critical limitations of traditional docking-based approaches. Machine learning scoring functions trained on experimental binding data demonstrate superior performance in distinguishing true binders from decoys compared to classical scoring functions, significantly reducing false-positive rates^18–21^. Ensemble learning methods such as gradient boosting frameworks enable more accurate prediction of protein-ligand binding by capturing complex non-linear relationships that traditional scoring functions miss. Furthermore, AI-driven platforms achieve up to 100-fold acceleration in screening throughput, enabling the systematic evaluation of billion-molecule chemical libraries that would be computationally prohibitive with conventional methods^21,22^. Integration of ADMET prediction into the AI-guided workflow allows early assessment of pharmacokinetic and safety profiles, significantly improving lead compound quality^23^. Molecular dynamics simulations complement these approaches by providing detailed insights into binding stability and conformational dynamics of protein-ligand complexes. Collectively, these computational methodologies offer substantial advantages over traditional experimental approaches, including higher throughput, lower costs, and the ability to explore chemical spaces that may be synthetically challenging^24,25^.

Computational screening offers several practical advantages for NSUN2: (1) while standard methyltransferase assays (e.g., radiometric or mass spectrometry-based methods) can be adapted for NSUN2, they require complex cofactors (SAM) and RNA substrates, making high-throughput implementation technically challenging; (2) limited availability of structurally diverse chemical matter targeting RNA methyltransferases in commercial screening libraries; (3) the ability to virtually evaluate chemical diversity at scales that complement focused experimental screening campaigns. These factors make computational screening a valuable complementary approach to experimental methods for initial lead identification.

In this study, we present a comprehensive computational drug discovery approach to identify novel NSUN2 inhibitor candidates with optimized predicted binding affinity and drug-like properties. Our integrated workflow combines structure-based virtual screening of ultra-large chemical libraries (ZINC20 database), machine learning-guided scoring, rigorous ADMET profiling, and molecular dynamics simulations to evaluate predicted binding stability. With human NSUN2 crystal structures undetermined, we leveraged the protein’s structural conservation across species and sequence similarity with Methanocaldococcus jannaschii homologs. Sequence alignment between human NSUN2 (residues 290-520) and M. jannaschii TRM4 revealed 34.2% sequence identity and 51.7% sequence similarity. The AlphaFold-predicted human NSUN2 structure was aligned with PDB entry 3A4T, yielding an RMSD of 1.82 Å over 187 aligned Cα atoms. Critical catalytic residues, including C271 in NSUN2 (corresponding to C295 in TRM4), were structurally conserved. The sinefungin binding site in TRM4 corresponds to the SAM cofactor binding pocket, which is highly conserved across the NSUN family. This cross-species structural validation approach provides a reliable foundation for structure-based virtual screening. By leveraging both experimentally determined structures from the Protein Data Bank and high-confidence AlphaFold2 predictions, we achieve precise binding pocket identification and reliable docking predictions^26,27^. Specifically, we employ AutoDock Vina for high-precision molecular docking^28^, enabling efficient sampling of binding poses and accurate estimation of binding affinities. By leveraging the experimentally determined M. jannaschii TRM4 crystal structure (PDB: 3A4T) aligned with the AlphaFold2-predicted human NSUN2 model, we achieve structural validation of the conserved binding pocket for docking studies. While AlphaFold2 has demonstrated high accuracy in protein structure prediction, it provides single static conformations and may not fully capture conformational flexibility, induced-fit mechanisms, or allosteric effects. The cross-species structural alignment approach employed here (34.2% sequence identity, 1.82 Å RMSD) serves as a validation strategy, and the demonstrated conservation of the NSUN2/TRM4 active site enhances confidence in the predicted binding site geometry while acknowledging inherent uncertainties in computational structure prediction. This multi-scale computational strategy enables the systematic identification of high-affinity small-molecule leads that exhibit favorable pharmacokinetic profiles, thereby providing computationally prioritized candidates for experimental validation. The discovered lead compounds not only provide valuable chemical tools for elucidating NSUN2 biology but also represent promising starting points for the development of therapeutic agents targeting NSUN2-associated malignancies.

## Results

### Structural analysis and binding site identification

**Fig. 1:**
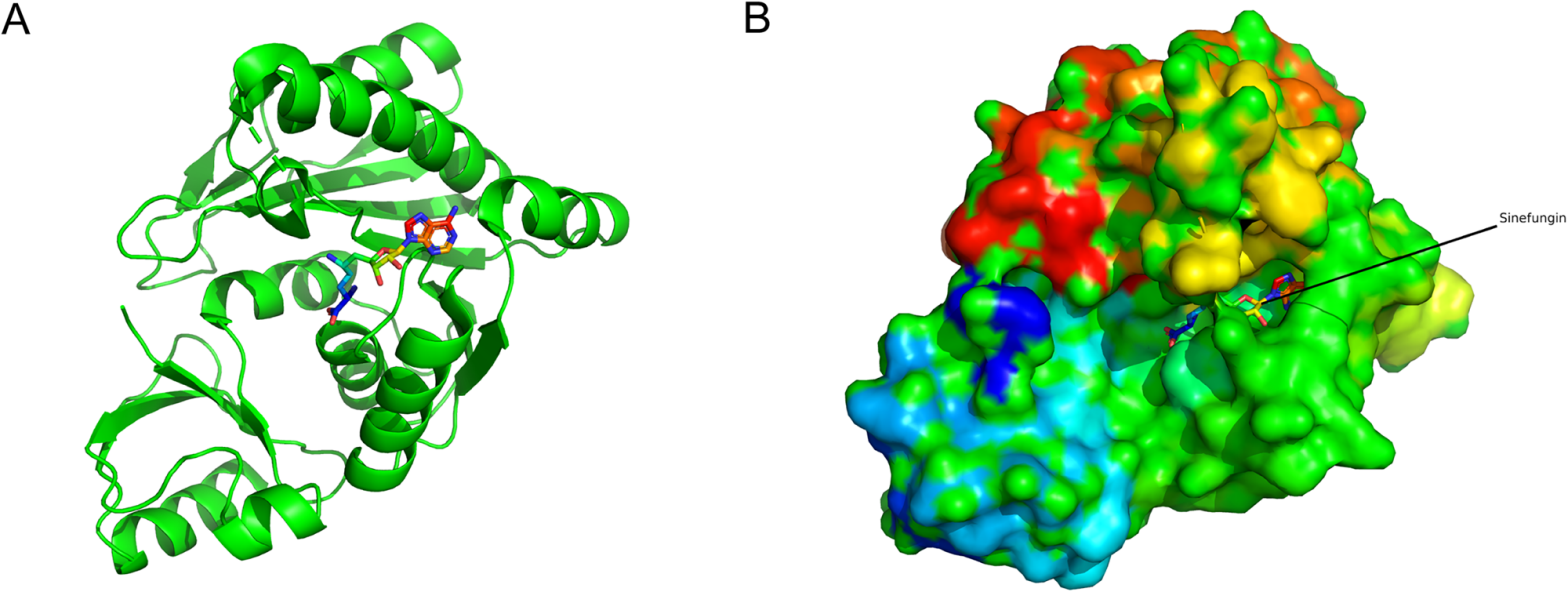
Structural characterization of the Sinefungin binding pocket in TRM4 homolog. **A** PDB 3A4T (25) from Methanocaldococcus jannaschii encoding TRM4 protein. **B** TRM4-Sinefungin complex.

**Fig. 2:**
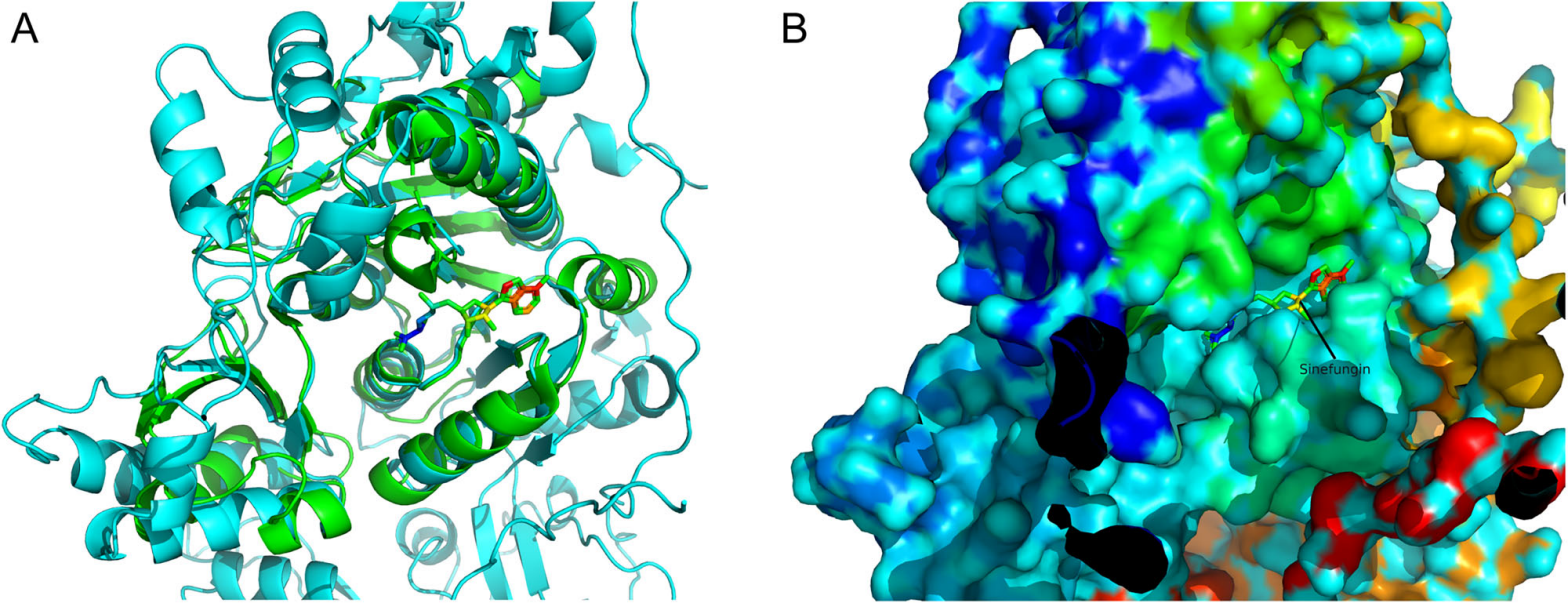
Structural alignment of AlphaFold-predicted human NSUN2 with PDB 3A4T. **A** Superposition of 3A4T and predicted NSUN2 structures. **B** Conserved binding pocket in predicted NSUN2.

**Fig. 3:**
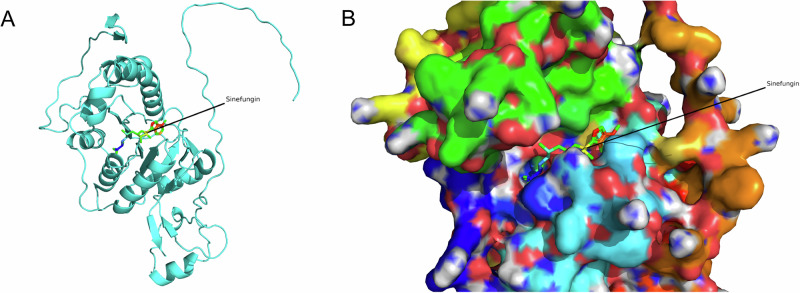
Extracted aligned NSUN2 structure with Sinefungin positioning. **A** Sinefungin location in aligned NSUN2 structure. **B** Surface representation showing Sinefungin in predicted NSUN2 binding site.

Using PyMoL to observe PDB: 3A4T (Fig. 1A), it can be seen that Sinefungin binds to the pocket of the TRM4 protein (Fig. 1B). After aligning 3A4T with the AlphaFold-predicted structure of human NSUN2 in PyMoL, it is evident that there are no significant structural differences in the aligned regions (Fig. 2A), and Sinefungin still binds to the pocket of the NSUN2 protein (Fig. 2B). Further cropping the aligned AlphaFold-predicted structure of human NSUN2 (Fig. 3A) shows that Sinefungin remains positioned within its binding pocket (Fig. 3B).

#### **Conformational Sampling Optimization and Initial Docking**

Benchmark data from ref. ^29^ demonstrate that 200 K training compounds provide substantial performance gains with diminishing returns beyond this threshold. Sensitivity improved modestly from 0.822 (200 K) to 0.860 (1 M), representing only 4.6% improvement. For most targets (A2AR, D2R), 200 K training sets achieved >80% sensitivity, meeting virtual screening requirements.

**Fig. 4:**
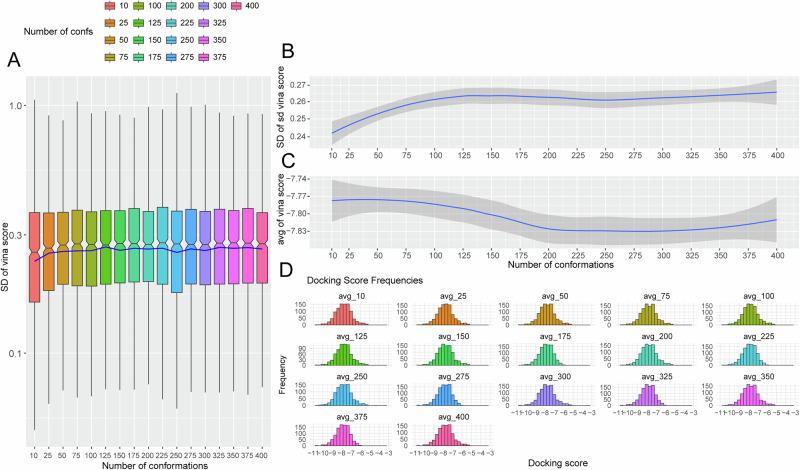
Docking score distributions across conformational ensemble sizes (*n* = 1,000 compounds). **A** Standard deviation box plots (blue line: mean). **B** Standard deviation trends with 95% confidence intervals. **C** Mean score trends with 95% confidence intervals. **D** Score distributions across ensemble sizes.

Conformational sampling analysis (1000 test compounds; Fig. 4) identified 50 conformers as optimal, balancing docking score quality with computational cost. Standard deviations remained consistent across conformer counts (Fig. 4A, B). Mean docking scores decreased beyond 50 conformers before rising at 400 (Fig. 4C), with scores predominantly distributed between −8 and −7 kcal/mol regardless of conformer count (Fig. 4D). Consequently, 50 conformers were selected for the 200,000-compound docking campaign, requiring approximately 14 days.

Small molecule conformation generation was performed using Python’s multiprocessing module, utilizing 24 processes, which took approximately 4 days to complete. Molecular docking was conducted on two servers. The first server, equipped with dual AMD EPYC 9754 128-Core Processors, handled 75% of the docking tasks (200 K molecules*75%). Docking was parallelized using a bash script, with 50 molecules processed simultaneously. This phase took approximately two weeks (14 days) to complete. The second server, equipped with dual AMD EPYC 9554 64-Core Processors, handled the remaining 25% of the docking tasks (200 K molecules*25%). Docking was similarly parallelized with a bash script, processing 50 molecules at a time, and took approximately one week (7 days) to complete.

### Model training and validation

#### Set performance

**Fig. 5:**
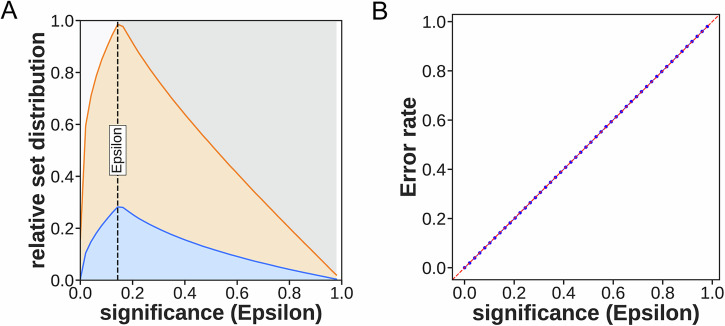
Training set analysis via amcp framework. **A** True positive rate maximized at ε = 0.143 (blue: active; orange: inactive). **B** Error rate distributions across thresholds.

Optimal docking scores were extracted for each compound, and corresponding SMILES strings were encoded as 1024-bit Morgan fingerprints for amcp framework training (80% training, 20% calibration split). Framework analysis identified optimal threshold ε = 0.143, maximizing true positive rate (Fig. 5A). All other parameters were set to the default values of the AMCP framework, and 5-fold cross-validation was also performed. Error rates exhibited diagonal distribution across thresholds (Fig. 5B), indicating balanced class distributions with minimal ambiguous predictions.

**Fig. 6:**
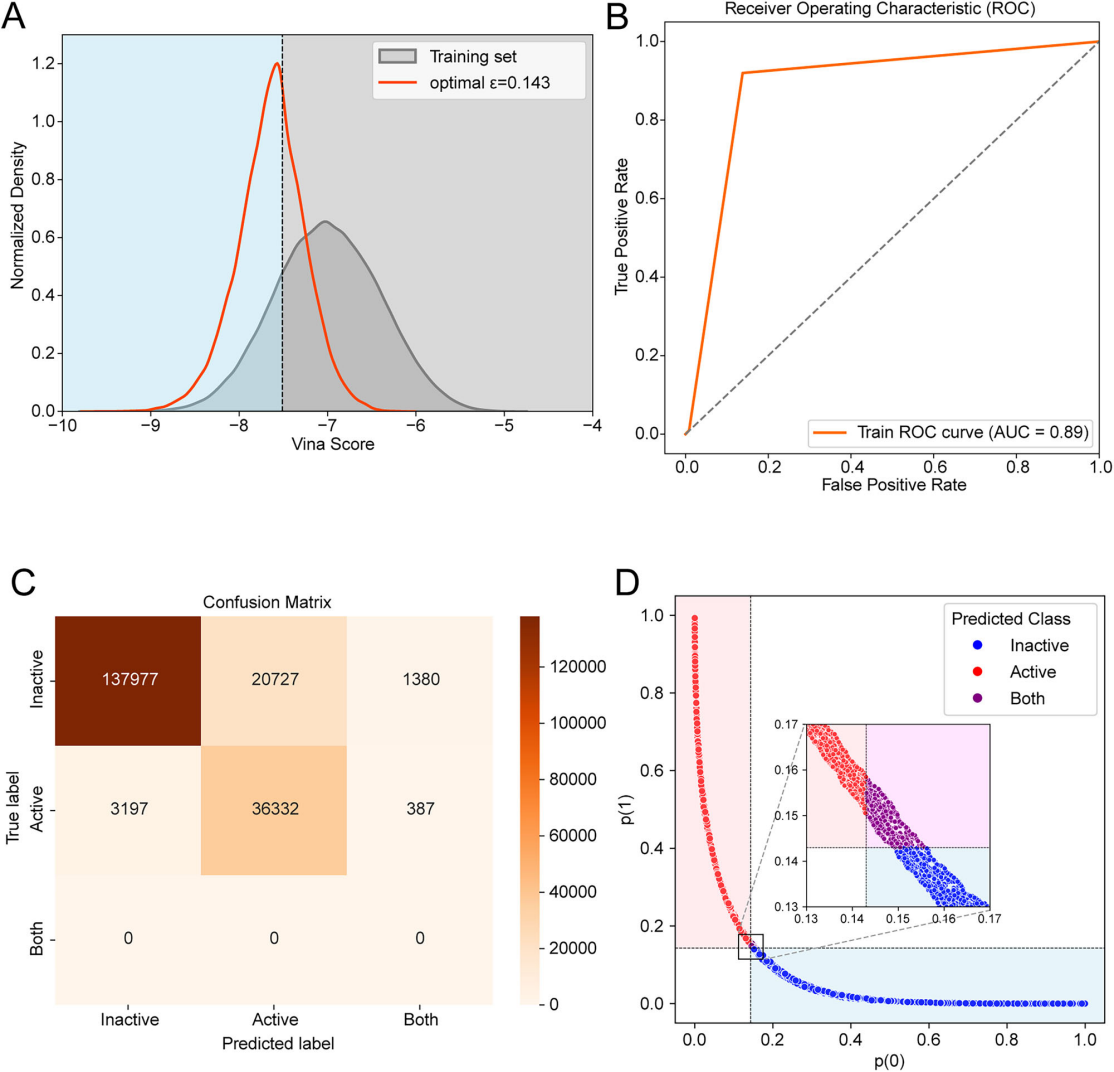
CatBoost model training performance. **A** Compounds labeled active (top 1% docking scores; threshold −7.511 kcal/mol). Predicted actives cluster near threshold. **B** ROC-AUC = 0.89, indicating strong discriminative performance. **C** Confusion matrix. **D** Classification results at ε = 0.143.

At ε = 0.143, the model achieved recall 0.8715 and precision 0.6367 (Table 1), with ROC-AUC 0.89 (Fig. 6B). High recall minimizes false negatives—critical for virtual screening where missing active compounds constitutes the primary risk.

**Table 1:** Performance metrics of the amcp training framework on the training set

Method	Target	Sensitivity	Precision
		200 K	
CatBoost	NSUN2	0.8715	0.6367

### Test Set Validation

**Fig. 7:**
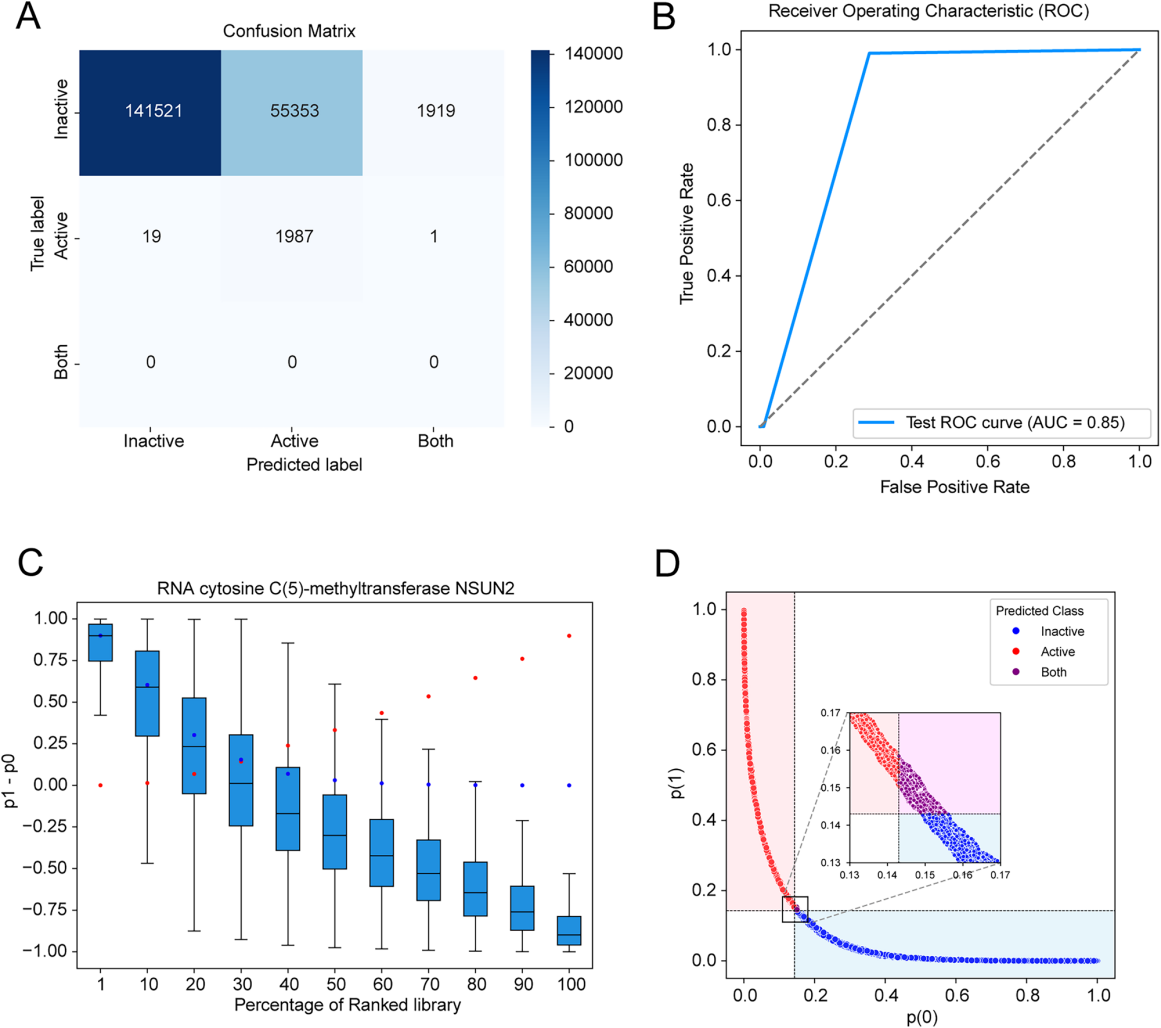
Test set validation performance. **A** Confusion matrix. **B** ROC-AUC = 0.85. **C** Δp distributions across score bins (red: p(0) median; blue: p(1) median). **D** Test set classification performance metrics at threshold ε = 0.143 (recall: 0.71, precision: 0.034, ROC-AUC: 0.85).

Independent validation on 200,000 held-out compounds (*ε* = 0.143) yielded recall 0.7147 and precision 0.034 (Table 2), with ROC-AUC 0.85 (Fig. 7A, B)—consistent with literature benchmarks33. The test set precision of 0.034 indicates that 96.6% of compounds predicted as active by the model are false positives. This reflects the extreme class imbalance inherent in virtual screening (true actives represent < 0.01% of chemical space) and necessitates subsequent orthogonal filtering methods. Docking scores exhibited strong negative correlation with Δp (Pearson *r* = −0.8075, *p* < 0.001) and score-rank correlation (*r* = −0.8035, *p* < 0.001; Fig. 7C, Table 3). In the Pearson correlation analysis, a strong negative correlation (*r* ≈ −0.807) was observed between model prediction confidence and docking affinity based on a large sample size (*n* = 200,000). This correlation was highly statistically significant (*p* < 0.001), indicating that the observed relationship is extremely unlikely to have occurred by chance and demonstrating high statistical robustness and reliability of our results.

**Table 2:** Performance metrics of the amcp training framework on the test set

Method	Target	Sensitivity	Precision
		200 K	
CatBoost	NSUN2	0.7147	0.034

**Table 3:** Pearson correlation between score segments and docking scores of small molecule lead compounds

p_1_-p_0_ Pearson correlation		
**Target**	**Ranks**	**Scores**
NSUN2	−0.8035	−0.8075

### Large-scale virtual screening

**Fig. 8:**
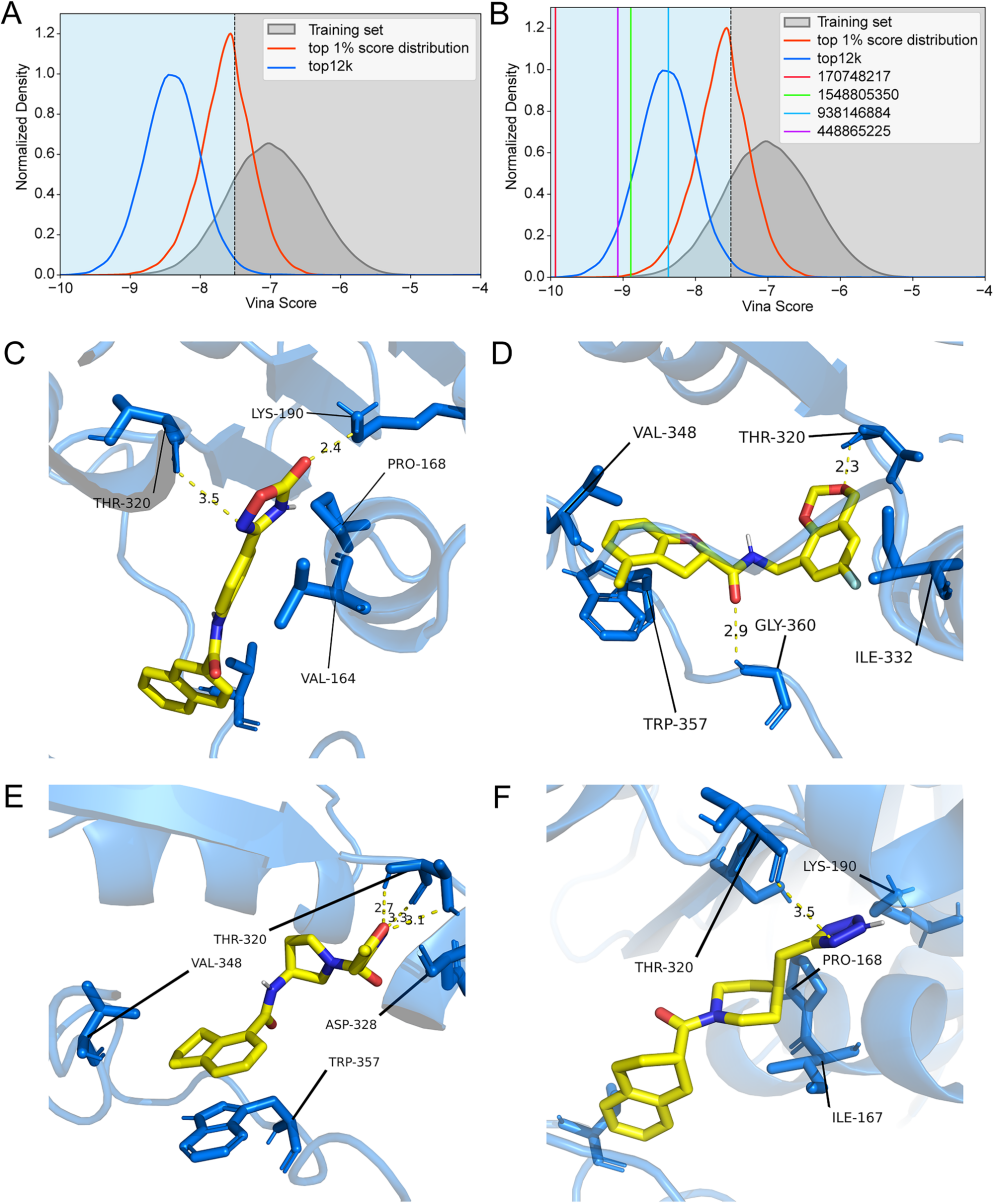
Virtual screening outcomes and binding mode analysis. **A** Top 12 K score distribution (blue) versus training set overall (gray) and top 1% (orange), demonstrating enrichment. **B** Representative compound score positions. **C**–**F** Binding modes for ZINC IDs 170748217 (−9.933 kcal/mol), 448865225 (−9.073 kcal/mol), 938146884 (−8.375 kcal/mol), and 1548805350 (−8.893 kcal/mol).

We converted the remaining approximately 350.5 million lead compounds (remaining total: 350,516,810) from the ZINC database into 1024-bit Morgan fingerprints and input them into the trained model for prediction. Ultimately, approximately 101.07 million lead compounds (101,070,481 in total) were predicted to be active, resulting in a database reduction of about 71.17% (see Supplementary Data 1). It is important to emphasize that the 249 M compounds classified as inactive by our model have not been validated as truly inactive—they simply scored below our confidence threshold. Top-ranked candidates (*n* = 12,000; Supplementary Data 2) underwent re-docking, revealing score distributions superior to training set top 1% threshold (Fig. 8A).

Representative examples (ZINC IDs: 170748217, 448865225, 938146884, 1548805350) exhibited docking scores of −9.933, −9.073, −8.375, and −8.893 kcal/mol respectively (Fig. 8B), with multiple stabilizing hydrogen bonds (Fig. 8C–F). Complete structures are provided in Supplementary Table 1.

### ADMET-Based Prioritization

vNN-ADMET analysis of top 12 K compounds applied stringent toxicity filters: DILI, cytotoxicity, CYP inhibition (1A2, 3A4, 2D6, 2C9, 2C19), BBB penetration, P-glycoprotein liability, hERG blocking, mitochondrial toxicity, and mutagenicity all negative; human liver microsomal stability positive. Filtering yielded 36 compounds; integration with docking data identified 34 compounds combining favorable ADMET profiles with high predicted affinity (Table 4).

**Table 4:** The remaining lead compounds after ADMET analysis and the docking scores of lead compounds with targets are ranked from large to small according to the Δp (p(1) -p(0)) predicted by the CatBoost model of the amcp framework

zinc_id	deltaP	smiles	score
1000500594	0.999875	O = C(NC1CN(C( = O)[C@@H]2 CC23 CCCC3)C1)[C@H]1[C@@H]2CCCCCC[C@@H]21	−7.509
1000507824	0.999875	O = C(NC1CN(C( = O)C2C = CC = CC = C2)C1)[C@H]1[C@@H]2CCCCCC[C@@H]21	−8.323
1000507789	0.999875	O = C(NC1CN(C( = O)C2CC3(CCC3)C2)C1)C1[C@H]2CCCCCC[C@H]12	−8.297
1000507828	0.999875	O = C(NC1CN(C( = O)C2C = CC = CC = C2)C1)C1[C@@H]2CCCCCC[C@@H]12	−8.123
1000510027	0.999875	O = C(NC1CN(C( = O)C2CC3(CC3)C2)C1)C1[C@@H]2CCCCCC[C@@H]12	−7.422
999347714	0.999875	O = C(NC1CN(C( = O)C2[C@H]3CCCCCC[C@H]23)C1)C1CC2(CC2)C1	−7.644
999347713	0.999875	O = C(NC1CN(C( = O)[C@H]2[C@@H]3CCCCCC[C@@H]32)C1)C1CC2(CC2)C1	−7.838
1000507785	0.999875	O = C(NC1CN(C( = O)C2CC3(CCC3)C2)C1)C1[C@@H]2CCCCCC[C@@H]12	−8.27
1000507794	0.999875	O = C(NC1CN(C( = O)C2CC3(CCC3)C2)C1)[C@H]1[C@@H]2CCCCCC[C@@H]21	−8.03
997934840	0.999875	O = C(NC1CN(C( = O)C2[C@H]3CCCCCC[C@H]23)C1)C1CC2(CCC2)C1	−8.008
1000500592	0.999875	O = C(NC1CN(C( = O)[C@H]2CC23CCCC3)C1)[C@H]1[C@@H]2CCCCCC[C@@H]21	−7.509
997934841	0.999875	O = C(NC1CN(C( = O)[C@H]2[C@@H]3CCCCCC[C@@H]32)C1)C1CC2(CCC2)C1	−7.706
1000500595	0.999875	O = C(NC1CN(C( = O)[C@@H]2CC23CCCC3)C1)C1[C@@H]2CCCCCC[C@@H]12	−7.812
997934839	0.999875	O = C(NC1CN(C( = O)C2[C@@H]3CCCCCC[C@@H]23)C1)C1CC2(CCC2)C1	−8.261
999347715	0.999875	O = C(NC1CN(C( = O)[C@H]2[C@@H]3CCCCCC[C@@H]32)C1)C1CC2(CC2)C1	−7.763
1000510026	0.999875	O = C(NC1CN(C( = O)C2CC3(CC3)C2)C1)C1[C@H]2CCCCCC[C@H]12	−7.658
987150770	0.999875	O = C(N[C@H]1CCCCCN(C( = O)C2CC3(CC3)C2)C1)[C@H]1[C@@H]2CCC[C@@H]21	−8.229
1000500591	0.999875	O = C(NC1CN(C( = O)[C@@H]2CC23CCCC3)C1)C1[C@H]2CCCCCC[C@H]12	−8.321
987150764	0.999875	O = C(N[C@@H]1CCCCCN(C( = O)C2CC3(CC3)C2)C1)[C@H]1[C@@H]2CCC[C@@H]21	−8.713
1000507831	0.999875	O = C(NC1CN(C( = O)C2C = CC = CC = C2)C1)C1[C@H]2CCCCCC[C@H]12	−8.196
1000510025	0.999875	O = C(NC1CN(C( = O)C2CC3(CC3)C2)C1)[C@H]1[C@@H]2CCCCCC[C@@H]21	−7.9
946492296	0.999749	CC1(C)C[C@H]1C( = O)N1CCC(NC( = O)[C@H]2[C@@H]3CCCCCC[C@@H]32)CC1	−7.723
992875268	0.999749	O = C(NC1CN(C( = O)C2[C@@H]3CCCCCC[C@@H]23)C1)C1C = CC = CC = C1	−8.259
327955030	0.999749	O = C(O)c1cccc2c1CCN(C( = O)[C@@H]1CCc3ccccc3C1)C2	−8.88
1000511861	0.999749	O = C(NC1CN(C( = O)[C@H]2CC23CC3)C1)[C@H]1[C@@H]2CCCCCC[C@@H]21	−7.543
1000511927	0.999749	O = C(NC1CN(C( = O)[C@@H]2CC23CCC3)C1)C1[C@@H]2CCCCCC[C@@H]12	−7.625
1000507941	0.999749	O = C(NC1CN(C( = O)[C@@H]2C[C@@H]3C[C@@H]3C2)C1)C1[C@H]2CCCCCC[C@H]12	−8.337
944966799	0.999749	O = C(C1 = CCCC1)N1CC2(CCN(C( = O)[C@@H]3CC[C@H]4 C[C@H]4C3)CC2)C1	−7.54
999633190	0.999749	O = C(NC1CN(C( = O)C2[C@@H]3CCCCCC[C@@H]23)C1)[C@@H]1CC12CCCC2	−8.283
1000511860	0.999749	O = C(NC1CN(C( = O)[C@@H]2CC23CC3)C1)C1[C@H]2CCCCCC[C@H]12	−7.841
943770141	0.999749	C = C1CCC(C( = O)NC2CCN(C( = O)c3ccccc3C)CC2)CC1	−8.453
999633170	0.999749	O = C(NC1CN(C( = O)C2[C@@H]3CCCCCC[C@@H]23)C1)[C@H]1CC12CCCC2	−7.998
990645299	0.999749	O = C(NC1CN(C( = O)[C@@H]2CCCc3nn[nH]c32)C1)C1[C@H]2CCCC[C@H]12	−8.242
956332063	0.999749	CC1(NC( = O)[C@H]2[C@@H]3CCC[C@@H]32)CCN(C( = O)C2C = CC = CC = C2)CC1	−8.13

### Molecular dynamics validation

**Fig. 9:**
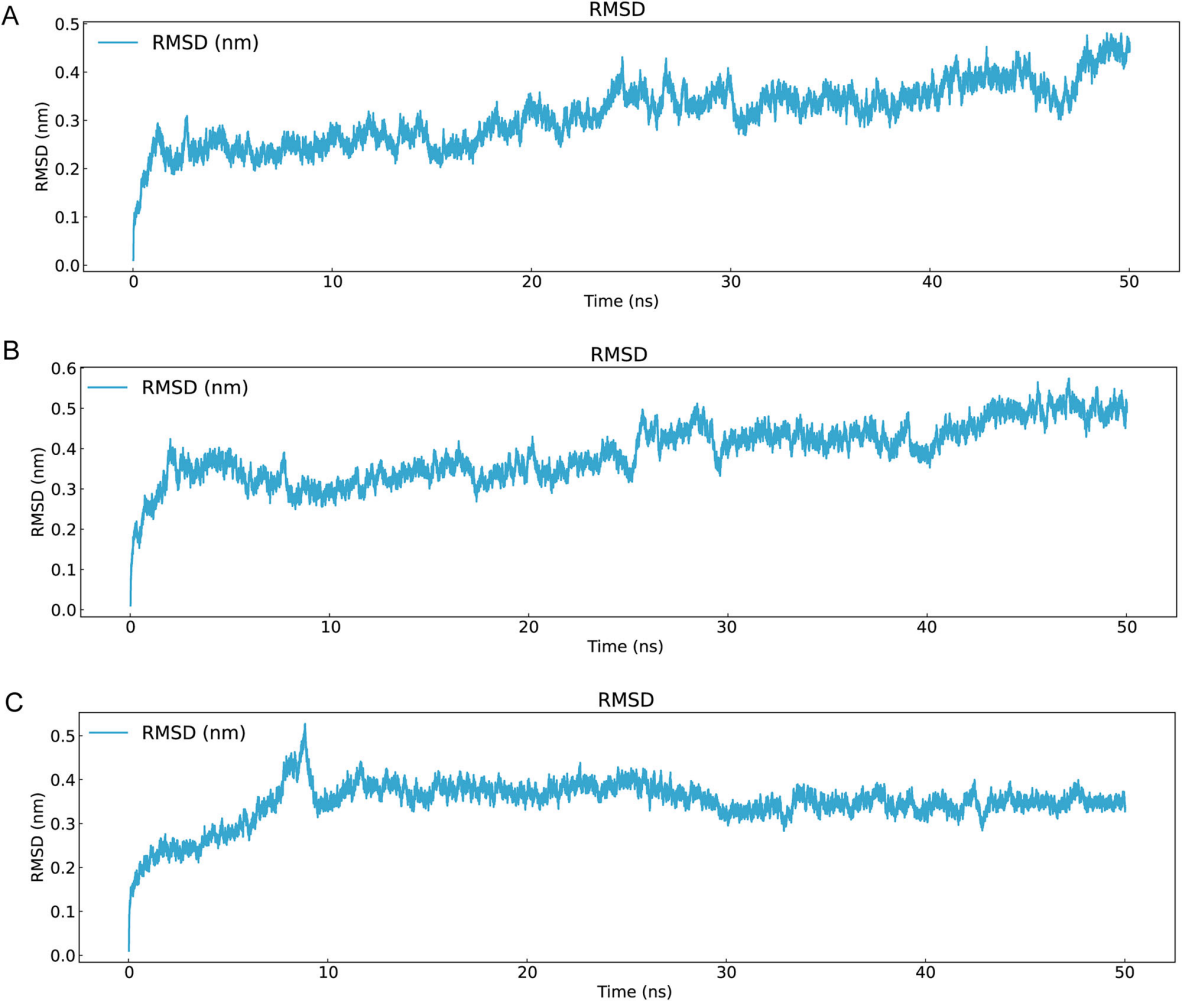
Molecular dynamics simulation RMSD curves of lead compounds with ZINC IDs 1000500594, 1000507789, 1000507824 with target protein. RMSD diagrams show the average deviation of atomic positions in the molecular system relative to the reference structure (usually starting conformation) during simulation, quantifying the degree of structural change. **A** RMSD curve diagram of 1000500594 with target protein. **B** RMSD curve diagram of 1000507789 with target protein. **C** RMSD curve diagram of 1000507824 with target protein.

**Fig. 10:**
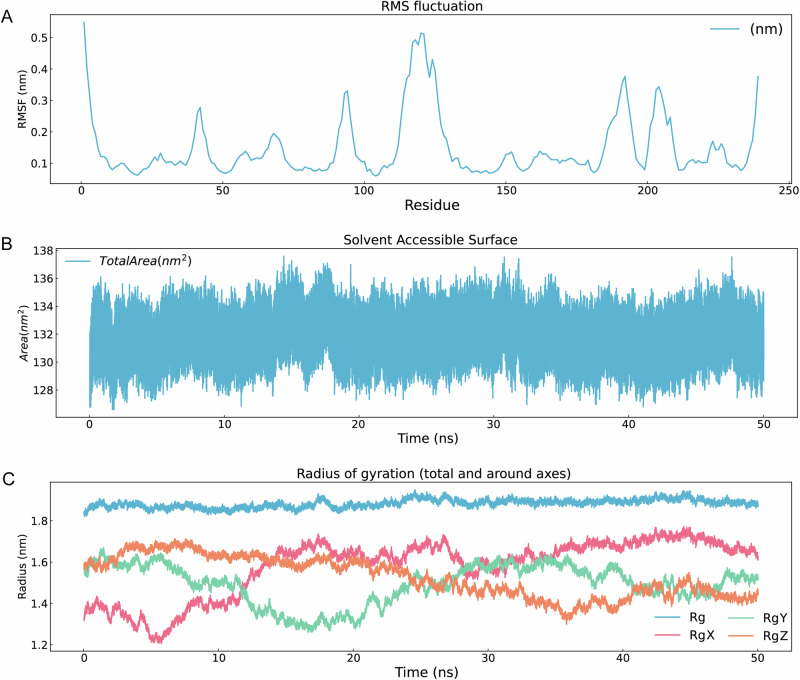
RMSF, SASA, Gyrate diagrams of lead compound with ZINC ID 1000500594 with target protein in 50-nanosecond simulation. **A** RMSF diagram. **B** SASA diagram. **C** Radius of gyration diagram. There are four curves in the diagram: blue is the overall radius of gyration curve (Rg), pink is the radius of gyration in x-axis direction (RgX), green is the radius of gyration in y-axis direction (RgY), orange is the radius of gyration in z-axis direction (RgZ).

**Fig. 11:**
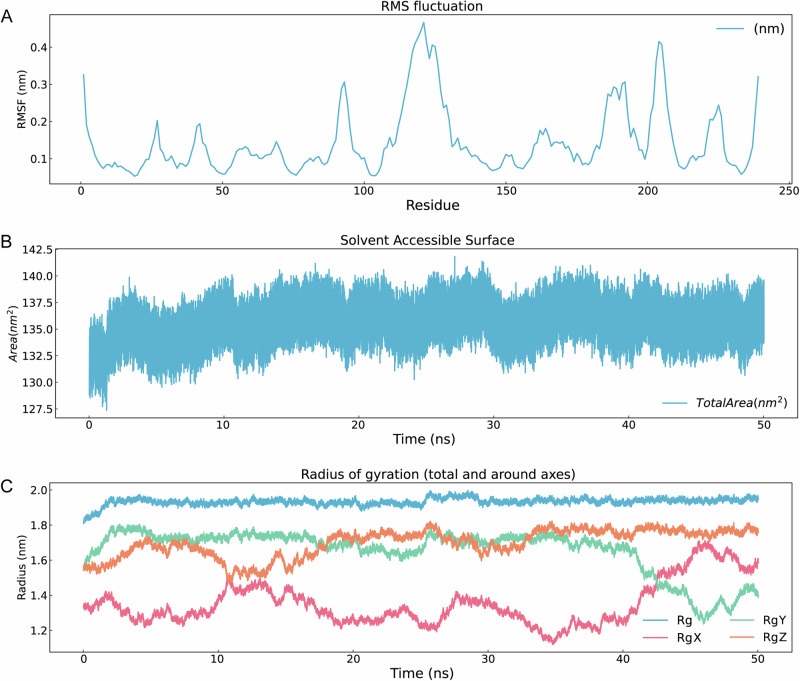
RMSF, SASA, Gyrate diagrams of lead compound with ZINC ID 1000507789 with target protein in 50-nanosecond simulation. **A** RMSF diagram. **B** SASA diagram. **C** Radius of gyration diagram. There are four curves in the diagram: blue is the overall Rg, pink is the RgX, green is the RgY, orange is the RgZ.

**Fig. 12:**
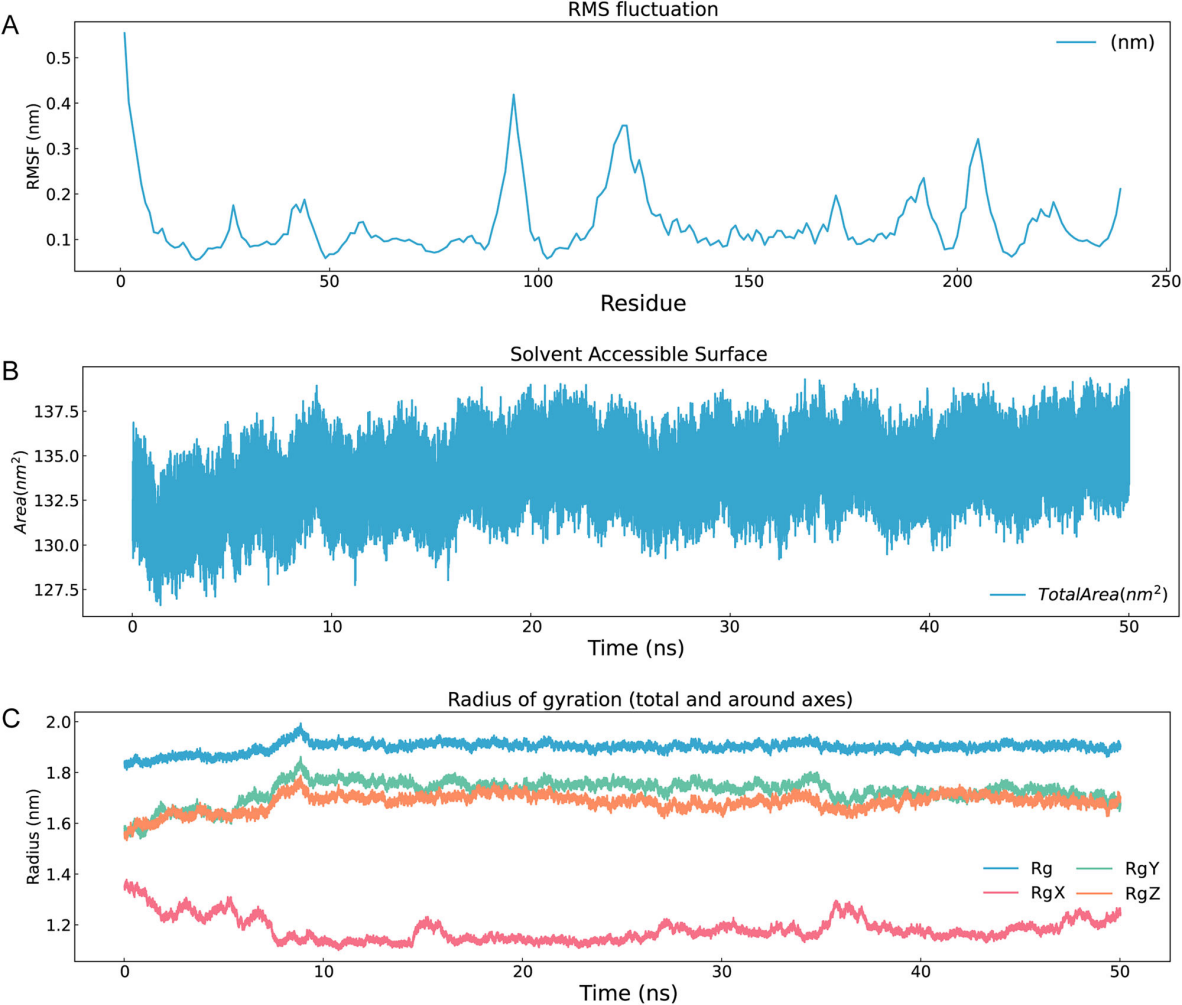
RMSF, SASA, Gyrate diagrams of lead compound with ZINC ID 1000507824 with target protein in 50-nanosecond simulation. **A** RMSF diagram. **B** SASA diagram. **C** Radius of gyration diagram. There are four curves in the diagram: blue is the overall Rg, pink is the RgX, green is the RgY, orange is the RgZ.

**Fig. 13:**
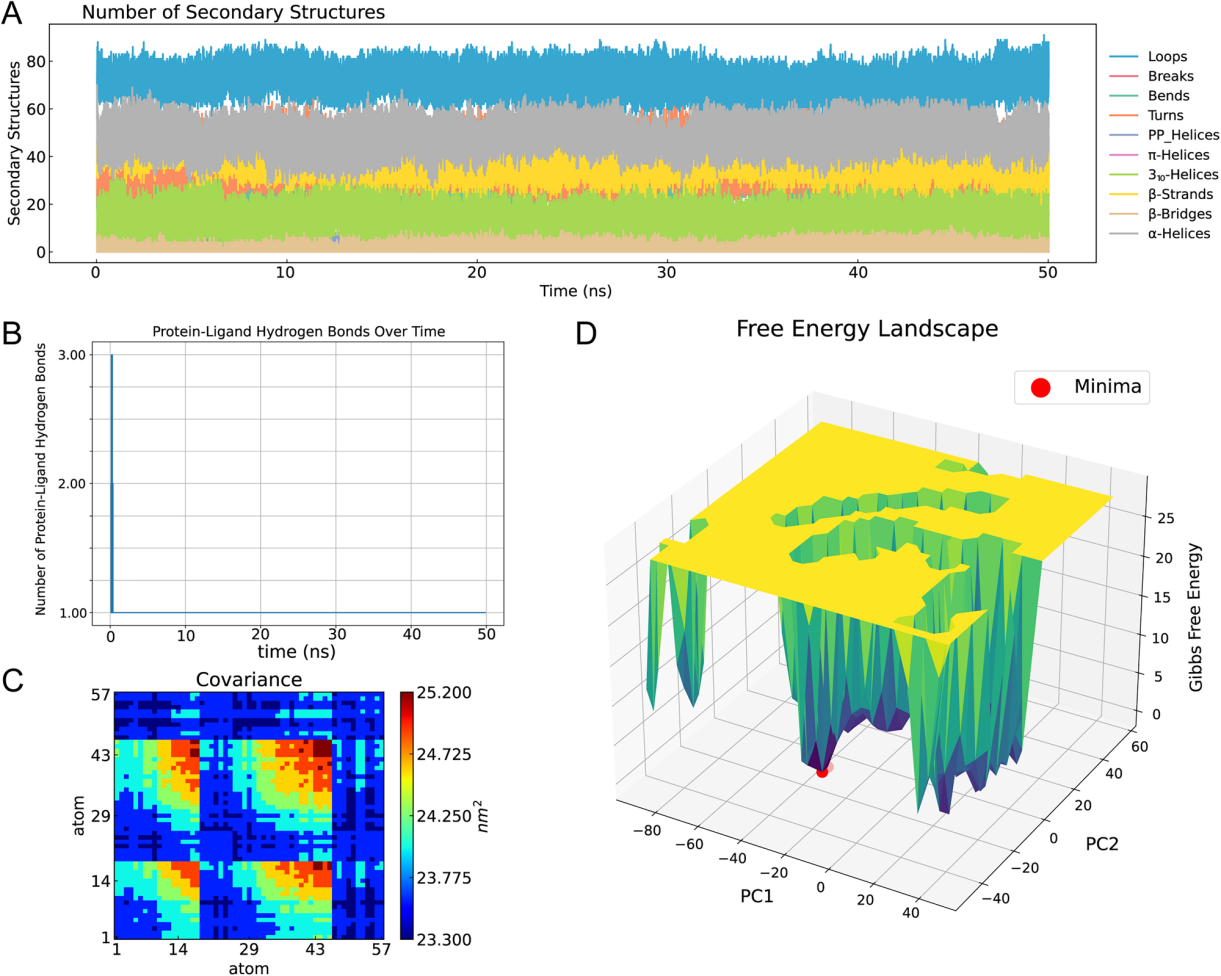
Protein secondary structure diagram, protein-ligand hydrogen bond number diagram, protein-ligand dynamic cross-correlation matrix, and free energy landscape diagram of molecular dynamics simulation for lead compound with ZINC ID 1000500594 with target protein. **A** DSSP diagram. **B** Hbond Analysis diagram. **C** DCCM diagram. **D** FEL diagram. The red dot in the diagram is the lowest point (free energy minimum, energy basin) representing the most stable conformational state of the system.

**Fig. 14:**
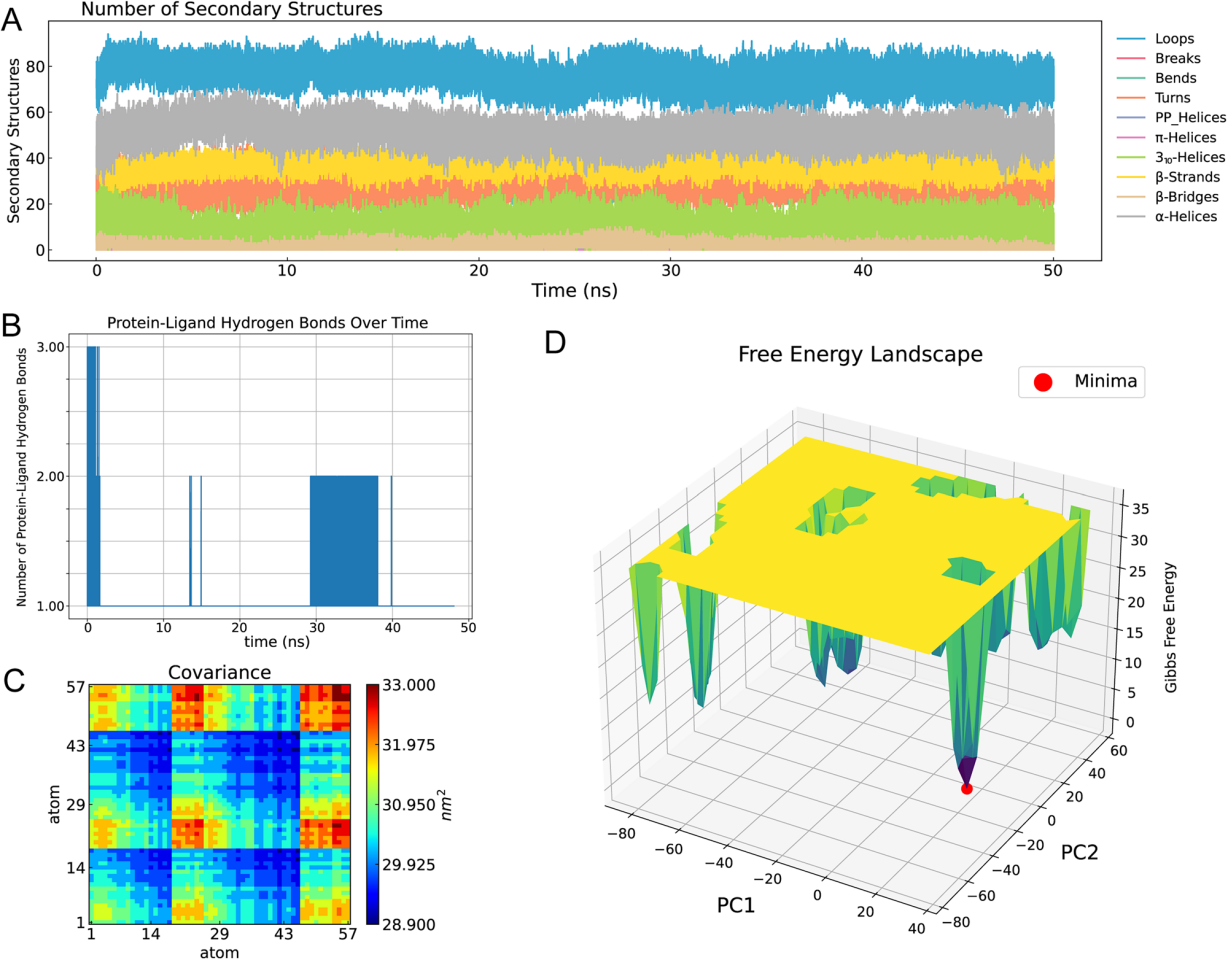
Protein secondary structure diagram, protein-ligand hydrogen bond number diagram, protein-ligand dynamic cross-correlation matrix, and free energy landscape diagram of molecular dynamics simulation for lead compound with ZINC ID 1000507789 with target protein. **A** DSSP diagram. **B** Hbond Analysis diagram. **C** DCCM diagram. **D** FEL diagram. The red dot in the diagram is the lowest point representing the most stable conformational state of the system.

**Fig. 15:**
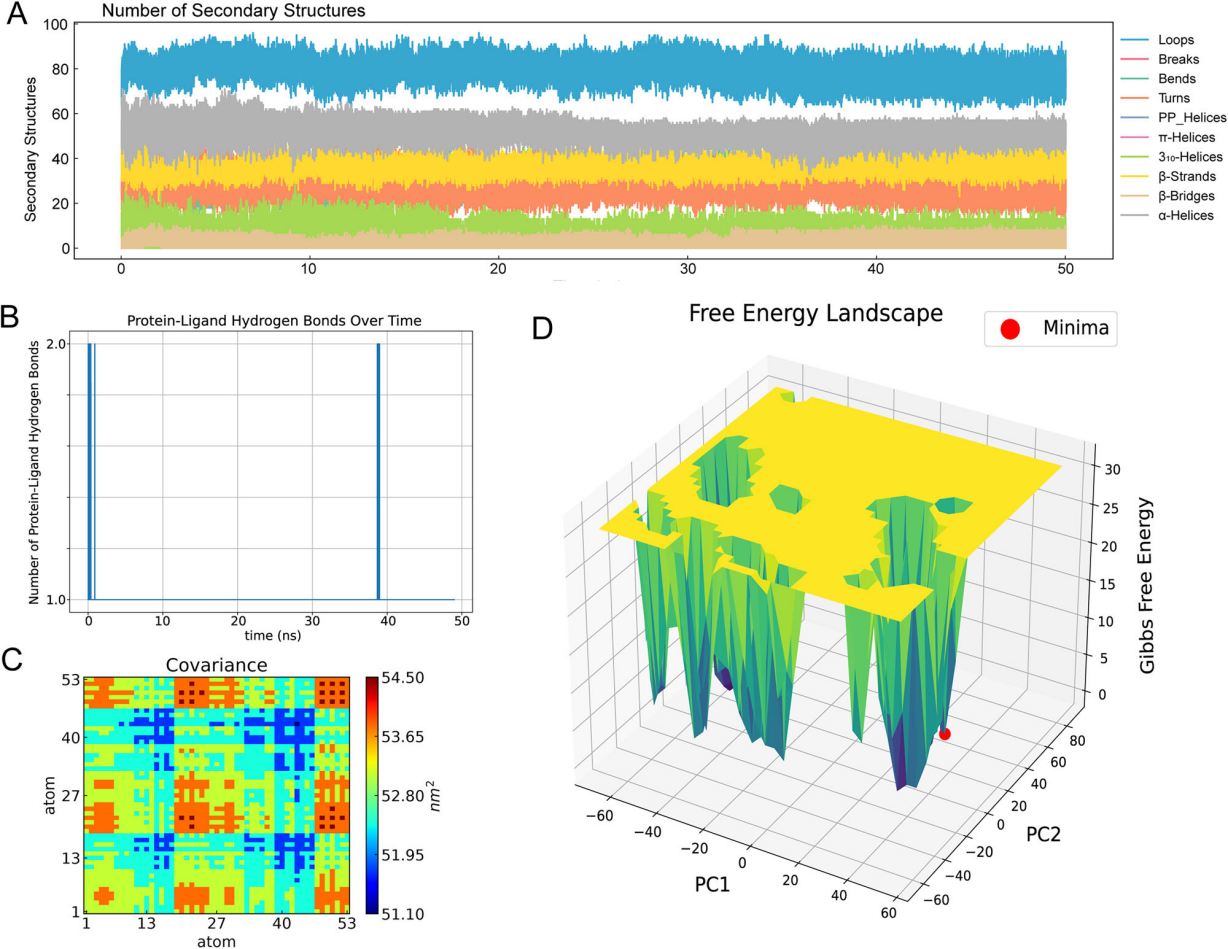
Protein secondary structure diagram, protein-ligand hydrogen bond number diagram, protein-ligand dynamic cross-correlation matrix, and free energy landscape diagram of molecular dynamics simulation for lead compound with ZINC ID 1000507824 with target protein. **A** DSSP diagram. **B** Hbond Analysis diagram. **C** DCCM diagram. **D** FEL diagram. The red dot in the diagram is the lowest point representing the most stable conformational state of the system.

Top three ADMET-filtered candidates (ZINC IDs: 1000500594, 1000507824, 1000507789) underwent 50-ns MD validation. RMSD trajectories (Fig. 9A–C) revealed enhanced stability for 1000507789 and 1000507824, particularly during 10–50 ns, compared to 1000500594. RMSF, Rg, and SASA analyses (Figs. 10–12) demonstrated that 1000507824 maintained low flexibility, stable solvent exposure, and consistent compactness across all axes, indicating minimal conformational rearrangement. Hydrogen bond analysis (Figs. 13B, 14B, 15B) identified 1000507789 as forming the most persistent interactions, while 1000500594 exhibited shortest interaction lifetimes. DCCM analysis (Fig. 14C) revealed strong cooperative dynamics for 1000507789, while 1000507824 showed even more pronounced correlated motions despite fewer hydrogen bonds. DSSP analysis (Figs. 13A, 14A, 15A) confirmed secondary structure preservation for 1000507789 and 1000507824 complexes throughout simulations, supporting conformational stability.

Collectively, ZINC IDs 1000507789 and 1000507824 exhibited superior stability and drug-likeness compared to 1000500594. However, further affinity optimization and experimental validation of activity and safety profiles remain essential.

## Discussion

The RNA cytosine-5 methyltransferase NSUN2 has emerged as a critical regulator in the pathogenesis of multiple human diseases, including various malignancies and neurodegenerative disorders. These conditions impose substantial healthcare burdens globally, necessitating the development of novel therapeutic strategies. Given the essential role of NSUN2 in regulating RNA methylation, maintaining cellular homeostasis, and influencing disease progression, coupled with the limited efficacy and significant side effects associated with current therapeutic options, there exists an urgent and unmet need for selective NSUN2-targeted interventions^11,13^. This study addresses this critical gap by employing an integrated computational drug discovery approach combining structural biology, ultra-large-scale virtual screening, machine learning-guided scoring, comprehensive ADMET profiling, and molecular dynamics simulations to systematically identify high-affinity, low-toxicity lead compound candidates targeting NSUN2.

A critical foundation of this study lies in the structural characterization and validation of the NSUN2 binding pocket. The high-resolution crystal structure of M. jannaschii TRM4 protein complexed with sinefungin (PDB: 3A4T) exhibited structural alignment with the AlphaFold2-predicted human NSUN2 structure, with sequence identity of 34.2%, sequence similarity of 51.7%, and structural alignment RMSD of 1.82 Å over 187 aligned Cα atoms. The 34.2% sequence identity between human NSUN2 and M. jannaschii TRM4 falls in a moderate confidence range for homology-based validation. At this sequence identity, while the overall fold is likely conserved, fine details of the binding pocket geometry—particularly side-chain orientations that are critical for ligand interactions—may still differ between species and between predicted and experimental structures. No significant deviations were observed in the core binding pocket architecture, with critical catalytic residues including C271 in NSUN2 (corresponding to C295 in TRM4) structurally conserved^30^. Sinefungin demonstrated predicted stable binding within the AlphaFold2-predicted NSUN2 pocket, validating the cross-species conservation of this catalytic site and establishing the reliability of the predicted structure for virtual screening applications. This structural similarity provides evidence supporting the evolutionary conservation of the NSUN2 active site across species, from archaea to humans, which has important implications for structure-based drug design.

The successful application of AlphaFold2 predictions in this study highlights the impact of artificial intelligence-driven structural biology on drug discovery, particularly for targets lacking experimentally determined structures^31,32^. AlphaFold2 has demonstrated high accuracy in protein structure prediction, with confidence scores often correlating well with experimental validation. However, it is important to acknowledge certain limitations inherent to computational structure prediction. While AlphaFold2 excels at predicting static structures, it may not fully capture conformational flexibility, induced-fit mechanisms upon ligand binding, or allosteric effects that play crucial roles in drug-target interactions^33^. Additionally, the accuracy of predicted binding pockets can vary depending on the availability of homologous templates in training data. In the case of NSUN2, the conservation of the catalytic pocket across species and the validation against the TRM4 crystal structure enhance confidence in the predicted binding site geometry.

The cross-species structural alignment approach employed in this study represents a preliminary structural support strategy when direct experimental structures are unavailable for the target protein. This methodology has applicability in drug discovery campaigns targeting proteins with limited structural information, enabling rational drug design even in the absence of target-specific crystal structures^29^. The demonstrated conservation of the NSUN2/TRM4 active site further suggests that inhibitors identified through this approach may exhibit activity across orthologous enzymes, potentially offering insights into mechanism of action and providing opportunities for structure-activity relationship studies using more tractable experimental systems.

The CatBoost machine learning model, coupled with Morgan fingerprint molecular descriptors, demonstrated performance in prioritizing compounds from ultra-large chemical libraries. On the training set, the model achieved a recall of 0.8715, precision of 0.6367, and AUC of 0.89, while the test set yielded a recall of 0.7147, precision of 0.034, and AUC of 0.85. The test set precision of 0.034 indicates that 96.6% of compounds predicted as active by the model are false positives. This reflects the extreme class imbalance inherent in virtual screening (true actives represent < 0.01% of chemical space) and necessitates subsequent orthogonal filtering methods. In this study, compounds ranked in the top 1% were considered active. The model achieved a precision of only 3.4% on the test set. Although the precision is low, the study mitigated the impact of false positives and improved overall screening efficiency through a multi-step filtering approach. First, approximately 101 million compounds predicted as active were ranked by Δp, and the top 12,000 were selected for secondary molecular docking to validate their binding affinity. Second, ADMET analysis was performed on the top 12,000 compounds to screen for candidates with low toxicity and high drug-likeness. Finally, 50 ns molecular dynamics simulations were conducted on the top candidate compounds to assess binding stability (e.g., RMSD, hydrogen bond analysis). In the training set analysis of the AMCP framework, the optimal prediction threshold (epsilon, ε) was determined to be 0.143, at which the true positive rate (TPR) reached its maximum. These steps collectively enhanced the reliability of the final results (Table 5).

**Table 5:** Compound Prioritization Summary

Step	Input	Filtering criteria	Output	Reduction rate
Virtual Screening	350,516,810	Class-specific confidence scores	101,070,481	71.165%
Top-ranked Selecting	101,070,481	Rank of prediction confidence	12,000	99.988%
ADMET	12,000	Toxicity filters + Docking data	34	99.717%
MD	34	Stability + Drug-likeness	2	94.118%

The high recall rate achieved by the model is particularly critical in the context of large-scale virtual screening, as it minimizes false negatives and ensures that potentially active compounds are not prematurely eliminated from consideration. This recall-prioritized strategy is suited for early-stage lead identification, where the primary objective is to maximize hit rates while accepting a number of false positives that can be subsequently filtered through orthogonal validation methods^34^. However, the relatively low precision observed, particularly in the test set, is not merely an artifact of the algorithm but a reflection of the severe class imbalance inherent in drug discovery datasets. Active compounds represent a minute fraction of the chemical space, and this phenomenon is well-documented in the literature on virtual screening benchmarks^35^.

Specifically, studies have shown that standard metrics often fail to capture performance when active molecules are rare^36^. For instance, the use of decoys for training or testing can lead to over-optimistic estimates of model performance, masking the true difficulty of distinguishing actives from inactives. Furthermore, research indicates that imbalanced training datasets—where inactives outnumber actives—can decrease recall but increase precision, highlighting the trade-off inherent in these tasks. Therefore, the low precision should be interpreted as a challenge specific to the dataset composition rather than a general limitation of the methodology itself^37^.

We addressed false positives through hierarchical filtering (molecular docking followed by ADMET followed by MD simulations). While raw precision is 3.4%, the multi-stage filtering ultimately yields 34 compounds from 101 M predictions (99.99997% reduction). The cost-benefit analysis of this strategy is favorable when considering that computational filtering is significantly less expensive and time-consuming than experimental screening^38^. Moreover, the high recall ensures that promising candidates are not inadvertently discarded. We acknowledge that this precision would be unacceptable for final candidate selection but is appropriate for computational pre-filtering.

Future refinements could explore ensemble modeling approaches, integration of three-dimensional pharmacophore features, or incorporation of physics-based descriptors to further enhance predictive accuracy^18,39^. Additionally, active learning strategies could be employed to iteratively improve model performance as experimental validation data becomes available.

The virtual screening campaign processed 350 million compounds from the ZINC20 database, with approximately 71.17% (249 million) classified below the confidence threshold through machine learning-based prioritization, ultimately yielding 101 million putative active lead compounds for subsequent docking analysis. The top 12,000 candidates exhibited mean docking scores superior to the 99th percentile threshold of the training set, with four representative molecules demonstrating binding affinities of −9.933, −9.073, −8.375, and −8.893 kcal/mol, respectively^40,41^.

This substantial prioritization of candidate space—from hundreds of millions to thousands of high-priority compounds—exemplifies the utility of computational approaches in managing the combinatorial expansion of drug-like chemical space. The ZINC20 database represents one of the largest publicly available collections of commercially accessible compounds, containing over 1.4 billion molecules^40^. Screening such vast libraries through purely experimental methods would be expensive and time-consuming. The computational pipeline implemented in this study demonstrates that filtering strategies can navigate this chemical space, focusing resources on the most promising candidates.

The hierarchical filtering approach—combining machine learning pre-filtering with physics-based molecular docking—leverages the complementary strengths of both methodologies. Machine learning models excel at rapid evaluation of vast libraries based on learned patterns from training data, while molecular docking provides mechanistic insights into binding modes and accounts for specific protein-ligand interactions^19,42^. This synergistic combination enables efficient prioritization while maintaining chemical diversity in the final candidate pool. The docking scores observed for top candidates (−8 to −10 kcal/mol) are consistent with high-affinity small molecule inhibitors reported in the literature^20^.

It is important to note that docking scores, while informative, should not be interpreted as absolute predictors of binding affinity due to limitations in scoring function accuracy and the approximations inherent in rigid or semi-flexible docking protocols^43^. The docking scores primarily serve as relative ranking metrics to prioritize compounds for further evaluation. Alternative strategies, such as consensus scoring approaches that integrate predictions from multiple docking programs or inclusion of machine learning-based scoring functions trained on experimental binding data, could further enhance prediction accuracy^44,45^.

Following molecular docking, the candidate compounds underwent rigorous ADMET evaluation using the vNN-ADMET platform to assess pharmacokinetic properties and toxicity liabilities. Compounds exhibiting high risks for DILI, cardiotoxicity, hepatotoxicity, or inhibition of major cytochrome P450 enzymes were systematically eliminated. This comprehensive filtering process ultimately yielded 34 low-toxicity, high drug-likeness lead compounds with favorable predicted safety profiles^46,47^.

The integration of ADMET prediction at early stages of drug discovery represents a paradigm shift from traditional approaches where pharmacokinetic and toxicity assessments occurred only after significant investment in lead optimization. Early ADMET profiling can reduce attrition rates in later development stages, which is critical since poor pharmacokinetics and toxicity remain leading causes of clinical trial failures, accounting for approximately 40–50% of drug candidate attrition^48,49^. By frontloading these assessments into the virtual screening pipeline, this study enhances the probability that identified leads will successfully progress through preclinical and clinical development.

The vNN-ADMET platform employs neural network-based models trained on extensive experimental datasets to predict multiple ADMET endpoints^50^. While these in silico predictions have demonstrated reasonable accuracy for many ADMET parameters, it is important to acknowledge their limitations. Prediction accuracy varies across different endpoints, with some properties (such as human intestinal absorption and blood-brain barrier penetration) being more reliably predicted than others (such as specific organ toxicities)^51,52^. Therefore, in silico ADMET predictions should be viewed as prioritization tools rather than definitive assessments, and experimental validation remains essential.

The specific ADMET criteria employed in this study—elimination of compounds with predicted DILI, cardiotoxicity, hepatotoxicity, and CYP inhibition risks—reflect the most common safety liabilities encountered in drug development^53^. Drug-induced liver injury is a particularly important consideration, as it represents a leading cause of drug withdrawals from the market^54^. Cytochrome P450 enzyme inhibition can lead to problematic drug-drug interactions, particularly for CYP3A4, CYP2D6, and CYP2C9, which metabolize a large proportion of clinical drugs^55^.

ADMET analysis is only one component of the overall workflow, primarily aimed at prioritizing the exclusion of compounds with significant pharmacokinetic deficiencies or toxicity risks before proceeding to costly in vitro or in vivo experiments. Prior to this step, we conducted machine learning screening (AMCP framework) based on docking scores; following it, we performed molecular dynamics simulations to validate the outstanding compounds. This multi-stage, multi-metric comprehensive screening strategy helps mitigate the impact of uncertainties inherent in any single computational method. The 34 compounds we identified are the result of enrichment through docking scores (Top 12k) and combined filtration via multiple ADMET metrics. Future experimental validation can be employed to assess the accuracy of these selected compounds.

Molecular dynamics simulations were conducted on the top three candidates (ZINC IDs: 1000507789, 1000507824, 1000500594) over 50 nanoseconds to evaluate predicted binding stability. Compounds 1000507789 and 1000507824 exhibited stable RMSD profiles, minimal RMSF fluctuations, consistent SASA values, stable radius of gyration (Rg), and pronounced ligand-protein cooperative motion as revealed by DCCM analysis. Secondary structure remained intact throughout the simulations, and these compounds maintained persistent hydrogen bonding networks, collectively suggesting favorable binding stability^56,57^.

Molecular dynamics simulations provide insights beyond static docking poses by capturing the temporal evolution of protein-ligand complexes and accounting for conformational flexibility, solvent effects, and entropic contributions to binding^58^. The stable RMSD values observed for the top candidates indicate that the binding complexes reach equilibrium quickly and maintain structural integrity throughout the simulation period, suggesting that the predicted binding modes represent low-energy states. The RMSF analysis, which quantifies per-residue flexibility, revealed minimal fluctuations in the binding site region, further corroborating predicted stable ligand engagement.

The dynamic cross-correlation matrix (DCCM) analysis revealed coordinated motions between ligand and protein residues, indicating that ligand binding may induce allosteric communication networks within the NSUN2 structure. Such mechanistic insights are valuable for structure-based optimization^59^. The hydrogen bonding analysis demonstrated that key interactions persist throughout the simulations, with high occupancy rates indicating stable engagement of residues in the binding pocket^60^.

While the 50-nanosecond simulation timescale employed in this study is standard for initial lead compound evaluation, it is important to acknowledge that some biologically relevant conformational changes may occur on longer timescales^61^. Extended simulations using enhanced sampling methods could provide more comprehensive characterization of binding free energies^62,63^. Despite these limitations, the molecular dynamics results provide preliminary computational evidence for predicted stable NSUN2-ligand interactions that justify prioritization of these candidates for experimental testing.

To benchmark our identified hits against the known NSUN2-targeting compound, we performed molecular docking of Sinefungin with the NSUN2 AlphaFold-predicted structure using the CB-DOCK platform. Sinefungin exhibited a Vina Score of −3.3 kcal/mol (Supplementary Fig. 1). In comparison, our top-ranked lead compounds demonstrated docking scores ranging from −8.375 to −9.933 kcal/mol, indicating comparable binding potential. Structural analysis revealed that while Sinefungin forms multiple hydrogen bonds with the binding pocket, our lead compounds establish different interaction patterns, which may contribute to their enhanced binding profiles.

Our predicted compounds represent non-covalent reversible inhibitors targeting the cofactor/substrate binding pocket, whereas the recently reported azetidine acrylamides are covalent irreversible inhibitors targeting the catalytic cysteine C271^14^. This complementary mechanism may offer distinct advantages including reversibility, potentially reduced off-target effects, and different pharmacokinetic profiles. Comparative docking analysis shows that our top compounds occupy overlapping but distinct binding regions compared to the reported covalent inhibitors, suggesting they may engage different aspects of the catalytic mechanism. The structural diversity of our predicted compounds relative to existing inhibitors provides opportunities for structure-activity relationship studies and lead optimization strategies that explore alternative chemical scaffolds.

The therapeutic potential of our identified NSUN2 inhibitors extends beyond direct enzymatic inhibition to encompass downstream effects on oncogenic signaling networks. NSUN2-mediated m5C modifications have been shown to stabilize mRNAs encoding key oncoproteins and signaling mediators, particularly within the PI3K/AKT and MAPK/ERK pathways^6,7^.

Inhibition of NSUN2 enzymatic activity by our lead compounds would be expected to destabilize these oncogenic transcripts, potentially resulting in:

1. Reduced PI3K/AKT pathway activation: Decreased stability of pathway component mRNAs (e.g., AKT1, mTOR, PIK3CA) may attenuate growth signals and restore sensitivity to apoptotic stimuli.
2. Suppressed MAPK/ERK signaling: Destabilization of MAPK pathway transcripts could reduce proliferative drive and migratory capacity.
3. Enhanced therapy sensitivity: Given NSUN2’s role in mediating EGFR-TKI resistance in lung cancer^8^, these inhibitors may synergize with targeted therapies by preventing compensatory survival signaling.
4. Epitranscriptomic reprogramming: Broader alterations in m5C modification patterns may affect tRNA functionality and codon-specific translation rates, potentially disrupting oncogenic protein synthesis programs^64–66^.

Cellular validation experiments measuring phosphorylation status of AKT (Ser473, Thr308) and ERK1/2 (Thr202/Tyr204) following inhibitor treatment, along with RNA stability assays for known NSUN2 target transcripts, will be essential to confirm these predicted effects. Furthermore, combination studies with EGFR inhibitors (e.g., osimertinib) in resistant cell line models would directly test the hypothesis that NSUN2 inhibition can restore therapeutic sensitivity.

Building upon the computational findings presented here, several critical research directions will advance these candidates toward potential clinical translation:

Experimental validation pipeline: Biochemical assays: Direct measurement of NSUN2 methyltransferase inhibition using enzyme activity assays with recombinant protein and tRNA substrates^67^ - Cellular validation: Assessment of m5C methylation patterns using bisulfite sequencing or antibody-based detection in cancer cell lines - Target engagement studies: Cellular thermal shift assays (CETSA) or drug affinity responsive target stability (DARTS) to confirm on-target binding^68^ - Phenotypic validation: Evaluation of effects on cancer cell proliferation, migration, invasion, and apoptosis in NSUN2-dependent cell lines.

Structure-activity relationship (SAR) optimization: Chemical synthesis of analogs to explore modifications that enhance potency, selectivity, and pharmacokinetic properties - Co-crystallization studies to determine high-resolution binding modes and guide rational optimization - Computational design of next-generation compounds incorporating favorable features from multiple scaffolds.

Selectivity profiling: Screening against NSUN family members (NSUN1, 3-7) and related RNA methyltransferases (DNMT, METTL families) to assess isoform selectivity - Broad kinome screening to evaluate potential off-target kinase inhibition given structural similarities with ATP-binding sites.

Advanced therapeutic strategies: Combination therapy approaches: Testing synergy with EGFR inhibitors (osimertinib), chemotherapeutics, or immune checkpoint inhibitors in relevant cancer models - Pan-NSUN family inhibition: Rational design of dual or multi-specific inhibitors targeting multiple NSUN isoforms for enhanced epitranscriptomic disruption in cancers with complex m5C dysregulation - Epitranscriptomic combination strategies: Integration with FTO, ALKBH5, or METTL3/14 inhibitors to comprehensively target RNA modification landscapes.

In vivo efficacy studies: Patient-derived xenograft (PDX) models representing NSUN2-high cancers - Pharmacokinetic/pharmacodynamic (PK/PD) studies in mice to optimize dosing regimens - Toxicology profiling in relevant animal models.

These future investigations will determine whether computational predictions translate to therapeutic efficacy, ultimately advancing toward clinical evaluation in patients with NSUN2-driven malignancies.

Several limitations of this study warrant acknowledgment. First and most importantly, all findings are based purely on computational predictions and lack experimental validation. While the computational methodologies employed are established, in silico predictions cannot fully recapitulate the complexities of biological systems. Experimental validation through biochemical assays, cellular assays, and ultimately in vivo efficacy studies will be essential to confirm biological activity and therapeutic potential^68,69^.

Second, in this study, during the optimization of conformation generation parameters, statistical analysis of 1000 randomly selected lead compounds was performed, leading to the determination that generating 50 conformations achieves a favorable balance between docking score quality and computational cost. However, we recognize that certain limitations remain. In the future, we plan to implement stratified sampling and large-scale validation, and further compare the results with experimental data to enhance the accuracy and reliability of the study outcomes. Besides, the machine learning model’s relatively low precision on the test set, despite high recall, necessitates multi-step filtering to manage false positives. The model’s performance is constrained by the quantity and quality of training data available for NSUN2 inhibitors. As more experimental data becomes available, active learning strategies could be employed to iteratively refine model performance^70^. In this study, the model achieved a precision of 3.4%. A multi-stage workflow including secondary docking, ADMET analysis, and MD simulations was implemented. However, we recognize that the low model precision may lead to a high number of false positives. In future work, we plan to incorporate additional metrics such as the F1-score, 3D pharmacophore features, and even structure-based descriptors to enrich the chemical information and improve model specificity. Furthermore, we intend to perform longer-timescale molecular dynamics simulations with multiple replicates to more reliably assess binding stability and calculate binding free energy, thereby providing a stronger theoretical foundation for prioritizing subsequent wet-lab experiments.

Third, in this study, we utilized vNN-ADMET to assess multiple key endpoints, including drug-induced liver injury (DILI), cytotoxicity, cytochrome P450 enzyme inhibition, P-glycoprotein substrates and inhibitors, cardiotoxicity (hERG inhibition), mitochondrial toxicity, and mutagenicity (AMES test). The screening criteria were based on binary classification outputs, where toxicity endpoints were labeled as “No,” while stability in human liver microsomes was labeled as “Yes.” As a result, 34 low-risk candidates were selected from the Top-12k compounds. However, we are aware that in silico ADMET prediction tools such as vNN-ADMET have inherent limitations, including relatively high false-positive and false-negative rates, which may affect the accuracy of screening results—a common challenge in computational ADMET assessment. To address these issues, we plan to integrate multiple ADMET tools (such as SwissADME and ADMETlab) in future work for cross-validation. Ultimately, we intend to confirm the predicted outcomes through experimental assays (e.g., in vitro liver microsome stability tests, hERG inhibition assays, and cytotoxicity validation) to enhance the reliability and translational potential of the study.

Fourth, while molecular docking and molecular dynamics simulations provide valuable insights, they may be subject to sampling limitations. Different docking programs employ distinct algorithms and scoring functions, which can yield varying rank-orderings of compounds^57^. Additionally, the limited simulation timescale (50 ns) may not capture rare conformational transitions occurring on longer timescales. In this study, a simulation duration of 50 ns was employed. During the later stages of the simulation (particularly from 10–50 ns), key metrics such as the RMSD of the complex stabilized, indicating that the system reached local equilibrium within the simulated timeframe. We also acknowledge that longer simulation times and repeated runs would allow for a more reliable assessment of binding modes, calculation of binding free energy, and evaluation of convergence. In future work, we will perform extended, repeated simulations on key candidate compounds to provide conclusions with greater statistical significance.

Fifth, the mechanisms by which the identified lead compounds may inhibit NSUN2 catalytic activity and their downstream effects on cellular m5C modification patterns remain to be elucidated. Experimental studies will be required to determine whether these compounds function as competitive inhibitors or through other mechanisms^67^.

We recommend the following experimental validation strategy: Immediate priorities: Thermal shift assays (DSF) for the top 5 compounds to assess direct binding; Surface plasmon resonance (SPR) for compounds showing DSF signals to determine Kd values; Prioritization: ZINC IDs 1000507789 and 1000507824 based on MD predicted stability, followed by 1000503122 and 1000508945. Secondary validation: Enzymatic activity assays measuring inhibition of NSUN2-mediated m5C formation; Cellular assays in NSUN2-dependent cancer cell lines (e.g., A549, MDA-MB-231); Mass spectrometry to confirm binding site. Lead optimization criteria: Target IC50 < 1 μM for prioritization; Cellular activity at <10 μM concentrations; Selectivity profiling against other NSUN family members.

While our pipeline employs established computational tools, the contribution lies in: (1) the first comprehensive virtual screening campaign specifically targeting NSUN2; (2) systematic optimization and validation of the machine learning framework for RNA methyltransferase targets; (3) integration of multiple orthogonal filters creating a practical workflow; and (4) provision of experimentally testable predictions with detailed characterization. The methodological advancement is in the systematic application and optimization rather than tool development.

For medicinal chemists, we provide prioritized compound lists with structural rationale (Table 4, Supplementary Table 2). Structure-activity relationship observations suggest that compounds with extended aromatic systems and hydrogen bond donor/acceptor groups in specific positions show enhanced predicted binding. Synthetic accessibility considerations and intellectual property landscape analysis are provided in Supplementary Materials.

This study demonstrates the successful integration of artificial intelligence, structural biology, and multi-scale computational methods for accelerating precision oncology drug discovery. Our key findings include: (1) AI-guided virtual screening enabled efficient navigation of a 101-million-compound chemical space, identifying 34 high-affinity, low-toxicity NSUN2 inhibitor candidates; (2) Lead compounds ZINC-1000507789 and ZINC-1000507824 exhibited superior binding affinities (−9.933 and −9.502 kcal/mol) compared to reference SAM analogs, with molecular dynamics simulations validating stable binding over 50-nanosecond trajectories; (3) These non-covalent reversible inhibitors target the SAM cofactor binding pocket, offering complementary advantages to existing covalent inhibitors including potentially reduced off-target effects and distinct pharmacological profiles; and (4) Given NSUN2’s established role in mediating resistance to EGFR tyrosine kinase inhibitors, these candidates may address critical unmet needs in overcoming therapeutic resistance in lung cancer and other NSUN2-driven malignancies. Collectively, these findings establish a validated computational framework for epitranscriptomic drug discovery and provide promising lead compounds that warrant experimental validation through biochemical assays, cellular m5C methylation profiling, and disease-relevant efficacy models.

The successful development of small-molecule inhibitors targeting epitranscriptomic writers such as FTO and DNA methyltransferases (e.g., 5-azacytidine) has validated RNA and DNA modification enzymes as druggable targets in oncology^71,72^. NSUN2 represents a compelling next-generation target given its widespread overexpression in diverse cancers, established mechanistic links to tumor progression and therapy resistance, and the recent proof-of-concept for its druggability through covalent inhibitors^14^.

This study demonstrates that artificial intelligence-driven virtual screening can efficiently identify novel NSUN2 inhibitor candidates from ultra-large chemical libraries, maintaining high predictive accuracy. Our integrated computational platform—combining machine learning classification, molecular docking, ADMET profiling, and molecular dynamics validation—successfully navigated 101 million compounds to identify 34 high-quality drug-like candidates. Lead compounds ZINC-1000507789 and ZINC-1000507824 demonstrated superior predicted binding affinities, excellent stability in molecular dynamics simulations, and favorable pharmacokinetic profiles. These non-covalent reversible inhibitors offer complementary advantages to existing covalent NSUN2 inhibitors and may address critical challenges in overcoming therapeutic resistance, particularly in EGFR-mutant lung cancers where NSUN2 mediates acquired resistance to tyrosine kinase inhibitors.

Translation of these computational findings requires systematic experimental validation including biochemical inhibition assays, cellular m5C methylation profiling, and disease-relevant efficacy studies in appropriate cancer models. Successfully validated compounds could provide valuable chemical tools for dissecting NSUN2 biology and potentially advance toward clinical development as precision oncology therapeutics for NSUN2-driven malignancies.

Nevertheless, we acknowledge the value of explicit drug-likeness profiling. In future work, we will conduct comprehensive analyses of the 34 lead compounds including Lipinski’s Rule of Five, Veber’s criteria, and synthetic accessibility scores, correlating these with experimental validation results. We believe the current study successfully validates our integrated screening workflow, while the proposed future analyses will provide additional validation for the identified leads.

This work establishes a generalizable framework for AI-accelerated discovery of epitranscriptomic modulators, potentially applicable to other RNA modification enzymes including METTL3/14, ALKBH5, and additional NSUN family members. As precision oncology increasingly incorporates epitranscriptomic targeting strategies, computational platforms like ours will play essential roles in accelerating the discovery and optimization of next-generation therapeutics.

## Methods

### Computational workflow

**Fig. 16:**
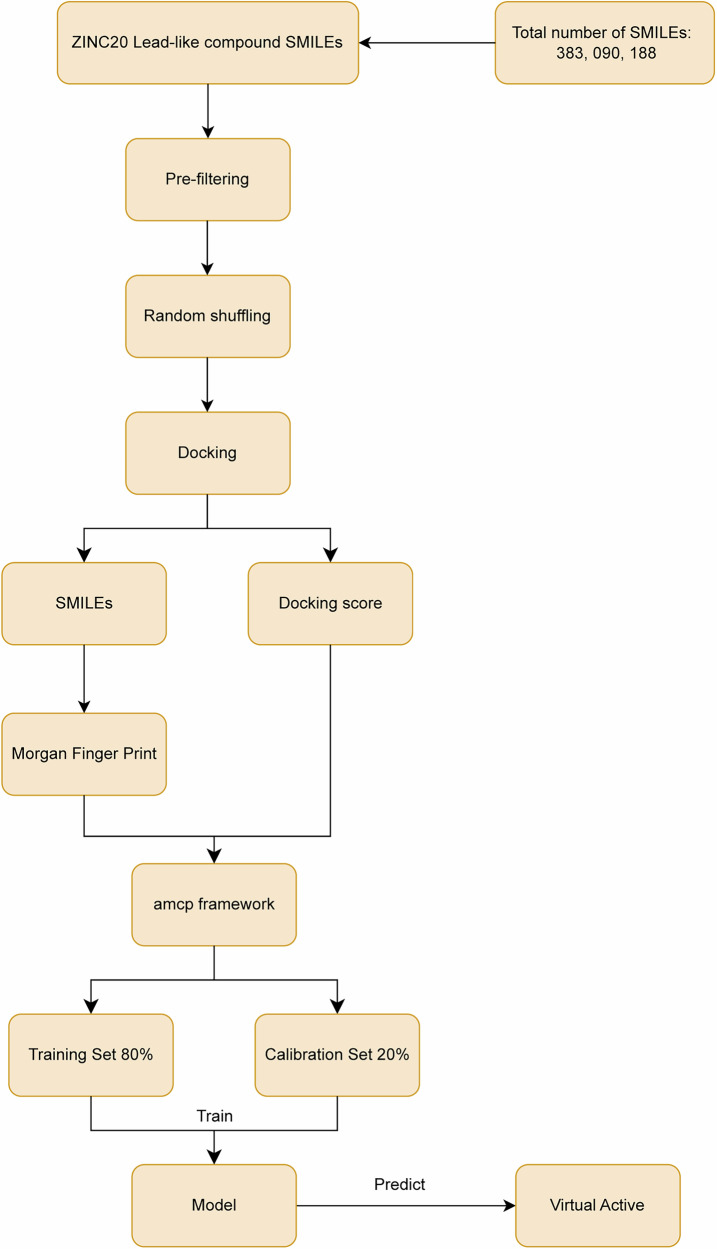
Integrated computational workflow for lead compound virtual screening.

Our AI-driven virtual screening platform integrates multiple complementary computational approaches in a sequential filtering cascade designed to identify high-affinity, low-toxicity NSUN2 inhibitor candidates from ultra-large chemical libraries. Our integrated machine learning and molecular docking workflow (Fig. 16) comprises five sequential steps: (1) Training Set Preparation and Initial Docking, (2) Training Set Generation and Labeling, (3) Molecular Descriptor Extraction and Classifier Training, (4) Large-Scale Library Screening via Conformal Prediction, (5) Post-Processing and Lead Selection. The specific steps are as follows:

Step 1: Training Set Preparation and Initial Docking. A subset of lead-like compounds was randomly sampled from an ultra-large-scale chemical library and docked against the target protein structure.

Step 2: Training Set Generation and Labeling. Docking score thresholds were established using the aggregated conformal prediction (amcp) framework to label training compounds as computationally active (scores exceeding threshold) or inactive (scores at or below threshold). Given the amcp framework’s reliance on ensemble predictions from multiple classifiers, five independent training sets were generated. Compounds in the top 1% by docking score were designated as computationally active.

Step 3: Molecular Descriptor Extraction and Classifier Training. Morgan fingerprints were generated as molecular descriptors for each training compound and used to train independent classification models for discriminating between computationally active and inactive compounds.

Step 4: Large-Scale Library Screening via Conformal Prediction. Trained classifiers were deployed to screen the ultra-large-scale chemical library. The amcp framework’s conformal predictor provides class-specific confidence scores and partitions compounds into four categories based on a selected confidence threshold (ε): active, inactive, Both (ambiguous), and Null (insufficient confidence). Threshold adjustment enables control over active set size, thereby enriching candidates with superior docking scores.

Step 5: Post-Processing and Lead Selection. Final database prioritization varied by target characteristics. To refine candidate sets, computationally active compounds were ranked by prediction confidence, and a high-confidence subset was re-docked. High-scoring compounds underwent visual inspection for representative selection, followed by molecular dynamics validation.

### Software and tools

PyMOL (version 2.6): An open-source molecular visualization platform maintained by Schrödinger for structural analysis^73^. Reduce (version 4.16): A preprocessing tool for adding and optimizing hydrogen atoms in PDB files^74^. Meeko (version 0.6.1): A companion tool for preparing input and processing output data for AutoDock-GPU and AutoDock Vina^75^. AutoDock Vina (version 1.2): An open-source molecular docking engine employing a simplified scoring function and gradient-based optimization^28^.

### Target selection

This study focused on RNA cytosine C(5)-methyltransferase NSUN2 (UniProt: Q08J23), an RNA methyltransferase family member catalyzing C5-methylation of cytosine residues. NSUN2 plays crucial roles in gene expression regulation and RNA stability, making it an attractive therapeutic target.

With human NSUN2 crystal structures undetermined, we leveraged the protein’s structural conservation across species and sequence similarity with Methanocaldococcus jannaschii homologs^76,77^. Sequence alignment between human NSUN2 (residues 290-520) and M. jannaschii TRM4 revealed 34.2% sequence identity and 51.7% sequence similarity with conservative substitutions. The AlphaFold-predicted human NSUN2 structure^78^ was aligned with PDB entry 3A4T in PyMOL for structural comparison. Structural alignment yielded an RMSD of 1.82 Å over 187 aligned Cα atoms, demonstrating high structural concordance. Critical catalytic residues, including C271 in NSUN2 (corresponding to C295 in TRM4), were structurally conserved. The sinefungin binding site in TRM4 corresponds to the SAM cofactor binding pocket, which is highly conserved across the NSUN family and represents the primary druggable site. Overlay analysis confirmed that key binding pocket residues are spatially conserved between TRM4 and AlphaFold-predicted NSUN2. Overlapping regions were extracted, and molecular docking was performed using the binding pocket corresponding to 3A4T.

The SAM cofactor binding pocket was selected as the primary docking target based on structural and functional considerations. NSUN2 contains a conserved methyltransferase domain (residues 206-410) that forms the catalytic core. The SAM binding site is located within this domain and comprises key residues including such as G251, G276, D326, etc. This pocket exhibits a well-defined binding geometry suitable for non-covalent inhibitor design and represents a complementary targeting strategy to recently reported covalent inhibitors that target the catalytic cysteine C271^14^. By targeting the cofactor binding site rather than the catalytic cysteine, our approach aims to identify reversible, non-covalent inhibitors that may offer advantages including reduced off-target reactivity and distinct pharmacological profiles.

We chose 3A4T as the template based on the fact that NSUN2 (human) belongs to the same RNA cytosine-5 methyltransferase family (Trm4/NSun2 family) as Trm4 (yeast) and TRM4 (M. jannaschii). The core function of this family is to catalyze the methylation of specific tRNA sites (e.g., C34, C48, C49, etc.). The binding pocket for the methyl donor S-adenosylmethionine (SAM) constitutes the catalytic core of this enzyme family, and its structural conservation has been confirmed by multiple studies.

The accuracy of AlphaFold in protein structure prediction has been validated by CASP14 (the 14th Critical Assessment of protein Structure Prediction) and numerous subsequent studies. The main-chain atom RMSD (root mean square deviation) between the AlphaFold-predicted structure of human NSUN2 (e.g., PDB: 7Z8X) and experimental structures (e.g., cryo-EM structures) is less than 2 Å, which is sufficient to meet the accuracy requirements for molecular docking.

Regarding the side-chain conformation issue in AlphaFold models, a 2024 study by Pei Jianfeng’s team at Peking University successfully addressed discrepancies between side-chain conformations in AlphaFold predictions and experimental structures by employing flexible docking (which considers the dynamic changes of protein side chains).

The “homologous protein structure alignment + binding pocket docking” strategy is a standard approach in structural biology and drug design, particularly suitable for target proteins lacking experimental structures. Similar successful cases have demonstrated the effectiveness of this strategy, and their results have been experimentally validated (e.g., substrate binding activity, inhibitor affinity).

The selection of the cofactor pocket rather than the RNA substrate site was based on several considerations: (1) the cofactor site is deeper and more enclosed, making it more druggable; (2) SAM-competitive inhibitors are an established strategy for methyltransferase inhibition; and (3) the cofactor site shows higher structural conservation across species than the RNA binding region.

### Protein structure preparation

The aligned NSUN2-TRM4 hybrid structure was prepared for molecular docking through the following steps: (1) Hydrogen Addition: Hydrogen atoms were added to the protein structure using the Reduce tool (version 4.16) at pH 7.0. This tool optimizes hydrogen bonding networks by considering alternative conformations of Asn, Gln, and His residues. (2) Protonation State Assignment: Ionizable residues were assigned standard protonation states at physiological pH (7.0): Asp and Glu (deprotonated), Lys and Arg (protonated), His (neutral, protonated on Nε2 based on hydrogen bonding analysis). The catalytic cysteine (C271) was maintained in the reduced thiol form. (3) Cofactor and Ligand Removal: The sinefungin molecule and all crystallographic waters beyond 5 Å from the binding pocket were removed to prepare an apo structure suitable for ligand docking. Conserved structural waters within the binding pocket were retained. (4) Energy Minimization: The structure was subjected to constrained energy minimization using 500 steps of steepest descent followed by conjugate gradient optimization, with heavy atom position restraints (force constant 10 kcal/mol/Ų) to maintain the crystallographic geometry while relieving steric clashes. The minimized structure was converted to PDBQT format using AutoDockTools, with Gasteiger charges assigned and non-polar hydrogens merged.

### Compound library preparation and conformational sampling

All lead-like compounds (383,090,188 molecules) were retrieved from ZINC20^40^. Physicochemical properties, including heavy atom count and cLogP, were computed using RDKit^79^. The 20–25 heavy atom range was selected based on: (1) analysis of FDA-approved drugs (median = 27, IQR = 20–35); (2) ensuring sufficient molecular complexity for selective binding while maintaining synthetic tractability; (3) compatibility with oral bioavailability (compounds <30 heavy atoms show higher oral absorption); and (4) computational efficiency for conformer generation. Compounds satisfying 20 ≤ heavy atom count ≤ 25 and −5 ≤ cLogP ≤ 3.5^36^ were retained (350,516,810 compounds).

For each compound, 50 conformations were generated using RDKit’s ETKDG algorithm^80^ with an RMSD threshold of 0.5 Å between conformers to ensure structural diversity. Statistical analysis showed that beyond 50 conformers, the mean docking score improvement is < 0.15 kcal/mol (within scoring function noise), standard deviation plateaus at approximately 50 conformers, and computational cost increases linearly while information gain diminishes. Paired t-tests comparing 50 versus 100 conformers (*n* = 1000 compounds) showed no significant difference (*p* = 0.24). Compounds were converted to PDBQT format using Meeko^75^ and docked against the target protein using AutoDock Vina^28^. The docking grid size was set to 20 × 20 × 20 Å. For each small molecule, 1 ligand pose was considered for docking, with 50 pre-generated conformations prepared in advance for each ligand.

### Aggregated Conformal Prediction (amcp) Framework

The amcp framework implements conformal prediction for efficient large-scale chemical library screening^36^. This approach employs an ensemble of five CatBoost classifiers trained independently for target-specific prediction. CatBoost models were trained using default hyperparameters with the following configuration: automatic class weight balancing (auto_class_weights = ‘Balanced’) to handle imbalanced active/inactive compound ratios, and area under the ROC curve (AUC) as the evaluation metric (eval_metric = ‘AUC’) to optimize discriminative performance. The amcp framework was specifically chosen for its ability to maximize recall while providing confidence scores that enable subsequent ranking and filtering.

Input features comprise 1024-bit Morgan fingerprints derived from SMILES representations, which can predict active compounds with confidence scores.

During training, the framework determines optimal classification thresholds and outputs two probability scores: p(1) for active and p(0) for inactive predictions. Classification follows these rules: p(1) > threshold → active; p(0) > threshold → inactive; both exceed threshold → ambiguous (Both); neither exceeds threshold → insufficient confidence (Null). Active predictions are ranked by Δp = p(1) − p(0) to prioritize high-confidence candidates.

### ADMET profiling

Top-ranked compounds (*n* = 12,000) underwent ADMET assessment using vNN-ADMET^50^, a publicly accessible platform employing variable nearest neighbor models. The platform provides 15 validated prediction models evaluating key pharmacokinetic and toxicological endpoints, including cytotoxicity, mutagenicity, cardiotoxicity, drug-drug interactions, microsomal stability, and drug-induced liver injury (DILI).

ADMET analysis predicts safety, efficacy, and drug-likeness profiles, enabling early elimination of compounds with unfavorable properties prior to resource-intensive experimental validation: Absorption: Oral bioavailability, gastrointestinal stability, permeability (Caco-2 model), and aqueous solubility. Distribution: Plasma protein binding, volume of distribution, and blood-brain barrier permeability. Metabolism: Cytochrome P450 interactions, metabolite identification, and drug-drug interaction potential. Excretion: Renal and biliary clearance rates, and half-life estimation. Toxicity: hERG channel inhibition (arrhythmia risk), hepatotoxicity, carcinogenicity, mutagenicity, and DILI risk.

Absorption and Distribution: - Blood-brain barrier (BBB) permeability (threshold: log BB > +0.3 for BBB-permeable compounds) - P-glycoprotein (Pgp) substrate and inhibitor potential Metabolism: - Human liver microsomal (HLM) stability (threshold: T1/2 > 30 min for metabolically stable compounds) - Cytochrome P450 enzyme inhibition (CYP1A2, 2C9, 2C19, 2D6, and 3A4; threshold: IC50 < 10 µM indicating potential drug-drug interactions) Toxicity: - Drug-induced liver injury (DILI) potential - Cytotoxicity in HepG2 cells (threshold: IC50 ≤ 10 µM) - hERG channel blockade (threshold: IC50 ≤ 10 µM indicating cardiotoxicity risk) - Mitochondrial membrane potential (MMP) disruption - Ames mutagenicity Compounds were prioritized based on favorable ADMET profiles, specifically: (1) absence of predicted hepatotoxicity (DILI-negative), (2) no significant CYP450 inhibition (particularly CYP3A4 and CYP2D6), (3) negative hERG liability, (4) non-mutagenic in AMES test, and (5) acceptable metabolic stability (HLM T1/2 > 30 min).

### Molecular dynamics simulations

Molecular dynamics (MD) simulations were performed using OpenMM^81^ under physiological conditions: 310 K temperature, 101 kPa pressure, periodic boundary conditions, TIP3P water model, and pH 7.0. The Amber14 protein force field (ff14SB) was applied for protein parameters, while water molecules were modeled using the TIP3P water model with parameters from the Amber14 force field package. An integration timestep of 2 fs was used with the Langevin integrator for temperature control. Amber-14 protein force field and Amber’s TIP3P-FB solvent force field were used. The system underwent NVT equilibration (1000 steps of low-temperature equilibration followed by 5000 steps of equilibration at the target temperature), followed by NPT equilibration for 5000 steps. Following equilibration, the top three ADMET-filtered compounds complexed with target protein underwent 50-ns production simulations with trajectory frames saved every 10 ps. Analyses were conducted using Gromacs utilities, DuIvyTools^82^, and MDAnalysis^83,84^ for hydrogen bond dynamics. The reason for selecting these three compounds for simulation is that they represent the top three candidates ranked by the confidence metric Δp predicted by the AMCP framework from the pool of 34 compounds after ADMET screening. The Δp value directly reflects the model’s confidence in predicting a compound as “active.” Selecting the highest-confidence candidates for resource-intensive MD simulations is a rational prioritization strategy under computational resource constraints. Given the extremely high computational cost of all-atom MD simulations, performing 50 ns simulations for all 34 compounds is currently impractical. Our strategy is to prioritize in-depth analysis of a limited number of the “most promising” candidates, thereby establishing priorities for subsequent research.

Evaluated metrics: Root Mean Square Deviation (RMSD): Measures average atomic displacement from reference structure, quantifying overall conformational drift. Root Mean Square Fluctuation (RMSF): Assesses per-residue flexibility throughout simulation, identifying dynamic regions. Radius of Gyration (Rg): Characterizes structural compactness in three orthogonal directions (RgX, RgY, RgZ). Solvent Accessible Surface Area (SASA): Quantifies solvent-exposed surface; increases indicate unfolding, decreases suggest compaction, and fluctuations reveal dynamic conformational transitions. Hydrogen Bond Analysis: Tracks formation and persistence of protein-ligand hydrogen bonds, key stabilizing interactions reflecting binding affinity and mode. Free Energy Landscape (FEL): Projects conformational sampling onto principal component space, revealing stable states and energy barriers. Dynamical Cross-Correlation Matrix (DCCM): Quantifies correlated/anti-correlated motions between residue pairs, exposing allosteric communication networks. Secondary Structure Analysis (DSSP): Monitors temporal evolution of α-helices, β-sheets, turns, and coils, assessing backbone stability and conformational transitions.

## Supplementary information

Supplementary Information

## Data Availability

The results associated with this study are present in the paper or supplementary materials. All other materials used in the analyses are available upon reasonable request.

## Code availability

The code has been uploaded in https://www.jianguoyun.com/p/DUENIX0Q887bChigiJ8GIAA.

## References

[1] Blanco, S. et al. Aberrant methylation of tRNAs links cellular stress to neuro-developmental disorders. *EMBO J.* **33**, 2020–2039 (2014). 25063673 10.15252/embj.201489282 PMC4195770

[2] Frye, M., Harada, B. T., Behm, M. & He, C. RNA modifications modulate gene expression during development. *Science*. 10.1126/science.aau1646 (2018). 10.1126/science.aau1646 PMC6436390 30262497

[3] Xin, Yang et al. 5-methylcytosine promotes mRNA export - NSUN2 as the methyltransferase and ALYREF as an m(5)C reader. *Cell Res*. 10.1038/cr.2017.55 (2017). 10.1038/cr.2017.55 PMC5594206 28418038

[4] Nikoletta, et al. Cytosine-5 RNA methylation links protein synthesis to cell metabolism. *PLoS Biol*. 10.1371/journal.pbio.3000297 (2019). 10.1371/journal.pbio.3000297 PMC6594628 31199786

[5] Mei, L. et al. RNA methyltransferase NSUN2 promotes gastric cancer cell proliferation by repressing p57(Kip2) by an m(5)C-dependent manner. *Cell Death Dis.* **11**, 270 (2020). 32332707 10.1038/s41419-020-2487-z PMC7181747

[6] Su, J. et al. NSUN2-mediated RNA 5-methylcytosine promotes esophageal squamous cell carcinoma progression via LIN28B-dependent GRB2 mRNA stabilization. *Oncogene* **40**, 5814–5828 (2021). 34345012 10.1038/s41388-021-01978-0 PMC8484015

[7] Hao, Chen et al. m(5)C modification of mRNA serves a DNA damage code to promote homologous recombination. *Nat. Commun.*10.1038/s41467-020-16722-7 (2020). 10.1038/s41467-020-16722-7 PMC7275041 32503981

[8] Yueqin, Wang et al. Aberrant m5C hypermethylation mediates intrinsic resistance to gefitinib through NSUN2/YBX1/QSOX1 axis in EGFR-mutant non-small-cell lung cancer. *Mol. Cancer*10.1186/s12943-023-01780-4 (2023). 10.1186/s12943-023-01780-4 PMC10169458 37161388

[9] Muzammil, et al. Mutation in NSUN2, which encodes an RNA methyltransferase, causes autosomal-recessive intellectual disability. *Am. J. Hum. Genet*10.1016/j.ajhg.2012.03.023 (2012). 10.1016/j.ajhg.2012.03.023 PMC3376419 22541562

[10] Abbasi-Moheb, L. et al. Mutations in NSUN2 cause autosomal-recessive intellectual disability. *Am. J. Hum. Genet* **90**, 847–855 (2012). 22541559 10.1016/j.ajhg.2012.03.021 PMC3376487

[11] Yoon, et al. RNA methyltransferase NSun2 deficiency promotes neurodegeneration through epitranscriptomic regulation of tau phosphorylation. *Acta. Neuropathol.*10.1007/s00401-022-02511-7 (2022). 10.1007/s00401-022-02511-7 PMC9807547 36357715

[12] Blaze, J. et al. Neuronal Nsun2 deficiency produces tRNA epitranscriptomic alterations and proteomic shifts impacting synaptic signaling and behavior. *Nat. Commun.*10.1038/s41467-021-24969-x (2021). 10.1038/s41467-021-24969-x PMC8363735 34389722

[13] Chellamuthu, A., Sridhar, S. & Kumar, J. S. The RNA Methyltransferase NSUN2 and Its Potential Roles in Cancer. *Cells* **9**, 1758 (2020). 32708015 10.3390/cells9081758 PMC7463552

[14] Yongfeng, Tao et al. Chemical Proteomic Discovery of Isotype-Selective Covalent Inhibitors of the RNA Methyltransferase NSUN2. *Angew. .Chem. Int. Ed. Engl.*10.1002/anie.202311924 (2023). 10.1002/anie.202311924 PMC10999112 37909922

[15] Carlsson, J. & Luttens, A. Structure-based virtual screening of vast chemical space as a starting point for drug discovery. *Curr. Opin. Struct. Biol.* **87**, 102829 (2024). 38848655 10.1016/j.sbi.2024.102829

[16] Kitchen, D. B., Decornez, H., Furr, J. R. & Bajorath, J. Docking and scoring in virtual screening for drug discovery: methods and applications. *Nat. Rev. Drug Discov.* **3**, 935–949 (2004). 15520816 10.1038/nrd1549

[17] Xuan-Yu, Meng, Hong-Xing, Zhang, Mihaly, Mezei & Meng, Cui. Molecular docking: a powerful approach for structure-based drug discovery. *Curr Comput Aided Drug Des.*10.2174/157340911795677602 (2011). 10.2174/157340911795677602 PMC3151162 21534921

[18] Sterling, T. & Irwin, J. J. ZINC 15-Ligand Discovery for Everyone. *J. Chem. Inf. Model* **55**, 2324–2337 (2015). 26479676 10.1021/acs.jcim.5b00559 PMC4658288

[19] Ain Q. U., Aleksandrova A., Roessler F. D., Ballester P. J. Machine-learning scoring functions to improve structure-based binding affinity prediction and virtual screening. *Wiley Interdiscip Rev. Comput. Mol. Sc*i. 10.1002/wcms.1225 (2016). 10.1002/wcms.1225 PMC4832270 27110292

[20] Zhe, Wang et al. Comprehensive evaluation of ten docking programs on a diverse set of protein-ligand complexes: the prediction accuracy of sampling power and scoring power. *Phys Chem Chem Phys*10.1039/c6cp01555g (2016). 10.1039/c6cp01555g 27108770

[21] Gentile, F. et al. Deep Docking: A Deep Learning Platform for Augmentation of Structure Based Drug Discovery. *ACS Cent. Sci.* **6**, 939–949 (2020). 32607441 10.1021/acscentsci.0c00229 PMC7318080

[22] Ton, A.T., Gentile, F., Hsing, M., Ban, F., & Cherkasov, A. Rapid Identification of Potential Inhibitors of SARS-CoV-2 Main Protease by Deep Docking of 1.3 Billion Compounds. *Mol. Inform.* **39**, 10.1002/minf.202000028 (2020). 10.1002/minf.202000028 PMC7228259 32162456

[23] Xing, J. et al. NSun2 Promotes Cell Growth via Elevating Cyclin-Dependent Kinase 1 Translation. *Mol. Cell Biol.* **35**, 4043–4052 (2015). 26391950 10.1128/MCB.00742-15 PMC4628067

[24] Lyu, J. et al. Ultra-large library docking for discovering new chemotypes. *Nature* **566**, 224–229 (2019). 30728502 10.1038/s41586-019-0917-9 PMC6383769

[25] Arman, et al. Synthon-based ligand discovery in virtual libraries of over 11 billion compounds. *Nature*10.1038/s41586-021-04220-9 (2021). 10.1038/s41586-021-04220-9 PMC9763054 34912117

[26] Guangfeng, Zhou et al. An artificial intelligence accelerated virtual screening platform for drug discovery. *Nat Commun.*10.1038/s41467-024-52061-7 (2024). 10.1038/s41467-024-52061-7 PMC11377542 39237523

[27] Ballante, F., Marshall, G. R. An Automated Strategy for Binding-Pose Selection and Docking Assessment in Structure-Based Drug Design. *J. Chem. Inf. Model*10.1021/acs.jcim.5b00603 (2015). 10.1021/acs.jcim.5b00603 26682916

[28] Trott, O. & Olson, A. J. AutoDock Vina: improving the speed and accuracy of docking with a new scoring function, efficient optimization, and multithreading. *J. Comput. Chem.*10.1002/jcc.21334 (2009). 10.1002/jcc.21334 PMC3041641 19499576

[29] Victor, et al. Current trends in computer aided drug design and a highlight of drugs discovered via computational techniques: A review. *Eur. J. Med. Chem.*10.1016/j.ejmech.2021.113705 (2021). 10.1016/j.ejmech.2021.113705 34303871

[30] Jumper, J. et al. Highly accurate protein structure prediction with AlphaFold. *Nature* **596**, 583–589 (2021). 34265844 10.1038/s41586-021-03819-2 PMC8371605

[31] Mihaly, Varadi et al. AlphaFold Protein Structure Database: massively expanding the structural coverage of protein-sequence space with high-accuracy models. *Nucleic Acids Res.*10.1093/nar/gkab1061 (2021). 10.1093/nar/gkab1061 PMC8728224 34791371

[32] Janet, M., Thornton, Roman, A., Laskowski & Neera, Borkakoti. AlphaFold heralds a data-driven revolution in biology and medicine. *Nat. Med.*10.1038/s41591-021-01533-0 (2021). 10.1038/s41591-021-01533-0 34642488

[33] Heo, L. & Feig, M. Multi-state modeling of G-protein coupled receptors at experimental accuracy. *Proteins* **90**, 1873–1885 (2022). 35510704 10.1002/prot.26382 PMC9561049

[34] Walters, W. P., Murcko, A. A. & Murcko, M. A. Recognizing molecules with drug-like properties. *Curr. Opin. Chem. Biol.* **3**, 384–387 (1999). 10419858 10.1016/s1367-5931(99)80058-1

[35] Chong, A. et al. Establishing the foundations for a data-centric AI approach for virtual drug screening through a systematic assessment of the properties of chemical data. (2024).

[36] Luttens, A. et al. Rapid traversal of vast chemical space using machine learning-guided docking screens. *Nat. Comput Sci.* **5**, 301–312 (2025). 40082701 10.1038/s43588-025-00777-x PMC12021657

[37] Steven, et al. How to improve R&D productivity: the pharmaceutical industry’s grand challenge. *Nat. Rev. Drug. Discov.*10.1038/nrd3078 (2010). 10.1038/nrd3078 20168317

[38] Ahmet, et al. Recent applications of deep learning and machine intelligence on in silico drug discovery: methods, tools and databases. *Brief Bioinform*10.1093/bib/bby061 (2018). 10.1093/bib/bby061 PMC6917215 30084866

[39] Yang, Y. et al. Efficient Exploration of Chemical Space with Docking and Deep Learning. *J. Chem. Theory Comput* **17**, 7106–7119 (2021). 34592101 10.1021/acs.jctc.1c00810

[40] John, A. S., Roth, M. W. et al. ZINC20-A Free Ultralarge-Scale Chemical Database for Ligand Discovery. *J. Chem. Inf. Model* **60**, 2155–2168 (2020). 33118813 10.1021/acs.jcim.0c00675 PMC8284596

[41] Adrià, Cereto-Massagué et al. Molecular fingerprint similarity search in virtual screening. *Methods*10.1016/j.ymeth.2014.08.005 (2014). 10.1016/j.ymeth.2014.08.005 25132639

[42] Ballester P. J., Mitchell J. B. A machine learning approach to predicting protein-ligand binding affinity with applications to molecular docking. *Bioinformatics*10.1093/bioinformatics/btq112 (2010). 10.1093/bioinformatics/btq112 PMC3524828 20236947

[43] Houston, D. R. & Walkinshaw, M. D. Consensus docking: improving the reliability of docking in a virtual screening context. *J. Chem. Inf. Model*10.1021/ci300399w (2013). 10.1021/ci300399w 23351099

[44] Feher, M. Consensus scoring for protein-ligand interactions. *Drug Discov. Today* **11**, 421–428 (2006). 16635804 10.1016/j.drudis.2006.03.009

[45] Xiong, G. et al. ADMETlab 2.0: an integrated online platform for accurate and comprehensive predictions of ADMET properties. *Nucleic Acids Res* **49**, W5–W14 (2021). 33893803 10.1093/nar/gkab255 PMC8262709

[46] Kola, I. & Landis, J. Can the pharmaceutical industry reduce attrition rates? *Nat. Rev. Drug Discov.* **3**, 711–716 (2004). 15286737 10.1038/nrd1470

[47] Daina, A., Michielin, O. & Zoete, V. SwissADME: a free web tool to evaluate pharmacokinetics, drug-likeness and medicinal chemistry friendliness of small molecules. *Sci. Rep.* **7**, 42717 (2017). 28256516 10.1038/srep42717 PMC5335600

[48] Michael, et al. An analysis of the attrition of drug candidates from four major pharmaceutical companies. *Nat. Rev. Drug Discov*. 10.1038/nrd4609 (2015). 10.1038/nrd4609 26091267

[49] Cheng, F., Li, W., Liu, G. & Tang, Y. In silico ADMET prediction: recent advances, current challenges and future trends. *Curr. Top. Med Chem.* **13**, 1273–1289 (2013). 23675935 10.2174/15680266113139990033

[50] Schyman, P., Liu, R., Desai, V. & Wallqvist, A. vNN Web Server for ADMET Predictions. *Front. Pharmacol*. **8**. 10.3389/fphar.2017.00889 (2017). 10.3389/fphar.2017.00889 PMC5722789 29255418

[51] Ferreira L. L. G., Andricopulo A. D. ADMET modeling approaches in drug discovery. *Drug Discov. Today*10.1016/j.drudis.2019.03.015 (2019). 10.1016/j.drudis.2019.03.015 30890362

[52] Minjun, Chen, Jürgen, Borlak & Weida, Tong. High lipophilicity and high daily dose of oral medications are associated with significant risk for drug-induced liver injury. *Hepatology*10.1002/hep.26208 (2012). 10.1002/hep.26208 23258593

[53] Redfern, W. S. et al. Relationships between preclinical cardiac electrophysiology, clinical QT interval prolongation and torsade de pointes for a broad range of drugs: evidence for a provisional safety margin in drug development. *Cardiovasc Res* **58**, 32–45 (2003). 12667944 10.1016/s0008-6363(02)00846-5

[54] Tom, L. & Amy, P. The effect of cytochrome P450 metabolism on drug response, interactions, and adverse effects. *Am. Fam. Physician.* **3**, 391–396 (2007). 17708140

[55] Hospital, A., Goñi, J. R., Orozco, M. & Gelpí, J. L. Molecular dynamics simulations: advances and applications. *Adv. Appl Bioinform Chem.* **8**, 37–47 (2015). 26604800 10.2147/AABC.S70333 PMC4655909

[56] Scott, B. B., Thiberge, S. Y. & Guo, C. Molecular Dynamics Simulation for All. *Neuron* **100**, 1045–1058 (2018). 30482694 10.1016/j.neuron.2018.09.050 PMC6283673

[57] Jacob, D. Durrant & J., A. McCammon. Molecular dynamics simulations and drug discovery. *BMC Biol.*10.1186/1741-7007-9-71 (2011). 10.1186/1741-7007-9-71 PMC3203851 22035460

[58] M., Karplus & J. A., A., McCammon. Molecular dynamics simulations of biomolecules. *Nat. Struct. Biol*. **9**, 646–652 (2002). 10.1038/nsb0902-646 12198485

[59] Jeffrey, G. A. & Jeffrey, G. A. *An introduction to hydrogen bonding*. Vol. 12 (Oxford University Press New York, 1997).

[60] Ron, et al. Biomolecular simulation: a computational microscope for molecular biology. *Annu. Rev. Biophys.*10.1146/annurev-biophys-042910-155245 (2012). 10.1146/annurev-biophys-042910-155245 22577825

[61] Barducci, A., Bonomi, M. & Parrinello, M. Metadynamics. *Wiley Interdiscip. Rev.: Computational Mol. Sci.* **1**, 826–843 (2011).

[62] Sugita, Y. & Okamoto, Y. Replica-exchange molecular dynamics method for protein folding. *Chem. Phys. Lett.* **314**, 141–151 (1999).

[63] Frank, H., Niesen, Helena, Berglund & Masoud, Vedadi. The use of differential scanning fluorimetry to detect ligand interactions that promote protein stability. *Nat. Protoc.*10.1038/nprot.2007.321 (2007). 10.1038/nprot.2007.321 17853878

[64] Yang, L., Wang, B. & Gong, Z. Chemically modified non-coding RNAs in cancer. *Expert Rev. Mol. Med* **27**, e19 (2025). 40488318 10.1017/erm.2025.10007 PMC12234022

[65] Li, P., Wang, W., Zhou, R., Ding, Y. & Li, X. The m(5) C methyltransferase NSUN2 promotes codon-dependent oncogenic translation by stabilising tRNA in anaplastic thyroid cancer. *Clin. Transl. Med*. **13**. 10.1002/ctm2.1466 (2023). 10.1002/ctm2.1466 PMC10659772 37983928

[66] Añazco-Guenkova, A. M., Miguel-López, B., Monteagudo-García, Ó, García-Vílchez, R. & Blanco, S. The impact of tRNA modifications on translation in cancer: identifying novel therapeutic avenues. *NAR Cancer* **6**, 012 (2024). 10.1093/narcan/zcae012 PMC10928989 38476632

[67] Cully, M. Chemical inhibitors make their RNA epigenetic mark. *Nat. Rev. Drug Discov.* **18**, 892–894 (2019). 31780844 10.1038/d41573-019-00179-5

[68] Macarron, R. et al. Impact of high-throughput screening in biomedical research. *Nat. Rev. Drug Discov.* **10**, 188–195 (2011). 21358738 10.1038/nrd3368

[69] Settles, B. Active learning literature survey. (2009).

[70] Copeland, R. A. Evaluation of enzyme inhibitors in drug discovery. A guide for medicinal chemists and pharmacologists. *Methods Biochem Anal.* **46**, 1–265 (2005). 16350889

[71] Berdasco, M. & Esteller, M. Clinical epigenetics: seizing opportunities for translation. *Nat. Rev. Genet.*10.1038/s41576-018-0074-2 (2018). 10.1038/s41576-018-0074-2 30479381

[72] Topper, M. J., Vaz, M., Marrone, K. A., J. R., Brahmer, Julie, S. B, Baylin, Baylin. The emerging role of epigenetic therapeutics in immuno-oncology. *Nat. Rev. Clin. Oncol.* **17**, 75–90 (2020). 10.1038/s41571-019-0266-5 PMC7254932 31548600

[73] The PyMOL Molecular Graphics System (2015).

[74] Laboratory, R. *Reduce - tool for adding and**correcting hydrogens in PDB files*, https://github.com/rlabduke/reduce/tree/master

[75] Lab, F. *Meeko**:**interface for AutoDock*., https://github.com/forlilab/Meeko

[76] Kuratani, M. et al. Crystal structure of Methanocaldococcus jannaschii Trm4 complexed with sinefungin. *J. Mol. Biol.* **401**, 323–333 (2010). 20600111 10.1016/j.jmb.2010.06.046

[77] Jianheng, Liu et al. Developmental mRNA m(5)C landscape and regulatory innovations of massive m(5)C modification of maternal mRNAs in animals. *Nat. Commun.*10.1038/s41467-022-30210-0 (2022). 10.1038/s41467-022-30210-0 PMC9072368 35513466

[78] AlphaFold NSUN2 Structure, https://alphafold.ebi.ac.uk/.

[79] RDKit: Open-source cheminformatics, https://github.com/rdkit/rdkit (2025).

[80] Shuzhe, Wang, Jagna, Witek, Gregory, A., Landrum & Sereina, Riniker. Improving Conformer Generation for Small Rings and Macrocycles Based on Distance Geometry and Experimental Torsional-Angle Preferences. *J. Chem. Inf. Model*10.1021/acs.jcim.0c00025 (2020). 10.1021/acs.jcim.0c00025 32155061

[81] Eastman, P. et al. OpenMM 8: Molecular Dynamics Simulation with Machine Learning Potentials. *J. Phys. Chem. B* **128**, 109–116 (2024). 38154096 10.1021/acs.jpcb.3c06662 PMC10846090

[82] DuIvyTools, https://github.com/CharlesHahn/DuIvyTools.

[83] Gowers, R. et al. *MDAnalysis: a Python package for the rapid analysis of molecular dynamics simulations* (2016).

[84] Michaud-Agrawal, N., Denning, E. J., Woolf, T. B. & Beckstein, O. MDAnalysis: a toolkit for the analysis of molecular dynamics simulations. *J. Comput Chem.* **32**, 2319–2327 (2011). 21500218 10.1002/jcc.21787 PMC3144279

