# Supplementary material for: AI-driven virtual screening platform identifies novel NSUN2 inhibitor candidates for targeted cancer therapy: a computational drug discovery approach
Source: NPJ Precis Oncol. 2026 Jan 30;10:98. doi: 10.1038/s41698-026-01296-2 (PMC12960686; doi:10.1038/s41698-026-01296-2)
Supplement: Supplementary file 1 — Supplementary Information [file 41698_2026_1296_MOESM1_ESM.pdf]

Supplementary Table 1. Two-Dimensional Structures and Properties of Top 12,000 Lead Compounds

Chemical Structure	SMILEs	ZINC ID	Heavy Atom Count	Mol Weight	cLogP
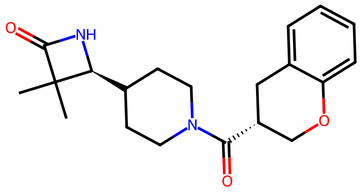	CC1(C)C(=O)N[C@H]1C1CCN(C(=O)[C@H]2COc3ccccc3C2)CC1	294107802	25	342.19	2.0
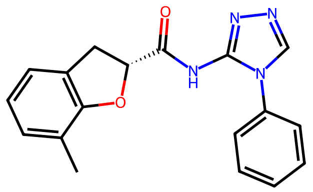	Cc1cccc2c1O[C@@H](C(=O)Nc1nncn1-c1ccccc1)C2	1446344312	24	320.13	2.52
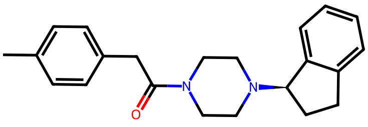	Cc1ccc(CC(=O)N2CCN([C@@H]3CCc4ccccc43)CC2)cc1	682077057	25	334.2	3.37
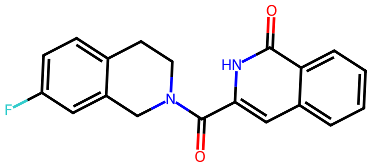	O=C(c1cc2ccccc2c(=O)[nH]1)N1CCc2ccc(F)cc2C1	121596933	24	322.11	2.87
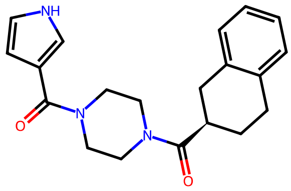	O=C(c1cc[nH]c1)N1CCN(C(=O)[C@@H]2CCc3ccccc3C2)CC1	1011246930	25	337.18	2.1
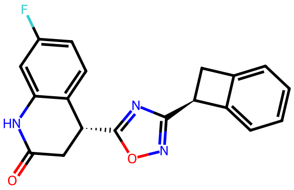	O=C1C[C@@H](c2nc([C@H]3Cc4ccccc43)no2)c2ccc(F)cc2N1	546296570	25	335.11	3.37
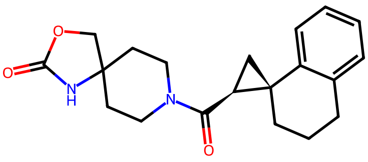	O=C1NC2(CCN(C(=O)[C@H]3C[C@@]34CCCc3ccccc34)CC2)CO1	495140530	25	340.18	2.38
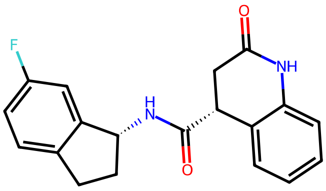	O=C1C[C@@H](C(=O)N[C@@H]2CCc3ccc(F)cc32)c2ccccc2N1	106680925	24	324.13	3.06

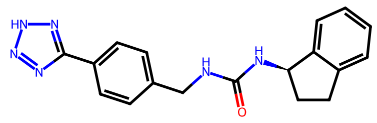	O=C(NCc1ccc(-c2nn[nH]n2)cc1)N[C@@H]1CCc2ccccc21	1582546242	25	334.15	2.35
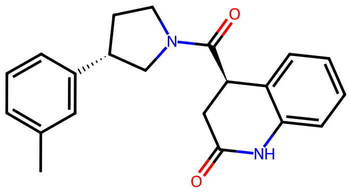	Cc1cccc([C@@H]2CCN(C(=O)[C@@H]3CC(=O)Nc4ccccc43)C2)c1	273124494	25	334.17	3.44
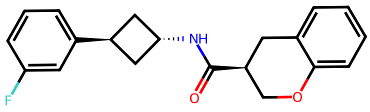	O=C(N[C@H]1C[C@H](c2cccc(F)c2)C1)[C@@H]1COc2ccccc2C1	437442536	24	325.15	3.44
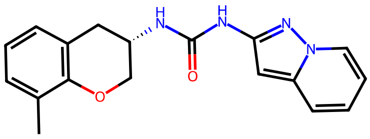	Cc1cccc2c1OC[C@@H](NC(=O)Nc1cc3ccccn3n1)C2	685655158	24	322.14	2.77
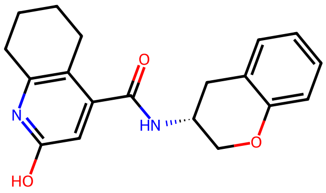	O=C(N[C@H]1COc2ccccc2C1)c1cc(O)nc2c1CCCC2	930240386	24	324.15	2.4
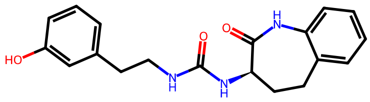	O=C(NCCc1cccc(O)c1)N[C@@H]1CCc2ccccc2NC1=O	654421564	25	339.16	2.19
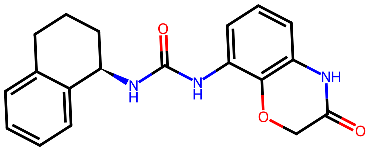	O=C1COc2c(cccc2NC(=O)N[C@@H]2CCCc3ccccc32)N1	290296367	25	337.14	3.22
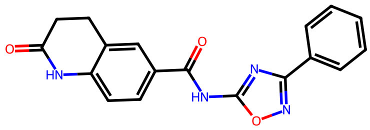	O=C1CCc2cc(C(=O)Nc3nc(-c4ccccc4)no3)ccc2N1	1654274420	25	334.11	2.87

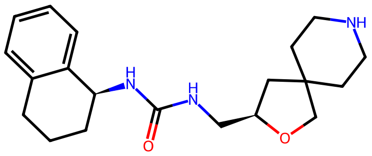	O=C(NC[C@H]1CC2(CCNCC2)CO1)N[C@H]1CCCc2ccccc21	874429510	25	343.23	2.52
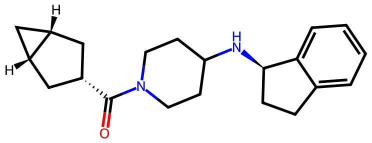	O=C([C@@H]1C[C@@H]2C[C@@H]2C1)N1CCC(N[C@@H]2CCc3ccccc32)CC1	996900509	24	324.22	3.3
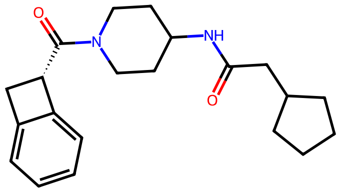	O=C(CC1CCCC1)NC1CCN(C(=O)[C@H]2Cc3ccccc32)CC1	358475982	25	340.22	3.01
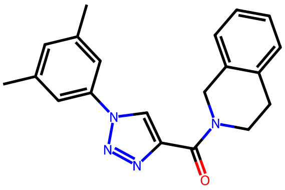	Cc1cc(C)cc(-n2cc(C(=O)N3CCc4ccccc4C3)nn2)c1	71765023	25	332.16	3.08
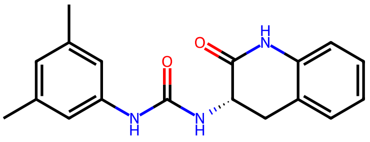	Cc1cc(C)cc(NC(=O)N[C@H]2Cc3ccccc3NC2=O)c1	1202850233	23	309.15	2.99
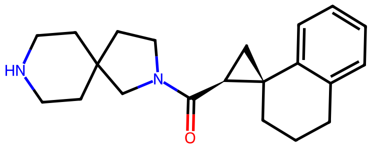	O=C([C@H]1C[C@@]12CCCc1ccccc12)N1CCC2(CCNCC2)C1	378404698	24	324.22	2.88
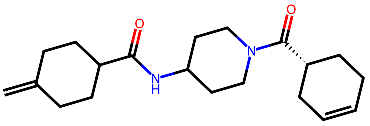	C=C1CCC(C(=O)NC2CCN(C(=O)[C@H]3CC=CCC3)CC2)CC1	944105477	24	330.23	3.2
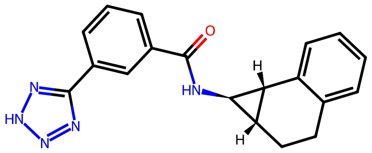	O=C(N[C@@H]1[C@H]2CCc3ccccc3[C@H]21)c1cccc(-c2nn[nH]n2)c1	823145341	25	331.14	2.32

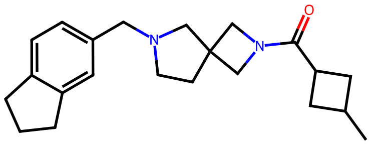	CC1CC(C(=O)N2CC3(CCN(Cc4ccc5c(c4)CCC5)C3)C2)C1	1072606792	25	338.24	3.26
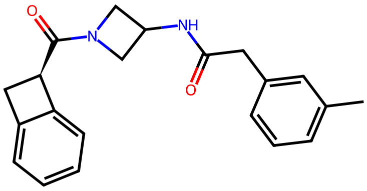	Cc1cccc(CC(=O)NC2CN(C(=O)[C@@H]3Cc4ccccc43)C2)c1	998723771	25	334.17	2.2
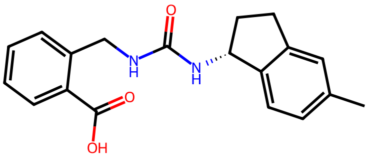	Cc1ccc2c(c1)CC[C@H]2NC(=O)NCc1ccccc1C(=O)O	874387876	24	324.15	3.18
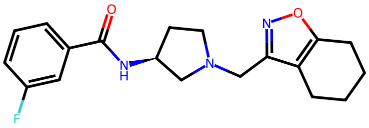	O=C(N[C@H]1CCN(Cc2noc3c2CCCC3)C1)c1cccc(F)c1	1009984293	25	343.17	2.7
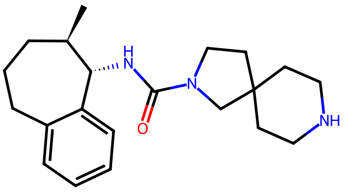	C[C@@H]1CCCc2ccccc2[C@H]1NC(=O)N1CCC2(CCNCC2)C1	418018813	25	341.25	3.49
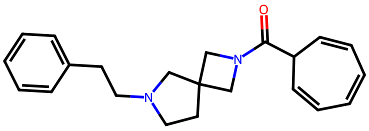	O=C(C1C=CC=CC=C1)N1CC2(CCN(CCc3ccccc3)C2)C1	1072785162	25	334.2	3.06
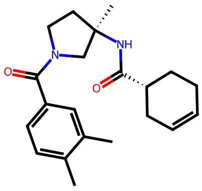	Cc1ccc(C(=O)N2CC[C@@](C)(NC(=O)[C@H]3CC=CCC3)C2)cc1C	956691097	25	340.22	3.38
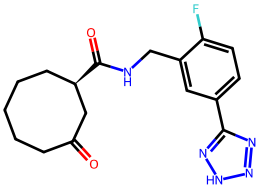	O=C1CCCCC[C@@H](C(=O)NCc2cc(-c3nn[nH]n3)ccc2F)C1	1607045310	25	345.16	2.16

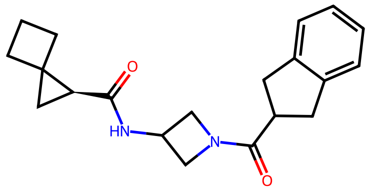	O=C(NC1CN(C(=O)C2Cc3ccccc3C2)C1)[C@H]1CC12CCC2	991550188	24	324.18	1.92
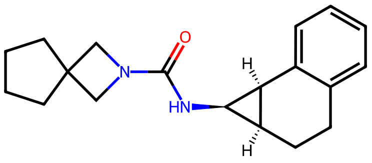	O=C(N[C@@H]1[C@@H]2CCc3ccccc3[C@@H]21)N1CC2(CCCC2)C1	430359223	22	296.19	3.3
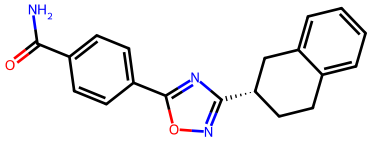	NC(=O)c1ccc(-c2nc([C@H]3CCc4ccccc4C3)no2)cc1	770887949	24	319.13	3.11
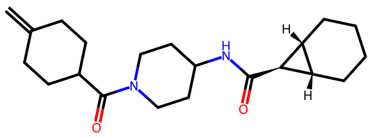	C=C1CCC(C(=O)N2CCC(NC(=O)[C@@H]3[C@H]4CCCC[C@H]43)CC2)CC1	948190104	25	344.25	3.28
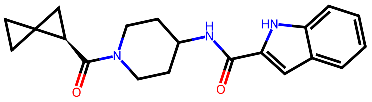	O=C(NC1CCN(C(=O)[C@@H]2CC23CC3)CC1)c1cc2ccccc2[nH]1	948558470	25	337.18	2.69
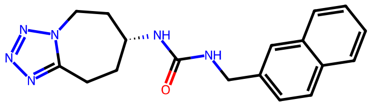	O=C(NCc1ccc2ccccc2c1)N[C@@H]1CCc2nnnn2CC1	643378532	25	336.17	2.03
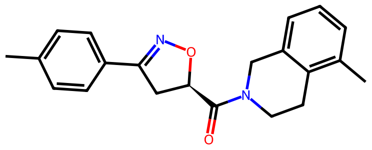	Cc1ccc(C2=NO[C@@H](C(=O)N3CCc4c(C)cccc4C3)C2)cc1	1125996858	25	334.17	3.38
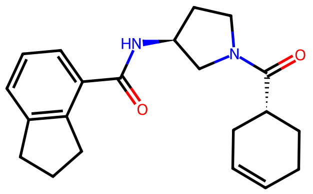	O=C(N[C@H]1CCN(C(=O)[C@H]2CC=CCC2)C1)c1cccc2c1CCC2	938148028	25	338.2	2.86

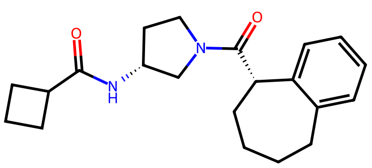	O=C(N[C@@H]1CCN(C(=O)[C@H]2CCCCc3ccccc32)C1)C1CCC1	939032585	25	340.22	3.01
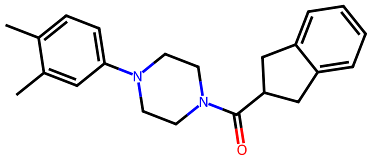	Cc1ccc(N2CCN(C(=O)C3Cc4ccccc4C3)CC2)cc1C	762189055	25	334.2	3.37
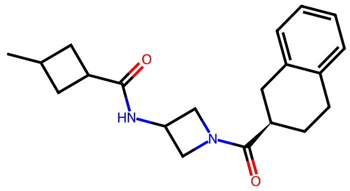	CC1CC(C(=O)NC2CN(C(=O)[C@@H]3CCc4ccccc4C3)C2)C1	990755012	24	326.2	2.16
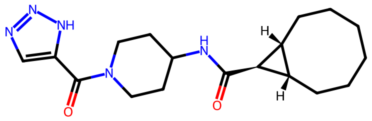	O=C(NC1CCN(C(=O)c2cnn[nH]2)CC1)[C@@H]1[C@H]2CCCCCC[C@H]21	947373279	25	345.22	1.74
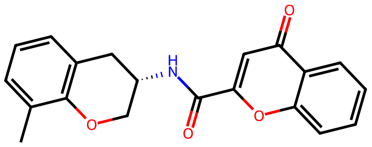	Cc1cccc2c1OC[C@@H](NC(=O)c1cc(=O)c3ccccc3o1)C2	813305067	25	335.12	2.83
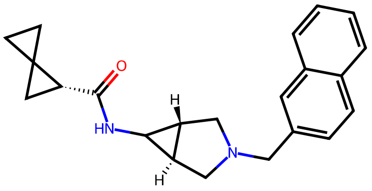	O=C(NC1[C@@H]2CN(Cc3ccc4ccccc4c3)C[C@@H]12)[C@@H]1CC12CC2	961888347	25	332.19	3.19
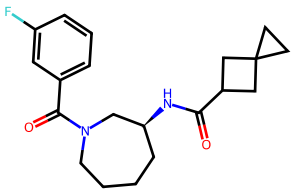	O=C(N[C@H]1CCCCN(C(=O)c2cccc(F)c2)C1)C1CC2(CC2)C1	943514080	25	344.19	3.13
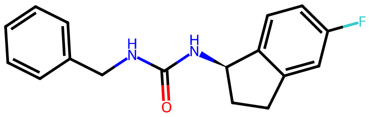	O=C(NCc1ccccc1)N[C@@H]1CCc2cc(F)ccc21	56515622	21	284.13	3.31

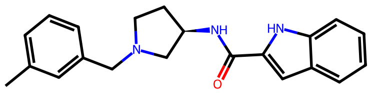	Cc1cccc(CN2CC[C@@H](NC(=O)c3cc4ccccc4[nH]3)C2)c1	1016209898	25	333.18	3.48
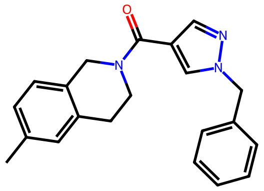	Cc1ccc2c(c1)CCN(C(=O)c1cnn(Cc3ccccc3)c1)C2	360221505	25	331.17	3.44
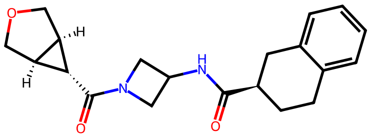	O=C(NC1CN(C(=O)[C@@H]2[C@H]3COC[C@H]32)C1)[C@@H]1CCc2ccccc2C1	1000545006	25	340.18	1.01
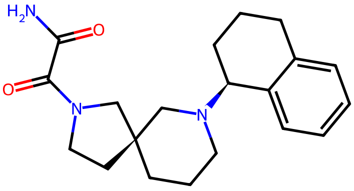	NC(=O)C(=O)N1CC[C@@]2(CCCN([C@H]3CCCc4ccccc43)C2)C1	1040371018	25	341.21	1.86
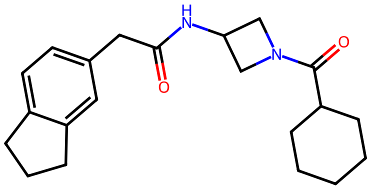	O=C(Cc1ccc2c(c1)CCC2)NC1CN(C(=O)C2CCCCC2)C1	991383974	25	340.22	2.63
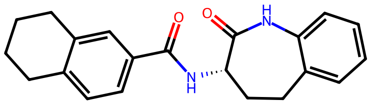	O=C(N[C@H]1CCc2ccccc2NC1=O)c1ccc2c(c1)CCCC2	654005263	25	334.17	3.25
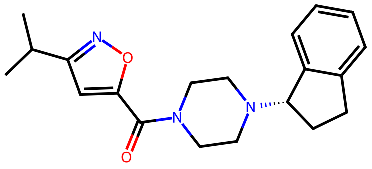	CC(C)c1cc(C(=O)N2CCN([C@H]3CCc4ccccc43)CC2)on1	951188810	25	339.19	3.24
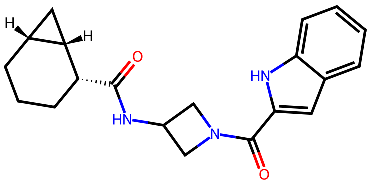	O=C(NC1CN(C(=O)c2cc3ccccc3[nH]2)C1)[C@@H]1CCC[C@@H]2C[C@@H]21	994727577	25	337.18	2.54

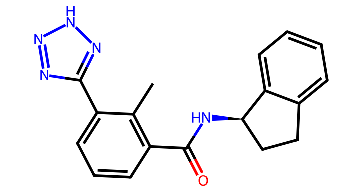	Cc1c(C(=O)N[C@@H]2CCc3ccccc32)cccc1-c1nn[nH]n1	1580547029	24	319.14	2.59
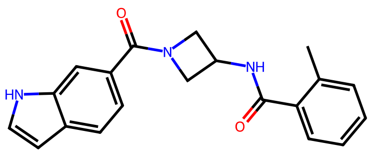	Cc1ccccc1C(=O)NC1CN(C(=O)c2ccc3cc[nH]c3c2)C1	990111483	25	333.15	2.73
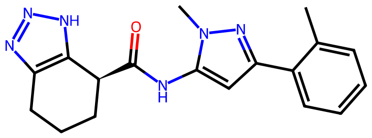	Cc1ccccc1-c1cc(NC(=O)[C@H]2CCCc3nn[nH]c32)n(C)n1	646888847	25	336.17	2.57
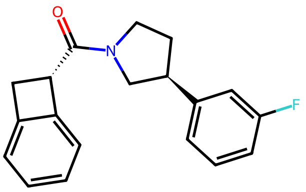	O=C([C@H]1Cc2ccccc21)N1CC[C@@H](c2cccc(F)c2)C1	460157387	22	295.14	3.48
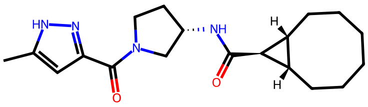	Cc1cc(C(=O)N2CC[C@H](NC(=O)[C@@H]3[C@H]4CCCCCC[C@H]43)C2)n[nH]1	938971658	25	344.22	2.27
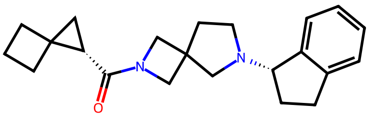	O=C([C@H]1CC12CCC2)N1CC2(CCN([C@H]3CCc4ccccc43)C2)C1	1073018964	25	336.22	3.4
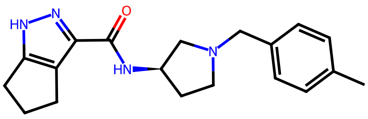	Cc1ccc(CN2CC[C@@H](NC(=O)c3n[nH]c4c3CCC4)C2)cc1	634455791	24	324.2	2.21
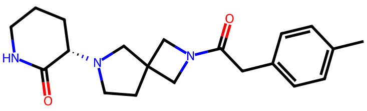	Cc1ccc(CC(=O)N2CC3(CCN([C@H]4CCCNC4=O)C3)C2)cc1	1072902725	25	341.21	1.35

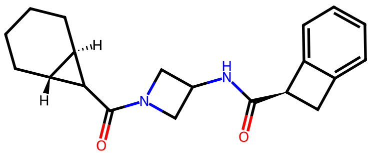	O=C(NC1CN(C(=O)C2[C@@H]3CCCC[C@@H]23)C1)[C@@H]1Cc2ccccc21	998680558	24	324.18	2.09
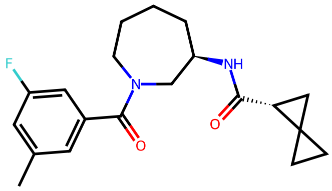	Cc1cc(F)cc(C(=O)N2CCCC[C@@H](NC(=O)[C@@H]3CC34CC4)C2)c1	943574026	25	344.19	3.05
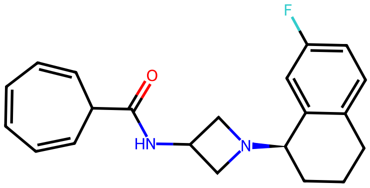	O=C(NC1CN([C@@H]2CCCc3ccc(F)cc32)C1)C1C=CC=CC=C1	1030381028	25	338.18	3.3
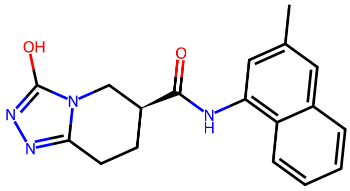	Cc1cc(NC(=O)[C@H]2CCc3nnc(O)n3C2)c2ccccc2c1	1335979128	24	322.14	2.65
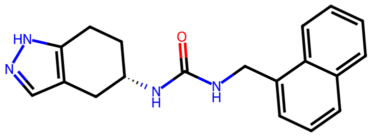	O=C(NCc1cccc2ccccc12)N[C@H]1CCc2[nH]ncc2C1	165676021	24	320.16	2.92
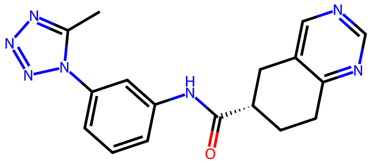	Cc1nnnn1-c1cccc(NC(=O)[C@H]2CCc3ncncc3C2)c1	648242508	25	335.15	1.5
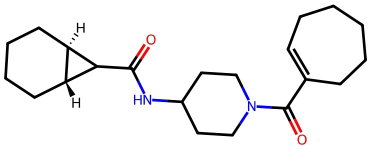	O=C(NC1CCN(C(=O)C2=CCCCCC2)CC1)C1[C@@H]2CCCC[C@@H]12	946548926	25	344.25	3.42
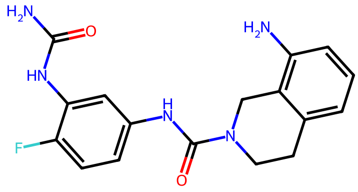	NC(=O)Nc1cc(NC(=O)N2CCc3cccc(N)c3C2)ccc1F	1580097713	25	343.14	2.49

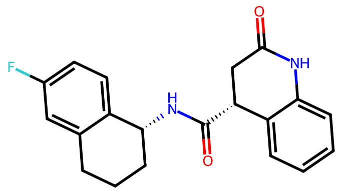	O=C1C[C@@H](C(=O)N[C@@H]2CCCc3cc(F)ccc32)c2ccccc2N1	157618477	25	338.14	3.45
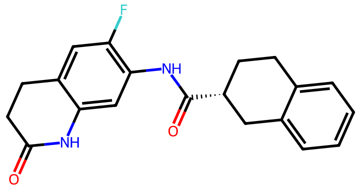	O=C1CCc2cc(F)c(NC(=O)[C@@H]3CCc4ccccc4C3)cc2N1	569582398	25	338.14	3.45
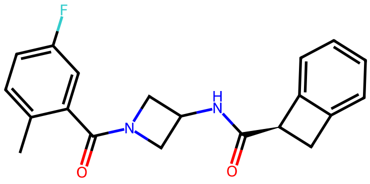	Cc1ccc(F)cc1C(=O)N1CC(NC(=O)[C@@H]2Cc3ccccc32)C1	998688189	25	338.14	2.41
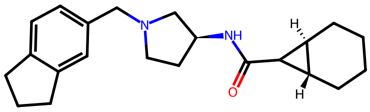	O=C(N[C@H]1CCN(Cc2ccc3c(c2)CCC3)C1)C1[C@H]2CCCC[C@H]12	1032825057	25	338.24	3.3
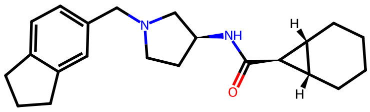	O=C(N[C@H]1CCN(Cc2ccc3c(c2)CCC3)C1)[C@@H]1[C@H]2CCCC[C@H]21	1010439989	25	338.24	3.3
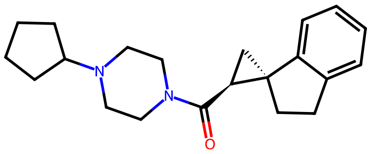	O=C([C@H]1C[C@]12CCc1ccccc12)N1CCN(C2CCCC2)CC1	175260511	24	324.22	2.98
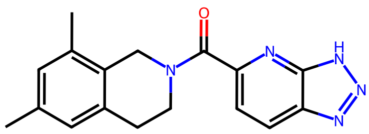	Cc1cc(C)c2c(c1)CCN(C(=O)c1ccc3nn[nH]c3n1)C2	1160052030	23	307.14	2.17
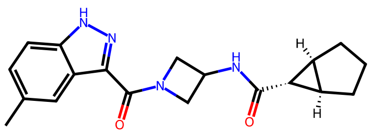	Cc1ccc2[nH]nc(C(=O)N3CC(NC(=O)[C@@H]4[C@H]5CCC[C@H]54)C3)c2c1	990705762	25	338.17	1.86

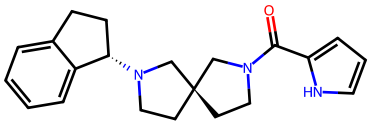	O=C(c1ccc[nH]1)N1CC[C@]2(CCN([C@H]3CCc4ccccc43)C2)C1	1041066487	25	335.2	3.24
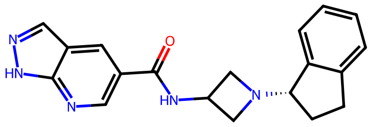	O=C(NC1CN([C@H]2CCc3ccccc32)C1)c1cnc2[nH]ncc2c1	1030499651	25	333.16	2.06
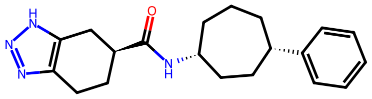	O=C(N[C@@H]1CCC[C@H](c2ccccc2)CC1)[C@H]1CCc2nn[nH]c2C1	888905279	25	338.21	3.14
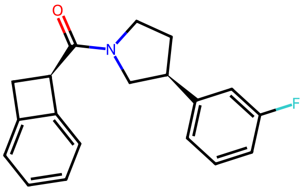	O=C([C@@H]1Cc2ccccc21)N1CC[C@@H](c2cccc(F)c2)C1	460157385	22	295.14	3.48
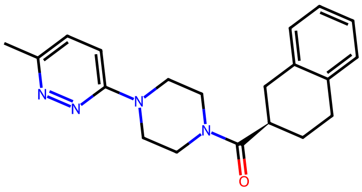	Cc1ccc(N2CCN(C(=O)[C@@H]3CCc4ccccc4C3)CC2)nn1	1056040251	25	336.2	2.24
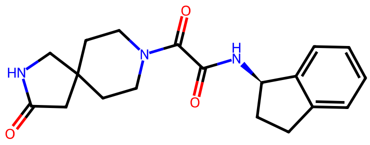	O=C1CC2(CCN(C(=O)C(=O)N[C@@H]3CCc4ccccc43)CC2)CN1	1261724478	25	341.17	0.92
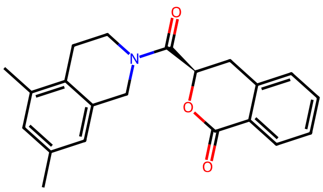	Cc1cc(C)c2c(c1)CN(C(=O)[C@H]1Cc3ccccc3C(=O)O1)CC2	187733045	25	335.15	2.97
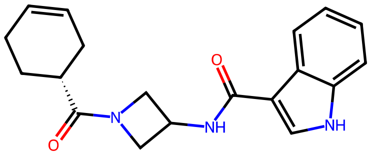	O=C(NC1CN(C(=O)[C@H]2CC=CCC2)C1)c1c[nH]c2ccccc12	995030690	24	323.16	2.46

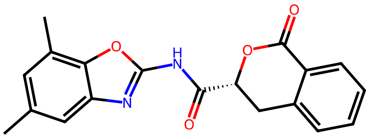	Cc1cc(C)c2oc(NC(=O)[C@H]3Cc4ccccc4C(=O)O3)nc2c1	749155373	25	336.11	3.16
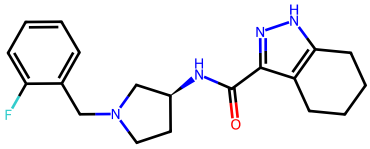	O=C(N[C@H]1CCN(Cc2ccccc2F)C1)c1n[nH]c2c1CCCC2	1015077906	25	342.19	2.43
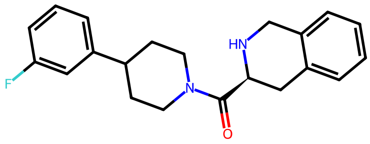	O=C([C@@H]1Cc2ccccc2CN1)N1CCC(c2cccc(F)c2)CC1	1343800127	25	338.18	3.25
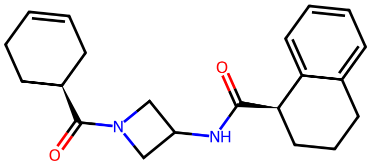	O=C(NC1CN(C(=O)[C@@H]2CC=CCC2)C1)[C@@H]1CCCc2ccccc21	995071184	25	338.2	2.79
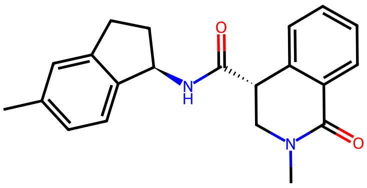	Cc1ccc2c(c1)CC[C@H]2NC(=O)[C@H]1CN(C)C(=O)c2ccccc21	345861737	25	334.17	2.97
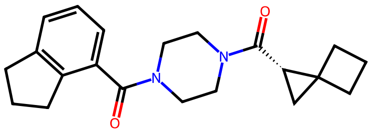	O=C(c1cccc2c1CCC2)N1CCN(C(=O)[C@H]2CC23CCC3)CC1	1011144435	25	338.2	2.65
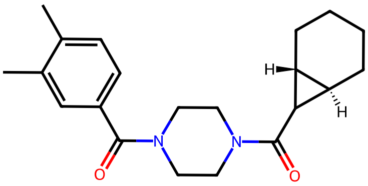	Cc1ccc(C(=O)N2CCN(C(=O)C3[C@@H]4CCCC[C@@H]34)CC2)cc1C	1010902189	25	340.22	3.02
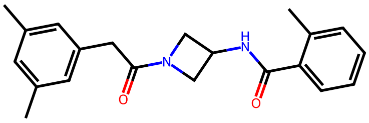	Cc1cc(C)cc(CC(=O)N2CC(NC(=O)c3ccccc3C)C2)c1	990111669	25	336.18	2.8

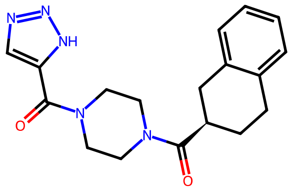	O=C(c1cnn[nH]1)N1CCN(C(=O)[C@@H]2CCc3ccccc3C2)CC1	1011448434	25	339.17	0.89
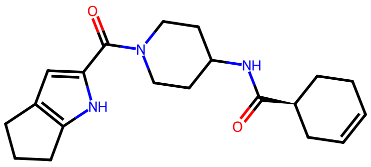	O=C(NC1CCN(C(=O)c2cc3c([nH]2)CCC3)CC1)[C@@H]1CC=CCC1	947446025	25	341.21	2.58
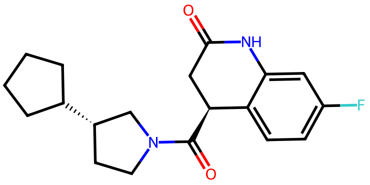	O=C1C[C@H](C(=O)N2CC[C@H](C3CCCC3)C2)c2ccc(F)cc2N1	421644331	24	330.17	3.29
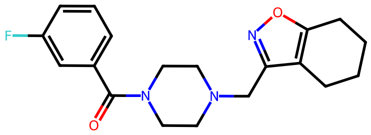	O=C(c1cccc(F)c1)N1CCN(Cc2noc3c2CCCC3)CC1	108852485	25	343.17	2.65
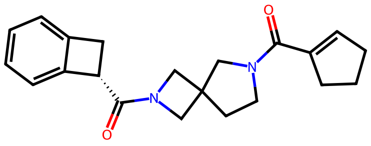	O=C(C1=CCCC1)N1CCC2(C1)CN(C(=O)[C@H]1Cc3ccccc31)C2	1019349649	25	336.18	2.5
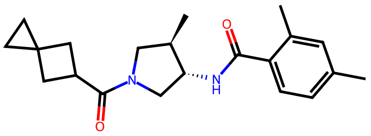	Cc1ccc(C(=O)N[C@@H]2CN(C(=O)C3CC4(CC4)C3)C[C@H]2C)c(C)c1	940141318	25	340.22	3.07
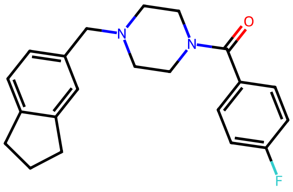	O=C(c1ccc(F)cc1)N1CCN(Cc2ccc3c(c2)CCC3)CC1	349855424	25	338.18	3.27
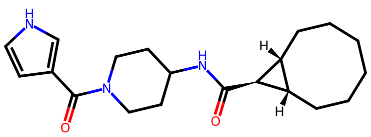	O=C(NC1CCN(C(=O)c2cc[nH]c2)CC1)[C@@H]1[C@H]2CCCCCC[C@H]21	947020499	25	343.23	2.95

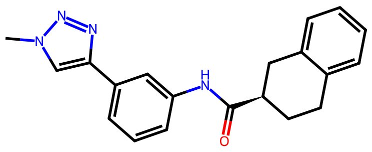	Cn1cc(-c2cccc(NC(=O)[C@@H]3CCc4ccccc4C3)c2)nn1	620270390	25	332.16	3.23
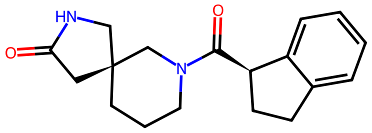	O=C1C[C@@]2(CCCN(C(=O)[C@@H]3CCc4ccccc43)C2)CN1	336402781	22	298.17	1.85
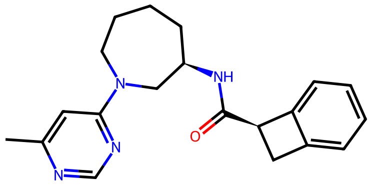	Cc1cc(N2CCCC[C@@H](NC(=O)[C@@H]3Cc4ccccc43)C2)ncn1	1063227758	25	336.2	2.6
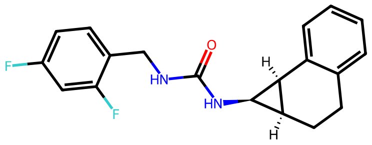	O=C(NCc1ccc(F)cc1F)N[C@@H]1[C@@H]2CCc3ccccc3[C@@H]21	435771853	24	328.14	3.49
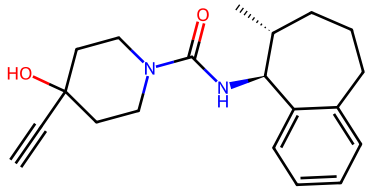	C#CC1(O)CCN(C(=O)N[C@@H]2c3ccccc3CCC[C@H]2C)CC1	1345843216	24	326.2	2.87
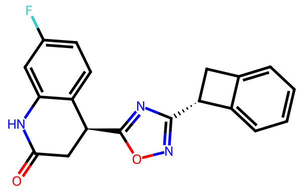	O=C1C[C@H](c2nc([C@@H]3Cc4ccccc43)no2)c2ccc(F)cc2N1	546296567	25	335.11	3.37
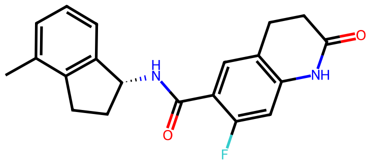	Cc1cccc2c1CC[C@H]2NC(=O)c1cc2c(cc1F)NC(=O)CC2	122788473	25	338.14	3.44
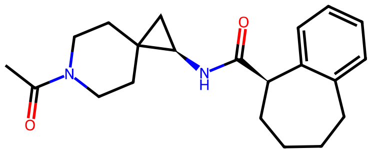	CC(=O)N1CCC2(CC1)C[C@H]2NC(=O)[C@@H]1CCCCc2ccccc21	964029776	25	340.22	3.01

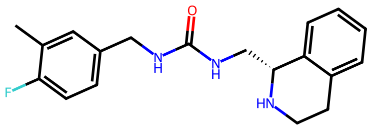	Cc1cc(CNC(=O)NC[C@H]2NCCc3ccccc32)ccc1F	516260984	24	327.17	2.82
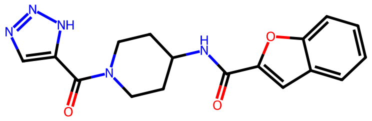	O=C(NC1CCN(C(=O)c2cnn[nH]2)CC1)c1cc2ccccc2o1	947370871	25	339.13	1.59
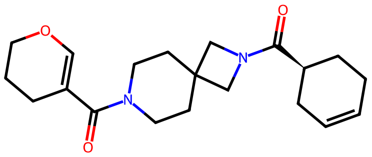	O=C(C1=COCCC1)N1CCC2(CC1)CN(C(=O)[C@@H]1CC=CCC1)C2	944878475	25	344.21	2.49
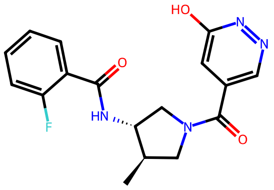	C[C@@H]1CN(C(=O)c2cnnc(O)c2)C[C@H]1NC(=O)c1ccccc1F	950032376	25	344.13	1.21
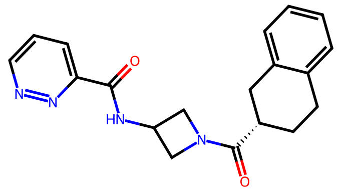	O=C(NC1CN(C(=O)[C@H]2CCc3ccccc3C2)C1)c1cccnn1	998298768	25	336.16	1.22
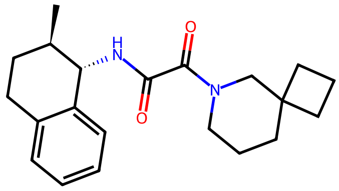	C[C@@H]1CCc2ccccc2[C@H]1NC(=O)C(=O)N1CCCC2(CCC2)C1	359648126	25	340.22	3.22
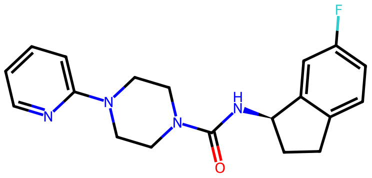	O=C(N[C@@H]1CCc2ccc(F)cc21)N1CCN(c2ccccn2)CC1	106576866	25	340.17	2.74
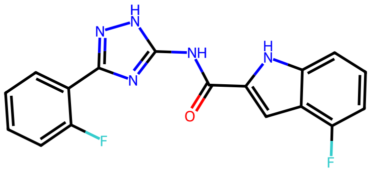	O=C(Nc1nc(-c2ccccc2F)n[nH]1)c1cc2c(F)cccc2[nH]1	345666808	25	339.09	3.48

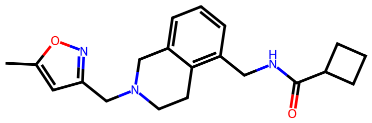	Cc1cc(CN2CCc3c(CNC(=O)C4CCC4)cccc3C2)no1	1051274420	25	339.19	2.96
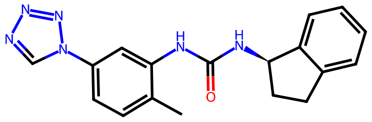	Cc1ccc(-n2cnnn2)cc1NC(=O)N[C@@H]1CCc2ccccc21	47926460	25	334.15	2.78
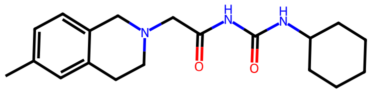	Cc1ccc2c(c1)CCN(CC(=O)NC(=O)NC1CCCCC1)C2	361848126	24	329.21	2.51
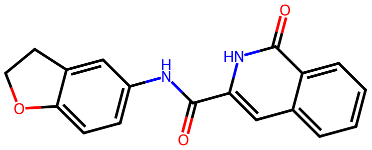	O=C(Nc1ccc2c(c1)CCO2)c1cc2ccccc2c(=O)[nH]1	30371704	23	306.1	2.72
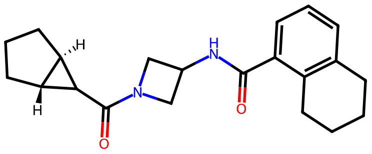	O=C(NC1CN(C(=O)C2[C@@H]3CCC[C@@H]23)C1)c1cccc2c1CCCC2	991900251	25	338.2	2.55
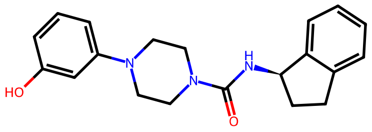	O=C(N[C@@H]1CCc2ccccc21)N1CCN(c2cccc(O)c2)CC1	121641639	25	337.18	2.91
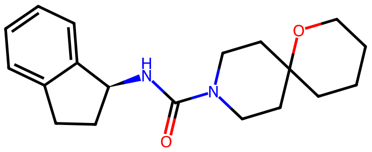	O=C(N[C@H]1CCc2ccccc21)N1CCC2(CCCCO2)CC1	883086790	23	314.2	3.42
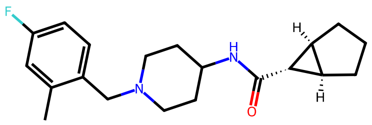	Cc1cc(F)ccc1CN1CCC(NC(=O)[C@@H]2[C@H]3CCC[C@H]32)CC1	1002562210	24	330.21	3.26

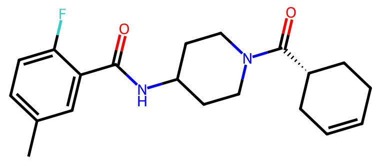	Cc1ccc(F)c(C(=O)NC2CCN(C(=O)[C@H]3CC=CCC3)CC2)c1	944127773	25	344.19	3.21
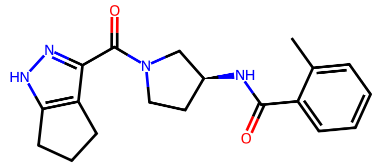	Cc1ccccc1C(=O)N[C@H]1CCN(C(=O)c2n[nH]c3c2CCC3)C1	937629567	25	338.17	1.85
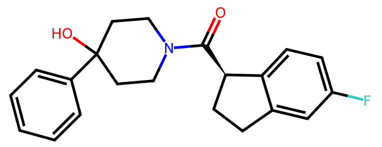	O=C([C@@H]1CCc2cc(F)ccc21)N1CCC(O)(c2ccccc2)CC1	832266297	25	339.16	3.37
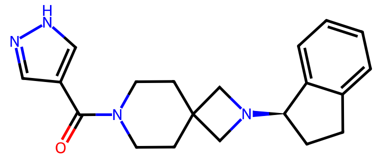	O=C(c1cn[nH]c1)N1CCC2(CC1)CN([C@@H]1CCc3ccccc31)C2	1035657923	25	336.2	2.64
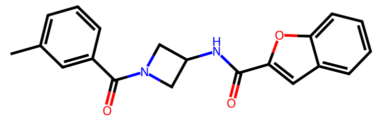	Cc1cccc(C(=O)N2CC(NC(=O)c3cc4ccccc4o3)C2)c1	1000051034	25	334.13	3.0
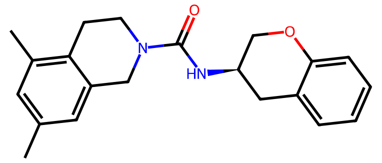	Cc1cc(C)c2c(c1)CN(C(=O)N[C@H]1COc3ccccc3C1)CC2	1676551321	25	336.18	3.37
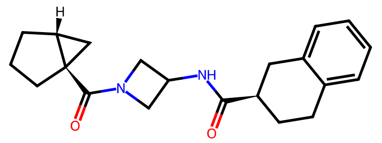	O=C(NC1CN(C(=O)[C@@]23CCC[C@@H]2C3)C1)[C@@H]1CCc2ccccc2C1	1000537984	25	338.2	2.31
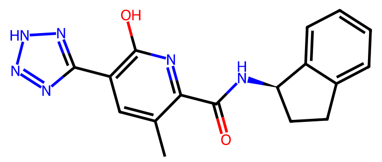	Cc1cc(-c2nn[nH]n2)c(O)nc1C(=O)N[C@@H]1CCc2ccccc21	1580606052	25	336.13	1.69

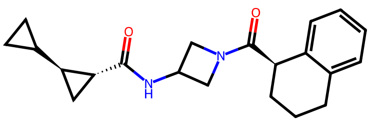	O=C(NC1CN(C(=O)[C@@H]2CCCc3ccccc32)C1)[C@@H]1C[C@H]1C1CC1	992001852	25	338.2	2.48
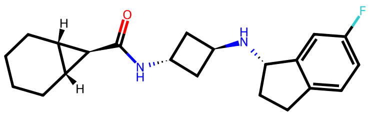	O=C(N[C@H]1C[C@H](N[C@H]2CCc3ccc(F)cc32)C1)[C@@H]1[C@H]2CCCC[C@H]21	1020047966	25	342.21	3.49
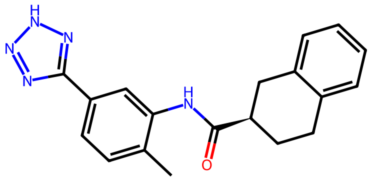	Cc1ccc(-c2nn[nH]n2)cc1NC(=O)[C@@H]1CCc2ccccc2C1	1605795136	25	333.16	2.92
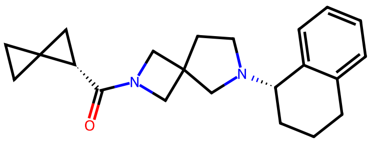	O=C([C@H]1CC12CC2)N1CC2(CCN([C@H]3CCCc4ccccc43)C2)C1	1073012101	25	336.22	3.4
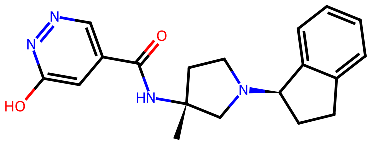	C[C@@]1(NC(=O)c2cnnc(O)c2)CCN([C@@H]2CCc3ccccc32)C1	1046399303	25	338.17	2.06
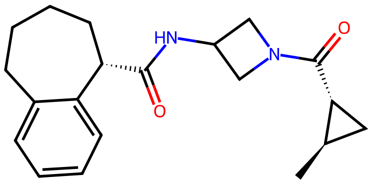	C[C@@H]1C[C@H]1C(=O)N1CC(NC(=O)[C@H]2CCCCc3ccccc32)C1	994323848	24	326.2	2.48
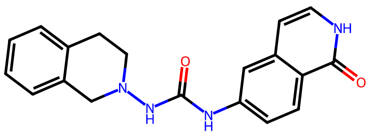	O=C(Nc1ccc2c(=O)[nH]ccc2c1)NN1CCc2ccccc2C1	1679094255	25	334.14	2.62
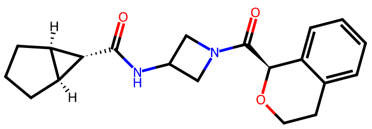	O=C(NC1CN(C(=O)[C@@H]2OCCc3ccccc32)C1)[C@@H]1[C@H]2CCC[C@H]21	990713000	25	340.18	1.67

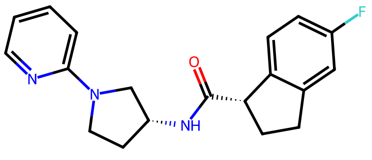	O=C(N[C@@H]1CCN(c2ccccn2)C1)[C@H]1CCc2cc(F)ccc21	612904287	24	325.16	2.65
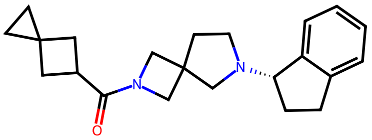	O=C(C1CC2(CC2)C1)N1CC2(CCN([C@H]3CCc4ccccc43)C2)C1	1072972244	25	336.22	3.4
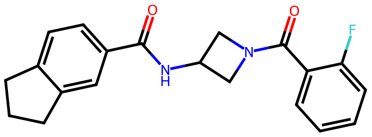	O=C(NC1CN(C(=O)c2ccccc2F)C1)c1ccc2c(c1)CCC2	991353935	25	338.14	2.57
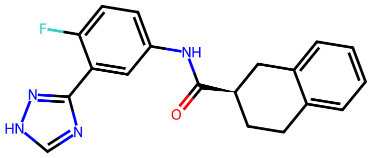	O=C(Nc1ccc(F)c(-c2nc[nH]n2)c1)[C@@H]1CCc2ccccc2C1	1128485513	25	336.14	3.35
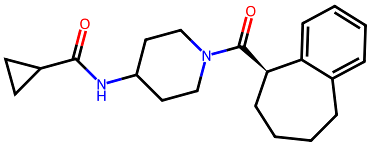	O=C(NC1CCN(C(=O)[C@@H]2CCCCc3ccccc32)CC1)C1CC1	947145464	25	340.22	3.01
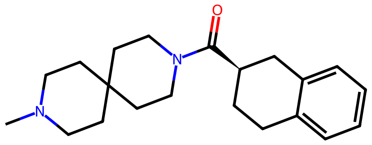	CN1CCC2(CC1)CCN(C(=O)[C@@H]1CCc3ccccc3C1)CC2	1118988301	24	326.24	3.13
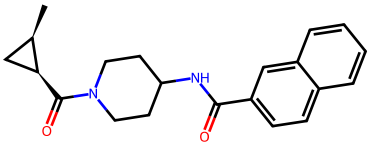	C[C@@H]1C[C@@H]1C(=O)N1CCC(NC(=O)c2ccc3ccccc3c2)CC1	943836346	25	336.18	3.22
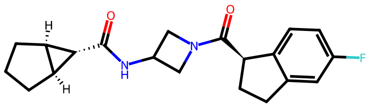	O=C(NC1CN(C(=O)[C@@H]2CCc3cc(F)ccc32)C1)[C@@H]1[C@H]2CCC[C@H]21	990701879	25	342.17	2.23

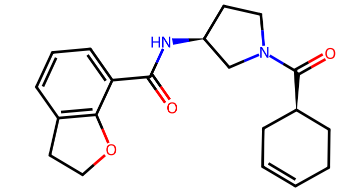	O=C(N[C@H]1CCN(C(=O)[C@@H]2CC=CCC2)C1)c1cccc2c1OCC2	938951934	25	340.18	2.31
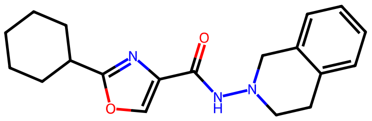	O=C(NN1CCc2ccccc2C1)c1coc(C2CCCCC2)n1	1628725895	24	325.18	3.43
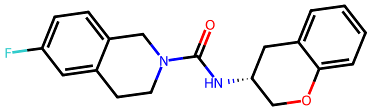	O=C(N[C@H]1COc2ccccc2C1)N1CCc2cc(F)ccc2C1	92187682	24	326.14	2.9
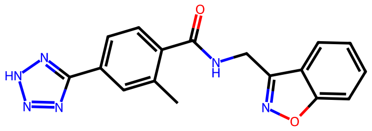	Cc1cc(-c2nn[nH]n2)ccc1C(=O)NCc1noc2ccccc12	1605536736	25	334.12	2.25
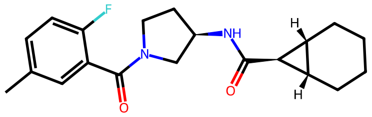	Cc1ccc(F)c(C(=O)N2CC[C@@H](NC(=O)[C@@H]3[C@H]4CCCC[C@H]43)C2)c1	936274000	25	344.19	2.9
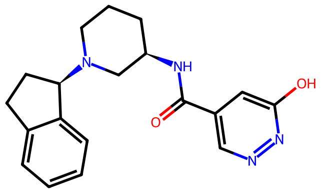	O=C(N[C@@H]1CCCN([C@@H]2CCc3ccccc32)C1)c1cnnc(O)c1	1007549053	25	338.17	2.06
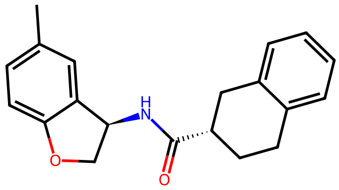	Cc1ccc2c(c1)[C@@H](NC(=O)[C@H]1CCc3ccccc3C1)CO2	567392395	23	307.16	3.35
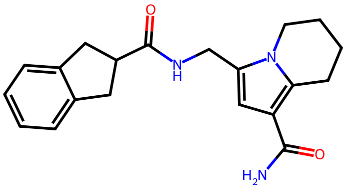	NC(=O)c1cc(CNC(=O)C2Cc3ccccc3C2)n2c1CCCC2	864326925	25	337.18	1.95

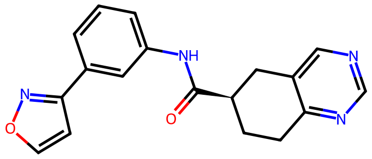	O=C(Nc1cccc(-c2ccon2)c1)[C@@H]1<br>CCc2ncncc2C1	1536209960	24	320.13	2.88
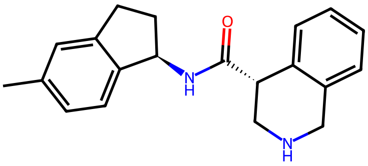	Cc1ccc2c(c1)CC[C@H]2NC(=O)[C@<br>H]1CNCc2ccccc21	515837421	23	306.17	2.99
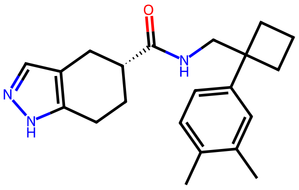	Cc1ccc(C2(CNC(=O)[C@@H]3CCc4[<br>nH]ncc4C3)CCC2)cc1C	278794230	25	337.22	3.37
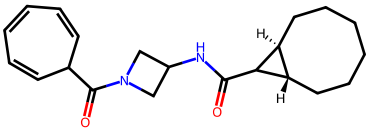	O=C(NC1CN(C(=O)C2C=CC=CC=C2)<br>C1)C1[C@H]2CCCCCC[C@H]12	1000507831	25	340.22	2.83
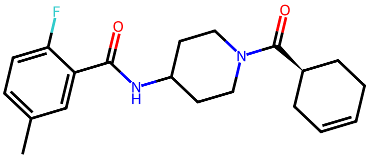	Cc1ccc(F)c(C(=O)NC2CCN(C(=O)[C<br>@@H]3CC=CCC3)CC2)c1	944127774	25	344.19	3.21
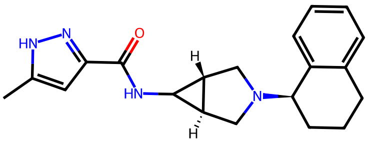	Cc1cc(C(=O)NC2[C@@H]3CN([C@<br>@H]4CCCc5ccccc54)C[C@@H]23)n[<br>nH]1	958332178	25	336.2	2.46
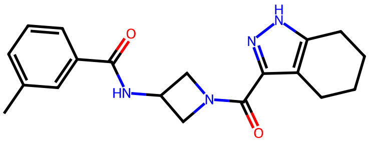	Cc1cccc(C(=O)NC2CN(C(=O)c3n[nH]<br>c4c3CCCC4)C2)c1	990121074	25	338.17	1.85
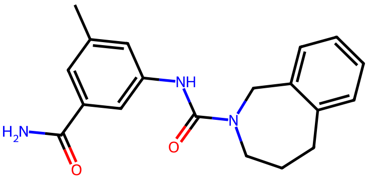	Cc1cc(NC(=O)N2CCCc3ccccc3C2)cc(<br>C(N)=O)c1	455850771	24	323.16	3.07

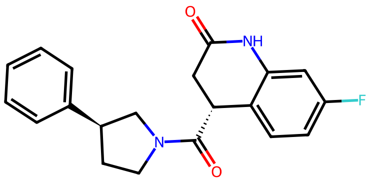	O=C1C[C@@H](C(=O)N2CC[C@@H](c3ccccc3)C2)c2ccc(F)cc2N1	121578657	25	338.14	3.27
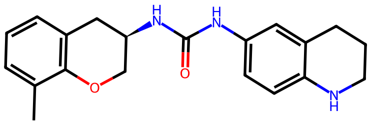	Cc1cccc2c1OC[C@H](NC(=O)Nc1ccc3c(c1)CCCN3)C2	415867450	25	337.18	3.48
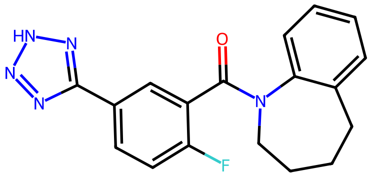	O=C(c1cc(-c2nn[nH]n2)ccc1F)N1CCCCc2ccccc21	1607003842	25	337.13	2.99
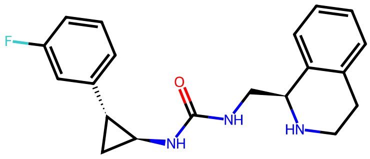	O=C(NC[C@@H]1NCCc2ccccc21)N[C@H]1C[C@@H]1c1ccc(F)c1	572825141	25	339.17	2.87
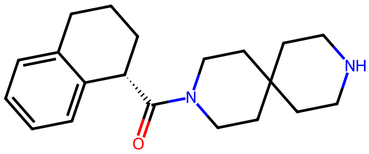	O=C([C@H]1CCCc2ccccc21)N1CCC2(CCNCC2)CC1	261613454	23	312.22	3.1
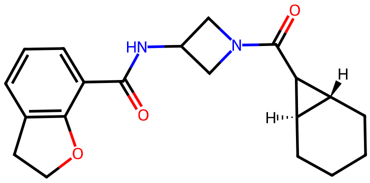	O=C(NC1CN(C(=O)C2[C@@H]3CCCC[C@@H]23)C1)c1cccc2c1OCC2	1000416623	25	340.18	2.0
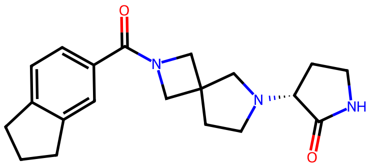	O=C1NCC[C@H]1N1CCC2(CN(C(=O)c3ccc4c(c3)CCC4)C2)C1	1073418218	25	339.19	1.21
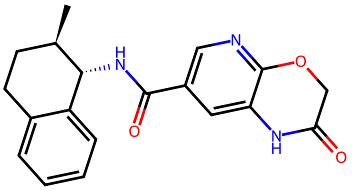	C[C@@H]1CCc2ccccc2[C@H]1NC(=O)c1cnc2c(c1)NC(=O)CO2	271422753	25	337.14	2.47

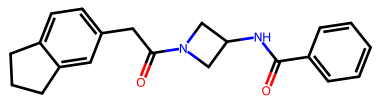	O=C(NC1CN(C(=O)Cc2ccc3c(c2)CCC3)C1)c1ccccc1	990095854	25	334.17	2.36
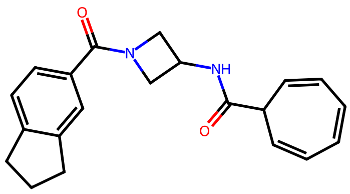	O=C(NC1CN(C(=O)c2ccc3c(c2)CCC3)C1)C1C=CC=CC=C1	992876221	25	334.17	2.41
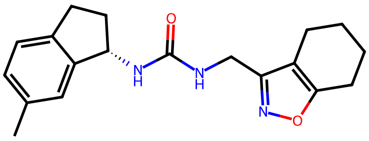	Cc1ccc2c(c1)[C@@H](NC(=O)NCc1noc3c1CCCC3)CC2	672840989	24	325.18	3.35
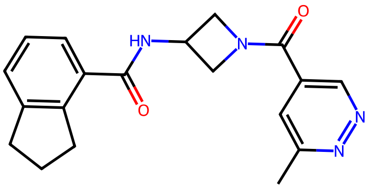	Cc1cc(C(=O)N2CC(NC(=O)c3cccc4c3CCC4)C2)cnn1	994403314	25	336.16	1.53
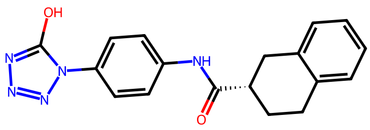	O=C(Nc1ccc(-n2nnnc2O)cc1)[C@H]1CCc2ccccc2C1	1466902254	25	335.14	2.11
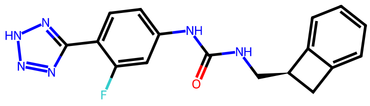	O=C(NC[C@@H]1Cc2ccccc21)Nc1ccc(-c2nn[nH]n2)c(F)c1	1609546885	25	338.13	2.47
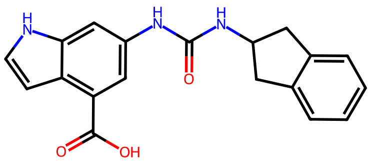	O=C(Nc1cc(C(=O)O)c2cc[nH]c2c1)NC1Cc2ccccc2C1	1601584115	25	335.13	3.16
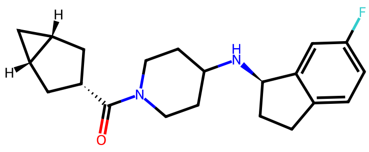	O=C([C@@H]1C[C@@H]2C[C@@H]2C1)N1CCC(N[C@@H]2CCc3ccc(F)cc32)CC1	996911103	25	342.21	3.44

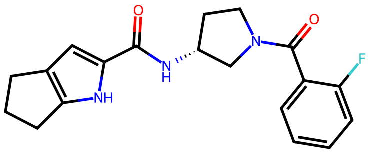	O=C(N[C@@H]1CCN(C(=O)c2ccccc2<br>F)C1)c1cc2c([nH]1)CCC2	941583740	25	341.15	2.29
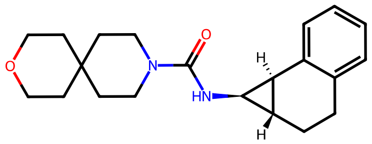	O=C(N[C@@H]1[C@H]2CCc3ccccc3[<br>C@@H]21)N1CCC2(CCOCC2)CC1	289303139	25	340.22	3.32
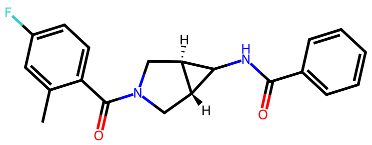	Cc1cc(F)ccc1C(=O)N1C[C@H]2C(NC<br>(=O)c3ccccc3)[C@@H]2C1	1012708884	25	338.14	2.63
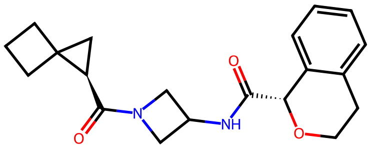	O=C(NC1CN(C(=O)[C@@H]2CC23C<br>CC3)C1)[C@H]1OCCc2ccccc21	997326405	25	340.18	1.82
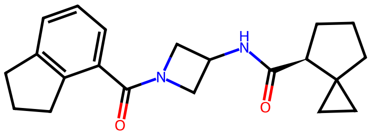	O=C(NC1CN(C(=O)c2cccc3c2CCC3)<br>C1)[C@H]1CCCC12CC2	994862721	25	338.2	2.7
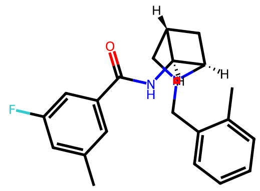	Cc1cc(F)cc(C(=O)N[C@H]2[C@H]3C[<br>C@H]2N(Cc2ccccc2C)C3)c1	1037588406	25	338.18	3.45
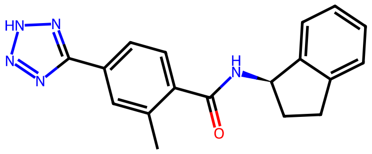	Cc1cc(-c2nn[nH]n2)ccc1C(=O)N[C@<br>@H]1CCc2ccccc21	1591863398	24	319.14	2.59
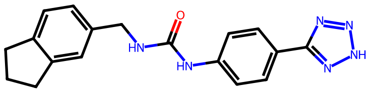	O=C(NCc1ccc2c(c1)CCC2)Nc1ccc(-c<br>2nn[nH]n2)cc1	1606832923	25	334.15	2.68

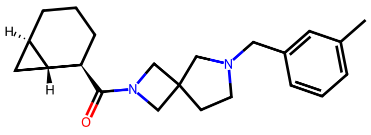	Cc1cccc(CN2CCC3(C2)CN(C(=O)[C@@H]2CCC[C@@H]4C[C@H]42)C3)c1	1072875665	25	338.24	3.47
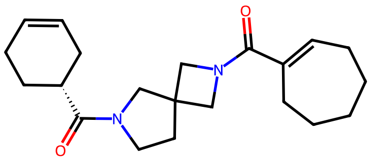	O=C(C1=CCCCCC1)N1CC2(CCN(C(=O)[C@H]3CC=CCC3)C2)C1	1018967205	25	342.23	3.29
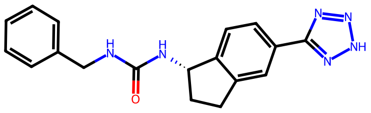	O=C(NCc1ccccc1)N[C@H]1CCc2cc(-c3nn[nH]n3)ccc21	1593143911	25	334.15	2.35
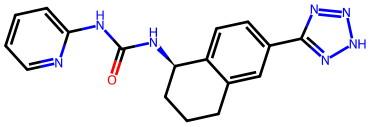	O=C(Nc1ccccn1)N[C@@H]1CCCc2cc(-c3nn[nH]n3)ccc21	1593254198	25	335.15	2.46
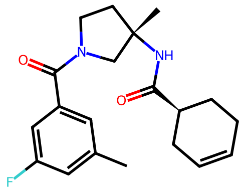	Cc1cc(F)cc(C(=O)N2CC[C@](C)(NC(=O)[C@@H]3CC=CCC3)C2)c1	956689890	25	344.19	3.21
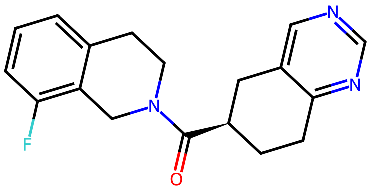	O=C([C@@H]1CCc2ncncc2C1)N1CCc2cccc(F)c2C1	648342283	23	311.14	2.31
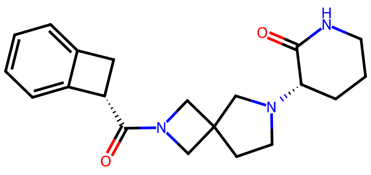	O=C1NCCC[C@@H]1N1CCC2(CN(C(=O)[C@H]3Cc4ccccc43)C2)C1	1072931285	25	339.19	1.14
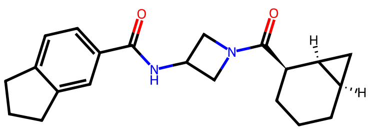	O=C(NC1CN(C(=O)[C@@H]2CCC[C@@H]3C[C@@H]32)C1)c1ccc2c(c1)CCC2	991370394	25	338.2	2.55

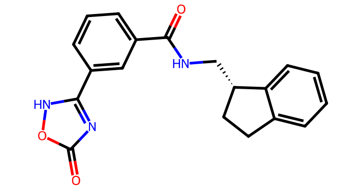	O=C(NC[C@H]1CCc2ccccc21)c1cccc(-c2nc(=O)o[nH]2)c1	1586081319	25	335.13	2.49
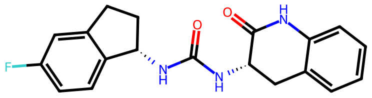	O=C(N[C@H]1Cc2ccccc2NC1=O)N[C@H]1CCc2cc(F)ccc21	636635620	25	339.14	2.68
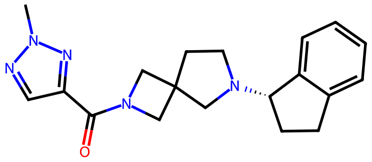	Cn1ncc(C(=O)N2CC3(CCN([C@H]4CCc5ccccc54)C3)C2)n1	1073056134	25	337.19	1.65
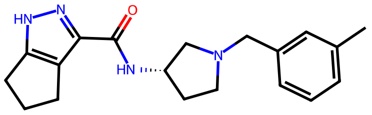	Cc1cccc(CN2CC[C@H](NC(=O)c3n[nH]c4c3CCC4)C2)c1	634273283	24	324.2	2.21
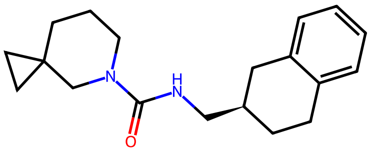	O=C(NC[C@@H]1CCc2ccccc2C1)N1CCCC2(CC2)C1	681160708	22	298.2	3.38
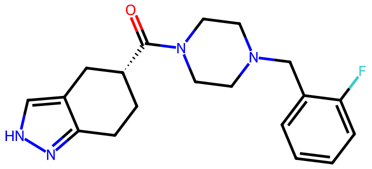	O=C([C@@H]1CCc2n[nH]cc2C1)N1CCN(Cc2ccccc2F)CC1	957308897	25	342.19	2.0
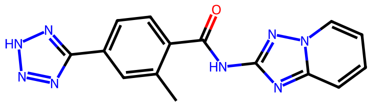	Cc1cc(-c2nn[nH]n2)ccc1C(=O)Nc1nc2ccccn2n1	826251235	24	320.11	1.47
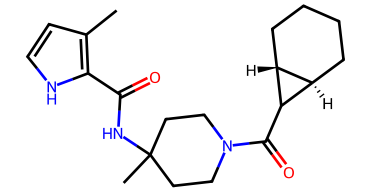	Cc1cc[nH]c1C(=O)NC1(C)CCN(C(=O)C2[C@@H]3CCCC[C@@H]23)CC1	956354975	25	343.23	2.87

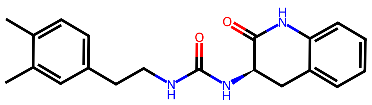	Cc1ccc(CCNC(=O)N[C@@H]2Cc3ccccc3NC2=O)cc1C	1414694720	25	337.18	2.71
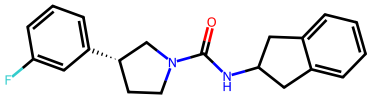	O=C(NC1Cc2ccccc2C1)N1CC[C@H](c2cccc(F)c2)C1	576915760	24	324.16	3.49
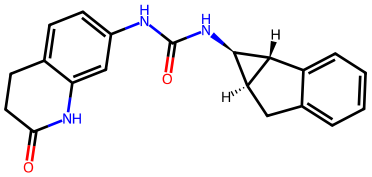	O=C1CCc2ccc(NC(=O)N[C@H]3[C@@H]4c5ccccc5C[C@H]43)cc2N1	151354058	25	333.15	3.03
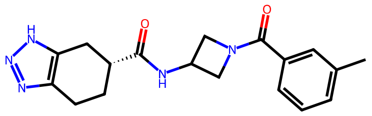	Cc1cccc(C(=O)N2CC(NC(=O)[C@@H]3CCc4nn[nH]c4C3)C2)c1	996390340	25	339.17	0.86
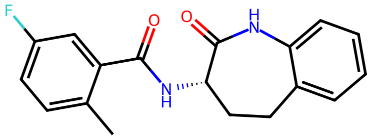	Cc1ccc(F)cc1C(=O)N[C@H]1CCc2ccccc2NC1=O	1433512883	23	312.13	2.82
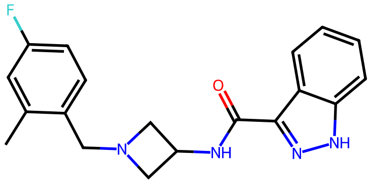	Cc1cc(F)ccc1CN1CC(NC(=O)c2n[nH]c3ccccc23)C1	1031342099	25	338.15	2.62
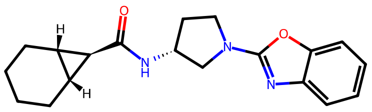	O=C(N[C@@H]1CCN(c2nc3ccccc3o2)C1)[C@@H]1[C@H]2CCCC[C@H]21	1058334094	24	325.18	2.96
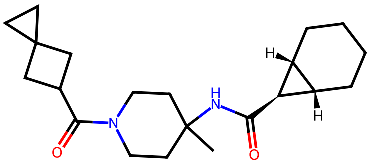	CC1(NC(=O)[C@@H]2[C@H]3CCCC[C@H]32)CCN(C(=O)C2CC3(CC3)C2)CC1	956331529	25	344.25	3.11

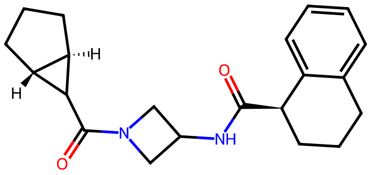	O=C(NC1CN(C(=O)C2[C@@H]3CCC[C@@H]23)C1)[C@@H]1CCCc2ccccc21	995069926	25	338.2	2.48
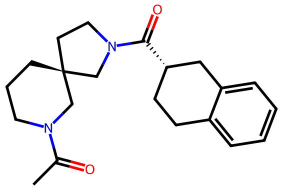	CC(=O)N1CCC[C@]2(CCN(C(=O)[C@@H]3CCc4ccccc4C3)C2)C1	952958018	25	340.22	2.65
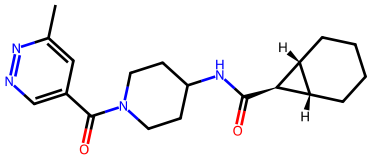	Cc1cc(C(=O)N2CCC(NC(=O)[C@@H]3[C@H]4CCCC[C@H]43)CC2)cnn1	946563660	25	342.21	1.94
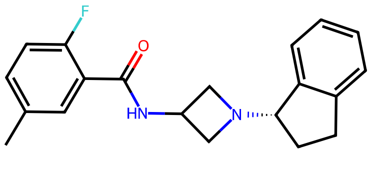	Cc1ccc(F)c(C(=O)NC2CN([C@H]3CCc4ccccc43)C2)c1	1031011775	24	324.16	3.24
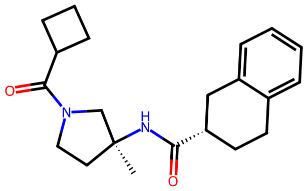	C[C@]1(NC(=O)[C@H]2CCc3ccccc3C2)CCN(C(=O)C2CCC2)C1	957727572	25	340.22	2.7
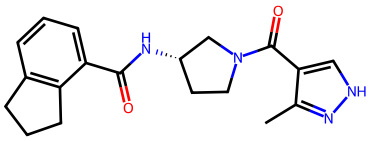	Cc1n[nH]cc1C(=O)N1CC[C@H](NC(=O)c2cccc3c2CCC3)C1	938149739	25	338.17	1.85
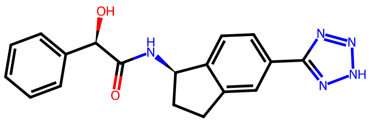	O=C(N[C@@H]1CCc2cc(-c3nn[nH]n3)ccc21)[C@H](O)c1ccccc1	1606683633	25	335.14	1.7
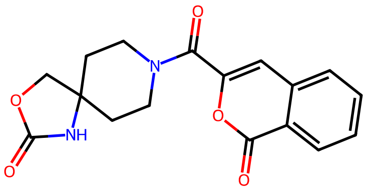	O=C1NC2(CCN(C(=O)c3cc4ccccc4c(=O)o3)CC2)CO1	496606897	24	328.11	1.51

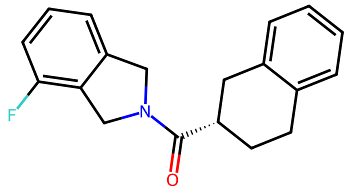	O=C([C@H]1CCc2ccccc2C1)N1Cc2cccc(F)c2C1	570284511	22	295.14	3.47
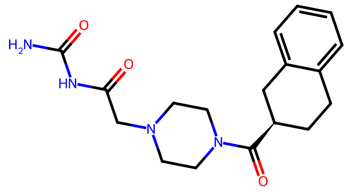	NC(=O)NC(=O)CN1CCN(C(=O)[C@@H]2CCc3ccccc3C2)CC1	1113381900	25	344.18	0.13
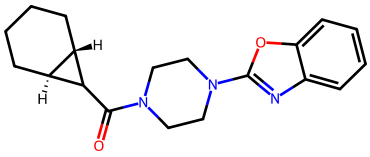	O=C(C1[C@H]2CCCC[C@H]12)N1CCN(c2nc3ccccc3o2)CC1	175067438	24	325.18	2.91
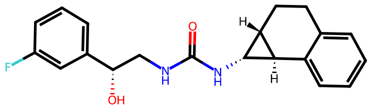	O=C(NC[C@H](O)c1cccc(F)c1)N[C@H]1[C@@H]2c3ccccc3CC[C@H]21	130679494	25	340.16	2.89
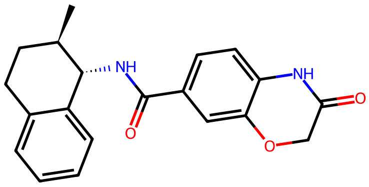	C[C@@H]1CCc2ccccc2[C@H]1NC(=O)c1ccc2c(c1)OCC(=O)N2	179463725	25	336.15	3.07
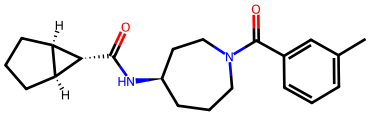	Cc1cccc(C(=O)N2CCC[C@@H](NC(=O)[C@@H]3[C@H]4CCC[C@H]43)CC2)c1	964820045	25	340.22	3.15
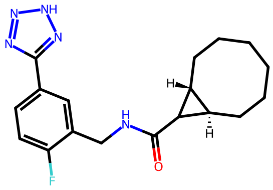	O=C(NCc1cc(-c2nn[nH]n2)ccc1F)C1[C@@H]2CCCCCC[C@@H]12	1606814842	25	343.18	2.84
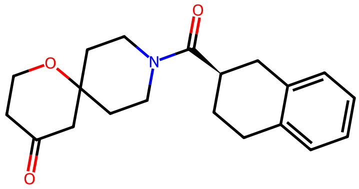	O=C1CCOC2(CCN(C(=O)[C@@H]3CCc4ccccc4C3)CC2)C1	1551858590	24	327.18	2.53

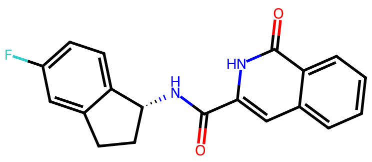	O=C(N[C@@H]1CCc2cc(F)ccc21)c1cc2ccccc2c(=O)[nH]1	56314792	24	322.11	3.08
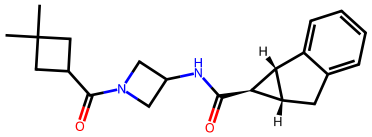	CC1(C)CC(C(=O)N2CC(NC(=O)[C@@H]3[C@H]4Cc5ccccc5[C@H]43)C2)C1	991644362	25	338.2	2.34
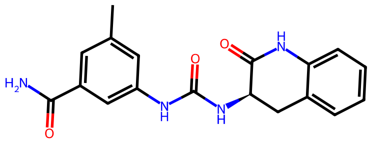	Cc1cc(NC(=O)N[C@@H]2Cc3ccccc3NC2=O)cc(C(N)=O)c1	1365201193	25	338.14	1.78
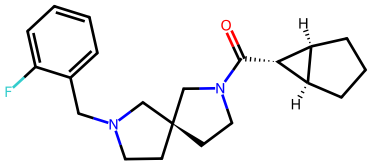	O=C([C@@H]1[C@H]2CCC[C@H]21)N1CC[C@]2(CCN(Cc3ccccc3F)C2)C1	1041176988	25	342.21	3.3
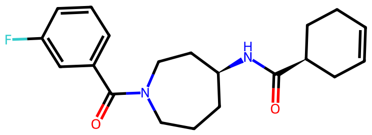	O=C(N[C@H]1CCCN(C(=O)c2cccc(F)c2)CC1)[C@@H]1CC=CCC1	964722417	25	344.19	3.29
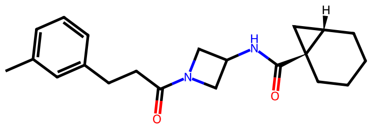	Cc1cccc(CCC(=O)N2CC(NC(=O)[C@]34CCCC[C@H]3C4)C2)c1	999283800	25	340.22	2.83
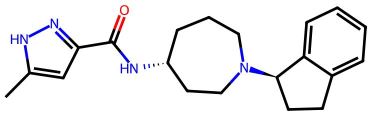	Cc1cc(C(=O)N[C@@H]2CCCN([C@@H]3CCc4ccccc43)CC2)n[nH]1	1052236599	25	338.21	2.99
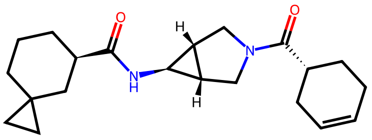	O=C(N[C@H]1[C@@H]2CN(C(=O)[C@H]3CC=CCC3)C[C@@H]21)[C@@H]1CCCC2(CC2)C1	976510614	25	342.23	2.89

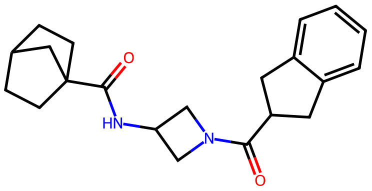	O=C(C1Cc2ccccc2C1)N1CC(NC(=O)C23CCC(CC2)C3)C1	995093526	25	338.2	2.31
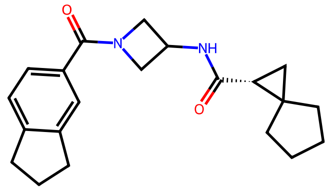	O=C(NC1CN(C(=O)c2ccc3c(c2)CCC3)C1)[C@@H]1CC12CCCC2	999630995	25	338.2	2.7
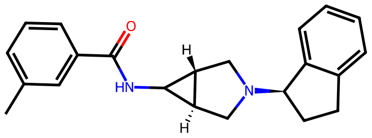	Cc1cccc(C(=O)NC2[C@@H]3CN([C@@H]4CCc5ccccc54)C[C@@H]23)c1	958155123	25	332.19	3.34
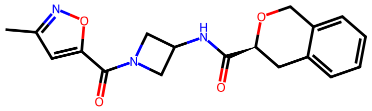	Cc1cc(C(=O)N2CC(NC(=O)[C@@H]3Cc4ccccc4CO3)C2)on1	997728421	25	341.14	1.07
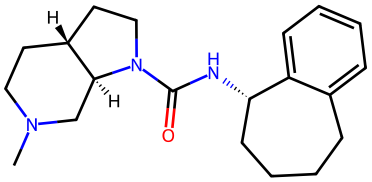	CN1CC[C@@H]2CCN(C(=O)N[C@H]3CCCCc4ccccc43)[C@H]2C1	542777813	24	327.23	3.19
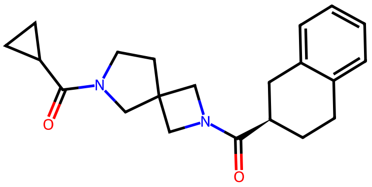	O=C(C1CC1)N1CCC2(C1)CN(C(=O)[C@@H]1CCc3ccccc3C1)C2	1019492035	25	338.2	2.26
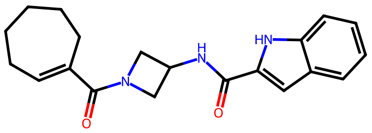	O=C(NC1CN(C(=O)C2=CCCCCC2)C1)c1cc2ccccc2[nH]1	1000143212	25	337.18	3.0
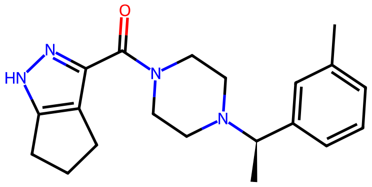	Cc1cccc([C@@H](C)N2CCN(C(=O)c3n[nH]c4c3CCC4)CC2)c1	949063014	25	338.21	2.73

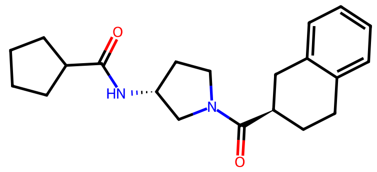	O=C(N[C@@H]1CCN(C(=O)[C@@H]2CCc3ccccc3C2)C1)C1CCCC1	936274782	25	340.22	2.7
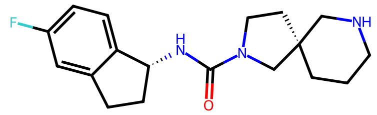	O=C(N[C@@H]1CCc2cc(F)ccc21)N1CC[C@]2(CCCNC2)C1	583311485	23	317.19	2.6
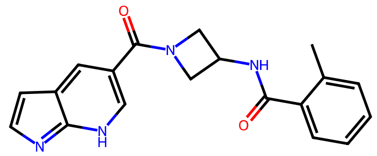	Cc1ccccc1C(=O)NC1CN(C(=O)c2c[nH]c3nccc-3c2)C1	990111651	25	334.14	2.08
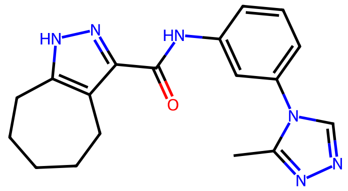	Cc1nncn1-c1cccc(NC(=O)c2n[nH]c3c2CCCCC3)c1	190905131	25	336.17	2.82
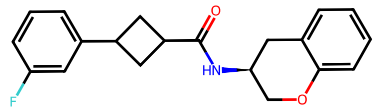	O=C(N[C@@H]1COc2ccccc2C1)C1CC(c2cccc(F)c2)C1	522905106	24	325.15	3.44
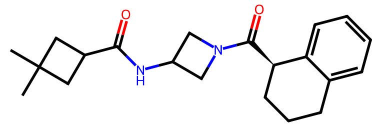	CC1(C)CC(C(=O)NC2CN(C(=O)[C@@H]3CCCc4ccccc43)C2)C1	991138272	25	340.22	2.87
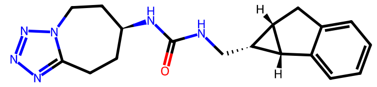	O=C(NC[C@H]1[C@H]2c3ccccc3C[C@H]21)N[C@H]1CCc2nnnn2CC1	333637563	25	338.19	1.26
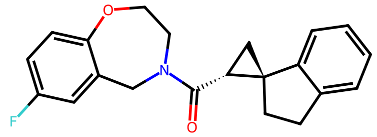	O=C([C@@H]1C[C@@]12CCc1ccccc12)N1CCOc2ccc(F)cc2C1	470582325	25	337.15	3.45

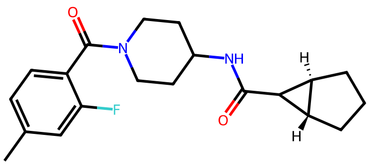	Cc1ccc(C(=O)N2CCC(NC(=O)C3[C@H]4CCC[C@H]34)CC2)c(F)c1	948559461	25	344.19	2.9
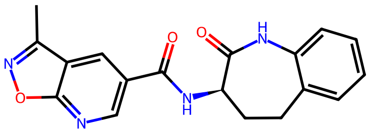	Cc1noc2ncc(C(=O)N[C@@H]3CCc4ccccc4NC3=O)cc12	1639525001	25	336.12	2.21
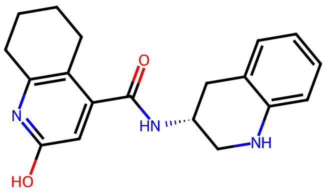	O=C(N[C@H]1CNc2ccccc2C1)c1cc(O)nc2c1CCCC2	1580622125	24	323.16	2.43
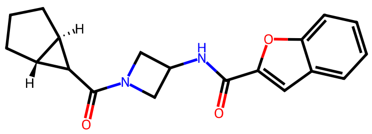	O=C(NC1CN(C(=O)C2[C@@H]3CCC[C@@H]23)C1)c1cc2ccccc2o1	1000052951	24	324.15	2.42
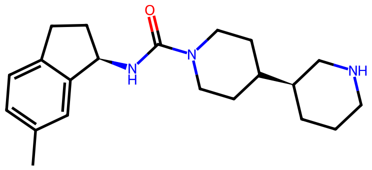	Cc1ccc2c(c1)[C@H](NC(=O)N1CCC([C@@H]3CCCNC3)CC1)CC2	659453061	25	341.25	3.4
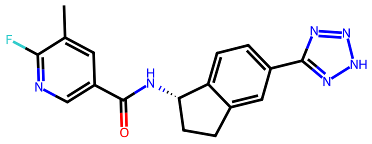	Cc1cc(C(=O)N[C@H]2CCc3cc(-c4nn[nH]n4)ccc32)cnc1F	1580840605	25	338.13	2.13
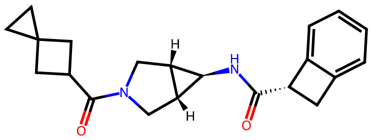	O=C(N[C@H]1[C@@H]2CN(C(=O)C3CC4(CC4)C3)C[C@@H]21)[C@H]1Cc2ccccc21	977080413	25	336.18	2.09
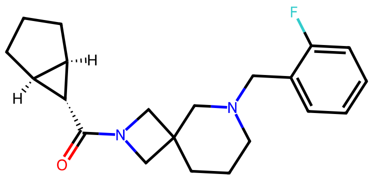	O=C([C@@H]1[C@H]2CCC[C@H]21)N1CC2(CCCN(Cc3ccccc3F)C2)C1	981694631	25	342.21	3.3

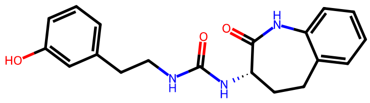	O=C(NCCc1cccc(O)c1)N[C@H]1CCc2ccccc2NC1=O	654421563	25	339.16	2.19
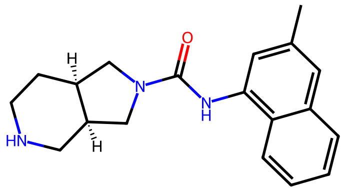	Cc1cc(NC(=O)N2C[C@@H]3CCNC[C@@H]3C2)c2ccccc2c1	415803330	23	309.18	3.22
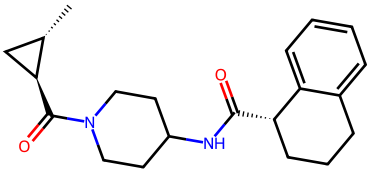	C[C@H]1C[C@@H]1C(=O)N1CCC(NC(=O)[C@H]2CCCc3ccccc32)CC1	947836970	25	340.22	2.87
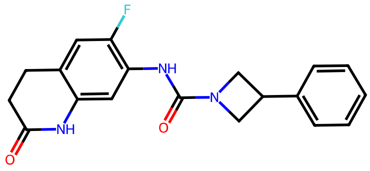	O=C1CCc2cc(F)c(NC(=O)N3CC(c4ccccc4)C3)cc2N1	639522855	25	339.14	3.34
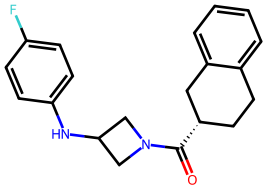	O=C([C@H]1CCc2ccccc2C1)N1CC(Nc2ccc(F)cc2)C1	1170240582	24	324.16	3.25
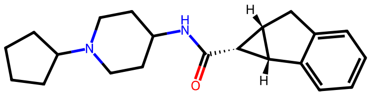	O=C(NC1CCN(C2CCCC2)CC1)[C@H]1[C@H]2c3ccccc3C[C@H]21	625994421	24	324.22	3.1
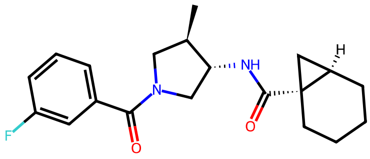	C[C@@H]1CN(C(=O)c2cccc(F)c2)C[C@H]1NC(=O)[C@@]12CCCC[C@@H]1C2	939980506	25	344.19	2.98
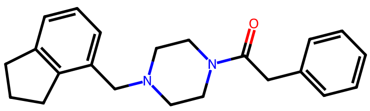	O=C(Cc1ccccc1)N1CCN(Cc2cccc3c2CCC3)CC1	1249458801	25	334.2	3.06

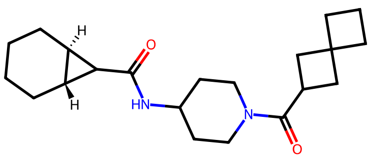	O=C(NC1CCN(C(=O)C2CC3(CCC3)C2)CC1)C1[C@@H]2CCCC[C@@H]12	947882835	25	344.25	3.11
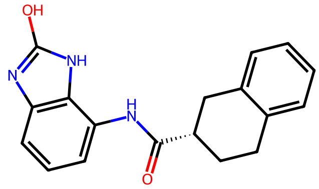	O=C(Nc1cccc2nc(O)[nH]c12)[C@H]1CCc2ccccc2C1	1641882708	23	307.13	3.01
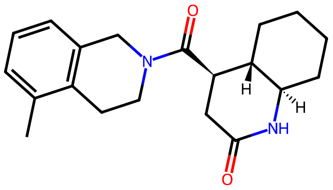	Cc1cccc2c1CCN(C(=O)[C@@H]1CC(=O)N[C@@H]3CCCC[C@H]31)C2	1552535743	24	326.2	2.57
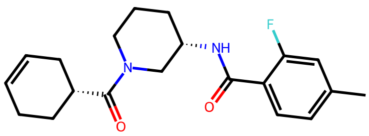	Cc1ccc(C(=O)N[C@H]2CCCN(C(=O)[C@H]3CC=CCC3)C2)c(F)c1	949975407	25	344.19	3.21
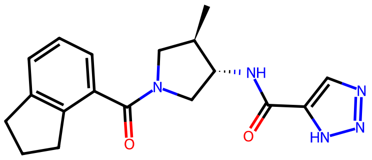	C[C@@H]1CN(C(=O)c2cccc3c2CCC3)C[C@H]1NC(=O)c1cnn[nH]1	950514678	25	339.17	1.18
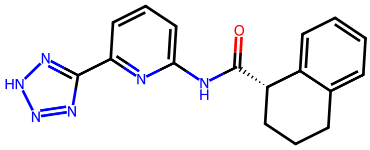	O=C(Nc1cccc(-c2nn[nH]n2)n1)[C@H]1CCCc2ccccc21	1593243958	24	320.14	2.32
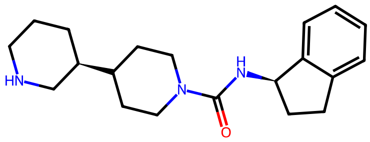	O=C(N[C@@H]1CCc2ccccc21)N1CCC([C@@H]2CCCNC2)CC1	659595933	24	327.23	3.1
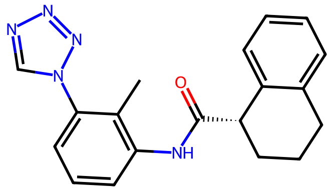	Cc1c(NC(=O)[C@H]2CCCc3ccccc32)cccc1-n1cnnn1	641916822	25	333.16	3.03

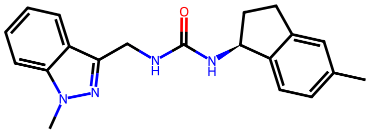	Cc1ccc2c(c1)CC[C@@H]2NC(=O)NCc1nn(C)c2ccccc12	680551579	25	334.18	3.37
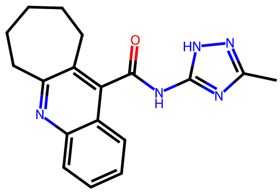	Cc1n[nH]c(NC(=O)c2c3c(nc4ccccc24)CCCCC3)n1	1610939438	24	321.16	3.18
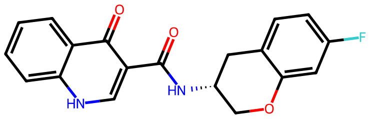	O=C(N[C@H]1COc2cc(F)ccc2C1)c1c[nH]c2ccccc2c1=O	1131189142	25	338.11	2.4
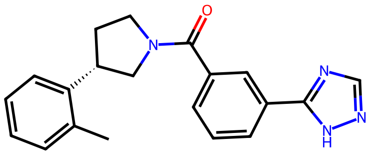	Cc1ccccc1[C@@H]1CCN(C(=O)c2cccc(-c3ncn[nH]3)c2)C1	271282855	25	332.16	3.41
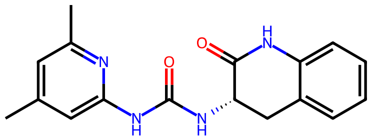	Cc1cc(C)nc(NC(=O)N[C@H]2Cc3ccccc3NC2=O)c1	1364105969	23	310.14	2.38
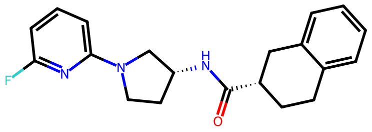	O=C(N[C@@H]1CCN(c2cccc(F)n2)C1)[C@H]1CCc2ccccc2C1	1058560167	25	339.17	2.72
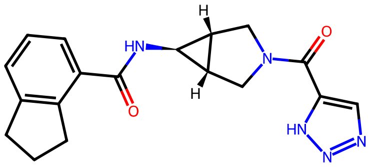	O=C(N[C@H]1[C@@H]2CN(C(=O)c3cnn[nH]3)C[C@@H]21)c1cccc2c1CCC2	976709253	25	337.15	0.79
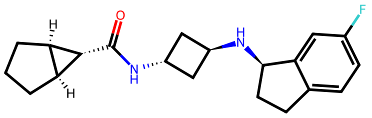	O=C(N[C@H]1C[C@H](N[C@@H]2CCc3ccc(F)cc32)C1)[C@@H]1[C@H]2CCC[C@H]21	1020055531	24	328.2	3.1

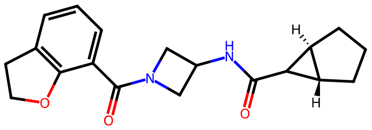	O=C(NC1CN(C(=O)c2cccc3c2OCC3)C1)C1[C@H]2CCC[C@H]12	990711778	24	326.16	1.61
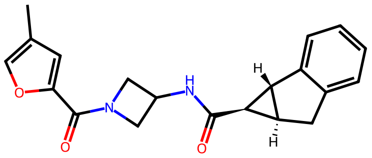	Cc1coc(C(=O)N2CC(NC(=O)[C@@H]3[C@@H]4Cc5ccccc5[C@H]43)C2)c1	991647553	25	336.15	2.11
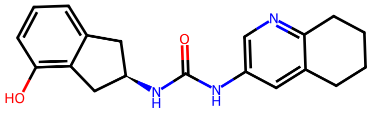	O=C(Nc1cnc2c(c1)CCCC2)N[C@@H]1Cc2cccc(O)c2C1	562383494	24	323.16	2.95
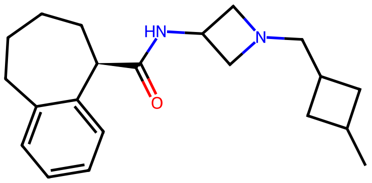	CC1CC(CN2CC(NC(=O)[C@@H]3CCCCc4ccccc43)C2)C1	1031451496	24	326.24	3.34
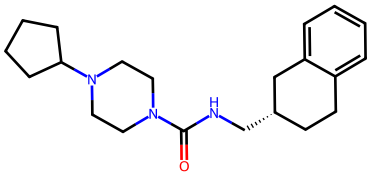	O=C(NC[C@H]1CCc2ccccc2C1)N1CCN(C2CCCC2)CC1	1633732247	25	341.25	3.06
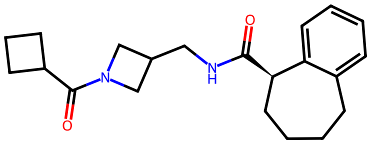	O=C(NCC1CN(C(=O)C2CCC2)C1)[C@@H]1CCCCc2ccccc21	1002646849	25	340.22	2.87
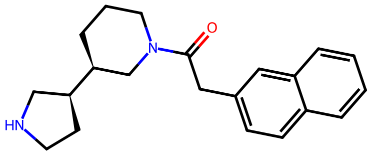	O=C(Cc1ccc2ccccc2c1)N1CCC[C@H]([C@H]2CCNC2)C1	449196486	24	322.2	3.23
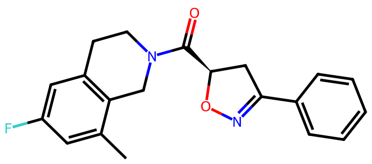	Cc1cc(F)cc2c1CN(C(=O)[C@H]1CC(c3ccccc3)=NO1)CC2	800245490	25	338.14	3.21

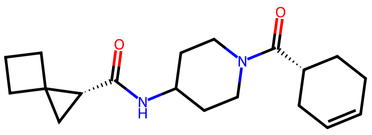	O=C(NC1CCN(C(=O)[C@H]2CC=CCC2)CC1)[C@@H]1CC12CCC2	944127605	23	316.22	2.64
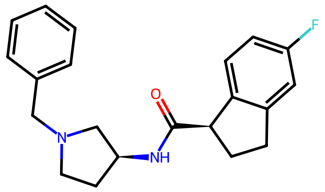	O=C(N[C@H]1CCN(Cc2ccccc2)C1)[C@@H]1CCc2cc(F)ccc21	665576577	25	338.18	3.25
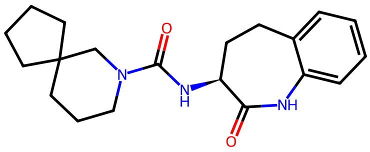	O=C1Nc2ccccc2CC[C@@H]1NC(=O)N1CCCC2(CCCC2)C1	847396978	25	341.21	3.31
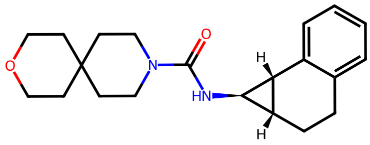	O=C(N[C@@H]1[C@H]2CCc3ccccc3[C@H]21)N1CCC2(CCOCC2)CC1	289303142	25	340.22	3.32
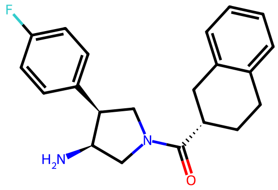	N[C@@H]1CN(C(=O)[C@H]2CCc3ccccc3C2)C[C@@H]1c1ccc(F)cc1	1549122619	25	338.18	2.88
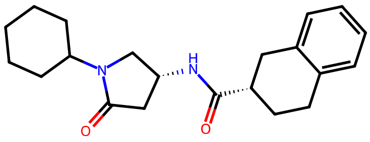	O=C(N[C@@H]1CC(=O)N(C2CCCCC2)C1)[C@H]1CCc2ccccc2C1	1362286755	25	340.22	2.84
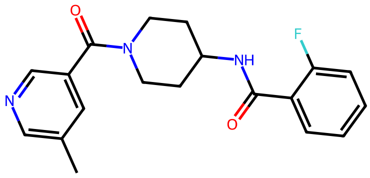	Cc1cncc(C(=O)N2CCC(NC(=O)c3ccccc3F)CC2)c1	79550277	25	341.15	2.56
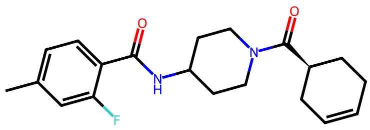	Cc1ccc(C(=O)NC2CCN(C(=O)[C@@H]3CC=CCC3)CC2)c(F)c1	944127886	25	344.19	3.21

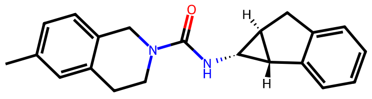	Cc1ccc2c(c1)CCN(C(=O)N[C@@H]1[C@H]3Cc4ccccc4[C@@H]31)C2	481264537	24	318.17	3.4
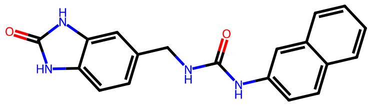	O=C(NCc1ccc2[nH]c(=O)[nH]c2c1)Nc1ccc2ccccc2c1	193707953	25	332.13	3.33
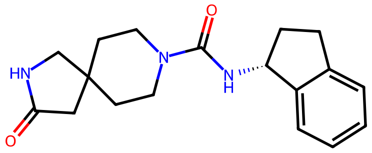	O=C1CC2(CCN(C(=O)N[C@@H]3CCc4ccccc43)CC2)CN1	73560789	23	313.18	1.99
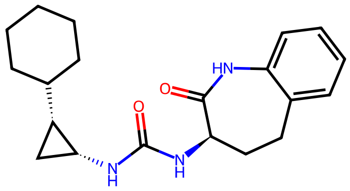	O=C(N[C@@H]1CCc2ccccc2NC1=O)N[C@@H]1C[C@@H]1C1CCCCC1	1469095513	25	341.21	3.21
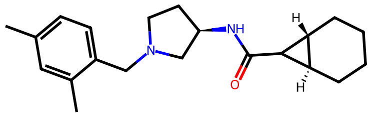	Cc1ccc(CN2CC[C@@H](NC(=O)C3[C@@H]4CCCC[C@@H]34)C2)c(C)c1	1032834764	24	326.24	3.43
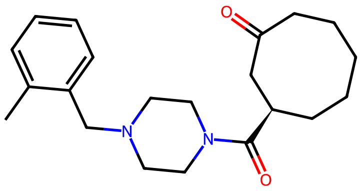	Cc1ccccc1CN1CCN(C(=O)[C@@H]2CCCCCC(=O)C2)CC1	362470410	25	342.23	3.18
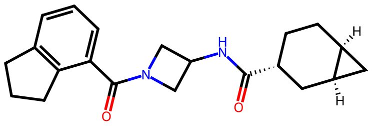	O=C(NC1CN(C(=O)c2cccc3c2CCC3)C1)[C@@H]1CC[C@H]2C[C@H]2C1	994672921	25	338.2	2.55
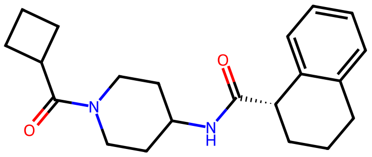	O=C(NC1CCN(C(=O)C2CCC2)CC1)[C@H]1CCCc2ccccc21	943328056	25	340.22	3.01

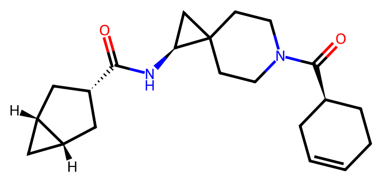	O=C(N[C@H]1CC12CCN(C(=O)[C@@H]1CC=CCC1)CC2)[C@@H]1C[C@@H]2C[C@@H]2C1	964770639	25	342.23	2.89
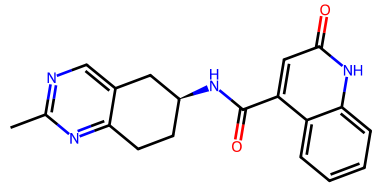	Cc1ncc2c(n1)CC[C@H](NC(=O)c1cc(=O)[nH]c3ccccc13)C2	29313493	25	334.14	1.91
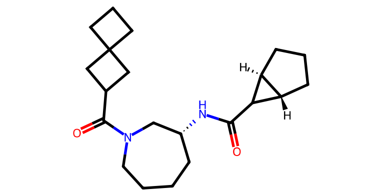	O=C(N[C@@H]1CCCCN(C(=O)C2CC3(CCC3)C2)C1)C1[C@H]2CCC[C@H]12	943169092	25	344.25	3.11
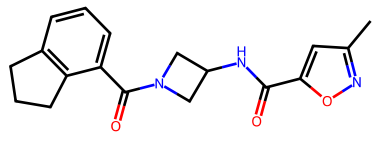	Cc1cc(C(=O)NC2CN(C(=O)c3cccc4c3CCC4)C2)on1	990218191	24	325.14	1.73
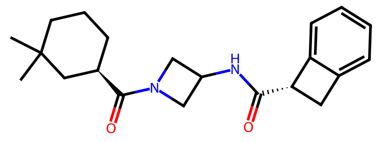	CC1(C)CCC[C@@H](C(=O)N2CC(NC(=O)[C@H]3Cc4ccccc43)C2)C1	998686754	25	340.22	2.87
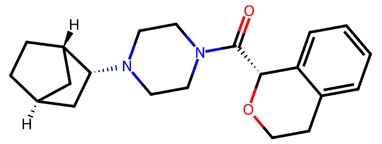	O=C([C@H]1OCCc2ccccc21)N1CCN([C@@H]2C[C@H]3CC[C@H]2C3)CC1	441469677	25	340.22	2.63
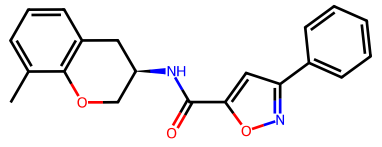	Cc1cccc2c1OC[C@H](NC(=O)c1cc(-c3ccccc3)no1)C2	484938523	25	334.13	3.38
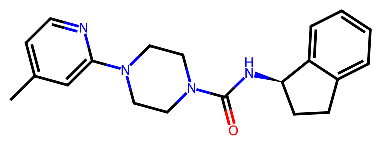	Cc1ccnc(N2CCN(C(=O)N[C@@H]3CCc4ccccc43)CC2)c1	136608005	25	336.2	2.91

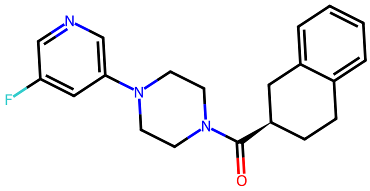	O=C([C@@H]1CCc2ccccc2C1)N1CCN(c2cncc(F)c2)CC1	655033325	25	339.17	2.67
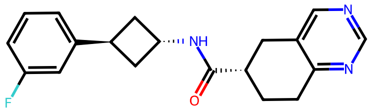	O=C(N[C@H]1C[C@H](c2cccc(F)c2)C1)[C@H]1CCc2ncncc2C1	648890801	24	325.16	2.78
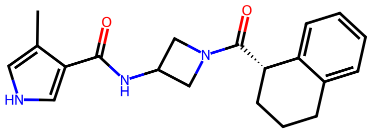	Cc1c[nH]cc1C(=O)NC1CN(C(=O)[C@H]2CCCc3ccccc32)C1	991711930	25	337.18	2.38
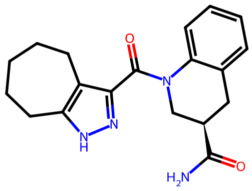	NC(=O)[C@@H]1Cc2ccccc2N(C(=O)c2n[nH]c3c2CCCCC3)C1	276470139	25	338.17	1.98
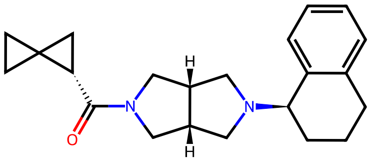	O=C([C@H]1CC12CC2)N1C[C@@H]2CN([C@@H]3CCCc4ccccc43)C[C@@H]2C1	1049082783	25	336.22	3.25
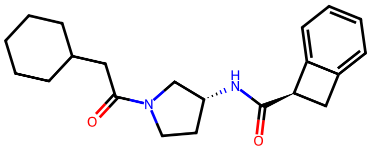	O=C(N[C@@H]1CCN(C(=O)CC2CCCCC2)C1)[C@@H]1Cc2ccccc21	942304997	25	340.22	3.01
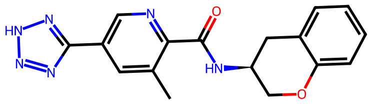	Cc1cc(-c2nn[nH]n2)cnc1C(=O)N[C@@H]1COc2ccccc2C1	1591877879	25	336.13	1.3
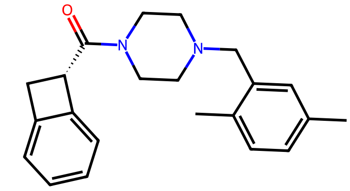	Cc1ccc(C)c(CN2CCN(C(=O)[C@H]3Cc4ccccc43)CC2)c1	949948881	25	334.2	3.29

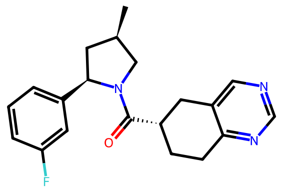	C[C@@H]1C[C@H](c2cccc(F)c2)N(C(=O)[C@H]2CCc3ncncc3C2)C1	648798077	25	339.17	3.33
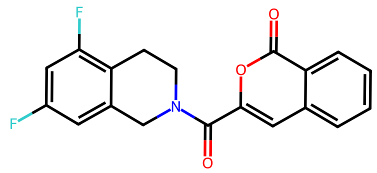	O=C(c1cc2ccccc2c(=O)o1)N1CCc2c(F)cc(F)cc2C1	337105121	25	341.09	3.27
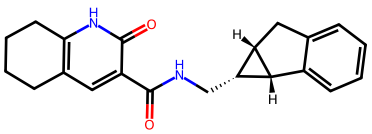	O=C(NC[C@H]1[C@H]2c3ccccc3C[C@H]21)c1cc2c([nH]c1=O)CCCC2	152424340	25	334.17	2.57
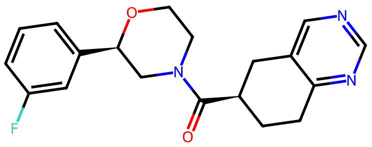	O=C([C@@H]1CCc2ncncc2C1)N1CCO[C@H](c2cccc(F)c2)C1	648764902	25	341.15	2.32
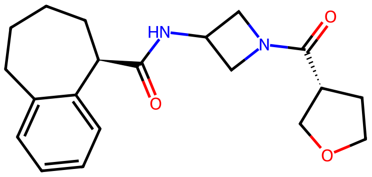	O=C(NC1CN(C(=O)[C@@H]2CCOC2)C1)[C@@H]1CCCCc2ccccc21	994305000	25	342.19	1.86
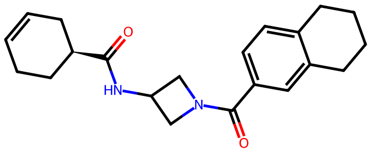	O=C(NC1CN(C(=O)c2ccc3c(c2)CCCC3)C1)[C@@H]1CC=CCC1	990214389	25	338.2	2.86
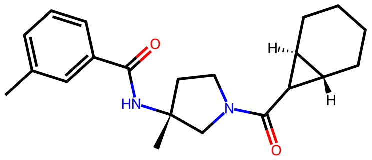	Cc1cccc(C(=O)N[C@]2(C)CCN(C(=O)C3[C@H]4CCCC[C@H]34)C2)c1	956661924	25	340.22	3.15
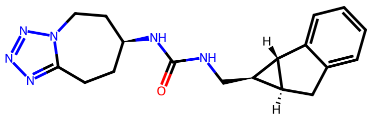	O=C(NC[C@@H]1[C@@H]2Cc3ccccc3[C@@H]12)N[C@H]1CCc2nnnn2CC1	333637565	25	338.19	1.26

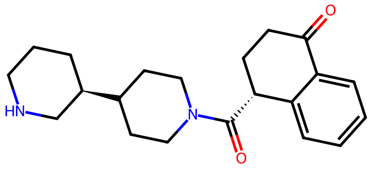	O=C1CC[C@@H](C(=O)N2CCC([C@@H]3CCCNC3)CC2)c2ccccc21	662587758	25	340.22	2.98
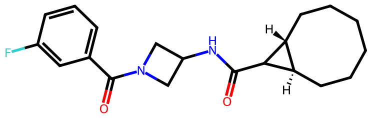	O=C(NC1CN(C(=O)c2cccc(F)c2)C1)C1[C@@H]2CCCCCC[C@@H]12	1000465993	25	344.19	2.98
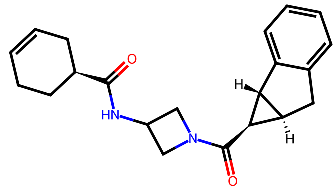	O=C(NC1CN(C(=O)[C@@H]2[C@@H]3Cc4ccccc4[C@H]32)C1)[C@@H]1CC=CCC1	990220873	25	336.18	2.26
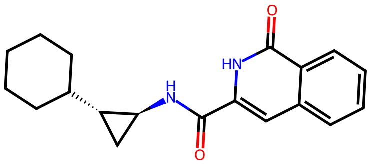	O=C(N[C@H]1C[C@@H]1C1CCCCC1)c1cc2ccccc2c(=O)[nH]1	152714882	23	310.17	3.23
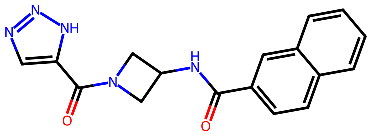	O=C(NC1CN(C(=O)c2cnn[nH]2)C1)c1ccc2ccccc2c1	999443961	24	321.12	1.21
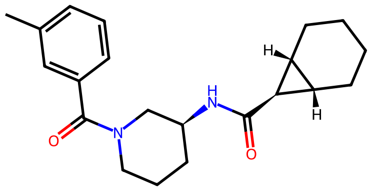	Cc1cccc(C(=O)N2CCC[C@H](NC(=O)[C@@H]3[C@H]4CCCC[C@H]43)C2)c1	949869536	25	340.22	3.15
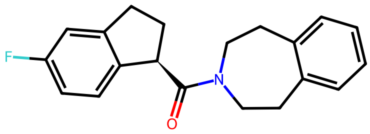	O=C([C@@H]1CCc2cc(F)ccc21)N1CCc2ccccc2CC1	668830822	23	309.15	3.48
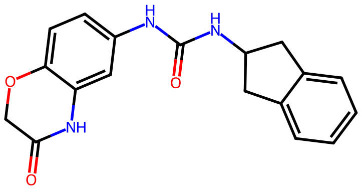	O=C1COc2ccc(NC(=O)NC3Cc4ccccc4C3)cc2N1	680831270	24	323.13	2.31

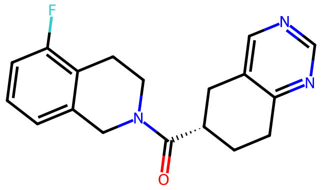	O=C([C@H]1CCc2ncncc2C1)N1CCc2c(F)cccc2C1	843570460	23	311.14	2.31
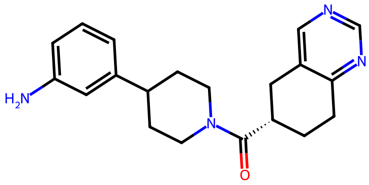	Nc1cccc(C2CCN(C(=O)[C@H]3CCc4ncncc4C3)CC2)c1	648178668	25	336.2	2.57
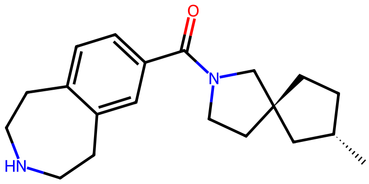	C[C@H]1CC[C@]2(CCN(C(=O)c3ccc4c(c3)CCNCC4)C2)C1	423891011	23	312.22	3.03
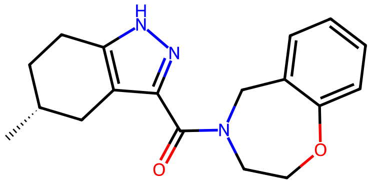	C[C@@H]1CCc2[nH]nc(C(=O)N3CCOc4ccccc4C3)c2C1	895807409	23	311.16	2.57
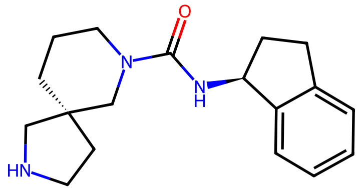	O=C(N[C@H]1CCc2ccccc21)N1CCC[C@@]2(CCNC2)C1	335612082	22	299.2	2.46
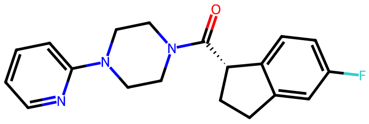	O=C([C@H]1CCc2cc(F)ccc21)N1CCN(c2ccccn2)CC1	665153896	24	325.16	2.6
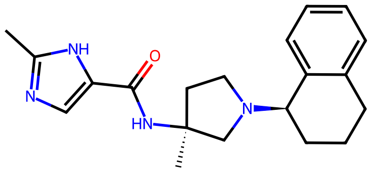	Cc1ncc(C(=O)N[C@@]2(C)CCN([C@@H]3CCCc4ccccc43)C2)[nH]1	1046199612	25	338.21	2.99
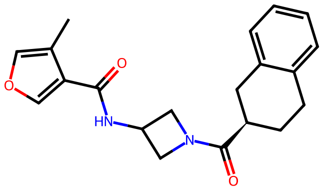	Cc1cocc1C(=O)NC1CN(C(=O)[C@@H]2CCc3ccccc3C2)C1	997833647	25	338.16	2.33

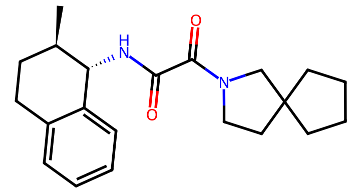	C[C@@H]1CCc2ccccc2[C@H]1NC(=O)C(=O)N1CCC2(CCCC2)C1	807691451	25	340.22	3.22
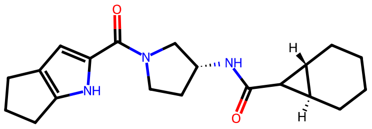	O=C(N[C@@H]1CCN(C(=O)c2cc3c([nH]2)CCC3)C1)C1[C@@H]2CCCC[C@@H]12	939895592	25	341.21	2.27
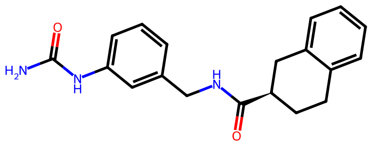	NC(=O)Nc1cccc(CNC(=O)[C@@H]2CCc3ccccc3C2)c1	572166689	24	323.16	2.6
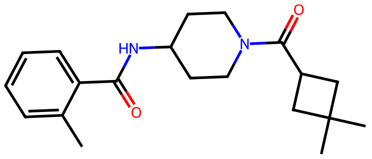	Cc1ccccc1C(=O)NC1CCN(C(=O)C2CC(C)(C)C2)CC1	375641876	24	328.22	3.15
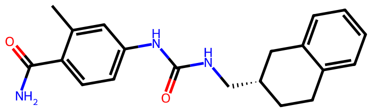	Cc1cc(NC(=O)NC[C@H]2CCc3ccccc3C2)ccc1C(N)=O	605032665	25	337.18	3.02
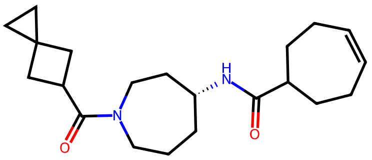	O=C(N[C@@H]1CCCN(C(=O)C2CC3(CC3)C2)CC1)C1CCC=CCC1	964914255	25	344.25	3.42
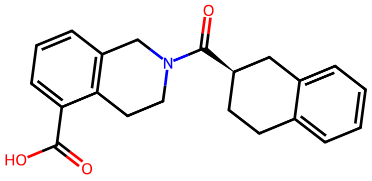	O=C(O)c1cccc2c1CCN(C(=O)[C@@H]1CCc3ccccc3C1)C2	327955030	25	335.15	3.07
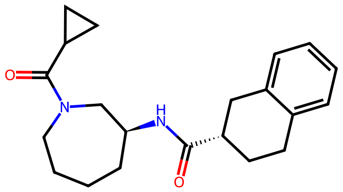	O=C(N[C@H]1CCCCN(C(=O)C2CC2)C1)[C@H]1CCc2ccccc2C1	943573731	25	340.22	2.7

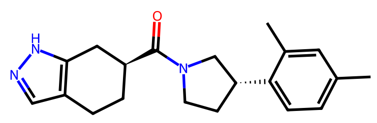	Cc1ccc([C@@H]2CCN(C(=O)[C@H]3CCc4cn[nH]c4C3)C2)c(C)c1	280254642	24	323.2	3.15
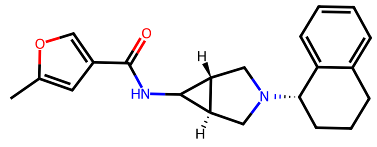	Cc1cc(C(=O)NC2[C@@H]3CN([C@H]4CCCc5ccccc54)C[C@@H]23)co1	958672208	25	336.18	3.33
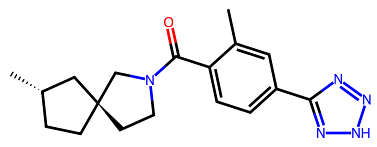	Cc1cc(-c2nn[nH]n2)ccc1C(=O)N1CC[C@]2(CC[C@H](C)C2)C1	826245098	24	325.19	2.83
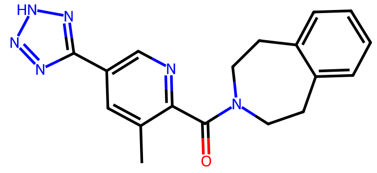	Cc1cc(-c2nn[nH]n2)cnc1C(=O)N1CCc2ccccc2CC1	1591875954	25	334.15	1.81
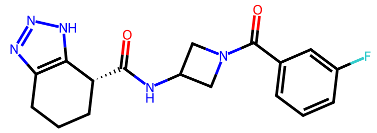	O=C(NC1CN(C(=O)c2cccc(F)c2)C1)[C@@H]1CCCc2nn[nH]c21	998567719	25	343.14	1.0
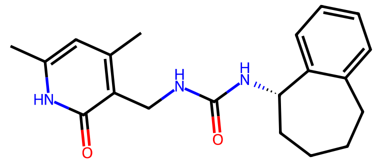	Cc1cc(C)c(CNC(=O)N[C@H]2CCCCc3ccccc32)c(=O)[nH]1	69657186	25	339.19	3.26
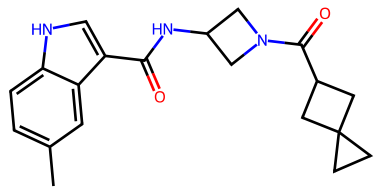	Cc1ccc2[nH]cc(C(=O)NC3CN(C(=O)C4CC5(CC5)C4)C3)c2c1	994290148	25	337.18	2.61
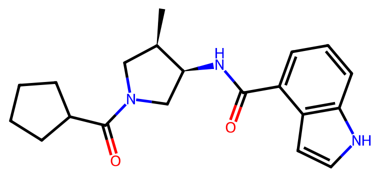	C[C@@H]1CN(C(=O)C2CCCC2)C[C@@H]1NC(=O)c1cccc2[nH]ccc12	966038009	25	339.19	2.93

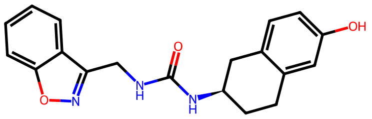	O=C(NCc1noc2ccccc12)N[C@@H]1CCc2cc(O)ccc2C1	847362307	25	337.14	2.89
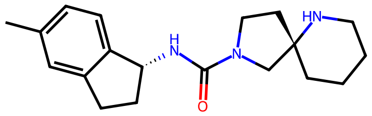	Cc1ccc2c(c1)CC[C@H]2NC(=O)N1CC[C@@]2(CCCCN2)C1	1553438238	23	313.22	2.91
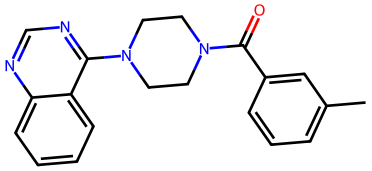	Cc1cccc(C(=O)N2CCN(c3ncnc4ccccc34)CC2)c1	1055707054	25	332.16	2.9
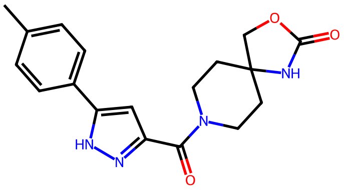	Cc1ccc(-c2cc(C(=O)N3CCC4(CC3)COC(=O)N4)n[nH]2)cc1	495302410	25	340.15	2.1
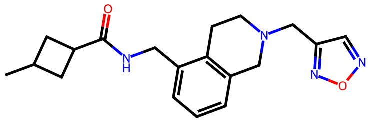	CC1CC(C(=O)NCc2cccc3c2CCN(Cc2cnon2)C3)C1	1051283559	25	340.19	2.29
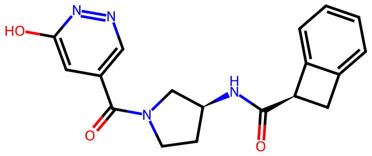	O=C(N[C@H]1CCN(C(=O)c2cnnc(O)c2)C1)[C@@H]1Cc2ccccc21	938576959	25	338.14	0.85
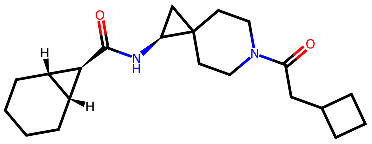	O=C(N[C@H]1CC12CCN(C(=O)CC1CCC1)CC2)[C@@H]1[C@H]2CCCC[C@H]21	963448118	25	344.25	3.11
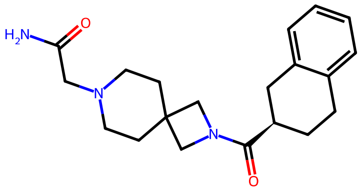	NC(=O)CN1CCC2(CC1)CN(C(=O)[C@@H]1CCc3ccccc3C1)C2	1050682352	25	341.21	1.2

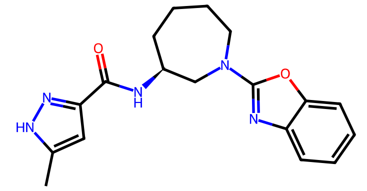	Cc1cc(C(=O)N[C@H]2CCCCN(c3nc4ccccc4o3)C2)n[nH]1	1063166428	25	339.17	2.65
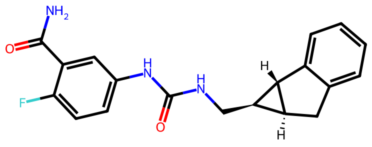	NC(=O)c1cc(NC(=O)NC[C@@H]2[C@@H]3Cc4ccccc4[C@@H]23)ccc1F	121679965	25	339.14	2.63
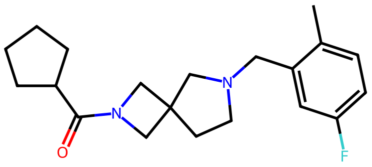	Cc1ccc(F)cc1CN1CCC2(C1)CN(C(=O)C1CCCC1)C2	1072383864	24	330.21	3.36
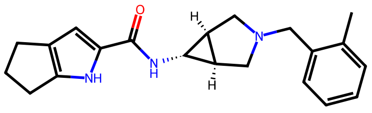	Cc1ccccc1CN1C[C@H]2[C@H](NC(=O)c3cc4c([nH]3)CCC4)[C@H]2C1	962276291	25	335.2	2.67
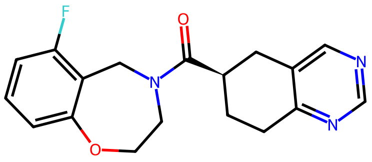	O=C([C@@H]1CCc2ncncc2C1)N1CCOc2cccc(F)c2C1	648893901	24	327.14	2.14
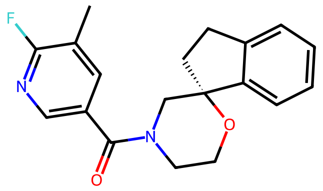	Cc1cc(C(=O)N2CCO[C@@]3(CCc4ccccc43)C2)cnc1F	857714298	24	326.14	2.84
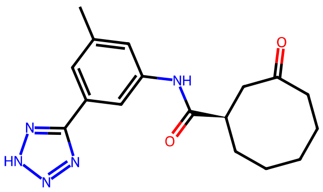	Cc1cc(NC(=O)[C@@H]2CCCCCC(=O)C2)cc(-c2nn[nH]n2)c1	1609406286	24	327.17	2.65
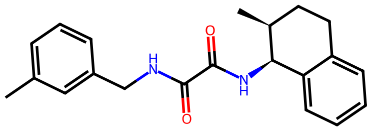	Cc1cccc(CNC(=O)C(=O)N[C@@H]2c3ccccc3CC[C@@H]2C)c1	340069336	25	336.18	3.05

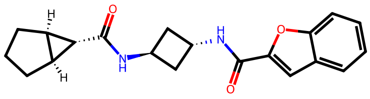	O=C(N[C@H]1C[C@H](NC(=O)[C@@H]2[C@H]3CCC[C@H]32)C1)c1cc2ccccc2o1	973152177	25	338.16	2.86
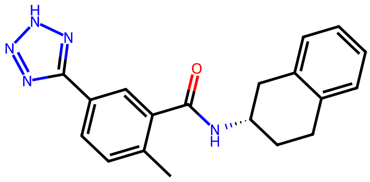	Cc1ccc(-c2nn[nH]n2)cc1C(=O)N[C@H]1CCc2ccccc2C1	1592098410	25	333.16	2.46
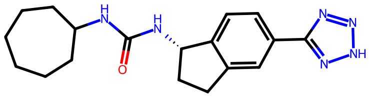	O=C(NC1CCCCCC1)N[C@H]1CCc2cc(-c3nn[nH]n3)ccc21	1592976559	25	340.2	2.88
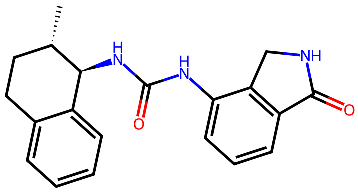	C[C@H]1CCc2ccccc2[C@@H]1NC(=O)Nc1cccc2c1CNC2=O	287460860	25	335.16	3.38
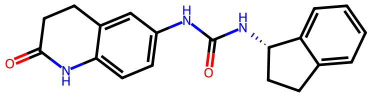	O=C1CCc2cc(NC(=O)N[C@H]3CCc4ccccc43)ccc2N1	76103461	24	321.15	3.38
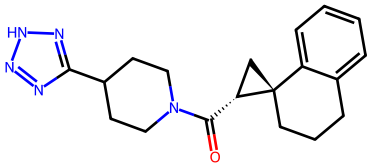	O=C([C@@H]1C[C@@]12CCCc1ccccc12)N1CCC(c2nn[nH]n2)CC1	913743894	25	337.19	2.2
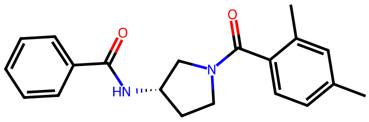	Cc1ccc(C(=O)N2CC[C@H](NC(=O)c3ccccc3)C2)c(C)c1	937627924	24	322.17	2.95
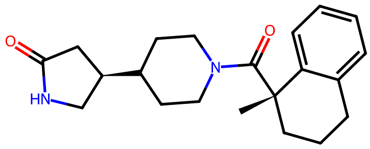	C[C@]1(C(=O)N2CCC([C@H]3CNC(=O)C3)CC2)CCCc2ccccc21	1551708989	25	340.22	2.66

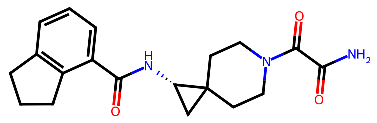	NC(=O)C(=O)N1CCC2(CC1)C[C@@H]2NC(=O)c1cccc2c1CCC2	964032352	25	341.17	0.77
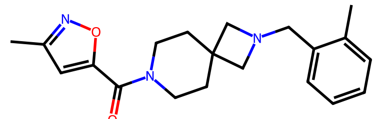	Cc1cc(C(=O)N2CCC3(CC2)CN(Cc2ccccc2C)C3)on1	1035648645	25	339.19	3.03
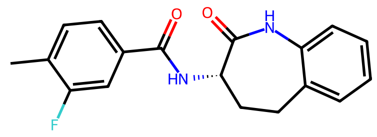	Cc1ccc(C(=O)N[C@H]2CCc3ccccc3NC2=O)cc1F	1361647504	23	312.13	2.82
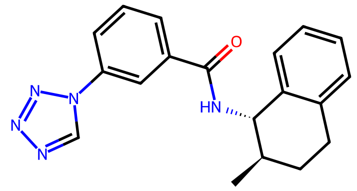	C[C@@H]1CCc2ccccc2[C@H]1NC(=O)c1cccc(-n2cnnn2)c1	122724399	25	333.16	2.72
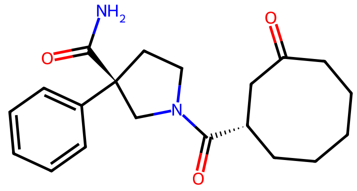	NC(=O)[C@@]1(c2ccccc2)CCN(C(=O)[C@H]2CCCCCC(=O)C2)C1	665993669	25	342.19	2.18
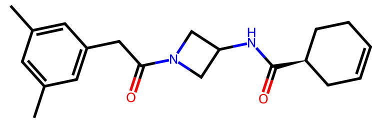	Cc1cc(C)cc(CC(=O)N2CC(NC(=O)[C@@H]3CC=CCC3)C2)c1	990214250	24	326.2	2.53
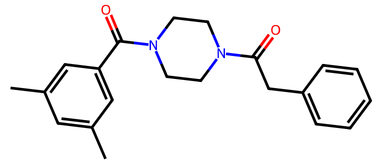	Cc1cc(C)cc(C(=O)N2CCN(C(=O)Cc3ccccc3)CC2)c1	29096640	25	336.18	2.83
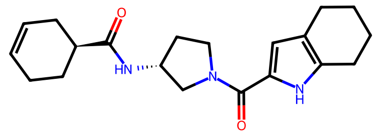	O=C(N[C@@H]1CCN(C(=O)c2cc3c([nH]2)CCCC3)C1)[C@@H]1CC=CCC1	939379924	25	341.21	2.58

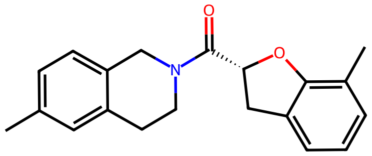	Cc1ccc2c(c1)CCN(C(=O)[C@H]1Cc3cccc(C)c3O1)C2	451136008	23	307.16	3.19
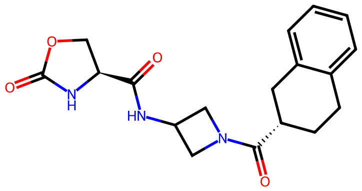	O=C1N[C@H](C(=O)NC2CN(C(=O)[C@H]3CCc4ccccc4C3)C2)CO1	991125991	25	343.15	0.23
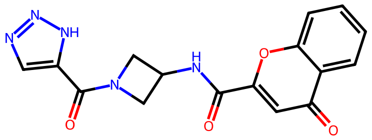	O=C(NC1CN(C(=O)c2cnn[nH]2)C1)c1cc(=O)c2ccccc2o1	999522559	25	339.1	0.17
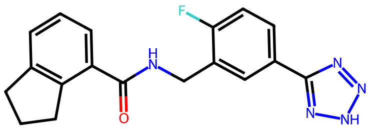	O=C(NCc1cc(-c2nn[nH]n2)ccc1F)c1cccc2c1CCC2	823241669	25	337.13	2.42
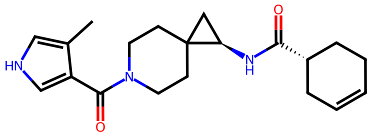	Cc1c[nH]cc1C(=O)N1CCC2(CC1)C[C@H]2NC(=O)[C@H]1CC=CCC1	963026530	25	341.21	2.79
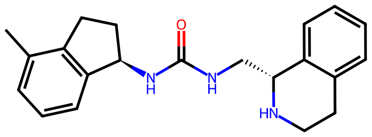	Cc1cccc2c1CC[C@H]2NC(=O)NC[C@H]1NCCc2ccccc21	563570256	25	335.2	3.17
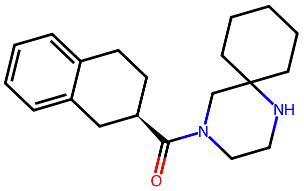	O=C([C@@H]1CCc2ccccc2C1)N1CCNC2(CCCCC2)C1	400766518	23	312.22	2.93
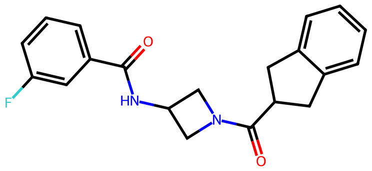	O=C(NC1CN(C(=O)C2Cc3ccccc3C2)C1)c1cccc(F)c1	990118611	25	338.14	2.18

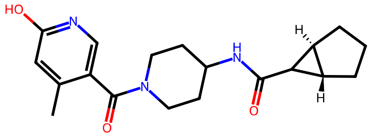	Cc1cc(O)ncc1C(=O)N1CCC(NC(=O)C2[C@H]3CCC[C@H]23)CC1	948305873	25	343.19	1.86
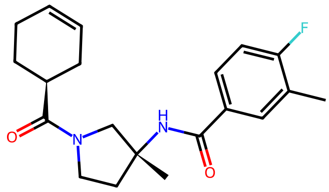	Cc1cc(C(=O)N[C@]2(C)CCN(C(=O)[C@@H]3CC=CCC3)C2)ccc1F	957460119	25	344.19	3.21
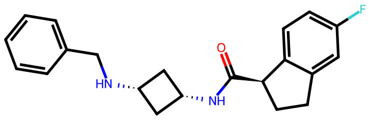	O=C(N[C@H]1C[C@@H](NCc2ccccc2)C1)[C@@H]1CCc2cc(F)ccc21	1022439051	25	338.18	3.29
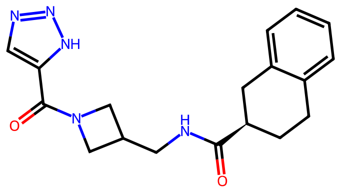	O=C(NCC1CN(C(=O)c2cnn[nH]2)C1)[C@@H]1CCc2ccccc2C1	936099473	25	339.17	0.8
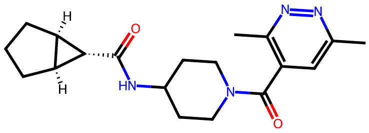	Cc1cc(C(=O)N2CCC(NC(=O)[C@@H]3[C@H]4CCC[C@H]43)CC2)c(C)nn1	946854670	25	342.21	1.86
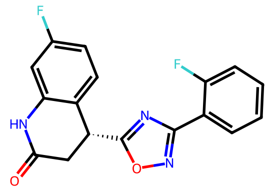	O=C1C[C@@H](c2nc(-c3ccccc3F)no2)c2ccc(F)cc2N1	371925154	24	327.08	3.49
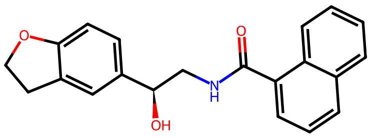	O=C(NC[C@@H](O)c1ccc2c(c1)CCO2)c1cccc2ccccc12	85559093	25	333.14	3.24
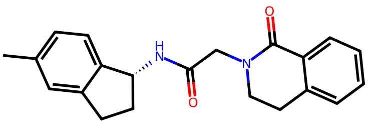	Cc1ccc2c(c1)CC[C@H]2NC(=O)CN1CCc2ccccc2C1=O	278870645	25	334.17	2.8

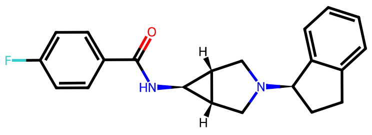	O=C(N[C@H]1[C@@H]2CN([C@@H]3CCc4ccccc43)C[C@@H]21)c1ccc(F)cc1	958085101	25	336.16	3.17
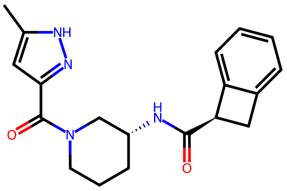	Cc1cc(C(=O)N2CCC[C@@H](NC(=O)[C@@H]3Cc4ccccc43)C2)n[nH]1	950158511	25	338.17	1.78
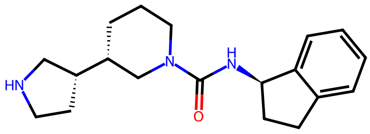	O=C(N[C@@H]1CCc2ccccc21)N1CCC[C@@H]([C@@H]2CCNC2)C1	416929906	23	313.22	2.71
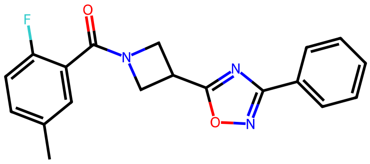	Cc1ccc(F)c(C(=O)N2CC(c3nc(-c4ccccc4)no3)C2)c1	1456143158	25	337.12	3.42
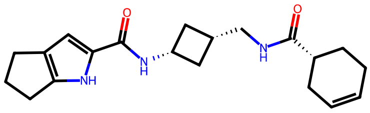	O=C(N[C@H]1C[C@@H](CNC(=O)[C@H]2CC=CCC2)C1)c1cc2c([nH]1)CCC2	978582751	25	341.21	2.48
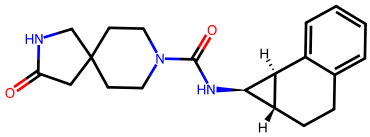	O=C1CC2(CCN(C(=O)N[C@@H]3[C@H]4CCc5ccccc5[C@@H]43)CC2)CN1	269450140	25	339.19	2.03
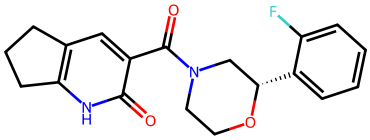	O=C(c1cc2c([nH]c1=O)CCC2)N1CCO[C@@H](c2ccccc2F)C1	66689670	25	342.14	2.22
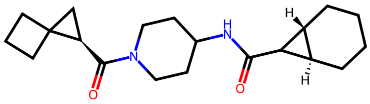	O=C(NC1CCN(C(=O)[C@@H]2CC23CCC3)CC1)C1[C@@H]2CCCC[C@@H]12	946763517	24	330.23	2.72

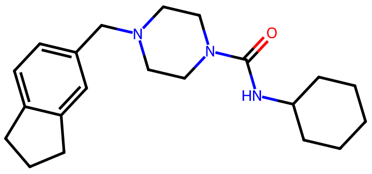	O=C(NC1CCCCC1)N1CCN(Cc2ccc3c(c2)CCC3)CC1	1652738752	25	341.25	3.34
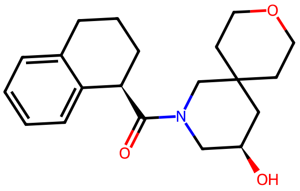	O=C([C@@H]1CCCc2ccccc21)N1C[C@H](O)CC2(CCOCC2)C1	882968353	24	329.2	2.5
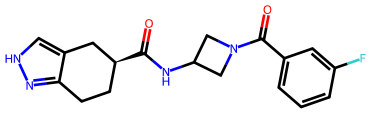	O=C(NC1CN(C(=O)c2cccc(F)c2)C1)[C@H]1CCc2n[nH]cc2C1	996396455	25	342.15	1.29
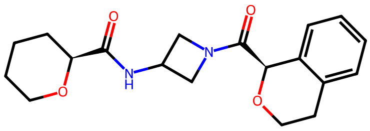	O=C(NC1CN(C(=O)[C@@H]2OCCc3ccccc32)C1)[C@@H]1CCCCO1	990854096	25	344.17	1.2
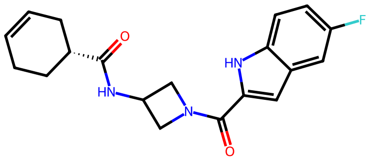	O=C(NC1CN(C(=O)c2cc3cc(F)ccc3[nH]2)C1)[C@H]1CC=CCC1	990215091	25	341.15	2.6
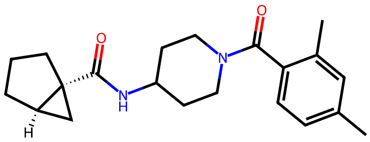	Cc1ccc(C(=O)N2CCC(NC(=O)[C@@]34CCC[C@@H]3C4)CC2)c(C)c1	948346728	25	340.22	3.21
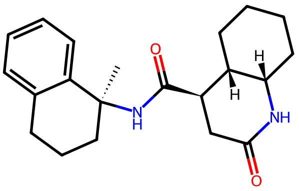	C[C@]1(NC(=O)[C@@H]2CC(=O)N[C@H]3CCCC[C@H]32)CCCc2ccccc21	667870539	25	340.22	3.05
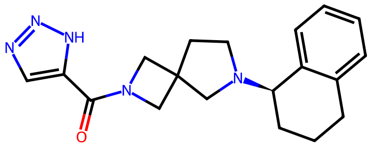	O=C(c1cnn[nH]1)N1CC2(CCN([C@@H]3CCCc4ccccc43)C2)C1	1072892331	25	337.19	2.03

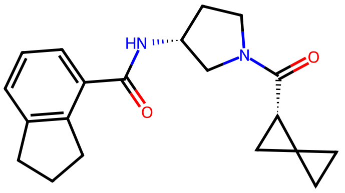	O=C(N[C@@H]1CCN(C(=O)[C@H]2CC23CC3)C1)c1cccc2c1CCC2	936273652	24	324.18	2.31
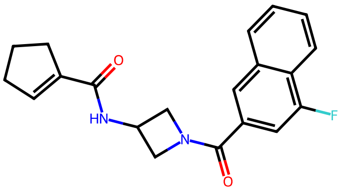	O=C(NC1CN(C(=O)c2cc(F)c3ccccc3c2)C1)C1=CCCC1	990810626	25	338.14	3.03
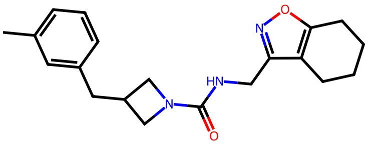	Cc1cccc(CC2CN(C(=O)NCc3noc4c3CCCC4)C2)c1	673235028	25	339.19	3.25
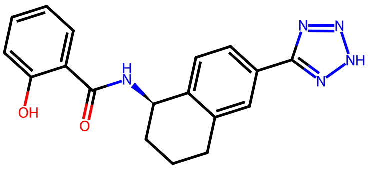	O=C(N[C@@H]1CCCc2cc(-c3nn[nH]n3)ccc21)c1ccccc1O	1606671370	25	335.14	2.38
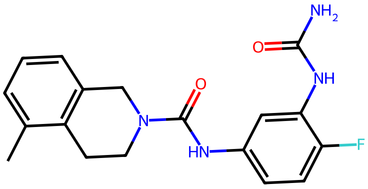	Cc1cccc2c1CCN(C(=O)Nc1ccc(F)c(NC(N)=O)c1)C2	1218363014	25	342.15	3.21
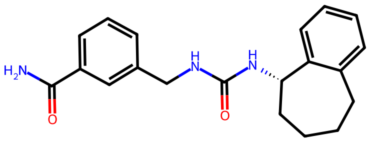	NC(=O)c1cccc(CNC(=O)N[C@H]2CCCCc3ccccc32)c1	69416241	25	337.18	3.05
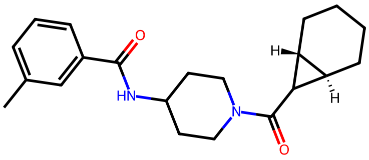	Cc1cccc(C(=O)NC2CCN(C(=O)C3[C@@H]4CCCC[C@@H]34)CC2)c1	870834265	25	340.22	3.15
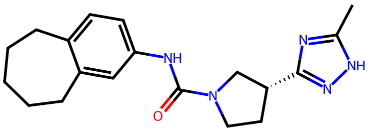	Cc1nc([C@@H]2CCN(C(=O)Nc3ccc4c(c3)CCCCC4)C2)n[nH]1	650425581	25	339.21	3.4

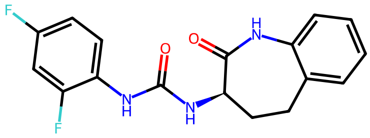	O=C(Nc1ccc(F)cc1F)N[C@@H]1CCc2ccccc2NC1=O	1202028557	24	331.11	3.04
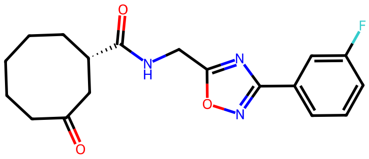	O=C1CCCCC[C@H](C(=O)NCc2nc(-c3cccc(F)c3)no2)C1	583077467	25	345.15	3.03
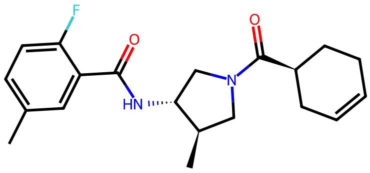	Cc1ccc(F)c(C(=O)N[C@@H]2CN(C(=O)[C@@H]3CC=CCC3)C[C@H]2C)c1	940685447	25	344.19	3.07
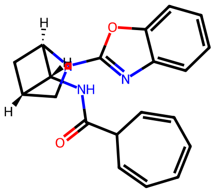	O=C(N[C@@H]1[C@H]2C[C@H]1N(c1nc3ccccc3o1)C2)C1C=CC=CC=C1	1064298938	25	333.15	2.82
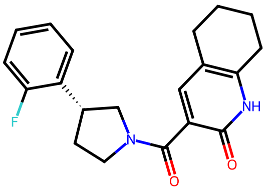	O=C(c1cc2c([nH]c1=O)CCCC2)N1CC[C@H](c2ccccc2F)C1	68107609	25	340.16	3.02
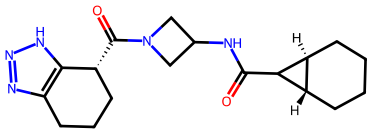	O=C(NC1CN(C(=O)[C@@H]2CCCc3nn[nH]c32)C1)C1[C@H]2CCCC[C@H]12	990645299	25	343.2	0.99
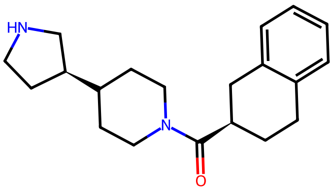	O=C([C@@H]1CCc2ccccc2C1)N1CCC([C@H]2CCNC2)CC1	582613851	23	312.22	2.64
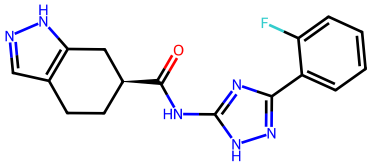	O=C(Nc1nc(-c2ccccc2F)n[nH]1)[C@H]1CCc2cn[nH]c2C1	266486532	24	326.13	2.08

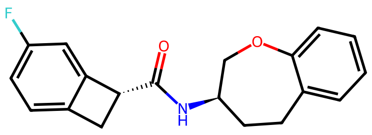	O=C(N[C@@H]1CCc2ccccc2OC1)[C@@H]1Cc2ccc(F)cc21	801019480	23	311.13	2.98
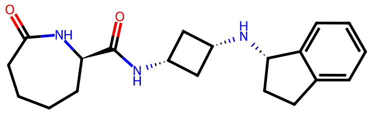	O=C1CCCC[C@H](C(=O)N[C@H]2C[C@@H](N[C@H]3CCc4ccccc43)C2)N1	1022693879	25	341.21	1.97
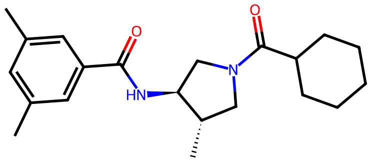	Cc1cc(C)cc(C(=O)N[C@H]2CN(C(=O)C3CCCCC3)C[C@@H]2C)c1	966097720	25	342.23	3.46
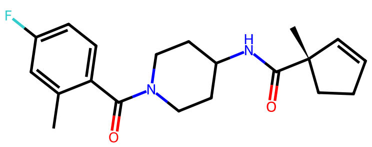	Cc1cc(F)ccc1C(=O)N1CCC(NC(=O)[C@]2(C)C=CCC2)CC1	947646066	25	344.19	3.21
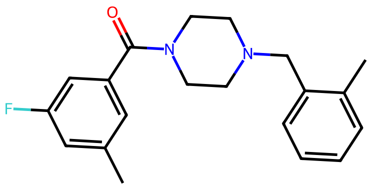	Cc1cc(F)cc(C(=O)N2CCN(Cc3ccccc3C)CC2)c1	361607389	24	326.18	3.4
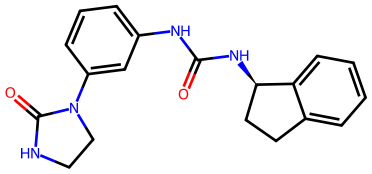	O=C(Nc1cccc(N2CCNC2=O)c1)N[C@@H]1CCc2ccccc21	47891607	25	336.16	3.03
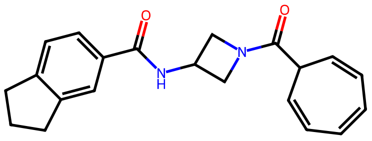	O=C(NC1CN(C(=O)C2C=CC=CC=C2)C1)c1ccc2c(c1)CCC2	991355011	25	334.17	2.41
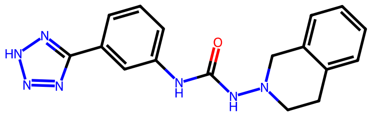	O=C(Nc1cccc(-c2nn[nH]n2)c1)NN1CCc2ccccc2C1	1606932358	25	335.15	1.96

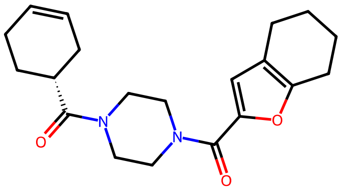	O=C(c1cc2c(o1)CCCC2)N1CCN(C(=O)[C@H]2CC=CCC2)CC1	1010303842	25	342.19	2.8
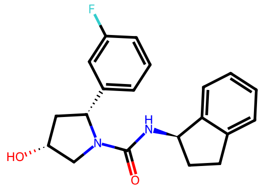	O=C(N[C@@H]1CCc2ccccc21)N1C[C@H](O)C[C@@H]1c1cccc(F)c1	283110722	25	340.16	3.33
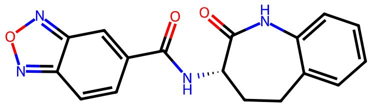	O=C(N[C@H]1CCc2ccccc2NC1=O)c1ccc2nonc2c1	1640486057	24	322.11	1.91
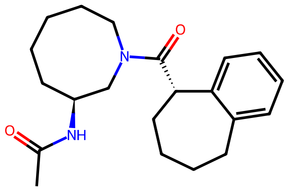	CC(=O)N[C@H]1CCCCCN(C(=O)[C@H]2CCCCc3ccccc32)C1	987064379	25	342.23	3.4
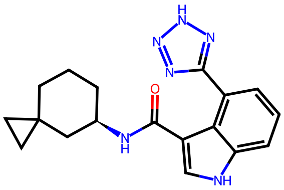	O=C(N[C@@H]1CCCC2(CC2)C1)c1c[nH]c2cccc(-c3nn[nH]n3)c12	1606667814	25	336.17	2.8
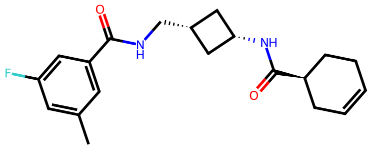	Cc1cc(F)cc(C(=O)NC[C@H]2C[C@@H](NC(=O)[C@@H]3CC=CCC3)C2)c1	979359246	25	344.19	3.12
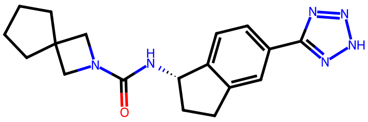	O=C(N[C@H]1CCc2cc(-c3nn[nH]n3)ccc21)N1CC2(CCCC2)C1	1606686761	25	338.19	2.44
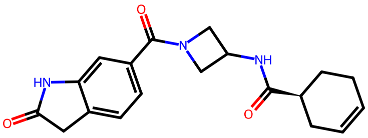	O=C1Cc2ccc(C(=O)N3CC(NC(=O)[C@@H]4CC=CCC4)C3)cc2N1	990217326	25	339.16	1.48

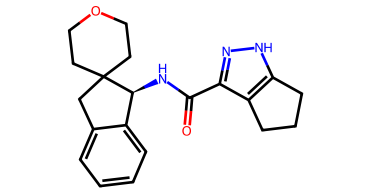	O=C(N[C@H]1c2ccccc2CC12CCOCC2)c1n[nH]c2c1CCC2	290579139	25	337.18	2.72
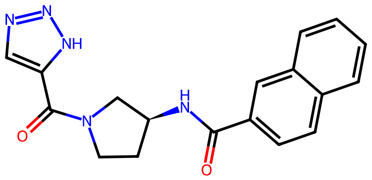	O=C(N[C@H]1CCN(C(=O)c2cnn[nH]2)C1)c1ccc2ccccc2c1	938738450	25	335.14	1.6
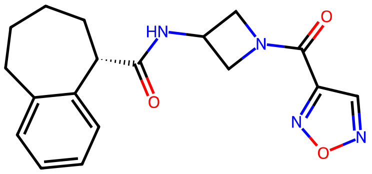	O=C(NC1CN(C(=O)c2cnon2)C1)[C@H]1CCCCc2ccccc21	994323955	25	340.15	1.52
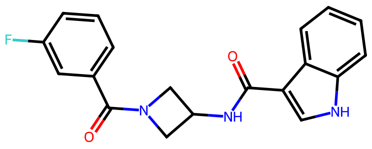	O=C(NC1CN(C(=O)c2cccc(F)c2)C1)c1c[nH]c2ccccc12	995032882	25	337.12	2.56
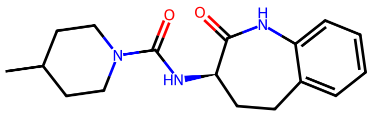	CC1CCN(C(=O)N[C@@H]2CCc3ccccc3NC2=O)CC1	654630329	22	301.18	2.38
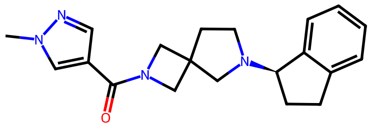	Cn1cc(C(=O)N2CC3(CCN([C@@H]4CCc5ccccc54)C3)C2)cn1	1072453040	25	336.2	2.26
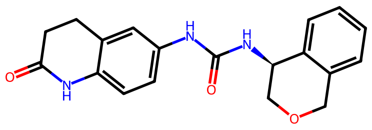	O=C1CCc2cc(NC(=O)N[C@@H]3COCc4ccccc43)ccc2N1	562787928	25	337.14	2.96
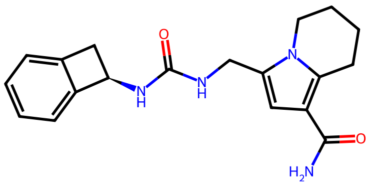	NC(=O)c1cc(CNC(=O)N[C@@H]2Cc3ccccc32)n2c1CCCC2	1551273686	25	338.17	2.02

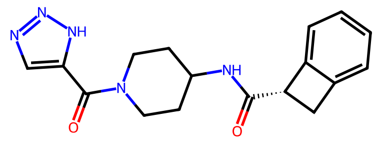	O=C(NC1CCN(C(=O)c2cnn[nH]2)CC1)[C@H]1Cc2ccccc21	947370889	24	325.15	0.87
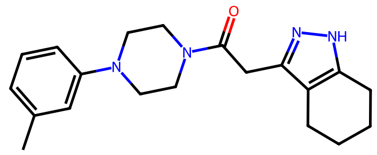	Cc1cccc(N2CCN(C(=O)Cc3n[nH]c4c3CCCC4)CC2)c1	188310972	25	338.21	2.49
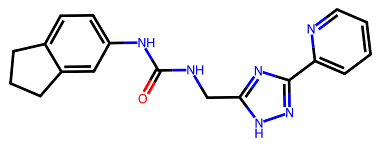	O=C(NCc1nc(-c2ccccn2)n[nH]1)Nc1ccc2c(c1)CCC2	1465058089	25	334.15	2.68
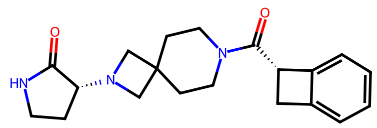	O=C1NCC[C@H]1N1CC2(CCN(C(=O)[C@H]3Cc4ccccc43)CC2)C1	1035780105	25	339.19	1.14
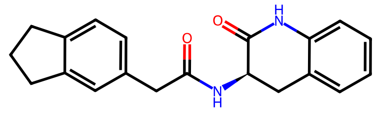	O=C(Cc1ccc2c(c1)CCC2)N[C@@H]1Cc2ccccc2NC1=O	529950641	24	320.15	2.4
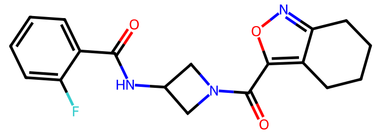	O=C(NC1CN(C(=O)c2onc3c2CCCC3)C1)c1ccccc1F	990098172	25	343.13	1.95
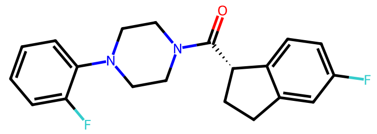	O=C([C@H]1CCc2cc(F)ccc21)N1CCN(c2ccccc2F)CC1	1463626498	25	342.15	3.34
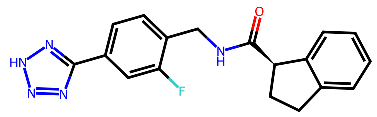	O=C(NCc1ccc(-c2nn[nH]n2)cc1F)[C@@H]1CCc2ccccc21	738100554	25	337.13	2.35

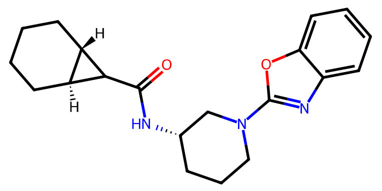	O=C(N[C@H]1CCCN(c2nc3ccccc3o2)C1)C1[C@H]2CCCC[C@H]12	1062375552	25	339.19	3.35
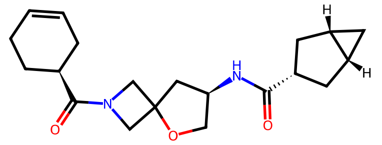	O=C(N[C@H]1COC2(C1)CN(C(=O)[C@@H]1CC=CCC1)C2)[C@@H]1C[C@@H]2C[C@@H]2C1	965785062	25	344.21	1.87
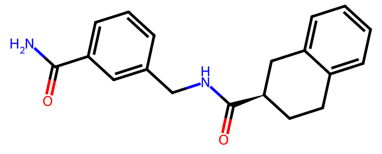	NC(=O)c1cccc(CNC(=O)[C@@H]2CCc3ccccc3C2)c1	573925368	23	308.15	2.21
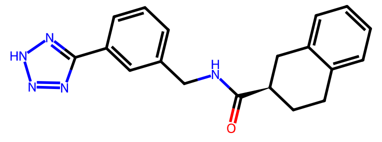	O=C(NCc1cccc(-c2nn[nH]n2)c1)[C@@H]1CCc2ccccc2C1	1609555788	25	333.16	2.29
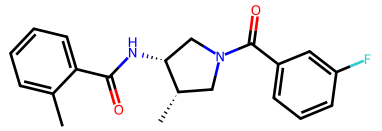	Cc1ccccc1C(=O)N[C@@H]1CN(C(=O)c2cccc(F)c2)C[C@@H]1C	966102941	25	340.16	3.02
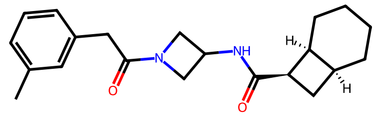	Cc1cccc(CC(=O)N2CC(NC(=O)[C@@H]3C[C@@H]4CCCC[C@@H]43)C2)c1	997652576	25	340.22	2.69
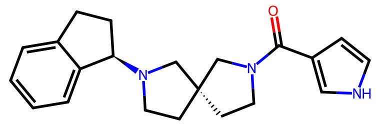	O=C(c1cc[nH]c1)N1CC[C@@]2(CCN([C@@H]3CCc4ccccc43)C2)C1	1041312086	25	335.2	3.24
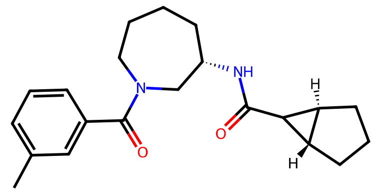	Cc1cccc(C(=O)N2CCCC[C@H](NC(=O)C3[C@H]4CCC[C@H]34)C2)c1	943169641	25	340.22	3.15

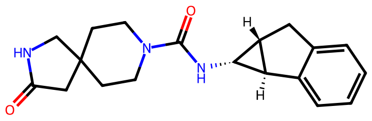	O=C1CC2(CCN(C(=O)N[C@H]3[C@@H]4c5ccccc5C[C@H]43)CC2)CN1	268681925	24	325.18	1.64
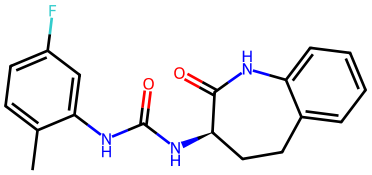	Cc1ccc(F)cc1NC(=O)N[C@@H]1CCc2ccccc2NC1=O	1191428704	24	327.14	3.21
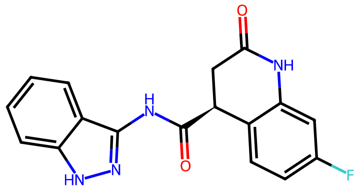	O=C1C[C@H](C(=O)Nc2n[nH]c3ccccc23)c2ccc(F)cc2N1	914531601	24	324.1	2.77
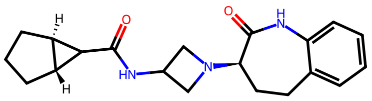	O=C(NC1CN([C@@H]2CCc3ccccc3NC2=O)C1)C1[C@@H]2CCC[C@@H]12	1030202505	25	339.19	1.79
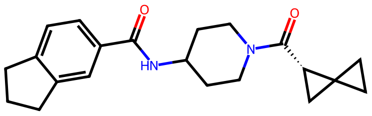	O=C(NC1CCN(C(=O)[C@H]2CC23CC3)CC1)c1ccc2c(c1)CCC2	948557025	25	338.2	2.7
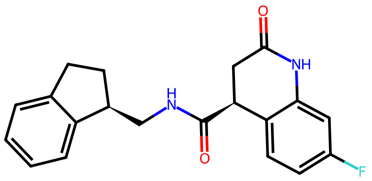	O=C1C[C@H](C(=O)NC[C@@H]2CCc3ccccc32)c2ccc(F)cc2N1	110378225	25	338.14	3.1
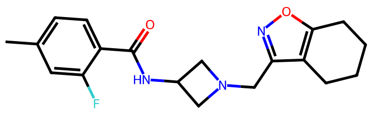	Cc1ccc(C(=O)NC2CN(Cc3noc4c3CCCC4)C2)c(F)c1	1031004608	25	343.17	2.62
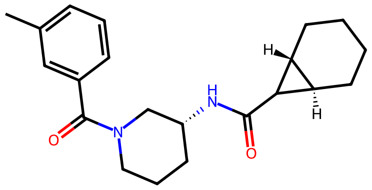	Cc1cccc(C(=O)N2CCC[C@@H](NC(=O)C3[C@@H]4CCCC[C@@H]34)C2)c1	965889391	25	340.22	3.15

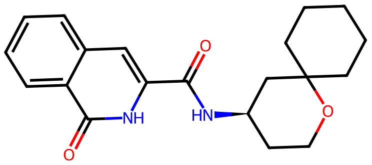	O=C(N[C@@H]1CCOC2(CCCCC2)C1)c1cc2ccccc2c(=O)[nH]1	161530610	25	340.18	3.14
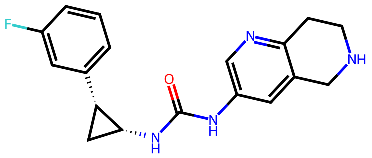	O=C(Nc1cnc2c(c1)CNCC2)N[C@@H]1C[C@@H]1c1cccc(F)c1	667804650	24	326.15	2.54
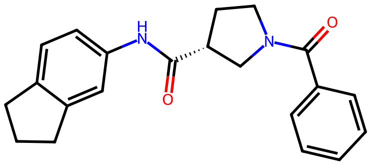	O=C(Nc1ccc2c(c1)CCC2)[C@@H]1CCN(C(=O)c2ccccc2)C1	623416543	25	334.17	3.28
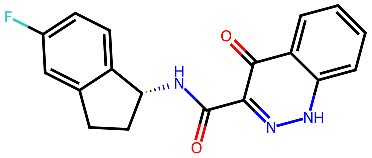	O=C(N[C@@H]1CCc2cc(F)ccc21)c1n[nH]c2ccccc2c1=O	111579023	24	323.11	2.48
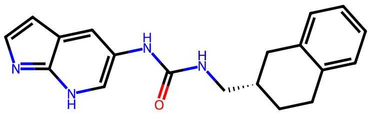	O=C(NC[C@H]1CCc2ccccc2C1)Nc1c[nH]c2ncc-2c1	1563261848	24	320.16	3.44
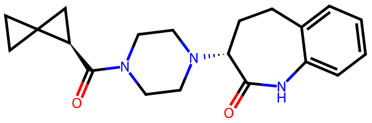	O=C1Nc2ccccc2CC[C@H]1N1CCN(C(=O)[C@@H]2CC23CC3)CC1	950707451	25	339.19	1.88
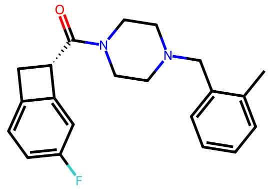	Cc1ccccc1CN1CCN(C(=O)[C@H]2Cc3ccc(F)cc32)CC1	684362003	25	338.18	3.12
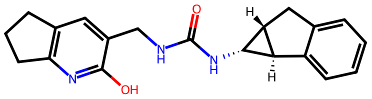	O=C(NCc1cc2c(nc1O)CCC2)N[C@H]1[C@@H]2c3ccccc3C[C@H]21	675712913	25	335.16	2.41

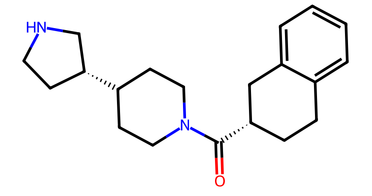	O=C([C@H]1CCc2ccccc2C1)N1CCC([C@@H]2CCNC2)CC1	582613856	23	312.22	2.64
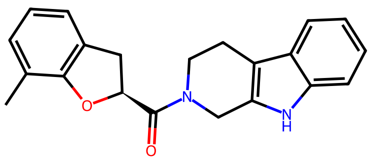	Cc1cccc2c1O[C@H](C(=O)N1CCc3c([nH]c4ccccc34)C1)C2	450276638	25	332.15	3.36
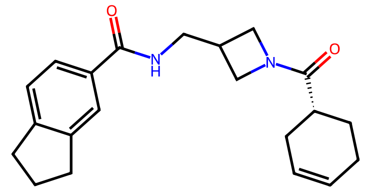	O=C(NCC1CN(C(=O)[C@H]2CC=CCC2)C1)c1ccc2c(c1)CCC2	1001830619	25	338.2	2.72
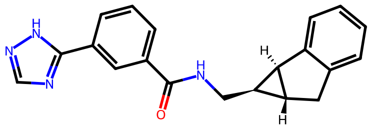	O=C(NC[C@@H]1[C@H]2Cc3ccccc3[C@@H]21)c1cccc(-c2ncn[nH]2)c1	157757901	25	330.15	2.79
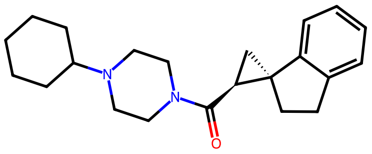	O=C([C@H]1C[C@]12CCc1ccccc12)N1CCN(C2CCCCC2)CC1	482064726	25	338.24	3.37
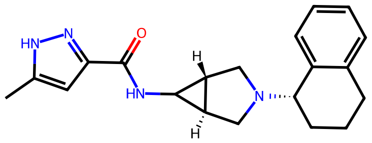	Cc1cc(C(=O)NC2[C@@H]3CN([C@H]4CCCc5ccccc54)C[C@@H]23)n[nH]1	958332180	25	336.2	2.46
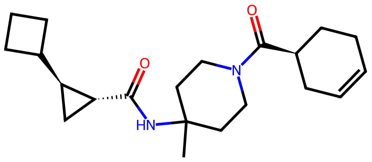	CC1(NC(=O)[C@@H]2C[C@H]2C2CCC2)CCN(C(=O)[C@@H]2CC=CCC2)CC1	956382556	25	344.25	3.28
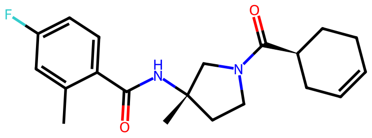	Cc1cc(F)ccc1C(=O)N[C@@]1(C)CCN(C(=O)[C@@H]2CC=CCC2)C1	957256366	25	344.19	3.21

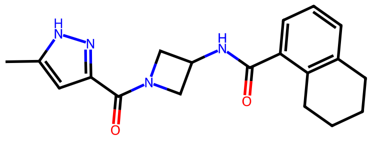	Cc1cc(C(=O)N2CC(NC(=O)c3cccc4c3CCCC4)C2)n[nH]1	991899416	25	338.17	1.85
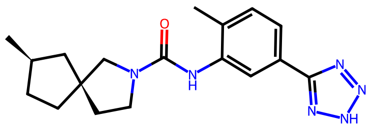	Cc1ccc(-c2nn[nH]n2)cc1NC(=O)N1CC[C@]2(CC[C@@H](C)C2)C1	826277987	25	340.2	3.22
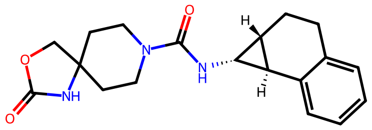	O=C1NC2(CCN(C(=O)N[C@H]3[C@@H]4c5ccccc5CC[C@H]43)CC2)CO1	495156345	25	341.17	2.0
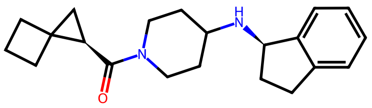	O=C([C@@H]1CC12CCC2)N1CCC(N[C@@H]2CCc3ccccc32)CC1	997041663	24	324.22	3.44
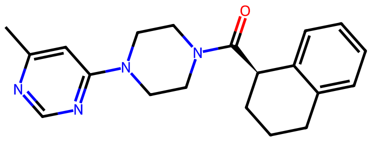	Cc1cc(N2CCN(C(=O)[C@@H]3CCCc4ccccc43)CC2)ncn1	1056074129	25	336.2	2.55
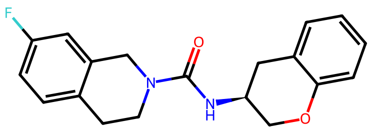	O=C(N[C@@H]1COc2ccccc2C1)N1CCc2ccc(F)cc2C1	178428812	24	326.14	2.9
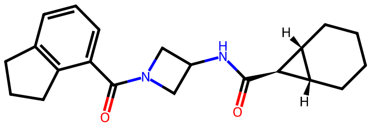	O=C(NC1CN(C(=O)c2cccc3c2CCC3)C1)[C@@H]1[C@H]2CCCC[C@H]21	990656530	25	338.2	2.55
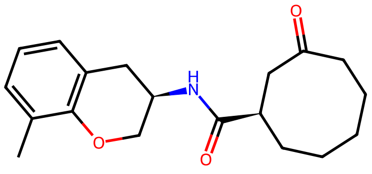	Cc1cccc2c1OC[C@H](NC(=O)[C@@H]1CCCCCC(=O)C1)C2	517228905	23	315.18	2.95

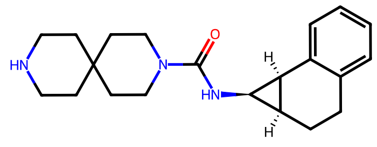	O=C(N[C@@H]1[C@@H]2CCc3ccccc3[C@@H]21)N1CCC2(CCNCC2)CC1	417916846	25	339.23	2.89
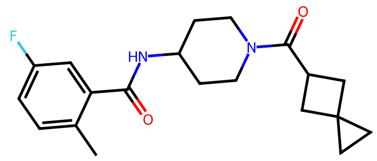	Cc1ccc(F)cc1C(=O)NC1CCN(C(=O)C2CC3(CC3)C2)CC1	948308466	25	344.19	3.05
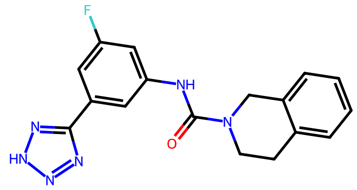	O=C(Nc1cc(F)cc(-c2nn[nH]n2)c1)N1CCc2ccccc2C1	1606902945	25	338.13	2.6
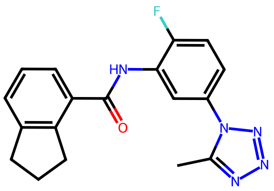	Cc1nnnn1-c1ccc(F)c(NC(=O)c2cccc3c2CCC3)c1	108170827	25	337.13	2.85
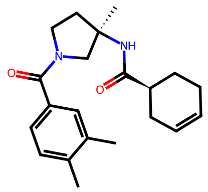	Cc1ccc(C(=O)N2CC[C@@](C)(NC(=O)[C@@H]3CC=CCC3)C2)cc1C	956691095	25	340.22	3.38
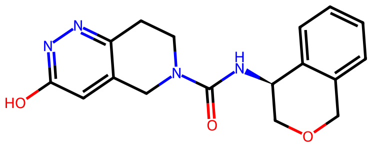	O=C(N[C@@H]1COCc2ccccc21)N1CCc2nnc(O)cc2C1	1470568660	24	326.14	1.52
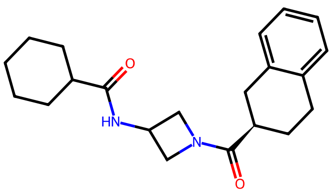	O=C(NC1CN(C(=O)[C@@H]2CCc3ccccc3C2)C1)C1CCCCC1	990100028	25	340.22	2.7
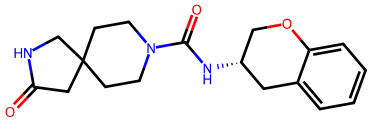	O=C1CC2(CCN(C(=O)N[C@@H]3COc4ccccc4C3)CC2)CN1	267854402	24	329.17	1.3

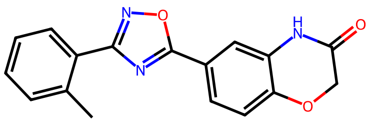	Cc1ccccc1-c1noc(-c2ccc3c(c2)NC(=O)CO3)n1	113730771	23	307.1	3.04
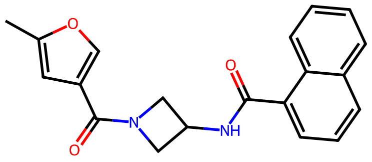	Cc1cc(C(=O)N2CC(NC(=O)c3cccc4ccccc34)C2)co1	999541837	25	334.13	3.0
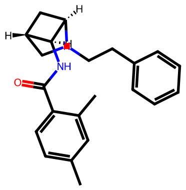	Cc1ccc(C(=O)N[C@H]2[C@H]3C[C@H]2N(CCc2ccccc2)C3)c(C)c1	1037698833	25	334.2	3.35
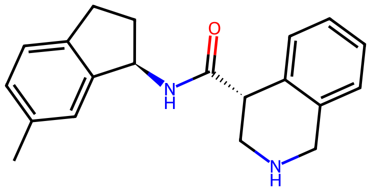	Cc1ccc2c(c1)[C@H](NC(=O)[C@H]1CNCc3ccccc31)CC2	645946688	23	306.17	2.99
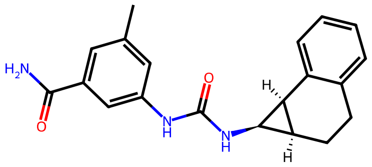	Cc1cc(NC(=O)N[C@@H]2[C@@H]3CCc4ccccc4[C@@H]32)cc(C(N)=O)c1	565994176	25	335.16	2.94
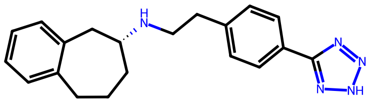	c1ccc2c(c1)CCC[C@@H](NCCc1ccc(-c3nn[nH]n3)cc1)C2	1593580216	25	333.2	2.95
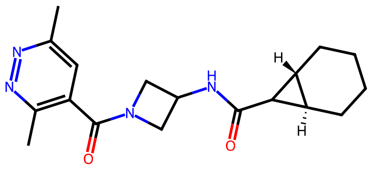	Cc1cc(C(=O)N2CC(NC(=O)C3[C@@H]4CCCC[C@@H]34)C2)c(C)nn1	990644248	24	328.19	1.47
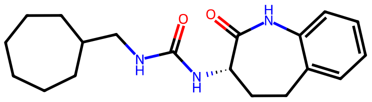	O=C(NCC1CCCCCC1)N[C@H]1CCc2ccccc2NC1=O	1686479902	24	329.21	3.21

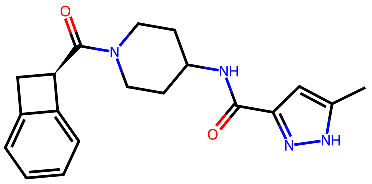	Cc1cc(C(=O)NC2CCN(C(=O)[C@@H]3Cc4ccccc43)CC2)n[nH]1	948059683	25	338.17	1.78
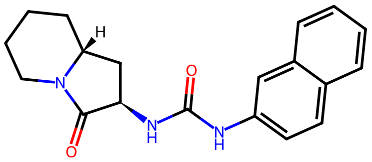	O=C(Nc1ccc2ccccc2c1)N[C@@H]1C[C@H]2CCCCN2C1=O	836189428	24	323.16	3.11
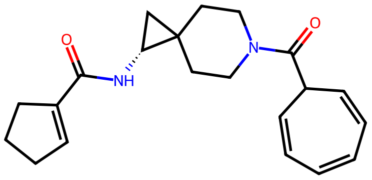	O=C(N[C@@H]1CC12CCN(C(=O)C1C=CC=CC=C1)CC2)C1=CCCC1	963563133	25	338.2	2.89
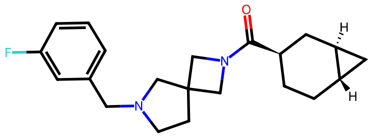	O=C([C@@H]1CC[C@H]2C[C@@H]2C1)N1CC2(CCN(Cc3cccc(F)c3)C2)C1	1072879729	25	342.21	3.3
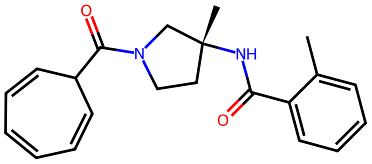	Cc1ccccc1C(=O)N[C@]1(C)CCN(C(=O)C2C=CC=CC=C2)C1	956656983	25	336.18	3.01
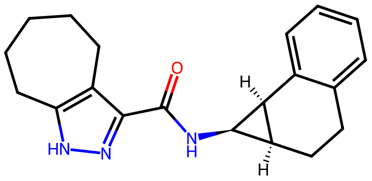	O=C(N[C@@H]1[C@@H]2CCc3ccccc3[C@@H]21)c1n[nH]c2c1CCCCC2	190775835	24	321.18	3.14
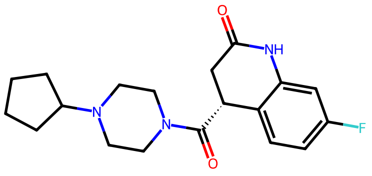	O=C1C[C@@H](C(=O)N2CCN(C3CCCC3)CC2)c2ccc(F)cc2N1	45085763	25	345.19	2.34
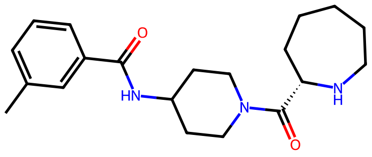	Cc1cccc(C(=O)NC2CCN(C(=O)[C@@H]3CCCCCN3)CC2)c1	1550101317	25	343.23	2.25

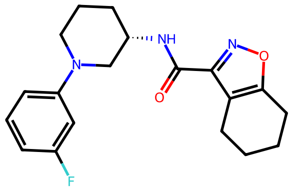	O=C(N[C@H]1CCCN(c2cccc(F)c2)C1)c1noc2c1CCCC2	116107326	25	343.17	3.09
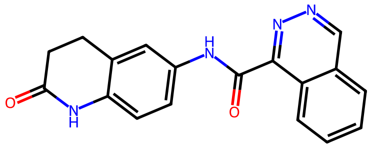	O=C1CCc2cc(NC(=O)c3nncc4ccccc34)ccc2N1	1526549766	24	318.11	2.77
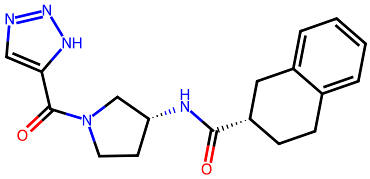	O=C(N[C@@H]1CCN(C(=O)c2cnn[nH]2)C1)[C@H]1CCc2ccccc2C1	942507407	25	339.17	0.94
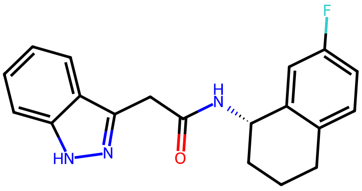	O=C(Cc1n[nH]c2ccccc12)N[C@H]1CCCc2ccc(F)cc21	89392171	24	323.14	3.44
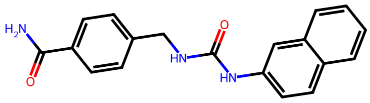	NC(=O)c1ccc(CNC(=O)Nc2ccc3ccccc3c2)cc1	119829915	24	319.13	3.26
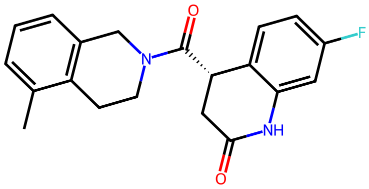	Cc1cccc2c1CCN(C(=O)[C@H]1CC(=O)Nc3cc(F)ccc31)C2	171540370	25	338.14	3.14
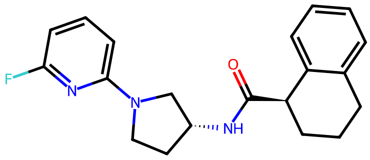	O=C(N[C@@H]1CCN(c2cccc(F)n2)C1)[C@@H]1CCCc2ccccc21	1058592032	25	339.17	3.04
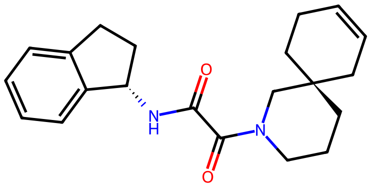	O=C(N[C@H]1CCc2ccccc21)C(=O)N1CCC[C@@]2(CC=CCC2)C1	339872978	25	338.2	3.14

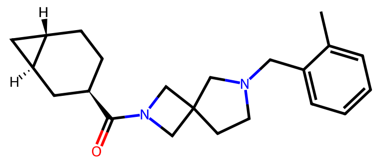	Cc1ccccc1CN1CCC2(C1)CN(C(=O)[C@@H]1CC[C@H]3C[C@@H]3C1)C2	1072879086	25	338.24	3.47
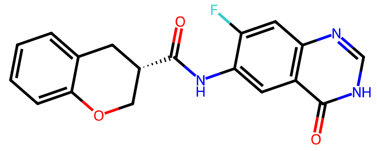	O=C(Nc1cc2c(=O)[nH]cnc2cc1F)[C@@H]1COc2ccccc2C1	192497046	25	339.1	2.25
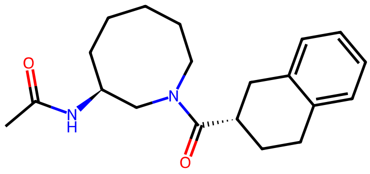	CC(=O)N[C@H]1CCCCCN(C(=O)[C@H]2CCc3ccccc3C2)C1	987173049	24	328.22	2.7
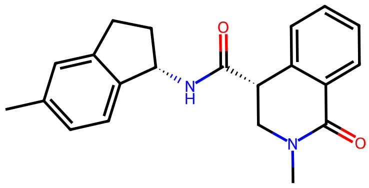	Cc1ccc2c(c1)CC[C@@H]2NC(=O)[C@H]1CN(C)C(=O)c2ccccc21	345861734	25	334.17	2.97
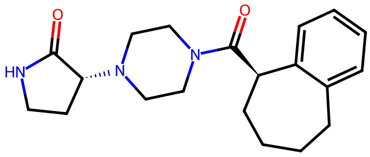	O=C1NCC[C@H]1N1CCN(C(=O)[C@@H]2CCCCc3ccccc32)CC1	957819263	25	341.21	1.53
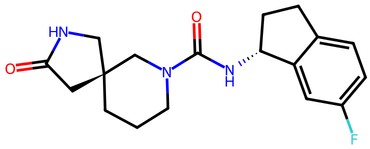	O=C1C[C@@]2(CCCN(C(=O)N[C@@H]3CCc4ccc(F)cc43)C2)CN1	608426948	24	331.17	2.12
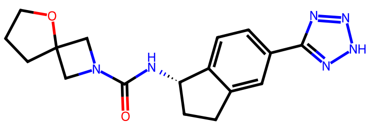	O=C(N[C@H]1CCc2cc(-c3nn[nH]n3)ccc21)N1CC2(CCCO2)C1	1606684538	25	340.16	1.43
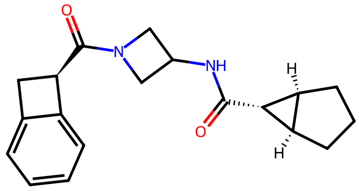	O=C(NC1CN(C(=O)[C@@H]2Cc3ccccc32)C1)[C@@H]1[C@H]2CCC[C@H]21	990691356	23	310.17	1.7

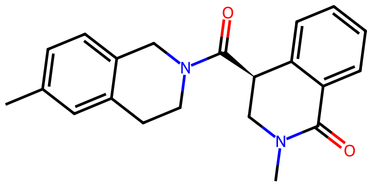	Cc1ccc2c(c1)CCN(C(=O)[C@@H]1CN(C)C(=O)c3ccccc31)C2	362506233	25	334.17	2.75
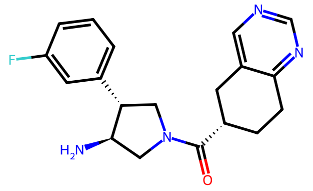	N[C@@H]1CN(C(=O)[C@H]2CCc3ncncc3C2)C[C@H]1c1cccc(F)c1	648178045	25	340.17	1.67
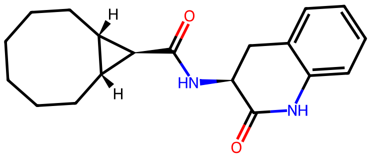	O=C1Nc2ccccc2C[C@@H]1NC(=O)[C@@H]1[C@H]2CCCCCC[C@H]21	628943770	23	312.18	2.88
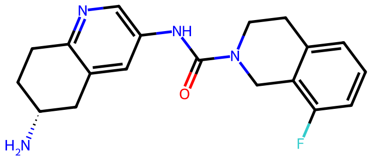	N[C@@H]1CCc2ncc(NC(=O)N3CCc4cccc(F)c4C3)cc2C1	333349661	25	340.17	2.63
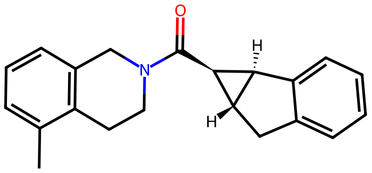	Cc1cccc2c1CCN(C(=O)[C@@H]1[C@H]3Cc4ccccc4[C@@H]31)C2	859211922	23	303.16	3.47
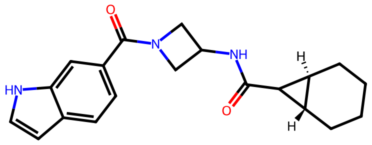	O=C(NC1CN(C(=O)c2ccc3cc[nH]c3c2)C1)C1[C@H]2CCCC[C@H]12	990664131	25	337.18	2.54
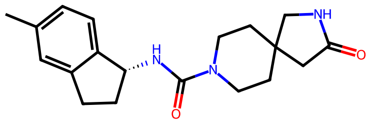	Cc1ccc2c(c1)CC[C@H]2NC(=O)N1CCC2(CC1)CNC(=O)C2	267790530	24	327.19	2.29
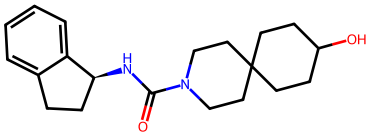	O=C(N[C@H]1CCc2ccccc21)N1CCC2(CCC(O)CC2)CC1	297566095	24	328.22	3.4

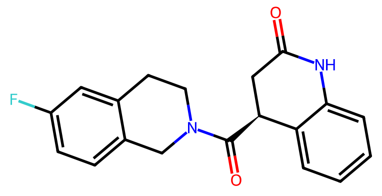	O=C1C[C@H](C(=O)N2CCc3cc(F)ccc3C2)c2ccccc2N1	92528534	24	324.13	2.84
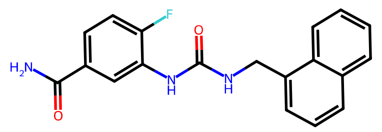	NC(=O)c1ccc(F)c(NC(=O)NCc2cccc3ccccc23)c1	194570889	25	337.12	3.4
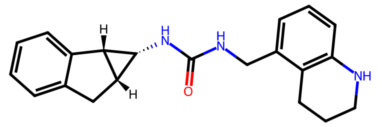	O=C(NCc1cccc2c1CCCN2)N[C@@H]1[C@@H]2Cc3ccccc3[C@@H]21	573701147	25	333.18	3.18
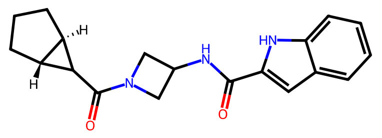	O=C(NC1CN(C(=O)C2[C@@H]3CCC[C@@H]23)C1)c1cc2ccccc2[nH]1	1000142146	24	323.16	2.15
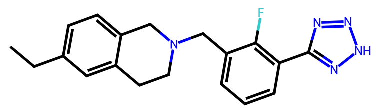	CCc1ccc2c(c1)CCN(Cc1cccc(-c3nn[nH]n3)c1F)C2	1590892037	25	337.17	3.13
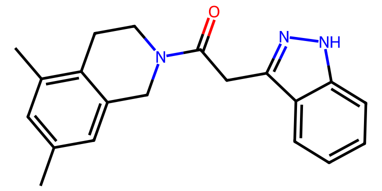	Cc1cc(C)c2c(c1)CN(C(=O)Cc1n[nH]c3ccccc13)CC2	189243499	24	319.17	3.31
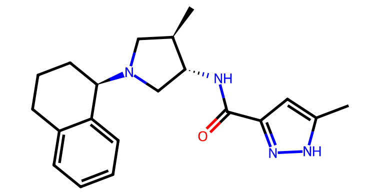	Cc1cc(C(=O)N[C@@H]2CN([C@@H]3CCCc4ccccc43)C[C@H]2C)n[nH]1	1079462674	25	338.21	2.85
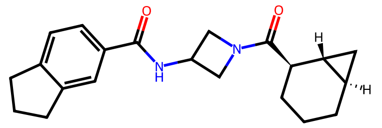	O=C(NC1CN(C(=O)[C@@H]2CCC[C@@H]3C[C@H]32)C1)c1ccc2c(c1)CCC2	991370360	25	338.2	2.55

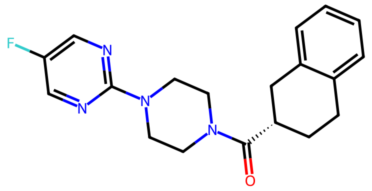	O=C([C@H]1CCc2ccccc2C1)N1CCN(c2ncc(F)cn2)CC1	565425976	25	340.17	2.07
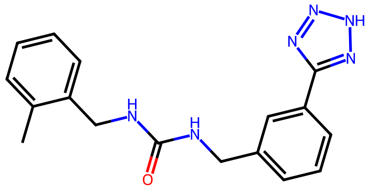	Cc1ccccc1CNC(=O)NCc1cccc(-c2nn[nH]n2)c1	1592257043	24	322.15	2.17
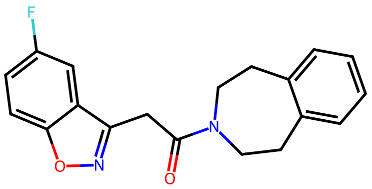	O=C(Cc1noc2ccc(F)cc12)N1CCc2ccccc2CC1	563457179	24	324.13	3.14
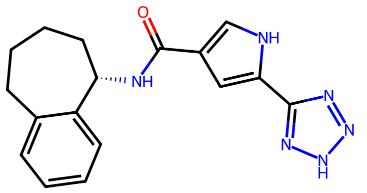	O=C(N[C@H]1CCCCc2ccccc21)c1c[nH]c(-c2nn[nH]n2)c1	826429865	24	322.15	2.39
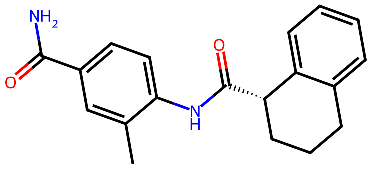	Cc1cc(C(N)=O)ccc1NC(=O)[C@H]1CCCc2ccccc21	75612151	23	308.15	3.15
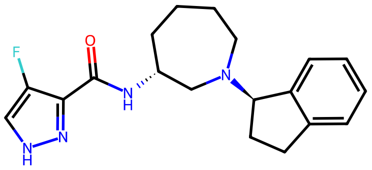	O=C(N[C@@H]1CCCCN([C@@H]2CCc3ccccc32)C1)c1n[nH]cc1F	1034381092	25	342.19	2.82
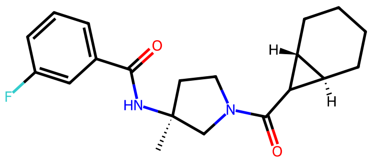	C[C@]1(NC(=O)c2cccc(F)c2)CCN(C(=O)C2[C@@H]3CCCC[C@@H]23)C1	956654837	25	344.19	2.98
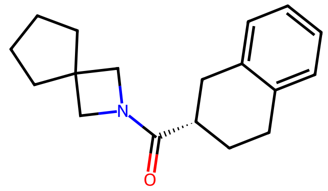	O=C([C@H]1CCc2ccccc2C1)N1CC2(CCCC2)C1	580718823	20	269.18	3.19

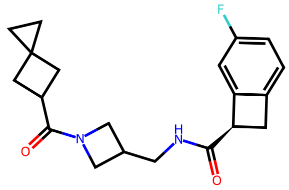	O=C(NCC1CN(C(=O)C2CC3(CC3)C2)C1)[C@@H]1Cc2ccc(F)cc21	1003886983	25	342.17	2.23
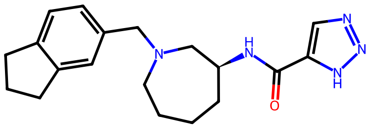	O=C(N[C@H]1CCCCN(Cc2ccc3c(c2)CCC3)C1)c1cnn[nH]1	1034286864	25	339.21	2.08
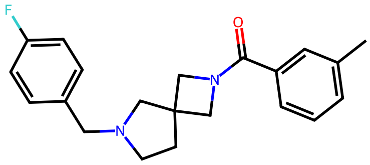	Cc1cccc(C(=O)N2CC3(CCN(Cc4ccc(F)cc4)C3)C2)c1	1072434174	25	338.18	3.48
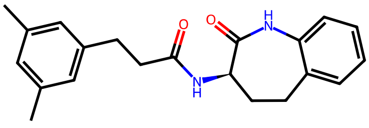	Cc1cc(C)cc(CCC(=O)N[C@@H]2CCc3ccccc3NC2=O)c1	653999904	25	336.18	3.31
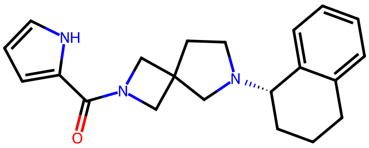	O=C(c1ccc[nH]1)N1CC2(CCN([C@H]3CCCc4ccccc43)C2)C1	1072466019	25	335.2	3.24
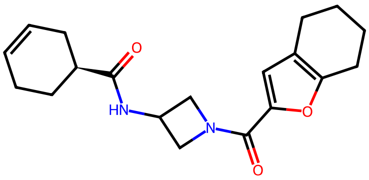	O=C(NC1CN(C(=O)c2cc3c(o2)CCCC3)C1)[C@@H]1CC=CCC1	990214444	24	328.18	2.46
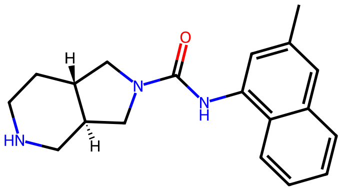	Cc1cc(NC(=O)N2C[C@H]3CCNC[C@@H]3C2)c2ccccc2c1	415803327	23	309.18	3.22
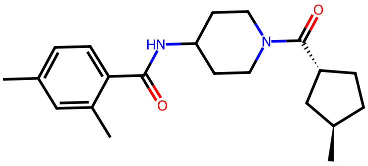	Cc1ccc(C(=O)NC2CCN(C(=O)[C@@H]3CC[C@@H](C)C3)CC2)c(C)c1	946734176	25	342.23	3.46

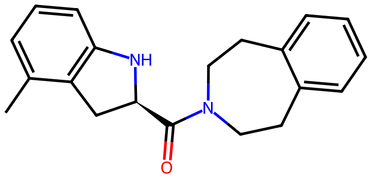	Cc1cccc2c1C[C@H](C(=O)N1CCc3ccccc3CC1)N2	637996881	23	306.17	2.96
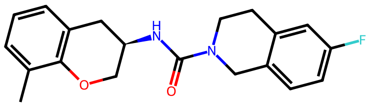	Cc1cccc2c1OC[C@H](NC(=O)N1CCc3cc(F)ccc3C1)C2	462615351	25	340.16	3.21
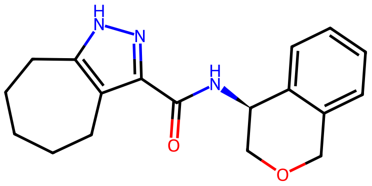	O=C(N[C@@H]1COCc2ccccc21)c1n[nH]c2c1CCCCC2	416272808	23	311.16	2.68
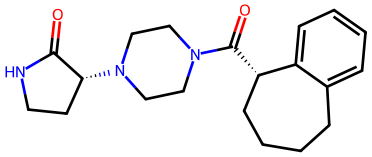	O=C1NCC[C@H]1N1CCN(C(=O)[C@H]2CCCCc3ccccc32)CC1	957819265	25	341.21	1.53
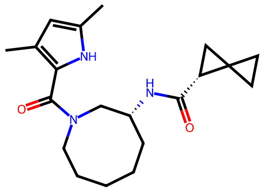	Cc1cc(C)c(C(=O)N2CCCCC[C@@H](NC(=O)[C@@H]3CC34CC4)C2)[nH]1	987074925	25	343.23	2.93
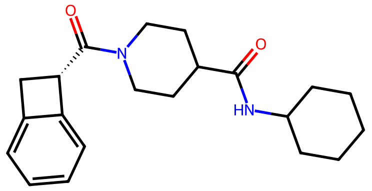	O=C(NC1CCCCC1)C1CCN(C(=O)[C@H]2Cc3ccccc32)CC1	358191264	25	340.22	3.01
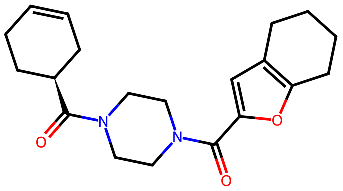	O=C(c1cc2c(o1)CCCC2)N1CCN(C(=O)[C@@H]2CC=CCC2)CC1	1010303846	25	342.19	2.8
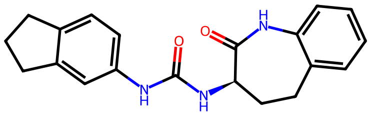	O=C(Nc1ccc2c(c1)CCC2)N[C@@H]1CCc2ccccc2NC1=O	653926049	25	335.16	3.25

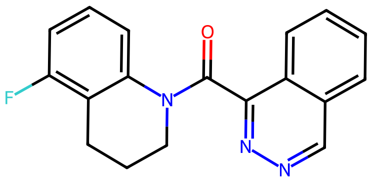	O=C(c1nncc2ccccc12)N1CCCc2c(F)cccc21	1126754860	23	307.11	3.36
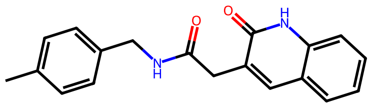	Cc1ccc(CNC(=O)Cc2cc3ccccc3[nH]c2=O)cc1	78349371	23	306.14	2.7
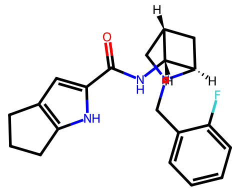	O=C(N[C@@H]1[C@H]2C[C@H]1N(Cc1ccccc1F)C2)c1cc2c([nH]1)CCC2	1037675946	25	339.17	2.65
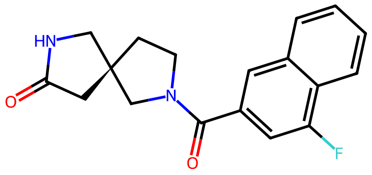	O=C1C[C@]2(CCN(C(=O)c3cc(F)c4ccccc4c3)C2)CN1	351838390	23	312.13	2.33
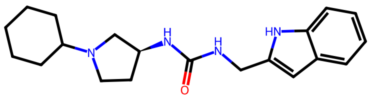	O=C(NCc1cc2ccccc2[nH]1)N[C@H]1CCN(C2CCCCC2)C1	661853367	25	340.23	3.37
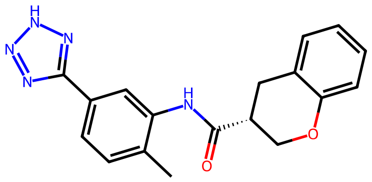	Cc1ccc(-c2nn[nH]n2)cc1NC(=O)[C@H]1COc2ccccc2C1	1592103995	25	335.14	2.36
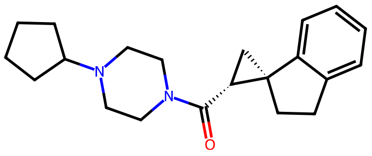	O=C([C@@H]1C[C@]12CCc1ccccc12)N1CCN(C2CCCC2)CC1	175260552	24	324.22	2.98
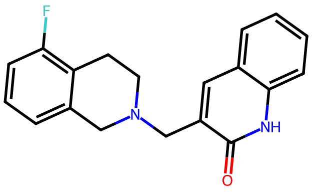	O=c1[nH]c2ccccc2cc1CN1CCc2c(F)cccc2C1	152689288	23	308.13	3.23

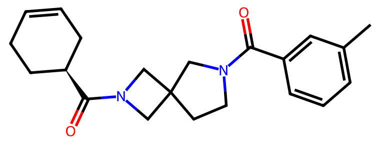	Cc1cccc(C(=O)N2CCC3(C2)CN(C(=O)[C@@H]2CC=CCC2)C3)c1	1018770696	25	338.2	3.03
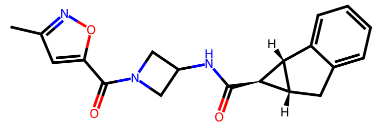	Cc1cc(C(=O)N2CC(NC(=O)[C@@H]3[C@H]4Cc5ccccc5[C@H]43)C2)on1	991645902	25	337.14	1.51
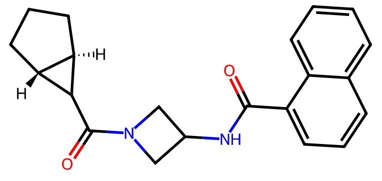	O=C(NC1CN(C(=O)C2[C@@H]3CCC[C@H]23)C1)c1cccc2ccccc12	999540251	25	334.17	2.83
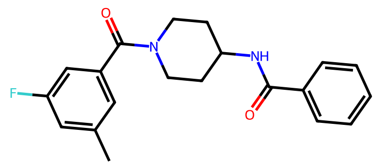	Cc1cc(F)cc(C(=O)N2CCC(NC(=O)c3ccccc3)CC2)c1	79383985	25	340.16	3.17
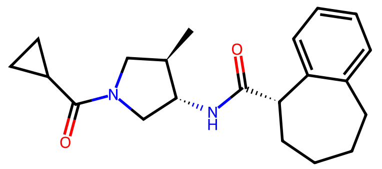	C[C@@H]1CN(C(=O)C2CC2)C[C@H]1NC(=O)[C@H]1CCCCc2ccccc21	947037173	25	340.22	2.87
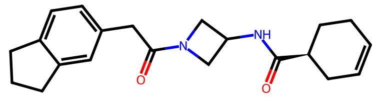	O=C(NC1CN(C(=O)Cc2ccc3c(c2)CCC3)C1)[C@@H]1CC=CCC1	990222332	25	338.2	2.4
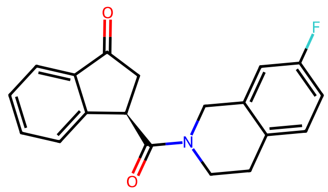	O=C1C[C@@H](C(=O)N2CCc3ccc(F)cc3C2)c2ccccc21	277280534	23	309.12	3.08
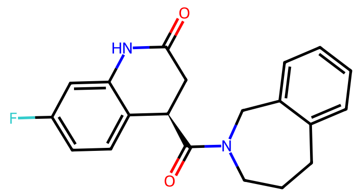	O=C1C[C@@H](C(=O)N2CCCc3cccc3C2)c2ccc(F)cc2N1	106713999	25	338.14	3.23

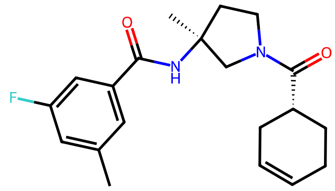	Cc1cc(F)cc(C(=O)N[C@@]2(C)CCN(C(=O)[C@H]3CC=CCC3)C2)c1	957700735	25	344.19	3.21
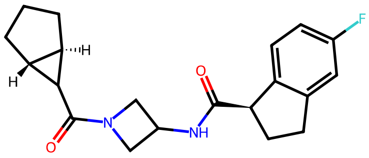	O=C(NC1CN(C(=O)C2[C@@H]3CCC[C@@H]23)C1)[C@@H]1CCc2cc(F)ccc21	996098993	25	342.17	2.23
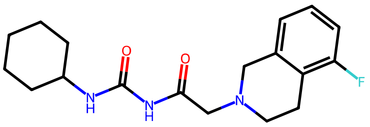	O=C(CN1CCc2c(F)cccc2C1)NC(=O)NC1CCCCC1	152848522	24	333.19	2.34
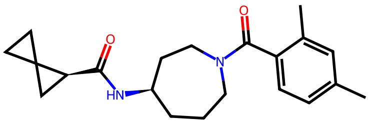	Cc1ccc(C(=O)N2CCC[C@@H](NC(=O)[C@H]3CC34CC4)CC2)c(C)c1	965217521	25	340.22	3.21
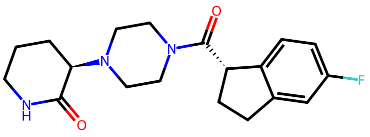	O=C1NCCC[C@H]1N1CCN(C(=O)[C@H]2CCc3cc(F)ccc32)CC1	949321334	25	345.19	1.28
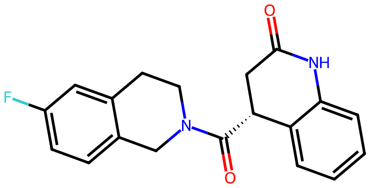	O=C1C[C@@H](C(=O)N2CCc3cc(F)ccc3C2)c2ccccc2N1	92528532	24	324.13	2.84
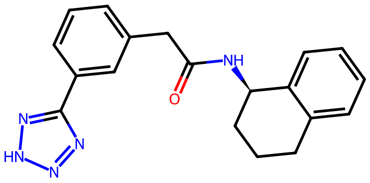	O=C(Cc1cccc(-c2nn[nH]n2)c1)N[C@@H]1CCCc2ccccc21	823093115	25	333.16	2.6
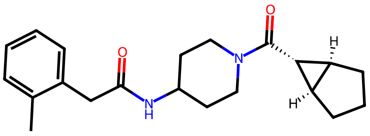	Cc1ccccc1CC(=O)NC1CCN(C(=O)[C@@H]2[C@H]3CCC[C@H]32)CC1	946091834	25	340.22	2.69

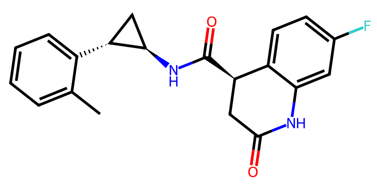	Cc1ccccc1[C@@H]1C[C@H]1NC(=O)[C@@H]1CC(=O)Nc2cc(F)ccc21	126160212	25	338.14	3.23
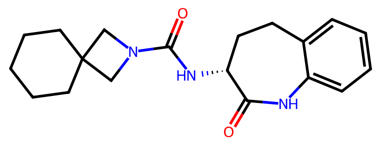	O=C1Nc2ccccc2CC[C@H]1NC(=O)N1CC2(CCCCC2)C1	654628865	24	327.19	2.92
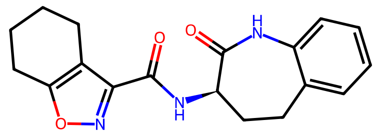	O=C(N[C@@H]1CCc2ccccc2NC1=O)c1noc2c1CCCC2	654005097	24	325.14	2.24
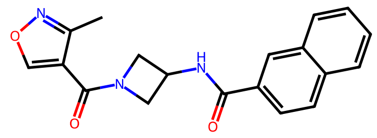	Cc1nocc1C(=O)N1CC(NC(=O)c2ccc3ccccc3c2)C1	999451528	25	335.13	2.39
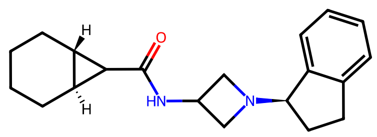	O=C(NC1CN([C@@H]2CCc3ccccc32)C1)C1[C@H]2CCCC[C@H]12	1030183260	23	310.2	2.91
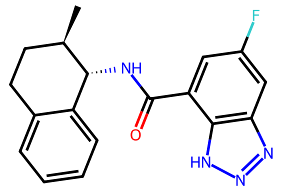	C[C@@H]1CCc2ccccc2[C@H]1NC(=O)c1cc(F)cc2nn[nH]c12	337463267	24	324.14	3.15
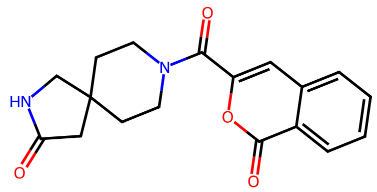	O=C1CC2(CCN(C(=O)c3cc4ccccc4c(=O)o3)CC2)CN1	408152313	24	326.13	1.54
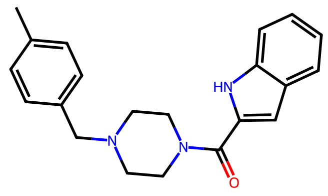	Cc1ccc(CN2CCN(C(=O)c3cc4ccccc4[nH]3)CC2)cc1	72073743	25	333.18	3.43

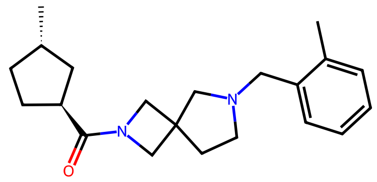	Cc1ccccc1CN1CCC2(C1)CN(C(=O)[C@H]1CC[C@H](C)C1)C2	1073033273	24	326.24	3.47
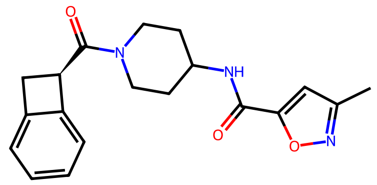	Cc1cc(C(=O)NC2CCN(C(=O)[C@@H]3Cc4ccccc43)CC2)on1	948059504	25	339.16	2.04
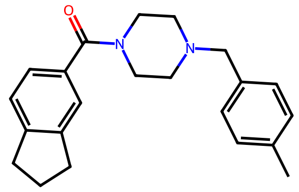	Cc1ccc(CN2CCN(C(=O)c3ccc4c(c3)CCC4)CC2)cc1	957610222	25	334.2	3.44
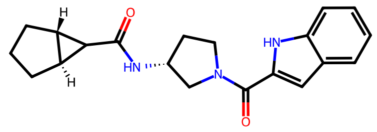	O=C(N[C@@H]1CCN(C(=O)c2cc3ccccc3[nH]2)C1)C1[C@H]2CCC[C@H]12	936158054	25	337.18	2.54
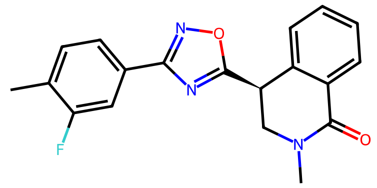	Cc1ccc(-c2noc([C@@H]3CN(C)C(=O)c4ccccc43)n2)cc1F	365993149	25	337.12	3.4
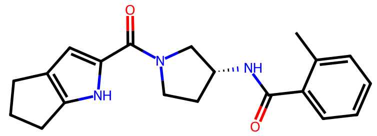	Cc1ccccc1C(=O)N[C@@H]1CCN(C(=O)c2cc3c([nH]2)CCC3)C1	939206695	25	337.18	2.46
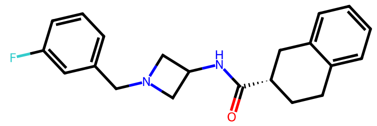	O=C(NC1CN(Cc2cccc(F)c2)C1)[C@H]1CCc2ccccc2C1	1031012237	25	338.18	2.93
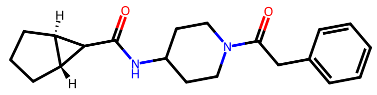	O=C(NC1CCN(C(=O)Cc2ccccc2)CC1)C1[C@@H]2CCC[C@@H]12	947344841	24	326.2	2.38

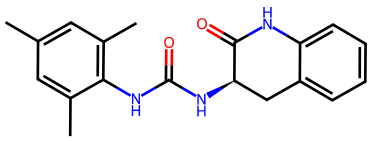	Cc1cc(C)c(NC(=O)N[C@@H]2Cc3ccccc3NC2=O)c(C)c1	528849298	24	323.16	3.3
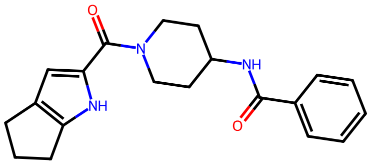	O=C(NC1CCN(C(=O)c2cc3c([nH]2)CCC3)CC1)c1ccccc1	947445821	25	337.18	2.54
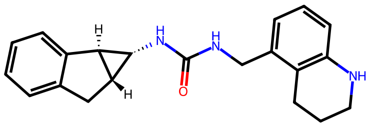	O=C(NCc1cccc2c1CCCN2)N[C@H]1[C@@H]2c3ccccc3C[C@H]21	573701144	25	333.18	3.18
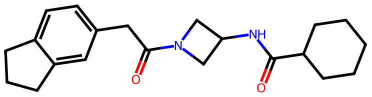	O=C(NC1CN(C(=O)Cc2ccc3c(c2)CCC3)C1)C1CCCCC1	990100662	25	340.22	2.63
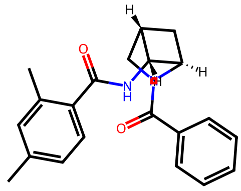	Cc1ccc(C(=O)N[C@@H]2[C@H]3C[C@H]2N(C(=O)c2ccccc2)C3)c(C)c1	948057884	25	334.17	2.95
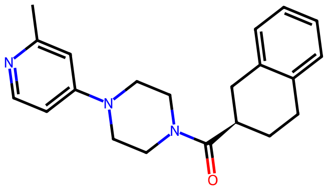	Cc1cc(N2CCN(C(=O)[C@@H]3CCc4ccccc4C3)CC2)ccn1	223350249	25	335.2	2.84
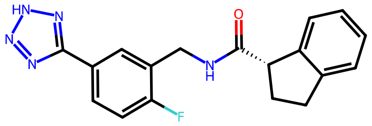	O=C(NCc1cc(-c2nn[nH]n2)ccc1F)[C@H]1CCc2ccccc21	823235475	25	337.13	2.35
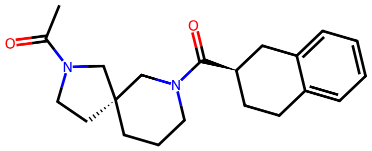	CC(=O)N1CC[C@]2(CCCN(C(=O)[C@@H]3CCc4ccccc4C3)C2)C1	952819502	25	340.22	2.65

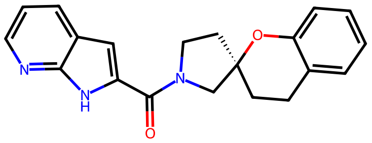	O=C(c1cc2cccnc2[nH]1)N1CC[C@]2(CCc3ccccc3O2)C1	571860168	25	333.15	3.17
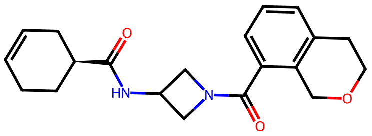	O=C(NC1CN(C(=O)c2cccc3c2COCC3)C1)[C@@H]1CC=CCC1	990214419	25	340.18	2.06
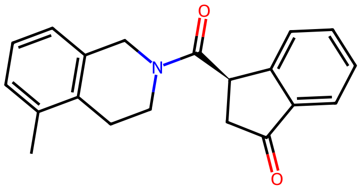	Cc1cccc2c1CCN(C(=O)[C@@H]1CC(=O)c3ccccc31)C2	789644477	23	305.14	3.25
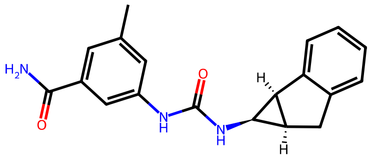	Cc1cc(NC(=O)N[C@@H]2[C@@H]3Cc4ccccc4[C@@H]32)cc(C(N)=O)c1	576560113	24	321.15	2.55
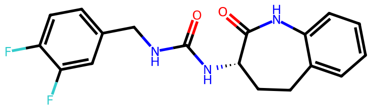	O=C(NCc1ccc(F)c(F)c1)N[C@H]1CCc2ccccc2NC1=O	1468253594	25	345.13	2.72
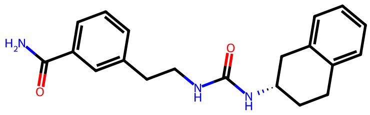	NC(=O)c1cccc(CCNC(=O)N[C@H]2CCc3ccccc3C2)c1	69591057	25	337.18	2.18
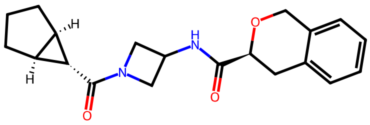	O=C(NC1CN(C(=O)[C@@H]2[C@H]3CCC[C@H]32)C1)[C@@H]1Cc2ccccc2CO1	997733896	25	340.18	1.5
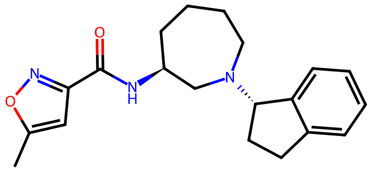	Cc1cc(C(=O)N[C@H]2CCCCN([C@H]3CCc4ccccc43)C2)no1	1034088273	25	339.19	3.25

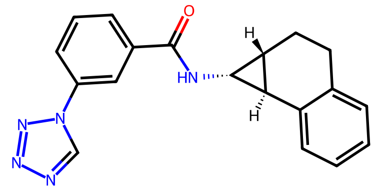	O=C(N[C@H]1[C@@H]2c3ccccc3CC[C@H]21)c1cccc(-n2cnnn2)c1	153854754	25	331.14	2.12
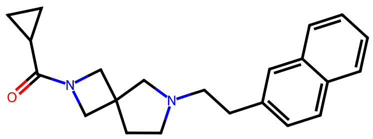	O=C(C1CC1)N1CC2(CCN(CCc3ccc4ccccc4c3)C2)C1	1072378348	25	334.2	3.33
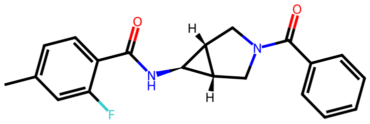	Cc1ccc(C(=O)N[C@H]2[C@@H]3CN(C(=O)c4ccccc4)C[C@@H]32)c(F)c1	979492577	25	338.14	2.63
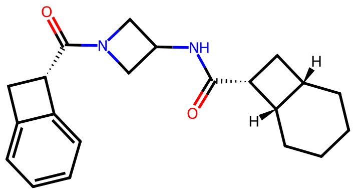	O=C(NC1CN(C(=O)[C@H]2Cc3ccccc32)C1)[C@@H]1C[C@@H]2CCCC[C@@H]21	997651796	25	338.2	2.48
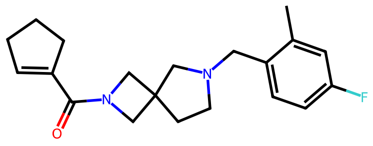	Cc1cc(F)ccc1CN1CCC2(C1)CN(C(=O)C1=CCCC1)C2	1072615277	24	328.2	3.28
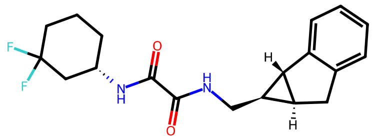	O=C(NC[C@@H]1[C@@H]2Cc3ccccc3[C@@H]12)C(=O)N[C@H]1CCCC(F)(F)C1	1329679179	25	348.16	2.38
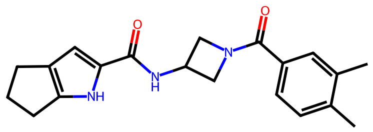	Cc1ccc(C(=O)N2CC(NC(=O)c3cc4c([nH]3)CCC4)C2)cc1C	995964302	25	337.18	2.37
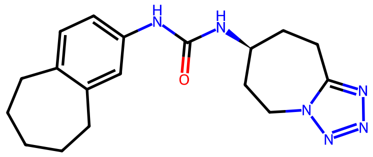	O=C(Nc1ccc2c(c1)CCCCC2)N[C@H]1CCc2nnnn2CC1	650426047	25	340.2	2.47

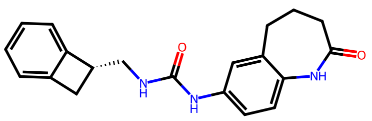	O=C1CCCc2cc(NC(=O)NC[C@@H]3Cc4ccccc43)ccc2N1	171523291	25	335.16	3.42
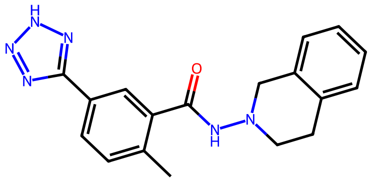	Cc1ccc(-c2nn[nH]n2)cc1C(=O)NN1CCc2ccccc2C1	1581134573	25	334.15	1.88
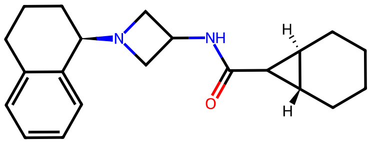	O=C(NC1CN([C@@H]2CCCc3ccccc32)C1)C1[C@H]2CCCC[C@H]12	1030187229	24	324.22	3.3
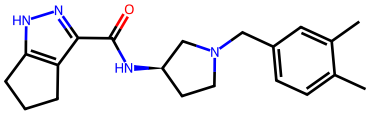	Cc1ccc(CN2CC[C@@H](NC(=O)c3n[nH]c4c3CCC4)C2)cc1C	1015071467	25	338.21	2.52
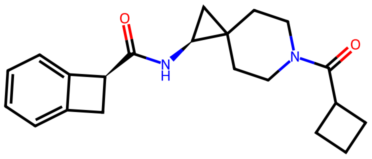	O=C(N[C@H]1CC12CCN(C(=O)C1CCC1)CC2)[C@H]1Cc2ccccc21	965003312	25	338.2	2.62
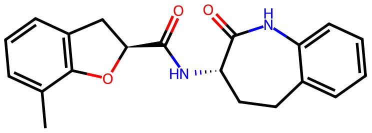	Cc1cccc2c1O[C@H](C(=O)N[C@H]1CCc3ccccc3NC1=O)C2	653996571	25	336.15	2.37
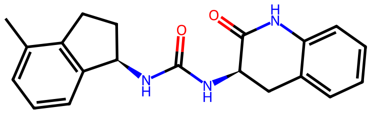	Cc1cccc2c1CC[C@H]2NC(=O)N[C@@H]1Cc2ccccc2NC1=O	636735161	25	335.16	2.84
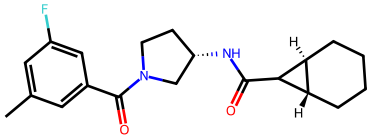	Cc1cc(F)cc(C(=O)N2CC[C@H](NC(=O)C3[C@H]4CCCC[C@H]34)C2)c1	937794555	25	344.19	2.9

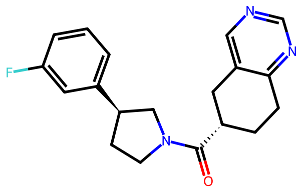	O=C([C@H]1CCc2ncncc2C1)N1CC[C@@H](c2cccc(F)c2)C1	648277073	24	325.16	2.74
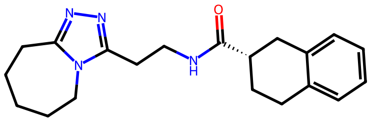	O=C(NCCc1nnc2n1CCCCC2)[C@H]1CCc2ccccc2C1	580204261	25	338.21	2.47
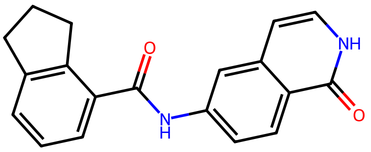	O=C(Nc1ccc2c(=O)[nH]ccc2c1)c1cccc2c1CCC2	1628908089	23	304.12	3.27
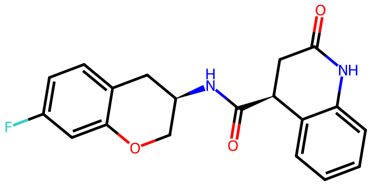	O=C1C[C@H](C(=O)N[C@H]2COc3cc(F)ccc3C2)c2ccccc2N1	1361448746	25	340.12	2.37
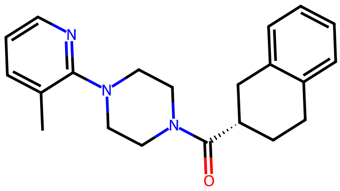	Cc1cccnc1N1CCN(C(=O)[C@H]2CCc3ccccc3C2)CC1	223598586	25	335.2	2.84
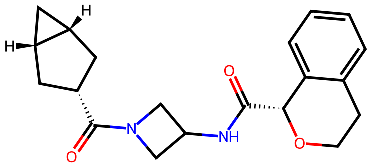	O=C(NC1CN(C(=O)[C@@H]2C[C@@H]3C[C@@H]3C2)C1)[C@H]1OCCc2ccccc21	997321241	25	340.18	1.67
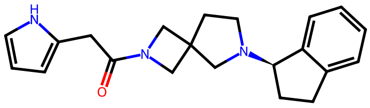	O=C(Cc1ccc[nH]1)N1CC2(CCN([C@@H]3CCc4ccccc43)C2)C1	1073447149	25	335.2	2.78
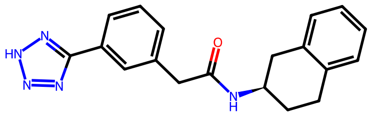	O=C(Cc1cccc(-c2nn[nH]n2)c1)N[C@@H]1CCc2ccccc2C1	823093435	25	333.16	2.08

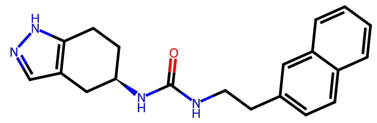	O=C(NCCc1ccc2ccccc2c1)N[C@@H]1CCc2[nH]ncc2C1	177948401	25	334.18	2.96
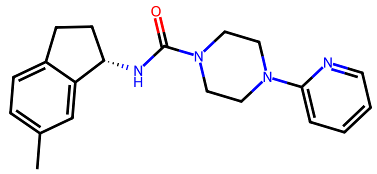	Cc1ccc2c(c1)[C@@H](NC(=O)N1CCN(c3ccccn3)CC1)CC2	592548166	25	336.2	2.91
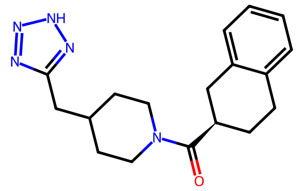	O=C([C@@H]1CCc2ccccc2C1)N1CCC(Cc2nn[nH]n2)CC1	1548805351	24	325.19	1.79
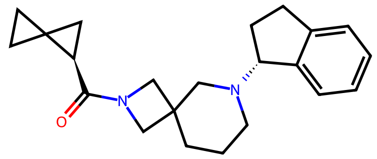	O=C([C@@H]1CC12CC2)N1CC2(CCCN([C@@H]3CCc4ccccc43)C2)C1	982038872	25	336.22	3.4
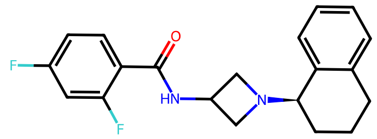	O=C(NC1CN([C@@H]2CCCc3ccccc32)C1)c1ccc(F)cc1F	1031336916	25	342.15	3.46
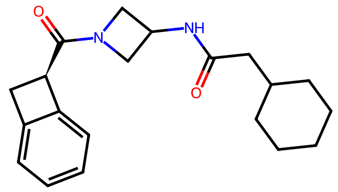	O=C(CC1CCCCC1)NC1CN(C(=O)[C@@H]2Cc3ccccc32)C1	990168976	24	326.2	2.62
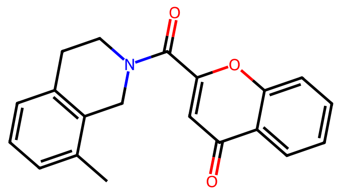	Cc1cccc2c1CN(C(=O)c1cc(=O)c3ccccc3o1)CC2	759829836	24	319.12	3.3
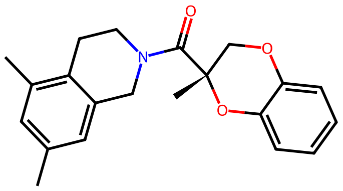	Cc1cc(C)c2c(c1)CN(C(=O)[C@]1(C)COc3ccccc3O1)CC2	1435943008	25	337.17	3.42

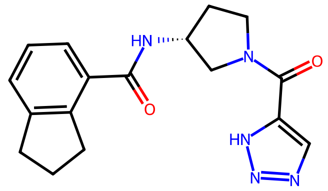	O=C(N[C@@H]1CCN(C(=O)c2cnn[nH]2)C1)c1cccc2c1CCC2	941157223	24	325.15	0.94
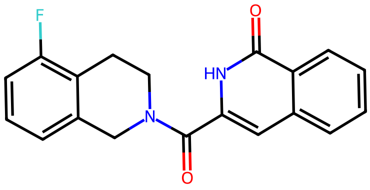	O=C(c1cc2ccccc2c(=O)[nH]1)N1CCc2c(F)cccc2C1	160788882	24	322.11	2.87
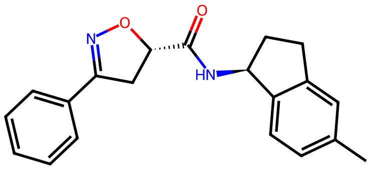	Cc1ccc2c(c1)CC[C@@H]2NC(=O)[C@@H]1CC(c2ccccc2)=NO1	56317061	24	320.15	3.29
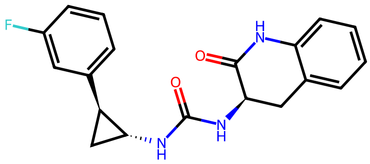	O=C(N[C@@H]1Cc2ccccc2NC1=O)N[C@@H]1C[C@H]1c1cccc(F)c1	636661396	25	339.14	2.54
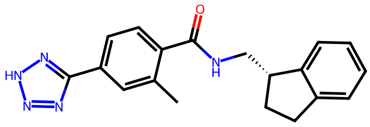	Cc1cc(-c2nn[nH]n2)ccc1C(=O)NC[C@H]1CCc2ccccc21	826248287	25	333.16	2.63
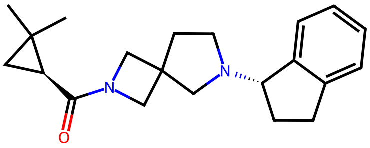	CC1(C)C[C@@H]1C(=O)N1CC2(CCN([C@H]3CCc4ccccc43)C2)C1	1072637818	24	324.22	3.25
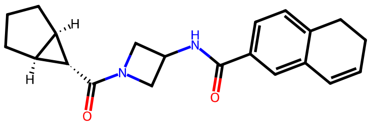	O=C(NC1CN(C(=O)[C@@H]2[C@H]3CCC[C@H]32)C1)c1ccc2c(c1)C=CCC2	995372070	25	336.18	2.63
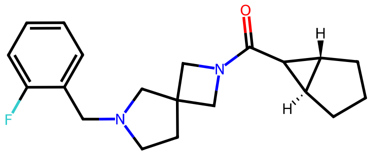	O=C(C1[C@@H]2CCC[C@@H]12)N1CC2(CCN(Cc3ccccc3F)C2)C1	1072590899	24	328.2	2.91

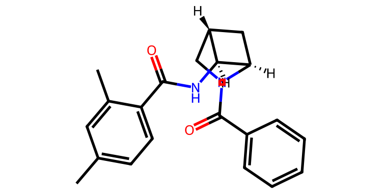	Cc1ccc(C(=O)N[C@H]2[C@H]3C[C@H]2N(C(=O)c2ccccc2)C3)c(C)c1	948057886	25	334.17	2.95
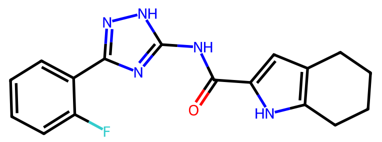	O=C(Nc1nc(-c2ccccc2F)n[nH]1)c1cc2c([nH]1)CCCC2	1648490772	24	325.13	3.07
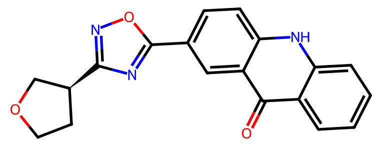	O=c1c2ccccc2[nH]c2ccc(-c3nc([C@H]4CCOC4)no3)cc12	435993599	25	333.11	3.24
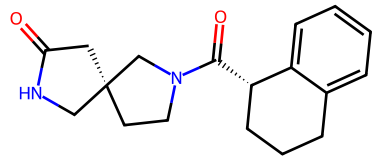	O=C1C[C@]2(CCN(C(=O)[C@H]3CCCc4ccccc43)C2)CN1	279456497	22	298.17	1.85
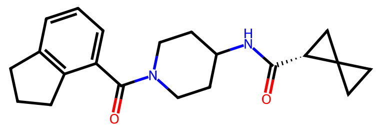	O=C(NC1CCN(C(=O)c2cccc3c2CCC3)CC1)[C@@H]1CC12CC2	947164291	25	338.2	2.7
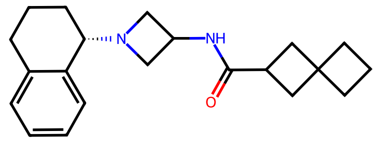	O=C(NC1CN([C@H]2CCCc3ccccc32)C1)C1CC2(CCC2)C1	1030814621	24	324.22	3.44
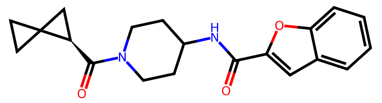	O=C(NC1CCN(C(=O)[C@@H]2CC23CC3)CC1)c1cc2ccccc2o1	948558952	25	338.16	2.95
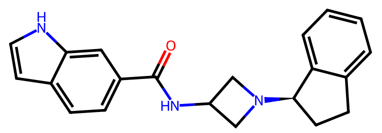	O=C(NC1CN([C@@H]2CCc3ccccc32)C1)c1ccc2cc[nH]c2c1	1031013640	25	331.17	3.27

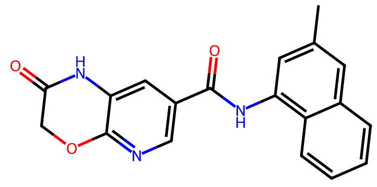	Cc1cc(NC(=O)c2cnc3c(c2)NC(=O)CO3)c2ccccc2c1	1569275328	25	333.11	3.13
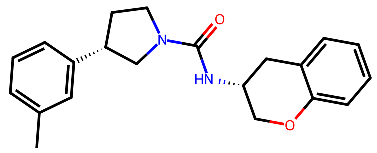	Cc1cccc([C@@H]2CCN(C(=O)N[C@H]3COc4ccccc4C3)C2)c1	542426546	25	336.18	3.5
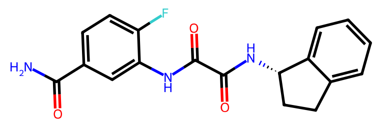	NC(=O)c1ccc(F)c(NC(=O)C(=O)N[C@H]2CCc3ccccc32)c1	80159894	25	341.12	1.67
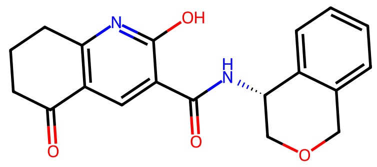	O=C(N[C@H]1COCc2ccccc21)c1cc2c(nc1O)CCCC2=O	1413133160	25	338.13	2.31
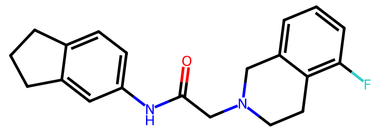	O=C(CN1CCc2c(F)cccc2C1)Nc1ccc2c(c1)CCC2	339783953	24	324.16	3.31
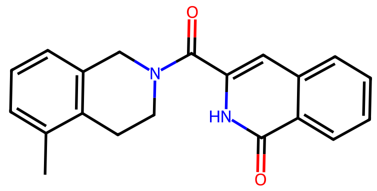	Cc1cccc2c1CCN(C(=O)c1cc3ccccc3c(=O)[nH]1)C2	171539533	24	318.14	3.04
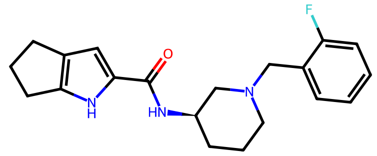	O=C(N[C@@H]1CCCN(Cc2ccccc2F)C1)c1cc2c([nH]1)CCC2	1009164620	25	341.19	3.04
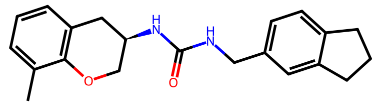	Cc1cccc2c1OC[C@H](NC(=O)NCc1ccc3c(c1)CCC3)C2	1467860265	25	336.18	3.29

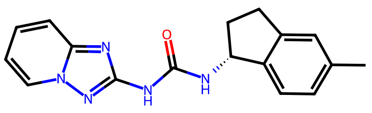	Cc1ccc2c(c1)CC[C@H]2NC(=O)Nc1nc2ccccn2n1	353914958	23	307.14	2.85
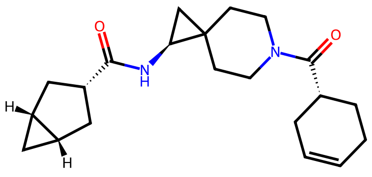	O=C(N[C@H]1CC12CCN(C(=O)[C@H]1CC=CCC1)CC2)[C@@H]1C[C@@H]2C[C@@H]2C1	964770630	25	342.23	2.89
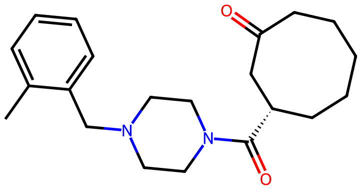	Cc1ccccc1CN1CCN(C(=O)[C@H]2CCCCCC(=O)C2)CC1	362470415	25	342.23	3.18
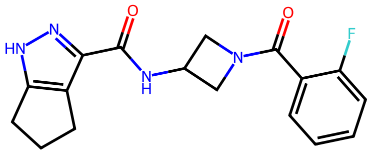	O=C(NC1CN(C(=O)c2ccccc2F)C1)c1n[nH]c2c1CCC2	995163594	24	328.13	1.29
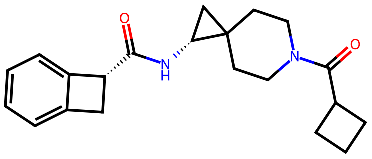	O=C(N[C@@H]1CC12CCN(C(=O)C1CCC1)CC2)[C@@H]1Cc2ccccc21	965003311	25	338.2	2.62
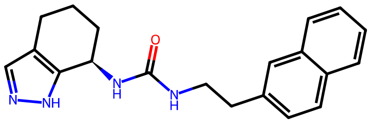	O=C(NCCc1ccc2ccccc2c1)N[C@@H]1CCCc2cn[nH]c21	268880483	25	334.18	3.48
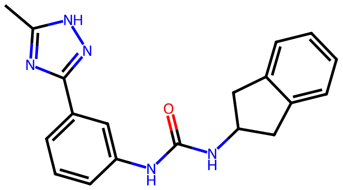	Cc1nc(-c2cccc(NC(=O)NC3Cc4ccccc4C3)c2)n[nH]1	184203010	25	333.16	3.07
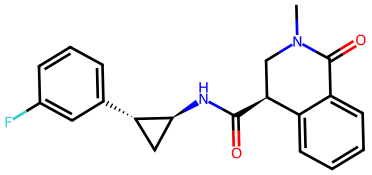	CN1C[C@H](C(=O)N[C@H]2C[C@@H]2c2cccc(F)c2)c2ccccc2C1=O	345478093	25	338.14	2.67

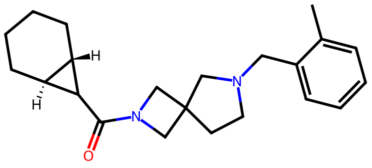	Cc1ccccc1CN1CCC2(C1)CN(C(=O)C1[C@H]3CCCC[C@H]13)C2	1072580465	25	338.24	3.47
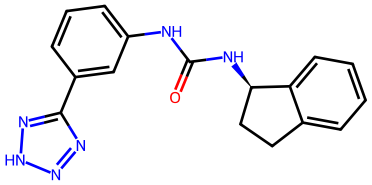	O=C(Nc1cccc(-c2nn[nH]n2)c1)N[C@@H]1CCc2ccccc21	1606932449	24	320.14	2.68
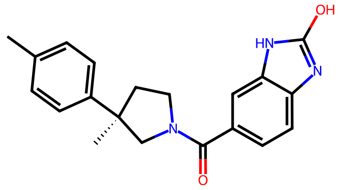	Cc1ccc([C@@]2(C)CCN(C(=O)c3ccc4nc(O)[nH]c4c3)C2)cc1	364388367	25	335.16	3.38
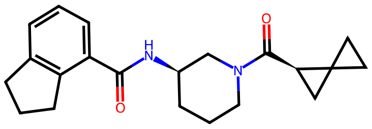	O=C(N[C@@H]1CCCN(C(=O)[C@@H]2CC23CC3)C1)c1cccc2c1CCC2	952141830	25	338.2	2.7
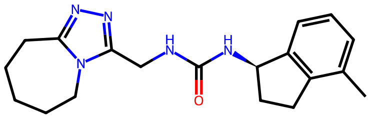	Cc1cccc2c1CC[C@H]2NC(=O)NCc1nnc2n1CCCCC2	519171263	25	339.21	2.8
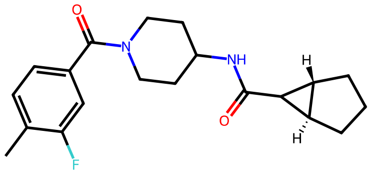	Cc1ccc(C(=O)N2CCC(NC(=O)C3[C@@H]4CCC[C@@H]34)CC2)cc1F	947403366	25	344.19	2.9
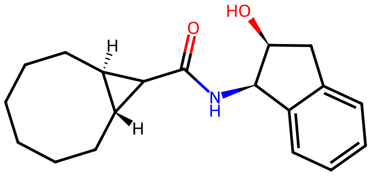	O=C(N[C@@H]1c2ccccc2C[C@@H]1O)C1[C@@H]2CCCCCC[C@@H]12	612657932	22	299.19	2.98
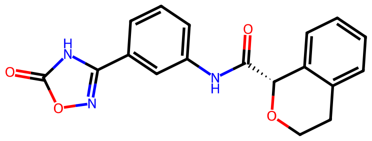	O=C(Nc1cccc(-c2noc(=O)[nH]2)c1)[C@H]1OCCc2ccccc21	119260506	25	337.11	2.28

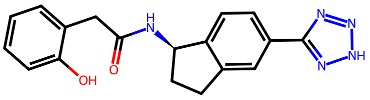	O=C(Cc1ccccc1O)N[C@@H]1CCc2cc(-c3nn[nH]n3)ccc21	1609536633	25	335.14	1.92
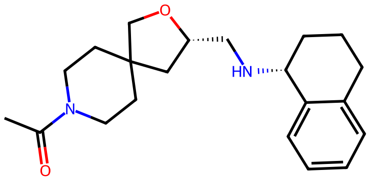	CC(=O)N1CCC2(CC1)CO[C@H](CN[C@@H]1CCCc3ccccc31)C2	982866013	25	342.23	3.07
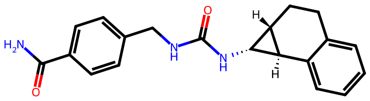	NC(=O)c1ccc(CNC(=O)N[C@H]2[C@@H]3c4ccccc4CC[C@H]32)cc1	126356634	25	335.16	2.31
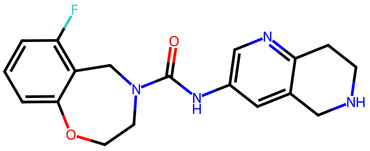	O=C(Nc1cnc2c(c1)CNCC2)N1CCOc2cccc(F)c2C1	667802620	25	342.15	2.29
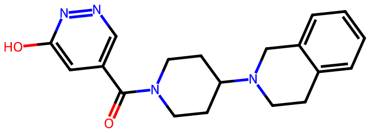	O=C(c1cnnc(O)c1)N1CCC(N2CCc3ccccc3C2)CC1	667385614	25	338.17	1.85
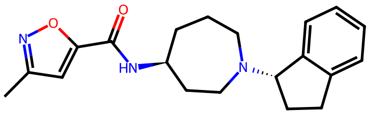	Cc1cc(C(=O)N[C@H]2CCCN([C@H]3CCc4ccccc43)CC2)on1	1052198730	25	339.19	3.25
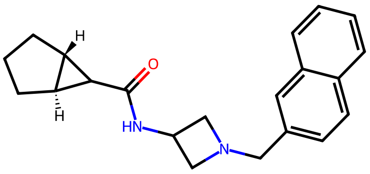	O=C(NC1CN(Cc2ccc3ccccc3c2)C1)C1[C@H]2CCC[C@H]12	1030192601	24	320.19	3.19
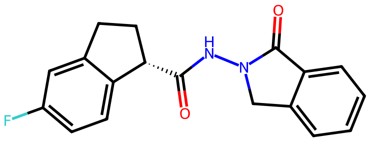	O=C(NN1Cc2ccccc2C1=O)[C@H]1CCc2cc(F)ccc21	1412818516	23	310.11	2.54

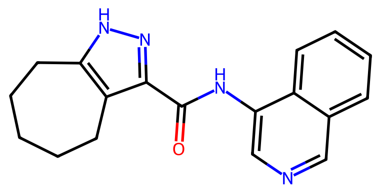	O=C(Nc1cncc2ccccc12)c1n[nH]c2c1CCCCC2	530401265	23	306.15	3.48
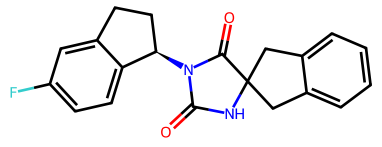	O=C1NC2(Cc3ccccc3C2)C(=O)N1[C@@H]1CCc2cc(F)ccc21	530320837	25	336.13	2.9
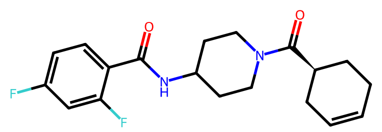	O=C(NC1CCN(C(=O)[C@@H]2CC=CCC2)CC1)c1ccc(F)cc1F	944105587	25	348.16	3.04
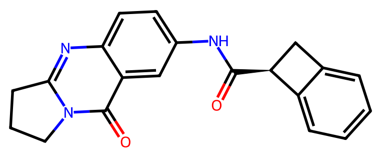	O=C(Nc1ccc2nc3n(c(=O)c2c1)CCC3)[C@H]1Cc2ccccc21	607790916	25	331.13	2.62
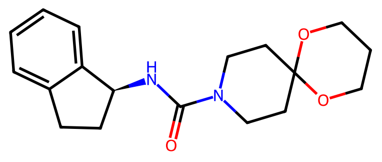	O=C(N[C@H]1CCc2ccccc21)N1CCC2(CC1)OCCCO2	297589732	23	316.18	2.61
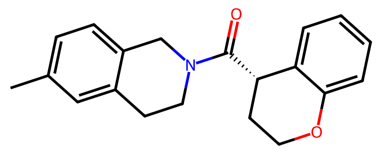	Cc1ccc2c(c1)CCN(C(=O)[C@H]1CCOc3ccccc31)C2	360227459	23	307.16	3.45
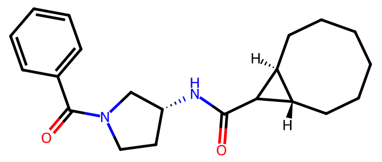	O=C(N[C@@H]1CCN(C(=O)c2ccccc2)C1)C1[C@H]2CCCCCC[C@H]12	942504707	25	340.22	3.23
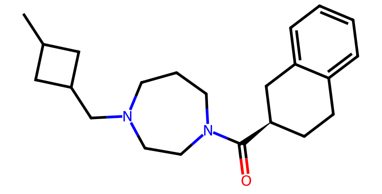	CC1CC(CN2CCCN(C(=O)[C@@H]3CCc4ccccc4C3)CC2)C1	982033644	25	340.25	3.37

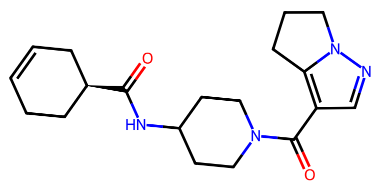	O=C(NC1CCN(C(=O)c2cnn3c2CCC3)CC1)[C@@H]1CC=CCC1	947115007	25	342.21	1.91
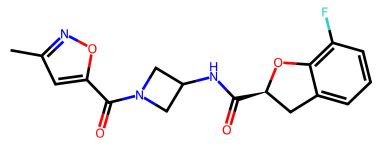	Cc1cc(C(=O)N2CC(NC(=O)[C@@H]3Cc4cccc(F)c4O3)C2)on1	992514039	25	345.11	1.07
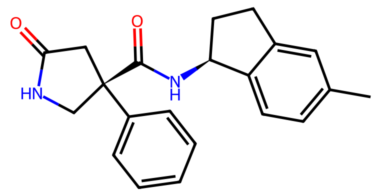	Cc1ccc2c(c1)CC[C@@H]2NC(=O)[C@@]1(c2ccccc2)CNC(=O)C1	567246679	25	334.17	2.56
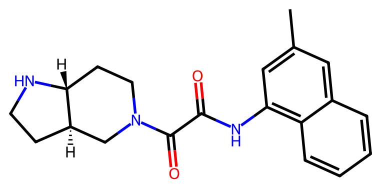	Cc1cc(NC(=O)C(=O)N2CC[C@H]3NCC[C@@H]3C2)c2ccccc2c1	667627794	25	337.18	2.3
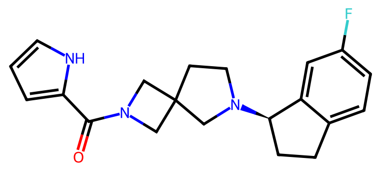	O=C(c1ccc[nH]1)N1CC2(CCN([C@@H]3CCc4cc(F)cc43)C2)C1	1072466044	25	339.17	2.99
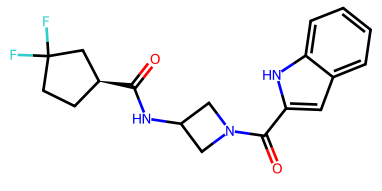	O=C(NC1CN(C(=O)c2cc3ccccc3[nH]2)C1)[C@H]1CCC(F)(F)C1	994164879	25	347.14	2.54
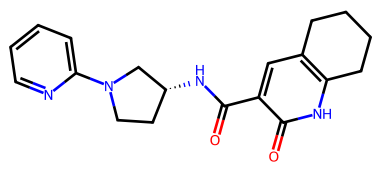	O=C(N[C@@H]1CCN(c2ccccn2)C1)c1cc2c([nH]c1=O)CCCC2	152499507	25	338.17	1.66
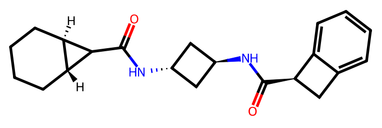	O=C(N[C@H]1C[C@H](NC(=O)[C@@H]2Cc3ccccc32)C1)C1[C@@H]2CCCC[C@@H]12	973135558	25	338.2	2.53

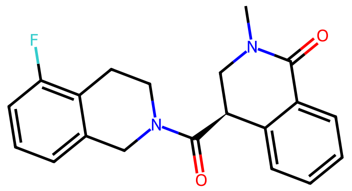	CN1C[C@H](C(=O)N2CCc3c(F)cccc3C2)c2ccccc2C1=O	346382391	25	338.14	2.58
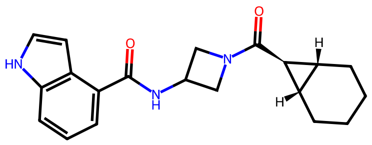	O=C(NC1CN(C(=O)[C@@H]2[C@H]3CCCC[C@H]32)C1)c1cccc2[nH]ccc12	1000403527	25	337.18	2.54
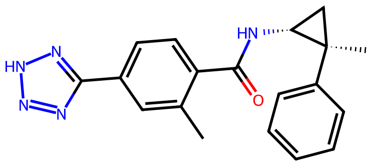	Cc1cc(-c2nn[nH]n2)ccc1C(=O)N[C@@H]1C[C@@]1(C)c1ccccc1	826246933	25	333.16	2.64
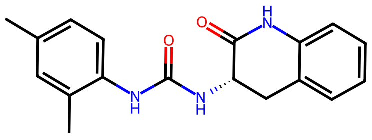	Cc1ccc(NC(=O)N[C@H]2Cc3ccccc3NC2=O)c(C)c1	1202751078	23	309.15	2.99
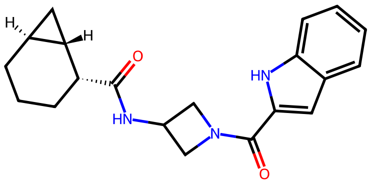	O=C(NC1CN(C(=O)c2cc3ccccc3[nH]2)C1)[C@@H]1CCC[C@H]2C[C@@H]21	994727572	25	337.18	2.54
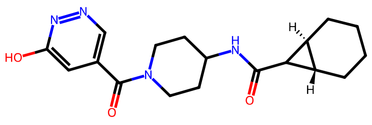	O=C(NC1CCN(C(=O)c2cnnc(O)c2)CC1)C1[C@H]2CCCC[C@H]12	947096280	25	344.18	1.34
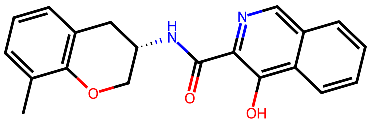	Cc1cccc2c1OC[C@@H](NC(=O)c1ncc3ccccc3c1O)C2	1141747559	25	334.13	2.98
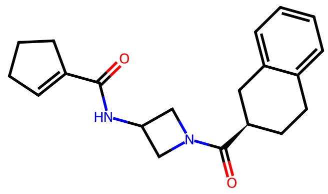	O=C(NC1CN(C(=O)[C@@H]2CCc3ccccc3C2)C1)C1=CCCC1	990798133	24	324.18	2.23

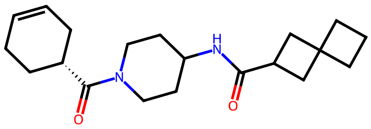	O=C(NC1CCN(C(=O)[C@H]2CC=CC C2)CC1)C1CC2(CCC2)C1	944127168	24	330.23	3.03
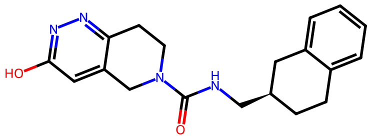	O=C(NC[C@@H]1CCc2ccccc2C1)N1 CCc2nnc(O)cc2C1	681519416	25	338.17	2.05
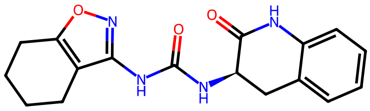	O=C(Nc1noc2c1CCCC2)N[C@@H]1C c2ccccc2NC1=O	934347991	24	326.14	2.24
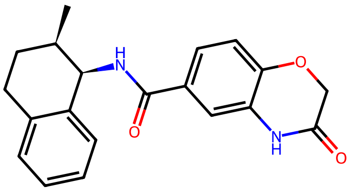	C[C@@H]1CCc2ccccc2[C@@H]1NC(=O)c1ccc2c(c1)NC(=O)CO2	122492106	25	336.15	3.07
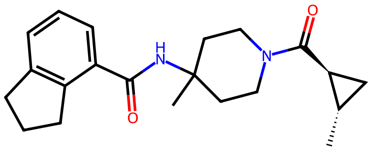	C[C@H]1C[C@@H]1C(=O)N1CCC(C) (NC(=O)c2cccc3c2CCC3)CC1	956400307	25	340.22	2.94
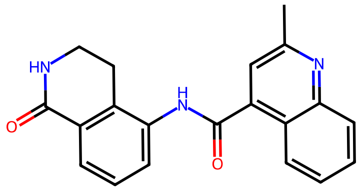	Cc1cc(C(=O)Nc2cccc3c2CCNC3=O)c 2ccccc2n1	1129703200	25	331.13	3.08
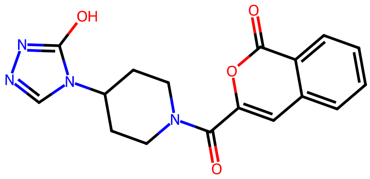	O=C(c1cc2ccccc2c(=O)o1)N1CCC(n2 cnnc2O)CC1	646045239	25	340.12	1.57
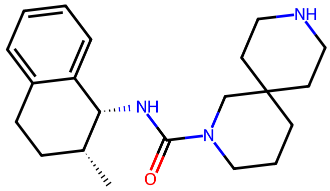	C[C@@H]1CCc2ccccc2[C@@H]1NC(=O)N1CCCC2(CCNCC2)C1	1325365407	25	341.25	3.49

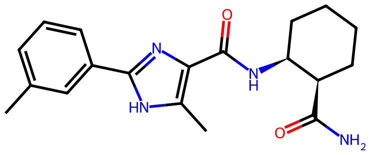	Cc1cccc(-c2nc(C(=O)N[C@H]3CCCC[C@H]3C(N)=O)c(C)[nH]2)c1	270385601	25	340.19	2.47
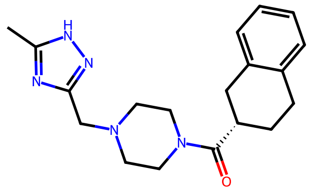	Cc1nc(CN2CCN(C(=O)[C@H]3CCc4ccccc4C3)CC2)n[nH]1	573495316	25	339.21	1.56
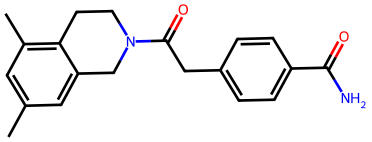	Cc1cc(C)c2c(c1)CN(C(=O)Cc1ccc(C(N)=O)cc1)CC2	276939523	24	322.17	2.53
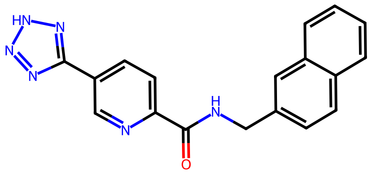	O=C(NCc1ccc2ccccc2c1)c1ccc(-c2nn[nH]n2)cn1	738113607	25	330.12	2.34
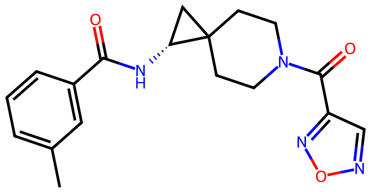	Cc1cccc(C(=O)N[C@@H]2CC23CCN(C(=O)c2cnon2)CC3)c1	962879156	25	340.15	1.8
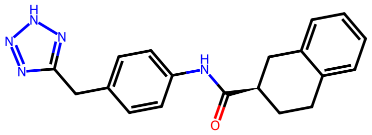	O=C(Nc1ccc(Cc2nn[nH]n2)cc1)[C@@H]1CCc2ccccc2C1	568025071	25	333.16	2.53
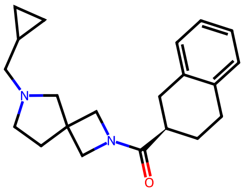	O=C([C@@H]1CCc2ccccc2C1)N1CC2(CCN(CC3CC3)C2)C1	1073014872	24	324.22	2.74
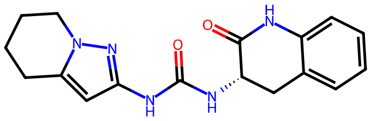	O=C(Nc1cc2n(n1)CCCC2)N[C@H]1Cc2ccccc2NC1=O	529098891	24	325.15	1.9

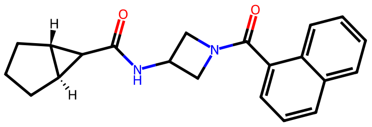	O=C(NC1CN(C(=O)c2cccc3ccccc23)C1)C1[C@H]2CCC[C@H]12	990710648	25	334.17	2.83
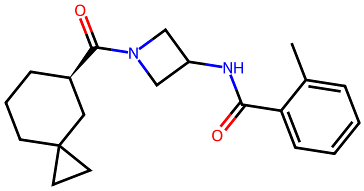	Cc1ccccc1C(=O)NC1CN(C(=O)[C@@H]2CCCC3(CC3)C2)C1	990111009	24	326.2	2.91
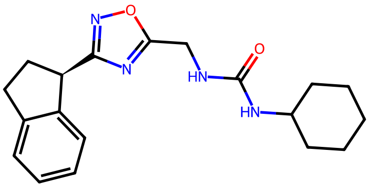	O=C(NCc1nc([C@@H]2CCc3ccccc32)no1)NC1CCCCC1	680747913	25	340.19	3.28
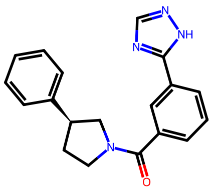	O=C(c1cccc(-c2ncn[nH]2)c1)N1CC[C@@H](c2ccccc2)C1	119729210	24	318.15	3.1
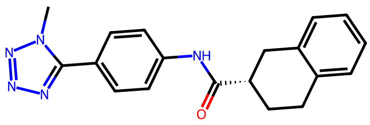	Cn1nnnc1-c1ccc(NC(=O)[C@H]2CCc3ccccc3C2)cc1	1131225312	25	333.16	2.62
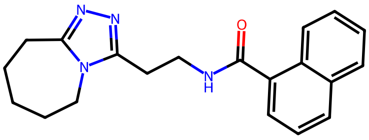	O=C(NCCc1nnc2n1CCCCC2)c1cccc2ccccc12	48303657	25	334.18	3.13
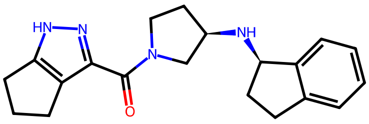	O=C(c1n[nH]c2c1CCC2)N1CC[C@@H](N[C@@H]2CCc3ccccc32)C1	969180678	25	336.2	2.39
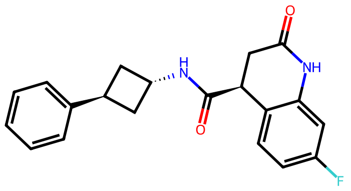	O=C1C[C@H](C(=O)N[C@H]2C[C@H](c3ccccc3)C2)c2ccc(F)cc2N1	280724921	25	338.14	3.31

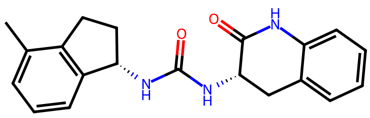	Cc1cccc2c1CC[C@@H]2NC(=O)N[C@H]1Cc2ccccc2NC1=O	636735162	25	335.16	2.84
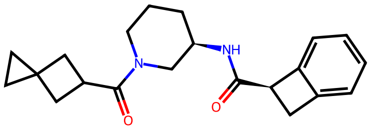	O=C(N[C@@H]1CCCN(C(=O)C2CC3(CC3)C2)C1)[C@@H]1Cc2ccccc21	951964981	25	338.2	2.62
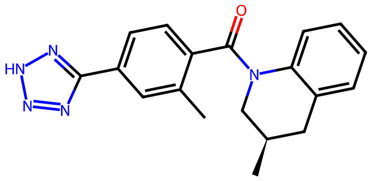	Cc1cc(-c2nn[nH]n2)ccc1C(=O)N1C[C@H](C)Cc2ccccc21	826244645	25	333.16	3.01
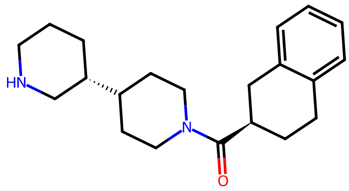	O=C([C@@H]1CCc2ccccc2C1)N1CCC([C@H]2CCCNC2)CC1	658960445	24	326.24	3.03
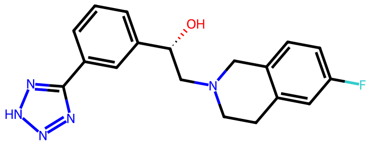	O[C@H](CN1CCc2cc(F)ccc2C1)c1cccc(-c2nn[nH]n2)c1	1582809683	25	339.15	2.1
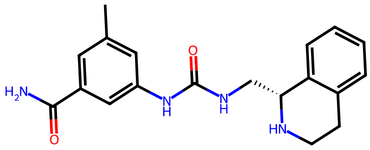	Cc1cc(NC(=O)NC[C@H]2NCCc3ccccc32)cc(C(N)=O)c1	415790309	25	338.17	2.1
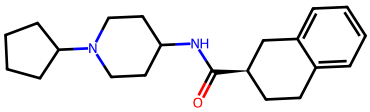	O=C(NC1CCN(C2CCCC2)CC1)[C@@H]1CCc2ccccc2C1	651445316	24	326.24	3.31
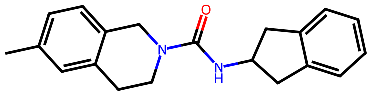	Cc1ccc2c(c1)CCN(C(=O)NC1Cc3ccccc3C1)C2	479783631	23	306.17	3.23

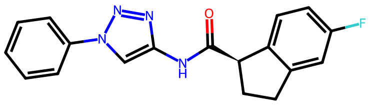	O=C(Nc1cn(-c2ccccc2)nn1)[C@@H]1CCc2cc(F)ccc21	1540217150	24	322.12	3.07
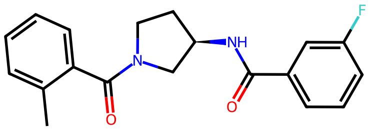	Cc1ccccc1C(=O)N1CC[C@@H](NC(=O)c2cccc(F)c2)C1	939202031	24	326.14	2.78
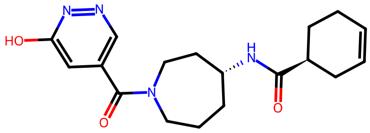	O=C(N[C@@H]1CCCN(C(=O)c2cnnc(O)c2)CC1)[C@@H]1CC=CCC1	964749781	25	344.18	1.65
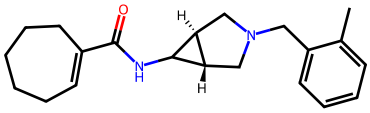	Cc1ccccc1CN1C[C@@H]2C(NC(=O)C3=CCCCCC3)[C@H]2C1	958655634	24	324.22	3.43
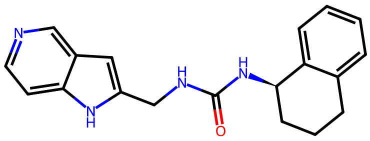	O=C(NCc1cc2cnccc2[nH]1)N[C@@H]1CCCc2ccccc21	1875326145	24	320.16	3.44
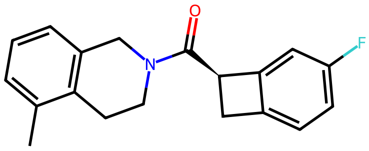	Cc1cccc2c1CCN(C(=O)[C@@H]1Cc3ccc(F)cc31)C2	793444942	22	295.14	3.36
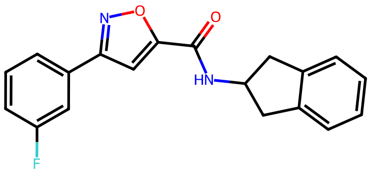	O=C(NC1Cc2ccccc2C1)c1cc(-c2cccc(F)c2)no1	545830500	24	322.11	3.38
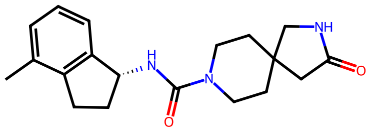	Cc1cccc2c1CC[C@H]2NC(=O)N1CCC2(CC1)CNC(=O)C2	74108014	24	327.19	2.29

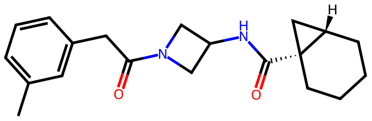	Cc1cccc(CC(=O)N2CC(NC(=O)[C@@]34CCCC[C@H]3C4)C2)c1	999285587	24	326.2	2.44
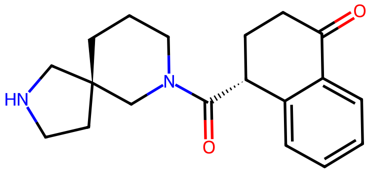	O=C1CC[C@@H](C(=O)N2CCC[C@]3(CCNC3)C2)c2ccccc21	423636572	23	312.18	2.35
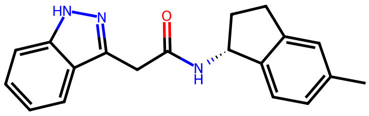	Cc1ccc2c(c1)CC[C@H]2NC(=O)Cc1n[nH]c2ccccc12	50610422	23	305.15	3.22
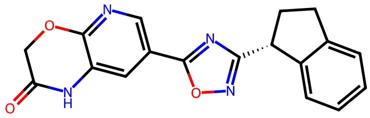	O=C1COc2ncc(-c3nc([C@@H]4CCc5ccccc54)no3)cc2N1	356608041	25	334.11	2.54
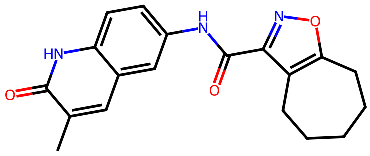	Cc1cc2cc(NC(=O)c3noc4c3CCCCC4)ccc2[nH]c1=O	908458098	25	337.14	3.35
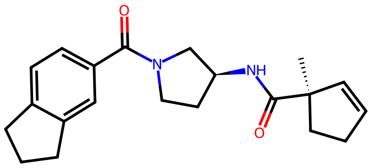	C[C@]1(C(=O)N[C@H]2CCN(C(=O)c3ccc4c(c3)CCC4)C2)C=CCC1	938461255	25	338.2	2.86
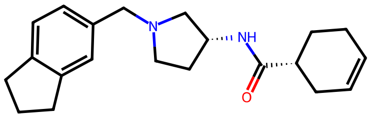	O=C(N[C@@H]1CCN(Cc2ccc3c(c2)CCC3)C1)[C@H]1CC=CCC1	1010080041	24	324.22	3.22
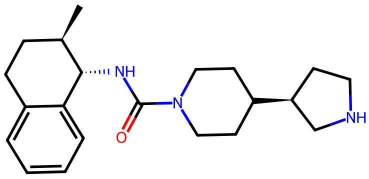	C[C@@H]1CCc2ccccc2[C@H]1NC(=O)N1CCC([C@H]2CCNC2)CC1	417962165	25	341.25	3.34

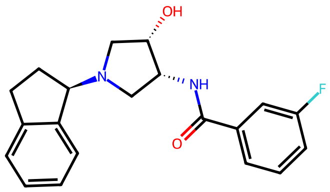	O=C(N[C@@H]1CN([C@@H]2CCc3ccccc32)C[C@@H]1O)c1cccc(F)c1	1083318502	25	340.16	2.29
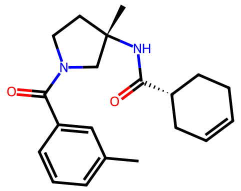	Cc1cccc(C(=O)N2CC[C@](C)(NC(=O)[C@H]3CC=CCC3)C2)c1	956700418	24	326.2	3.07
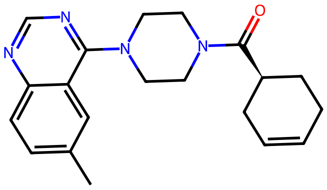	Cc1ccc2ncnc(N3CCN(C(=O)[C@@H]4CC=CCC4)CC3)c2c1	1055721913	25	336.2	2.94
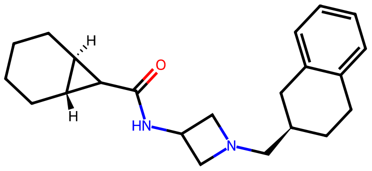	O=C(NC1CN(C[C@@H]2CCc3ccccc3C2)C1)C1[C@@H]2CCCC[C@@H]12	1030182368	25	338.24	3.03
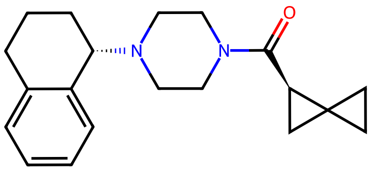	O=C([C@@H]1CC12CC2)N1CCN([C@H]2CCCc3ccccc32)CC1	950710788	23	310.2	3.01
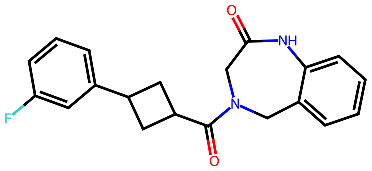	O=C1CN(C(=O)C2CC(c3cccc(F)c3)C2)Cc2ccccc2N1	584122779	25	338.14	3.3
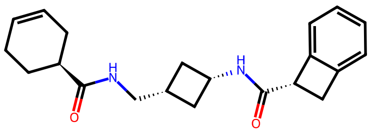	O=C(NC[C@H]1C[C@@H](NC(=O)[C@@H]2Cc3ccccc32)C1)[C@@H]1CC=CCC1	978582713	25	338.2	2.69
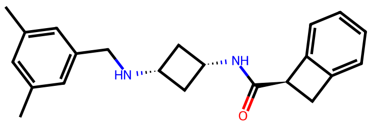	Cc1cc(C)cc(CN[C@H]2C[C@@H](NC(=O)[C@@H]3Cc4ccccc43)C2)c1	1022625721	25	334.2	3.38

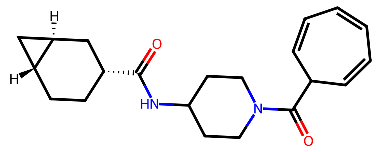	O=C(NC1CCN(C(=O)C2C=CC=CC=C2)CC1)[C@@H]1CC[C@@H]2C[C@H]2C1	946950341	25	340.22	2.83
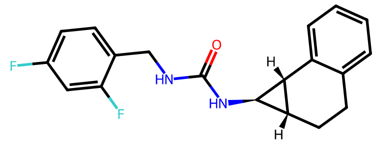	O=C(NCc1ccc(F)cc1F)N[C@@H]1[C@H]2CCc3ccccc3[C@H]21	435771854	24	328.14	3.49
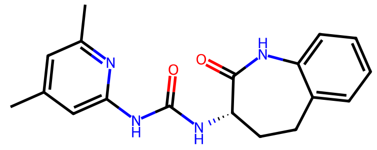	Cc1cc(C)nc(NC(=O)N[C@H]2CCc3ccccc3NC2=O)c1	654039082	24	324.16	2.77
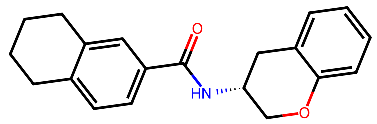	O=C(N[C@H]1COc2ccccc2C1)c1ccc2c(c1)CCCC2	613757889	23	307.16	3.3
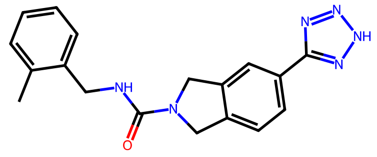	Cc1ccccc1CNC(=O)N1Cc2ccc(-c3nn[nH]n3)cc2C1	1581259877	25	334.15	2.4
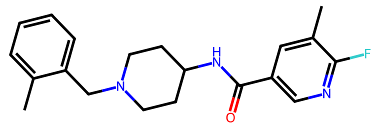	Cc1ccccc1CN1CCC(NC(=O)c2cnc(F)c(C)c2)CC1	857709279	25	341.19	3.23
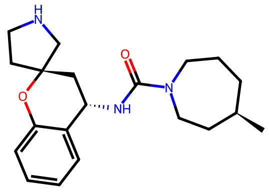	C[C@@H]1CCCN(C(=O)N[C@H]2C[C@]3(CCNC3)Oc3ccccc32)CC1	1337005307	25	343.23	3.07
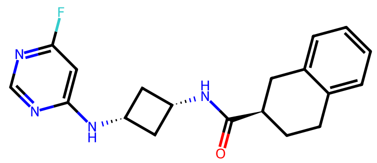	O=C(N[C@H]1C[C@@H](Nc2cc(F)ncn2)C1)[C@@H]1CCc2ccccc2C1	1059840105	25	340.17	2.48

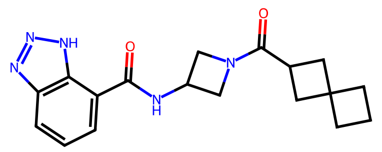	O=C(NC1CN(C(=O)C2CC3(CCC3)C2)C1)c1cccc2nn[nH]c12	991901257	25	339.17	1.48
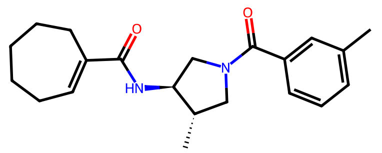	Cc1cccc(C(=O)N2C[C@H](C)[C@@H](NC(=O)C3=CCCCCC3)C2)c1	966109440	25	340.22	3.46
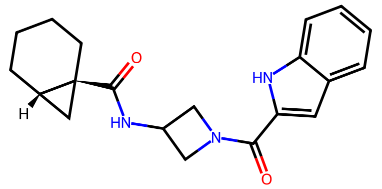	O=C(c1cc2ccccc2[nH]1)N1CC(NC(=O)[C@]23CCCC[C@H]2C3)C1	999281469	25	337.18	2.69
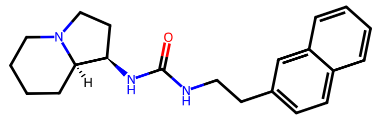	O=C(NCCc1ccc2ccccc2c1)N[C@@H]1CCN2CCCC[C@H]12	47066192	25	337.22	3.31
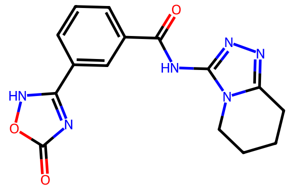	O=C(Nc1nnc2n1CCCC2)c1cccc(-c2nc(=O)o[nH]2)c1	831885913	24	326.11	1.21
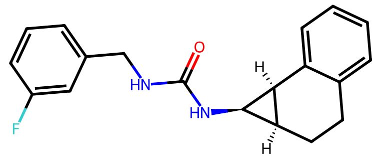	O=C(NCc1cccc(F)c1)N[C@@H]1[C@@H]2CCc3ccccc3[C@@H]21	126057640	23	310.15	3.35
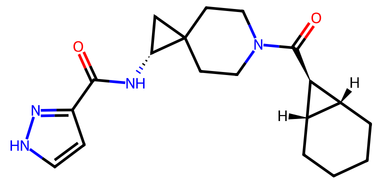	O=C(N[C@@H]1CC12CCN(C(=O)[C@@H]1[C@H]3CCCC[C@H]31)CC2)c1cc[nH]n1	963249368	25	342.21	1.96
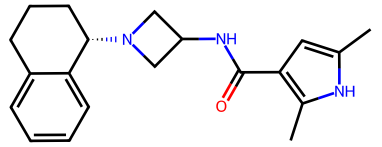	Cc1cc(C(=O)NC2CN([C@H]3CCCc4ccccc43)C2)c(C)[nH]1	1031019027	24	323.2	3.12

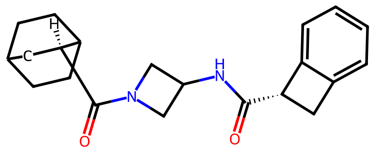	O=C(NC1CN(C(=O)[C@H]2CC3CCC2CC3)C1)[C@H]1Cc2ccccc21	998687505	25	338.2	2.48
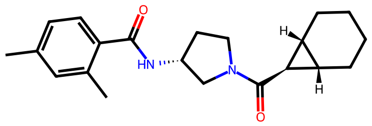	Cc1ccc(C(=O)N[C@@H]2CCN(C(=O)[C@@H]3[C@H]4CCCC[C@H]43)C2)c(C)c1	942396924	25	340.22	3.07
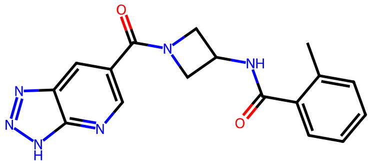	Cc1ccccc1C(=O)NC1CN(C(=O)c2cnc3[nH]nnc3c2)C1	990107363	25	336.13	0.92
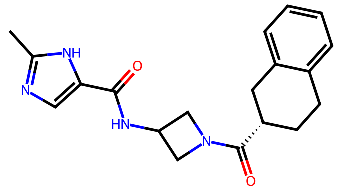	Cc1ncc(C(=O)NC2CN(C(=O)[C@H]3CCc4ccccc4C3)C2)[nH]1	990503000	25	338.17	1.46
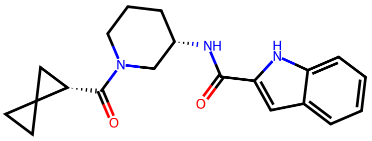	O=C(N[C@H]1CCCN(C(=O)[C@H]2CC23CC3)C1)c1cc2ccccc2[nH]1	952144131	25	337.18	2.69
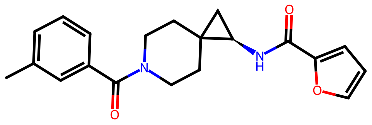	Cc1cccc(C(=O)N2CCC3(CC2)C[C@H]3NC(=O)c2ccco2)c1	962734086	25	338.16	3.01
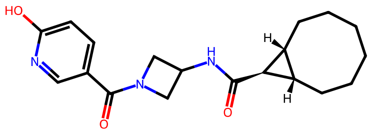	O=C(NC1CN(C(=O)c2ccc(O)nc2)C1)[C@@H]1[C@H]2CCCCCC[C@H]21	1000510905	25	343.19	1.94
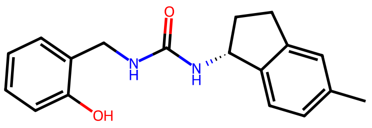	Cc1ccc2c(c1)CC[C@H]2NC(=O)NCc1ccccc1O	263744979	22	296.15	3.19

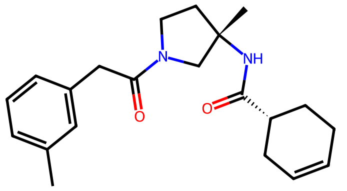	Cc1cccc(CC(=O)N2CC[C@](C)(NC(=O)[C@H]3CC=CCC3)C2)c1	956691331	25	340.22	3.0
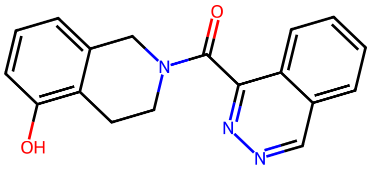	O=C(c1nncc2ccccc12)N1CCc2c(O)cccc2C1	1635081444	23	305.12	2.53
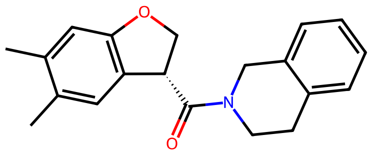	Cc1cc2c(cc1C)[C@@H](C(=O)N1CCc3ccccc3C1)CO2	184537880	23	307.16	3.36
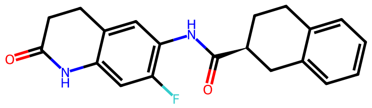	O=C1CCc2cc(NC(=O)[C@H]3CCc4ccccc4C3)c(F)cc2N1	514791628	25	338.14	3.45
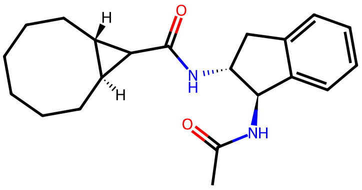	CC(=O)N[C@@H]1c2ccccc2C[C@H]1NC(=O)C1[C@H]2CCCCCC[C@H]12	1001967776	25	340.22	3.12
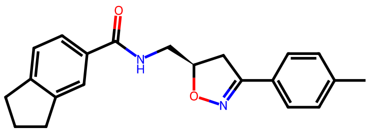	Cc1ccc(C2=NO[C@@H](CNC(=O)c3ccc4c(c3)CCC4)C2)cc1	800676686	25	334.17	3.41
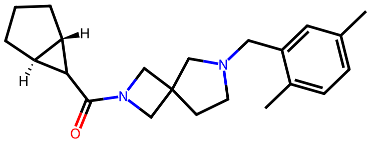	Cc1ccc(C)c(CN2CCC3(C2)CN(C(=O)C2[C@H]4CCC[C@H]24)C3)c1	1072591940	25	338.24	3.38
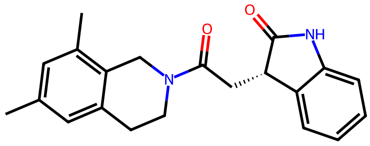	Cc1cc(C)c2c(c1)CCN(C(=O)C[C@@H]1C(=O)Nc3ccccc31)C2	188054322	25	334.17	3.31

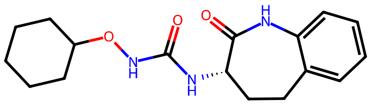	O=C(NOC1CCCCC1)N[C@H]1CCc2ccccc2NC1=O	847358182	23	317.17	2.5
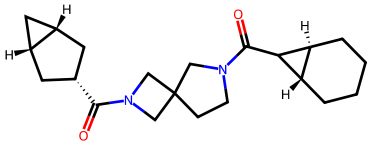	O=C(C1[C@H]2CCCC[C@H]12)N1CCC2(C1)CN(C(=O)[C@@H]1C[C@@H]3C[C@@H]3C1)C2	1019309516	25	342.23	2.53
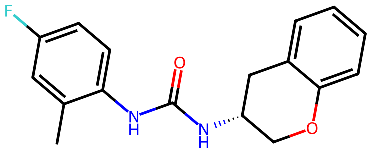	Cc1cc(F)ccc1NC(=O)N[C@H]1COc2ccccc2C1	47722326	22	300.13	3.26
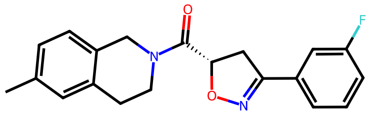	Cc1ccc2c(c1)CCN(C(=O)[C@@H]1CC(c3cccc(F)c3)=NO1)C2	790990767	25	338.14	3.21
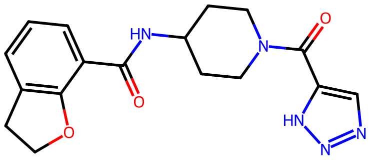	O=C(NC1CCN(C(=O)c2cnn[nH]2)CC1)c1cccc2c1OCC2	947372933	25	341.15	0.77
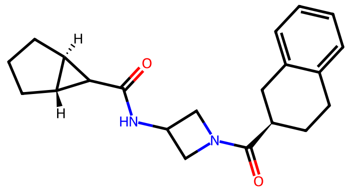	O=C(NC1CN(C(=O)[C@@H]2CCc3ccccc3C2)C1)C1[C@@H]2CCC[C@@H]12	990718588	25	338.2	2.16
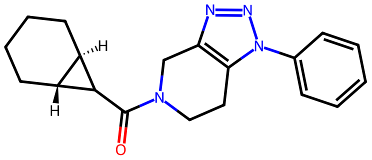	O=C(C1[C@@H]2CCCC[C@@H]12)N1CCc2c(nnn2-c2ccccc2)C1	329403511	24	322.18	2.59
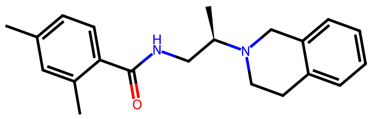	Cc1ccc(C(=O)NC[C@@H](C)N2CCc3ccccc3C2)c(C)c1	25634074	24	322.2	3.48

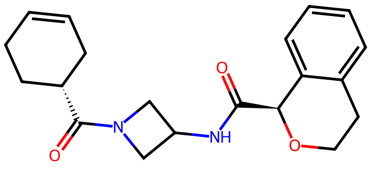	O=C(NC1CN(C(=O)[C@H]2CC=CCC2)C1)[C@@H]1OCCc2ccccc21	997322106	25	340.18	1.98
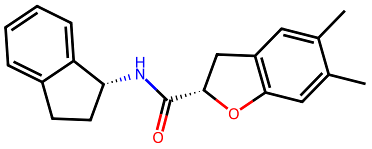	Cc1cc2c(cc1C)O[C@H](C(=O)N[C@@H]1CCc3ccccc31)C2	463350912	23	307.16	3.41
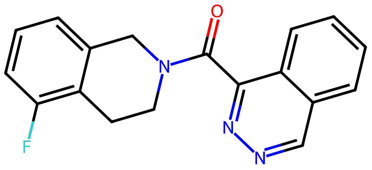	O=C(c1nncc2ccccc12)N1CCc2c(F)cccc2C1	1127292346	23	307.11	2.97
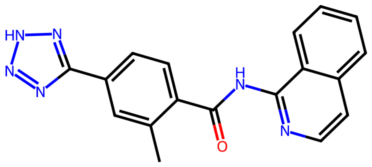	Cc1cc(-c2nn[nH]n2)ccc1C(=O)Nc1nccc2ccccc12	1580621341	25	330.12	2.98
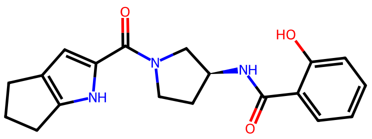	O=C(N[C@H]1CCN(C(=O)c2cc3c([nH]2)CCC3)C1)c1ccccc1O	937695974	25	339.16	1.85
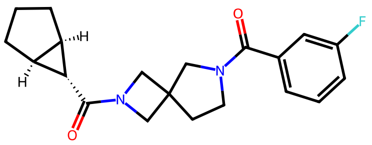	O=C(c1cccc(F)c1)N1CCC2(C1)CN(C(=O)[C@@H]1[C@H]3CCC[C@H]31)C2	1018909662	25	342.17	2.55
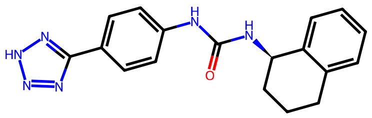	O=C(Nc1ccc(-c2nn[nH]n2)cc1)N[C@@H]1CCCc2ccccc21	1593216627	25	334.15	3.07
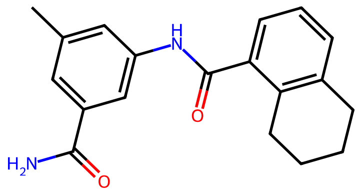	Cc1cc(NC(=O)c2cccc3c2CCCC3)cc(C(N)=O)c1	456166781	23	308.15	3.23

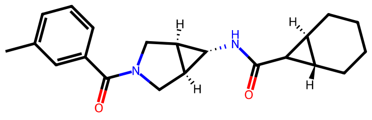	Cc1cccc(C(=O)N2C[C@H]3[C@H](NC(=O)C4[C@H]5CCCC[C@H]45)[C@H]3C2)c1	976306514	25	338.2	2.62
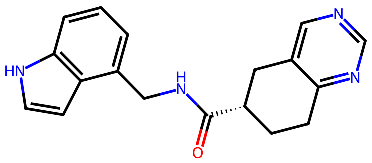	O=C(NCc1cccc2[nH]ccc12)[C@H]1CCc2ncncc2C1	636365907	23	306.15	2.38
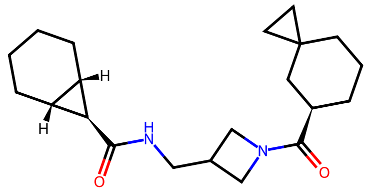	O=C(NCC1CN(C(=O)[C@@H]2CCCC3(CC3)C2)C1)[C@@H]1[C@H]2CCCC[C@H]21	1001323001	25	344.25	2.97
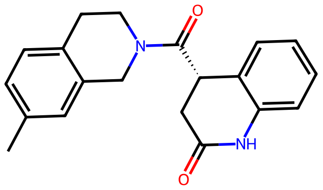	Cc1ccc2c(c1)CN(C(=O)[C@H]1CC(=O)Nc3ccccc31)CC2	1130662837	24	320.15	3.01
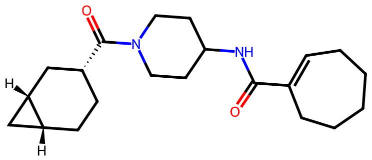	O=C(NC1CCN(C(=O)[C@@H]2CC[C@@H]3C[C@@H]3C2)CC1)C1=CCCCCC1	947222902	25	344.25	3.42
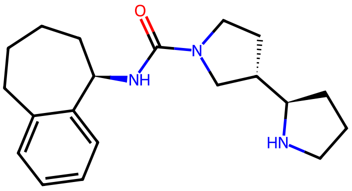	O=C(N[C@@H]1CCCCc2ccccc21)N1CC[C@H]([C@H]2CCCN2)C1	1553443968	24	327.23	3.24
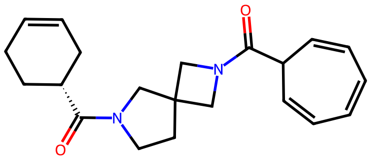	O=C(C1C=CC=CC=C1)N1CC2(CCN(C(=O)[C@H]3CC=CCC3)C2)C1	1019080586	25	338.2	2.7
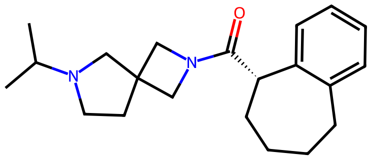	CC(C)N1CCC2(CN(C(=O)[C@H]3CCCc4ccccc43)C2)C1	1073469635	24	326.24	3.44

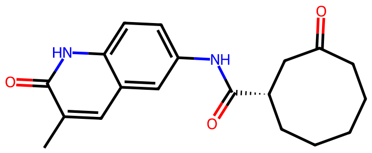	Cc1cc2cc(NC(=O)[C@H]3CCCCCC(=O)C3)ccc2[nH]c1=O	908456672	24	326.16	3.31
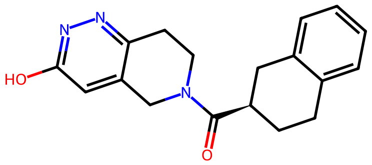	O=C([C@@H]1CCc2ccccc2C1)N1CCc2nnc(O)cc2C1	563891980	23	309.15	1.87
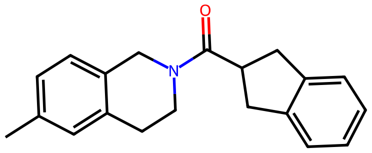	Cc1ccc2c(c1)CCN(C(=O)C1Cc3ccccc3C1)C2	432144110	22	291.16	3.29
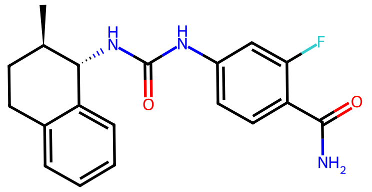	C[C@@H]1CCc2ccccc2[C@H]1NC(=O)Nc1ccc(C(N)=O)c(F)c1	179538971	25	341.15	3.37
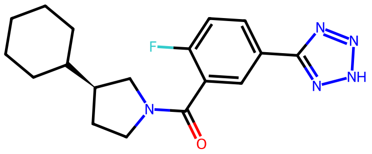	O=C(c1cc(-c2nn[nH]n2)ccc1F)N1CC[C@@H](C2CCCCC2)C1	1593323931	25	343.18	3.05
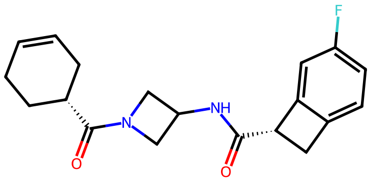	O=C(NC1CN(C(=O)[C@H]2CC=CCC2)C1)[C@H]1Cc2ccc(F)cc21	998485774	24	328.16	2.15
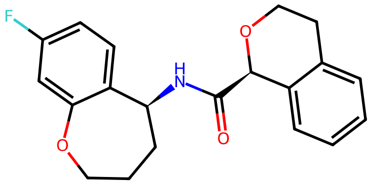	O=C(N[C@H]1CCCOc2cc(F)ccc21)[C@H]1OCCc2ccccc21	182818315	25	341.14	3.47
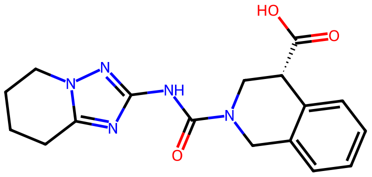	O=C(O)[C@H]1CN(C(=O)Nc2nc3n(n2)CCCC3)Cc2ccccc21	1601844678	25	341.15	1.83

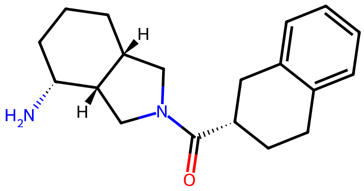	N[C@@H]1CCC[C@@H]2CN(C(=O)[C@H]3CCc4ccccc4C3)C[C@@H]21	318639108	22	298.2	2.38
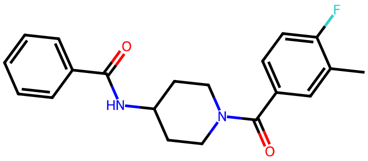	Cc1cc(C(=O)N2CCC(NC(=O)c3ccccc3)CC2)ccc1F	264102603	25	340.16	3.17
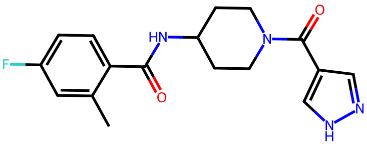	Cc1cc(F)ccc1C(=O)NC1CCN(C(=O)c2cn[nH]c2)CC1	944674241	24	330.15	1.89
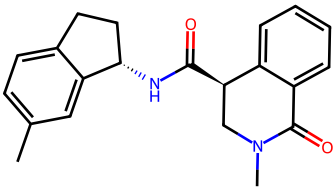	Cc1ccc2c(c1)[C@@H](NC(=O)[C@@H]1CN(C)C(=O)c3ccccc31)CC2	683741560	25	334.17	2.97
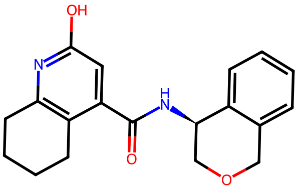	O=C(N[C@@H]1COCc2ccccc21)c1cc(O)nc2c1CCCC2	822915786	24	324.15	2.67
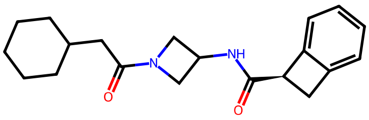	O=C(NC1CN(C(=O)CC2CCCCC2)C1)[C@@H]1Cc2ccccc21	998678945	24	326.2	2.62
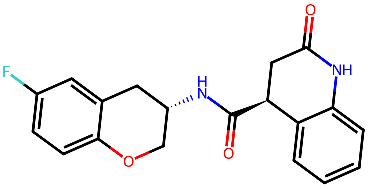	O=C1C[C@H](C(=O)N[C@@H]2COc3ccc(F)cc3C2)c2ccccc2N1	290681427	25	340.12	2.37
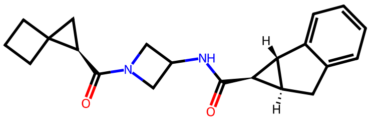	O=C(NC1CN(C(=O)[C@@H]2CC23CCC3)C1)[C@@H]1[C@@H]2Cc3ccccc3[C@H]21	991647440	25	336.18	2.09

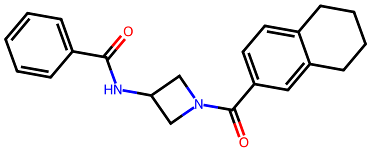	O=C(NC1CN(C(=O)c2ccc3c(c2)CCCC3)C1)c1ccccc1	990094713	25	334.17	2.82
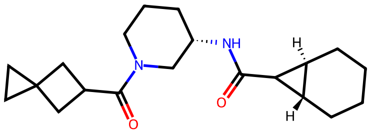	O=C(N[C@H]1CCCN(C(=O)C2CC3(CC3)C2)C1)C1[C@H]2CCCC[C@H]12	989307419	24	330.23	2.72
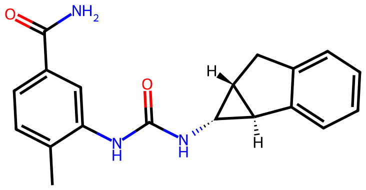	Cc1ccc(C(N)=O)cc1NC(=O)N[C@H]1[C@@H]2c3ccccc3C[C@H]21	248294342	24	321.15	2.55
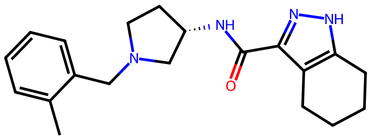	Cc1ccccc1CN1CC[C@H](NC(=O)c2n[nH]c3c2CCCC3)C1	1015072818	25	338.21	2.6
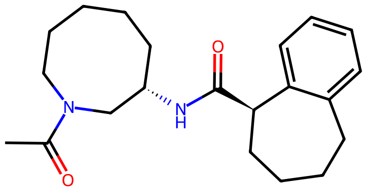	CC(=O)N1CCCCC[C@H](NC(=O)[C@@H]2CCCCc3ccccc32)C1	986790178	25	342.23	3.4
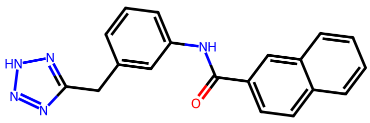	O=C(Nc1cccc(Cc2nn[nH]n2)c1)c1ccc2ccccc2c1	1570107245	25	329.13	3.2
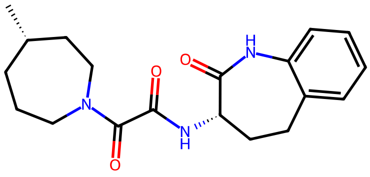	C[C@H]1CCCN(C(=O)C(=O)N[C@H]2CCc3ccccc3NC2=O)CC1	1327237239	25	343.19	1.7
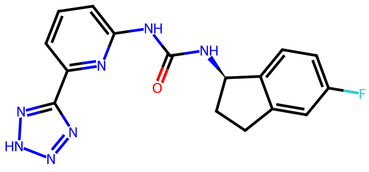	O=C(Nc1cccc(-c2nn[nH]n2)n1)N[C@@H]1CCc2cc(F)ccc21	1582645467	25	339.12	2.21

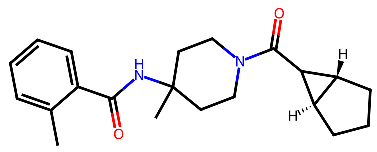	Cc1ccccc1C(=O)NC1(C)CCN(C(=O)C2[C@@H]3CCC[C@@H]23)CC1	956273158	25	340.22	3.15
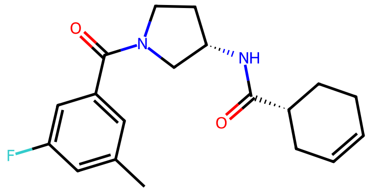	Cc1cc(F)cc(C(=O)N2CC[C@H](NC(=O)[C@H]3CC=CCC3)C2)c1	937668425	24	330.17	2.82
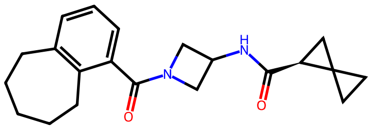	O=C(NC1CN(C(=O)c2cccc3c2CCCCC3)C1)[C@H]1CC12CC2	1000513810	25	338.2	2.7
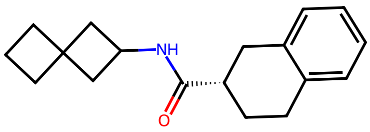	O=C(NC1CC2(CCC2)C1)[C@H]1CCc2ccccc2C1	652968652	20	269.18	3.24
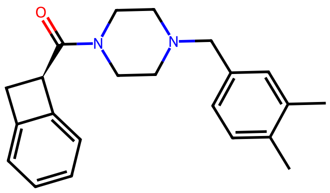	Cc1ccc(CN2CCN(C(=O)[C@@H]3Cc4ccccc43)CC2)cc1C	949957788	25	334.2	3.29
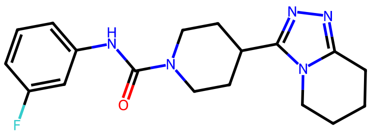	O=C(Nc1cccc(F)c1)N1CCC(c2nnc3n2CCCC3)CC1	274758787	25	343.18	3.17
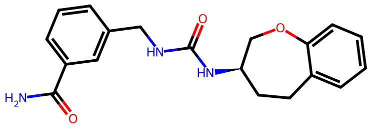	NC(=O)c1cccc(CNC(=O)N[C@@H]2CCc3ccccc3OC2)c1	374507574	25	339.16	1.98
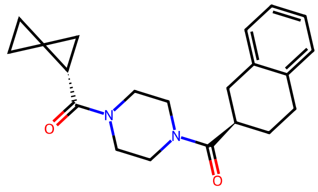	O=C([C@H]1CC12CC2)N1CCN(C(=O)[C@@H]2CCc3ccccc3C2)CC1	1012616214	25	338.2	2.26

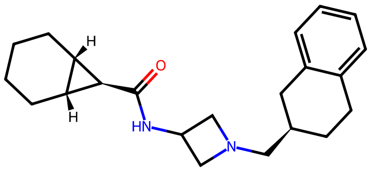	O=C(NC1CN(C[C@@H]2CCc3ccccc3C2)C1)[C@@H]1[C@H]2CCCC[C@H]21	1030182370	25	338.24	3.03
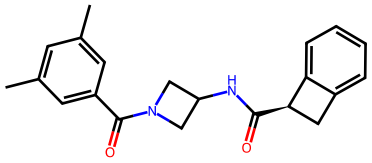	Cc1cc(C)cc(C(=O)N2CC(NC(=O)[C@@H]3Cc4ccccc43)C2)c1	998692631	25	334.17	2.58
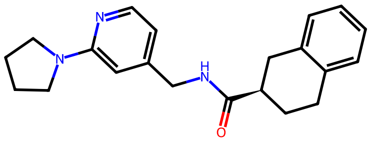	O=C(NCc1ccnc(N2CCCC2)c1)[C@@H]1CCc2ccccc2C1	570806119	25	335.2	3.1
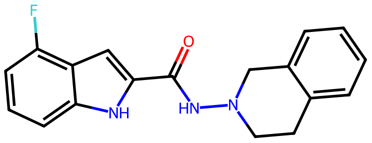	O=C(NN1CCc2ccccc2C1)c1cc2c(F)cccc2[nH]1	1583867139	23	309.13	3.01
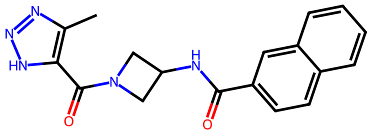	Cc1nn[nH]c1C(=O)N1CC(NC(=O)c2ccc3ccccc3c2)C1	999457034	25	335.14	1.52
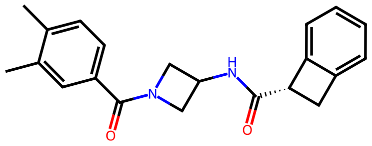	Cc1ccc(C(=O)N2CC(NC(=O)[C@H]3Cc4ccccc43)C2)cc1C	998692379	25	334.17	2.58
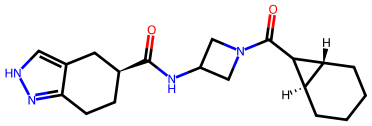	O=C(NC1CN(C(=O)C2[C@@H]3CCCC[C@@H]23)C1)[C@H]1CCc2n[nH]cc2C1	996397679	25	342.21	1.28
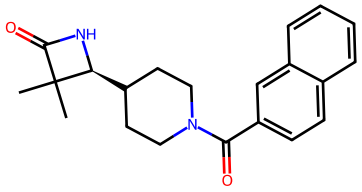	CC1(C)C(=O)N[C@H]1C1CCN(C(=O)c2ccc3ccccc3c2)CC1	294768511	25	336.18	3.22

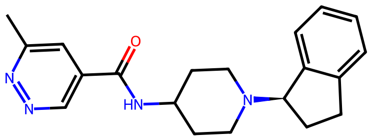	Cc1cc(C(=O)NC2CCN([C@@H]3CCc4ccccc43)CC2)cnn1	1002682703	25	336.2	2.67
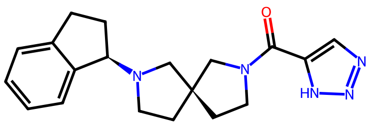	O=C(c1cnn[nH]1)N1CC[C@]2(CCN([C@@H]3CCc4ccccc43)C2)C1	1041434228	25	337.19	2.03
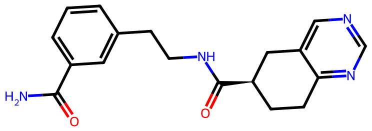	NC(=O)c1cccc(CCNC(=O)[C@@H]2CCc3ncncc3C2)c1	648268877	24	324.16	1.04
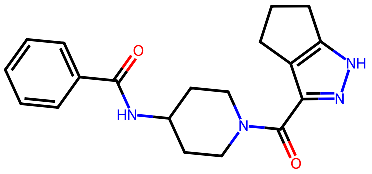	O=C(NC1CCN(C(=O)c2n[nH]c3c2CCC3)CC1)c1ccccc1	120732290	25	338.17	1.93
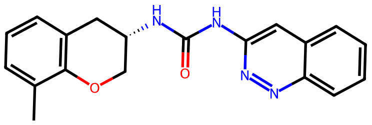	Cc1cccc2c1OC[C@@H](NC(=O)Nc1cc3ccccc3nn1)C2	1206803564	25	334.14	3.06
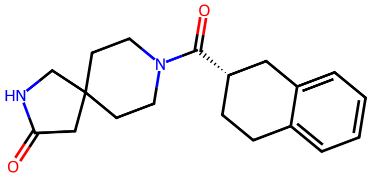	O=C1CC2(CCN(C(=O)[C@H]3CCc4ccccc4C3)CC2)CN1	77541663	23	312.18	1.92
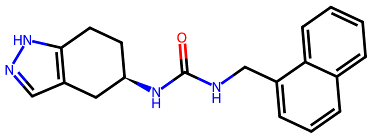	O=C(NCc1cccc2ccccc12)N[C@@H]1CCc2[nH]ncc2C1	165675896	24	320.16	2.92
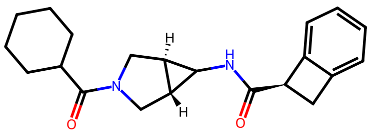	O=C(NC1[C@@H]2CN(C(=O)C3CCCCC3)C[C@@H]12)[C@@H]1Cc2ccccc21	1013681255	25	338.2	2.48

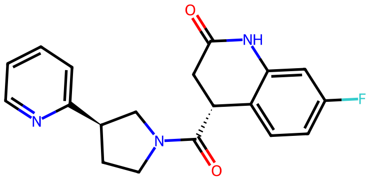	O=C1C[C@@H](C(=O)N2CC[C@@H](c3ccccn3)C2)c2ccc(F)cc2N1	798720350	25	339.14	2.66
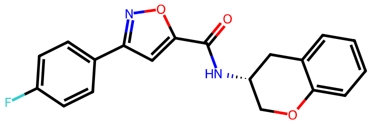	O=C(N[C@H]1COc2ccccc2C1)c1cc(-c2ccc(F)cc2)no1	1625690990	25	338.11	3.21
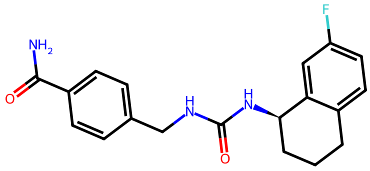	NC(=O)c1ccc(CNC(=O)N[C@@H]2CCCc3ccc(F)cc32)cc1	93355068	25	341.15	2.8
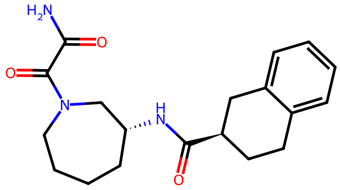	NC(=O)C(=O)N1CCCC[C@@H](NC(=O)[C@@H]2CCc3ccccc3C2)C1	943575400	25	343.19	0.77
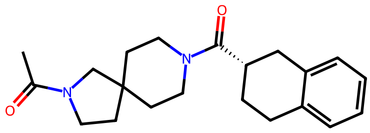	CC(=O)N1CCC2(CCN(C(=O)[C@H]3CCc4ccccc4C3)CC2)C1	944428795	25	340.22	2.65
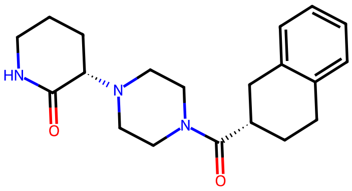	O=C1NCCC[C@@H]1N1CCN(C(=O)[C@H]2CCc3ccccc3C2)CC1	950725695	25	341.21	1.21
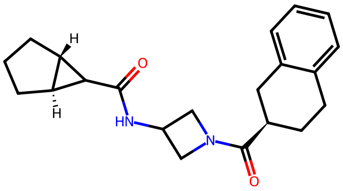	O=C(NC1CN(C(=O)[C@@H]2CCc3ccccc3C2)C1)C1[C@H]2CCC[C@H]12	990718593	25	338.2	2.16
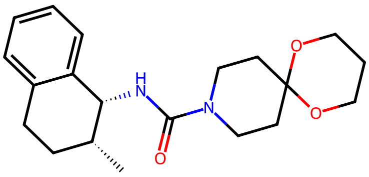	C[C@@H]1CCc2ccccc2[C@@H]1NC(=O)N1CCC2(CC1)OCCCO2	838533225	25	344.21	3.25

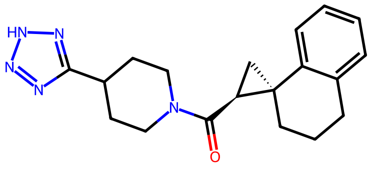	O=C([C@H]1C[C@]12CCCc1ccccc12)N1CCC(c2nn[nH]n2)CC1	913743892	25	337.19	2.2
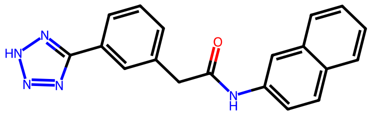	O=C(Cc1cccc(-c2nn[nH]n2)c1)Nc1ccc2ccccc2c1	823108775	25	329.13	3.2
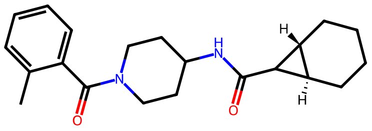	Cc1ccccc1C(=O)N1CCC(NC(=O)C2[C@@H]3CCCC[C@@H]23)CC1	943772871	25	340.22	3.15
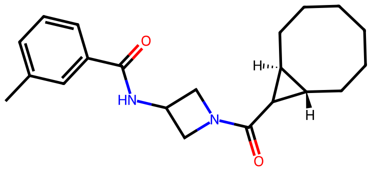	Cc1cccc(C(=O)NC2CN(C(=O)C3[C@H]4CCCCCC[C@H]34)C2)c1	990138099	25	340.22	3.15
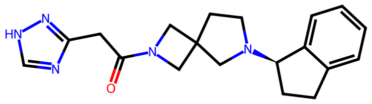	O=C(Cc1nc[nH]n1)N1CC2(CCN([C@@H]3CCc4ccccc43)C2)C1	1073113636	25	337.19	1.57
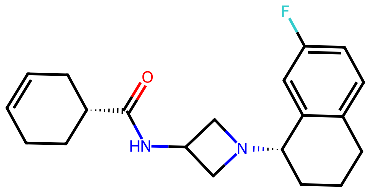	O=C(NC1CN([C@H]2CCCc3ccc(F)cc32)C1)[C@H]1CC=CCC1	1030098613	24	328.2	3.36
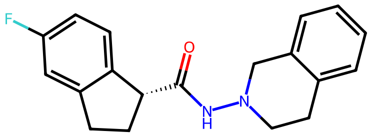	O=C(NN1CCc2ccccc2C1)[C@@H]1CCc2cc(F)ccc21	855707454	23	310.15	2.95
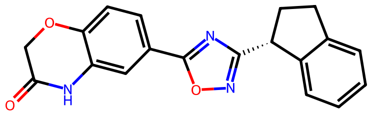	O=C1COc2ccc(-c3nc([C@@H]4CCc5ccccc54)no3)cc2N1	356605616	25	333.11	3.15

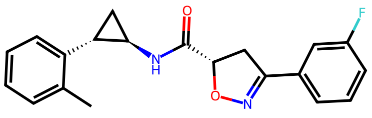	Cc1ccccc1[C@@H]1C[C@H]1NC(=O)[C@@H]1CC(c2cccc(F)c2)=NO1	1527705037	25	338.14	3.3
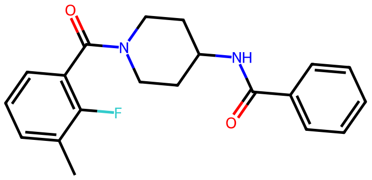	Cc1cccc(C(=O)N2CCC(NC(=O)c3ccccc3)CC2)c1F	119170124	25	340.16	3.17
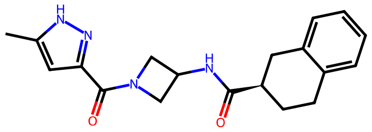	Cc1cc(C(=O)N2CC(NC(=O)[C@@H]3CCc4ccccc4C3)C2)n[nH]1	1000537644	25	338.17	1.46
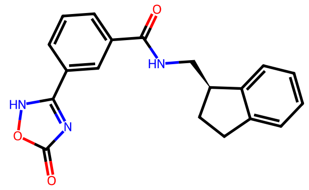	O=C(NC[C@@H]1CCc2ccccc21)c1cccc(-c2nc(=O)o[nH]2)c1	1586081318	25	335.13	2.49
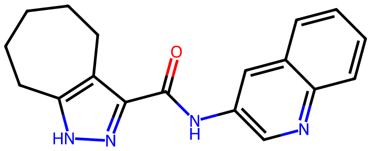	O=C(Nc1cnc2ccccc2c1)c1n[nH]c2c1CCCCC2	190049675	23	306.15	3.48
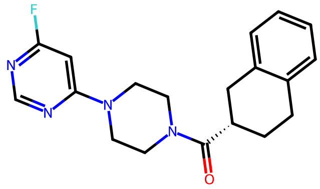	O=C([C@H]1CCc2ccccc2C1)N1CCN(c2cc(F)ncn2)CC1	908885508	25	340.17	2.07
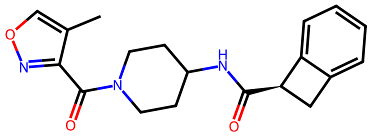	Cc1conc1C(=O)N1CCC(NC(=O)[C@@H]2Cc3ccccc32)CC1	948376863	25	339.16	2.04
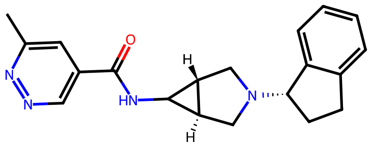	Cc1cc(C(=O)NC2[C@@H]3CN([C@H]4CCc5ccccc54)C[C@@H]23)cnn1	958675597	25	334.18	2.13

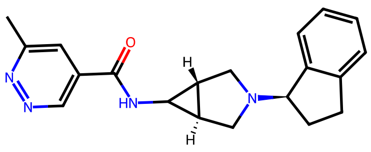	Cc1cc(C(=O)NC2[C@@H]3CN([C@@H]4CCc5ccccc54)C[C@@H]23)cnn1	958675598	25	334.18	2.13
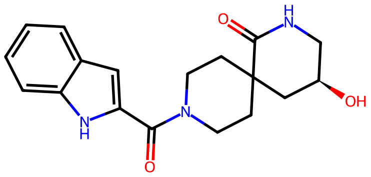	O=C(c1cc2ccccc2[nH]1)N1CCC2(CC1)C[C@H](O)CNC2=O	885024748	24	327.16	1.27
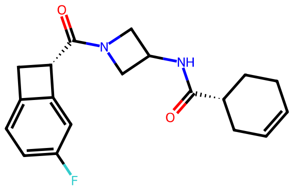	O=C(NC1CN(C(=O)[C@H]2Cc3ccc(F)cc32)C1)[C@H]1CC=CCC1	990200915	24	328.16	2.15
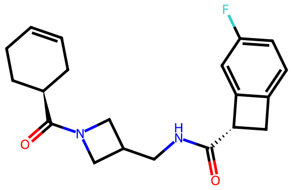	O=C(NCC1CN(C(=O)[C@@H]2CC=CCC2)C1)[C@H]1Cc2ccc(F)cc21	1003882954	25	342.17	2.4
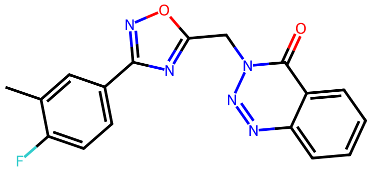	Cc1cc(-c2noc(Cn3nnc4ccccc4c3=O)n2)ccc1F	109558349	25	337.1	2.34
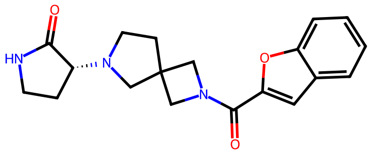	O=C1NCC[C@H]1N1CCC2(CN(C(=O)c3cc4ccccc4o3)C2)C1	1073403948	25	339.16	1.47
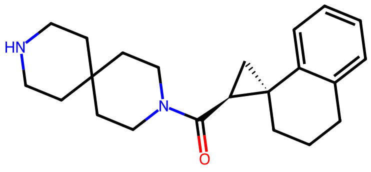	O=C([C@H]1C[C@]12CCCc1ccccc12)N1CCC2(CCNCC2)CC1	357887480	25	338.24	3.27
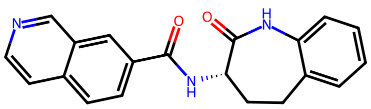	O=C(N[C@H]1CCc2ccccc2NC1=O)c1ccc2ccncc2c1	1611425624	25	331.13	2.92

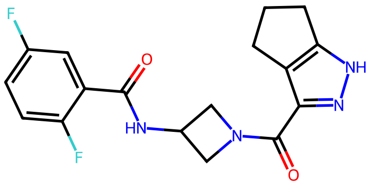	O=C(NC1CN(C(=O)c2n[nH]c3c2CCC3)C1)c1cc(F)ccc1F	993972225	25	346.12	1.43
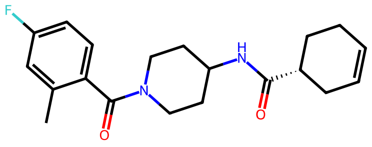	Cc1cc(F)ccc1C(=O)N1CCC(NC(=O)[C@H]2CC=CCC2)CC1	947642495	25	344.19	3.21
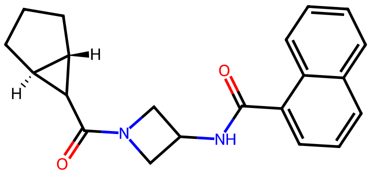	O=C(NC1CN(C(=O)C2[C@H]3CCC[C@H]23)C1)c1cccc2ccccc12	999540253	25	334.17	2.83
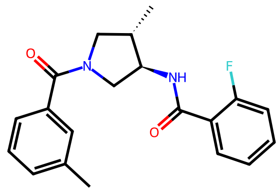	Cc1cccc(C(=O)N2C[C@H](C)[C@@H](NC(=O)c3ccccc3F)C2)c1	966118394	25	340.16	3.02
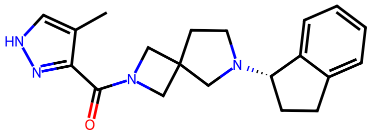	Cc1c[nH]nc1C(=O)N1CC2(CCN([C@H]3CCc4ccccc43)C2)C1	1072713744	25	336.2	2.55
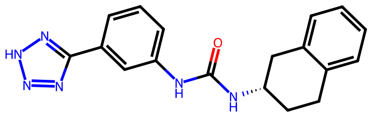	O=C(Nc1cccc(-c2nn[nH]n2)c1)N[C@H]1CCc2ccccc2C1	1606932514	25	334.15	2.55
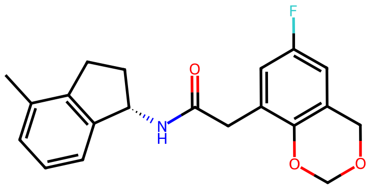	Cc1cccc2c1CC[C@@H]2NC(=O)Cc1cc(F)cc2c1OCOC2	517835882	25	341.14	3.35
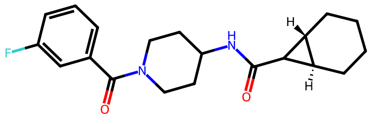	O=C(NC1CCN(C(=O)c2cccc(F)c2)CC1)C1[C@@H]2CCCC[C@@H]12	943774550	25	344.19	2.98

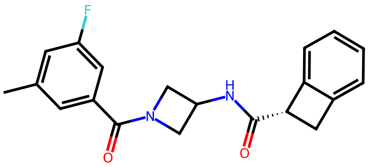	Cc1cc(F)cc(C(=O)N2CC(NC(=O)[C@H]3Cc4ccccc43)C2)c1	998694515	25	338.14	2.41
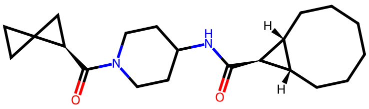	O=C(NC1CCN(C(=O)[C@@H]2CC23CC3)CC1)[C@@H]1[C@H]2CCCCCC[C@H]21	948557991	25	344.25	3.11
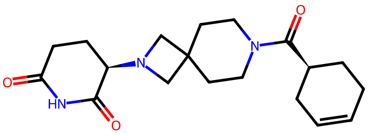	O=C1CC[C@@H](N2CC3(CCN(C(=O)[C@@H]4CC=CCC4)CC3)C2)C(=O)N1	1035648625	25	345.21	1.07
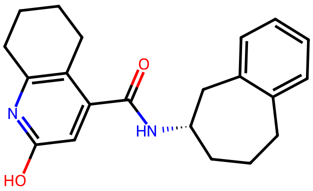	O=C(N[C@H]1CCCc2ccccc2C1)c1cc(O)nc2c1CCCC2	888244308	25	336.18	3.34
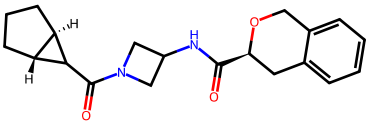	O=C(NC1CN(C(=O)C2[C@@H]3CCC[C@@H]23)C1)[C@@H]1Cc2ccccc2CO1	997733898	25	340.18	1.5
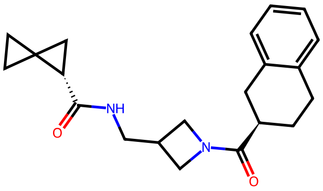	O=C(NCC1CN(C(=O)[C@@H]2CCc3ccccc3C2)C1)[C@H]1CC12CC2	936091181	25	338.2	2.17
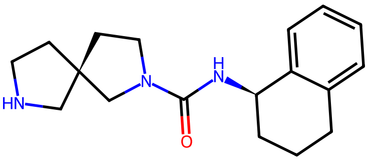	O=C(N[C@@H]1CCCc2ccccc21)N1CC[C@@]2(CCNC2)C1	335270126	22	299.2	2.46
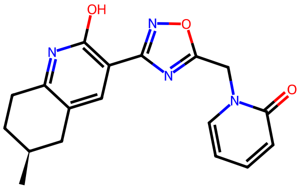	C[C@H]1CCc2nc(O)c(-c3noc(Cn4cccc4=O)n3)cc2C1	897277874	25	338.14	2.17

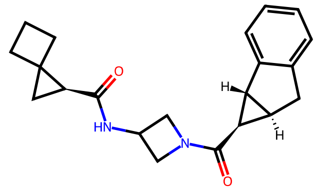	O=C(NC1CN(C(=O)[C@@H]2[C@@H]3Cc4ccccc4[C@H]32)C1)[C@H]1CC12CCC2	991554843	25	336.18	2.09
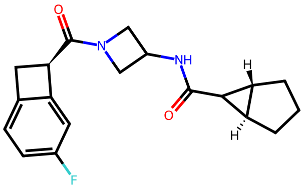	O=C(NC1CN(C(=O)[C@@H]2Cc3ccc(F)cc32)C1)C1[C@@H]2CCC[C@@H]12	990707694	24	328.16	1.84
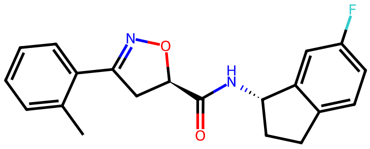	Cc1ccccc1C1=NO[C@@H](C(=O)N[C@H]2CCc3ccc(F)cc32)C1	1360016919	25	338.14	3.43
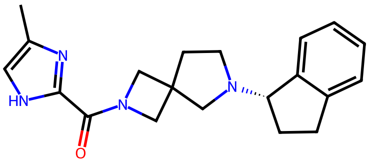	Cc1c[nH]c(C(=O)N2CC3(CCN([C@H]4CCc5ccccc54)C3)C2)n1	1073046013	25	336.2	2.55
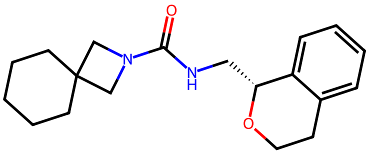	O=C(NC[C@H]1OCCc2ccccc21)N1CC2(CCCCC2)C1	680642983	23	314.2	3.28
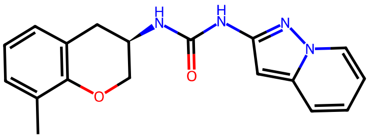	Cc1cccc2c1OC[C@H](NC(=O)Nc1cc3ccccn3n1)C2	685655159	24	322.14	2.77
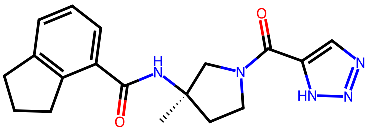	C[C@@]1(NC(=O)c2cccc3c2CCC3)CCN(C(=O)c2cnn[nH]2)C1	957143926	25	339.17	1.33
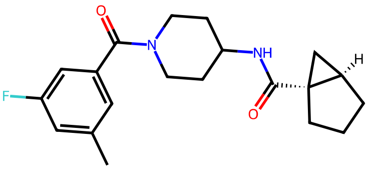	Cc1cc(F)cc(C(=O)N2CCC(NC(=O)[C@@]34CCC[C@@H]3C4)CC2)c1	948549870	25	344.19	3.05

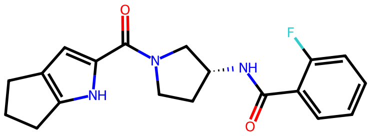	O=C(N[C@@H]1CCN(C(=O)c2cc3c([nH]2)CCC3)C1)c1ccccc1F	939173218	25	341.15	2.29
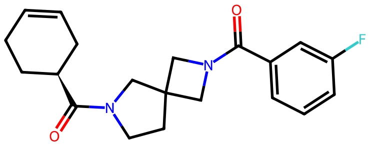	O=C(c1cccc(F)c1)N1CC2(CCN(C(=O)[C@@H]3CC=CCC3)C2)C1	1018726017	25	342.17	2.86
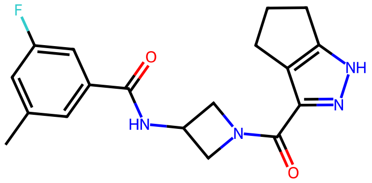	Cc1cc(F)cc(C(=O)NC2CN(C(=O)c3n[nH]c4c3CCC4)C2)c1	1000440137	25	342.15	1.6
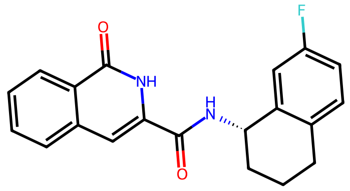	O=C(N[C@H]1CCCc2ccc(F)cc21)c1cc2ccccc2c(=O)[nH]1	93062912	25	336.13	3.47
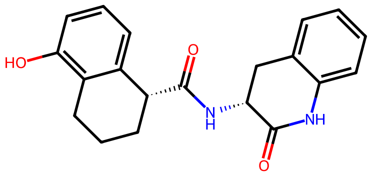	O=C1Nc2ccccc2C[C@H]1NC(=O)[C@@H]1CCCc2c(O)cccc21	529949074	25	336.15	2.49
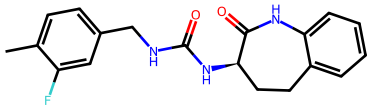	Cc1ccc(CNC(=O)N[C@@H]2CCc3ccccc3NC2=O)cc1F	847278772	25	341.15	2.89
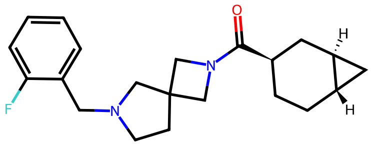	O=C([C@@H]1CC[C@H]2C[C@@H]2C1)N1CC2(CCN(Cc3ccccc3F)C2)C1	1072879373	25	342.21	3.3
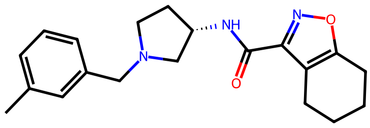	Cc1cccc(CN2CC[C@H](NC(=O)c3noc4c3CCCC4)C2)c1	634270821	25	339.19	2.87

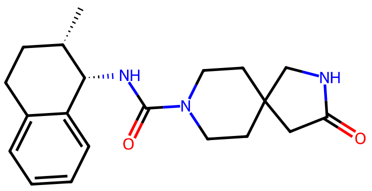	C[C@H]1CCc2ccccc2[C@H]1NC(=O)N1CCC2(CC1)CNC(=O)C2	270729627	25	341.21	2.62
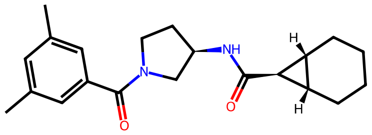	Cc1cc(C)cc(C(=O)N2CC[C@@H](NC(=O)[C@@H]3[C@H]4CCCC[C@H]43)C2)c1	939895352	25	340.22	3.07
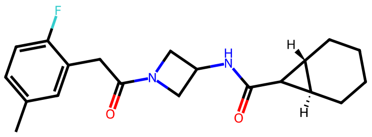	Cc1ccc(F)c(CC(=O)N2CC(NC(=O)C3[C@@H]4CCCC[C@@H]34)C2)c1	990643901	25	344.19	2.44
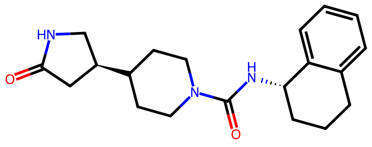	O=C1C[C@H](C2CCN(C(=O)N[C@H]3CCCc4ccccc43)CC2)CN1	1553351036	25	341.21	2.62
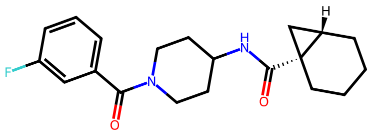	O=C(c1cccc(F)c1)N1CCC(NC(=O)[C@@]23CCCC[C@H]2C3)CC1	943776521	25	344.19	3.13
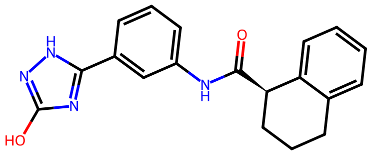	O=C(Nc1cccc(-c2nc(O)n[nH]2)c1)[C@@H]1CCCc2ccccc21	356019178	25	334.14	3.24
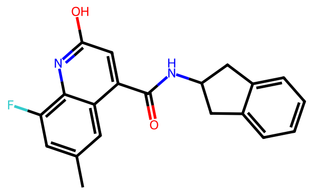	Cc1cc(F)c2nc(O)cc(C(=O)NC3Cc4ccccc4C3)c2c1	547240264	25	336.13	3.29
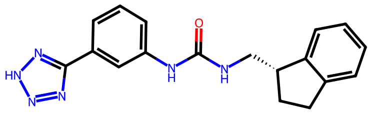	O=C(NC[C@H]1CCc2ccccc21)Nc1cccc(-c2nn[nH]n2)c1	738051364	25	334.15	2.72

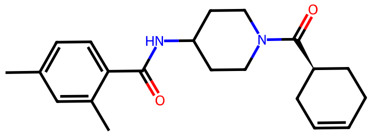	Cc1ccc(C(=O)NC2CCN(C(=O)[C@@H]3CC=CCC3)CC2)c(C)c1	944107485	25	340.22	3.38
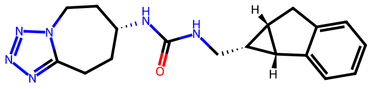	O=C(NC[C@H]1[C@H]2c3ccccc3C[C@H]21)N[C@@H]1CCc2nnnn2CC1	333637564	25	338.19	1.26
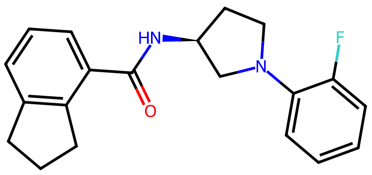	O=C(N[C@H]1CCN(c2ccccc2F)C1)c1cccc2c1CCC2	345247246	24	324.16	3.32
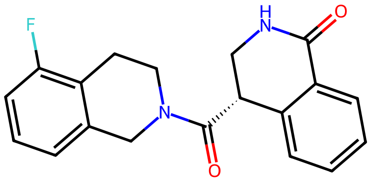	O=C1NC[C@@H](C(=O)N2CCc3c(F)cccc3C2)c2ccccc21	860435420	24	324.13	2.24
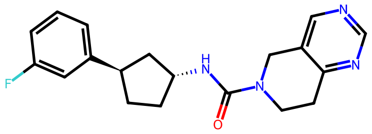	O=C(N[C@@H]1CC[C@@H](c2cccc(F)c2)C1)N1CCc2ncncc2C1	883620713	25	340.17	3.02
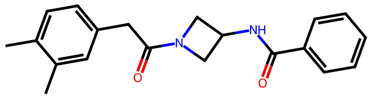	Cc1ccc(CC(=O)N2CC(NC(=O)c3ccccc3)C2)cc1C	990095178	24	322.17	2.49
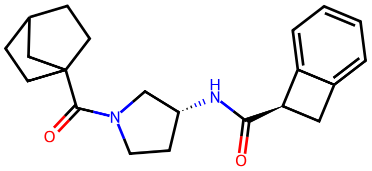	O=C(N[C@@H]1CCN(C(=O)C23CCC(CC2)C3)C1)[C@@H]1Cc2ccccc21	942298237	25	338.2	2.62
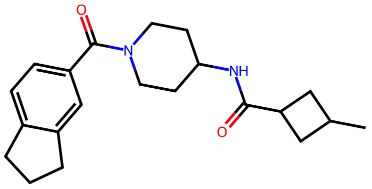	CC1CC(C(=O)NC2CCN(C(=O)c3ccc4c(c3)CCC4)CC2)C1	946720877	25	340.22	2.94

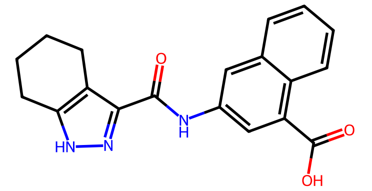	O=C(Nc1cc(C(=O)O)c2ccccc2c1)c1n[nH]c2c1CCCC2	1601582405	25	335.13	3.39
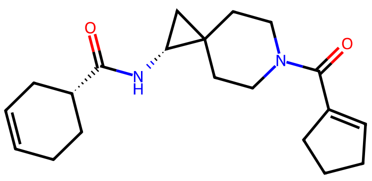	O=C(N[C@@H]1CC12CCN(C(=O)C1=CCCC1)CC2)[C@H]1CC=CCC1	963030451	24	328.22	2.95
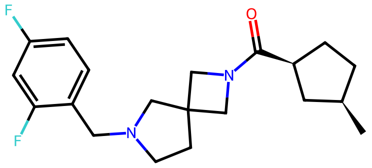	C[C@@H]1CC[C@H](C(=O)N2CC3(CCN(Cc4ccc(F)cc4F)C3)C2)C1	1073031348	25	348.2	3.44
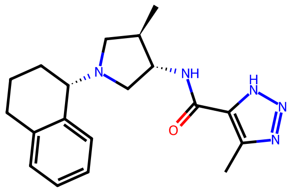	Cc1nn[nH]c1C(=O)N[C@@H]1CN([C@H]2CCCc3ccccc32)C[C@H]1C	1080246086	25	339.21	2.24
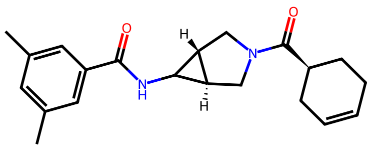	Cc1cc(C)cc(C(=O)NC2[C@@H]3CN(C(=O)[C@@H]4CC=CCC4)C[C@@H]23)c1	1013768582	25	338.2	2.85
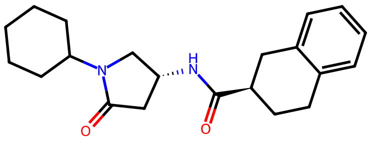	O=C(N[C@@H]1CC(=O)N(C2CCCCC2)C1)[C@@H]1CCc2ccccc2C1	1362286748	25	340.22	2.84
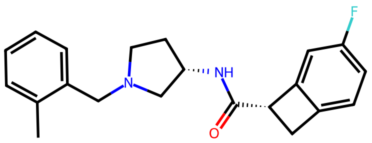	Cc1ccccc1CN1CC[C@H](NC(=O)[C@H]2Cc3ccc(F)cc32)C1	1015615716	25	338.18	3.16
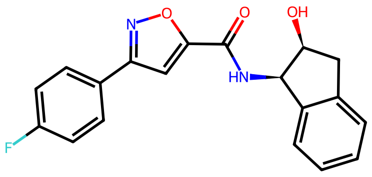	O=C(N[C@@H]1c2ccccc2C[C@@H]1O)c1cc(-c2ccc(F)cc2)no1	1445721538	25	338.11	2.87

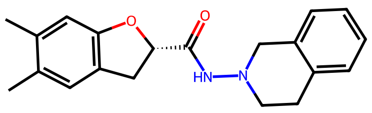	Cc1cc2c(cc1C)O[C@H](C(=O)NN1CCc3ccccc3C1)C2	1469893065	24	322.17	2.7
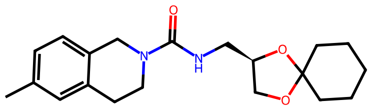	Cc1ccc2c(c1)CCN(C(=O)NC[C@@H]1COC3(CCCCC3)O1)C2	790452786	25	344.21	3.14
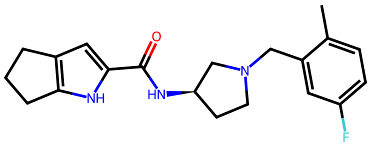	Cc1ccc(F)cc1CN1CC[C@@H](NC(=O)c2cc3c([nH]2)CCC3)C1	1015980968	25	341.19	2.96
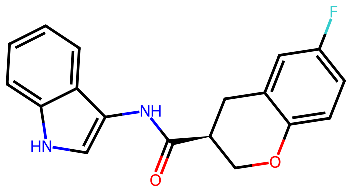	O=C(Nc1c[nH]c2ccccc12)[C@@H]1COc2ccc(F)cc2C1	1157066354	23	310.11	3.5
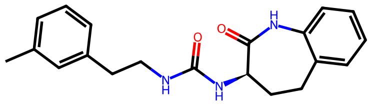	Cc1cccc(CCNC(=O)N[C@@H]2CCc3ccccc3NC2=O)c1	654413783	25	337.18	2.79
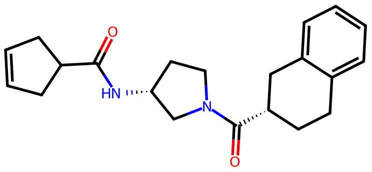	O=C(N[C@@H]1CCN(C(=O)[C@H]2CCc3ccccc3C2)C1)C1CC=CC1	936274792	25	338.2	2.47
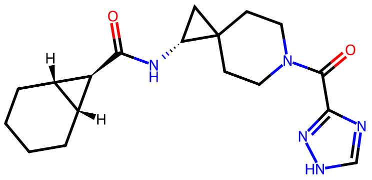	O=C(N[C@@H]1CC12CCN(C(=O)c1nc[nH]n1)CC2)[C@@H]1[C@H]2CCCC[C@H]21	963443517	25	343.2	1.35
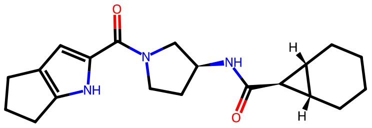	O=C(N[C@H]1CCN(C(=O)c2cc3c([nH]2)CCC3)C1)[C@@H]1[C@H]2CCCC[C@H]21	937788574	25	341.21	2.27

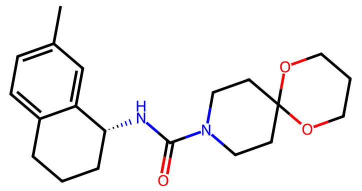	Cc1ccc2c(c1)[C@H](NC(=O)N1CCC3(CC1)OCCCO3)CCC2	298032525	25	344.21	3.31
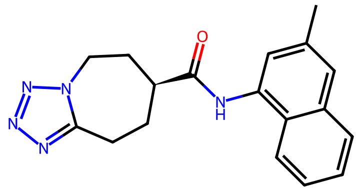	Cc1cc(NC(=O)[C@H]2CCc3nnnn3CC2)c2ccccc2c1	576166586	24	321.16	2.73
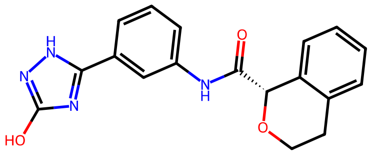	O=C(Nc1cccc(-c2nc(O)n[nH]2)c1)[C@H]1OCCc2ccccc21	367739070	25	336.12	2.43
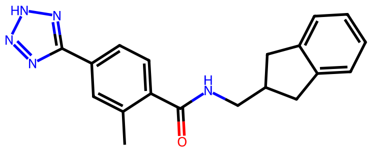	Cc1cc(-c2nn[nH]n2)ccc1C(=O)NCC1Cc2ccccc2C1	826248252	25	333.16	2.32
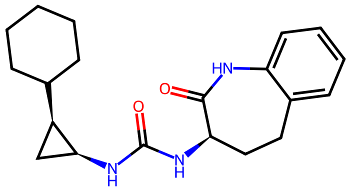	O=C(N[C@H]1C[C@H]1C1CCCCC1)N[C@@H]1CCc2ccccc2NC1=O	1469095512	25	341.21	3.21
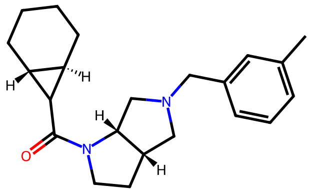	Cc1cccc(CN2C[C@@H]3CCN(C(=O)C4[C@@H]5CCCC[C@@H]45)[C@@H]3C2)c1	1075650437	25	338.24	3.46
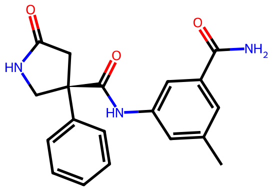	Cc1cc(NC(=O)[C@@]2(c3ccccc3)CNC(=O)C2)cc(C(N)=O)c1	1361676787	25	337.14	1.49
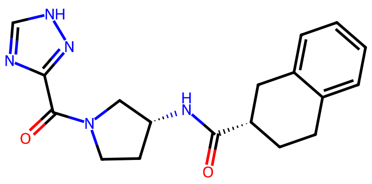	O=C(N[C@@H]1CCN(C(=O)c2nc[nH]n2)C1)[C@H]1CCc2ccccc2C1	942507231	25	339.17	0.94

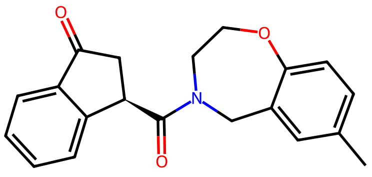	Cc1ccc2c(c1)CN(C(=O)[C@@H]1CC(=O)c3ccccc31)CCO2	287984660	24	321.14	3.09
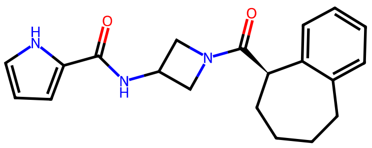	O=C(NC1CN(C(=O)[C@@H]2CCCCc3ccccc32)C1)c1ccc[nH]1	990227687	25	337.18	2.47
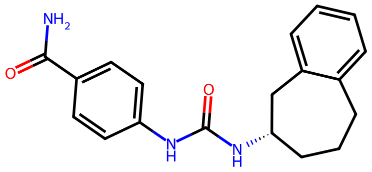	NC(=O)c1ccc(NC(=O)N[C@H]2CCCc3ccccc3C2)cc1	929401709	24	323.16	2.85
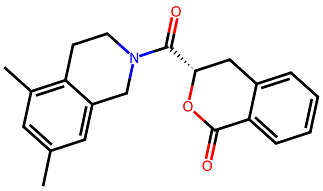	Cc1cc(C)c2c(c1)CN(C(=O)[C@@H]1Cc3ccccc3C(=O)O1)CC2	187733023	25	335.15	2.97
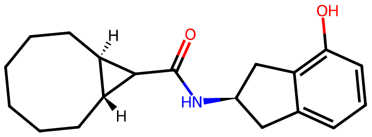	O=C(N[C@@H]1Cc2cccc(O)c2C1)C1[C@@H]2CCCCCC[C@@H]12	613999384	22	299.19	3.19
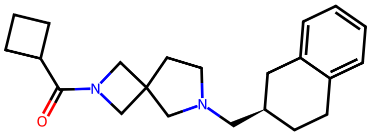	O=C(C1CCC1)N1CC2(CCN(C[C@@H]3CCc4ccccc4C3)C2)C1	1072389139	25	338.24	3.13
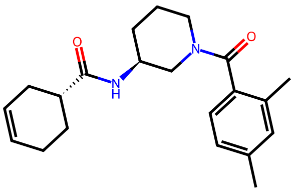	Cc1ccc(C(=O)N2CCC[C@H](NC(=O)[C@H]3CC=CCC3)C2)c(C)c1	951996657	25	340.22	3.38
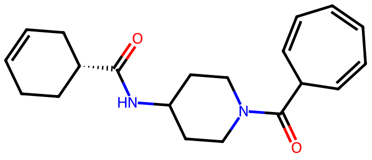	O=C(NC1CCN(C(=O)C2C=CC=CC=C2)CC1)[C@H]1CC=CCC1	946948849	24	326.2	2.75

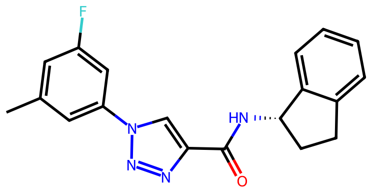	Cc1cc(F)cc(-n2cc(C(=O)N[C@H]3CCc4ccccc43)nn2)c1	339958806	25	336.14	3.13
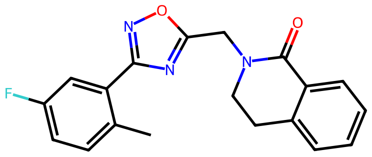	Cc1ccc(F)cc1-c1noc(CN2CCc3ccccc3C2=O)n1	345934867	25	337.12	3.38
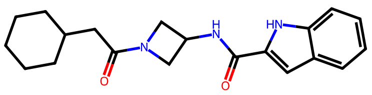	O=C(NC1CN(C(=O)CC2CCCCC2)C1)c1cc2ccccc2[nH]1	1000142218	25	339.19	3.08
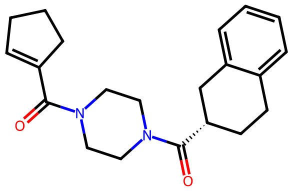	O=C(C1=CCCC1)N1CCN(C(=O)[C@H]2CCc3ccccc3C2)CC1	1010991439	25	338.2	2.57
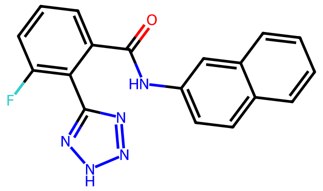	O=C(Nc1ccc2ccccc2c1)c1cccc(F)c1-c1nn[nH]n1	1606929623	25	333.1	3.41
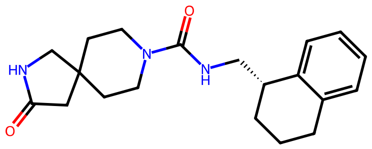	O=C1CC2(CCN(C(=O)NC[C@H]3CCCc4ccccc43)CC2)CN1	73713392	25	341.21	2.42
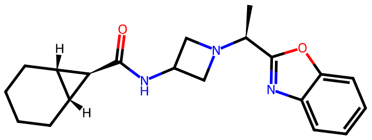	C[C@@H](c1nc2ccccc2o1)N1CC(NC(=O)[C@@H]2[C@H]3CCCC[C@H]32)C1	1030188566	25	339.19	3.13
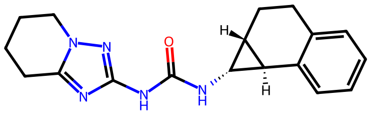	O=C(Nc1nc2n(n1)CCCC2)N[C@H]1[C@@H]2c3ccccc3CC[C@H]21	333621117	24	323.17	2.46

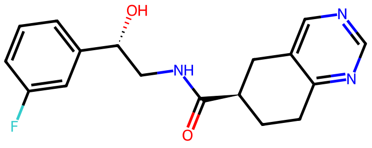	O=C(NC[C@@H](O)c1cccc(F)c1)[C@@H]1CCc2ncncc2C1	843824870	23	315.14	1.57
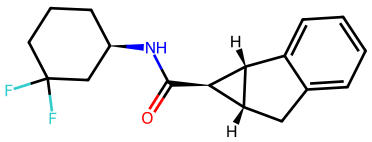	O=C(N[C@@H]1CCCC(F)(F)C1)[C@@H]1[C@H]2Cc3ccccc3[C@H]21	626188115	21	291.14	3.27
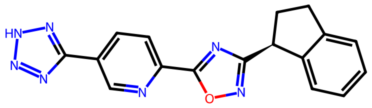	c1ccc2c(c1)CC[C@@H]2c1noc(-c2ccc(-c3nn[nH]n3)cn2)n1	1593580981	25	331.12	2.39
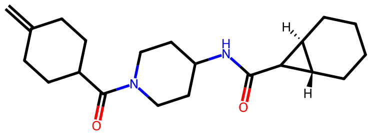	C=C1CCC(C(=O)N2CCC(NC(=O)C3[C@H]4CCCC[C@H]34)CC2)CC1	948190107	25	344.25	3.28
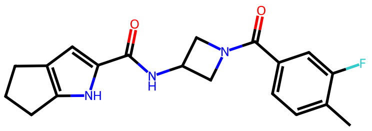	Cc1ccc(C(=O)N2CC(NC(=O)c3cc4c([nH]3)CCC4)C2)cc1F	995956435	25	341.15	2.21
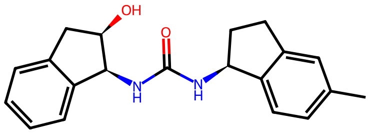	Cc1ccc2c(c1)CC[C@@H]2NC(=O)N[C@H]1c2ccccc2C[C@H]1O	263792552	24	322.17	2.94
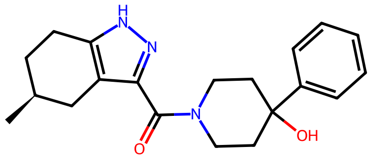	C[C@H]1CCc2[nH]nc(C(=O)N3CCC(O)(c4ccccc4)CC3)c2C1	897412295	25	339.19	2.66
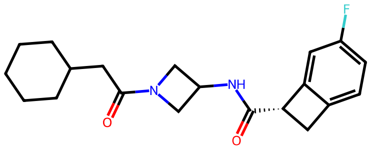	O=C(NC1CN(C(=O)CC2CCCCC2)C1)[C@H]1Cc2ccc(F)cc21	998489075	25	344.19	2.76

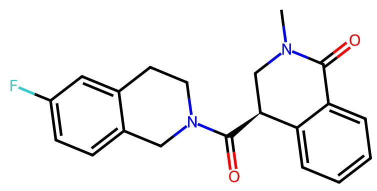	CN1C[C@H](C(=O)N2CCc3cc(F)ccc3C2)c2ccccc2C1=O	365967985	25	338.14	2.58
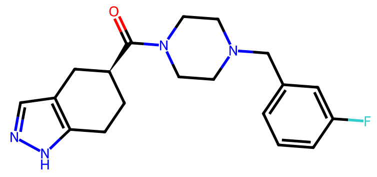	O=C([C@H]1CCc2[nH]ncc2C1)N1CCN(Cc2cccc(F)c2)CC1	270260310	25	342.19	2.0
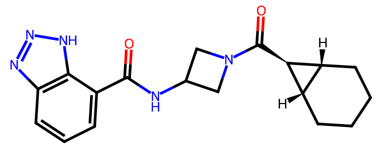	O=C(NC1CN(C(=O)[C@@H]2[C@H]3CCCC[C@H]32)C1)c1cccc2nn[nH]c12	991895257	25	339.17	1.33
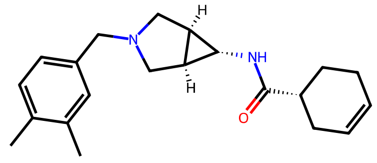	Cc1ccc(CN2C[C@H]3[C@H](NC(=O)[C@H]4CC=CCC4)[C@H]3C2)cc1C	958208039	24	324.22	3.21
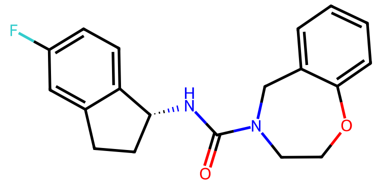	O=C(N[C@@H]1CCc2cc(F)ccc21)N1CCOc2ccccc2C1	619389131	24	326.14	3.42
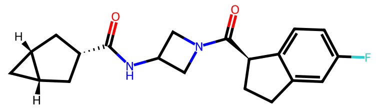	O=C(NC1CN(C(=O)[C@@H]2CCc3cc(F)ccc32)C1)[C@@H]1C[C@@H]2C[C@@H]2C1	998008107	25	342.17	2.23
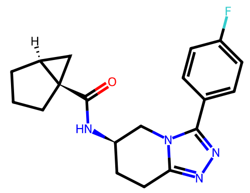	O=C(N[C@@H]1CCc2nnc(-c3ccc(F)cc3)n2C1)[C@@]12CCC[C@H]1C2	570831972	25	340.17	2.71
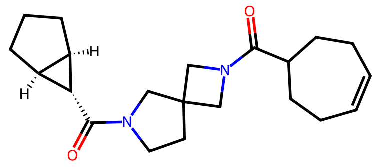	O=C(C1CCC=CCC1)N1CC2(CCN(C(=O)[C@@H]3[C@H]4CCC[C@H]43)C2)C1	1019044164	25	342.23	2.84

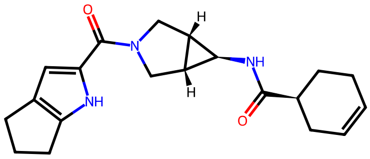	O=C(N[C@H]1[C@@H]2CN(C(=O)c3cc4c([nH]3)CCC4)C[C@@H]21)[C@@H]1CC=CCC1	976180658	25	339.19	2.05
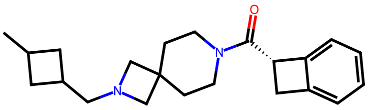	CC1CC(CN2CC3(CCN(C(=O)[C@H]4Cc5ccccc54)CC3)C2)C1	1035779835	25	338.24	3.3
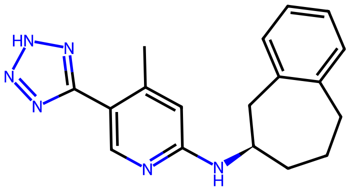	Cc1cc(N[C@@H]2CCCc3ccccc3C2)ncc1-c1nn[nH]n1	1592038430	24	320.17	2.93
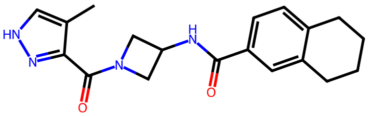	Cc1c[nH]nc1C(=O)N1CC(NC(=O)c2ccc3c(c2)CCCC3)C1	1000056820	25	338.17	1.85
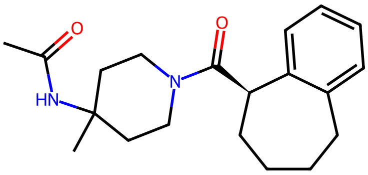	CC(=O)NC1(C)CCN(C(=O)[C@@H]2CCCCc3ccccc32)CC1	956253736	24	328.22	3.01
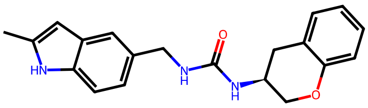	Cc1cc2cc(CNC(=O)N[C@@H]3COc4ccccc4C3)ccc2[nH]1	515321212	25	335.16	3.28
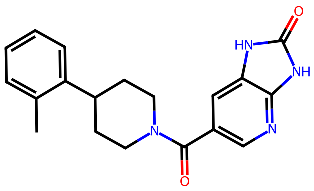	Cc1ccccc1C1CCN(C(=O)c2cnc3[nH]c(=O)[nH]c3c2)CC1	289116278	25	336.16	2.58
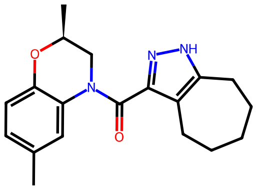	Cc1ccc2c(c1)N(C(=O)c1n[nH]c3c1CCCCC3)C[C@H](C)O2	190576009	24	325.18	3.41

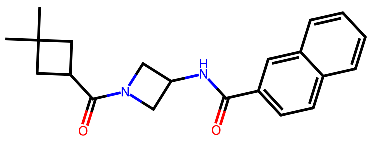	CC1(C)CC(C(=O)N2CC(NC(=O)c3ccc4ccccc4c3)C2)C1	999446927	25	336.18	3.22
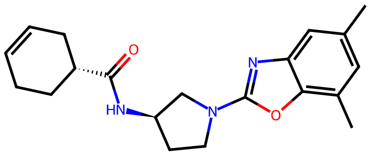	Cc1cc(C)c2oc(N3CC[C@@H](NC(=O)[C@H]4CC=CCC4)C3)nc2c1	1058297460	25	339.19	3.5
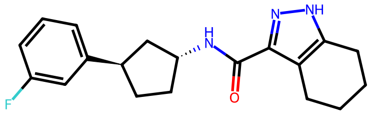	O=C(N[C@@H]1CC[C@@H](c2cccc(F)c2)C1)c1n[nH]c2c1CCCC2	684513719	24	327.17	3.49
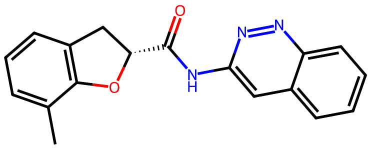	Cc1cccc2c1O[C@@H](C(=O)Nc1cc3ccccc3nn1)C2	1461175979	23	305.12	2.88
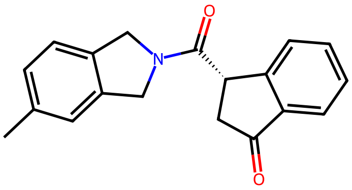	Cc1ccc2c(c1)CN(C(=O)[C@H]1CC(=O)c3ccccc31)C2	361331474	22	291.13	3.21
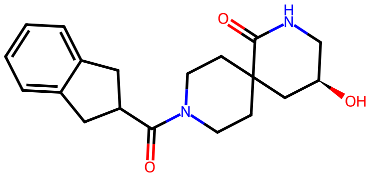	O=C(C1Cc2ccccc2C1)N1CCC2(CC1)C[C@H](O)CNC2=O	885016146	24	328.18	0.89
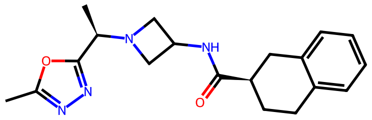	Cc1nnc([C@@H](C)N2CC(NC(=O)[C@@H]3CCc4ccccc4C3)C2)o1	1031012294	25	340.19	2.04
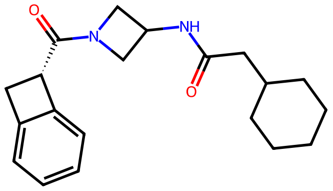	O=C(CC1CCCCC1)NC1CN(C(=O)[C@H]2Cc3ccccc32)C1	990168978	24	326.2	2.62

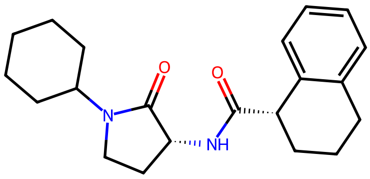	O=C(N[C@@H]1CCN(C2CCCCC2)C1=O)[C@H]1CCCc2ccccc21	675913095	25	340.22	3.16
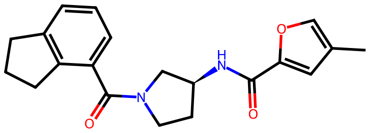	Cc1coc(C(=O)N[C@H]2CCN(C(=O)c3cccc4c3CCC4)C2)c1	938475105	25	338.16	2.72
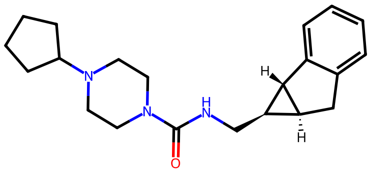	O=C(NC[C@@H]1[C@@H]2Cc3ccccc3[C@@H]12)N1CCN(C2CCCC2)CC1	155467129	25	339.23	2.84
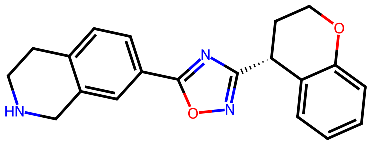	c1ccc2c(c1)OCC[C@H]2c1noc(-c2ccc3c(c2)CNCC3)n1	327002248	25	333.15	3.3
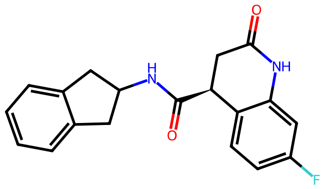	O=C1C[C@H](C(=O)NC2Cc3ccccc3C2)c2ccc(F)cc2N1	185727607	24	324.13	2.54
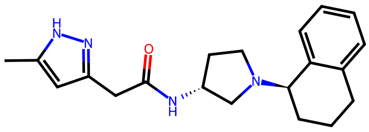	Cc1cc(CC(=O)N[C@@H]2CCN([C@@H]3CCCc4ccccc43)C2)n[nH]1	1010510340	25	338.21	2.53
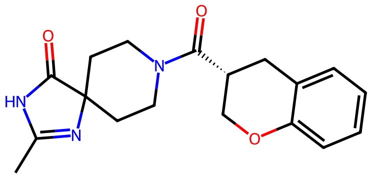	CC1=NC2(CCN(C(=O)[C@H]3COc4ccccc4C3)CC2)C(=O)N1	244788042	24	327.16	1.15
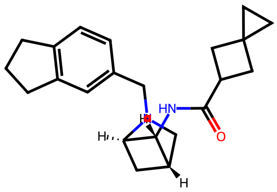	O=C(N[C@@H]1[C@H]2C[C@H]1N(Cc1ccc3c(c1)CCC3)C2)C1CC2(CC2)C1	1037578540	25	336.22	3.05

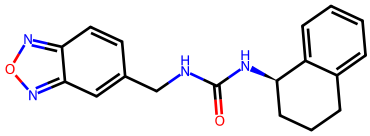	O=C(NCc1ccc2nonc2c1)N[C@@H]1CCCc2ccccc21	891452194	24	322.14	3.1
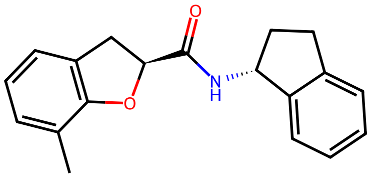	Cc1cccc2c1O[C@H](C(=O)N[C@@H]1CCc3ccccc31)C2	449205624	22	293.14	3.1
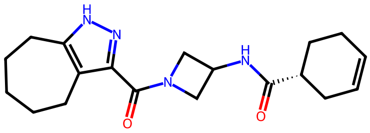	O=C(NC1CN(C(=O)c2n[nH]c3c2CCCCC3)C1)[C@H]1CC=CCC1	990222282	25	342.21	1.98
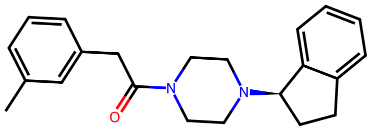	Cc1cccc(CC(=O)N2CCN([C@@H]3CCc4ccccc43)CC2)c1	949979819	25	334.2	3.37
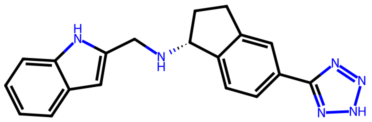	c1ccc2[nH]c(CN[C@@H]3CCc4cc(-c5nn[nH]n5)ccc43)cc2c1	1593577873	25	330.16	3.13
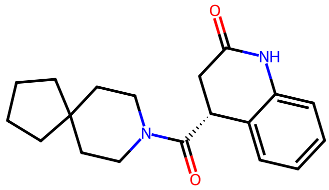	O=C1C[C@@H](C(=O)N2CCC3(CCCC3)CC2)c2ccccc2N1	150865991	23	312.18	3.3
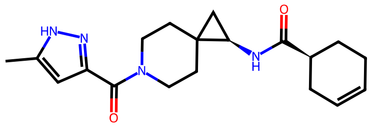	Cc1cc(C(=O)N2CCC3(CC2)C[C@H]3NC(=O)[C@@H]2CC=CCC2)n[nH]1	963030427	25	342.21	2.19
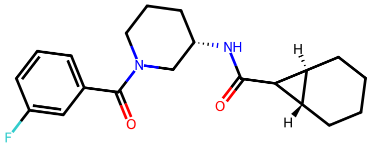	O=C(N[C@H]1CCCN(C(=O)c2cccc(F)c2)C1)C1[C@H]2CCCC[C@H]12	988719878	25	344.19	2.98

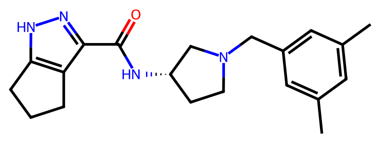	Cc1cc(C)cc(CN2CC[C@H](NC(=O)c3n[nH]c4c3CCC4)C2)c1	1015069672	25	338.21	2.52
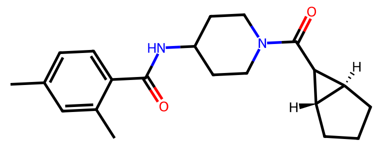	Cc1ccc(C(=O)NC2CCN(C(=O)C3[C@H]4CCC[C@H]34)CC2)c(C)c1	946079806	25	340.22	3.07
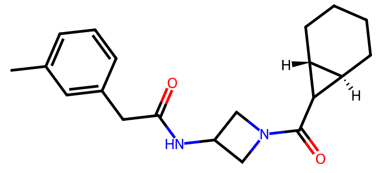	Cc1cccc(CC(=O)NC2CN(C(=O)C3[C@@H]4CCCC[C@@H]34)C2)c1	998709753	24	326.2	2.3
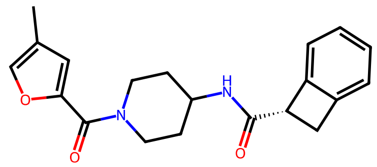	Cc1coc(C(=O)N2CCC(NC(=O)[C@H]3Cc4ccccc43)CC2)c1	947897292	25	338.16	2.65
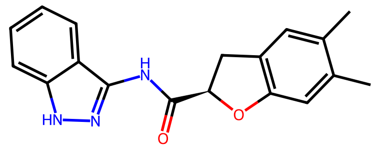	Cc1cc2c(cc1C)O[C@@H](C(=O)Nc1n[nH]c3ccccc13)C2	79464101	23	307.13	3.12
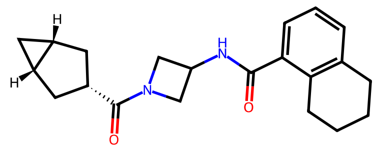	O=C(NC1CN(C(=O)[C@@H]2C[C@@H]3C[C@@H]3C2)C1)c1cccc2c1CCCC2	991900449	25	338.2	2.55
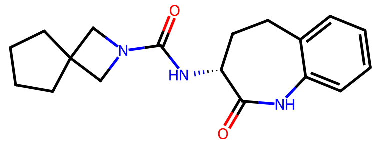	O=C1Nc2ccccc2CC[C@H]1NC(=O)N1CC2(CCCC2)C1	654631742	23	313.18	2.53
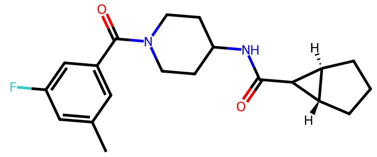	Cc1cc(F)cc(C(=O)N2CCC(NC(=O)C3[C@H]4CCC[C@H]34)CC2)c1	948549846	25	344.19	2.9

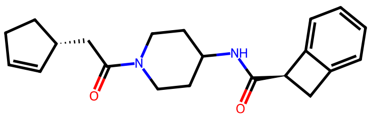	O=C(NC1CCN(C(=O)C[C@@H]2C=CCC2)CC1)[C@@H]1Cc2ccccc21	944065173	25	338.2	2.79
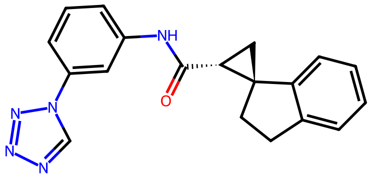	O=C(Nc1cccc(-n2cnnn2)c1)[C@@H]1C[C@@]12CCc1ccccc12	174630313	25	331.14	2.5
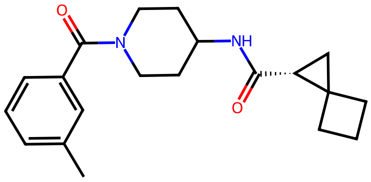	Cc1cccc(C(=O)N2CCC(NC(=O)[C@@H]3CC34CCC4)CC2)c1	943836219	24	326.2	2.91
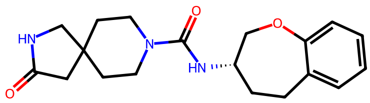	O=C1CC2(CCN(C(=O)N[C@H]3CCc4ccccc4OC3)CC2)CN1	374967944	25	343.19	1.69
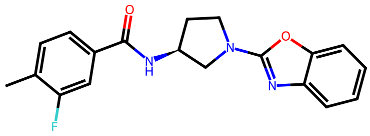	Cc1ccc(C(=O)N[C@H]2CCN(c3nc4ccccc4o3)C2)cc1F	1058485365	25	339.14	3.28
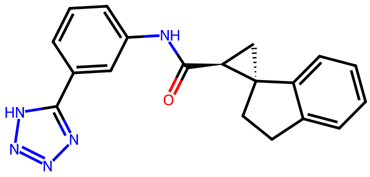	O=C(Nc1cccc(-c2nnn[nH]2)c1)[C@H]1C[C@]12CCc1ccccc12	266615504	25	331.14	2.71
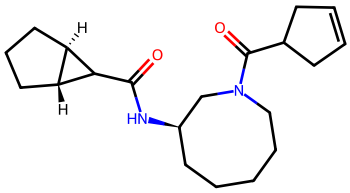	O=C(N[C@@H]1CCCCCN(C(=O)C2CC=CC2)C1)C1[C@@H]2CCC[C@@H]12	986948038	24	330.23	2.89
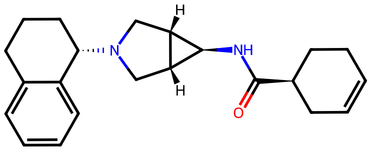	O=C(N[C@H]1[C@@H]2CN([C@H]3CCCc4ccccc43)C[C@@H]21)[C@@H]1CC=CCC1	1025675252	25	336.22	3.47

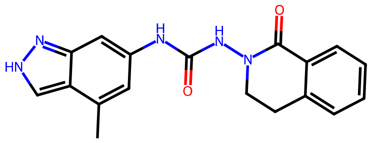	Cc1cc(NC(=O)NN2CCc3ccccc3C2=O)cc2n[nH]cc12	1310444663	25	335.14	2.61
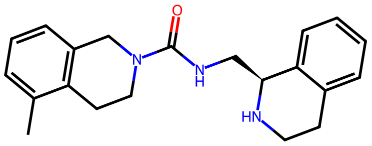	Cc1cccc2c1CCN(C(=O)NC[C@@H]1NCCc3ccccc31)C2	841996569	25	335.2	2.95
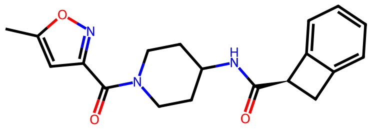	Cc1cc(C(=O)N2CCC(NC(=O)[C@@H]3Cc4ccccc43)CC2)no1	943979727	25	339.16	2.04
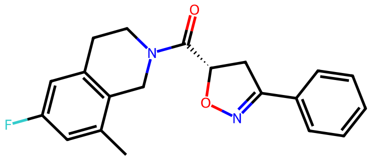	Cc1cc(F)cc2c1CN(C(=O)[C@@H]1CC(c3ccccc3)=NO1)CC2	800245489	25	338.14	3.21
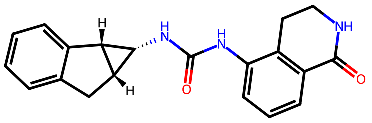	O=C(Nc1cccc2c1CCNC2=O)N[C@@H]1[C@@H]2Cc3ccccc3[C@@H]21	557142757	25	333.15	2.43
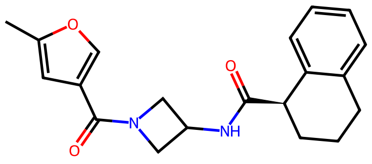	Cc1cc(C(=O)N2CC(NC(=O)[C@@H]3CCCc4ccccc43)C2)co1	995068544	25	338.16	2.65
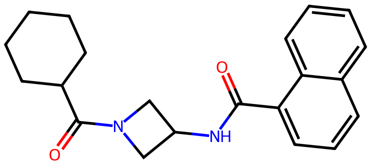	O=C(NC1CN(C(=O)C2CCCCC2)C1)c1cccc2ccccc12	999530479	25	336.18	3.36
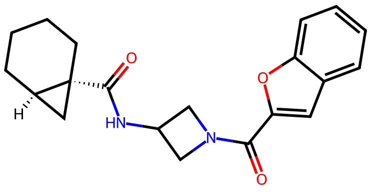	O=C(c1cc2ccccc2o1)N1CC(NC(=O)[C@@]23CCCC[C@@H]2C3)C1	999284287	25	338.16	2.95

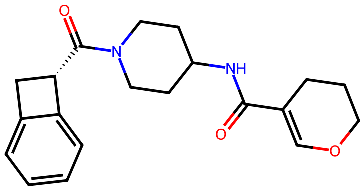	O=C(NC1CCN(C(=O)[C@H]2Cc3ccccc32)CC1)C1=COCCC1	948060100	25	340.18	2.13
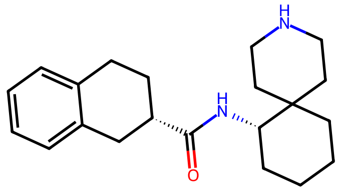	O=C(N[C@H]1CCCCC12CCNCC2)[C@H]1CCc2ccccc2C1	877406994	24	326.24	3.22
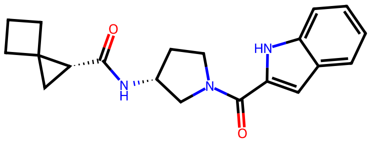	O=C(N[C@@H]1CCN(C(=O)c2cc3ccccc3[nH]2)C1)[C@@H]1CC12CCC2	936158247	25	337.18	2.69
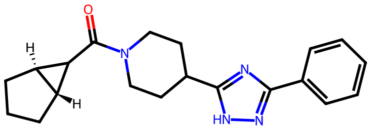	O=C(C1[C@@H]2CCC[C@@H]12)N1CCC(c2nc(-c3ccccc3)n[nH]2)CC1	267379063	25	336.2	3.22
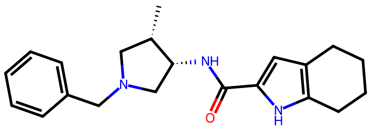	C[C@H]1CN(Cc2ccccc2)C[C@H]1NC(=O)c1cc2c([nH]1)CCCC2	1296043437	25	337.22	3.14
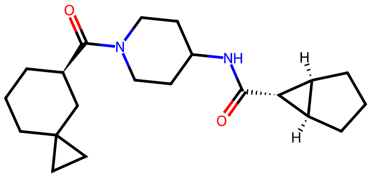	O=C(NC1CCN(C(=O)[C@@H]2CCCC3(CC3)C2)CC1)[C@@H]1[C@H]2CCC[C@H]21	946752527	25	344.25	3.11
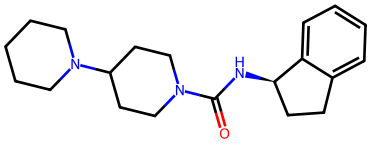	O=C(N[C@@H]1CCc2ccccc21)N1CCC(N2CCCCC2)CC1	45305100	24	327.23	3.33
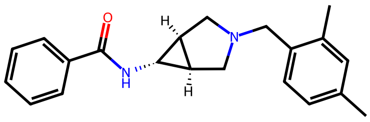	Cc1ccc(CN2C[C@H]3[C@H](NC(=O)c4ccccc4)[C@H]3C2)c(C)c1	958115686	24	320.19	3.16

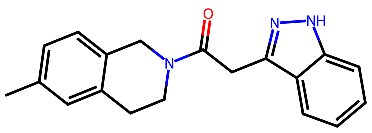	Cc1ccc2c(c1)CCN(C(=O)Cc1n[nH]c3ccccc13)C2	357171062	23	305.15	3.0
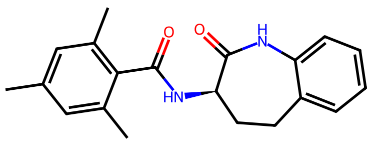	Cc1cc(C)c(C(=O)N[C@@H]2CCc3ccccc3NC2=O)c(C)c1	654006004	24	322.17	3.3
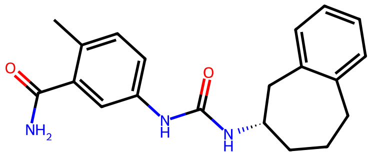	Cc1ccc(NC(=O)N[C@H]2CCCc3ccccc3C2)cc1C(N)=O	930145035	25	337.18	3.16
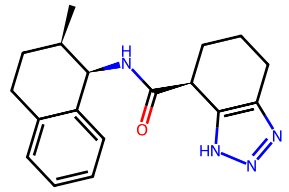	C[C@@H]1CCc2ccccc2[C@@H]1NC(=O)[C@H]1CCCc2nn[nH]c21	613124552	23	310.18	2.66
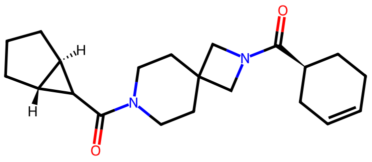	O=C(C1[C@@H]2CCC[C@@H]12)N1CCC2(CC1)CN(C(=O)[C@H]1CC=CCC1)C2	944903091	25	342.23	2.84
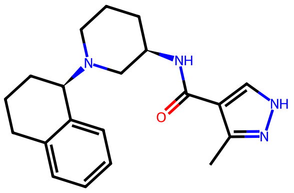	Cc1n[nH]cc1C(=O)N[C@@H]1CCCN([C@@H]2CCCc3ccccc32)C1	1008126843	25	338.21	2.99
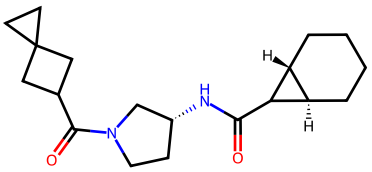	O=C(N[C@@H]1CCN(C(=O)C2CC3(CC3)C2)C1)C1[C@@H]2CCCC[C@@H]12	939897019	23	316.22	2.33
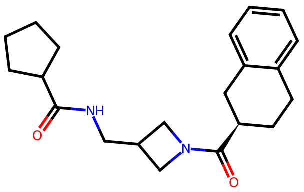	O=C(NCC1CN(C(=O)[C@@H]2CCc3ccccc3C2)C1)C1CCCC1	1000635984	25	340.22	2.56

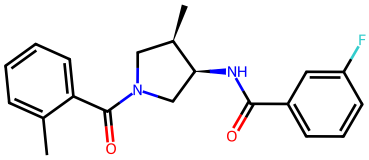	Cc1ccccc1C(=O)N1C[C@@H](C)[C@@H](NC(=O)c2cccc(F)c2)C1	966095831	25	340.16	3.02
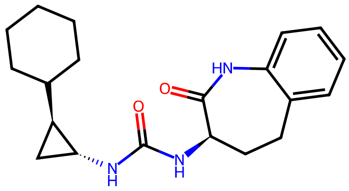	O=C(N[C@@H]1CCc2ccccc2NC1=O)N[C@@H]1C[C@H]1C1CCCCC1	1469095511	25	341.21	3.21
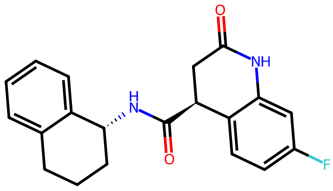	O=C1C[C@H](C(=O)N[C@@H]2CCCc3ccccc32)c2ccc(F)cc2N1	120391407	25	338.14	3.45
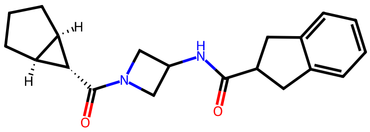	O=C(NC1CN(C(=O)[C@@H]2[C@H]3CCC[C@H]32)C1)C1Cc2ccccc2C1	1000449335	24	324.18	1.77
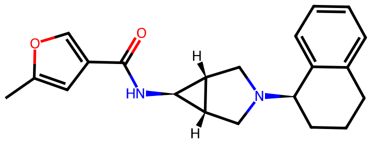	Cc1cc(C(=O)N[C@H]2[C@@H]3CN([C@@H]4CCCc5ccccc54)C[C@@H]32)co1	958672209	25	336.18	3.33
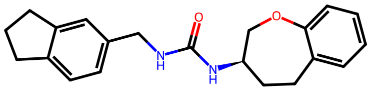	O=C(NCc1ccc2c(c1)CCC2)N[C@@H]1CCc2ccccc2OC1	1629078225	25	336.18	3.37
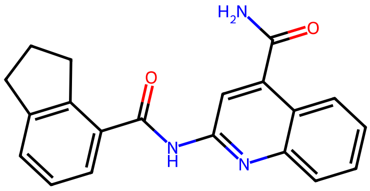	NC(=O)c1cc(NC(=O)c2cccc3c2CCC3)nc2ccccc12	1131361201	25	331.13	3.07
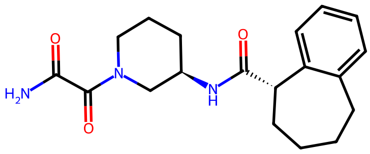	NC(=O)C(=O)N1CCC[C@@H](NC(=O)[C@H]2CCCCc3ccccc32)C1	951861438	25	343.19	1.09

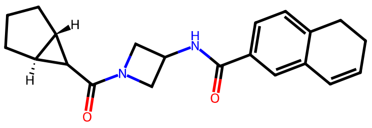	O=C(NC1CN(C(=O)C2[C@H]3CCC[C@H]23)C1)c1ccc2c(c1)C=CCC2	995372074	25	336.18	2.63
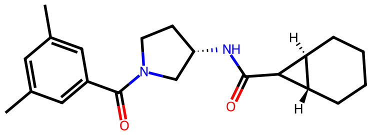	Cc1cc(C)cc(C(=O)N2CC[C@H](NC(=O)C3[C@H]4CCCC[C@H]34)C2)c1	937789039	25	340.22	3.07
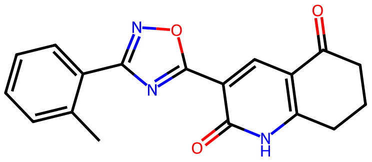	Cc1ccccc1-c1noc(-c2cc3c([nH]c2=O)CCCC3=O)n1	113729505	24	321.11	2.92
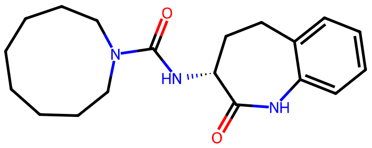	O=C1Nc2ccccc2CC[C@H]1NC(=O)N1CCCCCCCC1	1230517025	24	329.21	3.31
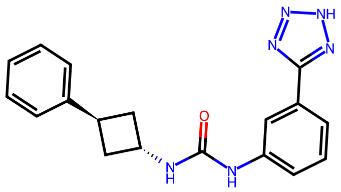	O=C(Nc1cccc(-c2nn[nH]n2)c1)N[C@H]1C[C@H](c2ccccc2)C1	1609566689	25	334.15	2.93
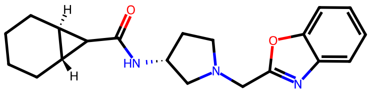	O=C(N[C@@H]1CCN(Cc2nc3ccccc3o2)C1)C1[C@@H]2CCCC[C@@H]12	1010437588	25	339.19	2.95
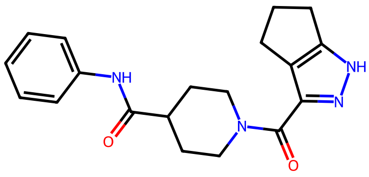	O=C(Nc1ccccc1)C1CCN(C(=O)c2n[nH]c3c2CCC3)CC1	298649993	25	338.17	2.39
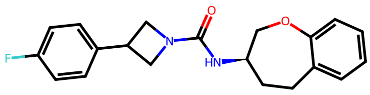	O=C(N[C@@H]1CCc2ccccc2OC1)N1CC(c2ccc(F)cc2)C1	1326770334	25	340.16	3.33

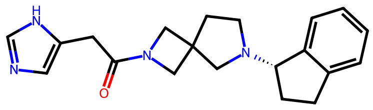	O=C(Cc1cnc[nH]1)N1CC2(CCN([C@H]3CCc4ccccc43)C2)C1	1073019268	25	336.2	2.17
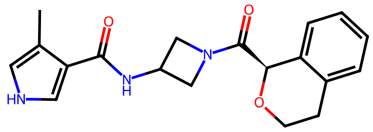	Cc1c[nH]cc1C(=O)NC1CN(C(=O)[C@@H]2OCCc3ccccc32)C1	991710102	25	339.16	1.58
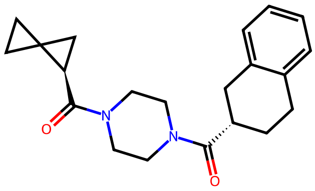	O=C([C@H]1CCc2ccccc2C1)N1CCN(C(=O)[C@@H]2CC23CC3)CC1	1012616217	25	338.2	2.26
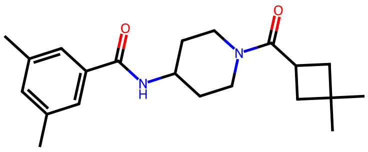	Cc1cc(C)cc(C(=O)NC2CCN(C(=O)C3CC(C)(C)C3)CC2)c1	946627506	25	342.23	3.46
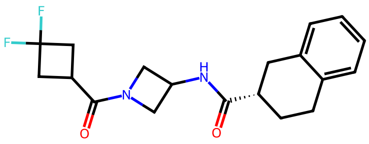	O=C(NC1CN(C(=O)C2CC(F)(F)C2)C1)[C@H]1CCc2ccccc2C1	1000536896	25	348.16	2.16
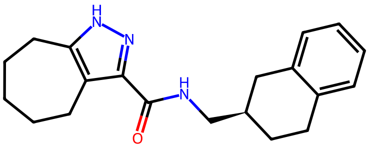	O=C(NC[C@@H]1CCc2ccccc2C1)c1n[nH]c2c1CCCCC2	1632926304	24	323.2	3.21
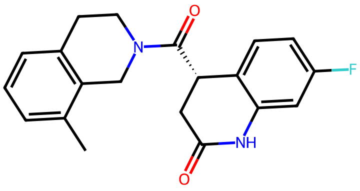	Cc1cccc2c1CN(C(=O)[C@H]1CC(=O)Nc3cc(F)ccc31)CC2	277064281	25	338.14	3.14
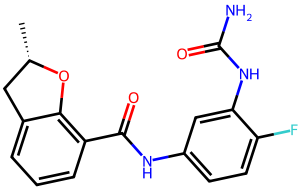	C[C@H]1Cc2cccc(C(=O)Nc3ccc(F)c(NC(N)=O)c3)c2O1	1413021931	24	329.12	2.89

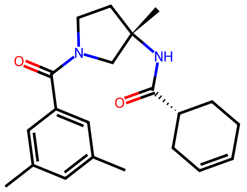	Cc1cc(C)cc(C(=O)N2CC[C@](C)(NC(=O)[C@H]3CC=CCC3)C2)c1	956688349	25	340.22	3.38
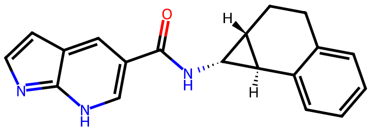	O=C(N[C@H]1[C@@H]2c3ccccc3CC[C@H]21)c1c[nH]c2nccc-2c1	563783482	23	303.14	2.97
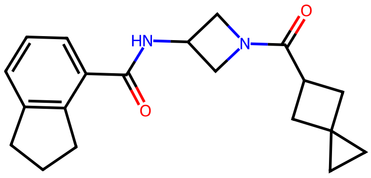	O=C(NC1CN(C(=O)C2CC3(CC3)C2)C1)c1cccc2c1CCC2	994396485	24	324.18	2.31
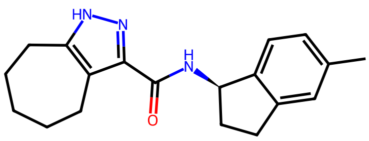	Cc1ccc2c(c1)CC[C@H]2NC(=O)c1n[nH]c2c1CCCCC2	473776370	23	309.18	3.4
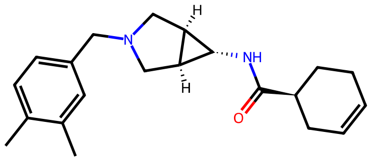	Cc1ccc(CN2C[C@H]3[C@H](NC(=O)[C@@H]4CC=CCC4)[C@H]3C2)cc1C	958208040	24	324.22	3.21
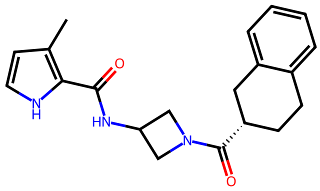	Cc1cc[nH]c1C(=O)NC1CN(C(=O)[C@H]2CCc3ccccc3C2)C1	991099751	25	337.18	2.07
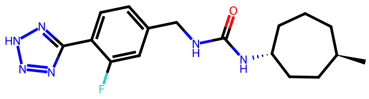	C[C@@H]1CCC[C@@H](NC(=O)NCc2ccc(-c3nn[nH]n3)c(F)c2)CC1	1609162745	25	346.19	2.77
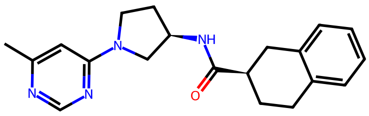	Cc1cc(N2CC[C@@H](NC(=O)[C@@H]3CCc4ccccc4C3)C2)ncn1	1058559922	25	336.2	2.29

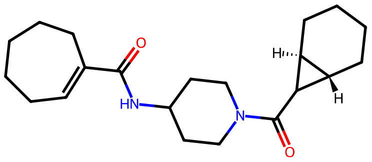	O=C(NC1CCN(C(=O)C2[C@H]3CCCC[C@H]23)CC1)C1=CCCCCC1	945957345	25	344.25	3.42
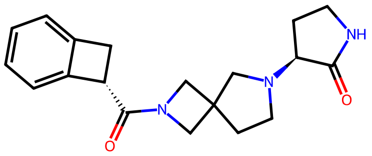	O=C1NCC[C@@H]1N1CCC2(CN(C(=O)[C@H]3Cc4ccccc43)C2)C1	1072931217	24	325.18	0.75
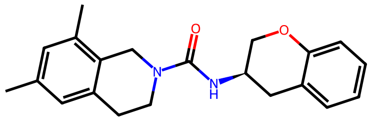	Cc1cc(C)c2c(c1)CCN(C(=O)N[C@H]1COc3ccccc3C1)C2	1666237928	25	336.18	3.37
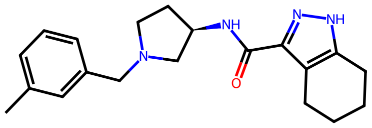	Cc1cccc(CN2CC[C@@H](NC(=O)c3n[nH]c4c3CCCC4)C2)c1	1015075253	25	338.21	2.6
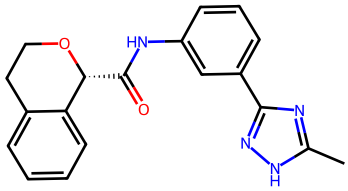	Cc1nc(-c2cccc(NC(=O)[C@H]3OCCc4ccccc43)c2)n[nH]1	137729905	25	334.14	3.03
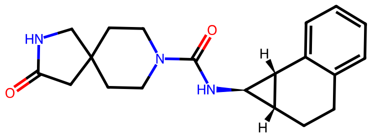	O=C1CC2(CCN(C(=O)N[C@@H]3[C@H]4CCc5ccccc5[C@H]43)CC2)CN1	269450126	25	339.19	2.03
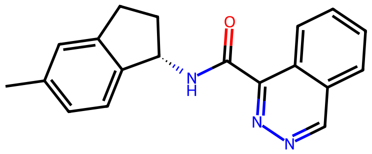	Cc1ccc2c(c1)CC[C@@H]2NC(=O)c1nncc2ccccc12	1356042778	23	303.14	3.36
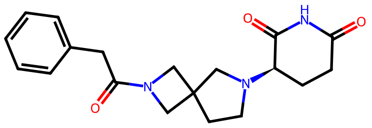	O=C1CC[C@@H](N2CCC3(CN(C(=O)Cc4ccccc4)C3)C2)C(=O)N1	1072862054	25	341.17	0.57

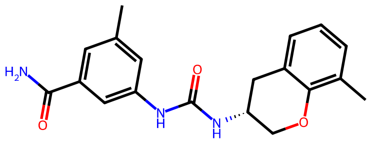	Cc1cc(NC(=O)N[C@H]2COc3c(C)cccc3C2)cc(C(N)=O)c1	455824989	25	339.16	2.53
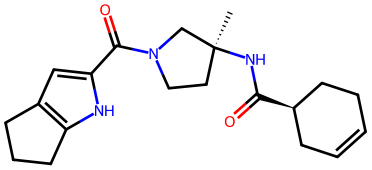	C[C@]1(NC(=O)[C@@H]2CC=CCC2)CCN(C(=O)c2cc3c([nH]2)CCC3)C1	956689633	25	341.21	2.58
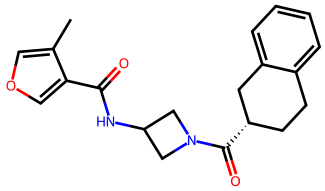	Cc1cocc1C(=O)NC1CN(C(=O)[C@H]2CCc3ccccc3C2)C1	997833646	25	338.16	2.33
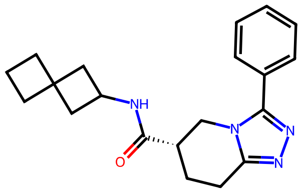	O=C(NC1CC2(CCC2)C1)[C@H]1CCc2nnc(-c3ccccc3)n2C1	652968079	25	336.2	2.96
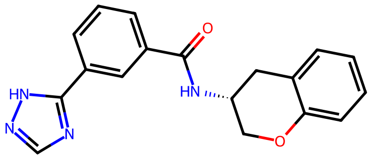	O=C(N[C@H]1COc2ccccc2C1)c1cccc(-c2ncn[nH]2)c1	179323030	24	320.13	2.21
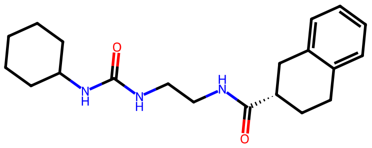	O=C(NCCNC(=O)[C@H]1CCc2ccccc2C1)NC1CCCCC1	566330202	25	343.23	2.54
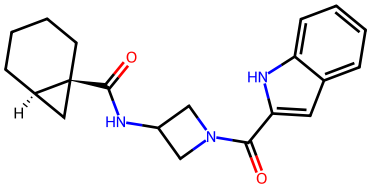	O=C(c1cc2ccccc2[nH]1)N1CC(NC(=O)[C@]23CCCC[C@@H]2C3)C1	999281468	25	337.18	2.69
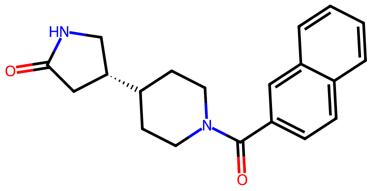	O=C1C[C@@H](C2CCN(C(=O)c3ccc4ccccc4c3)CC2)CN1	1551707942	24	322.17	2.83

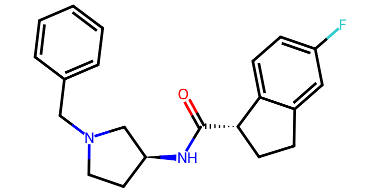	O=C(N[C@H]1CCN(Cc2ccccc2)C1)[C@H]1CCc2cc(F)ccc21	665576578	25	338.18	3.25
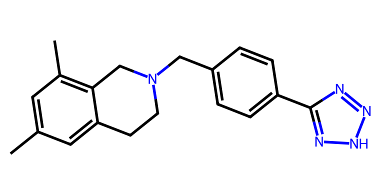	Cc1cc(C)c2c(c1)CCN(Cc1ccc(-c3nn[nH]n3)cc1)C2	1591950524	24	319.18	3.04
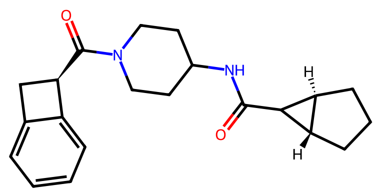	O=C(NC1CCN(C(=O)[C@@H]2Cc3ccccc32)CC1)C1[C@H]2CCC[C@H]12	948059459	25	338.2	2.48
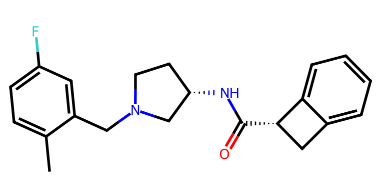	Cc1ccc(F)cc1CN1CC[C@H](NC(=O)[C@H]2Cc3ccccc32)C1	1015636085	25	338.18	3.16
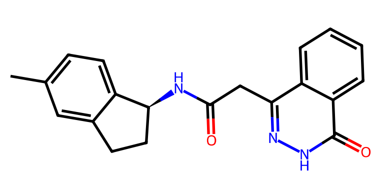	Cc1ccc2c(c1)CC[C@@H]2NC(=O)Cc1n[nH]c(=O)c2ccccc12	55659428	25	333.15	2.58
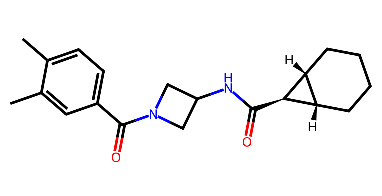	Cc1ccc(C(=O)N2CC(NC(=O)[C@@H]3[C@H]4CCCC[C@H]43)C2)cc1C	990666520	24	326.2	2.68
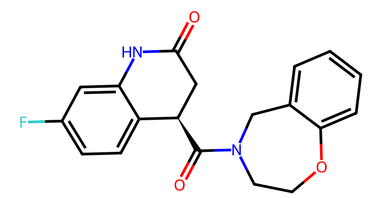	O=C1C[C@@H](C(=O)N2CCOc3ccccc3C2)c2ccc(F)cc2N1	376617183	25	340.12	2.67
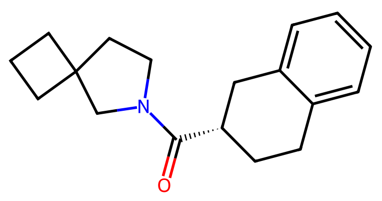	O=C([C@H]1CCc2ccccc2C1)N1CCC2(CCC2)C1	571450474	20	269.18	3.19

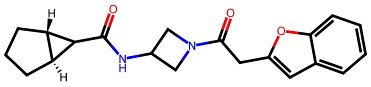	O=C(NC1CN(C(=O)Cc2cc3ccccc3o2)C1)C1[C@H]2CCC[C@H]12	990708135	25	338.16	2.35
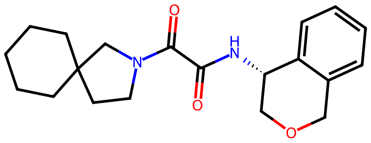	O=C(N[C@H]1COCc2ccccc21)C(=O)N1CCC2(CCCCC2)C1	1329006202	25	342.19	2.56
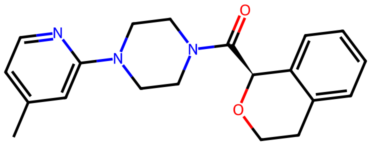	Cc1ccnc(N2CCN(C(=O)[C@@H]3OCCc4ccccc43)CC2)c1	124813392	25	337.18	2.35
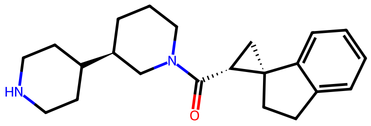	O=C([C@@H]1C[C@]12CCc1ccccc12)N1CCC[C@H](C2CCNCC2)C1	662596889	25	338.24	3.13
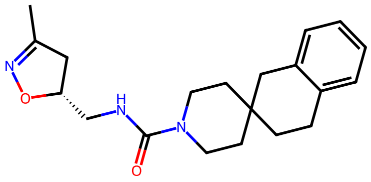	CC1=NO[C@@H](CNC(=O)N2CCC3(CCc4ccccc4C3)CC2)C1	1470999611	25	341.21	3.13
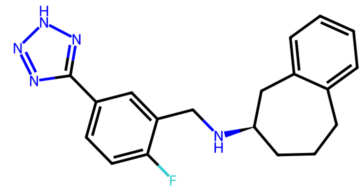	Fc1ccc(-c2nn[nH]n2)cc1CN[C@@H]1CCCc2ccccc2C1	1606316007	25	337.17	3.04
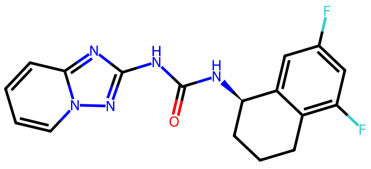	O=C(Nc1nc2ccccn2n1)N[C@@H]1CCCc2c(F)cc(F)cc21	1670721231	25	343.12	3.21
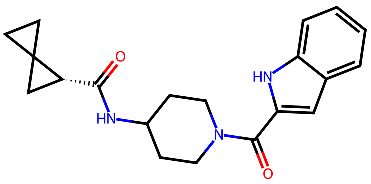	O=C(NC1CCN(C(=O)c2cc3ccccc3[nH]2)CC1)[C@@H]1CC12CC2	948490779	25	337.18	2.69

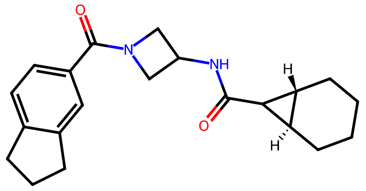	O=C(NC1CN(C(=O)c2ccc3c(c2)CCC3)C1)C1[C@@H]2CCCC[C@@H]12	990665618	25	338.2	2.55
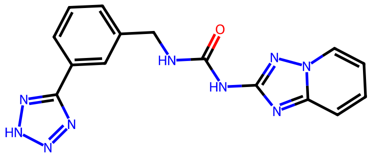	O=C(NCc1cccc(-c2nn[nH]n2)c1)Nc1nc2ccccn2n1	826442993	25	335.12	1.23
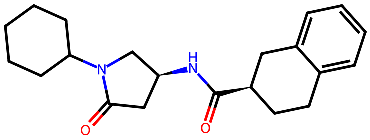	O=C(N[C@H]1CC(=O)N(C2CCCCC2)C1)[C@@H]1CCc2ccccc2C1	1362286746	25	340.22	2.84
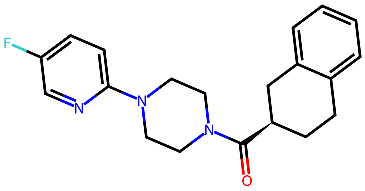	O=C([C@@H]1CCc2ccccc2C1)N1CCN(c2ccc(F)cn2)CC1	519759990	25	339.17	2.67
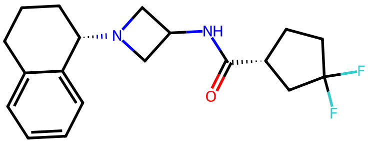	O=C(NC1CN([C@H]2CCCc3ccccc32)C1)[C@@H]1CCC(F)(F)C1	1030412203	24	334.19	3.3
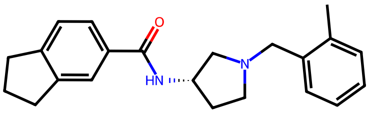	Cc1ccccc1CN1CC[C@H](NC(=O)c2ccc3c(c2)CCC3)C1	1016222446	25	334.2	3.49
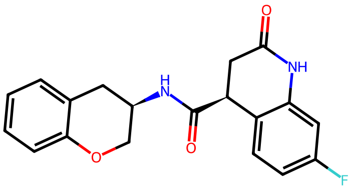	O=C1C[C@H](C(=O)N[C@H]2COc3ccccc3C2)c2ccc(F)cc2N1	53045178	25	340.12	2.37
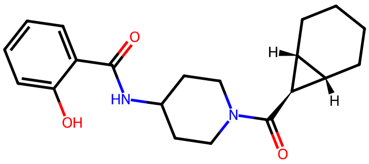	O=C(NC1CCN(C(=O)[C@@H]2[C@H]3CCCC[C@H]32)CC1)c1ccccc1O	945957382	25	342.19	2.55

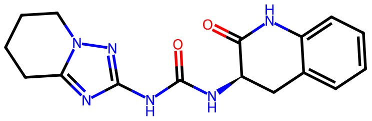	O=C(Nc1nc2n(n1)CCCC2)N[C@@H]1Cc2ccccc2NC1=O	529116720	24	326.15	1.3
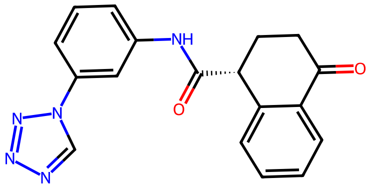	O=C1CC[C@@H](C(=O)Nc2cccc(-n3cnnn3)c2)c2ccccc21	339836157	25	333.12	2.36
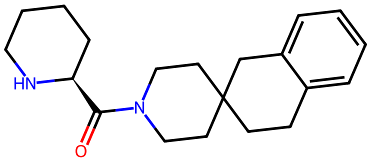	O=C([C@@H]1CCCCN1)N1CCC2(CCc3ccccc3C2)CC1	653178588	23	312.22	2.93
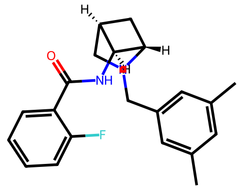	Cc1cc(C)cc(CN2C[C@H]3C[C@H]2[C@H]3NC(=O)c2ccccc2F)c1	1037280083	25	338.18	3.45
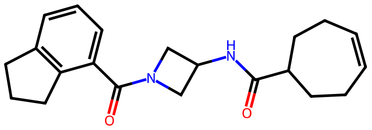	O=C(NC1CN(C(=O)c2cccc3c2CCC3)C1)C1CCC=CCC1	991817796	25	338.2	2.86
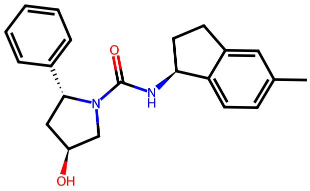	Cc1ccc2c(c1)CC[C@@H]2NC(=O)N1C[C@@H](O)C[C@@H]1c1ccccc1	413162451	25	336.18	3.5
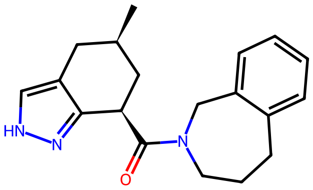	C[C@@H]1Cc2c[nH]nc2[C@H](C(=O)N2CCCc3ccccc3C2)C1	623191803	23	309.18	3.05
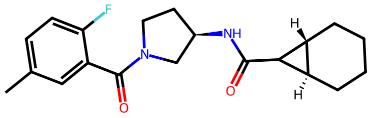	Cc1ccc(F)c(C(=O)N2CC[C@@H](NC(=O)C3[C@@H]4CCCC[C@@H]34)C2)c1	936274003	25	344.19	2.9

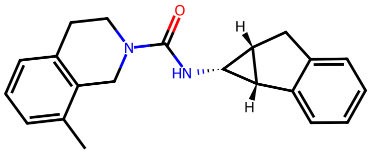	Cc1cccc2c1CN(C(=O)N[C@@H]1[C@@H]3Cc4ccccc4[C@@H]31)CC2	429102463	24	318.17	3.4
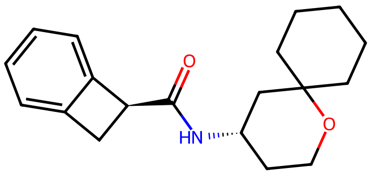	O=C(N[C@H]1CCOC2(CCCCC2)C1)[C@H]1Cc2ccccc21	335892036	22	299.19	3.32
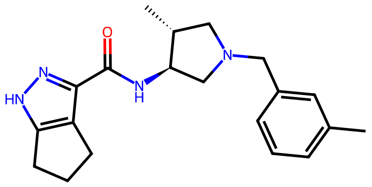	Cc1cccc(CN2C[C@@H](NC(=O)c3n[nH]c4c3CCC4)[C@H](C)C2)c1	1080027549	25	338.21	2.46
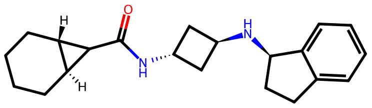	O=C(N[C@H]1C[C@H](N[C@@H]2CCc3ccccc32)C1)C1[C@H]2CCCC[C@H]12	1020052289	24	324.22	3.35
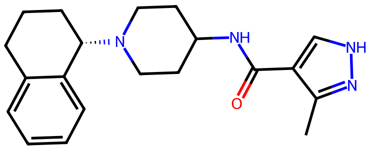	Cc1n[nH]cc1C(=O)NC1CCN([C@H]2CCCc3ccccc32)CC1	1003408612	25	338.21	2.99
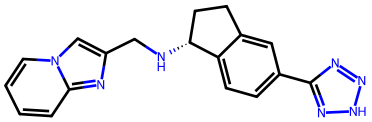	c1ccn2cc(CN[C@@H]3CCc4cc(-c5nn[nH]n5)ccc43)nc2c1	1593582565	25	331.15	2.29
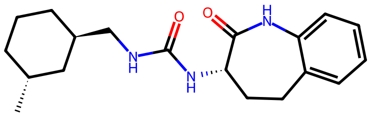	C[C@@H]1CCC[C@@H](CNC(=O)N[C@H]2CCc3ccccc3NC2=O)C1	1563768274	24	329.21	3.07
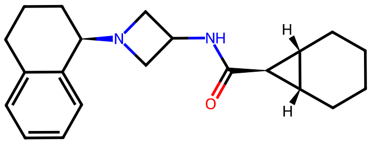	O=C(NC1CN([C@@H]2CCCc3ccccc32)C1)[C@@H]1[C@H]2CCCC[C@H]21	1030187234	24	324.22	3.3

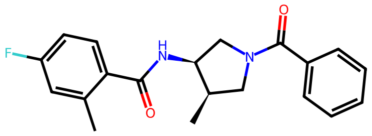	Cc1cc(F)ccc1C(=O)N[C@H]1CN(C(=O)c2ccccc2)C[C@H]1C	966090810	25	340.16	3.02
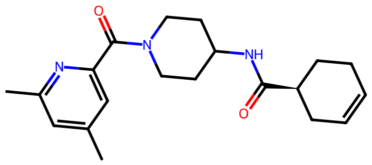	Cc1cc(C)nc(C(=O)N2CCC(NC(=O)[C@@H]3CC=CCC3)CC2)c1	947307783	25	341.21	2.78
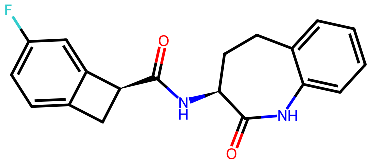	O=C1Nc2ccccc2CC[C@@H]1NC(=O)[C@H]1Cc2ccc(F)cc21	653998861	24	324.13	2.54
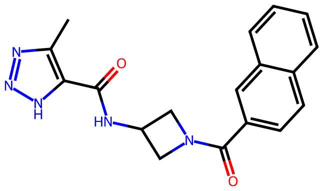	Cc1nn[nH]c1C(=O)NC1CN(C(=O)c2ccc3ccccc3c2)C1	997340816	25	335.14	1.52
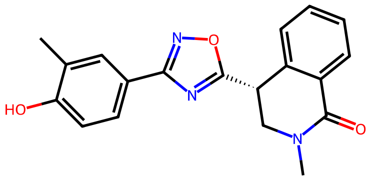	Cc1cc(-c2noc([C@H]3CN(C)C(=O)c4ccccc43)n2)ccc1O	350605548	25	335.13	2.97
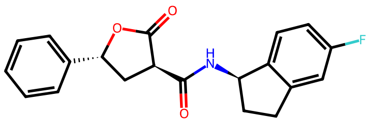	O=C(N[C@@H]1CCc2cc(F)ccc21)[C@H]1C[C@H](c2ccccc2)OC1=O	290409578	25	339.13	3.23
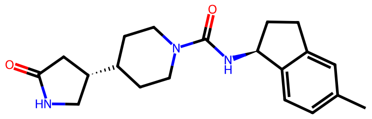	Cc1ccc2c(c1)CC[C@@H]2NC(=O)N1CCC([C@@H]2CNC(=O)C2)CC1	1553385646	25	341.21	2.54
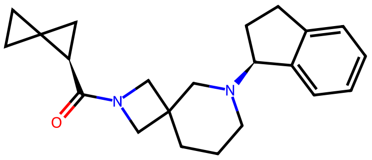	O=C([C@@H]1CC12CC2)N1CC2(CCCN([C@H]3CCc4ccccc43)C2)C1	982038871	25	336.22	3.4

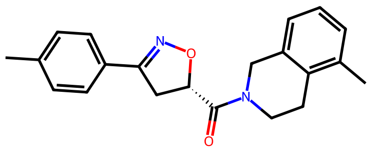	Cc1ccc(C2=NO[C@H](C(=O)N3CCc4c(C)cccc4C3)C2)cc1	1125996856	25	334.17	3.38
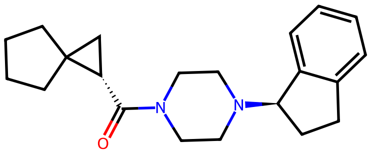	O=C([C@H]1CC12CCCC2)N1CCN([C@@H]2CCc3ccccc32)CC1	957078631	24	324.22	3.4
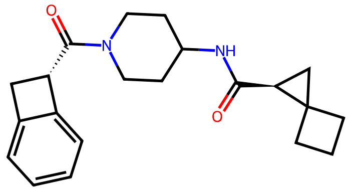	O=C(NC1CCN(C(=O)[C@H]2Cc3ccccc32)CC1)[C@H]1CC12CCC2	948062511	25	338.2	2.62
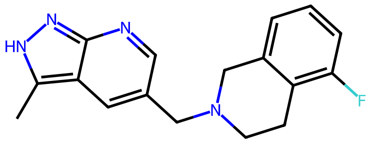	Cc1[nH]nc2ncc(CN3CCc4c(F)cccc4C3)cc12	353530495	22	296.14	2.96
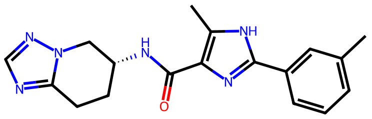	Cc1cccc(-c2nc(C(=O)N[C@@H]3CCc4ncnn4C3)c(C)[nH]2)c1	270259549	25	336.17	2.03
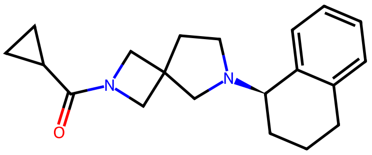	O=C(C1CC1)N1CC2(CCN([C@@H]3CCCc4ccccc43)C2)C1	1072378385	23	310.2	3.01
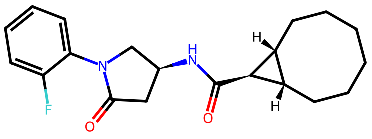	O=C(N[C@H]1CC(=O)N(c2ccccc2F)C1)[C@@H]1[C@H]2CCCCCC[C@H]21	1460432396	25	344.19	3.26
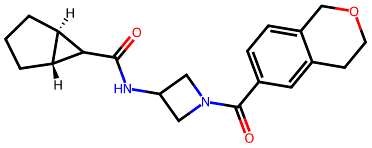	O=C(NC1CN(C(=O)c2ccc3c(c2)CCOC3)C1)C1[C@@H]2CCC[C@@H]12	990699728	25	340.18	1.75

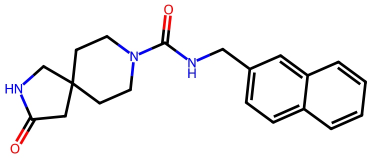	O=C1CC2(CCN(C(=O)NCc3ccc4cccc<br>c4c3)CC2)CN1	74103042	25	337.18	2.65
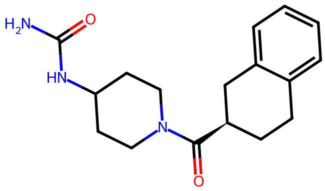	NC(=O)NC1CCN(C(=O)[C@@H]2CC<br>c3ccccc3C2)CC1	565073192	22	301.18	1.45
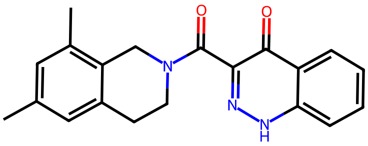	Cc1cc(C)c2c(c1)CCN(C(=O)c1n[nH]c3<br>ccccc3c1=O)C2	186386354	25	333.15	2.74
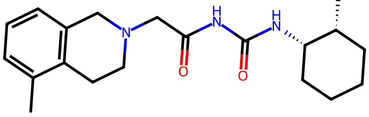	Cc1cccc2c1CCN(CC(=O)NC(=O)N[C<br>@H]1CCCC[C@H]1C)C2	729478533	25	343.23	2.76
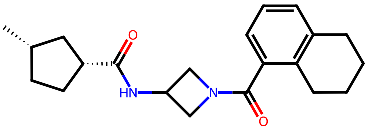	C[C@H]1CC[C@@H](C(=O)NC2CN(<br>C(=O)c3cccc4c3CCCC4)C2)C1	991435436	25	340.22	2.94
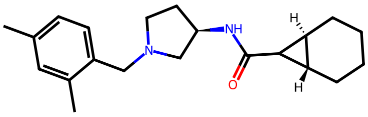	Cc1ccc(CN2CC[C@@H](NC(=O)C3[C<br>@H]4CCCC[C@H]34)C2)c(C)c1	1032834765	24	326.24	3.43
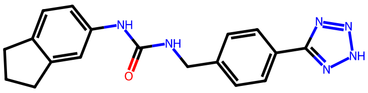	O=C(NCc1ccc(-c2nn[nH]n2)cc1)Nc1cc<br>c2c(c1)CCC2	1593128786	25	334.15	2.68
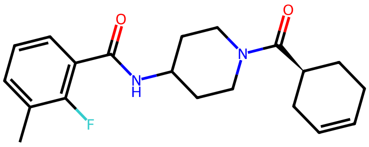	Cc1cccc(C(=O)NC2CCN(C(=O)[C@@<br>H]3CC=CCC3)CC2)c1F	944126941	25	344.19	3.21

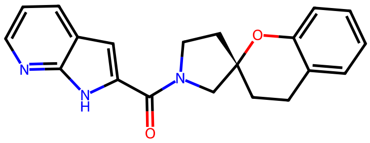	O=C(c1cc2cccnc2[nH]1)N1CC[C@@]2(CCc3ccccc3O2)C1	571860167	25	333.15	3.17
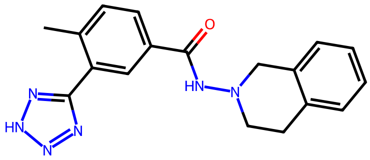	Cc1ccc(C(=O)NN2CCc3ccccc3C2)cc1-c1nn[nH]n1	1581170843	25	334.15	1.88
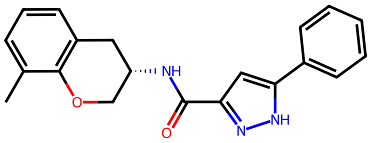	Cc1cccc2c1OC[C@@H](NC(=O)c1cc(-c3ccccc3)[nH]n1)C2	355598344	25	333.15	3.12
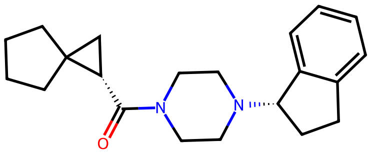	O=C([C@H]1CC12CCCC2)N1CCN([C@H]2CCc3ccccc32)CC1	957078625	24	324.22	3.4
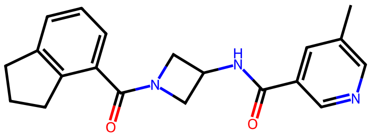	Cc1cncc(C(=O)NC2CN(C(=O)c3cccc4c3CCC4)C2)c1	990617521	25	335.16	2.13
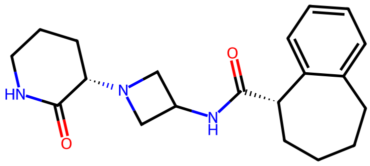	O=C(NC1CN([C@H]2CCCNC2=O)C1)[C@H]1CCCCc2ccccc21	1031452579	25	341.21	1.58
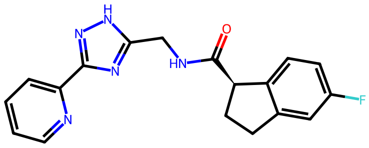	O=C(NCc1nc(-c2ccccn2)n[nH]1)[C@@H]1CCc2cc(F)ccc21	1468061323	25	337.13	2.35
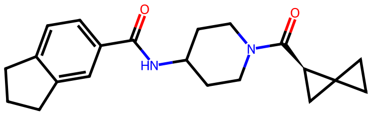	O=C(NC1CCN(C(=O)[C@@H]2CC23CC3)CC1)c1ccc2c(c1)CCC2	948557026	25	338.2	2.7

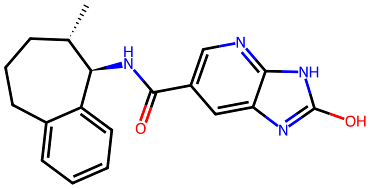	C[C@H]1CCCc2ccccc2[C@@H]1NC(=O)c1cnc2[nH]c(O)nc2c1	572563545	25	336.16	3.11
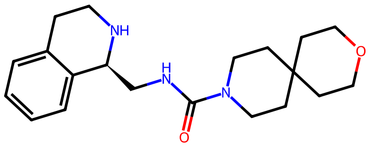	O=C(NC[C@@H]1NCCc2ccccc21)N1CCC2(CCOCC2)CC1	841992785	25	343.23	2.48
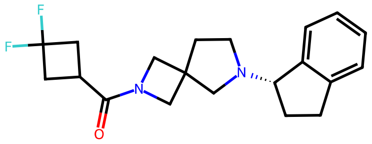	O=C(C1CC(F)(F)C1)N1CC2(CCN([C@H]3CCc4ccccc43)C2)C1	1072604708	25	346.19	3.25
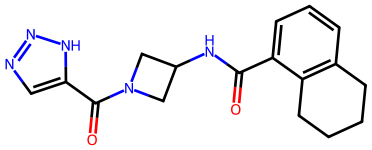	O=C(NC1CN(C(=O)c2cnn[nH]2)C1)c1cccc2c1CCCC2	991902962	24	325.15	0.94
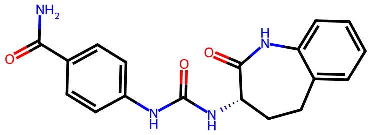	NC(=O)c1ccc(NC(=O)N[C@H]2CCc3ccccc3NC2=O)cc1	1365009987	25	338.14	1.86
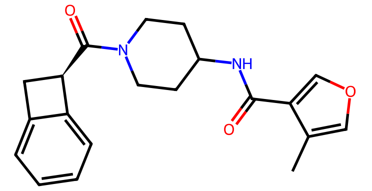	Cc1cocc1C(=O)NC1CCN(C(=O)[C@@H]2Cc3ccccc32)CC1	948063437	25	338.16	2.65
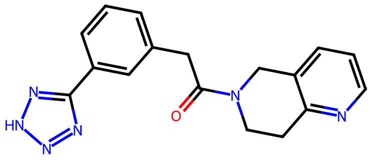	O=C(Cc1cccc(-c2nn[nH]n2)c1)N1CCc2ncccc2C1	826422558	24	320.14	1.39
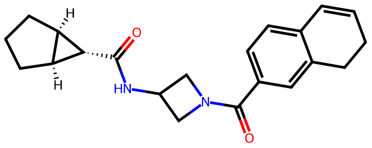	O=C(NC1CN(C(=O)c2ccc3c(c2)CCC=C3)C1)[C@@H]1[C@H]2CCC[C@H]21	990697146	25	336.18	2.63

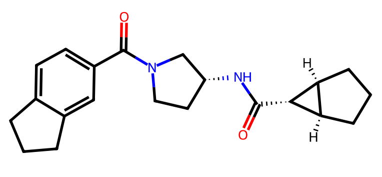	O=C(N[C@@H]1CCN(C(=O)c2ccc3c(c2)CCC3)C1)[C@@H]1[C@H]2CCC[C@H]21	939933092	25	338.2	2.55
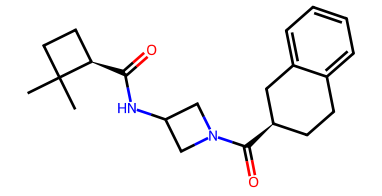	CC1(C)CC[C@H]1C(=O)NC1CN(C(=O)[C@@H]2CCc3ccccc3C2)C1	997966737	25	340.22	2.55
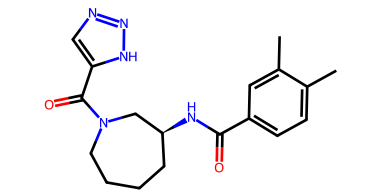	Cc1ccc(C(=O)N[C@H]2CCCCN(C(=O)c3cnn[nH]3)C2)cc1C	943526688	25	341.19	1.85
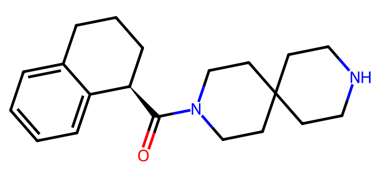	O=C([C@@H]1CCCc2ccccc21)N1CCC2(CCNCC2)CC1	261613451	23	312.22	3.1
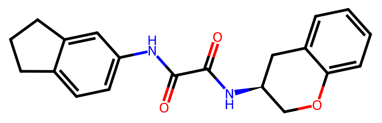	O=C(Nc1ccc2c(c1)CCC2)C(=O)N[C@@H]1COc2ccccc2C1	42050028	25	336.15	2.23
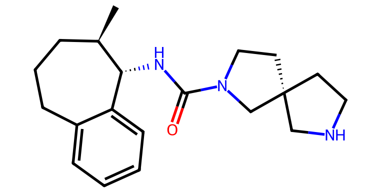	C[C@@H]1CCCc2ccccc2[C@H]1NC(=O)N1CC[C@@]2(CCNC2)C1	418017791	24	327.23	3.1
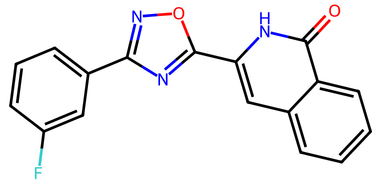	O=c1[nH]c(-c2nc(-c3cccc(F)c3)no2)cc2ccccc12	109603867	23	307.08	3.38
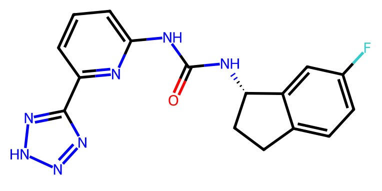	O=C(Nc1cccc(-c2nn[nH]n2)n1)N[C@H]1CCc2ccc(F)cc21	1582645403	25	339.12	2.21

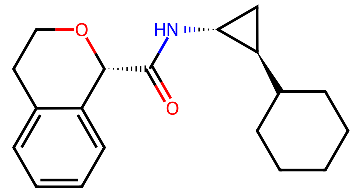	O=C(N[C@@H]1C[C@H]1C1CCCCC1)[C@H]1OCCc2ccccc21	441448740	22	299.19	3.39
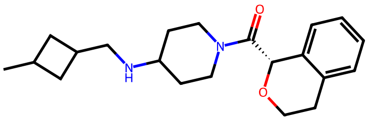	CC1CC(CNC2CCN(C(=O)[C@H]3OCCc4ccccc43)CC2)C1	996864493	25	342.23	2.93
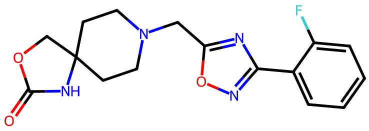	O=C1NC2(CCN(Cc3nc(-c4ccccc4F)no3)CC2)CO1	496911419	24	332.13	1.95
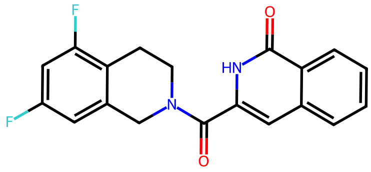	O=C(c1cc2ccccc2c(=O)[nH]1)N1CCc2c(F)cc(F)cc2C1	122584541	25	340.1	3.0
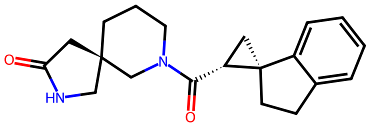	O=C1C[C@]2(CCCN(C(=O)[C@@H]3C[C@]34CCc3ccccc34)C2)CN1	341499940	24	324.18	2.02
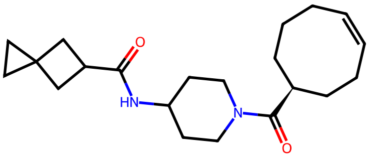	O=C(NC1CCN(C(=O)[C@@H]2CCC=CCCC2)CC1)C1CC2(CC2)C1	947247103	25	344.25	3.42
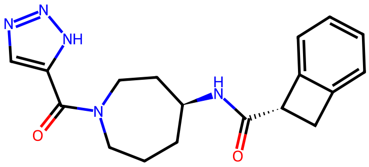	O=C(N[C@H]1CCCN(C(=O)c2cnn[nH]2)CC1)[C@H]1Cc2ccccc21	965142454	25	339.17	1.26
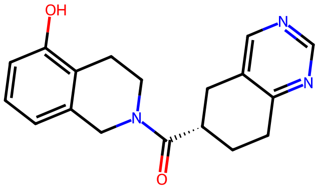	O=C([C@H]1CCc2ncncc2C1)N1CCc2c(O)cccc2C1	629152312	23	309.15	1.87

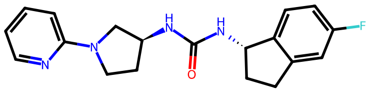	O=C(N[C@H]1CCN(c2ccccn2)C1)N[C@H]1CCc2cc(F)ccc21	245503432	25	340.17	2.79
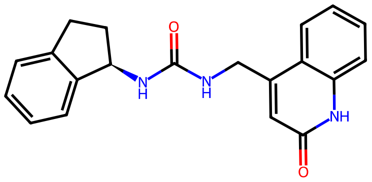	O=C(NCc1cc(=O)[nH]c2ccccc12)N[C@@H]1CCc2ccccc21	85388539	25	333.15	3.01
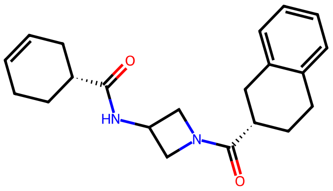	O=C(NC1CN(C(=O)[C@H]2CCc3ccccc3C2)C1)[C@H]1CC=CCC1	990221460	25	338.2	2.47
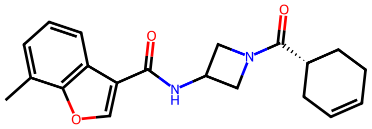	Cc1cccc2c(C(=O)NC3CN(C(=O)[C@H]4CC=CCC4)C3)coc12	994205437	25	338.16	3.04
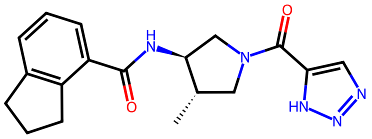	C[C@H]1CN(C(=O)c2cnn[nH]2)C[C@@H]1NC(=O)c1cccc2c1CCC2	966594942	25	339.17	1.18
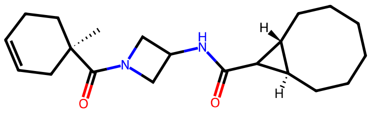	C[C@]1(C(=O)N2CC(NC(=O)C3[C@@H]4CCCCCC[C@@H]34)C2)CC=CCC1	1000504850	25	344.25	3.28
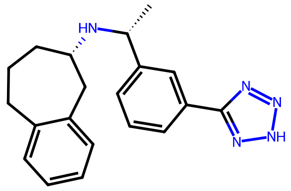	C[C@@H](N[C@H]1CCCc2ccccc2C1)c1cccc(-c2nn[nH]n2)c1	1603656509	25	333.2	3.46
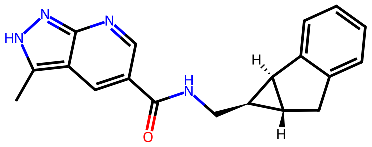	Cc1[nH]nc2ncc(C(=O)NC[C@@H]3[C@H]4Cc5ccccc5[C@@H]43)cc12	152515236	24	318.15	2.58

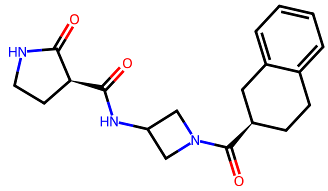	O=C1NCC[C@@H]1C(=O)NC1CN(C(=O)[C@@H]2CCc3ccccc3C2)C1	997424322	25	341.17	0.25
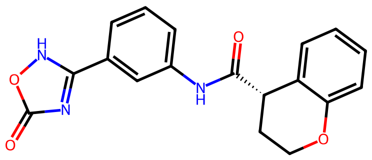	O=C(Nc1cccc(-c2nc(=O)o[nH]2)c1)[C@H]1CCOc2ccccc21	591188480	25	337.11	2.53
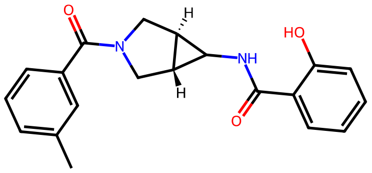	Cc1cccc(C(=O)N2C[C@H]3C(NC(=O)c4ccccc4O)[C@@H]3C2)c1	1012785408	25	336.15	2.2
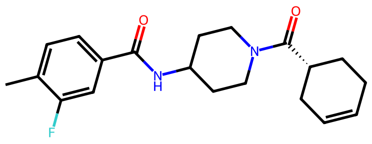	Cc1ccc(C(=O)NC2CCN(C(=O)[C@H]3CC=CCC3)CC2)cc1F	944127719	25	344.19	3.21
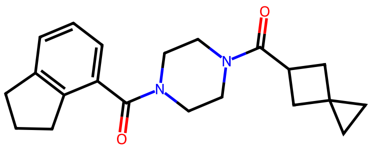	O=C(c1cccc2c1CCC2)N1CCN(C(=O)C2CC3(CC3)C2)CC1	1011292733	25	338.2	2.65
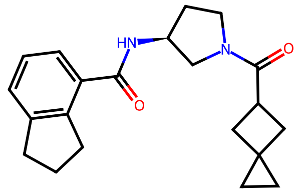	O=C(N[C@H]1CCN(C(=O)C2CC3(CC3)C2)C1)c1cccc2c1CCC2	938149373	25	338.2	2.7
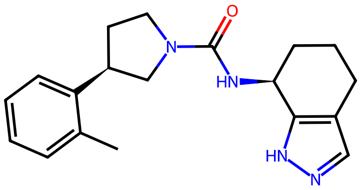	Cc1ccccc1[C@H]1CCN(C(=O)N[C@H]2CCCc3cn[nH]c32)C1	269028646	24	324.2	3.29
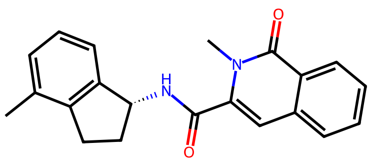	Cc1cccc2c1CC[C@H]2NC(=O)c1cc2ccccc2c(=O)n1C	556820120	25	332.15	3.26

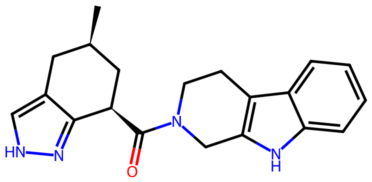	C[C@@H]1Cc2c[nH]nc2[C@H](C(=O)N2CCc3c([nH]c4ccccc34)C2)C1	623206518	25	334.18	3.14
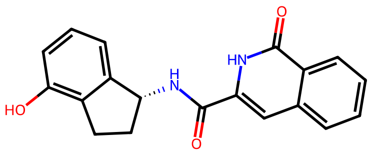	O=C(N[C@@H]1CCc2c(O)cccc21)c1cc2ccccc2c(=O)[nH]1	74141574	24	320.12	2.65
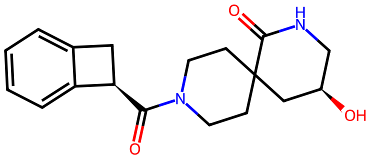	O=C([C@@H]1Cc2ccccc21)N1CCC2(CC1)C[C@H](O)CNC2=O	885019349	23	314.16	0.82
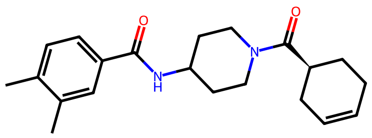	Cc1ccc(C(=O)NC2CCN(C(=O)[C@@H]3CC=CCC3)CC2)cc1C	944105721	25	340.22	3.38
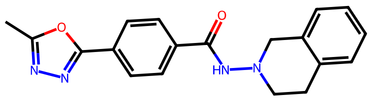	Cc1nnc(-c2ccc(C(=O)NN3CCc4ccccc4C3)cc2)o1	1300426999	25	334.14	2.75
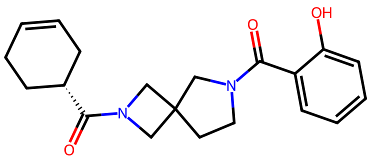	O=C(c1ccccc1O)N1CCC2(C1)CN(C(=O)[C@H]1CC=CCC1)C2	1018770284	25	340.18	2.42
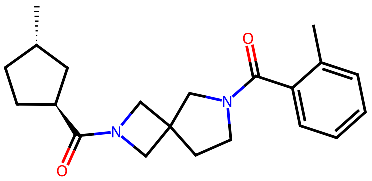	Cc1ccccc1C(=O)N1CCC2(C1)CN(C(=O)[C@H]1CC[C@H](C)C1)C2	1019019666	25	340.22	3.11
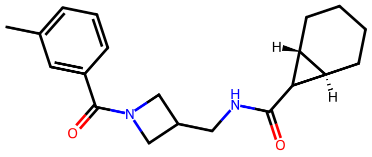	Cc1cccc(C(=O)N2CC(CNC(=O)C3[C@@H]4CCCC[C@@H]34)C2)c1	1001300700	24	326.2	2.62

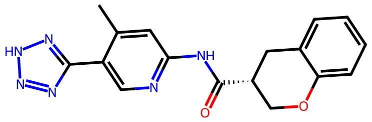	Cc1cc(NC(=O)[C@H]2COc3ccccc3C2)ncc1-c1nn[nH]n1	1580978797	25	336.13	1.76
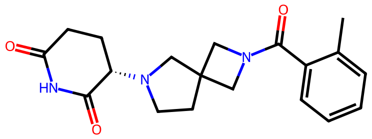	Cc1ccccc1C(=O)N1CC2(CCN([C@H]3CCC(=O)NC3=O)C2)C1	1072413828	25	341.17	0.95
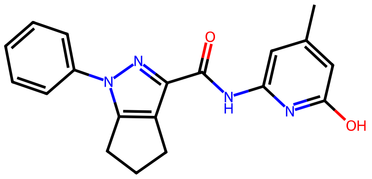	Cc1cc(O)nc(NC(=O)c2nn(-c3ccccc3)c3c2CCC3)c1	1469286674	25	334.14	3.02
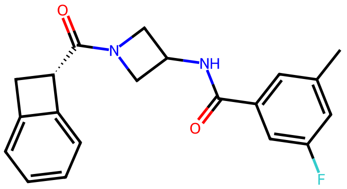	Cc1cc(F)cc(C(=O)NC2CN(C(=O)[C@H]3Cc4ccccc43)C2)c1	1000442068	25	338.14	2.41
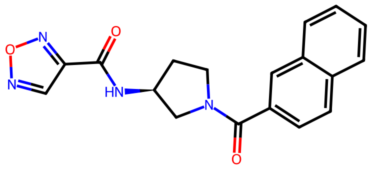	O=C(N[C@H]1CCN(C(=O)c2ccc3ccccc3c2)C1)c1cnon1	938508769	25	336.12	1.87
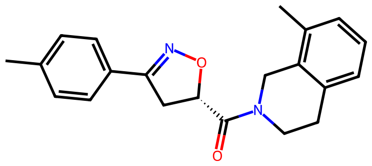	Cc1ccc(C2=NO[C@H](C(=O)N3CCc4cccc(C)c4C3)C2)cc1	1435877043	25	334.17	3.38
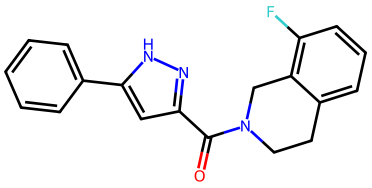	O=C(c1cc(-c2ccccc2)[nH]n1)N1CCc2cccc(F)c2C1	348355777	24	321.13	3.41
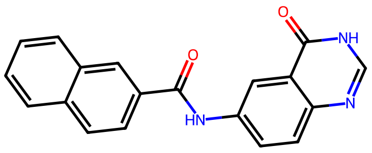	O=C(Nc1ccc2nc[nH]c(=O)c2c1)c1ccc2ccccc2c1	63829202	24	315.1	3.33

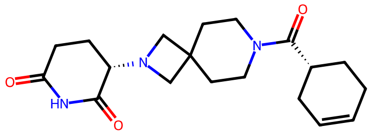	O=C1CC[C@H](N2CC3(CCN(C(=O)[C@H]4CC=CCC4)CC3)C2)C(=O)N1	1035648623	25	345.21	1.07
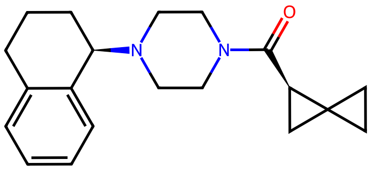	O=C([C@@H]1CC12CC2)N1CCN([C@@H]2CCCc3ccccc32)CC1	950710789	23	310.2	3.01
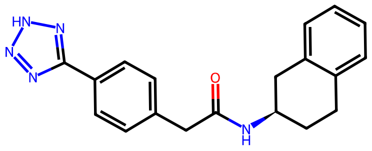	O=C(Cc1ccc(-c2nn[nH]n2)cc1)N[C@@H]1CCc2ccccc2C1	1592911608	25	333.16	2.08
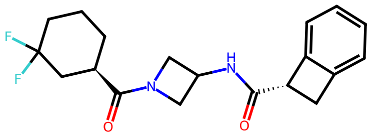	O=C(NC1CN(C(=O)[C@@H]2CCCC(F)(F)C2)C1)[C@H]1Cc2ccccc21	998689501	25	348.16	2.48
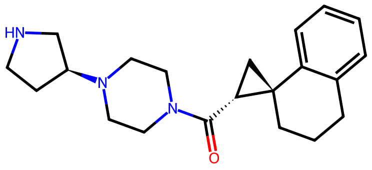	O=C([C@@H]1C[C@@]12CCCc1ccccc12)N1CCN([C@H]2CCNC2)CC1	343604813	25	339.23	1.79
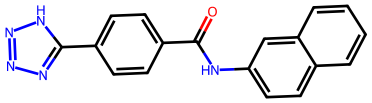	O=C(Nc1ccc2ccccc2c1)c1ccc(-c2nnn[nH]2)cc1	25376229	24	315.11	3.27
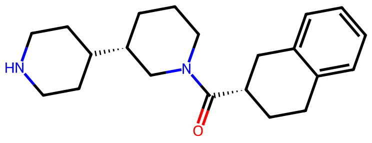	O=C([C@H]1CCc2ccccc2C1)N1CCC[C@@H](C2CCNCC2)C1	662590337	24	326.24	3.03
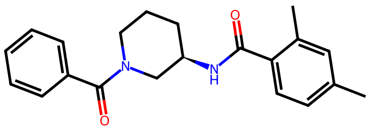	Cc1ccc(C(=O)N[C@@H]2CCCN(C(=O)c3ccccc3)C2)c(C)c1	949820411	25	336.18	3.34

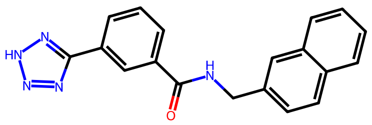	O=C(NCc1ccc2ccccc2c1)c1cccc(-c2nn[nH]n2)c1	1593141447	25	329.13	2.95
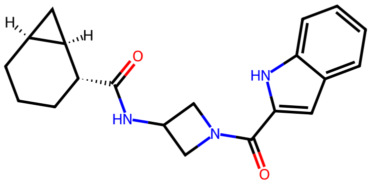	O=C(NC1CN(C(=O)c2cc3ccccc3[nH]2)C1)[C@@H]1CCC[C@H]2C[C@H]21	994727564	25	337.18	2.54
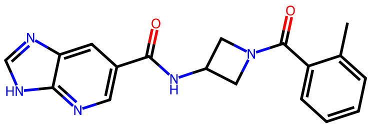	Cc1ccccc1C(=O)N1CC(NC(=O)c2cnc3[nH]cnc3c2)C1	992828353	25	335.14	1.52
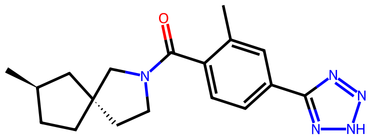	Cc1cc(-c2nn[nH]n2)ccc1C(=O)N1CC[C@@]2(CC[C@@H](C)C2)C1	826245097	24	325.19	2.83
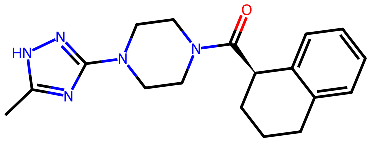	Cc1nc(N2CCN(C(=O)[C@@H]3CCCc4ccccc43)CC2)n[nH]1	898174726	24	325.19	1.88
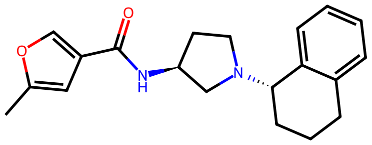	Cc1cc(C(=O)N[C@H]2CCN([C@H]3CCCc4ccccc43)C2)co1	1010768440	24	324.18	3.47
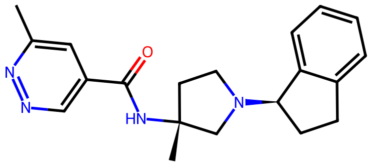	Cc1cc(C(=O)N[C@]2(C)CCN([C@@H]3CCc4ccccc43)C2)cnn1	1046269702	25	336.2	2.67
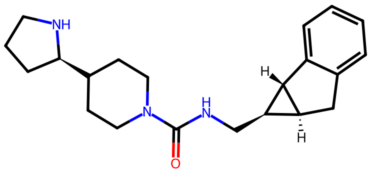	O=C(NC[C@@H]1[C@@H]2Cc3ccccc3[C@@H]12)N1CCC([C@H]2CCCN2)CC1	417915486	25	339.23	2.75

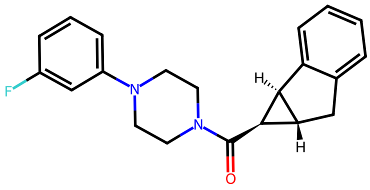	O=C([C@@H]1[C@H]2Cc3ccccc3[C@@H]21)N1CCN(c2cccc(F)c2)CC1	633894209	25	336.16	3.06
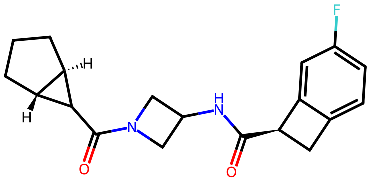	O=C(NC1CN(C(=O)C2[C@@H]3CCC[C@@H]23)C1)[C@@H]1Cc2ccc(F)cc21	998486441	24	328.16	1.84
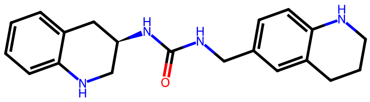	O=C(NCc1ccc2c(c1)CCCN2)N[C@H]1CNc2ccccc2C1	652220978	25	336.2	2.88
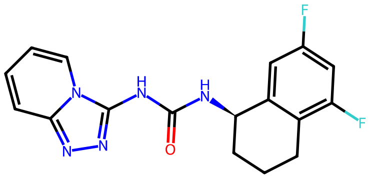	O=C(Nc1nnc2ccccn12)N[C@@H]1CCCc2c(F)cc(F)cc21	1310264962	25	343.12	3.21
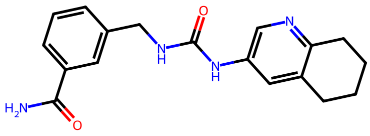	NC(=O)c1cccc(CNC(=O)Nc2cnc3c(c2)CCCC3)c1	542955699	24	324.16	2.38
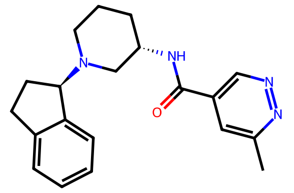	Cc1cc(C(=O)N[C@H]2CCCN([C@@H]3CCc4ccccc43)C2)cnn1	1007214562	25	336.2	2.67
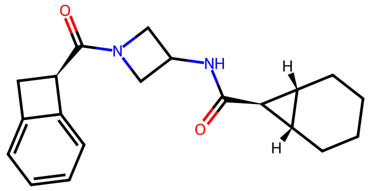	O=C(NC1CN(C(=O)[C@@H]2Cc3ccccc32)C1)[C@@H]1[C@H]2CCCC[C@H]21	990649263	24	324.18	2.09
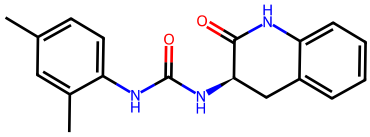	Cc1ccc(NC(=O)N[C@@H]2Cc3ccccc3NC2=O)c(C)c1	1202751081	23	309.15	2.99

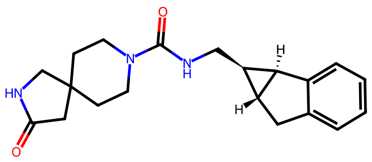	O=C1CC2(CCN(C(=O)NC[C@@H]3[C@H]4Cc5ccccc5[C@@H]43)CC2)CN1	269043558	25	339.19	1.88
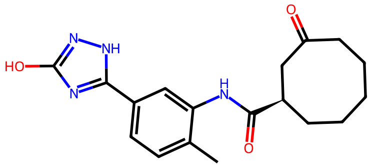	Cc1ccc(-c2nc(O)n[nH]2)cc1NC(=O)[C@@H]1CCCCCC(=O)C1	521897858	25	342.17	2.96
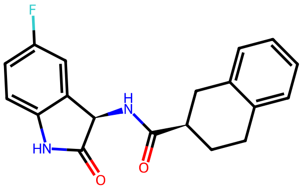	O=C(N[C@H]1C(=O)Nc2ccc(F)cc21)[C@@H]1CCc2ccccc2C1	19744049	24	324.13	2.74
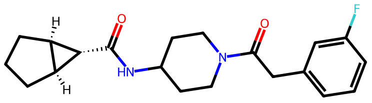	O=C(NC1CCN(C(=O)Cc2cccc(F)c2)CC1)[C@@H]1[C@H]2CCC[C@H]21	948481086	25	344.19	2.52
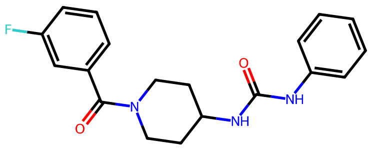	O=C(Nc1ccccc1)NC1CCN(C(=O)c2ccc(F)c2)CC1	55410902	25	341.15	3.25
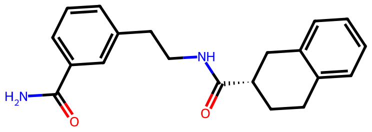	NC(=O)c1cccc(CCNC(=O)[C@H]2CCc3ccccc3C2)c1	521882938	24	322.17	2.25
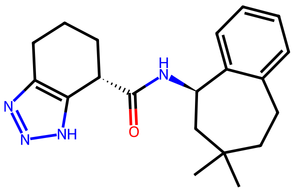	CC1(C)CCc2ccccc2[C@H](NC(=O)[C@@H]2CCCc3nn[nH]c32)C1	646966698	25	338.21	3.44
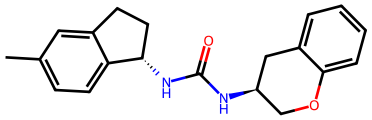	Cc1ccc2c(c1)CC[C@@H]2NC(=O)N[C@@H]1COc2ccccc2C1	516689632	24	322.17	3.29

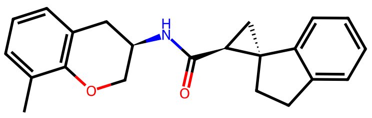	Cc1cccc2c1OC[C@H](NC(=O)[C@H]1C[C@]13CCc1ccccc13)C2	564516627	25	333.17	3.32
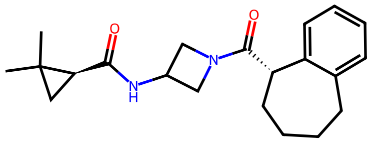	CC1(C)C[C@@H]1C(=O)NC1CN(C(=O)[C@H]2CCCCc3ccccc32)C1	990877262	25	340.22	2.87
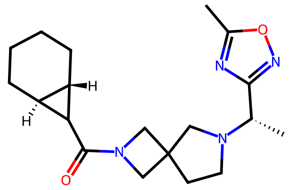	Cc1nc([C@H](C)N2CCC3(CN(C(=O)C4[C@H]5CCCC[C@H]45)C3)C2)no1	1072580901	25	344.22	2.41
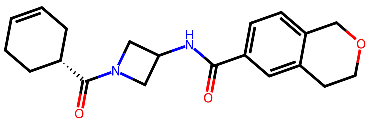	O=C(NC1CN(C(=O)[C@H]2CC=CCC2)C1)c1ccc2c(c1)CCOC2	996918765	25	340.18	2.06
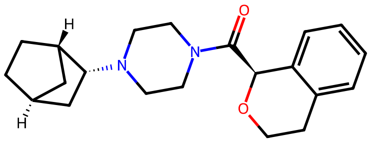	O=C([C@@H]1OCCc2ccccc21)N1CCN([C@@H]2C[C@H]3CC[C@H]2C3)CC1	441469650	25	340.22	2.63
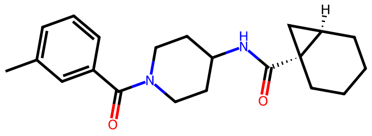	Cc1cccc(C(=O)N2CCC(NC(=O)[C@@]34CCCC[C@@H]3C4)CC2)c1	943834388	25	340.22	3.3
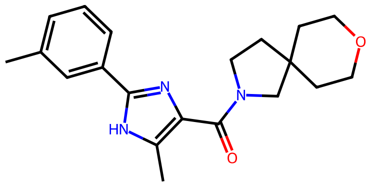	Cc1cccc(-c2nc(C(=O)N3CCC4(CCOCC4)C3)c(C)[nH]2)c1	270453714	25	339.19	3.34
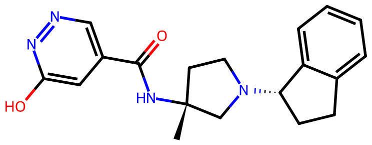	C[C@@]1(NC(=O)c2cnnc(O)c2)CCN([C@H]2CCc3ccccc32)C1	1046399302	25	338.17	2.06

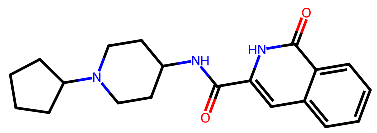	O=C(NC1CCN(C2CCCC2)CC1)c1cc2ccccc2c(=O)[nH]1	177454008	25	339.19	2.66
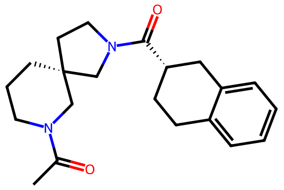	CC(=O)N1CCC[C@@]2(CCN(C(=O)[C@H]3CCc4ccccc4C3)C2)C1	952958019	25	340.22	2.65
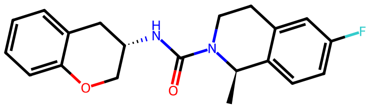	C[C@@H]1c2ccc(F)cc2CCN1C(=O)N[C@@H]1COc2ccccc2C1	606644260	25	340.16	3.46
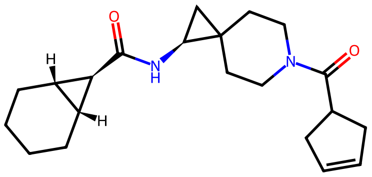	O=C(N[C@H]1CC12CCN(C(=O)C1CC=CC1)CC2)[C@@H]1[C@H]2CCCC[C@H]21	963446744	25	342.23	2.89
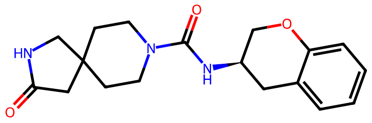	O=C1CC2(CCN(C(=O)N[C@H]3COc4ccccc4C3)CC2)CN1	267854403	24	329.17	1.3
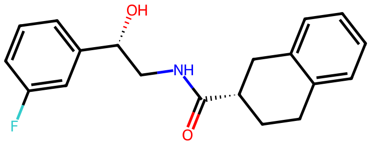	O=C(NC[C@@H](O)c1cccc(F)c1)[C@H]1CCc2ccccc2C1	845815058	23	313.15	2.78
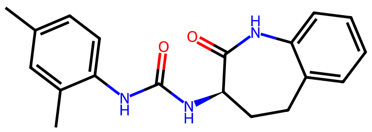	Cc1ccc(NC(=O)N[C@@H]2CCc3ccccc3NC2=O)c(C)c1	653914571	24	323.16	3.38
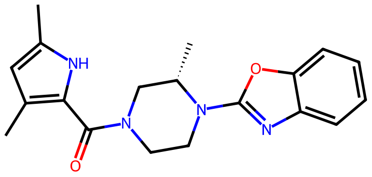	Cc1cc(C)c(C(=O)N2CCN(c3nc4ccccc4o3)[C@@H](C)C2)[nH]1	353649178	25	338.17	3.12

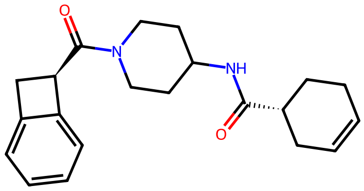	O=C(NC1CCN(C(=O)[C@@H]2Cc3ccccc32)CC1)[C@H]1CC=CCC1	948059698	25	338.2	2.79
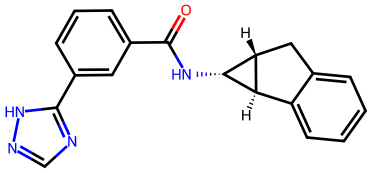	O=C(N[C@H]1[C@@H]2c3ccccc3C[C@H]21)c1cccc(-c2ncn[nH]2)c1	156440941	24	316.13	2.54
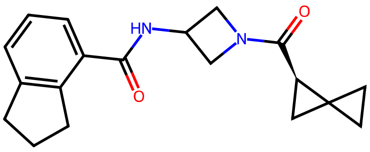	O=C(NC1CN(C(=O)[C@@H]2CC23CC3)C1)c1cccc2c1CCC2	994401849	23	310.17	1.92
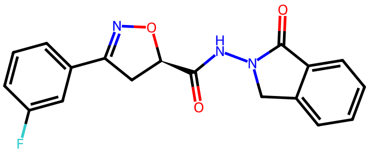	O=C(NN1Cc2ccccc2C1=O)[C@H]1CC(c2cccc(F)c2)=NO1	183823410	25	339.1	2.01
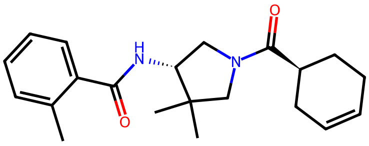	Cc1ccccc1C(=O)N[C@@H]1CN(C(=O)[C@@H]2CC=CCC2)CC1(C)C	1007830506	25	340.22	3.32
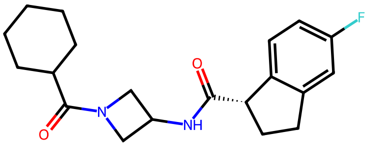	O=C(NC1CN(C(=O)C2CCCCC2)C1)[C@H]1CCc2cc(F)ccc21	996100650	25	344.19	2.76
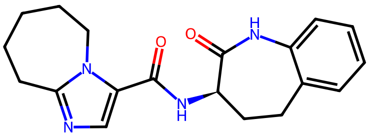	O=C(N[C@@H]1CCc2ccccc2NC1=O)c1cnc2n1CCCCC2	622128951	25	338.17	2.29
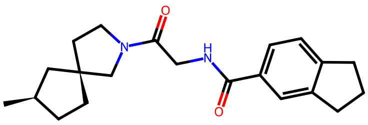	C[C@@H]1CC[C@]2(CCN(C(=O)CNC(=O)c3ccc4c(c3)CCC4)C2)C1	120444735	25	340.22	2.94

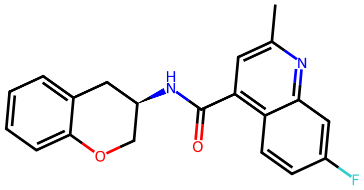	Cc1cc(C(=O)N[C@H]2COc3ccccc3C2)c2ccc(F)cc2n1	53046048	25	336.13	3.42
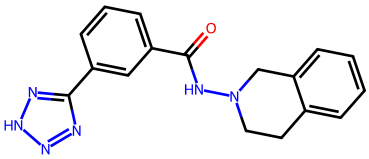	O=C(NN1CCc2ccccc2C1)c1cccc(-c2nn[nH]n2)c1	1582561524	24	320.14	1.57
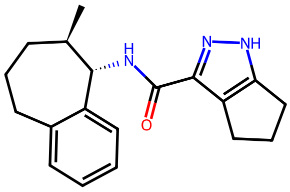	C[C@@H]1CCCc2ccccc2[C@H]1NC(=O)c1n[nH]c2c1CCC2	463033734	23	309.18	3.34
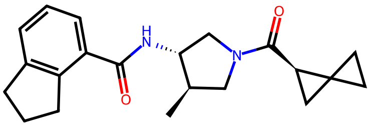	C[C@@H]1CN(C(=O)[C@@H]2CC23CC3)C[C@H]1NC(=O)c1cccc2c1CCC2	959763894	25	338.2	2.55
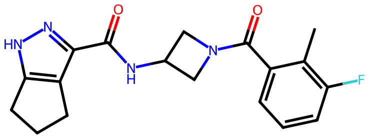	Cc1c(F)cccc1C(=O)N1CC(NC(=O)c2n[nH]c3c2CCC3)C1	995166882	25	342.15	1.6
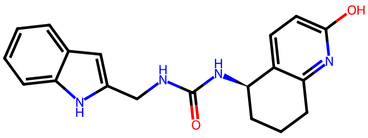	O=C(NCc1cc2ccccc2[nH]1)N[C@@H]1CCCc2nc(O)ccc21	299401006	25	336.16	3.15
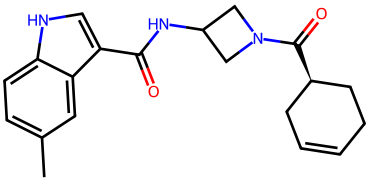	Cc1ccc2[nH]cc(C(=O)NC3CN(C(=O)[C@@H]4CC=CCC4)C3)c2c1	994274193	25	337.18	2.77
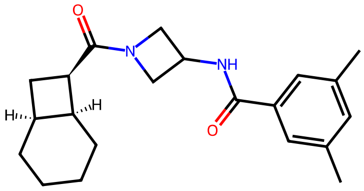	Cc1cc(C)cc(C(=O)NC2CN(C(=O)[C@@H]3C[C@@H]4CCCC[C@H]43)C2)c1	999485558	25	340.22	3.07

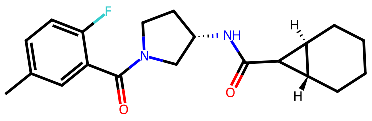	Cc1ccc(F)c(C(=O)N2CC[C@H](NC(=O)C3[C@H]4CCCC[C@H]34)C2)c1	937793519	25	344.19	2.9
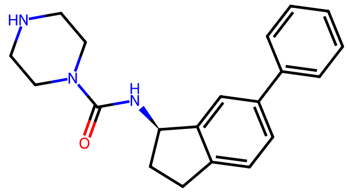	O=C(N[C@@H]1CCc2ccc(-c3ccccc3)cc21)N1CCNCC1	418047938	24	321.18	2.96
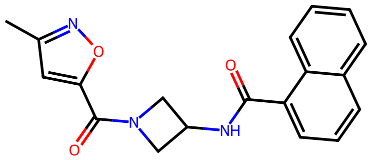	Cc1cc(C(=O)N2CC(NC(=O)c3cccc4ccccc34)C2)on1	999543582	25	335.13	2.39
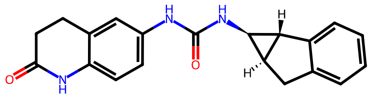	O=C1CCc2cc(NC(=O)N[C@H]3[C@@H]4c5ccccc5C[C@H]43)ccc2N1	133252453	25	333.15	3.03
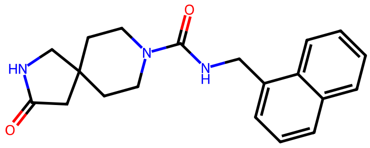	O=C1CC2(CCN(C(=O)NCc3cccc4ccccc34)CC2)CN1	73911872	25	337.18	2.65
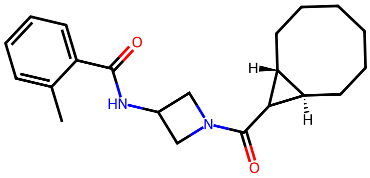	Cc1ccccc1C(=O)NC1CN(C(=O)C2[C@@H]3CCCCCC[C@@H]23)C1	990111562	25	340.22	3.15
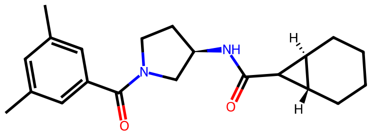	Cc1cc(C)cc(C(=O)N2CC[C@@H](NC(=O)C3[C@H]4CCCC[C@H]34)C2)c1	939895351	25	340.22	3.07
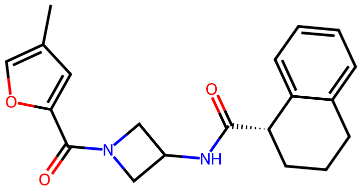	Cc1coc(C(=O)N2CC(NC(=O)[C@H]3CCCc4ccccc43)C2)c1	995075504	25	338.16	2.65

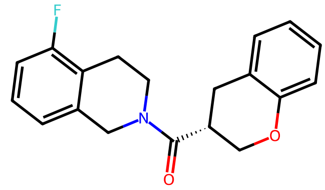	O=C([C@H]1COc2ccccc2C1)N1CCc2c(F)cccc2C1	270531821	23	311.13	2.96
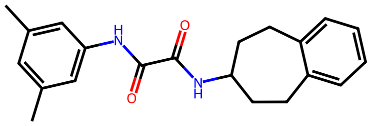	Cc1cc(C)cc(NC(=O)C(=O)NC2CCc3ccccc3CC2)c1	453965184	25	336.18	3.31
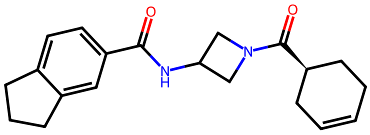	O=C(NC1CN(C(=O)[C@@H]2CC=CCC2)C1)c1ccc2c(c1)CCC2	991356441	24	324.18	2.47
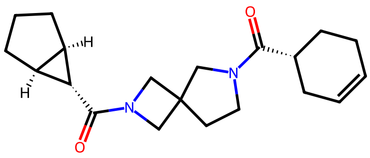	O=C([C@H]1CC=CCC1)N1CCC2(C1)CN(C(=O)[C@@H]1[C@H]3CCC[C@H]31)C2	1018909545	24	328.22	2.45
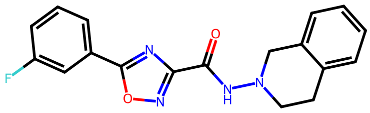	O=C(NN1CCc2ccccc2C1)c1noc(-c2cccc(F)c2)n1	1644634818	25	338.12	2.58
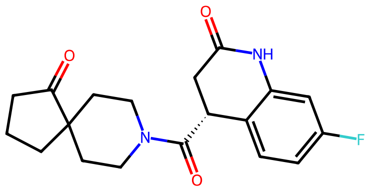	O=C1C[C@@H](C(=O)N2CCC3(CCCC3=O)CC2)c2ccc(F)cc2N1	619334238	25	344.15	2.61
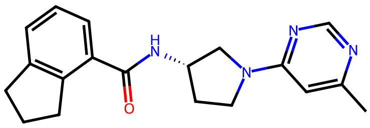	Cc1cc(N2CC[C@H](NC(=O)c3cccc4c3CCC4)C2)ncn1	1058385886	24	322.18	2.28
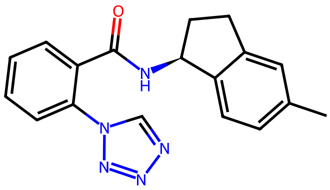	Cc1ccc2c(c1)CC[C@@H]2NC(=O)c1ccccc1-n1cnnn1	342858901	24	319.14	2.39

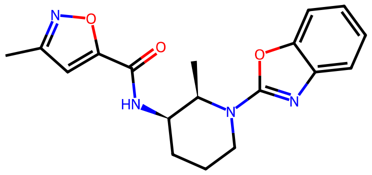	Cc1cc(C(=O)N[C@@H]2CCCN(c3nc4ccccc4o3)[C@@H]2C)on1	1069340656	25	340.15	2.91
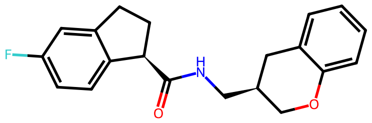	O=C(NC[C@@H]1COc2ccccc2C1)[C@@H]1CCc2cc(F)ccc21	854308945	24	325.15	3.22
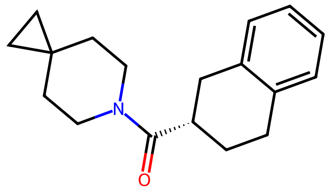	O=C([C@H]1CCc2ccccc2C1)N1CCC2(CC1)CC2	649715265	20	269.18	3.19
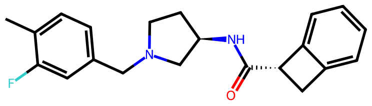	Cc1ccc(CN2CC[C@@H](NC(=O)[C@H]3Cc4ccccc43)C2)cc1F	1015634522	25	338.18	3.16
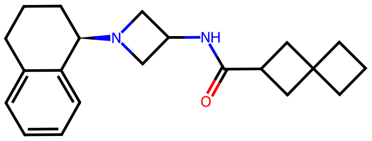	O=C(NC1CN([C@@H]2CCCc3ccccc32)C1)C1CC2(CCC2)C1	1030814622	24	324.22	3.44
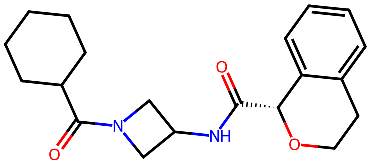	O=C(NC1CN(C(=O)C2CCCCC2)C1)[C@H]1OCCc2ccccc21	997320487	25	342.19	2.21
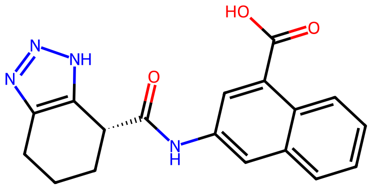	O=C(O)c1cc(NC(=O)[C@@H]2CCCc3nn[nH]c32)cc2ccccc12	1602280606	25	336.12	2.71
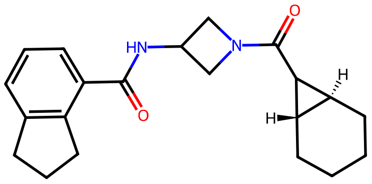	O=C(NC1CN(C(=O)C2[C@H]3CCCC[C@H]23)C1)c1cccc2c1CCC2	994404976	25	338.2	2.55

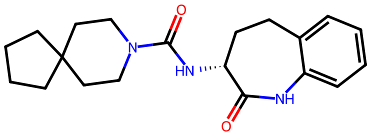	O=C1Nc2ccccc2CC[C@H]1NC(=O)N1CCC2(CCCC2)CC1	847397582	25	341.21	3.31
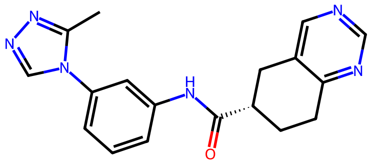	Cc1nncn1-c1cccc(NC(=O)[C@H]2CCc3ncncc3C2)c1	648339047	25	334.15	2.11
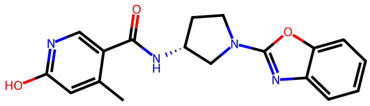	Cc1cc(O)ncc1C(=O)N[C@@H]1CCN(c2nc3ccccc3o2)C1	1058541307	25	338.14	2.25
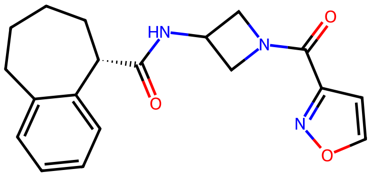	O=C(NC1CN(C(=O)c2ccon2)C1)[C@H]1CCCCc2ccccc21	994306605	25	339.16	2.13
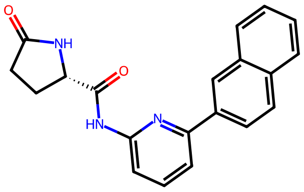	O=C1CC[C@@H](C(=O)Nc2cccc(-c3ccc4ccccc4c3)n2)N1	104380817	25	331.13	3.12
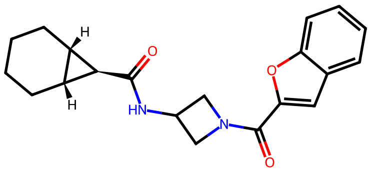	O=C(NC1CN(C(=O)c2cc3ccccc3o2)C1)[C@@H]1[C@H]2CCCC[C@H]21	990674363	25	338.16	2.81
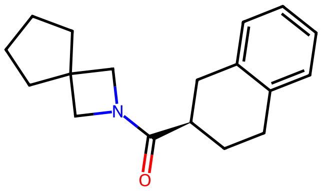	O=C([C@@H]1CCc2ccccc2C1)N1CC2(CCCC2)C1	580718824	20	269.18	3.19
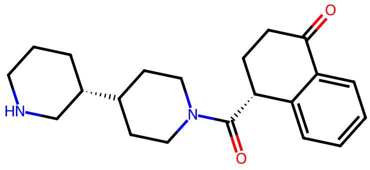	O=C1CC[C@@H](C(=O)N2CCC([C@H]3CCCNC3)CC2)c2ccccc21	662587757	25	340.22	2.98

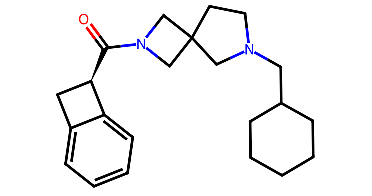	O=C([C@@H]1Cc2ccccc21)N1CC2(CCN(CC3CCCCC3)C2)C1	1072932440	25	338.24	3.44
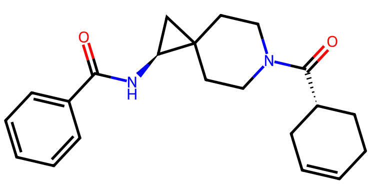	O=C(N[C@H]1CC12CCN(C(=O)[C@H]1CC=CCC1)CC2)c1ccccc1	962784351	25	338.2	3.15
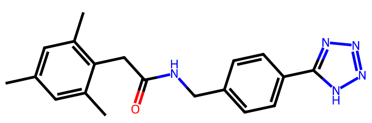	Cc1cc(C)c(CC(=O)NCc2ccc(-c3nnn[nH]3)cc2)c(C)c1	180083544	25	335.17	2.65
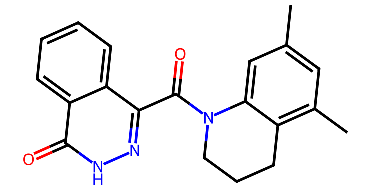	Cc1cc(C)c2c(c1)N(C(=O)c1n[nH]c(=O)c3ccccc13)CCC2	66733052	25	333.15	3.13
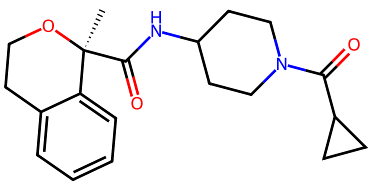	C[C@@]1(C(=O)NC2CCN(C(=O)C3CC3)CC2)OCCc2ccccc21	639493539	25	342.19	1.99
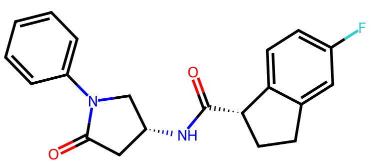	O=C(N[C@@H]1CC(=O)N(c2ccccc2)C1)[C@H]1CCc2cc(F)ccc21	667307696	25	338.14	2.78
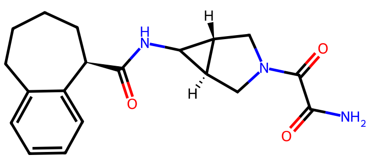	NC(=O)C(=O)N1C[C@H]2C(NC(=O)[C@@H]3CCCCc4ccccc43)[C@@H]2C1	1013287963	25	341.17	0.55
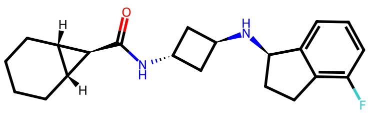	O=C(N[C@H]1C[C@H](N[C@@H]2CCc3c(F)cccc32)C1)[C@@H]1[C@H]2CCCC[C@H]21	1020048100	25	342.21	3.49

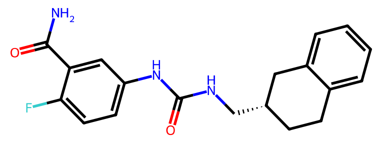	NC(=O)c1cc(NC(=O)NC[C@H]2CCc3ccccc3C2)ccc1F	605029357	25	341.15	2.85
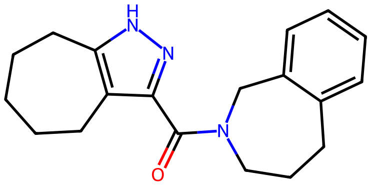	O=C(c1n[nH]c2c1CCCCC2)N1CCCc2ccccc2C1	188552738	23	309.18	3.27
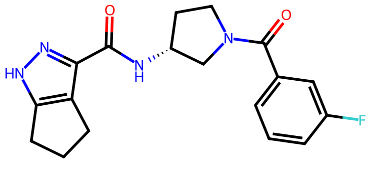	O=C(N[C@@H]1CCN(C(=O)c2cccc(F)c2)C1)c1n[nH]c2c1CCC2	941438811	25	342.15	1.68
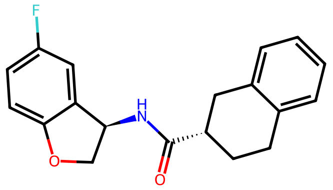	O=C(N[C@H]1COc2ccc(F)cc21)[C@H]1CCc2ccccc2C1	573807411	23	311.13	3.18
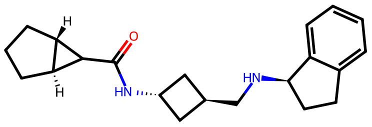	O=C(N[C@H]1C[C@H](CN[C@@H]2CCc3ccccc32)C1)C1[C@H]2CCC[C@H]12	983438693	24	324.22	3.2
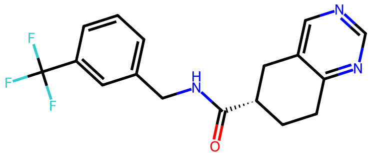	O=C(NCc1cccc(C(F)(F)F)c1)[C@H]1CCc2ncncc2C1	648217571	24	335.12	2.92
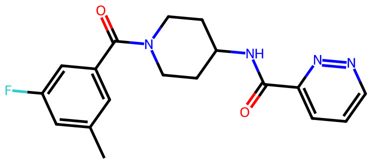	Cc1cc(F)cc(C(=O)N2CCC(NC(=O)c3cccnn3)CC2)c1	948550266	25	342.15	1.96
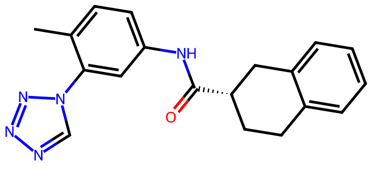	Cc1ccc(NC(=O)[C@H]2CCc3ccccc3C2)cc1-n1cnnn1	563221506	25	333.16	2.71

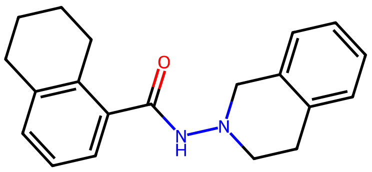	O=C(NN1CCc2ccccc2C1)c1cccc2c1CCCC2	1165603240	23	306.17	3.27
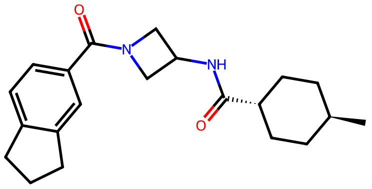	C[C@H]1CC[C@H](C(=O)NC2CN(C(=O)c3ccc4c(c3)CCC4)C2)CC1	997153578	25	340.22	2.94
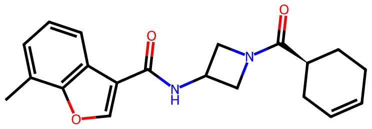	Cc1cccc2c(C(=O)NC3CN(C(=O)[C@@H]4CC=CCC4)C3)coc12	994205443	25	338.16	3.04
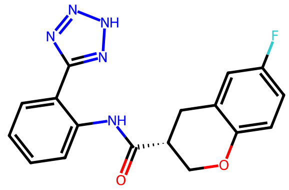	O=C(Nc1ccccc1-c1nn[nH]n1)[C@H]1COc2ccc(F)cc2C1	1557791618	25	339.11	2.2
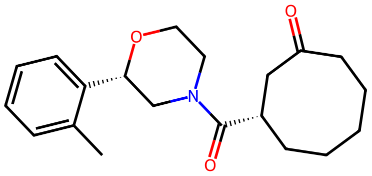	Cc1ccccc1[C@H]1CN(C(=O)[C@H]2CCCCCC(=O)C2)CCO1	563928420	24	329.2	3.43
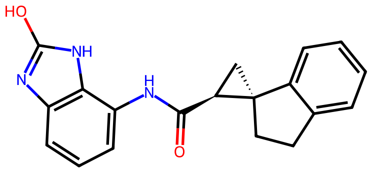	O=C(Nc1cccc2nc(O)[nH]c12)[C@H]1C[C@]12CCc1ccccc12	563395848	24	319.13	3.11
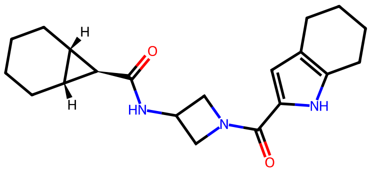	O=C(NC1CN(C(=O)c2cc3c([nH]2)CCCC3)C1)[C@@H]1[C@H]2CCCC[C@H]21	990649641	25	341.21	2.27
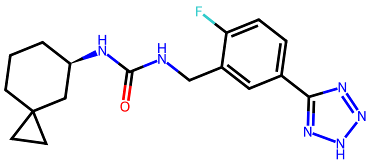	O=C(NCc1cc(-c2nn[nH]n2)ccc1F)N[C@@H]1CCCC2(CC2)C1	1593119845	25	344.18	2.53

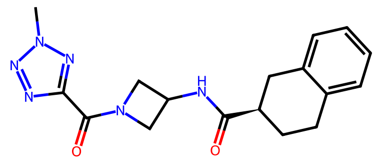	Cn1nnc(C(=O)N2CC(NC(=O)[C@@H]3CCc4ccccc4C3)C2)n1	1000545375	25	340.16	-0.04
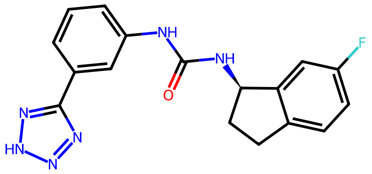	O=C(Nc1cccc(-c2nn[nH]n2)c1)N[C@@H]1CCc2ccc(F)cc21	738177621	25	338.13	2.81
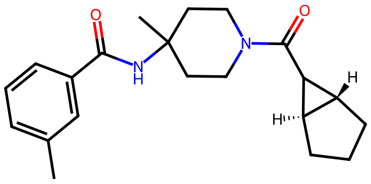	Cc1cccc(C(=O)NC2(C)CCN(C(=O)C3[C@@H]4CCC[C@@H]34)CC2)c1	956279571	25	340.22	3.15
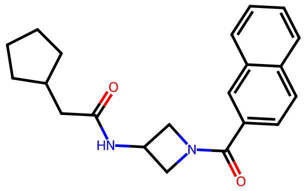	O=C(CC1CCCC1)NC1CN(C(=O)c2ccc3ccccc3c2)C1	990112775	25	336.18	3.36
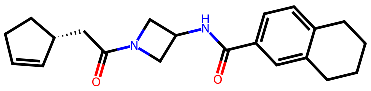	O=C(NC1CN(C(=O)C[C@@H]2C=CCC2)C1)c1ccc2c(c1)CCCC2	1000060451	25	338.2	2.86
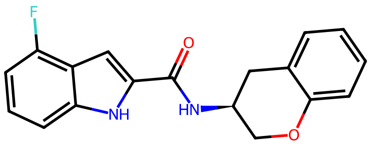	O=C(N[C@@H]1COc2ccccc2C1)c1cc2c(F)cccc2[nH]1	53045580	23	310.11	3.04
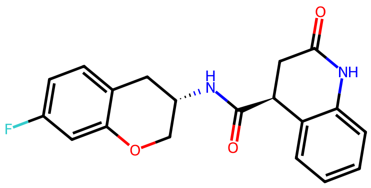	O=C1C[C@H](C(=O)N[C@@H]2COc3cc(F)ccc3C2)c2ccccc2N1	1361448748	25	340.12	2.37
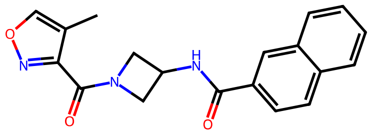	Cc1conc1C(=O)N1CC(NC(=O)c2ccc3ccccc3c2)C1	999452845	25	335.13	2.39

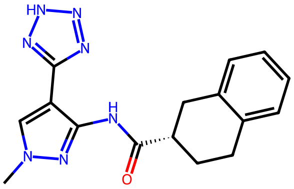	Cn1cc(-c2nn[nH]n2)c(NC(=O)[C@H]2CCc3ccccc3C2)n1	1592559088	24	323.15	1.34
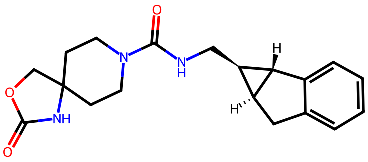	O=C1NC2(CCN(C(=O)NC[C@@H]3[C@@H]4Cc5ccccc5[C@@H]34)CC2)CO1	495141962	25	341.17	1.86
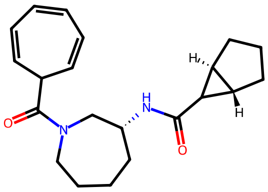	O=C(N[C@@H]1CCCCN(C(=O)C2C=CC=CC=C2)C1)C1[C@H]2CCC[C@H]12	943164489	25	340.22	2.83
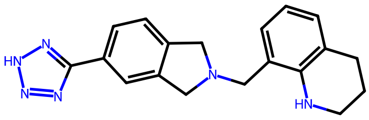	c1cc2c(c(CN3Cc4ccc(-c5nn[nH]n5)cc4C3)c1)NCCC2	1593566784	25	332.17	2.74
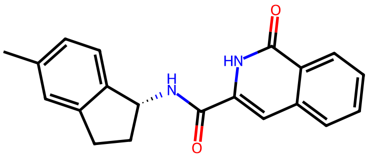	Cc1ccc2c(c1)CC[C@H]2NC(=O)c1cc2ccccc2c(=O)[nH]1	56316230	24	318.14	3.25
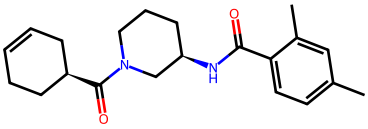	Cc1ccc(C(=O)N[C@@H]2CCCN(C(=O)[C@@H]3CC=CCC3)C2)c(C)c1	949983884	25	340.22	3.38
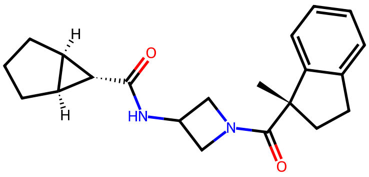	C[C@]1(C(=O)N2CC(NC(=O)[C@@H]3[C@H]4CCC[C@H]43)C2)CCc2ccccc21	990713329	25	338.2	2.26
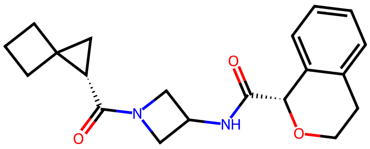	O=C(NC1CN(C(=O)[C@H]2CC23CCC3)C1)[C@H]1OCCc2ccccc21	997326408	25	340.18	1.82

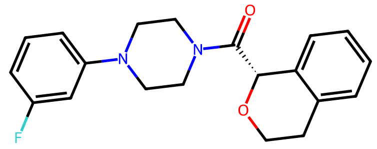	O=C([C@H]1OCCc2ccccc21)N1CCN(c2cccc(F)c2)CC1	676722372	25	340.16	2.79
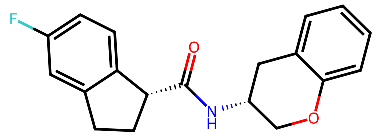	O=C(N[C@H]1COc2ccccc2C1)[C@@H]1CCc2cc(F)ccc21	666390977	23	311.13	2.98
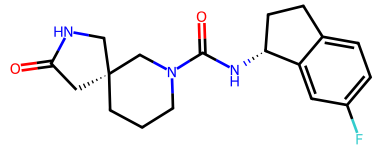	O=C1C[C@]2(CCCN(C(=O)N[C@@H]3CCc4ccc(F)cc43)C2)CN1	608426946	24	331.17	2.12
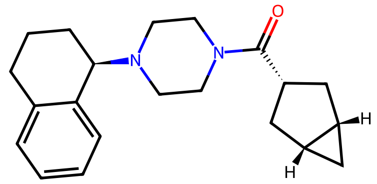	O=C([C@@H]1C[C@@H]2C[C@@H]2C1)N1CCN([C@@H]2CCCc3ccccc32)CC1	949778170	24	324.22	3.25
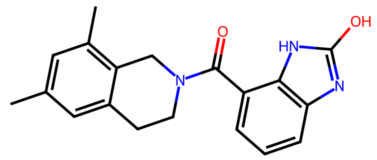	Cc1cc(C)c2c(c1)CCN(C(=O)c1cccc3nc(O)[nH]c13)C2	1305985251	24	321.15	3.08
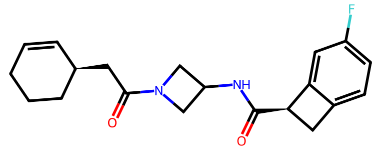	O=C(NC1CN(C(=O)C[C@@H]2C=CCCC2)C1)[C@@H]1Cc2ccc(F)cc21	998490093	25	342.17	2.54
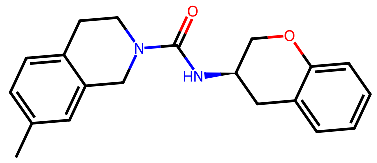	Cc1ccc2c(c1)CN(C(=O)N[C@H]1COc3ccccc3C1)CC2	1220856149	24	322.17	3.07
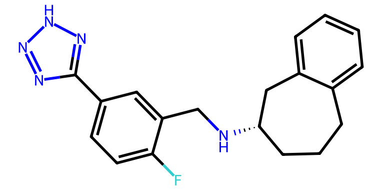	Fc1ccc(-c2nn[nH]n2)cc1CN[C@H]1CCc2ccccc2C1	1606316006	25	337.17	3.04

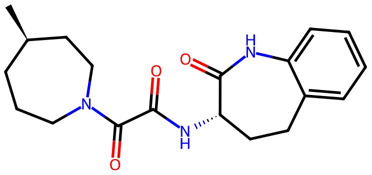	C[C@@H]1CCCN(C(=O)C(=O)N[C@H]2CCc3ccccc3NC2=O)CC1	1327237241	25	343.19	1.7
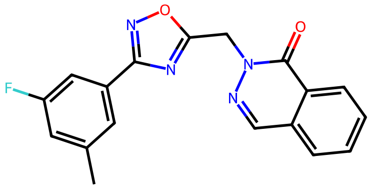	Cc1cc(F)cc(-c2noc(Cn3ncc4ccccc4c3=O)n2)c1	355870250	25	336.1	2.94
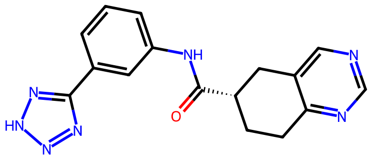	O=C(Nc1cccc(-c2nn[nH]n2)c1)[C@H]1CCc2ncncc2C1	648721288	24	321.13	1.4
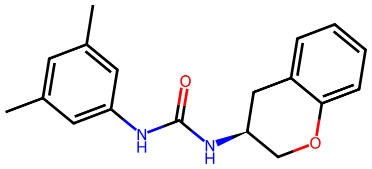	Cc1cc(C)cc(NC(=O)N[C@@H]2COc3ccccc3C2)c1	47701368	22	296.15	3.43
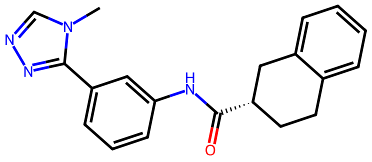	Cn1cnnc1-c1cccc(NC(=O)[C@H]2CCc3ccccc3C2)c1	649604086	25	332.16	3.23
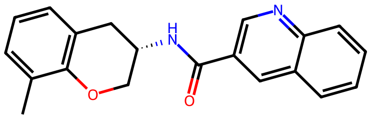	Cc1cccc2c1OC[C@@H](NC(=O)c1cnc3ccccc3c1)C2	1527464545	24	318.14	3.28
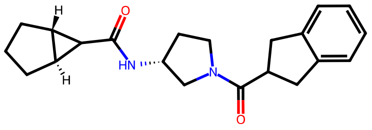	O=C(N[C@@H]1CCN(C(=O)C2Cc3ccccc3C2)C1)C1[C@H]2CCC[C@H]12	936250511	25	338.2	2.16
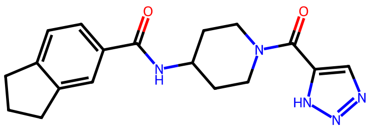	O=C(NC1CCN(C(=O)c2cnn[nH]2)CC1)c1ccc2c(c1)CCC2	947373359	25	339.17	1.33

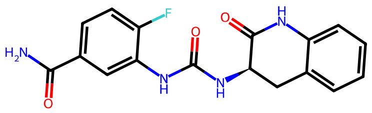	NC(=O)c1ccc(F)c(NC(=O)N[C@@H]2Cc3ccccc3NC2=O)c1	1365123970	25	342.11	1.61
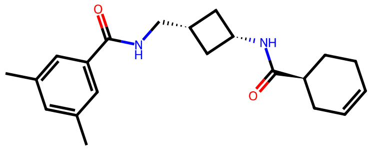	Cc1cc(C)cc(C(=O)NC[C@H]2C[C@@H](NC(=O)[C@@H]3CC=CCC3)C2)c1	979286538	25	340.22	3.28
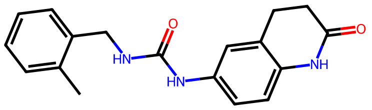	Cc1ccccc1CNC(=O)Nc1ccc2c(c1)CCC(=O)N2	76103595	23	309.15	3.2
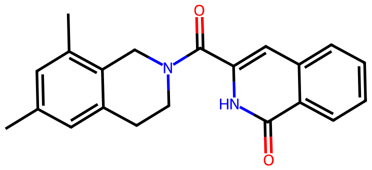	Cc1cc(C)c2c(c1)CCN(C(=O)c1cc3ccccc3c(=O)[nH]1)C2	186726809	25	332.15	3.34
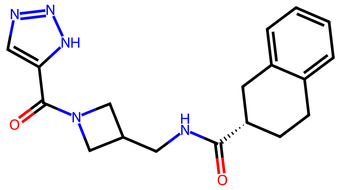	O=C(NCC1CN(C(=O)c2cnn[nH]2)C1)[C@H]1CCc2ccccc2C1	936099472	25	339.17	0.8
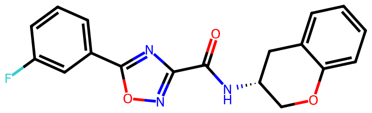	O=C(N[C@H]1COc2ccccc2C1)c1noc(-c2cccc(F)c2)n1	673639366	25	339.1	2.61
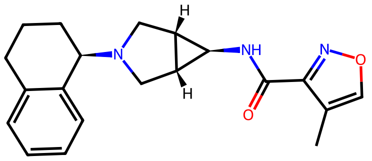	Cc1conc1C(=O)N[C@H]1[C@@H]2CN([C@@H]3CCCc4ccccc43)C[C@@H]21	962216966	25	337.18	2.72
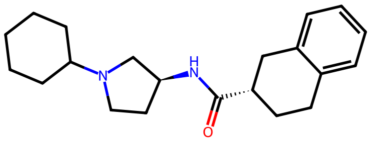	O=C(N[C@H]1CCN(C2CCCCC2)C1)[C@H]1CCc2ccccc2C1	618656946	24	326.24	3.31

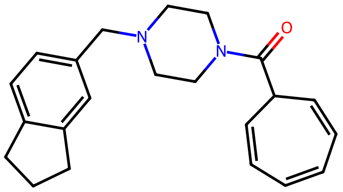	O=C(C1C=CC=CC=C1)N1CCN(Cc2ccc3c(c2)CCC3)CC1	948571289	25	334.2	3.12
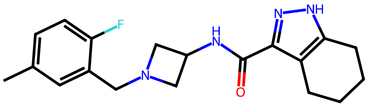	Cc1ccc(F)c(CN2CC(NC(=O)c3n[nH]c4c3CCCC4)C2)c1	1030599071	25	342.19	2.35
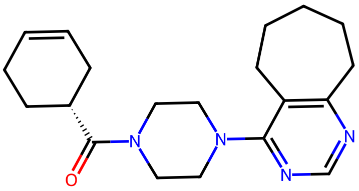	O=C([C@H]1CC=CCC1)N1CCN(c2ncnc3c2CCCCC3)CC1	1055720897	25	340.23	2.75
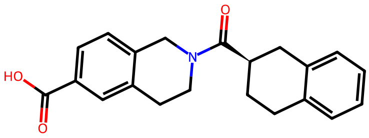	O=C(O)c1ccc2c(c1)CCN(C(=O)[C@@H]1CCc3ccccc3C1)C2	910500830	25	335.15	3.07
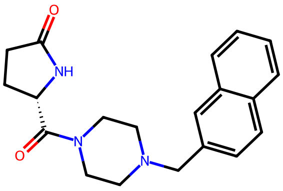	O=C1CC[C@@H](C(=O)N2CCN(Cc3ccc4ccccc4c3)CC2)N1	957388611	25	337.18	1.76
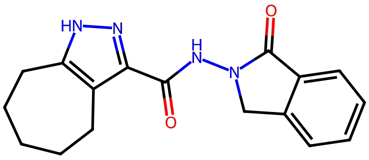	O=C(NN1Cc2ccccc2C1=O)c1n[nH]c2c1CCCCC2	191010304	23	310.14	1.98
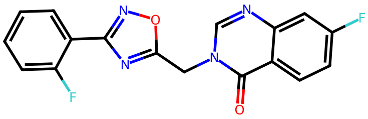	O=c1c2ccc(F)cc2ncn1Cc1nc(-c2ccccc2F)no1	548688438	25	340.08	2.77
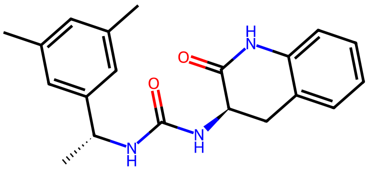	Cc1cc(C)cc([C@@H](C)NC(=O)N[C@@H]2Cc3ccccc3NC2=O)c1	636752806	25	337.18	3.23

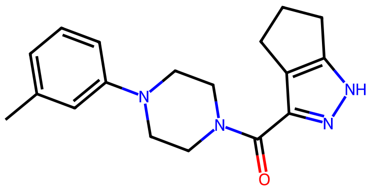	Cc1cccc(N2CCN(C(=O)c3n[nH]c4c3CCC4)CC2)c1	49679097	23	310.18	2.17
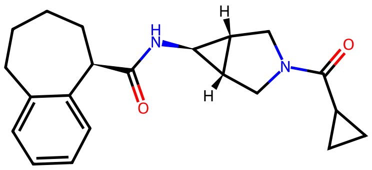	O=C(N[C@H]1[C@@H]2CN(C(=O)C3CC3)C[C@@H]21)[C@@H]1CCCCc2ccccc21	976705527	25	338.2	2.48
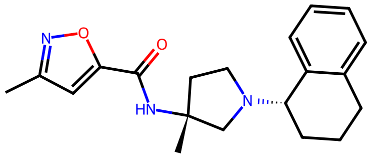	Cc1cc(C(=O)N[C@]2(C)CCN([C@H]3CCCc4ccccc43)C2)on1	1046139311	25	339.19	3.25
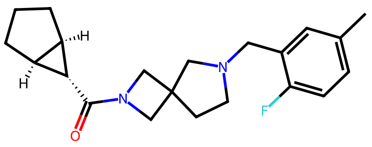	Cc1ccc(F)c(CN2CCC3(C2)CN(C(=O)[C@@H]2[C@H]4CCC[C@H]42)C3)c1	1072592886	25	342.21	3.21
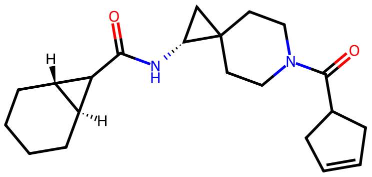	O=C(N[C@@H]1CC12CCN(C(=O)C1CC=CC1)CC2)C1[C@H]2CCCC[C@H]12	963446745	25	342.23	2.89
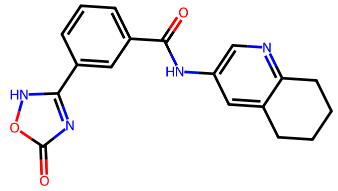	O=C(Nc1cnc2c(c1)CCCC2)c1cccc(-c2nc(=O)o[nH]2)c1	622502257	25	336.12	2.56
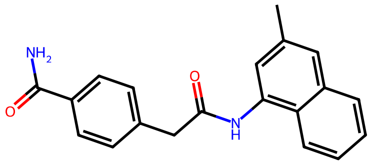	Cc1cc(NC(=O)Cc2ccc(C(N)=O)cc2)c2ccccc2c1	1521903164	24	318.14	3.43
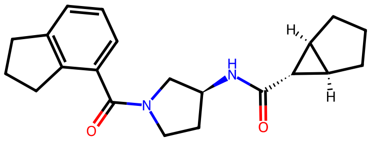	O=C(N[C@H]1CCN(C(=O)c2cccc3c2CCC3)C1)[C@@H]1[C@H]2CCC[C@H]21	937800259	25	338.2	2.55

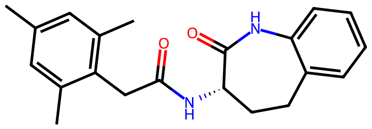	Cc1cc(C)c(CC(=O)N[C@H]2CCc3ccccc3NC2=O)c(C)c1	654000800	25	336.18	3.22
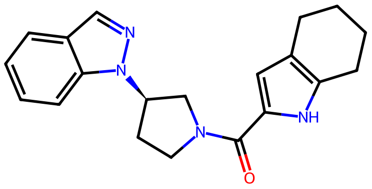	O=C(c1cc2c([nH]1)CCCC2)N1CC[C@@H](n2ncc3ccccc32)C1	560208145	25	334.18	3.33
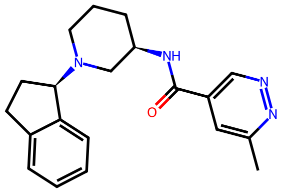	Cc1cc(C(=O)N[C@@H]2CCCN([C@@H]3CCc4ccccc43)C2)cnn1	1007214560	25	336.2	2.67
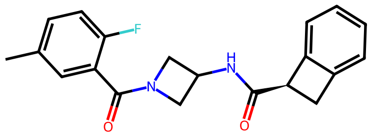	Cc1ccc(F)c(C(=O)N2CC(NC(=O)[C@@H]3Cc4ccccc43)C2)c1	998695380	25	338.14	2.41
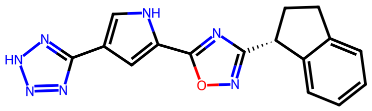	c1ccc2c(c1)CC[C@H]2c1noc(-c2cc(-c3nn[nH]n3)c[nH]2)n1	1593580262	24	319.12	2.32
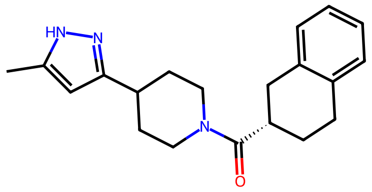	Cc1cc(C2CCN(C(=O)[C@H]3CCc4ccccc4C3)CC2)n[nH]1	579720013	24	323.2	3.23
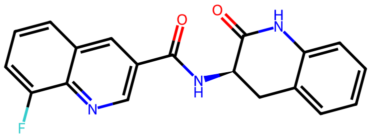	O=C(N[C@@H]1Cc2ccccc2NC1=O)c1cnc2c(F)cccc2c1	1648608978	25	335.11	2.67
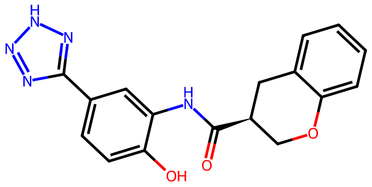	O=C(Nc1cc(-c2nn[nH]n2)ccc1O)[C@@H]1COc2ccccc2C1	823294503	25	337.12	1.76

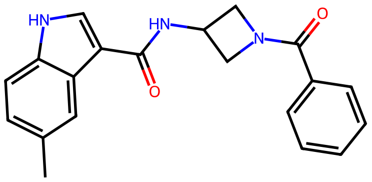	Cc1ccc2[nH]cc(C(=O)NC3CN(C(=O)c4ccccc4)C3)c2c1	994285799	25	333.15	2.73
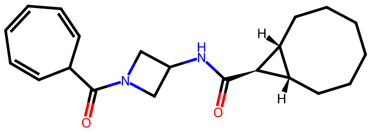	O=C(NC1CN(C(=O)C2C=CC=CC=C2)C1)[C@@H]1[C@H]2CCCCCC[C@H]21	1000507824	25	340.22	2.83
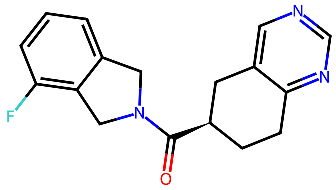	O=C([C@@H]1CCc2ncncc2C1)N1Cc2cccc(F)c2C1	648417193	22	297.13	2.26
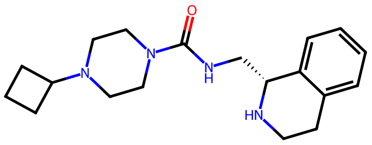	O=C(NC[C@H]1NCCc2ccccc21)N1CCN(C2CCC2)CC1	418642755	24	328.23	1.75
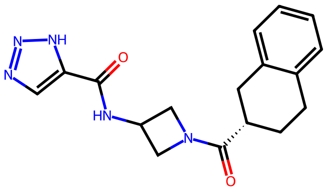	O=C(NC1CN(C(=O)[C@H]2CCc3ccccc3C2)C1)c1cnn[nH]1	995185856	24	325.15	0.55
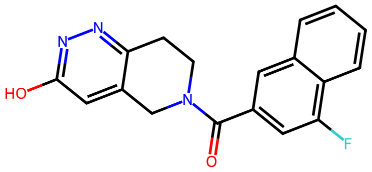	O=C(c1cc(F)c2ccccc2c1)N1CCc2nnc(O)cc2C1	347207823	24	323.11	2.67
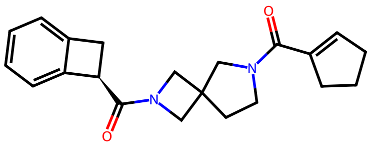	O=C(C1=CCCC1)N1CCC2(C1)CN(C(=O)[C@@H]1Cc3ccccc31)C2	1019349648	25	336.18	2.5
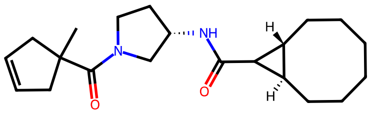	CC1(C(=O)N2CC[C@H](NC(=O)C3[C@@H]4CCCCCC[C@@H]34)C2)CC=CC1	938971350	25	344.25	3.28

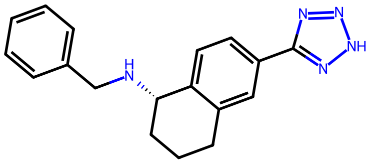	c1ccc(CN[C@H]2CCCc3cc(-c4nn[nH]n4)ccc32)cc1	826521390	23	305.16	3.03
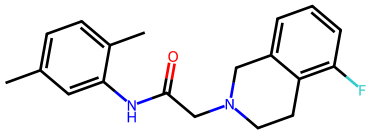	Cc1ccc(C)c(NC(=O)CN2CCc3c(F)cccc3C2)c1	152812623	23	312.16	3.44
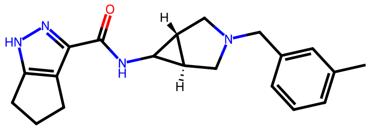	Cc1cccc(CN2C[C@H]3C(NC(=O)c4n[nH]c5c4CCC5)[C@@H]3C2)c1	960728411	25	336.2	2.07
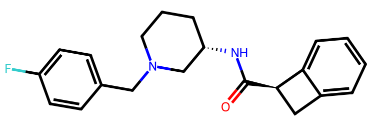	O=C(N[C@H]1CCCN(Cc2ccc(F)cc2)C1)[C@@H]1Cc2ccccc21	1008355300	25	338.18	3.25
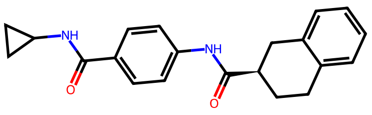	O=C(NC1CC1)c1ccc(NC(=O)[C@@H]2CCc3ccccc3C2)cc1	522005516	25	334.17	3.32
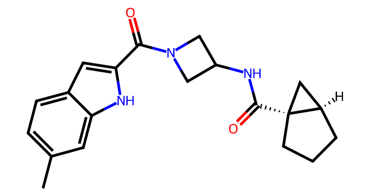	Cc1ccc2cc(C(=O)N3CC(NC(=O)[C@@]45CCC[C@@H]4C5)C3)[nH]c2c1	997554240	25	337.18	2.61
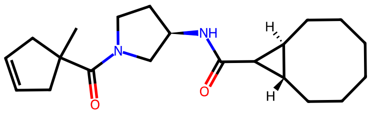	CC1(C(=O)N2CC[C@@H](NC(=O)C3[C@H]4CCCCCC[C@H]34)C2)CC=CC1	942510924	25	344.25	3.28
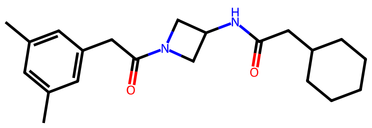	Cc1cc(C)cc(CC(=O)N2CC(NC(=O)CC3CCCCC3)C2)c1	990170759	25	342.23	3.14

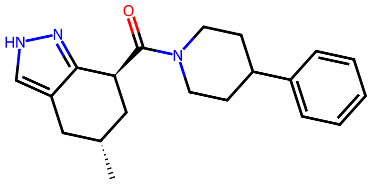	C[C@@H]1Cc2c[nH]nc2[C@@H](C(=O)N2CCC(c3ccccc3)CC2)C1	623178165	24	323.2	3.48
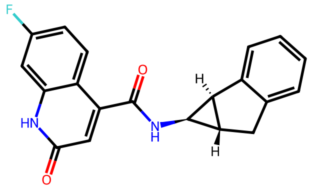	O=C(N[C@@H]1[C@H]2Cc3ccccc3[C@@H]21)c1cc(=O)[nH]c2cc(F)ccc12	152380605	25	334.11	2.74
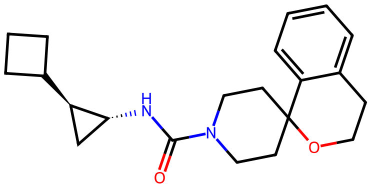	O=C(N[C@@H]1C[C@H]1C1CCC1)N1CCC2(CC1)OCCc1ccccc12	425604086	25	340.22	3.45
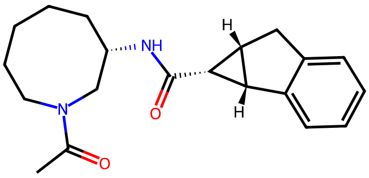	CC(=O)N1CCCCC[C@H](NC(=O)[C@H]2[C@H]3c4ccccc4C[C@H]32)C1	986792969	24	326.2	2.48
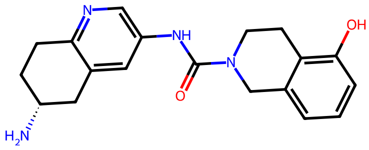	N[C@@H]1CCc2ncc(NC(=O)N3CCc4c(O)cccc4C3)cc2C1	625895757	25	338.17	2.19
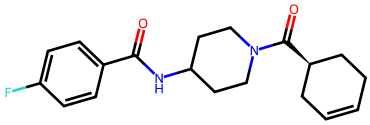	O=C(NC1CCN(C(=O)[C@@H]2CC=CCC2)CC1)c1ccc(F)cc1	29093633	24	330.17	2.9
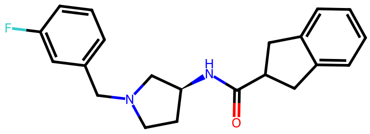	O=C(N[C@H]1CCN(Cc2cccc(F)c2)C1)C1Cc2ccccc2C1	1015748457	25	338.18	2.93
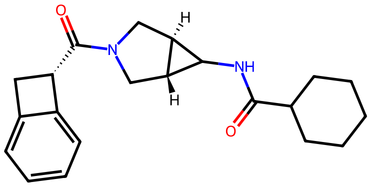	O=C(NC1[C@@H]2CN(C(=O)[C@H]3Cc4ccccc43)C[C@@H]12)C1CCCCC1	1012708698	25	338.2	2.48

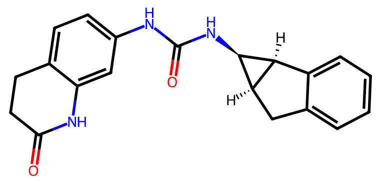	O=C1CCc2ccc(NC(=O)N[C@@H]3[C@@H]4Cc5ccccc5[C@@H]43)cc2N1	151354172	25	333.15	3.03
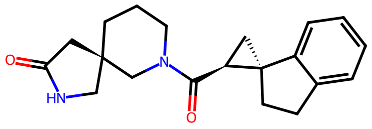	O=C1C[C@]2(CCCN(C(=O)[C@H]3C[C@]34CCc3ccccc34)C2)CN1	341499930	24	324.18	2.02
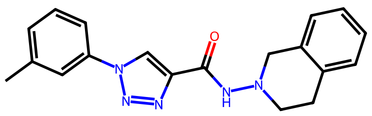	Cc1cccc(-n2cc(C(=O)NN3CCc4ccccc4C3)nn2)c1	1146331930	25	333.16	2.28
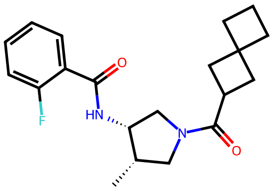	C[C@H]1CN(C(=O)C2CC3(CCC3)C2)C[C@H]1NC(=O)c1ccccc1F	966695525	25	344.19	2.98
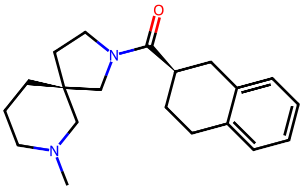	CN1CCC[C@]2(CCN(C(=O)[C@@H]3CCc4ccccc4C3)C2)C1	576286188	23	312.22	2.74
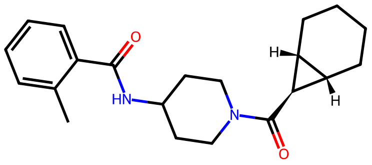	Cc1ccccc1C(=O)NC1CCN(C(=O)[C@@H]2[C@H]3CCCC[C@H]32)CC1	245492649	25	340.22	3.15
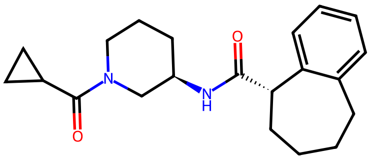	O=C(N[C@@H]1CCCN(C(=O)C2CC2)C1)[C@H]1CCCCc2ccccc21	949616516	25	340.22	3.01
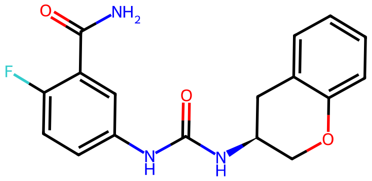	NC(=O)c1cc(NC(=O)N[C@@H]2COc3ccccc3C2)ccc1F	47886909	24	329.12	2.05

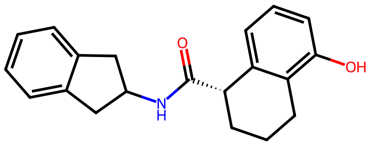	O=C(NC1Cc2ccccc2C1)[C@H]1CCCc2c(O)cccc21	191027862	23	307.16	3.1
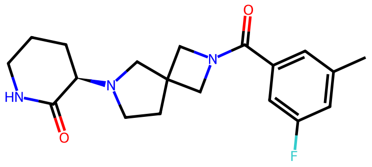	Cc1cc(F)cc(C(=O)N2CC3(CCN([C@@H]4CCCNC4=O)C3)C2)c1	1073007309	25	345.19	1.56
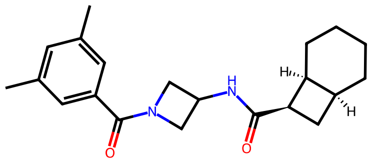	Cc1cc(C)cc(C(=O)N2CC(NC(=O)[C@@H]3C[C@@H]4CCCC[C@@H]43)C2)c1	997650857	25	340.22	3.07
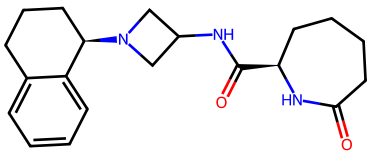	O=C1CCCC[C@H](C(=O)NC2CN([C@@H]3CCCc4ccccc43)C2)N1	1030937061	25	341.21	1.92
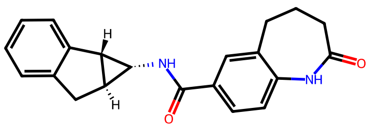	O=C1CCCc2cc(C(=O)N[C@@H]3[C@H]4Cc5ccccc5[C@@H]43)ccc2N1	675695910	25	332.15	3.03
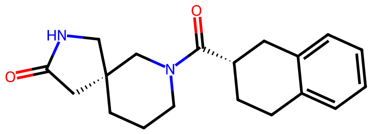	O=C1C[C@]2(CCCN(C(=O)[C@H]3CCc4ccccc4C3)C2)CN1	579013744	23	312.18	1.92
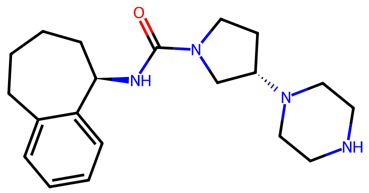	O=C(N[C@@H]1CCCCc2ccccc21)N1CC[C@H](N2CCNCC2)C1	577831477	25	342.24	2.14
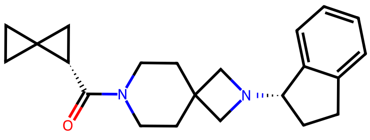	O=C([C@H]1CC12CC2)N1CCC2(CC1)CN([C@H]1CCc3ccccc31)C2	1035808192	25	336.22	3.4

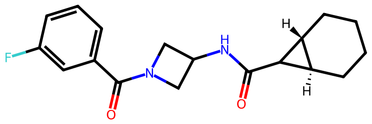	O=C(NC1CN(C(=O)c2cccc(F)c2)C1)C1[C@@H]2CCCC[C@@H]12	990640230	23	316.16	2.2
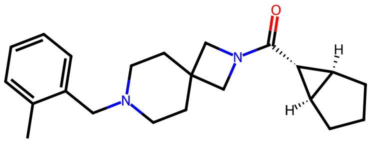	Cc1ccccc1CN1CCC2(CC1)CN(C(=O)[C@@H]1[C@H]3CCC[C@H]31)C2	1050476033	25	338.24	3.47
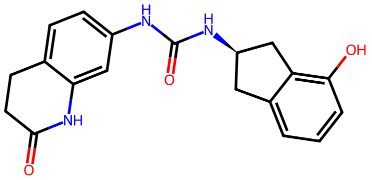	O=C1CCc2ccc(NC(=O)N[C@@H]3Cc4cccc(O)c4C3)cc2N1	429762359	25	337.14	2.57
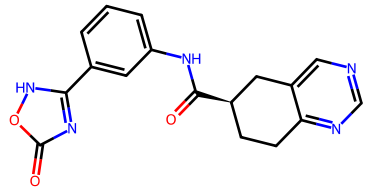	O=C(Nc1cccc(-c2nc(=O)o[nH]2)c1)[C@@H]1CCc2ncncc2C1	1358227897	25	337.12	1.56
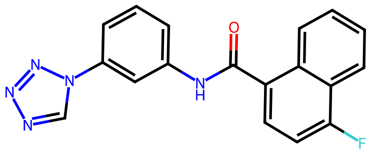	O=C(Nc1cccc(-n2cnnn2)c1)c1ccc(F)c2ccccc12	327526854	25	333.1	3.21
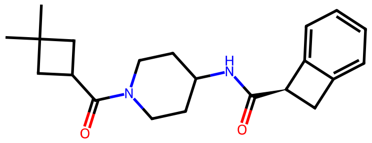	CC1(C)CC(C(=O)N2CCC(NC(=O)[C@@H]3Cc4ccccc43)CC2)C1	946627533	25	340.22	2.87
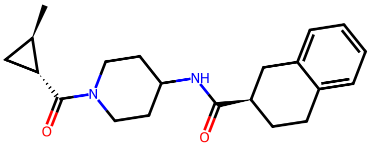	C[C@@H]1C[C@H]1C(=O)N1CCC(NC(=O)[C@@H]2CCc3ccccc3C2)CC1	947724159	25	340.22	2.55
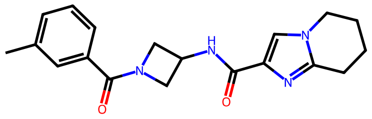	Cc1cccc(C(=O)N2CC(NC(=O)c3cn4c(n3)CCCC4)C2)c1	993118676	25	338.17	1.78

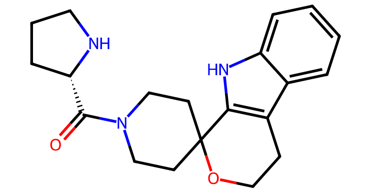	O=C([C@@H]1CCCN1)N1CCC2(CC1)OCCc1c2[nH]c2ccccc12	1704298975	25	339.19	2.31
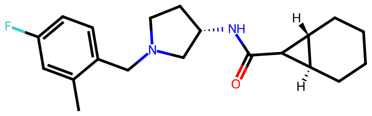	Cc1cc(F)ccc1CN1CC[C@H](NC(=O)C2[C@@H]3CCCC[C@@H]23)C1	1010439874	24	330.21	3.26
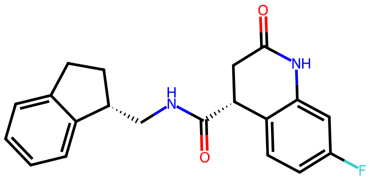	O=C1C[C@@H](C(=O)NC[C@H]2CCc3ccccc32)c2ccc(F)cc2N1	110378218	25	338.14	3.1
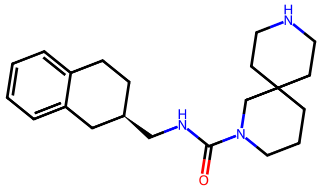	O=C(NC[C@@H]1CCc2ccccc2C1)N1CCCC2(CCNCC2)C1	1649836828	25	341.25	2.97
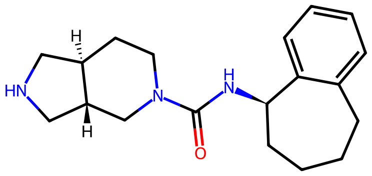	O=C(N[C@@H]1CCCCc2ccccc21)N1CC[C@@H]2CNC[C@H]2C1	578333973	23	313.22	2.71
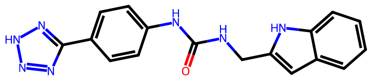	O=C(NCc1cc2ccccc2[nH]1)Nc1ccc(-c2nn[nH]n2)cc1	1593123504	25	333.13	2.67
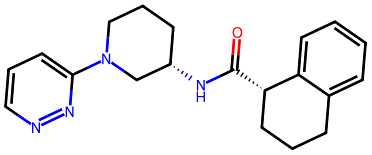	O=C(N[C@H]1CCCN(c2cccnn2)C1)[C@H]1CCCc2ccccc21	265515835	25	336.2	2.68
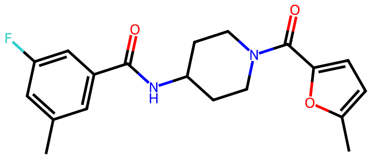	Cc1cc(F)cc(C(=O)NC2CCN(C(=O)c3ccc(C)o3)CC2)c1	943913558	25	344.15	3.07

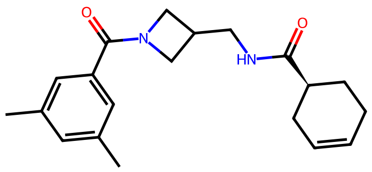	Cc1cc(C)cc(C(=O)N2CC(CNC(=O)[C@@H]3CC=CCC3)C2)c1	1000889738	24	326.2	2.85
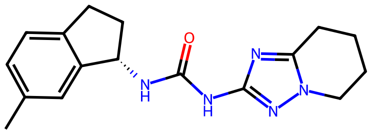	Cc1ccc2c(c1)[C@@H](NC(=O)Nc1nc3n(n1)CCCC3)CC2	653709305	23	311.17	2.73
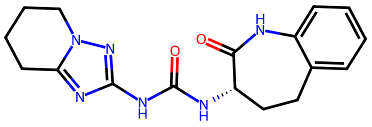	O=C(Nc1nc2n(n1)CCCC2)N[C@H]1CCc2ccccc2NC1=O	654107357	25	340.16	1.69
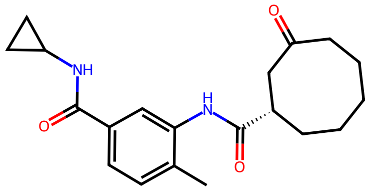	Cc1ccc(C(=O)NC2CC2)cc1NC(=O)[C@H]1CCCCCC(=O)C1	570568618	25	342.19	3.37
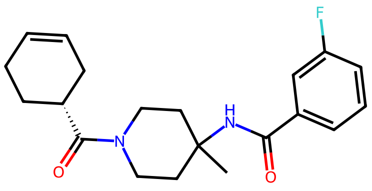	CC1(NC(=O)c2cccc(F)c2)CCN(C(=O)[C@H]2CC=CCC2)CC1	956271805	25	344.19	3.29
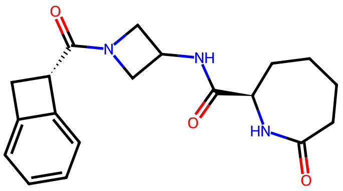	O=C1CCCC[C@H](C(=O)NC2CN(C(=O)[C@H]3Cc4ccccc43)C2)N1	999369230	25	341.17	0.71
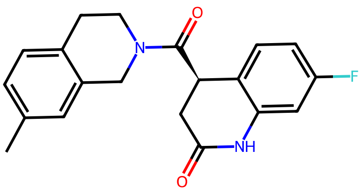	Cc1ccc2c(c1)CN(C(=O)[C@@H]1CC(=O)Nc3cc(F)ccc31)CC2	617602248	25	338.14	3.14
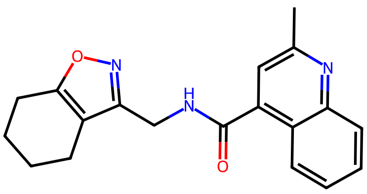	Cc1cc(C(=O)NCc2noc3c2CCCC3)c2ccccc2n1	288172682	24	321.15	3.34

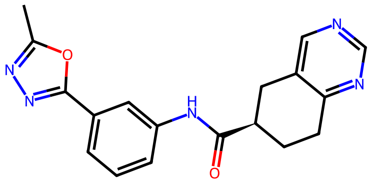	Cc1nnc(-c2cccc(NC(=O)[C@@H]3CCc4ncncc4C3)c2)o1	648302948	25	335.14	2.58
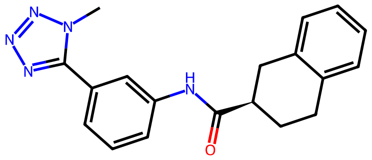	Cn1nnnc1-c1cccc(NC(=O)[C@@H]2CCc3ccccc3C2)c1	570283443	25	333.16	2.62
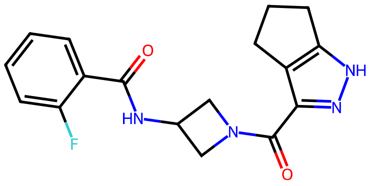	O=C(NC1CN(C(=O)c2n[nH]c3c2CCC3)C1)c1ccccc1F	990095044	24	328.13	1.29
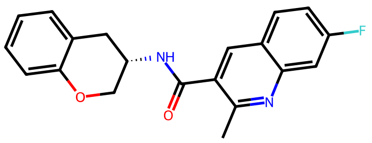	Cc1nc2cc(F)ccc2cc1C(=O)N[C@@H]1COc2ccccc2C1	45847445	25	336.13	3.42
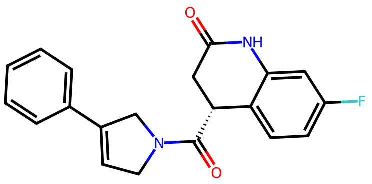	O=C1C[C@@H](C(=O)N2CC=C(c3ccccc3)C2)c2ccc(F)cc2N1	378511437	25	336.13	3.18
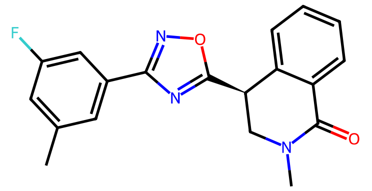	Cc1cc(F)cc(-c2noc([C@@H]3CN(C)C(=O)c4ccccc43)n2)c1	377438649	25	337.12	3.4
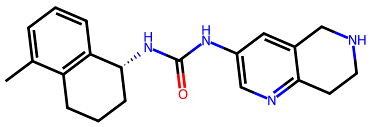	Cc1cccc2c1CCC[C@H]2NC(=O)Nc1cnc2c(c1)CNCC2	659019479	25	336.2	3.23
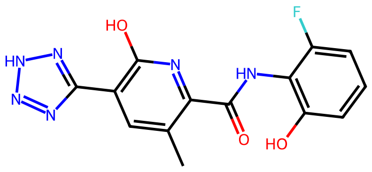	Cc1cc(-c2nn[nH]n2)c(O)nc1C(=O)Nc1c(O)cccc1F	1580607466	24	330.09	1.37

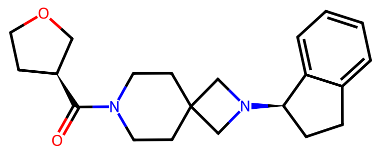	O=C([C@H]1CCOC1)N1CCC2(CC1)CN([C@@H]1CCc3ccccc31)C2	1035663240	25	340.22	2.63
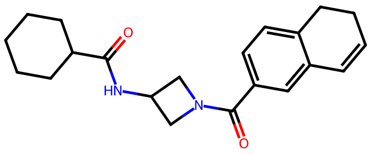	O=C(NC1CN(C(=O)c2ccc3c(c2)C=CCC3)C1)C1CCCCC1	990091843	25	338.2	3.17
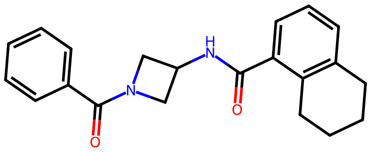	O=C(NC1CN(C(=O)c2ccccc2)C1)c1cccc2c1CCCC2	991901893	25	334.17	2.82
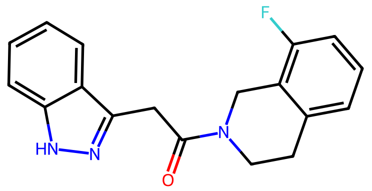	O=C(Cc1n[nH]c2ccccc12)N1CCc2cccc(F)c2C1	132646443	23	309.13	2.83
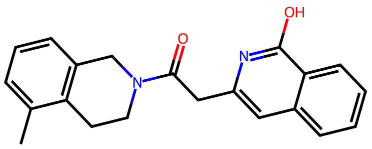	Cc1cccc2c1CCN(C(=O)Cc1cc3ccccc3c(O)n1)C2	1293135456	25	332.15	3.38
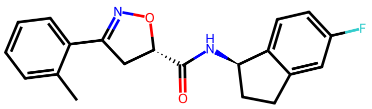	Cc1ccccc1C1=NO[C@H](C(=O)N[C@@H]2CCc3cc(F)ccc32)C1	1534972388	25	338.14	3.43
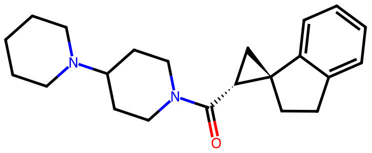	O=C([C@@H]1C[C@@]12CCc1ccccc12)N1CCC(N2CCCCC2)CC1	266541360	25	338.24	3.37
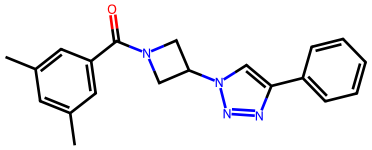	Cc1cc(C)cc(C(=O)N2CC(n3cc(-c4ccccc4)nn3)C2)c1	238928506	25	332.16	3.26

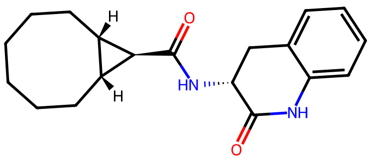	O=C1Nc2ccccc2C[C@H]1NC(=O)[C@@H]1[C@H]2CCCCCC[C@H]21	628943771	23	312.18	2.88
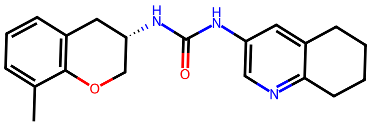	Cc1cccc2c1OC[C@@H](NC(=O)Nc1cnc3c(c1)CCCC3)C2	1660701419	25	337.18	3.39
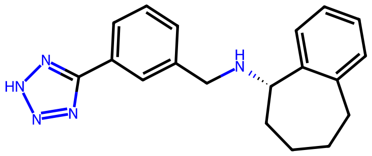	c1cc(CN[C@H]2CCCCc3ccccc32)cc(-c2nn[nH]n2)c1	738410792	24	319.18	3.42
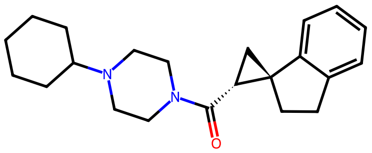	O=C([C@@H]1C[C@@]12CCc1ccccc12)N1CCN(C2CCCCC2)CC1	482064731	25	338.24	3.37
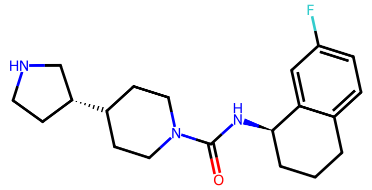	O=C(N[C@@H]1CCCc2ccc(F)cc21)N1CCC([C@@H]2CCNC2)CC1	418118516	25	345.22	3.23
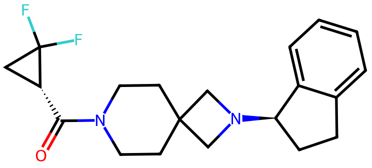	O=C([C@@H]1CC1(F)F)N1CCC2(CC1)CN([C@@H]1CCc3ccccc31)C2	1035700078	25	346.19	3.25
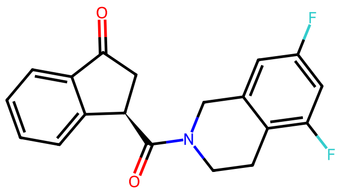	O=C1C[C@@H](C(=O)N2CCc3c(F)cc(F)cc3C2)c2ccccc21	355984198	24	327.11	3.22
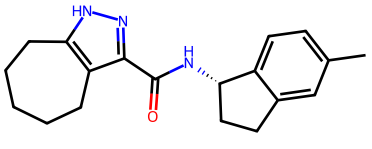	Cc1ccc2c(c1)CC[C@@H]2NC(=O)c1n[nH]c2c1CCCCC2	473776369	23	309.18	3.4

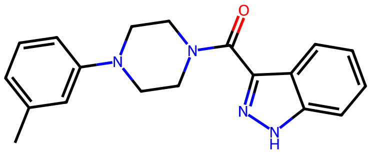	Cc1cccc(N2CCN(C(=O)c3n[nH]c4cccc c34)CC2)c1	12235285	24	320.16	2.83
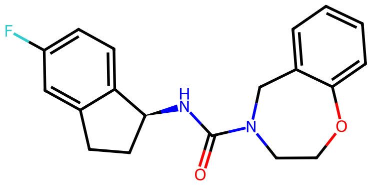	O=C(N[C@H]1CCc2cc(F)ccc21)N1CC Oc2ccccc2C1	619389130	24	326.14	3.42
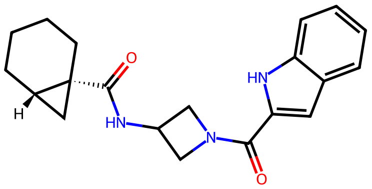	O=C(c1cc2ccccc2[nH]1)N1CC(NC(=O)[C@@]23CCCC[C@H]2C3)C1	999281465	25	337.18	2.69
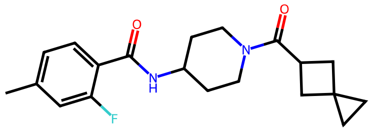	Cc1ccc(C(=O)NC2CCN(C(=O)C3CC4(CC4)C3)CC2)c(F)c1	948305759	25	344.19	3.05
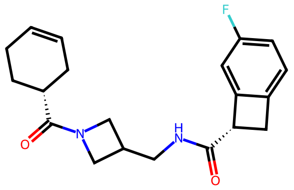	O=C(NCC1CN(C(=O)[C@H]2CC=CC C2)C1)[C@H]1Cc2ccc(F)cc21	1003882956	25	342.17	2.4
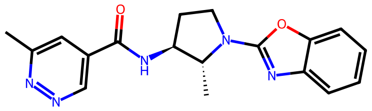	Cc1cc(C(=O)N[C@H]2CCN(c3nc4ccc cc4o3)[C@@H]2C)cnn1	1050190540	25	337.15	2.32
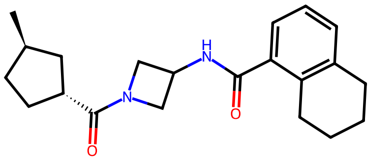	C[C@@H]1CC[C@@H](C(=O)N2CC(NC(=O)c3cccc4c3CCCC4)C2)C1	991904019	25	340.22	2.94
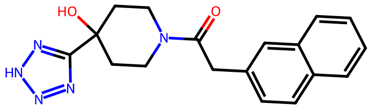	O=C(Cc1ccc2ccccc2c1)N1CCC(O)(c2 nn[nH]n2)CC1	798148640	25	337.15	1.41

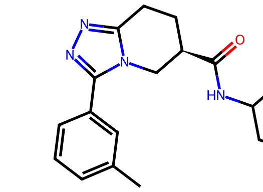	Cc1cccc(-c2nnc3n2C[C@H](C(=O)NC2CCCCC2)CC3)c1	562646181	25	338.21	3.26
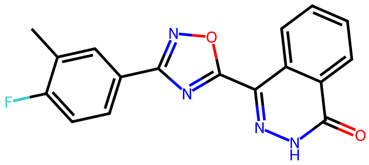	Cc1cc(-c2noc(-c3n[nH]c(=O)c4ccccc34)n2)ccc1F	109558408	24	322.09	3.09
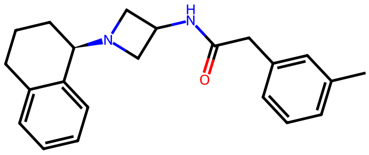	Cc1cccc(CC(=O)NC2CN([C@@H]3CCCc4ccccc43)C2)c1	1030884488	25	334.2	3.42
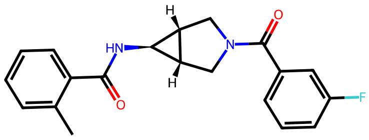	Cc1ccccc1C(=O)N[C@H]1[C@@H]2CN(C(=O)c3cccc(F)c3)C[C@@H]21	976081508	25	338.14	2.63
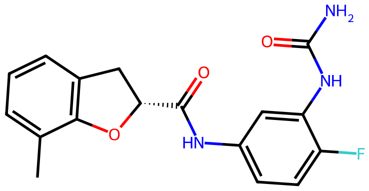	Cc1cccc2c1O[C@@H](C(=O)Nc1ccc(F)c(NC(N)=O)c1)C2	1413024261	24	329.12	2.57
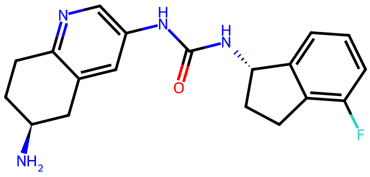	N[C@H]1CCc2ncc(NC(=O)N[C@H]3CCc4c(F)cccc43)cc2C1	333286792	25	340.17	2.85
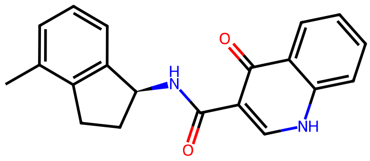	Cc1cccc2c1CC[C@@H]2NC(=O)c1c[nH]c2ccccc2c1=O	591313801	24	318.14	3.25
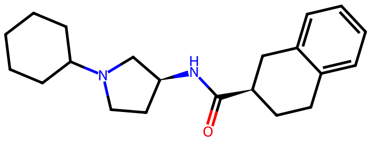	O=C(N[C@H]1CCN(C2CCCCC2)C1)[C@@H]1CCc2ccccc2C1	618656945	24	326.24	3.31

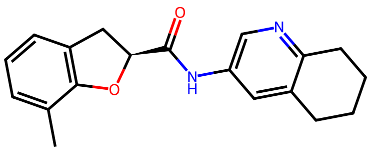	Cc1cccc2c1O[C@H](C(=O)Nc1cnc3c(c1)CCCC3)C2	333759406	23	308.15	3.21
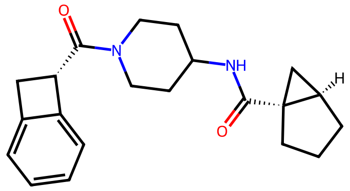	O=C([C@H]1Cc2ccccc21)N1CCC(NC(=O)[C@@]23CCC[C@@H]2C3)CC1	948059932	25	338.2	2.62
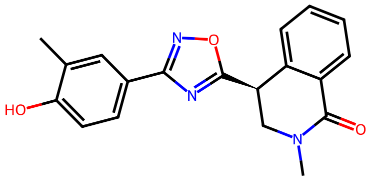	Cc1cc(-c2noc([C@@H]3CN(C)C(=O)c4ccccc43)n2)ccc1O	350605549	25	335.13	2.97
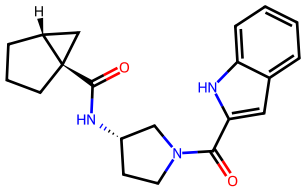	O=C(c1cc2ccccc2[nH]1)N1CC[C@H](NC(=O)[C@@]23CCC[C@@H]2C3)C1	938426134	25	337.18	2.69
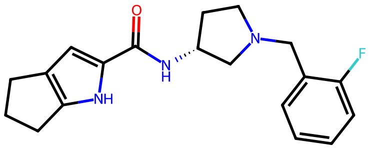	O=C(N[C@@H]1CCN(Cc2ccccc2F)C1)c1cc2c([nH]1)CCC2	1015978290	24	327.17	2.65
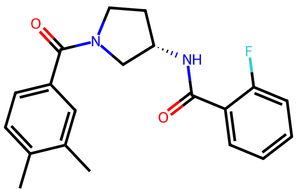	Cc1ccc(C(=O)N2CC[C@H](NC(=O)c3ccccc3F)C2)cc1C	937623557	25	340.16	3.09
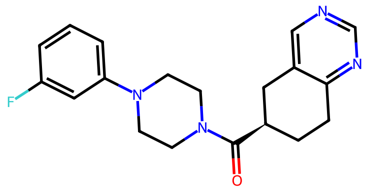	O=C([C@@H]1CCc2ncncc2C1)N1CCN(c2cccc(F)c2)CC1	648244029	25	340.17	2.07
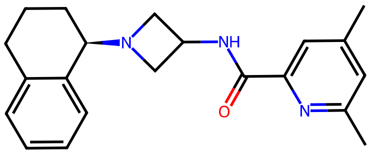	Cc1cc(C)nc(C(=O)NC2CN([C@@H]3CCCc4ccccc43)C2)c1	1030587117	25	335.2	3.19

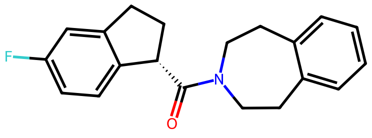	O=C([C@H]1CCc2cc(F)ccc21)N1CCc2ccccc2CC1	668830821	23	309.15	3.48
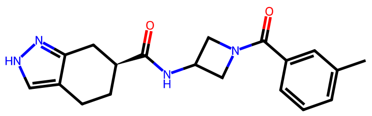	Cc1cccc(C(=O)N2CC(NC(=O)[C@H]3CCc4c[nH]nc4C3)C2)c1	992553699	25	338.17	1.46
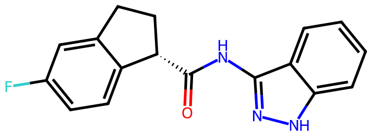	O=C(Nc1n[nH]c2ccccc12)[C@H]1CCc2cc(F)ccc21	1135394964	22	295.11	3.37
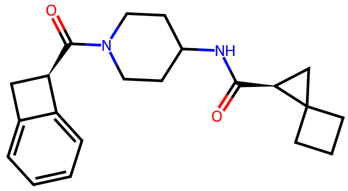	O=C(NC1CCN(C(=O)[C@@H]2Cc3ccccc32)CC1)[C@H]1CC12CCC2	948062508	25	338.2	2.62
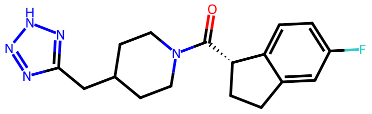	O=C([C@H]1CCc2cc(F)ccc21)N1CCC(Cc2nn[nH]n2)CC1	1548805478	24	329.17	1.85
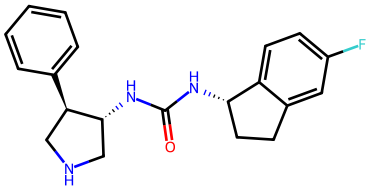	O=C(N[C@H]1CCc2cc(F)ccc21)N[C@@H]1CNC[C@H]1c1ccccc1	561174227	25	339.17	2.87
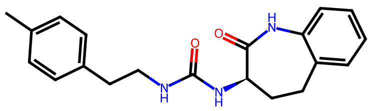	Cc1ccc(CCNC(=O)N[C@@H]2CCc3ccccc3NC2=O)cc1	654446382	25	337.18	2.79
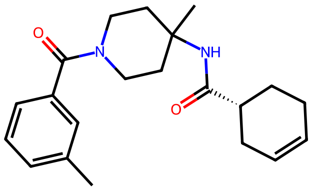	Cc1cccc(C(=O)N2CCC(C)(NC(=O)[C@H]3CC=CCC3)CC2)c1	956288784	25	340.22	3.46

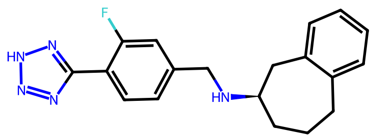	Fc1cc(CN[C@@H]2CCCc3ccccc3C2)ccc1-c1nn[nH]n1	1581853086	25	337.17	3.04
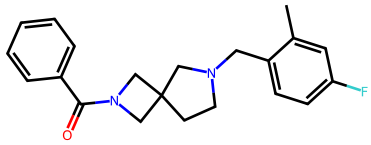	Cc1cc(F)ccc1CN1CCC2(C1)CN(C(=O)c1ccccc1)C2	1072408436	25	338.18	3.48
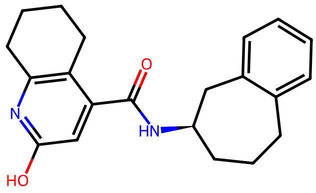	O=C(N[C@@H]1CCCc2ccccc2C1)c1cc(O)nc2c1CCCC2	888244309	25	336.18	3.34
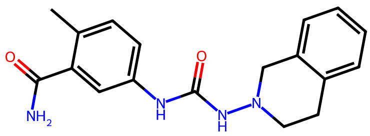	Cc1ccc(NC(=O)NN2CCc3ccccc3C2)cc1C(N)=O	1365488204	24	324.16	2.19
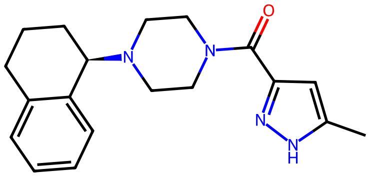	Cc1cc(C(=O)N2CCN([C@@H]3CCCc4ccccc43)CC2)n[nH]1	1480339065	24	324.2	2.55
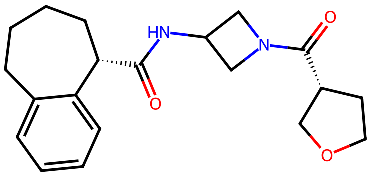	O=C(NC1CN(C(=O)[C@@H]2CCOC2)C1)[C@H]1CCCCc2ccccc21	994305237	25	342.19	1.86
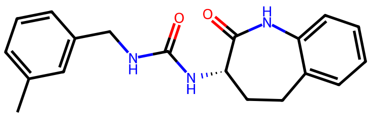	Cc1cccc(CNC(=O)N[C@H]2CCc3ccccc3NC2=O)c1	654366412	24	323.16	2.75
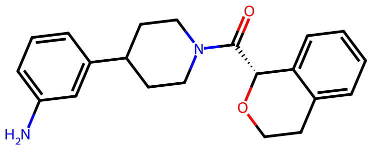	Nc1cccc(C2CCN(C(=O)[C@H]3OCCc4ccccc43)CC2)c1	424034183	25	336.18	3.29

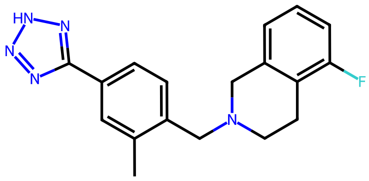	Cc1cc(-c2nn[nH]n2)ccc1CN1CCc2c(F)cccc2C1	1591865744	24	323.15	2.87
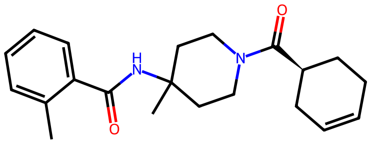	Cc1ccccc1C(=O)NC1(C)CCN(C(=O)[C@@H]2CC=CCC2)CC1	956273933	25	340.22	3.46
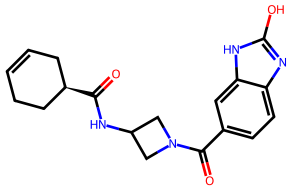	O=C(NC1CN(C(=O)c2ccc3nc(O)[nH]c3c2)C1)[C@@H]1CC=CCC1	990222549	25	340.15	1.57
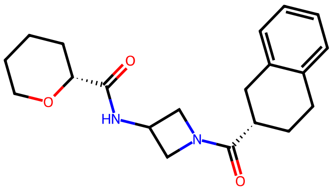	O=C(NC1CN(C(=O)[C@H]2CCc3ccccc3C2)C1)[C@H]1CCCCO1	990855876	25	342.19	1.69
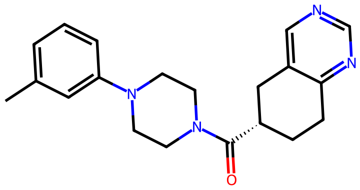	Cc1cccc(N2CCN(C(=O)[C@H]3CCc4ncncc4C3)CC2)c1	648226882	25	336.2	2.24
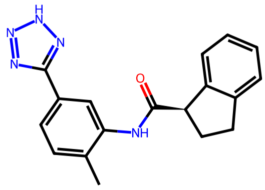	Cc1ccc(-c2nn[nH]n2)cc1NC(=O)[C@@H]1CCc2ccccc21	737291074	24	319.14	2.84
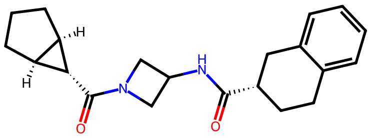	O=C(NC1CN(C(=O)[C@@H]2[C@H]3CCC[C@H]32)C1)[C@H]1CCc2ccccc2C1	1000537289	25	338.2	2.16
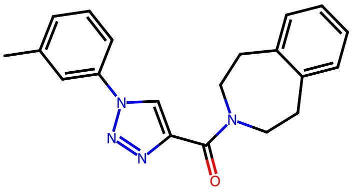	Cc1cccc(-n2cc(C(=O)N3CCc4ccccc4CC3)nn2)c1	116072120	25	332.16	2.82

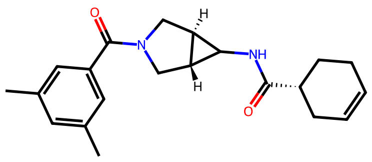	Cc1cc(C)cc(C(=O)N2C[C@H]3C(NC(=O)[C@H]4CC=CCC4)[C@@H]3C2)c1	1012767539	25	338.2	2.85
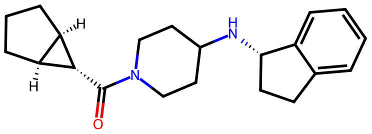	O=C([C@@H]1[C@H]2CCC[C@H]21)N1CCC(N[C@H]2CCc3ccccc32)CC1	990770130	24	324.22	3.3
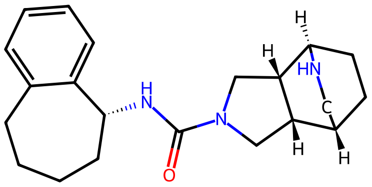	O=C(N[C@@H]1CCCCc2ccccc21)N1C[C@H]2[C@H]3CC[C@@H](NC3)[C@H]2C1	626961578	25	339.23	3.09
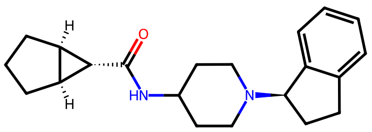	O=C(NC1CCN([C@@H]2CCc3ccccc32)CC1)[C@@H]1[C@H]2CCC[C@H]21	1002545295	24	324.22	3.3
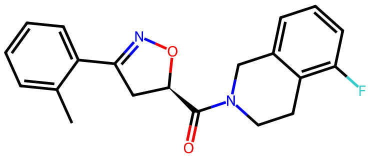	Cc1ccccc1C1=NO[C@@H](C(=O)N2CCc3c(F)cccc3C2)C1	1535713681	25	338.14	3.21
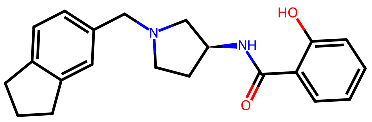	O=C(N[C@H]1CCN(Cc2ccc3c(c2)CCC3)C1)c1ccccc1O	1010275147	25	336.18	2.89
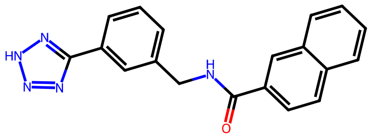	O=C(NCc1cccc(-c2nn[nH]n2)c1)c1ccc2ccccc2c1	1609556255	25	329.13	2.95
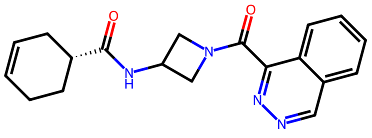	O=C(NC1CN(C(=O)c2nncc3ccccc23)C1)[C@H]1CC=CCC1	990212849	25	336.16	1.93

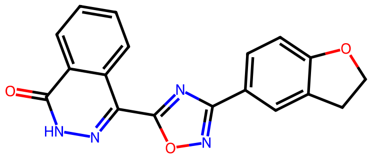	O=c1[nH]nc(-c2nc(-c3ccc4c(c3)CCO4)<br>no2)c2ccccc12	154839842	25	332.09	2.58
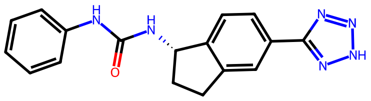	O=C(Nc1ccccc1)N[C@H]1CCc2cc(-c3<br>nn[nH]n3)ccc21	1593250466	24	320.14	2.68
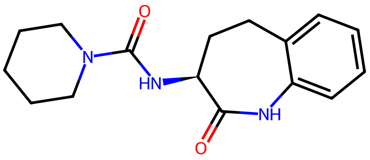	O=C1Nc2ccccc2CC[C@@H]1NC(=O)<br>N1CCCCC1	848596390	21	287.16	2.14
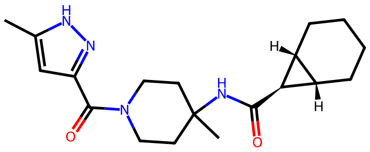	Cc1cc(C(=O)N2CCC(C)(NC(=O)[C@<br>@H]3[C@H]4CCCC[C@H]43)CC2)n[n<br>H]1	956325076	25	344.22	2.27
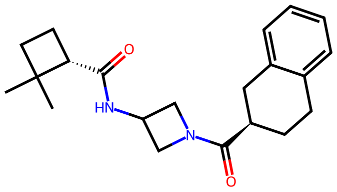	CC1(C)CC[C@@H]1C(=O)NC1CN(C(<br>=O)[C@@H]2CCc3ccccc3C2)C1	997966742	25	340.22	2.55
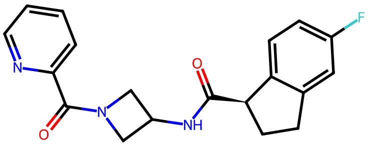	O=C(NC1CN(C(=O)c2ccccn2)C1)[C@<br>@H]1CCc2cc(F)ccc21	996099838	25	339.14	1.89
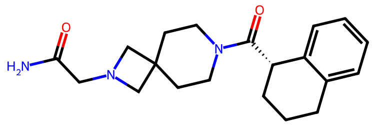	NC(=O)CN1CC2(CCN(C(=O)[C@H]3<br>CCCc4ccccc43)CC2)C1	1035734087	25	341.21	1.52
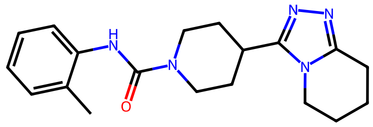	Cc1ccccc1NC(=O)N1CCC(c2nnc3n2C<br>CCC3)CC1	274789789	25	339.21	3.33

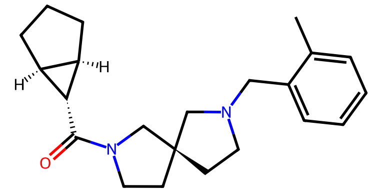	Cc1ccccc1CN1CC[C@]2(CCN(C(=O)[C@@H]3[C@H]4CCC[C@H]43)C2)C1	1041179211	25	338.24	3.47
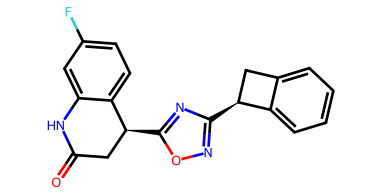	O=C1C[C@H](c2nc([C@H]3Cc4ccccc43)no2)c2ccc(F)cc2N1	546296568	25	335.11	3.37
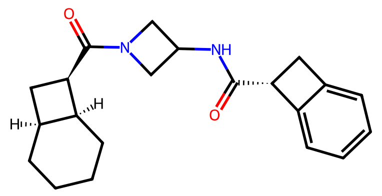	O=C(NC1CN(C(=O)[C@@H]2C[C@@H]3CCCC[C@@H]32)C1)[C@@H]1Cc2ccccc21	998681765	25	338.2	2.48
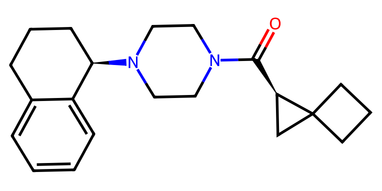	O=C([C@@H]1CC12CCC2)N1CCN([C@@H]2CCCc3ccccc32)CC1	950851297	24	324.22	3.4
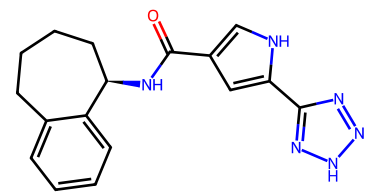	O=C(N[C@@H]1CCCCc2ccccc21)c1c[nH]c(-c2nn[nH]n2)c1	826429866	24	322.15	2.39
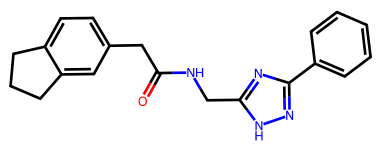	O=C(Cc1ccc2c(c1)CCC2)NCc1nc(-c2ccccc2)n[nH]1	726773687	25	332.16	2.82
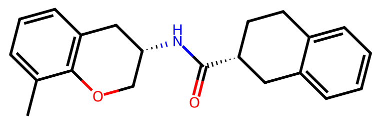	Cc1cccc2c1OC[C@@H](NC(=O)[C@@H]1CCc3ccccc3C1)C2	1129553627	24	321.17	3.22
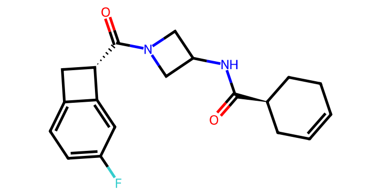	O=C(NC1CN(C(=O)[C@H]2Cc3ccc(F)cc32)C1)[C@@H]1CC=CCC1	990200910	24	328.16	2.15

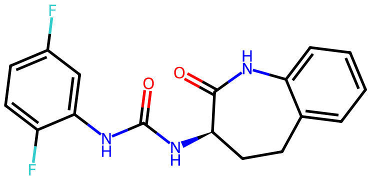	O=C(Nc1cc(F)ccc1F)N[C@@H]1CCc2ccccc2NC1=O	1202661716	24	331.11	3.04
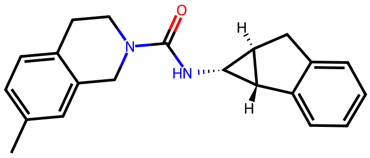	Cc1ccc2c(c1)CN(C(=O)N[C@@H]1[C@H]3Cc4ccccc4[C@@H]31)CC2	660914468	24	318.17	3.4
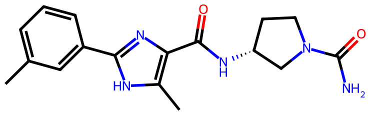	Cc1cccc(-c2nc(C(=O)N[C@@H]3CCN(C(N)=O)C3)c(C)[nH]2)c1	270282087	24	327.17	1.58
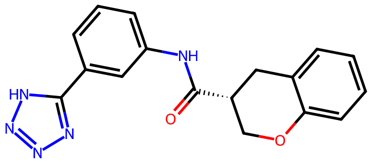	O=C(Nc1cccc(-c2nnn[nH]2)c1)[C@H]1COc2ccccc2C1	70710043	24	321.12	2.06
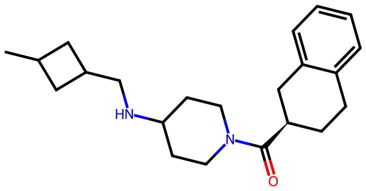	CC1CC(CNC2CCN(C(=O)[C@@H]3CCc4ccccc4C3)CC2)C1	997035962	25	340.25	3.42
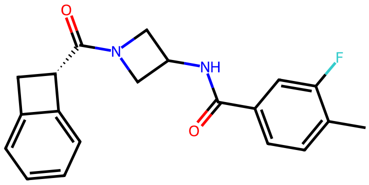	Cc1ccc(C(=O)NC2CN(C(=O)[C@H]3Cc4ccccc43)C2)cc1F	995442837	25	338.14	2.41
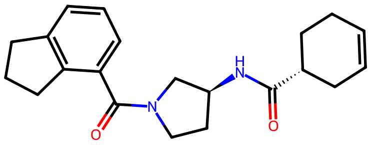	O=C(N[C@H]1CCN(C(=O)c2cccc3c2CCC3)C1)[C@H]1CC=CCC1	937664835	25	338.2	2.86
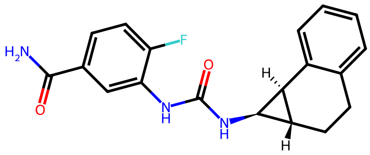	NC(=O)c1ccc(F)c(NC(=O)N[C@@H]2[C@H]3CCc4ccccc4[C@@H]32)c1	178923039	25	339.14	2.77

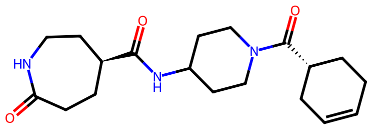	O=C1CC[C@H](C(=O)NC2CCN(C(=O)[C@H]3CC=CCC3)CC2)CCN1	944127269	25	347.22	1.37
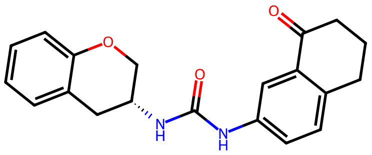	O=C(Nc1ccc2c(c1)C(=O)CCC2)N[C@H]1COc2ccccc2C1	177286425	25	336.15	3.33
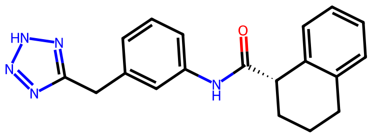	O=C(Nc1cccc(Cc2nn[nH]n2)c1)[C@H]1CCCc2ccccc21	910041803	25	333.16	2.85
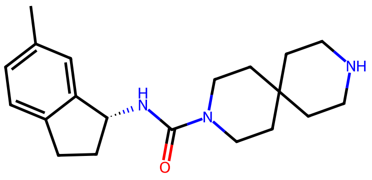	Cc1ccc2c(c1)[C@H](NC(=O)N1CCC3(CCNCC3)CC1)CC2	669466478	24	327.23	3.16
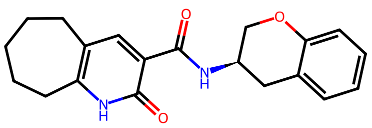	O=C(N[C@H]1COc2ccccc2C1)c1cc2c([nH]c1=O)CCCCC2	156149304	25	338.16	2.38
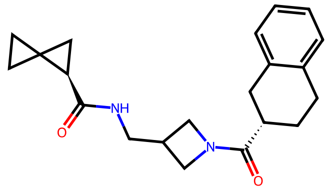	O=C(NCC1CN(C(=O)[C@H]2CCc3ccccc3C2)C1)[C@@H]1CC12CC2	936091182	25	338.2	2.17
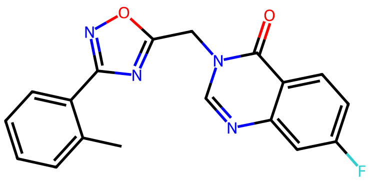	Cc1ccccc1-c1noc(Cn2cnc3cc(F)ccc3c2=O)n1	9691137	25	336.1	2.94
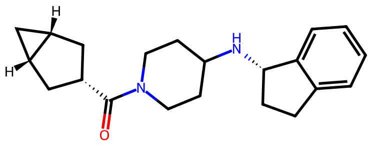	O=C([C@@H]1C[C@@H]2C[C@@H]2C1)N1CCC(N[C@H]2CCc3ccccc32)CC1	996900511	24	324.22	3.3

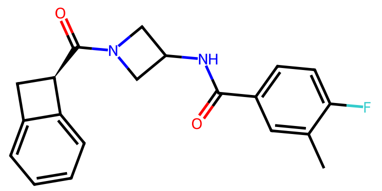	Cc1cc(C(=O)NC2CN(C(=O)[C@@H]3 Cc4ccccc43)C2)ccc1F	998697793	25	338.14	2.41
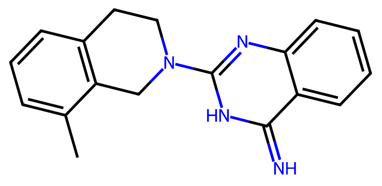	Cc1cccc2c1CN(c1nc3ccccc3c(=N)[nH] 1)CC2	572673347	22	290.15	2.91
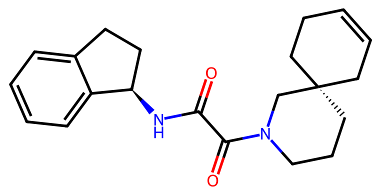	O=C(N[C@@H]1CCc2ccccc21)C(=O) N1CCC[C@]2(CC=CCC2)C1	339872979	25	338.2	3.14
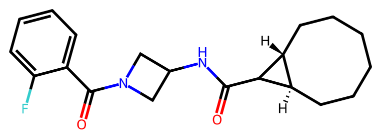	O=C(NC1CN(C(=O)c2ccccc2F)C1)C1[C@@H]2CCCCCC[C@@H]12	1000467940	25	344.19	2.98
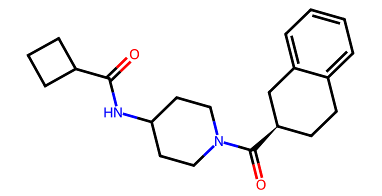	O=C(NC1CCN(C(=O)[C@@H]2CCc3 ccccc3C2)CC1)C1CCC1	948571834	25	340.22	2.7
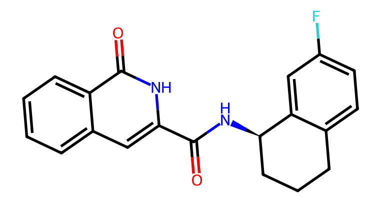	O=C(N[C@@H]1CCCc2ccc(F)cc21)c1 cc2ccccc2c(=O)[nH]1	93062911	25	336.13	3.47
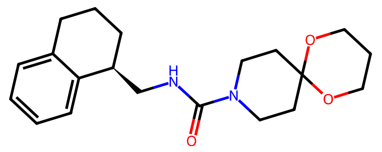	O=C(NC[C@@H]1CCCc2ccccc21)N1 CCC2(CC1)OCCCO2	836206132	25	344.21	3.05
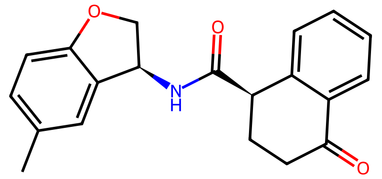	Cc1ccc2c(c1)[C@H](NC(=O)[C@@H] 1CCC(=O)c3ccccc31)CO2	375070910	24	321.14	3.31

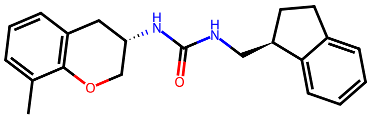	Cc1cccc2c1OC[C@@H](NC(=O)NC[C@H]1CCc3ccccc31)C2	1668963682	25	336.18	3.33
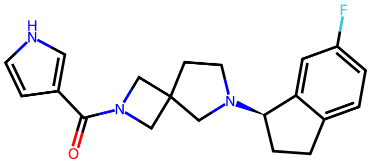	O=C(c1cc[nH]c1)N1CC2(CCN([C@@H]3CCc4ccc(F)cc43)C2)C1	1072771101	25	339.17	2.99
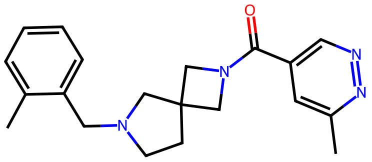	Cc1cc(C(=O)N2CC3(CCN(Cc4ccccc4C)C3)C2)cnn1	1072665359	25	336.2	2.44
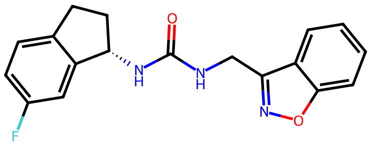	O=C(NCc1noc2ccccc12)N[C@H]1CCc2ccc(F)cc21	1668676237	24	325.12	3.45
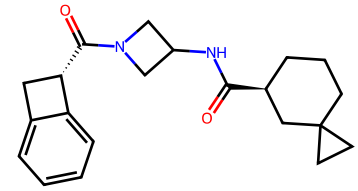	O=C(NC1CN(C(=O)[C@H]2Cc3ccccc32)C1)[C@H]1CCCC2(CC2)C1	991515482	25	338.2	2.62
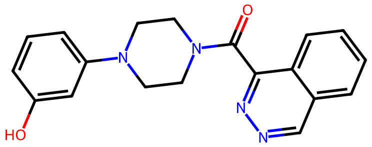	O=C(c1nncc2ccccc12)N1CCN(c2cccc(O)c2)CC1	1440706774	25	334.14	2.3
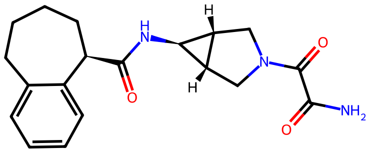	NC(=O)C(=O)N1C[C@H]2[C@H](NC(=O)[C@@H]3CCCCc4ccccc43)[C@H]2C1	976704993	25	341.17	0.55
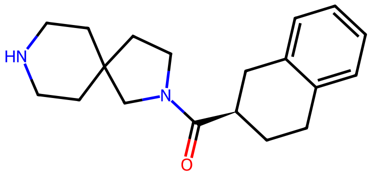	O=C([C@@H]1CCc2ccccc2C1)N1CCC2(CCNCC2)C1	319328200	22	298.2	2.39

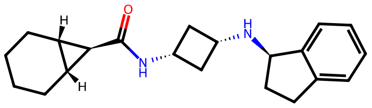	O=C(N[C@H]1C[C@@H](N[C@@H]2CCc3ccccc32)C1)[C@@H]1[C@H]2CCC[C@H]21	1021936286	24	324.22	3.35
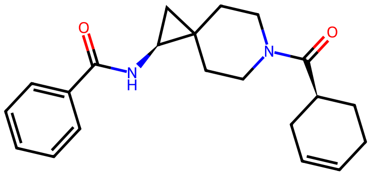	O=C(N[C@H]1CC12CCN(C(=O)[C@@H]1CC=CCC1)CC2)c1ccccc1	962784347	25	338.2	3.15
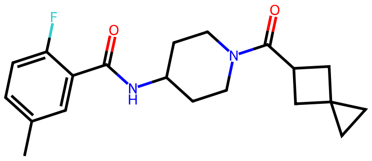	Cc1ccc(F)c(C(=O)NC2CCN(C(=O)C3CC4(CC4)C3)CC2)c1	948305797	25	344.19	3.05
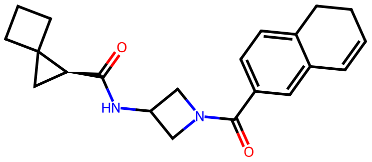	O=C(NC1CN(C(=O)c2ccc3c(c2)C=CCC3)C1)[C@H]1CC12CCC2	991534279	25	336.18	2.78
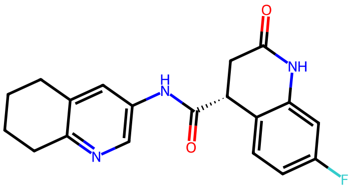	O=C1C[C@@H](C(=O)Nc2cnc3c(c2)CCCC3)c2ccc(F)cc2N1	543897805	25	339.14	3.16
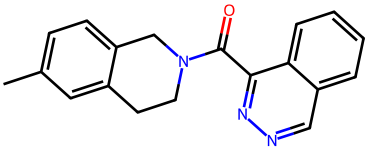	Cc1ccc2c(c1)CCN(C(=O)c1nncc3ccccc13)C2	1598092710	23	303.14	3.14
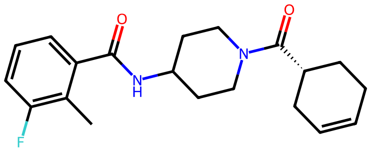	Cc1c(F)cccc1C(=O)NC1CCN(C(=O)[C@H]2CC=CCC2)CC1	944127517	25	344.19	3.21
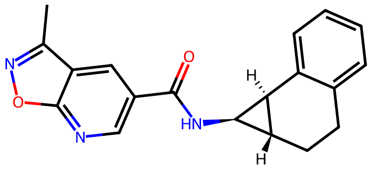	Cc1noc2ncc(C(=O)N[C@@H]3[C@H]4CCc5ccccc5[C@@H]43)cc12	121352093	24	319.13	2.99

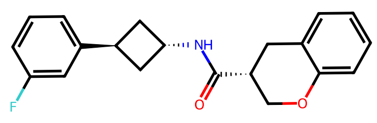	O=C(N[C@H]1C[C@H](c2cccc(F)c2)C1)[C@H]1COc2ccccc2C1	437442537	24	325.15	3.44
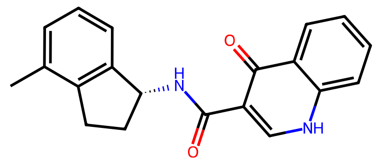	Cc1cccc2c1CC[C@H]2NC(=O)c1c[nH]c2ccccc2c1=O	591313800	24	318.14	3.25
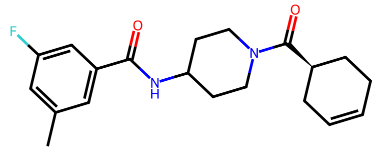	Cc1cc(F)cc(C(=O)NC2CCN(C(=O)[C@@H]3CC=CCC3)CC2)c1	944127960	25	344.19	3.21
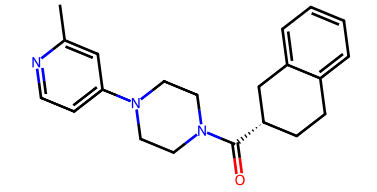	Cc1cc(N2CCN(C(=O)[C@H]3CCc4ccccc4C3)CC2)ccn1	223350304	25	335.2	2.84
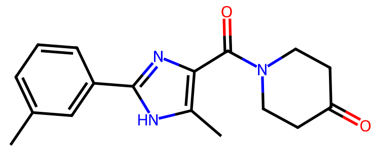	Cc1cccc(-c2nc(C(=O)N3CCC(=O)CC3)c(C)[nH]2)c1	272318096	22	297.15	2.5
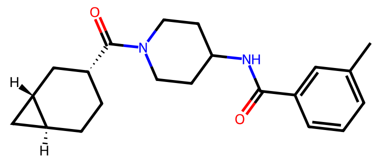	Cc1cccc(C(=O)NC2CCN(C(=O)[C@@H]3CC[C@H]4C[C@@H]4C3)CC2)c1	870837570	25	340.22	3.15
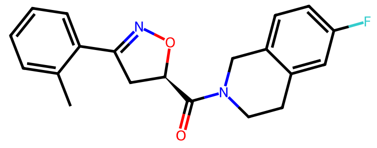	Cc1ccccc1C1=NO[C@@H](C(=O)N2CCc3cc(F)ccc3C2)C1	1127326394	25	338.14	3.21
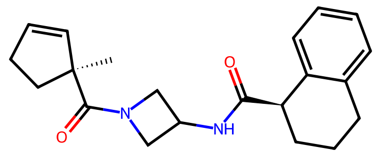	C[C@@]1(C(=O)N2CC(NC(=O)[C@@H]3CCCc4ccccc43)C2)C=CCC1	995076436	25	338.2	2.79

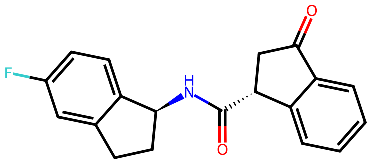	O=C1C[C@@H](C(=O)N[C@H]2CCc3cc(F)ccc32)c2ccccc21	277338897	23	309.12	3.3
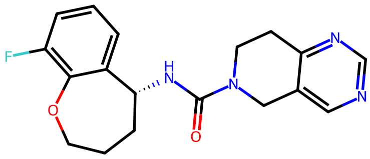	O=C(N[C@@H]1CCCOc2c(F)cccc21)N1CCc2ncncc2C1	883558932	25	342.15	2.6
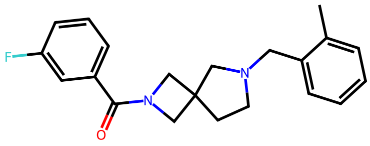	Cc1ccccc1CN1CCC2(C1)CN(C(=O)c1cccc(F)c1)C2	1072418919	25	338.18	3.48
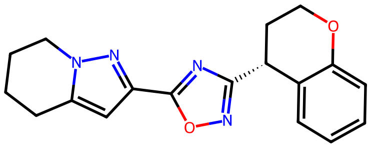	c1ccc2c(c1)OCC[C@H]2c1noc(-c2cc3n(n2)CCCC3)n1	351090234	24	322.14	3.18
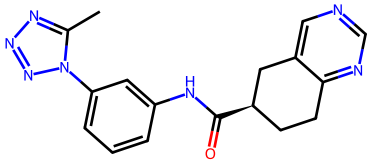	Cc1nnnn1-c1cccc(NC(=O)[C@@H]2CCc3ncncc3C2)c1	648242507	25	335.15	1.5
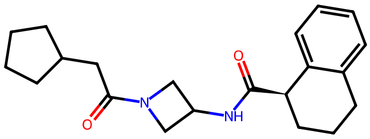	O=C(NC1CN(C(=O)CC2CCCC2)C1)[C@@H]1CCCc2ccccc21	995068151	25	340.22	3.01
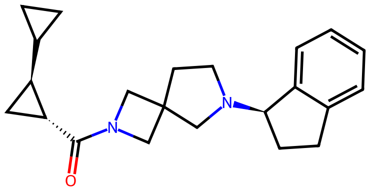	O=C([C@@H]1C[C@H]1C1CC1)N1CC2(CCN([C@@H]3CCc4ccccc43)C2)C1	1073047279	25	336.22	3.25
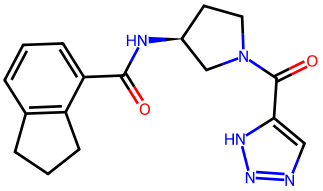	O=C(N[C@H]1CCN(C(=O)c2cnn[nH]2)C1)c1cccc2c1CCC2	938149699	24	325.15	0.94

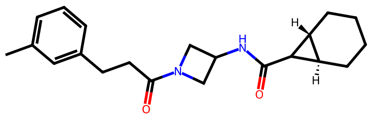	Cc1cccc(CCC(=O)N2CC(NC(=O)C3[C@@H]4CCCC[C@@H]34)C2)c1	990653227	25	340.22	2.69
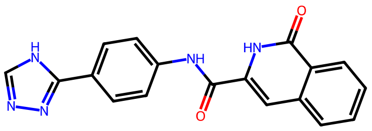	O=C(Nc1ccc(-c2nnc[nH]2)cc1)c1cc2ccccc2c(=O)[nH]1	111094903	25	331.11	2.57
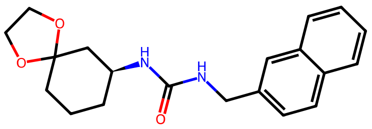	O=C(NCc1ccc2ccccc2c1)N[C@H]1CCCC2(C1)OCCO2	768323676	25	340.18	3.32
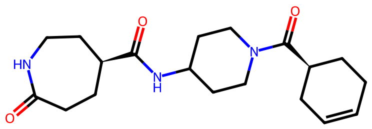	O=C1CC[C@H](C(=O)NC2CCN(C(=O)[C@@H]3CC=CCC3)CC2)CCN1	944127271	25	347.22	1.37
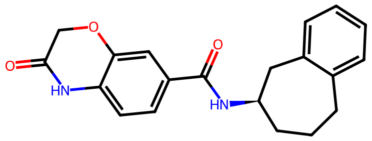	O=C1COc2cc(C(=O)N[C@@H]3CCCc4ccccc4C3)ccc2N1	888253539	25	336.15	2.69
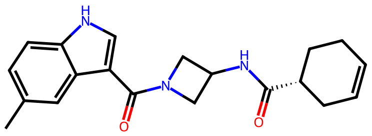	Cc1ccc2[nH]cc(C(=O)N3CC(NC(=O)[C@H]4CC=CCC4)C3)c2c1	990216950	25	337.18	2.77
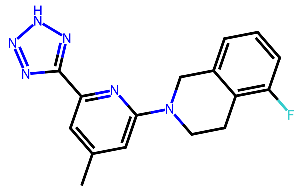	Cc1cc(-c2nn[nH]n2)nc(N2CCc3c(F)cccc3C2)c1	1591888073	23	310.13	2.27
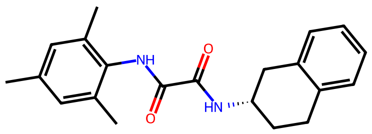	Cc1cc(C)c(NC(=O)C(=O)N[C@H]2CCc3ccccc3C2)c(C)c1	356713063	25	336.18	3.22

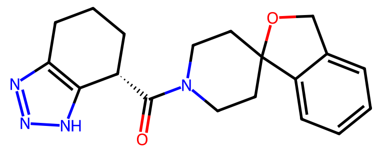	O=C([C@H]1CCCc2nn[nH]c21)N1CCC2(CC1)OCc1ccccc12	612378941	25	338.17	2.27
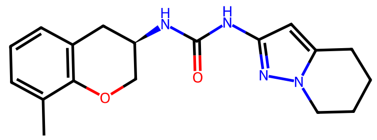	Cc1cccc2c1OC[C@H](NC(=O)Nc1cc3n(n1)CCCC3)C2	290726601	24	326.17	2.65
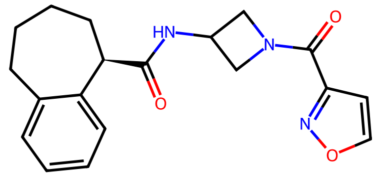	O=C(NC1CN(C(=O)c2ccon2)C1)[C@@H]1CCCCc2ccccc21	994306608	25	339.16	2.13
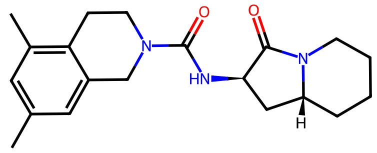	Cc1cc(C)c2c(c1)CN(C(=O)N[C@@H]1C[C@H]3CCCCN3C1=O)CC2	566780837	25	341.21	2.52
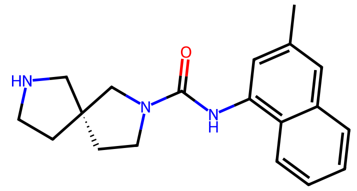	Cc1cc(NC(=O)N2CC[C@@]3(CCNC3)C2)c2ccccc2c1	415803839	23	309.18	3.37
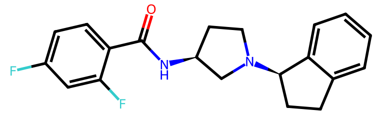	O=C(N[C@H]1CCN([C@@H]2CCc3ccccc32)C1)c1ccc(F)cc1F	1016150785	25	342.15	3.46
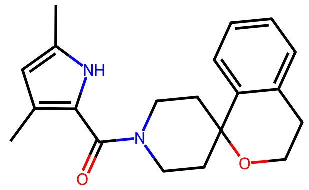	Cc1cc(C)c(C(=O)N2CCC3(CC2)OCCc2ccccc23)[nH]1	457133038	24	324.18	3.34
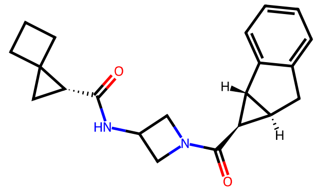	O=C(NC1CN(C(=O)[C@@H]2[C@@H]3Cc4ccccc4[C@H]32)C1)[C@@H]1CC12CCC2	991554855	25	336.18	2.09

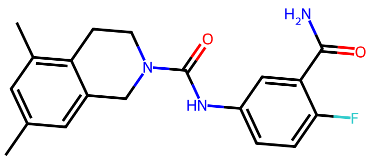	Cc1cc(C)c2c(c1)CN(C(=O)Nc1ccc(F)c(C(N)=O)c1)CC2	681609784	25	341.15	3.13
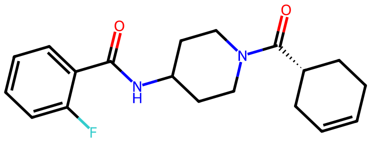	O=C(NC1CCN(C(=O)[C@H]2CC=CCC2)CC1)c1ccccc1F	361502619	24	330.17	2.9
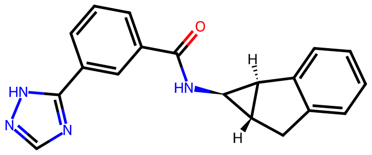	O=C(N[C@@H]1[C@H]2Cc3ccccc3[C@@H]21)c1cccc(-c2ncn[nH]2)c1	156441274	24	316.13	2.54
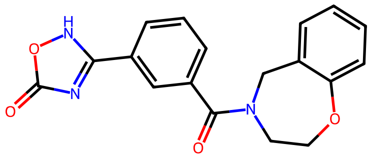	O=C(c1cccc(-c2nc(=O)o[nH]2)c1)N1CCOc2ccccc2C1	622319815	25	337.11	2.06
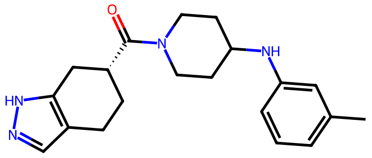	Cc1cccc(NC2CCN(C(=O)[C@@H]3CCc4cn[nH]c4C3)CC2)c1	80442356	25	338.21	2.93
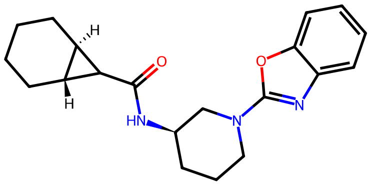	O=C(N[C@@H]1CCCN(c2nc3ccccc3o2)C1)C1[C@@H]2CCCC[C@@H]12	1058130131	25	339.19	3.35
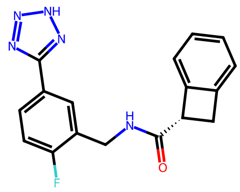	O=C(NCc1cc(-c2nn[nH]n2)ccc1F)[C@H]1Cc2ccccc21	823234814	24	323.12	1.96
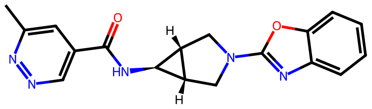	Cc1cc(C(=O)N[C@H]2[C@@H]3CN(c4nc5ccccc5o4)C[C@@H]32)cnn1	1056166506	25	335.14	1.79

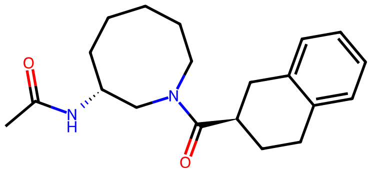	CC(=O)N[C@@H]1CCCCCN(C(=O)[C@@H]2CCc3ccccc3C2)C1	987173051	24	328.22	2.7
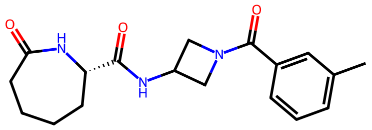	Cc1cccc(C(=O)N2CC(NC(=O)[C@@H]3CCCCC(=O)N3)C2)c1	999380454	24	329.17	0.99
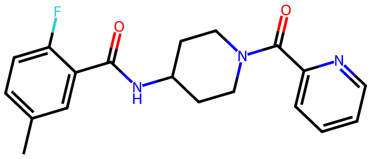	Cc1ccc(F)c(C(=O)NC2CCN(C(=O)c3ccccn3)CC2)c1	943598708	25	341.15	2.56
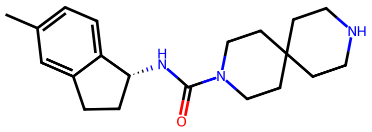	Cc1ccc2c(c1)CC[C@H]2NC(=O)N1CCC2(CCNCC2)CC1	417263226	24	327.23	3.16
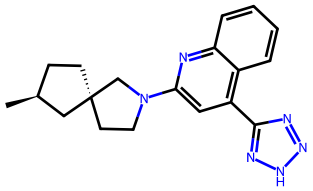	C[C@H]1CC[C@]2(CCN(c3cc(-c4nn[nH]n4)c4ccccc4n3)C2)C1	824917904	25	334.19	3.43
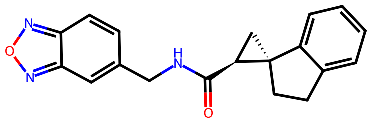	O=C(NCc1ccc2nonc2c1)[C@H]1C[C@]12CCc1ccccc12	889875006	24	319.13	2.74
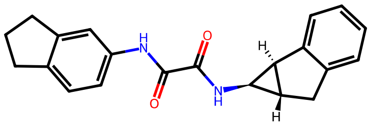	O=C(Nc1ccc2c(c1)CCC2)C(=O)N[C@@H]1[C@H]2Cc3ccccc3[C@@H]21	158862761	25	332.15	2.57
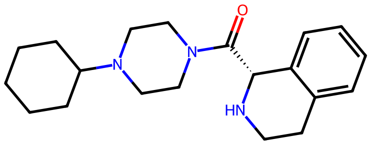	O=C([C@H]1NCCc2ccccc21)N1CCN(C2CCCCC2)CC1	564996760	24	327.23	2.35

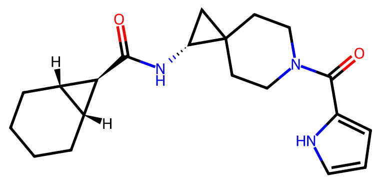	O=C(N[C@@H]1CC12CCN(C(=O)c1ccc[nH]1)CC2)[C@@H]1[C@H]2CCCC[C@H]21	963444121	25	341.21	2.56
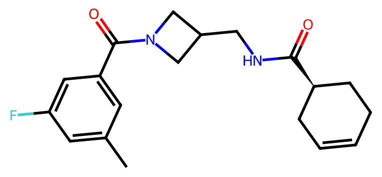	Cc1cc(F)cc(C(=O)N2CC(CNC(=O)[C@@H]3CC=CCC3)C2)c1	1000899289	24	330.17	2.68
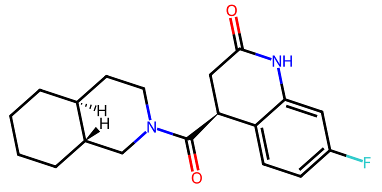	O=C1C[C@H](C(=O)N2CC[C@@H]3CCCC[C@H]3C2)c2ccc(F)cc2N1	1317731781	24	330.17	3.29
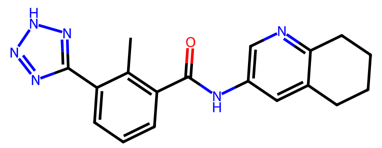	Cc1c(C(=O)Nc2cnc3c(c2)CCCC3)cccc1-c1nn[nH]n1	1591811367	25	334.15	2.7
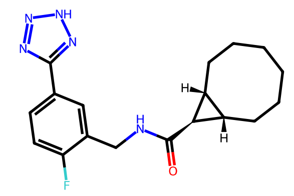	O=C(NCc1cc(-c2nn[nH]n2)ccc1F)[C@@H]1[C@H]2CCCCCC[C@H]21	1606814840	25	343.18	2.84
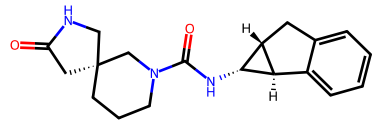	O=C1C[C@]2(CCCN(C(=O)N[C@H]3[C@@H]4c5ccccc5C[C@H]43)C2)CN1	332911908	24	325.18	1.64
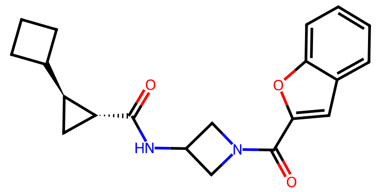	O=C(NC1CN(C(=O)c2cc3ccccc3o2)C1)[C@@H]1C[C@H]1C1CCC1	992014226	25	338.16	2.81
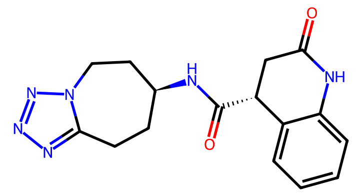	O=C1C[C@@H](C(=O)N[C@H]2CCc3nnnn3CC2)c2ccccc2N1	1418054922	24	326.15	0.62

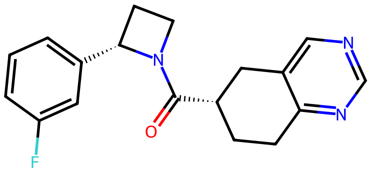	O=C([C@H]1CCc2ncncc2C1)N1CC[C@H]1c1cccc(F)c1	648801542	23	311.14	2.69
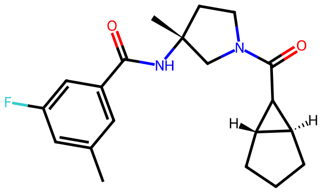	Cc1cc(F)cc(C(=O)N[C@]2(C)CCN(C(=O)C3[C@H]4CCC[C@H]34)C2)c1	957700248	25	344.19	2.9
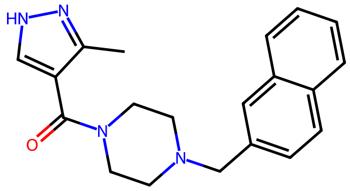	Cc1n[nH]cc1C(=O)N1CCN(Cc2ccc3ccccc3c2)CC1	949574366	25	334.18	2.83
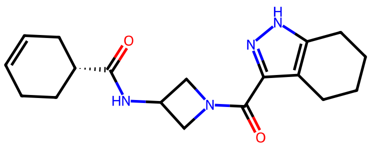	O=C(NC1CN(C(=O)c2n[nH]c3c2CCCC3)C1)[C@H]1CC=CCC1	990201020	24	328.19	1.59
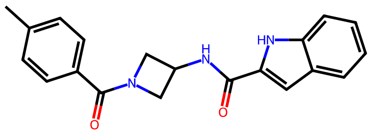	Cc1ccc(C(=O)N2CC(NC(=O)c3cc4ccccc4[nH]3)C2)cc1	1000140307	25	333.15	2.73
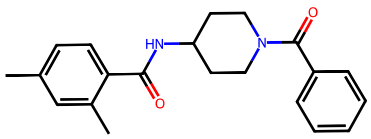	Cc1ccc(C(=O)NC2CCN(C(=O)c3ccccc3)CC2)c(C)c1	53392741	25	336.18	3.34
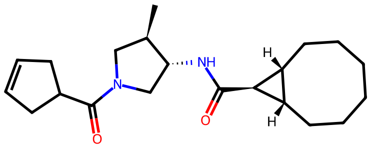	C[C@@H]1CN(C(=O)C2CC=CC2)C[C@H]1NC(=O)[C@@H]1[C@H]2CCCCCC[C@H]21	940656665	25	344.25	3.13
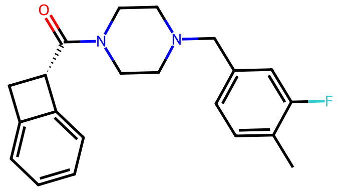	Cc1ccc(CN2CCN(C(=O)[C@H]3Cc4ccccc43)CC2)cc1F	949958809	25	338.18	3.12

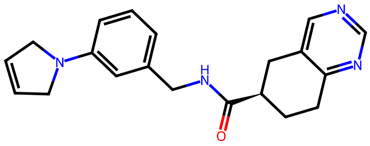	O=C(NCc1cccc(N2CC=CC2)c1)[C@@H]1CCc2ncncc2C1	648296093	25	334.18	2.27
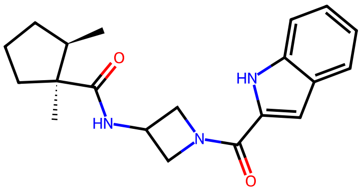	C[C@@H]1CCC[C@]1(C)C(=O)NC1CN(C(=O)c2cc3ccccc3[nH]2)C1	999175919	25	339.19	2.93
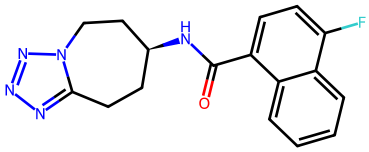	O=C(N[C@H]1CCc2nnnn2CC1)c1ccc(F)c2ccccc12	579107049	24	325.13	2.1
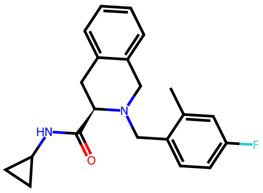	Cc1cc(F)ccc1CN1Cc2ccccc2C[C@@H]1C(=O)NC1CC1	1659303876	25	338.18	3.34
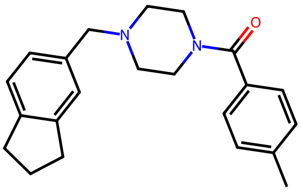	Cc1ccc(C(=O)N2CCN(Cc3ccc4c(c3)CCC4)CC2)cc1	957434843	25	334.2	3.44
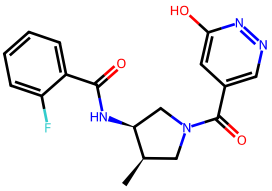	C[C@@H]1CN(C(=O)c2cnnc(O)c2)C[C@@H]1NC(=O)c1ccccc1F	966507124	25	344.13	1.21
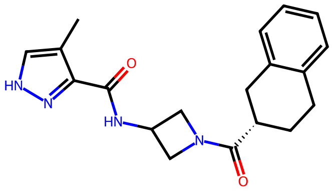	Cc1c[nH]nc1C(=O)NC1CN(C(=O)[C@H]2CCc3ccccc3C2)C1	991258153	25	338.17	1.46
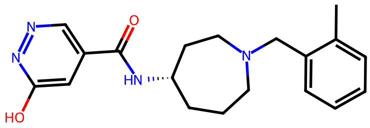	Cc1ccccc1CN1CCC[C@H](NC(=O)c2cnnc(O)c2)CC1	1052466779	25	340.19	2.28

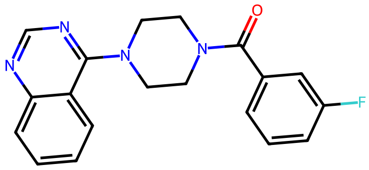	O=C(c1cccc(F)c1)N1CCN(c2ncnc3ccccc23)CC1	32803907	25	336.14	2.73
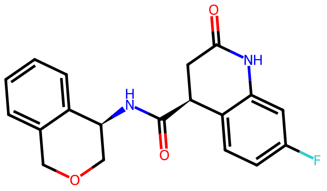	O=C1C[C@H](C(=O)N[C@H]2COCc3ccccc32)c2ccc(F)cc2N1	577364418	25	340.12	2.64
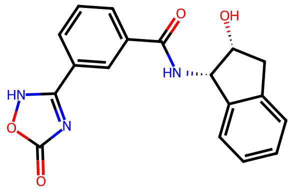	O=C(N[C@H]1c2ccccc2C[C@H]1O)c1cccc(-c2nc(=O)o[nH]2)c1	1588308491	25	337.11	1.42
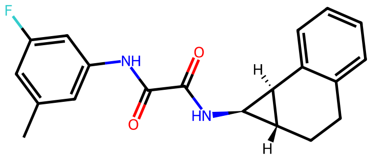	Cc1cc(F)cc(NC(=O)C(=O)N[C@@H]2[C@H]3CCc4ccccc4[C@@H]32)c1	180327623	25	338.14	2.92
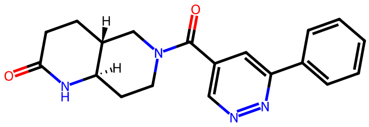	O=C1CC[C@@H]2CN(C(=O)c3cnnc(-c4ccccc4)c3)CC[C@H]2N1	618202611	25	336.16	1.88
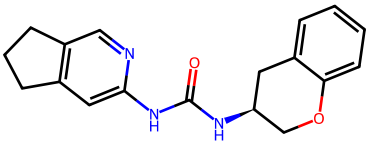	O=C(Nc1cc2c(cn1)CCC2)N[C@@H]1COc2ccccc2C1	570793888	23	309.15	2.7
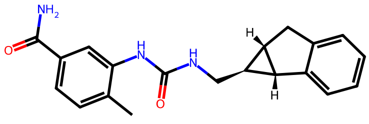	Cc1ccc(C(N)=O)cc1NC(=O)NC[C@@H]1[C@H]2c3ccccc3C[C@@H]12	123590541	25	335.16	2.8
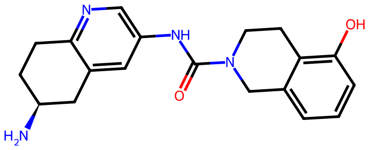	N[C@H]1CCc2ncc(NC(=O)N3CCc4c(O)cccc4C3)cc2C1	625895756	25	338.17	2.19

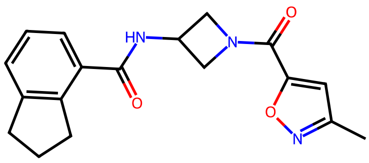	Cc1cc(C(=O)N2CC(NC(=O)c3cccc4c3CCC4)C2)on1	994401066	24	325.14	1.73
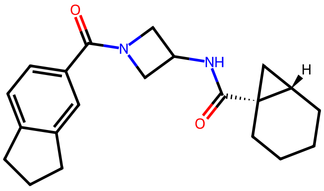	O=C(c1ccc2c(c1)CCC2)N1CC(NC(=O)[C@@]23CCCC[C@H]2C3)C1	999287389	25	338.2	2.7
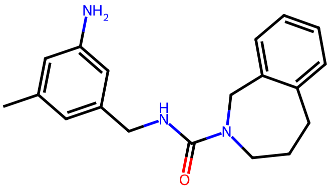	Cc1cc(N)cc(CNC(=O)N2CCCc3ccccc3C2)c1	418745858	23	309.18	3.24
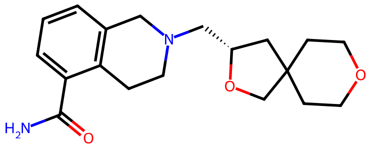	NC(=O)c1cccc2c1CCN(C[C@@H]1CC3(CCOCC3)CO1)C2	878999548	24	330.19	1.73
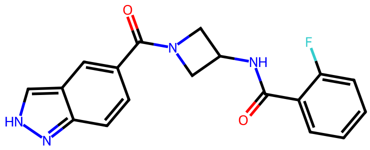	O=C(NC1CN(C(=O)c2ccc3n[nH]cc3c2)C1)c1ccccc1F	990091257	25	338.12	1.96
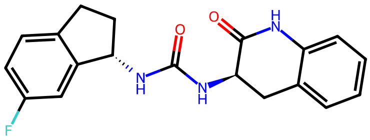	O=C(N[C@H]1CCc2ccc(F)cc21)N[C@@H]1Cc2ccccc2NC1=O	636743795	25	339.14	2.68
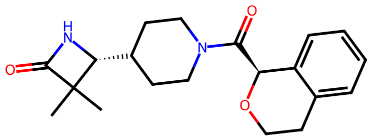	CC1(C)C(=O)N[C@@H]1C1CCN(C(=O)[C@@H]2OCCc3ccccc32)CC1	294717997	25	342.19	2.06
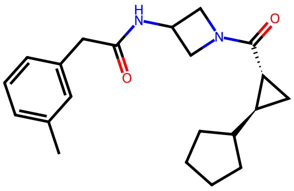	Cc1cccc(CC(=O)NC2CN(C(=O)[C@@H]3C[C@H]3C3CCCC3)C2)c1	998707020	25	340.22	2.69

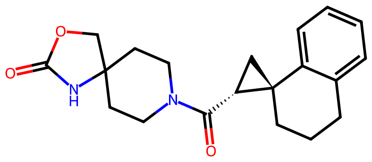	O=C1NC2(CCN(C(=O)[C@@H]3C[C@@]34CCCc3ccccc34)CC2)CO1	495140526	25	340.18	2.38
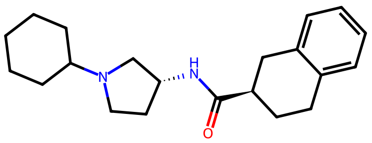	O=C(N[C@@H]1CCN(C2CCCCC2)C1)[C@@H]1CCc2ccccc2C1	618656948	24	326.24	3.31
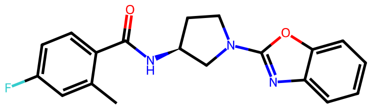	Cc1cc(F)ccc1C(=O)N[C@H]1CCN(c2nc3ccccc3o2)C1	1058411349	25	339.14	3.28
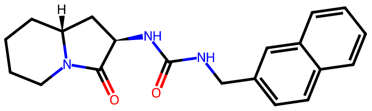	O=C(NCc1ccc2ccccc2c1)N[C@@H]1C[C@H]2CCCCN2C1=O	556740143	25	337.18	2.79
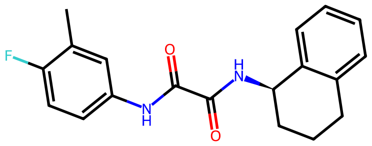	Cc1cc(NC(=O)C(=O)N[C@@H]2CCCc3ccccc32)ccc1F	103321982	24	326.14	3.27
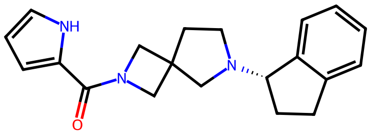	O=C(c1ccc[nH]1)N1CC2(CCN([C@H]3CCc4ccccc43)C2)C1	1072466313	24	321.18	2.85
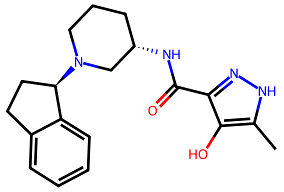	Cc1[nH]nc(C(=O)N[C@H]2CCCN([C@@H]3CCc4ccccc43)C2)c1O	1007132754	25	340.19	2.31
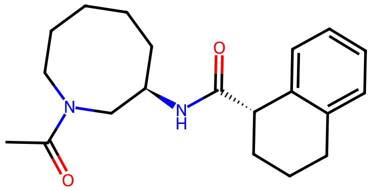	CC(=O)N1CCCCC[C@@H](NC(=O)[C@H]2CCCc3ccccc32)C1	986804243	24	328.22	3.01

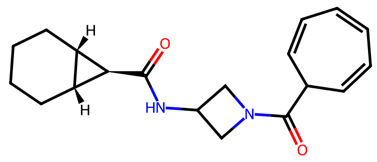	O=C(NC1CN(C(=O)C2C=CC=CC=C2)C1)[C@@H]1[C@H]2CCCC[C@H]21	990669213	23	312.18	2.05
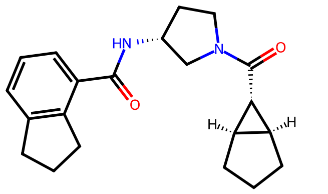	O=C(N[C@@H]1CCN(C(=O)[C@@H]2[C@H]3CCC[C@H]32)C1)c1cccc2c1CCC2	941156918	25	338.2	2.55
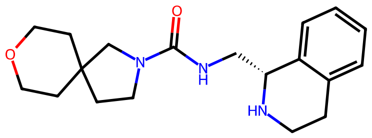	O=C(NC[C@H]1NCCc2ccccc21)N1CCC2(CCOCC2)C1	580293845	24	329.21	2.09
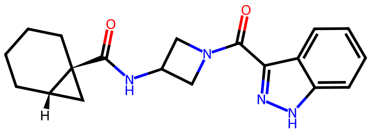	O=C(c1n[nH]c2ccccc12)N1CC(NC(=O)[C@]23CCCC[C@H]2C3)C1	999289168	25	338.17	2.08
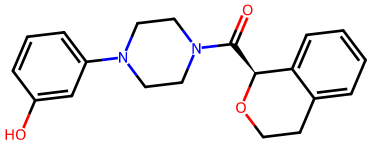	O=C([C@@H]1OCCc2ccccc21)N1CCN(c2cccc(O)c2)CC1	154555757	25	338.16	2.35
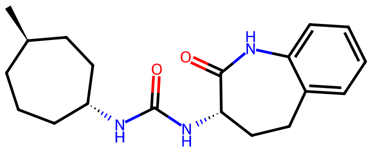	C[C@@H]1CCC[C@@H](NC(=O)N[C@H]2CCc3ccccc3NC2=O)CC1	1226349667	24	329.21	3.21
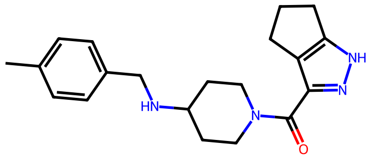	Cc1ccc(CNC2CCN(C(=O)c3n[nH]c4c3CCC4)CC2)cc1	996753942	25	338.21	2.6
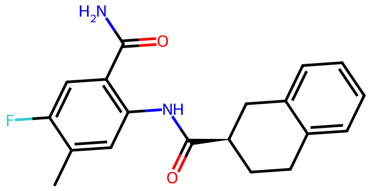	Cc1cc(NC(=O)[C@@H]2CCc3ccccc3C2)c(C(N)=O)cc1F	844396958	24	326.14	2.98

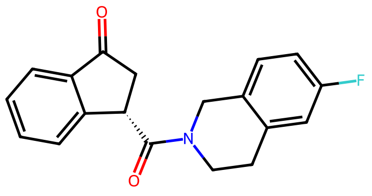	O=C1C[C@H](C(=O)N2CCc3cc(F)ccc3C2)c2ccccc21	277194098	23	309.12	3.08
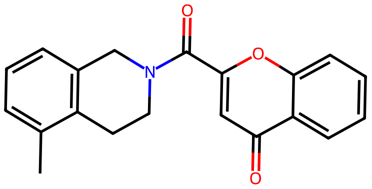	Cc1cccc2c1CCN(C(=O)c1cc(=O)c3ccccc3o1)C2	729411844	24	319.12	3.3
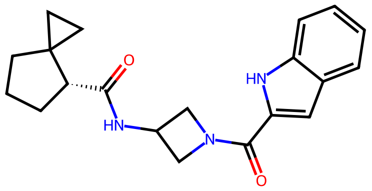	O=C(NC1CN(C(=O)c2cc3ccccc3[nH]2)C1)[C@@H]1CCCC12CC2	994866078	25	337.18	2.69
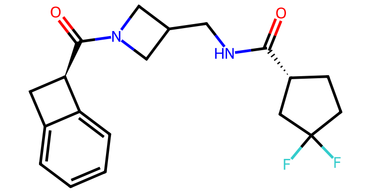	O=C(NCC1CN(C(=O)[C@@H]2Cc3ccccc32)C1)[C@@H]1CCC(F)(F)C1	1002598194	25	348.16	2.34
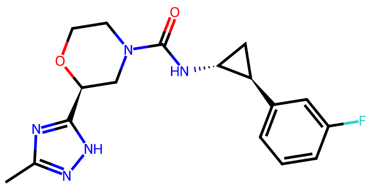	Cc1n[nH]c([C@@H]2CN(C(=O)N[C@@H]3C[C@H]3c3cccc(F)c3)CCO2)n1	195200558	25	345.16	1.89
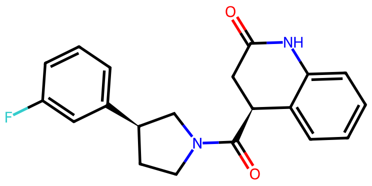	O=C1C[C@H](C(=O)N2CC[C@@H](c3cccc(F)c3)C2)c2ccccc2N1	264253023	25	338.14	3.27
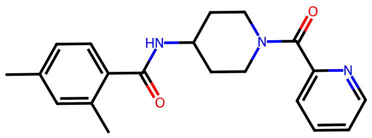	Cc1ccc(C(=O)NC2CCN(C(=O)c3ccccn3)CC2)c(C)c1	943590377	25	337.18	2.73
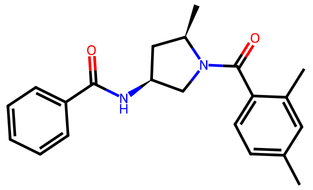	Cc1ccc(C(=O)N2C[C@@H](NC(=O)c3ccccc3)C[C@H]2C)c(C)c1	1014056026	25	336.18	3.34

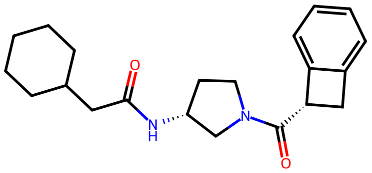	O=C(CC1CCCCC1)N[C@@H]1CCN(C(=O)[C@H]2Cc3ccccc32)C1	939295536	25	340.22	3.01
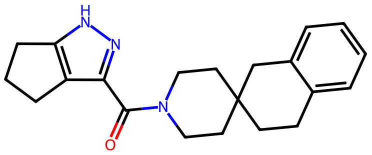	O=C(c1n[nH]c2c1CCC2)N1CCC2(CCc3ccccc3C2)CC1	652527017	25	335.2	3.31
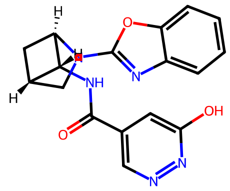	O=C(N[C@@H]1[C@H]2C[C@H]1N(c1nc3ccccc3o1)C2)c1cnnc(O)c1	1064310509	25	337.12	1.33
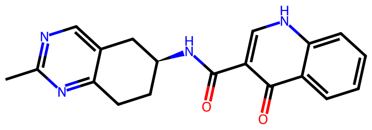	Cc1ncc2c(n1)CC[C@H](NC(=O)c1c[nH]c3ccccc3c1=O)C2	360568533	25	334.14	1.91
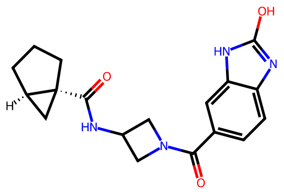	O=C(c1ccc2nc(O)[nH]c2c1)N1CC(NC(=O)[C@@]23CCC[C@@H]2C3)C1	997551477	25	340.15	1.4
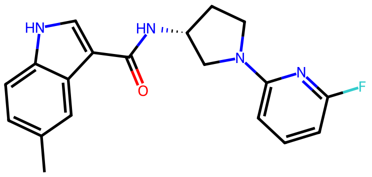	Cc1ccc2[nH]cc(C(=O)N[C@@H]3CCN(c4cccc(F)n4)C3)c2c1	1058381898	25	338.15	3.02
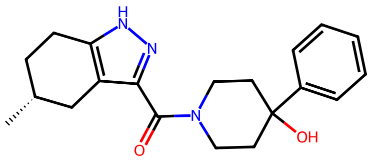	C[C@@H]1CCc2[nH]nc(C(=O)N3CCC(O)(c4ccccc4)CC3)c2C1	897412296	25	339.19	2.66
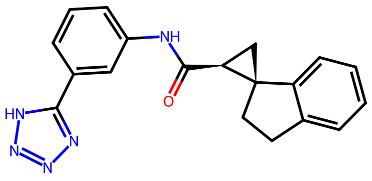	O=C(Nc1cccc(-c2nnn[nH]2)c1)[C@H]1C[C@@]12CCc1ccccc12	266615512	25	331.14	2.71

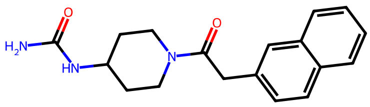	NC(=O)NC1CCN(C(=O)Cc2ccc3ccccc3c2)CC1	174401652	23	311.16	2.04
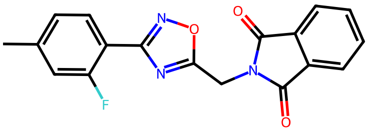	Cc1ccc(-c2noc(CN3C(=O)c4ccccc4C3=O)n2)c(F)c1	460819879	25	337.09	2.98
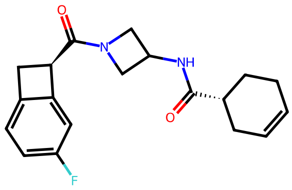	O=C(NC1CN(C(=O)[C@@H]2Cc3ccc(F)cc32)C1)[C@H]1CC=CCC1	990200920	24	328.16	2.15
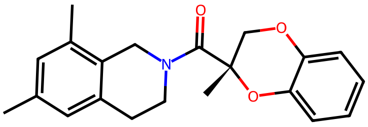	Cc1cc(C)c2c(c1)CCN(C(=O)[C@]1(C)COc3ccccc3O1)C2	1628060041	25	337.17	3.42
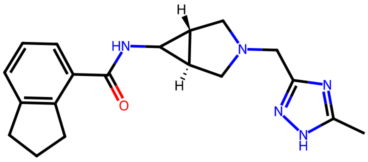	Cc1nc(CN2C[C@H]3C(NC(=O)c4cccc5c4CCC5)[C@@H]3C2)n[nH]1	962987666	25	337.19	1.46
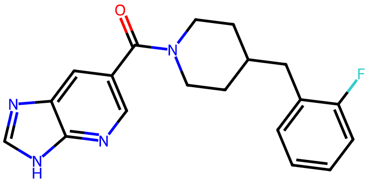	O=C(c1cnc2[nH]cnc2c1)N1CCC(Cc2ccccc2F)CC1	677887918	25	338.15	3.19
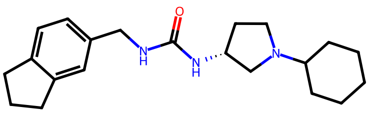	O=C(NCc1ccc2c(c1)CCC2)N[C@@H]1CCN(C2CCCCC2)C1	1666269787	25	341.25	3.38
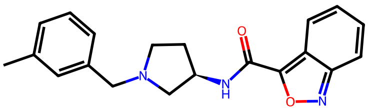	Cc1cccc(CN2CC[C@@H](NC(=O)c3onc4ccccc34)C2)c1	1301783412	25	335.16	3.14

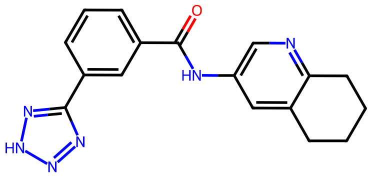	O=C(Nc1cnc2c(c1)CCCC2)c1cccc(-c2nn[nH]n2)c1	826459636	24	320.14	2.39
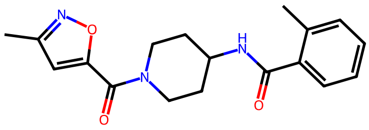	Cc1cc(C(=O)N2CCC(NC(=O)c3ccccc3C)CC2)on1	32306153	24	327.16	2.33
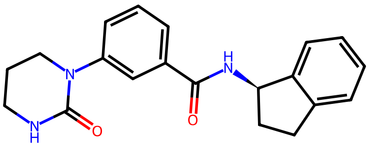	O=C(N[C@@H]1CCc2ccccc21)c1cccc(N2CCCNC2=O)c1	267162820	25	335.16	3.02
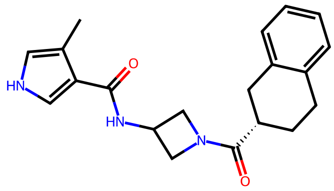	Cc1c[nH]cc1C(=O)NC1CN(C(=O)[C@H]2CCc3ccccc3C2)C1	991708281	25	337.18	2.07
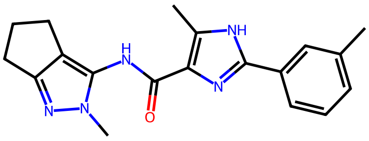	Cc1cccc(-c2nc(C(=O)Nc3c4c(nn3C)CCC4)c(C)[nH]2)c1	270257600	25	335.17	3.17
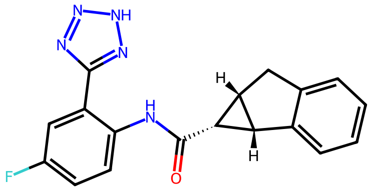	O=C(Nc1ccc(F)cc1-c1nn[nH]n1)[C@H]1[C@H]2c3ccccc3C[C@H]21	1593232015	25	335.12	2.53
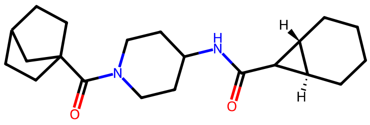	O=C(NC1CCN(C(=O)C23CCC(CC2)C3)CC1)C1[C@@H]2CCCC[C@@H]12	947332443	25	344.25	3.11
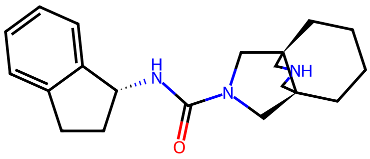	O=C(N[C@@H]1CCc2ccccc21)N1C[C@]23CCCC[C@@]2(CNC3)C1	1553507649	24	325.22	2.85

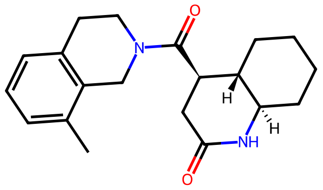	Cc1cccc2c1CN(C(=O)[C@@H]1CC(=O)N[C@@H]3CCCC[C@H]31)CC2	614003101	24	326.2	2.57
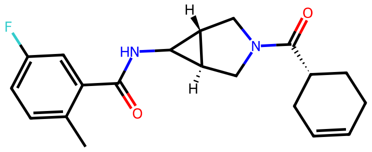	Cc1ccc(F)cc1C(=O)NC1[C@@H]2CN(C(=O)[C@H]3CC=CCC3)C[C@@H]12	1013468391	25	342.17	2.68
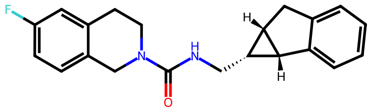	O=C(NC[C@H]1[C@H]2c3ccccc3C[C@H]21)N1CCc2cc(F)ccc2C1	91706800	25	336.16	3.48
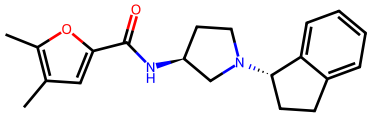	Cc1cc(C(=O)N[C@H]2CCN([C@H]3CCc4ccccc43)C2)oc1C	1010618759	24	324.18	3.39
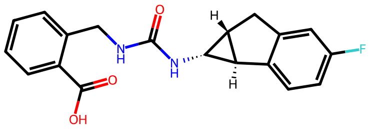	O=C(NCc1ccccc1C(=O)O)N[C@H]1[C@@H]2c3ccc(F)cc3C[C@H]21	874406872	25	340.12	2.66
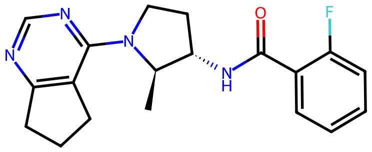	C[C@@H]1[C@@H](NC(=O)c2ccccc2F)CCN1c1ncnc2c1CCC2	1050000108	25	340.17	2.5
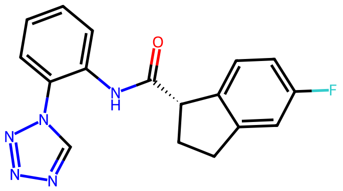	O=C(Nc1ccccc1-n1cnnn1)[C@H]1CCc2cc(F)ccc21	1355413947	24	323.12	2.47
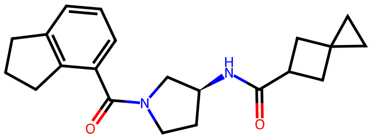	O=C(N[C@H]1CCN(C(=O)c2cccc3c2CCC3)C1)C1CC2(CC2)C1	938714612	25	338.2	2.7

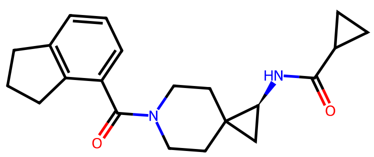	O=C(N[C@H]1CC12CCN(C(=O)c1cccc3c1CCC3)CC2)C1CC1	962425922	25	338.2	2.7
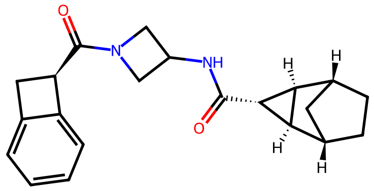	O=C(NC1CN(C(=O)[C@@H]2Cc3ccccc32)C1)[C@@H]1[C@H]2[C@@H]3CC[C@@H](C3)[C@H]21	994362267	25	336.18	1.95
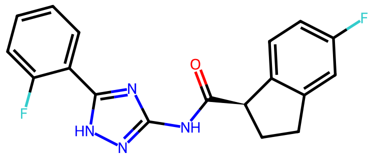	O=C(Nc1n[nH]c(-c2ccccc2F)n1)[C@@H]1CCc2cc(F)ccc21	1654249884	25	340.11	3.42
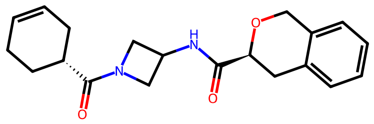	O=C(NC1CN(C(=O)[C@H]2CC=CCC2)C1)[C@@H]1Cc2ccccc2CO1	997728092	25	340.18	1.81
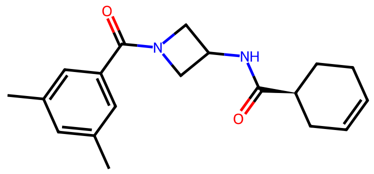	Cc1cc(C)cc(C(=O)N2CC(NC(=O)[C@@H]3CC=CCC3)C2)c1	990214587	23	312.18	2.6
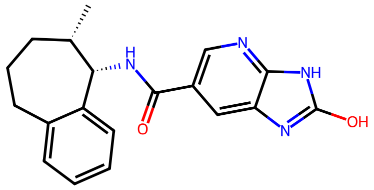	C[C@H]1CCCc2ccccc2[C@H]1NC(=O)c1cnc2[nH]c(O)nc2c1	572563546	25	336.16	3.11
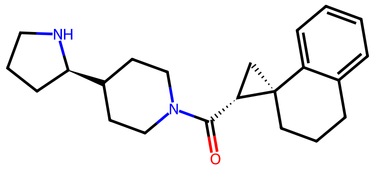	O=C([C@@H]1C[C@]12CCCc1ccccc12)N1CCC([C@H]2CCCN2)CC1	378224928	25	338.24	3.27
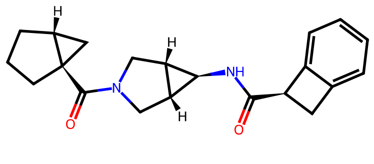	O=C(N[C@H]1[C@@H]2CN(C(=O)[C@@]34CCC[C@@H]3C4)C[C@@H]21)[C@@H]1Cc2ccccc21	977076009	25	336.18	2.09

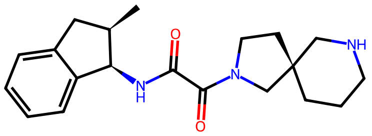	C[C@@H]1Cc2ccccc2[C@@H]1NC(=O)C(=O)N1CC[C@@]2(CCCNC2)C1	578982540	25	341.21	1.64
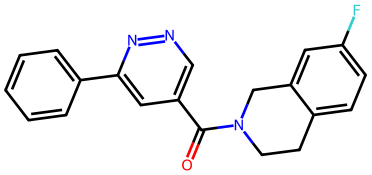	O=C(c1cnnc(-c2ccccc2)c1)N1CCc2ccc(F)cc2C1	355127571	25	333.13	3.48
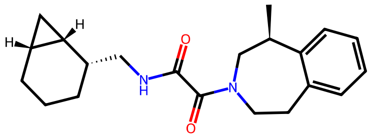	C[C@@H]1CN(C(=O)C(=O)NC[C@@H]2CCC[C@@H]3C[C@H]23)CCc2ccccc21	658783525	25	340.22	2.73
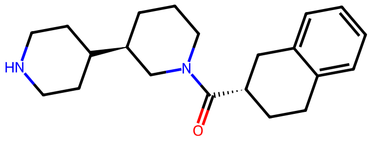	O=C([C@H]1CCc2ccccc2C1)N1CCC[C@H](C2CCNCC2)C1	662590331	24	326.24	3.03
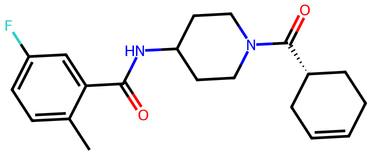	Cc1ccc(F)cc1C(=O)NC1CCN(C(=O)[C@H]2CC=CCC2)CC1	944126537	25	344.19	3.21
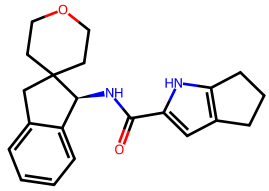	O=C(N[C@H]1c2ccccc2CC12CCOCC2)c1cc2c([nH]1)CCC2	1548838929	25	336.18	3.33
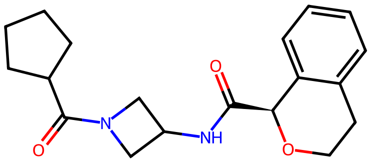	O=C(NC1CN(C(=O)C2CCCC2)C1)[C@@H]1OCCc2ccccc21	997326998	24	328.18	1.82
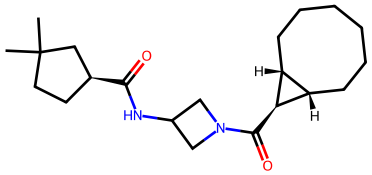	CC1(C)CC[C@H](C(=O)NC2CN(C(=O)[C@@H]3[C@H]4CCCCCC[C@H]43)C2)C1	999243773	25	346.26	3.36

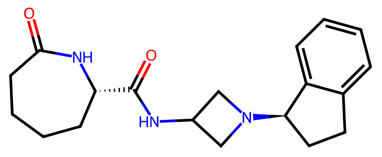	O=C1CCCC[C@@H](C(=O)NC2CN([C@@H]3CCc4ccccc43)C2)N1	1030937234	24	327.19	1.53
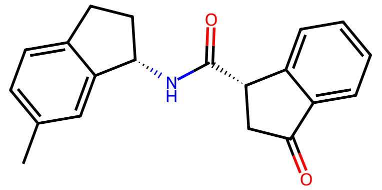	Cc1ccc2c(c1)[C@@H](NC(=O)[C@H]1CC(=O)c3ccccc31)CC2	682798010	23	305.14	3.47
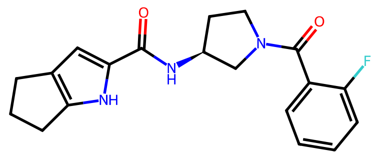	O=C(N[C@H]1CCN(C(=O)c2ccccc2F)C1)c1cc2c([nH]1)CCC2	938289286	25	341.15	2.29
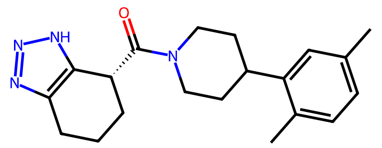	Cc1ccc(C)c(C2CCN(C(=O)[C@@H]3CCCc4nn[nH]c43)CC2)c1	1336628607	25	338.21	3.25
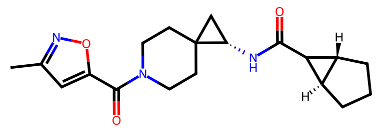	Cc1cc(C(=O)N2CCC3(CC2)C[C@@H]3NC(=O)C2[C@@H]3CCC[C@@H]23)on1	963462578	25	343.19	2.14
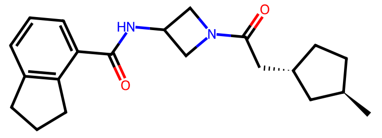	C[C@@H]1CC[C@@H](CC(=O)N2CC(NC(=O)c3cccc4c3CCC4)C2)C1	994395382	25	340.22	2.94
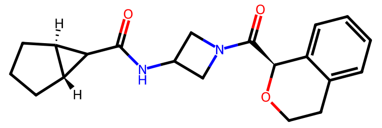	O=C(NC1CN(C(=O)[C@@H]2OCCc3ccccc32)C1)C1[C@@H]2CCC[C@@H]12	990712987	25	340.18	1.67
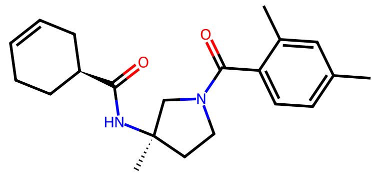	Cc1ccc(C(=O)N2CC[C@@](C)(NC(=O)[C@@H]3CC=CCC3)C2)c(C)c1	956689638	25	340.22	3.38

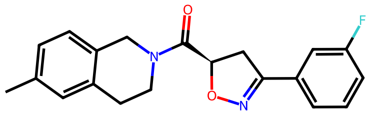	Cc1ccc2c(c1)CCN(C(=O)[C@H]1CC(c3cccc(F)c3)=NO1)C2	790990770	25	338.14	3.21
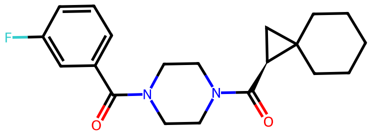	O=C(c1cccc(F)c1)N1CCN(C(=O)[C@H]2CC23CCCCC3)CC1	357267941	25	344.19	3.08
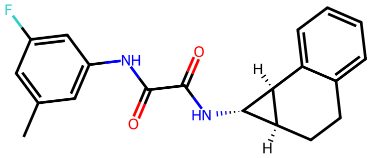	Cc1cc(F)cc(NC(=O)C(=O)N[C@H]2[C@@H]3CCc4ccccc4[C@H]23)c1	91422916	25	338.14	2.92
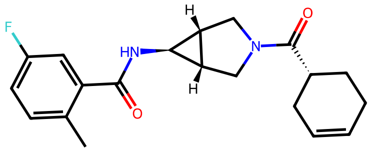	Cc1ccc(F)cc1C(=O)N[C@H]1[C@@H]2CN(C(=O)[C@H]3CC=CCC3)C[C@@H]21	976891388	25	342.17	2.68
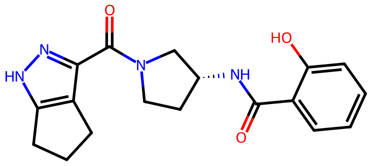	O=C(N[C@@H]1CCN(C(=O)c2n[nH]c3c2CCC3)C1)c1ccccc1O	939496708	25	340.15	1.25
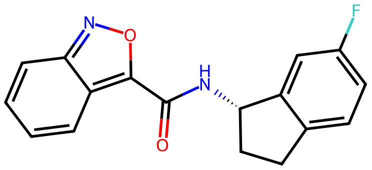	O=C(N[C@H]1CCc2ccc(F)cc21)c1onc2ccccc12	1464995906	22	296.1	3.38
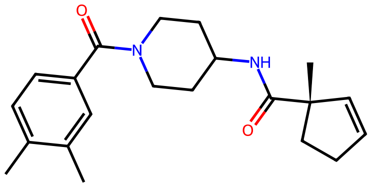	Cc1ccc(C(=O)N2CCC(NC(=O)[C@]3(C)C=CCC3)CC2)cc1C	948352134	25	340.22	3.38
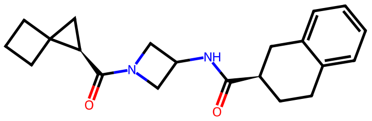	O=C(NC1CN(C(=O)[C@@H]2CC23CCC3)C1)[C@@H]1CCc2ccccc2C1	1000545877	25	338.2	2.31

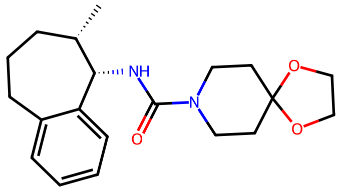	C[C@H]1CCCc2ccccc2[C@H]1NC(=O)N1CCC2(CC1)OCCO2	185616394	25	344.21	3.25
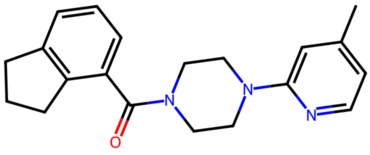	Cc1ccnc(N2CCN(C(=O)c3cccc4c3CCC4)CC2)c1	159009011	24	321.18	2.84
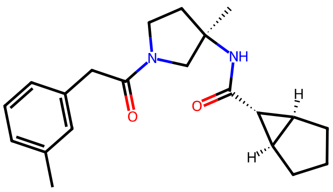	Cc1cccc(CC(=O)N2CC[C@@](C)(NC(=O)[C@@H]3[C@H]4CCC[C@H]43)C2)c1	956803743	25	340.22	2.69
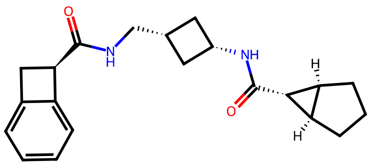	O=C(NC[C@H]1C[C@@H](NC(=O)[C@@H]2[C@H]3CCC[C@H]32)C1)[C@@H]1Cc2ccccc21	979226255	25	338.2	2.38
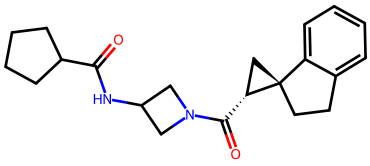	O=C(NC1CN(C(=O)[C@@H]2C[C@@]23CCc2ccccc23)C1)C1CCCC1	990036733	25	338.2	2.41
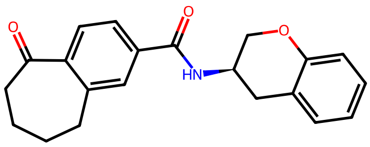	O=C(N[C@H]1COc2ccccc2C1)c1ccc2c(c1)CCCCC2=O	564789793	25	335.15	3.33
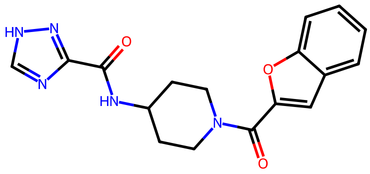	O=C(NC1CCN(C(=O)c2cc3ccccc3o2)CC1)c1nc[nH]n1	948481077	25	339.13	1.59
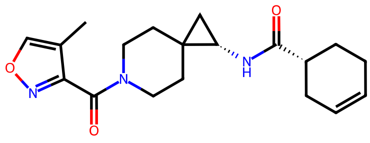	Cc1conc1C(=O)N1CCC2(CC1)C[C@@H]2NC(=O)[C@H]1CC=CCC1	963024872	25	343.19	2.45

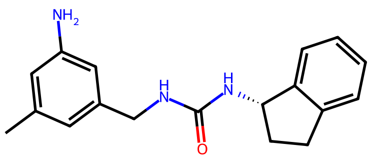	Cc1cc(N)cc(CNC(=O)N[C@H]2CCc3ccccc32)c1	335612063	22	295.17	3.06
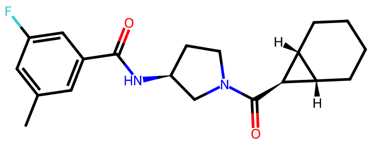	Cc1cc(F)cc(C(=O)N[C@H]2CCN(C(=O)[C@@H]3[C@H]4CCCC[C@H]43)C2)c1	938959827	25	344.19	2.9
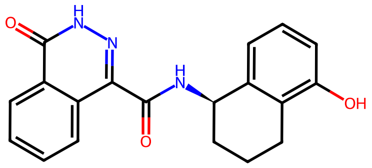	O=C(N[C@@H]1CCCc2c(O)cccc21)c1n[nH]c(=O)c2ccccc12	190202289	25	335.13	2.44
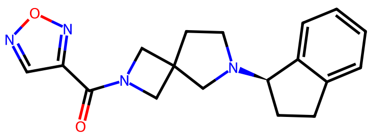	O=C(c1cnon1)N1CC2(CCN([C@@H]3CCc4ccccc43)C2)C1	1073068638	24	324.16	1.91
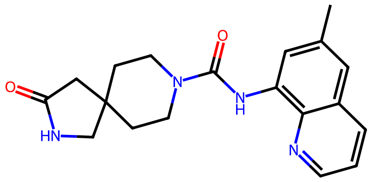	Cc1cc(NC(=O)N2CCC3(CC2)CNC(=O)C3)c2ncccc2c1	535082748	25	338.17	2.68
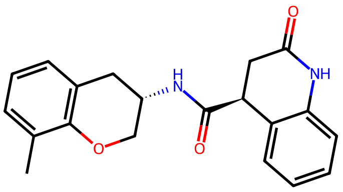	Cc1cccc2c1OC[C@@H](NC(=O)[C@H]1CC(=O)Nc3ccccc31)C2	291627691	25	336.15	2.54
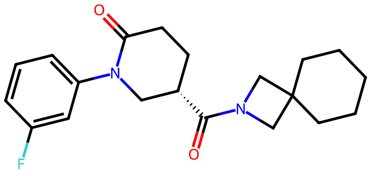	O=C([C@H]1CCC(=O)N(c2cccc(F)c2)C1)N1CC2(CCCCC2)C1	613342063	25	344.19	3.36
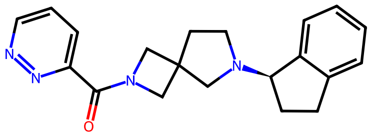	O=C(c1cccnn1)N1CC2(CCN([C@@H]3CCc4ccccc43)C2)C1	1073071410	25	334.18	2.31

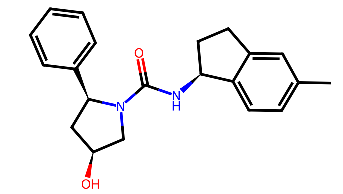	Cc1ccc2c(c1)CC[C@@H]2NC(=O)N1C[C@@H](O)C[C@H]1c1ccccc1	413162450	25	336.18	3.5
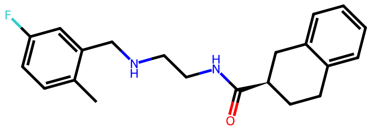	Cc1ccc(F)cc1CNCCNC(=O)[C@@H]1CCc2ccccc2C1	1134378222	25	340.2	3.15
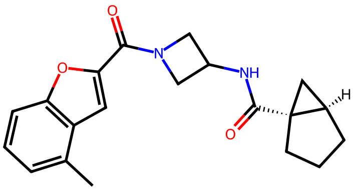	Cc1cccc2oc(C(=O)N3CC(NC(=O)[C@@]45CCC[C@@H]4C5)C3)cc12	997553640	25	338.16	2.87
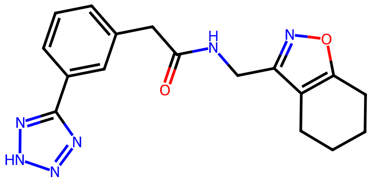	O=C(Cc1cccc(-c2nn[nH]n2)c1)NCc1noc2c1CCCC2	1606601515	25	338.15	1.59
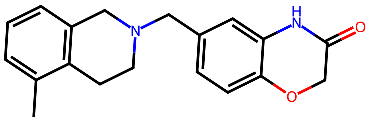	Cc1cccc2c1CCN(Cc1ccc3c(c1)NC(=O)CO3)C2	816505559	23	308.15	2.88
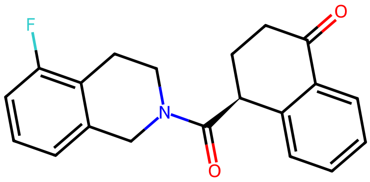	O=C1CC[C@H](C(=O)N2CCc3c(F)cccc3C2)c2ccccc21	285652303	24	323.13	3.47
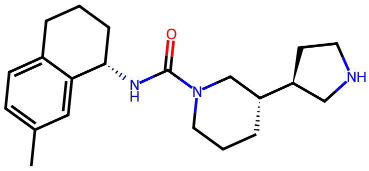	Cc1ccc2c(c1)[C@@H](NC(=O)N1CCC[C@@H]([C@H]3CCNC3)C1)CCC2	418189397	25	341.25	3.4
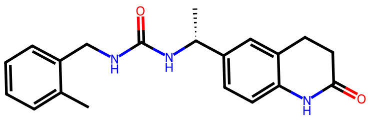	Cc1ccccc1CNC(=O)N[C@H](C)c1ccc2c(c1)CCC(=O)N2	43479582	25	337.18	3.44

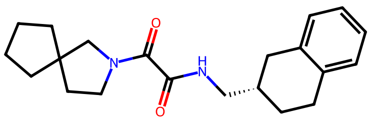	O=C(NC[C@H]1CCc2ccccc2C1)C(=O)N1CCC2(CCCC2)C1	807117645	25	340.22	2.7
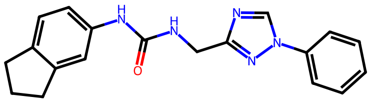	O=C(NCc1ncn(-c2ccccc2)n1)Nc1ccc2c(c1)CCC2	1672794996	25	333.16	3.08
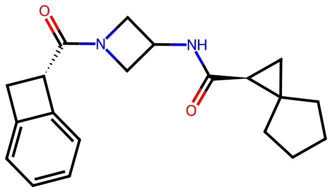	O=C(NC1CN(C(=O)[C@H]2Cc3ccccc32)C1)[C@H]1CC12CCCC2	999634035	24	324.18	2.23
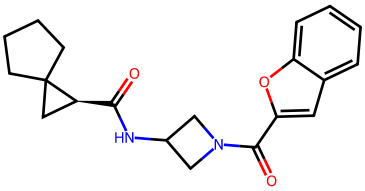	O=C(NC1CN(C(=O)c2cc3ccccc3o2)C1)[C@H]1CC12CCCC2	999636651	25	338.16	2.95
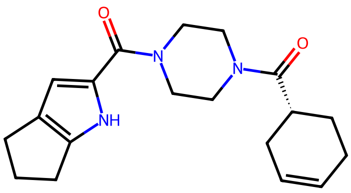	O=C(c1cc2c([nH]1)CCC2)N1CCN(C(=O)[C@H]2CC=CCC2)CC1	1010289609	24	327.19	2.14
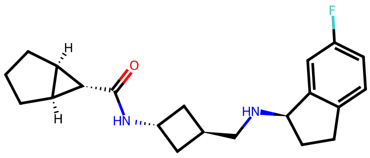	O=C(N[C@H]1C[C@H](CN[C@@H]2CCc3ccc(F)cc32)C1)[C@@H]1[C@H]2CCC[C@H]21	983422488	25	342.21	3.34
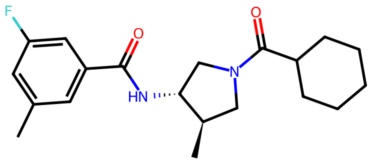	Cc1cc(F)cc(C(=O)N[C@@H]2CN(C(=O)C3CCCCC3)C[C@H]2C)c1	940618030	25	346.21	3.29
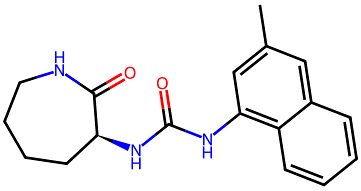	Cc1cc(NC(=O)N[C@H]2CCCCNC2=O)c2ccccc2c1	297385098	23	311.16	2.94

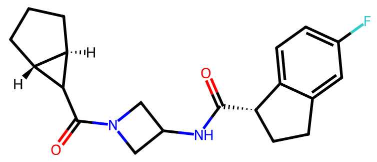	O=C(NC1CN(C(=O)C2[C@@H]3CCC[C@@H]23)C1)[C@H]1CCc2cc(F)ccc21	996099008	25	342.17	2.23
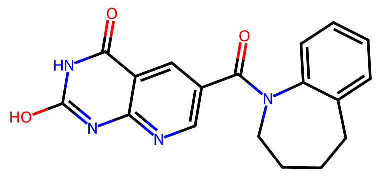	O=C(c1cnc2nc(O)[nH]c(=O)c2c1)N1CCCCc2ccccc21	71799403	25	336.12	2.01
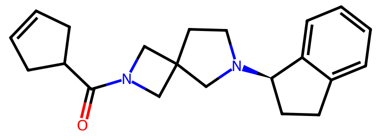	O=C(C1CC=CC1)N1CC2(CCN([C@@H]3CCc4ccccc43)C2)C1	1072568299	24	322.2	3.17
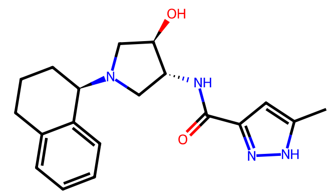	Cc1cc(C(=O)N[C@@H]2CN([C@@H]3CCCc4ccccc43)C[C@H]2O)n[nH]1	1076781981	25	340.19	1.57
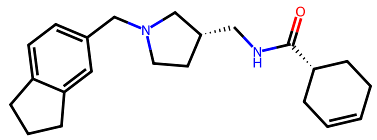	O=C(NC[C@@H]1CCN(Cc2ccc3c(c2)CCC3)C1)[C@H]1CC=CCC1	1028395295	25	338.24	3.47
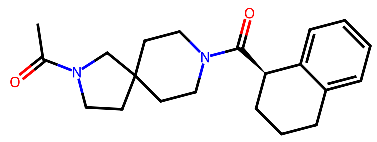	CC(=O)N1CCC2(CCN(C(=O)[C@@H]3CCCc4ccccc43)CC2)C1	944393134	25	340.22	2.97
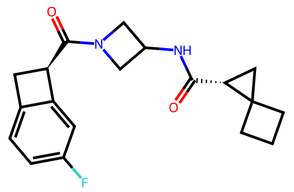	O=C(NC1CN(C(=O)[C@@H]2Cc3ccc(F)cc32)C1)[C@@H]1CC12CCC2	991553898	24	328.16	1.98
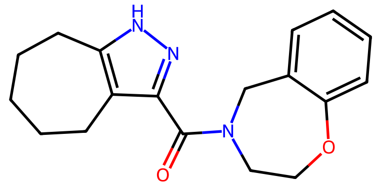	O=C(c1n[nH]c2c1CCCCC2)N1CCOc2ccccc2C1	682716236	23	311.16	2.71

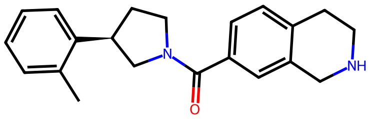	Cc1ccccc1[C@H]1CCN(C(=O)c2ccc3c(c2)CNCC3)C1	1326596206	24	320.19	3.27
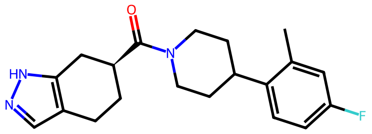	Cc1cc(F)ccc1C1CCN(C(=O)[C@H]2CCc3cn[nH]c3C2)CC1	292529795	25	341.19	3.37
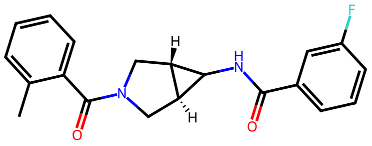	Cc1ccccc1C(=O)N1C[C@@H]2C(NC(=O)c3cccc(F)c3)[C@H]2C1	1012715989	25	338.14	2.63
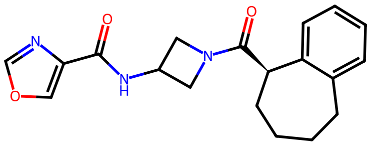	O=C(NC1CN(C(=O)[C@@H]2CCCCc3ccccc32)C1)c1cocn1	990480996	25	339.16	2.13
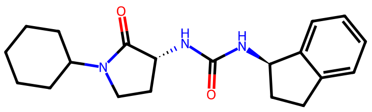	O=C(N[C@@H]1CCN(C2CCCCC2)C1=O)N[C@@H]1CCc2ccccc21	159001450	25	341.21	2.91
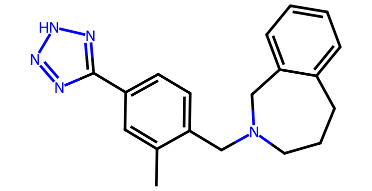	Cc1cc(-c2nn[nH]n2)ccc1CN1CCCc2ccccc2C1	1605540696	24	319.18	3.12
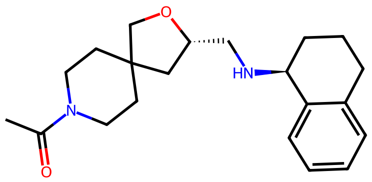	CC(=O)N1CCC2(CC1)CO[C@H](CN[C@H]1CCCc3ccccc31)C2	982866016	25	342.23	3.07
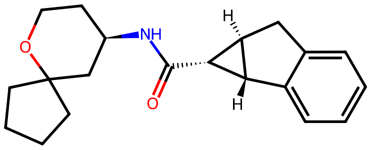	O=C(N[C@@H]1CCOC2(CCCC2)C1)[C@@H]1[C@H]2Cc3ccccc3[C@@H]21	626186263	23	311.19	3.18

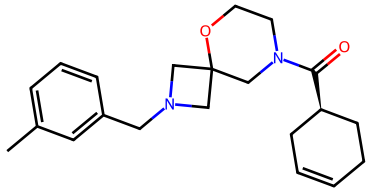	Cc1cccc(CN2CC3(C2)CN(C(=O)[C@@H]2CC=CCC2)CCO3)c1	1053152100	25	340.22	2.76
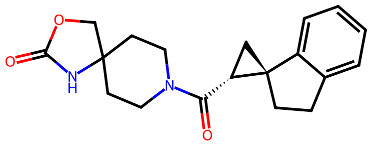	O=C1NC2(CCN(C(=O)[C@@H]3C[C@@]34CCc3ccccc34)CC2)CO1	495157009	24	326.16	1.99
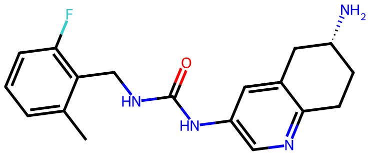	Cc1cccc(F)c1CNC(=O)Nc1cnc2c(c1)C[C@H](N)CC2	841323923	24	328.17	2.67
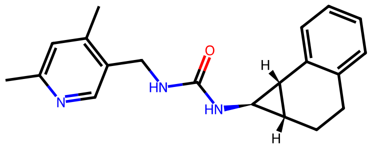	Cc1cc(C)c(CNC(=O)N[C@@H]2[C@H]3CCc4ccccc4[C@H]32)cn1	458562717	24	321.18	3.23
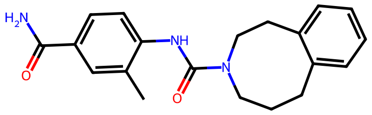	Cc1cc(C(N)=O)ccc1NC(=O)N1CCCc2ccccc2CC1	836276470	25	337.18	3.12
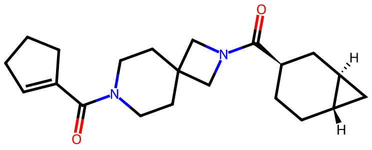	O=C(C1=CCCC1)N1CCC2(CC1)CN(C(=O)[C@@H]1CC[C@H]3C[C@@H]3C1)C2	944911893	25	342.23	2.98
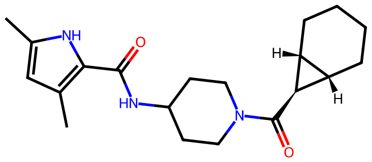	Cc1cc(C)c(C(=O)NC2CCN(C(=O)[C@@H]3[C@H]4CCCC[C@H]43)CC2)[nH]1	945956608	25	343.23	2.79
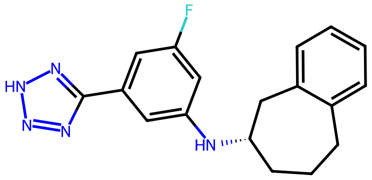	Fc1cc(N[C@H]2CCCc3ccccc3C2)cc(-c2nn[nH]n2)c1	1581852936	24	323.15	3.37

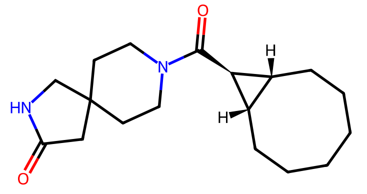	O=C1CC2(CCN(C(=O)[C@@H]3[C@H]4CCCCCC[C@H]43)CC2)CN1	829422959	22	304.22	2.33
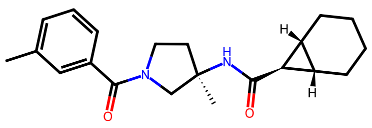	Cc1cccc(C(=O)N2CC[C@@](C)(NC(=O)[C@@H]3[C@H]4CCCC[C@H]43)C2)c1	956790397	25	340.22	3.15
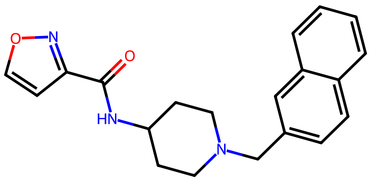	O=C(NC1CCN(Cc2ccc3ccccc3c2)CC1)c1ccon1	1002402946	25	335.16	3.22
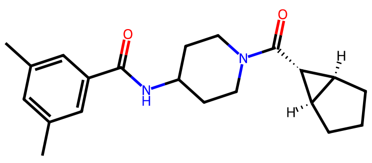	Cc1cc(C)cc(C(=O)NC2CCN(C(=O)[C@@H]3[C@H]4CCC[C@H]43)CC2)c1	946079772	25	340.22	3.07
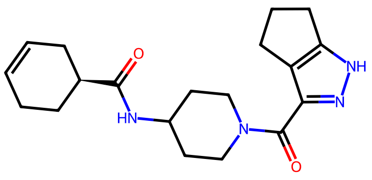	O=C(NC1CCN(C(=O)c2n[nH]c3c2CCC3)CC1)[C@@H]1CC=CCC1	947366675	25	342.21	1.98
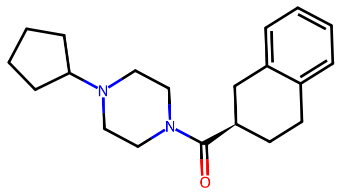	O=C([C@@H]1CCc2ccccc2C1)N1CCN(C2CCCC2)CC1	575993070	23	312.22	2.88
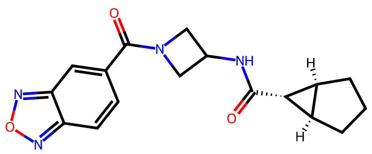	O=C(NC1CN(C(=O)c2ccc3nonc3c2)C1)[C@@H]1[C@H]2CCC[C@H]21	990695033	24	326.14	1.21
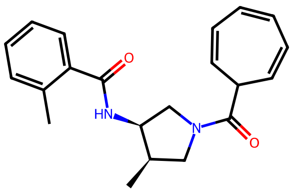	Cc1ccccc1C(=O)N[C@H]1CN(C(=O)C2C=CC=CC=C2)C[C@H]1C	966480711	25	336.18	2.87

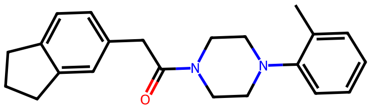	Cc1ccccc1N1CCN(C(=O)Cc2ccc3c(c2)CCC3)CC1	216304732	25	334.2	3.38
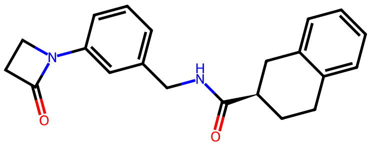	O=C(NCc1cccc(N2CCC2=O)c1)[C@@H]1CCc2ccccc2C1	566682097	25	334.17	2.84
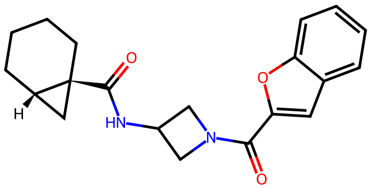	O=C(c1cc2ccccc2o1)N1CC(NC(=O)[C@]23CCCC[C@H]2C3)C1	999284298	25	338.16	2.95
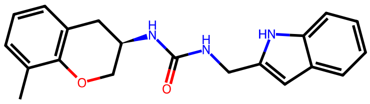	Cc1cccc2c1OC[C@H](NC(=O)NCc1cc3ccccc3[nH]1)C2	460160905	25	335.16	3.28
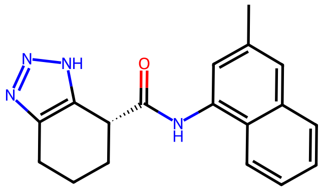	Cc1cc(NC(=O)[C@@H]2CCCc3nn[nH]c32)c2ccccc2c1	615678696	23	306.15	3.32
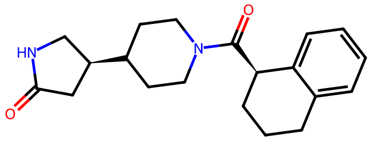	O=C1C[C@H](C2CCN(C(=O)[C@@H]3CCCc4ccccc43)CC2)CN1	1551707194	24	326.2	2.48
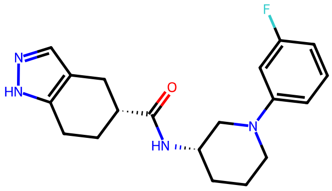	O=C(N[C@H]1CCCN(c2cccc(F)c2)C1)[C@@H]1CCc2[nH]ncc2C1	270282291	25	342.19	2.44
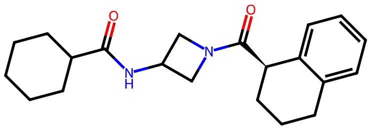	O=C(NC1CN(C(=O)[C@@H]2CCCc3ccccc32)C1)C1CCCCC1	990099260	25	340.22	3.01

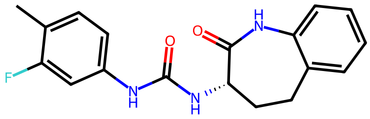	Cc1ccc(NC(=O)N[C@H]2CCc3ccccc3NC2=O)cc1F	1188002299	24	327.14	3.21
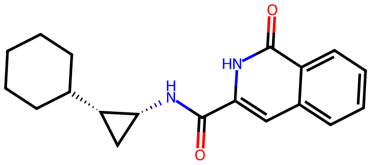	O=C(N[C@@H]1C[C@@H]1C1CCCCC1)c1cc2ccccc2c(=O)[nH]1	152714786	23	310.17	3.23
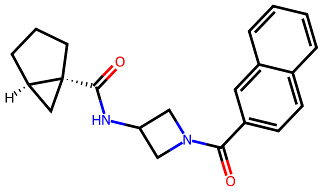	O=C(c1ccc2ccccc2c1)N1CC(NC(=O)[C@@]23CCC[C@@H]2C3)C1	997542331	25	334.17	2.97
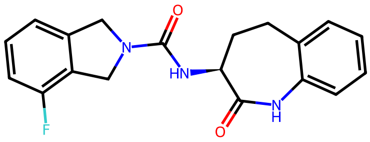	O=C1Nc2ccccc2CC[C@@H]1NC(=O)N1Cc2cccc(F)c2C1	654633503	25	339.14	2.8
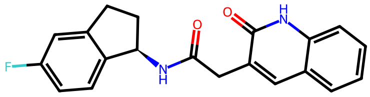	O=C(Cc1cc2ccccc2[nH]c1=O)N[C@@H]1CCc2cc(F)ccc21	55658384	25	336.13	3.01
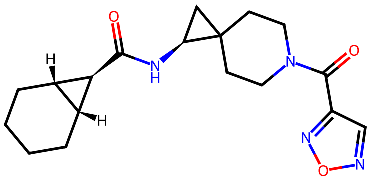	O=C(N[C@H]1CC12CCN(C(=O)c1cnon1)CC2)[C@@H]1[C@H]2CCCC[C@H]21	963448091	25	344.18	1.62
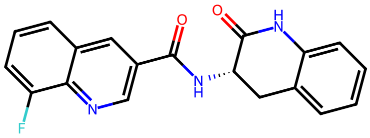	O=C(N[C@H]1Cc2ccccc2NC1=O)c1cnc2c(F)cccc2c1	1648608976	25	335.11	2.67
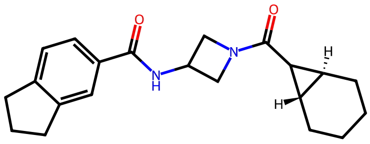	O=C(NC1CN(C(=O)C2[C@H]3CCCC[C@H]23)C1)c1ccc2c(c1)CCC2	991357687	25	338.2	2.55

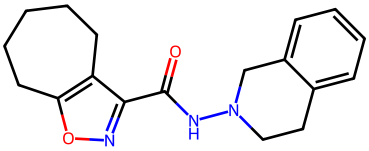	O=C(NN1CCc2ccccc2C1)c1noc2c1CCCCC2	855709802	23	311.16	2.65
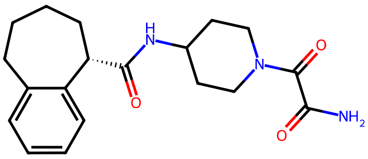	NC(=O)C(=O)N1CCC(NC(=O)[C@H]2CCCCc3ccccc32)CC1	948083720	25	343.19	1.09
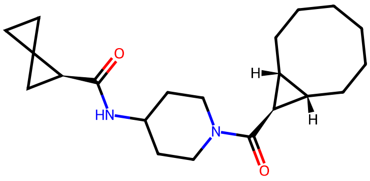	O=C(NC1CCN(C(=O)[C@@H]2[C@H]3CCCCCC[C@H]32)CC1)[C@H]1CC12CC2	948568087	25	344.25	3.11
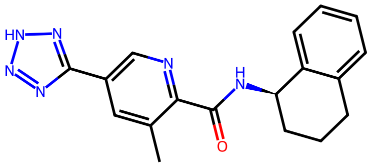	Cc1cc(-c2nn[nH]n2)cnc1C(=O)N[C@@H]1CCCc2ccccc21	1605552940	25	334.15	2.38
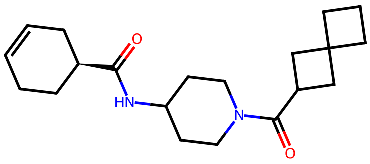	O=C(NC1CCN(C(=O)C2CC3(CCC3)C2)CC1)[C@@H]1CC=CCC1	947879590	24	330.23	3.03
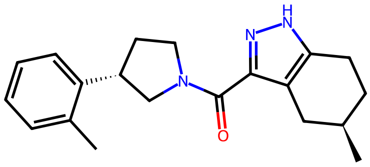	Cc1ccccc1[C@@H]1CCN(C(=O)c2n[nH]c3c2C[C@H](C)CC3)C1	1142810722	24	323.2	3.47
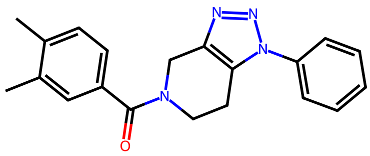	Cc1ccc(C(=O)N2CCc3c(nnn3-c3ccccc3)C2)cc1C	368346100	25	332.16	3.08
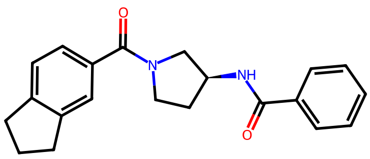	O=C(N[C@H]1CCN(C(=O)c2ccc3c(c2)CCC3)C1)c1ccccc1	937627827	25	334.17	2.82

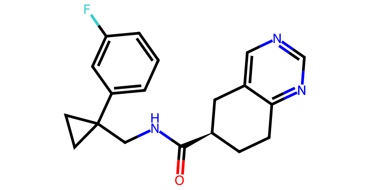	O=C(NCC1(c2cccc(F)c2)CC1)[C@@H]1CCc2ncncc2C1	648296659	24	325.16	2.57
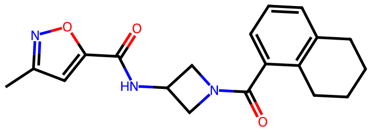	Cc1cc(C(=O)NC2CN(C(=O)c3cccc4c3CCCC4)C2)on1	990221748	25	339.16	2.12
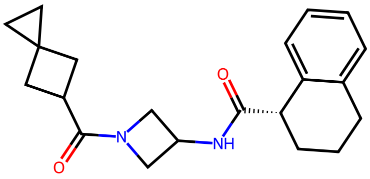	O=C(NC1CN(C(=O)C2CC3(CC3)C2)C1)[C@H]1CCCc2ccccc21	995072214	25	338.2	2.62
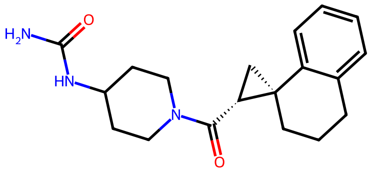	NC(=O)NC1CCN(C(=O)[C@@H]2C[C@]23CCCc2ccccc23)CC1	176063205	24	327.19	1.94
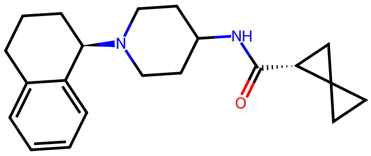	O=C(NC1CCN([C@@H]2CCCc3ccccc32)CC1)[C@@H]1CC12CC2	1003695571	24	324.22	3.44
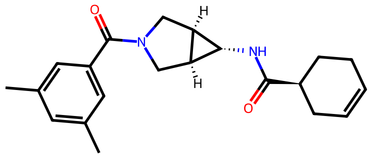	Cc1cc(C)cc(C(=O)N2C[C@H]3[C@H](NC(=O)[C@@H]4CC=CCC4)[C@H]3C2)c1	976177765	25	338.2	2.85
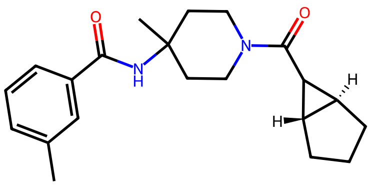	Cc1cccc(C(=O)NC2(C)CCN(C(=O)C3[C@H]4CCC[C@H]34)CC2)c1	956279576	25	340.22	3.15
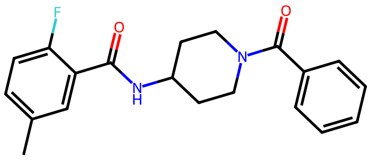	Cc1ccc(F)c(C(=O)NC2CCN(C(=O)c3ccccc3)CC2)c1	171359673	25	340.16	3.17

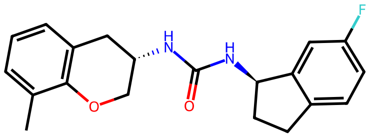	Cc1cccc2c1OC[C@@H](NC(=O)N[C@@H]1CCc3ccc(F)cc31)C2	573147371	25	340.16	3.42
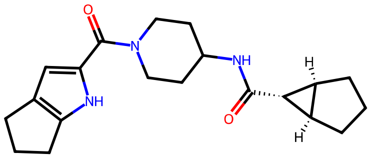	O=C(NC1CCN(C(=O)c2cc3c([nH]2)CCC3)CC1)[C@@H]1[C@H]2CCC[C@H]21	947445687	25	341.21	2.27
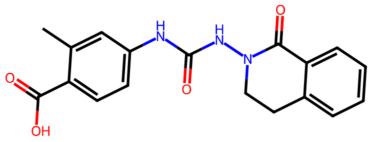	Cc1cc(NC(=O)NN2CCc3ccccc3C2=O)ccc1C(=O)O	1600270452	25	339.12	2.43
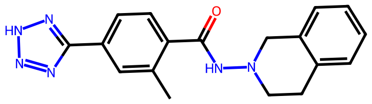	Cc1cc(-c2nn[nH]n2)ccc1C(=O)NN1CCc2ccccc2C1	1591863290	25	334.15	1.88
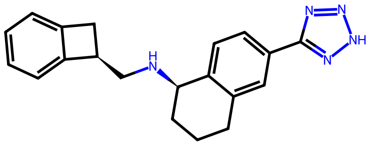	c1ccc2c(c1)C[C@H]2CN[C@@H]1CCCc2cc(-c3nn[nH]n3)ccc21	826523334	25	331.18	3.17
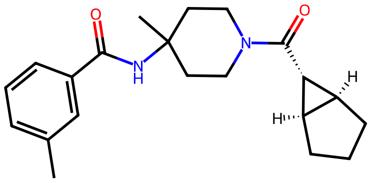	Cc1cccc(C(=O)NC2(C)CCN(C(=O)[C@@H]3[C@H]4CCC[C@H]43)CC2)c1	956279583	25	340.22	3.15
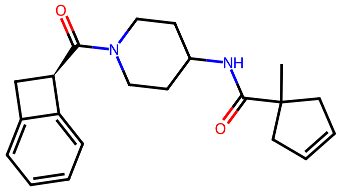	CC1(C(=O)NC2CCN(C(=O)[C@@H]3Cc4ccccc43)CC2)CC=CC1	948059493	25	338.2	2.79
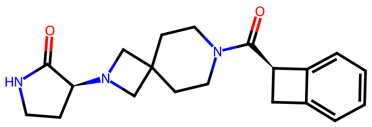	O=C1NCC[C@@H]1N1CC2(CCN(C(=O)[C@@H]3Cc4ccccc43)CC2)C1	1035780108	25	339.19	1.14

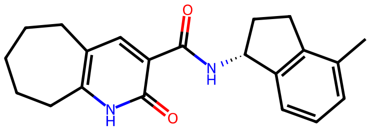	Cc1cccc2c1CC[C@H]2NC(=O)c1cc2c([nH]c1=O)CCCCC2	78116483	25	336.18	3.37
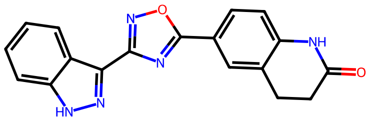	O=C1CCc2cc(-c3nc(-c4n[nH]c5ccccc45)no3)ccc2N1	356766955	25	331.11	3.16
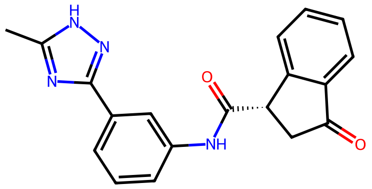	Cc1nc(-c2cccc(NC(=O)[C@H]3CC(=O)c4ccccc43)c2)n[nH]1	365155423	25	332.13	3.09
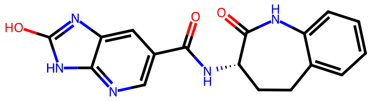	O=C(N[C@H]1CCc2ccccc2NC1=O)c1cnc2[nH]c(O)nc2c1	1437231498	25	337.12	1.35
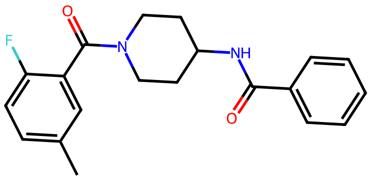	Cc1ccc(F)c(C(=O)N2CCC(NC(=O)c3ccccc3)CC2)c1	263947317	25	340.16	3.17
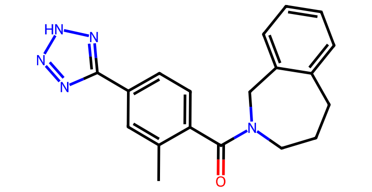	Cc1cc(-c2nn[nH]n2)ccc1C(=O)N1CCCc2ccccc2C1	826244849	25	333.16	2.76
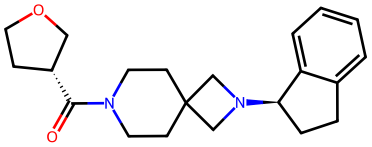	O=C([C@@H]1CCOC1)N1CCC2(CC1)CN([C@@H]1CCc3ccccc31)C2	1035663237	25	340.22	2.63
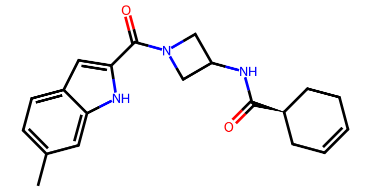	Cc1ccc2cc(C(=O)N3CC(NC(=O)[C@@H]4CC=CCC4)C3)[nH]c2c1	990212116	25	337.18	2.77

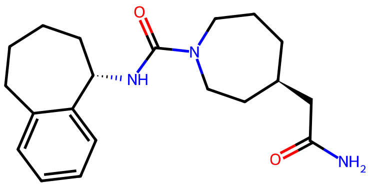	NC(=O)C[C@@H]1CCCN(C(=O)N[C@H]2CCCCc3ccccc32)CC1	671571535	25	343.23	3.14
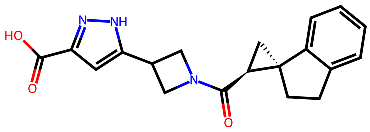	O=C(O)c1cc(C2CN(C(=O)[C@H]3C[C@]34CCc3ccccc34)C2)[nH]n1	1602253150	25	337.14	1.94
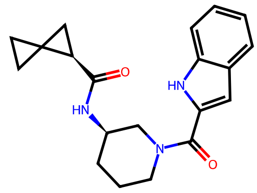	O=C(N[C@@H]1CCCN(C(=O)c2cc3ccccc3[nH]2)C1)[C@@H]1CC12CC2	952080977	25	337.18	2.69
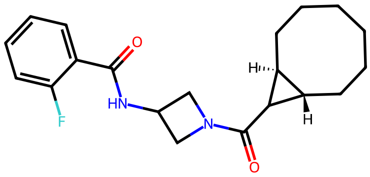	O=C(NC1CN(C(=O)C2[C@H]3CCCCCC[C@H]23)C1)c1ccccc1F	990092422	25	344.19	2.98
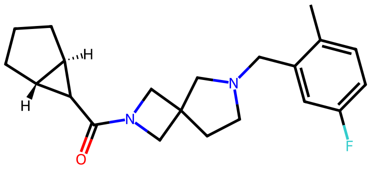	Cc1ccc(F)cc1CN1CCC2(C1)CN(C(=O)C1[C@@H]3CCC[C@@H]13)C2	1072590517	25	342.21	3.21
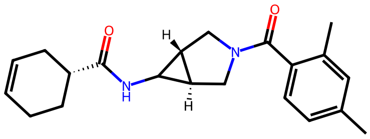	Cc1ccc(C(=O)N2C[C@H]3C(NC(=O)[C@H]4CC=CCC4)[C@@H]3C2)c(C)c1	1012771048	25	338.2	2.85
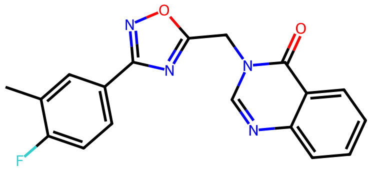	Cc1cc(-c2noc(Cn3cnc4ccccc4c3=O)n2)ccc1F	152251464	25	336.1	2.94
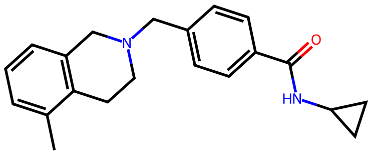	Cc1cccc2c1CCN(Cc1ccc(C(=O)NC3CC3)cc1)C2	769337024	24	320.19	3.45

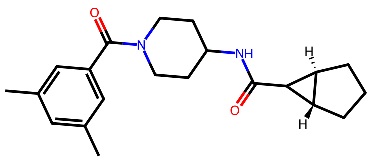	Cc1cc(C)cc(C(=O)N2CCC(NC(=O)C3[C@H]4CCC[C@H]34)CC2)c1	948338798	25	340.22	3.07
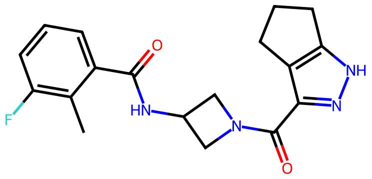	Cc1c(F)cccc1C(=O)NC1CN(C(=O)c2n[nH]c3c2CCC3)C1	993594580	25	342.15	1.6
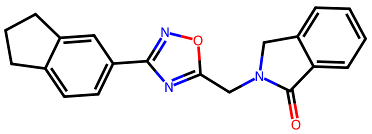	O=C1c2ccccc2CN1Cc1nc(-c2ccc3c(c2)CCC3)no1	289998421	25	331.13	3.38
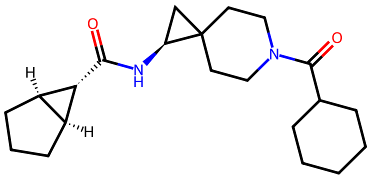	O=C(N[C@H]1CC12CCN(C(=O)C1CCCCC1)CC2)[C@@H]1[C@H]2CCC[C@H]21	963462683	25	344.25	3.11
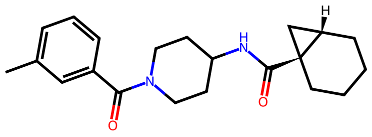	Cc1cccc(C(=O)N2CCC(NC(=O)[C@]34CCCC[C@H]3C4)CC2)c1	943834395	25	340.22	3.3
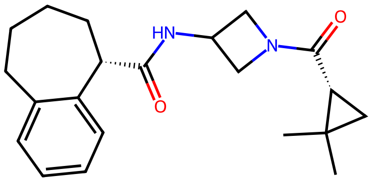	CC1(C)C[C@H]1C(=O)N1CC(NC(=O)[C@H]2CCCCc3ccccc32)C1	994311779	25	340.22	2.87
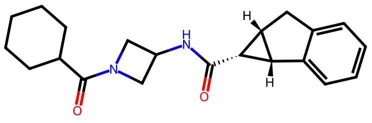	O=C(NC1CN(C(=O)C2CCCCC2)C1)[C@H]1[C@H]2c3ccccc3C[C@H]21	991647239	25	338.2	2.48
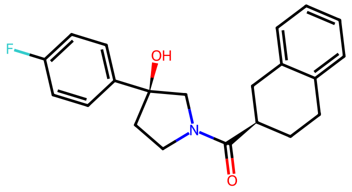	O=C([C@@H]1CCc2ccccc2C1)N1CC[C@](O)(c2ccc(F)cc2)C1	845919476	25	339.16	3.05

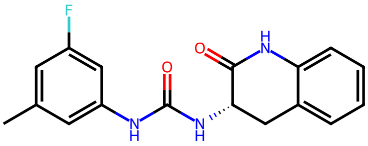	Cc1cc(F)cc(NC(=O)N[C@H]2Cc3ccccc3NC2=O)c1	1364318166	23	313.12	2.82
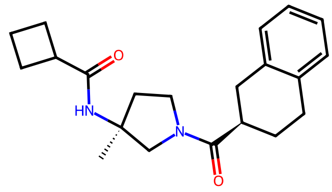	C[C@]1(NC(=O)C2CCC2)CCN(C(=O)[C@@H]2CCc3ccccc3C2)C1	956628603	25	340.22	2.7
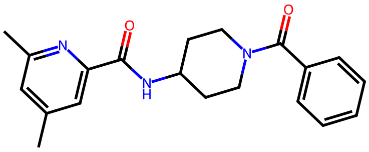	Cc1cc(C)nc(C(=O)NC2CCN(C(=O)c3ccccc3)CC2)c1	801114191	25	337.18	2.73
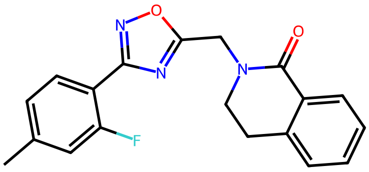	Cc1ccc(-c2noc(CN3CCc4ccccc4C3=O)n2)c(F)c1	346948557	25	337.12	3.38
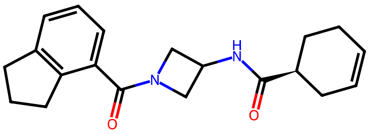	O=C(NC1CN(C(=O)c2cccc3c2CCC3)C1)[C@@H]1CC=CCC1	990212162	24	324.18	2.47
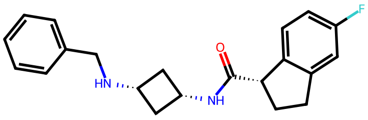	O=C(N[C@H]1C[C@@H](NCc2ccccc2)C1)[C@H]1CCc2cc(F)ccc21	1022439050	25	338.18	3.29
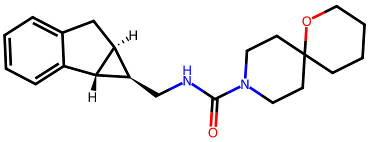	O=C(NC[C@@H]1[C@@H]2Cc3ccccc3[C@@H]12)N1CCC2(CCCCO2)CC1	883522629	25	340.22	3.32
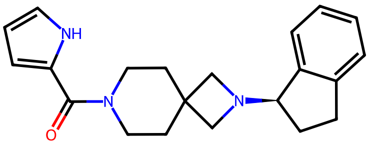	O=C(c1ccc[nH]1)N1CCC2(CC1)CN([C@@H]1CCc3ccccc31)C2	1035650355	25	335.2	3.24

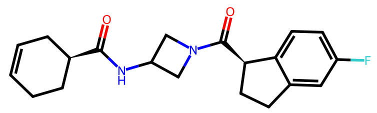	O=C(NC1CN(C(=O)[C@@H]2CCc3cc(F)ccc32)C1)[C@@H]1CC=CCC1	990217331	25	342.17	2.54
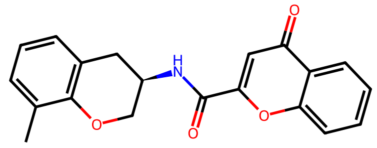	Cc1cccc2c1OC[C@H](NC(=O)c1cc(=O)c3ccccc3o1)C2	813305068	25	335.12	2.83
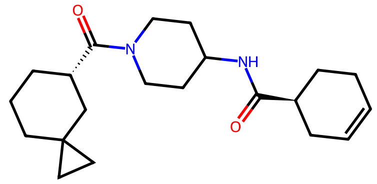	O=C(NC1CCN(C(=O)[C@H]2CCCC3(CC3)C2)CC1)[C@@H]1CC=CCC1	946752926	25	344.25	3.42
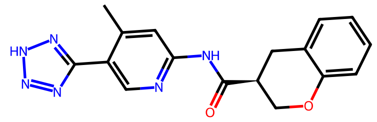	Cc1cc(NC(=O)[C@@H]2COc3ccccc3C2)ncc1-c1nn[nH]n1	1580978798	25	336.13	1.76
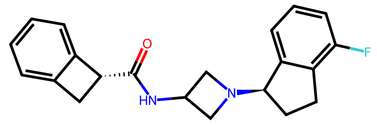	O=C(NC1CN([C@@H]2CCc3c(F)cccc32)C1)[C@@H]1Cc2ccccc21	1030881522	25	336.16	2.95
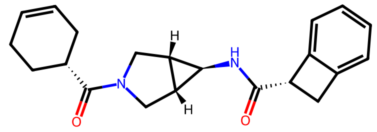	O=C(N[C@H]1[C@@H]2CN(C(=O)[C@H]3CC=CCC3)C[C@@H]21)[C@H]1Cc2ccccc21	977081556	25	336.18	2.26
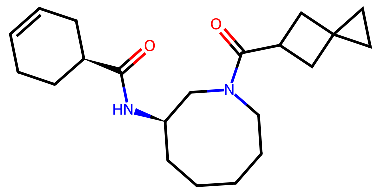	O=C(N[C@@H]1CCCCCN(C(=O)C2CC3(CC3)C2)C1)[C@@H]1CC=CCC1	987155347	25	344.25	3.42
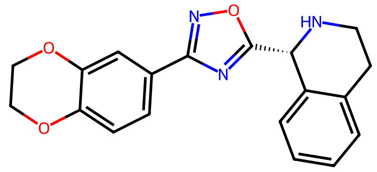	c1ccc2c(c1)CCN[C@H]2c1nc(-c2ccc3c(c2)OCCO3)no1	822728930	25	335.13	2.74

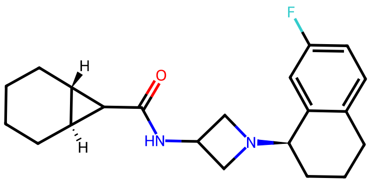	O=C(NC1CN([C@@H]2CCCc3ccc(F)cc32)C1)C1[C@H]2CCCC[C@H]12	1030189261	25	342.21	3.44
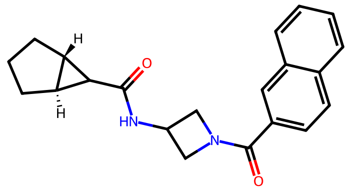	O=C(NC1CN(C(=O)c2ccc3ccccc3c2)C1)C1[C@H]2CCC[C@H]12	990691244	25	334.17	2.83
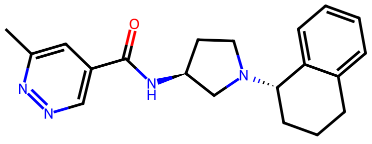	Cc1cc(C(=O)N[C@H]2CCN([C@H]3CCCc4ccccc43)C2)cnn1	1010758319	25	336.2	2.67
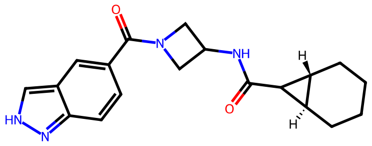	O=C(NC1CN(C(=O)c2ccc3n[nH]cc3c2)C1)C1[C@@H]2CCCC[C@@H]12	990643514	25	338.17	1.94
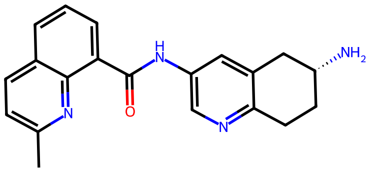	Cc1ccc2cccc(C(=O)Nc3cnc4c(c3)C[C@H](N)CC4)c2n1	349023169	25	332.16	3.01
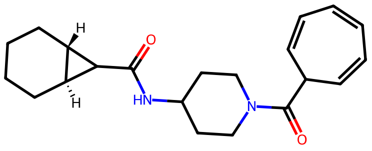	O=C(NC1CCN(C(=O)C2C=CC=CC=C2)CC1)C1[C@H]2CCCC[C@H]12	946949673	25	340.22	2.83
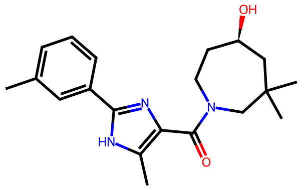	Cc1cccc(-c2nc(C(=O)N3CC[C@@H](O)CC(C)(C)C3)c(C)[nH]2)c1	296786927	25	341.21	3.32
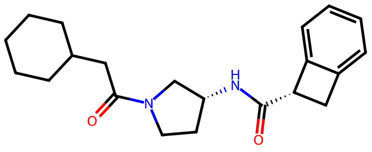	O=C(N[C@@H]1CCN(C(=O)CC2CCCCC2)C1)[C@H]1Cc2ccccc21	942304999	25	340.22	3.01

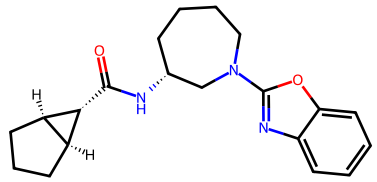	O=C(N[C@@H]1CCCCN(c2nc3ccccc3o2)C1)[C@@H]1[C@H]2CCC[C@H]21	1063182065	25	339.19	3.35
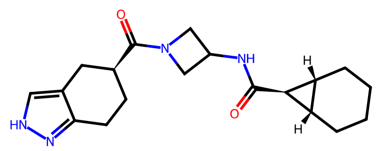	O=C(NC1CN(C(=O)[C@H]2CCc3n[nH]cc3C2)C1)[C@@H]1[C@H]2CCCC[C@H]21	990649352	25	342.21	1.28
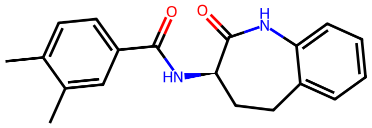	Cc1ccc(C(=O)N[C@@H]2CCc3ccccc3NC2=O)cc1C	654006175	23	308.15	2.99
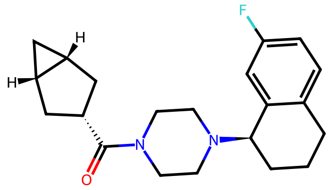	O=C([C@@H]1C[C@@H]2C[C@@H]2C1)N1CCN([C@@H]2CCCc3ccc(F)cc32)CC1	949788766	25	342.21	3.39
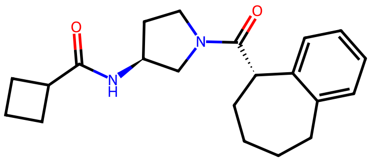	O=C(N[C@H]1CCN(C(=O)[C@H]2CCCCc3ccccc32)C1)C1CCC1	937592820	25	340.22	3.01
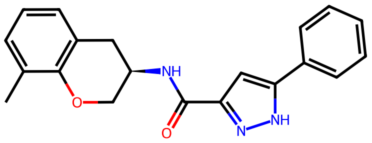	Cc1cccc2c1OC[C@H](NC(=O)c1cc(-c3ccccc3)[nH]n1)C2	355598387	25	333.15	3.12
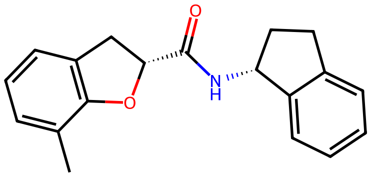	Cc1cccc2c1O[C@@H](C(=O)N[C@@H]1CCc3ccccc31)C2	449205622	22	293.14	3.1
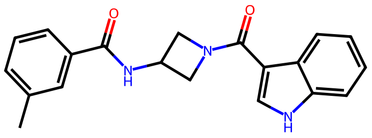	Cc1cccc(C(=O)NC2CN(C(=O)c3c[nH]c4ccccc34)C2)c1	990137261	25	333.15	2.73

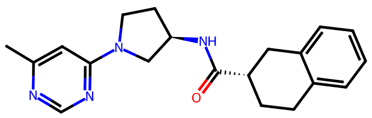	Cc1cc(N2CC[C@@H](NC(=O)[C@H]3CCc4ccccc4C3)C2)ncn1	1058559924	25	336.2	2.29
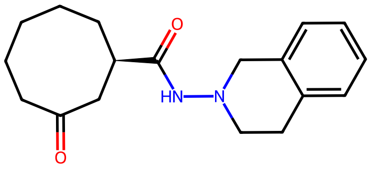	O=C1CCCCC[C@@H](C(=O)NN2CCc3ccccc3C2)C1	855712220	22	300.18	2.62
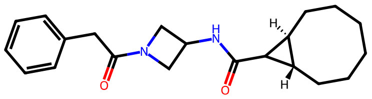	O=C(NC1CN(C(=O)Cc2ccccc2)C1)C1[C@H]2CCCCCC[C@H]12	1000507612	25	340.22	2.77
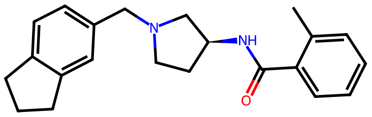	Cc1ccccc1C(=O)N[C@H]1CCN(Cc2ccc3c(c2)CCC3)C1	1009968996	25	334.2	3.49
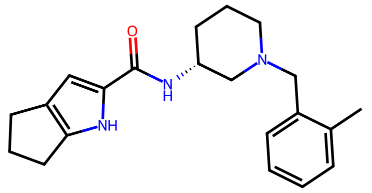	Cc1ccccc1CN1CCC[C@@H](NC(=O)c2cc3c([nH]2)CCC3)C1	1009163382	25	337.22	3.21
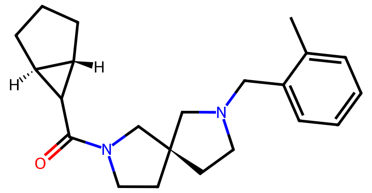	Cc1ccccc1CN1CC[C@]2(CCN(C(=O)C3[C@H]4CCC[C@H]34)C2)C1	1041179212	25	338.24	3.47
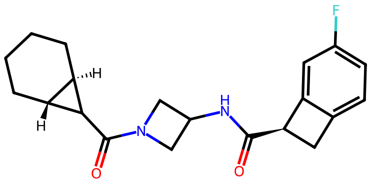	O=C(NC1CN(C(=O)C2[C@@H]3CCCC[C@@H]23)C1)[C@@H]1Cc2ccc(F)cc21	998488012	25	342.17	2.23
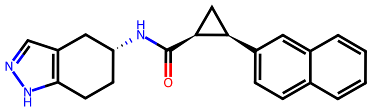	O=C(N[C@@H]1CCc2[nH]ncc2C1)[C@H]1C[C@H]1c1ccc2ccccc2c1	172212452	25	331.17	3.34

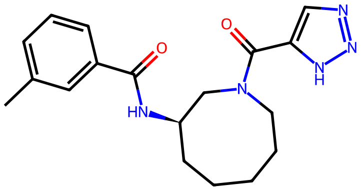	Cc1cccc(C(=O)N[C@@H]2CCCCCN(C(=O)c3cnn[nH]3)C2)c1	987073652	25	341.19	1.93
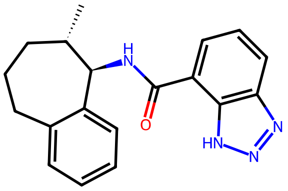	C[C@H]1CCCc2ccccc2[C@@H]1NC(=O)c1cccc2nn[nH]c12	1138085096	24	320.16	3.4
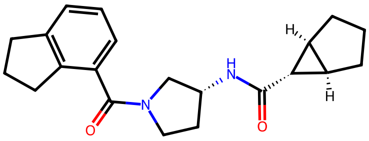	O=C(N[C@@H]1CCN(C(=O)c2cccc3c2CCC3)C1)[C@@H]1[C@H]2CCC[C@H]21	939930785	25	338.2	2.55
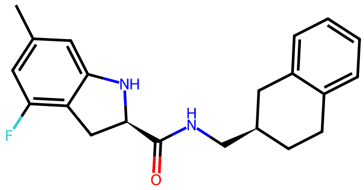	Cc1cc(F)c2c(c1)N[C@@H](C(=O)NC[C@@H]1CCc3ccccc3C1)C2	1579610644	25	338.18	3.39
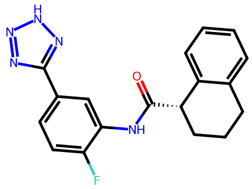	O=C(Nc1cc(-c2nn[nH]n2)ccc1F)[C@H]1CCCc2ccccc21	1584714592	25	337.13	3.06
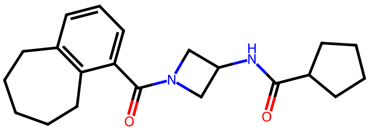	O=C(NC1CN(C(=O)c2cccc3c2CCCCC3)C1)C1CCCC1	990037204	25	340.22	3.09
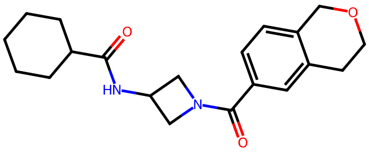	O=C(NC1CN(C(=O)c2ccc3c(c2)CCOC3)C1)C1CCCCC1	990091366	25	342.19	2.28
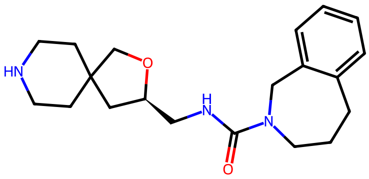	O=C(NC[C@H]1CC2(CCNCC2)CO1)N1CCCc2ccccc2C1	892768100	25	343.23	2.3

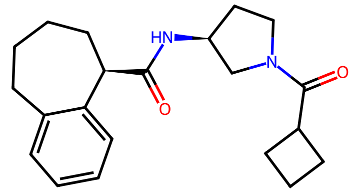	O=C(N[C@H]1CCN(C(=O)C2CCC2)C1)[C@@H]1CCCCc2ccccc21	938139963	25	340.22	3.01
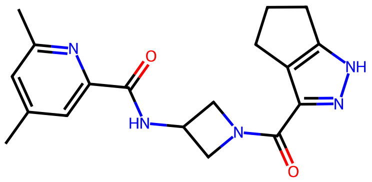	Cc1cc(C)nc(C(=O)NC2CN(C(=O)c3n[nH]c4c3CCC4)C2)c1	995017158	25	339.17	1.16
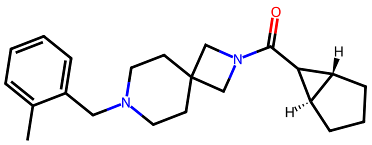	Cc1ccccc1CN1CCC2(CC1)CN(C(=O)C1[C@@H]3CCC[C@@H]13)C2	1050476035	25	338.24	3.47
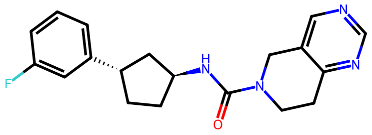	O=C(N[C@H]1CC[C@H](c2cccc(F)c2)C1)N1CCc2ncncc2C1	883620715	25	340.17	3.02
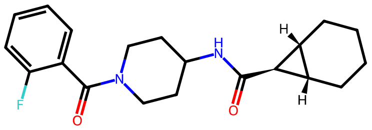	O=C(NC1CCN(C(=O)c2ccccc2F)CC1)[C@@H]1[C@H]2CCCC[C@H]21	245496965	25	344.19	2.98
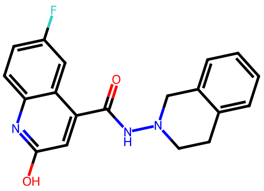	O=C(NN1CCc2ccccc2C1)c1cc(O)nc2ccc(F)cc12	1640002158	25	337.12	2.78
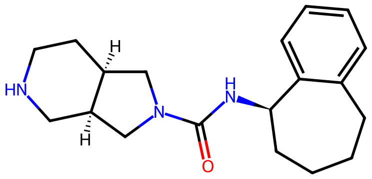	O=C(N[C@@H]1CCCCc2ccccc21)N1C[C@@H]2CCNC[C@@H]2C1	583252285	23	313.22	2.71
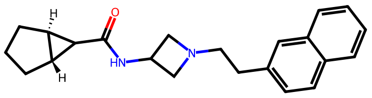	O=C(NC1CN(CCc2ccc3ccccc3c2)C1)C1[C@@H]2CCC[C@@H]12	1030190594	25	334.2	3.23

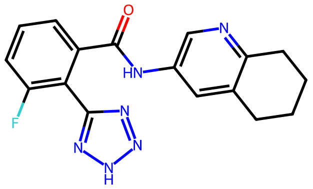	O=C(Nc1cnc2c(c1)CCCC2)c1cccc(F)c1-c1nn[nH]n1	1593258464	25	338.13	2.53
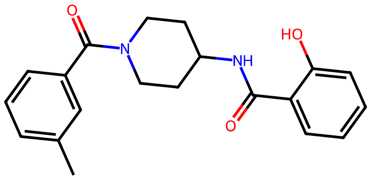	Cc1cccc(C(=O)N2CCC(NC(=O)c3ccccc3O)CC2)c1	169859507	25	338.16	2.74
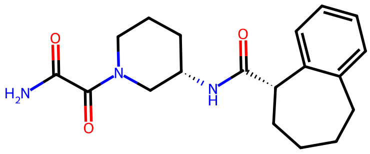	NC(=O)C(=O)N1CCC[C@H](NC(=O)[C@H]2CCCCc3ccccc32)C1	951861436	25	343.19	1.09
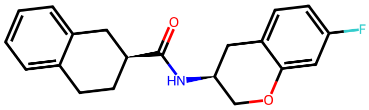	O=C(N[C@@H]1COc2cc(F)ccc2C1)[C@H]1CCc2ccccc2C1	618657625	24	325.15	3.05
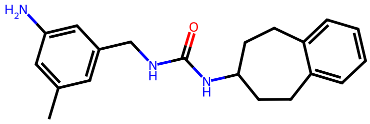	Cc1cc(N)cc(CNC(=O)NC2CCc3ccccc3CC2)c1	418243937	24	323.2	3.32
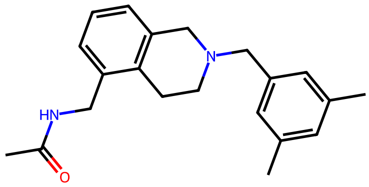	CC(=O)NCc1cccc2c1CCN(Cc1cc(C)cc(C)c1)C2	1051274445	24	322.2	3.5
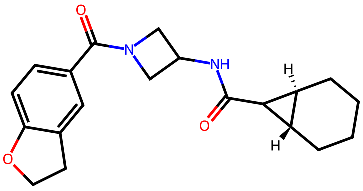	O=C(NC1CN(C(=O)c2ccc3c(c2)CCO3)C1)C1[C@H]2CCCC[C@H]12	990657178	25	340.18	2.0
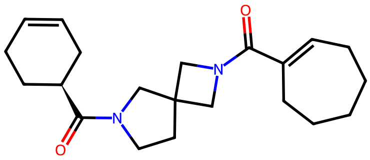	O=C(C1=CCCCCC1)N1CC2(CCN(C(=O)[C@@H]3CC=CCC3)C2)C1	1018967206	25	342.23	3.29

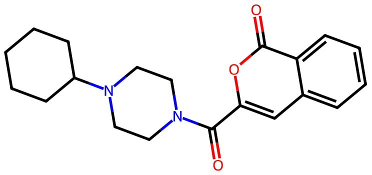	O=C(c1cc2ccccc2c(=O)o1)N1CCN(C2CCCCC2)CC1	280775754	25	340.18	2.88
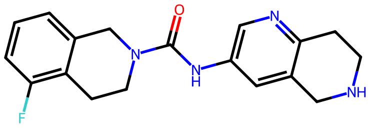	O=C(Nc1cnc2c(c1)CNCC2)N1CCc2c(F)cccc2C1	841321285	24	326.15	2.46
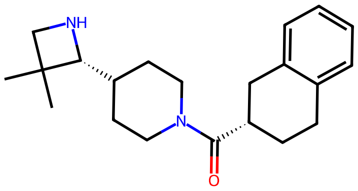	CC1(C)CN[C@@H]1C1CCN(C(=O)[C@H]2CCc3ccccc3C2)CC1	653164893	24	326.24	3.03
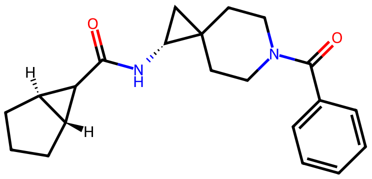	O=C(N[C@@H]1CC12CCN(C(=O)c1ccccc1)CC2)C1[C@@H]2CCC[C@@H]12	963462693	25	338.2	2.84
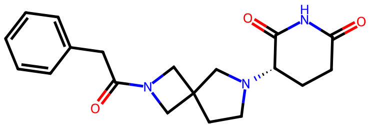	O=C1CC[C@H](N2CCC3(CN(C(=O)Cc4ccccc4)C3)C2)C(=O)N1	1072862053	25	341.17	0.57
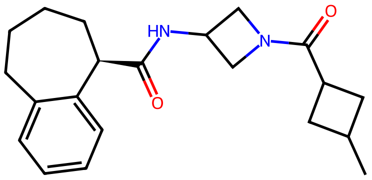	CC1CC(C(=O)N2CC(NC(=O)[C@@H]3CCCCc4ccccc43)C2)C1	994307260	25	340.22	2.87
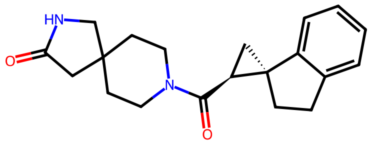	O=C1CC2(CCN(C(=O)[C@H]3C[C@]34CCc3ccccc34)CC2)CN1	267619876	24	324.18	2.02
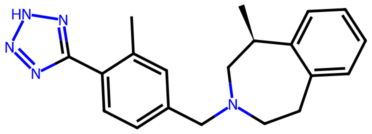	Cc1cc(CN2CCc3ccccc3[C@H](C)C2)ccc1-c1nn[nH]n1	1591964773	25	333.2	3.34

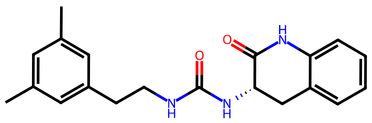	Cc1cc(C)cc(CCNC(=O)N[C@H]2Cc3ccccc3NC2=O)c1	1365110151	25	337.18	2.71
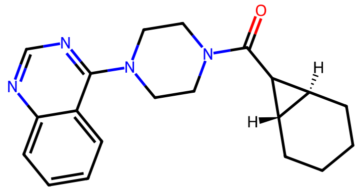	O=C(C1[C@H]2CCCC[C@H]12)N1CCN(c2ncnc3ccccc23)CC1	1055759616	25	336.2	2.71
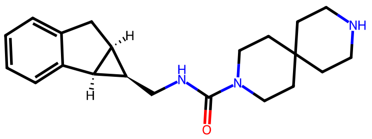	O=C(NC[C@H]1[C@H]2c3ccccc3C[C@H]21)N1CCC2(CCNCC2)CC1	417915133	25	339.23	2.75
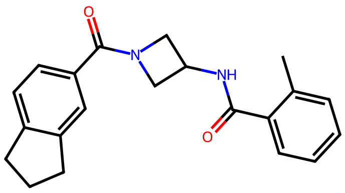	Cc1ccccc1C(=O)NC1CN(C(=O)c2ccc3c(c2)CCC3)C1	990110547	25	334.17	2.74
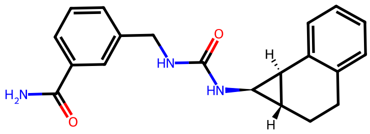	NC(=O)c1cccc(CNC(=O)N[C@@H]2[C@H]3CCc4ccccc4[C@@H]32)c1	126277995	25	335.16	2.31
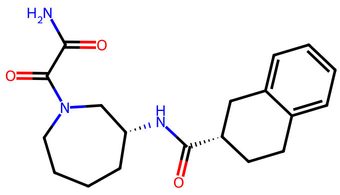	NC(=O)C(=O)N1CCCC[C@@H](NC(=O)[C@H]2CCc3ccccc3C2)C1	943575401	25	343.19	0.77
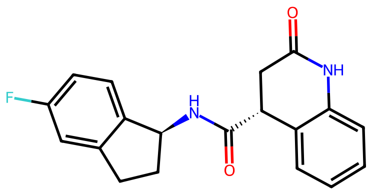	O=C1C[C@@H](C(=O)N[C@H]2CCc3cc(F)ccc32)c2ccccc2N1	56315393	24	324.13	3.06
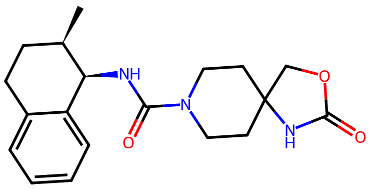	C[C@@H]1CCc2ccccc2[C@@H]1NC(=O)N1CCC2(CC1)COC(=O)N2	495297707	25	343.19	2.59

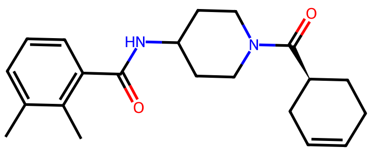	Cc1cccc(C(=O)NC2CCN(C(=O)[C@@H]3CC=CCC3)CC2)c1C	944107795	25	340.22	3.38
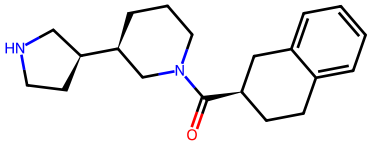	O=C([C@@H]1CCc2ccccc2C1)N1CCC[C@H]([C@H]2CCNC2)C1	573262483	23	312.22	2.64
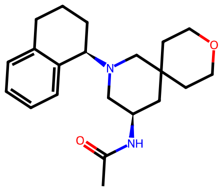	CC(=O)N[C@H]1CN([C@@H]2CCCc3ccccc32)CC2(CCOCC2)C1	1089916372	25	342.23	3.07
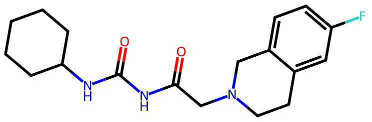	O=C(CN1CCc2cc(F)ccc2C1)NC(=O)NC1CCCCC1	91891986	24	333.19	2.34
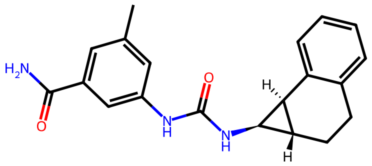	Cc1cc(NC(=O)N[C@@H]2[C@H]3CCc4ccccc4[C@@H]32)cc(C(N)=O)c1	565994178	25	335.16	2.94
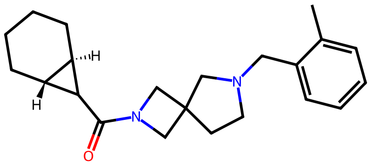	Cc1ccccc1CN1CCC2(C1)CN(C(=O)C1[C@@H]3CCCC[C@@H]13)C2	1072580467	25	338.24	3.47
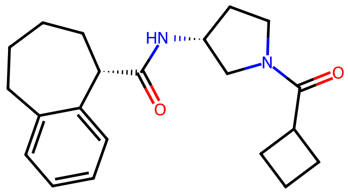	O=C(N[C@@H]1CCN(C(=O)C2CCC2)C1)[C@H]1CCCCc2ccccc21	941119889	25	340.22	3.01
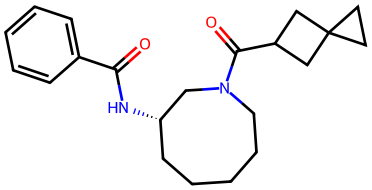	O=C(N[C@H]1CCCCCN(C(=O)C2CC3(CC3)C2)C1)c1ccccc1	987154150	25	340.22	3.38

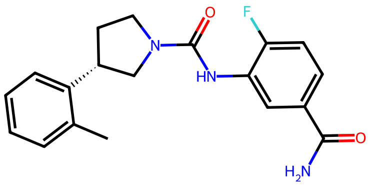	Cc1ccccc1[C@@H]1CCN(C(=O)Nc2cc(C(N)=O)ccc2F)C1	299679559	25	341.15	3.25
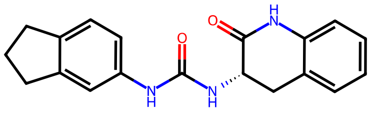	O=C(Nc1ccc2c(c1)CCC2)N[C@H]1Cc2ccccc2NC1=O	528866995	24	321.15	2.86
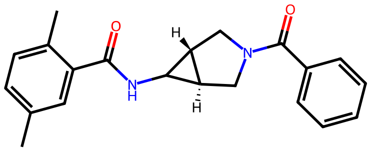	Cc1ccc(C)c(C(=O)NC2[C@@H]3CN(C(=O)c4ccccc4)C[C@@H]23)c1	1013772239	25	334.17	2.8
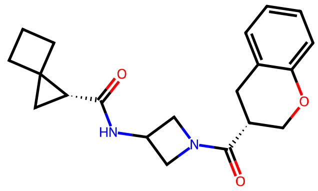	O=C(NC1CN(C(=O)[C@H]2COc3ccccc3C2)C1)[C@@H]1CC12CCC2	991556949	25	340.18	1.75
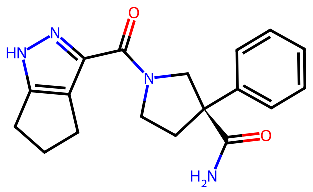	NC(=O)[C@@]1(c2ccccc2)CCN(C(=O)c2n[nH]c3c2CCC3)C1	412614121	24	324.16	1.17
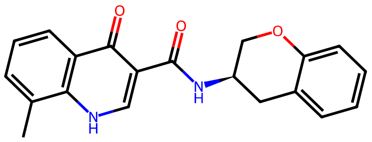	Cc1cccc2c(=O)c(C(=O)N[C@H]3COc4ccccc4C3)c[nH]c12	1472488417	25	334.13	2.57
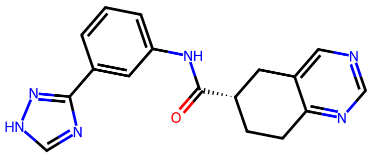	O=C(Nc1cccc(-c2nc[nH]n2)c1)[C@H]1CCc2ncncc2C1	1529324184	24	320.14	2.01
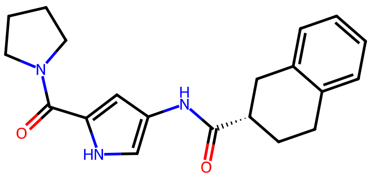	O=C(Nc1c[nH]c(C(=O)N2CCCC2)c1)[C@H]1CCc2ccccc2C1	1417704604	25	337.18	2.99

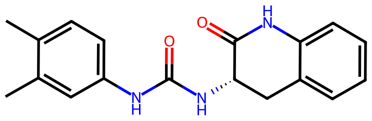	Cc1ccc(NC(=O)N[C@H]2Cc3ccccc3NC2=O)cc1C	1202830470	23	309.15	2.99
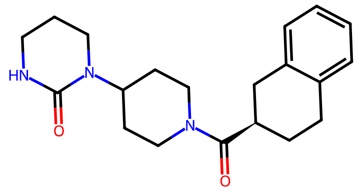	O=C([C@@H]1CCc2ccccc2C1)N1CCC(N2CCCNC2=O)CC1	565076121	25	341.21	2.2
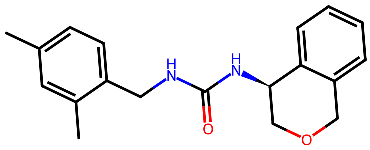	Cc1ccc(CNC(=O)N[C@@H]2COCc3ccccc32)c(C)c1	565823935	23	310.17	3.37
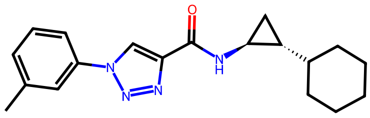	Cc1cccc(-n2cc(C(=O)N[C@H]3C[C@@H]3C3CCCCC3)nn2)c1	607593464	24	324.2	3.27
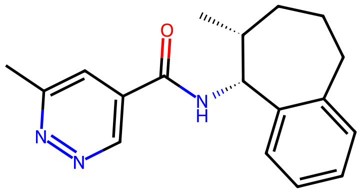	Cc1cc(C(=O)N[C@H]2c3ccccc3CCC[C@H]2C)cnn1	332917179	22	295.17	3.23
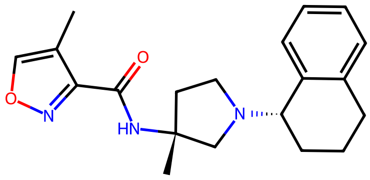	Cc1conc1C(=O)N[C@]1(C)CCN([C@H]2CCCc3ccccc32)C1	1046752856	25	339.19	3.25
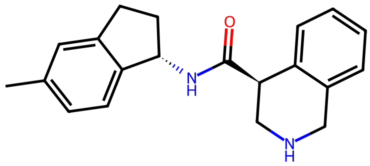	Cc1ccc2c(c1)CC[C@@H]2NC(=O)[C@@H]1CNCc2ccccc21	515837419	23	306.17	2.99
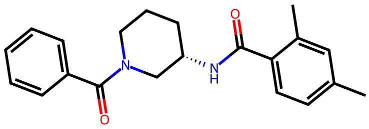	Cc1ccc(C(=O)N[C@H]2CCCN(C(=O)c3ccccc3)C2)c(C)c1	949820412	25	336.18	3.34

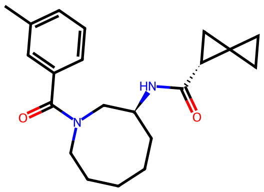	Cc1cccc(C(=O)N2CCCCCC[C@H](NC(=O)[C@@H]3CC34CC4)C2)c1	986868368	25	340.22	3.3
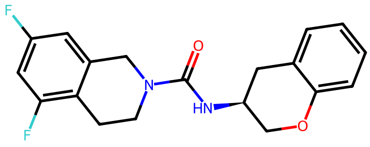	O=C(N[C@@H]1COc2ccccc2C1)N1CCc2c(F)cc(F)cc2C1	485172556	25	344.13	3.04
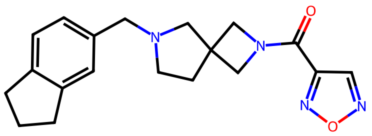	O=C(c1cnon1)N1CC2(CCN(Cc3ccc4c(c3)CCC4)C2)C1	1073068586	25	338.17	1.91
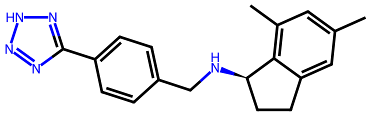	Cc1cc(C)c2c(c1)CC[C@H]2NCc1ccc(-c2nn[nH]n2)cc1	1591950669	24	319.18	3.26
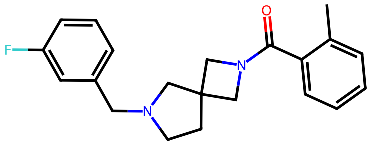	Cc1ccccc1C(=O)N1CC2(CCN(Cc3cccc(F)c3)C2)C1	1072413963	25	338.18	3.48
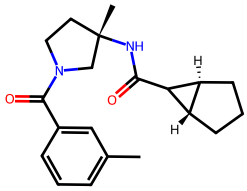	Cc1cccc(C(=O)N2CC[C@](C)(NC(=O)C3[C@H]4CCC[C@H]34)C2)c1	956799948	24	326.2	2.76
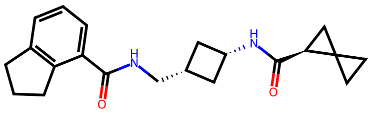	O=C(NC[C@H]1C[C@@H](NC(=O)[C@H]2CC23CC3)C1)c1cccc2c1CCC2	978947176	25	338.2	2.6
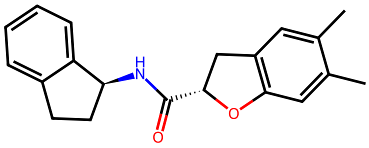	Cc1cc2c(cc1C)O[C@H](C(=O)N[C@H]1CCc3ccccc31)C2	463350910	23	307.16	3.41

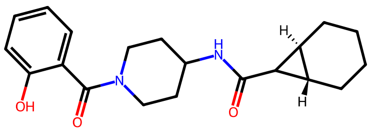	O=C(NC1CCN(C(=O)c2ccccc2O)CC1)C1[C@H]2CCCC[C@H]12	408409951	25	342.19	2.55
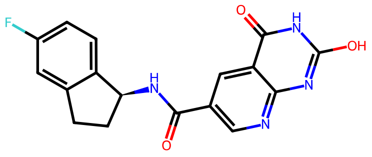	O=C(N[C@H]1CCc2cc(F)ccc21)c1cnc2nc(O)[nH]c(=O)c2c1	55658392	25	340.1	1.58
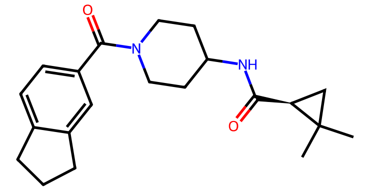	CC1(C)C[C@@H]1C(=O)NC1CCN(C(=O)c2ccc3c(c2)CCC3)CC1	946721195	25	340.22	2.94
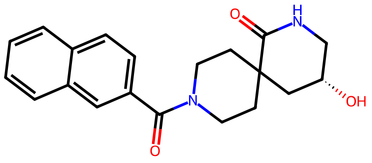	O=C(c1ccc2ccccc2c1)N1CCC2(CC1)C[C@@H](O)CNC2=O	885026769	25	338.16	1.94
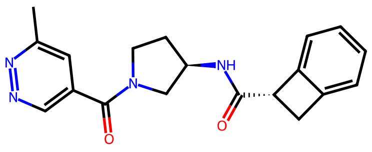	Cc1cc(C(=O)N2CC[C@@H](NC(=O)[C@H]3Cc4ccccc43)C2)cnn1	942304368	25	336.16	1.46
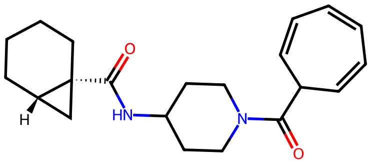	O=C(C1C=CC=CC=C1)N1CCC(NC(=O)[C@@]23CCCC[C@H]2C3)CC1	946950026	25	340.22	2.97
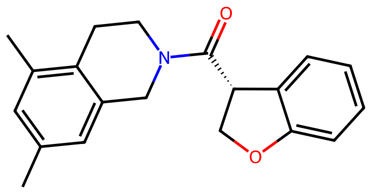	Cc1cc(C)c2c(c1)CN(C(=O)[C@H]1COc3ccccc31)CC2	548581444	23	307.16	3.36
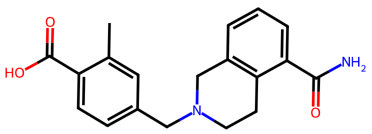	Cc1cc(CN2CCc3c(cccc3C(N)=O)C2)ccc1C(=O)O	1607642069	24	324.15	2.35

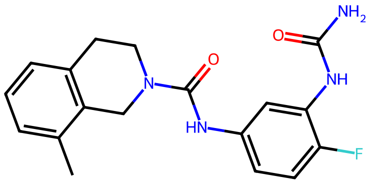	Cc1cccc2c1CN(C(=O)Nc1ccc(F)c(NC(N)=O)c1)CC2	1625013125	25	342.15	3.21
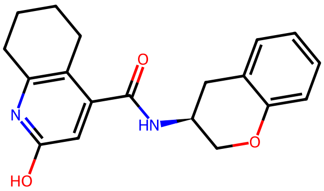	O=C(N[C@@H]1COc2ccccc2C1)c1cc(O)nc2c1CCCC2	930240387	24	324.15	2.4
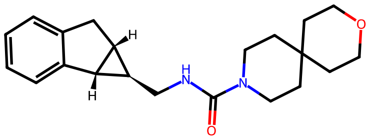	O=C(NC[C@@H]1[C@H]2Cc3ccccc3[C@H]21)N1CCC2(CCOCC2)CC1	285281111	25	340.22	3.17
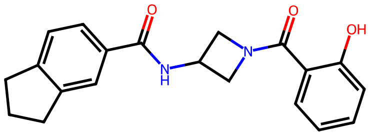	O=C(NC1CN(C(=O)c2ccccc2O)C1)c1ccc2c(c1)CCC2	991353972	25	336.15	2.14
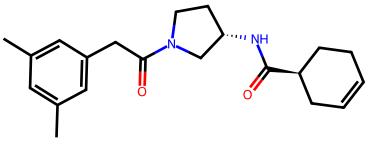	Cc1cc(C)cc(CC(=O)N2CC[C@H](NC(=O)[C@@H]3CC=CCC3)C2)c1	937667233	25	340.22	2.92
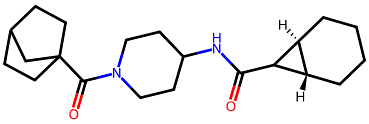	O=C(NC1CCN(C(=O)C23CCC(CC2)C3)CC1)C1[C@H]2CCCC[C@H]12	947332450	25	344.25	3.11
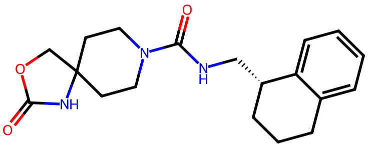	O=C1NC2(CCN(C(=O)NC[C@H]3CCCc4ccccc43)CC2)CO1	495296840	25	343.19	2.39
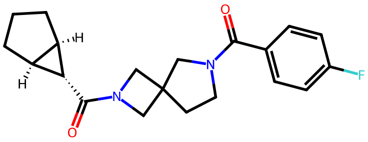	O=C(c1ccc(F)cc1)N1CCC2(C1)CN(C(=O)[C@@H]1[C@H]3CCC[C@H]31)C2	1018911105	25	342.17	2.55

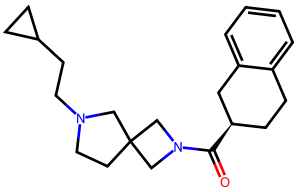	O=C([C@@H]1CCc2ccccc2C1)N1CC2(CCN(CCC3CC3)C2)C1	1073013423	25	338.24	3.13
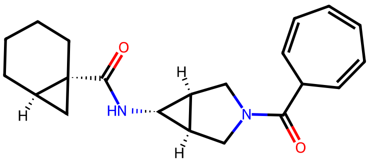	O=C(C1C=CC=CC=C1)N1C[C@H]2[C@H](NC(=O)[C@@]34CCCC[C@@H]3C4)[C@H]2C1	977157247	25	338.2	2.44
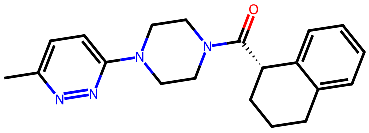	Cc1ccc(N2CCN(C(=O)[C@H]3CCCc4ccccc43)CC2)nn1	1056073597	25	336.2	2.55
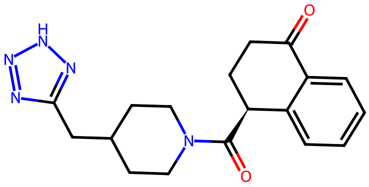	O=C1CC[C@H](C(=O)N2CCC(Cc3nn[nH]n3)CC2)c2ccccc21	1548805517	25	339.17	1.74
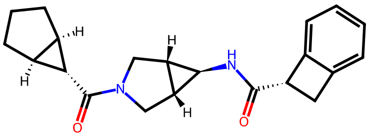	O=C(N[C@H]1[C@@H]2CN(C(=O)[C@@H]3[C@H]4CCC[C@H]43)C[C@@H]21)[C@H]1Cc2ccccc21	977081652	25	336.18	1.95
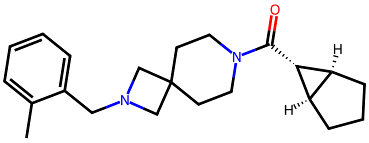	Cc1ccccc1CN1CC2(CCN(C(=O)[C@@H]3[C@H]4CCC[C@H]43)CC2)C1	1035680956	25	338.24	3.47
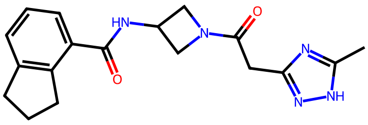	Cc1nc(CC(=O)N2CC(NC(=O)c3cccc4c3CCC4)C2)n[nH]1	994403451	25	339.17	0.79
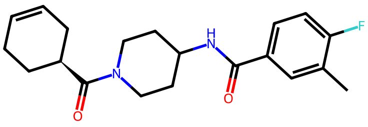	Cc1cc(C(=O)NC2CCN(C(=O)[C@@H]3CC=CCC3)CC2)ccc1F	944107530	25	344.19	3.21

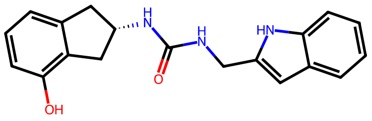	O=C(NCc1cc2ccccc2[nH]1)N[C@H]1Cc2cccc(O)c2C1	428554697	24	321.15	2.84
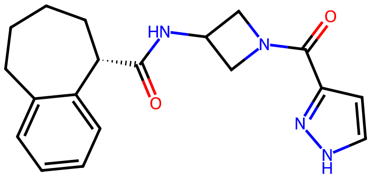	O=C(NC1CN(C(=O)c2cc[nH]n2)C1)[C@H]1CCCCc2ccccc21	994307223	25	338.17	1.86
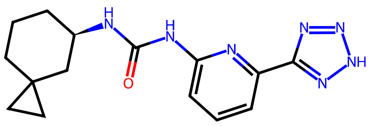	O=C(Nc1cccc(-c2nn[nH]n2)n1)N[C@@H]1CCCC2(CC2)C1	1593243546	23	313.17	2.11
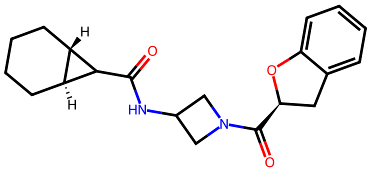	O=C(NC1CN(C(=O)[C@@H]2Cc3ccccc3O2)C1)C1[C@H]2CCCC[C@H]12	990659042	25	340.18	1.75
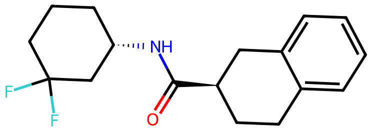	O=C(N[C@H]1CCCC(F)(F)C1)[C@@H]1CCc2ccccc2C1	649847876	21	293.16	3.49
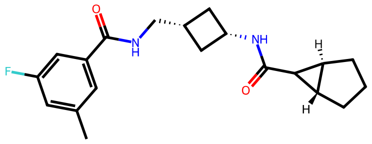	Cc1cc(F)cc(C(=O)NC[C@H]2C[C@@H](NC(=O)C3[C@H]4CCC[C@H]34)C2)c1	979358314	25	344.19	2.8
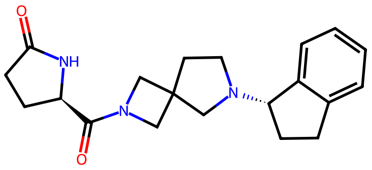	O=C1CC[C@H](C(=O)N2CC3(CCN([C@H]4CCc5ccccc54)C3)C2)N1	1072549954	25	339.19	1.49
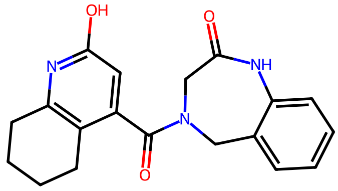	O=C1CN(C(=O)c2cc(O)nc3c2CCCC3)Cc2ccccc2N1	824322750	25	337.14	2.26

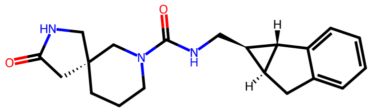	O=C1C[C@]2(CCCN(C(=O)NC[C@@H]3[C@@H]4Cc5ccccc5[C@@H]34)C2)CN1	332906345	25	339.19	1.88
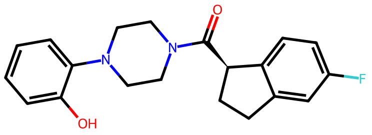	O=C([C@@H]1CCc2cc(F)ccc21)N1CCN(c2ccccc2O)CC1	665126377	25	340.16	2.91
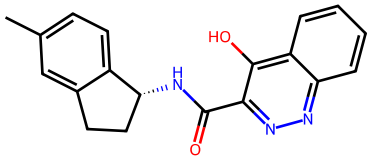	Cc1ccc2c(c1)CC[C@H]2NC(=O)c1nnc2ccccc2c1O	732276703	24	319.13	3.06
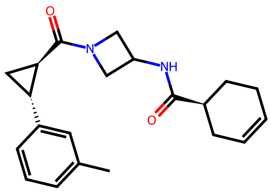	Cc1cccc([C@@H]2C[C@H]2C(=O)N2CC(NC(=O)[C@@H]3CC=CCC3)C2)c1	990213485	25	338.2	2.78
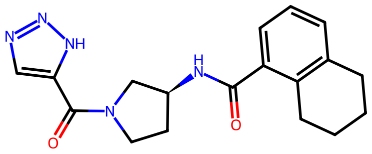	O=C(N[C@H]1CCN(C(=O)c2cnn[nH]2)C1)c1cccc2c1CCCC2	938005843	25	339.17	1.33
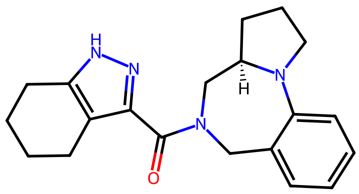	O=C(c1n[nH]c2c1CCCC2)N1Cc2ccccc2N2CCC[C@@H]2C1	882808591	25	336.2	2.91
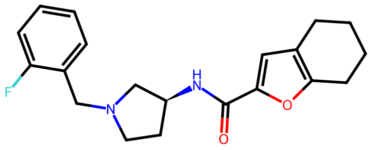	O=C(N[C@H]1CCN(Cc2ccccc2F)C1)c1cc2c(o1)CCCC2	1014676861	25	342.17	3.3
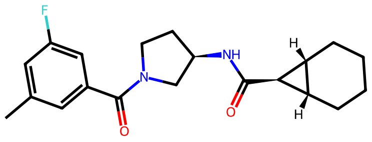	Cc1cc(F)cc(C(=O)N2CC[C@@H](NC(=O)[C@@H]3[C@H]4CCCC[C@H]43)C2)c1	936245947	25	344.19	2.9

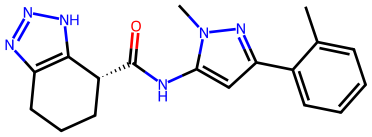	Cc1ccccc1-c1cc(NC(=O)[C@@H]2CCCc3nn[nH]c32)n(C)n1	646888846	25	336.17	2.57
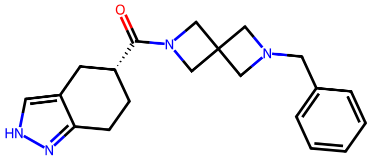	O=C([C@@H]1CCc2n[nH]cc2C1)N1CC2(CN(Cc3ccccc3)C2)C1	869815908	25	336.2	1.86
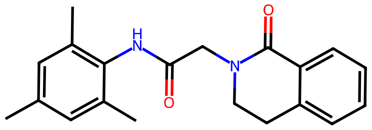	Cc1cc(C)c(NC(=O)CN2CCc3ccccc3C2=O)c(C)c1	278990821	24	322.17	3.25
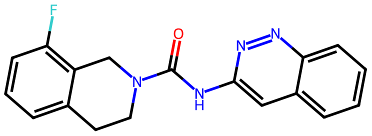	O=C(Nc1cc2ccccc2nn1)N1CCc2cccc(F)c2C1	1671259927	24	322.12	3.36
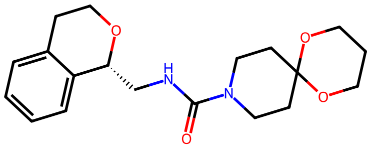	O=C(NC[C@H]1OCCc2ccccc21)N1CCC2(CC1)OCCCO2	838472160	25	346.19	2.24
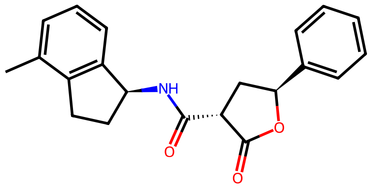	Cc1cccc2c1CC[C@@H]2NC(=O)[C@@H]1C[C@@H](c2ccccc2)OC1=O	809666559	25	335.15	3.4
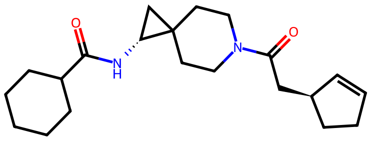	O=C(N[C@@H]1CC12CCN(C(=O)C[C@H]1C=CCC1)CC2)C1CCCCC1	962748515	25	344.25	3.42
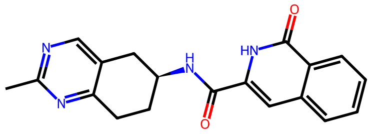	Cc1ncc2c(n1)CC[C@H](NC(=O)c1cc3ccccc3c(=O)[nH]1)C2	30387009	25	334.14	1.91

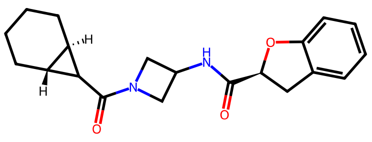	O=C(NC1CN(C(=O)C2[C@@H]3CCCC[C@@H]23)C1)[C@@H]1Cc2ccccc2O1	995414563	25	340.18	1.75
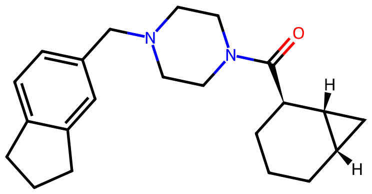	O=C([C@@H]1CCC[C@H]2C[C@H]21)N1CCN(Cc2ccc3c(c2)CCC3)CC1	948972718	25	338.24	3.26
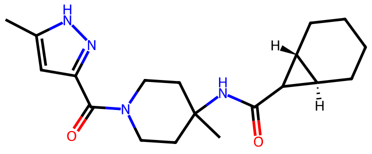	Cc1cc(C(=O)N2CCC(C)(NC(=O)C3[C@@H]4CCCC[C@@H]34)CC2)n[nH]1	956325078	25	344.22	2.27
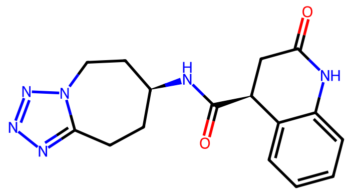	O=C1C[C@H](C(=O)N[C@H]2CCc3nnnn3CC2)c2ccccc2N1	1418054925	24	326.15	0.62
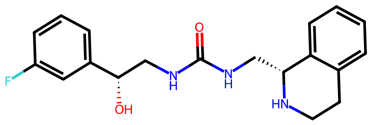	O=C(NC[C@H]1NCCc2ccccc21)NC[C@H](O)c1cccc(F)c1	841994888	25	343.17	2.05
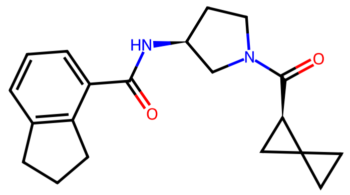	O=C(N[C@H]1CCN(C(=O)[C@@H]2CC23CC3)C1)c1cccc2c1CCC2	938149389	24	324.18	2.31
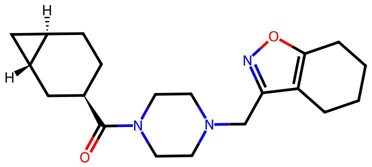	O=C([C@@H]1CC[C@@H]2C[C@H]2C1)N1CCN(Cc2noc3c2CCCC3)CC1	948979338	25	343.23	2.63
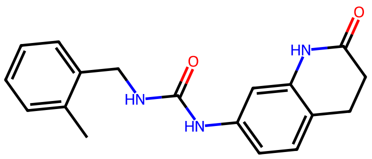	Cc1ccccc1CNC(=O)Nc1ccc2c(c1)NC(=O)CC2	71922254	23	309.15	3.2

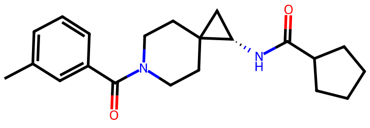	Cc1cccc(C(=O)N2CCC3(CC2)C[C@@H]3NC(=O)C2CCCC2)c1	962504722	25	340.22	3.3
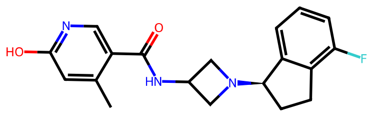	Cc1cc(O)ncc1C(=O)NC1CN([C@@H]2CCc3c(F)cccc32)C1	1030933888	25	341.15	2.34
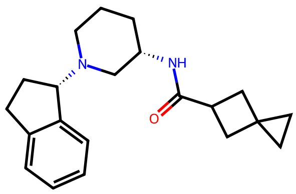	O=C(N[C@H]1CCCN([C@H]2CCc3ccccc32)C1)C1CC2(CC2)C1	1008480523	24	324.22	3.44
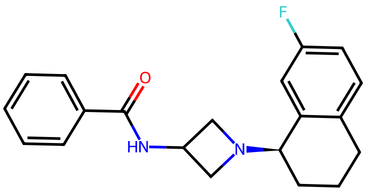	O=C(NC1CN([C@@H]2CCCc3ccc(F)cc32)C1)c1ccccc1	1030064211	24	324.16	3.32
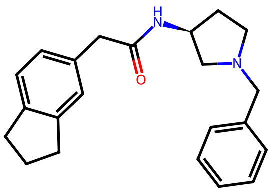	O=C(Cc1ccc2c(c1)CCC2)N[C@H]1CCN(Cc2ccccc2)C1	44720545	25	334.2	3.11
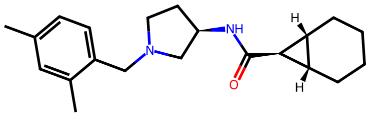	Cc1ccc(CN2CC[C@@H](NC(=O)[C@@H]3[C@H]4CCCC[C@H]43)C2)c(C)c1	1010438656	24	326.24	3.43
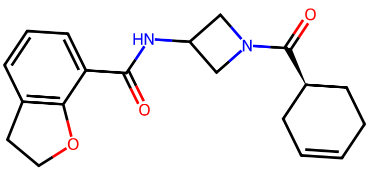	O=C(NC1CN(C(=O)[C@@H]2CC=CCC2)C1)c1cccc2c1OCC2	1000414709	24	326.16	1.92
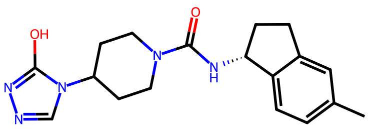	Cc1ccc2c(c1)CC[C@H]2NC(=O)N1CCC(n2cnnc2O)CC1	663796852	25	341.19	2.33

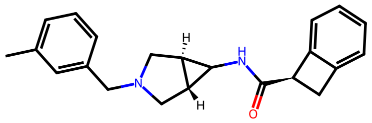	Cc1cccc(CN2C[C@H]3C(NC(=O)[C@@H]4Cc5ccccc54)[C@@H]3C2)c1	961627363	25	332.19	2.88
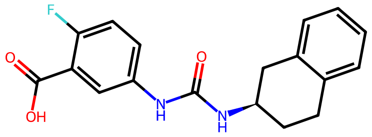	O=C(Nc1ccc(F)c(C(=O)O)c1)N[C@@H]1CCc2ccccc2C1	324398840	24	328.12	3.2
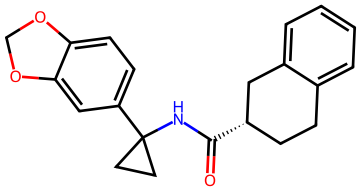	O=C(NC1(c2ccc3c(c2)OCO3)CC1)[C@H]1CCc2ccccc2C1	571331301	25	335.15	3.33
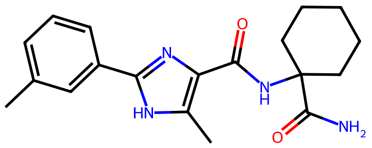	Cc1cccc(-c2nc(C(=O)NC3(C(N)=O)CCCC3)c(C)[nH]2)c1	270255940	25	340.19	2.61
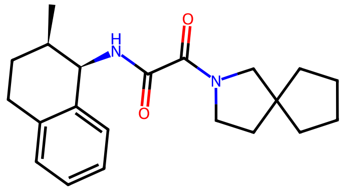	C[C@@H]1CCc2ccccc2[C@@H]1NC(=O)C(=O)N1CCC2(CCCC2)C1	807691449	25	340.22	3.22
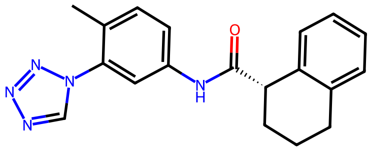	Cc1ccc(NC(=O)[C@H]2CCCc3ccccc32)cc1-n1cnnn1	46271146	25	333.16	3.03
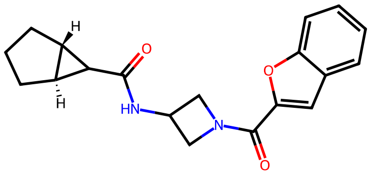	O=C(NC1CN(C(=O)c2cc3ccccc3o2)C1)C1[C@H]2CCC[C@H]12	990711483	24	324.15	2.42
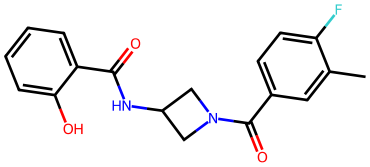	Cc1cc(C(=O)N2CC(NC(=O)c3ccccc3O)C2)ccc1F	990280066	24	328.12	2.09

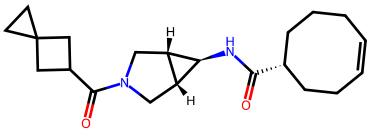	O=C(N[C@H]1[C@@H]2CN(C(=O)C3CC4(CC4)C3)C[C@@H]21)[C@H]1CCC=CCCC1	976758654	25	342.23	2.89
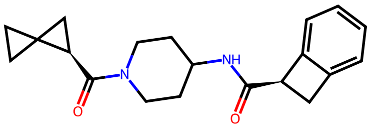	O=C(NC1CCN(C(=O)[C@@H]2CC23CC3)CC1)[C@@H]1Cc2ccccc21	948571713	24	324.18	2.23
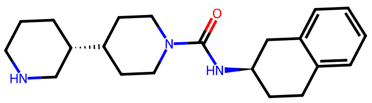	O=C(N[C@@H]1CCc2ccccc2C1)N1CCC([C@H]2CCCNC2)CC1	659447170	25	341.25	2.97
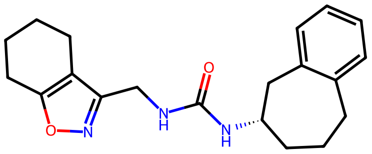	O=C(NCc1noc2c1CCCC2)N[C@H]1CCCc2ccccc2C1	894881402	25	339.19	3.3
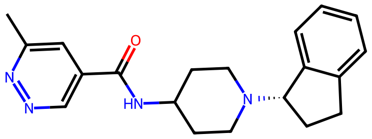	Cc1cc(C(=O)NC2CCN([C@H]3CCc4ccccc43)CC2)cnn1	1002682702	25	336.2	2.67
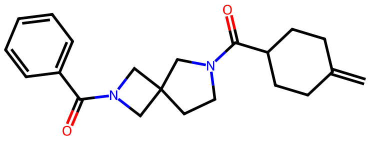	C=C1CCC(C(=O)N2CCC3(CN(C(=O)c4ccccc4)C3)C2)CC1	1018714484	25	338.2	3.11
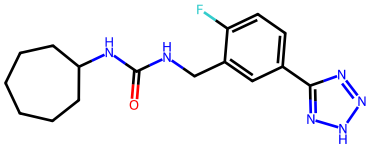	O=C(NCc1cc(-c2nn[nH]n2)ccc1F)NC1CCCCCC1	823232988	24	332.18	2.53
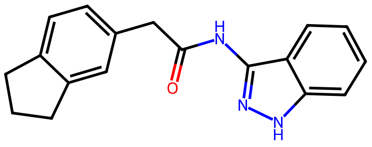	O=C(Cc1ccc2c(c1)CCC2)Nc1n[nH]c2ccccc12	75534530	22	291.14	3.23

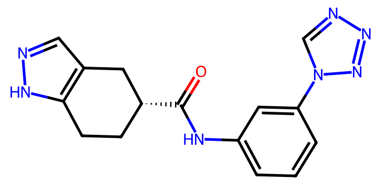	O=C(Nc1cccc(-n2cnnn2)c1)[C@@H]1CCc2[nH]ncc2C1	270181634	23	309.13	1.13
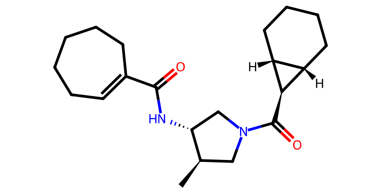	C[C@@H]1CN(C(=O)[C@@H]2[C@H]3CCCC[C@H]32)C[C@H]1NC(=O)C1=CCCCCC1	948158392	25	344.25	3.28
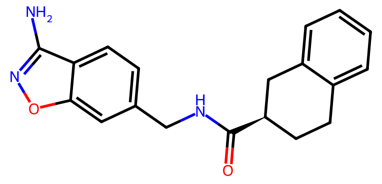	Nc1noc2cc(CNC(=O)[C@@H]3CCc4ccccc4C3)ccc12	627802722	24	321.15	2.83
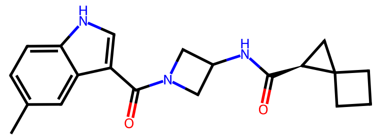	Cc1ccc2[nH]cc(C(=O)N3CC(NC(=O)[C@H]4CC45CCC5)C3)c2c1	991551803	25	337.18	2.61
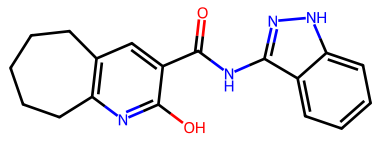	O=C(Nc1n[nH]c2ccccc12)c1cc2c(nc1O)CCCCC2	737791486	24	322.14	3.18
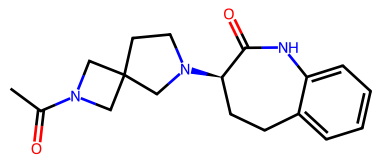	CC(=O)N1CC2(CCN([C@@H]3CCc4ccccc4NC3=O)C2)C1	1365729779	23	313.18	1.49
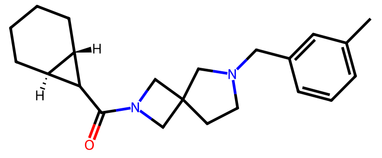	Cc1cccc(CN2CCC3(C2)CN(C(=O)C2[C@H]4CCCC[C@H]24)C3)c1	1072580643	25	338.24	3.47
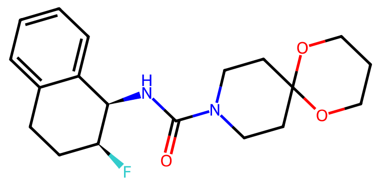	O=C(N[C@@H]1c2ccccc2CC[C@@H]1F)N1CCC2(CC1)OCCCO2	890176672	25	348.18	2.95

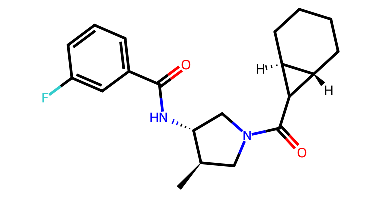	C[C@@H]1CN(C(=O)C2[C@H]3CCCC[C@H]23)C[C@H]1NC(=O)c1cccc(F)c1	948157809	25	344.19	2.84
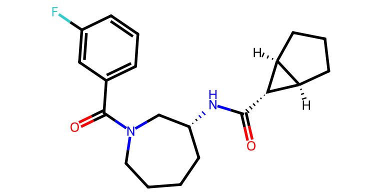	O=C(N[C@@H]1CCCCN(C(=O)c2cccc(F)c2)C1)[C@@H]1[C@H]2CCC[C@H]21	943168349	25	344.19	2.98
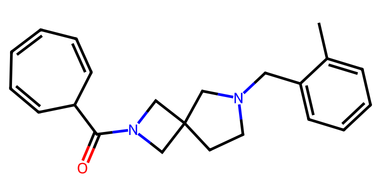	Cc1ccccc1CN1CCC2(C1)CN(C(=O)C1C=CC=CC=C1)C2	1072784783	25	334.2	3.33
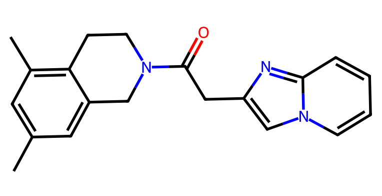	Cc1cc(C)c2c(c1)CN(C(=O)Cc1cn3ccccc3n1)CC2	187793005	24	319.17	3.08
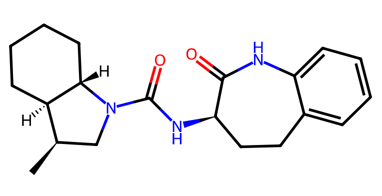	C[C@@H]1CN(C(=O)N[C@@H]2CCc3ccccc3NC2=O)[C@H]2CCCC[C@H]12	654629067	25	341.21	3.16
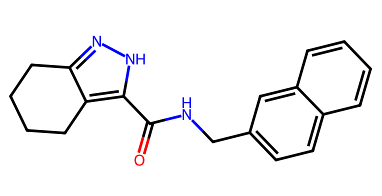	O=C(NCc1ccc2ccccc2c1)c1[nH]nc2c1CCCC2	195546722	23	305.15	3.37
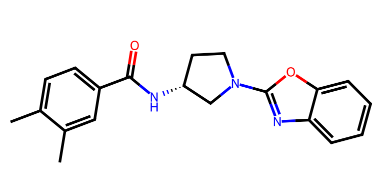	Cc1ccc(C(=O)N[C@@H]2CCN(c3nc4ccccc4o3)C2)cc1C	1058361626	25	335.16	3.45
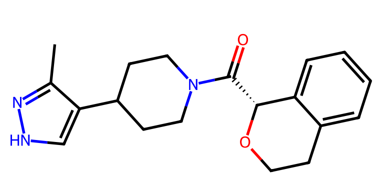	Cc1n[nH]cc1C1CCN(C(=O)[C@H]2OCCc3ccccc32)CC1	557158198	24	325.18	2.74

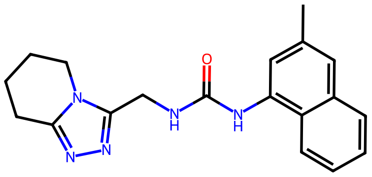	Cc1cc(NC(=O)NCc2nnc3n2CCCC3)c2ccccc2c1	297444149	25	335.17	3.4
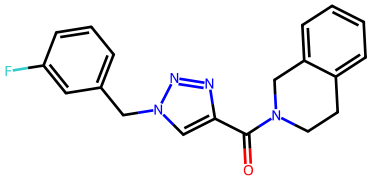	O=C(c1cn(Cc2cccc(F)c2)nn1)N1CCc2ccccc2C1	164075154	25	336.14	2.66
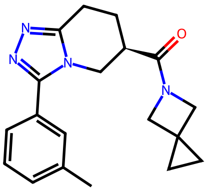	Cc1cccc(-c2nnc3n2C[C@H](C(=O)N2CC4(CC4)C2)CC3)c1	565815630	24	322.18	2.44
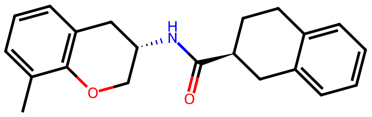	Cc1cccc2c1OC[C@@H](NC(=O)[C@H]1CCc3ccccc3C1)C2	1129553631	24	321.17	3.22
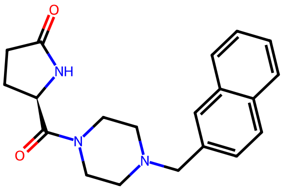	O=C1CC[C@H](C(=O)N2CCN(Cc3ccc4ccccc4c3)CC2)N1	1365628853	25	337.18	1.76
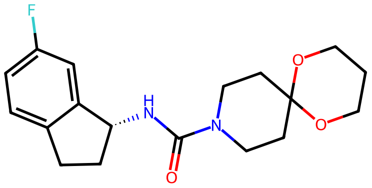	O=C(N[C@@H]1CCc2ccc(F)cc21)N1CCC2(CC1)OCCCO2	836288456	24	334.17	2.75
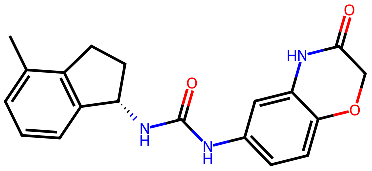	Cc1cccc2c1CC[C@@H]2NC(=O)Nc1ccc2c(c1)NC(=O)CO2	104985101	25	337.14	3.13
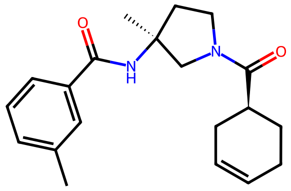	Cc1cccc(C(=O)N[C@@]2(C)CCN(C(=O)[C@@H]3CC=CCC3)C2)c1	956663622	24	326.2	3.07

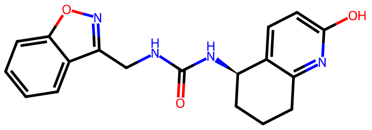	O=C(NCc1noc2ccccc12)N[C@@H]1CCCc2nc(O)ccc21	580343666	25	338.14	2.81
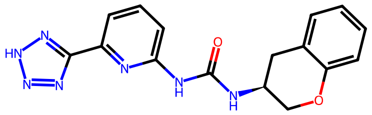	O=C(Nc1cccc(-c2nn[nH]n2)n1)N[C@@H]1COc2ccccc2C1	1582644577	25	337.13	1.39
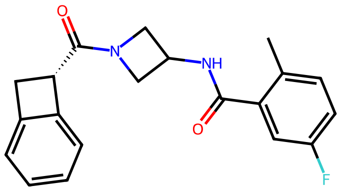	Cc1ccc(F)cc1C(=O)NC1CN(C(=O)[C@H]2Cc3ccccc32)C1	996641275	25	338.14	2.41
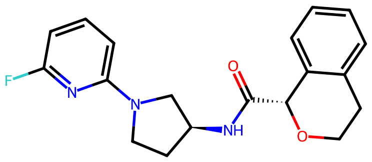	O=C(N[C@H]1CCN(c2cccc(F)n2)C1)[C@H]1OCCc2ccccc21	1058510755	25	341.15	2.23
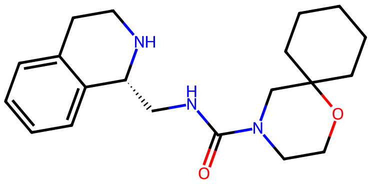	O=C(NC[C@H]1NCCc2ccccc21)N1CCOC2(CCCCC2)C1	563610702	25	343.23	2.62
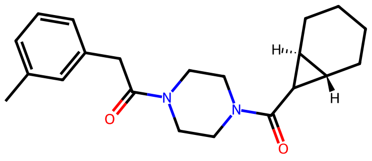	Cc1cccc(CC(=O)N2CCN(C(=O)C3[C@H]4CCCC[C@H]34)CC2)c1	1010903801	25	340.22	2.64
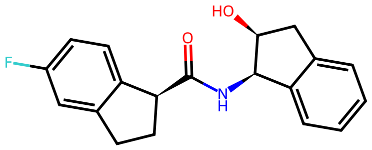	O=C(N[C@@H]1c2ccccc2C[C@@H]1O)[C@H]1CCc2cc(F)ccc21	612658579	23	311.13	2.63
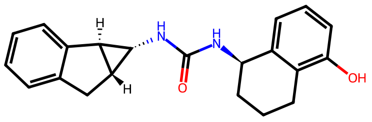	O=C(N[C@@H]1CCCc2c(O)cccc21)N[C@H]1[C@@H]2c3ccccc3C[C@H]21	191358785	25	334.17	3.41

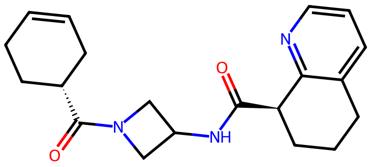	O=C(NC1CN(C(=O)[C@H]2CC=CCC2)C1)[C@@H]1CCCc2cccnc21	998042194	25	339.19	2.18
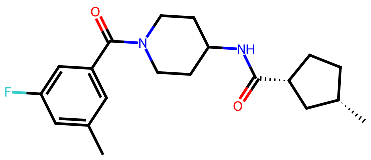	Cc1cc(F)cc(C(=O)N2CCC(NC(=O)[C@@H]3CC[C@H](C)C3)CC2)c1	948549819	25	346.21	3.29
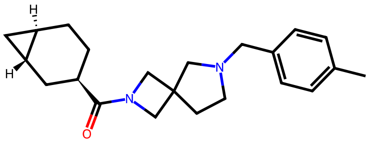	Cc1ccc(CN2CCC3(C2)CN(C(=O)[C@@H]2CC[C@@H]4C[C@H]4C2)C3)cc1	1072879508	25	338.24	3.47
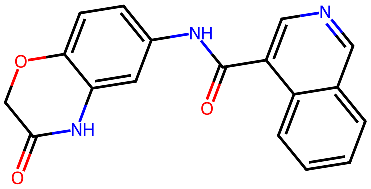	O=C1COc2ccc(NC(=O)c3cncc4ccccc34)cc2N1	614524947	24	319.1	2.82
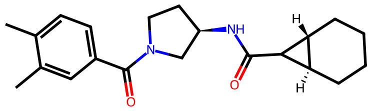	Cc1ccc(C(=O)N2CC[C@@H](NC(=O)C3[C@@H]4CCCC[C@@H]34)C2)cc1C	939903065	25	340.22	3.07
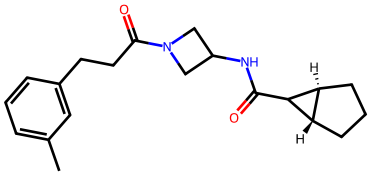	Cc1cccc(CCC(=O)N2CC(NC(=O)C3[C@H]4CCC[C@H]34)C2)c1	990707486	24	326.2	2.3
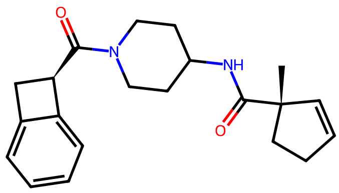	C[C@@]1(C(=O)NC2CCN(C(=O)[C@@H]3Cc4ccccc43)CC2)C=CCC1	948062447	25	338.2	2.79
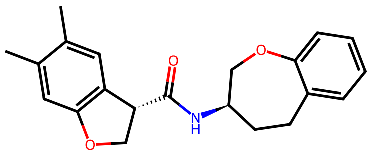	Cc1cc2c(cc1C)[C@H](C(=O)N[C@@H]1CCc3ccccc3OC1)CO2	511624754	25	337.17	3.29

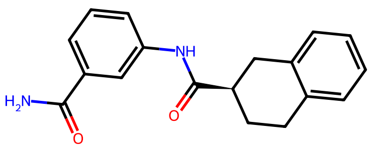	NC(=O)c1cccc(NC(=O)[C@@H]2CCc3ccccc3C2)c1	1412392606	22	294.14	2.53
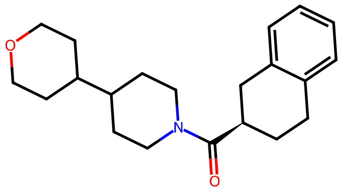	O=C([C@@H]1CCc2ccccc2C1)N1CCC(C2CCOCC2)CC1	519654963	24	327.22	3.46
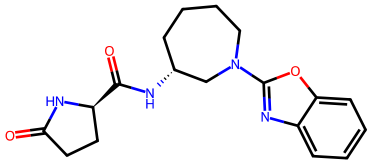	O=C1CC[C@H](C(=O)N[C@@H]2CCCCN(c3nc4ccccc4o3)C2)N1	1063173122	25	342.17	1.58
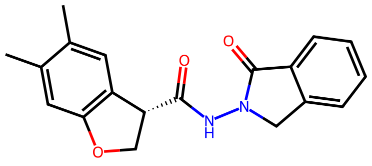	Cc1cc2c(cc1C)[C@H](C(=O)NN1Cc3ccccc3C1=O)CO2	186342079	24	322.13	2.47
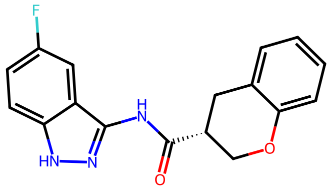	O=C(Nc1n[nH]c2ccc(F)cc12)[C@H]1COc2ccccc2C1	342950895	23	311.11	2.89
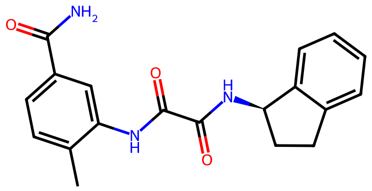	Cc1ccc(C(N)=O)cc1NC(=O)C(=O)N[C@@H]1CCc2ccccc21	63824639	25	337.14	1.84
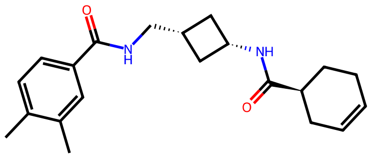	Cc1ccc(C(=O)NC[C@H]2C[C@@H](NC(=O)[C@@H]3CC=CCC3)C2)cc1C	979287058	25	340.22	3.28
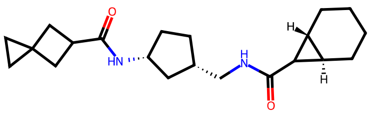	O=C(N[C@@H]1CC[C@H](CNC(=O)C2[C@@H]3CCCC[C@@H]23)C1)C1CC2(CC2)C1	981097858	25	344.25	3.01

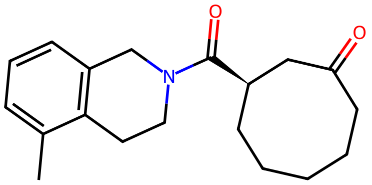	Cc1cccc2c1CCN(C(=O)[C@@H]1CCCCCC(=O)C1)C2	813441245	22	299.19	3.42
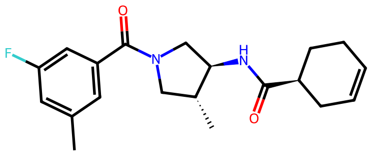	Cc1cc(F)cc(C(=O)N2C[C@@H](NC(=O)[C@@H]3CC=CCC3)[C@H](C)C2)c1	959723139	25	344.19	3.07
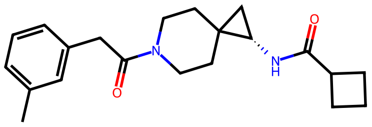	Cc1cccc(CC(=O)N2CCC3(CC2)C[C@@H]3NC(=O)C2CCC2)c1	962489655	25	340.22	2.83
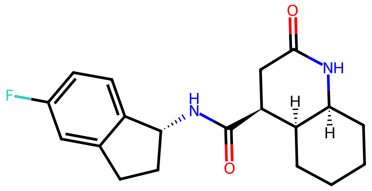	O=C1C[C@H](C(=O)N[C@@H]2CCc3cc(F)ccc32)[C@@H]2CCCC[C@@H]2N1	667094752	24	330.17	2.62
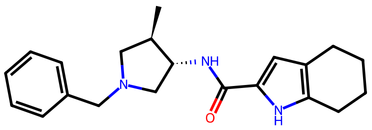	C[C@@H]1CN(Cc2ccccc2)C[C@H]1NC(=O)c1cc2c([nH]1)CCCC2	1080729505	25	337.22	3.14
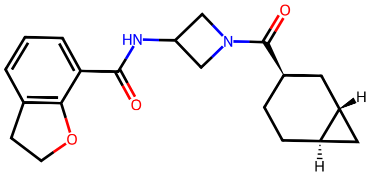	O=C(NC1CN(C(=O)[C@@H]2CC[C@@H]3C[C@H]3C2)C1)c1cccc2c1OCC2	1000416963	25	340.18	2.0
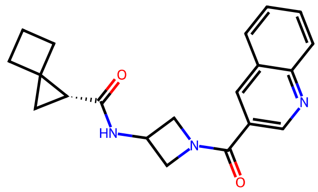	O=C(NC1CN(C(=O)c2cnc3ccccc3c2)C1)[C@@H]1CC12CCC2	991546481	25	335.16	2.37
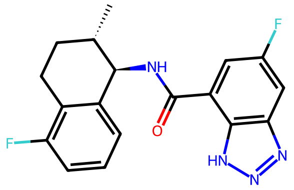	C[C@H]1CCc2c(F)cccc2[C@@H]1NC(=O)c1cc(F)cc2nn[nH]c12	433380022	25	342.13	3.29

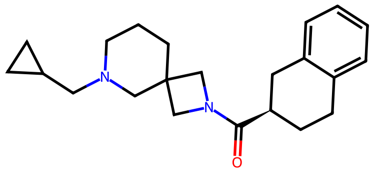	O=C([C@@H]1CCc2ccccc2C1)N1CC2(CCCN(CC3CC3)C2)C1	982037996	25	338.24	3.13
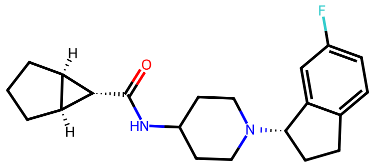	O=C(NC1CCN([C@H]2CCc3ccc(F)cc32)CC1)[C@@H]1[C@H]2CCC[C@H]21	1002545543	25	342.21	3.44
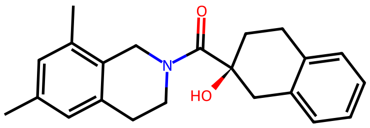	Cc1cc(C)c2c(c1)CCN(C(=O)[C@]1(O)CCc3ccccc3C1)C2	1457381153	25	335.19	3.11
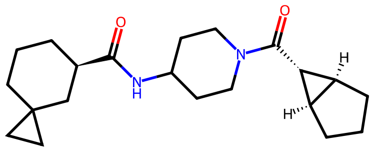	O=C(NC1CCN(C(=O)[C@@H]2[C@H]3CCC[C@H]32)CC1)[C@@H]1CCCC2(CC2)C1	946077834	25	344.25	3.11
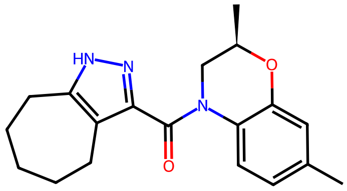	Cc1ccc2c(c1)O[C@H](C)CN2C(=O)c1n[nH]c2c1CCCCC2	190580189	24	325.18	3.41
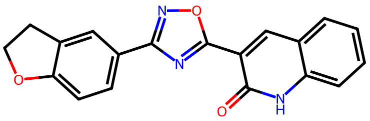	O=c1[nH]c2ccccc2cc1-c1nc(-c2ccc3c(c2)CCO3)no1	178927692	25	331.1	3.18
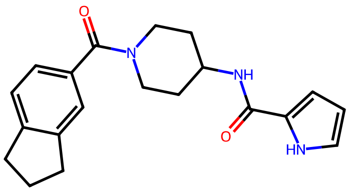	O=C(NC1CCN(C(=O)c2ccc3c(c2)CCC3)CC1)c1ccc[nH]1	363585387	25	337.18	2.54
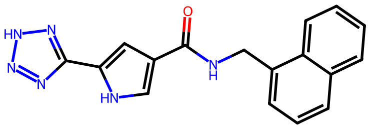	O=C(NCc1cccc2ccccc12)c1c[nH]c(-c2nn[nH]n2)c1	826442671	24	318.12	2.28

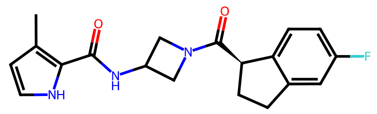	Cc1cc[nH]c1C(=O)NC1CN(C(=O)[C@@H]2CCc3cc(F)ccc32)C1	991100527	25	341.15	2.13
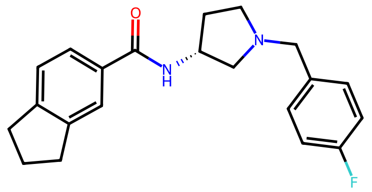	O=C(N[C@@H]1CCN(Cc2ccc(F)cc2)C1)c1ccc2c(c1)CCC2	1016223180	25	338.18	3.32
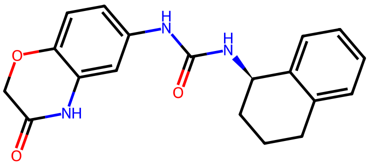	O=C1COc2ccc(NC(=O)N[C@@H]3CCCc4ccccc43)cc2N1	125121111	25	337.14	3.22
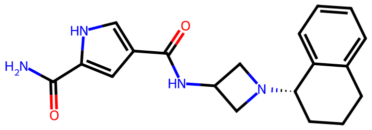	NC(=O)c1cc(C(=O)NC2CN([C@H]3CCCc4ccccc43)C2)c[nH]1	1031393536	25	338.17	1.61
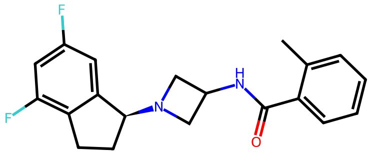	Cc1ccccc1C(=O)NC1CN([C@H]2CCc3c(F)cc(F)cc32)C1	1030067723	25	342.15	3.37
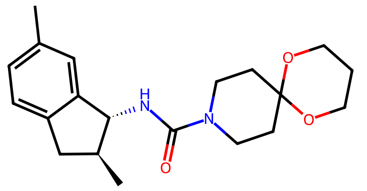	Cc1ccc2c(c1)[C@H](NC(=O)N1CCC3(CC1)OCCCO3)[C@@H](C)C2	297227896	25	344.21	3.17
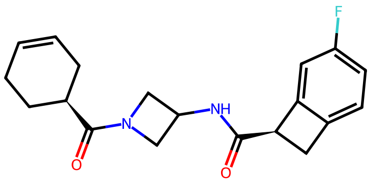	O=C(NC1CN(C(=O)[C@@H]2CC=CCC2)C1)[C@@H]1Cc2ccc(F)cc21	998485779	24	328.16	2.15
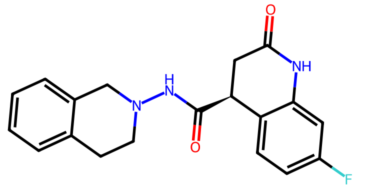	O=C1C[C@H](C(=O)NN2CCc3ccccc3C2)c2ccc(F)cc2N1	855708397	25	339.14	2.34

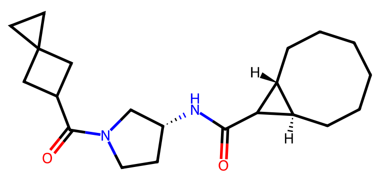	O=C(N[C@@H]1CCN(C(=O)C2CC3(CC3)C2)C1)C1[C@@H]2CCCCCC[C@@H]12	942510230	25	344.25	3.11
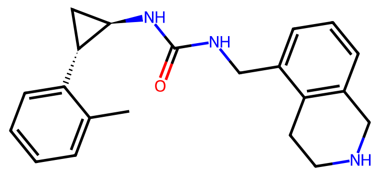	Cc1ccccc1[C@@H]1C[C@H]1NC(=O)NCc1cccc2c1CCNC2	892217693	25	335.2	3.0
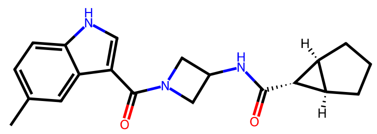	Cc1ccc2[nH]cc(C(=O)N3CC(NC(=O)[C@@H]4[C@H]5CCC[C@H]54)C3)c2c1	990697507	25	337.18	2.46
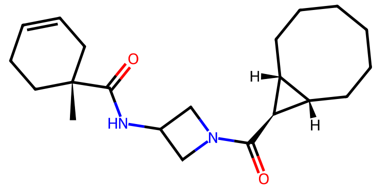	C[C@]1(C(=O)NC2CN(C(=O)[C@@H]3[C@H]4CCCCCC[C@H]43)C2)CC=CCC1	991916685	25	344.25	3.28
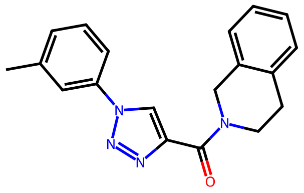	Cc1cccc(-n2cc(C(=O)N3CCc4ccccc4C3)nn2)c1	158836279	24	318.15	2.77
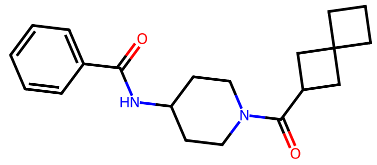	O=C(NC1CCN(C(=O)C2CC3(CCC3)C2)CC1)c1ccccc1	579233171	24	326.2	2.99
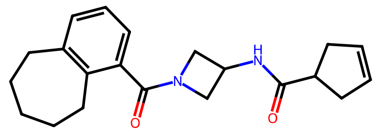	O=C(NC1CN(C(=O)c2cccc3c2CCCCC3)C1)C1CC=CC1	990586747	25	338.2	2.86
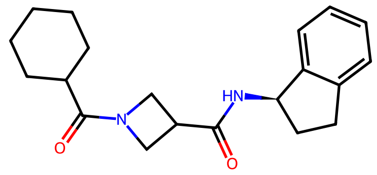	O=C(N[C@@H]1CCc2ccccc21)C1CN(C(=O)C2CCCCC2)C1	454415993	24	326.2	2.83

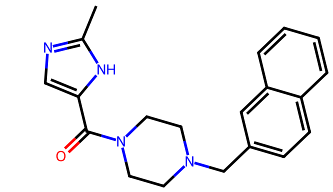	Cc1ncc(C(=O)N2CCN(Cc3ccc4ccccc4c3)CC2)[nH]1	1480342372	25	334.18	2.83
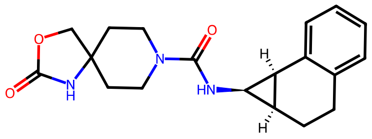	O=C1NC2(CCN(C(=O)N[C@@H]3[C@@H]4CCc5ccccc5[C@@H]43)CC2)CO1	495156347	25	341.17	2.0
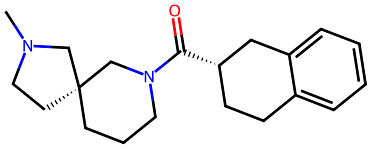	CN1CC[C@]2(CCCN(C(=O)[C@H]3CCc4ccccc4C3)C2)C1	615601226	23	312.22	2.74
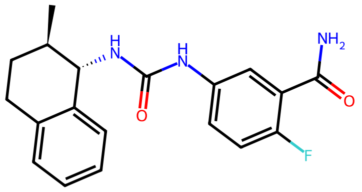	C[C@@H]1CCc2ccccc2[C@H]1NC(=O)Nc1ccc(F)c(C(N)=O)c1	677822194	25	341.15	3.37
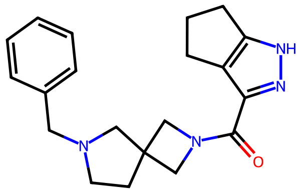	O=C(c1n[nH]c2c1CCC2)N1CC2(CCN(Cc3ccccc3)C2)C1	1072893925	25	336.2	2.25
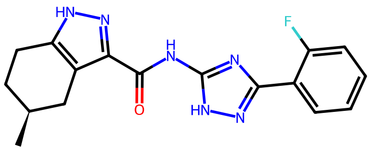	C[C@H]1CCc2[nH]nc(C(=O)Nc3nc(-c4ccccc4F)n[nH]3)c2C1	1648491058	25	340.14	2.71
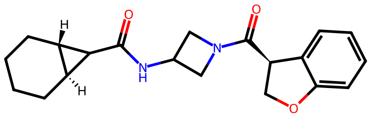	O=C(NC1CN(C(=O)[C@@H]2COc3ccccc32)C1)C1[C@H]2CCCC[C@H]12	990649050	25	340.18	1.93
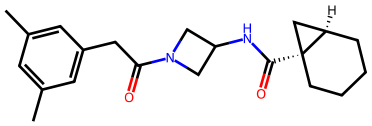	Cc1cc(C)cc(CC(=O)N2CC(NC(=O)[C@@]34CCCC[C@@H]3C4)C2)c1	999285197	25	340.22	2.75

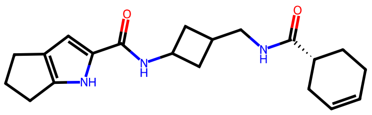	O=C(NC1CC(CNC(=O)[C@H]2CC=CCC2)C1)c1cc2c([nH]1)CCC2	991914970	25	341.21	2.48
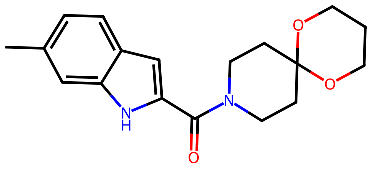	Cc1ccc2cc(C(=O)N3CCC4(CC3)OCCCO4)[nH]c2c1	293903786	23	314.16	2.85
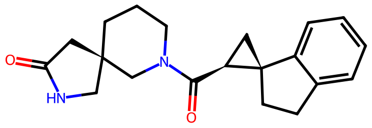	O=C1C[C@]2(CCCN(C(=O)[C@H]3C[C@@]34CCc3ccccc34)C2)CN1	341499945	24	324.18	2.02
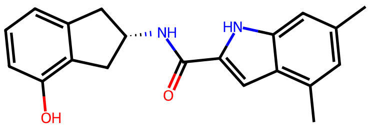	Cc1cc(C)c2cc(C(=O)N[C@H]3Cc4cccc(O)c4C3)[nH]c2c1	430497743	24	320.15	3.39
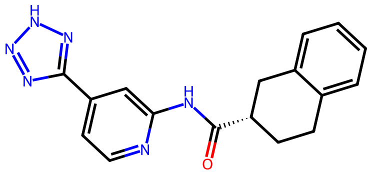	O=C(Nc1cc(-c2nn[nH]n2)ccn1)[C@H]1CCc2ccccc2C1	1582634554	24	320.14	2.01
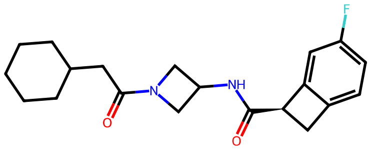	O=C(NC1CN(C(=O)CC2CCCCC2)C1)[C@@H]1Cc2ccc(F)cc21	998489073	25	344.19	2.76
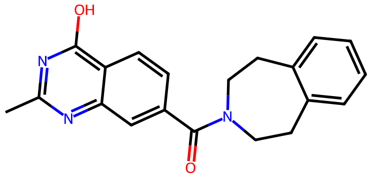	Cc1nc(O)c2ccc(C(=O)N3CCc4ccccc4CC3)cc2n1	889015167	25	333.15	2.88
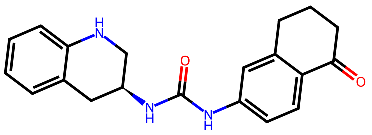	O=C(Nc1ccc2c(c1)CCCC2=O)N[C@@H]1CNc2ccccc2C1	652608303	25	335.16	3.36

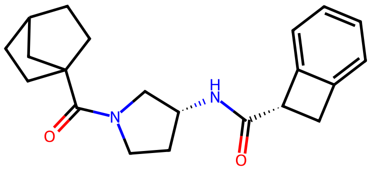	O=C(N[C@@H]1CCN(C(=O)C23CCC(CC2)C3)C1)[C@H]1Cc2ccccc21	942298228	25	338.2	2.62
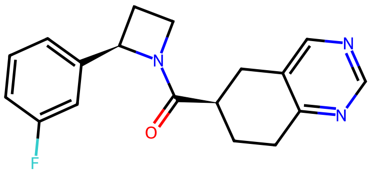	O=C([C@@H]1CCc2ncncc2C1)N1CC[C@@H]1c1cccc(F)c1	648801540	23	311.14	2.69
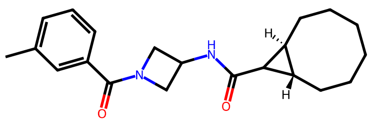	Cc1cccc(C(=O)N2CC(NC(=O)C3[C@H]4CCCCCC[C@H]34)C2)c1	1000468304	25	340.22	3.15
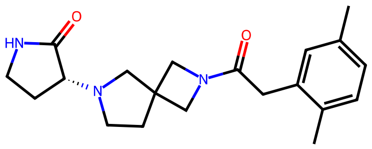	Cc1ccc(C)c(CC(=O)N2CC3(CCN([C@@H]4CCNC4=O)C3)C2)c1	1072944394	25	341.21	1.27
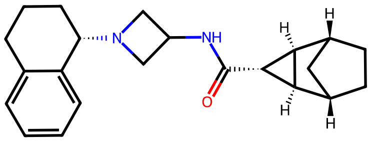	O=C(NC1CN([C@H]2CCCc3ccccc32)C1)[C@@H]1[C@H]2[C@@H]3CC[C@@H](C3)[C@H]21	1031463026	25	336.22	3.16
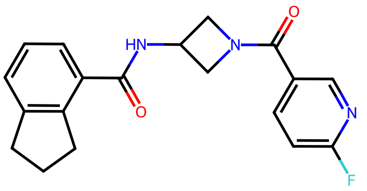	O=C(NC1CN(C(=O)c2ccc(F)nc2)C1)c1cccc2c1CCC2	994405799	25	339.14	1.96
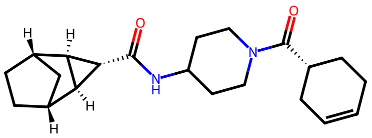	O=C(NC1CCN(C(=O)[C@H]2CC=CC2)CC1)[C@@H]1[C@H]2[C@@H]3CC[C@@H](C3)[C@H]21	944127034	25	342.23	2.74
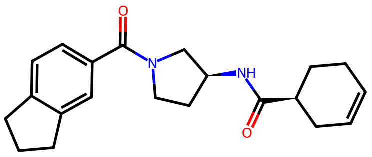	O=C(N[C@H]1CCN(C(=O)c2ccc3c(c2)CCC3)C1)[C@@H]1CC=CCC1	937668075	25	338.2	2.86

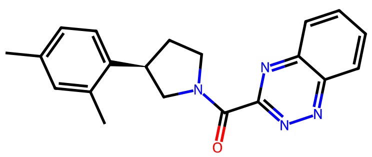	Cc1ccc([C@H]2CCN(C(=O)c3nnc4ccccc4n3)C2)c(C)c1	290775135	25	332.16	3.27
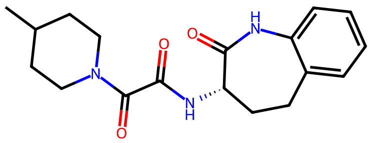	CC1CCN(C(=O)C(=O)N[C@H]2CCc3ccccc3NC2=O)CC1	655223994	24	329.17	1.31
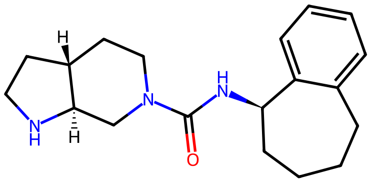	O=C(N[C@@H]1CCCCc2ccccc21)N1CC[C@H]2CCN[C@@H]2C1	669439939	23	313.22	2.85
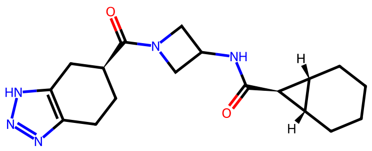	O=C(NC1CN(C(=O)[C@H]2CCc3nn[nH]c3C2)C1)[C@@H]1[C@H]2CCCC[C@H]21	990645362	25	343.2	0.67
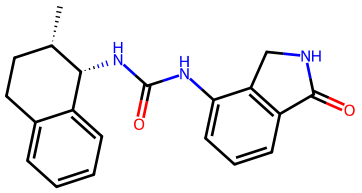	C[C@H]1CCc2ccccc2[C@H]1NC(=O)Nc1cccc2c1CNC2=O	287460891	25	335.16	3.38
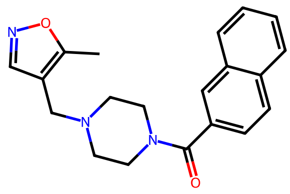	Cc1oncc1CN1CCN(C(=O)c2ccc3ccccc3c2)CC1	238923143	25	335.16	3.09
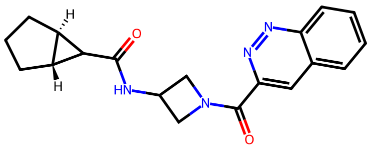	O=C(NC1CN(C(=O)c2cc3ccccc3nn2)C1)C1[C@@H]2CCC[C@@H]12	990706848	25	336.16	1.62
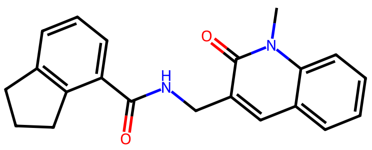	Cn1c(=O)c(CNC(=O)c2cccc3c2CCC3)cc2ccccc21	343383400	25	332.15	2.96

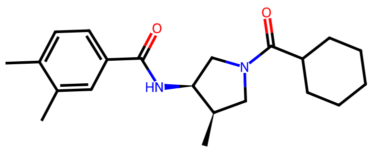	Cc1ccc(C(=O)N[C@H]2CN(C(=O)C3CCCCC3)C[C@H]2C)cc1C	966097708	25	342.23	3.46
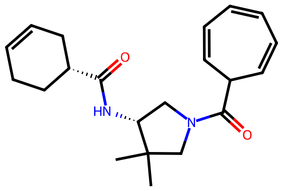	CC1(C)CN(C(=O)C2C=CC=CC=C2)C[C@H]1NC(=O)[C@H]1CC=CCC1	1007875288	25	340.22	2.99
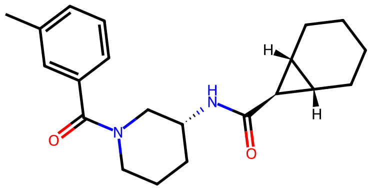	Cc1cccc(C(=O)N2CCC[C@@H](NC(=O)[C@@H]3[C@H]4CCCC[C@H]43)C2)c1	949869538	25	340.22	3.15
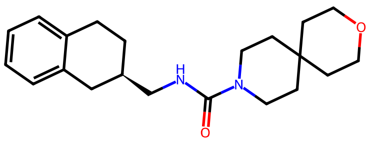	O=C(NC[C@@H]1CCc2ccccc2C1)N1CCC2(CCOCC2)CC1	671204513	25	342.23	3.39
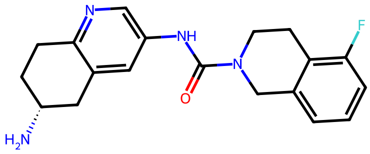	N[C@@H]1CCc2ncc(NC(=O)N3CCc4c(F)cccc4C3)cc2C1	333298115	25	340.17	2.63
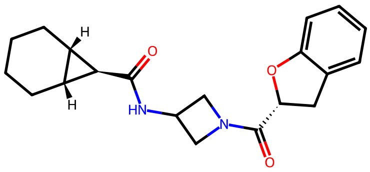	O=C(NC1CN(C(=O)[C@H]2Cc3ccccc3O2)C1)[C@@H]1[C@H]2CCCC[C@H]21	990659024	25	340.18	1.75
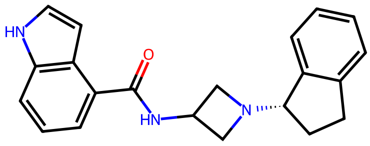	O=C(NC1CN([C@H]2CCc3ccccc32)C1)c1cccc2[nH]ccc12	1030995495	25	331.17	3.27
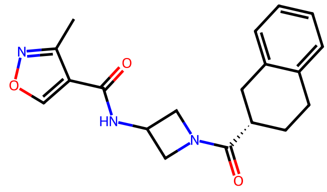	Cc1nocc1C(=O)NC1CN(C(=O)[C@H]2CCc3ccccc3C2)C1	998750382	25	339.16	1.73

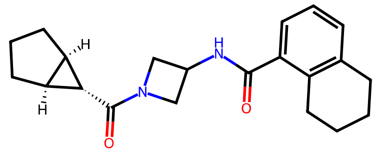	O=C(NC1CN(C(=O)[C@@H]2[C@H]3CCC[C@H]32)C1)c1cccc2c1CCCC2	991900248	25	338.2	2.55
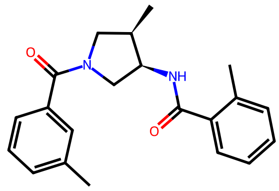	Cc1cccc(C(=O)N2C[C@@H](C)[C@@H](NC(=O)c3ccccc3C)C2)c1	966115728	25	336.18	3.19
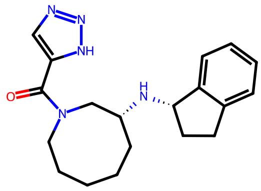	O=C(c1cnn[nH]1)N1CCCCC[C@@H](N[C@H]2CCc3ccccc32)C1	1088340450	25	339.21	2.47
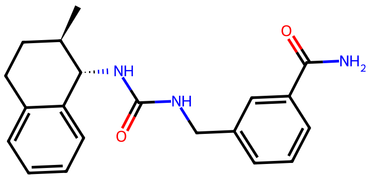	C[C@@H]1CCc2ccccc2[C@H]1NC(=O)NCc1cccc(C(N)=O)c1	179306757	25	337.18	2.91
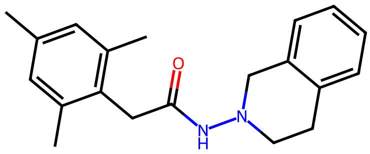	Cc1cc(C)c(CC(=O)NN2CCc3ccccc3C2)c(C)c1	1644634762	23	308.19	3.24
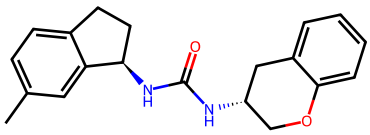	Cc1ccc2c(c1)[C@H](NC(=O)N[C@H]1COc3ccccc3C1)CC2	592499151	24	322.17	3.29
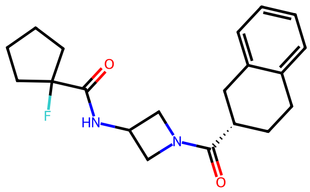	O=C([C@H]1CCc2ccccc2C1)N1CC(NC(=O)C2(F)CCCC2)C1	994992468	25	344.19	2.4
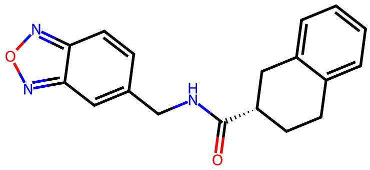	O=C(NCc1ccc2nonc2c1)[C@H]1CCc2ccccc2C1	889877999	23	307.13	2.64

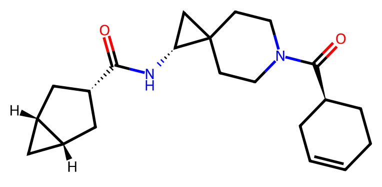	O=C(N[C@@H]1CC12CCN(C(=O)[C@@H]1CC=CCC1)CC2)[C@@H]1C[C@@H]2C[C@@H]2C1	964770633	25	342.23	2.89
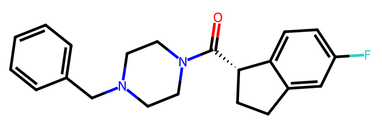	O=C([C@H]1CCc2cc(F)ccc21)N1CCN(Cc2ccccc2)CC1	949321874	25	338.18	3.2
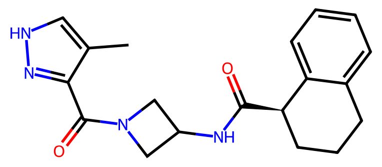	Cc1c[nH]nc1C(=O)N1CC(NC(=O)[C@@H]2CCCc3ccccc32)C1	995069887	25	338.17	1.78
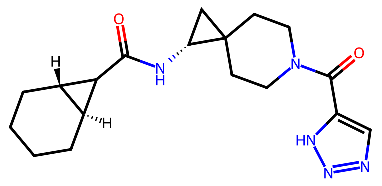	O=C(N[C@@H]1CC12CCN(C(=O)c1cnn[nH]1)CC2)C1[C@H]2CCCC[C@H]12	963448782	25	343.2	1.35
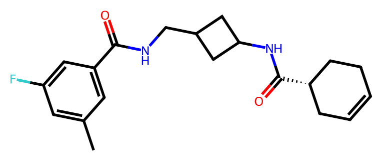	Cc1cc(F)cc(C(=O)NCC2CC(NC(=O)[C@H]3CC=CCC3)C2)c1	992431916	25	344.19	3.12
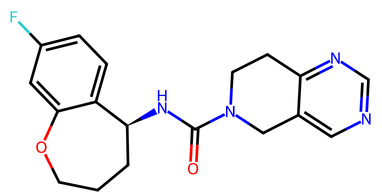	O=C(N[C@H]1CCCOc2cc(F)ccc21)N1CCc2ncncc2C1	883556283	25	342.15	2.6
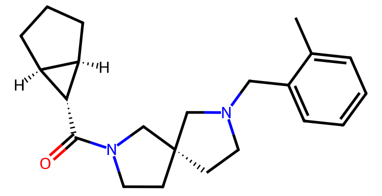	Cc1ccccc1CN1CC[C@@]2(CCN(C(=O)[C@@H]3[C@H]4CCC[C@H]43)C2)C1	1041179210	25	338.24	3.47
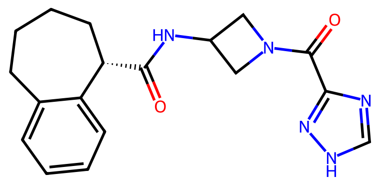	O=C(NC1CN(C(=O)c2nc[nH]n2)C1)[C@H]1CCCCc2ccccc21	994322189	25	339.17	1.26

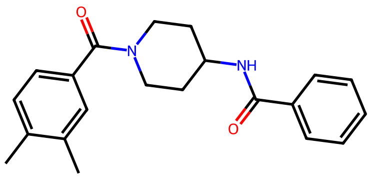	Cc1ccc(C(=O)N2CCC(NC(=O)c3ccccc3)CC2)cc1C	54128747	25	336.18	3.34
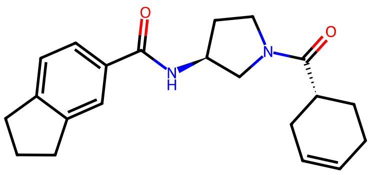	O=C(N[C@H]1CCN(C(=O)[C@H]2CC=CCC2)C1)c1ccc2c(c1)CCC2	937934051	25	338.2	2.86
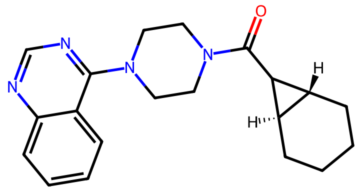	O=C(C1[C@@H]2CCCC[C@@H]12)N1CCN(c2ncnc3ccccc23)CC1	1055759617	25	336.2	2.71
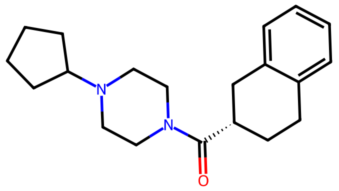	O=C([C@H]1CCc2ccccc2C1)N1CCN(C2CCCC2)CC1	575993071	23	312.22	2.88
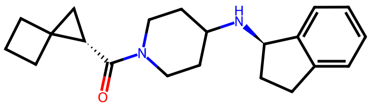	O=C([C@H]1CC12CCC2)N1CCC(N[C@@H]2CCc3ccccc32)CC1	997041665	24	324.22	3.44
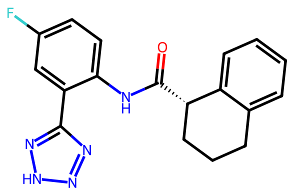	O=C(Nc1ccc(F)cc1-c1nn[nH]n1)[C@H]1CCCc2ccccc21	823344580	25	337.13	3.06
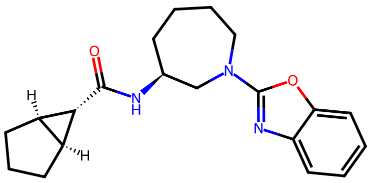	O=C(N[C@H]1CCCCN(c2nc3ccccc3o2)C1)[C@@H]1[C@H]2CCC[C@H]21	1063182067	25	339.19	3.35
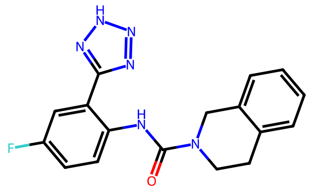	O=C(Nc1ccc(F)cc1-c1nn[nH]n1)N1CCc2ccccc2C1	1593234051	25	338.13	2.6

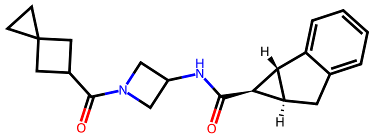	O=C(NC1CN(C(=O)C2CC3(CC3)C2)C1)[C@@H]1[C@@H]2Cc3ccccc3[C@H]21	991645994	25	336.18	2.09
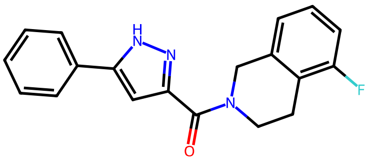	O=C(c1cc(-c2ccccc2)[nH]n1)N1CCc2c(F)cccc2C1	339844099	24	321.13	3.41
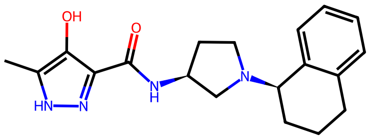	Cc1[nH]nc(C(=O)N[C@H]2CCN([C@@H]3CCCc4ccccc43)C2)c1O	1010645057	25	340.19	2.31
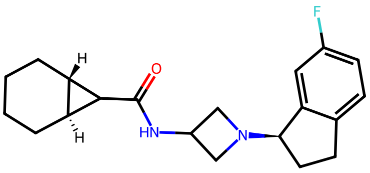	O=C(NC1CN([C@@H]2CCc3ccc(F)cc32)C1)C1[C@H]2CCCC[C@H]12	1030185902	24	328.2	3.05
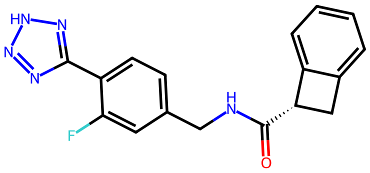	O=C(NCc1ccc(-c2nn[nH]n2)c(F)c1)[C@H]1Cc2ccccc21	1582545730	24	323.12	1.96
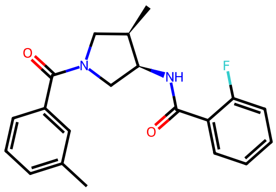	Cc1cccc(C(=O)N2C[C@@H](C)[C@@H](NC(=O)c3ccccc3F)C2)c1	966118390	25	340.16	3.02
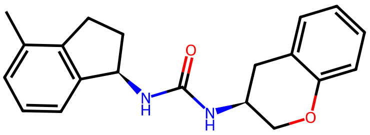	Cc1cccc2c1CC[C@H]2NC(=O)N[C@@H]1COc2ccccc2C1	517916003	24	322.17	3.29
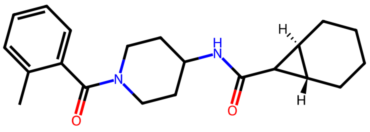	Cc1ccccc1C(=O)N1CCC(NC(=O)C2[C@H]3CCCC[C@H]23)CC1	943772872	25	340.22	3.15

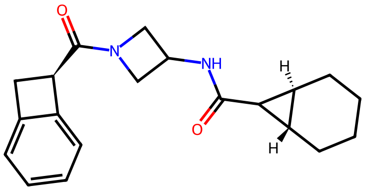	O=C(NC1CN(C(=O)[C@@H]2Cc3ccccc32)C1)C1[C@H]2CCCC[C@H]12	990649273	24	324.18	2.09
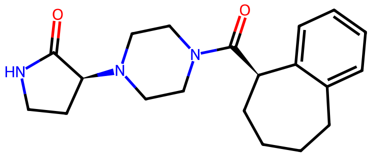	O=C1NCC[C@@H]1N1CCN(C(=O)[C@@H]2CCCCc3ccccc32)CC1	957819262	25	341.21	1.53
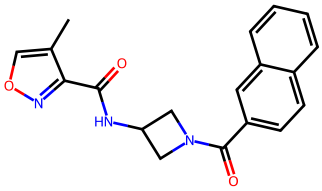	Cc1conc1C(=O)NC1CN(C(=O)c2ccc3ccccc3c2)C1	999666663	25	335.13	2.39
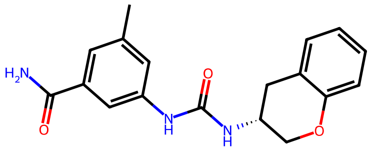	Cc1cc(NC(=O)N[C@H]2COc3ccccc3C2)cc(C(N)=O)c1	1365202069	24	325.14	2.22
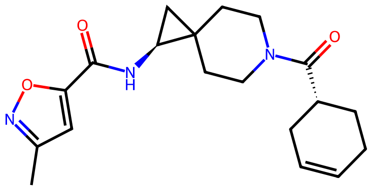	Cc1cc(C(=O)N[C@H]2CC23CCN(C(=O)[C@H]2CC=CCC2)CC3)on1	963022806	25	343.19	2.45
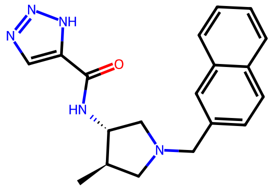	C[C@@H]1CN(Cc2ccc3ccccc3c2)C[C@H]1NC(=O)c1cnn[nH]1	1080029914	25	335.17	2.21
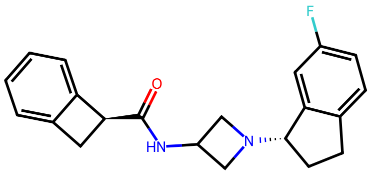	O=C(NC1CN([C@H]2CCc3ccc(F)cc32)C1)[C@H]1Cc2ccccc21	1030881225	25	336.16	2.95
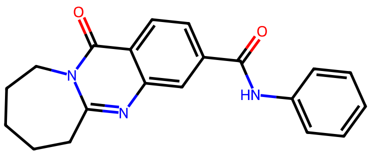	O=C(Nc1ccccc1)c1ccc2c(=O)n3c(nc2c1)CCCCC3	5558138	25	333.15	3.38

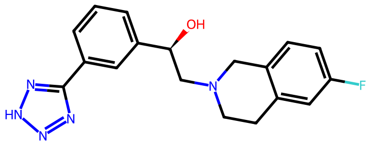	O[C@@H](CN1CCc2cc(F)ccc2C1)c1cccc(-c2nn[nH]n2)c1	1582809682	25	339.15	2.1
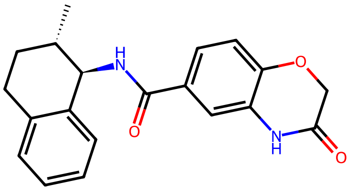	C[C@H]1CCc2ccccc2[C@@H]1NC(=O)c1ccc2c(c1)NC(=O)CO2	122492319	25	336.15	3.07
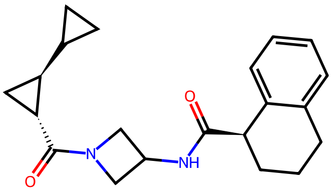	O=C(NC1CN(C(=O)[C@@H]2C[C@H]2C2CC2)C1)[C@@H]1CCCc2ccccc21	995075702	25	338.2	2.48
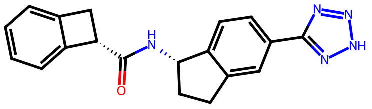	O=C(N[C@H]1CCc2cc(-c3nn[nH]n3)ccc21)[C@H]1Cc2ccccc21	1609543186	25	331.14	2.31
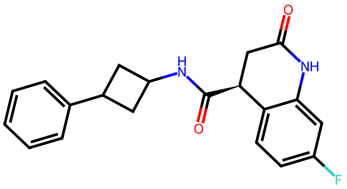	O=C1C[C@H](C(=O)NC2CC(c3ccccc3)C2)c2ccc(F)cc2N1	264006817	25	338.14	3.31
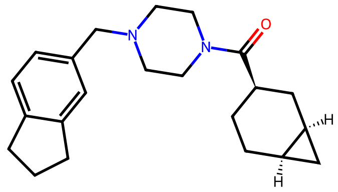	O=C([C@@H]1CC[C@@H]2C[C@@H]2C1)N1CCN(Cc2ccc3c(c2)CCC3)CC1	948952249	25	338.24	3.26
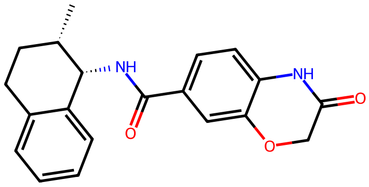	C[C@H]1CCc2ccccc2[C@H]1NC(=O)c1ccc2c(c1)OCC(=O)N2	179463732	25	336.15	3.07
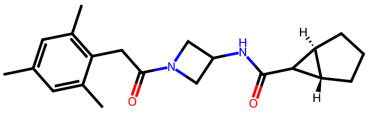	Cc1cc(C)c(CC(=O)N2CC(NC(=O)C3[C@H]4CCC[C@H]34)C2)c(C)c1	990690075	25	340.22	2.53

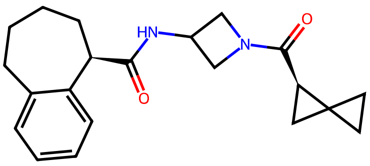	O=C(NC1CN(C(=O)[C@@H]2CC23CC3)C1)[C@@H]1CCCCc2ccccc21	994322762	25	338.2	2.62
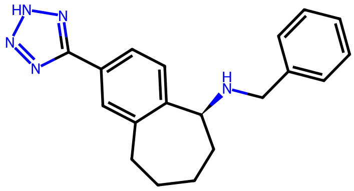	c1ccc(CN[C@H]2CCCCc3cc(-c4nn[nH]n4)ccc32)cc1	1609617305	24	319.18	3.42
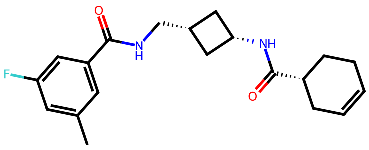	Cc1cc(F)cc(C(=O)NC[C@H]2C[C@@H](NC(=O)[C@H]3CC=CCC3)C2)c1	979359249	25	344.19	3.12
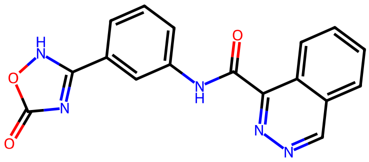	O=C(Nc1cccc(-c2nc(=O)o[nH]2)c1)c1nncc2ccccc12	1536712558	25	333.09	2.23
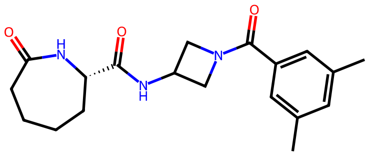	Cc1cc(C)cc(C(=O)N2CC(NC(=O)[C@@H]3CCCCC(=O)N3)C2)c1	999368348	25	343.19	1.3
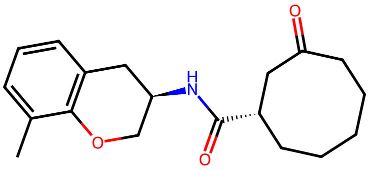	Cc1cccc2c1OC[C@H](NC(=O)[C@H]1CCCCCC(=O)C1)C2	517228906	23	315.18	2.95
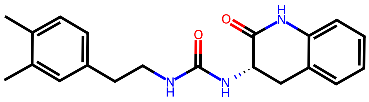	Cc1ccc(CCNC(=O)N[C@H]2Cc3ccccc3NC2=O)cc1C	1414694723	25	337.18	2.71
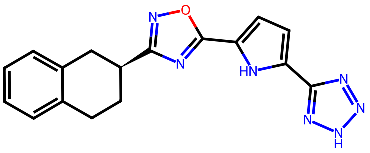	c1ccc2c(c1)CC[C@H](c1noc(-c3ccc(-c4nn[nH]n4)[nH]3)n1)C2	1607132324	25	333.13	2.52

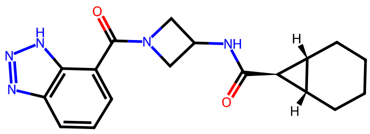	O=C(NC1CN(C(=O)c2cccc3nn[nH]c23)C1)[C@@H]1[C@H]2CCCC[C@H]21	990651875	25	339.17	1.33
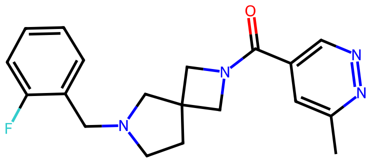	Cc1cc(C(=O)N2CC3(CCN(Cc4ccccc4F)C3)C2)cnn1	1072665493	25	340.17	2.27
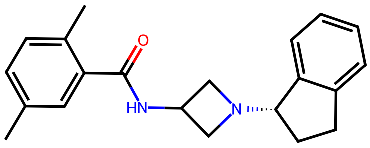	Cc1ccc(C)c(C(=O)NC2CN([C@H]3CCc4ccccc43)C2)c1	1031308127	24	320.19	3.4
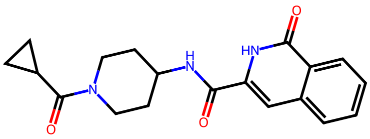	O=C(NC1CCN(C(=O)C2CC2)CC1)c1cc2ccccc2c(=O)[nH]1	43505717	25	339.16	1.66
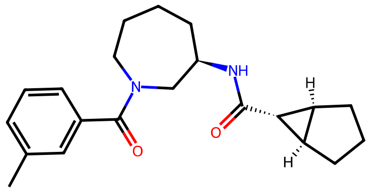	Cc1cccc(C(=O)N2CCCC[C@@H](NC(=O)[C@@H]3[C@H]4CCC[C@H]43)C2)c1	943169644	25	340.22	3.15
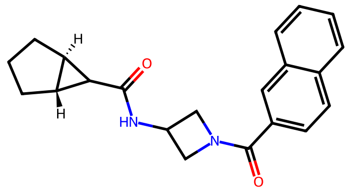	O=C(NC1CN(C(=O)c2ccc3ccccc3c2)C1)C1[C@@H]2CCC[C@@H]12	990691236	25	334.17	2.83
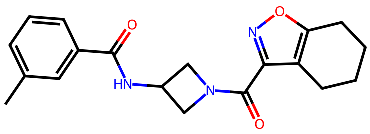	Cc1cccc(C(=O)NC2CN(C(=O)c3noc4c3CCCC4)C2)c1	990137044	25	339.16	2.12
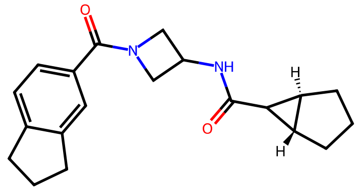	O=C(NC1CN(C(=O)c2ccc3c(c2)CCC3)C1)C1[C@H]2CCC[C@H]12	990707393	24	324.18	2.16

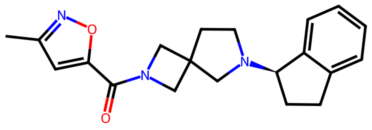	Cc1cc(C(=O)N2CC3(CCN([C@@H]4CCc5ccccc54)C3)C2)on1	1072462439	25	337.18	2.82
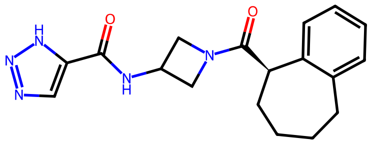	O=C(NC1CN(C(=O)[C@@H]2CCCCc3ccccc32)C1)c1cnn[nH]1	995177714	25	339.17	1.26
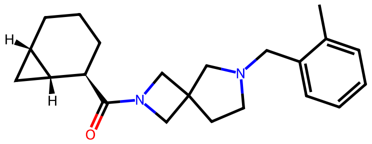	Cc1ccccc1CN1CCC2(C1)CN(C(=O)[C@@H]1CCC[C@H]3C[C@H]31)C2	1072874522	25	338.24	3.47
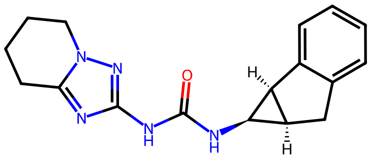	O=C(Nc1nc2n(n1)CCCC2)N[C@@H]1[C@@H]2Cc3ccccc3[C@@H]21	653706408	23	309.16	2.07
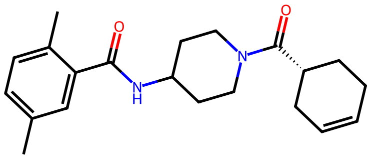	Cc1ccc(C)c(C(=O)NC2CCN(C(=O)[C@H]3CC=CCC3)CC2)c1	944106196	25	340.22	3.38
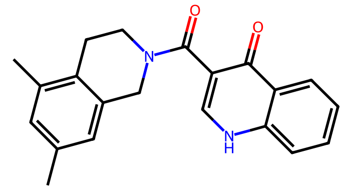	Cc1cc(C)c2c(c1)CN(C(=O)c1c[nH]c3ccccc3c1=O)CC2	188653511	25	332.15	3.34
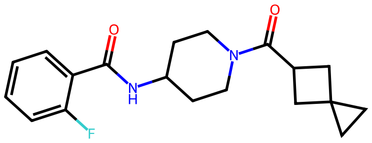	O=C(NC1CCN(C(=O)C2CC3(CC3)C2)CC1)c1ccccc1F	948300383	24	330.17	2.74
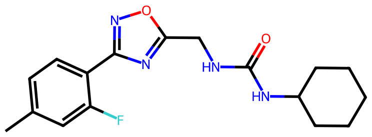	Cc1ccc(-c2noc(CNC(=O)NC3CCCCC3)n2)c(F)c1	273988112	24	332.16	3.32

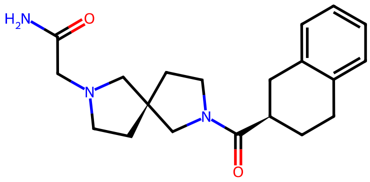	NC(=O)CN1CC[C@]2(CCN(C(=O)[C@@H]3CCc4ccccc4C3)C2)C1	1041710111	25	341.21	1.2
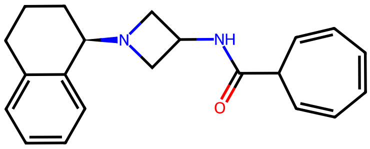	O=C(NC1CN([C@@H]2CCCc3ccccc32)C1)C1C=CC=CC=C1	1030382301	24	320.19	3.16
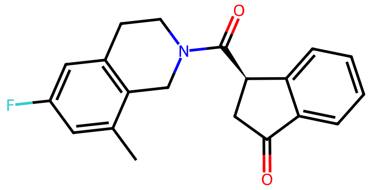	Cc1cc(F)cc2c1CN(C(=O)[C@@H]1CC(=O)c3ccccc31)CC2	337329418	24	323.13	3.39
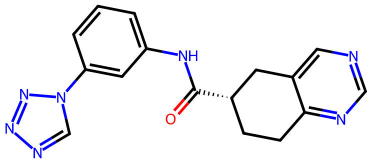	O=C(Nc1cccc(-n2cnnn2)c1)[C@H]1CCc2ncncc2C1	648220223	24	321.13	1.2
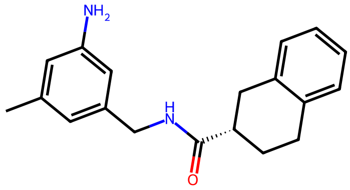	Cc1cc(N)cc(CNC(=O)[C@H]2CCc3ccccc3C2)c1	399044199	22	294.17	3.0
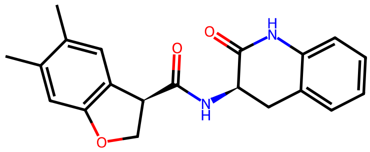	Cc1cc2c(cc1C)[C@@H](C(=O)N[C@@H]1Cc3ccccc3NC1=O)CO2	529946541	25	336.15	2.46
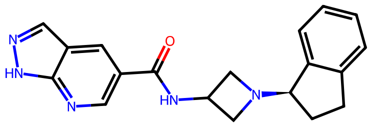	O=C(NC1CN([C@@H]2CCc3ccccc32)C1)c1cnc2[nH]ncc2c1	1030499652	25	333.16	2.06
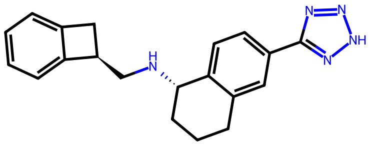	c1ccc2c(c1)C[C@H]2CN[C@H]1CCCc2cc(-c3nn[nH]n3)ccc21	826523333	25	331.18	3.17

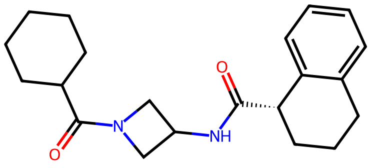	O=C(NC1CN(C(=O)C2CCCCC2)C1)[C@H]1CCCc2ccccc21	995068207	25	340.22	3.01
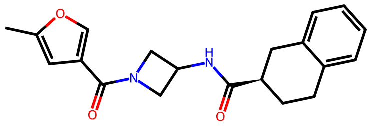	Cc1cc(C(=O)N2CC(NC(=O)[C@@H]3CCc4ccccc4C3)C2)co1	1000537669	25	338.16	2.33
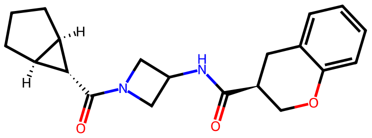	O=C(NC1CN(C(=O)[C@@H]2[C@H]3CCC[C@H]32)C1)[C@@H]1COc2ccccc2C1	995466377	25	340.18	1.61
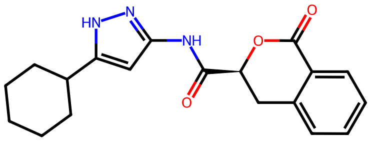	O=C1O[C@H](C(=O)Nc2cc(C3CCCCC3)[nH]n2)Cc2ccccc21	735929181	25	339.16	3.18
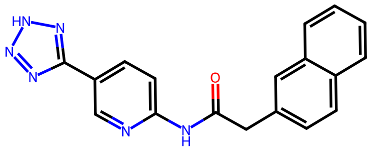	O=C(Cc1ccc2ccccc2c1)Nc1ccc(-c2nn[nH]n2)cn1	737966944	25	330.12	2.6
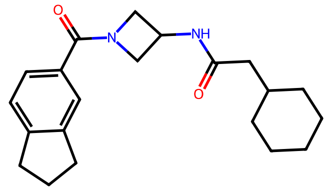	O=C(CC1CCCCC1)NC1CN(C(=O)c2ccc3c(c2)CCC3)C1	990170050	25	340.22	3.09
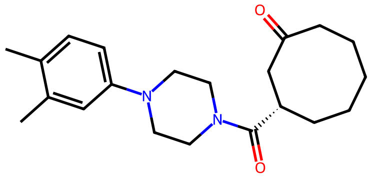	Cc1ccc(N2CCN(C(=O)[C@H]3CCCCCC(=O)C3)CC2)cc1C	813500357	25	342.23	3.49
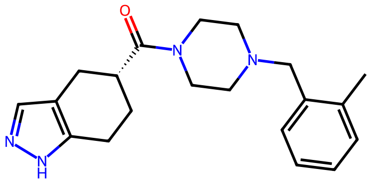	Cc1ccccc1CN1CCN(C(=O)[C@@H]2CCc3[nH]ncc3C2)CC1	270099181	25	338.21	2.17

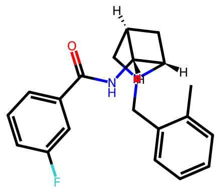	Cc1ccccc1CN1C[C@H]2C[C@H]1[C@@H]2NC(=O)c1cccc(F)c1	1037286837	24	324.16	3.14
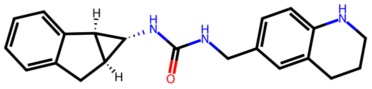	O=C(NCc1ccc2c(c1)CCCN2)N[C@@H]1[C@H]2Cc3ccccc3[C@H]21	572264078	25	333.18	3.18
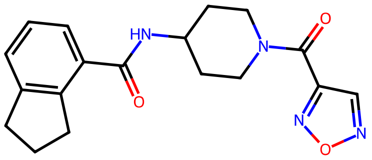	O=C(NC1CCN(C(=O)c2cnon2)CC1)c1cccc2c1CCC2	947970481	25	340.15	1.59
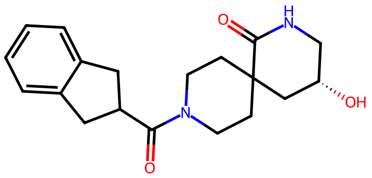	O=C(C1Cc2ccccc2C1)N1CCC2(CC1)C[C@@H](O)CNC2=O	885016147	24	328.18	0.89
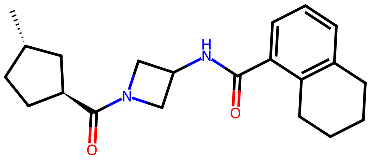	C[C@H]1CC[C@H](C(=O)N2CC(NC(=O)c3cccc4c3CCCC4)C2)C1	991904027	25	340.22	2.94
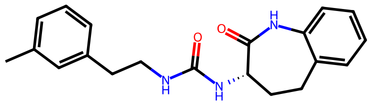	Cc1cccc(CCNC(=O)N[C@H]2CCc3ccccc3NC2=O)c1	654413784	25	337.18	2.79
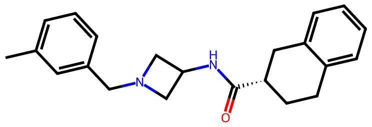	Cc1cccc(CN2CC(NC(=O)[C@H]3CCc4ccccc4C3)C2)c1	1031012443	25	334.2	3.1
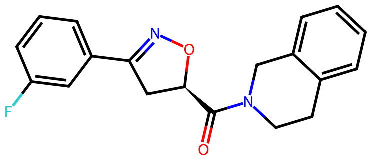	O=C([C@H]1CC(c2cccc(F)c2)=NO1)N1CCc2ccccc2C1	915045123	24	324.13	2.9

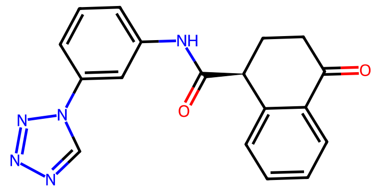	O=C1CC[C@H](C(=O)Nc2cccc(-n3cnnn3)c2)c2ccccc21	339836156	25	333.12	2.36
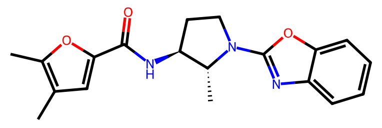	Cc1cc(C(=O)N[C@H]2CCN(c3nc4ccccc4o3)[C@@H]2C)oc1C	1050165251	25	339.16	3.43
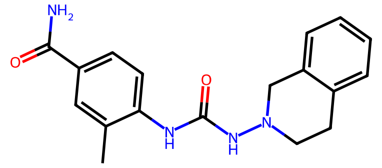	Cc1cc(C(N)=O)ccc1NC(=O)NN1CCc2ccccc2C1	1425222469	24	324.16	2.19
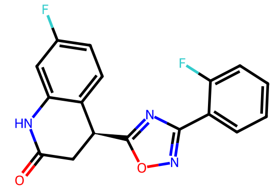	O=C1C[C@H](c2nc(-c3ccccc3F)no2)c2ccc(F)cc2N1	371925155	24	327.08	3.49
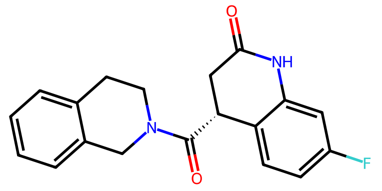	O=C1C[C@@H](C(=O)N2CCc3ccccc3C2)c2ccc(F)cc2N1	12939075	24	324.13	2.84
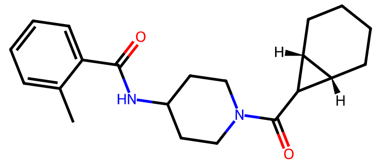	Cc1ccccc1C(=O)NC1CCN(C(=O)C2[C@H]3CCCC[C@@H]23)CC1	73698672	25	340.22	3.15
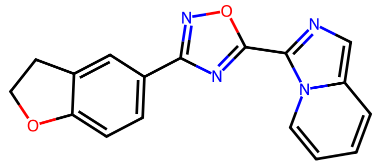	c1ccn2c(-c3nc(-c4ccc5c(c4)CCO5)no3)ncc2c1	904692049	23	304.1	2.99
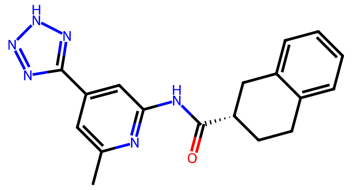	Cc1cc(-c2nn[nH]n2)cc(NC(=O)[C@H]2CCc3ccccc3C2)n1	1605529011	25	334.15	2.31

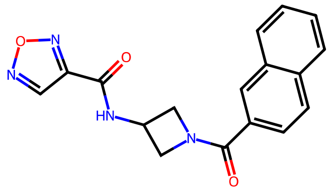	O=C(NC1CN(C(=O)c2ccc3ccccc3c2)C1)c1cnon1	998227611	24	322.11	1.48
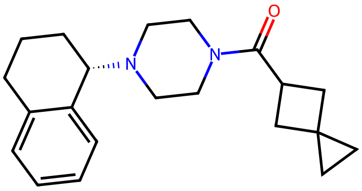	O=C(C1CC2(CC2)C1)N1CCN([C@H]2CCCc3ccccc32)CC1	950426505	24	324.22	3.4
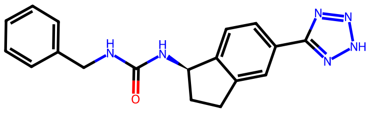	O=C(NCc1ccccc1)N[C@@H]1CCc2cc(-c3nn[nH]n3)ccc21	1593143913	25	334.15	2.35
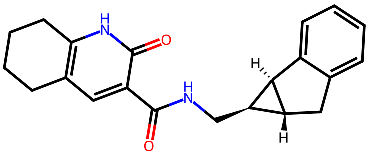	O=C(NC[C@@H]1[C@H]2Cc3ccccc3[C@@H]21)c1cc2c([nH]c1=O)CCCC2	152424437	25	334.17	2.57
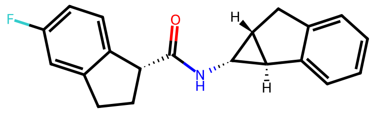	O=C(N[C@H]1[C@@H]2c3ccccc3C[C@H]21)[C@@H]1CCc2cc(F)ccc21	612495121	23	307.14	3.31
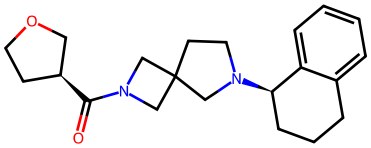	O=C([C@H]1CCOC1)N1CC2(CCN([C@@H]3CCCc4ccccc43)C2)C1	1072541616	25	340.22	2.63
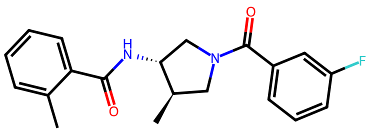	Cc1ccccc1C(=O)N[C@@H]1CN(C(=O)c2cccc(F)c2)C[C@H]1C	947284456	25	340.16	3.02
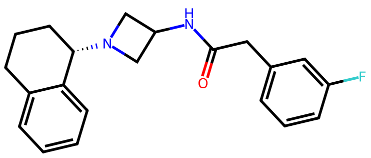	O=C(Cc1cccc(F)c1)NC1CN([C@H]2CCCc3ccccc32)C1	1031352775	25	338.18	3.25

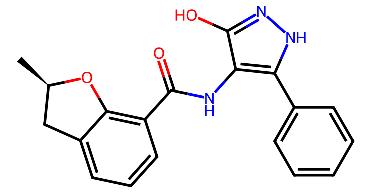	C[C@@H]1Cc2cccc(C(=O)Nc3c(O)n[nH]c3-c3ccccc3)c2O1	1298438533	25	335.13	3.36
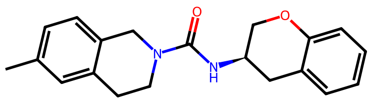	Cc1ccc2c(c1)CCN(C(=O)N[C@H]1COc3ccccc3C1)C2	478814388	24	322.17	3.07
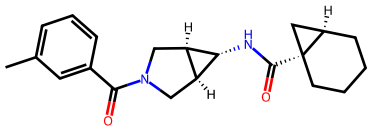	Cc1cccc(C(=O)N2C[C@H]3[C@H](NC(=O)[C@@]45CCCC[C@@H]4C5)[C@H]3C2)c1	977154240	25	338.2	2.76
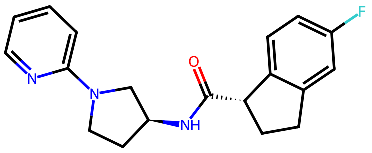	O=C(N[C@H]1CCN(c2ccccn2)C1)[C@H]1CCc2cc(F)ccc21	612904286	24	325.16	2.65
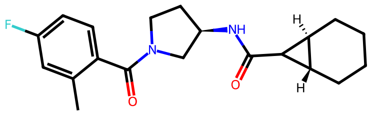	Cc1cc(F)ccc1C(=O)N1CC[C@@H](NC(=O)C2[C@H]3CCCC[C@H]23)C1	939894976	25	344.19	2.9
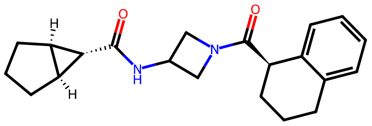	O=C(NC1CN(C(=O)[C@@H]2CCCc3ccccc32)C1)[C@@H]1[C@H]2CCC[C@H]21	990708377	25	338.2	2.48
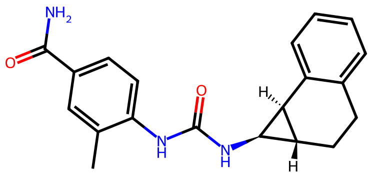	Cc1cc(C(N)=O)ccc1NC(=O)N[C@@H]1[C@H]2CCc3ccccc3[C@@H]21	772418281	25	335.16	2.94
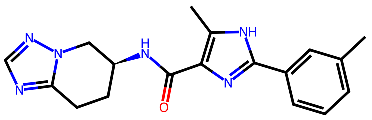	Cc1cccc(-c2nc(C(=O)N[C@H]3CCc4ncnn4C3)c(C)[nH]2)c1	270259547	25	336.17	2.03

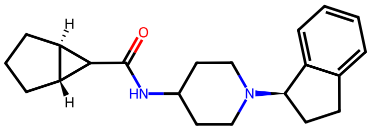	O=C(NC1CCN([C@@H]2CCc3ccccc3 2)CC1)C1[C@@H]2CCC[C@@H]12	1002545290	24	324.22	3.3
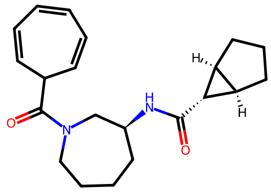	O=C(N[C@H]1CCCCN(C(=O)C2C=C C=CC=C2)C1)[C@@H]1[C@H]2CCC[C@H]21	943164486	25	340.22	2.83
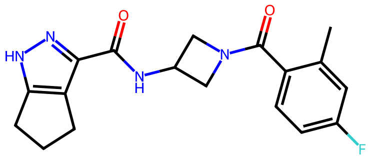	Cc1cc(F)ccc1C(=O)N1CC(NC(=O)c2n [nH]c3c2CCC3)C1	995171623	25	342.15	1.6
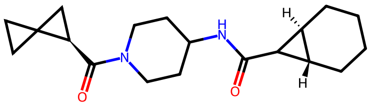	O=C(NC1CCN(C(=O)[C@@H]2CC23 CC3)CC1)C1[C@H]2CCCC[C@@H]12	948562706	23	316.22	2.33
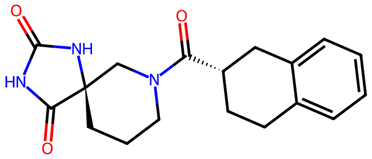	O=C1NC(=O)[C@]2(CCCN(C(=O)[C @H]3CCc4ccccc4C3)C2)N1	392626368	24	327.16	0.99
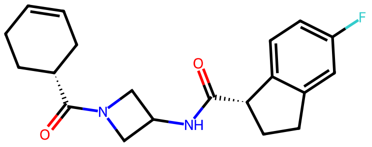	O=C(NC1CN(C(=O)[C@H]2CC=CCC 2)C1)[C@H]1CCc2cc(F)ccc21	996094487	25	342.17	2.54
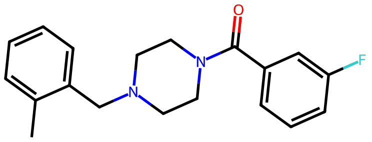	Cc1ccccc1CN1CCN(C(=O)c2cccc(F)c 2)CC1	19237222	23	312.16	3.09
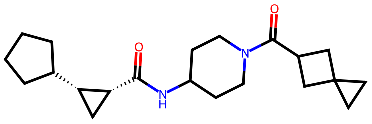	O=C(NC1CCN(C(=O)C2CC3(CC3)C2) CC1)[C@@H]1C[C@@H]1C1CCCC1	948308719	25	344.25	3.11

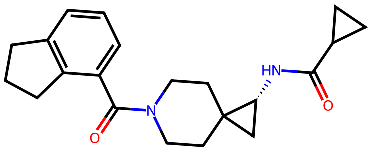	O=C(N[C@@H]1CC12CCN(C(=O)c1cccc3c1CCC3)CC2)C1CC1	962425918	25	338.2	2.7
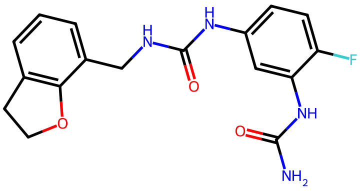	NC(=O)Nc1cc(NC(=O)NCc2cccc3c2OCC3)ccc1F	1413585283	25	344.13	2.57
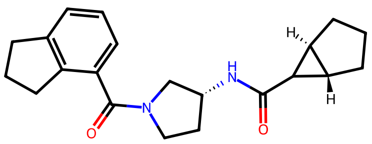	O=C(N[C@@H]1CCN(C(=O)c2cccc3c2CCC3)C1)C1[C@H]2CCC[C@H]12	939930786	25	338.2	2.55
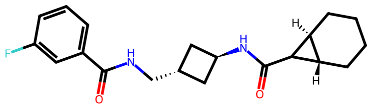	O=C(NC[C@H]1C[C@H](NC(=O)C2[C@H]3CCCC[C@H]23)C1)c1cccc(F)c1	979412184	25	344.19	2.89
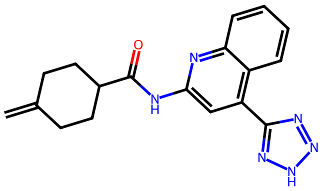	C=C1CCC(C(=O)Nc2cc(-c3nn[nH]n3)c3ccccc3n2)CC1	1588462037	25	334.15	3.1
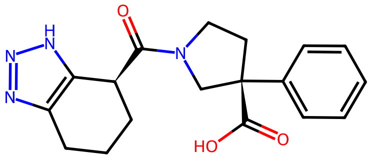	O=C([C@H]1CCCc2nn[nH]c21)N1CC[C@](C(=O)O)(c2ccccc2)C1	909857204	25	340.15	1.48
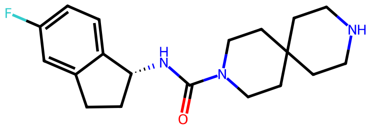	O=C(N[C@@H]1CCc2cc(F)ccc21)N1CCC2(CCNCC2)CC1	417273324	24	331.21	2.99
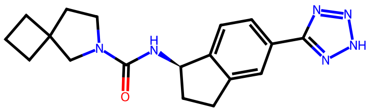	O=C(N[C@@H]1CCc2cc(-c3nn[nH]n3)ccc21)N1CCC2(CCC2)C1	1606684006	25	338.19	2.44

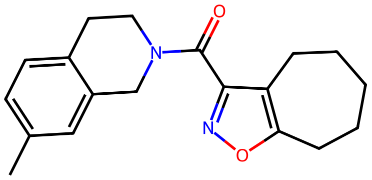	Cc1ccc2c(c1)CN(C(=O)c1noc3c1CCCCC3)CC2	617605598	23	310.17	3.45
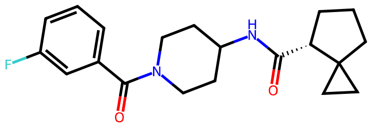	O=C(NC1CCN(C(=O)c2cccc(F)c2)CC1)[C@@H]1CCCC12CC2	943789676	25	344.19	3.13
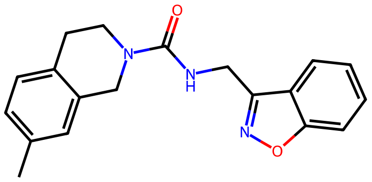	Cc1ccc2c(c1)CN(C(=O)NCc1noc3ccccc13)CC2	852405625	24	321.15	3.4
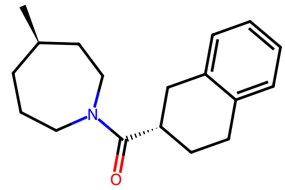	C[C@@H]1CCCN(C(=O)[C@H]2CCc3ccccc3C2)CC1	569008710	20	271.19	3.44
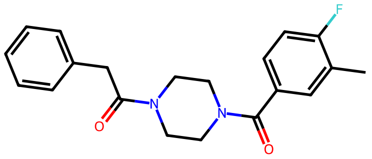	Cc1cc(C(=O)N2CCN(C(=O)Cc3ccccc3)CC2)ccc1F	75028197	25	340.16	2.66
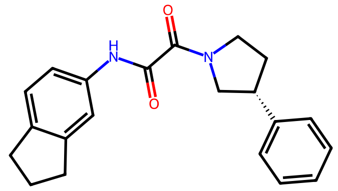	O=C(Nc1ccc2c(c1)CCC2)C(=O)N1CC[C@H](c2ccccc2)C1	126071099	25	334.17	3.13
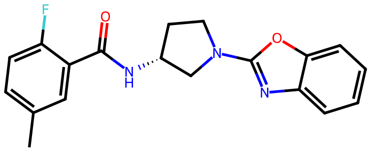	Cc1ccc(F)c(C(=O)N[C@@H]2CCN(c3nc4ccccc4o3)C2)c1	1058559899	25	339.14	3.28
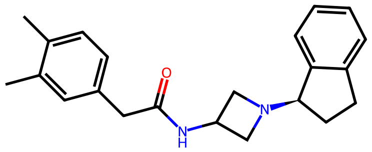	Cc1ccc(CC(=O)NC2CN([C@@H]3CCc4ccccc43)C2)cc1C	1031357547	25	334.2	3.33

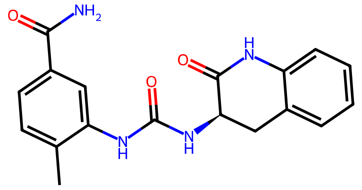	Cc1ccc(C(N)=O)cc1NC(=O)N[C@@H]1Cc2ccccc2NC1=O	1364252575	25	338.14	1.78
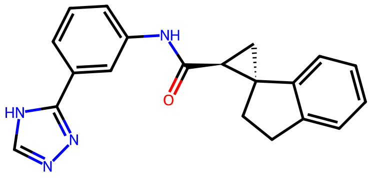	O=C(Nc1cccc(-c2nnc[nH]2)c1)[C@H]1C[C@]12CCc1ccccc12	266489977	25	330.15	3.31
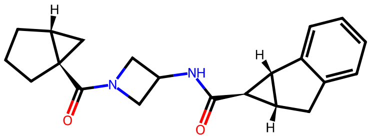	O=C(NC1CN(C(=O)[C@@]23CCC[C@@H]2C3)C1)[C@@H]1[C@H]2Cc3cccc3[C@H]21	991644157	25	336.18	2.09
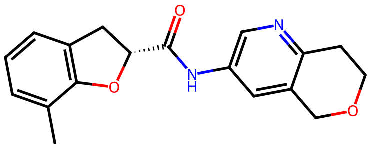	Cc1cccc2c1O[C@@H](C(=O)Nc1cnc3c(c1)COCC3)C2	649409279	23	310.13	2.4
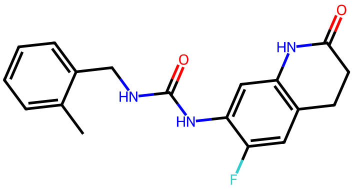	Cc1ccccc1CNC(=O)Nc1cc2c(cc1F)CCC(=O)N2	359626196	24	327.14	3.34
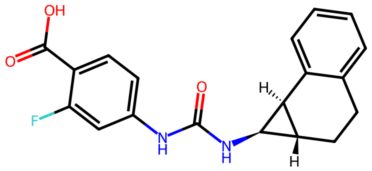	O=C(Nc1ccc(C(=O)O)c(F)c1)N[C@@H]1[C@H]2CCc3ccccc3[C@@H]21	833153662	25	340.12	3.37
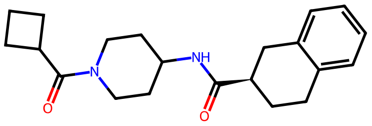	O=C(NC1CCN(C(=O)C2CCC2)CC1)[C@@H]1CCc2ccccc2C1	943344871	25	340.22	2.7
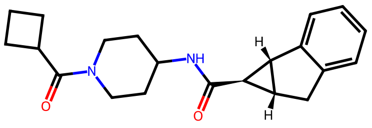	O=C(NC1CCN(C(=O)C2CCC2)CC1)[C@@H]1[C@H]2Cc3ccccc3[C@H]21	943344672	25	338.2	2.48

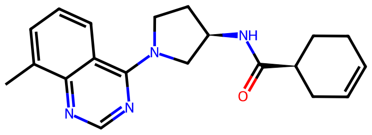	Cc1cccc2c(N3CC[C@@H](NC(=O)[C@@H]4CC=CCC4)C3)ncnc12	1058297483	25	336.2	2.99
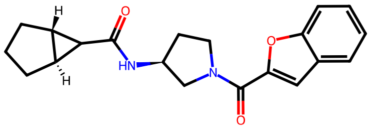	O=C(N[C@H]1CCN(C(=O)c2cc3ccccc3o2)C1)C1[C@H]2CCC[C@H]12	937805188	25	338.16	2.81
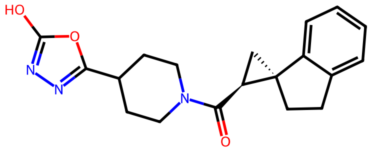	O=C([C@H]1C[C@]12CCc1ccccc12)N1CCC(c2nnc(O)o2)CC1	583080679	25	339.16	2.39
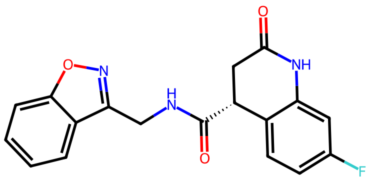	O=C1C[C@@H](C(=O)NCc2noc3ccccc23)c2ccc(F)cc2N1	565867239	25	339.1	2.71
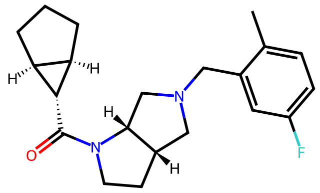	Cc1ccc(F)cc1CN1C[C@@H]2CCN(C(=O)[C@@H]3[C@H]4CCC[C@H]43)[C@@H]2C1	1075657313	25	342.21	3.21
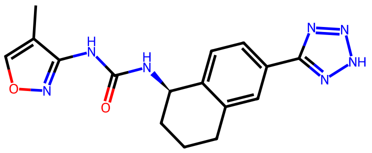	Cc1conc1NC(=O)N[C@@H]1CCCc2cc(-c3nn[nH]n3)ccc21	1605999003	25	339.14	2.36
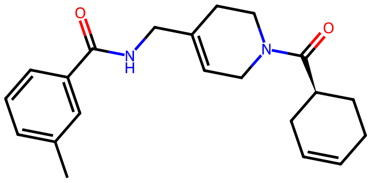	Cc1cccc(C(=O)NCC2=CCN(C(=O)[C@@H]3CC=CCC3)CC2)c1	1017268002	25	338.2	3.24
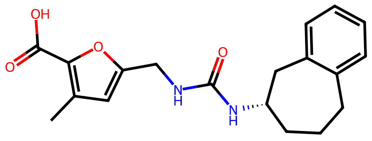	Cc1cc(CNC(=O)N[C@H]2CCCc3ccccc3C2)oc1C(=O)O	1600199304	25	342.16	3.03

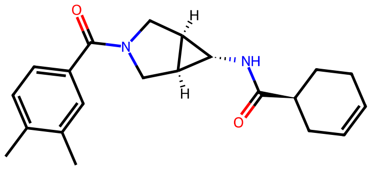	Cc1ccc(C(=O)N2C[C@H]3[C@H](NC(=O)[C@@H]4CC=CCC4)[C@H]3C2)cc1C	976173946	25	338.2	2.85
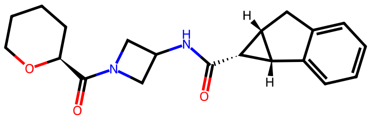	O=C(NC1CN(C(=O)[C@@H]2CCCCO2)C1)[C@H]1[C@H]2c3ccccc3C[C@H]21	991644331	25	340.18	1.47
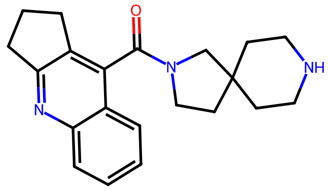	O=C(c1c2c(nc3ccccc13)CCC2)N1CCC2(CCNCC2)C1	358519037	25	335.2	2.94
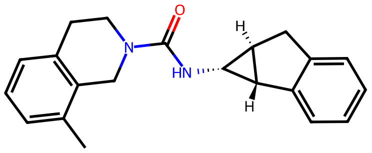	Cc1cccc2c1CN(C(=O)N[C@@H]1[C@H]3Cc4ccccc4[C@@H]31)CC2	429102462	24	318.17	3.4
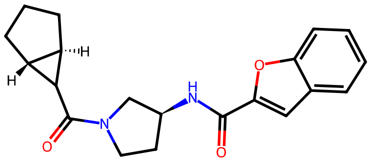	O=C(N[C@H]1CCN(C(=O)C2[C@@H]3CCC[C@@H]23)C1)c1cc2ccccc2o1	938870362	25	338.16	2.81
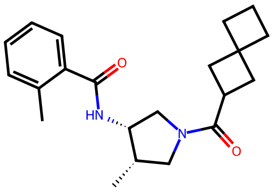	Cc1ccccc1C(=O)N[C@@H]1CN(C(=O)C2CC3(CCC3)C2)C[C@@H]1C	966688058	25	340.22	3.15
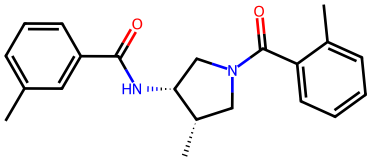	Cc1cccc(C(=O)N[C@@H]2CN(C(=O)c3ccccc3C)C[C@@H]2C)c1	966095717	25	336.18	3.19
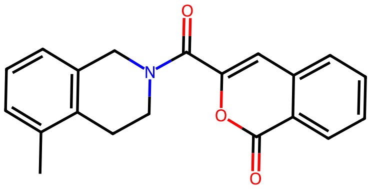	Cc1cccc2c1CCN(C(=O)c1cc3ccccc3c(=O)o1)C2	797721588	24	319.12	3.3

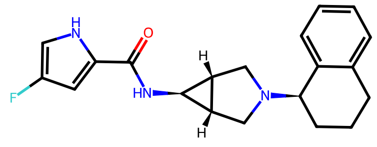	O=C(N[C@H]1[C@@H]2CN([C@@H]3CCCc4ccccc43)C[C@@H]21)c1cc(F)c[nH]1	961232253	25	339.17	2.89
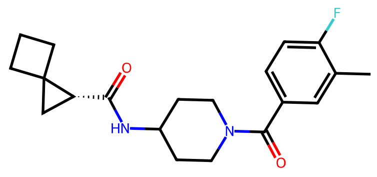	Cc1cc(C(=O)N2CCC(NC(=O)[C@@H]3CC34CCC4)CC2)ccc1F	948065682	25	344.19	3.05
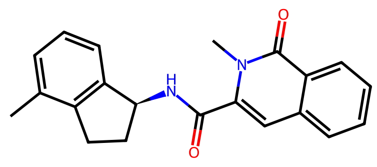	Cc1cccc2c1CC[C@@H]2NC(=O)c1cc2ccccc2c(=O)n1C	556820121	25	332.15	3.26
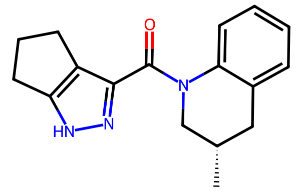	C[C@H]1Cc2ccccc2N(C(=O)c2n[nH]c3c2CCC3)C1	104523415	21	281.15	2.74
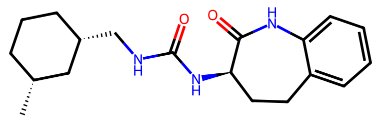	C[C@@H]1CCC[C@H](CNC(=O)N[C@@H]2CCc3ccccc3NC2=O)C1	1563768275	24	329.21	3.07
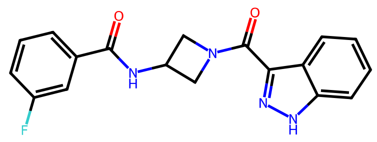	O=C(NC1CN(C(=O)c2n[nH]c3ccccc23)C1)c1cccc(F)c1	990119505	25	338.12	1.96
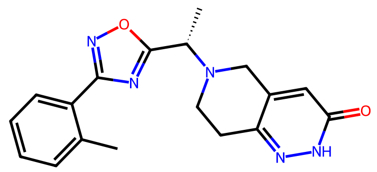	Cc1ccccc1-c1noc([C@H](C)N2CCc3n[nH]c(=O)cc3C2)n1	274982582	25	337.15	2.25
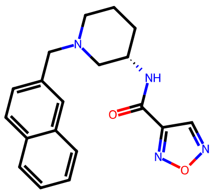	O=C(N[C@H]1CCCN(Cc2ccc3ccccc3c2)C1)c1cnon1	1008931721	25	336.16	2.62

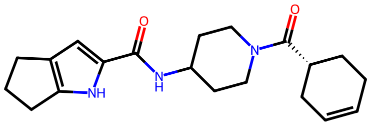	O=C(NC1CCN(C(=O)[C@H]2CC=CCC2)CC1)c1cc2c([nH]1)CCC2	944114341	25	341.21	2.58
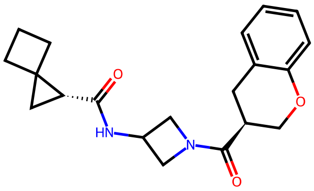	O=C(NC1CN(C(=O)[C@@H]2COc3ccccc3C2)C1)[C@@H]1CC12CCC2	991556945	25	340.18	1.75
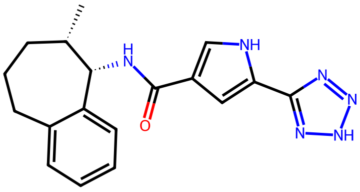	C[C@H]1CCCc2ccccc2[C@H]1NC(=O)c1c[nH]c(-c2nn[nH]n2)c1	1590263698	25	336.17	2.64
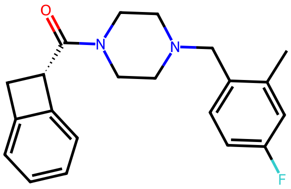	Cc1cc(F)ccc1CN1CCN(C(=O)[C@H]2Cc3ccccc32)CC1	949958849	25	338.18	3.12
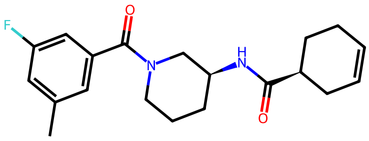	Cc1cc(F)cc(C(=O)N2CCC[C@H](NC(=O)[C@@H]3CC=CCC3)C2)c1	952126914	25	344.19	3.21
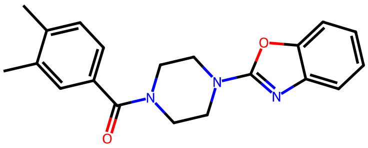	Cc1ccc(C(=O)N2CCN(c3nc4ccccc4o3)CC2)cc1C	172388485	25	335.16	3.41
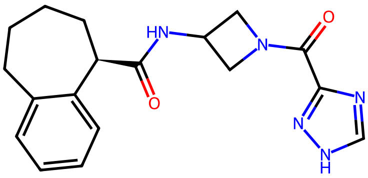	O=C(NC1CN(C(=O)c2nc[nH]n2)C1)[C@@H]1CCCCc2ccccc21	994322192	25	339.17	1.26
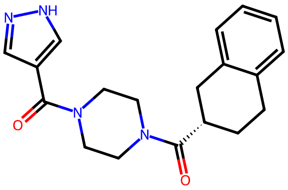	O=C(c1cn[nH]c1)N1CCN(C(=O)[C@H]2CCc3ccccc3C2)CC1	1010446605	25	338.17	1.5

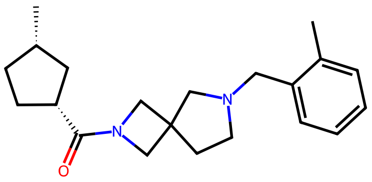	Cc1ccccc1CN1CCC2(C1)CN(C(=O)[C@@H]1CC[C@H](C)C1)C2	1073033271	24	326.24	3.47
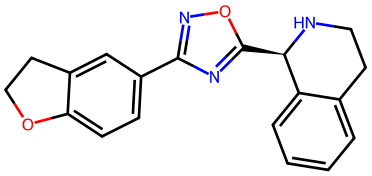	c1ccc2c(c1)CCN[C@@H]2c1nc(-c2ccc3c(c2)CCO3)no1	579584957	24	319.13	2.91
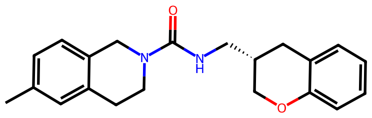	Cc1ccc2c(c1)CCN(C(=O)NC[C@H]1COc3ccccc3C1)C2	790125150	25	336.18	3.31
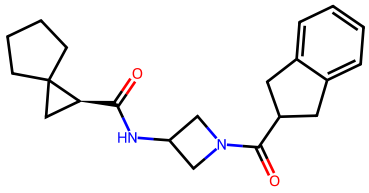	O=C(NC1CN(C(=O)C2Cc3ccccc3C2)C1)[C@H]1CC12CCCC2	999629669	25	338.2	2.31
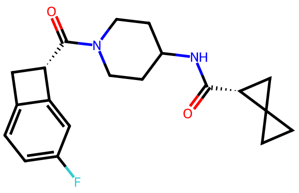	O=C(NC1CCN(C(=O)[C@H]2Cc3ccc(F)cc32)CC1)[C@@H]1CC12CC2	948026948	25	342.17	2.37
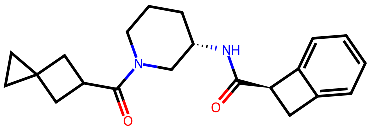	O=C(N[C@H]1CCCN(C(=O)C2CC3(CC3)C2)C1)[C@@H]1Cc2ccccc21	951964980	25	338.2	2.62
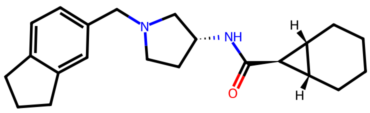	O=C(N[C@@H]1CCN(Cc2ccc3c(c2)CCC3)C1)[C@@H]1[C@H]2CCCC[C@H]21	1010439988	25	338.24	3.3
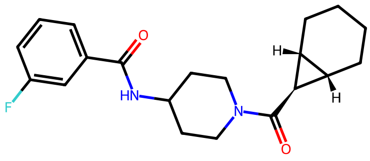	O=C(NC1CCN(C(=O)[C@@H]2[C@H]3CCCC[C@H]32)CC1)c1cccc(F)c1	945956143	25	344.19	2.98

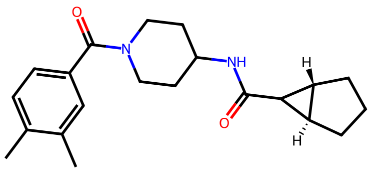	Cc1ccc(C(=O)N2CCC(NC(=O)C3[C@@H]4CCC[C@@H]34)CC2)cc1C	948347891	25	340.22	3.07
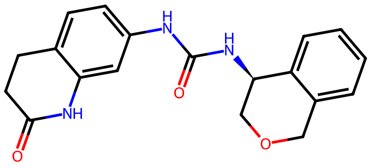	O=C1CCc2ccc(NC(=O)N[C@@H]3COCc4ccccc43)cc2N1	562792920	25	337.14	2.96
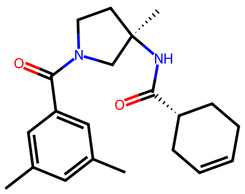	Cc1cc(C)cc(C(=O)N2CC[C@@](C)(NC(=O)[C@H]3CC=CCC3)C2)c1	956688347	25	340.22	3.38
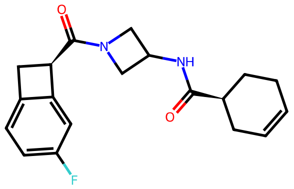	O=C(NC1CN(C(=O)[C@@H]2Cc3ccc(F)cc32)C1)[C@@H]1CC=CCC1	990200917	24	328.16	2.15
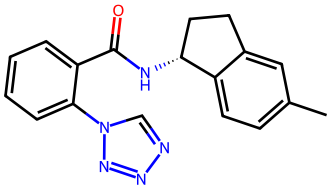	Cc1ccc2c(c1)CC[C@H]2NC(=O)c1ccccc1-n1cnnn1	342858902	24	319.14	2.39
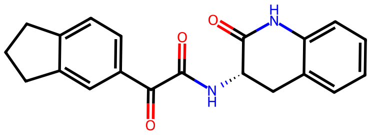	O=C(N[C@H]1Cc2ccccc2NC1=O)C(=O)c1ccc2c(c1)CCC2	529952617	25	334.13	2.04
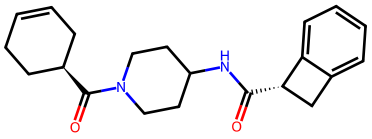	O=C(NC1CCN(C(=O)[C@@H]2CC=CCC2)CC1)[C@H]1Cc2ccccc21	944107488	25	338.2	2.79
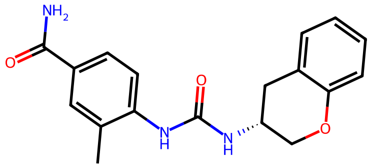	Cc1cc(C(N)=O)ccc1NC(=O)N[C@H]1COc2ccccc2C1	47887354	24	325.14	2.22

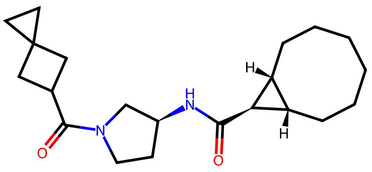	O=C(N[C@H]1CCN(C(=O)C2CC3(CC3)C2)C1)[C@@H]1[C@H]2CCCCCC[C@H]21	938980166	25	344.25	3.11
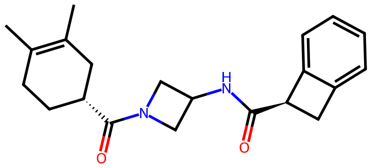	CC1=C(C)C[C@H](C(=O)N2CC(NC(=O)[C@@H]3Cc4ccccc43)C2)CC1	998686647	25	338.2	2.79
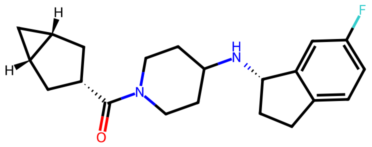	O=C([C@@H]1C[C@@H]2C[C@@H]2C1)N1CCC(N[C@H]2CCc3ccc(F)cc32)CC1	996911109	25	342.21	3.44
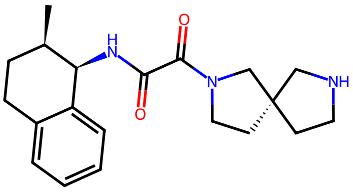	C[C@@H]1CCc2ccccc2[C@@H]1NC(=O)C(=O)N1CC[C@]2(CCNC2)C1	580764516	25	341.21	1.64
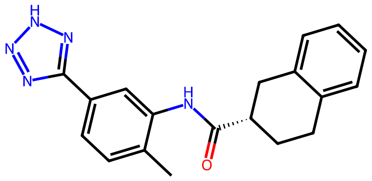	Cc1ccc(-c2nn[nH]n2)cc1NC(=O)[C@H]1CCc2ccccc2C1	1605795135	25	333.16	2.92
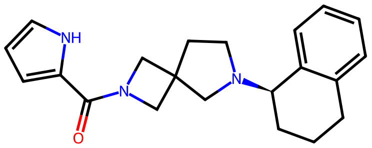	O=C(c1ccc[nH]1)N1CC2(CCN([C@@H]3CCCc4ccccc43)C2)C1	1072466020	25	335.2	3.24
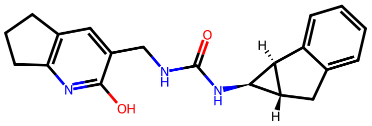	O=C(NCc1cc2c(nc1O)CCC2)N[C@@H]1[C@H]2Cc3ccccc3[C@@H]21	675712914	25	335.16	2.41
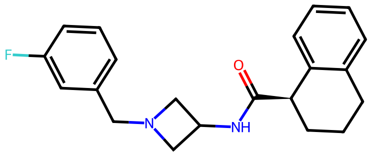	O=C(NC1CN(Cc2cccc(F)c2)C1)[C@@H]1CCCc2ccccc21	1030528645	25	338.18	3.25

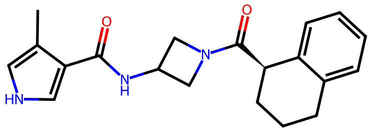	Cc1c[nH]cc1C(=O)NC1CN(C(=O)[C@@H]2CCCc3ccccc32)C1	991711932	25	337.18	2.38
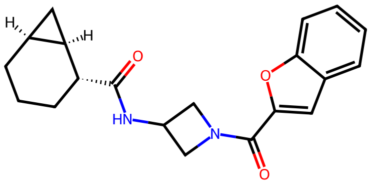	O=C(NC1CN(C(=O)c2cc3ccccc3o2)C1)[C@@H]1CCC[C@H]2C[C@H]21	994726078	25	338.16	2.81
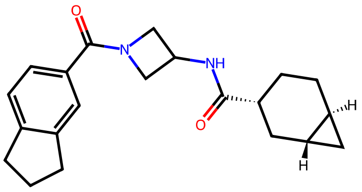	O=C(NC1CN(C(=O)c2ccc3c(c2)CCC3)C1)[C@@H]1CC[C@H]2C[C@@H]2C1	994697276	25	338.2	2.55
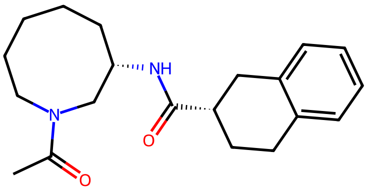	CC(=O)N1CCCCC[C@H](NC(=O)[C@H]2CCc3ccccc3C2)C1	986791970	24	328.22	2.7
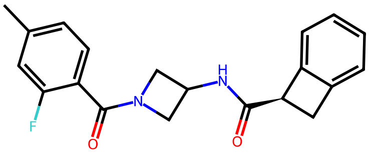	Cc1ccc(C(=O)N2CC(NC(=O)[C@@H]3Cc4ccccc43)C2)c(F)c1	998693606	25	338.14	2.41
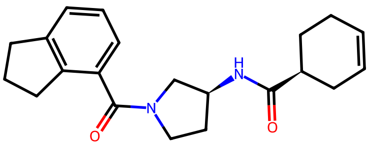	O=C(N[C@H]1CCN(C(=O)c2cccc3c2CCC3)C1)[C@@H]1CC=CCC1	937664836	25	338.2	2.86
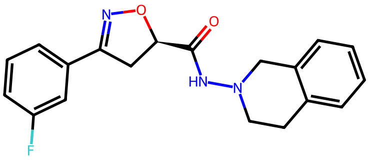	O=C(NN1CCc2ccccc2C1)[C@H]1CC(c2cccc(F)c2)=NO1	1418366058	25	339.14	2.41
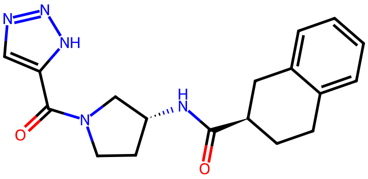	O=C(N[C@@H]1CCN(C(=O)c2cnn[nH]2)C1)[C@@H]1CCc2ccccc2C1	942507406	25	339.17	0.94

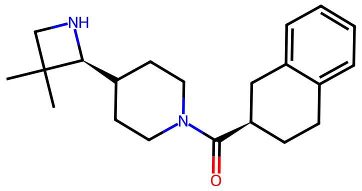	CC1(C)CN[C@H]1C1CCN(C(=O)[C@@H]2CCc3ccccc3C2)CC1	653164891	24	326.24	3.03
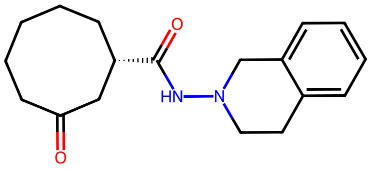	O=C1CCCCC[C@H](C(=O)NN2CCc3ccccc3C2)C1	855712219	22	300.18	2.62
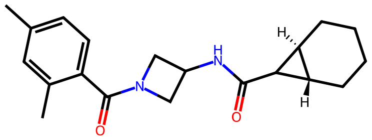	Cc1ccc(C(=O)N2CC(NC(=O)C3[C@H]4CCCC[C@H]34)C2)c(C)c1	990674054	24	326.2	2.68
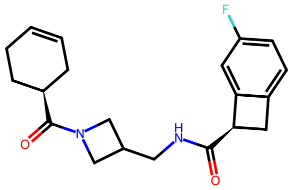	O=C(NCC1CN(C(=O)[C@@H]2CC=CCC2)C1)[C@@H]1Cc2ccc(F)cc21	1003882957	25	342.17	2.4
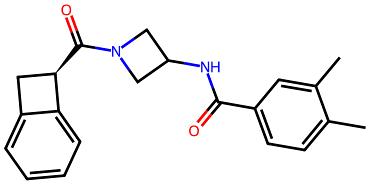	Cc1ccc(C(=O)NC2CN(C(=O)[C@@H]3Cc4ccccc43)C2)cc1C	999548890	25	334.17	2.58
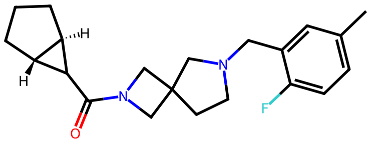	Cc1ccc(F)c(CN2CCC3(C2)CN(C(=O)C2[C@@H]4CCC[C@@H]24)C3)c1	1072592887	25	342.21	3.21
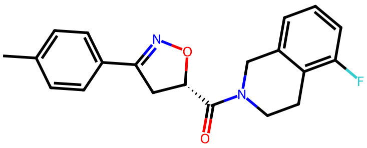	Cc1ccc(C2=NO[C@H](C(=O)N3CCc4c(F)cccc4C3)C2)cc1	1544806355	25	338.14	3.21
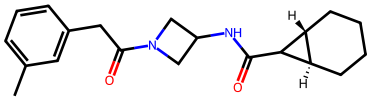	Cc1cccc(CC(=O)N2CC(NC(=O)C3[C@@H]4CCCC[C@@H]34)C2)c1	990648579	24	326.2	2.3

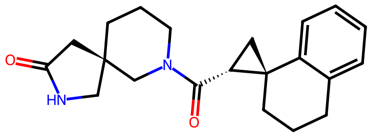	O=C1C[C@]2(CCCN(C(=O)[C@@H]3C[C@@]34CCCc3ccccc34)C2)CN1	341506614	25	338.2	2.41
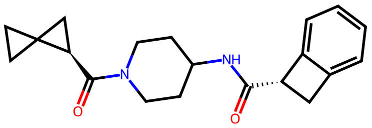	O=C(NC1CCN(C(=O)[C@@H]2CC23CC3)CC1)[C@H]1Cc2ccccc21	948571712	24	324.18	2.23
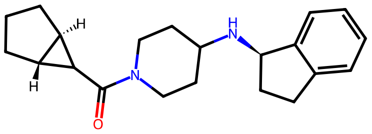	O=C(C1[C@@H]2CCC[C@@H]12)N1CCC(N[C@@H]2CCc3ccccc32)CC1	990770122	24	324.22	3.3
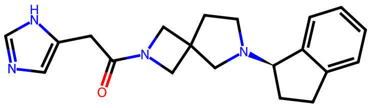	O=C(Cc1cnc[nH]1)N1CC2(CCN([C@@H]3CCc4ccccc43)C2)C1	1073019267	25	336.2	2.17
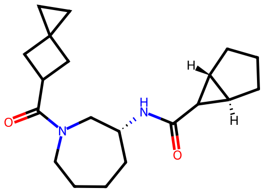	O=C(N[C@@H]1CCCCN(C(=O)C2CC3(CC3)C2)C1)C1[C@@H]2CCC[C@@H]12	943166811	24	330.23	2.72
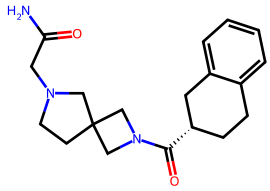	NC(=O)CN1CCC2(C1)CN(C(=O)[C@H]1CCc3ccccc3C1)C2	1073015042	24	327.19	0.81
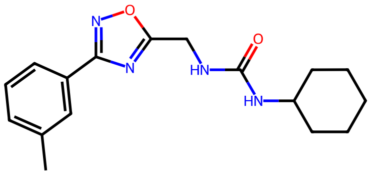	Cc1cccc(-c2noc(CNC(=O)NC3CCCCC3)n2)c1	4512750	23	314.17	3.18
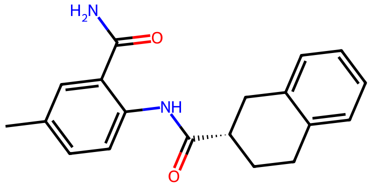	Cc1ccc(NC(=O)[C@H]2CCc3ccccc3C2)c(C(N)=O)c1	844624320	23	308.15	2.84

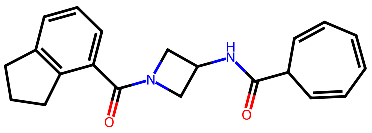	O=C(NC1CN(C(=O)c2cccc3c2CCC3)C1)C1C=CC=CC=C1	992868383	25	334.17	2.41
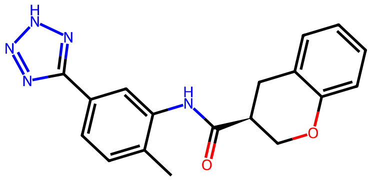	Cc1ccc(-c2nn[nH]n2)cc1NC(=O)[C@@H]1COc2ccccc2C1	1592103993	25	335.14	2.36
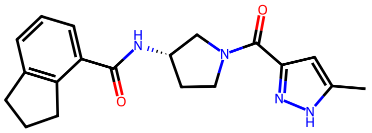	Cc1cc(C(=O)N2CC[C@H](NC(=O)c3cccc4c3CCC4)C2)n[nH]1	938146532	25	338.17	1.85
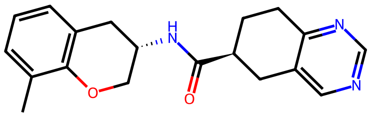	Cc1cccc2c1OC[C@@H](NC(=O)[C@H]1CCc3ncncc3C1)C2	648396896	24	323.16	2.01
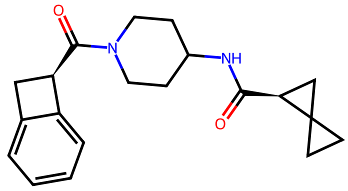	O=C(NC1CCN(C(=O)[C@@H]2Cc3ccccc32)CC1)[C@H]1CC12CC2	948059406	24	324.18	2.23
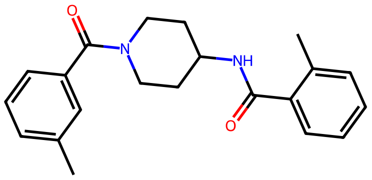	Cc1cccc(C(=O)N2CCC(NC(=O)c3ccccc3C)CC2)c1	32307883	25	336.18	3.34
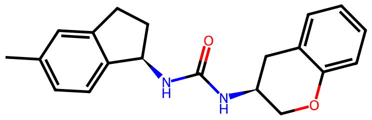	Cc1ccc2c(c1)CC[C@H]2NC(=O)N[C@@H]1COc2ccccc2C1	516689629	24	322.17	3.29
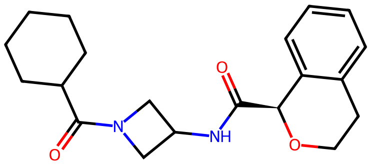	O=C(NC1CN(C(=O)C2CCCCC2)C1)[C@@H]1OCCc2ccccc21	997320484	25	342.19	2.21

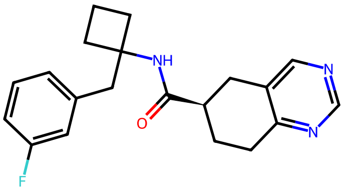	O=C(NC1(Cc2cccc(F)c2)CCC1)[C@@H]1CCc2ncncc2C1	648361279	25	339.17	3.0
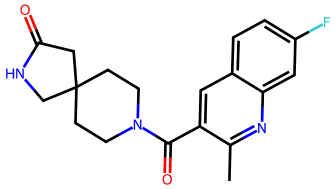	Cc1nc2cc(F)ccc2cc1C(=O)N1CCC2(CC1)CNC(=O)C2	267654186	25	341.15	2.42
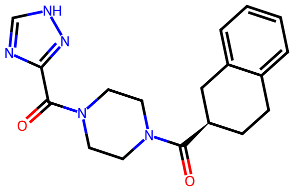	O=C(c1nc[nH]n1)N1CCN(C(=O)[C@@H]2CCc3ccccc3C2)CC1	1012033887	25	339.17	0.89
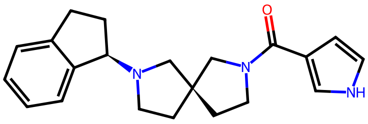	O=C(c1cc[nH]c1)N1CC[C@]2(CCN([C@@H]3CCc4ccccc43)C2)C1	1041312083	25	335.2	3.24
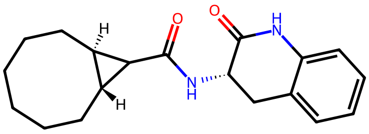	O=C(N[C@H]1Cc2ccccc2NC1=O)C1[C@@H]2CCCCCC[C@@H]12	628943769	23	312.18	2.88
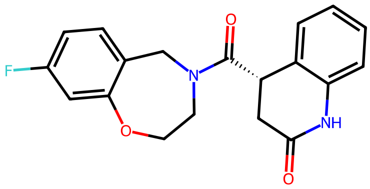	O=C1C[C@H](C(=O)N2CCOc3cc(F)ccc3C2)c2ccccc2N1	1434245366	25	340.12	2.67
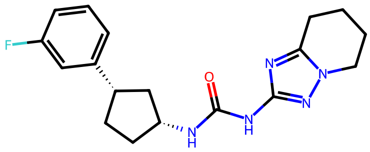	O=C(Nc1nc2n(n1)CCCC2)N[C@@H]1CC[C@H](c2cccc(F)c2)C1	580161369	25	343.18	3.21
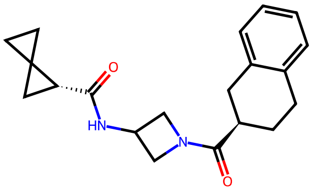	O=C(NC1CN(C(=O)[C@@H]2CCc3ccccc3C2)C1)[C@@H]1CC12CC2	1000508415	24	324.18	1.92

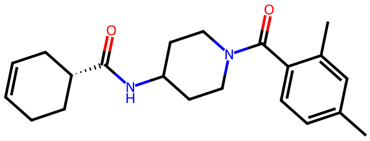	Cc1ccc(C(=O)N2CCC(NC(=O)[C@H]3CC=CCC3)CC2)c(C)c1	948346322	25	340.22	3.38
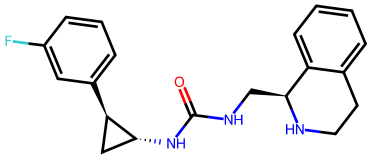	O=C(NC[C@@H]1NCCc2ccccc21)N[C@@H]1C[C@H]1c1cccc(F)c1	562812398	25	339.17	2.87
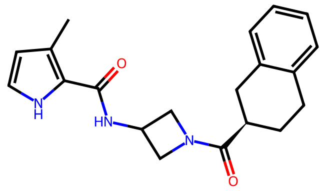	Cc1cc[nH]c1C(=O)NC1CN(C(=O)[C@@H]2CCc3ccccc3C2)C1	991099753	25	337.18	2.07
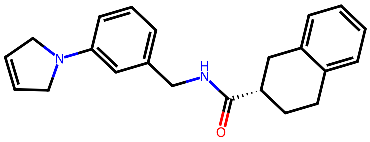	O=C(NCc1cccc(N2CC=CC2)c1)[C@H]1CCc2ccccc2C1	584056205	25	332.19	3.48
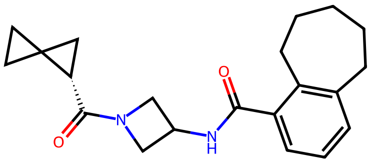	O=C(NC1CN(C(=O)[C@H]2CC23CC3)C1)c1cccc2c1CCCCC2	994066602	25	338.2	2.7
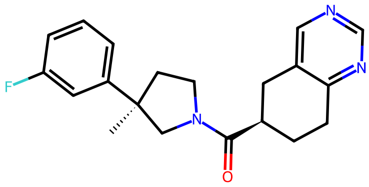	C[C@]1(c2cccc(F)c2)CCN(C(=O)[C@@H]2CCc3ncncc3C2)C1	650218237	25	339.17	2.91
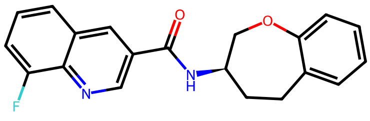	O=C(N[C@@H]1CCc2ccccc2OC1)c1cnc2c(F)cccc2c1	353143324	25	336.13	3.5
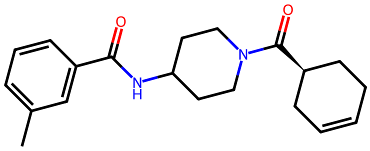	Cc1cccc(C(=O)NC2CCN(C(=O)[C@@H]3CC=CCC3)CC2)c1	870831844	24	326.2	3.07

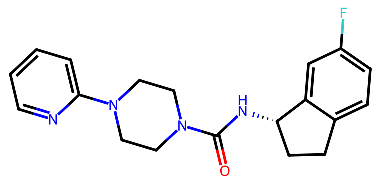	O=C(N[C@H]1CCc2ccc(F)cc21)N1CCN(c2ccccn2)CC1	106576867	25	340.17	2.74
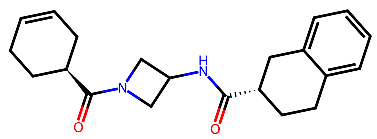	O=C(NC1CN(C(=O)[C@@H]2CC=CCC2)C1)[C@H]1CCc2ccccc2C1	1000537903	25	338.2	2.47
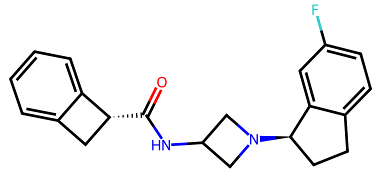	O=C(NC1CN([C@@H]2CCc3ccc(F)cc32)C1)[C@@H]1Cc2ccccc21	1030881228	25	336.16	2.95
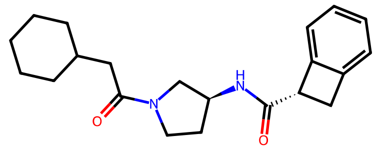	O=C(N[C@H]1CCN(C(=O)CC2CCCCC2)C1)[C@H]1Cc2ccccc21	938574995	25	340.22	3.01
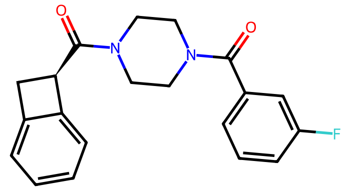	O=C(c1cccc(F)c1)N1CCN(C(=O)[C@@H]2Cc3ccccc32)CC1	358262252	25	338.14	2.45
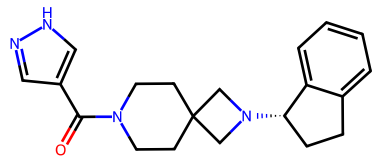	O=C(c1cn[nH]c1)N1CCC2(CC1)CN([C@H]1CCc3ccccc31)C2	1035657924	25	336.2	2.64
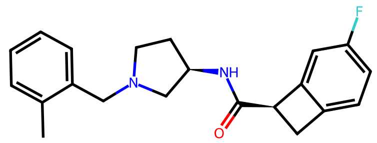	Cc1ccccc1CN1CC[C@@H](NC(=O)[C@@H]2Cc3ccc(F)cc32)C1	1015615718	25	338.18	3.16
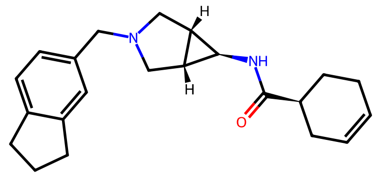	O=C(N[C@H]1[C@@H]2CN(Cc3ccc4c(c3)CCC4)C[C@@H]21)[C@@H]1CC=CCC1	958208418	25	336.22	3.08

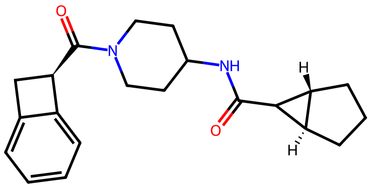	O=C(NC1CCN(C(=O)[C@@H]2Cc3ccccc32)CC1)C1[C@@H]2CCC[C@@H]12	948059458	25	338.2	2.48
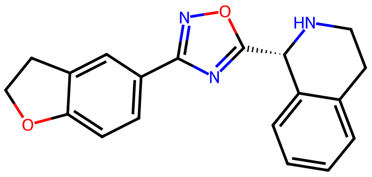	c1ccc2c(c1)CCN[C@H]2c1nc(-c2ccc3c(c2)CCO3)no1	579584958	24	319.13	2.91
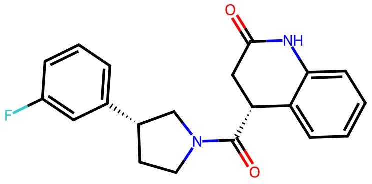	O=C1C[C@@H](C(=O)N2CC[C@H](c3cccc(F)c3)C2)c2ccccc2N1	264253025	25	338.14	3.27
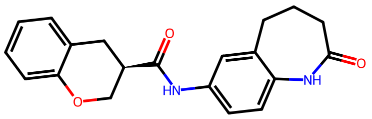	O=C1CCCc2cc(NC(=O)[C@H]3COc4ccccc4C3)ccc2N1	264084931	25	336.15	3.15
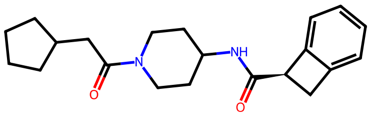	O=C(NC1CCN(C(=O)CC2CCCC2)CC1)[C@@H]1Cc2ccccc21	943770178	25	340.22	3.01
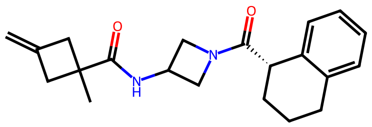	C=C1CC(C)(C(=O)NC2CN(C(=O)[C@H]3CCCc4ccccc43)C2)C1	999264917	25	338.2	2.79
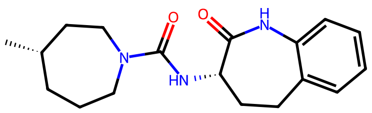	C[C@H]1CCCN(C(=O)N[C@H]2CCc3ccccc3NC2=O)CC1	654629900	23	315.19	2.77
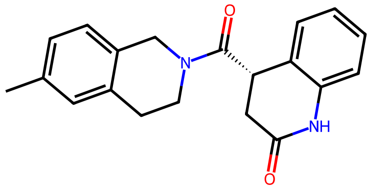	Cc1ccc2c(c1)CCN(C(=O)[C@H]1CC(=O)Nc3ccccc31)C2	360195823	24	320.15	3.01

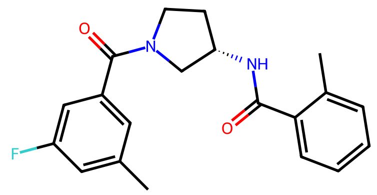	Cc1cc(F)cc(C(=O)N2CC[C@H](NC(=O)c3ccccc3C)C2)c1	937634969	25	340.16	3.09
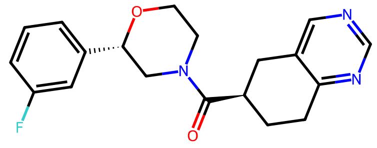	O=C([C@@H]1CCc2ncncc2C1)N1CCO[C@@H](c2cccc(F)c2)C1	648764904	25	341.15	2.32
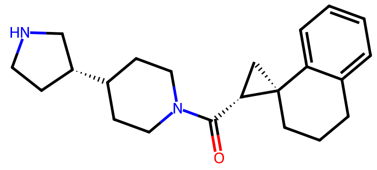	O=C([C@@H]1C[C@]12CCCc1ccccc12)N1CCC([C@@H]2CCNC2)CC1	377808349	25	338.24	3.13
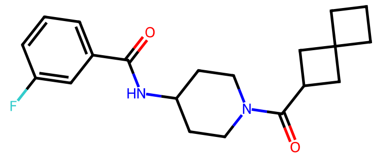	O=C(NC1CCN(C(=O)C2CC3(CCC3)C2)CC1)c1cccc(F)c1	947879504	25	344.19	3.13
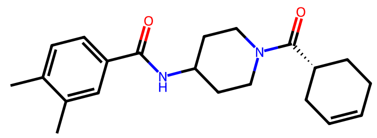	Cc1ccc(C(=O)NC2CCN(C(=O)[C@H]3CC=CCC3)CC2)cc1C	944105718	25	340.22	3.38
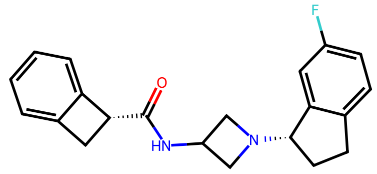	O=C(NC1CN([C@H]2CCc3ccc(F)cc32)C1)[C@@H]1Cc2ccccc21	1030881226	25	336.16	2.95
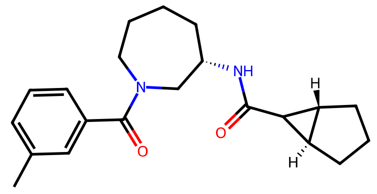	Cc1cccc(C(=O)N2CCCC[C@H](NC(=O)C3[C@@H]4CCC[C@@H]34)C2)c1	943169643	25	340.22	3.15
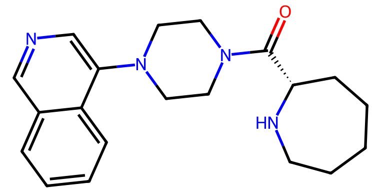	O=C([C@@H]1CCCCCN1)N1CCN(c2cncc3ccccc23)CC1	516868089	25	338.21	2.42

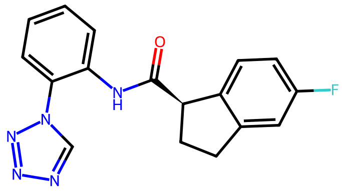	O=C(Nc1ccccc1-n1cnnn1)[C@@H]1CCc2cc(F)ccc21	1355413950	24	323.12	2.47
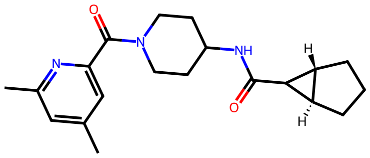	Cc1cc(C)nc(C(=O)N2CCC(NC(=O)C3[C@@H]4CCC[C@@H]34)CC2)c1	947307729	25	341.21	2.47
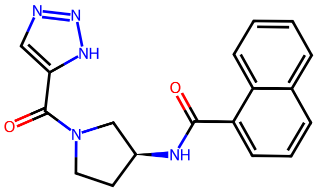	O=C(N[C@H]1CCN(C(=O)c2cnn[nH]2)C1)c1cccc2ccccc12	938760722	25	335.14	1.6
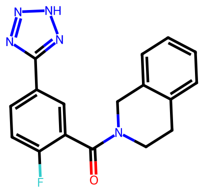	O=C(c1cc(-c2nn[nH]n2)ccc1F)N1CCc2ccccc2C1	1582684513	24	323.12	2.2
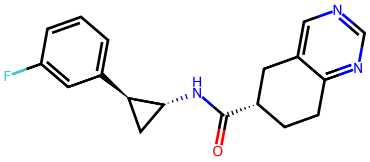	O=C(N[C@@H]1C[C@H]1c1cccc(F)c1)[C@H]1CCc2ncncc2C1	648260541	23	311.14	2.39
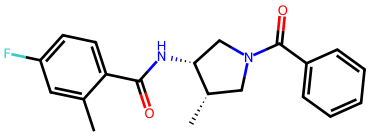	Cc1cc(F)ccc1C(=O)N[C@@H]1CN(C(=O)c2ccccc2)C[C@@H]1C	966090809	25	340.16	3.02
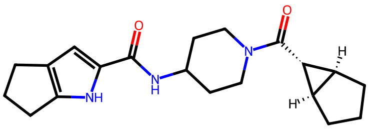	O=C(NC1CCN(C(=O)[C@@H]2[C@H]3CCC[C@H]32)CC1)c1cc2c([nH]1)CCC2	946062211	25	341.21	2.27
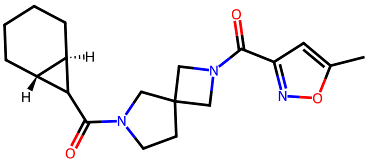	Cc1cc(C(=O)N2CC3(CCN(C(=O)C4[C@@H]5CCCC[C@@H]45)C3)C2)no1	1018756876	25	343.19	2.09

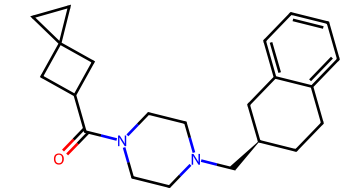	O=C(C1CC2(CC2)C1)N1CCN(C[C@@H]2CCc3ccccc3C2)CC1	950429939	25	338.24	3.13
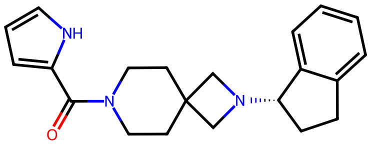	O=C(c1ccc[nH]1)N1CCC2(CC1)CN([C@H]1CCc3ccccc31)C2	1035650351	25	335.2	3.24
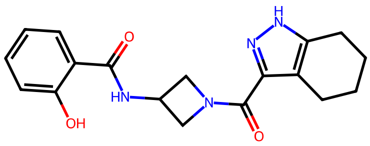	O=C(NC1CN(C(=O)c2n[nH]c3c2CCCC3)C1)c1ccccc1O	990275726	25	340.15	1.25
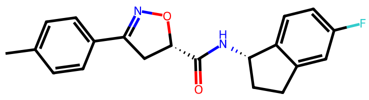	Cc1ccc(C2=NO[C@H](C(=O)N[C@H]3CCc4cc(F)ccc43)C2)cc1	1526637384	25	338.14	3.43
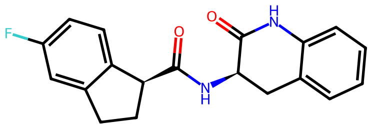	O=C(N[C@@H]1Cc2ccccc2NC1=O)[C@H]1CCc2cc(F)ccc21	529945398	24	324.13	2.54
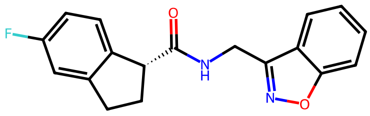	O=C(NCc1noc2ccccc12)[C@@H]1CCc2cc(F)ccc21	1597556442	23	310.11	3.31
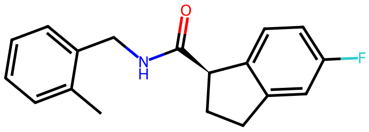	Cc1ccccc1CNC(=O)[C@@H]1CCc2cc(F)ccc21	665400387	21	283.14	3.48
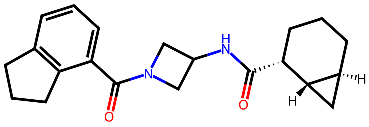	O=C(NC1CN(C(=O)c2cccc3c2CCC3)C1)[C@@H]1CCC[C@H]2C[C@@H]21	994664899	25	338.2	2.55

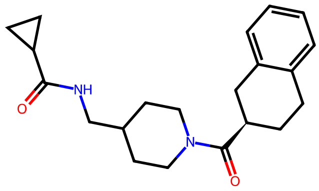	O=C(NCC1CCN(C(=O)[C@@H]2CCc3ccccc3C2)CC1)C1CC1	949549489	25	340.22	2.56
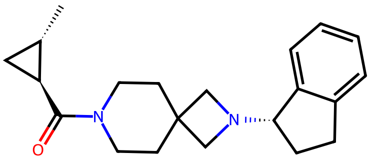	C[C@H]1C[C@@H]1C(=O)N1CCC2(CC1)CN([C@H]1CCc3ccccc31)C2	1035769336	24	324.22	3.25
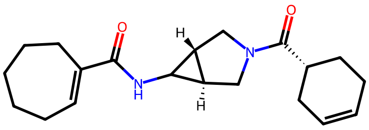	O=C(NC1[C@@H]2CN(C(=O)[C@H]3CC=CCC3)C[C@@H]12)C1=CCCCCC1	1013008252	24	328.22	2.81
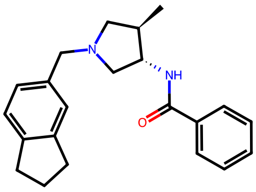	C[C@@H]1CN(Cc2ccc3c(c2)CCC3)C[C@H]1NC(=O)c1ccccc1	1079365819	25	334.2	3.43
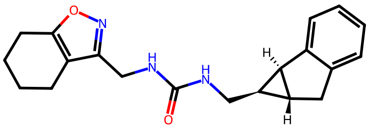	O=C(NCc1noc2c1CCCC2)NC[C@@H]1[C@H]2Cc3ccccc3[C@@H]21	289331228	25	337.18	2.94
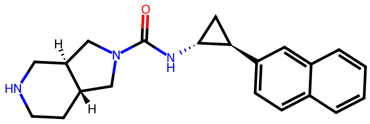	O=C(N[C@@H]1C[C@H]1c1ccc2ccccc2c1)N1C[C@@H]2CNCC[C@H]2C1	418136783	25	335.2	2.95
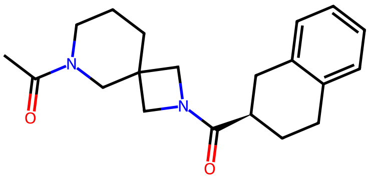	CC(=O)N1CCCC2(C1)CN(C(=O)[C@@H]1CCc3ccccc3C1)C2	1010896143	24	326.2	2.26
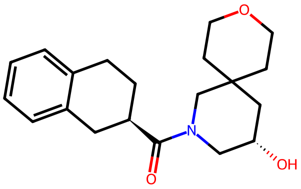	O=C([C@@H]1CCc2ccccc2C1)N1C[C@@H](O)CC2(CCOCC2)C1	882954969	24	329.2	2.18

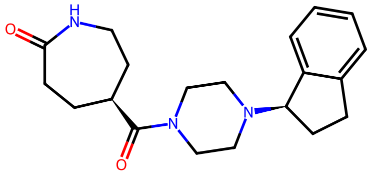	O=C1CC[C@H](C(=O)N2CCN([C@@H]3CCc4ccccc43)CC2)CCN1	949488674	25	341.21	1.73
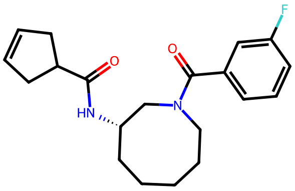	O=C(N[C@H]1CCCCCN(C(=O)c2cccc(F)c2)C1)C1CC=CC1	986854400	25	344.19	3.29
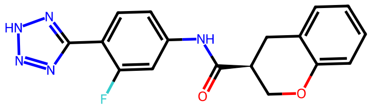	O=C(Nc1ccc(-c2nn[nH]n2)c(F)c1)[C@@H]1COc2ccccc2C1	1593212727	25	339.11	2.2
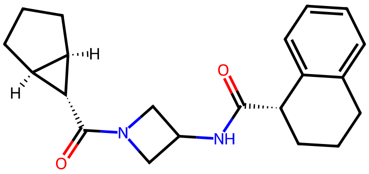	O=C(NC1CN(C(=O)[C@@H]2[C@H]3CCC[C@H]32)C1)[C@H]1CCCc2ccccc21	995069927	25	338.2	2.48
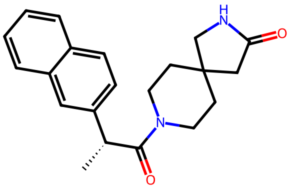	C[C@@H](C(=O)N1CCC2(CC1)CNC(=O)C2)c1ccc2ccccc2c1	267789078	25	336.18	3.07
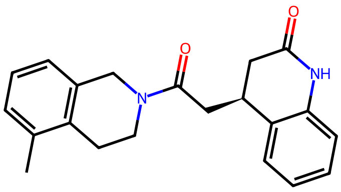	Cc1cccc2c1CCN(C(=O)C[C@H]1CC(=O)Nc3ccccc31)C2	932001820	25	334.17	3.4
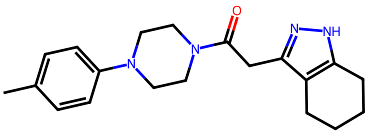	Cc1ccc(N2CCN(C(=O)Cc3n[nH]c4c3CCCC4)CC2)cc1	777197647	25	338.21	2.49
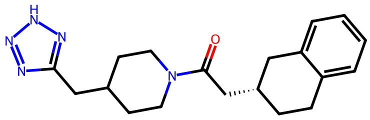	O=C(C[C@H]1CCc2ccccc2C1)N1CCC(Cc2nn[nH]n2)CC1	1548804245	25	339.21	2.18

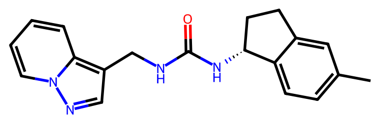	Cc1ccc2c(c1)CC[C@H]2NC(=O)NCc1cnn2ccccc12	571471357	24	320.16	3.13
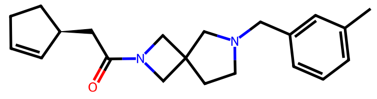	Cc1cccc(CN2CCC3(C2)CN(C(=O)C[C@H]2C=CCC2)C3)c1	1072455091	24	324.22	3.39
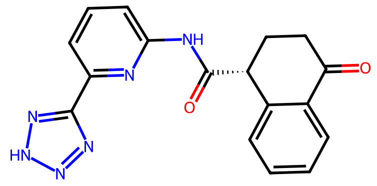	O=C1CC[C@@H](C(=O)Nc2cccc(-c3nn[nH]n3)n2)c2ccccc21	1582765225	25	334.12	1.96
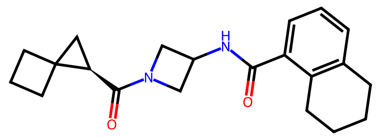	O=C(NC1CN(C(=O)[C@@H]2CC23CCC3)C1)c1cccc2c1CCCC2	991899252	25	338.2	2.7
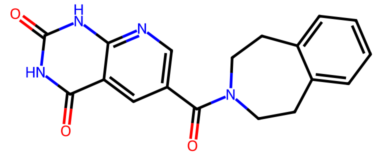	O=C(c1cnc2[nH]c(=O)[nH]c(=O)c2c1)N1CCc2ccccc2CC1	116217100	25	336.12	0.85
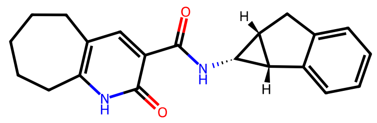	O=C(N[C@@H]1[C@@H]2Cc3ccccc3[C@@H]21)c1cc2c([nH]c1=O)CCCCC2	130041468	25	334.17	2.71
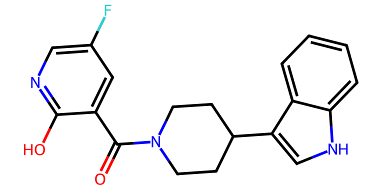	O=C(c1cc(F)cnc1O)N1CCC(c2c[nH]c3ccccc23)CC1	621351827	25	339.14	3.43
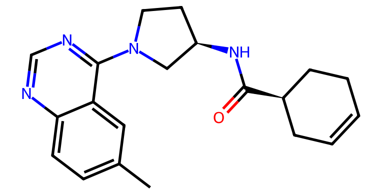	Cc1ccc2ncnc(N3CC[C@@H](NC(=O)[C@@H]4CC=CCC4)C3)c2c1	1058296056	25	336.2	2.99

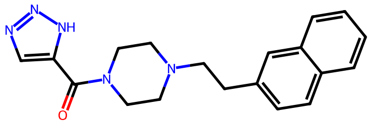	O=C(c1cnn[nH]1)N1CCN(CCc2ccc3ccccc3c2)CC1	949059806	25	335.17	1.96
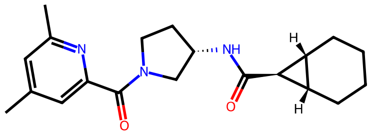	Cc1cc(C)nc(C(=O)N2CC[C@H](NC(=O)[C@@H]3[C@H]4CCCC[C@H]43)C2)c1	937790183	25	341.21	2.47
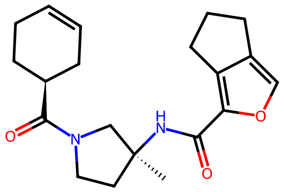	C[C@]1(NC(=O)c2occ3c2CCC3)CCN(C(=O)[C@@H]2CC=CCC2)C1	957226196	25	342.19	2.85
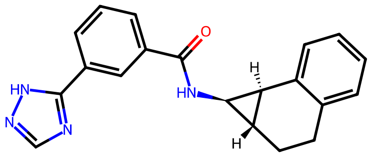	O=C(N[C@@H]1[C@H]2CCc3ccccc3[C@@H]21)c1cccc(-c2ncn[nH]2)c1	159113298	25	330.15	2.93
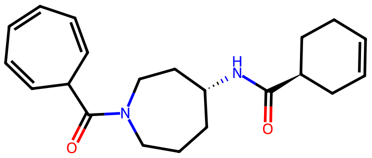	O=C(N[C@@H]1CCCN(C(=O)C2C=CC=CC=C2)CC1)[C@@H]1CC=CCC1	964740620	25	340.22	3.14
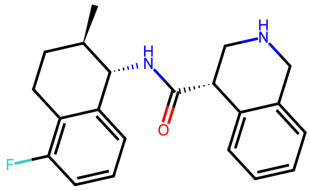	C[C@@H]1CCc2c(F)cccc2[C@H]1NC(=O)[C@@H]1CNCc2ccccc21	566190198	25	338.18	3.45
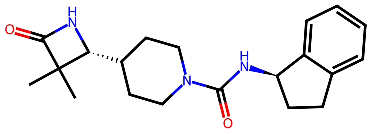	CC1(C)C(=O)N[C@@H]1C1CCN(C(=O)N[C@@H]2CCc3ccccc32)CC1	637275375	25	341.21	2.62
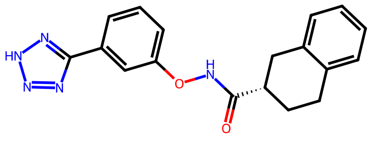	O=C(NOc1cccc(-c2nn[nH]n2)c1)[C@H]1CCc2ccccc2C1	1593171273	25	335.14	2.08

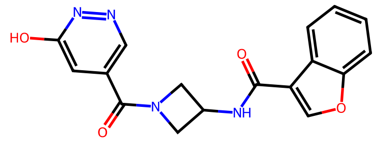	O=C(NC1CN(C(=O)c2cnnc(O)c2)C1)c1coc2ccccc12	994928018	25	338.1	1.18
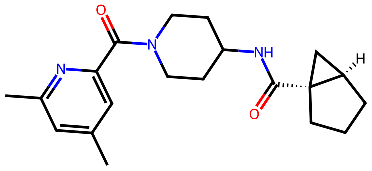	Cc1cc(C)nc(C(=O)N2CCC(NC(=O)[C@@]34CCC[C@@H]3C4)CC2)c1	947308010	25	341.21	2.61
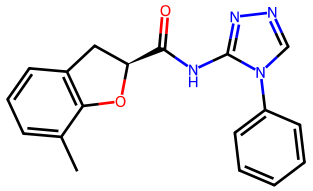	Cc1cccc2c1O[C@H](C(=O)Nc1nncn1-c1ccccc1)C2	1446344310	24	320.13	2.52
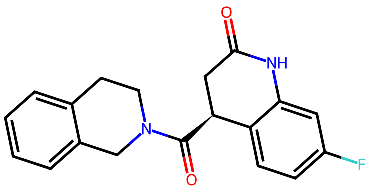	O=C1C[C@H](C(=O)N2CCc3ccccc3C2)c2ccc(F)cc2N1	12939069	24	324.13	2.84
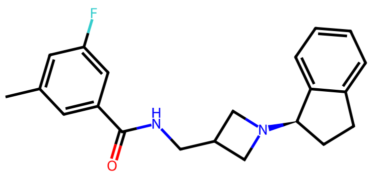	Cc1cc(F)cc(C(=O)NCC2CN([C@@H]3CCc4ccccc43)C2)c1	1031998504	25	338.18	3.48
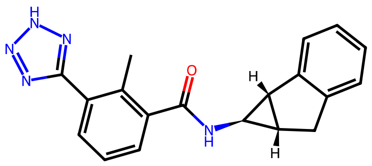	Cc1c(C(=O)N[C@@H]2[C@H]3Cc4ccccc4[C@H]32)cccc1-c1nn[nH]n1	1580547066	25	331.14	2.24
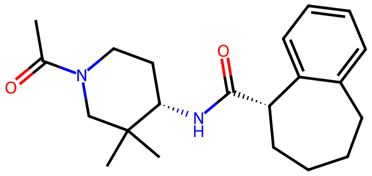	CC(=O)N1CC[C@H](NC(=O)[C@H]2CCCCc3ccccc32)C(C)(C)C1	967056850	25	342.23	3.26
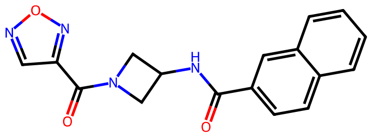	O=C(NC1CN(C(=O)c2cnon2)C1)c1ccc2ccccc2c1	999444863	24	322.11	1.48

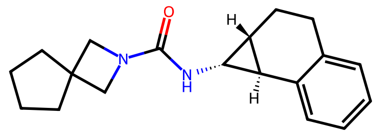	O=C(N[C@H]1[C@@H]2c3ccccc3CC[C@H]21)N1CC2(CCCC2)C1	430359215	22	296.19	3.3
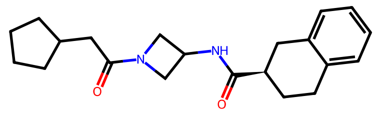	O=C(NC1CN(C(=O)CC2CCCC2)C1)[C@@H]1CCc2ccccc2C1	1000540138	25	340.22	2.7
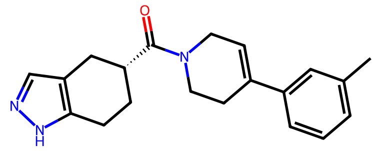	Cc1cccc(C2=CCN(C(=O)[C@@H]3CCc4[nH]ncc4C3)CC2)c1	271371374	24	321.18	3.14
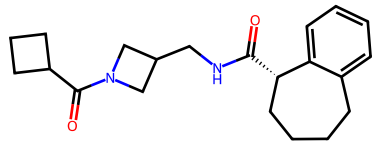	O=C(NCC1CN(C(=O)C2CCC2)C1)[C@H]1CCCCc2ccccc21	1002646847	25	340.22	2.87
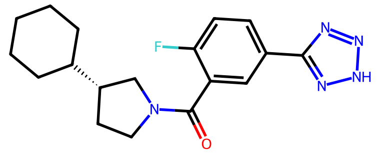	O=C(c1cc(-c2nn[nH]n2)ccc1F)N1CC[C@H](C2CCCCC2)C1	1593323929	25	343.18	3.05
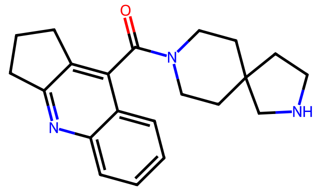	O=C(c1c2c(nc3ccccc13)CCC2)N1CCC2(CCNC2)CC1	261682395	25	335.2	2.94
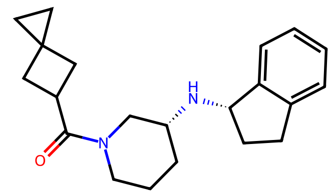	O=C(C1CC2(CC2)C1)N1CCC[C@@H](N[C@H]2CCc3ccccc32)C1	1000152534	24	324.22	3.44
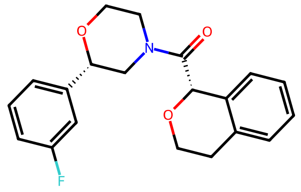	O=C([C@H]1OCCc2ccccc21)N1CCO[C@@H](c2cccc(F)c2)C1	178790646	25	341.14	3.04

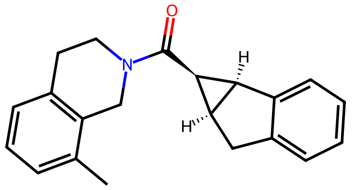	Cc1cccc2c1CN(C(=O)[C@H]1[C@H]3c4ccccc4C[C@H]31)CC2	626664576	23	303.16	3.47
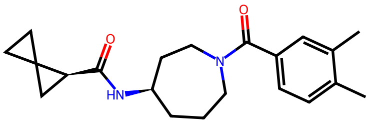	Cc1ccc(C(=O)N2CCC[C@@H](NC(=O)[C@H]3CC34CC4)CC2)cc1C	965221924	25	340.22	3.21
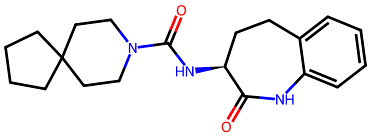	O=C1Nc2ccccc2CC[C@@H]1NC(=O)N1CCC2(CCCC2)CC1	847397580	25	341.21	3.31
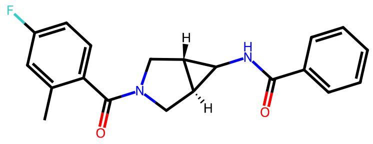	Cc1cc(F)ccc1C(=O)N1C[C@@H]2C(NC(=O)c3ccccc3)[C@H]2C1	1012708889	25	338.14	2.63
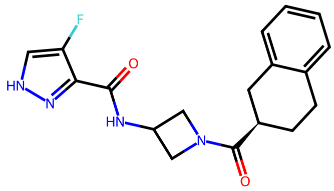	O=C(NC1CN(C(=O)[C@@H]2CCc3ccccc3C2)C1)c1n[nH]cc1F	999194463	25	342.15	1.29
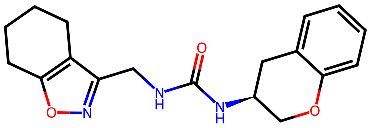	O=C(NCc1noc2c1CCCC2)N[C@@H]1COc2ccccc2C1	287376229	24	327.16	2.36
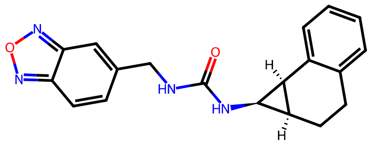	O=C(NCc1ccc2nonc2c1)N[C@@H]1[C@@H]2CCc3ccccc3[C@@H]21	893766474	25	334.14	2.75
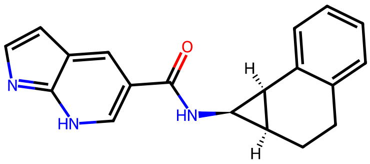	O=C(N[C@@H]1[C@@H]2CCc3ccccc3[C@@H]21)c1c[nH]c2nccc-2c1	563783495	23	303.14	2.97

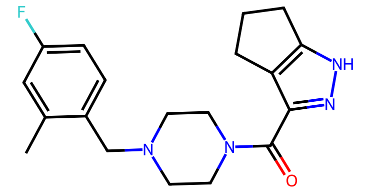	Cc1cc(F)ccc1CN1CCN(C(=O)c2n[nH]c3c2CCC3)CC1	949063049	25	342.19	2.3
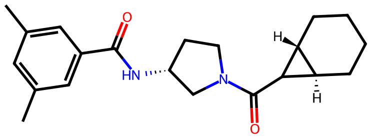	Cc1cc(C)cc(C(=O)N[C@@H]2CCN(C(=O)C3[C@@H]4CCCC[C@@H]34)C2)c1	942390857	25	340.22	3.07
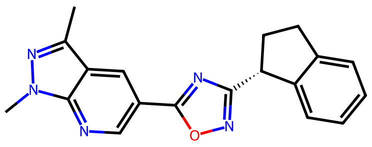	Cc1nn(C)c2ncc(-c3nc([C@@H]4CCc5ccccc54)no3)cc12	680749376	25	331.14	3.4
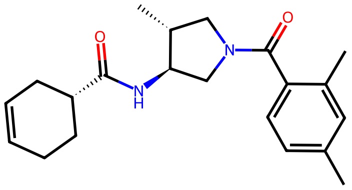	Cc1ccc(C(=O)N2C[C@@H](NC(=O)[C@H]3CC=CCC3)[C@H](C)C2)c(C)c1	959621217	25	340.22	3.24
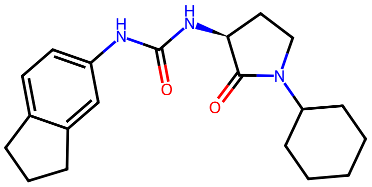	O=C(Nc1ccc2c(c1)CCC2)N[C@H]1CCN(C2CCCCC2)C1=O	153053592	25	341.21	3.23
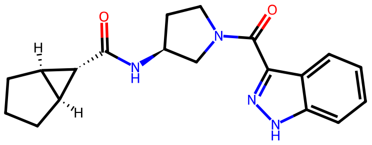	O=C(N[C@H]1CCN(C(=O)c2n[nH]c3ccccc23)C1)[C@@H]1[C@H]2CCC[C@H]21	937805140	25	338.17	1.94
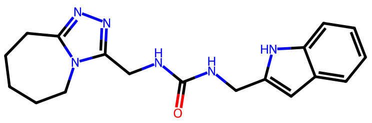	O=C(NCc1cc2ccccc2[nH]1)NCc1nnc2n1CCCCC2	58558057	25	338.19	2.49
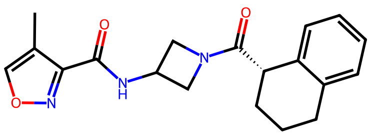	Cc1conc1C(=O)NC1CN(C(=O)[C@H]2CCCc3ccccc32)C1	999660681	25	339.16	2.04

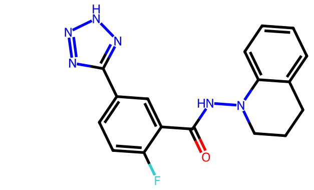	O=C(NN1CCCc2ccccc21)c1cc(-c2nn[nH]n2)ccc1F	1582561547	25	338.13	2.1
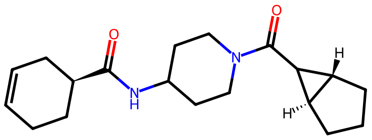	O=C(NC1CCN(C(=O)C2[C@@H]3CCC[C@@H]23)CC1)[C@@H]1CC=CCC1	946064268	23	316.22	2.5
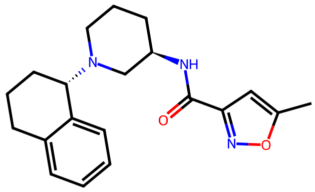	Cc1cc(C(=O)N[C@@H]2CCCN([C@H]3CCCc4ccccc43)C2)no1	1006670428	25	339.19	3.25
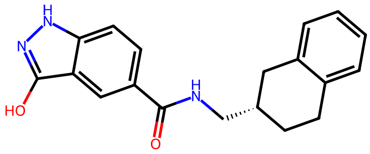	O=C(NC[C@H]1CCc2ccccc2C1)c1ccc2[nH]nc(O)c2c1	668860033	24	321.15	2.8
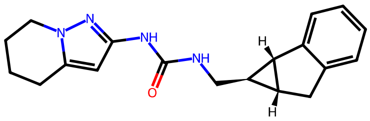	O=C(NC[C@@H]1[C@H]2Cc3ccccc3[C@H]21)Nc1cc2n(n1)CCCC2	287410899	24	322.18	2.93
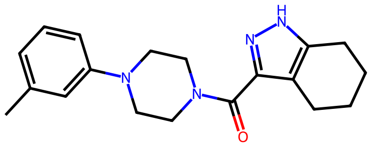	Cc1cccc(N2CCN(C(=O)c3n[nH]c4c3CCCC4)CC2)c1	51319531	24	324.2	2.56
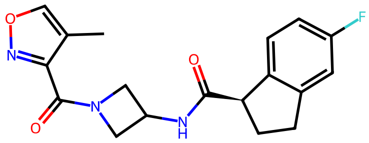	Cc1conc1C(=O)N1CC(NC(=O)[C@@H]2CCc3cc(F)ccc32)C1	996094412	25	343.13	1.79
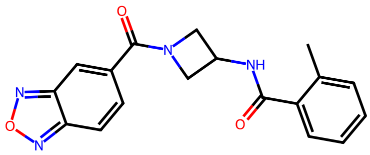	Cc1ccccc1C(=O)NC1CN(C(=O)c2ccc3nonc3c2)C1	990111819	25	336.12	1.79

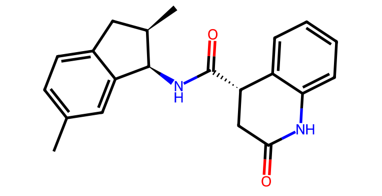	Cc1ccc2c(c1)[C@H](NC(=O)[C@H]1CC(=O)Nc3ccccc31)[C@H](C)C2	248607908	25	334.17	3.47
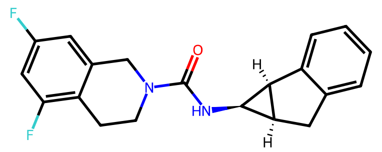	O=C(N[C@@H]1[C@@H]2Cc3ccccc3[C@@H]21)N1CCc2c(F)cc(F)cc2C1	152925980	25	340.14	3.37
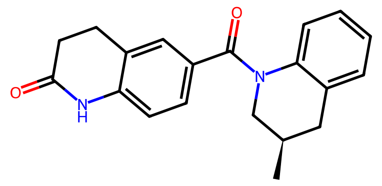	C[C@@H]1Cc2ccccc2N(C(=O)c2ccc3c(c2)CCC(=O)N3)C1	96735944	24	320.15	3.41
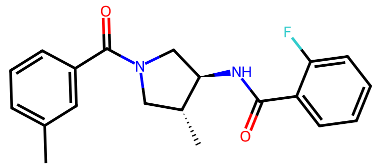	Cc1cccc(C(=O)N2C[C@@H](NC(=O)c3ccccc3F)[C@H](C)C2)c1	947313522	25	340.16	3.02
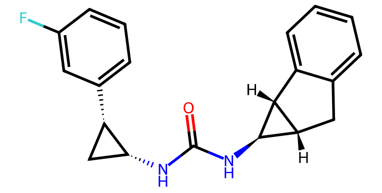	O=C(N[C@@H]1[C@H]2Cc3ccccc3[C@H]21)N[C@@H]1C[C@@H]1c1cccc(F)c1	177810729	24	322.15	3.32
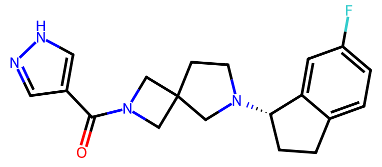	O=C(c1cn[nH]c1)N1CC2(CCN([C@H]3CCc4ccc(F)cc43)C2)C1	1072511740	25	340.17	2.38
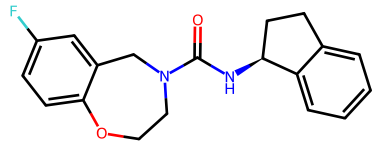	O=C(N[C@H]1CCc2ccccc21)N1CCOc2ccc(F)cc2C1	494191072	24	326.14	3.42
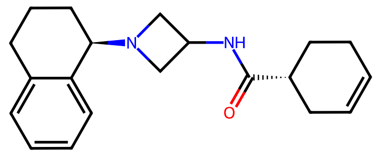	O=C(NC1CN([C@@H]2CCCc3ccccc32)C1)[C@H]1CC=CCC1	1030098628	23	310.2	3.22

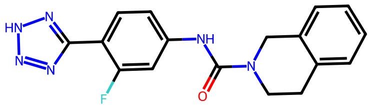	O=C(Nc1ccc(-c2nn[nH]n2)c(F)c1)N1CCc2ccccc2C1	1606909951	25	338.13	2.6
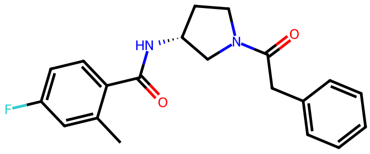	Cc1cc(F)ccc1C(=O)N[C@@H]1CCN(C(=O)Cc2ccccc2)C1	375270933	25	340.16	2.71
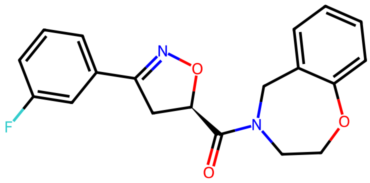	O=C([C@H]1CC(c2cccc(F)c2)=NO1)N1CCOc2ccccc2C1	918663606	25	340.12	2.74
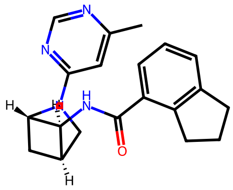	Cc1cc(N2C[C@H]3C[C@H]2[C@H]3NC(=O)c2cccc3c2CCC3)ncn1	1064268314	25	334.18	2.28
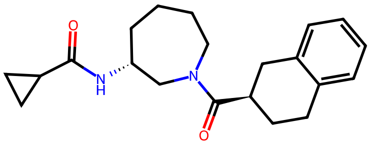	O=C(N[C@@H]1CCCCN(C(=O)[C@@H]2CCc3ccccc3C2)C1)C1CC1	943025790	25	340.22	2.7
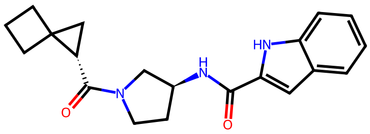	O=C(N[C@H]1CCN(C(=O)[C@H]2CC23CCC3)C1)c1cc2ccccc2[nH]1	938891340	25	337.18	2.69
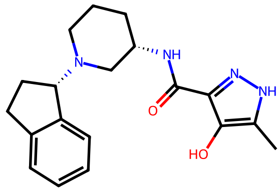	Cc1[nH]nc(C(=O)N[C@H]2CCCN([C@H]3CCc4ccccc43)C2)c1O	1007132756	25	340.19	2.31
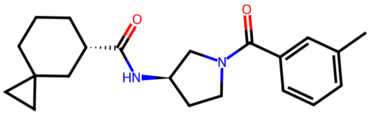	Cc1cccc(C(=O)N2CC[C@@H](NC(=O)[C@H]3CCCC4(CC4)C3)C2)c1	940429529	25	340.22	3.3

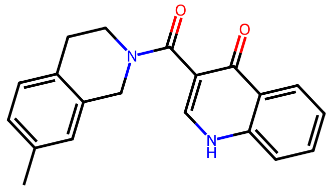	Cc1ccc2c(c1)CN(C(=O)c1c[nH]c3ccccc3c1=O)CC2	1585658853	24	318.14	3.04
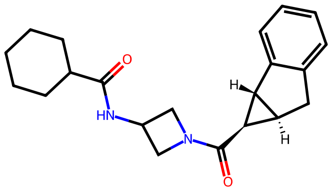	O=C(NC1CN(C(=O)[C@@H]2[C@@H]3Cc4ccccc4[C@H]32)C1)C1CCCCC1	990104218	25	338.2	2.48
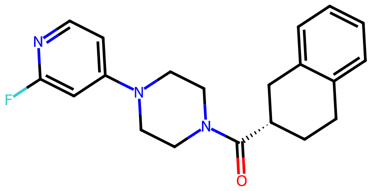	O=C([C@H]1CCc2ccccc2C1)N1CCN(c2ccnc(F)c2)CC1	844562240	25	339.17	2.67
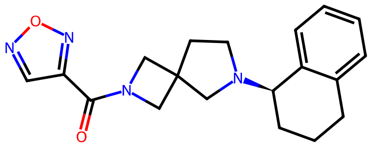	O=C(c1cnon1)N1CC2(CCN([C@@H]3CCCc4ccccc43)C2)C1	1073068422	25	338.17	2.3
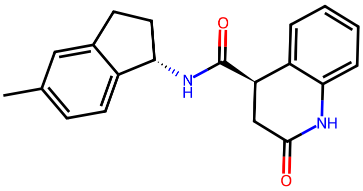	Cc1ccc2c(c1)CC[C@@H]2NC(=O)[C@@H]1CC(=O)Nc2ccccc21	56316910	24	320.15	3.22
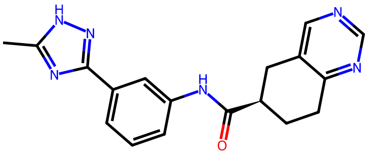	Cc1nc(-c2cccc(NC(=O)[C@@H]3CCc4ncncc4C3)c2)n[nH]1	648251187	25	334.15	2.31
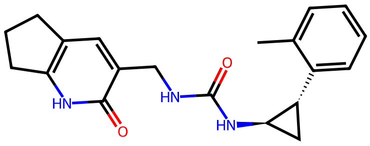	Cc1ccccc1[C@@H]1C[C@H]1NC(=O)NCc1cc2c([nH]c1=O)CCC2	77828331	25	337.18	2.53
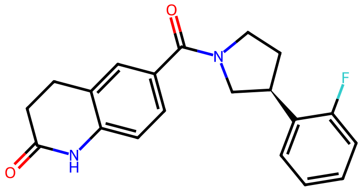	O=C1CCc2cc(C(=O)N3CC[C@@H](c4ccccc4F)C3)ccc2N1	264510564	25	338.14	3.34

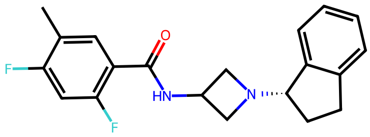	Cc1cc(C(=O)NC2CN([C@H]3CCc4cccc43)C2)c(F)cc1F	1030504188	25	342.15	3.37
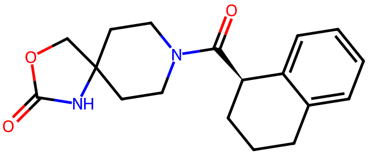	O=C1NC2(CCN(C(=O)[C@@H]3CCCc4ccccc43)CC2)CO1	495140512	23	314.16	2.21
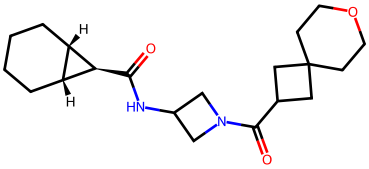	O=C(NC1CN(C(=O)C2CC3(CCOCC3)C2)C1)[C@@H]1[C@H]2CCCC[C@H]21	990644451	25	346.23	1.96
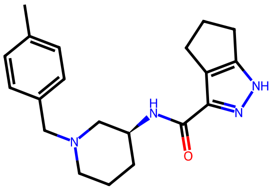	Cc1ccc(CN2CCC[C@H](NC(=O)c3n[nH]c4c3CCC4)C2)cc1	1007746312	25	338.21	2.6
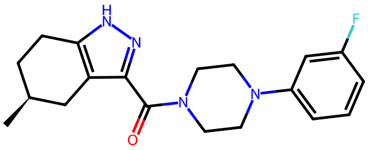	C[C@H]1CCc2[nH]nc(C(=O)N3CCN(c4cccc(F)c4)CC3)c2C1	899159141	25	342.19	2.64
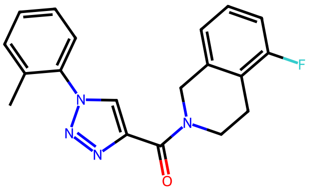	Cc1ccccc1-n1cc(C(=O)N2CCc3c(F)cccc3C2)nn1	1519655763	25	336.14	2.91
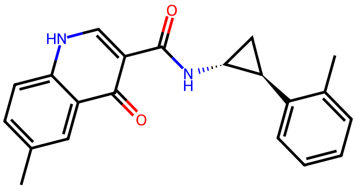	Cc1ccc2[nH]cc(C(=O)N[C@@H]3C[C@H]3c3ccccc3C)c(=O)c2c1	1641725020	25	332.15	3.43
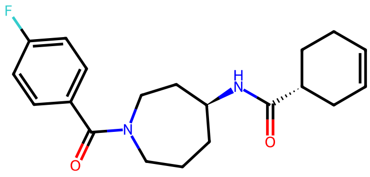	O=C(N[C@H]1CCCN(C(=O)c2ccc(F)cc2)CC1)[C@H]1CC=CCC1	964747731	25	344.19	3.29

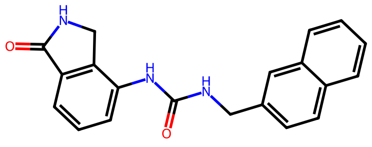	O=C(NCc1ccc2ccccc2c1)Nc1cccc2c1CNC2=O	353076123	25	331.13	3.4
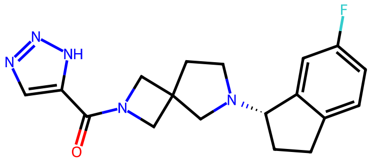	O=C(c1cnn[nH]1)N1CC2(CCN([C@H]3CCc4ccc(F)cc43)C2)C1	1072892303	25	341.17	1.78
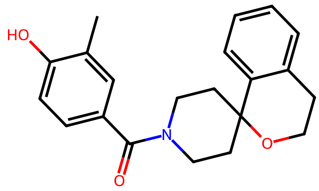	Cc1cc(C(=O)N2CCC3(CC2)OCCc2ccccc23)ccc1O	457144716	25	337.17	3.4
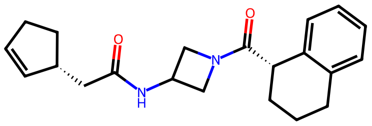	O=C(C[C@@H]1C=CCC1)NC1CN(C(=O)[C@H]2CCCc3ccccc32)C1	990184610	25	338.2	2.79
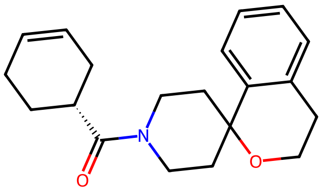	O=C([C@H]1CC=CCC1)N1CCC2(CC1)OCCc1ccccc12	457125594	23	311.19	3.43
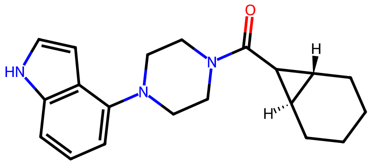	O=C(C1[C@@H]2CCCC[C@@H]12)N1CCN(c2cccc3[nH]ccc23)CC1	616751581	24	323.2	3.25
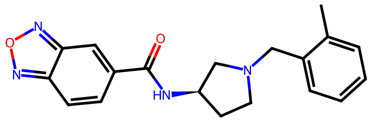	Cc1ccccc1CN1CC[C@@H](NC(=O)c2ccc3nonc3c2)C1	1015646774	25	336.16	2.54
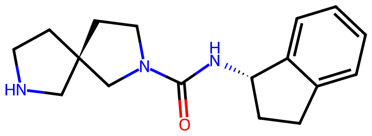	O=C(N[C@H]1CCc2ccccc21)N1CC[C@@]2(CCNC2)C1	335613186	21	285.18	2.07

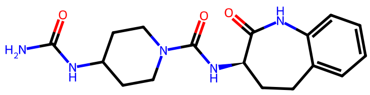	NC(=O)NC1CCN(C(=O)N[C@@H]2CCc3ccccc3NC2=O)CC1	654628513	25	345.18	0.78
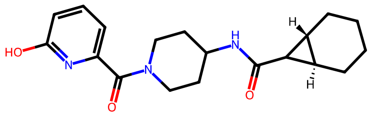	O=C(NC1CCN(C(=O)c2cccc(O)n2)CC1)C1[C@@H]2CCCC[C@@H]12	945980317	25	343.19	1.94
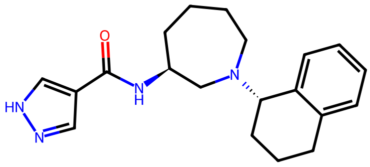	O=C(N[C@H]1CCCCN([C@H]2CCCc3ccccc32)C1)c1cn[nH]c1	1034116351	25	338.21	3.07
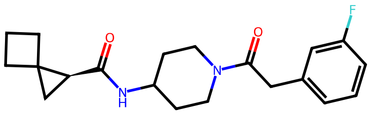	O=C(NC1CCN(C(=O)Cc2cccc(F)c2)CC1)[C@H]1CC12CCC2	948482474	25	344.19	2.67
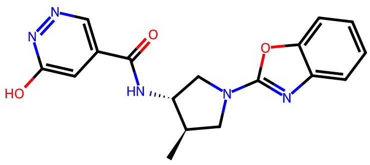	C[C@@H]1CN(c2nc3ccccc3o2)C[C@H]1NC(=O)c1cnnc(O)c1	1042286874	25	339.13	1.58
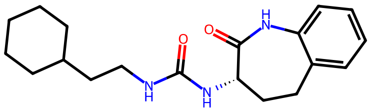	O=C(NCCC1CCCCC1)N[C@H]1CCc2ccccc2NC1=O	1220759028	24	329.21	3.21
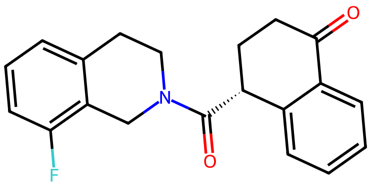	O=C1CC[C@@H](C(=O)N2CCc3cccc(F)c3C2)c2ccccc21	285795809	24	323.13	3.47
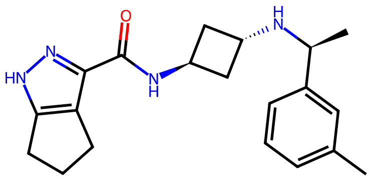	Cc1cccc([C@H](C)N[C@H]2C[C@H](NC(=O)c3n[nH]c4c3CCC4)C2)c1	1020344495	25	338.21	2.82

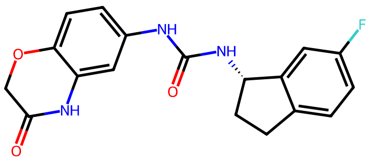	O=C1COc2ccc(NC(=O)N[C@H]3CCc4ccc(F)cc43)cc2N1	106577398	25	341.12	2.97
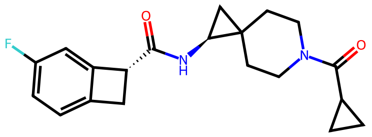	O=C(N[C@H]1CC12CCN(C(=O)C1CC1)CC2)[C@@H]1Cc2ccc(F)cc21	964977169	25	342.17	2.37
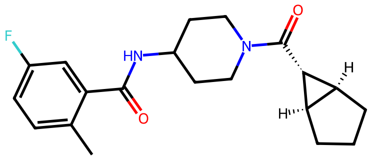	Cc1ccc(F)cc1C(=O)NC1CCN(C(=O)[C@@H]2[C@H]3CCC[C@H]32)CC1	946078695	25	344.19	2.9
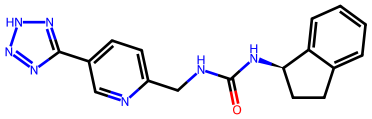	O=C(NCc1ccc(-c2nn[nH]n2)cn1)N[C@@H]1CCc2ccccc21	1582548778	25	335.15	1.75
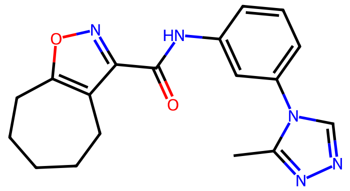	Cc1nncn1-c1cccc(NC(=O)c2noc3c2CCCCC3)c1	156960843	25	337.15	3.08
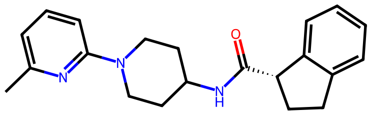	Cc1cccc(N2CCC(NC(=O)[C@H]3CCc4ccccc43)CC2)n1	342185499	25	335.2	3.21
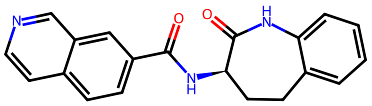	O=C(N[C@@H]1CCc2ccccc2NC1=O)c1ccc2ccncc2c1	1611425623	25	331.13	2.92
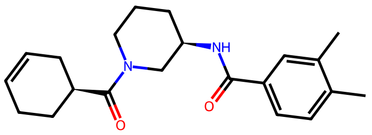	Cc1ccc(C(=O)N[C@@H]2CCCN(C(=O)[C@@H]3CC=CCC3)C2)cc1C	949984630	25	340.22	3.38

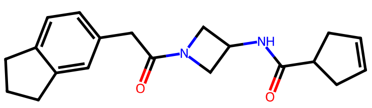	O=C(NC1CN(C(=O)Cc2ccc3c(c2)CCC3)C1)C1CC=CC1	990582769	24	324.18	2.01
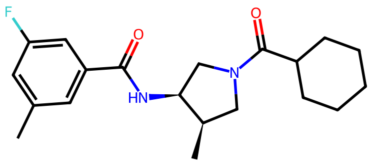	Cc1cc(F)cc(C(=O)N[C@H]2CN(C(=O)C3CCCCC3)C[C@H]2C)c1	966112307	25	346.21	3.29
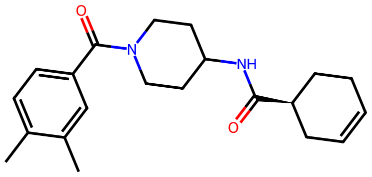	Cc1ccc(C(=O)N2CCC(NC(=O)[C@@H]3CC=CCC3)CC2)cc1C	948347926	25	340.22	3.38
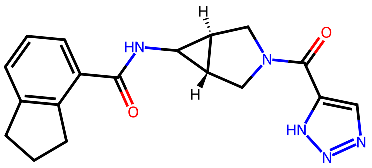	O=C(NC1[C@H]2CN(C(=O)c3cnn[nH]3)C[C@H]12)c1cccc2c1CCC2	1013292287	25	337.15	0.79
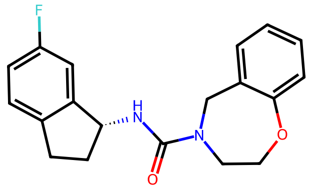	O=C(N[C@@H]1CCc2ccc(F)cc21)N1CCOc2ccccc2C1	592546943	24	326.14	3.42
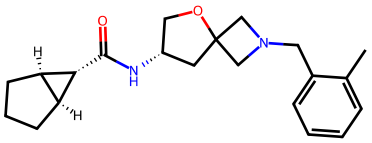	Cc1ccccc1CN1CC2(C[C@H](NC(=O)[C@@H]3[C@H]4CCC[C@H]43)CO2)C1	1053769413	25	340.22	2.5
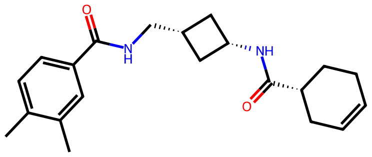	Cc1ccc(C(=O)NC[C@H]2C[C@@H](NC(=O)[C@H]3CC=CCC3)C2)cc1C	979287054	25	340.22	3.28
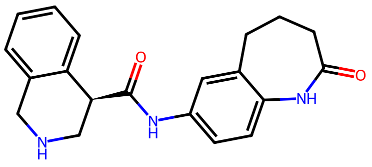	O=C1CCCc2cc(NC(=O)[C@H]3CNCc4ccccc43)ccc2N1	572997886	25	335.16	2.79

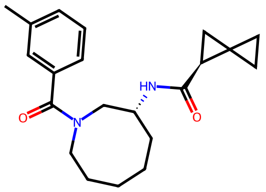	Cc1cccc(C(=O)N2CCCCCC[C@@H](NC(=O)[C@H]3CC34CC4)C2)c1	986868364	25	340.22	3.3
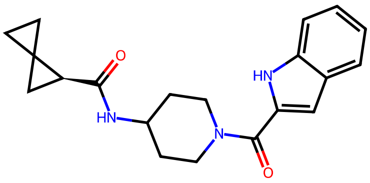	O=C(NC1CCN(C(=O)c2cc3ccccc3[nH]2)CC1)[C@H]1CC12CC2	948490780	25	337.18	2.69
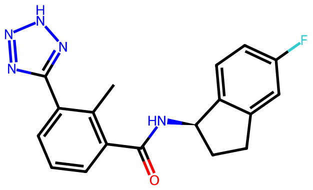	Cc1c(C(=O)N[C@@H]2CCc3cc(F)ccc32)cccc1-c1nn[nH]n1	1580546971	25	337.13	2.73
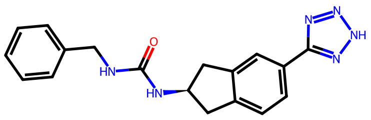	O=C(NCc1ccccc1)N[C@@H]1Cc2ccc(-c3nn[nH]n3)cc2C1	1593144217	25	334.15	1.83
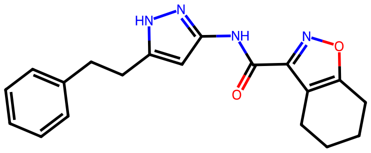	O=C(Nc1cc(CCc2ccccc2)[nH]n1)c1noc2c1CCCC2	607116343	25	336.16	3.31
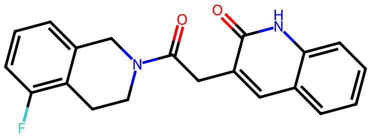	O=C(Cc1cc2ccccc2[nH]c1=O)N1CCc2c(F)cccc2C1	162775359	25	336.13	2.79
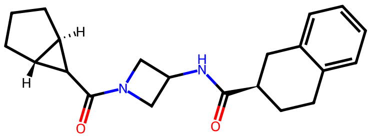	O=C(NC1CN(C(=O)C2[C@@H]3CCC[C@@H]23)C1)[C@@H]1CCc2ccccc2C1	1000537291	25	338.2	2.16
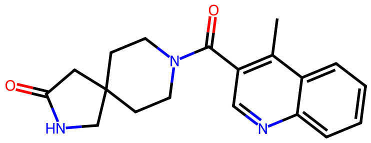	Cc1c(C(=O)N2CCC3(CC2)CNC(=O)C3)cnc2ccccc12	408345289	24	323.16	2.29

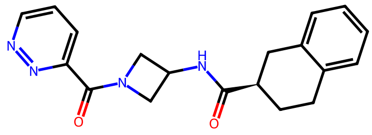	O=C(NC1CN(C(=O)c2cccnn2)C1)[C@@H]1CCc2ccccc2C1	1000537854	25	336.16	1.22
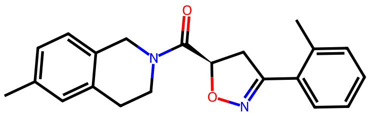	Cc1ccc2c(c1)CCN(C(=O)[C@H]1CC(c3ccccc3C)=NO1)C2	1536809073	25	334.17	3.38
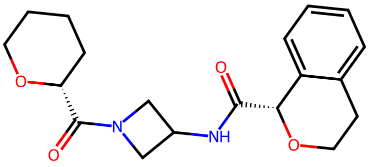	O=C(NC1CN(C(=O)[C@H]2CCCCO2)C1)[C@H]1OCCc2ccccc21	997321178	25	344.17	1.2
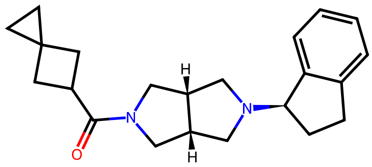	O=C(C1CC2(CC2)C1)N1C[C@@H]2CN([C@@H]3CCc4ccccc43)C[C@@H]2C1	1049049152	25	336.22	3.25
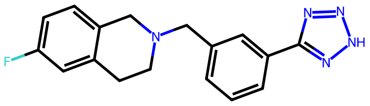	Fc1ccc2c(c1)CCN(Cc1cccc(-c3nn[nH]n3)c1)C2	826376013	23	309.14	2.56
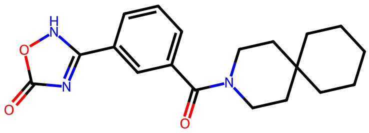	O=C(c1cccc(-c2nc(=O)o[nH]2)c1)N1CCC2(CCCCC2)CC1	667922476	25	341.17	3.22
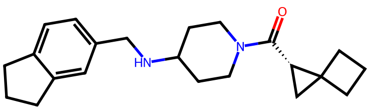	O=C([C@H]1CC12CCC2)N1CCC(NCc2ccc3c(c2)CCC3)CC1	997042479	25	338.24	3.45
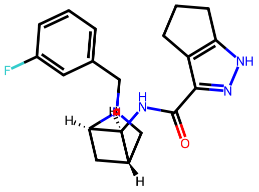	O=C(N[C@H]1[C@H]2C[C@H]1N(Cc1cccc(F)c1)C2)c1n[nH]c2c1CCC2	1037483699	25	340.17	2.04

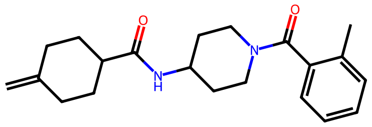	C=C1CCC(C(=O)NC2CCN(C(=O)c3ccccc3C)CC2)CC1	943770141	25	340.22	3.46
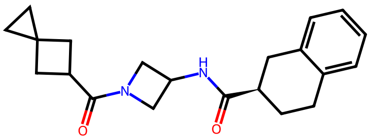	O=C(NC1CN(C(=O)C2CC3(CC3)C2)C1)[C@@H]1CCc2ccccc2C1	1000537329	25	338.2	2.31
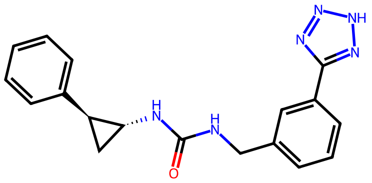	O=C(NCc1cccc(-c2nn[nH]n2)c1)N[C@@H]1C[C@H]1c1ccccc1	1593142562	25	334.15	2.22
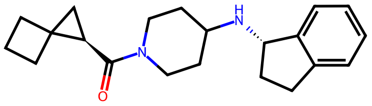	O=C([C@@H]1CC12CCC2)N1CCC(N[C@H]2CCc3ccccc32)CC1	997041657	24	324.22	3.44
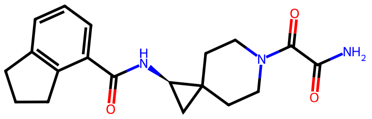	NC(=O)C(=O)N1CCC2(CC1)C[C@H]2NC(=O)c1cccc2c1CCC2	964032355	25	341.17	0.77
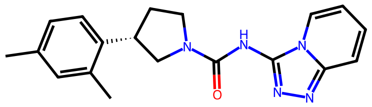	Cc1ccc([C@@H]2CCN(C(=O)Nc3nnc4ccccn34)C2)c(C)c1	373410830	25	335.17	3.37
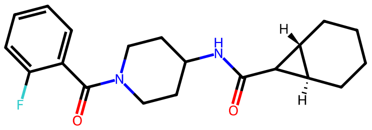	O=C(NC1CCN(C(=O)c2ccccc2F)CC1)C1[C@@H]2CCCC[C@@H]12	174637613	25	344.19	2.98
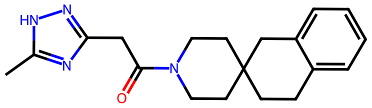	Cc1nc(CC(=O)N2CCC3(CCc4ccccc4C3)CC2)n[nH]1	616511387	24	324.2	2.45

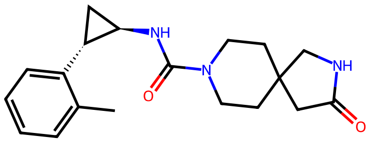	Cc1ccccc1[C@@H]1C[C@H]1NC(=O)N1CCC2(CC1)CNC(=O)C2	74408582	24	327.19	2.16
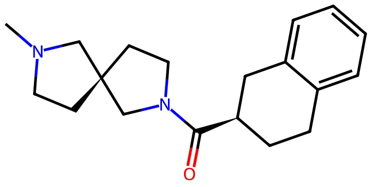	CN1CC[C@]2(CCN(C(=O)[C@@H]3CCc4ccccc4C3)C2)C1	569130527	22	298.2	2.35
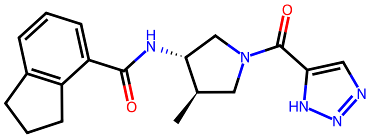	C[C@@H]1CN(C(=O)c2cnn[nH]2)C[C@H]1NC(=O)c1cccc2c1CCC2	951195953	25	339.17	1.18
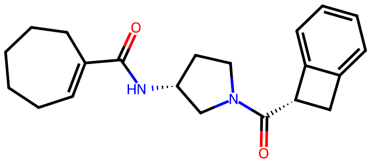	O=C(N[C@@H]1CCN(C(=O)[C@H]2Cc3ccccc32)C1)C1=CCCCCC1	940159292	25	338.2	2.93
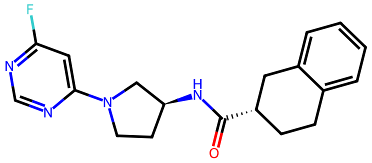	O=C(N[C@H]1CCN(c2cc(F)ncn2)C1)[C@H]1CCc2ccccc2C1	1058559468	25	340.17	2.12
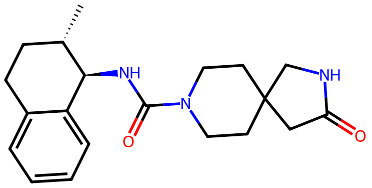	C[C@H]1CCc2ccccc2[C@@H]1NC(=O)N1CCC2(CC1)CNC(=O)C2	270729618	25	341.21	2.62
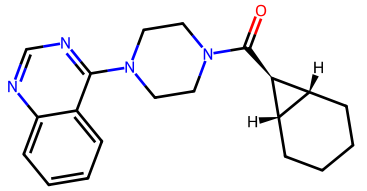	O=C([C@@H]1[C@H]2CCCC[C@H]21)N1CCN(c2ncnc3ccccc23)CC1	1055759618	25	336.2	2.71
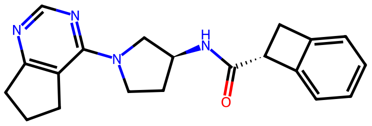	O=C(N[C@H]1CCN(c2ncnc3c2CCC3)C1)[C@@H]1Cc2ccccc21	1058531179	25	334.18	2.0

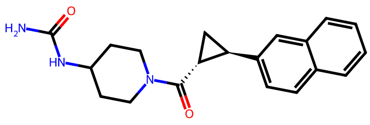	NC(=O)NC1CCN(C(=O)[C@@H]2C[C@H]2c2ccc3ccccc3c2)CC1	176057401	25	337.18	2.6
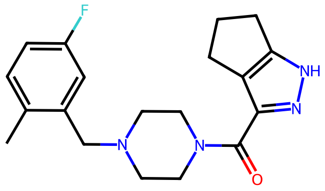	Cc1ccc(F)cc1CN1CCN(C(=O)c2n[nH]c3c2CCC3)CC1	949066523	25	342.19	2.3
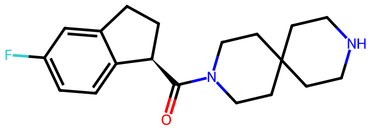	O=C([C@@H]1CCc2cc(F)ccc21)N1CCC2(CCNCC2)CC1	659322246	23	316.2	2.85
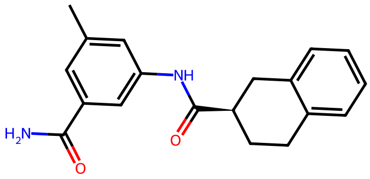	Cc1cc(NC(=O)[C@@H]2CCc3ccccc3C2)cc(C(N)=O)c1	583634455	23	308.15	2.84
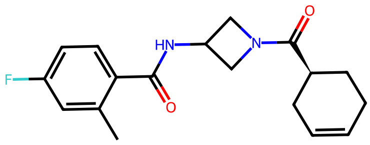	Cc1cc(F)ccc1C(=O)NC1CN(C(=O)[C@@H]2CC=CCC2)C1	997173597	23	316.16	2.43
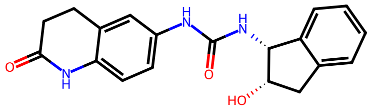	O=C1CCc2cc(NC(=O)N[C@@H]3c4ccccc4C[C@@H]3O)ccc2N1	69029728	25	337.14	2.35
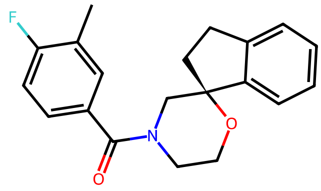	Cc1cc(C(=O)N2CCO[C@]3(CCc4ccccc43)C2)ccc1F	158521116	24	325.15	3.45
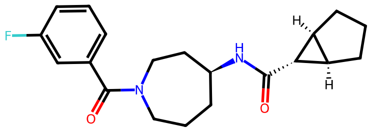	O=C(N[C@H]1CCCN(C(=O)c2cccc(F)c2)CC1)[C@@H]1[C@H]2CCC[C@H]21	964819278	25	344.19	2.98

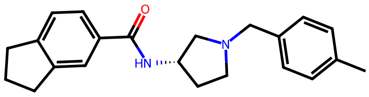	Cc1ccc(CN2CC[C@H](NC(=O)c3ccc4c(c3)CCC4)C2)cc1	1016227136	25	334.2	3.49
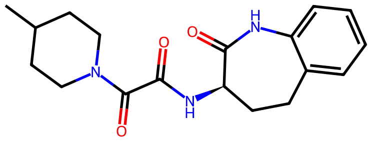	CC1CCN(C(=O)C(=O)N[C@@H]2CCc3ccccc3NC2=O)CC1	655223997	24	329.17	1.31
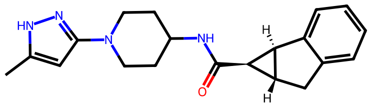	Cc1cc(N2CCC(NC(=O)[C@@H]3[C@H]4Cc5ccccc5[C@@H]43)CC2)n[nH]1	626603639	25	336.2	2.39
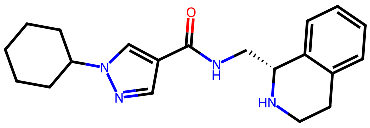	O=C(NC[C@H]1NCCc2ccccc21)c1cnn(C2CCCCC2)c1	1325856317	25	338.21	3.01
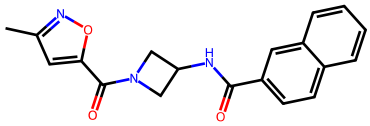	Cc1cc(C(=O)N2CC(NC(=O)c3ccc4ccccc4c3)C2)on1	999446231	25	335.13	2.39
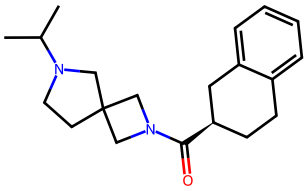	CC(C)N1CCC2(CN(C(=O)[C@@H]3CCc4ccccc4C3)C2)C1	1073014296	23	312.22	2.73
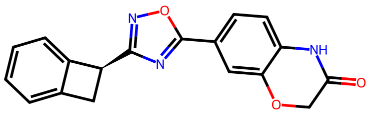	O=C1COc2cc(-c3nc([C@H]4Cc5ccccc54)no3)ccc2N1	546292841	24	319.1	2.76
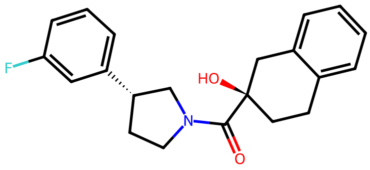	O=C(N1CC[C@H](c2cccc(F)c2)C1)[C@@]1(O)CCc2ccccc2C1	786105130	25	339.16	3.06

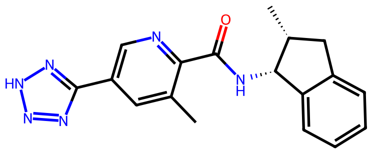	Cc1cc(-c2nn[nH]n2)cnc1C(=O)N[C@H]1c2ccccc2C[C@H]1C	1591879514	25	334.15	2.23
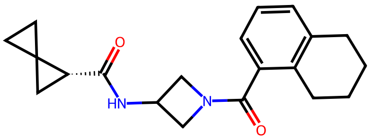	O=C(NC1CN(C(=O)c2cccc3c2CCCC3)C1)[C@@H]1CC12CC2	1000500535	24	324.18	2.31
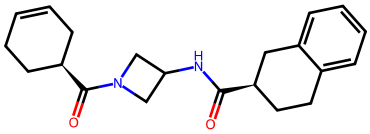	O=C(NC1CN(C(=O)[C@@H]2CC=CCC2)C1)[C@@H]1CCc2ccccc2C1	1000537906	25	338.2	2.47
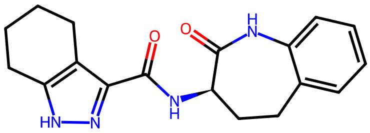	O=C(N[C@@H]1CCc2ccccc2NC1=O)c1n[nH]c2c1CCCC2	655223504	24	324.16	1.97
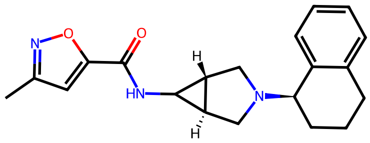	Cc1cc(C(=O)NC2[C@@H]3CN([C@@H]4CCCc5ccccc54)C[C@@H]23)on1	958216847	25	337.18	2.72
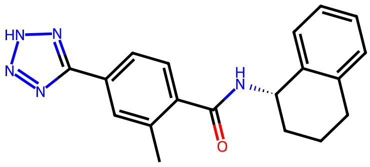	Cc1cc(-c2nn[nH]n2)ccc1C(=O)N[C@H]1CCCc2ccccc21	826247225	25	333.16	2.98
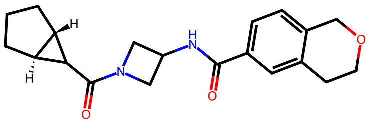	O=C(NC1CN(C(=O)C2[C@H]3CCC[C@H]23)C1)c1ccc2c(c1)CCOC2	996918927	25	340.18	1.75
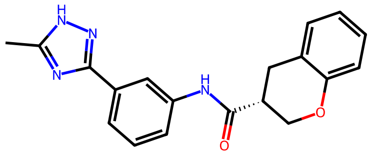	Cc1nc(-c2cccc(NC(=O)[C@H]3COc4ccccc4C3)c2)n[nH]1	363216398	25	334.14	2.97

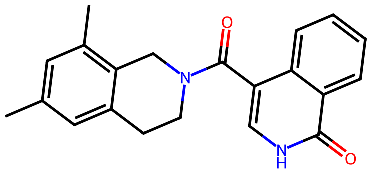	Cc1cc(C)c2c(c1)CCN(C(=O)c1c[nH]c(=O)c3ccccc13)C2	189987159	25	332.15	3.34
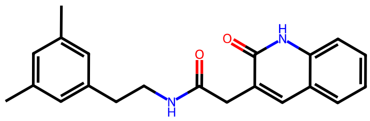	Cc1cc(C)cc(CCNC(=O)Cc2cc3ccccc3[nH]c2=O)c1	155174167	25	334.17	3.05
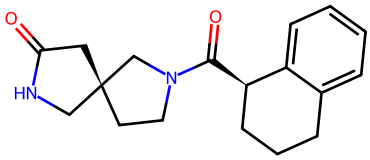	O=C1C[C@@]2(CCN(C(=O)[C@@H]3CCCc4ccccc43)C2)CN1	279456500	22	298.17	1.85
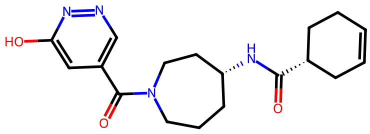	O=C(N[C@@H]1CCCN(C(=O)c2cnnc(O)c2)CC1)[C@H]1CC=CCC1	964749777	25	344.18	1.65
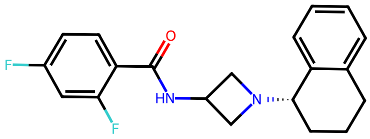	O=C(NC1CN([C@H]2CCCc3ccccc32)C1)c1ccc(F)cc1F	1031336915	25	342.15	3.46
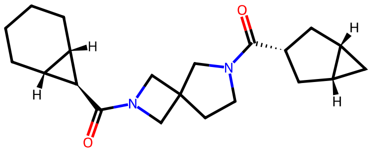	O=C([C@@H]1C[C@@H]2C[C@@H]2C1)N1CCC2(C1)CN(C(=O)[C@@H]1[C@H]3CCCC[C@H]31)C2	1018906043	25	342.23	2.53
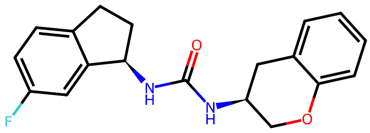	O=C(N[C@@H]1COc2ccccc2C1)N[C@@H]1CCc2ccc(F)cc21	106536589	24	326.14	3.12
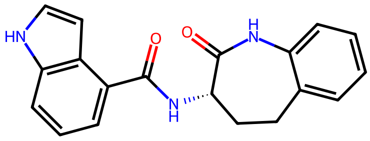	O=C(N[C@H]1CCc2ccccc2NC1=O)c1cccc2[nH]ccc12	1537778048	24	319.13	2.85

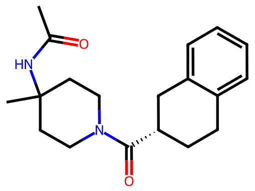	CC(=O)NC1(C)CCN(C(=O)[C@H]2CCc3ccccc3C2)CC1	956255062	23	314.2	2.31
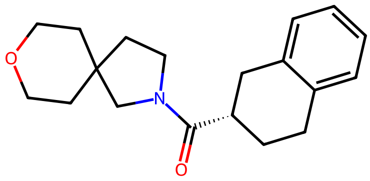	O=C([C@H]1CCc2ccccc2C1)N1CCC2(CCOCC2)C1	577400635	22	299.19	2.82
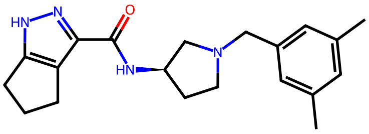	Cc1cc(C)cc(CN2CC[C@@H](NC(=O)c3n[nH]c4c3CCC4)C2)c1	1015069676	25	338.21	2.52
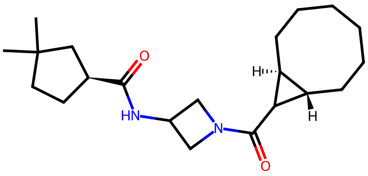	CC1(C)CC[C@H](C(=O)NC2CN(C(=O)C3[C@H]4CCCCCC[C@H]34)C2)C1	999244221	25	346.26	3.36
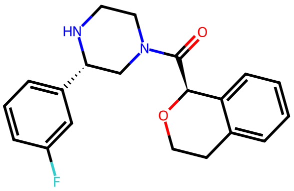	O=C([C@@H]1OCCc2ccccc21)N1CCN[C@@H](c2cccc(F)c2)C1	337614784	25	340.16	2.61
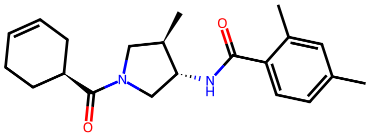	Cc1ccc(C(=O)N[C@@H]2CN(C(=O)[C@@H]3CC=CCC3)C[C@H]2C)c(C)c1	940136098	25	340.22	3.24
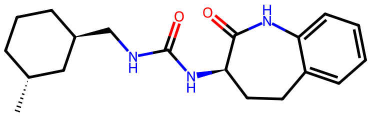	C[C@@H]1CCC[C@@H](CNC(=O)N[C@@H]2CCc3ccccc3NC2=O)C1	1563768273	24	329.21	3.07
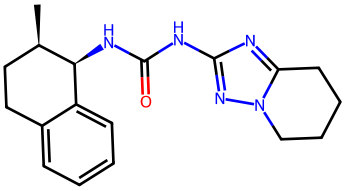	C[C@@H]1CCc2ccccc2[C@@H]1NC(=O)Nc1nc2n(n1)CCCC2	333657450	24	325.19	3.06

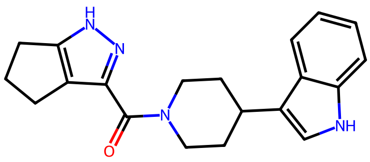	O=C(c1n[nH]c2c1CCC2)N1CCC(c2c[nH]c3ccccc23)CC1	84587995	25	334.18	3.4
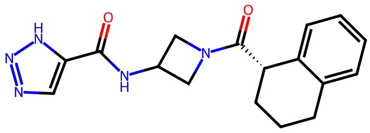	O=C(NC1CN(C(=O)[C@H]2CCCc3ccccc32)C1)c1cnn[nH]1	995173856	24	325.15	0.87
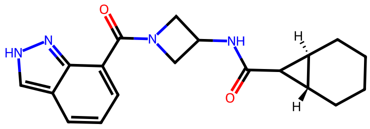	O=C(NC1CN(C(=O)c2cccc3c[nH]nc23)C1)C1[C@H]2CCCC[C@H]12	990655585	25	338.17	1.94
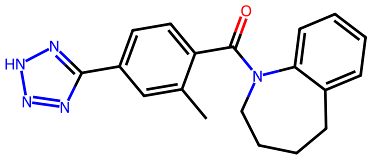	Cc1cc(-c2nn[nH]n2)ccc1C(=O)N1CCCCc2ccccc21	826245486	25	333.16	3.16
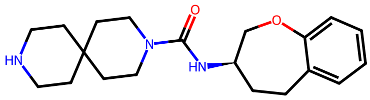	O=C(N[C@@H]1CCc2ccccc2OC1)N1CCC2(CCNCC2)CC1	418164493	25	343.23	2.56
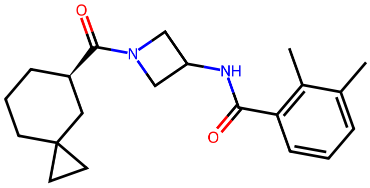	Cc1cccc(C(=O)NC2CN(C(=O)[C@@H]3CCCC4(CC4)C3)C2)c1C	999515778	25	340.22	3.21
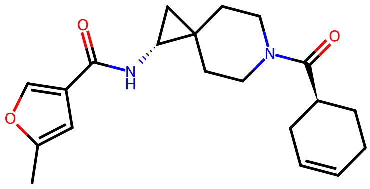	Cc1cc(C(=O)N[C@@H]2CC23CCN(C(=O)[C@@H]2CC=CCC2)CC3)co1	963729800	25	342.19	3.06
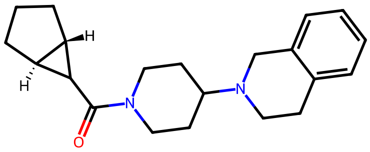	O=C(C1[C@H]2CCC[C@H]12)N1CCC(N2CCc3ccccc3C2)CC1	267319615	24	324.22	3.08

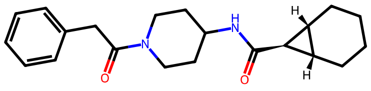	O=C(NC1CCN(C(=O)Cc2ccccc2)CC1)[C@@H]1[C@H]2CCCC[C@H]21	947344908	25	340.22	2.77
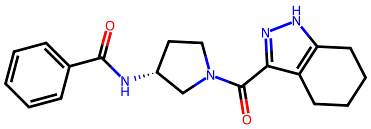	O=C(N[C@@H]1CCN(C(=O)c2n[nH]c3c2CCCC3)C1)c1ccccc1	939169976	25	338.17	1.93
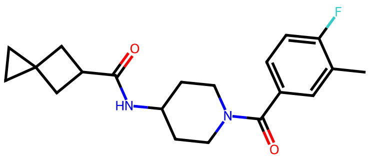	Cc1cc(C(=O)N2CCC(NC(=O)C3CC4(CC4)C3)CC2)ccc1F	948068721	25	344.19	3.05
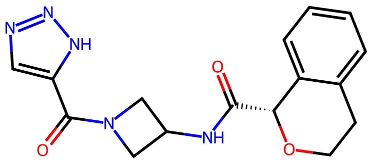	O=C(NC1CN(C(=O)c2cnn[nH]2)C1)[C@H]1OCCc2ccccc21	997325553	24	327.13	0.06
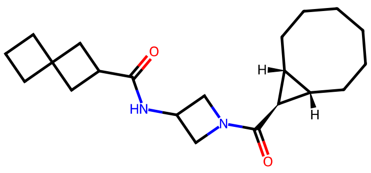	O=C(NC1CN(C(=O)[C@@H]2[C@H]3CCCCCC[C@H]32)C1)C1CC2(CCC2)C1	997934841	25	344.25	3.11
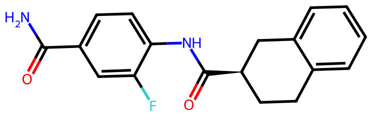	NC(=O)c1ccc(NC(=O)[C@@H]2CCc3ccccc3C2)c(F)c1	1456911928	23	312.13	2.67
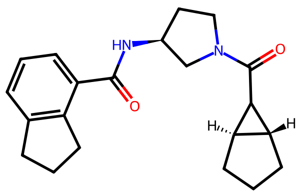	O=C(N[C@H]1CCN(C(=O)C2[C@@H]3CCC[C@@H]23)C1)c1cccc2c1CCC2	938149275	25	338.2	2.55
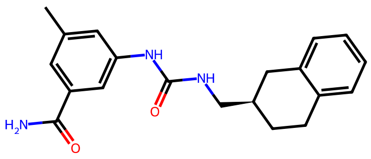	Cc1cc(NC(=O)NC[C@@H]2CCc3ccccc3C2)cc(C(N)=O)c1	653587401	25	337.18	3.02

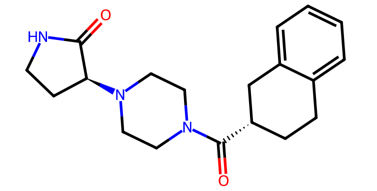	O=C1NCC[C@@H]1N1CCN(C(=O)[C@H]2CCc3ccccc3C2)CC1	950725683	24	327.19	0.82
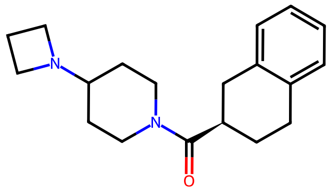	O=C([C@@H]1CCc2ccccc2C1)N1CCC(N2CCC2)CC1	912870472	22	298.2	2.49
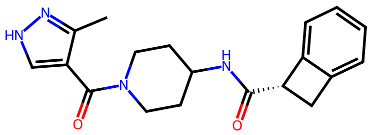	Cc1n[nH]cc1C(=O)N1CCC(NC(=O)[C@H]2Cc3ccccc32)CC1	947668663	25	338.17	1.78
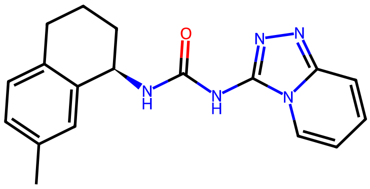	Cc1ccc2c(c1)[C@H](NC(=O)Nc1nnc3ccccn13)CCC2	572819931	24	321.16	3.24
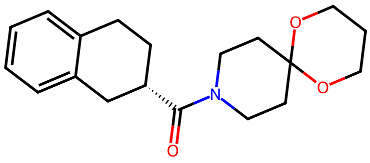	O=C([C@H]1CCc2ccccc2C1)N1CCC2(CC1)OCCCO2	846227730	23	315.18	2.55
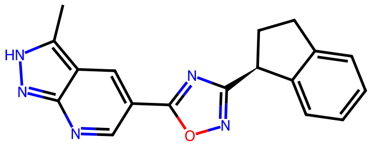	Cc1[nH]nc2ncc(-c3nc([C@H]4CCc5ccccc54)no3)cc12	356593965	24	317.13	3.39
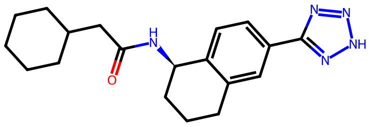	O=C(CC1CCCCC1)N[C@@H]1CCCc2cc(-c3nn[nH]n3)ccc21	1606503767	25	339.21	3.33
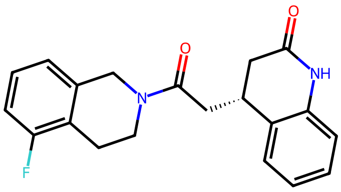	O=C1C[C@@H](CC(=O)N2CCc3c(F)cccc3C2)c2ccccc2N1	935103819	25	338.14	3.23

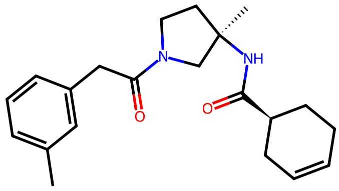	Cc1cccc(CC(=O)N2CC[C@@](C)(NC(=O)[C@@H]3CC=CCC3)C2)c1	956691333	25	340.22	3.0
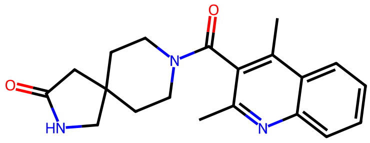	Cc1nc2ccccc2c(C)c1C(=O)N1CCC2(CC1)CNC(=O)C2	1362348401	25	337.18	2.59
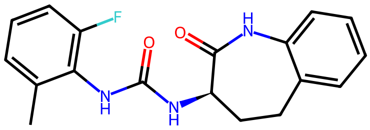	Cc1cccc(F)c1NC(=O)N[C@@H]1CCc2ccccc2NC1=O	1669775036	24	327.14	3.21
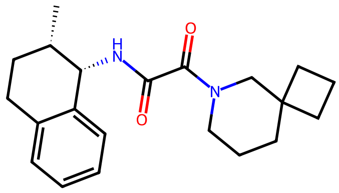	C[C@H]1CCc2ccccc2[C@H]1NC(=O)C(=O)N1CCCC2(CCC2)C1	359648128	25	340.22	3.22
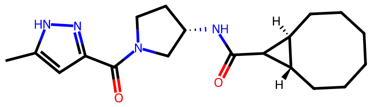	Cc1cc(C(=O)N2CC[C@H](NC(=O)C3[C@H]4CCCCCC[C@H]34)C2)n[nH]1	938971657	25	344.22	2.27
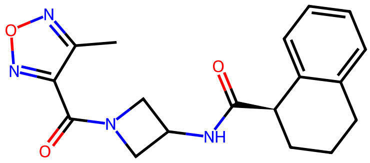	Cc1nonc1C(=O)N1CC(NC(=O)[C@@H]2CCCc3ccccc32)C1	995075591	25	340.15	1.44
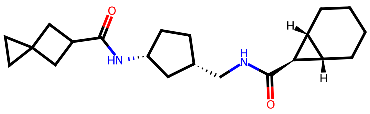	O=C(N[C@@H]1CC[C@H](CNC(=O)[C@@H]2[C@H]3CCCC[C@H]32)C1)C1CC2(CC2)C1	981097856	25	344.25	3.01
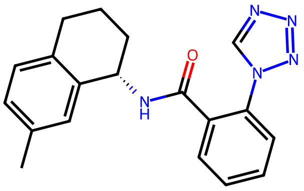	Cc1ccc2c(c1)[C@@H](NC(=O)c1ccccc1-n1cnnn1)CCC2	179596244	25	333.16	2.78

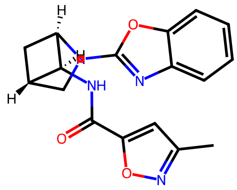	Cc1cc(C(=O)N[C@H]2[C@H]3C[C@H]2N(c2nc4ccccc4o2)C3)on1	1064205534	24	324.12	2.13
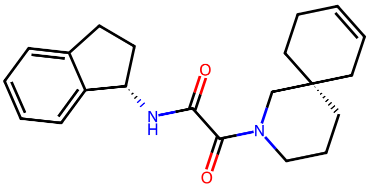	O=C(N[C@H]1CCc2ccccc21)C(=O)N1CCC[C@]2(CC=CCC2)C1	339872976	25	338.2	3.14
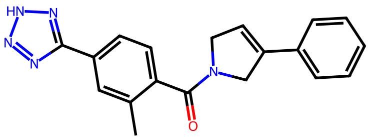	Cc1cc(-c2nn[nH]n2)ccc1C(=O)N1CC=C(c2ccccc2)C1	826244609	25	331.14	2.71
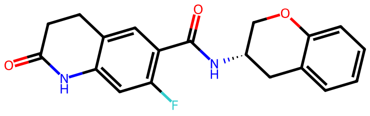	O=C1CCc2cc(C(=O)N[C@@H]3COc4ccccc4C3)c(F)cc2N1	178490599	25	340.12	2.44
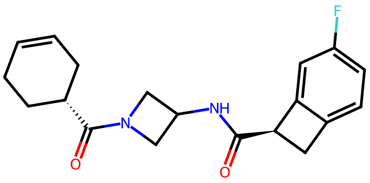	O=C(NC1CN(C(=O)[C@H]2CC=CCC2)C1)[C@@H]1Cc2ccc(F)cc21	998485782	24	328.16	2.15
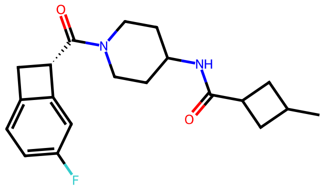	CC1CC(C(=O)NC2CCN(C(=O)[C@H]3Cc4ccc(F)cc43)CC2)C1	948024521	25	344.19	2.62
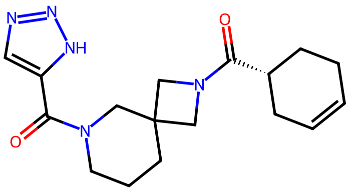	O=C(c1cnn[nH]1)N1CCCC2(C1)CN(C(=O)[C@H]1CC=CCC1)C2	1010525442	24	329.19	1.23
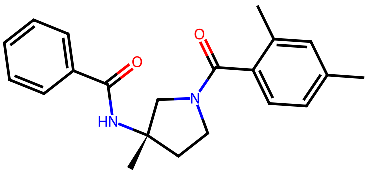	Cc1ccc(C(=O)N2CC[C@](C)(NC(=O)c3ccccc3)C2)c(C)c1	956657311	25	336.18	3.34

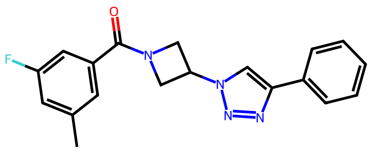	Cc1cc(F)cc(C(=O)N2CC(n3cc(-c4ccccc4)nn3)C2)c1	354709468	25	336.14	3.09
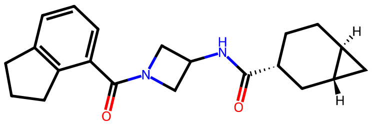	O=C(NC1CN(C(=O)c2cccc3c2CCC3)C1)[C@@H]1CC[C@H]2C[C@@H]2C1	994672891	25	338.2	2.55
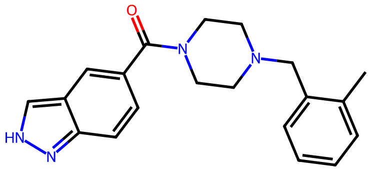	Cc1ccccc1CN1CCN(C(=O)c2ccc3n[nH]cc3c2)CC1	522507506	25	334.18	2.83
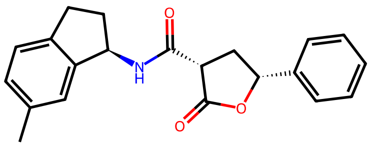	Cc1ccc2c(c1)[C@H](NC(=O)[C@@H]1C[C@H](c3ccccc3)OC1=O)CC2	809668252	25	335.15	3.4
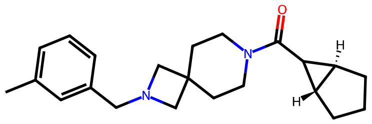	Cc1cccc(CN2CC3(CCN(C(=O)C4[C@H]5CCC[C@H]45)CC3)C2)c1	1035680614	25	338.24	3.47
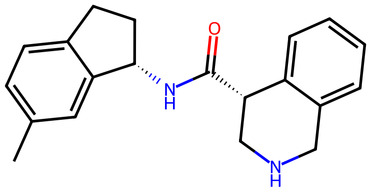	Cc1ccc2c(c1)[C@@H](NC(=O)[C@H]1CNCc3ccccc31)CC2	645946687	23	306.17	2.99
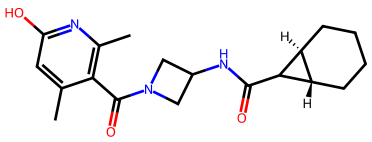	Cc1cc(O)nc(C)c1C(=O)N1CC(NC(=O)C2[C@H]3CCCC[C@H]23)C1	990656041	25	343.19	1.78
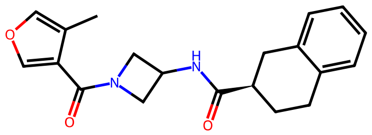	Cc1cocc1C(=O)N1CC(NC(=O)[C@@H]2CCc3ccccc3C2)C1	1000542419	25	338.16	2.33

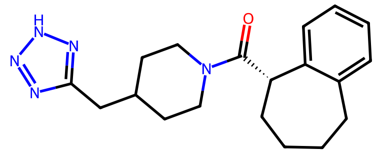	O=C([C@H]1CCCCc2ccccc21)N1CCC(Cc2nn[nH]n2)CC1	1548805742	25	339.21	2.49
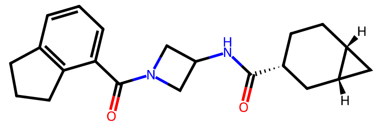	O=C(NC1CN(C(=O)c2cccc3c2CCC3)C1)[C@@H]1CC[C@@H]2C[C@@H]2C1	994672904	25	338.2	2.55
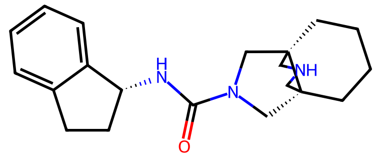	O=C(N[C@@H]1CCc2ccccc21)N1C[C@@]23CCCC[C@]2(CNC3)C1	1553507645	24	325.22	2.85
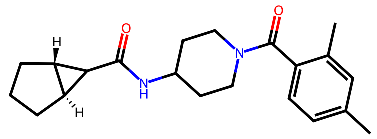	Cc1ccc(C(=O)N2CCC(NC(=O)C3[C@H]4CCC[C@H]34)CC2)c(C)c1	948346349	25	340.22	3.07
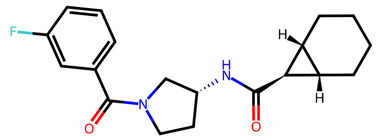	O=C(N[C@@H]1CCN(C(=O)c2cccc(F)c2)C1)[C@@H]1[C@H]2CCCC[C@H]21	939881792	24	330.17	2.59
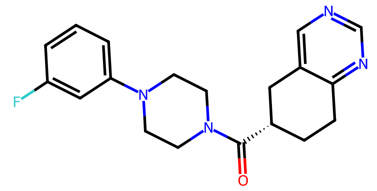	O=C([C@H]1CCc2ncncc2C1)N1CCN(c2cccc(F)c2)CC1	648244028	25	340.17	2.07
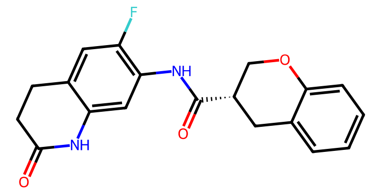	O=C1CCc2cc(F)c(NC(=O)[C@@H]3COc4ccccc4C3)cc2N1	361393179	25	340.12	2.9
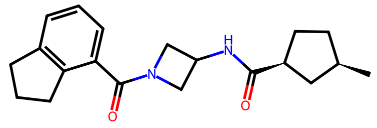	C[C@@H]1CC[C@H](C(=O)NC2CN(C(=O)c3cccc4c3CCC4)C2)C1	991429639	24	326.2	2.55

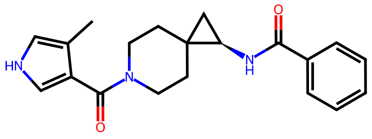	Cc1c[nH]cc1C(=O)N1CCC2(CC1)C[C@H]2NC(=O)c1ccccc1	962779711	25	337.18	2.75
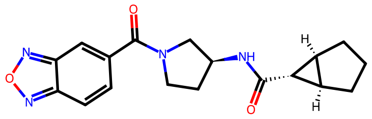	O=C(N[C@H]1CCN(C(=O)c2ccc3nonc3c2)C1)[C@@H]1[C@H]2CCC[C@H]21	937803614	25	340.15	1.6
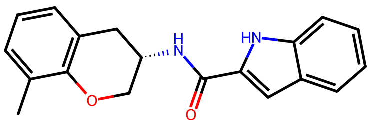	Cc1cccc2c1OC[C@@H](NC(=O)c1cc3ccccc3[nH]1)C2	484926136	23	306.14	3.21
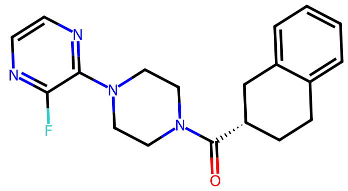	O=C([C@H]1CCc2ccccc2C1)N1CCN(c2nccnc2F)CC1	1056034083	25	340.17	2.07
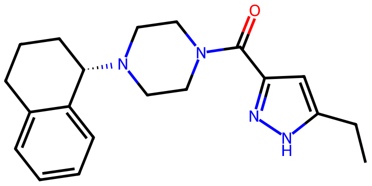	CCc1cc(C(=O)N2CCN([C@H]3CCCc4ccccc43)CC2)n[nH]1	1415818139	25	338.21	2.81
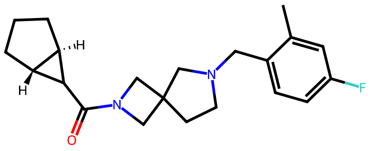	Cc1cc(F)ccc1CN1CCC2(C1)CN(C(=O)C1[C@@H]3CCC[C@@H]13)C2	1072590436	25	342.21	3.21
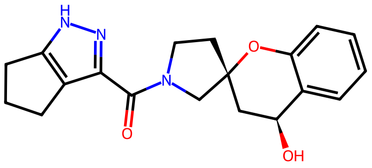	O=C(c1n[nH]c2c1CCC2)N1CC[C@@]2(C[C@H](O)c3ccccc3O2)C1	818408524	25	339.16	2.0
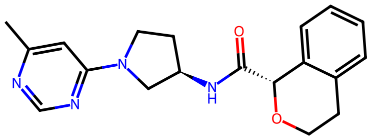	Cc1cc(N2CC[C@@H](NC(=O)[C@H]3OCCc4ccccc43)C2)ncn1	1058511103	25	338.17	1.79

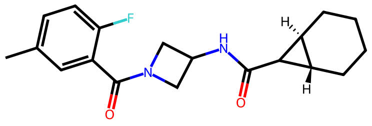	Cc1ccc(F)c(C(=O)N2CC(NC(=O)C3[C<br>@H]4CCCC[C@H]34)C2)c1	990659652	24	330.17	2.51
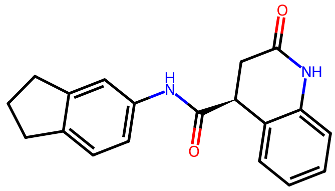	O=C1C[C@H](C(=O)Nc2ccc3c(c2)CC<br>C3)c2ccccc2N1	10642691	23	306.14	3.24
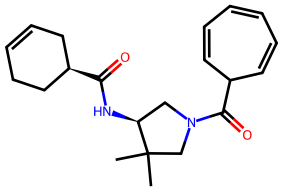	CC1(C)CN(C(=O)C2C=CC=CC=C2)C[<br>C@@H]1NC(=O)[C@@H]1CC=CCC1	1007875252	25	340.22	2.99
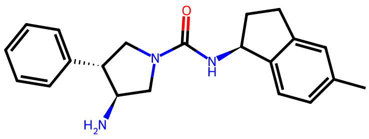	Cc1ccc2c(c1)CC[C@@H]2NC(=O)N1<br>C[C@@H](N)[C@H](c2ccccc2)C1	333947903	25	335.2	3.12
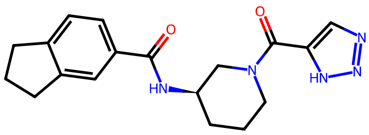	O=C(N[C@@H]1CCCN(C(=O)c2cnn[n<br>H]2)C1)c1ccc2c(c1)CCC2	951311273	25	339.17	1.33
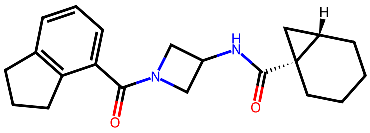	O=C(c1cccc2c1CCC2)N1CC(NC(=O)[<br>C@@]23CCCC[C@H]2C3)C1	999281314	25	338.2	2.7
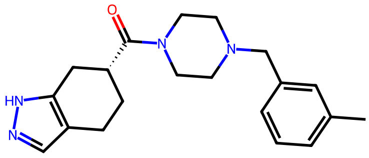	Cc1cccc(CN2CCN(C(=O)[C@@H]3C<br>Cc4cn[nH]c4C3)CC2)c1	79302586	25	338.21	2.17
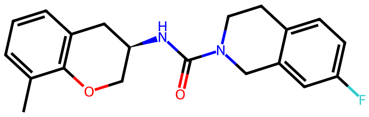	Cc1cccc2c1OC[C@H](NC(=O)N1CCc<br>3ccc(F)cc3C1)C2	462662011	25	340.16	3.21

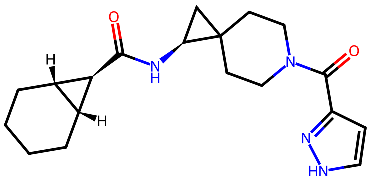	O=C(N[C@H]1CC12CCN(C(=O)c1cc[nH]n1)CC2)[C@@H]1[C@H]2CCCC[C@H]21	963448165	25	342.21	1.96
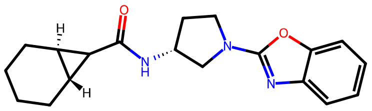	O=C(N[C@@H]1CCN(c2nc3ccccc3o2)C1)C1[C@@H]2CCCC[C@@H]12	1058334091	24	325.18	2.96
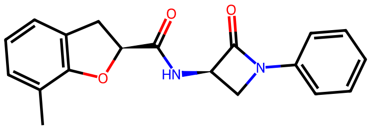	Cc1cccc2c1O[C@H](C(=O)N[C@@H]1CN(c3ccccc3)C1=O)C2	562693059	24	322.13	1.83
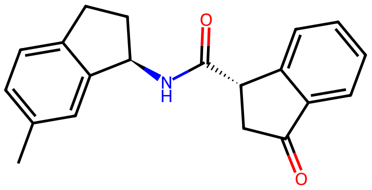	Cc1ccc2c(c1)[C@H](NC(=O)[C@H]1CC(=O)c3ccccc31)CC2	682798011	23	305.14	3.47
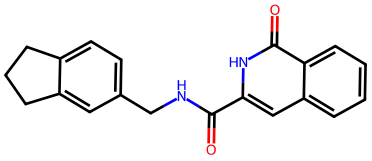	O=C(NCc1ccc2c(c1)CCC2)c1cc2ccccc2c(=O)[nH]1	60488838	24	318.14	2.95
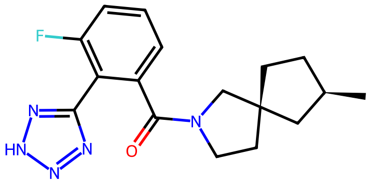	C[C@@H]1CC[C@]2(CCN(C(=O)c3cccc(F)c3-c3nn[nH]n3)C2)C1	1603882988	24	329.17	2.66
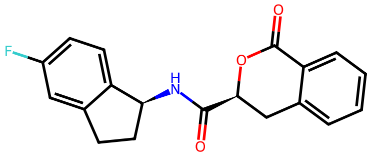	O=C1O[C@H](C(=O)N[C@H]2CCc3cc(F)ccc32)Cc2ccccc21	55657742	24	325.11	2.71
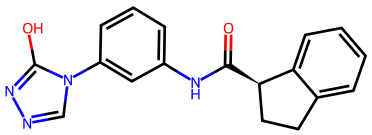	O=C(Nc1cccc(-n2cnnc2O)c1)[C@@H]1CCc2ccccc21	354599729	24	320.13	2.64

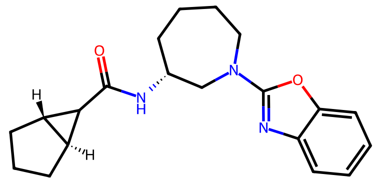	O=C(N[C@@H]1CCCCN(c2nc3ccccc3o2)C1)C1[C@H]2CCC[C@H]12	1063182066	25	339.19	3.35
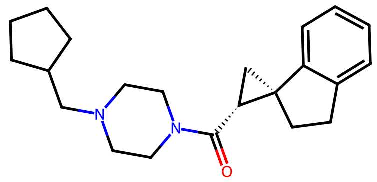	O=C([C@@H]1C[C@]12CCc1ccccc12)N1CCN(CC2CCCC2)CC1	956974909	25	338.24	3.22
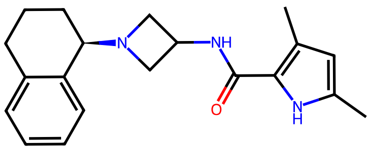	Cc1cc(C)c(C(=O)NC2CN([C@@H]3CCCc4ccccc43)C2)[nH]1	1030535686	24	323.2	3.12
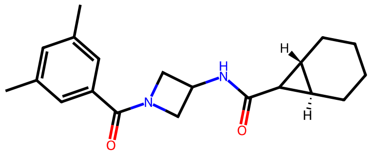	Cc1cc(C)cc(C(=O)N2CC(NC(=O)C3[C@@H]4CCCC[C@@H]34)C2)c1	990651966	24	326.2	2.68
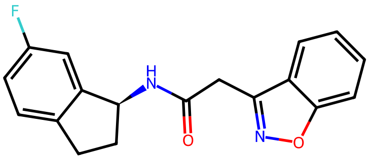	O=C(Cc1noc2ccccc12)N[C@H]1CCc2ccc(F)cc21	106680954	23	310.11	3.31
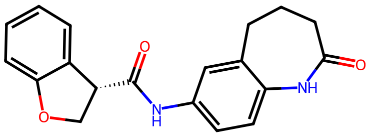	O=C1CCCc2cc(NC(=O)[C@@H]3COc4ccccc43)ccc2N1	113049060	24	322.13	3.08
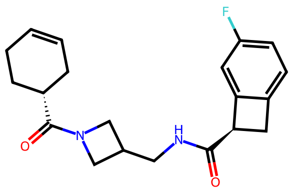	O=C(NCC1CN(C(=O)[C@H]2CC=CCC2)C1)[C@@H]1Cc2ccc(F)cc21	1003882946	25	342.17	2.4
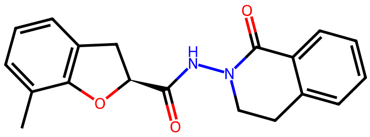	Cc1cccc2c1O[C@H](C(=O)NN1CCc3ccccc3C1=O)C2	827689282	24	322.13	2.03

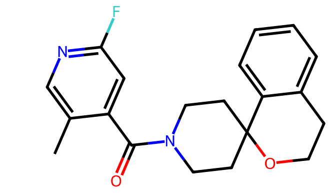	Cc1cnc(F)cc1C(=O)N1CCC2(CC1)OCCc1ccccc12	891397673	25	340.16	3.23
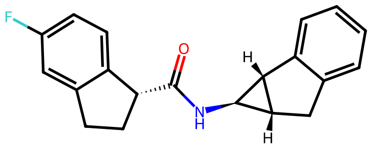	O=C(N[C@@H]1[C@H]2Cc3ccccc3[C@H]21)[C@@H]1CCc2cc(F)ccc21	926765034	23	307.14	3.31
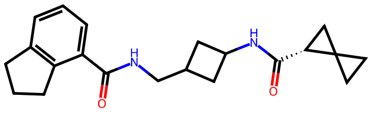	O=C(NCC1CC(NC(=O)[C@@H]2CC23CC3)C1)c1cccc2c1CCC2	992200894	25	338.2	2.6
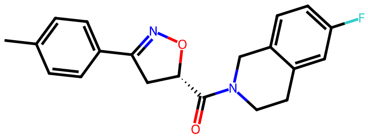	Cc1ccc(C2=NO[C@H](C(=O)N3CCc4cc(F)ccc4C3)C2)cc1	1127241527	25	338.14	3.21
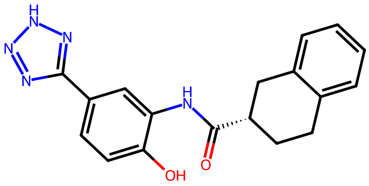	O=C(Nc1cc(-c2nn[nH]n2)ccc1O)[C@H]1CCc2ccccc2C1	1606897793	25	335.14	2.32
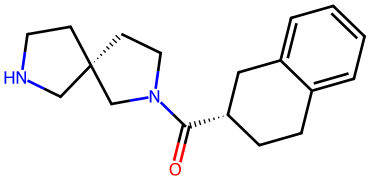	O=C([C@H]1CCc2ccccc2C1)N1CC[C@]2(CCNC2)C1	314203823	21	284.19	2.0
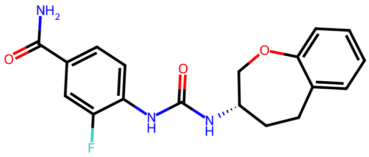	NC(=O)c1ccc(NC(=O)N[C@H]2CCc3ccccc3OC2)c(F)c1	338404306	25	343.13	2.44
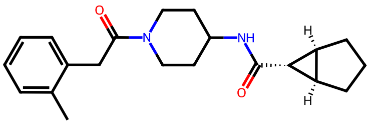	Cc1ccccc1CC(=O)N1CCC(NC(=O)[C@@H]2[C@H]3CCC[C@H]32)CC1	946717793	25	340.22	2.69

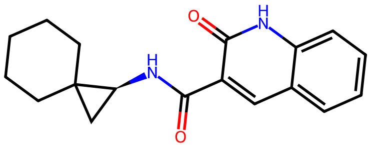	O=C(N[C@H]1CC12CCCCC2)c1cc2ccccc2[nH]c1=O	180794726	22	296.15	2.98
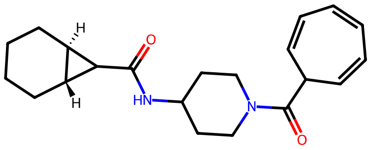	O=C(NC1CCN(C(=O)C2C=CC=CC=C2)CC1)C1[C@@H]2CCCC[C@@H]12	946949672	25	340.22	2.83
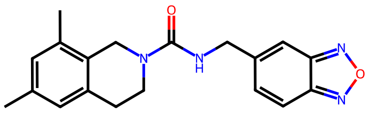	Cc1cc(C)c2c(c1)CCN(C(=O)NCc1ccc3nonc3c1)C2	1685599334	25	336.16	3.11
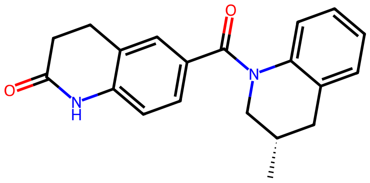	C[C@H]1Cc2ccccc2N(C(=O)c2ccc3c(c2)CCC(=O)N3)C1	96735945	24	320.15	3.41
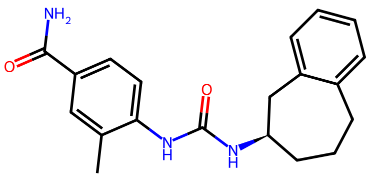	Cc1cc(C(N)=O)ccc1NC(=O)N[C@@H]1CCCc2ccccc2C1	929641614	25	337.18	3.16
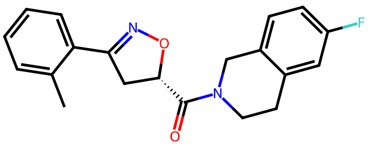	Cc1ccccc1C1=NO[C@H](C(=O)N2CCc3cc(F)ccc3C2)C1	1127326392	25	338.14	3.21
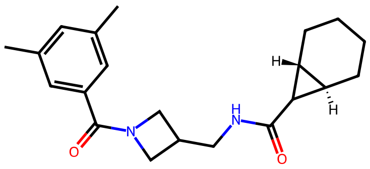	Cc1cc(C)cc(C(=O)N2CC(CNC(=O)C3[C@@H]4CCCC[C@@H]34)C2)c1	1001322780	25	340.22	2.93
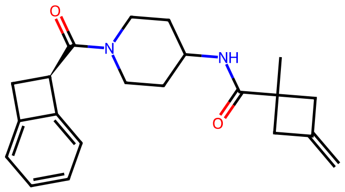	C=C1CC(C)(C(=O)NC2CCN(C(=O)[C@@H]3Cc4ccccc43)CC2)C1	948060109	25	338.2	2.79

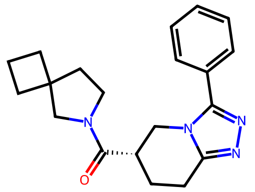	O=C([C@H]1CCc2nnc(-c3ccccc3)n2C1)N1CCC2(CCC2)C1	564287935	25	336.2	2.91
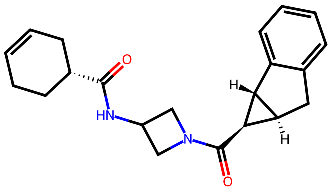	O=C(NC1CN(C(=O)[C@@H]2[C@@H]3Cc4ccccc4[C@H]32)C1)[C@H]1CC=CCC1	990220876	25	336.18	2.26
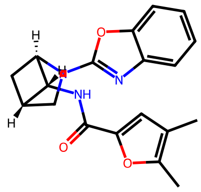	Cc1cc(C(=O)N[C@@H]2[C@H]3C[C@H]2N(c2nc4ccccc4o2)C3)oc1C	1064243225	25	337.14	3.04
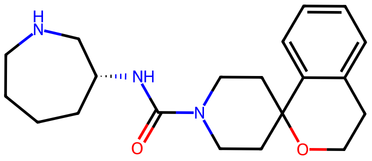	O=C(N[C@@H]1CCCCNC1)N1CCC2(CC1)OCCc1ccccc12	561207824	25	343.23	2.4
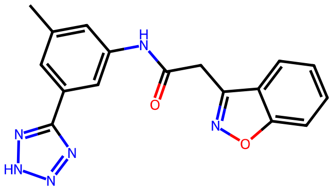	Cc1cc(NC(=O)Cc2noc3ccccc23)cc(-c2nn[nH]n2)c1	1605707976	25	334.12	2.5
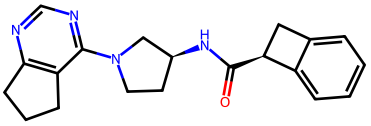	O=C(N[C@H]1CCN(c2ncnc3c2CCC3)C1)[C@H]1Cc2ccccc21	1058531180	25	334.18	2.0
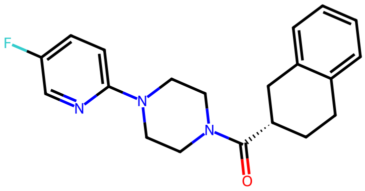	O=C([C@H]1CCc2ccccc2C1)N1CCN(c2ccc(F)cn2)CC1	519759987	25	339.17	2.67
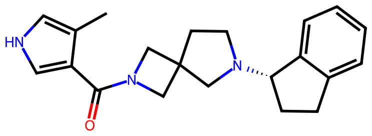	Cc1c[nH]cc1C(=O)N1CC2(CCN([C@H]3CCc4ccccc43)C2)C1	1073428024	25	335.2	3.16

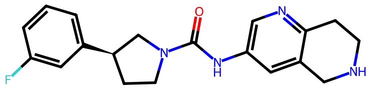	O=C(Nc1cnc2c(c1)CNCC2)N1CC[C@@H](c2cccc(F)c2)C1	667795524	25	340.17	2.89
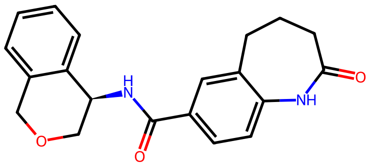	O=C1CCCc2cc(C(=O)N[C@H]3COCc4ccccc43)ccc2N1	667902076	25	336.15	2.96
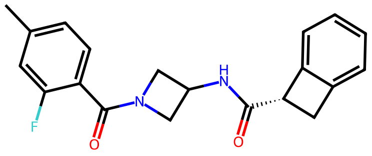	Cc1ccc(C(=O)N2CC(NC(=O)[C@H]3Cc4ccccc43)C2)c(F)c1	998693603	25	338.14	2.41
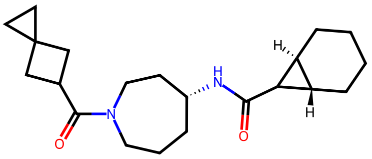	O=C(N[C@@H]1CCCN(C(=O)C2CC3(CC3)C2)CC1)C1[C@H]2CCCC[C@H]12	964809582	25	344.25	3.11
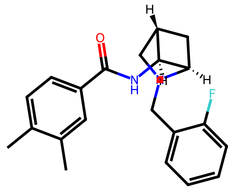	Cc1ccc(C(=O)N[C@H]2[C@H]3C[C@H]2N(Cc2ccccc2F)C3)cc1C	1037711758	25	338.18	3.45
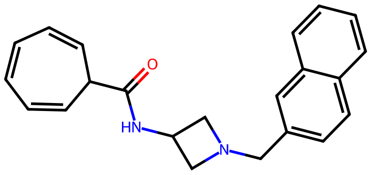	O=C(NC1CN(Cc2ccc3ccccc3c2)C1)C1C=CC=CC=C1	1030383007	25	330.17	3.44
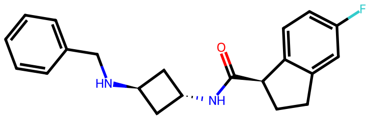	O=C(N[C@H]1C[C@H](NCc2ccccc2)C1)[C@@H]1CCc2cc(F)ccc21	1020399749	25	338.18	3.29
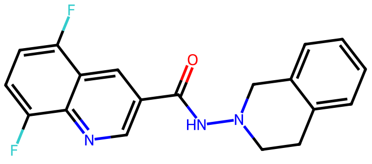	O=C(NN1CCc2ccccc2C1)c1cnc2c(F)ccc(F)c2c1	1640002837	25	339.12	3.22

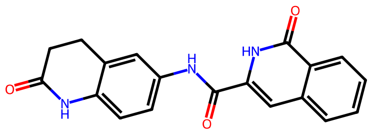	O=C1CCc2cc(NC(=O)c3cc4ccccc4c(=O)[nH]3)ccc2N1	33066584	25	333.11	2.67
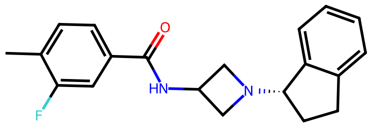	Cc1ccc(C(=O)NC2CN([C@H]3CCc4ccccc43)C2)cc1F	1030638017	24	324.16	3.24
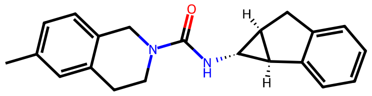	Cc1ccc2c(c1)CCN(C(=O)N[C@@H]1[C@H]3Cc4ccccc4[C@H]31)C2	481264539	24	318.17	3.4
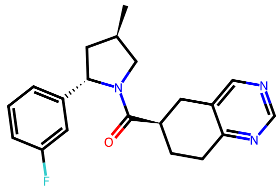	C[C@@H]1C[C@@H](c2cccc(F)c2)N(C(=O)[C@@H]2CCc3ncncc3C2)C1	648798078	25	339.17	3.33
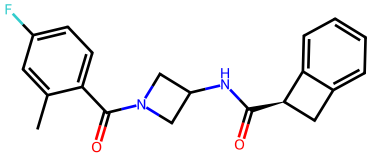	Cc1cc(F)ccc1C(=O)N1CC(NC(=O)[C@@H]2Cc3ccccc32)C1	998682198	25	338.14	2.41
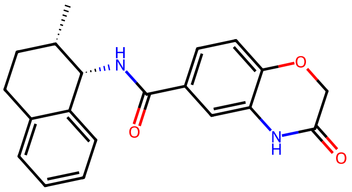	C[C@H]1CCc2ccccc2[C@H]1NC(=O)c1ccc2c(c1)NC(=O)CO2	122492781	25	336.15	3.07
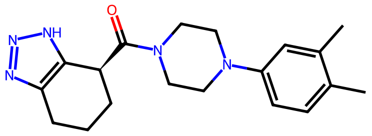	Cc1ccc(N2CCN(C(=O)[C@H]3CCCc4nn[nH]c43)CC2)cc1C	831330534	25	339.21	2.19
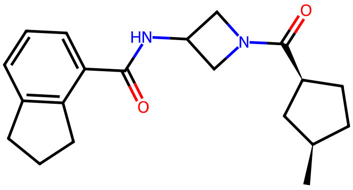	C[C@@H]1CC[C@H](C(=O)N2CC(NC(=O)c3cccc4c3CCC4)C2)C1	994398913	24	326.2	2.55

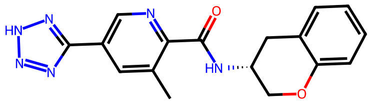	Cc1cc(-c2nn[nH]n2)cnc1C(=O)N[C@H]1COc2ccccc2C1	1591877874	25	336.13	1.3
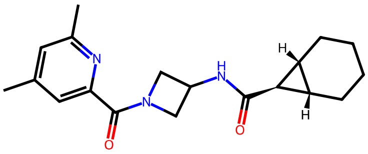	Cc1cc(C)nc(C(=O)N2CC(NC(=O)[C@@H]3[C@H]4CCCC[C@H]43)C2)c1	990657251	24	327.19	2.08
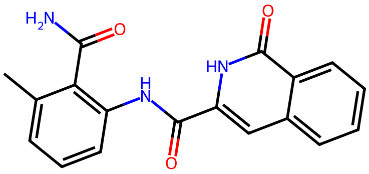	Cc1cccc(NC(=O)c2cc3ccccc3c(=O)[nH]2)c1C(N)=O	78829248	24	321.11	2.19
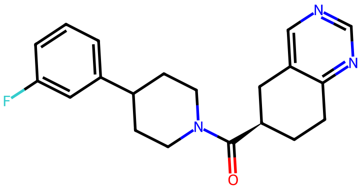	O=C([C@@H]1CCc2ncncc2C1)N1CCC(c2cccc(F)c2)CC1	843561294	25	339.17	3.13
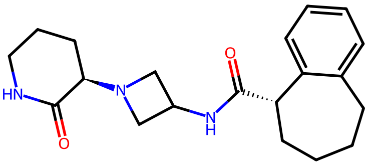	O=C(NC1CN([C@@H]2CCCNC2=O)C1)[C@H]1CCCCc2ccccc21	1031452581	25	341.21	1.58
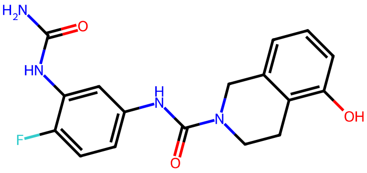	NC(=O)Nc1cc(NC(=O)N2CCc3c(O)cccc3C2)ccc1F	1365212043	25	344.13	2.61
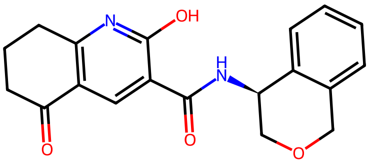	O=C(N[C@@H]1COCc2ccccc21)c1cc2c(nc1O)CCCC2=O	1413133162	25	338.13	2.31
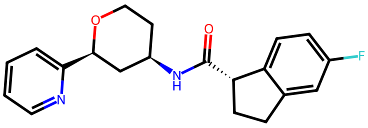	O=C(N[C@@H]1CCO[C@H](c2ccccn2)C1)[C@H]1CCc2cc(F)ccc21	1549391063	25	340.16	3.29

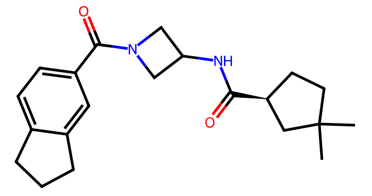	CC1(C)CC[C@H](C(=O)NC2CN(C(=O)c3ccc4c(c3)CCC4)C2)C1	999237373	25	340.22	2.94
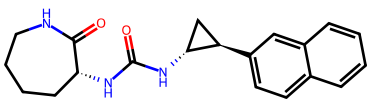	O=C(N[C@@H]1CCCCNC1=O)N[C@@H]1C[C@H]1c1ccc2ccccc2c1	333145510	25	337.18	2.66
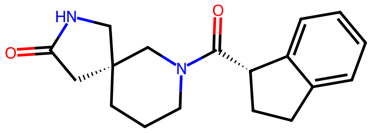	O=C1C[C@]2(CCCN(C(=O)[C@H]3CCc4ccccc43)C2)CN1	336402780	22	298.17	1.85
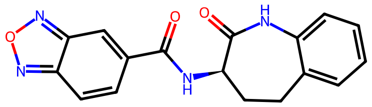	O=C(N[C@@H]1CCc2ccccc2NC1=O)c1ccc2nonc2c1	1640486058	24	322.11	1.91
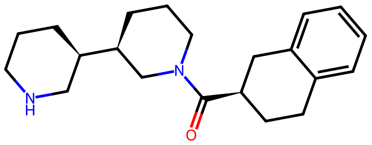	O=C([C@@H]1CCc2ccccc2C1)N1CCC[C@H]([C@@H]2CCCNC2)C1	653143383	24	326.24	3.03
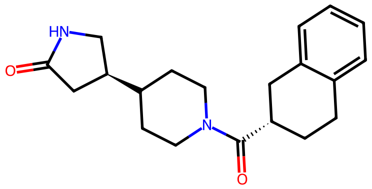	O=C1C[C@H](C2CCN(C(=O)[C@H]3CCc4ccccc4C3)CC2)CN1	1551708578	24	326.2	2.17
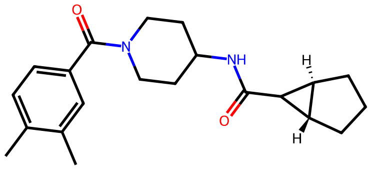	Cc1ccc(C(=O)N2CCC(NC(=O)C3[C@H]4CCC[C@H]34)CC2)cc1C	948347892	25	340.22	3.07
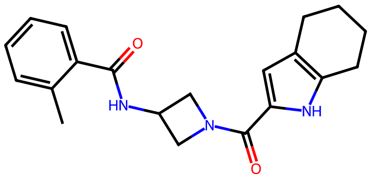	Cc1ccccc1C(=O)NC1CN(C(=O)c2cc3c([nH]2)CCCC3)C1	990109647	25	337.18	2.46

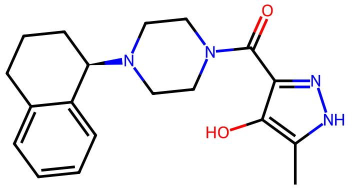	Cc1[nH]nc(C(=O)N2CCN([C@@H]3CCCc4ccccc43)CC2)c1O	1365692705	25	340.19	2.26
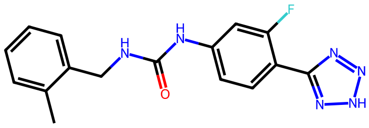	Cc1ccccc1CNC(=O)Nc1ccc(-c2nn[nH]n2)c(F)c1	1592257591	24	326.13	2.64
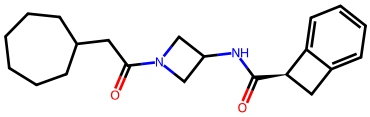	O=C(NC1CN(C(=O)CC2CCCCCC2)C1)[C@@H]1Cc2ccccc21	998694136	25	340.22	3.01
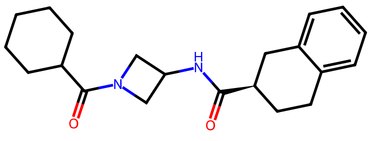	O=C(NC1CN(C(=O)C2CCCCC2)C1)[C@@H]1CCc2ccccc2C1	1000538586	25	340.22	2.7
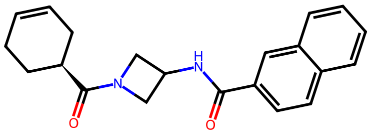	O=C(NC1CN(C(=O)[C@@H]2CC=CCC2)C1)c1ccc2ccccc2c1	999448739	25	334.17	3.14
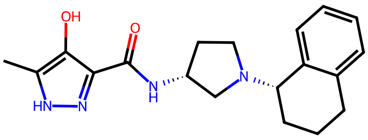	Cc1[nH]nc(C(=O)N[C@@H]2CCN([C@H]3CCCc4ccccc43)C2)c1O	1010645500	25	340.19	2.31
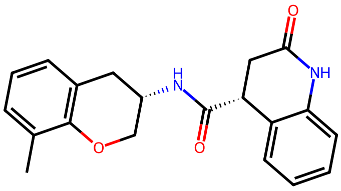	Cc1cccc2c1OC[C@@H](NC(=O)[C@@H]1CC(=O)Nc3ccccc31)C2	291627687	25	336.15	2.54
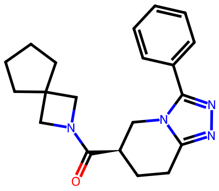	O=C([C@@H]1CCc2nnc(-c3ccccc3)n2C1)N1CC2(CCCC2)C1	576194240	25	336.2	2.91

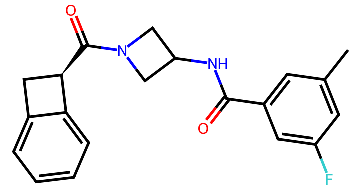	Cc1cc(F)cc(C(=O)NC2CN(C(=O)[C@@H]3Cc4ccccc43)C2)c1	1000442066	25	338.14	2.41
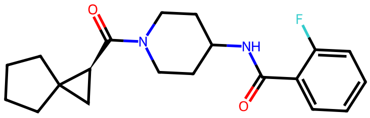	O=C(NC1CCN(C(=O)[C@H]2CC23CCCC3)CC1)c1ccccc1F	948360708	25	344.19	3.13
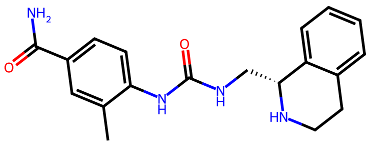	Cc1cc(C(N)=O)ccc1NC(=O)NC[C@H]1NCCc2ccccc21	841038917	25	338.17	2.1
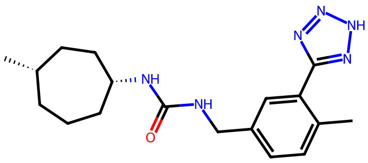	Cc1ccc(CNC(=O)N[C@@H]2CCC[C@H](C)CC2)cc1-c1nn[nH]n1	1605852043	25	342.22	2.94
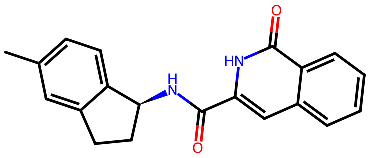	Cc1ccc2c(c1)CC[C@@H]2NC(=O)c1cc2ccccc2c(=O)[nH]1	56316228	24	318.14	3.25
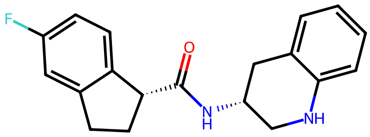	O=C(N[C@H]1CNc2ccccc2C1)[C@@H]1CCc2cc(F)ccc21	658941630	23	310.15	3.01
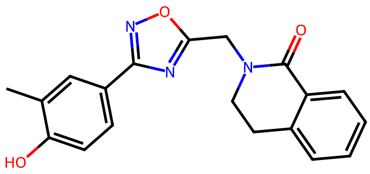	Cc1cc(-c2noc(CN3CCc4ccccc4C3=O)n2)ccc1O	580739136	25	335.13	2.95
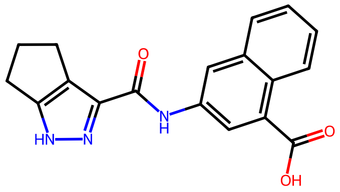	O=C(Nc1cc(C(=O)O)c2ccccc2c1)c1n[nH]c2c1CCC2	1608499262	24	321.11	3.0

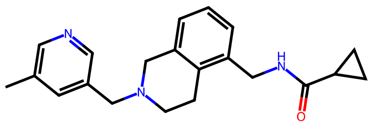	Cc1cncc(CN2CCc3c(CNC(=O)C4CC4)cccc3C2)c1	1051274017	25	335.2	2.97
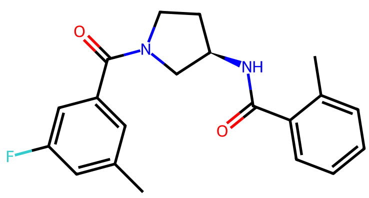	Cc1cc(F)cc(C(=O)N2CC[C@@H](NC(=O)c3ccccc3C)C2)c1	936242551	25	340.16	3.09
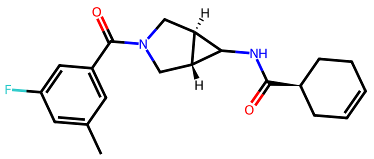	Cc1cc(F)cc(C(=O)N2C[C@H]3C(NC(=O)[C@@H]4CC=CCC4)[C@@H]3C2)c1	1012765984	25	342.17	2.68
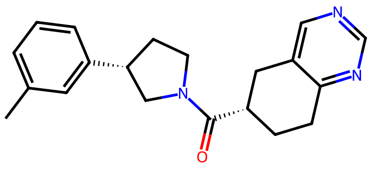	Cc1cccc([C@@H]2CCN(C(=O)[C@H]3CCc4ncncc4C3)C2)c1	648353006	24	321.18	2.91
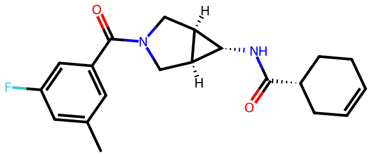	Cc1cc(F)cc(C(=O)N2C[C@H]3[C@H](NC(=O)[C@H]4CC=CCC4)[C@H]3C2)c1	976175656	25	342.17	2.68
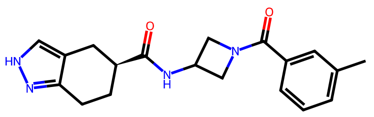	Cc1cccc(C(=O)N2CC(NC(=O)[C@H]3CCc4n[nH]cc4C3)C2)c1	996395992	25	338.17	1.46
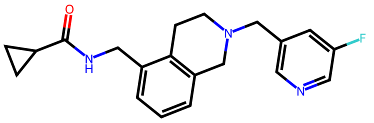	O=C(NCc1cccc2c1CCN(Cc1cncc(F)c1)C2)C1CC1	1051275845	25	339.17	2.81
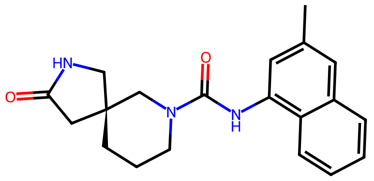	Cc1cc(NC(=O)N2CCC[C@@]3(CNC(=O)C3)C2)c2ccccc2c1	455746635	25	337.18	3.28

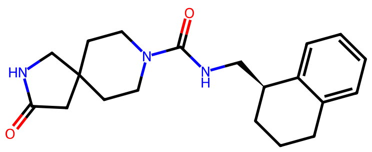	O=C1CC2(CCN(C(=O)NC[C@@H]3CCCc4ccccc43)CC2)CN1	73713397	25	341.21	2.42
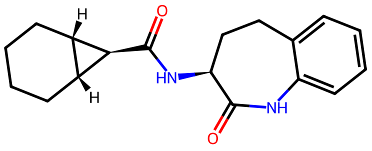	O=C1Nc2ccccc2CC[C@@H]1NC(=O)[C@@H]1[C@H]2CCCC[C@H]21	654002298	22	298.17	2.49
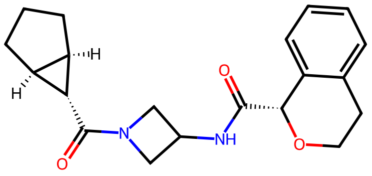	O=C(NC1CN(C(=O)[C@@H]2[C@H]3CCC[C@H]32)C1)[C@H]1OCCc2ccccc21	997327091	25	340.18	1.67
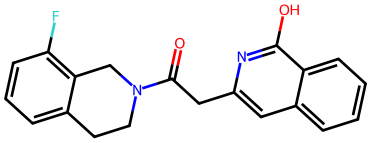	O=C(Cc1cc2ccccc2c(O)n1)N1CCc2ccc(F)c2C1	1598406388	25	336.13	3.21
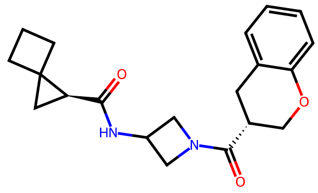	O=C(NC1CN(C(=O)[C@H]2COc3ccccc3C2)C1)[C@H]1CC12CCC2	991556374	25	340.18	1.75
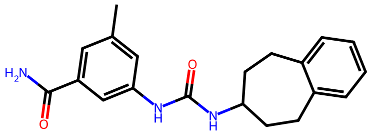	Cc1cc(NC(=O)NC2CCc3ccccc3CC2)cc(C(N)=O)c1	520519157	25	337.18	3.16
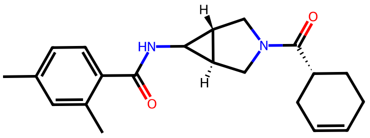	Cc1ccc(C(=O)NC2[C@@H]3CN(C(=O)[C@H]4CC=CCC4)C[C@@H]23)c(C)c1	1013775082	25	338.2	2.85
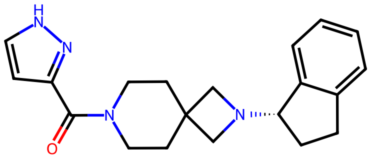	O=C(c1cc[nH]n1)N1CCC2(CC1)CN([C@H]1CCc3ccccc31)C2	1035657912	25	336.2	2.64

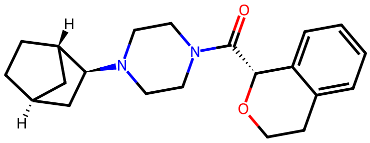	O=C([C@H]1OCCc2ccccc21)N1CCN([C@H]2C[C@H]3CC[C@H]2C3)CC1	441469669	25	340.22	2.63
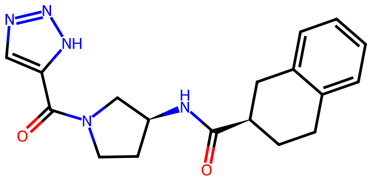	O=C(N[C@H]1CCN(C(=O)c2cnn[nH]2)C1)[C@@H]1CCc2ccccc2C1	938982826	25	339.17	0.94
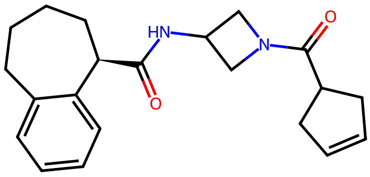	O=C(NC1CN(C(=O)C2CC=CC2)C1)[C@@H]1CCCCc2ccccc21	994305268	25	338.2	2.79
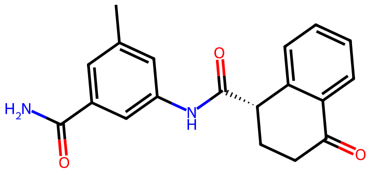	Cc1cc(NC(=O)[C@H]2CCC(=O)c3ccccc32)cc(C(N)=O)c1	1412124003	24	322.13	2.79
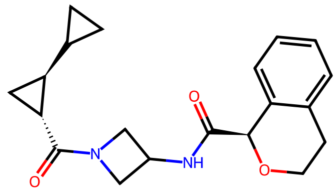	O=C(NC1CN(C(=O)[C@@H]2C[C@H]2C2CC2)C1)[C@@H]1OCCc2ccccc21	997322820	25	340.18	1.67
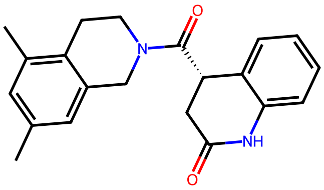	Cc1cc(C)c2c(c1)CN(C(=O)[C@H]1CC(=O)Nc3ccccc31)CC2	187793704	25	334.17	3.31
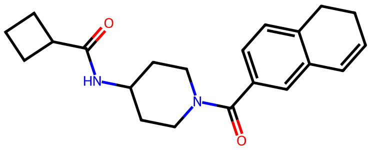	O=C(NC1CCN(C(=O)c2ccc3c(c2)C=CCC3)CC1)C1CCC1	947394907	25	338.2	3.17
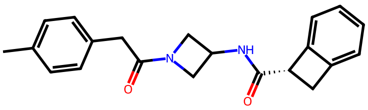	Cc1ccc(CC(=O)N2CC(NC(=O)[C@H]3Cc4ccccc43)C2)cc1	998694551	25	334.17	2.2

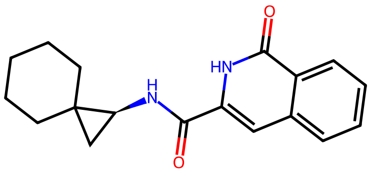	O=C(N[C@H]1CC12CCCCC2)c1cc2ccccc2c(=O)[nH]1	180793668	22	296.15	2.98
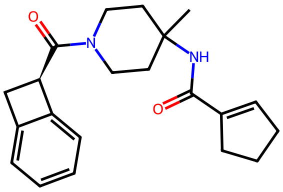	CC1(NC(=O)C2=CCCC2)CCN(C(=O)[C@@H]2Cc3ccccc32)CC1	956334983	25	338.2	2.93
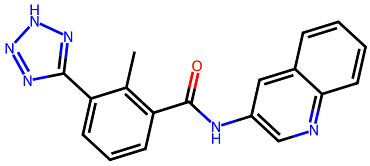	Cc1c(C(=O)Nc2cnc3ccccc3c2)cccc1-c1nn[nH]n1	1605481895	25	330.12	2.98
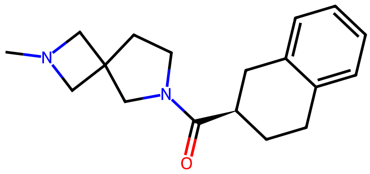	CN1CC2(CCN(C(=O)[C@@H]3CCc4ccccc4C3)C2)C1	614033424	21	284.19	1.96
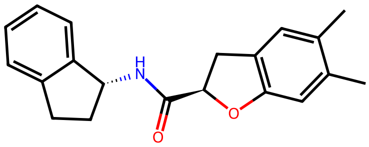	Cc1cc2c(cc1C)O[C@@H](C(=O)N[C@@H]1CCc3ccccc31)C2	463350911	23	307.16	3.41
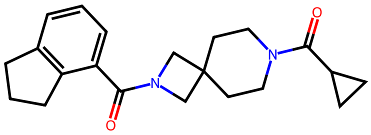	O=C(c1cccc2c1CCC2)N1CC2(CCN(C(=O)C3CC3)CC2)C1	944817996	25	338.2	2.65
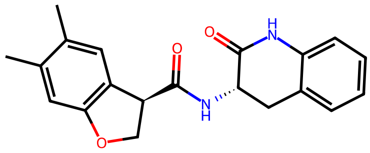	Cc1cc2c(cc1C)[C@@H](C(=O)N[C@H]1Cc3ccccc3NC1=O)CO2	529946544	25	336.15	2.46
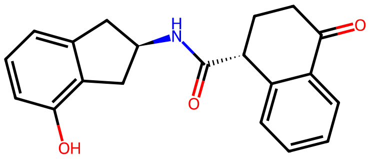	O=C1CC[C@@H](C(=O)N[C@@H]2Cc3cccc(O)c3C2)c2ccccc21	560679752	24	321.14	2.74

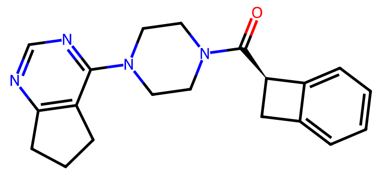	O=C([C@@H]1Cc2ccccc21)N1CCN(c2ncnc3c2CCC3)CC1	1055998639	25	334.18	1.95
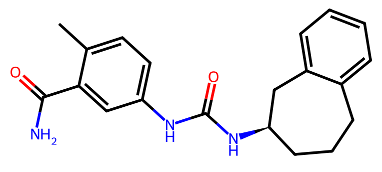	Cc1ccc(NC(=O)N[C@@H]2CCCc3ccccc3C2)cc1C(N)=O	930145037	25	337.18	3.16
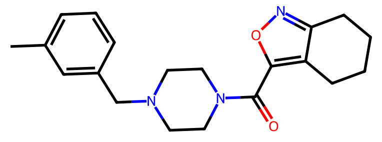	Cc1cccc(CN2CCN(C(=O)c3onc4c3CCCC4)CC2)c1	665397523	25	339.19	2.82
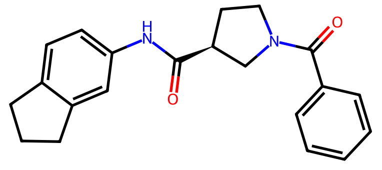	O=C(Nc1ccc2c(c1)CCC2)[C@H]1CCN(C(=O)c2ccccc2)C1	623416544	25	334.17	3.28
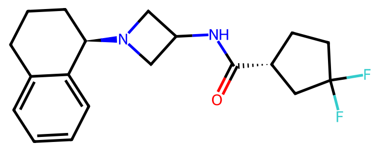	O=C(NC1CN([C@@H]2CCCc3ccccc32)C1)[C@@H]1CCC(F)(F)C1	1030412204	24	334.19	3.3
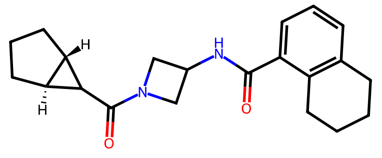	O=C(NC1CN(C(=O)C2[C@H]3CCC[C@H]23)C1)c1cccc2c1CCCC2	991900253	25	338.2	2.55
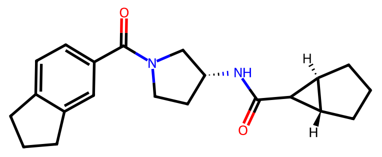	O=C(N[C@@H]1CCN(C(=O)c2ccc3c(c2)CCC3)C1)C1[C@H]2CCC[C@H]12	939933091	25	338.2	2.55
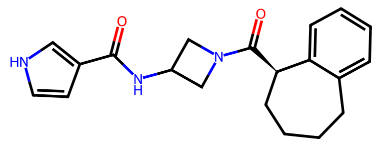	O=C(NC1CN(C(=O)[C@@H]2CCCCc3ccccc32)C1)c1cc[nH]c1	993723642	25	337.18	2.47

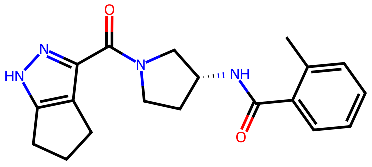	Cc1ccccc1C(=O)N[C@@H]1CCN(C(=O)c2n[nH]c3c2CCC3)C1	939208337	25	338.17	1.85
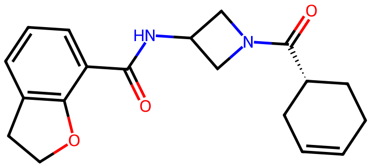	O=C(NC1CN(C(=O)[C@H]2CC=CCC2)C1)c1cccc2c1OCC2	1000414209	24	326.16	1.92
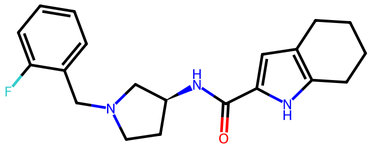	O=C(N[C@H]1CCN(Cc2ccccc2F)C1)c1cc2c([nH]1)CCCC2	1015953769	25	341.19	3.04
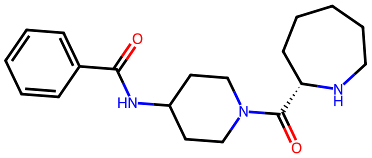	O=C(NC1CCN(C(=O)[C@@H]2CCCCCN2)CC1)c1ccccc1	573491611	24	329.21	1.94
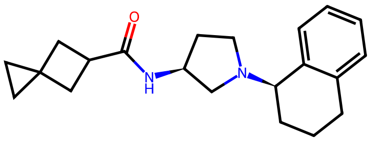	O=C(N[C@H]1CCN([C@@H]2CCCc3ccccc32)C1)C1CC2(CC2)C1	1015681203	24	324.22	3.44
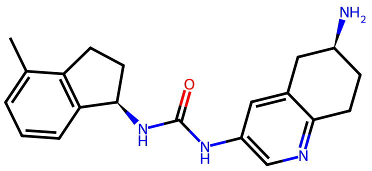	Cc1cccc2c1CC[C@H]2NC(=O)Nc1cnc2c(c1)C[C@@H](N)CC2	579450947	25	336.2	3.02
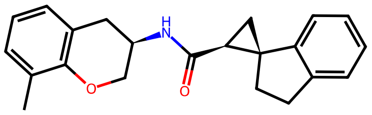	Cc1cccc2c1OC[C@H](NC(=O)[C@H]1C[C@@]13CCc1ccccc13)C2	564516633	25	333.17	3.32
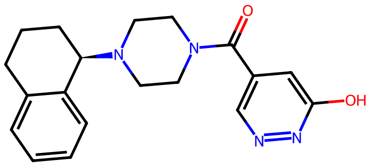	O=C(c1cnnc(O)c1)N1CCN([C@@H]2CCCc3ccccc32)CC1	1374093824	25	338.17	2.02

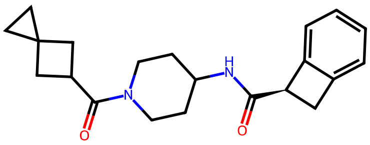	O=C(NC1CCN(C(=O)C2CC3(CC3)C2)CC1)[C@@H]1Cc2ccccc21	948308651	25	338.2	2.62
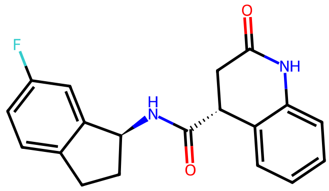	O=C1C[C@@H](C(=O)N[C@H]2CCc3ccc(F)cc32)c2ccccc2N1	106680930	24	324.13	3.06
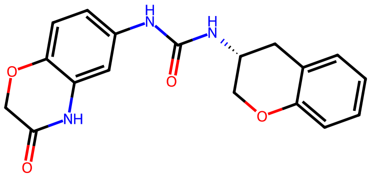	O=C1COc2ccc(NC(=O)N[C@H]3COc4ccccc4C3)cc2N1	47777240	25	339.12	2.14
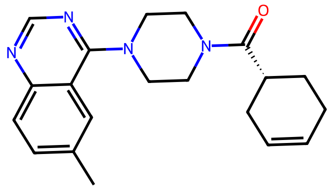	Cc1ccc2ncnc(N3CCN(C(=O)[C@H]4CC=CCC4)CC3)c2c1	1055721910	25	336.2	2.94
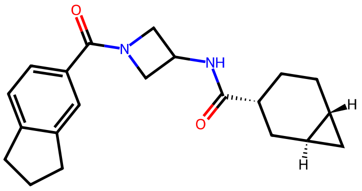	O=C(NC1CN(C(=O)c2ccc3c(c2)CCC3)C1)[C@@H]1CC[C@@H]2C[C@H]2C1	994697280	25	338.2	2.55
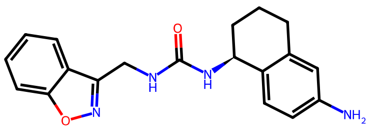	Nc1ccc2c(c1)CCC[C@@H]2NC(=O)NCc1noc2ccccc12	568245788	25	336.16	3.29
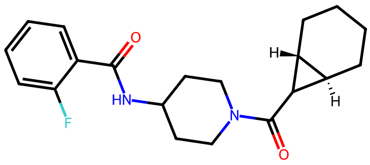	O=C(NC1CCN(C(=O)C2[C@@H]3CCCC[C@@H]23)CC1)c1ccccc1F	369941367	25	344.19	2.98
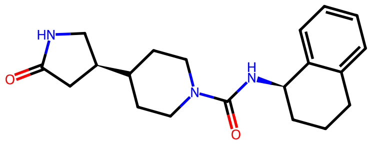	O=C1C[C@H](C2CCN(C(=O)N[C@@H]3CCCc4ccccc43)CC2)CN1	1553351028	25	341.21	2.62

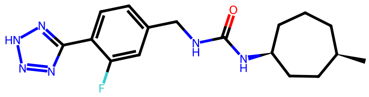	C[C@@H]1CCC[C@H](NC(=O)NCc2ccc(-c3nn[nH]n3)c(F)c2)CC1	1609162747	25	346.19	2.77
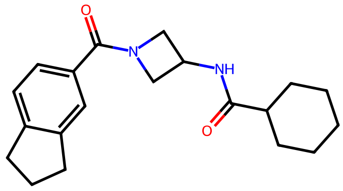	O=C(NC1CN(C(=O)c2ccc3c(c2)CCC3)C1)C1CCCCC1	990099899	24	326.2	2.7
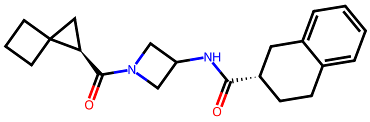	O=C(NC1CN(C(=O)[C@@H]2CC23CCC3)C1)[C@H]1CCc2ccccc2C1	1000545884	25	338.2	2.31
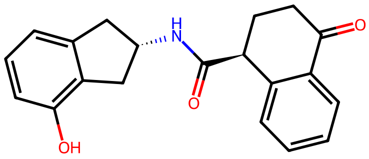	O=C1CC[C@H](C(=O)N[C@H]2Cc3cccc(O)c3C2)c2ccccc21	560679749	24	321.14	2.74
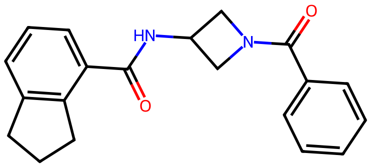	O=C(NC1CN(C(=O)c2ccccc2)C1)c1cccc2c1CCC2	994399078	24	320.15	2.43
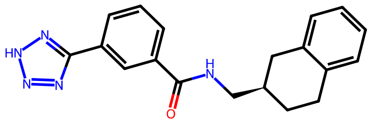	O=C(NC[C@@H]1CCc2ccccc2C1)c1cccc(-c2nn[nH]n2)c1	738050892	25	333.16	2.4
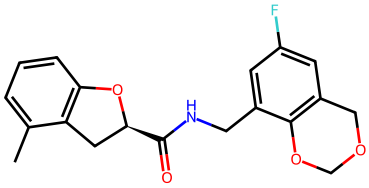	Cc1cccc2c1C[C@H](C(=O)NCc1cc(F)cc3c1OCOC3)O2	448865226	25	343.12	2.62
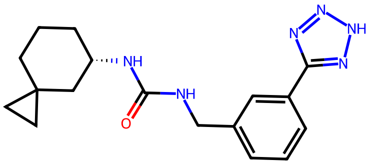	O=C(NCc1cccc(-c2nn[nH]n2)c1)N[C@H]1CCCC2(CC2)C1	1593142909	24	326.19	2.39

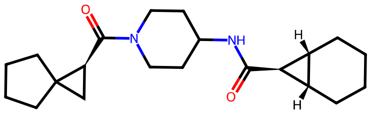	O=C(NC1CCN(C(=O)[C@H]2CC23CCCC3)CC1)[C@@H]1[C@H]2CCCC[C@H]21	948360789	25	344.25	3.11
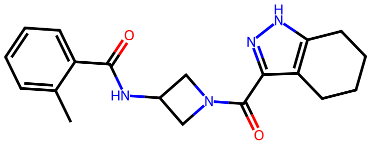	Cc1ccccc1C(=O)NC1CN(C(=O)c2n[nH]c3c2CCCC3)C1	990105923	25	338.17	1.85
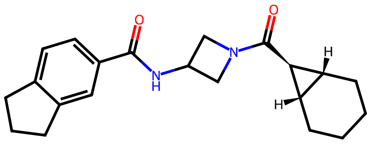	O=C(NC1CN(C(=O)[C@@H]2[C@H]3CCCC[C@H]32)C1)c1ccc2c(c1)CCC2	991357678	25	338.2	2.55
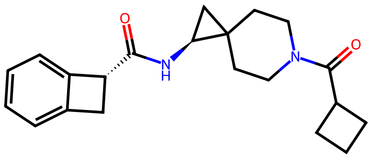	O=C(N[C@H]1CC12CCN(C(=O)C1CCC1)CC2)[C@@H]1Cc2ccccc21	965003310	25	338.2	2.62
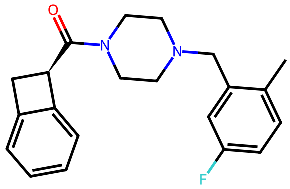	Cc1ccc(F)cc1CN1CCN(C(=O)[C@@H]2Cc3ccccc32)CC1	949948640	25	338.18	3.12
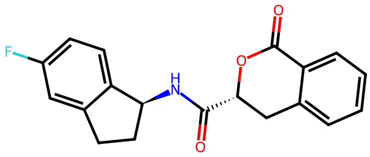	O=C1O[C@@H](C(=O)N[C@H]2CCc3cc(F)ccc32)Cc2ccccc21	55657741	24	325.11	2.71
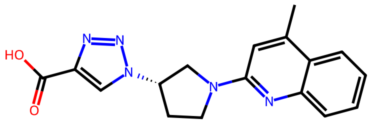	Cc1cc(N2CC[C@H](n3cc(C(=O)O)nn3)C2)nc2ccccc12	1346104987	24	323.14	2.28
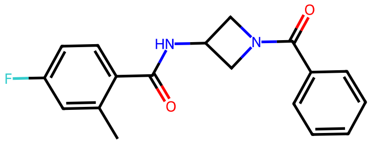	Cc1cc(F)ccc1C(=O)NC1CN(C(=O)c2ccccc2)C1	997173627	23	312.13	2.39

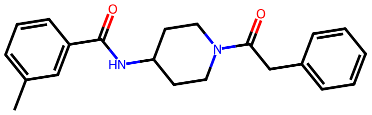	Cc1cccc(C(=O)NC2CCN(C(=O)Cc3ccccc3)CC2)c1	75058283	25	336.18	2.96
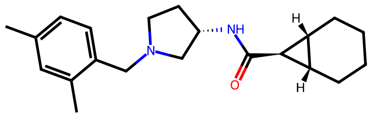	Cc1ccc(CN2CC[C@H](NC(=O)[C@@H]3[C@H]4CCCC[C@H]43)C2)c(C)c1	1010438652	24	326.24	3.43
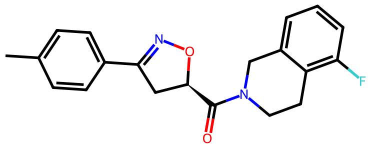	Cc1ccc(C2=NO[C@@H](C(=O)N3CCc4c(F)cccc4C3)C2)cc1	1544806356	25	338.14	3.21
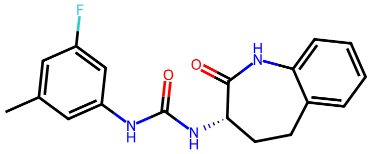	Cc1cc(F)cc(NC(=O)N[C@H]2CCc3ccccc3NC2=O)c1	1667871767	24	327.14	3.21
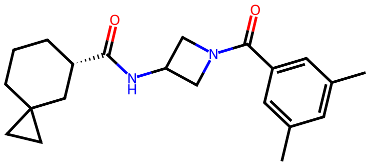	Cc1cc(C)cc(C(=O)N2CC(NC(=O)[C@H]3CCCC4(CC4)C3)C2)c1	991515300	25	340.22	3.21
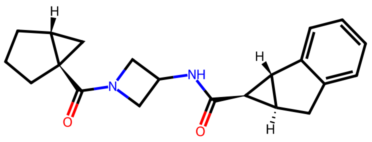	O=C(NC1CN(C(=O)[C@@]23CCC[C@@H]2C3)C1)[C@@H]1[C@@H]2Cc3ccccc3[C@H]21	991644150	25	336.18	2.09
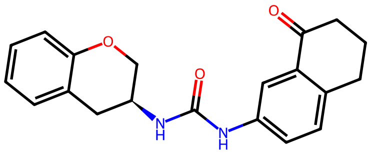	O=C(Nc1ccc2c(c1)C(=O)CCC2)N[C@@H]1COc2ccccc2C1	177286414	25	336.15	3.33
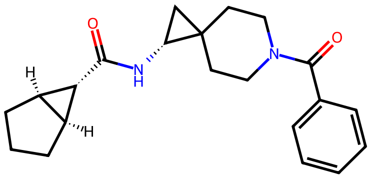	O=C(N[C@@H]1CC12CCN(C(=O)c1ccccc1)CC2)[C@@H]1[C@H]2CCC[C@H]21	963462694	25	338.2	2.84

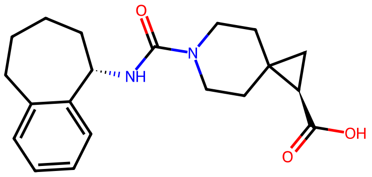	O=C(O)[C@@H]1CC12CCN(C(=O)N[C@H]1CCCCc3ccccc31)CC2	739527320	25	342.19	3.35
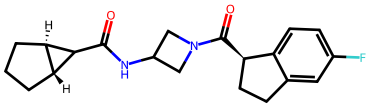	O=C(NC1CN(C(=O)[C@@H]2CCc3cc(F)ccc32)C1)C1[C@@H]2CCC[C@@H]12	990701878	25	342.17	2.23
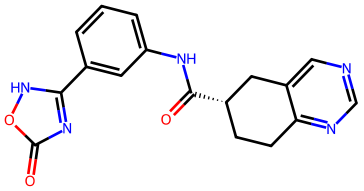	O=C(Nc1cccc(-c2nc(=O)o[nH]2)c1)[C@H]1CCc2ncncc2C1	1358227898	25	337.12	1.56
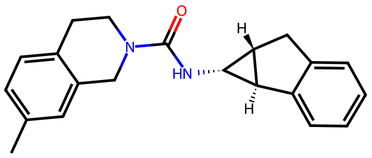	Cc1ccc2c(c1)CN(C(=O)N[C@H]1[C@@H]3c4ccccc4C[C@H]31)CC2	660914469	24	318.17	3.4
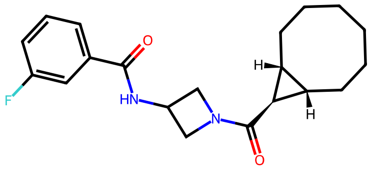	O=C(NC1CN(C(=O)[C@@H]2[C@H]3CCCCCC[C@H]32)C1)c1cccc(F)c1	990118130	25	344.19	2.98
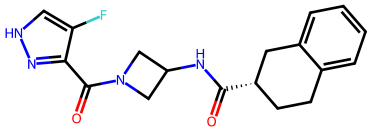	O=C(NC1CN(C(=O)c2n[nH]cc2F)C1)[C@H]1CCc2ccccc2C1	1000537204	25	342.15	1.29
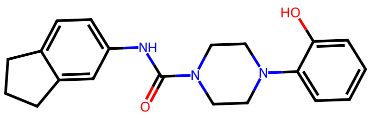	O=C(Nc1ccc2c(c1)CCC2)N1CCN(c2ccccc2O)CC1	43390716	25	337.18	3.24
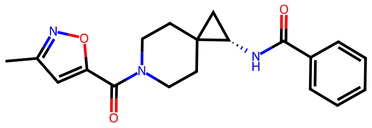	Cc1cc(C(=O)N2CCC3(CC2)C[C@@H]3NC(=O)c2ccccc2)on1	962778540	25	339.16	2.41

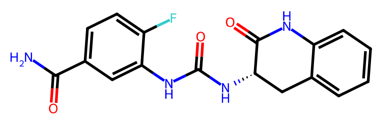	NC(=O)c1ccc(F)c(NC(=O)N[C@H]2Cc3ccccc3NC2=O)c1	1365123969	25	342.11	1.61
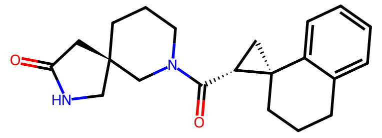	O=C1C[C@]2(CCCN(C(=O)[C@@H]3C[C@]34CCCc3ccccc34)C2)CN1	341506616	25	338.2	2.41
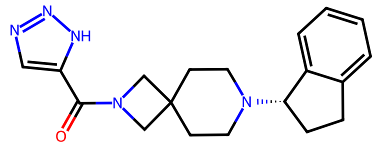	O=C(c1cnn[nH]1)N1CC2(CCN([C@H]3CCc4ccccc43)CC2)C1	1050578018	25	337.19	2.03
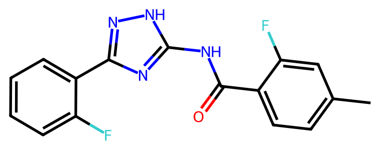	Cc1ccc(C(=O)Nc2nc(-c3ccccc3F)n[nH]2)c(F)c1	175260540	23	314.1	3.31
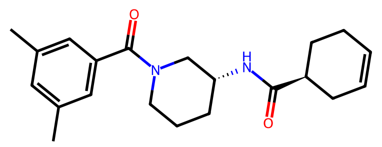	Cc1cc(C)cc(C(=O)N2CCC[C@@H](NC(=O)[C@@H]3CC=CCC3)C2)c1	951988424	25	340.22	3.38
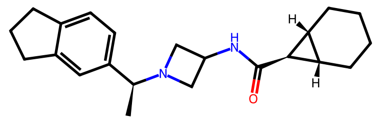	C[C@@H](c1ccc2c(c1)CCC2)N1CC(NC(=O)[C@@H]2[C@H]3CCCC[C@H]32)C1	1030186195	25	338.24	3.47
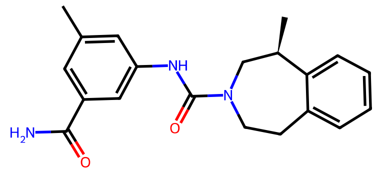	Cc1cc(NC(=O)N2CCc3ccccc3[C@H](C)C2)cc(C(N)=O)c1	650236367	25	337.18	3.29
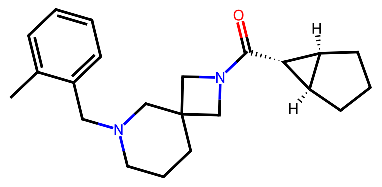	Cc1ccccc1CN1CCCC2(C1)CN(C(=O)[C@@H]1[C@H]3CCC[C@H]31)C2	981696755	25	338.24	3.47

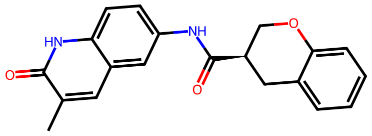	Cc1cc2cc(NC(=O)[C@H]3COc4ccccc4C3)ccc2[nH]c1=O	1556731998	25	334.13	3.03
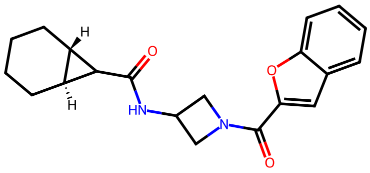	O=C(NC1CN(C(=O)c2cc3ccccc3o2)C1)C1[C@H]2CCCC[C@H]12	990674359	25	338.16	2.81
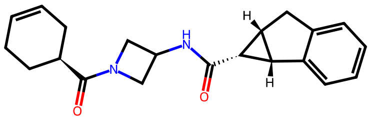	O=C(NC1CN(C(=O)[C@@H]2CC=CCC2)C1)[C@H]1[C@H]2c3ccccc3C[C@H]21	991655685	25	336.18	2.26
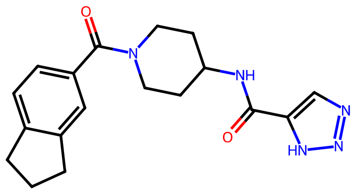	O=C(NC1CCN(C(=O)c2ccc3c(c2)CCC3)CC1)c1cnn[nH]1	946723193	25	339.17	1.33
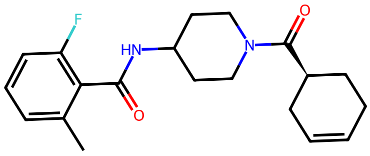	Cc1cccc(F)c1C(=O)NC1CCN(C(=O)[C@@H]2CC=CCC2)CC1	944127911	25	344.19	3.21
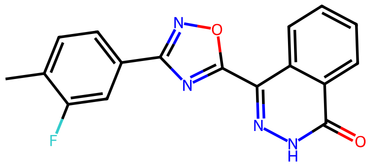	Cc1ccc(-c2noc(-c3n[nH]c(=O)c4ccccc34)n2)cc1F	109507480	24	322.09	3.09
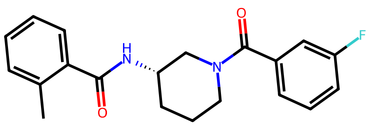	Cc1ccccc1C(=O)N[C@H]1CCCN(C(=O)c2cccc(F)c2)C1	585989373	25	340.16	3.17
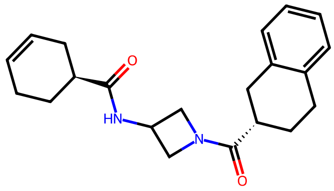	O=C(NC1CN(C(=O)[C@H]2CCc3ccccc3C2)C1)[C@@H]1CC=CCC1	990221457	25	338.2	2.47

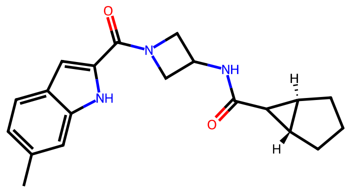	Cc1ccc2cc(C(=O)N3CC(NC(=O)C4[C@H]5CCC[C@H]45)C3)[nH]c2c1	990713237	25	337.18	2.46
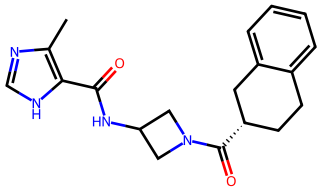	Cc1nc[nH]c1C(=O)NC1CN(C(=O)[C@H]2CCc3ccccc3C2)C1	995253922	25	338.17	1.46
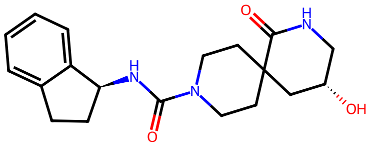	O=C(N[C@H]1CCc2ccccc21)N1CCC2(CC1)C[C@@H](O)CNC2=O	913965887	25	343.19	1.35
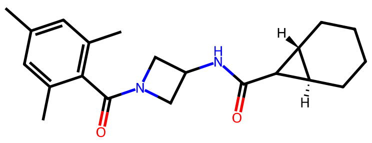	Cc1cc(C)c(C(=O)N2CC(NC(=O)C3[C@@H]4CCCC[C@@H]34)C2)c(C)c1	990673980	25	340.22	2.99
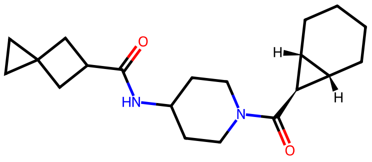	O=C(NC1CCN(C(=O)[C@@H]2[C@H]3CCCC[C@H]32)CC1)C1CC2(CC2)C1	945958564	24	330.23	2.72
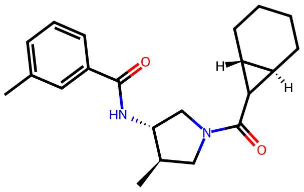	Cc1cccc(C(=O)N[C@@H]2CN(C(=O)C3[C@@H]4CCCC[C@@H]34)C[C@H]2C)c1	948157461	25	340.22	3.01
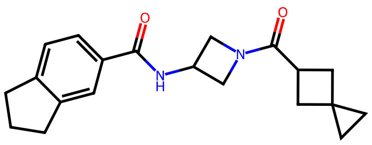	O=C(NC1CN(C(=O)C2CC3(CC3)C2)C1)c1ccc2c(c1)CCC2	991368459	24	324.18	2.31
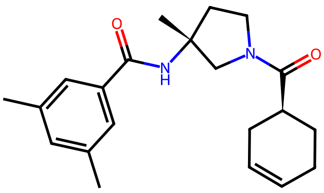	Cc1cc(C)cc(C(=O)N[C@]2(C)CCN(C(=O)[C@@H]3CC=CCC3)C2)c1	957560536	25	340.22	3.38

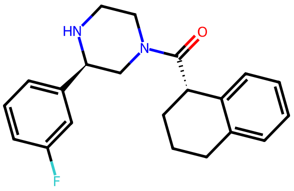	O=C([C@H]1CCCc2ccccc21)N1CCN[C@H](c2cccc(F)c2)C1	337589463	25	338.18	3.42
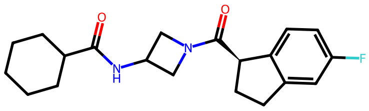	O=C(NC1CN(C(=O)[C@@H]2CCc3cc(F)ccc32)C1)C1CCCCC1	990101961	25	344.19	2.76
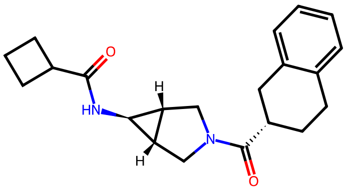	O=C(N[C@H]1[C@@H]2CN(C(=O)[C@H]3CCc4ccccc4C3)C[C@@H]21)C1CCC1	976033257	25	338.2	2.16
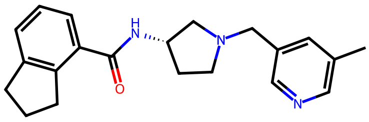	Cc1cncc(CN2CC[C@H](NC(=O)c3cccc4c3CCC4)C2)c1	1016380446	25	335.2	2.88
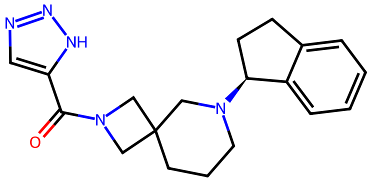	O=C(c1cnn[nH]1)N1CC2(CCCN([C@H]3CCc4ccccc43)C2)C1	981867501	25	337.19	2.03
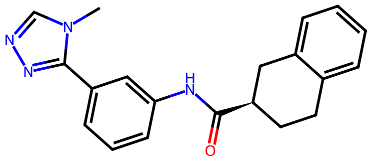	Cn1cnnc1-c1cccc(NC(=O)[C@@H]2CCc3ccccc3C2)c1	649604087	25	332.16	3.23
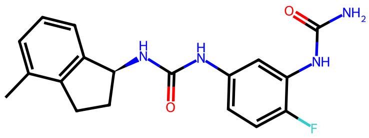	Cc1cccc2c1CC[C@@H]2NC(=O)Nc1cc(F)c(NC(N)=O)c1	1671610821	25	342.15	3.43
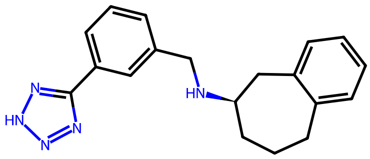	c1cc(CN[C@@H]2CCCc3ccccc3C2)cc(-c2nn[nH]n2)c1	1607118753	24	319.18	2.9

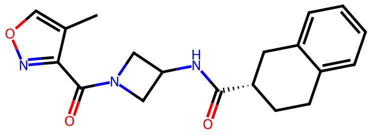	Cc1conc1C(=O)N1CC(NC(=O)[C@H]2CCc3ccccc3C2)C1	1000538490	25	339.16	1.73
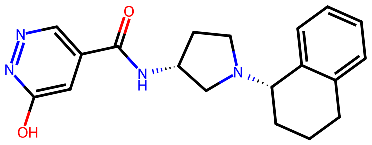	O=C(N[C@@H]1CCN([C@H]2CCCc3ccccc32)C1)c1cnnc(O)c1	1014829876	25	338.17	2.06
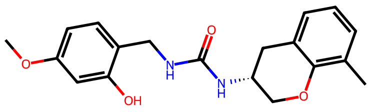	COc1ccc(CNC(=O)N[C@H]2COc3c(C)cccc3C2)c(O)c1	366068751	25	342.16	2.51
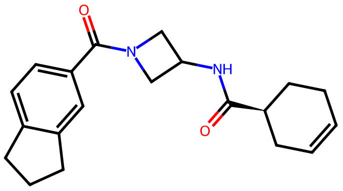	O=C(NC1CN(C(=O)c2ccc3c(c2)CCC3)C1)[C@@H]1CC=CCC1	990222652	24	324.18	2.47
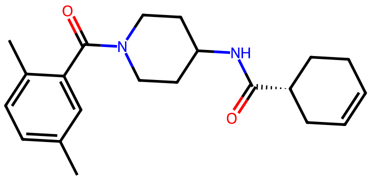	Cc1ccc(C)c(C(=O)N2CCC(NC(=O)[C@H]3CC=CCC3)CC2)c1	948336349	25	340.22	3.38
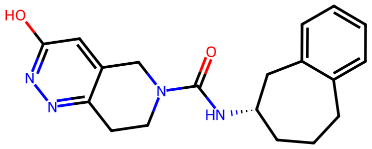	O=C(N[C@H]1CCCc2ccccc2C1)N1CCc2nnc(O)cc2C1	897612452	25	338.17	2.2
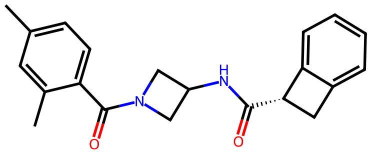	Cc1ccc(C(=O)N2CC(NC(=O)[C@H]3Cc4ccccc43)C2)c(C)c1	998685951	25	334.17	2.58
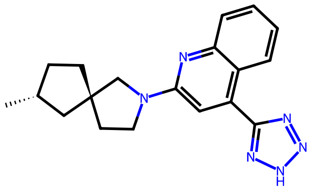	C[C@@H]1CC[C@@]2(CCN(c3cc(-c4nn[nH]n4)c4ccccc4n3)C2)C1	824917903	25	334.19	3.43

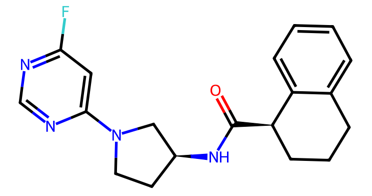	O=C(N[C@H]1CCN(c2cc(F)ncn2)C1)[C@@H]1CCCc2ccccc21	1058591964	25	340.17	2.43
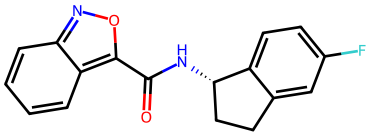	O=C(N[C@H]1CCc2cc(F)ccc21)c1onc2ccccc12	1292043988	22	296.1	3.38
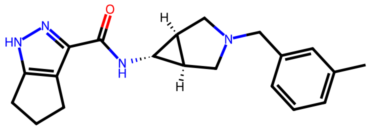	Cc1cccc(CN2C[C@H]3[C@H](NC(=O)c4n[nH]c5c4CCC5)[C@H]3C2)c1	960728413	25	336.2	2.07
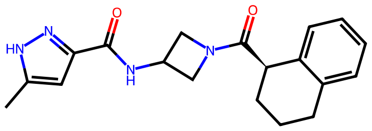	Cc1cc(C(=O)NC2CN(C(=O)[C@@H]3CCCc4ccccc43)C2)n[nH]1	990367605	25	338.17	1.78
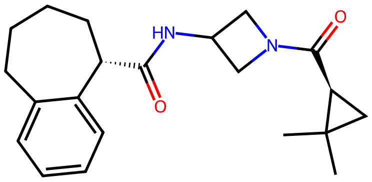	CC1(C)C[C@@H]1C(=O)N1CC(NC(=O)[C@H]2CCCCc3ccccc32)C1	994311783	25	340.22	2.87
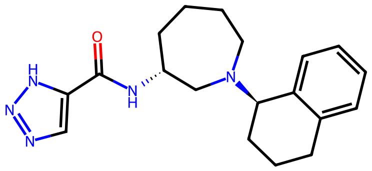	O=C(N[C@@H]1CCCCN([C@@H]2CCCc3ccccc32)C1)c1cnn[nH]1	1034291871	25	339.21	2.47
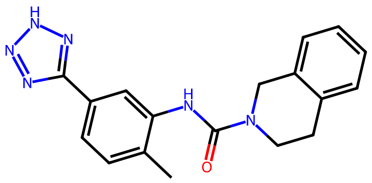	Cc1ccc(-c2nn[nH]n2)cc1NC(=O)N1CCc2ccccc2C1	1592105673	25	334.15	2.77
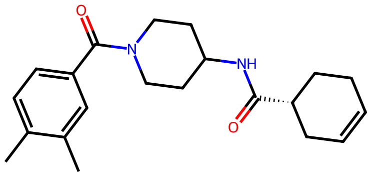	Cc1ccc(C(=O)N2CCC(NC(=O)[C@H]3CC=CCC3)CC2)cc1C	948347925	25	340.22	3.38

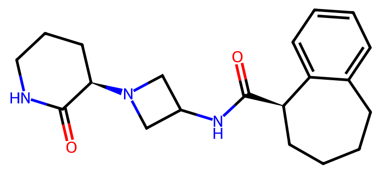	O=C(NC1CN([C@@H]2CCCNC2=O)C1)[C@@H]1CCCCc2ccccc21	1031452577	25	341.21	1.58
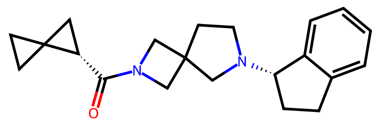	O=C([C@H]1CC12CC2)N1CC2(CCN([C@H]3CCc4ccccc43)C2)C1	1073013326	24	322.2	3.01
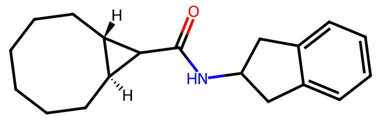	O=C(NC1Cc2ccccc2C1)C1[C@H]2CCCCCC[C@H]12	1127809049	21	283.19	3.49
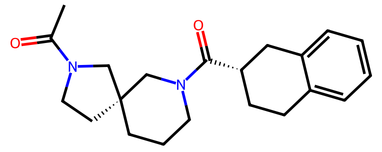	CC(=O)N1CC[C@]2(CCCN(C(=O)[C@H]3CCc4ccccc4C3)C2)C1	952819501	25	340.22	2.65
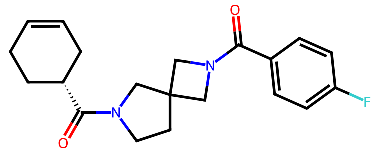	O=C(c1ccc(F)cc1)N1CC2(CCN(C(=O)[C@H]3CC=CCC3)C2)C1	1018699510	25	342.17	2.86
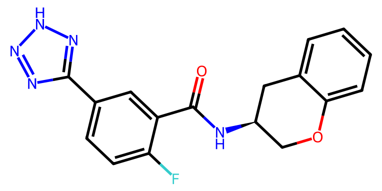	O=C(N[C@@H]1COc2ccccc2C1)c1cc(-c2nn[nH]n2)ccc1F	1582343784	25	339.11	1.74
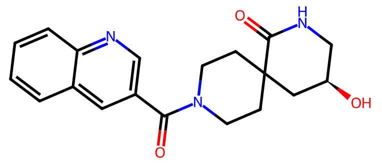	O=C(c1cnc2ccccc2c1)N1CCC2(CC1)C[C@H](O)CNC2=O	885015337	25	339.16	1.34
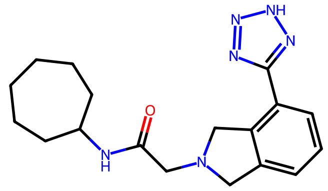	O=C(CN1Cc2cccc(-c3nn[nH]n3)c2C1)NC1CCCCCC1	1609527127	25	340.2	2.02

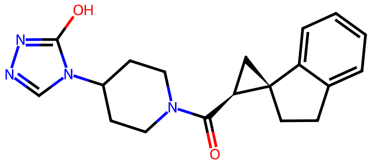	O=C([C@H]1C[C@@]12CCc1ccccc12)N1CCC(n2cnnc2O)CC1	517586136	25	338.17	2.05
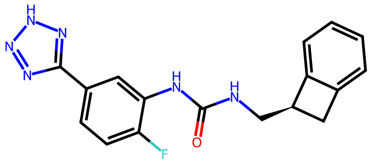	O=C(NC[C@@H]1Cc2ccccc21)Nc1cc(-c2nn[nH]n2)ccc1F	1606753413	25	338.13	2.47
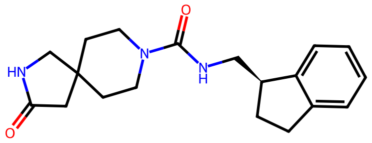	O=C1CC2(CCN(C(=O)NC[C@@H]3CCc4ccccc43)CC2)CN1	371839496	24	327.19	2.03
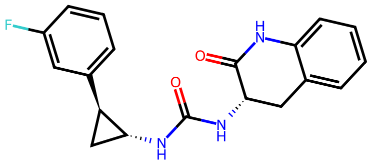	O=C(N[C@H]1Cc2ccccc2NC1=O)N[C@@H]1C[C@H]1c1cccc(F)c1	637097932	25	339.14	2.54
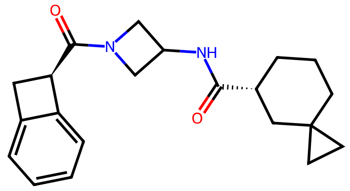	O=C(NC1CN(C(=O)[C@@H]2Cc3ccccc32)C1)[C@@H]1CCCC2(CC2)C1	991515466	25	338.2	2.62
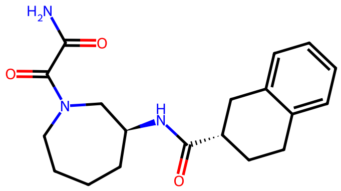	NC(=O)C(=O)N1CCCC[C@H](NC(=O)[C@H]2CCc3ccccc3C2)C1	943575399	25	343.19	0.77
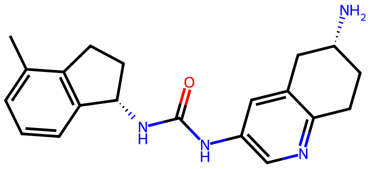	Cc1cccc2c1CC[C@@H]2NC(=O)Nc1cnc2c(c1)C[C@H](N)CC2	579450948	25	336.2	3.02
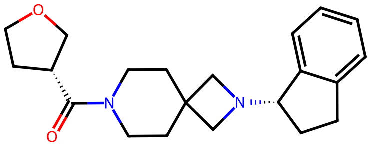	O=C([C@@H]1CCOC1)N1CCC2(CC1)CN([C@H]1CCc3ccccc31)C2	1035663238	25	340.22	2.63

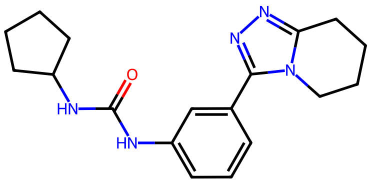	O=C(Nc1cccc(-c2nnc3n2CCCC3)c1)NC1CCCC1	169771907	24	325.19	3.35
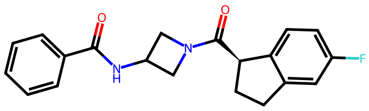	O=C(NC1CN(C(=O)[C@@H]2CCc3cc(F)ccc32)C1)c1ccccc1	990097772	25	338.14	2.5
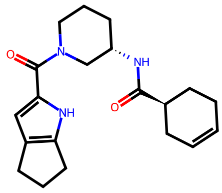	O=C(N[C@H]1CCCN(C(=O)c2cc3c([nH]2)CCC3)C1)[C@@H]1CC=CCC1	951367693	25	341.21	2.58
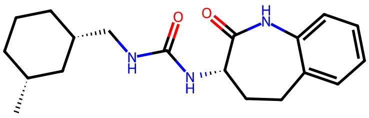	C[C@@H]1CCC[C@H](CNC(=O)N[C@H]2CCc3ccccc3NC2=O)C1	1563768276	24	329.21	3.07
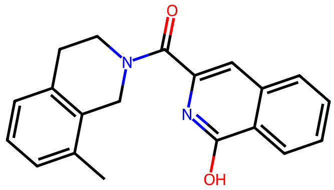	Cc1cccc2c1CN(C(=O)c1cc3ccccc3c(O)n1)CC2	346923694	24	318.14	3.45
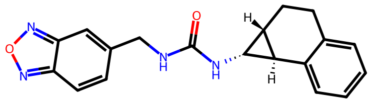	O=C(NCc1ccc2nonc2c1)N[C@H]1[C@@H]2c3ccccc3CC[C@H]21	893766476	25	334.14	2.75
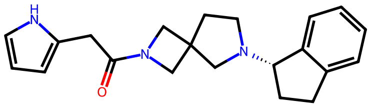	O=C(Cc1ccc[nH]1)N1CC2(CCN([C@H]3CCc4ccccc43)C2)C1	1073447148	25	335.2	2.78
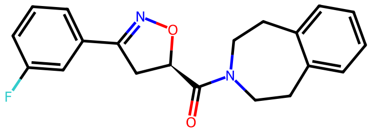	O=C([C@H]1CC(c2cccc(F)c2)=NO1)N1CCc2ccccc2CC1	734783627	25	338.14	2.95

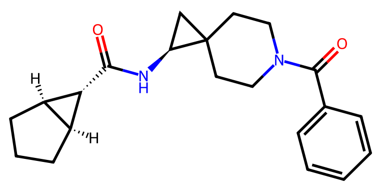	O=C(N[C@H]1CC12CCN(C(=O)c1ccccc1)CC2)[C@@H]1[C@H]2CCC[C@H]21	963462695	25	338.2	2.84
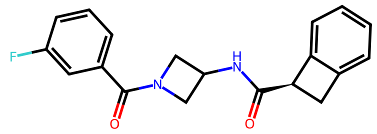	O=C(NC1CN(C(=O)c2cccc(F)c2)C1)[C@@H]1Cc2ccccc21	998685609	24	324.13	2.11
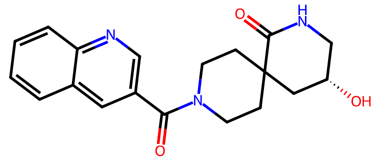	O=C(c1cnc2ccccc2c1)N1CCC2(CC1)C[C@@H](O)CNC2=O	885015338	25	339.16	1.34
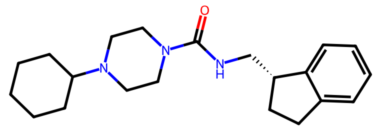	O=C(NC[C@H]1CCc2ccccc21)N1CCN(C2CCCCC2)CC1	1468803855	25	341.25	3.38
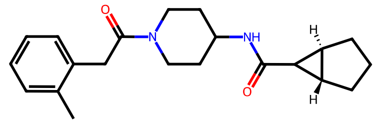	Cc1ccccc1CC(=O)N1CCC(NC(=O)C2[C@H]3CCC[C@H]23)CC1	946717792	25	340.22	2.69
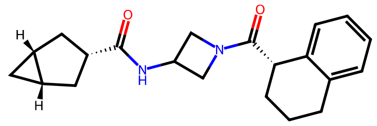	O=C(NC1CN(C(=O)[C@H]2CCCc3ccccc32)C1)[C@@H]1C[C@@H]2C[C@@H]2C1	998005789	25	338.2	2.48
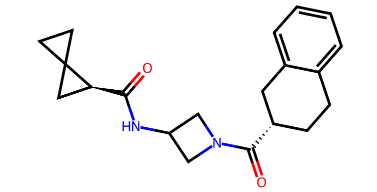	O=C(NC1CN(C(=O)[C@H]2CCc3ccccc3C2)C1)[C@H]1CC12CC2	1000508411	24	324.18	1.92
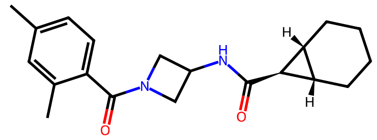	Cc1ccc(C(=O)N2CC(NC(=O)[C@@H]3[C@H]4CCCC[C@H]43)C2)c(C)c1	990674055	24	326.2	2.68

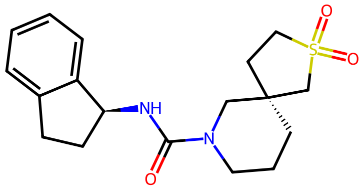	O=C(N[C@H]1CCc2ccccc21)N1CCC[C@@]2(CCS(=O)(=O)C2)C1	333819579	24	348.15	2.28
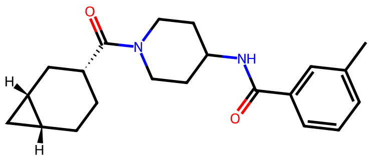	Cc1cccc(C(=O)NC2CCN(C(=O)[C@@H]3CC[C@@H]4C[C@@H]4C3)CC2)c1	870837572	25	340.22	3.15
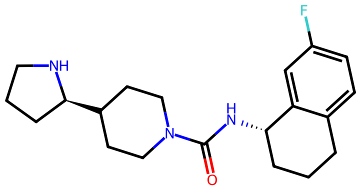	O=C(N[C@H]1CCCc2ccc(F)cc21)N1CCC([C@H]2CCCN2)CC1	418114487	25	345.22	3.38
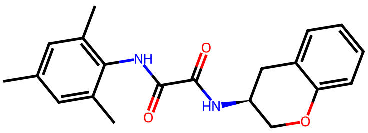	Cc1cc(C)c(NC(=O)C(=O)N[C@@H]2COc3ccccc3C2)c(C)c1	42049454	25	338.16	2.67
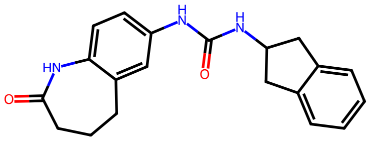	O=C1CCCc2cc(NC(=O)NC3Cc4ccccc4C3)ccc2N1	184203938	25	335.16	3.25
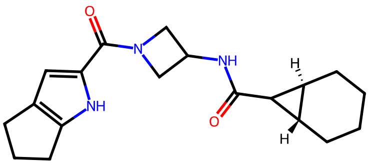	O=C(NC1CN(C(=O)c2cc3c([nH]2)CCC3)C1)C1[C@H]2CCCC[C@H]12	990651546	24	327.19	1.88
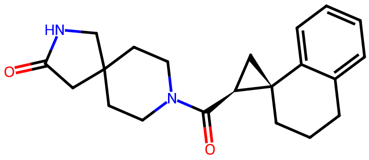	O=C1CC2(CCN(C(=O)[C@H]3C[C@@]34CCCc3ccccc34)CC2)CN1	374098579	25	338.2	2.41
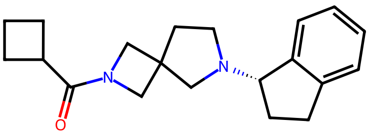	O=C(C1CCC1)N1CC2(CCN([C@H]3CCc4ccccc43)C2)C1	1072375566	23	310.2	3.01

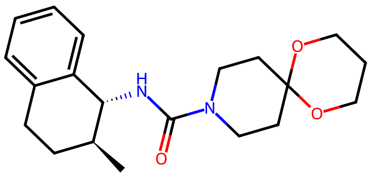	C[C@H]1CCc2ccccc2[C@@H]1NC(=O)N1CCC2(CC1)OCCCO2	838533231	25	344.21	3.25
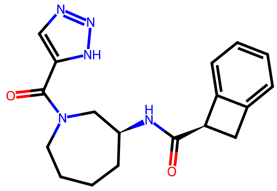	O=C(N[C@H]1CCCCN(C(=O)c2cnn[nH]2)C1)[C@@H]1Cc2ccccc21	943485505	25	339.17	1.26
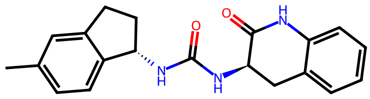	Cc1ccc2c(c1)CC[C@@H]2NC(=O)N[C@@H]1Cc2ccccc2NC1=O	636635274	25	335.16	2.84
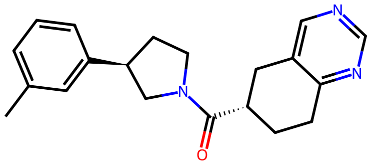	Cc1cccc([C@H]2CCN(C(=O)[C@H]3CCc4ncncc4C3)C2)c1	648353008	24	321.18	2.91
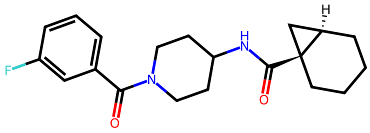	O=C(c1cccc(F)c1)N1CCC(NC(=O)[C@]23CCCC[C@@H]2C3)CC1	943776522	25	344.19	3.13
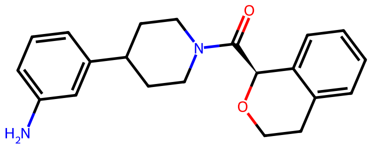	Nc1cccc(C2CCN(C(=O)[C@@H]3OCCc4ccccc43)CC2)c1	424034184	25	336.18	3.29
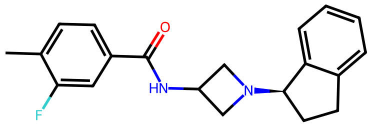	Cc1ccc(C(=O)NC2CN([C@@H]3CCc4ccccc43)C2)cc1F	1030638018	24	324.16	3.24
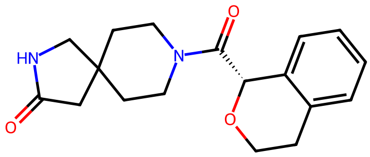	O=C1CC2(CCN(C(=O)[C@H]3OCCc4ccccc43)CC2)CN1	269752050	23	314.16	1.43

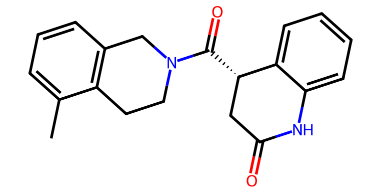	Cc1cccc2c1CCN(C(=O)[C@H]1CC(=O)Nc3ccccc31)C2	171540284	24	320.15	3.01
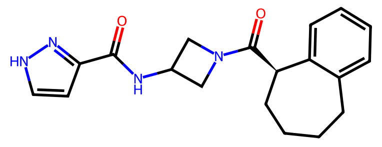	O=C(NC1CN(C(=O)[C@@H]2CCCCc3ccccc32)C1)c1cc[nH]n1	990296172	25	338.17	1.86
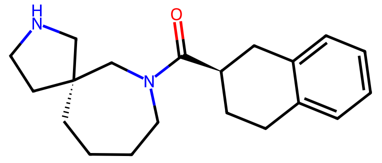	O=C([C@@H]1CCc2ccccc2C1)N1CCCC[C@@]2(CCNC2)C1	1550236248	23	312.22	2.78
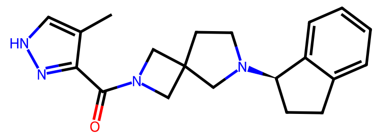	Cc1c[nH]nc1C(=O)N1CC2(CCN([C@@H]3CCc4ccccc43)C2)C1	1072713745	25	336.2	2.55
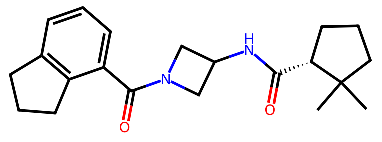	CC1(C)CCC[C@H]1C(=O)NC1CN(C(=O)c2cccc3c2CCC3)C1	997894717	25	340.22	2.94
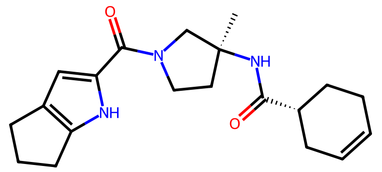	C[C@]1(NC(=O)[C@H]2CC=CCC2)CCN(C(=O)c2cc3c([nH]2)CCC3)C1	956689634	25	341.21	2.58
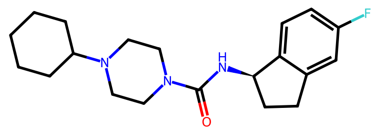	O=C(N[C@@H]1CCc2cc(F)ccc21)N1CCN(C2CCCCC2)CC1	485155811	25	345.22	3.47
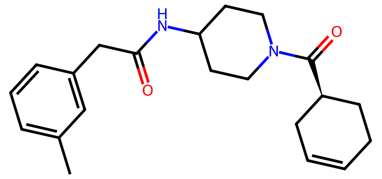	Cc1cccc(CC(=O)NC2CCN(C(=O)[C@@H]3CC=CCC3)CC2)c1	944105668	25	340.22	3.0

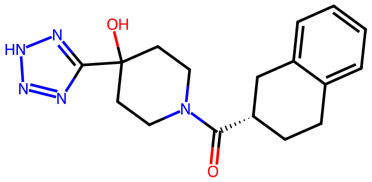	O=C([C@H]1CCc2ccccc2C1)N1CCC(O)(c2nn[nH]n2)CC1	827763584	24	327.17	0.81
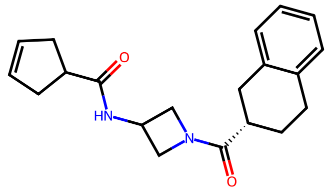	O=C(NC1CN(C(=O)[C@H]2CCc3ccccc3C2)C1)C1CC=CC1	990587766	24	324.18	2.08
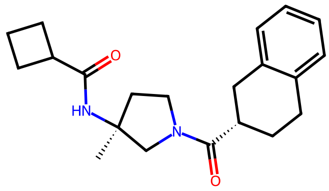	C[C@]1(NC(=O)C2CCC2)CCN(C(=O)[C@H]2CCc3ccccc3C2)C1	956628611	25	340.22	2.7
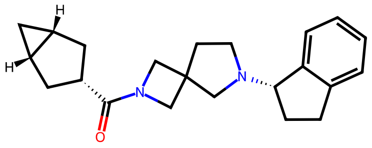	O=C([C@@H]1C[C@@H]2C[C@@H]2C1)N1CC2(CCN([C@H]3CCc4ccccc43)C2)C1	1072907616	25	336.22	3.25
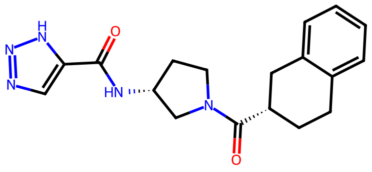	O=C(N[C@@H]1CCN(C(=O)[C@H]2CCc3ccccc3C2)C1)c1cnn[nH]1	936267210	25	339.17	0.94
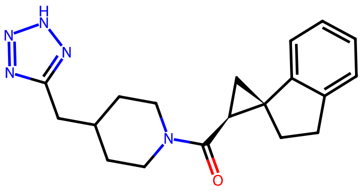	O=C([C@H]1C[C@@]12CCc1ccccc12)N1CCC(Cc2nn[nH]n2)CC1	1548804794	25	337.19	1.88
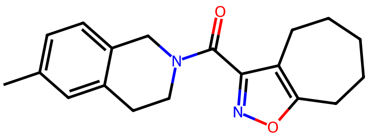	Cc1ccc2c(c1)CCN(C(=O)c1noc3c1CCCCC3)C2	480980482	23	310.17	3.45
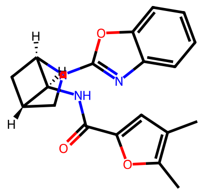	Cc1cc(C(=O)N[C@H]2[C@H]3C[C@H]2N(c2nc4ccccc4o2)C3)oc1C	1064243226	25	337.14	3.04

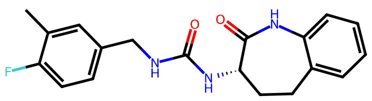	Cc1cc(CNC(=O)N[C@H]2CCc3ccccc3NC2=O)ccc1F	654360877	25	341.15	2.89
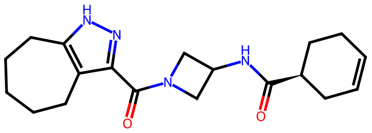	O=C(NC1CN(C(=O)c2n[nH]c3c2CCCCC3)C1)[C@@H]1CC=CCC1	990222281	25	342.21	1.98
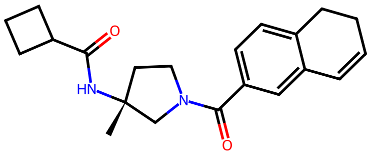	C[C@@]1(NC(=O)C2CCC2)CCN(C(=O)c2ccc3c(c2)C=CCC3)C1	956624638	25	338.2	3.17
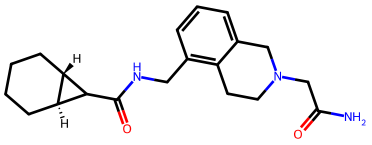	NC(=O)CN1CCc2c(CNC(=O)C3[C@H]4CCCC[C@H]34)cccc2C1	1051284066	25	341.21	1.58
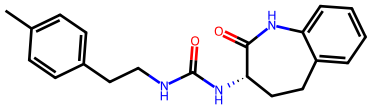	Cc1ccc(CCNC(=O)N[C@H]2CCc3ccccc3NC2=O)cc1	654446383	25	337.18	2.79
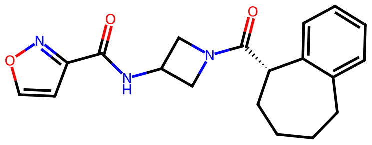	O=C(NC1CN(C(=O)[C@H]2CCCCc3ccccc32)C1)c1ccon1	990363547	25	339.16	2.13
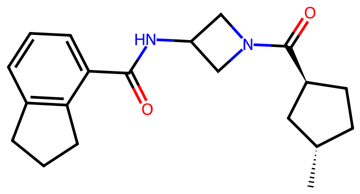	C[C@H]1CC[C@H](C(=O)N2CC(NC(=O)c3cccc4c3CCC4)C2)C1	994398922	24	326.2	2.55
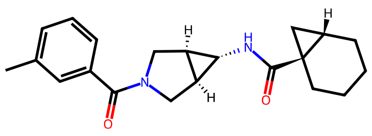	Cc1cccc(C(=O)N2C[C@H]3[C@H](NC(=O)[C@]45CCCC[C@H]4C5)[C@H]3C2)c1	977154238	25	338.2	2.76

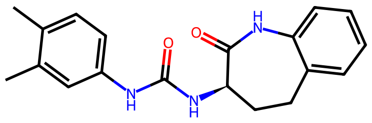	Cc1ccc(NC(=O)N[C@@H]2CCc3cccc c3NC2=O)cc1C	653914131	24	323.16	3.38
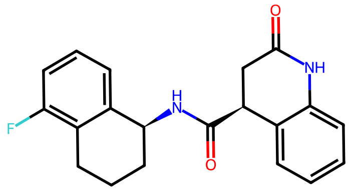	O=C1C[C@H](C(=O)N[C@H]2CCCc3 c(F)cccc32)c2ccccc2N1	452198317	25	338.14	3.45
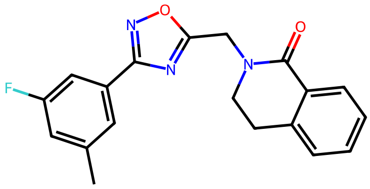	Cc1cc(F)cc(-c2noc(CN3CCc4ccccc4C 3=O)n2)c1	345894184	25	337.12	3.38
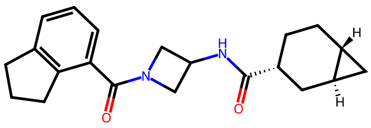	O=C(NC1CN(C(=O)c2cccc3c2CCC3) C1)[C@@H]1CC[C@@H]2C[C@H]2C 1	994672917	25	338.2	2.55
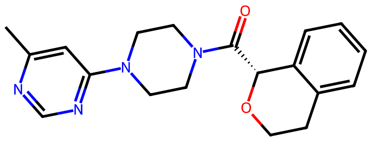	Cc1cc(N2CCN(C(=O)[C@H]3OCCc4c cccc43)CC2)ncn1	1055978669	25	338.17	1.75
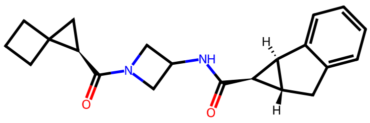	O=C(NC1CN(C(=O)[C@@H]2CC23C CC3)C1)[C@@H]1[C@H]2Cc3ccccc3[C@@H]21	991647436	25	336.18	2.09
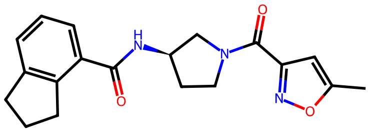	Cc1cc(C(=O)N2CC[C@@H](NC(=O)c 3cccc4c3CCC4)C2)no1	941157053	25	339.16	2.12
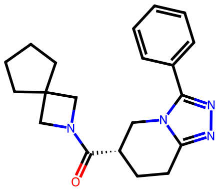	O=C([C@H]1CCc2nnc(-c3ccccc3)n2C 1)N1CC2(CCCC2)C1	576194239	25	336.2	2.91

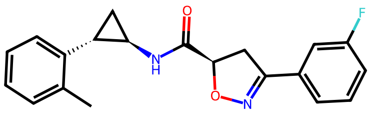	Cc1ccccc1[C@@H]1C[C@H]1NC(=O)[C@H]1CC(c2cccc(F)c2)=NO1	1527705035	25	338.14	3.3
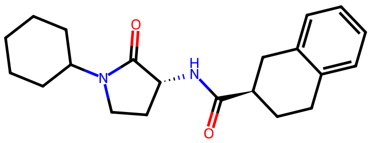	O=C(N[C@@H]1CCN(C2CCCCC2)C1=O)[C@@H]1CCc2ccccc2C1	649844873	25	340.22	2.84
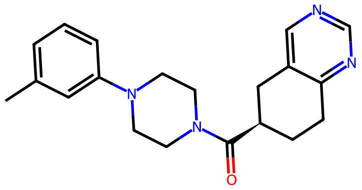	Cc1cccc(N2CCN(C(=O)[C@@H]3CCc4ncncc4C3)CC2)c1	648226883	25	336.2	2.24
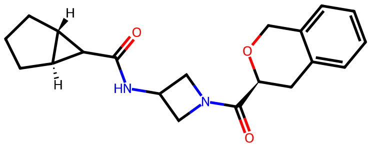	O=C(NC1CN(C(=O)[C@@H]2Cc3ccccc3CO2)C1)C1[C@H]2CCC[C@H]12	990698939	25	340.18	1.5
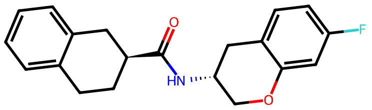	O=C(N[C@H]1COc2cc(F)ccc2C1)[C@H]1CCc2ccccc2C1	618657623	24	325.15	3.05
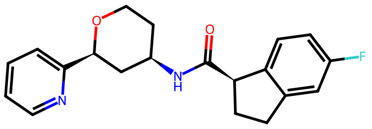	O=C(N[C@@H]1CCO[C@H](c2ccccn2)C1)[C@@H]1CCc2cc(F)ccc21	1549391065	25	340.16	3.29
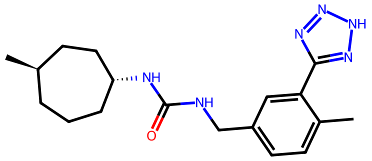	Cc1ccc(CNC(=O)N[C@@H]2CCC[C@@H](C)CC2)cc1-c1nn[nH]n1	1605852041	25	342.22	2.94
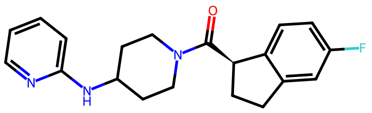	O=C([C@@H]1CCc2cc(F)ccc21)N1CCC(Nc2ccccn2)CC1	927074707	25	339.17	3.35

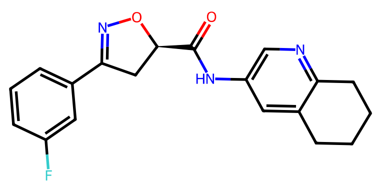	O=C(Nc1cnc2c(c1)CCCC2)[C@H]1CC(c2cccc(F)c2)=NO1	782513923	25	339.14	3.23
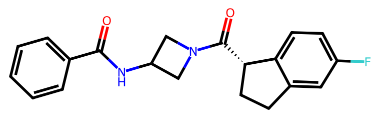	O=C(NC1CN(C(=O)[C@H]2CCc3cc(F)ccc32)C1)c1ccccc1	990097773	25	338.14	2.5
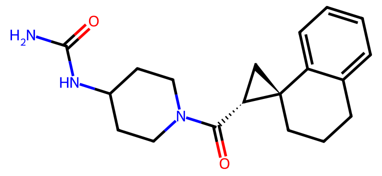	NC(=O)NC1CCN(C(=O)[C@@H]2C[C@@]23CCCc2ccccc23)CC1	176063187	24	327.19	1.94
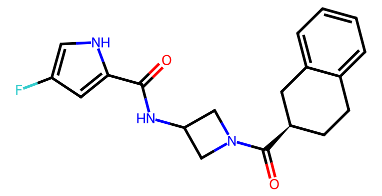	O=C(NC1CN(C(=O)[C@@H]2CCc3ccccc3C2)C1)c1cc(F)c[nH]1	996235887	25	341.15	1.9
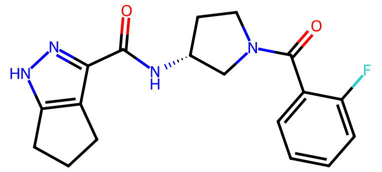	O=C(N[C@@H]1CCN(C(=O)c2ccccc2F)C1)c1n[nH]c2c1CCC2	941439220	25	342.15	1.68
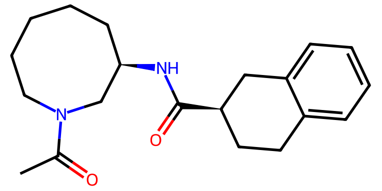	CC(=O)N1CCCCC[C@@H](NC(=O)[C@@H]2CCc3ccccc3C2)C1	986791975	24	328.22	2.7
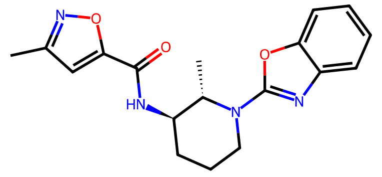	Cc1cc(C(=O)N[C@@H]2CCCN(c3nc4ccccc4o3)[C@H]2C)on1	1069340658	25	340.15	2.91
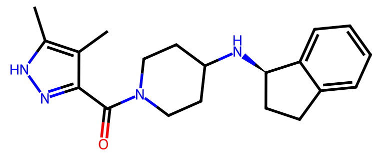	Cc1[nH]nc(C(=O)N2CCC(N[C@@H]3CCc4ccccc43)CC2)c1C	990403571	25	338.21	2.91

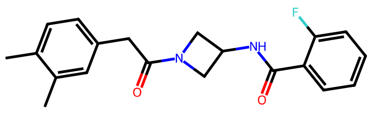	Cc1ccc(CC(=O)N2CC(NC(=O)c3ccccc3F)C2)cc1C	990093655	25	340.16	2.63
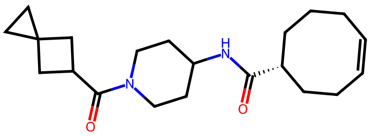	O=C(NC1CCN(C(=O)C2CC3(CC3)C2)CC1)[C@H]1CCC=CCCC1	948306147	25	344.25	3.42
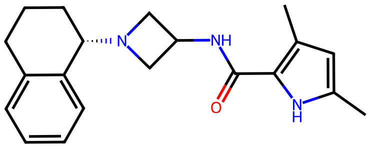	Cc1cc(C)c(C(=O)NC2CN([C@H]3CCCc4ccccc43)C2)[nH]1	1030535687	24	323.2	3.12
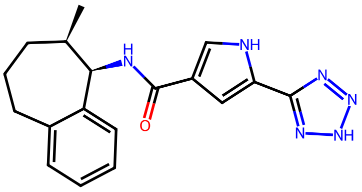	C[C@@H]1CCCc2ccccc2[C@@H]1NC(=O)c1c[nH]c(-c2nn[nH]n2)c1	1590263693	25	336.17	2.64
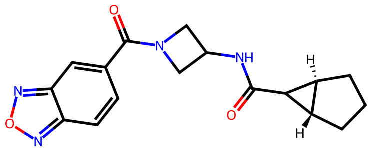	O=C(NC1CN(C(=O)c2ccc3nonc3c2)C1)C1[C@H]2CCC[C@H]12	990695036	24	326.14	1.21
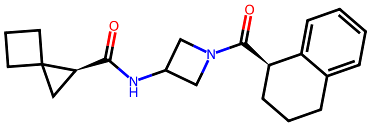	O=C(NC1CN(C(=O)[C@@H]2CCCc3ccccc32)C1)[C@H]1CC12CCC2	991554312	25	338.2	2.62
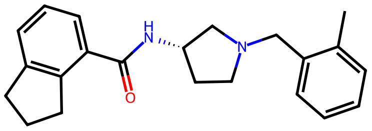	Cc1ccccc1CN1CC[C@H](NC(=O)c2cccc3c2CCC3)C1	1016382005	25	334.2	3.49
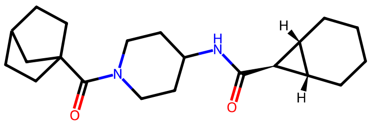	O=C(NC1CCN(C(=O)C23CCC(CC2)C3)CC1)[C@@H]1[C@H]2CCCC[C@H]21	947332447	25	344.25	3.11

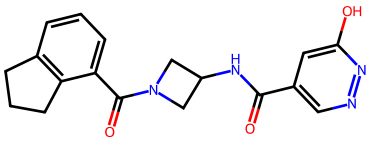	O=C(NC1CN(C(=O)c2cccc3c2CCC3)C1)c1cnnc(O)c1	994038395	25	338.14	0.93
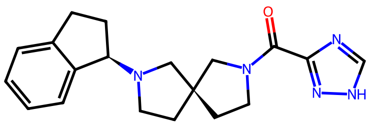	O=C(c1nc[nH]n1)N1CC[C@]2(CCN([C@@H]3CCc4ccccc43)C2)C1	1041748545	25	337.19	2.03
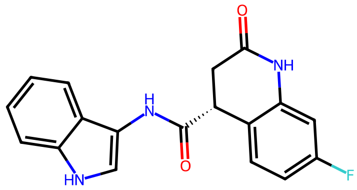	O=C1C[C@@H](C(=O)Nc2c[nH]c3ccccc23)c2ccc(F)cc2N1	1645449070	24	323.11	3.37
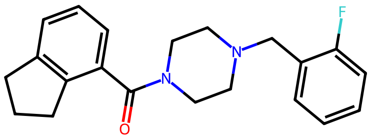	O=C(c1cccc2c1CCC2)N1CCN(Cc2ccccc2F)CC1	957870820	25	338.18	3.27
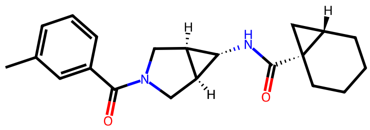	Cc1cccc(C(=O)N2C[C@H]3[C@H](NC(=O)[C@@]45CCCC[C@H]4C5)[C@H]3C2)c1	977154237	25	338.2	2.76
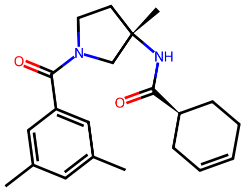	Cc1cc(C)cc(C(=O)N2CC[C@](C)(NC(=O)[C@@H]3CC=CCC3)C2)c1	956688348	25	340.22	3.38
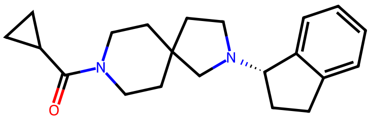	O=C(C1CC1)N1CCC2(CC1)CCN([C@H]1CCc3ccccc31)C2	1035148951	24	324.22	3.4
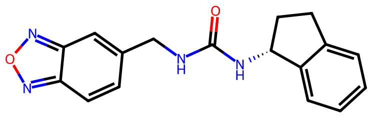	O=C(NCc1ccc2nonc2c1)N[C@@H]1CCc2ccccc21	1193003681	23	308.13	2.71

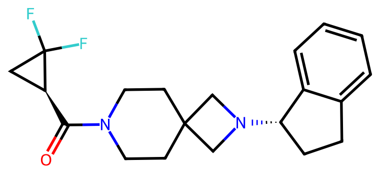	O=C([C@H]1CC1(F)F)N1CCC2(CC1)CN([C@H]1CCc3ccccc31)C2	1035700084	25	346.19	3.25
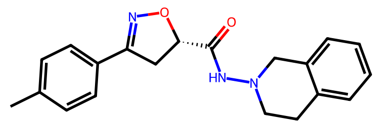	Cc1ccc(C2=NO[C@H](C(=O)NN3CCc4ccccc4C3)C2)cc1	1418389462	25	335.16	2.58
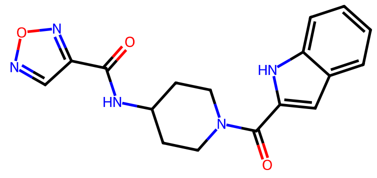	O=C(NC1CCN(C(=O)c2cc3ccccc3[nH]2)CC1)c1cnon1	948488696	25	339.13	1.59
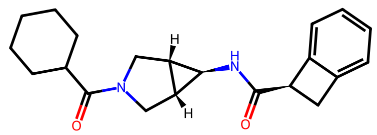	O=C(N[C@H]1[C@@H]2CN(C(=O)C3CCCCC3)C[C@@H]21)[C@@H]1Cc2ccccc21	977084528	25	338.2	2.48
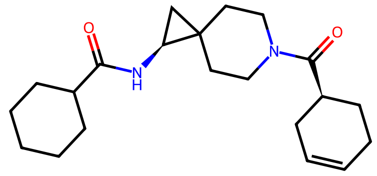	O=C(N[C@H]1CC12CCN(C(=O)[C@@H]1CC=CCC1)CC2)C1CCCCC1	962750261	25	344.25	3.42
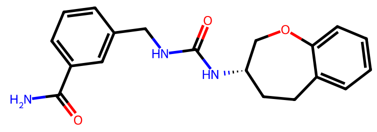	NC(=O)c1cccc(CNC(=O)N[C@H]2CCc3ccccc3OC2)c1	374507573	25	339.16	1.98
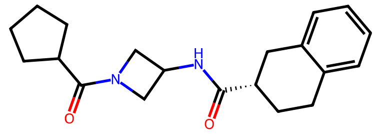	O=C(NC1CN(C(=O)C2CCCC2)C1)[C@H]1CCc2ccccc2C1	1000539389	24	326.2	2.31
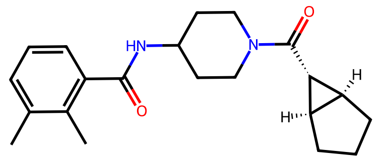	Cc1cccc(C(=O)NC2CCN(C(=O)[C@@H]3[C@H]4CCC[C@H]43)CC2)c1C	946079485	25	340.22	3.07

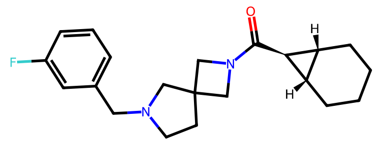	O=C([C@@H]1[C@H]2CCCC[C@H]21)N1CC2(CCN(Cc3cccc(F)c3)C2)C1	1072580947	25	342.21	3.3
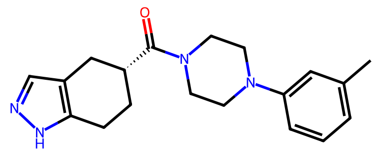	Cc1cccc(N2CCN(C(=O)[C@@H]3CCc4[nH]ncc4C3)CC2)c1	270170310	24	324.2	2.17
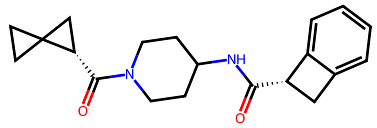	O=C(NC1CCN(C(=O)[C@H]2CC23CC3)CC1)[C@H]1Cc2ccccc21	948571710	24	324.18	2.23
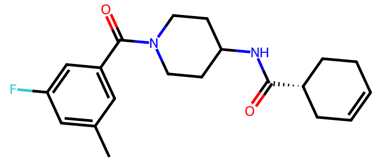	Cc1ccc(F)cc(C(=O)N2CCC(NC(=O)[C@H]3CC=CCC3)CC2)c1	948545980	25	344.19	3.21
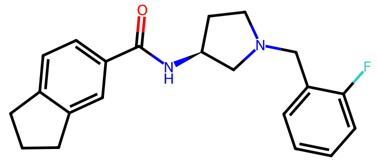	O=C(N[C@H]1CCN(Cc2ccccc2F)C1)c1ccc2c(c1)CCC2	1016225819	25	338.18	3.32
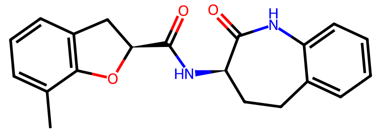	Cc1cccc2c1O[C@H](C(=O)N[C@@H]1CCc3ccccc3NC1=O)C2	653996572	25	336.15	2.37
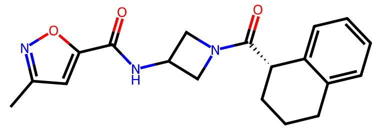	Cc1cc(C(=O)NC2CN(C(=O)[C@H]3CCc4ccccc43)C2)on1	990222963	25	339.16	2.04
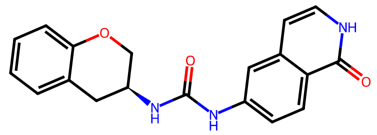	O=C(Nc1ccc2c(=O)[nH]ccc2c1)N[C@@H]1COc2ccccc2C1	1626189849	25	335.13	2.65

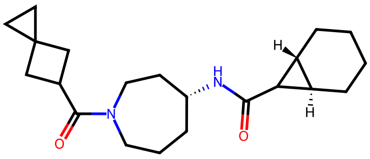	O=C(N[C@@H]1CCCN(C(=O)C2CC3(CC3)C2)CC1)C1[C@@H]2CCCC[C@@H]12	964809581	25	344.25	3.11
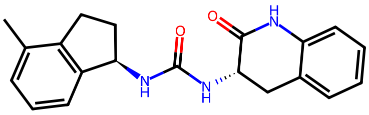	Cc1cccc2c1CC[C@H]2NC(=O)N[C@H]1Cc2ccccc2NC1=O	636735160	25	335.16	2.84
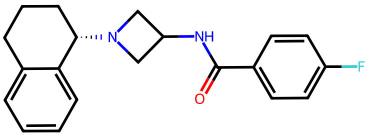	O=C(NC1CN([C@H]2CCCc3ccccc32)C1)c1ccc(F)cc1	1030054500	24	324.16	3.32
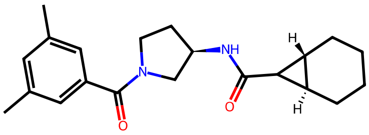	Cc1cc(C)cc(C(=O)N2CC[C@@H](NC(=O)C3[C@@H]4CCCC[C@@H]34)C2)c1	939895350	25	340.22	3.07
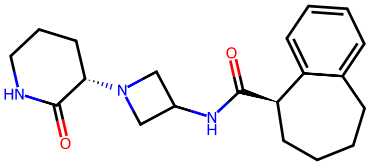	O=C1NCCC[C@@H]1N1CC(NC(=O)[C@@H]2CCCCc3ccccc32)C1	1031452576	25	341.21	1.58
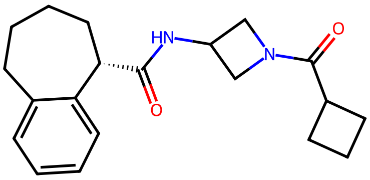	O=C(NC1CN(C(=O)C2CCC2)C1)[C@H]1CCCCc2ccccc21	994322804	24	326.2	2.62
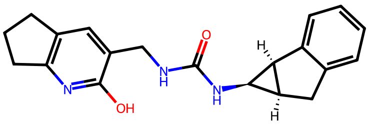	O=C(NCc1cc2c(nc1O)CCC2)N[C@@H]1[C@@H]2Cc3ccccc3[C@@H]21	675712911	25	335.16	2.41
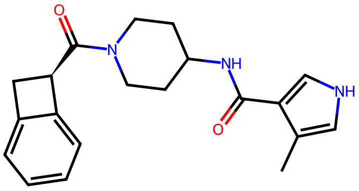	Cc1c[nH]cc1C(=O)NC1CCN(C(=O)[C@@H]2Cc3ccccc32)CC1	948063047	25	337.18	2.38

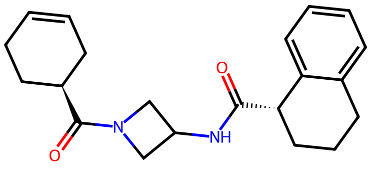	O=C(NC1CN(C(=O)[C@@H]2CC=CCC2)C1)[C@H]1CCCc2ccccc21	995071177	25	338.2	2.79
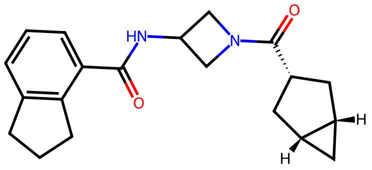	O=C(NC1CN(C(=O)[C@@H]2C[C@@H]3C[C@@H]3C2)C1)c1cccc2c1CCC2	994397416	24	324.18	2.16
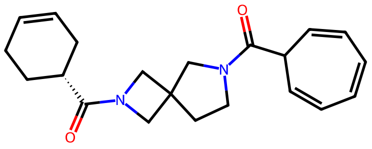	O=C(C1C=CC=CC=C1)N1CCC2(C1)CN(C(=O)[C@H]1CC=CCC1)C2	1018772876	25	338.2	2.7
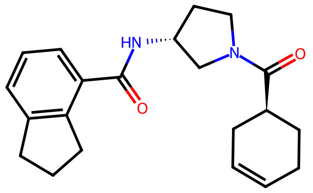	O=C(N[C@@H]1CCN(C(=O)[C@@H]2CC=CCC2)C1)c1cccc2c1CCC2	941157603	25	338.2	2.86
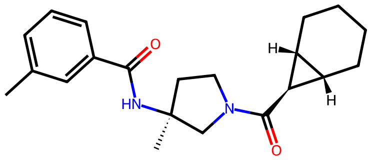	Cc1cccc(C(=O)N[C@@]2(C)CCN(C(=O)[C@@H]3[C@H]4CCCC[C@H]43)C2)c1	956661920	25	340.22	3.15
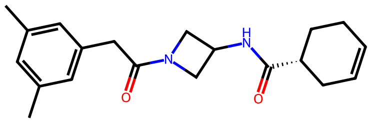	Cc1cc(C)cc(CC(=O)N2CC(NC(=O)[C@H]3CC=CCC3)C2)c1	990214249	24	326.2	2.53
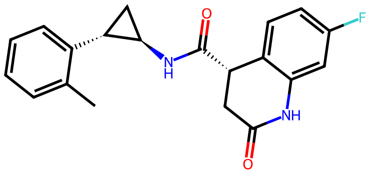	Cc1ccccc1[C@@H]1C[C@H]1NC(=O)[C@H]1CC(=O)Nc2cc(F)ccc21	650387456	25	338.14	3.23
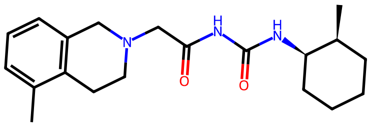	Cc1cccc2c1CCN(CC(=O)NC(=O)N[C@@H]1CCCC[C@@H]1C)C2	729478532	25	343.23	2.76

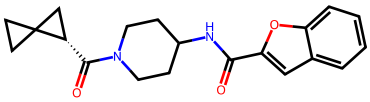	O=C(NC1CCN(C(=O)[C@H]2CC23CC3)CC1)c1cc2ccccc2o1	948558954	25	338.16	2.95
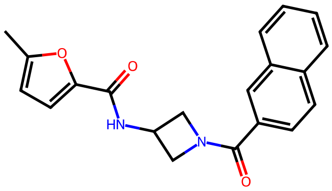	Cc1ccc(C(=O)NC2CN(C(=O)c3ccc4ccccc4c3)C2)o1	990151387	25	334.13	3.0
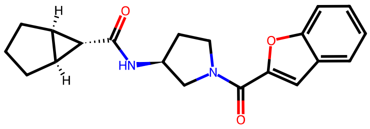	O=C(N[C@H]1CCN(C(=O)c2cc3ccccc3o2)C1)[C@@H]1[C@H]2CCC[C@H]21	937805189	25	338.16	2.81
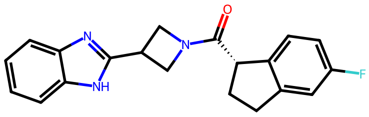	O=C([C@H]1CCc2cc(F)ccc21)N1CC(c2nc3ccccc3[nH]2)C1	612235060	25	335.14	3.36
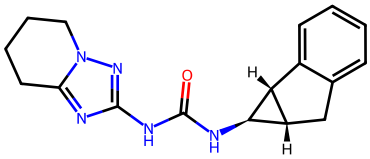	O=C(Nc1nc2n(n1)CCCC2)N[C@@H]1[C@H]2Cc3ccccc3[C@H]21	653706407	23	309.16	2.07
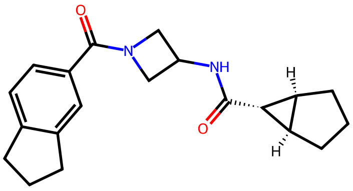	O=C(NC1CN(C(=O)c2ccc3c(c2)CCC3)C1)[C@@H]1[C@H]2CCC[C@H]21	990707401	24	324.18	2.16
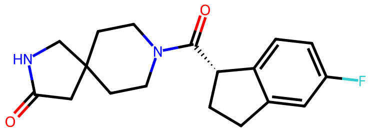	O=C1CC2(CCN(C(=O)[C@H]3CCc4cc(F)ccc43)CC2)CN1	829422898	23	316.16	1.98
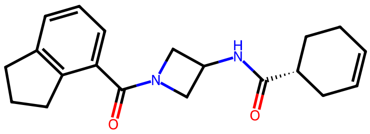	O=C(NC1CN(C(=O)c2cccc3c2CCC3)C1)[C@H]1CC=CCC1	990212161	24	324.18	2.47

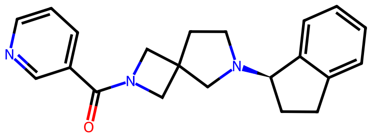	O=C(c1cccnc1)N1CC2(CCN([C@@H]3CCc4ccccc43)C2)C1	1072400268	25	333.18	2.92
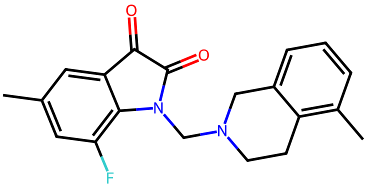	Cc1cc(F)c2c(c1)C(=O)C(=O)N2CN1CCc2c(C)cccc2C1	729465401	25	338.14	2.99
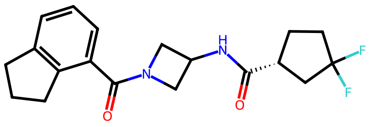	O=C(NC1CN(C(=O)c2cccc3c2CCC3)C1)[C@@H]1CCC(F)(F)C1	994170608	25	348.16	2.55
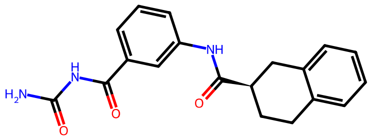	NC(=O)NC(=O)c1cccc(NC(=O)[C@@H]2CCc3ccccc3C2)c1	1412344533	25	337.14	2.24
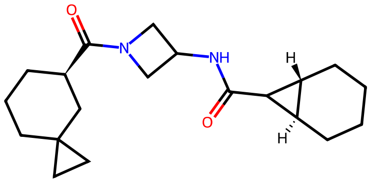	O=C(NC1CN(C(=O)[C@@H]2CCCC3(CC3)C2)C1)C1[C@@H]2CCCC[C@@H]12	990651871	24	330.23	2.72
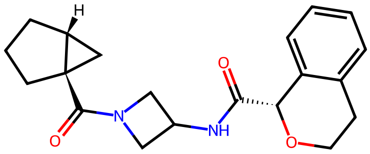	O=C(NC1CN(C(=O)[C@@]23CCC[C@@H]2C3)C1)[C@H]1OCCc2ccccc21	997321318	25	340.18	1.82
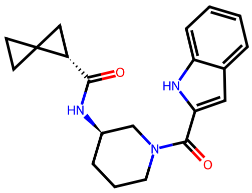	O=C(N[C@@H]1CCCN(C(=O)c2cc3ccccc3[nH]2)C1)[C@H]1CC12CC2	952080982	25	337.18	2.69
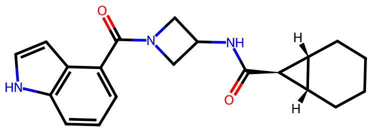	O=C(NC1CN(C(=O)c2cccc3[nH]ccc23)C1)[C@@H]1[C@H]2CCCC[C@H]21	990659250	25	337.18	2.54

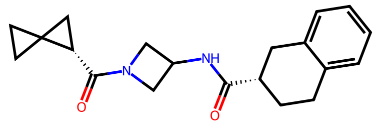	O=C(NC1CN(C(=O)[C@H]2CC23CC3)C1)[C@H]1CCc2ccccc2C1	1000545200	24	324.18	1.92
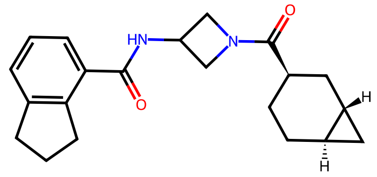	O=C(NC1CN(C(=O)[C@@H]2CC[C@@H]3C[C@H]3C2)C1)c1cccc2c1CCC2	994400585	25	338.2	2.55
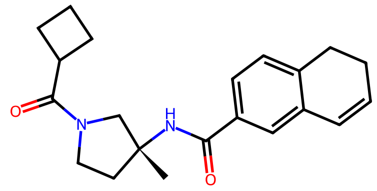	C[C@@]1(NC(=O)c2ccc3c(c2)C=CCC3)CCN(C(=O)C2CCC2)C1	957193867	25	338.2	3.17
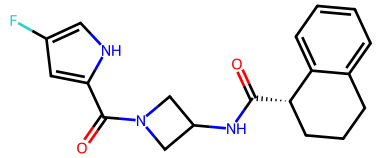	O=C(NC1CN(C(=O)c2cc(F)c[nH]2)C1)[C@H]1CCCc2ccccc21	995075651	25	341.15	2.21
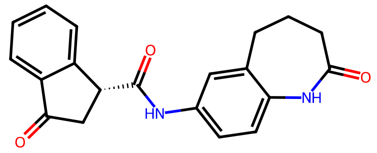	O=C1CCCc2cc(NC(=O)[C@@H]3CC(=O)c4ccccc43)ccc2N1	277177340	25	334.13	3.27
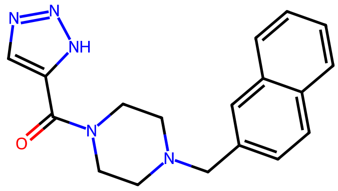	O=C(c1cnn[nH]1)N1CCN(Cc2ccc3ccccc3c2)CC1	949060348	24	321.16	1.92
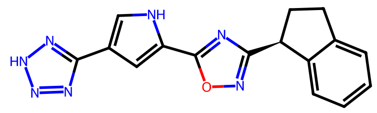	c1ccc2c(c1)CC[C@@H]2c1noc(-c2cc(-c3nn[nH]n3)c[nH]2)n1	1593580263	24	319.12	2.32
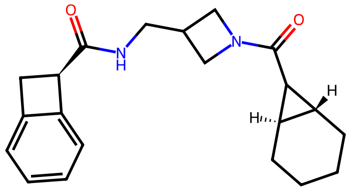	O=C(NCC1CN(C(=O)C2[C@@H]3CCCC[C@@H]23)C1)[C@@H]1Cc2ccccc21	1003949088	25	338.2	2.34

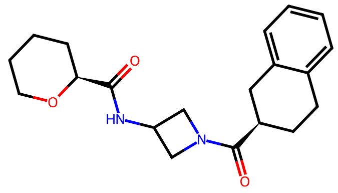	O=C(NC1CN(C(=O)[C@@H]2CCc3ccccc3C2)C1)[C@@H]1CCCCO1	990855873	25	342.19	1.69
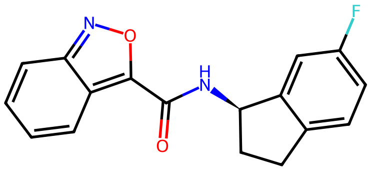	O=C(N[C@@H]1CCc2ccc(F)cc21)c1onc2ccccc12	1464995909	22	296.1	3.38
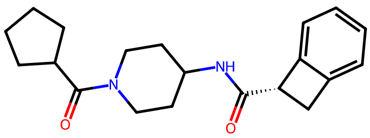	O=C(NC1CCN(C(=O)C2CCCC2)CC1)[C@H]1Cc2ccccc21	943376428	24	326.2	2.62
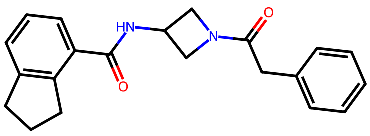	O=C(NC1CN(C(=O)Cc2ccccc2)C1)c1cccc2c1CCC2	994393969	25	334.17	2.36
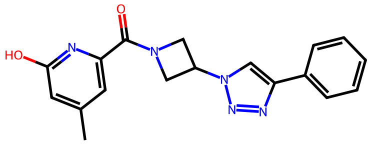	Cc1cc(O)nc(C(=O)N2CC(n3cc(-c4ccccc4)nn3)C2)c1	345876116	25	335.14	2.05
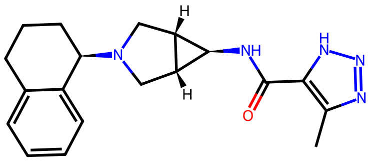	Cc1nn[nH]c1C(=O)N[C@H]1[C@@H]2CN([C@@H]3CCCc4ccccc43)C[C@@H]21	961448320	25	337.19	1.85
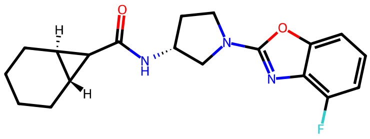	O=C(N[C@@H]1CCN(c2nc3c(F)cccc3o2)C1)C1[C@@H]2CCCC[C@@H]12	1058334380	25	343.17	3.1
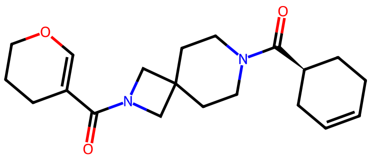	O=C(C1=COCCC1)N1CC2(CCN(C(=O)[C@@H]3CC=CCC3)CC2)C1	944851863	25	344.21	2.49

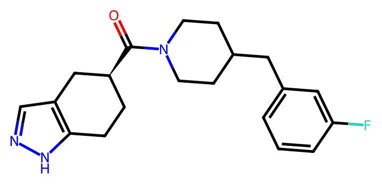	O=C([C@H]1CCc2[nH]ncc2C1)N1CCC(Cc2cccc(F)c2)CC1	270280585	25	341.19	3.14
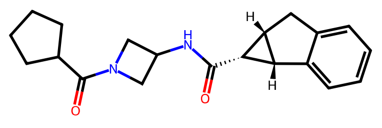	O=C(NC1CN(C(=O)C2CCCC2)C1)[C@H]1[C@H]2c3ccccc3C[C@H]21	991647481	24	324.18	2.09
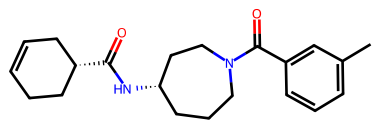	Cc1cccc(C(=O)N2CCC[C@H](NC(=O)[C@H]3CC=CCC3)CC2)c1	964717484	25	340.22	3.46
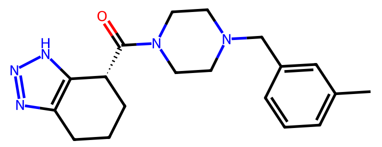	Cc1cccc(CN2CCN(C(=O)[C@@H]3CCCc4nn[nH]c43)CC2)c1	646585140	25	339.21	1.88
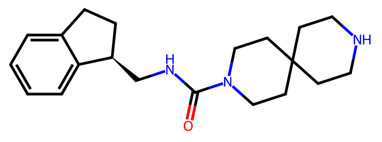	O=C(NC[C@@H]1CCc2ccccc21)N1CCC2(CCNCC2)CC1	417587668	24	327.23	2.89
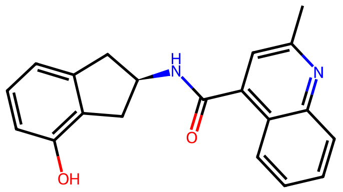	Cc1cc(C(=O)N[C@@H]2Cc3cccc(O)c3C2)c2ccccc2n1	430239333	24	318.14	3.15
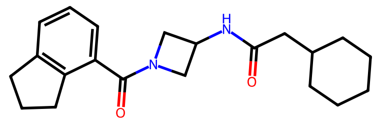	O=C(CC1CCCCC1)NC1CN(C(=O)c2cccc3c2CCC3)C1	990158631	25	340.22	3.09
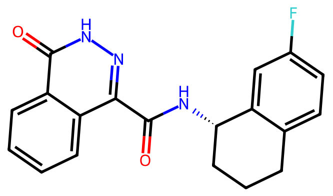	O=C(N[C@H]1CCCc2ccc(F)cc21)c1n[nH]c(=O)c2ccccc12	93190711	25	337.12	2.87

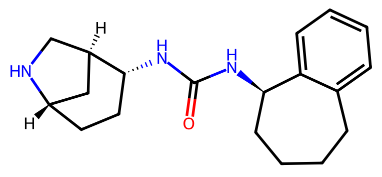	O=C(N[C@@H]1CCCCc2ccccc21)N[C@@H]1CC[C@H]2C[C@H]1CN2	1551523064	23	313.22	2.89
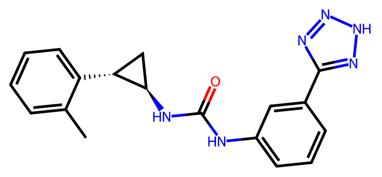	Cc1ccccc1[C@@H]1C[C@H]1NC(=O)Nc1cccc(-c2nn[nH]n2)c1	1581261075	25	334.15	2.85
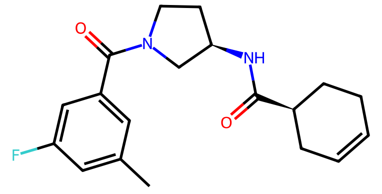	Cc1cc(F)cc(C(=O)N2CC[C@@H](NC(=O)[C@@H]3CC=CCC3)C2)c1	936241816	24	330.17	2.82
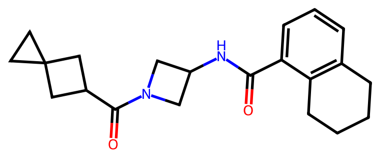	O=C(NC1CN(C(=O)C2CC3(CC3)C2)C1)c1cccc2c1CCCC2	991900857	25	338.2	2.7
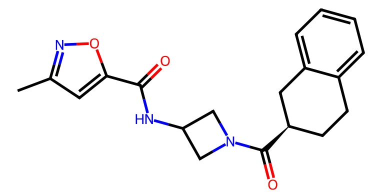	Cc1cc(C(=O)NC2CN(C(=O)[C@@H]3CCc4ccccc4C3)C2)on1	990217901	25	339.16	1.73
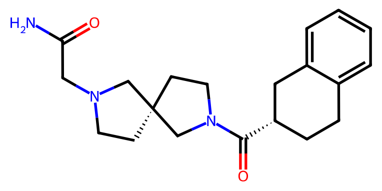	NC(=O)CN1CC[C@@]2(CCN(C(=O)[C@H]3CCc4ccccc4C3)C2)C1	1041710105	25	341.21	1.2
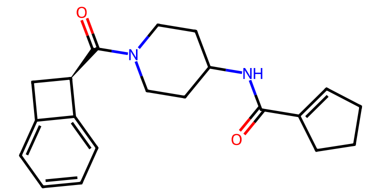	O=C(NC1CCN(C(=O)[C@@H]2Cc3ccccc32)CC1)C1=CCCC1	948060129	24	324.18	2.54
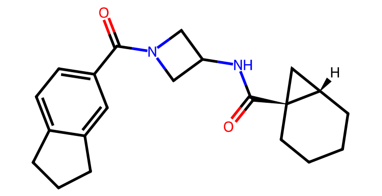	O=C(c1ccc2c(c1)CCC2)N1CC(NC(=O)[C@]23CCCC[C@H]2C3)C1	999287394	25	338.2	2.7

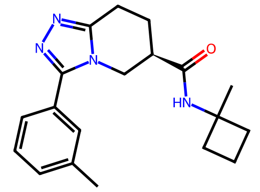	Cc1cccc(-c2nnc3n2C[C@H](C(=O)NC2(C)CCC2)CC3)c1	580618930	24	324.2	2.87
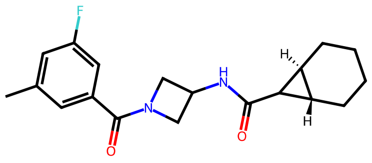	Cc1cc(F)cc(C(=O)N2CC(NC(=O)C3[C@H]4CCCC[C@H]34)C2)c1	990663142	24	330.17	2.51
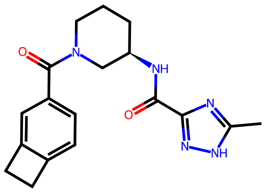	Cc1nc(C(=O)N[C@@H]2CCCN(C(=O)c3ccc4c(c3)CC4)C2)n[nH]1	1875387889	25	339.17	1.25
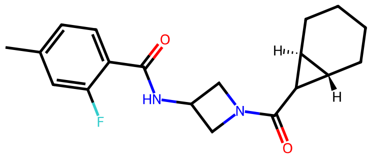	Cc1ccc(C(=O)NC2CN(C(=O)C3[C@H]4CCCC[C@H]34)C2)c(F)c1	1000482848	24	330.17	2.51
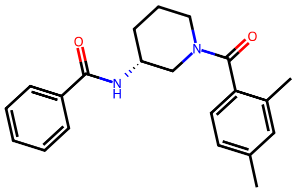	Cc1ccc(C(=O)N2CCC[C@@H](NC(=O)c3ccccc3)C2)c(C)c1	951996399	25	336.18	3.34
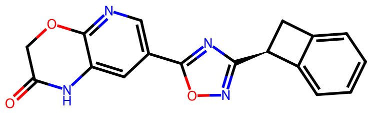	O=C1COc2ncc(-c3nc([C@H]4Cc5ccccc54)no3)cc2N1	547002462	24	320.09	2.15
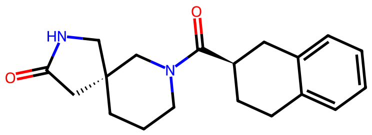	O=C1C[C@]2(CCCN(C(=O)[C@@H]3CCc4ccccc4C3)C2)CN1	579013745	23	312.18	1.92
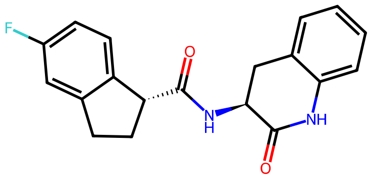	O=C1Nc2ccccc2C[C@@H]1NC(=O)[C@@H]1CCc2cc(F)ccc21	529945401	24	324.13	2.54

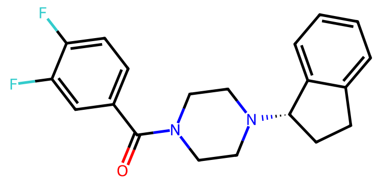	O=C(c1ccc(F)c(F)c1)N1CCN([C@H]2CCc3ccccc32)CC1	49719958	25	342.15	3.41
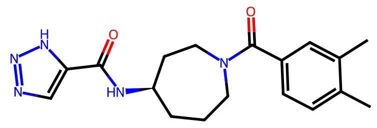	Cc1ccc(C(=O)N2CCC[C@@H](NC(=O)c3cnn[nH]3)CC2)cc1C	965005776	25	341.19	1.85
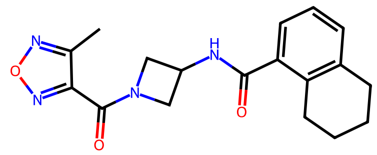	Cc1nonc1C(=O)N1CC(NC(=O)c2cccc3c2CCCC3)C1	991897814	25	340.15	1.51
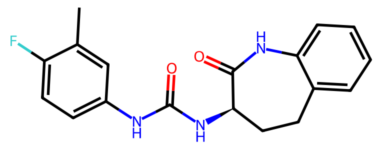	Cc1cc(NC(=O)N[C@@H]2CCc3ccccc3NC2=O)ccc1F	1192407371	24	327.14	3.21
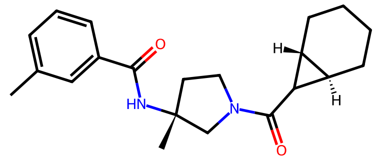	Cc1cccc(C(=O)N[C@]2(C)CCN(C(=O)C3[C@@H]4CCCC[C@@H]34)C2)c1	956661909	25	340.22	3.15
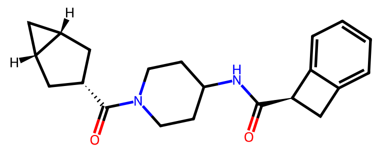	O=C(NC1CCN(C(=O)[C@@H]2C[C@@H]3C[C@@H]3C2)CC1)[C@@H]1Cc2ccccc21	947908750	25	338.2	2.48
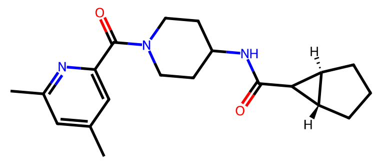	Cc1cc(C)nc(C(=O)N2CCC(NC(=O)C3[C@H]4CCC[C@H]34)CC2)c1	947307728	25	341.21	2.47
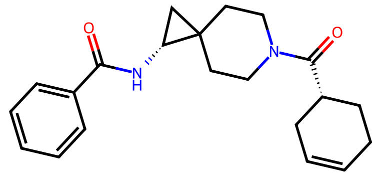	O=C(N[C@@H]1CC12CCN(C(=O)[C@@H]1CC=CCC1)CC2)c1ccccc1	962784353	25	338.2	3.15

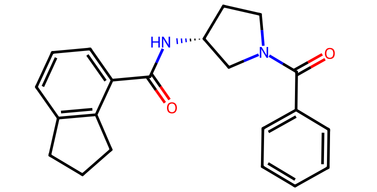	O=C(N[C@@H]1CCN(C(=O)c2ccccc2)C1)c1cccc2c1CCC2	941157250	25	334.17	2.82
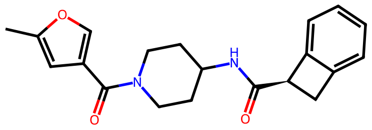	Cc1cc(C(=O)N2CCC(NC(=O)[C@@H]3Cc4ccccc43)CC2)co1	946557088	25	338.16	2.65
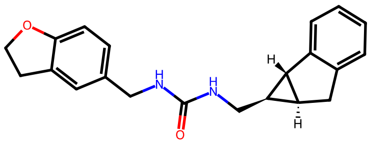	O=C(NCc1ccc2c(c1)CCO2)NC[C@@H]1[C@@H]2Cc3ccccc3[C@@H]12	532616624	25	334.17	3.01
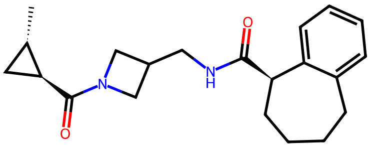	C[C@H]1C[C@@H]1C(=O)N1CC(CNC(=O)[C@@H]2CCCCc3ccccc32)C1	1002643983	25	340.22	2.73
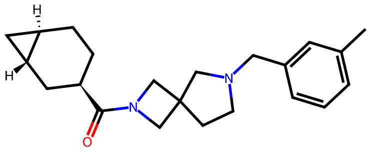	Cc1cccc(CN2CCC3(C2)CN(C(=O)[C@@H]2CC[C@@H]4C[C@H]4C2)C3)c1	1072878996	25	338.24	3.47
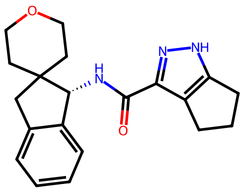	O=C(N[C@@H]1c2ccccc2CC12CCOCC2)c1n[nH]c2c1CCC2	290579132	25	337.18	2.72
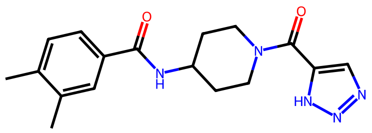	Cc1ccc(C(=O)NC2CCN(C(=O)c3cnn[nH]3)CC2)cc1C	947369938	24	327.17	1.46
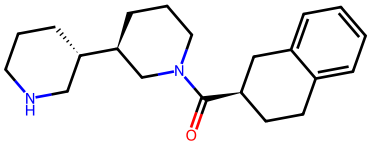	O=C([C@@H]1CCc2ccccc2C1)N1CCC[C@H]([C@@H]2CCCNC2)C1	653143382	24	326.24	3.03

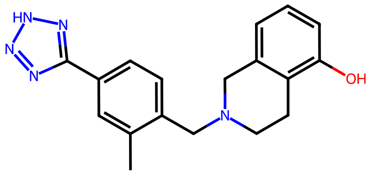	Cc1cc(-c2nn[nH]n2)ccc1CN1CCc2c(O)cccc2C1	1591866879	24	321.16	2.44
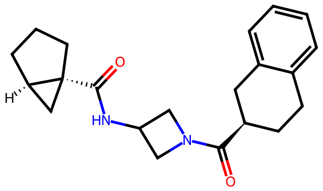	O=C([C@@H]1CCc2ccccc2C1)N1CC(NC(=O)[C@@]23CCC[C@@H]2C3)C1	997549127	25	338.2	2.31
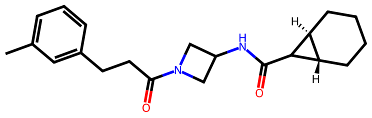	Cc1cccc(CCC(=O)N2CC(NC(=O)C3[C@H]4CCCC[C@H]34)C2)c1	990653230	25	340.22	2.69
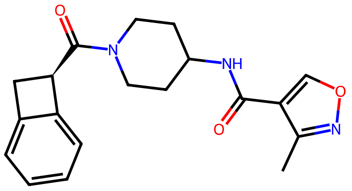	Cc1nocc1C(=O)NC1CCN(C(=O)[C@@H]2Cc3ccccc32)CC1	948059486	25	339.16	2.04
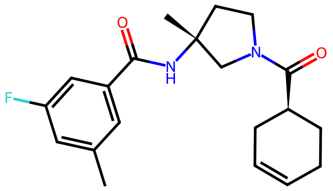	Cc1cc(F)cc(C(=O)N[C@]2(C)CCN(C(=O)[C@@H]3CC=CCC3)C2)c1	957700732	25	344.19	3.21
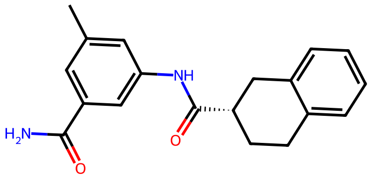	Cc1cc(NC(=O)[C@H]2CCc3ccccc3C2)cc(C(N)=O)c1	583634454	23	308.15	2.84
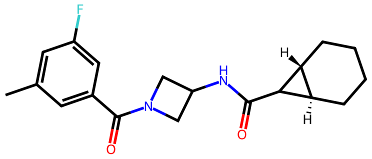	Cc1cc(F)cc(C(=O)N2CC(NC(=O)C3[C@@H]4CCCC[C@@H]34)C2)c1	990663148	24	330.17	2.51
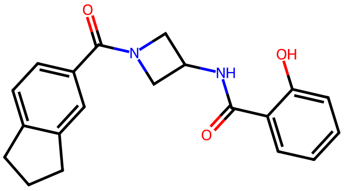	O=C(NC1CN(C(=O)c2ccc3c(c2)CCC3)C1)c1ccccc1O	990277891	25	336.15	2.14

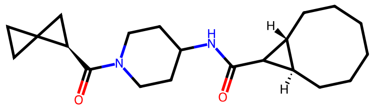	O=C(NC1CCN(C(=O)[C@@H]2CC23CC3)CC1)C1[C@@H]2CCCCCC[C@@H]12	948557992	25	344.25	3.11
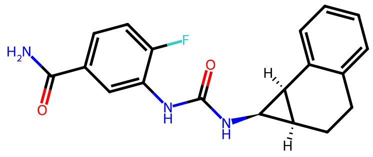	NC(=O)c1ccc(F)c(NC(=O)N[C@@H]2[C@@H]3CCc4ccccc4[C@@H]32)c1	178923029	25	339.14	2.77
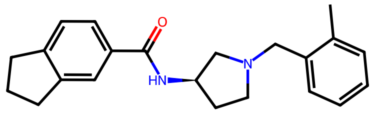	Cc1ccccc1CN1CC[C@@H](NC(=O)c2ccc3c(c2)CCC3)C1	1016222449	25	334.2	3.49
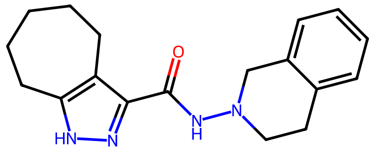	O=C(NN1CCc2ccccc2C1)c1n[nH]c2c1CCCCC2	855709684	23	310.18	2.38
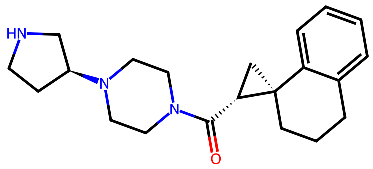	O=C([C@@H]1C[C@]12CCCc1ccccc12)N1CCN([C@H]2CCNC2)CC1	343604811	25	339.23	1.79
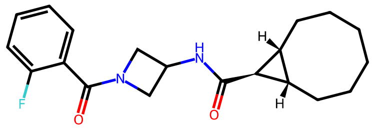	O=C(NC1CN(C(=O)c2ccccc2F)C1)[C@@H]1[C@H]2CCCCCC[C@H]21	1000467941	25	344.19	2.98
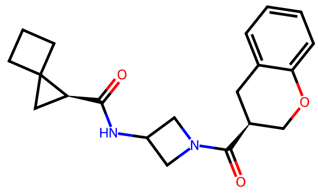	O=C(NC1CN(C(=O)[C@@H]2COc3ccccc3C2)C1)[C@H]1CC12CCC2	991556377	25	340.18	1.75
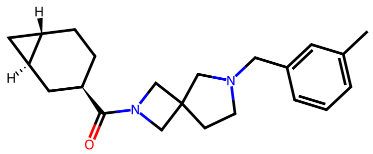	Cc1cccc(CN2CCC3(C2)CN(C(=O)[C@@H]2CC[C@H]4C[C@@H]4C2)C3)c1	1072878998	25	338.24	3.47

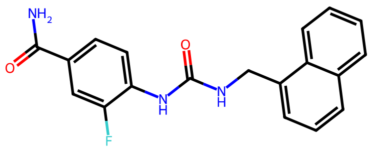	NC(=O)c1ccc(NC(=O)NCc2cccc3cccc c23)c(F)c1	355824145	25	337.12	3.4
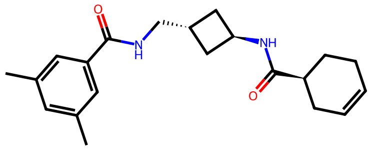	Cc1cc(C)cc(C(=O)NC[C@H]2C[C@H] (NC(=O)[C@@H]3CC=CCC3)C2)c1	980498376	25	340.22	3.28
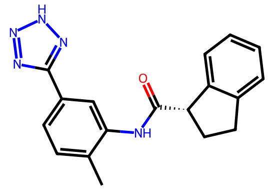	Cc1ccc(-c2nn[nH]n2)cc1NC(=O)[C@H]1CCc2ccccc21	737291073	24	319.14	2.84
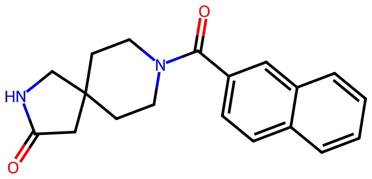	O=C1CC2(CCN(C(=O)c3ccc4ccccc4c 3)CC2)CN1	267644605	23	308.15	2.58
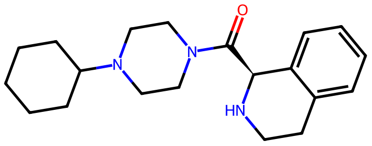	O=C([C@@H]1NCCc2ccccc21)N1CC N(C2CCCCC2)CC1	564996761	24	327.23	2.35
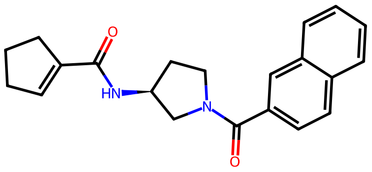	O=C(N[C@H]1CCN(C(=O)c2ccc3cccc c3c2)C1)C1=CCCC1	937820274	25	334.17	3.28
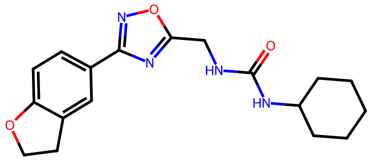	O=C(NCc1nc(-c2ccc3c(c2)CCO3)no1) NC1CCCCC1	154754652	25	342.17	2.8
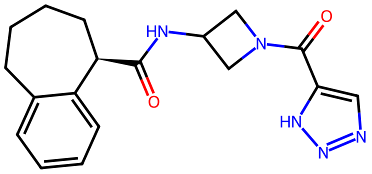	O=C(NC1CN(C(=O)c2cnn[nH]2)C1)[C @@H]1CCCCc2ccccc21	994322939	25	339.17	1.26

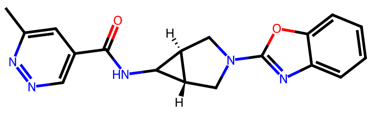	Cc1cc(C(=O)NC2[C@H]3CN(c4nc5ccccc5o4)C[C@H]23)cnn1	1056166505	25	335.14	1.79
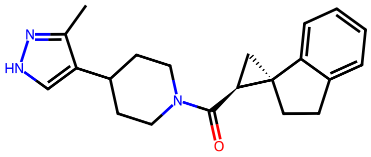	Cc1n[nH]cc1C1CCN(C(=O)[C@H]2C[C@]23CCc2ccccc23)CC1	375990266	25	335.2	3.33
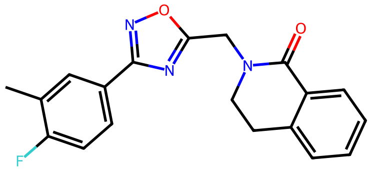	Cc1cc(-c2noc(CN3CCc4ccccc4C3=O)n2)ccc1F	345743788	25	337.12	3.38
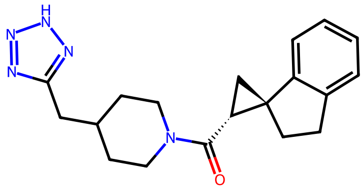	O=C([C@@H]1C[C@@]12CCc1ccccc12)N1CCC(Cc2nn[nH]n2)CC1	1548804793	25	337.19	1.88
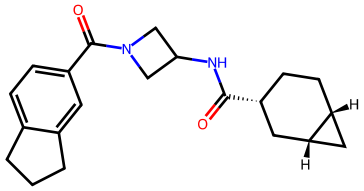	O=C(NC1CN(C(=O)c2ccc3c(c2)CCC3)C1)[C@@H]1CC[C@@H]2C[C@@H]2C1	994697261	25	338.2	2.55
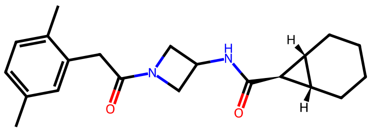	Cc1ccc(C)c(CC(=O)N2CC(NC(=O)[C@@H]3[C@H]4CCCC[C@H]43)C2)c1	990647992	25	340.22	2.61
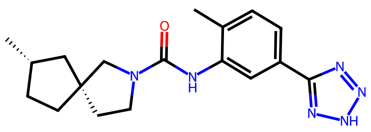	Cc1ccc(-c2nn[nH]n2)cc1NC(=O)N1CC[C@@]2(CC[C@H](C)C2)C1	826277984	25	340.2	3.22
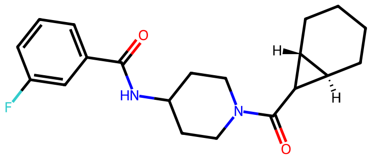	O=C(NC1CCN(C(=O)C2[C@@H]3CCCC[C@@H]23)CC1)c1cccc(F)c1	945956140	25	344.19	2.98

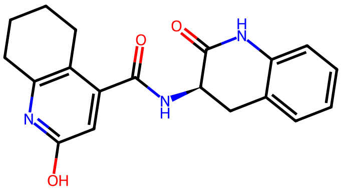	O=C(N[C@@H]1Cc2ccccc2NC1=O)c1cc(O)nc2c1CCCC2	827439120	25	337.14	1.96
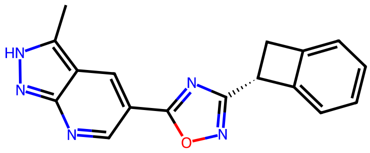	Cc1[nH]nc2ncc(-c3nc([C@@H]4Cc5cccc54)no3)cc12	546287067	23	303.11	3.0
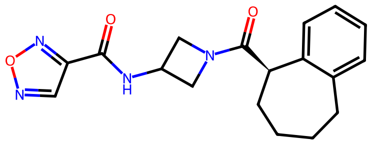	O=C(NC1CN(C(=O)[C@@H]2CCCCc3ccccc32)C1)c1cnon1	998237913	25	340.15	1.52
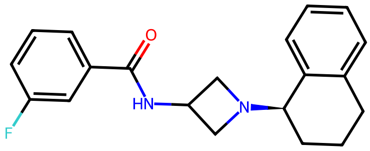	O=C(NC1CN([C@@H]2CCCc3ccccc32)C1)c1cccc(F)c1	1030067647	24	324.16	3.32
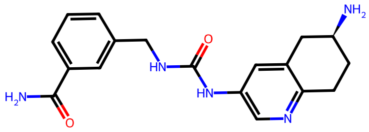	NC(=O)c1cccc(CNC(=O)Nc2cnc3c(c2)C[C@@H](N)CC3)c1	415957137	25	339.17	1.32
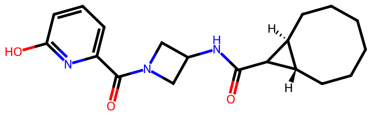	O=C(NC1CN(C(=O)c2cccc(O)n2)C1)C1[C@H]2CCCCCC[C@H]12	1000482551	25	343.19	1.94
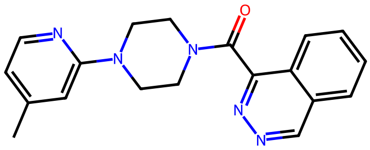	Cc1ccnc(N2CCN(C(=O)c3nncc4ccccc34)CC2)c1	934895794	25	333.16	2.3
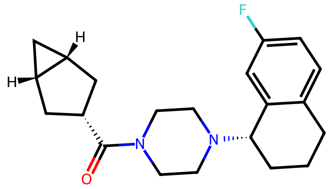	O=C([C@@H]1C[C@@H]2C[C@@H]2C1)N1CCN([C@H]2CCCc3ccc(F)cc32)CC1	949788767	25	342.21	3.39

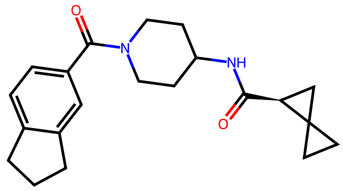	O=C(NC1CCN(C(=O)c2ccc3c(c2)CCC3)CC1)[C@H]1CC12CC2	946723472	25	338.2	2.7
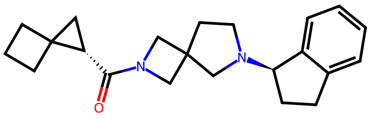	O=C([C@H]1CC12CCC2)N1CC2(CCN([C@@H]3CCc4ccccc43)C2)C1	1073018967	25	336.22	3.4
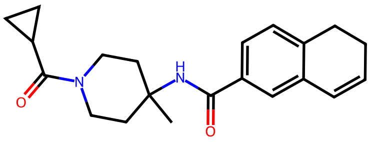	CC1(NC(=O)c2ccc3c(c2)C=CCC3)CCN(C(=O)C2CC2)CC1	956423089	25	338.2	3.17
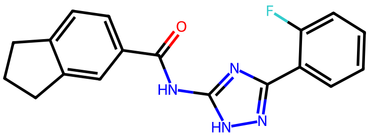	O=C(Nc1nc(-c2ccccc2F)n[nH]1)c1ccc2c(c1)CCC2	342152422	24	322.12	3.35
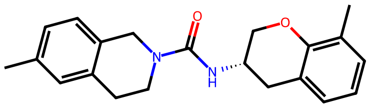	Cc1ccc2c(c1)CCN(C(=O)N[C@@H]1COc3c(C)cccc3C1)C2	1229550429	25	336.18	3.37
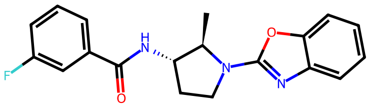	C[C@@H]1[C@@H](NC(=O)c2cccc(F)c2)CCN1c1nc2ccccc2o1	1050024899	25	339.14	3.36
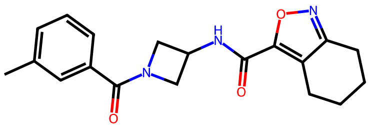	Cc1cccc(C(=O)N2CC(NC(=O)c3onc4c3CCCC4)C2)c1	995575993	25	339.16	2.12
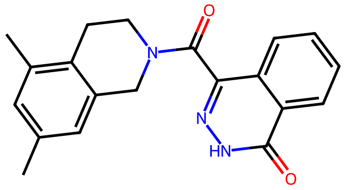	Cc1cc(C)c2c(c1)CN(C(=O)c1n[nH]c(=O)c3ccccc13)CC2	187757702	25	333.15	2.74

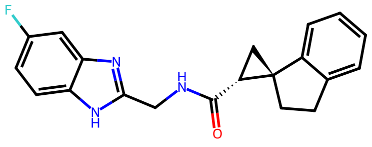	O=C(NCc1nc2cc(F)ccc2[nH]1)[C@@H]1C[C@@]12CCc1ccccc12	176114656	25	335.14	3.22
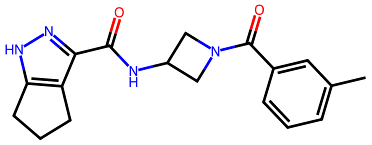	Cc1cccc(C(=O)N2CC(NC(=O)c3n[nH]c4c3CCC4)C2)c1	995164509	24	324.16	1.46
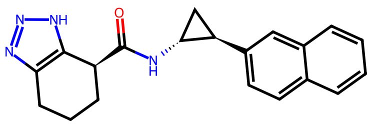	O=C(N[C@@H]1C[C@H]1c1ccc2ccccc2c1)[C@H]1CCCc2nn[nH]c21	614366881	25	332.16	3.05
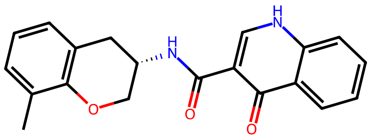	Cc1cccc2c1OC[C@@H](NC(=O)c1c[nH]c3ccccc3c1=O)C2	644643177	25	334.13	2.57
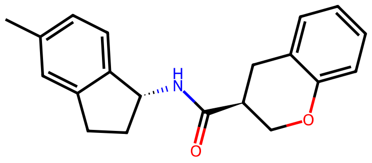	Cc1ccc2c(c1)CC[C@H]2NC(=O)[C@@H]1COc2ccccc2C1	501961034	23	307.16	3.35
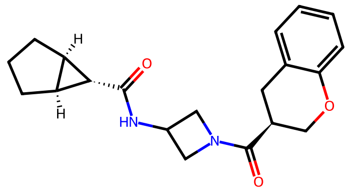	O=C(NC1CN(C(=O)[C@@H]2COc3ccccc3C2)C1)[C@@H]1[C@H]2CCC[C@H]21	990708414	25	340.18	1.61
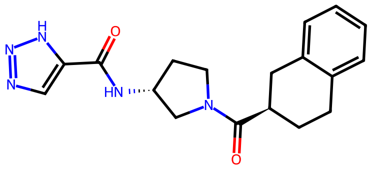	O=C(N[C@@H]1CCN(C(=O)[C@@H]2CCc3ccccc3C2)C1)c1cnn[nH]1	936267206	25	339.17	0.94
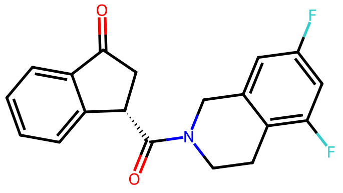	O=C1C[C@H](C(=O)N2CCc3c(F)cc(F)cc3C2)c2ccccc21	355984196	24	327.11	3.22

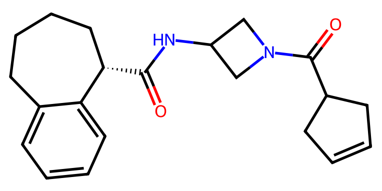	O=C(NC1CN(C(=O)C2CC=CC2)C1)[C@H]1CCCCc2ccccc21	994305267	25	338.2	2.79
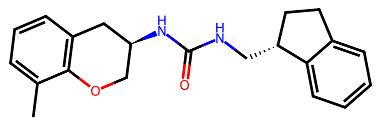	Cc1cccc2c1OC[C@H](NC(=O)NC[C@@H]1CCc3ccccc31)C2	1668963685	25	336.18	3.33
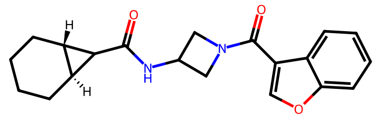	O=C(NC1CN(C(=O)c2coc3ccccc23)C1)C1[C@H]2CCCC[C@H]12	990655456	25	338.16	2.81
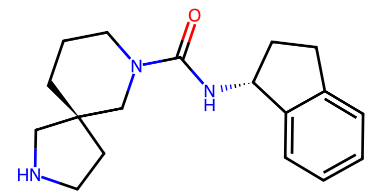	O=C(N[C@@H]1CCc2ccccc21)N1CCC[C@]2(CCNC2)C1	335612081	22	299.2	2.46
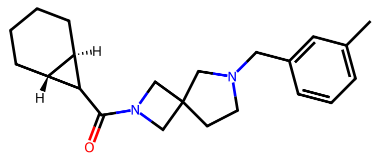	Cc1cccc(CN2CCC3(C2)CN(C(=O)C2[C@@H]4CCCC[C@@H]24)C3)c1	1072580645	25	338.24	3.47
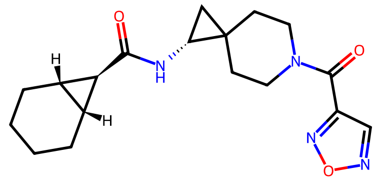	O=C(N[C@@H]1CC12CCN(C(=O)c1cnon1)CC2)[C@@H]1[C@H]2CCCC[C@H]21	963448092	25	344.18	1.62
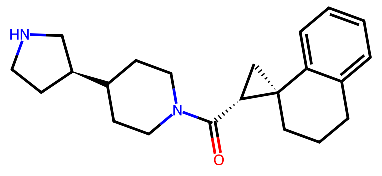	O=C([C@@H]1C[C@]12CCCc1ccccc12)N1CCC([C@H]2CCNC2)CC1	377808352	25	338.24	3.13
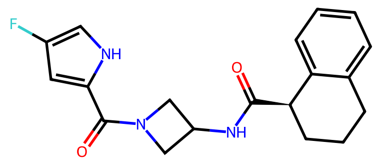	O=C(NC1CN(C(=O)c2cc(F)c[nH]2)C1)[C@@H]1CCCc2ccccc21	995075652	25	341.15	2.21

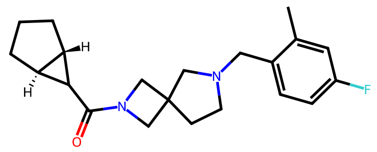	Cc1cc(F)ccc1CN1CCC2(C1)CN(C(=O)C1[C@H]3CCC[C@H]13)C2	1072590438	25	342.21	3.21
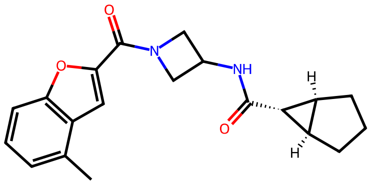	Cc1cccc2oc(C(=O)N3CC(NC(=O)[C@@H]4[C@H]5CCC[C@H]54)C3)cc12	990700291	25	338.16	2.73
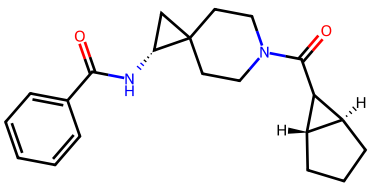	O=C(N[C@@H]1CC12CCN(C(=O)C1[C@H]3CCC[C@H]13)CC2)c1ccccc1	962781716	25	338.2	2.84
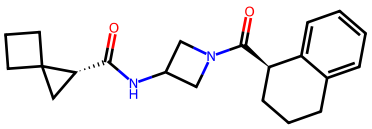	O=C(NC1CN(C(=O)[C@@H]2CCCc3ccccc32)C1)[C@@H]1CC12CCC2	991554307	25	338.2	2.62
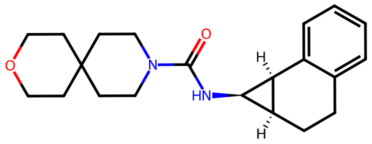	O=C(N[C@@H]1[C@@H]2CCc3ccccc3[C@@H]21)N1CCC2(CCOCC2)CC1	289303138	25	340.22	3.32
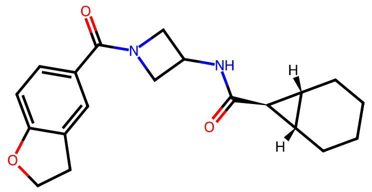	O=C(NC1CN(C(=O)c2ccc3c(c2)CCO3)C1)[C@@H]1[C@H]2CCCC[C@H]21	990657202	25	340.18	2.0
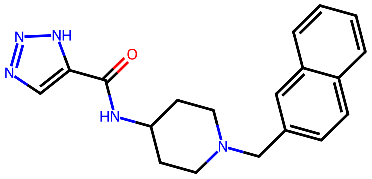	O=C(NC1CCN(Cc2ccc3ccccc3c2)CC1)c1cnn[nH]1	1003236070	25	335.17	2.35
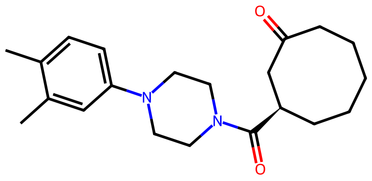	Cc1ccc(N2CCN(C(=O)[C@@H]3CCCCCC(=O)C3)CC2)cc1C	813500356	25	342.23	3.49

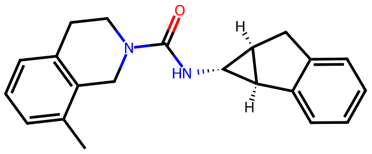	Cc1cccc2c1CN(C(=O)N[C@@H]1[C@H]3Cc4ccccc4[C@H]31)CC2	429102460	24	318.17	3.4
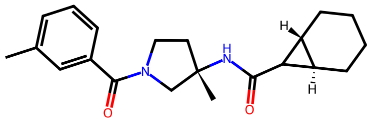	Cc1cccc(C(=O)N2CC[C@](C)(NC(=O)C3[C@@H]4CCCC[C@@H]34)C2)c1	956790396	25	340.22	3.15
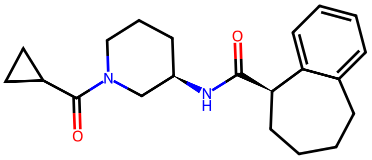	O=C(N[C@@H]1CCCN(C(=O)C2CC2)C1)[C@@H]1CCCCc2ccccc21	949616515	25	340.22	3.01
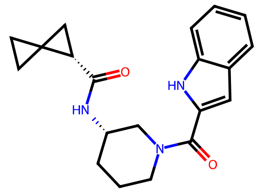	O=C(N[C@H]1CCCN(C(=O)c2cc3ccccc3[nH]2)C1)[C@H]1CC12CC2	952080984	25	337.18	2.69
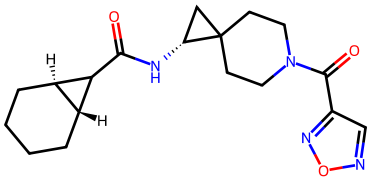	O=C(N[C@@H]1CC12CCN(C(=O)c1conon1)CC2)C1[C@@H]2CCCC[C@@H]12	963448094	25	344.18	1.62
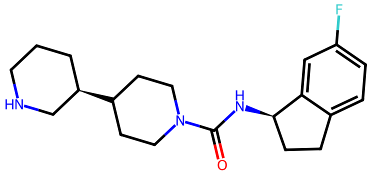	O=C(N[C@@H]1CCc2ccc(F)cc21)N1CCC([C@@H]2CCCNC2)CC1	659452480	25	345.22	3.23
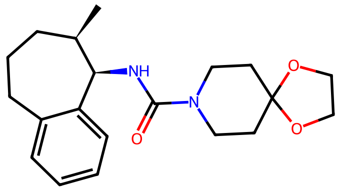	C[C@@H]1CCCc2ccccc2[C@@H]1NC(=O)N1CCC2(CC1)OCCO2	185616343	25	344.21	3.25
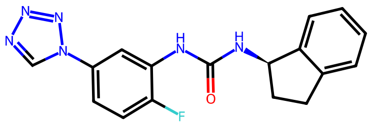	O=C(Nc1cc(-n2cnnn2)ccc1F)N[C@@H]1CCc2ccccc21	76516671	25	338.13	2.61

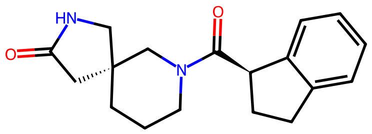	O=C1C[C@]2(CCCN(C(=O)[C@@H]3CCc4ccccc43)C2)CN1	336402779	22	298.17	1.85
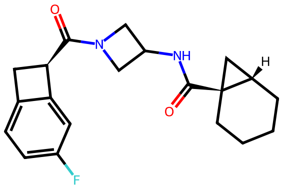	O=C([C@@H]1Cc2ccc(F)cc21)N1CC(NC(=O)[C@]23CCCC[C@H]2C3)C1	999232967	25	342.17	2.37
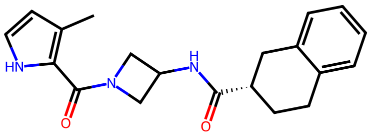	Cc1cc[nH]c1C(=O)N1CC(NC(=O)[C@H]2CCc3ccccc3C2)C1	1000536796	25	337.18	2.07
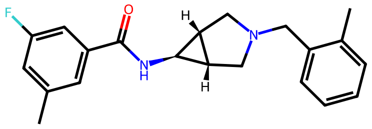	Cc1cc(F)cc(C(=O)N[C@H]2[C@@H]3CN(Cc4ccccc4C)C[C@@H]32)c1	961852751	25	338.18	3.3
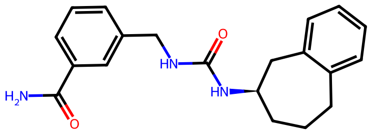	NC(=O)c1cccc(CNC(=O)N[C@@H]2CCCc3ccccc3C2)c1	1551219724	25	337.18	2.53
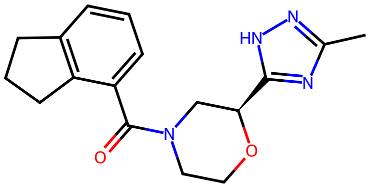	Cc1n[nH]c([C@@H]2CN(C(=O)c3cccc4c3CCC4)CCO2)n1	173529996	23	312.16	1.82
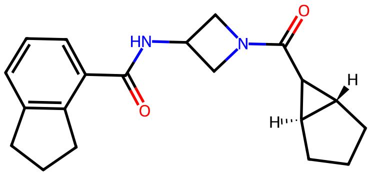	O=C(NC1CN(C(=O)C2[C@@H]3CCC[C@@H]23)C1)c1cccc2c1CCC2	994404494	24	324.18	2.16
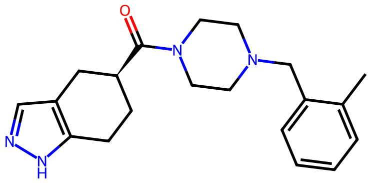	Cc1ccccc1CN1CCN(C(=O)[C@H]2CCc3[nH]ncc3C2)CC1	270099177	25	338.21	2.17

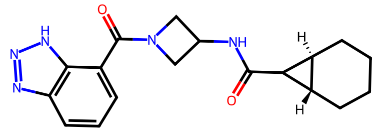	O=C(NC1CN(C(=O)c2cccc3nn[nH]c23)C1)C1[C@H]2CCCC[C@H]12	990651877	25	339.17	1.33
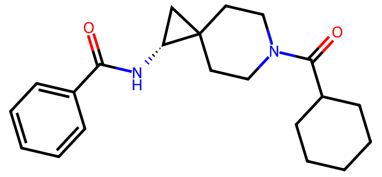	O=C(N[C@@H]1CC12CCN(C(=O)C1CCCCC1)CC2)c1ccccc1	962786784	25	340.22	3.38
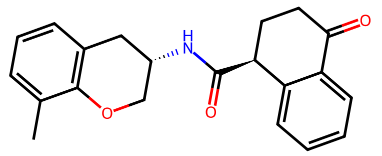	Cc1cccc2c1OC[C@@H](NC(=O)[C@H]1CCC(=O)c3ccccc31)C2	291649067	25	335.15	3.18
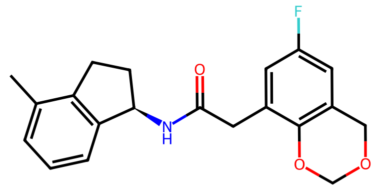	Cc1cccc2c1CC[C@H]2NC(=O)Cc1cc(F)cc2c1OCOC2	517835881	25	341.14	3.35
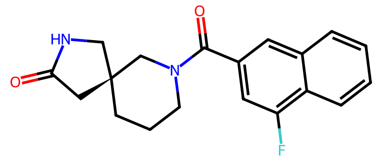	O=C1C[C@@]2(CCCN(C(=O)c3cc(F)c4ccccc4c3)C2)CN1	341215960	24	326.14	2.72
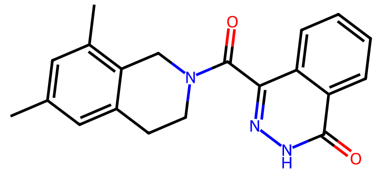	Cc1cc(C)c2c(c1)CCN(C(=O)c1n[nH]c(=O)c3ccccc13)C2	186714205	25	333.15	2.74
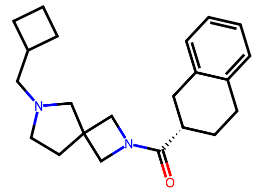	O=C([C@H]1CCc2ccccc2C1)N1CC2(CCN(CC3CCC3)C2)C1	1073013572	25	338.24	3.13
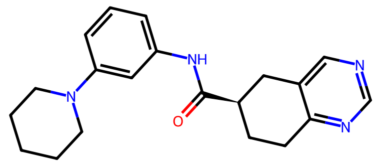	O=C(Nc1cccc(N2CCCCC2)c1)[C@@H]1CCc2ncncc2C1	843536684	25	336.2	3.21

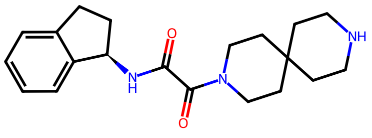	O=C(N[C@@H]1CCc2ccccc21)C(=O)N1CCC2(CCNCC2)CC1	1347085587	25	341.21	1.78
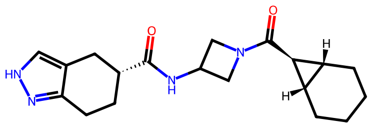	O=C(NC1CN(C(=O)[C@@H]2[C@H]3CCCC[C@H]32)C1)[C@@H]1CCc2n[nH]cc2C1	996397675	25	342.21	1.28
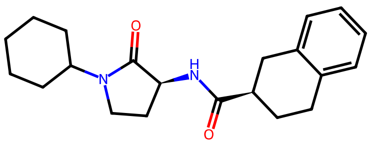	O=C(N[C@H]1CCN(C2CCCCC2)C1=O)[C@@H]1CCc2ccccc2C1	649844874	25	340.22	2.84
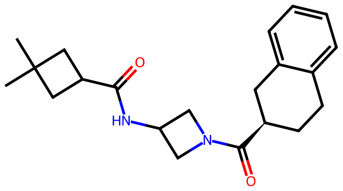	CC1(C)CC(C(=O)NC2CN(C(=O)[C@@H]3CCc4ccccc4C3)C2)C1	991151700	25	340.22	2.55
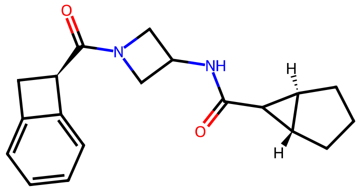	O=C(NC1CN(C(=O)[C@@H]2Cc3ccccc32)C1)C1[C@H]2CCC[C@H]12	990691342	23	310.17	1.7
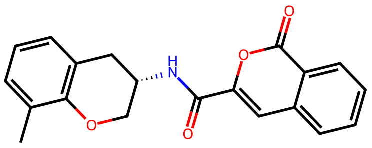	Cc1cccc2c1OC[C@@H](NC(=O)c1cc3ccccc3c(=O)o1)C2	365990013	25	335.12	2.83
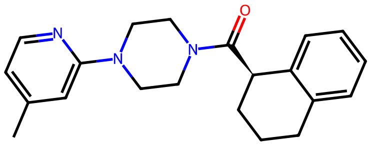	Cc1ccnc(N2CCN(C(=O)[C@@H]3CCCc4ccccc43)CC2)c1	361928737	25	335.2	3.16
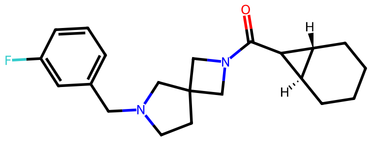	O=C(C1[C@@H]2CCCC[C@@H]12)N1CC2(CCN(Cc3cccc(F)c3)C2)C1	1072580948	25	342.21	3.3

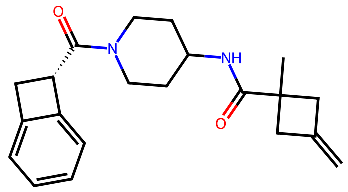	C=C1CC(C)(C(=O)NC2CCN(C(=O)[C@H]3Cc4ccccc43)CC2)C1	948060110	25	338.2	2.79
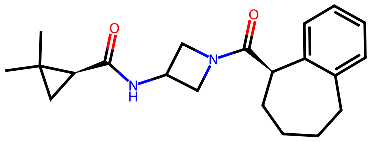	CC1(C)C[C@@H]1C(=O)NC1CN(C(=O)[C@@H]2CCCCc3ccccc32)C1	990877255	25	340.22	2.87
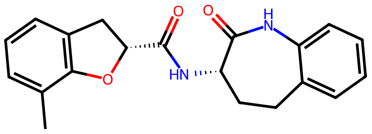	Cc1cccc2c1O[C@@H](C(=O)N[C@H]1CCc3ccccc3NC1=O)C2	653996569	25	336.15	2.37
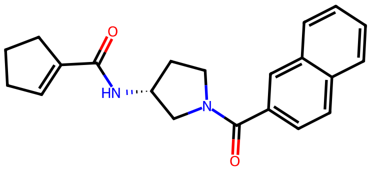	O=C(N[C@@H]1CCN(C(=O)c2ccc3ccccc3c2)C1)C1=CCCC1	940000591	25	334.17	3.28
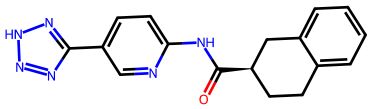	O=C(Nc1ccc(-c2nn[nH]n2)cn1)[C@@H]1CCc2ccccc2C1	1582638387	24	320.14	2.01
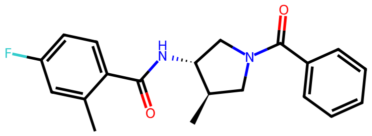	Cc1cc(F)ccc1C(=O)N[C@@H]1CN(C(=O)c2ccccc2)C[C@H]1C	947229973	25	340.16	3.02
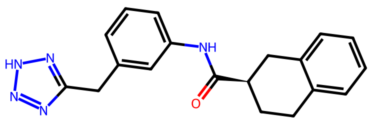	O=C(Nc1cccc(Cc2nn[nH]n2)c1)[C@@H]1CCc2ccccc2C1	910048434	25	333.16	2.53
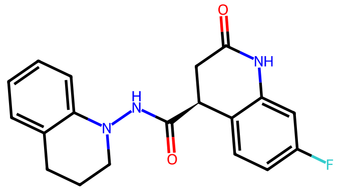	O=C1C[C@H](C(=O)NN2CCCc3ccccc32)c2ccc(F)cc2N1	767058104	25	339.14	2.74

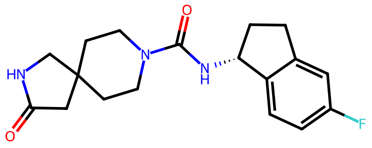	O=C1CC2(CCN(C(=O)N[C@@H]3CCc4cc(F)ccc43)CC2)CN1	73834870	24	331.17	2.12
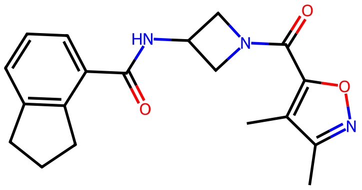	Cc1noc(C(=O)N2CC(NC(=O)c3cccc4c3CCC4)C2)c1C	994396996	25	339.16	2.03
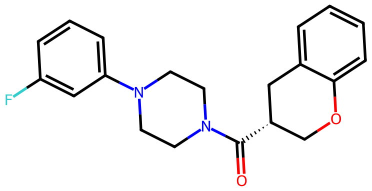	O=C([C@H]1COc2ccccc2C1)N1CCN(c2cccc(F)c2)CC1	618147880	25	340.16	2.73
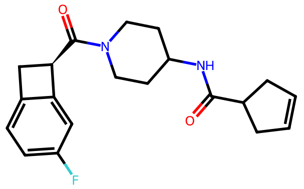	O=C(NC1CCN(C(=O)[C@@H]2Cc3ccc(F)cc32)CC1)C1CC=CC1	948024590	25	342.17	2.54
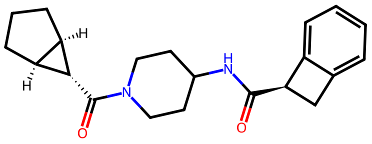	O=C(NC1CCN(C(=O)[C@@H]2[C@H]3CCC[C@H]32)CC1)[C@@H]1Cc2ccccc21	946079925	25	338.2	2.48
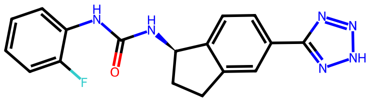	O=C(Nc1ccccc1F)N[C@@H]1CCc2cc(-c3nn[nH]n3)ccc21	1606940107	25	338.13	2.81
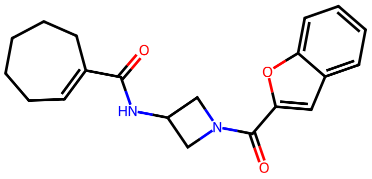	O=C(NC1CN(C(=O)c2cc3ccccc3o2)C1)C1=CCCCCC1	991008188	25	338.16	3.26
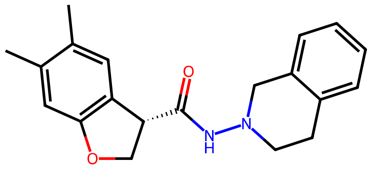	Cc1cc2c(cc1C)[C@H](C(=O)NN1CCc3ccccc3C1)CO2	1469893545	24	322.17	2.87

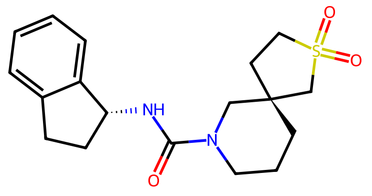	O=C(N[C@@H]1CCc2ccccc21)N1CCC[C@]2(CCS(=O)(=O)C2)C1	333819578	24	348.15	2.28
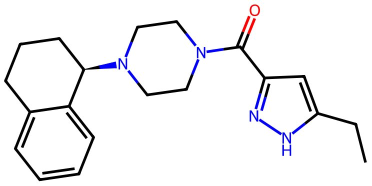	CCc1cc(C(=O)N2CCN([C@@H]3CCCc4ccccc43)CC2)n[nH]1	1415818140	25	338.21	2.81
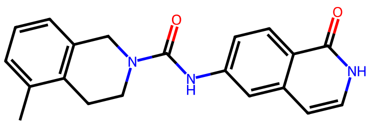	Cc1cccc2c1CCN(C(=O)Nc1ccc3c(=O)[nH]ccc3c1)C2	1310978729	25	333.15	3.43
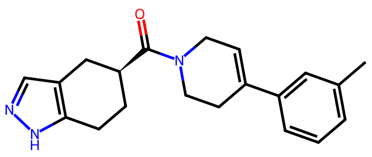	Cc1cccc(C2=CCN(C(=O)[C@H]3CCc4[nH]ncc4C3)CC2)c1	271371373	24	321.18	3.14
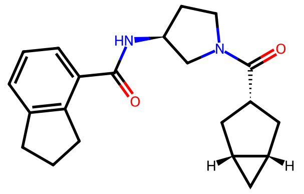	O=C(N[C@H]1CCN(C(=O)[C@@H]2C[C@@H]3C[C@@H]3C2)C1)c1cccc2c1CCC2	938149353	25	338.2	2.55
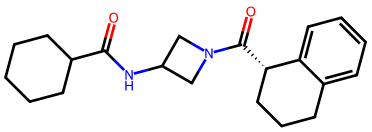	O=C(NC1CN(C(=O)[C@H]2CCCc3ccccc32)C1)C1CCCCC1	990099259	25	340.22	3.01
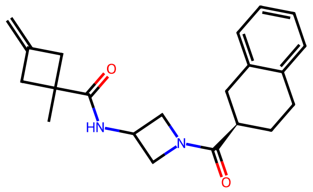	C=C1CC(C)(C(=O)NC2CN(C(=O)[C@@H]3CCc4ccccc4C3)C2)C1	999293131	25	338.2	2.47
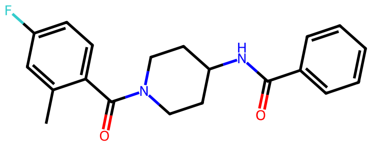	Cc1cc(F)ccc1C(=O)N1CCC(NC(=O)c2ccccc2)CC1	137092982	25	340.16	3.17

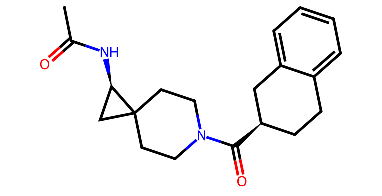	CC(=O)N[C@@H]1CC12CCN(C(=O)[C@@H]1CCc3ccccc3C1)CC2	962445461	24	326.2	2.31
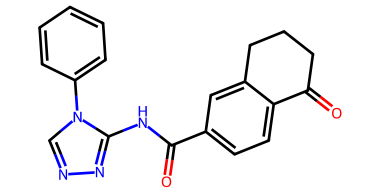	O=C(Nc1nncn1-c1ccccc1)c1ccc2c(c1)CCCC2=O	1636586983	25	332.13	3.04
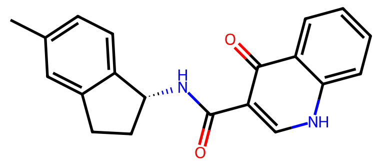	Cc1ccc2c(c1)CC[C@H]2NC(=O)c1c[nH]c2ccccc2c1=O	55661578	24	318.14	3.25
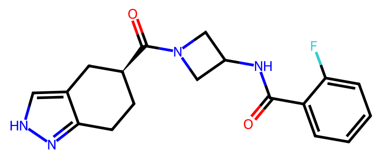	O=C(NC1CN(C(=O)[C@H]2CCc3n[nH]cc3C2)C1)c1ccccc1F	990089458	25	342.15	1.29
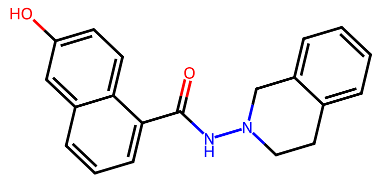	O=C(NN1CCc2ccccc2C1)c1cccc2cc(O)ccc12	1644635166	24	318.14	3.25
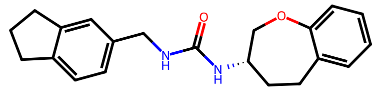	O=C(NCc1ccc2c(c1)CCC2)N[C@H]1CCc2ccccc2OC1	1629078224	25	336.18	3.37
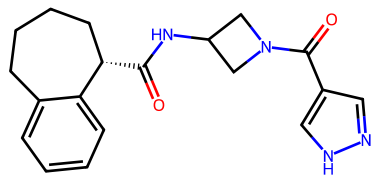	O=C(NC1CN(C(=O)c2cn[nH]c2)C1)[C@H]1CCCCc2ccccc21	994303443	25	338.17	1.86
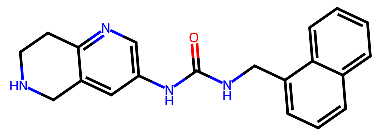	O=C(NCc1cccc2ccccc12)Nc1cnc2c(c1)CNCC2	1580721629	25	332.16	3.2

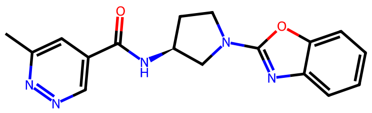	Cc1cc(C(=O)N[C@H]2CCN(c3nc4ccccc4o3)C2)cnn1	1058340428	24	323.14	1.93
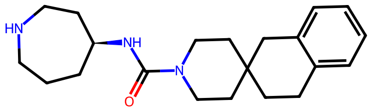	O=C(N[C@H]1CCCNCC1)N1CCC2(CCc3ccccc3C2)CC1	652215094	25	341.25	3.11
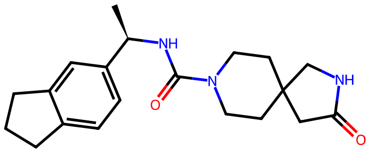	C[C@@H](NC(=O)N1CCC2(CC1)CNC(=O)C2)c1ccc2c(c1)CCC2	1364598780	25	341.21	2.55
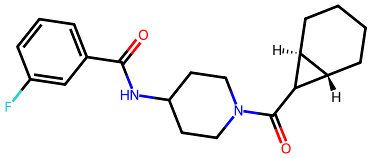	O=C(NC1CCN(C(=O)C2[C@H]3CCCC[C@H]23)CC1)c1cccc(F)c1	945956142	25	344.19	2.98
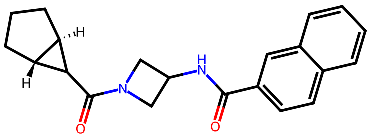	O=C(NC1CN(C(=O)C2[C@@H]3CCC[C@@H]23)C1)c1ccc2ccccc2c1	999446196	25	334.17	2.83
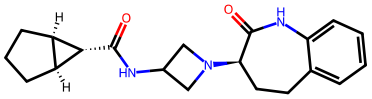	O=C(NC1CN([C@@H]2CCc3ccccc3NC2=O)C1)[C@@H]1[C@H]2CCC[C@H]21	1030202504	25	339.19	1.79
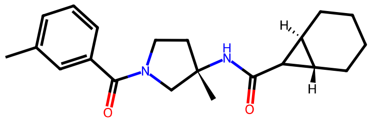	Cc1cccc(C(=O)N2CC[C@](C)(NC(=O)C3[C@H]4CCCC[C@H]34)C2)c1	956790395	25	340.22	3.15
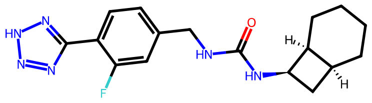	O=C(NCc1ccc(-c2nn[nH]n2)c(F)c1)N[C@@H]1C[C@@H]2CCCC[C@@H]21	1593126444	25	344.18	2.38

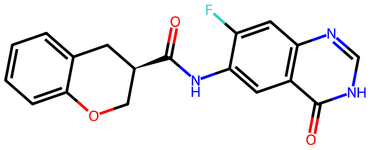	O=C(Nc1cc2c(=O)[nH]cnc2cc1F)[C@H]1COc2ccccc2C1	192497064	25	339.1	2.25
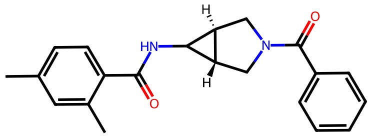	Cc1ccc(C(=O)NC2[C@H]3CN(C(=O)c4ccccc4)C[C@H]23)c(C)c1	1013775348	25	334.17	2.8
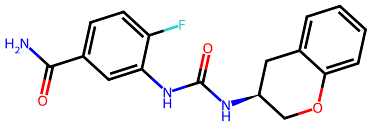	NC(=O)c1ccc(F)c(NC(=O)N[C@@H]2COc3ccccc3C2)c1	47875652	24	329.12	2.05
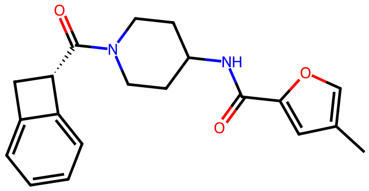	Cc1coc(C(=O)NC2CCN(C(=O)[C@H]3Cc4ccccc43)CC2)c1	948063253	25	338.16	2.65
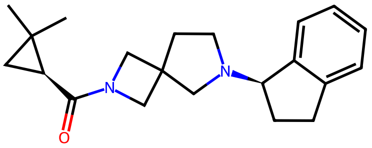	CC1(C)C[C@@H]1C(=O)N1CC2(CCN([C@@H]3CCc4ccccc43)C2)C1	1072637819	24	324.22	3.25
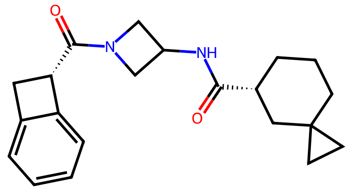	O=C(NC1CN(C(=O)[C@H]2Cc3ccccc32)C1)[C@@H]1CCCC2(CC2)C1	991515477	25	338.2	2.62
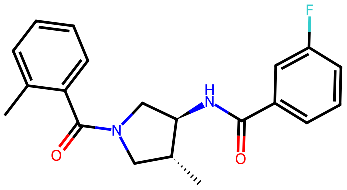	Cc1ccccc1C(=O)N1C[C@@H](NC(=O)c2cccc(F)c2)[C@H](C)C1	947268571	25	340.16	3.02
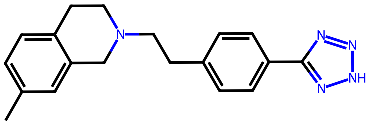	Cc1ccc2c(c1)CN(CCc1ccc(-c3nn[nH]n3)cc1)CC2	1605888898	24	319.18	2.78

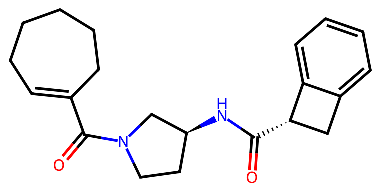	O=C(N[C@H]1CCN(C(=O)C2=CCCCCC2)C1)[C@H]1Cc2ccccc21	938576866	25	338.2	2.93
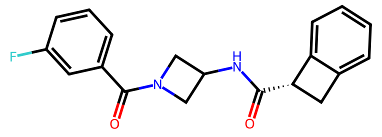	O=C(NC1CN(C(=O)c2cccc(F)c2)C1)[C@H]1Cc2ccccc21	998685607	24	324.13	2.11
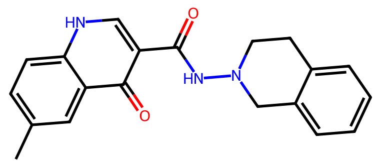	Cc1ccc2[nH]cc(C(=O)NN3CCc4ccccc4C3)c(=O)c2c1	1559010782	25	333.15	2.54
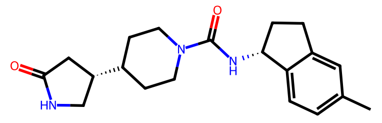	Cc1ccc2c(c1)CC[C@H]2NC(=O)N1CCC([C@@H]2CNC(=O)C2)CC1	1553385644	25	341.21	2.54
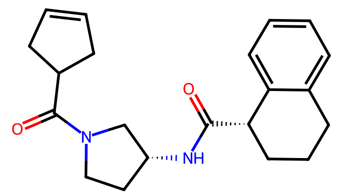	O=C(N[C@@H]1CCN(C(=O)C2CC=CC2)C1)[C@H]1CCCc2ccccc21	941386878	25	338.2	2.79
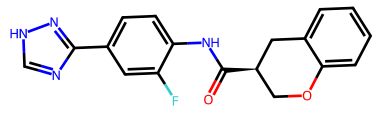	O=C(Nc1ccc(-c2nc[nH]n2)cc1F)[C@@H]1COc2ccccc2C1	344290310	25	338.12	2.8
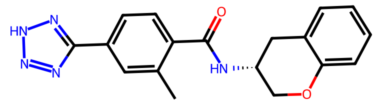	Cc1cc(-c2nn[nH]n2)ccc1C(=O)N[C@H]1COc2ccccc2C1	826247841	25	335.14	1.91
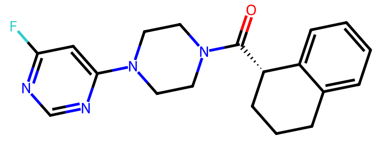	O=C([C@H]1CCCc2ccccc21)N1CCN(c2cc(F)ncn2)CC1	908912258	25	340.17	2.38

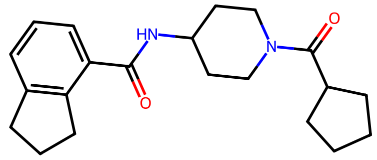	O=C(NC1CCN(C(=O)C2CCCC2)CC1)c1cccc2c1CCC2	943401949	25	340.22	3.09
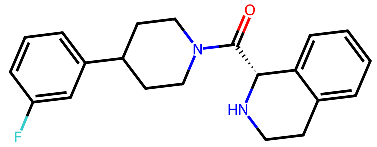	O=C([C@H]1NCCc2ccccc21)N1CCC(c2cccc(F)c2)CC1	1340470613	25	338.18	3.42
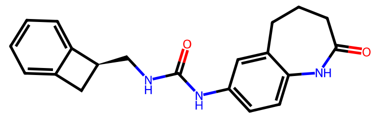	O=C1CCCc2cc(NC(=O)NC[C@H]3Cc4ccccc43)ccc2N1	171523287	25	335.16	3.42
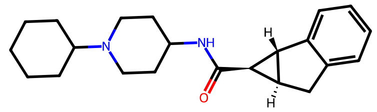	O=C(NC1CCN(C2CCCCC2)CC1)[C@@H]1[C@@H]2Cc3ccccc3[C@H]21	625970885	25	338.24	3.49
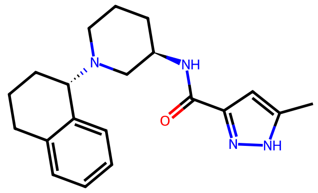	Cc1cc(C(=O)N[C@@H]2CCCN([C@H]3CCCc4ccccc43)C2)n[nH]1	1006857941	25	338.21	2.99
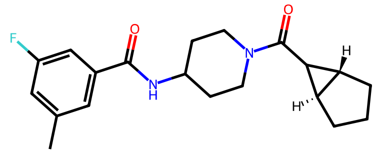	Cc1cc(F)cc(C(=O)NC2CCN(C(=O)C3[C@@H]4CCC[C@@H]34)CC2)c1	946094225	25	344.19	2.9
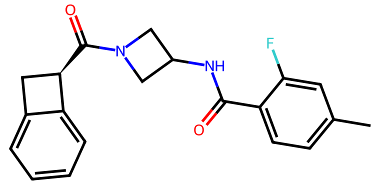	Cc1ccc(C(=O)NC2CN(C(=O)[C@@H]3Cc4ccccc43)C2)c(F)c1	1000493051	25	338.14	2.41
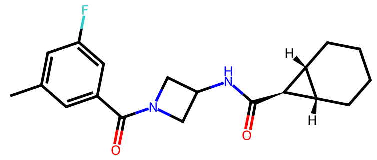	Cc1cc(F)cc(C(=O)N2CC(NC(=O)[C@@H]3[C@H]4CCCC[C@H]43)C2)c1	990663139	24	330.17	2.51

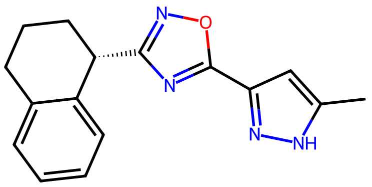	Cc1cc(-c2nc([C@H]3CCCc4ccccc43)no2)n[nH]1	273815102	21	280.13	3.24
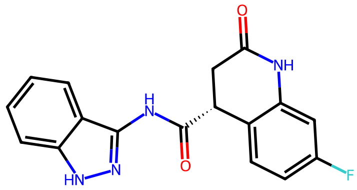	O=C1C[C@@H](C(=O)Nc2n[nH]c3ccccc23)c2ccc(F)cc2N1	914531600	24	324.1	2.77
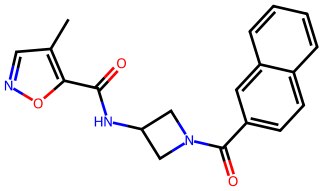	Cc1cnoc1C(=O)NC1CN(C(=O)c2ccc3ccccc3c2)C1	998975832	25	335.13	2.39
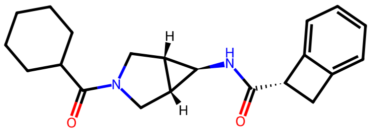	O=C(N[C@H]1[C@@H]2CN(C(=O)C3CCCCC3)C[C@@H]21)[C@H]1Cc2ccccc21	977084525	25	338.2	2.48
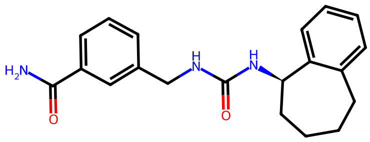	NC(=O)c1cccc(CNC(=O)N[C@@H]2CCCCc3ccccc32)c1	69416243	25	337.18	3.05
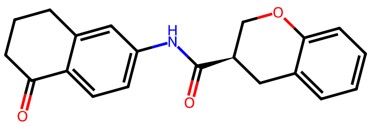	O=C1CCCc2cc(NC(=O)[C@H]3COc4ccccc4C3)ccc21	556365823	24	321.14	3.4
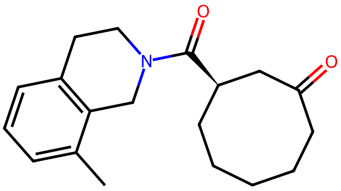	Cc1cccc2c1CN(C(=O)[C@@H]1CCCCCC(=O)C1)CC2	487632245	22	299.19	3.42
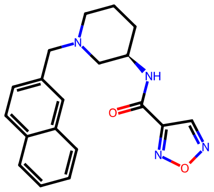	O=C(N[C@@H]1CCCN(Cc2ccc3ccccc3c2)C1)c1cnon1	1008931723	25	336.16	2.62

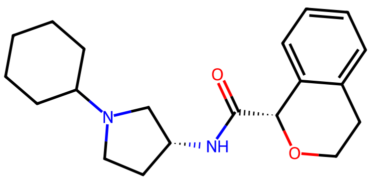	O=C(N[C@@H]1CCN(C2CCCCC2)C1)[C@H]1OCCc2ccccc21	618663739	24	328.22	2.82
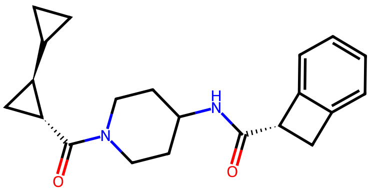	O=C(NC1CCN(C(=O)[C@@H]2C[C@H]2C2CC2)CC1)[C@H]1Cc2ccccc21	946878001	25	338.2	2.48
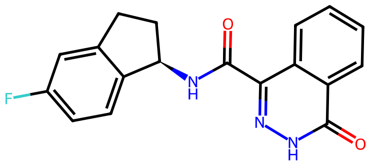	O=C(N[C@@H]1CCc2cc(F)ccc21)c1n[nH]c(=O)c2ccccc12	72022051	24	323.11	2.48
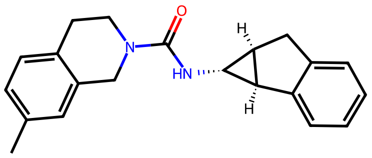	Cc1ccc2c(c1)CN(C(=O)N[C@@H]1[C@H]3Cc4ccccc4[C@H]31)CC2	660914470	24	318.17	3.4
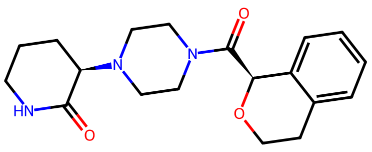	O=C1NCCC[C@H]1N1CCN(C(=O)[C@@H]2OCCc3ccccc32)CC1	949590069	25	343.19	0.72
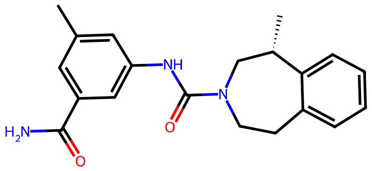	Cc1cc(NC(=O)N2CCc3ccccc3[C@@H](C)C2)cc(C(N)=O)c1	650236366	25	337.18	3.29
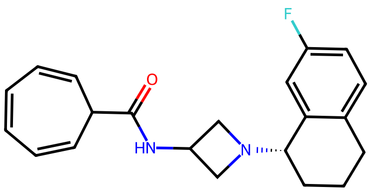	O=C(NC1CN([C@H]2CCCc3ccc(F)cc32)C1)C1C=CC=CC=C1	1030381027	25	338.18	3.3
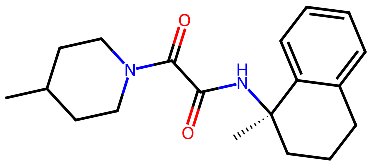	CC1CCN(C(=O)C(=O)N[C@]2(C)CCCc3ccccc32)CC1	444144274	23	314.2	2.61

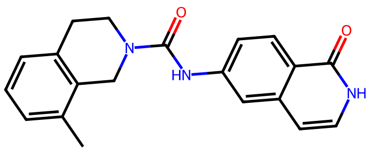	Cc1cccc2c1CN(C(=O)Nc1ccc3c(=O)[nH]ccc3c1)CC2	1671580343	25	333.15	3.43
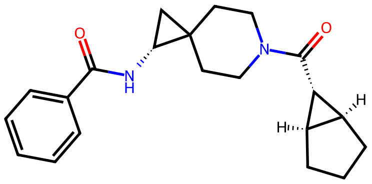	O=C(N[C@@H]1CC12CCN(C(=O)[C@@H]1[C@H]3CCC[C@H]31)CC2)c1ccccc1	962781711	25	338.2	2.84
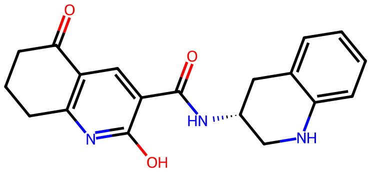	O=C(N[C@H]1CNc2ccccc2C1)c1cc2c(nc1O)CCCC2=O	653206246	25	337.14	2.07
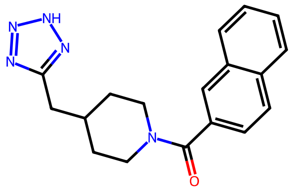	O=C(c1ccc2ccccc2c1)N1CCC(Cc2nn[nH]n2)CC1	1352696754	24	321.16	2.45
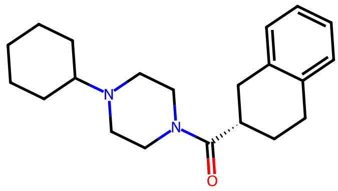	O=C([C@H]1CCc2ccccc2C1)N1CCN(C2CCCCC2)CC1	1583138608	24	326.24	3.27
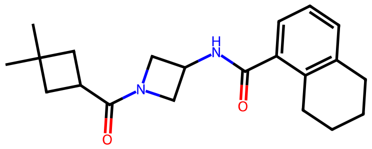	CC1(C)CC(C(=O)N2CC(NC(=O)c3cccc4c3CCCC4)C2)C1	991901702	25	340.22	2.94
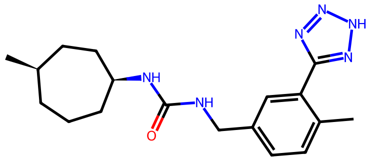	Cc1ccc(CNC(=O)N[C@H]2CCC[C@@H](C)CC2)cc1-c1nn[nH]n1	1605852040	25	342.22	2.94
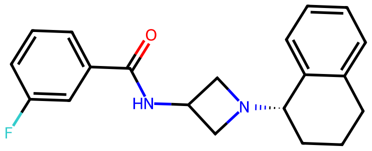	O=C(NC1CN([C@H]2CCCc3ccccc32)C1)c1cccc(F)c1	1030067644	24	324.16	3.32

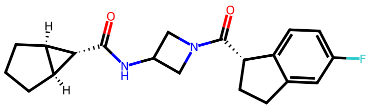	O=C(NC1CN(C(=O)[C@H]2CCc3cc(F)ccc32)C1)[C@@H]1[C@H]2CCC[C@H]21	990701881	25	342.17	2.23
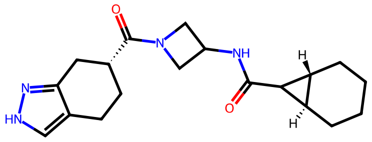	O=C(NC1CN(C(=O)[C@@H]2CCc3c[nH]nc3C2)C1)C1[C@@H]2CCCC[C@@H]12	990647991	25	342.21	1.28
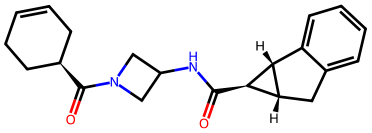	O=C(NC1CN(C(=O)[C@@H]2CC=CCC2)C1)[C@@H]1[C@H]2Cc3ccccc3[C@H]21	991655690	25	336.18	2.26
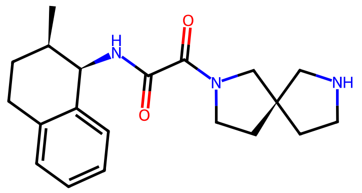	C[C@@H]1CCc2ccccc2[C@@H]1NC(=O)C(=O)N1CC[C@@]2(CCNC2)C1	580764515	25	341.21	1.64
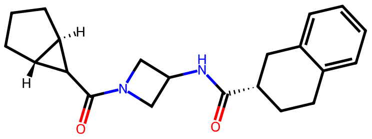	O=C(NC1CN(C(=O)C2[C@@H]3CCC[C@@H]23)C1)[C@H]1CCc2ccccc2C1	1000537286	25	338.2	2.16
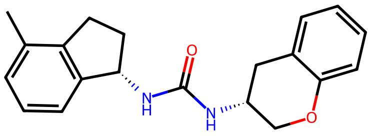	Cc1cccc2c1CC[C@@H]2NC(=O)N[C@H]1COc2ccccc2C1	517916005	24	322.17	3.29
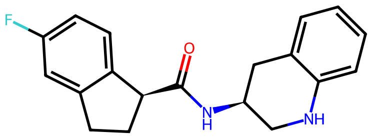	O=C(N[C@@H]1CNc2ccccc2C1)[C@H]1CCc2cc(F)ccc21	658941631	23	310.15	3.01
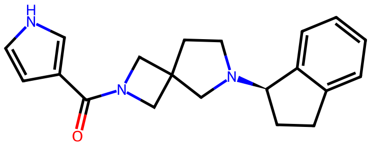	O=C(c1cc[nH]c1)N1CC2(CCN([C@@H]3CCc4ccccc43)C2)C1	1072769007	24	321.18	2.85

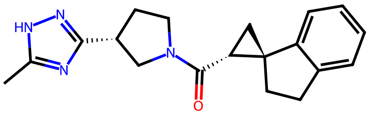	Cc1nc([C@@H]2CCN(C(=O)[C@@H]3C[C@@]34CCc3ccccc34)C2)n[nH]1	333996520	24	322.18	2.33
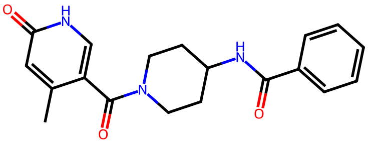	Cc1cc(=O)[nH]cc1C(=O)N1CCC(NC(=O)c2ccccc2)CC1	164864306	25	339.16	1.72
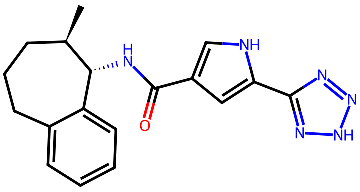	C[C@@H]1CCCc2ccccc2[C@H]1NC(=O)c1c[nH]c(-c2nn[nH]n2)c1	1590263695	25	336.17	2.64
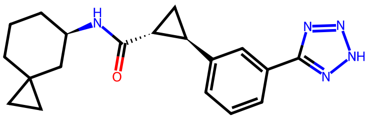	O=C(N[C@@H]1CCCC2(CC2)C1)[C@@H]1C[C@H]1c1cccc(-c2nn[nH]n2)c1	1592980096	25	337.19	2.81
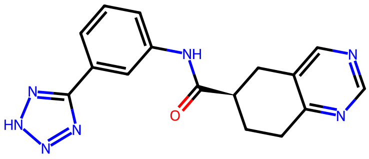	O=C(Nc1cccc(-c2nn[nH]n2)c1)[C@@H]1CCc2ncncc2C1	648721287	24	321.13	1.4
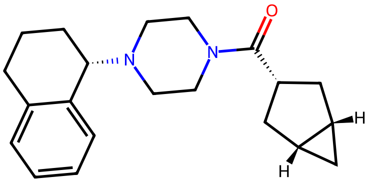	O=C([C@@H]1C[C@@H]2C[C@@H]2C1)N1CCN([C@H]2CCCc3ccccc32)CC1	949778171	24	324.22	3.25
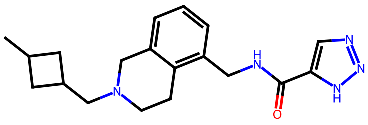	CC1CC(CN2CCc3c(CNC(=O)c4cnn[nH]4)cccc3C2)C1	1051292379	25	339.21	2.14
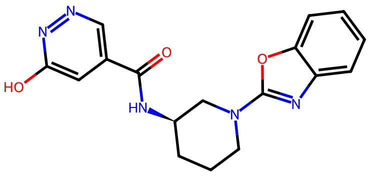	O=C(N[C@@H]1CCCN(c2nc3ccccc3o2)C1)c1cnnc(O)c1	1058205918	25	339.13	1.72

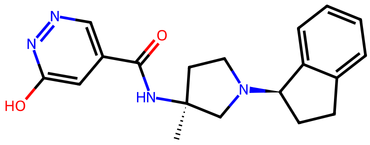	C[C@]1(NC(=O)c2cnnc(O)c2)CCN([C@@H]2CCc3ccccc32)C1	1046399300	25	338.17	2.06
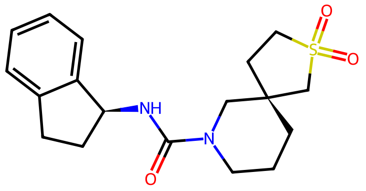	O=C(N[C@H]1CCc2ccccc21)N1CCC[C@]2(CCS(=O)(=O)C2)C1	333819581	24	348.15	2.28
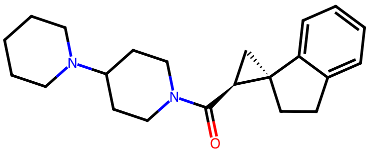	O=C([C@H]1C[C@]12CCc1ccccc12)N1CCC(N2CCCCC2)CC1	266541352	25	338.24	3.37
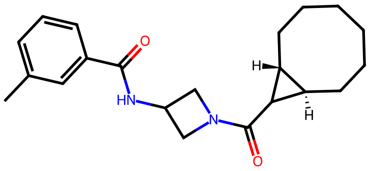	Cc1cccc(C(=O)NC2CN(C(=O)C3[C@@H]4CCCCCC[C@@H]34)C2)c1	990138096	25	340.22	3.15
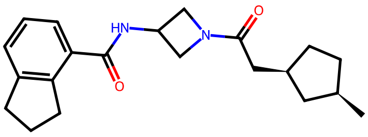	C[C@@H]1CC[C@H](CC(=O)N2CC(NC(=O)c3cccc4c3CCC4)C2)C1	994395374	25	340.22	2.94
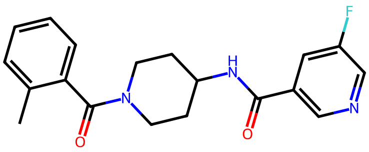	Cc1ccccc1C(=O)N1CCC(NC(=O)c2cncc(F)c2)CC1	943773038	25	341.15	2.56
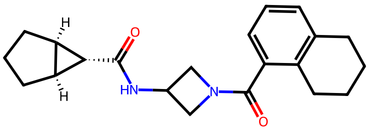	O=C(NC1CN(C(=O)c2cccc3c2CCCC3)C1)[C@@H]1[C@H]2CCC[C@H]21	990696641	25	338.2	2.55
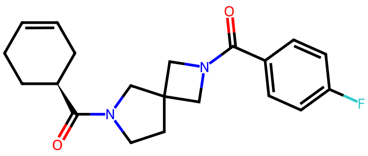	O=C(c1ccc(F)cc1)N1CC2(CCN(C(=O)[C@@H]3CC=CCC3)C2)C1	1018699511	25	342.17	2.86

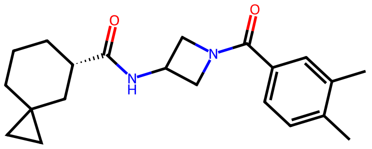	Cc1ccc(C(=O)N2CC(NC(=O)[C@H]3CCCC4(CC4)C3)C2)cc1C	991516699	25	340.22	3.21
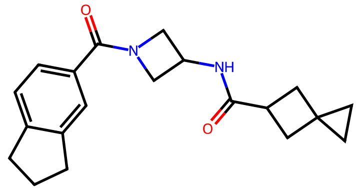	O=C(NC1CN(C(=O)c2ccc3c(c2)CCC3)C1)C1CC2(CC2)C1	999346796	24	324.18	2.31
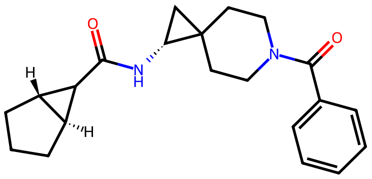	O=C(N[C@@H]1CC12CCN(C(=O)c1ccccc1)CC2)C1[C@H]2CCC[C@H]12	963462696	25	338.2	2.84
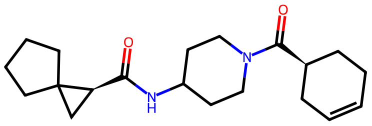	O=C(NC1CCN(C(=O)[C@@H]2CC=CCC2)CC1)[C@H]1CC12CCCC2	944105936	24	330.23	3.03
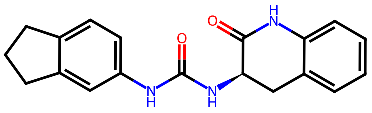	O=C(Nc1ccc2c(c1)CCC2)N[C@@H]1Cc2ccccc2NC1=O	528866998	24	321.15	2.86
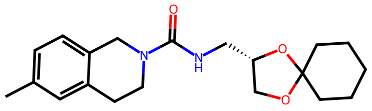	Cc1ccc2c(c1)CCN(C(=O)NC[C@H]1COC3(CCCCC3)O1)C2	790452787	25	344.21	3.14
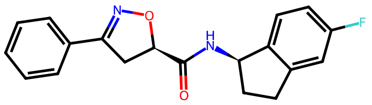	O=C(N[C@@H]1CCc2cc(F)ccc21)[C@H]1CC(c2ccccc2)=NO1	56318604	24	324.13	3.12
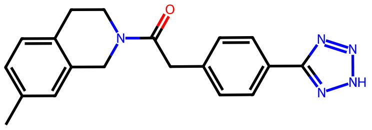	Cc1ccc2c(c1)CN(C(=O)Cc1ccc(-c3nn[nH]n3)cc1)CC2	1605888635	25	333.16	2.3

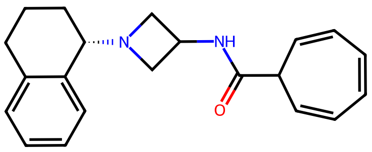	O=C(NC1CN([C@H]2CCCc3ccccc32)C1)C1C=CC=CC=C1	1030382300	24	320.19	3.16
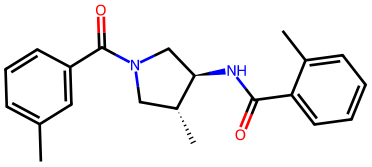	Cc1cccc(C(=O)N2C[C@@H](NC(=O)c3ccccc3C)[C@H](C)C2)c1	947313222	25	336.18	3.19
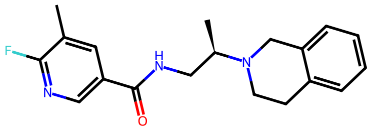	Cc1cc(C(=O)NC[C@@H](C)N2CCc3ccccc3C2)cnc1F	857576662	24	327.17	2.71
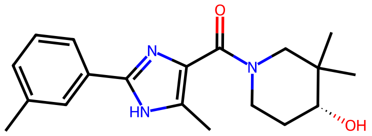	Cc1cccc(-c2nc(C(=O)N3CC[C@@H](O)C(C)(C)C3)c(C)[nH]2)c1	275766353	24	327.19	2.93
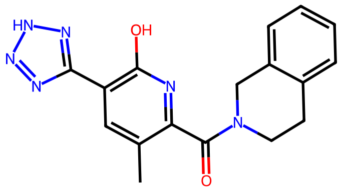	Cc1cc(-c2nn[nH]n2)c(O)nc1C(=O)N1CCc2ccccc2C1	1580600448	25	336.13	1.47
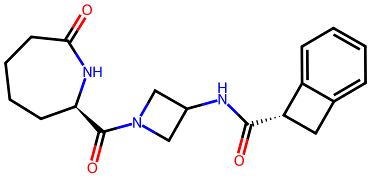	O=C1CCCC[C@H](C(=O)N2CC(NC(=O)[C@H]3Cc4ccccc43)C2)N1	998690535	25	341.17	0.71
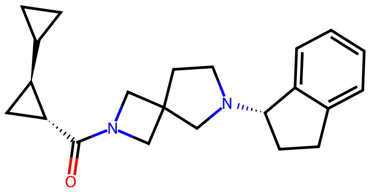	O=C([C@@H]1C[C@H]1C1CC1)N1CC2(CCN([C@H]3CCc4ccccc43)C2)C1	1073047280	25	336.22	3.25
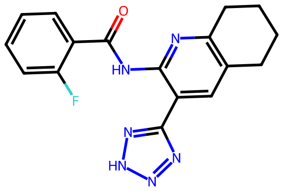	O=C(Nc1nc2c(cc1-c1nn[nH]n1)CCCC2)c1ccccc1F	826460103	25	338.13	2.53

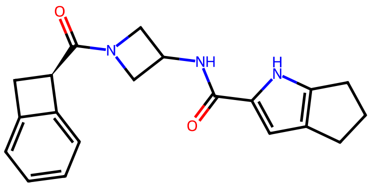	O=C(NC1CN(C(=O)[C@@H]2Cc3ccccc32)C1)c1cc2c([nH]1)CCC2	995964325	25	335.16	1.78
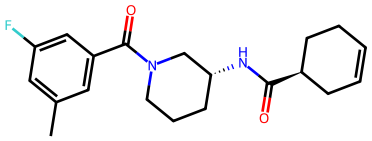	Cc1cc(F)cc(C(=O)N2CCC[C@@H](NC(=O)[C@@H]3CC=CCC3)C2)c1	952126912	25	344.19	3.21
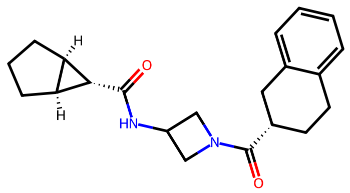	O=C(NC1CN(C(=O)[C@H]2CCc3ccccc3C2)C1)[C@@H]1[C@H]2CCC[C@H]21	990718584	25	338.2	2.16
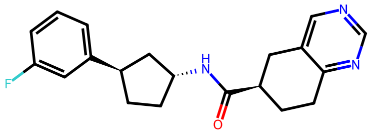	O=C(N[C@@H]1CC[C@@H](c2cccc(F)c2)C1)[C@@H]1CCc2ncncc2C1	648378420	25	339.17	3.17
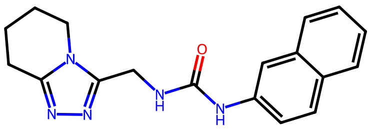	O=C(NCc1nnc2n1CCCC2)Nc1ccc2ccccc2c1	193365948	24	321.16	3.09
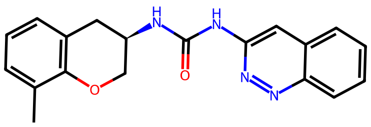	Cc1cccc2c1OC[C@H](NC(=O)Nc1cc3ccccc3nn1)C2	1206803567	25	334.14	3.06
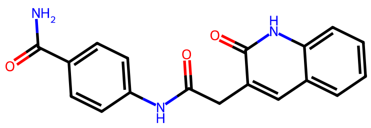	NC(=O)c1ccc(NC(=O)Cc2cc3ccccc3[nH]c2=O)cc1	47074966	24	321.11	1.81
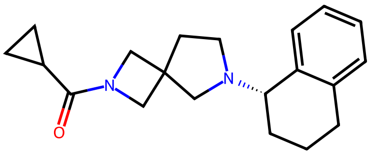	O=C(C1CC1)N1CC2(CCN([C@H]3CCCc4ccccc43)C2)C1	1072378386	23	310.2	3.01

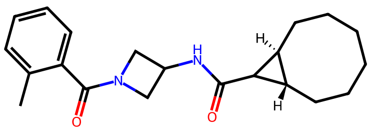	Cc1ccccc1C(=O)N1CC(NC(=O)C2[C@H]3CCCCCC[C@H]23)C1	1000466637	25	340.22	3.15
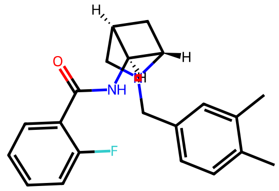	Cc1ccc(CN2C[C@H]3C[C@H]2[C@H]3NC(=O)c2ccccc2F)cc1C	1037286207	25	338.18	3.45
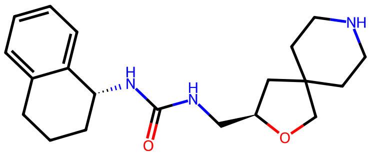	O=C(NC[C@H]1CC2(CCNCC2)CO1)N[C@@H]1CCCc2ccccc21	874429512	25	343.23	2.52
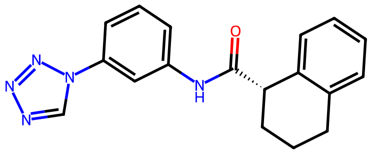	O=C(Nc1cccc(-n2cnnn2)c1)[C@H]1CCCc2ccccc21	44363281	24	319.14	2.72
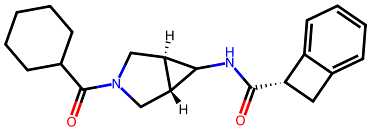	O=C(NC1[C@@H]2CN(C(=O)C3CCCCC3)C[C@@H]12)[C@H]1Cc2ccccc21	1013681256	25	338.2	2.48
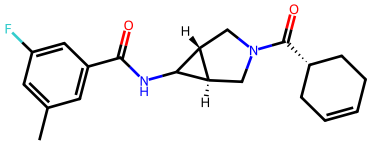	Cc1cc(F)cc(C(=O)NC2[C@@H]3CN(C(=O)[C@H]4CC=CCC4)C[C@@H]23)c1	1013862333	25	342.17	2.68
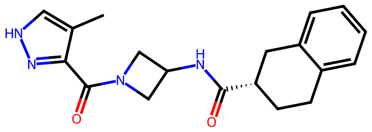	Cc1c[nH]nc1C(=O)N1CC(NC(=O)[C@H]2CCc3ccccc3C2)C1	1000536773	25	338.17	1.46
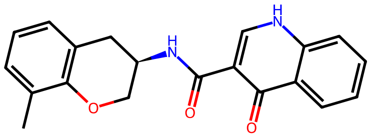	Cc1cccc2c1OC[C@H](NC(=O)c1c[nH]c3ccccc3c1=O)C2	644643178	25	334.13	2.57

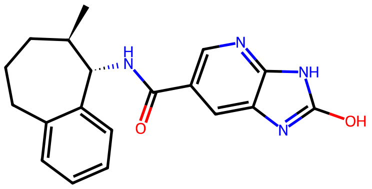	C[C@@H]1CCCc2ccccc2[C@H]1NC(=O)c1cnc2[nH]c(O)nc2c1	572563544	25	336.16	3.11
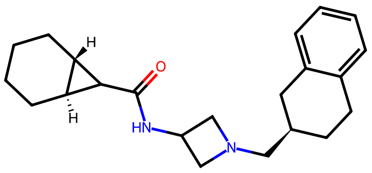	O=C(NC1CN(C[C@@H]2CCc3ccccc3C2)C1)C1[C@H]2CCCC[C@H]12	1030182374	25	338.24	3.03
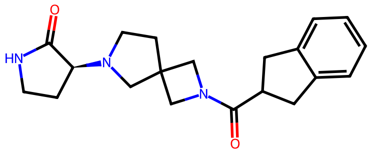	O=C1NCC[C@@H]1N1CCC2(CN(C(=O)C3Cc4ccccc4C3)C2)C1	1073009955	25	339.19	0.82
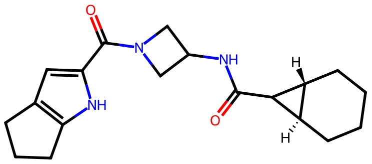	O=C(NC1CN(C(=O)c2cc3c([nH]2)CCC3)C1)C1[C@@H]2CCCC[C@@H]12	990651552	24	327.19	1.88
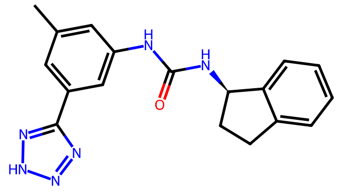	Cc1cc(NC(=O)N[C@@H]2CCc3ccccc32)cc(-c2nn[nH]n2)c1	1580983508	25	334.15	2.98
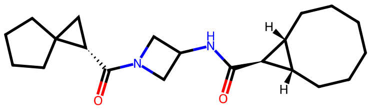	O=C(NC1CN(C(=O)[C@H]2CC23CCCC3)C1)[C@@H]1[C@H]2CCCCCC[C@H]21	1000500592	25	344.25	3.11
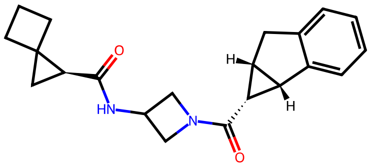	O=C(NC1CN(C(=O)[C@H]2[C@H]3c4ccccc4C[C@H]32)C1)[C@H]1CC12CCC2	991554862	25	336.18	2.09
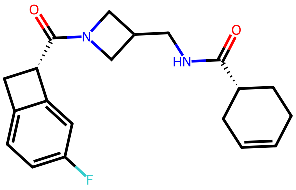	O=C(NCC1CN(C(=O)[C@H]2Cc3ccc(F)cc32)C1)[C@H]1CC=CCC1	1000868605	25	342.17	2.4

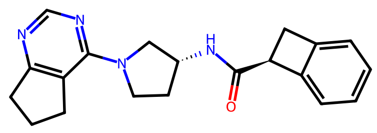	O=C(N[C@@H]1CCN(c2ncnc3c2CCC3)C1)[C@H]1Cc2ccccc21	1058531177	25	334.18	2.0
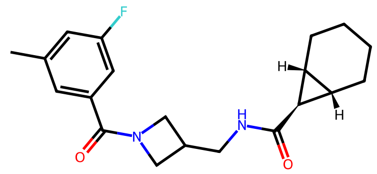	Cc1cc(F)cc(C(=O)N2CC(CNC(=O)[C@@H]3[C@H]4CCCC[C@H]43)C2)c1	1001335774	25	344.19	2.76
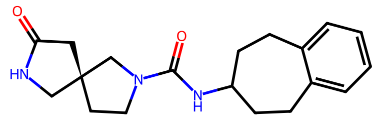	O=C1C[C@@]2(CCN(C(=O)NC3CCc4ccccc4CC3)C2)CN1	656205151	24	327.19	1.86
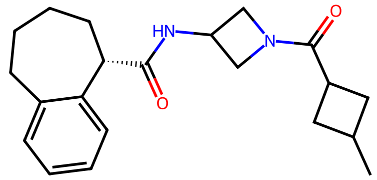	CC1CC(C(=O)N2CC(NC(=O)[C@H]3CCCCc4ccccc43)C2)C1	994307262	25	340.22	2.87
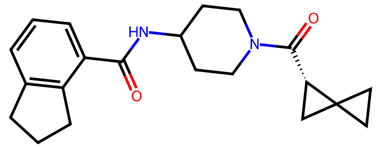	O=C(NC1CCN(C(=O)[C@H]2CC23CC3)CC1)c1cccc2c1CCC2	948567155	25	338.2	2.7
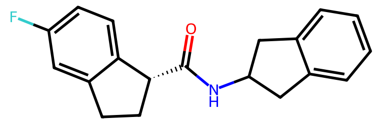	O=C(NC1Cc2ccccc2C1)[C@@H]1CCc2cc(F)ccc21	613521217	22	295.14	3.14
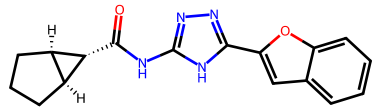	O=C(Nc1nnc(-c2cc3ccccc3o2)[nH]1)[C@@H]1[C@H]2CCC[C@H]21	276777949	23	308.13	3.2
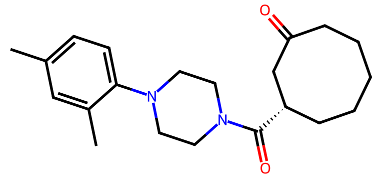	Cc1ccc(N2CCN(C(=O)[C@H]3CCCCCC(=O)C3)CC2)c(C)c1	1557625167	25	342.23	3.49

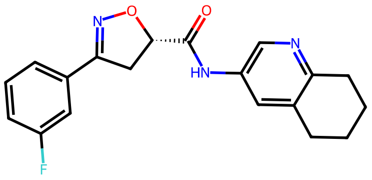	O=C(Nc1cnc2c(c1)CCCC2)[C@@H]1CC(c2cccc(F)c2)=NO1	782513924	25	339.14	3.23
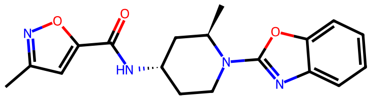	Cc1cc(C(=O)N[C@H]2CCN(c3nc4ccccc4o3)[C@H](C)C2)on1	1067507717	25	340.15	2.91
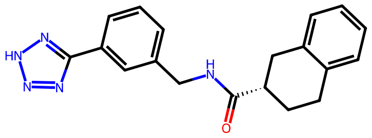	O=C(NCc1cccc(-c2nn[nH]n2)c1)[C@H]1CCc2ccccc2C1	1609555787	25	333.16	2.29
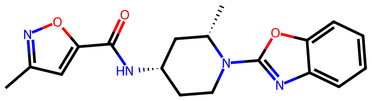	Cc1cc(C(=O)N[C@H]2CCN(c3nc4ccccc4o3)[C@@H](C)C2)on1	1067507719	25	340.15	2.91
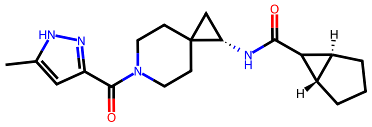	Cc1cc(C(=O)N2CCC3(CC2)C[C@@H]3NC(=O)C2[C@H]3CCC[C@H]23)n[nH]1	963468261	25	342.21	1.88
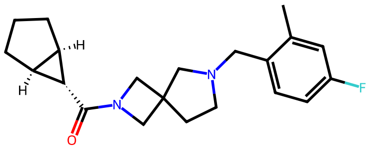	Cc1cc(F)ccc1CN1CCC2(C1)CN(C(=O)[C@@H]1[C@H]3CCC[C@H]31)C2	1072590433	25	342.21	3.21
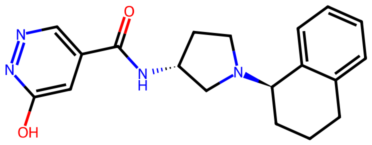	O=C(N[C@@H]1CCN([C@@H]2CCCc3ccccc32)C1)c1cnnc(O)c1	1014829874	25	338.17	2.06
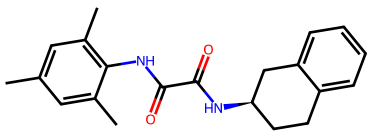	Cc1cc(C)c(NC(=O)C(=O)N[C@@H]2CCc3ccccc3C2)c(C)c1	356713062	25	336.18	3.22

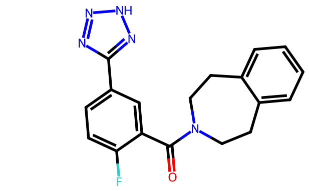	O=C(c1cc(-c2nn[nH]n2)ccc1F)N1CCc2ccccc2CC1	1582685473	25	337.13	2.25
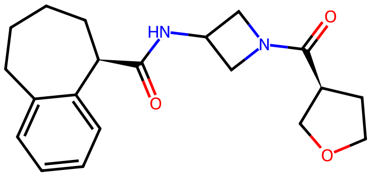	O=C(NC1CN(C(=O)[C@H]2CCOC2)C1)[C@@H]1CCCCc2ccccc21	994305003	25	342.19	1.86
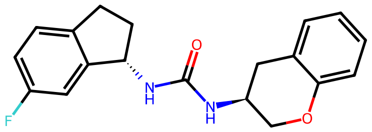	O=C(N[C@@H]1COc2ccccc2C1)N[C@H]1CCc2ccc(F)cc21	106536592	24	326.14	3.12
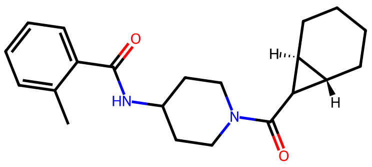	Cc1ccccc1C(=O)NC1CCN(C(=O)C2[C@H]3CCCC[C@H]23)CC1	174637949	25	340.22	3.15
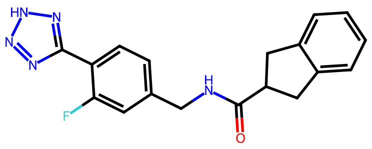	O=C(NCc1ccc(-c2nn[nH]n2)c(F)c1)C1Cc2ccccc2C1	1582545485	25	337.13	2.04
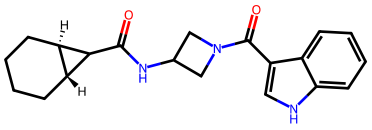	O=C(NC1CN(C(=O)c2c[nH]c3ccccc23)C1)C1[C@@H]2CCCC[C@@H]12	990665767	25	337.18	2.54
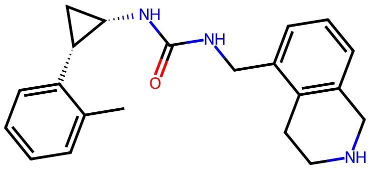	Cc1ccccc1[C@@H]1C[C@@H]1NC(=O)NCc1cccc2c1CCNC2	1550270206	25	335.2	3.0
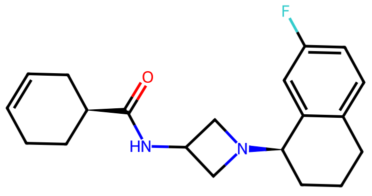	O=C(NC1CN([C@@H]2CCCc3ccc(F)cc32)C1)[C@@H]1CC=CCC1	1030098611	24	328.2	3.36

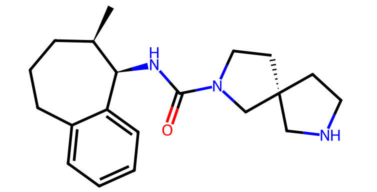	C[C@@H]1CCCc2ccccc2[C@@H]1NC(=O)N1CC[C@@]2(CCNC2)C1	418017792	24	327.23	3.1
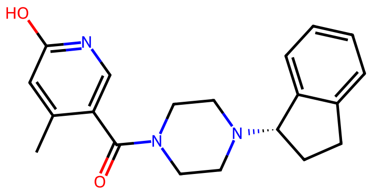	Cc1cc(O)ncc1C(=O)N1CCN([C@H]2CCc3ccccc32)CC1	950426530	25	337.18	2.54
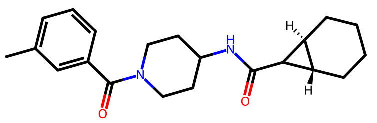	Cc1cccc(C(=O)N2CCC(NC(=O)C3[C@H]4CCCC[C@H]34)CC2)c1	943805879	25	340.22	3.15
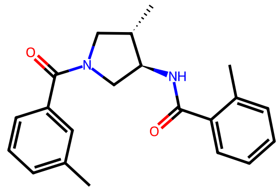	Cc1cccc(C(=O)N2C[C@H](C)[C@@H](NC(=O)c3ccccc3C)C2)c1	966115733	25	336.18	3.19
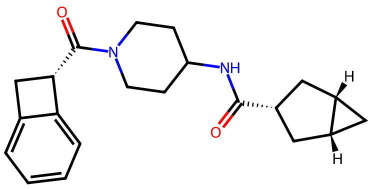	O=C(NC1CCN(C(=O)[C@H]2Cc3ccccc32)CC1)[C@@H]1C[C@@H]2C[C@@H]2C1	948063061	25	338.2	2.48
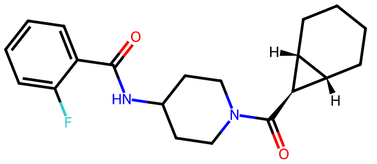	O=C(NC1CCN(C(=O)[C@@H]2[C@H]3CCCC[C@H]32)CC1)c1ccccc1F	369941368	25	344.19	2.98
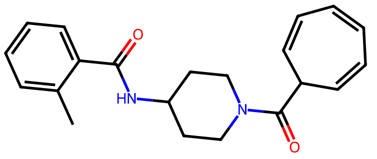	Cc1ccccc1C(=O)NC1CCN(C(=O)C2C=CC=CC=C2)CC1	367395039	25	336.18	3.01
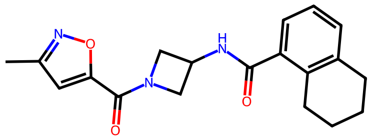	Cc1cc(C(=O)N2CC(NC(=O)c3cccc4c3CCCC4)C2)on1	991903971	25	339.16	2.12

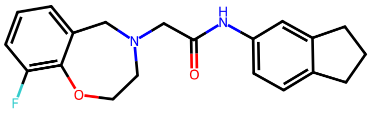	O=C(CN1CCOc2c(F)cccc2C1)Nc1ccc2c(c1)CCC2	434623144	25	340.16	3.15
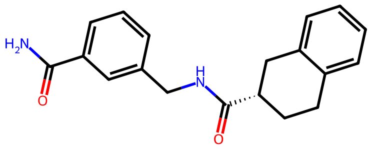	NC(=O)c1cccc(CNC(=O)[C@H]2CCc3ccccc3C2)c1	573925367	23	308.15	2.21
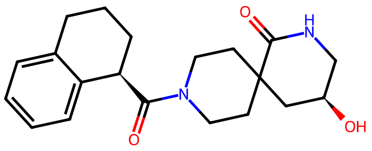	O=C([C@@H]1CCCc2ccccc21)N1CCC2(CC1)C[C@H](O)CNC2=O	885024776	25	342.19	1.6
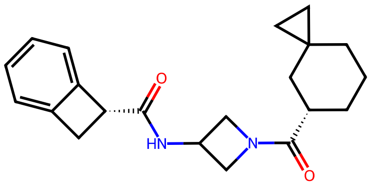	O=C(NC1CN(C(=O)[C@H]2CCCC3(CC3)C2)C1)[C@@H]1Cc2ccccc21	998688751	25	338.2	2.62
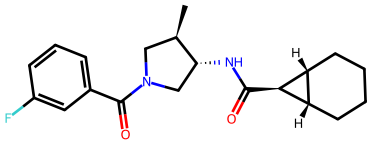	C[C@@H]1CN(C(=O)c2cccc(F)c2)C[C@H]1NC(=O)[C@@H]1[C@H]2CCCC[C@H]21	947284526	25	344.19	2.84
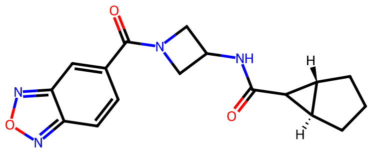	O=C(NC1CN(C(=O)c2ccc3nonc3c2)C1)C1[C@@H]2CCC[C@@H]12	990695039	24	326.14	1.21
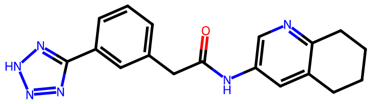	O=C(Cc1cccc(-c2nn[nH]n2)c1)Nc1cnc2c(c1)CCCC2	826423327	25	334.15	2.32
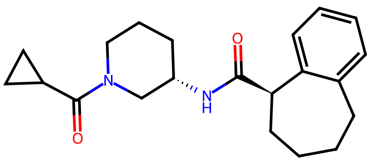	O=C(N[C@H]1CCCN(C(=O)C2CC2)C1)[C@@H]1CCCCc2ccccc21	949616517	25	340.22	3.01

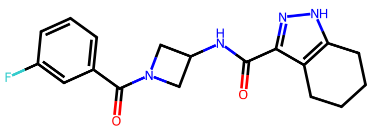	O=C(NC1CN(C(=O)c2cccc(F)c2)C1)c1n[nH]c2c1CCCC2	995172268	25	342.15	1.68
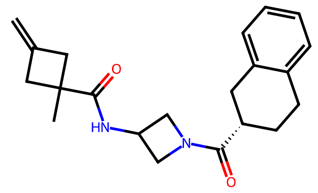	C=C1CC(C)(C(=O)NC2CN(C(=O)[C@H]3CCc4ccccc4C3)C2)C1	999293130	25	338.2	2.47
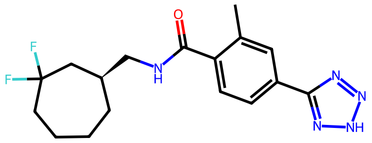	Cc1cc(-c2nn[nH]n2)ccc1C(=O)NC[C@H]1CCCCC(F)(F)C1	1591862436	25	349.17	3.12
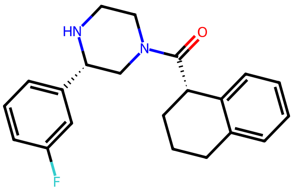	O=C([C@H]1CCCc2ccccc21)N1CCN[C@@H](c2cccc(F)c2)C1	337589469	25	338.18	3.42
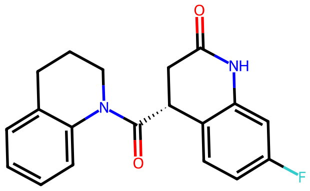	O=C1C[C@@H](C(=O)N2CCCc3ccccc32)c2ccc(F)cc2N1	25894711	24	324.13	3.23
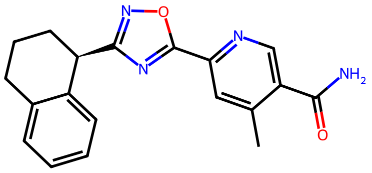	Cc1cc(-c2nc([C@@H]3CCCc4ccccc43)no2)ncc1C(N)=O	580284872	25	334.14	3.01
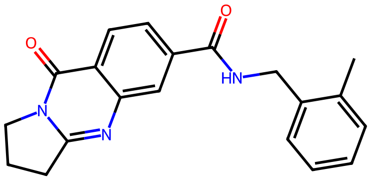	Cc1ccccc1CNC(=O)c1ccc2c(=O)n3c(nc2c1)CCC3	8367267	25	333.15	2.58
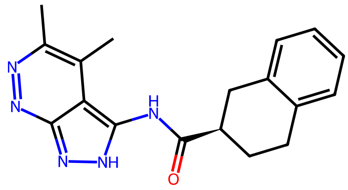	Cc1nnc2n[nH]c(NC(=O)[C@@H]3CCc4ccccc4C3)c2c1C	614650146	24	321.16	2.71

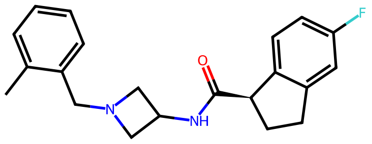	Cc1ccccc1CN1CC(NC(=O)[C@@H]2CCc3cc(F)ccc32)C1	1030686256	25	338.18	3.16
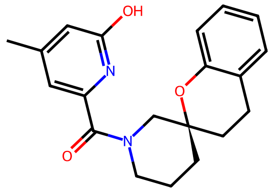	Cc1cc(O)nc(C(=O)N2CCC[C@@]3(CCc4ccccc4O3)C2)c1	529905949	25	338.16	3.1
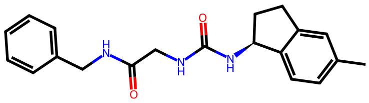	Cc1ccc2c(c1)CC[C@@H]2NC(=O)NCC(=O)NCc1ccccc1	56843769	25	337.18	2.6
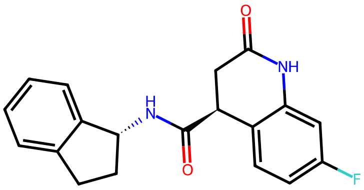	O=C1C[C@H](C(=O)N[C@@H]2CCc3ccccc32)c2ccc(F)cc2N1	78434372	24	324.13	3.06
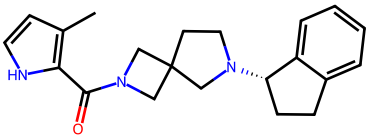	Cc1cc[nH]c1C(=O)N1CC2(CCN([C@H]3CCc4ccccc43)C2)C1	1072683703	25	335.2	3.16
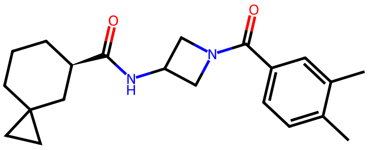	Cc1ccc(C(=O)N2CC(NC(=O)[C@@H]3CCCC4(CC4)C3)C2)cc1C	991516705	25	340.22	3.21
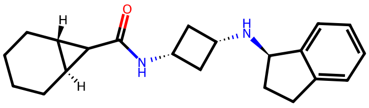	O=C(N[C@H]1C[C@@H](N[C@@H]2CCc3ccccc32)C1)C1[C@H]2CCCC[C@H]12	1021936284	24	324.22	3.35
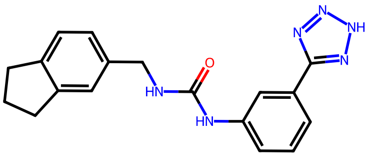	O=C(NCc1ccc2c(c1)CCC2)Nc1cccc(-c2nn[nH]n2)c1	1593139797	25	334.15	2.68

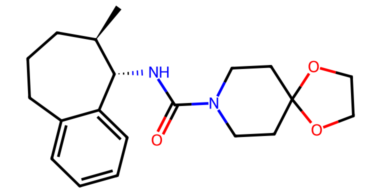	C[C@@H]1CCCc2ccccc2[C@H]1NC(=O)N1CCC2(CC1)OCCO2	185616378	25	344.21	3.25
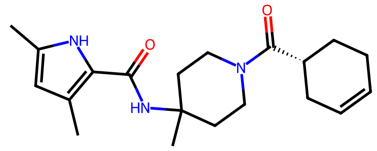	Cc1cc(C)c(C(=O)NC2(C)CCN(C(=O)[C@H]3CC=CCC3)CC2)[nH]1	956419980	25	343.23	3.1
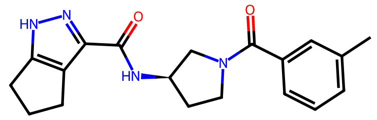	Cc1cccc(C(=O)N2CC[C@@H](NC(=O)c3n[nH]c4c3CCC4)C2)c1	941439277	25	338.17	1.85
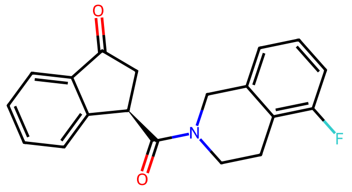	O=C1C[C@@H](C(=O)N2CCc3c(F)cccc3C2)c2ccccc21	277349616	23	309.12	3.08
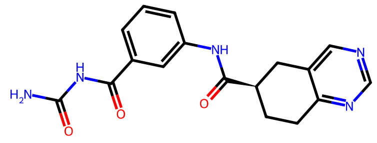	NC(=O)NC(=O)c1cccc(NC(=O)[C@@H]2CCc3ncncc3C2)c1	1463414759	25	339.13	1.03
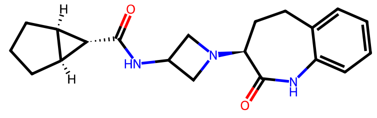	O=C1Nc2ccccc2CC[C@@H]1N1CC(NC(=O)[C@@H]2[C@H]3CCC[C@H]32)C1	1030202506	25	339.19	1.79
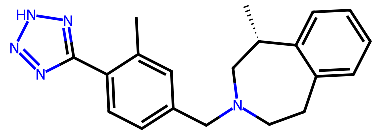	Cc1cc(CN2CCc3ccccc3[C@@H](C)C2)ccc1-c1nn[nH]n1	1591964772	25	333.2	3.34
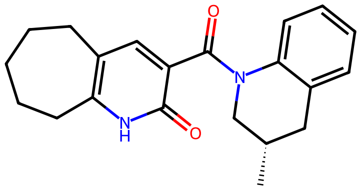	C[C@H]1Cc2ccccc2N(C(=O)c2cc3c([nH]c2=O)CCCCC3)C1	78116260	25	336.18	3.48

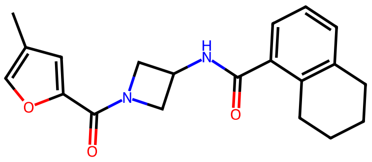	Cc1coc(C(=O)N2CC(NC(=O)c3cccc4c3CCCC4)C2)c1	991901814	25	338.16	2.72
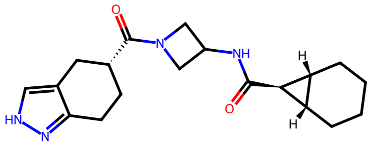	O=C(NC1CN(C(=O)[C@@H]2CCc3n[nH]cc3C2)C1)[C@@H]1[C@H]2CCCC[C@H]21	990649344	25	342.21	1.28
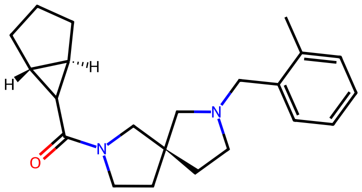	Cc1ccccc1CN1CC[C@]2(CCN(C(=O)C3[C@@H]4CCC[C@@H]34)C2)C1	1041179213	25	338.24	3.47
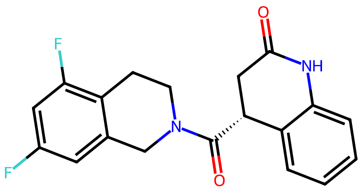	O=C1C[C@@H](C(=O)N2CCc3c(F)cc(F)cc3C2)c2ccccc2N1	134279790	25	342.12	2.98
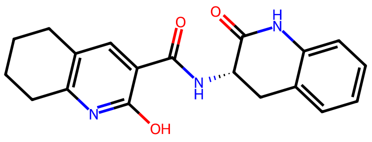	O=C(N[C@H]1Cc2ccccc2NC1=O)c1cc2c(nc1O)CCCC2	529953970	25	337.14	1.96
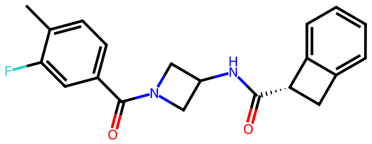	Cc1ccc(C(=O)N2CC(NC(=O)[C@H]3Cc4ccccc43)C2)cc1F	998686458	25	338.14	2.41
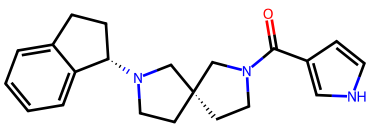	O=C(c1cc[nH]c1)N1CC[C@@]2(CCN([C@H]3CCc4ccccc43)C2)C1	1041312085	25	335.2	3.24
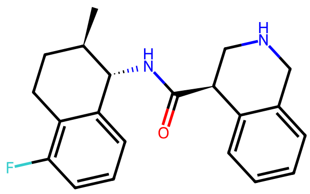	C[C@@H]1CCc2c(F)cccc2[C@H]1NC(=O)[C@H]1CNCc2ccccc21	566190196	25	338.18	3.45

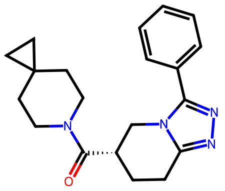	O=C([C@H]1CCc2nnc(-c3ccccc3)n2C1)N1CCC2(CC1)CC2	648924080	25	336.2	2.91
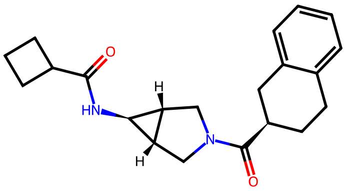	O=C(N[C@H]1[C@@H]2CN(C(=O)[C@@H]3CCc4ccccc4C3)C[C@@H]21)C1CCC1	976033263	25	338.2	2.16
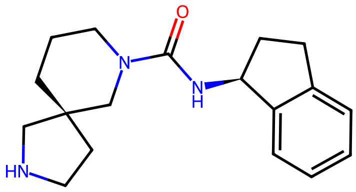	O=C(N[C@H]1CCc2ccccc21)N1CCC[C@]2(CCNC2)C1	335612080	22	299.2	2.46
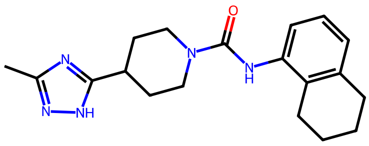	Cc1n[nH]c(C2CCN(C(=O)Nc3cccc4c3CCCC4)CC2)n1	95524738	25	339.21	3.4
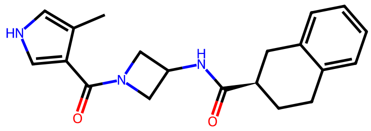	Cc1c[nH]cc1C(=O)N1CC(NC(=O)[C@@H]2CCc3ccccc3C2)C1	1000543290	25	337.18	2.07
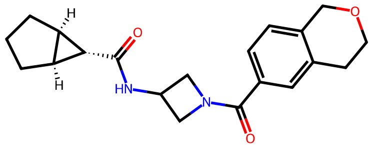	O=C(NC1CN(C(=O)c2ccc3c(c2)CCOC3)C1)[C@@H]1[C@H]2CCC[C@H]21	990699746	25	340.18	1.75
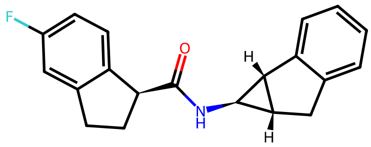	O=C(N[C@@H]1[C@H]2Cc3ccccc3[C@H]21)[C@H]1CCc2cc(F)ccc21	926765035	23	307.14	3.31
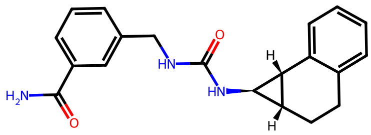	NC(=O)c1cccc(CNC(=O)N[C@@H]2[C@H]3CCc4ccccc4[C@H]32)c1	126277526	25	335.16	2.31

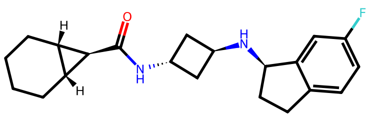	O=C(N[C@H]1C[C@H](N[C@@H]2CCc3ccc(F)cc32)C1)[C@@H]1[C@H]2CCCC[C@H]21	1020047964	25	342.21	3.49
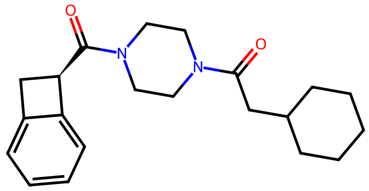	O=C(CC1CCCCC1)N1CCN(C(=O)[C@@H]2Cc3ccccc32)CC1	358329158	25	340.22	2.97
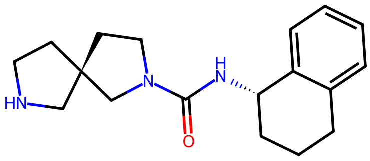	O=C(N[C@H]1CCCc2ccccc21)N1CC[C@@]2(CCNC2)C1	335270124	22	299.2	2.46
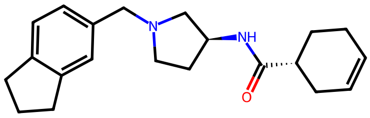	O=C(N[C@H]1CCN(Cc2ccc3c(c2)CCC3)C1)[C@H]1CC=CCC1	1010080044	24	324.22	3.22
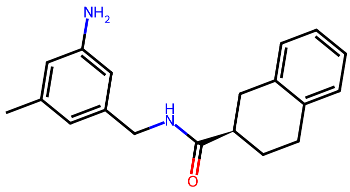	Cc1cc(N)cc(CNC(=O)[C@@H]2CCc3ccccc3C2)c1	399044200	22	294.17	3.0
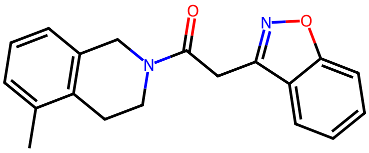	Cc1cccc2c1CCN(C(=O)Cc1noc3ccccc13)C2	171538997	23	306.14	3.26
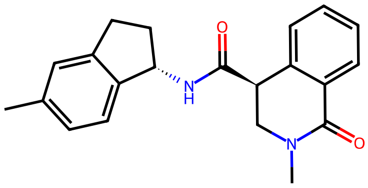	Cc1ccc2c(c1)CC[C@@H]2NC(=O)[C@@H]1CN(C)C(=O)c2ccccc21	345861732	25	334.17	2.97
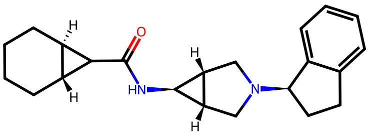	O=C(N[C@H]1[C@@H]2CN([C@@H]3CCc4ccccc43)C[C@@H]21)C1[C@@H]2CCCC[C@@H]12	1025723610	25	336.22	3.16

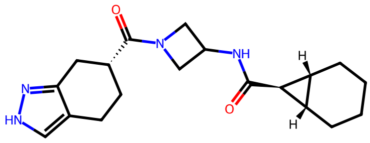	O=C(NC1CN(C(=O)[C@@H]2CCc3c[nH]nc3C2)C1)[C@@H]1[C@H]2CCCC[C@H]21	990647995	25	342.21	1.28
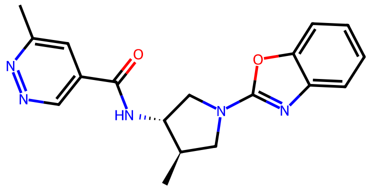	Cc1cc(C(=O)N[C@@H]2CN(c3nc4ccccc4o3)C[C@H]2C)cnn1	1041846286	25	337.15	2.18
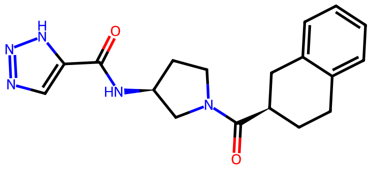	O=C(N[C@H]1CCN(C(=O)[C@@H]2CCc3ccccc3C2)C1)c1cnn[nH]1	938241445	25	339.17	0.94
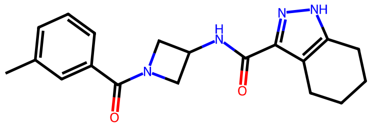	Cc1cccc(C(=O)N2CC(NC(=O)c3n[nH]c4c3CCCC4)C2)c1	995171231	25	338.17	1.85
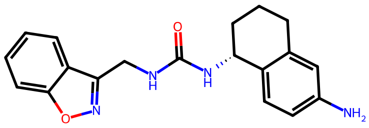	Nc1ccc2c(c1)CCC[C@H]2NC(=O)NCc1noc2ccccc12	568245789	25	336.16	3.29
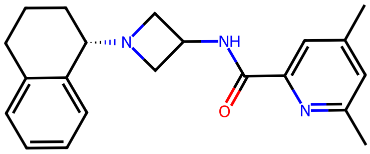	Cc1cc(C)nc(C(=O)NC2CN([C@H]3CCCc4ccccc43)C2)c1	1030587116	25	335.2	3.19
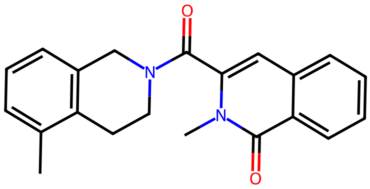	Cc1cccc2c1CCN(C(=O)c1cc3ccccc3c(=O)n1C)C2	796161317	25	332.15	3.05
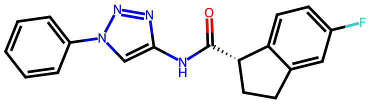	O=C(Nc1cn(-c2ccccc2)nn1)[C@H]1CCc2cc(F)ccc21	1540217149	24	322.12	3.07

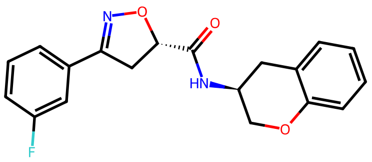	O=C(N[C@@H]1COc2ccccc2C1)[C@@H]1CC(c2cccc(F)c2)=NO1	194461030	25	340.12	2.44
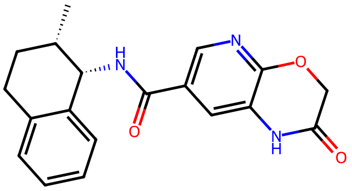	C[C@H]1CCc2ccccc2[C@H]1NC(=O)c1cnc2c(c1)NC(=O)CO2	271422754	25	337.14	2.47
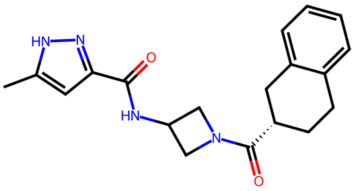	Cc1cc(C(=O)NC2CN(C(=O)[C@H]3CCc4ccccc4C3)C2)n[nH]1	990382109	25	338.17	1.46
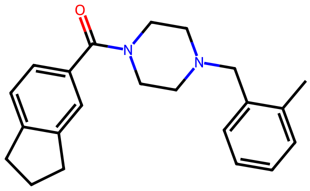	Cc1ccccc1CN1CCN(C(=O)c2ccc3c(c2)CCC3)CC1	32483761	25	334.2	3.44
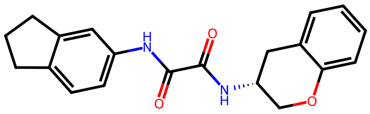	O=C(Nc1ccc2c(c1)CCC2)C(=O)N[C@H]1COc2ccccc2C1	42050030	25	336.15	2.23
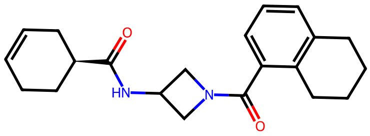	O=C(NC1CN(C(=O)c2cccc3c2CCCC3)C1)[C@@H]1CC=CCC1	990215527	25	338.2	2.86
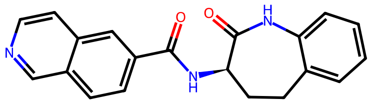	O=C(N[C@@H]1CCc2ccccc2NC1=O)c1ccc2cnccc2c1	1537777751	25	331.13	2.92
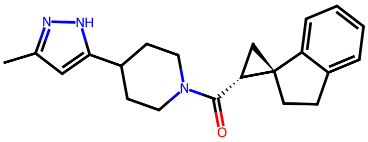	Cc1cc(C2CCN(C(=O)[C@@H]3C[C@@]34CCc3ccccc34)CC2)[nH]n1	273565901	25	335.2	3.33

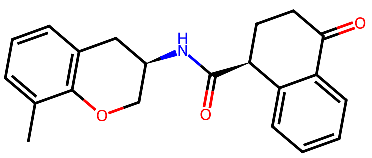	Cc1cccc2c1OC[C@H](NC(=O)[C@H]1CCC(=O)c3ccccc31)C2	291649062	25	335.15	3.18
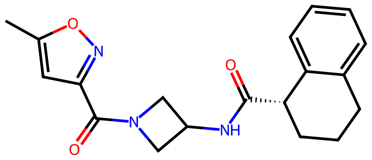	Cc1cc(C(=O)N2CC(NC(=O)[C@H]3CCCc4ccccc43)C2)no1	995071056	25	339.16	2.04
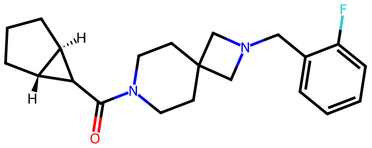	O=C(C1[C@@H]2CCC[C@@H]12)N1CCC2(CC1)CN(Cc1ccccc1F)C2	1035679278	25	342.21	3.3
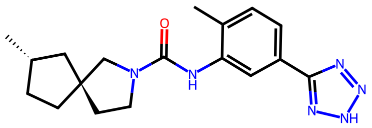	Cc1ccc(-c2nn[nH]n2)cc1NC(=O)N1CC[C@]2(CC[C@H](C)C2)C1	826277986	25	340.2	3.22
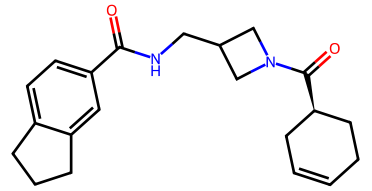	O=C(NCC1CN(C(=O)[C@@H]2CC=CCC2)C1)c1ccc2c(c1)CCC2	1001830620	25	338.2	2.72
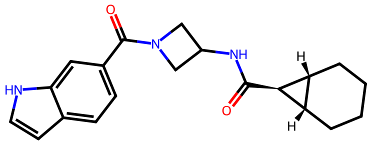	O=C(NC1CN(C(=O)c2ccc3cc[nH]c3c2)C1)[C@@H]1[C@H]2CCCC[C@H]21	990664149	25	337.18	2.54
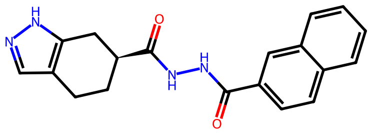	O=C(NNC(=O)[C@H]1CCc2cn[nH]c2C1)c1ccc2ccccc2c1	79629028	25	334.14	2.13
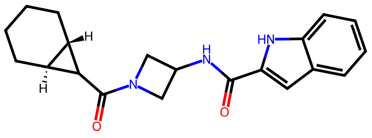	O=C(NC1CN(C(=O)C2[C@H]3CCCC[C@H]23)C1)c1cc2ccccc2[nH]1	1000142239	25	337.18	2.54

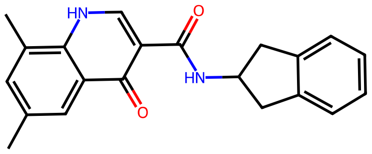	Cc1cc(C)c2[nH]cc(C(=O)NC3Cc4cccc c4C3)c(=O)c2c1	1161127649	25	332.15	3.04
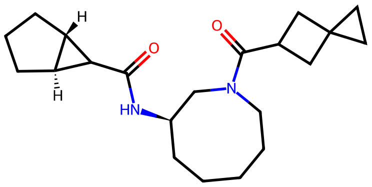	O=C(N[C@@H]1CCCCCN(C(=O)C2C C3(CC3)C2)C1)C1[C@H]2CCC[C@H] 12	987150768	25	344.25	3.11
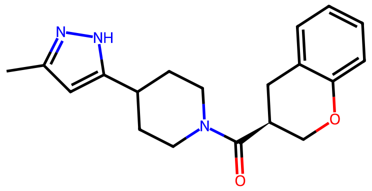	Cc1cc(C2CCN(C(=O)[C@@H]3COc4 ccccc4C3)CC2)[nH]n1	273643525	24	325.18	2.68
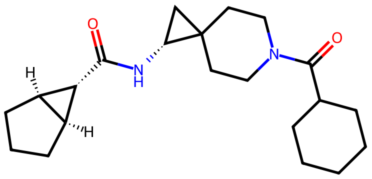	O=C(N[C@@H]1CC12CCN(C(=O)C1 CCCCC1)CC2)[C@@H]1[C@H]2CCC [C@H]21	963462682	25	344.25	3.11
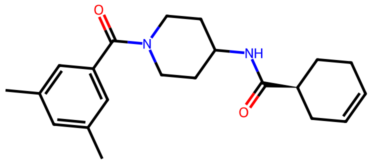	Cc1cc(C)cc(C(=O)N2CCC(NC(=O)[C @@H]3CC=CCC3)CC2)c1	948338269	25	340.22	3.38
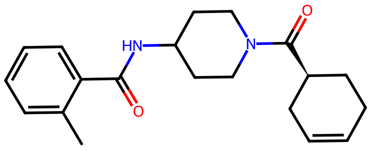	Cc1ccccc1C(=O)NC1CCN(C(=O)[C@ @H]2CC=CCC2)CC1	32306062	24	326.2	3.07
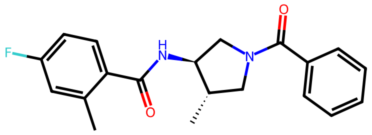	Cc1cc(F)ccc1C(=O)N[C@H]1CN(C(= O)c2ccccc2)C[C@@H]1C	966090811	25	340.16	3.02
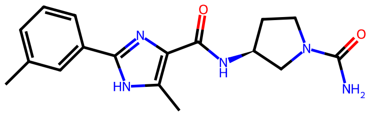	Cc1cccc(-c2nc(C(=O)N[C@H]3CCN(C (N)=O)C3)c(C)[nH]2)c1	270282090	24	327.17	1.58

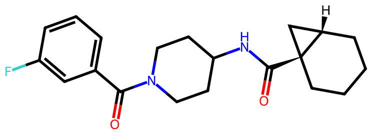	O=C(c1cccc(F)c1)N1CCC(NC(=O)[C@]23CCCC[C@H]2C3)CC1	943776523	25	344.19	3.13
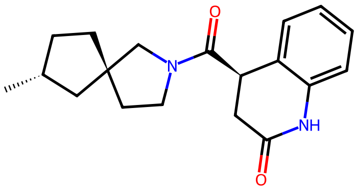	C[C@@H]1CC[C@@]2(CCN(C(=O)[C@@H]3CC(=O)Nc4ccccc43)C2)C1	359157418	23	312.18	3.15
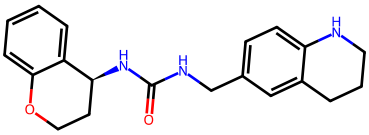	O=C(NCc1ccc2c(c1)CCCN2)N[C@H]1CCOc2ccccc21	668620533	25	337.18	3.37
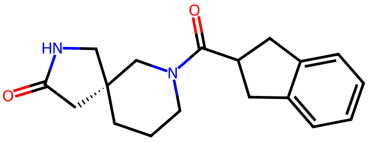	O=C1C[C@]2(CCCN(C(=O)C3Cc4ccccc4C3)C2)CN1	334709972	22	298.17	1.53
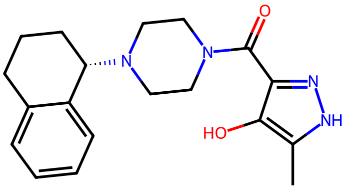	Cc1[nH]nc(C(=O)N2CCN([C@H]3CCCc4ccccc43)CC2)c1O	1365692454	25	340.19	2.26
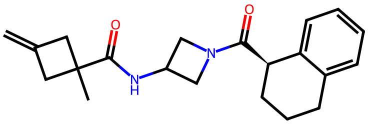	C=C1CC(C)(C(=O)NC2CN(C(=O)[C@@H]3CCCc4ccccc43)C2)C1	999264914	25	338.2	2.79
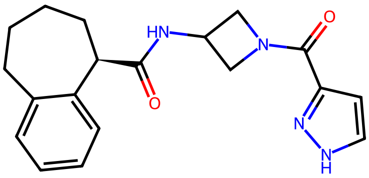	O=C(NC1CN(C(=O)c2cc[nH]n2)C1)[C@@H]1CCCCc2ccccc21	994307226	25	338.17	1.86
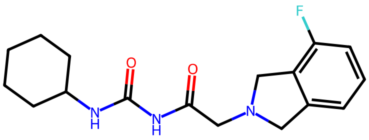	O=C(CN1Cc2cccc(F)c2C1)NC(=O)NC1CCCCC1	459764995	23	319.17	2.3

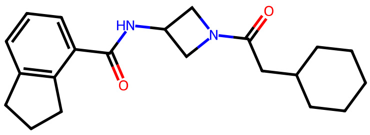	O=C(NC1CN(C(=O)CC2CCCCC2)C1)c1cccc2c1CCC2	994400278	25	340.22	3.09
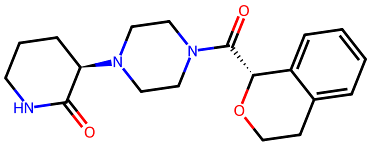	O=C1NCCC[C@H]1N1CCN(C(=O)[C@H]2OCCc3ccccc32)CC1	949590071	25	343.19	0.72
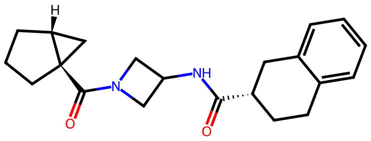	O=C(NC1CN(C(=O)[C@@]23CCC[C@@H]2C3)C1)[C@H]1CCc2ccccc2C1	1000537993	25	338.2	2.31
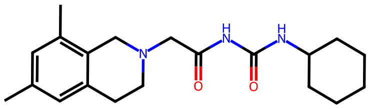	Cc1cc(C)c2c(c1)CCN(CC(=O)NC(=O)NC1CCCCC1)C2	1363857066	25	343.23	2.82
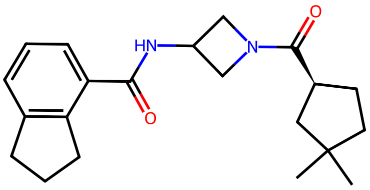	CC1(C)CC[C@H](C(=O)N2CC(NC(=O)c3cccc4c3CCC4)C2)C1	994399971	25	340.22	2.94
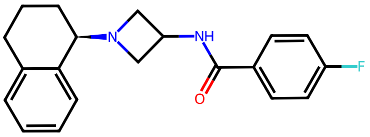	O=C(NC1CN([C@@H]2CCCc3ccccc32)C1)c1ccc(F)cc1	1030054501	24	324.16	3.32
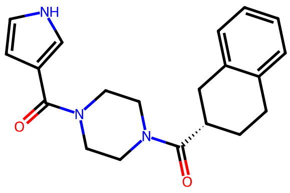	O=C(c1cc[nH]c1)N1CCN(C(=O)[C@H]2CCc3ccccc3C2)CC1	1011246929	25	337.18	2.1
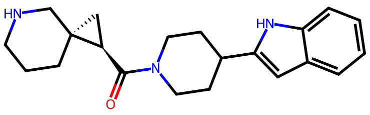	O=C([C@@H]1C[C@@]12CCCNC2)N1CCC(c2cc3ccccc3[nH]2)CC1	848397460	25	337.22	3.26

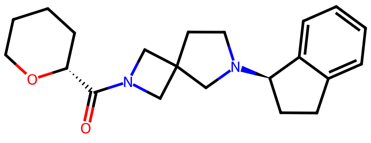	O=C([C@H]1CCCCO1)N1CC2(CCN([C@@H]3CCc4ccccc43)C2)C1	1072628540	25	340.22	2.78
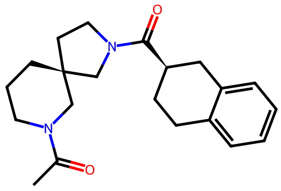	CC(=O)N1CCC[C@]2(CCN(C(=O)[C@@H]3CCc4ccccc4C3)C2)C1	952958017	25	340.22	2.65
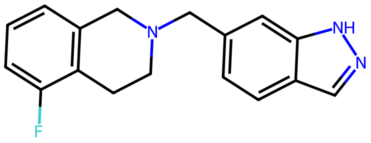	Fc1cccc2c1CCN(Cc1ccc3cn[nH]c3c1)C2	123791807	21	281.13	3.26
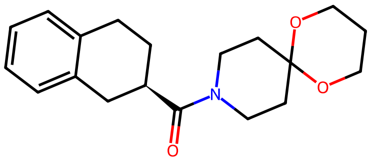	O=C([C@@H]1CCc2ccccc2C1)N1CCC2(CC1)OCCCO2	846227732	23	315.18	2.55
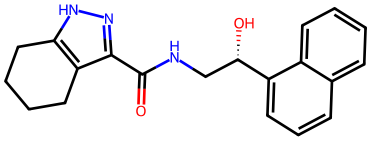	O=C(NC[C@H](O)c1cccc2ccccc12)c1n[nH]c2c1CCCC2	855486657	25	335.16	2.91
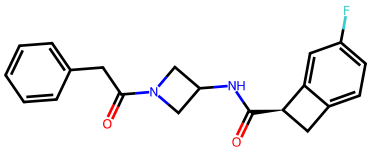	O=C(NC1CN(C(=O)Cc2ccccc2)C1)[C@@H]1Cc2ccc(F)cc21	998494328	25	338.14	2.04
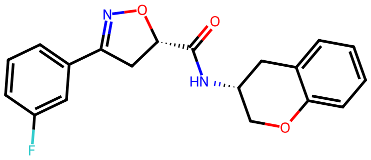	O=C(N[C@H]1COc2ccccc2C1)[C@@H]1CC(c2cccc(F)c2)=NO1	194461047	25	340.12	2.44
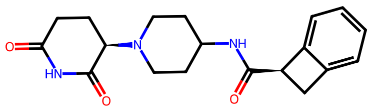	O=C1CC[C@@H](N2CCC(NC(=O)[C@@H]3Cc4ccccc43)CC2)C(=O)N1	1003535051	25	341.17	0.71

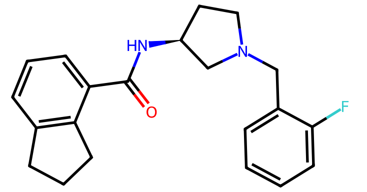	O=C(N[C@H]1CCN(Cc2ccccc2F)C1)c1cccc2c1CCC2	1016382193	25	338.18	3.32
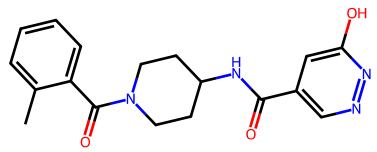	Cc1ccccc1C(=O)N1CCC(NC(=O)c2cnnc(O)c2)CC1	943766109	25	340.15	1.53
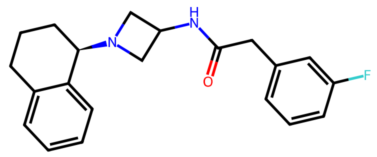	O=C(Cc1cccc(F)c1)NC1CN([C@@H]2CCCc3ccccc32)C1	1031352776	25	338.18	3.25
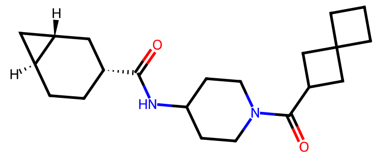	O=C(NC1CCN(C(=O)C2CC3(CCC3)C2)CC1)[C@@H]1CC[C@H]2C[C@@H]2C1	947886366	25	344.25	3.11
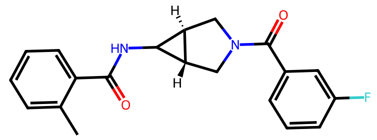	Cc1ccccc1C(=O)NC1[C@H]2CN(C(=O)c3cccc(F)c3)C[C@H]12	1012712400	25	338.14	2.63
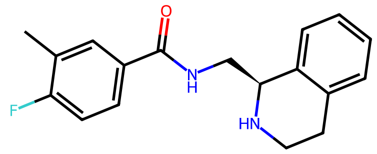	Cc1cc(C(=O)NC[C@@H]2NCCc3ccccc32)ccc1F	262510630	22	298.15	2.75
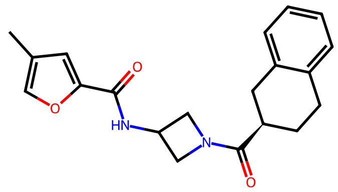	Cc1coc(C(=O)NC2CN(C(=O)[C@@H]3CCc4ccccc4C3)C2)c1	997963344	25	338.16	2.33
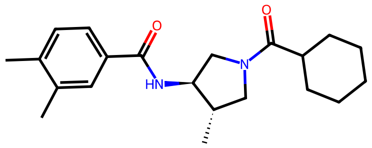	Cc1ccc(C(=O)N[C@H]2CN(C(=O)C3CCCCC3)C[C@@H]2C)cc1C	966097711	25	342.23	3.46

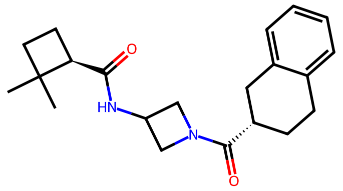	CC1(C)CC[C@H]1C(=O)NC1CN(C(=O)[C@H]2CCc3ccccc3C2)C1	997966747	25	340.22	2.55
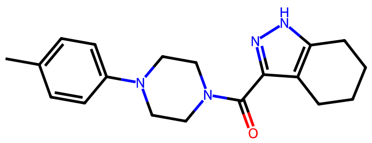	Cc1ccc(N2CCN(C(=O)c3n[nH]c4c3CCCC4)CC2)cc1	51319548	24	324.2	2.56
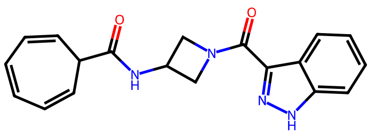	O=C(NC1CN(C(=O)c2n[nH]c3ccccc23)C1)C1C=CC=CC=C1	992882053	25	334.14	1.8
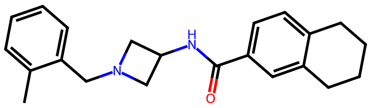	Cc1ccccc1CN1CC(NC(=O)c2ccc3c(c2)CCCC3)C1	1031351180	25	334.2	3.49
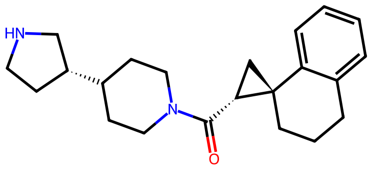	O=C([C@@H]1C[C@@]12CCCc1ccccc12)N1CCC([C@@H]2CCNC2)CC1	377808350	25	338.24	3.13
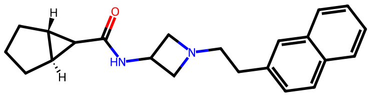	O=C(NC1CN(CCc2ccc3ccccc3c2)C1)C1[C@H]2CCC[C@H]12	1030190593	25	334.2	3.23
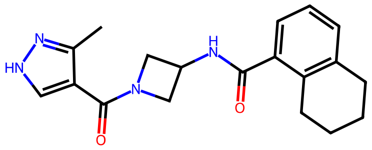	Cc1n[nH]cc1C(=O)N1CC(NC(=O)c2cccc3c2CCCC3)C1	991898350	25	338.17	1.85
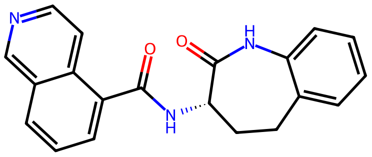	O=C(N[C@H]1CCc2ccccc2NC1=O)c1cccc2cnccc12	1130541385	25	331.13	2.92

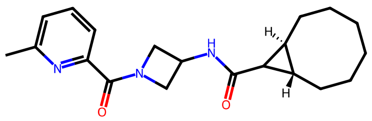	Cc1cccc(C(=O)N2CC(NC(=O)C3[C@H]4CCCCCC[C@H]34)C2)n1	1000481145	25	341.21	2.55
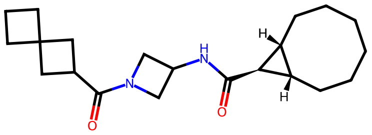	O=C(NC1CN(C(=O)C2CC3(CCC3)C2)C1)[C@@H]1[C@H]2CCCCCC[C@H]21	1000507794	25	344.25	3.11
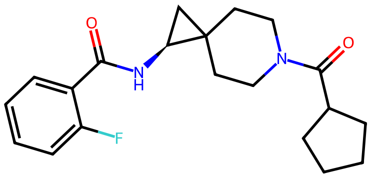	O=C(N[C@H]1CC12CCN(C(=O)C1CCCC1)CC2)c1ccccc1F	962750798	25	344.19	3.13
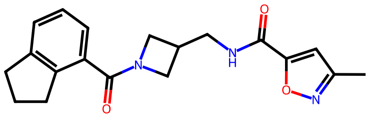	Cc1cc(C(=O)NCC2CN(C(=O)c3cccc4c3CCC4)C2)on1	1000916630	25	339.16	1.97
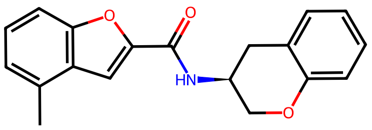	Cc1cccc2oc(C(=O)N[C@@H]3COc4ccccc4C3)cc12	1540826542	23	307.12	3.47
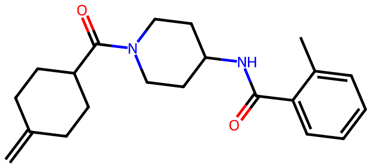	C=C1CCC(C(=O)N2CCC(NC(=O)c3ccccc3C)CC2)CC1	888191523	25	340.22	3.46
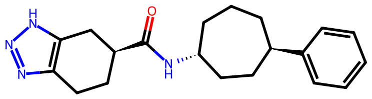	O=C(N[C@@H]1CCC[C@@H](c2ccccc2)CC1)[C@H]1CCc2nn[nH]c2C1	888905278	25	338.21	3.14
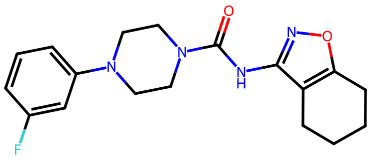	O=C(Nc1noc2c1CCCC2)N1CCN(c2cccc(F)c2)CC1	934351100	25	344.16	3.05

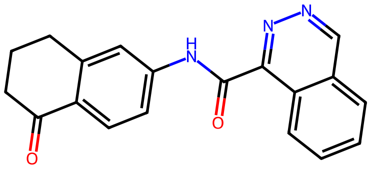	O=C1CCCc2cc(NC(=O)c3nncc4ccccc34)ccc21	1523548224	24	317.12	3.4
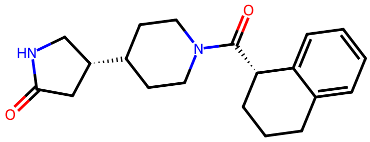	O=C1C[C@@H](C2CCN(C(=O)[C@H]3CCCc4ccccc43)CC2)CN1	1551707193	24	326.2	2.48
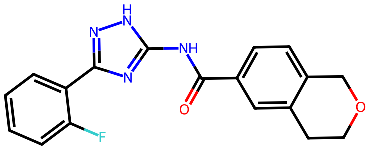	O=C(Nc1nc(-c2ccccc2F)n[nH]1)c1ccc2c(c1)CCOC2	1472496153	25	338.12	2.94
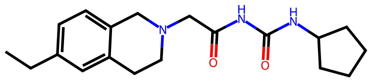	CCc1ccc2c(c1)CCN(CC(=O)NC(=O)NC1CCCC1)C2	1364533844	24	329.21	2.38
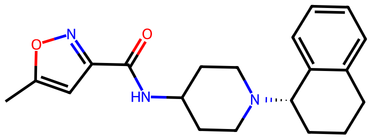	Cc1cc(C(=O)NC2CCN([C@H]3CCCc4ccccc43)CC2)no1	65954113	25	339.19	3.25
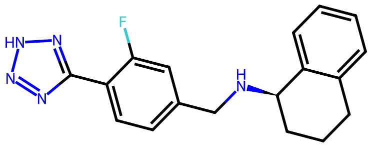	Fc1cc(CN[C@@H]2CCCc3ccccc32)ccc1-c1nn[nH]n1	1581853298	24	323.15	3.17
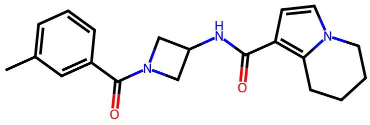	Cc1cccc(C(=O)N2CC(NC(=O)c3ccn4c3CCCC4)C2)c1	994277553	25	337.18	2.39
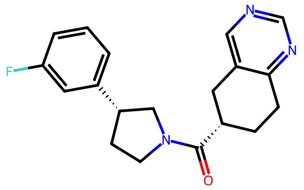	O=C([C@H]1CCc2ncncc2C1)N1CC[C@H](c2cccc(F)c2)C1	648277071	24	325.16	2.74

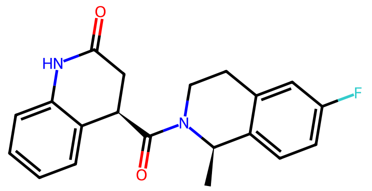	C[C@@H]1c2ccc(F)cc2CCN1C(=O)[C@@H]1CC(=O)Nc2ccccc21	1133322570	25	338.14	3.4
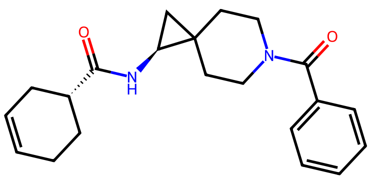	O=C(N[C@H]1CC12CCN(C(=O)c1ccccc1)CC2)[C@H]1CC=CCC1	963029857	25	338.2	3.15
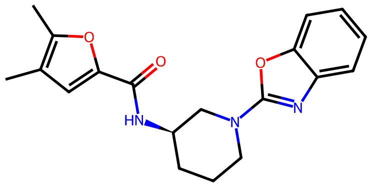	Cc1cc(C(=O)N[C@@H]2CCCN(c3nc4ccccc4o3)C2)oc1C	1058140524	25	339.16	3.44
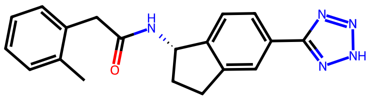	Cc1ccccc1CC(=O)N[C@H]1CCc2cc(-c3nn[nH]n3)ccc21	1592253953	25	333.16	2.52
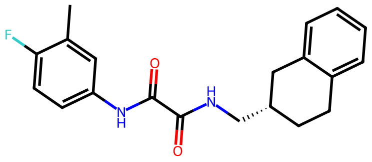	Cc1cc(NC(=O)C(=O)NC[C@H]2CCc3ccccc3C2)ccc1F	605068819	25	340.16	2.99
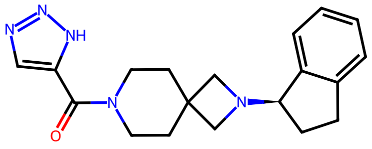	O=C(c1cnn[nH]1)N1CCC2(CC1)CN([C@@H]1CCc3ccccc31)C2	1035740359	25	337.19	2.03
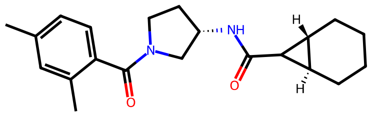	Cc1ccc(C(=O)N2CC[C@H](NC(=O)C3[C@@H]4CCCC[C@@H]34)C2)c(C)c1	937789966	25	340.22	3.07
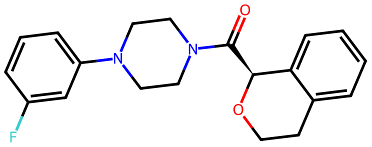	O=C([C@@H]1OCCc2ccccc21)N1CCN(c2cccc(F)c2)CC1	676722373	25	340.16	2.79

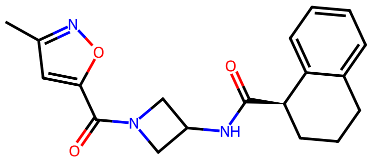	Cc1cc(C(=O)N2CC(NC(=O)[C@@H]3CCCc4ccccc43)C2)on1	995071670	25	339.16	2.04
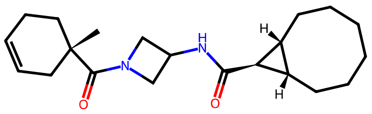	C[C@@]1(C(=O)N2CC(NC(=O)[C@@H]3[C@H]4CCCCCC[C@H]43)C2)CC=CCC1	1000504852	25	344.25	3.28
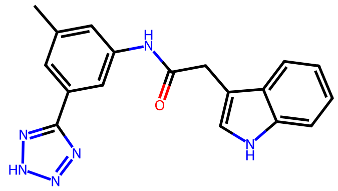	Cc1cc(NC(=O)Cc2c[nH]c3ccccc23)cc(-c2nn[nH]n2)c1	1592018878	25	332.14	2.84
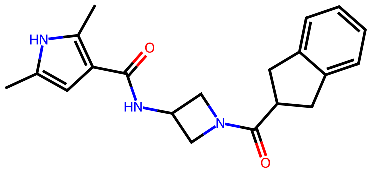	Cc1cc(C(=O)NC2CN(C(=O)C3Cc4ccccc4C3)C2)c(C)[nH]1	991546997	25	337.18	1.99
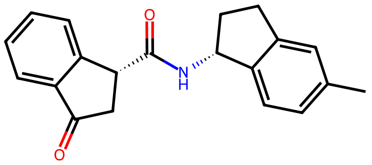	Cc1ccc2c(c1)CC[C@H]2NC(=O)[C@@H]1CC(=O)c2ccccc21	355158831	23	305.14	3.47
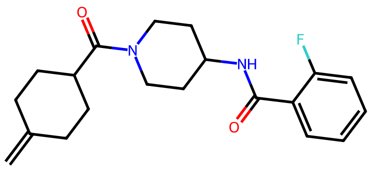	C=C1CCC(C(=O)N2CCC(NC(=O)c3ccccc3F)CC2)CC1	948189610	25	344.19	3.29
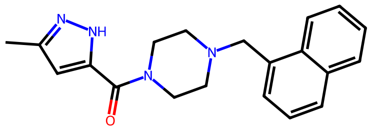	Cc1cc(C(=O)N2CCN(Cc3cccc4ccccc34)CC2)[nH]n1	54130088	25	334.18	2.83
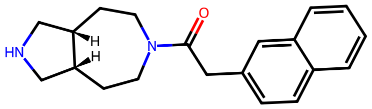	O=C(Cc1ccc2ccccc2c1)N1CC[C@@H]2CNC[C@@H]2CC1	261915658	23	308.19	2.84

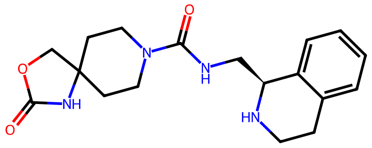	O=C1NC2(CCN(C(=O)NC[C@@H]3NCCc4ccccc43)CC2)CO1	497132409	25	344.18	1.16
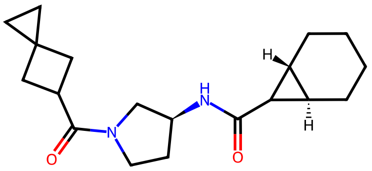	O=C(N[C@H]1CCN(C(=O)C2CC3(CC3)C2)C1)C1[C@@H]2CCCC[C@@H]12	937791685	23	316.22	2.33
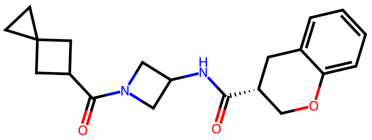	O=C(NC1CN(C(=O)C2CC3(CC3)C2)C1)[C@H]1COc2ccccc2C1	995467813	25	340.18	1.75
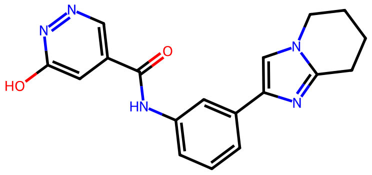	O=C(Nc1cccc(-c2cn3c(n2)CCCC3)c1)c1cnnc(O)c1	1142021401	25	335.14	2.63
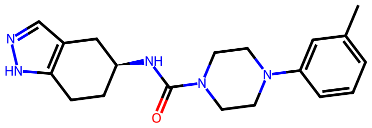	Cc1cccc(N2CCN(C(=O)N[C@H]3CCc4[nH]ncc4C3)CC2)c1	153656691	25	339.21	2.11
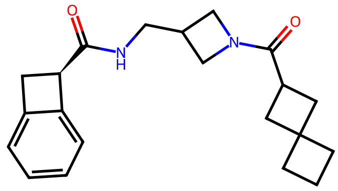	O=C(NCC1CN(C(=O)C2CC3(CCC3)C2)C1)[C@@H]1Cc2ccccc21	1003947820	25	338.2	2.48
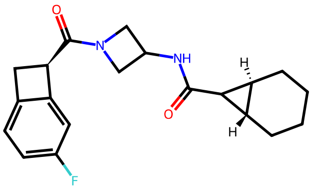	O=C(NC1CN(C(=O)[C@@H]2Cc3ccc(F)cc32)C1)C1[C@H]2CCCC[C@H]12	990662687	25	342.17	2.23
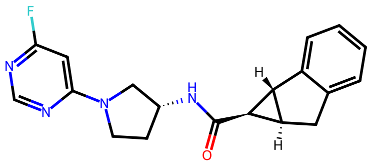	O=C(N[C@@H]1CCN(c2cc(F)ncn2)C1)[C@@H]1[C@@H]2Cc3ccccc3[C@H]21	1058566670	25	338.15	1.9

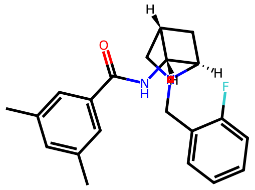	Cc1cc(C)cc(C(=O)N[C@@H]2[C@H]3C[C@H]2N(Cc2ccccc2F)C3)c1	1037694009	25	338.18	3.45
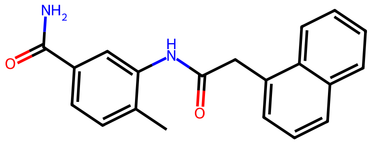	Cc1ccc(C(N)=O)cc1NC(=O)Cc1cccc2ccccc12	63947363	24	318.14	3.43
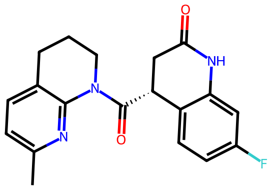	Cc1ccc2c(n1)N(C(=O)[C@@H]1CC(=O)Nc3cc(F)ccc31)CCC2	876491222	25	339.14	2.93
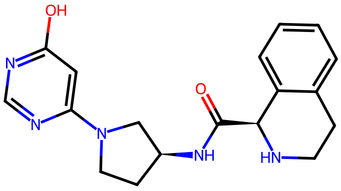	O=C(N[C@H]1CCN(c2cc(O)ncn2)C1)[C@@H]1NCCc2ccccc21	1580613266	25	339.17	0.76
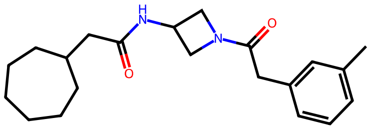	Cc1cccc(CC(=O)N2CC(NC(=O)CC3CCCCCC3)C2)c1	1000583824	25	342.23	3.23
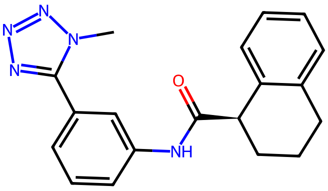	Cn1nnnc1-c1cccc(NC(=O)[C@@H]2CCCc3ccccc32)c1	248031405	25	333.16	2.94
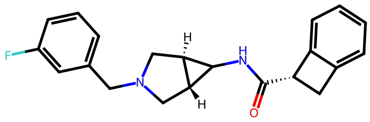	O=C(NC1[C@@H]2CN(Cc3cccc(F)c3)C[C@@H]12)[C@H]1Cc2ccccc21	961627505	25	336.16	2.71
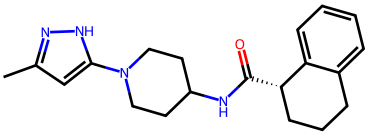	Cc1cc(N2CCC(NC(=O)[C@H]3CCCc4ccccc43)CC2)[nH]n1	275993224	25	338.21	2.92

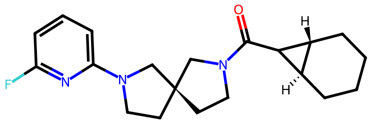	O=C(C1[C@@H]2CCCC[C@@H]12)N1CC[C@]2(CCN(c3cccc(F)n3)C2)C1	1064610228	25	343.21	3.09
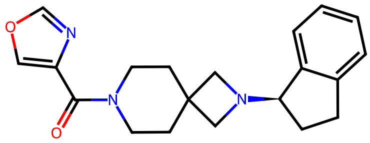	O=C(c1cocn1)N1CCC2(CC1)CN([C@@H]1CCc3ccccc31)C2	1035668547	25	337.18	2.9
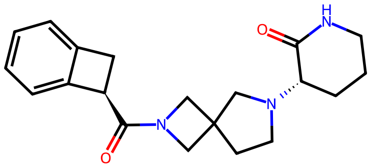	O=C1NCCC[C@@H]1N1CCC2(CN(C(=O)[C@@H]3Cc4ccccc43)C2)C1	1072931286	25	339.19	1.14
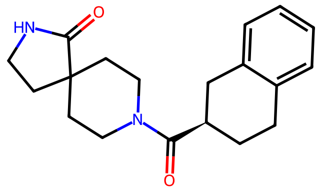	O=C([C@@H]1CCc2ccccc2C1)N1CCC2(CCNC2=O)CC1	831767323	23	312.18	1.92
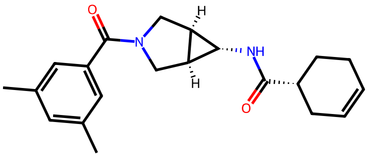	Cc1cc(C)cc(C(=O)N2C[C@H]3[C@H](NC(=O)[C@H]4CC=CCC4)[C@H]3C2)c1	976177764	25	338.2	2.85
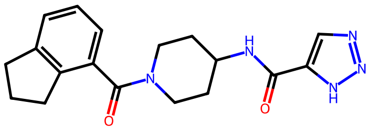	O=C(NC1CCN(C(=O)c2cccc3c2CCC3)CC1)c1cnn[nH]1	947161999	25	339.17	1.33
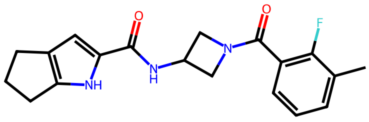	Cc1cccc(C(=O)N2CC(NC(=O)c3cc4c([nH]3)CCC4)C2)c1F	995957066	25	341.15	2.21
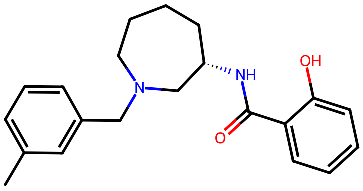	Cc1cccc(CN2CCCC[C@H](NC(=O)c3ccccc3O)C2)c1	1034102847	25	338.2	3.49

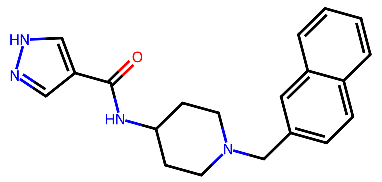	O=C(NC1CCN(Cc2ccc3ccccc3c2)CC1)c1cn[nH]c1	1002397107	25	334.18	2.96
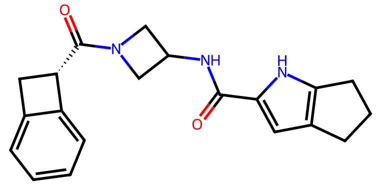	O=C(NC1CN(C(=O)[C@H]2Cc3ccccc32)C1)c1cc2c([nH]1)CCC2	995964321	25	335.16	1.78
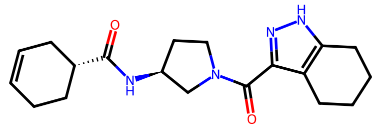	O=C(N[C@H]1CCN(C(=O)c2n[nH]c3c2CCCC3)C1)[C@H]1CC=CCC1	937664998	25	342.21	1.98
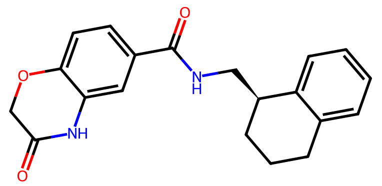	O=C1COc2ccc(C(=O)NC[C@@H]3CCCc4ccccc43)cc2N1	44623545	25	336.15	2.87
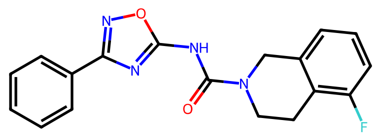	O=C(Nc1nc(-c2ccccc2)no1)N1CCc2c(F)cccc2C1	1623496867	25	338.12	3.47
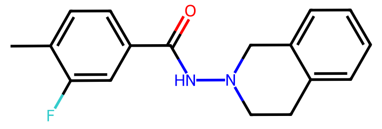	Cc1ccc(C(=O)NN2CCc3ccccc3C2)cc1F	1469892863	21	284.13	2.84
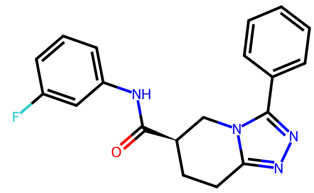	O=C(Nc1cccc(F)c1)[C@@H]1CCc2nnc(-c3ccccc3)n2C1	1521389977	25	336.14	3.29
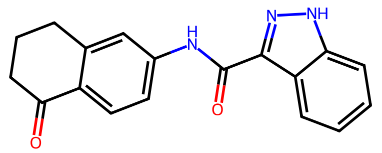	O=C1CCCc2cc(NC(=O)c3n[nH]c4ccccc34)ccc21	1299842020	23	305.12	3.33

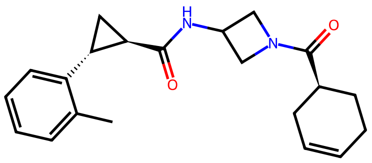	Cc1ccccc1[C@@H]1C[C@H]1C(=O)NC1CN(C(=O)[C@@H]2CC=CCC2)C1	992024297	25	338.2	2.78
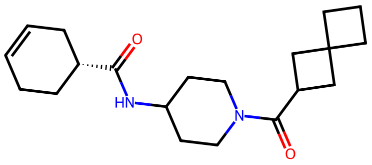	O=C(NC1CCN(C(=O)C2CC3(CCC3)C2)CC1)[C@H]1CC=CCC1	947879589	24	330.23	3.03
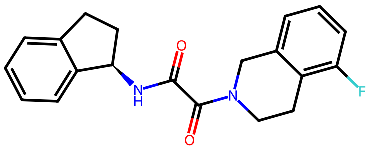	O=C(N[C@@H]1CCc2ccccc21)C(=O)N1CCc2c(F)cccc2C1	339869926	25	338.14	2.51
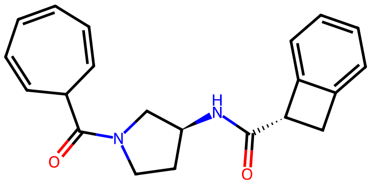	O=C(N[C@H]1CCN(C(=O)C2C=CC=CC=C2)C1)[C@H]1Cc2ccccc21	938576871	25	334.17	2.34
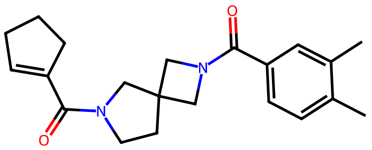	Cc1ccc(C(=O)N2CC3(CCN(C(=O)C4=CCCC4)C3)C2)cc1C	1019428155	25	338.2	3.09
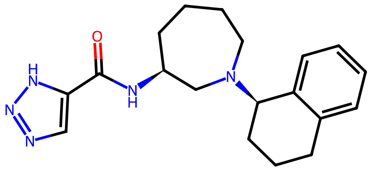	O=C(N[C@H]1CCCCN([C@@H]2CCCc3ccccc32)C1)c1cnn[nH]1	1034291872	25	339.21	2.47
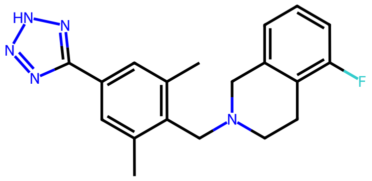	Cc1cc(-c2nn[nH]n2)cc(C)c1CN1CCc2c(F)cccc2C1	1591846978	25	337.17	3.18
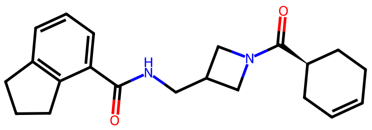	O=C(NCC1CN(C(=O)[C@@H]2CC=CCC2)C1)c1cccc2c1CCC2	1002713139	25	338.2	2.72

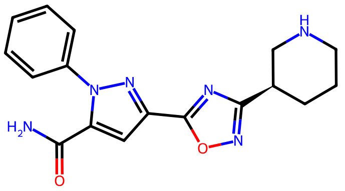	NC(=O)c1cc(-c2nc([C@@H]3CCCNC3)no2)nn1-c1ccccc1	842669753	25	338.15	1.49
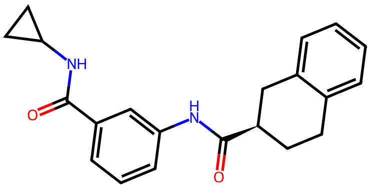	O=C(NC1CC1)c1cccc(NC(=O)[C@@H]2CCc3ccccc3C2)c1	576163157	25	334.17	3.32
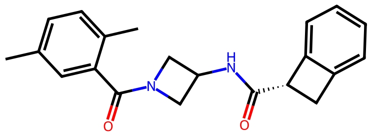	Cc1ccc(C)c(C(=O)N2CC(NC(=O)[C@H]3Cc4ccccc43)C2)c1	998691936	25	334.17	2.58
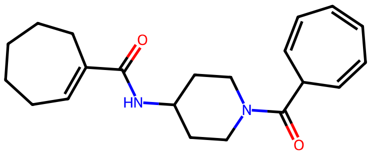	O=C(NC1CCN(C(=O)C2C=CC=CC=C2)CC1)C1=CCCCCC1	946950034	25	340.22	3.28
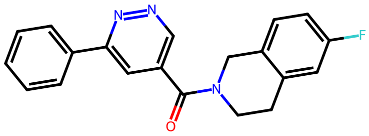	O=C(c1cnnc(-c2ccccc2)c1)N1CCc2cc(F)ccc2C1	355138310	25	333.13	3.48
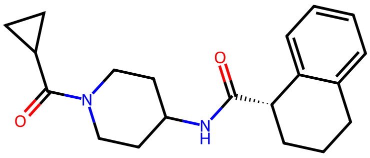	O=C(NC1CCN(C(=O)C2CC2)CC1)[C@H]1CCCc2ccccc21	344576049	24	326.2	2.62
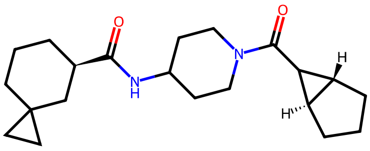	O=C(NC1CCN(C(=O)C2[C@@H]3CCC[C@@H]23)CC1)[C@@H]1CCCC2(CC2)C1	946077835	25	344.25	3.11
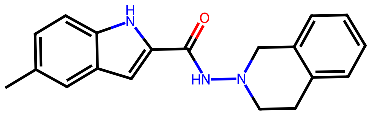	Cc1ccc2[nH]c(C(=O)NN3CCc4ccccc4C3)cc2c1	1628725918	23	305.15	3.18

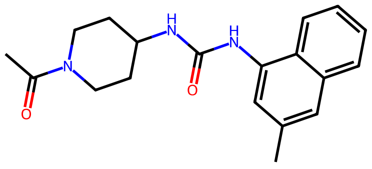	CC(=O)N1CCC(NC(=O)Nc2cc(C)cc3ccccc23)CC1	455753645	24	325.18	3.28
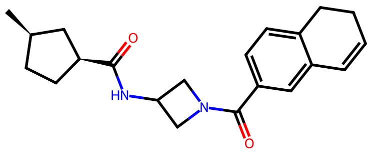	C[C@@H]1CC[C@H](C(=O)NC2CN(C(=O)c3ccc4c(c3)C=CCC4)C2)C1	991406023	25	338.2	3.02
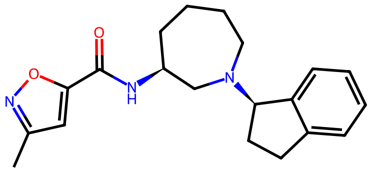	Cc1cc(C(=O)N[C@H]2CCCCN([C@@H]3CCc4ccccc43)C2)on1	1034099132	25	339.19	3.25
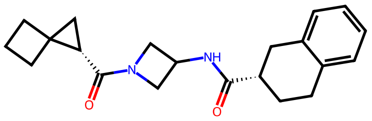	O=C(NC1CN(C(=O)[C@H]2CC23CCC3)C1)[C@H]1CCc2ccccc2C1	1000545879	25	338.2	2.31
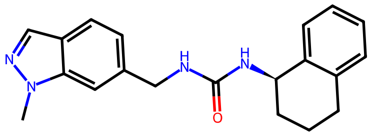	Cn1ncc2ccc(CNC(=O)N[C@@H]3CCCc4ccccc43)cc21	570869713	25	334.18	3.45
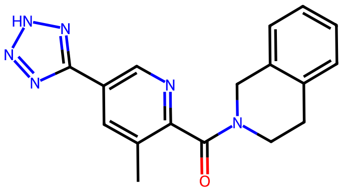	Cc1cc(-c2nn[nH]n2)cnc1C(=O)N1CCc2ccccc2C1	1591875498	24	320.14	1.77
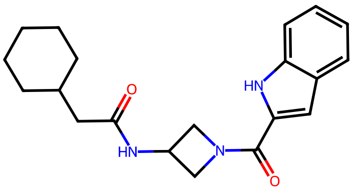	O=C(CC1CCCCC1)NC1CN(C(=O)c2cc3ccccc3[nH]2)C1	990170057	25	339.19	3.08
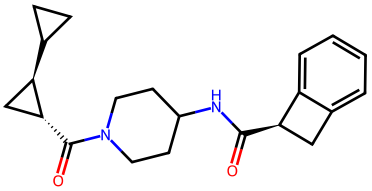	O=C(NC1CCN(C(=O)[C@@H]2C[C@H]2C2CC2)CC1)[C@@H]1Cc2ccccc21	946878003	25	338.2	2.48

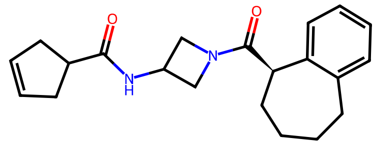	O=C(NC1CN(C(=O)[C@@H]2CCCCc3ccccc32)C1)C1CC=CC1	990587078	25	338.2	2.79
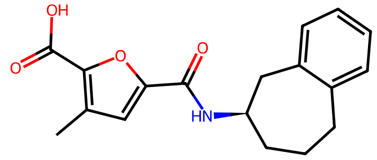	Cc1cc(C(=O)N[C@@H]2CCCc3ccccc3C2)oc1C(=O)O	1607577643	23	313.13	2.96
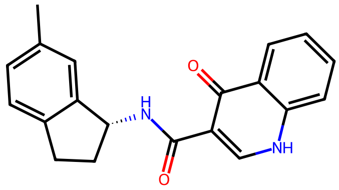	Cc1ccc2c(c1)[C@H](NC(=O)c1c[nH]c3ccccc3c1=O)CC2	592785700	24	318.14	3.25
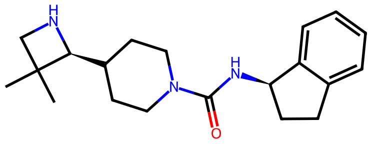	CC1(C)CN[C@H]1C1CCN(C(=O)N[C@@H]2CCc3ccccc32)CC1	416902508	24	327.23	3.09
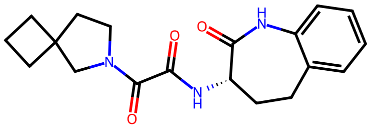	O=C(N[C@H]1CCc2ccccc2NC1=O)C(=O)N1CCC2(CCC2)C1	1327234001	25	341.17	1.46
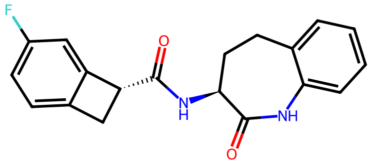	O=C1Nc2ccccc2CC[C@@H]1NC(=O)[C@@H]1Cc2ccc(F)cc21	653998862	24	324.13	2.54
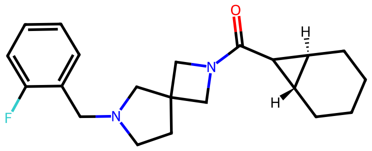	O=C(C1[C@H]2CCCC[C@H]12)N1CC2(CCN(Cc3ccccc3F)C2)C1	1072581110	25	342.21	3.3
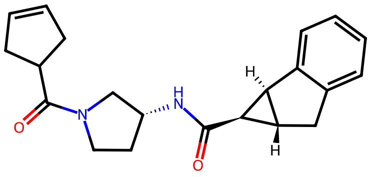	O=C(N[C@@H]1CCN(C(=O)C2CC=CC2)C1)[C@@H]1[C@H]2Cc3ccccc3[C@@H]21	940494203	25	336.18	2.26

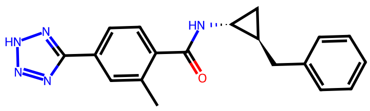	Cc1cc(-c2nn[nH]n2)ccc1C(=O)N[C@@H]1C[C@H]1Cc1ccccc1	826246777	25	333.16	2.54
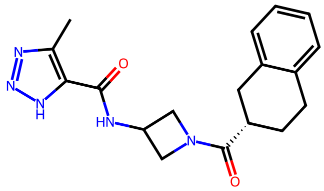	Cc1nn[nH]c1C(=O)NC1CN(C(=O)[C@H]2CCc3ccccc3C2)C1	997352830	25	339.17	0.86
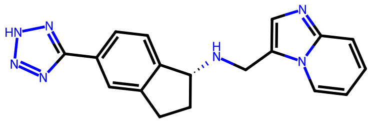	c1ccn2c(CN[C@@H]3CCc4cc(-c5nn[nH]n5)ccc43)cnc2c1	1607133421	25	331.15	2.29
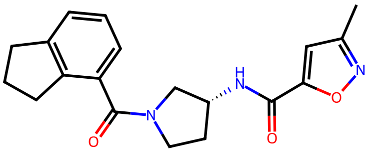	Cc1cc(C(=O)N[C@@H]2CCN(C(=O)c3cccc4c3CCC4)C2)on1	939386493	25	339.16	2.12
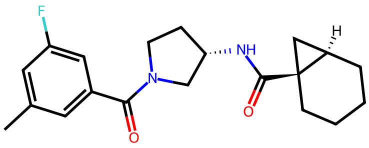	Cc1cc(F)cc(C(=O)N2CC[C@H](NC(=O)[C@]34CCCC[C@@H]3C4)C2)c1	938694731	25	344.19	3.05
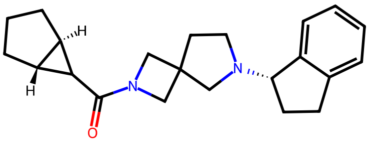	O=C(C1[C@@H]2CCC[C@@H]12)N1CC2(CCN([C@H]3CCc4ccccc43)C2)C1	1072591520	25	336.22	3.25
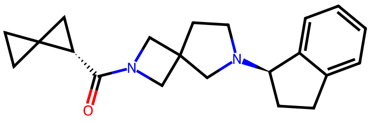	O=C([C@H]1CC12CC2)N1CC2(CCN([C@@H]3CCc4ccccc43)C2)C1	1073013327	24	322.2	3.01
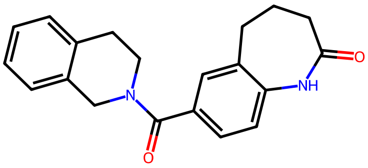	O=C1CCCc2cc(C(=O)N3CCc4ccccc4C3)ccc2N1	642254532	24	320.15	3.16

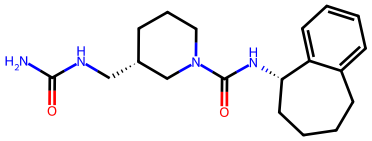	NC(=O)NC[C@@H]1CCCN(C(=O)N[C@H]2CCCCc3ccccc32)C1	72230554	25	344.22	2.54
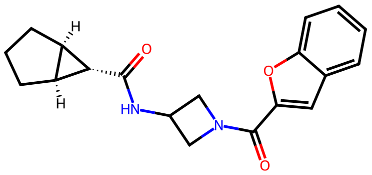	O=C(NC1CN(C(=O)c2cc3ccccc3o2)C1)[C@@H]1[C@H]2CCC[C@H]21	990711482	24	324.15	2.42
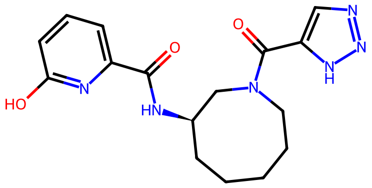	O=C(N[C@@H]1CCCCCN(C(=O)c2cnn[nH]2)C1)c1cccc(O)n1	987079202	25	344.16	0.72
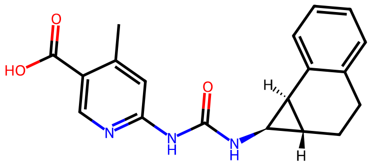	Cc1cc(NC(=O)N[C@@H]2[C@H]3CCc4ccccc4[C@@H]32)ncc1C(=O)O	1600266461	25	337.14	2.94
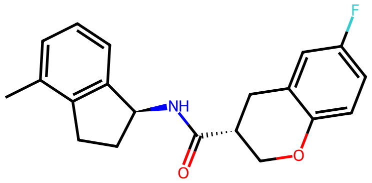	Cc1cccc2c1CC[C@@H]2NC(=O)[C@H]1COc2ccc(F)cc2C1	1341287800	24	325.15	3.49
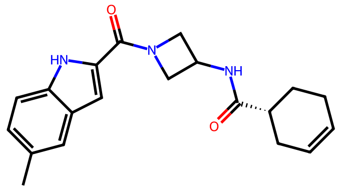	Cc1ccc2[nH]c(C(=O)N3CC(NC(=O)[C@H]4CC=CCC4)C3)cc2c1	990216943	25	337.18	2.77
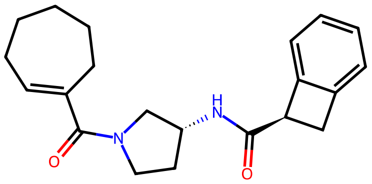	O=C(N[C@@H]1CCN(C(=O)C2=CCCCCC2)C1)[C@@H]1Cc2ccccc21	942304981	25	338.2	2.93
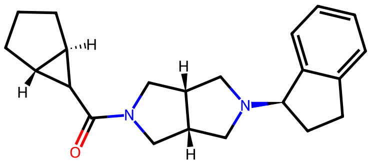	O=C(C1[C@@H]2CCC[C@@H]12)N1C[C@@H]2CN([C@@H]3CCc4ccccc43)C[C@@H]2C1	1048746901	25	336.22	3.11

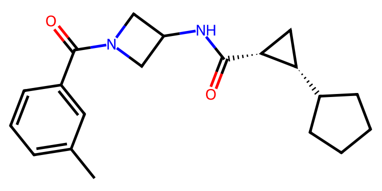	Cc1cccc(C(=O)N2CC(NC(=O)[C@@H]3C[C@@H]3C3CCCC3)C2)c1	998808327	24	326.2	2.76
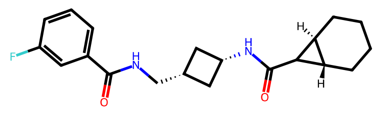	O=C(NC[C@H]1C[C@@H](NC(=O)C2[C@H]3CCCC[C@H]23)C1)c1cccc(F)c1	978536106	25	344.19	2.89
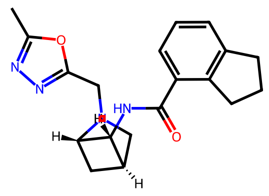	Cc1nnc(CN2C[C@H]3C[C@H]2[C@@H]3NC(=O)c2cccc3c2CCC3)o1	1037747245	25	338.17	1.87
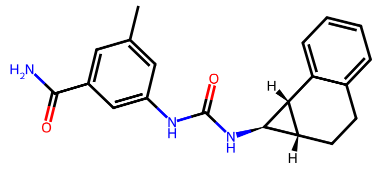	Cc1cc(NC(=O)N[C@@H]2[C@H]3CCc4ccccc4[C@H]32)cc(C(N)=O)c1	565994179	25	335.16	2.94
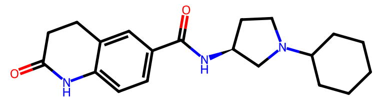	O=C1CCc2cc(C(=O)N[C@H]3CCN(C4CCCCC4)C3)ccc2N1	618663529	25	341.21	2.71
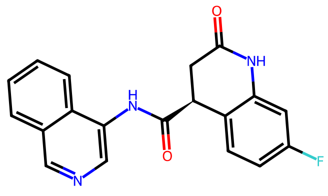	O=C1C[C@H](C(=O)Nc2cncc3ccccc23)c2ccc(F)cc2N1	89626870	25	335.11	3.44
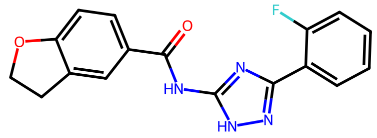	O=C(Nc1nc(-c2ccccc2F)n[nH]1)c1ccc2c(c1)CCO2	109625461	24	324.1	2.8
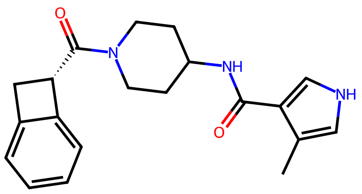	Cc1c[nH]cc1C(=O)NC1CCN(C(=O)[C@H]2Cc3ccccc32)CC1	948063045	25	337.18	2.38

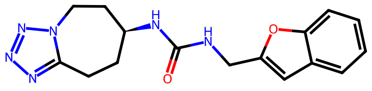	O=C(NCc1cc2ccccc2o1)N[C@H]1CCc2nnnn2CC1	569592068	24	326.15	1.62
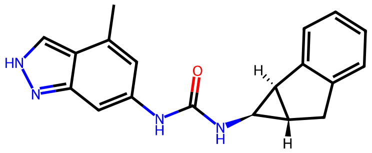	Cc1cc(NC(=O)N[C@@H]2[C@H]3Cc4ccccc4[C@@H]32)cc2n[nH]cc12	347338255	24	318.15	3.33
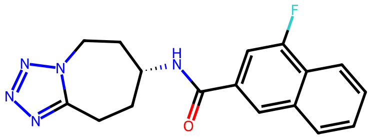	O=C(N[C@@H]1CCc2nnnn2CC1)c1cc(F)c2ccccc2c1	580180784	24	325.13	2.1
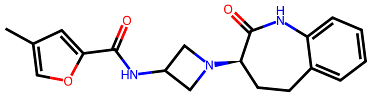	Cc1coc(C(=O)NC2CN([C@@H]3CCc4ccccc4NC3=O)C2)c1	1030816131	25	339.16	1.96
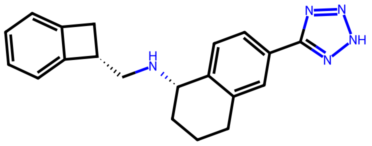	c1ccc2c(c1)C[C@@H]2CN[C@H]1CCCc2cc(-c3nn[nH]n3)ccc21	826523332	25	331.18	3.17
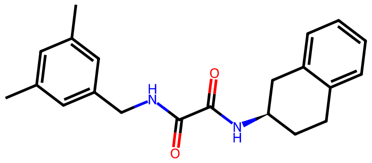	Cc1cc(C)cc(CNC(=O)C(=O)N[C@@H]2CCc3ccccc3C2)c1	668380008	25	336.18	2.59
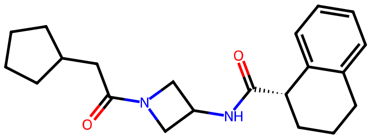	O=C(NC1CN(C(=O)CC2CCCC2)C1)[C@H]1CCCc2ccccc21	995068137	25	340.22	3.01
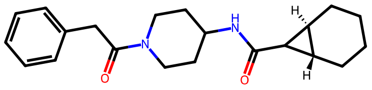	O=C(NC1CCN(C(=O)Cc2ccccc2)CC1)C1[C@H]2CCCC[C@H]12	947344907	25	340.22	2.77

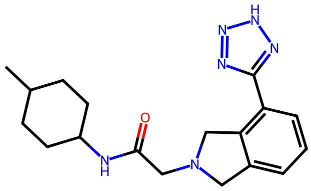	CC1CCC(NC(=O)CN2Cc3cccc(-c4nn[nH]n4)c3C2)CC1	1590233949	25	340.2	1.88
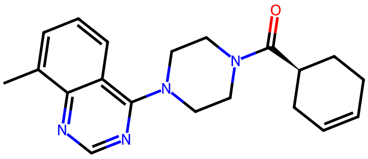	Cc1cccc2c(N3CCN(C(=O)[C@@H]4CC=CCC4)CC3)ncnc12	1055721595	25	336.2	2.94
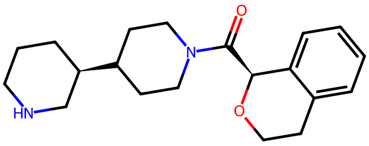	O=C([C@@H]1OCCc2ccccc21)N1CCC([C@@H]2CCCNC2)CC1	658957119	24	328.22	2.54
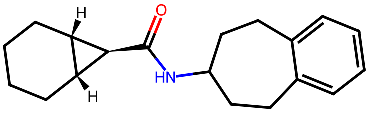	O=C(NC1CCc2ccccc2CC1)[C@@H]1[C@H]2CCCC[C@H]21	456463502	21	283.19	3.49
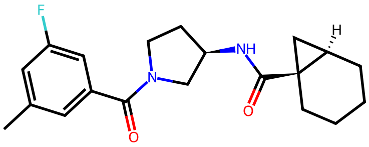	Cc1cc(F)cc(C(=O)N2CC[C@@H](NC(=O)[C@]34CCCC[C@@H]3C4)C2)c1	936243680	25	344.19	3.05
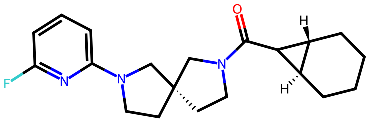	O=C(C1[C@@H]2CCCC[C@@H]12)N1CC[C@@]2(CCN(c3cccc(F)n3)C2)C1	1064610227	25	343.21	3.09
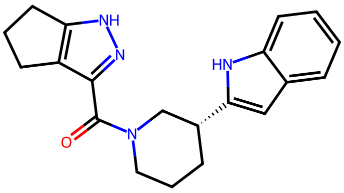	O=C(c1n[nH]c2c1CCC2)N1CCC[C@@H](c2cc3ccccc3[nH]2)C1	618122235	25	334.18	3.4
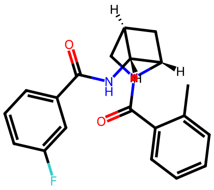	Cc1ccccc1C(=O)N1C[C@H]2C[C@H]1[C@@H]2NC(=O)c1cccc(F)c1	946608565	25	338.14	2.78

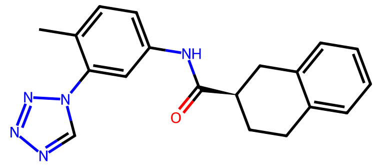	Cc1ccc(NC(=O)[C@@H]2CCc3ccccc3C2)cc1-n1cnnn1	563221511	25	333.16	2.71
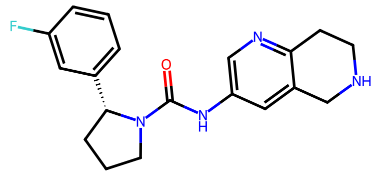	O=C(Nc1cnc2c(c1)CNCC2)N1CCC[C@@H]1c1cccc(F)c1	667793883	25	340.17	3.24
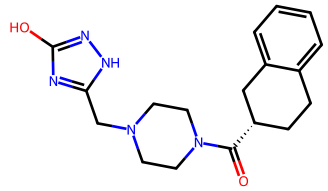	O=C([C@H]1CCc2ccccc2C1)N1CCN(Cc2nc(O)n[nH]2)CC1	950725780	25	341.19	0.96
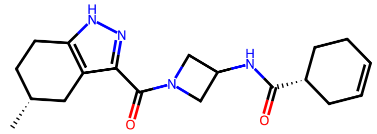	C[C@@H]1CCc2[nH]nc(C(=O)N3CC(NC(=O)[C@H]4CC=CCC4)C3)c2C1	990211344	25	342.21	1.83
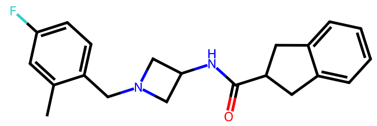	Cc1cc(F)ccc1CN1CC(NC(=O)C2Cc3ccccc3C2)C1	1031001048	25	338.18	2.85
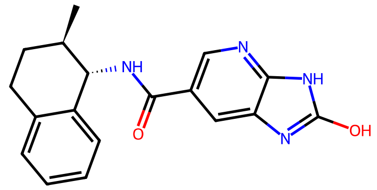	C[C@@H]1CCc2ccccc2[C@H]1NC(=O)c1cnc2[nH]c(O)nc2c1	558416261	24	322.14	2.72
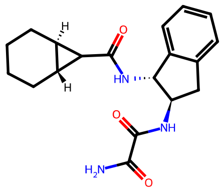	NC(=O)C(=O)N[C@@H]1Cc2ccccc2[C@H]1NC(=O)C1[C@@H]2CCCC[C@@H]12	1001957196	25	341.17	0.81
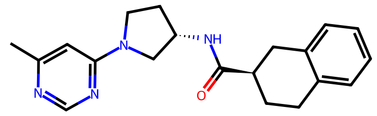	Cc1cc(N2CC[C@H](NC(=O)[C@@H]3CCc4ccccc4C3)C2)ncn1	1058559921	25	336.2	2.29

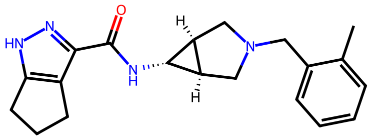	Cc1ccccc1CN1C[C@H]2[C@H](NC(=O)c3n[nH]c4c3CCC4)[C@H]2C1	960730879	25	336.2	2.07
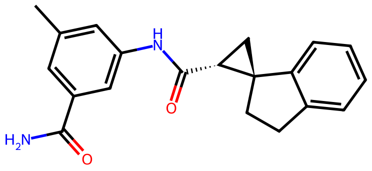	Cc1cc(NC(=O)[C@@H]2C[C@@]23CCc2ccccc23)cc(C(N)=O)c1	456173197	24	320.15	2.94
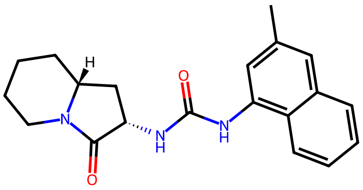	Cc1cc(NC(=O)N[C@H]2C[C@H]3CCCCN3C2=O)c2ccccc2c1	576594480	25	337.18	3.42
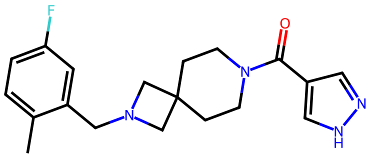	Cc1ccc(F)cc1CN1CC2(CCN(C(=O)c3cn[nH]c3)CC2)C1	1035657970	25	342.19	2.6
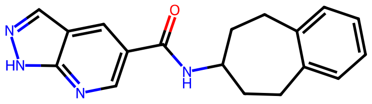	O=C(NC1CCc2ccccc2CC1)c1cnc2[nH]ncc2c1	1548714999	23	306.15	2.64
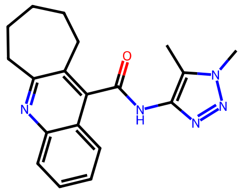	Cc1c(NC(=O)c2c3c(nc4ccccc24)CCCCC3)nnn1C	333352335	25	335.17	3.19
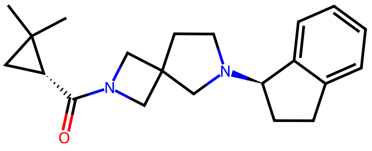	CC1(C)C[C@H]1C(=O)N1CC2(CCN([C@@H]3CCc4ccccc43)C2)C1	1072637824	24	324.22	3.25
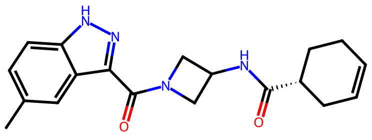	Cc1ccc2[nH]nc(C(=O)N3CC(NC(=O)[C@H]4CC=CCC4)C3)c2c1	990213446	25	338.17	2.17

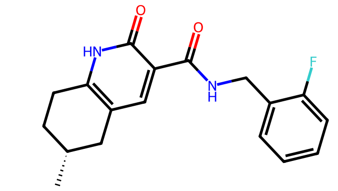	C[C@@H]1CCc2[nH]c(=O)c(C(=O)NC<br>c3ccccc3F)cc2C1	49551229	23	314.14	2.57
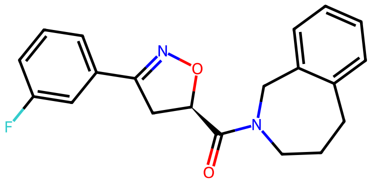	O=C([C@H]1CC(c2cccc(F)c2)=NO1)N<br>1CCCc2ccccc2C1	730202639	25	338.14	3.29
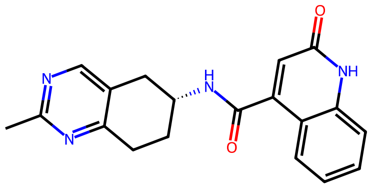	Cc1ncc2c(n1)CC[C@@H](NC(=O)c1c<br>c(=O)[nH]c3ccccc13)C2	29313499	25	334.14	1.91
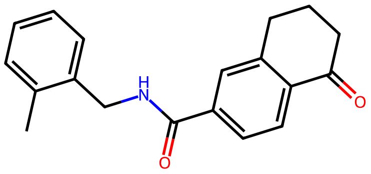	Cc1ccccc1CNC(=O)c1ccc2c(c1)CCCC<br>2=O	338580155	22	293.14	3.44
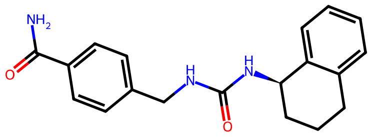	NC(=O)c1ccc(CNC(=O)N[C@@H]2C<br>CCc3ccccc32)cc1	47622416	24	323.16	2.66
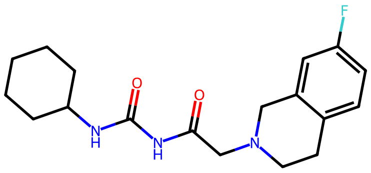	O=C(CN1CCc2ccc(F)cc2C1)NC(=O)N<br>C1CCCCC1	134034530	24	333.19	2.34
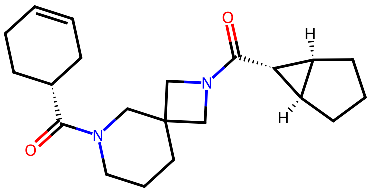	O=C([C@H]1CC=CCC1)N1CCCC2(C<br>1)CN(C(=O)[C@@H]1[C@H]3CCC[C<br>@H]31)C2	1010600533	25	342.23	2.84
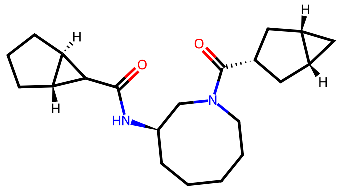	O=C(N[C@@H]1CCCCCN(C(=O)[C@<br>@H]2C[C@@H]3C[C@@H]3C2)C1)C<br>1[C@@H]2CCC[C@@H]12	987114381	25	344.25	2.97

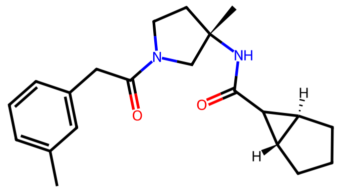	Cc1cccc(CC(=O)N2CC[C@](C)(NC(=O)C3[C@H]4CCC[C@H]34)C2)c1	956803741	25	340.22	2.69
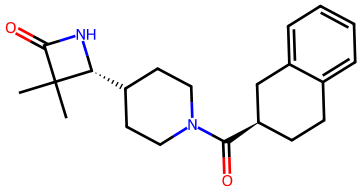	CC1(C)C(=O)N[C@@H]1C1CCN(C(=O)[C@@H]2CCc3ccccc3C2)CC1	577184881	25	340.22	2.55
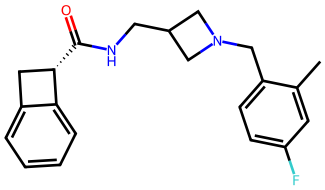	Cc1cc(F)ccc1CN1CC(CNC(=O)[C@H]2Cc3ccccc32)C1	1031944521	25	338.18	3.02
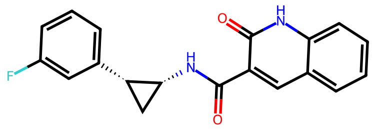	O=C(N[C@@H]1C[C@@H]1c1cccc(F)c1)c1cc2ccccc2[nH]c1=O	67333488	24	322.11	2.95
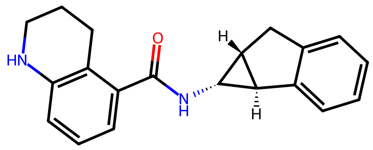	O=C(N[C@H]1[C@@H]2c3ccccc3C[C@H]21)c1cccc2c1CCCN2	130261082	23	304.16	3.11
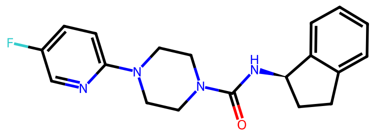	O=C(N[C@@H]1CCc2ccccc21)N1CCN(c2ccc(F)cn2)CC1	91599569	25	340.17	2.74
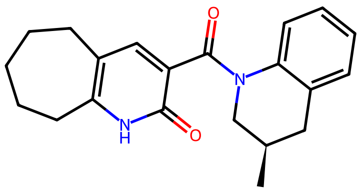	C[C@@H]1Cc2ccccc2N(C(=O)c2cc3c([nH]c2=O)CCCCC3)C1	78116257	25	336.18	3.48
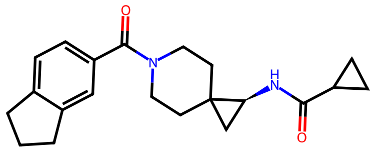	O=C(N[C@H]1CC12CCN(C(=O)c1ccc3c(c1)CCC3)CC2)C1CC1	962485208	25	338.2	2.7

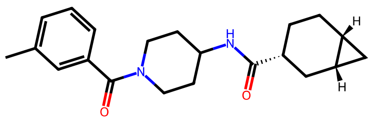	Cc1cccc(C(=O)N2CCC(NC(=O)[C@@H]3CC[C@@H]4C[C@@H]4C3)CC2)c1	943836125	25	340.22	3.15
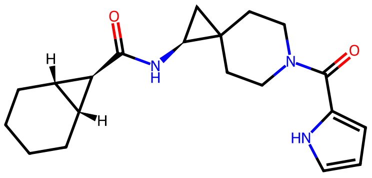	O=C(N[C@H]1CC12CCN(C(=O)c1ccc[nH]1)CC2)[C@@H]1[C@H]2CCCC[C@H]21	963444117	25	341.21	2.56
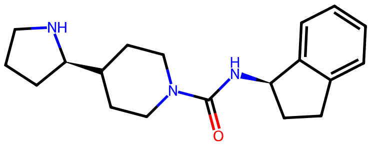	O=C(N[C@@H]1CCc2ccccc21)N1CCC([C@H]2CCCN2)CC1	416929458	23	313.22	2.85
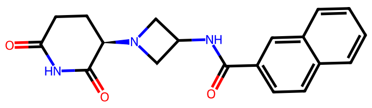	O=C1CC[C@@H](N2CC(NC(=O)c3ccc4ccccc4c3)C2)C(=O)N1	1031304402	25	337.14	1.06
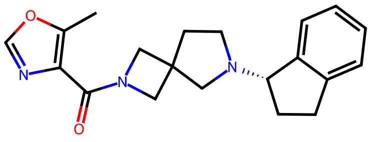	Cc1ocnc1C(=O)N1CC2(CCN([C@H]3CCc4ccccc43)C2)C1	1072528631	25	337.18	2.82
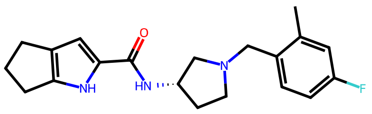	Cc1cc(F)ccc1CN1CC[C@H](NC(=O)c2cc3c([nH]2)CCC3)C1	1015979913	25	341.19	2.96
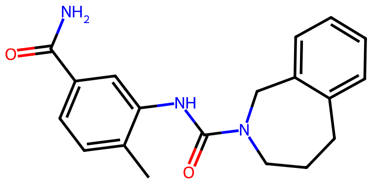	Cc1ccc(C(N)=O)cc1NC(=O)N1CCCc2ccccc2C1	171637222	24	323.16	3.07
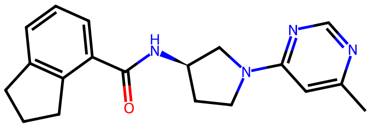	Cc1cc(N2CC[C@@H](NC(=O)c3cccc4c3CCC4)C2)ncn1	1058385887	24	322.18	2.28

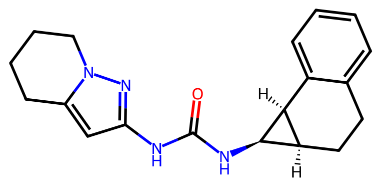	O=C(Nc1cc2n(n1)CCCC2)N[C@@H]1[C@@H]2CCc3ccccc3[C@@H]21	287406809	24	322.18	3.07
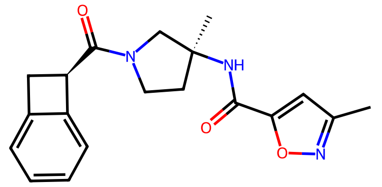	Cc1cc(C(=O)N[C@@]2(C)CCN(C(=O)[C@@H]3Cc4ccccc43)C2)on1	956691858	25	339.16	2.04
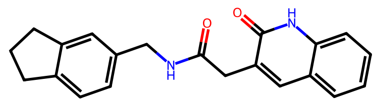	O=C(Cc1cc2ccccc2[nH]c1=O)NCc1ccc2c(c1)CCC2	76385076	25	332.15	2.88
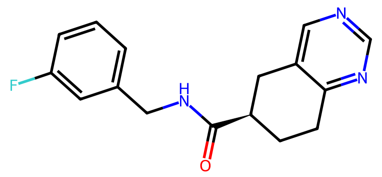	O=C(NCc1cccc(F)c1)[C@@H]1CCc2ncncc2C1	648233827	21	285.13	2.04
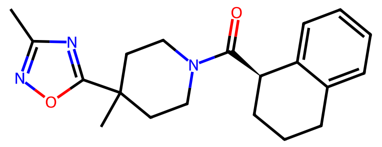	Cc1noc(C2(C)CCN(C(=O)[C@@H]3CCCc4ccccc43)CC2)n1	364698921	25	339.19	3.38
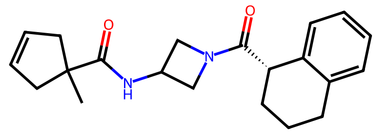	CC1(C(=O)NC2CN(C(=O)[C@H]3CCCc4ccccc43)C2)CC=CC1	999071986	25	338.2	2.79
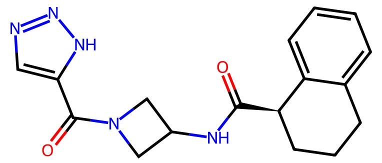	O=C(NC1CN(C(=O)c2cnn[nH]2)C1)[C@@H]1CCCc2ccccc21	995078858	24	325.15	0.87
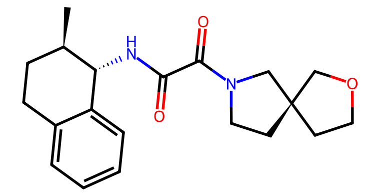	C[C@@H]1CCc2ccccc2[C@H]1NC(=O)C(=O)N1CC[C@@]2(CCOC2)C1	673299613	25	342.19	2.07

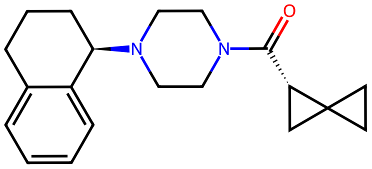	O=C([C@H]1CC12CC2)N1CCN([C@@H]2CCCc3ccccc32)CC1	950710787	23	310.2	3.01
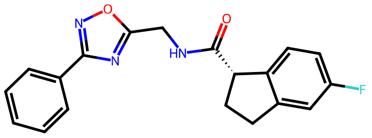	O=C(NCc1nc(-c2ccccc2)no1)[C@H]1CCc2cc(F)ccc21	1611332507	25	337.12	3.22
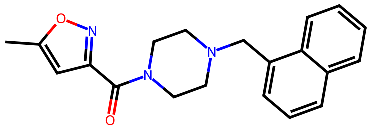	Cc1cc(C(=O)N2CCN(Cc3cccc4ccccc34)CC2)no1	58391633	25	335.16	3.09
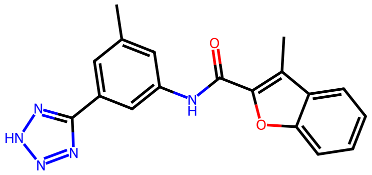	Cc1cc(NC(=O)c2oc3ccccc3c2C)cc(-c2nn[nH]n2)c1	1592034170	25	333.12	3.48
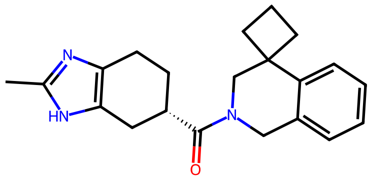	Cc1nc2c([nH]1)C[C@@H](C(=O)N1Cc3ccccc3C3(CCC3)C1)CC2	1549382918	25	335.2	3.29
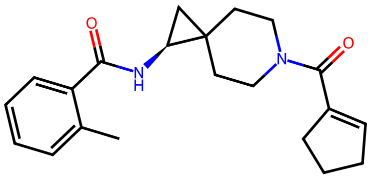	Cc1ccccc1C(=O)N[C@H]1CC12CCN(C(=O)C1=CCCC1)CC2	962812879	25	338.2	3.22
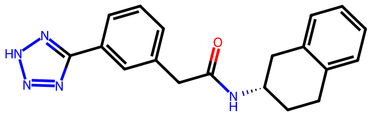	O=C(Cc1cccc(-c2nn[nH]n2)c1)N[C@H]1CCc2ccccc2C1	823093434	25	333.16	2.08
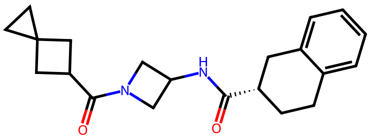	O=C(NC1CN(C(=O)C2CC3(CC3)C2)C1)[C@H]1CCc2ccccc2C1	1000537327	25	338.2	2.31

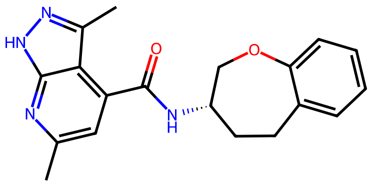	Cc1cc(C(=O)N[C@H]2CCc3ccccc3OC2)c2c(C)n[nH]c2n1	338041885	25	336.16	2.7
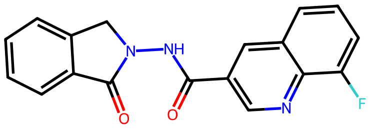	O=C(NN1Cc2ccccc2C1=O)c1cnc2c(F)cccc2c1	816038643	24	321.09	2.67
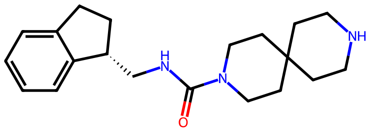	O=C(NC[C@H]1CCc2ccccc21)N1CCC2(CCNCC2)CC1	417587669	24	327.23	2.89
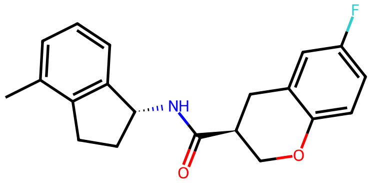	Cc1cccc2c1CC[C@H]2NC(=O)[C@@H]1COc2ccc(F)cc2C1	1341287798	24	325.15	3.49
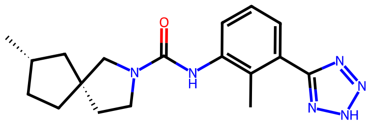	Cc1c(NC(=O)N2CC[C@@]3(CC[C@H](C)C3)C2)cccc1-c1nn[nH]n1	826241893	25	340.2	3.22
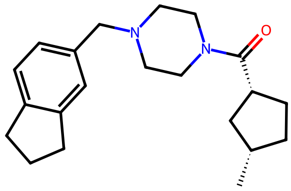	C[C@H]1CC[C@@H](C(=O)N2CCN(Cc3ccc4c(c3)CCC4)CC2)C1	951176083	24	326.24	3.26
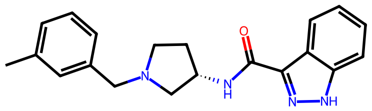	Cc1cccc(CN2CC[C@H](NC(=O)c3n[nH]c4ccccc34)C2)c1	1016160154	25	334.18	2.88
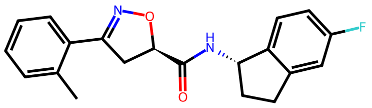	Cc1ccccc1C1=NO[C@@H](C(=O)N[C@H]2CCc3cc(F)ccc32)C1	1534972390	25	338.14	3.43

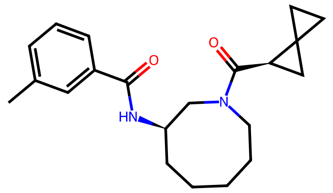	Cc1cccc(C(=O)N[C@@H]2CCCCCN(C(=O)[C@@H]3CC34CC4)C2)c1	987168600	25	340.22	3.3
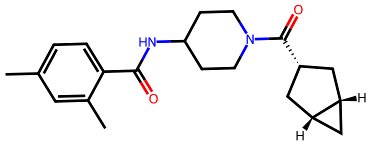	Cc1ccc(C(=O)NC2CCN(C(=O)[C@@H]3C[C@@H]4C[C@@H]4C3)CC2)c(C)c1	947909315	25	340.22	3.07
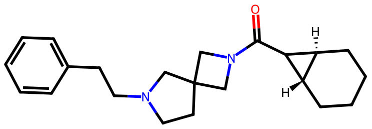	O=C(C1[C@H]2CCCC[C@H]12)N1CC2(CCN(CCc3ccccc3)C2)C1	1072581280	25	338.24	3.2
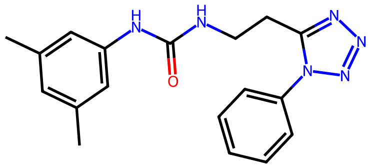	Cc1cc(C)cc(NC(=O)NCCc2nnnn2-c2ccccc2)c1	337822863	25	336.17	2.64
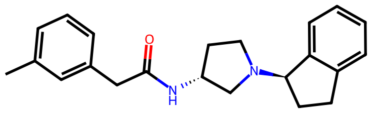	Cc1cccc(CC(=O)N[C@@H]2CCN([C@@H]3CCc4ccccc43)C2)c1	1015644415	25	334.2	3.42
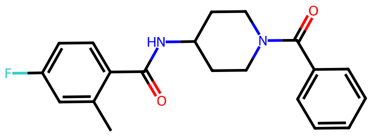	Cc1cc(F)ccc1C(=O)NC1CCN(C(=O)c2ccccc2)CC1	178893749	25	340.16	3.17
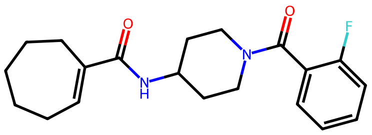	O=C(NC1CCN(C(=O)c2ccccc2F)CC1)C1=CCCCCC1	128960102	25	344.19	3.44
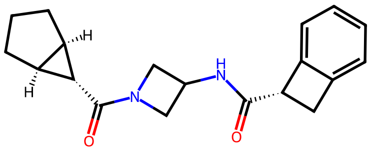	O=C(NC1CN(C(=O)[C@@H]2[C@H]3CCC[C@H]32)C1)[C@H]1Cc2ccccc21	998685012	23	310.17	1.7

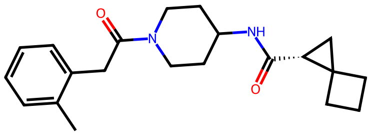	Cc1ccccc1CC(=O)N1CCC(NC(=O)[C@@H]2CC23CCC3)CC1	946717923	25	340.22	2.83
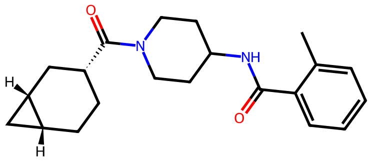	Cc1ccccc1C(=O)NC1CCN(C(=O)[C@@H]2CC[C@@H]3C[C@@H]3C2)CC1	421425416	25	340.22	3.15
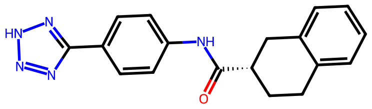	O=C(Nc1ccc(-c2nn[nH]n2)cc1)[C@H]1CCc2ccccc2C1	1464618341	24	319.14	2.61
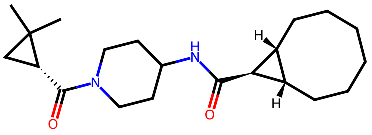	CC1(C)C[C@H]1C(=O)N1CCC(NC(=O)[C@@H]2[C@H]3CCCCCC[C@H]32)CC1	946492296	25	346.26	3.36
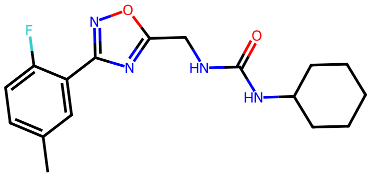	Cc1ccc(F)c(-c2noc(CNC(=O)NC3CCCCC3)n2)c1	278125133	24	332.16	3.32
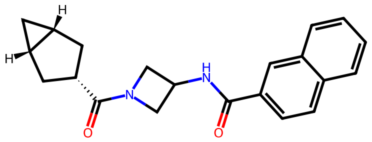	O=C(NC1CN(C(=O)[C@@H]2C[C@@H]3C[C@@H]3C2)C1)c1ccc2ccccc2c1	999453754	25	334.17	2.83
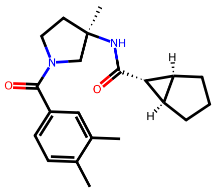	Cc1ccc(C(=O)N2CC[C@@](C)(NC(=O)[C@@H]3[C@H]4CCC[C@H]43)C2)cc1C	956803589	25	340.22	3.07
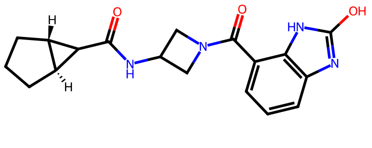	O=C(NC1CN(C(=O)c2cccc3nc(O)[nH]c23)C1)C1[C@H]2CCC[C@H]12	990699338	25	340.15	1.26

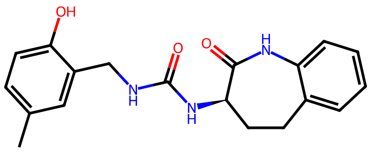	Cc1ccc(O)c(CNC(=O)N[C@@H]2CCc3ccccc3NC2=O)c1	875702480	25	339.16	2.45
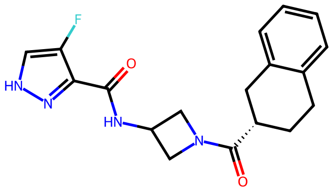	O=C(NC1CN(C(=O)[C@H]2CCc3ccccc3C2)C1)c1n[nH]cc1F	999194464	25	342.15	1.29
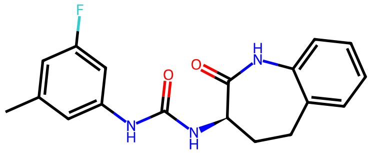	Cc1cc(F)cc(NC(=O)N[C@@H]2CCc3ccccc3NC2=O)c1	1667871769	24	327.14	3.21
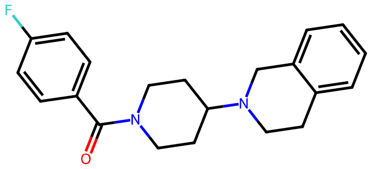	O=C(c1ccc(F)cc1)N1CCC(N2CCc3ccccc3C2)CC1	352713317	25	338.18	3.49
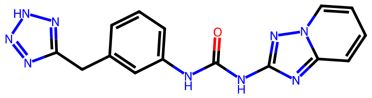	O=C(Nc1cccc(Cc2nn[nH]n2)c1)Nc1nc2ccccn2n1	1216670277	25	335.12	1.48
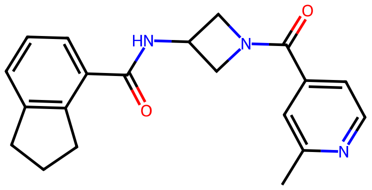	Cc1cc(C(=O)N2CC(NC(=O)c3cccc4c3CCC4)C2)ccn1	994403063	25	335.16	2.13
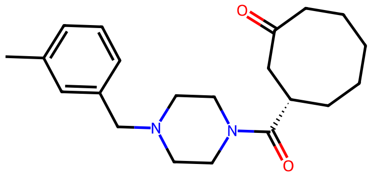	Cc1cccc(CN2CCN(C(=O)[C@H]3CCCCCC(=O)C3)CC2)c1	362467972	25	342.23	3.18
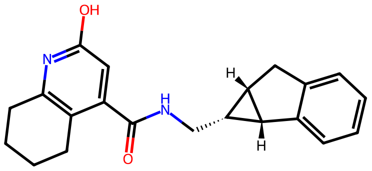	O=C(NC[C@H]1[C@H]2c3ccccc3C[C@H]21)c1cc(O)nc2c1CCCC2	934913625	25	334.17	2.98

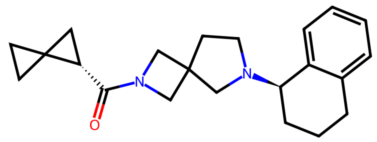	O=C([C@H]1CC12CC2)N1CC2(CCN([C@@H]3CCCc4ccccc43)C2)C1	1073012104	25	336.22	3.4
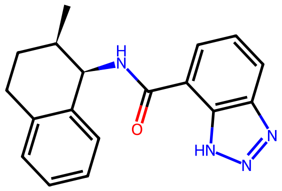	C[C@@H]1CCc2ccccc2[C@@H]1NC(=O)c1cccc2nn[nH]c12	526119683	23	306.15	3.01
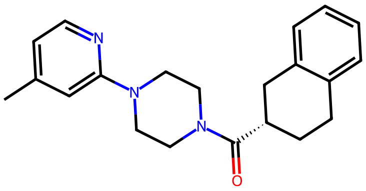	Cc1ccnc(N2CCN(C(=O)[C@H]3CCc4ccccc4C3)CC2)c1	514733995	25	335.2	2.84
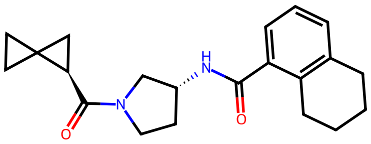	O=C(N[C@@H]1CCN(C(=O)[C@@H]2CC23CC3)C1)c1cccc2c1CCCC2	936263758	25	338.2	2.7
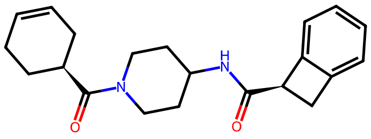	O=C(NC1CCN(C(=O)[C@@H]2CC=CCC2)CC1)[C@@H]1Cc2ccccc21	944107489	25	338.2	2.79
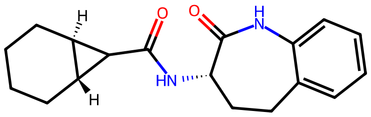	O=C(N[C@H]1CCc2ccccc2NC1=O)C1[C@@H]2CCCC[C@@H]12	654002299	22	298.17	2.49
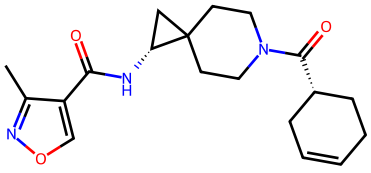	Cc1nocc1C(=O)N[C@@H]1CC12CCN(C(=O)[C@H]1CC=CCC1)CC2	965008923	25	343.19	2.45
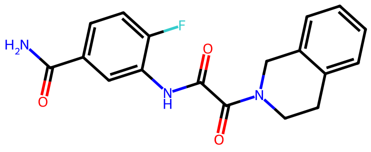	NC(=O)c1ccc(F)c(NC(=O)C(=O)N2CCc3ccccc3C2)c1	80217850	25	341.12	1.45

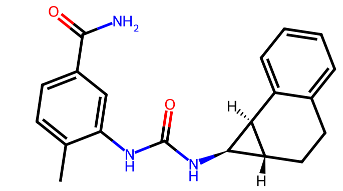	Cc1ccc(C(N)=O)cc1NC(=O)N[C@@H]1[C@H]2CCc3ccccc3[C@@H]21	155460015	25	335.16	2.94
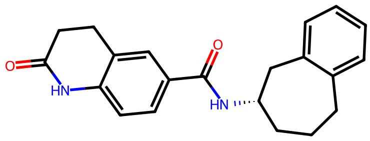	O=C1CCc2cc(C(=O)N[C@H]3CCCc4ccccc4C3)ccc2N1	888254384	25	334.17	3.25
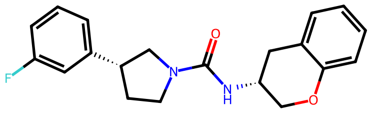	O=C(N[C@H]1COc2ccccc2C1)N1CC[C@H](c2cccc(F)c2)C1	552634903	25	340.16	3.33
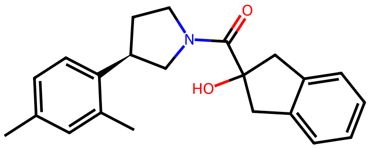	Cc1ccc([C@H]2CCN(C(=O)C3(O)Cc4ccccc4C3)C2)c(C)c1	1321062341	25	335.19	3.15
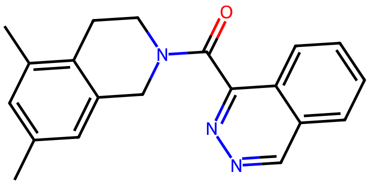	Cc1cc(C)c2c(c1)CN(C(=O)c1nncc3ccccc13)CC2	935476367	24	317.15	3.45
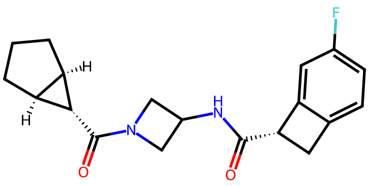	O=C(NC1CN(C(=O)[C@@H]2[C@H]3CCC[C@H]32)C1)[C@H]1Cc2ccc(F)cc21	998486439	24	328.16	1.84
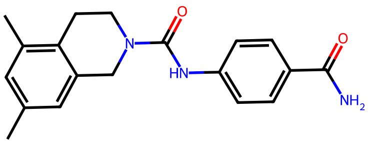	Cc1cc(C)c2c(c1)CN(C(=O)Nc1ccc(C(N)=O)cc1)CC2	186496337	24	323.16	2.99
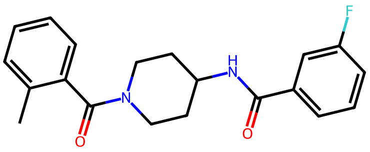	Cc1ccccc1C(=O)N1CCC(NC(=O)c2cccc(F)c2)CC1	75057866	25	340.16	3.17

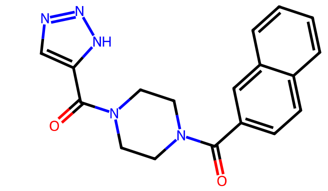	O=C(c1ccc2ccccc2c1)N1CCN(C(=O)c2cnn[nH]2)CC1	1011445309	25	335.14	1.56
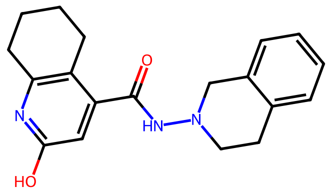	O=C(NN1CCc2ccccc2C1)c1cc(O)nc2c1CCCC2	825237895	24	323.16	2.37
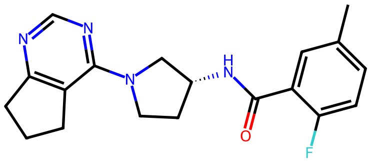	Cc1ccc(F)c(C(=O)N[C@@H]2CCN(c3ncnc4c3CCC4)C2)c1	1058560095	25	340.17	2.42
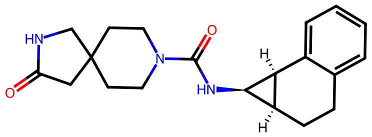	O=C1CC2(CCN(C(=O)N[C@@H]3[C@@H]4CCc5ccccc5[C@@H]43)CC2)CN1	269450120	25	339.19	2.03
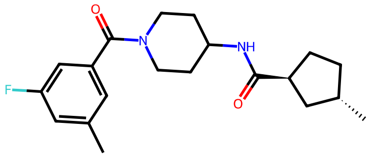	Cc1cc(F)cc(C(=O)N2CCC(NC(=O)[C@H]3CC[C@H](C)C3)CC2)c1	948549822	25	346.21	3.29
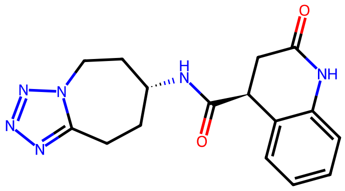	O=C1C[C@H](C(=O)N[C@@H]2CCc3nnnn3CC2)c2ccccc2N1	1418054928	24	326.15	0.62
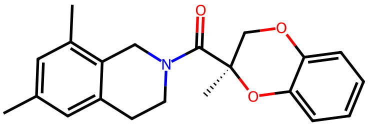	Cc1cc(C)c2c(c1)CCN(C(=O)[C@@]1(C)COc3ccccc3O1)C2	1628060042	25	337.17	3.42
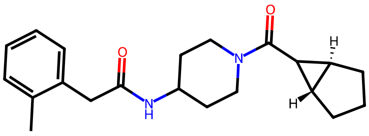	Cc1ccccc1CC(=O)NC1CCN(C(=O)C2[C@H]3CCC[C@H]23)CC1	946091832	25	340.22	2.69

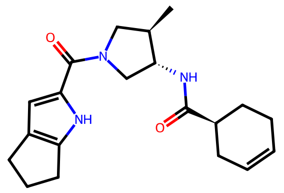	C[C@@H]1CN(C(=O)c2cc3c([nH]2)CCC3)C[C@H]1NC(=O)[C@@H]1CC=CCC1	952380391	25	341.21	2.44
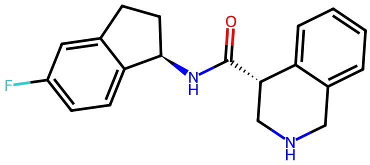	O=C(N[C@@H]1CCc2cc(F)ccc21)[C@H]1CNCc2ccccc21	580069636	23	310.15	2.82
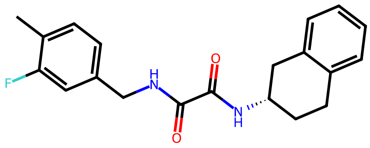	Cc1ccc(CNC(=O)C(=O)N[C@H]2CCc3ccccc3C2)cc1F	339700947	25	340.16	2.42
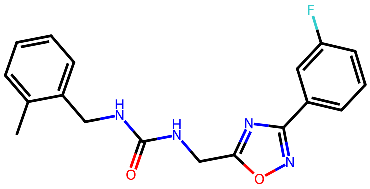	Cc1ccccc1CNC(=O)NCc1nc(-c2cccc(F)c2)no1	365127598	25	340.13	3.18
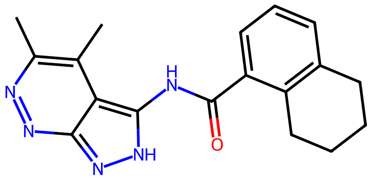	Cc1nnc2n[nH]c(NC(=O)c3cccc4c3CCCC4)c2c1C	614655120	24	321.16	3.1
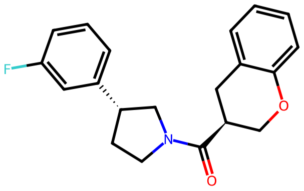	O=C([C@@H]1COc2ccccc2C1)N1CC[C@H](c2cccc(F)c2)C1	591365900	24	325.15	3.39
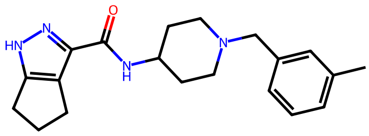	Cc1cccc(CN2CCC(NC(=O)c3n[nH]c4c3CCC4)CC2)c1	120335094	25	338.21	2.6
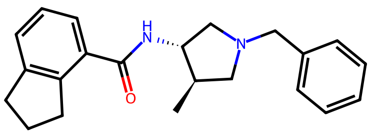	C[C@@H]1CN(Cc2ccccc2)C[C@H]1NC(=O)c1cccc2c1CCC2	1081028427	25	334.2	3.43

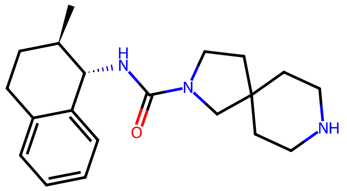	C[C@@H]1CCc2ccccc2[C@H]1NC(=O)N1CCC2(CCNCC2)C1	417962760	24	327.23	3.1
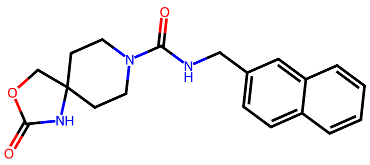	O=C1NC2(CCN(C(=O)NCc3ccc4ccccc4c3)CC2)CO1	495050606	25	339.16	2.62
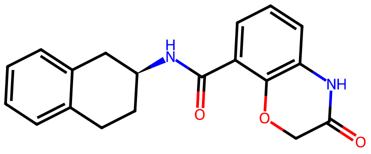	O=C1COc2c(cccc2C(=O)N[C@H]2CCc3ccccc3C2)N1	354061398	24	322.13	2.3
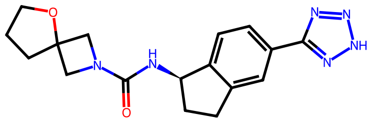	O=C(N[C@@H]1CCc2cc(-c3nn[nH]n3)ccc21)N1CC2(CCCO2)C1	1606684539	25	340.16	1.43
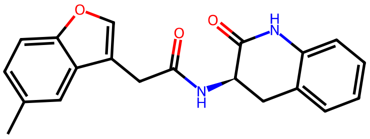	Cc1ccc2occ(CC(=O)N[C@@H]3Cc4ccccc4NC3=O)c2c1	1643362028	25	334.13	2.96
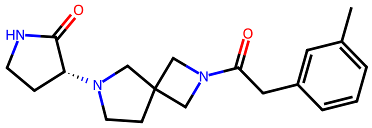	Cc1cccc(CC(=O)N2CC3(CCN([C@@H]4CCNC4=O)C3)C2)c1	1072928439	24	327.19	0.96
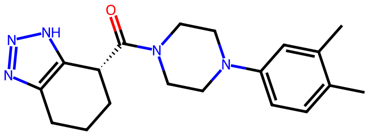	Cc1ccc(N2CCN(C(=O)[C@@H]3CCCc4nn[nH]c43)CC2)cc1C	831330535	25	339.21	2.19
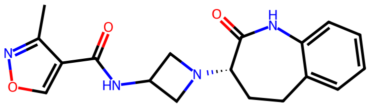	Cc1nocc1C(=O)NC1CN([C@H]2CCc3ccccc3NC2=O)C1	1030887254	25	340.15	1.35

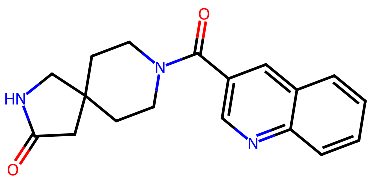	O=C1CC2(CCN(C(=O)c3cnc4ccccc4c3)CC2)CN1	324326925	23	309.15	1.98
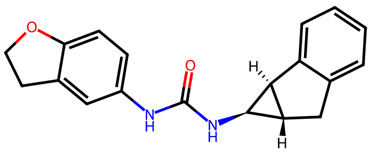	O=C(Nc1ccc2c(c1)CCO2)N[C@@H]1[C@H]2Cc3ccccc3[C@@H]21	178198923	23	306.14	3.08
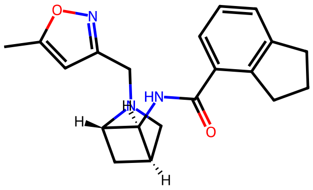	Cc1cc(CN2C[C@H]3C[C@H]2[C@H]3NC(=O)c2cccc3c2CCC3)no1	1037741334	25	337.18	2.47
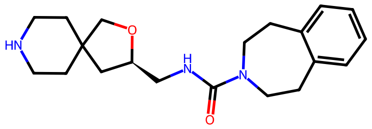	O=C(NC[C@H]1CC2(CCNCC2)CO1)N1CCc2ccccc2CC1	892776445	25	343.23	1.96
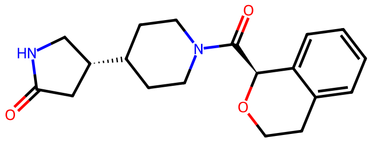	O=C1C[C@@H](C2CCN(C(=O)[C@@H]3OCCc4ccccc43)CC2)CN1	1551706744	24	328.18	1.68
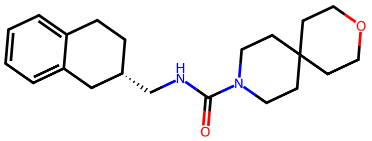	O=C(NC[C@H]1CCc2ccccc2C1)N1CCC2(CCOCC2)CC1	671204512	25	342.23	3.39
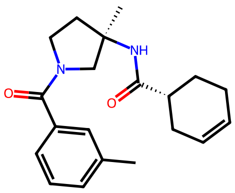	Cc1cccc(C(=O)N2CC[C@@](C)(NC(=O)[C@H]3CC=CCC3)C2)c1	956700419	24	326.2	3.07
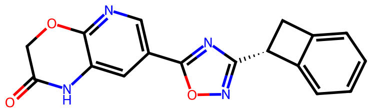	O=C1COc2ncc(-c3nc([C@@H]4Cc5ccccc54)no3)cc2N1	547002461	24	320.09	2.15

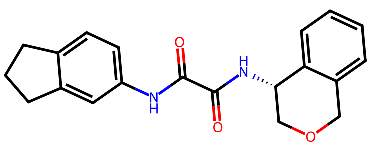	O=C(Nc1ccc2c(c1)CCC2)C(=O)N[C@H]1COCc2ccccc21	419778291	25	336.15	2.5
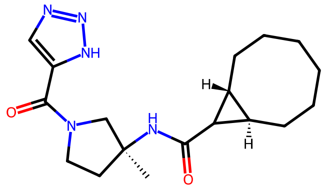	C[C@]1(NC(=O)C2[C@@H]3CCCCCC[C@@H]23)CCN(C(=O)c2cnn[nH]2)C1	957717973	25	345.22	1.74
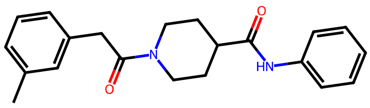	Cc1cccc(CC(=O)N2CCC(C(=O)Nc3ccccc3)CC2)c1	242783400	25	336.18	3.41
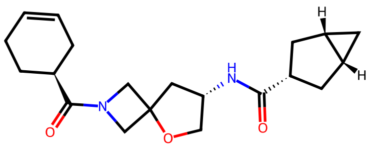	O=C(N[C@@H]1COC2(C1)CN(C(=O)[C@@H]1CC=CCC1)C2)[C@@H]1C[C@@H]2C[C@@H]2C1	965785059	25	344.21	1.87
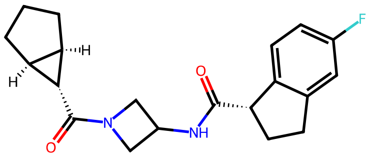	O=C(NC1CN(C(=O)[C@@H]2[C@H]3CCC[C@H]32)C1)[C@H]1CCc2cc(F)ccc21	996099001	25	342.17	2.23
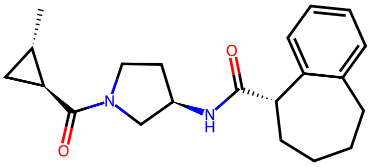	C[C@H]1C[C@@H]1C(=O)N1CC[C@@H](NC(=O)[C@H]2CCCCc3ccccc32)C1	941123890	25	340.22	2.87
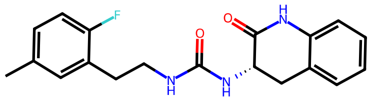	Cc1ccc(F)c(CCNC(=O)N[C@H]2Cc3ccccc3NC2=O)c1	637196137	25	341.15	2.54
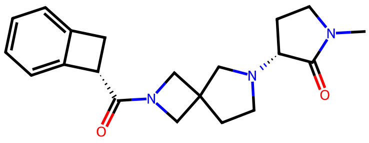	CN1CC[C@@H](N2CCC3(CN(C(=O)[C@H]4Cc5ccccc54)C3)C2)C1=O	1072932698	25	339.19	1.09

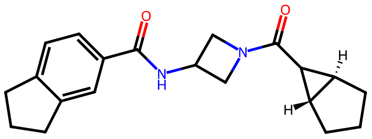	O=C(NC1CN(C(=O)C2[C@H]3CCC[C@H]23)C1)c1ccc2c(c1)CCC2	991359638	24	324.18	2.16
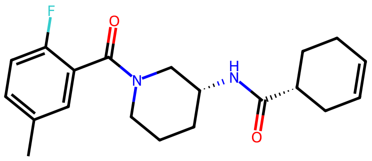	Cc1ccc(F)c(C(=O)N2CCC[C@@H](NC(=O)[C@H]3CC=CCC3)C2)c1	952146891	25	344.19	3.21
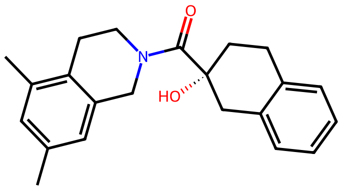	Cc1cc(C)c2c(c1)CN(C(=O)[C@@]1(O)CCc3ccccc3C1)CC2	803125018	25	335.19	3.11
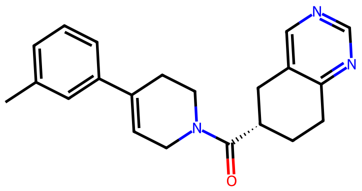	Cc1cccc(C2=CCN(C(=O)[C@H]3CCc4ncncc4C3)CC2)c1	648343373	25	333.18	3.21
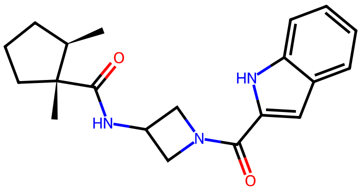	C[C@@H]1CCC[C@@]1(C)C(=O)NC1CN(C(=O)c2cc3ccccc3[nH]2)C1	999175921	25	339.19	2.93
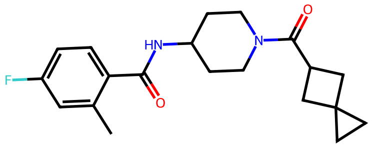	Cc1cc(F)ccc1C(=O)NC1CCN(C(=O)C2CC3(CC3)C2)CC1	948308410	25	344.19	3.05
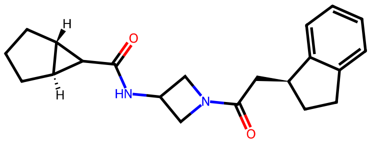	O=C(NC1CN(C(=O)C[C@@H]2CCc3ccccc32)C1)C1[C@H]2CCC[C@H]12	990704475	25	338.2	2.48
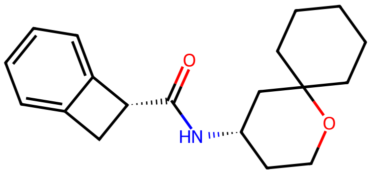	O=C(N[C@H]1CCOC2(CCCCC2)C1)[C@@H]1Cc2ccccc21	335892032	22	299.19	3.32

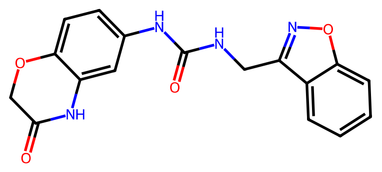	O=C1COc2ccc(NC(=O)NCc3noc4cccc c34)cc2N1	1622733410	25	338.1	2.48
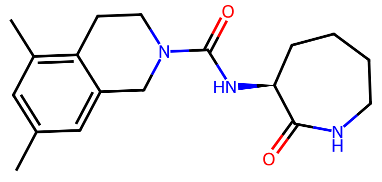	Cc1cc(C)c2c(c1)CN(C(=O)N[C@H]1C CCCNC1=O)CC2	185964250	23	315.19	2.04
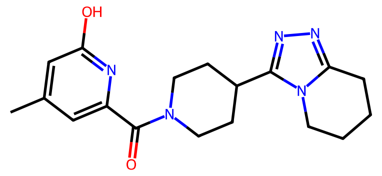	Cc1cc(O)nc(C(=O)N2CCC(c3nnc4n3C CCC4)CC2)c1	329064358	25	341.19	2.04
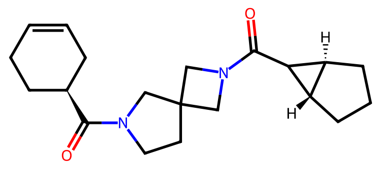	O=C(C1[C@H]2CCC[C@H]12)N1CC2 (CCN(C(=O)[C@@H]3CC=CCC3)C2) C1	1018909547	24	328.22	2.45
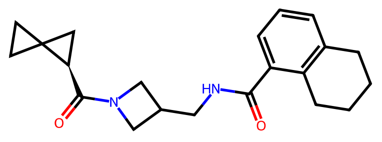	O=C(NCC1CN(C(=O)[C@@H]2CC23 CC3)C1)c1cccc2c1CCCC2	1002097863	25	338.2	2.55
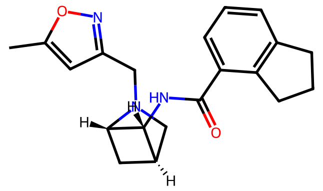	Cc1cc(CN2C[C@H]3C[C@H]2[C@@ H]3NC(=O)c2cccc3c2CCC3)no1	1037741336	25	337.18	2.47
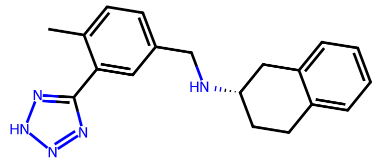	Cc1ccc(CN[C@H]2CCc3ccccc3C2)cc 1-c1nn[nH]n1	1581199103	24	319.18	2.82
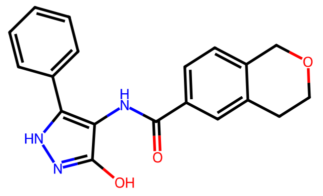	O=C(Nc1c(O)n[nH]c1-c1ccccc1)c1ccc 2c(c1)CCOC2	1141388802	25	335.13	3.11

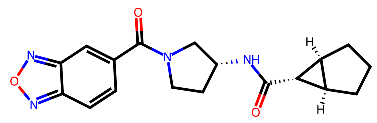	O=C(N[C@@H]1CCN(C(=O)c2ccc3nonc3c2)C1)[C@@H]1[C@H]2CCC[C@H]21	939926411	25	340.15	1.6
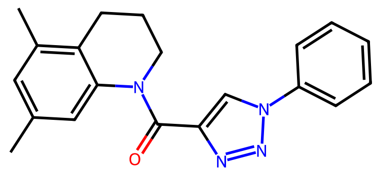	Cc1cc(C)c2c(c1)N(C(=O)c1cn(-c3ccccc3)nn1)CCC2	589389862	25	332.16	3.48
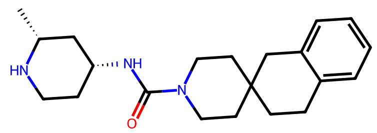	C[C@@H]1C[C@H](NC(=O)N2CCC3(CCc4ccccc4C3)CC2)CCN1	652216708	25	341.25	3.11
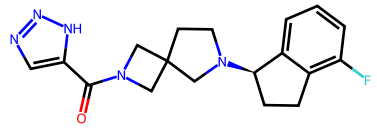	O=C(c1cnn[nH]1)N1CC2(CCN([C@@H]3CCc4c(F)cccc43)C2)C1	1072892464	25	341.17	1.78
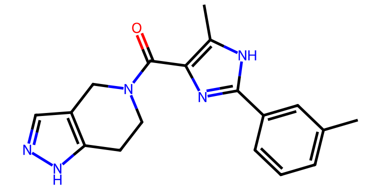	Cc1cccc(-c2nc(C(=O)N3CCc4[nH]ncc4C3)c(C)[nH]2)c1	270163731	24	321.16	2.62
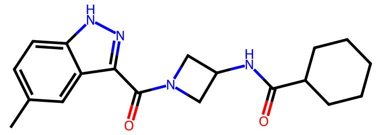	Cc1ccc2[nH]nc(C(=O)N3CC(NC(=O)C4CCCCC4)C3)c2c1	990093730	25	340.19	2.39
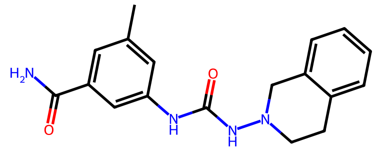	Cc1cc(NC(=O)NN2CCc3ccccc3C2)cc(C(N)=O)c1	1365057162	24	324.16	2.19
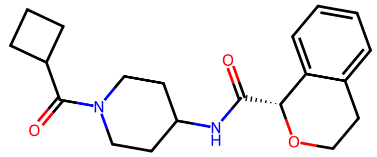	O=C(NC1CCN(C(=O)C2CCC2)CC1)[C@H]1OCCc2ccccc21	943315000	25	342.19	2.21

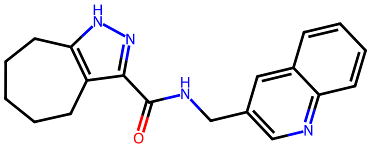	O=C(NCc1cnc2ccccc2c1)c1n[nH]c2c1CCCCC2	356144250	24	320.16	3.16
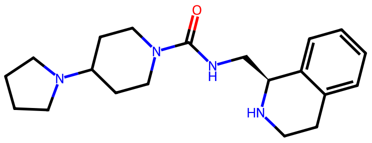	O=C(NC[C@@H]1NCCc2ccccc21)N1CCC(N2CCCC2)CC1	418636231	25	342.24	2.14
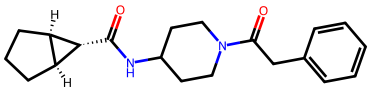	O=C(NC1CCN(C(=O)Cc2ccccc2)CC1)[C@@H]1[C@H]2CCC[C@H]21	947344840	24	326.2	2.38
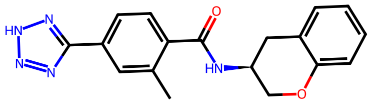	Cc1cc(-c2nn[nH]n2)ccc1C(=O)N[C@@H]1COc2ccccc2C1	826247840	25	335.14	1.91
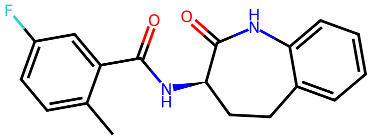	Cc1ccc(F)cc1C(=O)N[C@@H]1CCc2ccccc2NC1=O	1433512884	23	312.13	2.82
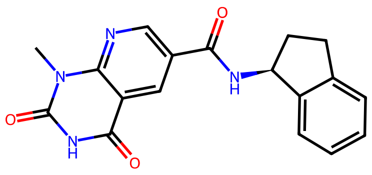	Cn1c(=O)[nH]c(=O)c2cc(C(=O)N[C@H]3CCc4ccccc43)cnc21	43481571	25	336.12	1.04
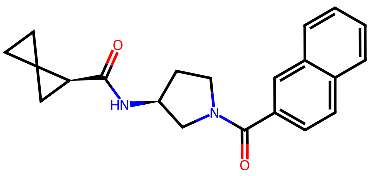	O=C(N[C@H]1CCN(C(=O)c2ccc3ccccc3c2)C1)[C@H]1CC12CC2	938977527	25	334.17	2.97
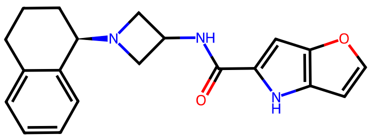	O=C(NC1CN([C@@H]2CCCc3ccccc32)C1)c1cc2occc2[nH]1	1030387367	25	335.16	3.25

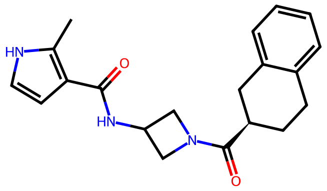	Cc1[nH]ccc1C(=O)NC1CN(C(=O)[C@@H]2CCc3ccccc3C2)C1	990735683	25	337.18	2.07
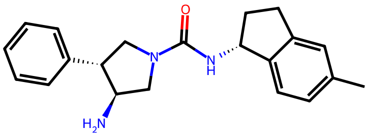	Cc1ccc2c(c1)CC[C@H]2NC(=O)N1C[C@@H](N)[C@H](c2ccccc2)C1	333947902	25	335.2	3.12
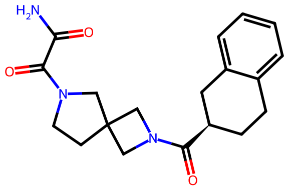	NC(=O)C(=O)N1CCC2(C1)CN(C(=O)[C@@H]1CCc3ccccc3C1)C2	1019491961	25	341.17	0.34
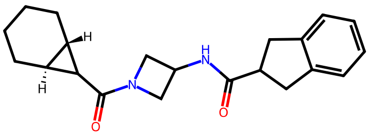	O=C(NC1CN(C(=O)C2[C@H]3CCCC[C@H]23)C1)C1Cc2ccccc2C1	1000449249	25	338.2	2.16
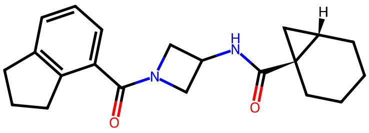	O=C(c1cccc2c1CCC2)N1CC(NC(=O)[C@]23CCCC[C@H]2C3)C1	999281309	25	338.2	2.7
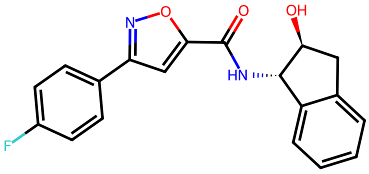	O=C(N[C@H]1c2ccccc2C[C@@H]1O)c1cc(-c2ccc(F)cc2)no1	1144246807	25	338.11	2.87
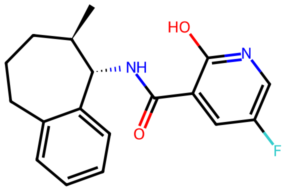	C[C@@H]1CCCc2ccccc2[C@H]1NC(=O)c1cc(F)cnc1O	1633565922	23	314.14	3.37
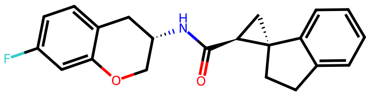	O=C(N[C@@H]1COc2cc(F)ccc2C1)[C@H]1C[C@]12CCc1ccccc12	618651805	25	337.15	3.15

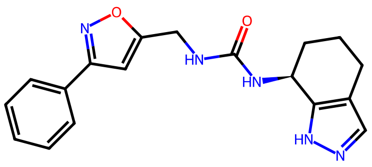	O=C(NCc1cc(-c2ccccc2)no1)N[C@H]1CCCc2cn[nH]c21	268907793	25	337.15	2.94
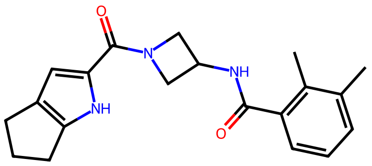	Cc1cccc(C(=O)NC2CN(C(=O)c3cc4c([nH]3)CCC4)C2)c1C	999519377	25	337.18	2.37
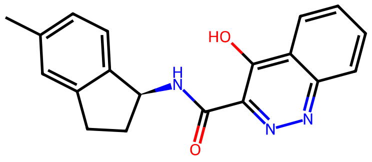	Cc1ccc2c(c1)CC[C@@H]2NC(=O)c1nnc2ccccc2c1O	732276702	24	319.13	3.06
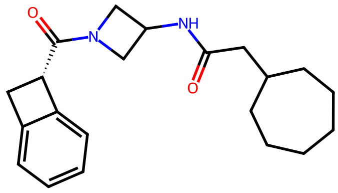	O=C(CC1CCCCCC1)NC1CN(C(=O)[C@H]2Cc3ccccc32)C1	1000581624	25	340.22	3.01
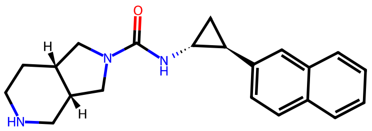	O=C(N[C@@H]1C[C@H]1c1ccc2ccccc2c1)N1C[C@H]2CCNC[C@H]2C1	418136781	25	335.2	2.95
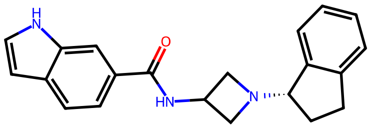	O=C(NC1CN([C@H]2CCc3ccccc32)C1)c1ccc2cc[nH]c2c1	1031013635	25	331.17	3.27
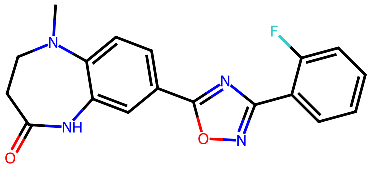	CN1CCC(=O)Nc2cc(-c3nc(-c4ccccc4F)no3)ccc21	363955961	25	338.12	3.32
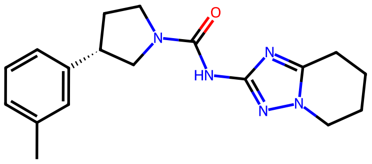	Cc1cccc([C@@H]2CCN(C(=O)Nc3nc4n(n3)CCCC4)C2)c1	333735435	24	325.19	2.94

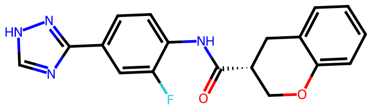	O=C(Nc1ccc(-c2nc[nH]n2)cc1F)[C@H]1COc2ccccc2C1	344290308	25	338.12	2.8
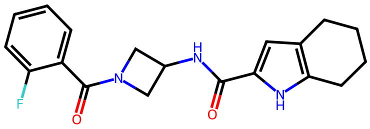	O=C(NC1CN(C(=O)c2ccccc2F)C1)c1cc2c([nH]1)CCCC2	999656709	25	341.15	2.29
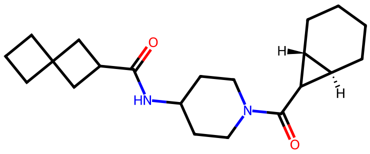	O=C(NC1CCN(C(=O)C2[C@@H]3CCCC[C@@H]23)CC1)C1CC2(CCC2)C1	945956235	25	344.25	3.11
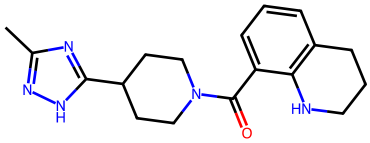	Cc1n[nH]c(C2CCN(C(=O)c3cccc4c3NCCC4)CC2)n1	299766242	24	325.19	2.49
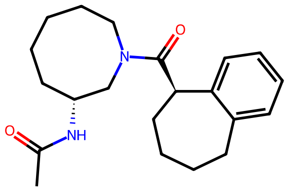	CC(=O)N[C@@H]1CCCCCN(C(=O)[C@@H]2CCCCc3ccccc32)C1	987064384	25	342.23	3.4
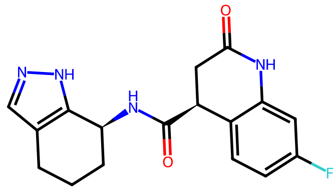	O=C1C[C@H](C(=O)N[C@H]2CCCc3cn[nH]c32)c2ccc(F)cc2N1	268992689	24	328.13	2.17
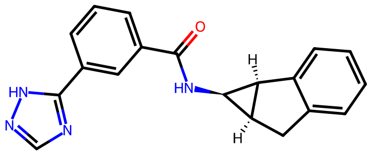	O=C(N[C@@H]1[C@@H]2Cc3ccccc3[C@@H]21)c1cccc(-c2ncn[nH]2)c1	156441175	24	316.13	2.54
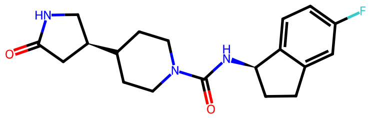	O=C1C[C@H](C2CCN(C(=O)N[C@@H]3CCc4cc(F)ccc43)CC2)CN1	1553384568	25	345.19	2.37

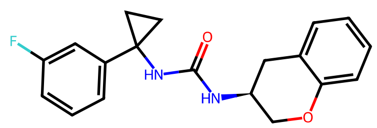	O=C(N[C@@H]1COc2ccccc2C1)NC1(c2cccc(F)c2)CC1	172476609	24	326.14	3.12
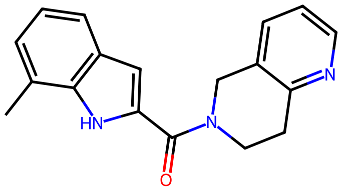	Cc1cccc2cc(C(=O)N3CCc4ncccc4C3)[nH]c12	1875252407	22	291.14	3.07
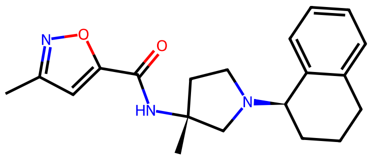	Cc1cc(C(=O)N[C@]2(C)CCN([C@@H]3CCCc4ccccc43)C2)on1	1046139314	25	339.19	3.25
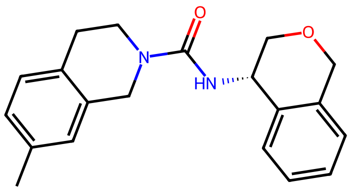	Cc1ccc2c(c1)CN(C(=O)N[C@@H]1COCc3ccccc31)CC2	665161702	24	322.17	3.33
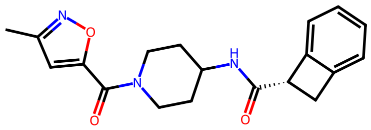	Cc1cc(C(=O)N2CCC(NC(=O)[C@H]3Cc4ccccc43)CC2)on1	944152632	25	339.16	2.04
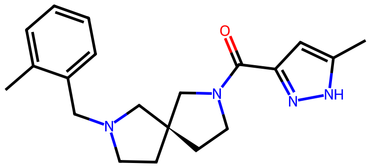	Cc1cc(C(=O)N2CC[C@]3(CCN(Cc4ccccc4C)C3)C2)n[nH]1	1041107755	25	338.21	2.76
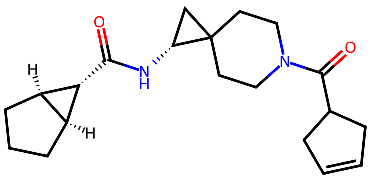	O=C(N[C@@H]1CC12CCN(C(=O)C1CC=CC1)CC2)[C@@H]1[C@H]2CCC[C@H]21	963462080	24	328.22	2.5
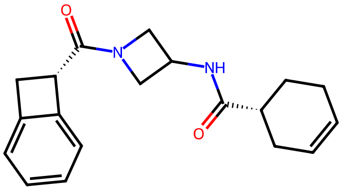	O=C(NC1CN(C(=O)[C@H]2Cc3ccccc32)C1)[C@H]1CC=CCC1	990214630	23	310.17	2.01

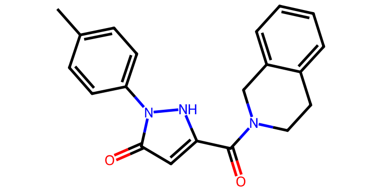	Cc1ccc(-n2[nH]c(C(=O)N3CCc4ccccc4C3)cc2=O)cc1	174345386	25	333.15	2.67
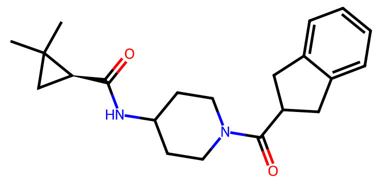	CC1(C)C[C@@H]1C(=O)NC1CCN(C(=O)C2Cc3ccccc3C2)CC1	948550224	25	340.22	2.55
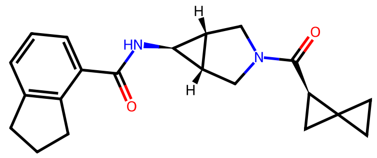	O=C(N[C@H]1[C@@H]2CN(C(=O)[C@@H]3CC34CC4)C[C@@H]21)c1cccc2c1CCC2	976708836	25	336.18	2.16
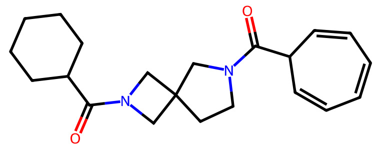	O=C(C1C=CC=CC=C1)N1CCC2(C1)CN(C(=O)C1CCCCC1)C2	1018720774	25	340.22	2.93
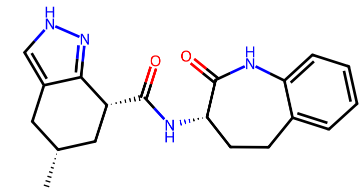	C[C@@H]1Cc2c[nH]nc2[C@H](C(=O)N[C@H]2CCc3ccccc3NC2=O)C1	623225357	25	338.17	2.15
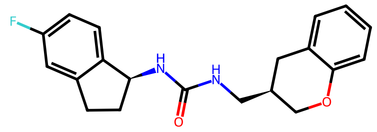	O=C(NC[C@@H]1COc2ccccc2C1)N[C@H]1CCc2cc(F)ccc21	56839438	25	340.16	3.36
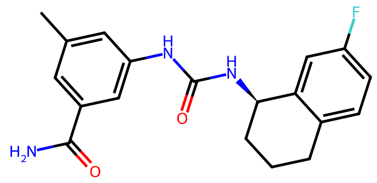	Cc1cc(NC(=O)N[C@@H]2CCCc3ccc(F)cc32)cc(C(N)=O)c1	653586125	25	341.15	3.43
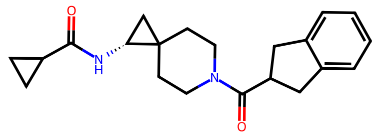	O=C(N[C@@H]1CC12CCN(C(=O)C1Cc3ccccc3C1)CC2)C1CC1	962488210	25	338.2	2.31

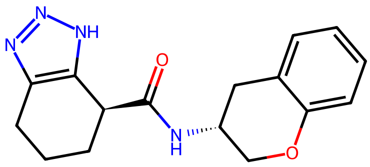	O=C(N[C@H]1COc2ccccc2C1)[C@H]1CCCc2nn[nH]c21	666388928	22	298.14	1.34
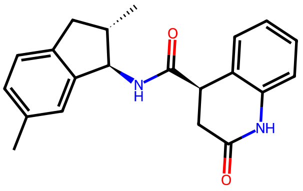	Cc1ccc2c(c1)[C@H](NC(=O)[C@@H]1CC(=O)Nc3ccccc31)[C@@H](C)C2	182193783	25	334.17	3.47
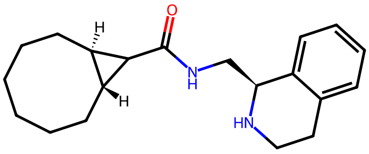	O=C(NC[C@@H]1NCCc2ccccc21)C1[C@@H]2CCCCCC[C@@H]12	852878882	23	312.22	3.21
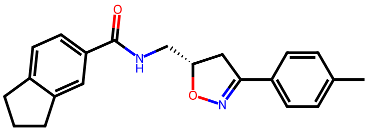	Cc1ccc(C2=NO[C@H](CNC(=O)c3ccc4c(c3)CCC4)C2)cc1	800676685	25	334.17	3.41
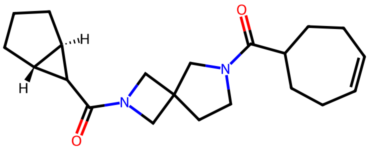	O=C(C1CCC=CCC1)N1CCC2(C1)CN(C(=O)C1[C@@H]3CCC[C@@H]13)C2	1018914020	25	342.23	2.84
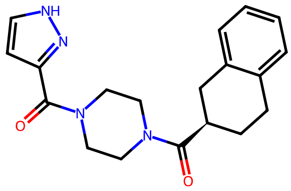	O=C(c1cc[nH]n1)N1CCN(C(=O)[C@@H]2CCc3ccccc3C2)CC1	1010422966	25	338.17	1.5
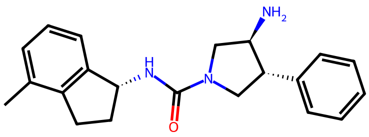	Cc1cccc2c1CC[C@H]2NC(=O)N1C[C@@H](N)[C@H](c2ccccc2)C1	515522024	25	335.2	3.12
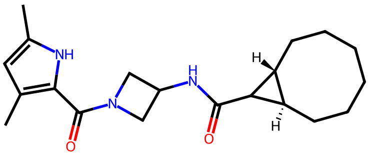	Cc1cc(C)c(C(=O)N2CC(NC(=O)C3[C@@H]4CCCCCC[C@@H]34)C2)[nH]1	1000507609	25	343.23	2.79

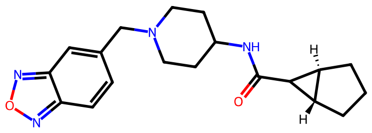	O=C(NC1CCN(Cc2ccc3nonc3c2)CC1)C1[C@H]2CCC[C@H]12	1002546165	25	340.19	2.35
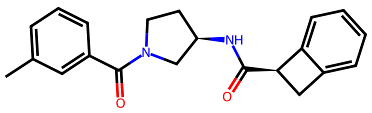	Cc1cccc(C(=O)N2CC[C@@H](NC(=O)[C@@H]3Cc4ccccc43)C2)c1	942302748	25	334.17	2.67
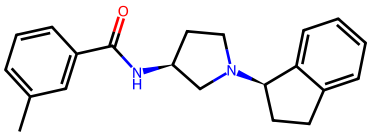	Cc1cccc(C(=O)N[C@H]2CCN([C@@H]3CCc4ccccc43)C2)c1	1010012694	24	320.19	3.49
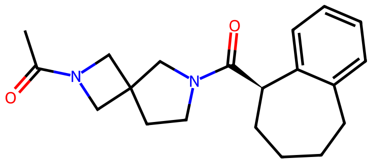	CC(=O)N1CC2(CCN(C(=O)[C@@H]3CCCCc4ccccc43)C2)C1	1018657130	24	326.2	2.58
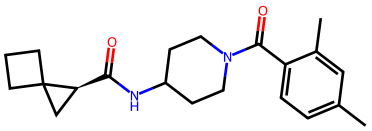	Cc1ccc(C(=O)N2CCC(NC(=O)[C@H]3CC34CCC4)CC2)c(C)c1	948347845	25	340.22	3.21
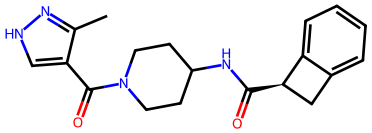	Cc1n[nH]cc1C(=O)N1CCC(NC(=O)[C@@H]2Cc3ccccc32)CC1	947668664	25	338.17	1.78
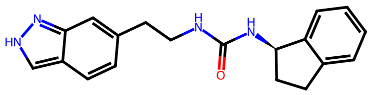	O=C(NCCc1ccc2c[nH]nc2c1)N[C@@H]1CCc2ccccc21	349836800	24	320.16	3.09
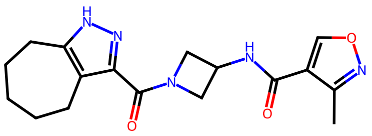	Cc1nocc1C(=O)NC1CN(C(=O)c2n[nH]c3c2CCCCC3)C1	998749957	25	343.16	1.23

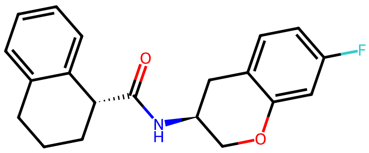	O=C(N[C@@H]1COc2cc(F)ccc2C1)[C@@H]1CCCc2ccccc21	1618175294	24	325.15	3.37
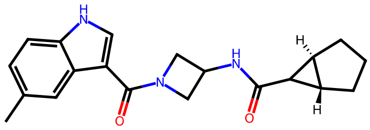	Cc1ccc2[nH]cc(C(=O)N3CC(NC(=O)C4[C@H]5CCC[C@H]45)C3)c2c1	990697502	25	337.18	2.46
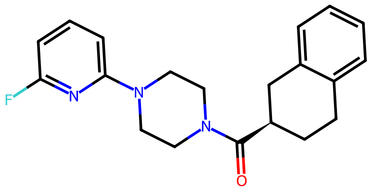	O=C([C@@H]1CCc2ccccc2C1)N1CCN(c2cccc(F)n2)CC1	844560830	25	339.17	2.67
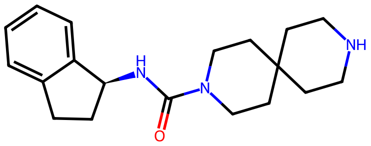	O=C(N[C@H]1CCc2ccccc21)N1CCC2(CCNCC2)CC1	416928696	23	313.22	2.85
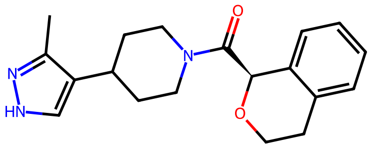	Cc1n[nH]cc1C1CCN(C(=O)[C@@H]2OCCc3ccccc32)CC1	557158197	24	325.18	2.74
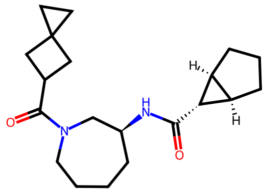	O=C(N[C@H]1CCCCN(C(=O)C2CC3(CC3)C2)C1)[C@@H]1[C@H]2CCC[C@H]21	943166814	24	330.23	2.72
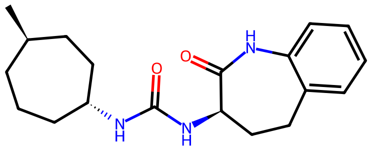	C[C@@H]1CCC[C@@H](NC(=O)N[C@@H]2CCc3ccccc3NC2=O)CC1	1226349664	24	329.21	3.21
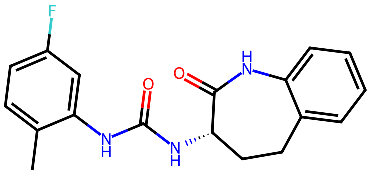	Cc1ccc(F)cc1NC(=O)N[C@H]1CCc2ccccc2NC1=O	1191428703	24	327.14	3.21

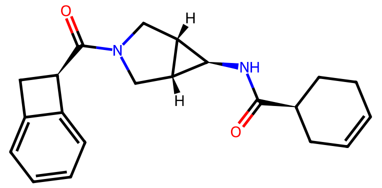	O=C(N[C@H]1[C@@H]2CN(C(=O)[C@@H]3Cc4ccccc43)C[C@@H]21)[C@@H]1CC=CCC1	976178665	25	336.18	2.26
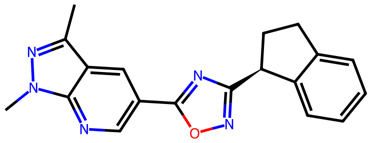	Cc1nn(C)c2ncc(-c3nc([C@H]4CCc5ccccc54)no3)cc12	680749373	25	331.14	3.4
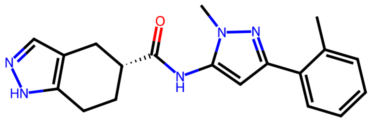	Cc1ccccc1-c1cc(NC(=O)[C@@H]2CCc3[nH]ncc3C2)n(C)n1	270161175	25	335.17	2.86
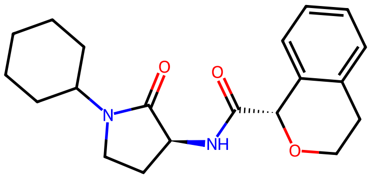	O=C(N[C@H]1CCN(C2CCCCC2)C1=O)[C@H]1OCCc2ccccc21	124801874	25	342.19	2.35
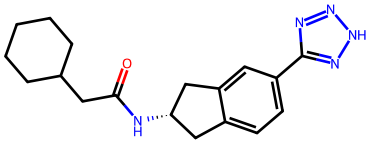	O=C(CC1CCCCC1)N[C@H]1Cc2ccc(-c3nn[nH]n3)cc2C1	1606503771	24	325.19	2.42
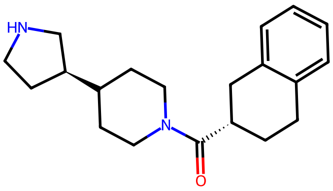	O=C([C@H]1CCc2ccccc2C1)N1CCC([C@H]2CCNC2)CC1	582613855	23	312.22	2.64
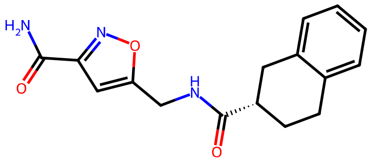	NC(=O)c1cc(CNC(=O)[C@H]2CCc3ccccc3C2)on1	566331734	22	299.13	1.19
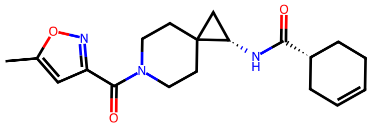	Cc1cc(C(=O)N2CCC3(CC2)C[C@@H]3NC(=O)[C@H]2CC=CCC2)no1	963026993	25	343.19	2.45

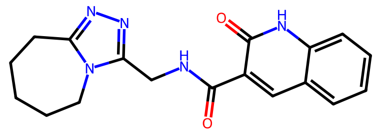	O=C(NCc1nnc2n1CCCCC2)c1cc2ccccc2[nH]c1=O	192983683	25	337.15	1.78
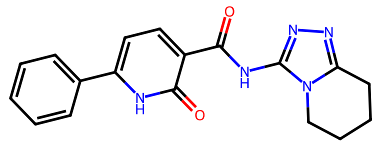	O=C(Nc1nnc2n1CCCC2)c1ccc(-c2ccccc2)[nH]c1=O	180886257	25	335.14	2.22
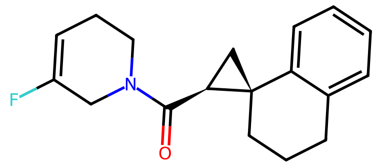	O=C([C@H]1C[C@@]12CCCc1ccccc12)N1CCC=C(F)C1	376854286	21	285.15	3.37
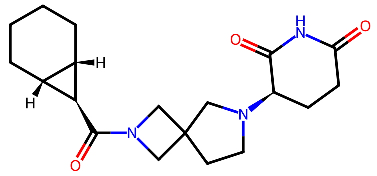	O=C1CC[C@@H](N2CCC3(CN(C(=O)[C@@H]4[C@H]5CCCC[C@H]54)C3)C2)C(=O)N1	1072581010	25	345.21	0.76
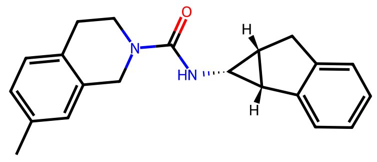	Cc1ccc2c(c1)CN(C(=O)N[C@@H]1[C@@H]3Cc4ccccc4[C@@H]31)CC2	660914467	24	318.17	3.4
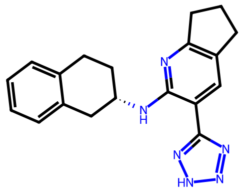	c1ccc2c(c1)CC[C@H](Nc1nc3c(cc1-c1nn[nH]n1)CCC3)C2	1607132408	25	332.17	2.72
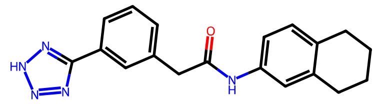	O=C(Cc1cccc(-c2nn[nH]n2)c1)Nc1ccc2c(c1)CCCC2	1609536498	25	333.16	2.93
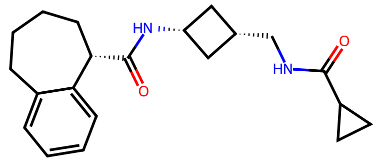	O=C(NC[C@H]1C[C@@H](NC(=O)[C@H]2CCCCc3ccccc32)C1)C1CC1	978496757	25	340.22	2.92

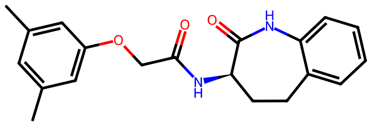	Cc1cc(C)cc(OCC(=O)N[C@@H]2CCc3ccccc3NC2=O)c1	654005974	25	338.16	2.75
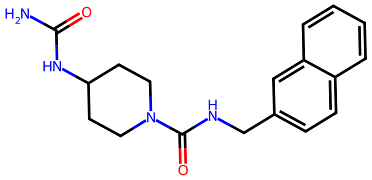	NC(=O)NC1CCN(C(=O)NCc2ccc3ccccc3c2)CC1	120530807	24	326.17	2.18
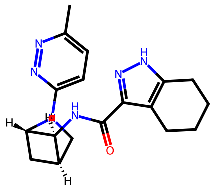	Cc1ccc(N2C[C@H]3C[C@H]2[C@H]3NC(=O)c2n[nH]c3c2CCCC3)nn1	1064360983	25	338.19	1.39
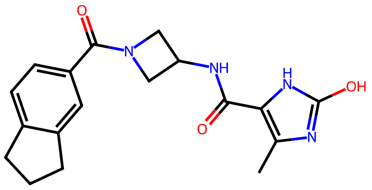	Cc1nc(O)[nH]c1C(=O)NC1CN(C(=O)c2ccc3c(c2)CCC3)C1	994052078	25	340.15	1.17
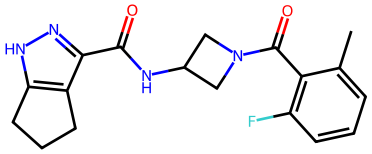	Cc1cccc(F)c1C(=O)N1CC(NC(=O)c2n[nH]c3c2CCC3)C1	995167312	25	342.15	1.6
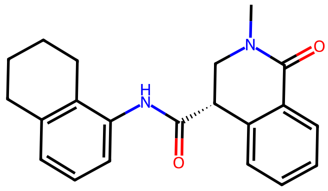	CN1C[C@@H](C(=O)Nc2cccc3c2CCCC3)c2ccccc2C1=O	362809483	25	334.17	3.37
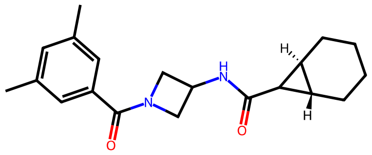	Cc1cc(C)cc(C(=O)N2CC(NC(=O)C3[C@H]4CCCC[C@H]34)C2)c1	990651968	24	326.2	2.68
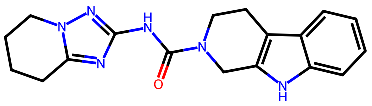	O=C(Nc1nc2n(n1)CCCC2)N1CCc2c([nH]c3ccccc23)C1	566662614	25	336.17	2.69

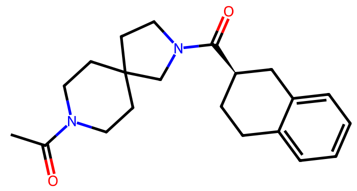	CC(=O)N1CCC2(CC1)CCN(C(=O)[C@@H]1CCc3ccccc3C1)C2	944337434	25	340.22	2.65
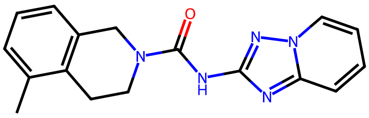	Cc1cccc2c1CCN(C(=O)Nc1nc3ccccn3n1)C2	760032467	23	307.14	2.63
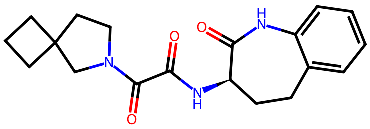	O=C(N[C@@H]1CCc2ccccc2NC1=O)C(=O)N1CCC2(CCC2)C1	1327234002	25	341.17	1.46
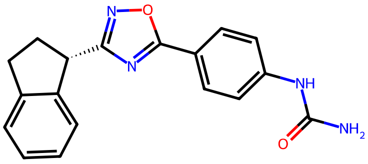	NC(=O)Nc1ccc(-c2nc([C@H]3CCc4ccccc43)no2)cc1	356583664	24	320.13	3.31
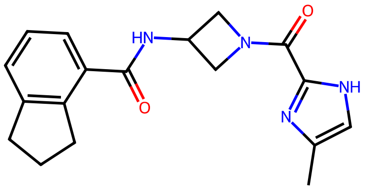	Cc1c[nH]c(C(=O)N2CC(NC(=O)c3cccc4c3CCC4)C2)n1	994399006	24	324.16	1.46
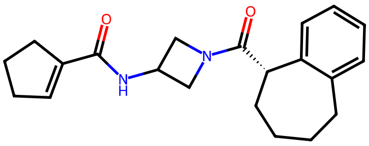	O=C(NC1CN(C(=O)[C@H]2CCCCc3ccccc32)C1)C1=CCCC1	990810472	25	338.2	2.93
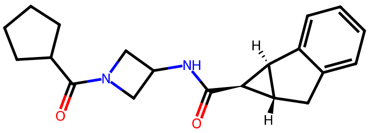	O=C(NC1CN(C(=O)C2CCCC2)C1)[C@@H]1[C@H]2Cc3ccccc3[C@@H]21	991647489	24	324.18	2.09
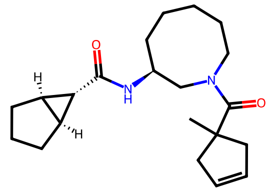	CC1(C(=O)N2CCCCC[C@H](NC(=O)[C@@H]3[C@H]4CCC[C@H]43)C2)CC=CC1	987141367	25	344.25	3.28

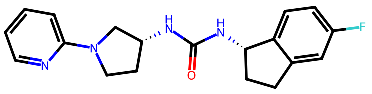	O=C(N[C@H]1CCc2cc(F)ccc21)N[C@@H]1CCN(c2ccccn2)C1	245503434	25	340.17	2.79
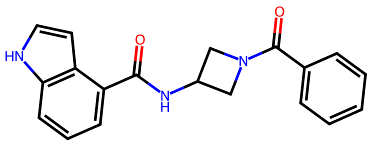	O=C(NC1CN(C(=O)c2ccccc2)C1)c1cccc2[nH]ccc12	1000405242	24	319.13	2.42
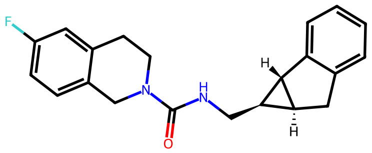	O=C(NC[C@@H]1[C@@H]2Cc3ccccc3[C@@H]12)N1CCc2cc(F)ccc2C1	129719511	25	336.16	3.48
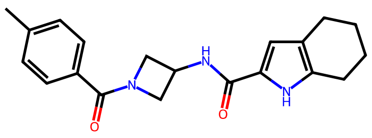	Cc1ccc(C(=O)N2CC(NC(=O)c3cc4c([nH]3)CCCC4)C2)cc1	999678028	25	337.18	2.46
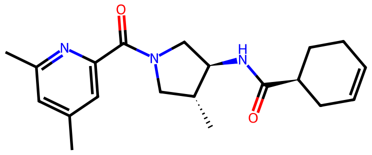	Cc1cc(C)nc(C(=O)N2C[C@@H](NC(=O)[C@@H]3CC=CCC3)[C@H](C)C2)c1	950897324	25	341.21	2.63
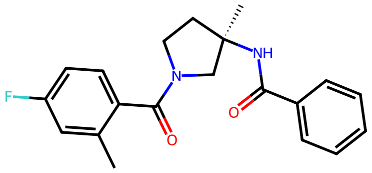	Cc1cc(F)ccc1C(=O)N1CC[C@@](C)(NC(=O)c2ccccc2)C1	956655972	25	340.16	3.17
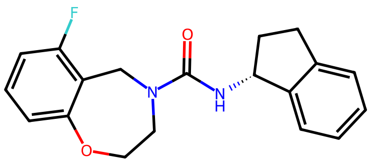	O=C(N[C@@H]1CCc2ccccc21)N1CCOc2cccc(F)c2C1	510867841	24	326.14	3.42
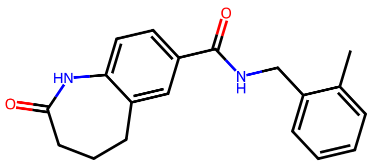	Cc1ccccc1CNC(=O)c1ccc2c(c1)CCCC(=O)N2	642255417	23	308.15	3.2

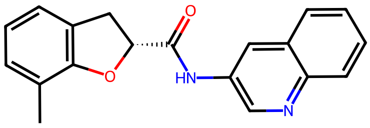	Cc1cccc2c1O[C@@H](C(=O)Nc1cnc3ccccc3c1)C2	1357479611	23	304.12	3.49
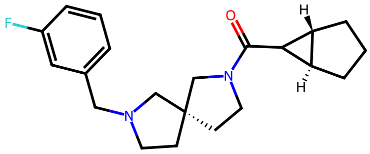	O=C(C1[C@@H]2CCC[C@@H]12)N1CC[C@@]2(CCN(Cc3cccc(F)c3)C2)C1	1041179160	25	342.21	3.3
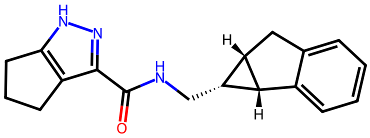	O=C(NC[C@H]1[C@H]2c3ccccc3C[C@H]21)c1n[nH]c2c1CCC2	152397957	22	293.15	2.21
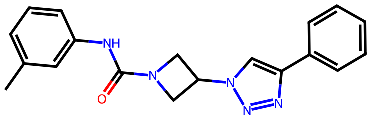	Cc1cccc(NC(=O)N2CC(n3cc(-c4ccccc4)nn3)C2)c1	238879131	25	333.16	3.34
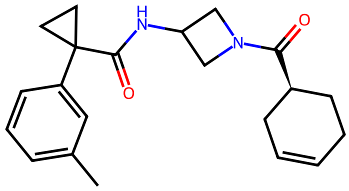	Cc1cccc(C2(C(=O)NC3CN(C(=O)[C@@H]4CC=CCC4)C3)CC2)c1	993893400	25	338.2	2.71
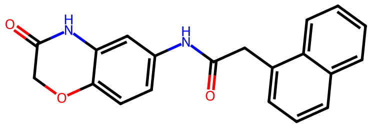	O=C(Cc1cccc2ccccc12)Nc1ccc2c(c1)NC(=O)CO2	27580903	25	332.12	3.35
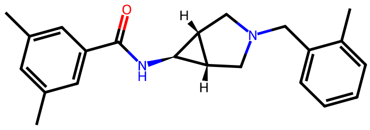	Cc1cc(C)cc(C(=O)N[C@H]2[C@@H]3CN(Cc4ccccc4C)C[C@@H]32)c1	962393705	25	334.2	3.47
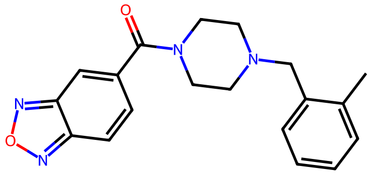	Cc1ccccc1CN1CCN(C(=O)c2ccc3nonc3c2)CC1	949989304	25	336.16	2.49

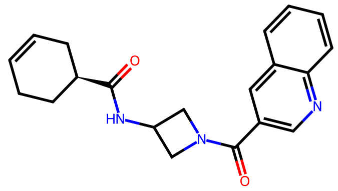	O=C(NC1CN(C(=O)c2cnc3ccccc3c2)C1)[C@@H]1CC=CCC1	990221490	25	335.16	2.53
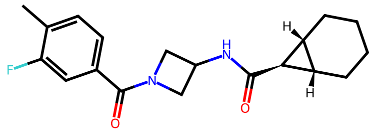	Cc1ccc(C(=O)N2CC(NC(=O)[C@@H]3[C@H]4CCCC[C@H]43)C2)cc1F	990648296	24	330.17	2.51
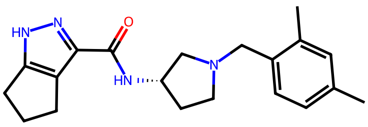	Cc1ccc(CN2CC[C@H](NC(=O)c3n[nH]c4c3CCC4)C2)c(C)c1	1015071498	25	338.21	2.52
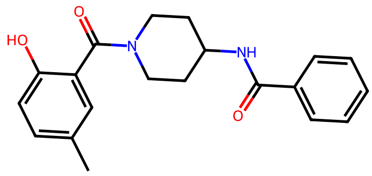	Cc1ccc(O)c(C(=O)N2CCC(NC(=O)c3ccccc3)CC2)c1	10777305	25	338.16	2.74
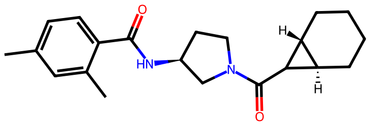	Cc1ccc(C(=O)N[C@H]2CCN(C(=O)C3[C@@H]4CCCC[C@@H]34)C2)c(C)c1	938757797	25	340.22	3.07
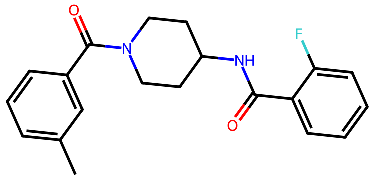	Cc1cccc(C(=O)N2CCC(NC(=O)c3ccccc3F)CC2)c1	75057902	25	340.16	3.17
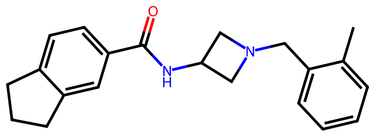	Cc1ccccc1CN1CC(NC(=O)c2ccc3c(c2)CCC3)C1	1031371607	24	320.19	3.1
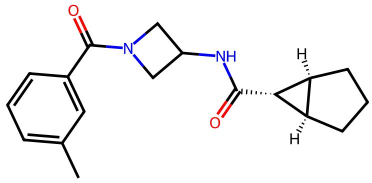	Cc1cccc(C(=O)N2CC(NC(=O)[C@@H]3[C@H]4CCC[C@H]43)C2)c1	990684520	22	298.17	1.98

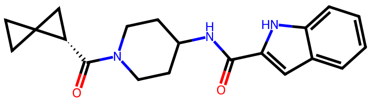	O=C(NC1CCN(C(=O)[C@H]2CC23CC3)CC1)c1cc2ccccc2[nH]1	948558472	25	337.18	2.69
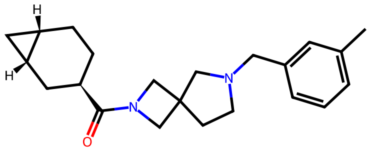	Cc1cccc(CN2CCC3(C2)CN(C(=O)[C@@H]2CC[C@H]4C[C@H]4C2)C3)c1	1072878999	25	338.24	3.47
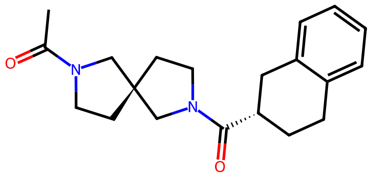	CC(=O)N1CC[C@]2(CCN(C(=O)[C@H]3CCc4ccccc4C3)C2)C1	953028186	24	326.2	2.26
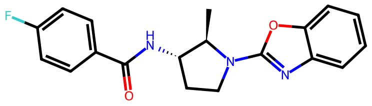	C[C@@H]1[C@@H](NC(=O)c2ccc(F)cc2)CCN1c1nc2ccccc2o1	1049992743	25	339.14	3.36
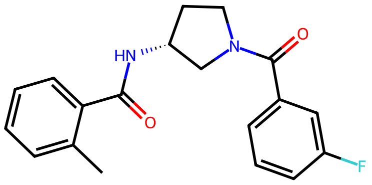	Cc1ccccc1C(=O)N[C@@H]1CCN(C(=O)c2cccc(F)c2)C1	939206506	24	326.14	2.78
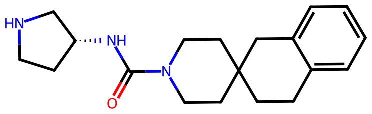	O=C(N[C@@H]1CCNC1)N1CCC2(CCc3ccccc3C2)CC1	627251878	23	313.22	2.33
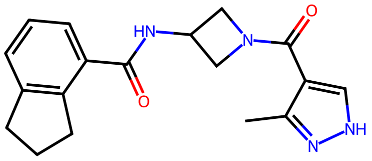	Cc1n[nH]cc1C(=O)N1CC(NC(=O)c2cccc3c2CCC3)C1	994393794	24	324.16	1.46
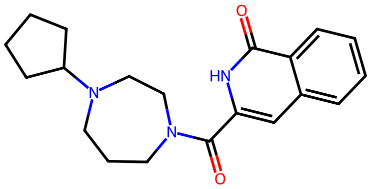	O=C(c1cc2ccccc2c(=O)[nH]1)N1CCCN(C2CCCC2)CC1	172419326	25	339.19	2.62

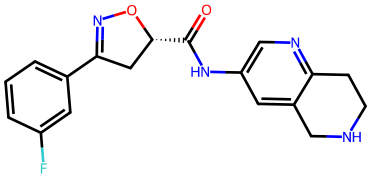	O=C(Nc1cnc2c(c1)CNCC2)[C@@H]1CC(c2cccc(F)c2)=NO1	846675502	25	340.13	2.0
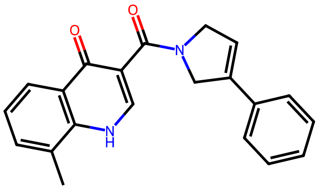	Cc1cccc2c(=O)c(C(=O)N3CC=C(c4ccccc4)C3)c[nH]c12	361674713	25	330.14	3.38
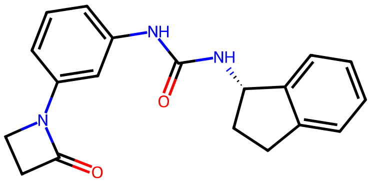	O=C(Nc1cccc(N2CCC2=O)c1)N[C@H]1CCc2ccccc21	280402877	24	321.15	3.23
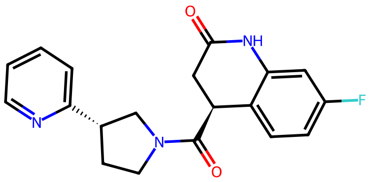	O=C1C[C@H](C(=O)N2CC[C@H](c3ccccn3)C2)c2ccc(F)cc2N1	798720352	25	339.14	2.66
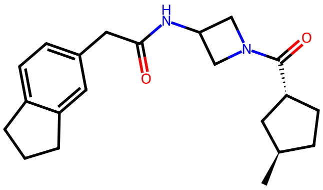	C[C@@H]1CC[C@@H](C(=O)N2CC(NC(=O)Cc3ccc4c(c3)CCC4)C2)C1	991384204	25	340.22	2.48
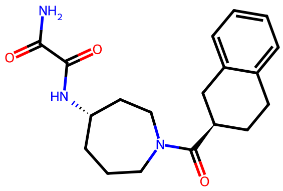	NC(=O)C(=O)N[C@H]1CCCN(C(=O)[C@@H]2CCc3ccccc3C2)CC1	965146138	25	343.19	0.77
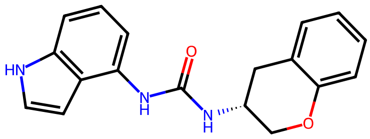	O=C(Nc1cccc2[nH]ccc12)N[C@H]1COc2ccccc2C1	171393635	23	307.13	3.29
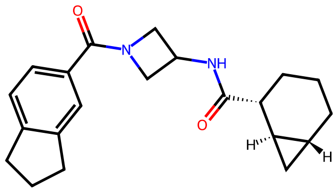	O=C(NC1CN(C(=O)c2ccc3c(c2)CCC3)C1)[C@@H]1CCC[C@@H]2C[C@H]21	994719218	25	338.2	2.55

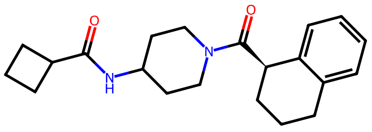	O=C(NC1CCN(C(=O)[C@@H]2CCCc3ccccc32)CC1)C1CCC1	363598621	25	340.22	3.01
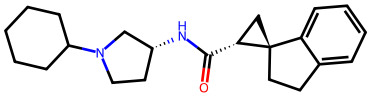	O=C(N[C@@H]1CCN(C2CCCCC2)C1)[C@@H]1C[C@@]12CCc1ccccc12	618663748	25	338.24	3.41
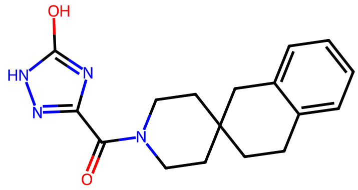	O=C(c1n[nH]c(O)n1)N1CCC2(CCc3ccccc3C2)CC1	653037206	23	312.16	1.92
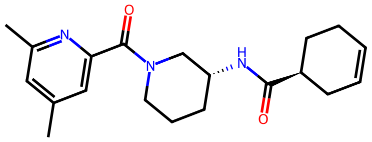	Cc1cc(C)nc(C(=O)N2CCC[C@@H](NC(=O)[C@@H]3CC=CCC3)C2)c1	951271190	25	341.21	2.78
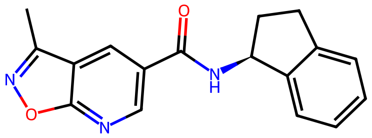	Cc1noc2ncc(C(=O)N[C@H]3CCc4ccccc43)cc12	43077374	22	293.12	2.95
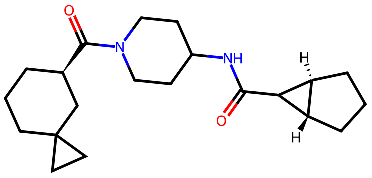	O=C(NC1CCN(C(=O)[C@@H]2CCCC3(CC3)C2)CC1)C1[C@H]2CCC[C@H]12	946752530	25	344.25	3.11
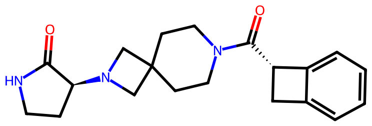	O=C1NCC[C@@H]1N1CC2(CCN(C(=O)[C@H]3Cc4ccccc43)CC2)C1	1035780107	25	339.19	1.14
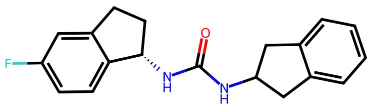	O=C(NC1Cc2ccccc2C1)N[C@H]1CCc2cc(F)ccc21	186516276	23	310.15	3.28

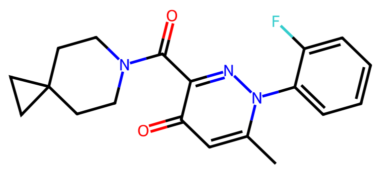	Cc1cc(=O)c(C(=O)N2CCC3(CC2)CC3)nn1-c1ccccc1F	370877397	25	341.15	2.7
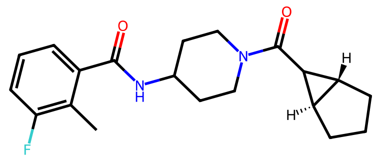	Cc1c(F)cccc1C(=O)NC1CCN(C(=O)C2[C@@H]3CCC[C@@H]23)CC1	946094478	25	344.19	2.9
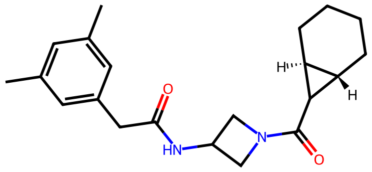	Cc1cc(C)cc(CC(=O)NC2CN(C(=O)C3[C@H]4CCCC[C@H]34)C2)c1	998753260	25	340.22	2.61
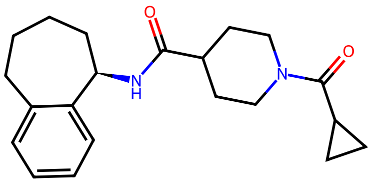	O=C(N[C@@H]1CCCCc2ccccc21)C1CCN(C(=O)C2CC2)CC1	58393026	25	340.22	3.22
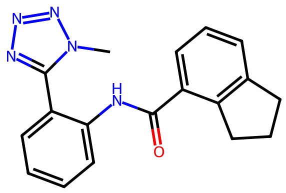	Cn1nnnc1-c1ccccc1NC(=O)c1cccc2c1CCC2	1438707773	24	319.14	2.62
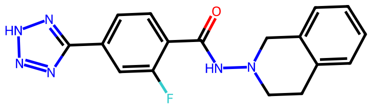	O=C(NN1CCc2ccccc2C1)c1ccc(-c2nn[nH]n2)cc1F	1582561774	25	338.13	1.71
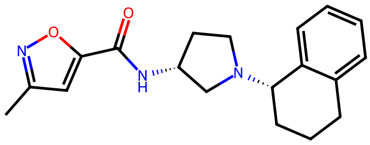	Cc1cc(C(=O)N[C@@H]2CCN([C@H]3CCCc4ccccc43)C2)on1	1010138731	24	325.18	2.86
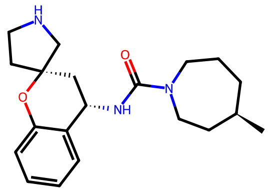	C[C@@H]1CCCN(C(=O)N[C@H]2C[C@@]3(CCNC3)Oc3ccccc32)CC1	1337005306	25	343.23	3.07

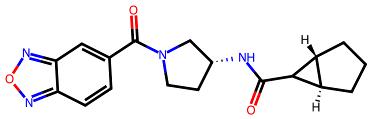	O=C(N[C@@H]1CCN(C(=O)c2ccc3nonc3c2)C1)C1[C@@H]2CCC[C@@H]12	939926412	25	340.15	1.6
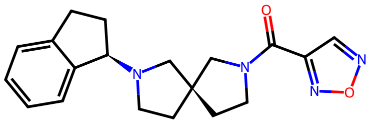	O=C(c1cnon1)N1CC[C@]2(CCN([C@@H]3CCc4ccccc43)C2)C1	1041748429	25	338.17	2.3
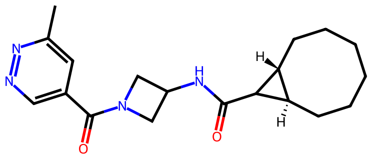	Cc1cc(C(=O)N2CC(NC(=O)C3[C@@H]4CCCCCC[C@@H]34)C2)cnn1	1000494739	25	342.21	1.94
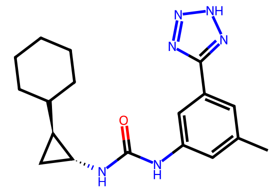	Cc1cc(NC(=O)N[C@@H]2C[C@H]2C2CCCCC2)cc(-c2nn[nH]n2)c1	1580982961	25	340.2	3.27
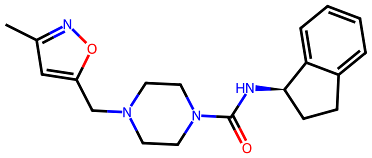	Cc1cc(CN2CCN(C(=O)N[C@@H]3CCc4ccccc43)CC2)on1	45596362	25	340.19	2.5
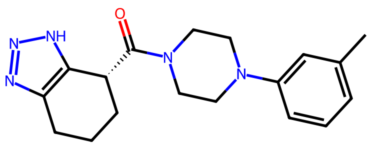	Cc1cccc(N2CCN(C(=O)[C@@H]3CCCc4nn[nH]c43)CC2)c1	646595020	24	325.19	1.88
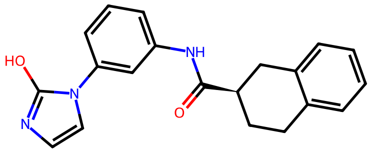	O=C(Nc1cccc(-n2ccnc2O)c1)[C@@H]1CCc2ccccc2C1	1517430192	25	333.15	3.32
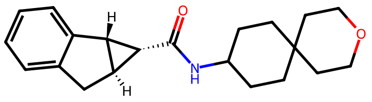	O=C(NC1CCC2(CCOCC2)CC1)[C@@H]1[C@H]2Cc3ccccc3[C@@H]21	1329627692	24	325.2	3.43

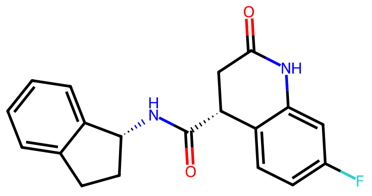	O=C1C[C@@H](C(=O)N[C@@H]2CCc3ccccc32)c2ccc(F)cc2N1	78434359	24	324.13	3.06
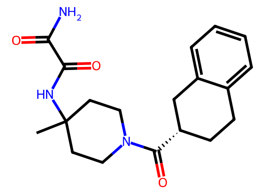	CC1(NC(=O)C(N)=O)CCN(C(=O)[C@H]2CCc3ccccc3C2)CC1	1073275774	25	343.19	0.77
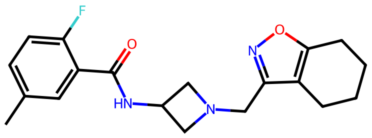	Cc1ccc(F)c(C(=O)NC2CN(Cc3noc4c3CCCC4)C2)c1	1031012316	25	343.17	2.62
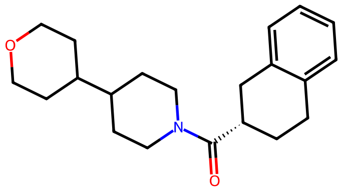	O=C([C@H]1CCc2ccccc2C1)N1CCC(C2CCOCC2)CC1	519654955	24	327.22	3.46
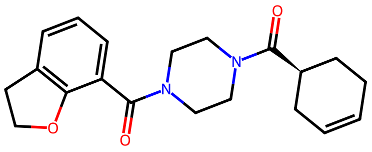	O=C(c1cccc2c1OCC2)N1CCN(C(=O)[C@@H]2CC=CCC2)CC1	1010295262	25	340.18	2.26
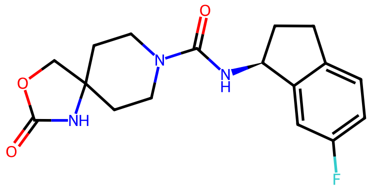	O=C1NC2(CCN(C(=O)N[C@H]3CCc4ccc(F)cc43)CC2)CO1	495032701	24	333.15	2.1
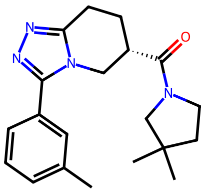	Cc1cccc(-c2nnc3n2C[C@@H](C(=O)N2CCC(C)(C)C2)CC3)c1	517502661	25	338.21	3.07
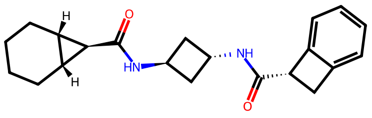	O=C(N[C@H]1C[C@H](NC(=O)[C@@H]2[C@H]3CCCC[C@H]32)C1)[C@H]1Cc2ccccc21	973135560	25	338.2	2.53

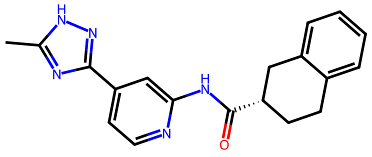	Cc1nc(-c2ccnc(NC(=O)[C@H]3CCc4ccccc4C3)c2)n[nH]1	910100315	25	333.16	2.92
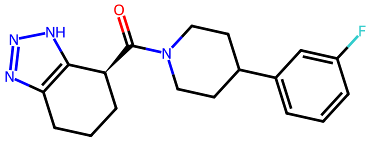	O=C([C@H]1CCCc2nn[nH]c21)N1CCC(c2cccc(F)c2)CC1	1646594794	24	328.17	2.77
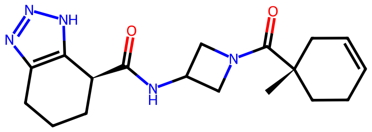	C[C@@]1(C(=O)N2CC(NC(=O)[C@H]3CCCc4nn[nH]c43)C2)CC=CCC1	998572547	25	343.2	1.3
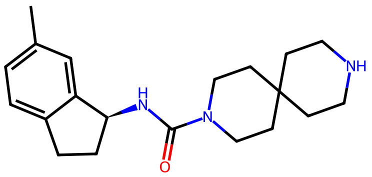	Cc1ccc2c(c1)[C@@H](NC(=O)N1CCC3(CCNCC3)CC1)CC2	669466479	24	327.23	3.16
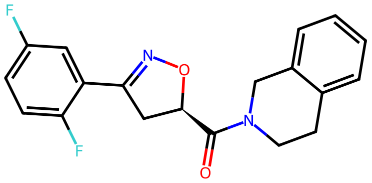	O=C([C@H]1CC(c2cc(F)ccc2F)=NO1)N1CCc2ccccc2C1	732627088	25	342.12	3.04
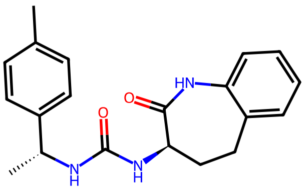	Cc1ccc([C@@H](C)NC(=O)N[C@@H]2CCc3ccccc3NC2=O)cc1	654389855	25	337.18	3.31
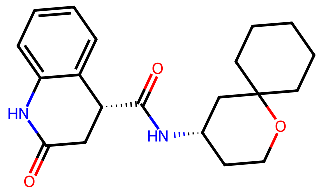	O=C1C[C@@H](C(=O)N[C@H]2CCOC3(CCCCC3)C2)c2ccccc2N1	162335522	25	342.19	3.11
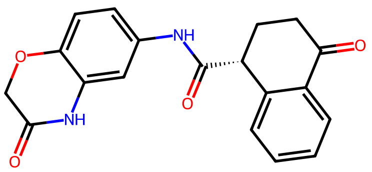	O=C1COc2ccc(NC(=O)[C@@H]3CCC(=O)c4ccccc43)cc2N1	670755735	25	336.11	2.72

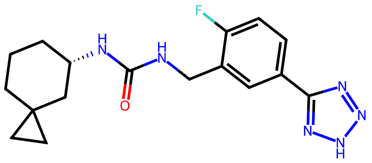	O=C(NCc1cc(-c2nn[nH]n2)ccc1F)N[C@H]1CCCC2(CC2)C1	1593119846	25	344.18	2.53
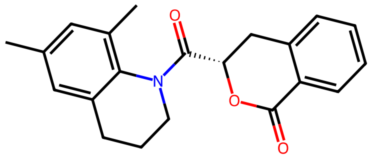	Cc1cc(C)c2c(c1)CCCN2C(=O)[C@@H]1Cc2ccccc2C(=O)O1	747308217	25	335.15	3.36
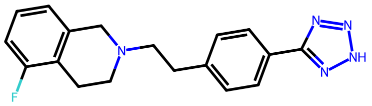	Fc1cccc2c1CCN(CCc1ccc(-c3nn[nH]n3)cc1)C2	1592658430	24	323.15	2.61
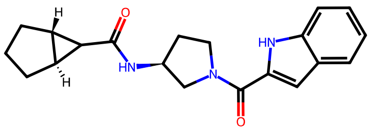	O=C(N[C@H]1CCN(C(=O)c2cc3ccccc3[nH]2)C1)C1[C@H]2CCC[C@H]12	937804030	25	337.18	2.54
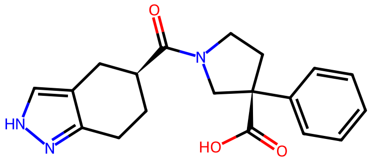	O=C([C@H]1CCc2n[nH]cc2C1)N1CC[C@](C(=O)O)(c2ccccc2)C1	909861361	25	339.16	1.77
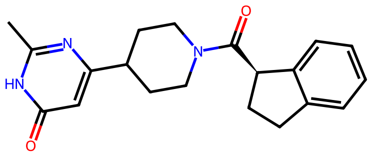	Cc1nc(C2CCN(C(=O)[C@@H]3CCc4ccccc43)CC2)cc(=O)[nH]1	82120282	25	337.18	2.51
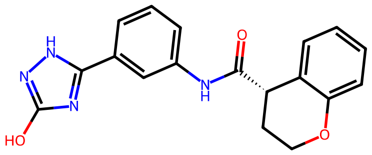	O=C(Nc1cccc(-c2nc(O)n[nH]2)c1)[C@H]1CCOc2ccccc21	370576356	25	336.12	2.68
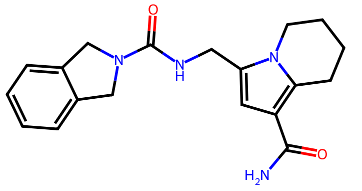	NC(=O)c1cc(CNC(=O)N2Cc3ccccc3C2)n2c1CCCC2	864358818	25	338.17	2.15

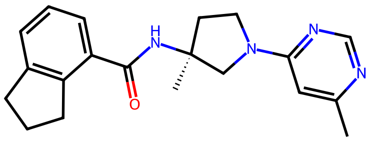	Cc1cc(N2CC[C@](C)(NC(=O)c3cccc4c3CCC4)C2)ncn1	1065262055	25	336.2	2.67
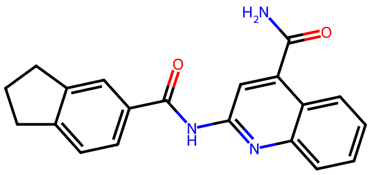	NC(=O)c1cc(NC(=O)c2ccc3c(c2)CCC3)nc2ccccc12	1618573343	25	331.13	3.07
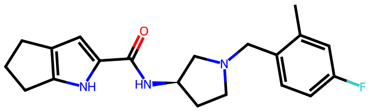	Cc1cc(F)ccc1CN1CC[C@@H](NC(=O)c2cc3c([nH]2)CCC3)C1	1015979914	25	341.19	2.96
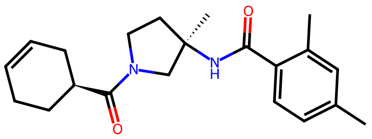	Cc1ccc(C(=O)N[C@]2(C)CCN(C(=O)[C@@H]3CC=CCC3)C2)c(C)c1	957565969	25	340.22	3.38
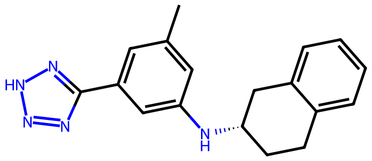	Cc1cc(N[C@H]2CCc3ccccc3C2)cc(-c2nn[nH]n2)c1	1592038216	23	305.16	3.14
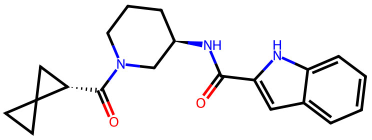	O=C(N[C@@H]1CCCN(C(=O)[C@H]2CC23CC3)C1)c1cc2ccccc2[nH]1	952144130	25	337.18	2.69
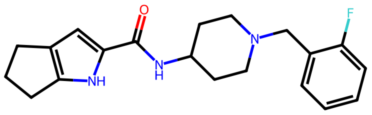	O=C(NC1CCN(Cc2ccccc2F)CC1)c1cc2c([nH]1)CCC2	1003945428	25	341.19	3.04
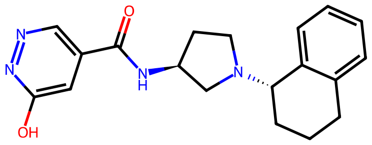	O=C(N[C@H]1CCN([C@H]2CCCc3ccccc32)C1)c1cnnc(O)c1	1014829875	25	338.17	2.06

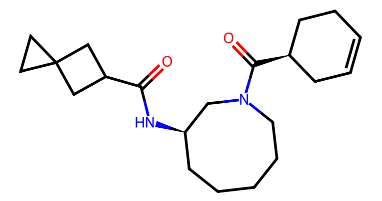	O=C(N[C@@H]1CCCCCN(C(=O)[C@@H]2CC=CCC2)C1)C1CC2(CC2)C1	986884555	25	344.25	3.42
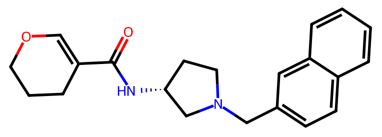	O=C(N[C@@H]1CCN(Cc2ccc3ccccc3c2)C1)C1=COCCC1	1010370548	25	336.18	3.22
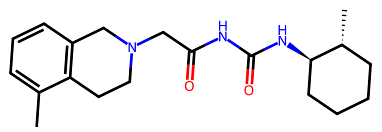	Cc1cccc2c1CCN(CC(=O)NC(=O)N[C@@H]1CCCC[C@H]1C)C2	729478531	25	343.23	2.76
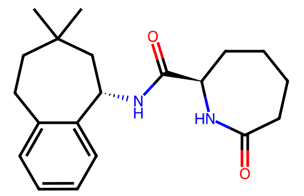	CC1(C)CCc2ccccc2[C@@H](NC(=O)[C@H]2CCCCC(=O)N2)C1	332712847	24	328.22	3.27
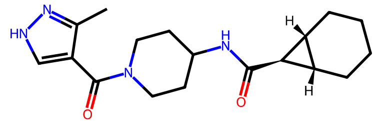	Cc1n[nH]cc1C(=O)N1CCC(NC(=O)[C@@H]2[C@H]3CCCC[C@H]32)CC1	947668623	24	330.21	1.88
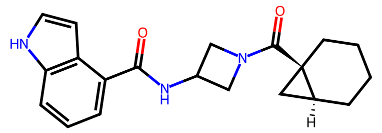	O=C(NC1CN(C(=O)[C@@]23CCCC[C@H]2C3)C1)c1cccc2[nH]ccc12	1000410744	25	337.18	2.69
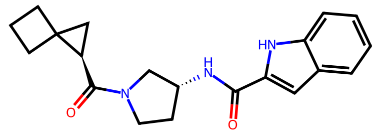	O=C(N[C@@H]1CCN(C(=O)[C@@H]2CC23CCC3)C1)c1cc2ccccc2[nH]1	942468055	25	337.18	2.69
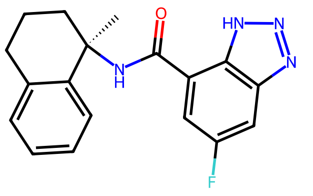	C[C@@]1(NC(=O)c2cc(F)cc3nn[nH]c23)CCCc2ccccc21	337449013	24	324.14	3.08

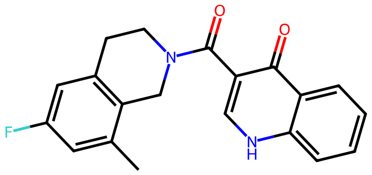	Cc1cc(F)cc2c1CN(C(=O)c1c[nH]c3ccccc3c1=O)CC2	337519873	25	336.13	3.17
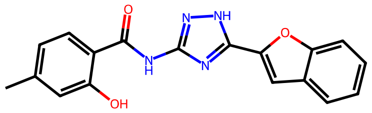	Cc1ccc(C(=O)Nc2n[nH]c(-c3cc4ccccc4o3)n2)c(O)c1	1161280100	25	334.11	3.48
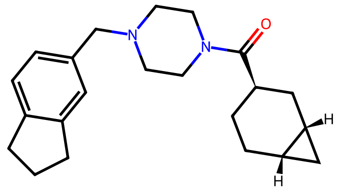	O=C([C@@H]1CC[C@H]2C[C@H]2C1)N1CCN(Cc2ccc3c(c2)CCC3)CC1	948952248	25	338.24	3.26
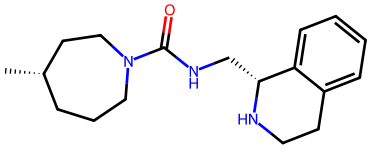	C[C@H]1CCCN(C(=O)NC[C@H]2NCCc3ccccc32)CC1	418647849	22	301.22	2.71
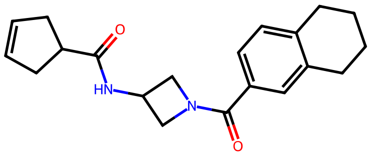	O=C(NC1CN(C(=O)c2ccc3c(c2)CCCC3)C1)C1CC=CC1	990582808	24	324.18	2.47
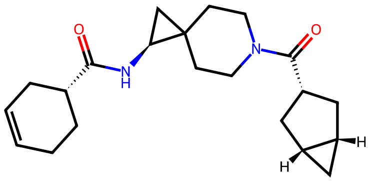	O=C(N[C@H]1CC12CCN(C(=O)[C@@H]1C[C@@H]3C[C@@H]3C1)CC2)[C@H]1CC=CCC1	963023545	25	342.23	2.89
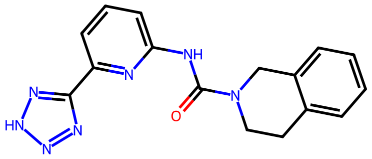	O=C(Nc1cccc(-c2nn[nH]n2)n1)N1CCc2ccccc2C1	1582644582	24	321.13	1.85
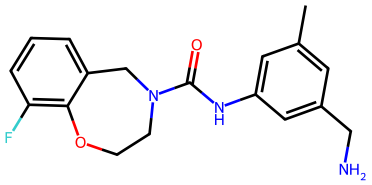	Cc1cc(CN)cc(NC(=O)N2CCOc3c(F)cccc3C2)c1	424426410	24	329.15	3.02

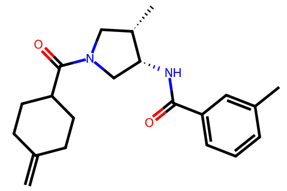	C=C1CCC(C(=O)N2C[C@H](C)[C@H](NC(=O)c3cccc(C)c3)C2)CC1	966790453	25	340.22	3.32
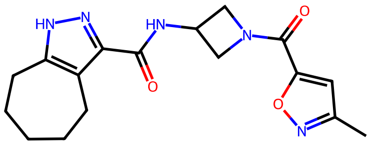	Cc1cc(C(=O)N2CC(NC(=O)c3n[nH]c4c3CCCCC4)C2)on1	991764069	25	343.16	1.23
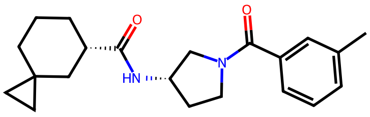	Cc1cccc(C(=O)N2CC[C@H](NC(=O)[C@H]3CCCC4(CC4)C3)C2)c1	937948926	25	340.22	3.3
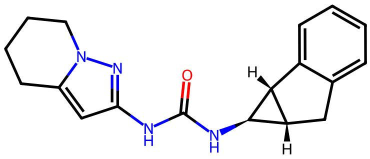	O=C(Nc1cc2n(n1)CCCC2)N[C@@H]1[C@H]2Cc3ccccc3[C@H]21	287275931	23	308.16	2.68
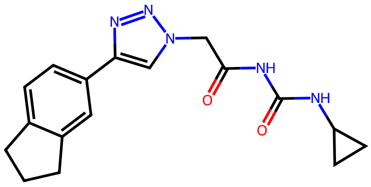	O=C(Cn1cc(-c2ccc3c(c2)CCC3)nn1)NC(=O)NC1CC1	603180255	24	325.15	1.42
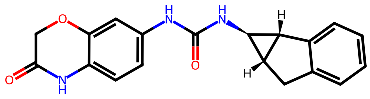	O=C1COc2cc(NC(=O)N[C@@H]3[C@H]4Cc5ccccc5[C@H]43)ccc2N1	150784675	25	335.13	2.48
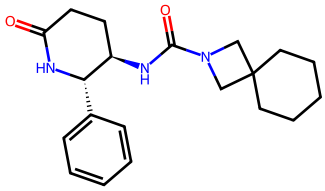	O=C1CC[C@@H](NC(=O)N2CC3(CCCCC3)C2)[C@H](c2ccccc2)N1	334127283	25	341.21	2.98
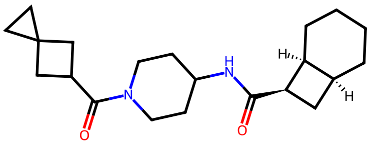	O=C(NC1CCN(C(=O)C2CC3(CC3)C2)CC1)[C@@H]1C[C@@H]2CCCC[C@@H]21	948308259	25	344.25	3.11

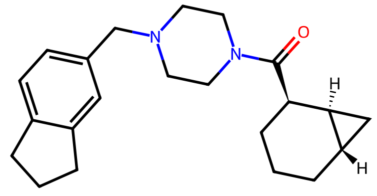	O=C([C@@H]1CCC[C@H]2C[C@@H]21)N1CCN(Cc2ccc3c(c2)CCC3)CC1	948972710	25	338.24	3.26
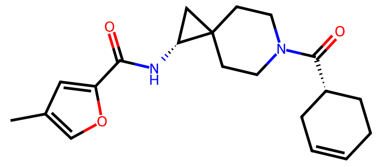	Cc1coc(C(=O)N[C@@H]2CC23CCN(C(=O)[C@H]2CC=CCC2)CC3)c1	964714522	25	342.19	3.06
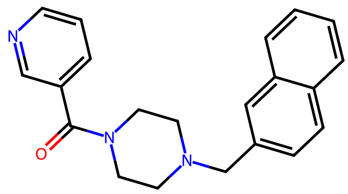	O=C(c1cccnc1)N1CCN(Cc2ccc3ccccc3c2)CC1	19399620	25	331.17	3.19
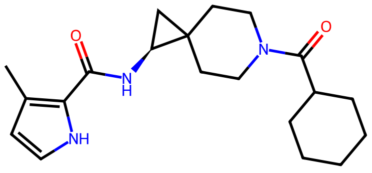	Cc1cc[nH]c1C(=O)N[C@H]1CC12CCN(C(=O)C1CCCCC1)CC2	963755801	25	343.23	3.01
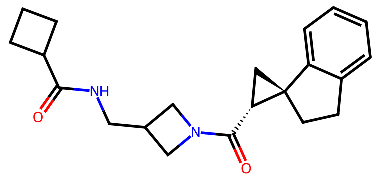	O=C(NCC1CN(C(=O)[C@@H]2C[C@@]23CCc2ccccc23)C1)C1CCC1	1000613613	25	338.2	2.27
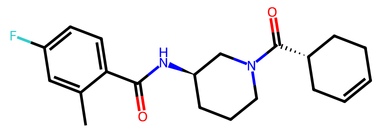	Cc1cc(F)ccc1C(=O)N[C@@H]1CCCN(C(=O)[C@H]2CC=CCC2)C1	949984229	25	344.19	3.21
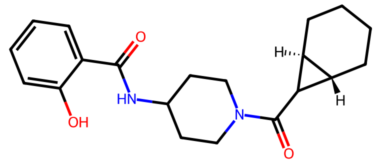	O=C(NC1CCN(C(=O)C2[C@H]3CCCC[C@H]23)CC1)c1ccccc1O	945957380	25	342.19	2.55
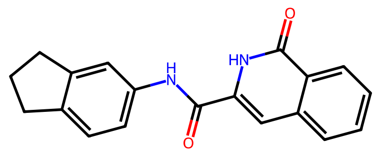	O=C(Nc1ccc2c(c1)CCC2)c1cc2ccccc2c(=O)[nH]1	7899919	23	304.12	3.27

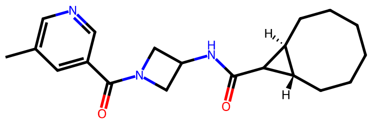	Cc1cncc(C(=O)N2CC(NC(=O)C3[C@H]4CCCCCC[C@H]34)C2)c1	1000486570	25	341.21	2.55
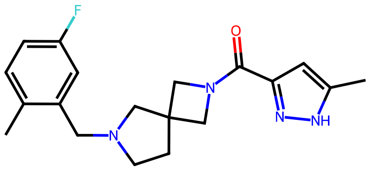	Cc1cc(C(=O)N2CC3(CCN(Cc4cc(F)ccc4C)C3)C2)n[nH]1	1072519857	25	342.19	2.51
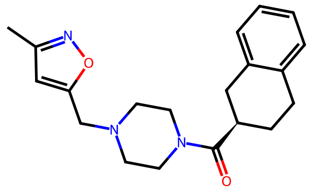	Cc1cc(CN2CCN(C(=O)[C@@H]3CCc4ccccc4C3)CC2)on1	569814636	25	339.19	2.43
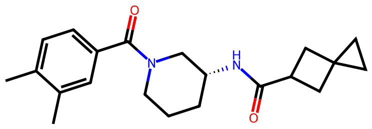	Cc1ccc(C(=O)N2CCC[C@@H](NC(=O)C3CC4(CC4)C3)C2)cc1C	951998531	25	340.22	3.21
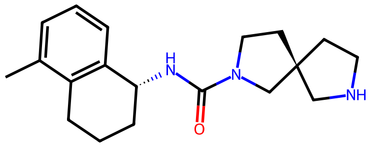	Cc1cccc2c1CCC[C@H]2NC(=O)N1CC[C@]2(CCNC2)C1	659628758	23	313.22	2.77
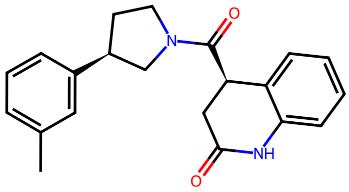	Cc1cccc([C@H]2CCN(C(=O)[C@@H]3CC(=O)Nc4ccccc43)C2)c1	273124493	25	334.17	3.44
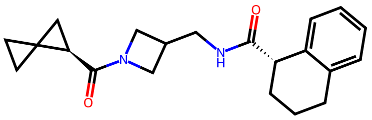	O=C(NCC1CN(C(=O)[C@@H]2CC23CC3)C1)[C@H]1CCCc2ccccc21	1003035024	25	338.2	2.48
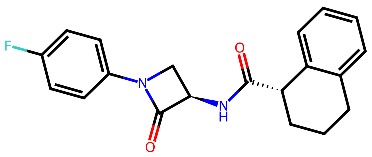	O=C(N[C@@H]1CN(c2ccc(F)cc2)C1=O)[C@H]1CCCc2ccccc21	360848567	25	338.14	2.78

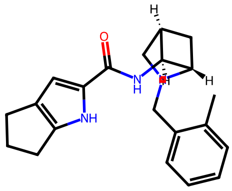	Cc1ccccc1CN1C[C@H]2C[C@H]1[C@H]2NC(=O)c1cc2c([nH]1)CCC2	1037675228	25	335.2	2.81
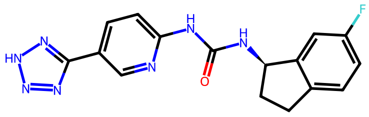	O=C(Nc1ccc(-c2nn[nH]n2)cn1)N[C@@H]1CCc2ccc(F)cc21	1606916184	25	339.12	2.21
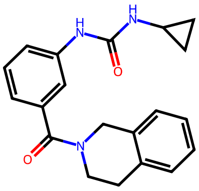	O=C(Nc1cccc(C(=O)N2CCc3ccccc3C2)c1)NC1CC1	54799939	25	335.16	3.17
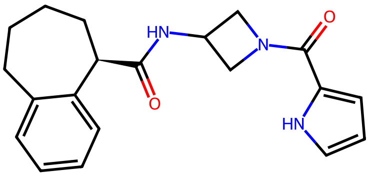	O=C(NC1CN(C(=O)c2ccc[nH]2)C1)[C@@H]1CCCCc2ccccc21	994304879	25	337.18	2.47
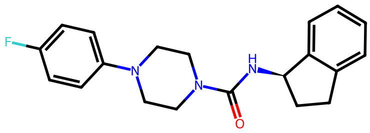	O=C(N[C@@H]1CCc2ccccc21)N1CCN(c2ccc(F)cc2)CC1	45596295	25	339.17	3.34
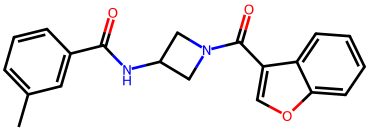	Cc1cccc(C(=O)NC2CN(C(=O)c3coc4ccccc34)C2)c1	990141475	25	334.13	3.0
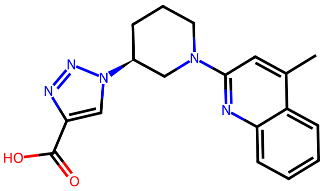	Cc1cc(N2CCC[C@H](n3cc(C(=O)O)nn3)C2)nc2ccccc12	1346113622	25	337.15	2.67
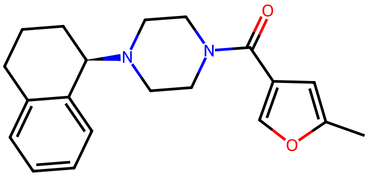	Cc1cc(C(=O)N2CCN([C@@H]3CCCc4ccccc43)CC2)co1	1565501958	24	324.18	3.42

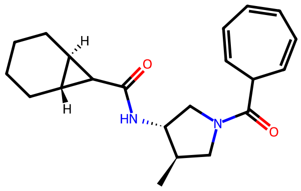	C[C@@H]1CN(C(=O)C2C=CC=CC=C2)C[C@H]1NC(=O)C1[C@@H]2CCCC[C@@H]12	949636606	25	340.22	2.68
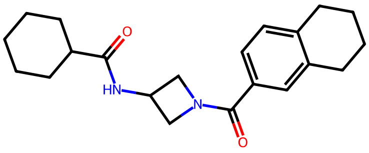	O=C(NC1CN(C(=O)c2ccc3c(c2)CCCC3)C1)C1CCCCC1	990100205	25	340.22	3.09
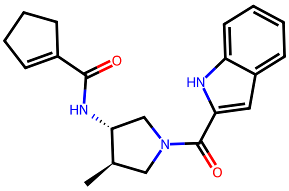	C[C@@H]1CN(C(=O)c2cc3ccccc3[nH]2)C[C@H]1NC(=O)C1=CCCC1	959693522	25	337.18	2.85
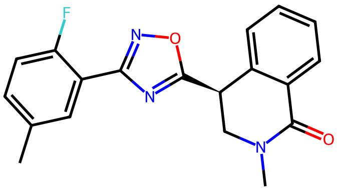	Cc1ccc(F)c(-c2noc([C@@H]3CN(C)C(=O)c4ccccc43)n2)c1	377463017	25	337.12	3.4
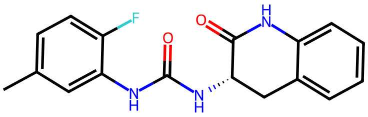	Cc1ccc(F)c(NC(=O)N[C@H]2Cc3ccccc3NC2=O)c1	1185268866	23	313.12	2.82
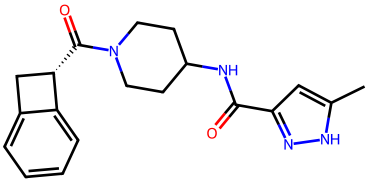	Cc1cc(C(=O)NC2CCN(C(=O)[C@H]3Cc4ccccc43)CC2)n[nH]1	948059682	25	338.17	1.78
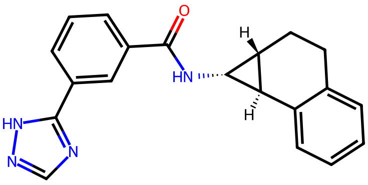	O=C(N[C@H]1[C@@H]2c3ccccc3CC[C@H]21)c1cccc(-c2ncn[nH]2)c1	159112919	25	330.15	2.93
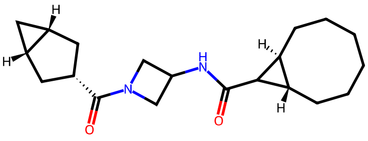	O=C(NC1CN(C(=O)[C@@H]2C[C@@H]3C[C@@H]3C2)C1)C1[C@H]2CCCCCC[C@H]12	1000507941	24	330.23	2.58

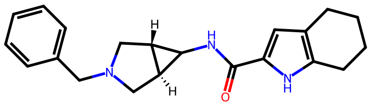	O=C(NC1[C@H]2CN(Cc3ccccc3)C[C@H]12)c1cc2c([nH]1)CCCC2	962225414	25	335.2	2.75
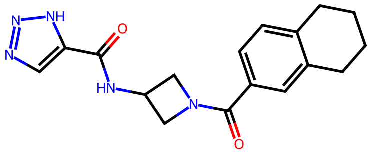	O=C(NC1CN(C(=O)c2ccc3c(c2)CCCC3)C1)c1cnn[nH]1	995184727	24	325.15	0.94
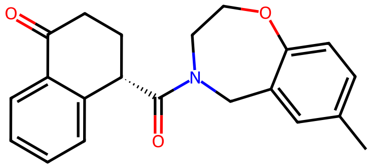	Cc1ccc2c(c1)CN(C(=O)[C@H]1CCC(=O)c3ccccc31)CCO2	376412632	25	335.15	3.48
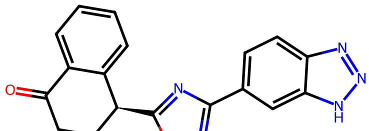	O=C1CC[C@H](c2nc(-c3ccc4nn[nH]c4c3)no2)c2ccccc21	350488877	25	331.11	3.12
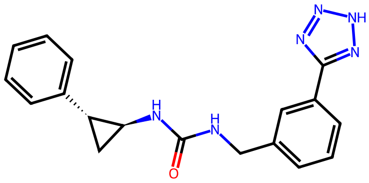	O=C(NCc1cccc(-c2nn[nH]n2)c1)N[C@H]1C[C@@H]1c1ccccc1	1593142559	25	334.15	2.22
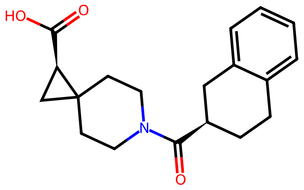	O=C(O)[C@@H]1CC12CCN(C(=O)[C@@H]1CCc3ccccc3C1)CC2	405366292	23	313.17	2.5
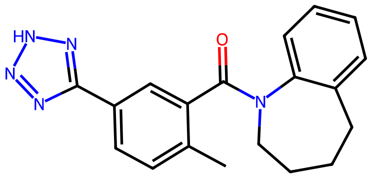	Cc1ccc(-c2nn[nH]n2)cc1C(=O)N1CCCCc2ccccc21	1581135076	25	333.16	3.16
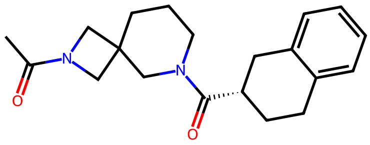	CC(=O)N1CC2(CCCN(C(=O)[C@H]3CCc4ccccc4C3)C2)C1	1010449272	24	326.2	2.26

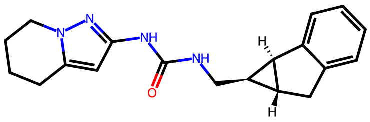	O=C(NC[C@@H]1[C@H]2Cc3ccccc3[C@@H]21)Nc1cc2n(n1)CCCC2	287410920	24	322.18	2.93
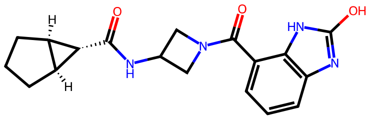	O=C(NC1CN(C(=O)c2cccc3nc(O)[nH]c23)C1)[C@@H]1[C@H]2CCC[C@H]21	990699339	25	340.15	1.26
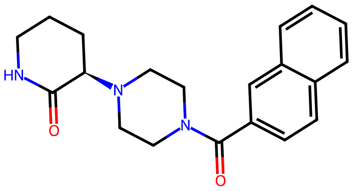	O=C1NCCC[C@H]1N1CCN(C(=O)c2ccc3ccccc3c2)CC1	957443427	25	337.18	1.88
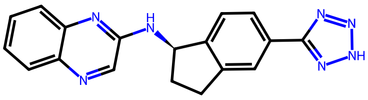	c1ccc2nc(N[C@@H]3CCc4cc(-c5nn[nH]n5)ccc43)cnc2c1	1593582598	25	329.14	2.91
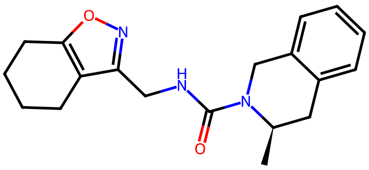	C[C@@H]1Cc2ccccc2CN1C(=O)NCc1noc2c1CCCC2	580471147	24	325.18	3.21
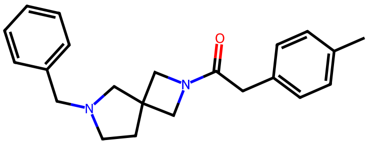	Cc1ccc(CC(=O)N2CC3(CCN(Cc4ccccc4)C3)C2)cc1	1072902360	25	334.2	3.27
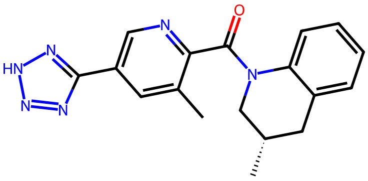	Cc1cc(-c2nn[nH]n2)cnc1C(=O)N1C[C@@H](C)Cc2ccccc21	1591875295	25	334.15	2.41
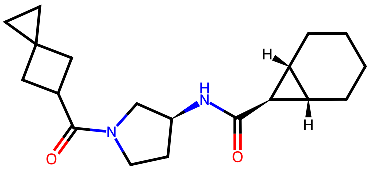	O=C(N[C@H]1CCN(C(=O)C2CC3(CC3)C2)C1)[C@@H]1[C@H]2CCCC[C@H]21	937791683	23	316.22	2.33

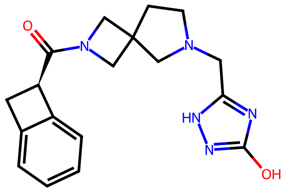	O=C([C@@H]1Cc2ccccc21)N1CC2(CCN(Cc3nc(O)n[nH]3)C2)C1	1072931458	25	339.17	0.88
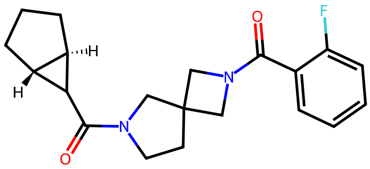	O=C(c1ccccc1F)N1CC2(CCN(C(=O)C3[C@@H]4CCC[C@@H]34)C2)C1	1018712131	25	342.17	2.55
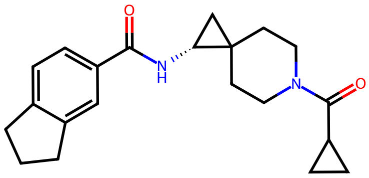	O=C(N[C@@H]1CC12CCN(C(=O)C1CC1)CC2)c1ccc2c(c1)CCC2	963835191	25	338.2	2.7
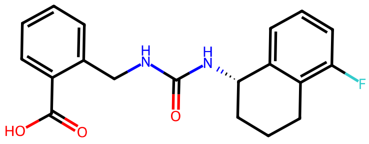	O=C(NCc1ccccc1C(=O)O)N[C@H]1CCCc2c(F)cccc21	874402627	25	342.14	3.4
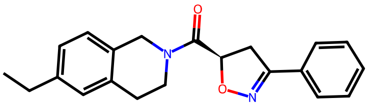	CCc1ccc2c(c1)CCN(C(=O)[C@H]1CC(c3ccccc3)=NO1)C2	1440188148	25	334.17	3.33
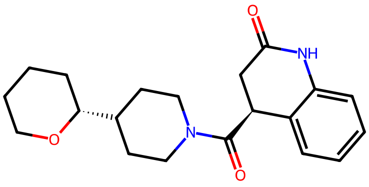	O=C1C[C@H](C(=O)N2CCC([C@H]3CCCCO3)CC2)c2ccccc2N1	1413278225	25	342.19	2.92
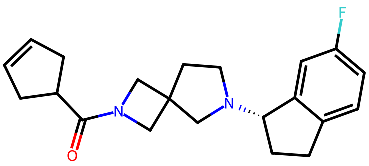	O=C(C1CC=CC1)N1CC2(CCN([C@H]3CCc4ccc(F)cc43)C2)C1	1072571338	25	340.2	3.31
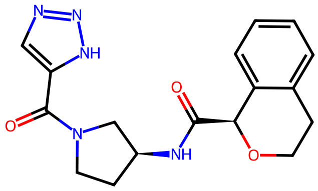	O=C(N[C@H]1CCN(C(=O)c2cnn[nH]2)C1)[C@@H]1OCCc2ccccc21	938396882	25	341.15	0.45

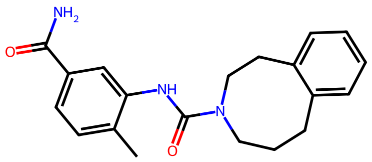	Cc1ccc(C(N)=O)cc1NC(=O)N1CCCc2<br>ccccc2CC1	562850994	25	337.18	3.12
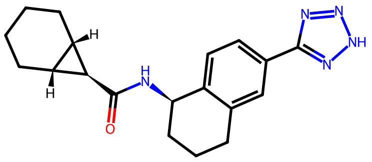	O=C(N[C@@H]1CCCc2cc(-c3nn[nH]n<br>3)ccc21)[C@@H]1[C@H]2CCCC[C@<br>H]21	1592985268	25	337.19	2.8
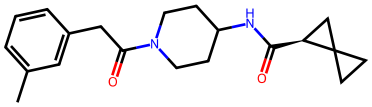	Cc1cccc(CC(=O)N2CCC(NC(=O)[C@<br>H]3CC34CC4)CC2)c1	948062673	24	326.2	2.44
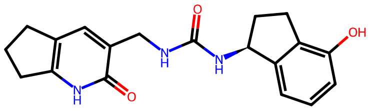	O=C(NCc1cc2c([nH]c1=O)CCC2)N[C<br>@H]1CCc2c(O)cccc21	74207309	25	339.16	2.06
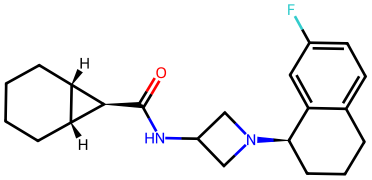	O=C(NC1CN([C@@H]2CCCc3ccc(F)<br>cc32)C1)[C@@H]1[C@H]2CCCC[C@<br>H]21	1030189258	25	342.21	3.44
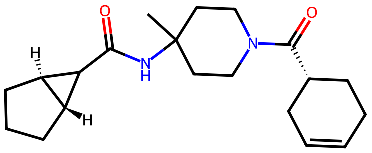	CC1(NC(=O)C2[C@@H]3CCC[C@@<br>H]23)CCN(C(=O)[C@H]2CC=CCC2)C<br>C1	956327126	24	330.23	2.89
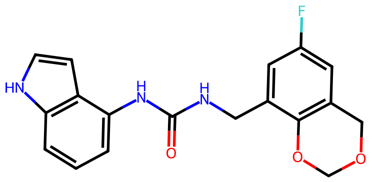	O=C(NCc1cc(F)cc2c1OCOC2)Nc1ccc<br>c2[nH]ccc12	171392727	25	341.12	3.5
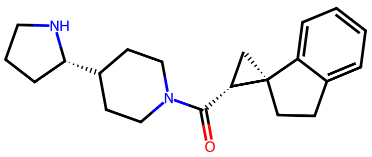	O=C([C@@H]1C[C@]12CCc1ccccc12<br>)N1CCC([C@@H]2CCCN2)CC1	353052301	24	324.22	2.88

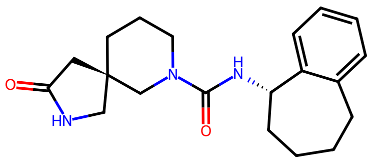	O=C1C[C@]2(CCCN(C(=O)N[C@H]3CCCCc4ccccc43)C2)CN1	525766801	25	341.21	2.77
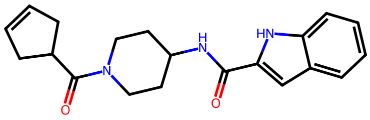	O=C(NC1CCN(C(=O)C2CC=CC2)CC1)c1cc2ccccc2[nH]1	945717399	25	337.18	2.85
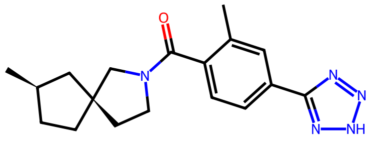	Cc1cc(-c2nn[nH]n2)ccc1C(=O)N1CC[C@]2(CC[C@@H](C)C2)C1	826245099	24	325.19	2.83
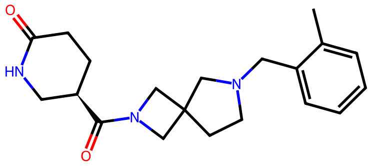	Cc1ccccc1CN1CCC2(C1)CN(C(=O)[C@@H]1CCC(=O)NC1)C2	1072886102	25	341.21	1.56
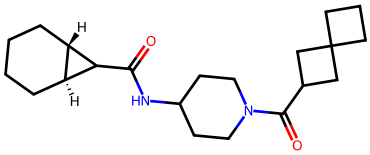	O=C(NC1CCN(C(=O)C2CC3(CCC3)C2)CC1)C1[C@H]2CCCC[C@H]12	947882836	25	344.25	3.11
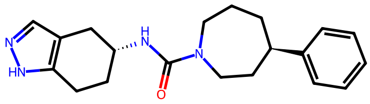	O=C(N[C@@H]1CCc2[nH]ncc2C1)N1CCC[C@@H](c2ccccc2)CC1	154092030	25	338.21	3.25
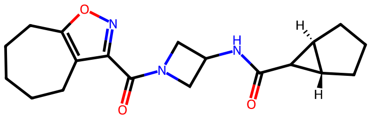	O=C(NC1CN(C(=O)c2noc3c2CCCCC3)C1)C1[C@H]2CCC[C@H]12	990712012	25	343.19	1.93
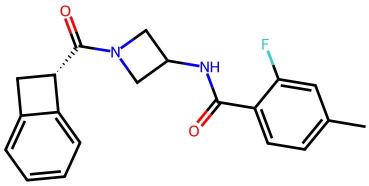	Cc1ccc(C(=O)NC2CN(C(=O)[C@H]3Cc4ccccc43)C2)c(F)c1	1000493049	25	338.14	2.41

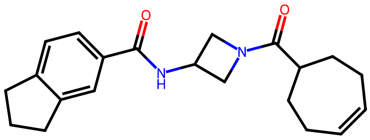	O=C(NC1CN(C(=O)C2CCC=CCC2)C1)c1ccc2c(c1)CCC2	991371463	25	338.2	2.86
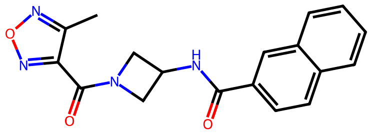	Cc1nonc1C(=O)N1CC(NC(=O)c2ccc3ccccc3c2)C1	999453716	25	336.12	1.79
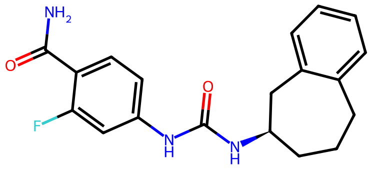	NC(=O)c1ccc(NC(=O)N[C@@H]2CCCc3ccccc3C2)cc1F	930183414	25	341.15	2.99
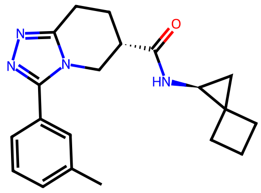	Cc1cccc(-c2nnc3n2C[C@@H](C(=O)N[C@H]2CC24CCC4)CC3)c1	874777758	25	336.2	2.87
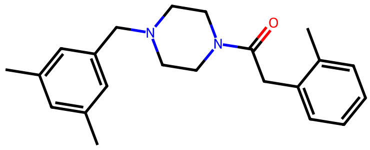	Cc1cc(C)cc(CN2CCN(C(=O)Cc3ccccc3C)CC2)c1	523370945	25	336.22	3.5
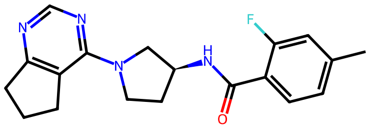	Cc1ccc(C(=O)N[C@H]2CCN(c3ncnc4c3CCC4)C2)c(F)c1	1058558318	25	340.17	2.42
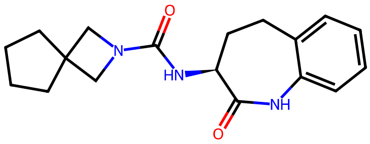	O=C1Nc2ccccc2CC[C@@H]1NC(=O)N1CC2(CCCC2)C1	654631741	23	313.18	2.53
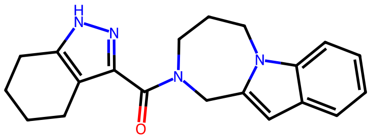	O=C(c1n[nH]c2c1CCCC2)N1CCCn2c(cc3ccccc32)C1	377192044	25	334.18	3.29

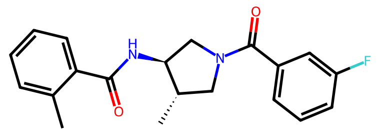	Cc1ccccc1C(=O)N[C@H]1CN(C(=O)c2cccc(F)c2)C[C@@H]1C	966102943	25	340.16	3.02
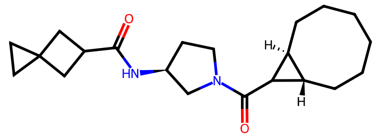	O=C(N[C@H]1CCN(C(=O)C2[C@H]3CCCCCC[C@H]23)C1)C1CC2(CC2)C1	938711873	25	344.25	3.11
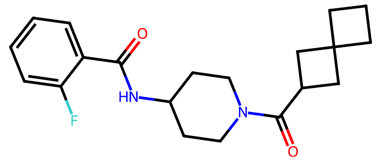	O=C(NC1CCN(C(=O)C2CC3(CCC3)C2)CC1)c1ccccc1F	947879998	25	344.19	3.13
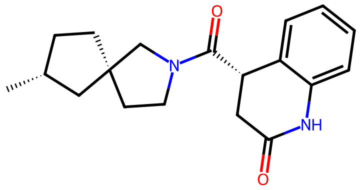	C[C@@H]1CC[C@]2(CCN(C(=O)[C@H]3CC(=O)Nc4ccccc43)C2)C1	359157417	23	312.18	3.15
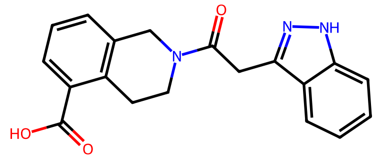	O=C(O)c1cccc2c1CCN(C(=O)Cc1n[nH]c3ccccc13)C2	1602456008	25	335.13	2.39
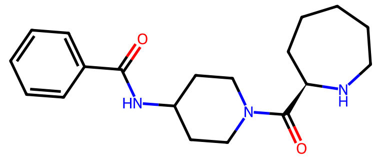	O=C(NC1CCN(C(=O)[C@H]2CCCCCN2)CC1)c1ccccc1	573491612	24	329.21	1.94
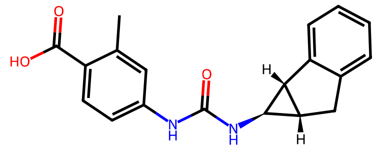	Cc1cc(NC(=O)N[C@@H]2[C@H]3Cc4ccccc4[C@H]32)ccc1C(=O)O	1594323248	24	322.13	3.15
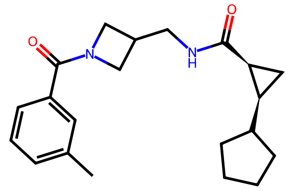	Cc1cccc(C(=O)N2CC(CNC(=O)[C@H]3C[C@H]3C3CCCC3)C2)c1	1004004898	25	340.22	3.01

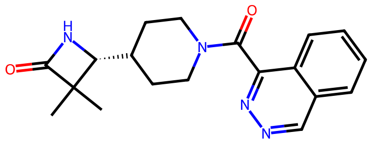	CC1(C)C(=O)N[C@@H]1C1CCN(C(=O)c2nncc3ccccc23)CC1	820746690	25	338.17	2.01
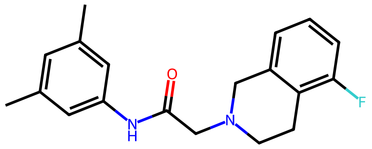	Cc1cc(C)cc(NC(=O)CN2CCc3c(F)cccc3C2)c1	757149636	23	312.16	3.44
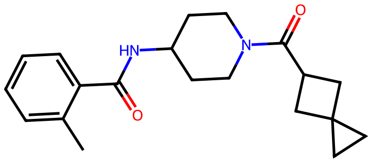	Cc1ccccc1C(=O)NC1CCN(C(=O)C2CC3(CC3)C2)CC1	666397091	24	326.2	2.91
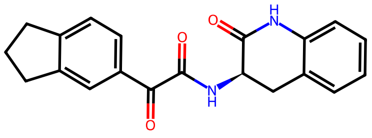	O=C(N[C@@H]1Cc2ccccc2NC1=O)C(=O)c1ccc2c(c1)CCC2	529952618	25	334.13	2.04
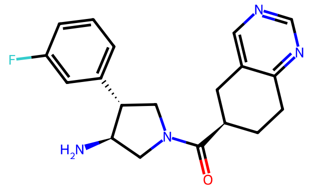	N[C@@H]1CN(C(=O)[C@@H]2CCc3ncncc3C2)C[C@H]1c1cccc(F)c1	648178046	25	340.17	1.67
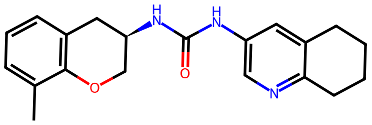	Cc1cccc2c1OC[C@H](NC(=O)Nc1cnc3c(c1)CCCC3)C2	1660701420	25	337.18	3.39
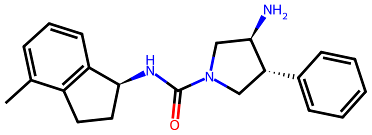	Cc1cccc2c1CC[C@@H]2NC(=O)N1C[C@@H](N)[C@H](c2ccccc2)C1	515522029	25	335.2	3.12
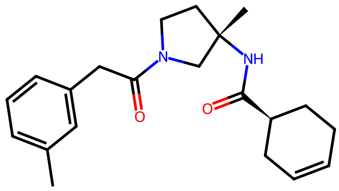	Cc1cccc(CC(=O)N2CC[C@](C)(NC(=O)[C@@H]3CC=CCC3)C2)c1	956691329	25	340.22	3.0

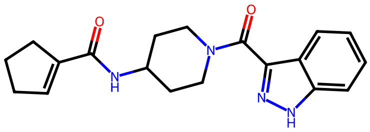	O=C(NC1CCN(C(=O)c2n[nH]c3ccccc23)CC1)C1=CCCC1	948347834	25	338.17	2.39
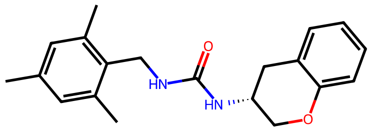	Cc1cc(C)c(CNC(=O)N[C@H]2COc3ccccc3C2)c(C)c1	195733412	24	324.18	3.41
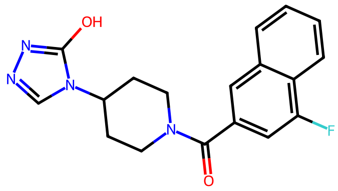	O=C(c1cc(F)c2ccccc2c1)N1CCC(n2cnnc2O)CC1	565664213	25	340.13	2.75
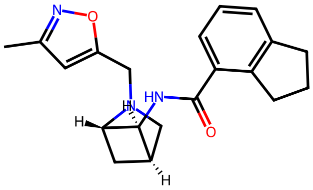	Cc1cc(CN2C[C@H]3C[C@H]2[C@H]3NC(=O)c2cccc3c2CCC3)on1	1037747786	25	337.18	2.47
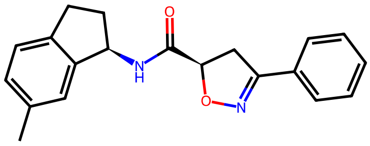	Cc1ccc2c(c1)[C@H](NC(=O)[C@H]1CC(c3ccccc3)=NO1)CC2	730191322	24	320.15	3.29
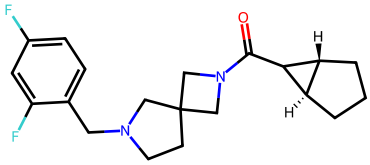	O=C(C1[C@@H]2CCC[C@@H]12)N1CC2(CCN(Cc3ccc(F)cc3F)C2)C1	1072591993	25	346.19	3.05
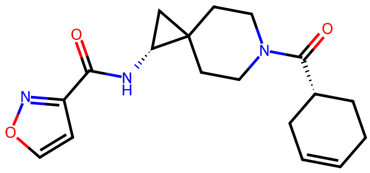	O=C(N[C@@H]1CC12CCN(C(=O)[C@H]1CC=CCC1)CC2)c1ccon1	963275469	24	329.17	2.14
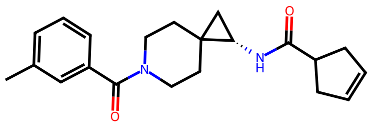	Cc1cccc(C(=O)N2CCC3(CC2)C[C@@H]3NC(=O)C2CC=CC2)c1	963377260	25	338.2	3.07

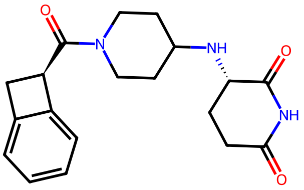	O=C1CC[C@H](NC2CCN(C(=O)[C@@H]3Cc4ccccc43)CC2)C(=O)N1	996931443	25	341.17	0.71
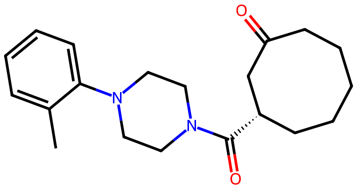	Cc1ccccc1N1CCN(C(=O)[C@H]2CCCCCC(=O)C2)CC1	362460093	24	328.22	3.18
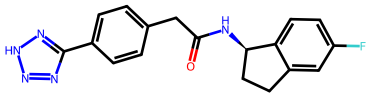	O=C(Cc1ccc(-c2nn[nH]n2)cc1)N[C@@H]1CCc2cc(F)ccc21	1592910993	25	337.13	2.35
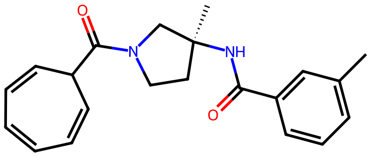	Cc1cccc(C(=O)N[C@@]2(C)CCN(C(=O)C3C=CC=CC=C3)C2)c1	956667483	25	336.18	3.01
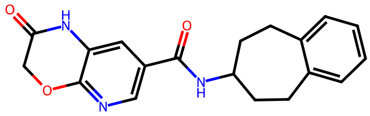	O=C1COc2ncc(C(=O)NC3CCc4ccccc4CC3)cc2N1	516954200	25	337.14	2.09
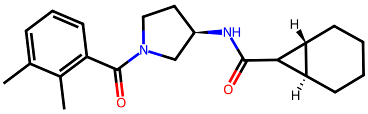	Cc1cccc(C(=O)N2CC[C@@H](NC(=O)C3[C@@H]4CCCC[C@@H]34)C2)c1C	939895627	25	340.22	3.07
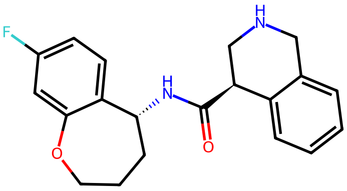	O=C(N[C@@H]1CCCOc2cc(F)ccc21)[C@H]1CNCc2ccccc21	569691033	25	340.16	3.04
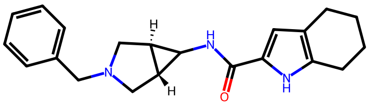	O=C(NC1[C@@H]2CN(Cc3ccccc3)C[C@@H]12)c1cc2c([nH]1)CCCC2	962225412	25	335.2	2.75

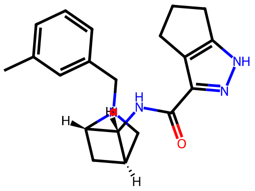	Cc1cccc(CN2C[C@H]3C[C@H]2[C@@H]3NC(=O)c2n[nH]c3c2CCC3)c1	1037484517	25	336.2	2.21
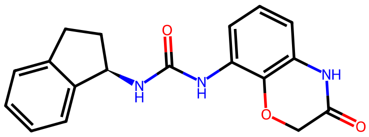	O=C1COc2c(cccc2NC(=O)N[C@@H]2CCc3ccccc32)N1	368097880	24	323.13	2.83
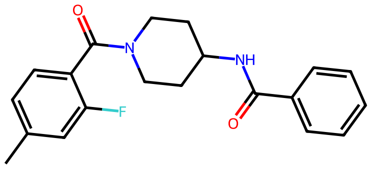	Cc1ccc(C(=O)N2CCC(NC(=O)c3ccccc3)CC2)c(F)c1	175438006	25	340.16	3.17
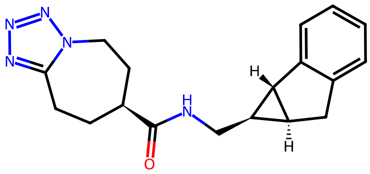	O=C(NC[C@@H]1[C@@H]2Cc3ccccc3[C@@H]12)[C@H]1CCc2nnnn2CC1	651535134	24	323.17	1.33
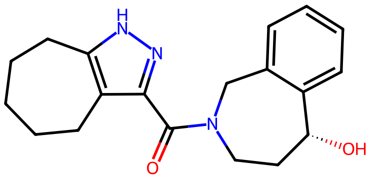	O=C(c1n[nH]c2c1CCCCC2)N1CC[C@@H](O)c2ccccc2C1	375466908	24	325.18	2.76
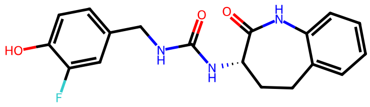	O=C(NCc1ccc(O)c(F)c1)N[C@H]1CCc2ccccc2NC1=O	1434776840	25	343.13	2.28
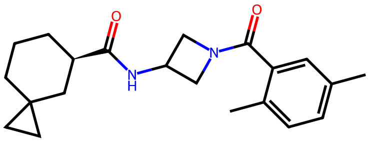	Cc1ccc(C)c(C(=O)N2CC(NC(=O)[C@@H]3CCCC4(CC4)C3)C2)c1	991515204	25	340.22	3.21
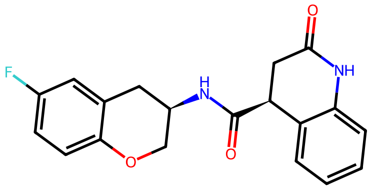	O=C1C[C@H](C(=O)N[C@H]2COc3ccc(F)cc3C2)c2ccccc2N1	290681419	25	340.12	2.37

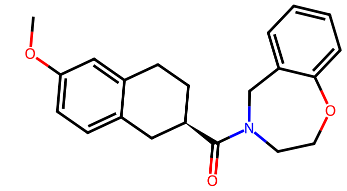	COc1ccc2c(c1)CC[C@@H](C(=O)N1CCOc3ccccc3C1)C2	878345111	25	337.17	3.22
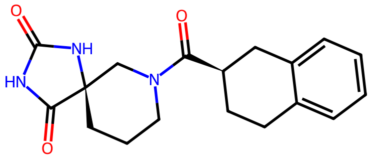	O=C1NC(=O)[C@]2(CCCN(C(=O)[C@@H]3CCc4ccccc4C3)C2)N1	392626373	24	327.16	0.99
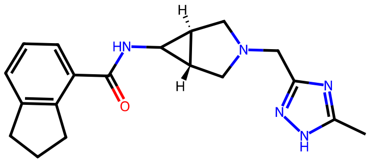	Cc1nc(CN2C[C@@H]3C(NC(=O)c4cccc5c4CCC5)[C@H]3C2)n[nH]1	962987663	25	337.19	1.46
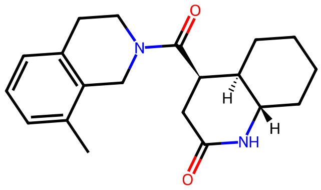	Cc1cccc2c1CN(C(=O)[C@@H]1CC(=O)N[C@H]3CCCC[C@@H]31)CC2	614003102	24	326.2	2.57
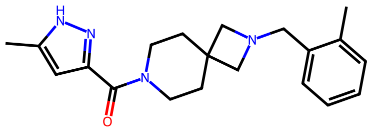	Cc1cc(C(=O)N2CCC3(CC2)CN(Cc2ccccc2C)C3)n[nH]1	1035663526	25	338.21	2.76
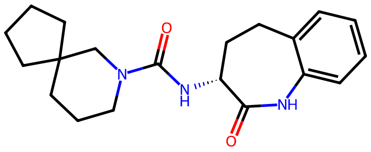	O=C1Nc2ccccc2CC[C@H]1NC(=O)N1CCCC2(CCCC2)C1	847396979	25	341.21	3.31
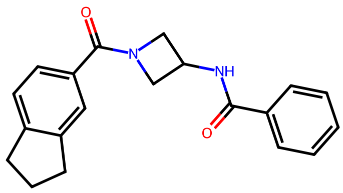	O=C(NC1CN(C(=O)c2ccc3c(c2)CCC3)C1)c1ccccc1	990096188	24	320.15	2.43
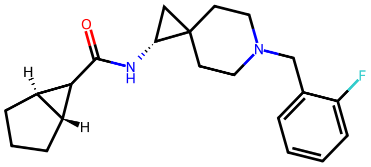	O=C(N[C@@H]1CC12CCN(Cc1ccccc1F)CC2)C1[C@@H]2CCC[C@@H]12	1016565289	25	342.21	3.34

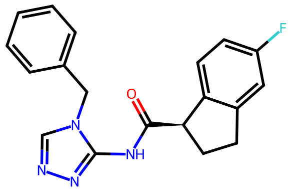	O=C(Nc1nncn1Cc1ccccc1)[C@@H]1CCc2cc(F)ccc21	1618159648	25	336.14	3.13
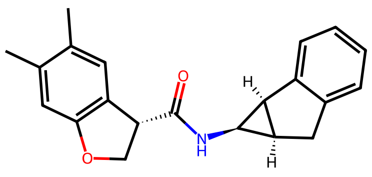	Cc1cc2c(cc1C)[C@H](C(=O)N[C@@H]1[C@@H]3Cc4ccccc4[C@@H]31)CO2	248634222	24	319.16	3.23
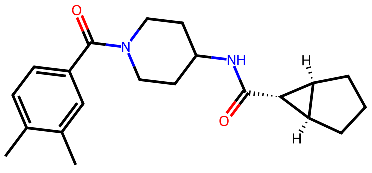	Cc1ccc(C(=O)N2CCC(NC(=O)[C@@H]3[C@H]4CCC[C@H]43)CC2)cc1C	948347890	25	340.22	3.07
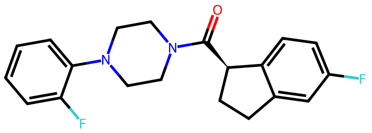	O=C([C@@H]1CCc2cc(F)ccc21)N1CCN(c2ccccc2F)CC1	1463626500	25	342.15	3.34
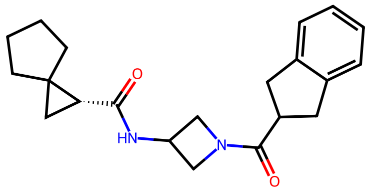	O=C(NC1CN(C(=O)C2Cc3ccccc3C2)C1)[C@@H]1CC12CCCC2	999629673	25	338.2	2.31
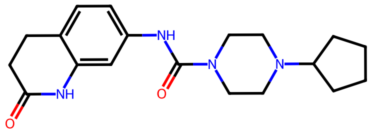	O=C1CCc2ccc(NC(=O)N3CCN(C4CCCC4)CC3)cc2N1	173756395	25	342.21	2.66
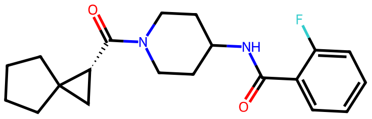	O=C(NC1CCN(C(=O)[C@@H]2CC23CCCC3)CC1)c1ccccc1F	948360707	25	344.19	3.13
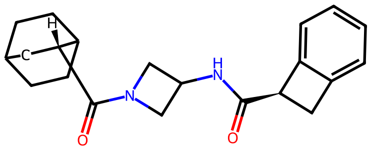	O=C(NC1CN(C(=O)[C@@H]2CC3CCC2CC3)C1)[C@@H]1Cc2ccccc21	998687514	25	338.2	2.48

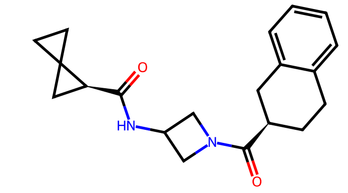	O=C(NC1CN(C(=O)[C@@H]2CCc3ccccc3C2)C1)[C@H]1CC12CC2	1000508413	24	324.18	1.92
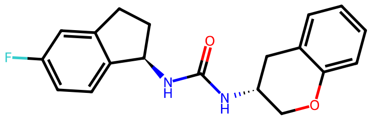	O=C(N[C@H]1COc2ccccc2C1)N[C@@H]1CCc2cc(F)ccc21	193788746	24	326.14	3.12
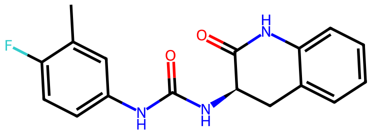	Cc1cc(NC(=O)N[C@@H]2Cc3ccccc3NC2=O)ccc1F	1192400784	23	313.12	2.82
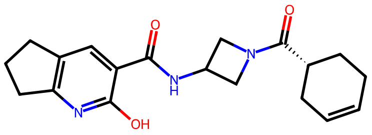	O=C(NC1CN(C(=O)[C@H]2CC=CCC2)C1)c1cc2c(nc1O)CCC2	991734732	25	341.17	1.57
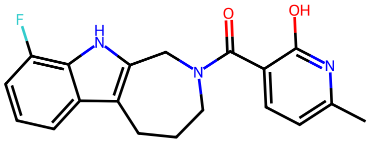	Cc1ccc(C(=O)N2CCCc3c([nH]c4c(F)cccc34)C2)c(O)n1	374111367	25	339.14	3.3
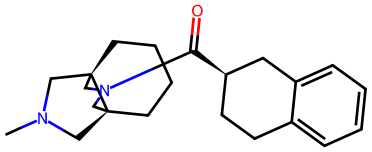	CN1C[C@]23CCCC[C@]2(C1)CN(C(=O)[C@@H]1CCc2ccccc2C1)C3	1337791352	25	338.24	3.13
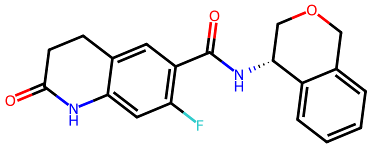	O=C1CCc2cc(C(=O)N[C@@H]3COCc4ccccc43)c(F)cc2N1	577364626	25	340.12	2.71
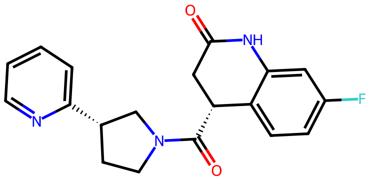	O=C1C[C@@H](C(=O)N2CC[C@H](c3ccccn3)C2)c2ccc(F)cc2N1	798720351	25	339.14	2.66

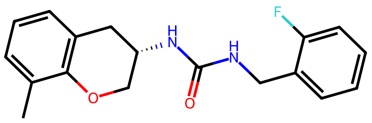	Cc1cccc2c1OC[C@@H](NC(=O)NCc1ccccc1F)C2	357445634	23	314.14	2.94
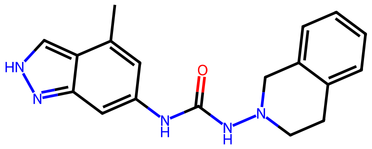	Cc1cc(NC(=O)NN2CCc3ccccc3C2)cc2n[nH]cc12	1669490914	24	321.16	2.97
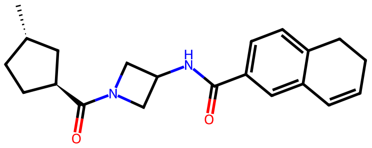	C[C@H]1CC[C@H](C(=O)N2CC(NC(=O)c3ccc4c(c3)C=CCC4)C2)C1	995375363	25	338.2	3.02
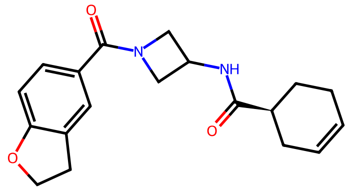	O=C(NC1CN(C(=O)c2ccc3c(c2)CCO3)C1)[C@@H]1CC=CCC1	990219830	24	326.16	1.92
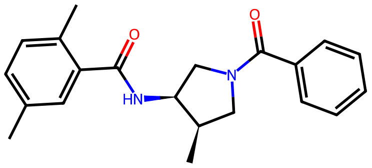	Cc1ccc(C)c(C(=O)N[C@H]2CN(C(=O)c3ccccc3)C[C@H]2C)c1	966089244	25	336.18	3.19
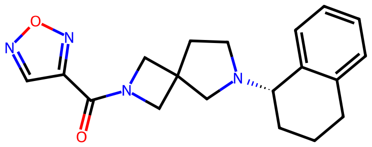	O=C(c1cnon1)N1CC2(CCN([C@H]3CCCc4ccccc43)C2)C1	1073068421	25	338.17	2.3
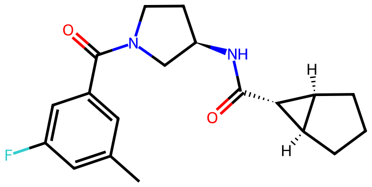	Cc1cc(F)cc(C(=O)N2CC[C@@H](NC(=O)[C@@H]3[C@H]4CCC[C@H]43)C2)c1	936245387	24	330.17	2.51
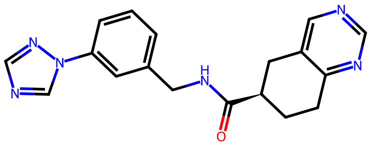	O=C(NCc1cccc(-n2cncn2)c1)[C@@H]1CCc2ncncc2C1	648292397	25	334.15	1.48

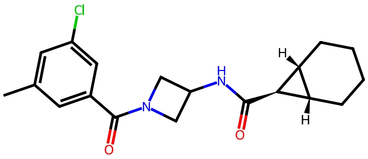	Cc1cc(Cl)cc(C(=O)N2CC(NC(=O)[C@@H]3[C@H]4CCCC[C@H]43)C2)c1	990653532	24	346.14	3.03
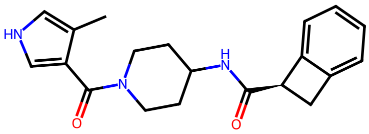	Cc1c[nH]cc1C(=O)N1CCC(NC(=O)[C@@H]2Cc3ccccc32)CC1	946791144	25	337.18	2.38
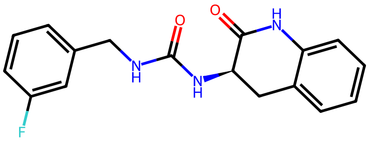	O=C(NCc1cccc(F)c1)N[C@@H]1Cc2ccccc2NC1=O	1185841344	23	313.12	2.19
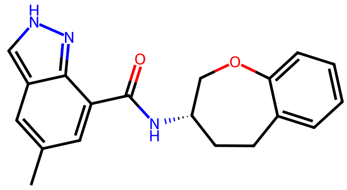	Cc1cc(C(=O)N[C@H]2CCc3ccccc3OC2)c2n[nH]cc2c1	338280038	24	321.15	2.99
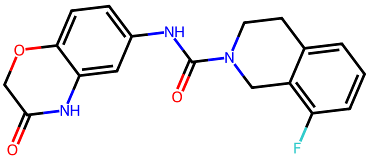	O=C1COc2ccc(NC(=O)N3CCc4cccc(F)c4C3)cc2N1	159028311	25	341.12	2.75
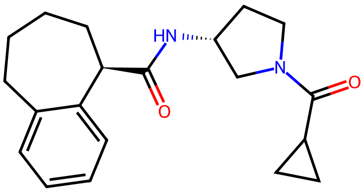	O=C(N[C@@H]1CCN(C(=O)C2CC2)C1)[C@@H]1CCCCc2ccccc21	941119654	24	326.2	2.62
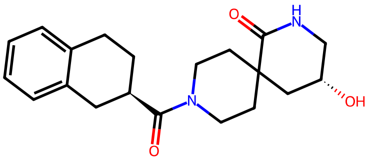	O=C([C@@H]1CCc2ccccc2C1)N1CCC2(CC1)C[C@@H](O)CNC2=O	885016105	25	342.19	1.28
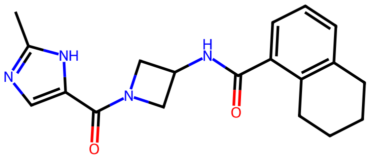	Cc1ncc(C(=O)N2CC(NC(=O)c3cccc4c3CCCC4)C2)[nH]1	991903984	25	338.17	1.85

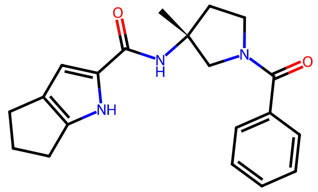	C[C@@]1(NC(=O)c2cc3c([nH]2)CCC3)CCN(C(=O)c2ccccc2)C1	957207577	25	337.18	2.54
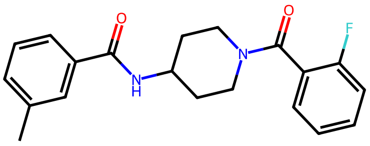	Cc1cccc(C(=O)NC2CCN(C(=O)c3ccccc3F)CC2)c1	32926455	25	340.16	3.17
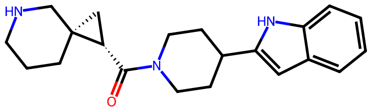	O=C([C@H]1C[C@@]12CCCNC2)N1CCC(c2cc3ccccc3[nH]2)CC1	848397459	25	337.22	3.26
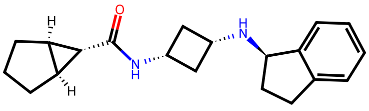	O=C(N[C@H]1C[C@@H](N[C@@H]2CCc3ccccc32)C1)[C@@H]1[C@H]2CCC[C@H]21	1021946329	23	310.2	2.96
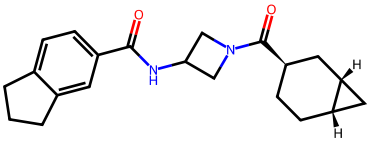	O=C(NC1CN(C(=O)[C@@H]2CC[C@H]3C[C@H]3C2)C1)c1ccc2c(c1)CCC2	991373577	25	338.2	2.55
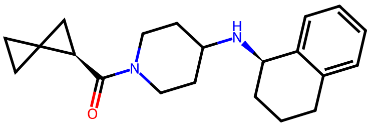	O=C([C@@H]1CC12CC2)N1CCC(N[C@@H]2CCCc3ccccc32)CC1	997040920	24	324.22	3.44
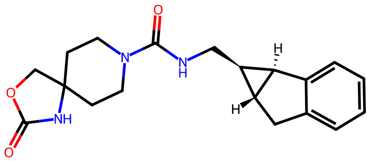	O=C1NC2(CCN(C(=O)NC[C@@H]3[C@H]4Cc5ccccc5[C@@H]43)CC2)CO1	495141964	25	341.17	1.86
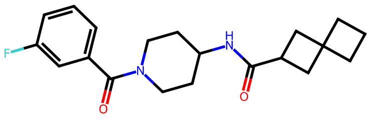	O=C(NC1CCN(C(=O)c2cccc(F)c2)CC1)C1CC2(CCC2)C1	943789268	25	344.19	3.13

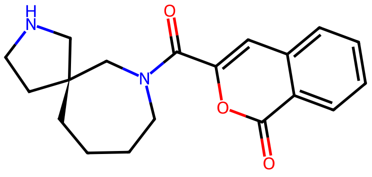	O=C(c1cc2ccccc2c(=O)o1)N1CCCC[C@]2(CCNC2)C1	1580813468	24	326.16	2.4
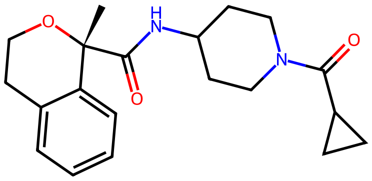	C[C@]1(C(=O)NC2CCN(C(=O)C3CC3)CC2)OCCc2ccccc21	639493538	25	342.19	1.99
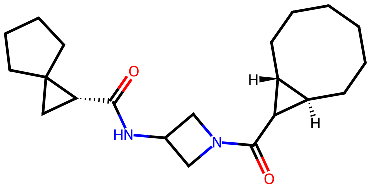	O=C(NC1CN(C(=O)C2[C@@H]3CCCCCC[C@@H]23)C1)[C@@H]1CC12CCC2	999633190	25	344.25	3.11
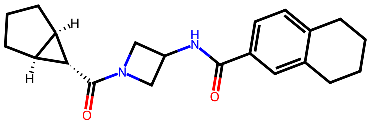	O=C(NC1CN(C(=O)[C@@H]2[C@H]3CCC[C@H]32)C1)c1ccc2c(c1)CCCC2	1000057094	25	338.2	2.55
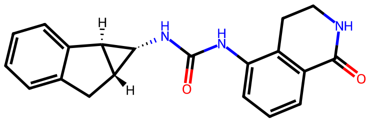	O=C(Nc1cccc2c1CCNC2=O)N[C@H]1[C@@H]2c3ccccc3C[C@H]21	557142763	25	333.15	2.43
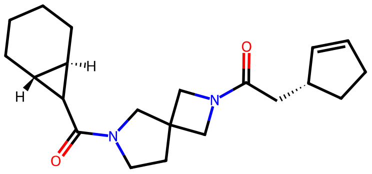	O=C(C[C@@H]1C=CCC1)N1CC2(CCN(C(=O)C3[C@@H]4CCCC[C@@H]34)C2)C1	1018760229	25	342.23	2.84
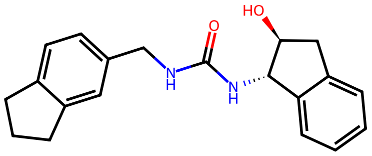	O=C(NCc1ccc2c(c1)CCC2)N[C@H]1c2ccccc2C[C@@H]1O	79158204	24	322.17	2.63
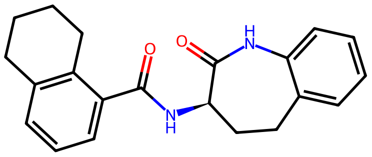	O=C(N[C@@H]1CCc2ccccc2NC1=O)c1cccc2c1CCCC2	653998895	25	334.17	3.25

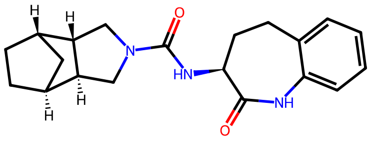	O=C1Nc2ccccc2CC[C@@H]1NC(=O)N1C[C@H]2[C@H]3CC[C@@H](C3)[C@@H]2C1	654633850	25	339.19	2.63
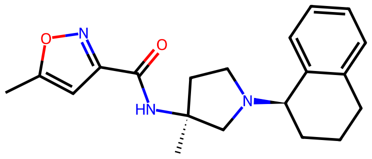	Cc1cc(C(=O)N[C@@]2(C)CCN([C@@H]3CCCc4ccccc43)C2)no1	1046121932	25	339.19	3.25
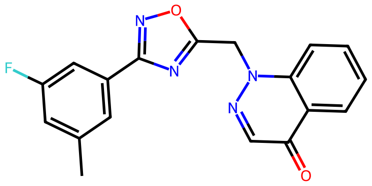	Cc1cc(F)cc(-c2noc(Cn3ncc(=O)c4ccccc43)n2)c1	355856300	25	336.1	2.94
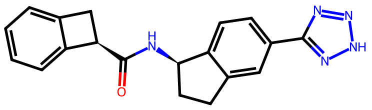	O=C(N[C@@H]1CCc2cc(-c3nn[nH]n3)ccc21)[C@@H]1Cc2ccccc21	1609543185	25	331.14	2.31
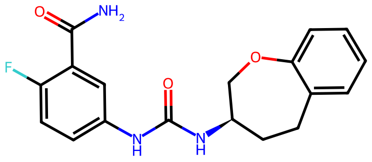	NC(=O)c1cc(NC(=O)N[C@@H]2CCc3ccccc3OC2)ccc1F	801613706	25	343.13	2.44
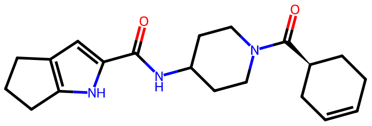	O=C(NC1CCN(C(=O)[C@@H]2CC=CCC2)CC1)c1cc2c([nH]1)CCC2	944114342	25	341.21	2.58
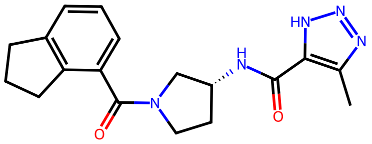	Cc1nn[nH]c1C(=O)N[C@@H]1CCN(C(=O)c2cccc3c2CCC3)C1	941977408	25	339.17	1.25
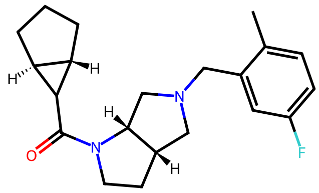	Cc1ccc(F)cc1CN1C[C@@H]2CCN(C(=O)C3[C@H]4CCC[C@H]34)[C@@H]2C1	1075657315	25	342.21	3.21

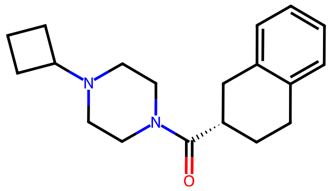	O=C([C@H]1CCc2ccccc2C1)N1CCN(C2CCC2)CC1	563243556	22	298.2	2.49
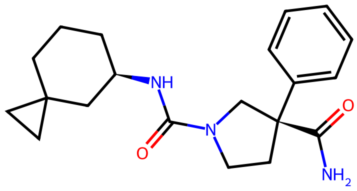	NC(=O)[C@@]1(c2ccccc2)CCN(C(=O)N[C@@H]2CCCC3(CC3)C2)C1	897767822	25	341.21	2.55
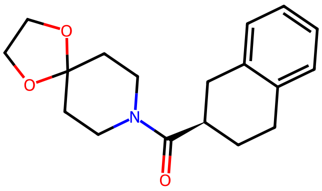	O=C([C@@H]1CCc2ccccc2C1)N1CCC2(CC1)OCCO2	388806831	22	301.17	2.16
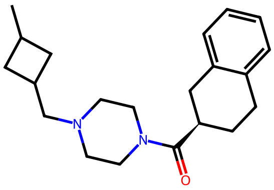	CC1CC(CN2CCN(C(=O)[C@@H]3CCc4ccccc4C3)CC2)C1	950721533	24	326.24	2.98
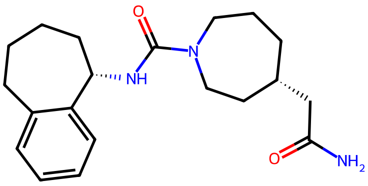	NC(=O)C[C@H]1CCCN(C(=O)N[C@H]2CCCCc3ccccc32)CC1	671571536	25	343.23	3.14
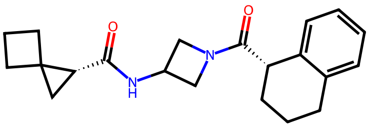	O=C(NC1CN(C(=O)[C@H]2CCCc3ccccc32)C1)[C@@H]1CC12CCC2	991554315	25	338.2	2.62
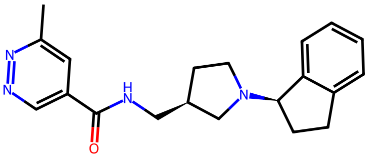	Cc1cc(C(=O)NC[C@H]2CCN([C@@H]3CCc4ccccc43)C2)cnn1	1028505441	25	336.2	2.52
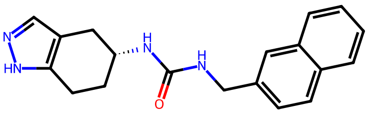	O=C(NCc1ccc2ccccc2c1)N[C@@H]1CCc2[nH]ncc2C1	138959708	24	320.16	2.92

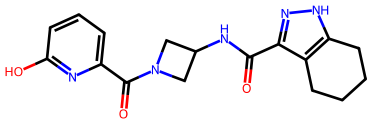	O=C(NC1CN(C(=O)c2cccc(O)n2)C1)c1n[nH]c2c1CCCC2	995172715	25	341.15	0.64
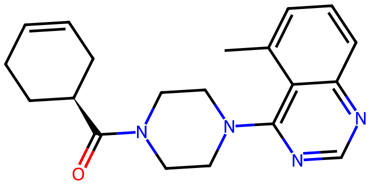	Cc1cccc2ncnc(N3CCN(C(=O)[C@@H]4CC=CCC4)CC3)c12	1055721821	25	336.2	2.94
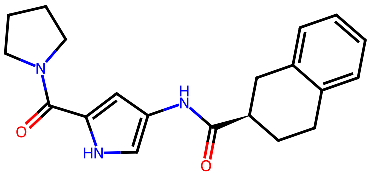	O=C(Nc1c[nH]c(C(=O)N2CCCC2)c1)[C@@H]1CCc2ccccc2C1	1417704605	25	337.18	2.99
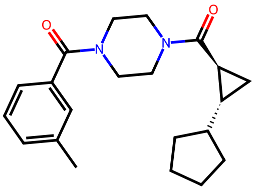	Cc1cccc(C(=O)N2CCN(C(=O)[C@H]3C[C@@H]3C3CCCC3)CC2)c1	1010099024	25	340.22	3.11
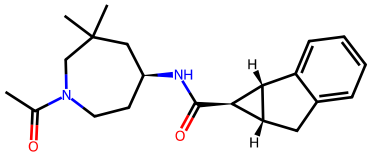	CC(=O)N1CC[C@H](NC(=O)[C@@H]2[C@H]3Cc4ccccc4[C@H]32)CC(C)(C)C1	990702229	25	340.22	2.73
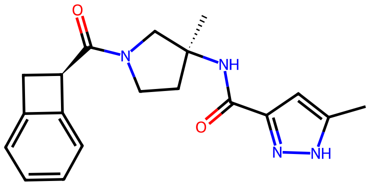	Cc1cc(C(=O)N[C@@]2(C)CCN(C(=O)[C@@H]3Cc4ccccc43)C2)n[nH]1	956743465	25	338.17	1.78
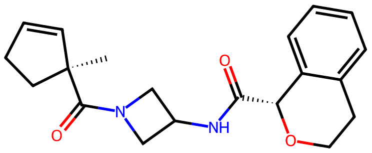	C[C@@]1(C(=O)N2CC(NC(=O)[C@H]3OCCc4ccccc43)C2)C=CCC1	997327026	25	340.18	1.98
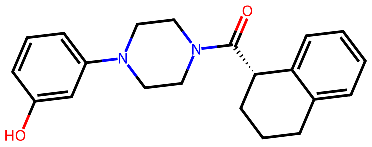	O=C([C@H]1CCCc2ccccc21)N1CCN(c2cccc(O)c2)CC1	432515403	25	336.18	3.16

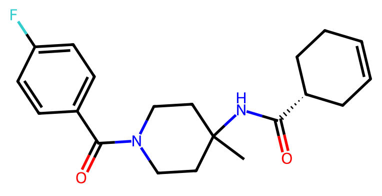	CC1(NC(=O)[C@H]2CC=CCC2)CCN(C(=O)c2ccc(F)cc2)CC1	956289267	25	344.19	3.29
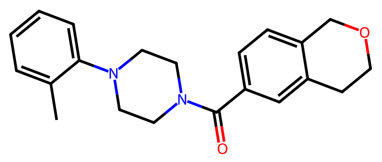	Cc1ccccc1N1CCN(C(=O)c2ccc3c(c2)CCOC3)CC1	665376572	25	336.18	3.03
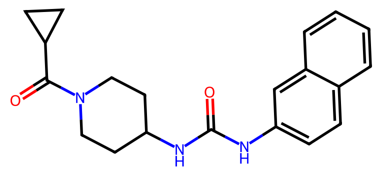	O=C(Nc1ccc2ccccc2c1)NC1CCN(C(=O)C2CC2)CC1	915303259	25	337.18	3.36
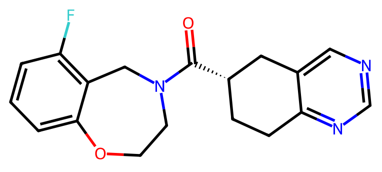	O=C([C@H]1CCc2ncncc2C1)N1CCOc2cccc(F)c2C1	648893902	24	327.14	2.14
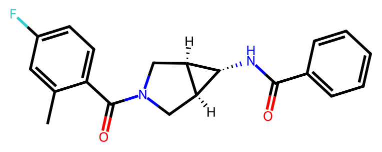	Cc1cc(F)ccc1C(=O)N1C[C@H]2[C@H](NC(=O)c3ccccc3)[C@H]2C1	976086413	25	338.14	2.63
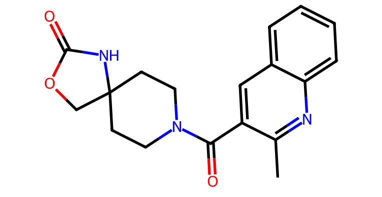	Cc1nc2ccccc2cc1C(=O)N1CCC2(CC1)COC(=O)N2	496126903	24	325.14	2.26
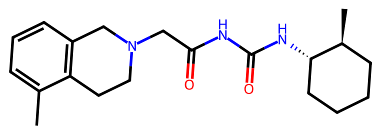	Cc1cccc2c1CCN(CC(=O)NC(=O)N[C@H]1CCCC[C@@H]1C)C2	729478534	25	343.23	2.76
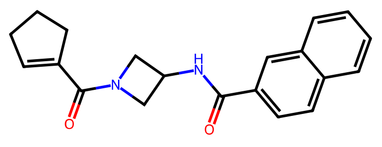	O=C(NC1CN(C(=O)C2=CCCC2)C1)c1ccc2ccccc2c1	999449348	24	320.15	2.89

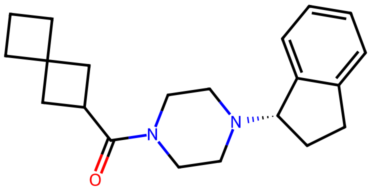	O=C(C1CC2(CCC2)C1)N1CCN([C@H]2CCc3ccccc32)CC1	949753743	24	324.22	3.4
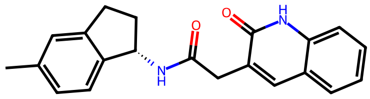	Cc1ccc2c(c1)CC[C@@H]2NC(=O)Cc1cc2ccccc2[nH]c1=O	55661729	25	332.15	3.18
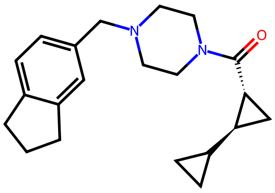	O=C([C@@H]1C[C@H]1C1CC1)N1CCN(Cc2ccc3c(c2)CCC3)CC1	951617126	24	324.22	2.87
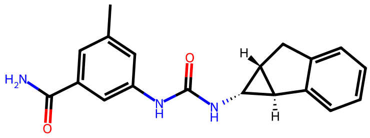	Cc1cc(NC(=O)N[C@H]2[C@@H]3c4ccccc4C[C@H]32)cc(C(N)=O)c1	576560110	24	321.15	2.55
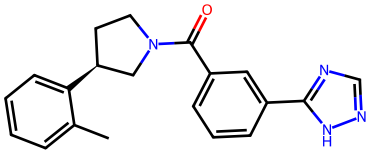	Cc1ccccc1[C@H]1CCN(C(=O)c2cccc(-c3ncn[nH]3)c2)C1	271282854	25	332.16	3.41
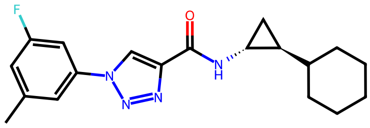	Cc1cc(F)cc(-n2cc(C(=O)N[C@@H]3C[C@H]3C3CCCCC3)nn2)c1	1270676695	25	342.19	3.41
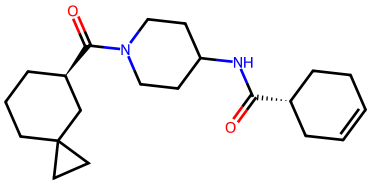	O=C(NC1CCN(C(=O)[C@@H]2CCCC3(CC3)C2)CC1)[C@H]1CC=CCC1	946752923	25	344.25	3.42
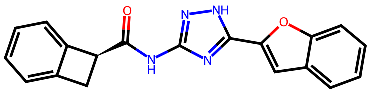	O=C(Nc1n[nH]c(-c2cc3ccccc3o2)n1)[C@H]1Cc2ccccc21	362244773	25	330.11	3.5

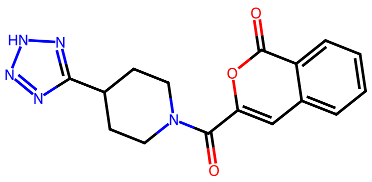	O=C(c1cc2ccccc2c(=O)o1)N1CCC(c2nn[nH]n2)CC1	1632650886	24	325.12	1.33
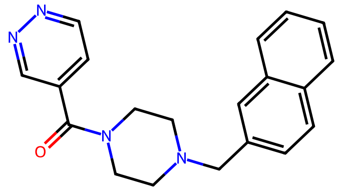	O=C(c1ccnnc1)N1CCN(Cc2ccc3ccccc3c2)CC1	950703601	25	332.16	2.59
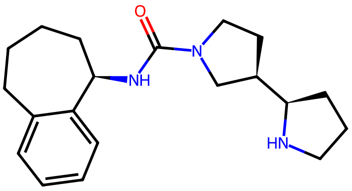	O=C(N[C@@H]1CCCCc2ccccc21)N1CC[C@@H]([C@H]2CCCN2)C1	1553443964	24	327.23	3.24
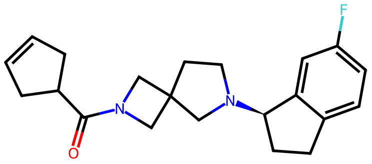	O=C(C1CC=CC1)N1CC2(CCN([C@@H]3CCc4ccc(F)cc43)C2)C1	1072571340	25	340.2	3.31
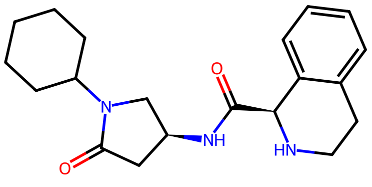	O=C(N[C@H]1CC(=O)N(C2CCCCC2)C1)[C@@H]1NCCc2ccccc21	576228607	25	341.21	1.92
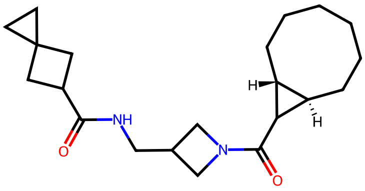	O=C(NCC1CN(C(=O)C2[C@@H]3CCCCCC[C@@H]23)C1)C1CC2(CC2)C1	1004216838	25	344.25	2.97
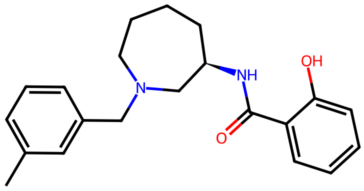	Cc1cccc(CN2CCCC[C@@H](NC(=O)c3ccccc3O)C2)c1	1034102846	25	338.2	3.49
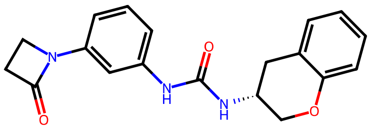	O=C(Nc1cccc(N2CCC2=O)c1)N[C@H]1COc2ccccc2C1	279815631	25	337.14	2.55

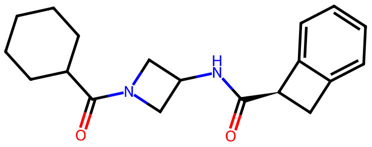	O=C(NC1CN(C(=O)C2CCCCC2)C1)[C@@H]1Cc2ccccc21	998679404	23	312.18	2.23
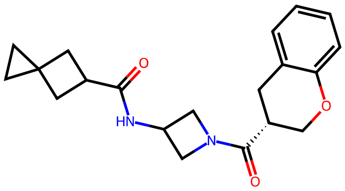	O=C(NC1CN(C(=O)[C@H]2COc3ccccc3C2)C1)C1CC2(CC2)C1	999347685	25	340.18	1.75
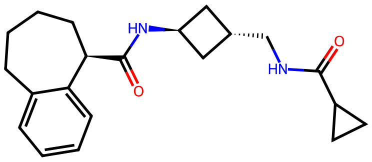	O=C(NC[C@H]1C[C@H](NC(=O)[C@@H]2CCCCc3ccccc32)C1)C1CC1	979391332	25	340.22	2.92
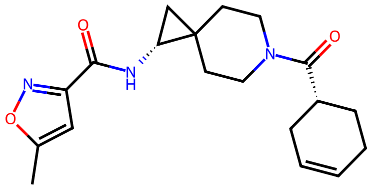	Cc1cc(C(=O)N[C@@H]2CC23CCN(C(=O)[C@H]2CC=CCC2)CC3)no1	962875839	25	343.19	2.45
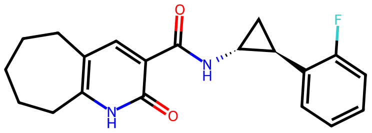	O=C(N[C@@H]1C[C@H]1c1ccccc1F)c1cc2c([nH]c1=O)CCCCC2	78116151	25	340.16	3.07
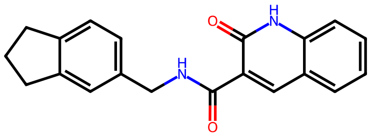	O=C(NCc1ccc2c(c1)CCC2)c1cc2ccccc2[nH]c1=O	60487802	24	318.14	2.95
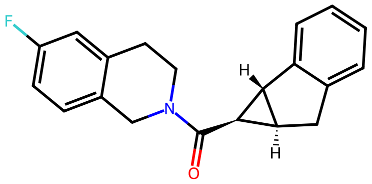	O=C([C@@H]1[C@@H]2Cc3ccccc3[C@H]21)N1CCc2cc(F)ccc2C1	626333518	23	307.14	3.3
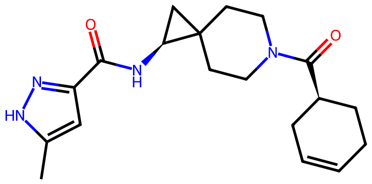	Cc1cc(C(=O)N[C@H]2CC23CCN(C(=O)[C@@H]2CC=CCC2)CC3)n[nH]1	963292649	25	342.21	2.19

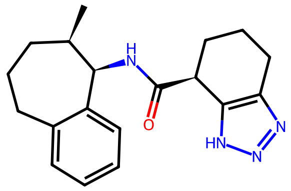	C[C@@H]1CCCc2ccccc2[C@@H]1NC(=O)[C@H]1CCCc2nn[nH]c21	613537323	24	324.2	3.05
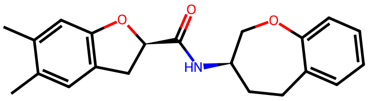	Cc1cc2c(cc1C)O[C@@H](C(=O)N[C@@H]1CCc3ccccc3OC1)C2	511632005	25	337.17	3.12
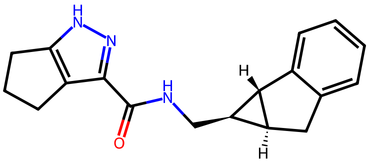	O=C(NC[C@@H]1[C@@H]2Cc3ccccc3[C@@H]12)c1n[nH]c2c1CCC2	152397786	22	293.15	2.21
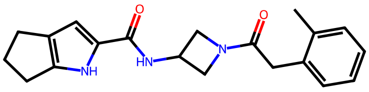	Cc1ccccc1CC(=O)N1CC(NC(=O)c2cc3c([nH]2)CCC3)C1	995943972	25	337.18	2.0
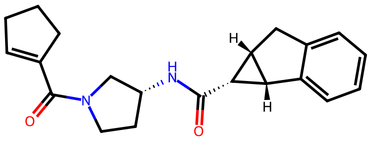	O=C(N[C@@H]1CCN(C(=O)C2=CCCC2)C1)[C@H]1[C@H]2c3ccccc3C[C@H]21	940495252	25	336.18	2.4
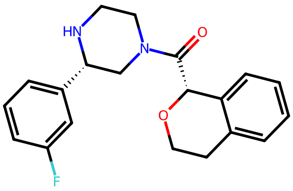	O=C([C@H]1OCCc2ccccc21)N1CCN[C@@H](c2cccc(F)c2)C1	337614770	25	340.16	2.61
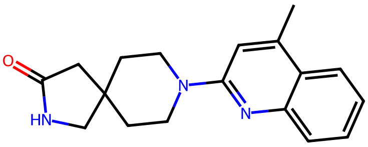	Cc1cc(N2CCC3(CC2)CNC(=O)C3)nc2ccccc12	75065755	22	295.17	2.65
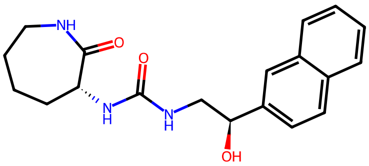	O=C(NC[C@H](O)c1ccc2ccccc2c1)N[C@@H]1CCCCNC1=O	109149080	25	341.17	1.84

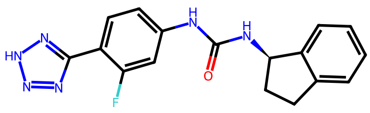	O=C(Nc1ccc(-c2nn[nH]n2)c(F)c1)N[C@@H]1CCc2ccccc21	1593215067	25	338.13	2.81
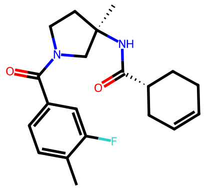	Cc1ccc(C(=O)N2CC[C@@](C)(NC(=O)[C@H]3CC=CCC3)C2)cc1F	956688035	25	344.19	3.21
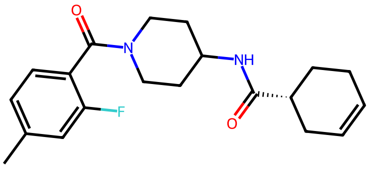	Cc1ccc(C(=O)N2CCC(NC(=O)[C@H]3CC=CCC3)CC2)c(F)c1	948557603	25	344.19	3.21
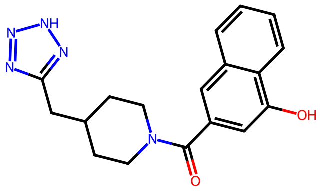	O=C(c1cc(O)c2ccccc2c1)N1CCC(Cc2nn[nH]n2)CC1	1352696342	25	337.15	2.15
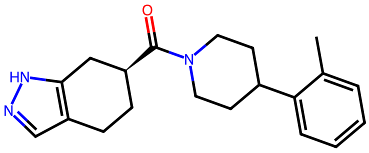	Cc1ccccc1C1CCN(C(=O)[C@H]2CCc3cn[nH]c3C2)CC1	80085861	24	323.2	3.23
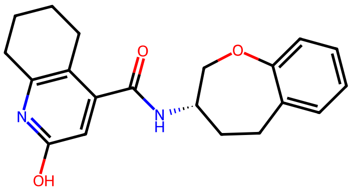	O=C(N[C@H]1CCc2ccccc2OC1)c1cc(O)nc2c1CCCC2	935851457	25	338.16	2.79
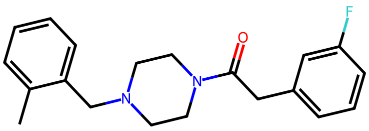	Cc1ccccc1CN1CCN(C(=O)Cc2cccc(F)c2)CC1	45291266	24	326.18	3.02
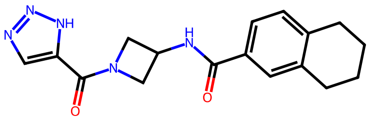	O=C(NC1CN(C(=O)c2cnn[nH]2)C1)c1ccc2c(c1)CCCC2	1000052411	24	325.15	0.94

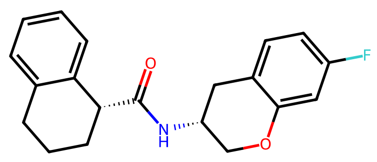	O=C(N[C@H]1COc2cc(F)ccc2C1)[C@@H]1CCCc2ccccc21	1618175295	24	325.15	3.37
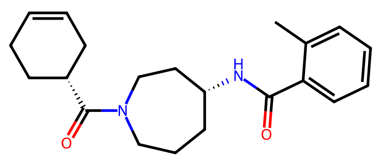	Cc1ccccc1C(=O)N[C@@H]1CCCN(C(=O)[C@H]2CC=CCC2)CC1	964705540	25	340.22	3.46
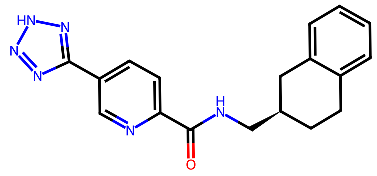	O=C(NC[C@@H]1CCc2ccccc2C1)c1ccc(-c2nn[nH]n2)cn1	738050678	25	334.15	1.8
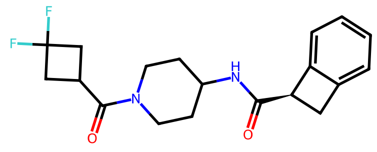	O=C(NC1CCN(C(=O)C2CC(F)(F)C2)CC1)[C@@H]1Cc2ccccc21	946288664	25	348.16	2.48
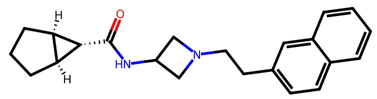	O=C(NC1CN(CCc2ccc3ccccc3c2)C1)[C@@H]1[C@H]2CCC[C@H]21	1030190591	25	334.2	3.23
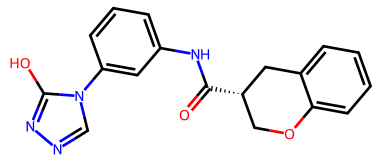	O=C(Nc1cccc(-n2cnnc2O)c1)[C@H]1COc2ccccc2C1	364282614	25	336.12	2.16
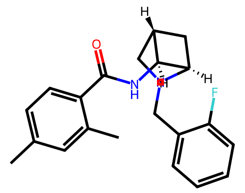	Cc1ccc(C(=O)N[C@H]2[C@H]3C[C@H]2N(Cc2ccccc2F)C3)c(C)c1	1037703838	25	338.18	3.45
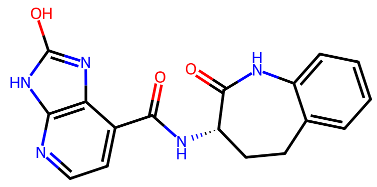	O=C(N[C@H]1CCc2ccccc2NC1=O)c1ccnc2[nH]c(O)nc12	1631673857	25	337.12	1.35

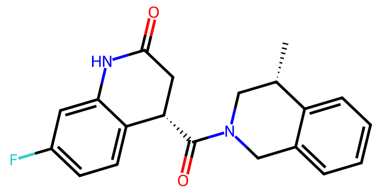	C[C@H]1CN(C(=O)[C@H]2CC(=O)Nc3cc(F)ccc32)Cc2ccccc21	837285788	25	338.14	3.4
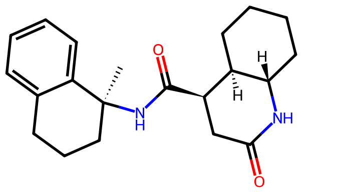	C[C@]1(NC(=O)[C@@H]2CC(=O)N[C@H]3CCCC[C@@H]32)CCCc2ccccc21	667870542	25	340.22	3.05
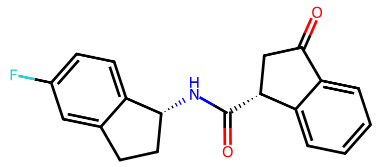	O=C1C[C@@H](C(=O)N[C@@H]2CCc3cc(F)ccc32)c2ccccc21	277338890	23	309.12	3.3
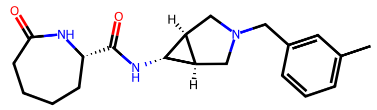	Cc1cccc(CN2C[C@H]3[C@H](NC(=O)[C@@H]4CCCCC(=O)N4)[C@H]3C2)c1	961764075	25	341.21	1.6
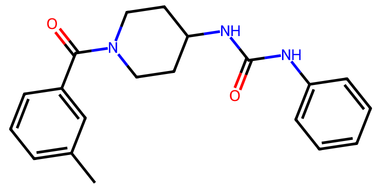	Cc1cccc(C(=O)N2CCC(NC(=O)Nc3ccccc3)CC2)c1	55411024	25	337.18	3.42
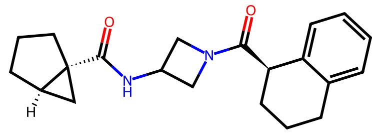	O=C([C@@H]1CCCc2ccccc21)N1CC(NC(=O)[C@@]23CCC[C@@H]2C3)C1	997551964	25	338.2	2.62
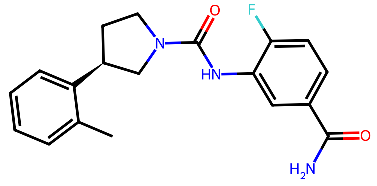	Cc1ccccc1[C@H]1CCN(C(=O)Nc2cc(C(N)=O)ccc2F)C1	299679557	25	341.15	3.25
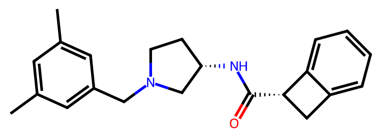	Cc1cc(C)cc(CN2CC[C@H](NC(=O)[C@H]3Cc4ccccc43)C2)c1	1015632850	25	334.2	3.33

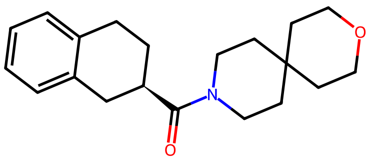	O=C([C@@H]1CCc2ccccc2C1)N1CCC2(CCOCC2)CC1	650011910	23	313.2	3.21
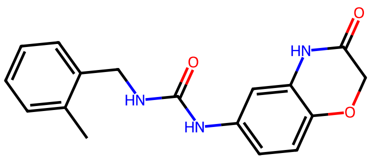	Cc1ccccc1CNC(=O)Nc1ccc2c(c1)NC(=O)CO2	43690992	23	311.13	2.65
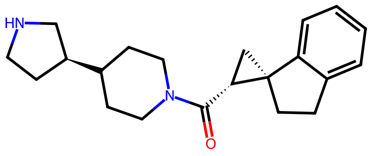	O=C([C@@H]1C[C@]12CCc1ccccc12)N1CCC([C@H]2CCNC2)CC1	290276661	24	324.22	2.74
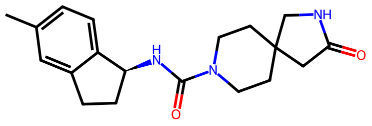	Cc1ccc2c(c1)CC[C@@H]2NC(=O)N1CCC2(CC1)CNC(=O)C2	267790529	24	327.19	2.29
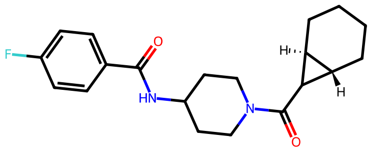	O=C(NC1CCN(C(=O)C2[C@H]3CCCC[C@H]23)CC1)c1ccc(F)cc1	468508928	25	344.19	2.98
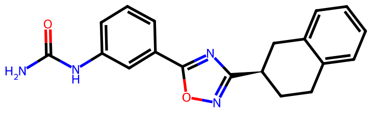	NC(=O)Nc1cccc(-c2nc([C@@H]3CCc4ccccc4C3)no2)c1	680770256	25	334.14	3.5
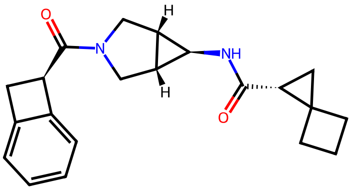	O=C(N[C@H]1[C@@H]2CN(C(=O)[C@@H]3Cc4ccccc43)C[C@@H]21)[C@@H]1CC12CCC2	976531514	25	336.18	2.09
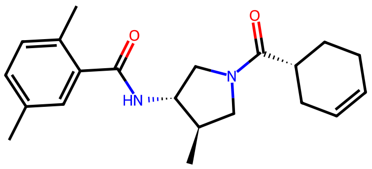	Cc1ccc(C)c(C(=O)N[C@@H]2CN(C(=O)[C@H]3CC=CCC3)C[C@H]2C)c1	940103858	25	340.22	3.24

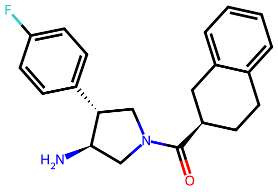	N[C@@H]1CN(C(=O)[C@@H]2CCc3ccccc3C2)C[C@H]1c1ccc(F)cc1	1549122622	25	338.18	2.88
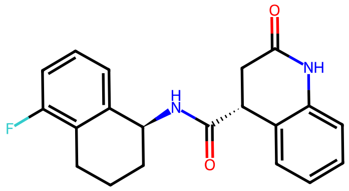	O=C1C[C@@H](C(=O)N[C@H]2CCCc3c(F)cccc32)c2ccccc2N1	452198315	25	338.14	3.45
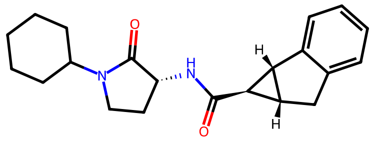	O=C(N[C@@H]1CCN(C2CCCCC2)C1=O)[C@@H]1[C@H]2Cc3ccccc3[C@H]21	626161294	25	338.2	2.62
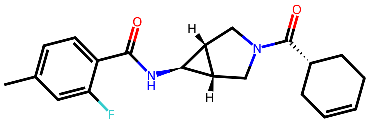	Cc1ccc(C(=O)N[C@H]2[C@@H]3CN(C(=O)[C@H]4CC=CCC4)C[C@@H]32)c(F)c1	979488612	25	342.17	2.68
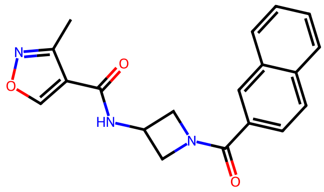	Cc1nocc1C(=O)NC1CN(C(=O)c2ccc3ccccc3c2)C1	998739973	25	335.13	2.39
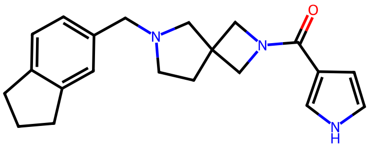	O=C(c1cc[nH]c1)N1CC2(CCN(Cc3ccc4c(c3)CCC4)C2)C1	1072766122	25	335.2	2.85
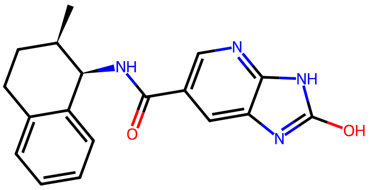	C[C@@H]1CCc2ccccc2[C@@H]1NC(=O)c1cnc2[nH]c(O)nc2c1	558416263	24	322.14	2.72
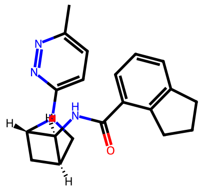	Cc1ccc(N2C[C@H]3C[C@H]2[C@H]3NC(=O)c2cccc3c2CCC3)nn1	1064268009	25	334.18	2.28

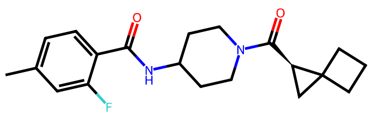	Cc1ccc(C(=O)NC2CCN(C(=O)[C@@H]3CC34CCC4)CC2)c(F)c1	946755734	25	344.19	3.05
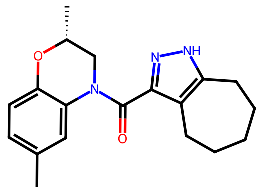	Cc1ccc2c(c1)N(C(=O)c1n[nH]c3c1CCCCC3)C[C@@H](C)O2	190576028	24	325.18	3.41
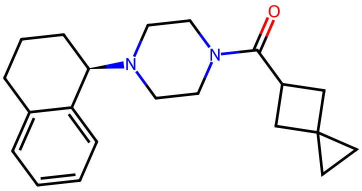	O=C(C1CC2(CC2)C1)N1CCN([C@@H]2CCCc3ccccc32)CC1	950426506	24	324.22	3.4
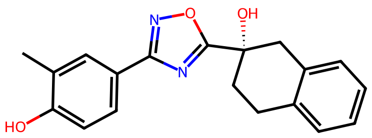	Cc1cc(-c2noc([C@]3(O)CCc4ccccc4C3)n2)ccc1O	809471532	24	322.13	3.13
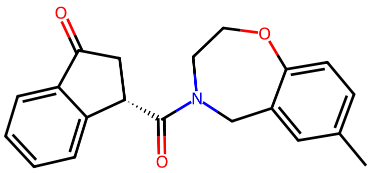	Cc1ccc2c(c1)CN(C(=O)[C@H]1CC(=O)c3ccccc31)CCO2	287984657	24	321.14	3.09
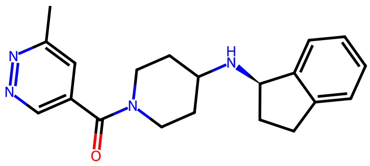	Cc1cc(C(=O)N2CCC(N[C@@H]3CCc4ccccc43)CC2)cnn1	991759572	25	336.2	2.67
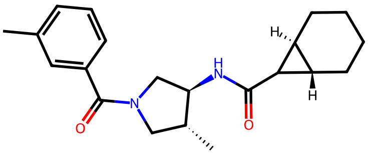	Cc1cccc(C(=O)N2C[C@@H](NC(=O)C3[C@H]4CCCC[C@H]34)[C@H](C)C2)c1	947313693	25	340.22	3.01
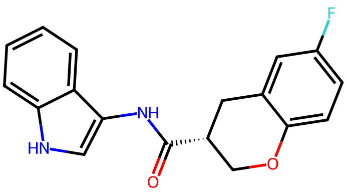	O=C(Nc1c[nH]c2ccccc12)[C@H]1COc2ccc(F)cc2C1	1157066352	23	310.11	3.5

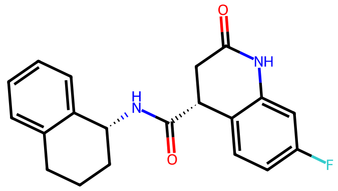	O=C1C[C@@H](C(=O)N[C@@H]2CCCc3ccccc32)c2ccc(F)cc2N1	120391139	25	338.14	3.45
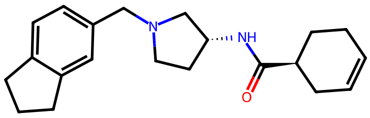	O=C(N[C@@H]1CCN(Cc2ccc3c(c2)CCC3)C1)[C@@H]1CC=CCC1	1010080050	24	324.22	3.22
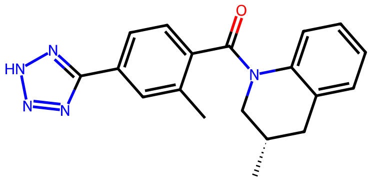	Cc1cc(-c2nn[nH]n2)ccc1C(=O)N1C[C@@H](C)Cc2ccccc21	826244646	25	333.16	3.01
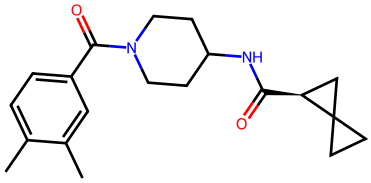	Cc1ccc(C(=O)N2CCC(NC(=O)[C@H]3CC34CC4)CC2)cc1C	948351649	24	326.2	2.82
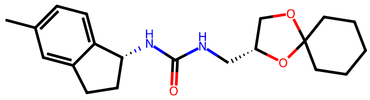	Cc1ccc2c(c1)CC[C@H]2NC(=O)NC[C@@H]1COC2(CCCCC2)O1	775727364	25	344.21	3.36
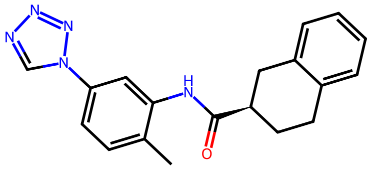	Cc1ccc(-n2cnnn2)cc1NC(=O)[C@@H]1CCc2ccccc2C1	574088514	25	333.16	2.71
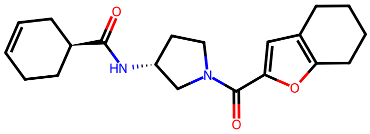	O=C(N[C@@H]1CCN(C(=O)c2cc3c(o2)CCCC3)C1)[C@@H]1CC=CCC1	939379939	25	342.19	2.85
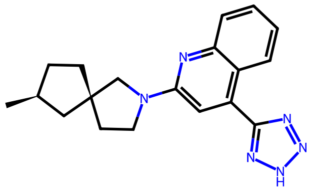	C[C@H]1CC[C@@]2(CCN(c3cc(-c4nn[nH]n4)c4ccccc4n3)C2)C1	824917905	25	334.19	3.43

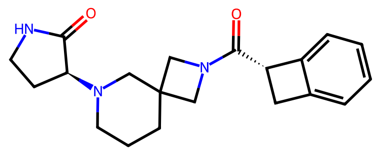	O=C1NCC[C@@H]1N1CCCC2(CN(C(=O)[C@H]3Cc4ccccc43)C2)C1	981976709	25	339.19	1.14
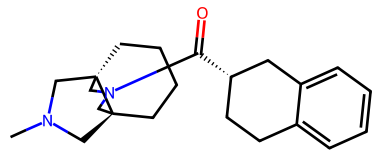	CN1C[C@]23CCCC[C@@]2(C1)CN(C(=O)[C@H]1CCc2ccccc2C1)C3	1337791354	25	338.24	3.13
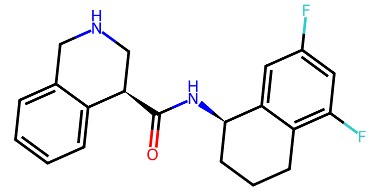	O=C(N[C@@H]1CCCc2c(F)cc(F)cc21)[C@@H]1CNCc2ccccc21	1580742428	25	342.15	3.35
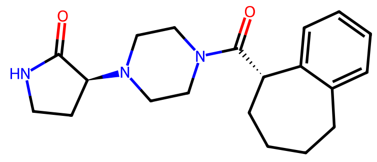	O=C1NCC[C@@H]1N1CCN(C(=O)[C@H]2CCCCc3ccccc32)CC1	957819264	25	341.21	1.53
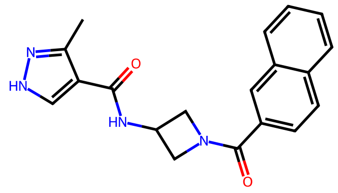	Cc1n[nH]cc1C(=O)NC1CN(C(=O)c2ccc3ccccc3c2)C1	997295346	25	334.14	2.13
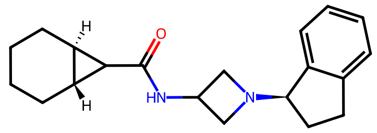	O=C(NC1CN([C@@H]2CCc3ccccc32)C1)C1[C@@H]2CCCC[C@@H]12	1030183257	23	310.2	2.91
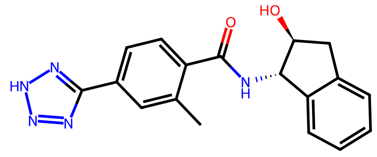	Cc1cc(-c2nn[nH]n2)ccc1C(=O)N[C@H]1c2ccccc2C[C@@H]1O	826247865	25	335.14	1.56
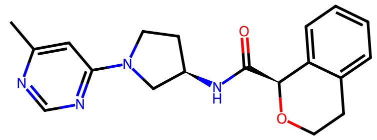	Cc1cc(N2CC[C@@H](NC(=O)[C@@H]3OCCc4ccccc43)C2)ncn1	1058511101	25	338.17	1.79

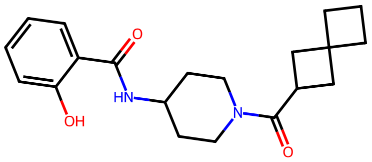	O=C(NC1CCN(C(=O)C2CC3(CCC3)C2)CC1)c1ccccc1O	947879723	25	342.19	2.69
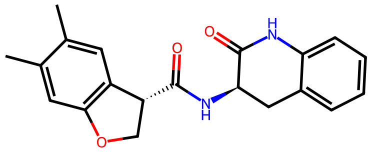	Cc1cc2c(cc1C)[C@H](C(=O)N[C@@H]1Cc3ccccc3NC1=O)CO2	529946542	25	336.15	2.46
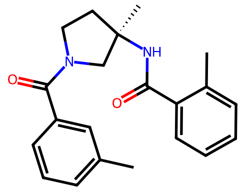	Cc1cccc(C(=O)N2CC[C@@](C)(NC(=O)c3ccccc3C)C2)c1	956660133	25	336.18	3.34
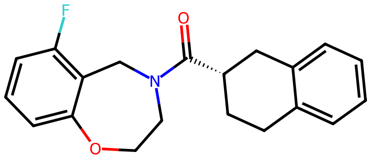	O=C([C@H]1CCc2ccccc2C1)N1CCOc2cccc(F)c2C1	652001189	24	325.15	3.35
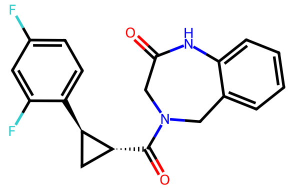	O=C1CN(C(=O)[C@@H]2C[C@H]2c2ccc(F)cc2F)Cc2ccccc2N1	655315438	25	342.12	3.05
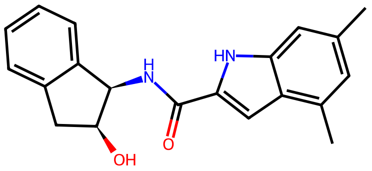	Cc1cc(C)c2cc(C(=O)N[C@@H]3c4ccccc4C[C@@H]3O)[nH]c2c1	81706145	24	320.15	3.17
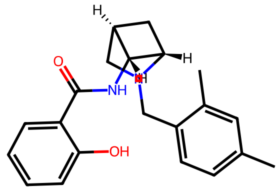	Cc1ccc(CN2C[C@H]3C[C@H]2[C@@H]3NC(=O)c2ccccc2O)c(C)c1	1037323777	25	336.18	3.01
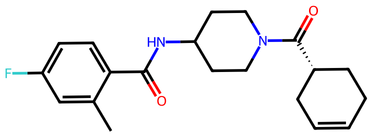	Cc1cc(F)ccc1C(=O)NC1CCN(C(=O)[C@H]2CC=CCC2)CC1	944113608	25	344.19	3.21

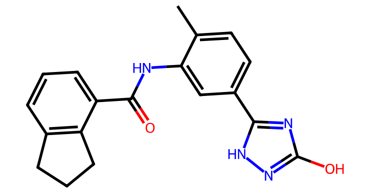	Cc1ccc(-c2nc(O)n[nH]2)cc1NC(=O)c1cccc2c1CCC2	1296860823	25	334.14	3.23
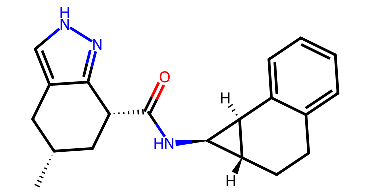	C[C@@H]1Cc2c[nH]nc2[C@H](C(=O)N[C@@H]2[C@H]3CCc4ccccc4[C@@H]32)C1	646925196	24	321.18	2.92
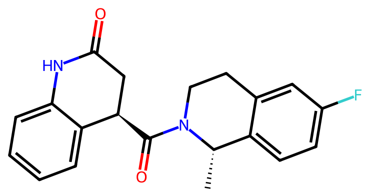	C[C@H]1c2ccc(F)cc2CCN1C(=O)[C@@H]1CC(=O)Nc2ccccc21	1133322571	25	338.14	3.4
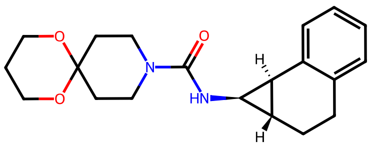	O=C(N[C@@H]1[C@H]2CCc3ccccc3[C@@H]21)N1CCC2(CC1)OCCCO2	297365844	25	342.19	2.65
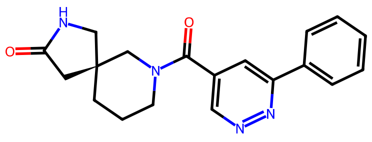	O=C1C[C@@]2(CCCN(C(=O)c3cnnc(-c4ccccc4)c3)C2)CN1	580252101	25	336.16	1.89
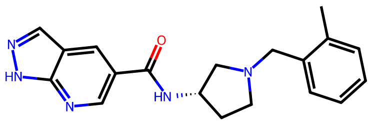	Cc1ccccc1CN1CC[C@H](NC(=O)c2cnc3[nH]ncc3c2)C1	1014893883	25	335.17	2.27
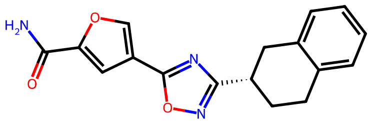	NC(=O)c1cc(-c2nc([C@H]3CCc4ccccc4C3)no2)co1	680769517	23	309.11	2.7
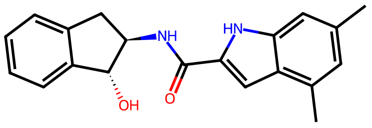	Cc1cc(C)c2cc(C(=O)N[C@@H]3Cc4ccccc4[C@H]3O)[nH]c2c1	178359631	24	320.15	3.17

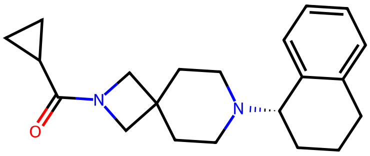	O=C(C1CC1)N1CC2(CCN([C@H]3CCCc4ccccc43)CC2)C1	1050401408	24	324.22	3.4
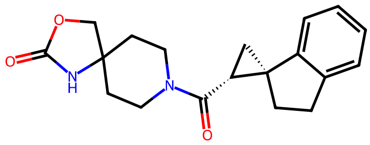	O=C1NC2(CCN(C(=O)[C@@H]3C[C@]34CCc3ccccc34)CC2)CO1	495157010	24	326.16	1.99
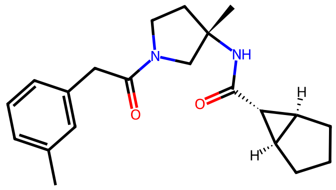	Cc1cccc(CC(=O)N2CC[C@](C)(NC(=O)[C@@H]3[C@H]4CCC[C@H]43)C2)c1	956803740	25	340.22	2.69
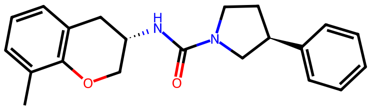	Cc1cccc2c1OC[C@@H](NC(=O)N1CC[C@@H](c3ccccc3)C1)C2	462665451	25	336.18	3.5
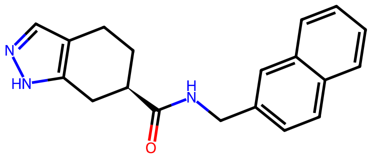	O=C(NCc1ccc2ccccc2c1)[C@@H]1CCc2cn[nH]c2C1	79483080	23	305.15	2.98
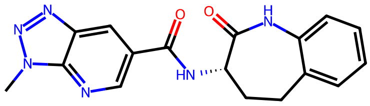	Cn1nnc2cc(C(=O)N[C@H]3CCc4ccccc4NC3=O)cnc21	1277685939	25	336.13	1.05
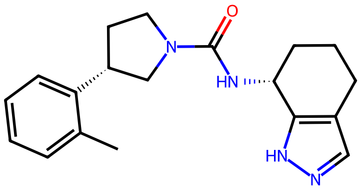	Cc1ccccc1[C@@H]1CCN(C(=O)N[C@@H]2CCCc3cn[nH]c32)C1	269028644	24	324.2	3.29
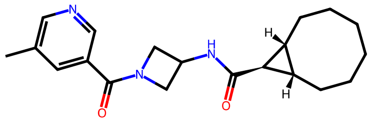	Cc1cncc(C(=O)N2CC(NC(=O)[C@@H]3[C@H]4CCCCCC[C@H]43)C2)c1	1000486572	25	341.21	2.55

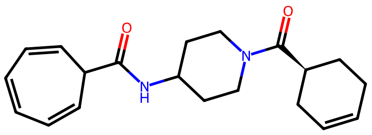	O=C(NC1CCN(C(=O)[C@@H]2CC=CCC2)CC1)C1C=CC=CC=C1	944107813	24	326.2	2.75
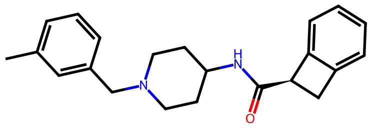	Cc1cccc(CN2CCC(NC(=O)[C@@H]3Cc4ccccc43)CC2)c1	1003535670	25	334.2	3.42
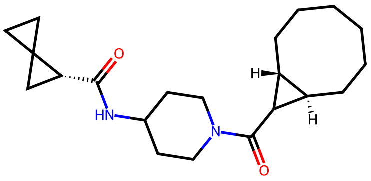	O=C(NC1CCN(C(=O)C2[C@@H]3CCCCCC[C@@H]23)CC1)[C@@H]1CC12CC2	948568089	25	344.25	3.11
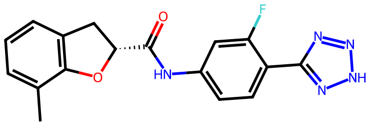	Cc1cccc2c1O[C@@H](C(=O)Nc1ccc(-c3nn[nH]n3)c(F)c1)C2	1605928818	25	339.11	2.26
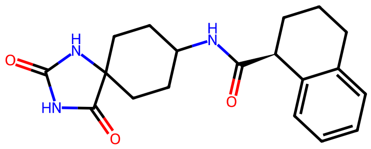	O=C1NC(=O)C2(CCC(NC(=O)[C@H]3CCCc4ccccc43)CC2)N1	286210888	25	341.17	1.74
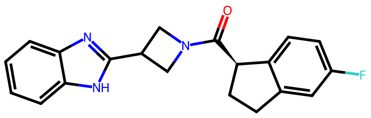	O=C([C@@H]1CCc2cc(F)ccc21)N1CC(c2nc3ccccc3[nH]2)C1	612235062	25	335.14	3.36
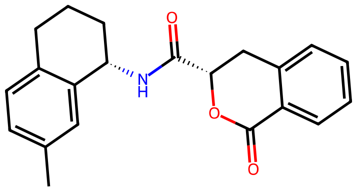	Cc1ccc2c(c1)[C@@H](NC(=O)[C@@H]1Cc3ccccc3C(=O)O1)CCC2	807898156	25	335.15	3.27
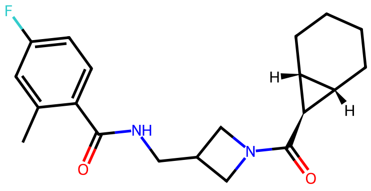	Cc1cc(F)ccc1C(=O)NCC1CN(C(=O)[C@@H]2[C@H]3CCCC[C@H]32)C1	1003487532	25	344.19	2.76

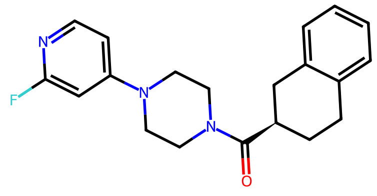	O=C([C@@H]1CCc2ccccc2C1)N1CCN(c2ccnc(F)c2)CC1	844562241	25	339.17	2.67
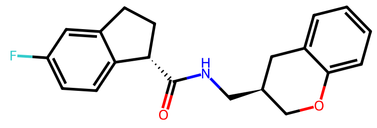	O=C(NC[C@@H]1COc2ccccc2C1)[C@H]1CCc2cc(F)ccc21	854308943	24	325.15	3.22
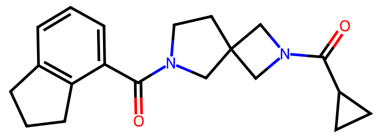	O=C(c1cccc2c1CCC2)N1CCC2(C1)CN(C(=O)C1CC1)C2	1018662310	24	324.18	2.26
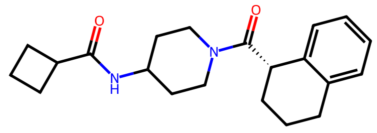	O=C(NC1CCN(C(=O)[C@H]2CCCc3ccccc32)CC1)C1CCC1	363598620	25	340.22	3.01
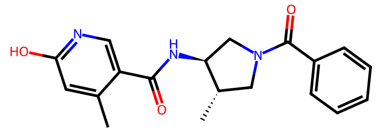	Cc1cc(O)ncc1C(=O)N[C@H]1CN(C(=O)c2ccccc2)C[C@@H]1C	966087931	25	339.16	1.99
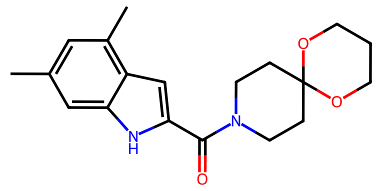	Cc1cc(C)c2cc(C(=O)N3CCC4(CC3)OCCCO4)[nH]c2c1	818406290	24	328.18	3.15
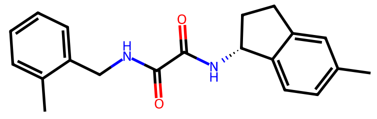	Cc1ccc2c(c1)CC[C@H]2NC(=O)C(=O)NCc1ccccc1C	1327313480	24	322.17	2.72
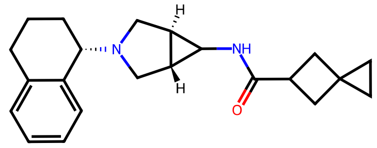	O=C(NC1[C@@H]2CN([C@H]3CCCc4ccccc43)C[C@@H]12)C1CC2(CC2)C1	961728546	25	336.22	3.3

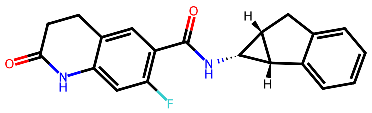	O=C1CCc2cc(C(=O)N[C@@H]3[C@@H]4Cc5ccccc5[C@@H]43)c(F)cc2N1	133693331	25	336.13	2.78
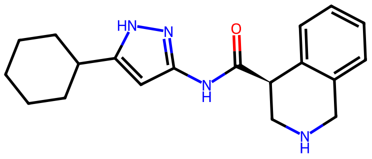	O=C(Nc1cc(C2CCCCC2)[nH]n1)[C@@H]1CNCc2ccccc21	582179414	24	324.2	3.28
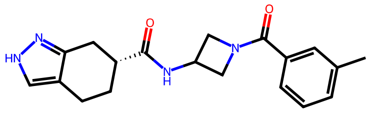	Cc1cccc(C(=O)N2CC(NC(=O)[C@@H]3CCc4c[nH]nc4C3)C2)c1	992553696	25	338.17	1.46
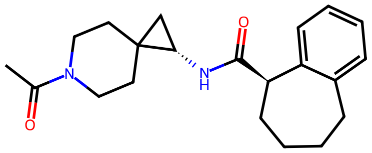	CC(=O)N1CCC2(CC1)C[C@@H]2NC(=O)[C@@H]1CCCCc2ccccc21	964029773	25	340.22	3.01
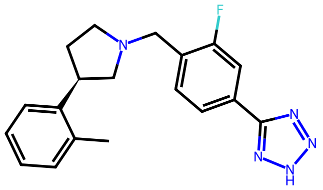	Cc1ccccc1[C@H]1CCN(Cc2ccc(-c3nn[nH]n3)cc2F)C1	737420307	25	337.17	3.3
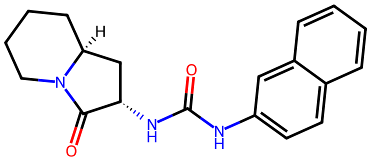	O=C(Nc1ccc2ccccc2c1)N[C@H]1C[C@@H]2CCCCN2C1=O	836189430	24	323.16	3.11
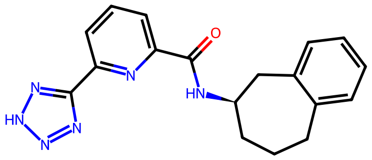	O=C(N[C@@H]1CCCc2ccccc2C1)c1cccc(-c2nn[nH]n2)n1	1592985276	25	334.15	1.94
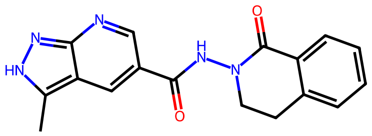	Cc1[nH]nc2ncc(C(=O)NN3CCc4ccccc4C3=O)cc12	762654537	24	321.12	1.61

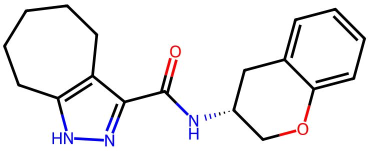	O=C(N[C@H]1COc2ccccc2C1)c1n[nH]c2c1CCCCC2	191299797	23	311.16	2.41
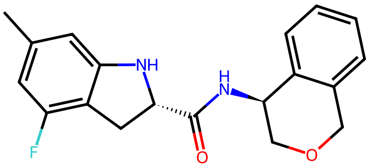	Cc1cc(F)c2c(c1)N[C@H](C(=O)N[C@@H]1COCc3ccccc31)C2	1579611866	24	326.14	2.86
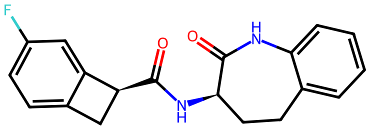	O=C(N[C@@H]1CCc2ccccc2NC1=O)[C@H]1Cc2ccc(F)cc21	653998859	24	324.13	2.54
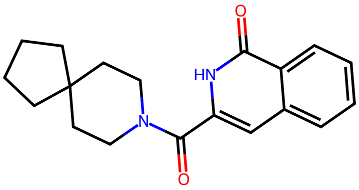	O=C(c1cc2ccccc2c(=O)[nH]1)N1CCC2(CCCC2)CC1	150912450	23	310.17	3.32
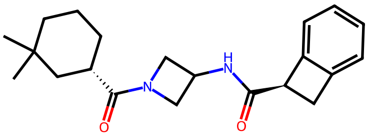	CC1(C)CCC[C@H](C(=O)N2CC(NC(=O)[C@@H]3Cc4ccccc43)C2)C1	998686753	25	340.22	2.87
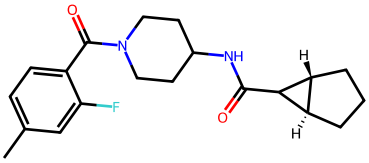	Cc1ccc(C(=O)N2CCC(NC(=O)C3[C@@H]4CCC[C@@H]34)CC2)c(F)c1	948559463	25	344.19	2.9
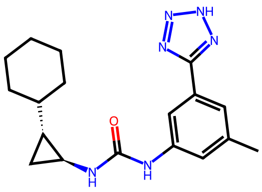	Cc1cc(NC(=O)N[C@H]2C[C@@H]2C2CCCCC2)cc(-c2nn[nH]n2)c1	1580982958	25	340.2	3.27
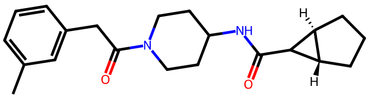	Cc1cccc(CC(=O)N2CCC(NC(=O)C3[C@H]4CCC[C@H]34)CC2)c1	948068520	25	340.22	2.69

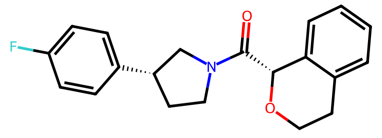	O=C([C@H]1OCCc2ccccc21)N1CC[C@H](c2ccc(F)cc2)C1	478209418	24	325.15	3.46
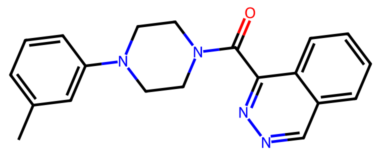	Cc1cccc(N2CCN(C(=O)c3nncc4ccccc34)CC2)c1	929568730	25	332.16	2.9
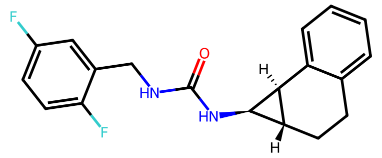	O=C(NCc1cc(F)ccc1F)N[C@@H]1[C@H]2CCc3ccccc3[C@@H]21	435770701	24	328.14	3.49
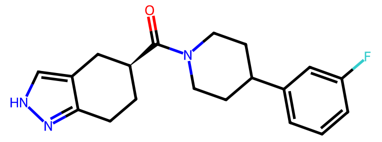	O=C([C@H]1CCc2n[nH]cc2C1)N1CCC(c2cccc(F)c2)CC1	371088103	24	327.17	3.06
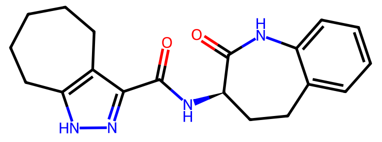	O=C(N[C@@H]1CCc2ccccc2NC1=O)c1n[nH]c2c1CCCCC2	655225016	25	338.17	2.36
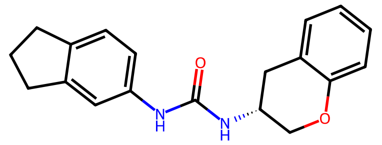	O=C(Nc1ccc2c(c1)CCC2)N[C@H]1COc2ccccc2C1	47720565	23	308.15	3.3
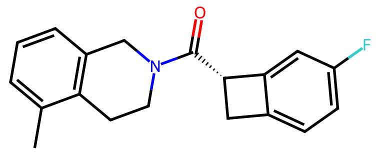	Cc1cccc2c1CCN(C(=O)[C@H]1Cc3ccc(F)cc31)C2	793444969	22	295.14	3.36
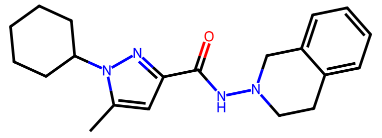	Cc1cc(C(=O)NN2CCc3ccccc3C2)nn1C1CCCCC1	855710475	25	338.21	3.4

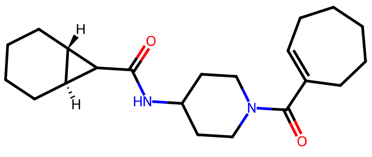	O=C(NC1CCN(C(=O)C2=CCCCCC2)CC1)C1[C@H]2CCCC[C@H]12	946548927	25	344.25	3.42
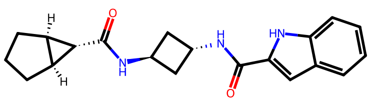	O=C(N[C@H]1C[C@H](NC(=O)[C@@H]2[C@H]3CCC[C@H]32)C1)c1cc2ccccc2[nH]1	973140974	25	337.18	2.59
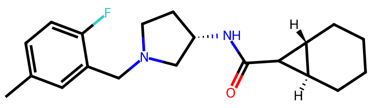	Cc1ccc(F)c(CN2CC[C@H](NC(=O)C3[C@@H]4CCCC[C@@H]34)C2)c1	1010442060	24	330.21	3.26
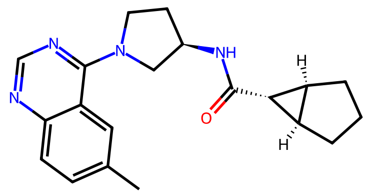	Cc1ccc2ncnc(N3CC[C@@H](NC(=O)[C@@H]4[C@H]5CCC[C@H]54)C3)c2c1	1058323108	25	336.2	2.68
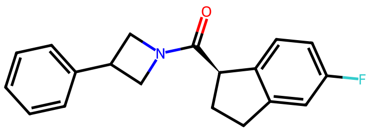	O=C([C@@H]1CCc2cc(F)ccc21)N1CC(c2ccccc2)C1	633815243	22	295.14	3.48
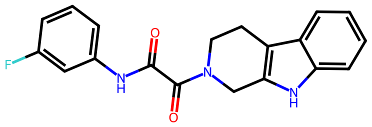	O=C(Nc1cccc(F)c1)C(=O)N1CCc2c([nH]c3ccccc23)C1	135544335	25	337.12	2.83
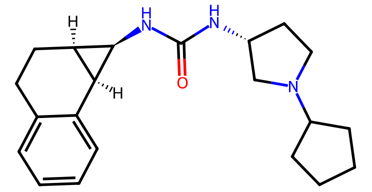	O=C(N[C@@H]1CCN(C2CCCC2)C1)N[C@@H]1[C@@H]2CCc3ccccc3[C@@H]21	128831650	25	339.23	3.03
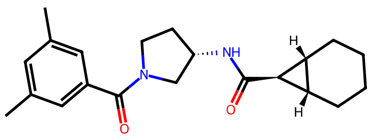	Cc1cc(C)cc(C(=O)N2CC[C@H](NC(=O)[C@@H]3[C@H]4CCCC[C@H]43)C2)c1	937789040	25	340.22	3.07

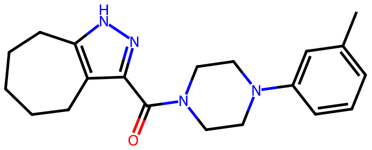	Cc1cccc(N2CCN(C(=O)c3n[nH]c4c3CCCCC4)CC2)c1	190122465	25	338.21	2.95
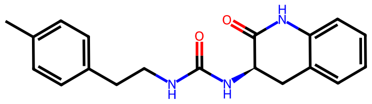	Cc1ccc(CCNC(=O)N[C@@H]2Cc3ccccc3NC2=O)cc1	636793497	24	323.16	2.4
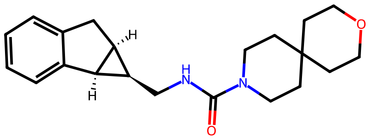	O=C(NC[C@H]1[C@H]2c3ccccc3C[C@H]21)N1CCC2(CCOCC2)CC1	285281119	25	340.22	3.17
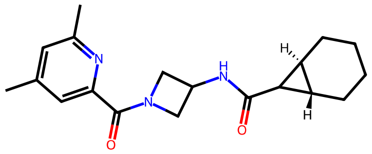	Cc1cc(C)nc(C(=O)N2CC(NC(=O)C3[C@H]4CCCC[C@H]34)C2)c1	990657234	24	327.19	2.08
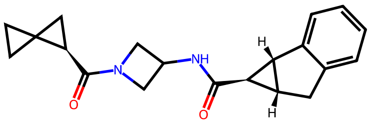	O=C(NC1CN(C(=O)[C@@H]2CC23CC3)C1)[C@@H]1[C@H]2Cc3ccccc3[C@H]21	991649517	24	322.17	1.7
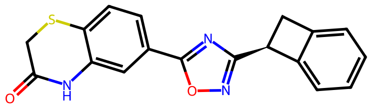	O=C1CSc2ccc(-c3nc([C@H]4Cc5ccccc54)no3)cc2N1	544985489	24	335.07	3.47
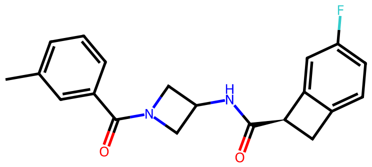	Cc1cccc(C(=O)N2CC(NC(=O)[C@@H]3Cc4ccc(F)cc43)C2)c1	998488647	25	338.14	2.41
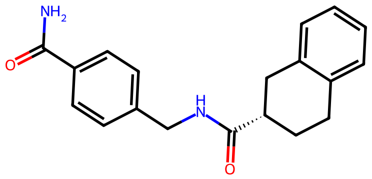	NC(=O)c1ccc(CNC(=O)[C@H]2CCc3ccccc3C2)cc1	584722440	23	308.15	2.21

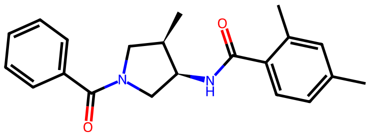	Cc1ccc(C(=O)N[C@H]2CN(C(=O)c3ccccc3)C[C@H]2C)c(C)c1	966093561	25	336.18	3.19
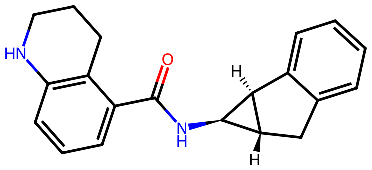	O=C(N[C@@H]1[C@H]2Cc3ccccc3[C@@H]21)c1cccc2c1CCCN2	130261768	23	304.16	3.11
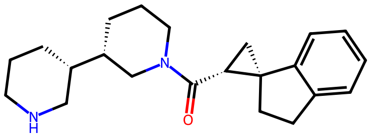	O=C([C@@H]1C[C@]12CCc1ccccc12)N1CCC[C@@H]([C@H]2CCCNC2)C1	646381700	25	338.24	3.13
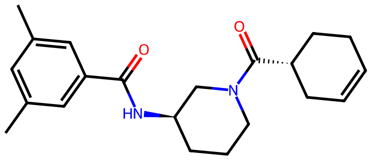	Cc1cc(C)cc(C(=O)N[C@@H]2CCCN(C(=O)[C@H]3CC=CCC3)C2)c1	949985077	25	340.22	3.38
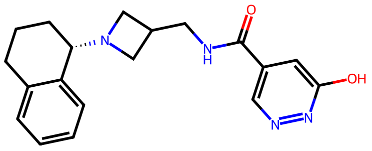	O=C(NCC1CN([C@H]2CCCc3ccccc32)C1)c1cnnc(O)c1	1031751159	25	338.17	1.92
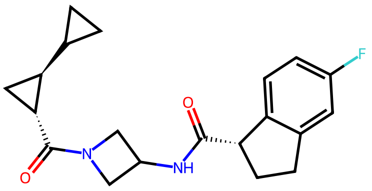	O=C(NC1CN(C(=O)[C@@H]2C[C@H]2C2CC2)C1)[C@H]1CCc2cc(F)ccc21	996082150	25	342.17	2.23
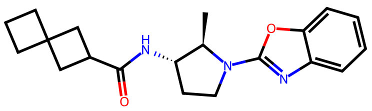	C[C@@H]1[C@@H](NC(=O)C2CC3(CCC3)C2)CCN1c1nc2ccccc2o1	1050875395	25	339.19	3.49
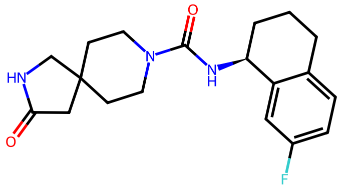	O=C1CC2(CCN(C(=O)N[C@H]3CCCc4ccc(F)cc43)CC2)CN1	279656075	25	345.19	2.51

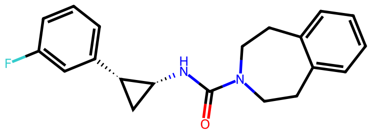	O=C(N[C@@H]1C[C@@H]1c1cccc(F)c1)N1CCc2ccccc2CC1	580597604	24	324.16	3.49
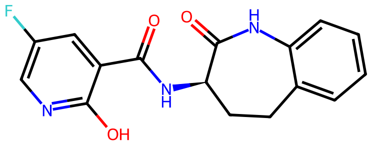	O=C(N[C@@H]1CCc2ccccc2NC1=O)c1cc(F)cnc1O	1434149413	23	315.1	1.61
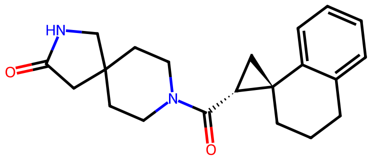	O=C1CC2(CCN(C(=O)[C@@H]3C[C@@]34CCCc3ccccc34)CC2)CN1	374098580	25	338.2	2.41
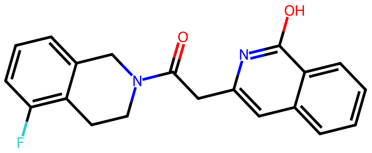	O=C(Cc1cc2ccccc2c(O)n1)N1CCc2c(F)cccc2C1	1137590528	25	336.13	3.21
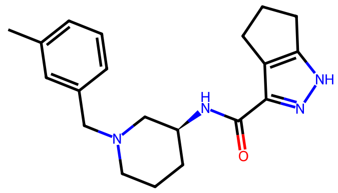	Cc1cccc(CN2CCC[C@H](NC(=O)c3n[nH]c4c3CCC4)C2)c1	1007748360	25	338.21	2.6
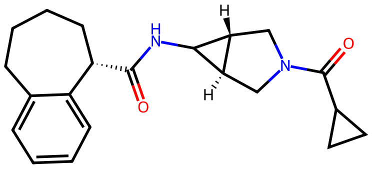	O=C(NC1[C@@H]2CN(C(=O)C3CC3)C[C@@H]12)[C@H]1CCCCc2ccccc21	1013288180	25	338.2	2.48
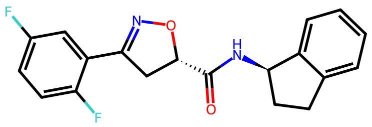	O=C(N[C@@H]1CCc2ccccc21)[C@@H]1CC(c2cc(F)ccc2F)=NO1	1461411722	25	342.12	3.26
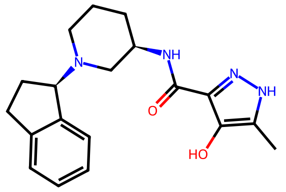	Cc1[nH]nc(C(=O)N[C@@H]2CCCN([C@@H]3CCc4ccccc43)C2)c1O	1007132752	25	340.19	2.31

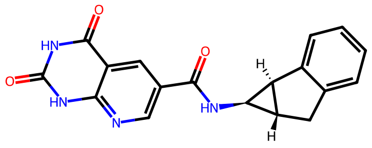	O=C(N[C@@H]1[C@H]2Cc3ccccc3[C@@H]21)c1cnc2[nH]c(=O)[nH]c(=O)c2c1	152227841	25	334.11	0.68
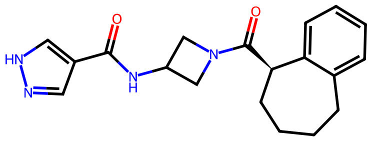	O=C(NC1CN(C(=O)[C@@H]2CCCCc3ccccc32)C1)c1cn[nH]c1	990323613	25	338.17	1.86
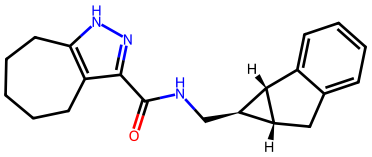	O=C(NC[C@@H]1[C@H]2Cc3ccccc3[C@H]21)c1n[nH]c2c1CCCCC2	190709864	24	321.18	2.99
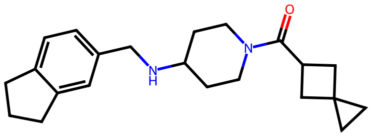	O=C(C1CC2(CC2)C1)N1CCC(NCc2ccc3c(c2)CCC3)CC1	996982805	25	338.24	3.45
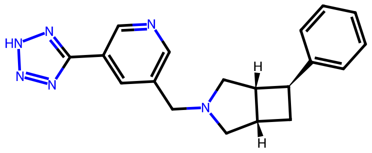	c1ccc([C@H]2C[C@@H]3CN(Cc4cncc(-c5nn[nH]n5)c4)C[C@@H]32)cc1	1593578044	25	332.17	2.5
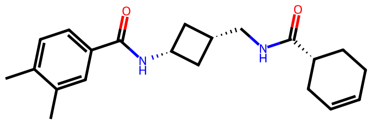	Cc1ccc(C(=O)N[C@H]2C[C@@H](CNC(=O)[C@H]3CC=CCC3)C2)cc1C	978571571	25	340.22	3.28
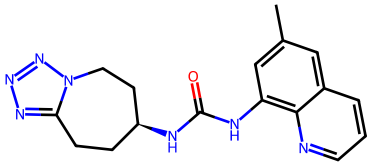	Cc1cc(NC(=O)N[C@H]2CCc3nnnn3CC2)c2ncccc2c1	563879394	25	337.17	2.06
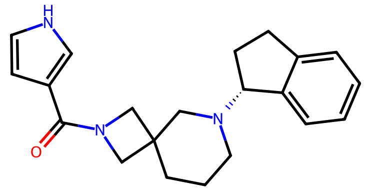	O=C(c1cc[nH]c1)N1CC2(CCCN([C@@H]3CCc4ccccc43)C2)C1	981784192	25	335.2	3.24

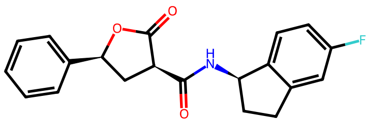	O=C(N[C@@H]1CCc2cc(F)ccc21)[C@H]1C[C@@H](c2ccccc2)OC1=O	290409577	25	339.13	3.23
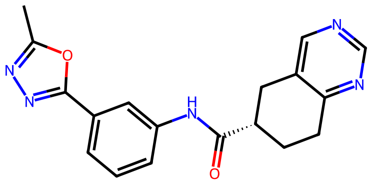	Cc1nnc(-c2cccc(NC(=O)[C@H]3CCc4ncncc4C3)c2)o1	648302949	25	335.14	2.58
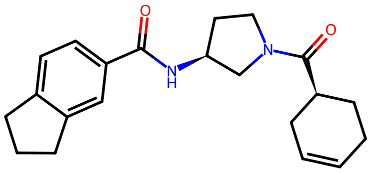	O=C(N[C@H]1CCN(C(=O)[C@@H]2CC=CCC2)C1)c1ccc2c(c1)CCC2	937934050	25	338.2	2.86
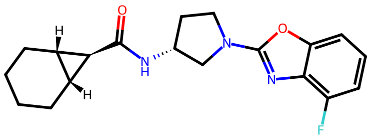	O=C(N[C@@H]1CCN(c2nc3c(F)cccc3o2)C1)[C@@H]1[C@H]2CCCC[C@H]21	1058334379	25	343.17	3.1
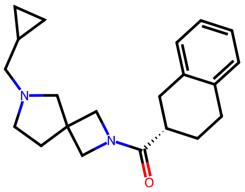	O=C([C@H]1CCc2ccccc2C1)N1CC2(CCN(CC3CC3)C2)C1	1073014871	24	324.22	2.74
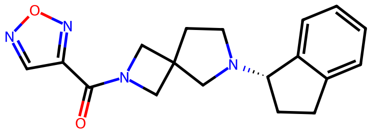	O=C(c1cnon1)N1CC2(CCN([C@H]3CCc4ccccc43)C2)C1	1073068637	24	324.16	1.91
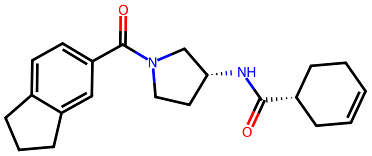	O=C(N[C@@H]1CCN(C(=O)c2ccc3c(c2)CCC3)C1)[C@H]1CC=CCC1	939379576	25	338.2	2.86
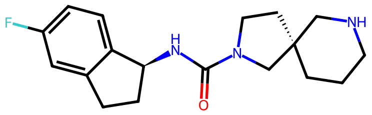	O=C(N[C@H]1CCc2cc(F)ccc21)N1CC[C@]2(CCCNC2)C1	583311488	23	317.19	2.6

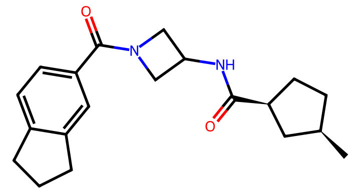	C[C@@H]1CC[C@H](C(=O)NC2CN(C(=O)c3ccc4c(c3)CCC4)C2)C1	991418251	24	326.2	2.55
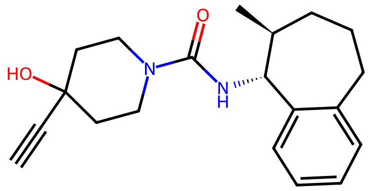	C#CC1(O)CCN(C(=O)N[C@H]2c3ccccc3CCC[C@@H]2C)CC1	1345843215	24	326.2	2.87
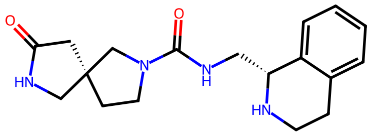	O=C1C[C@]2(CCN(C(=O)NC[C@H]3NCCc4ccccc43)C2)CN1	572825554	24	328.19	0.8
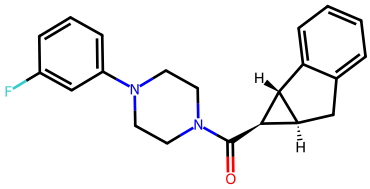	O=C([C@@H]1[C@@H]2Cc3ccccc3[C@H]21)N1CCN(c2cccc(F)c2)CC1	633894210	25	336.16	3.06
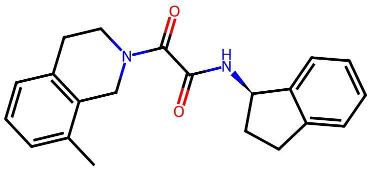	Cc1cccc2c1CN(C(=O)C(=O)N[C@@H]1CCc3ccccc31)CC2	339875265	25	334.17	2.68
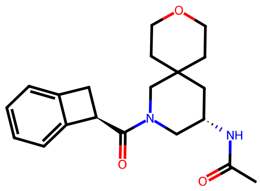	CC(=O)N[C@@H]1CN(C(=O)[C@@H]2Cc3ccccc32)CC2(CCOCC2)C1	991113938	25	342.19	1.86
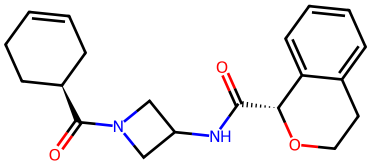	O=C(NC1CN(C(=O)[C@@H]2CC=CCC2)C1)[C@H]1OCCc2ccccc21	997322114	25	340.18	1.98
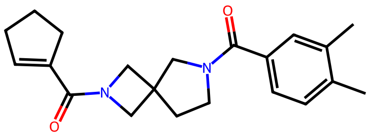	Cc1ccc(C(=O)N2CCC3(CN(C(=O)C4=CCCC4)C3)C2)cc1C	1018938093	25	338.2	3.09

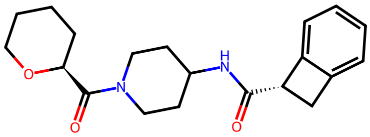	O=C(NC1CCN(C(=O)[C@@H]2CCCCO2)CC1)[C@H]1Cc2ccccc21	946472635	25	342.19	2.0
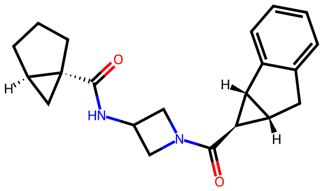	O=C([C@@H]1[C@H]2Cc3ccccc3[C@H]21)N1CC(NC(=O)[C@@]23CCC[C@@H]2C3)C1	997549114	25	336.18	2.09
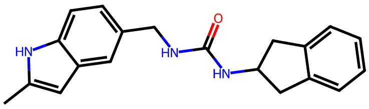	Cc1cc2cc(CNC(=O)NC3Cc4ccccc4C3)ccc2[nH]1	577088315	24	319.17	3.44
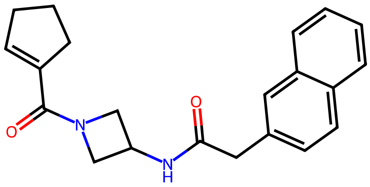	O=C(Cc1ccc2ccccc2c1)NC1CN(C(=O)C2=CCCC2)C1	991323437	25	334.17	2.82
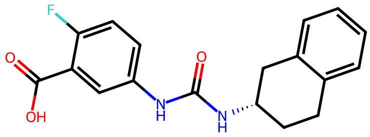	O=C(Nc1ccc(F)c(C(=O)O)c1)N[C@H]1CCc2ccccc2C1	324398841	24	328.12	3.2
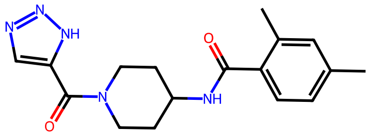	Cc1ccc(C(=O)NC2CCN(C(=O)c3cnn[nH]3)CC2)c(C)c1	947370598	24	327.17	1.46
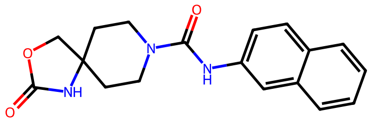	O=C1NC2(CCN(C(=O)Nc3ccc4ccccc4c3)CC2)CO1	495297186	24	325.14	2.95
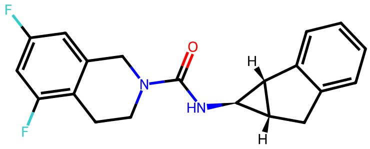	O=C(N[C@@H]1[C@H]2Cc3ccccc3[C@H]21)N1CCc2c(F)cc(F)cc2C1	152925884	25	340.14	3.37

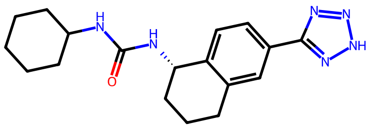	O=C(NC1CCCCC1)N[C@H]1CCCc2cc(-c3nn[nH]n3)ccc21	1609540654	25	340.2	2.88
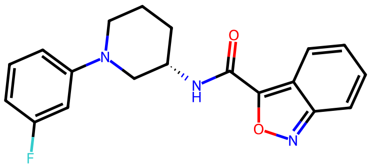	O=C(N[C@H]1CCCN(c2cccc(F)c2)C1)c1onc2ccccc12	1465081298	25	339.14	3.37
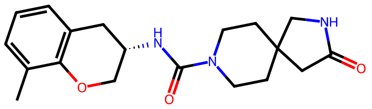	Cc1cccc2c1OC[C@@H](NC(=O)N1CCC3(CC1)CNC(=O)C3)C2	291277317	25	343.19	1.61
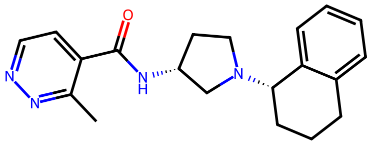	Cc1nnccc1C(=O)N[C@@H]1CCN([C@H]2CCCc3ccccc32)C1	1015115064	25	336.2	2.67
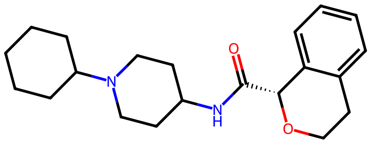	O=C(NC1CCN(C2CCCCC2)CC1)[C@H]1OCCc2ccccc21	123827291	25	342.23	3.21
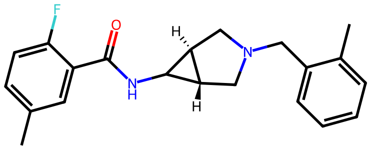	Cc1ccc(F)c(C(=O)NC2[C@H]3CN(Cc4ccccc4C)C[C@H]23)c1	961878414	25	338.18	3.3
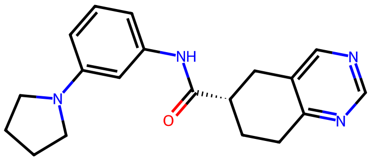	O=C(Nc1cccc(N2CCCC2)c1)[C@H]1CCc2ncncc2C1	648249865	24	322.18	2.82
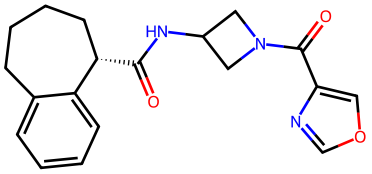	O=C(NC1CN(C(=O)c2cocn2)C1)[C@H]1CCCCc2ccccc21	994305257	25	339.16	2.13

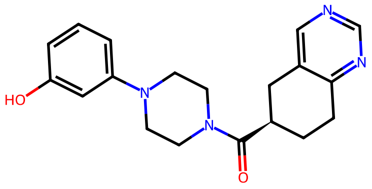	O=C([C@@H]1CCc2ncncc2C1)N1CCN(c2cccc(O)c2)CC1	648720741	25	338.17	1.64
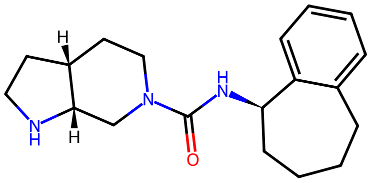	O=C(N[C@@H]1CCCCc2ccccc21)N1CC[C@H]2CCN[C@H]2C1	669439940	23	313.22	2.85
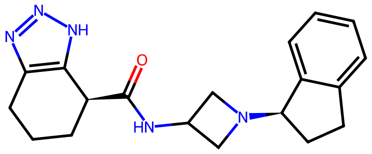	O=C(NC1CN([C@@H]2CCc3ccccc32)C1)[C@H]1CCCc2nn[nH]c21	1030861579	25	337.19	1.71
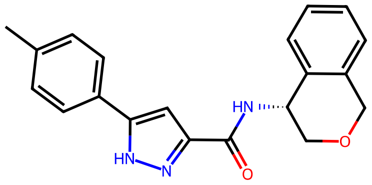	Cc1ccc(-c2cc(C(=O)N[C@H]3COCc4ccccc43)n[nH]2)cc1	1638320975	25	333.15	3.39
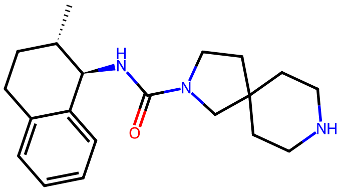	C[C@H]1CCc2ccccc2[C@@H]1NC(=O)N1CCC2(CCNCC2)C1	417962758	24	327.23	3.1
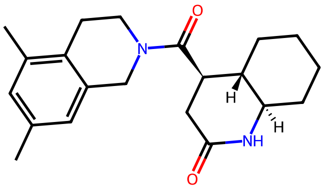	Cc1cc(C)c2c(c1)CN(C(=O)[C@@H]1CC(=O)N[C@@H]3CCCC[C@H]31)CC2	613651633	25	340.22	2.88
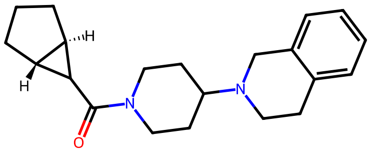	O=C(C1[C@@H]2CCC[C@@H]12)N1CCC(N2CCc3ccccc3C2)CC1	267319617	24	324.22	3.08
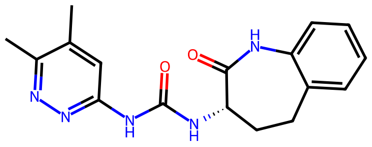	Cc1cc(NC(=O)N[C@H]2CCc3ccccc3NC2=O)nnc1C	653987529	24	325.15	2.17

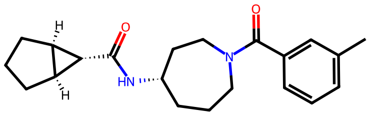	Cc1cccc(C(=O)N2CCC[C@H](NC(=O)[C@@H]3[C@H]4CCC[C@H]43)CC2)c1	964820036	25	340.22	3.15
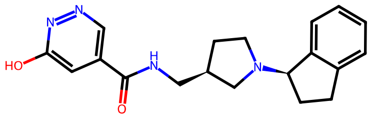	O=C(NC[C@H]1CCN([C@@H]2CCc3ccccc32)C1)c1cnnc(O)c1	1028612971	25	338.17	1.92
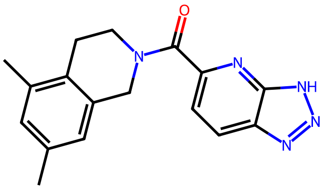	Cc1cc(C)c2c(c1)CN(C(=O)c1ccc3nn[nH]c3n1)CC2	416212135	23	307.14	2.17
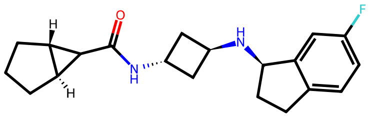	O=C(N[C@H]1C[C@H](N[C@@H]2CCc3ccc(F)cc32)C1)C1[C@H]2CCC[C@H]12	1020055532	24	328.2	3.1
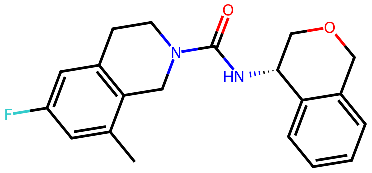	Cc1cc(F)cc2c1CN(C(=O)N[C@@H]1COCc3ccccc31)CC2	419544398	25	340.16	3.47
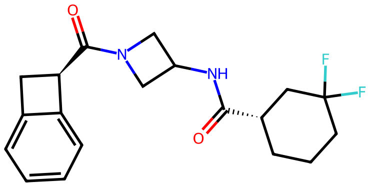	O=C(NC1CN(C(=O)[C@@H]2Cc3ccccc32)C1)[C@H]1CCCC(F)(F)C1	999754426	25	348.16	2.48
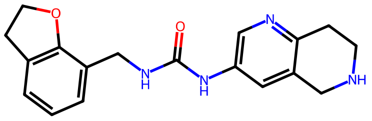	O=C(NCc1cccc2c1OCC2)Nc1cnc2c(c1)CNCC2	926162607	24	324.16	1.98
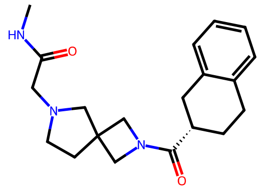	CNC(=O)CN1CCC2(C1)CN(C(=O)[C@H]1CCc3ccccc3C1)C2	1073014968	25	341.21	1.07

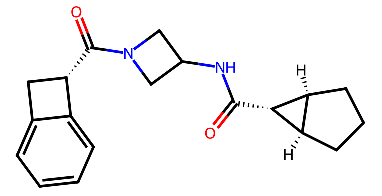	O=C(NC1CN(C(=O)[C@H]2Cc3ccccc32)C1)[C@@H]1[C@H]2CCC[C@H]21	990691368	23	310.17	1.7
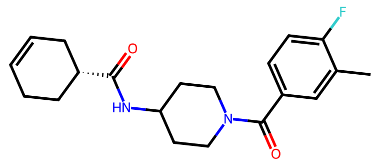	Cc1cc(C(=O)N2CCC(NC(=O)[C@H]3CC=CCC3)CC2)ccc1F	948053324	25	344.19	3.21
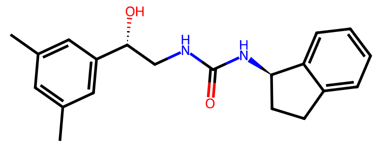	Cc1cc(C)cc([C@H](O)CNC(=O)N[C@@H]2CCc3ccccc32)c1	800988971	24	324.18	3.32
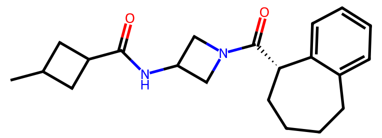	CC1CC(C(=O)NC2CN(C(=O)[C@H]3CCCCc4ccccc43)C2)C1	990758787	25	340.22	2.87
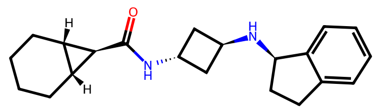	O=C(N[C@H]1C[C@H](N[C@@H]2CCc3ccccc32)C1)[C@@H]1[C@H]2CCCC[C@H]21	1020052287	24	324.22	3.35
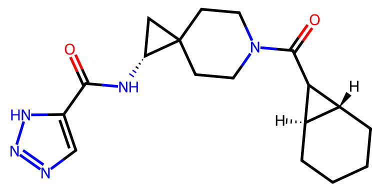	O=C(N[C@@H]1CC12CCN(C(=O)C1[C@@H]3CCCC[C@@H]13)CC2)c1cnn[nH]1	964246211	25	343.2	1.35
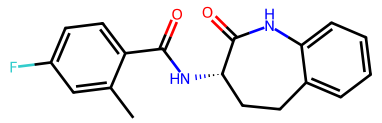	Cc1cc(F)ccc1C(=O)N[C@H]1CCc2ccccc2NC1=O	1411464144	23	312.13	2.82
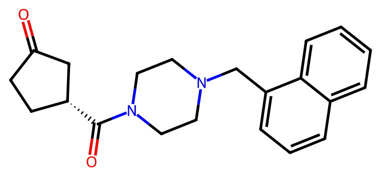	O=C1CC[C@@H](C(=O)N2CCN(Cc3cccc4ccccc34)CC2)C1	519768562	25	336.18	2.85

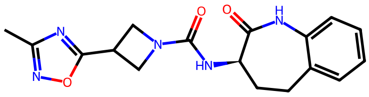	Cc1noc(C2CN(C(=O)N[C@@H]3CCc4ccccc4NC3=O)C2)n1	847396918	25	341.15	1.44
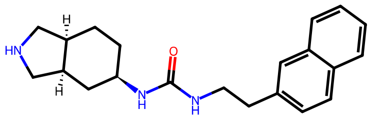	O=C(NCCc1ccc2ccccc2c1)N[C@@H]1CC[C@@H]2CNC[C@@H]2C1	569440313	25	337.22	3.07
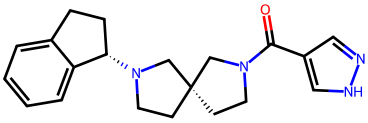	O=C(c1cn[nH]c1)N1CC[C@@]2(CCN([C@H]3CCc4ccccc43)C2)C1	1041104342	25	336.2	2.64
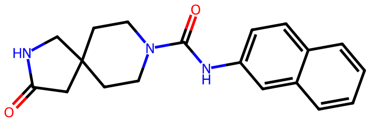	O=C1CC2(CCN(C(=O)Nc3ccc4ccccc4c3)CC2)CN1	267775400	24	323.16	2.97
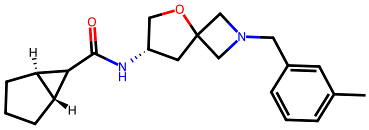	Cc1cccc(CN2CC3(C[C@H](NC(=O)C4[C@@H]5CCC[C@@H]45)CO3)C2)c1	1053769755	25	340.22	2.5
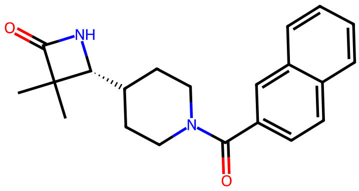	CC1(C)C(=O)N[C@@H]1C1CCN(C(=O)c2ccc3ccccc3c2)CC1	294768510	25	336.18	3.22
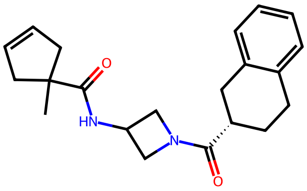	CC1(C(=O)NC2CN(C(=O)[C@H]3CCc4ccccc4C3)C2)CC=CC1	999079763	25	338.2	2.47
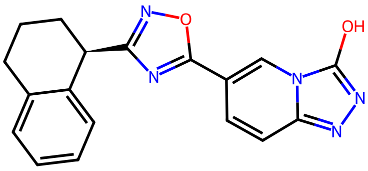	Oc1nnc2ccc(-c3nc([C@@H]4CCCc5ccccc54)no3)cn12	355884905	25	333.12	2.95

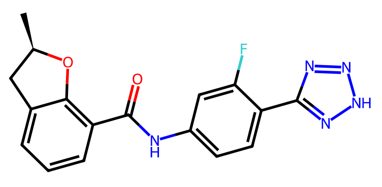	C[C@@H]1Cc2cccc(C(=O)Nc3ccc(-c4nn[nH]n4)c(F)c3)c2O1	1590332071	25	339.11	2.58
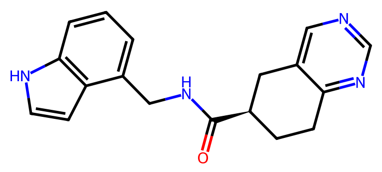	O=C(NCc1cccc2[nH]ccc12)[C@@H]1CCc2ncncc2C1	636365906	23	306.15	2.38
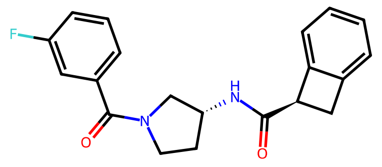	O=C(N[C@@H]1CCN(C(=O)c2cccc(F)c2)C1)[C@@H]1Cc2ccccc21	942303322	25	338.14	2.5
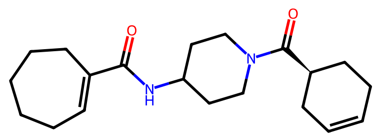	O=C(NC1CCN(C(=O)[C@@H]2CC=CCC2)CC1)C1=CCCCCC1	944113724	24	330.23	3.34
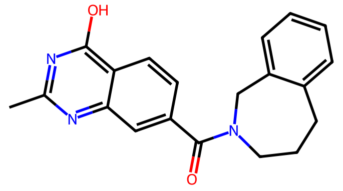	Cc1nc(O)c2ccc(C(=O)N3CCCc4ccccc4C3)cc2n1	888842488	25	333.15	3.23
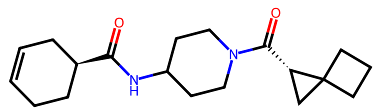	O=C(NC1CCN(C(=O)[C@H]2CC23CCC3)CC1)[C@@H]1CC=CCC1	946763926	23	316.22	2.64
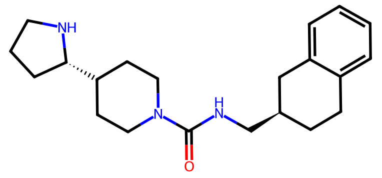	O=C(NC[C@@H]1CCc2ccccc2C1)N1CCC([C@@H]2CCCN2)CC1	669551598	25	341.25	2.97
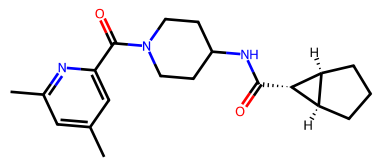	Cc1cc(C)nc(C(=O)N2CCC(NC(=O)[C@@H]3[C@H]4CCC[C@H]43)CC2)c1	947307730	25	341.21	2.47

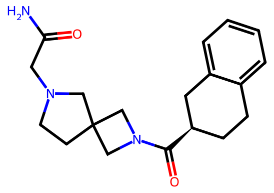	NC(=O)CN1CCC2(C1)CN(C(=O)[C@@H]1CCc3ccccc3C1)C2	1073015043	24	327.19	0.81
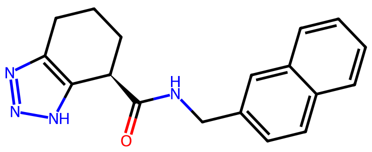	O=C(NCc1ccc2ccccc2c1)[C@@H]1CCCc2nn[nH]c21	821045033	23	306.15	2.69
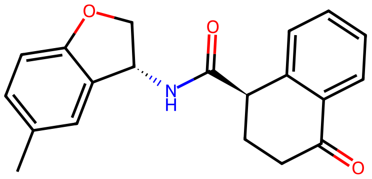	Cc1ccc2c(c1)[C@@H](NC(=O)[C@@H]1CCC(=O)c3ccccc31)CO2	375070914	24	321.14	3.31
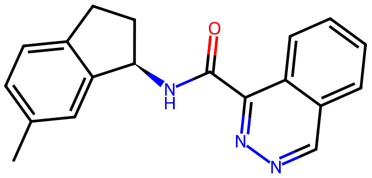	Cc1ccc2c(c1)[C@H](NC(=O)c1nncc3ccccc13)CC2	1527353727	23	303.14	3.36
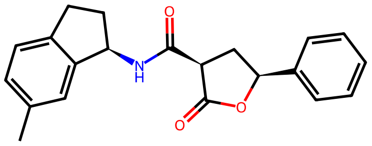	Cc1ccc2c(c1)[C@H](NC(=O)[C@H]1C[C@@H](c3ccccc3)OC1=O)CC2	809668254	25	335.15	3.4
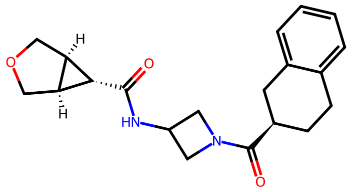	O=C(NC1CN(C(=O)[C@@H]2CCc3ccccc3C2)C1)[C@@H]1[C@H]2COC[C@H]21	997944923	25	340.18	1.01
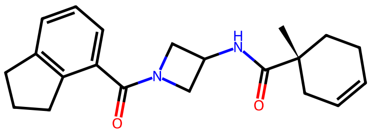	C[C@]1(C(=O)NC2CN(C(=O)c3cccc4c3CCC4)C2)CC=CCC1	991917950	25	338.2	2.86
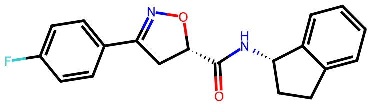	O=C(N[C@H]1CCc2ccccc21)[C@@H]1CC(c2ccc(F)cc2)=NO1	1875346314	24	324.13	3.12

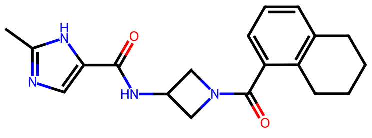	Cc1ncc(C(=O)NC2CN(C(=O)c3cccc4c3CCCC4)C2)[nH]1	990501564	25	338.17	1.85
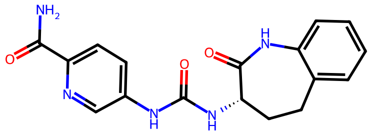	NC(=O)c1ccc(NC(=O)N[C@H]2CCc3ccccc3NC2=O)cn1	1364318119	25	339.13	1.26
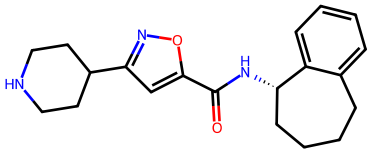	O=C(N[C@H]1CCCCc2ccccc21)c1cc(C2CCNCC2)no1	662434871	25	339.19	3.34
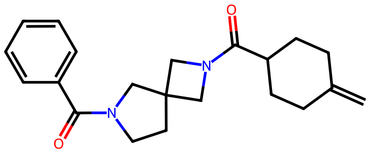	C=C1CCC(C(=O)N2CC3(CCN(C(=O)c4ccccc4)C3)C2)CC1	1019391267	25	338.2	3.11
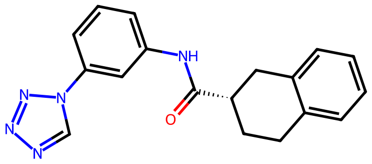	O=C(Nc1cccc(-n2cnnn2)c1)[C@H]1CCc2ccccc2C1	1349147301	24	319.14	2.41
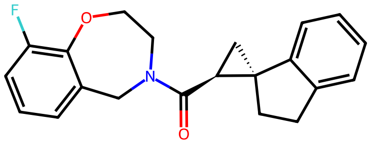	O=C([C@H]1C[C@]12CCc1ccccc12)N1CCOc2c(F)cccc2C1	375927259	25	337.15	3.45
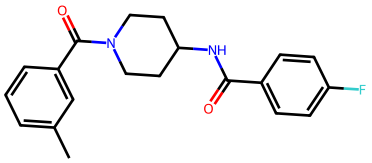	Cc1cccc(C(=O)N2CCC(NC(=O)c3ccc(F)cc3)CC2)c1	29092426	25	340.16	3.17
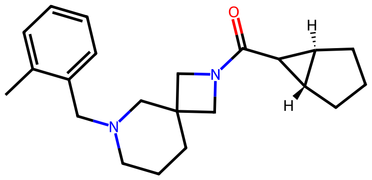	Cc1ccccc1CN1CCCC2(C1)CN(C(=O)C1[C@H]3CCC[C@H]13)C2	981696749	25	338.24	3.47

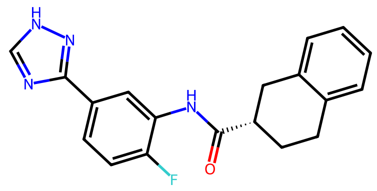	O=C(Nc1cc(-c2nc[nH]n2)ccc1F)[C@H]1CCc2ccccc2C1	1524729844	25	336.14	3.35
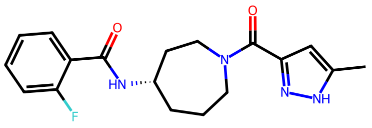	Cc1cc(C(=O)N2CCC[C@H](NC(=O)c3ccccc3F)CC2)n[nH]1	964691161	25	344.16	2.28
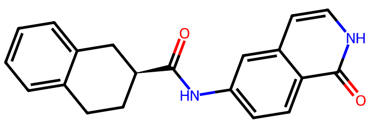	O=C(Nc1ccc2c(=O)[nH]ccc2c1)[C@H]1CCc2ccccc2C1	1453071411	24	318.14	3.27
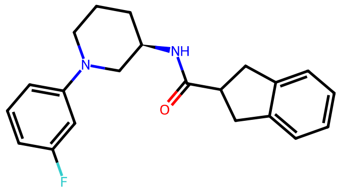	O=C(N[C@@H]1CCCN(c2cccc(F)c2)C1)C1Cc2ccccc2C1	469434096	25	338.18	3.33
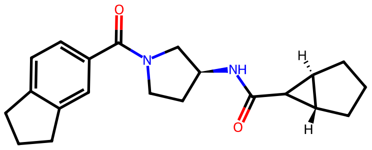	O=C(N[C@H]1CCN(C(=O)c2ccc3c(c2)CCC3)C1)C1[C@H]2CCC[C@H]12	937805348	25	338.2	2.55
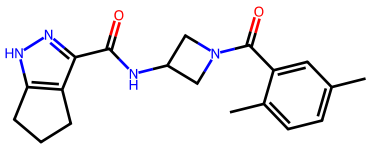	Cc1ccc(C)c(C(=O)N2CC(NC(=O)c3n[nH]c4c3CCC4)C2)c1	995166285	25	338.17	1.77
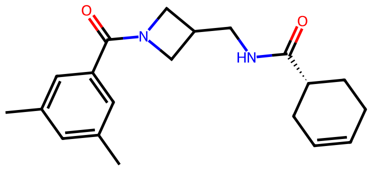	Cc1cc(C)cc(C(=O)N2CC(CNC(=O)[C@H]3CC=CCC3)C2)c1	1000889735	24	326.2	2.85
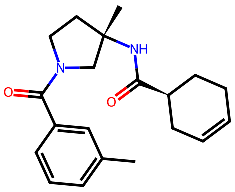	Cc1cccc(C(=O)N2CC[C@](C)(NC(=O)[C@@H]3CC=CCC3)C2)c1	956700421	24	326.2	3.07

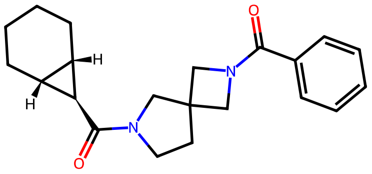	O=C(c1ccccc1)N1CC2(CCN(C(=O)[C@@H]3[C@H]4CCCC[C@H]43)C2)C1	1018711942	25	338.2	2.8
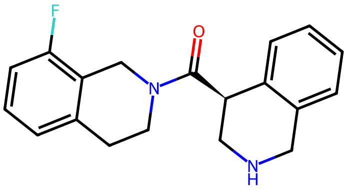	O=C([C@@H]1CNCc2ccccc21)N1CCc2cccc(F)c2C1	584409935	23	310.15	2.6
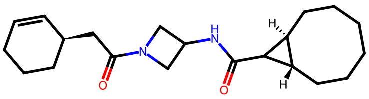	O=C(NC1CN(C(=O)C[C@@H]2C=CCCC2)C1)C1[C@H]2CCCCCC[C@H]12	1000512924	25	344.25	3.28
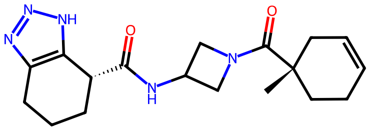	C[C@@]1(C(=O)N2CC(NC(=O)[C@@H]3CCCc4nn[nH]c43)C2)CC=CCC1	998572545	25	343.2	1.3
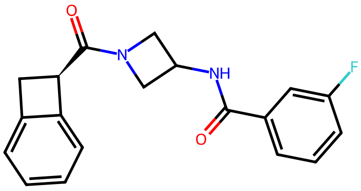	O=C(NC1CN(C(=O)[C@@H]2Cc3ccccc32)C1)c1cccc(F)c1	990113633	24	324.13	2.11
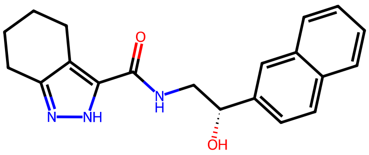	O=C(NC[C@@H](O)c1ccc2ccccc2c1)c1[nH]nc2c1CCCC2	303982454	25	335.16	2.91
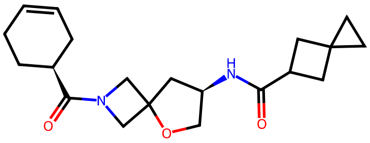	O=C(N[C@H]1COC2(C1)CN(C(=O)[C@@H]1CC=CCC1)C2)C1CC2(CC2)C1	965802619	25	344.21	2.02
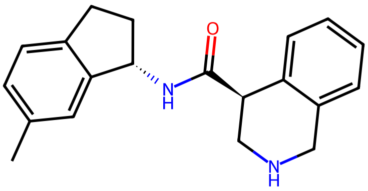	Cc1ccc2c(c1)[C@@H](NC(=O)[C@@H]1CNCc3ccccc31)CC2	645946686	23	306.17	2.99

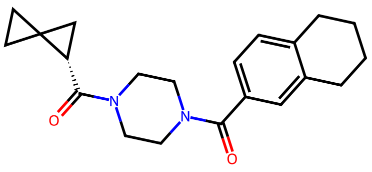	O=C(c1ccc2c(c1)CCCC2)N1CCN(C(=O)[C@H]2CC23CC3)CC1	1012579261	25	338.2	2.65
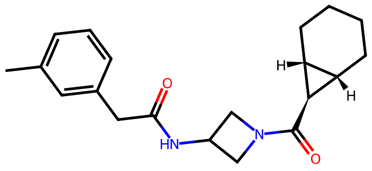	Cc1cccc(CC(=O)NC2CN(C(=O)[C@@H]3[C@H]4CCCC[C@H]43)C2)c1	998709743	24	326.2	2.3
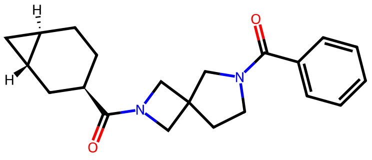	O=C(c1ccccc1)N1CCC2(C1)CN(C(=O)[C@@H]1CC[C@@H]3C[C@H]3C1)C2	1019141889	25	338.2	2.8
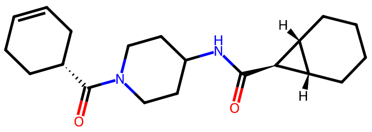	O=C(NC1CCN(C(=O)[C@H]2CC=CCC2)CC1)[C@@H]1[C@H]2CCCC[C@H]21	944107224	24	330.23	2.89
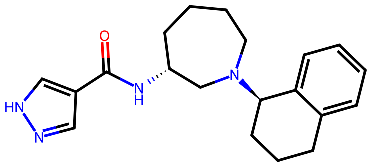	O=C(N[C@@H]1CCCCN([C@@H]2CCCc3ccccc32)C1)c1cn[nH]c1	1034116354	25	338.21	3.07
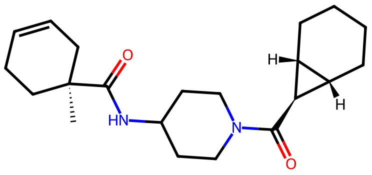	C[C@@]1(C(=O)NC2CCN(C(=O)[C@@H]3[C@H]4CCCC[C@H]43)CC2)CC=CCC1	945956989	25	344.25	3.28
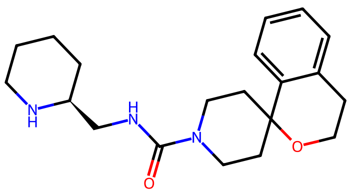	O=C(NC[C@@H]1CCCCN1)N1CCC2(CC1)OCCc1ccccc12	634269509	25	343.23	2.4
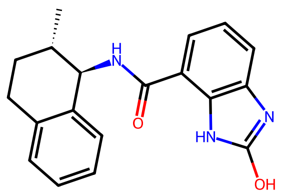	C[C@H]1CCc2ccccc2[C@@H]1NC(=O)c1cccc2nc(O)[nH]c12	654972077	24	321.15	3.32

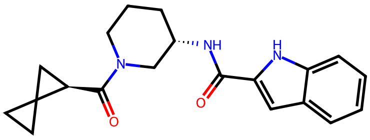	O=C(N[C@H]1CCCN(C(=O)[C@@H]2CC23CC3)C1)c1cc2ccccc2[nH]1	952144129	25	337.18	2.69
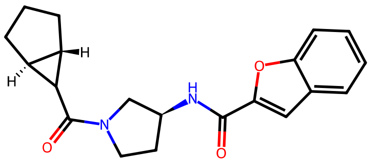	O=C(N[C@H]1CCN(C(=O)C2[C@H]3CCC[C@H]23)C1)c1cc2ccccc2o1	938870360	25	338.16	2.81
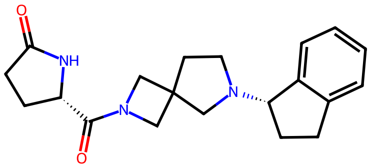	O=C1CC[C@@H](C(=O)N2CC3(CCN([C@H]4CCc5ccccc54)C3)C2)N1	1073137653	25	339.19	1.49
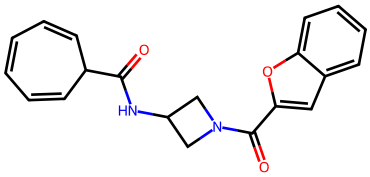	O=C(NC1CN(C(=O)c2cc3ccccc3o2)C1)C1C=CC=CC=C1	992875454	25	334.13	2.67
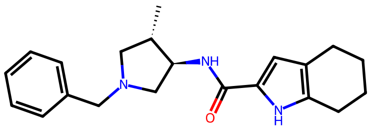	C[C@H]1CN(Cc2ccccc2)C[C@@H]1NC(=O)c1cc2c([nH]1)CCCC2	1296043439	25	337.22	3.14
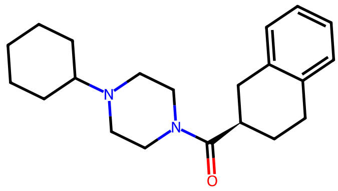	O=C([C@@H]1CCc2ccccc2C1)N1CCN(C2CCCCC2)CC1	1583138609	24	326.24	3.27
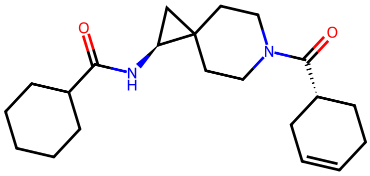	O=C(N[C@H]1CC12CCN(C(=O)[C@H]1CC=CCC1)CC2)C1CCCCC1	962750263	25	344.25	3.42
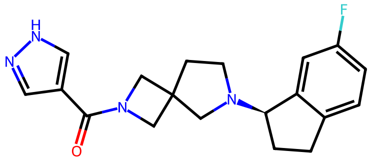	O=C(c1cn[nH]c1)N1CC2(CCN([C@@H]3CCc4ccc(F)cc43)C2)C1	1072511741	25	340.17	2.38

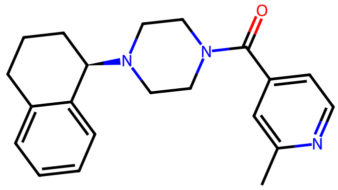	Cc1cc(C(=O)N2CCN([C@@H]3CCCc4ccccc43)CC2)ccn1	952349336	25	335.2	3.23
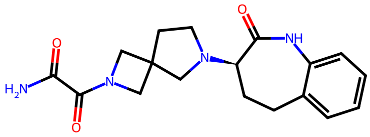	NC(=O)C(=O)N1CC2(CCN([C@@H]3CCc4ccccc4NC3=O)C2)C1	1072935840	25	342.17	-0.04
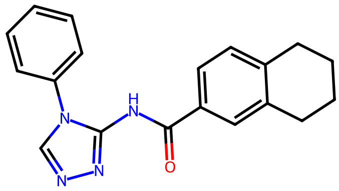	O=C(Nc1nncn1-c1ccccc1)c1ccc2c(c1)CCCC2	355757142	24	318.15	3.4
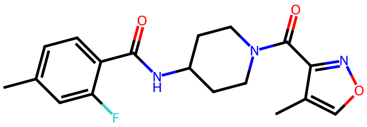	Cc1ccc(C(=O)NC2CCN(C(=O)c3nocc3C)CC2)c(F)c1	948372460	25	345.15	2.47
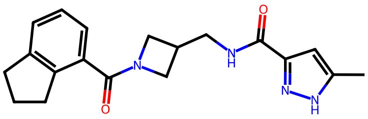	Cc1cc(C(=O)NCC2CN(C(=O)c3cccc4c3CCC4)C2)n[nH]1	1001099654	25	338.17	1.71
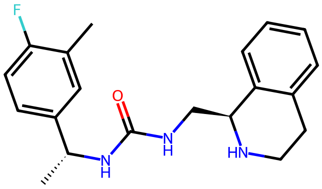	Cc1cc([C@@H](C)NC(=O)NC[C@@H]2NCCc3ccccc32)ccc1F	574623648	25	341.19	3.38
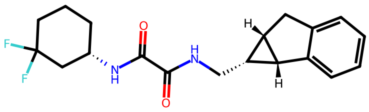	O=C(NC[C@H]1[C@H]2c3ccccc3C[C@H]21)C(=O)N[C@H]1CCCC(F)(F)C1	1329679183	25	348.16	2.38
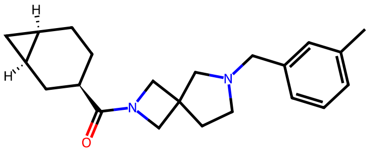	Cc1cccc(CN2CCC3(C2)CN(C(=O)[C@@H]2CC[C@@H]4C[C@@H]4C2)C3)c1	1072878997	25	338.24	3.47

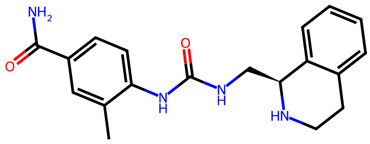	Cc1cc(C(N)=O)ccc1NC(=O)NC[C@@H]1NCCc2ccccc21	841038916	25	338.17	2.1
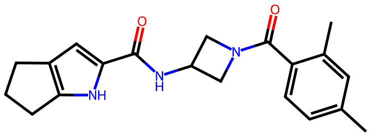	Cc1ccc(C(=O)N2CC(NC(=O)c3cc4c([nH]3)CCC4)C2)c(C)c1	995962360	25	337.18	2.37
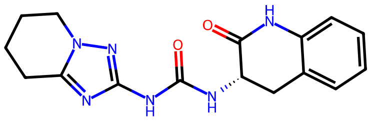	O=C(Nc1nc2n(n1)CCCC2)N[C@H]1Cc2ccccc2NC1=O	529116721	24	326.15	1.3
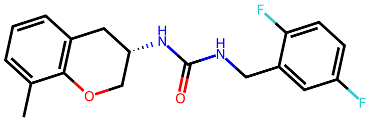	Cc1cccc2c1OC[C@@H](NC(=O)NCc1cc(F)ccc1F)C2	358724268	24	332.13	3.08
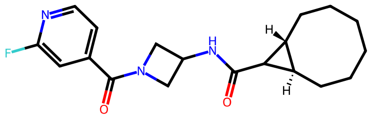	O=C(NC1CN(C(=O)c2ccnc(F)c2)C1)C1[C@@H]2CCCCCC[C@@H]12	1000484299	25	345.19	2.38
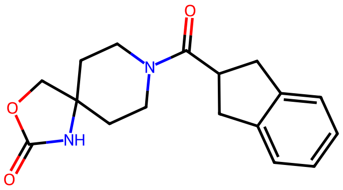	O=C1NC2(CCN(C(=O)C3Cc4ccccc4C3)CC2)CO1	496241479	22	300.15	1.5
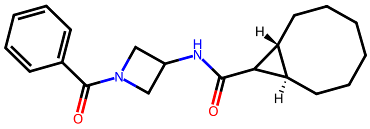	O=C(NC1CN(C(=O)c2ccccc2)C1)C1[C@@H]2CCCCCC[C@@H]12	1000465389	24	326.2	2.84
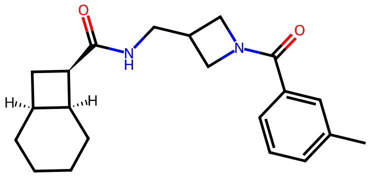	Cc1cccc(C(=O)N2CC(CNC(=O)[C@@H]3C[C@@H]4CCCC[C@@H]43)C2)c1	1003625242	25	340.22	3.01

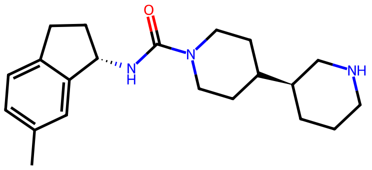	Cc1ccc2c(c1)[C@@H](NC(=O)N1CCC([C@@H]3CCCNC3)CC1)CC2	659453060	25	341.25	3.4
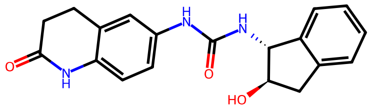	O=C1CCc2cc(NC(=O)N[C@@H]3c4ccccc4C[C@H]3O)ccc2N1	193243251	25	337.14	2.35
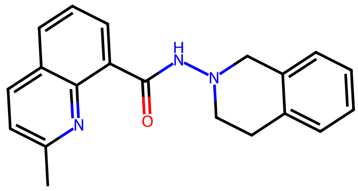	Cc1ccc2cccc(C(=O)NN3CCc4ccccc4C3)c2n1	1165594715	24	317.15	3.25
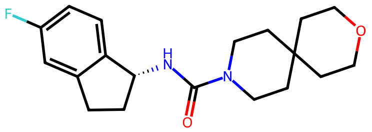	O=C(N[C@@H]1CCc2cc(F)ccc21)N1CCC2(CCOCC2)CC1	285265992	24	332.19	3.42
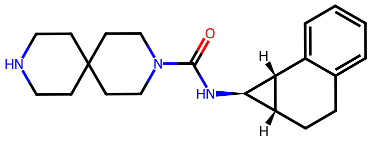	O=C(N[C@@H]1[C@H]2CCc3ccccc3[C@H]21)N1CCC2(CCNCC2)CC1	417916849	25	339.23	2.89
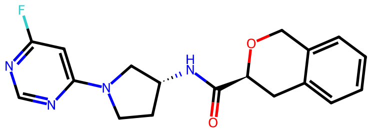	O=C(N[C@@H]1CCN(c2cc(F)ncn2)C1)[C@@H]1Cc2ccccc2CO1	1058419171	25	342.15	1.45
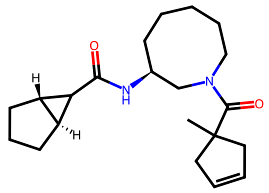	CC1(C(=O)N2CCCCC[C@H](NC(=O)C3[C@H]4CCC[C@H]34)C2)CC=CC1	987141364	25	344.25	3.28
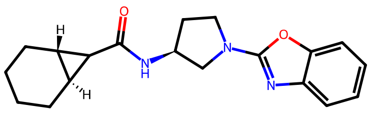	O=C(N[C@H]1CCN(c2nc3ccccc3o2)C1)C1[C@H]2CCCC[C@H]12	1062633411	24	325.18	2.96

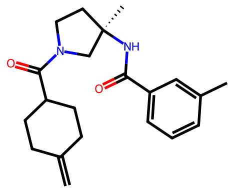	C=C1CCC(C(=O)N2CC[C@@](C)(NC(=O)c3cccc(C)c3)C2)CC1	956667946	25	340.22	3.46
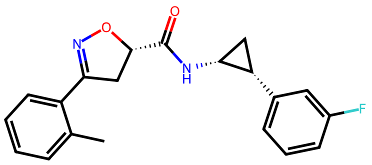	Cc1ccccc1C1=NO[C@H](C(=O)N[C@@H]2C[C@@H]2c2cccc(F)c2)C1	1524709798	25	338.14	3.3
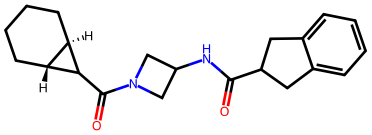	O=C(NC1CN(C(=O)C2[C@@H]3CCCC[C@@H]23)C1)C1Cc2ccccc2C1	1000449247	25	338.2	2.16
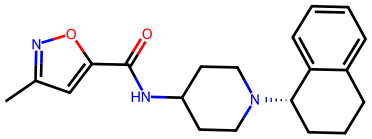	Cc1cc(C(=O)NC2CCN([C@H]3CCCc4ccccc43)CC2)on1	1002333125	25	339.19	3.25
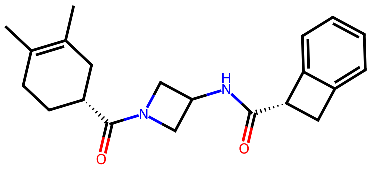	CC1=C(C)C[C@H](C(=O)N2CC(NC(=O)[C@H]3Cc4ccccc43)C2)CC1	998686642	25	338.2	2.79
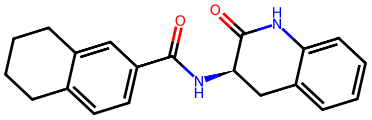	O=C(N[C@@H]1Cc2ccccc2NC1=O)c1ccc2c(c1)CCCC2	529951880	24	320.15	2.86
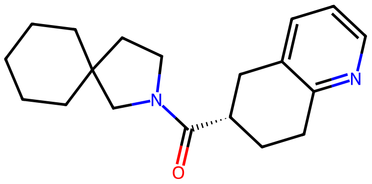	O=C([C@H]1CCc2ncccc2C1)N1CCC2(CCCCC2)C1	1875307445	22	298.2	3.37
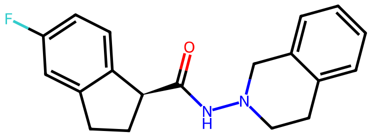	O=C(NN1CCc2ccccc2C1)[C@H]1CCc2cc(F)ccc21	855707456	23	310.15	2.95

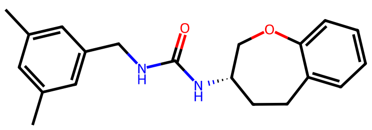	Cc1cc(C)cc(CNC(=O)N[C@H]2CCc3ccccc3OC2)c1	1235747812	24	324.18	3.5
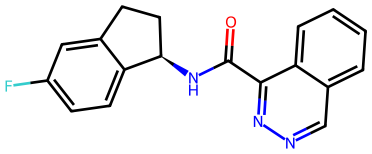	O=C(N[C@@H]1CCc2cc(F)ccc21)c1nncc2ccccc12	1526637153	23	307.11	3.19
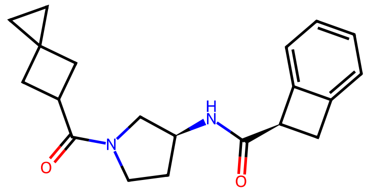	O=C(N[C@H]1CCN(C(=O)C2CC3(CC3)C2)C1)[C@@H]1Cc2ccccc21	938576463	24	324.18	2.23
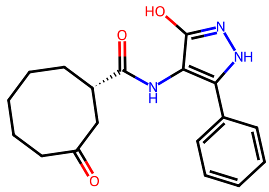	O=C1CCCCC[C@H](C(=O)Nc2c(O)n[nH]c2-c2ccccc2)C1	615282201	24	327.16	3.26
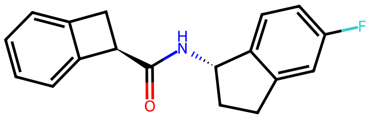	O=C(N[C@H]1CCc2cc(F)ccc21)[C@@H]1Cc2ccccc21	353844972	21	281.12	3.27
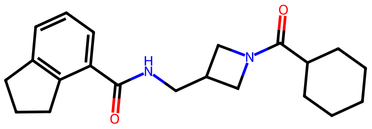	O=C(NCC1CN(C(=O)C2CCCCC2)C1)c1cccc2c1CCC2	1002708871	25	340.22	2.94
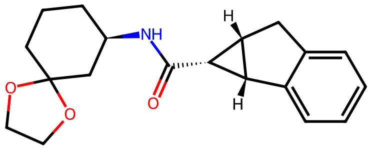	O=C(N[C@@H]1CCCC2(C1)OCCO2)[C@H]1[C@H]2c3ccccc3C[C@H]21	860695406	23	313.17	2.37
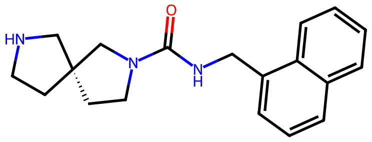	O=C(NCc1cccc2ccccc12)N1CC[C@@]2(CCNC2)C1	417528185	23	309.18	2.73

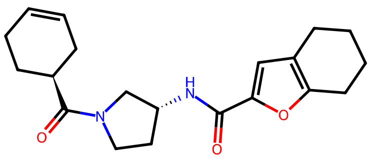	O=C(N[C@@H]1CCN(C(=O)[C@@H]2CC=CCC2)C1)c1cc2c(o1)CCCC2	940868622	25	342.19	2.85
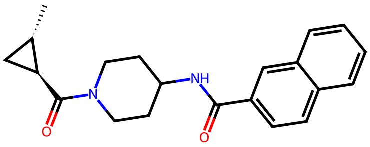	C[C@H]1C[C@@H]1C(=O)N1CCC(NC(=O)c2ccc3ccccc3c2)CC1	943836347	25	336.18	3.22
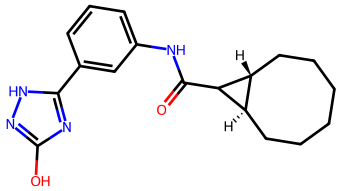	O=C(Nc1cccc(-c2nc(O)n[nH]2)c1)C1[C@@H]2CCCCCC[C@@H]12	614561442	24	326.17	3.33
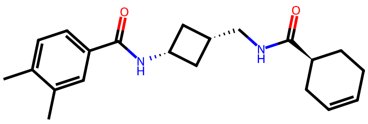	Cc1ccc(C(=O)N[C@H]2C[C@@H](CNC(=O)[C@@H]3CC=CCC3)C2)cc1C	978571574	25	340.22	3.28
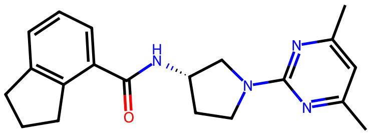	Cc1cc(C)nc(N2CC[C@H](NC(=O)c3cccc4c3CCC4)C2)n1	1058385390	25	336.2	2.59
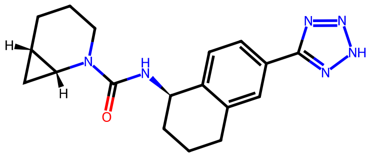	O=C(N[C@@H]1CCCc2cc(-c3nn[nH]n3)ccc21)N1CCC[C@H]2C[C@H]21	1592982690	25	338.19	2.44
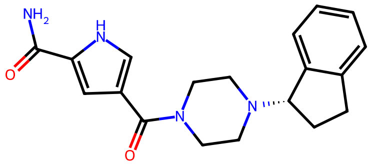	NC(=O)c1cc(C(=O)N2CCN([C@H]3CCc4ccccc43)CC2)c[nH]1	957670060	25	338.17	1.56
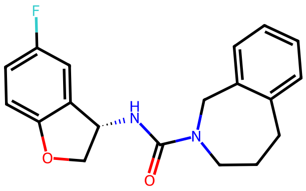	O=C(N[C@@H]1COc2ccc(F)cc21)N1CCCc2ccccc2C1	468463128	24	326.14	3.42

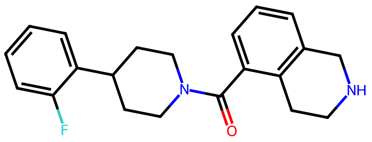	O=C(c1cccc2c1CCNC2)N1CCC(c2ccccc2F)CC1	662617376	25	338.18	3.49
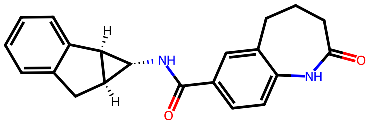	O=C1CCCc2cc(C(=O)N[C@@H]3[C@H]4Cc5ccccc5[C@H]43)ccc2N1	675695920	25	332.15	3.03
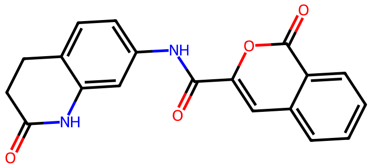	O=C1CCc2ccc(NC(=O)c3cc4ccccc4c(=O)o3)cc2N1	354070786	25	334.1	2.93
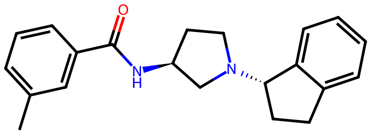	Cc1cccc(C(=O)N[C@H]2CCN([C@H]3CCc4ccccc43)C2)c1	1010012695	24	320.19	3.49
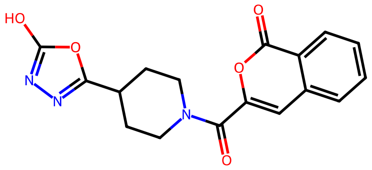	O=C(c1cc2ccccc2c(=O)o1)N1CCC(c2nnc(O)o2)CC1	351861858	25	341.1	1.9
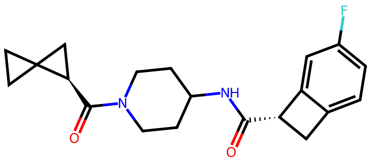	O=C(NC1CCN(C(=O)[C@@H]2CC23CC3)CC1)[C@H]1Cc2ccc(F)cc21	948568397	25	342.17	2.37
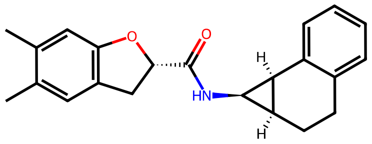	Cc1cc2c(cc1C)O[C@H](C(=O)N[C@@H]1[C@@H]3CCc4ccccc4[C@@H]31)C2	435966332	25	333.17	3.45
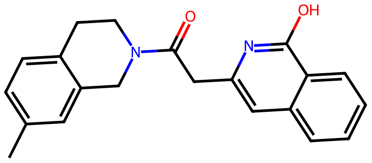	Cc1ccc2c(c1)CN(C(=O)Cc1cc3ccccc3c(O)n1)CC2	1632295648	25	332.15	3.38

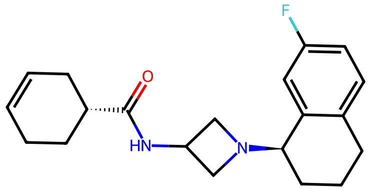	O=C(NC1CN([C@@H]2CCCc3ccc(F)cc32)C1)[C@H]1CC=CCC1	1030098612	24	328.2	3.36
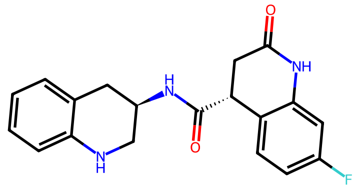	O=C1C[C@@H](C(=O)N[C@H]2CNc3ccccc3C2)c2ccc(F)cc2N1	653205578	25	339.14	2.4
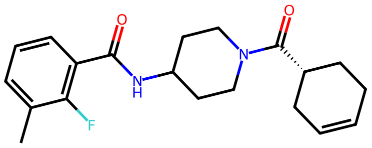	Cc1cccc(C(=O)NC2CCN(C(=O)[C@H]3CC=CCC3)CC2)c1F	944126940	25	344.19	3.21
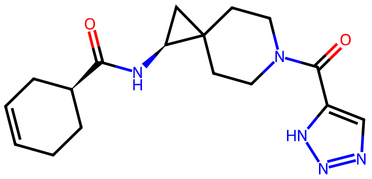	O=C(N[C@H]1CC12CCN(C(=O)c1cnn[nH]1)CC2)[C@@H]1CC=CCC1	962970778	24	329.19	1.27
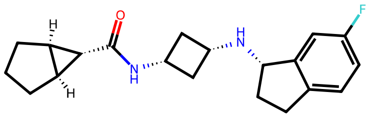	O=C(N[C@H]1C[C@@H](N[C@H]2CCc3ccc(F)cc32)C1)[C@@H]1[C@H]2CCC[C@H]21	1021941879	24	328.2	3.1
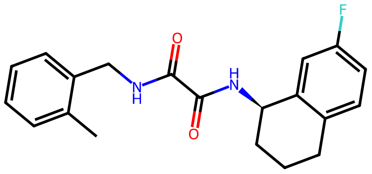	Cc1ccccc1CNC(=O)C(=O)N[C@@H]1CCCc2ccc(F)cc21	1321361069	25	340.16	2.94
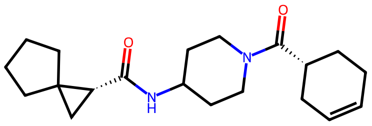	O=C(NC1CCN(C(=O)[C@H]2CC=CCC2)CC1)[C@@H]1CC12CCCC2	944105938	24	330.23	3.03
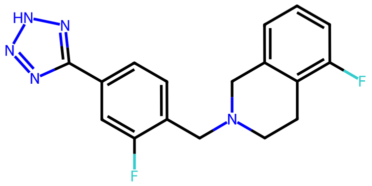	Fc1cc(-c2nn[nH]n2)ccc1CN1CCc2c(F)cccc2C1	826371723	24	327.13	2.7

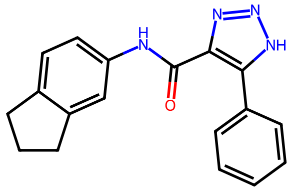	O=C(Nc1ccc2c(c1)CCC2)c1nn[nH]c1-c1ccccc1	188927448	23	304.13	3.21
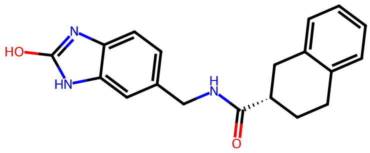	O=C(NCc1ccc2nc(O)[nH]c2c1)[C@H]1CCc2ccccc2C1	521053326	24	321.15	2.69
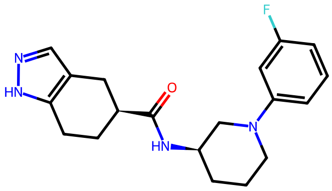	O=C(N[C@@H]1CCCN(c2cccc(F)c2)C1)[C@H]1CCc2[nH]ncc2C1	270282297	25	342.19	2.44
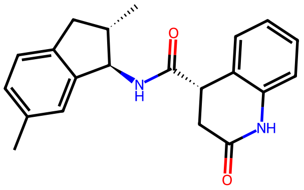	Cc1ccc2c(c1)[C@H](NC(=O)[C@H]1CC(=O)Nc3ccccc31)[C@@H](C)C2	248607912	25	334.17	3.47
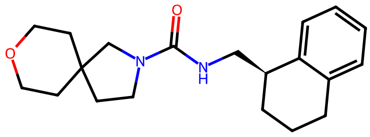	O=C(NC[C@@H]1CCCc2ccccc21)N1CCC2(CCOCC2)C1	76084709	24	328.22	3.32
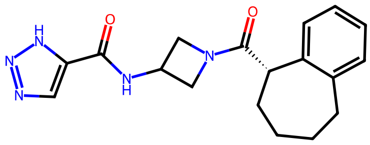	O=C(NC1CN(C(=O)[C@H]2CCCCc3ccccc32)C1)c1cnn[nH]1	995177712	25	339.17	1.26
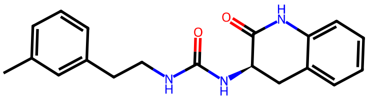	Cc1cccc(CCNC(=O)N[C@@H]2Cc3ccccc3NC2=O)c1	636697260	24	323.16	2.4
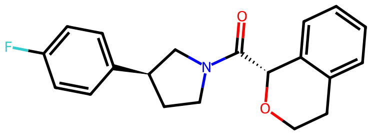	O=C([C@H]1OCCc2ccccc21)N1CC[C@@H](c2ccc(F)cc2)C1	478209416	24	325.15	3.46

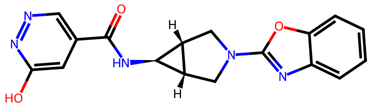	O=C(N[C@H]1[C@@H]2CN(c3nc4ccccc4o3)C[C@@H]21)c1cnnc(O)c1	1056333221	25	337.12	1.19
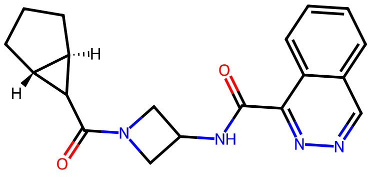	O=C(NC1CN(C(=O)C2[C@@H]3CCC[C@@H]23)C1)c1nncc2ccccc12	996893138	25	336.16	1.62
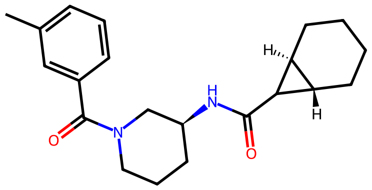	Cc1cccc(C(=O)N2CCC[C@H](NC(=O)C3[C@H]4CCCC[C@H]34)C2)c1	949869535	25	340.22	3.15
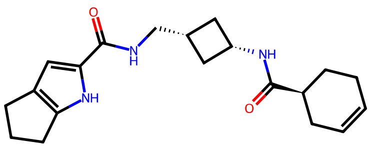	O=C(NC[C@H]1C[C@@H](NC(=O)[C@@H]2CC=CCC2)C1)c1cc2c([nH]1)CCC2	979027101	25	341.21	2.48
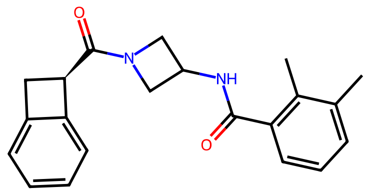	Cc1cccc(C(=O)NC2CN(C(=O)[C@@H]3Cc4ccccc43)C2)c1C	999516473	25	334.17	2.58
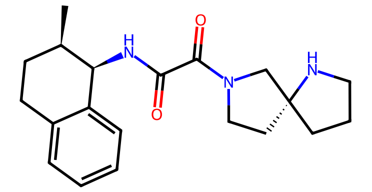	C[C@@H]1CCc2ccccc2[C@@H]1NC(=O)C(=O)N1CC[C@]2(CCCN2)C1	842188219	25	341.21	1.78
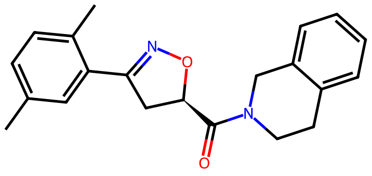	Cc1ccc(C)c(C2=NO[C@@H](C(=O)N3CCc4ccccc4C3)C2)c1	1264069376	25	334.17	3.38
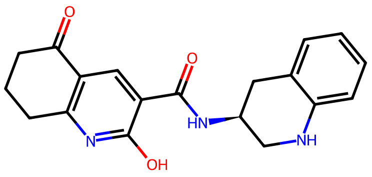	O=C(N[C@@H]1CNc2ccccc2C1)c1cc2c(nc1O)CCCC2=O	653206247	25	337.14	2.07

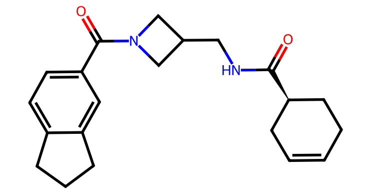	O=C(NCC1CN(C(=O)c2ccc3c(c2)CCC3)C1)[C@@H]1CC=CCC1	1000899204	25	338.2	2.72
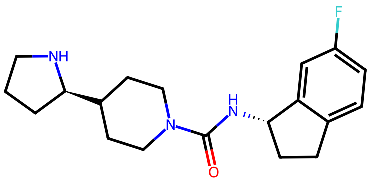	O=C(N[C@H]1CCc2ccc(F)cc21)N1CCC([C@H]2CCCN2)CC1	518497265	24	331.21	2.99
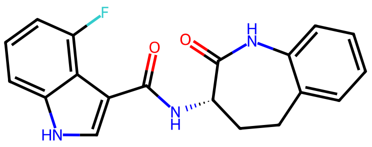	O=C(N[C@H]1CCc2ccccc2NC1=O)c1c[nH]c2cccc(F)c12	1300147211	25	337.12	2.99
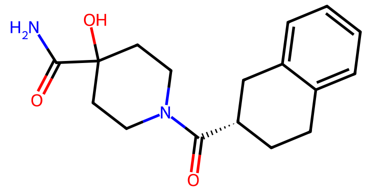	NC(=O)C1(O)CCN(C(=O)[C@H]2CCc3ccccc3C2)CC1	319300441	22	302.16	0.63
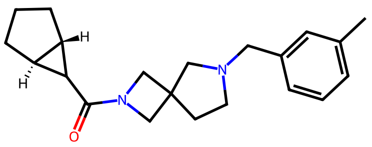	Cc1cccc(CN2CCC3(C2)CN(C(=O)C2[C@H]4CCC[C@H]24)C3)c1	1072591325	24	324.22	3.08
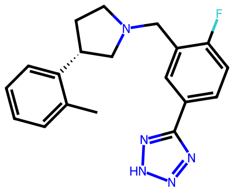	Cc1ccccc1[C@@H]1CCN(Cc2cc(-c3nn[nH]n3)ccc2F)C1	737420221	25	337.17	3.3
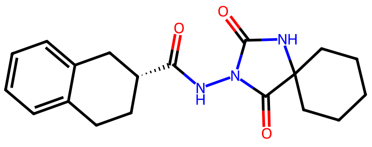	O=C(NN1C(=O)NC2(CCCCC2)C1=O)[C@@H]1CCc2ccccc2C1	845121001	25	341.17	2.08
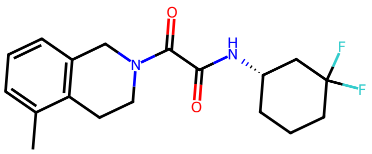	Cc1cccc2c1CCN(C(=O)C(=O)N[C@H]1CCCC(F)(F)C1)C2	1323200835	24	336.16	2.57

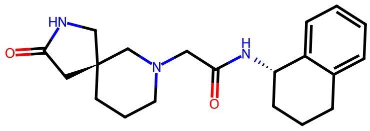	O=C1C[C@@]2(CCCN(CC(=O)N[C@H]3CCCc4ccccc43)C2)CN1	362409355	25	341.21	1.78
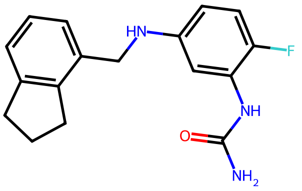	NC(=O)Nc1cc(NCc2cccc3c2CCC3)ccc1F	1308420119	22	299.14	3.42
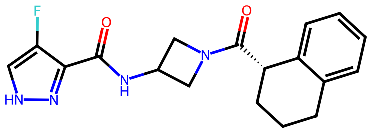	O=C(NC1CN(C(=O)[C@H]2CCCc3ccccc32)C1)c1n[nH]cc1F	999175689	25	342.15	1.61
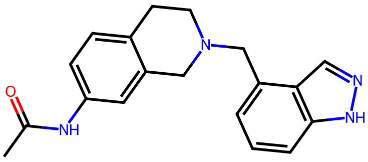	CC(=O)Nc1ccc2c(c1)CN(Cc1cccc3[nH]ncc13)CC2	877816978	24	320.16	3.08
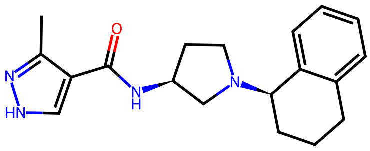	Cc1n[nH]cc1C(=O)N[C@H]1CCN([C@@H]2CCCc3ccccc32)C1	1015462723	24	324.2	2.6
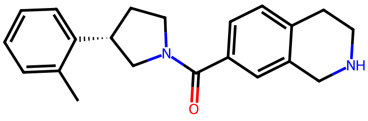	Cc1ccccc1[C@@H]1CCN(C(=O)c2ccc3c(c2)CNCC3)C1	1326596203	24	320.19	3.27
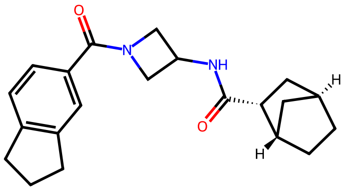	O=C(NC1CN(C(=O)c2ccc3c(c2)CCC3)C1)[C@@H]1C[C@H]2CC[C@H]1C2	990251330	25	338.2	2.55
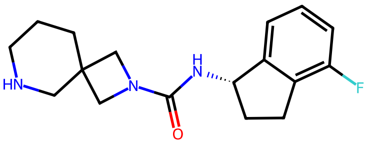	O=C(N[C@H]1CCc2c(F)cccc21)N1CC2(CCCNC2)C1	1580754232	22	303.17	2.21

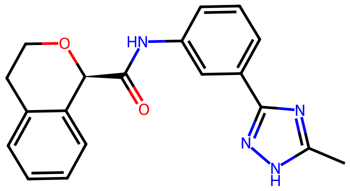	Cc1nc(-c2cccc(NC(=O)[C@@H]3OCCc4ccccc43)c2)n[nH]1	137729982	25	334.14	3.03
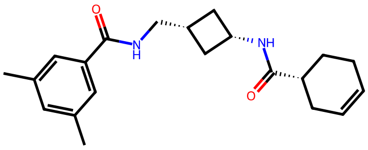	Cc1cc(C)cc(C(=O)NC[C@H]2C[C@@H](NC(=O)[C@H]3CC=CCC3)C2)c1	979286537	25	340.22	3.28
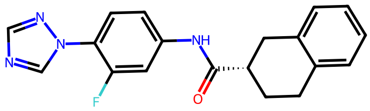	O=C(Nc1ccc(-n2cncn2)c(F)c1)[C@H]1CCc2ccccc2C1	1125341545	25	336.14	3.15
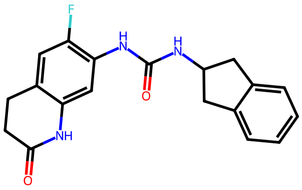	O=C1CCc2cc(F)c(NC(=O)NC3Cc4ccccc4C3)cc2N1	358477555	25	339.14	3.0
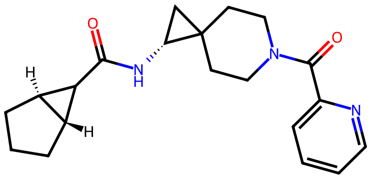	O=C(N[C@@H]1CC12CCN(C(=O)c1ccccn1)CC2)C1[C@@H]2CCC[C@@H]12	963471475	25	339.19	2.24
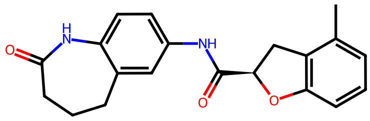	Cc1cccc2c1C[C@H](C(=O)Nc1ccc3c(c1)CCCC(=O)N3)O2	448904060	25	336.15	3.21
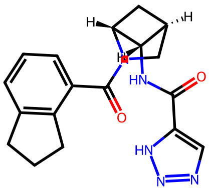	O=C(N[C@H]1[C@H]2C[C@H]1N(C(=O)c1cccc3c1CCC3)C2)c1cnn[nH]1	947492380	25	337.15	0.94
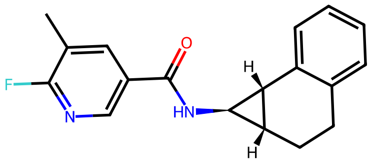	Cc1cc(C(=O)N[C@@H]2[C@H]3CCc4ccccc4[C@H]32)cnc1F	857714539	22	296.13	2.99

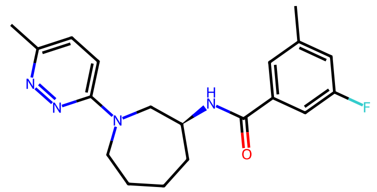	Cc1cc(F)cc(C(=O)N[C@H]2CCCCN(c3ccc(C)nn3)C2)c1	1063233996	25	342.19	3.02
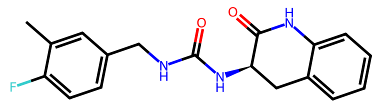	Cc1cc(CNC(=O)N[C@@H]2Cc3ccccc3NC2=O)ccc1F	1426581393	24	327.14	2.5
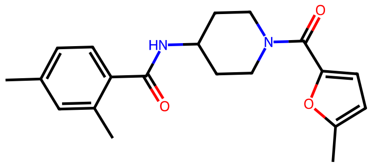	Cc1ccc(C(=O)NC2CCN(C(=O)c3ccc(C)o3)CC2)c(C)c1	943921108	25	340.18	3.24
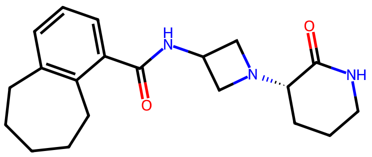	O=C(NC1CN([C@H]2CCCNC2=O)C1)c1cccc2c1CCCCC2	1030487204	25	341.21	1.65
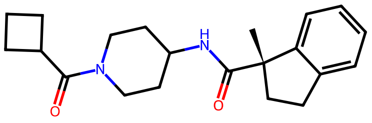	C[C@]1(C(=O)NC2CCN(C(=O)C3CCC3)CC2)CCc2ccccc21	943314904	25	340.22	2.8
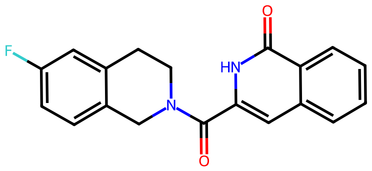	O=C(c1cc2ccccc2c(=O)[nH]1)N1CCc2cc(F)ccc2C1	91687615	24	322.11	2.87
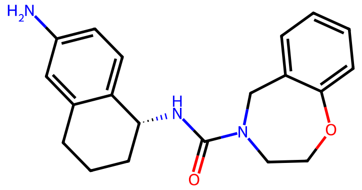	Nc1ccc2c(c1)CCC[C@H]2NC(=O)N1CCOc2ccccc2C1	640884742	25	337.18	3.25
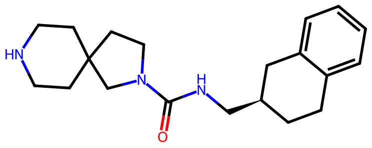	O=C(NC[C@@H]1CCc2ccccc2C1)N1CCC2(CCNCC2)C1	841868346	24	327.23	2.58

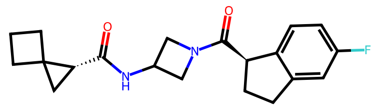	O=C(NC1CN(C(=O)[C@@H]2CCc3cc(F)ccc32)C1)[C@@H]1CC12CCC2	991555101	25	342.17	2.37
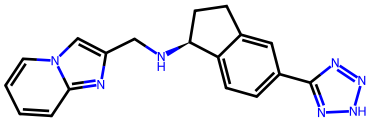	c1ccn2cc(CN[C@H]3CCc4cc(-c5nn[nH]n5)ccc43)nc2c1	1593582564	25	331.15	2.29
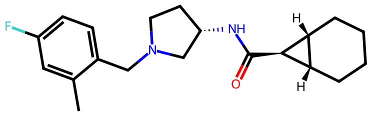	Cc1cc(F)ccc1CN1CC[C@H](NC(=O)[C@@H]2[C@H]3CCCC[C@H]32)C1	1010439873	24	330.21	3.26
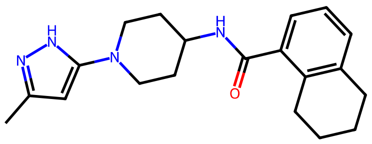	Cc1cc(N2CCC(NC(=O)c3cccc4c3CCCC4)CC2)[nH]n1	295708135	25	338.21	3.0
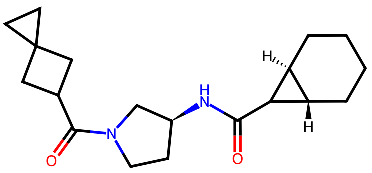	O=C(N[C@H]1CCN(C(=O)C2CC3(CC3)C2)C1)C1[C@H]2CCCC[C@H]12	937791684	23	316.22	2.33
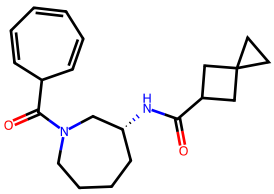	O=C(N[C@@H]1CCCCN(C(=O)C2C=CC=CC=C2)C1)C1CC2(CC2)C1	943518197	25	340.22	2.97
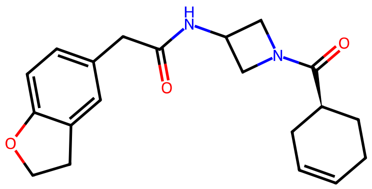	O=C(Cc1ccc2c(c1)CCO2)NC1CN(C(=O)[C@@H]2CC=CCC2)C1	996708005	25	340.18	1.85
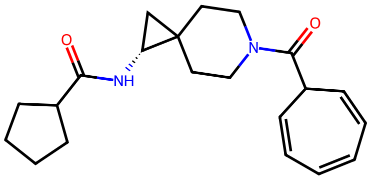	O=C(N[C@@H]1CC12CCN(C(=O)C1C=CC=CC=C1)CC2)C1CCCC1	962543436	25	340.22	2.97

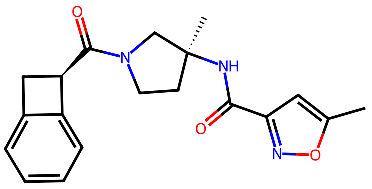	Cc1cc(C(=O)N[C@@]2(C)CCN(C(=O)[C@@H]3Cc4ccccc43)C2)no1	956677646	25	339.16	2.04
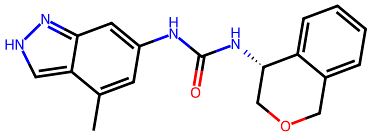	Cc1cc(NC(=O)N[C@H]2COCc3ccccc32)cc2n[nH]cc12	1562512904	24	322.14	3.26
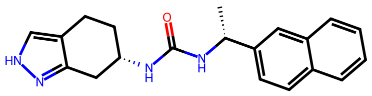	C[C@@H](NC(=O)N[C@H]1CCc2c[nH]nc2C1)c1ccc2ccccc2c1	363938594	25	334.18	3.48
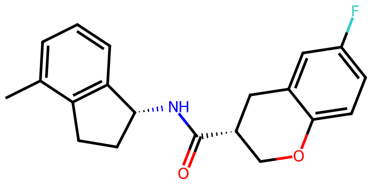	Cc1cccc2c1CC[C@H]2NC(=O)[C@H]1COc2ccc(F)cc2C1	1341287804	24	325.15	3.49
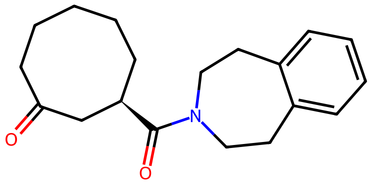	O=C1CCCCC[C@@H](C(=O)N2CCc3ccccc3CC2)C1	643988892	22	299.19	3.15
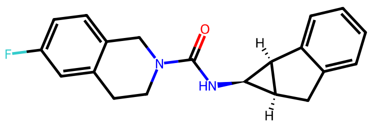	O=C(N[C@@H]1[C@@H]2Cc3ccccc3[C@@H]21)N1CCc2cc(F)ccc2C1	91706209	24	322.15	3.23
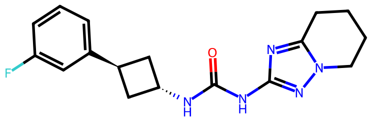	O=C(Nc1nc2n(n1)CCCC2)N[C@H]1C[C@H](c2cccc(F)c2)C1	571616734	24	329.17	2.82
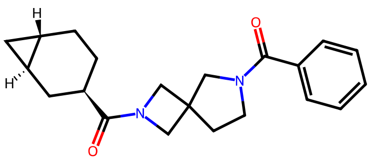	O=C(c1ccccc1)N1CCC2(C1)CN(C(=O)[C@@H]1CC[C@H]3C[C@@H]3C1)C2	1019141891	25	338.2	2.8

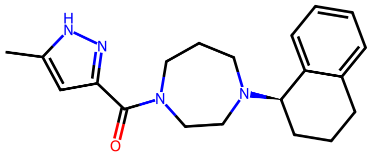	Cc1cc(C(=O)N2CCCN([C@@H]3CCCc4ccccc43)CC2)n[nH]1	980772501	25	338.21	2.94
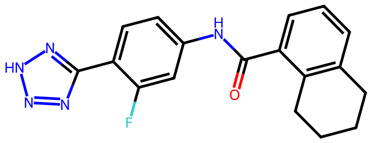	O=C(Nc1ccc(-c2nn[nH]n2)c(F)c1)c1cccc2c1CCCC2	1582636686	25	337.13	3.14
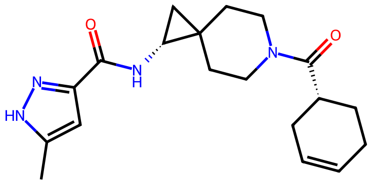	Cc1cc(C(=O)N[C@@H]2CC23CCN(C(=O)[C@H]2CC=CCC2)CC3)n[nH]1	963292647	25	342.21	2.19
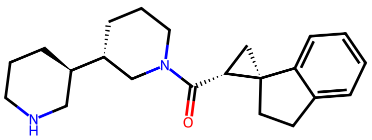	O=C([C@@H]1C[C@]12CCc1ccccc12)N1CCC[C@@H]([C@@H]2CCCNC2)C1	646381701	25	338.24	3.13
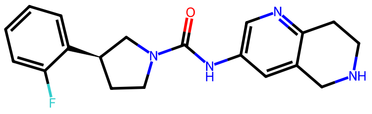	O=C(Nc1cnc2c(c1)CNCC2)N1CC[C@@H](c2ccccc2F)C1	667795543	25	340.17	2.89
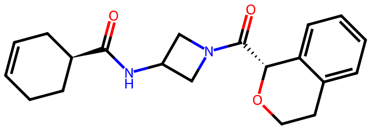	O=C(NC1CN(C(=O)[C@H]2OCCc3ccccc32)C1)[C@@H]1CC=CCC1	990218192	25	340.18	1.98
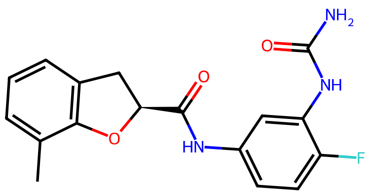	Cc1cccc2c1O[C@H](C(=O)Nc1ccc(F)c(NC(N)=O)c1)C2	1413024251	24	329.12	2.57
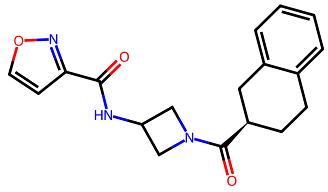	O=C(NC1CN(C(=O)[C@@H]2CCc3ccccc3C2)C1)c1ccon1	990357513	24	325.14	1.42

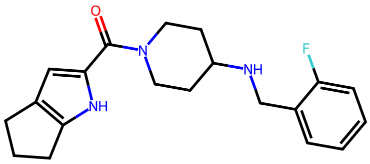	O=C(c1cc2c([nH]1)CCC2)N1CCC(NCc2ccccc2F)CC1	997174219	25	341.19	3.04
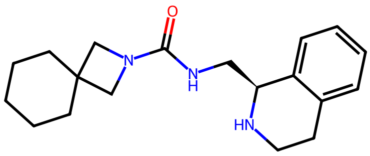	O=C(NC[C@@H]1NCCc2ccccc21)N1CC2(CCCCC2)C1	418638632	23	313.22	2.85
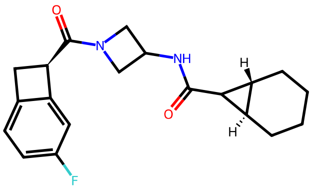	O=C(NC1CN(C(=O)[C@@H]2Cc3ccc(F)cc32)C1)C1[C@@H]2CCCC[C@@H]12	990662683	25	342.17	2.23
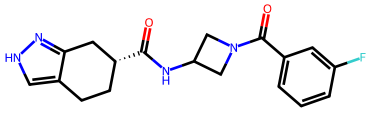	O=C(NC1CN(C(=O)c2cccc(F)c2)C1)[C@@H]1CCc2c[nH]nc2C1	992552819	25	342.15	1.29
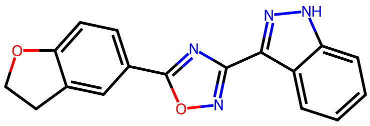	c1ccc2c(-c3noc(-c4ccc5c(c4)CCO5)n3)n[nH]c2c1	547125031	23	304.1	3.21
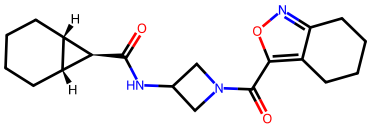	O=C(NC1CN(C(=O)c2onc3c2CCCC3)C1)[C@@H]1[C@H]2CCCC[C@H]21	990647302	25	343.19	1.93
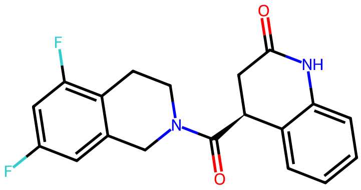	O=C1C[C@H](C(=O)N2CCc3c(F)cc(F)cc3C2)c2ccccc2N1	134279817	25	342.12	2.98
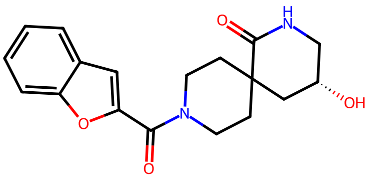	O=C(c1cc2ccccc2o1)N1CCC2(CC1)C[C@@H](O)CNC2=O	885025573	24	328.14	1.54

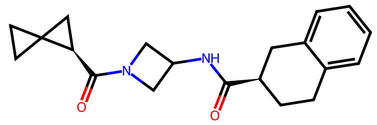	O=C(NC1CN(C(=O)[C@@H]2CC23CC3)C1)[C@@H]1CCc2ccccc2C1	1000545208	24	324.18	1.92
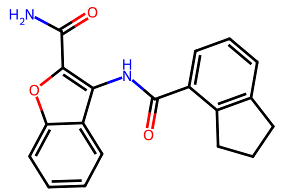	NC(=O)c1oc2ccccc2c1NC(=O)c1cccc2c1CCC2	194325396	24	320.12	3.27
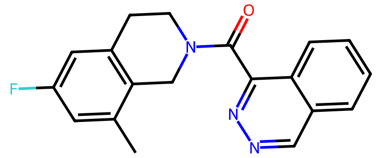	Cc1cc(F)cc2c1CN(C(=O)c1nncc3cccc13)CC2	1530240432	24	321.13	3.28
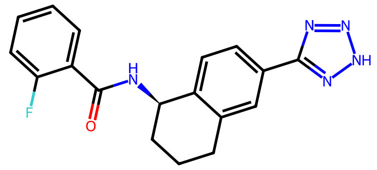	O=C(N[C@@H]1CCCc2cc(-c3nn[nH]n3)ccc21)c1ccccc1F	1592986098	25	337.13	2.81
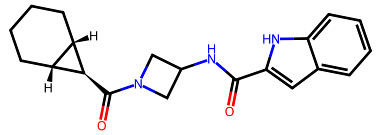	O=C(NC1CN(C(=O)[C@@H]2[C@H]3CCCC[C@H]32)C1)c1cc2ccccc2[nH]1	1000142243	25	337.18	2.54
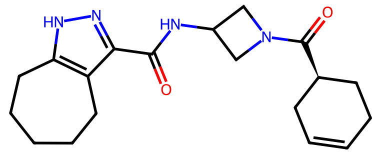	O=C(NC1CN(C(=O)[C@@H]2CC=CCC2)C1)c1n[nH]c2c1CCCCC2	991765789	25	342.21	1.98
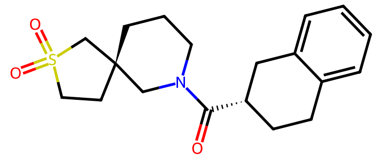	O=C([C@H]1CCc2ccccc2C1)N1CCC[C@]2(CCS(=O)(=O)C2)C1	576640525	24	347.16	2.22
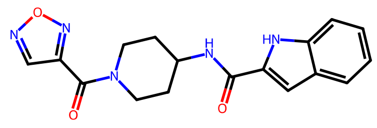	O=C(NC1CCN(C(=O)c2cnon2)CC1)c1cc2ccccc2[nH]1	947961046	25	339.13	1.59

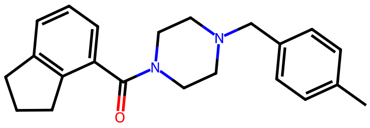	Cc1ccc(CN2CCN(C(=O)c3cccc4c3CCC4)CC2)cc1	957870422	25	334.2	3.44
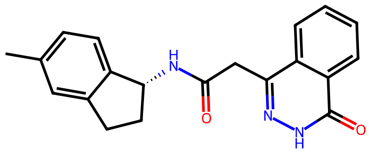	Cc1ccc2c(c1)CC[C@H]2NC(=O)Cc1n[nH]c(=O)c2ccccc12	55659429	25	333.15	2.58
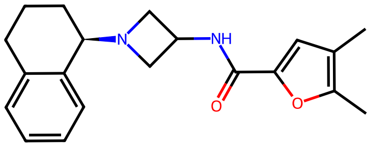	Cc1cc(C(=O)NC2CN([C@@H]3CCCc4ccccc43)C2)oc1C	1030217207	24	324.18	3.39
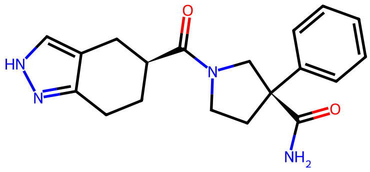	NC(=O)[C@@]1(c2ccccc2)CCN(C(=O)[C@H]2CCc3n[nH]cc3C2)C1	1363444252	25	338.17	1.17
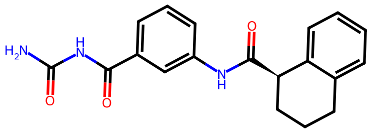	NC(=O)NC(=O)c1cccc(NC(=O)[C@@H]2CCCc3ccccc32)c1	338744773	25	337.14	2.55
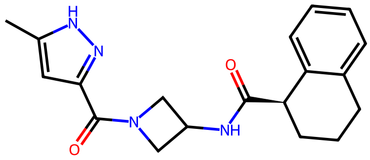	Cc1cc(C(=O)N2CC(NC(=O)[C@@H]3CCCc4ccccc43)C2)n[nH]1	995069781	25	338.17	1.78
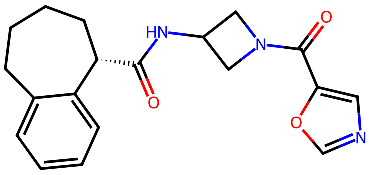	O=C(NC1CN(C(=O)c2cnco2)C1)[C@H]1CCCCc2ccccc21	994320610	25	339.16	2.13
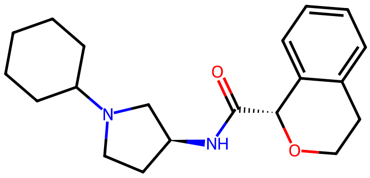	O=C(N[C@H]1CCN(C2CCCCC2)C1)[C@H]1OCCc2ccccc21	618663740	24	328.22	2.82

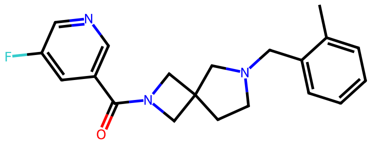	Cc1ccccc1CN1CCC2(C1)CN(C(=O)c1cncc(F)c1)C2	1072607860	25	339.17	2.88
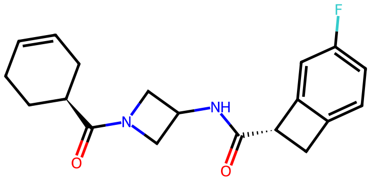	O=C(NC1CN(C(=O)[C@@H]2CC=CCC2)C1)[C@H]1Cc2ccc(F)cc21	998485771	24	328.16	2.15
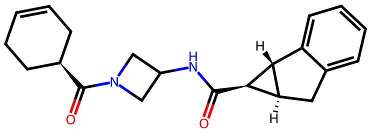	O=C(NC1CN(C(=O)[C@@H]2CC=CCC2)C1)[C@@H]1[C@@H]2Cc3ccccc3[C@H]21	991655680	25	336.18	2.26
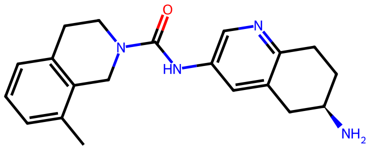	Cc1cccc2c1CN(C(=O)Nc1cnc3c(c1)C[C@H](N)CC3)CC2	333378513	25	336.2	2.8
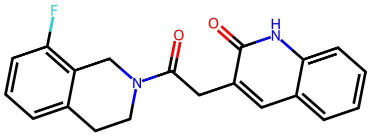	O=C(Cc1cc2ccccc2[nH]c1=O)N1CCc2cccc(F)c2C1	132328256	25	336.13	2.79
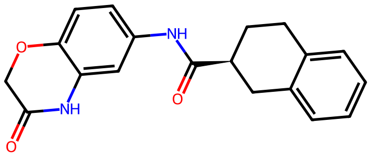	O=C1COc2ccc(NC(=O)[C@H]3CCc4ccccc4C3)cc2N1	649531974	24	322.13	2.76
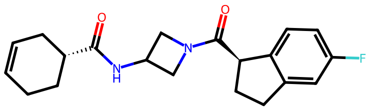	O=C(NC1CN(C(=O)[C@@H]2CCc3cc(F)ccc32)C1)[C@H]1CC=CCC1	990217332	25	342.17	2.54
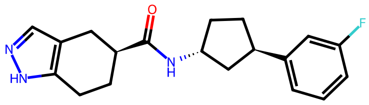	O=C(N[C@@H]1CC[C@@H](c2cccc(F)c2)C1)[C@H]1CCc2[nH]ncc2C1	280364742	24	327.17	3.11

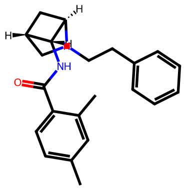	Cc1ccc(C(=O)N[C@@H]2[C@H]3C[C@H]2N(CCc2ccccc2)C3)c(C)c1	1037698829	25	334.2	3.35
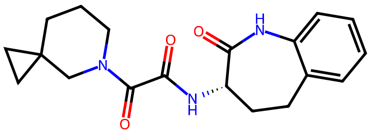	O=C(N[C@H]1CCc2ccccc2NC1=O)C(=O)N1CCCC2(CC2)C1	1326935543	25	341.17	1.46
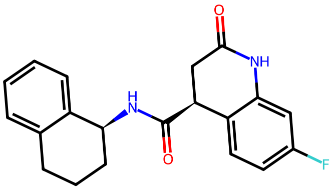	O=C1C[C@H](C(=O)N[C@H]2CCCc3ccccc32)c2ccc(F)cc2N1	120391266	25	338.14	3.45
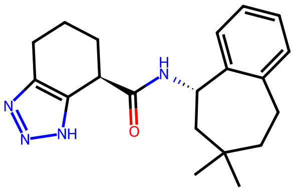	CC1(C)CCc2ccccc2[C@@H](NC(=O)[C@@H]2CCCc3nn[nH]c32)C1	646966701	25	338.21	3.44
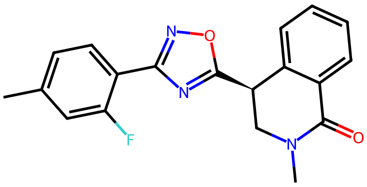	Cc1ccc(-c2noc([C@@H]3CN(C)C(=O)c4ccccc43)n2)c(F)c1	377470553	25	337.12	3.4
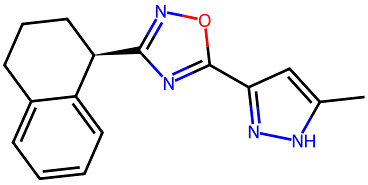	Cc1cc(-c2nc([C@@H]3CCCc4ccccc43)no2)n[nH]1	273815097	21	280.13	3.24
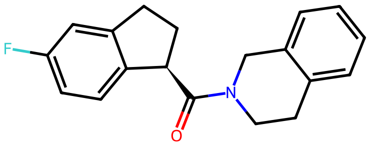	O=C([C@@H]1CCc2cc(F)ccc21)N1CCc2ccccc2C1	665059914	22	295.14	3.44
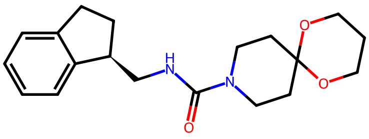	O=C(NC[C@@H]1CCc2ccccc21)N1CCC2(CC1)OCCCO2	838428819	24	330.19	2.66

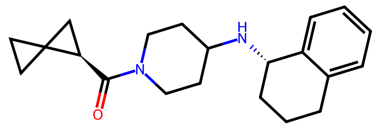	O=C([C@@H]1CC12CC2)N1CCC(N[C@H]2CCCc3ccccc32)CC1	997040912	24	324.22	3.44
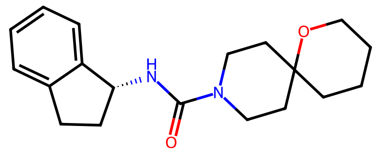	O=C(N[C@@H]1CCc2ccccc21)N1CCC2(CCCCO2)CC1	883086791	23	314.2	3.42
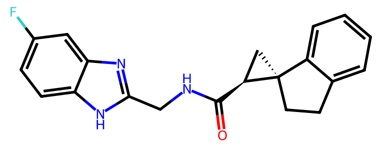	O=C(NCc1nc2cc(F)ccc2[nH]1)[C@H]1C[C@]12CCc1ccccc12	176114636	25	335.14	3.22
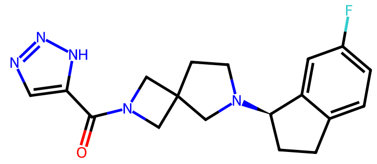	O=C(c1cnn[nH]1)N1CC2(CCN([C@@H]3CCc4ccc(F)cc43)C2)C1	1072892304	25	341.17	1.78
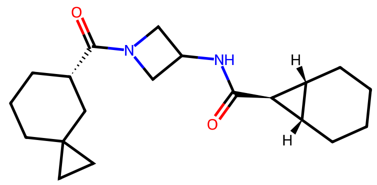	O=C(NC1CN(C(=O)[C@H]2CCCC3(CC3)C2)C1)[C@@H]1[C@H]2CCCC[C@H]21	990651873	24	330.23	2.72
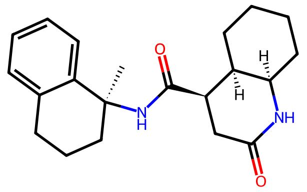	C[C@]1(NC(=O)[C@@H]2CC(=O)N[C@@H]3CCCC[C@@H]32)CCCc2ccccc21	667870541	25	340.22	3.05
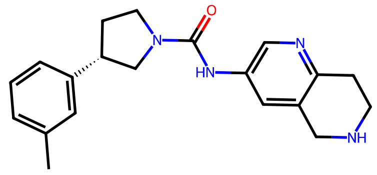	Cc1cccc([C@@H]2CCN(C(=O)Nc3cnc4c(c3)CNCC4)C2)c1	667803986	25	336.2	3.06
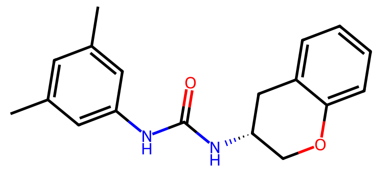	Cc1cc(C)cc(NC(=O)N[C@H]2COc3ccccc3C2)c1	47701366	22	296.15	3.43

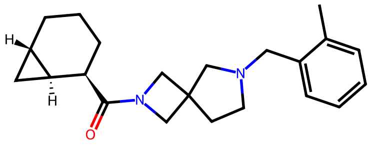	Cc1ccccc1CN1CCC2(C1)CN(C(=O)[C@@H]1CCC[C@H]3C[C@@H]31)C2	1072874525	25	338.24	3.47
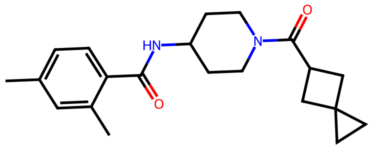	Cc1ccc(C(=O)NC2CCN(C(=O)C3CC4(CC4)C3)CC2)c(C)c1	948306145	25	340.22	3.21
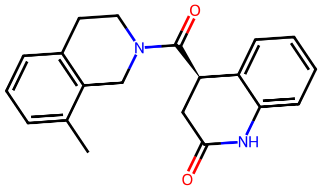	Cc1cccc2c1CN(C(=O)[C@@H]1CC(=O)Nc3ccccc31)CC2	365403255	24	320.15	3.01
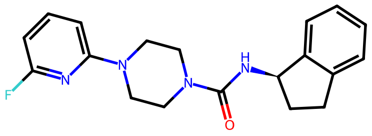	O=C(N[C@@H]1CCc2ccccc21)N1CCN(c2cccc(F)n2)CC1	822050614	25	340.17	2.74
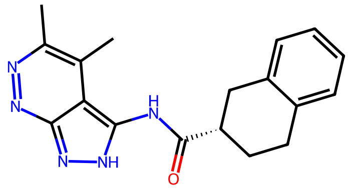	Cc1nnc2n[nH]c(NC(=O)[C@H]3CCc4ccccc4C3)c2c1C	614650144	24	321.16	2.71
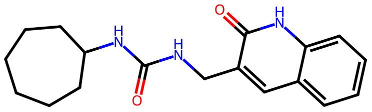	O=C(NCc1cc2ccccc2[nH]c1=O)NC1CCCCCC1	96709098	23	313.18	3.05
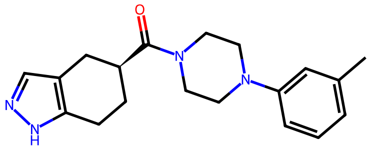	Cc1cccc(N2CCN(C(=O)[C@H]3CCc4[nH]ncc4C3)CC2)c1	270170309	24	324.2	2.17
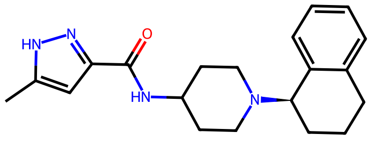	Cc1cc(C(=O)NC2CCN([C@@H]3CCCc4ccccc43)CC2)n[nH]1	1002410393	25	338.21	2.99

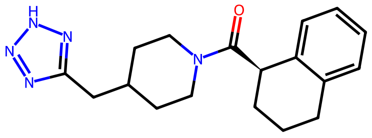	O=C([C@@H]1CCCc2ccccc21)N1CCC(Cc2nn[nH]n2)CC1	1548805093	24	325.19	2.1
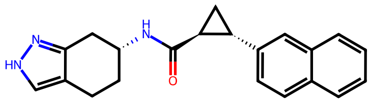	O=C(N[C@@H]1CCc2c[nH]nc2C1)[C@H]1C[C@@H]1c1ccc2ccccc2c1	332914340	25	331.17	3.34
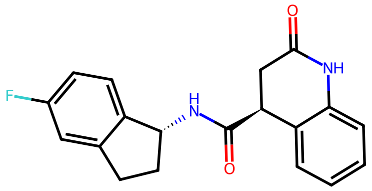	O=C1C[C@H](C(=O)N[C@@H]2CCc3cc(F)ccc32)c2ccccc2N1	56315391	24	324.13	3.06
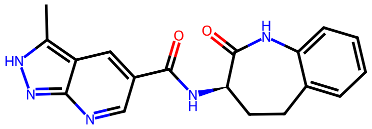	Cc1[nH]nc2ncc(C(=O)N[C@@H]3CCc4ccccc4NC3=O)cc12	1144236792	25	335.14	1.95
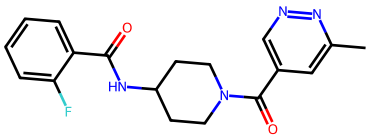	Cc1cc(C(=O)N2CCC(NC(=O)c3ccccc3F)CC2)cnn1	946562876	25	342.15	1.96
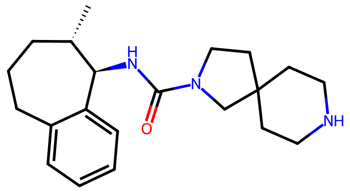	C[C@H]1CCCc2ccccc2[C@@H]1NC(=O)N1CCC2(CCNCC2)C1	418018815	25	341.25	3.49
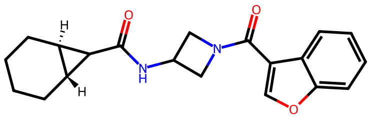	O=C(NC1CN(C(=O)c2coc3ccccc23)C1)C1[C@@H]2CCCC[C@@H]12	990655465	25	338.16	2.81
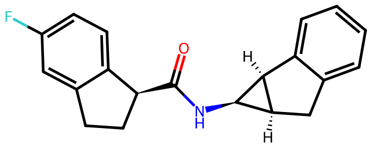	O=C(N[C@@H]1[C@@H]2Cc3ccccc3[C@@H]21)[C@H]1CCc2cc(F)ccc21	612495123	23	307.14	3.31

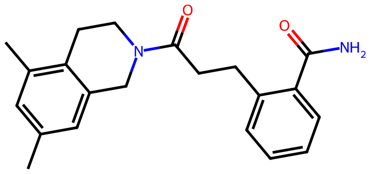	Cc1cc(C)c2c(c1)CN(C(=O)CCc1ccccc1C(N)=O)CC2	280075734	25	336.18	2.92
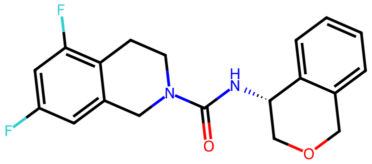	O=C(N[C@H]1COCc2ccccc21)N1CCc2c(F)cc(F)cc2C1	419556378	25	344.13	3.3
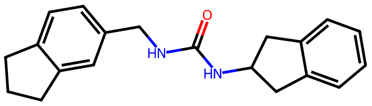	O=C(NCc1ccc2c(c1)CCC2)NC1Cc2ccccc2C1	1337939744	23	306.17	3.14
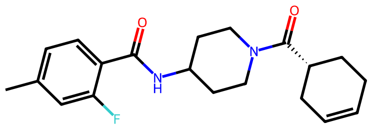	Cc1ccc(C(=O)NC2CCN(C(=O)[C@H]3CC=CCC3)CC2)c(F)c1	944127885	25	344.19	3.21
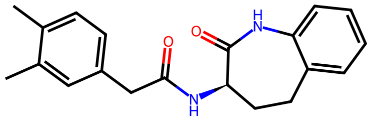	Cc1ccc(CC(=O)N[C@@H]2CCc3ccccc3NC2=O)cc1C	654005792	24	322.17	2.92
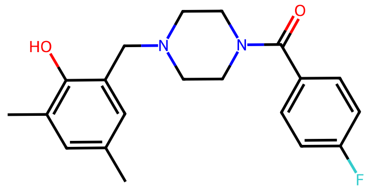	Cc1cc(C)c(O)c(CN2CCN(C(=O)c3ccc(F)cc3)CC2)c1	163939791	25	342.17	3.11
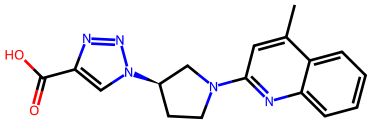	Cc1cc(N2CC[C@@H](n3cc(C(=O)O)nn3)C2)nc2ccccc12	1346104986	24	323.14	2.28
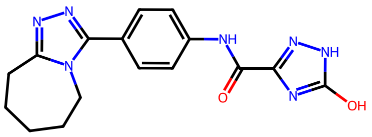	O=C(Nc1ccc(-c2nnc3n2CCCCC3)cc1)c1n[nH]c(O)n1	616983455	25	339.14	1.75

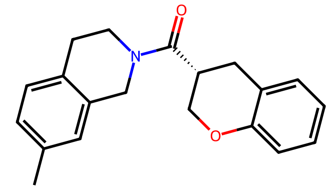	Cc1ccc2c(c1)CN(C(=O)[C@H]1COc3ccccc3C1)CC2	617602250	23	307.16	3.13
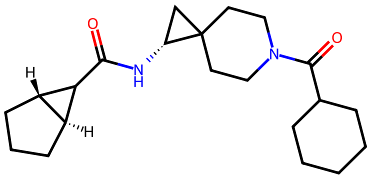	O=C(N[C@@H]1CC12CCN(C(=O)C1CCCCC1)CC2)C1[C@H]2CCC[C@H]12	963462684	25	344.25	3.11
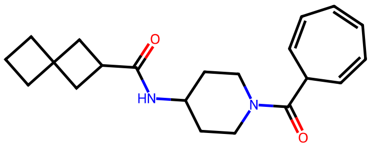	O=C(NC1CCN(C(=O)C2C=CC=CC=C2)CC1)C1CC2(CCC2)C1	946948897	25	340.22	2.97
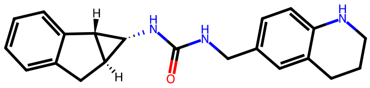	O=C(NCc1ccc2c(c1)CCCN2)N[C@@H]1[C@H]2Cc3ccccc3[C@@H]21	572264080	25	333.18	3.18
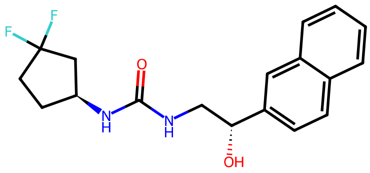	O=C(NC[C@@H](O)c1ccc2ccccc2c1)N[C@H]1CCC(F)(F)C1	763708735	24	334.15	3.36
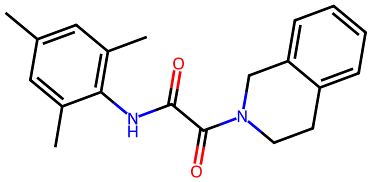	Cc1cc(C)c(NC(=O)C(=O)N2CCc3ccccc3C2)c(C)c1	170355945	24	322.17	3.14
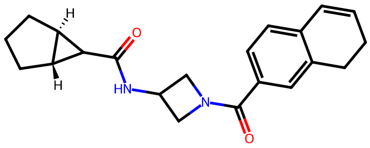	O=C(NC1CN(C(=O)c2ccc3c(c2)CCC=C3)C1)C1[C@@H]2CCC[C@@H]12	990697148	25	336.18	2.63
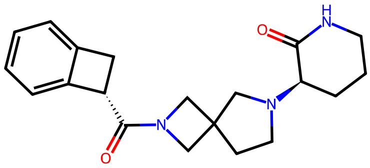	O=C1NCCC[C@H]1N1CCC2(CN(C(=O)[C@H]3Cc4ccccc43)C2)C1	1072931283	25	339.19	1.14

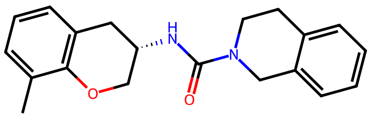	Cc1cccc2c1OC[C@@H](NC(=O)N1CCc3ccccc3C1)C2	571459173	24	322.17	3.07
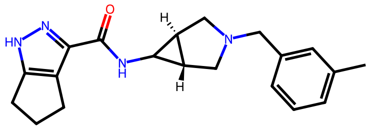	Cc1cccc(CN2C[C@@H]3C(NC(=O)c4n[nH]c5c4CCC5)[C@H]3C2)c1	960728407	25	336.2	2.07
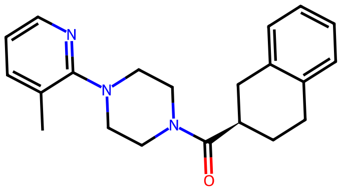	Cc1cccnc1N1CCN(C(=O)[C@@H]2CCc3ccccc3C2)CC1	223598525	25	335.2	2.84
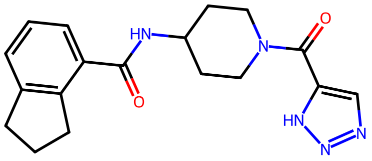	O=C(NC1CCN(C(=O)c2cnn[nH]2)CC1)c1cccc2c1CCC2	947367753	25	339.17	1.33
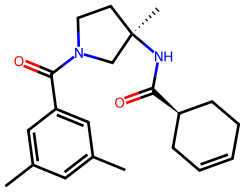	Cc1cc(C)cc(C(=O)N2CC[C@@](C)(NC(=O)[C@@H]3CC=CCC3)C2)c1	956688346	25	340.22	3.38
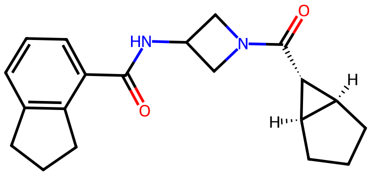	O=C(NC1CN(C(=O)[C@@H]2[C@H]3CCC[C@H]32)C1)c1cccc2c1CCC2	994404495	24	324.18	2.16
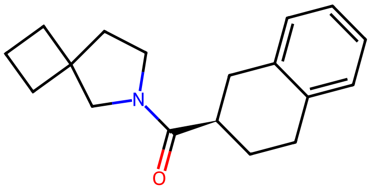	O=C([C@@H]1CCc2ccccc2C1)N1CCC2(CCC2)C1	571450475	20	269.18	3.19
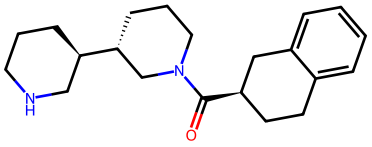	O=C([C@@H]1CCc2ccccc2C1)N1CCC[C@@H]([C@@H]2CCCNC2)C1	653143385	24	326.24	3.03

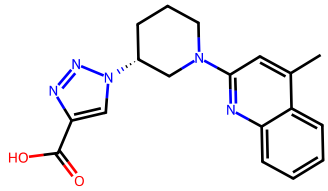	Cc1cc(N2CCC[C@@H](n3cc(C(=O)O)nn3)C2)nc2ccccc12	1346113614	25	337.15	2.67
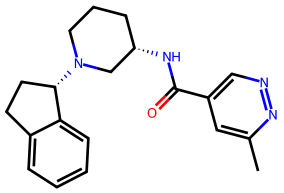	Cc1cc(C(=O)N[C@H]2CCCN([C@H]3CCc4ccccc43)C2)cnn1	1007214559	25	336.2	2.67
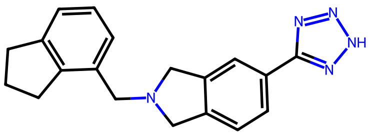	c1cc2c(c(CN3Cc4ccc(-c5nn[nH]n5)cc4C3)c1)CCC2	1607123731	24	317.16	2.87
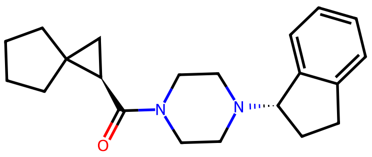	O=C([C@@H]1CC12CCCC2)N1CCN([C@H]2CCc3ccccc32)CC1	957078628	24	324.22	3.4
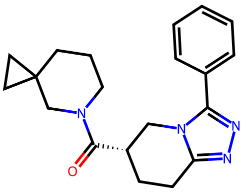	O=C([C@H]1CCc2nnc(-c3ccccc3)n2C1)N1CCCC2(CC2)C1	649420498	25	336.2	2.91
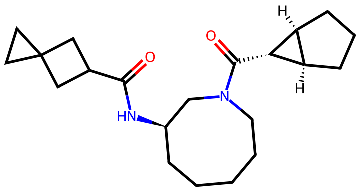	O=C(N[C@@H]1CCCCCN(C(=O)[C@@H]2[C@H]3CCC[C@H]32)C1)C1CC2(CC2)C1	986974512	25	344.25	3.11
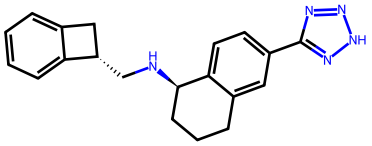	c1ccc2c(c1)C[C@@H]2CN[C@@H]1CCCc2cc(-c3nn[nH]n3)ccc21	826523335	25	331.18	3.17
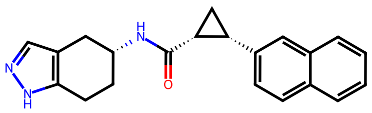	O=C(N[C@@H]1CCc2[nH]ncc2C1)[C@@H]1C[C@@H]1c1ccc2ccccc2c1	172212463	25	331.17	3.34

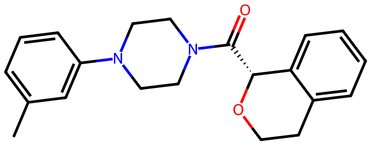	Cc1cccc(N2CCN(C(=O)[C@H]3OCCc4ccccc43)CC2)c1	136930974	25	336.18	2.96
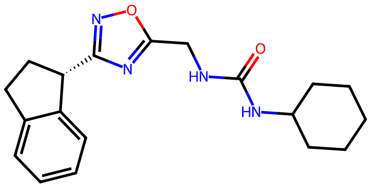	O=C(NCc1nc([C@H]2CCc3ccccc32)no1)NC1CCCCC1	680747911	25	340.19	3.28
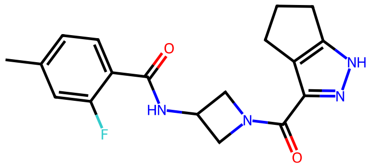	Cc1ccc(C(=O)NC2CN(C(=O)c3n[nH]c4c3CCC4)C2)c(F)c1	1000481487	25	342.15	1.6
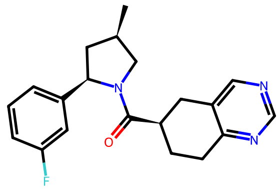	C[C@@H]1C[C@H](c2cccc(F)c2)N(C(=O)[C@@H]2CCc3ncncc3C2)C1	648798080	25	339.17	3.33
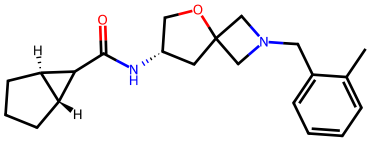	Cc1ccccc1CN1CC2(C[C@H](NC(=O)C3[C@@H]4CCC[C@@H]34)CO2)C1	1053769414	25	340.22	2.5
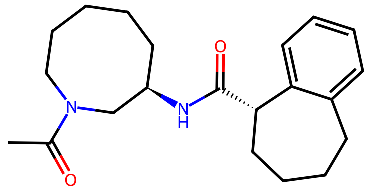	CC(=O)N1CCCCC[C@@H](NC(=O)[C@H]2CCCCc3ccccc32)C1	986790183	25	342.23	3.4
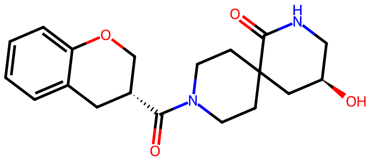	O=C([C@H]1COc2ccccc2C1)N1CCC2(CC1)C[C@H](O)CNC2=O	885026439	25	344.17	0.73
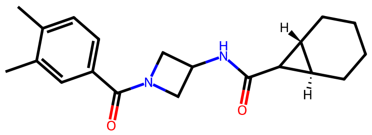	Cc1ccc(C(=O)N2CC(NC(=O)C3[C@@H]4CCCC[C@@H]34)C2)cc1C	990666517	24	326.2	2.68

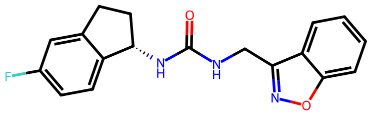	O=C(NCc1noc2ccccc12)N[C@H]1CCc2cc(F)ccc21	1224628609	24	325.12	3.45
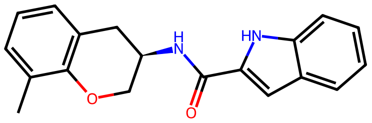	Cc1cccc2c1OC[C@H](NC(=O)c1cc3ccccc3[nH]1)C2	484926137	23	306.14	3.21
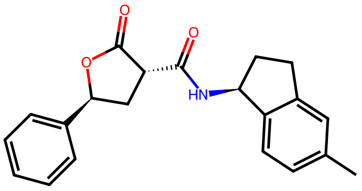	Cc1ccc2c(c1)CC[C@@H]2NC(=O)[C@@H]1C[C@@H](c2ccccc2)OC1=O	290211520	25	335.15	3.4
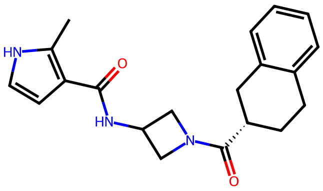	Cc1[nH]ccc1C(=O)NC1CN(C(=O)[C@H]2CCc3ccccc3C2)C1	990735661	25	337.18	2.07
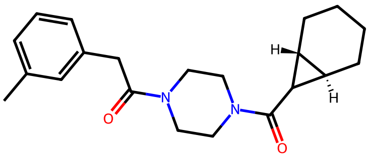	Cc1cccc(CC(=O)N2CCN(C(=O)C3[C@@H]4CCCC[C@@H]34)CC2)c1	1010903798	25	340.22	2.64
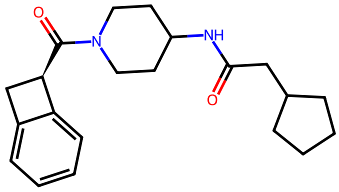	O=C(CC1CCCC1)NC1CCN(C(=O)[C@@H]2Cc3ccccc32)CC1	358475983	25	340.22	3.01
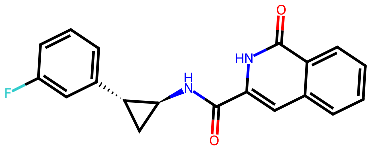	O=C(N[C@H]1C[C@@H]1c1cccc(F)c1)c1cc2ccccc2c(=O)[nH]1	67333741	24	322.11	2.95
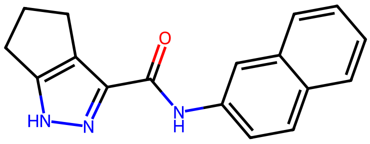	O=C(Nc1ccc2ccccc2c1)c1n[nH]c2c1CCC2	127126245	21	277.12	3.3

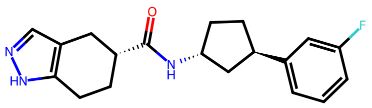	O=C(N[C@@H]1CC[C@@H](c2cccc(F)c2)C1)[C@@H]1CCc2[nH]ncc2C1	280364733	24	327.17	3.11
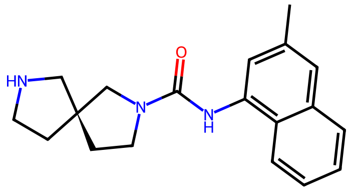	Cc1cc(NC(=O)N2CC[C@]3(CCNC3)C2)c2ccccc2c1	415803838	23	309.18	3.37
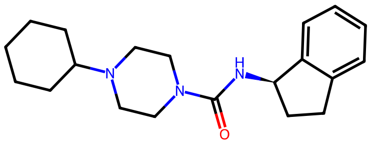	O=C(N[C@@H]1CCc2ccccc21)N1CCN(C2CCCCC2)CC1	485294077	24	327.23	3.33
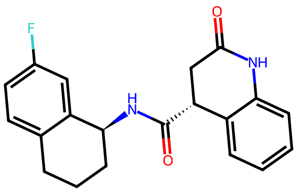	O=C1C[C@@H](C(=O)N[C@H]2CCCc3ccc(F)cc32)c2ccccc2N1	93062920	25	338.14	3.45
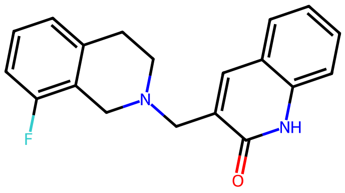	O=c1[nH]c2ccccc2cc1CN1CCc2cccc(F)c2C1	158091135	23	308.13	3.23
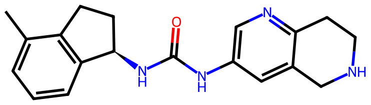	Cc1cccc2c1CC[C@H]2NC(=O)Nc1cnc2c(c1)CNCC2	667795904	24	322.18	2.84
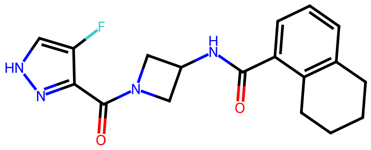	O=C(NC1CN(C(=O)c2n[nH]cc2F)C1)c1cccc2c1CCCC2	991900378	25	342.15	1.68
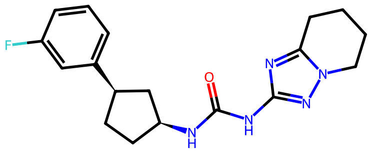	O=C(Nc1nc2n(n1)CCCC2)N[C@H]1CC[C@@H](c2cccc(F)c2)C1	580161370	25	343.18	3.21

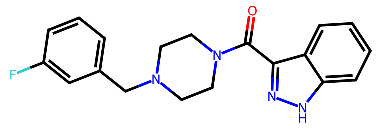	O=C(c1n[nH]c2ccccc12)N1CCN(Cc2cccc(F)c2)CC1	69010414	25	338.15	2.66
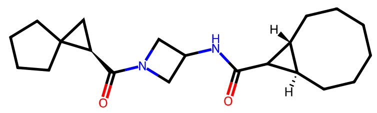	O=C(NC1CN(C(=O)[C@@H]2CC23CCCC3)C1)C1[C@@H]2CCCCCC[C@@H]12	1000500595	25	344.25	3.11
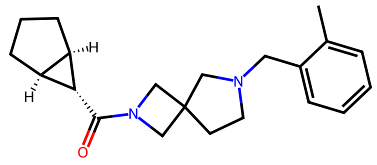	Cc1ccccc1CN1CCC2(C1)CN(C(=O)[C@@H]1[C@H]3CCC[C@H]31)C2	1072590578	24	324.22	3.08
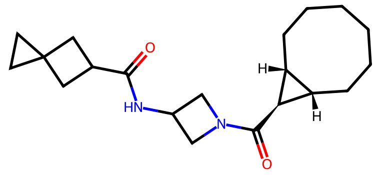	O=C(NC1CN(C(=O)[C@@H]2[C@H]3CCCCCC[C@H]32)C1)C1CC2(CC2)C1	999347713	24	330.23	2.72
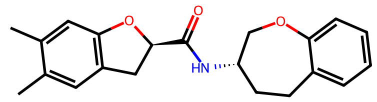	Cc1cc2c(cc1C)O[C@@H](C(=O)N[C@H]1CCc3ccccc3OC1)C2	511631997	25	337.17	3.12
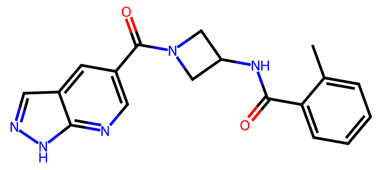	Cc1ccccc1C(=O)NC1CN(C(=O)c2cnc3[nH]ncc3c2)C1	990112474	25	335.14	1.52
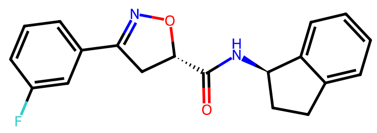	O=C(N[C@@H]1CCc2ccccc21)[C@@H]1CC(c2cccc(F)c2)=NO1	47904456	24	324.13	3.12
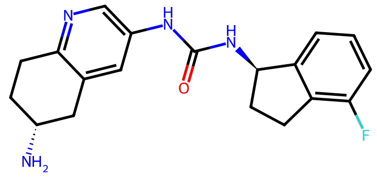	N[C@@H]1CCc2ncc(NC(=O)N[C@@H]3CCc4c(F)cccc43)cc2C1	333286789	25	340.17	2.85

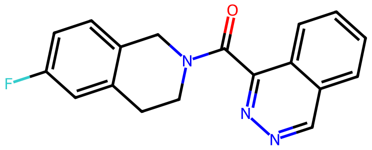	O=C(c1nncc2ccccc12)N1CCc2cc(F)ccc2C1	1458088951	23	307.11	2.97
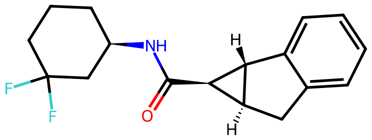	O=C(N[C@@H]1CCCC(F)(F)C1)[C@@H]1[C@@H]2Cc3ccccc3[C@H]21	626188116	21	291.14	3.27
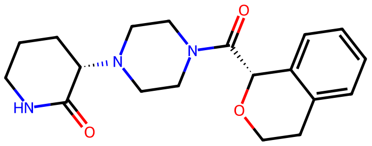	O=C1NCCC[C@@H]1N1CCN(C(=O)[C@H]2OCCc3ccccc32)CC1	949590070	25	343.19	0.72
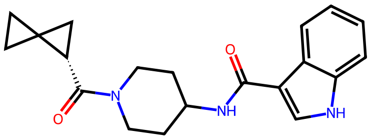	O=C(NC1CCN(C(=O)[C@H]2CC23CC3)CC1)c1c[nH]c2ccccc12	948558532	25	337.18	2.69
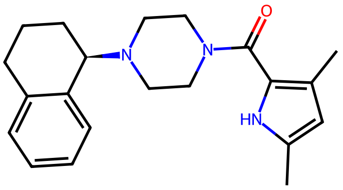	Cc1cc(C)c(C(=O)N2CCN([C@@H]3CCCc4ccccc43)CC2)[nH]1	948892700	25	337.22	3.47
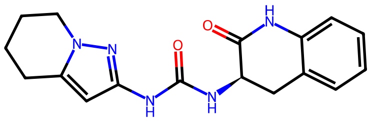	O=C(Nc1cc2n(n1)CCCC2)N[C@@H]1Cc2ccccc2NC1=O	529098892	24	325.15	1.9
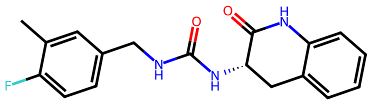	Cc1cc(CNC(=O)N[C@H]2Cc3ccccc3NC2=O)ccc1F	1426581388	24	327.14	2.5
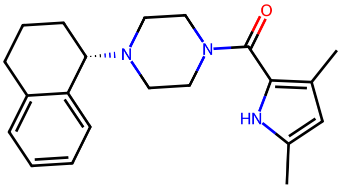	Cc1cc(C)c(C(=O)N2CCN([C@H]3CCCc4ccccc43)CC2)[nH]1	948892699	25	337.22	3.47

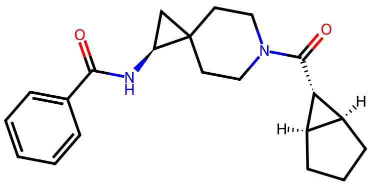	O=C(N[C@H]1CC12CCN(C(=O)[C@@H]1[C@H]3CCC[C@H]31)CC2)c1ccccc1	962781714	25	338.2	2.84
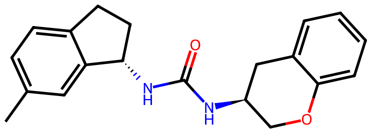	Cc1ccc2c(c1)[C@@H](NC(=O)N[C@@H]1COc3ccccc3C1)CC2	592499152	24	322.17	3.29
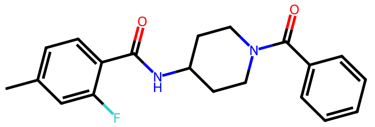	Cc1ccc(C(=O)NC2CCN(C(=O)c3ccccc3)CC2)c(F)c1	175820221	25	340.16	3.17
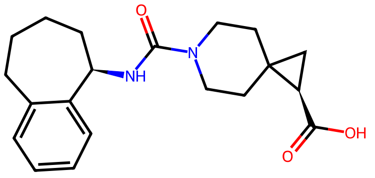	O=C(O)[C@@H]1CC12CCN(C(=O)N[C@@H]1CCCCc3ccccc31)CC2	739527312	25	342.19	3.35
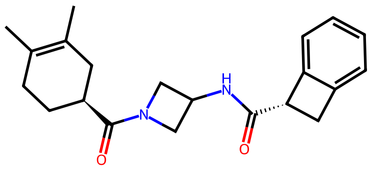	CC1=C(C)C[C@@H](C(=O)N2CC(NC(=O)[C@H]3Cc4ccccc43)C2)CC1	998686644	25	338.2	2.79
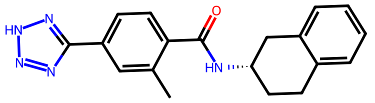	Cc1cc(-c2nn[nH]n2)ccc1C(=O)N[C@H]1CCc2ccccc2C1	826247899	25	333.16	2.46
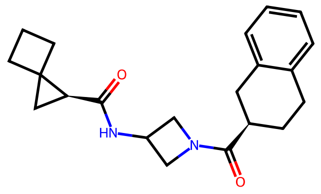	O=C(NC1CN(C(=O)[C@@H]2CCc3ccccc3C2)C1)[C@H]1CC12CCC2	991549040	25	338.2	2.31
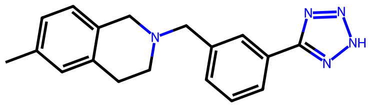	Cc1ccc2c(c1)CCN(Cc1cccc(-c3nn[nH]n3)c1)C2	1139857792	23	305.16	2.73

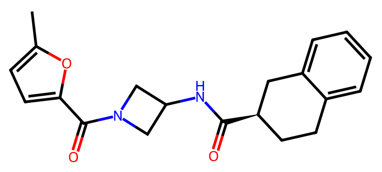	Cc1ccc(C(=O)N2CC(NC(=O)[C@@H]3CCc4ccccc4C3)C2)o1	1000540021	25	338.16	2.33
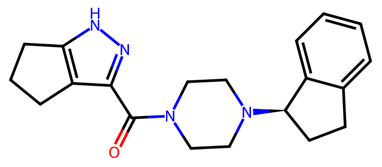	O=C(c1n[nH]c2c1CCC2)N1CCN([C@@H]2CCc3ccccc32)CC1	949062967	25	336.2	2.34
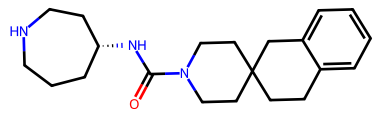	O=C(N[C@@H]1CCCNCC1)N1CCC2(CCc3ccccc3C2)CC1	652215095	25	341.25	3.11
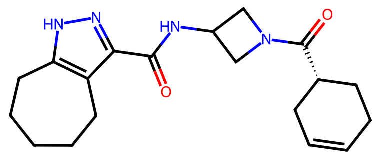	O=C(NC1CN(C(=O)[C@H]2CC=CCC2)C1)c1n[nH]c2c1CCCCC2	991765790	25	342.21	1.98
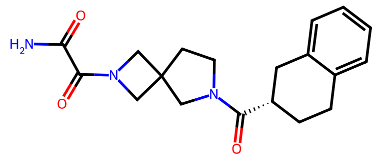	NC(=O)C(=O)N1CC2(CCN(C(=O)[C@H]3CCc4ccccc4C3)C2)C1	1019358191	25	341.17	0.34
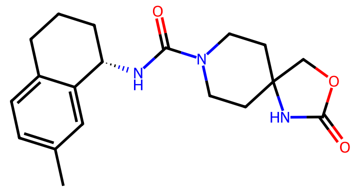	Cc1ccc2c(c1)[C@@H](NC(=O)N1CCC3(CC1)COC(=O)N3)CCC2	496655639	25	343.19	2.66
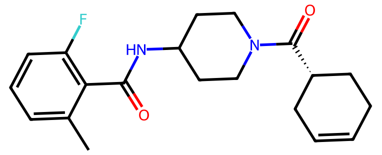	Cc1cccc(F)c1C(=O)NC1CCN(C(=O)[C@H]2CC=CCC2)CC1	944127910	25	344.19	3.21
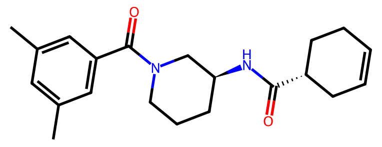	Cc1cc(C)cc(C(=O)N2CCC[C@H](NC(=O)[C@H]3CC=CCC3)C2)c1	951988423	25	340.22	3.38

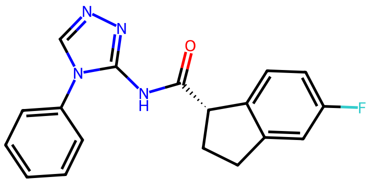	O=C(Nc1nncn1-c1ccccc1)[C@H]1CCc2cc(F)ccc21	1151337354	24	322.12	3.07
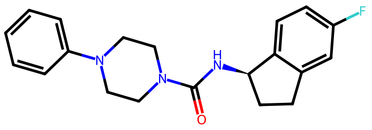	O=C(N[C@@H]1CCc2cc(F)ccc21)N1CCN(c2ccccc2)CC1	56838592	25	339.17	3.34
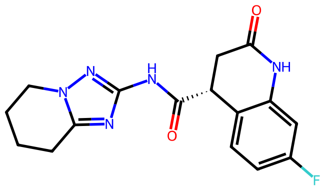	O=C1C[C@@H](C(=O)Nc2nc3n(n2)CCCC3)c2ccc(F)cc2N1	333579238	24	329.13	1.82
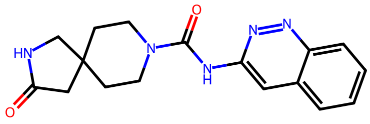	O=C1CC2(CCN(C(=O)Nc3cc4ccccc4nn3)CC2)CN1	888608670	24	325.15	1.76
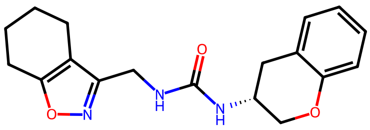	O=C(NCc1noc2c1CCCC2)N[C@H]1COc2ccccc2C1	287376227	24	327.16	2.36
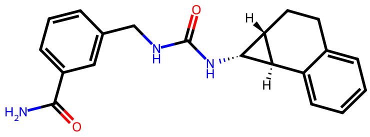	NC(=O)c1cccc(CNC(=O)N[C@H]2[C@@H]3c4ccccc4CC[C@H]32)c1	126277304	25	335.16	2.31
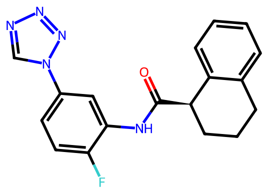	O=C(Nc1cc(-n2cnnn2)ccc1F)[C@@H]1CCCc2ccccc21	47180238	25	337.13	2.86
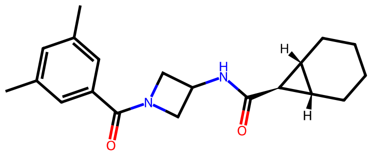	Cc1cc(C)cc(C(=O)N2CC(NC(=O)[C@@H]3[C@H]4CCCC[C@H]43)C2)c1	990651971	24	326.2	2.68

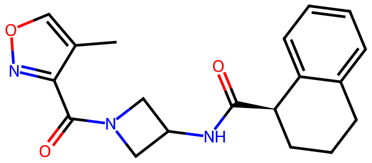	Cc1conc1C(=O)N1CC(NC(=O)[C@@H]2CCCc3ccccc32)C1	995071662	25	339.16	2.04
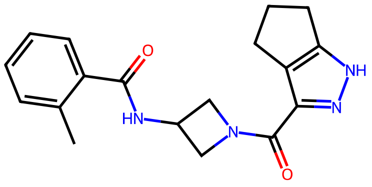	Cc1ccccc1C(=O)NC1CN(C(=O)c2n[nH]c3c2CCC3)C1	990104534	24	324.16	1.46
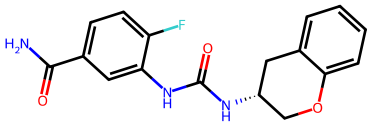	NC(=O)c1ccc(F)c(NC(=O)N[C@H]2COc3ccccc3C2)c1	47875653	24	329.12	2.05
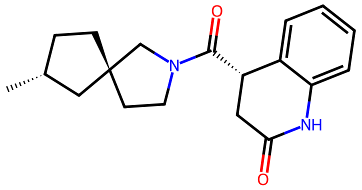	C[C@@H]1CC[C@@]2(CCN(C(=O)[C@H]3CC(=O)Nc4ccccc43)C2)C1	359157413	23	312.18	3.15
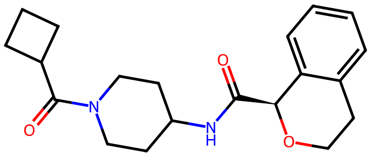	O=C(NC1CCN(C(=O)C2CCC2)CC1)[C@@H]1OCCc2ccccc21	943314999	25	342.19	2.21
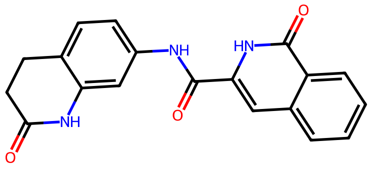	O=C1CCc2ccc(NC(=O)c3cc4ccccc4c(=O)[nH]3)cc2N1	150968065	25	333.11	2.67
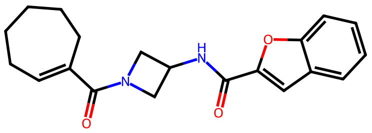	O=C(NC1CN(C(=O)C2=CCCCCC2)C1)c1cc2ccccc2o1	1000053009	25	338.16	3.26
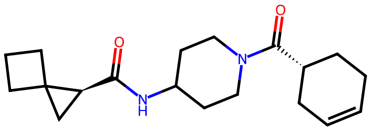	O=C(NC1CCN(C(=O)[C@H]2CC=CCC2)CC1)[C@H]1CC12CCC2	944127604	23	316.22	2.64

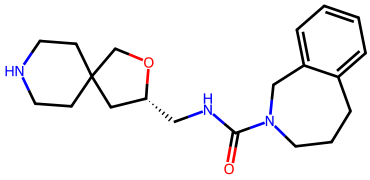	O=C(NC[C@@H]1CC2(CCNCC2)CO1)N1CCCc2ccccc2C1	892768101	25	343.23	2.3
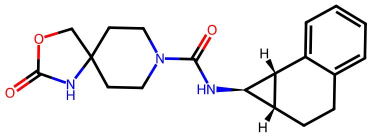	O=C1NC2(CCN(C(=O)N[C@@H]3[C@H]4CCc5ccccc5[C@H]43)CC2)CO1	495156344	25	341.17	2.0
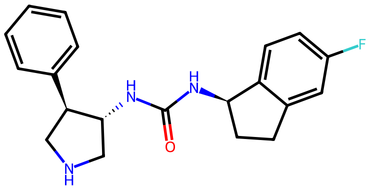	O=C(N[C@@H]1CNC[C@H]1c1ccccc1)N[C@@H]1CCc2cc(F)ccc21	561174229	25	339.17	2.87
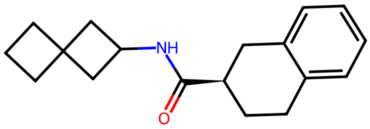	O=C(NC1CC2(CCC2)C1)[C@@H]1CCc2ccccc2C1	652968653	20	269.18	3.24
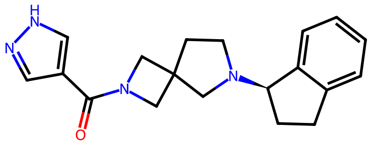	O=C(c1cn[nH]c1)N1CC2(CCN([C@@H]3CCc4ccccc43)C2)C1	1072511713	24	322.18	2.25
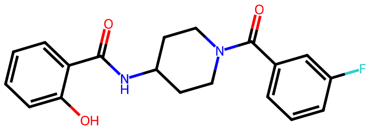	O=C(NC1CCN(C(=O)c2cccc(F)c2)CC1)c1ccccc1O	169859551	25	342.14	2.57
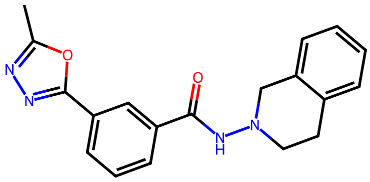	Cc1nnc(-c2cccc(C(=O)NN3CCc4ccccc4C3)c2)o1	1557395599	25	334.14	2.75
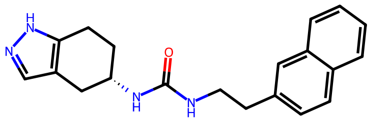	O=C(NCCc1ccc2ccccc2c1)N[C@H]1CCc2[nH]ncc2C1	177948412	25	334.18	2.96

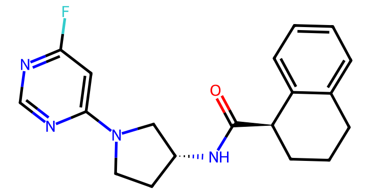	O=C(N[C@@H]1CCN(c2cc(F)ncn2)C1)[C@@H]1CCCc2ccccc21	1058591963	25	340.17	2.43
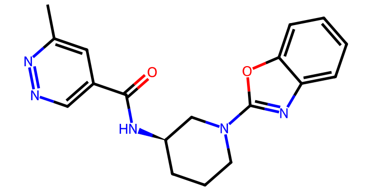	Cc1cc(C(=O)N[C@@H]2CCCN(c3nc4ccccc4o3)C2)cnn1	1058149144	25	337.15	2.33
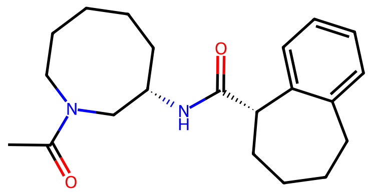	CC(=O)N1CCCCC[C@H](NC(=O)[C@H]2CCCCc3ccccc32)C1	986790180	25	342.23	3.4
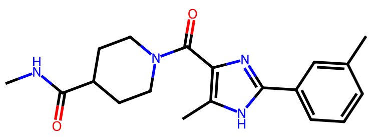	CNC(=O)C1CCN(C(=O)c2nc(-c3cccc(C)c3)[nH]c2C)CC1	270173635	25	340.19	2.29
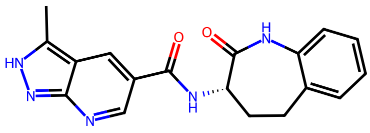	Cc1[nH]nc2ncc(C(=O)N[C@H]3CCc4ccccc4NC3=O)cc12	1144236791	25	335.14	1.95
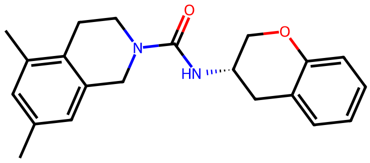	Cc1cc(C)c2c(c1)CN(C(=O)N[C@@H]1COc3ccccc3C1)CC2	1676551322	25	336.18	3.37
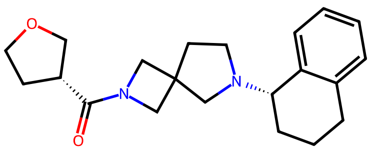	O=C([C@@H]1CCOC1)N1CC2(CCN([C@H]3CCCc4ccccc43)C2)C1	1072541615	25	340.22	2.63
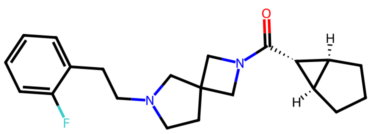	O=C([C@@H]1[C@H]2CCC[C@H]21)N1CC2(CCN(CCc3ccccc3F)C2)C1	1072590712	25	342.21	2.95

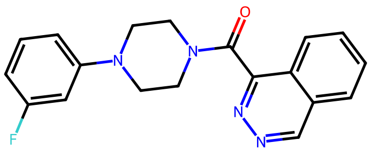	O=C(c1nncc2ccccc12)N1CCN(c2cccc(F)c2)CC1	1515148009	25	336.14	2.73
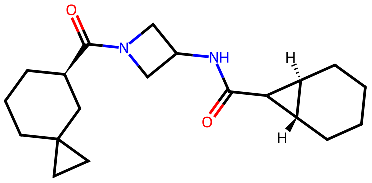	O=C(NC1CN(C(=O)[C@@H]2CCCC3(CC3)C2)C1)C1[C@H]2CCCC[C@H]12	990651872	24	330.23	2.72
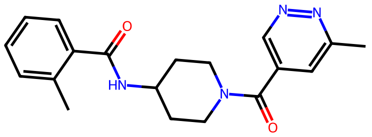	Cc1cc(C(=O)N2CCC(NC(=O)c3ccccc3C)CC2)cnn1	276076044	25	338.17	2.13
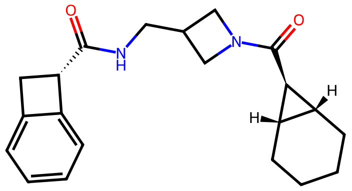	O=C(NCC1CN(C(=O)[C@@H]2[C@H]3CCCC[C@H]32)C1)[C@H]1Cc2ccccc21	1003949091	25	338.2	2.34
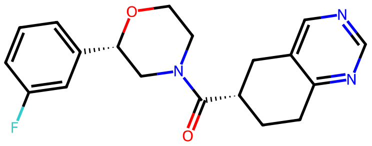	O=C([C@H]1CCc2ncncc2C1)N1CCO[C@@H](c2cccc(F)c2)C1	648764901	25	341.15	2.32
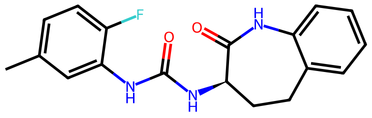	Cc1ccc(F)c(NC(=O)N[C@@H]2CCc3ccccc3NC2=O)c1	1185274967	24	327.14	3.21
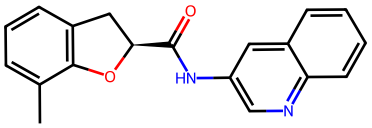	Cc1cccc2c1O[C@H](C(=O)Nc1cnc3ccccc3c1)C2	1357479610	23	304.12	3.49
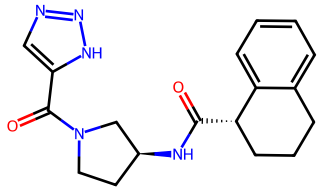	O=C(N[C@H]1CCN(C(=O)c2cnn[nH]2)C1)[C@H]1CCCc2ccccc21	938222196	25	339.17	1.26

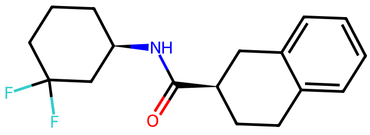	O=C(N[C@@H]1CCCC(F)(F)C1)[C@@H]1CCc2ccccc2C1	649847878	21	293.16	3.49
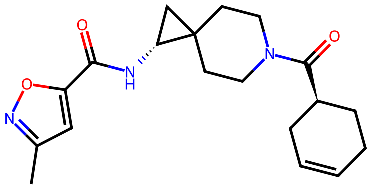	Cc1cc(C(=O)N[C@@H]2CC23CCN(C(=O)[C@@H]2CC=CCC2)CC3)on1	963022795	25	343.19	2.45
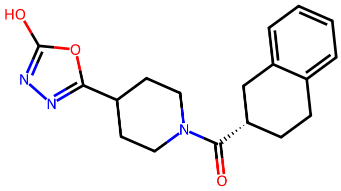	O=C([C@H]1CCc2ccccc2C1)N1CCC(c2nnc(O)o2)CC1	577071852	24	327.16	2.29
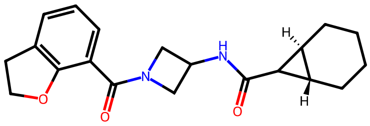	O=C(NC1CN(C(=O)c2cccc3c2OCC3)C1)C1[C@H]2CCCC[C@H]12	990662701	25	340.18	2.0
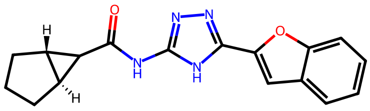	O=C(Nc1nnc(-c2cc3ccccc3o2)[nH]1)C1[C@H]2CCC[C@H]12	276777942	23	308.13	3.2
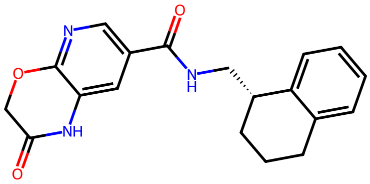	O=C1COc2ncc(C(=O)NC[C@H]3CCCc4ccccc43)cc2N1	271239166	25	337.14	2.26
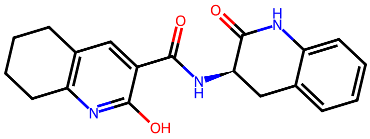	O=C(N[C@@H]1Cc2ccccc2NC1=O)c1cc2c(nc1O)CCCC2	529953983	25	337.14	1.96
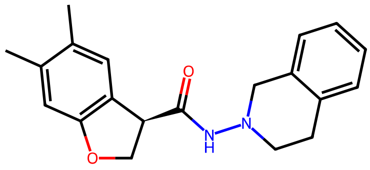	Cc1cc2c(cc1C)[C@@H](C(=O)NN1CCc3ccccc3C1)CO2	1469893548	24	322.17	2.87

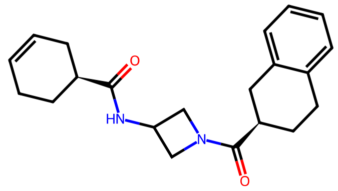	O=C(NC1CN(C(=O)[C@@H]2CCc3cc ccc3C2)C1)[C@@H]1CC=CCC1	990221459	25	338.2	2.47
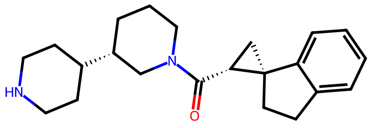	O=C([C@@H]1C[C@]12CCc1ccccc12)N1CCC[C@@H](C2CCNCC2)C1	662596881	25	338.24	3.13
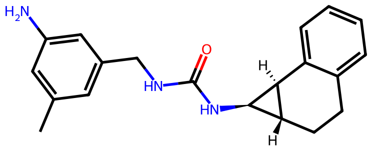	Cc1cc(N)cc(CNC(=O)N[C@@H]2[C@ H]3CCc4ccccc4[C@@H]32)c1	417916902	24	321.18	3.1
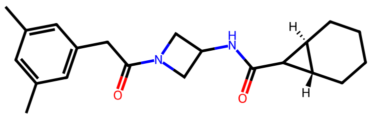	Cc1cc(C)cc(CC(=O)N2CC(NC(=O)C3[C@H]4CCCC[C@H]34)C2)c1	990650123	25	340.22	2.61
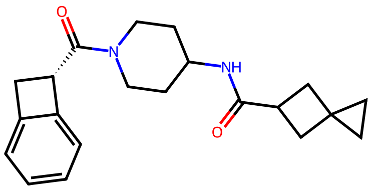	O=C(NC1CCN(C(=O)[C@H]2Cc3cccc c32)CC1)C1CC2(CC2)C1	948060207	25	338.2	2.62
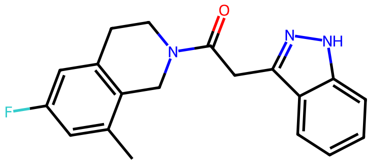	Cc1cc(F)cc2c1CN(C(=O)Cc1n[nH]c3c cccc13)CC2	337526284	24	323.14	3.14
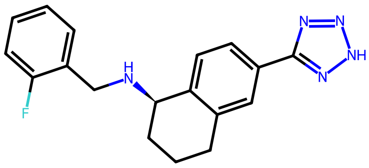	Fc1ccccc1CN[C@@H]1CCCc2cc(-c3 nn[nH]n3)ccc21	826378266	24	323.15	3.17
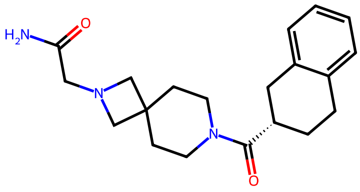	NC(=O)CN1CC2(CCN(C(=O)[C@H]3 CCc4ccccc4C3)CC2)C1	1035805239	25	341.21	1.2

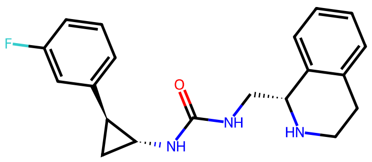	O=C(NC[C@H]1NCCc2ccccc21)N[C@@H]1C[C@H]1c1cccc(F)c1	562812397	25	339.17	2.87
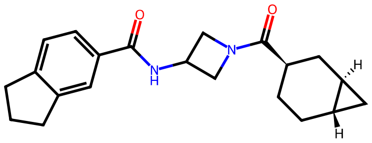	O=C(NC1CN(C(=O)[C@@H]2CC[C@H]3C[C@@H]3C2)C1)c1ccc2c(c1)CCC2	991373573	25	338.2	2.55
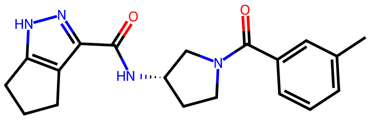	Cc1cccc(C(=O)N2CC[C@H](NC(=O)c3n[nH]c4c3CCC4)C2)c1	938240954	25	338.17	1.85
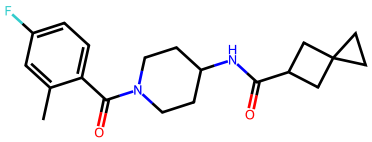	Cc1cc(F)ccc1C(=O)N1CCC(NC(=O)C2CC3(CC3)C2)CC1	947646214	25	344.19	3.05
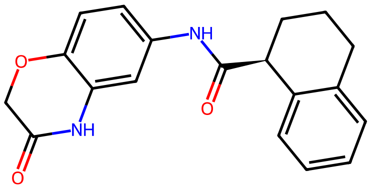	O=C1COc2ccc(NC(=O)[C@H]3CCCc4ccccc43)cc2N1	44336013	24	322.13	3.08
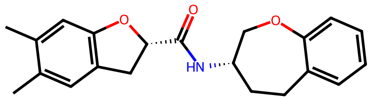	Cc1cc2c(cc1C)O[C@H](C(=O)N[C@H]1CCc3ccccc3OC1)C2	511632001	25	337.17	3.12
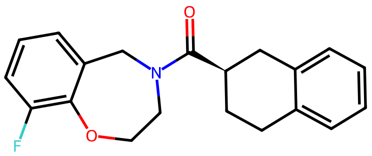	O=C([C@@H]1CCc2ccccc2C1)N1CCOc2c(F)cccc2C1	570728359	24	325.15	3.35
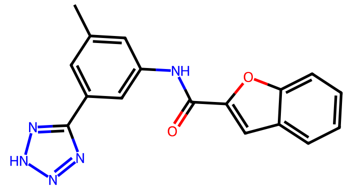	Cc1cc(NC(=O)c2cc3ccccc3o2)cc(-c2nn[nH]n2)c1	1592030021	24	319.11	3.17

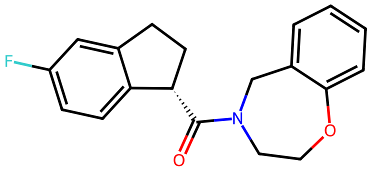	O=C([C@H]1CCc2cc(F)ccc21)N1CCOc2ccccc2C1	666868275	23	311.13	3.28
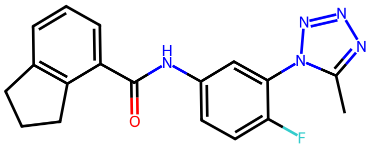	Cc1nnnn1-c1cc(NC(=O)c2cccc3c2CCC3)ccc1F	171170907	25	337.13	2.85
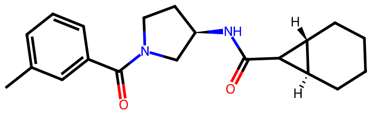	Cc1cccc(C(=O)N2CC[C@@H](NC(=O)C3[C@@H]4CCCC[C@@H]34)C2)c1	939880442	24	326.2	2.76
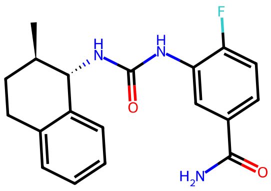	C[C@@H]1CCc2ccccc2[C@H]1NC(=O)Nc1cc(C(N)=O)ccc1F	179534128	25	341.15	3.37
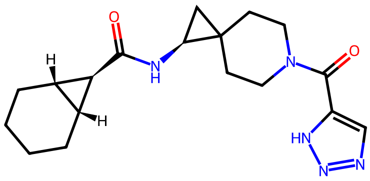	O=C(N[C@H]1CC12CCN(C(=O)c1cnn[nH]1)CC2)[C@@H]1[C@H]2CCCC[C@H]21	963448779	25	343.2	1.35
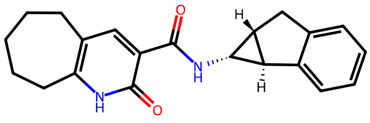	O=C(N[C@H]1[C@@H]2c3ccccc3C[C@H]21)c1cc2c([nH]c1=O)CCCCC2	130041057	25	334.17	2.71
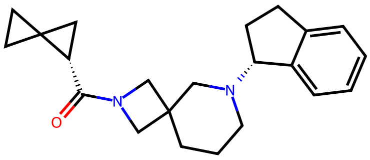	O=C([C@H]1CC12CC2)N1CC2(CCCN([C@@H]3CCc4ccccc43)C2)C1	982038873	25	336.22	3.4
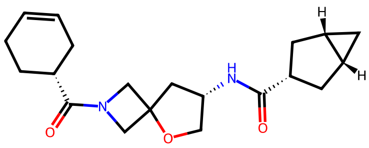	O=C(N[C@@H]1COC2(C1)CN(C(=O)[C@H]1CC=CCC1)C2)[C@@H]1C[C@@H]2C[C@@H]2C1	965785060	25	344.21	1.87

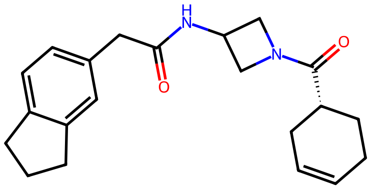	O=C(Cc1ccc2c(c1)CCC2)NC1CN(C(=O)[C@H]2CC=CCC2)C1	991386942	25	338.2	2.4
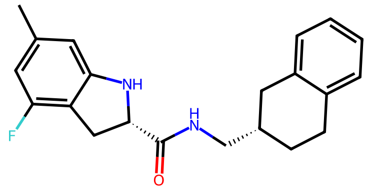	Cc1cc(F)c2c(c1)N[C@H](C(=O)NC[C@H]1CCc3ccccc3C1)C2	1579610647	25	338.18	3.39
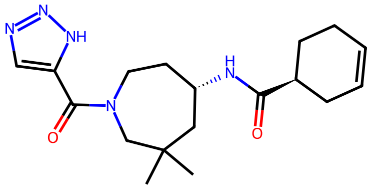	CC1(C)C[C@@H](NC(=O)[C@@H]2CC=CCC2)CCN(C(=O)c2cnn[nH]2)C1	990660099	25	345.22	1.91
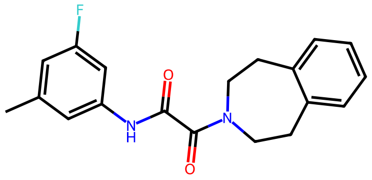	Cc1cc(F)cc(NC(=O)C(=O)N2CCc3ccccc3CC2)c1	153344673	24	326.14	2.7
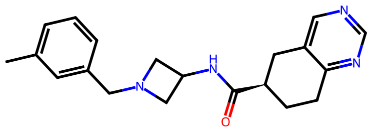	Cc1cccc(CN2CC(NC(=O)[C@@H]3CCc4ncncc4C3)C2)c1	1030426384	25	336.2	1.89
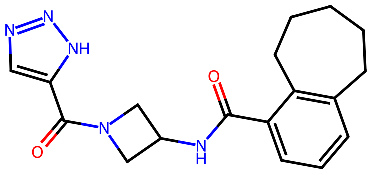	O=C(NC1CN(C(=O)c2cnn[nH]2)C1)c1cccc2c1CCCCC2	994065033	25	339.17	1.33
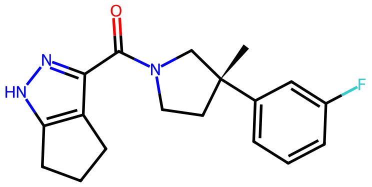	C[C@@]1(c2cccc(F)c2)CCN(C(=O)c2n[nH]c3c2CCC3)C1	652618500	23	313.16	2.84
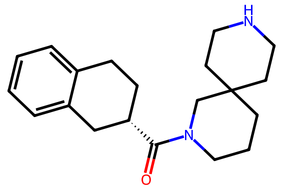	O=C([C@H]1CCc2ccccc2C1)N1CCCC2(CCNCC2)C1	1647255897	23	312.22	2.78

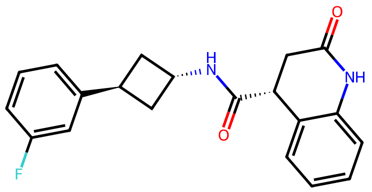	O=C1C[C@@H](C(=O)N[C@H]2C[C@H](c3cccc(F)c3)C2)c2ccccc2N1	280609378	25	338.14	3.31
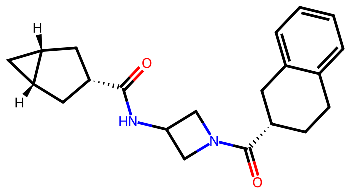	O=C(NC1CN(C(=O)[C@H]2CCc3ccccc3C2)C1)[C@@H]1C[C@@H]2C[C@@H]2C1	998010175	25	338.2	2.16
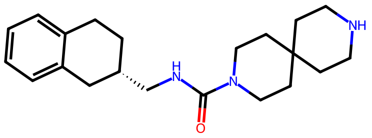	O=C(NC[C@H]1CCc2ccccc2C1)N1CCC2(CCNCC2)CC1	669551295	25	341.25	2.97
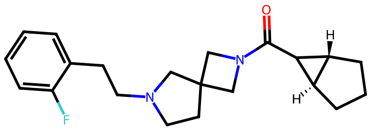	O=C(C1[C@@H]2CCC[C@@H]12)N1CC2(CCN(CCc3ccccc3F)C2)C1	1072590713	25	342.21	2.95
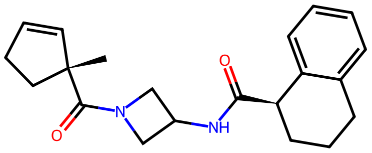	C[C@]1(C(=O)N2CC(NC(=O)[C@@H]3CCCc4ccccc43)C2)C=CCC1	995076434	25	338.2	2.79
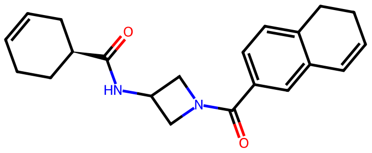	O=C(NC1CN(C(=O)c2ccc3c(c2)C=CCC3)C1)[C@@H]1CC=CCC1	990209133	25	336.18	2.94
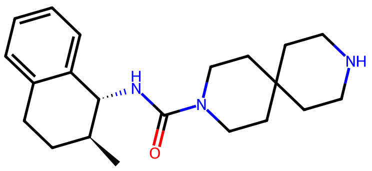	C[C@H]1CCc2ccccc2[C@@H]1NC(=O)N1CCC2(CCNCC2)CC1	417945397	25	341.25	3.49
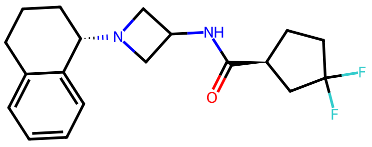	O=C(NC1CN([C@H]2CCCc3ccccc32)C1)[C@H]1CCC(F)(F)C1	1030412206	24	334.19	3.3

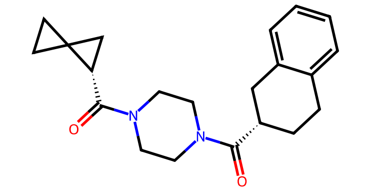	O=C([C@H]1CCc2ccccc2C1)N1CCN(C(=O)[C@H]2CC23CC3)CC1	1012616220	25	338.2	2.26
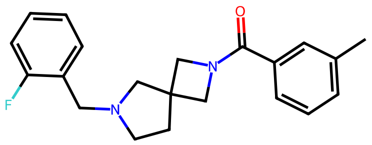	Cc1cccc(C(=O)N2CC3(CCN(Cc4ccccc4F)C3)C2)c1	1072434408	25	338.18	3.48
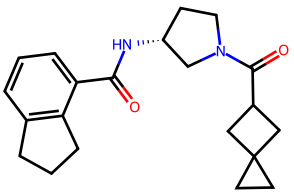	O=C(N[C@@H]1CCN(C(=O)C2CC3(CC3)C2)C1)c1cccc2c1CCC2	941159576	25	338.2	2.7
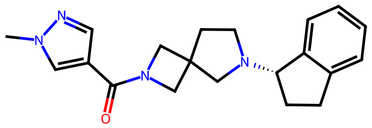	Cn1cc(C(=O)N2CC3(CCN([C@H]4CCc5ccccc54)C3)C2)cn1	1072453041	25	336.2	2.26
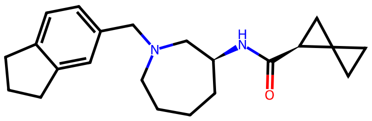	O=C(N[C@H]1CCCCN(Cc2ccc3c(c2)CCC3)C1)[C@H]1CC12CC2	1034416883	25	338.24	3.45
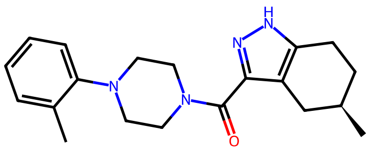	Cc1ccccc1N1CCN(C(=O)c2n[nH]c3c2C[C@H](C)CC3)CC1	1362163742	25	338.21	2.81
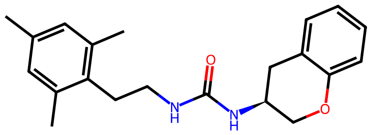	Cc1cc(C)c(CCNC(=O)N[C@@H]2COc3ccccc3C2)c(C)c1	1563551123	25	338.2	3.46
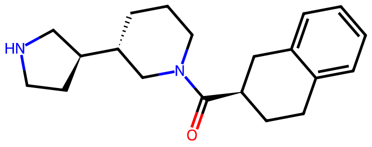	O=C([C@@H]1CCc2ccccc2C1)N1CCC[C@@H]([C@H]2CCNC2)C1	573262480	23	312.22	2.64

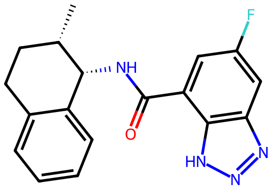	C[C@H]1CCc2ccccc2[C@H]1NC(=O)<br>c1cc(F)cc2nn[nH]c12	337463285	24	324.14	3.15
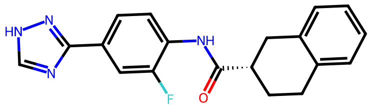	O=C(Nc1ccc(-c2nc[nH]n2)cc1F)[C@H<br>]1CCc2ccccc2C1	1270853399	25	336.14	3.35
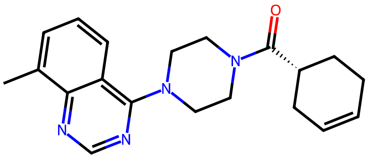	Cc1cccc2c(N3CCN(C(=O)[C@H]4CC<br>=CCC4)CC3)ncnc12	1055721592	25	336.2	2.94
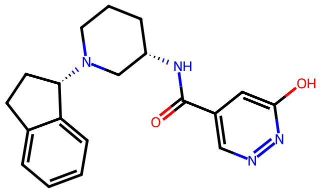	O=C(N[C@H]1CCCN([C@H]2CCc3cc<br>ccc32)C1)c1cnnc(O)c1	1007549055	25	338.17	2.06
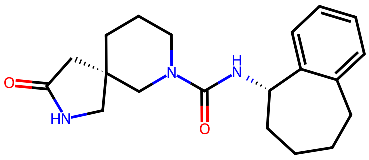	O=C1C[C@@]2(CCCN(C(=O)N[C@H<br>]3CCCCc4ccccc43)C2)CN1	525766802	25	341.21	2.77
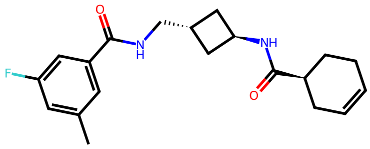	Cc1cc(F)cc(C(=O)NC[C@H]2C[C@H](<br>NC(=O)[C@@H]3CC=CCC3)C2)c1	980623040	25	344.19	3.12
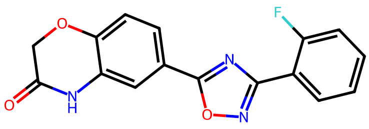	O=C1COc2ccc(-c3nc(-c4ccccc4F)no3)<br>cc2N1	355771904	23	311.07	2.87
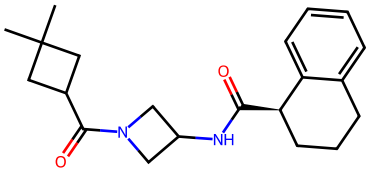	CC1(C)CC(C(=O)N2CC(NC(=O)[C@<br>@H]3CCCc4ccccc43)C2)C1	995072103	25	340.22	2.87

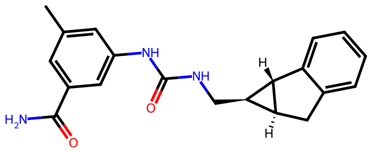	Cc1cc(NC(=O)NC[C@@H]2[C@@H]3Cc4ccccc4[C@@H]23)cc(C(N)=O)c1	583608645	25	335.16	2.8
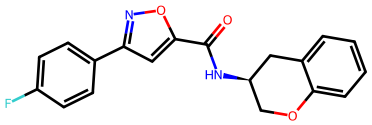	O=C(N[C@@H]1COc2ccccc2C1)c1cc(-c2ccc(F)cc2)no1	1625690989	25	338.11	3.21
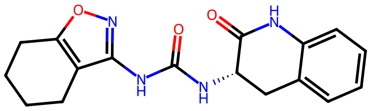	O=C(Nc1noc2c1CCCC2)N[C@H]1Cc2ccccc2NC1=O	934347994	24	326.14	2.24
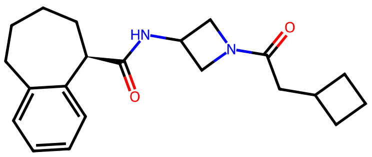	O=C(NC1CN(C(=O)CC2CCC2)C1)[C@@H]1CCCCc2ccccc21	994306592	25	340.22	3.01
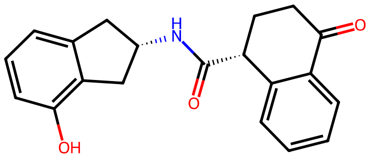	O=C1CC[C@@H](C(=O)N[C@H]2Cc3cccc(O)c3C2)c2ccccc21	560679753	24	321.14	2.74
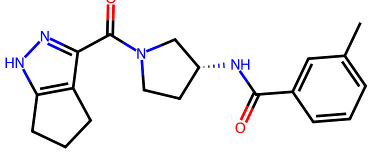	Cc1cccc(C(=O)N[C@@H]2CCN(C(=O)c3n[nH]c4c3CCC4)C2)c1	939225875	25	338.17	1.85
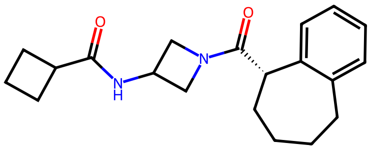	O=C(NC1CN(C(=O)[C@H]2CCCCc3ccccc32)C1)C1CCC1	990028717	24	326.2	2.62
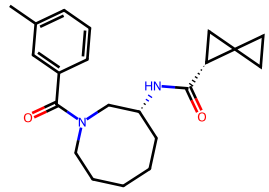	Cc1cccc(C(=O)N2CCCCC[C@@H](NC(=O)[C@@H]3CC34CC4)C2)c1	986868361	25	340.22	3.3

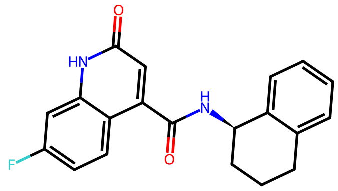	O=C(N[C@@H]1CCCc2ccccc21)c1cc(=O)[nH]c2cc(F)ccc12	130190479	25	336.13	3.47
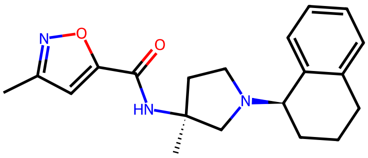	Cc1cc(C(=O)N[C@@]2(C)CCN([C@@H]3CCCc4ccccc43)C2)on1	1046139313	25	339.19	3.25
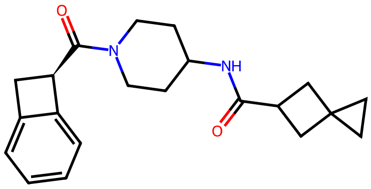	O=C(NC1CCN(C(=O)[C@@H]2Cc3ccccc32)CC1)C1CC2(CC2)C1	948060206	25	338.2	2.62
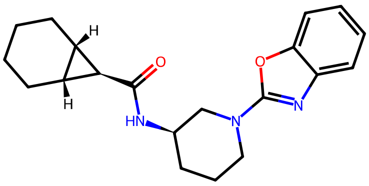	O=C(N[C@@H]1CCCN(c2nc3ccccc3o2)C1)[C@@H]1[C@H]2CCCC[C@H]21	1058130128	25	339.19	3.35
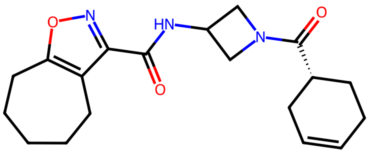	O=C(NC1CN(C(=O)[C@H]2CC=CCC2)C1)c1noc2c1CCCCC2	991590038	25	343.19	2.24
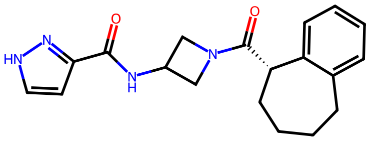	O=C(NC1CN(C(=O)[C@H]2CCCCc3ccccc32)C1)c1cc[nH]n1	990296171	25	338.17	1.86
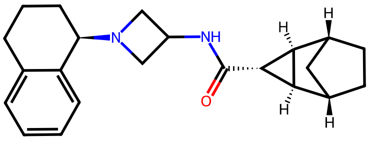	O=C(NC1CN([C@@H]2CCCc3ccccc32)C1)[C@@H]1[C@H]2[C@@H]3CC[C@@H](C3)[C@H]21	1031462918	25	336.22	3.16
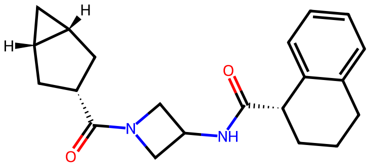	O=C(NC1CN(C(=O)[C@@H]2C[C@@H]3C[C@@H]3C2)C1)[C@H]1CCCc2ccccc21	995073268	25	338.2	2.48

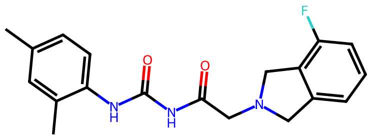	Cc1ccc(NC(=O)NC(=O)CN2Cc3cccc(F)c3C2)c(C)c1	459770722	25	341.15	3.11
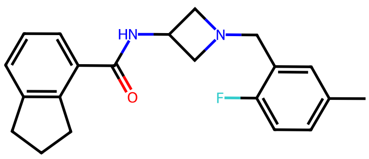	Cc1ccc(F)c(CN2CC(NC(=O)c3cccc4c3CCC4)C2)c1	1031468391	25	338.18	3.24
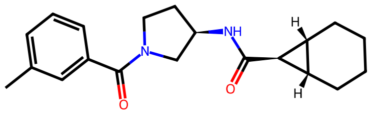	Cc1cccc(C(=O)N2CC[C@@H](NC(=O)[C@@H]3[C@H]4CCCC[C@H]43)C2)c1	939880441	24	326.2	2.76
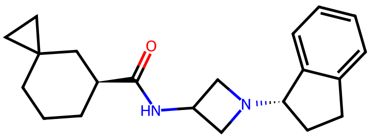	O=C(NC1CN([C@H]2CCc3ccccc32)C1)[C@H]1CCCC2(CC2)C1	1030307333	24	324.22	3.44
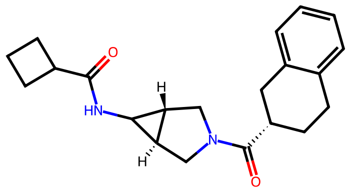	O=C(NC1[C@@H]2CN(C(=O)[C@H]3CCc4ccccc4C3)C[C@@H]12)C1CCC1	1012656074	25	338.2	2.16
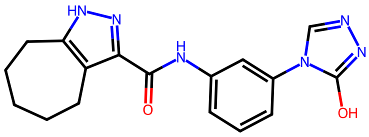	O=C(Nc1cccc(-n2cnnc2O)c1)c1n[nH]c2c1CCCCC2	365098466	25	338.15	2.22
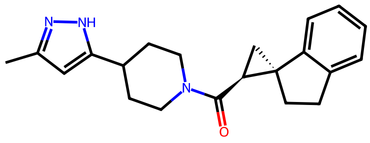	Cc1cc(C2CCN(C(=O)[C@H]3C[C@]34CCc3ccccc34)CC2)[nH]n1	273565904	25	335.2	3.33
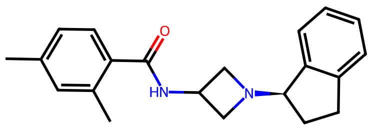	Cc1ccc(C(=O)NC2CN([C@@H]3CCc4ccccc43)C2)c(C)c1	1031339667	24	320.19	3.4

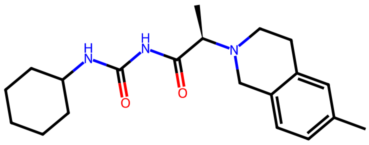	Cc1ccc2c(c1)CCN([C@H](C)C(=O)NC(=O)NC1CCCCC1)C2	361841069	25	343.23	2.9
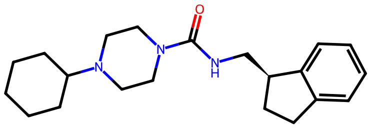	O=C(NC[C@@H]1CCc2ccccc21)N1CCN(C2CCCCC2)CC1	1468803857	25	341.25	3.38
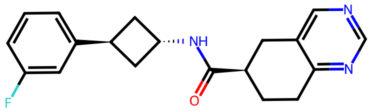	O=C(N[C@H]1C[C@H](c2cccc(F)c2)C1)[C@@H]1CCc2ncncc2C1	648890800	24	325.16	2.78
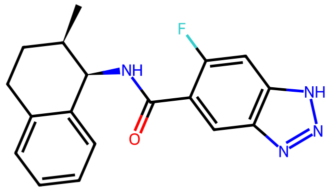	C[C@@H]1CCc2ccccc2[C@@H]1NC(=O)c1cc2nn[nH]c2cc1F	781961677	24	324.14	3.15
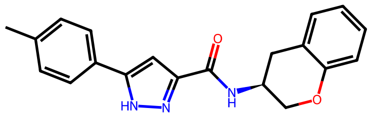	Cc1ccc(-c2cc(C(=O)N[C@@H]3COc4ccccc4C3)n[nH]2)cc1	516105194	25	333.15	3.12
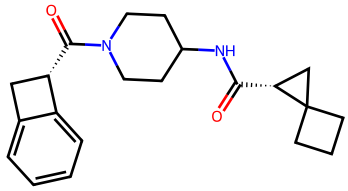	O=C(NC1CCN(C(=O)[C@H]2Cc3ccccc32)CC1)[C@@H]1CC12CCC2	948062510	25	338.2	2.62
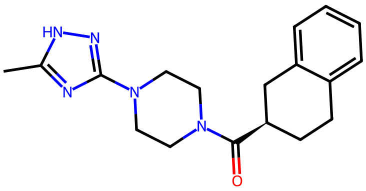	Cc1nc(N2CCN(C(=O)[C@@H]3CCc4ccccc4C3)CC2)n[nH]1	898184623	24	325.19	1.57
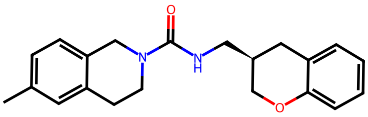	Cc1ccc2c(c1)CCN(C(=O)NC[C@@H]1COc3ccccc3C1)C2	790125151	25	336.18	3.31

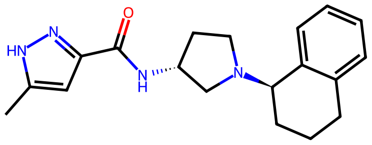	Cc1cc(C(=O)N[C@@H]2CCN([C@@H]3CCCc4ccccc43)C2)n[nH]1	1010333898	24	324.2	2.6
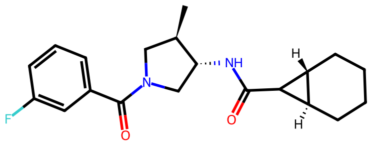	C[C@@H]1CN(C(=O)c2cccc(F)c2)C[C@H]1NC(=O)C1[C@@H]2CCCC[C@@H]12	947284525	25	344.19	2.84
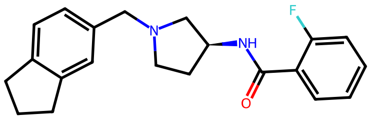	O=C(N[C@H]1CCN(Cc2ccc3c(c2)CCC3)C1)c1ccccc1F	1009951655	25	338.18	3.32
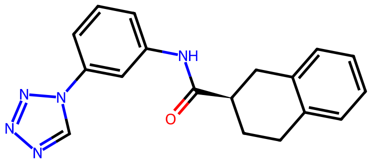	O=C(Nc1cccc(-n2cnnn2)c1)[C@@H]1CCc2ccccc2C1	1349147302	24	319.14	2.41
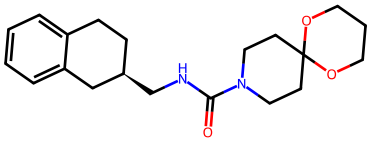	O=C(NC[C@@H]1CCc2ccccc2C1)N1CCC2(CC1)OCCCO2	836309959	25	344.21	2.73
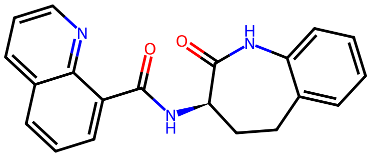	O=C(N[C@@H]1CCc2ccccc2NC1=O)c1cccc2cccnc12	1130318489	25	331.13	2.92
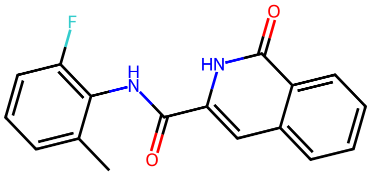	Cc1cccc(F)c1NC(=O)c1cc2ccccc2c(=O)[nH]1	181668253	22	296.1	3.23
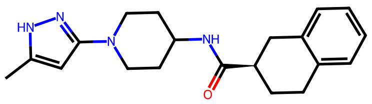	Cc1cc(N2CCC(NC(=O)[C@@H]3CCc4ccccc4C3)CC2)n[nH]1	579966436	25	338.21	2.61

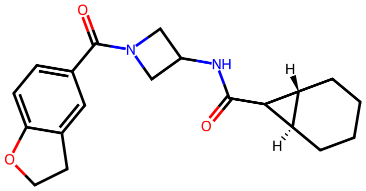	O=C(NC1CN(C(=O)c2ccc3c(c2)CCO3)C1)C1[C@@H]2CCCC[C@@H]12	990657188	25	340.18	2.0
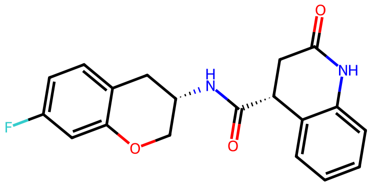	O=C1C[C@@H](C(=O)N[C@@H]2COc3cc(F)ccc3C2)c2ccccc2N1	1361448745	25	340.12	2.37
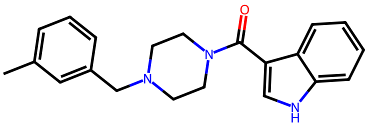	Cc1cccc(CN2CCN(C(=O)c3c[nH]c4ccccc34)CC2)c1	949143335	25	333.18	3.43
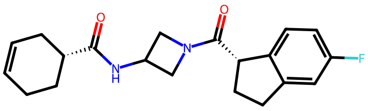	O=C(NC1CN(C(=O)[C@H]2CCc3cc(F)ccc32)C1)[C@H]1CC=CCC1	990217333	25	342.17	2.54
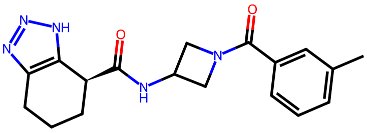	Cc1cccc(C(=O)N2CC(NC(=O)[C@H]3CCCc4nn[nH]c43)C2)c1	998569506	25	339.17	1.17
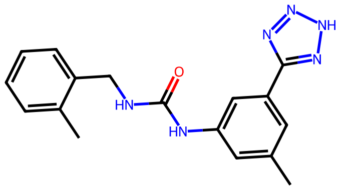	Cc1cc(NC(=O)NCc2ccccc2C)cc(-c2nn[nH]n2)c1	1605713357	24	322.15	2.81
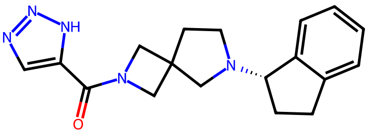	O=C(c1cnn[nH]1)N1CC2(CCN([C@H]3CCc4ccccc43)C2)C1	1072888569	24	323.17	1.64
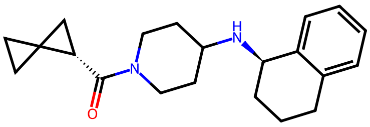	O=C([C@H]1CC12CC2)N1CCC(N[C@@H]2CCCc3ccccc32)CC1	997040930	24	324.22	3.44

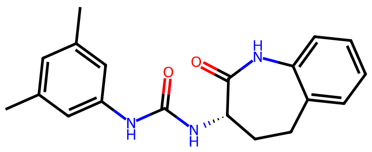	Cc1cc(C)cc(NC(=O)N[C@H]2CCc3ccccc3NC2=O)c1	653917648	24	323.16	3.38
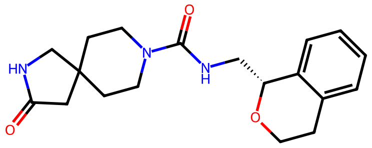	O=C1CC2(CCN(C(=O)NC[C@H]3OCCc4ccccc43)CC2)CN1	362888504	25	343.19	1.61
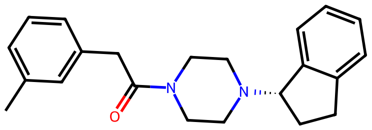	Cc1cccc(CC(=O)N2CCN([C@H]3CCc4ccccc43)CC2)c1	949979818	25	334.2	3.37
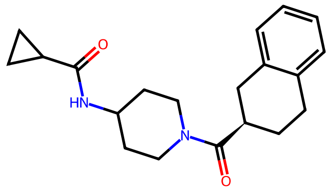	O=C(NC1CCN(C(=O)[C@@H]2CCc3ccccc3C2)CC1)C1CC1	580717855	24	326.2	2.31
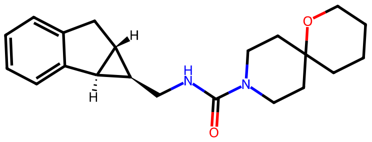	O=C(NC[C@@H]1[C@H]2Cc3ccccc3[C@@H]21)N1CCC2(CCCCO2)CC1	883522631	25	340.22	3.32
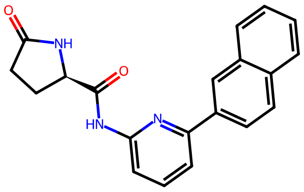	O=C1CC[C@H](C(=O)Nc2cccc(-c3ccc4ccccc4c3)n2)N1	104380820	25	331.13	3.12
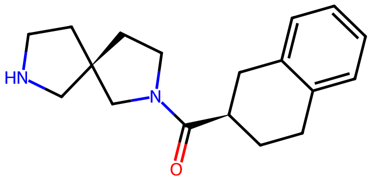	O=C([C@@H]1CCc2ccccc2C1)N1CC[C@@]2(CCNC2)C1	314203821	21	284.19	2.0
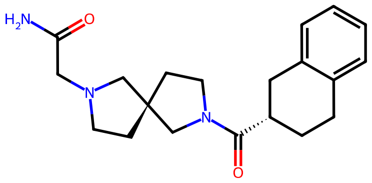	NC(=O)CN1CC[C@]2(CCN(C(=O)[C@H]3CCc4ccccc4C3)C2)C1	1041710108	25	341.21	1.2

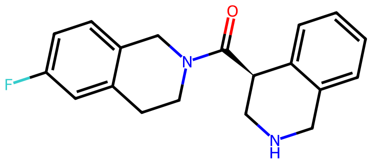	O=C([C@@H]1CNCc2ccccc21)N1CCc2cc(F)ccc2C1	646286705	23	310.15	2.6
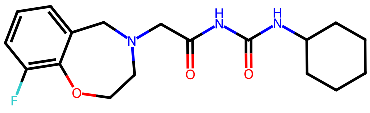	O=C(CN1CCOc2c(F)cccc2C1)NC(=O)NC1CCCCC1	562449649	25	349.18	2.18
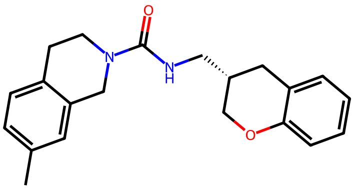	Cc1ccc2c(c1)CN(C(=O)NC[C@H]1COc3ccccc3C1)CC2	851972737	25	336.18	3.31
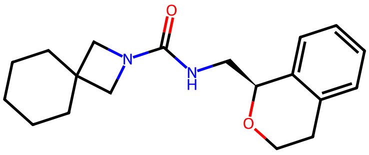	O=C(NC[C@@H]1OCCc2ccccc21)N1CC2(CCCCC2)C1	680642981	23	314.2	3.28
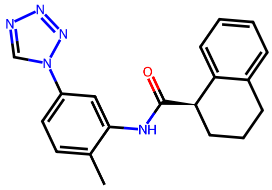	Cc1ccc(-n2cnnn2)cc1NC(=O)[C@@H]1CCCc2ccccc21	44601202	25	333.16	3.03
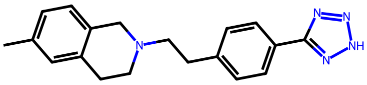	Cc1ccc2c(c1)CCN(CCc1ccc(-c3nn[nH]n3)cc1)C2	1592203634	24	319.18	2.78
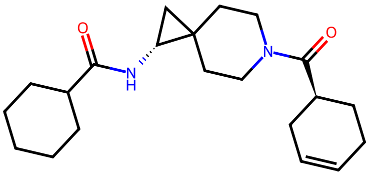	O=C(N[C@@H]1CC12CCN(C(=O)[C@@H]1CC=CCC1)CC2)C1CCCCC1	962750262	25	344.25	3.42
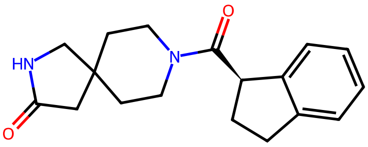	O=C1CC2(CCN(C(=O)[C@@H]3CCc4ccccc43)CC2)CN1	336042468	22	298.17	1.85

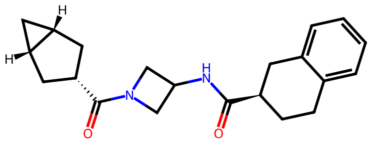	O=C(NC1CN(C(=O)[C@@H]2C[C@@H]3C[C@@H]3C2)C1)[C@@H]1CCc2ccccc2C1	1000543122	25	338.2	2.16
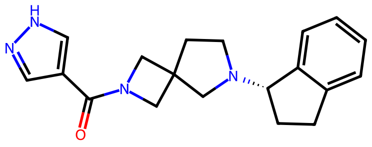	O=C(c1cn[nH]c1)N1CC2(CCN([C@H]3CCc4ccccc43)C2)C1	1072511712	24	322.18	2.25
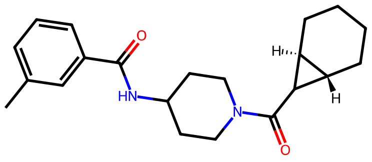	Cc1cccc(C(=O)NC2CCN(C(=O)C3[C@H]4CCCC[C@H]34)CC2)c1	870834266	25	340.22	3.15
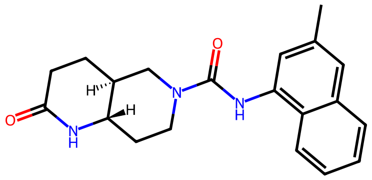	Cc1cc(NC(=O)N2CC[C@@H]3NC(=O)CC[C@H]3C2)c2ccccc2c1	667170965	25	337.18	3.28
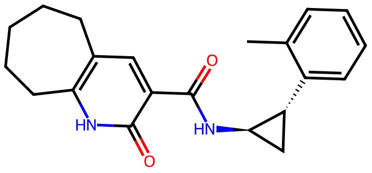	Cc1ccccc1[C@@H]1C[C@H]1NC(=O)c1cc2c([nH]c1=O)CCCCC2	126886887	25	336.18	3.24
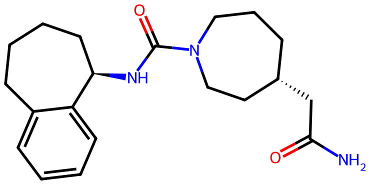	NC(=O)C[C@H]1CCCN(C(=O)N[C@@H]2CCCCc3ccccc32)CC1	671571534	25	343.23	3.14
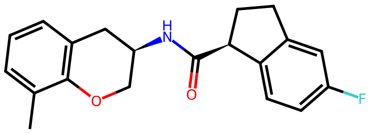	Cc1cccc2c1OC[C@H](NC(=O)[C@H]1CCc3cc(F)ccc31)C2	615059812	24	325.15	3.28
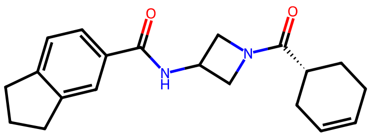	O=C(NC1CN(C(=O)[C@H]2CC=CCC2)C1)c1ccc2c(c1)CCC2	991356439	24	324.18	2.47

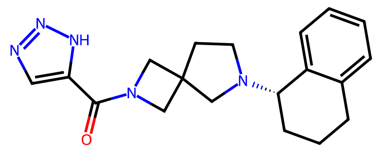	O=C(c1cnn[nH]1)N1CC2(CCN([C@H]3CCCc4ccccc43)C2)C1	1072892330	25	337.19	2.03
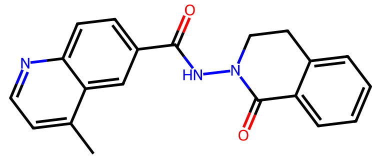	Cc1ccnc2ccc(C(=O)NN3CCc4ccccc4C3=O)cc12	803074620	25	331.13	2.89
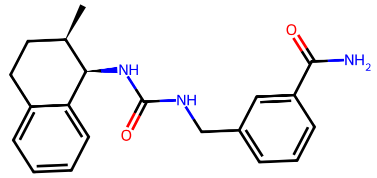	C[C@@H]1CCc2ccccc2[C@@H]1NC(=O)NCc1cccc(C(N)=O)c1	179306739	25	337.18	2.91
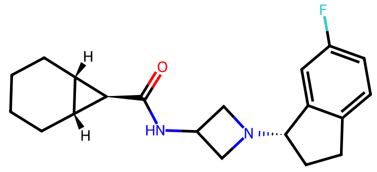	O=C(NC1CN([C@H]2CCc3ccc(F)cc32)C1)[C@@H]1[C@H]2CCCC[C@H]21	1030185899	24	328.2	3.05
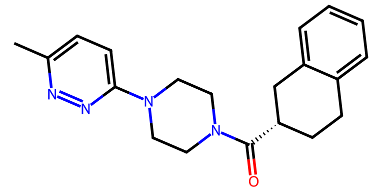	Cc1ccc(N2CCN(C(=O)[C@H]3CCc4ccccc4C3)CC2)nn1	1056040252	25	336.2	2.24
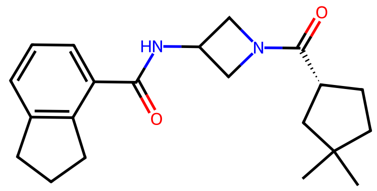	CC1(C)CC[C@@H](C(=O)N2CC(NC(=O)c3cccc4c3CCC4)C2)C1	994399972	25	340.22	2.94
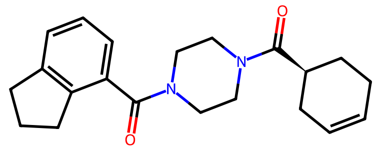	O=C(c1cccc2c1CCC2)N1CCN(C(=O)[C@@H]2CC=CCC2)CC1	1010310355	25	338.2	2.82
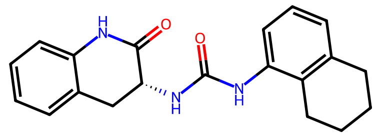	O=C(Nc1cccc2c1CCCC2)N[C@@H]1Cc2ccccc2NC1=O	528860109	25	335.16	3.25

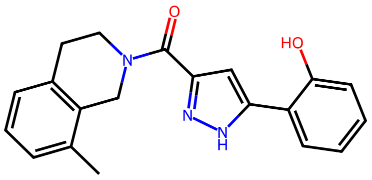	Cc1cccc2c1CN(C(=O)c1cc(-c3ccccc3O)[nH]n1)CC2	1556186469	25	333.15	3.29
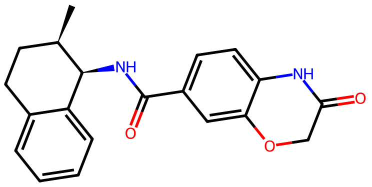	C[C@@H]1CCc2ccccc2[C@@H]1NC(=O)c1ccc2c(c1)OCC(=O)N2	179463708	25	336.15	3.07
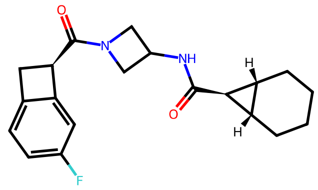	O=C(NC1CN(C(=O)[C@@H]2Cc3ccc(F)cc32)C1)[C@@H]1[C@H]2CCCC[C@H]21	990662680	25	342.17	2.23
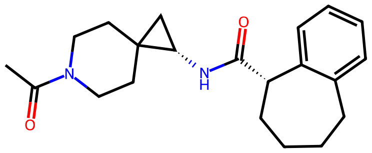	CC(=O)N1CCC2(CC1)C[C@@H]2NC(=O)[C@H]1CCCCc2ccccc21	964029774	25	340.22	3.01
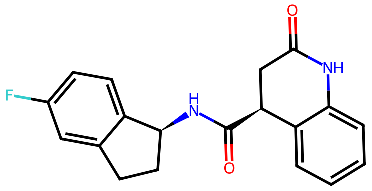	O=C1C[C@H](C(=O)N[C@H]2CCc3c(F)ccc32)c2ccccc2N1	56315395	24	324.13	3.06
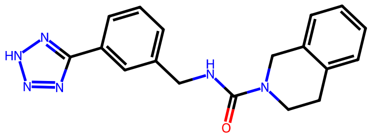	O=C(NCc1cccc(-c2nn[nH]n2)c1)N1CCc2ccccc2C1	1593143004	25	334.15	2.13
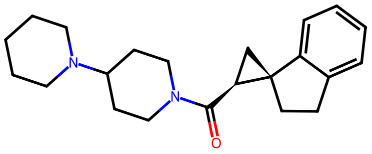	O=C([C@H]1C[C@@]12CCc1ccccc12)N1CCC(N2CCCCC2)CC1	266541344	25	338.24	3.37
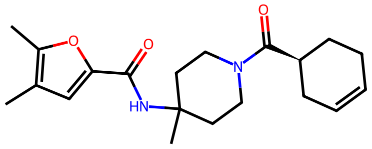	Cc1cc(C(=O)NC2(C)CCN(C(=O)[C@@H]3CC=CCC3)CC2)oc1C	956339954	25	344.21	3.36

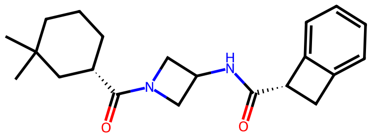	CC1(C)CCC[C@H](C(=O)N2CC(NC(=O)[C@H]3Cc4ccccc43)C2)C1	998686755	25	340.22	2.87
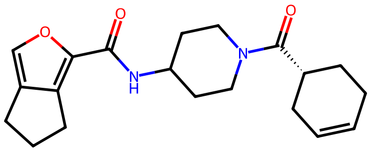	O=C(NC1CCN(C(=O)[C@H]2CC=CCC2)CC1)c1occ2c1CCC2	944127311	25	342.19	2.85
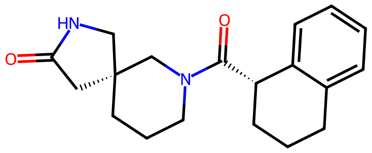	O=C1C[C@]2(CCCN(C(=O)[C@H]3CCc4ccccc43)C2)CN1	362206875	23	312.18	2.24
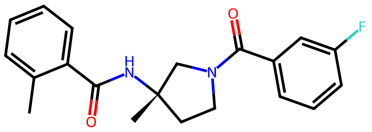	Cc1ccccc1C(=O)N[C@@]1(C)CCN(C(=O)c2cccc(F)c2)C1	956660120	25	340.16	3.17
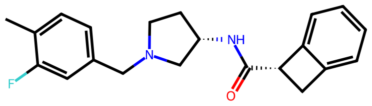	Cc1ccc(CN2CC[C@H](NC(=O)[C@H]3Cc4ccccc43)C2)cc1F	1015634519	25	338.18	3.16
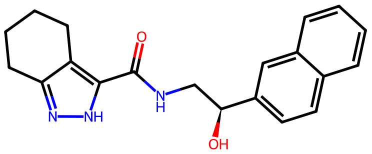	O=C(NC[C@H](O)c1ccc2ccccc2c1)c1[nH]nc2c1CCCC2	303982455	25	335.16	2.91
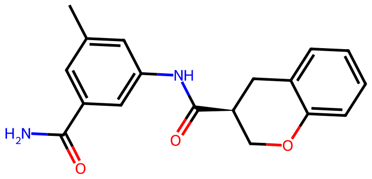	Cc1cc(NC(=O)[C@@H]2COc3ccccc3C2)cc(C(N)=O)c1	1361836850	23	310.13	2.28
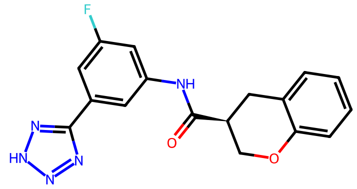	O=C(Nc1cc(F)cc(-c2nn[nH]n2)c1)[C@@H]1COc2ccccc2C1	1582635002	25	339.11	2.2

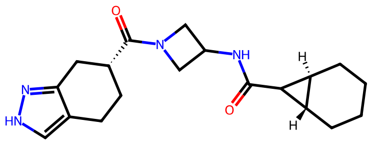	O=C(NC1CN(C(=O)[C@@H]2CCc3c[nH]nc3C2)C1)C1[C@H]2CCCC[C@H]12	990647999	25	342.21	1.28
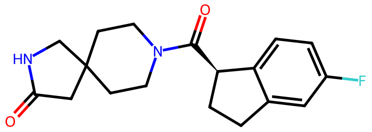	O=C1CC2(CCN(C(=O)[C@@H]3CCc4cc(F)ccc43)CC2)CN1	829422899	23	316.16	1.98
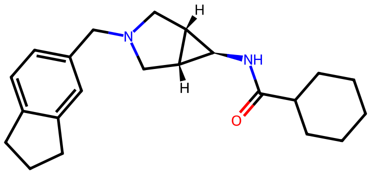	O=C(N[C@H]1[C@@H]2CN(Cc3ccc4c(c3)CCC4)C[C@@H]21)C1CCCCC1	958102799	25	338.24	3.3
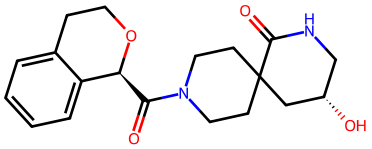	O=C([C@@H]1OCCc2ccccc21)N1CCC2(CC1)C[C@@H](O)CNC2=O	885019519	25	344.17	0.79
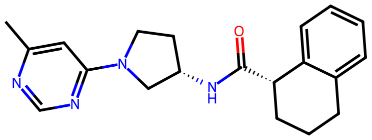	Cc1cc(N2CC[C@H](NC(=O)[C@H]3CCCc4ccccc43)C2)ncn1	1058592336	25	336.2	2.6
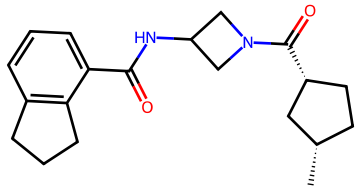	C[C@H]1CC[C@@H](C(=O)N2CC(NC(=O)c3cccc4c3CCC4)C2)C1	994398908	24	326.2	2.55
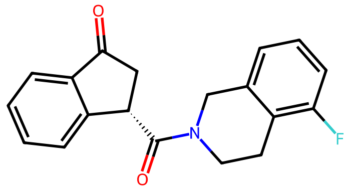	O=C1C[C@H](C(=O)N2CCc3c(F)ccc c3C2)c2ccccc21	277349610	23	309.12	3.08
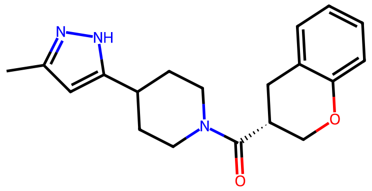	Cc1cc(C2CCN(C(=O)[C@H]3COc4ccccc4C3)CC2)[nH]n1	273643530	24	325.18	2.68

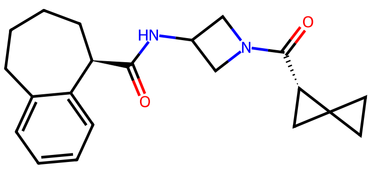	O=C(NC1CN(C(=O)[C@H]2CC23CC3)C1)[C@@H]1CCCCc2ccccc21	994322764	25	338.2	2.62
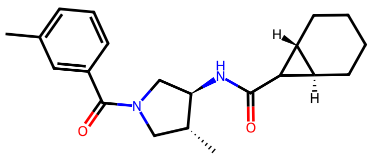	Cc1cccc(C(=O)N2C[C@@H](NC(=O)C3[C@@H]4CCCC[C@@H]34)[C@H](C)C2)c1	947313696	25	340.22	3.01
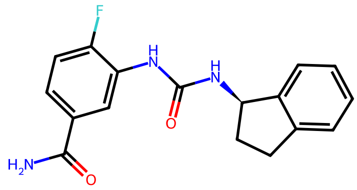	NC(=O)c1ccc(F)c(NC(=O)N[C@@H]2CCc3ccccc32)c1	47874815	23	313.12	2.73
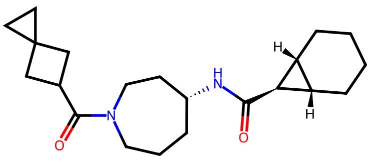	O=C(N[C@@H]1CCCN(C(=O)C2CC3(CC3)C2)CC1)[C@@H]1[C@H]2CCCC[C@H]21	964809579	25	344.25	3.11
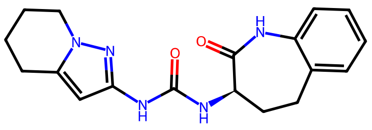	O=C(Nc1cc2n(n1)CCCC2)N[C@@H]1CCc2ccccc2NC1=O	654073754	25	339.17	2.29
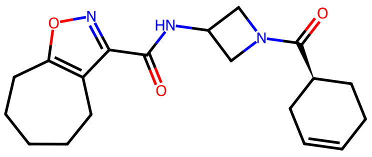	O=C(NC1CN(C(=O)[C@@H]2CC=CCC2)C1)c1noc2c1CCCCC2	991590033	25	343.19	2.24
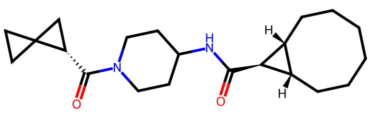	O=C(NC1CCN(C(=O)[C@H]2CC23CC3)CC1)[C@@H]1[C@H]2CCCCCC[C@H]21	948557990	25	344.25	3.11
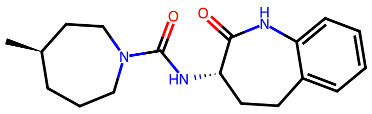	C[C@@H]1CCCN(C(=O)N[C@H]2CCc3ccccc3NC2=O)CC1	654629901	23	315.19	2.77

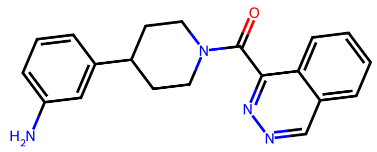	Nc1cccc(C2CCN(C(=O)c3nncc4ccccc34)CC2)c1	1263129825	25	332.16	3.23
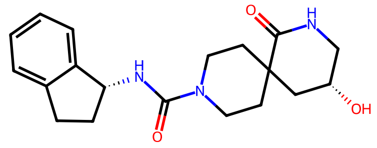	O=C(N[C@@H]1CCc2ccccc21)N1CCC2(CC1)C[C@@H](O)CNC2=O	913965884	25	343.19	1.35
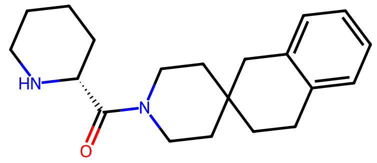	O=C([C@H]1CCCCN1)N1CCC2(CCc3ccccc3C2)CC1	653178587	23	312.22	2.93
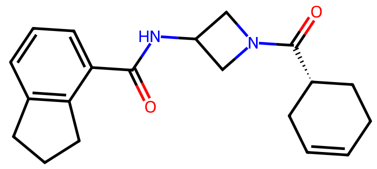	O=C(NC1CN(C(=O)[C@H]2CC=CCC2)C1)c1cccc2c1CCC2	994397742	24	324.18	2.47
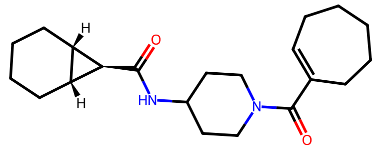	O=C(NC1CCN(C(=O)C2=CCCCCC2)CC1)[C@@H]1[C@H]2CCCC[C@H]21	946548925	25	344.25	3.42
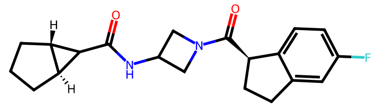	O=C(NC1CN(C(=O)[C@@H]2CCc3cc(F)ccc32)C1)C1[C@H]2CCC[C@H]12	990701883	25	342.17	2.23
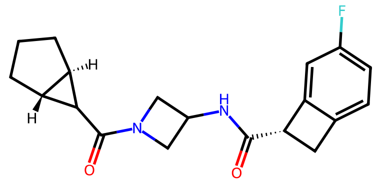	O=C(NC1CN(C(=O)C2[C@@H]3CCC[C@@H]23)C1)[C@H]1Cc2ccc(F)cc21	998486438	24	328.16	1.84
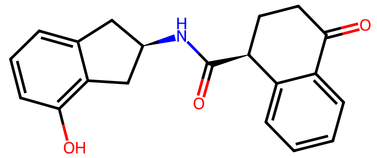	O=C1CC[C@H](C(=O)N[C@@H]2Cc3cccc(O)c3C2)c2ccccc21	560679750	24	321.14	2.74

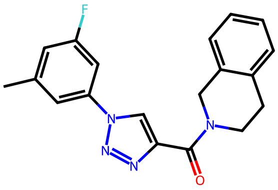	Cc1cc(F)cc(-n2cc(C(=O)N3CCc4ccccc4C3)nn2)c1	344656285	25	336.14	2.91
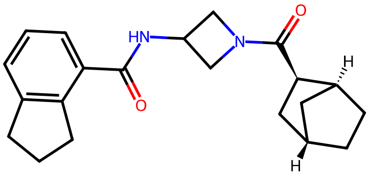	O=C(NC1CN(C(=O)[C@@H]2C[C@H]3CC[C@H]2C3)C1)c1cccc2c1CCC2	994399600	25	338.2	2.55
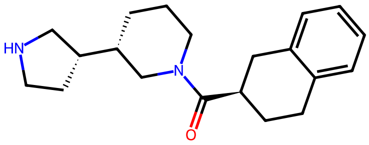	O=C([C@@H]1CCc2ccccc2C1)N1CCC[C@@H]([C@@H]2CCNC2)C1	573262481	23	312.22	2.64
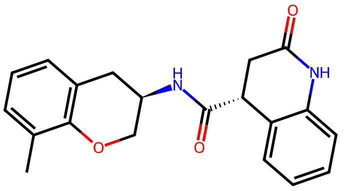	Cc1cccc2c1OC[C@H](NC(=O)[C@@H]1CC(=O)Nc3ccccc31)C2	291627681	25	336.15	2.54
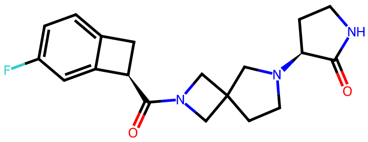	O=C1NCC[C@@H]1N1CCC2(CN(C(=O)[C@@H]3Cc4ccc(F)cc43)C2)C1	1072918204	25	343.17	0.89
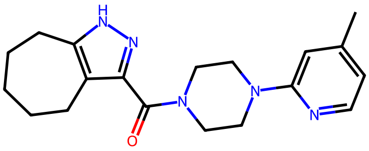	Cc1ccnc(N2CCN(C(=O)c3n[nH]c4c3CCCCC4)CC2)c1	353617391	25	339.21	2.34
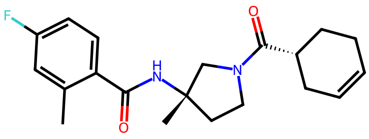	Cc1cc(F)ccc1C(=O)N[C@@]1(C)CCN(C(=O)[C@H]2CC=CCC2)C1	957256367	25	344.19	3.21
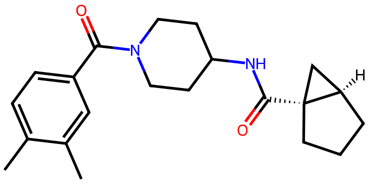	Cc1ccc(C(=O)N2CCC(NC(=O)[C@@]34CCC[C@@H]3C4)CC2)cc1C	948347781	25	340.22	3.21

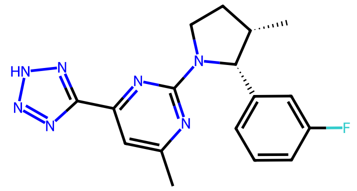	Cc1cc(-c2nn[nH]n2)nc(N2CC[C@H](C)[C@@H]2c2cccc(F)c2)n1	1609391682	25	339.16	2.69
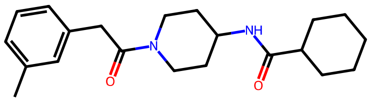	Cc1cccc(CC(=O)N2CCC(NC(=O)C3CCCCC3)CC2)c1	75066932	25	342.23	3.23
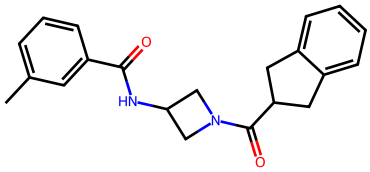	Cc1cccc(C(=O)NC2CN(C(=O)C3Cc4ccccc4C3)C2)c1	990136617	25	334.17	2.35
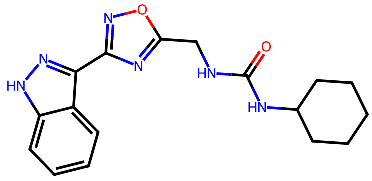	O=C(NCc1nc(-c2n[nH]c3ccccc23)no1)NC1CCCCC1	546425707	25	340.16	2.74
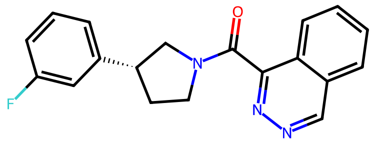	O=C(c1nncc2ccccc12)N1CC[C@H](c2cccc(F)c2)C1	1519843545	24	321.13	3.4
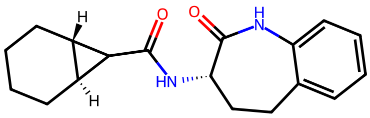	O=C(N[C@H]1CCc2ccccc2NC1=O)C1[C@H]2CCCC[C@H]12	654002300	22	298.17	2.49
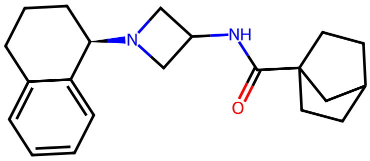	O=C(NC1CN([C@@H]2CCCc3ccccc32)C1)C12CCC(CC1)C2	1030536479	24	324.22	3.44
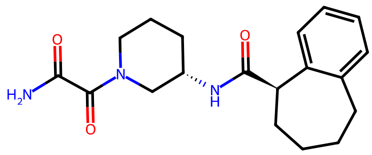	NC(=O)C(=O)N1CCC[C@H](NC(=O)[C@@H]2CCCCc3ccccc32)C1	951861434	25	343.19	1.09

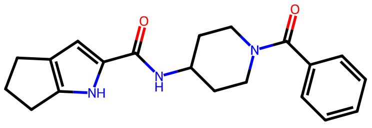	O=C(NC1CCN(C(=O)c2ccccc2)CC1)c1cc2c([nH]1)CCC2	943713927	25	337.18	2.54
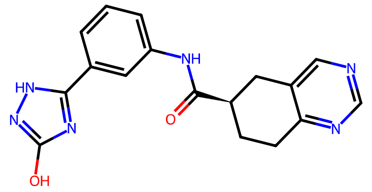	O=C(Nc1cccc(-c2nc(O)n[nH]2)c1)[C@@H]1CCc2ncncc2C1	1449246647	25	336.13	1.71
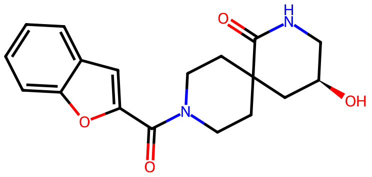	O=C(c1cc2ccccc2o1)N1CCC2(CC1)C[C@H](O)CNC2=O	885025577	24	328.14	1.54
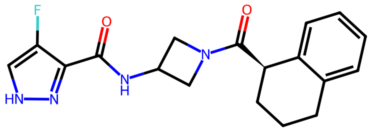	O=C(NC1CN(C(=O)[C@@H]2CCCc3ccccc32)C1)c1n[nH]cc1F	999175692	25	342.15	1.61
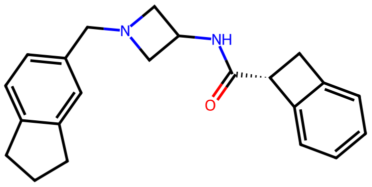	O=C(NC1CN(Cc2ccc3c(c2)CCC3)C1)[C@@H]1Cc2ccccc21	1030876491	25	332.19	2.82
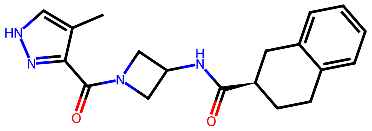	Cc1c[nH]nc1C(=O)N1CC(NC(=O)[C@@H]2CCc3ccccc3C2)C1	1000536774	25	338.17	1.46
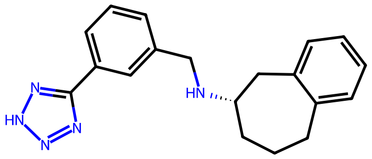	c1cc(CN[C@H]2CCCc3ccccc3C2)cc(-c2nn[nH]n2)c1	1607118754	24	319.18	2.9
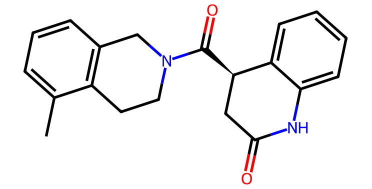	Cc1cccc2c1CCN(C(=O)[C@@H]1CC(=O)Nc3ccccc31)C2	171540280	24	320.15	3.01

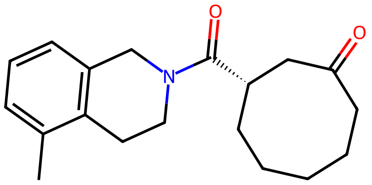	Cc1cccc2c1CCN(C(=O)[C@H]1CCCCCC(=O)C1)C2	813441244	22	299.19	3.42
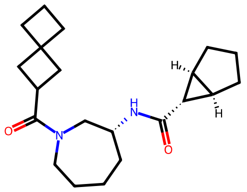	O=C(N[C@@H]1CCCCN(C(=O)C2CC3(CCC3)C2)C1)[C@@H]1[C@H]2CCC[C@H]21	943169090	25	344.25	3.11
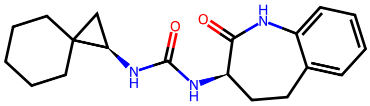	O=C(N[C@@H]1CCc2ccccc2NC1=O)N[C@@H]1CC12CCCCC2	656111466	24	327.19	2.96
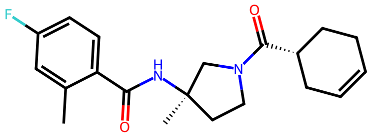	Cc1cc(F)ccc1C(=O)N[C@]1(C)CCN(C(=O)[C@H]2CC=CCC2)C1	957256365	25	344.19	3.21
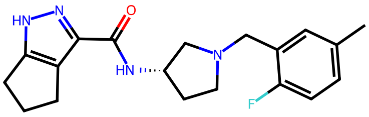	Cc1ccc(F)c(CN2CC[C@H](NC(=O)c3n[nH]c4c3CCC4)C2)c1	1015068256	25	342.19	2.35
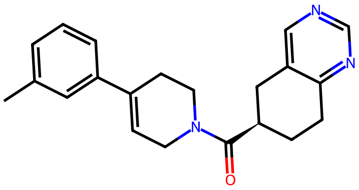	Cc1cccc(C2=CCN(C(=O)[C@@H]3CCc4ncncc4C3)CC2)c1	648343372	25	333.18	3.21
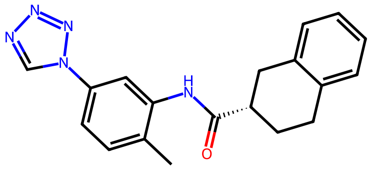	Cc1ccc(-n2cnnn2)cc1NC(=O)[C@H]1CCc2ccccc2C1	574088513	25	333.16	2.71
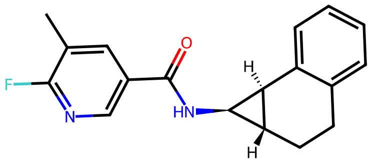	Cc1cc(C(=O)N[C@@H]2[C@H]3CCc4ccccc4[C@@H]32)cnc1F	857714541	22	296.13	2.99

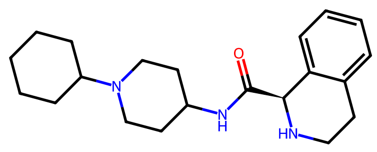	O=C(NC1CCN(C2CCCCC2)CC1)[C@@H]1NCCc2ccccc21	237990123	25	341.25	2.79
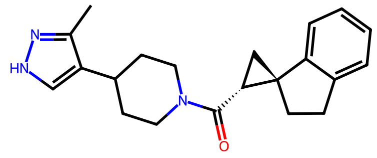	Cc1n[nH]cc1C1CCN(C(=O)[C@@H]2C[C@@]23CCc2ccccc23)CC1	375990271	25	335.2	3.33
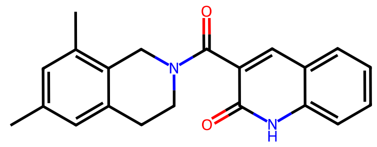	Cc1cc(C)c2c(c1)CCN(C(=O)c1cc3ccccc3[nH]c1=O)C2	186710254	25	332.15	3.34
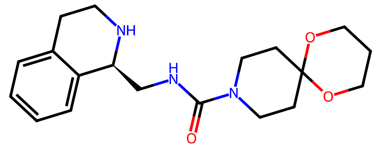	O=C(NC[C@@H]1NCCc2ccccc21)N1CCC2(CC1)OCCCO2	841998908	25	345.21	1.81
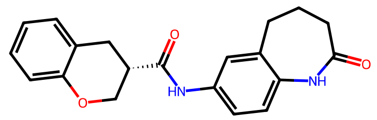	O=C1CCCc2cc(NC(=O)[C@@H]3COc4ccccc4C3)ccc2N1	264084929	25	336.15	3.15
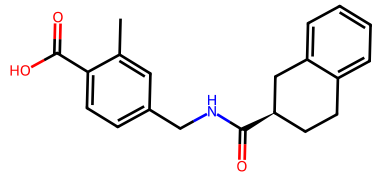	Cc1cc(CNC(=O)[C@@H]2CCc3ccccc3C2)ccc1C(=O)O	1777422647	24	323.15	3.11
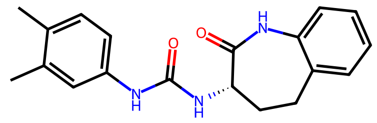	Cc1ccc(NC(=O)N[C@H]2CCc3ccccc3NC2=O)cc1C	653914130	24	323.16	3.38
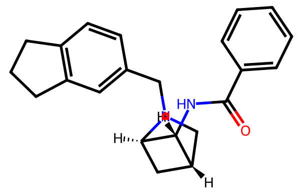	O=C(N[C@@H]1[C@H]2C[C@H]1N(Cc1ccc3c(c1)CCC3)C2)c1ccccc1	1037284704	25	332.19	3.18

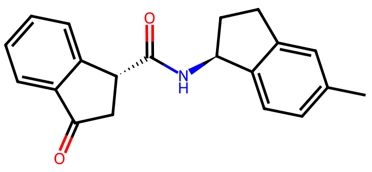	Cc1ccc2c(c1)CC[C@@H]2NC(=O)[C@@H]1CC(=O)c2ccccc21	355158830	23	305.14	3.47
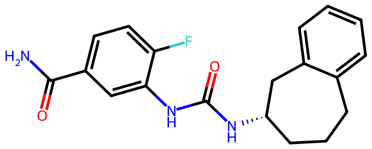	NC(=O)c1ccc(F)c(NC(=O)N[C@H]2CCCc3ccccc3C2)c1	929838757	25	341.15	2.99
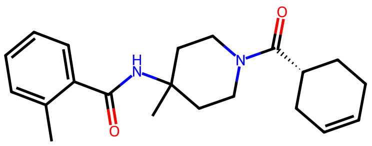	Cc1ccccc1C(=O)NC1(C)CCN(C(=O)[C@H]2CC=CCC2)CC1	956273932	25	340.22	3.46
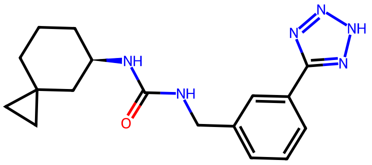	O=C(NCc1cccc(-c2nn[nH]n2)c1)N[C@@H]1CCCC2(CC2)C1	1593142910	24	326.19	2.39
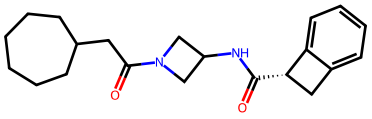	O=C(NC1CN(C(=O)CC2CCCCCC2)C1)[C@H]1Cc2ccccc21	998694133	25	340.22	3.01
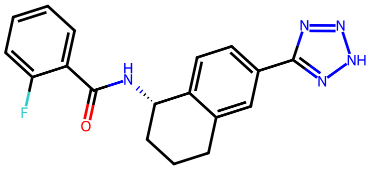	O=C(N[C@H]1CCCc2cc(-c3nn[nH]n3)ccc21)c1ccccc1F	1592986097	25	337.13	2.81
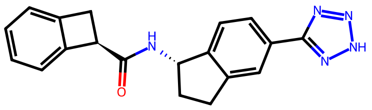	O=C(N[C@H]1CCc2cc(-c3nn[nH]n3)ccc21)[C@@H]1Cc2ccccc21	1609543187	25	331.14	2.31
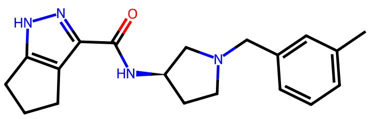	Cc1cccc(CN2CC[C@@H](NC(=O)c3n[nH]c4c3CCC4)C2)c1	634273284	24	324.2	2.21

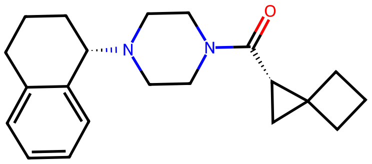	O=C([C@H]1CC12CCC2)N1CCN([C@H]2CCCc3ccccc32)CC1	950851295	24	324.22	3.4
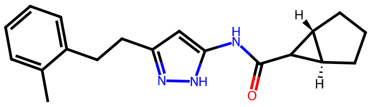	Cc1ccccc1CCc1cc(NC(=O)C2[C@@H]3CCC[C@@H]23)[nH]n1	182888214	23	309.18	3.49
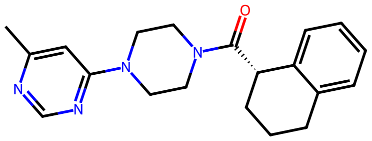	Cc1cc(N2CCN(C(=O)[C@H]3CCCc4ccccc43)CC2)ncn1	1056074130	25	336.2	2.55
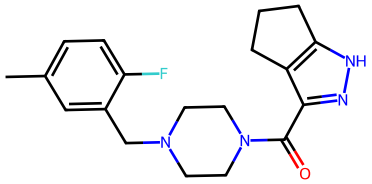	Cc1ccc(F)c(CN2CCN(C(=O)c3n[nH]c4c3CCC4)CC2)c1	949062907	25	342.19	2.3
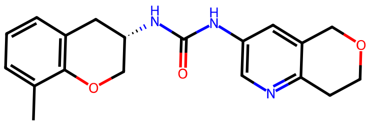	Cc1cccc2c1OC[C@@H](NC(=O)Nc1cnc3c(c1)COCC3)C2	685642160	25	339.16	2.59
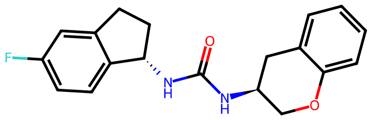	O=C(N[C@@H]1COc2ccccc2C1)N[C@H]1CCc2cc(F)ccc21	193788764	24	326.14	3.12
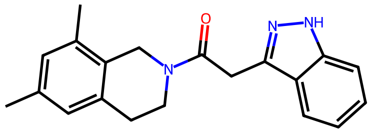	Cc1cc(C)c2c(c1)CCN(C(=O)Cc1n[nH]c3ccccc13)C2	190767259	24	319.17	3.31
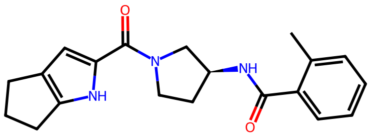	Cc1ccccc1C(=O)N[C@H]1CCN(C(=O)c2cc3c([nH]2)CCC3)C1	937632347	25	337.18	2.46

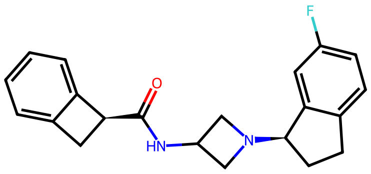	O=C(NC1CN([C@@H]2CCc3ccc(F)cc32)C1)[C@H]1Cc2ccccc21	1030881227	25	336.16	2.95
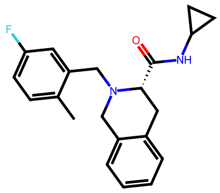	Cc1ccc(F)cc1CN1Cc2ccccc2C[C@H]1C(=O)NC1CC1	1559934974	25	338.18	3.34
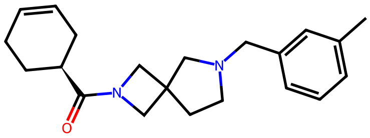	Cc1cccc(CN2CCC3(C2)CN(C(=O)[C@@H]2CC=CCC2)C3)c1	1072458054	24	324.22	3.39
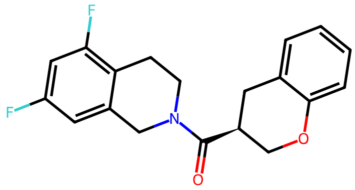	O=C([C@@H]1COc2ccccc2C1)N1CCc2c(F)cc(F)cc2C1	361059065	24	329.12	3.1
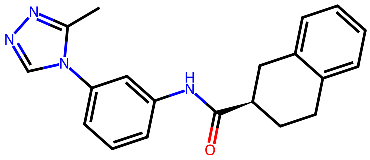	Cc1nncn1-c1cccc(NC(=O)[C@@H]2CCc3ccccc3C2)c1	565045841	25	332.16	3.32
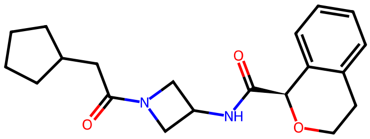	O=C(NC1CN(C(=O)CC2CCCC2)C1)[C@@H]1OCCc2ccccc21	997322863	25	342.19	2.21
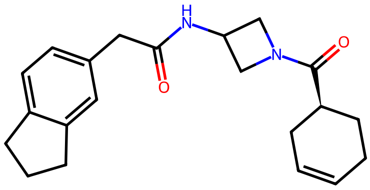	O=C(Cc1ccc2c(c1)CCC2)NC1CN(C(=O)[C@@H]2CC=CCC2)C1	991386947	25	338.2	2.4
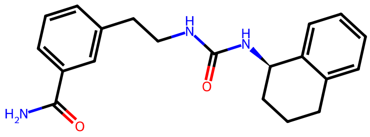	NC(=O)c1cccc(CCNC(=O)N[C@@H]2CCCc3ccccc32)c1	263813591	25	337.18	2.7

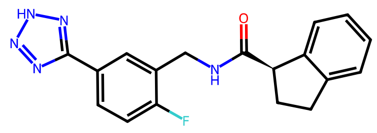	O=C(NCc1cc(-c2nn[nH]n2)ccc1F)[C@@H]1CCc2ccccc21	823235479	25	337.13	2.35
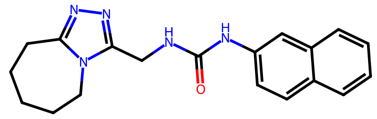	O=C(NCc1nnc2n1CCCCC2)Nc1ccc2ccccc2c1	122601230	25	335.17	3.48
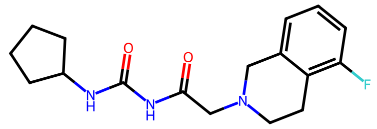	O=C(CN1CCc2c(F)cccc2C1)NC(=O)NC1CCCC1	757144866	23	319.17	1.95
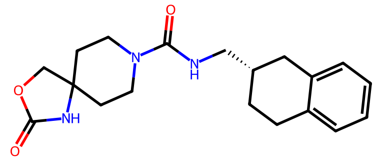	O=C1NC2(CCN(C(=O)NC[C@H]3CCc4ccccc4C3)CC2)CO1	784327057	25	343.19	2.08
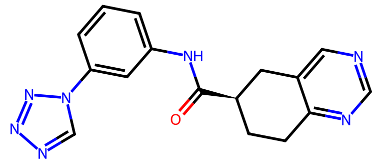	O=C(Nc1cccc(-n2cnnn2)c1)[C@@H]1CCc2ncncc2C1	648220222	24	321.13	1.2
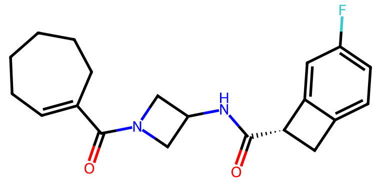	O=C(NC1CN(C(=O)C2=CCCCCC2)C1)[C@H]1Cc2ccc(F)cc21	998489150	25	342.17	2.68
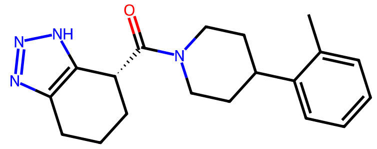	Cc1ccccc1C1CCN(C(=O)[C@@H]2CCCc3nn[nH]c32)CC1	668384717	24	324.2	2.94
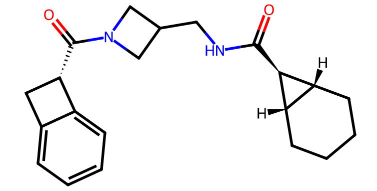	O=C(NCC1CN(C(=O)[C@H]2Cc3ccccc32)C1)[C@@H]1[C@H]2CCCC[C@H]21	1001321386	25	338.2	2.34

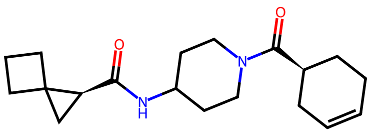	O=C(NC1CCN(C(=O)[C@@H]2CC=CCC2)CC1)[C@H]1CC12CCC2	944127602	23	316.22	2.64
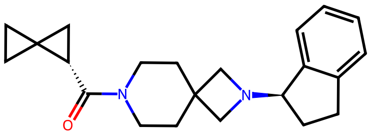	O=C([C@H]1CC12CC2)N1CCC2(CC1)CN([C@@H]1CCc3ccccc31)C2	1035808195	25	336.22	3.4
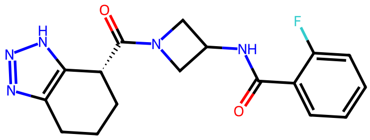	O=C(NC1CN(C(=O)[C@@H]2CCCc3nn[nH]c32)C1)c1ccccc1F	990089867	25	343.14	1.0
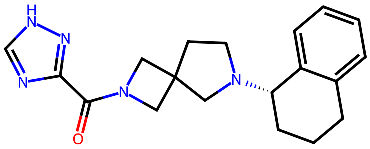	O=C(c1nc[nH]n1)N1CC2(CCN([C@H]3CCCc4ccccc43)C2)C1	1073067071	25	337.19	2.03
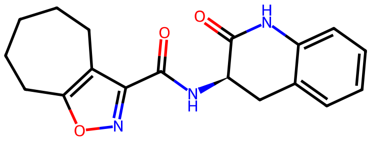	O=C(N[C@@H]1Cc2ccccc2NC1=O)c1noc2c1CCCCC2	529947989	24	325.14	2.24
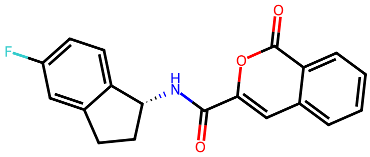	O=C(N[C@@H]1CCc2cc(F)ccc21)c1cc2ccccc2c(=O)o1	352463795	24	323.1	3.35
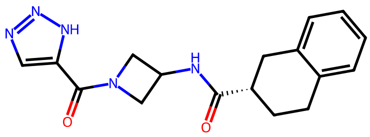	O=C(NC1CN(C(=O)c2cnn[nH]2)C1)[C@H]1CCc2ccccc2C1	1000545764	24	325.15	0.55
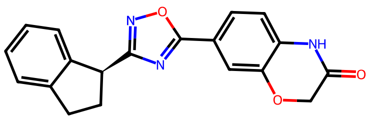	O=C1COc2cc(-c3nc([C@H]4CCc5ccccc54)no3)ccc2N1	356595879	25	333.11	3.15

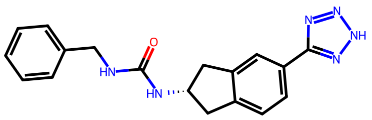	O=C(NCc1ccccc1)N[C@H]1Cc2ccc(-c3nn[nH]n3)cc2C1	1593144215	25	334.15	1.83
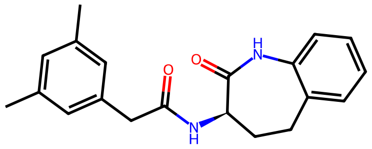	Cc1cc(C)cc(CC(=O)N[C@@H]2CCc3ccccc3NC2=O)c1	616692073	24	322.17	2.92
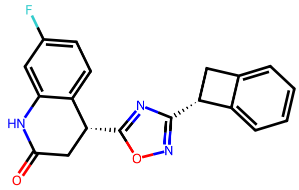	O=C1C[C@@H](c2nc([C@@H]3Cc4ccccc43)no2)c2ccc(F)cc2N1	546296569	25	335.11	3.37
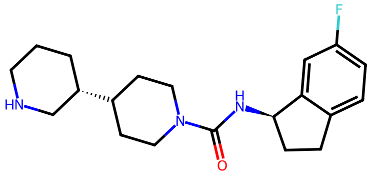	O=C(N[C@@H]1CCc2ccc(F)cc21)N1CCC([C@H]2CCCNC2)CC1	659452477	25	345.22	3.23
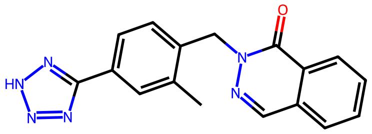	Cc1cc(-c2nn[nH]n2)ccc1Cn1ncc2ccccc2c1=O	1609388847	24	318.12	1.93
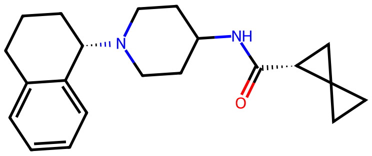	O=C(NC1CCN([C@H]2CCCc3ccccc32)CC1)[C@@H]1CC12CC2	1003695569	24	324.22	3.44
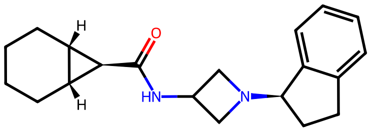	O=C(NC1CN([C@@H]2CCc3ccccc32)C1)[C@@H]1[C@H]2CCCC[C@H]21	1030183256	23	310.2	2.91
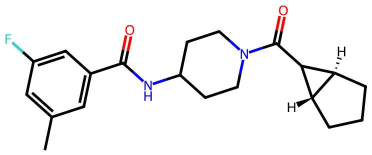	Cc1cc(F)cc(C(=O)NC2CCN(C(=O)C3[C@H]4CCC[C@H]34)CC2)c1	946094226	25	344.19	2.9

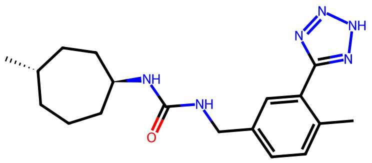	Cc1ccc(CNC(=O)N[C@H]2CCC[C@H](C)CC2)cc1-c1nn[nH]n1	1605852045	25	342.22	2.94
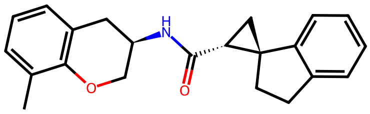	Cc1cccc2c1OC[C@H](NC(=O)[C@@H]1C[C@@]13CCc1ccccc13)C2	564516634	25	333.17	3.32
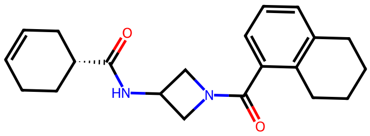	O=C(NC1CN(C(=O)c2cccc3c2CCCC3)C1)[C@H]1CC=CCC1	990215526	25	338.2	2.86
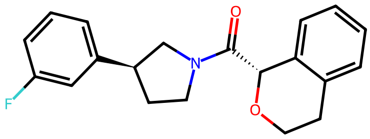	O=C([C@H]1OCCc2ccccc21)N1CC[C@@H](c2cccc(F)c2)C1	490666382	24	325.15	3.46
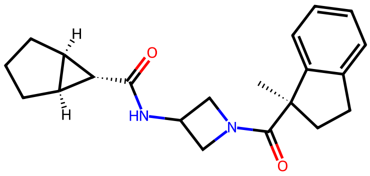	C[C@@]1(C(=O)N2CC(NC(=O)[C@@H]3[C@H]4CCC[C@H]43)C2)CCc2ccccc21	990713325	25	338.2	2.26
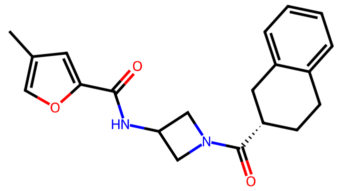	Cc1coc(C(=O)NC2CN(C(=O)[C@H]3CCc4ccccc4C3)C2)c1	997963341	25	338.16	2.33
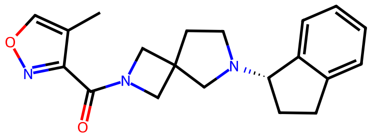	Cc1conc1C(=O)N1CC2(CCN([C@H]3CCc4ccccc43)C2)C1	1073107677	25	337.18	2.82
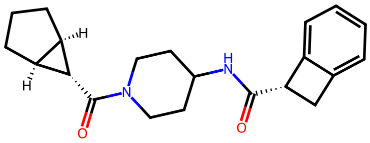	O=C(NC1CCN(C(=O)[C@@H]2[C@H]3CCC[C@H]32)CC1)[C@H]1Cc2ccccc21	946079928	25	338.2	2.48

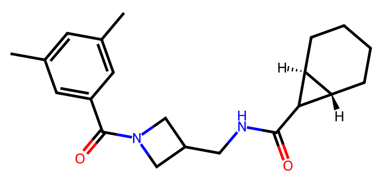	Cc1cc(C)cc(C(=O)N2CC(CNC(=O)C3[C@H]4CCCC[C@H]34)C2)c1	1001322782	25	340.22	2.93
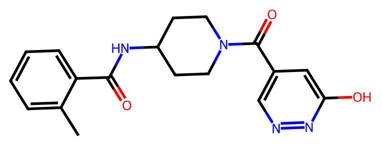	Cc1ccccc1C(=O)NC1CCN(C(=O)c2cnnc(O)c2)CC1	947096266	25	340.15	1.53
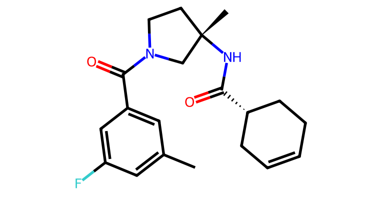	Cc1cc(F)cc(C(=O)N2CC[C@](C)(NC(=O)[C@H]3CC=CCC3)C2)c1	956689891	25	344.19	3.21
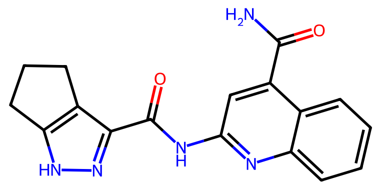	NC(=O)c1cc(NC(=O)c2n[nH]c3c2CCC3)nc2ccccc12	1654748093	24	321.12	1.8
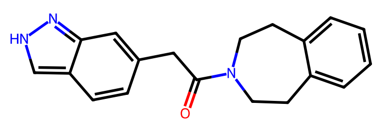	O=C(Cc1ccc2c[nH]nc2c1)N1CCc2ccccc2CC1	554510888	23	305.15	2.73
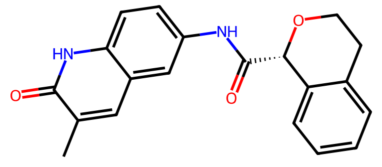	Cc1cc2cc(NC(=O)[C@@H]3OCCc4ccccc43)ccc2[nH]c1=O	1569493787	25	334.13	3.09
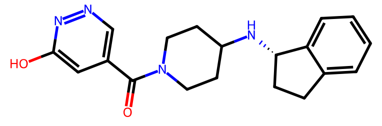	O=C(c1cnnc(O)c1)N1CCC(N[C@H]2CCc3ccccc32)CC1	996701166	25	338.17	2.06
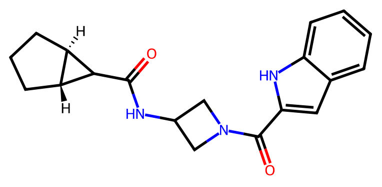	O=C(NC1CN(C(=O)c2cc3ccccc3[nH]2)C1)C1[C@@H]2CCC[C@@H]12	990707910	24	323.16	2.15

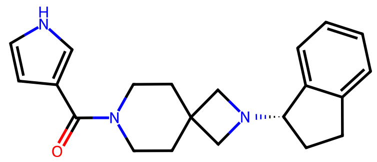	O=C(c1cc[nH]c1)N1CCC2(CC1)CN([C@H]1CCc3ccccc31)C2	1035711817	25	335.2	3.24
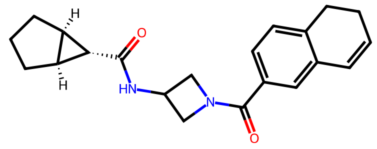	O=C(NC1CN(C(=O)c2ccc3c(c2)C=CCC3)C1)[C@@H]1[C@H]2CCC[C@H]21	990702813	25	336.18	2.63
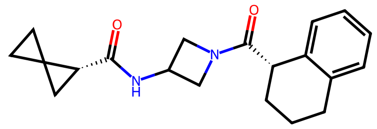	O=C(NC1CN(C(=O)[C@H]2CCCc3ccccc32)C1)[C@@H]1CC12CC2	1000518193	24	324.18	2.23
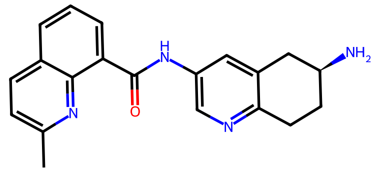	Cc1ccc2cccc(C(=O)Nc3cnc4c(c3)C[C@@H](N)CC4)c2n1	349023167	25	332.16	3.01
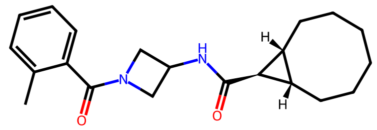	Cc1ccccc1C(=O)N1CC(NC(=O)[C@@H]2[C@H]3CCCCCC[C@H]32)C1	1000466633	25	340.22	3.15
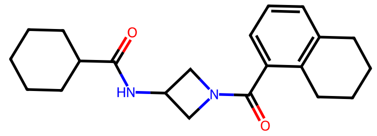	O=C(NC1CN(C(=O)c2cccc3c2CCCC3)C1)C1CCCCC1	990106860	25	340.22	3.09
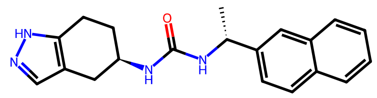	C[C@@H](NC(=O)N[C@@H]1CCc2[nH]ncc2C1)c1ccc2ccccc2c1	154163837	25	334.18	3.48
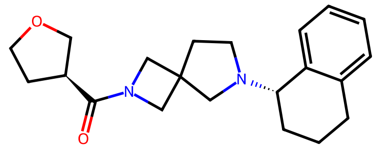	O=C([C@H]1CCOC1)N1CC2(CCN([C@H]3CCCc4ccccc43)C2)C1	1072541613	25	340.22	2.63

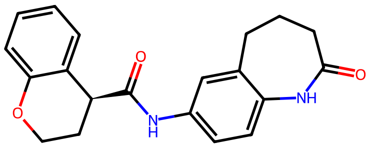	O=C1CCCc2cc(NC(=O)[C@H]3CCOc4ccccc43)ccc2N1	171249909	25	336.15	3.47
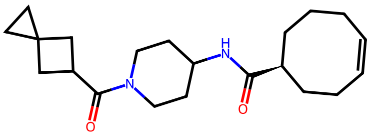	O=C(NC1CCN(C(=O)C2CC3(CC3)C2)CC1)[C@@H]1CCC=CCCC1	948306149	25	344.25	3.42
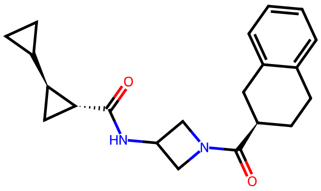	O=C(NC1CN(C(=O)[C@@H]2CCc3ccccc3C2)C1)[C@@H]1C[C@H]1C1CC1	992004870	25	338.2	2.16
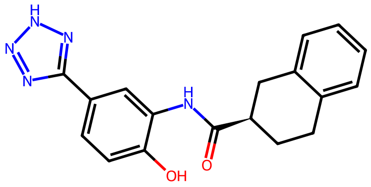	O=C(Nc1cc(-c2nn[nH]n2)ccc1O)[C@@H]1CCc2ccccc2C1	1606897794	25	335.14	2.32
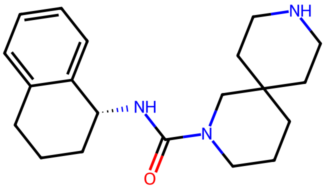	O=C(N[C@@H]1CCCc2ccccc21)N1CCCC2(CCNCC2)C1	1239257788	24	327.23	3.24
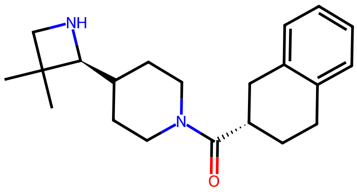	CC1(C)CN[C@H]1C1CCN(C(=O)[C@H]2CCc3ccccc3C2)CC1	653164892	24	326.24	3.03
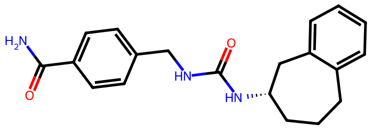	NC(=O)c1ccc(CNC(=O)N[C@H]2CCCc3ccccc3C2)cc1	891954654	25	337.18	2.53
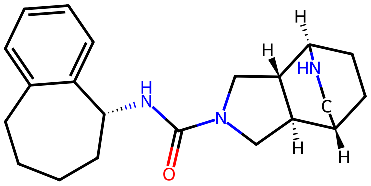	O=C(N[C@@H]1CCCCc2ccccc21)N1C[C@@H]2[C@H]3CC[C@@H](NC3)[C@H]2C1	626961577	25	339.23	3.09

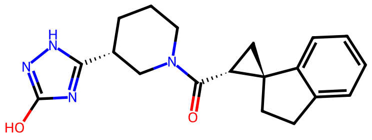	O=C([C@@H]1C[C@@]12CCc1ccccc12)N1CCC[C@@H](c2nc(O)n[nH]2)C1	334018333	25	338.17	2.12
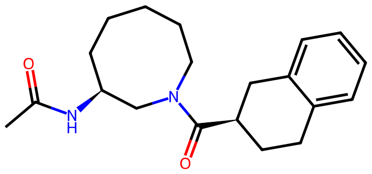	CC(=O)N[C@H]1CCCCCN(C(=O)[C@@H]2CCc3ccccc3C2)C1	987173048	24	328.22	2.7
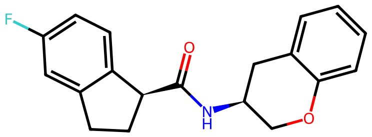	O=C(N[C@@H]1COc2ccccc2C1)[C@H]1CCc2cc(F)ccc21	666390978	23	311.13	2.98
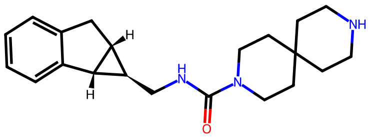	O=C(NC[C@@H]1[C@H]2Cc3ccccc3[C@H]21)N1CCC2(CCNCC2)CC1	417915131	25	339.23	2.75
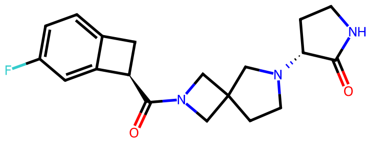	O=C1NCC[C@H]1N1CCC2(CN(C(=O)[C@@H]3Cc4ccc(F)cc43)C2)C1	1072918206	25	343.17	0.89
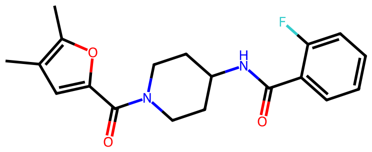	Cc1cc(C(=O)N2CCC(NC(=O)c3ccccc3F)CC2)oc1C	134976840	25	344.15	3.07
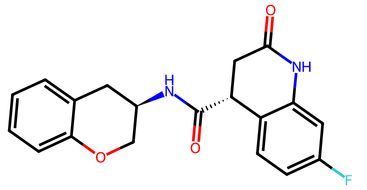	O=C1C[C@@H](C(=O)N[C@H]2COc3ccccc3C2)c2ccc(F)cc2N1	53045173	25	340.12	2.37
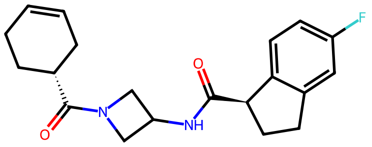	O=C(NC1CN(C(=O)[C@H]2CC=CCC2)C1)[C@@H]1CCc2cc(F)ccc21	996094480	25	342.17	2.54

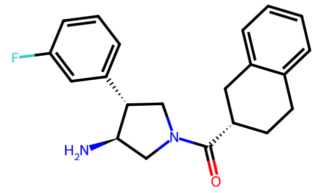	N[C@@H]1CN(C(=O)[C@H]2CCc3ccccc3C2)C[C@H]1c1cccc(F)c1	653164218	25	338.18	2.88
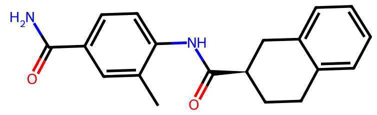	Cc1cc(C(N)=O)ccc1NC(=O)[C@@H]1CCc2ccccc2C1	844329025	23	308.15	2.84
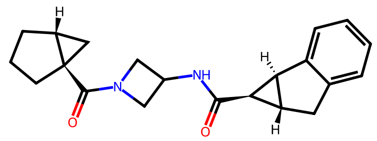	O=C(NC1CN(C(=O)[C@@]23CCC[C@@H]2C3)C1)[C@@H]1[C@H]2Cc3cccc3[C@@H]21	991644144	25	336.18	2.09
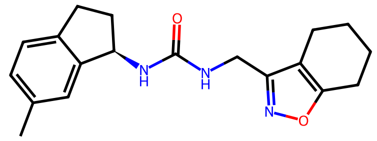	Cc1ccc2c(c1)[C@H](NC(=O)NCc1noc3c1CCCC3)CC2	672840990	24	325.18	3.35
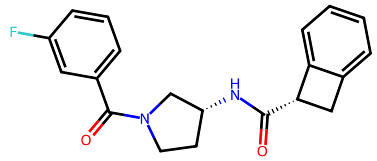	O=C(N[C@@H]1CCN(C(=O)c2cccc(F)c2)C1)[C@H]1Cc2ccccc21	942303319	25	338.14	2.5
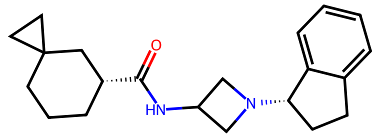	O=C(NC1CN([C@H]2CCc3ccccc32)C1)[C@@H]1CCCC2(CC2)C1	1030307332	24	324.22	3.44
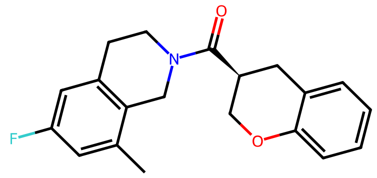	Cc1cc(F)cc2c1CN(C(=O)[C@@H]1COc3ccccc3C1)CC2	501221699	24	325.15	3.27
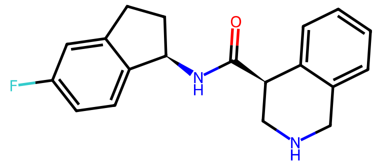	O=C(N[C@@H]1CCc2cc(F)ccc21)[C@@H]1CNCc2ccccc21	580069634	23	310.15	2.82

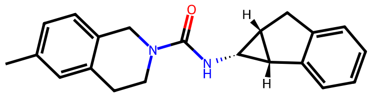	Cc1ccc2c(c1)CCN(C(=O)N[C@@H]1[C@@H]3Cc4ccccc4[C@@H]31)C2	481264536	24	318.17	3.4
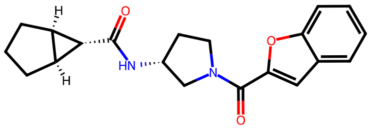	O=C(N[C@@H]1CCN(C(=O)c2cc3ccccc3o2)C1)[C@@H]1[C@H]2CCC[C@H]21	936129672	25	338.16	2.81
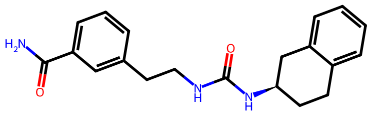	NC(=O)c1cccc(CCNC(=O)N[C@@H]2CCc3ccccc3C2)c1	69591063	25	337.18	2.18
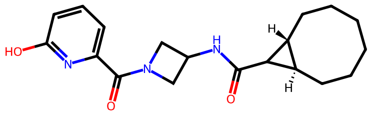	O=C(NC1CN(C(=O)c2cccc(O)n2)C1)C1[C@@H]2CCCCCC[C@@H]12	1000482553	25	343.19	1.94
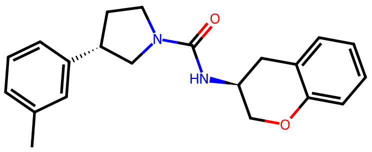	Cc1cccc([C@@H]2CCN(C(=O)N[C@@H]3COc4ccccc4C3)C2)c1	542426543	25	336.18	3.5
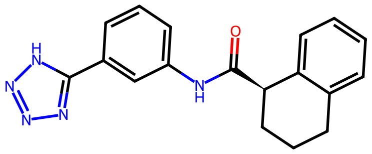	O=C(Nc1cccc(-c2nnn[nH]2)c1)[C@@H]1CCCc2ccccc21	74045546	24	319.14	2.93
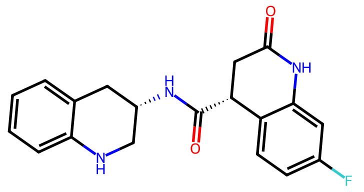	O=C1C[C@@H](C(=O)N[C@@H]2CNc3ccccc3C2)c2ccc(F)cc2N1	653205576	25	339.14	2.4
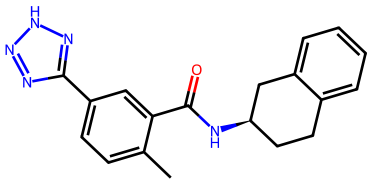	Cc1ccc(-c2nn[nH]n2)cc1C(=O)N[C@@H]1CCc2ccccc2C1	1592098411	25	333.16	2.46

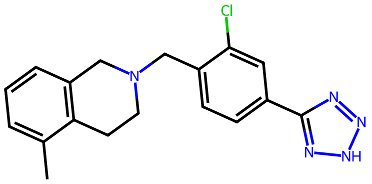	Cc1cccc2c1CCN(Cc1ccc(-c3nn[nH]n3)cc1Cl)C2	1592244355	24	339.13	3.39
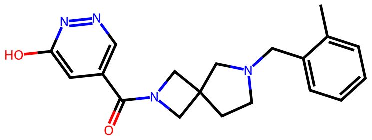	Cc1ccccc1CN1CCC2(C1)CN(C(=O)c1cnnc(O)c1)C2	1072835338	25	338.17	1.84
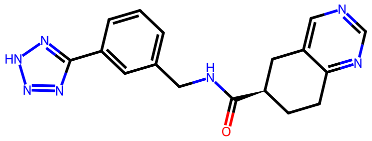	O=C(NCc1cccc(-c2nn[nH]n2)c1)[C@@H]1CCc2ncncc2C1	1593143018	25	335.15	1.08
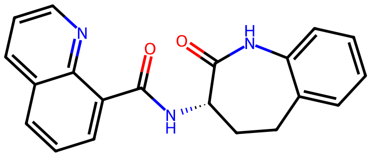	O=C(N[C@H]1CCc2ccccc2NC1=O)c1cccc2cccnc12	1130318488	25	331.13	2.92
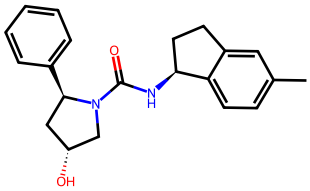	Cc1ccc2c(c1)CC[C@@H]2NC(=O)N1C[C@H](O)C[C@H]1c1ccccc1	413162449	25	336.18	3.5
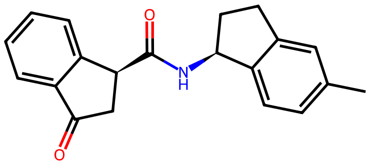	Cc1ccc2c(c1)CC[C@@H]2NC(=O)[C@H]1CC(=O)c2ccccc21	355158832	23	305.14	3.47
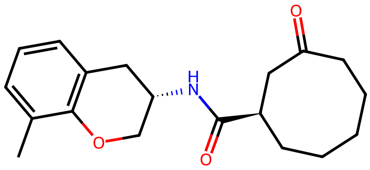	Cc1cccc2c1OC[C@@H](NC(=O)[C@@H]1CCCCCC(=O)C1)C2	517228902	23	315.18	2.95
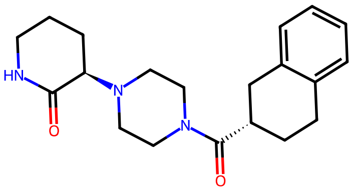	O=C1NCCC[C@H]1N1CCN(C(=O)[C@H]2CCc3ccccc3C2)CC1	950725696	25	341.21	1.21

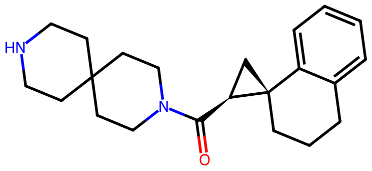	O=C([C@H]1C[C@@]12CCCCc1ccccc12)N1CCC2(CCNCC2)CC1	357887478	25	338.24	3.27
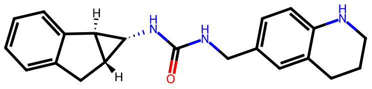	O=C(NCc1ccc2c(c1)CCCN2)N[C@H]1[C@@H]2c3ccccc3C[C@H]21	572264079	25	333.18	3.18
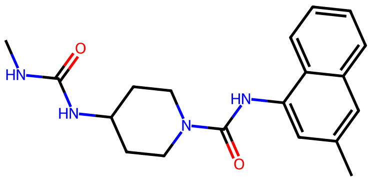	CNC(=O)NC1CCN(C(=O)Nc2cc(C)cc3ccccc23)CC1	417203013	25	340.19	3.07
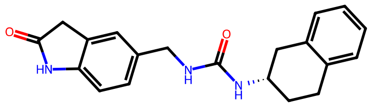	O=C1Cc2cc(CNC(=O)N[C@H]3CCc4ccccc4C3)ccc2N1	1875253024	25	335.16	2.54
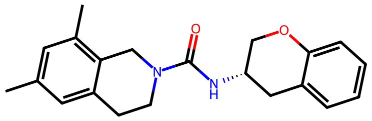	Cc1cc(C)c2c(c1)CCN(C(=O)N[C@@H]1COc3ccccc3C1)C2	1666237929	25	336.18	3.37
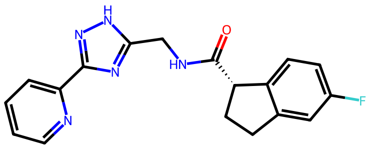	O=C(NCc1nc(-c2ccccn2)n[nH]1)[C@H]1CCc2cc(F)ccc21	1468061322	25	337.13	2.35
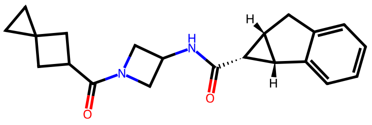	O=C(NC1CN(C(=O)C2CC3(CC3)C2)C1)[C@H]1[C@H]2c3ccccc3C[C@H]21	991645991	25	336.18	2.09
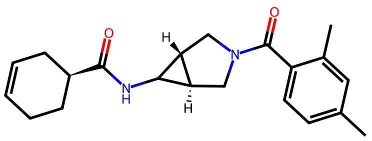	Cc1ccc(C(=O)N2C[C@H]3C(NC(=O)[C@@H]4CC=CCC4)[C@@H]3C2)c(C)c1	1012771052	25	338.2	2.85

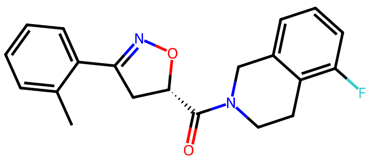	Cc1ccccc1C1=NO[C@H](C(=O)N2CC c3c(F)cccc3C2)C1	1535713680	25	338.14	3.21
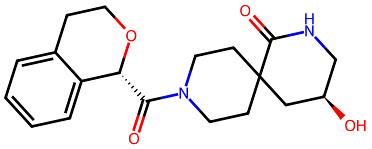	O=C([C@H]1OCCc2ccccc21)N1CCC2 (CC1)C[C@H](O)CNC2=O	885019521	25	344.17	0.79
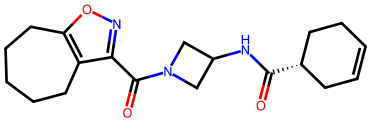	O=C(NC1CN(C(=O)c2noc3c2CCCCC 3)C1)[C@H]1CC=CCC1	990220829	25	343.19	2.24
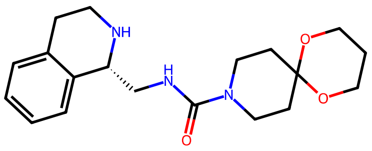	O=C(NC[C@H]1NCCc2ccccc21)N1C CC2(CC1)OCCCO2	841998909	25	345.21	1.81
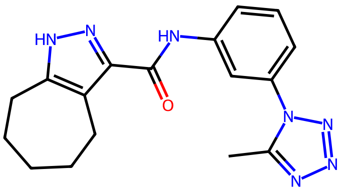	Cc1nnnn1-c1cccc(NC(=O)c2n[nH]c3c 2CCCCC3)c1	190121136	25	337.17	2.22
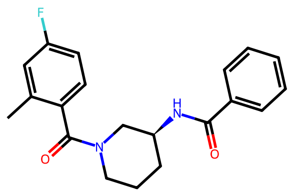	Cc1cc(F)ccc1C(=O)N1CCC[C@H](NC (=O)c2ccccc2)C1	951502821	25	340.16	3.17
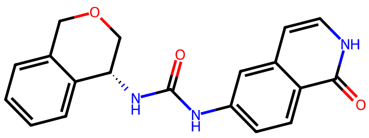	O=C(Nc1ccc2c(=O)[nH]ccc2c1)N[C@ H]1COCc2ccccc21	1672959178	25	335.13	2.92
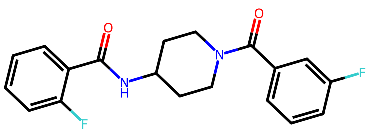	O=C(NC1CCN(C(=O)c2cccc(F)c2)CC 1)c1ccccc1F	75057962	25	344.13	3.0

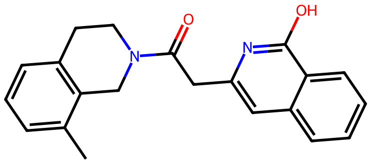	Cc1cccc2c1CN(C(=O)Cc1cc3ccccc3c(O)n1)CC2	1625114807	25	332.15	3.38
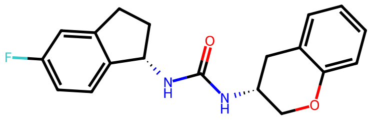	O=C(N[C@H]1CCc2cc(F)ccc21)N[C@H]1COc2ccccc2C1	193788777	24	326.14	3.12
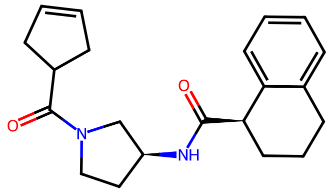	O=C(N[C@H]1CCN(C(=O)C2CC=CC2)C1)[C@@H]1CCCc2ccccc21	938221969	25	338.2	2.79
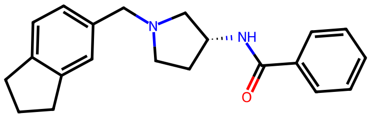	O=C(N[C@@H]1CCN(Cc2ccc3c(c2)CCC3)C1)c1ccccc1	1009949232	24	320.19	3.18
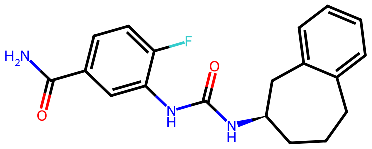	NC(=O)c1ccc(F)c(NC(=O)N[C@@H]2CCCc3ccccc3C2)c1	929838758	25	341.15	2.99
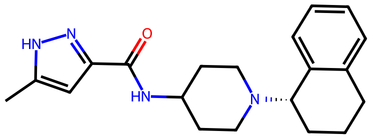	Cc1cc(C(=O)NC2CCN([C@H]3CCCc4ccccc43)CC2)n[nH]1	1002410382	25	338.21	2.99
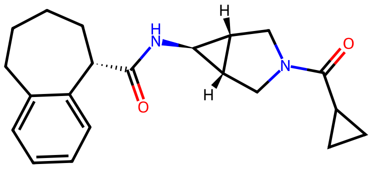	O=C(N[C@H]1[C@@H]2CN(C(=O)C3CC3)C[C@@H]21)[C@H]1CCCCc2ccccc21	976705526	25	338.2	2.48
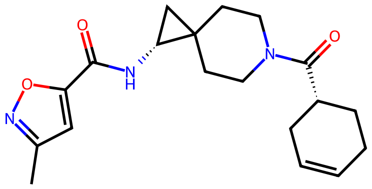	Cc1cc(C(=O)N[C@@H]2CC23CCN(C(=O)[C@H]2CC=CCC2)CC3)on1	963022802	25	343.19	2.45

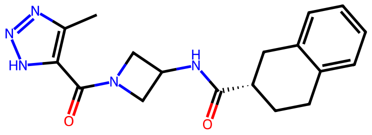	Cc1nn[nH]c1C(=O)N1CC(NC(=O)[C@H]2CCc3ccccc3C2)C1	1000542376	25	339.17	0.86
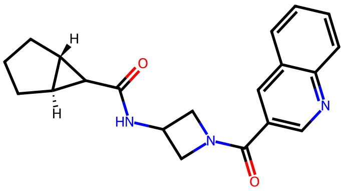	O=C(NC1CN(C(=O)c2cnc3ccccc3c2)C1)C1[C@H]2CCC[C@H]12	990713117	25	335.16	2.22
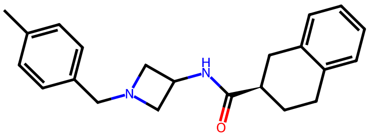	Cc1ccc(CN2CC(NC(=O)[C@@H]3CCc4ccccc4C3)C2)cc1	1031013761	25	334.2	3.1
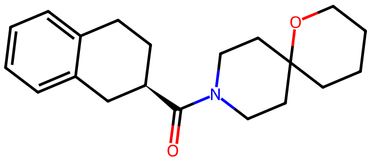	O=C([C@@H]1CCc2ccccc2C1)N1CCC2(CCCCO2)CC1	882441584	23	313.2	3.35
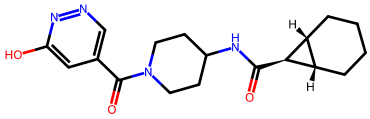	O=C(NC1CCN(C(=O)c2cnnc(O)c2)CC1)[C@@H]1[C@H]2CCCC[C@H]21	947096279	25	344.18	1.34
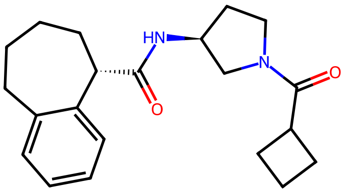	O=C(N[C@H]1CCN(C(=O)C2CCC2)C1)[C@H]1CCCCc2ccccc21	938139962	25	340.22	3.01
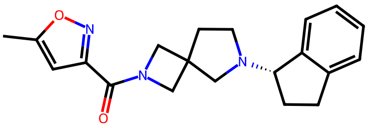	Cc1cc(C(=O)N2CC3(CCN([C@H]4CCc5ccccc54)C3)C2)no1	1072449118	25	337.18	2.82
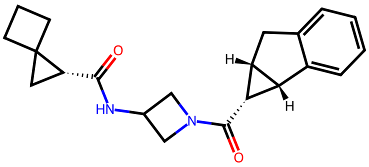	O=C(NC1CN(C(=O)[C@H]2[C@H]3c4ccccc4C[C@H]32)C1)[C@@H]1CC12CCC2	991554849	25	336.18	2.09

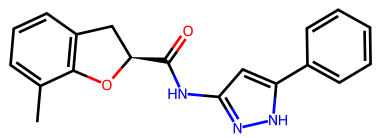	Cc1cccc2c1O[C@H](C(=O)Nc1cc(-c3ccccc3)[nH]n1)C2	1143768601	24	319.13	3.33
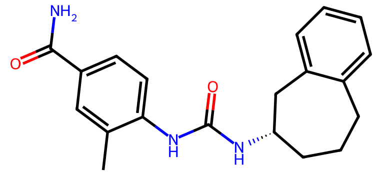	Cc1cc(C(N)=O)ccc1NC(=O)N[C@H]1CCCc2ccccc2C1	929641613	25	337.18	3.16
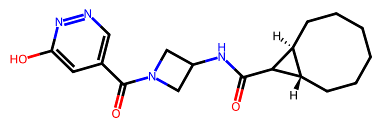	O=C(NC1CN(C(=O)c2cnnc(O)c2)C1)C1[C@H]2CCCCCC[C@H]12	1000510107	25	344.18	1.34
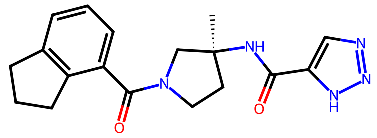	C[C@]1(NC(=O)c2cnn[nH]2)CCN(C(=O)c2cccc3c2CCC3)C1	957187271	25	339.17	1.33
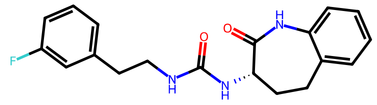	O=C(NCCc1cccc(F)c1)N[C@H]1CCc2ccccc2NC1=O	654356871	25	341.15	2.62
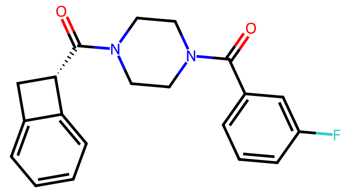	O=C(c1cccc(F)c1)N1CCN(C(=O)[C@H]2Cc3ccccc32)CC1	358262253	25	338.14	2.45
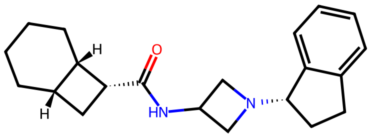	O=C(NC1CN([C@H]2CCc3ccccc32)C1)[C@@H]1C[C@@H]2CCCC[C@@H]21	1030724047	24	324.22	3.3
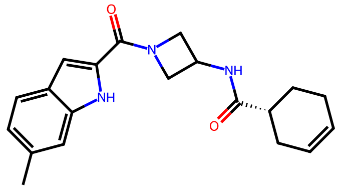	Cc1ccc2cc(C(=O)N3CC(NC(=O)[C@H]4CC=CCC4)C3)[nH]c2c1	990212114	25	337.18	2.77

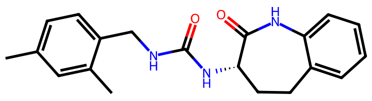	Cc1ccc(CNC(=O)N[C@H]2CCc3ccccc3NC2=O)c(C)c1	654389061	25	337.18	3.06
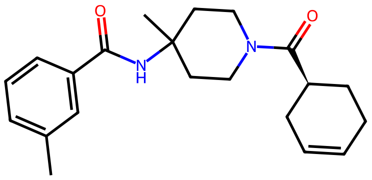	Cc1cccc(C(=O)NC2(C)CCN(C(=O)[C@@H]3CC=CCC3)CC2)c1	956279302	25	340.22	3.46
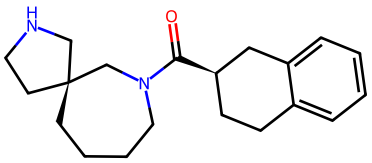	O=C([C@@H]1CCc2ccccc2C1)N1CCCC[C@]2(CCNC2)C1	1550236251	23	312.22	2.78
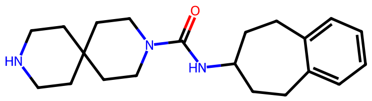	O=C(NC1CCc2ccccc2CC1)N1CCC2(CCNCC2)CC1	418261103	25	341.25	3.11
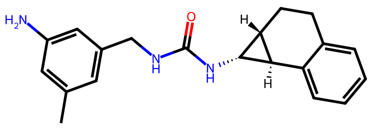	Cc1cc(N)cc(CNC(=O)N[C@H]2[C@@H]3c4ccccc4CC[C@H]32)c1	417916903	24	321.18	3.1
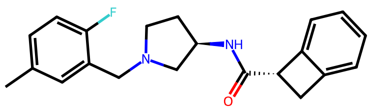	Cc1ccc(F)c(CN2CC[C@@H](NC(=O)[C@H]3Cc4ccccc43)C2)c1	1015632563	25	338.18	3.16
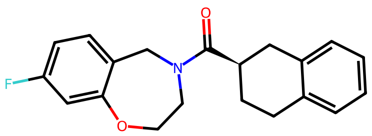	O=C([C@@H]1CCc2ccccc2C1)N1CCOc2cc(F)ccc2C1	629852067	24	325.15	3.35
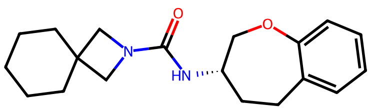	O=C(N[C@H]1CCc2ccccc2OC1)N1CC2(CCCCC2)C1	802404941	23	314.2	3.36

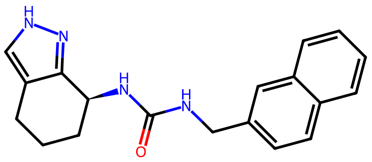	O=C(NCc1ccc2ccccc2c1)N[C@H]1CCCc2c[nH]nc21	372018596	24	320.16	3.44
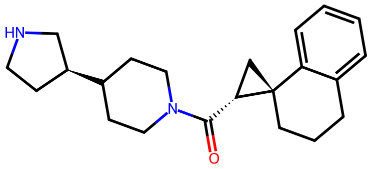	O=C([C@@H]1C[C@@]12CCCc1ccccc12)N1CCC([C@H]2CCNC2)CC1	377808351	25	338.24	3.13
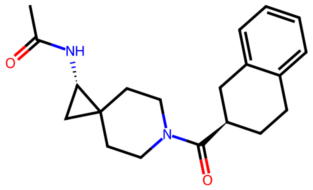	CC(=O)N[C@H]1CC12CCN(C(=O)[C@@H]1CCc3ccccc3C1)CC2	962445458	24	326.2	2.31
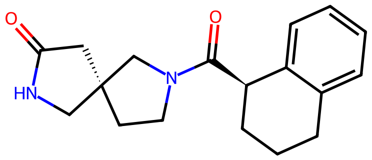	O=C1C[C@]2(CCN(C(=O)[C@@H]3CCCc4ccccc43)C2)CN1	279456493	22	298.17	1.85
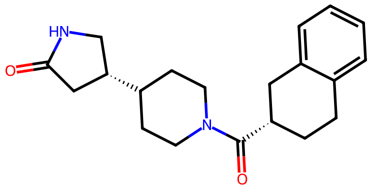	O=C1C[C@@H](C2CCN(C(=O)[C@H]3CCc4ccccc4C3)CC2)CN1	1551708580	24	326.2	2.17
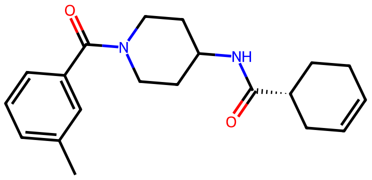	Cc1cccc(C(=O)N2CCC(NC(=O)[C@H]3CC=CCC3)CC2)c1	943794160	24	326.2	3.07
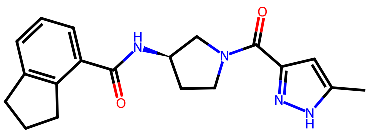	Cc1cc(C(=O)N2CC[C@@H](NC(=O)c3cccc4c3CCC4)C2)n[nH]1	941156705	25	338.17	1.85
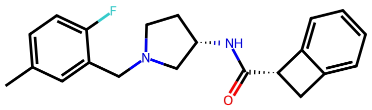	Cc1ccc(F)c(CN2CC[C@H](NC(=O)[C@H]3Cc4ccccc43)C2)c1	1015632554	25	338.18	3.16

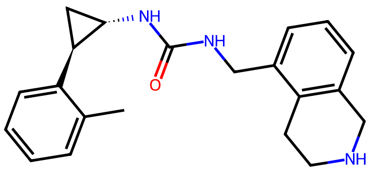	Cc1ccccc1[C@H]1C[C@@H]1NC(=O)NCc1cccc2c1CCNC2	1550270208	25	335.2	3.0
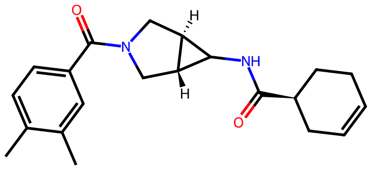	Cc1ccc(C(=O)N2C[C@H]3C(NC(=O)[C@@H]4CC=CCC4)[C@@H]3C2)cc1C	1012765473	25	338.2	2.85
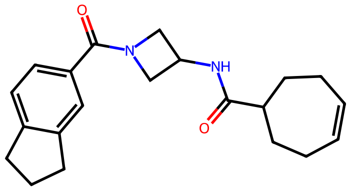	O=C(NC1CN(C(=O)c2ccc3c(c2)CCC3)C1)C1CCC=CCC1	991815125	25	338.2	2.86
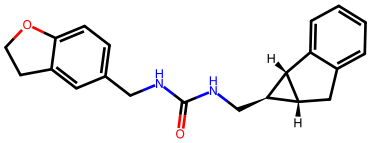	O=C(NCc1ccc2c(c1)CCO2)NC[C@@H]1[C@H]2Cc3ccccc3[C@H]21	532616626	25	334.17	3.01
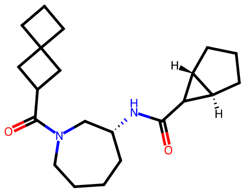	O=C(N[C@@H]1CCCCN(C(=O)C2CC3(CCC3)C2)C1)C1[C@@H]2CCC[C@@H]12	943169091	25	344.25	3.11
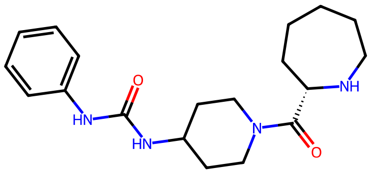	O=C(Nc1ccccc1)NC1CCN(C(=O)[C@@H]2CCCCCN2)CC1	364461116	25	344.22	2.33
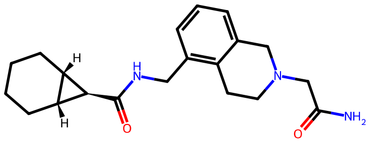	NC(=O)CN1CCc2c(CNC(=O)[C@@H]3[C@H]4CCCC[C@H]43)cccc2C1	1051284064	25	341.21	1.58
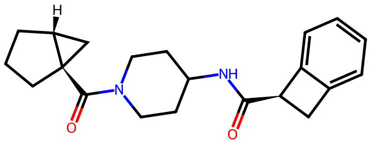	O=C(NC1CCN(C(=O)[C@@]23CCC[C@@H]2C3)CC1)[C@@H]1Cc2ccccc21	947751925	25	338.2	2.62

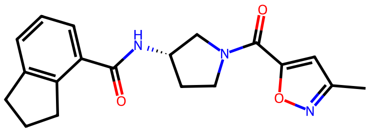	Cc1cc(C(=O)N2CC[C@H](NC(=O)c3cccc4c3CCC4)C2)on1	938146529	25	339.16	2.12
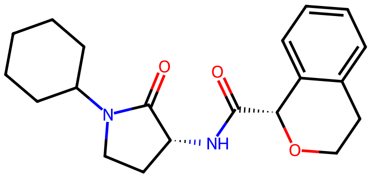	O=C(N[C@@H]1CCN(C2CCCCC2)C1=O)[C@H]1OCCc2ccccc21	124801452	25	342.19	2.35
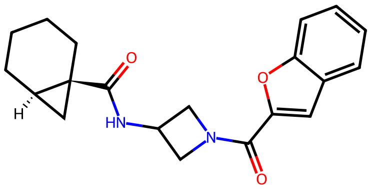	O=C(c1cc2ccccc2o1)N1CC(NC(=O)[C@]23CCCC[C@@H]2C3)C1	999284294	25	338.16	2.95
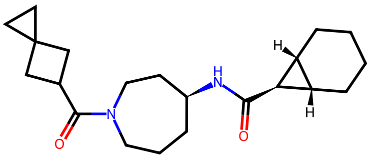	O=C(N[C@H]1CCCN(C(=O)C2CC3(CC3)C2)CC1)[C@@H]1[C@H]2CCCC[C@H]21	964809583	25	344.25	3.11
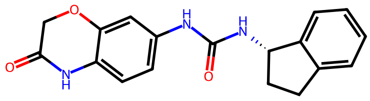	O=C1COc2cc(NC(=O)N[C@H]3CCc4ccccc43)ccc2N1	97463937	24	323.13	2.83
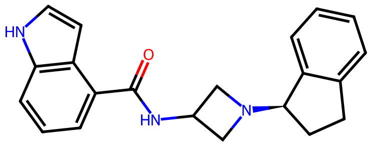	O=C(NC1CN([C@@H]2CCc3ccccc32)C1)c1cccc2[nH]ccc12	1030995496	25	331.17	3.27
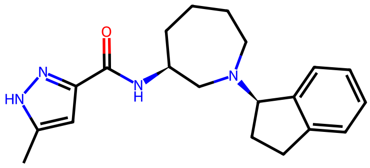	Cc1cc(C(=O)N[C@H]2CCCCN([C@@H]3CCc4ccccc43)C2)n[nH]1	1034123585	25	338.21	2.99
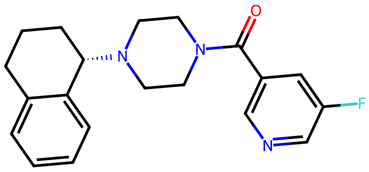	O=C(c1cncc(F)c1)N1CCN([C@H]2CCCc3ccccc32)CC1	1688692837	25	339.17	3.06

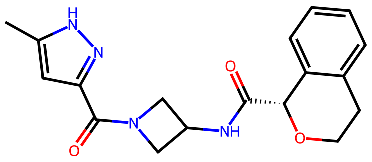	Cc1cc(C(=O)N2CC(NC(=O)[C@H]3OCCc4ccccc43)C2)n[nH]1	997325484	25	340.15	0.97
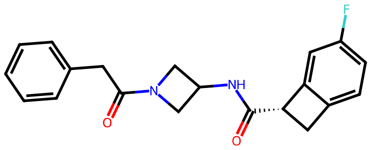	O=C(NC1CN(C(=O)Cc2ccccc2)C1)[C@H]1Cc2ccc(F)cc21	998494330	25	338.14	2.04
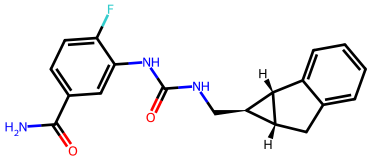	NC(=O)c1ccc(F)c(NC(=O)NC[C@@H]2[C@H]3Cc4ccccc4[C@H]32)c1	178121497	25	339.14	2.63
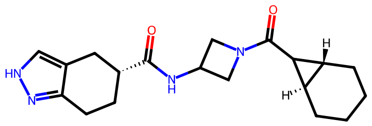	O=C(NC1CN(C(=O)C2[C@@H]3CCCC[C@@H]23)C1)[C@@H]1CCc2n[nH]cc2C1	996397669	25	342.21	1.28
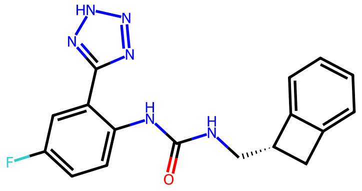	O=C(NC[C@H]1Cc2ccccc21)Nc1ccc(F)cc1-c1nn[nH]n1	823183187	25	338.13	2.47
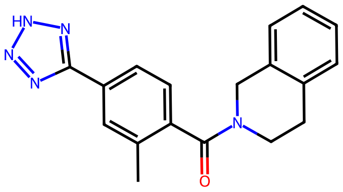	Cc1cc(-c2nn[nH]n2)ccc1C(=O)N1CCc2ccccc2C1	826246130	24	319.14	2.37
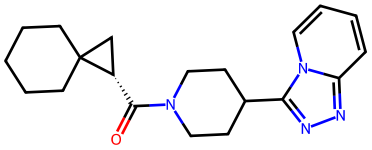	O=C([C@H]1CC12CCCCC2)N1CCC(c2nnc3ccccn23)CC1	370568829	25	338.21	3.41
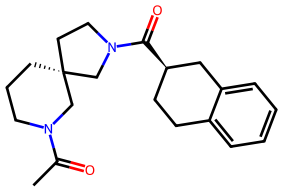	CC(=O)N1CCC[C@@]2(CCN(C(=O)[C@@H]3CCc4ccccc4C3)C2)C1	952958020	25	340.22	2.65

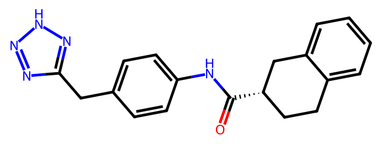	O=C(Nc1ccc(Cc2nn[nH]n2)cc1)[C@H]1CCc2ccccc2C1	568025070	25	333.16	2.53
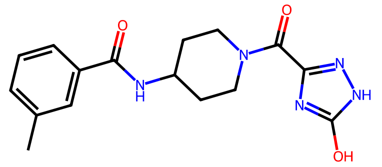	Cc1cccc(C(=O)NC2CCN(C(=O)c3n[nH]c(O)n3)CC2)c1	1364515914	24	329.15	0.85
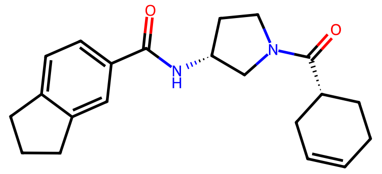	O=C(N[C@@H]1CCN(C(=O)[C@H]2CC=CCC2)C1)c1ccc2c(c1)CCC2	940375756	25	338.2	2.86
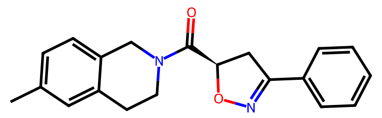	Cc1ccc2c(c1)CCN(C(=O)[C@H]1CC(c3ccccc3)=NO1)C2	790990147	24	320.15	3.07
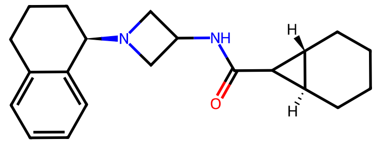	O=C(NC1CN([C@@H]2CCCc3ccccc32)C1)C1[C@@H]2CCCC[C@@H]12	1030187232	24	324.22	3.3
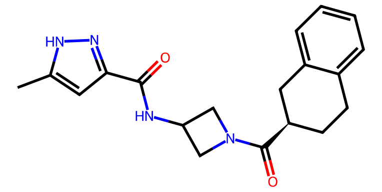	Cc1cc(C(=O)NC2CN(C(=O)[C@@H]3CCc4ccccc4C3)C2)n[nH]1	990382111	25	338.17	1.46
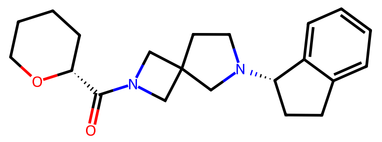	O=C([C@H]1CCCCO1)N1CC2(CCN([C@H]3CCc4ccccc43)C2)C1	1072628539	25	340.22	2.78
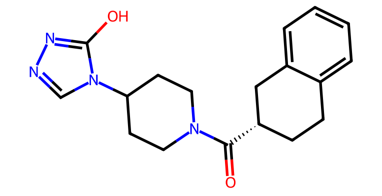	O=C([C@H]1CCc2ccccc2C1)N1CCC(n2cnnc2O)CC1	655042318	24	326.17	1.95

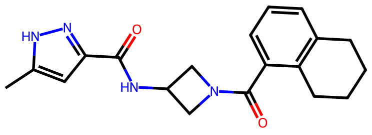	Cc1cc(C(=O)NC2CN(C(=O)c3cccc4c3CCCC4)C2)n[nH]1	990365208	25	338.17	1.85
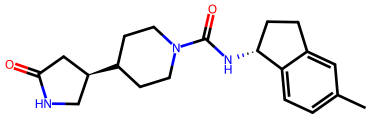	Cc1ccc2c(c1)CC[C@H]2NC(=O)N1CCC([C@H]2CNC(=O)C2)CC1	1553385645	25	341.21	2.54
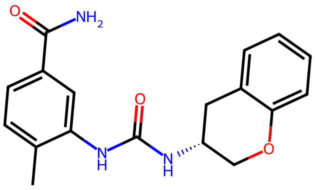	Cc1ccc(C(N)=O)cc1NC(=O)N[C@H]1COc2ccccc2C1	194578170	24	325.14	2.22
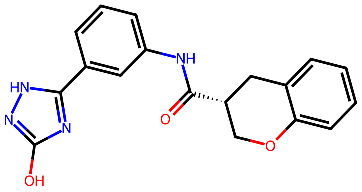	O=C(Nc1cccc(-c2nc(O)n[nH]2)c1)[C@H]1COc2ccccc2C1	377134938	25	336.12	2.37
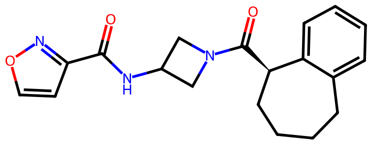	O=C(NC1CN(C(=O)[C@@H]2CCCCc3ccccc32)C1)c1ccon1	990363549	25	339.16	2.13
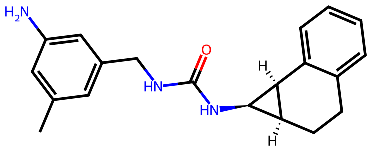	Cc1cc(N)cc(CNC(=O)N[C@@H]2[C@@H]3CCc4ccccc4[C@@H]32)c1	417916901	24	321.18	3.1
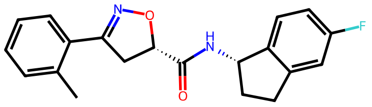	Cc1ccccc1C1=NO[C@H](C(=O)N[C@H]2CCc3cc(F)ccc32)C1	1534972386	25	338.14	3.43
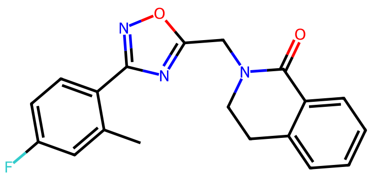	Cc1cc(F)ccc1-c1noc(CN2CCc3ccccc3C2=O)n1	346405534	25	337.12	3.38

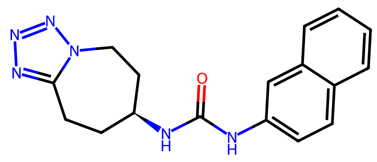	O=C(Nc1ccc2ccccc2c1)N[C@H]1CCc2nnnn2CC1	817296028	24	322.15	2.35
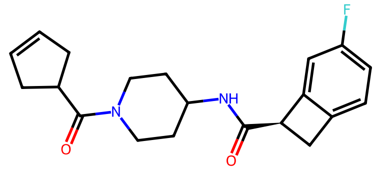	O=C(NC1CCN(C(=O)C2CC=CC2)CC1)[C@@H]1Cc2ccc(F)cc21	945689793	25	342.17	2.54
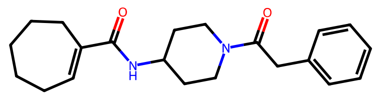	O=C(NC1CCN(C(=O)Cc2ccccc2)CC1)C1=CCCCCC1	947344796	25	340.22	3.23
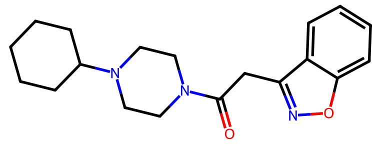	O=C(Cc1noc2ccccc12)N1CCN(C2CCCCC2)CC1	29749468	24	327.19	2.85
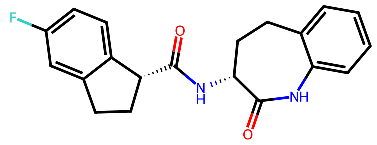	O=C1Nc2ccccc2CC[C@H]1NC(=O)[C@@H]1CCc2cc(F)ccc21	616691829	25	338.14	2.93
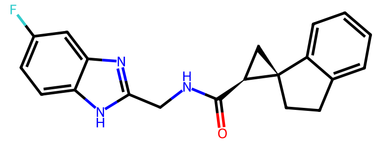	O=C(NCc1nc2cc(F)ccc2[nH]1)[C@H]1C[C@@]12CCc1ccccc12	176114616	25	335.14	3.22
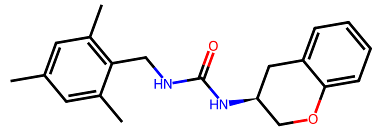	Cc1cc(C)c(CNC(=O)N[C@@H]2COc3ccccc3C2)c(C)c1	195733411	24	324.18	3.41
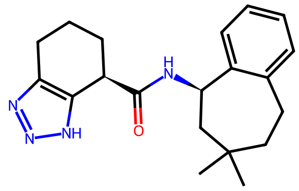	CC1(C)CCc2ccccc2[C@H](NC(=O)[C@@H]2CCCc3nn[nH]c32)C1	646966699	25	338.21	3.44

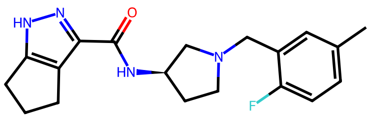	Cc1ccc(F)c(CN2CC[C@@H](NC(=O)c3n[nH]c4c3CCC4)C2)c1	1015068258	25	342.19	2.35
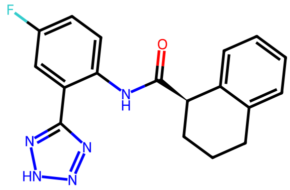	O=C(Nc1ccc(F)cc1-c1nn[nH]n1)[C@@H]1CCCc2ccccc21	823344581	25	337.13	3.06
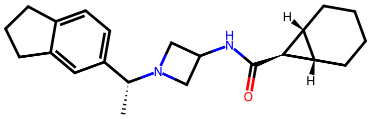	C[C@H](c1ccc2c(c1)CCC2)N1CC(NC(=O)[C@@H]2[C@H]3CCCC[C@H]32)C1	1030186205	25	338.24	3.47
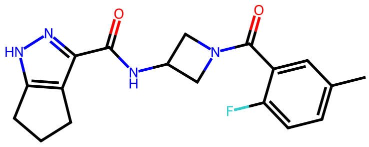	Cc1ccc(F)c(C(=O)N2CC(NC(=O)c3n[nH]c4c3CCC4)C2)c1	995168252	25	342.15	1.6
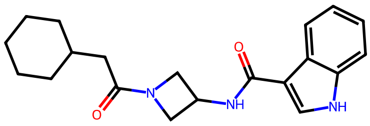	O=C(NC1CN(C(=O)CC2CCCCC2)C1)c1c[nH]c2ccccc12	995032856	25	339.19	3.08
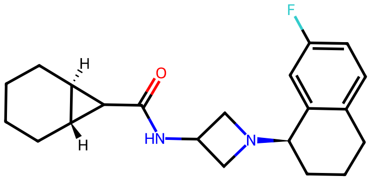	O=C(NC1CN([C@@H]2CCCc3ccc(F)cc32)C1)C1[C@@H]2CCCC[C@@H]12	1030189265	25	342.21	3.44
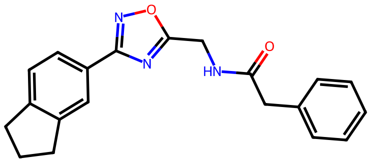	O=C(Cc1ccccc1)NCc1nc(-c2ccc3c(c2)CCC3)no1	576034772	25	333.15	3.08
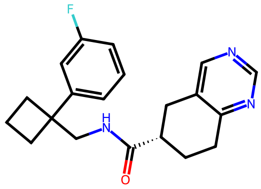	O=C(NCC1(c2cccc(F)c2)CCC1)[C@H]1CCc2ncncc2C1	830870517	25	339.17	2.96

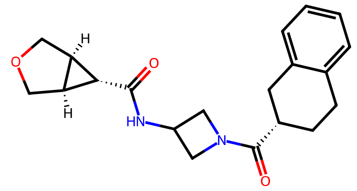	O=C(NC1CN(C(=O)[C@H]2CCc3ccccc3C2)C1)[C@@H]1[C@H]2COC[C@H]21	997944927	25	340.18	1.01
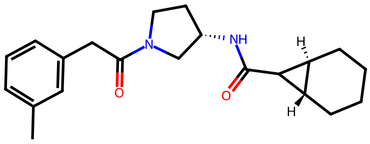	Cc1cccc(CC(=O)N2CC[C@H](NC(=O)C3[C@H]4CCCC[C@H]34)C2)c1	937788591	25	340.22	2.69
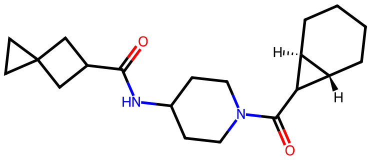	O=C(NC1CCN(C(=O)C2[C@H]3CCCC[C@H]23)CC1)C1CC2(CC2)C1	945958565	24	330.23	2.72
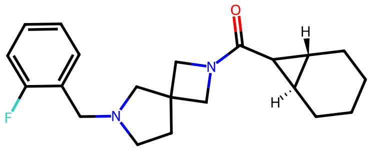	O=C(C1[C@@H]2CCCC[C@@H]12)N1CC2(CCN(Cc3ccccc3F)C2)C1	1072581109	25	342.21	3.3
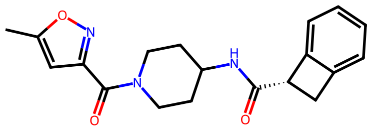	Cc1cc(C(=O)N2CCC(NC(=O)[C@H]3Cc4ccccc43)CC2)no1	943979726	25	339.16	2.04
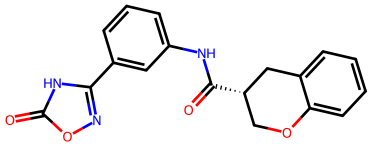	O=C(Nc1cccc(-c2noc(=O)[nH]2)c1)[C@H]1COc2ccccc2C1	69808717	25	337.11	2.22
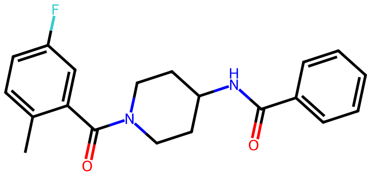	Cc1ccc(F)cc1C(=O)N1CCC(NC(=O)c2ccccc2)CC1	27120311	25	340.16	3.17
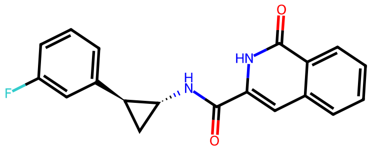	O=C(N[C@@H]1C[C@H]1c1cccc(F)c1)c1cc2ccccc2c(=O)[nH]1	67333743	24	322.11	2.95

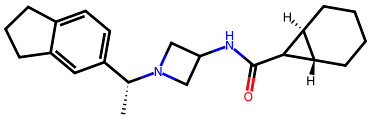	C[C@H](c1ccc2c(c1)CCC2)N1CC(NC(=O)C2[C@H]3CCCC[C@H]23)C1	1030186212	25	338.24	3.47
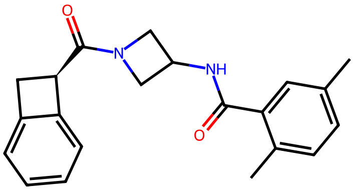	Cc1ccc(C)c(C(=O)NC2CN(C(=O)[C@@H]3Cc4ccccc43)C2)c1	999483658	25	334.17	2.58
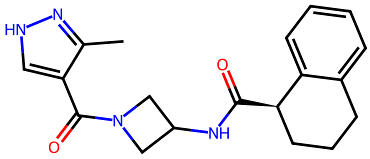	Cc1n[nH]cc1C(=O)N1CC(NC(=O)[C@@H]2CCCc3ccccc32)C1	995075724	25	338.17	1.78
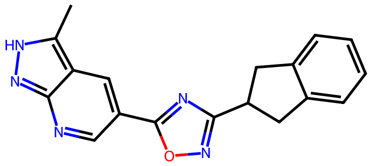	Cc1[nH]nc2ncc(-c3nc(C4Cc5ccccc5C4)no3)cc12	546384871	24	317.13	3.2
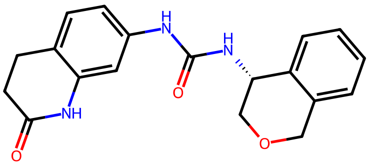	O=C1CCc2ccc(NC(=O)N[C@H]3COCc4ccccc43)cc2N1	562792919	25	337.14	2.96
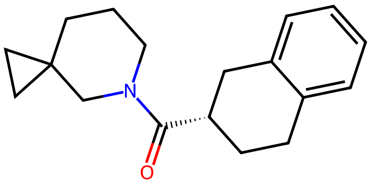	O=C([C@H]1CCc2ccccc2C1)N1CCCC2(CC2)C1	569369612	20	269.18	3.19
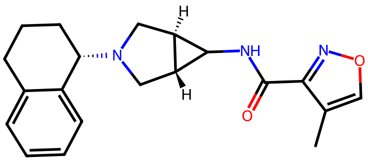	Cc1conc1C(=O)NC1[C@@H]2CN([C@H]3CCCc4ccccc43)C[C@@H]12	962216967	25	337.18	2.72
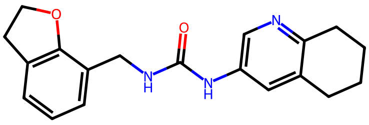	O=C(NCc1cccc2c1OCC2)Nc1cnc2c(c1)CCCC2	1670731120	24	323.16	3.22

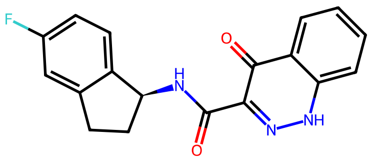	O=C(N[C@H]1CCc2cc(F)ccc21)c1n[nH]c2ccccc2c1=O	111579025	24	323.11	2.48
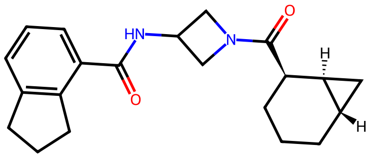	O=C(NC1CN(C(=O)[C@@H]2CCC[C@H]3C[C@@H]32)C1)c1cccc2c1CCC2	994397998	25	338.2	2.55
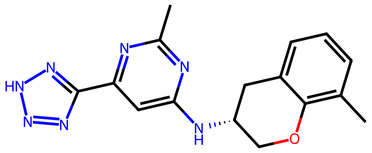	Cc1nc(N[C@H]2COc3c(C)cccc3C2)cc(-c2nn[nH]n2)n1	1606066132	24	323.15	1.69
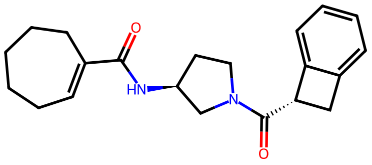	O=C(N[C@H]1CCN(C(=O)[C@H]2Cc3ccccc32)C1)C1=CCCCCC1	937872714	25	338.2	2.93
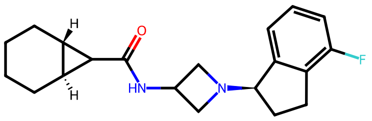	O=C(NC1CN([C@@H]2CCc3c(F)cccc32)C1)C1[C@H]2CCCC[C@H]12	1030184230	24	328.2	3.05
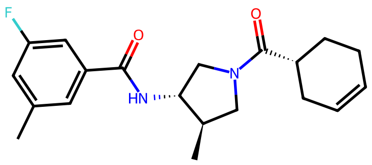	Cc1cc(F)cc(C(=O)N[C@@H]2CN(C(=O)[C@H]3CC=CCC3)C[C@H]2C)c1	940619129	25	344.19	3.07
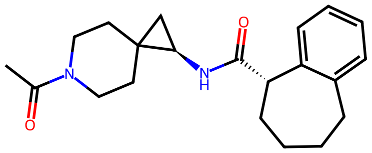	CC(=O)N1CCC2(CC1)C[C@H]2NC(=O)[C@H]1CCCCc2ccccc21	964029775	25	340.22	3.01
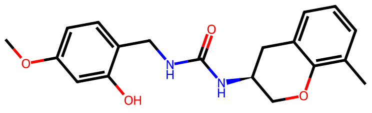	COc1ccc(CNC(=O)N[C@@H]2COc3c(C)cccc3C2)c(O)c1	366068750	25	342.16	2.51

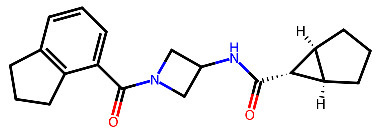	O=C(NC1CN(C(=O)c2cccc3c2CCC3)C1)[C@@H]1[C@H]2CCC[C@H]21	990710312	24	324.18	2.16
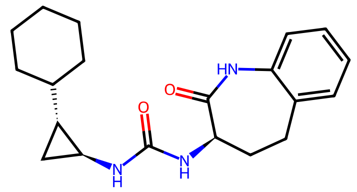	O=C(N[C@H]1C[C@@H]1C1CCCCC1)N[C@@H]1CCc2ccccc2NC1=O	1469095514	25	341.21	3.21
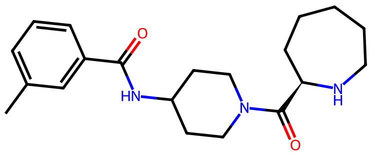	Cc1cccc(C(=O)NC2CCN(C(=O)[C@H]3CCCCCN3)CC2)c1	1550101315	25	343.23	2.25
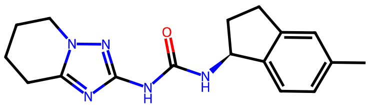	Cc1ccc2c(c1)CC[C@@H]2NC(=O)Nc1nc2n(n1)CCCC2	333229708	23	311.17	2.73
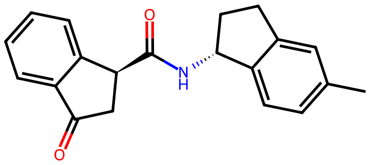	Cc1ccc2c(c1)CC[C@H]2NC(=O)[C@H]1CC(=O)c2ccccc21	355158833	23	305.14	3.47
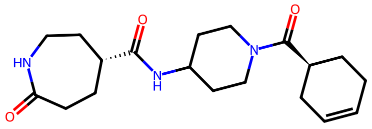	O=C1CC[C@@H](C(=O)NC2CCN(C(=O)[C@@H]3CC=CCC3)CC2)CCN1	944127270	25	347.22	1.37
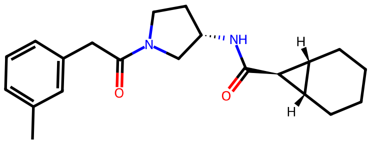	Cc1cccc(CC(=O)N2CC[C@H](NC(=O)[C@@H]3[C@H]4CCCC[C@H]43)C2)c1	937788592	25	340.22	2.69
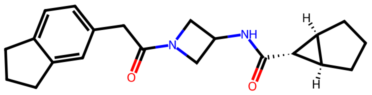	O=C(NC1CN(C(=O)Cc2ccc3c(c2)CCC3)C1)[C@@H]1[C@H]2CCC[C@H]21	990710935	25	338.2	2.09

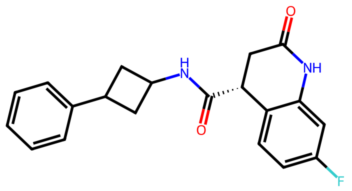	O=C1C[C@@H](C(=O)NC2CC(c3ccccc3)C2)c2ccc(F)cc2N1	264006818	25	338.14	3.31
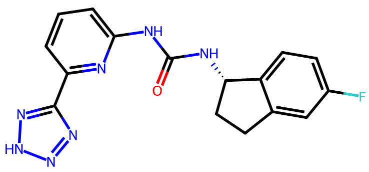	O=C(Nc1cccc(-c2nn[nH]n2)n1)N[C@H]1CCc2cc(F)ccc21	1582645468	25	339.12	2.21
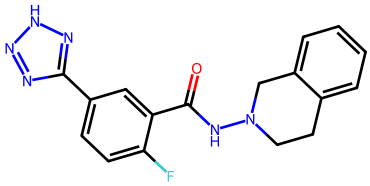	O=C(NN1CCc2ccccc2C1)c1cc(-c2nn[nH]n2)ccc1F	1582561413	25	338.13	1.71
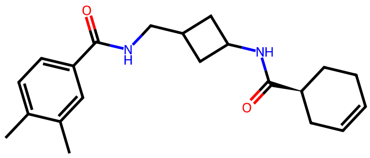	Cc1ccc(C(=O)NCC2CC(NC(=O)[C@@H]3CC=CCC3)C2)cc1C	992390610	25	340.22	3.28
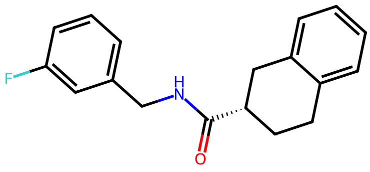	O=C(NCc1cccc(F)c1)[C@H]1CCc2ccccc2C1	583503197	21	283.14	3.25
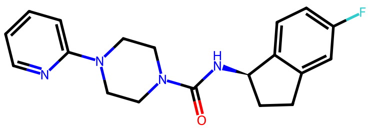	O=C(N[C@@H]1CCc2cc(F)ccc21)N1CCN(c2ccccn2)CC1	56838684	25	340.17	2.74
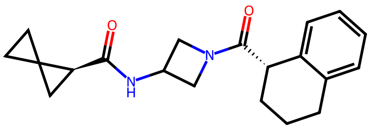	O=C(NC1CN(C(=O)[C@H]2CCCc3ccccc32)C1)[C@H]1CC12CC2	1000518191	24	324.18	2.23
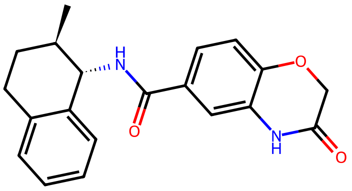	C[C@@H]1CCc2ccccc2[C@H]1NC(=O)c1ccc2c(c1)NC(=O)CO2	122492544	25	336.15	3.07

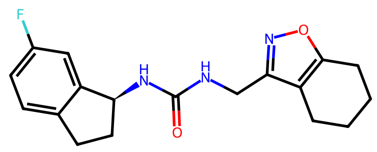	O=C(NCc1noc2c1CCCC2)N[C@H]1CCc2ccc(F)cc21	565617191	24	329.15	3.18
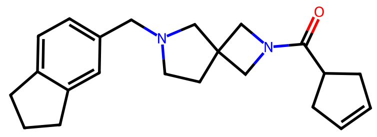	O=C(C1CC=CC1)N1CC2(CCN(Cc3ccc4c(c3)CCC4)C2)C1	1072568500	25	336.22	3.18
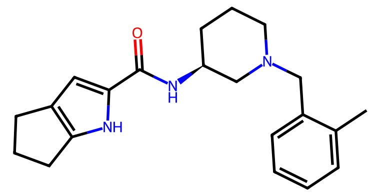	Cc1ccccc1CN1CCC[C@H](NC(=O)c2cc3c([nH]2)CCC3)C1	1009163384	25	337.22	3.21
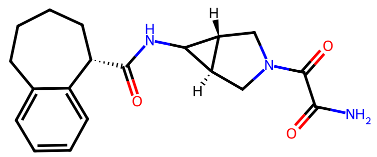	NC(=O)C(=O)N1C[C@H]2C(NC(=O)[C@H]3CCCCc4ccccc43)[C@@H]2C1	1013287964	25	341.17	0.55
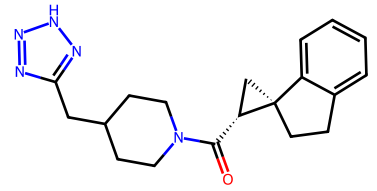	O=C([C@@H]1C[C@]12CCc1ccccc12)N1CCC(Cc2nn[nH]n2)CC1	1548804792	25	337.19	1.88
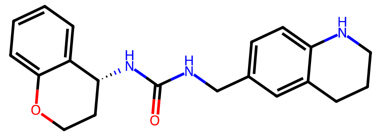	O=C(NCc1ccc2c(c1)CCCN2)N[C@@H]1CCOc2ccccc21	668620532	25	337.18	3.37
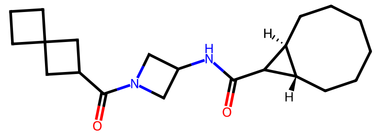	O=C(NC1CN(C(=O)C2CC3(CCC3)C2)C1)C1[C@H]2CCCCCC[C@H]12	1000507789	25	344.25	3.11
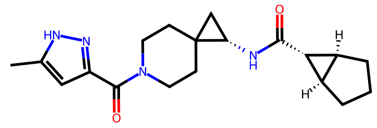	Cc1cc(C(=O)N2CCC3(CC2)C[C@@H]3NC(=O)[C@@H]2[C@H]3CCC[C@H]32)n[nH]1	963468256	25	342.21	1.88

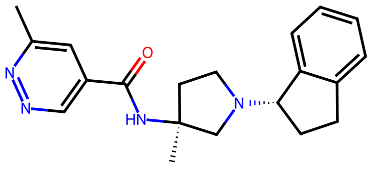	Cc1cc(C(=O)N[C@@]2(C)CCN([C@H]3CCc4ccccc43)C2)cnn1	1046269700	25	336.2	2.67
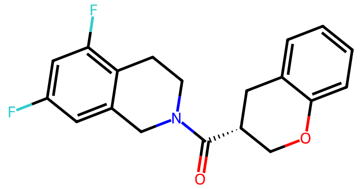	O=C([C@H]1COc2ccccc2C1)N1CCc2c(F)cc(F)cc2C1	361059066	24	329.12	3.1
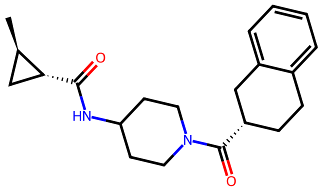	C[C@@H]1C[C@H]1C(=O)NC1CCN(C(=O)[C@H]2CCc3ccccc3C2)CC1	948572112	25	340.22	2.55
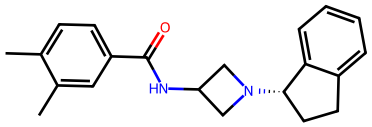	Cc1ccc(C(=O)NC2CN([C@H]3CCc4ccccc43)C2)cc1C	1031344390	24	320.19	3.4
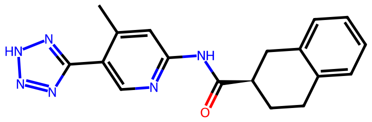	Cc1cc(NC(=O)[C@@H]2CCc3ccccc3C2)ncc1-c1nn[nH]n1	1592018414	25	334.15	2.31
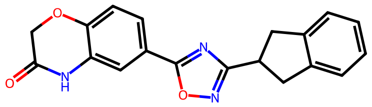	O=C1COc2ccc(-c3nc(C4Cc5ccccc5C4)no3)cc2N1	544557553	25	333.11	2.95
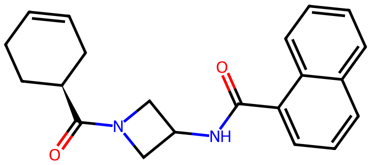	O=C(NC1CN(C(=O)[C@@H]2CC=CCC2)C1)c1cccc2ccccc12	999544541	25	334.17	3.14
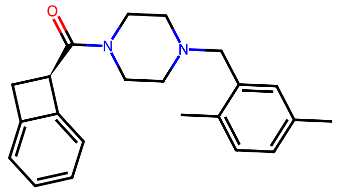	Cc1ccc(C)c(CN2CCN(C(=O)[C@@H]3Cc4ccccc43)CC2)c1	949948882	25	334.2	3.29

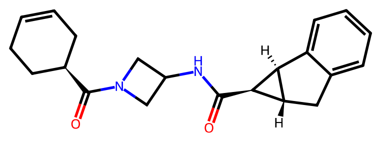	O=C(NC1CN(C(=O)[C@@H]2CC=CCC2)C1)[C@@H]1[C@H]2Cc3ccccc3[C@@H]21	991655693	25	336.18	2.26
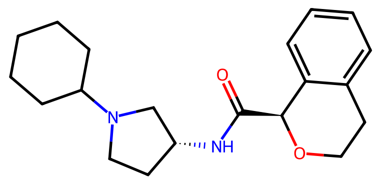	O=C(N[C@@H]1CCN(C2CCCCC2)C1)[C@@H]1OCCc2ccccc21	618663742	24	328.22	2.82
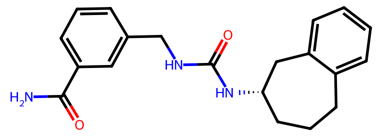	NC(=O)c1cccc(CNC(=O)N[C@H]2CCCc3ccccc3C2)c1	1551219726	25	337.18	2.53
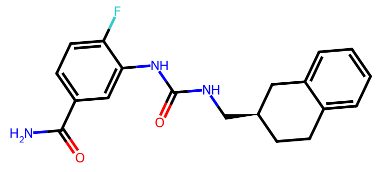	NC(=O)c1ccc(F)c(NC(=O)NC[C@@H]2CCc3ccccc3C2)c1	605030401	25	341.15	2.85
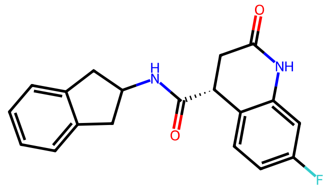	O=C1C[C@@H](C(=O)NC2Cc3ccccc3C2)c2ccc(F)cc2N1	185727584	24	324.13	2.54
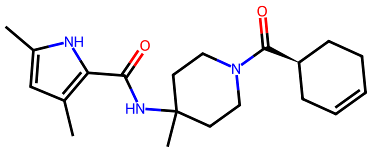	Cc1cc(C)c(C(=O)NC2(C)CCN(C(=O)[C@@H]3CC=CCC3)CC2)[nH]1	956419981	25	343.23	3.1
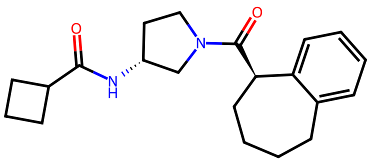	O=C(N[C@@H]1CCN(C(=O)[C@@H]2CCCCc3ccccc32)C1)C1CCC1	939032584	25	340.22	3.01
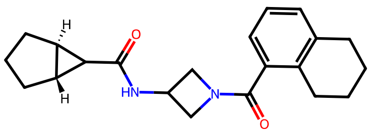	O=C(NC1CN(C(=O)c2cccc3c2CCCC3)C1)C1[C@@H]2CCC[C@@H]12	990696643	25	338.2	2.55

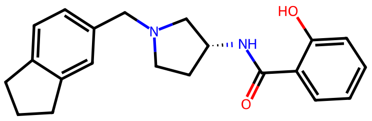	O=C(N[C@@H]1CCN(Cc2ccc3c(c2)CCC3)C1)c1ccccc1O	1010275146	25	336.18	2.89
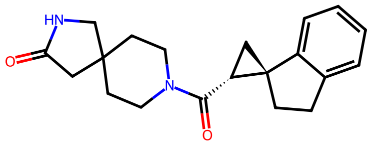	O=C1CC2(CCN(C(=O)[C@@H]3C[C@@]34CCc3ccccc34)CC2)CN1	267619867	24	324.18	2.02
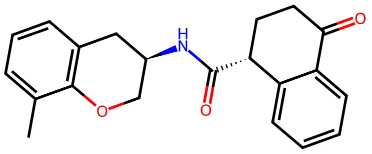	Cc1cccc2c1OC[C@H](NC(=O)[C@@H]1CCC(=O)c3ccccc31)C2	291649053	25	335.15	3.18
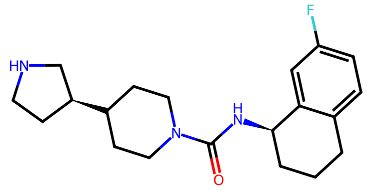	O=C(N[C@@H]1CCCc2ccc(F)cc21)N1CCC([C@H]2CCNC2)CC1	418118511	25	345.22	3.23
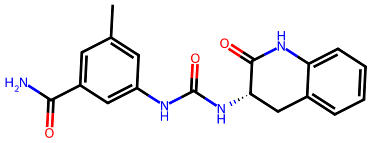	Cc1cc(NC(=O)N[C@H]2Cc3ccccc3NC2=O)cc(C(N)=O)c1	1365201181	25	338.14	1.78
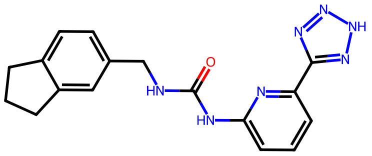	O=C(NCc1ccc2c(c1)CCC2)Nc1cccc(-c2nn[nH]n2)n1	1582551876	25	335.15	2.07
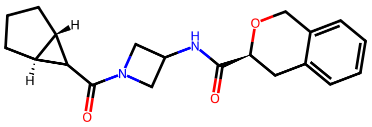	O=C(NC1CN(C(=O)C2[C@H]3CCC[C@H]23)C1)[C@@H]1Cc2ccccc2CO1	997733894	25	340.18	1.5
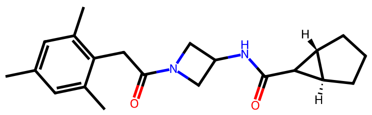	Cc1cc(C)c(CC(=O)N2CC(NC(=O)C3[C@@H]4CCC[C@@H]34)C2)c(C)c1	990690071	25	340.22	2.53

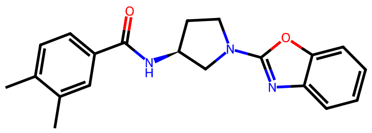	Cc1ccc(C(=O)N[C@H]2CCN(c3nc4ccccc4o3)C2)cc1C	1058361625	25	335.16	3.45
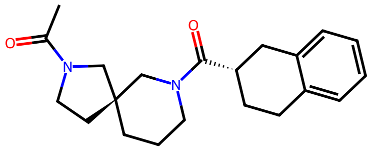	CC(=O)N1CC[C@@]2(CCCN(C(=O)[C@H]3CCc4ccccc4C3)C2)C1	952819500	25	340.22	2.65
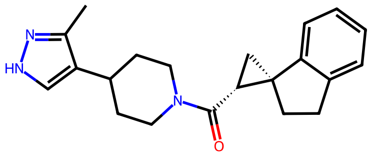	Cc1n[nH]cc1C1CCN(C(=O)[C@@H]2C[C@]23CCc2ccccc23)CC1	375990269	25	335.2	3.33
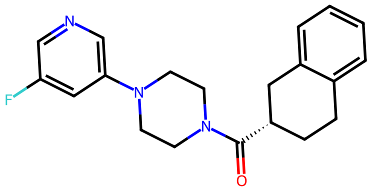	O=C([C@H]1CCc2ccccc2C1)N1CCN(c2cncc(F)c2)CC1	655033324	25	339.17	2.67
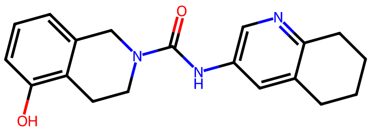	O=C(Nc1cnc2c(c1)CCCC2)N1CCc2c(O)cccc2C1	626489596	24	323.16	3.26
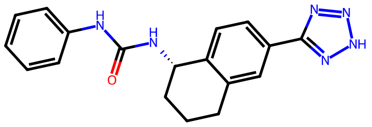	O=C(Nc1ccccc1)N[C@H]1CCCc2cc(-c3nn[nH]n3)ccc21	1593251334	25	334.15	3.07
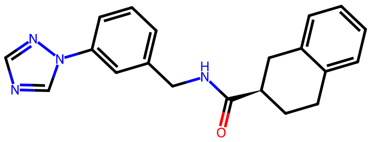	O=C(NCc1cccc(-n2cncn2)c1)[C@@H]1CCc2ccccc2C1	573254256	25	332.16	2.69
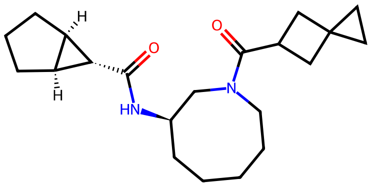	O=C(N[C@@H]1CCCCCN(C(=O)C2CC3(CC3)C2)C1)[C@@H]1[C@H]2CCC[C@H]21	987150764	25	344.25	3.11

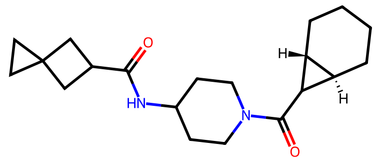	O=C(NC1CCN(C(=O)C2[C@@H]3CCCC[C@@H]23)CC1)C1CC2(CC2)C1	945958566	24	330.23	2.72
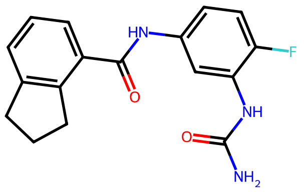	NC(=O)Nc1cc(NC(=O)c2cccc3c2CCC3)ccc1F	350285872	23	313.12	3.06
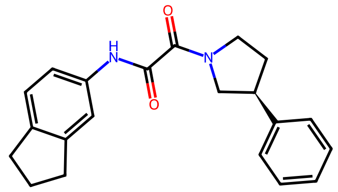	O=C(Nc1ccc2c(c1)CCC2)C(=O)N1CC[C@@H](c2ccccc2)C1	126070956	25	334.17	3.13
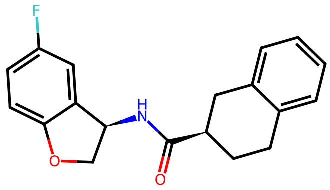	O=C(N[C@H]1COc2ccc(F)cc21)[C@@H]1CCc2ccccc2C1	573807408	23	311.13	3.18
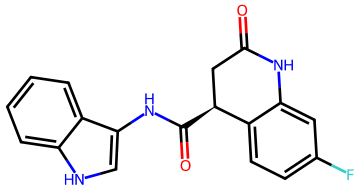	O=C1C[C@H](C(=O)Nc2c[nH]c3ccccc23)c2ccc(F)cc2N1	1645449071	24	323.11	3.37
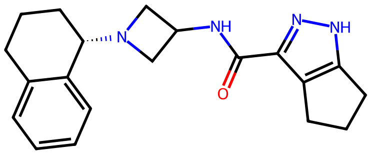	O=C(NC1CN([C@H]2CCCc3ccccc32)C1)c1n[nH]c2c1CCC2	1030597967	25	336.2	2.39
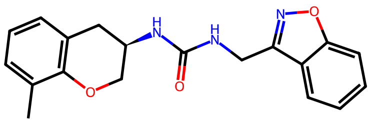	Cc1cccc2c1OC[C@H](NC(=O)NCc1noc3ccccc13)C2	654568390	25	337.14	2.94
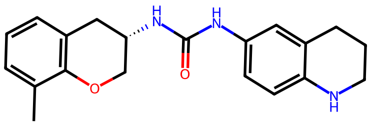	Cc1cccc2c1OC[C@@H](NC(=O)Nc1ccc3c(c1)CCCN3)C2	415867449	25	337.18	3.48

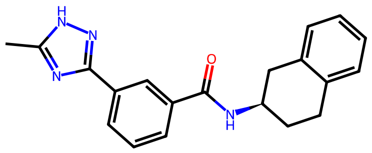	Cc1nc(-c2cccc(C(=O)N[C@@H]3CCc4ccccc4C3)c2)n[nH]1	81112571	25	332.16	3.07
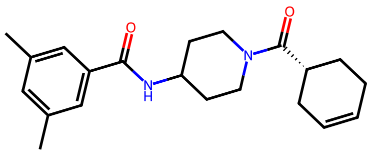	Cc1cc(C)cc(C(=O)NC2CCN(C(=O)[C@H]3CC=CCC3)CC2)c1	944106875	25	340.22	3.38
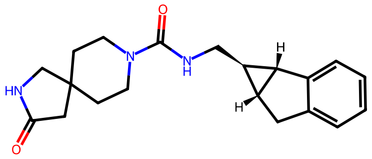	O=C1CC2(CCN(C(=O)NC[C@@H]3[C@H]4Cc5ccccc5[C@H]43)CC2)CN1	269043545	25	339.19	1.88
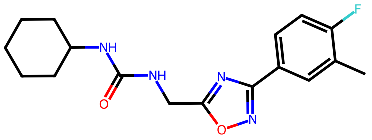	Cc1cc(-c2noc(CNC(=O)NC3CCCCC3)n2)ccc1F	109558209	24	332.16	3.32
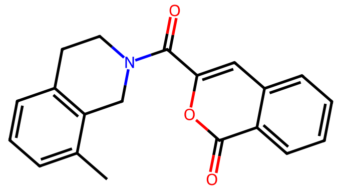	Cc1cccc2c1CN(C(=O)c1cc3ccccc3c(=O)o1)CC2	337011067	24	319.12	3.3
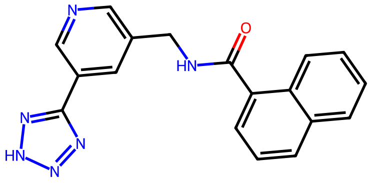	O=C(NCc1cncc(-c2nn[nH]n2)c1)c1cccc2ccccc12	1593148624	25	330.12	2.34
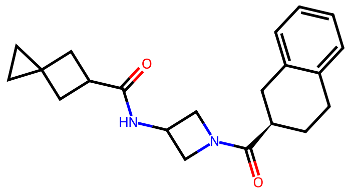	O=C(NC1CN(C(=O)[C@@H]2CCc3ccccc3C2)C1)C1CC2(CC2)C1	999346679	25	338.2	2.31
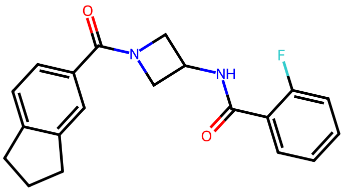	O=C(NC1CN(C(=O)c2ccc3c(c2)CCC3)C1)c1ccccc1F	990095004	25	338.14	2.57

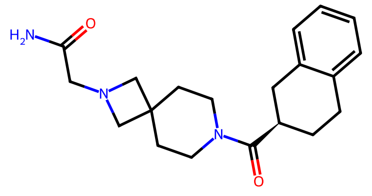	NC(=O)CN1CC2(CCN(C(=O)[C@@H]3CCc4ccccc4C3)CC2)C1	1035805243	25	341.21	1.2
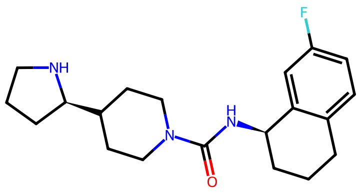	O=C(N[C@@H]1CCCc2ccc(F)cc21)N1CCC([C@H]2CCCN2)CC1	418114492	25	345.22	3.38
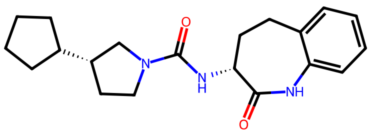	O=C1Nc2ccccc2CC[C@H]1NC(=O)N1CC[C@H](C2CCCC2)C1	654631104	25	341.21	3.16
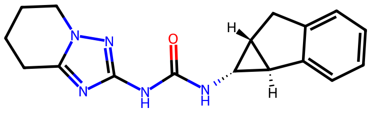	O=C(Nc1nc2n(n1)CCCC2)N[C@H]1[C@@H]2c3ccccc3C[C@H]21	653706406	23	309.16	2.07
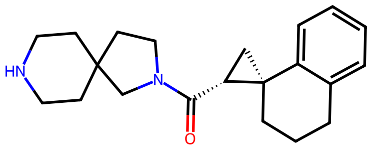	O=C([C@@H]1C[C@]12CCCc1ccccc12)N1CCC2(CCNCC2)C1	378404695	24	324.22	2.88
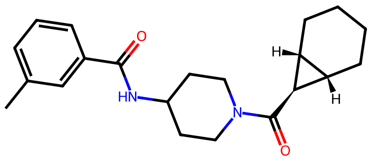	Cc1cccc(C(=O)NC2CCN(C(=O)[C@@H]3[C@H]4CCCC[C@H]43)CC2)c1	870834264	25	340.22	3.15
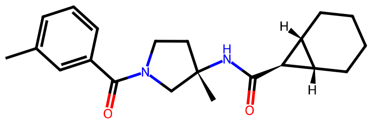	Cc1cccc(C(=O)N2CC[C@](C)(NC(=O)[C@@H]3[C@H]4CCCC[C@H]43)C2)c1	956790398	25	340.22	3.15
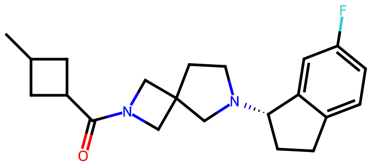	CC1CC(C(=O)N2CC3(CCN([C@H]4CCc5ccc(F)cc54)C3)C2)C1	1072606825	25	342.21	3.39

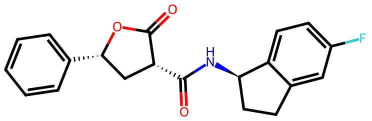	O=C(N[C@@H]1CCc2cc(F)ccc21)[C@@H]1C[C@H](c2ccccc2)OC1=O	290409579	25	339.13	3.23
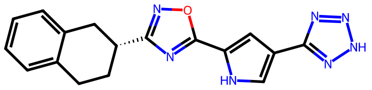	c1ccc2c(c1)CC[C@@H](c1noc(-c3cc(-c4nn[nH]n4)c[nH]3)n1)C2	1607133036	25	333.13	2.52
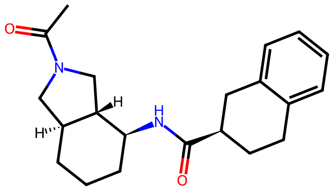	CC(=O)N1C[C@@H]2CCC[C@H](NC(=O)[C@@H]3CCc4ccccc4C3)[C@H]2C1	574919481	25	340.22	2.55
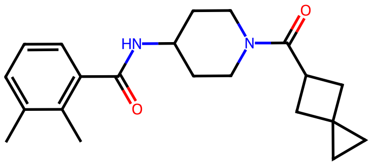	Cc1cccc(C(=O)NC2CCN(C(=O)C3CC4(CC4)C3)CC2)c1C	948305514	25	340.22	3.21
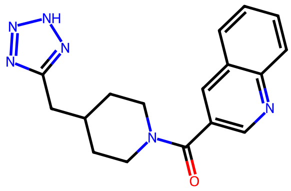	O=C(c1cnc2ccccc2c1)N1CCC(Cc2nn[nH]n2)CC1	1352696073	24	322.15	1.84
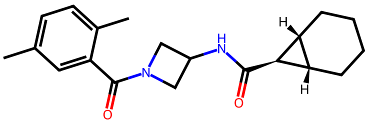	Cc1ccc(C)c(C(=O)N2CC(NC(=O)[C@@H]3[C@H]4CCCC[C@H]43)C2)c1	990644744	24	326.2	2.68
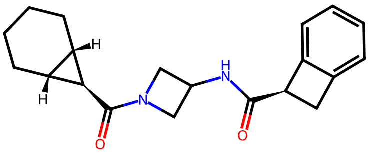	O=C(NC1CN(C(=O)[C@@H]2[C@H]3CCCC[C@H]32)C1)[C@@H]1Cc2ccccc21	998680562	24	324.18	2.09
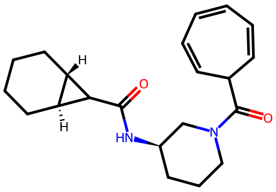	O=C(N[C@@H]1CCCN(C(=O)C2C=CC=CC=C2)C1)C1[C@H]2CCCC[C@H]12	951022597	25	340.22	2.83

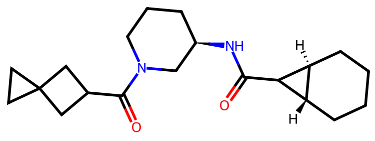	O=C(N[C@@H]1CCCN(C(=O)C2CC3(CC3)C2)C1)C1[C@H]2CCCC[C@H]12	951964992	24	330.23	2.72
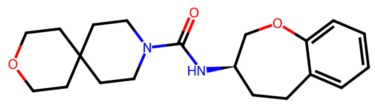	O=C(N[C@@H]1CCc2ccccc2OC1)N1CCC2(CCOCC2)CC1	790848821	25	344.21	2.98
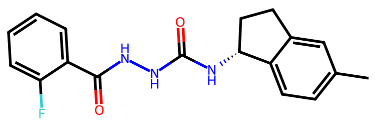	Cc1ccc2c(c1)CC[C@H]2NC(=O)NNC(=O)c1ccccc1F	56841867	24	327.14	2.77
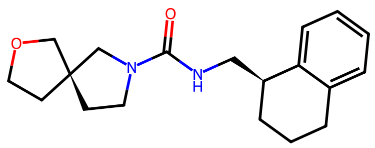	O=C(NC[C@@H]1CCCc2ccccc21)N1CC[C@]2(CCOC2)C1	156690520	23	314.2	2.93
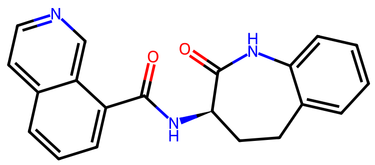	O=C(N[C@@H]1CCc2ccccc2NC1=O)c1cccc2ccncc12	1526999709	25	331.13	2.92
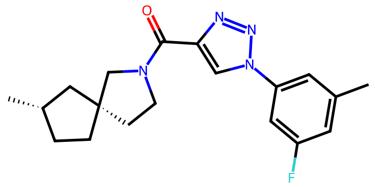	Cc1cc(F)cc(-n2cc(C(=O)N3CC[C@@]4(CC[C@H](C)C4)C3)nn2)c1	329866401	25	342.19	3.37
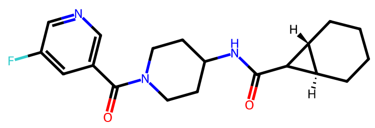	O=C(NC1CCN(C(=O)c2cncc(F)c2)CC1)C1[C@@H]2CCCC[C@@H]12	946431497	25	345.19	2.38
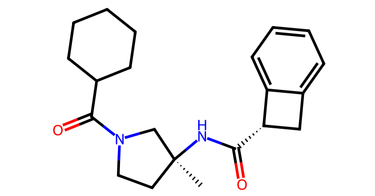	C[C@]1(NC(=O)[C@H]2Cc3ccccc32)CCN(C(=O)C2CCCCC2)C1	957455723	25	340.22	3.01

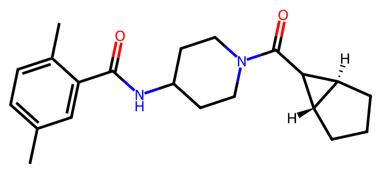	Cc1ccc(C)c(C(=O)NC2CCN(C(=O)C3[C@H]4CCC[C@H]34)CC2)c1	946079868	25	340.22	3.07
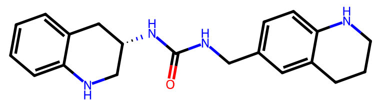	O=C(NCc1ccc2c(c1)CCCN2)N[C@@H]1CNc2ccccc2C1	652220977	25	336.2	2.88
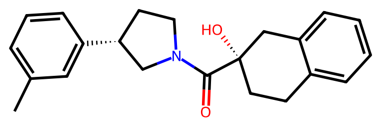	Cc1cccc([C@@H]2CCN(C(=O)[C@]3(O)CCc4ccccc4C3)C2)c1	803102743	25	335.19	3.23
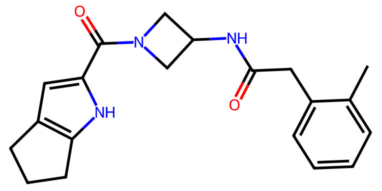	Cc1ccccc1CC(=O)NC1CN(C(=O)c2cc3c([nH]2)CCC3)C1	991356434	25	337.18	2.0
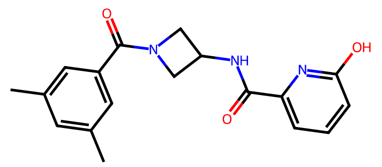	Cc1cc(C)cc(C(=O)N2CC(NC(=O)c3cccc(O)n3)C2)c1	990645069	24	325.14	1.66
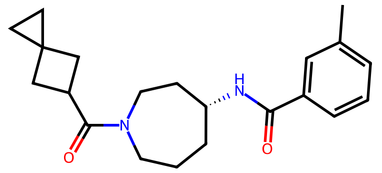	Cc1cccc(C(=O)N[C@@H]2CCCN(C(=O)C3CC4(CC4)C3)CC2)c1	964703146	25	340.22	3.3
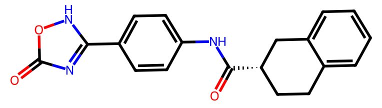	O=C(Nc1ccc(-c2nc(=O)o[nH]2)cc1)[C@H]1CCc2ccccc2C1	1128069457	25	335.13	2.77
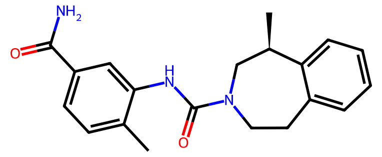	Cc1ccc(C(N)=O)cc1NC(=O)N1CCc2ccccc2[C@H](C)C1	650014980	25	337.18	3.29

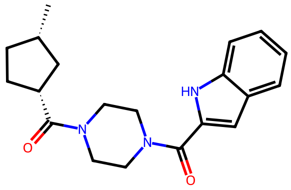	C[C@H]1CC[C@@H](C(=O)N2CCN(C(=O)c3cc4ccccc4[nH]3)CC2)C1	678946394	25	339.19	2.89
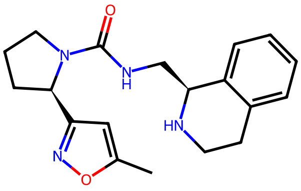	Cc1cc([C@H]2CCCN2C(=O)NC[C@@H]2NCCc3ccccc32)no1	841998688	25	340.19	2.72
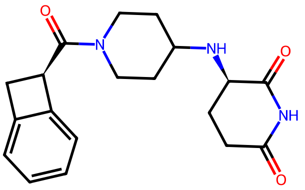	O=C1CC[C@@H](NC2CCN(C(=O)[C@@H]3Cc4ccccc43)CC2)C(=O)N1	996931449	25	341.17	0.71
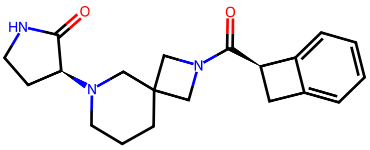	O=C1NCC[C@@H]1N1CCCC2(CN(C(=O)[C@@H]3Cc4ccccc43)C2)C1	981976710	25	339.19	1.14
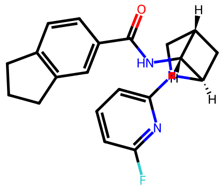	O=C(N[C@@H]1[C@H]2C[C@H]1N(c1cccc(F)n1)C2)c1ccc2c(c1)CCC2	1064259367	25	337.16	2.72
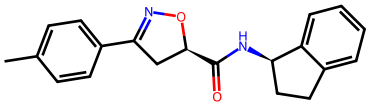	Cc1ccc(C2=NO[C@@H](C(=O)N[C@@H]3CCc4ccccc43)C2)cc1	1527932344	24	320.15	3.29
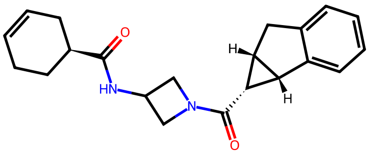	O=C(NC1CN(C(=O)[C@H]2[C@H]3c4ccccc4C[C@H]32)C1)[C@@H]1CC=CCC1	990220875	25	336.18	2.26
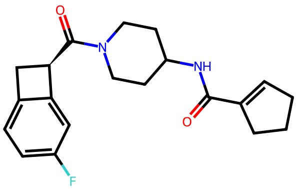	O=C(NC1CCN(C(=O)[C@@H]2Cc3ccc(F)cc32)CC1)C1=CCCC1	948024618	25	342.17	2.68

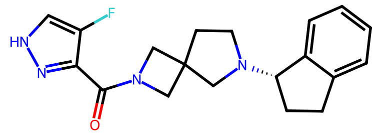	O=C(c1n[nH]cc1F)N1CC2(CCN([C@H]3CCc4ccccc43)C2)C1	1072958931	25	340.17	2.38
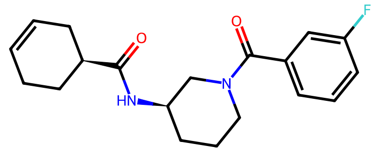	O=C(N[C@@H]1CCCN(C(=O)c2cccc(F)c2)C1)[C@@H]1CC=CCC1	949850582	24	330.17	2.9
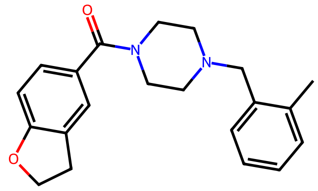	Cc1ccccc1CN1CCN(C(=O)c2ccc3c(c2)CCO3)CC1	32080665	25	336.18	2.89
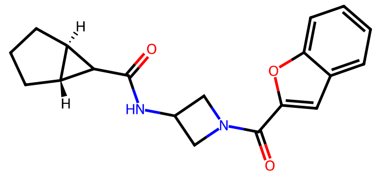	O=C(NC1CN(C(=O)c2cc3ccccc3o2)C1)C1[C@@H]2CCC[C@@H]12	990711486	24	324.15	2.42
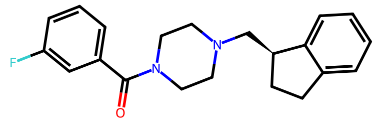	O=C(c1cccc(F)c1)N1CCN(C[C@@H]2CCc3ccccc32)CC1	780287362	25	338.18	3.31
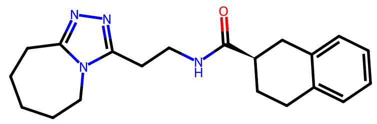	O=C(NCCc1nnc2n1CCCCC2)[C@@H]1CCc2ccccc2C1	580204262	25	338.21	2.47
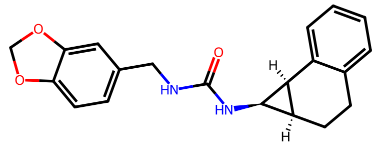	O=C(NCc1ccc2c(c1)OCO2)N[C@@H]1[C@@H]2CCc3ccccc3[C@@H]21	152941263	25	336.15	2.94
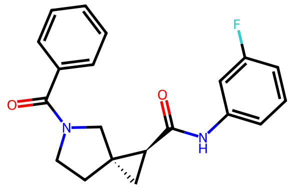	O=C(Nc1cccc(F)c1)[C@H]1C[C@@]12CCN(C(=O)c1ccccc1)C2	96227237	25	338.14	3.32

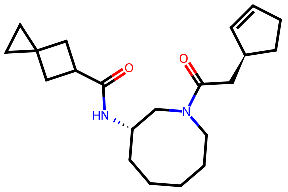	O=C(N[C@H]1CCCCCN(C(=O)C[C@H]2C=CCC2)C1)C1CC2(CC2)C1	986878694	25	344.25	3.42
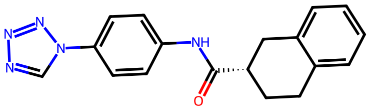	O=C(Nc1ccc(-n2cnnn2)cc1)[C@H]1CCc2ccccc2C1	1351946504	24	319.14	2.41
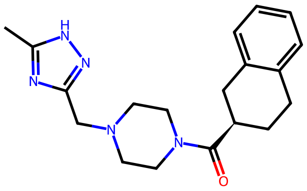	Cc1nc(CN2CCN(C(=O)[C@@H]3CCc4ccccc4C3)CC2)n[nH]1	573495324	25	339.21	1.56
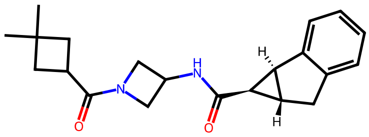	CC1(C)CC(C(=O)N2CC(NC(=O)[C@@H]3[C@H]4Cc5ccccc5[C@@H]43)C2)C1	991644357	25	338.2	2.34
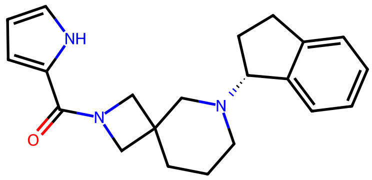	O=C(c1ccc[nH]1)N1CC2(CCCN([C@@H]3CCc4ccccc43)C2)C1	981619678	25	335.2	3.24
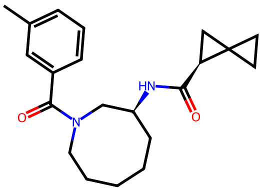	Cc1cccc(C(=O)N2CCCCC[C@H](NC(=O)[C@H]3CC34CC4)C2)c1	986868372	25	340.22	3.3
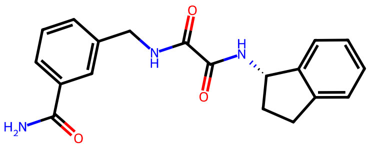	NC(=O)c1cccc(CNC(=O)C(=O)N[C@H]2CCc3ccccc32)c1	339874281	25	337.14	1.21
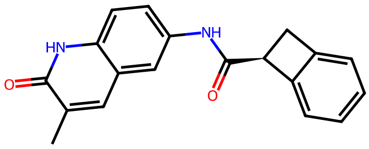	Cc1cc2cc(NC(=O)[C@H]3Cc4ccccc43)ccc2[nH]c1=O	1569494292	23	304.12	3.11

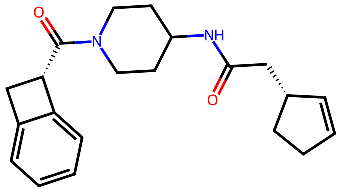	O=C(C[C@@H]1C=CCC1)NC1CCN(C(=O)[C@H]2Cc3ccccc32)CC1	948059825	25	338.2	2.79
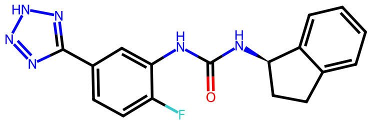	O=C(Nc1cc(-c2nn[nH]n2)ccc1F)N[C@@H]1CCc2ccccc21	1593202317	25	338.13	2.81
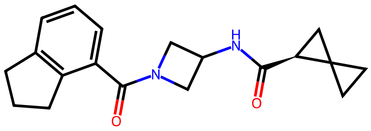	O=C(NC1CN(C(=O)c2cccc3c2CCC3)C1)[C@H]1CC12CC2	1000519989	23	310.17	1.92
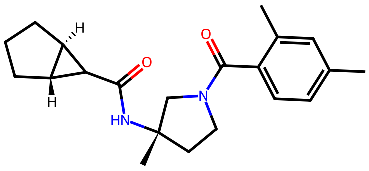	Cc1ccc(C(=O)N2CC[C@](C)(NC(=O)C3[C@@H]4CCC[C@@H]34)C2)c(C)c1	956803640	25	340.22	3.07
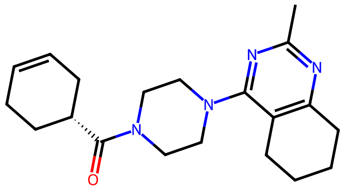	Cc1nc2c(c(N3CCN(C(=O)[C@H]4CC=CCC4)CC3)n1)CCCC2	1055721287	25	340.23	2.67
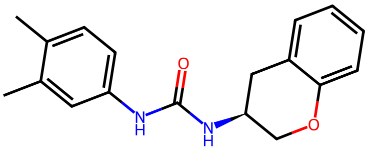	Cc1ccc(NC(=O)N[C@@H]2COc3ccccc3C2)cc1C	21101597	22	296.15	3.43
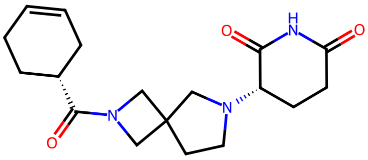	O=C1CC[C@H](N2CCC3(CN(C(=O)[C@H]4CC=CCC4)C3)C2)C(=O)N1	1072457643	24	331.19	0.68
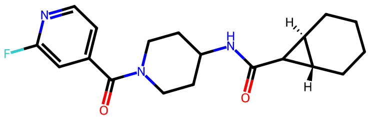	O=C(NC1CCN(C(=O)c2ccnc(F)c2)CC1)C1[C@H]2CCCC[C@H]12	945048712	25	345.19	2.38

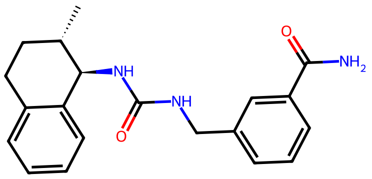	C[C@H]1CCc2ccccc2[C@@H]1NC(=O)NCc1cccc(C(N)=O)c1	179306745	25	337.18	2.91
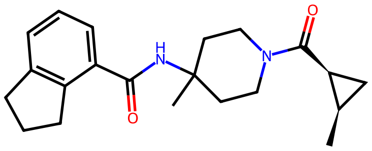	C[C@@H]1C[C@@H]1C(=O)N1CCC(C)(NC(=O)c2cccc3c2CCC3)CC1	956401998	25	340.22	2.94
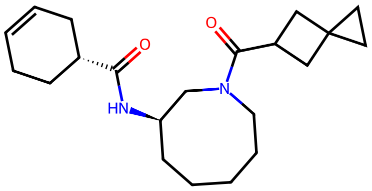	O=C(N[C@@H]1CCCCCN(C(=O)C2CC3(CC3)C2)C1)[C@H]1CC=CCC1	987155346	25	344.25	3.42
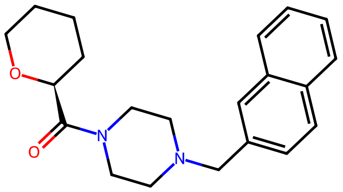	O=C([C@@H]1CCCCO1)N1CCN(Cc2ccc3ccccc3c2)CC1	1313444488	25	338.2	3.05
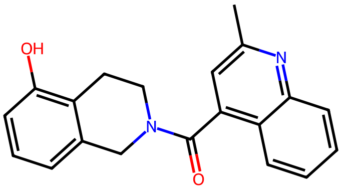	Cc1cc(C(=O)N2CCc3c(O)cccc3C2)c2ccccc2n1	1631559245	24	318.14	3.45
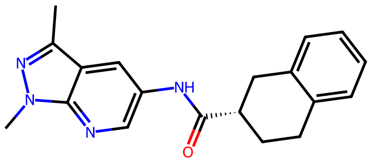	Cc1nn(C)c2ncc(NC(=O)[C@H]3CCc4ccccc4C3)cc12	649565472	24	320.16	3.02
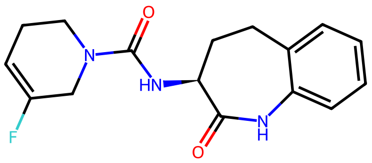	O=C1Nc2ccccc2CC[C@@H]1NC(=O)N1CCC=C(F)C1	654629376	22	303.14	2.21
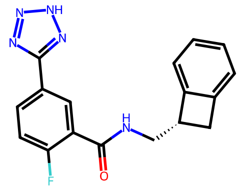	O=C(NC[C@H]1Cc2ccccc21)c1cc(-c2nn[nH]n2)ccc1F	1582490338	24	323.12	2.08

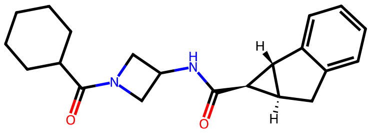	O=C(NC1CN(C(=O)C2CCCCC2)C1)[C@@H]1[C@@H]2Cc3ccccc3[C@H]21	991647250	25	338.2	2.48
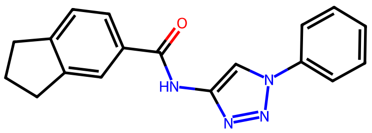	O=C(Nc1cn(-c2ccccc2)nn1)c1ccc2c(c1)CCC2	1540298856	23	304.13	3.01
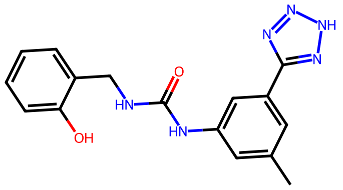	Cc1cc(NC(=O)NCc2ccccc2O)cc(-c2nn[nH]n2)c1	1592027303	24	324.13	2.2
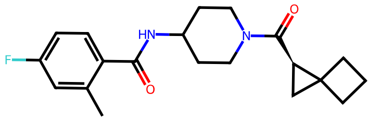	Cc1cc(F)ccc1C(=O)NC1CCN(C(=O)[C@@H]2CC23CCC3)CC1	946760917	25	344.19	3.05
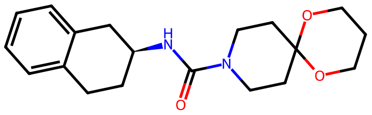	O=C(N[C@H]1CCc2ccccc2C1)N1CCC2(CC1)OCCCO2	297678149	24	330.19	2.48
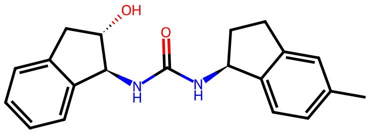	Cc1ccc2c(c1)CC[C@@H]2NC(=O)N[C@H]1c2ccccc2C[C@@H]1O	299183320	24	322.17	2.94
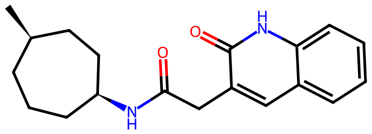	C[C@@H]1CCC[C@H](NC(=O)Cc2cc3ccccc3[nH]c2=O)CC1	188923968	23	312.18	3.16
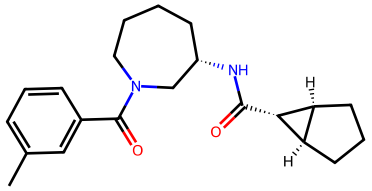	Cc1cccc(C(=O)N2CCCC[C@H](NC(=O)[C@@H]3[C@H]4CCC[C@H]43)C2)c1	943169642	25	340.22	3.15

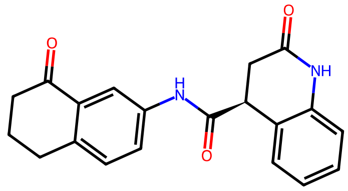	O=C1C[C@H](C(=O)Nc2ccc3c(c2)C(=O)CCC3)c2ccccc2N1	177992050	25	334.13	3.27
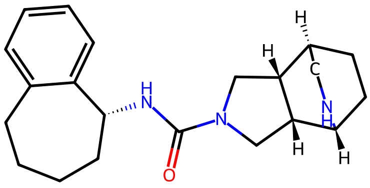	O=C(N[C@@H]1CCCCc2ccccc21)N1C[C@H]2[C@H]3CC[C@@H](CN3)[C@H]2C1	626961579	25	339.23	3.09
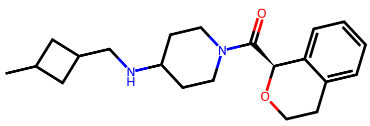	CC1CC(CNC2CCN(C(=O)[C@@H]3OCCc4ccccc43)CC2)C1	996864492	25	342.23	2.93
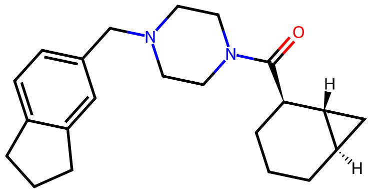	O=C([C@@H]1CCC[C@@H]2C[C@H]21)N1CCN(Cc2ccc3c(c2)CCC3)CC1	948972713	25	338.24	3.26
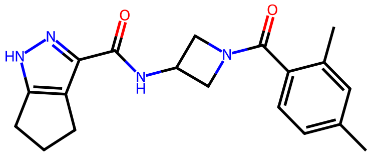	Cc1ccc(C(=O)N2CC(NC(=O)c3n[nH]c4c3CCC4)C2)c(C)c1	995167765	25	338.17	1.77
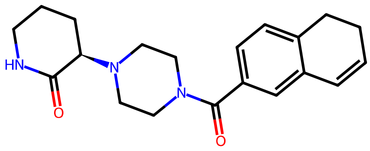	O=C1NCCC[C@H]1N1CCN(C(=O)c2ccc3c(c2)C=CCC3)CC1	951703629	25	339.19	1.68
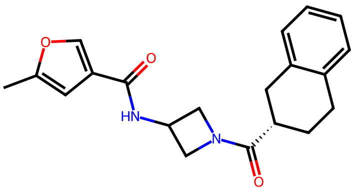	Cc1cc(C(=O)NC2CN(C(=O)[C@H]3CCc4ccccc4C3)C2)co1	991028492	25	338.16	2.33
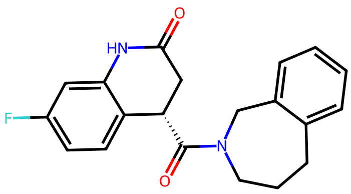	O=C1C[C@H](C(=O)N2CCCc3ccccc3C2)c2ccc(F)cc2N1	106714001	25	338.14	3.23

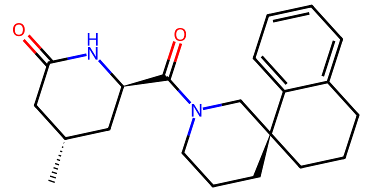	C[C@@H]1CC(=O)N[C@@H](C(=O)N2CCC[C@@]3(CCCc4ccccc43)C2)C1	342512938	25	340.22	2.8
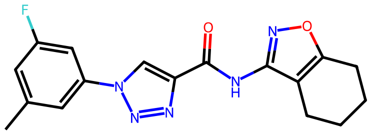	Cc1cc(F)cc(-n2cc(C(=O)Nc3noc4c3CCCC4)nn2)c1	900786432	25	341.13	2.83
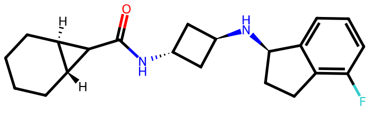	O=C(N[C@H]1C[C@H](N[C@@H]2CCc3c(F)cccc32)C1)C1[C@@H]2CCCC[C@@H]12	1020048097	25	342.21	3.49
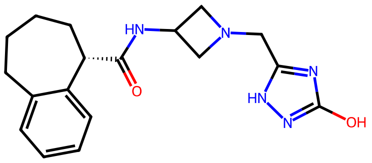	O=C(NC1CN(Cc2nc(O)n[nH]2)C1)[C@H]1CCCCc2ccccc21	1031451491	25	341.19	1.32
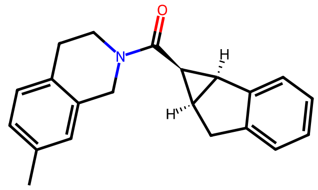	Cc1ccc2c(c1)CN(C(=O)[C@H]1[C@H]3c4ccccc4C[C@H]31)CC2	628058148	23	303.16	3.47
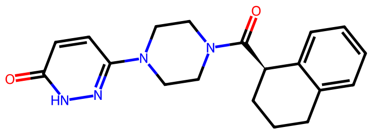	O=C([C@@H]1CCCc2ccccc21)N1CCN(c2ccc(=O)[nH]n2)CC1	69626093	25	338.17	1.54
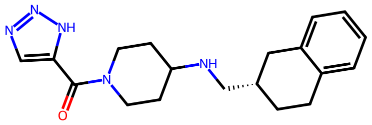	O=C(c1cnn[nH]1)N1CCC(NC[C@H]2CCc3ccccc3C2)CC1	996758011	25	339.21	1.8
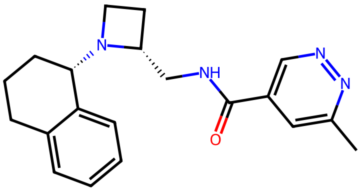	Cc1cc(C(=O)NC[C@H]2CCN2[C@H]2CCCc3ccccc32)cnn1	1038200705	25	336.2	2.67

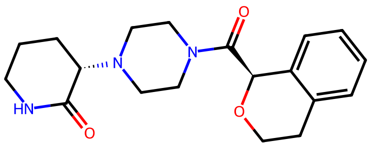	O=C1NCCC[C@@H]1N1CCN(C(=O)[C@@H]2OCCc3ccccc32)CC1	949590068	25	343.19	0.72
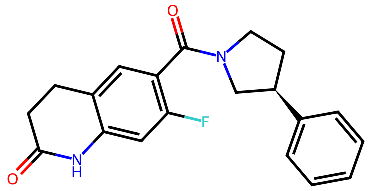	O=C1CCc2cc(C(=O)N3CC[C@@H](c4ccccc4)C3)c(F)cc2N1	136103309	25	338.14	3.34
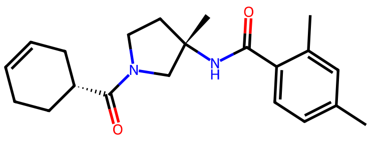	Cc1ccc(C(=O)N[C@@]2(C)CCN(C(=O)[C@H]3CC=CCC3)C2)c(C)c1	957565975	25	340.22	3.38
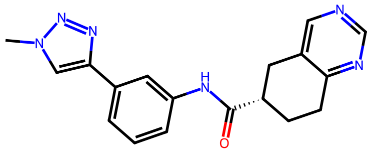	Cn1cc(-c2cccc(NC(=O)[C@H]3CCc4ncncc4C3)c2)nn1	620270341	25	334.15	2.02
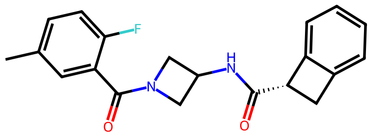	Cc1ccc(F)c(C(=O)N2CC(NC(=O)[C@H]3Cc4ccccc43)C2)c1	998695376	25	338.14	2.41
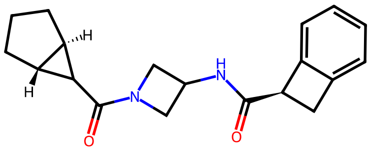	O=C(NC1CN(C(=O)C2[C@@H]3CCC[C@@H]23)C1)[C@@H]1Cc2ccccc21	998685005	23	310.17	1.7
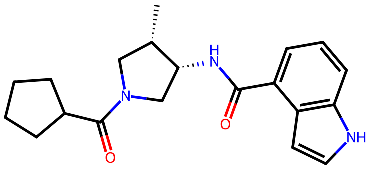	C[C@H]1CN(C(=O)C2CCCC2)C[C@H]1NC(=O)c1cccc2[nH]ccc12	966038011	25	339.19	2.93
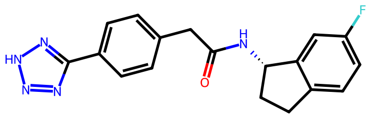	O=C(Cc1ccc(-c2nn[nH]n2)cc1)N[C@H]1CCc2ccc(F)cc21	737960924	25	337.13	2.35

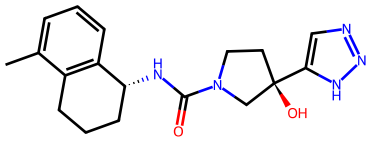	Cc1cccc2c1CCC[C@H]2NC(=O)N1CC[C@](O)(c2cnn[nH]2)C1	823844232	25	341.19	1.79
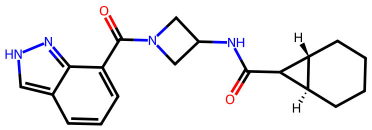	O=C(NC1CN(C(=O)c2cccc3c[nH]nc23)C1)C1[C@@H]2CCCC[C@@H]12	990655716	25	338.17	1.94
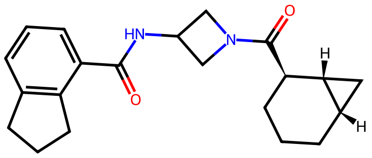	O=C(NC1CN(C(=O)[C@@H]2CCC[C@H]3C[C@H]32)C1)c1cccc2c1CCC2	994397920	25	338.2	2.55
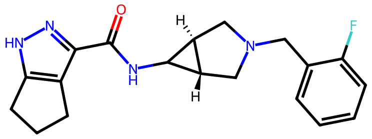	O=C(NC1[C@H]2CN(Cc3ccccc3F)C[C@H]12)c1n[nH]c2c1CCC2	960729987	25	340.17	1.9
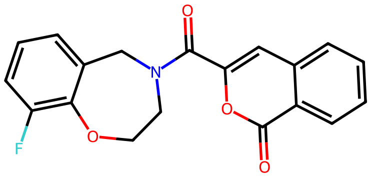	O=C(c1cc2ccccc2c(=O)o1)N1CCOc2c(F)cccc2C1	370554478	25	339.09	2.97
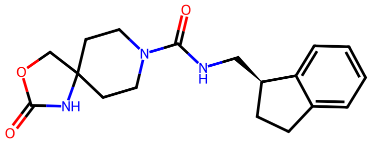	O=C1NC2(CCN(C(=O)NC[C@@H]3CCc4ccccc43)CC2)CO1	495152904	24	329.17	2.0
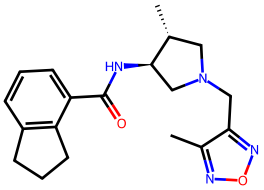	Cc1nonc1CN1C[C@@H](NC(=O)c2cccc3c2CCC3)[C@H](C)C1	1081028311	25	340.19	2.12
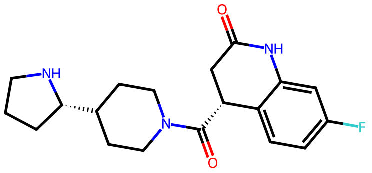	O=C1C[C@@H](C(=O)N2CCC([C@@H]3CCCN3)CC2)c2ccc(F)cc2N1	353042949	25	345.19	2.24

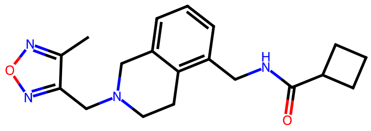	Cc1nonc1CN1CCc2c(CNC(=O)C3CCC3)cccc2C1	1051274910	25	340.19	2.35
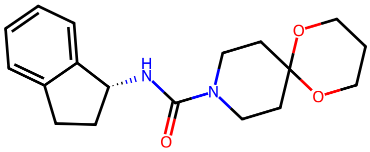	O=C(N[C@@H]1CCc2ccccc21)N1CCC2(CC1)OCCCO2	297589743	23	316.18	2.61
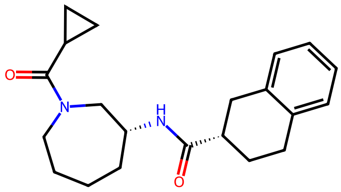	O=C(N[C@@H]1CCCCN(C(=O)C2CC2)C1)[C@H]1CCc2ccccc2C1	943573732	25	340.22	2.7
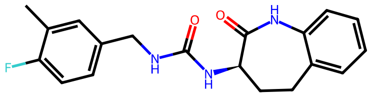	Cc1cc(CNC(=O)N[C@@H]2CCc3ccccc3NC2=O)ccc1F	654360878	25	341.15	2.89
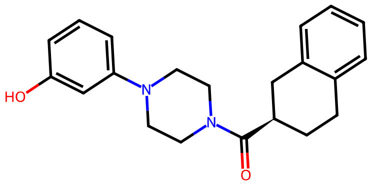	O=C([C@@H]1CCc2ccccc2C1)N1CCN(c2cccc(O)c2)CC1	565065054	25	336.18	2.85
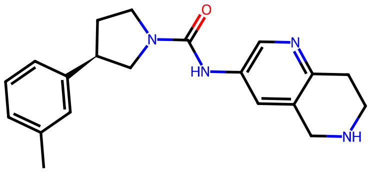	Cc1cccc([C@H]2CCN(C(=O)Nc3cnc4c(c3)CNCC4)C2)c1	667803984	25	336.2	3.06
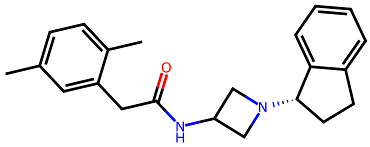	Cc1ccc(C)c(CC(=O)NC2CN([C@H]3CCc4ccccc43)C2)c1	1030891590	25	334.2	3.33
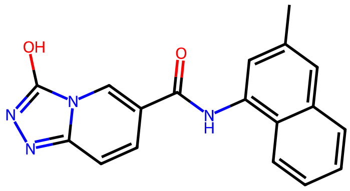	Cc1cc(NC(=O)c2ccc3nnc(O)n3c2)c2ccccc2c1	1298116970	24	318.11	3.15

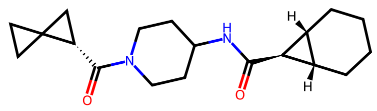	O=C(NC1CCN(C(=O)[C@H]2CC23CC3)CC1)[C@@H]1[C@H]2CCCC[C@H]21	948562707	23	316.22	2.33
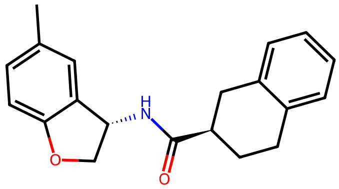	Cc1ccc2c(c1)[C@H](NC(=O)[C@@H]1CCc3ccccc3C1)CO2	567392394	23	307.16	3.35
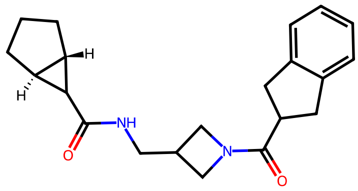	O=C(NCC1CN(C(=O)C2Cc3ccccc3C2)C1)C1[C@H]2CCC[C@H]12	1001349585	25	338.2	2.02
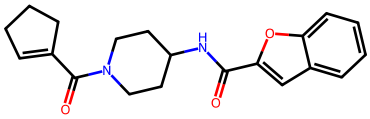	O=C(NC1CCN(C(=O)C2=CCCC2)CC1)c1cc2ccccc2o1	946449423	25	338.16	3.26
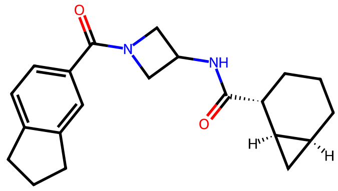	O=C(NC1CN(C(=O)c2ccc3c(c2)CCC3)C1)[C@@H]1CCC[C@H]2C[C@H]21	994719224	25	338.2	2.55
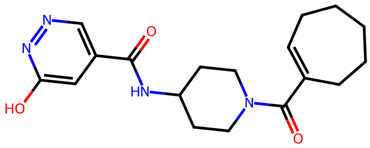	O=C(NC1CCN(C(=O)C2=CCCCCC2)CC1)c1cnnc(O)c1	946554184	25	344.18	1.79
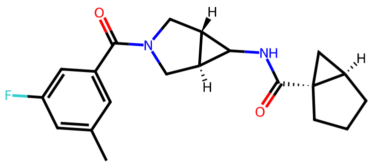	Cc1cc(F)cc(C(=O)N2C[C@@H]3C(NC(=O)[C@@]45CCC[C@@H]4C5)[C@H]3C2)c1	1013561699	25	342.17	2.51
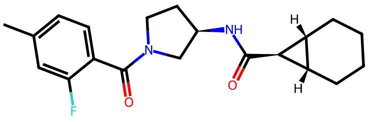	Cc1ccc(C(=O)N2CC[C@@H](NC(=O)[C@@H]3[C@H]4CCCC[C@H]43)C2)c(F)c1	936257255	25	344.19	2.9

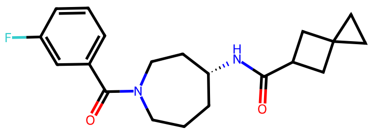	O=C(N[C@@H]1CCCN(C(=O)c2cccc(F)c2)CC1)C1CC2(CC2)C1	965182825	25	344.19	3.13
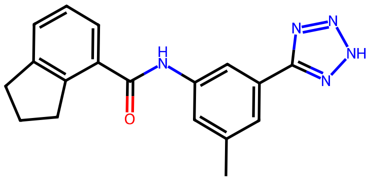	Cc1cc(NC(=O)c2cccc3c2CCC3)cc(-c2nn[nH]n2)c1	1592031749	24	319.14	2.92
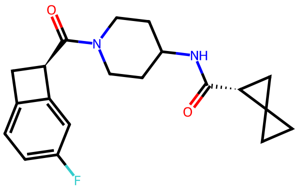	O=C(NC1CCN(C(=O)[C@@H]2Cc3ccc(F)cc32)CC1)[C@@H]1CC12CC2	948026949	25	342.17	2.37
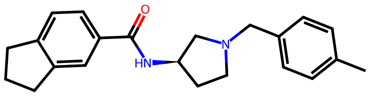	Cc1ccc(CN2CC[C@@H](NC(=O)c3ccc4c(c3)CCC4)C2)cc1	1016227139	25	334.2	3.49
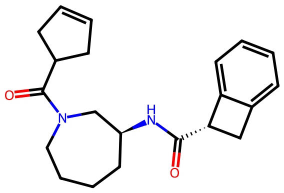	O=C(N[C@H]1CCCCN(C(=O)C2CC=CC2)C1)[C@H]1Cc2ccccc21	943498266	25	338.2	2.79
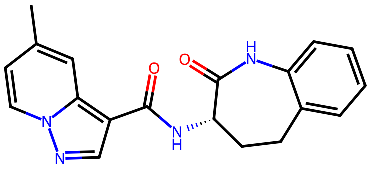	Cc1ccn2ncc(C(=O)N[C@H]3CCc4ccccc4NC3=O)c2c1	1557295921	25	334.14	2.33
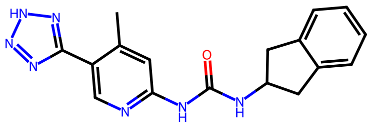	Cc1cc(NC(=O)NC2Cc3ccccc3C2)ncc1-c1nn[nH]n1	1580982571	25	335.15	1.86
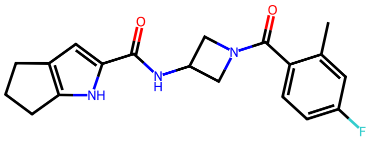	Cc1cc(F)ccc1C(=O)N1CC(NC(=O)c2cc3c([nH]2)CCC3)C1	995914226	25	341.15	2.21

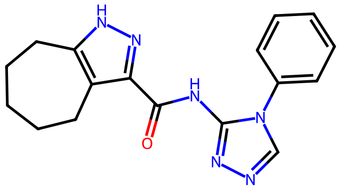	O=C(Nc1nncn1-c1ccccc1)c1n[nH]c2c1CCCCC2	188165427	24	322.15	2.51
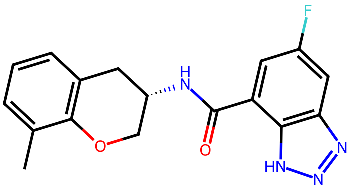	Cc1cccc2c1OC[C@@H](NC(=O)c1cc(F)cc3nn[nH]c13)C2	427988199	24	326.12	2.14
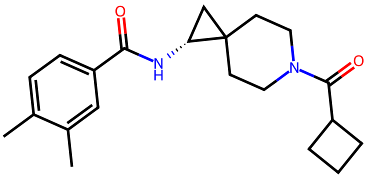	Cc1ccc(C(=O)N[C@@H]2CC23CCN(C(=O)C2CCC2)CC3)cc1C	965360968	25	340.22	3.21
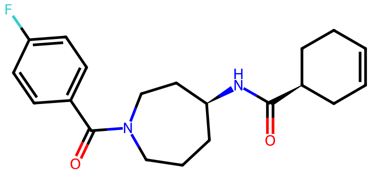	O=C(N[C@H]1CCCN(C(=O)c2ccc(F)cc2)CC1)[C@@H]1CC=CCC1	964747730	25	344.19	3.29
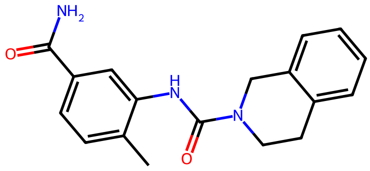	Cc1ccc(C(N)=O)cc1NC(=O)N1CCc2ccccc2C1	58450985	23	309.15	2.68
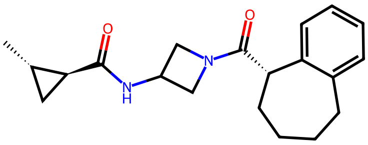	C[C@H]1C[C@@H]1C(=O)NC1CN(C(=O)[C@H]2CCCCc3ccccc32)C1	997826812	24	326.2	2.48
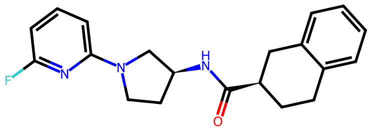	O=C(N[C@H]1CCN(c2cccc(F)n2)C1)[C@@H]1CCc2ccccc2C1	1058560170	25	339.17	2.72
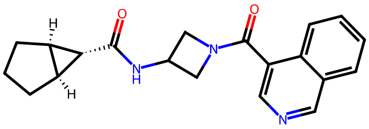	O=C(NC1CN(C(=O)c2cncc3ccccc23)C1)[C@@H]1[C@H]2CCC[C@H]21	990700059	25	335.16	2.22

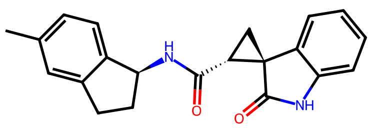	Cc1ccc2c(c1)CC[C@@H]2NC(=O)[C@@H]1C[C@@]12C(=O)Nc1ccccc12	930567635	25	332.15	3.01
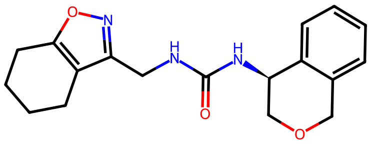	O=C(NCc1noc2c1CCCC2)N[C@@H]1COCc2ccccc21	569558259	24	327.16	2.62
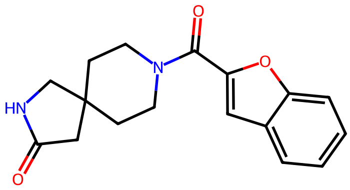	O=C1CC2(CCN(C(=O)c3cc4ccccc4o3)CC2)CN1	267608054	22	298.13	2.18
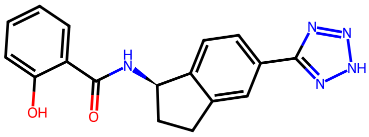	O=C(N[C@@H]1CCc2cc(-c3nn[nH]n3)ccc21)c1ccccc1O	1606684168	24	321.12	1.99
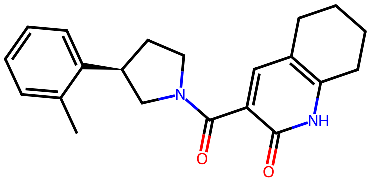	Cc1ccccc1[C@H]1CCN(C(=O)c2cc3c([nH]c2=O)CCCC3)C1	68093373	25	336.18	3.19
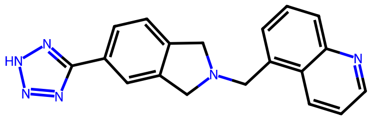	c1cc(CN2Cc3ccc(-c4nn[nH]n4)cc3C2)c2cccnc2c1	1582816408	25	328.14	2.93
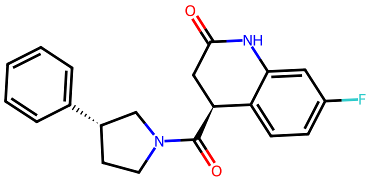	O=C1C[C@H](C(=O)N2CC[C@H](c3ccccc3)C2)c2ccc(F)cc2N1	121579146	25	338.14	3.27
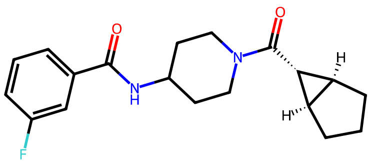	O=C(NC1CCN(C(=O)[C@@H]2[C@H]3CCC[C@H]32)CC1)c1cccc(F)c1	946066008	24	330.17	2.59

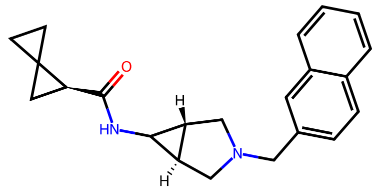	O=C(NC1[C@@H]2CN(Cc3ccc4ccccc4c3)C[C@@H]12)[C@H]1CC12CC2	961888345	25	332.19	3.19
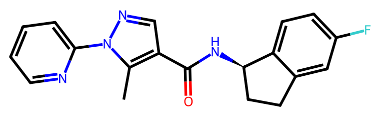	Cc1c(C(=O)N[C@@H]2CCc3cc(F)ccc32)cnn1-c1ccccn1	55657476	25	336.14	3.13
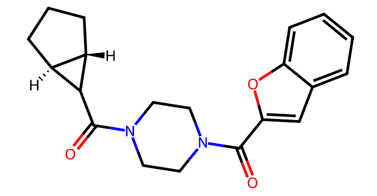	O=C(c1cc2ccccc2o1)N1CCN(C(=O)C2[C@H]3CCC[C@H]23)CC1	1010925354	25	338.16	2.76
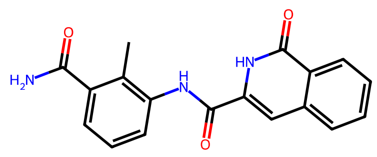	Cc1c(NC(=O)c2cc3ccccc3c(=O)[nH]2)cccc1C(N)=O	27249460	24	321.11	2.19
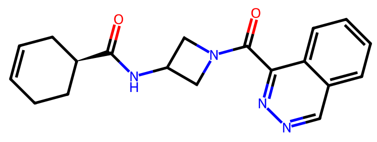	O=C(NC1CN(C(=O)c2nncc3ccccc23)C1)[C@@H]1CC=CCC1	990212848	25	336.16	1.93
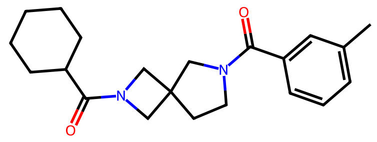	Cc1cccc(C(=O)N2CCC3(C2)CN(C(=O)C2CCCCC2)C3)c1	1018718251	25	340.22	3.25
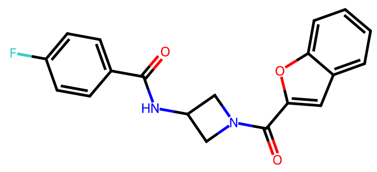	O=C(NC1CN(C(=O)c2cc3ccccc3o2)C1)c1ccc(F)cc1	990073975	25	338.11	2.83
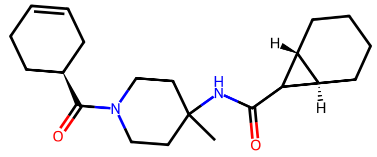	CC1(NC(=O)C2[C@@H]3CCCC[C@@H]23)CCN(C(=O)[C@@H]2CC=CCC2)CC1	956326923	25	344.25	3.28

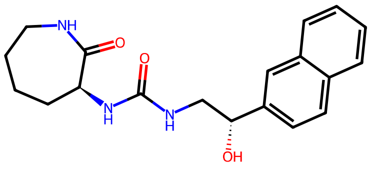	O=C(NC[C@@H](O)c1ccc2ccccc2c1)N[C@H]1CCCCNC1=O	109149082	25	341.17	1.84
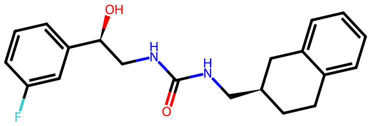	O=C(NC[C@@H]1CCc2ccccc2C1)NC[C@H](O)c1cccc(F)c1	117410649	25	342.17	2.96
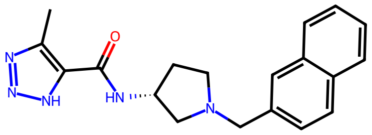	Cc1nn[nH]c1C(=O)N[C@@H]1CCN(Cc2ccc3ccccc3c2)C1	1015490355	25	335.17	2.27
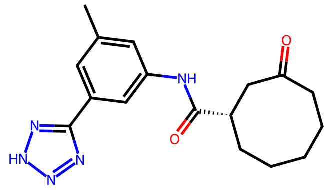	Cc1cc(NC(=O)[C@H]2CCCCCC(=O)C2)cc(-c2nn[nH]n2)c1	1609406287	24	327.17	2.65
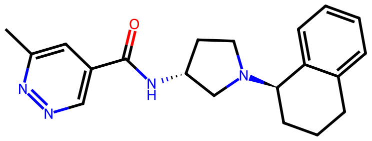	Cc1cc(C(=O)N[C@@H]2CCN([C@@H]3CCCc4ccccc43)C2)cnn1	1010758322	25	336.2	2.67
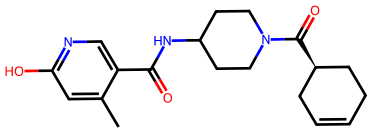	Cc1cc(O)ncc1C(=O)NC1CCN(C(=O)[C@@H]2CC=CCC2)CC1	944107016	25	343.19	2.17
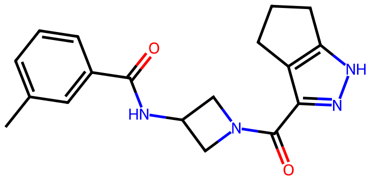	Cc1cccc(C(=O)NC2CN(C(=O)c3n[nH]c4c3CCC4)C2)c1	990120719	24	324.16	1.46
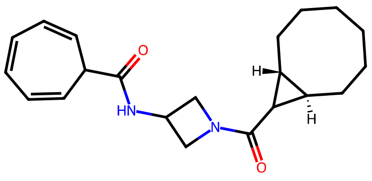	O=C(NC1CN(C(=O)C2[C@@H]3CCCCCC[C@@H]23)C1)C1C=CC=CC=C1	992875268	25	340.22	2.83

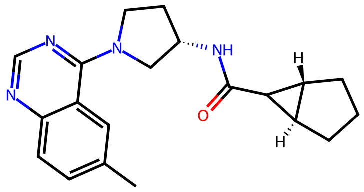	Cc1ccc2ncnc(N3CC[C@H](NC(=O)C4[C@@H]5CCC[C@@H]45)C3)c2c1	1058323111	25	336.2	2.68
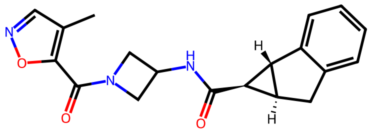	Cc1cnoc1C(=O)N1CC(NC(=O)[C@@H]2[C@@H]3Cc4ccccc4[C@H]32)C1	991649776	25	337.14	1.51
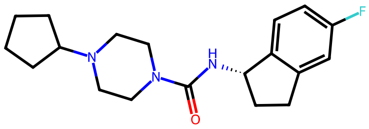	O=C(N[C@H]1CCc2cc(F)ccc21)N1CCN(C2CCCC2)CC1	56839965	24	331.21	3.08
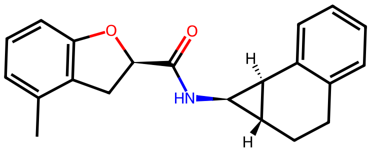	Cc1cccc2c1C[C@H](C(=O)N[C@@H]1[C@H]3CCc4ccccc4[C@@H]31)O2	450174479	24	319.16	3.14
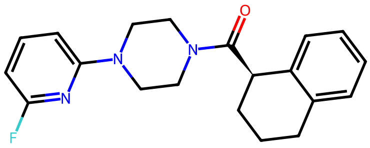	O=C([C@@H]1CCCc2ccccc21)N1CCN(c2cccc(F)n2)CC1	1056073890	25	339.17	2.99
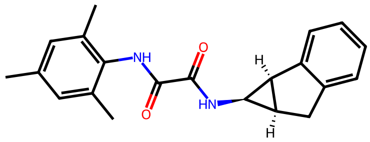	Cc1cc(C)c(NC(=O)C(=O)N[C@@H]2[C@@H]3Cc4ccccc4[C@@H]32)c(C)c1	158776665	25	334.17	3.0
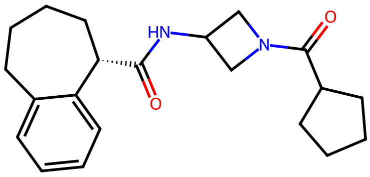	O=C(NC1CN(C(=O)C2CCCC2)C1)[C@H]1CCCCc2ccccc21	994322328	25	340.22	3.01
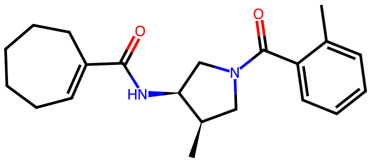	Cc1ccccc1C(=O)N1C[C@@H](C)[C@@H](NC(=O)C2=CCCCCC2)C1	966097431	25	340.22	3.46

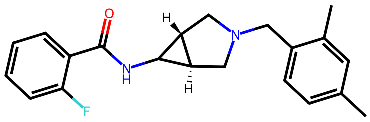	Cc1ccc(CN2C[C@H]3C(NC(=O)c4ccccc4F)[C@@H]3C2)c(C)c1	958107003	25	338.18	3.3
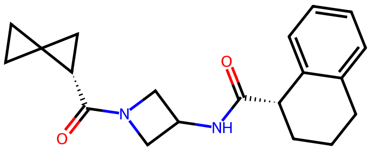	O=C(NC1CN(C(=O)[C@H]2CC23CC3)C1)[C@H]1CCCc2ccccc21	995074645	24	324.18	2.23
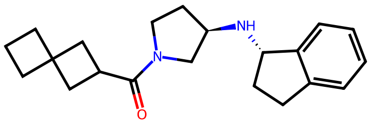	O=C(C1CC2(CCC2)C1)N1CC[C@@H](N[C@H]2CCc3ccccc32)C1	969646950	24	324.22	3.44
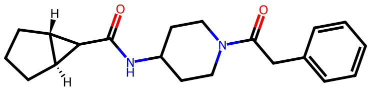	O=C(NC1CCN(C(=O)Cc2ccccc2)CC1)C1[C@H]2CCC[C@H]12	947344842	24	326.2	2.38
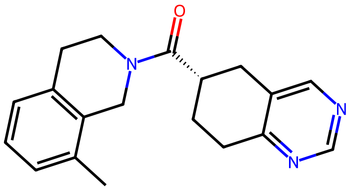	Cc1cccc2c1CN(C(=O)[C@H]1CCc3ncncc3C1)CC2	648371612	23	307.17	2.47
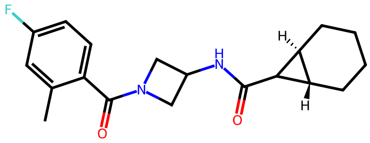	Cc1cc(F)ccc1C(=O)N1CC(NC(=O)C2[C@H]3CCCC[C@H]23)C1	990644882	24	330.17	2.51
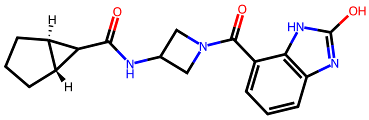	O=C(NC1CN(C(=O)c2cccc3nc(O)[nH]c23)C1)C1[C@@H]2CCC[C@@H]12	990699337	25	340.15	1.26
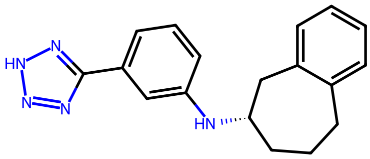	c1cc(N[C@H]2CCCc3ccccc3C2)cc(-c2nn[nH]n2)c1	1593559274	23	305.16	3.23

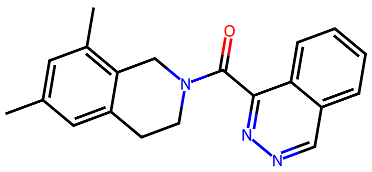	Cc1cc(C)c2c(c1)CCN(C(=O)c1nncc3ccccc13)C2	1121225767	24	317.15	3.45
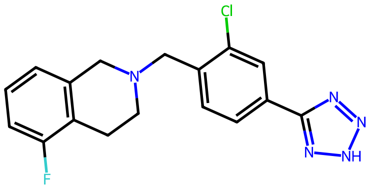	Fc1cccc2c1CCN(Cc1ccc(-c3nn[nH]n3)cc1Cl)C2	1609492221	24	343.1	3.22
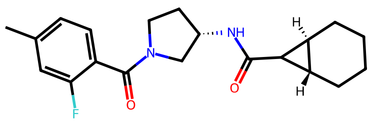	Cc1ccc(C(=O)N2CC[C@H](NC(=O)C3[C@H]4CCCC[C@H]34)C2)c(F)c1	937795361	25	344.19	2.9
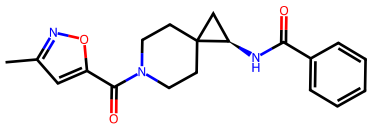	Cc1cc(C(=O)N2CCC3(CC2)C[C@H]3NC(=O)c2ccccc2)on1	962778581	25	339.16	2.41
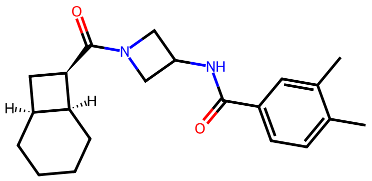	Cc1ccc(C(=O)NC2CN(C(=O)[C@@H]3C[C@@H]4CCCC[C@@H]43)C2)cc1C	999541708	25	340.22	3.07
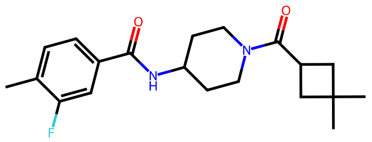	Cc1ccc(C(=O)NC2CCN(C(=O)C3CC(C)(C)C3)CC2)cc1F	946627843	25	346.21	3.29
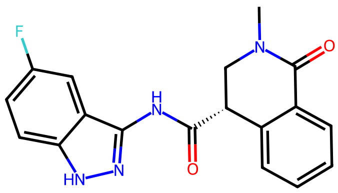	CN1C[C@@H](C(=O)Nc2n[nH]c3ccc(F)cc23)c2ccccc2C1=O	426467075	25	338.12	2.51
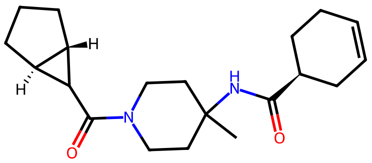	CC1(NC(=O)[C@@H]2CC=CCC2)CCN(C(=O)C2[C@H]3CCC[C@H]23)CC1	956293338	24	330.23	2.89

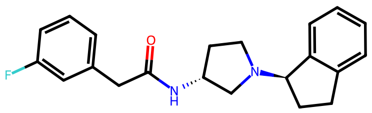	O=C(Cc1cccc(F)c1)N[C@@H]1CCN([C@@H]2CCc3ccccc32)C1	1016184467	25	338.18	3.25
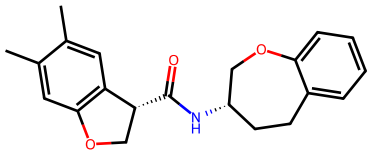	Cc1cc2c(cc1C)[C@H](C(=O)N[C@H]1CCc3ccccc3OC1)CO2	511624753	25	337.17	3.29
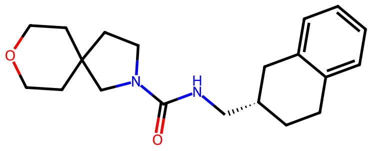	O=C(NC[C@H]1CCc2ccccc2C1)N1CCC2(CCOCC2)C1	608436100	24	328.22	3.0
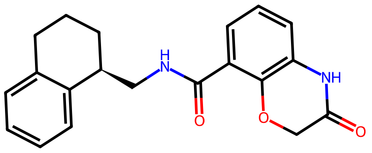	O=C1COc2c(cccc2C(=O)NC[C@@H]2CCCc3ccccc32)N1	956892698	25	336.15	2.87
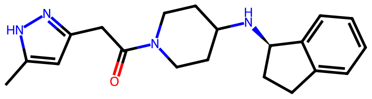	Cc1cc(CC(=O)N2CCC(N[C@@H]3CCc4ccccc43)CC2)n[nH]1	990974756	25	338.21	2.53
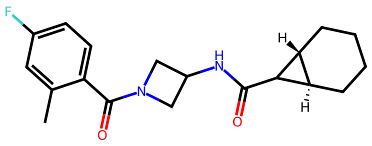	Cc1cc(F)ccc1C(=O)N1CC(NC(=O)C2[C@@H]3CCCC[C@@H]23)C1	990644884	24	330.17	2.51
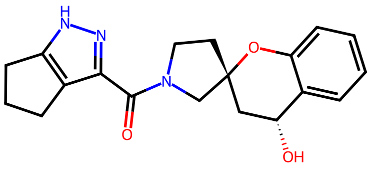	O=C(c1n[nH]c2c1CCC2)N1CC[C@@]2(C[C@@H](O)c3ccccc3O2)C1	818408522	25	339.16	2.0
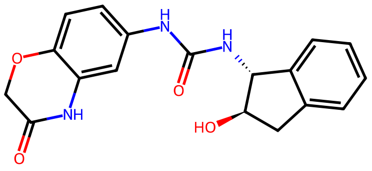	O=C1COc2ccc(NC(=O)N[C@@H]3c4ccccc4C[C@H]3O)cc2N1	612393301	25	339.12	1.8

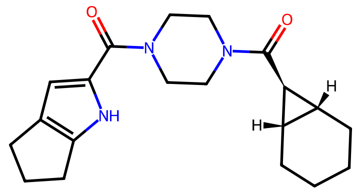	O=C(c1cc2c([nH]1)CCC2)N1CCN(C(=O)[C@@H]2[C@H]3CCCC[C@H]32)CC1	1010894002	25	341.21	2.22
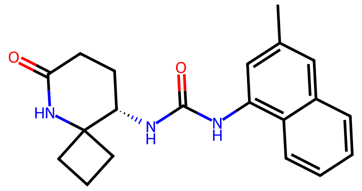	Cc1cc(NC(=O)N[C@H]2CCC(=O)NC23CCC3)c2ccccc2c1	297524481	25	337.18	3.47
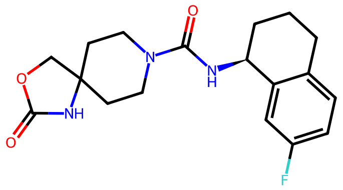	O=C1NC2(CCN(C(=O)N[C@H]3CCCc4ccc(F)cc43)CC2)CO1	492275597	25	347.16	2.49
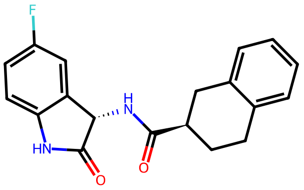	O=C1Nc2ccc(F)cc2[C@@H]1NC(=O)[C@@H]1CCc2ccccc2C1	102324743	24	324.13	2.74
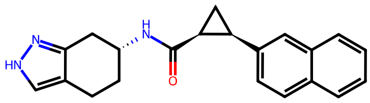	O=C(N[C@@H]1CCc2c[nH]nc2C1)[C@H]1C[C@H]1c1ccc2ccccc2c1	332914341	25	331.17	3.34
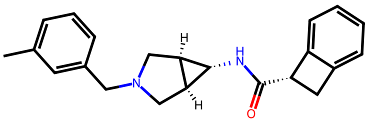	Cc1cccc(CN2C[C@H]3[C@H](NC(=O)[C@H]4Cc5ccccc54)[C@H]3C2)c1	961627361	25	332.19	2.88
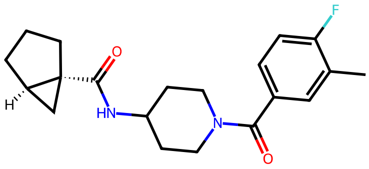	Cc1cc(C(=O)N2CCC(NC(=O)[C@@]34CCC[C@@H]3C4)CC2)ccc1F	948068046	25	344.19	3.05
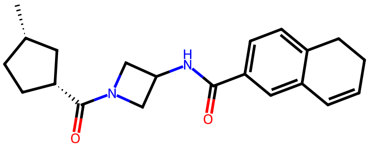	C[C@H]1CC[C@@H](C(=O)N2CC(NC(=O)c3ccc4c(c3)C=CCC4)C2)C1	995375357	25	338.2	3.02

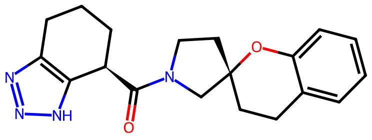	O=C([C@@H]1CCCc2nn[nH]c21)N1CC[C@@]2(CCc3ccccc3O2)C1	854502420	25	338.17	2.22
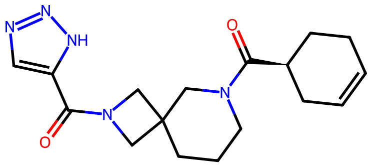	O=C(c1cnn[nH]1)N1CC2(CCCN(C(=O)[C@@H]3CC=CCC3)C2)C1	1010732461	24	329.19	1.23
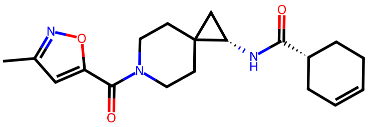	Cc1cc(C(=O)N2CCC3(CC2)C[C@@H]3NC(=O)[C@H]2CC=CCC2)on1	963030514	25	343.19	2.45
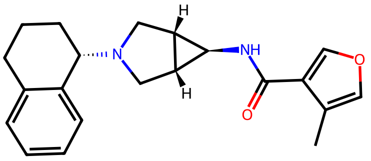	Cc1cocc1C(=O)N[C@H]1[C@@H]2CN([C@H]3CCCc4ccccc43)C[C@@H]21	961521043	25	336.18	3.33
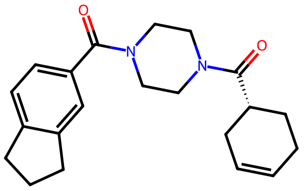	O=C(c1ccc2c(c1)CCC2)N1CCN(C(=O)[C@H]2CC=CCC2)CC1	1010297135	25	338.2	2.82
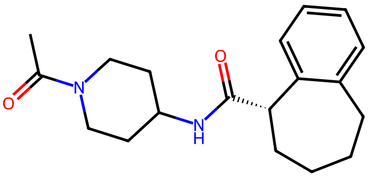	CC(=O)N1CCC(NC(=O)[C@H]2CCCCc3ccccc32)CC1	943035176	23	314.2	2.62
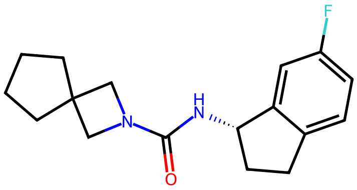	O=C(N[C@H]1CCc2ccc(F)cc21)N1CC2(CCCC2)C1	548329714	21	288.16	3.4
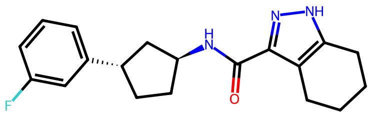	O=C(N[C@H]1CC[C@H](c2cccc(F)c2)C1)c1n[nH]c2c1CCCC2	684513721	24	327.17	3.49

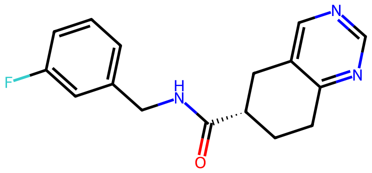	O=C(NCc1cccc(F)c1)[C@H]1CCc2ncncc2C1	648233828	21	285.13	2.04
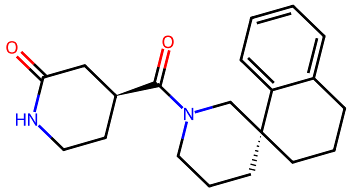	O=C1C[C@@H](C(=O)N2CCC[C@]3(CCCc4ccccc43)C2)CCN1	338962412	24	326.2	2.41
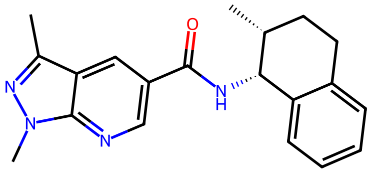	Cc1nn(C)c2ncc(C(=O)N[C@H]3c4ccccc4CC[C@H]3C)cc12	179440808	25	334.18	3.33
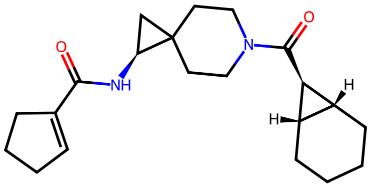	O=C(N[C@H]1CC12CCN(C(=O)[C@@H]1[C@H]3CCCC[C@H]31)CC2)C1=CCCC1	963562474	25	342.23	3.03
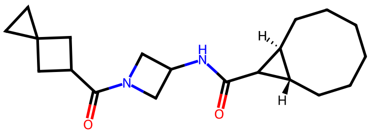	O=C(NC1CN(C(=O)C2CC3(CC3)C2)C1)C1[C@H]2CCCCCC[C@H]12	1000510026	24	330.23	2.72
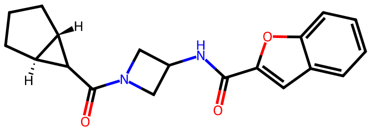	O=C(NC1CN(C(=O)C2[C@H]3CCC[C@H]23)C1)c1cc2ccccc2o1	1000052949	24	324.15	2.42
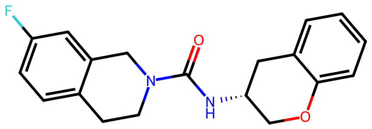	O=C(N[C@H]1COc2ccccc2C1)N1CCc2ccc(F)cc2C1	178428822	24	326.14	2.9
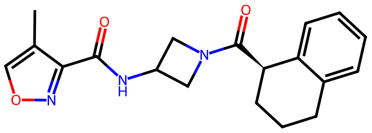	Cc1conc1C(=O)NC1CN(C(=O)[C@@H]2CCCc3ccccc32)C1	999660685	25	339.16	2.04

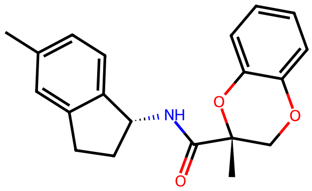	Cc1ccc2c(c1)CC[C@H]2NC(=O)[C@@]1(C)COc2ccccc2O1	1357412386	24	323.15	3.33
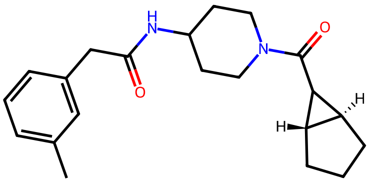	Cc1cccc(CC(=O)NC2CCN(C(=O)C3[C@H]4CCC[C@H]34)CC2)c1	946078305	25	340.22	2.69
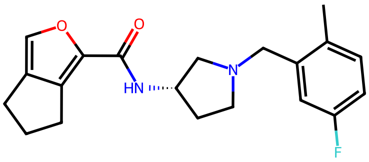	Cc1ccc(F)cc1CN1CC[C@H](NC(=O)c2occ3c2CCC3)C1	1015404393	25	342.17	3.22
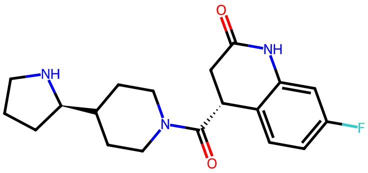	O=C1C[C@@H](C(=O)N2CCC([C@H]3CCCN3)CC2)c2ccc(F)cc2N1	353042958	25	345.19	2.24
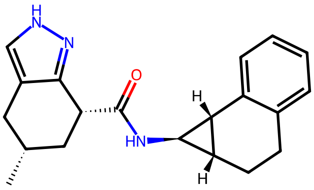	C[C@@H]1Cc2c[nH]nc2[C@H](C(=O)N[C@@H]2[C@H]3CCc4ccccc4[C@H]32)C1	646925201	24	321.18	2.92
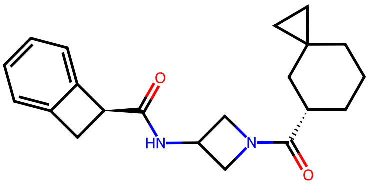	O=C(NC1CN(C(=O)[C@H]2CCCC3(CC3)C2)C1)[C@H]1Cc2ccccc21	998688759	25	338.2	2.62
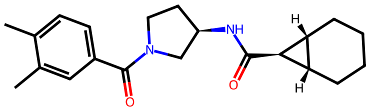	Cc1ccc(C(=O)N2CC[C@@H](NC(=O)[C@@H]3[C@H]4CCCC[C@H]43)C2)cc1C	939903067	25	340.22	3.07
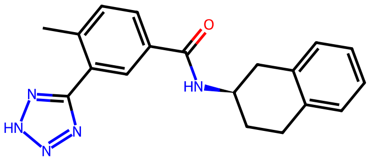	Cc1ccc(C(=O)N[C@@H]2CCc3ccccc3C2)cc1-c1nn[nH]n1	1609418846	25	333.16	2.46

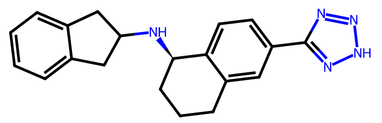	c1ccc2c(c1)CC(N[C@@H]1CCCc3cc(-c4nn[nH]n4)ccc31)C2	1593580218	25	331.18	3.0
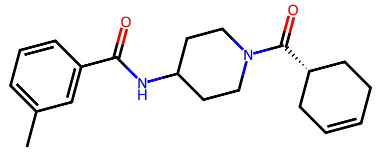	Cc1cccc(C(=O)NC2CCN(C(=O)[C@H]3CC=CCC3)CC2)c1	870831843	24	326.2	3.07
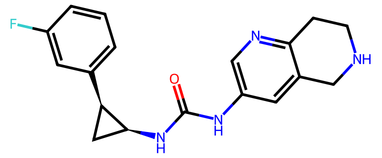	O=C(Nc1cnc2c(c1)CNCC2)N[C@H]1C[C@H]1c1cccc(F)c1	667804651	24	326.15	2.54
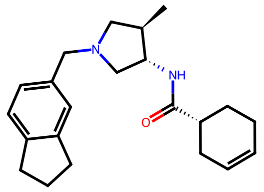	C[C@@H]1CN(Cc2ccc3c(c2)CCC3)C[C@H]1NC(=O)[C@H]1CC=CCC1	1079399990	25	338.24	3.47
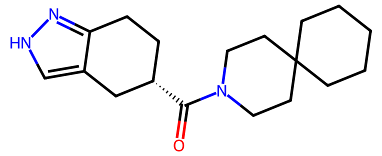	O=C([C@H]1CCc2n[nH]cc2C1)N1CCC2(CCCCC2)CC1	366760373	22	301.22	3.09
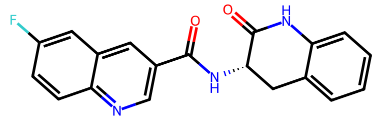	O=C(N[C@H]1Cc2ccccc2NC1=O)c1cnc2ccc(F)cc2c1	1471013993	25	335.11	2.67
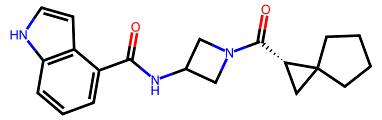	O=C(NC1CN(C(=O)[C@H]2CC23CCCC3)C1)c1cccc2[nH]ccc12	1000410687	25	337.18	2.69
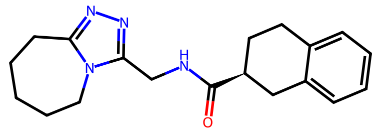	O=C(NCc1nnc2n1CCCCC2)[C@H]1CCc2ccccc2C1	575992863	24	324.2	2.43

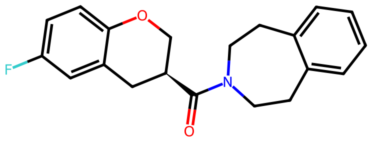	O=C([C@@H]1COc2ccc(F)cc2C1)N1CCc2ccccc2CC1	90419266	24	325.15	3.0
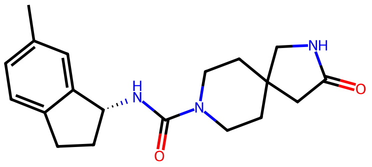	Cc1ccc2c(c1)[C@H](NC(=O)N1CCC3(CC1)CNC(=O)C3)CC2	752009388	24	327.19	2.29
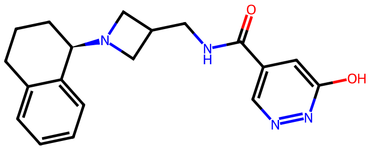	O=C(NCC1CN([C@@H]2CCCc3ccccc32)C1)c1cnnc(O)c1	1031751160	25	338.17	1.92
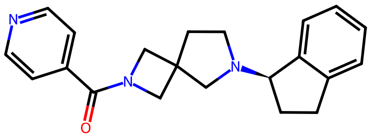	O=C(c1ccncc1)N1CC2(CCN([C@@H]3CCc4ccccc43)C2)C1	1072394585	25	333.18	2.92
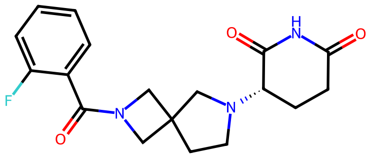	O=C1CC[C@H](N2CCC3(CN(C(=O)c4ccccc4F)C3)C2)C(=O)N1	1072408668	25	345.15	0.78
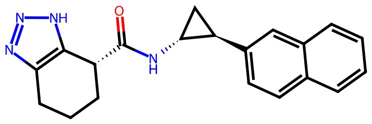	O=C(N[C@@H]1C[C@H]1c1ccc2ccccc2c1)[C@@H]1CCCc2nn[nH]c21	614366880	25	332.16	3.05
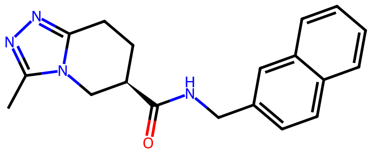	Cc1nnc2n1C[C@H](C(=O)NCc1ccc3ccccc3c1)CC2	750720035	24	320.16	2.62
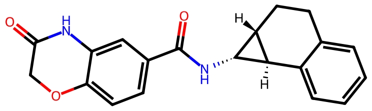	O=C1COc2ccc(C(=O)N[C@H]3[C@@H]4c5ccccc5CC[C@H]43)cc2N1	121385264	25	334.13	2.48

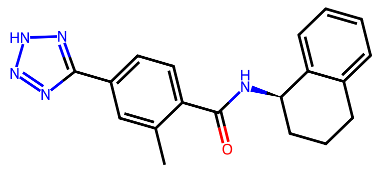	Cc1cc(-c2nn[nH]n2)ccc1C(=O)N[C@@H]1CCCc2ccccc21	826247226	25	333.16	2.98
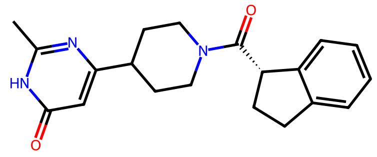	Cc1nc(C2CCN(C(=O)[C@H]3CCc4ccccc43)CC2)cc(=O)[nH]1	82120283	25	337.18	2.51
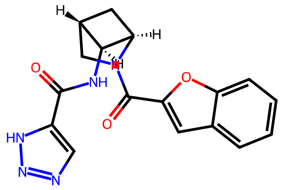	O=C(N[C@H]1[C@H]2C[C@H]1N(C(=O)c1cc3ccccc3o1)C2)c1cnn[nH]1	947492602	25	337.12	1.19
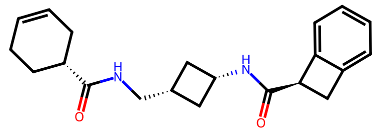	O=C(NC[C@H]1C[C@@H](NC(=O)[C@@H]2Cc3ccccc32)C1)[C@H]1CC=CCC1	978582711	25	338.2	2.69
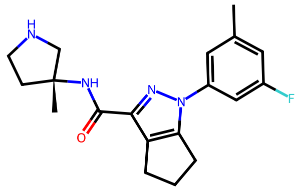	Cc1cc(F)cc(-n2nc(C(=O)N[C@]3(C)CCNC3)c3c2CCC3)c1	663013272	25	342.19	2.29
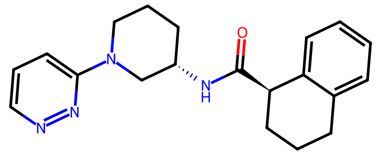	O=C(N[C@H]1CCCN(c2cccnn2)C1)[C@@H]1CCCc2ccccc21	265515829	25	336.2	2.68
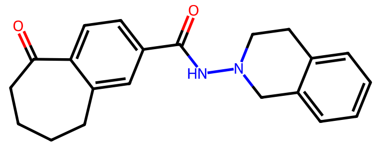	O=C(NN1CCc2ccccc2C1)c1ccc2c(c1)CCCCC2=O	1146331135	25	334.17	3.3
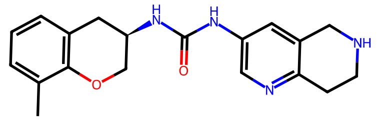	Cc1cccc2c1OC[C@H](NC(=O)Nc1cnc3c(c1)CNCC3)C2	667802545	25	338.17	2.16

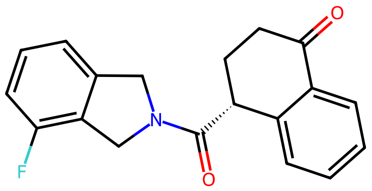	O=C1CC[C@@H](C(=O)N2Cc3cccc(F)c3C2)c2ccccc21	458133140	23	309.12	3.43
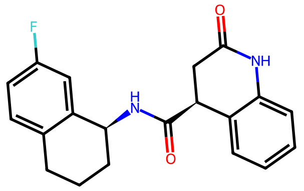	O=C1C[C@H](C(=O)N[C@H]2CCCc3ccc(F)cc32)c2ccccc2N1	93062921	25	338.14	3.45
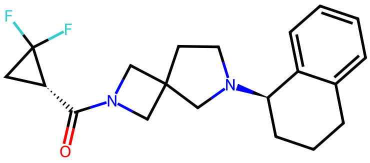	O=C([C@@H]1CC1(F)F)N1CC2(CCN([C@@H]3CCCc4ccccc43)C2)C1	1072707906	25	346.19	3.25
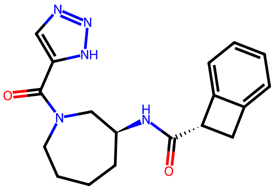	O=C(N[C@H]1CCCCN(C(=O)c2cnn[nH]2)C1)[C@H]1Cc2ccccc21	943485512	25	339.17	1.26
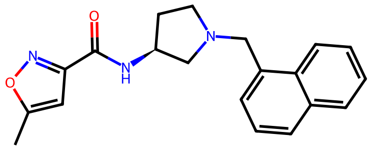	Cc1cc(C(=O)N[C@H]2CCN(Cc3cccc4ccccc34)C2)no1	1010022524	25	335.16	3.14
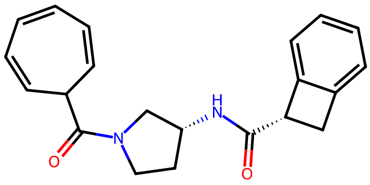	O=C(N[C@@H]1CCN(C(=O)C2C=CC=CC=C2)C1)[C@H]1Cc2ccccc21	942304991	25	334.17	2.34
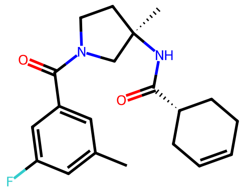	Cc1cc(F)cc(C(=O)N2CC[C@@](C)(NC(=O)[C@H]3CC=CCC3)C2)c1	956689820	25	344.19	3.21
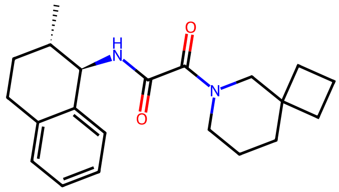	C[C@H]1CCc2ccccc2[C@@H]1NC(=O)C(=O)N1CCCC2(CCC2)C1	359648125	25	340.22	3.22

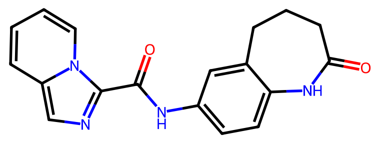	O=C1CCCc2cc(NC(=O)c3ncc4ccccn34)ccc2N1	1125910482	24	320.13	2.86
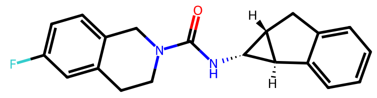	O=C(N[C@H]1[C@@H]2c3ccccc3C[C@H]21)N1CCc2cc(F)ccc2C1	129690350	24	322.15	3.23
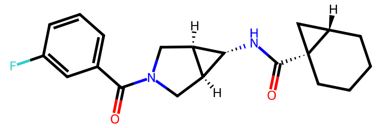	O=C(c1cccc(F)c1)N1C[C@H]2[C@H](NC(=O)[C@@]34CCCC[C@H]3C4)[C@H]2C1	977151378	25	342.17	2.59
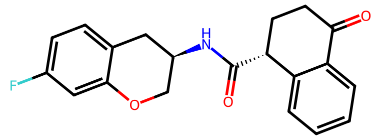	O=C1CC[C@@H](C(=O)N[C@H]2COc3cc(F)ccc3C2)c2ccccc21	618649057	25	339.13	3.01
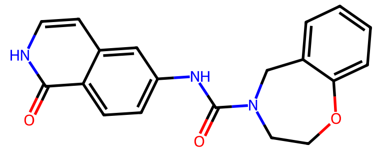	O=C(Nc1ccc2c(=O)[nH]ccc2c1)N1CCOc2ccccc2C1	1679089521	25	335.13	2.95
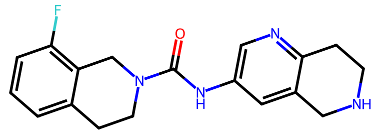	O=C(Nc1cnc2c(c1)CNCC2)N1CCc2cccc(F)c2C1	667800873	24	326.15	2.46
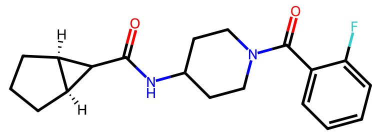	O=C(NC1CCN(C(=O)c2ccccc2F)CC1)C1[C@H]2CCC[C@@H]12	177666966	24	330.17	2.59
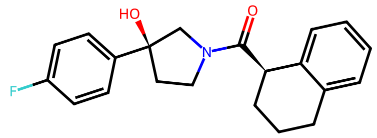	O=C([C@@H]1CCCc2ccccc21)N1CC[C@](O)(c2ccc(F)cc2)C1	777488735	25	339.16	3.37

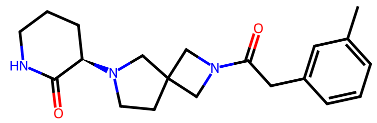	Cc1cccc(CC(=O)N2CC3(CCN([C@@H]4CCCNC4=O)C3)C2)c1	1072927848	25	341.21	1.35
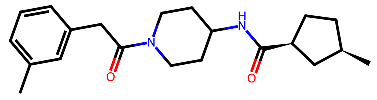	Cc1cccc(CC(=O)N2CCC(NC(=O)[C@H]3CC[C@@H](C)C3)CC2)c1	948063065	25	342.23	3.08
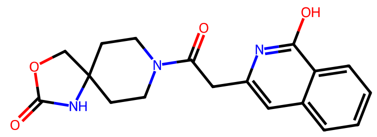	O=C1NC2(CCN(C(=O)Cc3cc4ccccc4c(O)n3)CC2)CO1	1412723842	25	341.14	1.58
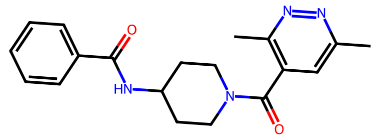	Cc1cc(C(=O)N2CCC(NC(=O)c3ccccc3)CC2)c(C)nn1	678042263	25	338.17	2.13
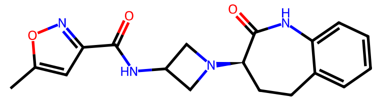	Cc1cc(C(=O)NC2CN([C@@H]3CCc4ccccc4NC3=O)C2)no1	1030083453	25	340.15	1.35
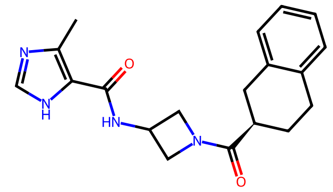	Cc1nc[nH]c1C(=O)NC1CN(C(=O)[C@@H]2CCc3ccccc3C2)C1	995253924	25	338.17	1.46
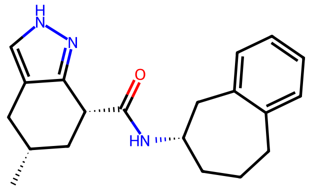	C[C@@H]1Cc2c[nH]nc2[C@H](C(=O)N[C@H]2CCCc3ccccc3C2)C1	888245438	24	323.2	3.14
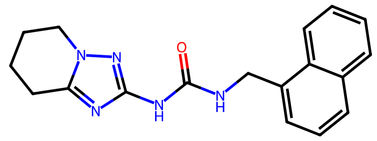	O=C(NCc1cccc2ccccc12)Nc1nc2n(n1)CCCC2	569563508	24	321.16	3.09

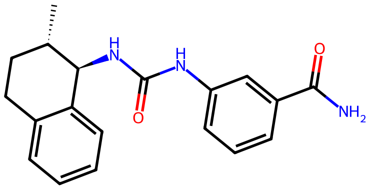	C[C@H]1CCc2ccccc2[C@@H]1NC(=O)Nc1cccc(C(N)=O)c1	179360361	24	323.16	3.23
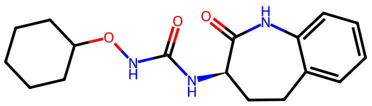	O=C(NOC1CCCCC1)N[C@@H]1CCc2ccccc2NC1=O	847358183	23	317.17	2.5
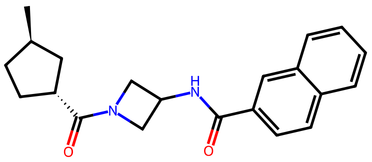	C[C@@H]1CC[C@@H](C(=O)N2CC(NC(=O)c3ccc4ccccc4c3)C2)C1	999456175	25	336.18	3.22
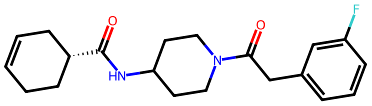	O=C(NC1CCN(C(=O)Cc2cccc(F)c2)CC1)[C@H]1CC=CCC1	948480568	25	344.19	2.83
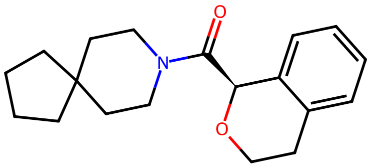	O=C([C@@H]1OCCc2ccccc21)N1CCC2(CCCC2)CC1	133781662	22	299.19	3.48
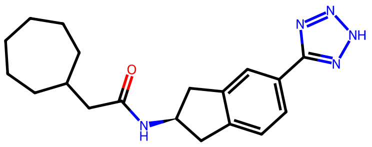	O=C(CC1CCCCCC1)N[C@@H]1Cc2ccc(-c3nn[nH]n3)cc2C1	1581984844	25	339.21	2.81
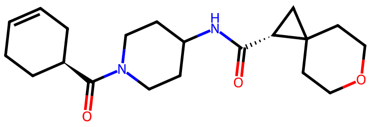	O=C(NC1CCN(C(=O)[C@@H]2CC=CCC2)CC1)[C@@H]1CC12CCOCC2	944127944	25	346.23	2.27
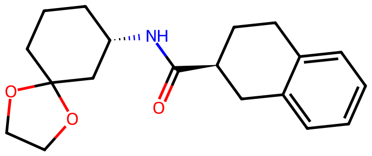	O=C(N[C@H]1CCCC2(C1)OCCO2)[C@@H]1CCc2ccccc2C1	715634483	23	315.18	2.59

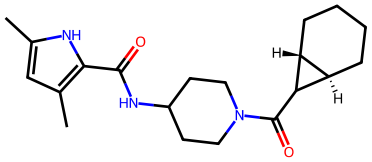	Cc1cc(C)c(C(=O)NC2CCN(C(=O)C3[C@@H]4CCCC[C@@H]34)CC2)[nH]1	945956612	25	343.23	2.79
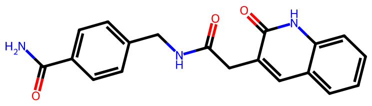	NC(=O)c1ccc(CNC(=O)Cc2cc3ccccc3[nH]c2=O)cc1	47045805	25	335.13	1.49
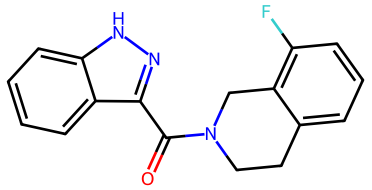	O=C(c1n[nH]c2ccccc12)N1CCc2cccc(F)c2C1	128040680	22	295.11	2.9
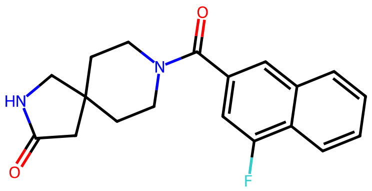	O=C1CC2(CCN(C(=O)c3cc(F)c4ccccc4c3)CC2)CN1	341681113	24	326.14	2.72
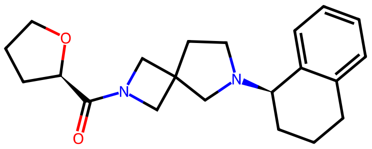	O=C([C@H]1CCCO1)N1CC2(CCN([C@@H]3CCCc4ccccc43)C2)C1	1072446566	25	340.22	2.78
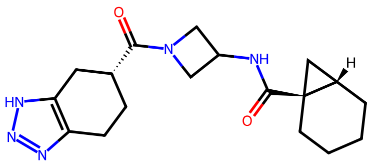	O=C([C@@H]1CCc2nn[nH]c2C1)N1CC(NC(=O)[C@]23CCCC[C@H]2C3)C1	999276241	25	343.2	0.82
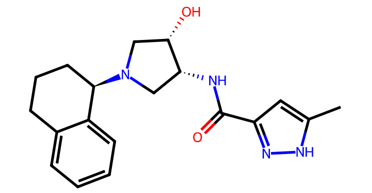	Cc1cc(C(=O)N[C@@H]2CN([C@@H]3CCCc4ccccc43)C[C@@H]2O)n[nH]1	1083357119	25	340.19	1.57
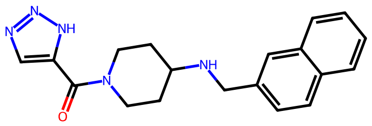	O=C(c1cnn[nH]1)N1CCC(NCc2ccc3ccccc3c2)CC1	996751214	25	335.17	2.35

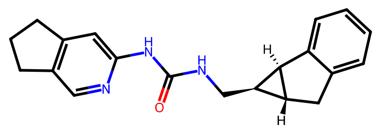	O=C(NC[C@@H]1[C@H]2Cc3ccccc3[C@@H]21)Nc1cc2c(cn1)CCC2	583483634	24	319.17	3.28
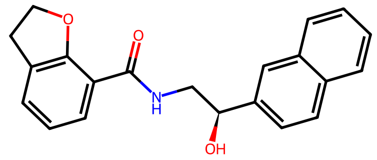	O=C(NC[C@H](O)c1ccc2ccccc2c1)c1cccc2c1OCC2	1137389095	25	333.14	3.24
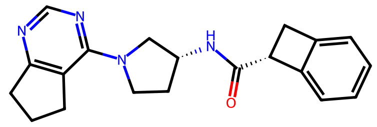	O=C(N[C@@H]1CCN(c2ncnc3c2CCC3)C1)[C@@H]1Cc2ccccc21	1058531178	25	334.18	2.0
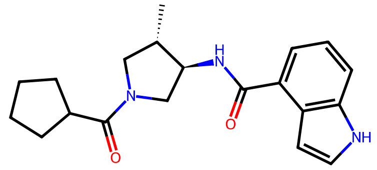	C[C@H]1CN(C(=O)C2CCCC2)C[C@@H]1NC(=O)c1cccc2[nH]ccc12	966038012	25	339.19	2.93
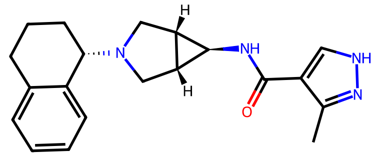	Cc1n[nH]cc1C(=O)N[C@H]1[C@@H]2CN([C@H]3CCCc4ccccc43)C[C@@H]21	961428524	25	336.2	2.46
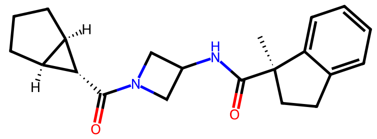	C[C@@]1(C(=O)NC2CN(C(=O)[C@@H]3[C@H]4CCC[C@H]43)C2)CCc2ccccc21	994372925	25	338.2	2.26
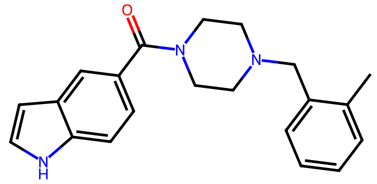	Cc1ccccc1CN1CCN(C(=O)c2ccc3[nH]ccc3c2)CC1	337419685	25	333.18	3.43
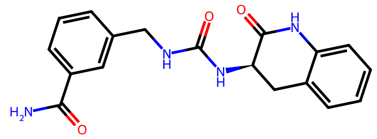	NC(=O)c1cccc(CNC(=O)N[C@@H]2Cc3ccccc3NC2=O)c1	1414530161	25	338.14	1.15

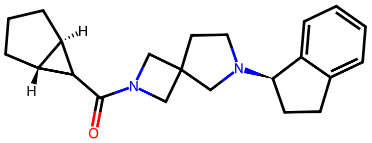	O=C(C1[C@@H]2CCC[C@@H]12)N1CC2(CCN([C@@H]3CCc4ccccc43)C2)C1	1072591517	25	336.22	3.25
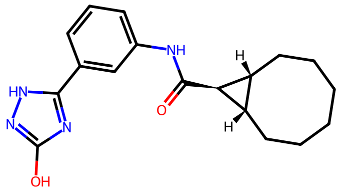	O=C(Nc1cccc(-c2nc(O)n[nH]2)c1)[C@@H]1[C@H]2CCCCCC[C@H]21	614561438	24	326.17	3.33
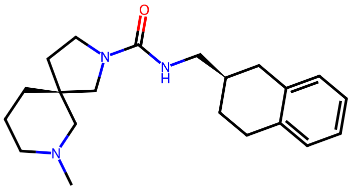	CN1CCC[C@]2(CCN(C(=O)NC[C@@H]3CCc4ccccc4C3)C2)C1	655562944	25	341.25	2.92
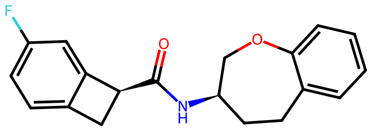	O=C(N[C@@H]1CCc2ccccc2OC1)[C@H]1Cc2ccc(F)cc21	801019482	23	311.13	2.98
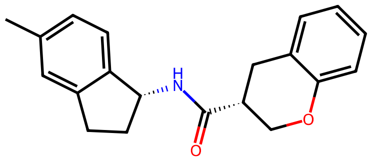	Cc1ccc2c(c1)CC[C@H]2NC(=O)[C@H]1COc2ccccc2C1	501961037	23	307.16	3.35
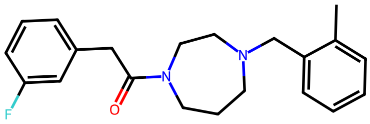	Cc1ccccc1CN1CCCN(C(=O)Cc2cccc(F)c2)CC1	41315450	25	340.2	3.41
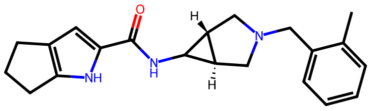	Cc1ccccc1CN1C[C@H]2C(NC(=O)c3cc4c([nH]3)CCC4)[C@@H]2C1	962276287	25	335.2	2.67
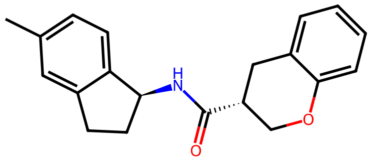	Cc1ccc2c(c1)CC[C@@H]2NC(=O)[C@@H]1COc2ccccc2C1	501961032	23	307.16	3.35

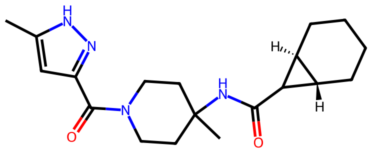	Cc1cc(C(=O)N2CCC(C)(NC(=O)C3[C@H]4CCCC[C@H]34)CC2)n[nH]1	956325077	25	344.22	2.27
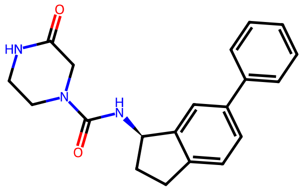	O=C1CN(C(=O)N[C@@H]2CCc3ccc(-c4ccccc4)cc32)CCN1	188095057	25	335.16	2.48
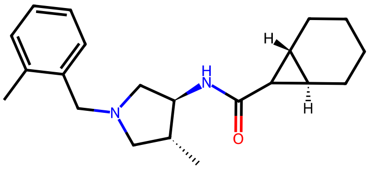	Cc1ccccc1CN1C[C@@H](NC(=O)C2[C@@H]3CCCC[C@@H]23)[C@H](C)C1	1079530451	24	326.24	3.37
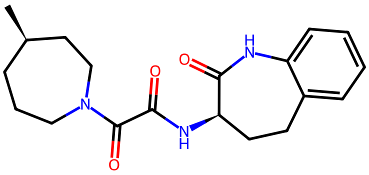	C[C@@H]1CCCN(C(=O)C(=O)N[C@@H]2CCc3ccccc3NC2=O)CC1	1327237243	25	343.19	1.7
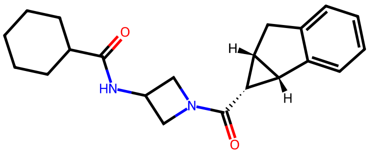	O=C(NC1CN(C(=O)[C@H]2[C@H]3c4ccccc4C[C@H]32)C1)C1CCCCC1	990104211	25	338.2	2.48
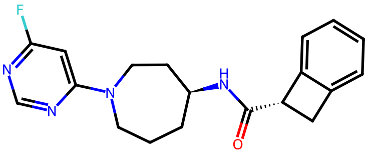	O=C(N[C@H]1CCCN(c2cc(F)ncn2)CC1)[C@H]1Cc2ccccc21	1066778781	25	340.17	2.43
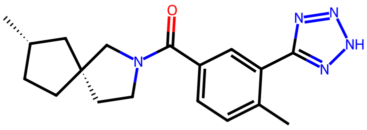	Cc1ccc(C(=O)N2CC[C@@]3(CC[C@H](C)C3)C2)cc1-c1nn[nH]n1	1592119546	24	325.19	2.83
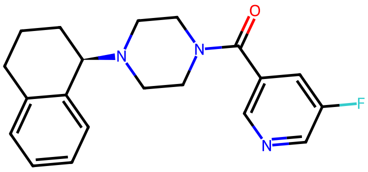	O=C(c1cncc(F)c1)N1CCN([C@@H]2CCCc3ccccc32)CC1	1688692838	25	339.17	3.06

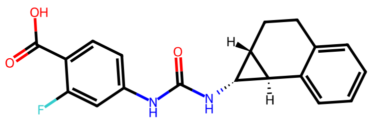	O=C(Nc1ccc(C(=O)O)c(F)c1)N[C@H]1[C@@H]2c3ccccc3CC[C@H]21	833153664	25	340.12	3.37
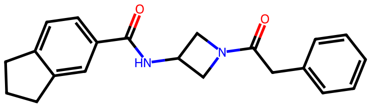	O=C(NC1CN(C(=O)Cc2ccccc2)C1)c1ccc2c(c1)CCC2	991366121	25	334.17	2.36
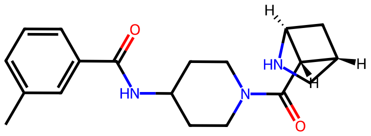	Cc1cccc(C(=O)NC2CCN(C(=O)[C@H]3[C@H]4CN[C@H]3C4)CC2)c1	1550189828	24	327.19	1.32
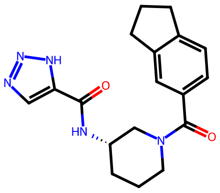	O=C(N[C@H]1CCCN(C(=O)c2ccc3c(c2)CCC3)C1)c1cnn[nH]1	950782057	25	339.17	1.33
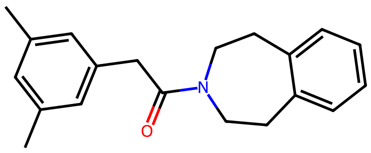	Cc1cc(C)cc(CC(=O)N2CCc3ccccc3CC2)c1	1538204693	22	293.18	3.47
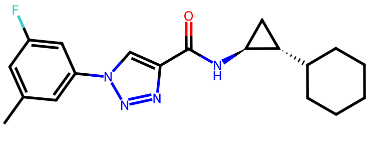	Cc1cc(F)cc(-n2cc(C(=O)N[C@H]3C[C@@H]3C3CCCCC3)nn2)c1	1354729632	25	342.19	3.41
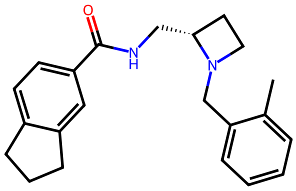	Cc1ccccc1CN1CC[C@H]1CNC(=O)c1ccc2c(c1)CCC2	1039003105	25	334.2	3.49
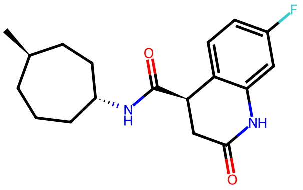	C[C@@H]1CCC[C@@H](NC(=O)[C@@H]2CC(=O)Nc3cc(F)ccc32)CC1	188848039	23	318.17	3.34

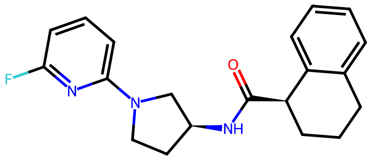	O=C(N[C@H]1CCN(c2cccc(F)n2)C1)[C@@H]1CCCc2ccccc21	1058592031	25	339.17	3.04
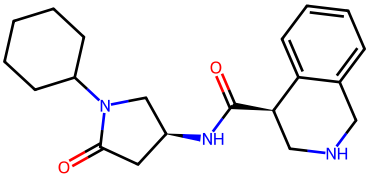	O=C(N[C@H]1CC(=O)N(C2CCCCC2)C1)[C@@H]1CNCc2ccccc21	574037347	25	341.21	1.92
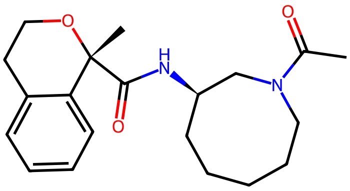	CC(=O)N1CCCCC[C@@H](NC(=O)[C@@]2(C)OCCc3ccccc32)C1	986795754	25	344.21	2.38
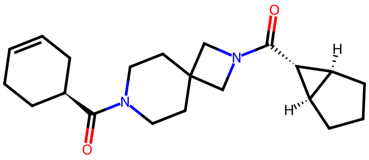	O=C([C@@H]1CC=CCC1)N1CCC2(CC1)CN(C(=O)[C@@H]1[C@H]3CCC[C@H]31)C2	944849225	25	342.23	2.84
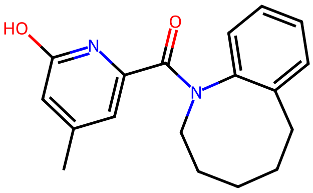	Cc1cc(O)nc(C(=O)N2CCCCCc3ccccc32)c1	328892263	22	296.15	3.47
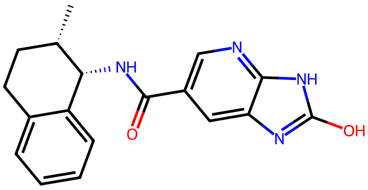	C[C@H]1CCc2ccccc2[C@H]1NC(=O)c1cnc2[nH]c(O)nc2c1	558416262	24	322.14	2.72
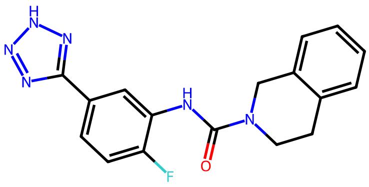	O=C(Nc1cc(-c2nn[nH]n2)ccc1F)N1CCc2ccccc2C1	1593201445	25	338.13	2.6
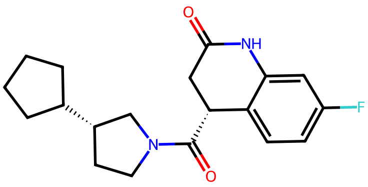	O=C1C[C@@H](C(=O)N2CC[C@H](C3CCCC3)C2)c2ccc(F)cc2N1	421644334	24	330.17	3.29

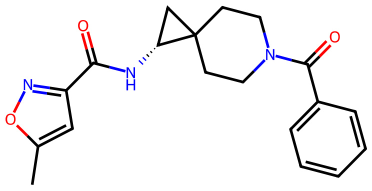	Cc1cc(C(=O)N[C@@H]2CC23CCN(C(=O)c2ccccc2)CC3)no1	962878909	25	339.16	2.41
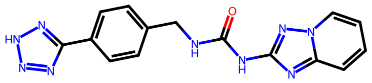	O=C(NCc1ccc(-c2nn[nH]n2)cc1)Nc1nc2ccccn2n1	1593129838	25	335.12	1.23
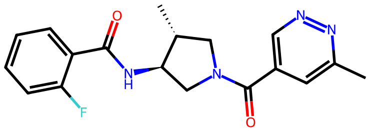	Cc1cc(C(=O)N2C[C@@H](NC(=O)c3ccccc3F)[C@H](C)C2)cnn1	948696628	25	342.15	1.81
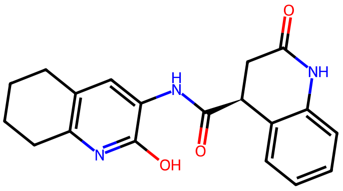	O=C1C[C@H](C(=O)Nc2cc3c(nc2O)CCC3)c2ccccc2N1	1461364357	25	337.14	2.73
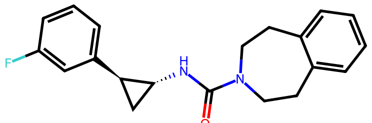	O=C(N[C@@H]1C[C@H]1c1cccc(F)c1)N1CCc2ccccc2CC1	559538508	24	324.16	3.49
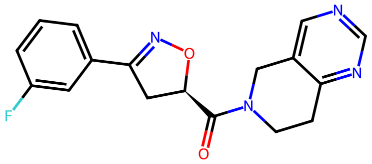	O=C([C@H]1CC(c2cccc(F)c2)=NO1)N1CCc2ncncc2C1	883032031	24	326.12	1.69
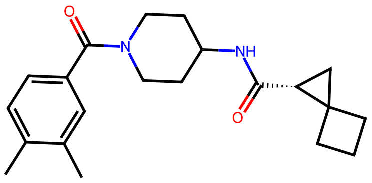	Cc1ccc(C(=O)N2CCC(NC(=O)[C@@H]3CC34CCC4)CC2)cc1C	948352104	25	340.22	3.21
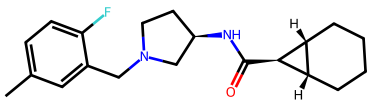	Cc1ccc(F)c(CN2CC[C@@H](NC(=O)[C@@H]3[C@H]4CCCC[C@H]43)C2)c1	1010442067	24	330.21	3.26

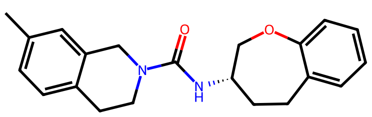	Cc1ccc2c(c1)CN(C(=O)N[C@H]1CCc3ccccc3OC1)CC2	1469949643	25	336.18	3.46
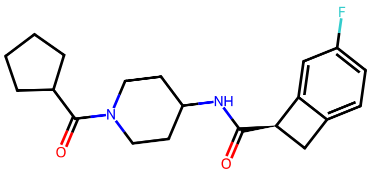	O=C(NC1CCN(C(=O)C2CCCC2)CC1)[C@@H]1Cc2ccc(F)cc21	943375810	25	344.19	2.76
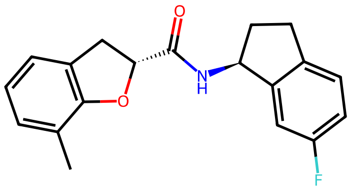	Cc1cccc2c1O[C@@H](C(=O)N[C@H]1CCc3cc(F)cc31)C2	516950697	23	311.13	3.24
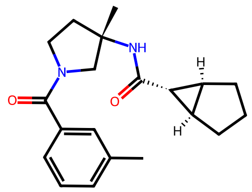	Cc1cccc(C(=O)N2CC[C@](C)(NC(=O)[C@@H]3[C@H]4CCC[C@H]43)C2)c1	956799952	24	326.2	2.76
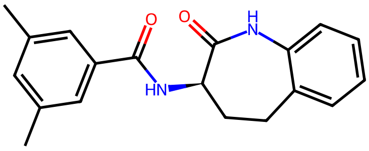	Cc1cc(C)cc(C(=O)N[C@@H]2CCc3ccccc3NC2=O)c1	654006203	23	308.15	2.99
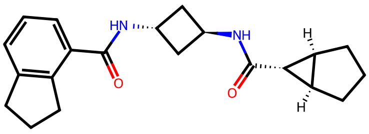	O=C(N[C@H]1C[C@H](NC(=O)[C@@H]2[C@H]3CCC[C@H]32)C1)c1cccc2c1CCC2	973143455	25	338.2	2.6
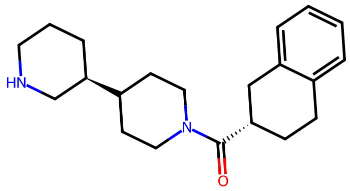	O=C([C@H]1CCc2ccccc2C1)N1CCC([C@@H]2CCCNC2)CC1	658960446	24	326.24	3.03
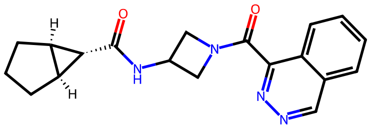	O=C(NC1CN(C(=O)c2nncc3ccccc23)C1)[C@@H]1[C@H]2CCC[C@H]21	990705936	25	336.16	1.62

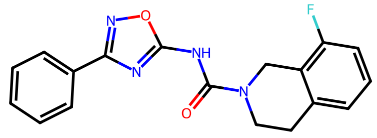	O=C(Nc1nc(-c2ccccc2)no1)N1CCc2cccc(F)c2C1	1623496822	25	338.12	3.47
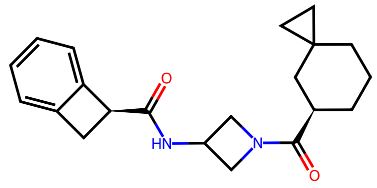	O=C(NC1CN(C(=O)[C@@H]2CCCC3(CC3)C2)C1)[C@H]1Cc2ccccc21	998688754	25	338.2	2.62
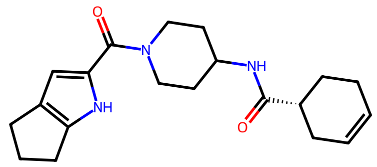	O=C(NC1CCN(C(=O)c2cc3c([nH]2)CCC3)CC1)[C@H]1CC=CCC1	947446024	25	341.21	2.58
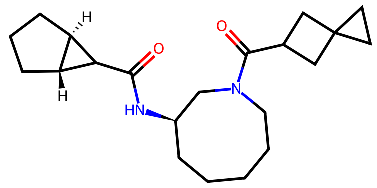	O=C(N[C@@H]1CCCCCN(C(=O)C2CC3(CC3)C2)C1)C1[C@@H]2CCC[C@@H]12	987150766	25	344.25	3.11
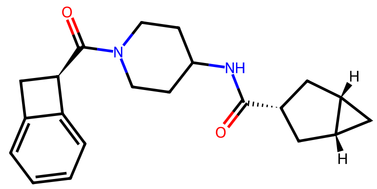	O=C(NC1CCN(C(=O)[C@@H]2Cc3ccccc32)CC1)[C@@H]1C[C@@H]2C[C@@H]2C1	948063063	25	338.2	2.48
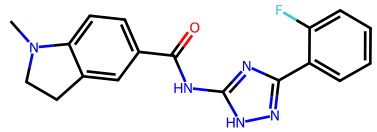	CN1CCc2cc(C(=O)Nc3nc(-c4ccccc4F)n[nH]3)ccc21	779380244	25	337.13	2.86
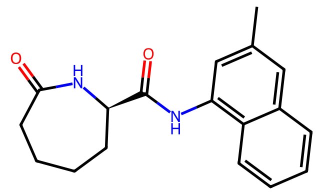	Cc1cc(NC(=O)[C@H]2CCCCC(=O)N2)c2ccccc2c1	296331446	22	296.15	3.15
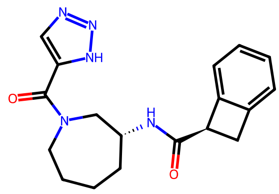	O=C(N[C@@H]1CCCCN(C(=O)c2cnn[nH]2)C1)[C@@H]1Cc2ccccc21	943485510	25	339.17	1.26

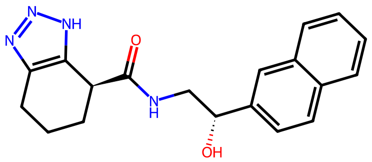	O=C(NC[C@@H](O)c1ccc2ccccc2c1)[C@H]1CCCc2nn[nH]c21	816922508	25	336.16	2.23
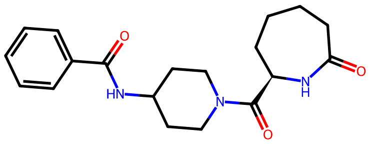	O=C1CCCC[C@H](C(=O)N2CCC(NC(=O)c3ccccc3)CC2)N1	193732960	25	343.19	1.47
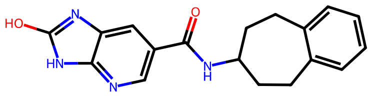	O=C(NC1CCc2ccccc2CC1)c1cnc2[nH]c(O)nc2c1	560465338	24	322.14	2.34
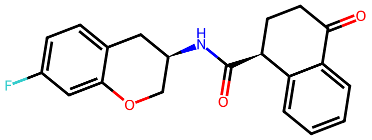	O=C1CC[C@H](C(=O)N[C@H]2COc3cc(F)ccc3C2)c2ccccc21	618649056	25	339.13	3.01
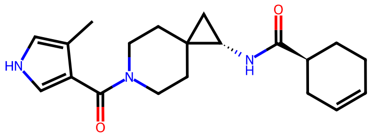	Cc1c[nH]cc1C(=O)N1CCC2(CC1)C[C@@H]2NC(=O)[C@@H]1CC=CCC1	963026921	25	341.21	2.79
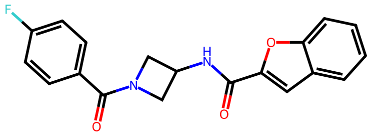	O=C(NC1CN(C(=O)c2ccc(F)cc2)C1)c1cc2ccccc2o1	1000050391	25	338.11	2.83
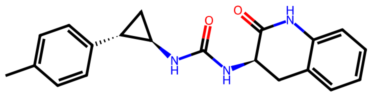	Cc1ccc([C@@H]2C[C@H]2NC(=O)N[C@@H]2Cc3ccccc3NC2=O)cc1	637240909	25	335.16	2.71
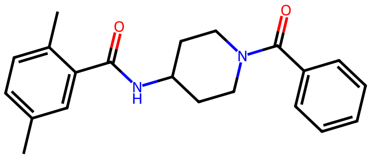	Cc1ccc(C)c(C(=O)NC2CCN(C(=O)c3ccccc3)CC2)c1	53392698	25	336.18	3.34

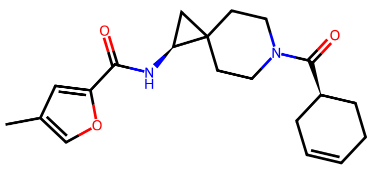	Cc1coc(C(=O)N[C@H]2CC23CCN(C(=O)[C@@H]2CC=CCC2)CC3)c1	964714530	25	342.19	3.06
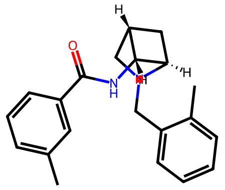	Cc1cccc(C(=O)N[C@@H]2[C@H]3C[C@H]2N(Cc2ccccc2C)C3)c1	1037295275	24	320.19	3.31
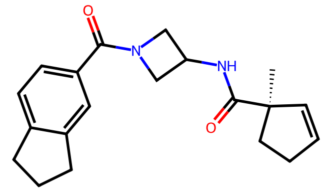	C[C@]1(C(=O)NC2CN(C(=O)c3ccc4c(c3)CCC4)C2)C=CCC1	997856650	24	324.18	2.47
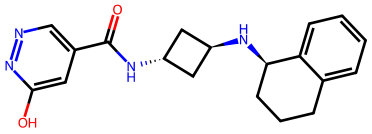	O=C(N[C@H]1C[C@H](N[C@@H]2CCCc3ccccc32)C1)c1cnnc(O)c1	1020267249	25	338.17	2.11
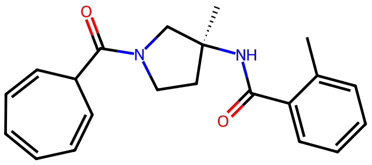	Cc1ccccc1C(=O)N[C@@]1(C)CCN(C(=O)C2C=CC=CC=C2)C1	956656982	25	336.18	3.01
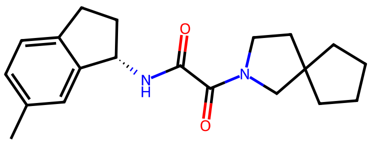	Cc1ccc2c(c1)[C@@H](NC(=O)C(=O)N1CCC3(CCCC3)C1)CC2	805055876	24	326.2	2.89
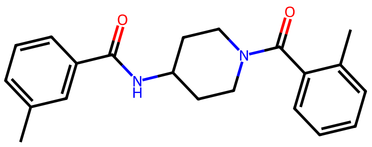	Cc1cccc(C(=O)NC2CCN(C(=O)c3ccccc3C)CC2)c1	75057863	25	336.18	3.34
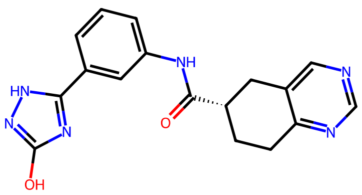	O=C(Nc1cccc(-c2nc(O)n[nH]2)c1)[C@H]1CCc2ncncc2C1	1449246652	25	336.13	1.71

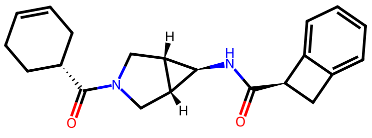	O=C(N[C@H]1[C@@H]2CN(C(=O)[C@H]3CC=CCC3)C[C@@H]21)[C@@H]1Cc2ccccc21	977081577	25	336.18	2.26
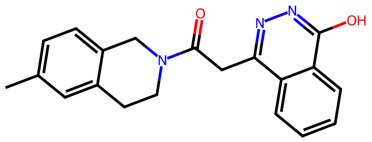	Cc1ccc2c(c1)CCN(C(=O)Cc1nnc(O)c3ccccc13)C2	571468673	25	333.15	2.77
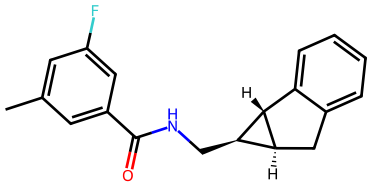	Cc1cc(F)cc(C(=O)NC[C@@H]2[C@@H]3Cc4ccccc4[C@@H]23)c1	488712636	22	295.14	3.45
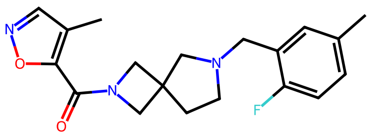	Cc1ccc(F)c(CN2CCC3(C2)CN(C(=O)c2oncc2C)C3)c1	1073100560	25	343.17	2.78
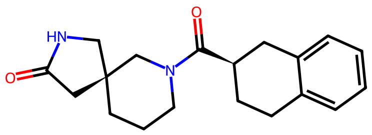	O=C1C[C@@]2(CCCN(C(=O)[C@@H]3CCc4ccccc4C3)C2)CN1	579013742	23	312.18	1.92
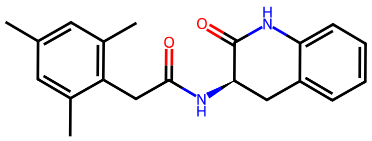	Cc1cc(C)c(CC(=O)N[C@@H]2Cc3ccccc3NC2=O)c(C)c1	529948094	24	322.17	2.83
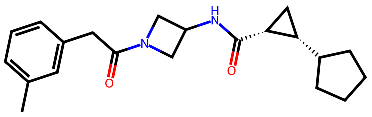	Cc1cccc(CC(=O)N2CC(NC(=O)[C@@H]3C[C@@H]3C3CCCC3)C2)c1	998828636	25	340.22	2.69
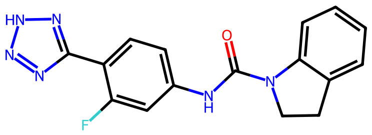	O=C(Nc1ccc(-c2nn[nH]n2)c(F)c1)N1CCc2ccccc21	1606910164	24	324.11	2.6

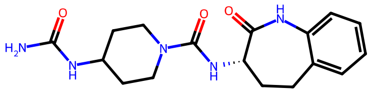	NC(=O)NC1CCN(C(=O)N[C@H]2CCc3ccccc3NC2=O)CC1	654628512	25	345.18	0.78
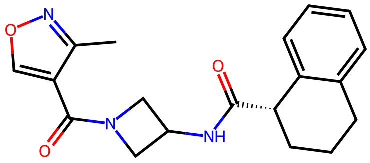	Cc1nocc1C(=O)N1CC(NC(=O)[C@H]2CCCc3ccccc32)C1	995071497	25	339.16	2.04
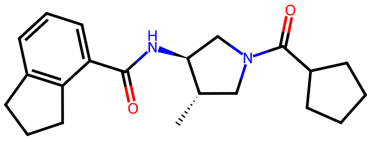	C[C@H]1CN(C(=O)C2CCCC2)C[C@@H]1NC(=O)c1cccc2c1CCC2	966038508	25	340.22	2.94
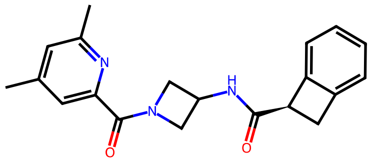	Cc1cc(C)nc(C(=O)N2CC(NC(=O)[C@@H]3Cc4ccccc43)C2)c1	998693640	25	335.16	1.98
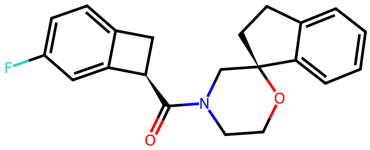	O=C([C@@H]1Cc2ccc(F)cc21)N1CCO[C@]2(CCc3ccccc32)C1	684466782	25	337.15	3.17
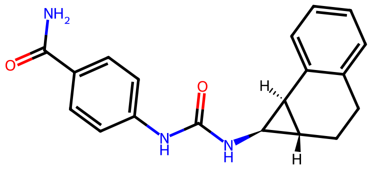	NC(=O)c1ccc(NC(=O)N[C@@H]2[C@H]3CCc4ccccc4[C@@H]32)cc1	178810991	24	321.15	2.64
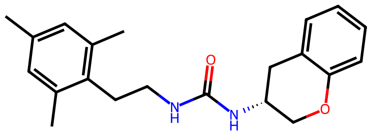	Cc1cc(C)c(CCNC(=O)N[C@H]2COc3ccccc3C2)c(C)c1	1563551122	25	338.2	3.46
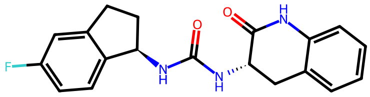	O=C(N[C@H]1Cc2ccccc2NC1=O)N[C@@H]1CCc2cc(F)ccc21	636635621	25	339.14	2.68

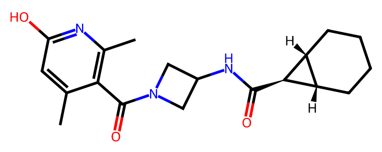	Cc1cc(O)nc(C)c1C(=O)N1CC(NC(=O)[C@@H]2[C@H]3CCCC[C@H]32)C1	990656036	25	343.19	1.78
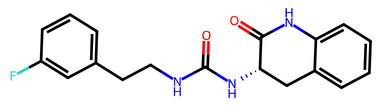	O=C(NCCc1cccc(F)c1)N[C@H]1Cc2ccccc2NC1=O	1365316112	24	327.14	2.23
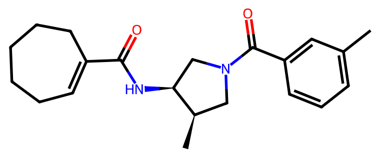	Cc1cccc(C(=O)N2C[C@@H](C)[C@@H](NC(=O)C3=CCCCCC3)C2)c1	966109439	25	340.22	3.46
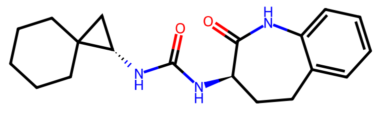	O=C(N[C@H]1CC12CCCCC2)N[C@@H]1CCc2ccccc2NC1=O	656111464	24	327.19	2.96
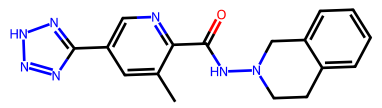	Cc1cc(-c2nn[nH]n2)cnc1C(=O)NN1CCc2ccccc2C1	1580626224	25	335.15	1.27
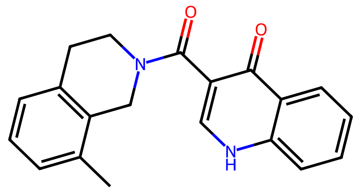	Cc1cccc2c1CN(C(=O)c1c[nH]c3ccccc3c1=O)CC2	430341745	24	318.14	3.04
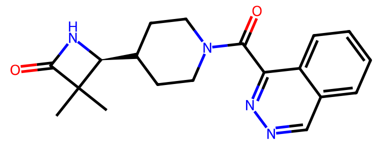	CC1(C)C(=O)N[C@H]1C1CCN(C(=O)c2nncc3ccccc23)CC1	820746689	25	338.17	2.01
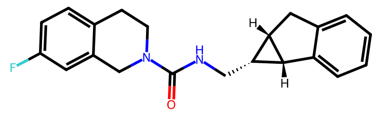	O=C(NC[C@H]1[C@H]2c3ccccc3C[C@H]21)N1CCc2ccc(F)cc2C1	136625663	25	336.16	3.48

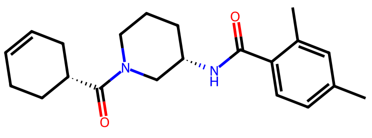	Cc1ccc(C(=O)N[C@H]2CCCN(C(=O)[C@H]3CC=CCC3)C2)c(C)c1	949983885	25	340.22	3.38
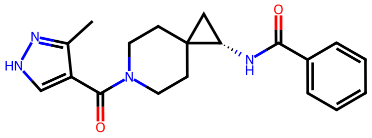	Cc1n[nH]cc1C(=O)N1CCC2(CC1)C[C@@H]2NC(=O)c1ccccc1	962780819	25	338.17	2.14
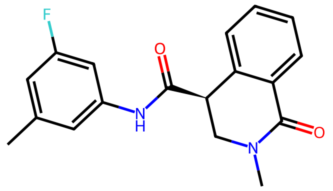	Cc1cc(F)cc(NC(=O)[C@@H]2CN(C)C(=O)c3ccccc32)c1	377536295	23	312.13	2.94
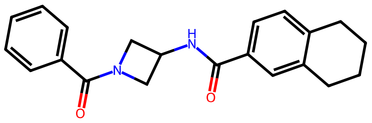	O=C(NC1CN(C(=O)c2ccccc2)C1)c1ccc2c(c1)CCCC2	1000058223	25	334.17	2.82
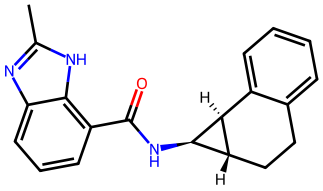	Cc1nc2cccc(C(=O)N[C@@H]3[C@H]4CCc5ccccc5[C@@H]43)c2[nH]1	332724220	24	317.15	3.33
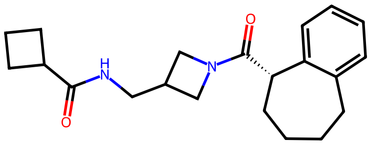	O=C(NCC1CN(C(=O)[C@H]2CCCCc3ccccc32)C1)C1CCC1	1000617615	25	340.22	2.87
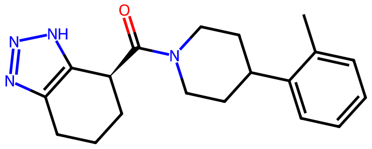	Cc1ccccc1C1CCN(C(=O)[C@H]2CCCc3nn[nH]c32)CC1	668384716	24	324.2	2.94
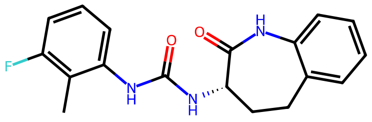	Cc1c(F)cccc1NC(=O)N[C@H]1CCc2ccccc2NC1=O	1189070974	24	327.14	3.21

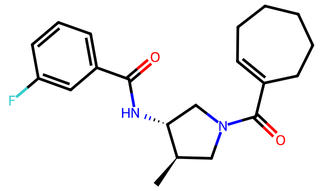	C[C@@H]1CN(C(=O)C2=CCCCCC2)C[C@H]1NC(=O)c1cccc(F)c1	948662875	25	344.19	3.29
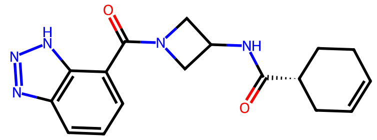	O=C(NC1CN(C(=O)c2cccc3nn[nH]c23)C1)[C@H]1CC=CCC1	990215949	24	325.15	1.25
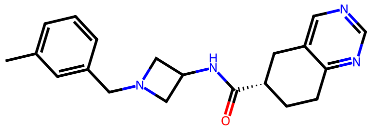	Cc1cccc(CN2CC(NC(=O)[C@H]3CCc4ncncc4C3)C2)c1	1030426383	25	336.2	1.89
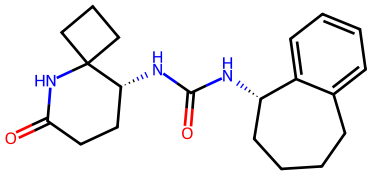	O=C1CC[C@@H](NC(=O)N[C@H]2CCCCc3ccccc32)C2(CCC2)N1	567252849	25	341.21	2.95
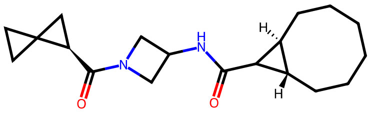	O=C(NC1CN(C(=O)[C@@H]2CC23CC3)C1)C1[C@H]2CCCCCC[C@H]12	1000511860	23	316.22	2.33
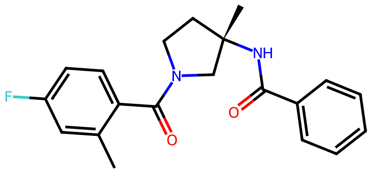	Cc1cc(F)ccc1C(=O)N1CC[C@](C)(NC(=O)c2ccccc2)C1	956655971	25	340.16	3.17
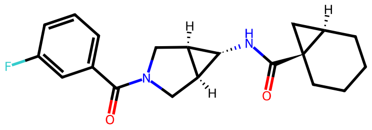	O=C(c1cccc(F)c1)N1C[C@H]2[C@H](NC(=O)[C@]34CCCC[C@@H]3C4)[C@H]2C1	977151372	25	342.17	2.59
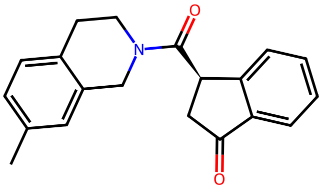	Cc1ccc2c(c1)CN(C(=O)[C@@H]1CC(=O)c3ccccc31)CC2	617605442	23	305.14	3.25

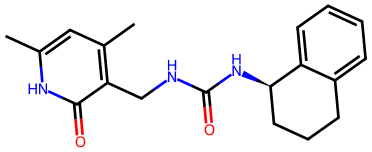	Cc1cc(C)c(CNC(=O)N[C@@H]2CCCc3ccccc32)c(=O)[nH]1	72305732	24	325.18	2.87
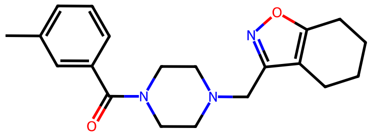	Cc1cccc(C(=O)N2CCN(Cc3noc4c3CCCC4)CC2)c1	1433404036	25	339.19	2.82
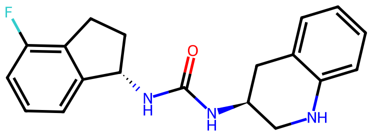	O=C(N[C@@H]1CNc2ccccc2C1)N[C@H]1CCc2c(F)cccc21	652138808	24	325.16	3.15
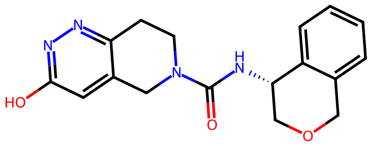	O=C(N[C@H]1COCc2ccccc21)N1CCc2nnc(O)cc2C1	1470568658	24	326.14	1.52
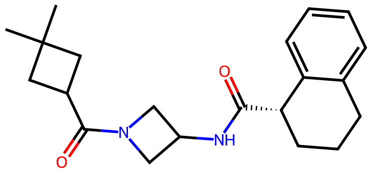	CC1(C)CC(C(=O)N2CC(NC(=O)[C@H]3CCCc4ccccc43)C2)C1	995072106	25	340.22	2.87
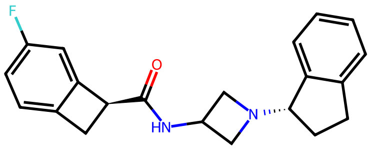	O=C(NC1CN([C@H]2CCc3ccccc32)C1)[C@H]1Cc2ccc(F)cc21	1030847157	25	336.16	2.95
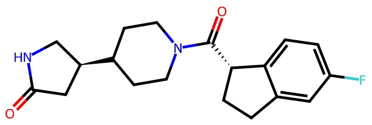	O=C1C[C@H](C2CCN(C(=O)[C@H]3CCc4cc(F)ccc43)CC2)CN1	1551710041	24	330.17	2.23
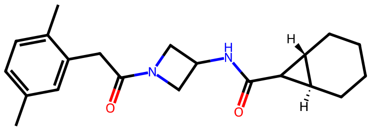	Cc1ccc(C)c(CC(=O)N2CC(NC(=O)C3[C@@H]4CCCC[C@@H]34)C2)c1	990647984	25	340.22	2.61

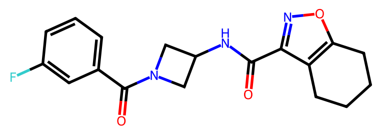	O=C(NC1CN(C(=O)c2cccc(F)c2)C1)c1noc2c1CCCC2	1000453334	25	343.13	1.95
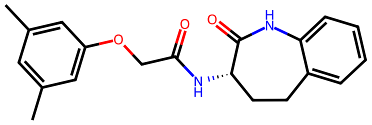	Cc1cc(C)cc(OCC(=O)N[C@H]2CCc3ccccc3NC2=O)c1	654005975	25	338.16	2.75
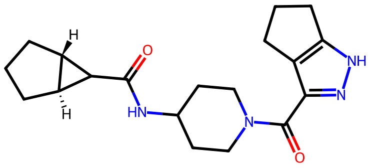	O=C(NC1CCN(C(=O)c2n[nH]c3c2CCC3)CC1)C1[C@H]2CCC[C@H]12	947366931	25	342.21	1.67
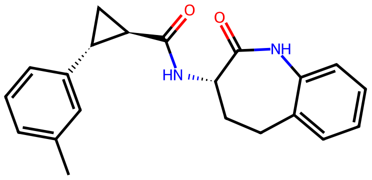	Cc1cccc([C@@H]2C[C@H]2C(=O)N[C@H]2CCc3ccccc3NC2=O)c1	628014805	25	334.17	3.17
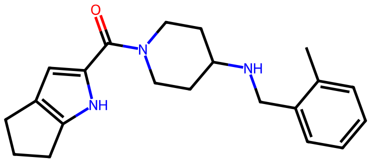	Cc1ccccc1CNC1CCN(C(=O)c2cc3c([nH]2)CCC3)CC1	997175935	25	337.22	3.21
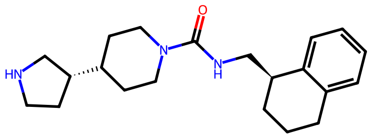	O=C(NC[C@@H]1CCCc2ccccc21)N1CCC([C@@H]2CCNC2)CC1	417070620	25	341.25	3.14
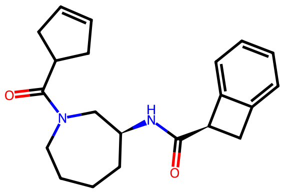	O=C(N[C@H]1CCCCN(C(=O)C2CC=CC2)C1)[C@@H]1Cc2ccccc21	943498269	25	338.2	2.79
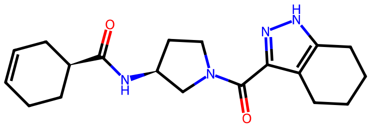	O=C(N[C@H]1CCN(C(=O)c2n[nH]c3c2CCCC3)C1)[C@@H]1CC=CCC1	937664999	25	342.21	1.98

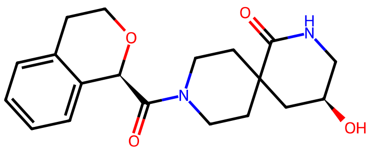	O=C([C@@H]1OCCc2ccccc21)N1CCC2(CC1)C[C@H](O)CNC2=O	885019520	25	344.17	0.79
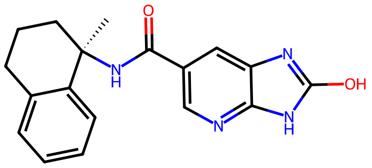	C[C@@]1(NC(=O)c2cnc3[nH]c(O)nc3c2)CCCc2ccccc21	564884052	24	322.14	2.65
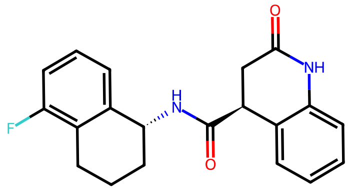	O=C1C[C@H](C(=O)N[C@@H]2CCCc3c(F)cccc32)c2ccccc2N1	452198312	25	338.14	3.45
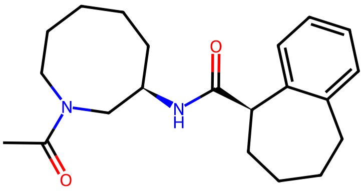	CC(=O)N1CCCCC[C@@H](NC(=O)[C@@H]2CCCCc3ccccc32)C1	986790185	25	342.23	3.4
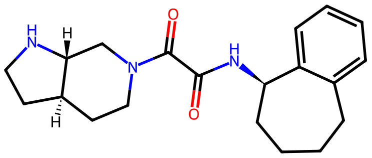	O=C(N[C@@H]1CCCCc2ccccc21)C(=O)N1CC[C@H]2CCN[C@@H]2C1	642645097	25	341.21	1.78
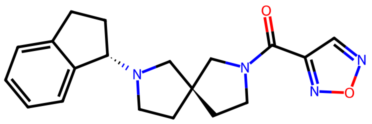	O=C(c1cnon1)N1CC[C@]2(CCN([C@H]3CCc4ccccc43)C2)C1	1041748424	25	338.17	2.3
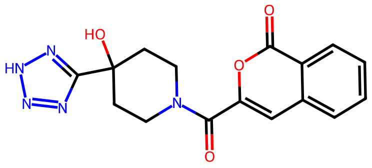	O=C(c1cc2ccccc2c(=O)o1)N1CCC(O)(c2nn[nH]n2)CC1	1339389683	25	341.11	0.43
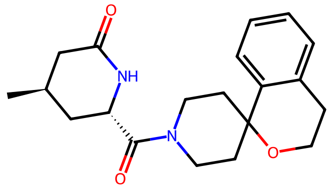	C[C@H]1CC(=O)N[C@H](C(=O)N2CCC3(CC2)OCCc2ccccc23)C1	1549808517	25	342.19	1.99

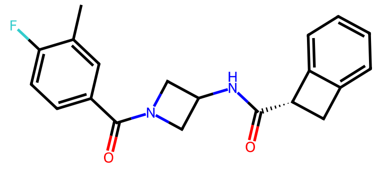	Cc1cc(C(=O)N2CC(NC(=O)[C@H]3Cc4ccccc43)C2)ccc1F	998692423	25	338.14	2.41
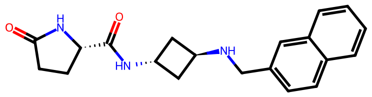	O=C1CC[C@@H](C(=O)N[C@H]2C[C@H](NCc3ccc4ccccc4c3)C2)N1	1020949019	25	337.18	1.86
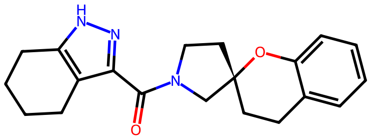	O=C(c1n[nH]c2c1CCCC2)N1CC[C@@]2(CCc3ccccc3O2)C1	377974222	25	337.18	2.9
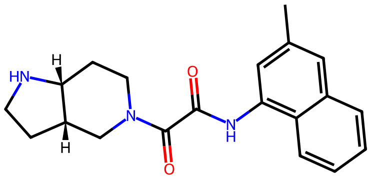	Cc1cc(NC(=O)C(=O)N2CC[C@H]3NCC[C@H]3C2)c2ccccc2c1	667627793	25	337.18	2.3
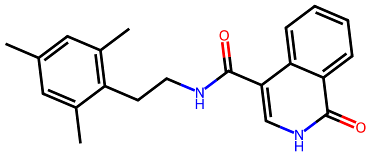	Cc1cc(C)c(CCNC(=O)c2c[nH]c(=O)c3ccccc23)c(C)c1	189974974	25	334.17	3.43
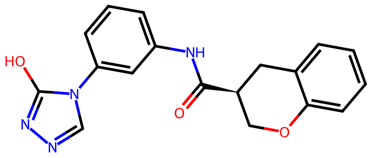	O=C(Nc1cccc(-n2cnnc2O)c1)[C@@H]1COc2ccccc2C1	364282615	25	336.12	2.16
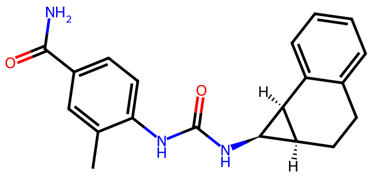	Cc1cc(C(N)=O)ccc1NC(=O)N[C@@H]1[C@@H]2CCc3ccccc3[C@@H]21	772418264	25	335.16	2.94
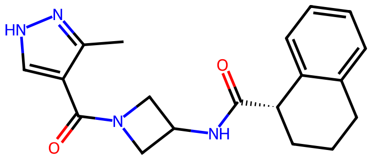	Cc1n[nH]cc1C(=O)N1CC(NC(=O)[C@H]2CCCc3ccccc32)C1	995075723	25	338.17	1.78

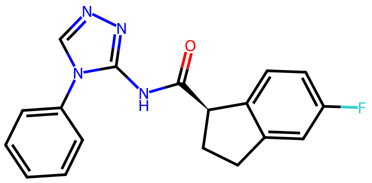	O=C(Nc1nncn1-c1ccccc1)[C@@H]1CCc2cc(F)ccc21	1151337355	24	322.12	3.07
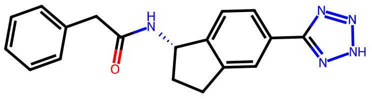	O=C(Cc1ccccc1)N[C@H]1CCc2cc(-c3nn[nH]n3)ccc21	1592923064	24	319.14	2.21
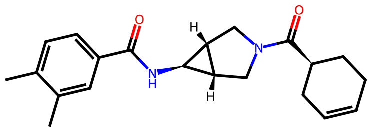	Cc1ccc(C(=O)N[C@H]2[C@@H]3CN(C(=O)[C@@H]4CC=CCC4)C[C@@H]32)cc1C	977179892	25	338.2	2.85
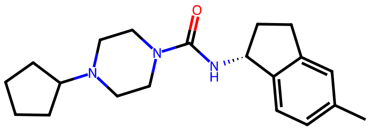	Cc1ccc2c(c1)CC[C@H]2NC(=O)N1CCN(C2CCCC2)CC1	56842893	24	327.23	3.25
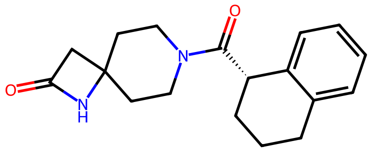	O=C1CC2(CCN(C(=O)[C@H]3CCCc4ccccc43)CC2)N1	1325280060	22	298.17	1.99
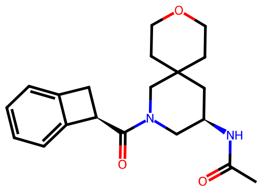	CC(=O)N[C@H]1CN(C(=O)[C@@H]2Cc3ccccc32)CC2(CCOCC2)C1	991113943	25	342.19	1.86
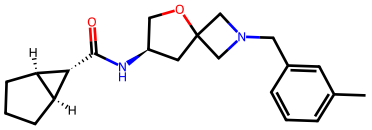	Cc1cccc(CN2CC3(C[C@@H](NC(=O)[C@@H]4[C@H]5CCC[C@H]54)CO3)C2)c1	1053769756	25	340.22	2.5
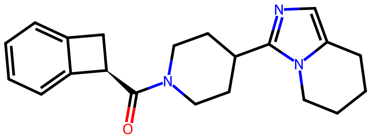	O=C([C@@H]1Cc2ccccc21)N1CCC(c2ncc3n2CCCC3)CC1	345473406	25	335.2	3.27

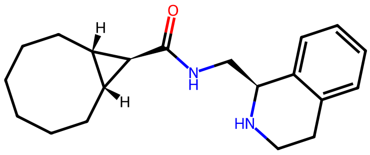	O=C(NC[C@@H]1NCCc2ccccc21)[C@@H]1[C@H]2CCCCCC[C@H]21	852878887	23	312.22	3.21
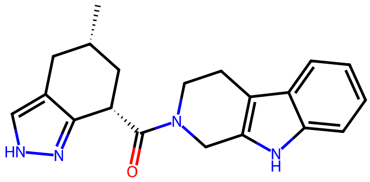	C[C@H]1Cc2c[nH]nc2[C@@H](C(=O)N2CCc3c([nH]c4ccccc34)C2)C1	623206517	25	334.18	3.14
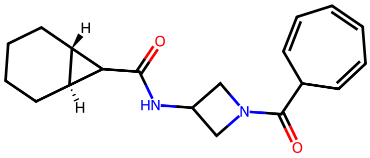	O=C(NC1CN(C(=O)C2C=CC=CC=C2)C1)C1[C@H]2CCCC[C@H]12	990669216	23	312.18	2.05
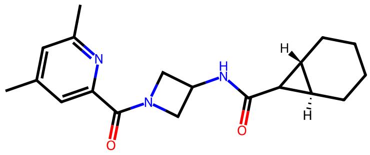	Cc1cc(C)nc(C(=O)N2CC(NC(=O)C3[C@@H]4CCCC[C@@H]34)C2)c1	990657240	24	327.19	2.08
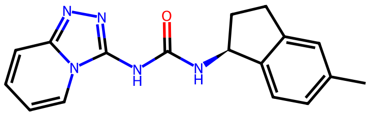	Cc1ccc2c(c1)CC[C@@H]2NC(=O)Nc1nnc2ccccn12	344739510	23	307.14	2.85
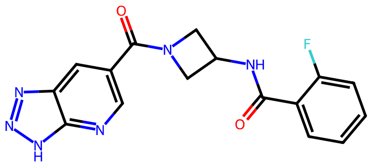	O=C(NC1CN(C(=O)c2cnc3[nH]nnc3c2)C1)c1ccccc1F	990096270	25	340.11	0.75
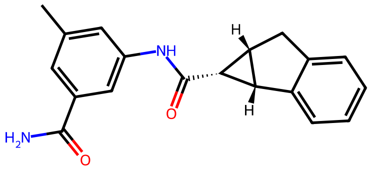	Cc1cc(NC(=O)[C@H]2[C@H]3c4ccccc4C[C@H]32)cc(C(N)=O)c1	627634616	23	306.14	2.62
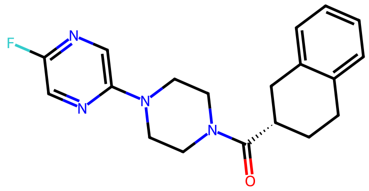	O=C([C@H]1CCc2ccccc2C1)N1CCN(c2cnc(F)cn2)CC1	1056040121	25	340.17	2.07

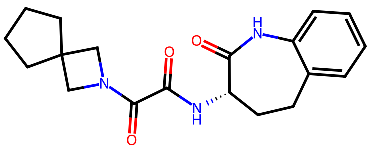	O=C(N[C@H]1CCc2ccccc2NC1=O)C(=O)N1CC2(CCCC2)C1	1118409457	25	341.17	1.46
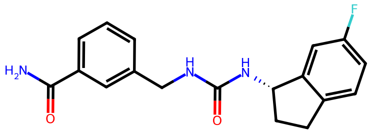	NC(=O)c1cccc(CNC(=O)N[C@H]2CCc3ccc(F)cc32)c1	592492276	24	327.14	2.41
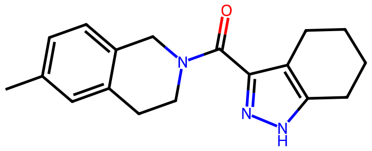	Cc1ccc2c(c1)CCN(C(=O)c1n[nH]c3c1CCCC3)C2	682942132	22	295.17	2.8
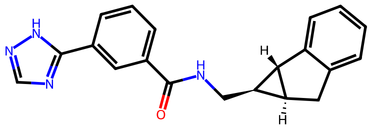	O=C(NC[C@@H]1[C@@H]2Cc3ccccc3[C@@H]12)c1cccc(-c2ncn[nH]2)c1	157757489	25	330.15	2.79
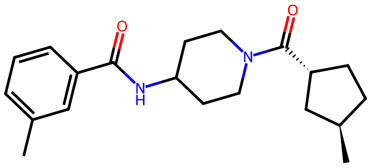	Cc1cccc(C(=O)NC2CCN(C(=O)[C@@H]3CC[C@@H](C)C3)CC2)c1	870835794	24	328.22	3.15
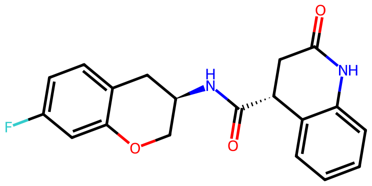	O=C1C[C@@H](C(=O)N[C@H]2COc3cc(F)ccc3C2)c2ccccc2N1	1361448747	25	340.12	2.37
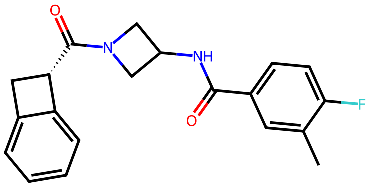	Cc1cc(C(=O)NC2CN(C(=O)[C@H]3Cc4ccccc43)C2)ccc1F	998697792	25	338.14	2.41
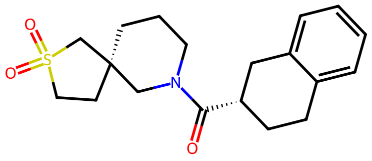	O=C([C@H]1CCc2ccccc2C1)N1CCC[C@@]2(CCS(=O)(=O)C2)C1	576640528	24	347.16	2.22

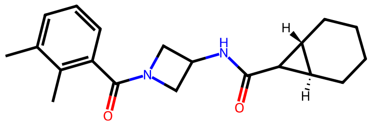	Cc1cccc(C(=O)N2CC(NC(=O)C3[C@@H]4CCCC[C@@H]34)C2)c1C	990644682	24	326.2	2.68
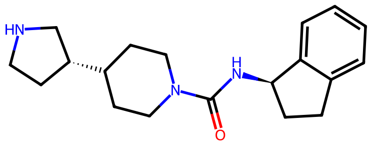	O=C(N[C@@H]1CCc2ccccc21)N1CCC([C@@H]2CCNC2)CC1	416904019	23	313.22	2.71
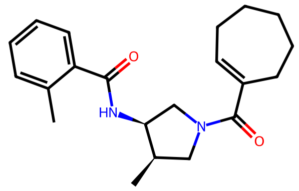	Cc1ccccc1C(=O)N[C@H]1CN(C(=O)C2=CCCCCC2)C[C@H]1C	966352286	25	340.22	3.46
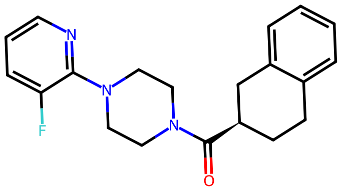	O=C([C@@H]1CCc2ccccc2C1)N1CCN(c2ncccc2F)CC1	570520266	25	339.17	2.67
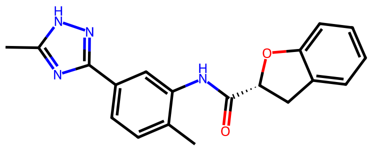	Cc1nc(-c2ccc(C)c(NC(=O)[C@H]3Cc4ccccc4O3)c2)n[nH]1	361289121	25	334.14	3.03
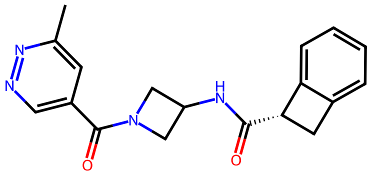	Cc1cc(C(=O)N2CC(NC(=O)[C@H]3Cc4ccccc43)C2)cnn1	998685782	24	322.14	1.07
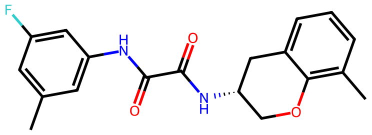	Cc1cc(F)cc(NC(=O)C(=O)N[C@H]2COc3c(C)cccc3C2)c1	290433667	25	342.14	2.5
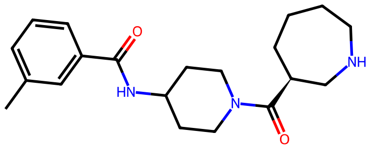	Cc1cccc(C(=O)NC2CCN(C(=O)[C@H]3CCCCNC3)CC2)c1	1550101450	25	343.23	2.11

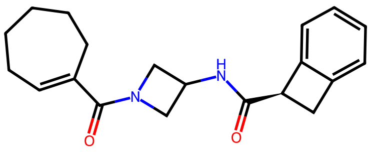	O=C(NC1CN(C(=O)C2=CCCCCC2)C1)[C@@H]1Cc2ccccc21	998682320	24	324.18	2.54
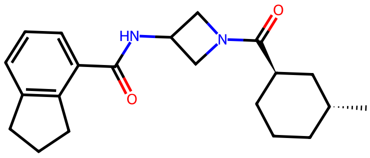	C[C@@H]1CCC[C@@H](C(=O)N2CC(NC(=O)c3cccc4c3CCC4)C2)C1	994404450	25	340.22	2.94
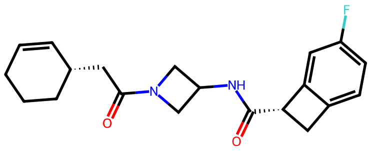	O=C(NC1CN(C(=O)C[C@H]2C=CCCC2)C1)[C@H]1Cc2ccc(F)cc21	998490097	25	342.17	2.54
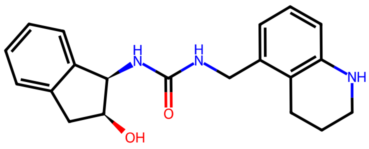	O=C(NCc1cccc2c1CCCN2)N[C@@H]1c2ccccc2C[C@@H]1O	418655739	25	337.18	2.5
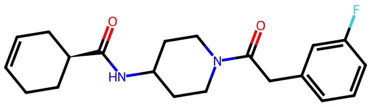	O=C(NC1CCN(C(=O)Cc2cccc(F)c2)CC1)[C@@H]1CC=CCC1	948480569	25	344.19	2.83
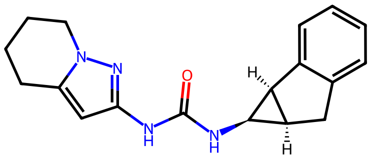	O=C(Nc1cc2n(n1)CCCC2)N[C@@H]1[C@@H]2Cc3ccccc3[C@@H]21	287275925	23	308.16	2.68
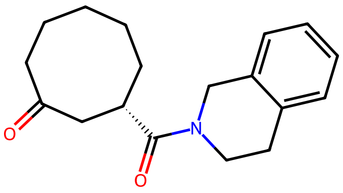	O=C1CCCCC[C@H](C(=O)N2CCc3ccccc3C2)C1	336330248	21	285.17	3.11
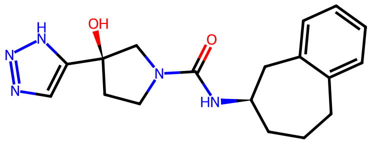	O=C(N[C@@H]1CCCc2ccccc2C1)N1CC[C@](O)(c2cnn[nH]2)C1	897608369	25	341.19	1.36

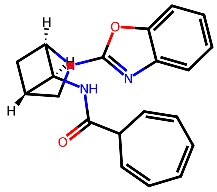	O=C(N[C@H]1[C@H]2C[C@H]1N(c1nc3ccccc3o1)C2)C1C=CC=CC=C1	1064298937	25	333.15	2.82
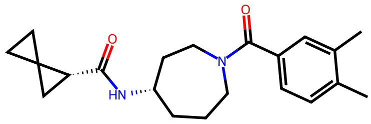	Cc1ccc(C(=O)N2CCC[C@H](NC(=O)[C@@H]3CC34CC4)CC2)cc1C	965221927	25	340.22	3.21
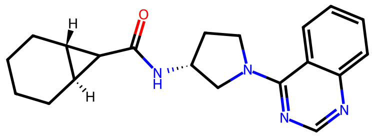	O=C(N[C@@H]1CCN(c2ncnc3ccccc23)C1)C1[C@H]2CCCC[C@H]12	1058334652	25	336.2	2.76
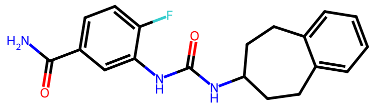	NC(=O)c1ccc(F)c(NC(=O)NC2CCc3ccccc3CC2)c1	565464089	25	341.15	2.99
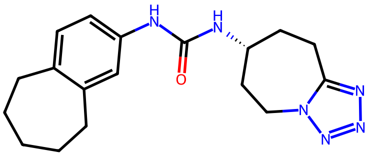	O=C(Nc1ccc2c(c1)CCCCC2)N[C@@H]1CCc2nnnn2CC1	650426048	25	340.2	2.47
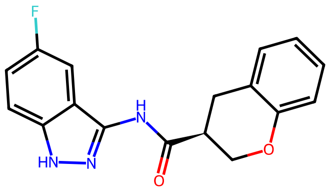	O=C(Nc1n[nH]c2ccc(F)cc12)[C@@H]1COc2ccccc2C1	342950896	23	311.11	2.89
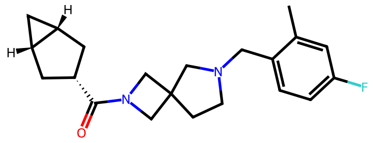	Cc1cc(F)ccc1CN1CCC2(C1)CN(C(=O)[C@@H]1C[C@@H]3C[C@@H]3C1)C2	1072907807	25	342.21	3.21
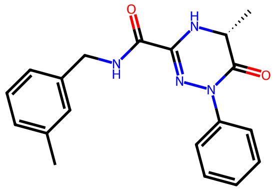	Cc1cccc(CNC(=O)C2=NN(c3ccccc3)C(=O)[C@@H](C)N2)c1	33002810	25	336.16	1.95

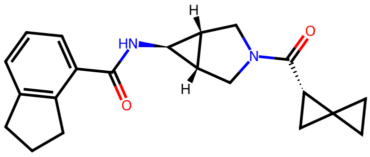	O=C(N[C@H]1[C@@H]2CN(C(=O)[C@H]3CC34CC4)C[C@@H]21)c1cccc2c1CCC2	976708834	25	336.18	2.16
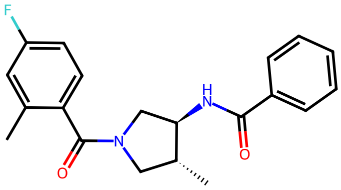	Cc1cc(F)ccc1C(=O)N1C[C@@H](NC(=O)c2ccccc2)[C@H](C)C1	957492405	25	340.16	3.02
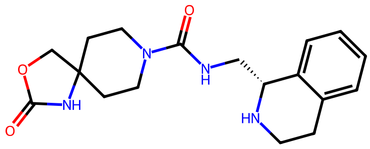	O=C1NC2(CCN(C(=O)NC[C@H]3NCCc4ccccc43)CC2)CO1	497132416	25	344.18	1.16
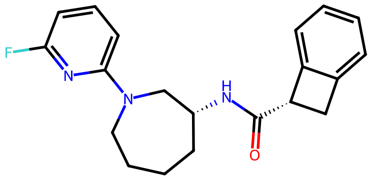	O=C(N[C@@H]1CCCCN(c2cccc(F)n2)C1)[C@H]1Cc2ccccc21	1063228355	25	339.17	3.04
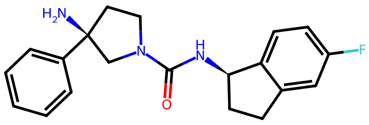	N[C@@]1(c2ccccc2)CCN(C(=O)N[C@@H]2CCc3cc(F)ccc32)C1	1551528925	25	339.17	3.08
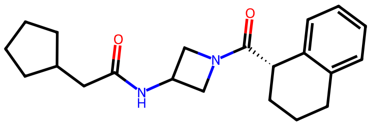	O=C(CC1CCCC1)NC1CN(C(=O)[C@H]2CCCc3ccccc32)C1	990104672	25	340.22	3.01
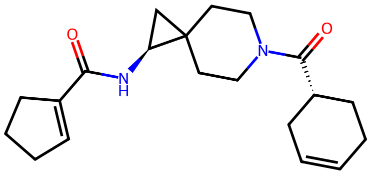	O=C(N[C@H]1CC12CCN(C(=O)[C@H]1CC=CCC1)CC2)C1=CCCC1	963569104	24	328.22	2.95
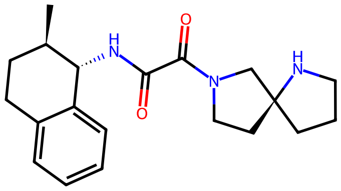	C[C@@H]1CCc2ccccc2[C@H]1NC(=O)C(=O)N1CC[C@@]2(CCCN2)C1	842188216	25	341.21	1.78

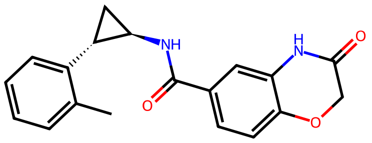	Cc1ccccc1[C@@H]1C[C@H]1NC(=O)c1ccc2c(c1)NC(=O)CO2	126830700	24	322.13	2.61
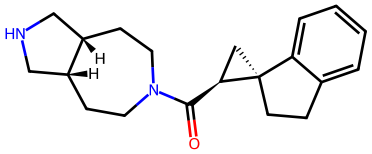	O=C([C@H]1C[C@]12CCc1ccccc12)N1CC[C@@H]2CNC[C@@H]2CC1	261680098	23	310.2	2.35
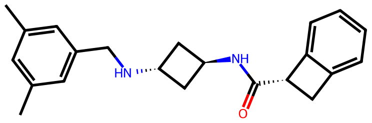	Cc1cc(C)cc(CN[C@H]2C[C@H](NC(=O)[C@H]3Cc4ccccc43)C2)c1	1020529000	25	334.2	3.38
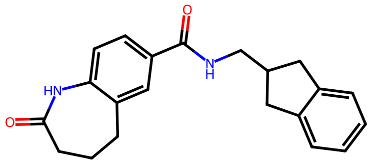	O=C1CCCc2cc(C(=O)NCC3Cc4ccccc4C3)ccc2N1	735259698	25	334.17	3.11
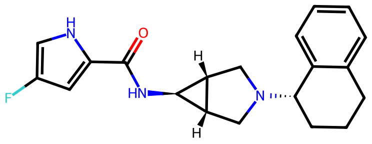	O=C(N[C@H]1[C@@H]2CN([C@H]3CCCc4ccccc43)C[C@@H]21)c1cc(F)c[nH]1	961232261	25	339.17	2.89
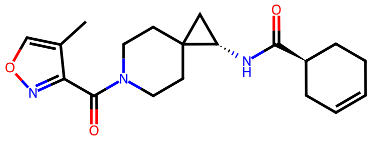	Cc1conc1C(=O)N1CCC2(CC1)C[C@@H]2NC(=O)[C@@H]1CC=CCC1	963024871	25	343.19	2.45
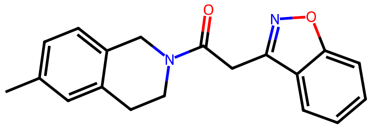	Cc1ccc2c(c1)CCN(C(=O)Cc1noc3ccccc13)C2	360216172	23	306.14	3.26
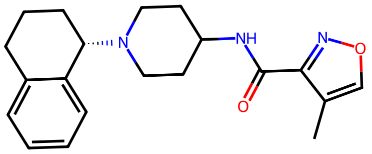	Cc1conc1C(=O)NC1CCN([C@H]2CCCc3ccccc32)CC1	1003917045	25	339.19	3.25

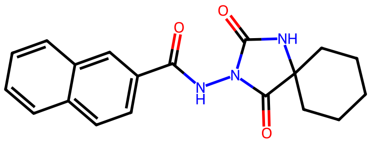	O=C(NN1C(=O)NC2(CCCCC2)C1=O)c1ccc2ccccc2c1	7758229	25	337.14	2.74
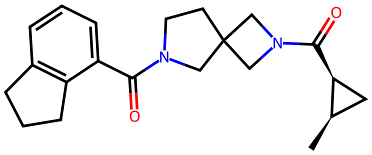	C[C@@H]1C[C@@H]1C(=O)N1CC2(CCN(C(=O)c3cccc4c3CCC4)C2)C1	1018732217	25	338.2	2.51
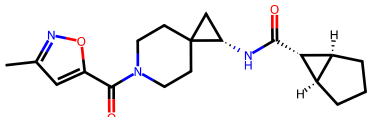	Cc1cc(C(=O)N2CCC3(CC2)C[C@@H]3NC(=O)[C@@H]2[C@H]3CCC[C@H]32)on1	963462577	25	343.19	2.14
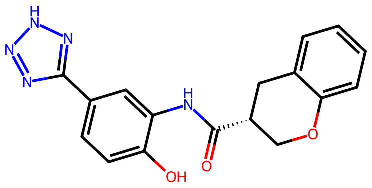	O=C(Nc1cc(-c2nn[nH]n2)ccc1O)[C@H]1COc2ccccc2C1	823294502	25	337.12	1.76
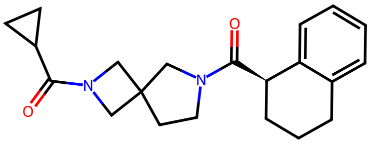	O=C(C1CC1)N1CC2(CCN(C(=O)[C@@H]3CCCc4ccccc43)C2)C1	1018665057	25	338.2	2.58
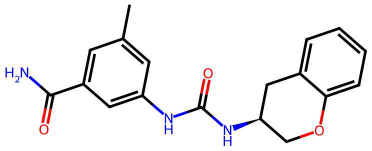	Cc1cc(NC(=O)N[C@@H]2COc3ccccc3C2)cc(C(N)=O)c1	1365202068	24	325.14	2.22
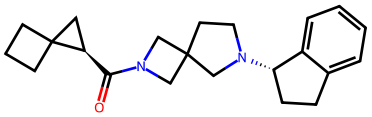	O=C([C@@H]1CC12CCC2)N1CC2(CCN([C@H]3CCc4ccccc43)C2)C1	1073018965	25	336.22	3.4
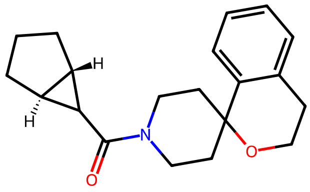	O=C(C1[C@H]2CCC[C@H]12)N1CCC2(CC1)OCCc1ccccc12	457135520	23	311.19	3.12

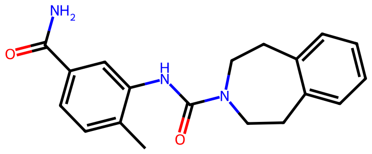	Cc1ccc(C(N)=O)cc1NC(=O)N1CCc2ccccc2CC1	116493882	24	323.16	2.73
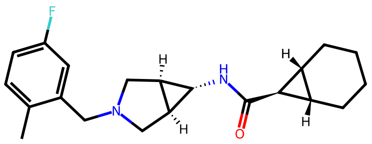	Cc1ccc(F)cc1CN1C[C@H]2[C@H](NC(=O)[C@@H]3[C@H]4CCCC[C@H]43)[C@H]2C1	1025723254	25	342.21	3.12
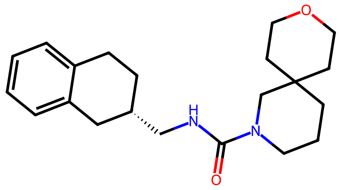	O=C(NC[C@H]1CCc2ccccc2C1)N1CCCC2(CCOCC2)C1	608689494	25	342.23	3.39
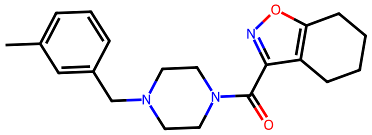	Cc1cccc(CN2CCN(C(=O)c3noc4c3CCCC4)CC2)c1	56153574	25	339.19	2.82
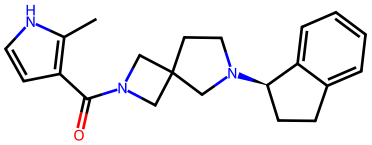	Cc1[nH]ccc1C(=O)N1CC2(CCN([C@@H]3CCc4ccccc43)C2)C1	1072597884	25	335.2	3.16
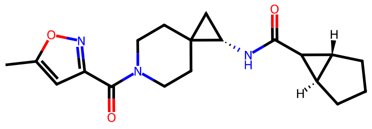	Cc1cc(C(=O)N2CCC3(CC2)C[C@@H]3NC(=O)C2[C@@H]3CCC[C@@H]23)no1	963468231	25	343.19	2.14
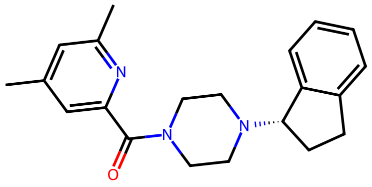	Cc1cc(C)nc(C(=O)N2CCN([C@H]3CCc4ccccc43)CC2)c1	949026260	25	335.2	3.14
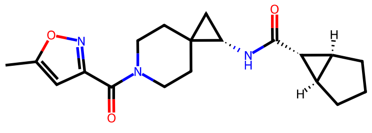	Cc1cc(C(=O)N2CCC3(CC2)C[C@@H]3NC(=O)[C@@H]2[C@H]3CCC[C@H]32)no1	963468228	25	343.19	2.14

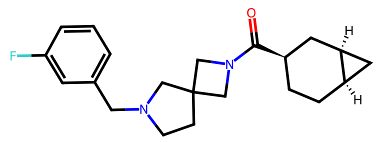	O=C([C@@H]1CC[C@@H]2C[C@@H]2C1)N1CC2(CCN(Cc3cccc(F)c3)C2)C1	1072879728	25	342.21	3.3
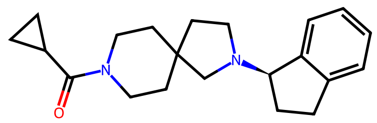	O=C(C1CC1)N1CCC2(CC1)CCN([C@@H]1CCc3ccccc31)C2	1035148955	24	324.22	3.4
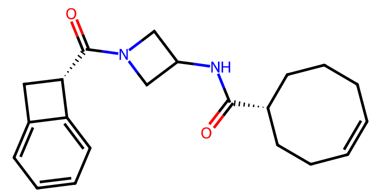	O=C(NC1CN(C(=O)[C@H]2Cc3ccccc32)C1)[C@H]1CCC=CCCC1	994834608	25	338.2	2.79
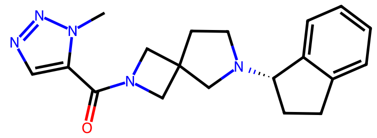	Cn1nncc1C(=O)N1CC2(CCN([C@H]3CCc4ccccc43)C2)C1	1072686928	25	337.19	1.65
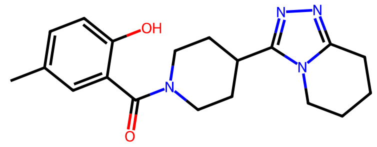	Cc1ccc(O)c(C(=O)N2CCC(c3nnc4n3CCCC4)CC2)c1	274722679	25	340.19	2.65
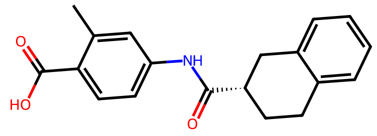	Cc1cc(NC(=O)[C@H]2CCc3ccccc3C2)ccc1C(=O)O	324502478	23	309.14	3.44
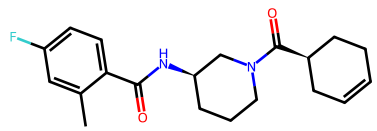	Cc1cc(F)ccc1C(=O)N[C@@H]1CCCN(C(=O)[C@@H]2CC=CCC2)C1	949984231	25	344.19	3.21
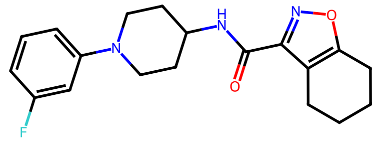	O=C(NC1CCN(c2cccc(F)c2)CC1)c1noc2c1CCCC2	363352938	25	343.17	3.09

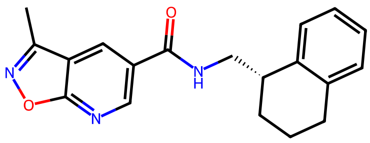	Cc1noc2ncc(C(=O)NC[C@H]3CCCc4ccccc43)cc12	29110058	24	321.15	3.38
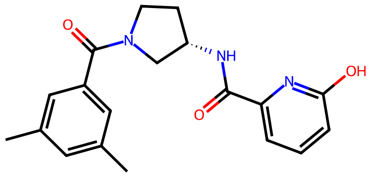	Cc1cc(C)cc(C(=O)N2CC[C@H](NC(=O)c3cccc(O)n3)C2)c1	937789271	25	339.16	2.05
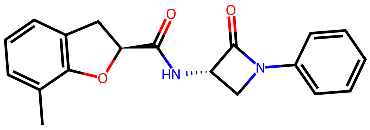	Cc1cccc2c1O[C@H](C(=O)N[C@H]1CN(c3ccccc3)C1=O)C2	562693060	24	322.13	1.83
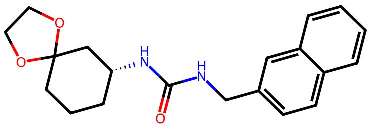	O=C(NCc1ccc2ccccc2c1)N[C@@H]1CCCC2(C1)OCCO2	768323668	25	340.18	3.32
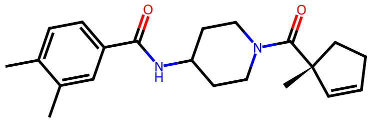	Cc1ccc(C(=O)NC2CCN(C(=O)[C@@]3(C)C=CCC3)CC2)cc1C	947853330	25	340.22	3.38
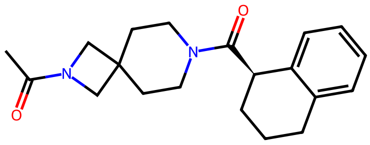	CC(=O)N1CC2(CCN(C(=O)[C@@H]3CCCc4ccccc43)CC2)C1	944974442	24	326.2	2.58
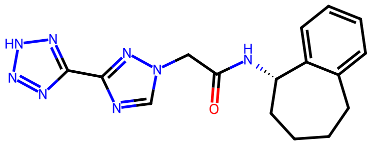	O=C(Cn1cnc(-c2nn[nH]n2)n1)N[C@H]1CCCCc2ccccc21	823127243	25	338.16	1.04
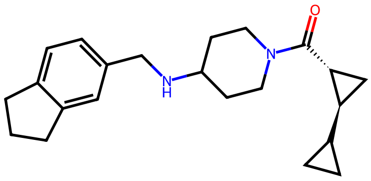	O=C([C@@H]1C[C@H]1C1CC1)N1CCC(NCc2ccc3c(c2)CCC3)CC1	997090204	25	338.24	3.3

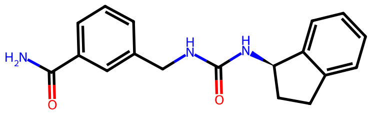	NC(=O)c1cccc(CNC(=O)N[C@@H]2CCc3ccccc32)c1	337364328	23	309.15	2.27
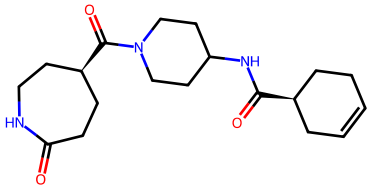	O=C1CC[C@H](C(=O)N2CCC(NC(=O)[C@@H]3CC=CCC3)CC2)CCN1	947528314	25	347.22	1.37
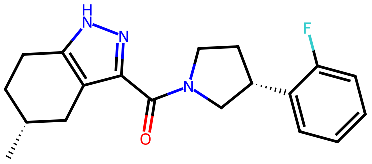	C[C@@H]1CCc2[nH]nc(C(=O)N3CC[C@H](c4ccccc4F)C3)c2C1	896244611	24	327.17	3.3
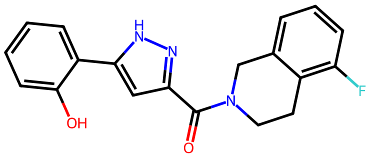	O=C(c1cc(-c2ccccc2O)[nH]n1)N1CCc2c(F)cccc2C1	1566443467	25	337.12	3.12
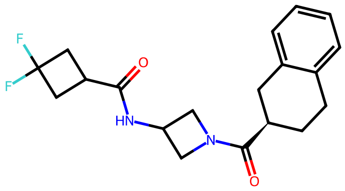	O=C(NC1CN(C(=O)[C@@H]2CCc3ccccc3C2)C1)C1CC(F)(F)C1	990757038	25	348.16	2.16
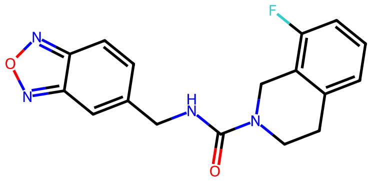	O=C(NCc1ccc2nonc2c1)N1CCc2cccc(F)c2C1	1471539242	24	326.12	2.63
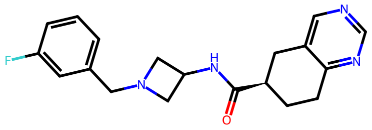	O=C(NC1CN(Cc2cccc(F)c2)C1)[C@@H]1CCc2ncncc2C1	1030428229	25	340.17	1.72
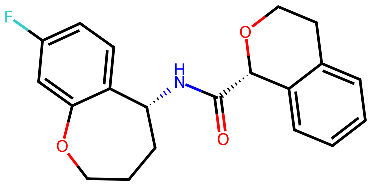	O=C(N[C@@H]1CCCOc2cc(F)ccc21)[C@@H]1OCCc2ccccc21	182818297	25	341.14	3.47

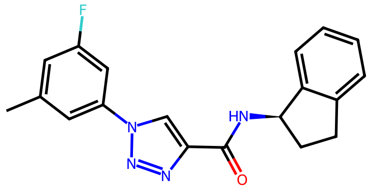	Cc1cc(F)cc(-n2cc(C(=O)N[C@@H]3CCc4ccccc43)nn2)c1	339958807	25	336.14	3.13
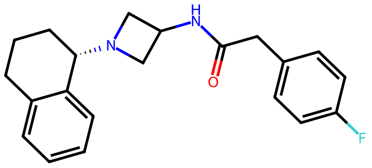	O=C(Cc1ccc(F)cc1)NC1CN([C@H]2CCCc3ccccc32)C1	1031337228	25	338.18	3.25
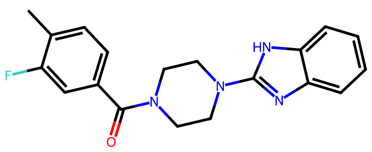	Cc1ccc(C(=O)N2CCN(c3nc4ccccc4[nH]3)CC2)cc1F	299690761	25	338.15	2.97
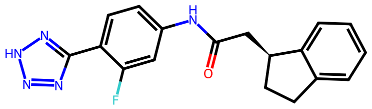	O=C(C[C@@H]1CCc2ccccc21)Nc1ccc(-c2nn[nH]n2)c(F)c1	1606507306	25	337.13	3.06
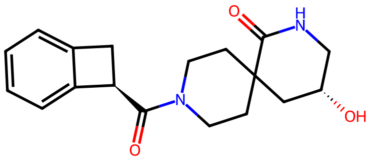	O=C([C@@H]1Cc2ccccc21)N1CCC2(CC1)C[C@@H](O)CNC2=O	885019351	23	314.16	0.82
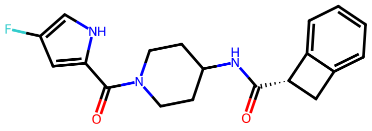	O=C(NC1CCN(C(=O)c2cc(F)c[nH]2)CC1)[C@H]1Cc2ccccc21	947461561	25	341.15	2.21
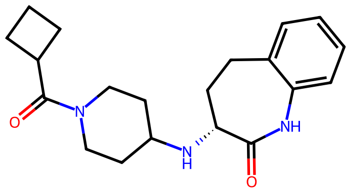	O=C1Nc2ccccc2CC[C@H]1NC1CCN(C(=O)C2CCC2)CC1	989843765	25	341.21	2.32
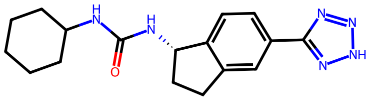	O=C(NC1CCCCC1)N[C@H]1CCc2cc(-c3nn[nH]n3)ccc21	1609541099	24	326.19	2.49

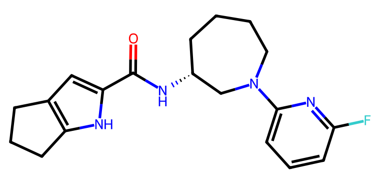	O=C(N[C@@H]1CCCCN(c2cccc(F)n2)C1)c1cc2c([nH]1)CCC2	1063198360	25	342.19	2.83
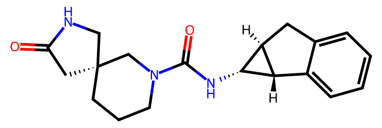	O=C1C[C@]2(CCCN(C(=O)N[C@@H]3[C@H]4Cc5ccccc5[C@@H]43)C2)CN1	332911905	24	325.18	1.64
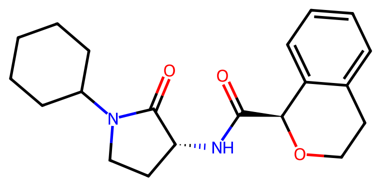	O=C(N[C@@H]1CCN(C2CCCCC2)C1=O)[C@@H]1OCCc2ccccc21	124801639	25	342.19	2.35
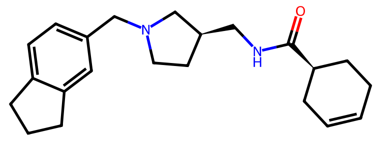	O=C(NC[C@H]1CCN(Cc2ccc3c(c2)CCC3)C1)[C@@H]1CC=CCC1	1028395297	25	338.24	3.47
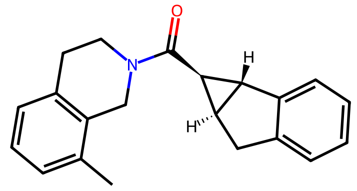	Cc1cccc2c1CN(C(=O)[C@@H]1[C@@H]3Cc4ccccc4[C@H]31)CC2	626664574	23	303.16	3.47
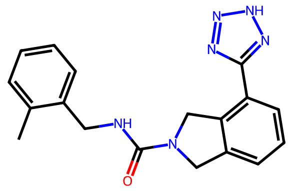	Cc1ccccc1CNC(=O)N1Cc2cccc(-c3nn[nH]n3)c2C1	1592256739	25	334.15	2.4
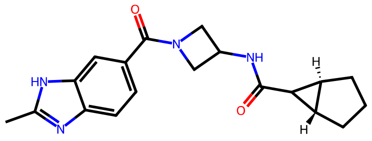	Cc1nc2ccc(C(=O)N3CC(NC(=O)C4[C@H]5CCC[C@H]45)C3)cc2[nH]1	990704866	25	338.17	1.86
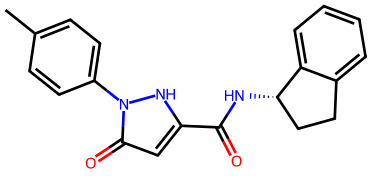	Cc1ccc(-n2[nH]c(C(=O)N[C@H]3CCc4ccccc43)cc2=O)cc1	360170828	25	333.15	2.89

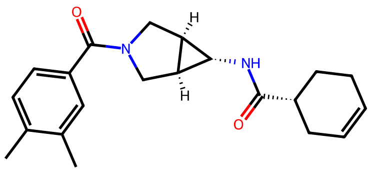	Cc1ccc(C(=O)N2C[C@H]3[C@H](NC(=O)[C@H]4CC=CCC4)[C@H]3C2)cc1C	976173943	25	338.2	2.85
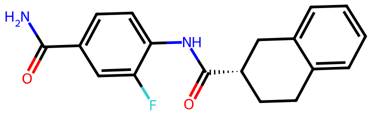	NC(=O)c1ccc(NC(=O)[C@H]2CCc3ccccc3C2)c(F)c1	1456911926	23	312.13	2.67
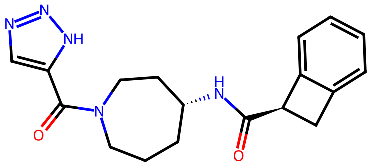	O=C(N[C@@H]1CCCN(C(=O)c2cnn[nH]2)CC1)[C@@H]1Cc2ccccc21	965142453	25	339.17	1.26
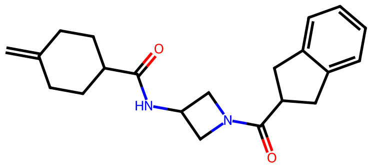	C=C1CCC(C(=O)NC2CN(C(=O)C3Cc4ccccc4C3)C2)CC1	999108675	25	338.2	2.47
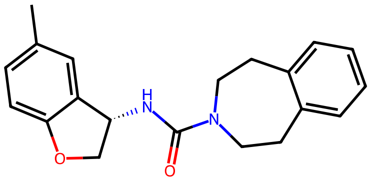	Cc1ccc2c(c1)[C@H](NC(=O)N1CCc3ccccc3CC1)CO2	532188904	24	322.17	3.24
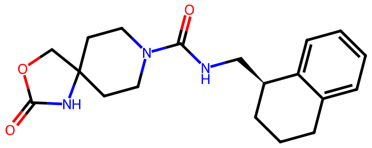	O=C1NC2(CCN(C(=O)NC[C@@H]3CCCc4ccccc43)CC2)CO1	495296839	25	343.19	2.39
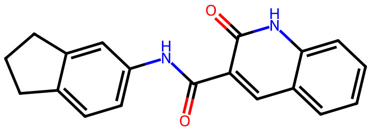	O=C(Nc1ccc2c(c1)CCC2)c1cc2ccccc2[nH]c1=O	10894978	23	304.12	3.27
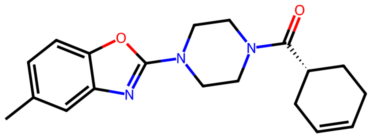	Cc1ccc2oc(N3CCN(C(=O)[C@H]4CC=CCC4)CC3)nc2c1	112264533	24	325.18	3.14

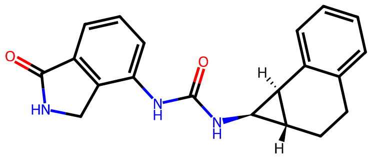	O=C(Nc1cccc2c1CNC2=O)N[C@@H]1[C@H]2CCc3ccccc3[C@@H]21	287828597	25	333.15	2.78
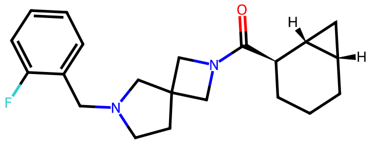	O=C([C@@H]1CCC[C@H]2C[C@H]21)N1CC2(CCN(Cc3ccccc3F)C2)C1	1072875759	25	342.21	3.3
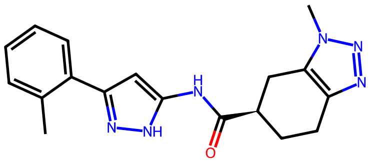	Cc1ccccc1-c1cc(NC(=O)[C@@H]2CCc3nnn(C)c3C2)[nH]n1	290323163	25	336.17	2.26
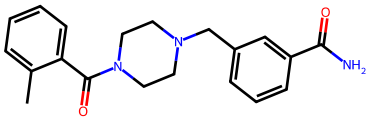	Cc1ccccc1C(=O)N1CCN(Cc2cccc(C(N)=O)c2)CC1	1237699301	25	337.18	2.05
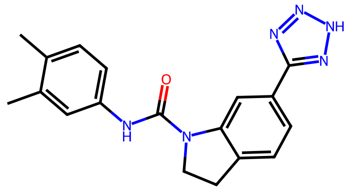	Cc1ccc(NC(=O)N2CCc3ccc(-c4nn[nH]n4)cc32)cc1C	1605870078	25	334.15	3.08
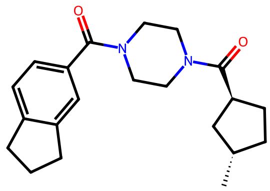	C[C@H]1CC[C@H](C(=O)N2CCN(C(=O)c3ccc4c(c3)CCC4)CC2)C1	1011121616	25	340.22	2.9
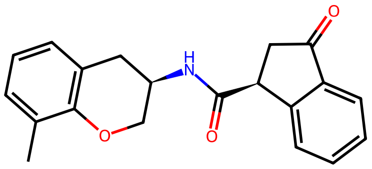	Cc1cccc2c1OC[C@H](NC(=O)[C@H]1CC(=O)c3ccccc31)C2	291803995	24	321.14	2.78
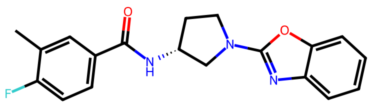	Cc1cc(C(=O)N[C@@H]2CCN(c3nc4ccccc4o3)C2)ccc1F	1058528461	25	339.14	3.28

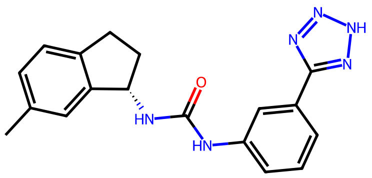	Cc1ccc2c(c1)[C@@H](NC(=O)Nc1cccc(-c3nn[nH]n3)c1)CC2	737371821	25	334.15	2.98
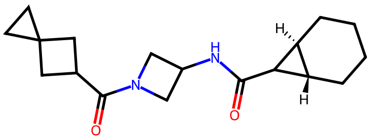	O=C(NC1CN(C(=O)C2CC3(CC3)C2)C1)C1[C@H]2CCCC[C@H]12	990666506	22	302.2	1.94
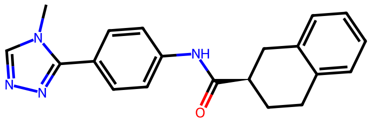	Cn1cnnc1-c1ccc(NC(=O)[C@@H]2CCc3ccccc3C2)cc1	565053225	25	332.16	3.23
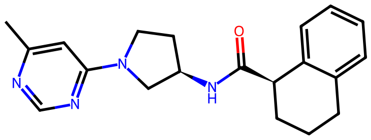	Cc1cc(N2CC[C@@H](NC(=O)[C@@H]3CCCc4ccccc43)C2)ncn1	1058592335	25	336.2	2.6
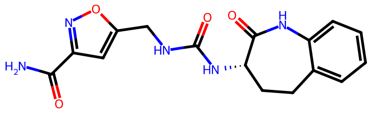	NC(=O)c1cc(CNC(=O)N[C@H]2CCc3ccccc3NC2=O)on1	654502479	25	343.13	0.53
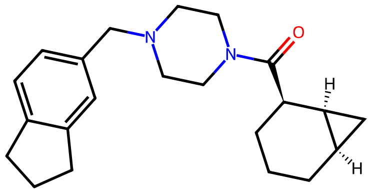	O=C([C@@H]1CCC[C@@H]2C[C@@H]21)N1CCN(Cc2ccc3c(c2)CCC3)CC1	948972715	25	338.24	3.26
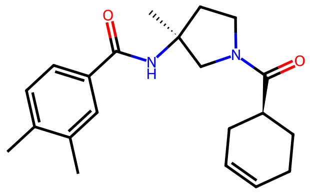	Cc1ccc(C(=O)N[C@@]2(C)CCN(C(=O)[C@@H]3CC=CCC3)C2)cc1C	957569114	25	340.22	3.38
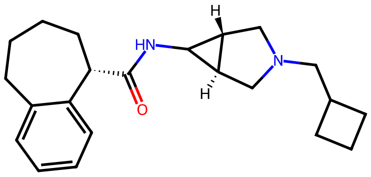	O=C(NC1[C@@H]2CN(CC3CCC3)C[C@@H]12)[C@H]1CCCCc2ccccc21	962850119	25	338.24	3.34

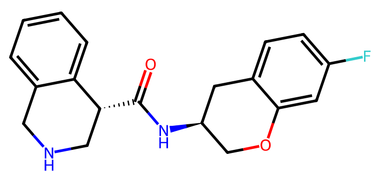	O=C(N[C@@H]1COc2cc(F)ccc2C1)[C@@H]1CNCc2ccccc21	662875986	24	326.14	2.13
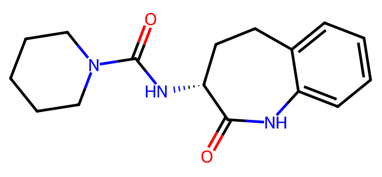	O=C1Nc2ccccc2CC[C@H]1NC(=O)N1CCCCC1	848596392	21	287.16	2.14
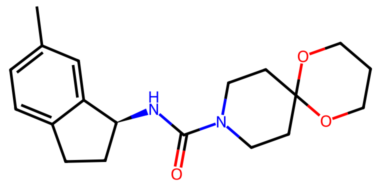	Cc1ccc2c(c1)[C@@H](NC(=O)N1CCC3(CC1)OCCCO3)CC2	836289275	24	330.19	2.92
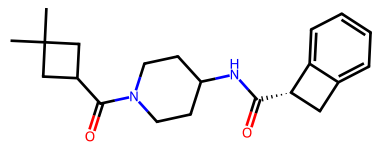	CC1(C)CC(C(=O)N2CCC(NC(=O)[C@H]3Cc4ccccc43)CC2)C1	946627534	25	340.22	2.87
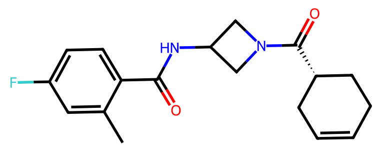	Cc1cc(F)ccc1C(=O)NC1CN(C(=O)[C@H]2CC=CCC2)C1	997173600	23	316.16	2.43
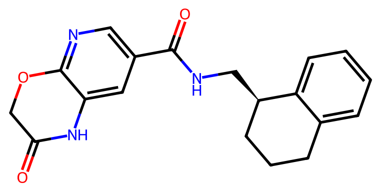	O=C1COc2ncc(C(=O)NC[C@@H]3CCCc4ccccc43)cc2N1	271239168	25	337.14	2.26
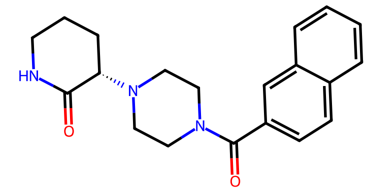	O=C1NCCC[C@@H]1N1CCN(C(=O)c2ccc3ccccc3c2)CC1	957443428	25	337.18	1.88
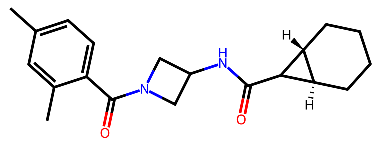	Cc1ccc(C(=O)N2CC(NC(=O)C3[C@@H]4CCCC[C@@H]34)C2)c(C)c1	990674052	24	326.2	2.68

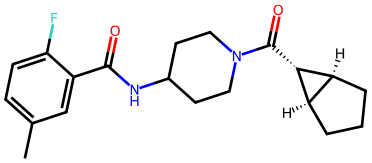	Cc1ccc(F)c(C(=O)NC2CCN(C(=O)[C@@H]3[C@H]4CCC[C@H]43)CC2)c1	946092784	25	344.19	2.9
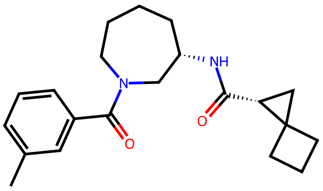	Cc1cccc(C(=O)N2CCCC[C@H](NC(=O)[C@@H]3CC34CCC4)C2)c1	943256164	25	340.22	3.3
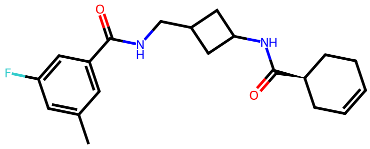	Cc1cc(F)cc(C(=O)NCC2CC(NC(=O)[C@@H]3CC=CCC3)C2)c1	992431921	25	344.19	3.12
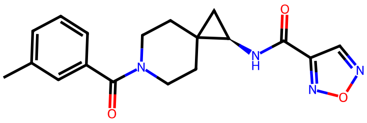	Cc1cccc(C(=O)N2CCC3(CC2)C[C@H]3NC(=O)c2cnon2)c1	964830761	25	340.15	1.8
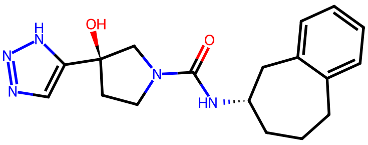	O=C(N[C@H]1CCCc2ccccc2C1)N1CC[C@](O)(c2cnn[nH]2)C1	897608370	25	341.19	1.36
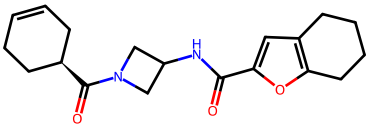	O=C(NC1CN(C(=O)[C@@H]2CC=CCC2)C1)c1cc2c(o1)CCCC2	993284909	24	328.18	2.46
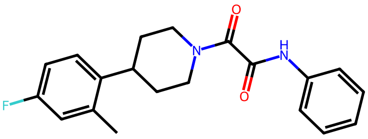	Cc1cc(F)ccc1C1CCN(C(=O)C(=O)Nc2ccccc2)CC1	340538729	25	340.16	3.48
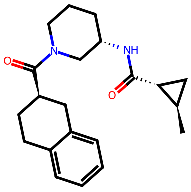	C[C@@H]1C[C@H]1C(=O)N[C@H]1CCCN(C(=O)[C@@H]2CCc3ccccc3C2)C1	989371573	25	340.22	2.55

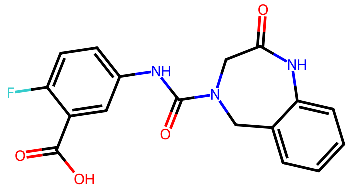	O=C1CN(C(=O)Nc2ccc(F)c(C(=O)O)c2)Cc2ccccc2N1	1609040736	25	343.1	2.51
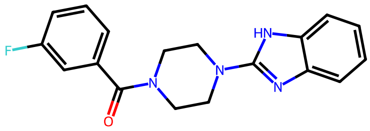	O=C(c1cccc(F)c1)N1CCN(c2nc3ccccc3[nH]2)CC1	301155211	24	324.14	2.66
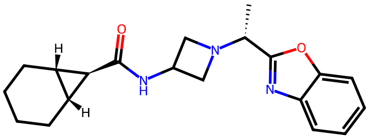	C[C@H](c1nc2ccccc2o1)N1CC(NC(=O)[C@@H]2[C@H]3CCCC[C@H]32)C1	1030188571	25	339.19	3.13
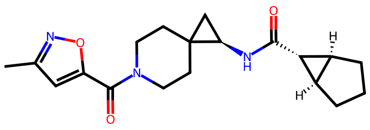	Cc1cc(C(=O)N2CCC3(CC2)C[C@H]3NC(=O)[C@@H]2[C@H]3CCC[C@H]32)on1	963462576	25	343.19	2.14
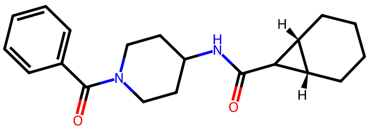	O=C(NC1CCN(C(=O)c2ccccc2)CC1)C1[C@H]2CCCC[C@@H]12	173743203	24	326.2	2.84
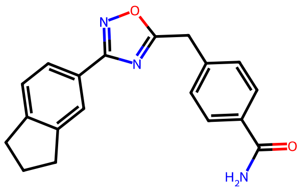	NC(=O)c1ccc(Cc2nc(-c3ccc4c(c3)CCC4)no2)cc1	354042809	24	319.13	2.92
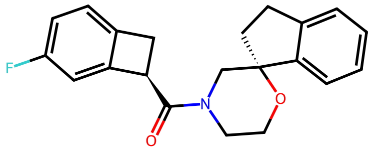	O=C([C@@H]1Cc2ccc(F)cc21)N1CCO[C@@]2(CCc3ccccc32)C1	684466780	25	337.15	3.17
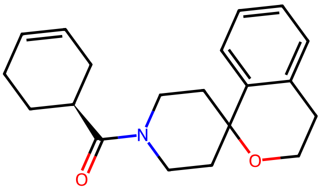	O=C([C@@H]1CC=CCC1)N1CCC2(CC1)OCCc1ccccc12	457125595	23	311.19	3.43

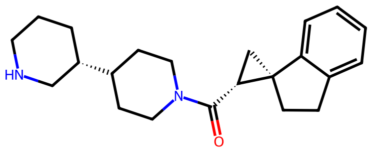	O=C([C@@H]1C[C@]12CCc1ccccc12)N1CCC([C@H]2CCCNC2)CC1	658956686	25	338.24	3.13
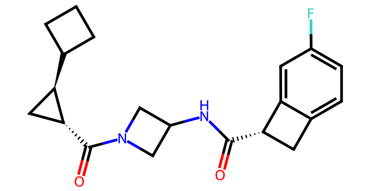	O=C(NC1CN(C(=O)[C@@H]2C[C@H]2C2CCC2)C1)[C@H]1Cc2ccc(F)cc21	998492704	25	342.17	2.23
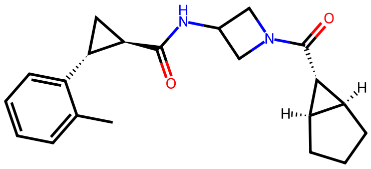	Cc1ccccc1[C@@H]1C[C@H]1C(=O)NC1CN(C(=O)[C@@H]2[C@H]3CCC[C@H]32)C1	992025842	25	338.2	2.47
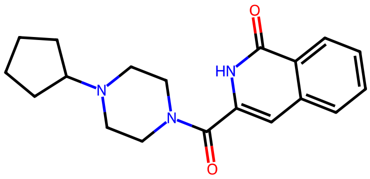	O=C(c1cc2ccccc2c(=O)[nH]1)N1CCN(C2CCCC2)CC1	47630714	24	325.18	2.23
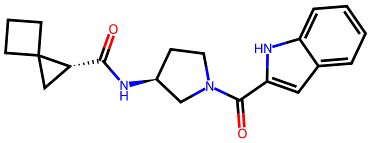	O=C(N[C@H]1CCN(C(=O)c2cc3ccccc3[nH]2)C1)[C@@H]1CC12CCC2	937953980	25	337.18	2.69
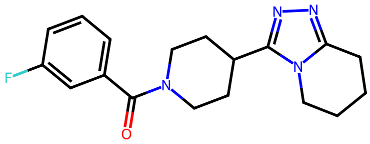	O=C(c1cccc(F)c1)N1CCC(c2nnc3n2CCCC3)CC1	274649559	24	328.17	2.77
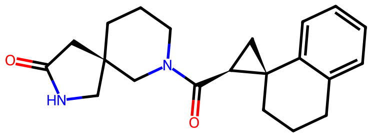	O=C1C[C@]2(CCCN(C(=O)[C@H]3C[C@@]34CCCc3ccccc34)C2)CN1	341506613	25	338.2	2.41
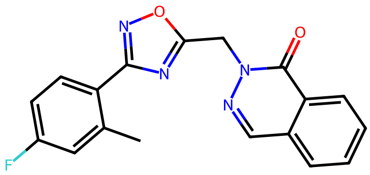	Cc1cc(F)ccc1-c1noc(Cn2ncc3ccccc3c2=O)n1	356716144	25	336.1	2.94

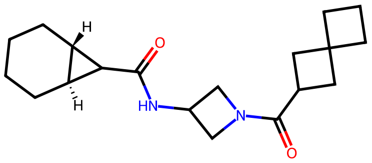	O=C(NC1CN(C(=O)C2CC3(CCC3)C2)C1)C1[C@H]2CCCC[C@H]12	990659939	23	316.22	2.33
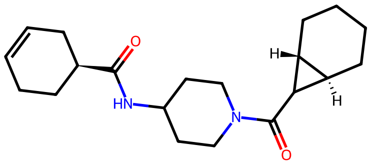	O=C(NC1CCN(C(=O)C2[C@@H]3CCCC[C@@H]23)CC1)[C@@H]1CC=CCC1	945956644	24	330.23	2.89
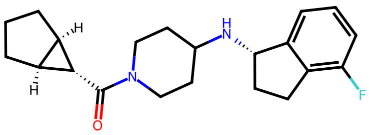	O=C([C@@H]1[C@H]2CCC[C@H]21)N1CCC(N[C@H]2CCc3c(F)cccc32)CC1	990788990	25	342.21	3.44
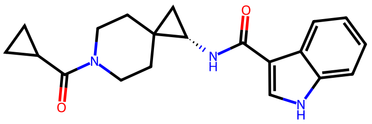	O=C(N[C@H]1CC12CCN(C(=O)C1CC1)CC2)c1c[nH]c2ccccc12	964187950	25	337.18	2.69
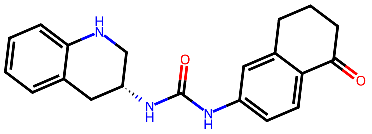	O=C(Nc1ccc2c(c1)CCCC2=O)N[C@H]1CNc2ccccc2C1	652608304	25	335.16	3.36
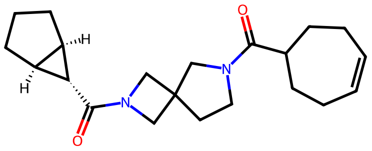	O=C(C1CCC=CCC1)N1CCC2(C1)CN(C(=O)[C@@H]1[C@H]3CCC[C@H]31)C2	1018914022	25	342.23	2.84
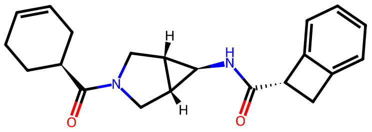	O=C(N[C@H]1[C@@H]2CN(C(=O)[C@@H]3CC=CCC3)C[C@@H]21)[C@H]1Cc2ccccc21	977081569	25	336.18	2.26
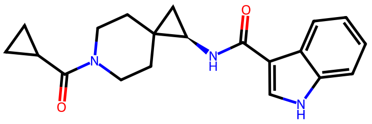	O=C(N[C@@H]1CC12CCN(C(=O)C1CC1)CC2)c1c[nH]c2ccccc12	964187955	25	337.18	2.69

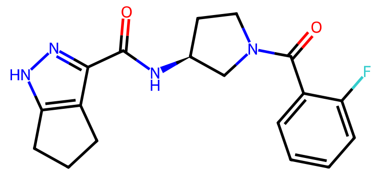	O=C(N[C@H]1CCN(C(=O)c2ccccc2F)C1)c1n[nH]c2c1CCC2	938240872	25	342.15	1.68
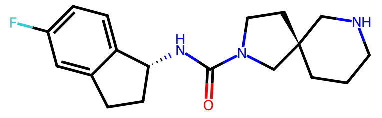	O=C(N[C@@H]1CCc2cc(F)ccc21)N1CC[C@@]2(CCCNC2)C1	583311487	23	317.19	2.6
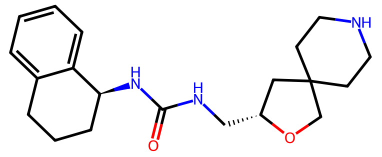	O=C(NC[C@@H]1CC2(CCNCC2)CO1)N[C@H]1CCCc2ccccc21	874429513	25	343.23	2.52
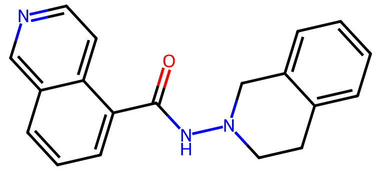	O=C(NN1CCc2ccccc2C1)c1cccc2cnccc12	1640002166	23	303.14	2.94
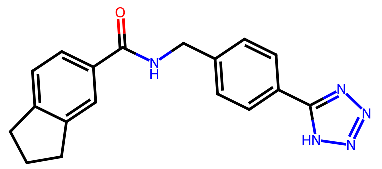	O=C(NCc1ccc(-c2nnn[nH]2)cc1)c1ccc2c(c1)CCC2	68973758	24	319.14	2.29
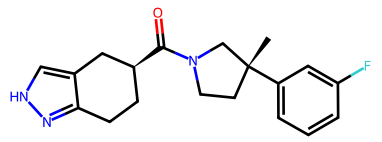	C[C@@]1(c2cccc(F)c2)CCN(C(=O)[C@H]2CCc3n[nH]cc3C2)C1	652619210	24	327.17	2.84
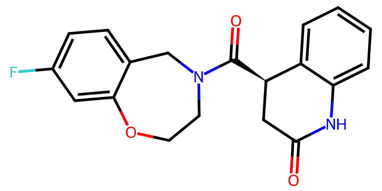	O=C1C[C@@H](C(=O)N2CCOc3cc(F)ccc3C2)c2ccccc2N1	1434245369	25	340.12	2.67
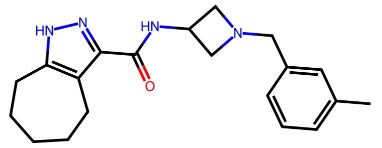	Cc1cccc(CN2CC(NC(=O)c3n[nH]c4c3CCCCC4)C2)c1	1031390091	25	338.21	2.6

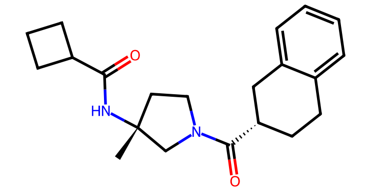	C[C@@]1(NC(=O)C2CCC2)CCN(C(=O)[C@H]2CCc3ccccc3C2)C1	956628596	25	340.22	2.7
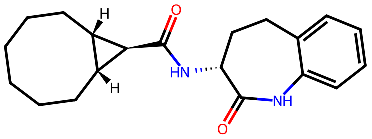	O=C1Nc2ccccc2CC[C@H]1NC(=O)[C@@H]1[C@H]2CCCCCC[C@H]21	616693211	24	326.2	3.27
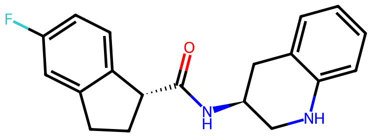	O=C(N[C@@H]1CNc2ccccc2C1)[C@@H]1CCc2cc(F)ccc21	658941629	23	310.15	3.01
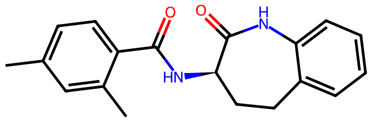	Cc1ccc(C(=O)N[C@@H]2CCc3ccccc3NC2=O)c(C)c1	654005917	23	308.15	2.99
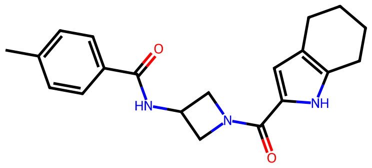	Cc1ccc(C(=O)NC2CN(C(=O)c3cc4c([nH]3)CCCC4)C2)cc1	999443057	25	337.18	2.46
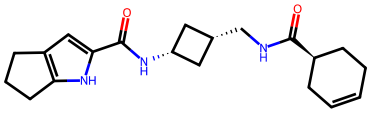	O=C(N[C@H]1C[C@@H](CNC(=O)[C@@H]2CC=CCC2)C1)c1cc2c([nH]1)CCC2	978582750	25	341.21	2.48
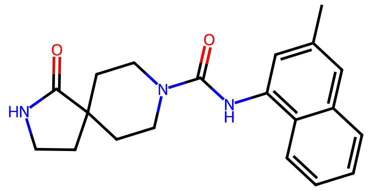	Cc1cc(NC(=O)N2CCC3(CCNC3=O)CC2)c2ccccc2c1	879724674	25	337.18	3.28
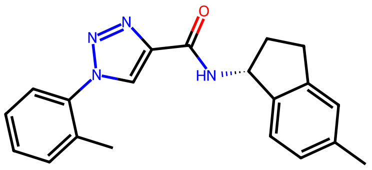	Cc1ccc2c(c1)CC[C@H]2NC(=O)c1cn(-c2ccccc2C)nn1	1264783715	25	332.16	3.3

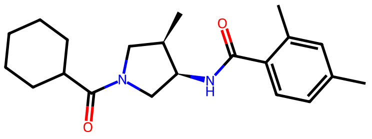	Cc1ccc(C(=O)N[C@H]2CN(C(=O)C3CCCCC3)C[C@H]2C)c(C)c1	966119198	25	342.23	3.46
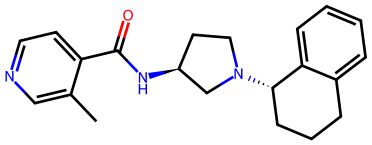	Cc1cnccc1C(=O)N[C@H]1CCN([C@H]2CCCc3ccccc32)C1	1014357460	25	335.2	3.27
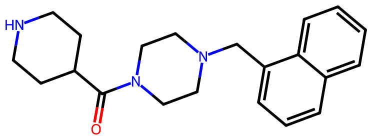	O=C(C1CCNCC1)N1CCN(Cc2cccc3ccccc23)CC1	238109042	25	337.22	2.48
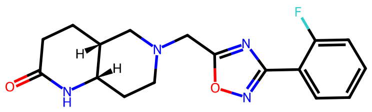	O=C1CC[C@@H]2CN(Cc3nc(-c4ccccc4F)no3)CC[C@@H]2N1	661599916	24	330.15	1.98
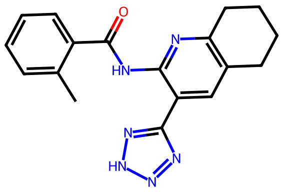	Cc1ccccc1C(=O)Nc1nc2c(cc1-c1nn[nH]n1)CCCC2	826306344	25	334.15	2.7
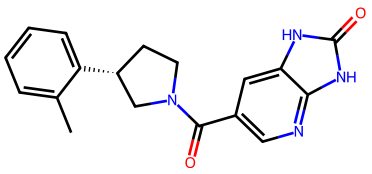	Cc1ccccc1[C@@H]1CCN(C(=O)c2cnc3[nH]c(=O)[nH]c3c2)C1	289065929	24	322.14	2.19
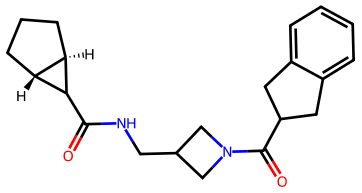	O=C(NCC1CN(C(=O)C2Cc3ccccc3C2)C1)C1[C@@H]2CCC[C@@H]12	1001349584	25	338.2	2.02
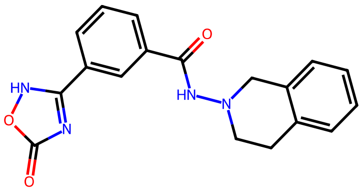	O=C(NN1CCc2ccccc2C1)c1cccc(-c2nc(=O)o[nH]2)c1	1583871482	25	336.12	1.73

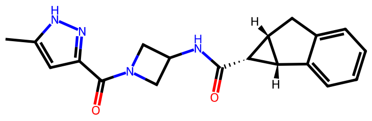	Cc1cc(C(=O)N2CC(NC(=O)[C@H]3[C@H]4c5ccccc5C[C@H]43)C2)n[nH]1	991653594	25	336.16	1.24
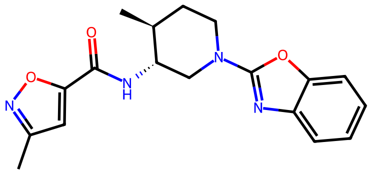	Cc1cc(C(=O)N[C@H]2CN(c3nc4ccccc4o3)CC[C@@H]2C)on1	1067934670	25	340.15	2.77
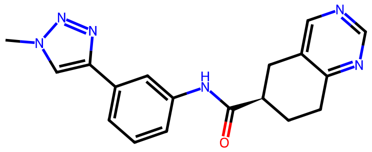	Cn1cc(-c2cccc(NC(=O)[C@@H]3CCc4ncncc4C3)c2)nn1	620270340	25	334.15	2.02
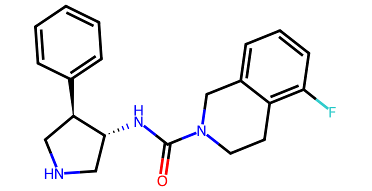	O=C(N[C@@H]1CNC[C@H]1c1ccccc1)N1CCc2c(F)cccc2C1	418689119	25	339.17	2.65
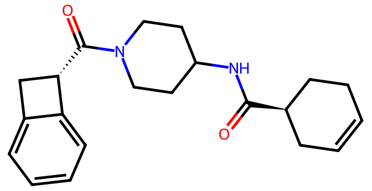	O=C(NC1CCN(C(=O)[C@H]2Cc3ccccc32)CC1)[C@@H]1CC=CCC1	948059696	25	338.2	2.79
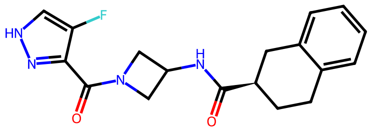	O=C(NC1CN(C(=O)c2n[nH]cc2F)C1)[C@@H]1CCc2ccccc2C1	1000536998	25	342.15	1.29
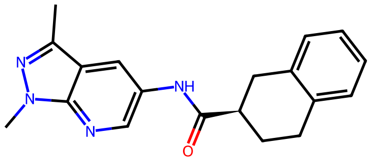	Cc1nn(C)c2ncc(NC(=O)[C@@H]3CCc4ccccc4C3)cc12	649565473	24	320.16	3.02
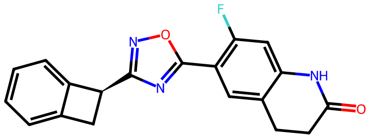	O=C1CCc2cc(-c3nc([C@H]4Cc5ccccc54)no3)c(F)cc2N1	544988222	25	335.11	3.45

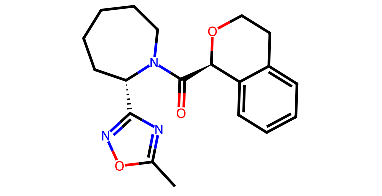	Cc1nc([C@@H]2CCCCCN2C(=O)[C@H]2OCCc3ccccc32)no1	329379479	25	341.17	3.14
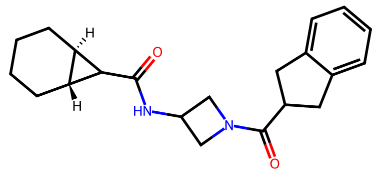	O=C(NC1CN(C(=O)C2Cc3ccccc3C2)C1)C1[C@@H]2CCCC[C@@H]12	990659121	25	338.2	2.16
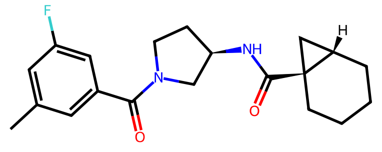	Cc1cc(F)cc(C(=O)N2CC[C@@H](NC(=O)[C@]34CCCC[C@H]3C4)C2)c1	936243684	25	344.19	3.05
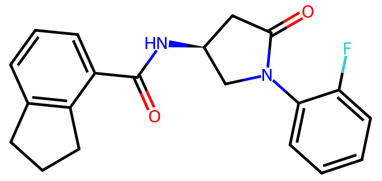	O=C(N[C@H]1CC(=O)N(c2ccccc2F)C1)c1cccc2c1CCC2	360583465	25	338.14	2.85
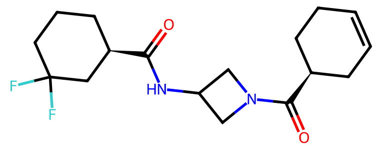	O=C(NC1CN(C(=O)[C@@H]2CC=CCC2)C1)[C@@H]1CCCC(F)(F)C1	999743939	23	326.18	2.5
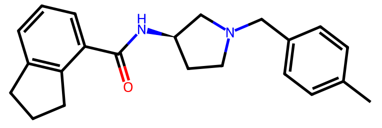	Cc1ccc(CN2CC[C@@H](NC(=O)c3cccc4c3CCC4)C2)cc1	1016380142	25	334.2	3.49
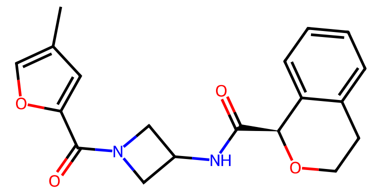	Cc1coc(C(=O)N2CC(NC(=O)[C@@H]3OCCc4ccccc43)C2)c1	997322001	25	340.14	1.84
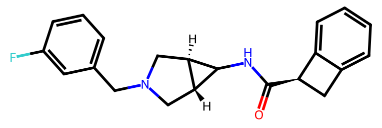	O=C(NC1[C@@H]2CN(Cc3cccc(F)c3)C[C@@H]12)[C@@H]1Cc2ccccc21	961627504	25	336.16	2.71

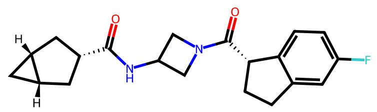	O=C(NC1CN(C(=O)[C@H]2CCc3cc(F)ccc32)C1)[C@@H]1C[C@@H]2C[C@@H]2C1	998008108	25	342.17	2.23
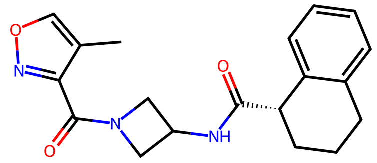	Cc1conc1C(=O)N1CC(NC(=O)[C@H]2CCCc3ccccc32)C1	995071658	25	339.16	2.04
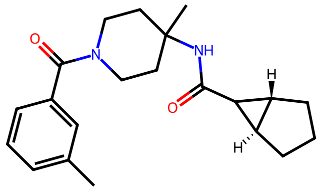	Cc1cccc(C(=O)N2CCC(C)(NC(=O)C3[C@@H]4CCC[C@@H]34)CC2)c1	956326536	25	340.22	3.15
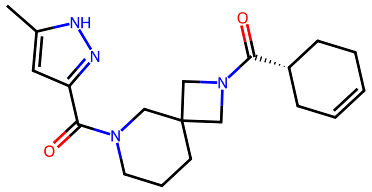	Cc1cc(C(=O)N2CCCC3(C2)CN(C(=O)[C@H]2CC=CCC2)C3)n[nH]1	1010529652	25	342.21	2.14
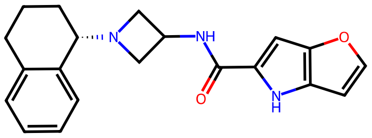	O=C(NC1CN([C@H]2CCCc3ccccc32)C1)c1cc2occc2[nH]1	1030387368	25	335.16	3.25
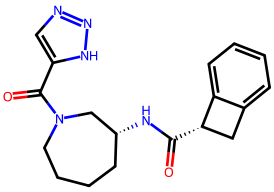	O=C(N[C@@H]1CCCCN(C(=O)c2cnn[nH]2)C1)[C@H]1Cc2ccccc21	943485507	25	339.17	1.26
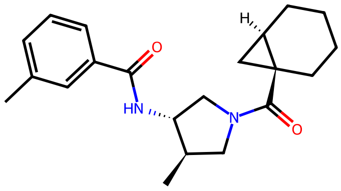	Cc1cccc(C(=O)N[C@@H]2CN(C(=O)[C@]34CCCC[C@@H]3C4)C[C@H]2C)c1	958643416	25	340.22	3.15
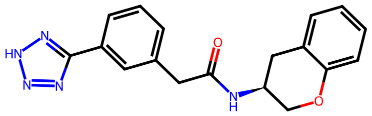	O=C(Cc1cccc(-c2nn[nH]n2)c1)N[C@@H]1COc2ccccc2C1	823093655	25	335.14	1.53

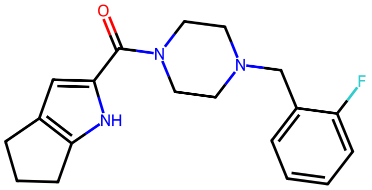	O=C(c1cc2c([nH]1)CCC2)N1CCN(Cc2<br>ccccc2F)CC1	957293151	24	327.17	2.6
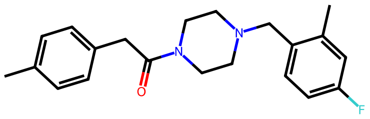	Cc1ccc(CC(=O)N2CCN(Cc3ccc(F)cc3<br>C)CC2)cc1	949157178	25	340.2	3.33
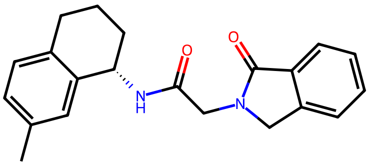	Cc1ccc2c(c1)[C@@H](NC(=O)CN1Cc<br>3ccccc3C1=O)CCC2	344698828	25	334.17	3.14
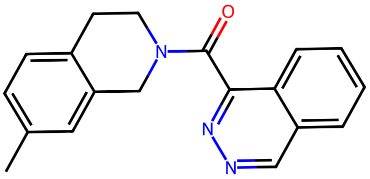	Cc1ccc2c(c1)CN(C(=O)c1nncc3ccccc<br>13)CC2	1437445369	23	303.14	3.14
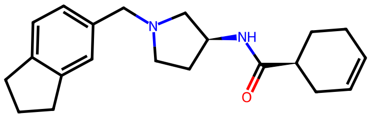	O=C(N[C@H]1CCN(Cc2ccc3c(c2)CC<br>C3)C1)[C@@H]1CC=CCC1	1010080047	24	324.22	3.22
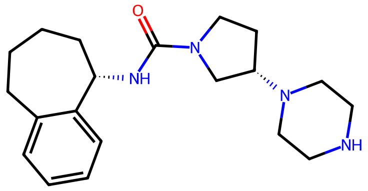	O=C(N[C@H]1CCCCc2ccccc21)N1C<br>C[C@H](N2CCNCC2)C1	577831474	25	342.24	2.14
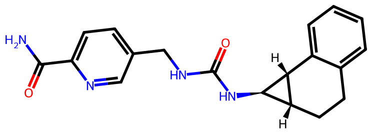	NC(=O)c1ccc(CNC(=O)N[C@@H]2[C<br>@H]3CCc4ccccc4[C@H]32)cn1	435771432	25	336.16	1.71
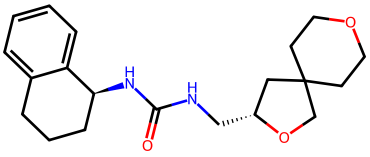	O=C(NC[C@@H]1CC2(CCOCC2)CO<br>1)N[C@H]1CCCc2ccccc21	869032391	25	344.21	2.95

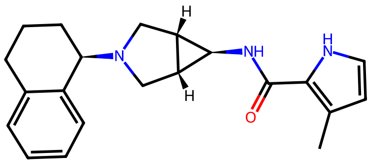	Cc1cc[nH]c1C(=O)N[C@H]1[C@@H]2CN([C@@H]3CCCc4ccccc43)C[C@@H]21	958741899	25	335.2	3.06
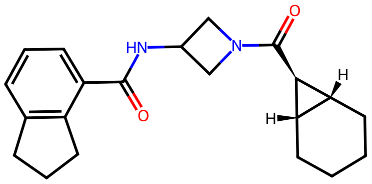	O=C(NC1CN(C(=O)[C@@H]2[C@H]3CCCC[C@H]32)C1)c1cccc2c1CCC2	994404974	25	338.2	2.55
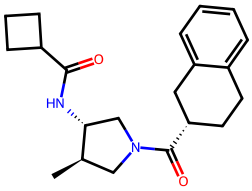	C[C@@H]1CN(C(=O)[C@H]2CCc3ccccc3C2)C[C@H]1NC(=O)C1CCC1	959765140	25	340.22	2.55
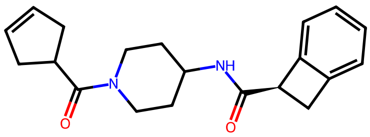	O=C(NC1CCN(C(=O)C2CC=CC2)CC1)[C@@H]1Cc2ccccc21	945756471	24	324.18	2.4
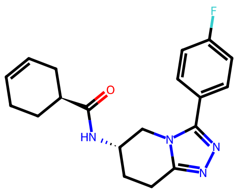	O=C(N[C@H]1CCc2nnc(-c3ccc(F)cc3)n2C1)[C@@H]1CC=CCC1	332855341	25	340.17	2.87
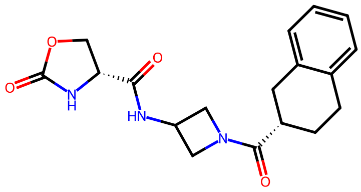	O=C1N[C@@H](C(=O)NC2CN(C(=O)[C@H]3CCc4ccccc4C3)C2)CO1	991125988	25	343.15	0.23
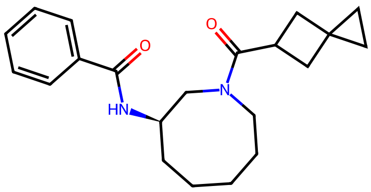	O=C(N[C@@H]1CCCCCN(C(=O)C2CC3(CC3)C2)C1)c1ccccc1	987154148	25	340.22	3.38
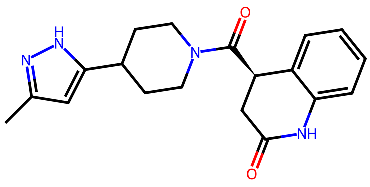	Cc1cc(C2CCN(C(=O)[C@@H]3CC(=O)Nc4ccccc43)CC2)[nH]n1	272208885	25	338.17	2.55

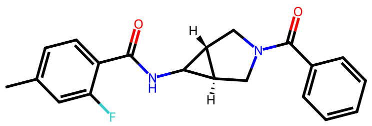	Cc1ccc(C(=O)NC2[C@@H]3CN(C(=O)c4ccccc4)C[C@@H]23)c(F)c1	1013871843	25	338.14	2.63
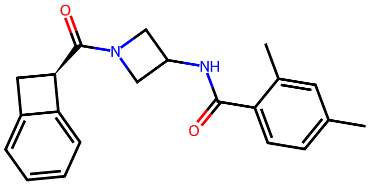	Cc1ccc(C(=O)NC2CN(C(=O)[C@@H]3Cc4ccccc43)C2)c(C)c1	999538163	25	334.17	2.58
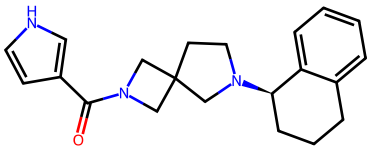	O=C(c1cc[nH]c1)N1CC2(CCN([C@@H]3CCCc4ccccc43)C2)C1	1072771016	25	335.2	3.24
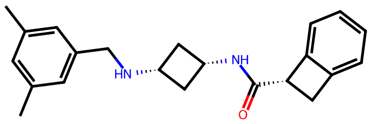	Cc1cc(C)cc(CN[C@H]2C[C@@H](NC(=O)[C@H]3Cc4ccccc43)C2)c1	1022625722	25	334.2	3.38
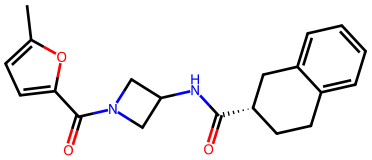	Cc1ccc(C(=O)N2CC(NC(=O)[C@H]3CCc4ccccc4C3)C2)o1	1000540017	25	338.16	2.33
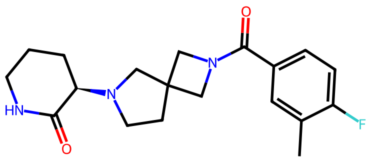	Cc1cc(C(=O)N2CC3(CCN([C@@H]4CCCNC4=O)C3)C2)ccc1F	1072929212	25	345.19	1.56
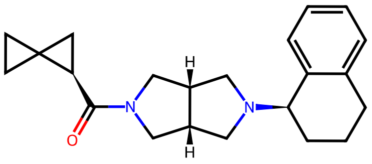	O=C([C@@H]1CC12CC2)N1C[C@@H]2CN([C@@H]3CCCc4ccccc43)C[C@@H]2C1	1049082782	25	336.22	3.25
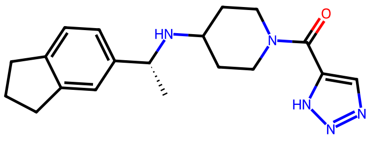	C[C@@H](NC1CCN(C(=O)c2cnn[nH]2)CC1)c1ccc2c(c1)CCC2	996754468	25	339.21	2.25

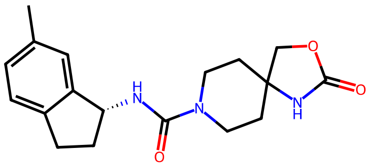	Cc1ccc2c(c1)[C@H](NC(=O)N1CCC3(CC1)COC(=O)N3)CC2	784301196	24	329.17	2.27
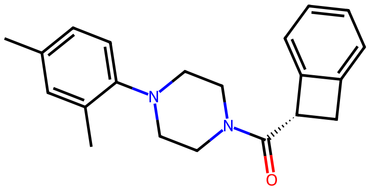	Cc1ccc(N2CCN(C(=O)[C@H]3Cc4ccccc43)CC2)c(C)c1	1167633143	24	320.19	3.29
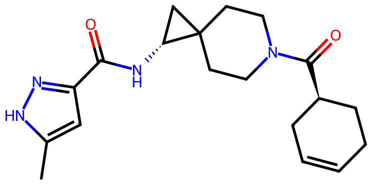	Cc1cc(C(=O)N[C@@H]2CC23CCN(C(=O)[C@@H]2CC=CCC2)CC3)n[nH]1	963292646	25	342.21	2.19
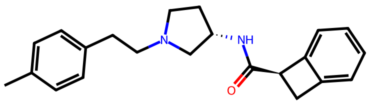	Cc1ccc(CCN2CC[C@H](NC(=O)[C@@H]3Cc4ccccc43)C2)cc1	1015636008	25	334.2	3.07
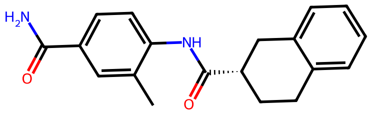	Cc1cc(C(N)=O)ccc1NC(=O)[C@H]1CCc2ccccc2C1	844329023	23	308.15	2.84
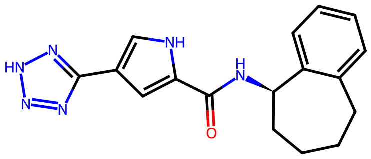	O=C(N[C@@H]1CCCCc2ccccc21)c1cc(-c2nn[nH]n2)c[nH]1	823150011	24	322.15	2.39
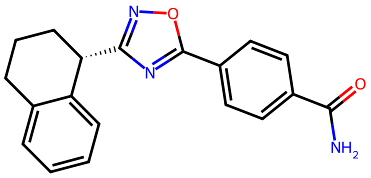	NC(=O)c1ccc(-c2nc([C@H]3CCCc4ccccc43)no2)cc1	358517741	24	319.13	3.3
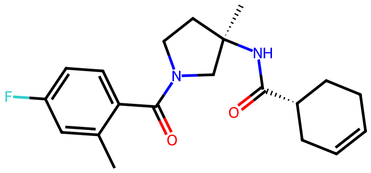	Cc1cc(F)ccc1C(=O)N1CC[C@@](C)(NC(=O)[C@H]2CC=CCC2)C1	956686673	25	344.19	3.21

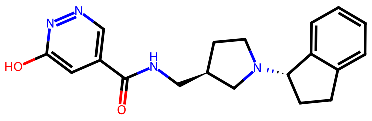	O=C(NC[C@H]1CCN([C@H]2CCc3ccccc32)C1)c1cnnc(O)c1	1028612972	25	338.17	1.92
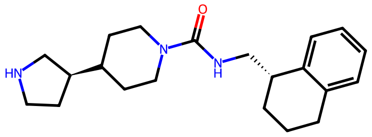	O=C(NC[C@H]1CCCc2ccccc21)N1CCC([C@H]2CCNC2)CC1	417070630	25	341.25	3.14
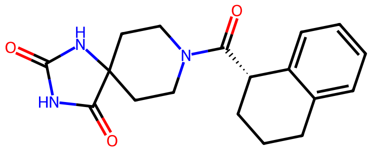	O=C1NC(=O)C2(CCN(C(=O)[C@H]3CCCc4ccccc43)CC2)N1	44385255	24	327.16	1.31
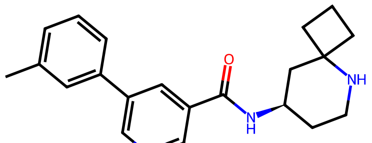	Cc1cccc(-c2cncc(C(=O)N[C@@H]3CCNC4(CCC4)C3)c2)c1	649370612	25	335.2	3.46
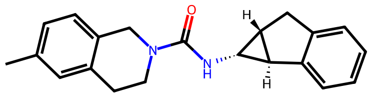	Cc1ccc2c(c1)CCN(C(=O)N[C@H]1[C@@H]3c4ccccc4C[C@H]31)C2	481264538	24	318.17	3.4
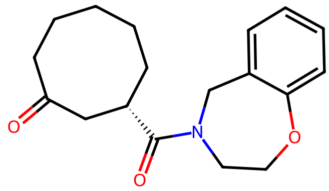	O=C1CCCCC[C@H](C(=O)N2CCOc3ccccc3C2)C1	643956185	22	301.17	2.95
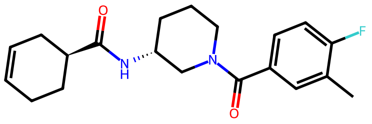	Cc1cc(C(=O)N2CCC[C@@H](NC(=O)[C@@H]3CC=CCC3)C2)ccc1F	951836767	25	344.19	3.21
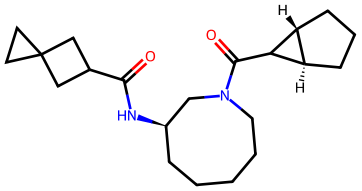	O=C(N[C@@H]1CCCCCN(C(=O)C2[C@@H]3CCC[C@@H]23)C1)C1CC2(CC2)C1	986974520	25	344.25	3.11

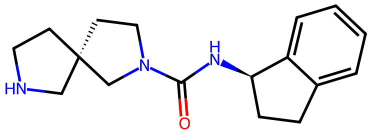	O=C(N[C@@H]1CCc2ccccc21)N1CC[C@]2(CCNC2)C1	335613189	21	285.18	2.07
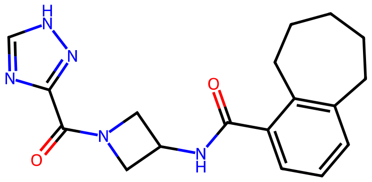	O=C(NC1CN(C(=O)c2nc[nH]n2)C1)c1cccc2c1CCCCC2	994061200	25	339.17	1.33
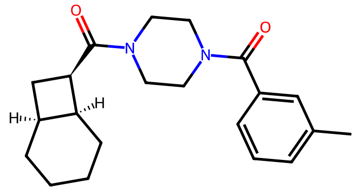	Cc1cccc(C(=O)N2CCN(C(=O)[C@@H]3C[C@@H]4CCCC[C@@H]43)CC2)c1	1010072712	25	340.22	3.11
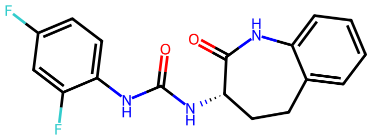	O=C(Nc1ccc(F)cc1F)N[C@H]1CCc2ccccc2NC1=O	1202028556	24	331.11	3.04
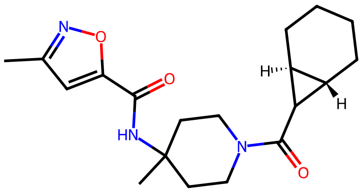	Cc1cc(C(=O)NC2(C)CCN(C(=O)C3[C@H]4CCCC[C@H]34)CC2)on1	956287245	25	345.21	2.53
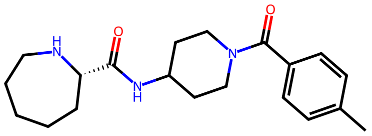	Cc1ccc(C(=O)N2CCC(NC(=O)[C@@H]3CCCCCN3)CC2)cc1	579124426	25	343.23	2.25
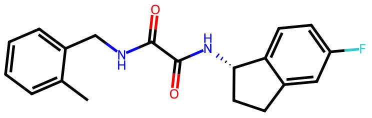	Cc1ccccc1CNC(=O)C(=O)N[C@H]1CCc2cc(F)ccc21	1116994848	24	326.14	2.55
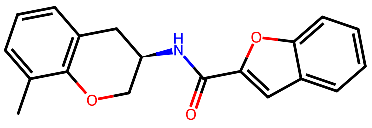	Cc1cccc2c1OC[C@H](NC(=O)c1cc3ccccc3o1)C2	685822559	23	307.12	3.47

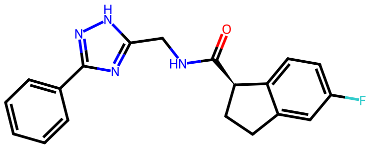	O=C(NCc1nc(-c2ccccc2)n[nH]1)[C@@H]1CCc2cc(F)ccc21	1443014455	25	336.14	2.96
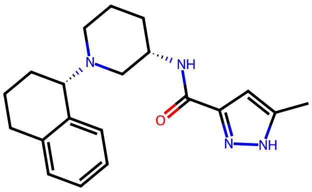	Cc1cc(C(=O)N[C@H]2CCCN([C@H]3CCCc4ccccc43)C2)n[nH]1	1006857943	25	338.21	2.99
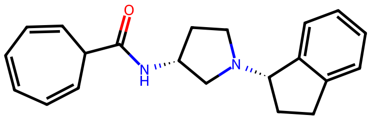	O=C(N[C@@H]1CCN([C@H]2CCc3ccccc32)C1)C1C=CC=CC=C1	1014598415	24	320.19	3.16
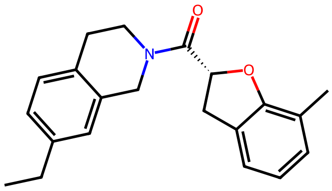	CCc1ccc2c(c1)CN(C(=O)[C@H]1Cc3cccc(C)c3O1)CC2	1540295172	24	321.17	3.45
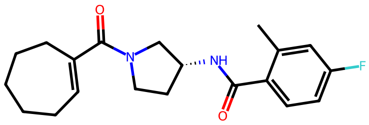	Cc1cc(F)ccc1C(=O)N[C@@H]1CCN(C(=O)C2=CCCCCC2)C1	941910045	25	344.19	3.36
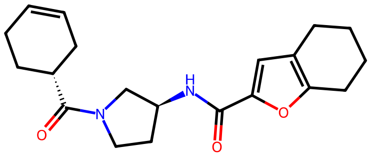	O=C(N[C@H]1CCN(C(=O)[C@H]2CC=CCC2)C1)c1cc2c(o1)CCCC2	938068511	25	342.19	2.85
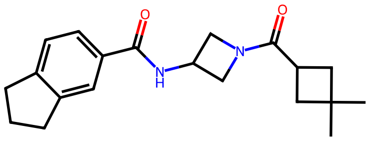	CC1(C)CC(C(=O)N2CC(NC(=O)c3ccc4c(c3)CCC4)C2)C1	991353993	24	326.2	2.55
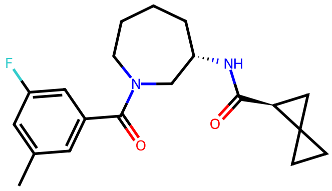	Cc1cc(F)cc(C(=O)N2CCCC[C@H](NC(=O)[C@H]3CC34CC4)C2)c1	943574022	25	344.19	3.05

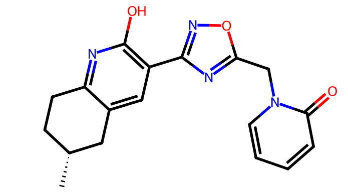	C[C@@H]1CCc2nc(O)c(-c3noc(Cn4ccccc4=O)n3)cc2C1	897277872	25	338.14	2.17
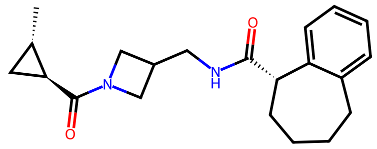	C[C@H]1C[C@@H]1C(=O)N1CC(CNC(=O)[C@H]2CCCCc3ccccc32)C1	1002643982	25	340.22	2.73
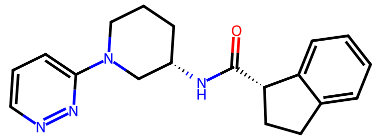	O=C(N[C@H]1CCCN(c2cccnn2)C1)[C@H]1CCc2ccccc21	332877291	24	322.18	2.29
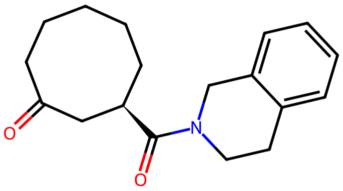	O=C1CCCCC[C@@H](C(=O)N2CCc3ccccc3C2)C1	336330249	21	285.17	3.11
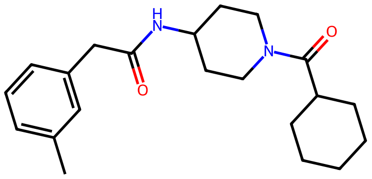	Cc1cccc(CC(=O)NC2CCN(C(=O)C3CCCCC3)CC2)c1	75057350	25	342.23	3.23
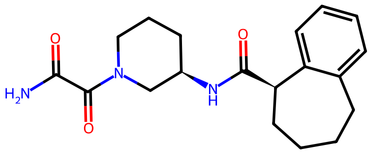	NC(=O)C(=O)N1CCC[C@@H](NC(=O)[C@@H]2CCCCc3ccccc32)C1	951861432	25	343.19	1.09
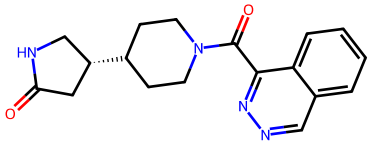	O=C1C[C@@H](C2CCN(C(=O)c3nnc c4ccccc34)CC2)CN1	1551699212	24	324.16	1.62
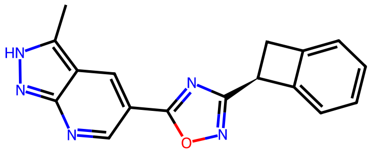	Cc1[nH]nc2ncc(-c3nc([C@H]4Cc5ccccc54)no3)cc12	546287066	23	303.11	3.0

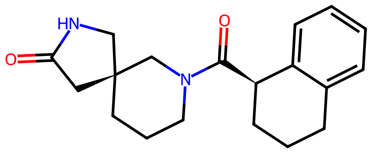	O=C1C[C@@]2(CCCN(C(=O)[C@@H]3CCCc4ccccc43)C2)CN1	362206873	23	312.18	2.24
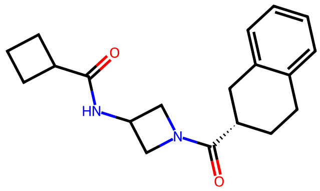	O=C(NC1CN(C(=O)[C@H]2CCc3ccccc3C2)C1)C1CCC1	517574517	23	312.18	1.92
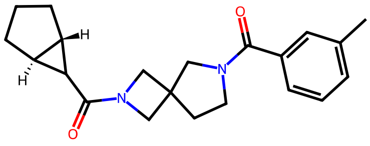	Cc1cccc(C(=O)N2CCC3(C2)CN(C(=O)C2[C@H]4CCC[C@H]24)C3)c1	1018911085	25	338.2	2.72
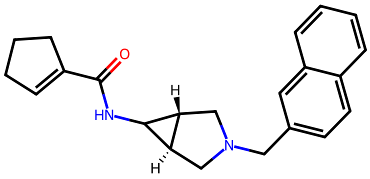	O=C(NC1[C@@H]2CN(Cc3ccc4ccccc4c3)C[C@@H]12)C1=CCCC1	958559998	25	332.19	3.5
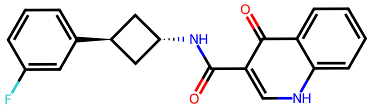	O=C(N[C@H]1C[C@H](c2cccc(F)c2)C1)c1c[nH]c2ccccc2c1=O	366717756	25	336.13	3.34
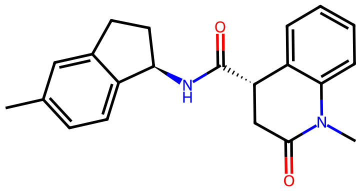	Cc1ccc2c(c1)CC[C@H]2NC(=O)[C@H]1CC(=O)N(C)c2ccccc21	1115889197	25	334.17	3.25
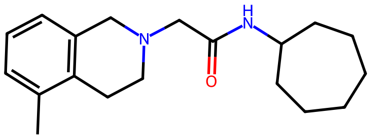	Cc1cccc2c1CCN(CC(=O)NC1CCCCCC1)C2	729478500	22	300.22	3.19
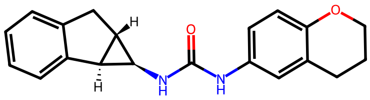	O=C(Nc1ccc2c(c1)CCCO2)N[C@@H]1[C@H]2Cc3ccccc3[C@@H]21	178217994	24	320.15	3.47

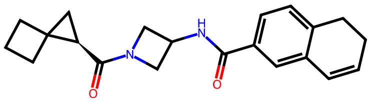	O=C(NC1CN(C(=O)[C@@H]2CC23CCC3)C1)c1ccc2c(c1)C=CCC2	995371335	25	336.18	2.78
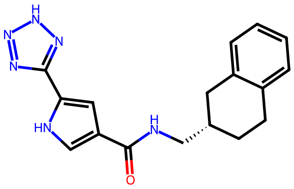	O=C(NC[C@H]1CCc2ccccc2C1)c1c[nH]c(-c2nn[nH]n2)c1	826435480	24	322.15	1.73
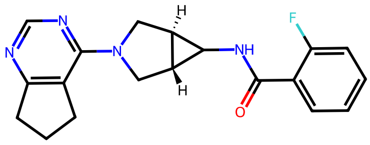	O=C(NC1[C@@H]2CN(c3ncnc4c3CCC4)C[C@@H]12)c1ccccc1F	1056107605	25	338.15	1.97
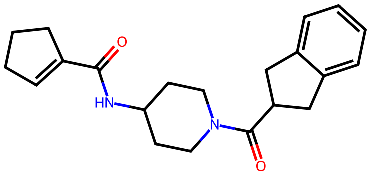	O=C(NC1CCN(C(=O)C2Cc3ccccc3C2)CC1)C1=CCCC1	948549910	25	338.2	2.62
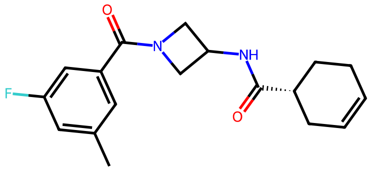	Cc1cc(F)cc(C(=O)N2CC(NC(=O)[C@H]3CC=CCC3)C2)c1	990222826	23	316.16	2.43
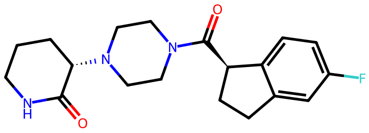	O=C1NCCC[C@@H]1N1CCN(C(=O)[C@@H]2CCc3cc(F)ccc32)CC1	949321337	25	345.19	1.28
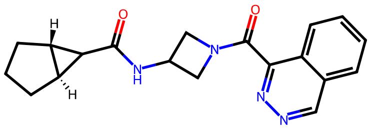	O=C(NC1CN(C(=O)c2nncc3ccccc23)C1)C1[C@H]2CCC[C@H]12	990705786	25	336.16	1.62
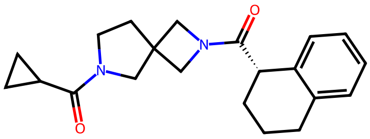	O=C(C1CC1)N1CCC2(C1)CN(C(=O)[C@H]1CCCc3ccccc31)C2	1019168003	25	338.2	2.58

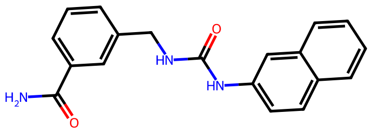	NC(=O)c1cccc(CNC(=O)Nc2ccc3ccccc3c2)c1	97305926	24	319.13	3.26
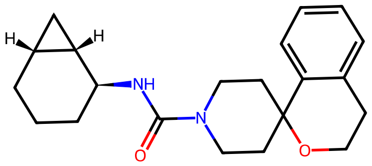	O=C(N[C@H]1CCC[C@@H]2C[C@H]12)N1CCC2(CC1)OCCc1ccccc12	459284396	25	340.22	3.45
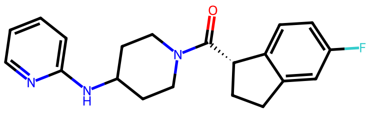	O=C([C@H]1CCc2cc(F)ccc21)N1CCC(Nc2ccccn2)CC1	927074708	25	339.17	3.35
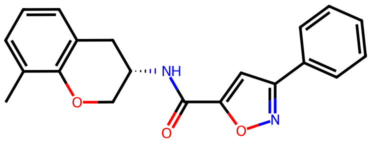	Cc1cccc2c1OC[C@@H](NC(=O)c1cc(-c3ccccc3)no1)C2	484938521	25	334.13	3.38
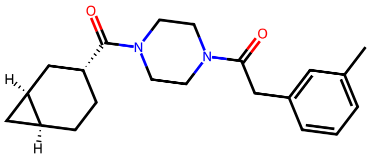	Cc1cccc(CC(=O)N2CCN(C(=O)[C@@H]3CC[C@H]4C[C@H]4C3)CC2)c1	1011312965	25	340.22	2.64
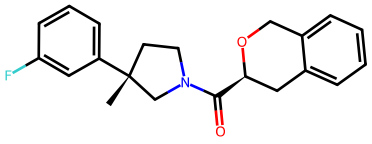	C[C@@]1(c2cccc(F)c2)CCN(C(=O)[C@@H]2Cc3ccccc3CO2)C1	855011921	25	339.16	3.46
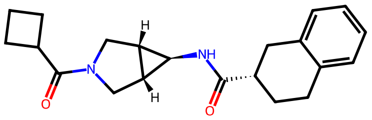	O=C(N[C@H]1[C@@H]2CN(C(=O)C3CCC3)C[C@@H]21)[C@H]1CCc2ccccc2C1	979518814	25	338.2	2.16
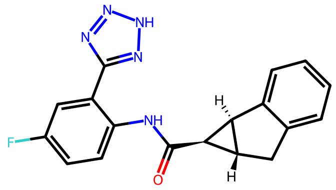	O=C(Nc1ccc(F)cc1-c1nn[nH]n1)[C@@H]1[C@H]2Cc3ccccc3[C@@H]21	1593232012	25	335.12	2.53

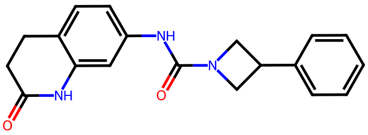	O=C1CCc2ccc(NC(=O)N3CC(c4ccccc4)C3)cc2N1	639718222	24	321.15	3.2
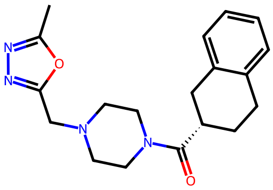	Cc1nnc(CN2CCN(C(=O)[C@H]3CCc4ccccc4C3)CC2)o1	950725124	25	340.19	1.83
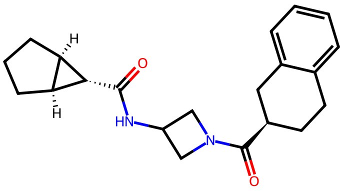	O=C(NC1CN(C(=O)[C@@H]2CCc3ccccc3C2)C1)[C@@H]1[C@H]2CCC[C@H]21	990718599	25	338.2	2.16
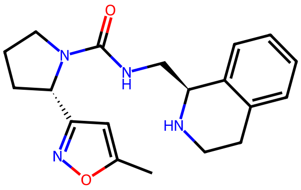	Cc1cc([C@@H]2CCCN2C(=O)NC[C@@H]2NCCc3ccccc32)no1	841998686	25	340.19	2.72
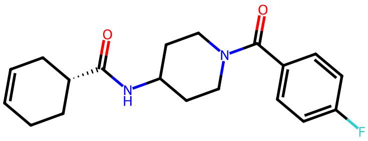	O=C(NC1CCN(C(=O)c2ccc(F)cc2)CC1)[C@H]1CC=CCC1	943597832	24	330.17	2.9
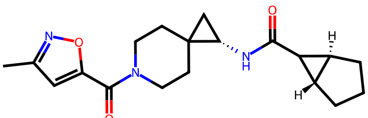	Cc1cc(C(=O)N2CCC3(CC2)C[C@@H]3NC(=O)C2[C@H]3CCC[C@H]23)on1	963462579	25	343.19	2.14
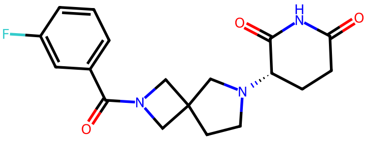	O=C1CC[C@H](N2CCC3(CN(C(=O)c4cccc(F)c4)C3)C2)C(=O)N1	1072419249	25	345.15	0.78
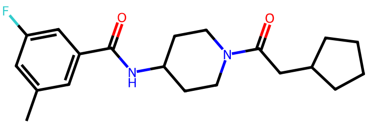	Cc1cc(F)cc(C(=O)NC2CCN(C(=O)CC3CCCC3)CC2)c1	943769253	25	346.21	3.44

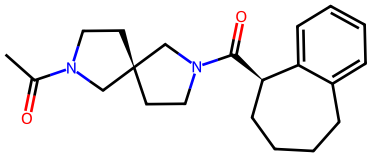	CC(=O)N1CC[C@@]2(CCN(C(=O)[C@@H]3CCCCc4ccccc43)C2)C1	953023750	25	340.22	2.97
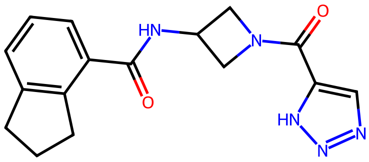	O=C(NC1CN(C(=O)c2cnn[nH]2)C1)c1cccc2c1CCC2	994393200	23	311.14	0.55
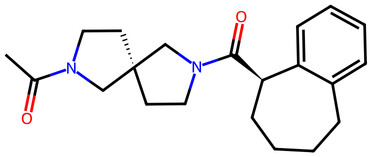	CC(=O)N1CC[C@]2(CCN(C(=O)[C@@H]3CCCCc4ccccc43)C2)C1	953023747	25	340.22	2.97
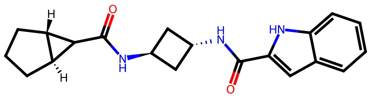	O=C(N[C@H]1C[C@H](NC(=O)C2[C@H]3CCC[C@H]23)C1)c1cc2ccccc2[nH]1	973140959	25	337.18	2.59
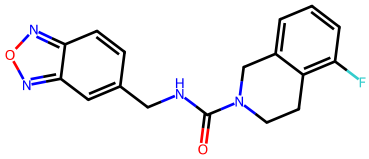	O=C(NCc1ccc2nonc2c1)N1CCc2c(F)cccc2C1	1684589698	24	326.12	2.63
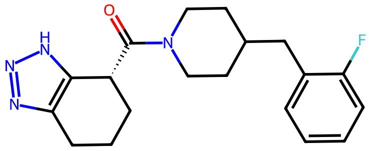	O=C([C@@H]1CCCc2nn[nH]c21)N1CCC(Cc2ccccc2F)CC1	668611637	25	342.19	2.85
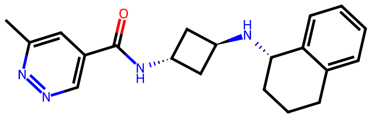	Cc1cc(C(=O)N[C@H]2C[C@H](N[C@H]3CCCc4ccccc43)C2)cnn1	1020107298	25	336.2	2.71
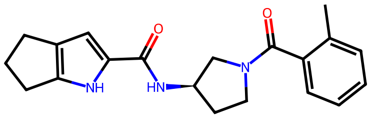	Cc1ccccc1C(=O)N1CC[C@@H](NC(=O)c2cc3c([nH]2)CCC3)C1	941583021	25	337.18	2.46

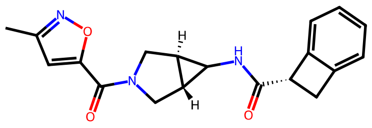	Cc1cc(C(=O)N2C[C@H]3C(NC(=O)[C@H]4Cc5ccccc54)[C@@H]3C2)on1	1013683715	25	337.14	1.51
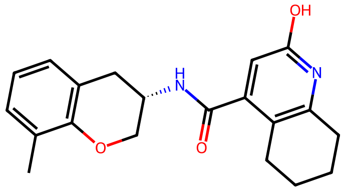	Cc1cccc2c1OC[C@@H](NC(=O)c1cc(O)nc3c1CCCC3)C2	936056842	25	338.16	2.71
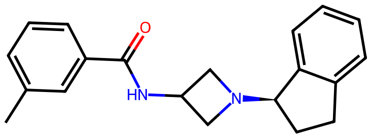	Cc1cccc(C(=O)NC2CN([C@@H]3CCc4ccccc43)C2)c1	1030074248	23	306.17	3.1
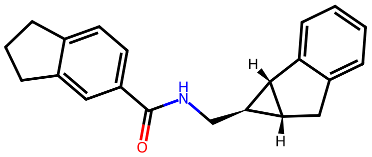	O=C(NC[C@@H]1[C@H]2Cc3ccccc3[C@H]21)c1ccc2c(c1)CCC2	488689591	23	303.16	3.49
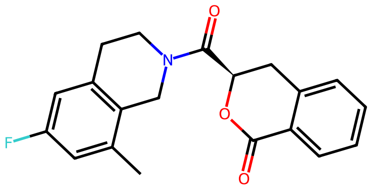	Cc1cc(F)cc2c1CN(C(=O)[C@H]1Cc3ccccc3C(=O)O1)CC2	800246407	25	339.13	2.8
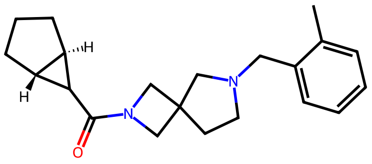	Cc1ccccc1CN1CCC2(C1)CN(C(=O)C1[C@@H]3CCC[C@@H]13)C2	1072590579	24	324.22	3.08
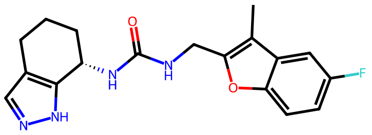	Cc1c(CNC(=O)N[C@H]2CCCc3cn[nH]c32)oc2ccc(F)cc12	268868685	25	342.15	3.48
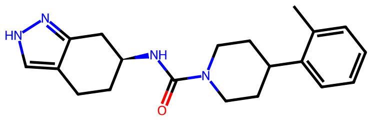	Cc1ccccc1C1CCN(C(=O)N[C@H]2CCc3c[nH]nc3C2)CC1	374574138	25	338.21	3.16

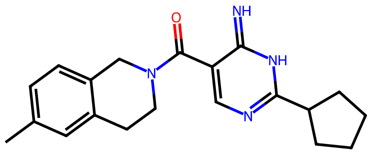	Cc1ccc2c(c1)CCN(C(=O)c1cnc(C3CCCC3)[nH]c1=N)C2	329516068	25	336.2	3.05
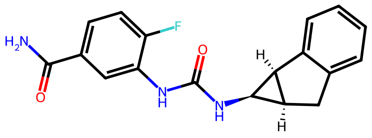	NC(=O)c1ccc(F)c(NC(=O)N[C@@H]2[C@@H]3Cc4ccccc4[C@@H]32)c1	178210318	24	325.12	2.38
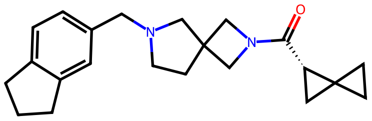	O=C([C@H]1CC12CC2)N1CC2(CCN(Cc3ccc4c(c3)CCC4)C2)C1	1073012272	25	336.22	3.01
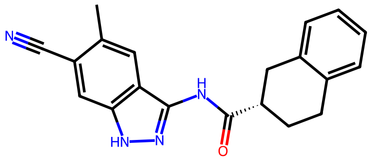	Cc1cc2c(NC(=O)[C@H]3CCc4ccccc4C3)n[nH]c2cc1C#N	1144854428	25	330.15	3.49
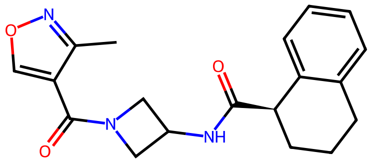	Cc1nocc1C(=O)N1CC(NC(=O)[C@@H]2CCCc3ccccc32)C1	995071500	25	339.16	2.04
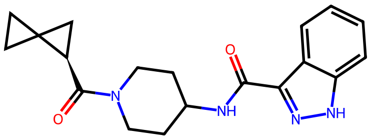	O=C(NC1CCN(C(=O)[C@@H]2CC23CC3)CC1)c1n[nH]c2ccccc12	948559448	25	338.17	2.08
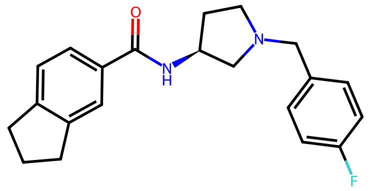	O=C(N[C@H]1CCN(Cc2ccc(F)cc2)C1)c1ccc2c(c1)CCC2	1016223177	25	338.18	3.32
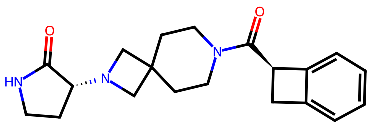	O=C1NCC[C@H]1N1CC2(CCN(C(=O)[C@@H]3Cc4ccccc43)CC2)C1	1035780106	25	339.19	1.14

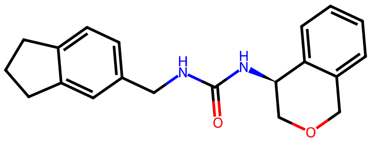	O=C(NCc1ccc2c(c1)CCC2)N[C@@H]1COCc2ccccc21	1325350869	24	322.17	3.25
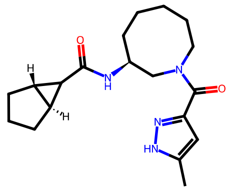	Cc1cc(C(=O)N2CCCCC[C@H](NC(=O)C3[C@H]4CCC[C@H]34)C2)n[nH]1	986925907	25	344.22	2.27
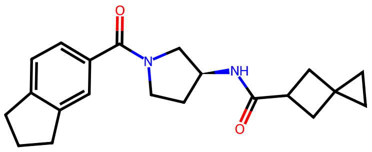	O=C(N[C@H]1CCN(C(=O)c2ccc3c(c2)CCC3)C1)C1CC2(CC2)C1	938710160	25	338.2	2.7
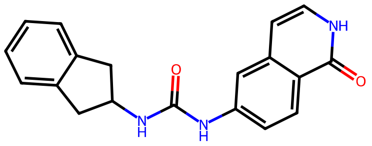	O=C(Nc1ccc2c(=O)[nH]ccc2c1)NC1Cc2ccccc2C1	1673420402	24	319.13	2.82
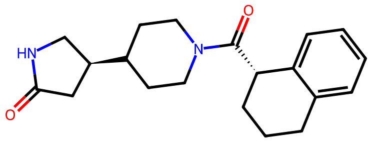	O=C1C[C@H](C2CCN(C(=O)[C@H]3CCCc4ccccc43)CC2)CN1	1551707198	24	326.2	2.48
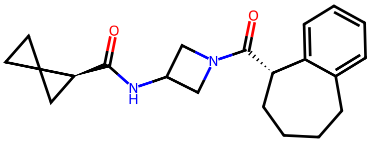	O=C(NC1CN(C(=O)[C@H]2CCCCc3ccccc32)C1)[C@H]1CC12CC2	1000511988	25	338.2	2.62
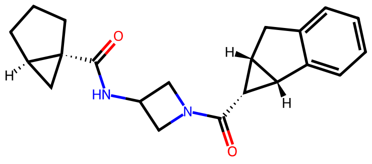	O=C([C@H]1[C@H]2c3ccccc3C[C@H]21)N1CC(NC(=O)[C@@]23CCC[C@@H]2C3)C1	997549107	25	336.18	2.09
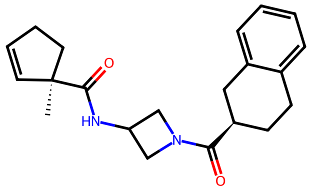	C[C@]1(C(=O)NC2CN(C(=O)[C@@H]3CCc4ccccc4C3)C2)C=CCC1	997849494	25	338.2	2.47

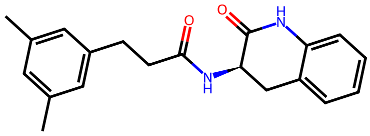	Cc1cc(C)cc(CCC(=O)N[C@@H]2Cc3ccccc3NC2=O)c1	529946729	24	322.17	2.92
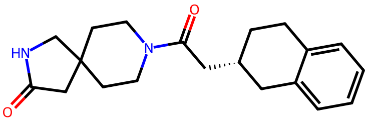	O=C1CC2(CCN(C(=O)C[C@@H]3CCc4ccccc4C3)CC2)CN1	267606349	24	326.2	2.31
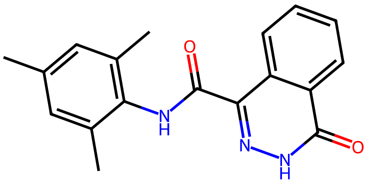	Cc1cc(C)c(NC(=O)c2n[nH]c(=O)c3ccccc23)c(C)c1	78225577	23	307.13	3.1
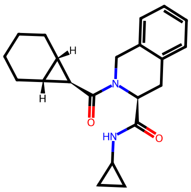	O=C(NC1CC1)[C@@H]1Cc2ccccc2CN1C(=O)[C@@H]1[C@H]2CCCC[C@H]21	648507716	25	338.2	2.65
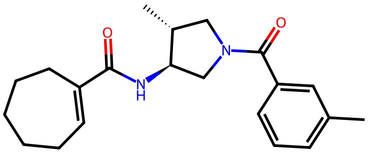	Cc1cccc(C(=O)N2C[C@@H](NC(=O)C3=CCCCCC3)[C@H](C)C2)c1	947313201	25	340.22	3.46
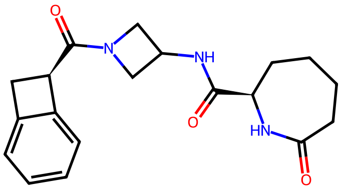	O=C1CCCC[C@H](C(=O)NC2CN(C(=O)[C@@H]3Cc4ccccc43)C2)N1	999369229	25	341.17	0.71
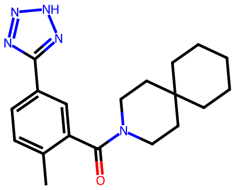	Cc1ccc(-c2nn[nH]n2)cc1C(=O)N1CCC2(CCCCC2)CC1	1592097730	25	339.21	3.36
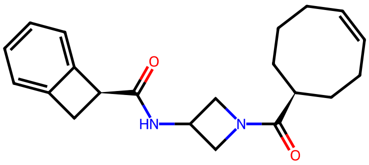	O=C(NC1CN(C(=O)[C@@H]2CCC=CCCC2)C1)[C@H]1Cc2ccccc21	998691292	25	338.2	2.79

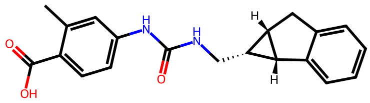	Cc1cc(NC(=O)NC[C@H]2[C@H]3c4ccccc4C[C@H]32)ccc1C(=O)O	832895474	25	336.15	3.4
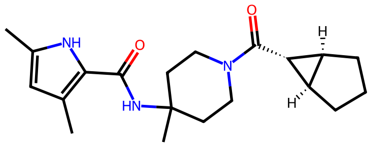	Cc1cc(C)c(C(=O)NC2(C)CCN(C(=O)[C@@H]3[C@H]4CCC[C@H]43)CC2)[nH]1	956421108	25	343.23	2.79
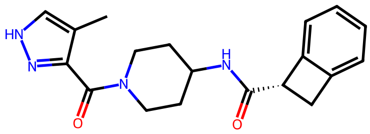	Cc1c[nH]nc1C(=O)N1CCC(NC(=O)[C@H]2Cc3ccccc32)CC1	946682548	25	338.17	1.78
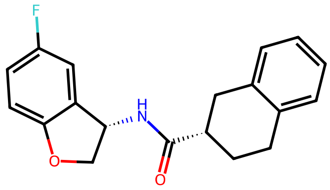	O=C(N[C@@H]1COc2ccc(F)cc21)[C@H]1CCc2ccccc2C1	573807410	23	311.13	3.18
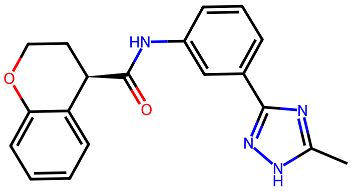	Cc1nc(-c2cccc(NC(=O)[C@@H]3CCOc4ccccc43)c2)n[nH]1	368947435	25	334.14	3.28
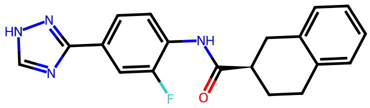	O=C(Nc1ccc(-c2nc[nH]n2)cc1F)[C@@H]1CCc2ccccc2C1	1270853400	25	336.14	3.35
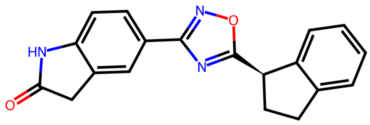	O=C1Cc2cc(-c3noc([C@@H]4CCc5ccccc54)n3)ccc2N1	355980762	24	317.12	3.31
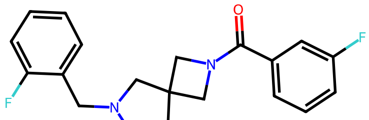	O=C(c1cccc(F)c1)N1CC2(CCN(Cc3ccccc3F)C2)C1	1072418806	25	342.15	3.31

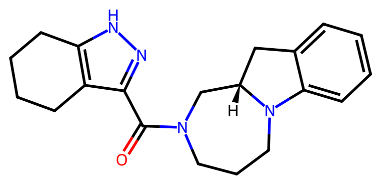	O=C(c1n[nH]c2c1CCCC2)N1CCCN2c3ccccc3C[C@H]2C1	378009179	25	336.2	2.57
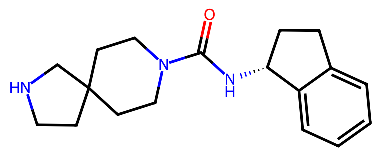	O=C(N[C@@H]1CCc2ccccc21)N1CCC2(CCNC2)CC1	335614168	22	299.2	2.46
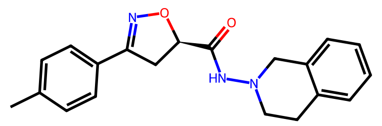	Cc1ccc(C2=NO[C@@H](C(=O)NN3CCc4ccccc4C3)C2)cc1	1418389466	25	335.16	2.58
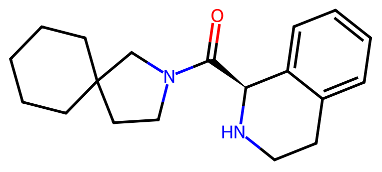	O=C([C@@H]1NCCc2ccccc21)N1CCC2(CCCCC2)C1	237964983	22	298.2	3.06
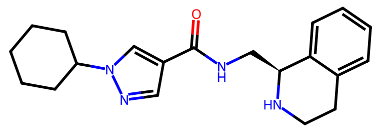	O=C(NC[C@@H]1NCCc2ccccc21)c1cnn(C2CCCCC2)c1	1325856316	25	338.21	3.01
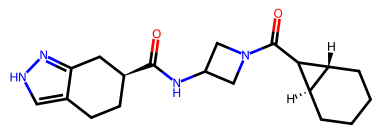	O=C(NC1CN(C(=O)C2[C@@H]3CCCC[C@@H]23)C1)[C@H]1CCc2c[nH]nc2C1	992573981	25	342.21	1.28
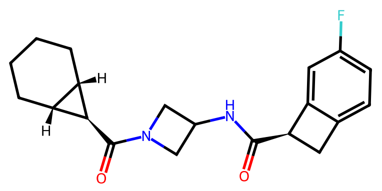	O=C(NC1CN(C(=O)[C@@H]2[C@H]3CCCC[C@H]32)C1)[C@@H]1Cc2ccc(F)cc21	998488011	25	342.17	2.23
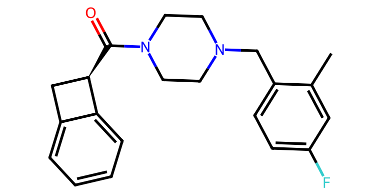	Cc1cc(F)ccc1CN1CCN(C(=O)[C@@H]2Cc3ccccc32)CC1	949958850	25	338.18	3.12

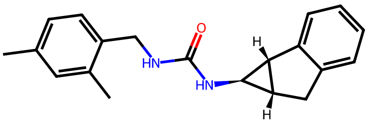	Cc1ccc(CNC(=O)N[C@@H]2[C@H]3Cc4ccccc4[C@H]32)c(C)c1	248121259	23	306.17	3.44
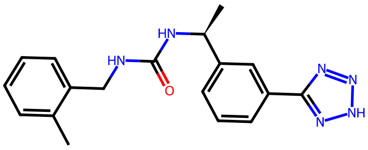	Cc1ccccc1CNC(=O)N[C@@H](C)c1cccc(-c2nn[nH]n2)c1	822361882	25	336.17	2.74
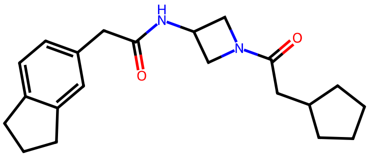	O=C(Cc1ccc2c(c1)CCC2)NC1CN(C(=O)CC2CCCC2)C1	991386826	25	340.22	2.63
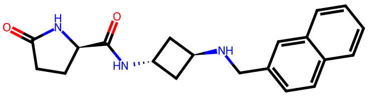	O=C1CC[C@H](C(=O)N[C@H]2C[C@H](NCc3ccc4ccccc4c3)C2)N1	1020027118	25	337.18	1.86
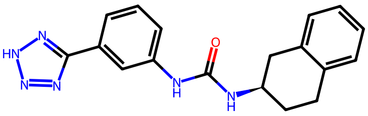	O=C(Nc1cccc(-c2nn[nH]n2)c1)N[C@@H]1CCc2ccccc2C1	1606932515	25	334.15	2.55
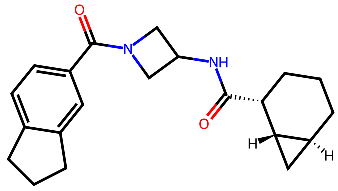	O=C(NC1CN(C(=O)c2ccc3c(c2)CCC3)C1)[C@@H]1CCC[C@H]2C[C@@H]21	994719220	25	338.2	2.55
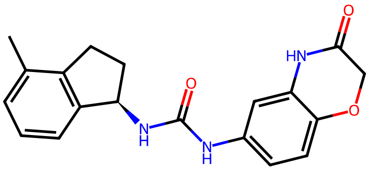	Cc1cccc2c1CC[C@H]2NC(=O)Nc1ccc2c(c1)NC(=O)CO2	104985106	25	337.14	3.13
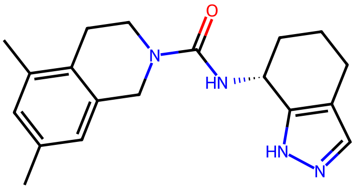	Cc1cc(C)c2c(c1)CN(C(=O)N[C@@H]1CCCc3cn[nH]c31)CC2	275226071	24	324.2	3.17

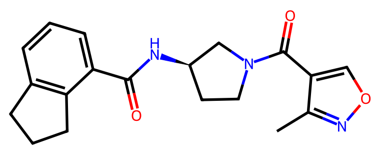	Cc1nocc1C(=O)N1CC[C@@H](NC(=O)c2cccc3c2CCC3)C1	941160166	25	339.16	2.12
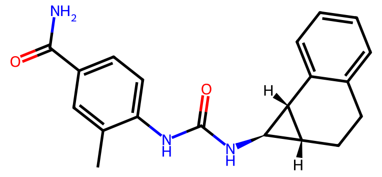	Cc1cc(C(N)=O)ccc1NC(=O)N[C@@H]1[C@H]2CCc3ccccc3[C@H]21	772418267	25	335.16	2.94
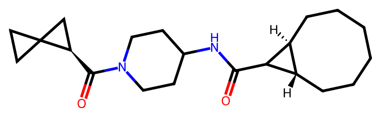	O=C(NC1CCN(C(=O)[C@@H]2CC23CC3)CC1)C1[C@H]2CCCCCC[C@H]12	948557989	25	344.25	3.11
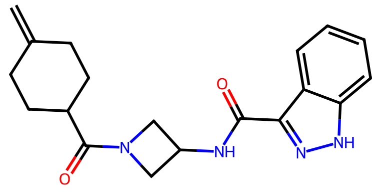	C=C1CCC(C(=O)N2CC(NC(=O)c3n[nH]c4ccccc34)C2)CC1	999529371	25	338.17	2.25
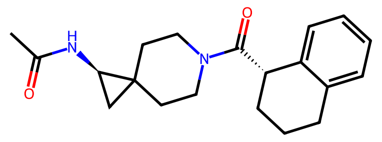	CC(=O)N[C@@H]1CC12CCN(C(=O)[C@H]1CCCc3ccccc31)CC2	962318204	24	326.2	2.62
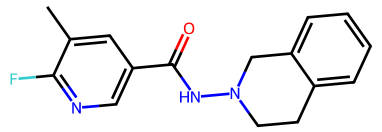	Cc1cc(C(=O)NN2CCc3ccccc3C2)cnc1F	857724576	21	285.13	2.23
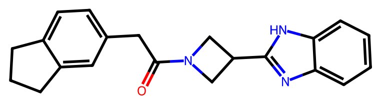	O=C(Cc1ccc2c(c1)CCC2)N1CC(c2nc3ccccc3[nH]2)C1	675526742	25	331.17	3.22
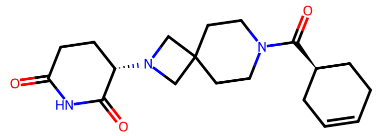	O=C1CC[C@H](N2CC3(CCN(C(=O)[C@@H]4CC=CCC4)CC3)C2)C(=O)N1	1035648621	25	345.21	1.07

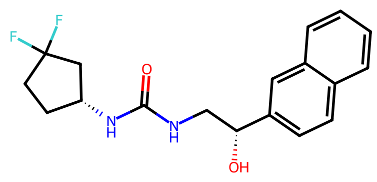	O=C(NC[C@@H](O)c1ccc2ccccc2c1)N[C@@H]1CCC(F)(F)C1	763708734	24	334.15	3.36
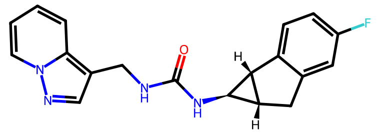	O=C(NCc1cnn2ccccc12)N[C@@H]1[C@H]2Cc3cc(F)ccc3[C@H]21	664876622	25	336.14	2.61
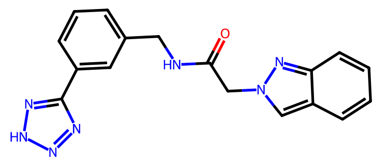	O=C(Cn1cc2ccccc2n1)NCc1cccc(-c2nn[nH]n2)c1	823123345	25	333.13	1.53
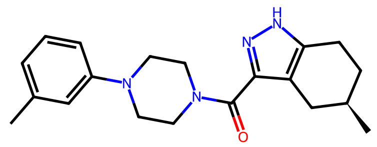	Cc1cccc(N2CCN(C(=O)c3n[nH]c4c3C[C@H](C)CC4)CC2)c1	1412383572	25	338.21	2.81
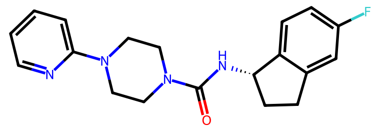	O=C(N[C@H]1CCc2cc(F)ccc21)N1CCN(c2ccccn2)CC1	56838685	25	340.17	2.74
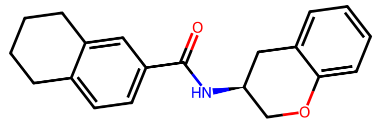	O=C(N[C@@H]1COc2ccccc2C1)c1ccc2c(c1)CCCC2	613757890	23	307.16	3.3
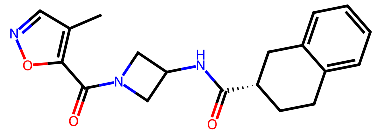	Cc1cnoc1C(=O)N1CC(NC(=O)[C@H]2CCc3ccccc3C2)C1	1000541765	25	339.16	1.73
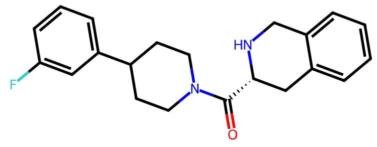	O=C([C@H]1Cc2ccccc2CN1)N1CCC(c2cccc(F)c2)CC1	1343800128	25	338.18	3.25

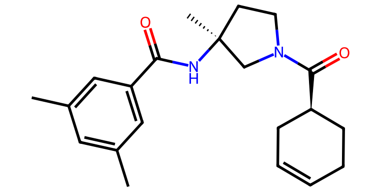	Cc1cc(C)cc(C(=O)N[C@@]2(C)CCN(C(=O)[C@@H]3CC=CCC3)C2)c1	957560538	25	340.22	3.38
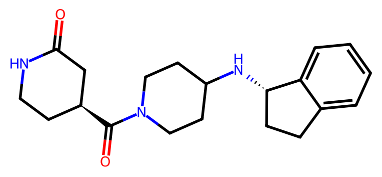	O=C1C[C@@H](C(=O)N2CCC(N[C@H]3CCc4ccccc43)CC2)CCN1	991030219	25	341.21	1.78
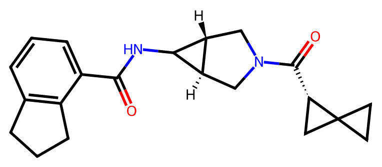	O=C(NC1[C@@H]2CN(C(=O)[C@H]3CC34CC4)C[C@@H]12)c1cccc2c1CCC2	1013293653	25	336.18	2.16
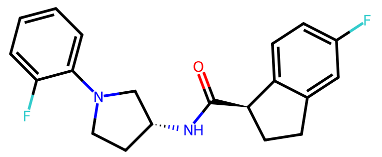	O=C(N[C@@H]1CCN(c2ccccc2F)C1)[C@@H]1CCc2cc(F)ccc21	615196902	25	342.15	3.39
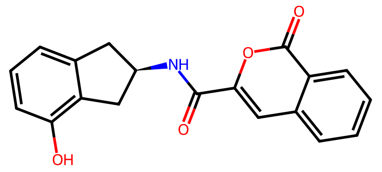	O=C(N[C@@H]1Cc2cccc(O)c2C1)c1cc2ccccc2c(=O)o1	357178760	24	321.1	2.4
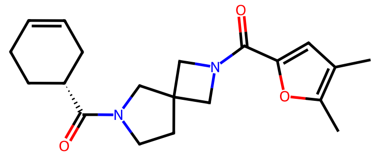	Cc1cc(C(=O)N2CC3(CCN(C(=O)[C@H]4CC=CCC4)C3)C2)oc1C	1018942524	25	342.19	2.93
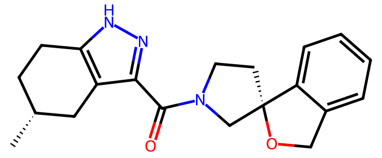	C[C@@H]1CCc2[nH]nc(C(=O)N3CC[C@@]4(C3)OCc3ccccc34)c2C1	897417531	25	337.18	2.81
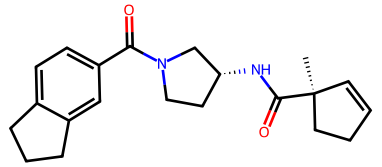	C[C@]1(C(=O)N[C@@H]2CCN(C(=O)c3ccc4c(c3)CCC4)C2)C=CCC1	942190908	25	338.2	2.86

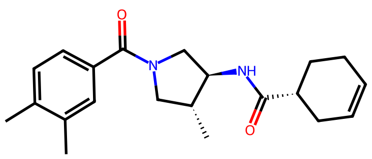	Cc1ccc(C(=O)N2C[C@@H](NC(=O)[C@H]3CC=CCC3)[C@H](C)C2)cc1C	959624519	25	340.22	3.24
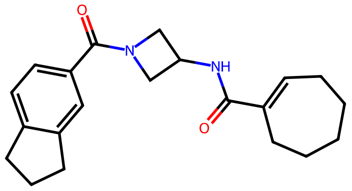	O=C(NC1CN(C(=O)c2ccc3c(c2)CCC3)C1)C1=CCCCCC1	991014197	25	338.2	3.01
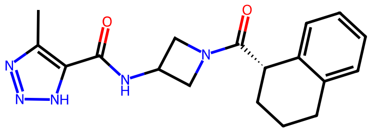	Cc1nn[nH]c1C(=O)NC1CN(C(=O)[C@H]2CCCc3ccccc32)C1	997347199	25	339.17	1.17
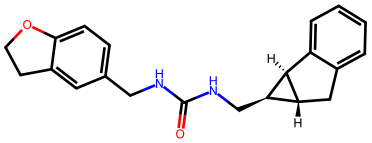	O=C(NCc1ccc2c(c1)CCO2)NC[C@@H]1[C@H]2Cc3ccccc3[C@@H]21	532616623	25	334.17	3.01
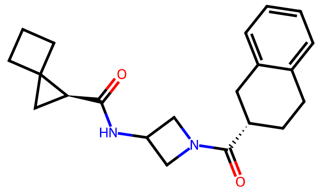	O=C(NC1CN(C(=O)[C@H]2CCc3ccccc3C2)C1)[C@H]1CC12CCC2	991549038	25	338.2	2.31
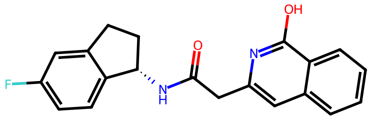	O=C(Cc1cc2ccccc2c(O)n1)N[C@H]1CCc2cc(F)ccc21	1626654691	25	336.13	3.43
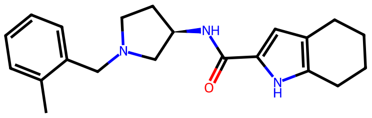	Cc1ccccc1CN1CC[C@@H](NC(=O)c2cc3c([nH]2)CCCC3)C1	1015954001	25	337.22	3.21
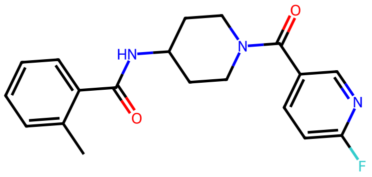	Cc1ccccc1C(=O)NC1CCN(C(=O)c2ccc(F)nc2)CC1	74219123	25	341.15	2.56

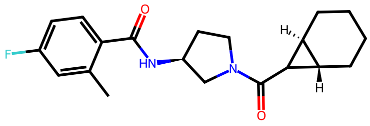	Cc1cc(F)ccc1C(=O)N[C@H]1CCN(C(=O)C2[C@H]3CCCC[C@H]23)C1	938381221	25	344.19	2.9
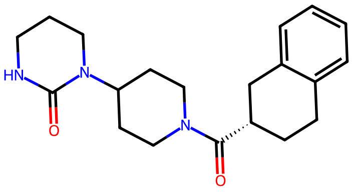	O=C([C@H]1CCc2ccccc2C1)N1CCC(N2CCCNC2=O)CC1	565076119	25	341.21	2.2
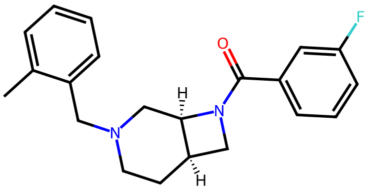	Cc1ccccc1CN1CC[C@@H]2CN(C(=O)c3cccc(F)c3)[C@@H]2C1	1084164519	25	338.18	3.48
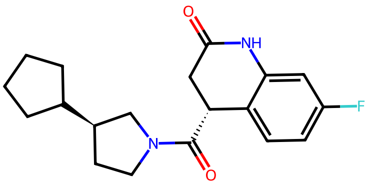	O=C1C[C@@H](C(=O)N2CC[C@@H](C3CCCC3)C2)c2ccc(F)cc2N1	421644333	24	330.17	3.29
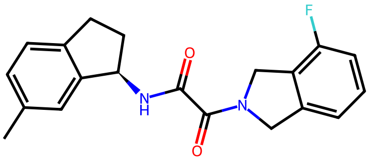	Cc1ccc2c(c1)[C@H](NC(=O)C(=O)N1Cc3cccc(F)c3C1)CC2	643395196	25	338.14	2.78
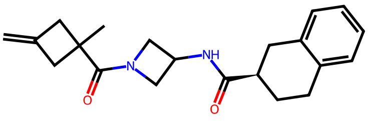	C=C1CC(C)(C(=O)N2CC(NC(=O)[C@@H]3CCc4ccccc4C3)C2)C1	1000537898	25	338.2	2.47
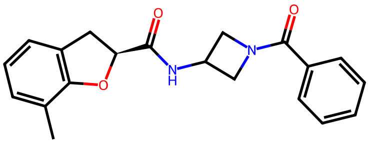	Cc1cccc2c1O[C@H](C(=O)NC1CN(C(=O)c3ccccc3)C1)C2	995326796	25	336.15	1.94
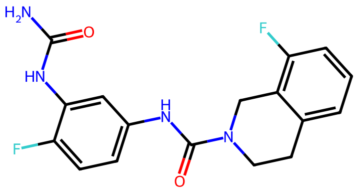	NC(=O)Nc1cc(NC(=O)N2CCc3cccc(F)c3C2)ccc1F	1562840105	25	346.12	3.05

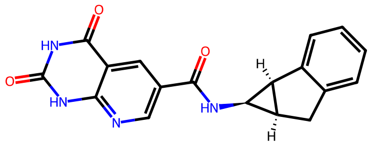	O=C(N[C@@H]1[C@@H]2Cc3ccccc3[C@@H]21)c1cnc2[nH]c(=O)[nH]c(=O)c2c1	152227743	25	334.11	0.68
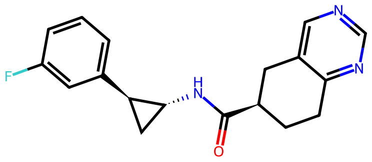	O=C(N[C@@H]1C[C@H]1c1cccc(F)c1)[C@@H]1CCc2ncncc2C1	648260538	23	311.14	2.39
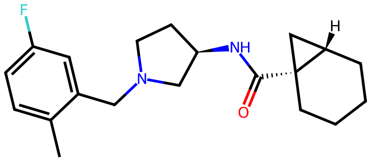	Cc1ccc(F)cc1CN1CC[C@@H](NC(=O)[C@@]23CCCC[C@H]2C3)C1	1032840880	24	330.21	3.4
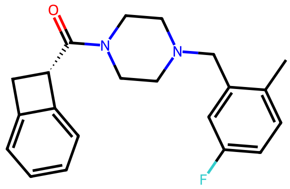	Cc1ccc(F)cc1CN1CCN(C(=O)[C@H]2Cc3ccccc32)CC1	949948639	25	338.18	3.12
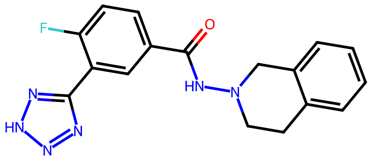	O=C(NN1CCc2ccccc2C1)c1ccc(F)c(-c2nn[nH]n2)c1	1582561767	25	338.13	1.71
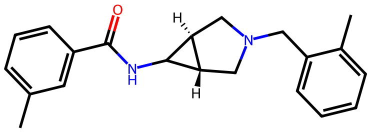	Cc1cccc(C(=O)NC2[C@H]3CN(Cc4ccccc4C)C[C@H]23)c1	958151331	24	320.19	3.16
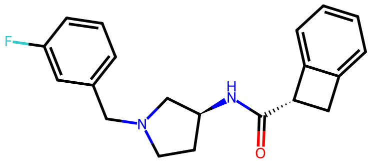	O=C(N[C@H]1CCN(Cc2cccc(F)c2)C1)[C@H]1Cc2ccccc21	1015632438	24	324.16	2.86
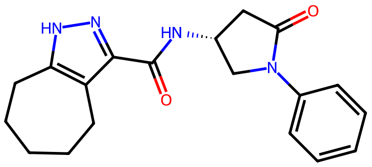	O=C(N[C@@H]1CC(=O)N(c2ccccc2)C1)c1n[nH]c2c1CCCCC2	191451389	25	338.17	2.21

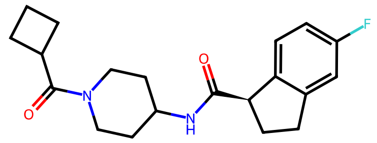	O=C(NC1CCN(C(=O)C2CCC2)CC1)[C@@H]1CCc2cc(F)ccc21	943344971	25	344.19	2.76
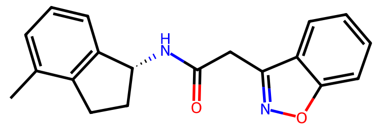	Cc1cccc2c1CC[C@H]2NC(=O)Cc1noc2ccccc12	591312807	23	306.14	3.48
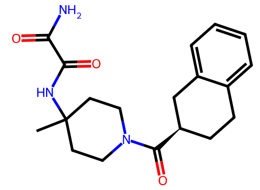	CC1(NC(=O)C(N)=O)CCN(C(=O)[C@@H]2CCc3ccccc3C2)CC1	1073275775	25	343.19	0.77
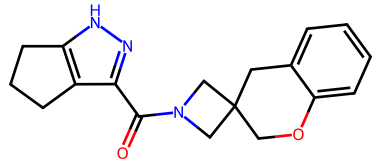	O=C(c1n[nH]c2c1CCC2)N1CC2(COc3ccccc3C2)C1	2100555592	23	309.15	1.98
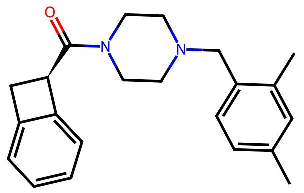	Cc1ccc(CN2CCN(C(=O)[C@@H]3Cc4ccccc43)CC2)c(C)c1	949958620	25	334.2	3.29
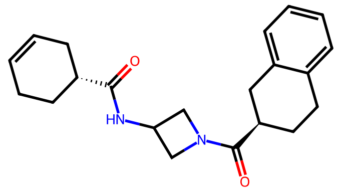	O=C(NC1CN(C(=O)[C@@H]2CCc3ccccc3C2)C1)[C@H]1CC=CCC1	990221458	25	338.2	2.47
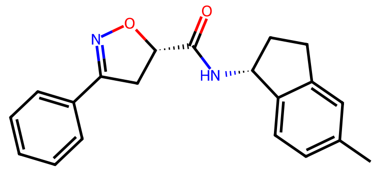	Cc1ccc2c(c1)CC[C@H]2NC(=O)[C@@H]1CC(c2ccccc2)=NO1	56317062	24	320.15	3.29
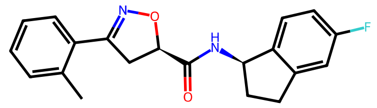	Cc1ccccc1C1=NO[C@@H](C(=O)N[C@@H]2CCc3cc(F)ccc32)C1	1534972387	25	338.14	3.43

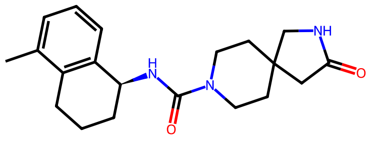	Cc1cccc2c1CCC[C@@H]2NC(=O)N1CCC2(CC1)CNC(=O)C2	823849739	25	341.21	2.68
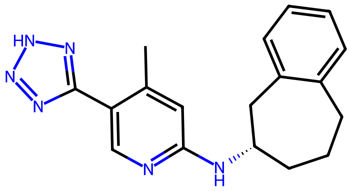	Cc1cc(N[C@H]2CCCc3ccccc3C2)ncc1-c1nn[nH]n1	1592038429	24	320.17	2.93
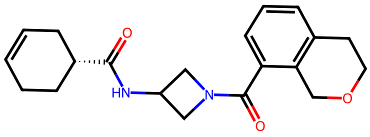	O=C(NC1CN(C(=O)c2cccc3c2COCC3)C1)[C@H]1CC=CCC1	990214418	25	340.18	2.06
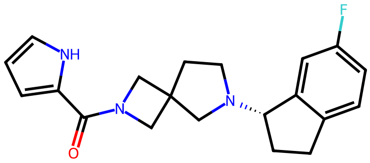	O=C(c1ccc[nH]1)N1CC2(CCN([C@H]3CCc4ccc(F)cc43)C2)C1	1072466045	25	339.17	2.99
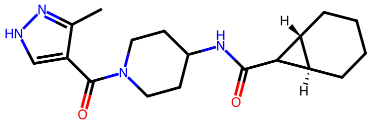	Cc1n[nH]cc1C(=O)N1CCC(NC(=O)C2[C@@H]3CCCC[C@@H]23)CC1	947668625	24	330.21	1.88
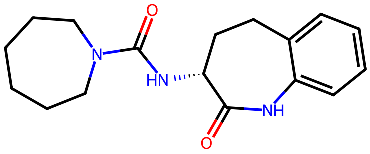	O=C1Nc2ccccc2CC[C@H]1NC(=O)N1CCCCCC1	654630001	22	301.18	2.53
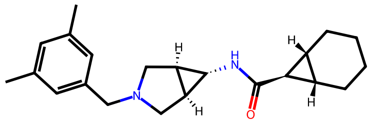	Cc1cc(C)cc(CN2C[C@H]3[C@H](NC(=O)[C@@H]4[C@H]5CCCC[C@H]54)[C@H]3C2)c1	1025720699	25	338.24	3.29
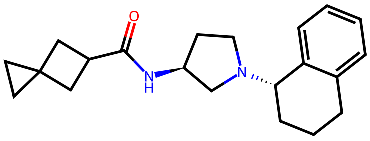	O=C(N[C@H]1CCN([C@H]2CCCc3ccccc32)C1)C1CC2(CC2)C1	1015681200	24	324.22	3.44

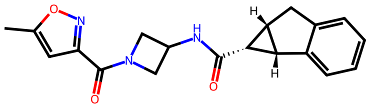	Cc1cc(C(=O)N2CC(NC(=O)[C@H]3[C@H]4c5ccccc5C[C@H]43)C2)no1	991655558	25	337.14	1.51
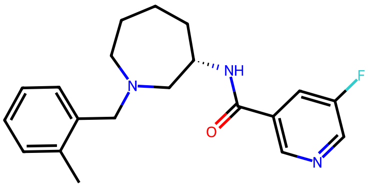	Cc1ccccc1CN1CCCC[C@H](NC(=O)c2cncc(F)c2)C1	1034155927	25	341.19	3.31
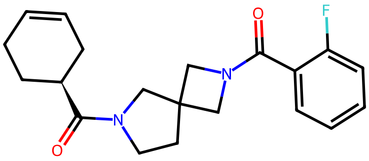	O=C(c1ccccc1F)N1CC2(CCN(C(=O)[C@@H]3CC=CCC3)C2)C1	1018713422	25	342.17	2.86
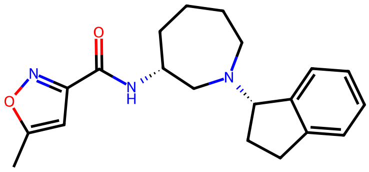	Cc1cc(C(=O)N[C@@H]2CCCCN([C@H]3CCc4ccccc43)C2)no1	1034088272	25	339.19	3.25
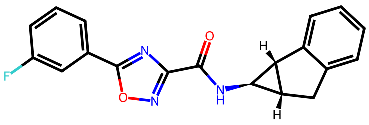	O=C(N[C@@H]1[C@H]2Cc3ccccc3[C@H]21)c1noc(-c2cccc(F)c2)n1	675696371	25	335.11	2.94
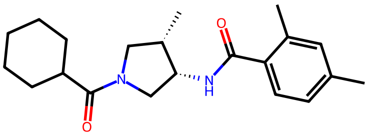	Cc1ccc(C(=O)N[C@@H]2CN(C(=O)C3CCCCC3)C[C@@H]2C)c(C)c1	966119201	25	342.23	3.46
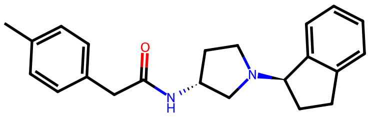	Cc1ccc(CC(=O)N[C@@H]2CCN([C@@H]3CCc4ccccc43)C2)cc1	1015154482	25	334.2	3.42
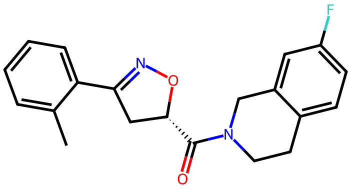	Cc1ccccc1C1=NO[C@H](C(=O)N2CCc3ccc(F)cc3C2)C1	1357172320	25	338.14	3.21

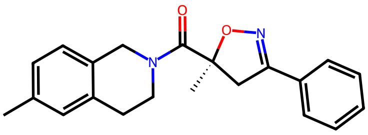	Cc1ccc2c(c1)CCN(C(=O)[C@]1(C)CC(c3ccccc3)=NO1)C2	1643180339	25	334.17	3.46
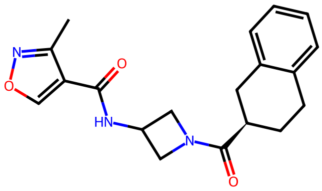	Cc1nocc1C(=O)NC1CN(C(=O)[C@@H]2CCc3ccccc3C2)C1	998750385	25	339.16	1.73
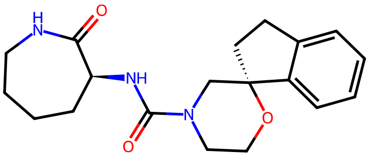	O=C1NCCCC[C@@H]1NC(=O)N1CCO[C@@]2(CCc3ccccc32)C1	119486272	25	343.19	1.54
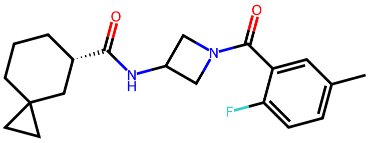	Cc1ccc(F)c(C(=O)N2CC(NC(=O)[C@H]3CCCC4(CC4)C3)C2)c1	991517229	25	344.19	3.05
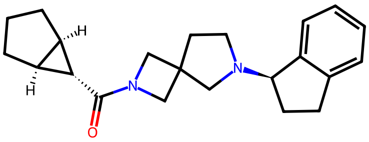	O=C([C@@H]1[C@H]2CCC[C@H]21)N1CC2(CCN([C@@H]3CCc4ccccc43)C2)C1	1072591523	25	336.22	3.25
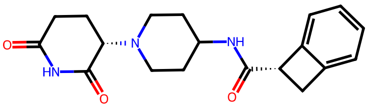	O=C1CC[C@H](N2CCC(NC(=O)[C@H]3Cc4ccccc43)CC2)C(=O)N1	1003535045	25	341.17	0.71
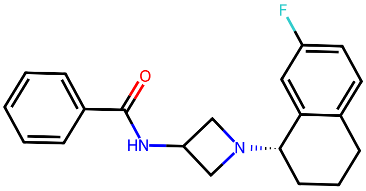	O=C(NC1CN([C@H]2CCCc3ccc(F)cc32)C1)c1ccccc1	1030064210	24	324.16	3.32
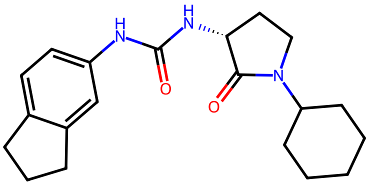	O=C(Nc1ccc2c(c1)CCC2)N[C@@H]1CCN(C2CCCCC2)C1=O	153053490	25	341.21	3.23

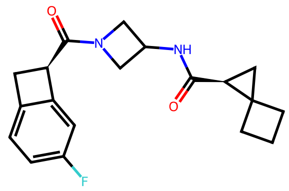	O=C(NC1CN(C(=O)[C@@H]2Cc3ccc(F)cc32)C1)[C@H]1CC12CCC2	991553904	24	328.16	1.98
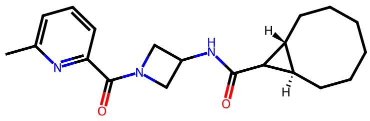	Cc1cccc(C(=O)N2CC(NC(=O)C3[C@@H]4CCCCCC[C@@H]34)C2)n1	1000481146	25	341.21	2.55
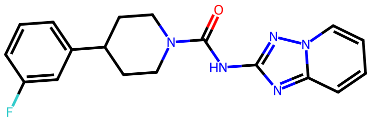	O=C(Nc1nc2ccccn2n1)N1CCC(c2cccc(F)c2)CC1	369636716	25	339.15	3.28
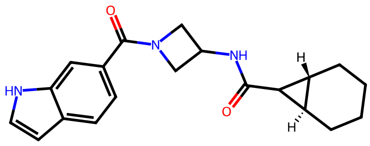	O=C(NC1CN(C(=O)c2ccc3cc[nH]c3c2)C1)C1[C@@H]2CCCC[C@@H]12	990664142	25	337.18	2.54
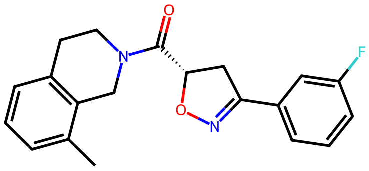	Cc1cccc2c1CN(C(=O)[C@@H]1CC(c3cccc(F)c3)=NO1)CC2	759803649	25	338.14	3.21
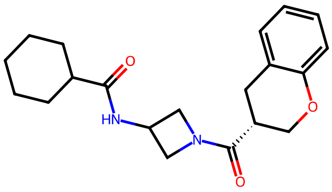	O=C(NC1CN(C(=O)[C@H]2COc3ccccc3C2)C1)C1CCCCC1	990104452	25	342.19	2.14
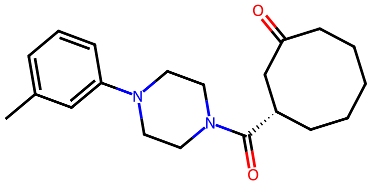	Cc1cccc(N2CCN(C(=O)[C@H]3CCCCCC(=O)C3)CC2)c1	362449488	24	328.22	3.18
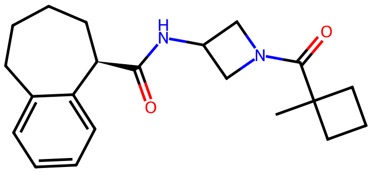	CC1(C(=O)N2CC(NC(=O)[C@@H]3CCCCc4ccccc43)C2)CCC1	994325505	25	340.22	3.01

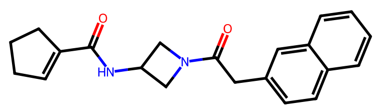	O=C(NC1CN(C(=O)Cc2ccc3ccccc3c2)C1)C1=CCCC1	990797075	25	334.17	2.82
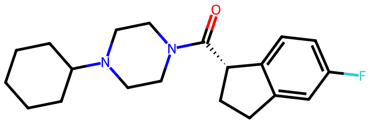	O=C([C@H]1CCc2cc(F)ccc21)N1CCN(C2CCCCC2)CC1	1127416144	24	330.21	3.33
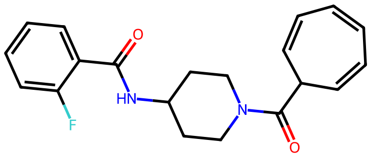	O=C(NC1CCN(C(=O)C2C=CC=CC=C2)CC1)c1ccccc1F	946947651	25	340.16	2.84
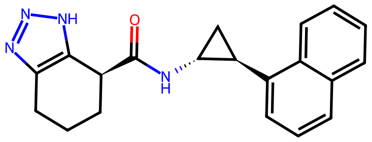	O=C(N[C@@H]1C[C@H]1c1cccc2ccccc12)[C@H]1CCCc2nn[nH]c21	614356567	25	332.16	3.05
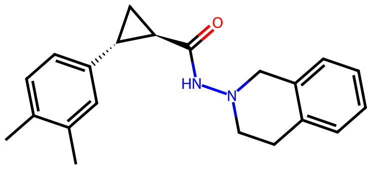	Cc1ccc([C@@H]2C[C@H]2C(=O)NN2CCc3ccccc3C2)cc1C	1640002856	24	320.19	3.5
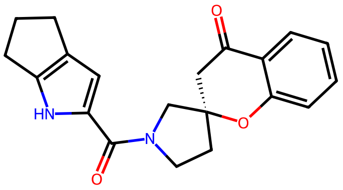	O=C1C[C@@]2(CCN(C(=O)c3cc4c([nH]3)CCC4)C2)Oc2ccccc21	1548917721	25	336.15	2.75
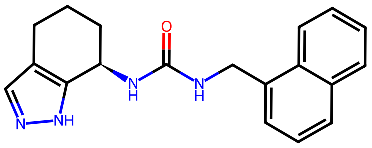	O=C(NCc1cccc2ccccc12)N[C@@H]1CCCc2cn[nH]c21	268940222	24	320.16	3.44
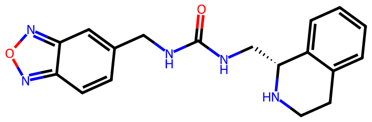	O=C(NCc1ccc2nonc2c1)NC[C@H]1NCCc2ccccc21	1336922556	25	337.15	1.91

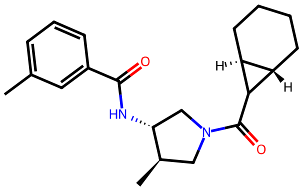	Cc1cccc(C(=O)N[C@@H]2CN(C(=O)C3[C@H]4CCCC[C@H]34)C[C@H]2C)c1	948157460	25	340.22	3.01
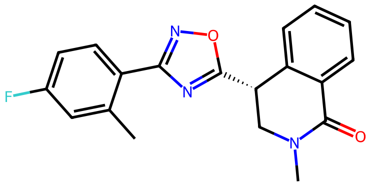	Cc1cc(F)ccc1-c1noc([C@H]2CN(C)C(=O)c3ccccc32)n1	377514949	25	337.12	3.4
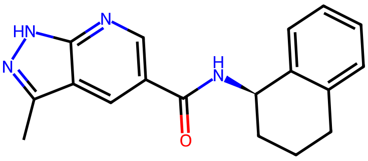	Cc1n[nH]c2ncc(C(=O)N[C@@H]3CCCc4ccccc43)cc12	47381919	23	306.15	3.07
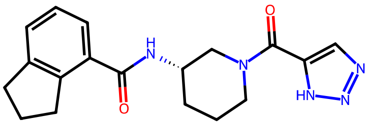	O=C(N[C@H]1CCCN(C(=O)c2cnn[nH]2)C1)c1cccc2c1CCC2	951314337	25	339.17	1.33
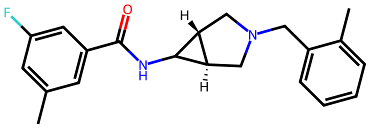	Cc1cc(F)cc(C(=O)NC2[C@@H]3CN(Cc4ccccc4C)C[C@@H]23)c1	961852754	25	338.18	3.3
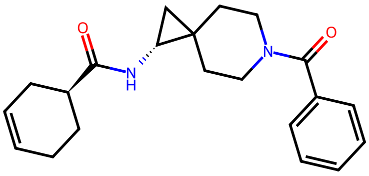	O=C(N[C@@H]1CC12CCN(C(=O)c1ccccc1)CC2)[C@@H]1CC=CCC1	963029854	25	338.2	3.15
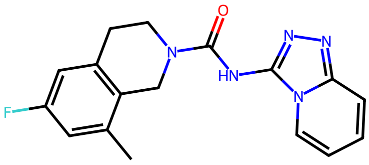	Cc1cc(F)cc2c1CN(C(=O)Nc1nnc3ccccn13)CC2	337410091	24	325.13	2.77
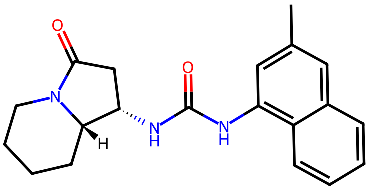	Cc1cc(NC(=O)N[C@H]2CC(=O)N3CCCC[C@@H]23)c2ccccc2c1	566722110	25	337.18	3.42

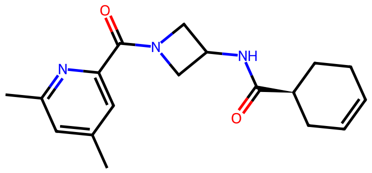	Cc1cc(C)nc(C(=O)N2CC(NC(=O)[C@@H]3CC=CCC3)C2)c1	990222091	23	313.18	2.0
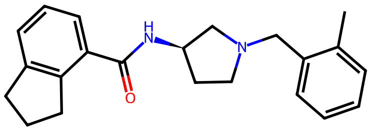	Cc1ccccc1CN1CC[C@@H](NC(=O)c2cccc3c2CCC3)C1	1016382006	25	334.2	3.49
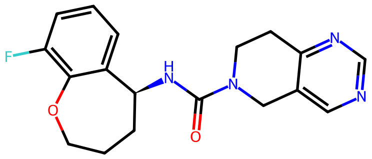	O=C(N[C@H]1CCCOc2c(F)cccc21)N1CCc2ncncc2C1	883558931	25	342.15	2.6
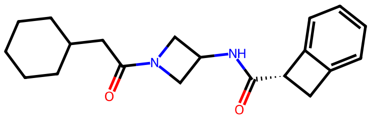	O=C(NC1CN(C(=O)CC2CCCCC2)C1)[C@H]1Cc2ccccc21	998678946	24	326.2	2.62
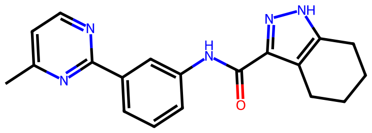	Cc1ccnc(-c2cccc(NC(=O)c3n[nH]c4c3CCCC4)c2)n1	605517353	25	333.16	3.31
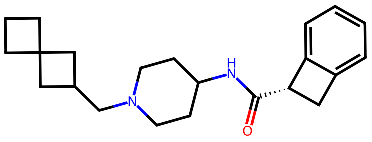	O=C(NC1CCN(CC2CC3(CCC3)C2)CC1)[C@H]1Cc2ccccc21	1003534074	25	338.24	3.49
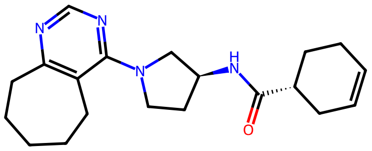	O=C(N[C@H]1CCN(c2ncnc3c2CCCCC3)C1)[C@H]1CC=CCC1	1058297536	25	340.23	2.8
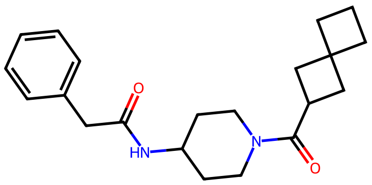	O=C(Cc1ccccc1)NC1CCN(C(=O)C2CC3(CCC3)C2)CC1	947877398	25	340.22	2.92

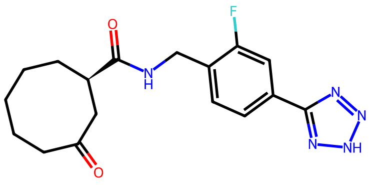	O=C1CCCCC[C@@H](C(=O)NCc2ccc(-c3nn[nH]n3)cc2F)C1	1593392981	25	345.16	2.16
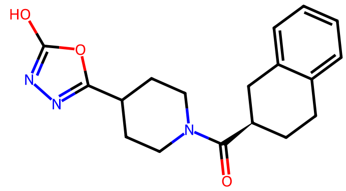	O=C([C@@H]1CCc2ccccc2C1)N1CCC(c2nnc(O)o2)CC1	577071853	24	327.16	2.29
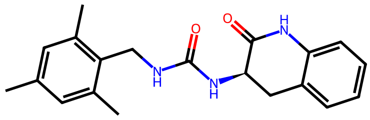	Cc1cc(C)c(CNC(=O)N[C@@H]2Cc3ccccc3NC2=O)c(C)c1	1365100494	25	337.18	2.97
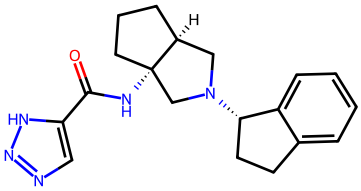	O=C(N[C@]12CCC[C@H]1CN([C@H]1CCc3ccccc31)C2)c1cnn[nH]1	1098919307	25	337.19	2.08
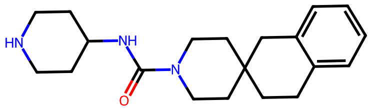	O=C(NC1CCNCC1)N1CCC2(CCc3ccccc3C2)CC1	652200215	24	327.23	2.72
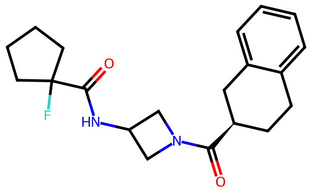	O=C([C@@H]1CCc2ccccc2C1)N1CC(NC(=O)C2(F)CCCC2)C1	994992470	25	344.19	2.4
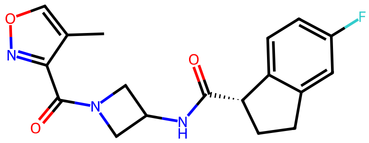	Cc1conc1C(=O)N1CC(NC(=O)[C@H]2CCc3cc(F)ccc32)C1	996094403	25	343.13	1.79
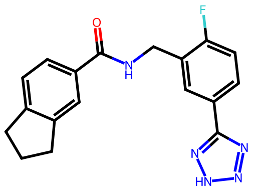	O=C(NCc1cc(-c2nn[nH]n2)ccc1F)c1ccc2c(c1)CCC2	823242199	25	337.13	2.42

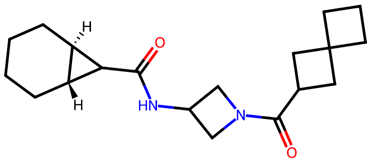	O=C(NC1CN(C(=O)C2CC3(CCC3)C2)C1)C1[C@@H]2CCCC[C@@H]12	990659940	23	316.22	2.33
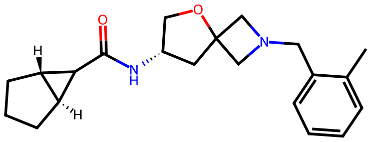	Cc1ccccc1CN1CC2(C[C@H](NC(=O)C3[C@H]4CCC[C@H]34)CO2)C1	1053769412	25	340.22	2.5
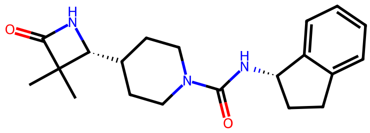	CC1(C)C(=O)N[C@@H]1C1CCN(C(=O)N[C@H]2CCc3ccccc32)CC1	875031272	25	341.21	2.62
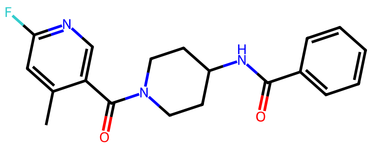	Cc1cc(F)ncc1C(=O)N1CCC(NC(=O)c2ccccc2)CC1	864662522	25	341.15	2.56
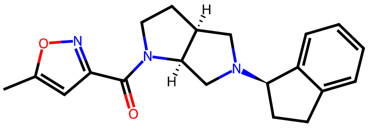	Cc1cc(C(=O)N2CC[C@H]3CN([C@@H]4CCc5ccccc54)C[C@H]32)no1	1075526030	25	337.18	2.82
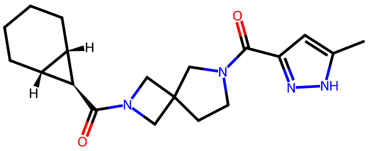	Cc1cc(C(=O)N2CCC3(C2)CN(C(=O)[C@@H]2[C@H]4CCCC[C@H]42)C3)n[nH]1	1018904670	25	342.21	1.83
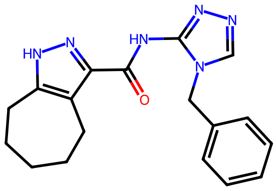	O=C(Nc1nncn1Cc1ccccc1)c1n[nH]c2c1CCCCC2	530508520	25	336.17	2.57
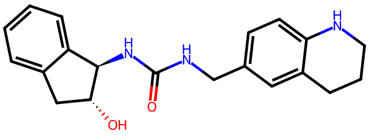	O=C(NCc1ccc2c(c1)CCCN2)N[C@@H]1c2ccccc2C[C@H]1O	418725620	25	337.18	2.5

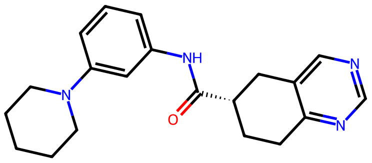	O=C(Nc1cccc(N2CCCCC2)c1)[C@H]1CCc2ncncc2C1	843536683	25	336.2	3.21
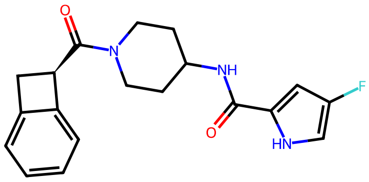	O=C(NC1CCN(C(=O)[C@@H]2Cc3ccccc32)CC1)c1cc(F)c[nH]1	948063468	25	341.15	2.21
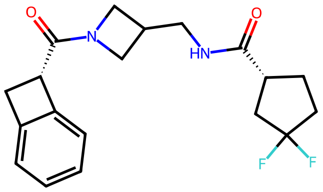	O=C(NCC1CN(C(=O)[C@H]2Cc3ccccc32)C1)[C@@H]1CCC(F)(F)C1	1002598199	25	348.16	2.34
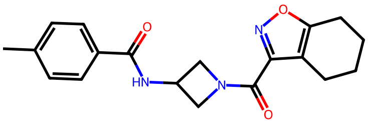	Cc1ccc(C(=O)NC2CN(C(=O)c3noc4c3CCCC4)C2)cc1	999445691	25	339.16	2.12
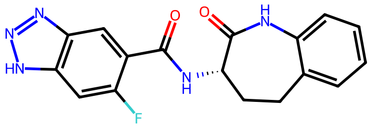	O=C(N[C@H]1CCc2ccccc2NC1=O)c1cc2nn[nH]c2cc1F	1583191232	25	339.11	1.78
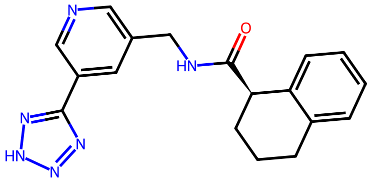	O=C(NCc1cncc(-c2nn[nH]n2)c1)[C@@H]1CCCc2ccccc21	1582557436	25	334.15	2.0
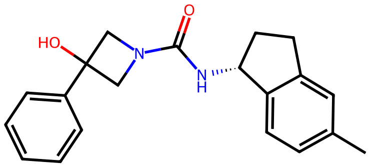	Cc1ccc2c(c1)CC[C@H]2NC(=O)N1CC(O)(c2ccccc2)C1	812012732	24	322.17	2.9
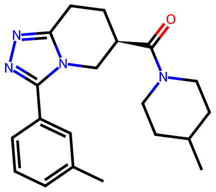	Cc1cccc(-c2nnc3n2C[C@H](C(=O)N2CCC(C)CC2)CC3)c1	578411327	25	338.21	3.07

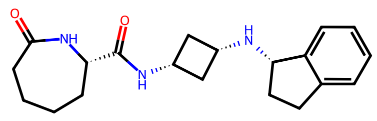	O=C1CCCC[C@@H](C(=O)N[C@H]2C[C@@H](N[C@H]3CCc4ccccc43)C2)N1	1022693881	25	341.21	1.97
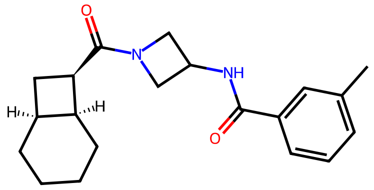	Cc1cccc(C(=O)NC2CN(C(=O)[C@@H]3C[C@H]4CCCC[C@@H]43)C2)c1	990140117	24	326.2	2.76
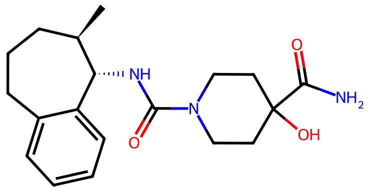	C[C@@H]1CCCc2ccccc2[C@H]1NC(=O)N1CCC(O)(C(N)=O)CC1	921984931	25	345.21	1.72
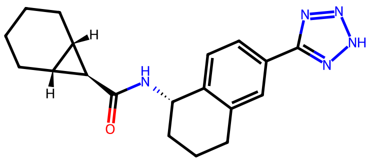	O=C(N[C@H]1CCCc2cc(-c3nn[nH]n3)ccc21)[C@@H]1[C@H]2CCCC[C@H]21	1592985270	25	337.19	2.8
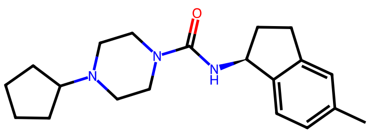	Cc1ccc2c(c1)CC[C@@H]2NC(=O)N1CCN(C2CCCC2)CC1	56842892	24	327.23	3.25
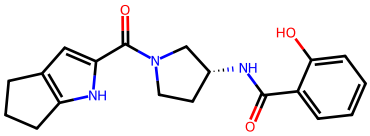	O=C(N[C@@H]1CCN(C(=O)c2cc3c([nH]2)CCC3)C1)c1ccccc1O	939496001	25	339.16	1.85
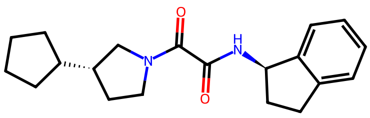	O=C(N[C@@H]1CCc2ccccc21)C(=O)N1CC[C@H](C2CCCC2)C1	421055152	24	326.2	2.83
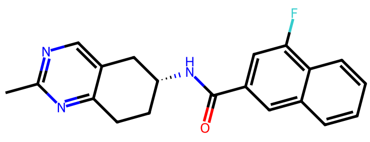	Cc1ncc2c(n1)CC[C@@H](NC(=O)c1cc(F)c3ccccc3c1)C2	549216359	25	335.14	3.36

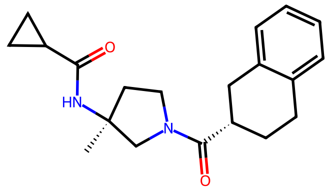	C[C@]1(NC(=O)C2CC2)CCN(C(=O)[C@H]2CCc3ccccc3C2)C1	956623547	24	326.2	2.31
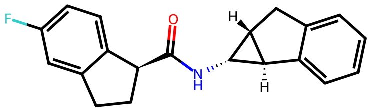	O=C(N[C@H]1[C@@H]2c3ccccc3C[C@H]21)[C@H]1CCc2cc(F)ccc21	612495122	23	307.14	3.31
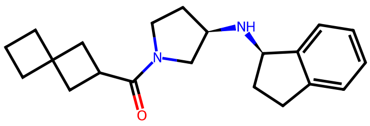	O=C(C1CC2(CCC2)C1)N1CC[C@@H](N[C@@H]2CCc3ccccc32)C1	969646944	24	324.22	3.44
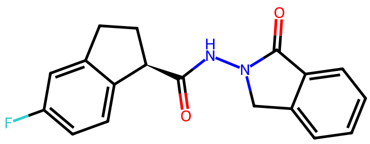	O=C(NN1Cc2ccccc2C1=O)[C@@H]1CCc2cc(F)ccc21	1412818520	23	310.11	2.54
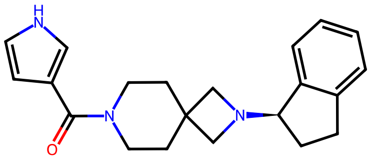	O=C(c1cc[nH]c1)N1CCC2(CC1)CN([C@@H]1CCc3ccccc31)C2	1035711819	25	335.2	3.24
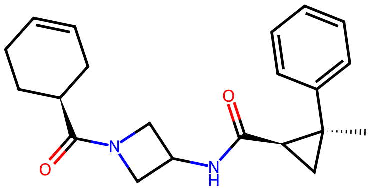	C[C@]1(c2ccccc2)C[C@H]1C(=O)NC1CN(C(=O)[C@@H]2CC=CCC2)C1	997530382	25	338.2	2.65
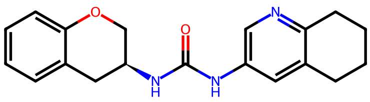	O=C(Nc1cnc2c(c1)CCCC2)N[C@@H]1COc2ccccc2C1	360372394	24	323.16	3.09
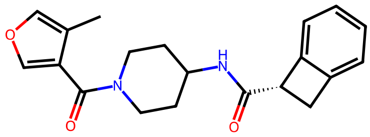	Cc1cocc1C(=O)N1CCC(NC(=O)[C@H]2Cc3ccccc32)CC1	947843009	25	338.16	2.65

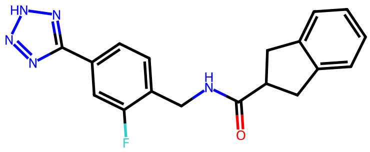	O=C(NCc1ccc(-c2nn[nH]n2)cc1F)C1Cc2ccccc2C1	1606825405	25	337.13	2.04
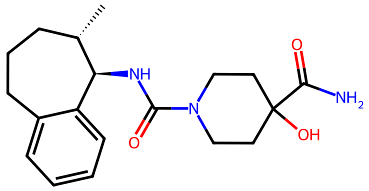	C[C@H]1CCCc2ccccc2[C@@H]1NC(=O)N1CCC(O)(C(N)=O)CC1	921984935	25	345.21	1.72
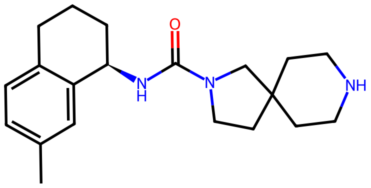	Cc1ccc2c(c1)[C@H](NC(=O)N1CCC3(CCNCC3)C1)CCC2	418218462	24	327.23	3.16
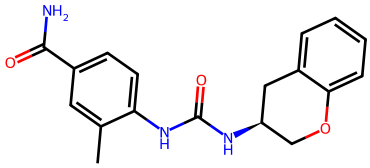	Cc1cc(C(N)=O)ccc1NC(=O)N[C@@H]1COc2ccccc2C1	47887356	24	325.14	2.22
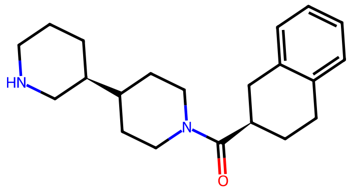	O=C([C@@H]1CCc2ccccc2C1)N1CCC([C@@H]2CCCNC2)CC1	658960447	24	326.24	3.03
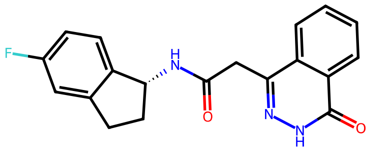	O=C(Cc1n[nH]c(=O)c2ccccc12)N[C@@H]1CCc2cc(F)ccc21	55656473	25	337.12	2.41
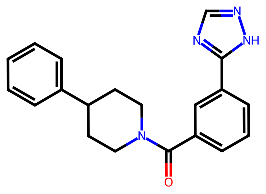	O=C(c1cccc(-c2ncn[nH]2)c1)N1CCC(c2ccccc2)CC1	158573223	25	332.16	3.49
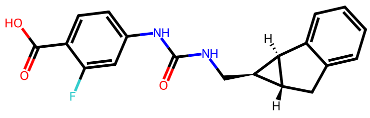	O=C(NC[C@@H]1[C@H]2Cc3ccccc3[C@@H]21)Nc1ccc(C(=O)O)c(F)c1	833141550	25	340.12	3.23

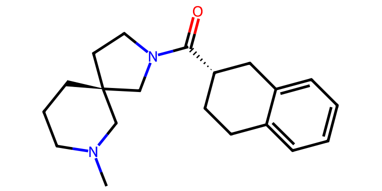	CN1CCC[C@]2(CCN(C(=O)[C@H]3CCc4ccccc4C3)C2)C1	576286187	23	312.22	2.74
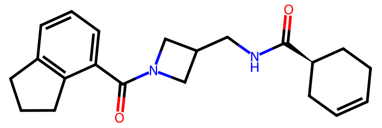	O=C(NCC1CN(C(=O)c2cccc3c2CCC3)C1)[C@@H]1CC=CCC1	1000895386	25	338.2	2.72
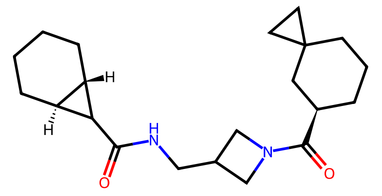	O=C(NCC1CN(C(=O)[C@@H]2CCCC3(CC3)C2)C1)C1[C@H]2CCCC[C@H]12	1001323006	25	344.25	2.97
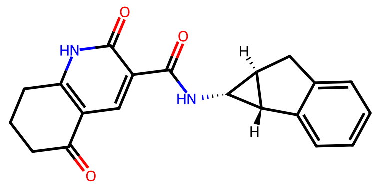	O=C1CCCc2[nH]c(=O)c(C(=O)N[C@@H]3[C@H]4Cc5ccccc5[C@@H]43)cc21	152375106	25	334.13	1.96
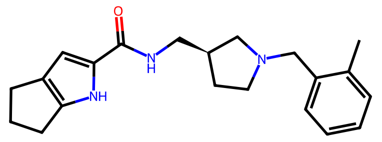	Cc1ccccc1CN1CC[C@@H](CNC(=O)c2cc3c([nH]2)CCC3)C1	1028922969	25	337.22	3.06
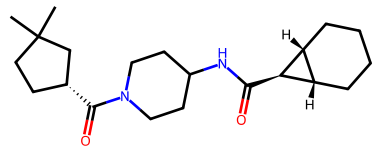	CC1(C)CC[C@@H](C(=O)N2CCC(NC(=O)[C@@H]3[C@H]4CCCC[C@H]43)CC2)C1	948254805	25	346.26	3.36
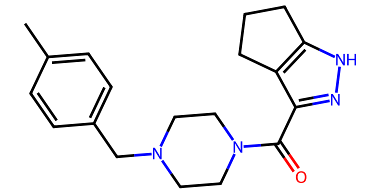	Cc1ccc(CN2CCN(C(=O)c3n[nH]c4c3CCC4)CC2)cc1	949066312	24	324.2	2.16
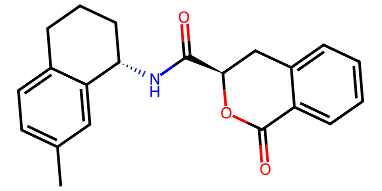	Cc1ccc2c(c1)[C@@H](NC(=O)[C@H]1Cc3ccccc3C(=O)O1)CCC2	807898157	25	335.15	3.27

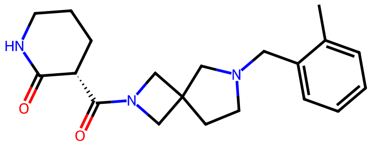	Cc1ccccc1CN1CCC2(C1)CN(C(=O)[C@H]1CCCNC1=O)C2	1072831195	25	341.21	1.56
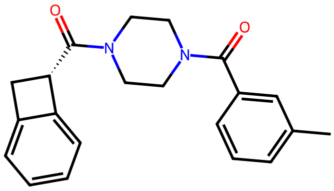	Cc1cccc(C(=O)N2CCN(C(=O)[C@H]3Cc4ccccc43)CC2)c1	1010099818	25	334.17	2.62
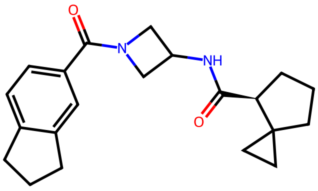	O=C(NC1CN(C(=O)c2ccc3c(c2)CCC3)C1)[C@H]1CCCC12CC2	994865653	25	338.2	2.7
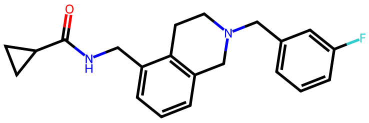	O=C(NCc1cccc2c1CCN(Cc1cccc(F)c1)C2)C1CC1	1051275232	25	338.18	3.41
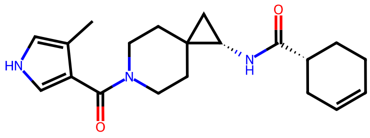	Cc1c[nH]cc1C(=O)N1CCC2(CC1)C[C@@H]2NC(=O)[C@H]1CC=CCC1	963026919	25	341.21	2.79
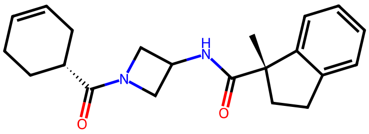	C[C@]1(C(=O)NC2CN(C(=O)[C@H]3CC=CCC3)C2)CCc2ccccc21	994376605	25	338.2	2.57
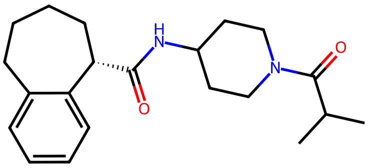	CC(C)C(=O)N1CCC(NC(=O)[C@H]2CCCCc3ccccc32)CC1	944377586	25	342.23	3.26
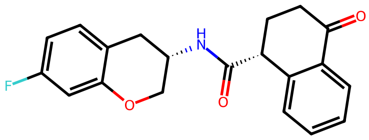	O=C1CC[C@@H](C(=O)N[C@@H]2COc3cc(F)ccc3C2)c2ccccc21	618649058	25	339.13	3.01

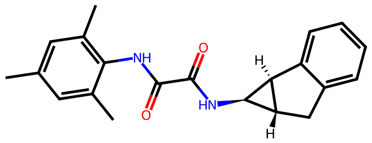	Cc1cc(C)c(NC(=O)C(=O)N[C@@H]2[C@H]3Cc4ccccc4[C@@H]32)c(C)c1	158776420	25	334.17	3.0
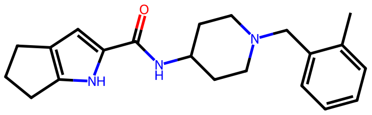	Cc1ccccc1CN1CCC(NC(=O)c2cc3c([nH]2)CCC3)CC1	1003945994	25	337.22	3.21
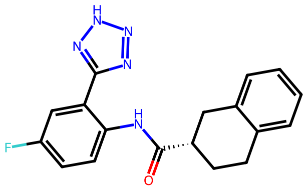	O=C(Nc1ccc(F)cc1-c1nn[nH]n1)[C@H]1CCc2ccccc2C1	1606922160	25	337.13	2.75
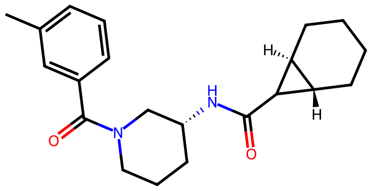	Cc1cccc(C(=O)N2CCC[C@@H](NC(=O)C3[C@H]4CCCC[C@H]34)C2)c1	965889393	25	340.22	3.15
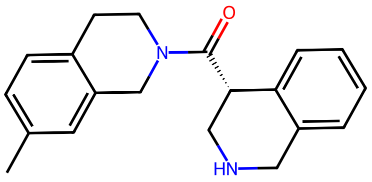	Cc1ccc2c(c1)CN(C(=O)[C@H]1CNCc3ccccc31)CC2	662626082	23	306.17	2.77
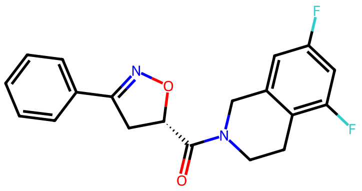	O=C([C@@H]1CC(c2ccccc2)=NO1)N1CCc2c(F)cc(F)cc2C1	134277850	25	342.12	3.04
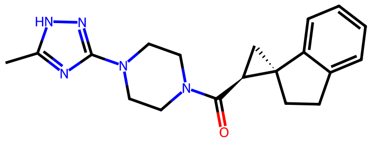	Cc1nc(N2CCN(C(=O)[C@H]3C[C@]34CCc3ccccc34)CC2)n[nH]1	898178784	25	337.19	1.67
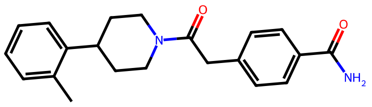	Cc1ccccc1C1CCN(C(=O)Cc2ccc(C(N)=O)cc2)CC1	276895959	25	336.18	3.04

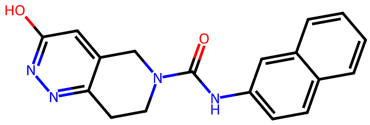	O=C(Nc1ccc2ccccc2c1)N1CCc2nnc(O)cc2C1	775419460	24	320.13	2.93
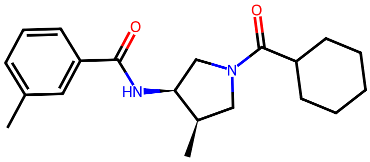	Cc1cccc(C(=O)N[C@H]2CN(C(=O)C3CCCCC3)C[C@H]2C)c1	966111591	24	328.22	3.15
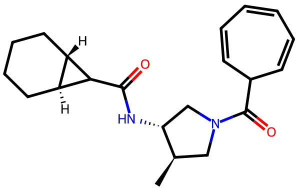	C[C@@H]1CN(C(=O)C2C=CC=CC=C2)C[C@H]1NC(=O)C1[C@H]2CCCC[C@H]12	949636604	25	340.22	2.68
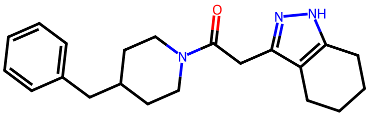	O=C(Cc1n[nH]c2c1CCCC2)N1CCC(Cc2ccccc2)CC1	188280834	25	337.22	3.31
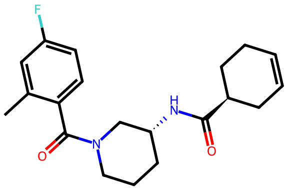	Cc1cc(F)ccc1C(=O)N1CCC[C@@H](NC(=O)[C@@H]2CC=CCC2)C1	951503190	25	344.19	3.21
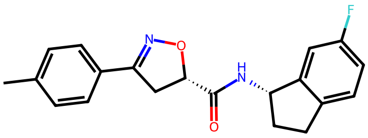	Cc1ccc(C2=NO[C@H](C(=O)N[C@H]3CCc4ccc(F)cc43)C2)cc1	1435102123	25	338.14	3.43
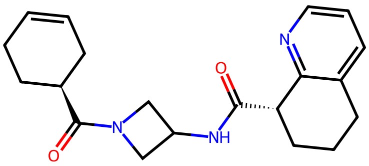	O=C(NC1CN(C(=O)[C@@H]2CC=CCC2)C1)[C@H]1CCCc2cccnc21	998042198	25	339.19	2.18
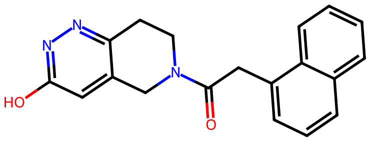	O=C(Cc1cccc2ccccc12)N1CCc2nnc(O)cc2C1	356112257	24	319.13	2.46

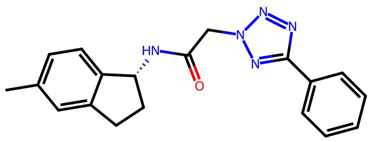	Cc1ccc2c(c1)CC[C@H]2NC(=O)Cn1nnc(-c2ccccc2)n1	56316765	25	333.16	2.45
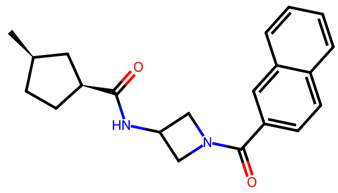	C[C@@H]1CC[C@H](C(=O)NC2CN(C(=O)c3ccc4ccccc4c3)C2)C1	991435505	25	336.18	3.22
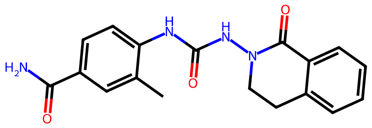	Cc1cc(C(N)=O)ccc1NC(=O)NN1CCc2ccccc2C1=O	1365764838	25	338.14	1.83
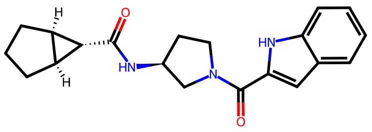	O=C(N[C@H]1CCN(C(=O)c2cc3ccccc3[nH]2)C1)[C@@H]1[C@H]2CCC[C@H]21	937804031	25	337.18	2.54
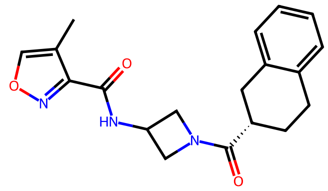	Cc1conc1C(=O)NC1CN(C(=O)[C@H]2CCc3ccccc3C2)C1	999667489	25	339.16	1.73
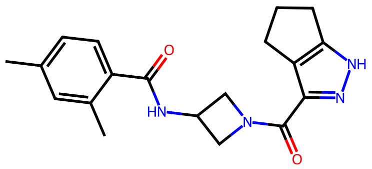	Cc1ccc(C(=O)NC2CN(C(=O)c3n[nH]c4c3CCC4)C2)c(C)c1	999532289	25	338.17	1.77
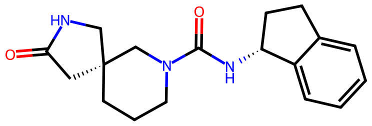	O=C1C[C@]2(CCCN(C(=O)N[C@@H]3CCc4ccccc43)C2)CN1	340919522	23	313.18	1.99
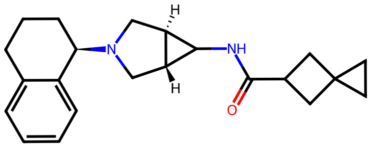	O=C(NC1[C@@H]2CN([C@@H]3CCCc4ccccc43)C[C@@H]12)C1CC2(CC2)C1	961728512	25	336.22	3.3

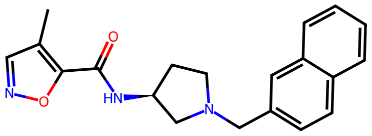	Cc1cnoc1C(=O)N[C@H]1CCN(Cc2ccc3ccccc3c2)C1	1015936665	25	335.16	3.14
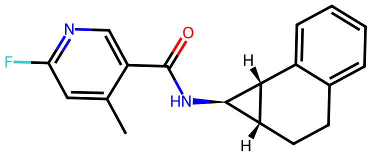	Cc1cc(F)ncc1C(=O)N[C@@H]1[C@H]2CCc3ccccc3[C@H]21	866831347	22	296.13	2.99
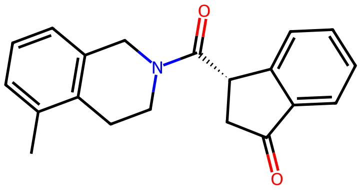	Cc1cccc2c1CCN(C(=O)[C@H]1CC(=O)c3ccccc31)C2	789644478	23	305.14	3.25
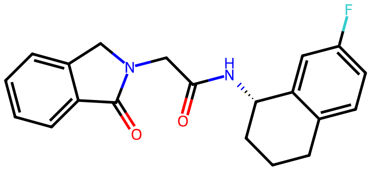	O=C(CN1Cc2ccccc2C1=O)N[C@H]1CCCc2ccc(F)cc21	357453824	25	338.14	2.98
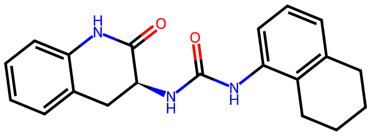	O=C(Nc1cccc2c1CCCC2)N[C@H]1Cc2ccccc2NC1=O	528860113	25	335.16	3.25
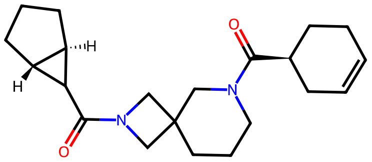	O=C(C1[C@@H]2CCC[C@@H]12)N1CC2(CCCN(C(=O)[C@@H]3CC=CCC3)C2)C1	1010600531	25	342.23	2.84
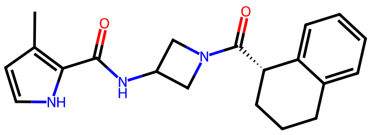	Cc1cc[nH]c1C(=O)NC1CN(C(=O)[C@H]2CCCc3ccccc32)C1	991103375	25	337.18	2.38
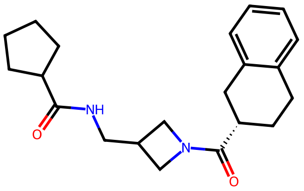	O=C(NCC1CN(C(=O)[C@H]2CCc3ccccc3C2)C1)C1CCCC1	1000635983	25	340.22	2.56

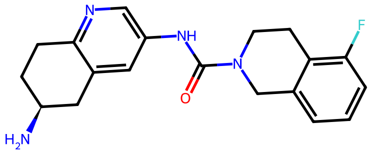	N[C@H]1CCc2ncc(NC(=O)N3CCc4c(F)cccc4C3)cc2C1	333298114	25	340.17	2.63
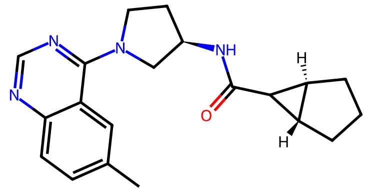	Cc1ccc2ncnc(N3CC[C@@H](NC(=O)C4[C@H]5CCC[C@H]45)C3)c2c1	1062761112	25	336.2	2.68
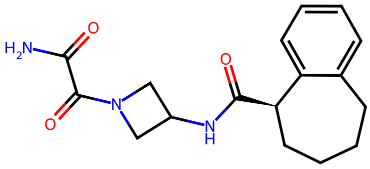	NC(=O)C(=O)N1CC(NC(=O)[C@@H]2CCCCc3ccccc32)C1	994322066	23	315.16	0.31
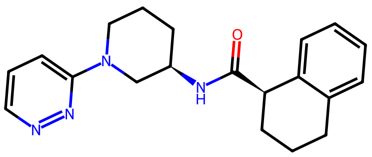	O=C(N[C@@H]1CCCN(c2cccnn2)C1)[C@@H]1CCCc2ccccc21	265515833	25	336.2	2.68
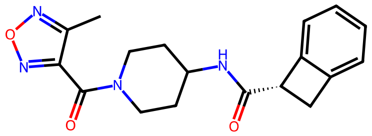	Cc1nonc1C(=O)N1CCC(NC(=O)[C@H]2Cc3ccccc32)CC1	947285252	25	340.15	1.44
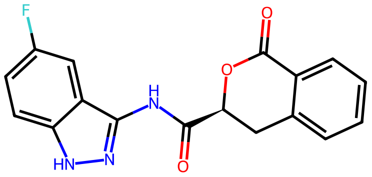	O=C1O[C@H](C(=O)Nc2n[nH]c3ccc(F)cc23)Cc2ccccc21	728284689	24	325.09	2.42
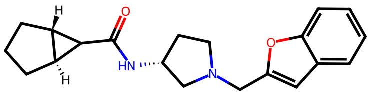	O=C(N[C@@H]1CCN(Cc2cc3ccccc3o2)C1)C1[C@H]2CCC[C@H]12	1010475726	24	324.18	3.17
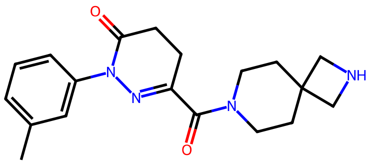	Cc1cccc(N2N=C(C(=O)N3CCC4(CC3)CNC4)CCC2=O)c1	1646768763	25	340.19	1.69

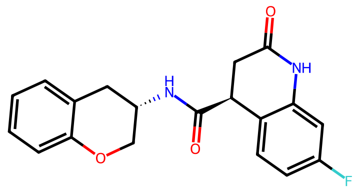	O=C1C[C@H](C(=O)N[C@@H]2COc3ccccc3C2)c2ccc(F)cc2N1	53045175	25	340.12	2.37
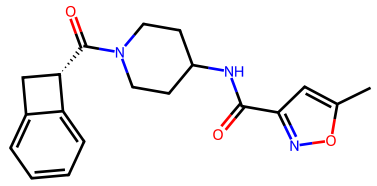	Cc1cc(C(=O)NC2CCN(C(=O)[C@H]3Cc4ccccc43)CC2)no1	948059621	25	339.16	2.04
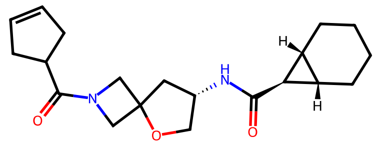	O=C(N[C@@H]1COC2(C1)CN(C(=O)C1CC=CC1)C2)[C@@H]1[C@H]2CCCC[C@H]21	965651936	25	344.21	1.87
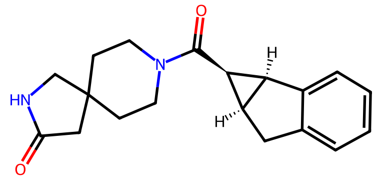	O=C1CC2(CCN(C(=O)[C@H]3[C@H]4c5ccccc5C[C@H]43)CC2)CN1	859774219	23	310.17	1.7
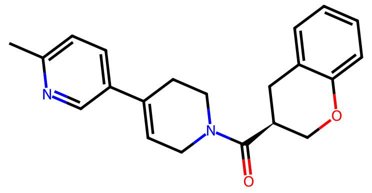	Cc1ccc(C2=CCN(C(=O)[C@@H]3COc4ccccc4C3)CC2)cn1	1137848599	25	334.17	3.26
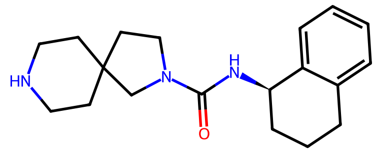	O=C(N[C@@H]1CCCc2ccccc21)N1CCC2(CCNCC2)C1	416792191	23	313.22	2.85
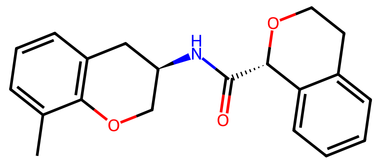	Cc1cccc2c1OC[C@H](NC(=O)[C@@H]1OCCc3ccccc31)C2	291713055	24	323.15	2.73
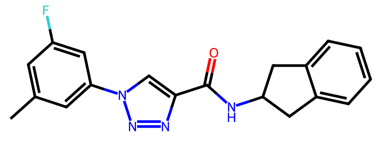	Cc1cc(F)cc(-n2cc(C(=O)NC3Cc4ccccc4C3)nn2)c1	345263323	25	336.14	2.61

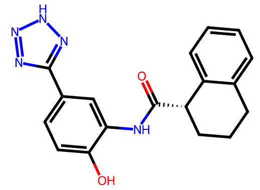	O=C(Nc1cc(-c2nn[nH]n2)ccc1O)[C@H]1CCCc2ccccc21	826450823	25	335.14	2.63
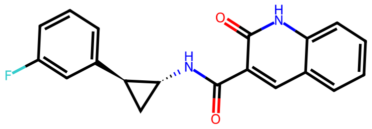	O=C(N[C@@H]1C[C@H]1c1cccc(F)c1)c1cc2ccccc2[nH]c1=O	67333491	24	322.11	2.95
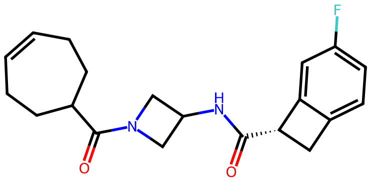	O=C(NC1CN(C(=O)C2CCC=CCC2)C1)[C@H]1Cc2ccc(F)cc21	998495169	25	342.17	2.54
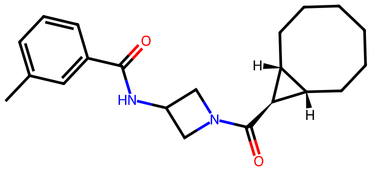	Cc1cccc(C(=O)NC2CN(C(=O)[C@@H]3[C@H]4CCCCCC[C@H]43)C2)c1	990138092	25	340.22	3.15
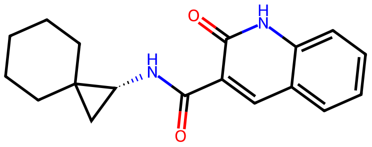	O=C(N[C@@H]1CC12CCCCC2)c1cc2ccccc2[nH]c1=O	180794705	22	296.15	2.98
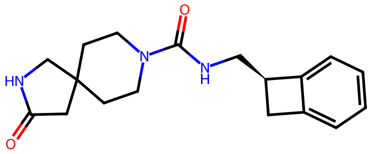	O=C1CC2(CCN(C(=O)NC[C@@H]3Cc4ccccc43)CC2)CN1	752888744	23	313.18	1.64
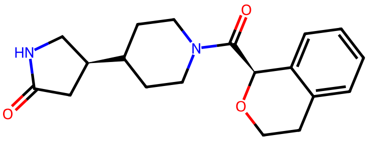	O=C1C[C@H](C2CCN(C(=O)[C@@H]3OCCc4ccccc43)CC2)CN1	1551706745	24	328.18	1.68
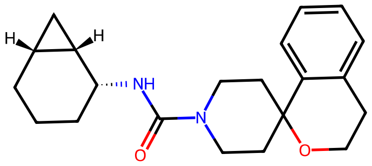	O=C(N[C@@H]1CCC[C@@H]2C[C@@H]21)N1CCC2(CC1)OCCc1ccccc12	459284397	25	340.22	3.45

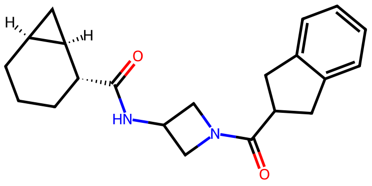	O=C(NC1CN(C(=O)C2Cc3ccccc3C2)C1)[C@@H]1CCC[C@H]2C[C@H]21	994727266	25	338.2	2.16
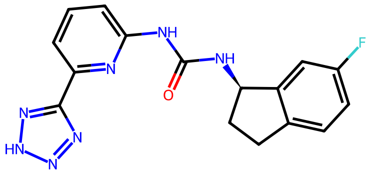	O=C(Nc1cccc(-c2nn[nH]n2)n1)N[C@@H]1CCc2ccc(F)cc21	1582645402	25	339.12	2.21
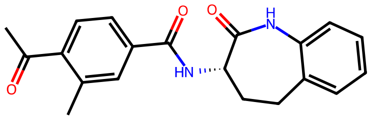	CC(=O)c1ccc(C(=O)N[C@H]2CCc3ccccc3NC2=O)cc1C	912046399	25	336.15	2.88
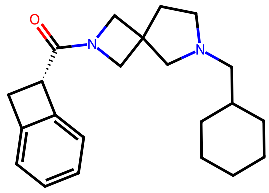	O=C([C@H]1Cc2ccccc21)N1CC2(CCN(CC3CCCCC3)C2)C1	1072932439	25	338.24	3.44
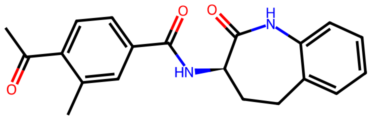	CC(=O)c1ccc(C(=O)N[C@@H]2CCc3ccccc3NC2=O)cc1C	912046391	25	336.15	2.88
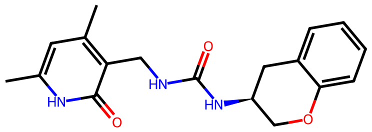	Cc1cc(C)c(CNC(=O)N[C@@H]2COc3ccccc3C2)c(=O)[nH]1	75117965	24	327.16	1.79
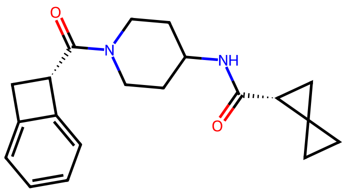	O=C(NC1CCN(C(=O)[C@H]2Cc3ccccc32)CC1)[C@@H]1CC12CC2	948059407	24	324.18	2.23
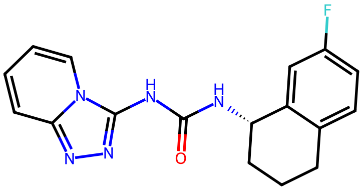	O=C(Nc1nnc2ccccn12)N[C@H]1CCCc2ccc(F)cc21	93256293	24	325.13	3.07

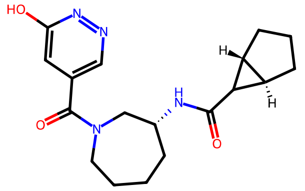	O=C(N[C@@H]1CCCCN(C(=O)c2cnnc(O)c2)C1)C1[C@@H]2CCC[C@@H]12	943166096	25	344.18	1.34
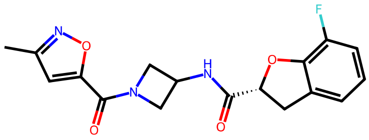	Cc1cc(C(=O)N2CC(NC(=O)[C@H]3Cc4cccc(F)c4O3)C2)on1	992514036	25	345.11	1.07
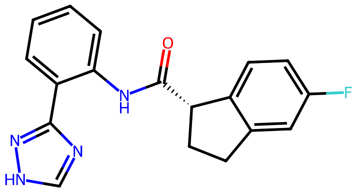	O=C(Nc1ccccc1-c1nc[nH]n1)[C@H]1CCc2cc(F)ccc21	1596581282	24	322.12	3.28
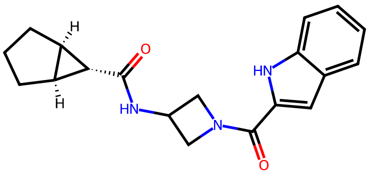	O=C(NC1CN(C(=O)c2cc3ccccc3[nH]2)C1)[C@@H]1[C@H]2CCC[C@H]21	990707902	24	323.16	2.15
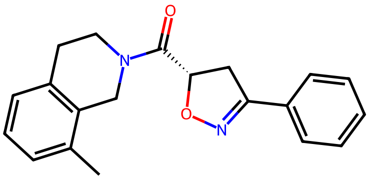	Cc1cccc2c1CN(C(=O)[C@@H]1CC(c3ccccc3)=NO1)CC2	759801855	24	320.15	3.07
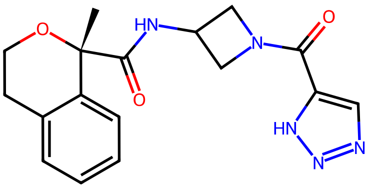	C[C@]1(C(=O)NC2CN(C(=O)c3cnn[nH]3)C2)OCCc2ccccc21	994911584	25	341.15	0.23
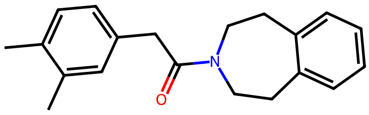	Cc1ccc(CC(=O)N2CCc3ccccc3CC2)cc1C	523812370	22	293.18	3.47
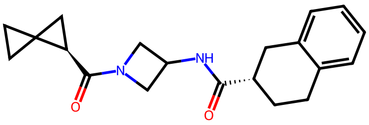	O=C(NC1CN(C(=O)[C@@H]2CC23CC3)C1)[C@H]1CCc2ccccc2C1	1000545205	24	324.18	1.92

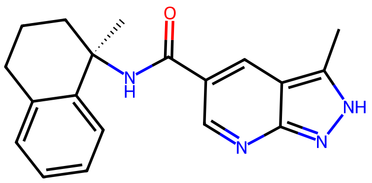	Cc1[nH]nc2ncc(C(=O)N[C@]3(C)CCCc4ccccc43)cc12	337580879	24	320.16	3.25
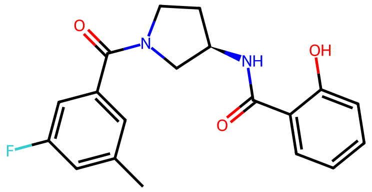	Cc1cc(F)cc(C(=O)N2CC[C@@H](NC(=O)c3ccccc3O)C2)c1	936241683	25	342.14	2.48
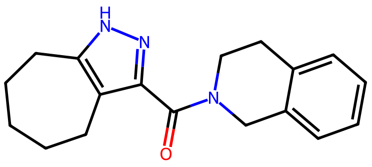	O=C(c1n[nH]c2c1CCCCC2)N1CCc2ccccc2C1	189860743	22	295.17	2.88
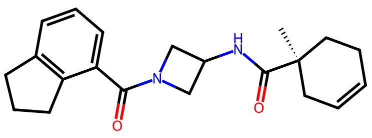	C[C@@]1(C(=O)NC2CN(C(=O)c3cccc4c3CCC4)C2)CC=CCC1	991917952	25	338.2	2.86
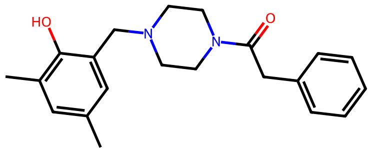	Cc1cc(C)c(O)c(CN2CCN(C(=O)Cc3ccccc3)CC2)c1	178858219	25	338.2	2.9
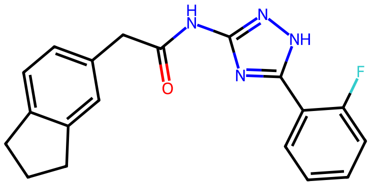	O=C(Cc1ccc2c(c1)CCC2)Nc1n[nH]c(-c2ccccc2F)n1	345669090	25	336.14	3.28
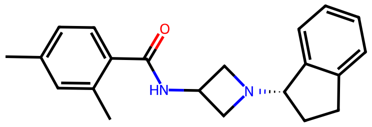	Cc1ccc(C(=O)NC2CN([C@H]3CCc4ccccc43)C2)c(C)c1	1031339658	24	320.19	3.4
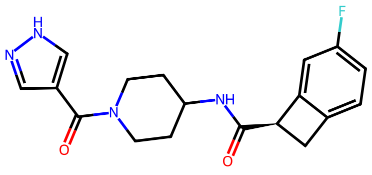	O=C(NC1CCN(C(=O)c2cn[nH]c2)CC1)[C@@H]1Cc2ccc(F)cc21	944593064	25	342.15	1.61

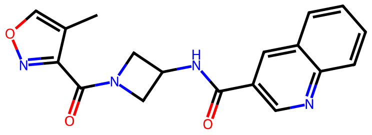	Cc1conc1C(=O)N1CC(NC(=O)c2cnc3ccccc3c2)C1	1000556324	25	336.12	1.79
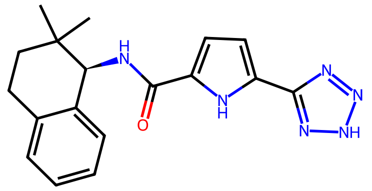	CC1(C)CCc2ccccc2[C@@H]1NC(=O)c1ccc(-c2nn[nH]n2)[nH]1	824808184	25	336.17	2.64
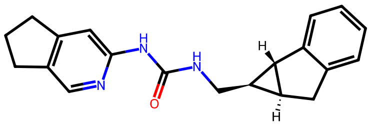	O=C(NC[C@@H]1[C@@H]2Cc3ccccc3[C@@H]12)Nc1cc2c(cn1)CCC2	583483636	24	319.17	3.28
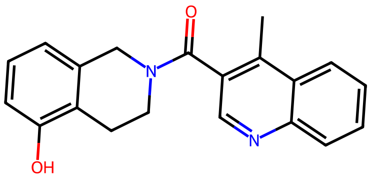	Cc1c(C(=O)N2CCc3c(O)cccc3C2)cnc2ccccc12	1635082234	24	318.14	3.45
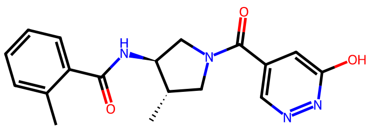	Cc1ccccc1C(=O)N[C@H]1CN(C(=O)c2cnnc(O)c2)C[C@@H]1C	966506564	25	340.15	1.38
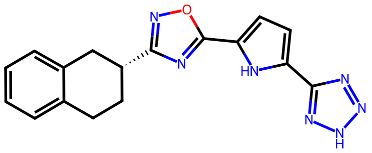	c1ccc2c(c1)CC[C@@H](c1noc(-c3ccc(-c4nn[nH]n4)[nH]3)n1)C2	1607132323	25	333.13	2.52
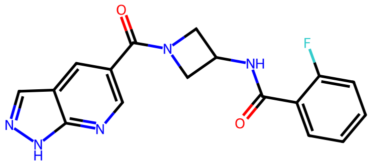	O=C(NC1CN(C(=O)c2cnc3[nH]ncc3c2)C1)c1ccccc1F	990092465	25	339.11	1.35
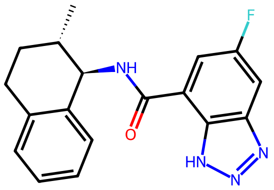	C[C@H]1CCc2ccccc2[C@@H]1NC(=O)c1cc(F)cc2nn[nH]c12	337463282	24	324.14	3.15

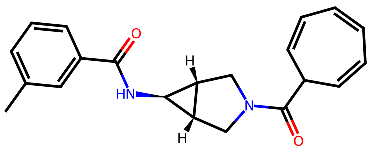	Cc1cccc(C(=O)N[C@H]2[C@@H]3CN(C(=O)C4C=CC=CC=C4)C[C@@H]32)c1	976105743	25	334.17	2.48
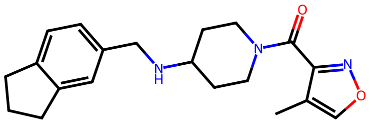	Cc1conc1C(=O)N1CCC(NCc2ccc3c(c2)CCC3)CC1	997151543	25	339.19	2.87
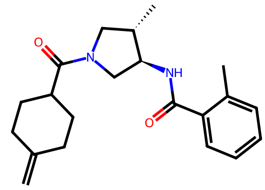	C=C1CCC(C(=O)N2C[C@H](C)[C@@H](NC(=O)c3ccccc3C)C2)CC1	966792588	25	340.22	3.32
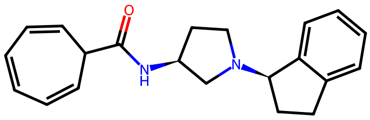	O=C(N[C@H]1CCN([C@@H]2CCc3ccccc32)C1)C1C=CC=CC=C1	1014598403	24	320.19	3.16
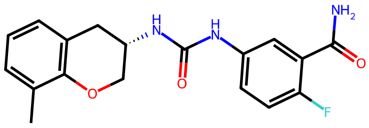	Cc1cccc2c1OC[C@@H](NC(=O)Nc1cc(F)c(C(N)=O)c1)C2	685624524	25	343.13	2.36
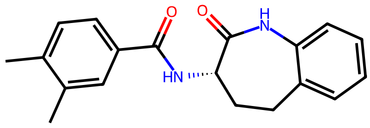	Cc1ccc(C(=O)N[C@H]2CCc3ccccc3NC2=O)cc1C	654006174	23	308.15	2.99
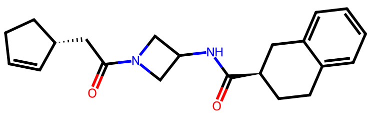	O=C(NC1CN(C(=O)C[C@@H]2C=CCC2)C1)[C@@H]1CCc2ccccc2C1	1000541689	25	338.2	2.47
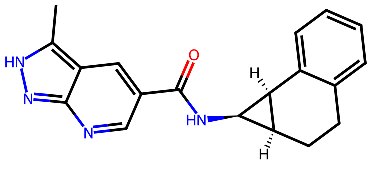	Cc1[nH]nc2ncc(C(=O)N[C@@H]3[C@@H]4CCc5ccccc5[C@@H]43)cc12	120195048	24	318.15	2.72

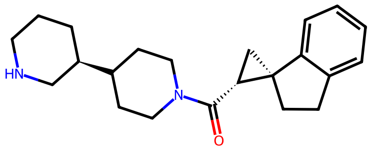	O=C([C@@H]1C[C@]12CCc1ccccc12)N1CCC([C@@H]2CCCNC2)CC1	658956683	25	338.24	3.13
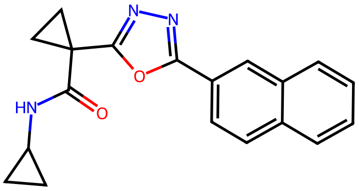	O=C(NC1CC1)C1(c2nnc(-c3ccc4ccccc4c3)o2)CC1	1322950490	24	319.13	3.2
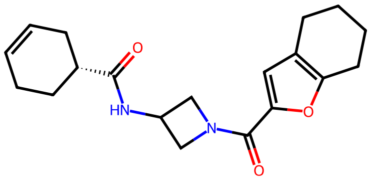	O=C(NC1CN(C(=O)c2cc3c(o2)CCCC3)C1)[C@H]1CC=CCC1	990214445	24	328.18	2.46
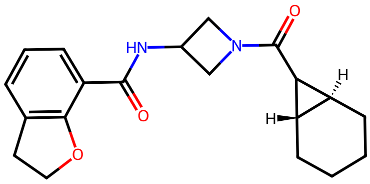	O=C(NC1CN(C(=O)C2[C@H]3CCCC[C@H]23)C1)c1cccc2c1OCC2	1000416622	25	340.18	2.0
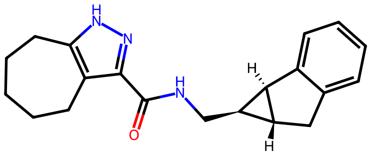	O=C(NC[C@@H]1[C@H]2Cc3ccccc3[C@@H]21)c1n[nH]c2c1CCCCC2	190709900	24	321.18	2.99
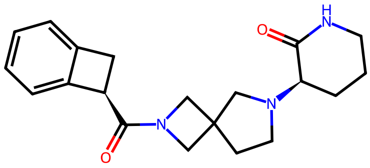	O=C1NCCC[C@H]1N1CCC2(CN(C(=O)[C@@H]3Cc4ccccc43)C2)C1	1072931284	25	339.19	1.14
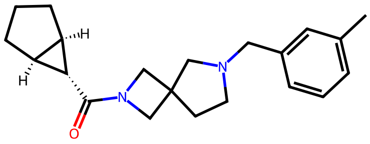	Cc1cccc(CN2CCC3(C2)CN(C(=O)[C@@H]2[C@H]4CCC[C@H]42)C3)c1	1072591321	24	324.22	3.08
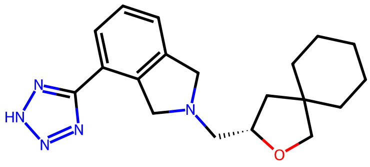	c1cc2c(c(-c3nn[nH]n3)c1)CN(C[C@@H]1CC3(CCCCC3)CO1)C2	1593566501	25	339.21	2.92

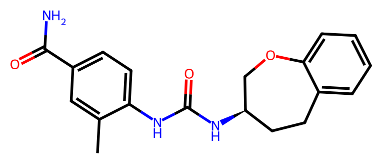	Cc1cc(C(N)=O)ccc1NC(=O)N[C@@H]1CCc2ccccc2OC1	801613492	25	339.16	2.61
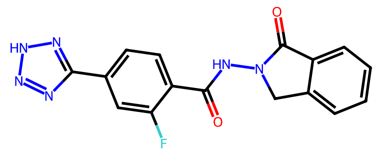	O=C(NN1Cc2ccccc2C1=O)c1ccc(-c2nn[nH]n2)cc1F	1606850657	25	338.09	1.31
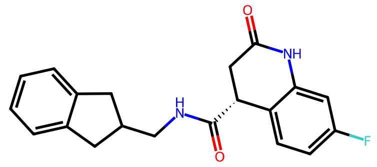	O=C1C[C@@H](C(=O)NCC2Cc3ccccc3C2)c2ccc(F)cc2N1	343616683	25	338.14	2.78
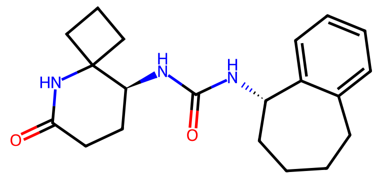	O=C1CC[C@H](NC(=O)N[C@H]2CCCCc3ccccc32)C2(CCC2)N1	567252851	25	341.21	2.95
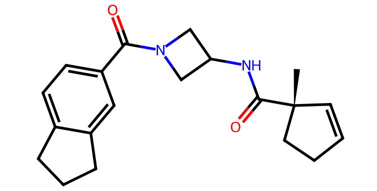	C[C@@]1(C(=O)NC2CN(C(=O)c3ccc4c(c3)CCC4)C2)C=CCC1	997856655	24	324.18	2.47
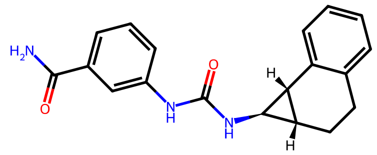	NC(=O)c1cccc(NC(=O)N[C@@H]2[C@H]3CCc4ccccc4[C@H]32)c1	178811837	24	321.15	2.64
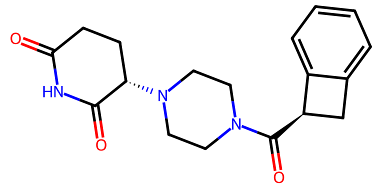	O=C1CC[C@H](N2CCN(C(=O)[C@@H]3Cc4ccccc43)CC2)C(=O)N1	949958813	24	327.16	0.28
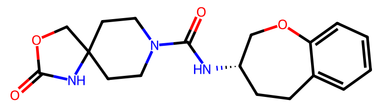	O=C1NC2(CCN(C(=O)N[C@H]3CCc4ccccc4OC3)CC2)CO1	801067063	25	345.17	1.66

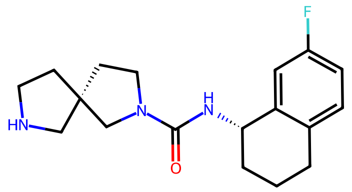	O=C(N[C@H]1CCCc2ccc(F)cc21)N1CC[C@]2(CCNC2)C1	418112471	23	317.19	2.6
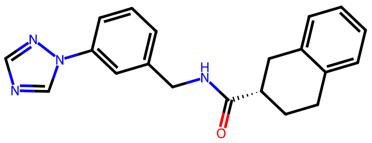	O=C(NCc1cccc(-n2cncn2)c1)[C@H]1CCc2ccccc2C1	573254257	25	332.16	2.69
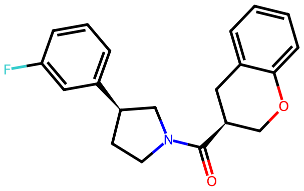	O=C([C@@H]1COc2ccccc2C1)N1CC[C@@H](c2cccc(F)c2)C1	591365901	24	325.15	3.39
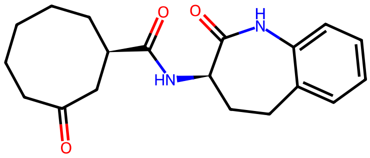	O=C1CCCCC[C@@H](C(=O)N[C@@H]2CCc3ccccc3NC2=O)C1	655225665	24	328.18	2.6
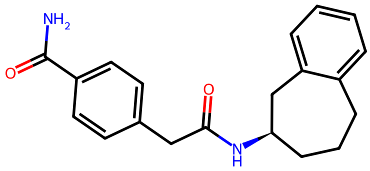	NC(=O)c1ccc(CC(=O)N[C@@H]2CCCc3ccccc3C2)cc1	888250194	24	322.17	2.39
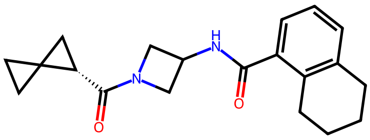	O=C(NC1CN(C(=O)[C@H]2CC23CC3)C1)c1cccc2c1CCCC2	991899053	24	324.18	2.31
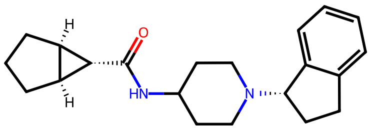	O=C(NC1CCN([C@H]2CCc3ccccc32)CC1)[C@@H]1[C@H]2CCC[C@H]21	1002545301	24	324.22	3.3
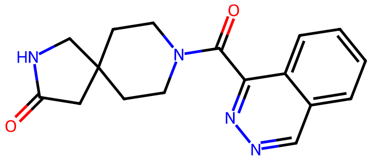	O=C1CC2(CCN(C(=O)c3nncc4ccccc34)CC2)CN1	1646333091	23	310.14	1.37

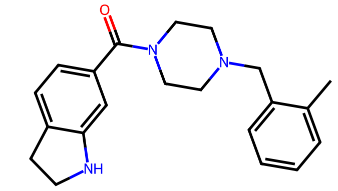	Cc1ccccc1CN1CCN(C(=O)c2ccc3c(c2)NCC3)CC1	1579802205	25	335.2	2.92
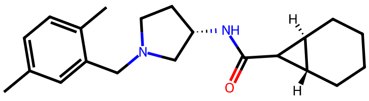	Cc1ccc(C)c(CN2CC[C@H](NC(=O)C3[C@H]4CCCC[C@H]34)C2)c1	1010438884	24	326.24	3.43
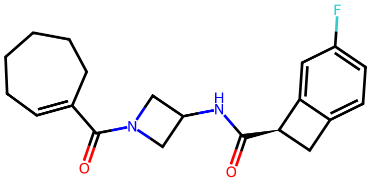	O=C(NC1CN(C(=O)C2=CCCCCC2)C1)[C@@H]1Cc2ccc(F)cc21	998489148	25	342.17	2.68
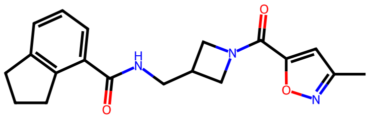	Cc1cc(C(=O)N2CC(CNC(=O)c3cccc4c3CCC4)C2)on1	1002714937	25	339.16	1.97
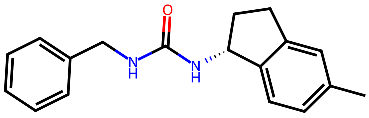	Cc1ccc2c(c1)CC[C@H]2NC(=O)NCc1ccccc1	56515738	21	280.16	3.48
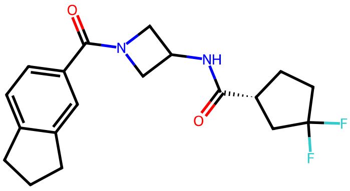	O=C(NC1CN(C(=O)c2ccc3c(c2)CCC3)C1)[C@@H]1CCC(F)(F)C1	994162078	25	348.16	2.55
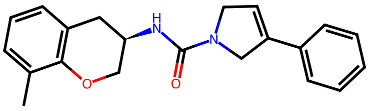	Cc1cccc2c1OC[C@H](NC(=O)N1CC=C(c3ccccc3)C1)C2	462618830	25	334.17	3.41
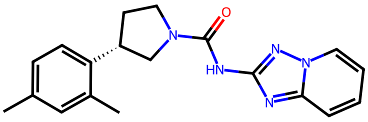	Cc1ccc([C@@H]2CCN(C(=O)Nc3nc4ccccn4n3)C2)c(C)c1	279487614	25	335.17	3.37

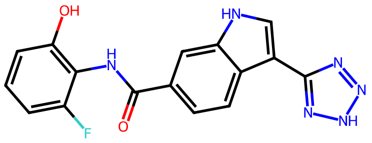	O=C(Nc1c(O)cccc1F)c1ccc2c(-c3nn[nH]n3)c[nH]c2c1	1606894215	25	338.09	2.44
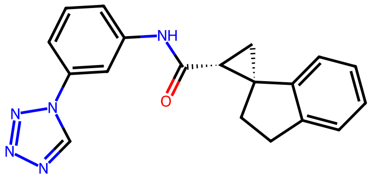	O=C(Nc1cccc(-n2cnnn2)c1)[C@@H]1C[C@]12CCc1ccccc12	174630326	25	331.14	2.5
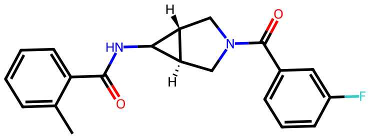	Cc1ccccc1C(=O)NC1[C@@H]2CN(C(=O)c3cccc(F)c3)C[C@@H]12	1012712397	25	338.14	2.63
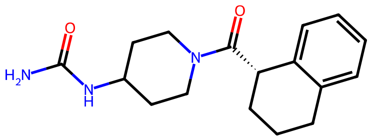	NC(=O)NC1CCN(C(=O)[C@H]2CCCc3ccccc32)CC1	266733931	22	301.18	1.77
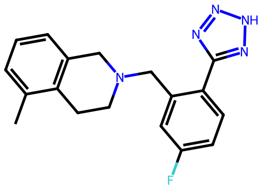	Cc1cccc2c1CCN(Cc1cc(F)ccc1-c1nn[nH]n1)C2	822340902	24	323.15	2.87
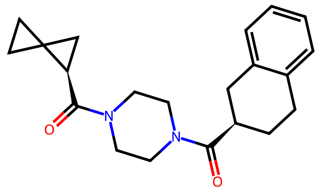	O=C([C@@H]1CCc2ccccc2C1)N1CCN(C(=O)[C@@H]2CC23CC3)CC1	1012616210	25	338.2	2.26
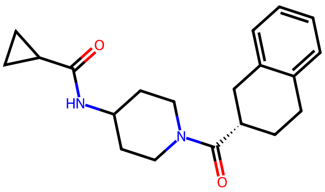	O=C(NC1CCN(C(=O)[C@H]2CCc3ccccc3C2)CC1)C1CC1	580717854	24	326.2	2.31
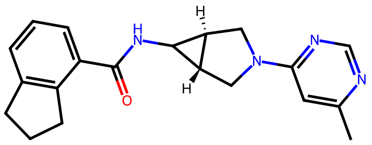	Cc1cc(N2C[C@@H]3C(NC(=O)c4cccc5c4CCC5)[C@H]3C2)ncn1	1056240431	25	334.18	2.14

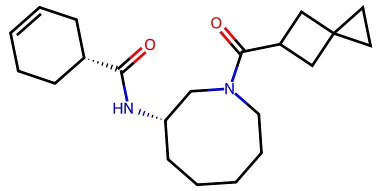	O=C(N[C@H]1CCCCCN(C(=O)C2CC3(CC3)C2)C1)[C@H]1CC=CCC1	987155348	25	344.25	3.42
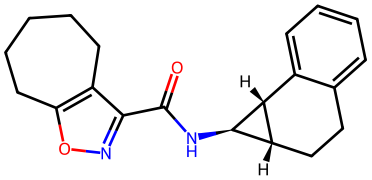	O=C(N[C@@H]1[C@H]2CCc3ccccc3[C@H]21)c1noc2c1CCCCC2	156492264	24	322.17	3.4
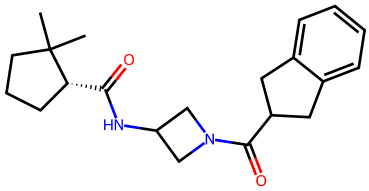	CC1(C)CCC[C@H]1C(=O)NC1CN(C(=O)C2Cc3ccccc3C2)C1	997879493	25	340.22	2.55
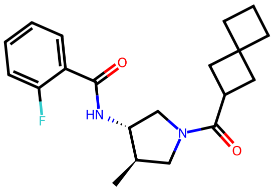	C[C@@H]1CN(C(=O)C2CC3(CCC3)C2)C[C@H]1NC(=O)c1ccccc1F	957879188	25	344.19	2.98
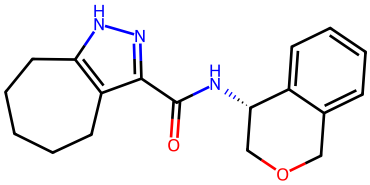	O=C(N[C@H]1COCc2ccccc21)c1n[nH]c2c1CCCCC2	416272807	23	311.16	2.68
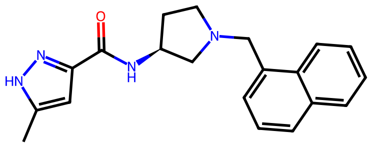	Cc1cc(C(=O)N[C@H]2CCN(Cc3cccc4ccccc34)C2)n[nH]1	1010343434	25	334.18	2.88
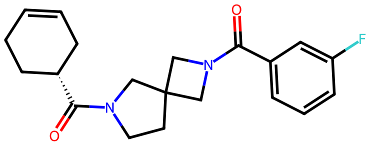	O=C(c1cccc(F)c1)N1CC2(CCN(C(=O)[C@H]3CC=CCC3)C2)C1	1018726015	25	342.17	2.86
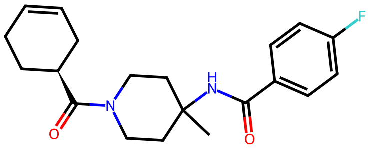	CC1(NC(=O)c2ccc(F)cc2)CCN(C(=O)[C@@H]2CC=CCC2)CC1	956270907	25	344.19	3.29

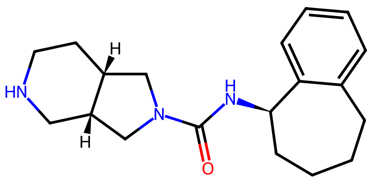	O=C(N[C@@H]1CCCCc2ccccc21)N1C[C@H]2CCNC[C@H]2C1	583252286	23	313.22	2.71
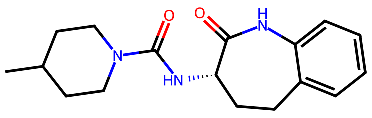	CC1CCN(C(=O)N[C@H]2CCc3ccccc3NC2=O)CC1	654630328	22	301.18	2.38
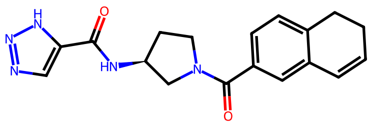	O=C(N[C@H]1CCN(C(=O)c2ccc3c(c2)C=CCC3)C1)c1cnn[nH]1	938238503	25	337.15	1.41
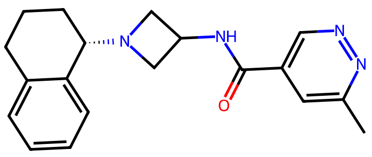	Cc1cc(C(=O)NC2CN([C@H]3CCCc4ccccc43)C2)cnn1	1030249760	24	322.18	2.28
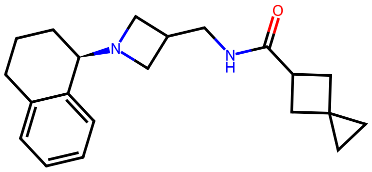	O=C(NCC1CN([C@@H]2CCCc3ccccc32)C1)C1CC2(CC2)C1	1031967790	24	324.22	3.3
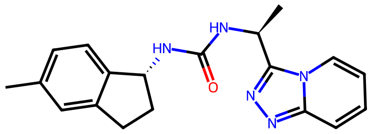	Cc1ccc2c(c1)CC[C@H]2NC(=O)N[C@@H](C)c1nnc2ccccn12	56842539	25	335.17	3.09
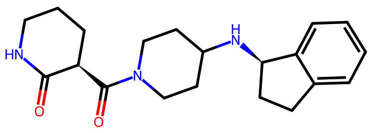	O=C1NCCC[C@H]1C(=O)N1CCC(N[C@@H]2CCc3ccccc32)CC1	996695262	25	341.21	1.78
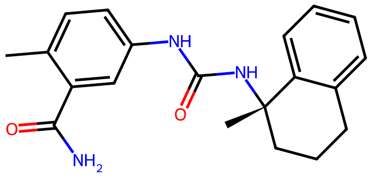	Cc1ccc(NC(=O)N[C@@]2(C)CCCc3ccccc32)cc1C(N)=O	359121814	25	337.18	3.47

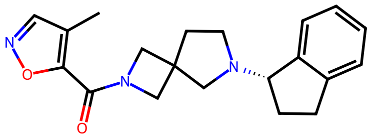	Cc1cnoc1C(=O)N1CC2(CCN([C@H]3CCc4ccccc43)C2)C1	1073100352	25	337.18	2.82
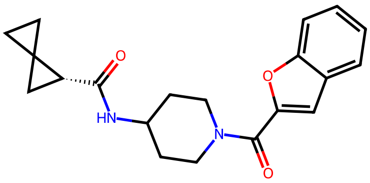	O=C(NC1CCN(C(=O)c2cc3ccccc3o2)CC1)[C@@H]1CC12CC2	948481341	25	338.16	2.95
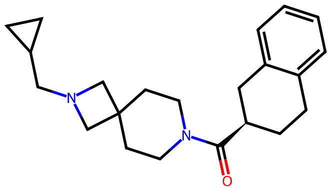	O=C([C@@H]1CCc2ccccc2C1)N1CCC2(CC1)CN(CC1CC1)C2	1035807126	25	338.24	3.13
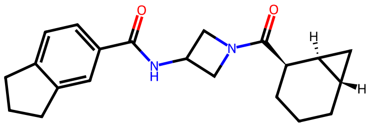	O=C(NC1CN(C(=O)[C@@H]2CCC[C@H]3C[C@@H]32)C1)c1ccc2c(c1)CCC2	991370372	25	338.2	2.55
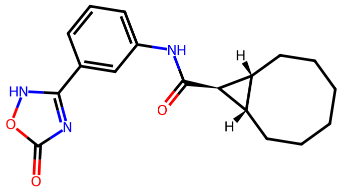	O=C(Nc1cccc(-c2nc(=O)o[nH]2)c1)[C@@H]1[C@H]2CCCCCC[C@H]21	667992452	24	327.16	3.18
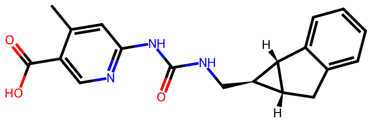	Cc1cc(NC(=O)NC[C@@H]2[C@H]3Cc4ccccc4[C@H]32)ncc1C(=O)O	1600268867	25	337.14	2.8
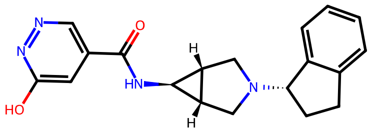	O=C(N[C@H]1[C@@H]2CN([C@H]3CCc4ccccc43)C[C@@H]21)c1cnnc(O)c1	960411888	25	336.16	1.53
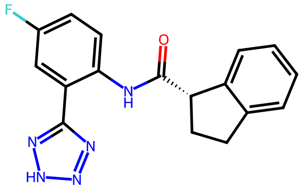	O=C(Nc1ccc(F)cc1-c1nn[nH]n1)[C@H]1CCc2ccccc21	823343805	24	323.12	2.67

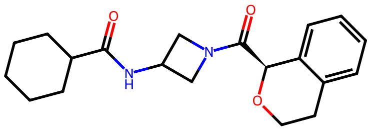	O=C(NC1CN(C(=O)[C@@H]2OCCc3ccccc32)C1)C1CCCCC1	990099371	25	342.19	2.21
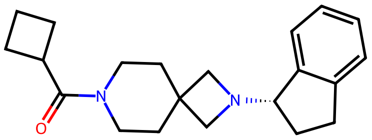	O=C(C1CCC1)N1CCC2(CC1)CN([C@H]1CCc3ccccc31)C2	1035635238	24	324.22	3.4
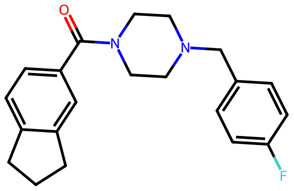	O=C(c1ccc2c(c1)CCC2)N1CCN(Cc2ccc(F)cc2)CC1	957607549	25	338.18	3.27
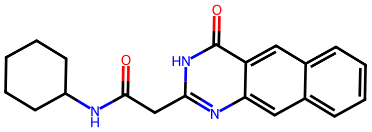	O=C(Cc1nc2cc3ccccc3cc2c(=O)[nH]1)NC1CCCCC1	77256570	25	335.16	3.07
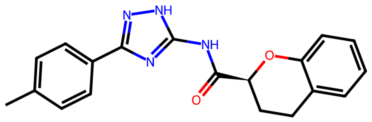	Cc1ccc(-c2n[nH]c(NC(=O)[C@@H]3CCc4ccccc4O3)n2)cc1	1654185297	25	334.14	3.11
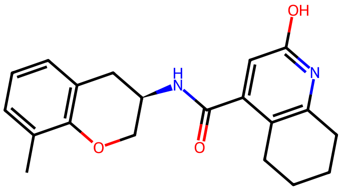	Cc1cccc2c1OC[C@H](NC(=O)c1cc(O)nc3c1CCCC3)C2	936056843	25	338.16	2.71
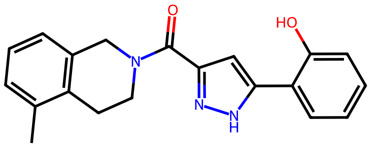	Cc1cccc2c1CCN(C(=O)c1cc(-c3ccccc3O)[nH]n1)C2	1293135710	25	333.15	3.29
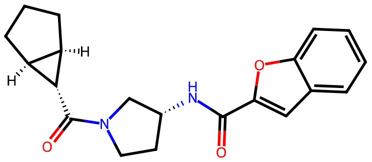	O=C(N[C@@H]1CCN(C(=O)[C@@H]2[C@H]3CCC[C@H]32)C1)c1cc2ccccc2o1	942453623	25	338.16	2.81

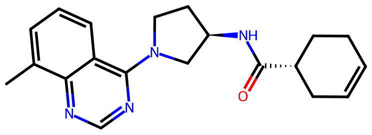	Cc1cccc2c(N3CC[C@@H](NC(=O)[C@H]4CC=CCC4)C3)ncnc12	1058297484	25	336.2	2.99
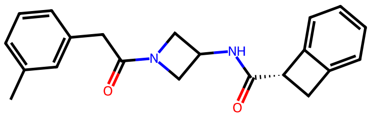	Cc1cccc(CC(=O)N2CC(NC(=O)[C@H]3Cc4ccccc43)C2)c1	998691927	25	334.17	2.2
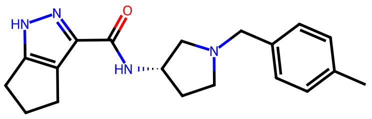	Cc1ccc(CN2CC[C@H](NC(=O)c3n[nH]c4c3CCC4)C2)cc1	634455790	24	324.2	2.21
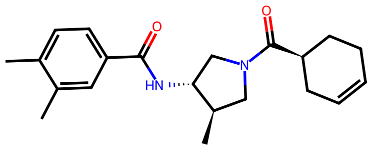	Cc1ccc(C(=O)N[C@@H]2CN(C(=O)[C@@H]3CC=CCC3)C[C@H]2C)cc1C	940146251	25	340.22	3.24
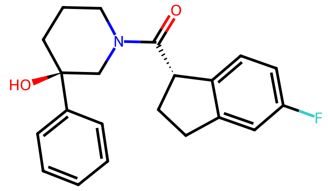	O=C([C@H]1CCc2cc(F)ccc21)N1CCC[C@@](O)(c2ccccc2)C1	862795753	25	339.16	3.37
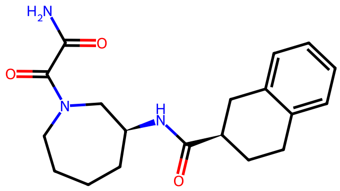	NC(=O)C(=O)N1CCCC[C@H](NC(=O)[C@@H]2CCc3ccccc3C2)C1	943575398	25	343.19	0.77
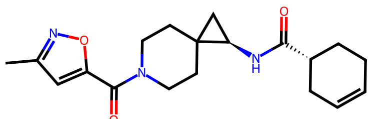	Cc1cc(C(=O)N2CCC3(CC2)C[C@H]3NC(=O)[C@H]2CC=CCC2)on1	963030513	25	343.19	2.45
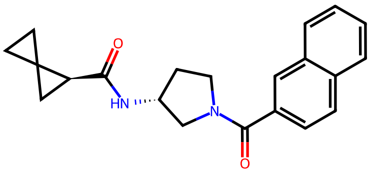	O=C(N[C@@H]1CCN(C(=O)c2ccc3ccccc3c2)C1)[C@H]1CC12CC2	942508063	25	334.17	2.97

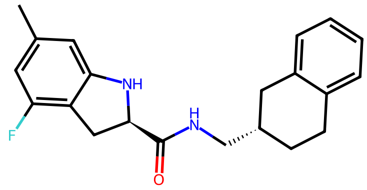	Cc1cc(F)c2c(c1)N[C@@H](C(=O)NC[C@H]1CCc3ccccc3C1)C2	1579610645	25	338.18	3.39
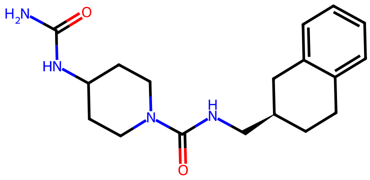	NC(=O)NC1CCN(C(=O)NC[C@@H]2CCc3ccccc3C2)CC1	674984365	24	330.21	1.63
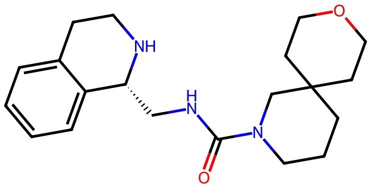	O=C(NC[C@H]1NCCc2ccccc21)N1CCCC2(CCOCC2)C1	561212450	25	343.23	2.48
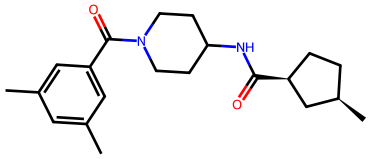	Cc1cc(C)cc(C(=O)N2CCC(NC(=O)[C@H]3CC[C@@H](C)C3)CC2)c1	948337768	25	342.23	3.46
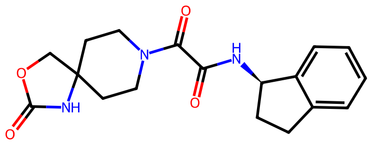	O=C1NC2(CCN(C(=O)C(=O)N[C@@H]3CCc4ccccc43)CC2)CO1	1350899193	25	343.15	0.89
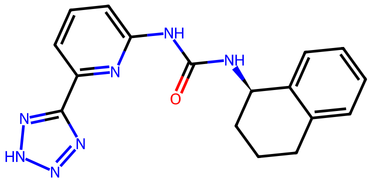	O=C(Nc1cccc(-c2nn[nH]n2)n1)N[C@@H]1CCCc2ccccc21	1582644766	25	335.15	2.46
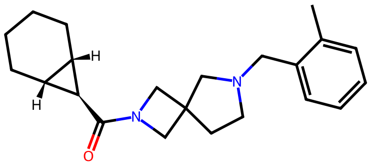	Cc1ccccc1CN1CCC2(C1)CN(C(=O)[C@@H]1[C@H]3CCCC[C@H]31)C2	1072580466	25	338.24	3.47
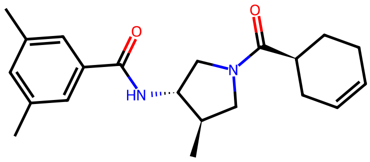	Cc1cc(C)cc(C(=O)N[C@@H]2CN(C(=O)[C@@H]3CC=CCC3)C[C@H]2C)c1	940110029	25	340.22	3.24

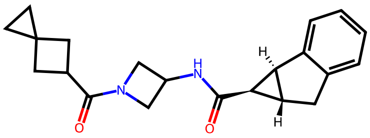	O=C(NC1CN(C(=O)C2CC3(CC3)C2)C1)[C@@H]1[C@H]2Cc3ccccc3[C@@H]21	991646000	25	336.18	2.09
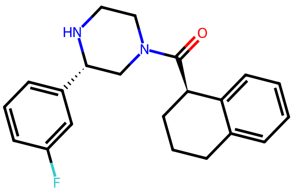	O=C([C@@H]1CCCc2ccccc21)N1CCN[C@@H](c2cccc(F)c2)C1	337589471	25	338.18	3.42
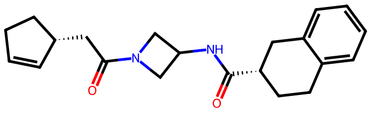	O=C(NC1CN(C(=O)C[C@@H]2C=CCC2)C1)[C@H]1CCc2ccccc2C1	1000541700	25	338.2	2.47
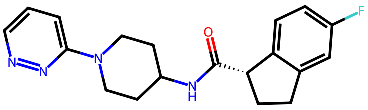	O=C(NC1CCN(c2cccnn2)CC1)[C@H]1CCc2cc(F)ccc21	614003197	25	340.17	2.43
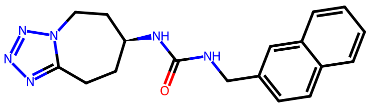	O=C(NCc1ccc2ccccc2c1)N[C@H]1CCc2nnnn2CC1	643378531	25	336.17	2.03
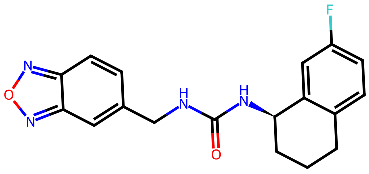	O=C(NCc1ccc2nonc2c1)N[C@@H]1CCCc2ccc(F)cc21	1470059382	25	340.13	3.24
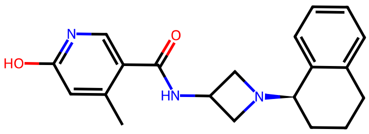	Cc1cc(O)ncc1C(=O)NC1CN([C@@H]2CCCc3ccccc32)C1	1030933556	25	337.18	2.59
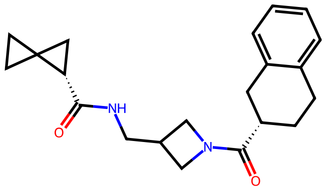	O=C(NCC1CN(C(=O)[C@H]2CCc3ccccc3C2)C1)[C@H]1CC12CC2	936091183	25	338.2	2.17

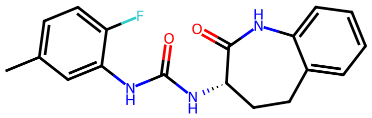	Cc1ccc(F)c(NC(=O)N[C@H]2CCc3ccccc3NC2=O)c1	1185274966	24	327.14	3.21
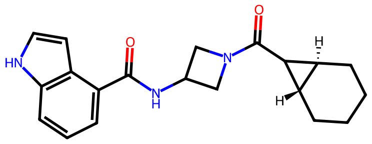	O=C(NC1CN(C(=O)C2[C@H]3CCCC[C@H]23)C1)c1cccc2[nH]ccc12	1000403521	25	337.18	2.54
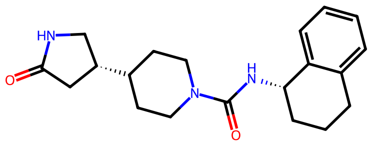	O=C1C[C@@H](C2CCN(C(=O)N[C@H]3CCCc4ccccc43)CC2)CN1	1553351025	25	341.21	2.62
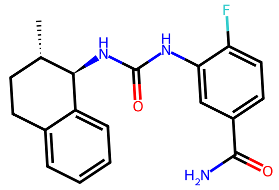	C[C@H]1CCc2ccccc2[C@@H]1NC(=O)Nc1cc(C(N)=O)ccc1F	179534117	25	341.15	3.37
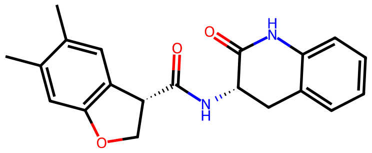	Cc1cc2c(cc1C)[C@H](C(=O)N[C@H]1Cc3ccccc3NC1=O)CO2	529946546	25	336.15	2.46
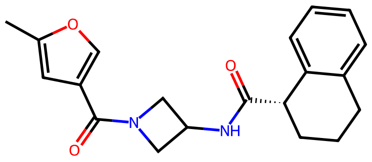	Cc1cc(C(=O)N2CC(NC(=O)[C@H]3CCCc4ccccc43)C2)co1	995068545	25	338.16	2.65
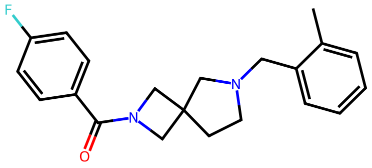	Cc1ccccc1CN1CCC2(C1)CN(C(=O)c1ccc(F)cc1)C2	1072398007	25	338.18	3.48
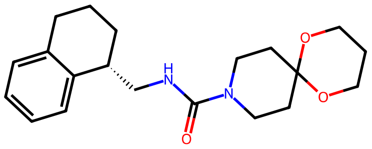	O=C(NC[C@H]1CCCc2ccccc21)N1CCC2(CC1)OCCCO2	836206133	25	344.21	3.05

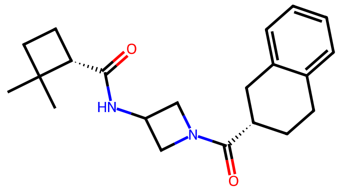	CC1(C)CC[C@@H]1C(=O)NC1CN(C(=O)[C@H]2CCc3ccccc3C2)C1	997966740	25	340.22	2.55
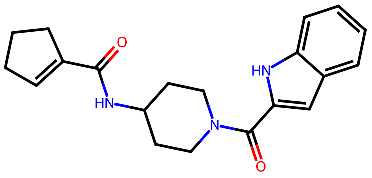	O=C(NC1CCN(C(=O)c2cc3ccccc3[nH]2)CC1)C1=CCCC1	948490837	25	337.18	3.0
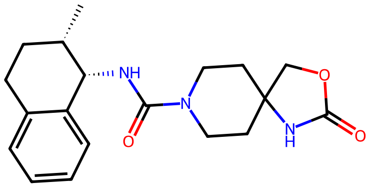	C[C@H]1CCc2ccccc2[C@H]1NC(=O)N1CCC2(CC1)COC(=O)N2	495297705	25	343.19	2.59
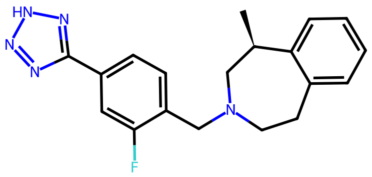	C[C@@H]1CN(Cc2ccc(-c3nn[nH]n3)cc2F)CCc2ccccc21	1576361018	25	337.17	3.17
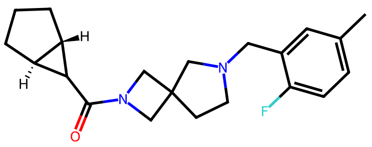	Cc1ccc(F)c(CN2CCC3(C2)CN(C(=O)C2[C@H]4CCC[C@H]24)C3)c1	1072592888	25	342.21	3.21
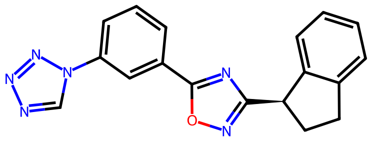	c1cc(-c2nc([C@@H]3CCc4ccccc43)no2)cc(-n2cnnn2)c1	356654147	25	330.12	2.79
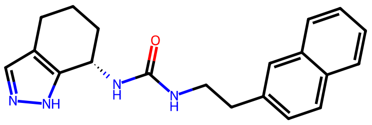	O=C(NCCc1ccc2ccccc2c1)N[C@H]1CCCc2cn[nH]c21	268880485	25	334.18	3.48
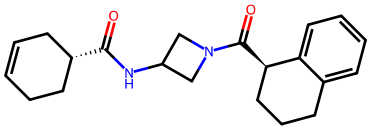	O=C(NC1CN(C(=O)[C@@H]2CCCc3ccccc32)C1)[C@H]1CC=CCC1	990202681	25	338.2	2.79

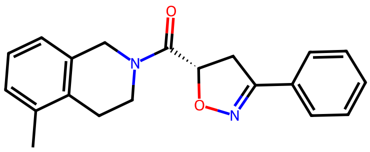	Cc1cccc2c1CCN(C(=O)[C@@H]1CC(c3ccccc3)=NO1)C2	729415259	24	320.15	3.07
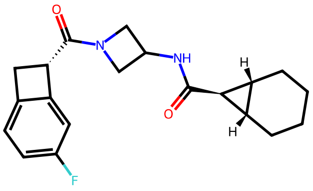	O=C(NC1CN(C(=O)[C@H]2Cc3ccc(F)cc32)C1)[C@@H]1[C@H]2CCCC[C@H]21	990662684	25	342.17	2.23
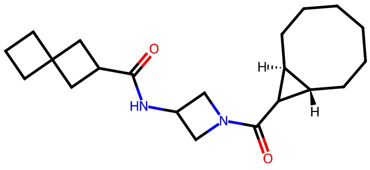	O=C(NC1CN(C(=O)C2[C@H]3CCCCCC[C@H]23)C1)C1CC2(CCC2)C1	997934840	25	344.25	3.11
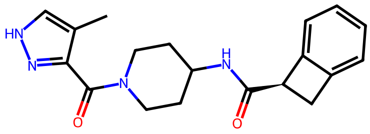	Cc1c[nH]nc1C(=O)N1CCC(NC(=O)[C@@H]2Cc3ccccc32)CC1	946682549	25	338.17	1.78
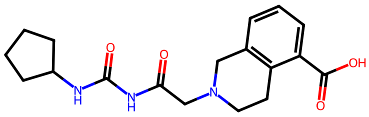	O=C(CN1CCc2c(cccc2C(=O)O)C1)NC(=O)NC1CCCC1	328038177	25	345.17	1.51
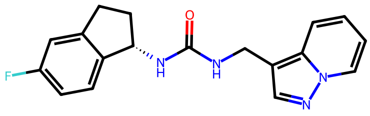	O=C(NCc1cnn2ccccc12)N[C@H]1CCc2cc(F)ccc21	344430380	24	324.14	2.96
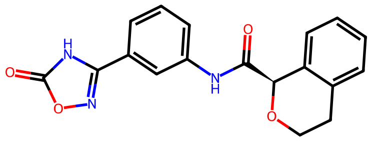	O=C(Nc1cccc(-c2noc(=O)[nH]2)c1)[C@@H]1OCCc2ccccc21	119260616	25	337.11	2.28
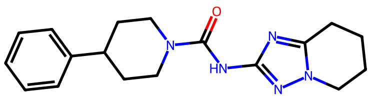	O=C(Nc1nc2n(n1)CCCC2)N1CCC(c2ccccc2)CC1	562926833	24	325.19	3.03

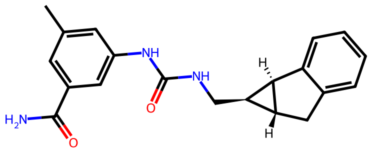	Cc1cc(NC(=O)NC[C@@H]2[C@H]3Cc4ccccc4[C@@H]32)cc(C(N)=O)c1	583608643	25	335.16	2.8
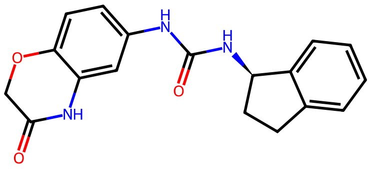	O=C1COc2ccc(NC(=O)N[C@@H]3CCc4ccccc43)cc2N1	43690779	24	323.13	2.83
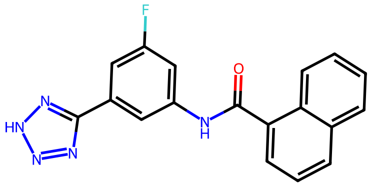	O=C(Nc1cc(F)cc(-c2nn[nH]n2)c1)c1cccc2ccccc12	1582636371	25	333.1	3.41
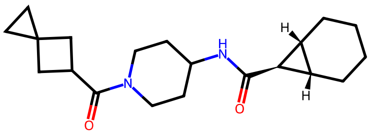	O=C(NC1CCN(C(=O)C2CC3(CC3)C2)CC1)[C@@H]1[C@H]2CCCC[C@H]21	948299945	24	330.23	2.72
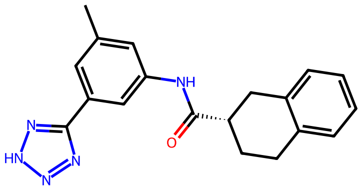	Cc1cc(NC(=O)[C@H]2CCc3ccccc3C2)cc(-c2nn[nH]n2)c1	1592017602	25	333.16	2.92
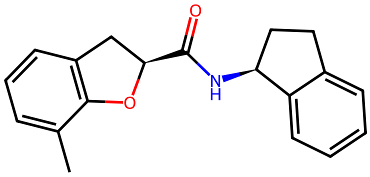	Cc1cccc2c1O[C@H](C(=O)N[C@H]1CCc3ccccc31)C2	449205623	22	293.14	3.1
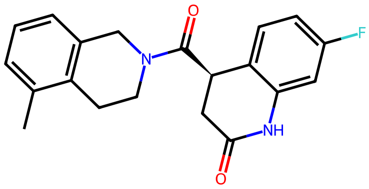	Cc1cccc2c1CCN(C(=O)[C@@H]1CC(=O)Nc3cc(F)ccc31)C2	171540365	25	338.14	3.14
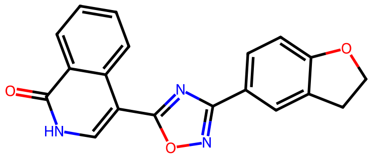	O=c1[nH]cc(-c2nc(-c3ccc4c(c3)CCO4)no2)c2ccccc12	189191005	25	331.1	3.18

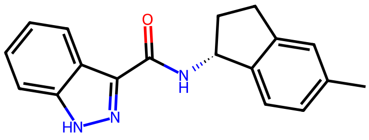	Cc1ccc2c(c1)CC[C@H]2NC(=O)c1n[nH]c2ccccc12	55658791	22	291.14	3.29
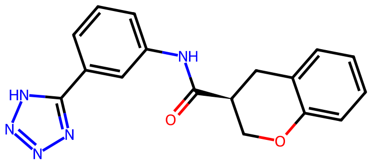	O=C(Nc1cccc(-c2nnn[nH]2)c1)[C@@H]1COc2ccccc2C1	70710040	24	321.12	2.06
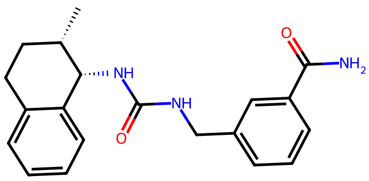	C[C@H]1CCc2ccccc2[C@H]1NC(=O)NCc1cccc(C(N)=O)c1	179306762	25	337.18	2.91
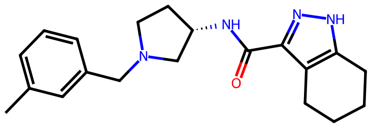	Cc1cccc(CN2CC[C@H](NC(=O)c3n[nH]c4c3CCCC4)C2)c1	1015075252	25	338.21	2.6
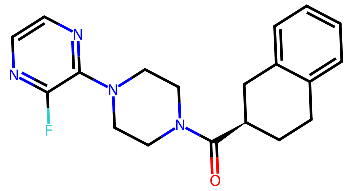	O=C([C@@H]1CCc2ccccc2C1)N1CCN(c2nccnc2F)CC1	1056034084	25	340.17	2.07
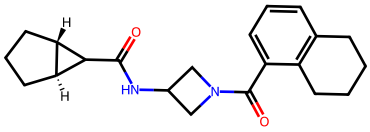	O=C(NC1CN(C(=O)c2cccc3c2CCCC3)C1)C1[C@H]2CCC[C@H]12	990696645	25	338.2	2.55
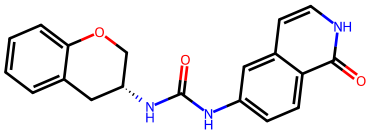	O=C(Nc1ccc2c(=O)[nH]ccc2c1)N[C@H]1COc2ccccc2C1	1626189853	25	335.13	2.65
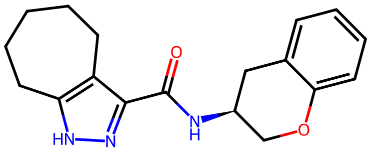	O=C(N[C@@H]1COc2ccccc2C1)c1n[nH]c2c1CCCCC2	191299782	23	311.16	2.41

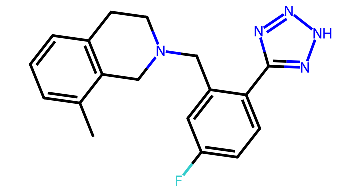	Cc1cccc2c1CN(Cc1cc(F)ccc1-c1nn[nH]n1)CC2	1609434687	24	323.15	2.87
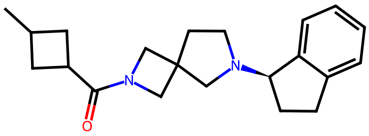	CC1CC(C(=O)N2CC3(CCN([C@@H]4CCc5ccccc54)C3)C2)C1	1072606577	24	324.22	3.25
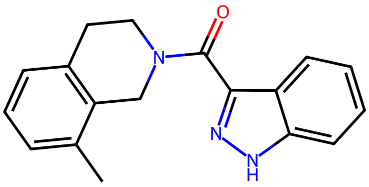	Cc1cccc2c1CN(C(=O)c1n[nH]c3ccccc13)CC2	346975831	22	291.14	3.07
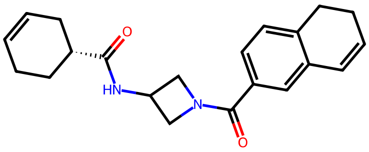	O=C(NC1CN(C(=O)c2ccc3c(c2)C=CCC3)C1)[C@H]1CC=CCC1	990209130	25	336.18	2.94
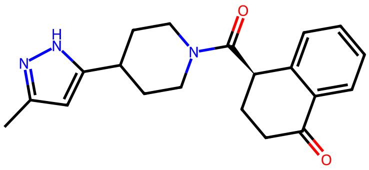	Cc1cc(C2CCN(C(=O)[C@@H]3CCC(=O)c4ccccc43)CC2)[nH]n1	285722560	25	337.18	3.18
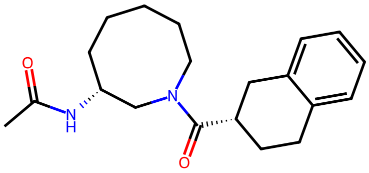	CC(=O)N[C@@H]1CCCCCN(C(=O)[C@H]2CCc3ccccc3C2)C1	987173050	24	328.22	2.7
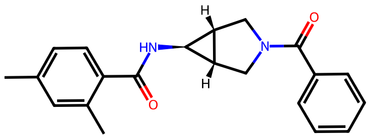	Cc1ccc(C(=O)N[C@H]2[C@@H]3CN(C(=O)c4ccccc4)C[C@@H]32)c(C)c1	977176371	25	334.17	2.8
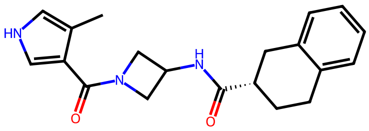	Cc1c[nH]cc1C(=O)N1CC(NC(=O)[C@H]2CCc3ccccc3C2)C1	1000543286	25	337.18	2.07

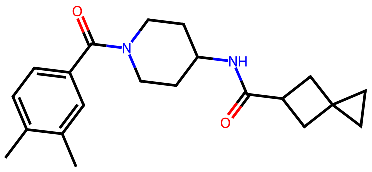	Cc1ccc(C(=O)N2CCC(NC(=O)C3CC4(CC4)C3)CC2)cc1C	948352117	25	340.22	3.21
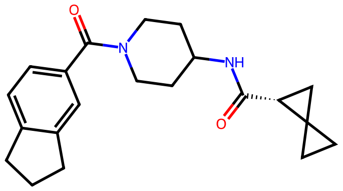	O=C(NC1CCN(C(=O)c2ccc3c(c2)CCC3)CC1)[C@@H]1CC12CC2	946723473	25	338.2	2.7
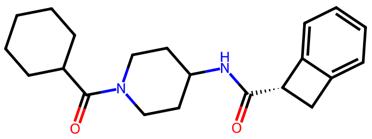	O=C(NC1CCN(C(=O)C2CCCCC2)CC1)[C@H]1Cc2ccccc21	943758130	25	340.22	3.01
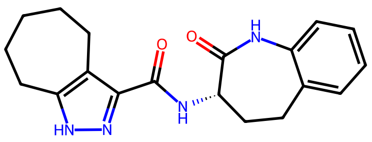	O=C(N[C@H]1CCc2ccccc2NC1=O)c1n[nH]c2c1CCCCC2	655225014	25	338.17	2.36
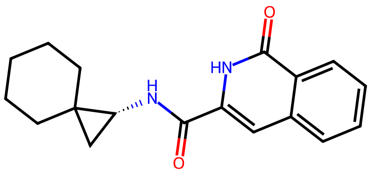	O=C(N[C@@H]1CC12CCCCC2)c1cc2ccccc2c(=O)[nH]1	180793652	22	296.15	2.98
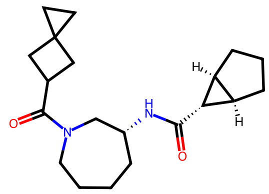	O=C(N[C@@H]1CCCCN(C(=O)C2CC3(CC3)C2)C1)[C@@H]1[C@H]2CCC[C@H]21	943166817	24	330.23	2.72
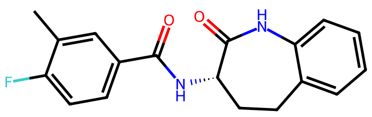	Cc1cc(C(=O)N[C@H]2CCc3ccccc3NC2=O)ccc1F	1417561930	23	312.13	2.82
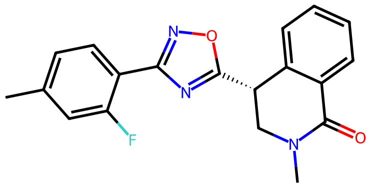	Cc1ccc(-c2noc([C@H]3CN(C)C(=O)c4ccccc43)n2)c(F)c1	377470552	25	337.12	3.4

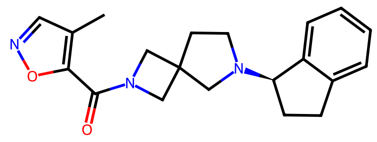	Cc1cnoc1C(=O)N1CC2(CCN([C@@H]3CCc4ccccc43)C2)C1	1073100353	25	337.18	2.82
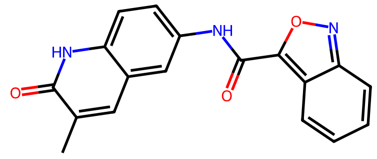	Cc1cc2cc(NC(=O)c3onc4ccccc34)ccc2[nH]c1=O	1468332487	24	319.1	3.23
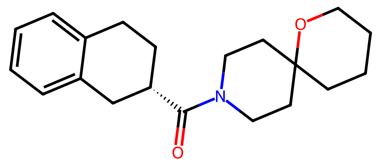	O=C([C@H]1CCc2ccccc2C1)N1CCC2(CCCCO2)CC1	882441585	23	313.2	3.35
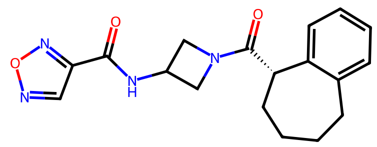	O=C(NC1CN(C(=O)[C@H]2CCCCc3ccccc32)C1)c1cnon1	998237908	25	340.15	1.52
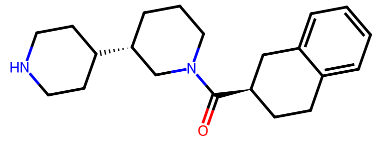	O=C([C@@H]1CCc2ccccc2C1)N1CCC[C@@H](C2CCNCC2)C1	662590339	24	326.24	3.03
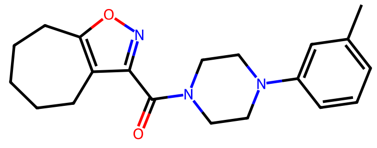	Cc1cccc(N2CCN(C(=O)c3noc4c3CCCCC4)CC2)c1	123917059	25	339.19	3.21
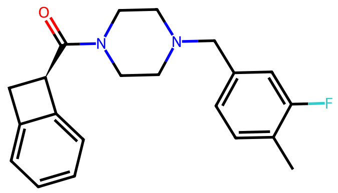	Cc1ccc(CN2CCN(C(=O)[C@@H]3Cc4ccccc43)CC2)cc1F	949958810	25	338.18	3.12
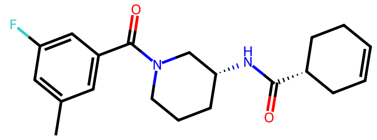	Cc1cc(F)cc(C(=O)N2CCC[C@@H](NC(=O)[C@H]3CC=CCC3)C2)c1	952126913	25	344.19	3.21

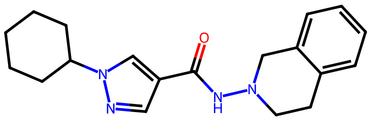	O=C(NN1CCc2ccccc2C1)c1cnn(C2CCCCC2)c1	1261753600	24	324.2	3.09
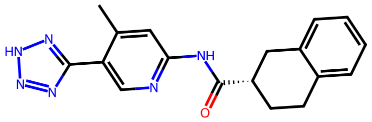	Cc1cc(NC(=O)[C@H]2CCc3ccccc3C2)ncc1-c1nn[nH]n1	1592018413	25	334.15	2.31
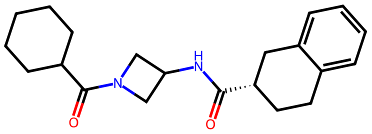	O=C(NC1CN(C(=O)C2CCCCC2)C1)[C@H]1CCc2ccccc2C1	1000538587	25	340.22	2.7
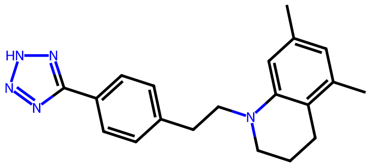	Cc1cc(C)c2c(c1)N(CCc1ccc(-c3nn[nH]n3)cc1)CCC2	1591950434	25	333.2	3.48
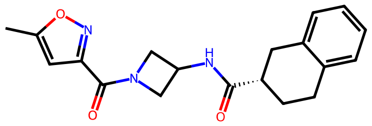	Cc1cc(C(=O)N2CC(NC(=O)[C@H]3CCc4ccccc4C3)C2)no1	1000540284	25	339.16	1.73
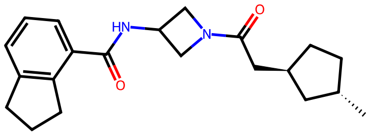	C[C@H]1CC[C@H](CC(=O)N2CC(NC(=O)c3cccc4c3CCC4)C2)C1	994395377	25	340.22	2.94
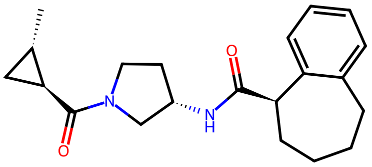	C[C@H]1C[C@@H]1C(=O)N1CC[C@H](NC(=O)[C@@H]2CCCCc3ccccc32)C1	938139003	25	340.22	2.87
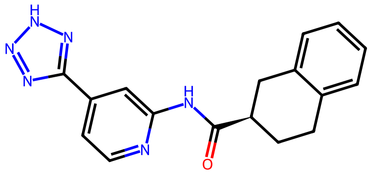	O=C(Nc1cc(-c2nn[nH]n2)ccn1)[C@@H]1CCc2ccccc2C1	1582634558	24	320.14	2.01

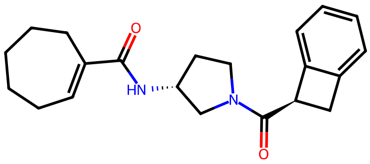	O=C(N[C@@H]1CCN(C(=O)[C@@H]2Cc3ccccc32)C1)C1=CCCCCC1	940159291	25	338.2	2.93
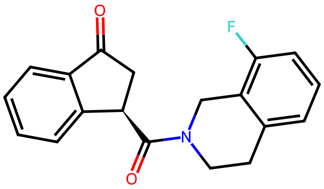	O=C1C[C@@H](C(=O)N2CCc3cccc(F)c3C2)c2ccccc21	277143753	23	309.12	3.08
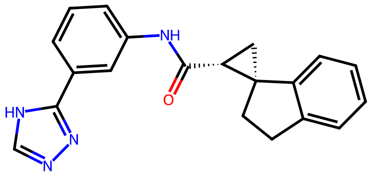	O=C(Nc1cccc(-c2nnc[nH]2)c1)[C@@H]1C[C@]12CCc1ccccc12	266489975	25	330.15	3.31
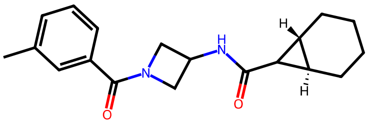	Cc1cccc(C(=O)N2CC(NC(=O)C3[C@@H]4CCCC[C@@H]34)C2)c1	990641546	23	312.18	2.37
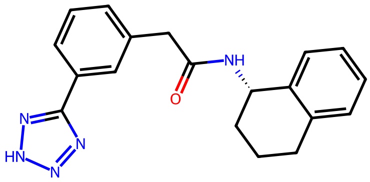	O=C(Cc1cccc(-c2nn[nH]n2)c1)N[C@H]1CCCc2ccccc21	823093114	25	333.16	2.6
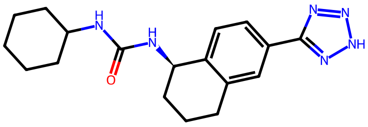	O=C(NC1CCCCC1)N[C@@H]1CCCc2cc(-c3nn[nH]n3)ccc21	1609540655	25	340.2	2.88
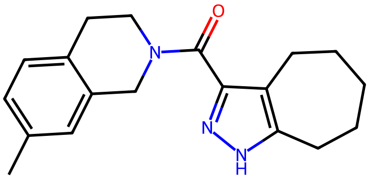	Cc1ccc2c(c1)CN(C(=O)c1n[nH]c3c1CCCCC3)CC2	617605334	23	309.18	3.19
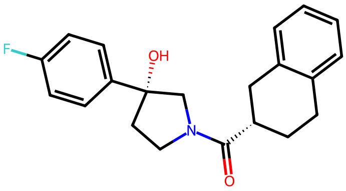	O=C([C@H]1CCc2ccccc2C1)N1CC[C@@](O)(c2ccc(F)cc2)C1	845919479	25	339.16	3.05

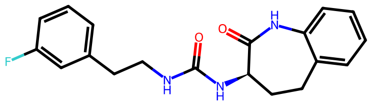	O=C(NCCc1cccc(F)c1)N[C@@H]1CCc2ccccc2NC1=O	654356872	25	341.15	2.62
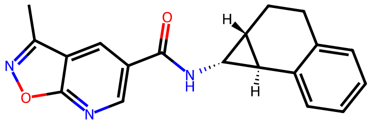	Cc1noc2ncc(C(=O)N[C@H]3[C@@H]4c5ccccc5CC[C@H]43)cc12	246203487	24	319.13	2.99
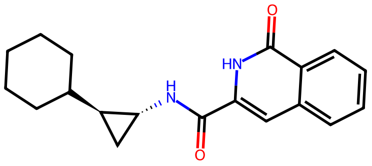	O=C(N[C@@H]1C[C@H]1C1CCCCC1)c1cc2ccccc2c(=O)[nH]1	152714964	23	310.17	3.23
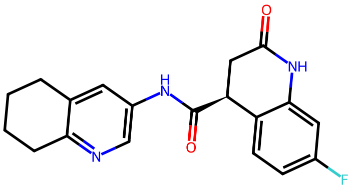	O=C1C[C@H](C(=O)Nc2cnc3c(c2)CCCC3)c2ccc(F)cc2N1	543897804	25	339.14	3.16
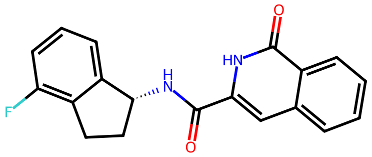	O=C(N[C@@H]1CCc2c(F)cccc21)c1cc2ccccc2c(=O)[nH]1	56325503	24	322.11	3.08
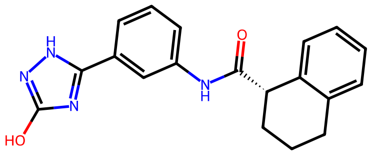	O=C(Nc1cccc(-c2nc(O)n[nH]2)c1)[C@H]1CCCc2ccccc21	356019168	25	334.14	3.24
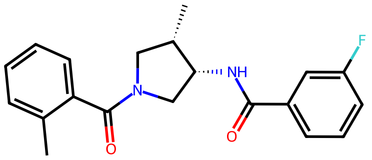	Cc1ccccc1C(=O)N1C[C@H](C)[C@H](NC(=O)c2cccc(F)c2)C1	966095828	25	340.16	3.02
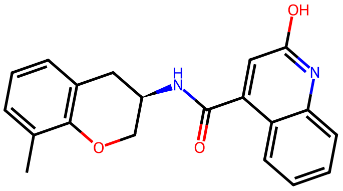	Cc1cccc2c1OC[C@H](NC(=O)c1cc(O)nc3ccccc13)C2	484928710	25	334.13	2.98

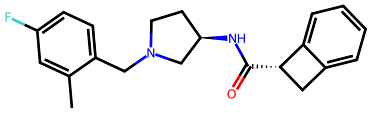	Cc1cc(F)ccc1CN1CC[C@@H](NC(=O)[C@H]2Cc3ccccc32)C1	1015636266	25	338.18	3.16
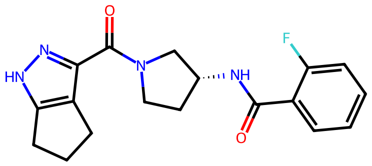	O=C(N[C@@H]1CCN(C(=O)c2n[nH]c3c2CCC3)C1)c1ccccc1F	939176078	25	342.15	1.68
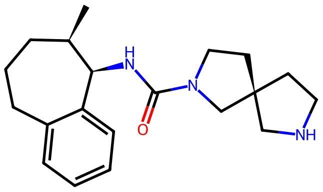	C[C@@H]1CCCc2ccccc2[C@@H]1NC(=O)N1CC[C@]2(CCNC2)C1	418017793	24	327.23	3.1
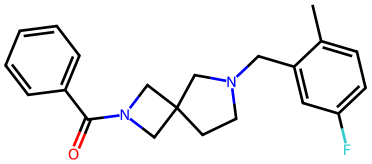	Cc1ccc(F)cc1CN1CCC2(C1)CN(C(=O)c1ccccc1)C2	1072408316	25	338.18	3.48
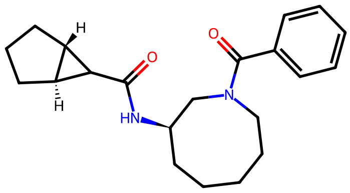	O=C(N[C@@H]1CCCCCN(C(=O)c2ccccc2)C1)C1[C@H]2CCC[C@H]12	986852255	25	340.22	3.23
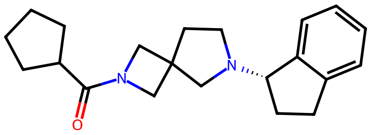	O=C(C1CCCC1)N1CC2(CCN([C@H]3CCc4ccccc43)C2)C1	1072386704	24	324.22	3.4
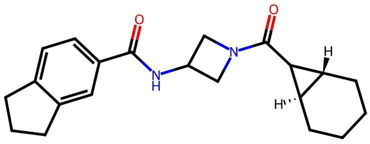	O=C(NC1CN(C(=O)C2[C@@H]3CCCC[C@@H]23)C1)c1ccc2c(c1)CCC2	991357683	25	338.2	2.55
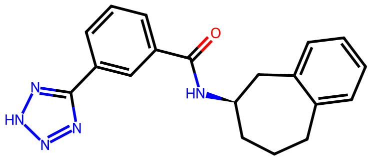	O=C(N[C@@H]1CCCc2ccccc2C1)c1cccc(-c2nn[nH]n2)c1	1592985826	25	333.16	2.54

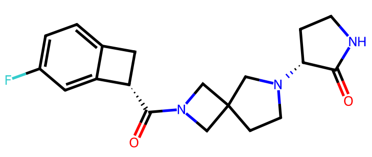	O=C1NCC[C@H]1N1CCC2(CN(C(=O)[C@H]3Cc4ccc(F)cc43)C2)C1	1072918207	25	343.17	0.89
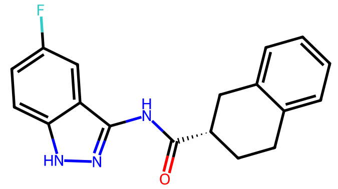	O=C(Nc1n[nH]c2ccc(F)cc12)[C@H]1CCc2ccccc2C1	1628081702	23	309.13	3.45
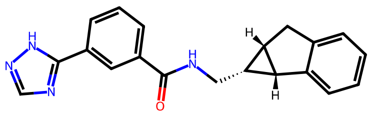	O=C(NC[C@H]1[C@H]2c3ccccc3C[C@H]21)c1cccc(-c2ncn[nH]2)c1	157757754	25	330.15	2.79
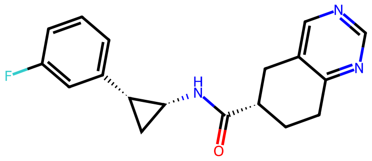	O=C(N[C@@H]1C[C@@H]1c1cccc(F)c1)[C@H]1CCc2ncncc2C1	648260540	23	311.14	2.39
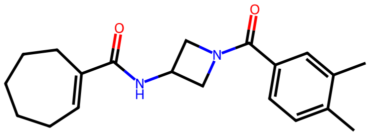	Cc1ccc(C(=O)N2CC(NC(=O)C3=CCCCCC3)C2)cc1C	991008946	24	326.2	3.13
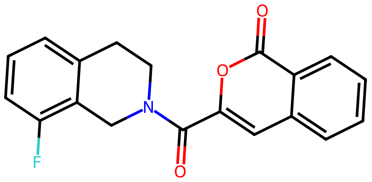	O=C(c1cc2ccccc2c(=O)o1)N1CCc2cccc(F)c2C1	337112614	24	323.1	3.13
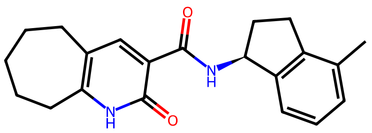	Cc1cccc2c1CC[C@@H]2NC(=O)c1cc2c([nH]c1=O)CCCCC2	78116481	25	336.18	3.37
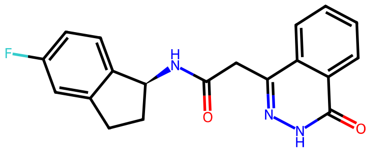	O=C(Cc1n[nH]c(=O)c2ccccc12)N[C@H]1CCc2cc(F)ccc21	55656474	25	337.12	2.41

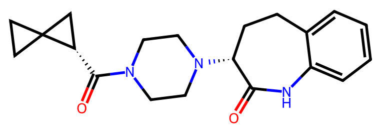	O=C1Nc2ccccc2CC[C@H]1N1CCN(C(=O)[C@H]2CC23CC3)CC1	950707452	25	339.19	1.88
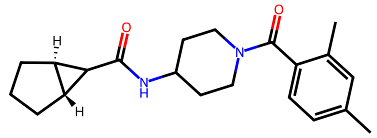	Cc1ccc(C(=O)N2CCC(NC(=O)C3[C@@H]4CCC[C@@H]34)CC2)c(C)c1	948346348	25	340.22	3.07
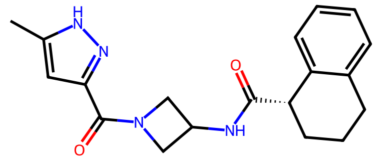	Cc1cc(C(=O)N2CC(NC(=O)[C@H]3CCCc4ccccc43)C2)n[nH]1	995069779	25	338.17	1.78
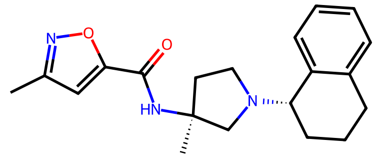	Cc1cc(C(=O)N[C@@]2(C)CCN([C@H]3CCCc4ccccc43)C2)on1	1046139312	25	339.19	3.25
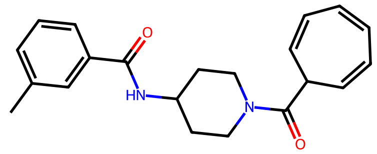	Cc1cccc(C(=O)NC2CCN(C(=O)C3C=CC=CC=C3)CC2)c1	946948571	25	336.18	3.01
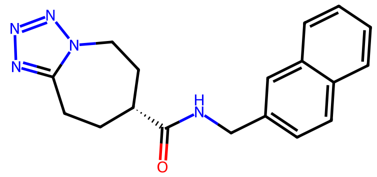	O=C(NCc1ccc2ccccc2c1)[C@@H]1CCc2nnnn2CC1	651074466	24	321.16	2.1
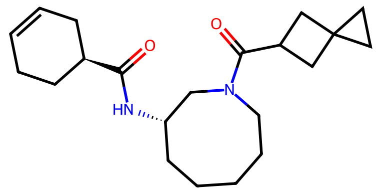	O=C(N[C@H]1CCCCCN(C(=O)C2CC3(CC3)C2)C1)[C@@H]1CC=CCC1	987155345	25	344.25	3.42
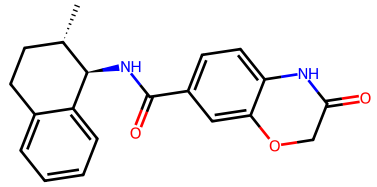	C[C@H]1CCc2ccccc2[C@@H]1NC(=O)c1ccc2c(c1)OCC(=O)N2	179463715	25	336.15	3.07

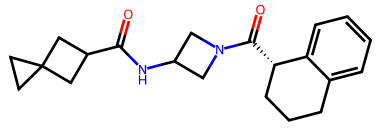	O=C(NC1CN(C(=O)[C@H]2CCCc3ccccc32)C1)C1CC2(CC2)C1	999344267	25	338.2	2.62
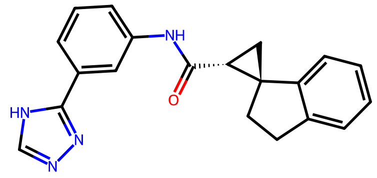	O=C(Nc1cccc(-c2nnc[nH]2)c1)[C@@H]1C[C@@]12CCc1ccccc12	266489973	25	330.15	3.31
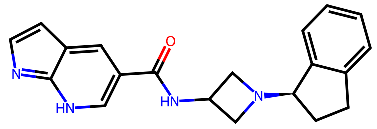	O=C(NC1CN([C@@H]2CCc3ccccc32)C1)c1c[nH]c2nccc-2c1	1030482100	25	332.16	2.62
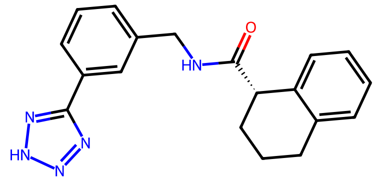	O=C(NCc1cccc(-c2nn[nH]n2)c1)[C@H]1CCCc2ccccc21	1606835304	25	333.16	2.6
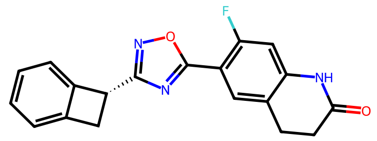	O=C1CCc2cc(-c3nc([C@@H]4Cc5ccccc54)no3)c(F)cc2N1	544988221	25	335.11	3.45
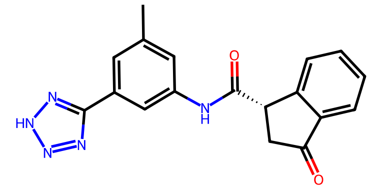	Cc1cc(NC(=O)[C@H]2CC(=O)c3ccccc32)cc(-c2nn[nH]n2)c1	1592016802	25	333.12	2.48
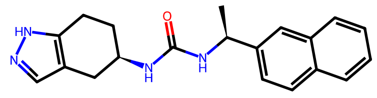	C[C@H](NC(=O)N[C@@H]1CCc2[nH]ncc2C1)c1ccc2ccccc2c1	154163713	25	334.18	3.48
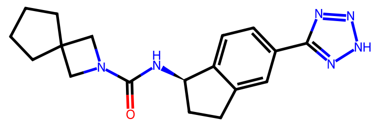	O=C(N[C@@H]1CCc2cc(-c3nn[nH]n3)ccc21)N1CC2(CCCC2)C1	1606686762	25	338.19	2.44

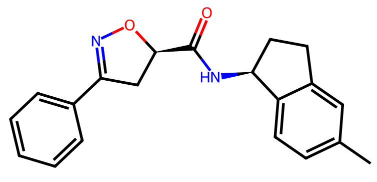	Cc1ccc2c(c1)CC[C@@H]2NC(=O)[C@H]1CC(c2ccccc2)=NO1	56317064	24	320.15	3.29
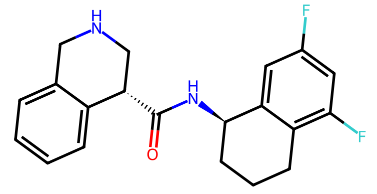	O=C(N[C@@H]1CCCc2c(F)cc(F)cc21)[C@H]1CNCc2ccccc21	1580742431	25	342.15	3.35
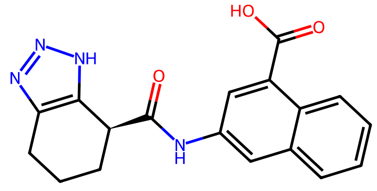	O=C(O)c1cc(NC(=O)[C@H]2CCCc3nn[nH]c32)cc2ccccc12	1602280607	25	336.12	2.71
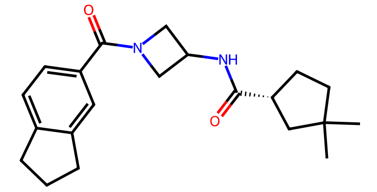	CC1(C)CC[C@@H](C(=O)NC2CN(C(=O)c3ccc4c(c3)CCC4)C2)C1	999237375	25	340.22	2.94
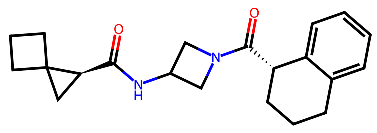	O=C(NC1CN(C(=O)[C@H]2CCCc3ccccc32)C1)[C@H]1CC12CCC2	991554318	25	338.2	2.62
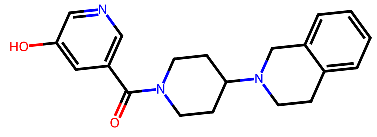	O=C(c1cncc(O)c1)N1CCC(N2CCc3ccccc3C2)CC1	264913550	25	337.18	2.45
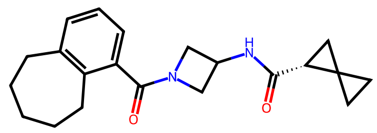	O=C(NC1CN(C(=O)c2cccc3c2CCCCC3)C1)[C@@H]1CC12CC2	1000513811	25	338.2	2.7
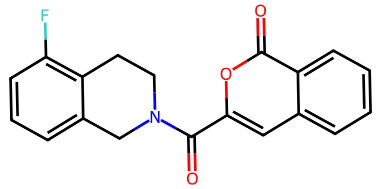	O=C(c1cc2ccccc2c(=O)o1)N1CCc2c(F)cccc2C1	337081806	24	323.1	3.13

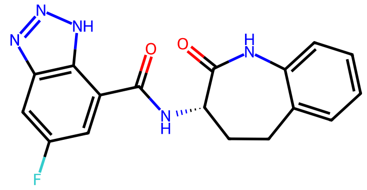	O=C(N[C@H]1CCc2ccccc2NC1=O)c1cc(F)cc2nn[nH]c12	1631674984	25	339.11	1.78
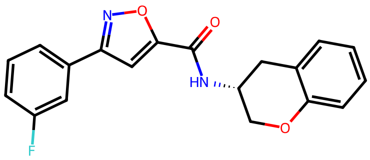	O=C(N[C@H]1COc2ccccc2C1)c1cc(-c2cccc(F)c2)no1	53046838	25	338.11	3.21
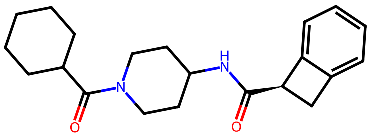	O=C(NC1CCN(C(=O)C2CCCCC2)CC1)[C@@H]1Cc2ccccc21	943758132	25	340.22	3.01
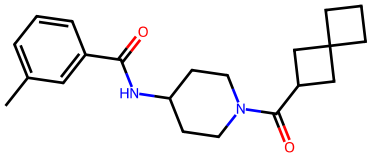	Cc1cccc(C(=O)NC2CCN(C(=O)C3CC4(CCC4)C3)CC2)c1	870838728	25	340.22	3.3
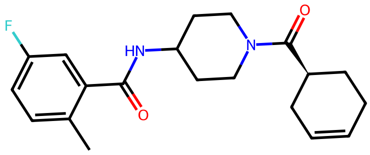	Cc1ccc(F)cc1C(=O)NC1CCN(C(=O)[C@@H]2CC=CCC2)CC1	944126539	25	344.19	3.21
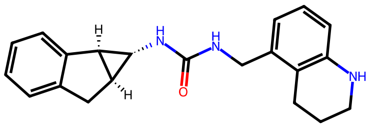	O=C(NCc1cccc2c1CCCN2)N[C@@H]1[C@H]2Cc3ccccc3[C@H]21	573701143	25	333.18	3.18
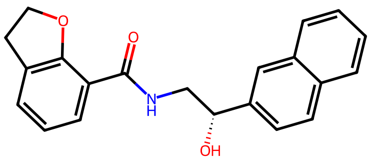	O=C(NC[C@@H](O)c1ccc2ccccc2c1)c1cccc2c1OCC2	1137389096	25	333.14	3.24
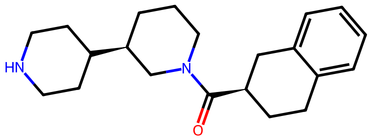	O=C([C@@H]1CCc2ccccc2C1)N1CCC[C@H](C2CCNCC2)C1	662590334	24	326.24	3.03

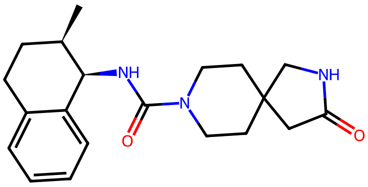	C[C@@H]1CCc2ccccc2[C@@H]1NC(=O)N1CCC2(CC1)CNC(=O)C2	270729631	25	341.21	2.62
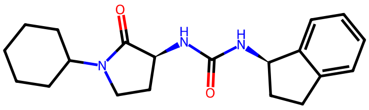	O=C(N[C@H]1CCN(C2CCCCC2)C1=O)N[C@@H]1CCc2ccccc21	159001745	25	341.21	2.91
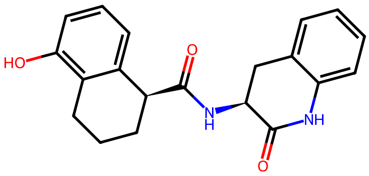	O=C1Nc2ccccc2C[C@@H]1NC(=O)[C@H]1CCCc2c(O)cccc21	529949076	25	336.15	2.49
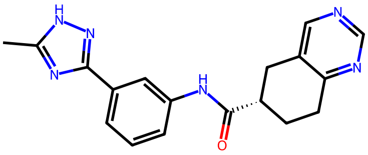	Cc1nc(-c2cccc(NC(=O)[C@H]3CCc4ncncc4C3)c2)n[nH]1	648251188	25	334.15	2.31
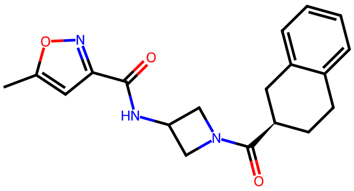	Cc1cc(C(=O)NC2CN(C(=O)[C@@H]3CCc4ccccc4C3)C2)no1	990175073	25	339.16	1.73
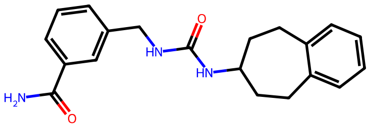	NC(=O)c1cccc(CNC(=O)NC2CCc3ccccc3CC2)c1	565758110	25	337.18	2.53
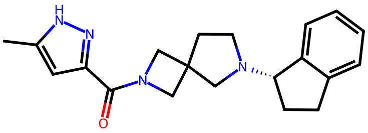	Cc1cc(C(=O)N2CC3(CCN([C@H]4CCc5ccccc54)C3)C2)n[nH]1	1072519902	25	336.2	2.55
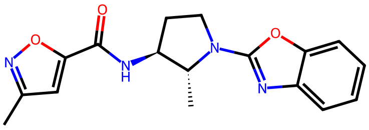	Cc1cc(C(=O)N[C@H]2CCN(c3nc4ccccc4o3)[C@@H]2C)on1	1050039836	24	326.14	2.52

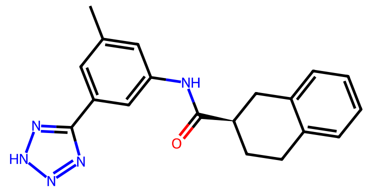	Cc1cc(NC(=O)[C@@H]2CCc3ccccc3C2)cc(-c2nn[nH]n2)c1	1592017604	25	333.16	2.92
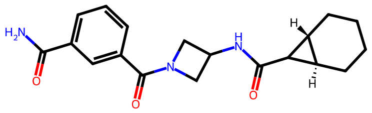	NC(=O)c1cccc(C(=O)N2CC(NC(=O)C3[C@@H]4CCCC[C@@H]34)C2)c1	990662974	25	341.17	1.16
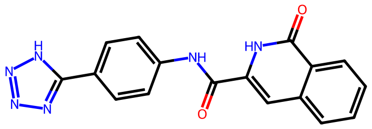	O=C(Nc1ccc(-c2nnn[nH]2)cc1)c1cc2ccccc2c(=O)[nH]1	121154817	25	332.1	1.96
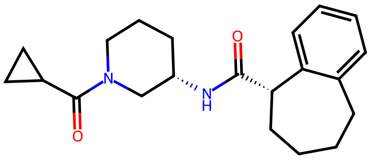	O=C(N[C@H]1CCCN(C(=O)C2CC2)C1)[C@H]1CCCCc2ccccc21	949616514	25	340.22	3.01
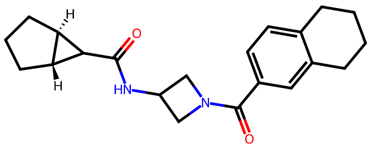	O=C(NC1CN(C(=O)c2ccc3c(c2)CCCC3)C1)C1[C@@H]2CCC[C@@H]12	990711840	25	338.2	2.55
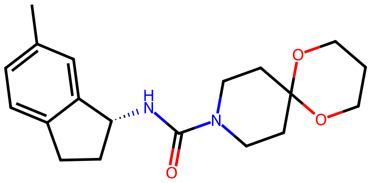	Cc1ccc2c(c1)[C@H](NC(=O)N1CCC3(CC1)OCCCO3)CC2	836289278	24	330.19	2.92
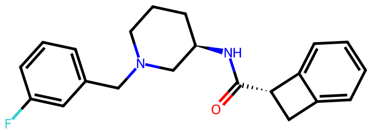	O=C(N[C@@H]1CCCN(Cc2cccc(F)c2)C1)[C@H]1Cc2ccccc21	1008359807	25	338.18	3.25
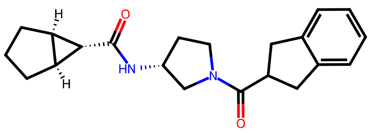	O=C(N[C@@H]1CCN(C(=O)C2Cc3ccccc3C2)C1)[C@@H]1[C@H]2CCC[C@@H]21	936250512	25	338.2	2.16

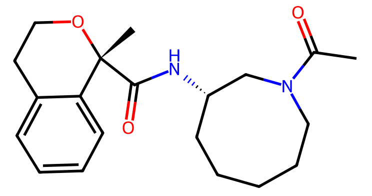	CC(=O)N1CCCCC[C@H](NC(=O)[C@@]2(C)OCCc3ccccc32)C1	986795753	25	344.21	2.38
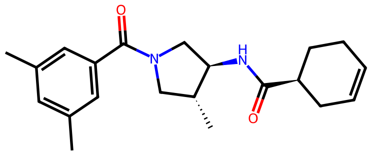	Cc1cc(C)cc(C(=O)N2C[C@@H](NC(=O)[C@@H]3CC=CCC3)[C@H](C)C2)c1	959616797	25	340.22	3.24
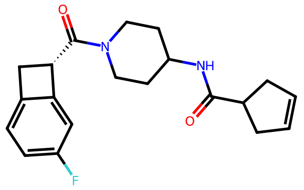	O=C(NC1CCN(C(=O)[C@H]2Cc3ccc(F)cc32)CC1)C1CC=CC1	948024591	25	342.17	2.54
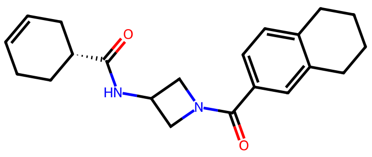	O=C(NC1CN(C(=O)c2ccc3c(c2)CCCC3)C1)[C@H]1CC=CCC1	990214388	25	338.2	2.86
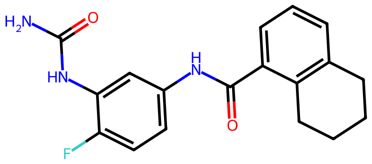	NC(=O)Nc1cc(NC(=O)c2cccc3c2CCCC3)ccc1F	1141542828	24	327.14	3.45
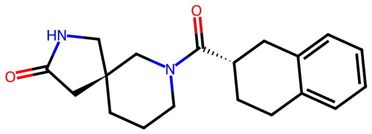	O=C1C[C@@]2(CCCN(C(=O)[C@H]3CCc4ccccc4C3)C2)CN1	579013743	23	312.18	1.92
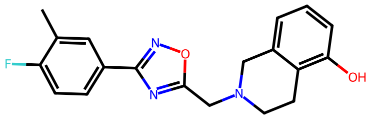	Cc1cc(-c2noc(CN3CCc4c(O)cccc4C3)n2)ccc1F	626027157	25	339.14	3.45
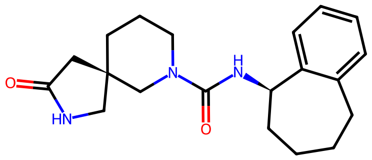	O=C1C[C@]2(CCCN(C(=O)N[C@@H]3CCCCc4ccccc43)C2)CN1	525766804	25	341.21	2.77

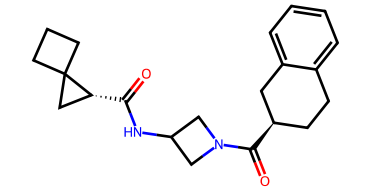	O=C(NC1CN(C(=O)[C@@H]2CCc3ccccc3C2)C1)[C@@H]1CC12CCC2	991549037	25	338.2	2.31
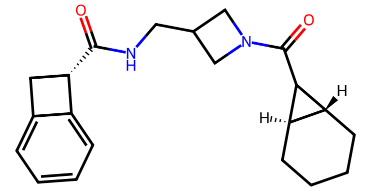	O=C(NCC1CN(C(=O)C2[C@@H]3CCCC[C@@H]23)C1)[C@H]1Cc2ccccc21	1003949083	25	338.2	2.34
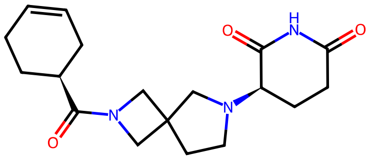	O=C1CC[C@@H](N2CCC3(CN(C(=O)[C@@H]4CC=CCC4)C3)C2)C(=O)N1	1072457646	24	331.19	0.68
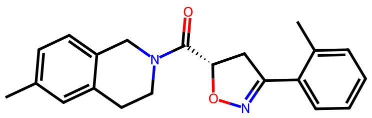	Cc1ccc2c(c1)CCN(C(=O)[C@@H]1CC(c3ccccc3C)=NO1)C2	1536809064	25	334.17	3.38
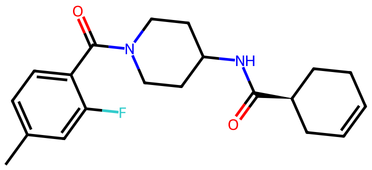	Cc1ccc(C(=O)N2CCC(NC(=O)[C@@H]3CC=CCC3)CC2)c(F)c1	948557605	25	344.19	3.21
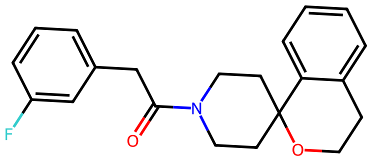	O=C(Cc1cccc(F)c1)N1CCC2(CC1)OCCc1ccccc12	457118367	25	339.16	3.46
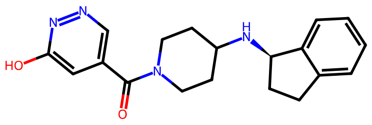	O=C(c1cnnc(O)c1)N1CCC(N[C@@H]2CCc3ccccc32)CC1	996701169	25	338.17	2.06
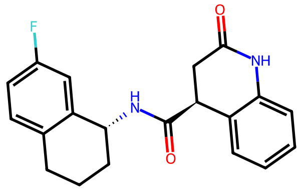	O=C1C[C@H](C(=O)N[C@@H]2CCCc3ccc(F)cc32)c2ccccc2N1	93062919	25	338.14	3.45

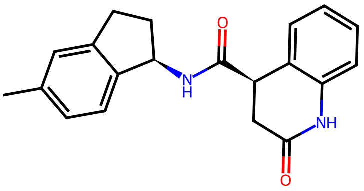	Cc1ccc2c(c1)CC[C@H]2NC(=O)[C@@H]1CC(=O)Nc2ccccc21	56316911	24	320.15	3.22
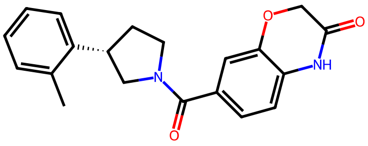	Cc1ccccc1[C@@H]1CCN(C(=O)c2ccc3c(c2)OCC(=O)N3)C1	299726619	25	336.15	2.96
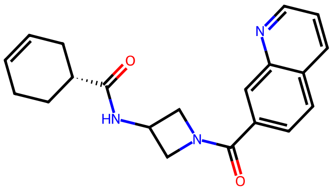	O=C(NC1CN(C(=O)c2ccc3cccnc3c2)C1)[C@H]1CC=CCC1	990211792	25	335.16	2.53
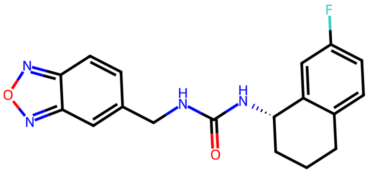	O=C(NCc1ccc2nonc2c1)N[C@H]1CCCc2ccc(F)cc21	1478565362	25	340.13	3.24
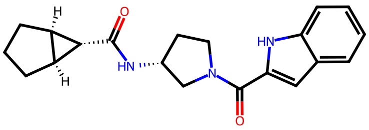	O=C(N[C@@H]1CCN(C(=O)c2cc3ccccc3[nH]2)C1)[C@@H]1[C@H]2CCC[C@H]21	936158053	25	337.18	2.54
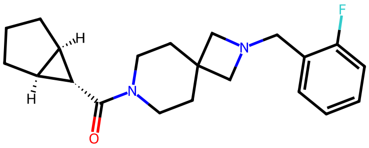	O=C([C@@H]1[C@H]2CCC[C@H]21)N1CCC2(CC1)CN(Cc1ccccc1F)C2	1035679276	25	342.21	3.3
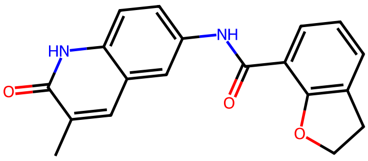	Cc1cc2cc(NC(=O)c3cccc4c3OCC4)ccc2[nH]c1=O	1159154612	24	320.12	3.02
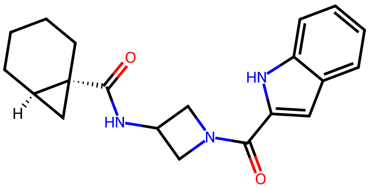	O=C(c1cc2ccccc2[nH]1)N1CC(NC(=O)[C@@]23CCCC[C@@H]2C3)C1	999281463	25	337.18	2.69

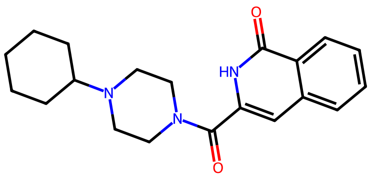	O=C(c1cc2ccccc2c(=O)[nH]1)N1CCN(C2CCCCC2)CC1	154538198	25	339.19	2.62
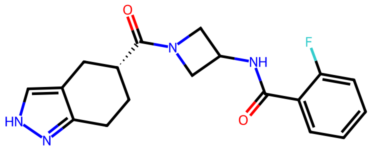	O=C(NC1CN(C(=O)[C@@H]2CCc3n[nH]cc3C2)C1)c1ccccc1F	990089457	25	342.15	1.29
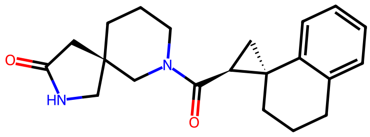	O=C1C[C@]2(CCCN(C(=O)[C@H]3C[C@]34CCCc3ccccc34)C2)CN1	341506615	25	338.2	2.41
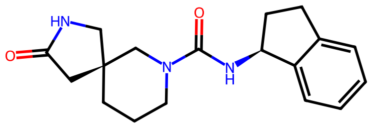	O=C1C[C@@]2(CCCN(C(=O)N[C@H]3CCc4ccccc43)C2)CN1	340919598	23	313.18	1.99
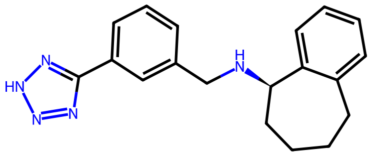	c1cc(CN[C@@H]2CCCCc3ccccc32)cc(-c2nn[nH]n2)c1	738410791	24	319.18	3.42
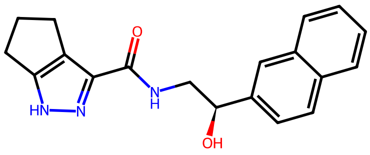	O=C(NC[C@H](O)c1ccc2ccccc2c1)c1n[nH]c2c1CCC2	109690459	24	321.15	2.52
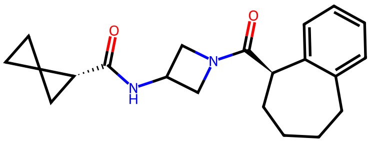	O=C(NC1CN(C(=O)[C@@H]2CCCCc3ccccc32)C1)[C@@H]1CC12CC2	1000511984	25	338.2	2.62
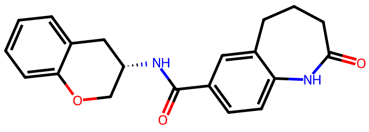	O=C1CCCc2cc(C(=O)N[C@@H]3COc4ccccc4C3)ccc2N1	613756983	25	336.15	2.69

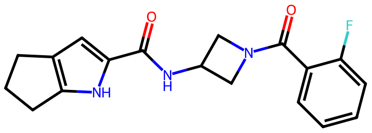	O=C(NC1CN(C(=O)c2ccccc2F)C1)c1cc2c([nH]1)CCC2	995911455	24	327.14	1.9
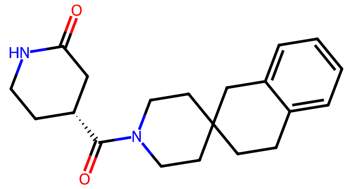	O=C1C[C@H](C(=O)N2CCC3(CCc4ccccc4C3)CC2)CCN1	652524944	24	326.2	2.31
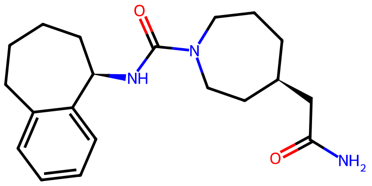	NC(=O)C[C@@H]1CCCN(C(=O)N[C@@H]2CCCCc3ccccc32)CC1	671571533	25	343.23	3.14
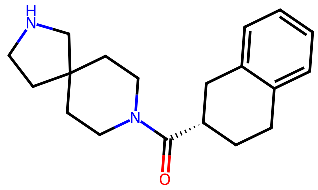	O=C([C@H]1CCc2ccccc2C1)N1CCC2(CCNC2)CC1	578759260	22	298.2	2.39
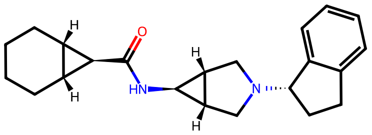	O=C(N[C@H]1[C@@H]2CN([C@H]3CCc4ccccc43)C[C@@H]21)[C@@H]1[C@H]2CCCC[C@H]21	1025723609	25	336.22	3.16
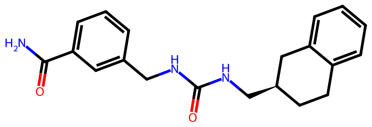	NC(=O)c1cccc(CNC(=O)NC[C@@H]2CCc3ccccc3C2)c1	605011865	25	337.18	2.39
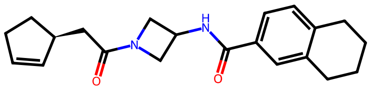	O=C(NC1CN(C(=O)C[C@H]2C=CCC2)C1)c1ccc2c(c1)CCCC2	1000060449	25	338.2	2.86
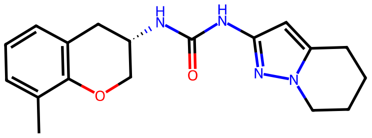	Cc1cccc2c1OC[C@@H](NC(=O)Nc1cc3n(n1)CCCC3)C2	290726599	24	326.17	2.65

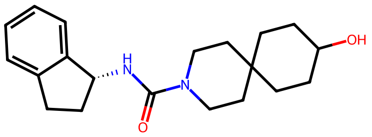	O=C(N[C@@H]1CCc2ccccc21)N1CCC2(CCC(O)CC2)CC1	297566094	24	328.22	3.4
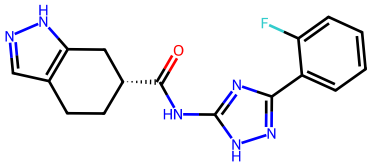	O=C(Nc1nc(-c2ccccc2F)n[nH]1)[C@@H]1CCc2cn[nH]c2C1	266486531	24	326.13	2.08
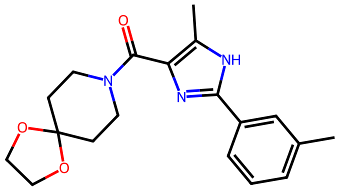	Cc1cccc(-c2nc(C(=O)N3CCC4(CC3)OCCO4)c(C)[nH]2)c1	270134203	25	341.17	2.67
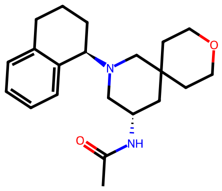	CC(=O)N[C@@H]1CN([C@@H]2CCCc3ccccc32)CC2(CCOCC2)C1	1089916368	25	342.23	3.07
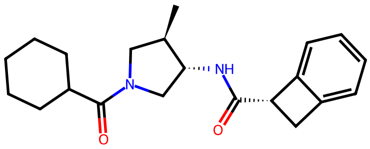	C[C@@H]1CN(C(=O)C2CCCCC2)C[C@H]1NC(=O)[C@H]1Cc2ccccc21	939656308	25	340.22	2.87
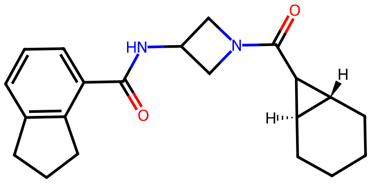	O=C(NC1CN(C(=O)C2[C@@H]3CCCC[C@@H]23)C1)c1cccc2c1CCC2	994404975	25	338.2	2.55
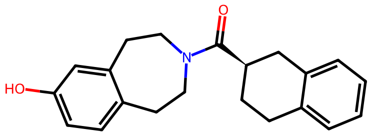	O=C([C@@H]1CCc2ccccc2C1)N1CCc2ccc(O)cc2CC1	653018831	24	321.17	3.12
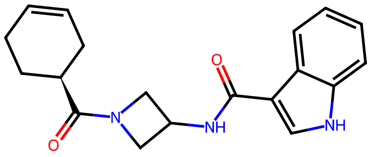	O=C(NC1CN(C(=O)[C@@H]2CC=CCC2)C1)c1c[nH]c2ccccc12	995030686	24	323.16	2.46

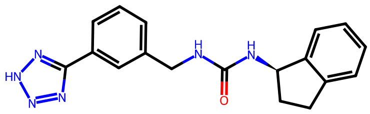	O=C(NCc1cccc(-c2nn[nH]n2)c1)N[C@@H]1CCc2ccccc21	1593143488	25	334.15	2.35
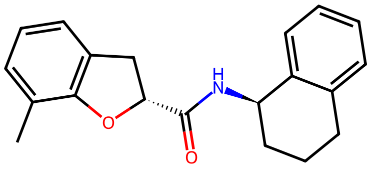	Cc1cccc2c1O[C@@H](C(=O)N[C@@H]1CCCc3ccccc31)C2	1616543289	23	307.16	3.49
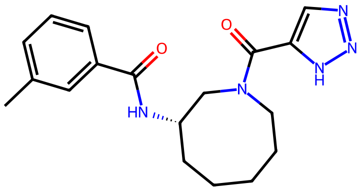	Cc1cccc(C(=O)N[C@H]2CCCCCN(C(=O)c3cnn[nH]3)C2)c1	987073651	25	341.19	1.93
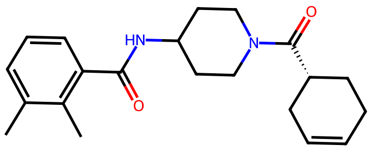	Cc1cccc(C(=O)NC2CCN(C(=O)[C@H]3CC=CCC3)CC2)c1C	944107794	25	340.22	3.38
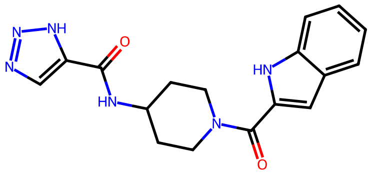	O=C(NC1CCN(C(=O)c2cc3ccccc3[nH]2)CC1)c1cnn[nH]1	948490617	25	338.15	1.32
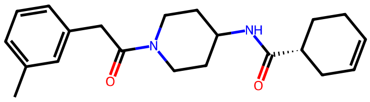	Cc1cccc(CC(=O)N2CCC(NC(=O)[C@H]3CC=CCC3)CC2)c1	948068620	25	340.22	3.0
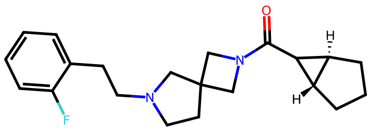	O=C(C1[C@H]2CCC[C@H]12)N1CC2(CCN(CCc3ccccc3F)C2)C1	1072590714	25	342.21	2.95
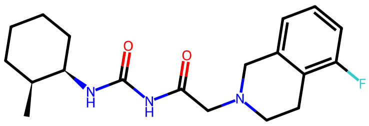	C[C@H]1CCCC[C@H]1NC(=O)NC(=O)CN1CCc2c(F)cccc2C1	339779917	25	347.2	2.59

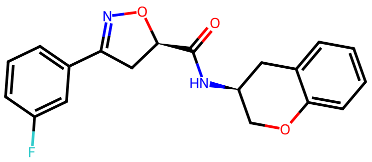	O=C(N[C@@H]1COc2ccccc2C1)[C@H]1CC(c2cccc(F)c2)=NO1	194461062	25	340.12	2.44
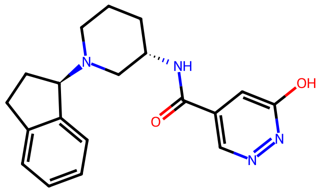	O=C(N[C@H]1CCCN([C@@H]2CCc3ccccc32)C1)c1cnnc(O)c1	1007549047	25	338.17	2.06
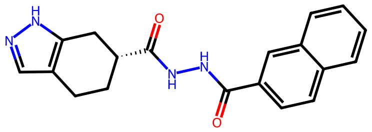	O=C(NNC(=O)[C@@H]1CCc2cn[nH]c2C1)c1ccc2ccccc2c1	79629025	25	334.14	2.13
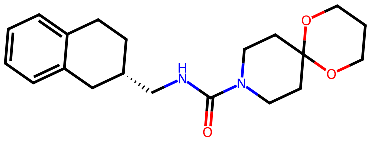	O=C(NC[C@H]1CCc2ccccc2C1)N1CCC2(CC1)OCCCO2	836309957	25	344.21	2.73
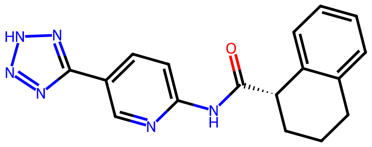	O=C(Nc1ccc(-c2nn[nH]n2)cn1)[C@H]1CCCc2ccccc21	738150189	24	320.14	2.32
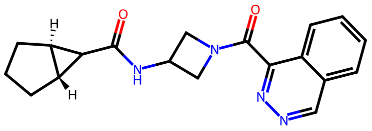	O=C(NC1CN(C(=O)c2nncc3ccccc23)C1)C1[C@@H]2CCC[C@@H]12	990705789	25	336.16	1.62
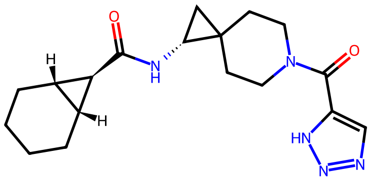	O=C(N[C@@H]1CC12CCN(C(=O)c1cnn[nH]1)CC2)[C@@H]1[C@H]2CCCC[C@H]21	963448780	25	343.2	1.35
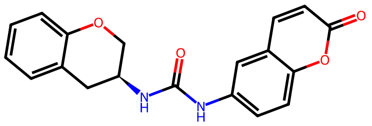	O=C(Nc1ccc2oc(=O)ccc2c1)N[C@@H]1COc2ccccc2C1	194784267	25	336.11	2.92

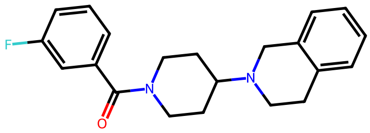	O=C(c1cccc(F)c1)N1CCC(N2CCc3ccccc3C2)CC1	264237014	25	338.18	3.49
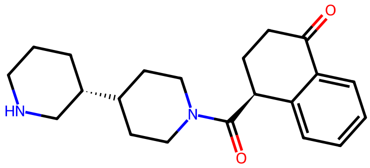	O=C1CC[C@H](C(=O)N2CCC([C@H]3CCCNC3)CC2)c2ccccc21	662587756	25	340.22	2.98
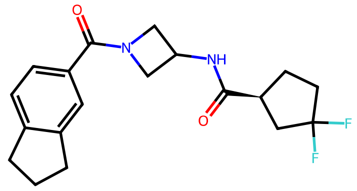	O=C(NC1CN(C(=O)c2ccc3c(c2)CCC3)C1)[C@H]1CCC(F)(F)C1	994162076	25	348.16	2.55
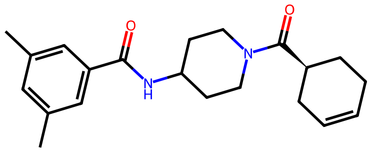	Cc1cc(C)cc(C(=O)NC2CCN(C(=O)[C@@H]3CC=CCC3)CC2)c1	944106877	25	340.22	3.38
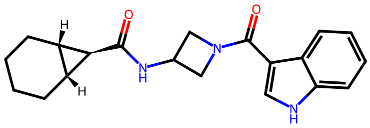	O=C(NC1CN(C(=O)c2c[nH]c3ccccc23)C1)[C@@H]1[C@H]2CCCC[C@H]21	990665771	25	337.18	2.54
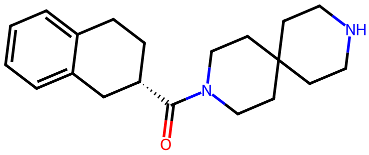	O=C([C@H]1CCc2ccccc2C1)N1CCC2(CCNCC2)CC1	324079811	23	312.22	2.78
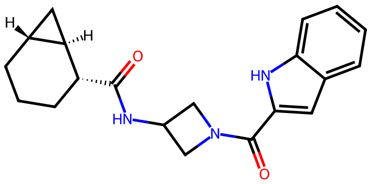	O=C(NC1CN(C(=O)c2cc3ccccc3[nH]2)C1)[C@@H]1CCC[C@@H]2C[C@H]21	994727568	25	337.18	2.54
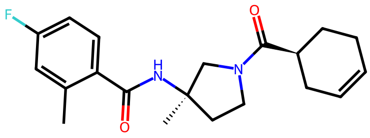	Cc1cc(F)ccc1C(=O)N[C@]1(C)CCN(C(=O)[C@@H]2CC=CCC2)C1	957256364	25	344.19	3.21

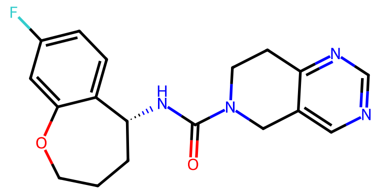	O=C(N[C@@H]1CCCOc2cc(F)ccc21)N1CCc2ncncc2C1	883556284	25	342.15	2.6
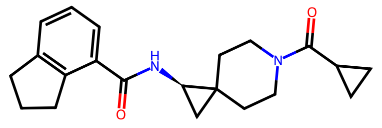	O=C(N[C@@H]1CC12CCN(C(=O)C1CC1)CC2)c1cccc2c1CCC2	964032702	25	338.2	2.7
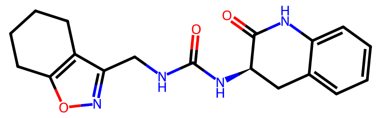	O=C(NCc1noc2c1CCCC2)N[C@@H]1Cc2ccccc2NC1=O	637150292	25	340.15	1.92
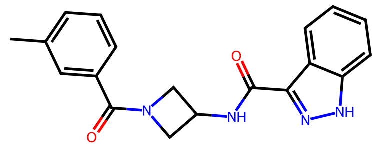	Cc1cccc(C(=O)N2CC(NC(=O)c3n[nH]c4ccccc34)C2)c1	999525617	25	334.14	2.13
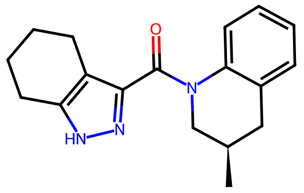	C[C@@H]1Cc2ccccc2N(C(=O)c2n[nH]c3c2CCCC3)C1	62222683	22	295.17	3.13
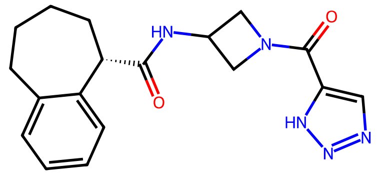	O=C(NC1CN(C(=O)c2cnn[nH]2)C1)[C@H]1CCCCc2ccccc21	994322937	25	339.17	1.26
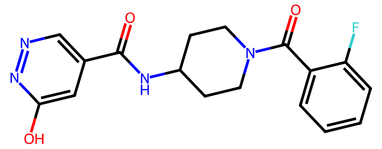	O=C(NC1CCN(C(=O)c2ccccc2F)CC1)c1cnnc(O)c1	943729895	25	344.13	1.36
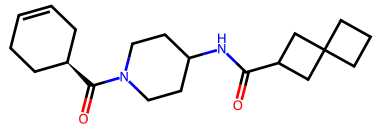	O=C(NC1CCN(C(=O)[C@@H]2CC=CCC2)CC1)C1CC2(CCC2)C1	944127169	24	330.23	3.03

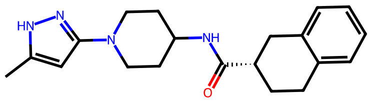	Cc1cc(N2CCC(NC(=O)[C@H]3CCc4ccccc4C3)CC2)n[nH]1	579966435	25	338.21	2.61
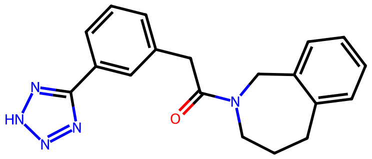	O=C(Cc1cccc(-c2nn[nH]n2)c1)N1CCCc2ccccc2C1	823087069	25	333.16	2.38
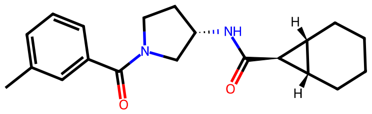	Cc1cccc(C(=O)N2CC[C@H](NC(=O)[C@@H]3[C@H]4CCCC[C@H]43)C2)c1	937787188	24	326.2	2.76
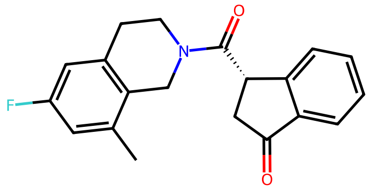	Cc1cc(F)cc2c1CN(C(=O)[C@H]1CC(=O)c3ccccc31)CC2	337329417	24	323.13	3.39
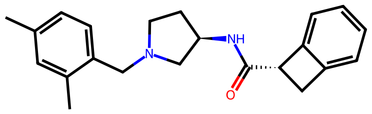	Cc1ccc(CN2CC[C@@H](NC(=O)[C@H]3Cc4ccccc43)C2)c(C)c1	1015634755	25	334.2	3.33
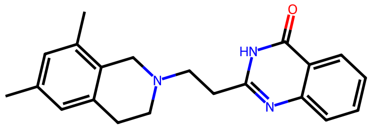	Cc1cc(C)c2c(c1)CCN(CCc1nc3ccccc3c(=O)[nH]1)C2	186460435	25	333.18	3.14
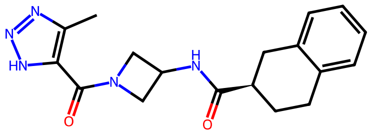	Cc1nn[nH]c1C(=O)N1CC(NC(=O)[C@@H]2CCc3ccccc3C2)C1	1000542377	25	339.17	0.86
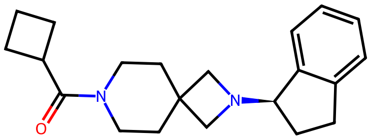	O=C(C1CCC1)N1CCC2(CC1)CN([C@@H]1CCc3ccccc31)C2	1035635245	24	324.22	3.4

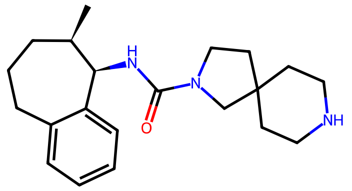	C[C@@H]1CCCc2ccccc2[C@@H]1NC(=O)N1CCC2(CCNCC2)C1	418018816	25	341.25	3.49
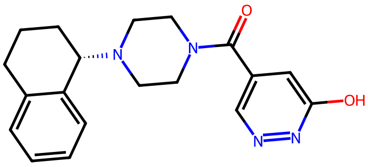	O=C(c1cnnc(O)c1)N1CCN([C@H]2CCCc3ccccc32)CC1	1374093823	25	338.17	2.02
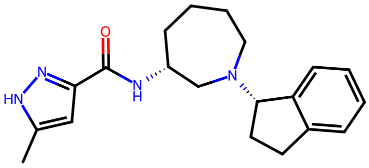	Cc1cc(C(=O)N[C@@H]2CCCCN([C@H]3CCc4ccccc43)C2)n[nH]1	1034123583	25	338.21	2.99
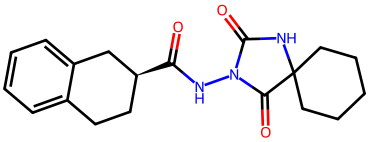	O=C(NN1C(=O)NC2(CCCCC2)C1=O)[C@H]1CCc2ccccc2C1	845120999	25	341.17	2.08
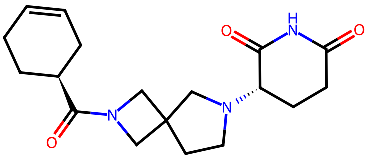	O=C1CC[C@H](N2CCC3(CN(C(=O)[C@@H]4CC=CCC4)C3)C2)C(=O)N1	1072457644	24	331.19	0.68
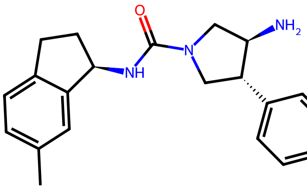	Cc1ccc2c(c1)[C@H](NC(=O)N1C[C@@H](N)[C@H](c3ccccc3)C1)CC2	841791975	25	335.2	3.12
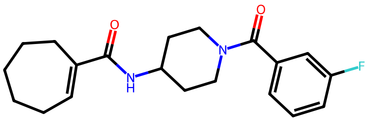	O=C(NC1CCN(C(=O)c2cccc(F)c2)CC1)C1=CCCCCC1	943774835	25	344.19	3.44
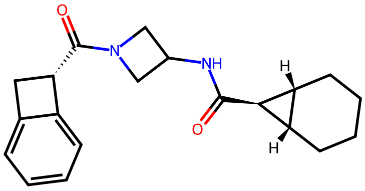	O=C(NC1CN(C(=O)[C@H]2Cc3ccccc32)C1)[C@@H]1[C@H]2CCCC[C@H]21	990649269	24	324.18	2.09

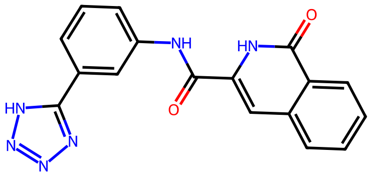	O=C(Nc1cccc(-c2nnn[nH]2)c1)c1cc2ccccc2c(=O)[nH]1	78341909	25	332.1	1.96
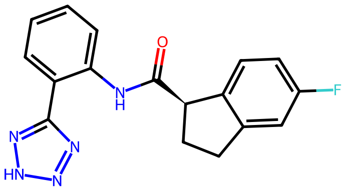	O=C(Nc1ccccc1-c1nn[nH]n1)[C@@H]1CCc2cc(F)ccc21	1586503160	24	323.12	2.67
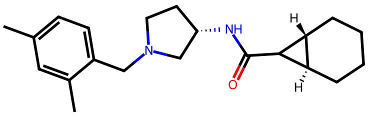	Cc1ccc(CN2CC[C@H](NC(=O)C3[C@@H]4CCCC[C@@H]34)C2)c(C)c1	1010438648	24	326.24	3.43
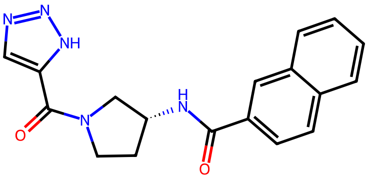	O=C(N[C@@H]1CCN(C(=O)c2cnn[nH]2)C1)c1ccc2ccccc2c1	942385905	25	335.14	1.6
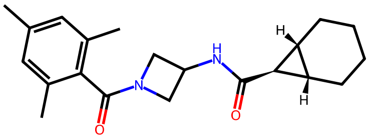	Cc1cc(C)c(C(=O)N2CC(NC(=O)[C@@H]3[C@H]4CCCC[C@H]43)C2)c(C)c1	990673973	25	340.22	2.99
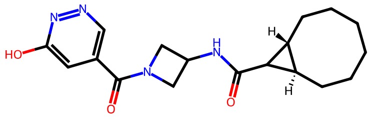	O=C(NC1CN(C(=O)c2cnnc(O)c2)C1)C1[C@@H]2CCCCCC[C@@H]12	1000510109	25	344.18	1.34
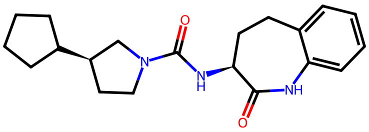	O=C1Nc2ccccc2CC[C@@H]1NC(=O)N1CC[C@@H](C2CCCC2)C1	654631103	25	341.21	3.16
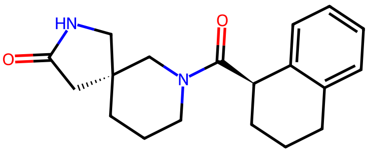	O=C1C[C@]2(CCCN(C(=O)[C@@H]3CCCc4ccccc43)C2)CN1	362206876	23	312.18	2.24

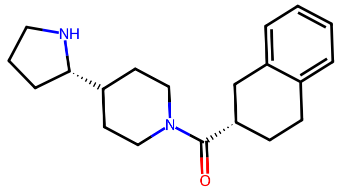	O=C([C@H]1CCc2ccccc2C1)N1CCC([C@@H]2CCCN2)CC1	324106683	23	312.22	2.78
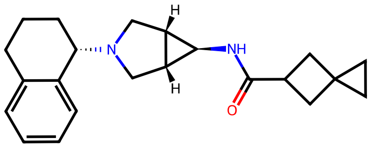	O=C(N[C@H]1[C@@H]2CN([C@H]3CCCc4ccccc43)C[C@@H]21)C1CC2(CC2)C1	961728550	25	336.22	3.3
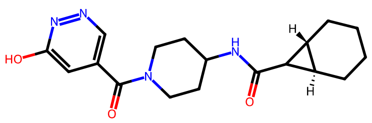	O=C(NC1CCN(C(=O)c2cnnc(O)c2)CC1)C1[C@@H]2CCCC[C@@H]12	947096281	25	344.18	1.34
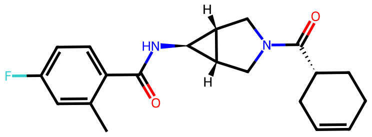	Cc1cc(F)ccc1C(=O)N[C@H]1[C@@H]2CN(C(=O)[C@H]3CC=CCC3)C[C@@H]21	976919007	25	342.17	2.68
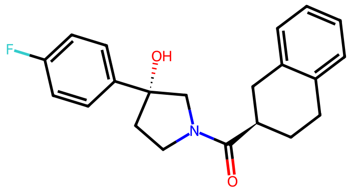	O=C([C@@H]1CCc2ccccc2C1)N1CC[C@@](O)(c2ccc(F)cc2)C1	845919478	25	339.16	3.05
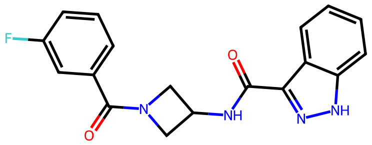	O=C(NC1CN(C(=O)c2cccc(F)c2)C1)c1n[nH]c2ccccc12	999527526	25	338.12	1.96
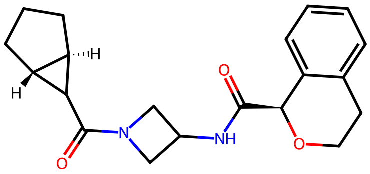	O=C(NC1CN(C(=O)C2[C@@H]3CCC[C@@H]23)C1)[C@@H]1OCCc2ccccc21	997327090	25	340.18	1.67
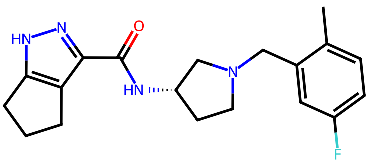	Cc1ccc(F)cc1CN1CC[C@H](NC(=O)c2n[nH]c3c2CCC3)C1	1015079415	25	342.19	2.35

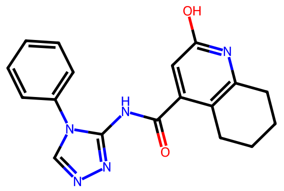	O=C(Nc1nncn1-c1ccccc1)c1cc(O)nc2c1CCCC2	1636588279	25	335.14	2.5
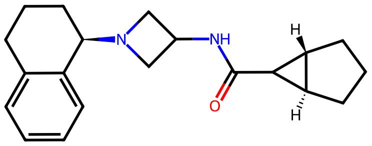	O=C(NC1CN([C@@H]2CCCc3ccccc32)C1)C1[C@@H]2CCC[C@@H]12	1030193408	23	310.2	2.91
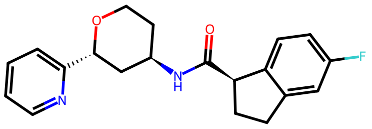	O=C(N[C@@H]1CCO[C@@H](c2ccccn2)C1)[C@@H]1CCc2cc(F)ccc21	615979991	25	340.16	3.29
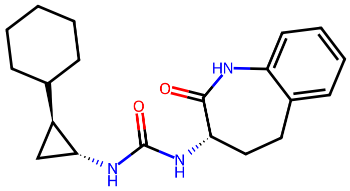	O=C(N[C@H]1CCc2ccccc2NC1=O)N[C@@H]1C[C@H]1C1CCCCC1	1686294303	25	341.21	3.21
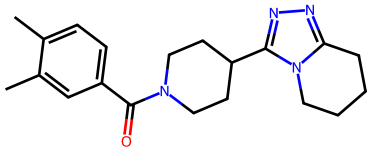	Cc1ccc(C(=O)N2CCC(c3nnc4n3CCCC4)CC2)cc1C	274687821	25	338.21	3.25
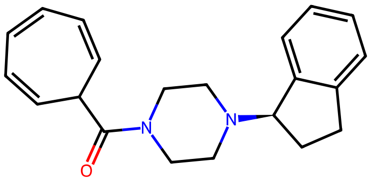	O=C(C1C=CC=CC=C1)N1CCN([C@@H]2CCc3ccccc32)CC1	948574726	24	320.19	3.12
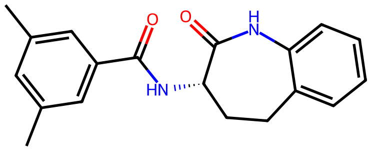	Cc1cc(C)cc(C(=O)N[C@H]2CCc3ccccc3NC2=O)c1	654006202	23	308.15	2.99
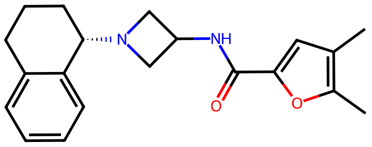	Cc1cc(C(=O)NC2CN([C@H]3CCCc4ccccc43)C2)oc1C	1030217209	24	324.18	3.39

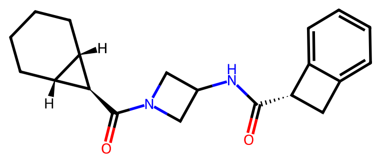	O=C(NC1CN(C(=O)[C@@H]2[C@H]3CCCC[C@H]32)C1)[C@H]1Cc2ccccc21	998680564	24	324.18	2.09
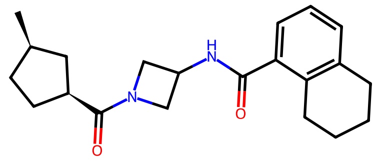	C[C@@H]1CC[C@H](C(=O)N2CC(NC(=O)c3cccc4c3CCCC4)C2)C1	991904032	25	340.22	2.94
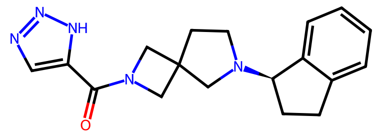	O=C(c1cnn[nH]1)N1CC2(CCN([C@@H]3CCc4ccccc43)C2)C1	1072888570	24	323.17	1.64
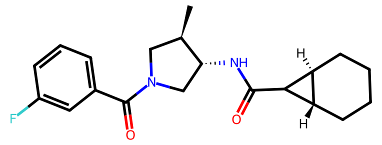	C[C@@H]1CN(C(=O)c2cccc(F)c2)C[C@H]1NC(=O)C1[C@H]2CCCC[C@H]12	947284527	25	344.19	2.84
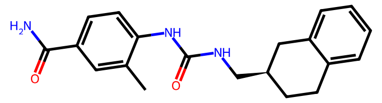	Cc1cc(C(N)=O)ccc1NC(=O)NC[C@@H]1CCc2ccccc2C1	735366960	25	337.18	3.02
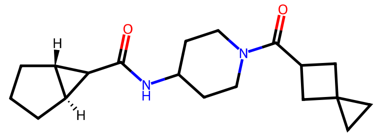	O=C(NC1CCN(C(=O)C2CC3(CC3)C2)CC1)C1[C@H]2CCC[C@H]12	948299969	23	316.22	2.33
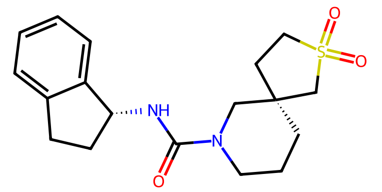	O=C(N[C@@H]1CCc2ccccc21)N1CCC[C@@]2(CCS(=O)(=O)C2)C1	333819580	24	348.15	2.28
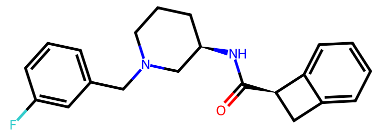	O=C(N[C@@H]1CCCN(Cc2cccc(F)c2)C1)[C@@H]1Cc2ccccc21	1008359811	25	338.18	3.25

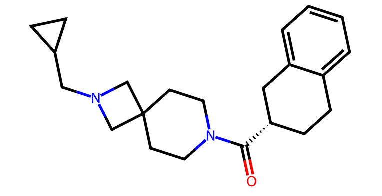	O=C([C@H]1CCc2ccccc2C1)N1CCC2(CC1)CN(CC1CC1)C2	1035807124	25	338.24	3.13
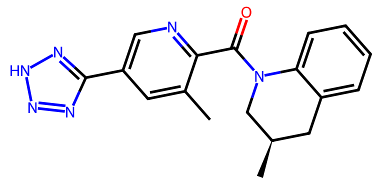	Cc1cc(-c2nn[nH]n2)cnc1C(=O)N1C[C@H](C)Cc2ccccc21	1591875294	25	334.15	2.41
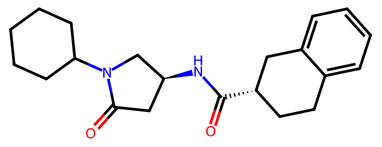	O=C(N[C@H]1CC(=O)N(C2CCCCC2)C1)[C@H]1CCc2ccccc2C1	1362286752	25	340.22	2.84
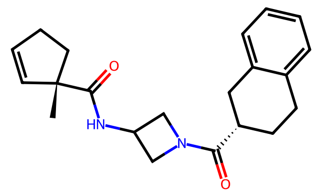	C[C@@]1(C(=O)NC2CN(C(=O)[C@H]3CCc4ccccc4C3)C2)C=CCC1	997849492	25	338.2	2.47
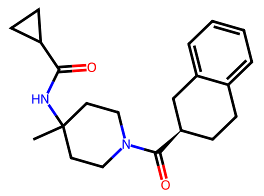	CC1(NC(=O)C2CC2)CCN(C(=O)[C@@H]2CCc3ccccc3C2)CC1	956263576	25	340.22	2.7
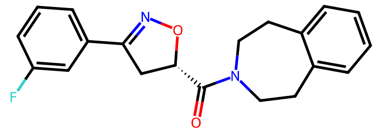	O=C([C@@H]1CC(c2cccc(F)c2)=NO1)N1CCc2ccccc2CC1	734783625	25	338.14	2.95
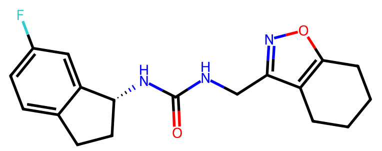	O=C(NCc1noc2c1CCCC2)N[C@@H]1CCc2ccc(F)cc21	565617192	24	329.15	3.18
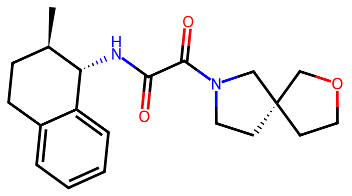	C[C@@H]1CCc2ccccc2[C@H]1NC(=O)C(=O)N1CC[C@]2(CCOC2)C1	673299621	25	342.19	2.07

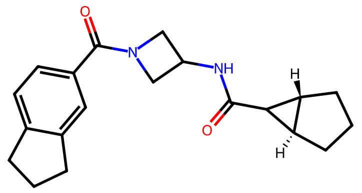	O=C(NC1CN(C(=O)c2ccc3c(c2)CCC3)C1)C1[C@@H]2CCC[C@@H]12	990707387	24	324.18	2.16
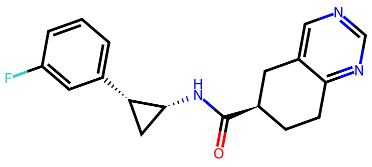	O=C(N[C@@H]1C[C@@H]1c1cccc(F)c1)[C@@H]1CCc2ncncc2C1	648260539	23	311.14	2.39
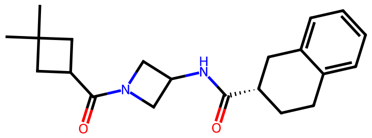	CC1(C)CC(C(=O)N2CC(NC(=O)[C@H]3CCc4ccccc4C3)C2)C1	1000538498	25	340.22	2.55
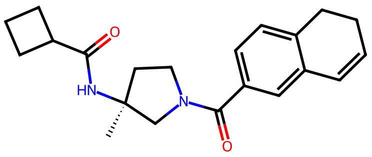	C[C@]1(NC(=O)C2CCC2)CCN(C(=O)c2ccc3c(c2)C=CCC3)C1	956624635	25	338.2	3.17
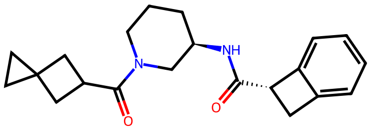	O=C(N[C@@H]1CCCN(C(=O)C2CC3(CC3)C2)C1)[C@H]1Cc2ccccc21	951964979	25	338.2	2.62
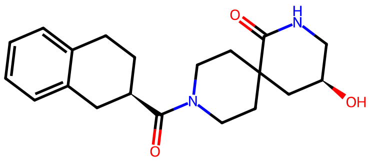	O=C([C@@H]1CCc2ccccc2C1)N1CCC2(CC1)C[C@H](O)CNC2=O	885016107	25	342.19	1.28
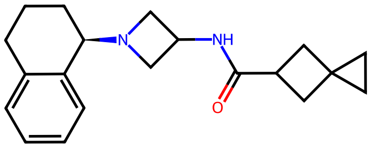	O=C(NC1CN([C@@H]2CCCc3ccccc32)C1)C1CC2(CC2)C1	1030932352	23	310.2	3.05
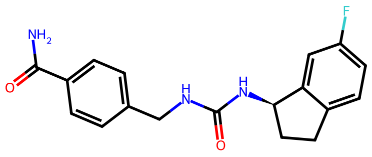	NC(=O)c1ccc(CNC(=O)N[C@@H]2CCc3ccc(F)cc32)cc1	106533269	24	327.14	2.41

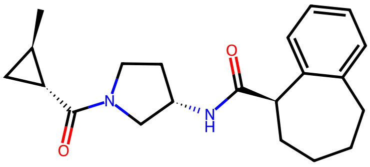	C[C@@H]1C[C@H]1C(=O)N1CC[C@H](NC(=O)[C@@H]2CCCCc3ccccc32)C1	938138994	25	340.22	2.87
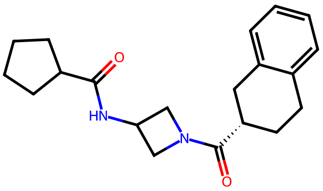	O=C(NC1CN(C(=O)[C@H]2CCc3ccccc3C2)C1)C1CCCC1	990040685	24	326.2	2.31
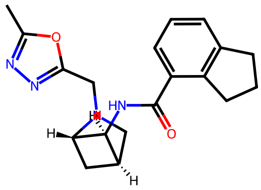	Cc1nnc(CN2C[C@H]3C[C@H]2[C@H]3NC(=O)c2cccc3c2CCC3)o1	1037747249	25	338.17	1.87
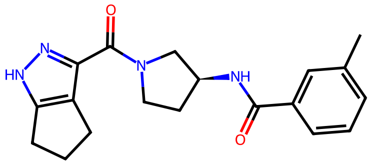	Cc1cccc(C(=O)N[C@H]2CCN(C(=O)c3n[nH]c4c3CCC4)C2)c1	937637744	25	338.17	1.85
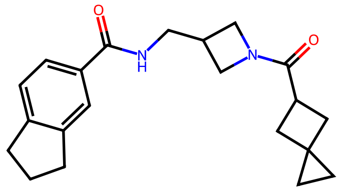	O=C(NCC1CN(C(=O)C2CC3(CC3)C2)C1)c1ccc2c(c1)CCC2	1001836083	25	338.2	2.55
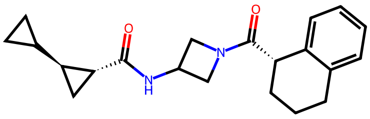	O=C(NC1CN(C(=O)[C@H]2CCCc3ccccc32)C1)[C@@H]1C[C@H]1C1CC1	992001854	25	338.2	2.48
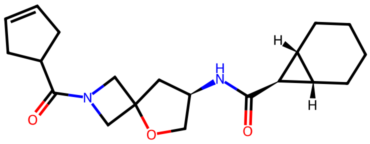	O=C(N[C@H]1COC2(C1)CN(C(=O)C1CC=CC1)C2)[C@@H]1[C@H]2CCCC[C@H]21	965651944	25	344.21	1.87
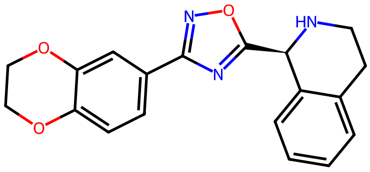	c1ccc2c(c1)CCN[C@@H]2c1nc(-c2ccc3c(c2)OCCO3)no1	822728929	25	335.13	2.74

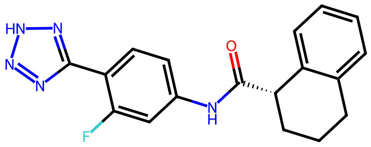	O=C(Nc1ccc(-c2nn[nH]n2)c(F)c1)[C@H]1CCCc2ccccc21	1593213305	25	337.13	3.06
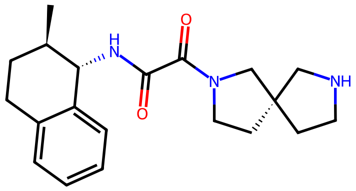	C[C@@H]1CCc2ccccc2[C@H]1NC(=O)C(=O)N1CC[C@]2(CCNC2)C1	580764514	25	341.21	1.64
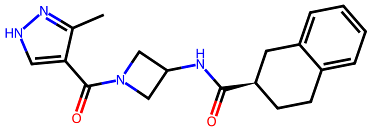	Cc1n[nH]cc1C(=O)N1CC(NC(=O)[C@@H]2CCc3ccccc3C2)C1	1000545000	25	338.17	1.46
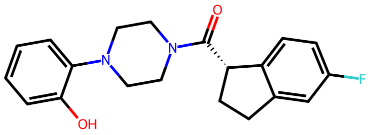	O=C([C@H]1CCc2cc(F)ccc21)N1CCN(c2ccccc2O)CC1	665126376	25	340.16	2.91
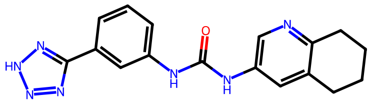	O=C(Nc1cccc(-c2nn[nH]n2)c1)Nc1cnc2c(c1)CCCC2	826456385	25	335.15	2.78
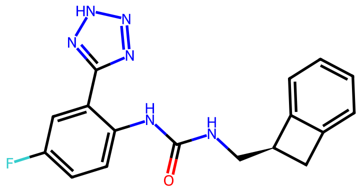	O=C(NC[C@@H]1Cc2ccccc21)Nc1ccc(F)cc1-c1nn[nH]n1	823183188	25	338.13	2.47
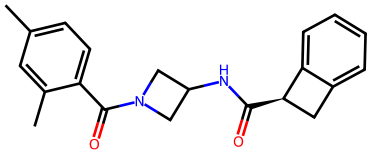	Cc1ccc(C(=O)N2CC(NC(=O)[C@@H]3Cc4ccccc43)C2)c(C)c1	998685956	25	334.17	2.58
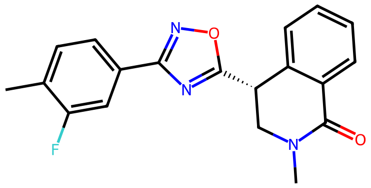	Cc1ccc(-c2noc([C@H]3CN(C)C(=O)c4ccccc43)n2)cc1F	365993148	25	337.12	3.4

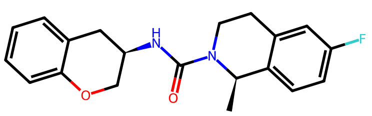	C[C@@H]1c2ccc(F)cc2CCN1C(=O)N[C@H]1COc2ccccc2C1	606644259	25	340.16	3.46
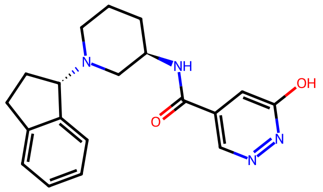	O=C(N[C@@H]1CCCN([C@H]2CCc3ccccc32)C1)c1cnnc(O)c1	1007549058	25	338.17	2.06
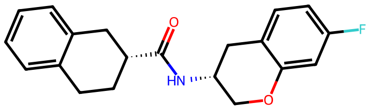	O=C(N[C@H]1COc2cc(F)ccc2C1)[C@@H]1CCc2ccccc2C1	618657624	24	325.15	3.05
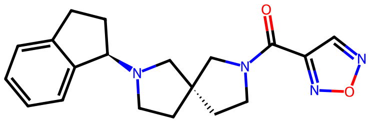	O=C(c1cnon1)N1CC[C@@]2(CCN([C@@H]3CCc4ccccc43)C2)C1	1041748427	25	338.17	2.3
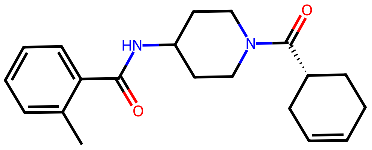	Cc1ccccc1C(=O)NC1CCN(C(=O)[C@H]2CC=CCC2)CC1	32306061	24	326.2	3.07
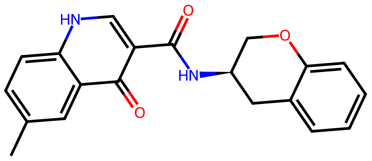	Cc1ccc2[nH]cc(C(=O)N[C@H]3COc4ccccc4C3)c(=O)c2c1	1641160324	25	334.13	2.57
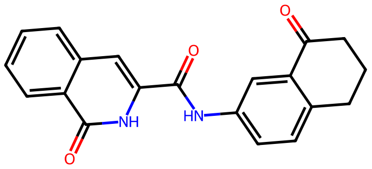	O=C(Nc1ccc2c(c1)C(=O)CCC2)c1cc2ccccc2c(=O)[nH]1	177976579	25	332.12	3.3
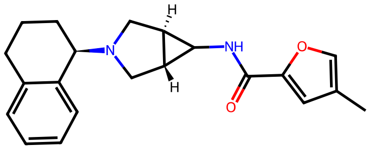	Cc1coc(C(=O)NC2[C@@H]3CN([C@@H]4CCCc5ccccc54)C[C@@H]23)c1	961517322	25	336.18	3.33

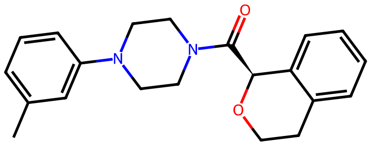	Cc1cccc(N2CCN(C(=O)[C@@H]3OCCc4ccccc43)CC2)c1	136931031	25	336.18	2.96
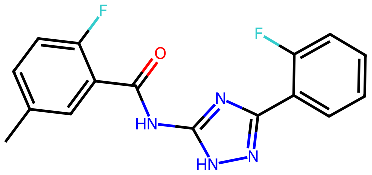	Cc1ccc(F)c(C(=O)Nc2nc(-c3ccccc3F)n[nH]2)c1	109625016	23	314.1	3.31
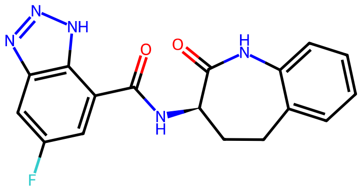	O=C(N[C@@H]1CCc2ccccc2NC1=O)c1cc(F)cc2nn[nH]c12	1631674983	25	339.11	1.78
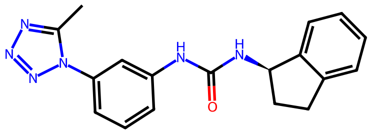	Cc1nnnn1-c1cccc(NC(=O)N[C@@H]2CCc3ccccc32)c1	45688790	25	334.15	2.78
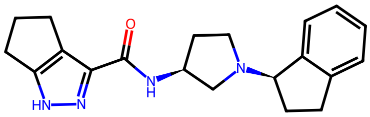	O=C(N[C@H]1CCN([C@@H]2CCc3ccccc32)C1)c1n[nH]c2c1CCC2	1015070829	25	336.2	2.39
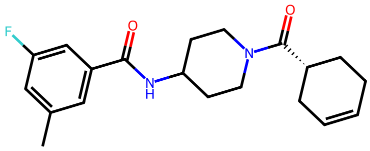	Cc1cc(F)cc(C(=O)NC2CCN(C(=O)[C@H]3CC=CCC3)CC2)c1	944127959	25	344.19	3.21
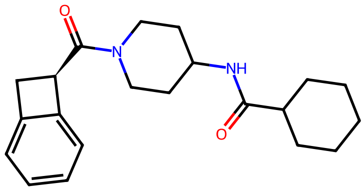	O=C(NC1CCN(C(=O)[C@@H]2Cc3ccccc32)CC1)C1CCCCC1	353779968	25	340.22	3.01
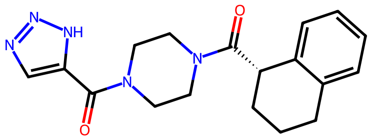	O=C(c1cnn[nH]1)N1CCN(C(=O)[C@H]2CCCc3ccccc32)CC1	1011403009	25	339.17	1.21

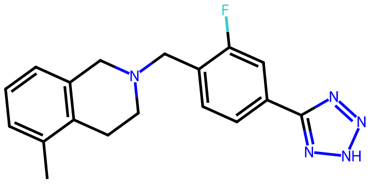	Cc1cccc2c1CCN(Cc1ccc(-c3nn[nH]n3)cc1F)C2	737408673	24	323.15	2.87
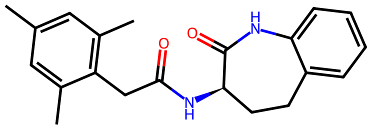	Cc1cc(C)c(CC(=O)N[C@@H]2CCc3ccccc3NC2=O)c(C)c1	654000801	25	336.18	3.22
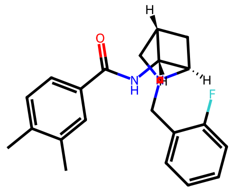	Cc1ccc(C(=O)N[C@@H]2[C@H]3C[C@H]2N(Cc2ccccc2F)C3)cc1C	1037711755	25	338.18	3.45
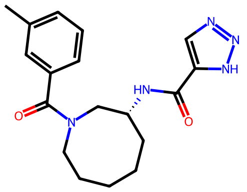	Cc1cccc(C(=O)N2CCCCC[C@@H](NC(=O)c3cnn[nH]3)C2)c1	986854648	25	341.19	1.93
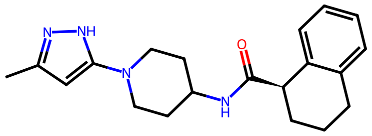	Cc1cc(N2CCC(NC(=O)[C@@H]3CCCc4ccccc43)CC2)[nH]n1	275993221	25	338.21	2.92
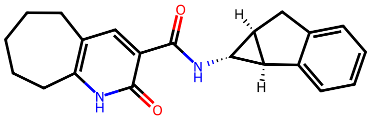	O=C(N[C@@H]1[C@H]2Cc3ccccc3[C@H]21)c1cc2c([nH]c1=O)CCCCC2	130041264	25	334.17	2.71
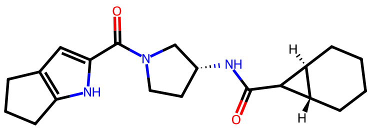	O=C(N[C@@H]1CCN(C(=O)c2cc3c([nH]2)CCC3)C1)C1[C@H]2CCCC[C@H]12	939895591	25	341.21	2.27
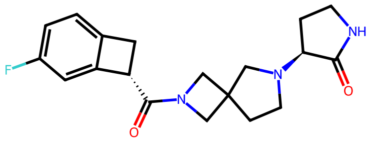	O=C1NCC[C@@H]1N1CCC2(CN(C(=O)[C@H]3Cc4ccc(F)cc43)C2)C1	1072918205	25	343.17	0.89

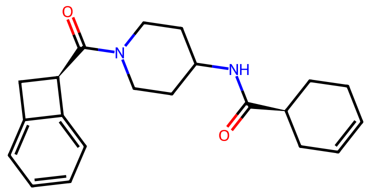	O=C(NC1CCN(C(=O)[C@@H]2Cc3ccccc32)CC1)[C@@H]1CC=CCC1	948059697	25	338.2	2.79
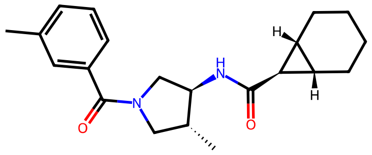	Cc1cccc(C(=O)N2C[C@@H](NC(=O)[C@@H]3[C@H]4CCCC[C@H]43)[C@H](C)C2)c1	947313692	25	340.22	3.01
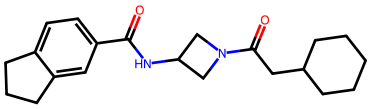	O=C(NC1CN(C(=O)CC2CCCCC2)C1)c1ccc2c(c1)CCC2	991359609	25	340.22	3.09
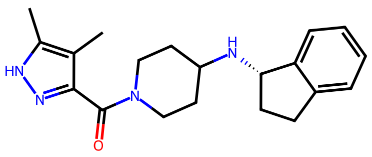	Cc1[nH]nc(C(=O)N2CCC(N[C@H]3CCc4ccccc43)CC2)c1C	990403573	25	338.21	2.91
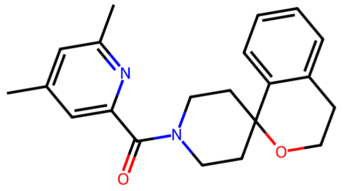	Cc1cc(C)nc(C(=O)N2CCC3(CC2)OCCc2ccccc23)c1	837213195	25	336.18	3.4
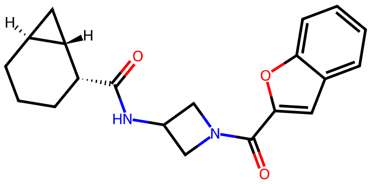	O=C(NC1CN(C(=O)c2cc3ccccc3o2)C1)[C@@H]1CCC[C@H]2C[C@@H]21	994726090	25	338.16	2.81
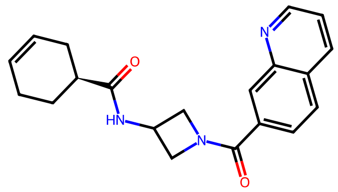	O=C(NC1CN(C(=O)c2ccc3cccnc3c2)C1)[C@@H]1CC=CCC1	990211793	25	335.16	2.53
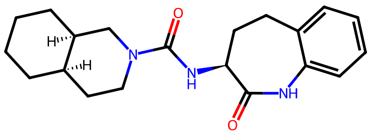	O=C1Nc2ccccc2CC[C@@H]1NC(=O)N1CC[C@H]2CCCC[C@H]2C1	1349786678	25	341.21	3.16

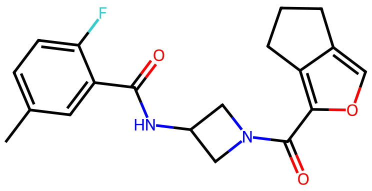	Cc1ccc(F)c(C(=O)NC2CN(C(=O)c3occ4c3CCC4)C2)c1	1000544964	25	342.14	2.47
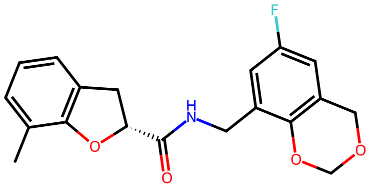	Cc1cccc2c1O[C@@H](C(=O)NCc1cc(F)cc3c1OCOC3)C2	449824932	25	343.12	2.62
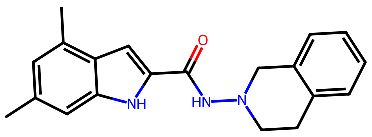	Cc1cc(C)c2cc(C(=O)NN3CCc4ccccc4C3)[nH]c2c1	1557393960	24	319.17	3.49
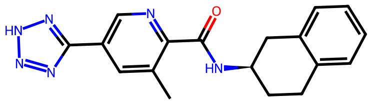	Cc1cc(-c2nn[nH]n2)cnc1C(=O)N[C@@H]1CCc2ccccc2C1	1605549019	25	334.15	1.86
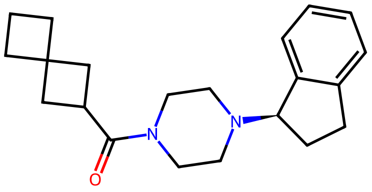	O=C(C1CC2(CCC2)C1)N1CCN([C@@H]2CCc3ccccc32)CC1	949753767	24	324.22	3.4
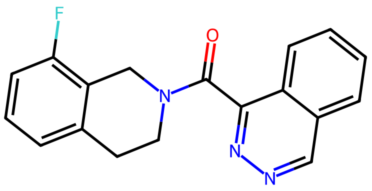	O=C(c1nncc2ccccc12)N1CCc2cccc(F)c2C1	1127728033	23	307.11	2.97
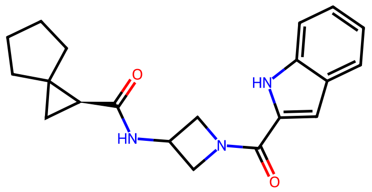	O=C(NC1CN(C(=O)c2cc3ccccc3[nH]2)C1)[C@@H]1CC12CCCC2	999628799	25	337.18	2.69
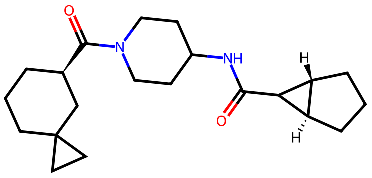	O=C(NC1CCN(C(=O)[C@@H]2CCCC3(CC3)C2)CC1)C1[C@@H]2CCC[C@@H]12	946752528	25	344.25	3.11

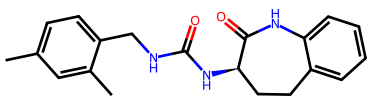	Cc1ccc(CNC(=O)N[C@@H]2CCc3ccccc3NC2=O)c(C)c1	654389060	25	337.18	3.06
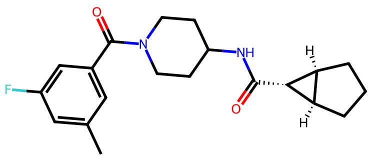	Cc1cc(F)cc(C(=O)N2CCC(NC(=O)[C@@H]3[C@H]4CCC[C@H]43)CC2)c1	948549847	25	344.19	2.9
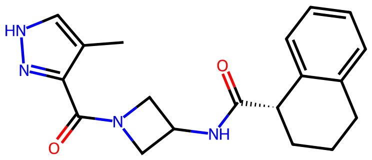	Cc1c[nH]nc1C(=O)N1CC(NC(=O)[C@H]2CCCc3ccccc32)C1	995069878	25	338.17	1.78
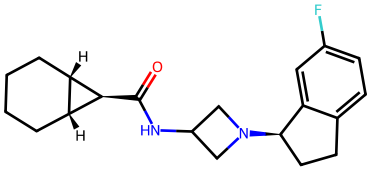	O=C(NC1CN([C@@H]2CCc3ccc(F)cc32)C1)[C@@H]1[C@H]2CCCC[C@H]21	1030185897	24	328.2	3.05
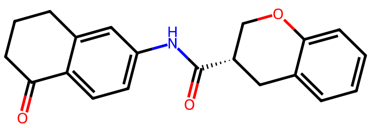	O=C1CCCc2cc(NC(=O)[C@@H]3COc4ccccc4C3)ccc21	556365822	24	321.14	3.4
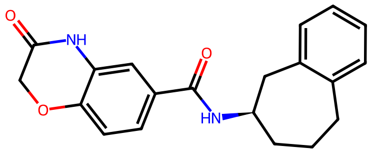	O=C1COc2ccc(C(=O)N[C@@H]3CCCc4ccccc4C3)cc2N1	888254505	25	336.15	2.69
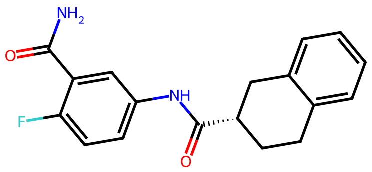	NC(=O)c1cc(NC(=O)[C@H]2CCc3ccccc3C2)ccc1F	1361333084	23	312.13	2.67
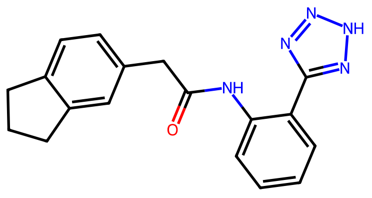	O=C(Cc1ccc2c(c1)CCC2)Nc1ccccc1-c1nn[nH]n1	823082208	24	319.14	2.54

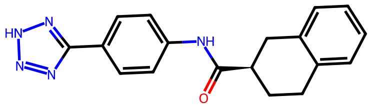	O=C(Nc1ccc(-c2nn[nH]n2)cc1)[C@@H]1CCc2ccccc2C1	1464618343	24	319.14	2.61
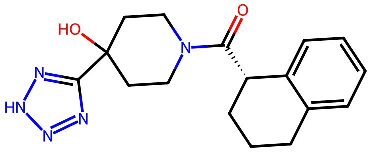	O=C([C@H]1CCCc2ccccc21)N1CCC(O)(c2nn[nH]n2)CC1	807992307	24	327.17	1.13
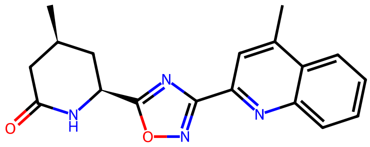	Cc1cc(-c2noc([C@@H]3C[C@H](C)CC(=O)N3)n2)nc2ccccc12	349694166	24	322.14	3.18
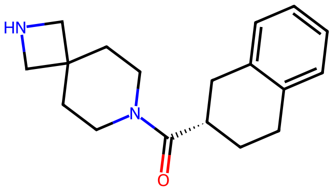	O=C([C@H]1CCc2ccccc2C1)N1CCC2(CC1)CNC2	1548506267	21	284.19	2.0
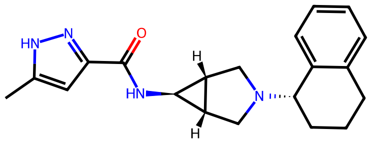	Cc1cc(C(=O)N[C@H]2[C@@H]3CN([C@H]4CCCc5ccccc54)C[C@@H]32)n[nH]1	958332181	25	336.2	2.46
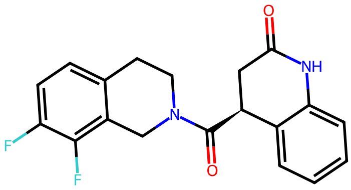	O=C1C[C@H](C(=O)N2CCc3ccc(F)c(F)c3C2)c2ccccc2N1	493905968	25	342.12	2.98
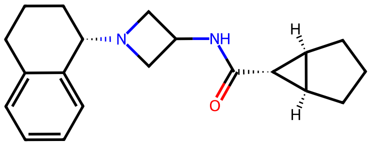	O=C(NC1CN([C@H]2CCCc3ccccc32)C1)[C@@H]1[C@H]2CCC[C@H]21	1030193411	23	310.2	2.91
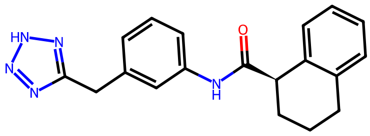	O=C(Nc1cccc(Cc2nn[nH]n2)c1)[C@@H]1CCCc2ccccc21	910041804	25	333.16	2.85

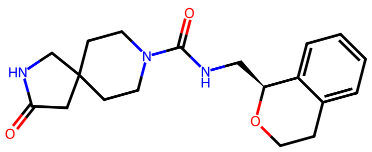	O=C1CC2(CCN(C(=O)NC[C@@H]3OCCc4ccccc43)CC2)CN1	362888503	25	343.19	1.61
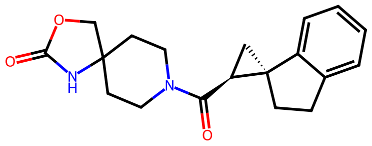	O=C1NC2(CCN(C(=O)[C@H]3C[C@]34CCc3ccccc34)CC2)CO1	495157007	24	326.16	1.99
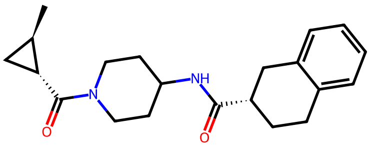	C[C@@H]1C[C@H]1C(=O)N1CCC(NC(=O)[C@H]2CCc3ccccc3C2)CC1	947724158	25	340.22	2.55
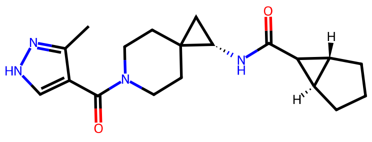	Cc1n[nH]cc1C(=O)N1CCC2(CC1)C[C@@H]2NC(=O)C1[C@@H]2CCC[C@@H]12	963486887	25	342.21	1.88
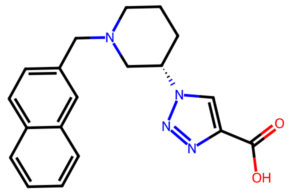	O=C(O)c1cn([C@H]2CCCN(Cc3ccc4ccccc4c3)C2)nn1	580778962	25	336.16	2.97
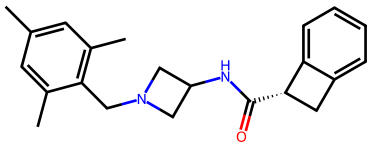	Cc1cc(C)c(CN2CC(NC(=O)[C@H]3Cc4ccccc43)C2)c(C)c1	1030875951	25	334.2	3.25
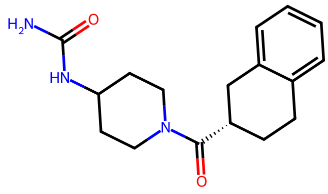	NC(=O)NC1CCN(C(=O)[C@H]2CCc3ccccc3C2)CC1	565073191	22	301.18	1.45
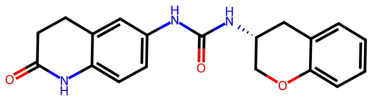	O=C1CCc2cc(NC(=O)N[C@H]3COc4ccccc4C3)ccc2N1	193011974	25	337.14	2.7

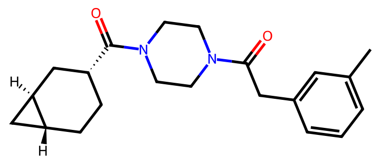	Cc1cccc(CC(=O)N2CCN(C(=O)[C@@H]3CC[C@@H]4C[C@H]4C3)CC2)c1	1011312966	25	340.22	2.64
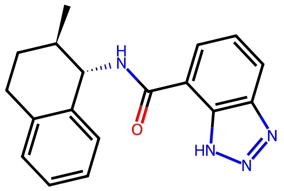	C[C@@H]1CCc2ccccc2[C@H]1NC(=O)c1cccc2nn[nH]c12	526119684	23	306.15	3.01
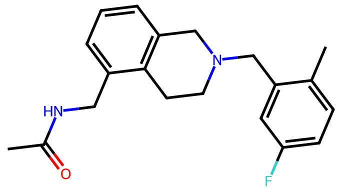	CC(=O)NCc1cccc2c1CCN(Cc1cc(F)ccc1C)C2	1051273585	24	326.18	3.33
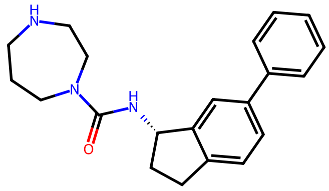	O=C(N[C@H]1CCc2ccc(-c3ccccc3)cc21)N1CCCNCC1	418039341	25	335.2	3.35
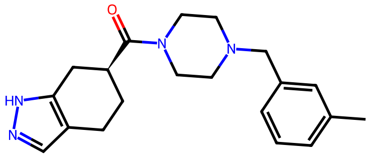	Cc1cccc(CN2CCN(C(=O)[C@H]3CCc4cn[nH]c4C3)CC2)c1	79302593	25	338.21	2.17
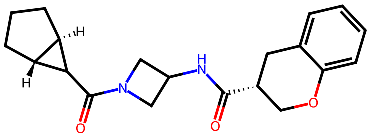	O=C(NC1CN(C(=O)C2[C@@H]3CCC[C@@H]23)C1)[C@H]1COc2ccccc2C1	995466374	25	340.18	1.61
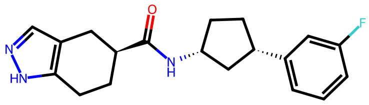	O=C(N[C@@H]1CC[C@H](c2cccc(F)c2)C1)[C@H]1CCc2[nH]ncc2C1	280364736	24	327.17	3.11
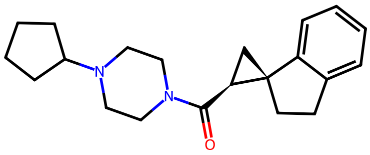	O=C([C@H]1C[C@@]12CCc1ccccc12)N1CCN(C2CCCC2)CC1	175260496	24	324.22	2.98

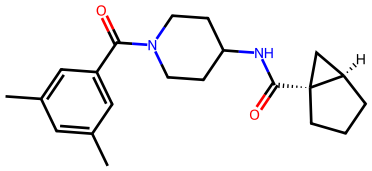	Cc1cc(C)cc(C(=O)N2CCC(NC(=O)[C@@]34CCC[C@@H]3C4)CC2)c1	948337958	25	340.22	3.21
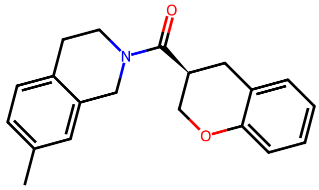	Cc1ccc2c(c1)CN(C(=O)[C@@H]1COc3ccccc3C1)CC2	617602251	23	307.16	3.13
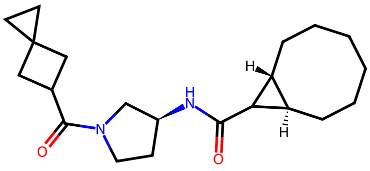	O=C(N[C@H]1CCN(C(=O)C2CC3(CC3)C2)C1)C1[C@@H]2CCCCCC[C@@H]12	938980164	25	344.25	3.11
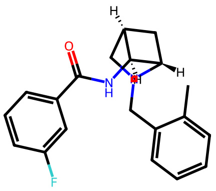	Cc1ccccc1CN1C[C@H]2C[C@H]1[C@H]2NC(=O)c1cccc(F)c1	1037286833	24	324.16	3.14
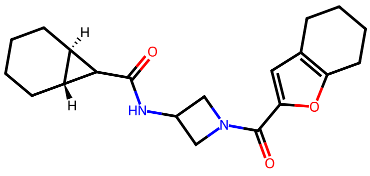	O=C(NC1CN(C(=O)c2cc3c(o2)CCCC3)C1)C1[C@@H]2CCCC[C@@H]12	990672032	25	342.19	2.54
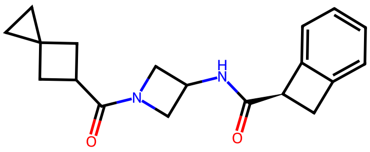	O=C(NC1CN(C(=O)C2CC3(CC3)C2)C1)[C@@H]1Cc2ccccc21	998688152	23	310.17	1.84
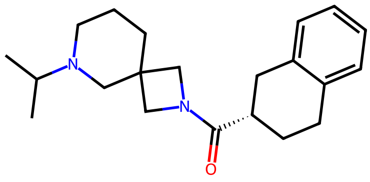	CC(C)N1CCCC2(CN(C(=O)[C@H]3CCc4ccccc4C3)C2)C1	982037368	24	326.24	3.12
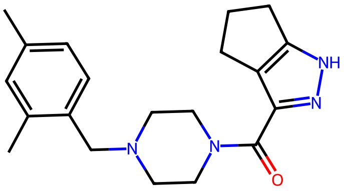	Cc1ccc(CN2CCN(C(=O)c3n[nH]c4c3CCC4)CC2)c(C)c1	949063020	25	338.21	2.47

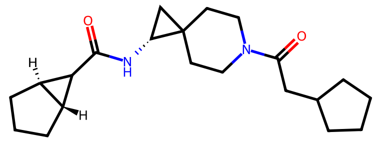	O=C(N[C@@H]1CC12CCN(C(=O)CC1CCCC1)CC2)C1[C@@H]2CCC[C@@H]12	963470277	25	344.25	3.11
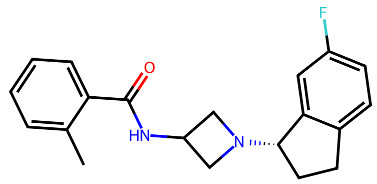	Cc1ccccc1C(=O)NC1CN([C@H]2CCc3ccc(F)cc32)C1	1030065620	24	324.16	3.24
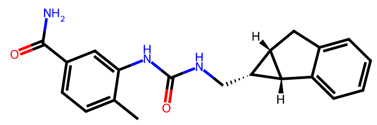	Cc1ccc(C(N)=O)cc1NC(=O)NC[C@H]1[C@H]2c3ccccc3C[C@H]21	123590326	25	335.16	2.8
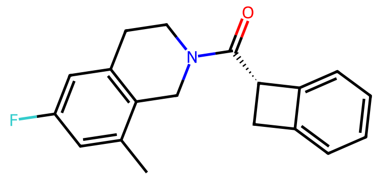	Cc1cc(F)cc2c1CN(C(=O)[C@H]1Cc3ccccc31)CC2	337328991	22	295.14	3.36
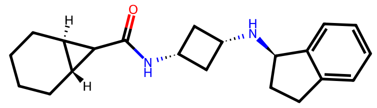	O=C(N[C@H]1C[C@@H](N[C@@H]2CCc3ccccc32)C1)C1[C@@H]2CCCC[C@@H]12	1021936283	24	324.22	3.35
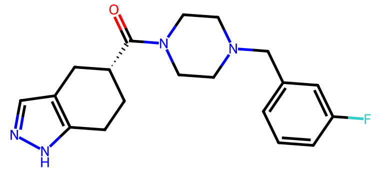	O=C([C@@H]1CCc2[nH]ncc2C1)N1CCN(Cc2cccc(F)c2)CC1	270260313	25	342.19	2.0
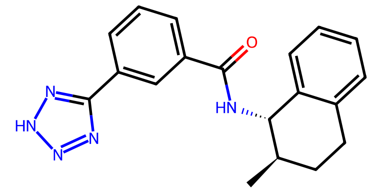	C[C@@H]1CCc2ccccc2[C@H]1NC(=O)c1cccc(-c2nn[nH]n2)c1	824970391	25	333.16	2.92
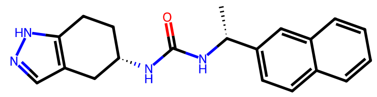	C[C@@H](NC(=O)N[C@H]1CCc2[nH]ncc2C1)c1ccc2ccccc2c1	154164078	25	334.18	3.48

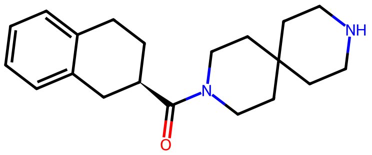	O=C([C@@H]1CCc2ccccc2C1)N1CCC2(CCNCC2)CC1	324079808	23	312.22	2.78
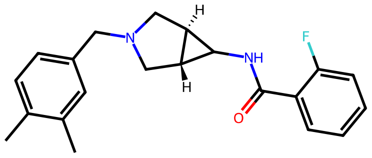	Cc1ccc(CN2C[C@H]3C(NC(=O)c4ccccc4F)[C@@H]3C2)cc1C	958107811	25	338.18	3.3
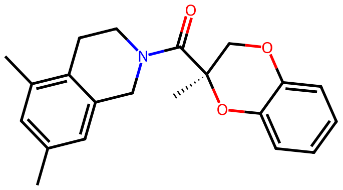	Cc1cc(C)c2c(c1)CN(C(=O)[C@@]1(C)COc3ccccc3O1)CC2	1435943013	25	337.17	3.42
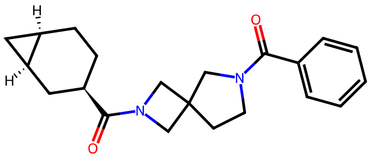	O=C(c1ccccc1)N1CCC2(C1)CN(C(=O)[C@@H]1CC[C@@H]3C[C@@H]3C1)C2	1019141890	25	338.2	2.8
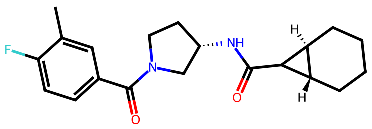	Cc1cc(C(=O)N2CC[C@H](NC(=O)C3[C@H]4CCCC[C@H]34)C2)ccc1F	937790188	25	344.19	2.9
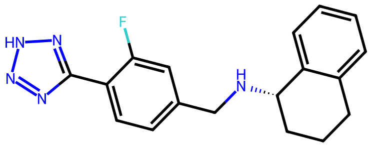	Fc1cc(CN[C@H]2CCCc3ccccc32)ccc1-c1nn[nH]n1	1606310279	24	323.15	3.17
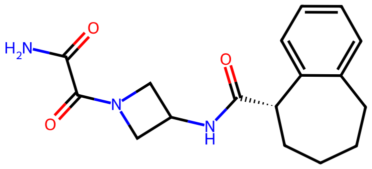	NC(=O)C(=O)N1CC(NC(=O)[C@H]2CCCCc3ccccc32)C1	994322063	23	315.16	0.31
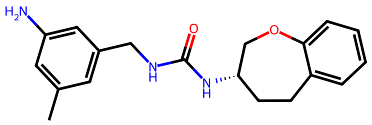	Cc1cc(N)cc(CNC(=O)N[C@H]2CCc3ccccc3OC2)c1	418152614	24	325.18	2.77

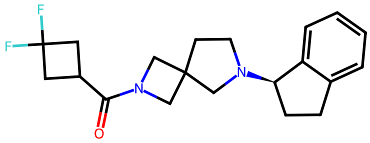	O=C(C1CC(F)(F)C1)N1CC2(CCN([C@@H]3CCc4ccccc43)C2)C1	1072604709	25	346.19	3.25
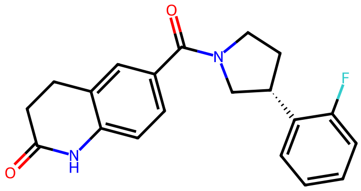	O=C1CCc2cc(C(=O)N3CC[C@H](c4ccccc4F)C3)ccc2N1	264510561	25	338.14	3.34
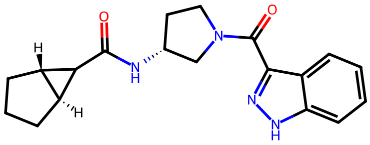	O=C(N[C@@H]1CCN(C(=O)c2n[nH]c3ccccc23)C1)C1[C@H]2CCC[C@H]12	939933029	25	338.17	1.94
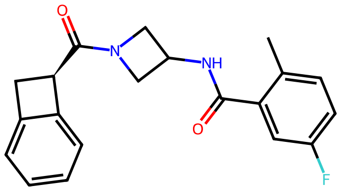	Cc1ccc(F)cc1C(=O)NC1CN(C(=O)[C@@H]2Cc3ccccc32)C1	996641271	25	338.14	2.41
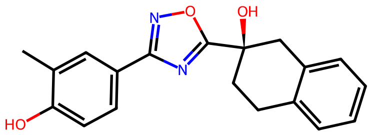	Cc1cc(-c2noc([C@@]3(O)CCc4ccccc4C3)n2)ccc1O	809471536	24	322.13	3.13
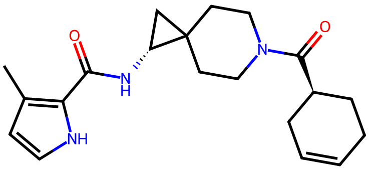	Cc1cc[nH]c1C(=O)N[C@@H]1CC12CCN(C(=O)[C@@H]1CC=CCC1)CC2	963754046	25	341.21	2.79
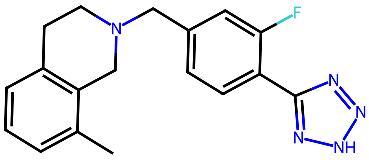	Cc1cccc2c1CN(Cc1ccc(-c3nn[nH]n3)c(F)c1)CC2	1592244943	24	323.15	2.87
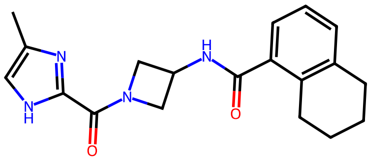	Cc1c[nH]c(C(=O)N2CC(NC(=O)c3cccc4c3CCCC4)C2)n1	991899111	25	338.17	1.85

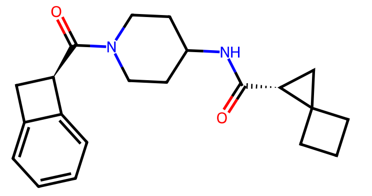	O=C(NC1CCN(C(=O)[C@@H]2Cc3ccccc32)CC1)[C@@H]1CC12CCC2	948062506	25	338.2	2.62
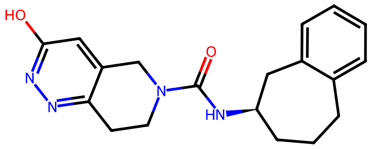	O=C(N[C@@H]1CCCc2ccccc2C1)N1CCc2nnc(O)cc2C1	897612453	25	338.17	2.2
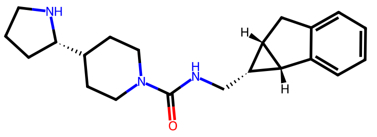	O=C(NC[C@H]1[C@H]2c3ccccc3C[C@H]21)N1CCC([C@@H]2CCCN2)CC1	417915488	25	339.23	2.75
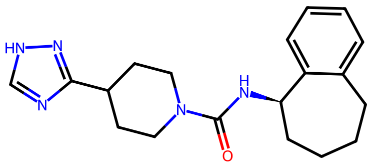	O=C(N[C@@H]1CCCCc2ccccc21)N1CCC(c2nc[nH]n2)CC1	83717105	25	339.21	3.16
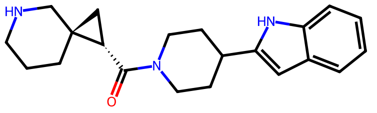	O=C([C@H]1C[C@]12CCCNC2)N1CCC(c2cc3ccccc3[nH]2)CC1	848397461	25	337.22	3.26
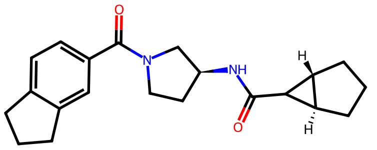	O=C(N[C@H]1CCN(C(=O)c2ccc3c(c2)CCC3)C1)C1[C@@H]2CCC[C@@H]12	937805351	25	338.2	2.55
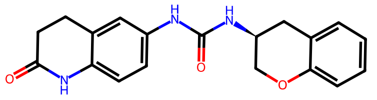	O=C1CCc2cc(NC(=O)N[C@@H]3COc4ccccc4C3)ccc2N1	193011963	25	337.14	2.7
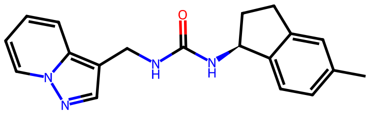	Cc1ccc2c(c1)CC[C@@H]2NC(=O)NCc1cnn2ccccc12	571471356	24	320.16	3.13

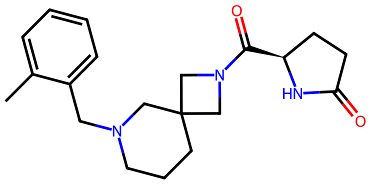	Cc1ccccc1CN1CCCC2(C1)CN(C(=O)[C@H]1CCC(=O)N1)C2	981668635	25	341.21	1.7
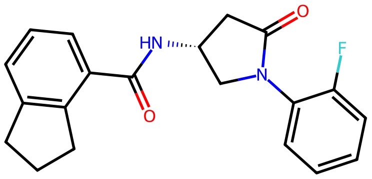	O=C(N[C@@H]1CC(=O)N(c2ccccc2F)C1)c1cccc2c1CCC2	360583467	25	338.14	2.85
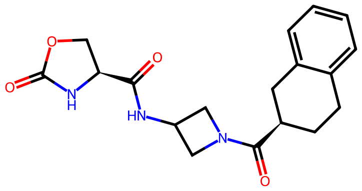	O=C1N[C@H](C(=O)NC2CN(C(=O)[C@@H]3CCc4ccccc4C3)C2)CO1	991125989	25	343.15	0.23
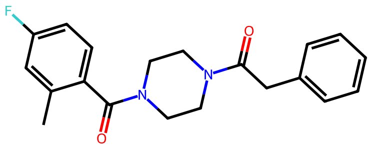	Cc1cc(F)ccc1C(=O)N1CCN(C(=O)Cc2ccccc2)CC1	178906981	25	340.16	2.66
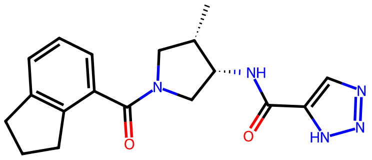	C[C@H]1CN(C(=O)c2cccc3c2CCC3)C[C@H]1NC(=O)c1cnn[nH]1	966534529	25	339.17	1.18
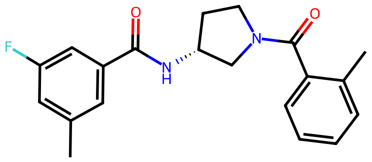	Cc1cc(F)cc(C(=O)N[C@@H]2CCN(C(=O)c3ccccc3C)C2)c1	942500401	25	340.16	3.09
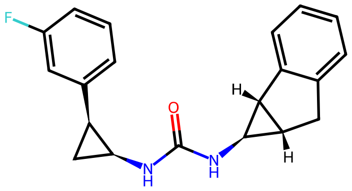	O=C(N[C@H]1C[C@H]1c1cccc(F)c1)N[C@@H]1[C@H]2Cc3ccccc3[C@H]21	915118934	24	322.15	3.32
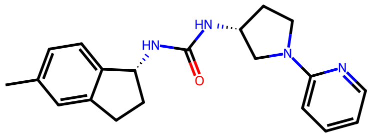	Cc1ccc2c(c1)CC[C@H]2NC(=O)N[C@@H]1CCN(c2ccccn2)C1	269903451	25	336.2	2.96

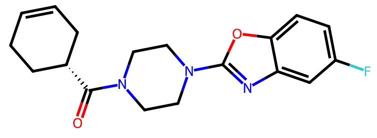	O=C([C@H]1CC=CCC1)N1CCN(c2nc3cc(F)ccc3o2)CC1	1055720868	24	329.15	2.97
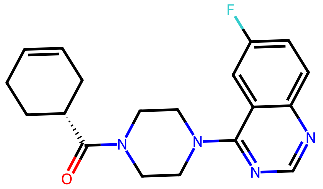	O=C([C@H]1CC=CCC1)N1CCN(c2ncnc3ccc(F)cc23)CC1	1055720919	25	340.17	2.77
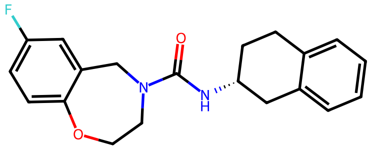	O=C(N[C@@H]1CCc2ccccc2C1)N1CCOc2ccc(F)cc2C1	494867086	25	340.16	3.29
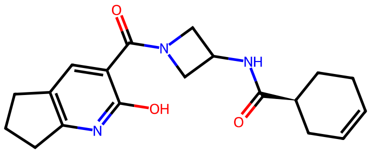	O=C(NC1CN(C(=O)c2cc3c(nc2O)CCC3)C1)[C@@H]1CC=CCC1	990221955	25	341.17	1.57
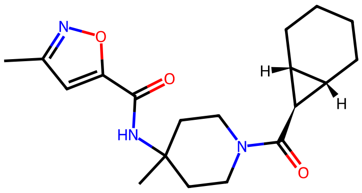	Cc1cc(C(=O)NC2(C)CCN(C(=O)[C@@H]3[C@H]4CCCC[C@H]43)CC2)on1	956287243	25	345.21	2.53
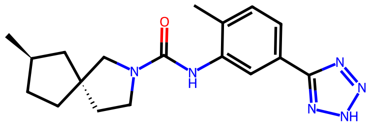	Cc1ccc(-c2nn[nH]n2)cc1NC(=O)N1CC[C@@]2(CC[C@@H](C)C2)C1	826277985	25	340.2	3.22
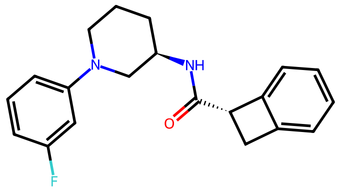	O=C(N[C@@H]1CCCN(c2cccc(F)c2)C1)[C@H]1Cc2ccccc21	358496086	24	324.16	3.25
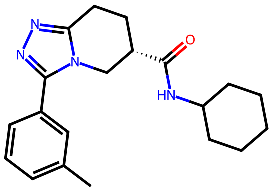	Cc1cccc(-c2nnc3n2C[C@@H](C(=O)NC2CCCCC2)CC3)c1	562646180	25	338.21	3.26

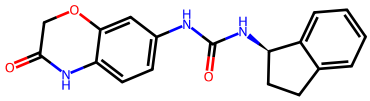	O=C1COc2cc(NC(=O)N[C@@H]3CCc4ccccc43)ccc2N1	97463938	24	323.13	2.83
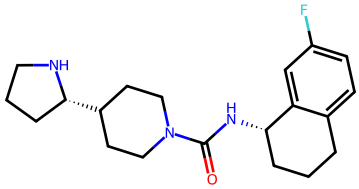	O=C(N[C@H]1CCCc2ccc(F)cc21)N1CCC([C@@H]2CCCN2)CC1	418114489	25	345.22	3.38
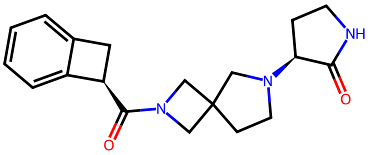	O=C1NCC[C@@H]1N1CCC2(CN(C(=O)[C@@H]3Cc4ccccc43)C2)C1	1072931216	24	325.18	0.75
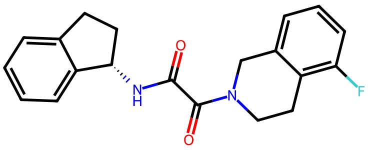	O=C(N[C@H]1CCc2ccccc21)C(=O)N1CCc2c(F)cccc2C1	339869927	25	338.14	2.51
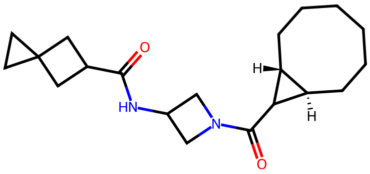	O=C(NC1CN(C(=O)C2[C@@H]3CCCCCC[C@@H]23)C1)C1CC2(CC2)C1	999347715	24	330.23	2.72
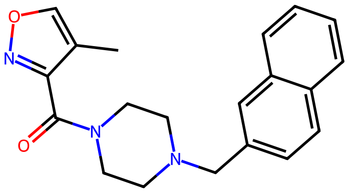	Cc1conc1C(=O)N1CCN(Cc2ccc3ccccc3c2)CC1	957090845	25	335.16	3.09
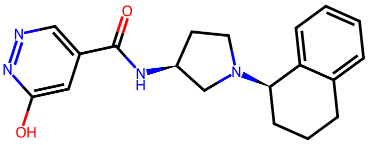	O=C(N[C@H]1CCN([C@@H]2CCCc3ccccc32)C1)c1cnnc(O)c1	1014829873	25	338.17	2.06
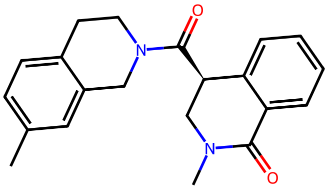	Cc1ccc2c(c1)CN(C(=O)[C@@H]1CN(C)C(=O)c3ccccc31)CC2	617605088	25	334.17	2.75

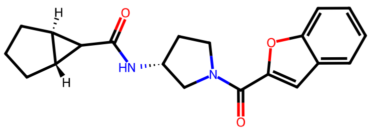	O=C(N[C@@H]1CCN(C(=O)c2cc3ccccc3o2)C1)C1[C@@H]2CCC[C@@H]12	936129674	25	338.16	2.81
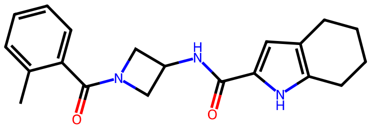	Cc1ccccc1C(=O)N1CC(NC(=O)c2cc3c([nH]2)CCCC3)C1	999653486	25	337.18	2.46
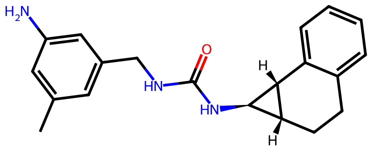	Cc1cc(N)cc(CNC(=O)N[C@@H]2[C@H]3CCc4ccccc4[C@H]32)c1	417916904	24	321.18	3.1
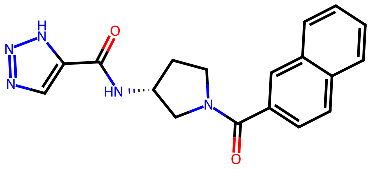	O=C(N[C@@H]1CCN(C(=O)c2ccc3ccccc3c2)C1)c1cnn[nH]1	941448025	25	335.14	1.6
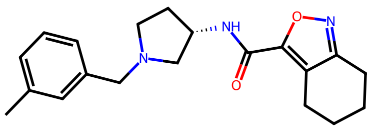	Cc1cccc(CN2CC[C@H](NC(=O)c3onc4c3CCCC4)C2)c1	634278079	25	339.19	2.87
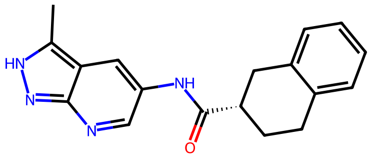	Cc1[nH]nc2ncc(NC(=O)[C@H]3CCc4ccccc4C3)cc12	572247815	23	306.15	3.01
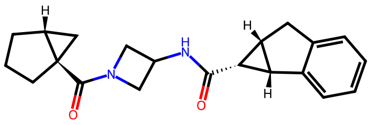	O=C(NC1CN(C(=O)[C@@]23CCC[C@@H]2C3)C1)[C@H]1[C@H]2c3ccccc3C[C@H]21	991644134	25	336.18	2.09
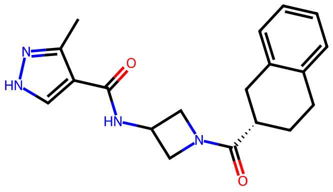	Cc1n[nH]cc1C(=O)NC1CN(C(=O)[C@H]2CCc3ccccc3C2)C1	997287160	25	338.17	1.46

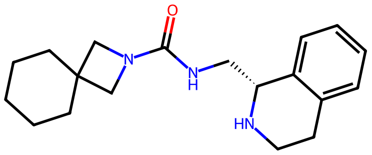	O=C(NC[C@H]1NCCc2ccccc21)N1CC2(CCCCC2)C1	418638634	23	313.22	2.85
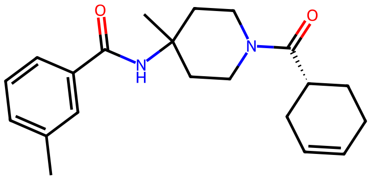	Cc1cccc(C(=O)NC2(C)CCN(C(=O)[C@H]3CC=CCC3)CC2)c1	956279301	25	340.22	3.46
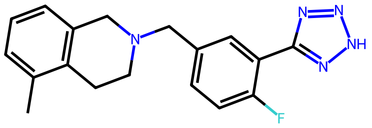	Cc1cccc2c1CCN(Cc1ccc(F)c(-c3nn[nH]n3)c1)C2	822340984	24	323.15	2.87
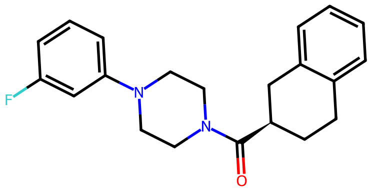	O=C([C@@H]1CCc2ccccc2C1)N1CCN(c2cccc(F)c2)CC1	1125059840	25	338.18	3.28
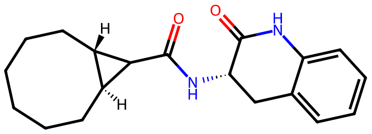	O=C(N[C@H]1Cc2ccccc2NC1=O)C1[C@H]2CCCCCC[C@H]12	628943768	23	312.18	2.88
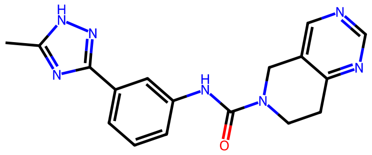	Cc1nc(-c2cccc(NC(=O)N3CCc4ncncc4C3)c2)n[nH]1	1199957155	25	335.15	2.16
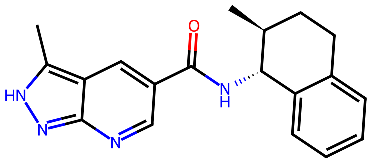	Cc1[nH]nc2ncc(C(=O)N[C@H]3c4ccccc4CC[C@@H]3C)cc12	342885249	24	320.16	3.32
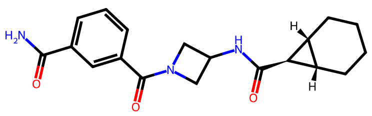	NC(=O)c1cccc(C(=O)N2CC(NC(=O)[C@@H]3[C@H]4CCCC[C@H]43)C2)c1	990662978	25	341.17	1.16

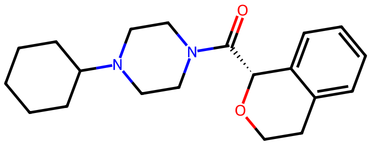	O=C([C@H]1OCCc2ccccc21)N1CCN(C2CCCCC2)CC1	124808379	24	328.22	2.78
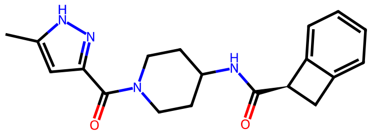	Cc1cc(C(=O)N2CCC(NC(=O)[C@@H]3Cc4ccccc43)CC2)n[nH]1	944822460	25	338.17	1.78
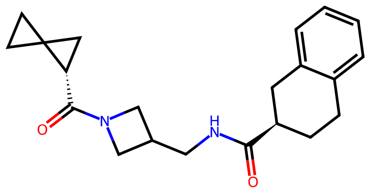	O=C(NCC1CN(C(=O)[C@H]2CC23CC3)C1)[C@@H]1CCc2ccccc2C1	936100054	25	338.2	2.17
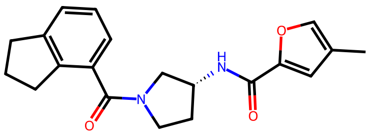	Cc1coc(C(=O)N[C@@H]2CCN(C(=O)c3cccc4c3CCC4)C2)c1	942223585	25	338.16	2.72
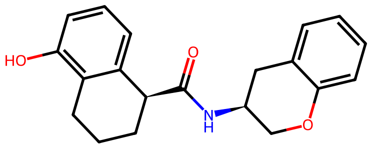	O=C(N[C@@H]1COc2ccccc2C1)[C@H]1CCCc2c(O)cccc21	190124934	24	323.15	2.93
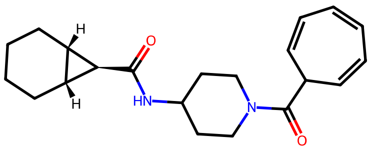	O=C(NC1CCN(C(=O)C2C=CC=CC=C2)CC1)[C@@H]1[C@H]2CCCC[C@H]21	946949671	25	340.22	2.83
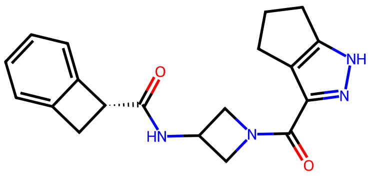	O=C(NC1CN(C(=O)c2n[nH]c3c2CCC3)C1)[C@@H]1Cc2ccccc21	998669183	25	336.16	1.18
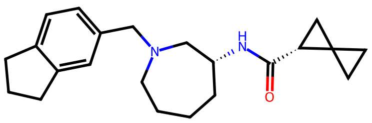	O=C(N[C@@H]1CCCCN(Cc2ccc3c(c2)CCC3)C1)[C@@H]1CC12CC2	1034416880	25	338.24	3.45

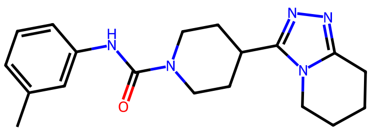	Cc1cccc(NC(=O)N2CCC(c3nnc4n3CCCC4)CC2)c1	274790640	25	339.21	3.33
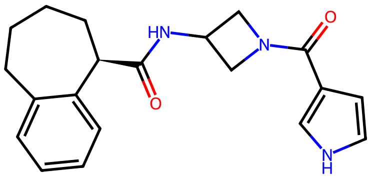	O=C(NC1CN(C(=O)c2cc[nH]c2)C1)[C@@H]1CCCCc2ccccc21	994322070	25	337.18	2.47
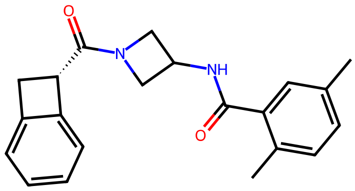	Cc1ccc(C)c(C(=O)NC2CN(C(=O)[C@H]3Cc4ccccc43)C2)c1	999483653	25	334.17	2.58
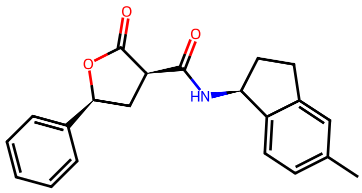	Cc1ccc2c(c1)CC[C@@H]2NC(=O)[C@H]1C[C@@H](c2ccccc2)OC1=O	290211532	25	335.15	3.4
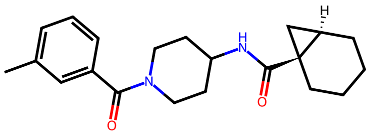	Cc1cccc(C(=O)N2CCC(NC(=O)[C@]34CCCC[C@@H]3C4)CC2)c1	943834393	25	340.22	3.3
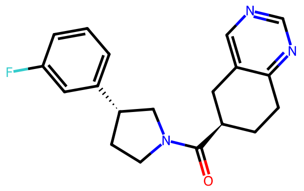	O=C([C@@H]1CCc2ncncc2C1)N1CC[C@H](c2cccc(F)c2)C1	648277070	24	325.16	2.74
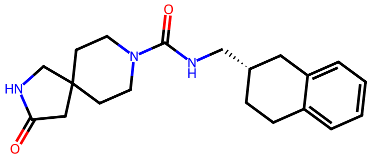	O=C1CC2(CCN(C(=O)NC[C@H]3CCc4ccccc4C3)CC2)CN1	752015746	25	341.21	2.1
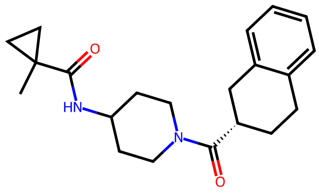	CC1(C(=O)NC2CCN(C(=O)[C@H]3CCc4ccccc4C3)CC2)CC1	948572412	25	340.22	2.7

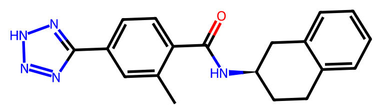	Cc1cc(-c2nn[nH]n2)ccc1C(=O)N[C@@H]1CCc2ccccc2C1	826247900	25	333.16	2.46
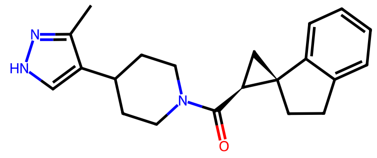	Cc1n[nH]cc1C1CCN(C(=O)[C@H]2C[C@@]23CCc2ccccc23)CC1	375990274	25	335.2	3.33
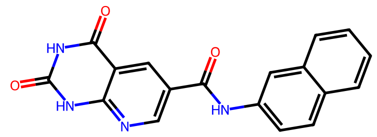	O=C(Nc1ccc2ccccc2c1)c1cnc2[nH]c(=O)[nH]c(=O)c2c1	124735776	25	332.09	2.02
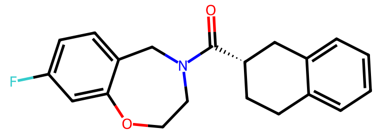	O=C([C@H]1CCc2ccccc2C1)N1CCOc2cc(F)ccc2C1	629852064	24	325.15	3.35
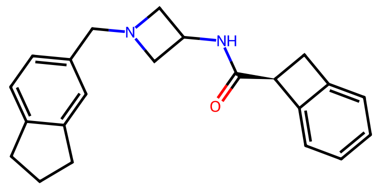	O=C(NC1CN(Cc2ccc3c(c2)CCC3)C1)[C@H]1Cc2ccccc21	1030876490	25	332.19	2.82
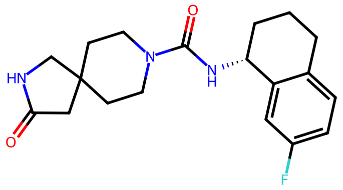	O=C1CC2(CCN(C(=O)N[C@@H]3CCCc4ccc(F)cc43)CC2)CN1	279656079	25	345.19	2.51
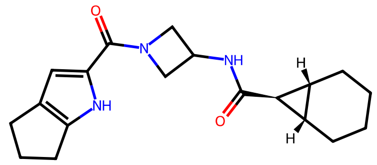	O=C(NC1CN(C(=O)c2cc3c([nH]2)CCC3)C1)[C@@H]1[C@H]2CCCC[C@H]21	990651540	24	327.19	1.88
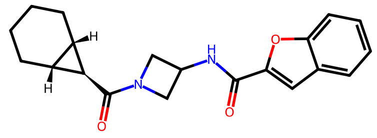	O=C(NC1CN(C(=O)[C@@H]2[C@H]3CCCC[C@H]32)C1)c1cc2ccccc2o1	1000050516	25	338.16	2.81

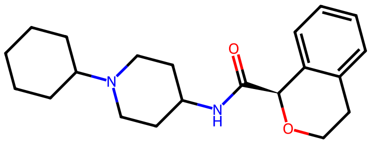	O=C(NC1CCN(C2CCCCC2)CC1)[C@@H]1OCCc2ccccc21	123827506	25	342.23	3.21
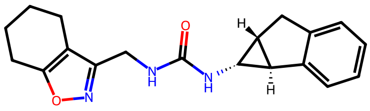	O=C(NCc1noc2c1CCCC2)N[C@H]1[C@@H]2c3ccccc3C[C@H]21	288004898	24	323.16	2.69
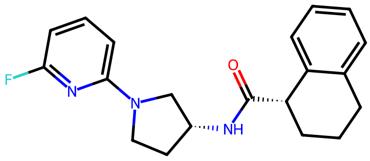	O=C(N[C@@H]1CCN(c2cccc(F)n2)C1)[C@H]1CCCc2ccccc21	1058592034	25	339.17	3.04
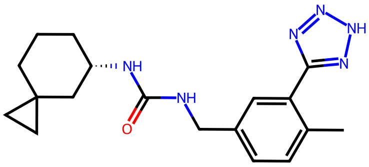	Cc1ccc(CNC(=O)N[C@H]2CCCC3(CC3)C2)cc1-c1nn[nH]n1	1592159825	25	340.2	2.7
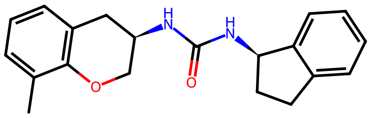	Cc1cccc2c1OC[C@H](NC(=O)N[C@@H]1CCc3ccccc31)C2	811996753	24	322.17	3.29
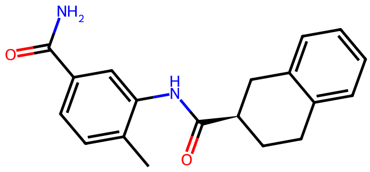	Cc1ccc(C(N)=O)cc1NC(=O)[C@@H]1CCc2ccccc2C1	566309156	23	308.15	2.84
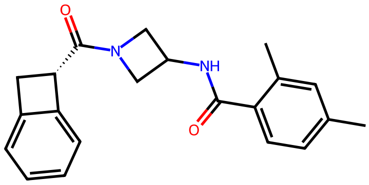	Cc1ccc(C(=O)NC2CN(C(=O)[C@H]3Cc4ccccc43)C2)c(C)c1	999538159	25	334.17	2.58
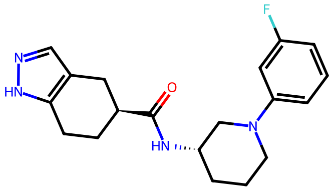	O=C(N[C@H]1CCCN(c2cccc(F)c2)C1)[C@H]1CCc2[nH]ncc2C1	270282288	25	342.19	2.44

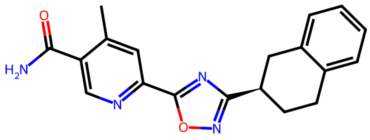	Cc1cc(-c2nc([C@@H]3CCc4ccccc4C3)no2)ncc1C(N)=O	651246072	25	334.14	2.81
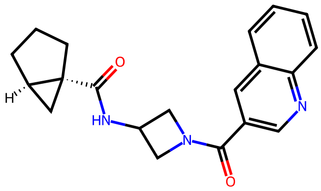	O=C(c1cnc2ccccc2c1)N1CC(NC(=O)[C@@]23CCC[C@@H]2C3)C1	997549305	25	335.16	2.37
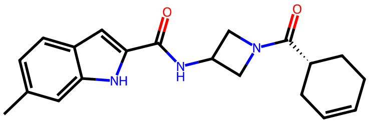	Cc1ccc2cc(C(=O)NC3CN(C(=O)[C@H]4CC=CCC4)C3)[nH]c2c1	996606202	25	337.18	2.77
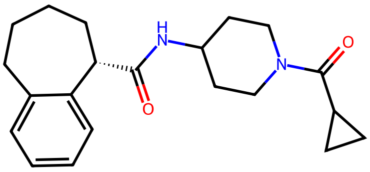	O=C(NC1CCN(C(=O)C2CC2)CC1)[C@H]1CCCCc2ccccc21	943234840	25	340.22	3.01
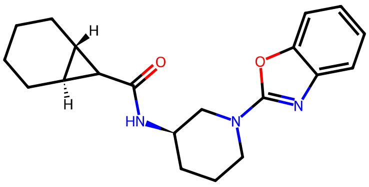	O=C(N[C@@H]1CCCN(c2nc3ccccc3o2)C1)C1[C@H]2CCCC[C@H]12	1058130129	25	339.19	3.35
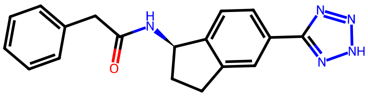	O=C(Cc1ccccc1)N[C@@H]1CCc2cc(-c3nn[nH]n3)ccc21	1592923065	24	319.14	2.21
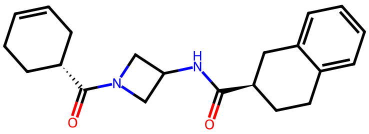	O=C(NC1CN(C(=O)[C@H]2CC=CCC2)C1)[C@@H]1CCc2ccccc2C1	1000537904	25	338.2	2.47
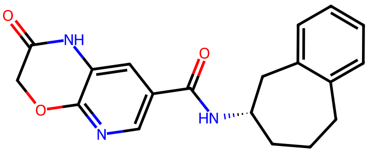	O=C1COc2ncc(C(=O)N[C@H]3CCCc4ccccc4C3)cc2N1	888250809	25	337.14	2.09

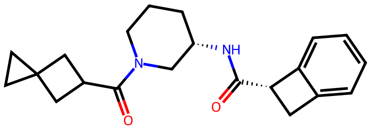	O=C(N[C@H]1CCCN(C(=O)C2CC3(CC3)C2)C1)[C@H]1Cc2ccccc21	951964982	25	338.2	2.62
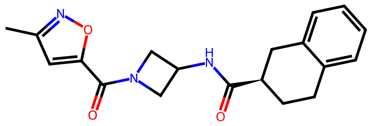	Cc1cc(C(=O)N2CC(NC(=O)[C@@H]3CCc4ccccc4C3)C2)on1	1000540035	25	339.16	1.73
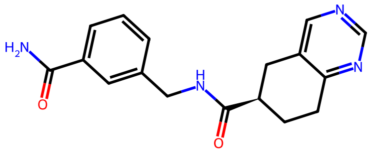	NC(=O)c1cccc(CNC(=O)[C@@H]2CCc3ncncc3C2)c1	648237570	23	310.14	1.0
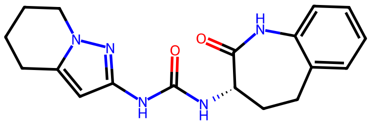	O=C(Nc1cc2n(n1)CCCC2)N[C@H]1CCc2ccccc2NC1=O	654073753	25	339.17	2.29
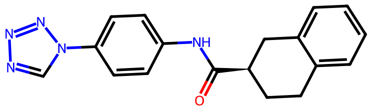	O=C(Nc1ccc(-n2cnnn2)cc1)[C@@H]1CCc2ccccc2C1	1351946506	24	319.14	2.41
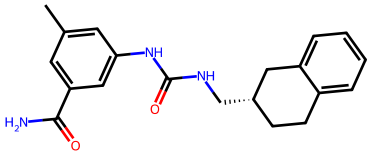	Cc1cc(NC(=O)NC[C@H]2CCc3ccccc3C2)cc(C(N)=O)c1	653587400	25	337.18	3.02
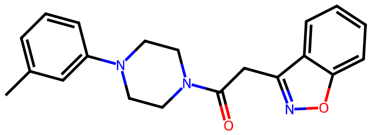	Cc1cccc(N2CCN(C(=O)Cc3noc4ccccc34)CC2)c1	16297500	25	335.16	3.03
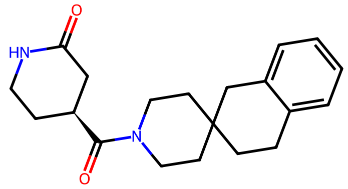	O=C1C[C@@H](C(=O)N2CCC3(CCc4ccccc4C3)CC2)CCN1	652524943	24	326.2	2.31

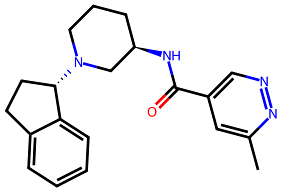	Cc1cc(C(=O)N[C@@H]2CCCN([C@H]3CCc4ccccc43)C2)cnn1	1007214561	25	336.2	2.67
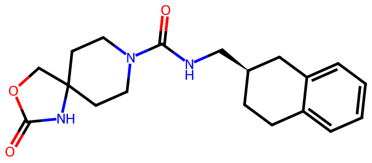	O=C1NC2(CCN(C(=O)NC[C@@H]3CCc4ccccc4C3)CC2)CO1	784327058	25	343.19	2.08
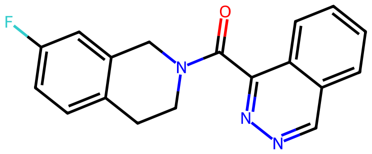	O=C(c1nncc2ccccc12)N1CCc2ccc(F)cc2C1	1458125255	23	307.11	2.97
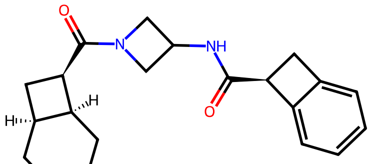	O=C(NC1CN(C(=O)[C@@H]2C[C@@H]3CCCC[C@@H]32)C1)[C@H]1Cc2ccccc21	998681760	25	338.2	2.48
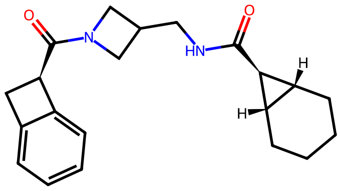	O=C(NCC1CN(C(=O)[C@@H]2Cc3ccccc32)C1)[C@@H]1[C@H]2CCCC[C@H]21	1001321382	25	338.2	2.34
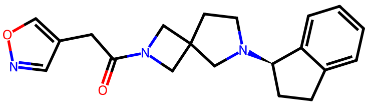	O=C(Cc1cnoc1)N1CC2(CCN([C@@H]3CCc4ccccc43)C2)C1	1072909145	25	337.18	2.44
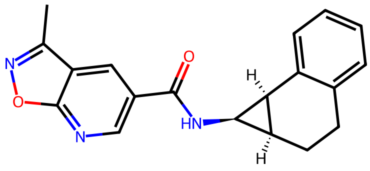	Cc1noc2ncc(C(=O)N[C@@H]3[C@@H]4CCc5ccccc5[C@@H]43)cc12	121352395	24	319.13	2.99
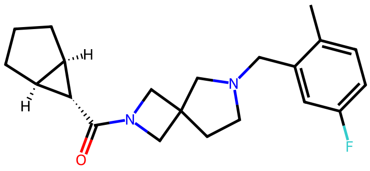	Cc1ccc(F)cc1CN1CCC2(C1)CN(C(=O)[C@@H]1[C@H]3CCC[C@H]31)C2	1072590516	25	342.21	3.21

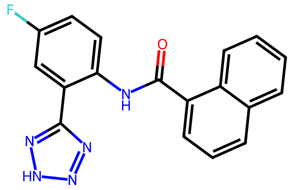	O=C(Nc1ccc(F)cc1-c1nn[nH]n1)c1cccc2ccccc12	823345635	25	333.1	3.41
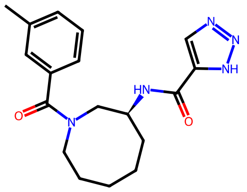	Cc1cccc(C(=O)N2CCCCC[C@H](NC(=O)c3cnn[nH]3)C2)c1	986854643	25	341.19	1.93
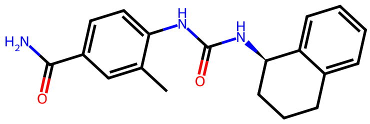	Cc1cc(C(N)=O)ccc1NC(=O)N[C@@H]1CCCc2ccccc21	45729497	24	323.16	3.29
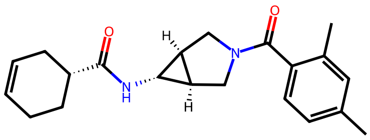	Cc1ccc(C(=O)N2C[C@H]3[C@H](NC(=O)[C@H]4CC=CCC4)[C@H]3C2)c(C)c1	976182678	25	338.2	2.85
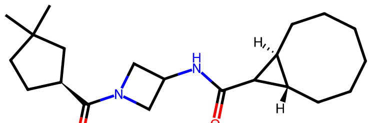	CC1(C)CC[C@H](C(=O)N2CC(NC(=O)C3[C@H]4CCCCCC[C@H]34)C2)C1	1000508402	25	346.26	3.36
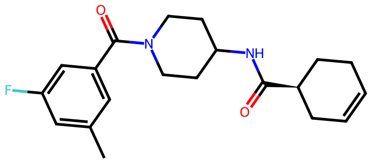	Cc1cc(F)cc(C(=O)N2CCC(NC(=O)[C@@H]3CC=CCC3)CC2)c1	948545981	25	344.19	3.21
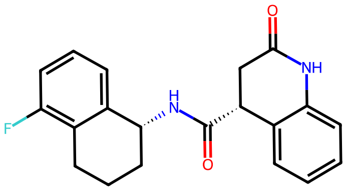	O=C1C[C@@H](C(=O)N[C@@H]2CCCc3c(F)cccc32)c2ccccc2N1	452198309	25	338.14	3.45
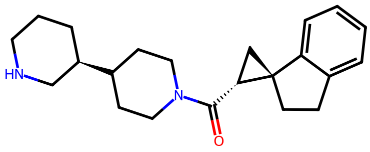	O=C([C@@H]1C[C@@]12CCc1ccccc12)N1CCC([C@@H]2CCCNC2)CC1	658956684	25	338.24	3.13

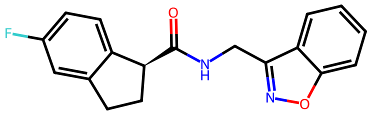	O=C(NCc1noc2ccccc12)[C@H]1CCc2cc(F)ccc21	1597556441	23	310.11	3.31
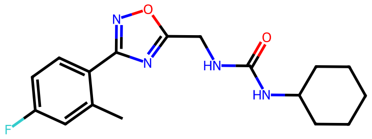	Cc1cc(F)ccc1-c1noc(CNC(=O)NC2CCCCC2)n1	274066299	24	332.16	3.32
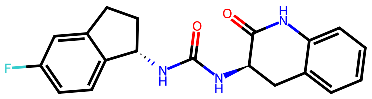	O=C(N[C@H]1CCc2cc(F)ccc21)N[C@@H]1Cc2ccccc2NC1=O	636635618	25	339.14	2.68
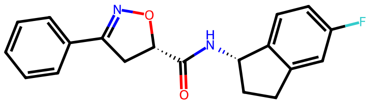	O=C(N[C@H]1CCc2cc(F)ccc21)[C@@H]1CC(c2ccccc2)=NO1	56318607	24	324.13	3.12
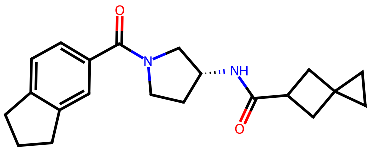	O=C(N[C@@H]1CCN(C(=O)c2ccc3c(c2)CCC3)C1)C1CC2(CC2)C1	942373489	25	338.2	2.7
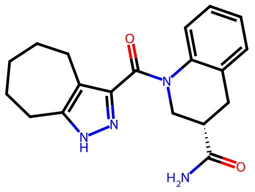	NC(=O)[C@H]1Cc2ccccc2N(C(=O)c2n[nH]c3c2CCCCC3)C1	276470146	25	338.17	1.98
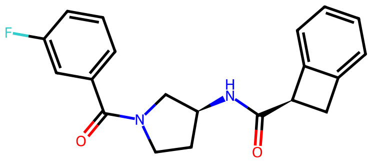	O=C(N[C@H]1CCN(C(=O)c2cccc(F)c2)C1)[C@@H]1Cc2ccccc21	938574406	25	338.14	2.5
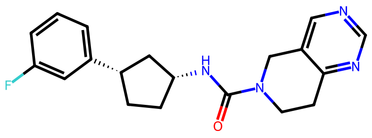	O=C(N[C@@H]1CC[C@H](c2cccc(F)c2)C1)N1CCc2ncncc2C1	883620714	25	340.17	3.02

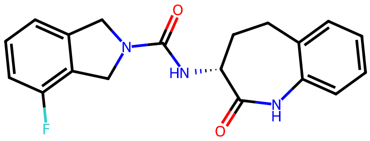	O=C1Nc2ccccc2CC[C@H]1NC(=O)N1Cc2cccc(F)c2C1	654633504	25	339.14	2.8
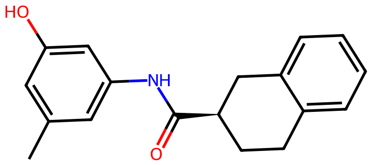	Cc1cc(O)cc(NC(=O)[C@@H]2CCc3ccccc3C2)c1	629629257	21	281.14	3.44
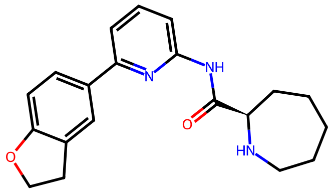	O=C(Nc1cccc(-c2ccc3c(c2)CCO3)n1)[C@H]1CCCCCN1	571926651	25	337.18	3.15
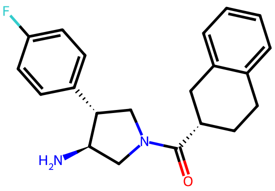	N[C@@H]1CN(C(=O)[C@H]2CCc3ccccc3C2)C[C@H]1c1ccc(F)cc1	1549122621	25	338.18	2.88
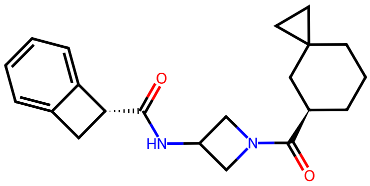	O=C(NC1CN(C(=O)[C@@H]2CCCC3(CC3)C2)C1)[C@@H]1Cc2ccccc21	998688750	25	338.2	2.62
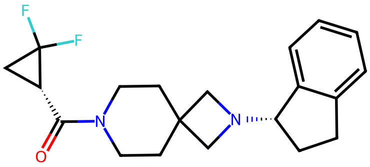	O=C([C@@H]1CC1(F)F)N1CCC2(CC1)CN([C@H]1CCc3ccccc31)C2	1035700082	25	346.19	3.25
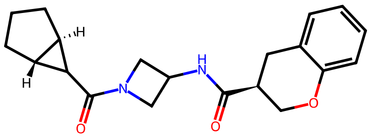	O=C(NC1CN(C(=O)C2[C@@H]3CCC[C@@H]23)C1)[C@@H]1COc2ccccc2C1	995466378	25	340.18	1.61
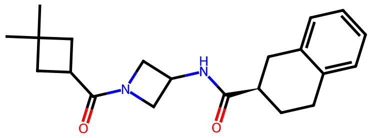	CC1(C)CC(C(=O)N2CC(NC(=O)[C@@H]3CCc4ccccc4C3)C2)C1	1000538499	25	340.22	2.55

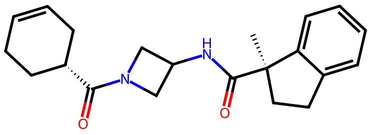	C[C@@]1(C(=O)NC2CN(C(=O)[C@H]3CC=CCC3)C2)CCc2ccccc21	994376600	25	338.2	2.57
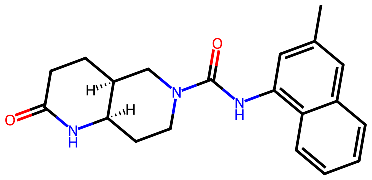	Cc1cc(NC(=O)N2CC[C@H]3NC(=O)CC[C@H]3C2)c2ccccc2c1	667170963	25	337.18	3.28
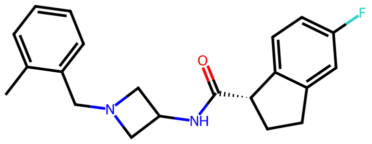	Cc1ccccc1CN1CC(NC(=O)[C@H]2CCc3cc(F)ccc32)C1	1030686255	25	338.18	3.16
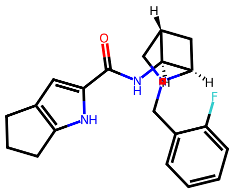	O=C(N[C@H]1[C@H]2C[C@H]1N(Cc1ccccc1F)C2)c1cc2c([nH]1)CCC2	1037675947	25	339.17	2.65
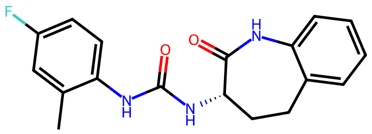	Cc1cc(F)ccc1NC(=O)N[C@H]1CCc2ccccc2NC1=O	1657919531	24	327.14	3.21
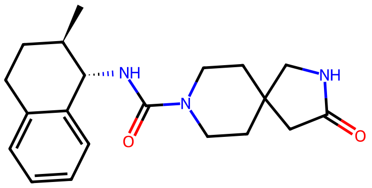	C[C@@H]1CCc2ccccc2[C@H]1NC(=O)N1CCC2(CC1)CNC(=O)C2	270729622	25	341.21	2.62
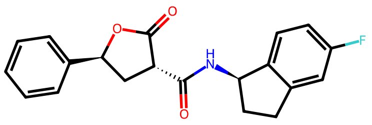	O=C(N[C@@H]1CCc2cc(F)ccc21)[C@@H]1C[C@@H](c2ccccc2)OC1=O	290409575	25	339.13	3.23
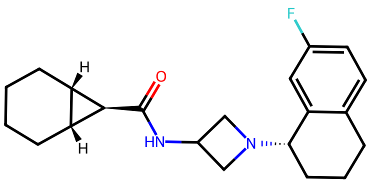	O=C(NC1CN([C@H]2CCCc3ccc(F)cc32)C1)[C@@H]1[C@H]2CCCC[C@H]21	1030189263	25	342.21	3.44

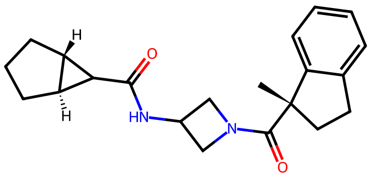	C[C@]1(C(=O)N2CC(NC(=O)C3[C@H]4CCC[C@H]34)C2)CCc2ccccc21	990713338	25	338.2	2.26
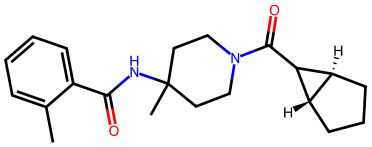	Cc1ccccc1C(=O)NC1(C)CCN(C(=O)C2[C@H]3CCC[C@H]23)CC1	956273159	25	340.22	3.15
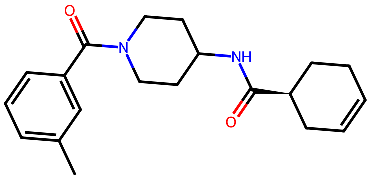	Cc1ccc(C(=O)N2CCC(NC(=O)[C@@H]3CC=CCC3)CC2)c1	943794161	24	326.2	3.07
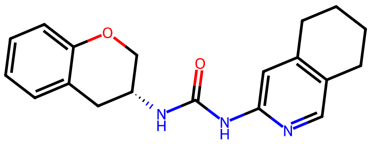	O=C(Nc1cc2c(cn1)CCCC2)N[C@H]1COc2ccccc2C1	361895631	24	323.16	3.09
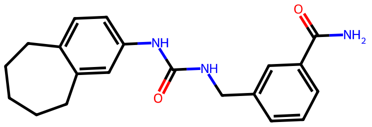	NC(=O)c1cccc(CNC(=O)Nc2ccc3c(c2)CCCCC3)c1	650421081	25	337.18	3.38
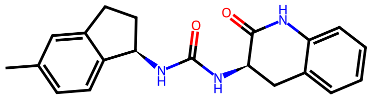	Cc1ccc2c(c1)CC[C@H]2NC(=O)N[C@@H]1Cc2ccccc2NC1=O	636635272	25	335.16	2.84
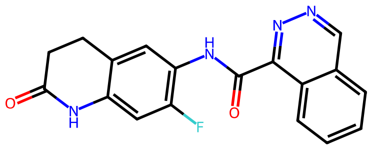	O=C1CCc2cc(NC(=O)c3nncc4ccccc34)c(F)cc2N1	1128349914	25	336.1	2.91
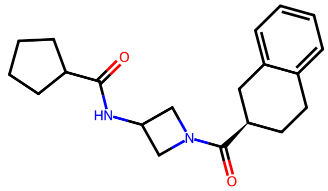	O=C(NC1CN(C(=O)[C@@H]2CCc3ccccc3C2)C1)C1CCCC1	990040688	24	326.2	2.31

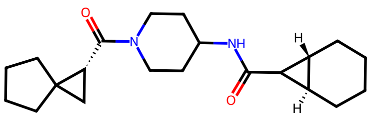	O=C(NC1CCN(C(=O)[C@@H]2CC23CCCC3)CC1)C1[C@@H]2CCCC[C@@H]12	948360791	25	344.25	3.11
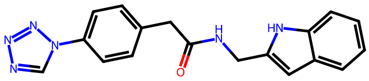	O=C(Cc1ccc(-n2cnnn2)cc1)NCc1cc2ccccc2[nH]1	58900985	25	332.14	2.0
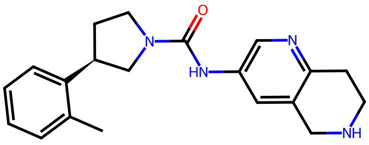	Cc1ccccc1[C@H]1CCN(C(=O)Nc2cnc3c(c2)CNCC3)C1	667795630	25	336.2	3.06
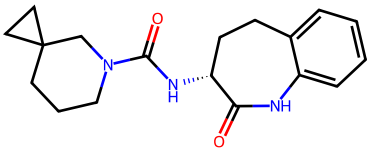	O=C1Nc2ccccc2CC[C@H]1NC(=O)N1CCCC2(CC2)C1	654632646	23	313.18	2.53
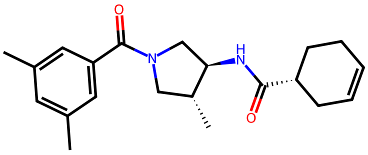	Cc1cc(C)cc(C(=O)N2C[C@@H](NC(=O)[C@H]3CC=CCC3)[C@H](C)C2)c1	959616798	25	340.22	3.24
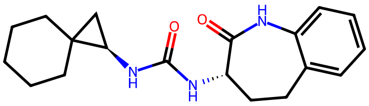	O=C(N[C@H]1CCc2ccccc2NC1=O)N[C@@H]1CC12CCCCC2	656111467	24	327.19	2.96
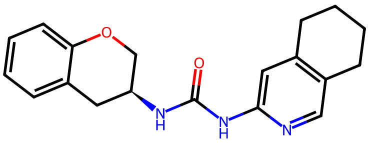	O=C(Nc1cc2c(cn1)CCCC2)N[C@@H]1COc2ccccc2C1	361895630	24	323.16	3.09
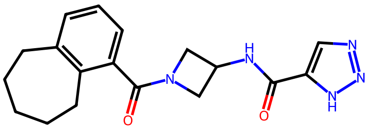	O=C(NC1CN(C(=O)c2cccc3c2CCCCC3)C1)c1cnn[nH]1	995183541	25	339.17	1.33

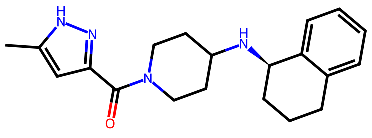	Cc1cc(C(=O)N2CCC(N[C@@H]3CCCc4ccccc43)CC2)n[nH]1	990066318	25	338.21	2.99
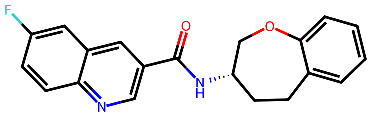	O=C(N[C@H]1CCc2ccccc2OC1)c1cnc2ccc(F)cc2c1	338058789	25	336.13	3.5
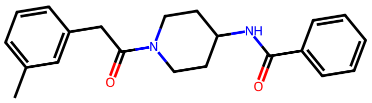	Cc1cccc(CC(=O)N2CCC(NC(=O)c3ccccc3)CC2)c1	75066941	25	336.18	2.96
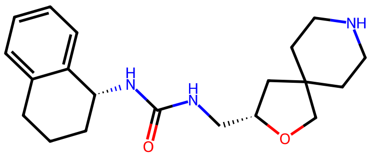	O=C(NC[C@@H]1CC2(CCNCC2)CO1)N[C@@H]1CCCc2ccccc21	874429511	25	343.23	2.52
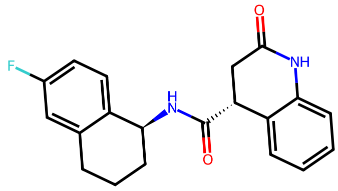	O=C1C[C@@H](C(=O)N[C@H]2CCCc3cc(F)ccc32)c2ccccc2N1	157618765	25	338.14	3.45
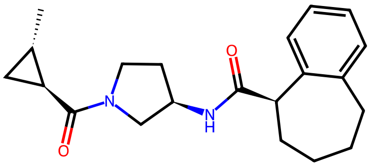	C[C@H]1C[C@@H]1C(=O)N1CC[C@@H](NC(=O)[C@@H]2CCCCc3ccccc32)C1	941123892	25	340.22	2.87
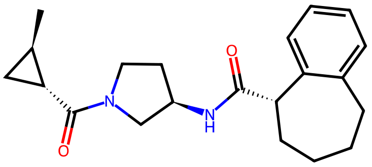	C[C@@H]1C[C@H]1C(=O)N1CC[C@@H](NC(=O)[C@H]2CCCCc3ccccc32)C1	941123891	25	340.22	2.87
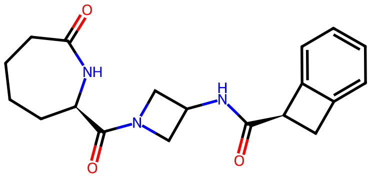	O=C1CCCC[C@H](C(=O)N2CC(NC(=O)[C@@H]3Cc4ccccc43)C2)N1	998690532	25	341.17	0.71

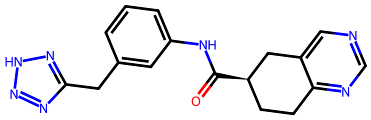	O=C(Nc1cccc(Cc2nn[nH]n2)c1)[C@@H]1CCc2ncncc2C1	1637393778	25	335.15	1.32
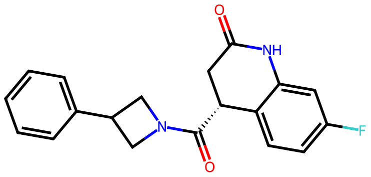	O=C1C[C@@H](C(=O)N2CC(c3ccccc3)C2)c2ccc(F)cc2N1	633806367	24	324.13	2.88
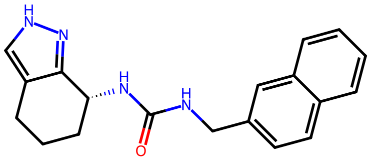	O=C(NCc1ccc2ccccc2c1)N[C@@H]1CCCc2c[nH]nc21	372018597	24	320.16	3.44
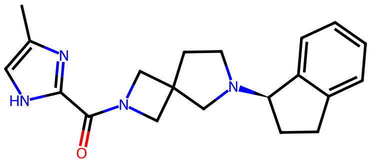	Cc1c[nH]c(C(=O)N2CC3(CCN([C@@H]4CCc5ccccc54)C3)C2)n1	1073046014	25	336.2	2.55
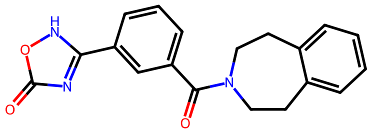	O=C(c1cccc(-c2nc(=O)o[nH]2)c1)N1CCc2ccccc2CC1	1628067040	25	335.13	2.27
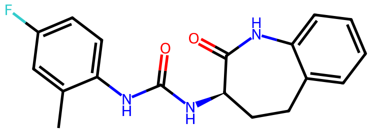	Cc1cc(F)ccc1NC(=O)N[C@@H]1CCc2ccccc2NC1=O	1657919532	24	327.14	3.21
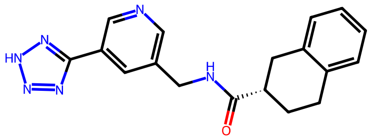	O=C(NCc1cncc(-c2nn[nH]n2)c1)[C@H]1CCc2ccccc2C1	1582557447	25	334.15	1.68
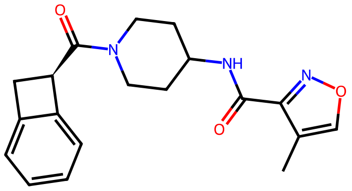	Cc1conc1C(=O)NC1CCN(C(=O)[C@@H]2Cc3ccccc32)CC1	948060015	25	339.16	2.04

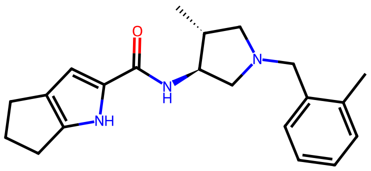	Cc1ccccc1CN1C[C@@H](NC(=O)c2cc3c([nH]2)CCC3)[C@H](C)C1	1080759873	25	337.22	3.06
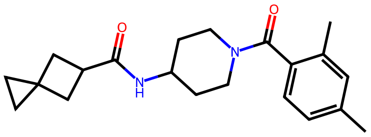	Cc1ccc(C(=O)N2CCC(NC(=O)C3CC4(CC4)C3)CC2)c(C)c1	948346886	25	340.22	3.21
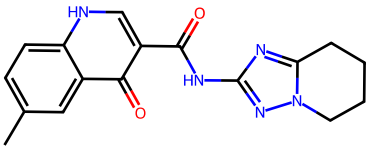	Cc1ccc2[nH]cc(C(=O)Nc3nc4n(n3)CCCC4)c(=O)c2c1	564539201	24	323.14	2.02
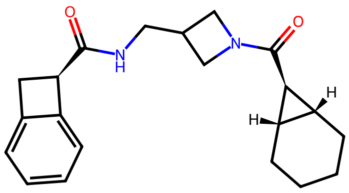	O=C(NCC1CN(C(=O)[C@@H]2[C@H]3CCCC[C@H]32)C1)[C@@H]1Cc2ccccc21	1003949079	25	338.2	2.34
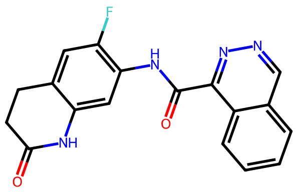	O=C1CCc2cc(F)c(NC(=O)c3nncc4ccccc34)cc2N1	1356626125	25	336.1	2.91
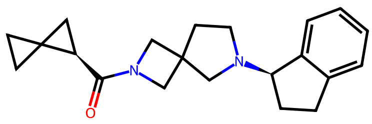	O=C([C@@H]1CC12CC2)N1CC2(CCN([C@@H]3CCc4ccccc43)C2)C1	1073013329	24	322.2	3.01
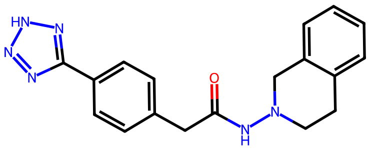	O=C(Cc1ccc(-c2nn[nH]n2)cc1)NN1CCc2ccccc2C1	1606592505	25	334.15	1.5
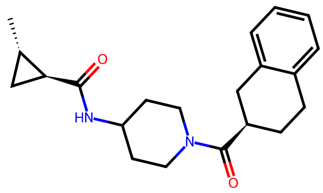	C[C@H]1C[C@@H]1C(=O)NC1CCN(C(=O)[C@@H]2CCc3ccccc3C2)CC1	948572115	25	340.22	2.55

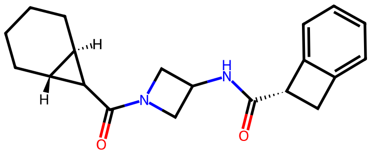	O=C(NC1CN(C(=O)C2[C@@H]3CCCC[C@@H]23)C1)[C@H]1Cc2ccccc21	998680568	24	324.18	2.09
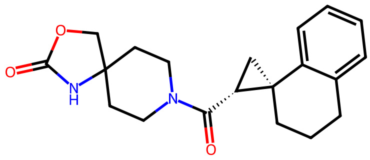	O=C1NC2(CCN(C(=O)[C@@H]3C[C@]34CCCc3ccccc34)CC2)CO1	495140524	25	340.18	2.38
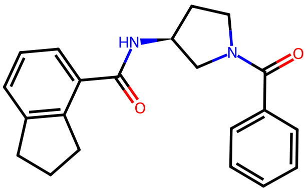	O=C(N[C@H]1CCN(C(=O)c2ccccc2)C1)c1cccc2c1CCC2	938147128	25	334.17	2.82
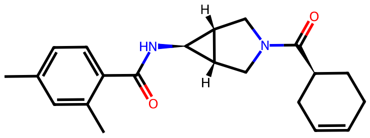	Cc1ccc(C(=O)N[C@H]2[C@@H]3CN(C(=O)[C@@H]4CC=CCC4)C[C@@H]32)c(C)c1	977175942	25	338.2	2.85
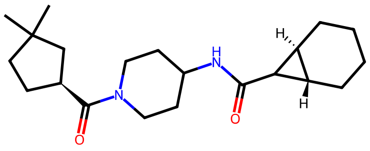	CC1(C)CC[C@H](C(=O)N2CCC(NC(=O)C3[C@H]4CCCC[C@H]34)CC2)C1	948254808	25	346.26	3.36
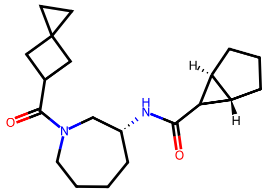	O=C(N[C@@H]1CCCCN(C(=O)C2CC3(CC3)C2)C1)C1[C@H]2CCC[C@H]12	943166819	24	330.23	2.72
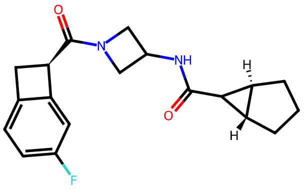	O=C(NC1CN(C(=O)[C@@H]2Cc3ccc(F)cc32)C1)C1[C@H]2CCC[C@H]12	990707695	24	328.16	1.84
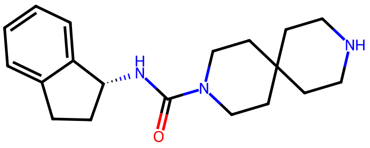	O=C(N[C@@H]1CCc2ccccc21)N1CCC2(CCNCC2)CC1	416928697	23	313.22	2.85

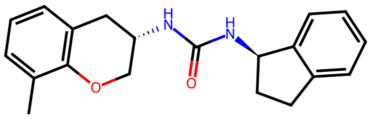	Cc1cccc2c1OC[C@@H](NC(=O)N[C@@H]1CCc3ccccc31)C2	811996752	24	322.17	3.29
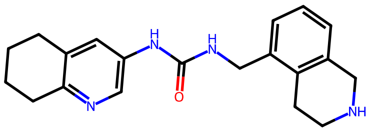	O=C(NCc1cccc2c1CCNC2)Nc1cnc2c(c1)CCCC2	632932025	25	336.2	2.93
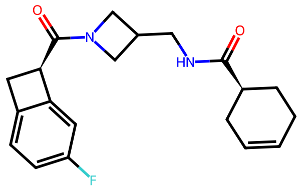	O=C(NCC1CN(C(=O)[C@@H]2Cc3ccc(F)cc32)C1)[C@@H]1CC=CCC1	1000868607	25	342.17	2.4
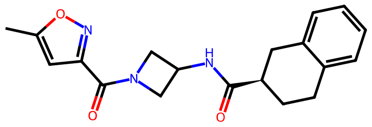	Cc1cc(C(=O)N2CC(NC(=O)[C@@H]3CCc4ccccc4C3)C2)no1	1000540847	25	339.16	1.73
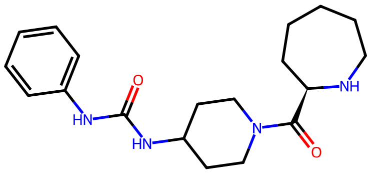	O=C(Nc1ccccc1)NC1CCN(C(=O)[C@H]2CCCCCN2)CC1	364461117	25	344.22	2.33
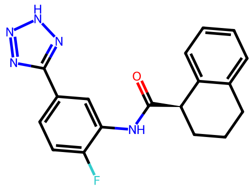	O=C(Nc1cc(-c2nn[nH]n2)ccc1F)[C@@H]1CCCc2ccccc21	1584714593	25	337.13	3.06
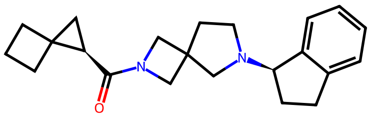	O=C([C@@H]1CC12CCC2)N1CC2(CCN([C@@H]3CCc4ccccc43)C2)C1	1073018966	25	336.22	3.4
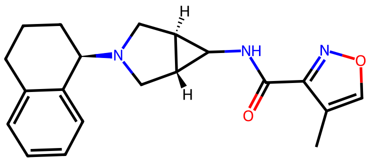	Cc1conc1C(=O)NC1[C@@H]2CN([C@@H]3CCCc4ccccc43)C[C@@H]12	962216964	25	337.18	2.72

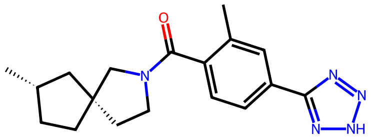	Cc1cc(-c2nn[nH]n2)ccc1C(=O)N1CC[C@@]2(CC[C@H](C)C2)C1	826245096	24	325.19	2.83
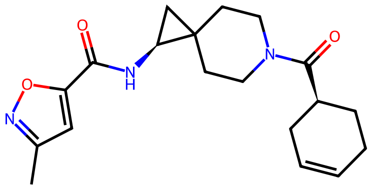	Cc1cc(C(=O)N[C@H]2CC23CCN(C(=O)[C@@H]2CC=CCC2)CC3)on1	963022808	25	343.19	2.45
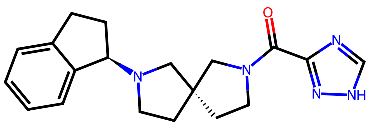	O=C(c1nc[nH]n1)N1CC[C@@]2(CCN([C@@H]3CCc4ccccc43)C2)C1	1041748570	25	337.19	2.03
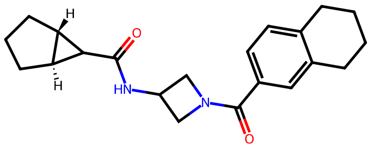	O=C(NC1CN(C(=O)c2ccc3c(c2)CCCC3)C1)C1[C@H]2CCC[C@H]12	990711848	25	338.2	2.55
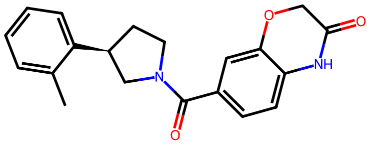	Cc1ccccc1[C@H]1CCN(C(=O)c2ccc3c(c2)OCC(=O)N3)C1	299726618	25	336.15	2.96
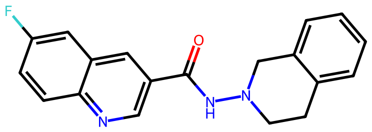	O=C(NN1CCc2ccccc2C1)c1cnc2ccc(F)cc2c1	1583871429	24	321.13	3.08
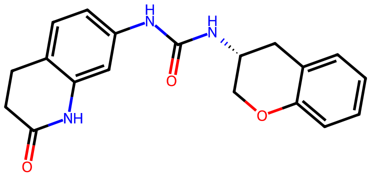	O=C1CCc2ccc(NC(=O)N[C@H]3COc4ccccc4C3)cc2N1	173739363	25	337.14	2.7
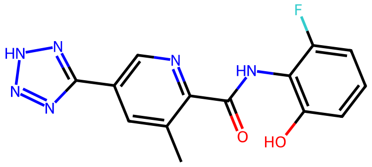	Cc1cc(-c2nn[nH]n2)cnc1C(=O)Nc1c(O)cccc1F	1591880381	23	314.09	1.67

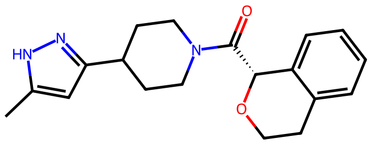	Cc1cc(C2CCN(C(=O)[C@H]3OCCc4ccccc43)CC2)n[nH]1	329521299	24	325.18	2.74
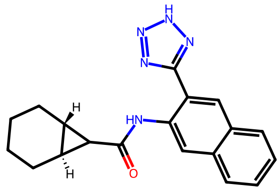	O=C(Nc1cc2ccccc2cc1-c1nn[nH]n1)C1[C@H]2CCCC[C@H]12	1593212523	25	333.16	3.39
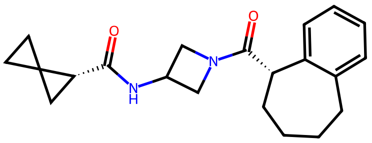	O=C(NC1CN(C(=O)[C@H]2CCCCc3ccccc32)C1)[C@@H]1CC12CC2	1000511986	25	338.2	2.62
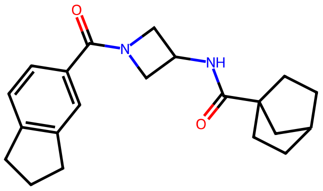	O=C(c1ccc2c(c1)CCC2)N1CC(NC(=O)C23CCC(CC2)C3)C1	995092449	25	338.2	2.7
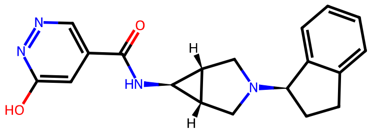	O=C(N[C@H]1[C@@H]2CN([C@@H]3CCc4ccccc43)C[C@@H]21)c1cnnc(O)c1	960411594	25	336.16	1.53
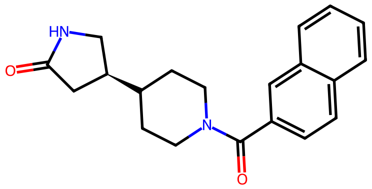	O=C1C[C@H](C2CCN(C(=O)c3ccc4ccccc4c3)CC2)CN1	1551707948	24	322.17	2.83
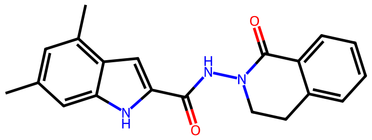	Cc1cc(C)c2cc(C(=O)NN3CCc4ccccc4C3=O)[nH]c2c1	770551651	25	333.15	3.13
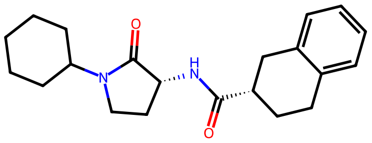	O=C(N[C@@H]1CCN(C2CCCCC2)C1=O)[C@H]1CCc2ccccc2C1	649844875	25	340.22	2.84

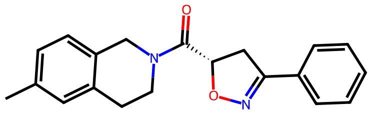	Cc1ccc2c(c1)CCN(C(=O)[C@@H]1CC(c3ccccc3)=NO1)C2	790990145	24	320.15	3.07
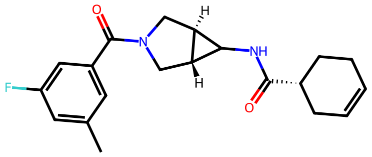	Cc1cc(F)cc(C(=O)N2C[C@H]3C(NC(=O)[C@H]4CC=CCC4)[C@@H]3C2)c1	1012765980	25	342.17	2.68
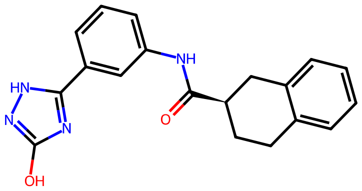	O=C(Nc1cccc(-c2nc(O)n[nH]2)c1)[C@@H]1CCc2ccccc2C1	1635088952	25	334.14	2.92
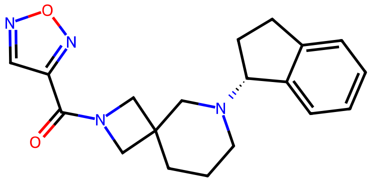	O=C(c1cnon1)N1CC2(CCCN([C@@H]3CCc4ccccc43)C2)C1	982077060	25	338.17	2.3
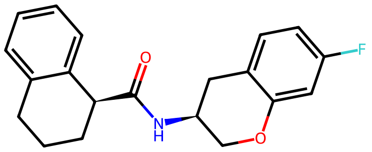	O=C(N[C@@H]1COc2cc(F)ccc2C1)[C@H]1CCCc2ccccc21	1618175296	24	325.15	3.37
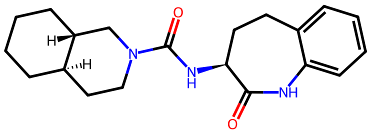	O=C1Nc2ccccc2CC[C@@H]1NC(=O)N1CC[C@H]2CCCC[C@@H]2C1	1349786679	25	341.21	3.16
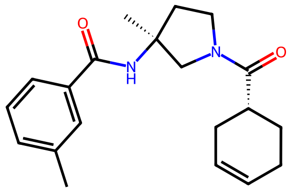	Cc1cccc(C(=O)N[C@@]2(C)CCN(C(=O)[C@H]3CC=CCC3)C2)c1	956663616	24	326.2	3.07
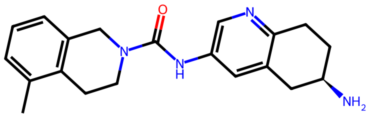	Cc1cccc2c1CCN(C(=O)Nc1cnc3c(c1)C[C@H](N)CC3)C2	841323387	25	336.2	2.8

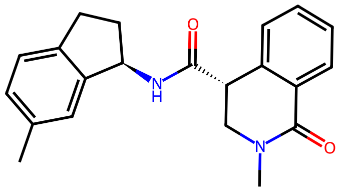	Cc1ccc2c(c1)[C@H](NC(=O)[C@H]1CN(C)C(=O)c3ccccc31)CC2	683741565	25	334.17	2.97
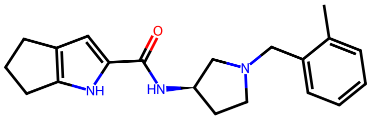	Cc1ccccc1CN1CC[C@@H](NC(=O)c2cc3c([nH]2)CCC3)C1	1015979531	24	323.2	2.82
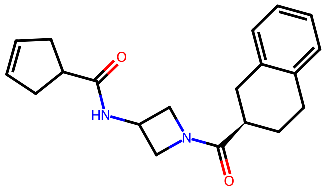	O=C(NC1CN(C(=O)[C@@H]2CCc3ccccc3C2)C1)C1CC=CC1	990587768	24	324.18	2.08
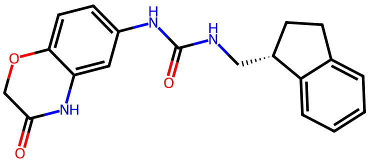	O=C1COc2ccc(NC(=O)NC[C@@H]3CCc4ccccc43)cc2N1	111714338	25	337.14	2.87
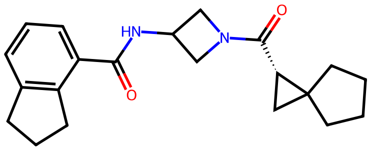	O=C(NC1CN(C(=O)[C@H]2CC23CCCC3)C1)c1cccc2c1CCC2	994404393	25	338.2	2.7
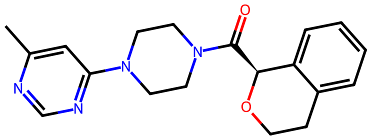	Cc1cc(N2CCN(C(=O)[C@@H]3OCCc4ccccc43)CC2)ncn1	1055978668	25	338.17	1.75
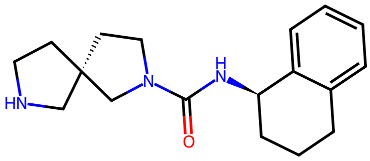	O=C(N[C@@H]1CCCc2ccccc21)N1CC[C@]2(CCNC2)C1	335270122	22	299.2	2.46
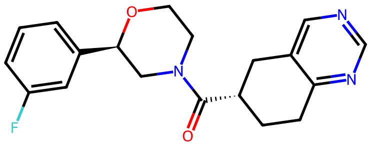	O=C([C@H]1CCc2ncncc2C1)N1CCO[C@H](c2cccc(F)c2)C1	648764903	25	341.15	2.32

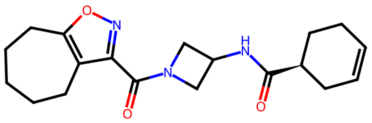	O=C(NC1CN(C(=O)c2noc3c2CCCCC3)C1)[C@@H]1CC=CCC1	990220828	25	343.19	2.24
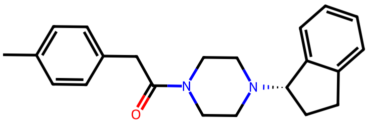	Cc1ccc(CC(=O)N2CCN([C@H]3CCc4ccccc43)CC2)cc1	682077056	25	334.2	3.37
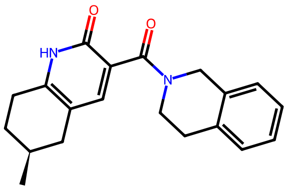	C[C@H]1CCc2[nH]c(=O)c(C(=O)N3CCc4ccccc4C3)cc2C1	49551331	24	322.17	2.7
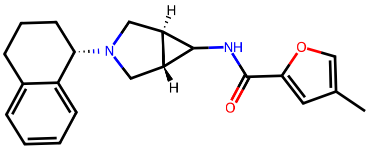	Cc1coc(C(=O)NC2[C@@H]3CN([C@H]4CCCc5ccccc54)C[C@@H]23)c1	961517321	25	336.18	3.33
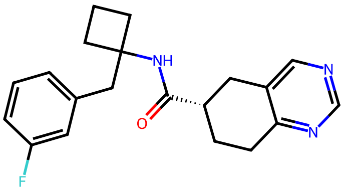	O=C(NC1(Cc2cccc(F)c2)CCC1)[C@H]1CCc2ncncc2C1	648361278	25	339.17	3.0
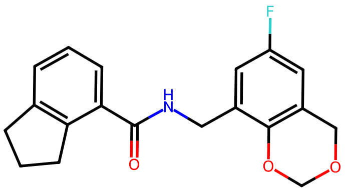	O=C(NCc1cc(F)cc2c1OCOC2)c1cccc2c1CCC2	170660844	24	327.13	3.11
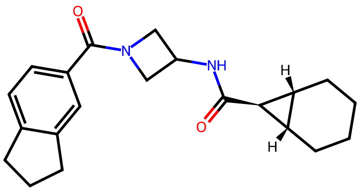	O=C(NC1CN(C(=O)c2ccc3c(c2)CCC3)C1)[C@@H]1[C@H]2CCCC[C@H]21	990665613	25	338.2	2.55
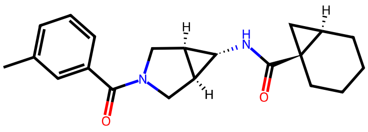	Cc1cccc(C(=O)N2C[C@H]3[C@H](NC(=O)[C@]45CCCC[C@@H]4C5)[C@H]3C2)c1	977154239	25	338.2	2.76

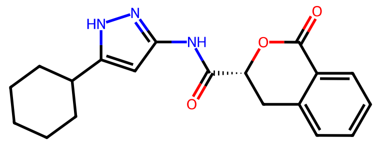	O=C1O[C@@H](C(=O)Nc2cc(C3CCCCC3)[nH]n2)Cc2ccccc21	735929183	25	339.16	3.18
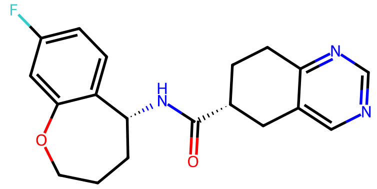	O=C(N[C@@H]1CCCOc2cc(F)ccc21)[C@@H]1CCc2ncncc2C1	648347054	25	341.15	2.75
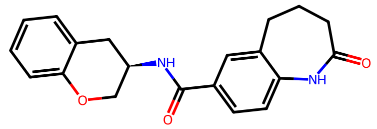	O=C1CCCc2cc(C(=O)N[C@H]3COc4ccccc4C3)ccc2N1	613756984	25	336.15	2.69
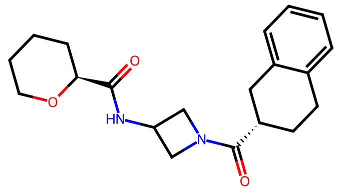	O=C(NC1CN(C(=O)[C@H]2CCc3ccccc3C2)C1)[C@@H]1CCCCO1	990855880	25	342.19	1.69
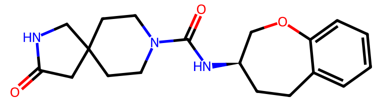	O=C1CC2(CCN(C(=O)N[C@@H]3CCc4ccccc4OC3)CC2)CN1	374967945	25	343.19	1.69
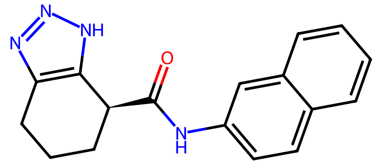	O=C(Nc1ccc2ccccc2c1)[C@H]1CCCc2nn[nH]c21	816810832	22	292.13	3.02
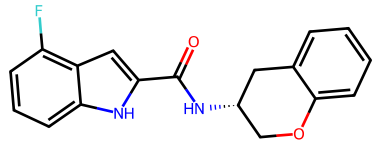	O=C(N[C@H]1COc2ccccc2C1)c1cc2c(F)cccc2[nH]1	53045581	23	310.11	3.04
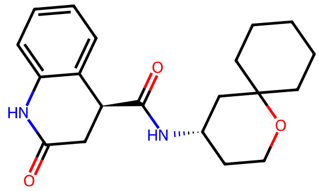	O=C1C[C@H](C(=O)N[C@H]2CCOC3(CCCCC3)C2)c2ccccc2N1	162335638	25	342.19	3.11

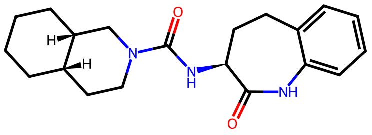	O=C1Nc2ccccc2CC[C@@H]1NC(=O)N1CC[C@@H]2CCCC[C@@H]2C1	1349786680	25	341.21	3.16
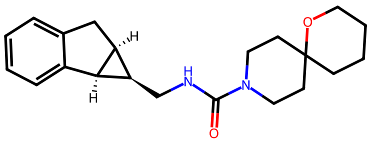	O=C(NC[C@H]1[C@H]2c3ccccc3C[C@H]21)N1CCC2(CCCCO2)CC1	883522630	25	340.22	3.32
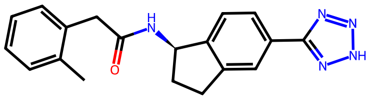	Cc1ccccc1CC(=O)N[C@@H]1CCc2cc(-c3nn[nH]n3)ccc21	1592253954	25	333.16	2.52
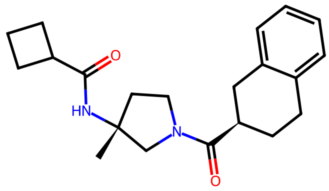	C[C@@]1(NC(=O)C2CCC2)CCN(C(=O)[C@@H]2CCc3ccccc3C2)C1	956628617	25	340.22	2.7
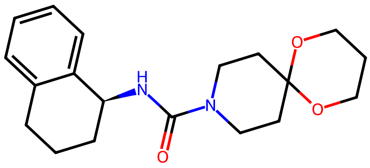	O=C(N[C@H]1CCCc2ccccc21)N1CCC2(CC1)OCCCO2	297426001	24	330.19	3.0
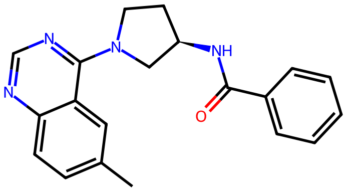	Cc1ccc2ncnc(N3CC[C@@H](NC(=O)c4ccccc4)C3)c2c1	1058285701	25	332.16	2.95
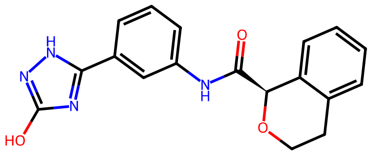	O=C(Nc1cccc(-c2nc(O)n[nH]2)c1)[C@@H]1OCCc2ccccc21	367739075	25	336.12	2.43
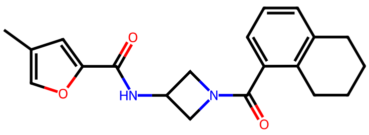	Cc1coc(C(=O)NC2CN(C(=O)c3cccc4c3CCCC4)C2)c1	997958426	25	338.16	2.72

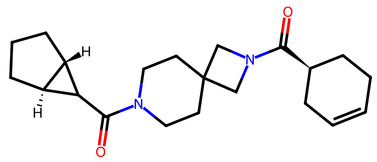	O=C(C1[C@H]2CCC[C@H]12)N1CCC2(CC1)CN(C(=O)[C@@H]1CC=CCC1)C2	944903092	25	342.23	2.84
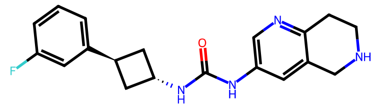	O=C(Nc1cnc2c(c1)CNCC2)N[C@H]1C[C@H](c2cccc(F)c2)C1	667805049	25	340.17	2.93
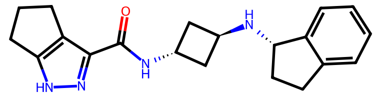	O=C(N[C@H]1C[C@H](N[C@H]2CCc3ccccc32)C1)c1n[nH]c2c1CCC2	1020345308	25	336.2	2.44
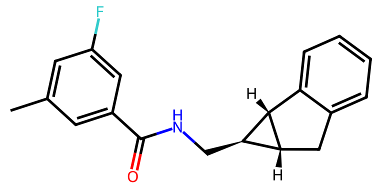	Cc1cc(F)cc(C(=O)NC[C@@H]2[C@H]3Cc4ccccc4[C@H]32)c1	488712635	22	295.14	3.45
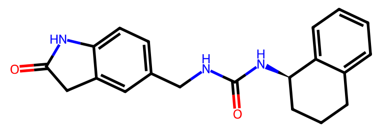	O=C1Cc2cc(CNC(=O)N[C@@H]3CCCc4ccccc43)ccc2N1	1186488472	25	335.16	3.06
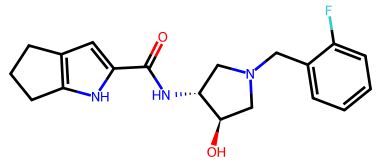	O=C(N[C@@H]1CN(Cc2ccccc2F)C[C@H]1O)c1cc2c([nH]1)CCC2	1077986313	25	343.17	1.62
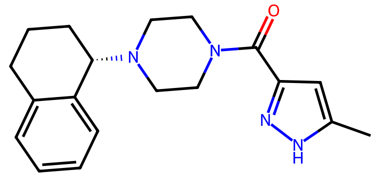	Cc1cc(C(=O)N2CCN([C@H]3CCCc4ccccc43)CC2)n[nH]1	1480339067	24	324.2	2.55
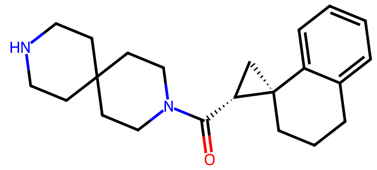	O=C([C@@H]1C[C@]12CCCc1ccccc12)N1CCC2(CCNCC2)CC1	357887482	25	338.24	3.27

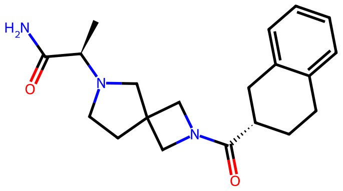	C[C@H](C(N)=O)N1CCC2(CN(C(=O)[C@H]3CCc4ccccc4C3)C2)C1	1073013591	25	341.21	1.2
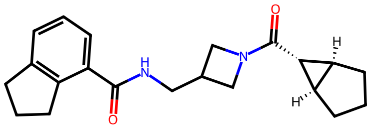	O=C(NCC1CN(C(=O)[C@@H]2[C@H]3CCC[C@H]32)C1)c1cccc2c1CCC2	1002702685	25	338.2	2.41
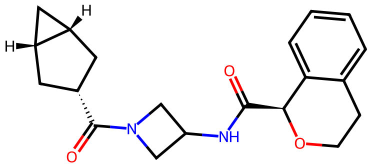	O=C(NC1CN(C(=O)[C@@H]2C[C@@H]3C[C@@H]3C2)C1)[C@@H]1OCCc2ccccc21	997321242	25	340.18	1.67
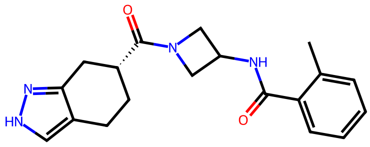	Cc1ccccc1C(=O)NC1CN(C(=O)[C@@H]2CCc3c[nH]nc3C2)C1	990112853	25	338.17	1.46
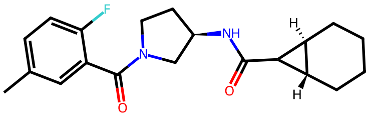	Cc1ccc(F)c(C(=O)N2CC[C@@H](NC(=O)C3[C@H]4CCCC[C@H]34)C2)c1	936274002	25	344.19	2.9
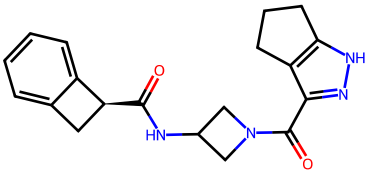	O=C(NC1CN(C(=O)c2n[nH]c3c2CCC3)C1)[C@H]1Cc2ccccc21	998669182	25	336.16	1.18
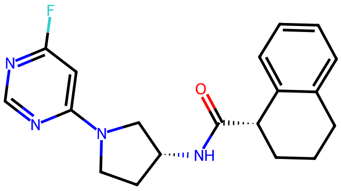	O=C(N[C@@H]1CCN(c2cc(F)ncn2)C1)[C@H]1CCCc2ccccc21	1058591966	25	340.17	2.43
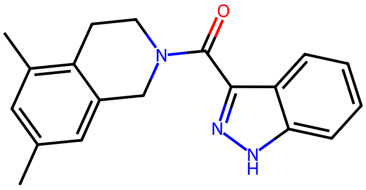	Cc1cc(C)c2c(c1)CN(C(=O)c1n[nH]c3ccccc13)CC2	362477228	23	305.15	3.38

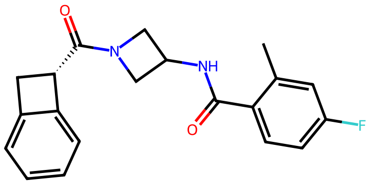	Cc1cc(F)ccc1C(=O)NC1CN(C(=O)[C@H]2Cc3ccccc32)C1	997181371	25	338.14	2.41
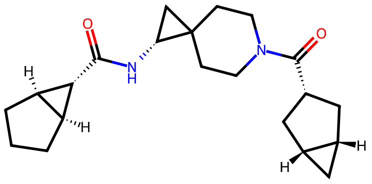	O=C(N[C@@H]1CC12CCN(C(=O)[C@@H]1C[C@@H]3C[C@@H]3C1)CC2)[C@@H]1[C@H]2CCC[C@H]21	963491143	25	342.23	2.58
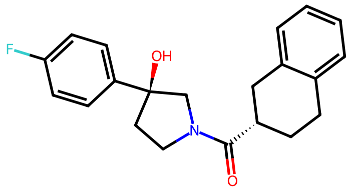	O=C([C@H]1CCc2ccccc2C1)N1CC[C@](O)(c2ccc(F)cc2)C1	845919477	25	339.16	3.05
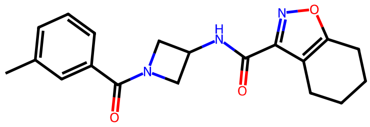	Cc1cccc(C(=O)N2CC(NC(=O)c3noc4c3CCCC4)C2)c1	1000452868	25	339.16	2.12
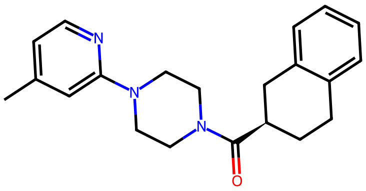	Cc1ccnc(N2CCN(C(=O)[C@@H]3CCc4ccccc4C3)CC2)c1	514733996	25	335.2	2.84
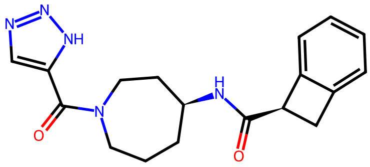	O=C(N[C@H]1CCCN(C(=O)c2cnn[nH]2)CC1)[C@@H]1Cc2ccccc21	965142451	25	339.17	1.26
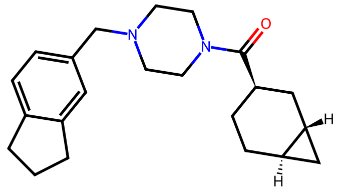	O=C([C@@H]1CC[C@@H]2C[C@H]2C1)N1CCN(Cc2ccc3c(c2)CCC3)CC1	948952250	25	338.24	3.26
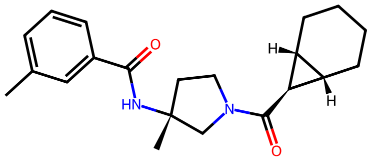	Cc1cccc(C(=O)N[C@]2(C)CCN(C(=O)[C@@H]3[C@H]4CCCC[C@H]43)C2)c1	956661914	25	340.22	3.15

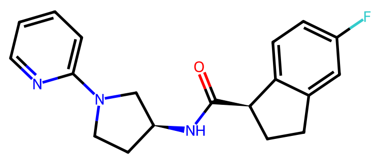	O=C(N[C@H]1CCN(c2ccccn2)C1)[C@@H]1CCc2cc(F)ccc21	612904284	24	325.16	2.65
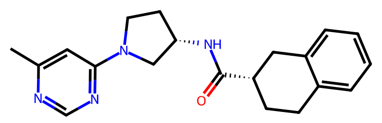	Cc1cc(N2CC[C@H](NC(=O)[C@H]3CCc4ccccc4C3)C2)ncn1	1058559923	25	336.2	2.29
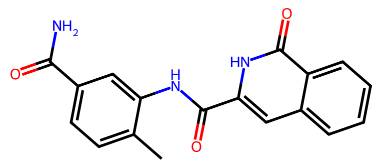	Cc1ccc(C(N)=O)cc1NC(=O)c1cc2ccccc2c(=O)[nH]1	58251187	24	321.11	2.19
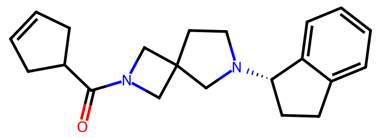	O=C(C1CC=CC1)N1CC2(CCN([C@H]3CCc4ccccc43)C2)C1	1072568298	24	322.2	3.17
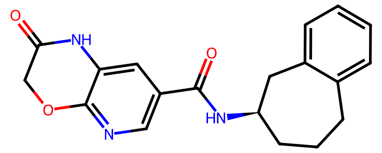	O=C1COc2ncc(C(=O)N[C@@H]3CCCc4ccccc4C3)cc2N1	888250810	25	337.14	2.09
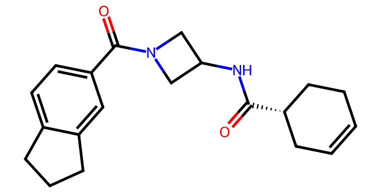	O=C(NC1CN(C(=O)c2ccc3c(c2)CCC3)C1)[C@H]1CC=CCC1	990222651	24	324.18	2.47
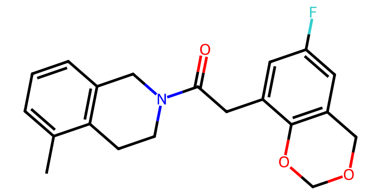	Cc1cccc2c1CCN(C(=O)Cc1cc(F)cc3c1OCOC3)C2	729413804	25	341.14	3.13
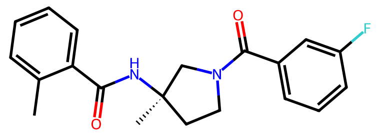	Cc1ccccc1C(=O)N[C@]1(C)CCN(C(=O)c2cccc(F)c2)C1	956660121	25	340.16	3.17

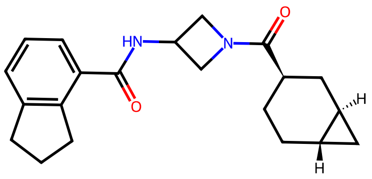	O=C(NC1CN(C(=O)[C@@H]2CC[C@H]3C[C@@H]3C2)C1)c1cccc2c1CCC2	994400576	25	338.2	2.55
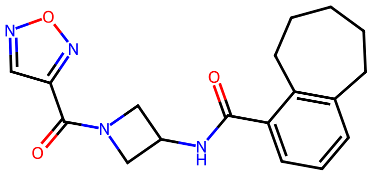	O=C(NC1CN(C(=O)c2cnon2)C1)c1cccc2c1CCCCC2	994064007	25	340.15	1.59
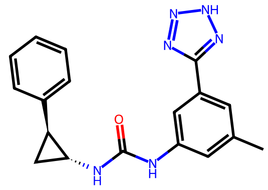	Cc1cc(NC(=O)N[C@@H]2C[C@H]2c2ccccc2)cc(-c2nn[nH]n2)c1	1592028040	25	334.15	2.85
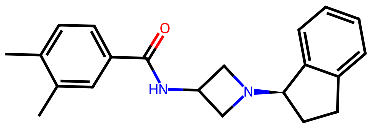	Cc1ccc(C(=O)NC2CN([C@@H]3CCc4ccccc43)C2)cc1C	1031344391	24	320.19	3.4
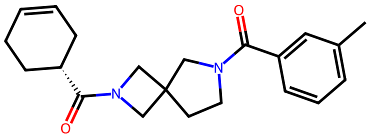	Cc1cccc(C(=O)N2CCC3(C2)CN(C(=O)[C@H]2CC=CCC2)C3)c1	1018770695	25	338.2	3.03
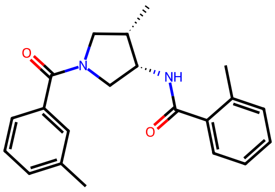	Cc1cccc(C(=O)N2C[C@H](C)[C@H](NC(=O)c3ccccc3C)C2)c1	966115737	25	336.18	3.19
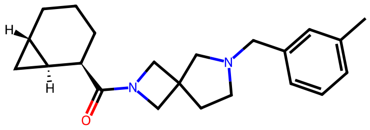	Cc1cccc(CN2CCC3(C2)CN(C(=O)[C@@H]2CCC[C@H]4C[C@@H]42)C3)c1	1072875667	25	338.24	3.47
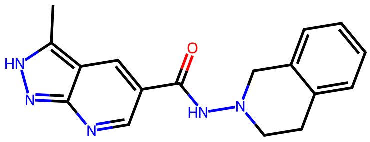	Cc1[nH]nc2ncc(C(=O)NN3CCc4ccccc4C3)cc12	1165596258	23	307.14	1.97

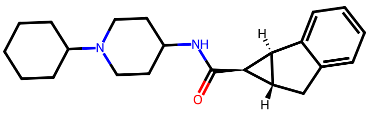	O=C(NC1CCN(C2CCCCC2)CC1)[C@@H]1[C@H]2Cc3ccccc3[C@@H]21	625970886	25	338.24	3.49
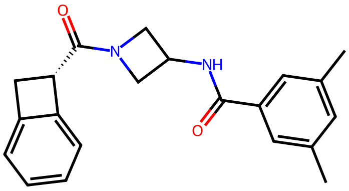	Cc1cc(C)cc(C(=O)NC2CN(C(=O)[C@H]3Cc4ccccc43)C2)c1	999493663	25	334.17	2.58
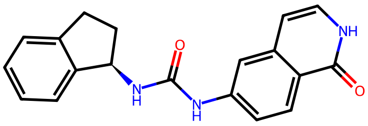	O=C(Nc1ccc2c(=O)[nH]ccc2c1)N[C@@H]1CCc2ccccc21	1192993097	24	319.13	3.34
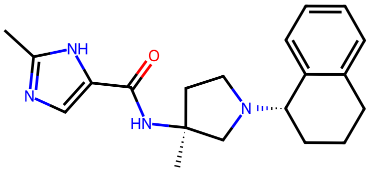	Cc1ncc(C(=O)N[C@@]2(C)CCN([C@H]3CCCc4ccccc43)C2)[nH]1	1046199610	25	338.21	2.99
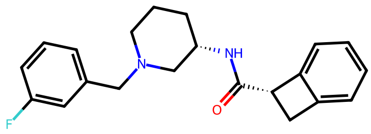	O=C(N[C@H]1CCCN(Cc2cccc(F)c2)C1)[C@H]1Cc2ccccc21	1008359802	25	338.18	3.25
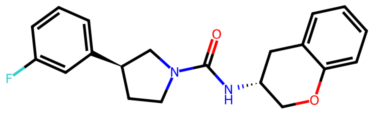	O=C(N[C@H]1COc2ccccc2C1)N1CC[C@@H](c2cccc(F)c2)C1	552634904	25	340.16	3.33
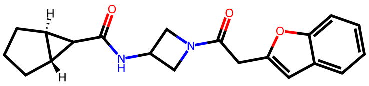	O=C(NC1CN(C(=O)Cc2cc3ccccc3o2)C1)C1[C@@H]2CCC[C@@H]12	990708143	25	338.16	2.35
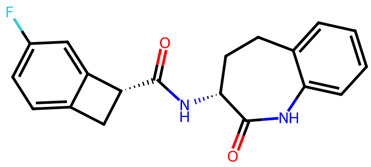	O=C1Nc2ccccc2CC[C@H]1NC(=O)[C@@H]1Cc2ccc(F)cc21	653998860	24	324.13	2.54

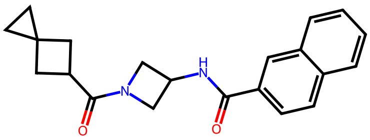	O=C(NC1CN(C(=O)C2CC3(CC3)C2)C1)c1ccc2ccccc2c1	999455906	25	334.17	2.97
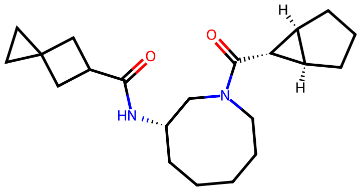	O=C(N[C@H]1CCCCCN(C(=O)[C@@H]2[C@H]3CCC[C@H]32)C1)C1CC2(CC2)C1	986974532	25	344.25	3.11
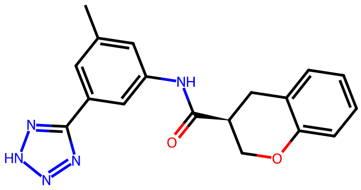	Cc1cc(NC(=O)[C@@H]2COc3ccccc3C2)cc(-c2nn[nH]n2)c1	1592017219	25	335.14	2.36
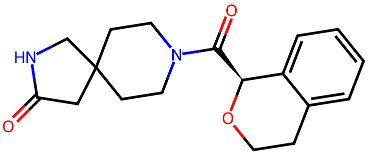	O=C1CC2(CCN(C(=O)[C@@H]3OCCc4ccccc43)CC2)CN1	269752052	23	314.16	1.43
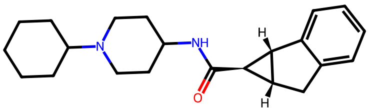	O=C(NC1CCN(C2CCCCC2)CC1)[C@@H]1[C@H]2Cc3ccccc3[C@H]21	625970887	25	338.24	3.49
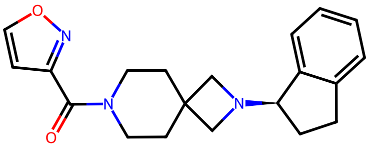	O=C(c1ccon1)N1CCC2(CC1)CN([C@@H]1CCc3ccccc31)C2	1035660881	25	337.18	2.9
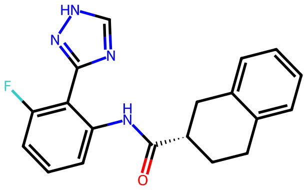	O=C(Nc1cccc(F)c1-c1nc[nH]n1)[C@H]1CCc2ccccc2C1	1639693333	25	336.14	3.35
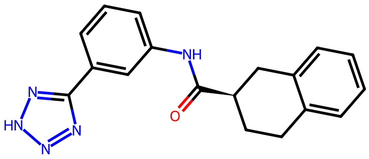	O=C(Nc1cccc(-c2nn[nH]n2)c1)[C@@H]1CCc2ccccc2C1	1540505403	24	319.14	2.61

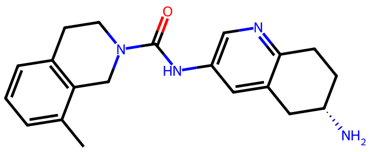	Cc1cccc2c1CN(C(=O)Nc1cnc3c(c1)C[C@@H](N)CC3)CC2	333378508	25	336.2	2.8
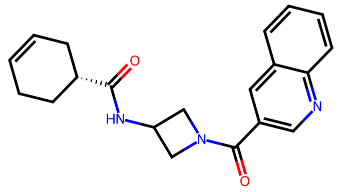	O=C(NC1CN(C(=O)c2cnc3ccccc3c2)C1)[C@H]1CC=CCC1	990221489	25	335.16	2.53
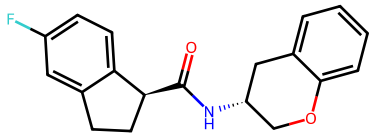	O=C(N[C@H]1COc2ccccc2C1)[C@H]1CCc2cc(F)ccc21	666390979	23	311.13	2.98
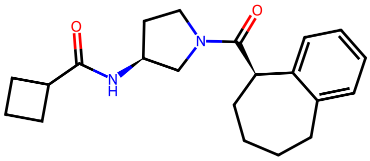	O=C(N[C@H]1CCN(C(=O)[C@@H]2CCCCc3ccccc32)C1)C1CCC1	937592821	25	340.22	3.01
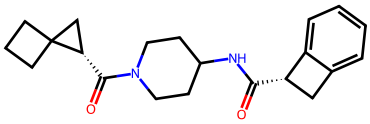	O=C(NC1CCN(C(=O)[C@H]2CC23CCC3)CC1)[C@H]1Cc2ccccc21	946759061	25	338.2	2.62
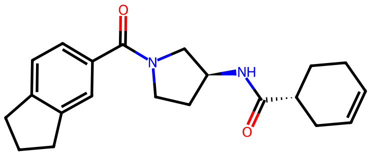	O=C(N[C@H]1CCN(C(=O)c2ccc3c(c2)CCC3)C1)[C@H]1CC=CCC1	937668076	25	338.2	2.86
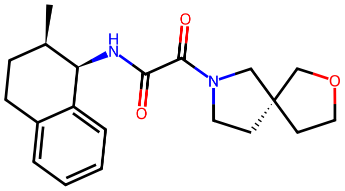	C[C@@H]1CCc2ccccc2[C@@H]1NC(=O)C(=O)N1CC[C@]2(CCOC2)C1	673299625	25	342.19	2.07
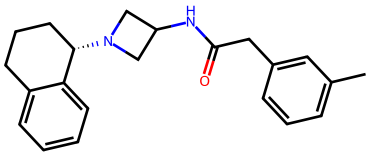	Cc1cccc(CC(=O)NC2CN([C@H]3CCCc4ccccc43)C2)c1	1030884487	25	334.2	3.42

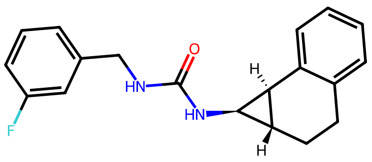	O=C(NCc1cccc(F)c1)N[C@@H]1[C@H]2CCc3ccccc3[C@@H]21	126057793	23	310.15	3.35
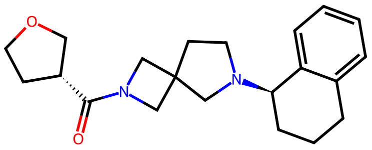	O=C([C@@H]1CCOC1)N1CC2(CCN([C@@H]3CCCc4ccccc43)C2)C1	1072541614	25	340.22	2.63
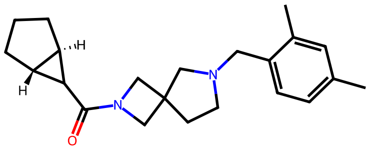	Cc1ccc(CN2CCC3(C2)CN(C(=O)C2[C@@H]4CCC[C@@H]24)C3)c(C)c1	1072592457	25	338.24	3.38
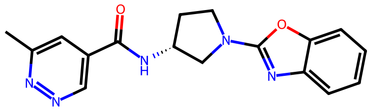	Cc1cc(C(=O)N[C@@H]2CCN(c3nc4ccccc4o3)C2)cnn1	1058340427	24	323.14	1.93
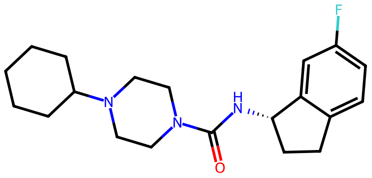	O=C(N[C@H]1CCc2ccc(F)cc21)N1CCN(C2CCCCC2)CC1	531996533	25	345.22	3.47
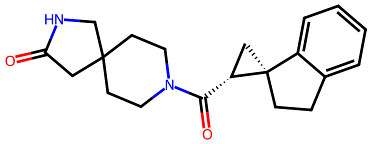	O=C1CC2(CCN(C(=O)[C@@H]3C[C@]34CCc3ccccc34)CC2)CN1	267619864	24	324.18	2.02
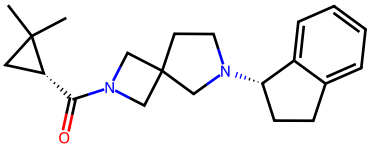	CC1(C)C[C@H]1C(=O)N1CC2(CCN([C@H]3CCc4ccccc43)C2)C1	1072637821	24	324.22	3.25
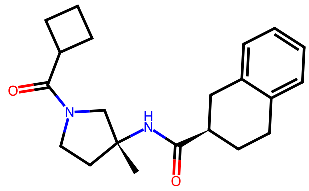	C[C@@]1(NC(=O)[C@@H]2CCc3ccccc3C2)CCN(C(=O)C2CCC2)C1	957727573	25	340.22	2.7

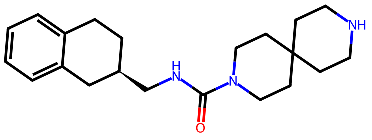	O=C(NC[C@@H]1CCc2ccccc2C1)N1CCC2(CCNCC2)CC1	669551296	25	341.25	2.97
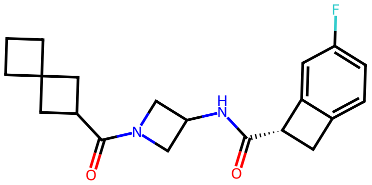	O=C(NC1CN(C(=O)C2CC3(CCC3)C2)C1)[C@H]1Cc2ccc(F)cc21	998490199	25	342.17	2.37
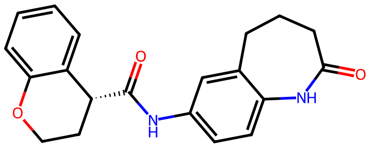	O=C1CCCc2cc(NC(=O)[C@@H]3CCOc4ccccc43)ccc2N1	171249905	25	336.15	3.47
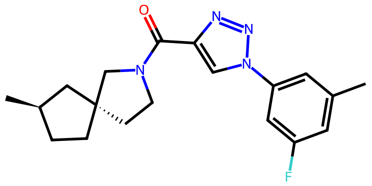	Cc1cc(F)cc(-n2cc(C(=O)N3CC[C@@]4(CC[C@@H](C)C4)C3)nn2)c1	329866399	25	342.19	3.37
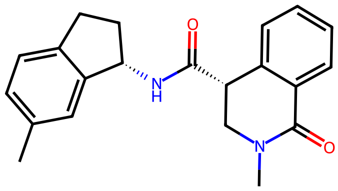	Cc1ccc2c(c1)[C@@H](NC(=O)[C@H]1CN(C)C(=O)c3ccccc31)CC2	683741561	25	334.17	2.97
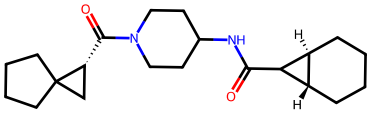	O=C(NC1CCN(C(=O)[C@@H]2CC23CCCC3)CC1)C1[C@H]2CCCC[C@H]12	948360788	25	344.25	3.11
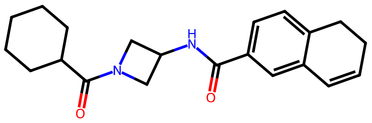	O=C(NC1CN(C(=O)C2CCCCC2)C1)c1ccc2c(c1)C=CCC2	995372852	25	338.2	3.17
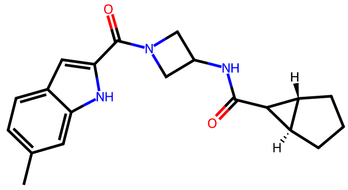	Cc1ccc2cc(C(=O)N3CC(NC(=O)C4[C@@H]5CCC[C@@H]45)C3)[nH]c2c1	990713247	25	337.18	2.46

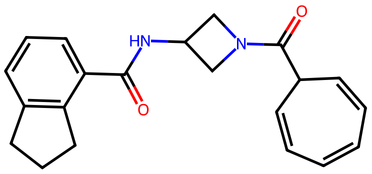	O=C(NC1CN(C(=O)C2C=CC=CC=C2)C1)c1cccc2c1CCC2	994405645	25	334.17	2.41
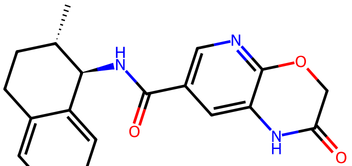	C[C@H]1CCc2ccccc2[C@@H]1NC(=O)c1cnc2c(c1)NC(=O)CO2	271422752	25	337.14	2.47
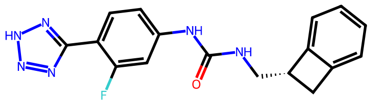	O=C(NC[C@H]1Cc2ccccc21)Nc1ccc(-c2nn[nH]n2)c(F)c1	1609546884	25	338.13	2.47
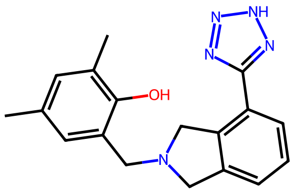	Cc1cc(C)c(O)c(CN2Cc3cccc(-c4nn[nH]n4)c3C2)c1	1605643841	24	321.16	2.7
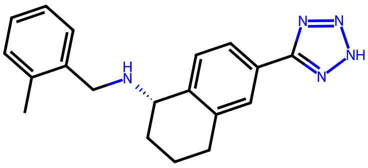	Cc1ccccc1CN[C@H]1CCCc2cc(-c3nn[nH]n3)ccc21	826307432	24	319.18	3.34
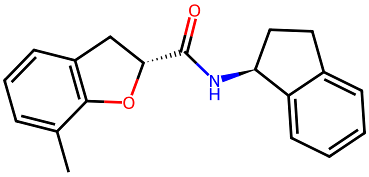	Cc1cccc2c1O[C@@H](C(=O)N[C@H]1CCc3ccccc31)C2	449205621	22	293.14	3.1
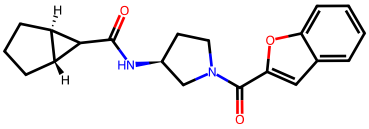	O=C(N[C@H]1CCN(C(=O)c2cc3ccccc3o2)C1)C1[C@@H]2CCC[C@@H]12	937805190	25	338.16	2.81
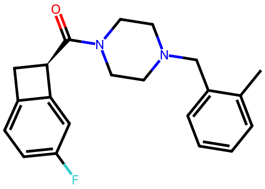	Cc1ccccc1CN1CCN(C(=O)[C@@H]2Cc3ccc(F)cc32)CC1	684362004	25	338.18	3.12

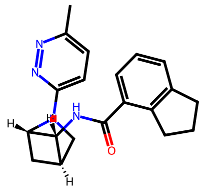	Cc1ccc(N2C[C@H]3C[C@H]2[C@@H]3NC(=O)c2cccc3c2CCC3)nn1	1064268008	25	334.18	2.28
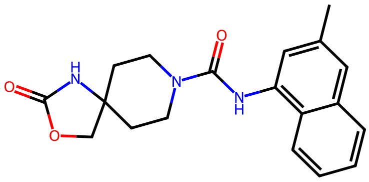	Cc1cc(NC(=O)N2CCC3(CC2)COC(=O)N3)c2ccccc2c1	496154035	25	339.16	3.25
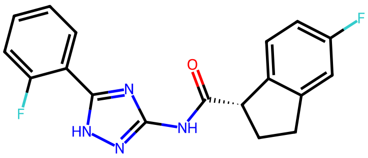	O=C(Nc1n[nH]c(-c2ccccc2F)n1)[C@H]1CCc2cc(F)ccc21	1654249885	25	340.11	3.42
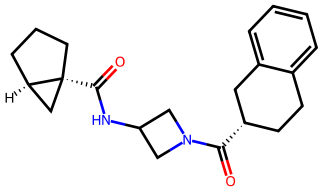	O=C([C@H]1CCc2ccccc2C1)N1CC(NC(=O)[C@@]23CCC[C@@H]2C3)C1	997549130	25	338.2	2.31
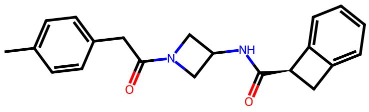	Cc1ccc(CC(=O)N2CC(NC(=O)[C@@H]3Cc4ccccc43)C2)cc1	998694553	25	334.17	2.2
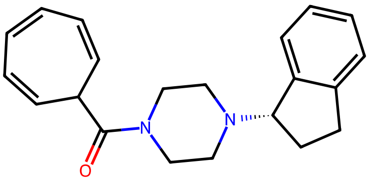	O=C(C1C=CC=CC=C1)N1CCN([C@H]2CCc3ccccc32)CC1	948574725	24	320.19	3.12
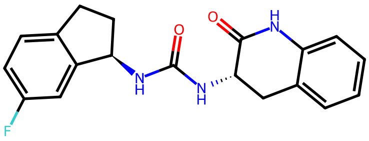	O=C(N[C@H]1Cc2ccccc2NC1=O)N[C@@H]1CCc2ccc(F)cc21	636743798	25	339.14	2.68
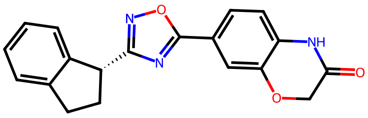	O=C1COc2cc(-c3nc([C@@H]4CCc5ccccc54)no3)ccc2N1	356595885	25	333.11	3.15

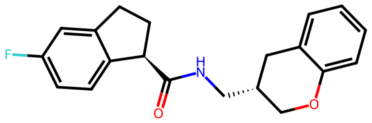	O=C(NC[C@H]1COc2ccccc2C1)[C@@H]1CCc2cc(F)ccc21	854308947	24	325.15	3.22
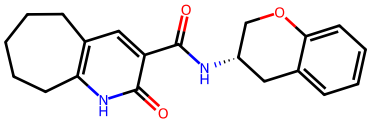	O=C(N[C@@H]1COc2ccccc2C1)c1cc2c([nH]c1=O)CCCCC2	156149165	25	338.16	2.38
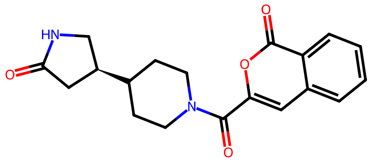	O=C1C[C@H](C2CCN(C(=O)c3cc4ccccc4c(=O)o3)CC2)CN1	1551707933	25	340.14	1.78
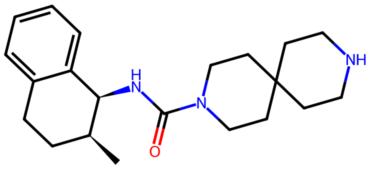	C[C@H]1CCc2ccccc2[C@H]1NC(=O)N1CCC2(CCNCC2)CC1	417945390	25	341.25	3.49
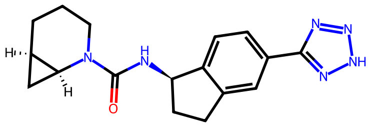	O=C(N[C@@H]1CCc2cc(-c3nn[nH]n3)ccc21)N1CCC[C@@H]2C[C@@H]21	1593000549	24	324.17	2.05
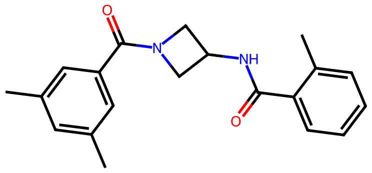	Cc1cc(C)cc(C(=O)N2CC(NC(=O)c3ccccc3C)C2)c1	990111522	24	322.17	2.87
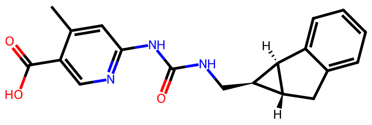	Cc1cc(NC(=O)NC[C@@H]2[C@H]3Cc4ccccc4[C@@H]32)ncc1C(=O)O	1600268872	25	337.14	2.8
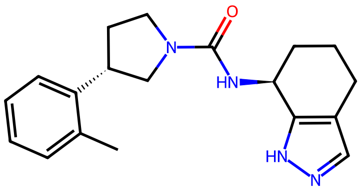	Cc1ccccc1[C@@H]1CCN(C(=O)N[C@@H]2CCCc3cn[nH]c32)C1	269028648	24	324.2	3.29

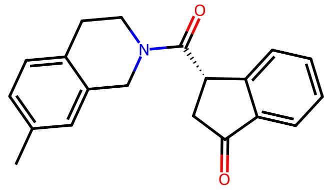	Cc1ccc2c(c1)CN(C(=O)[C@H]1CC(=O)c3ccccc31)CC2	617605443	23	305.14	3.25
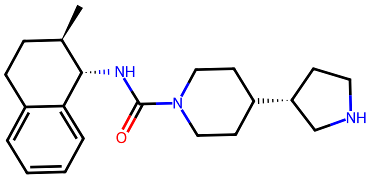	C[C@@H]1CCc2ccccc2[C@H]1NC(=O)N1CCC([C@@H]2CCNC2)CC1	417962169	25	341.25	3.34
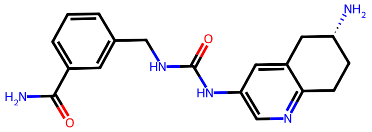	NC(=O)c1cccc(CNC(=O)Nc2cnc3c(c2)C[C@H](N)CC3)c1	415957138	25	339.17	1.32
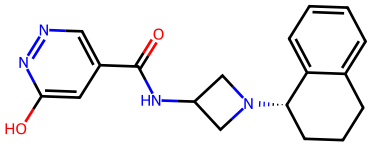	O=C(NC1CN([C@H]2CCCc3ccccc32)C1)c1cnnc(O)c1	1030485672	24	324.16	1.67
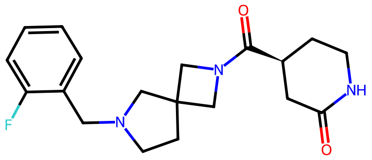	O=C1C[C@@H](C(=O)N2CC3(CCN(Cc4ccccc4F)C3)C2)CCN1	1072612544	25	345.19	1.39
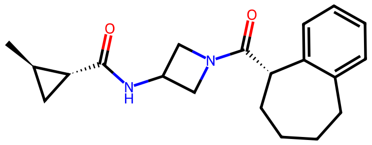	C[C@@H]1C[C@H]1C(=O)NC1CN(C(=O)[C@H]2CCCCc3ccccc32)C1	997466303	24	326.2	2.48
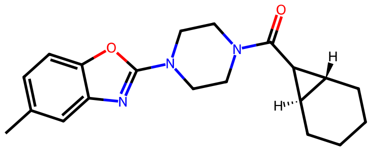	Cc1ccc2oc(N3CCN(C(=O)C4[C@@H]5CCCC[C@@H]45)CC3)nc2c1	174813422	25	339.19	3.22
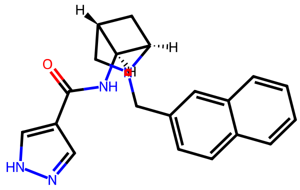	O=C(N[C@H]1[C@H]2C[C@H]1N(Cc1ccc3ccccc3c1)C2)c1cn[nH]c1	1037322147	25	332.16	2.57

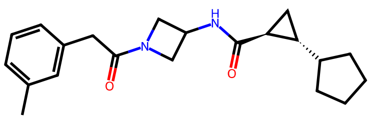	Cc1cccc(CC(=O)N2CC(NC(=O)[C@H]3C[C@@H]3C3CCCC3)C2)c1	998828633	25	340.22	2.69
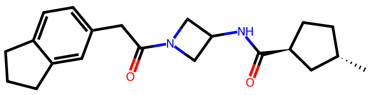	C[C@H]1CC[C@H](C(=O)NC2CN(C(=O)Cc3ccc4c(c3)CCC4)C2)C1	991428373	25	340.22	2.48
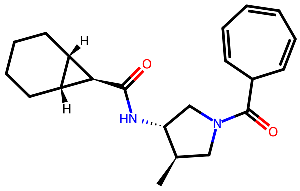	C[C@@H]1CN(C(=O)C2C=CC=CC=C2)C[C@H]1NC(=O)[C@@H]1[C@H]2CCCC[C@H]21	949636602	25	340.22	2.68
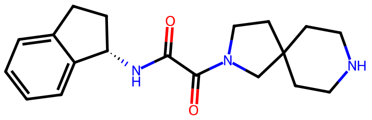	O=C(N[C@H]1CCc2ccccc21)C(=O)N1CCC2(CCNCC2)C1	419085572	24	327.19	1.39
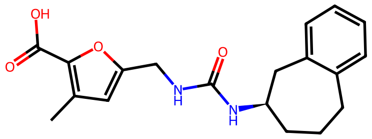	Cc1cc(CNC(=O)N[C@@H]2CCCc3ccccc3C2)oc1C(=O)O	1600199305	25	342.16	3.03
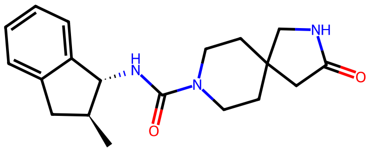	C[C@H]1Cc2ccccc2[C@@H]1NC(=O)N1CCC2(CC1)CNC(=O)C2	332928225	24	327.19	2.23
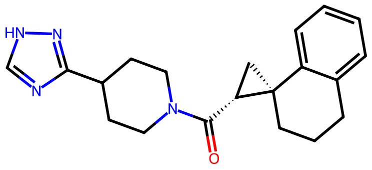	O=C([C@@H]1C[C@]12CCCc1ccccc12)N1CCC(c2nc[nH]n2)CC1	365913171	25	336.2	2.8
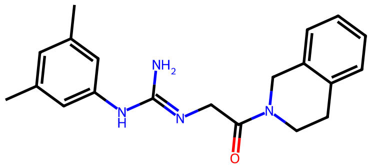	Cc1cc(C)cc(N/C(N)=N/CC(=O)N2CCc3ccccc3C2)c1	262024779	25	336.2	2.61

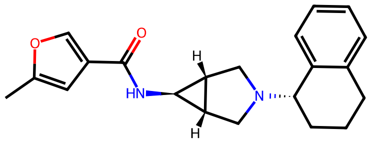	Cc1cc(C(=O)N[C@H]2[C@@H]3CN([C@H]4CCCc5ccccc54)C[C@@H]32)co1	958672210	25	336.18	3.33
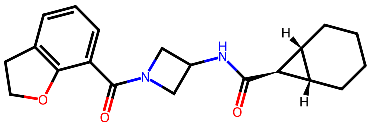	O=C(NC1CN(C(=O)c2cccc3c2OCC3)C1)[C@@H]1[C@H]2CCCC[C@H]21	990662708	25	340.18	2.0
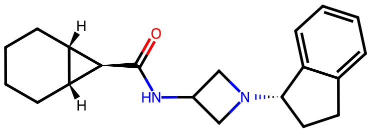	O=C(NC1CN([C@H]2CCc3ccccc32)C1)[C@@H]1[C@H]2CCCC[C@H]21	1030183261	23	310.2	2.91
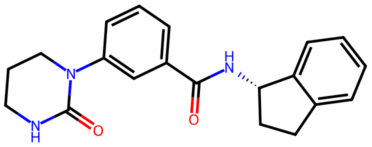	O=C(N[C@H]1CCc2ccccc21)c1cccc(N2CCCNC2=O)c1	267162823	25	335.16	3.02
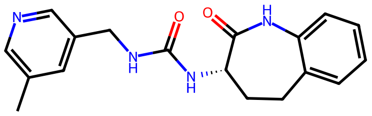	Cc1cncc(CNC(=O)N[C@H]2CCc3ccccc3NC2=O)c1	654510298	24	324.16	2.14
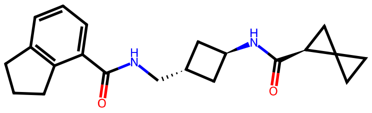	O=C(NC[C@H]1C[C@H](NC(=O)[C@H]2CC23CC3)C1)c1cccc2c1CCC2	979962669	25	338.2	2.6
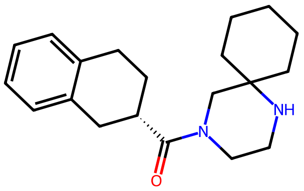	O=C([C@H]1CCc2ccccc2C1)N1CCNC2(CCCCC2)C1	400766519	23	312.22	2.93
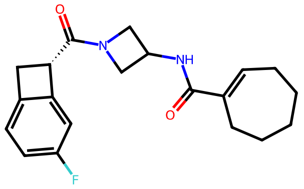	O=C(NC1CN(C(=O)[C@H]2Cc3ccc(F)cc32)C1)C1=CCCCCC1	991010060	25	342.17	2.68

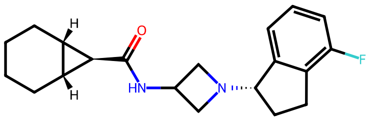	O=C(NC1CN([C@H]2CCc3c(F)cccc32)C1)[C@@H]1[C@H]2CCCC[C@H]21	1030184226	24	328.2	3.05
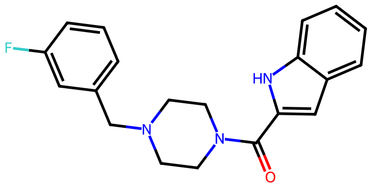	O=C(c1cc2ccccc2[nH]1)N1CCN(Cc2ccc(F)c2)CC1	337683022	25	337.16	3.27
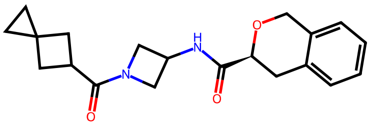	O=C(NC1CN(C(=O)C2CC3(CC3)C2)C1)[C@@H]1Cc2ccccc2CO1	997739716	25	340.18	1.65
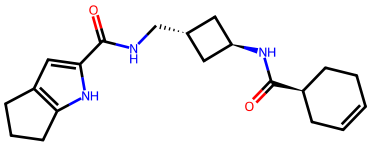	O=C(NC[C@H]1C[C@H](NC(=O)[C@@H]2CC=CCC2)C1)c1cc2c([nH]1)CCC2	980044223	25	341.21	2.48
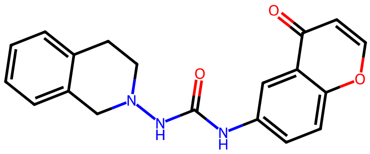	O=C(Nc1ccc2occc(=O)c2c1)NN1CCc2ccccc2C1	1477226607	25	335.13	2.89
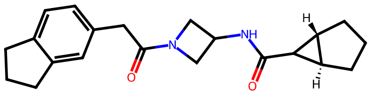	O=C(NC1CN(C(=O)Cc2ccc3c(c2)CCC3)C1)C1[C@@H]2CCC[C@@H]12	990710923	25	338.2	2.09
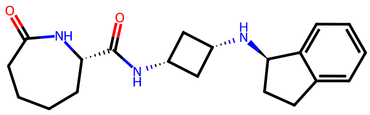	O=C1CCCC[C@@H](C(=O)N[C@H]2C[C@@H](N[C@@H]3CCc4ccccc43)C2)N1	1022693878	25	341.21	1.97
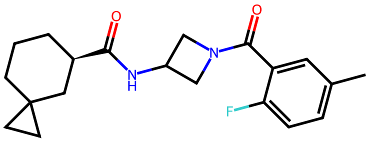	Cc1ccc(F)c(C(=O)N2CC(NC(=O)[C@@H]3CCCC4(CC4)C3)C2)c1	991517232	25	344.19	3.05

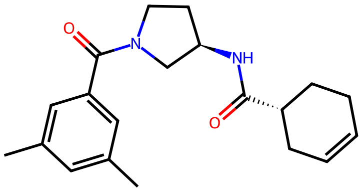	Cc1cc(C)cc(C(=O)N2CC[C@@H](NC(=O)[C@H]3CC=CCC3)C2)c1	939381293	24	326.2	2.99
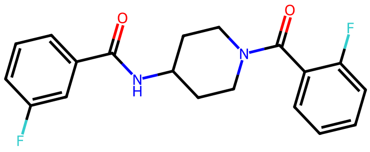	O=C(NC1CCN(C(=O)c2ccccc2F)CC1)c1cccc(F)c1	45779994	25	344.13	3.0
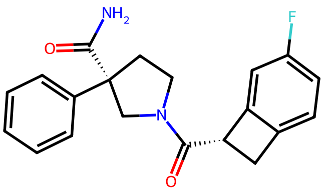	NC(=O)[C@]1(c2ccccc2)CCN(C(=O)[C@H]2Cc3ccc(F)cc32)C1	665647120	25	338.14	2.12
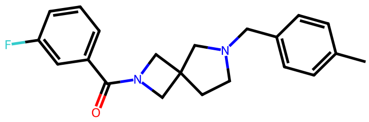	Cc1ccc(CN2CCC3(C2)CN(C(=O)c2cccc(F)c2)C3)cc1	1072418917	25	338.18	3.48
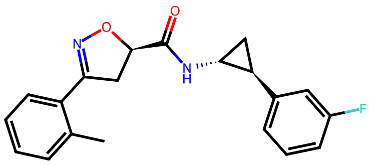	Cc1ccccc1C1=NO[C@@H](C(=O)N[C@@H]2C[C@H]2c2cccc(F)c2)C1	1128622827	25	338.14	3.3
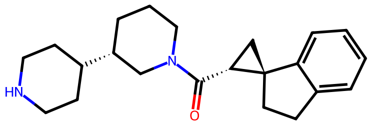	O=C([C@@H]1C[C@@]12CCc1ccccc12)N1CCC[C@@H](C2CCNCC2)C1	662596885	25	338.24	3.13
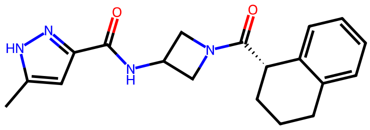	Cc1cc(C(=O)NC2CN(C(=O)[C@H]3CCc4ccccc43)C2)n[nH]1	990367603	25	338.17	1.78
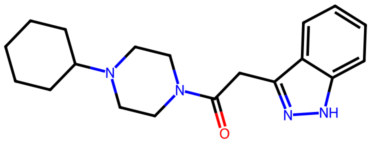	O=C(Cc1n[nH]c2ccccc12)N1CCN(C2CCCCC2)CC1	155008337	24	326.21	2.58

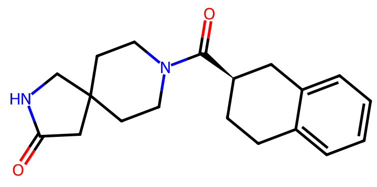	O=C1CC2(CCN(C(=O)[C@@H]3CCc4ccccc4C3)CC2)CN1	77541669	23	312.18	1.92
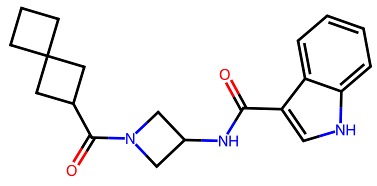	O=C(NC1CN(C(=O)C2CC3(CCC3)C2)C1)c1c[nH]c2ccccc12	995037345	25	337.18	2.69
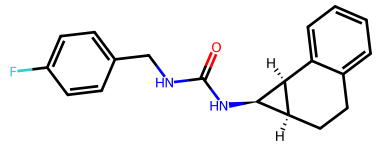	O=C(NCc1ccc(F)cc1)N[C@@H]1[C@@H]2CCc3ccccc3[C@@H]21	152958894	23	310.15	3.35
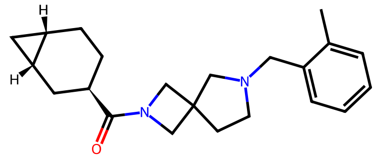	Cc1ccccc1CN1CCC2(C1)CN(C(=O)[C@@H]1CC[C@H]3C[C@H]3C1)C2	1072879087	25	338.24	3.47
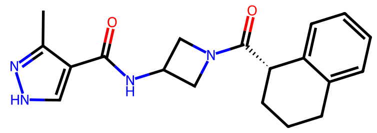	Cc1n[nH]cc1C(=O)NC1CN(C(=O)[C@H]2CCCc3ccccc32)C1	997295283	25	338.17	1.78
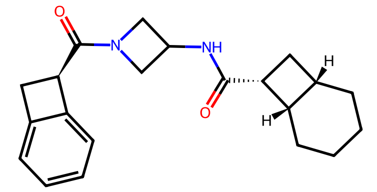	O=C(NC1CN(C(=O)[C@@H]2Cc3ccccc32)C1)[C@@H]1C[C@@H]2CCCC[C@@H]21	997651797	25	338.2	2.48
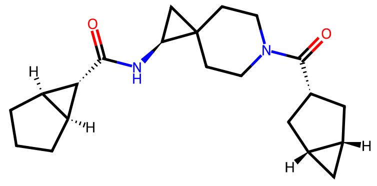	O=C(N[C@H]1CC12CCN(C(=O)[C@@H]1C[C@@H]3C[C@@H]3C1)CC2)[C@@H]1[C@H]2CCC[C@H]21	963491142	25	342.23	2.58
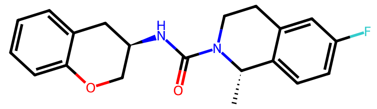	C[C@H]1c2ccc(F)cc2CCN1C(=O)N[C@H]1COc2ccccc2C1	606644262	25	340.16	3.46

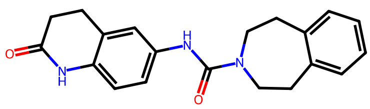	O=C1CCc2cc(NC(=O)N3CCc4ccccc4CC3)ccc2N1	116481943	25	335.16	3.2
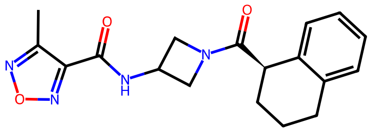	Cc1nonc1C(=O)NC1CN(C(=O)[C@@H]2CCCc3ccccc32)C1	994935858	25	340.15	1.44
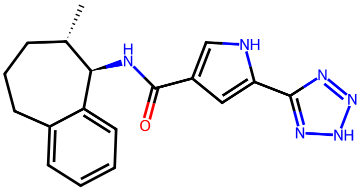	C[C@H]1CCCc2ccccc2[C@@H]1NC(=O)c1c[nH]c(-c2nn[nH]n2)c1	1590263696	25	336.17	2.64
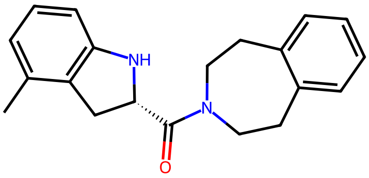	Cc1cccc2c1C[C@@H](C(=O)N1CCc3ccccc3CC1)N2	637996880	23	306.17	2.96
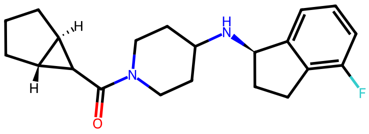	O=C(C1[C@@H]2CCC[C@@H]12)N1CCC(N[C@@H]2CCc3c(F)cccc32)CC1	990788995	25	342.21	3.44
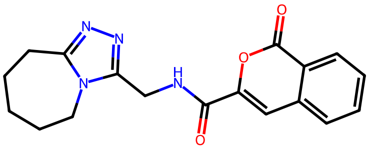	O=C(NCc1nnc2n1CCCCC2)c1cc2ccccc2c(=O)o1	278665544	25	338.14	2.04
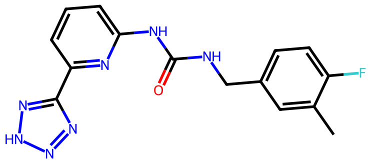	Cc1cc(CNC(=O)Nc2cccc(-c3nn[nH]n3)n2)ccc1F	1580925867	24	327.12	2.03
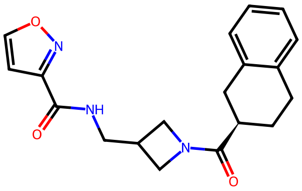	O=C(NCC1CN(C(=O)[C@@H]2CCc3ccccc3C2)C1)c1ccon1	1001077947	25	339.16	1.67

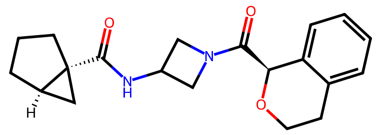	O=C([C@@H]1OCCc2ccccc21)N1CC(NC(=O)[C@@]23CCC[C@@H]2C3)C1	997552130	25	340.18	1.82
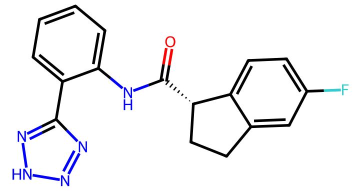	O=C(Nc1ccccc1-c1nn[nH]n1)[C@H]1CCc2cc(F)ccc21	1586503161	24	323.12	2.67
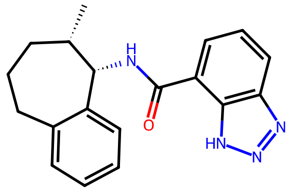	C[C@H]1CCCc2ccccc2[C@H]1NC(=O)c1cccc2nn[nH]c12	1138085094	24	320.16	3.4
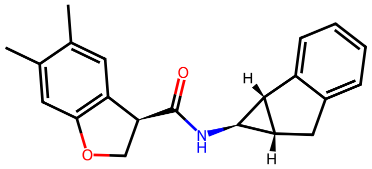	Cc1cc2c(cc1C)[C@@H](C(=O)N[C@@H]1[C@H]3Cc4ccccc4[C@H]31)CO2	926771979	24	319.16	3.23
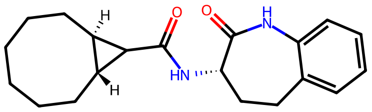	O=C(N[C@H]1CCc2ccccc2NC1=O)C1[C@@H]2CCCCCC[C@@H]12	616693213	24	326.2	3.27
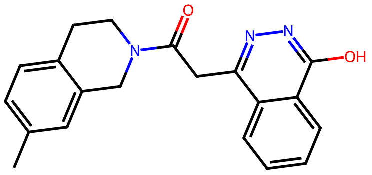	Cc1ccc2c(c1)CN(C(=O)Cc1nnc(O)c3ccccc13)CC2	855380124	25	333.15	2.77
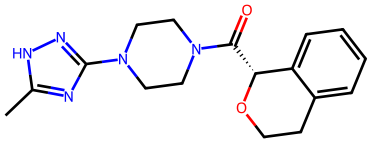	Cc1nc(N2CCN(C(=O)[C@H]3OCCc4ccccc43)CC2)n[nH]1	898179200	24	327.17	1.08
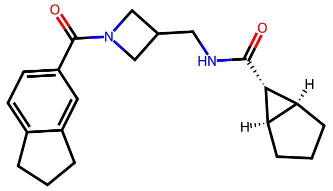	O=C(NCC1CN(C(=O)c2ccc3c(c2)CCC3)C1)[C@@H]1[C@H]2CCC[C@H]21	1001346070	25	338.2	2.41

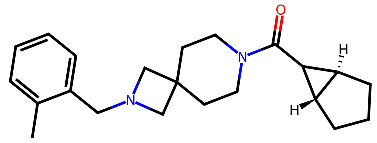	Cc1ccccc1CN1CC2(CCN(C(=O)C3[C@H]4CCC[C@H]34)CC2)C1	1035680955	25	338.24	3.47
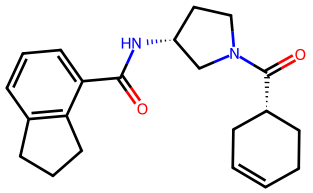	O=C(N[C@@H]1CCN(C(=O)[C@H]2CC=CCC2)C1)c1cccc2c1CCC2	941157602	25	338.2	2.86
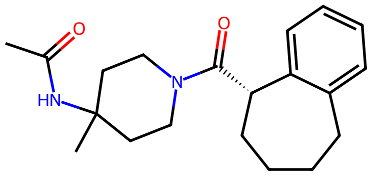	CC(=O)NC1(C)CCN(C(=O)[C@H]2CCCc3ccccc32)CC1	956253737	24	328.22	3.01
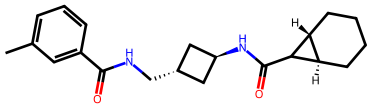	Cc1cccc(C(=O)NC[C@H]2C[C@H](NC(=O)C3[C@@H]4CCCC[C@@H]34)C2)c1	979453854	25	340.22	3.06
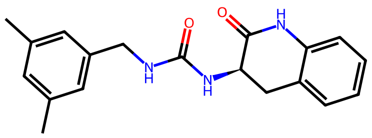	Cc1cc(C)cc(CNC(=O)N[C@@H]2Cc3ccccc3NC2=O)c1	637271360	24	323.16	2.67
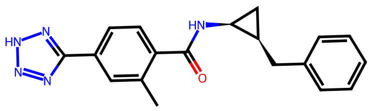	Cc1cc(-c2nn[nH]n2)ccc1C(=O)N[C@H]1C[C@H]1Cc1ccccc1	826246779	25	333.16	2.54
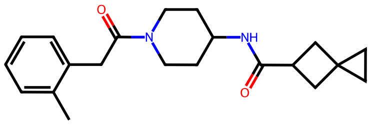	Cc1ccccc1CC(=O)N1CCC(NC(=O)C2CC3(CC3)C2)CC1	946717954	25	340.22	2.83
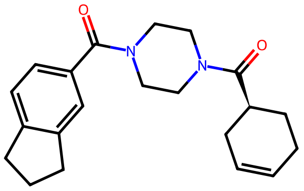	O=C(c1ccc2c(c1)CCC2)N1CCN(C(=O)[C@@H]2CC=CCC2)CC1	1010297140	25	338.2	2.82

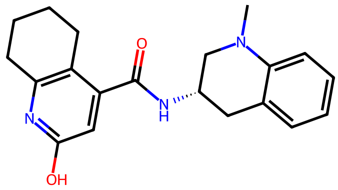	CN1C[C@@H](NC(=O)c2cc(O)nc3c2CCCC3)Cc2ccccc21	827345051	25	337.18	2.46
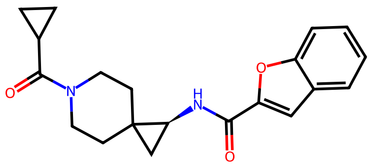	O=C(N[C@H]1CC12CCN(C(=O)C1CC1)CC2)c1cc2ccccc2o1	965492429	25	338.16	2.95
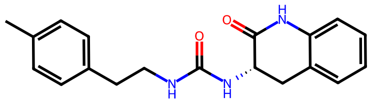	Cc1ccc(CCNC(=O)N[C@H]2Cc3ccccc3NC2=O)cc1	636793496	24	323.16	2.4
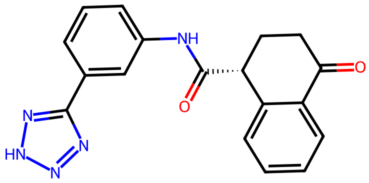	O=C1CC[C@@H](C(=O)Nc2cccc(-c3nn[nH]n3)c2)c2ccccc21	339838041	25	333.12	2.57
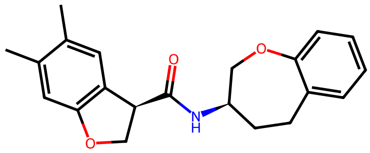	Cc1cc2c(cc1C)[C@@H](C(=O)N[C@@H]1CCc3ccccc3OC1)CO2	511624752	25	337.17	3.29
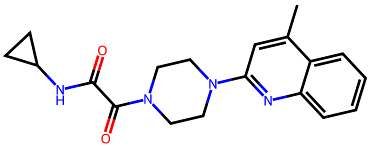	Cc1cc(N2CCN(C(=O)C(=O)NC3CC3)CC2)nc2ccccc12	52362253	25	338.17	1.47
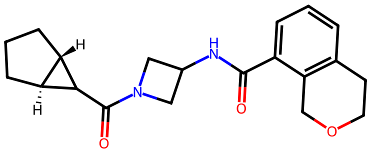	O=C(NC1CN(C(=O)C2[C@H]3CCC[C@H]23)C1)c1cccc2c1COCC2	993312541	25	340.18	1.75
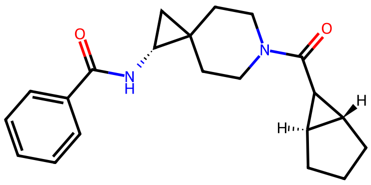	O=C(N[C@@H]1CC12CCN(C(=O)C1[C@@H]3CCC[C@@H]13)CC2)c1ccccc1	962781706	25	338.2	2.84

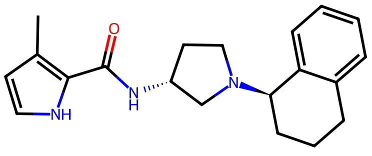	Cc1cc[nH]c1C(=O)N[C@@H]1CCN([C@@H]2CCCc3ccccc32)C1	1010820514	24	323.2	3.2
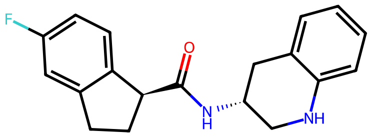	O=C(N[C@H]1CNc2ccccc2C1)[C@H]1CCc2cc(F)ccc21	658941632	23	310.15	3.01
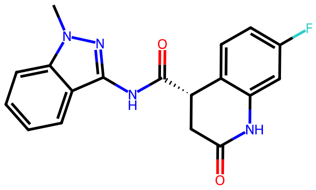	Cn1nc(NC(=O)[C@H]2CC(=O)Nc3cc(F)ccc32)c2ccccc21	1539706006	25	338.12	2.78
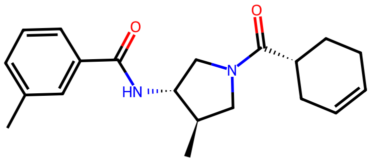	Cc1cccc(C(=O)N[C@@H]2CN(C(=O)[C@H]3CC=CCC3)C[C@H]2C)c1	947461491	24	326.2	2.93
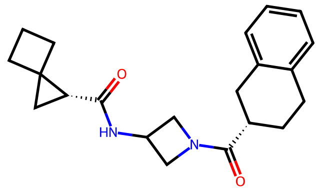	O=C(NC1CN(C(=O)[C@H]2CCc3ccccc3C2)C1)[C@@H]1CC12CCC2	991549043	25	338.2	2.31
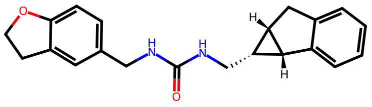	O=C(NCc1ccc2c(c1)CCO2)NC[C@H]1[C@H]2c3ccccc3C[C@H]21	532616625	25	334.17	3.01
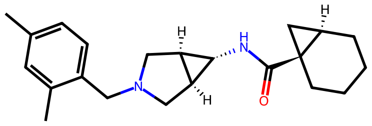	Cc1ccc(CN2C[C@H]3[C@H](NC(=O)[C@]45CCCC[C@@H]4C5)[C@H]3C2)c(C)c1	961732151	25	338.24	3.43
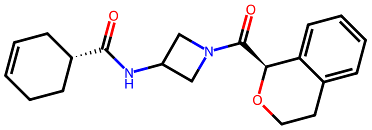	O=C(NC1CN(C(=O)[C@@H]2OCCc3ccccc32)C1)[C@H]1CC=CCC1	990218190	25	340.18	1.98

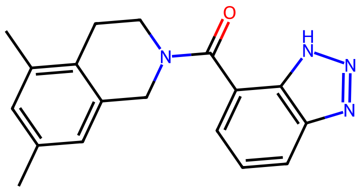	Cc1cc(C)c2c(c1)CN(C(=O)c1cccc3nn[nH]c13)CC2	188872970	23	306.15	2.77
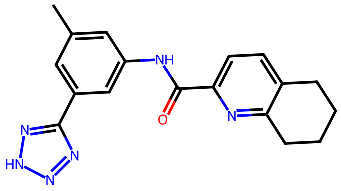	Cc1cc(NC(=O)c2ccc3c(n2)CCCC3)cc(-c2nn[nH]n2)c1	1609408609	25	334.15	2.7
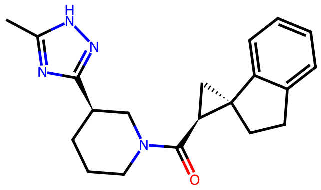	Cc1nc([C@@H]2CCCN(C(=O)[C@H]3C[C@]34CCc3ccccc34)C2)n[nH]1	539745233	25	336.2	2.72
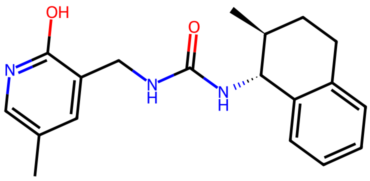	Cc1cnc(O)c(CNC(=O)N[C@H]2c3ccccc3CC[C@@H]2C)c1	1649287885	24	325.18	3.22
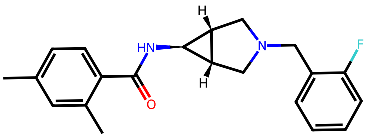	Cc1ccc(C(=O)N[C@H]2[C@@H]3CN(Cc4ccccc4F)C[C@@H]32)c(C)c1	962446017	25	338.18	3.3
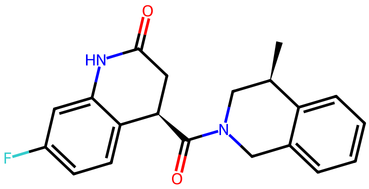	C[C@@H]1CN(C(=O)[C@@H]2CC(=O)Nc3cc(F)ccc32)Cc2ccccc21	837285791	25	338.14	3.4
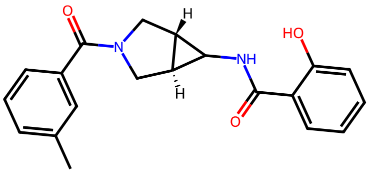	Cc1cccc(C(=O)N2C[C@@H]3C(NC(=O)c4ccccc4O)[C@H]3C2)c1	1012785405	25	336.15	2.2
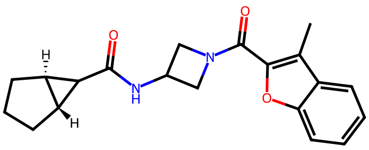	Cc1c(C(=O)N2CC(NC(=O)C3[C@@H]4CCC[C@@H]34)C2)oc2ccccc12	990708535	25	338.16	2.73

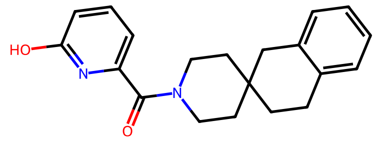	O=C(c1cccc(O)n1)N1CCC2(CCc3cccc c3C2)CC1	652525887	24	322.17	3.2
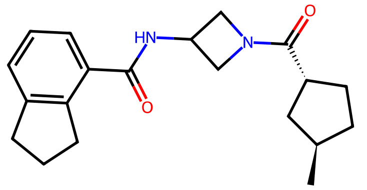	C[C@@H]1CC[C@@H](C(=O)N2CC(NC(=O)c3cccc4c3CCC4)C2)C1	994398917	24	326.2	2.55
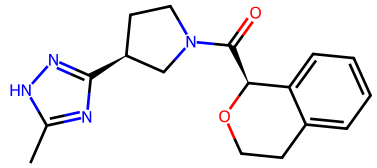	Cc1nc([C@H]2CCN(C(=O)[C@@H]3 OCCc4ccccc43)C2)n[nH]1	333999254	23	312.16	1.74
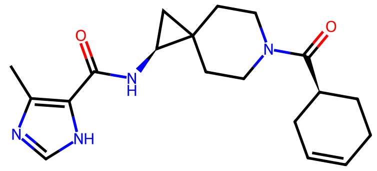	Cc1nc[nH]c1C(=O)N[C@H]1CC12CC N(C(=O)[C@@H]1CC=CCC1)CC2	964278533	25	342.21	2.19
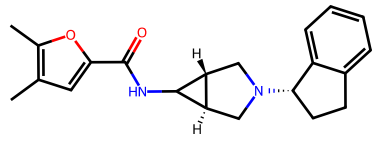	Cc1cc(C(=O)NC2[C@@H]3CN([C@H] 4CCc5ccccc54)C[C@@H]23)oc1C	958576795	25	336.18	3.24
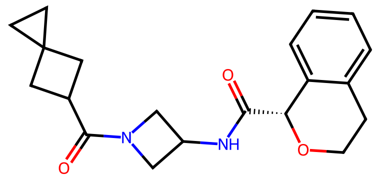	O=C(NC1CN(C(=O)C2CC3(CC3)C2)C 1)[C@H]1OCCc2ccccc21	997321943	25	340.18	1.82
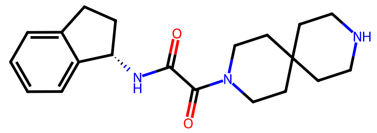	O=C(N[C@H]1CCc2ccccc21)C(=O)N1 CCC2(CCNCC2)CC1	1772504516	25	341.21	1.78
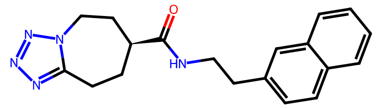	O=C(NCCc1ccc2ccccc2c1)[C@H]1CC c2nnnn2CC1	565368841	25	335.17	2.14

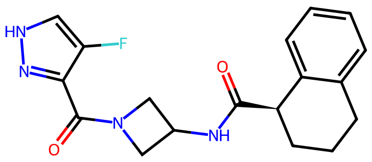	O=C(NC1CN(C(=O)c2n[nH]cc2F)C1)[C@@H]1CCCc2ccccc21	995072207	25	342.15	1.61
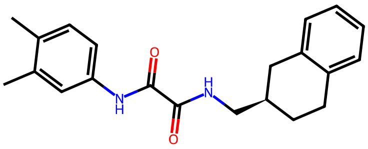	Cc1ccc(NC(=O)C(=O)NC[C@@H]2CCc3ccccc3C2)cc1C	605068700	25	336.18	3.16
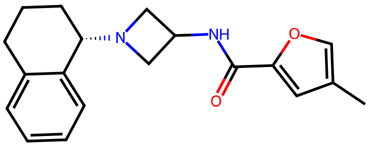	Cc1coc(C(=O)NC2CN([C@H]3CCCc4ccccc43)C2)c1	1030813237	23	310.17	3.08
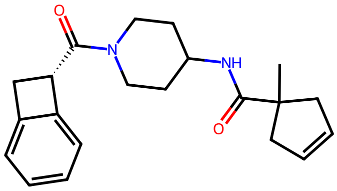	CC1(C(=O)NC2CCN(C(=O)[C@H]3Cc4ccccc43)CC2)CC=CC1	948059494	25	338.2	2.79
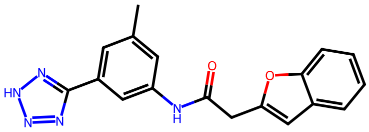	Cc1cc(NC(=O)Cc2cc3ccccc3o2)cc(-c2nn[nH]n2)c1	1580980530	25	333.12	3.1
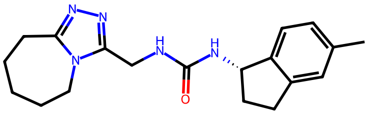	Cc1ccc2c(c1)CC[C@@H]2NC(=O)NCc1nnc2n1CCCCC2	56842300	25	339.21	2.8
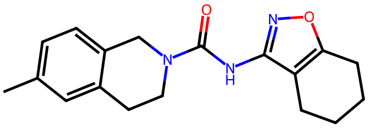	Cc1ccc2c(c1)CCN(C(=O)Nc1noc3c1CCCC3)C2	934346032	23	311.16	3.45
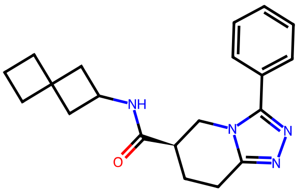	O=C(NC1CC2(CCC2)C1)[C@@H]1CCc2nnc(-c3ccccc3)n2C1	652968080	25	336.2	2.96

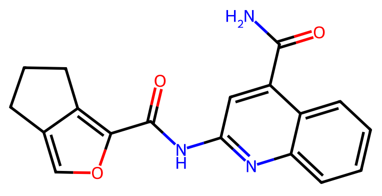	NC(=O)c1cc(NC(=O)c2occ3c2CCC3)nc2ccccc12	1533767459	24	321.11	2.67
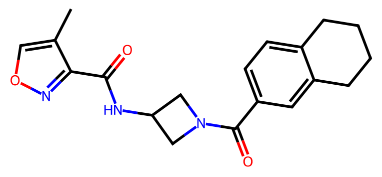	Cc1conc1C(=O)NC1CN(C(=O)c2ccc3c(c2)CCCC3)C1	999669341	25	339.16	2.12
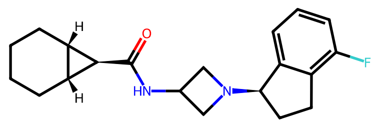	O=C(NC1CN([C@@H]2CCc3c(F)cccc32)C1)[C@@H]1[C@H]2CCCC[C@H]21	1030184221	24	328.2	3.05
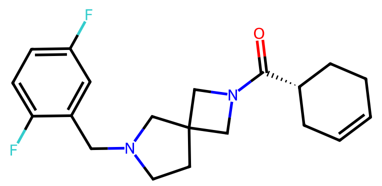	O=C([C@H]1CC=CCC1)N1CC2(CCN(Cc3cc(F)ccc3F)C2)C1	1072457828	25	346.19	3.36
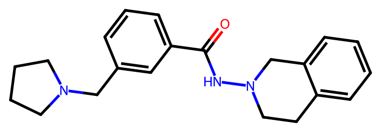	O=C(NN1CCc2ccccc2C1)c1cccc(CN2CCCC2)c1	855711022	25	335.2	2.99
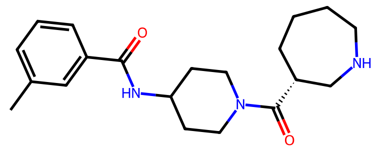	Cc1cccc(C(=O)NC2CCN(C(=O)[C@@H]3CCCCNC3)CC2)c1	1550101451	25	343.23	2.11
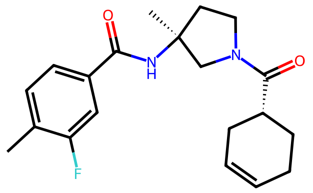	Cc1ccc(C(=O)N[C@@]2(C)CCN(C(=O)[C@H]3CC=CCC3)C2)cc1F	957196445	25	344.19	3.21
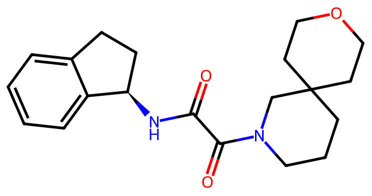	O=C(N[C@@H]1CCc2ccccc21)C(=O)N1CCCC2(CCOCC2)C1	1328176805	25	342.19	2.21

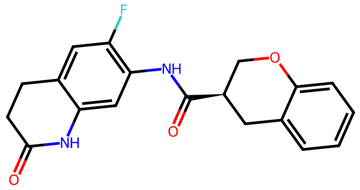	O=C1CCc2cc(F)c(NC(=O)[C@H]3COc4ccccc4C3)cc2N1	361393178	25	340.12	2.9
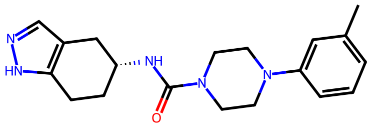	Cc1cccc(N2CCN(C(=O)N[C@@H]3CCc4[nH]ncc4C3)CC2)c1	153656559	25	339.21	2.11
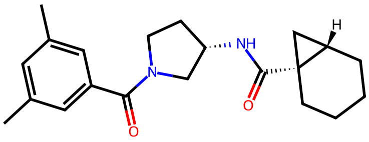	Cc1cc(C)cc(C(=O)N2CC[C@H](NC(=O)[C@@]34CCCC[C@H]3C4)C2)c1	938697139	25	340.22	3.21
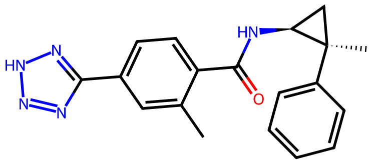	Cc1cc(-c2nn[nH]n2)ccc1C(=O)N[C@H]1C[C@@]1(C)c1ccccc1	826246934	25	333.16	2.64
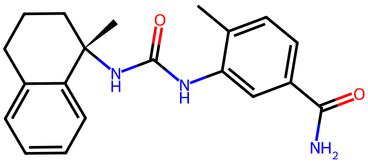	Cc1ccc(C(N)=O)cc1NC(=O)N[C@@]1(C)CCCc2ccccc21	359130521	25	337.18	3.47
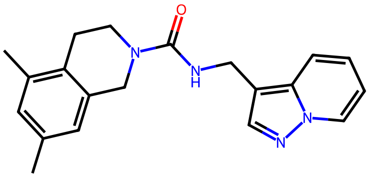	Cc1cc(C)c2c(c1)CN(C(=O)NCc1cnn3ccccc13)CC2	578309884	25	334.18	3.22
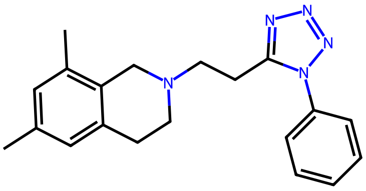	Cc1cc(C)c2c(c1)CCN(CCc1nnnn1-c1ccccc1)C2	1560241170	25	333.2	2.88
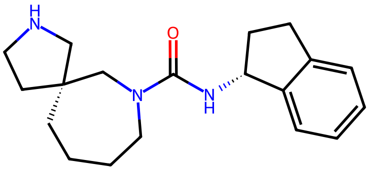	O=C(N[C@@H]1CCc2ccccc21)N1CCCC[C@@]2(CCNC2)C1	892256423	23	313.22	2.85

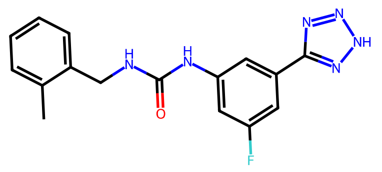	Cc1ccccc1CNC(=O)Nc1cc(F)cc(-c2nn[nH]n2)c1	1592256840	24	326.13	2.64
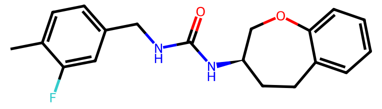	Cc1ccc(CNC(=O)N[C@@H]2CCc3ccccc3OC2)cc1F	509360657	24	328.16	3.33
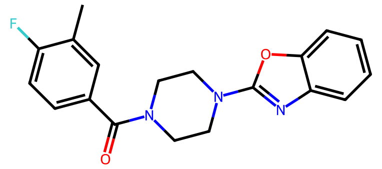	Cc1cc(C(=O)N2CCN(c3nc4ccccc4o3)CC2)ccc1F	172390997	25	339.14	3.24
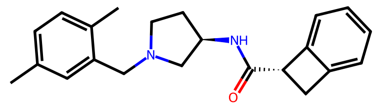	Cc1ccc(C)c(CN2CC[C@@H](NC(=O)[C@H]3Cc4ccccc43)C2)c1	1015635069	25	334.2	3.33
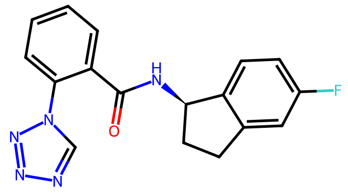	O=C(N[C@@H]1CCc2cc(F)ccc21)c1ccccc1-n1cnnn1	342858879	24	323.12	2.22
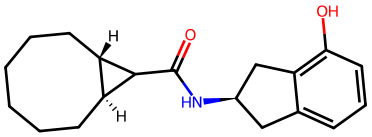	O=C(N[C@@H]1Cc2cccc(O)c2C1)C1[C@H]2CCCCCC[C@H]12	613999382	22	299.19	3.19
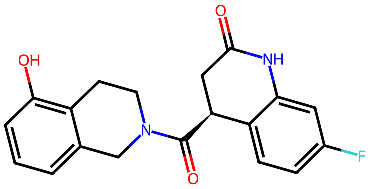	O=C1C[C@H](C(=O)N2CCc3c(O)cccc3C2)c2ccc(F)cc2N1	629158554	25	340.12	2.54
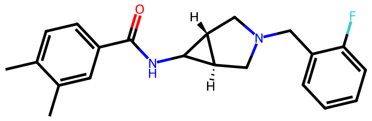	Cc1ccc(C(=O)NC2[C@@H]3CN(Cc4ccccc4F)C[C@@H]23)cc1C	962461921	25	338.18	3.3

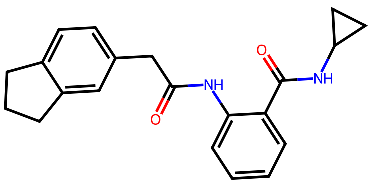	O=C(Cc1ccc2c(c1)CCC2)Nc1ccccc1C(=O)NC1CC1	23117695	25	334.17	3.25
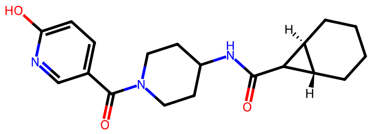	O=C(NC1CCN(C(=O)c2ccc(O)nc2)CC1)C1[C@H]2CCCC[C@H]12	948352228	25	343.19	1.94
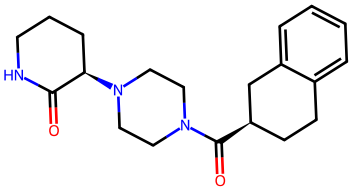	O=C1NCCC[C@H]1N1CCN(C(=O)[C@@H]2CCc3ccccc3C2)CC1	950725694	25	341.21	1.21
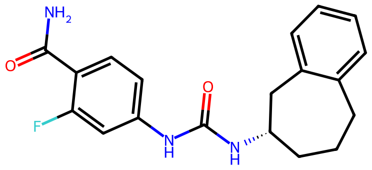	NC(=O)c1ccc(NC(=O)N[C@H]2CCCc3ccccc3C2)cc1F	930183413	25	341.15	2.99
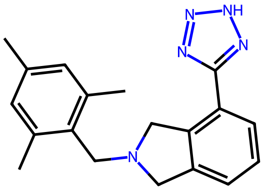	Cc1cc(C)c(CN2Cc3cccc(-c4nn[nH]n4)c3C2)c(C)c1	1591946806	24	319.18	3.31
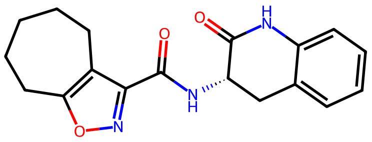	O=C(N[C@H]1Cc2ccccc2NC1=O)c1noc2c1CCCCC2	529947988	24	325.14	2.24
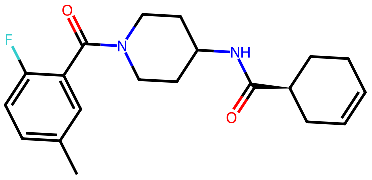	Cc1ccc(F)c(C(=O)N2CCC(NC(=O)[C@@H]3CC=CCC3)CC2)c1	948572043	25	344.19	3.21
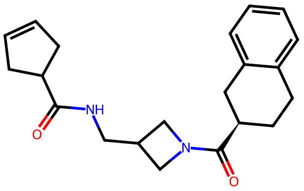	O=C(NCC1CN(C(=O)[C@@H]2CCc3ccccc3C2)C1)C1CC=CC1	1001264973	25	338.2	2.33

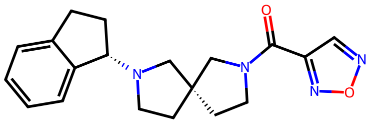	O=C(c1cnon1)N1CC[C@@]2(CCN([C@H]3CCc4ccccc43)C2)C1	1041748430	25	338.17	2.3
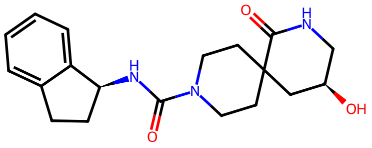	O=C(N[C@H]1CCc2ccccc21)N1CCC2(CC1)C[C@H](O)CNC2=O	913965886	25	343.19	1.35
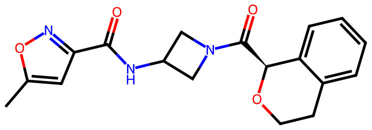	Cc1cc(C(=O)NC2CN(C(=O)[C@@H]3OCCc4ccccc43)C2)no1	990172831	25	341.14	1.24
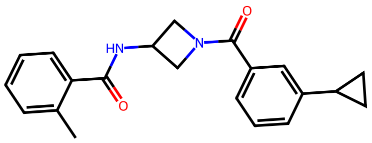	Cc1ccccc1C(=O)NC1CN(C(=O)c2cccc(C3CC3)c2)C1	990109264	25	334.17	3.13
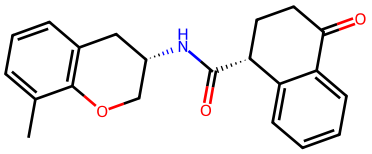	Cc1cccc2c1OC[C@@H](NC(=O)[C@@H]1CCC(=O)c3ccccc31)C2	291649058	25	335.15	3.18
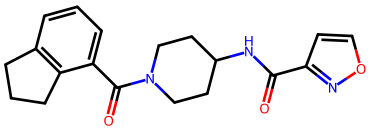	O=C(NC1CCN(C(=O)c2cccc3c2CCC3)CC1)c1ccon1	947161664	25	339.16	2.2
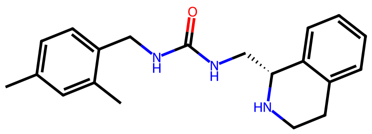	Cc1ccc(CNC(=O)NC[C@H]2NCCc3ccccc32)c(C)c1	570065887	24	323.2	2.99
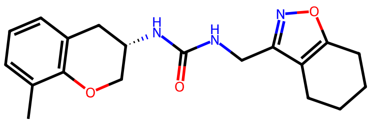	Cc1cccc2c1OC[C@@H](NC(=O)NCc1noc3c1CCCC3)C2	291466325	25	341.17	2.66

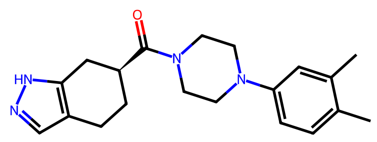	Cc1ccc(N2CCN(C(=O)[C@H]3CCc4cn[nH]c4C3)CC2)cc1C	153110069	25	338.21	2.48
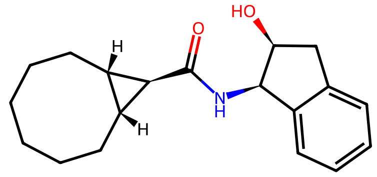	O=C(N[C@@H]1c2ccccc2C[C@@H]1O)[C@@H]1[C@H]2CCCCCC[C@H]21	612657927	22	299.19	2.98
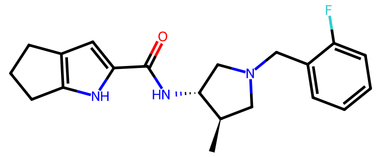	C[C@@H]1CN(Cc2ccccc2F)C[C@H]1NC(=O)c1cc2c([nH]1)CCC2	1080759939	25	341.19	2.89
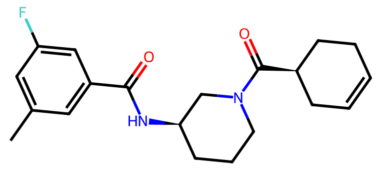	Cc1cc(F)cc(C(=O)N[C@@H]2CCCN(C(=O)[C@@H]3CC=CCC3)C2)c1	949975031	25	344.19	3.21
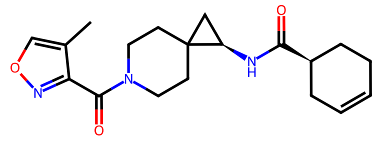	Cc1conc1C(=O)N1CCC2(CC1)C[C@H]2NC(=O)[C@@H]1CC=CCC1	963024873	25	343.19	2.45
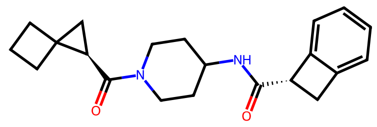	O=C(NC1CCN(C(=O)[C@@H]2CC23CCC3)CC1)[C@H]1Cc2ccccc21	946759059	25	338.2	2.62
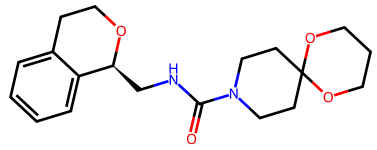	O=C(NC[C@@H]1OCCc2ccccc21)N1CCC2(CC1)OCCCO2	838472159	25	346.19	2.24
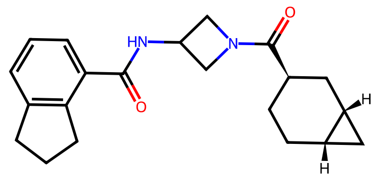	O=C(NC1CN(C(=O)[C@@H]2CC[C@H]3C[C@H]3C2)C1)c1cccc2c1CCC2	994400590	25	338.2	2.55

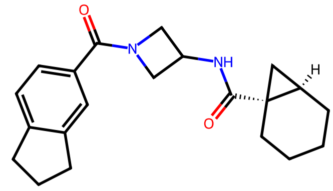	O=C(c1ccc2c(c1)CCC2)N1CC(NC(=O)[C@@]23CCCC[C@@H]2C3)C1	999287386	25	338.2	2.7
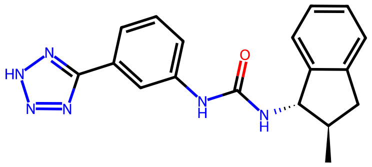	C[C@@H]1Cc2ccccc2[C@H]1NC(=O)Nc1cccc(-c2nn[nH]n2)c1	736299644	25	334.15	2.92
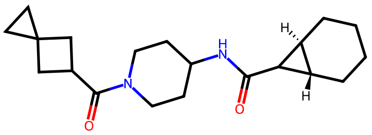	O=C(NC1CCN(C(=O)C2CC3(CC3)C2)CC1)C1[C@H]2CCCC[C@H]12	948299946	24	330.23	2.72
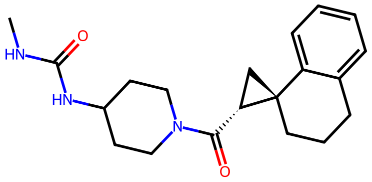	CNC(=O)NC1CCN(C(=O)[C@@H]2C[C@@]23CCCc2ccccc23)CC1	665669808	25	341.21	2.2
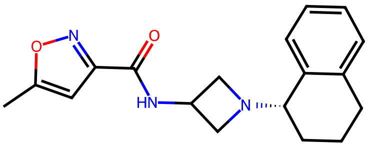	Cc1cc(C(=O)NC2CN([C@H]3CCCc4ccccc43)C2)no1	1030082610	23	311.16	2.47
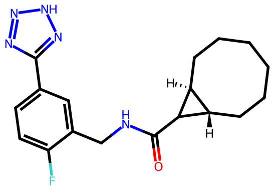	O=C(NCc1cc(-c2nn[nH]n2)ccc1F)C1[C@H]2CCCCCC[C@H]12	1606814841	25	343.18	2.84
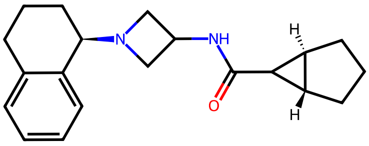	O=C(NC1CN([C@@H]2CCCc3ccccc32)C1)C1[C@H]2CCC[C@H]12	1030193410	23	310.2	2.91
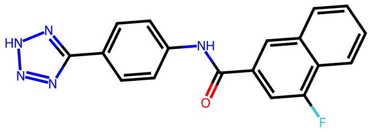	O=C(Nc1ccc(-c2nn[nH]n2)cc1)c1cc(F)c2ccccc2c1	550419465	25	333.1	3.41

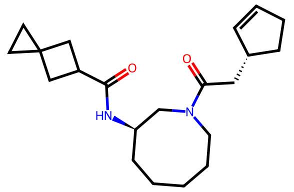	O=C(N[C@@H]1CCCCCN(C(=O)C[C@@H]2C=CCC2)C1)C1CC2(CC2)C1	986878691	25	344.25	3.42
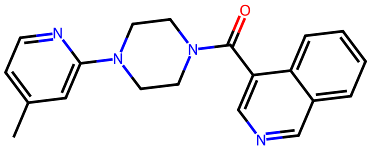	Cc1ccnc(N2CCN(C(=O)c3cncc4ccccc34)CC2)c1	162160707	25	332.16	2.9
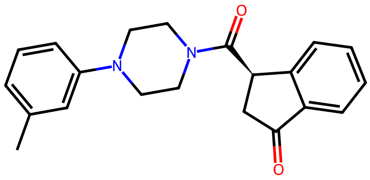	Cc1cccc(N2CCN(C(=O)[C@@H]3CC(=O)c4ccccc43)CC2)c1	355479142	25	334.17	3.01
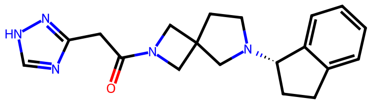	O=C(Cc1nc[nH]n1)N1CC2(CCN([C@H]3CCc4ccccc43)C2)C1	1073113635	25	337.19	1.57
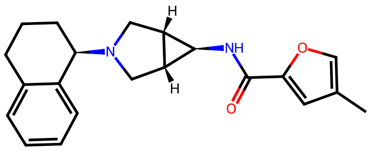	Cc1coc(C(=O)N[C@H]2[C@@H]3CN([C@@H]4CCCc5ccccc54)C[C@@H]32)c1	961517323	25	336.18	3.33
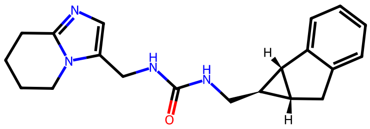	O=C(NCc1cnc2n1CCCC2)NC[C@@H]1[C@H]2Cc3ccccc3[C@H]21	375167980	25	336.2	2.6
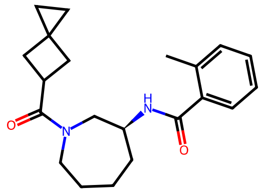	Cc1ccccc1C(=O)N[C@H]1CCCCN(C(=O)C2CC3(CC3)C2)C1	943058044	25	340.22	3.3
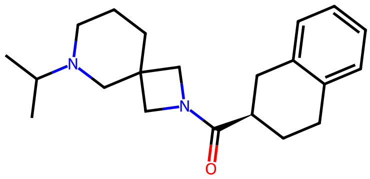	CC(C)N1CCCC2(CN(C(=O)[C@@H]3CCc4ccccc4C3)C2)C1	982037369	24	326.24	3.12

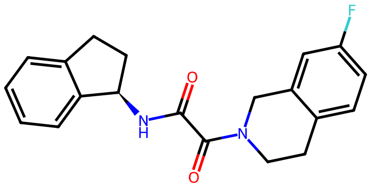	O=C(N[C@@H]1CCc2ccccc21)C(=O)N1CCc2ccc(F)cc2C1	339868703	25	338.14	2.51
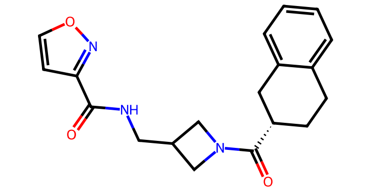	O=C(NCC1CN(C(=O)[C@H]2CCc3ccccc3C2)C1)c1ccon1	1001077937	25	339.16	1.67
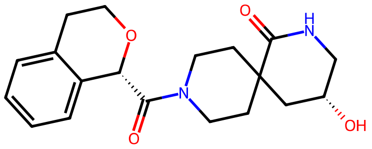	O=C([C@H]1OCCc2ccccc21)N1CCC2(CC1)C[C@@H](O)CNC2=O	885019522	25	344.17	0.79
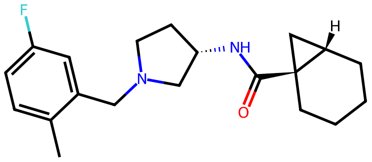	Cc1ccc(F)cc1CN1CC[C@H](NC(=O)[C@]23CCCC[C@H]2C3)C1	1015672621	24	330.21	3.4
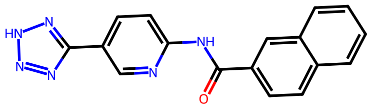	O=C(Nc1ccc(-c2nn[nH]n2)cn1)c1ccc2ccccc2c1	738149799	24	316.11	2.67
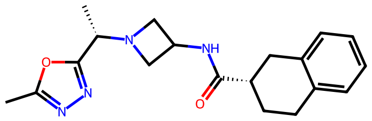	Cc1nnc([C@H](C)N2CC(NC(=O)[C@H]3CCc4ccccc4C3)C2)o1	1031012293	25	340.19	2.04
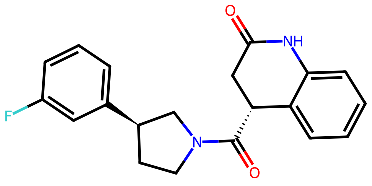	O=C1C[C@@H](C(=O)N2CC[C@@H](c3cccc(F)c3)C2)c2ccccc2N1	264253024	25	338.14	3.27
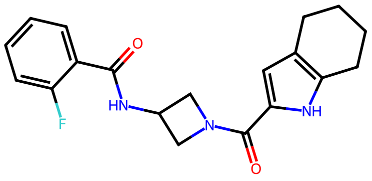	O=C(NC1CN(C(=O)c2cc3c([nH]2)CCCC3)C1)c1ccccc1F	990090288	25	341.15	2.29

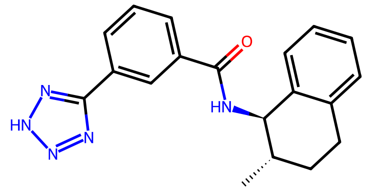	C[C@H]1CCc2ccccc2[C@@H]1NC(=O)c1cccc(-c2nn[nH]n2)c1	824970395	25	333.16	2.92
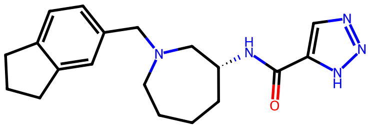	O=C(N[C@@H]1CCCCN(Cc2ccc3c(c2)CCC3)C1)c1cnn[nH]1	1034286865	25	339.21	2.08
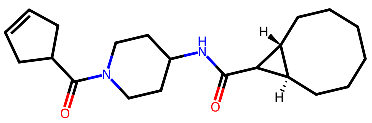	O=C(NC1CCN(C(=O)C2CC=CC2)CC1)C1[C@@H]2CCCCCC[C@@H]12	945717728	25	344.25	3.28
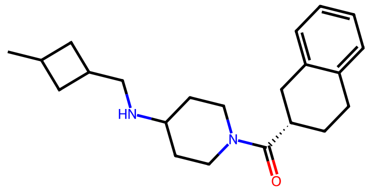	CC1CC(CNC2CCN(C(=O)[C@H]3CCc4ccccc4C3)CC2)C1	997035965	25	340.25	3.42
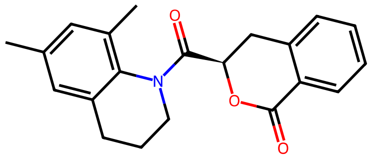	Cc1cc(C)c2c(c1)CCCN2C(=O)[C@H]1Cc2ccccc2C(=O)O1	747308216	25	335.15	3.36
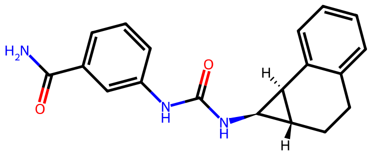	NC(=O)c1cccc(NC(=O)N[C@@H]2[C@H]3CCc4ccccc4[C@@H]32)c1	178811855	24	321.15	2.64
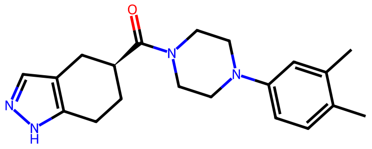	Cc1ccc(N2CCN(C(=O)[C@H]3CCc4[nH]ncc4C3)CC2)cc1C	270480708	25	338.21	2.48
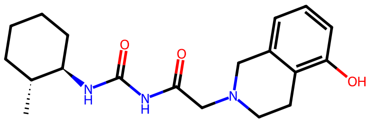	C[C@@H]1CCCC[C@H]1NC(=O)NC(=O)CN1CCc2c(O)cccc2C1	599648206	25	345.21	2.15

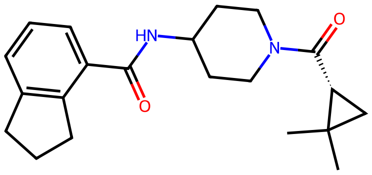	CC1(C)C[C@H]1C(=O)N1CCC(NC(=O)c2cccc3c2CCC3)CC1	946483390	25	340.22	2.94
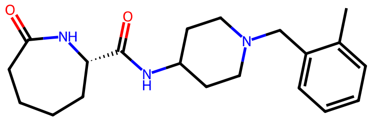	Cc1ccccc1CN1CCC(NC(=O)[C@@H]2CCCCC(=O)N2)CC1	193839322	25	343.23	2.13
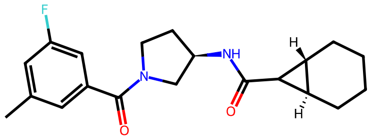	Cc1cc(F)cc(C(=O)N2CC[C@@H](NC(=O)C3[C@@H]4CCCC[C@@H]34)C2)c1	936245948	25	344.19	2.9
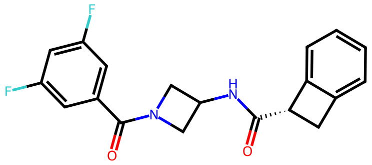	O=C(NC1CN(C(=O)c2cc(F)cc(F)c2)C1)[C@H]1Cc2ccccc21	998692612	25	342.12	2.25
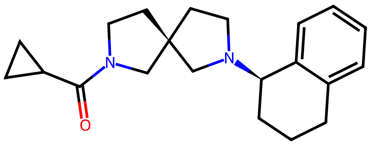	O=C(C1CC1)N1CC[C@]2(CCN([C@@H]3CCCc4ccccc43)C2)C1	1040991370	24	324.22	3.4
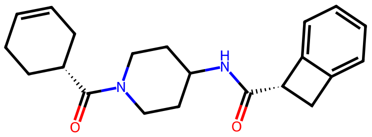	O=C(NC1CCN(C(=O)[C@H]2CC=CCC2)CC1)[C@H]1Cc2ccccc21	944107491	25	338.2	2.79
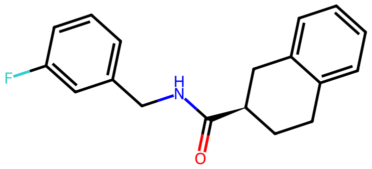	O=C(NCc1cccc(F)c1)[C@@H]1CCc2ccccc2C1	583503198	21	283.14	3.25
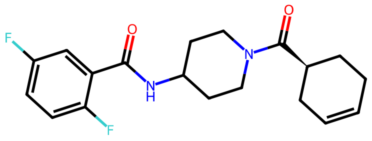	O=C(NC1CCN(C(=O)[C@@H]2CC=CCC2)CC1)c1cc(F)ccc1F	944127874	25	348.16	3.04

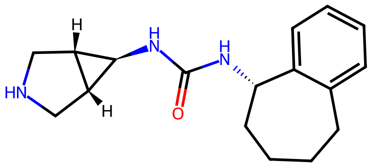	O=C(N[C@H]1CCCCc2ccccc21)N[C@H]1[C@@H]2CNC[C@@H]21	517337900	21	285.18	1.97
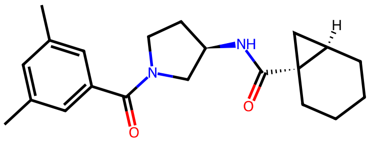	Cc1cc(C)cc(C(=O)N2CC[C@@H](NC(=O)[C@@]34CCCC[C@@H]3C4)C2)c1	942365900	25	340.22	3.21
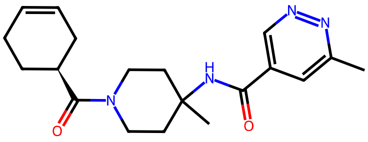	Cc1cc(C(=O)NC2(C)CCN(C(=O)[C@@H]3CC=CCC3)CC2)cnn1	956352916	25	342.21	2.25
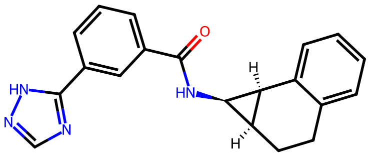	O=C(N[C@@H]1[C@@H]2CCc3ccccc3[C@@H]21)c1cccc(-c2ncn[nH]2)c1	159113163	25	330.15	2.93
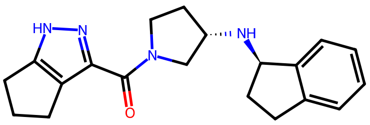	O=C(c1n[nH]c2c1CCC2)N1CC[C@H](N[C@@H]2CCc3ccccc32)C1	1019081131	25	336.2	2.39
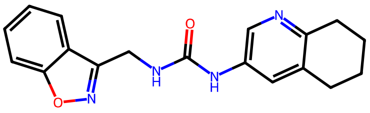	O=C(NCc1noc2ccccc12)Nc1cnc2c(c1)CCCC2	573456097	24	322.14	3.42
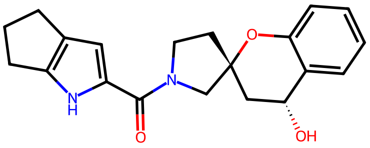	O=C(c1cc2c([nH]1)CCC2)N1CC[C@@]2(C[C@@H](O)c3ccccc3O2)C1	1550997111	25	338.16	2.6
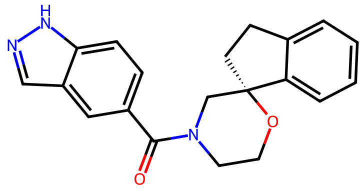	O=C(c1ccc2[nH]ncc2c1)N1CCO[C@@]2(CCc3ccccc32)C1	155521954	25	333.15	2.88

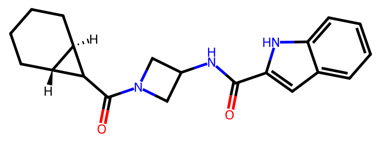	O=C(NC1CN(C(=O)C2[C@@H]3CCCC[C@@H]23)C1)c1cc2ccccc2[nH]1	1000142240	25	337.18	2.54
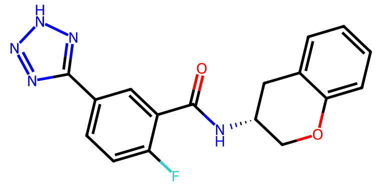	O=C(N[C@H]1COc2ccccc2C1)c1cc(-c2nn[nH]n2)ccc1F	1582343783	25	339.11	1.74
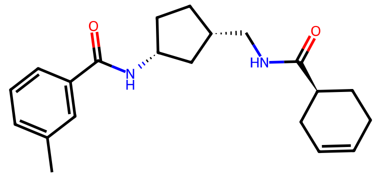	Cc1cccc(C(=O)N[C@@H]2CC[C@H](CNC(=O)[C@@H]3CC=CCC3)C2)c1	980895661	25	340.22	3.37
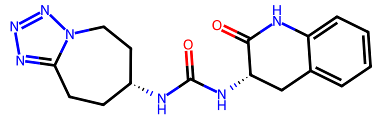	O=C(N[C@H]1Cc2ccccc2NC1=O)N[C@@H]1CCc2nnnn2CC1	1470562045	25	341.16	0.24
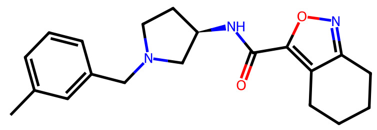	Cc1cccc(CN2CC[C@@H](NC(=O)c3onc4c3CCCC4)C2)c1	634278081	25	339.19	2.87
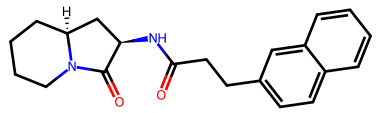	O=C(CCc1ccc2ccccc2c1)N[C@@H]1C[C@@H]2CCCCN2C1=O	457291743	25	336.18	3.04
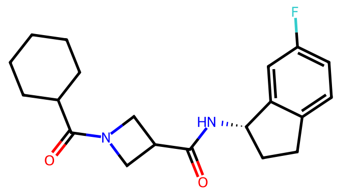	O=C(N[C@H]1CCc2ccc(F)cc21)C1CN(C(=O)C2CCCCC2)C1	566839853	25	344.19	2.97
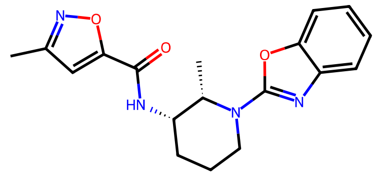	Cc1cc(C(=O)N[C@H]2CCCN(c3nc4ccccc4o3)[C@H]2C)on1	1069340659	25	340.15	2.91

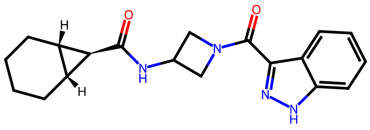	O=C(NC1CN(C(=O)c2n[nH]c3ccccc23)C1)[C@@H]1[C@H]2CCCC[C@H]21	990671045	25	338.17	1.94
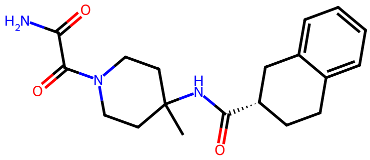	CC1(NC(=O)[C@H]2CCc3ccccc3C2)CCN(C(=O)C(N)=O)CC1	956515188	25	343.19	0.77
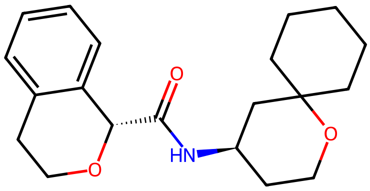	O=C(N[C@@H]1CCOC2(CCCCC2)C1)[C@@H]1OCCc2ccccc21	124894221	24	329.2	3.3
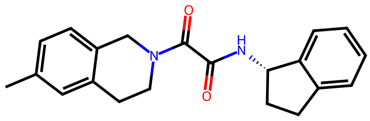	Cc1ccc2c(c1)CCN(C(=O)C(=O)N[C@H]1CCc3ccccc31)C2	339868579	25	334.17	2.68
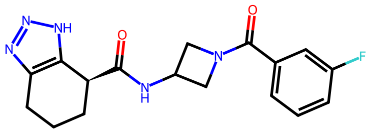	O=C(NC1CN(C(=O)c2cccc(F)c2)C1)[C@H]1CCCc2nn[nH]c21	998567721	25	343.14	1.0
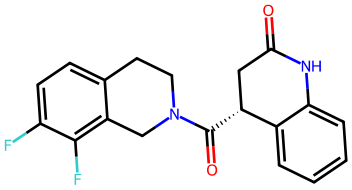	O=C1C[C@@H](C(=O)N2CCc3ccc(F)c(F)c3C2)c2ccccc2N1	493905967	25	342.12	2.98
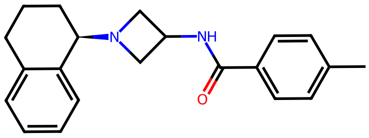	Cc1ccc(C(=O)NC2CN([C@@H]3CCCc4ccccc43)C2)cc1	1031300892	24	320.19	3.49
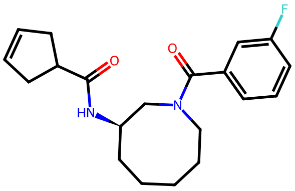	O=C(N[C@@H]1CCCCCN(C(=O)c2cccc(F)c2)C1)C1CC=CC1	986854392	25	344.19	3.29

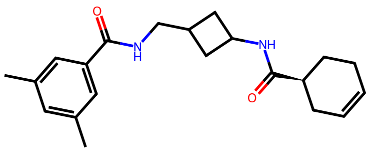	Cc1cc(C)cc(C(=O)NCC2CC(NC(=O)[C@@H]3CC=CCC3)C2)c1	992389207	25	340.22	3.28
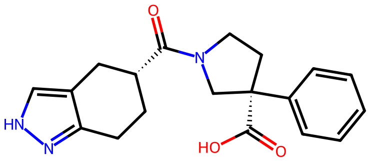	O=C([C@@H]1CCc2n[nH]cc2C1)N1CC[C@@](C(=O)O)(c2ccccc2)C1	909861362	25	339.16	1.77
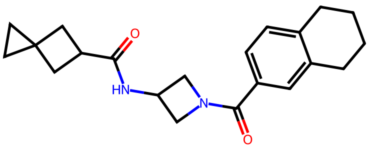	O=C(NC1CN(C(=O)c2ccc3c(c2)CCCC3)C1)C1CC2(CC2)C1	999337048	25	338.2	2.7
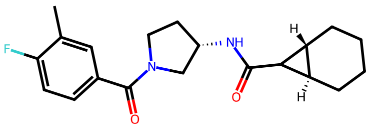	Cc1cc(C(=O)N2CC[C@H](NC(=O)C3[C@@H]4CCCC[C@@H]34)C2)ccc1F	937790187	25	344.19	2.9
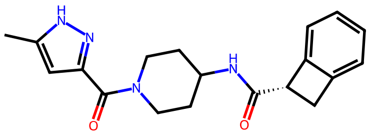	Cc1cc(C(=O)N2CCC(NC(=O)[C@H]3Cc4ccccc43)CC2)n[nH]1	944822459	25	338.17	1.78
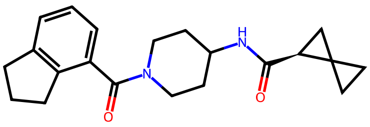	O=C(NC1CCN(C(=O)c2cccc3c2CCC3)CC1)[C@H]1CC12CC2	947164290	25	338.2	2.7
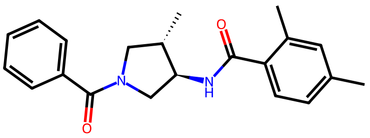	Cc1ccc(C(=O)N[C@H]2CN(C(=O)c3ccccc3)C[C@@H]2C)c(C)c1	966093563	25	336.18	3.19
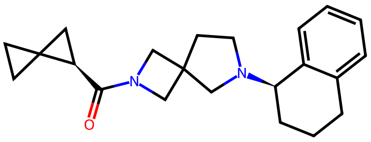	O=C([C@@H]1CC12CC2)N1CC2(CCN([C@@H]3CCCc4ccccc43)C2)C1	1073012103	25	336.22	3.4

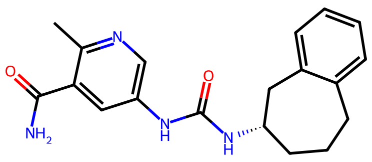	Cc1ncc(NC(=O)N[C@H]2CCCc3ccccc3C2)cc1C(N)=O	932142302	25	338.17	2.56
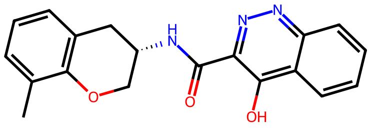	Cc1cccc2c1OC[C@@H](NC(=O)c1nnc3ccccc3c1O)C2	794168862	25	335.13	2.38
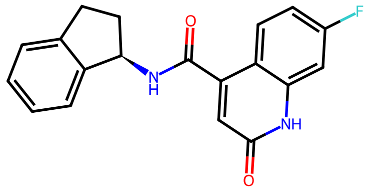	O=C(N[C@@H]1CCc2ccccc21)c1cc(=O)[nH]c2cc(F)ccc12	71916042	24	322.11	3.08
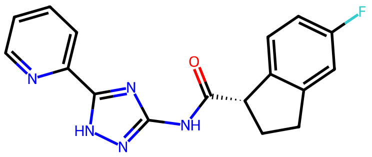	O=C(Nc1n[nH]c(-c2ccccn2)n1)[C@H]1CCc2cc(F)ccc21	1626420849	24	323.12	2.67
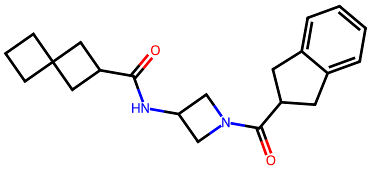	O=C(NC1CN(C(=O)C2Cc3ccccc3C2)C1)C1CC2(CCC2)C1	997938454	25	338.2	2.31
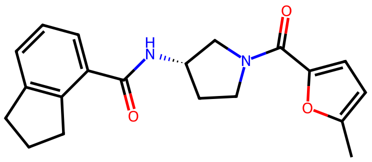	Cc1ccc(C(=O)N2CC[C@H](NC(=O)c3cccc4c3CCC4)C2)o1	938146538	25	338.16	2.72
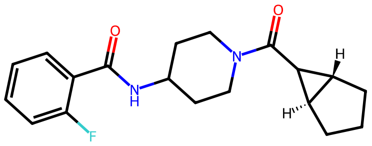	O=C(NC1CCN(C(=O)C2[C@@H]3CCC[C@@H]23)CC1)c1ccccc1F	365788066	24	330.17	2.59
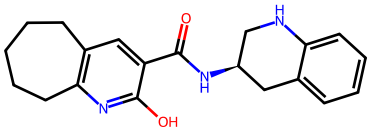	O=C(N[C@H]1CNc2ccccc2C1)c1cc2c(nc1O)CCCCC2	653208999	25	337.18	2.82

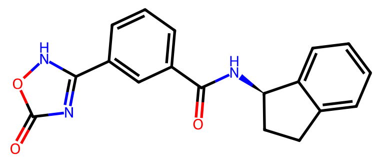	O=C(N[C@@H]1CCc2ccccc21)c1cccc(-c2nc(=O)o[nH]2)c1	1300453120	24	321.11	2.45
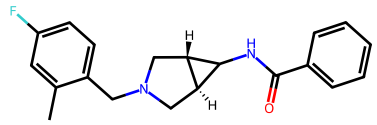	Cc1cc(F)ccc1CN1C[C@@H]2C(NC(=O)c3ccccc3)[C@H]2C1	958115675	24	324.16	2.99
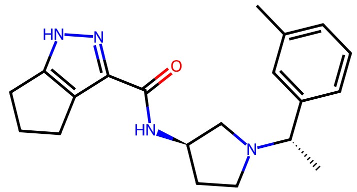	Cc1cccc([C@H](C)N2CC[C@@H](NC(=O)c3n[nH]c4c3CCC4)C2)c1	1015071070	25	338.21	2.77
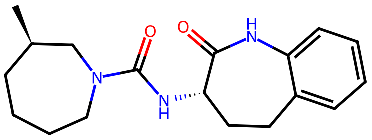	C[C@@H]1CCCCN(C(=O)N[C@H]2CCc3ccccc3NC2=O)C1	654631805	23	315.19	2.77
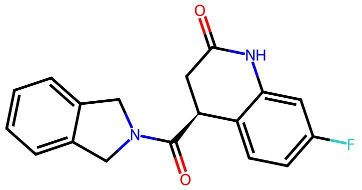	O=C1C[C@H](C(=O)N2Cc3ccccc3C2)c2ccc(F)cc2N1	105978526	23	310.11	2.79
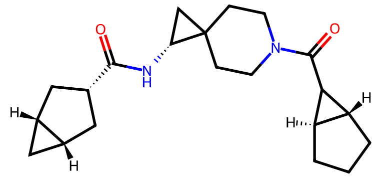	O=C(N[C@@H]1CC12CCN(C(=O)C1[C@@H]3CCC[C@@H]13)CC2)[C@@H]1C[C@@H]2C[C@@H]2C1	964766573	25	342.23	2.58
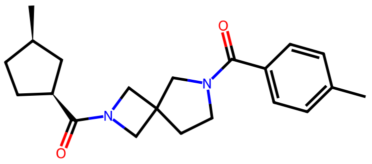	Cc1ccc(C(=O)N2CCC3(C2)CN(C(=O)[C@H]2CC[C@@H](C)C2)C3)cc1	1019021808	25	340.22	3.11
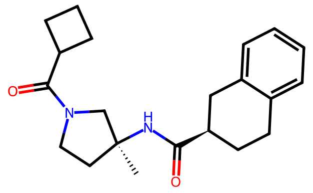	C[C@]1(NC(=O)[C@@H]2CCc3ccccc3C2)CCN(C(=O)C2CCC2)C1	957727571	25	340.22	2.7

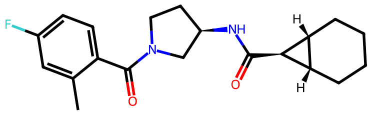	Cc1cc(F)ccc1C(=O)N1CC[C@@H](NC(=O)[C@@H]2[C@H]3CCCC[C@H]32)C1	939894974	25	344.19	2.9
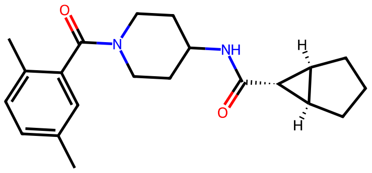	Cc1ccc(C)c(C(=O)N2CCC(NC(=O)[C@@H]3[C@H]4CCC[C@H]43)CC2)c1	948336082	25	340.22	3.07
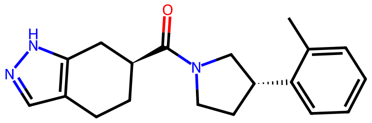	Cc1ccccc1[C@@H]1CCN(C(=O)[C@H]2CCc3cn[nH]c3C2)C1	266310218	23	309.18	2.84
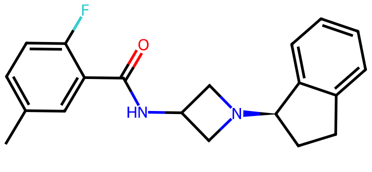	Cc1ccc(F)c(C(=O)NC2CN([C@@H]3CCc4ccccc43)C2)c1	1031011776	24	324.16	3.24
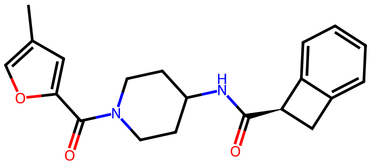	Cc1coc(C(=O)N2CCC(NC(=O)[C@@H]3Cc4ccccc43)CC2)c1	947897293	25	338.16	2.65
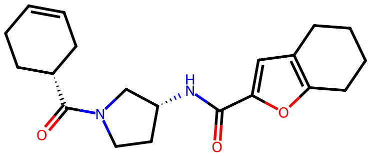	O=C(N[C@@H]1CCN(C(=O)[C@H]2CC=CCC2)C1)c1cc2c(o1)CCCC2	940868623	25	342.19	2.85
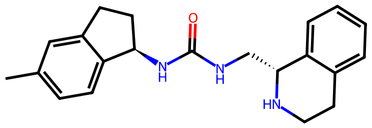	Cc1ccc2c(c1)CC[C@H]2NC(=O)NC[C@H]1NCCc2ccccc21	581359244	25	335.2	3.17
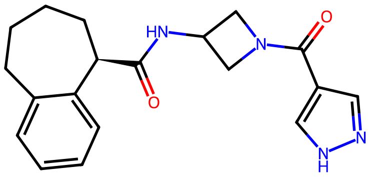	O=C(NC1CN(C(=O)c2cn[nH]c2)C1)[C@@H]1CCCCc2ccccc21	994303446	25	338.17	1.86

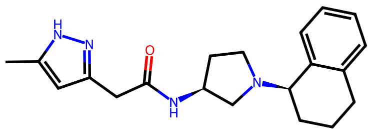	Cc1cc(CC(=O)N[C@H]2CCN([C@@H]3CCCc4ccccc43)C2)n[nH]1	1010510338	25	338.21	2.53
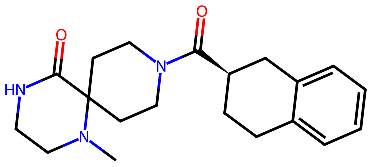	CN1CCNC(=O)C12CCN(C(=O)[C@@H]1CCc3ccccc3C1)CC2	67655127	25	341.21	1.21
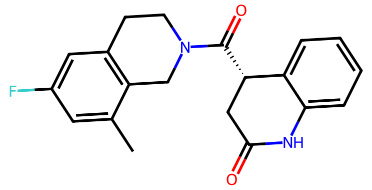	Cc1cc(F)cc2c1CN(C(=O)[C@H]1CC(=O)Nc3ccccc31)CC2	337236351	25	338.14	3.14
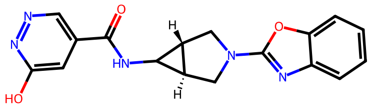	O=C(NC1[C@@H]2CN(c3nc4ccccc4o3)C[C@@H]12)c1cnnc(O)c1	1056333220	25	337.12	1.19
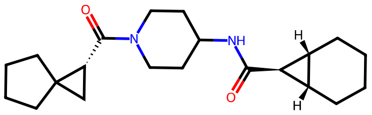	O=C(NC1CCN(C(=O)[C@@H]2CC23CCCC3)CC1)[C@@H]1[C@H]2CCCC[C@H]21	948360790	25	344.25	3.11
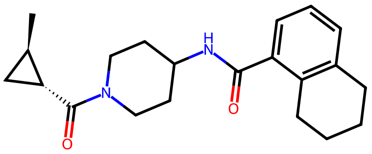	C[C@@H]1C[C@H]1C(=O)N1CCC(NC(=O)c2cccc3c2CCCC3)CC1	943825822	25	340.22	2.94
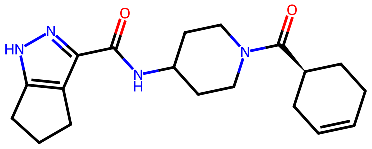	O=C(NC1CCN(C(=O)[C@@H]2CC=CCC2)CC1)c1n[nH]c2c1CCC2	944126513	25	342.21	1.98
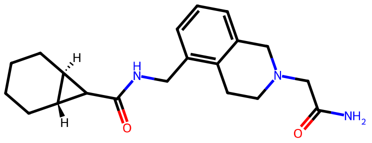	NC(=O)CN1CCc2c(CNC(=O)C3[C@@H]4CCCC[C@@H]34)cccc2C1	1051284065	25	341.21	1.58

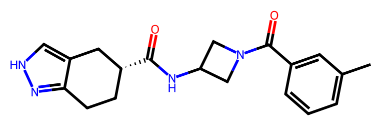	Cc1cccc(C(=O)N2CC(NC(=O)[C@@H]3CCc4n[nH]cc4C3)C2)c1	996395990	25	338.17	1.46
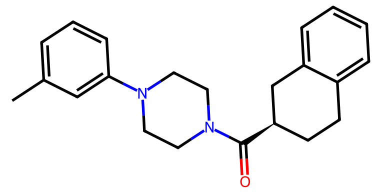	Cc1cccc(N2CCN(C(=O)[C@@H]3CCc4ccccc4C3)CC2)c1	1356301687	25	334.2	3.45
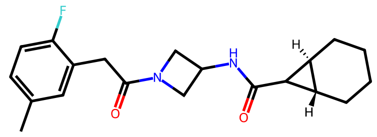	Cc1ccc(F)c(CC(=O)N2CC(NC(=O)C3[C@H]4CCCC[C@H]34)C2)c1	990643900	25	344.19	2.44
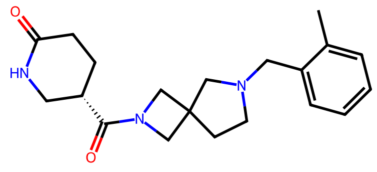	Cc1ccccc1CN1CCC2(C1)CN(C(=O)[C@H]1CCC(=O)NC1)C2	1072886101	25	341.21	1.56
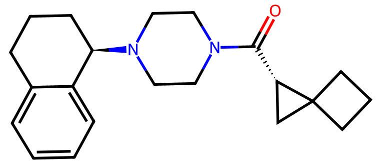	O=C([C@H]1CC12CCC2)N1CCN([C@@H]2CCCc3ccccc32)CC1	950851298	24	324.22	3.4
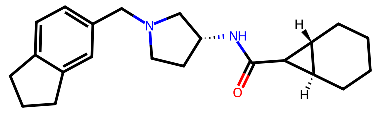	O=C(N[C@@H]1CCN(Cc2ccc3c(c2)CCC3)C1)C1[C@@H]2CCCC[C@@H]12	1010439990	25	338.24	3.3
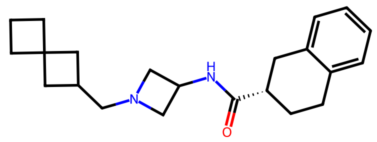	O=C(NC1CN(CC2CC3(CCC3)C2)C1)[C@H]1CCc2ccccc2C1	1031012183	25	338.24	3.17
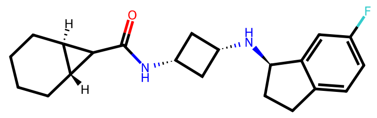	O=C(N[C@H]1C[C@@H](N[C@@H]2CCc3ccc(F)cc32)C1)C1[C@@H]2CCCC[C@@H]12	1021931913	25	342.21	3.49

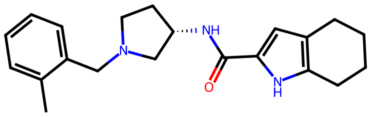	Cc1ccccc1CN1CC[C@H](NC(=O)c2cc3c([nH]2)CCCC3)C1	1015954003	25	337.22	3.21
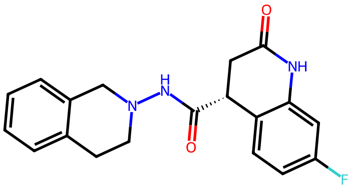	O=C1C[C@@H](C(=O)NN2CCc3ccccc3C2)c2ccc(F)cc2N1	855708398	25	339.14	2.34
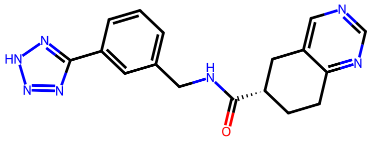	O=C(NCc1cccc(-c2nn[nH]n2)c1)[C@H]1CCc2ncncc2C1	1593143020	25	335.15	1.08
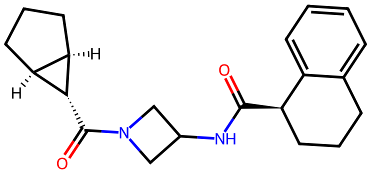	O=C(NC1CN(C(=O)[C@@H]2[C@H]3CCC[C@H]32)C1)[C@@H]1CCCc2ccccc21	995069921	25	338.2	2.48
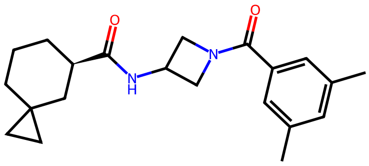	Cc1cc(C)cc(C(=O)N2CC(NC(=O)[C@@H]3CCCC4(CC4)C3)C2)c1	991515303	25	340.22	3.21
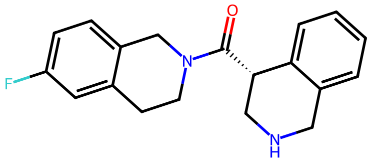	O=C([C@H]1CNCc2ccccc21)N1CCc2cc(F)ccc2C1	646286706	23	310.15	2.6
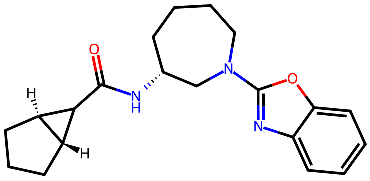	O=C(N[C@@H]1CCCCN(c2nc3ccccc3o2)C1)C1[C@@H]2CCC[C@@H]12	1063182068	25	339.19	3.35
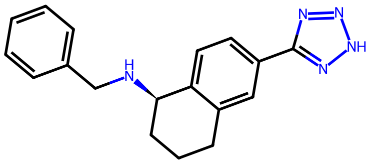	c1ccc(CN[C@@H]2CCCc3cc(-c4nn[nH]n4)ccc32)cc1	826521391	23	305.16	3.03

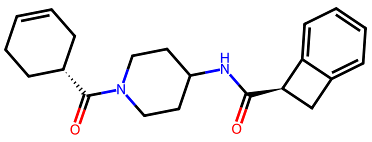	O=C(NC1CCN(C(=O)[C@H]2CC=CC C2)CC1)[C@@H]1Cc2ccccc21	944107490	25	338.2	2.79
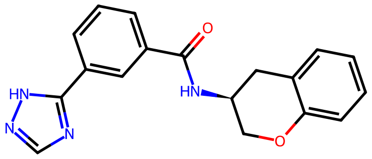	O=C(N[C@@H]1COc2ccccc2C1)c1cc cc(-c2ncn[nH]2)c1	179323022	24	320.13	2.21
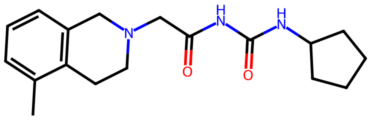	Cc1cccc2c1CCN(CC(=O)NC(=O)NC1 CCCC1)C2	170923031	23	315.19	2.12
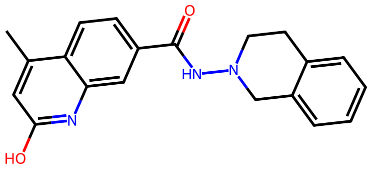	Cc1cc(O)nc2cc(C(=O)NN3CCc4ccccc 4C3)ccc12	1469893525	25	333.15	2.95
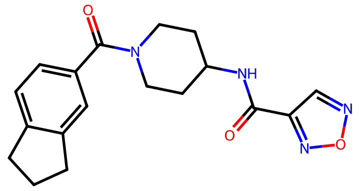	O=C(NC1CCN(C(=O)c2ccc3c(c2)CCC 3)CC1)c1cnon1	946720914	25	340.15	1.59
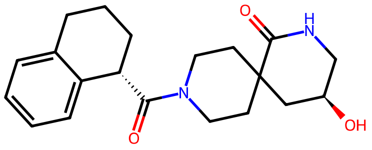	O=C([C@H]1CCCc2ccccc21)N1CCC2 (CC1)C[C@H](O)CNC2=O	885024779	25	342.19	1.6
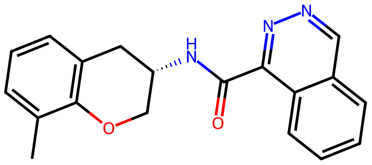	Cc1cccc2c1OC[C@@H](NC(=O)c1nn cc3ccccc13)C2	1584227572	24	319.13	2.67
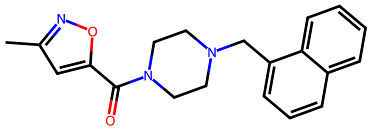	Cc1cc(C(=O)N2CCN(Cc3cccc4ccccc3 4)CC2)on1	23957250	25	335.16	3.09

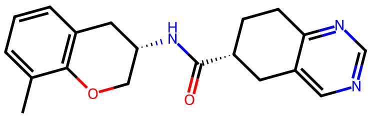	Cc1cccc2c1OC[C@@H](NC(=O)[C@@H]1CCc3ncncc3C1)C2	648396897	24	323.16	2.01
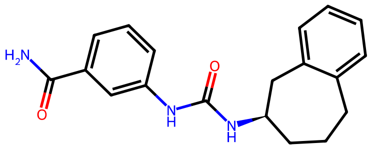	NC(=O)c1cccc(NC(=O)N[C@@H]2CCCc3ccccc3C2)c1	929406515	24	323.16	2.85
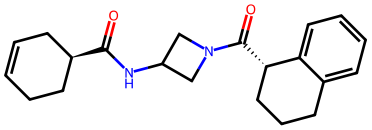	O=C(NC1CN(C(=O)[C@H]2CCCc3ccccc32)C1)[C@@H]1CC=CCC1	990202678	25	338.2	2.79
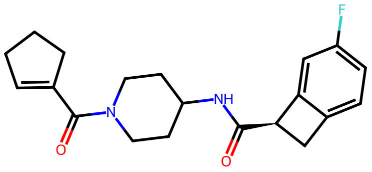	O=C(NC1CCN(C(=O)C2=CCCC2)CC1)[C@@H]1Cc2ccc(F)cc21	946449649	25	342.17	2.68
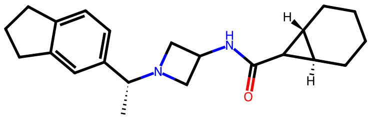	C[C@H](c1ccc2c(c1)CCC2)N1CC(NC(=O)C2[C@@H]3CCCC[C@@H]23)C1	1030186200	25	338.24	3.47
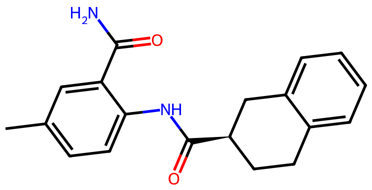	Cc1ccc(NC(=O)[C@@H]2CCc3ccccc3C2)c(C(N)=O)c1	844624327	23	308.15	2.84
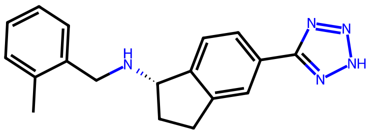	Cc1ccccc1CN[C@H]1CCc2cc(-c3nn[nH]n3)ccc21	1605939985	23	305.16	2.95
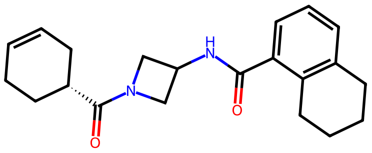	O=C(NC1CN(C(=O)[C@H]2CC=CCC2)C1)c1cccc2c1CCCC2	991900411	25	338.2	2.86

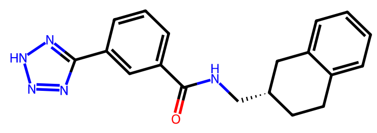	O=C(NC[C@H]1CCc2ccccc2C1)c1cccc(-c2nn[nH]n2)c1	738050893	25	333.16	2.4
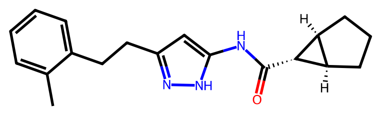	Cc1ccccc1CCc1cc(NC(=O)[C@@H]2[C@H]3CCC[C@H]32)[nH]n1	248487748	23	309.18	3.49
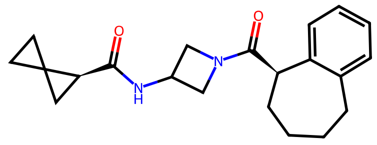	O=C(NC1CN(C(=O)[C@@H]2CCCCc3ccccc32)C1)[C@H]1CC12CC2	1000511985	25	338.2	2.62
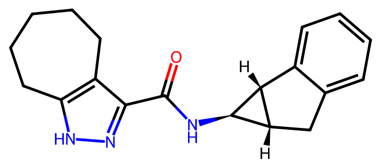	O=C(N[C@@H]1[C@H]2Cc3ccccc3[C@H]21)c1n[nH]c2c1CCCCC2	190676905	23	307.17	2.75
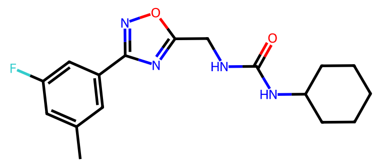	Cc1cc(F)cc(-c2noc(CNC(=O)NC3CCCCC3)n2)c1	273663342	24	332.16	3.32
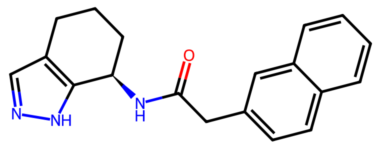	O=C(Cc1ccc2ccccc2c1)N[C@@H]1CCCc2cn[nH]c21	268990220	23	305.15	3.3
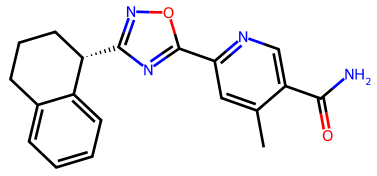	Cc1cc(-c2nc([C@H]3CCCc4ccccc43)no2)ncc1C(N)=O	580284871	25	334.14	3.01
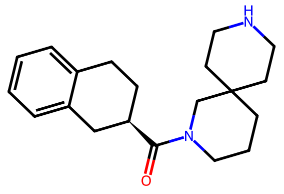	O=C([C@@H]1CCc2ccccc2C1)N1CCCC2(CCNCC2)C1	1647255896	23	312.22	2.78

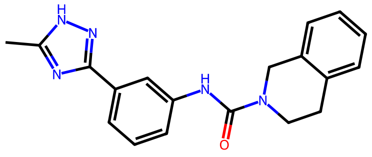	Cc1nc(-c2cccc(NC(=O)N3CCc4ccccc4C3)c2)n[nH]1	71799574	25	333.16	3.37
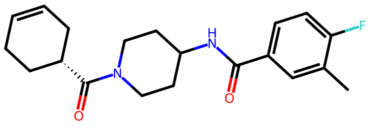	Cc1cc(C(=O)NC2CCN(C(=O)[C@H]3CC=CCC3)CC2)ccc1F	944107529	25	344.19	3.21
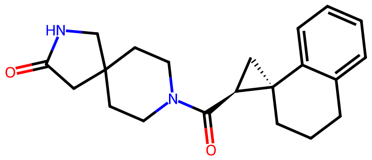	O=C1CC2(CCN(C(=O)[C@H]3C[C@]34CCCc3ccccc34)CC2)CN1	374098581	25	338.2	2.41
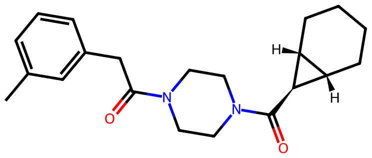	Cc1cccc(CC(=O)N2CCN(C(=O)[C@@H]3[C@H]4CCCC[C@H]43)CC2)c1	1010903804	25	340.22	2.64
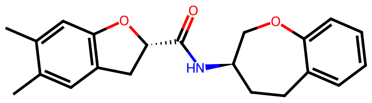	Cc1cc2c(cc1C)O[C@H](C(=O)N[C@@H]1CCc3ccccc3OC1)C2	511632003	25	337.17	3.12
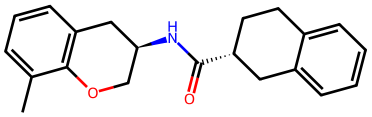	Cc1cccc2c1OC[C@H](NC(=O)[C@@H]1CCc3ccccc3C1)C2	1129553637	24	321.17	3.22
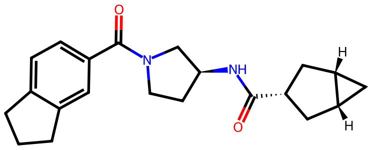	O=C(N[C@H]1CCN(C(=O)c2ccc3c(c2)CCC3)C1)[C@@H]1C[C@@H]2C[C@@H]2C1	938479781	25	338.2	2.55
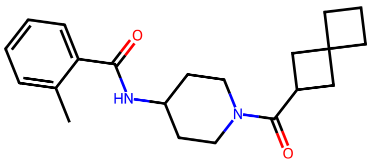	Cc1ccccc1C(=O)NC1CCN(C(=O)C2CC3(CCC3)C2)CC1	574042951	25	340.22	3.3

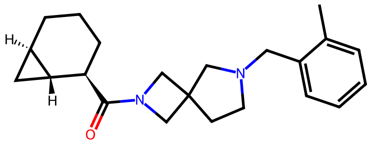	Cc1ccccc1CN1CCC2(C1)CN(C(=O)[C@@H]1CCC[C@@H]3C[C@H]31)C2	1072874523	25	338.24	3.47
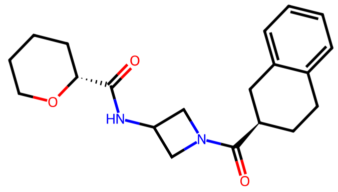	O=C(NC1CN(C(=O)[C@@H]2CCc3ccccc3C2)C1)[C@H]1CCCCO1	990855878	25	342.19	1.69
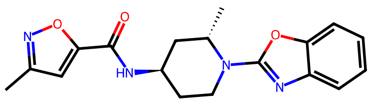	Cc1cc(C(=O)N[C@@H]2CCN(c3nc4ccccc4o3)[C@@H](C)C2)on1	1067507718	25	340.15	2.91
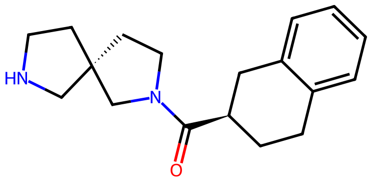	O=C([C@@H]1CCc2ccccc2C1)N1CC[C@]2(CCNC2)C1	314203818	21	284.19	2.0
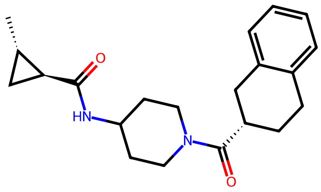	C[C@H]1C[C@@H]1C(=O)NC1CCN(C(=O)[C@H]2CCc3ccccc3C2)CC1	948572113	25	340.22	2.55
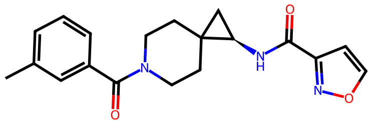	Cc1cccc(C(=O)N2CCC3(CC2)C[C@H]3NC(=O)c2ccon2)c1	963276049	25	339.16	2.41
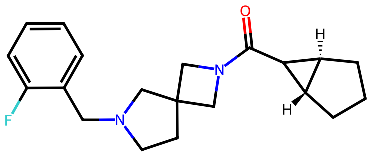	O=C(C1[C@H]2CCC[C@H]12)N1CC2(CCN(Cc3ccccc3F)C2)C1	1072590898	24	328.2	2.91
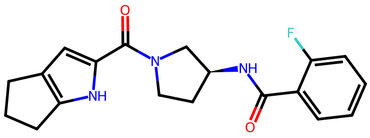	O=C(N[C@H]1CCN(C(=O)c2cc3c([nH]2)CCC3)C1)c1ccccc1F	937623554	25	341.15	2.29

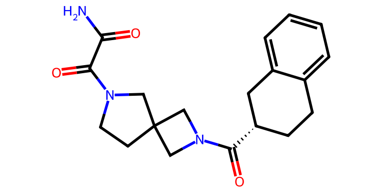	NC(=O)C(=O)N1CCC2(C1)CN(C(=O)[C@H]1CCc3ccccc3C1)C2	1019491960	25	341.17	0.34
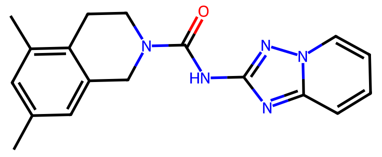	Cc1cc(C)c2c(c1)CN(C(=O)Nc1nc3ccccn3n1)CC2	187383441	24	321.16	2.94
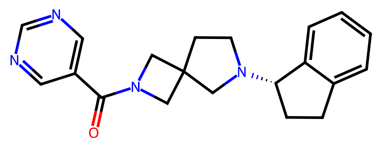	O=C(c1cncnc1)N1CC2(CCN([C@H]3CCc4ccccc43)C2)C1	1072656227	25	334.18	2.31
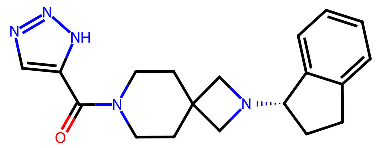	O=C(c1cnn[nH]1)N1CCC2(CC1)CN([C@H]1CCc3ccccc31)C2	1035740358	25	337.19	2.03
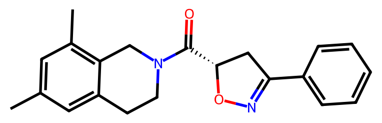	Cc1cc(C)c2c(c1)CCN(C(=O)[C@@H]1CC(c3ccccc3)=NO1)C2	1463500371	25	334.17	3.38
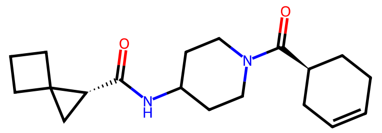	O=C(NC1CCN(C(=O)[C@@H]2CC=CCC2)CC1)[C@@H]1CC12CCC2	944127606	23	316.22	2.64
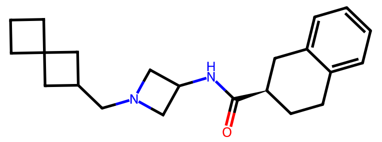	O=C(NC1CN(CC2CC3(CCC3)C2)C1)[C@@H]1CCc2ccccc2C1	1031012184	25	338.24	3.17
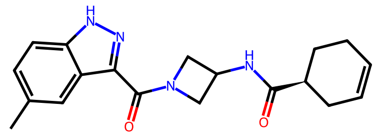	Cc1ccc2[nH]nc(C(=O)N3CC(NC(=O)[C@@H]4CC=CCC4)C3)c2c1	990213445	25	338.17	2.17

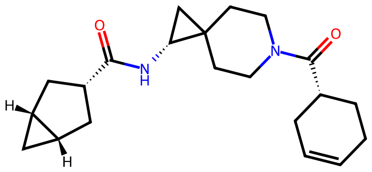	O=C(N[C@@H]1CC12CCN(C(=O)[C@H]1CC=CCC1)CC2)[C@@H]1C[C@@H]2C[C@@H]2C1	964770635	25	342.23	2.89
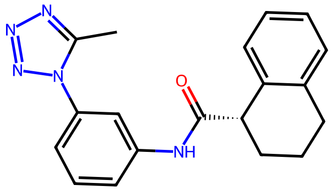	Cc1nnnn1-c1cccc(NC(=O)[C@H]2CCCc3ccccc32)c1	41985734	25	333.16	3.03
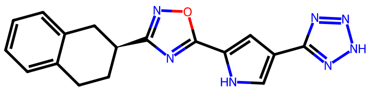	c1ccc2c(c1)CC[C@H](c1noc(-c3cc(-c4nn[nH]n4)c[nH]3)n1)C2	1607133037	25	333.13	2.52
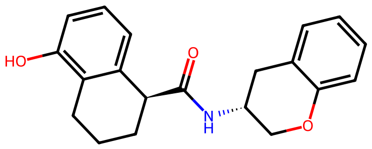	O=C(N[C@H]1COc2ccccc2C1)[C@H]1CCCc2c(O)cccc21	190124954	24	323.15	2.93
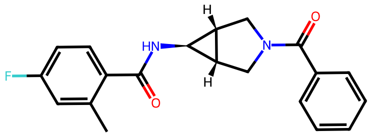	Cc1cc(F)ccc1C(=O)N[C@H]1[C@@H]2CN(C(=O)c3ccccc3)C[C@@H]21	976919515	25	338.14	2.63
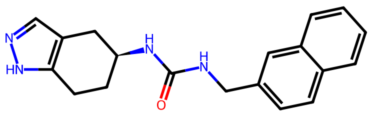	O=C(NCc1ccc2ccccc2c1)N[C@H]1CCc2[nH]ncc2C1	138959821	24	320.16	2.92
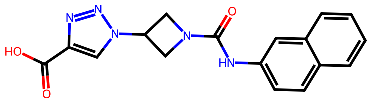	O=C(O)c1cn(C2CN(C(=O)Nc3ccc4ccccc4c3)C2)nn1	327170979	25	337.12	2.22
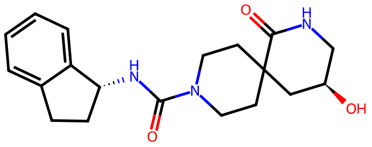	O=C(N[C@@H]1CCc2ccccc21)N1CCC2(CC1)C[C@H](O)CNC2=O	913965885	25	343.19	1.35

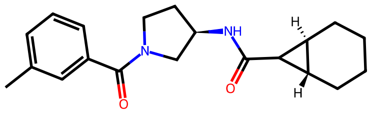	Cc1cccc(C(=O)N2CC[C@@H](NC(=O)C3[C@H]4CCCC[C@H]34)C2)c1	939880444	24	326.2	2.76
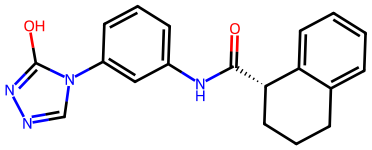	O=C(Nc1cccc(-n2cnnc2O)c1)[C@H]1CCCc2ccccc21	364279207	25	334.14	3.03
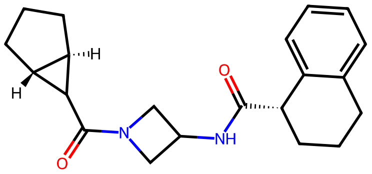	O=C(NC1CN(C(=O)C2[C@@H]3CCC[C@@H]23)C1)[C@H]1CCCc2ccccc21	995069923	25	338.2	2.48
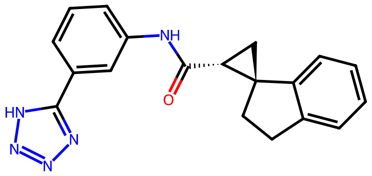	O=C(Nc1cccc(-c2nnn[nH]2)c1)[C@@H]1C[C@@]12CCc1ccccc12	266615509	25	331.14	2.71
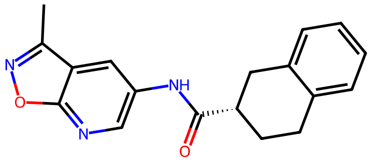	Cc1noc2ncc(NC(=O)[C@H]3CCc4ccccc4C3)cc12	581887537	23	307.13	3.27
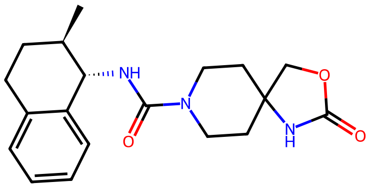	C[C@@H]1CCc2ccccc2[C@H]1NC(=O)N1CCC2(CC1)COC(=O)N2	495297706	25	343.19	2.59
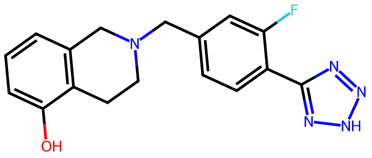	Oc1cccc2c1CCN(Cc1ccc(-c3nn[nH]n3)c(F)c1)C2	1582816177	24	325.13	2.27
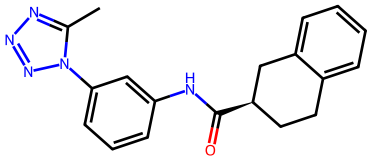	Cc1nnnn1-c1cccc(NC(=O)[C@@H]2CCc3ccccc3C2)c1	565541693	25	333.16	2.71

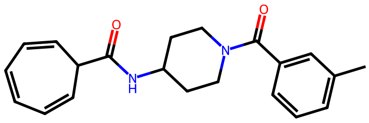	Cc1cccc(C(=O)N2CCC(NC(=O)C3C=CC=CC=C3)CC2)c1	943836258	25	336.18	3.01
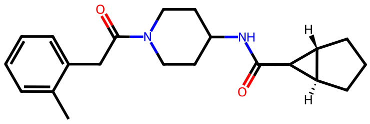	Cc1ccccc1CC(=O)N1CCC(NC(=O)C2[C@@H]3CCC[C@@H]23)CC1	946717791	25	340.22	2.69
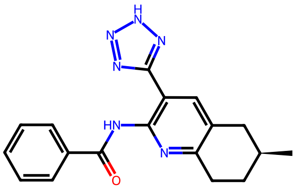	C[C@H]1CCc2nc(NC(=O)c3ccccc3)c(-c3nn[nH]n3)cc2C1	1590280476	25	334.15	2.64
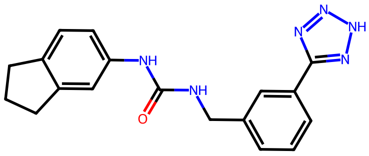	O=C(NCc1cccc(-c2nn[nH]n2)c1)Nc1ccc2c(c1)CCC2	1593143549	25	334.15	2.68
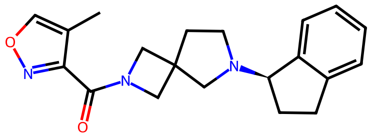	Cc1conc1C(=O)N1CC2(CCN([C@@H]3CCc4ccccc43)C2)C1	1073107678	25	337.18	2.82
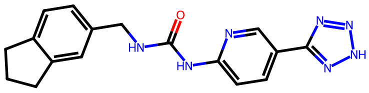	O=C(NCc1ccc2c(c1)CCC2)Nc1ccc(-c2nn[nH]n2)cn1	1582552102	25	335.15	2.07
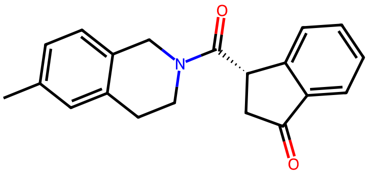	Cc1ccc2c(c1)CCN(C(=O)[C@H]1CC(=O)c3ccccc31)C2	360219343	23	305.14	3.25
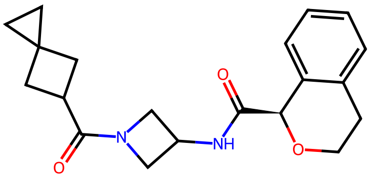	O=C(NC1CN(C(=O)C2CC3(CC3)C2)C1)[C@@H]1OCCc2ccccc21	997321956	25	340.18	1.82

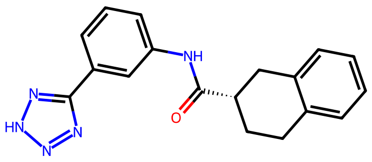	O=C(Nc1cccc(-c2nn[nH]n2)c1)[C@H]1CCc2ccccc2C1	1540505402	24	319.14	2.61
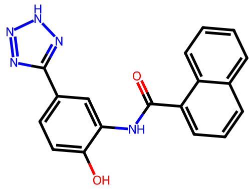	O=C(Nc1cc(-c2nn[nH]n2)ccc1O)c1cccc2ccccc12	826450783	25	331.11	2.98
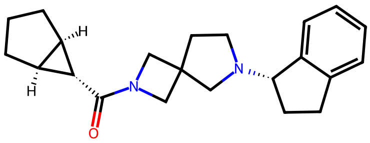	O=C([C@@H]1[C@H]2CCC[C@H]21)N1CC2(CCN([C@H]3CCc4ccccc43)C2)C1	1072591526	25	336.22	3.25
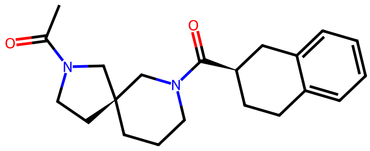	CC(=O)N1CC[C@@]2(CCCN(C(=O)[C@@H]3CCc4ccccc4C3)C2)C1	952819499	25	340.22	2.65
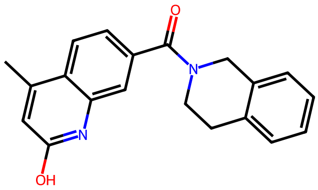	Cc1cc(O)nc2cc(C(=O)N3CCc4ccccc4C3)ccc12	360843652	24	318.14	3.45
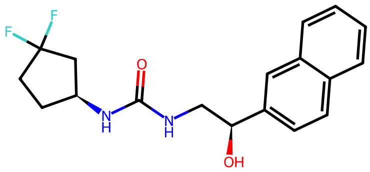	O=C(NC[C@H](O)c1ccc2ccccc2c1)N[C@H]1CCC(F)(F)C1	763708732	24	334.15	3.36
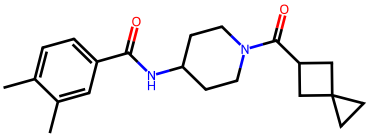	Cc1ccc(C(=O)NC2CCN(C(=O)C3CC4(CC4)C3)CC2)cc1C	948305757	25	340.22	3.21
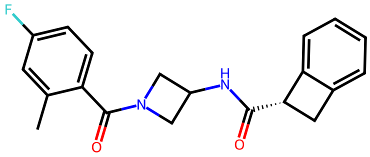	Cc1cc(F)ccc1C(=O)N1CC(NC(=O)[C@H]2Cc3ccccc32)C1	998682199	25	338.14	2.41

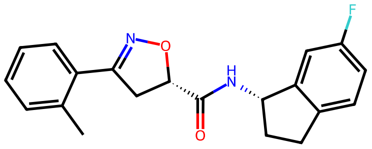	Cc1ccccc1C1=NO[C@H](C(=O)N[C@H]2CCc3ccc(F)cc32)C1	1360016916	25	338.14	3.43
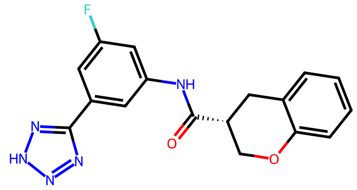	O=C(Nc1cc(F)cc(-c2nn[nH]n2)c1)[C@H]1COc2ccccc2C1	1582635001	25	339.11	2.2
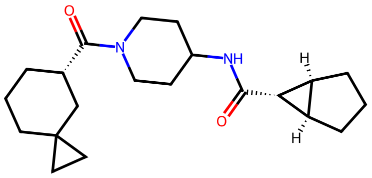	O=C(NC1CCN(C(=O)[C@H]2CCCC3(CC3)C2)CC1)[C@@H]1[C@H]2CCC[C@H]21	946752529	25	344.25	3.11
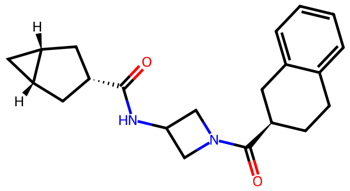	O=C(NC1CN(C(=O)[C@@H]2CCc3ccccc3C2)C1)[C@@H]1C[C@@H]2C[C@@H]2C1	998010170	25	338.2	2.16
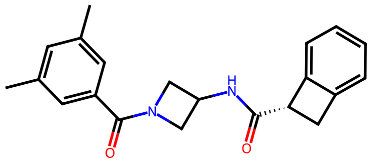	Cc1cc(C)cc(C(=O)N2CC(NC(=O)[C@H]3Cc4ccccc43)C2)c1	998692629	25	334.17	2.58
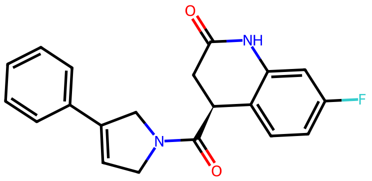	O=C1C[C@H](C(=O)N2CC=C(c3ccccc3)C2)c2ccc(F)cc2N1	378511435	25	336.13	3.18
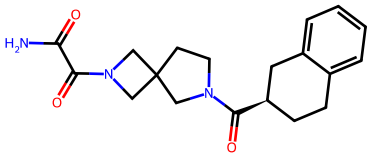	NC(=O)C(=O)N1CC2(CCN(C(=O)[C@@H]3CCc4ccccc4C3)C2)C1	1019358192	25	341.17	0.34
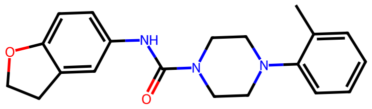	Cc1ccccc1N1CCN(C(=O)Nc2ccc3c(c2)CCO3)CC1	193029978	25	337.18	3.28

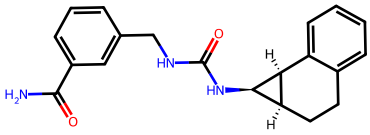	NC(=O)c1cccc(CNC(=O)N[C@@H]2[C@@H]3CCc4ccccc4[C@@H]32)c1	126277752	25	335.16	2.31
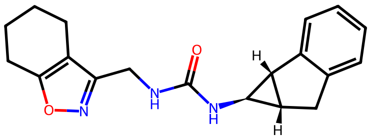	O=C(NCc1noc2c1CCCC2)N[C@@H]1[C@H]2Cc3ccccc3[C@H]21	288004886	24	323.16	2.69
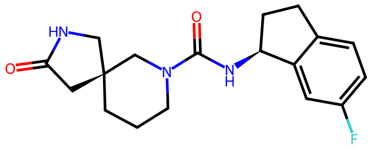	O=C1C[C@@]2(CCCN(C(=O)N[C@H]3CCc4ccc(F)cc43)C2)CN1	608426949	24	331.17	2.12
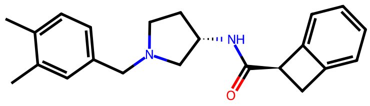	Cc1ccc(CN2CC[C@H](NC(=O)[C@@H]3Cc4ccccc43)C2)cc1C	1015636500	25	334.2	3.33
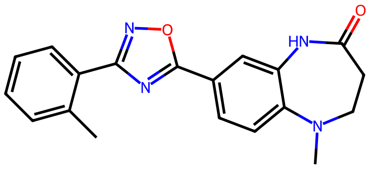	Cc1ccccc1-c1noc(-c2ccc3c(c2)NC(=O)CCN3C)n1	359900964	25	334.14	3.49
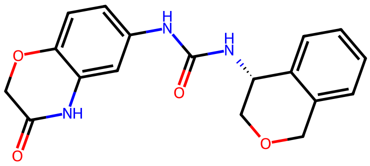	O=C1COc2ccc(NC(=O)N[C@H]3COCc4ccccc43)cc2N1	1464967623	25	339.12	2.41
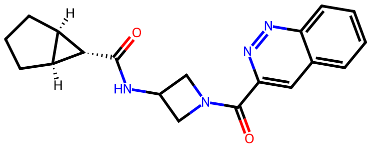	O=C(NC1CN(C(=O)c2cc3ccccc3nn2)C1)[C@@H]1[C@H]2CCC[C@H]21	990706844	25	336.16	1.62
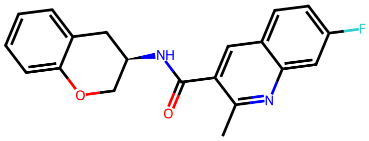	Cc1nc2cc(F)ccc2cc1C(=O)N[C@H]1COc2ccccc2C1	45847442	25	336.13	3.42

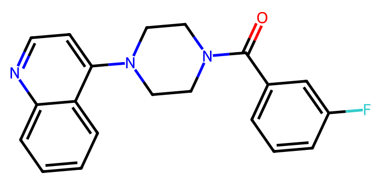	O=C(c1cccc(F)c1)N1CCN(c2ccnc3ccccc23)CC1	352764267	25	335.14	3.34
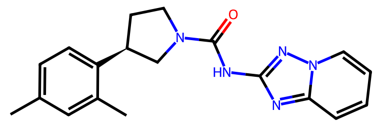	Cc1ccc([C@H]2CCN(C(=O)Nc3nc4ccccn4n3)C2)c(C)c1	279487611	25	335.17	3.37
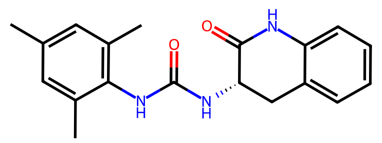	Cc1cc(C)c(NC(=O)N[C@H]2Cc3ccccc3NC2=O)c(C)c1	528849289	24	323.16	3.3
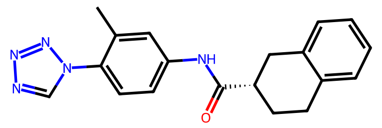	Cc1cc(NC(=O)[C@H]2CCc3ccccc3C2)ccc1-n1cnnn1	571332533	25	333.16	2.71
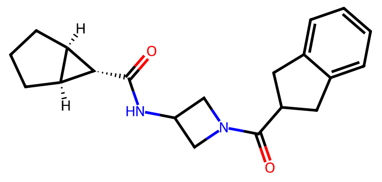	O=C(NC1CN(C(=O)C2Cc3ccccc3C2)C1)[C@@H]1[C@H]2CCC[C@H]21	990710896	24	324.18	1.77
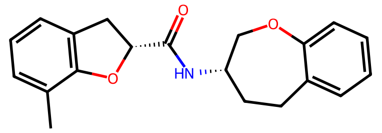	Cc1cccc2c1O[C@@H](C(=O)N[C@H]1CCc3ccccc3OC1)C2	451487667	24	323.15	2.81
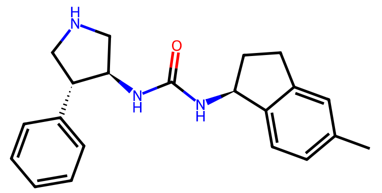	Cc1ccc2c(c1)CC[C@@H]2NC(=O)N[C@@H]1CNC[C@H]1c1ccccc1	567595560	25	335.2	3.04
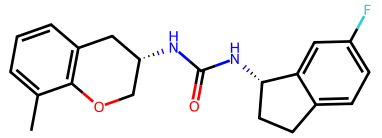	Cc1cccc2c1OC[C@@H](NC(=O)N[C@H]1CCc3ccc(F)cc31)C2	573147373	25	340.16	3.42

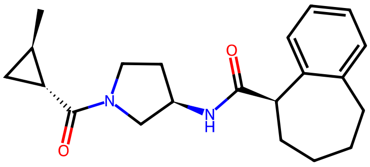	C[C@@H]1C[C@H]1C(=O)N1CC[C@@H](NC(=O)[C@@H]2CCCCc3ccccc32)C1	941123893	25	340.22	2.87
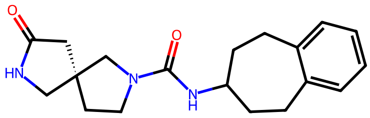	O=C1C[C@]2(CCN(C(=O)NC3CCc4ccccc4CC3)C2)CN1	656205152	24	327.19	1.86
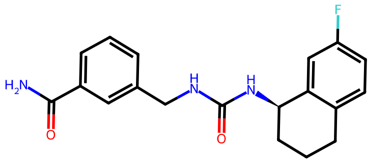	NC(=O)c1cccc(CNC(=O)N[C@@H]2CCCc3ccc(F)cc32)c1	93119744	25	341.15	2.8
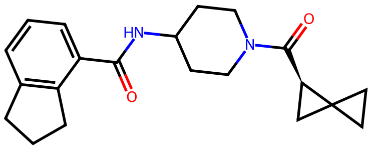	O=C(NC1CCN(C(=O)[C@@H]2CC23CC3)CC1)c1cccc2c1CCC2	948567157	25	338.2	2.7
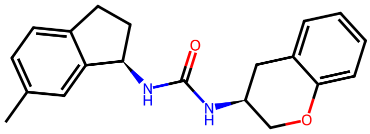	Cc1ccc2c(c1)[C@H](NC(=O)N[C@@H]1COc3ccccc3C1)CC2	592499150	24	322.17	3.29
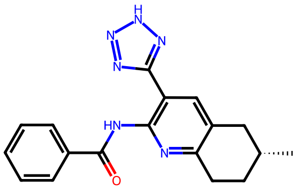	C[C@@H]1CCc2nc(NC(=O)c3ccccc3)c(-c3nn[nH]n3)cc2C1	1590280474	25	334.15	2.64
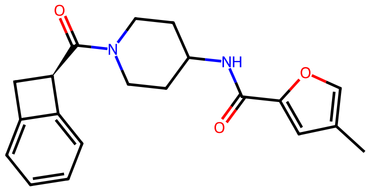	Cc1coc(C(=O)NC2CCN(C(=O)[C@@H]3Cc4ccccc43)CC2)c1	948063254	25	338.16	2.65
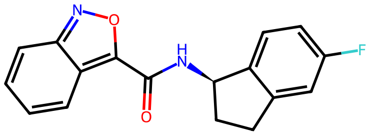	O=C(N[C@@H]1CCc2cc(F)ccc21)c1onc2ccccc12	1179853206	22	296.1	3.38

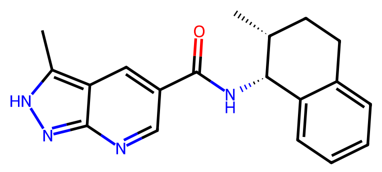	Cc1[nH]nc2ncc(C(=O)N[C@H]3c4cccc c4CC[C@H]3C)cc12	342885256	24	320.16	3.32
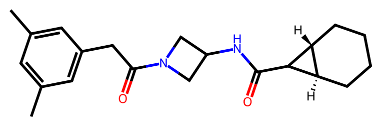	Cc1cc(C)cc(CC(=O)N2CC(NC(=O)C3[C@@H]4CCCC[C@@H]34)C2)c1	990650120	25	340.22	2.61
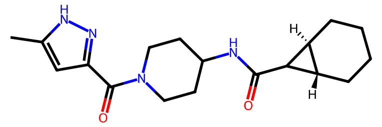	Cc1cc(C(=O)N2CCC(NC(=O)C3[C@H]4CCCC[C@H]34)CC2)n[nH]1	944822329	24	330.21	1.88
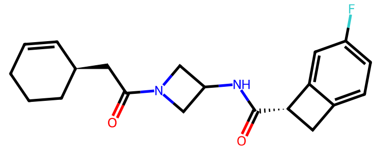	O=C(NC1CN(C(=O)C[C@@H]2C=CC CC2)C1)[C@H]1Cc2ccc(F)cc21	998490091	25	342.17	2.54
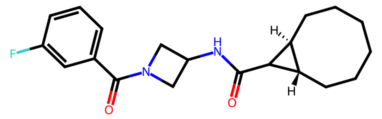	O=C(NC1CN(C(=O)c2cccc(F)c2)C1)C 1[C@H]2CCCCCC[C@H]12	1000465995	25	344.19	2.98
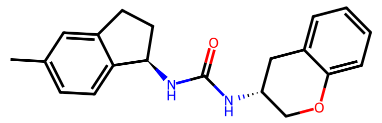	Cc1ccc2c(c1)CC[C@H]2NC(=O)N[C @H]1COc2ccccc2C1	516689630	24	322.17	3.29
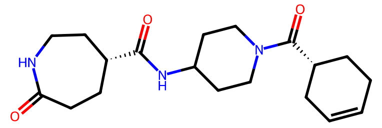	O=C1CC[C@@H](C(=O)NC2CCN(C(=O)[C@H]3CC=CCC3)CC2)CCN1	944127268	25	347.22	1.37
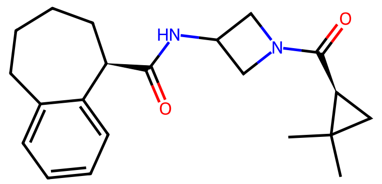	CC1(C)C[C@@H]1C(=O)N1CC(NC(= O)[C@@H]2CCCCc3ccccc32)C1	994311775	25	340.22	2.87

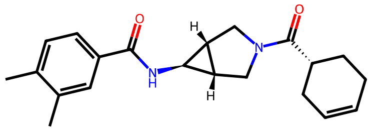	Cc1ccc(C(=O)N[C@H]2[C@@H]3CN(C(=O)[C@H]4CC=CCC4)C[C@@H]32)cc1C	977179891	25	338.2	2.85
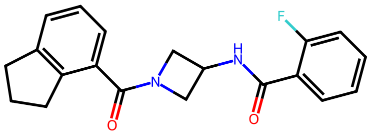	O=C(NC1CN(C(=O)c2cccc3c2CCC3)C1)c1ccccc1F	990093843	25	338.14	2.57
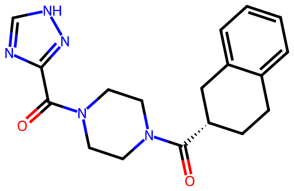	O=C(c1nc[nH]n1)N1CCN(C(=O)[C@H]2CCc3ccccc3C2)CC1	1012033884	25	339.17	0.89
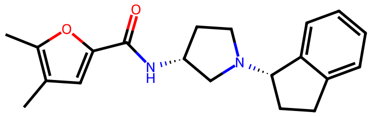	Cc1cc(C(=O)N[C@@H]2CCN([C@H]3CCc4ccccc43)C2)oc1C	1010618753	24	324.18	3.39
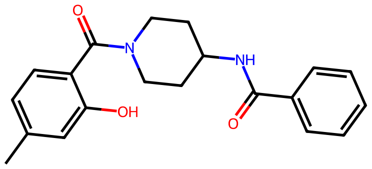	Cc1ccc(C(=O)N2CCC(NC(=O)c3ccccc3)CC2)c(O)c1	29776480	25	338.16	2.74
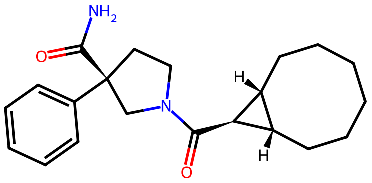	NC(=O)[C@@]1(c2ccccc2)CCN(C(=O)[C@@H]2[C@H]3CCCCCC[C@H]32)C1	616272795	25	340.22	2.86
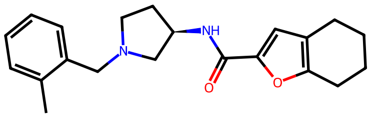	Cc1ccccc1CN1CC[C@@H](NC(=O)c2cc3c(o2)CCCC3)C1	1014682867	25	338.2	3.47
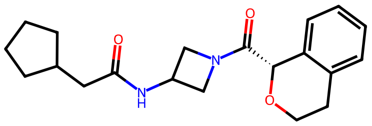	O=C(CC1CCCC1)NC1CN(C(=O)[C@H]2OCCc3ccccc32)C1	990107245	25	342.19	2.21

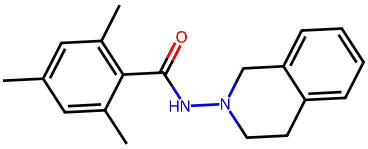	Cc1cc(C)c(C(=O)NN2CCc3ccccc3C2)c(C)c1	855709573	22	294.17	3.31
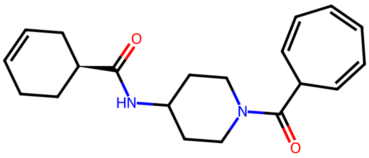	O=C(NC1CCN(C(=O)C2C=CC=CC=C2)CC1)[C@@H]1CC=CCC1	946948850	24	326.2	2.75
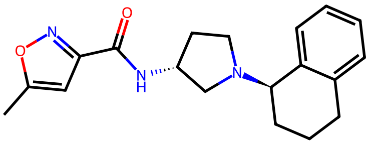	Cc1cc(C(=O)N[C@@H]2CCN([C@@H]3CCCc4ccccc43)C2)no1	1010071050	24	325.18	2.86
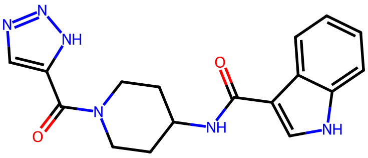	O=C(NC1CCN(C(=O)c2cnn[nH]2)CC1)c1c[nH]c2ccccc12	947373396	25	338.15	1.32
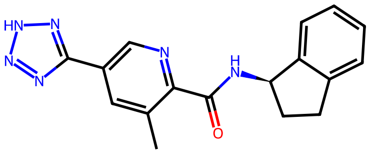	Cc1cc(-c2nn[nH]n2)cnc1C(=O)N[C@@H]1CCc2ccccc21	1580626210	24	320.14	1.99
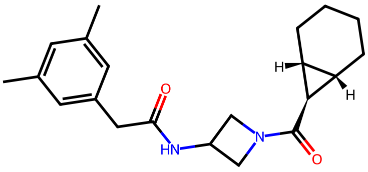	Cc1cc(C)cc(CC(=O)NC2CN(C(=O)[C@@H]3[C@H]4CCCC[C@H]43)C2)c1	998753272	25	340.22	2.61
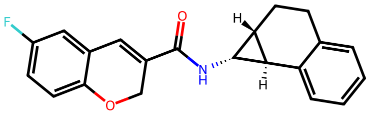	O=C(N[C@H]1[C@@H]2c3ccccc3CC[C@H]21)C1=Cc2cc(F)ccc2OC1	534213270	25	335.13	3.45
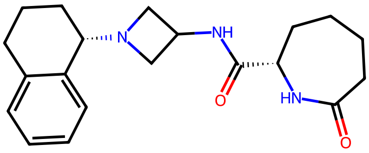	O=C1CCCC[C@@H](C(=O)NC2CN([C@H]3CCCc4ccccc43)C2)N1	1030937062	25	341.21	1.92

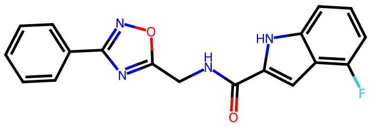	O=C(NCc1nc(-c2ccccc2)no1)c1cc2c(F)cccc2[nH]1	44780641	25	336.1	3.29
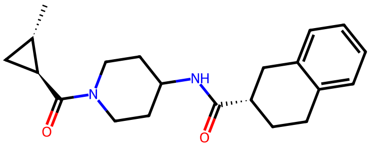	C[C@H]1C[C@@H]1C(=O)N1CCC(NC(=O)[C@H]2CCc3ccccc3C2)CC1	947839558	25	340.22	2.55
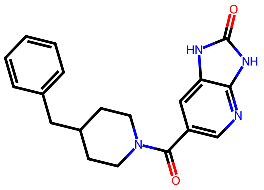	O=C(c1cnc2[nH]c(=O)[nH]c2c1)N1CCC(Cc2ccccc2)CC1	289152639	25	336.16	2.35
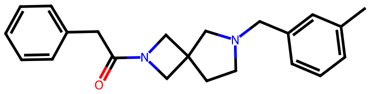	Cc1cccc(CN2CCC3(C2)CN(C(=O)Cc2ccccc2)C3)c1	1072862061	25	334.2	3.27
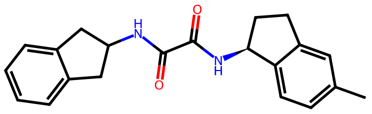	Cc1ccc2c(c1)CC[C@@H]2NC(=O)C(=O)NC1Cc2ccccc2C1	1323220251	25	334.17	2.38
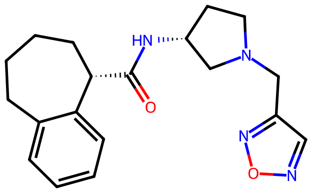	O=C(N[C@@H]1CCN(Cc2cnon2)C1)[C@H]1CCCCc2ccccc21	1016368459	25	340.19	2.27
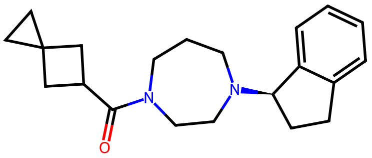	O=C(C1CC2(CC2)C1)N1CCCN([C@@H]2CCc3ccccc32)CC1	981933254	24	324.22	3.4
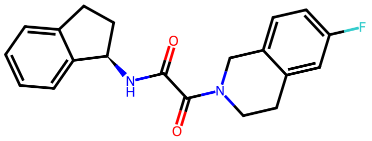	O=C(N[C@@H]1CCc2ccccc21)C(=O)N1CCc2cc(F)ccc2C1	339880387	25	338.14	2.51

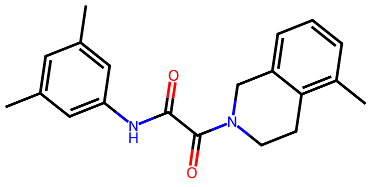	Cc1cc(C)cc(NC(=O)C(=O)N2CCc3c(C)cccc3C2)c1	729881934	24	322.17	3.14
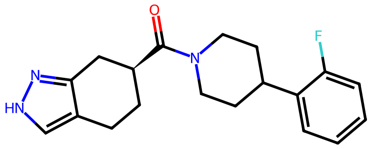	O=C([C@H]1CCc2c[nH]nc2C1)N1CCC(c2ccccc2F)CC1	608138213	24	327.17	3.06
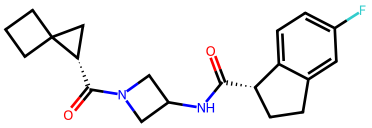	O=C(NC1CN(C(=O)[C@H]2CC23CCC3)C1)[C@H]1CCc2cc(F)ccc21	996091248	25	342.17	2.37
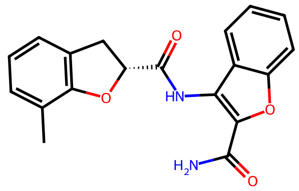	Cc1cccc2c1O[C@@H](C(=O)Nc1c(C(N)=O)oc3ccccc13)C2	1352452946	25	336.11	2.78
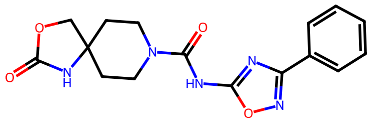	O=C1NC2(CCN(C(=O)Nc3nc(-c4ccccc4)no3)CC2)CO1	1364344014	25	343.13	1.84
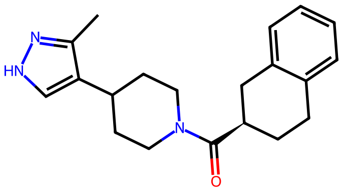	Cc1n[nH]cc1C1CCN(C(=O)[C@@H]2CCc3ccccc3C2)CC1	578242439	24	323.2	3.23
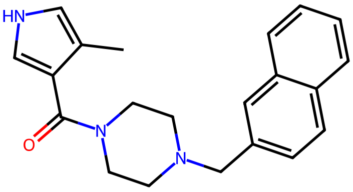	Cc1c[nH]cc1C(=O)N1CCN(Cc2ccc3ccccc3c2)CC1	957646834	25	333.18	3.43
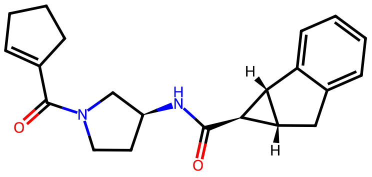	O=C(N[C@H]1CCN(C(=O)C2=CCCC2)C1)[C@@H]1[C@H]2Cc3ccccc3[C@H]21	937966868	25	336.18	2.4

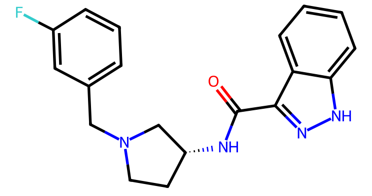	O=C(N[C@@H]1CCN(Cc2cccc(F)c2)C1)c1n[nH]c2ccccc12	1016160171	25	338.15	2.71
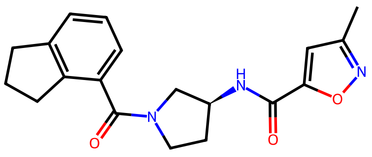	Cc1cc(C(=O)N[C@H]2CCN(C(=O)c3cccc4c3CCC4)C2)on1	937671852	25	339.16	2.12
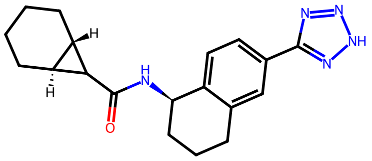	O=C(N[C@@H]1CCCc2cc(-c3nn[nH]n3)ccc21)C1[C@H]2CCCC[C@H]12	1592985266	25	337.19	2.8
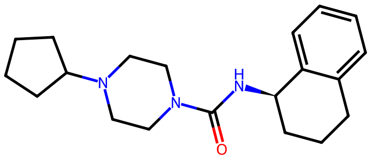	O=C(N[C@@H]1CCCc2ccccc21)N1CCN(C2CCCC2)CC1	107834137	24	327.23	3.33
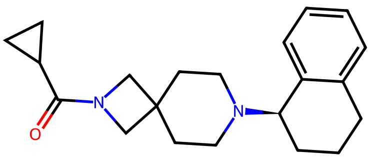	O=C(C1CC1)N1CC2(CCN([C@@H]3CCCc4ccccc43)CC2)C1	1050401409	24	324.22	3.4
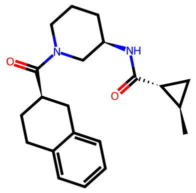	C[C@@H]1C[C@H]1C(=O)N[C@@H]1CCCN(C(=O)[C@@H]2CCc3ccccc3C2)C1	966006948	25	340.22	2.55
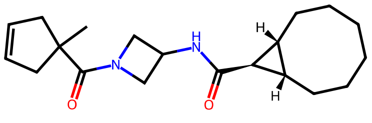	CC1(C(=O)N2CC(NC(=O)[C@@H]3[C@H]4CCCCCC[C@H]43)C2)CC=CC1	1000503306	24	330.23	2.89
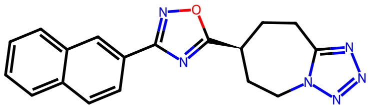	c1ccc2cc(-c3noc([C@H]4CCc5nnnn5CC4)n3)ccc2c1	896299951	25	332.14	3.0

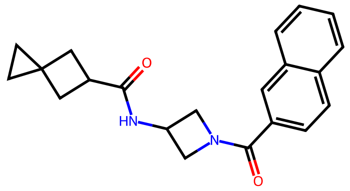	O=C(NC1CN(C(=O)c2ccc3ccccc3c2)C1)C1CC2(CC2)C1	999333135	25	334.17	2.97
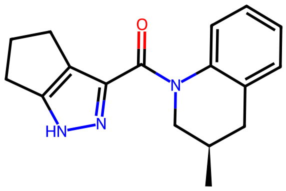	C[C@@H]1Cc2ccccc2N(C(=O)c2n[nH]c3c2CCC3)C1	104523408	21	281.15	2.74
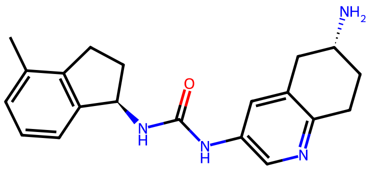	Cc1cccc2c1CC[C@H]2NC(=O)Nc1cnc2c(c1)C[C@H](N)CC2	579450946	25	336.2	3.02
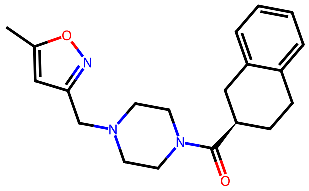	Cc1cc(CN2CCN(C(=O)[C@@H]3CCc4ccccc4C3)CC2)no1	583938292	25	339.19	2.43
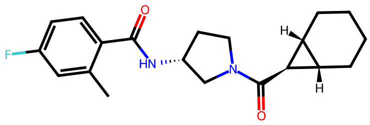	Cc1cc(F)ccc1C(=O)N[C@@H]1CCN(C(=O)[C@@H]2[C@H]3CCCC[C@H]32)C1	941909925	25	344.19	2.9
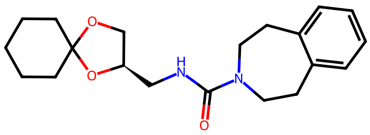	O=C(NC[C@@H]1COC2(CCCCC2)O1)N1CCc2ccccc2CC1	776062613	25	344.21	2.87
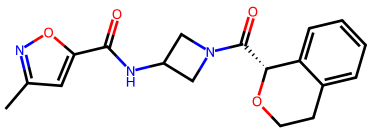	Cc1cc(C(=O)NC2CN(C(=O)[C@H]3OCCc4ccccc43)C2)on1	990218393	25	341.14	1.24
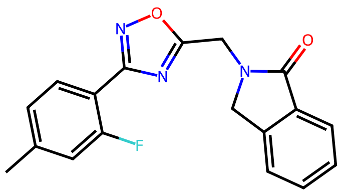	Cc1ccc(-c2noc(CN3Cc4ccccc4C3=O)n2)c(F)c1	359144553	24	323.11	3.34

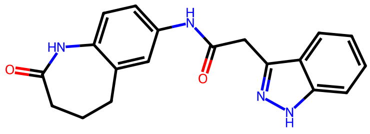	O=C(Cc1n[nH]c2ccccc12)Nc1ccc2c(c1)CCCC(=O)N2	171341883	25	334.14	3.02
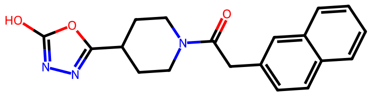	O=C(Cc1ccc2ccccc2c1)N1CCC(c2nnc(O)o2)CC1	339195845	25	337.14	2.88
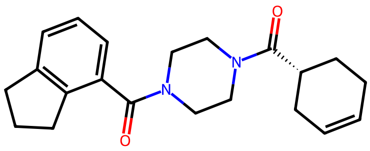	O=C(c1cccc2c1CCC2)N1CCN(C(=O)[C@H]2CC=CCC2)CC1	1010310352	25	338.2	2.82
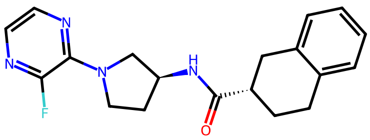	O=C(N[C@H]1CCN(c2nccnc2F)C1)[C@H]1CCc2ccccc2C1	1058559514	25	340.17	2.12
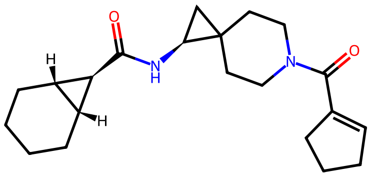	O=C(N[C@H]1CC12CCN(C(=O)C1=CCCC1)CC2)[C@@H]1[C@H]2CCCC[C@H]21	963447707	25	342.23	3.03
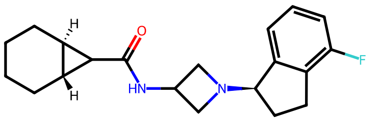	O=C(NC1CN([C@@H]2CCc3c(F)cccc32)C1)C1[C@@H]2CCCC[C@@H]12	1030184215	24	328.2	3.05
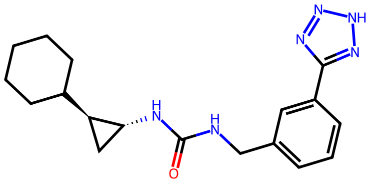	O=C(NCc1cccc(-c2nn[nH]n2)c1)N[C@@H]1C[C@H]1C1CCCCC1	823257230	25	340.2	2.63
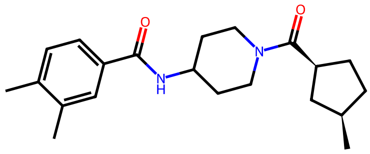	Cc1ccc(C(=O)NC2CCN(C(=O)[C@H]3CC[C@@H](C)C3)CC2)cc1C	946734331	25	342.23	3.46

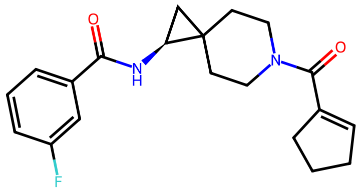	O=C(N[C@H]1CC12CCN(C(=O)C1=CCCC1)CC2)c1cccc(F)c1	962816866	25	342.17	3.05
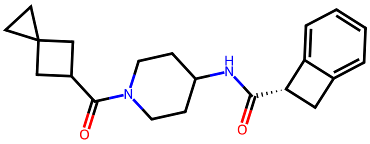	O=C(NC1CCN(C(=O)C2CC3(CC3)C2)CC1)[C@H]1Cc2ccccc21	948308652	25	338.2	2.62
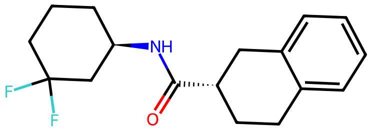	O=C(N[C@@H]1CCCC(F)(F)C1)[C@H]1CCc2ccccc2C1	649847879	21	293.16	3.49
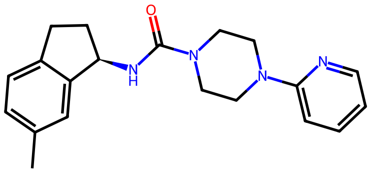	Cc1ccc2c(c1)[C@H](NC(=O)N1CCN(c3ccccn3)CC1)CC2	592548165	25	336.2	2.91
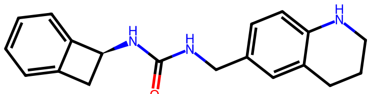	O=C(NCc1ccc2c(c1)CCCN2)N[C@H]1Cc2ccccc21	1548924000	23	307.17	3.14
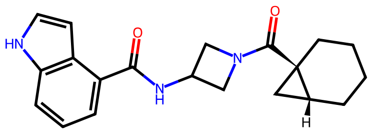	O=C(NC1CN(C(=O)[C@@]23CCCC[C@@H]2C3)C1)c1cccc2[nH]ccc12	1000410764	25	337.18	2.69
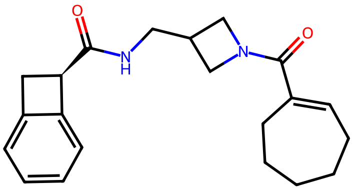	O=C(NCC1CN(C(=O)C2=CCCCCC2)C1)[C@@H]1Cc2ccccc21	1003957063	25	338.2	2.79
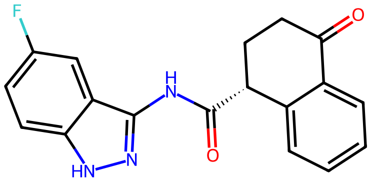	O=C1CC[C@@H](C(=O)Nc2n[nH]c3ccc(F)cc23)c2ccccc21	339922329	24	323.11	3.4

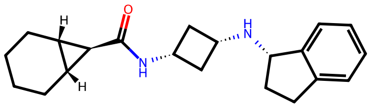	O=C(N[C@H]1C[C@@H](N[C@H]2CCc3ccccc32)C1)[C@@H]1[C@H]2CCCC[C@H]21	1021936285	24	324.22	3.35
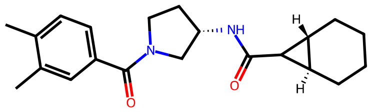	Cc1ccc(C(=O)N2CC[C@H](NC(=O)C3[C@@H]4CCCC[C@@H]34)C2)cc1C	937788008	25	340.22	3.07
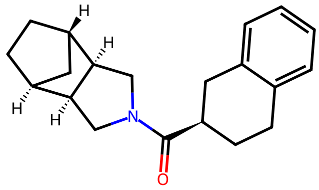	O=C([C@@H]1CCc2ccccc2C1)N1C[C@H]2[C@H]3CC[C@@H](C3)[C@H]2C1	519604064	22	295.19	3.3
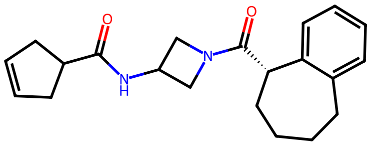	O=C(NC1CN(C(=O)[C@H]2CCCCc3ccccc32)C1)C1CC=CC1	990587076	25	338.2	2.79
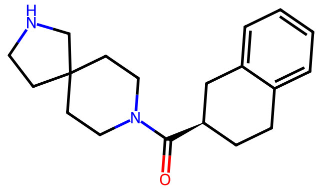	O=C([C@@H]1CCc2ccccc2C1)N1CCC2(CCNC2)CC1	578759261	22	298.2	2.39
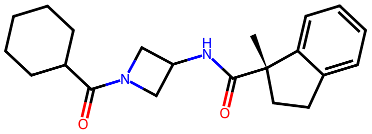	C[C@]1(C(=O)NC2CN(C(=O)C3CCCCC3)C2)CCc2ccccc21	994373046	25	340.22	2.8
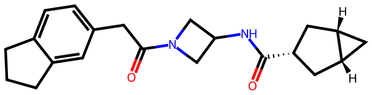	O=C(NC1CN(C(=O)Cc2ccc3c(c2)CCC3)C1)[C@@H]1C[C@@H]2C[C@@H]2C1	998010067	25	338.2	2.09
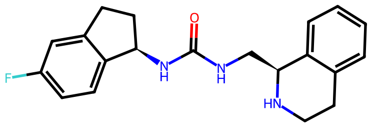	O=C(NC[C@@H]1NCCc2ccccc21)N[C@@H]1CCc2cc(F)ccc21	557162126	25	339.17	3.0

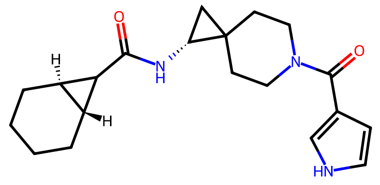	O=C(N[C@@H]1CC12CCN(C(=O)c1cc[nH]c1)CC2)C1[C@@H]2CCCC[C@@H]12	963443625	25	341.21	2.56
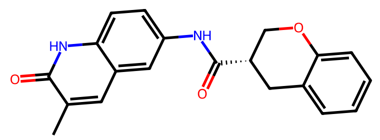	Cc1cc2cc(NC(=O)[C@@H]3COc4ccccc4C3)ccc2[nH]c1=O	1556731997	25	334.13	3.03
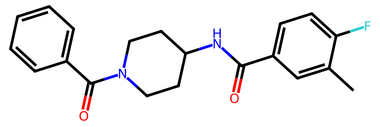	Cc1cc(C(=O)NC2CCN(C(=O)c3ccccc3)CC2)ccc1F	171523672	25	340.16	3.17
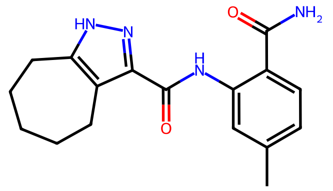	Cc1ccc(C(N)=O)c(NC(=O)c2n[nH]c3c2CCCCC3)c1	848346637	23	312.16	2.34
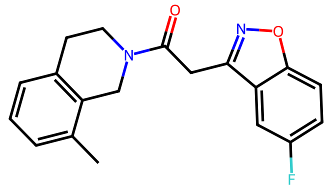	Cc1cccc2c1CN(C(=O)Cc1noc3ccc(F)cc13)CC2	415535776	24	324.13	3.4
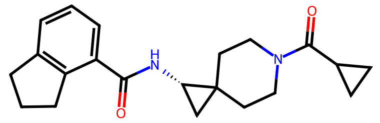	O=C(N[C@H]1CC12CCN(C(=O)C1CC1)CC2)c1cccc2c1CCC2	964032703	25	338.2	2.7
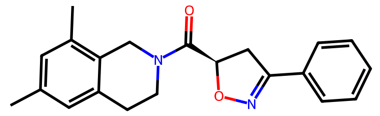	Cc1cc(C)c2c(c1)CCN(C(=O)[C@H]1CC(c3ccccc3)=NO1)C2	1463500386	25	334.17	3.38
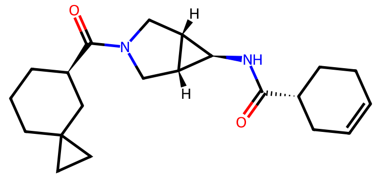	O=C(N[C@H]1[C@@H]2CN(C(=O)[C@@H]3CCCC4(CC4)C3)C[C@@H]21)[C@H]1CC=CCC1	976182621	25	342.23	2.89

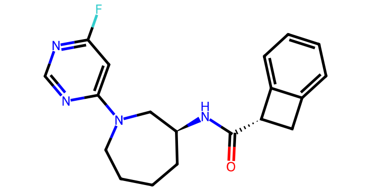	O=C(N[C@H]1CCCCN(c2cc(F)ncn2)C1)[C@H]1Cc2ccccc21	1063228264	25	340.17	2.43
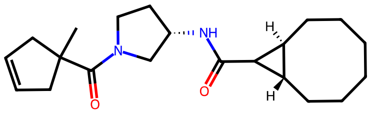	CC1(C(=O)N2CC[C@H](NC(=O)C3[C@H]4CCCCCC[C@H]34)C2)CC=CC1	938971351	25	344.25	3.28
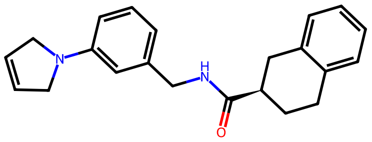	O=C(NCc1cccc(N2CC=CC2)c1)[C@@H]1CCc2ccccc2C1	584056206	25	332.19	3.48
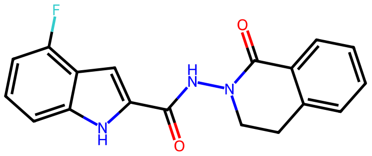	O=C(NN1CCc2ccccc2C1=O)c1cc2c(F)cccc2[nH]1	770552315	24	323.11	2.65
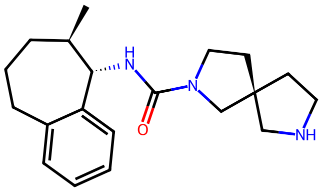	C[C@@H]1CCCc2ccccc2[C@H]1NC(=O)N1CC[C@]2(CCNC2)C1	418017790	24	327.23	3.1
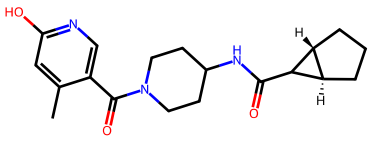	Cc1cc(O)ncc1C(=O)N1CCC(NC(=O)C2[C@@H]3CCC[C@@H]23)CC1	948305874	25	343.19	1.86
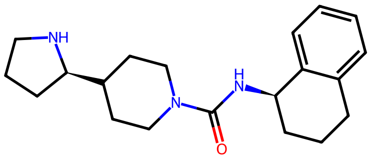	O=C(N[C@@H]1CCCc2ccccc21)N1CCC([C@H]2CCCN2)CC1	416803532	24	327.23	3.24
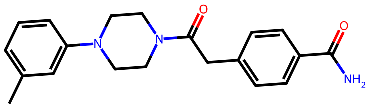	Cc1cccc(N2CCN(C(=O)Cc3ccc(C(N)=O)cc3)CC2)c1	362679068	25	337.18	1.99

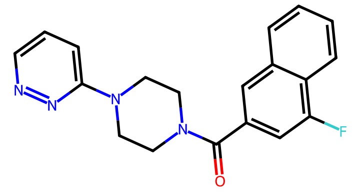	O=C(c1cc(F)c2ccccc2c1)N1CCN(c2cccnn2)CC1	339684610	25	336.14	2.73
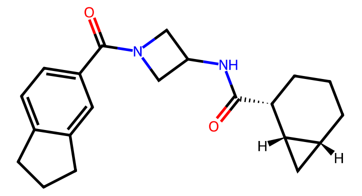	O=C(NC1CN(C(=O)c2ccc3c(c2)CCC3)C1)[C@@H]1CCC[C@@H]2C[C@@H]21	994719251	25	338.2	2.55
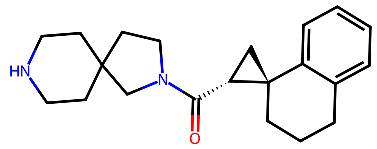	O=C([C@@H]1C[C@@]12CCCc1ccccc12)N1CCC2(CCNCC2)C1	378404693	24	324.22	2.88
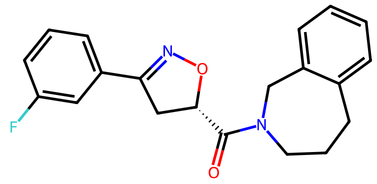	O=C([C@@H]1CC(c2cccc(F)c2)=NO1)N1CCCc2ccccc2C1	730202638	25	338.14	3.29
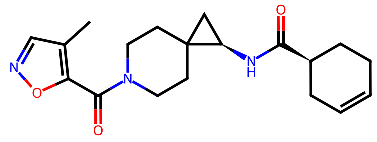	Cc1cnoc1C(=O)N1CCC2(CC1)C[C@H]2NC(=O)[C@@H]1CC=CCC1	963029841	25	343.19	2.45
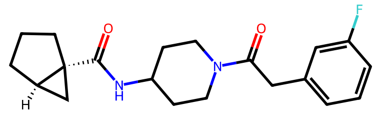	O=C(Cc1cccc(F)c1)N1CCC(NC(=O)[C@@]23CCC[C@@H]2C3)CC1	948482422	25	344.19	2.67
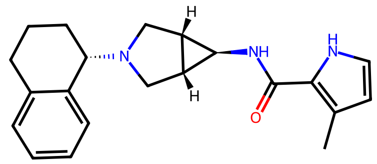	Cc1cc[nH]c1C(=O)N[C@H]1[C@@H]2CN([C@H]3CCCc4ccccc43)C[C@@H]21	958741896	25	335.2	3.06
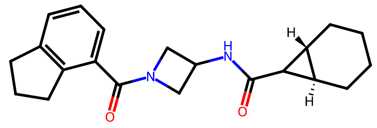	O=C(NC1CN(C(=O)c2cccc3c2CCC3)C1)C1[C@@H]2CCCC[C@@H]12	990656534	25	338.2	2.55

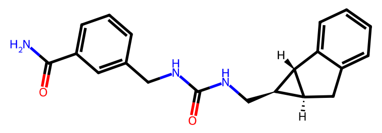	NC(=O)c1cccc(CNC(=O)NC[C@@H]2[C@@H]3Cc4ccccc4[C@@H]23)c1	137052489	25	335.16	2.17
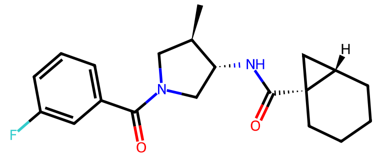	C[C@@H]1CN(C(=O)c2cccc(F)c2)C[C@H]1NC(=O)[C@@]12CCCC[C@H]1C2	939980508	25	344.19	2.98
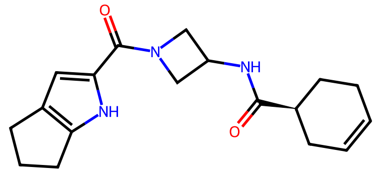	O=C(NC1CN(C(=O)c2cc3c([nH]2)CCC3)C1)[C@@H]1CC=CCC1	990213124	23	313.18	1.8
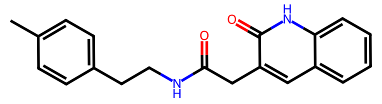	Cc1ccc(CCNC(=O)Cc2cc3ccccc3[nH]c2=O)cc1	116207528	24	320.15	2.74
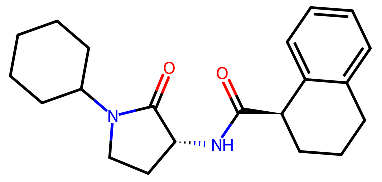	O=C(N[C@@H]1CCN(C2CCCCC2)C1=O)[C@@H]1CCCc2ccccc21	675913096	25	340.22	3.16
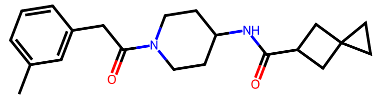	Cc1cccc(CC(=O)N2CCC(NC(=O)C3CC4(CC4)C3)CC2)c1	948062312	25	340.22	2.83
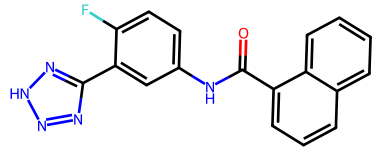	O=C(Nc1ccc(F)c(-c2nn[nH]n2)c1)c1cccc2ccccc12	738168309	25	333.1	3.41
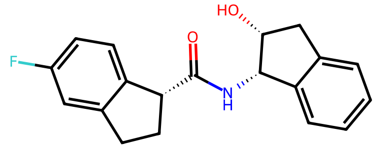	O=C(N[C@H]1c2ccccc2C[C@H]1O)[C@@H]1CCc2cc(F)ccc21	612660432	23	311.13	2.63

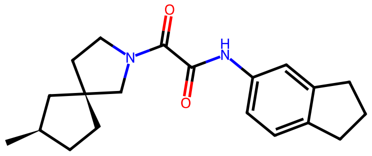	C[C@@H]1CC[C@]2(CCN(C(=O)C(=O)Nc3ccc4c(c3)CCC4)C2)C1	359461241	24	326.2	3.15
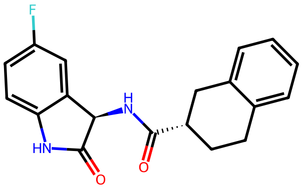	O=C(N[C@H]1C(=O)Nc2ccc(F)cc21)[C@H]1CCc2ccccc2C1	19744052	24	324.13	2.74
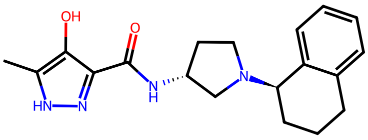	Cc1[nH]nc(C(=O)N[C@@H]2CCN([C@@H]3CCCc4ccccc43)C2)c1O	1010645053	25	340.19	2.31
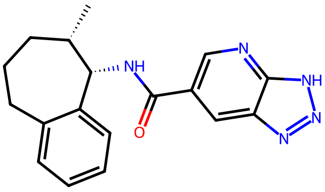	C[C@H]1CCCc2ccccc2[C@H]1NC(=O)c1cnc2[nH]nnc2c1	546616848	24	321.16	2.8
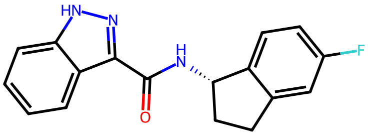	O=C(N[C@H]1CCc2cc(F)ccc21)c1n[nH]c2ccccc12	55655600	22	295.11	3.12
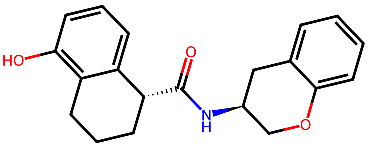	O=C(N[C@@H]1COc2ccccc2C1)[C@@H]1CCCc2c(O)cccc21	190124899	24	323.15	2.93
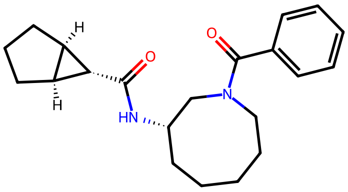	O=C(N[C@H]1CCCCCN(C(=O)c2ccccc2)C1)[C@@H]1[C@H]2CCC[C@H]21	986852253	25	340.22	3.23
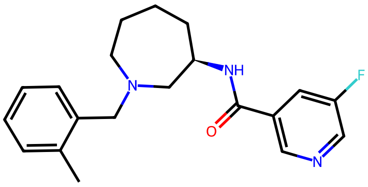	Cc1ccccc1CN1CCCC[C@@H](NC(=O)c2cncc(F)c2)C1	1034155928	25	341.19	3.31

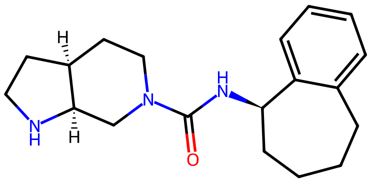	O=C(N[C@@H]1CCCCc2ccccc21)N1CC[C@@H]2CCN[C@@H]2C1	669439937	23	313.22	2.85
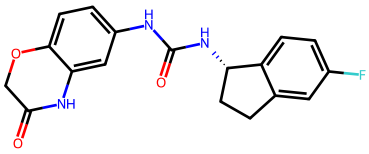	O=C1COc2ccc(NC(=O)N[C@H]3CCc4cc(F)ccc43)cc2N1	56575965	25	341.12	2.97
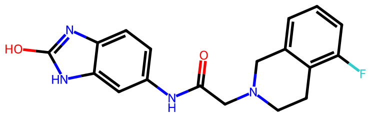	O=C(CN1CCc2c(F)cccc2C1)Nc1ccc2nc(O)[nH]c2c1	757151137	25	340.13	2.4
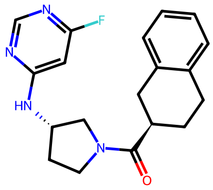	O=C([C@@H]1CCc2ccccc2C1)N1CC[C@H](Nc2cc(F)ncn2)C1	1059077468	25	340.17	2.43
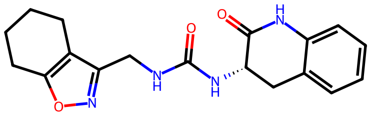	O=C(NCc1noc2c1CCCC2)N[C@H]1Cc2ccccc2NC1=O	637150290	25	340.15	1.92
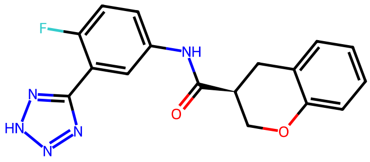	O=C(Nc1ccc(F)c(-c2nn[nH]n2)c1)[C@@H]1COc2ccccc2C1	738160807	25	339.11	2.2
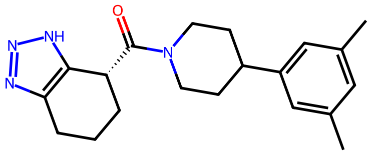	Cc1cc(C)cc(C2CCN(C(=O)[C@@H]3CCCc4nn[nH]c43)CC2)c1	891291205	25	338.21	3.25
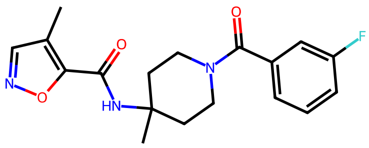	Cc1cnoc1C(=O)NC1(C)CCN(C(=O)c2cccc(F)c2)CC1	956478887	25	345.15	2.55

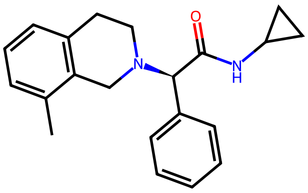	Cc1cccc2c1CN([C@@H](C(=O)NC1CC1)c1ccccc1)CC2	429574903	24	320.19	3.37
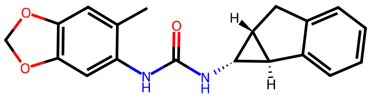	Cc1cc2c(cc1NC(=O)N[C@H]1[C@@H]3c4ccccc4C[C@H]31)OCO2	248390148	24	322.13	3.18
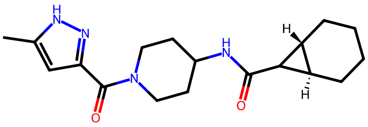	Cc1cc(C(=O)N2CCC(NC(=O)C3[C@@H]4CCCC[C@@H]34)CC2)n[nH]1	944822331	24	330.21	1.88
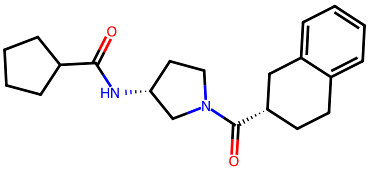	O=C(N[C@@H]1CCN(C(=O)[C@H]2CCc3ccccc3C2)C1)C1CCCC1	936274681	25	340.22	2.7
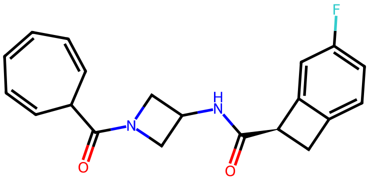	O=C(NC1CN(C(=O)C2C=CC=CC=C2)C1)[C@@H]1Cc2ccc(F)cc21	998494321	25	338.14	2.09
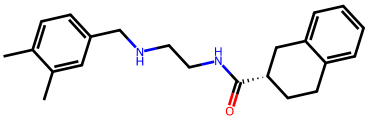	Cc1ccc(CNCCNC(=O)[C@H]2CCc3ccccc3C2)cc1C	1134378596	25	336.22	3.31
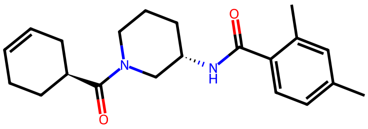	Cc1ccc(C(=O)N[C@H]2CCCN(C(=O)[C@@H]3CC=CCC3)C2)c(C)c1	949983883	25	340.22	3.38
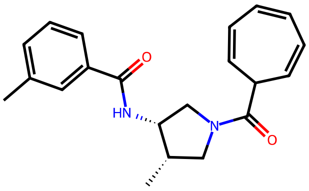	Cc1cccc(C(=O)N[C@@H]2CN(C(=O)C3C=CC=CC=C3)C[C@@H]2C)c1	966477400	25	336.18	2.87

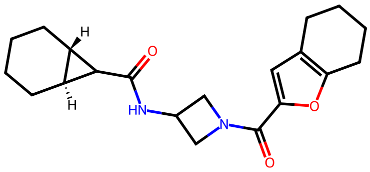	O=C(NC1CN(C(=O)c2cc3c(o2)CCCC3)C1)C1[C@H]2CCCC[C@H]12	990672025	25	342.19	2.54
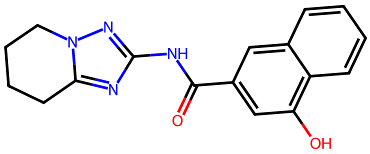	O=C(Nc1nc2n(n1)CCCC2)c1cc(O)c2ccccc2c1	1637956039	23	308.13	2.73
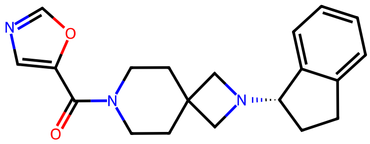	O=C(c1cnco1)N1CCC2(CC1)CN([C@H]1CCc3ccccc31)C2	1035763561	25	337.18	2.9
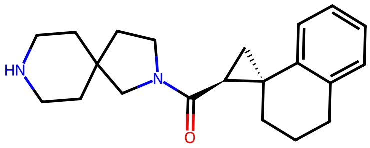	O=C([C@H]1C[C@]12CCCc1ccccc12)N1CCC2(CCNCC2)C1	378404694	24	324.22	2.88
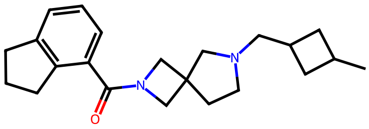	CC1CC(CN2CCC3(C2)CN(C(=O)c2cccc4c2CCC4)C3)C1	1073478813	25	338.24	3.37
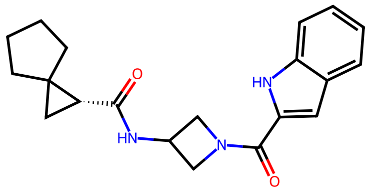	O=C(NC1CN(C(=O)c2cc3ccccc3[nH]2)C1)[C@@H]1CC12CCCC2	999628790	25	337.18	2.69
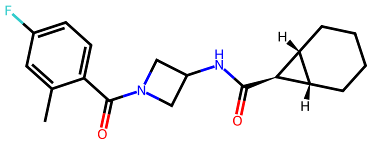	Cc1cc(F)ccc1C(=O)N1CC(NC(=O)[C@@H]2[C@H]3CCCC[C@H]32)C1	990644879	24	330.17	2.51
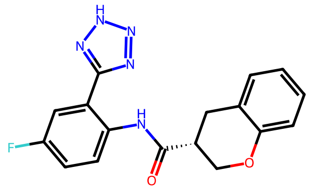	O=C(Nc1ccc(F)cc1-c1nn[nH]n1)[C@H]1COc2ccccc2C1	823343642	25	339.11	2.2

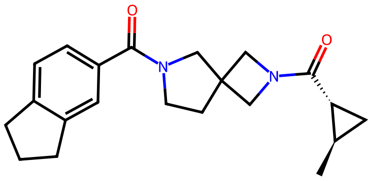	C[C@@H]1C[C@H]1C(=O)N1CC2(CCN(C(=O)c3ccc4c(c3)CCC4)C2)C1	1018737334	25	338.2	2.51
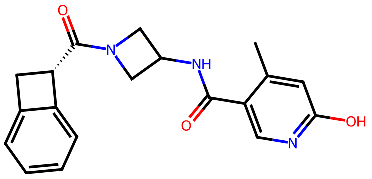	Cc1cc(O)ncc1C(=O)NC1CN(C(=O)[C@H]2Cc3ccccc32)C1	999343729	25	337.14	1.38
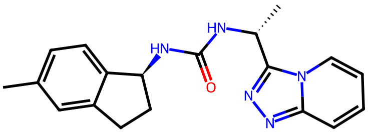	Cc1ccc2c(c1)CC[C@@H]2NC(=O)N[C@H](C)c1nnc2ccccn12	56842540	25	335.17	3.09
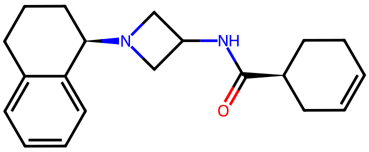	O=C(NC1CN([C@@H]2CCCc3ccccc32)C1)[C@@H]1CC=CCC1	1030098629	23	310.2	3.22
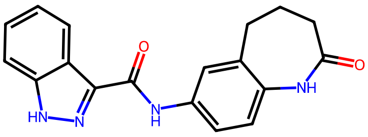	O=C1CCCc2cc(NC(=O)c3n[nH]c4ccccc34)ccc2N1	363623205	24	320.13	3.09
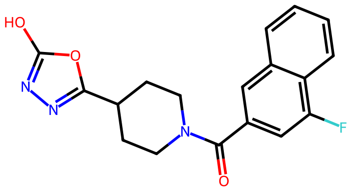	O=C(c1cc(F)c2ccccc2c1)N1CCC(c2nnc(O)o2)CC1	425321459	25	341.12	3.09
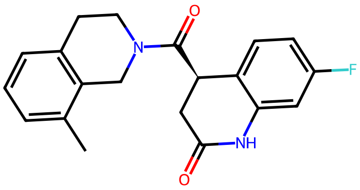	Cc1cccc2c1CN(C(=O)[C@@H]1CC(=O)Nc3cc(F)ccc31)CC2	277064288	25	338.14	3.14
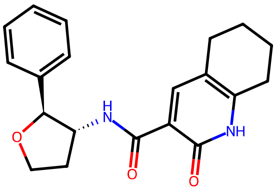	O=C(N[C@@H]1CCO[C@H]1c1ccccc1)c1cc2c([nH]c1=O)CCCC2	80981991	25	338.16	2.51

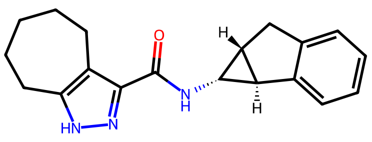	O=C(N[C@H]1[C@@H]2c3ccccc3C[C@H]21)c1n[nH]c2c1CCCCC2	190676883	23	307.17	2.75
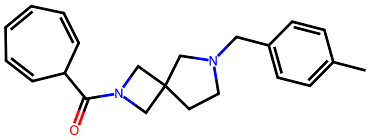	Cc1ccc(CN2CCC3(C2)CN(C(=O)C2C=CC=CC=C2)C3)cc1	1072786137	25	334.2	3.33
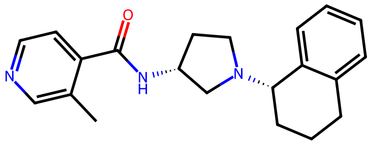	Cc1cnccc1C(=O)N[C@@H]1CCN([C@H]2CCCc3ccccc32)C1	1014357464	25	335.2	3.27
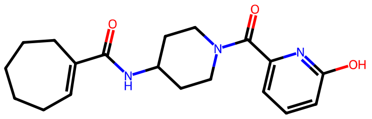	O=C(NC1CCN(C(=O)c2cccc(O)n2)CC1)C1=CCCCCC1	945981742	25	343.19	2.4
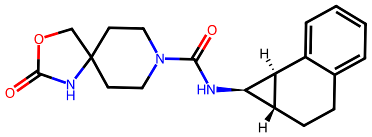	O=C1NC2(CCN(C(=O)N[C@@H]3[C@H]4CCc5ccccc5[C@@H]43)CC2)CO1	495156346	25	341.17	2.0
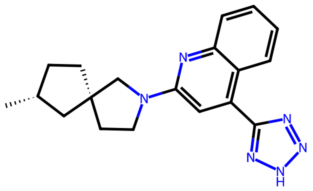	C[C@@H]1CC[C@]2(CCN(c3cc(-c4nn[nH]n4)c4ccccc4n3)C2)C1	824917906	25	334.19	3.43
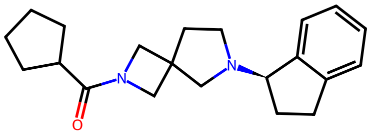	O=C(C1CCCC1)N1CC2(CCN([C@@H]3CCc4ccccc43)C2)C1	1072386705	24	324.22	3.4
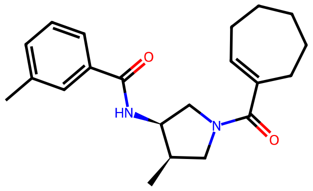	Cc1cccc(C(=O)N[C@H]2CN(C(=O)C3=CCCCCC3)C[C@H]2C)c1	966347764	25	340.22	3.46

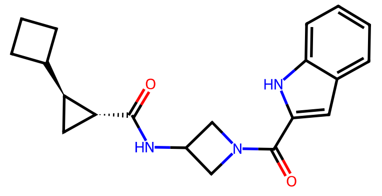	O=C(NC1CN(C(=O)c2cc3ccccc3[nH]2)C1)[C@@H]1C[C@H]1C1CCC1	992014494	25	337.18	2.54
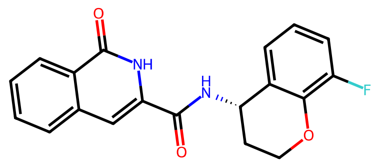	O=C(N[C@H]1CCOc2c(F)cccc21)c1cc2ccccc2c(=O)[nH]1	189594586	25	338.11	2.92
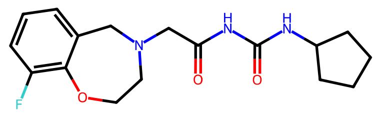	O=C(CN1CCOc2c(F)cccc2C1)NC(=O)NC1CCCC1	568950106	24	335.16	1.79
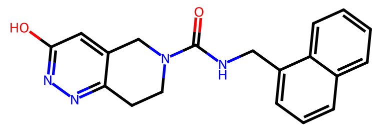	O=C(NCc1cccc2ccccc12)N1CCc2nnc(O)cc2C1	361370845	25	334.14	2.6
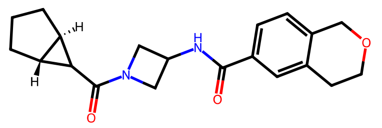	O=C(NC1CN(C(=O)C2[C@@H]3CCC[C@@H]23)C1)c1ccc2c(c1)CCOC2	996918924	25	340.18	1.75
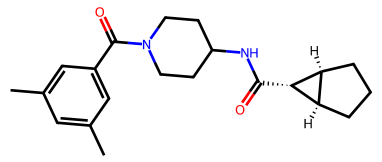	Cc1cc(C)cc(C(=O)N2CCC(NC(=O)[C@@H]3[C@H]4CCC[C@H]43)CC2)c1	948338794	25	340.22	3.07
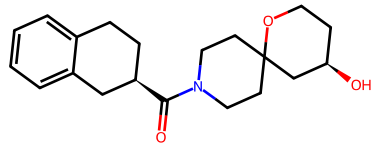	O=C([C@@H]1CCc2ccccc2C1)N1CCC2(CC1)C[C@H](O)CCO2	886103800	24	329.2	2.32
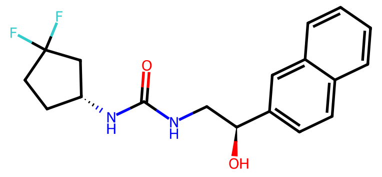	O=C(NC[C@H](O)c1ccc2ccccc2c1)N[C@@H]1CCC(F)(F)C1	763708730	24	334.15	3.36

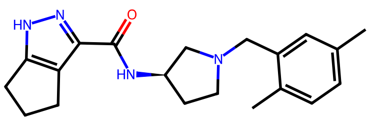	Cc1ccc(C)c(CN2CC[C@@H](NC(=O)c3n[nH]c4c3CCC4)C2)c1	1015069634	25	338.21	2.52
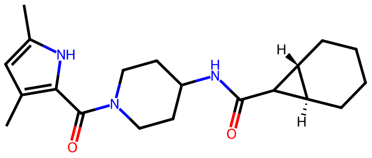	Cc1cc(C)c(C(=O)N2CCC(NC(=O)C3[C@@H]4CCCC[C@@H]34)CC2)[nH]1	947337099	25	343.23	2.79
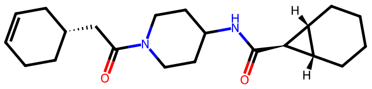	O=C(NC1CCN(C(=O)C[C@H]2CC=CCC2)CC1)[C@@H]1[C@H]2CCCC[C@@H]21	947728886	25	344.25	3.28
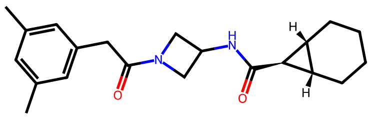	Cc1cc(C)cc(CC(=O)N2CC(NC(=O)[C@@H]3[C@H]4CCCC[C@H]43)C2)c1	990650124	25	340.22	2.61
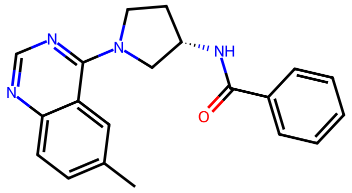	Cc1ccc2ncnc(N3CC[C@H](NC(=O)c4ccccc4)C3)c2c1	1058285700	25	332.16	2.95
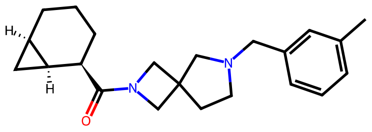	Cc1cccc(CN2CCC3(C2)CN(C(=O)[C@@H]2CCC[C@@H]4C[C@@H]42)C3)c1	1072875664	25	338.24	3.47
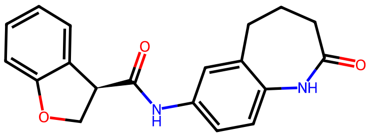	O=C1CCCc2cc(NC(=O)[C@H]3COc4ccccc43)ccc2N1	113049063	24	322.13	3.08
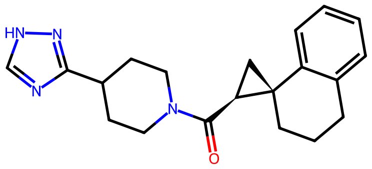	O=C([C@H]1C[C@@]12CCCc1ccccc12)N1CCC(c2nc[nH]n2)CC1	365913173	25	336.2	2.8

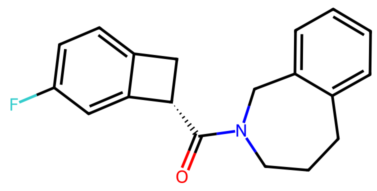	O=C([C@H]1Cc2ccc(F)cc21)N1CCCc2ccccc2C1	684435763	22	295.14	3.44
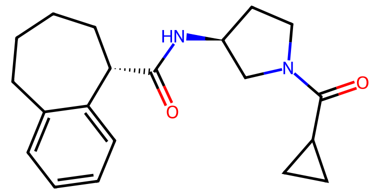	O=C(N[C@H]1CCN(C(=O)C2CC2)C1)[C@H]1CCCCc2ccccc21	938139032	24	326.2	2.62
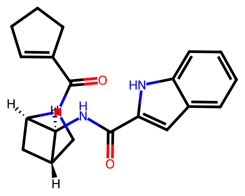	O=C(N[C@H]1[C@H]2C[C@H]1N(C(=O)C1=CCCC1)C2)c1cc2ccccc2[nH]1	948121772	25	335.16	2.61
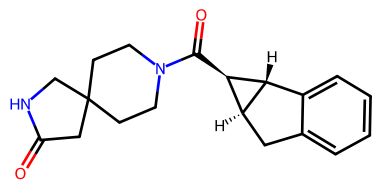	O=C1CC2(CCN(C(=O)[C@@H]3[C@@H]4Cc5ccccc5[C@H]43)CC2)CN1	859774218	23	310.17	1.7
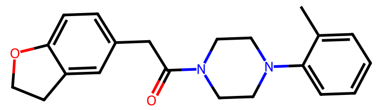	Cc1ccccc1N1CCN(C(=O)Cc2ccc3c(c2)CCO3)CC1	342921352	25	336.18	2.82
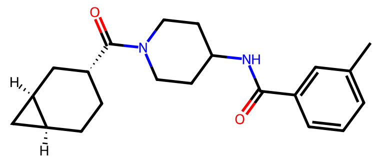	Cc1cccc(C(=O)NC2CCN(C(=O)[C@@H]3CC[C@H]4C[C@H]4C3)CC2)c1	870837571	25	340.22	3.15
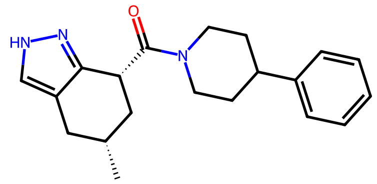	C[C@@H]1Cc2c[nH]nc2[C@H](C(=O)N2CCC(c3ccccc3)CC2)C1	623178167	24	323.2	3.48
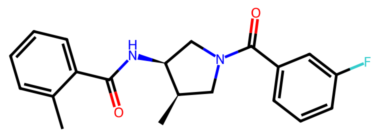	Cc1ccccc1C(=O)N[C@H]1CN(C(=O)c2cccc(F)c2)C[C@H]1C	966102942	25	340.16	3.02

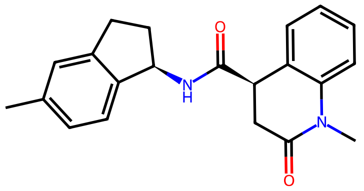	Cc1ccc2c(c1)CC[C@H]2NC(=O)[C@@H]1CC(=O)N(C)c2ccccc21	1115889195	25	334.17	3.25
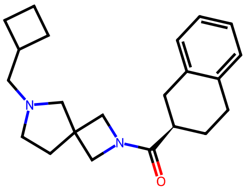	O=C([C@@H]1CCc2ccccc2C1)N1CC2(CCN(CC3CCC3)C2)C1	1073013573	25	338.24	3.13
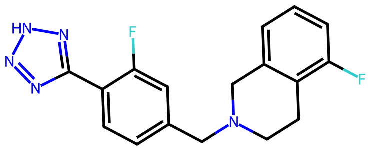	Fc1cc(CN2CCc3c(F)cccc3C2)ccc1-c1nn[nH]n1	1592642070	24	327.13	2.7
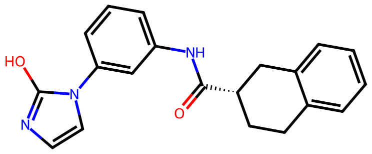	O=C(Nc1cccc(-n2ccnc2O)c1)[C@H]1CCc2ccccc2C1	1517430189	25	333.15	3.32
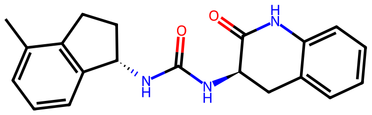	Cc1cccc2c1CC[C@@H]2NC(=O)N[C@@H]1Cc2ccccc2NC1=O	636735163	25	335.16	2.84
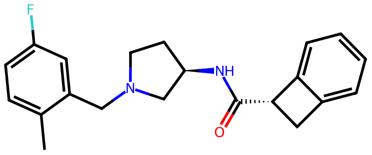	Cc1ccc(F)cc1CN1CC[C@@H](NC(=O)[C@H]2Cc3ccccc32)C1	1015636086	25	338.18	3.16
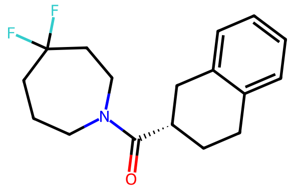	O=C([C@H]1CCc2ccccc2C1)N1CCCC(F)(F)CC1	628630709	21	293.16	3.44
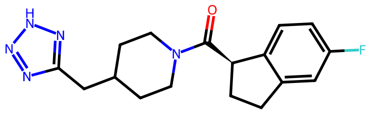	O=C([C@@H]1CCc2cc(F)ccc21)N1CCC(Cc2nn[nH]n2)CC1	1548805479	24	329.17	1.85

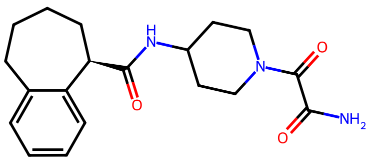	NC(=O)C(=O)N1CCC(NC(=O)[C@@H]2CCCCc3ccccc32)CC1	948083721	25	343.19	1.09
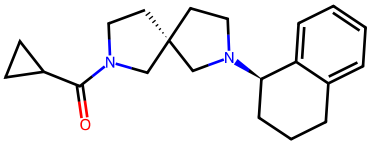	O=C(C1CC1)N1CC[C@@]2(CCN([C@@H]3CCCc4ccccc43)C2)C1	1040991373	24	324.22	3.4
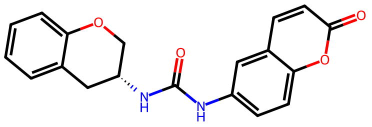	O=C(Nc1ccc2oc(=O)ccc2c1)N[C@H]1COc2ccccc2C1	194784282	25	336.11	2.92
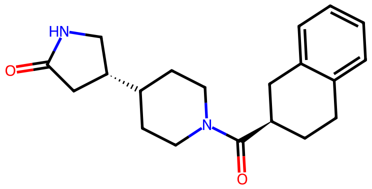	O=C1C[C@@H](C2CCN(C(=O)[C@@H]3CCc4ccccc4C3)CC2)CN1	1551708576	24	326.2	2.17
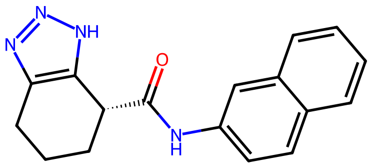	O=C(Nc1ccc2ccccc2c1)[C@@H]1CCCc2nn[nH]c21	816810833	22	292.13	3.02
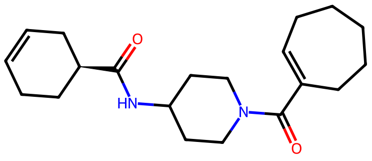	O=C(NC1CCN(C(=O)C2=CCCCCC2)CC1)[C@@H]1CC=CCC1	946549009	24	330.23	3.34
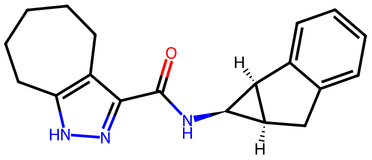	O=C(N[C@@H]1[C@@H]2Cc3ccccc3[C@@H]21)c1n[nH]c2c1CCCCC2	190676922	23	307.17	2.75
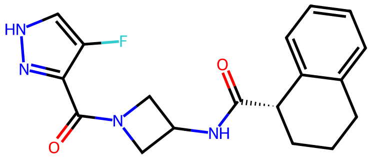	O=C(NC1CN(C(=O)c2n[nH]cc2F)C1)[C@H]1CCCc2ccccc21	995072205	25	342.15	1.61

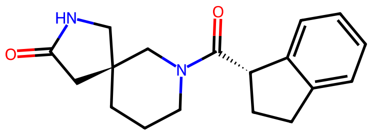	O=C1C[C@@]2(CCCN(C(=O)[C@H]3CCc4ccccc43)C2)CN1	336402782	22	298.17	1.85
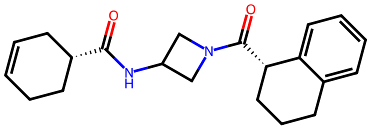	O=C(NC1CN(C(=O)[C@H]2CCCc3ccccc32)C1)[C@H]1CC=CCC1	990202680	25	338.2	2.79
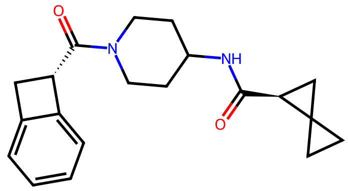	O=C(NC1CCN(C(=O)[C@H]2Cc3ccccc32)CC1)[C@H]1CC12CC2	948059405	24	324.18	2.23
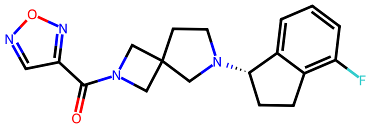	O=C(c1cnon1)N1CC2(CCN([C@H]3CCc4c(F)cccc43)C2)C1	1073068196	25	342.15	2.04
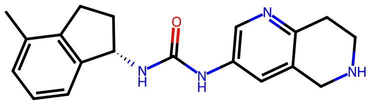	Cc1cccc2c1CC[C@@H]2NC(=O)Nc1cnc2c(c1)CNCC2	667795905	24	322.18	2.84
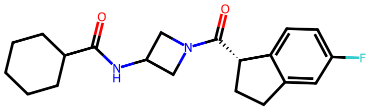	O=C(NC1CN(C(=O)[C@H]2CCc3cc(F)ccc32)C1)C1CCCCC1	990101962	25	344.19	2.76
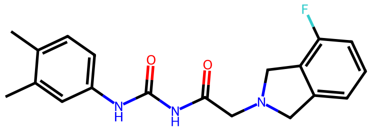	Cc1ccc(NC(=O)NC(=O)CN2Cc3cccc(F)c3C2)cc1C	459761875	25	341.15	3.11
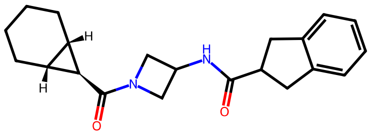	O=C(NC1CN(C(=O)[C@@H]2[C@H]3CCCC[C@H]32)C1)C1Cc2ccccc2C1	1000449244	25	338.2	2.16

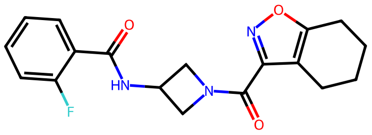	O=C(NC1CN(C(=O)c2noc3c2CCCC3)C1)c1ccccc1F	990093157	25	343.13	1.95
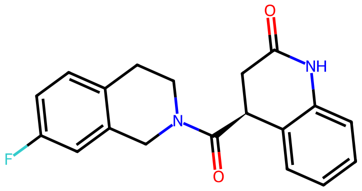	O=C1C[C@H](C(=O)N2CCc3ccc(F)cc3C2)c2ccccc2N1	151527349	24	324.13	2.84
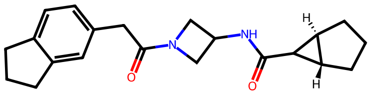	O=C(NC1CN(C(=O)Cc2ccc3c(c2)CCC3)C1)C1[C@H]2CCC[C@H]12	990710929	25	338.2	2.09
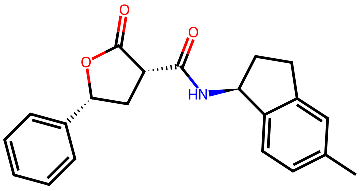	Cc1ccc2c(c1)CC[C@@H]2NC(=O)[C@@H]1C[C@H](c2ccccc2)OC1=O	290211526	25	335.15	3.4
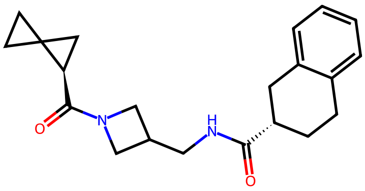	O=C(NCC1CN(C(=O)[C@@H]2CC23CC3)C1)[C@H]1CCc2ccccc2C1	936100055	25	338.2	2.17
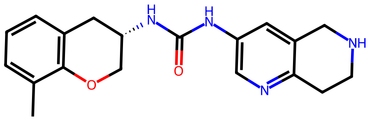	Cc1cccc2c1OC[C@@H](NC(=O)Nc1cnc3c(c1)CNCC3)C2	667802544	25	338.17	2.16
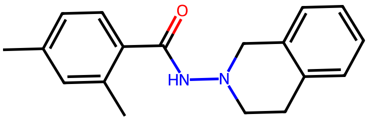	Cc1ccc(C(=O)NN2CCc3ccccc3C2)c(C)c1	855707689	21	280.16	3.01
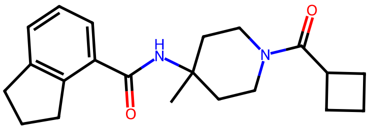	CC1(NC(=O)c2cccc3c2CCC3)CCN(C(=O)C2CCC2)CC1	956403255	25	340.22	3.09

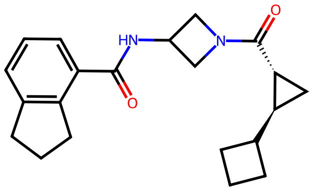	O=C(NC1CN(C(=O)[C@@H]2C[C@H]2C2CCC2)C1)c1cccc2c1CCC2	994400727	25	338.2	2.55
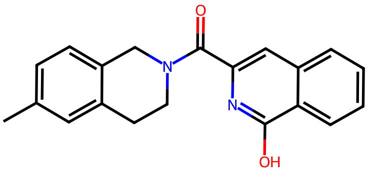	Cc1ccc2c(c1)CCN(C(=O)c1cc3ccccc3c(O)n1)C2	360196054	24	318.14	3.45
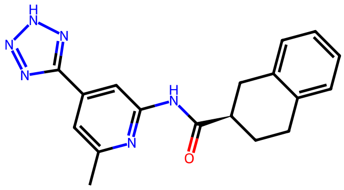	Cc1cc(-c2nn[nH]n2)cc(NC(=O)[C@@H]2CCc3ccccc3C2)n1	1605529012	25	334.15	2.31
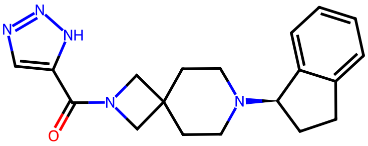	O=C(c1cnn[nH]1)N1CC2(CCN([C@@H]3CCc4ccccc43)CC2)C1	1050578019	25	337.19	2.03
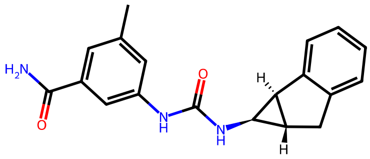	Cc1cc(NC(=O)N[C@@H]2[C@H]3Cc4ccccc4[C@@H]32)cc(C(N)=O)c1	576560115	24	321.15	2.55
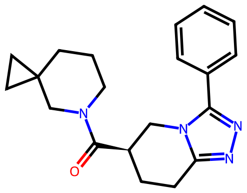	O=C([C@@H]1CCc2nnc(-c3ccccc3)n2C1)N1CCCC2(CC2)C1	649420499	25	336.2	2.91
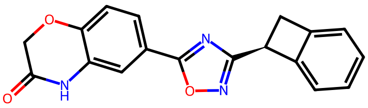	O=C1COc2ccc(-c3nc([C@H]4Cc5ccccc54)no3)cc2N1	546294160	24	319.1	2.76
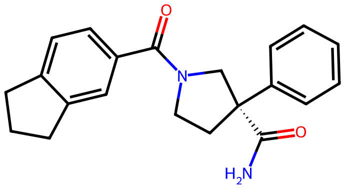	NC(=O)[C@]1(c2ccccc2)CCN(C(=O)c2ccc3c(c2)CCC3)C1	412601842	25	334.17	2.44

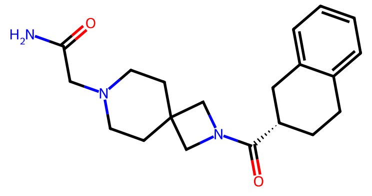	NC(=O)CN1CCC2(CC1)CN(C(=O)[C@H]1CCc3ccccc3C1)C2	1050682351	25	341.21	1.2
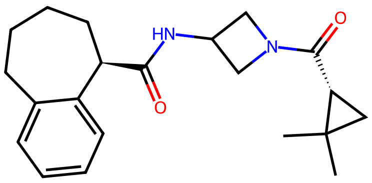	CC1(C)C[C@H]1C(=O)N1CC(NC(=O)[C@@H]2CCCCc3ccccc32)C1	994311772	25	340.22	2.87
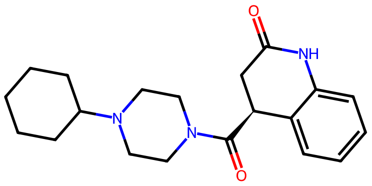	O=C1C[C@H](C(=O)N2CCN(C3CCCCC3)CC2)c2ccccc2N1	154449444	25	341.21	2.59
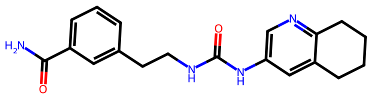	NC(=O)c1cccc(CCNC(=O)Nc2cnc3c(c2)CCCC3)c1	543886564	25	338.17	2.42
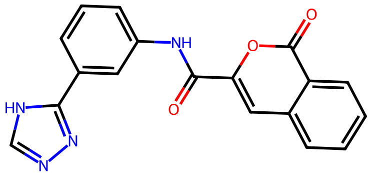	O=C(Nc1cccc(-c2nnc[nH]2)c1)c1cc2ccccc2c(=O)o1	253425416	25	332.09	2.83
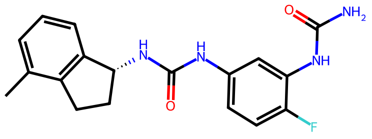	Cc1cccc2c1CC[C@H]2NC(=O)Nc1ccc(F)c(NC(N)=O)c1	1671610822	25	342.15	3.43
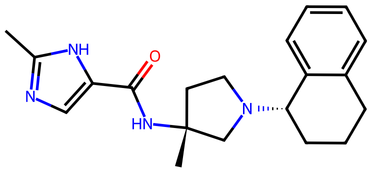	Cc1ncc(C(=O)N[C@]2(C)CCN([C@H]3CCCc4ccccc43)C2)[nH]1	1046199607	25	338.21	2.99
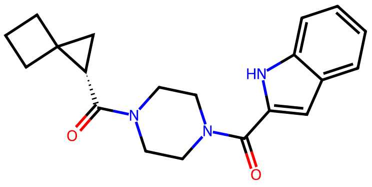	O=C(c1cc2ccccc2[nH]1)N1CCN(C(=O)[C@H]2CC23CCC3)CC1	675318809	25	337.18	2.64

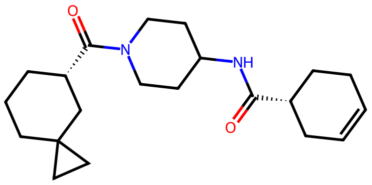	O=C(NC1CCN(C(=O)[C@H]2CCCC3(CC3)C2)CC1)[C@H]1CC=CCC1	946752925	25	344.25	3.42
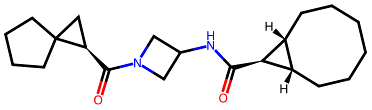	O=C(NC1CN(C(=O)[C@@H]2CC23CCCC3)C1)[C@@H]1[C@H]2CCCCCC[C@H]21	1000500594	25	344.25	3.11
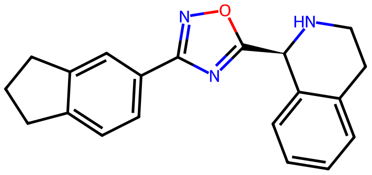	c1ccc2c(c1)CCN[C@@H]2c1nc(-c2ccc3c(c2)CCC3)no1	326754342	24	317.15	3.46
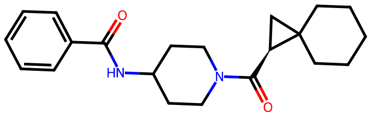	O=C(NC1CCN(C(=O)[C@H]2CC23CCCCC3)CC1)c1ccccc1	370570936	25	340.22	3.38
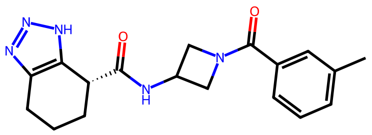	Cc1cccc(C(=O)N2CC(NC(=O)[C@@H]3CCCc4nn[nH]c43)C2)c1	998569507	25	339.17	1.17
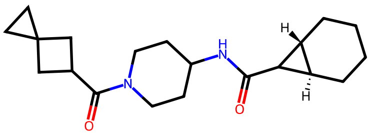	O=C(NC1CCN(C(=O)C2CC3(CC3)C2)CC1)C1[C@@H]2CCCC[C@@H]12	948299947	24	330.23	2.72
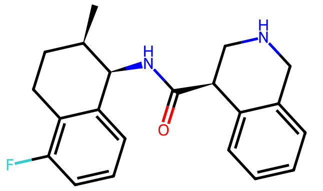	C[C@@H]1CCc2c(F)cccc2[C@@H]1NC(=O)[C@H]1CNCc2ccccc21	566190197	25	338.18	3.45
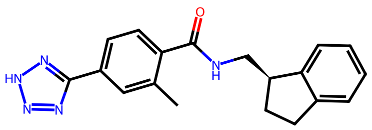	Cc1cc(-c2nn[nH]n2)ccc1C(=O)NC[C@@H]1CCc2ccccc21	826248286	25	333.16	2.63

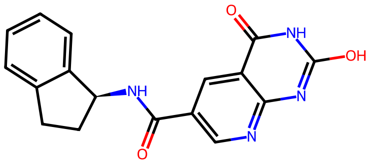	O=C(N[C@H]1CCc2ccccc21)c1cnc2nc(O)[nH]c(=O)c2c1	51184218	24	322.11	1.44
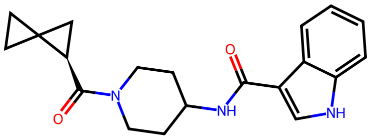	O=C(NC1CCN(C(=O)[C@@H]2CC23CC3)CC1)c1c[nH]c2ccccc12	948558529	25	337.18	2.69
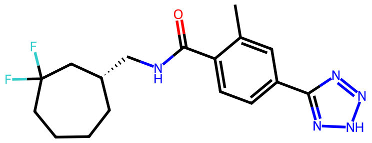	Cc1cc(-c2nn[nH]n2)ccc1C(=O)NC[C@@H]1CCCCC(F)(F)C1	1591862435	25	349.17	3.12
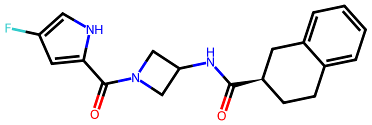	O=C(NC1CN(C(=O)c2cc(F)c[nH]2)C1)[C@@H]1CCc2ccccc2C1	1000543719	25	341.15	1.9
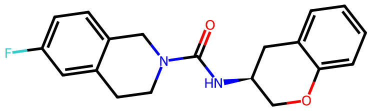	O=C(N[C@@H]1COc2ccccc2C1)N1CCc2cc(F)ccc2C1	92187681	24	326.14	2.9
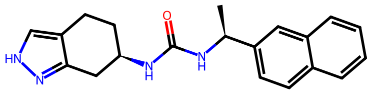	C[C@H](NC(=O)N[C@@H]1CCc2c[nH]nc2C1)c1ccc2ccccc2c1	363938596	25	334.18	3.48
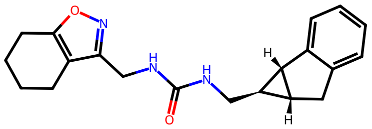	O=C(NCc1noc2c1CCCC2)NC[C@@H]1[C@H]2Cc3ccccc3[C@H]21	289331223	25	337.18	2.94
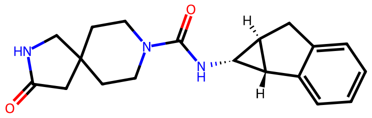	O=C1CC2(CCN(C(=O)N[C@@H]3[C@H]4Cc5ccccc5[C@@H]43)CC2)CN1	268681921	24	325.18	1.64

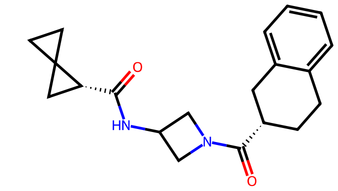	O=C(NC1CN(C(=O)[C@H]2CCc3ccccc3C2)C1)[C@@H]1CC12CC2	1000508414	24	324.18	1.92
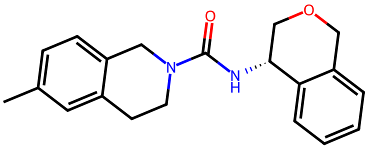	Cc1ccc2c(c1)CCN(C(=O)N[C@@H]1COCc3ccccc31)C2	419545929	24	322.17	3.33
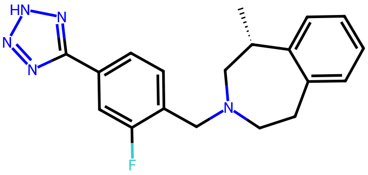	C[C@H]1CN(Cc2ccc(-c3nn[nH]n3)cc2F)CCc2ccccc21	1576361019	25	337.17	3.17
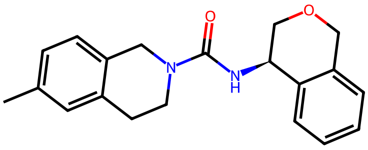	Cc1ccc2c(c1)CCN(C(=O)N[C@H]1COCc3ccccc31)C2	419545928	24	322.17	3.33
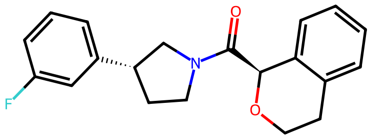	O=C([C@@H]1OCCc2ccccc21)N1CC[C@H](c2cccc(F)c2)C1	490666385	24	325.15	3.46
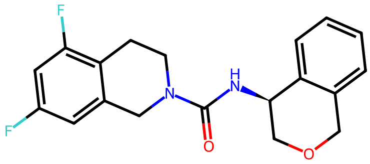	O=C(N[C@@H]1COCc2ccccc21)N1CCc2c(F)cc(F)cc2C1	419556377	25	344.13	3.3
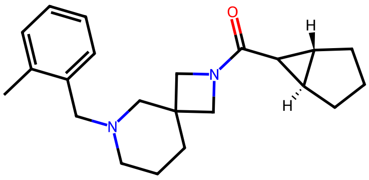	Cc1ccccc1CN1CCCC2(C1)CN(C(=O)C1[C@@H]3CCC[C@@H]13)C2	981696760	25	338.24	3.47
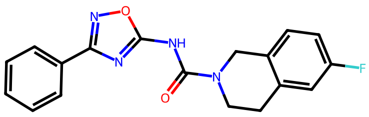	O=C(Nc1nc(-c2ccccc2)no1)N1CCc2cc(F)ccc2C1	1310515888	25	338.12	3.47

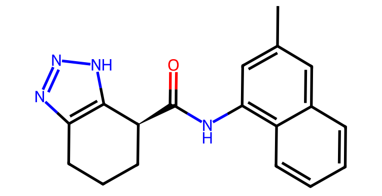	Cc1cc(NC(=O)[C@H]2CCCc3nn[nH]c32)c2ccccc2c1	615678695	23	306.15	3.32
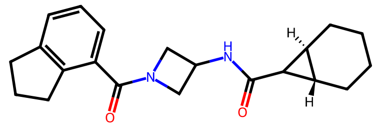	O=C(NC1CN(C(=O)c2cccc3c2CCC3)C1)C1[C@H]2CCCC[C@H]12	990656540	25	338.2	2.55
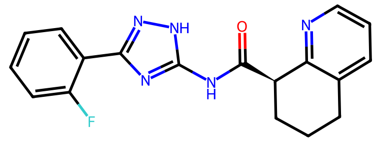	O=C(Nc1nc(-c2ccccc2F)n[nH]1)[C@@H]1CCCc2cccnc21	193217990	25	337.13	3.06
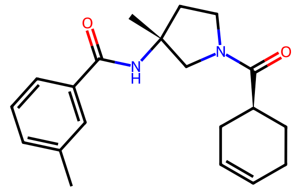	Cc1cccc(C(=O)N[C@]2(C)CCN(C(=O)[C@@H]3CC=CCC3)C2)c1	956663620	24	326.2	3.07
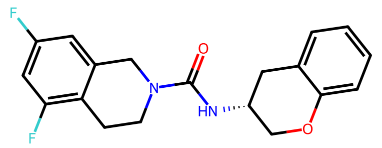	O=C(N[C@H]1COc2ccccc2C1)N1CCc2c(F)cc(F)cc2C1	485172557	25	344.13	3.04
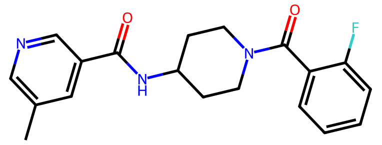	Cc1cncc(C(=O)NC2CCN(C(=O)c3ccccc3F)CC2)c1	79551303	25	341.15	2.56
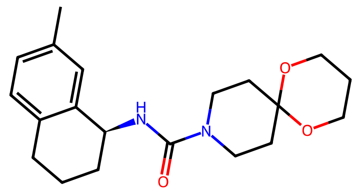	Cc1ccc2c(c1)[C@@H](NC(=O)N1CCC3(CC1)OCCCO3)CCC2	298032528	25	344.21	3.31
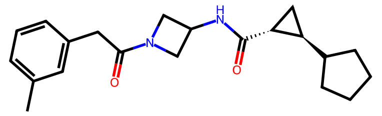	Cc1cccc(CC(=O)N2CC(NC(=O)[C@@H]3C[C@H]3C3CCCC3)C2)c1	998828635	25	340.22	2.69

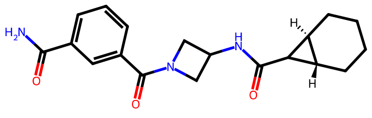	NC(=O)c1cccc(C(=O)N2CC(NC(=O)C3[C@H]4CCCC[C@H]34)C2)c1	990662970	25	341.17	1.16
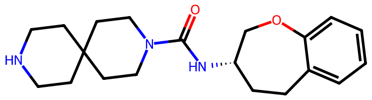	O=C(N[C@H]1CCc2ccccc2OC1)N1CCC2(CCNCC2)CC1	418164491	25	343.23	2.56
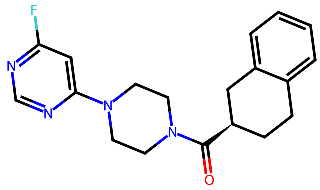	O=C([C@@H]1CCc2ccccc2C1)N1CCN(c2cc(F)ncn2)CC1	908885509	25	340.17	2.07
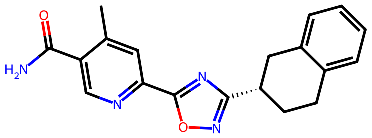	Cc1cc(-c2nc([C@H]3CCc4ccccc4C3)no2)ncc1C(N)=O	651246071	25	334.14	2.81
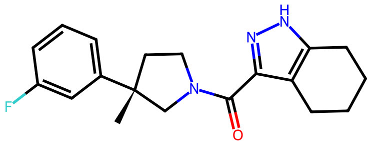	C[C@@]1(c2cccc(F)c2)CCN(C(=O)c2n[nH]c3c2CCCC3)C1	652618477	24	327.17	3.23
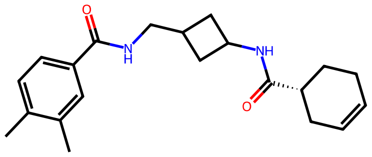	Cc1ccc(C(=O)NCC2CC(NC(=O)[C@H]3CC=CCC3)C2)cc1C	992390609	25	340.22	3.28
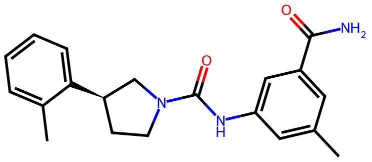	Cc1cc(NC(=O)N2CC[C@@H](c3ccccc3C)C2)cc(C(N)=O)c1	455809116	25	337.18	3.42
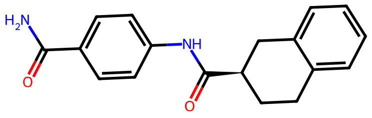	NC(=O)c1ccc(NC(=O)[C@@H]2CCc3ccccc3C2)cc1	1361319653	22	294.14	2.53

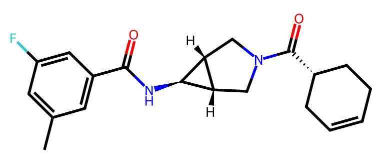	Cc1cc(F)cc(C(=O)N[C@H]2[C@@H]3CN(C(=O)[C@H]4CC=CCC4)C[C@@H]32)c1	977339009	25	342.17	2.68
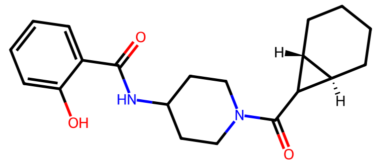	O=C(NC1CCN(C(=O)C2[C@@H]3CCCC[C@@H]23)CC1)c1ccccc1O	945957381	25	342.19	2.55
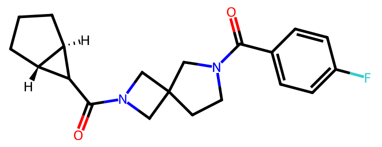	O=C(c1ccc(F)cc1)N1CCC2(C1)CN(C(=O)C1[C@@H]3CCC[C@@H]13)C2	1018911107	25	342.17	2.55
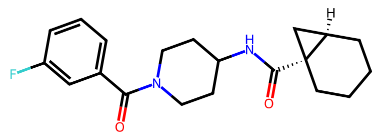	O=C(c1cccc(F)c1)N1CCC(NC(=O)[C@@]23CCCC[C@@H]2C3)CC1	943776520	25	344.19	3.13
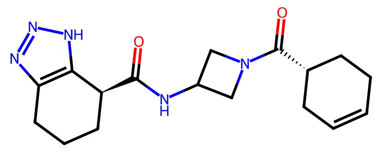	O=C(NC1CN(C(=O)[C@H]2CC=CCC2)C1)[C@H]1CCCc2nn[nH]c21	998568049	24	329.19	0.91
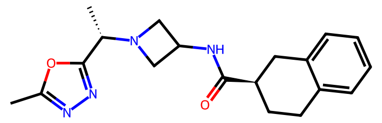	Cc1nnc([C@H](C)N2CC(NC(=O)[C@@H]3CCc4ccccc4C3)C2)o1	1031012292	25	340.19	2.04
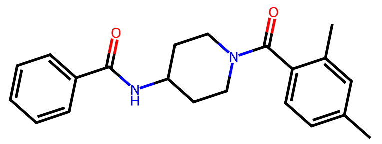	Cc1ccc(C(=O)N2CCC(NC(=O)c3ccccc3)CC2)c(C)c1	10780011	25	336.18	3.34
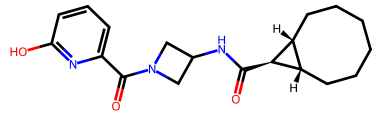	O=C(NC1CN(C(=O)c2cccc(O)n2)C1)[C@@H]1[C@H]2CCCCCC[C@H]21	1000482547	25	343.19	1.94

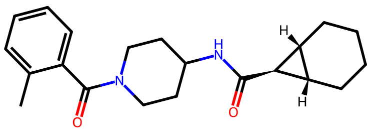	Cc1ccccc1C(=O)N1CCC(NC(=O)[C@@H]2[C@H]3CCCC[C@H]32)CC1	943772870	25	340.22	3.15
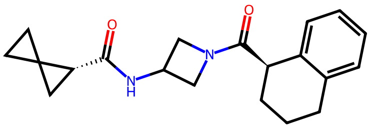	O=C(NC1CN(C(=O)[C@@H]2CCCc3ccccc32)C1)[C@@H]1CC12CC2	1000518194	24	324.18	2.23
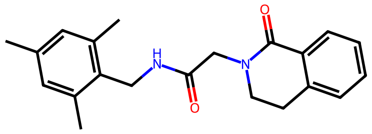	Cc1cc(C)c(CNC(=O)CN2CCc3ccccc3C2=O)c(C)c1	345022334	25	336.18	2.93
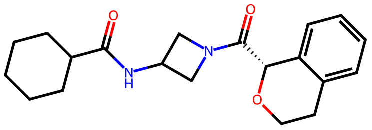	O=C(NC1CN(C(=O)[C@H]2OCCc3ccccc32)C1)C1CCCCC1	990099372	25	342.19	2.21
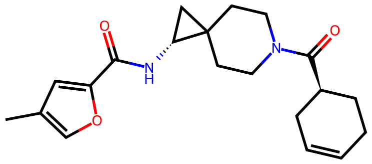	Cc1coc(C(=O)N[C@@H]2CC23CCN(C(=O)[C@@H]2CC=CCC2)CC3)c1	964714524	25	342.19	3.06
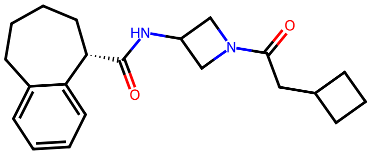	O=C(NC1CN(C(=O)CC2CCC2)C1)[C@H]1CCCCc2ccccc21	994306590	25	340.22	3.01
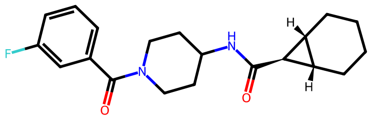	O=C(NC1CCN(C(=O)c2cccc(F)c2)CC1)[C@@H]1[C@H]2CCCC[C@H]21	943774552	25	344.19	2.98
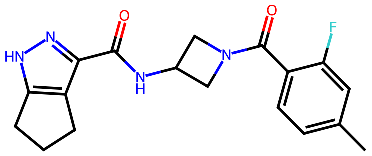	Cc1ccc(C(=O)N2CC(NC(=O)c3n[nH]c4c3CCC4)C2)c(F)c1	995165363	25	342.15	1.6

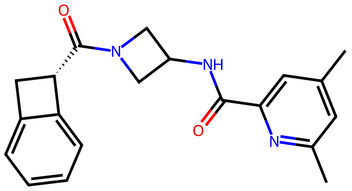	Cc1cc(C)nc(C(=O)NC2CN(C(=O)[C@H]3Cc4ccccc43)C2)c1	995014583	25	335.16	1.98
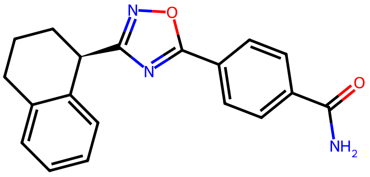	NC(=O)c1ccc(-c2nc([C@@H]3CCCc4ccccc43)no2)cc1	358517743	24	319.13	3.3
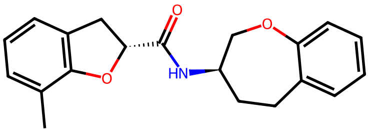	Cc1cccc2c1O[C@@H](C(=O)N[C@@H]1CCc3ccccc3OC1)C2	451487669	24	323.15	2.81
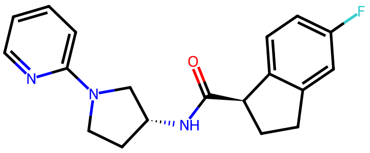	O=C(N[C@@H]1CCN(c2ccccn2)C1)[C@@H]1CCc2cc(F)ccc21	612904285	24	325.16	2.65
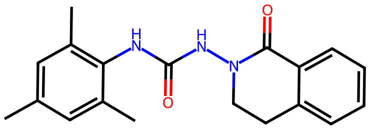	Cc1cc(C)c(NC(=O)NN2CCc3ccccc3C2=O)c(C)c1	1665732125	24	323.16	3.35
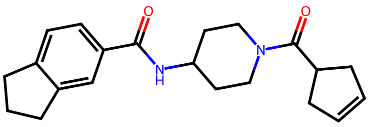	O=C(NC1CCN(C(=O)C2CC=CC2)CC1)c1ccc2c(c1)CCC2	945719006	25	338.2	2.86
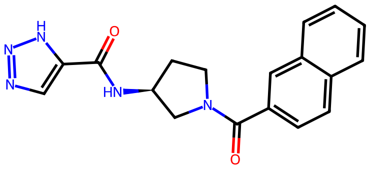	O=C(N[C@H]1CCN(C(=O)c2ccc3ccccc3c2)C1)c1cnn[nH]1	938240786	25	335.14	1.6
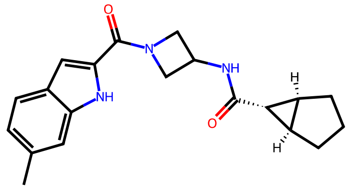	Cc1ccc2cc(C(=O)N3CC(NC(=O)[C@@H]4[C@H]5CCC[C@H]54)C3)[nH]c2c1	990713256	25	337.18	2.46

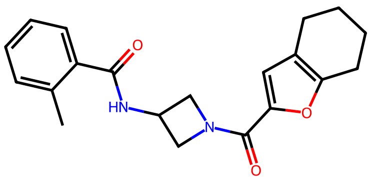	Cc1ccccc1C(=O)NC1CN(C(=O)c2cc3c(o2)CCCC3)C1	990111652	25	338.16	2.72
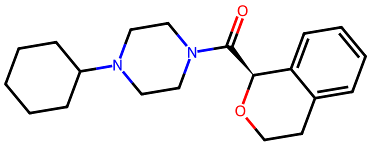	O=C([C@@H]1OCCc2ccccc21)N1CCN(C2CCCCC2)CC1	124808599	24	328.22	2.78
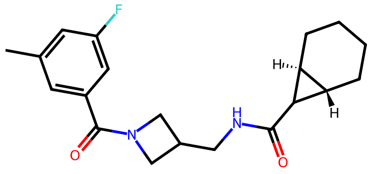	Cc1cc(F)cc(C(=O)N2CC(CNC(=O)C3[C@H]4CCCC[C@H]34)C2)c1	1001335772	25	344.19	2.76
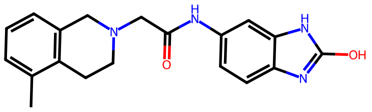	Cc1cccc2c1CCN(CC(=O)Nc1ccc3nc(O)[nH]c3c1)C2	729478571	25	336.16	2.57
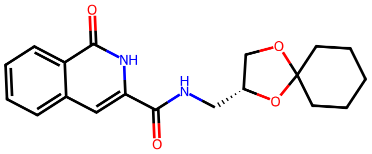	O=C(NC[C@@H]1COC2(CCCCC2)O1)c1cc2ccccc2c(=O)[nH]1	181699959	25	342.16	2.33
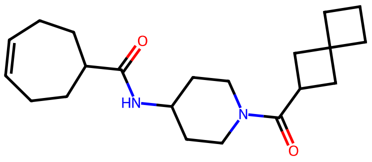	O=C(NC1CCN(C(=O)C2CC3(CCC3)C2)CC1)C1CCC=CCC1	947885740	25	344.25	3.42
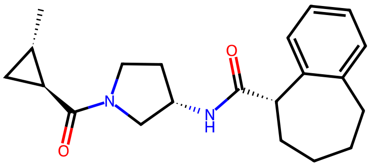	C[C@H]1C[C@@H]1C(=O)N1CC[C@H](NC(=O)[C@H]2CCCCc3ccccc32)C1	938138993	25	340.22	2.87
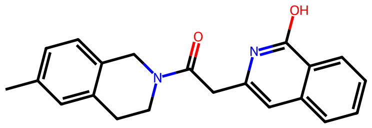	Cc1ccc2c(c1)CCN(C(=O)Cc1cc3ccccc3c(O)n1)C2	1643180601	25	332.15	3.38

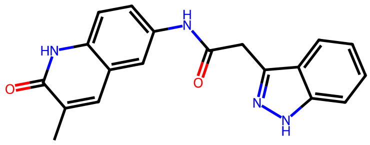	Cc1cc2cc(NC(=O)Cc3n[nH]c4ccccc34)ccc2[nH]c1=O	1468333949	25	332.13	2.89
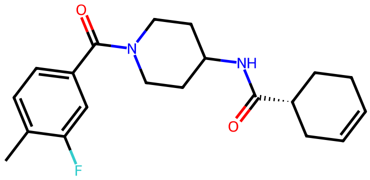	Cc1ccc(C(=O)N2CCC(NC(=O)[C@H]3CC=CCC3)CC2)cc1F	947403592	25	344.19	3.21
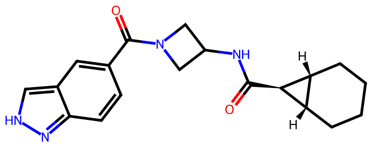	O=C(NC1CN(C(=O)c2ccc3n[nH]cc3c2)C1)[C@@H]1[C@H]2CCCC[C@H]21	990643515	25	338.17	1.94
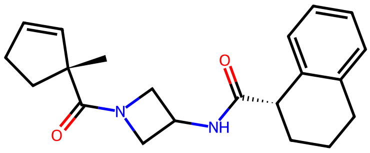	C[C@]1(C(=O)N2CC(NC(=O)[C@H]3CCCc4ccccc43)C2)C=CCC1	995076435	25	338.2	2.79
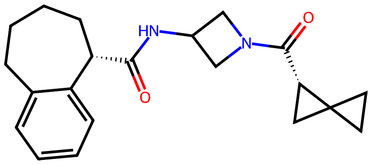	O=C(NC1CN(C(=O)[C@H]2CC23CC3)C1)[C@H]1CCCCc2ccccc21	994322767	25	338.2	2.62
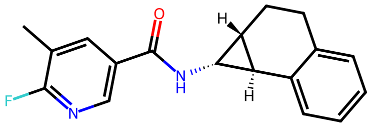	Cc1cc(C(=O)N[C@H]2[C@@H]3c4ccccc4CC[C@H]32)cnc1F	857714540	22	296.13	2.99
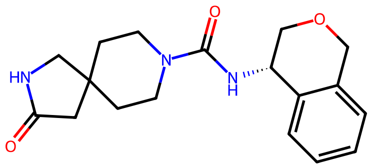	O=C1CC2(CCN(C(=O)N[C@@H]3COCc4ccccc43)CC2)CN1	1365204070	24	329.17	1.57
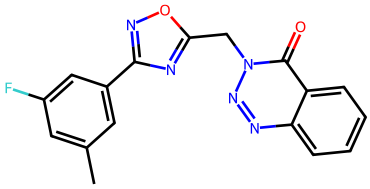	Cc1cc(F)cc(-c2noc(Cn3nnc4ccccc4c3=O)n2)c1	355855416	25	337.1	2.34

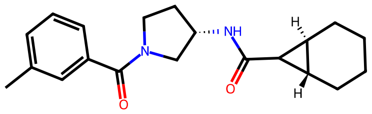	Cc1cccc(C(=O)N2CC[C@H](NC(=O)C3[C@H]4CCCC[C@H]34)C2)c1	937787187	24	326.2	2.76
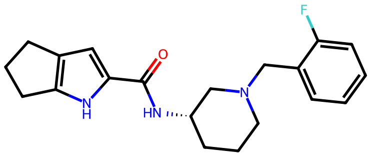	O=C(N[C@H]1CCCN(Cc2ccccc2F)C1)c1cc2c([nH]1)CCC2	1009164619	25	341.19	3.04
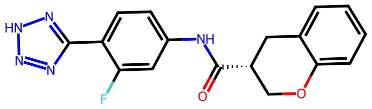	O=C(Nc1ccc(-c2nn[nH]n2)c(F)c1)[C@H]1COc2ccccc2C1	1593212725	25	339.11	2.2
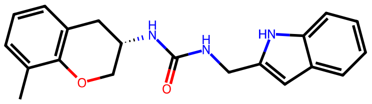	Cc1cccc2c1OC[C@@H](NC(=O)NCc1cc3ccccc3[nH]1)C2	460160904	25	335.16	3.28
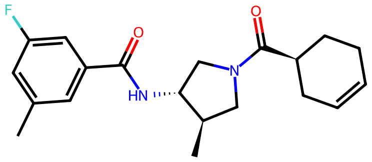	Cc1cc(F)cc(C(=O)N[C@@H]2CN(C(=O)[C@@H]3CC=CCC3)C[C@H]2C)c1	940619128	25	344.19	3.07
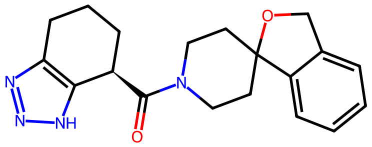	O=C([C@@H]1CCCc2nn[nH]c21)N1CCC2(CC1)OCc1ccccc12	612378943	25	338.17	2.27
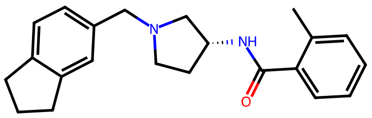	Cc1ccccc1C(=O)N[C@@H]1CCN(Cc2ccc3c(c2)CCC3)C1	1009968998	25	334.2	3.49
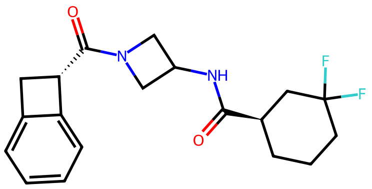	O=C(NC1CN(C(=O)[C@H]2Cc3ccccc32)C1)[C@@H]1CCCC(F)(F)C1	999754416	25	348.16	2.48

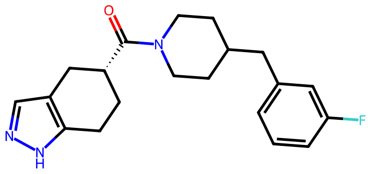	O=C([C@@H]1CCc2[nH]ncc2C1)N1CCC(Cc2cccc(F)c2)CC1	270280590	25	341.19	3.14
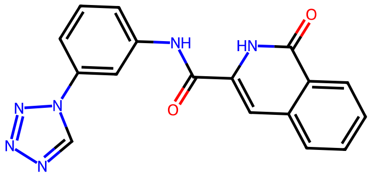	O=C(Nc1cccc(-n2cnnn2)c1)c1cc2ccccc2c(=O)[nH]1	56825568	25	332.1	1.76
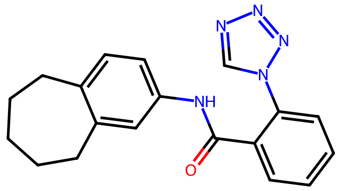	O=C(Nc1ccc2c(c1)CCCCC2)c1ccccc1-n1cnnn1	1585358086	25	333.16	3.18
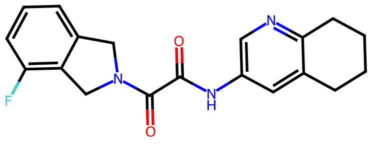	O=C(Nc1cnc2c(c1)CCCC2)C(=O)N1Cc2cccc(F)c2C1	411683248	25	339.14	2.58
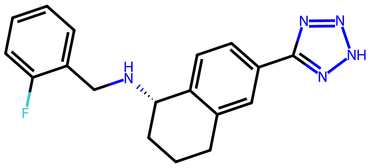	Fc1ccccc1CN[C@H]1CCCc2cc(-c3nn[nH]n3)ccc21	826378265	24	323.15	3.17
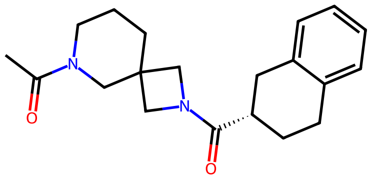	CC(=O)N1CCCC2(C1)CN(C(=O)[C@H]1CCc3ccccc3C1)C2	1010896144	24	326.2	2.26
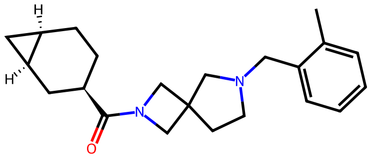	Cc1ccccc1CN1CCC2(C1)CN(C(=O)[C@@H]1CC[C@@H]3C[C@@H]3C1)C2	1072879088	25	338.24	3.47
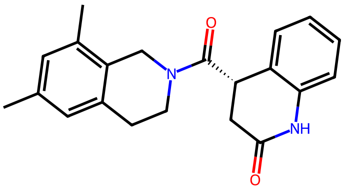	Cc1cc(C)c2c(c1)CCN(C(=O)[C@H]1CC(=O)Nc3ccccc31)C2	188052701	25	334.17	3.31

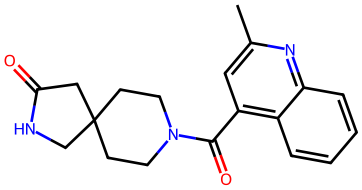	Cc1cc(C(=O)N2CCC3(CC2)CNC(=O)C3)c2ccccc2n1	267633184	24	323.16	2.29
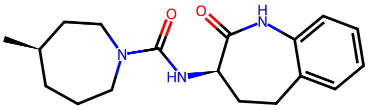	C[C@@H]1CCCN(C(=O)N[C@@H]2CCc3ccccc3NC2=O)CC1	654629902	23	315.19	2.77
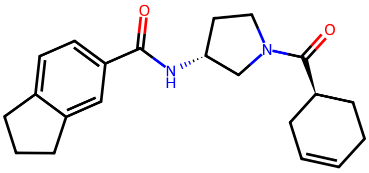	O=C(N[C@@H]1CCN(C(=O)[C@@H]2CC=CCC2)C1)c1ccc2c(c1)CCC2	940375758	25	338.2	2.86
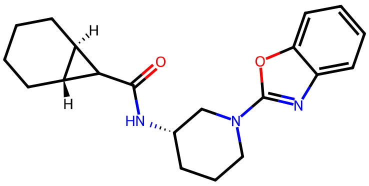	O=C(N[C@H]1CCCN(c2nc3ccccc3o2)C1)C1[C@@H]2CCCC[C@@H]12	1062375553	25	339.19	3.35
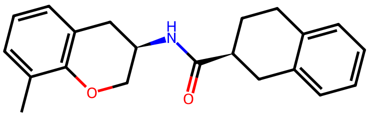	Cc1cccc2c1OC[C@H](NC(=O)[C@H]1CCc3ccccc3C1)C2	1129553634	24	321.17	3.22
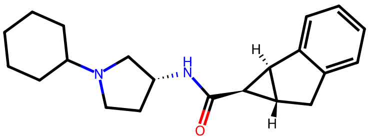	O=C(N[C@@H]1CCN(C2CCCCC2)C1)[C@@H]1[C@H]2Cc3ccccc3[C@@H]21	628092778	24	324.22	3.1
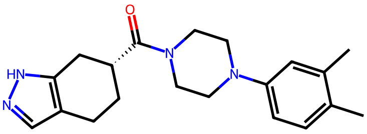	Cc1ccc(N2CCN(C(=O)[C@@H]3CCc4cn[nH]c4C3)CC2)cc1C	153109950	25	338.21	2.48
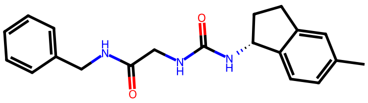	Cc1ccc2c(c1)CC[C@H]2NC(=O)NCC(=O)NCc1ccccc1	56843770	25	337.18	2.6

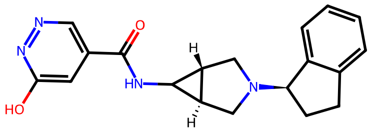	O=C(NC1[C@@H]2CN([C@@H]3CCc4ccccc43)C[C@@H]12)c1cnnc(O)c1	960411591	25	336.16	1.53
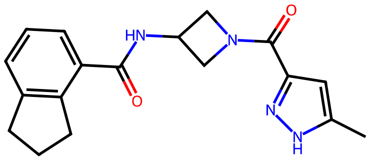	Cc1cc(C(=O)N2CC(NC(=O)c3cccc4c3CCC4)C2)n[nH]1	994405888	24	324.16	1.46
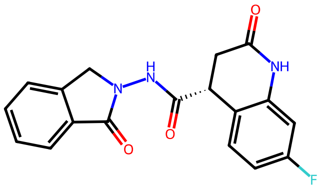	O=C1C[C@@H](C(=O)NN2Cc3ccccc3C2=O)c2ccc(F)cc2N1	183855476	25	339.1	1.94
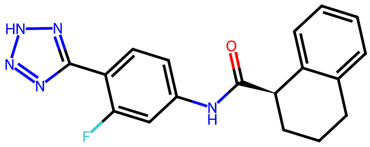	O=C(Nc1ccc(-c2nn[nH]n2)c(F)c1)[C@@H]1CCCc2ccccc21	1593213308	25	337.13	3.06
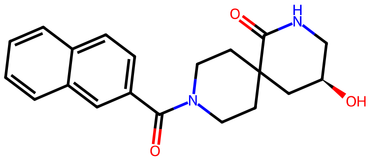	O=C(c1ccc2ccccc2c1)N1CCC2(CC1)C[C@H](O)CNC2=O	885026768	25	338.16	1.94
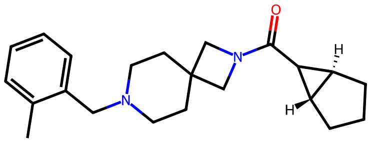	Cc1ccccc1CN1CCC2(CC1)CN(C(=O)C1[C@H]3CCC[C@H]13)C2	1050476031	25	338.24	3.47
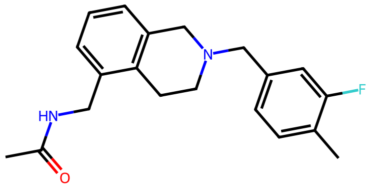	CC(=O)NCc1cccc2c1CCN(Cc1ccc(C)c(F)c1)C2	1051276782	24	326.18	3.33
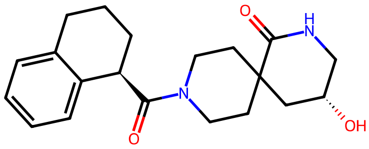	O=C([C@@H]1CCCc2ccccc21)N1CCC2(CC1)C[C@@H](O)CNC2=O	885024777	25	342.19	1.6

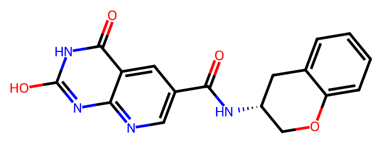	O=C(N[C@H]1COc2ccccc2C1)c1cnc2nc(O)[nH]c(=O)c2c1	53047094	25	338.1	0.76
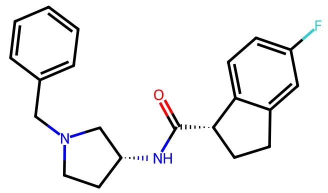	O=C(N[C@@H]1CCN(Cc2ccccc2)C1)[C@H]1CCc2cc(F)ccc21	665576579	25	338.18	3.25
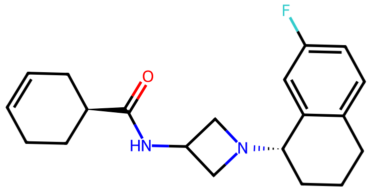	O=C(NC1CN([C@H]2CCCc3ccc(F)cc32)C1)[C@@H]1CC=CCC1	1030098610	24	328.2	3.36
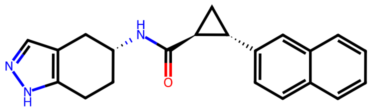	O=C(N[C@@H]1CCc2[nH]ncc2C1)[C@H]1C[C@@H]1c1ccc2ccccc2c1	172212456	25	331.17	3.34
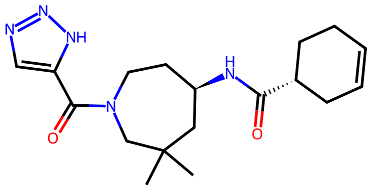	CC1(C)C[C@H](NC(=O)[C@H]2CC=CCC2)CCN(C(=O)c2cnn[nH]2)C1	990660100	25	345.22	1.91
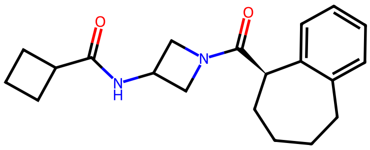	O=C(NC1CN(C(=O)[C@@H]2CCCCc3ccccc32)C1)C1CCC1	990028713	24	326.2	2.62
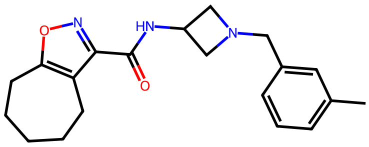	Cc1cccc(CN2CC(NC(=O)c3noc4c3CCCCC4)C2)c1	1031028473	25	339.19	2.87
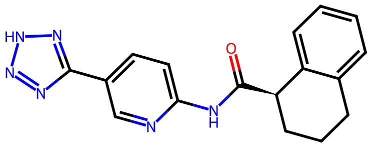	O=C(Nc1ccc(-c2nn[nH]n2)cn1)[C@@H]1CCCc2ccccc21	738150190	24	320.14	2.32

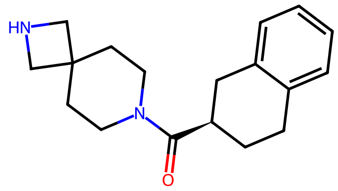	O=C([C@@H]1CCc2ccccc2C1)N1CCC2(CC1)CNC2	1548506271	21	284.19	2.0
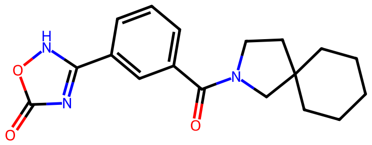	O=C(c1cccc(-c2nc(=O)o[nH]2)c1)N1CCC2(CCCCC2)C1	669221036	24	327.16	2.83
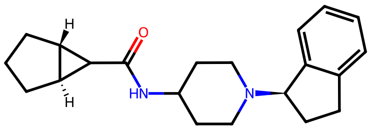	O=C(NC1CCN([C@@H]2CCc3ccccc32)CC1)C1[C@H]2CCC[C@H]12	1002545287	24	324.22	3.3
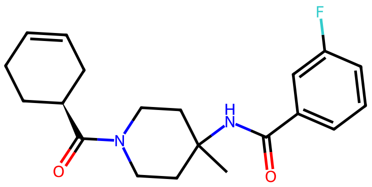	CC1(NC(=O)c2cccc(F)c2)CCN(C(=O)[C@@H]2CC=CCC2)CC1	956271806	25	344.19	3.29
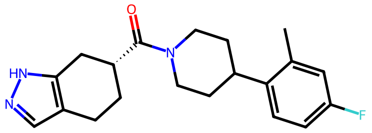	Cc1cc(F)ccc1C1CCN(C(=O)[C@@H]2CCc3cn[nH]c3C2)CC1	292529802	25	341.19	3.37
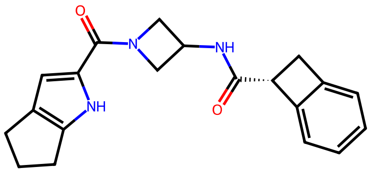	O=C(NC1CN(C(=O)c2cc3c([nH]2)CCC3)C1)[C@@H]1Cc2ccccc21	998680749	25	335.16	1.78
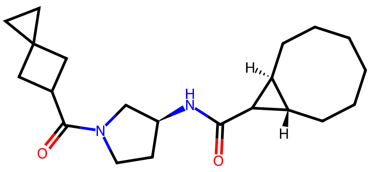	O=C(N[C@H]1CCN(C(=O)C2CC3(CC3)C2)C1)C1[C@H]2CCCCCC[C@H]12	938980165	25	344.25	3.11
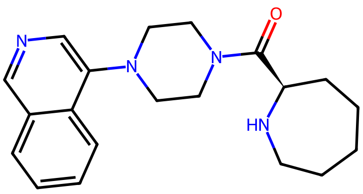	O=C([C@H]1CCCCCN1)N1CCN(c2cncc3ccccc23)CC1	516868088	25	338.21	2.42

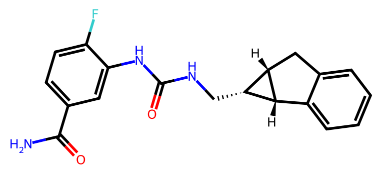	NC(=O)c1ccc(F)c(NC(=O)NC[C@H]2[C@H]3c4ccccc4C[C@H]32)c1	178121508	25	339.14	2.63
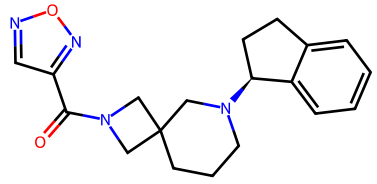	O=C(c1cnon1)N1CC2(CCCN([C@H]3CCc4ccccc43)C2)C1	982077057	25	338.17	2.3
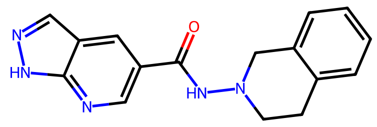	O=C(NN1CCc2ccccc2C1)c1cnc2[nH]ncc2c1	1469896692	22	293.13	1.66
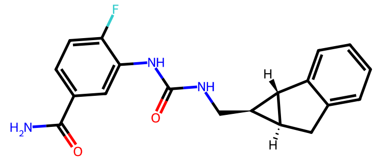	NC(=O)c1ccc(F)c(NC(=O)NC[C@@H]2[C@@H]3Cc4ccccc4[C@@H]23)c1	178121483	25	339.14	2.63
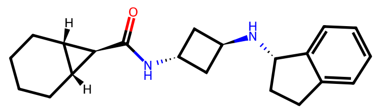	O=C(N[C@H]1C[C@H](N[C@H]2CCc3ccccc32)C1)[C@@H]1[C@H]2CCCC[C@H]21	1020052288	24	324.22	3.35
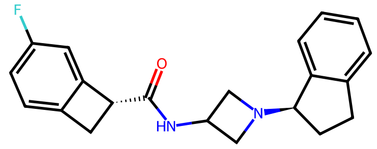	O=C(NC1CN([C@@H]2CCc3ccccc32)C1)[C@@H]1Cc2ccc(F)cc21	1030847160	25	336.16	2.95
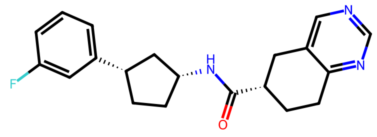	O=C(N[C@@H]1CC[C@H](c2cccc(F)c2)C1)[C@H]1CCc2ncncc2C1	648378421	25	339.17	3.17
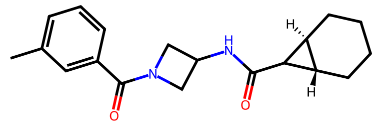	Cc1cccc(C(=O)N2CC(NC(=O)C3[C@H]4CCCC[C@H]34)C2)c1	990641541	23	312.18	2.37

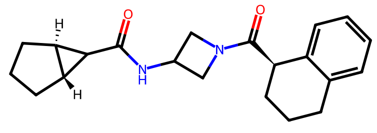	O=C(NC1CN(C(=O)[C@@H]2CCCc3ccccc32)C1)C1[C@@H]2CCC[C@@H]12	990708383	25	338.2	2.48
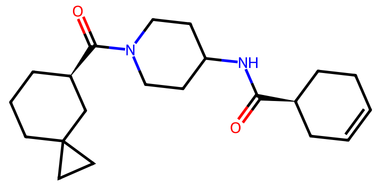	O=C(NC1CCN(C(=O)[C@@H]2CCCC3(CC3)C2)CC1)[C@@H]1CC=CCC1	946752924	25	344.25	3.42
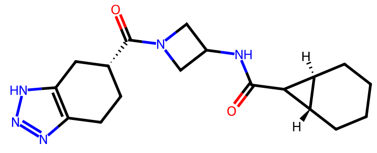	O=C(NC1CN(C(=O)[C@@H]2CCc3nn[nH]c3C2)C1)C1[C@H]2CCCC[C@H]12	990645376	25	343.2	0.67
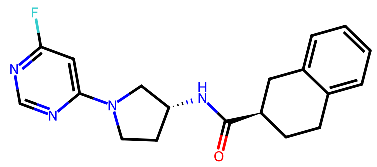	O=C(N[C@@H]1CCN(c2cc(F)ncn2)C1)[C@@H]1CCc2ccccc2C1	1058559469	25	340.17	2.12
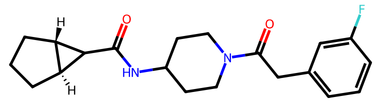	O=C(NC1CCN(C(=O)Cc2cccc(F)c2)CC1)C1[C@H]2CCC[C@H]12	948481088	25	344.19	2.52
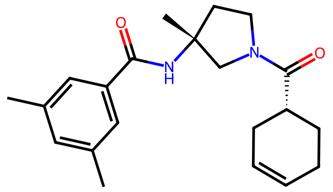	Cc1cc(C)cc(C(=O)N[C@]2(C)CCN(C(=O)[C@H]3CC=CCC3)C2)c1	957560537	25	340.22	3.38
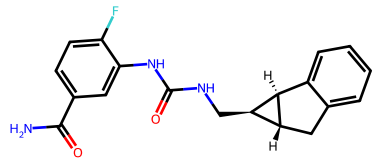	NC(=O)c1ccc(F)c(NC(=O)NC[C@@H]2[C@H]3Cc4ccccc4[C@@H]32)c1	178121528	25	339.14	2.63
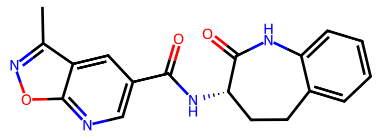	Cc1noc2ncc(C(=O)N[C@H]3CCc4ccccc4NC3=O)cc12	1639524985	25	336.12	2.21

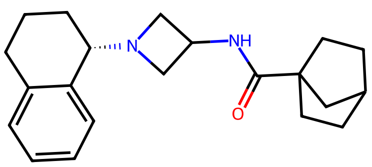	O=C(NC1CN([C@H]2CCCc3ccccc32)C1)C12CCC(CC1)C2	1030536480	24	324.22	3.44
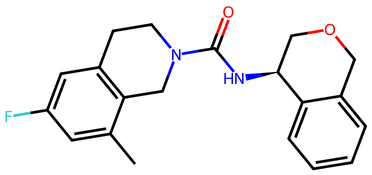	Cc1cc(F)cc2c1CN(C(=O)N[C@H]1COCc3ccccc31)CC2	419544397	25	340.16	3.47
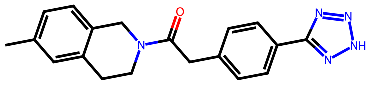	Cc1ccc2c(c1)CCN(C(=O)Cc1ccc(-c3nn[nH]n3)cc1)C2	1605888696	25	333.16	2.3
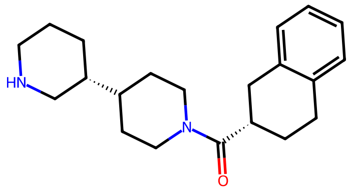	O=C([C@H]1CCc2ccccc2C1)N1CCC([C@H]2CCCNC2)CC1	658960444	24	326.24	3.03
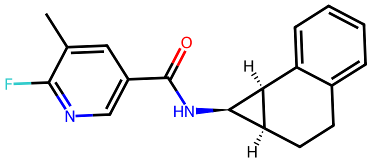	Cc1cc(C(=O)N[C@@H]2[C@@H]3CCc4ccccc4[C@@H]32)cnc1F	857714542	22	296.13	2.99
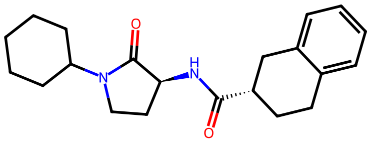	O=C(N[C@H]1CCN(C2CCCCC2)C1=O)[C@H]1CCc2ccccc2C1	649844872	25	340.22	2.84
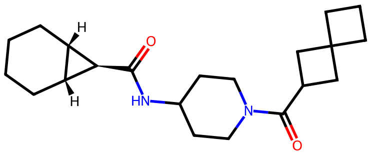	O=C(NC1CCN(C(=O)C2CC3(CCC3)C2)CC1)[C@@H]1[C@H]2CCCC[C@H]21	947882837	25	344.25	3.11
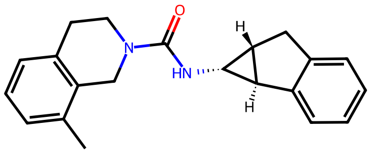	Cc1cccc2c1CN(C(=O)N[C@H]1[C@@H]3c4ccccc4C[C@H]31)CC2	429102461	24	318.17	3.4

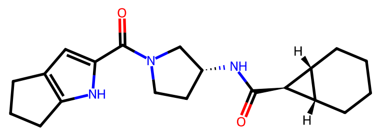	O=C(N[C@@H]1CCN(C(=O)c2cc3c([nH]2)CCC3)C1)[C@@H]1[C@H]2CCCC[C@H]21	939895590	25	341.21	2.27
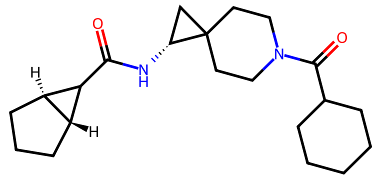	O=C(N[C@@H]1CC12CCN(C(=O)C1CCCCC1)CC2)C1[C@@H]2CCC[C@@H]12	963462681	25	344.25	3.11
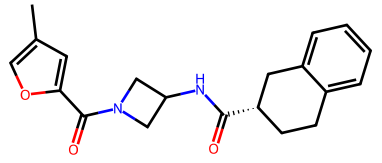	Cc1coc(C(=O)N2CC(NC(=O)[C@H]3CCc4ccccc4C3)C2)c1	1000544992	25	338.16	2.33
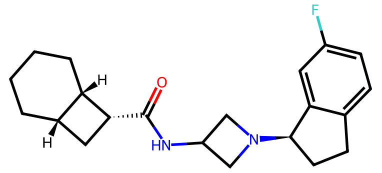	O=C(NC1CN([C@@H]2CCc3ccc(F)cc32)C1)[C@@H]1C[C@@H]2CCCC[C@@H]21	1030723339	25	342.21	3.44
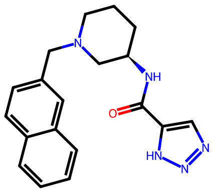	O=C(N[C@@H]1CCCN(Cc2ccc3ccccc3c2)C1)c1cnn[nH]1	1007757662	25	335.17	2.35
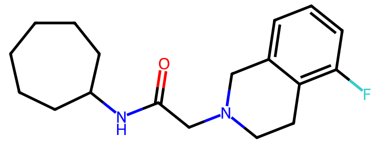	O=C(CN1CCc2c(F)cccc2C1)NC1CCCCCC1	757149753	22	304.2	3.02
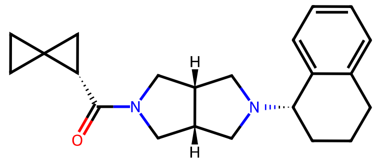	O=C([C@H]1CC12CC2)N1C[C@@H]2CN([C@H]3CCCc4ccccc43)C[C@@H]2C1	1049082780	25	336.22	3.25
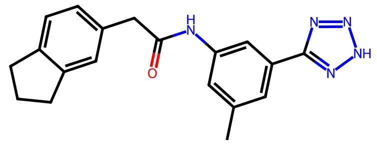	Cc1cc(NC(=O)Cc2ccc3c(c2)CCC3)cc(-c2nn[nH]n2)c1	1592018780	25	333.16	2.85

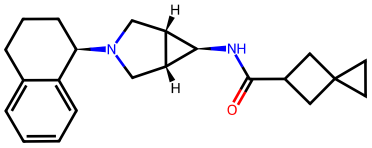	O=C(N[C@H]1[C@@H]2CN([C@@H]3CCCc4ccccc43)C[C@@H]21)C1CC2(CC2)C1	961728549	25	336.22	3.3
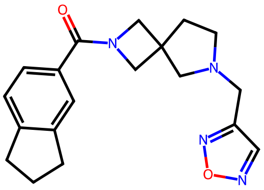	O=C(c1ccc2c(c1)CCC2)N1CC2(CCN(Cc3cnon3)C2)C1	1073417894	25	338.17	1.91
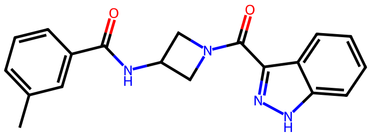	Cc1cccc(C(=O)NC2CN(C(=O)c3n[nH]c4ccccc34)C2)c1	990144273	25	334.14	2.13
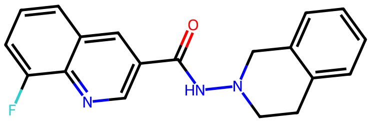	O=C(NN1CCc2ccccc2C1)c1cnc2c(F)cccc2c1	1300434191	24	321.13	3.08
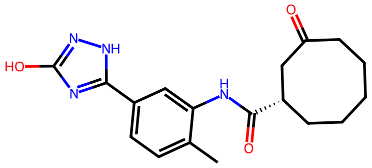	Cc1ccc(-c2nc(O)n[nH]2)cc1NC(=O)[C@H]1CCCCCC(=O)C1	521897859	25	342.17	2.96
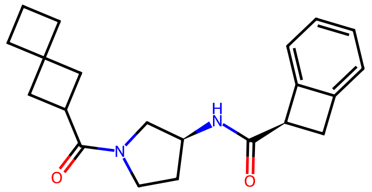	O=C(N[C@H]1CCN(C(=O)C2CC3(CCC3)C2)C1)[C@@H]1Cc2ccccc21	938570784	25	338.2	2.62
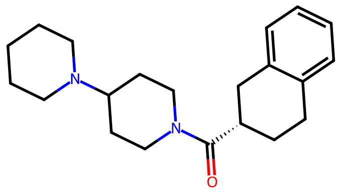	O=C([C@H]1CCc2ccccc2C1)N1CCC(N2CCCCC2)CC1	566324136	24	326.24	3.27
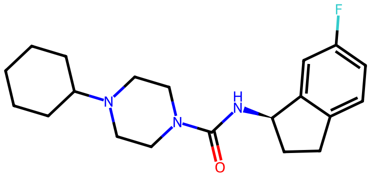	O=C(N[C@@H]1CCc2ccc(F)cc21)N1CCN(C2CCCCC2)CC1	531996534	25	345.22	3.47

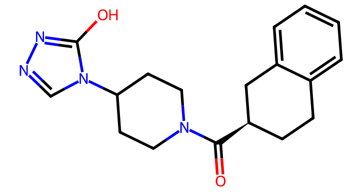	O=C([C@@H]1CCc2ccccc2C1)N1CCC(n2cnnc2O)CC1	655042319	24	326.17	1.95
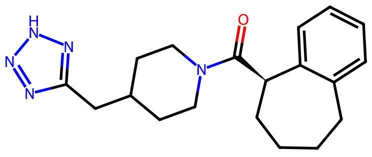	O=C([C@@H]1CCCCc2ccccc21)N1CCC(Cc2nn[nH]n2)CC1	1548805745	25	339.21	2.49
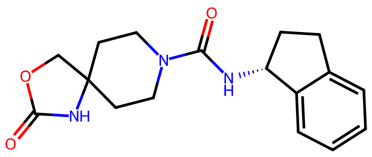	O=C1NC2(CCN(C(=O)N[C@@H]3CCc4ccccc43)CC2)CO1	784176885	23	315.16	1.96
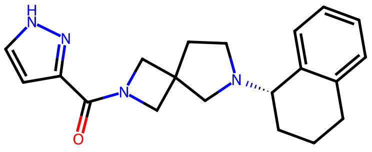	O=C(c1cc[nH]n1)N1CC2(CCN([C@H]3CCCc4ccccc43)C2)C1	1072500212	25	336.2	2.64
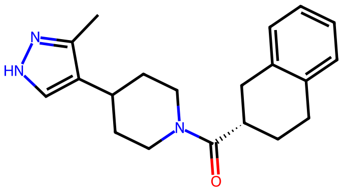	Cc1n[nH]cc1C1CCN(C(=O)[C@H]2CCc3ccccc3C2)CC1	578242440	24	323.2	3.23
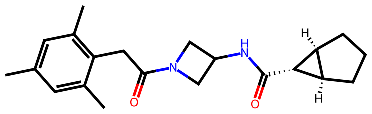	Cc1cc(C)c(CC(=O)N2CC(NC(=O)[C@@H]3[C@H]4CCC[C@H]43)C2)c(C)c1	990690085	25	340.22	2.53
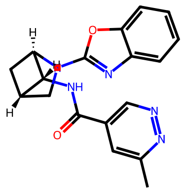	Cc1cc(C(=O)N[C@@H]2[C@H]3C[C@H]2N(c2nc4ccccc4o2)C3)cnn1	1064241262	25	335.14	1.93
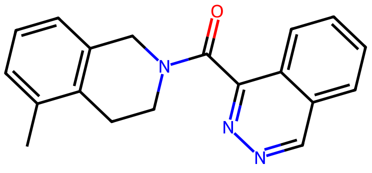	Cc1cccc2c1CCN(C(=O)c1nncc3ccccc13)C2	1456149779	23	303.14	3.14

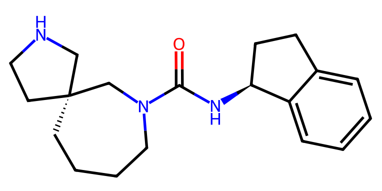	O=C(N[C@H]1CCc2ccccc21)N1CCCC[C@@]2(CCNC2)C1	1552956349	23	313.22	2.85
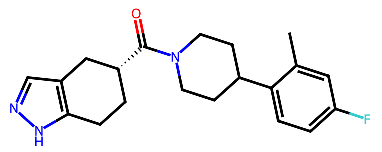	Cc1cc(F)ccc1C1CCN(C(=O)[C@@H]2CCc3[nH]ncc3C2)CC1	292954879	25	341.19	3.37
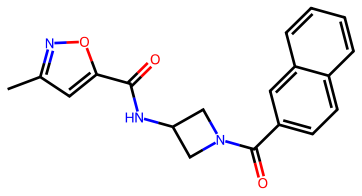	Cc1cc(C(=O)NC2CN(C(=O)c3ccc4ccccc4c3)C2)on1	990219933	25	335.13	2.39
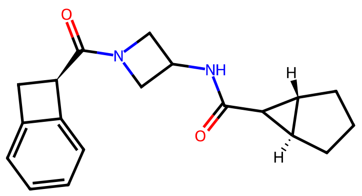	O=C(NC1CN(C(=O)[C@@H]2Cc3ccccc32)C1)C1[C@@H]2CCC[C@@H]12	990691338	23	310.17	1.7
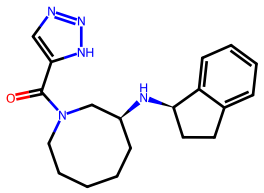	O=C(c1cnn[nH]1)N1CCCCC[C@H](N[C@@H]2CCc3ccccc32)C1	1088340447	25	339.21	2.47
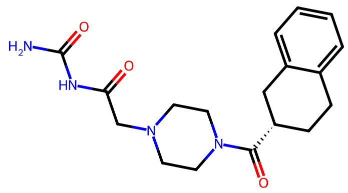	NC(=O)NC(=O)CN1CCN(C(=O)[C@H]2CCc3ccccc3C2)CC1	1113381899	25	344.18	0.13
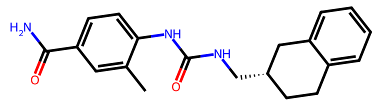	Cc1cc(C(N)=O)ccc1NC(=O)NC[C@H]1CCc2ccccc2C1	735366958	25	337.18	3.02
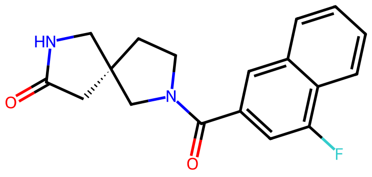	O=C1C[C@@]2(CCN(C(=O)c3cc(F)c4ccccc4c3)C2)CN1	351838391	23	312.13	2.33

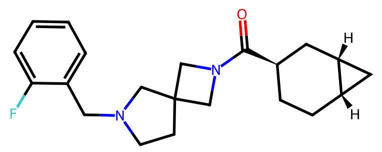	O=C([C@@H]1CC[C@H]2C[C@H]2C1)N1CC2(CCN(Cc3ccccc3F)C2)C1	1072879372	25	342.21	3.3
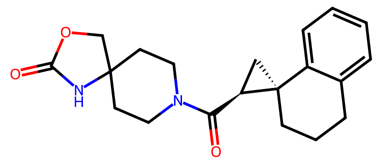	O=C1NC2(CCN(C(=O)[C@H]3C[C@]34CCCc3ccccc34)CC2)CO1	495140533	25	340.18	2.38
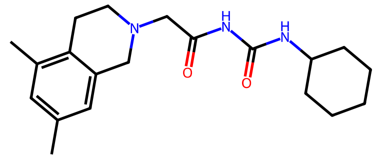	Cc1cc(C)c2c(c1)CN(CC(=O)NC(=O)NC1CCCCC1)CC2	1421717404	25	343.23	2.82
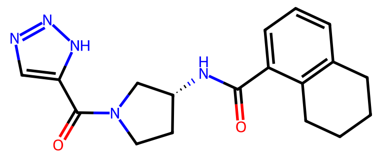	O=C(N[C@@H]1CCN(C(=O)c2cnn[nH]2)C1)c1cccc2c1CCCC2	940629964	25	339.17	1.33
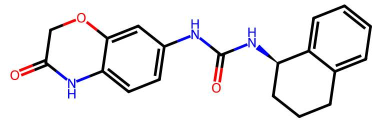	O=C1COc2cc(NC(=O)N[C@@H]3CCCc4ccccc43)ccc2N1	126625600	25	337.14	3.22
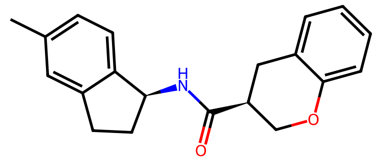	Cc1ccc2c(c1)CC[C@@H]2NC(=O)[C@@H]1COc2ccccc2C1	501961040	23	307.16	3.35
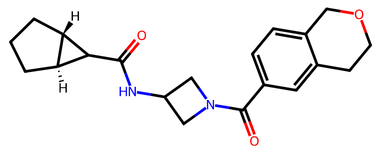	O=C(NC1CN(C(=O)c2ccc3c(c2)CCOC3)C1)C1[C@H]2CCC[C@H]12	990699740	25	340.18	1.75
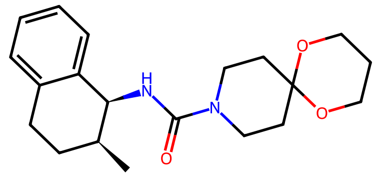	C[C@H]1CCc2ccccc2[C@H]1NC(=O)N1CCC2(CC1)OCCCO2	838533238	25	344.21	3.25

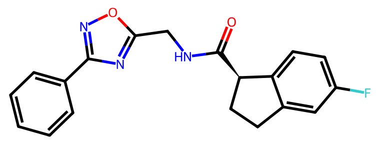	O=C(NCc1nc(-c2ccccc2)no1)[C@@H]1CCc2cc(F)ccc21	1611332508	25	337.12	3.22
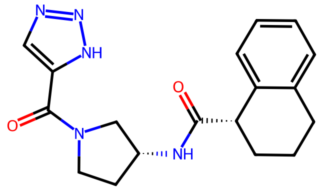	O=C(N[C@@H]1CCN(C(=O)c2cnn[nH]2)C1)[C@H]1CCCc2ccccc21	941389136	25	339.17	1.26
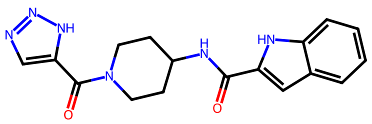	O=C(NC1CCN(C(=O)c2cnn[nH]2)CC1)c1cc2ccccc2[nH]1	947372651	25	338.15	1.32
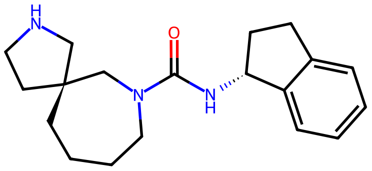	O=C(N[C@@H]1CCc2ccccc21)N1CCCC[C@]2(CCNC2)C1	892256422	23	313.22	2.85
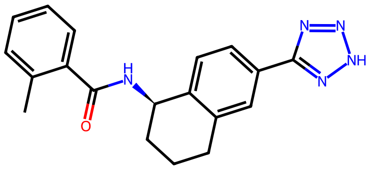	Cc1ccccc1C(=O)N[C@@H]1CCCc2cc(-c3nn[nH]n3)ccc21	1605932813	25	333.16	2.98
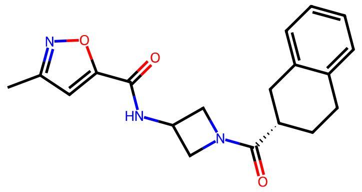	Cc1cc(C(=O)NC2CN(C(=O)[C@H]3CCc4ccccc4C3)C2)on1	990217899	25	339.16	1.73
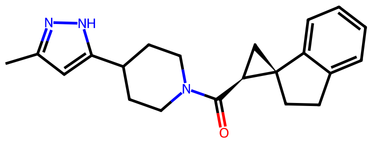	Cc1cc(C2CCN(C(=O)[C@H]3C[C@@]34CCc3ccccc34)CC2)[nH]n1	273565912	25	335.2	3.33
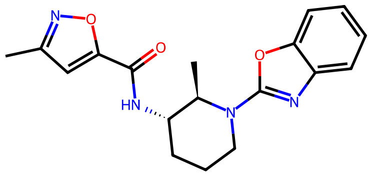	Cc1cc(C(=O)N[C@H]2CCCN(c3nc4ccccc4o3)[C@@H]2C)on1	1069340657	25	340.15	2.91

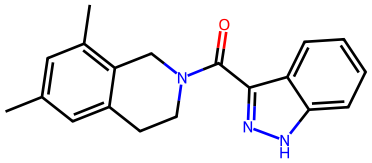	Cc1cc(C)c2c(c1)CCN(C(=O)c1n[nH]c3ccccc13)C2	1653982909	23	305.15	3.38
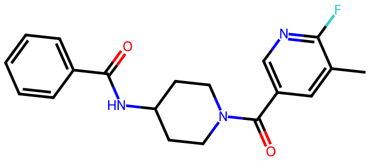	Cc1cc(C(=O)N2CCC(NC(=O)c3ccccc3)CC2)cnc1F	857573985	25	341.15	2.56
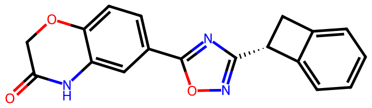	O=C1COc2ccc(-c3nc([C@@H]4Cc5ccccc54)no3)cc2N1	546294158	24	319.1	2.76
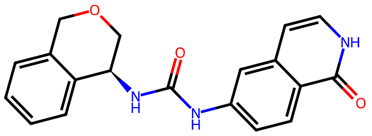	O=C(Nc1ccc2c(=O)[nH]ccc2c1)N[C@@H]1COCc2ccccc21	1672959179	25	335.13	2.92
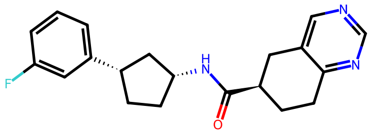	O=C(N[C@@H]1CC[C@H](c2cccc(F)c2)C1)[C@@H]1CCc2ncncc2C1	648378419	25	339.17	3.17
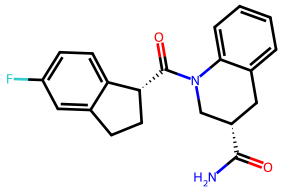	NC(=O)[C@H]1Cc2ccccc2N(C(=O)[C@@H]2CCc3cc(F)ccc32)C1	668055492	25	338.14	2.55
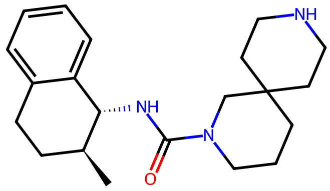	C[C@H]1CCc2ccccc2[C@@H]1NC(=O)N1CCCC2(CCNCC2)C1	1325365405	25	341.25	3.49
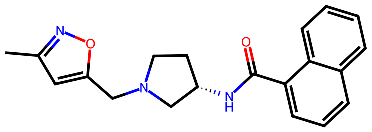	Cc1cc(CN2CC[C@H](NC(=O)c3cccc4ccccc34)C2)on1	1016151285	25	335.16	3.14

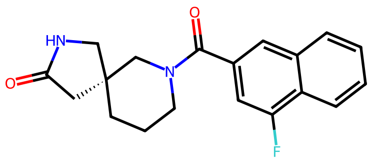	O=C1C[C@]2(CCCN(C(=O)c3cc(F)c4ccccc4c3)C2)CN1	341215962	24	326.14	2.72
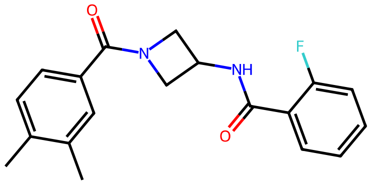	Cc1ccc(C(=O)N2CC(NC(=O)c3ccccc3F)C2)cc1C	990095809	24	326.14	2.7
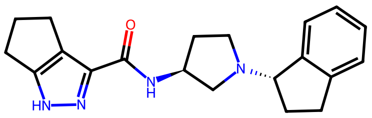	O=C(N[C@H]1CCN([C@H]2CCc3ccccc32)C1)c1n[nH]c2c1CCC2	1015070831	25	336.2	2.39
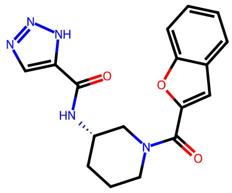	O=C(N[C@H]1CCCN(C(=O)c2cc3ccccc3o2)C1)c1cnn[nH]1	952071590	25	339.13	1.59
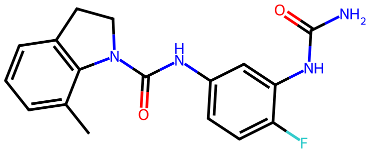	Cc1cccc2c1N(C(=O)Nc1ccc(F)c(NC(N)=O)c1)CC2	1677790314	24	328.13	3.22
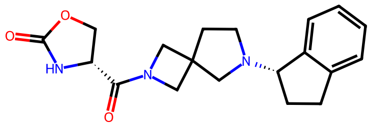	O=C1N[C@@H](C(=O)N2CC3(CCN([C@H]4CCc5ccccc54)C3)C2)CO1	1072686786	25	341.17	1.32
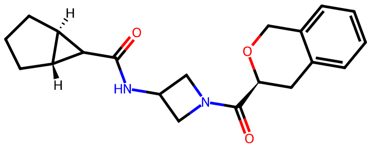	O=C(NC1CN(C(=O)[C@@H]2Cc3ccccc3CO2)C1)C1[C@@H]2CCC[C@@H]12	990698942	25	340.18	1.5
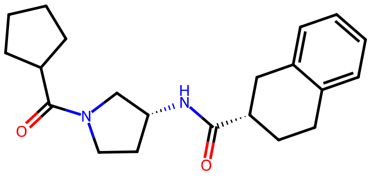	O=C(N[C@@H]1CCN(C(=O)C2CCCC2)C1)[C@H]1CCc2ccccc2C1	649842540	25	340.22	2.7

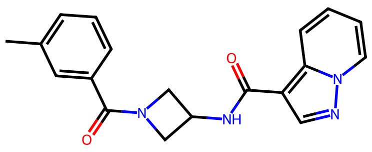	Cc1cccc(C(=O)N2CC(NC(=O)c3cnn4ccccc34)C2)c1	997759780	25	334.14	1.9
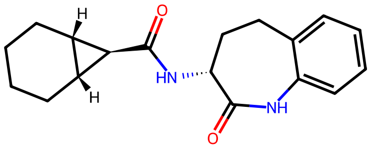	O=C1Nc2ccccc2CC[C@H]1NC(=O)[C@@H]1[C@H]2CCCC[C@H]21	654002297	22	298.17	2.49
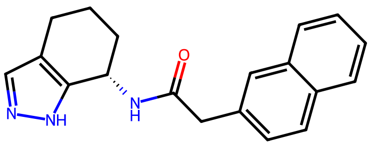	O=C(Cc1ccc2ccccc2c1)N[C@H]1CCCc2cn[nH]c21	268990218	23	305.15	3.3
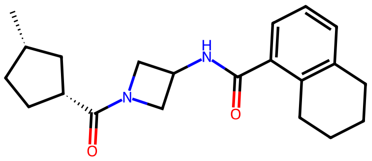	C[C@H]1CC[C@@H](C(=O)N2CC(NC(=O)c3cccc4c3CCCC4)C2)C1	991904023	25	340.22	2.94
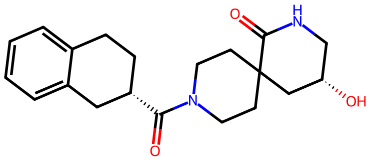	O=C([C@H]1CCc2ccccc2C1)N1CCC2(CC1)C[C@@H](O)CNC2=O	885016104	25	342.19	1.28
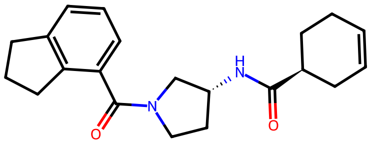	O=C(N[C@@H]1CCN(C(=O)c2cccc3c2CCC3)C1)[C@@H]1CC=CCC1	939378584	25	338.2	2.86
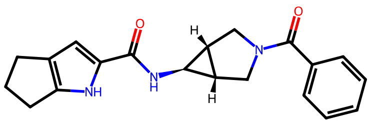	O=C(N[C@H]1[C@@H]2CN(C(=O)c3ccccc3)C[C@@H]21)c1cc2c([nH]1)CCC2	976838052	25	335.16	2.0
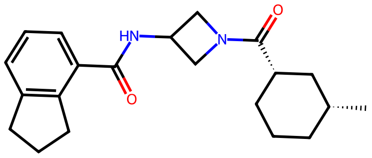	C[C@@H]1CCC[C@H](C(=O)N2CC(NC(=O)c3cccc4c3CCC4)C2)C1	994404455	25	340.22	2.94

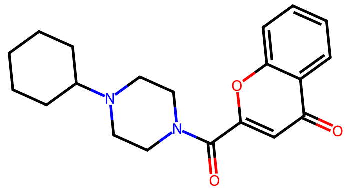	O=C(c1cc(=O)c2ccccc2o1)N1CCN(C2CCCCC2)CC1	154637295	25	340.18	2.88
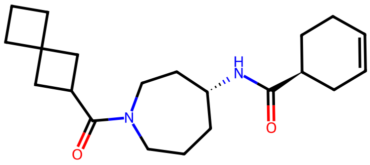	O=C(N[C@@H]1CCCN(C(=O)C2CC3(CCC3)C2)CC1)[C@@H]1CC=CCC1	964717428	25	344.25	3.42
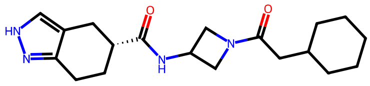	O=C(NC1CN(C(=O)CC2CCCCC2)C1)[C@@H]1CCc2n[nH]cc2C1	996395372	25	344.22	1.81
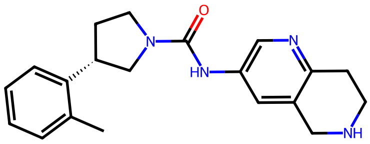	Cc1ccccc1[C@@H]1CCN(C(=O)Nc2cnc3c(c2)CNCC3)C1	667795631	25	336.2	3.06
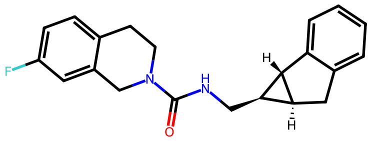	O=C(NC[C@@H]1[C@@H]2Cc3ccccc3[C@@H]12)N1CCc2ccc(F)cc2C1	136625567	25	336.16	3.48
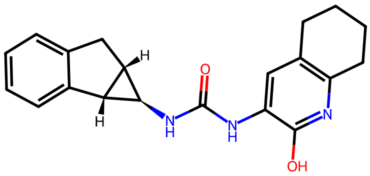	O=C(Nc1cc2c(nc1O)CCCC2)N[C@@H]1[C@H]2Cc3ccccc3[C@H]21	650270658	25	335.16	3.13
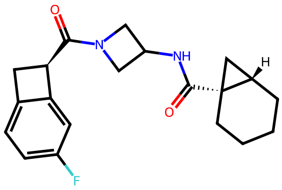	O=C([C@@H]1Cc2ccc(F)cc21)N1CC(NC(=O)[C@@]23CCCC[C@H]2C3)C1	999232965	25	342.17	2.37
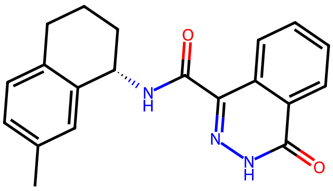	Cc1ccc2c(c1)[C@@H](NC(=O)c1n[nH]c(=O)c3ccccc13)CCC2	179473005	25	333.15	3.04

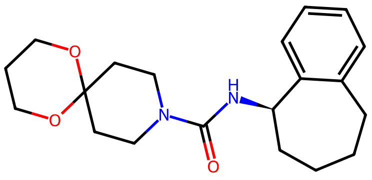	O=C(N[C@@H]1CCCCc2ccccc21)N1CCC2(CC1)OCCCO2	836269049	25	344.21	3.39
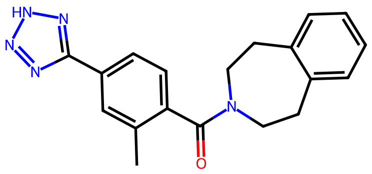	Cc1cc(-c2nn[nH]n2)ccc1C(=O)N1CCc2ccccc2CC1	826245976	25	333.16	2.42
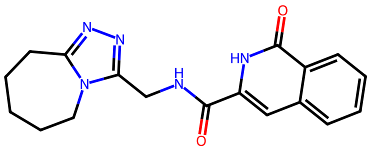	O=C(NCc1nnc2n1CCCCC2)c1cc2ccccc2c(=O)[nH]1	53282340	25	337.15	1.78
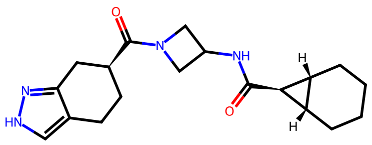	O=C(NC1CN(C(=O)[C@H]2CCc3c[nH]nc3C2)C1)[C@@H]1[C@H]2CCCC[C@H]21	990648006	25	342.21	1.28
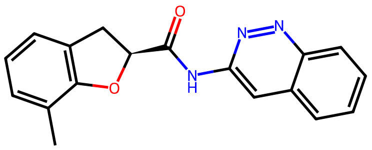	Cc1cccc2c1O[C@H](C(=O)Nc1cc3ccccc3nn1)C2	1461175978	23	305.12	2.88
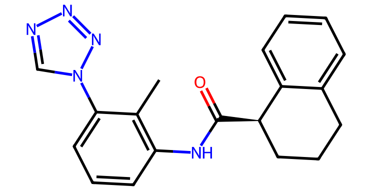	Cc1c(NC(=O)[C@@H]2CCCc3ccccc32)cccc1-n1cnnn1	641916823	25	333.16	3.03
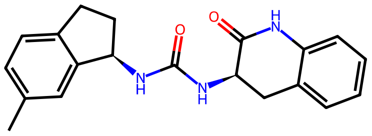	Cc1ccc2c(c1)[C@H](NC(=O)N[C@@H]1Cc3ccccc3NC1=O)CC2	636744413	25	335.16	2.84
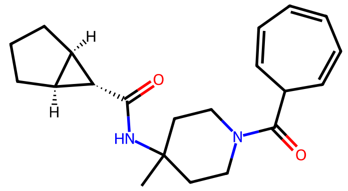	CC1(NC(=O)[C@@H]2[C@H]3CCC[C@H]32)CCN(C(=O)C2C=CC=CC=C2)CC1	956332063	25	340.22	2.83

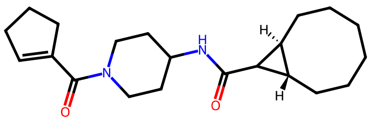	O=C(NC1CCN(C(=O)C2=CCCC2)CC1)C1[C@H]2CCCCCC[C@H]12	946453538	25	344.25	3.42
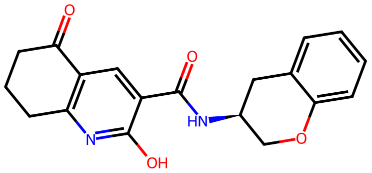	O=C(N[C@@H]1COc2ccccc2C1)c1cc2c(nc1O)CCCC2=O	299023617	25	338.13	2.04
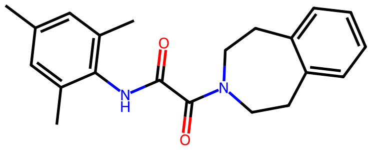	Cc1cc(C)c(NC(=O)C(=O)N2CCc3ccccc3CC2)c(C)c1	523900745	25	336.18	3.18
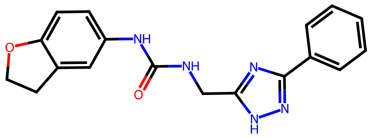	O=C(NCc1nc(-c2ccccc2)n[nH]1)Nc1ccc2c(c1)CCO2	735567317	25	335.14	2.73
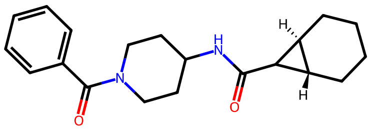	O=C(NC1CCN(C(=O)c2ccccc2)CC1)C1[C@H]2CCCC[C@H]12	173743186	24	326.2	2.84
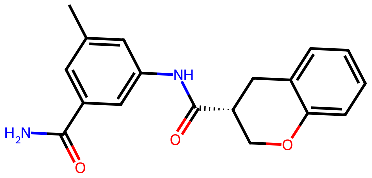	Cc1cc(NC(=O)[C@H]2COc3ccccc3C2)cc(C(N)=O)c1	1361836854	23	310.13	2.28
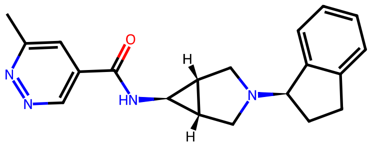	Cc1cc(C(=O)N[C@H]2[C@@H]3CN([C@@H]4CCc5ccccc54)C[C@@H]32)cnn1	958675596	25	334.18	2.13
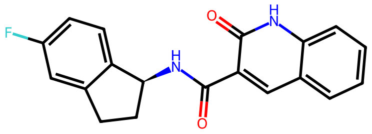	O=C(N[C@H]1CCc2cc(F)ccc21)c1cc2ccccc2[nH]c1=O	194277778	24	322.11	3.08

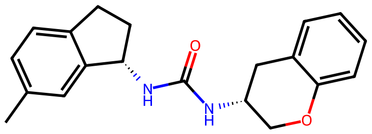	Cc1ccc2c(c1)[C@@H](NC(=O)N[C@H]1COc3ccccc3C1)CC2	592499149	24	322.17	3.29
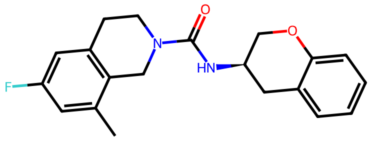	Cc1cc(F)cc2c1CN(C(=O)N[C@H]1COc3ccccc3C1)CC2	507260218	25	340.16	3.21
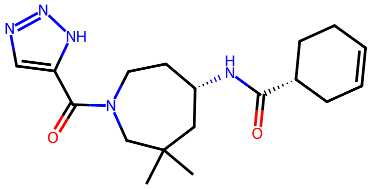	CC1(C)C[C@@H](NC(=O)[C@H]2CC=CCC2)CCN(C(=O)c2cnn[nH]2)C1	990660097	25	345.22	1.91
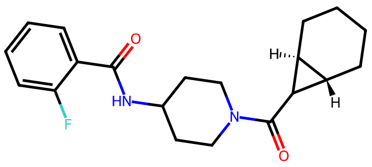	O=C(NC1CCN(C(=O)C2[C@H]3CCCC[C@H]23)CC1)c1ccccc1F	369941366	25	344.19	2.98
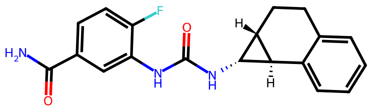	NC(=O)c1ccc(F)c(NC(=O)N[C@H]2[C@@H]3c4ccccc4CC[C@H]32)c1	178923007	25	339.14	2.77
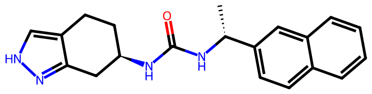	C[C@@H](NC(=O)N[C@@H]1CCc2c[nH]nc2C1)c1ccc2ccccc2c1	363938595	25	334.18	3.48
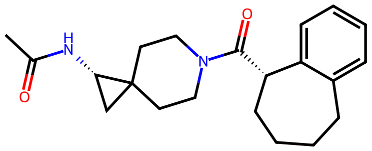	CC(=O)N[C@H]1CC12CCN(C(=O)[C@H]1CCCCc3ccccc31)CC2	962352475	25	340.22	3.01
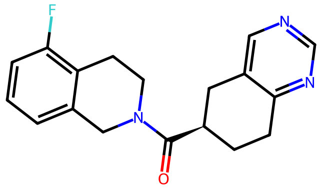	O=C([C@@H]1CCc2ncncc2C1)N1CCc2c(F)cccc2C1	843570457	23	311.14	2.31

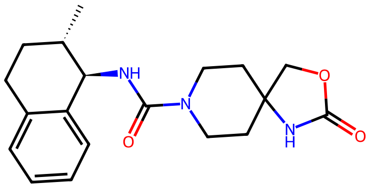	C[C@H]1CCc2ccccc2[C@@H]1NC(=O)N1CCC2(CC1)COC(=O)N2	495297708	25	343.19	2.59
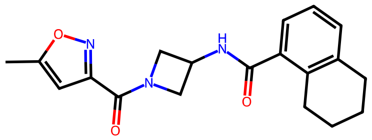	Cc1cc(C(=O)N2CC(NC(=O)c3cccc4c3CCCC4)C2)no1	991903767	25	339.16	2.12
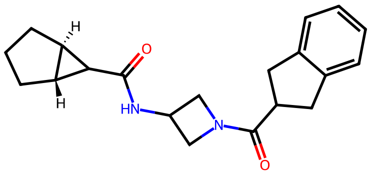	O=C(NC1CN(C(=O)C2Cc3ccccc3C2)C1)C1[C@@H]2CCC[C@@H]12	990710885	24	324.18	1.77
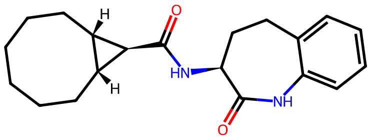	O=C1Nc2ccccc2CC[C@@H]1NC(=O)[C@@H]1[C@H]2CCCCCC[C@H]21	616693212	24	326.2	3.27
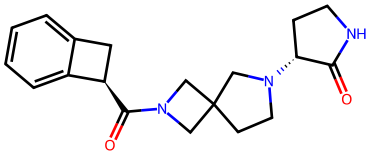	O=C1NCC[C@H]1N1CCC2(CN(C(=O)[C@@H]3Cc4ccccc43)C2)C1	1072931218	24	325.18	0.75
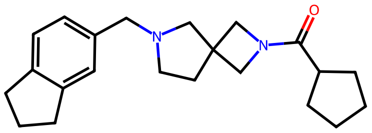	O=C(C1CCCC1)N1CC2(CCN(Cc3ccc4c(c3)CCC4)C2)C1	1072378329	25	338.24	3.4
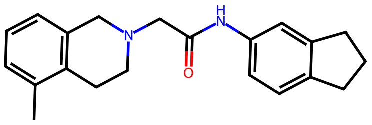	Cc1cccc2c1CCN(CC(=O)Nc1ccc3c(c1)CCC3)C2	729477187	24	320.19	3.48
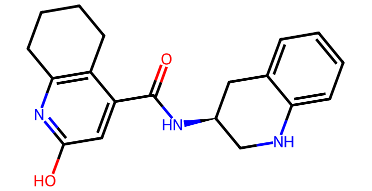	O=C(N[C@@H]1CNc2ccccc2C1)c1cc(O)nc2c1CCCC2	1580622127	24	323.16	2.43

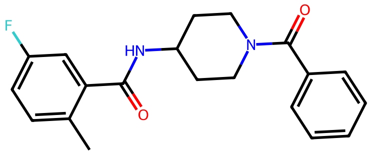	Cc1ccc(F)cc1C(=O)NC1CCN(C(=O)c2ccccc2)CC1	194571510	25	340.16	3.17
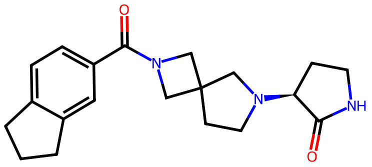	O=C1NCC[C@@H]1N1CCC2(CN(C(=O)c3ccc4c(c3)CCC4)C2)C1	1073418217	25	339.19	1.21
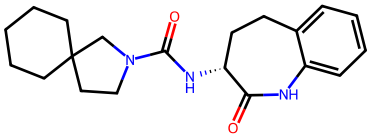	O=C1Nc2ccccc2CC[C@H]1NC(=O)N1CCC2(CCCCC2)C1	654628052	25	341.21	3.31
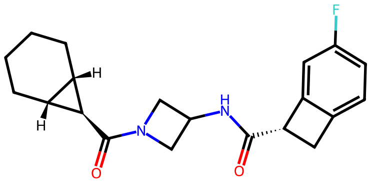	O=C(NC1CN(C(=O)[C@@H]2[C@H]3CCCC[C@H]32)C1)[C@H]1Cc2ccc(F)cc21	998488013	25	342.17	2.23
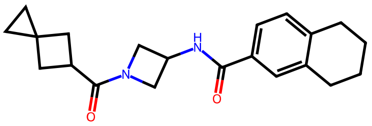	O=C(NC1CN(C(=O)C2CC3(CC3)C2)C1)c1ccc2c(c1)CCCC2	1000059695	25	338.2	2.7
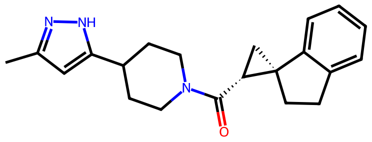	Cc1cc(C2CCN(C(=O)[C@@H]3C[C@]34CCc3ccccc34)CC2)[nH]n1	273565907	25	335.2	3.33
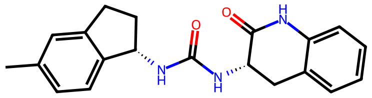	Cc1ccc2c(c1)CC[C@@H]2NC(=O)N[C@H]1Cc2ccccc2NC1=O	636635273	25	335.16	2.84
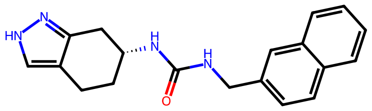	O=C(NCc1ccc2ccccc2c1)N[C@@H]1CCc2c[nH]nc2C1	372746171	24	320.16	2.92

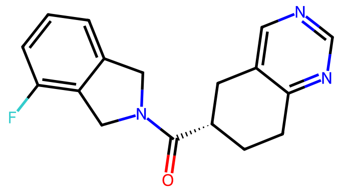	O=C([C@H]1CCc2ncncc2C1)N1Cc2cccc(F)c2C1	648417194	22	297.13	2.26
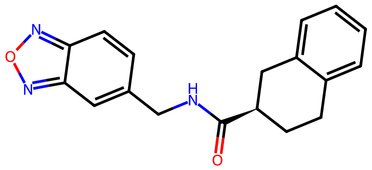	O=C(NCc1ccc2nonc2c1)[C@@H]1CCc2ccccc2C1	889878000	23	307.13	2.64
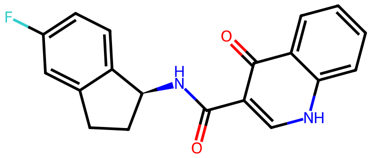	O=C(N[C@H]1CCc2cc(F)ccc21)c1c[nH]c2ccccc2c1=O	55658185	24	322.11	3.08
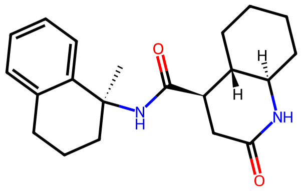	C[C@]1(NC(=O)[C@@H]2CC(=O)N[C@@H]3CCCC[C@H]32)CCCc2ccccc21	667870540	25	340.22	3.05
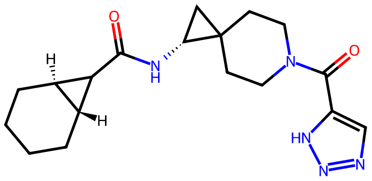	O=C(N[C@@H]1CC12CCN(C(=O)c1cnn[nH]1)CC2)C1[C@@H]2CCCC[C@@H]12	963448783	25	343.2	1.35
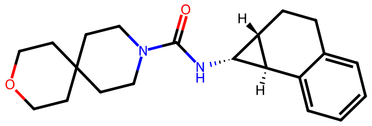	O=C(N[C@H]1[C@@H]2c3ccccc3CC[C@H]21)N1CCC2(CCOCC2)CC1	289303140	25	340.22	3.32
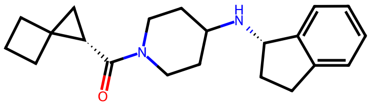	O=C([C@H]1CC12CCC2)N1CCC(N[C@@H]2CCc3ccccc32)CC1	997041655	24	324.22	3.44
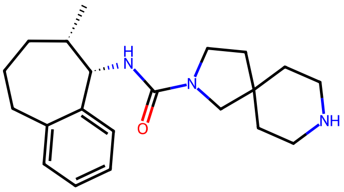	C[C@H]1CCCc2ccccc2[C@H]1NC(=O)N1CCC2(CCNCC2)C1	418018814	25	341.25	3.49

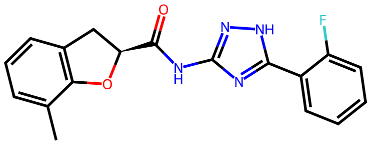	Cc1cccc2c1O[C@H](C(=O)Nc1n[nH]c(-c3ccccc3F)n1)C2	1558919660	25	338.12	2.86
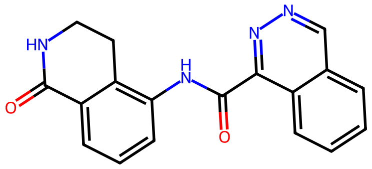	O=C1NCCc2c(NC(=O)c3nncc4ccccc34)cccc21	1525370593	24	318.11	2.17
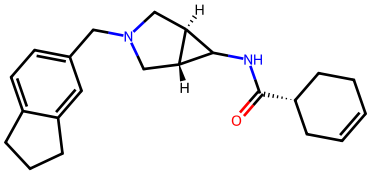	O=C(NC1[C@@H]2CN(Cc3ccc4c(c3)CCC4)C[C@@H]12)[C@H]1CC=CCC1	958208415	25	336.22	3.08
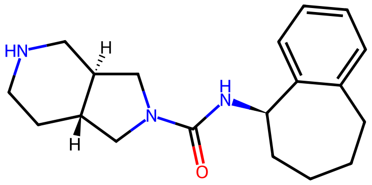	O=C(N[C@@H]1CCCCc2ccccc21)N1C[C@@H]2CNCC[C@H]2C1	583252287	23	313.22	2.71
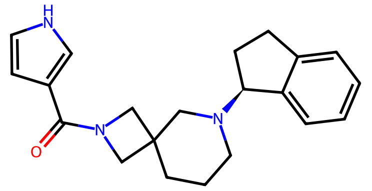	O=C(c1cc[nH]c1)N1CC2(CCCN([C@H]3CCc4ccccc43)C2)C1	981784190	25	335.2	3.24
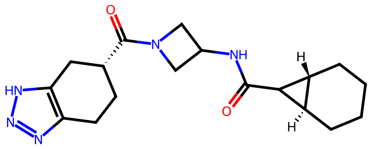	O=C(NC1CN(C(=O)[C@@H]2CCc3nn[nH]c3C2)C1)C1[C@@H]2CCCC[C@@H]12	990645372	25	343.2	0.67
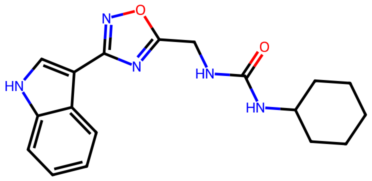	O=C(NCc1nc(-c2c[nH]c3ccccc23)no1)NC1CCCCC1	277901422	25	339.17	3.35
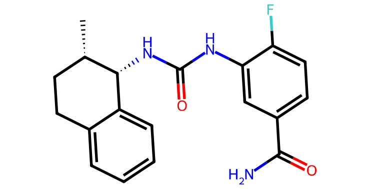	C[C@H]1CCc2ccccc2[C@H]1NC(=O)Nc1cc(C(N)=O)ccc1F	179534140	25	341.15	3.37

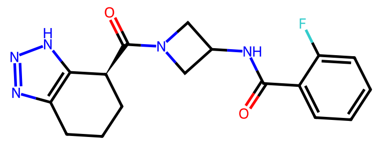	O=C(NC1CN(C(=O)[C@H]2CCCc3nn[nH]c32)C1)c1ccccc1F	990089868	25	343.14	1.0
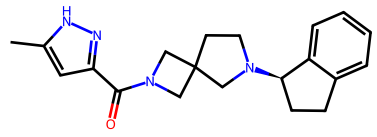	Cc1cc(C(=O)N2CC3(CCN([C@@H]4CCc5ccccc54)C3)C2)n[nH]1	1072519898	25	336.2	2.55
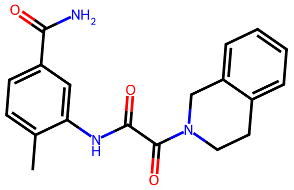	Cc1ccc(C(N)=O)cc1NC(=O)C(=O)N1CCc2ccccc2C1	57668875	25	337.14	1.62
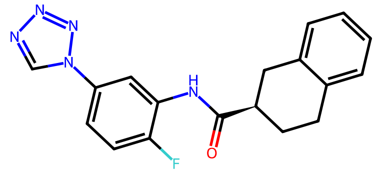	O=C(Nc1cc(-n2cnnn2)ccc1F)[C@@H]1CCc2ccccc2C1	1435715940	25	337.13	2.55
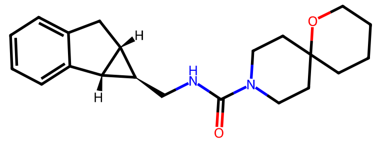	O=C(NC[C@@H]1[C@H]2Cc3ccccc3[C@H]21)N1CCC2(CCCCO2)CC1	883522628	25	340.22	3.32
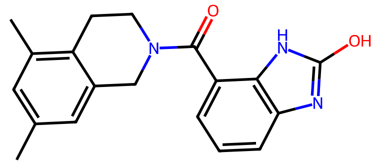	Cc1cc(C)c2c(c1)CN(C(=O)c1cccc3nc(O)[nH]c13)CC2	572133406	24	321.15	3.08
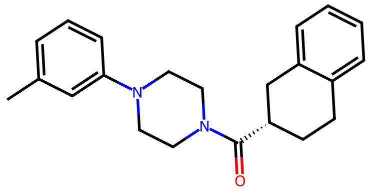	Cc1cccc(N2CCN(C(=O)[C@H]3CCc4ccccc4C3)CC2)c1	1356301686	25	334.2	3.45
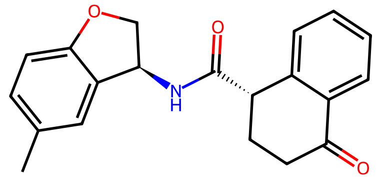	Cc1ccc2c(c1)[C@H](NC(=O)[C@H]1CCC(=O)c3ccccc31)CO2	375070908	24	321.14	3.31

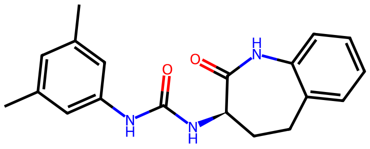	Cc1cc(C)cc(NC(=O)N[C@@H]2CCc3ccccc3NC2=O)c1	653917649	24	323.16	3.38
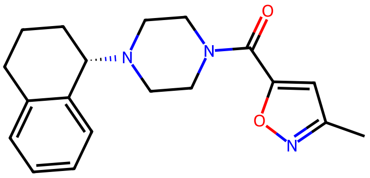	Cc1cc(C(=O)N2CCN([C@H]3CCCc4ccccc43)CC2)on1	65514819	24	325.18	2.82
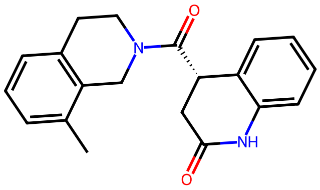	Cc1cccc2c1CN(C(=O)[C@H]1CC(=O)Nc3ccccc31)CC2	365403254	24	320.15	3.01
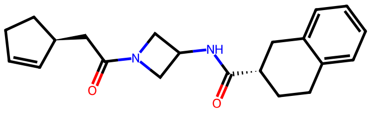	O=C(NC1CN(C(=O)C[C@H]2C=CCC2)C1)[C@H]1CCc2ccccc2C1	1000541707	25	338.2	2.47
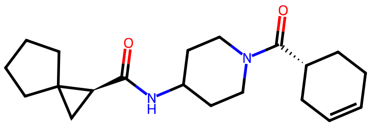	O=C(NC1CCN(C(=O)[C@H]2CC=CCC2)CC1)[C@H]1CC12CCCC2	944105934	24	330.23	3.03
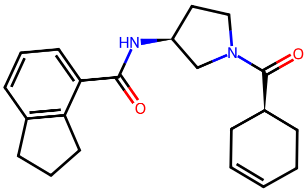	O=C(N[C@H]1CCN(C(=O)[C@@H]2CC=CCC2)C1)c1cccc2c1CCC2	938148031	25	338.2	2.86
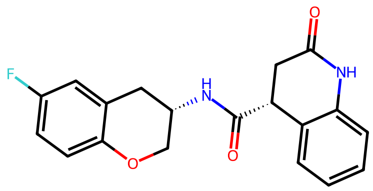	O=C1C[C@@H](C(=O)N[C@@H]2COc3ccc(F)cc3C2)c2ccccc2N1	290681415	25	340.12	2.37
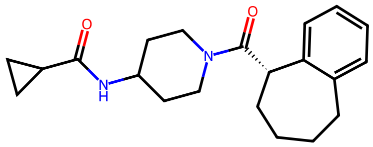	O=C(NC1CCN(C(=O)[C@H]2CCCCc3ccccc32)CC1)C1CC1	947145463	25	340.22	3.01

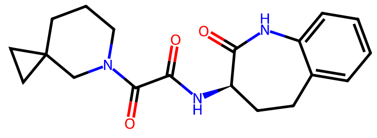	O=C(N[C@@H]1CCc2ccccc2NC1=O)C(=O)N1CCCC2(CC2)C1	1326935544	25	341.17	1.46
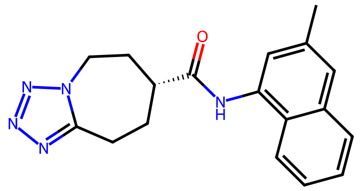	Cc1cc(NC(=O)[C@@H]2CCc3nnnn3CC2)c2ccccc2c1	576166585	24	321.16	2.73
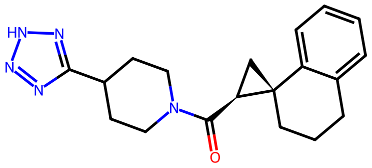	O=C([C@H]1C[C@@]12CCCc1ccccc12)N1CCC(c2nn[nH]n2)CC1	913743895	25	337.19	2.2
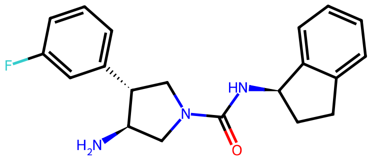	N[C@@H]1CN(C(=O)N[C@@H]2CCc3ccccc32)C[C@H]1c1cccc(F)c1	1580393352	25	339.17	2.95
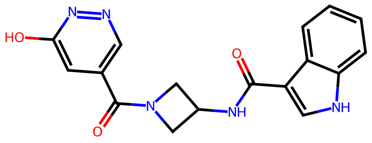	O=C(NC1CN(C(=O)c2cnnc(O)c2)C1)c1c[nH]c2ccccc12	995034640	25	337.12	0.92
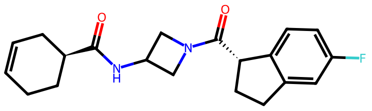	O=C(NC1CN(C(=O)[C@H]2CCc3cc(F)ccc32)C1)[C@@H]1CC=CCC1	990217330	25	342.17	2.54
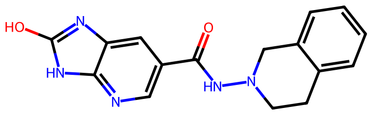	O=C(NN1CCc2ccccc2C1)c1cnc2[nH]c(O)nc2c1	1452970195	23	309.12	1.37
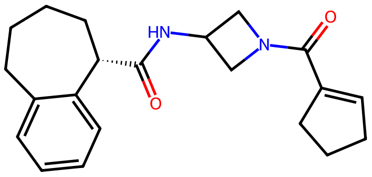	O=C(NC1CN(C(=O)C2=CCCC2)C1)[C@@H]1CCCCc2ccccc21	994304236	25	338.2	2.93

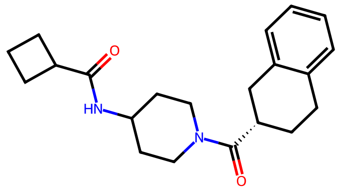	O=C(NC1CCN(C(=O)[C@H]2CCc3ccc cc3C2)CC1)C1CCC1	948571835	25	340.22	2.7
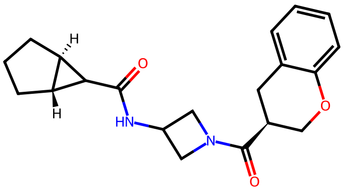	O=C(NC1CN(C(=O)[C@@H]2COc3cc ccc3C2)C1)C1[C@@H]2CCC[C@@H]12	990708418	25	340.18	1.61
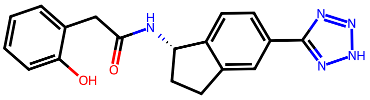	O=C(Cc1ccccc1O)N[C@H]1CCc2cc(- c3nn[nH]n3)ccc21	1609536632	25	335.14	1.92
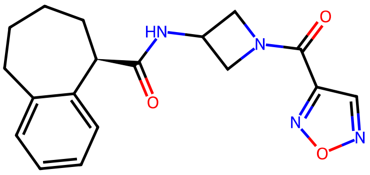	O=C(NC1CN(C(=O)c2cnon2)C1)[C@ @H]1CCCCc2ccccc21	994323960	25	340.15	1.52
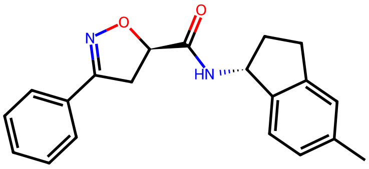	Cc1ccc2c(c1)CC[C@H]2NC(=O)[C@ H]1CC(c2ccccc2)=NO1	56317067	24	320.15	3.29
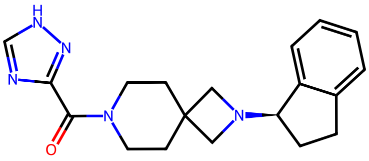	O=C(c1nc[nH]n1)N1CCC2(CC1)CN([C@@H]1CCc3ccccc31)C2	1035820224	25	337.19	2.03
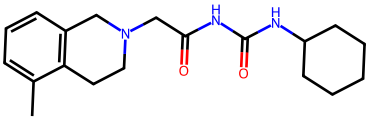	Cc1cccc2c1CCN(CC(=O)NC(=O)NC1 CCCCC1)C2	729480293	24	329.21	2.51
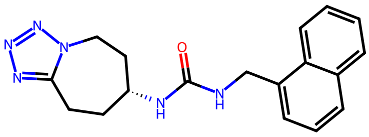	O=C(NCc1cccc2ccccc12)N[C@@H]1 CCc2nnnn2CC1	643377779	25	336.17	2.03

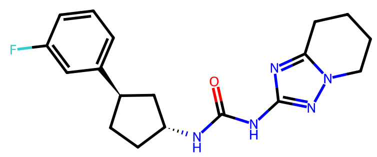	O=C(Nc1nc2n(n1)CCCC2)N[C@@H]1CC[C@@H](c2cccc(F)c2)C1	580161371	25	343.18	3.21
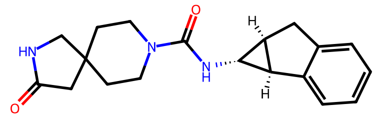	O=C1CC2(CCN(C(=O)N[C@@H]3[C@H]4Cc5ccccc5[C@H]43)CC2)CN1	268681933	24	325.18	1.64
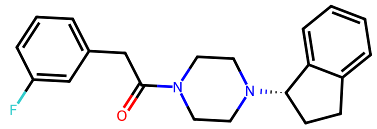	O=C(Cc1cccc(F)c1)N1CCN([C@H]2CCc3ccccc32)CC1	957558375	25	338.18	3.2
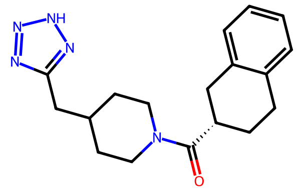	O=C([C@H]1CCc2ccccc2C1)N1CCC(Cc2nn[nH]n2)CC1	1548805350	24	325.19	1.79
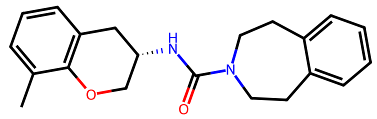	Cc1cccc2c1OC[C@@H](NC(=O)N1CCc3ccccc3CC1)C2	1682905669	25	336.18	3.11
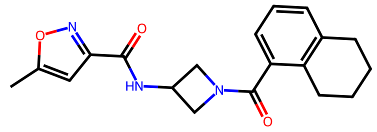	Cc1cc(C(=O)NC2CN(C(=O)c3cccc4c3CCCC4)C2)no1	990173508	25	339.16	2.12
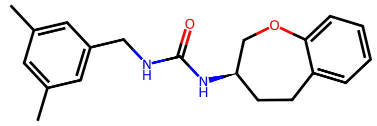	Cc1cc(C)cc(CNC(=O)N[C@@H]2CCc3ccccc3OC2)c1	1235747810	24	324.18	3.5
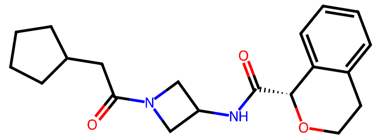	O=C(NC1CN(C(=O)CC2CCCC2)C1)[C@H]1OCCc2ccccc21	997322862	25	342.19	2.21

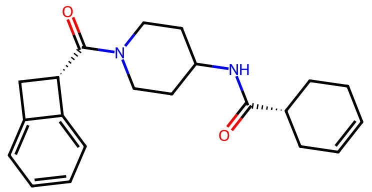	O=C(NC1CCN(C(=O)[C@H]2Cc3cccc c32)CC1)[C@H]1CC=CCC1	948059699	25	338.2	2.79
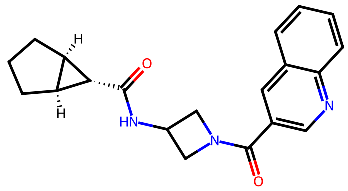	O=C(NC1CN(C(=O)c2cnc3ccccc3c2) C1)[C@@H]1[C@H]2CCC[C@H]21	990713128	25	335.16	2.22
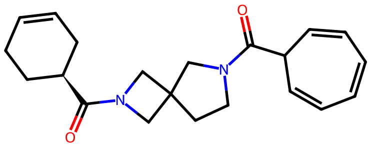	O=C(C1C=CC=CC=C1)N1CCC2(C1) CN(C(=O)[C@@H]1CC=CCC1)C2	1018772872	25	338.2	2.7
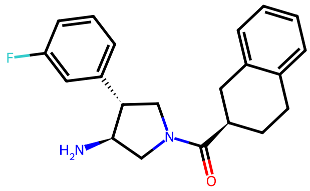	N[C@@H]1CN(C(=O)[C@@H]2CCc3 ccccc3C2)C[C@H]1c1cccc(F)c1	653164219	25	338.18	2.88
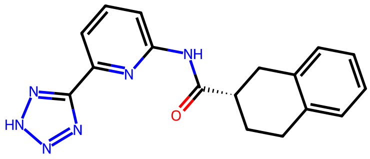	O=C(Nc1cccc(-c2nn[nH]n2)n1)[C@H] 1CCc2ccccc2C1	1582645299	24	320.14	2.01
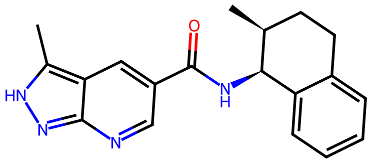	Cc1[nH]nc2ncc(C(=O)N[C@@H]3c4cc ccc4CC[C@@H]3C)cc12	342885253	24	320.16	3.32
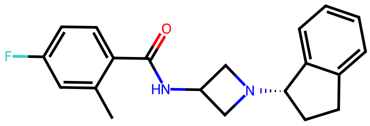	Cc1cc(F)ccc1C(=O)NC1CN([C@H]2C Cc3ccccc32)C1	1031287792	24	324.16	3.24
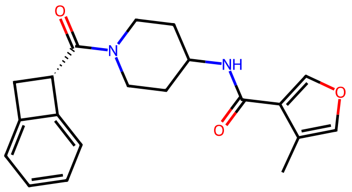	Cc1cocc1C(=O)NC1CCN(C(=O)[C@H]2Cc3ccccc32)CC1	948063436	25	338.16	2.65

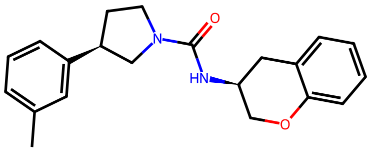	Cc1cccc([C@H]2CCN(C(=O)N[C@@H]3COc4ccccc4C3)C2)c1	542426544	25	336.18	3.5
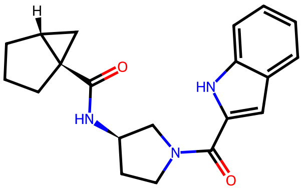	O=C(c1cc2ccccc2[nH]1)N1CC[C@@H](NC(=O)[C@@]23CCC[C@@H]2C3)C1	936158049	25	337.18	2.69
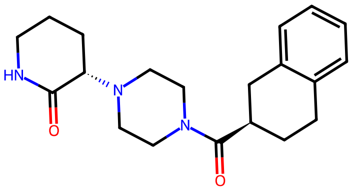	O=C1NCCC[C@@H]1N1CCN(C(=O)[C@@H]2CCc3ccccc3C2)CC1	950725693	25	341.21	1.21
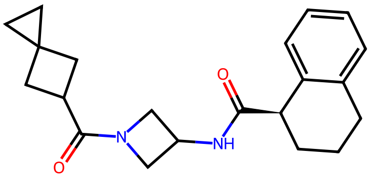	O=C(NC1CN(C(=O)C2CC3(CC3)C2)C1)[C@@H]1CCCc2ccccc21	995072212	25	338.2	2.62
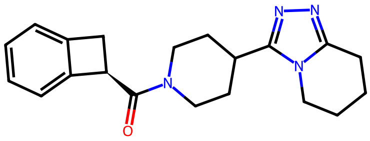	O=C([C@@H]1Cc2ccccc21)N1CCC(c2nnc3n2CCCC3)CC1	330011646	25	336.2	2.66
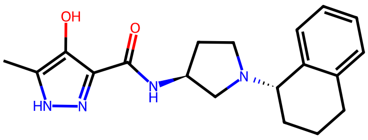	Cc1[nH]nc(C(=O)N[C@H]2CCN([C@H]3CCCc4ccccc43)C2)c1O	1010645499	25	340.19	2.31
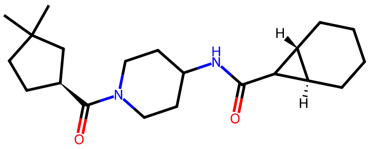	CC1(C)CC[C@H](C(=O)N2CCC(NC(=O)C3[C@@H]4CCCC[C@@H]34)CC2)C1	948254807	25	346.26	3.36
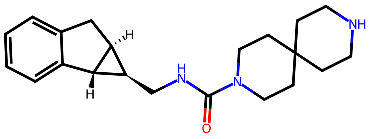	O=C(NC[C@@H]1[C@@H]2Cc3ccccc3[C@@H]12)N1CCC2(CCNCC2)CC1	417915132	25	339.23	2.75

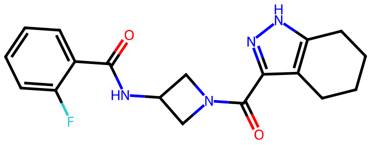	O=C(NC1CN(C(=O)c2n[nH]c3c2CCCC3)C1)c1ccccc1F	990093069	25	342.15	1.68
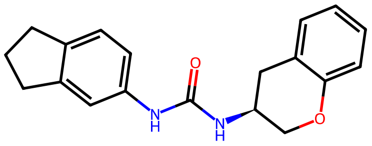	O=C(Nc1ccc2c(c1)CCC2)N[C@@H]1COc2ccccc2C1	47720564	23	308.15	3.3
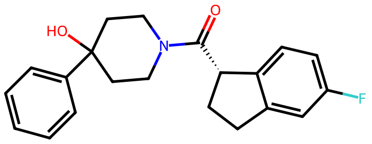	O=C([C@H]1CCc2cc(F)ccc21)N1CCC(O)(c2ccccc2)CC1	832266294	25	339.16	3.37
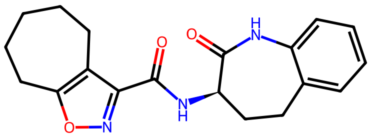	O=C(N[C@@H]1CCc2ccccc2NC1=O)c1noc2c1CCCCC2	653999688	25	339.16	2.63
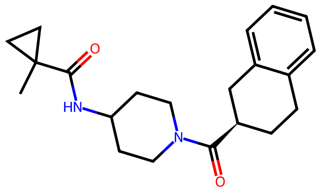	CC1(C(=O)NC2CCN(C(=O)[C@@H]3CCc4ccccc4C3)CC2)CC1	948572413	25	340.22	2.7
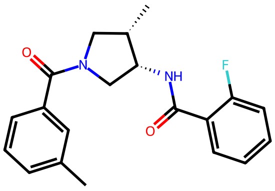	Cc1cccc(C(=O)N2C[C@H](C)[C@H](NC(=O)c3ccccc3F)C2)c1	966118386	25	340.16	3.02
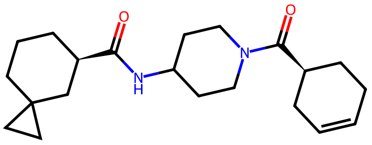	O=C(NC1CCN(C(=O)[C@@H]2CC=CCC2)CC1)[C@@H]1CCCC2(CC2)C1	944106678	25	344.25	3.42
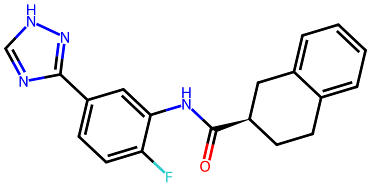	O=C(Nc1cc(-c2nc[nH]n2)ccc1F)[C@@H]1CCc2ccccc2C1	1524729928	25	336.14	3.35

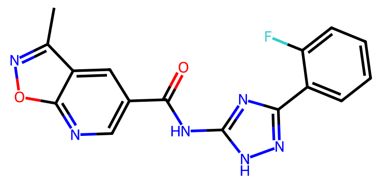	Cc1noc2ncc(C(=O)Nc3nc(-c4ccccc4F)n[nH]3)cc12	109625093	25	338.09	2.71
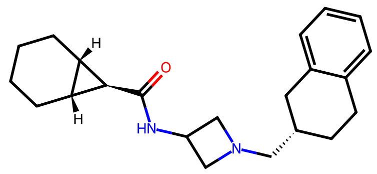	O=C(NC1CN(C[C@H]2CCc3ccccc3C2)C1)[C@@H]1[C@H]2CCCC[C@H]21	1030182372	25	338.24	3.03
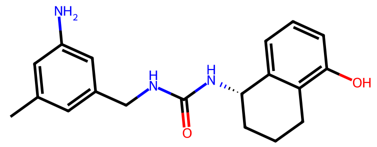	Cc1cc(N)cc(CNC(=O)N[C@H]2CCCc3c(O)cccc32)c1	418079992	24	325.18	3.16
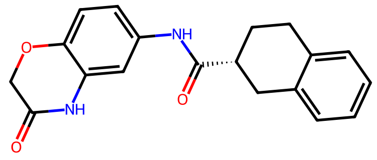	O=C1COc2ccc(NC(=O)[C@@H]3CCc4ccccc4C3)cc2N1	649531975	24	322.13	2.76
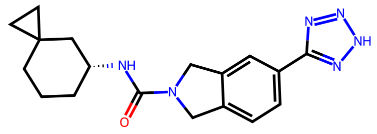	O=C(N[C@@H]1CCCC2(CC2)C1)N1Cc2ccc(-c3nn[nH]n3)cc2C1	1592979465	25	338.19	2.61
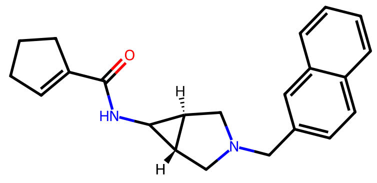	O=C(NC1[C@H]2CN(Cc3ccc4ccccc4c3)C[C@H]12)C1=CCCC1	958559997	25	332.19	3.5
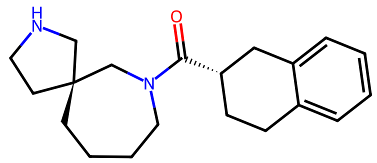	O=C([C@H]1CCc2ccccc2C1)N1CCCC[C@]2(CCNC2)C1	1550236240	23	312.22	2.78
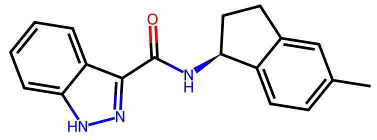	Cc1ccc2c(c1)CC[C@@H]2NC(=O)c1n[nH]c2ccccc12	55658790	22	291.14	3.29

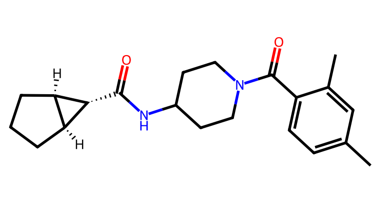	Cc1ccc(C(=O)N2CCC(NC(=O)[C@@H]3[C@H]4CCC[C@H]43)CC2)c(C)c1	948346347	25	340.22	3.07
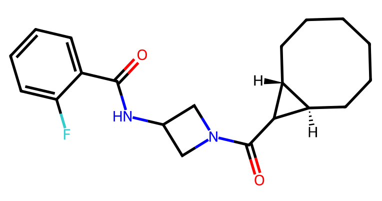	O=C(NC1CN(C(=O)C2[C@@H]3CCCCCC[C@@H]23)C1)c1ccccc1F	990092424	25	344.19	2.98
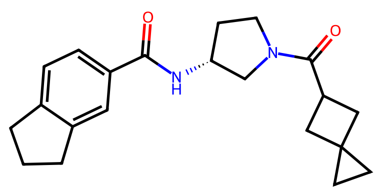	O=C(N[C@@H]1CCN(C(=O)C2CC3(CC3)C2)C1)c1ccc2c(c1)CCC2	940375823	25	338.2	2.7
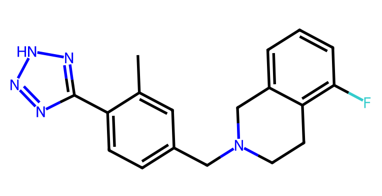	Cc1cc(CN2CCc3c(F)cccc3C2)ccc1-c1nn[nH]n1	1591964609	24	323.15	2.87
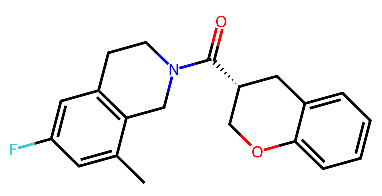	Cc1cc(F)cc2c1CN(C(=O)[C@H]1COc3ccccc3C1)CC2	501221700	24	325.15	3.27
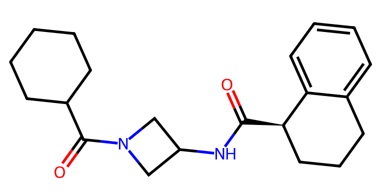	O=C(NC1CN(C(=O)C2CCCCC2)C1)[C@@H]1CCCc2ccccc21	995068214	25	340.22	3.01
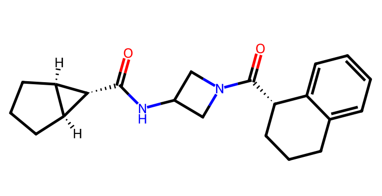	O=C(NC1CN(C(=O)[C@H]2CCCc3ccccc32)C1)[C@@H]1[C@H]2CCC[C@H]21	990708397	25	338.2	2.48
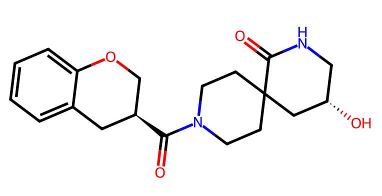	O=C([C@@H]1COc2ccccc2C1)N1CCC2(CC1)C[C@@H](O)CNC2=O	885026444	25	344.17	0.73

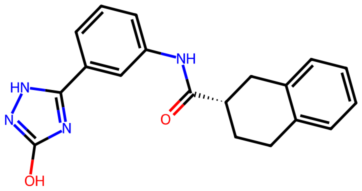	O=C(Nc1cccc(-c2nc(O)n[nH]2)c1)[C@H]1CCc2ccccc2C1	1635088950	25	334.14	2.92
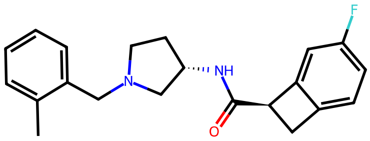	Cc1ccccc1CN1CC[C@H](NC(=O)[C@@H]2Cc3ccc(F)cc32)C1	1015615715	25	338.18	3.16
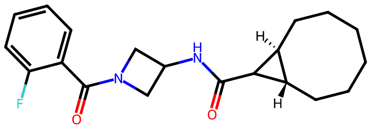	O=C(NC1CN(C(=O)c2ccccc2F)C1)C1[C@H]2CCCCCC[C@H]12	1000467939	25	344.19	2.98
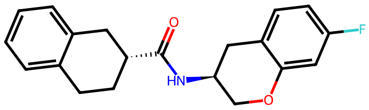	O=C(N[C@@H]1COc2cc(F)ccc2C1)[C@@H]1CCc2ccccc2C1	618657622	24	325.15	3.05
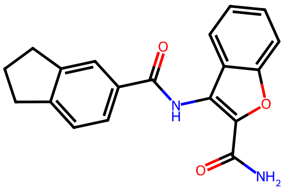	NC(=O)c1oc2ccccc2c1NC(=O)c1ccc2c(c1)CCC2	68535918	24	320.12	3.27
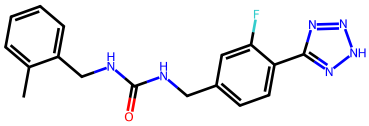	Cc1ccccc1CNC(=O)NCc1ccc(-c2nn[nH]n2)c(F)c1	1605939637	25	340.14	2.31
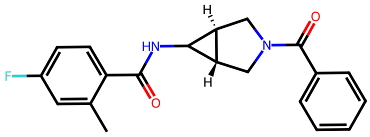	Cc1cc(F)ccc1C(=O)NC1[C@H]2CN(C(=O)c3ccccc3)C[C@H]12	1013518261	25	338.14	2.63
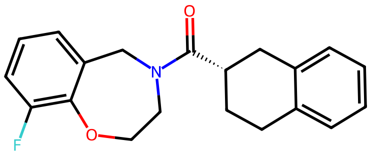	O=C([C@H]1CCc2ccccc2C1)N1CCOc2c(F)cccc2C1	570728358	24	325.15	3.35

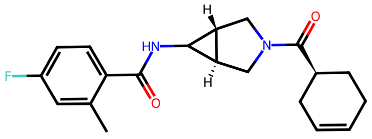	Cc1cc(F)ccc1C(=O)NC1[C@@H]2CN(C(=O)[C@@H]3CC=CCC3)C[C@@H]12	1013517354	25	342.17	2.68
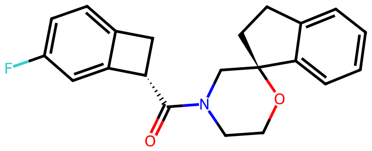	O=C([C@H]1Cc2ccc(F)cc21)N1CCO[C@]2(CCc3ccccc32)C1	684466779	25	337.15	3.17
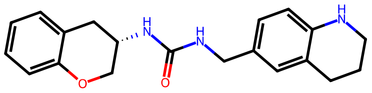	O=C(NCc1ccc2c(c1)CCCN2)N[C@@H]1COc2ccccc2C1	579452370	25	337.18	2.85
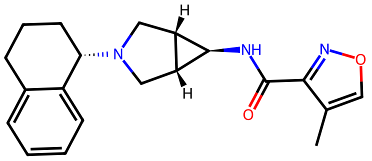	Cc1conc1C(=O)N[C@H]1[C@@H]2CN([C@H]3CCCc4ccccc43)C[C@@H]21	962216965	25	337.18	2.72
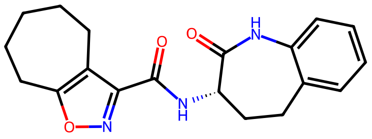	O=C(N[C@H]1CCc2ccccc2NC1=O)c1noc2c1CCCCC2	653999687	25	339.16	2.63
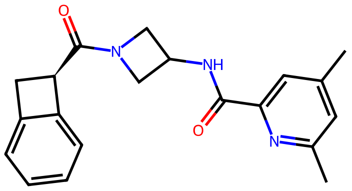	Cc1cc(C)nc(C(=O)NC2CN(C(=O)[C@@H]3Cc4ccccc43)C2)c1	995014584	25	335.16	1.98
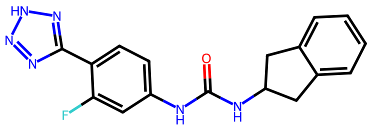	O=C(Nc1ccc(-c2nn[nH]n2)c(F)c1)NC1Cc2ccccc2C1	1609563028	25	338.13	2.29
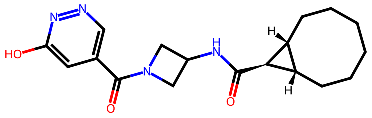	O=C(NC1CN(C(=O)c2cnnc(O)c2)C1)[C@@H]1[C@H]2CCCCCC[C@H]21	1000510105	25	344.18	1.34

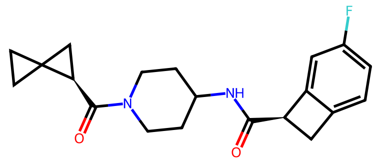	O=C(NC1CCN(C(=O)[C@@H]2CC23CC3)CC1)[C@@H]1Cc2ccc(F)cc21	948568398	25	342.17	2.37
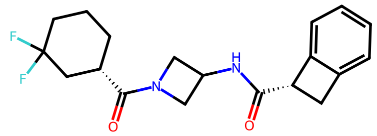	O=C(NC1CN(C(=O)[C@H]2CCCC(F)(F)C2)C1)[C@H]1Cc2ccccc21	998689500	25	348.16	2.48
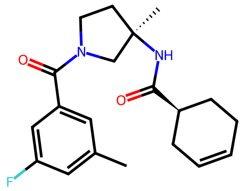	Cc1cc(F)cc(C(=O)N2CC[C@@](C)(NC(=O)[C@@H]3CC=CCC3)C2)c1	956689818	25	344.19	3.21
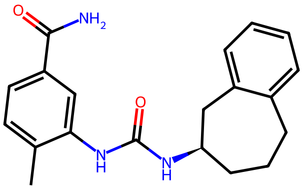	Cc1ccc(C(N)=O)cc1NC(=O)N[C@@H]1CCCc2ccccc2C1	930016466	25	337.18	3.16
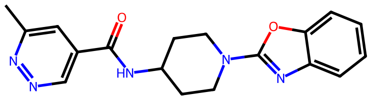	Cc1cc(C(=O)NC2CCN(c3nc4ccccc4o3)CC2)cnn1	1057862631	25	337.15	2.33
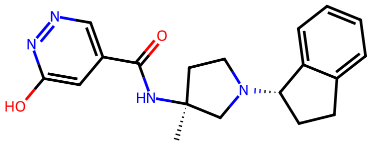	C[C@]1(NC(=O)c2cnnc(O)c2)CCN([C@H]2CCc3ccccc32)C1	1046399301	25	338.17	2.06
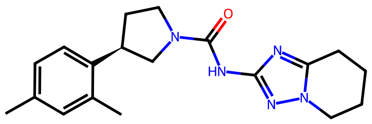	Cc1ccc([C@H]2CCN(C(=O)Nc3nc4n(n3)CCCC4)C2)c(C)c1	333723934	25	339.21	3.25
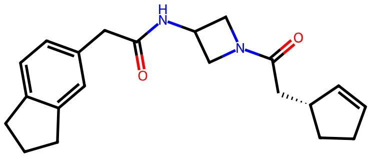	O=C(Cc1ccc2c(c1)CCC2)NC1CN(C(=O)C[C@@H]2C=CCC2)C1	991383285	25	338.2	2.4

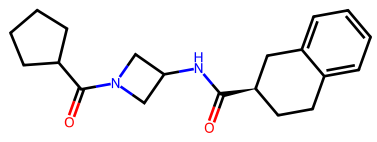	O=C(NC1CN(C(=O)C2CCCC2)C1)[C@@H]1CCc2ccccc2C1	1000539390	24	326.2	2.31
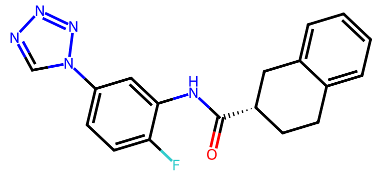	O=C(Nc1cc(-n2cnnn2)ccc1F)[C@H]1CCc2ccccc2C1	1435715939	25	337.13	2.55
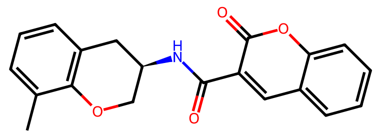	Cc1cccc2c1OC[C@H](NC(=O)c1cc3ccccc3oc1=O)C2	365645626	25	335.12	2.83
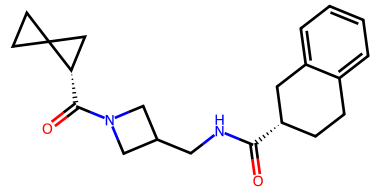	O=C(NCC1CN(C(=O)[C@H]2CC23CC3)C1)[C@H]1CCc2ccccc2C1	936100056	25	338.2	2.17
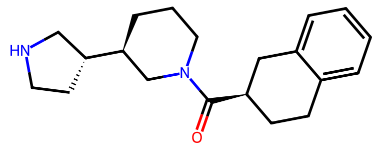	O=C([C@@H]1CCc2ccccc2C1)N1CCC[C@H]([C@@H]2CCNC2)C1	573262482	23	312.22	2.64
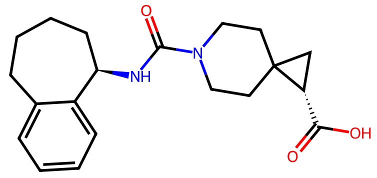	O=C(O)[C@H]1CC12CCN(C(=O)N[C@@H]1CCCCc3ccccc31)CC2	739527315	25	342.19	3.35
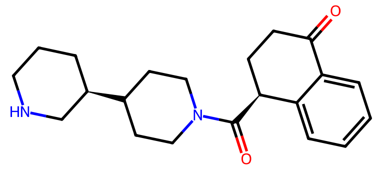	O=C1CC[C@H](C(=O)N2CCC([C@@H]3CCCNC3)CC2)c2ccccc21	662587755	25	340.22	2.98
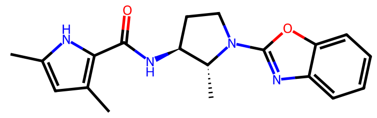	Cc1cc(C)c(C(=O)N[C@H]2CCN(c3nc4ccccc4o3)[C@@H]2C)[nH]1	1051215370	25	338.17	3.17

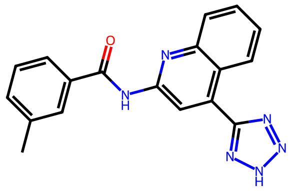	Cc1cccc(C(=O)Nc2cc(-c3nn[nH]n3)c3ccccc3n2)c1	1592221197	25	330.12	2.98
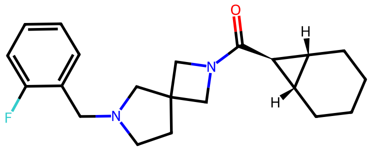	O=C([C@@H]1[C@H]2CCCC[C@H]21)N1CC2(CCN(Cc3ccccc3F)C2)C1	1072581108	25	342.21	3.3
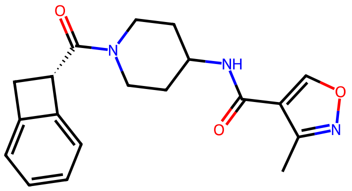	Cc1nocc1C(=O)NC1CCN(C(=O)[C@H]2Cc3ccccc32)CC1	948059485	25	339.16	2.04
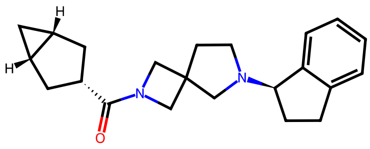	O=C([C@@H]1C[C@@H]2C[C@@H]2C1)N1CC2(CCN([C@@H]3CCc4ccccc43)C2)C1	1072907615	25	336.22	3.25
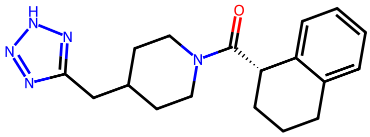	O=C([C@H]1CCCc2ccccc21)N1CCC(Cc2nn[nH]n2)CC1	1548805092	24	325.19	2.1
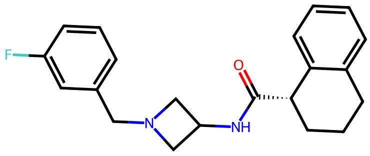	O=C(NC1CN(Cc2cccc(F)c2)C1)[C@H]1CCCc2ccccc21	1030528644	25	338.18	3.25
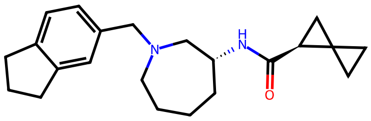	O=C(N[C@@H]1CCCCN(Cc2ccc3c(c2)CCC3)C1)[C@H]1CC12CC2	1034416884	25	338.24	3.45
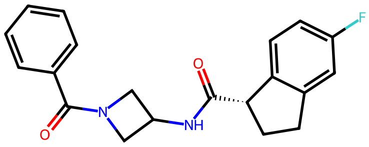	O=C(NC1CN(C(=O)c2ccccc2)C1)[C@H]1CCc2cc(F)ccc21	996099893	25	338.14	2.5

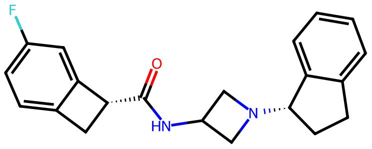	O=C(NC1CN([C@H]2CCc3ccccc32)C1)[C@@H]1Cc2ccc(F)cc21	1030847158	25	336.16	2.95
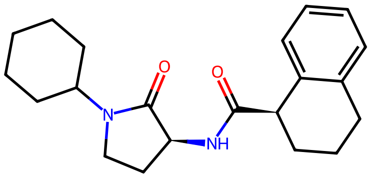	O=C(N[C@H]1CCN(C2CCCCC2)C1=O)[C@@H]1CCCc2ccccc21	675913093	25	340.22	3.16
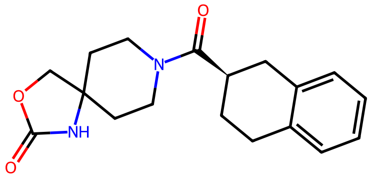	O=C1NC2(CCN(C(=O)[C@@H]3CCc4ccccc4C3)CC2)CO1	496739949	23	314.16	1.89
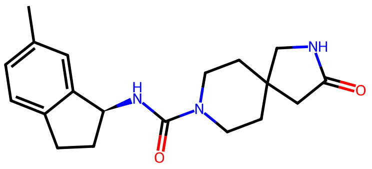	Cc1ccc2c(c1)[C@@H](NC(=O)N1CCC3(CC1)CNC(=O)C3)CC2	752009383	24	327.19	2.29
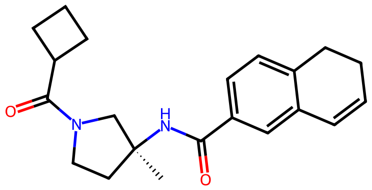	C[C@]1(NC(=O)c2ccc3c(c2)C=CCC3)CCN(C(=O)C2CCC2)C1	957193866	25	338.2	3.17
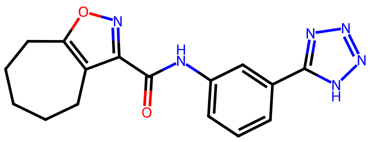	O=C(Nc1cccc(-c2nnn[nH]2)c1)c1noc2c1CCCCC2	275578895	24	324.13	2.38
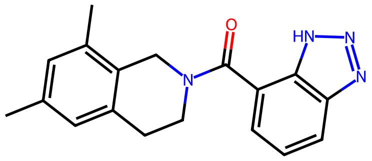	Cc1cc(C)c2c(c1)CCN(C(=O)c1cccc3n[nH]c13)C2	188876470	23	306.15	2.77
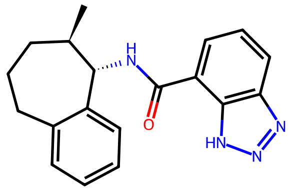	C[C@@H]1CCCc2ccccc2[C@H]1NC(=O)c1cccc2nn[nH]c12	1138085093	24	320.16	3.4

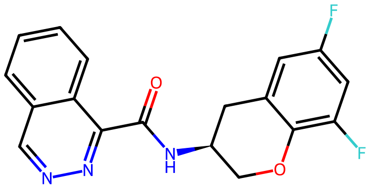	O=C(N[C@@H]1COc2c(F)cc(F)cc2C1)c1nncc2ccccc12	1640471898	25	341.1	2.64
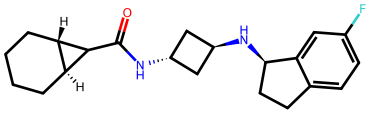	O=C(N[C@H]1C[C@H](N[C@@H]2CCc3ccc(F)cc32)C1)C1[C@H]2CCCC[C@H]12	1020047963	25	342.21	3.49
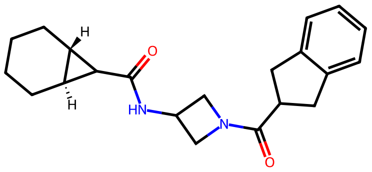	O=C(NC1CN(C(=O)C2Cc3ccccc3C2)C1)C1[C@H]2CCCC[C@H]12	990659125	25	338.2	2.16
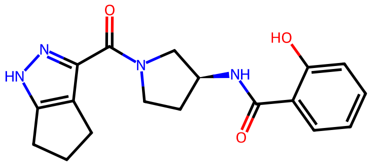	O=C(N[C@H]1CCN(C(=O)c2n[nH]c3c2CCC3)C1)c1ccccc1O	937691490	25	340.15	1.25
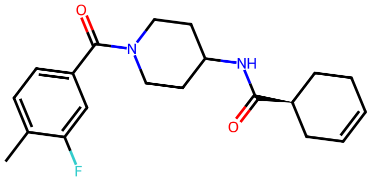	Cc1ccc(C(=O)N2CCC(NC(=O)[C@@H]3CC=CCC3)CC2)cc1F	947403593	25	344.19	3.21
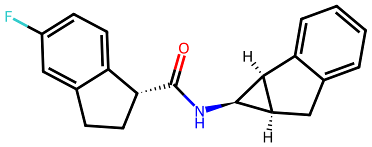	O=C(N[C@@H]1[C@@H]2Cc3ccccc3[C@@H]21)[C@@H]1CCc2cc(F)ccc21	612495120	23	307.14	3.31
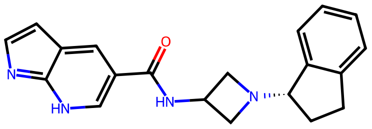	O=C(NC1CN([C@H]2CCc3ccccc32)C1)c1c[nH]c2nccc-2c1	1030482101	25	332.16	2.62
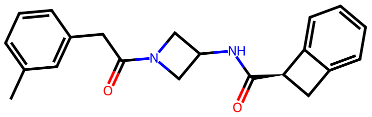	Cc1cccc(CC(=O)N2CC(NC(=O)[C@@H]3Cc4ccccc43)C2)c1	998691929	25	334.17	2.2

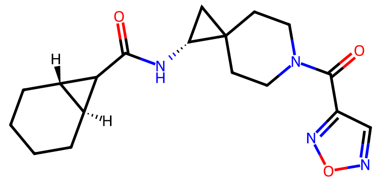	O=C(N[C@@H]1CC12CCN(C(=O)c1cnon1)CC2)C1[C@H]2CCCC[C@H]12	963448093	25	344.18	1.62
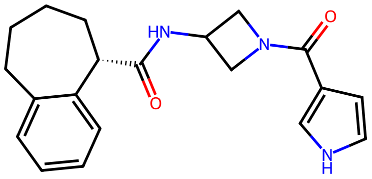	O=C(NC1CN(C(=O)c2cc[nH]c2)C1)[C@H]1CCCCc2ccccc21	994322074	25	337.18	2.47
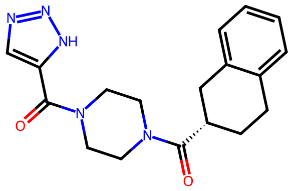	O=C(c1cnn[nH]1)N1CCN(C(=O)[C@H]2CCc3ccccc3C2)CC1	1011448433	25	339.17	0.89
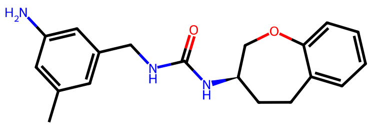	Cc1cc(N)cc(CNC(=O)N[C@@H]2CCc3ccccc3OC2)c1	418152618	24	325.18	2.77
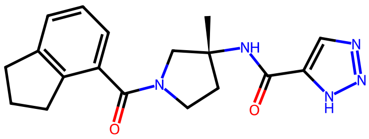	C[C@@]1(NC(=O)c2cnn[nH]2)CCN(C(=O)c2cccc3c2CCC3)C1	957187270	25	339.17	1.33
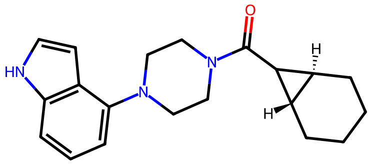	O=C(C1[C@H]2CCCC[C@H]12)N1CCN(c2cccc3[nH]ccc23)CC1	616751580	24	323.2	3.25
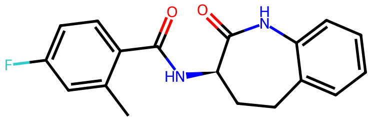	Cc1cc(F)ccc1C(=O)N[C@@H]1CCc2ccccc2NC1=O	1411464150	23	312.13	2.82
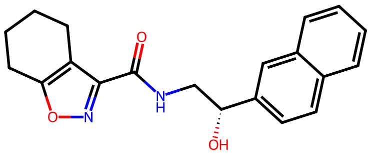	O=C(NC[C@@H](O)c1ccc2ccccc2c1)c1noc2c1CCCC2	109691392	25	336.15	3.17

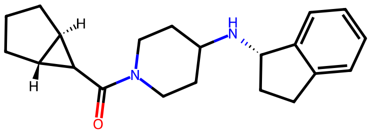	O=C(C1[C@@H]2CCC[C@@H]12)N1CCC(N[C@H]2CCc3ccccc32)CC1	990770124	24	324.22	3.3
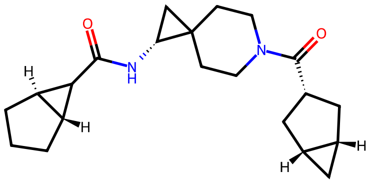	O=C(N[C@@H]1CC12CCN(C(=O)[C@@H]1C[C@@H]3C[C@@H]3C1)CC2)C1[C@@H]2CCC[C@@H]12	963491146	25	342.23	2.58
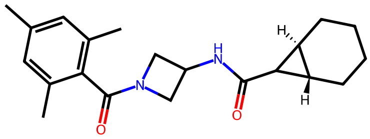	Cc1cc(C)c(C(=O)N2CC(NC(=O)C3[C@H]4CCCC[C@H]34)C2)c(C)c1	990673983	25	340.22	2.99
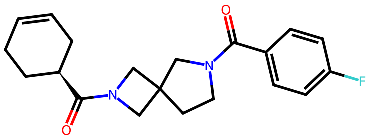	O=C(c1ccc(F)cc1)N1CCC2(C1)CN(C(=O)[C@@H]1CC=CCC1)C2	1018769166	25	342.17	2.86
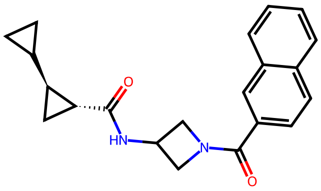	O=C(NC1CN(C(=O)c2ccc3ccccc3c2)C1)[C@@H]1C[C@H]1C1CC1	992000289	25	334.17	2.83
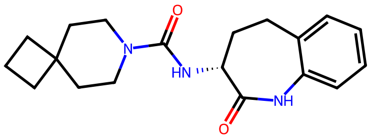	O=C1Nc2ccccc2CC[C@H]1NC(=O)N1CCC2(CCC2)CC1	654634281	24	327.19	2.92
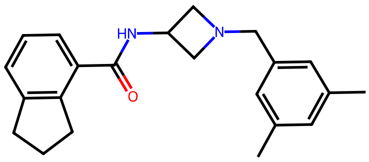	Cc1cc(C)cc(CN2CC(NC(=O)c3cccc4c3CCC4)C2)c1	1031464711	25	334.2	3.41
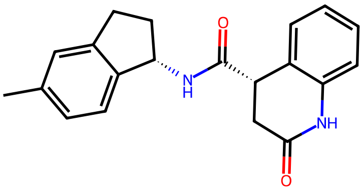	Cc1ccc2c(c1)CC[C@@H]2NC(=O)[C@@H]1CC(=O)Nc2ccccc21	56316914	24	320.15	3.22

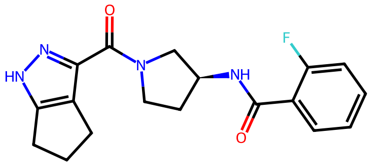	O=C(N[C@H]1CCN(C(=O)c2n[nH]c3c2CCC3)C1)c1ccccc1F	937625360	25	342.15	1.68
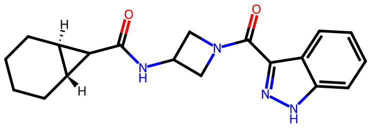	O=C(NC1CN(C(=O)c2n[nH]c3ccccc23)C1)C1[C@@H]2CCCC[C@@H]12	990670481	25	338.17	1.94
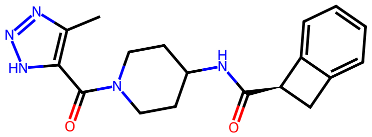	Cc1nn[nH]c1C(=O)N1CCC(NC(=O)[C@@H]2Cc3ccccc32)CC1	947680473	25	339.17	1.17
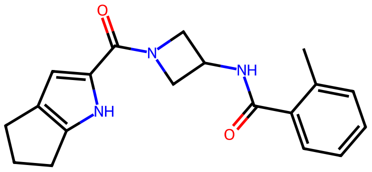	Cc1ccccc1C(=O)NC1CN(C(=O)c2cc3c([nH]2)CCC3)C1	990104917	24	323.16	2.07
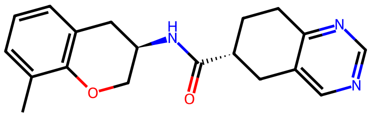	Cc1cccc2c1OC[C@H](NC(=O)[C@@H]1CCc3ncncc3C1)C2	648396894	24	323.16	2.01
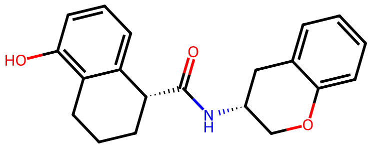	O=C(N[C@H]1COc2ccccc2C1)[C@@H]1CCCc2c(O)cccc21	190124914	24	323.15	2.93
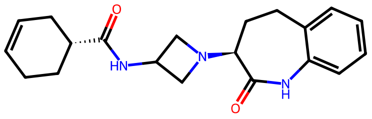	O=C1Nc2ccccc2CC[C@@H]1N1CC(NC(=O)[C@H]2CC=CCC2)C1	1030098288	25	339.19	2.1
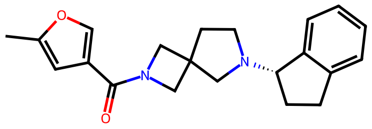	Cc1cc(C(=O)N2CC3(CCN([C@H]4CCc5ccccc54)C3)C2)co1	1072661188	25	336.18	3.42

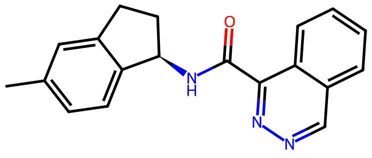	Cc1ccc2c(c1)CC[C@H]2NC(=O)c1nnc c2ccccc12	1356042781	23	303.14	3.36
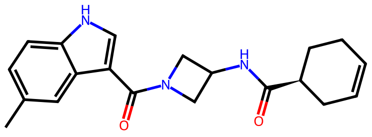	Cc1ccc2[nH]cc(C(=O)N3CC(NC(=O)[C@@H]4CC=CCC4)C3)c2c1	990216952	25	337.18	2.77
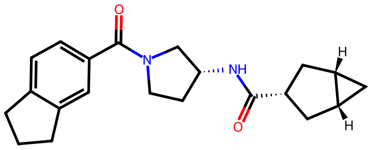	O=C(N[C@@H]1CCN(C(=O)c2ccc3c(c2)CCC3)C1)[C@@H]1C[C@@H]2C[C@@H]2C1	942231177	25	338.2	2.55
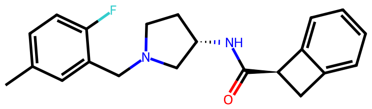	Cc1ccc(F)c(CN2CC[C@H](NC(=O)[C @@H]3Cc4ccccc43)C2)c1	1015632567	25	338.18	3.16
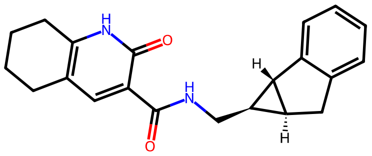	O=C(NC[C@@H]1[C@@H]2Cc3cccc c3[C@@H]12)c1cc2c([nH]c1=O)CCC C2	152424144	25	334.17	2.57
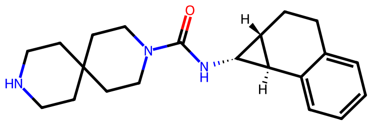	O=C(N[C@H]1[C@@H]2c3ccccc3CC[C@H]21)N1CCC2(CCNCC2)CC1	417916848	25	339.23	2.89
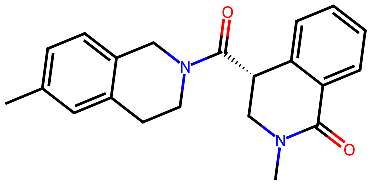	Cc1ccc2c(c1)CCN(C(=O)[C@H]1CN(C)C(=O)c3ccccc31)C2	362506232	25	334.17	2.75
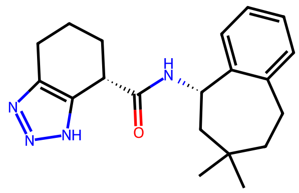	CC1(C)CCc2ccccc2[C@@H](NC(=O)[C@H]2CCCc3nn[nH]c32)C1	646966700	25	338.21	3.44

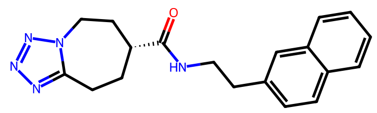	O=C(NCCc1ccc2ccccc2c1)[C@@H]1CCc2nnnn2CC1	565368842	25	335.17	2.14
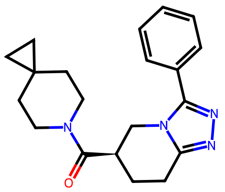	O=C([C@@H]1CCc2nnc(-c3ccccc3)n2C1)N1CCC2(CC1)CC2	648924081	25	336.2	2.91
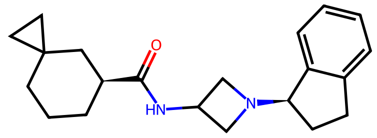	O=C(NC1CN([C@@H]2CCc3ccccc32)C1)[C@H]1CCCC2(CC2)C1	1030307334	24	324.22	3.44
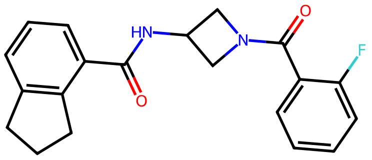	O=C(NC1CN(C(=O)c2ccccc2F)C1)c1cccc2c1CCC2	994399739	25	338.14	2.57
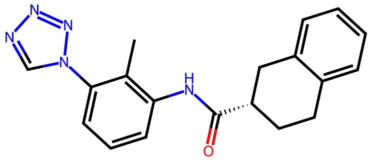	Cc1c(NC(=O)[C@H]2CCc3ccccc3C2)cccc1-n1cnnn1	566299266	25	333.16	2.71
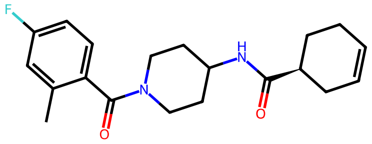	Cc1cc(F)ccc1C(=O)N1CCC(NC(=O)[C@@H]2CC=CCC2)CC1	947642496	25	344.19	3.21
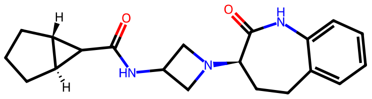	O=C(NC1CN([C@@H]2CCc3ccccc3NC2=O)C1)C1[C@H]2CCC[C@H]12	1030202507	25	339.19	1.79
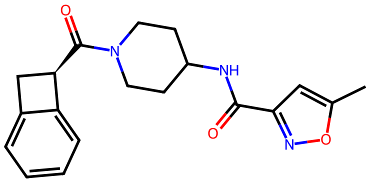	Cc1cc(C(=O)NC2CCN(C(=O)[C@@H]3Cc4ccccc43)CC2)no1	948059622	25	339.16	2.04

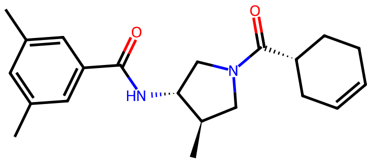	Cc1cc(C)cc(C(=O)N[C@@H]2CN(C(=O)[C@H]3CC=CCC3)C[C@H]2C)c1	940110031	25	340.22	3.24
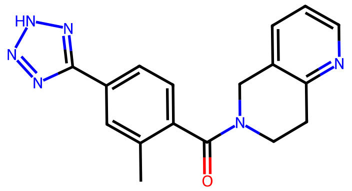	Cc1cc(-c2nn[nH]n2)ccc1C(=O)N1CCc2ncccc2C1	826245855	24	320.14	1.77
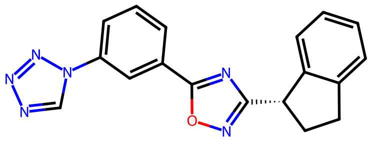	c1cc(-c2nc([C@H]3CCc4ccccc43)no2)cc(-n2cnnn2)c1	356654144	25	330.12	2.79
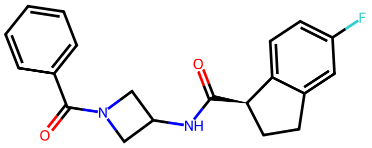	O=C(NC1CN(C(=O)c2ccccc2)C1)[C@@H]1CCc2cc(F)ccc21	996099891	25	338.14	2.5
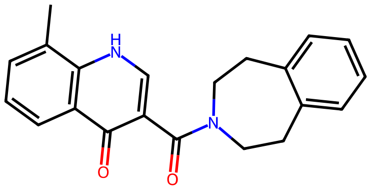	Cc1cccc2c(=O)c(C(=O)N3CCc4ccccc4CC3)c[nH]c12	547533369	25	332.15	3.08
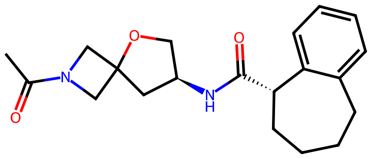	CC(=O)N1CC2(C[C@H](NC(=O)[C@H]3CCCCc4ccccc43)CO2)C1	965739652	25	342.19	2.0
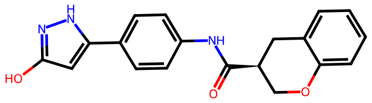	O=C(Nc1ccc(-c2cc(O)n[nH]2)cc1)[C@@H]1COc2ccccc2C1	339118673	25	335.13	2.97
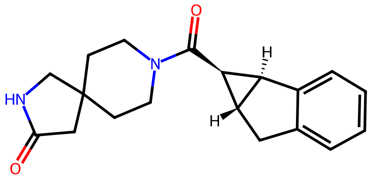	O=C1CC2(CCN(C(=O)[C@@H]3[C@H]4Cc5ccccc5[C@@H]43)CC2)CN1	859774217	23	310.17	1.7

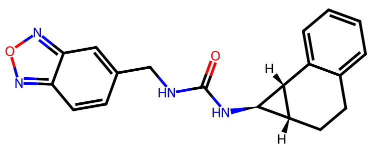	O=C(NCc1ccc2nonc2c1)N[C@@H]1[C@H]2CCc3ccccc3[C@H]21	893766473	25	334.14	2.75
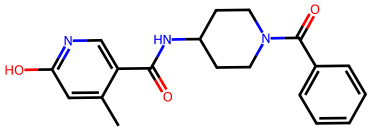	Cc1cc(O)ncc1C(=O)NC1CCN(C(=O)c2ccccc2)CC1	345577734	25	339.16	2.13
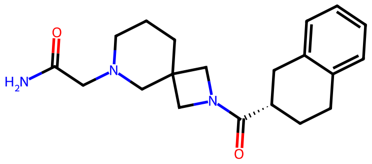	NC(=O)CN1CCCC2(C1)CN(C(=O)[C@H]1CCc3ccccc3C1)C2	982037133	25	341.21	1.2
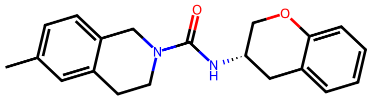	Cc1ccc2c(c1)CCN(C(=O)N[C@@H]1COc3ccccc3C1)C2	478814396	24	322.17	3.07
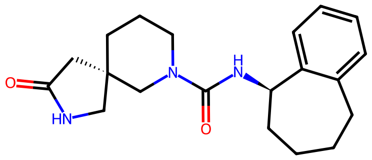	O=C1C[C@@]2(CCCN(C(=O)N[C@@H]3CCCCc4ccccc43)C2)CN1	525766803	25	341.21	2.77
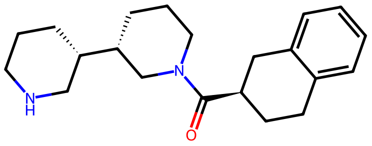	O=C([C@@H]1CCc2ccccc2C1)N1CCC[C@@H]([C@H]2CCCNC2)C1	653143384	24	326.24	3.03
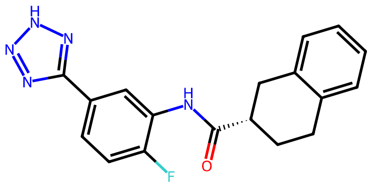	O=C(Nc1cc(-c2nn[nH]n2)ccc1F)[C@H]1CCc2ccccc2C1	1525064648	25	337.13	2.75
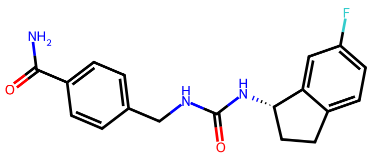	NC(=O)c1ccc(CNC(=O)N[C@H]2CCc3ccc(F)cc32)cc1	106533270	24	327.14	2.41

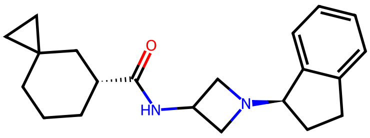	O=C(NC1CN([C@@H]2CCc3ccccc32)C1)[C@@H]1CCCC2(CC2)C1	1030307335	24	324.22	3.44
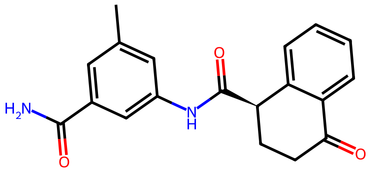	Cc1cc(NC(=O)[C@@H]2CCC(=O)c3ccccc32)cc(C(N)=O)c1	1412124009	24	322.13	2.79
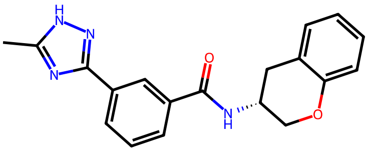	Cc1nc(-c2cccc(C(=O)N[C@H]3COc4ccccc4C3)c2)n[nH]1	96446770	25	334.14	2.51
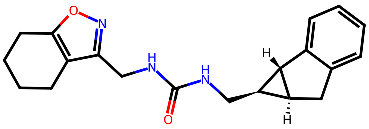	O=C(NCc1noc2c1CCCC2)NC[C@@H]1[C@@H]2Cc3ccccc3[C@@H]12	289331216	25	337.18	2.94
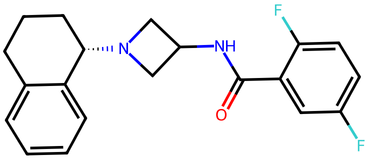	O=C(NC1CN([C@H]2CCCc3ccccc32)C1)c1cc(F)ccc1F	1030447216	25	342.15	3.46
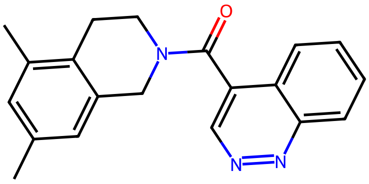	Cc1cc(C)c2c(c1)CN(C(=O)c1cnnc3ccccc13)CC2	529065921	24	317.15	3.45
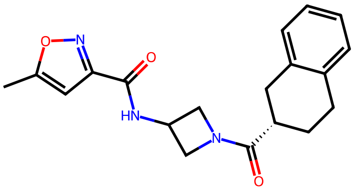	Cc1cc(C(=O)NC2CN(C(=O)[C@H]3CCc4ccccc4C3)C2)no1	990175072	25	339.16	1.73
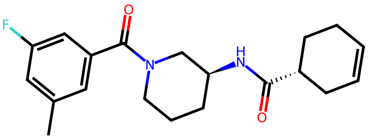	Cc1cc(F)cc(C(=O)N2CCC[C@H](NC(=O)[C@H]3CC=CCC3)C2)c1	952126915	25	344.19	3.21

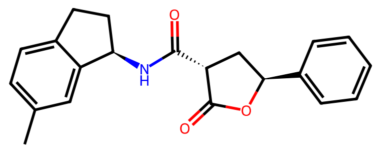	Cc1ccc2c(c1)[C@H](NC(=O)[C@@H]1C[C@@H](c3ccccc3)OC1=O)CC2	809668253	25	335.15	3.4
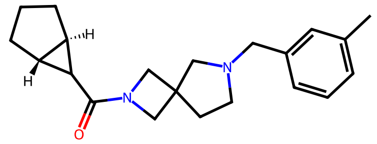	Cc1cccc(CN2CCC3(C2)CN(C(=O)C2[C@@H]4CCC[C@@H]24)C3)c1	1072591323	24	324.22	3.08
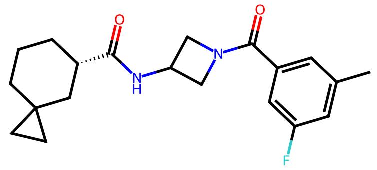	Cc1cc(F)cc(C(=O)N2CC(NC(=O)[C@H]3CCCC4(CC4)C3)C2)c1	991516948	25	344.19	3.05
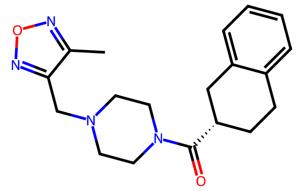	Cc1nonc1CN1CCN(C(=O)[C@H]2CCc3ccccc3C2)CC1	950725813	25	340.19	1.83
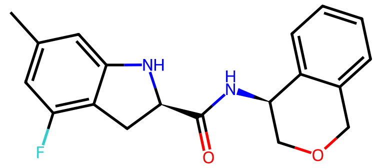	Cc1cc(F)c2c(c1)N[C@@H](C(=O)N[C@@H]1COCc3ccccc31)C2	1579611864	24	326.14	2.86
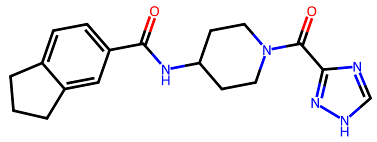	O=C(NC1CCN(C(=O)c2nc[nH]n2)CC1)c1ccc2c(c1)CCC2	947813417	25	339.17	1.33
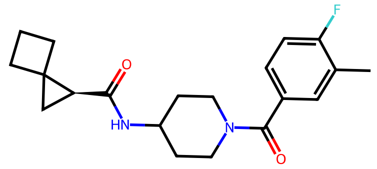	Cc1cc(C(=O)N2CCC(NC(=O)[C@H]3CC34CCC4)CC2)ccc1F	948065683	25	344.19	3.05
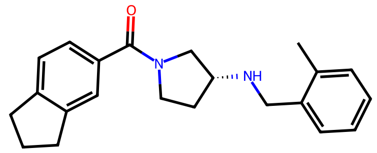	Cc1ccccc1CN[C@@H]1CCN(C(=O)c2ccc3c(c2)CCC3)C1	970683184	25	334.2	3.49

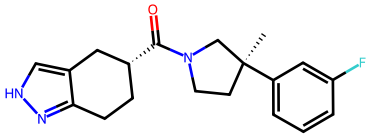	C[C@]1(c2cccc(F)c2)CCN(C(=O)[C@@H]2CCc3n[nH]cc3C2)C1	652619211	24	327.17	2.84
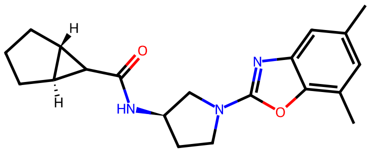	Cc1cc(C)c2oc(N3CC[C@@H](NC(=O)C4[C@H]5CCC[C@H]45)C3)nc2c1	1062761134	25	339.19	3.19
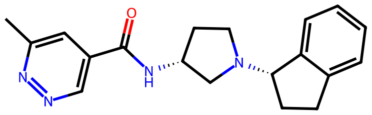	Cc1cc(C(=O)N[C@@H]2CCN([C@H]3CCc4ccccc43)C2)cnn1	1010769705	24	322.18	2.28
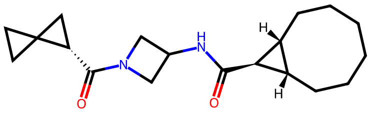	O=C(NC1CN(C(=O)[C@H]2CC23CC3)C1)[C@@H]1[C@H]2CCCCCC[C@H]21	1000511861	23	316.22	2.33
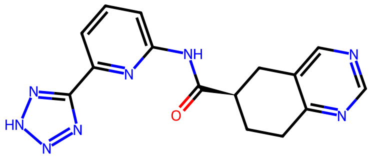	O=C(Nc1cccc(-c2nn[nH]n2)n1)[C@@H]1CCc2ncncc2C1	1609566997	24	322.13	0.8
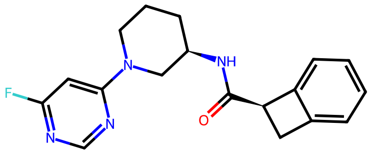	O=C(N[C@@H]1CCCN(c2cc(F)ncn2)C1)[C@@H]1Cc2ccccc21	1058231493	24	326.15	2.04
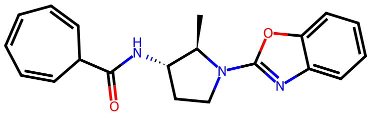	C[C@@H]1[C@@H](NC(=O)C2C=CC=CC=C2)CCN1c1nc2ccccc2o1	1050656190	25	335.16	3.21
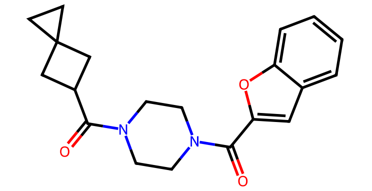	O=C(c1cc2ccccc2o1)N1CCN(C(=O)C2CC3(CC3)C2)CC1	1012531507	25	338.16	2.91

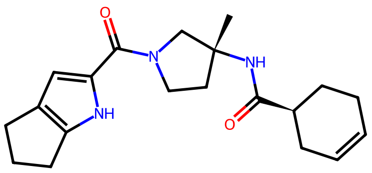	C[C@@]1(NC(=O)[C@@H]2CC=CCC2)CCN(C(=O)c2cc3c([nH]2)CCC3)C1	956689627	25	341.21	2.58
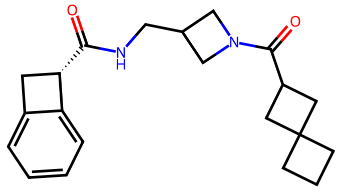	O=C(NCC1CN(C(=O)C2CC3(CCC3)C2)C1)[C@H]1Cc2ccccc21	1003947823	25	338.2	2.48
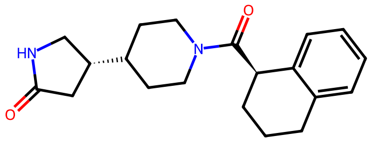	O=C1C[C@@H](C2CCN(C(=O)[C@@H]3CCCc4ccccc43)CC2)CN1	1551707196	24	326.2	2.48
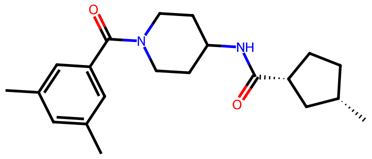	Cc1cc(C)cc(C(=O)N2CCC(NC(=O)[C@@H]3CC[C@H](C)C3)CC2)c1	948337770	25	342.23	3.46
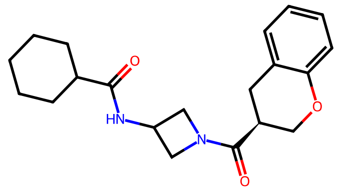	O=C(NC1CN(C(=O)[C@@H]2COc3ccccc3C2)C1)C1CCCCC1	990104451	25	342.19	2.14
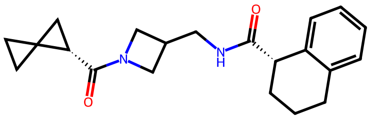	O=C(NCC1CN(C(=O)[C@H]2CC23CC3)C1)[C@H]1CCCc2ccccc21	1003035025	25	338.2	2.48
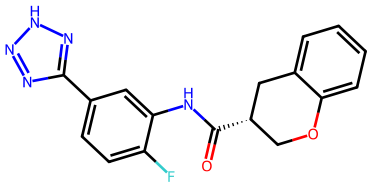	O=C(Nc1cc(-c2nn[nH]n2)ccc1F)[C@H]1COc2ccccc2C1	1275482837	25	339.11	2.2
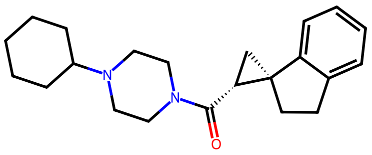	O=C([C@@H]1C[C@]12CCc1ccccc12)N1CCN(C2CCCCC2)CC1	482064736	25	338.24	3.37

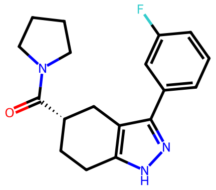	O=C([C@H]1CCc2[nH]nc(-c3cccc(F)c3)c2C1)N1CCCC1	306133091	23	313.16	2.94
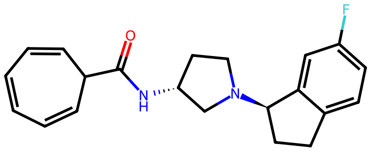	O=C(N[C@@H]1CCN([C@@H]2CCc3ccc(F)cc32)C1)C1C=CC=CC=C1	1014586054	25	338.18	3.3
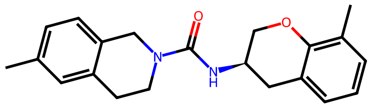	Cc1ccc2c(c1)CCN(C(=O)N[C@H]1COc3c(C)cccc3C1)C2	1229550430	25	336.18	3.37
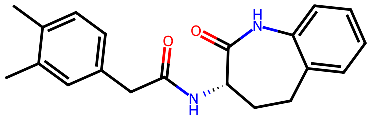	Cc1ccc(CC(=O)N[C@H]2CCc3ccccc3NC2=O)cc1C	654005793	24	322.17	2.92
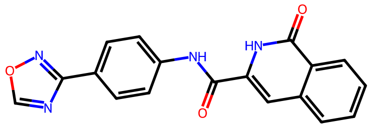	O=C(Nc1ccc(-c2ncon2)cc1)c1cc2ccccc2c(=O)[nH]1	171063102	25	332.09	2.83
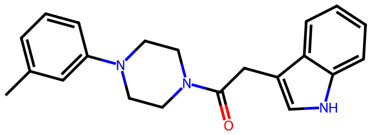	Cc1cccc(N2CCN(C(=O)Cc3c[nH]c4ccccc34)CC2)c1	16279082	25	333.18	3.37
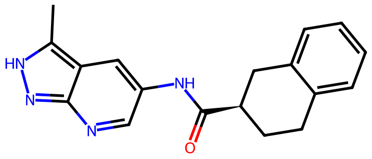	Cc1[nH]nc2ncc(NC(=O)[C@@H]3CCc4ccccc4C3)cc12	572247816	23	306.15	3.01
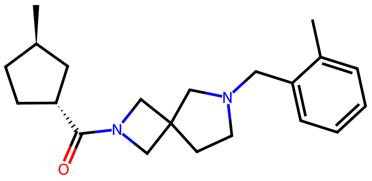	Cc1ccccc1CN1CCC2(C1)CN(C(=O)[C@@H]1CC[C@@H](C)C1)C2	1073033272	24	326.24	3.47

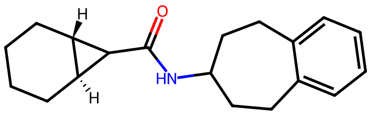	O=C(NC1CCc2ccccc2CC1)C1[C@H]2CCCC[C@H]12	456463500	21	283.19	3.49
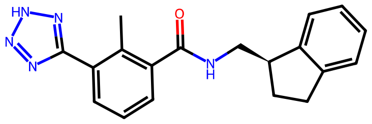	Cc1c(C(=O)NC[C@@H]2CCc3ccccc32)cccc1-c1nn[nH]n1	1591807564	25	333.16	2.63
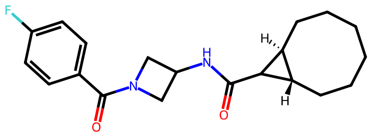	O=C(NC1CN(C(=O)c2ccc(F)cc2)C1)C1[C@H]2CCCCCC[C@H]12	1000467988	25	344.19	2.98
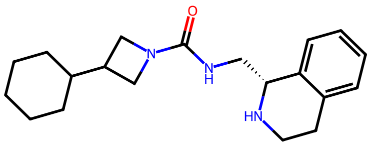	O=C(NC[C@H]1NCCc2ccccc21)N1CC(C2CCCCC2)C1	1580672534	24	327.23	3.1
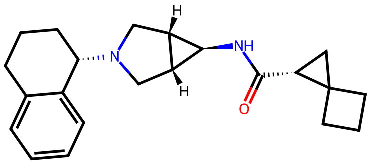	O=C(N[C@H]1[C@@H]2CN([C@H]3CCCc4ccccc43)C[C@@H]21)[C@@H]1CC12CCC2	1026078136	25	336.22	3.3
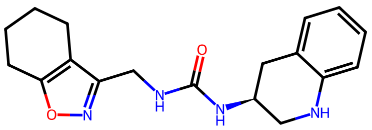	O=C(NCc1noc2c1CCCC2)N[C@@H]1CNc2ccccc2C1	652192762	24	326.17	2.39
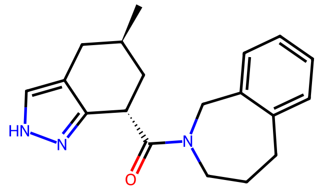	C[C@@H]1Cc2c[nH]nc2[C@@H](C(=O)N2CCCc3ccccc3C2)C1	623191804	23	309.18	3.05
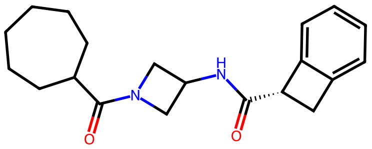	O=C(NC1CN(C(=O)C2CCCCCC2)C1)[C@H]1Cc2ccccc21	998678986	24	326.2	2.62

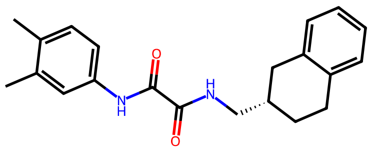	Cc1ccc(NC(=O)C(=O)NC[C@H]2CCc3ccccc3C2)cc1C	605068699	25	336.18	3.16
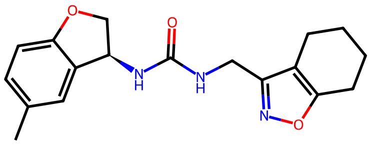	Cc1ccc2c(c1)[C@H](NC(=O)NCc1noc3c1CCCC3)CO2	376840099	24	327.16	2.79
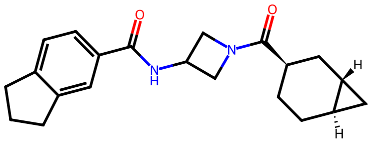	O=C(NC1CN(C(=O)[C@@H]2CC[C@@H]3C[C@H]3C2)C1)c1ccc2c(c1)CCC2	991373587	25	338.2	2.55
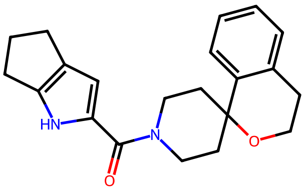	O=C(c1cc2c([nH]1)CCC2)N1CCC2(CC1)OCCc1ccccc12	1548887301	25	336.18	3.21
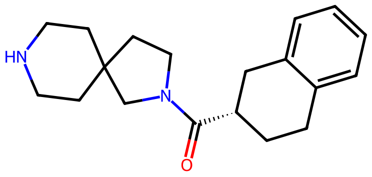	O=C([C@H]1CCc2ccccc2C1)N1CCC2(CCNCC2)C1	319328195	22	298.2	2.39
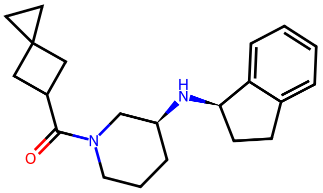	O=C(C1CC2(CC2)C1)N1CCC[C@H](N[C@@H]2CCc3ccccc32)C1	1000152536	24	324.22	3.44
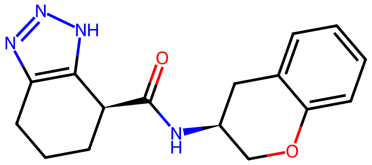	O=C(N[C@@H]1COc2ccccc2C1)[C@H]1CCCc2nn[nH]c21	666388930	22	298.14	1.34
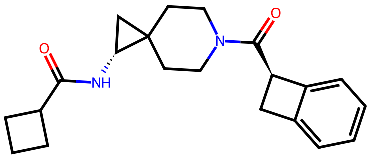	O=C(N[C@@H]1CC12CCN(C(=O)[C@@H]1Cc3ccccc31)CC2)C1CCC1	962485277	25	338.2	2.62

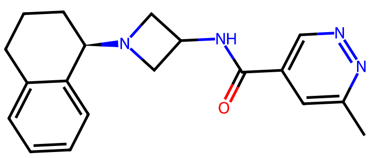	Cc1cc(C(=O)NC2CN([C@@H]3CCCc4ccccc43)C2)cnn1	1030249759	24	322.18	2.28
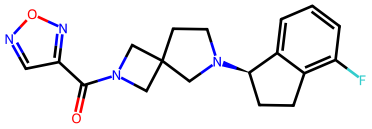	O=C(c1cnon1)N1CC2(CCN([C@@H]3CCc4c(F)cccc43)C2)C1	1073068197	25	342.15	2.04
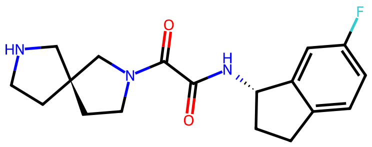	O=C(N[C@H]1CCc2ccc(F)cc21)C(=O)N1CC[C@]2(CCNC2)C1	573942739	24	331.17	1.14
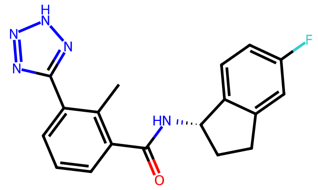	Cc1c(C(=O)N[C@H]2CCc3cc(F)ccc32)cccc1-c1nn[nH]n1	1591807170	25	337.13	2.73
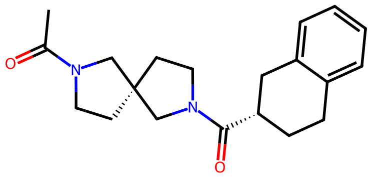	CC(=O)N1CC[C@@]2(CCN(C(=O)[C@H]3CCc4ccccc4C3)C2)C1	953028185	24	326.2	2.26
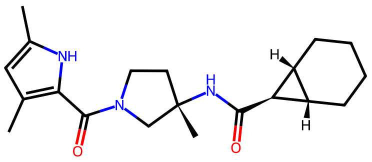	Cc1cc(C)c(C(=O)N2CC[C@](C)(NC(=O)[C@@H]3[C@H]4CCCC[C@H]43)C2)[nH]1	956795304	25	343.23	2.79
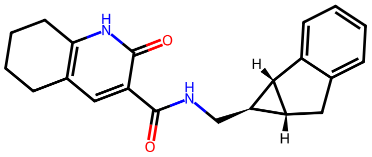	O=C(NC[C@@H]1[C@H]2Cc3ccccc3[C@H]21)c1cc2c([nH]c1=O)CCCC2	152424250	25	334.17	2.57
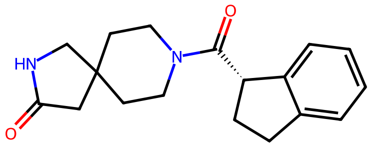	O=C1CC2(CCN(C(=O)[C@H]3CCc4ccccc43)CC2)CN1	336042467	22	298.17	1.85

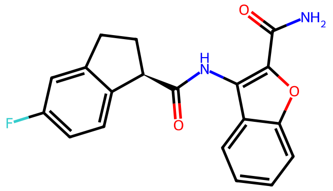	NC(=O)c1oc2ccccc2c1NC(=O)[C@@H]1CCc2cc(F)ccc21	1531551550	25	338.11	3.34
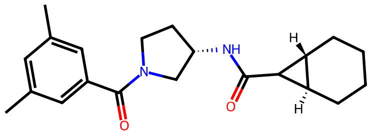	Cc1cc(C)cc(C(=O)N2CC[C@H](NC(=O)C3[C@@H]4CCCC[C@@H]34)C2)c1	937789041	25	340.22	3.07
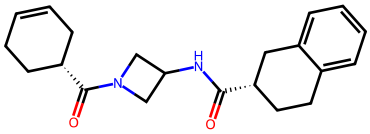	O=C(NC1CN(C(=O)[C@H]2CC=CCC2)C1)[C@H]1CCc2ccccc2C1	1000537908	25	338.2	2.47
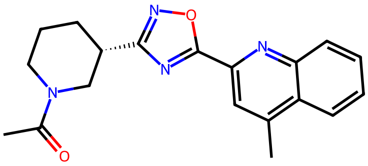	CC(=O)N1CCC[C@H](c2noc(-c3cc(C)c4ccccc4n3)n2)C1	1320212947	25	336.16	3.32
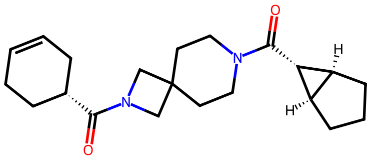	O=C([C@H]1CC=CCC1)N1CC2(CCN(C(=O)[C@@H]3[C@H]4CCC[C@H]43)CC2)C1	944903093	25	342.23	2.84
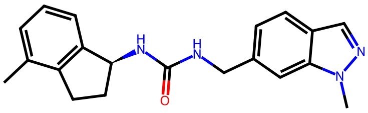	Cc1cccc2c1CC[C@@H]2NC(=O)NCc1ccc2cnn(C)c2c1	577570044	25	334.18	3.37
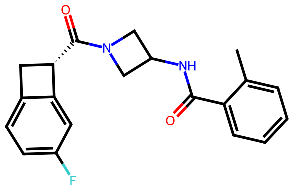	Cc1ccccc1C(=O)NC1CN(C(=O)[C@H]2Cc3ccc(F)cc32)C1	990110211	25	338.14	2.41
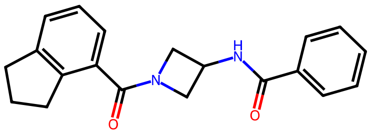	O=C(NC1CN(C(=O)c2cccc3c2CCC3)C1)c1ccccc1	990092876	24	320.15	2.43

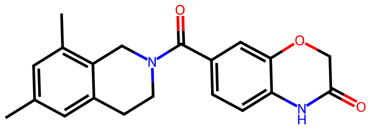	Cc1cc(C)c2c(c1)CCN(C(=O)c1ccc3c(c1)OCC(=O)N3)C2	190769503	25	336.15	2.83
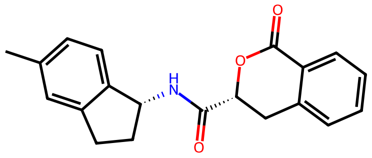	Cc1ccc2c(c1)CC[C@H]2NC(=O)[C@H]1Cc2ccccc2C(=O)O1	55661288	24	321.14	2.88
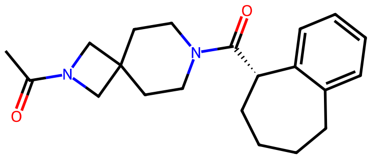	CC(=O)N1CC2(CCN(C(=O)[C@H]3CCCCc4ccccc43)CC2)C1	944963201	25	340.22	2.97
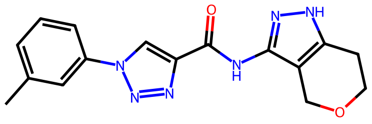	Cc1cccc(-n2cc(C(=O)Nc3n[nH]c4c3COCC4)nn2)c1	899257481	24	324.13	1.62
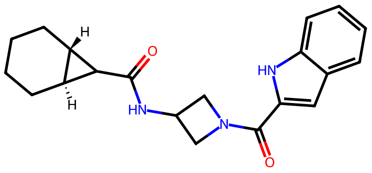	O=C(NC1CN(C(=O)c2cc3ccccc3[nH]2)C1)C1[C@H]2CCCC[C@H]12	990663282	25	337.18	2.54
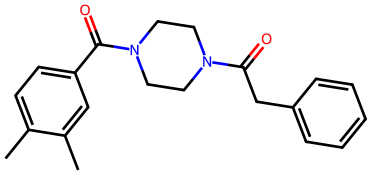	Cc1ccc(C(=O)N2CCN(C(=O)Cc3ccccc3)CC2)cc1C	41396565	25	336.18	2.83
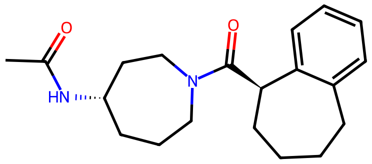	CC(=O)N[C@H]1CCCN(C(=O)[C@@H]2CCCCc3ccccc32)CC1	964647761	24	328.22	3.01
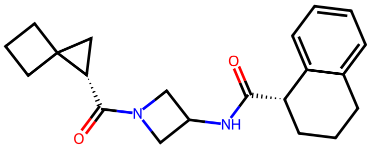	O=C(NC1CN(C(=O)[C@H]2CC23CCC3)C1)[C@H]1CCCc2ccccc21	995076531	25	338.2	2.62

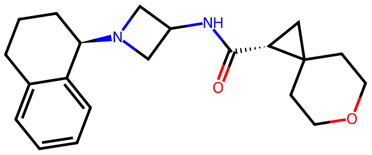	O=C(NC1CN([C@@H]2CCCc3ccccc32)C1)[C@@H]1CC12CCOCC2	1031494867	25	340.22	2.68
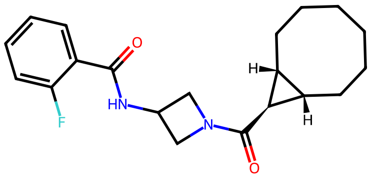	O=C(NC1CN(C(=O)[C@@H]2[C@H]3CCCCCC[C@H]32)C1)c1ccccc1F	990092425	25	344.19	2.98
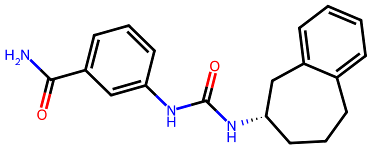	NC(=O)c1cccc(NC(=O)N[C@H]2CCCc3ccccc3C2)c1	929406514	24	323.16	2.85
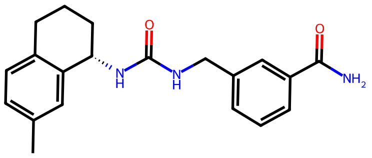	Cc1ccc2c(c1)[C@@H](NC(=O)NCc1cccc(C(N)=O)c1)CCC2	179298234	25	337.18	2.97
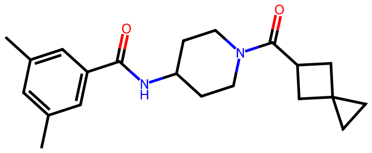	Cc1cc(C)cc(C(=O)NC2CCN(C(=O)C3CC4(CC4)C3)CC2)c1	948306324	25	340.22	3.21
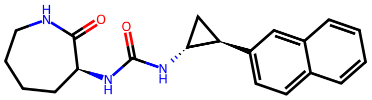	O=C(N[C@H]1CCCCNC1=O)N[C@@H]1C[C@H]1c1ccc2ccccc2c1	333145508	25	337.18	2.66
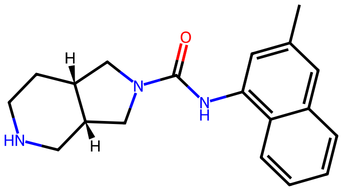	Cc1cc(NC(=O)N2C[C@H]3CCNC[C@H]3C2)c2ccccc2c1	415803322	23	309.18	3.22
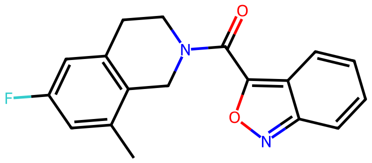	Cc1cc(F)cc2c1CN(C(=O)c1onc3ccccc13)CC2	1155505924	23	310.11	3.47

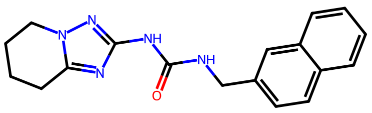	O=C(NCc1ccc2ccccc2c1)Nc1nc2n(n1)CCCC2	565405885	24	321.16	3.09
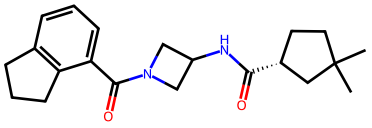	CC1(C)CC[C@@H](C(=O)NC2CN(C(=O)c3cccc4c3CCC4)C2)C1	999248587	25	340.22	2.94
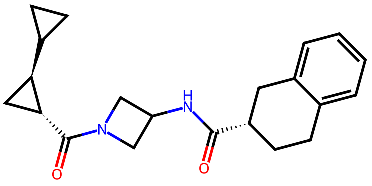	O=C(NC1CN(C(=O)[C@@H]2C[C@H]2C2CC2)C1)[C@H]1CCc2ccccc2C1	1000544531	25	338.2	2.16
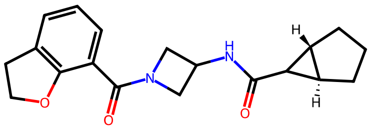	O=C(NC1CN(C(=O)c2cccc3c2OCC3)C1)C1[C@@H]2CCC[C@@H]12	990711782	24	326.16	1.61
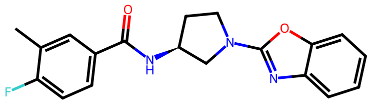	Cc1cc(C(=O)N[C@H]2CCN(c3nc4ccccc4o3)C2)ccc1F	1058528460	25	339.14	3.28
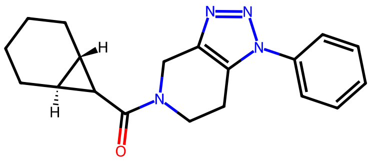	O=C(C1[C@H]2CCCC[C@H]12)N1CCc2c(nnn2-c2ccccc2)C1	329403510	24	322.18	2.59
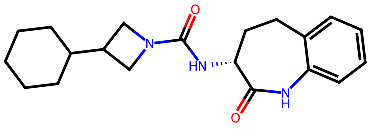	O=C1Nc2ccccc2CC[C@H]1NC(=O)N1CC(C2CCCCC2)C1	654634286	25	341.21	3.16
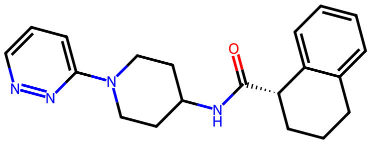	O=C(NC1CCN(c2cccnn2)CC1)[C@H]1CCCc2ccccc21	277182050	25	336.2	2.68

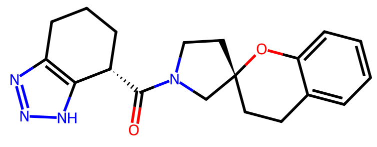	O=C([C@H]1CCCc2nn[nH]c21)N1CC[C@@]2(CCc3ccccc3O2)C1	854502421	25	338.17	2.22
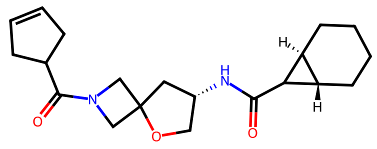	O=C(N[C@@H]1COC2(C1)CN(C(=O)C1CC=CC1)C2)C1[C@H]2CCCC[C@H]12	965651927	25	344.21	1.87
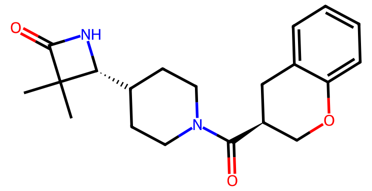	CC1(C)C(=O)N[C@@H]1C1CCN(C(=O)[C@@H]2COc3ccccc3C2)CC1	294107771	25	342.19	2.0
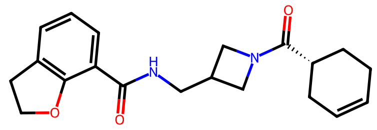	O=C(NCC1CN(C(=O)[C@H]2CC=CCC2)C1)c1cccc2c1OCC2	1004713296	25	340.18	2.17
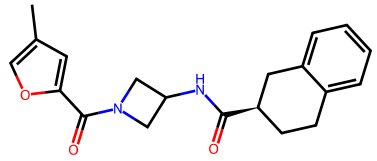	Cc1coc(C(=O)N2CC(NC(=O)[C@@H]3CCc4ccccc4C3)C2)c1	1000544993	25	338.16	2.33
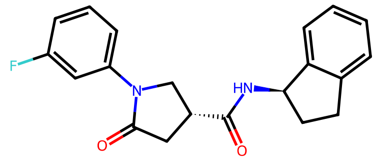	O=C(N[C@@H]1CCc2ccccc21)[C@@H]1CC(=O)N(c2cccc(F)c2)C1	1433522021	25	338.14	2.98
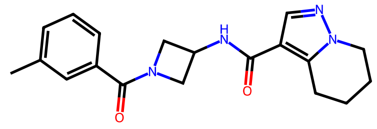	Cc1cccc(C(=O)N2CC(NC(=O)c3cnn4c3CCCC4)C2)c1	999642457	25	338.17	1.78
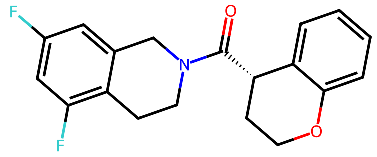	O=C([C@H]1CCOc2ccccc21)N1CCc2c(F)cc(F)cc2C1	134302582	24	329.12	3.42

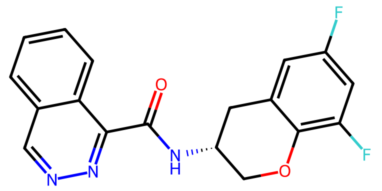	O=C(N[C@H]1COc2c(F)cc(F)cc2C1)c1nncc2ccccc12	1640471897	25	341.1	2.64
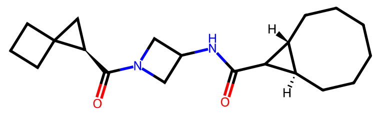	O=C(NC1CN(C(=O)[C@@H]2CC23CCC3)C1)C1[C@@H]2CCCCCC[C@@H]12	1000511927	24	330.23	2.72
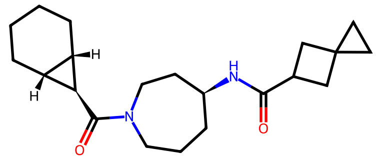	O=C(N[C@H]1CCCN(C(=O)[C@@H]2[C@H]3CCCC[C@H]32)CC1)C1CC2(CC2)C1	965188230	25	344.25	3.11
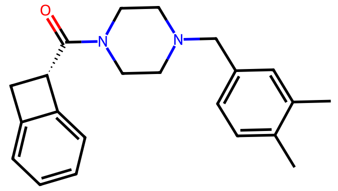	Cc1ccc(CN2CCN(C(=O)[C@H]3Cc4ccccc43)CC2)cc1C	949957787	25	334.2	3.29
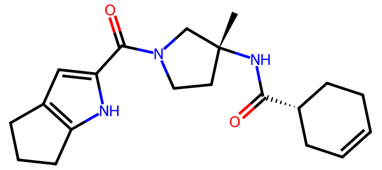	C[C@@]1(NC(=O)[C@H]2CC=CCC2)CCN(C(=O)c2cc3c([nH]2)CCC3)C1	956689630	25	341.21	2.58
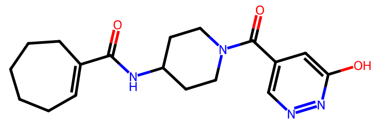	O=C(NC1CCN(C(=O)c2cnnc(O)c2)CC1)C1=CCCCCC1	947096236	25	344.18	1.79
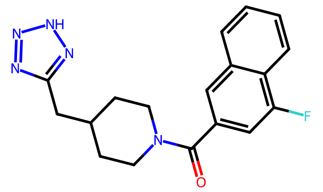	O=C(c1cc(F)c2ccccc2c1)N1CCC(Cc2nn[nH]n2)CC1	1548805489	25	339.15	2.59
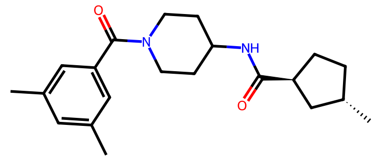	Cc1cc(C)cc(C(=O)N2CCC(NC(=O)[C@H]3CC[C@H](C)C3)CC2)c1	948337771	25	342.23	3.46

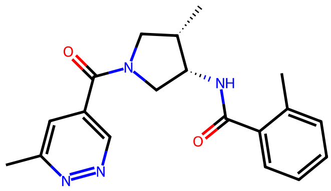	Cc1cc(C(=O)N2C[C@H](C)[C@H](NC(=O)c3ccccc3C)C2)cnn1	966347930	25	338.17	1.98
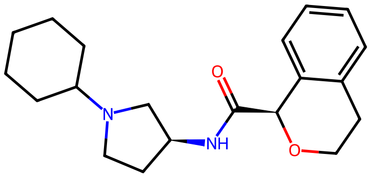	O=C(N[C@H]1CCN(C2CCCCC2)C1)[C@@H]1OCCc2ccccc21	618663741	24	328.22	2.82
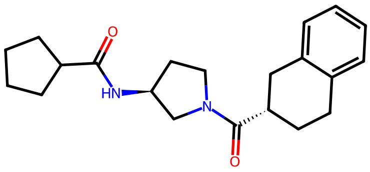	O=C(N[C@H]1CCN(C(=O)[C@H]2CCc3ccccc3C2)C1)C1CCCC1	937595681	25	340.22	2.7
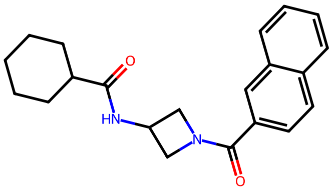	O=C(NC1CN(C(=O)c2ccc3ccccc3c2)C1)C1CCCCC1	990108122	25	336.18	3.36
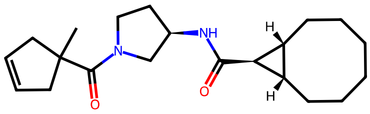	CC1(C(=O)N2CC[C@@H](NC(=O)[C@@H]3[C@H]4CCCCCC[C@H]43)C2)CC=CC1	942510920	25	344.25	3.28
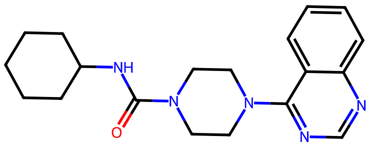	O=C(NC1CCCCC1)N1CCN(c2ncnc3ccccc23)CC1	26704550	25	339.21	2.79
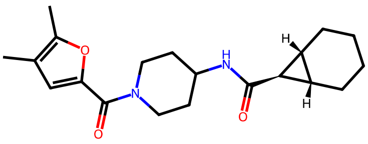	Cc1cc(C(=O)N2CCC(NC(=O)[C@@H]3[C@H]4CCCC[C@H]43)CC2)oc1C	946465096	25	344.21	3.05
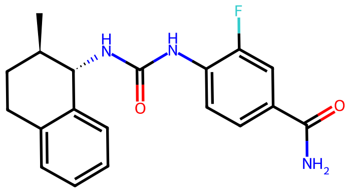	C[C@@H]1CCc2ccccc2[C@H]1NC(=O)Nc1ccc(C(N)=O)cc1F	342880173	25	341.15	3.37

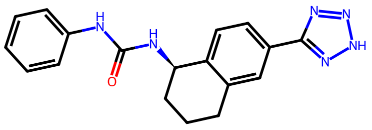	O=C(Nc1ccccc1)N[C@@H]1CCCc2cc(-c3nn[nH]n3)ccc21	1593251335	25	334.15	3.07
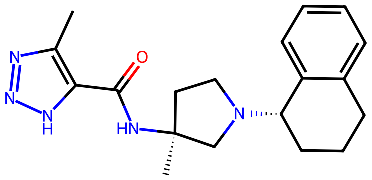	Cc1nn[nH]c1C(=O)N[C@@]1(C)CCN([C@H]2CCCc3ccccc32)C1	1046528746	25	339.21	2.38
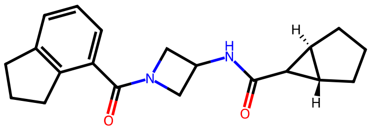	O=C(NC1CN(C(=O)c2cccc3c2CCC3)C1)C1[C@H]2CCC[C@H]12	990710294	24	324.18	2.16
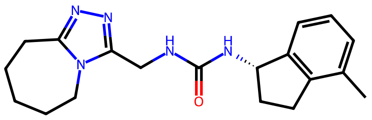	Cc1cccc2c1CC[C@@H]2NC(=O)NCc1nnc2n1CCCCC2	519171262	25	339.21	2.8
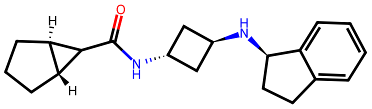	O=C(N[C@H]1C[C@H](N[C@@H]2CCc3ccccc32)C1)C1[C@@H]2CCC[C@@H]12	1020057327	23	310.2	2.96
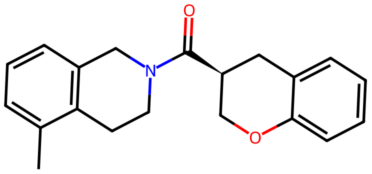	Cc1cccc2c1CCN(C(=O)[C@@H]1COc3ccccc3C1)C2	171539570	23	307.16	3.13
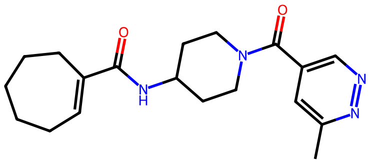	Cc1cc(C(=O)N2CCC(NC(=O)C3=CCCCCC3)CC2)cnn1	946562881	25	342.21	2.4
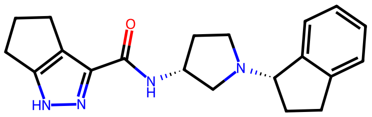	O=C(N[C@@H]1CCN([C@H]2CCc3ccccc32)C1)c1n[nH]c2c1CCC2	1015070830	25	336.2	2.39

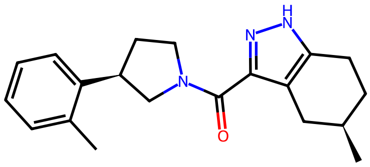	Cc1ccccc1[C@H]1CCN(C(=O)c2n[nH]c3c2C[C@H](C)CC3)C1	1142810721	24	323.2	3.47
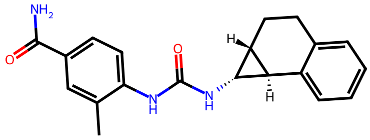	Cc1cc(C(N)=O)ccc1NC(=O)N[C@H]1[C@@H]2c3ccccc3CC[C@H]21	772418269	25	335.16	2.94
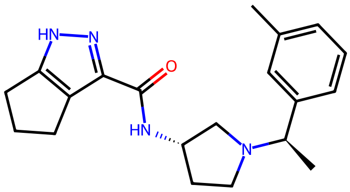	Cc1cccc([C@@H](C)N2CC[C@H](NC(=O)c3n[nH]c4c3CCC4)C2)c1	1015071072	25	338.21	2.77
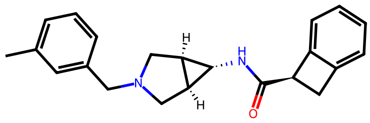	Cc1cccc(CN2C[C@H]3[C@H](NC(=O)[C@@H]4Cc5ccccc54)[C@H]3C2)c1	961627360	25	332.19	2.88
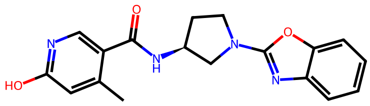	Cc1cc(O)ncc1C(=O)N[C@H]1CCN(c2nc3ccccc3o2)C1	1058541308	25	338.14	2.25
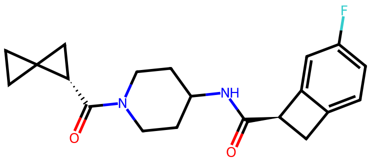	O=C(NC1CCN(C(=O)[C@H]2CC23CC3)CC1)[C@@H]1Cc2ccc(F)cc21	948568396	25	342.17	2.37
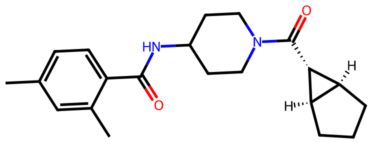	Cc1ccc(C(=O)NC2CCN(C(=O)[C@@H]3[C@H]4CCC[C@H]43)CC2)c(C)c1	946079798	25	340.22	3.07
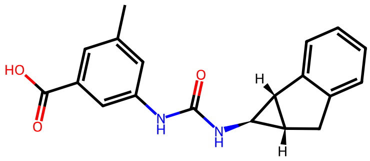	Cc1cc(NC(=O)N[C@@H]2[C@H]3Cc4ccccc4[C@H]32)cc(C(=O)O)c1	1600272065	24	322.13	3.15

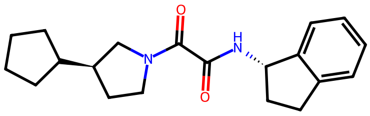	O=C(N[C@H]1CCc2ccccc21)C(=O)N1CC[C@@H](C2CCCC2)C1	421055150	24	326.2	2.83
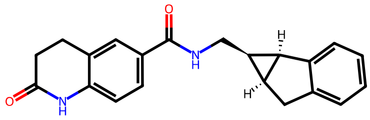	O=C1CCc2cc(C(=O)NC[C@H]3[C@H]4c5ccccc5C[C@H]43)ccc2N1	152503429	25	332.15	2.89
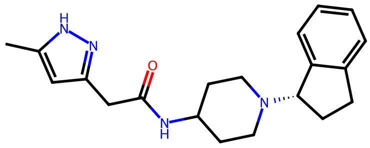	Cc1cc(CC(=O)NC2CCN([C@H]3CCc4ccccc43)CC2)n[nH]1	1002586331	25	338.21	2.53
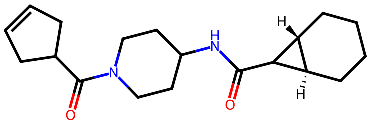	O=C(NC1CCN(C(=O)C2CC=CC2)CC1)C1[C@@H]2CCCC[C@@H]12	945718509	23	316.22	2.5
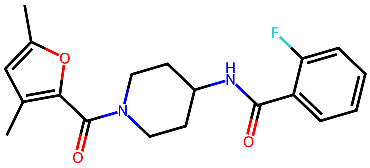	Cc1cc(C)c(C(=O)N2CCC(NC(=O)c3ccccc3F)CC2)o1	370153686	25	344.15	3.07
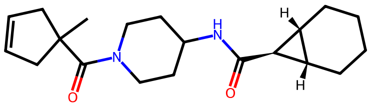	CC1(C(=O)N2CCC(NC(=O)[C@@H]3[C@H]4CCCC[C@H]43)CC2)CC=CC1	948178970	24	330.23	2.89
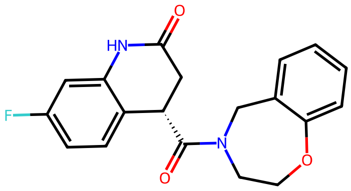	O=C1C[C@H](C(=O)N2CCOc3ccccc3C2)c2ccc(F)cc2N1	376617182	25	340.12	2.67
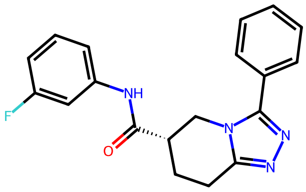	O=C(Nc1cccc(F)c1)[C@H]1CCc2nnc(-c3ccccc3)n2C1	1521389976	25	336.14	3.29

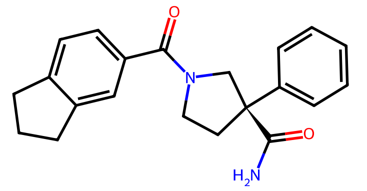	NC(=O)[C@@]1(c2ccccc2)CCN(C(=O)c2ccc3c(c2)CCC3)C1	412601841	25	334.17	2.44
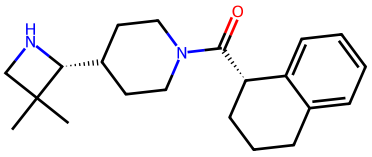	CC1(C)CN[C@@H]1C1CCN(C(=O)[C@H]2CCCc3ccccc32)CC1	424040252	24	326.24	3.34
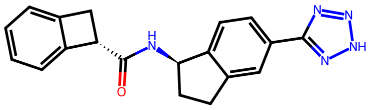	O=C(N[C@@H]1CCc2cc(-c3nn[nH]n3)ccc21)[C@H]1Cc2ccccc21	1609543184	25	331.14	2.31
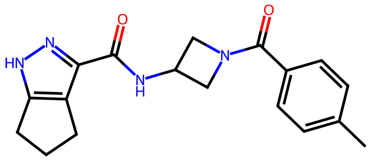	Cc1ccc(C(=O)N2CC(NC(=O)c3n[nH]c4c3CCC4)C2)cc1	995164854	24	324.16	1.46
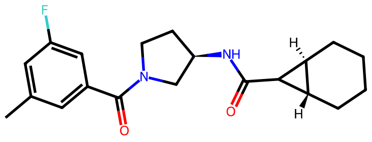	Cc1cc(F)cc(C(=O)N2CC[C@@H](NC(=O)C3[C@H]4CCCC[C@H]34)C2)c1	936245949	25	344.19	2.9
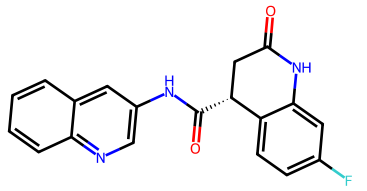	O=C1C[C@@H](C(=O)Nc2cnc3ccccc3c2)c2ccc(F)cc2N1	25533877	25	335.11	3.44
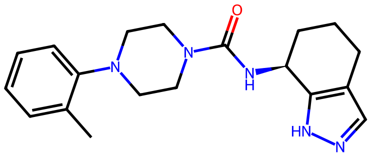	Cc1ccccc1N1CCN(C(=O)N[C@H]2CCCc3cn[nH]c32)CC1	269027467	25	339.21	2.63
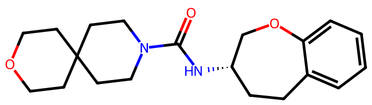	O=C(N[C@H]1CCc2ccccc2OC1)N1CCC2(CCOCC2)CC1	790848817	25	344.21	2.98

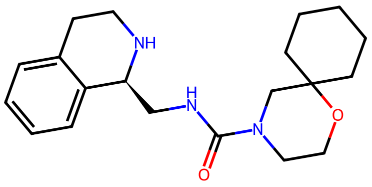	O=C(NC[C@@H]1NCCc2ccccc21)N1CCOC2(CCCCC2)C1	563610703	25	343.23	2.62
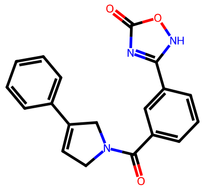	O=C(c1cccc(-c2nc(=O)o[nH]2)c1)N1CC=C(c2ccccc2)C1	1623840262	25	333.11	2.57
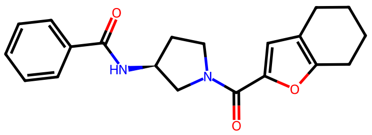	O=C(N[C@H]1CCN(C(=O)c2cc3c(o2)CCCC3)C1)c1ccccc1	937627369	25	338.16	2.8
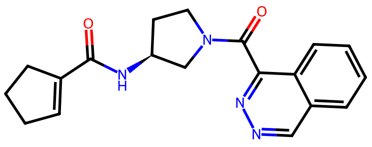	O=C(N[C@H]1CCN(C(=O)c2nncc3ccccc23)C1)C1=CCCC1	937819170	25	336.16	2.07
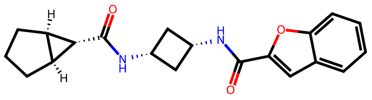	O=C(N[C@H]1C[C@@H](NC(=O)[C@@H]2[C@H]3CCC[C@H]32)C1)c1cc2ccccc2o1	974007053	25	338.16	2.86
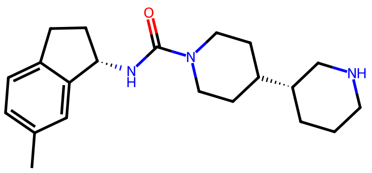	Cc1ccc2c(c1)[C@@H](NC(=O)N1CCC([C@H]3CCCNC3)CC1)CC2	659453059	25	341.25	3.4
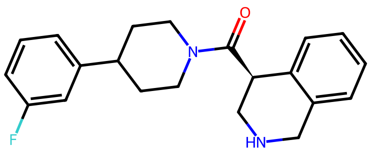	O=C([C@@H]1CNCc2ccccc21)N1CCC(c2cccc(F)c2)CC1	1341239465	25	338.18	3.42
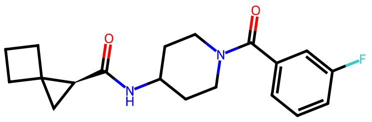	O=C(NC1CCN(C(=O)c2cccc(F)c2)CC1)[C@H]1CC12CCC2	943775913	24	330.17	2.74

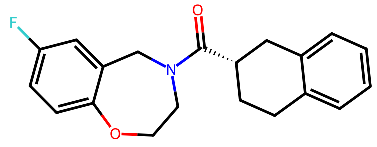	O=C([C@H]1CCc2ccccc2C1)N1CCOc2ccc(F)cc2C1	563259814	24	325.15	3.35
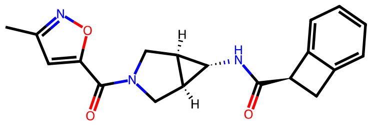	Cc1cc(C(=O)N2C[C@H]3[C@H](NC(=O)[C@@H]4Cc5ccccc54)[C@H]3C2)on1	977083038	25	337.14	1.51
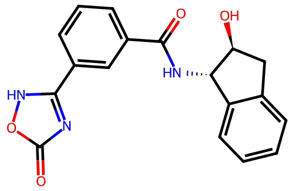	O=C(N[C@H]1c2ccccc2C[C@@H]1O)c1cccc(-c2nc(=O)o[nH]2)c1	1583199426	25	337.11	1.42
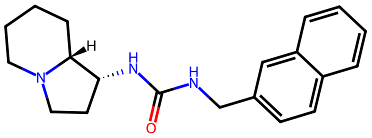	O=C(NCc1ccc2ccccc2c1)N[C@@H]1CCN2CCCC[C@H]12	356229345	24	323.2	3.27
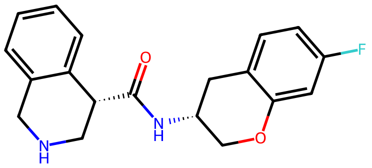	O=C(N[C@H]1COc2cc(F)ccc2C1)[C@@H]1CNCc2ccccc21	662875988	24	326.14	2.13
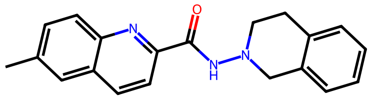	Cc1ccc2nc(C(=O)NN3CCc4ccccc4C3)ccc2c1	1628729894	24	317.15	3.25
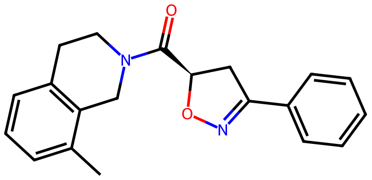	Cc1cccc2c1CN(C(=O)[C@H]1CC(c3ccccc3)=NO1)CC2	759801856	24	320.15	3.07
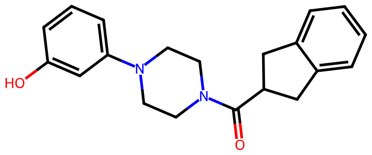	O=C(C1Cc2ccccc2C1)N1CCN(c2cccc(O)c2)CC1	277510245	24	322.17	2.46

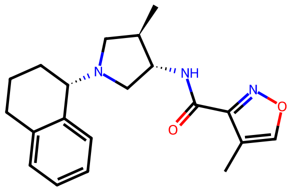	Cc1conc1C(=O)N[C@@H]1CN([C@H]2CCCc3ccccc32)C[C@H]1C	1080731415	25	339.19	3.11
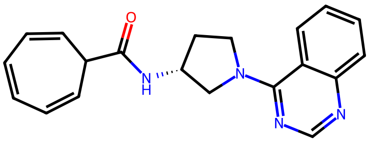	O=C(N[C@@H]1CCN(c2ncnc3ccccc23)C1)C1C=CC=CC=C1	1058461331	25	332.16	2.62
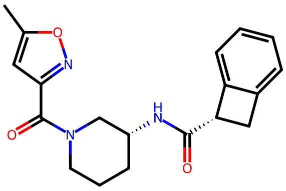	Cc1cc(C(=O)N2CCC[C@@H](NC(=O)[C@H]3Cc4ccccc43)C2)no1	949928565	25	339.16	2.04
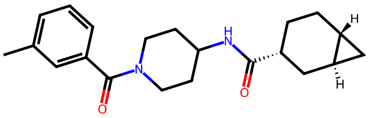	Cc1cccc(C(=O)N2CCC(NC(=O)[C@@H]3CC[C@@H]4C[C@H]4C3)CC2)c1	943836128	25	340.22	3.15
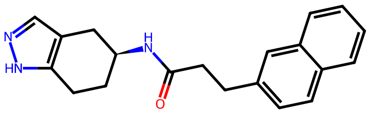	O=C(CCc1ccc2ccccc2c1)N[C@H]1CCc2[nH]ncc2C1	172037305	24	319.17	3.17
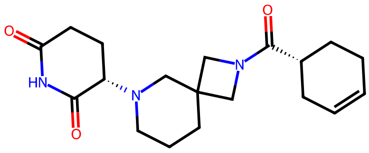	O=C1CC[C@H](N2CCCC3(CN(C(=O)[C@H]4CC=CCC4)C3)C2)C(=O)N1	981615370	25	345.21	1.07
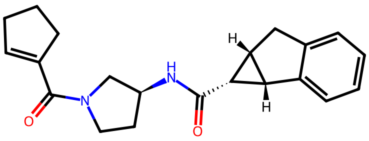	O=C(N[C@H]1CCN(C(=O)C2=CCCC2)C1)[C@H]1[C@H]2c3ccccc3C[C@H]21	937966870	25	336.18	2.4
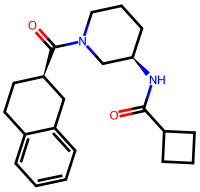	O=C(N[C@@H]1CCCN(C(=O)[C@@H]2CCc3ccccc3C2)C1)C1CCC1	952147405	25	340.22	2.7

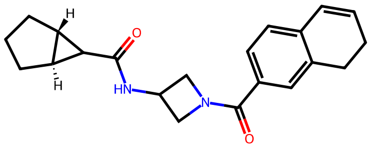	O=C(NC1CN(C(=O)c2ccc3c(c2)CCC=C3)C1)C1[C@H]2CCC[C@H]12	990697150	25	336.18	2.63
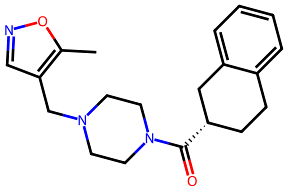	Cc1oncc1CN1CCN(C(=O)[C@H]2CCc3ccccc3C2)CC1	950721054	25	339.19	2.43
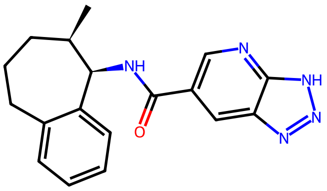	C[C@@H]1CCCc2ccccc2[C@@H]1NC(=O)c1cnc2[nH]nnc2c1	546616845	24	321.16	2.8
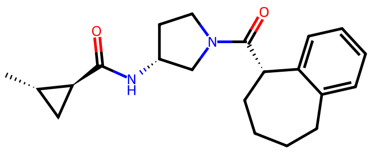	C[C@H]1C[C@@H]1C(=O)N[C@@H]1CCN(C(=O)[C@H]2CCCCc3ccccc32)C1	942181232	25	340.22	2.87
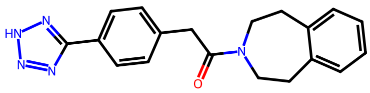	O=C(Cc1ccc(-c2nn[nH]n2)cc1)N1CCc2ccccc2CC1	737963810	25	333.16	2.04
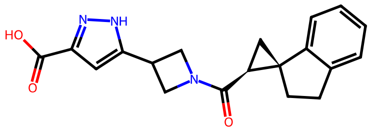	O=C(O)c1cc(C2CN(C(=O)[C@H]3C[C@@]34CCc3ccccc34)C2)[nH]n1	1602253147	25	337.14	1.94
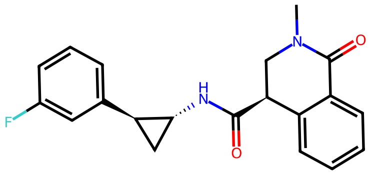	CN1C[C@H](C(=O)N[C@@H]2C[C@H]2c2cccc(F)c2)c2ccccc2C1=O	345478091	25	338.14	2.67
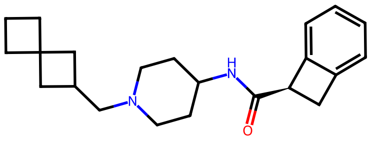	O=C(NC1CCN(CC2CC3(CCC3)C2)CC1)[C@@H]1Cc2ccccc21	1003534075	25	338.24	3.49

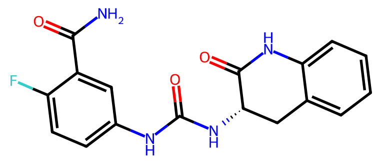	NC(=O)c1cc(NC(=O)N[C@H]2Cc3ccccc3NC2=O)ccc1F	1365753639	25	342.11	1.61
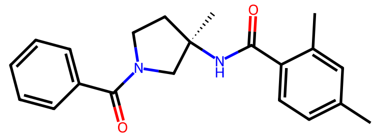	Cc1ccc(C(=O)N[C@]2(C)CCN(C(=O)c3ccccc3)C2)c(C)c1	957564626	25	336.18	3.34
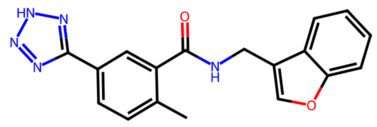	Cc1ccc(-c2nn[nH]n2)cc1C(=O)NCc1coc2ccccc12	1592100360	25	333.12	2.85
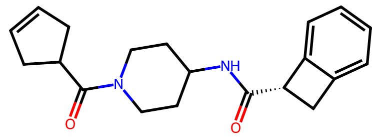	O=C(NC1CCN(C(=O)C2CC=CC2)CC1)[C@H]1Cc2ccccc21	945756472	24	324.18	2.4
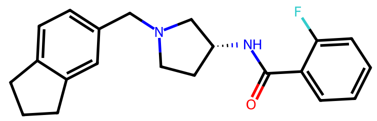	O=C(N[C@@H]1CCN(Cc2ccc3c(c2)CCC3)C1)c1ccccc1F	1009951654	25	338.18	3.32
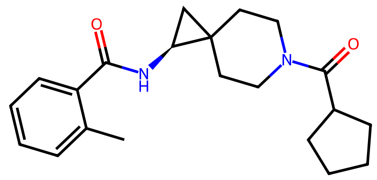	Cc1ccccc1C(=O)N[C@H]1CC12CCN(C(=O)C1CCCC1)CC2	962816810	25	340.22	3.3
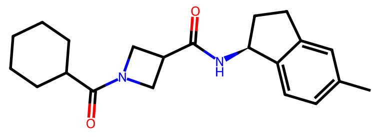	Cc1ccc2c(c1)CC[C@@H]2NC(=O)C1CN(C(=O)C2CCCCC2)C1	1264783742	25	340.22	3.14
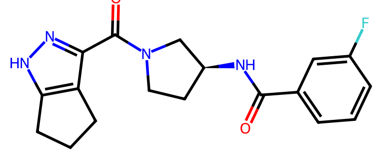	O=C(N[C@H]1CCN(C(=O)c2n[nH]c3c2CCC3)C1)c1cccc(F)c1	937631962	25	342.15	1.68

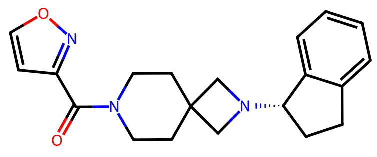	O=C(c1ccon1)N1CCC2(CC1)CN([C@H]1CCc3ccccc31)C2	1035660882	25	337.18	2.9
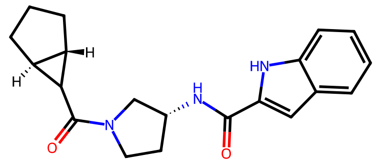	O=C(N[C@@H]1CCN(C(=O)C2[C@H]3CCC[C@H]23)C1)c1cc2ccccc2[nH]1	942463544	25	337.18	2.54
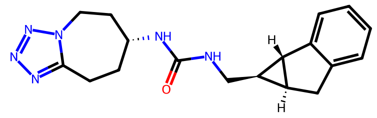	O=C(NC[C@@H]1[C@@H]2Cc3ccccc3[C@@H]12)N[C@@H]1CCc2nnnn2CC1	333637562	25	338.19	1.26
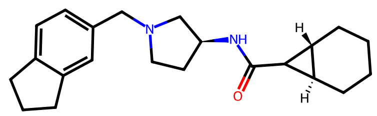	O=C(N[C@H]1CCN(Cc2ccc3c(c2)CCC3)C1)C1[C@@H]2CCCC[C@@H]12	1032825058	25	338.24	3.3
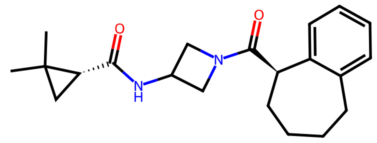	CC1(C)C[C@H]1C(=O)NC1CN(C(=O)[C@@H]2CCCCc3ccccc32)C1	990877252	25	340.22	2.87
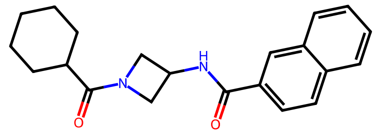	O=C(NC1CN(C(=O)C2CCCCC2)C1)c1ccc2ccccc2c1	999446331	25	336.18	3.36
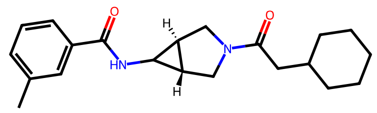	Cc1cccc(C(=O)NC2[C@H]3CN(C(=O)CC4CCCCC4)C[C@H]23)c1	1012721960	25	340.22	3.15
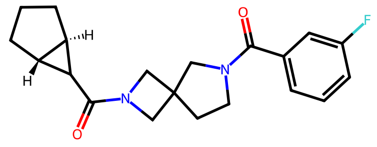	O=C(c1cccc(F)c1)N1CCC2(C1)CN(C(=O)C1[C@@H]3CCC[C@@H]13)C2	1018909664	25	342.17	2.55

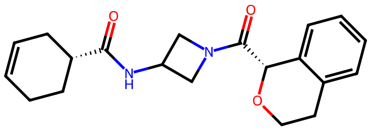	O=C(NC1CN(C(=O)[C@H]2OCCc3ccccc32)C1)[C@H]1CC=CCC1	990218195	25	340.18	1.98
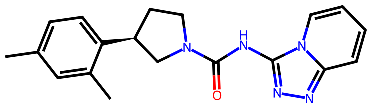	Cc1ccc([C@H]2CCN(C(=O)Nc3nnc4ccccn34)C2)c(C)c1	373410829	25	335.17	3.37
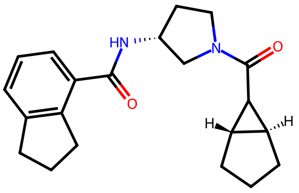	O=C(N[C@@H]1CCN(C(=O)C2[C@H]3CCC[C@H]23)C1)c1cccc2c1CCC2	941156919	25	338.2	2.55
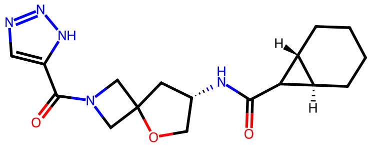	O=C(N[C@@H]1COC2(C1)CN(C(=O)c1cnn[nH]1)C2)C1[C@@H]2CCCC[C@@H]12	965674898	25	345.18	0.34
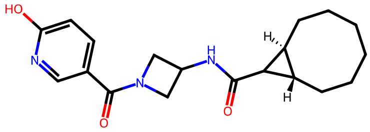	O=C(NC1CN(C(=O)c2ccc(O)nc2)C1)C1[C@H]2CCCCCC[C@H]12	1000510900	25	343.19	1.94
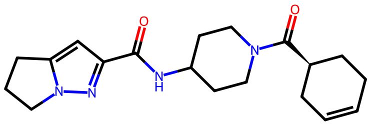	O=C(NC1CCN(C(=O)[C@@H]2CC=CCC2)CC1)c1cc2n(n1)CCC2	944127981	25	342.21	1.91
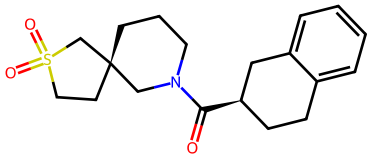	O=C([C@@H]1CCc2ccccc2C1)N1CCC[C@]2(CCS(=O)(=O)C2)C1	576640527	24	347.16	2.22
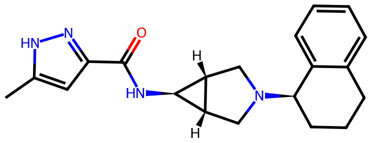	Cc1cc(C(=O)N[C@H]2[C@@H]3CN([C@@H]4CCCc5ccccc54)C[C@@H]32)n[nH]1	958332179	25	336.2	2.46

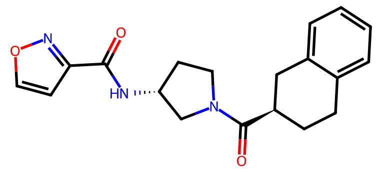	O=C(N[C@@H]1CCN(C(=O)[C@@H]2CCc3ccccc3C2)C1)c1ccon1	936274849	25	339.16	1.81
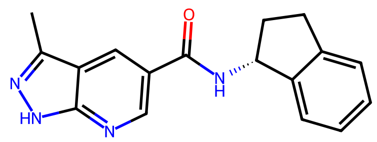	Cc1n[nH]c2ncc(C(=O)N[C@@H]3CCc4ccccc43)cc12	80051915	22	292.13	2.68
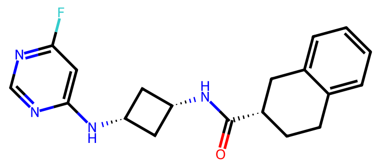	O=C(N[C@H]1C[C@@H](Nc2cc(F)ncn2)C1)[C@H]1CCc2ccccc2C1	1059840104	25	340.17	2.48
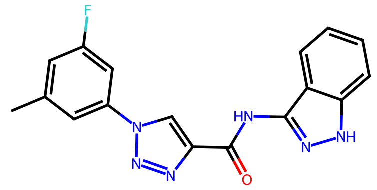	Cc1cc(F)cc(-n2cc(C(=O)Nc3n[nH]c4ccccc34)nn2)c1	789891959	25	336.11	2.84
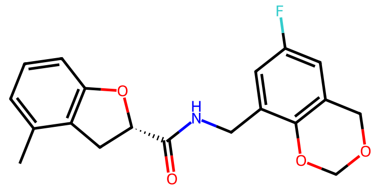	Cc1cccc2c1C[C@@H](C(=O)NCc1cc(F)cc3c1OCOC3)O2	448865225	25	343.12	2.62
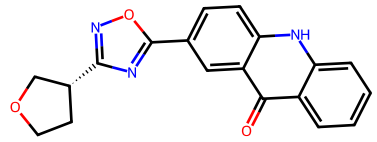	O=c1c2ccccc2[nH]c2ccc(-c3nc([C@@H]4CCOC4)no3)cc12	435993597	25	333.11	3.24
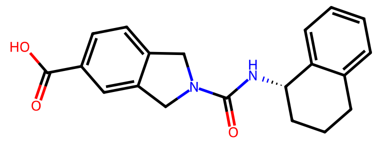	O=C(O)c1ccc2c(c1)CN(C(=O)N[C@H]1CCCc3ccccc31)C2	326988954	25	336.15	3.49
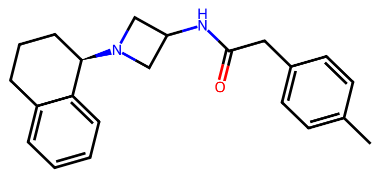	Cc1ccc(CC(=O)NC2CN([C@@H]3CCCc4ccccc43)C2)cc1	1030629125	25	334.2	3.42

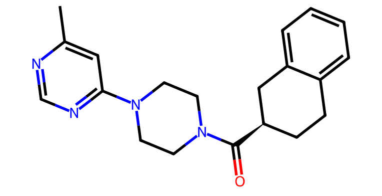	Cc1cc(N2CCN(C(=O)[C@@H]3CCc4ccccc4C3)CC2)ncn1	1056033865	25	336.2	2.24
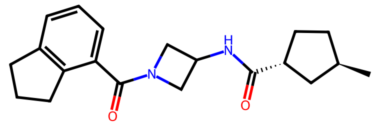	C[C@@H]1CC[C@@H](C(=O)NC2CN(C(=O)c3cccc4c3CCC4)C2)C1	991429641	24	326.2	2.55
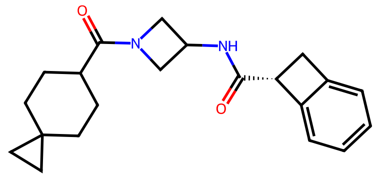	O=C(NC1CN(C(=O)C2CCC3(CC2)CC3)C1)[C@@H]1Cc2ccccc21	998684512	25	338.2	2.62
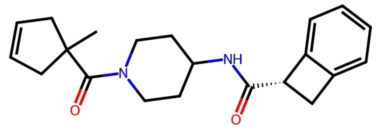	CC1(C(=O)N2CCC(NC(=O)[C@H]3Cc4ccccc43)CC2)CC=CC1	948190317	25	338.2	2.79
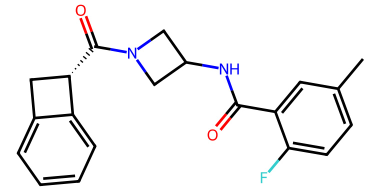	Cc1ccc(F)c(C(=O)NC2CN(C(=O)[C@H]3Cc4ccccc43)C2)c1	1000538487	25	338.14	2.41
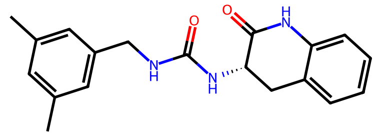	Cc1cc(C)cc(CNC(=O)N[C@H]2Cc3ccccc3NC2=O)c1	637271359	24	323.16	2.67
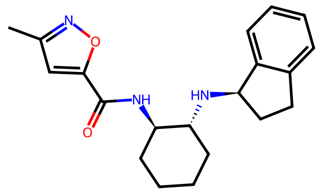	Cc1cc(C(=O)N[C@@H]2CCCC[C@H]2N[C@@H]2CCc3ccccc32)on1	1085132259	25	339.19	3.3
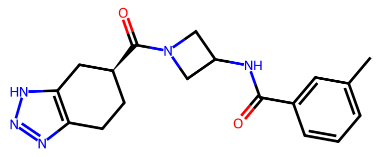	Cc1cccc(C(=O)NC2CN(C(=O)[C@H]3CCc4nn[nH]c4C3)C2)c1	990129377	25	339.17	0.86

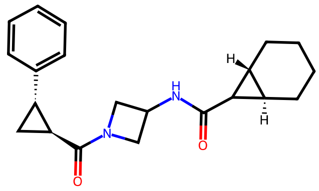	O=C(NC1CN(C(=O)[C@H]2C[C@@H]2c2ccccc2)C1)C1[C@@H]2CCCC[C@@H]12	990660837	25	338.2	2.55
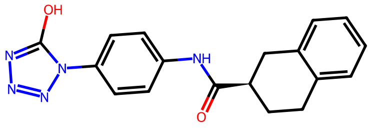	O=C(Nc1ccc(-n2nnnc2O)cc1)[C@@H]1CCc2ccccc2C1	1466902255	25	335.14	2.11
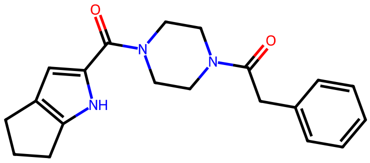	O=C(Cc1ccccc1)N1CCN(C(=O)c2cc3c([nH]2)CCC3)CC1	1011416922	25	337.18	2.03
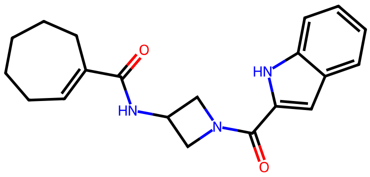	O=C(NC1CN(C(=O)c2cc3ccccc3[nH]2)C1)C1=CCCCCC1	991015151	25	337.18	3.0
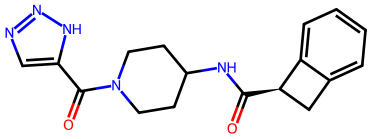	O=C(NC1CCN(C(=O)c2cnn[nH]2)CC1)[C@@H]1Cc2ccccc21	947370891	24	325.15	0.87
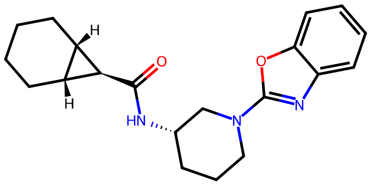	O=C(N[C@H]1CCCN(c2nc3ccccc3o2)C1)[C@@H]1[C@H]2CCCC[C@H]21	1058130130	25	339.19	3.35
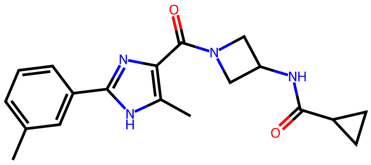	Cc1cccc(-c2nc(C(=O)N3CC(NC(=O)C4CC4)C3)c(C)[nH]2)c1	275097838	25	338.17	2.04
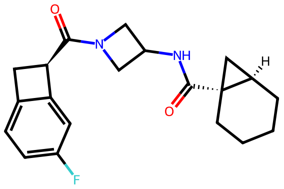	O=C([C@@H]1Cc2ccc(F)cc21)N1CC(NC(=O)[C@@]23CCCC[C@@H]2C3)C1	999232966	25	342.17	2.37

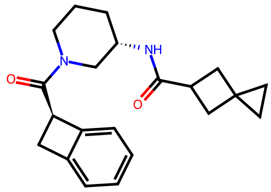	O=C(N[C@H]1CCCN(C(=O)[C@@H]2Cc3ccccc32)C1)C1CC2(CC2)C1	951842561	25	338.2	2.62
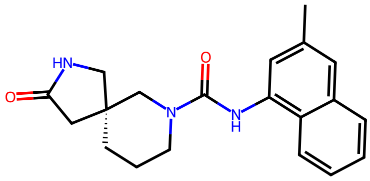	Cc1cc(NC(=O)N2CCC[C@]3(CNC(=O)C3)C2)c2ccccc2c1	455746631	25	337.18	3.28
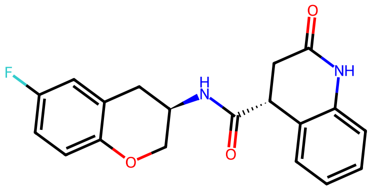	O=C1C[C@@H](C(=O)N[C@H]2COc3ccc(F)cc3C2)c2ccccc2N1	290681423	25	340.12	2.37
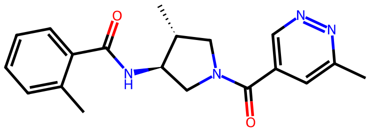	Cc1cc(C(=O)N2C[C@@H](NC(=O)c3ccccc3C)[C@H](C)C2)cnn1	948696695	25	338.17	1.98
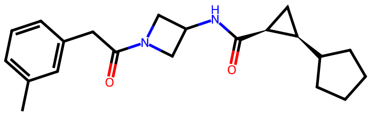	Cc1cccc(CC(=O)N2CC(NC(=O)[C@H]3C[C@H]3C3CCCC3)C2)c1	998828634	25	340.22	2.69
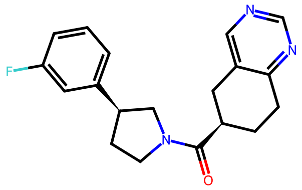	O=C([C@@H]1CCc2ncncc2C1)N1CC[C@@H](c2cccc(F)c2)C1	648277072	24	325.16	2.74
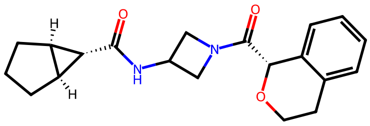	O=C(NC1CN(C(=O)[C@H]2OCCc3ccccc32)C1)[C@@H]1[C@H]2CCC[C@H]21	990712991	25	340.18	1.67
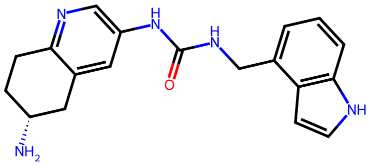	N[C@@H]1CCc2ncc(NC(=O)NCc3cccc4[nH]ccc34)cc2C1	632951337	25	335.17	2.7

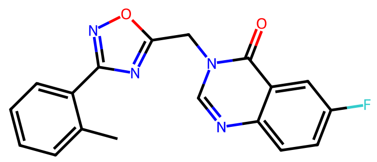	Cc1ccccc1-c1noc(Cn2cnc3ccc(F)cc3c2=O)n1	603639395	25	336.1	2.94
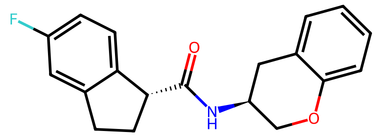	O=C(N[C@@H]1COc2ccccc2C1)[C@@H]1CCc2cc(F)ccc21	666390976	23	311.13	2.98
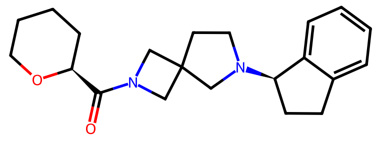	O=C([C@@H]1CCCCO1)N1CC2(CCN([C@@H]3CCc4ccccc43)C2)C1	1072628537	25	340.22	2.78
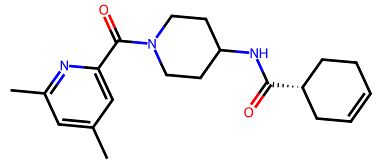	Cc1cc(C)nc(C(=O)N2CCC(NC(=O)[C@H]3CC=CCC3)CC2)c1	947307782	25	341.21	2.78
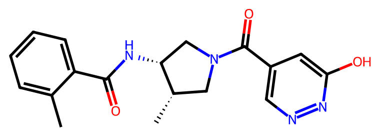	Cc1ccccc1C(=O)N[C@@H]1CN(C(=O)c2cnnc(O)c2)C[C@@H]1C	966506563	25	340.15	1.38
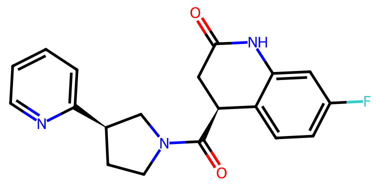	O=C1C[C@H](C(=O)N2CC[C@@H](c3ccccn3)C2)c2ccc(F)cc2N1	798720349	25	339.14	2.66
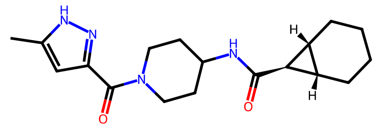	Cc1cc(C(=O)N2CCC(NC(=O)[C@@H]3[C@H]4CCCC[C@H]43)CC2)n[nH]1	944822327	24	330.21	1.88
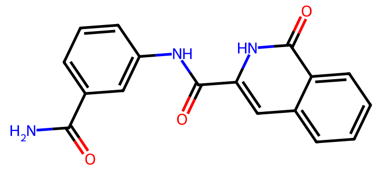	NC(=O)c1cccc(NC(=O)c2cc3ccccc3c(=O)[nH]2)c1	49419049	23	307.1	1.88

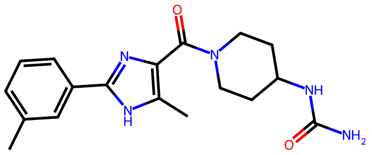	Cc1cccc(-c2nc(C(=O)N3CCC(NC(N)=O)CC3)c(C)[nH]2)c1	270437865	25	341.19	1.97
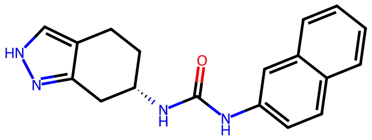	O=C(Nc1ccc2ccccc2c1)N[C@H]1CCc2c[nH]nc2C1	378106761	23	306.15	3.24
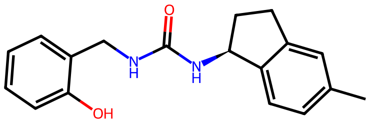	Cc1ccc2c(c1)CC[C@@H]2NC(=O)NCc1ccccc1O	263744977	22	296.15	3.19
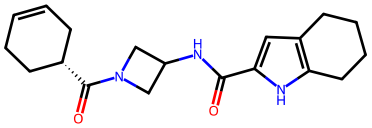	O=C(NC1CN(C(=O)[C@H]2CC=CCC2)C1)c1cc2c([nH]1)CCCC2	999657488	24	327.19	2.19
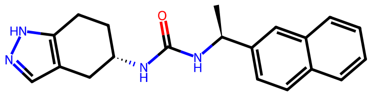	C[C@H](NC(=O)N[C@H]1CCc2[nH]ncc2C1)c1ccc2ccccc2c1	154163950	25	334.18	3.48
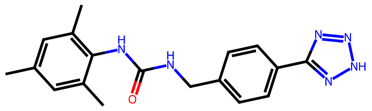	Cc1cc(C)c(NC(=O)NCc2ccc(-c3nn[nH]n3)cc2)c(C)c1	1591948656	25	336.17	3.11
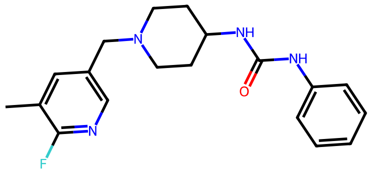	Cc1cc(CN2CCC(NC(=O)Nc3ccccc3)CC2)cnc1F	891802068	25	342.19	3.32
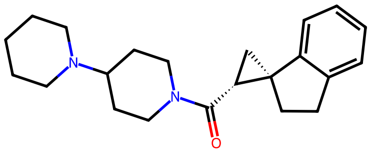	O=C([C@@H]1C[C@]12CCc1ccccc12)N1CCC(N2CCCCC2)CC1	266541368	25	338.24	3.37

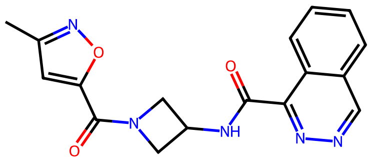	Cc1cc(C(=O)N2CC(NC(=O)c3nncc4ccccc34)C2)on1	996893074	25	337.12	1.18
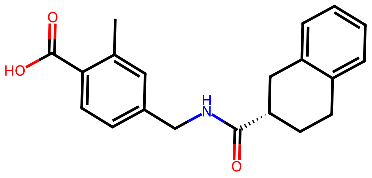	Cc1cc(CNC(=O)[C@H]2CCc3ccccc3C2)ccc1C(=O)O	1777422646	24	323.15	3.11
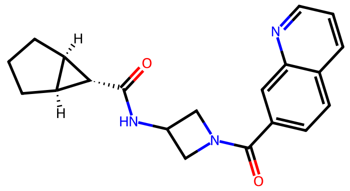	O=C(NC1CN(C(=O)c2ccc3cccnc3c2)C1)[C@@H]1[C@H]2CCC[C@H]21	990693171	25	335.16	2.22
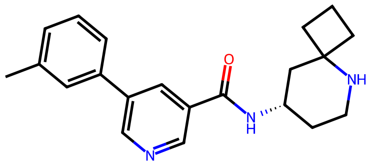	Cc1cccc(-c2cncc(C(=O)N[C@H]3CCNC4(CCC4)C3)c2)c1	649370611	25	335.2	3.46
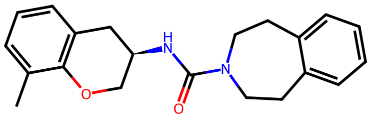	Cc1cccc2c1OC[C@H](NC(=O)N1CCc3ccccc3CC1)C2	1682905670	25	336.18	3.11
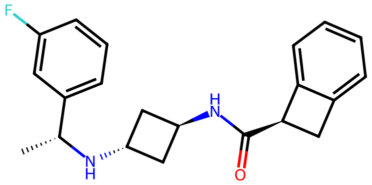	C[C@@H](N[C@H]1C[C@H](NC(=O)[C@@H]2Cc3ccccc32)C1)c1cccc(F)c1	1020529078	25	338.18	3.46
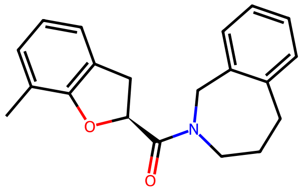	Cc1cccc2c1O[C@H](C(=O)N1CCCc3ccccc3C1)C2	449847829	23	307.16	3.27
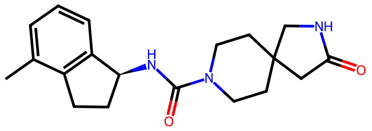	Cc1cccc2c1CC[C@@H]2NC(=O)N1CCC2(CC1)CNC(=O)C2	74108011	24	327.19	2.29

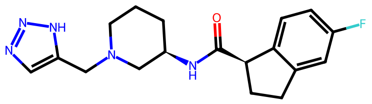	O=C(N[C@@H]1CCCN(Cc2cnn[nH]2)C1)[C@@H]1CCc2cc(F)ccc21	1578016644	25	343.18	1.75
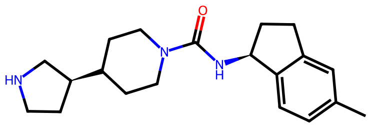	Cc1ccc2c(c1)CC[C@@H]2NC(=O)N1CCC([C@H]2CCNC2)CC1	417247194	24	327.23	3.01
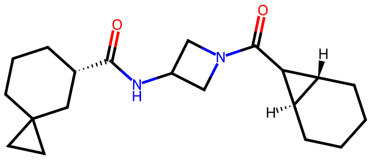	O=C(NC1CN(C(=O)C2[C@@H]3CCCC[C@@H]23)C1)[C@H]1CCCC2(CC2)C1	991507773	24	330.23	2.72
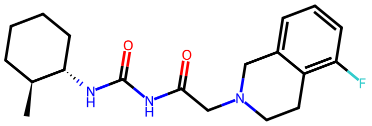	C[C@H]1CCCC[C@@H]1NC(=O)NC(=O)CN1CCc2c(F)cccc2C1	339779914	25	347.2	2.59
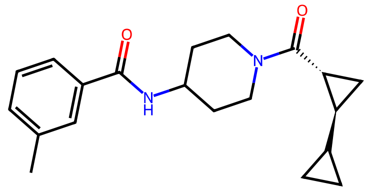	Cc1cccc(C(=O)NC2CCN(C(=O)[C@@H]3C[C@H]3C3CC3)CC2)c1	946872777	24	326.2	2.76
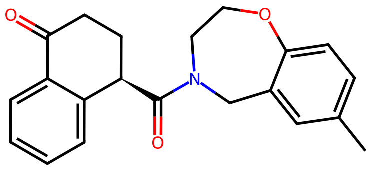	Cc1ccc2c(c1)CN(C(=O)[C@@H]1CCC(=O)c3ccccc31)CCO2	376412630	25	335.15	3.48
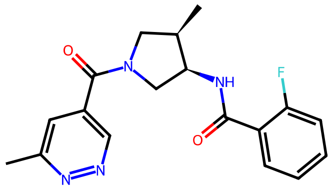	Cc1cc(C(=O)N2C[C@@H](C)[C@@H](NC(=O)c3ccccc3F)C2)cnn1	966347172	25	342.15	1.81
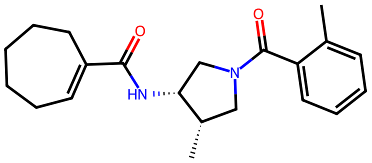	Cc1ccccc1C(=O)N1C[C@H](C)[C@H](NC(=O)C2=CCCCCC2)C1	966097433	25	340.22	3.46

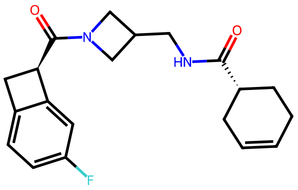	O=C(NCC1CN(C(=O)[C@@H]2Cc3ccc(F)cc32)C1)[C@H]1CC=CCC1	1000868601	25	342.17	2.4
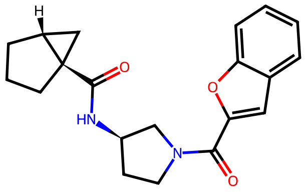	O=C(c1cc2ccccc2o1)N1CC[C@@H](NC(=O)[C@@]23CCC[C@@H]2C3)C1	936130302	25	338.16	2.95
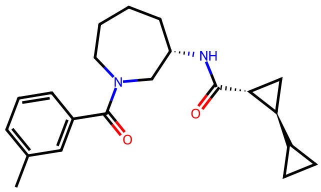	Cc1cccc(C(=O)N2CCCC[C@H](NC(=O)[C@@H]3C[C@H]3C3CC3)C2)c1	943281277	25	340.22	3.15
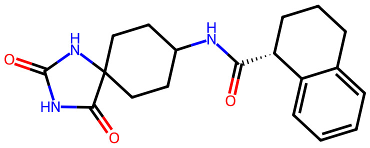	O=C1NC(=O)C2(CCC(NC(=O)[C@@H]3CCCc4ccccc43)CC2)N1	286210892	25	341.17	1.74
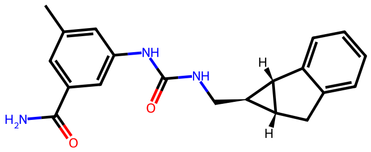	Cc1cc(NC(=O)NC[C@@H]2[C@H]3Cc4ccccc4[C@H]32)cc(C(N)=O)c1	583608644	25	335.16	2.8
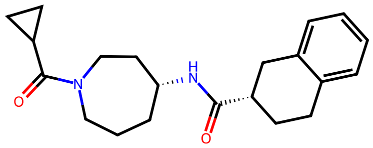	O=C(N[C@@H]1CCCN(C(=O)C2CC2)CC1)[C@H]1CCc2ccccc2C1	965224528	25	340.22	2.7
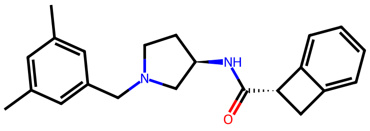	Cc1cc(C)cc(CN2CC[C@@H](NC(=O)[C@H]3Cc4ccccc43)C2)c1	1015632847	25	334.2	3.33
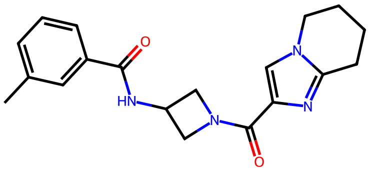	Cc1cccc(C(=O)NC2CN(C(=O)c3cn4c(n3)CCCC4)C2)c1	990142474	25	338.17	1.78

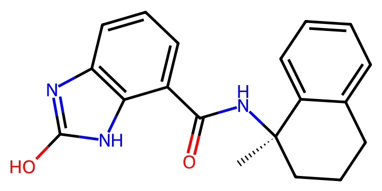	C[C@@]1(NC(=O)c2cccc3nc(O)[nH]c23)CCCc2ccccc21	516978204	24	321.15	3.25
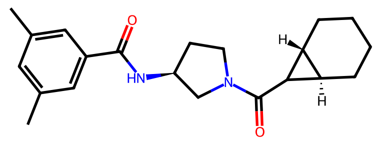	Cc1cc(C)cc(C(=O)N[C@H]2CCN(C(=O)C3[C@@H]4CCCC[C@@H]34)C2)c1	938749344	25	340.22	3.07
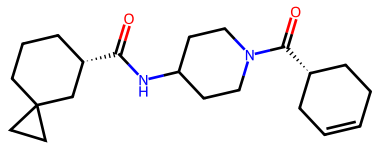	O=C(NC1CCN(C(=O)[C@H]2CC=CCC2)CC1)[C@H]1CCCC2(CC2)C1	944106679	25	344.25	3.42
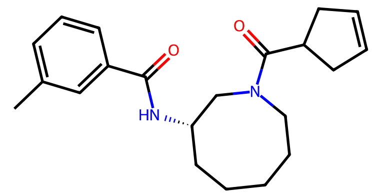	Cc1cccc(C(=O)N[C@H]2CCCCCN(C(=O)C3CC=CC3)C2)c1	986952480	25	340.22	3.46
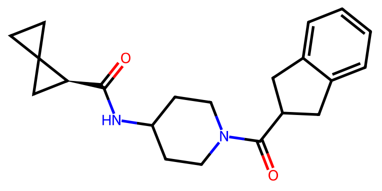	O=C(NC1CCN(C(=O)C2Cc3ccccc3C2)CC1)[C@H]1CC12CC2	948551342	25	338.2	2.31
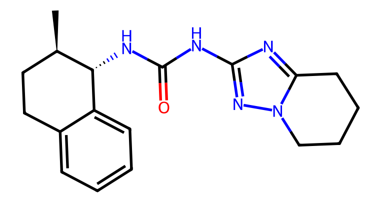	C[C@@H]1CCc2ccccc2[C@H]1NC(=O)Nc1nc2n(n1)CCCC2	333657451	24	325.19	3.06
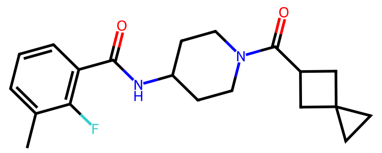	Cc1cccc(C(=O)NC2CCN(C(=O)C3CC4(CC4)C3)CC2)c1F	948305706	25	344.19	3.05
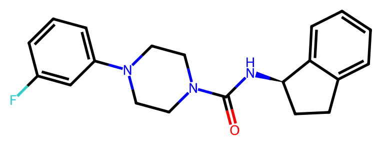	O=C(N[C@@H]1CCc2ccccc21)N1CCN(c2cccc(F)c2)CC1	1192985882	25	339.17	3.34

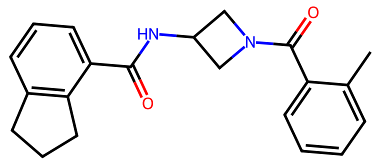	Cc1ccccc1C(=O)N1CC(NC(=O)c2cccc3c2CCC3)C1	994394912	25	334.17	2.74
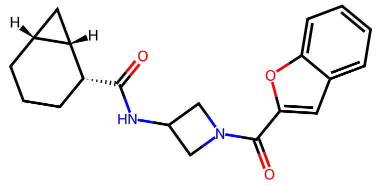	O=C(NC1CN(C(=O)c2cc3ccccc3o2)C1)[C@@H]1CCC[C@@H]2C[C@@H]21	994726095	25	338.16	2.81
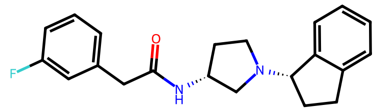	O=C(Cc1cccc(F)c1)N[C@@H]1CCN([C@H]2CCc3ccccc32)C1	1016184465	25	338.18	3.25
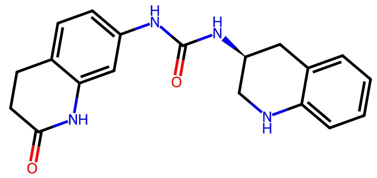	O=C1CCc2ccc(NC(=O)N[C@@H]3CNc4ccccc4C3)cc2N1	652580279	25	336.16	2.73
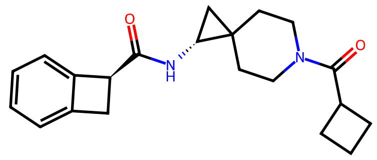	O=C(N[C@@H]1CC12CCN(C(=O)C1CCC1)CC2)[C@H]1Cc2ccccc21	965003313	25	338.2	2.62
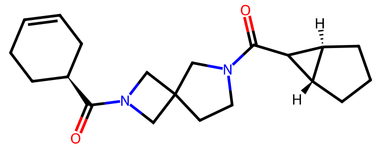	O=C(C1[C@H]2CCC[C@H]12)N1CCC2(C1)CN(C(=O)[C@@H]1CC=CCC1)C2	1018770035	24	328.22	2.45
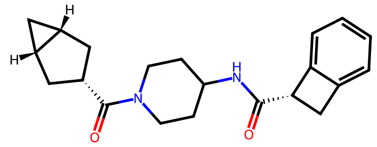	O=C(NC1CCN(C(=O)[C@@H]2C[C@@H]3C[C@@H]3C2)CC1)[C@H]1Cc2ccccc21	947908748	25	338.2	2.48
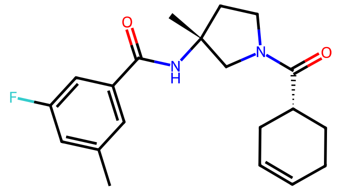	Cc1cc(F)cc(C(=O)N[C@]2(C)CCN(C(=O)[C@H]3CC=CCC3)C2)c1	957700733	25	344.19	3.21

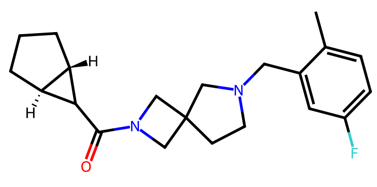	Cc1ccc(F)cc1CN1CCC2(C1)CN(C(=O)C1[C@H]3CCC[C@H]13)C2	1072590518	25	342.21	3.21
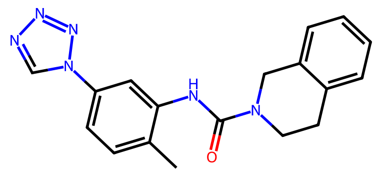	Cc1ccc(-n2cnnn2)cc1NC(=O)N1CCc2ccccc2C1	40540559	25	334.15	2.56
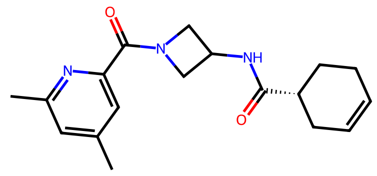	Cc1cc(C)nc(C(=O)N2CC(NC(=O)[C@H]3CC=CCC3)C2)c1	990222090	23	313.18	2.0
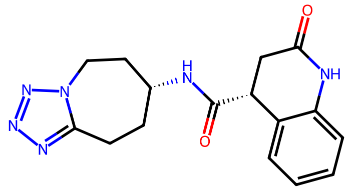	O=C1C[C@@H](C(=O)N[C@@H]2CCc3nnnn3CC2)c2ccccc2N1	1418054930	24	326.15	0.62
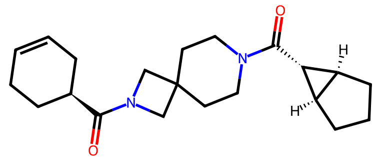	O=C([C@@H]1CC=CCC1)N1CC2(CCN(C(=O)[C@@H]3[C@H]4CCC[C@H]43)CC2)C1	944903090	25	342.23	2.84
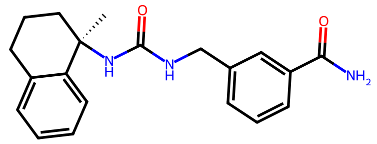	C[C@@]1(NC(=O)NCc2cccc(C(N)=O)c2)CCCc2ccccc21	359133145	25	337.18	2.84
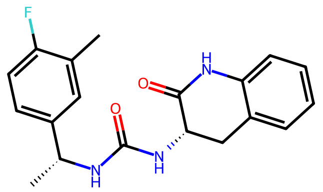	Cc1cc([C@@H](C)NC(=O)N[C@H]2Cc3ccccc3NC2=O)ccc1F	636903402	25	341.15	3.06
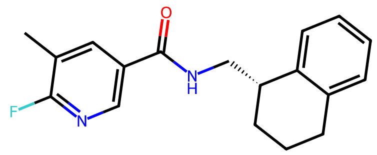	Cc1cc(C(=O)NC[C@H]2CCCc3ccccc32)cnc1F	857574268	22	298.15	3.38

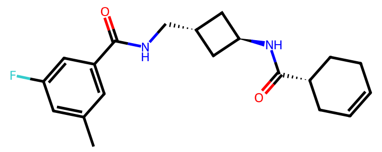	Cc1cc(F)cc(C(=O)NC[C@H]2C[C@H](NC(=O)[C@H]3CC=CCC3)C2)c1	980623041	25	344.19	3.12
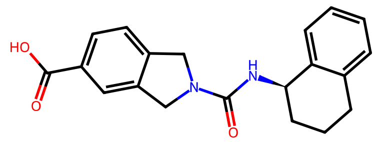	O=C(O)c1ccc2c(c1)CN(C(=O)N[C@@H]1CCCc3ccccc31)C2	326988953	25	336.15	3.49
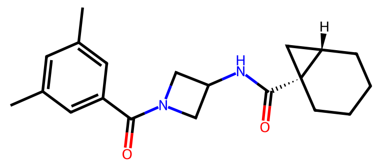	Cc1cc(C)cc(C(=O)N2CC(NC(=O)[C@@]34CCCC[C@H]3C4)C2)c1	999287932	24	326.2	2.82
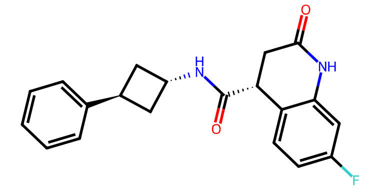	O=C1C[C@@H](C(=O)N[C@H]2C[C@H](c3ccccc3)C2)c2ccc(F)cc2N1	280724918	25	338.14	3.31
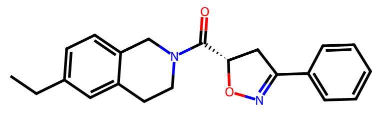	CCc1ccc2c(c1)CCN(C(=O)[C@@H]1CC(c3ccccc3)=NO1)C2	1440188141	25	334.17	3.33
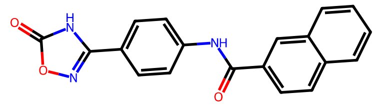	O=C(Nc1ccc(-c2noc(=O)[nH]2)cc1)c1ccc2ccccc2c1	170748217	25	331.1	3.44
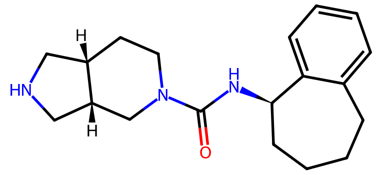	O=C(N[C@@H]1CCCCc2ccccc21)N1CC[C@H]2CNC[C@H]2C1	578333974	23	313.22	2.71
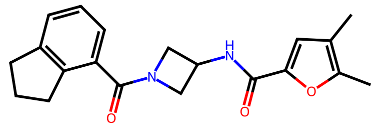	Cc1cc(C(=O)NC2CN(C(=O)c3cccc4c3CCC4)C2)oc1C	990846477	25	338.16	2.64

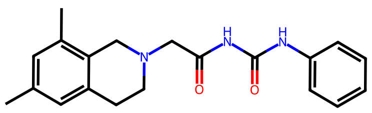	Cc1cc(C)c2c(c1)CCN(CC(=O)NC(=O)Nc1ccccc1)C2	1655153469	25	337.18	3.01
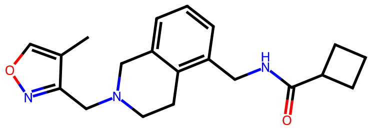	Cc1conc1CN1CCc2c(CNC(=O)C3CCC3)cccc2C1	1051274369	25	339.19	2.96
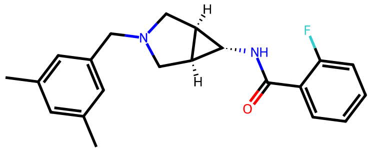	Cc1cc(C)cc(CN2C[C@H]3[C@H](NC(=O)c4ccccc4F)[C@H]3C2)c1	958088846	25	338.18	3.3
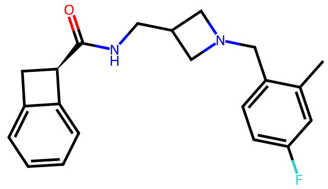	Cc1cc(F)ccc1CN1CC(CNC(=O)[C@@H]2Cc3ccccc32)C1	1031944522	25	338.18	3.02
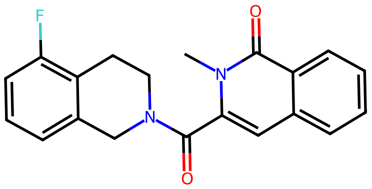	Cn1c(C(=O)N2CCc3c(F)cccc3C2)cc2ccccc2c1=O	354327458	25	336.13	2.88
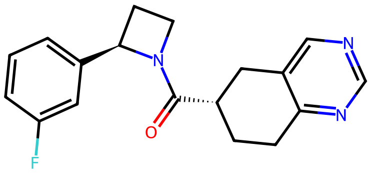	O=C([C@H]1CCc2ncncc2C1)N1CC[C@@H]1c1cccc(F)c1	648801543	23	311.14	2.69
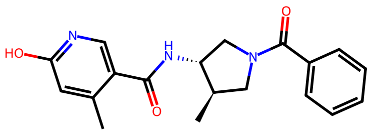	Cc1cc(O)ncc1C(=O)N[C@@H]1CN(C(=O)c2ccccc2)C[C@H]1C	940030516	25	339.16	1.99
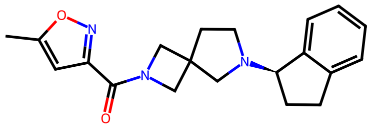	Cc1cc(C(=O)N2CC3(CCN([C@@H]4CCc5ccccc54)C3)C2)no1	1072449117	25	337.18	2.82

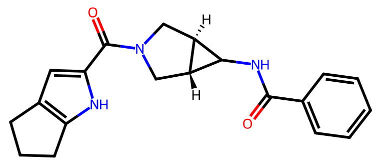	O=C(NC1[C@@H]2CN(C(=O)c3cc4c([nH]3)CCC4)C[C@@H]12)c1ccccc1	1012709257	25	335.16	2.0
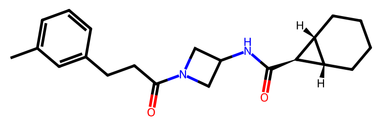	Cc1cccc(CCC(=O)N2CC(NC(=O)[C@@H]3[C@H]4CCCC[C@H]43)C2)c1	990653234	25	340.22	2.69
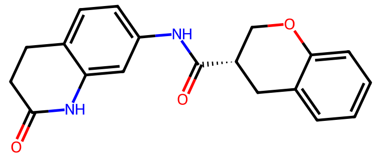	O=C1CCc2ccc(NC(=O)[C@@H]3COc4ccccc4C3)cc2N1	172658594	24	322.13	2.76
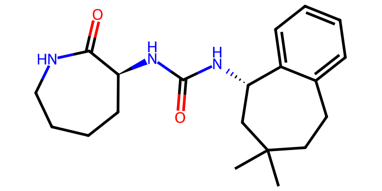	CC1(C)CCc2ccccc2[C@@H](NC(=O)N[C@H]2CCCCNC2=O)C1	440926762	25	343.23	3.06
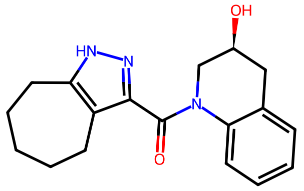	O=C(c1n[nH]c2c1CCCCC2)N1C[C@@H](O)Cc2ccccc21	279519732	23	311.16	2.24
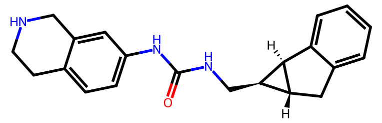	O=C(NC[C@@H]1[C@H]2Cc3ccccc3[C@@H]21)Nc1ccc2c(c1)CNCC2	563500347	25	333.18	3.04
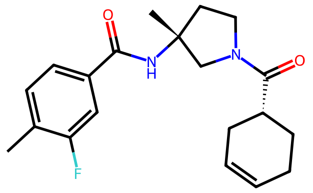	Cc1ccc(C(=O)N[C@]2(C)CCN(C(=O)[C@H]3CC=CCC3)C2)cc1F	957196443	25	344.19	3.21
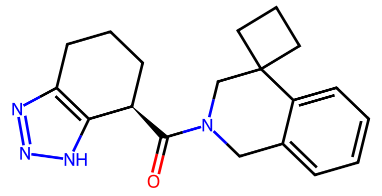	O=C([C@@H]1CCCc2nn[nH]c21)N1Cc2ccccc2C2(CCC2)C1	1549383380	24	322.18	2.69

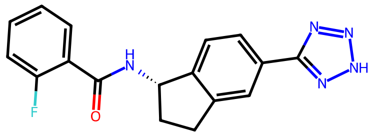	O=C(N[C@H]1CCc2cc(-c3nn[nH]n3)ccc21)c1ccccc1F	1606686401	24	323.12	2.42
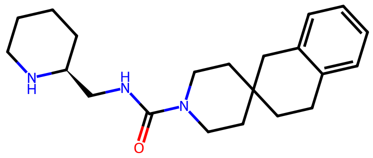	O=C(NC[C@@H]1CCCCN1)N1CCC2(CCc3ccccc3C2)CC1	1580647912	25	341.25	3.11
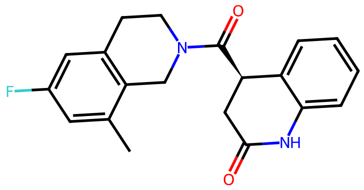	Cc1cc(F)cc2c1CN(C(=O)[C@@H]1CC(=O)Nc3ccccc31)CC2	337236355	25	338.14	3.14
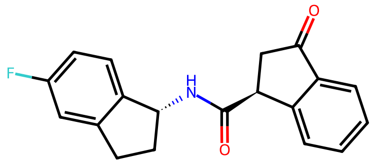	O=C1C[C@H](C(=O)N[C@@H]2CCc3cc(F)ccc32)c2ccccc21	277338887	23	309.12	3.3
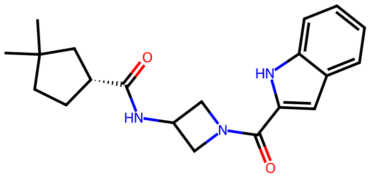	CC1(C)CC[C@@H](C(=O)NC2CN(C(=O)c3cc4ccccc4[nH]3)C2)C1	999237909	25	339.19	2.93
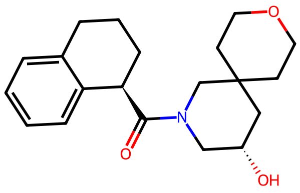	O=C([C@@H]1CCCc2ccccc21)N1C[C@@H](O)CC2(CCOCC2)C1	882968352	24	329.2	2.5
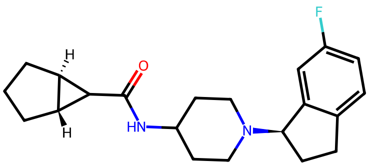	O=C(NC1CCN([C@@H]2CCc3ccc(F)cc32)CC1)C1[C@@H]2CCC[C@@H]12	1002545545	25	342.21	3.44
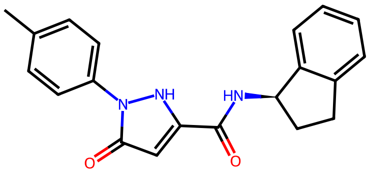	Cc1ccc(-n2[nH]c(C(=O)N[C@@H]3CCc4ccccc43)cc2=O)cc1	360170829	25	333.15	2.89

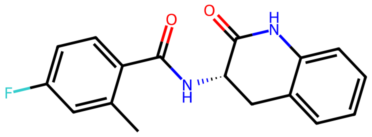	Cc1cc(F)ccc1C(=O)N[C@H]1Cc2ccccc2NC1=O	1364414171	22	298.11	2.43
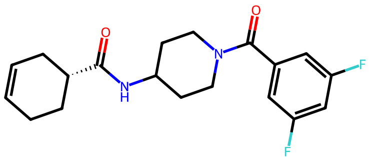	O=C(NC1CCN(C(=O)c2cc(F)cc(F)c2)CC1)[C@H]1CC=CCC1	948482226	25	348.16	3.04
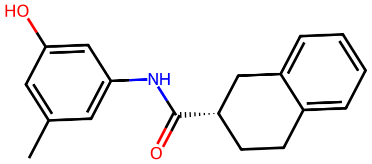	Cc1cc(O)cc(NC(=O)[C@H]2CCc3ccccc3C2)c1	629629255	21	281.14	3.44
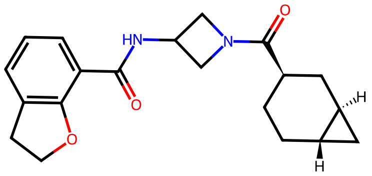	O=C(NC1CN(C(=O)[C@@H]2CC[C@H]3C[C@@H]3C2)C1)c1cccc2c1OCC2	1000416972	25	340.18	2.0
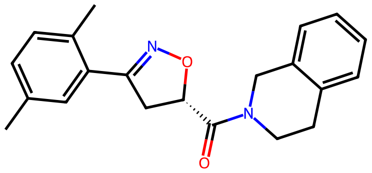	Cc1ccc(C)c(C2=NO[C@H](C(=O)N3CCc4ccccc4C3)C2)c1	1264069375	25	334.17	3.38
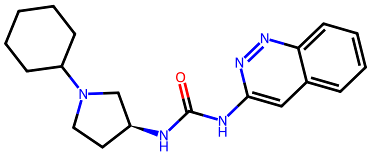	O=C(Nc1cc2ccccc2nn1)N[C@H]1CCN(C2CCCCC2)C1	931796734	25	339.21	3.16
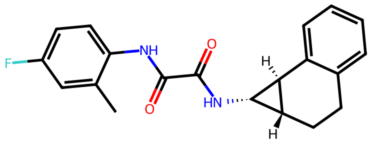	Cc1cc(F)ccc1NC(=O)C(=O)N[C@H]1[C@H]2CCc3ccccc3[C@@H]21	180334058	25	338.14	2.92
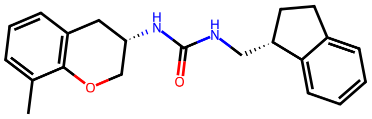	Cc1cccc2c1OC[C@@H](NC(=O)NC[C@@H]1CCc3ccccc31)C2	1668963684	25	336.18	3.33

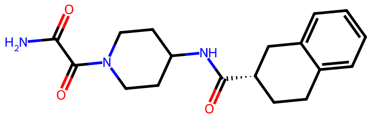	NC(=O)C(=O)N1CCC(NC(=O)[C@H]2CCc3ccccc3C2)CC1	948080692	24	329.17	0.38
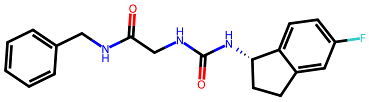	O=C(CNC(=O)N[C@H]1CCc2cc(F)ccc21)NCc1ccccc1	56840912	25	341.15	2.43
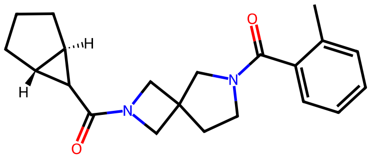	Cc1ccccc1C(=O)N1CCC2(C1)CN(C(=O)C1[C@@H]3CCC[C@@H]13)C2	1018909552	25	338.2	2.72
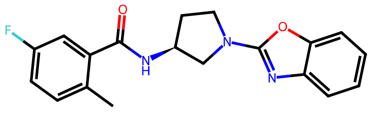	Cc1ccc(F)cc1C(=O)N[C@H]1CCN(c2nc3ccccc3o2)C1	1058502401	25	339.14	3.28
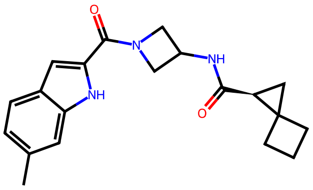	Cc1ccc2cc(C(=O)N3CC(NC(=O)[C@H]4CC45CCC5)C3)[nH]c2c1	991554414	25	337.18	2.61
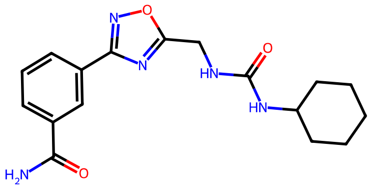	NC(=O)c1cccc(-c2noc(CNC(=O)NC3CCCCC3)n2)c1	680699959	25	343.16	1.97
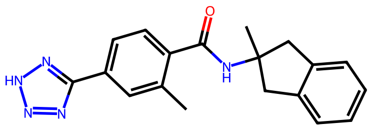	Cc1cc(-c2nn[nH]n2)ccc1C(=O)NC1(C)Cc2ccccc2C1	826246538	25	333.16	2.46
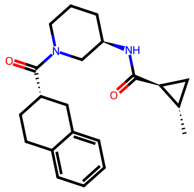	C[C@H]1C[C@@H]1C(=O)N[C@@H]1CCCN(C(=O)[C@H]2CCc3ccccc3C2)C1	966006944	25	340.22	2.55

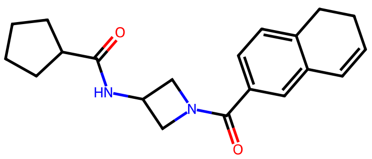	O=C(NC1CN(C(=O)c2ccc3c(c2)C=CCC3)C1)C1CCCC1	990039243	24	324.18	2.78
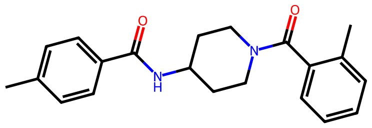	Cc1ccc(C(=O)NC2CCN(C(=O)c3ccccc3C)CC2)cc1	75057864	25	336.18	3.34
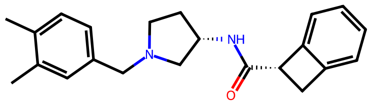	Cc1ccc(CN2CC[C@H](NC(=O)[C@H]3Cc4ccccc43)C2)cc1C	1015636499	25	334.2	3.33
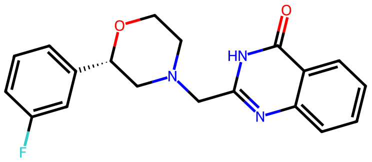	O=c1[nH]c(CN2CCO[C@@H](c3cccc(F)c3)C2)nc2ccccc12	66849104	25	339.14	2.64
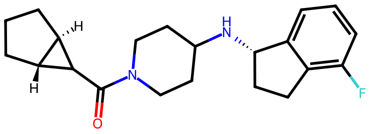	O=C(C1[C@@H]2CCC[C@@H]12)N1CCC(N[C@H]2CCc3c(F)cccc32)CC1	990788993	25	342.21	3.44
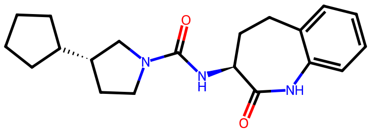	O=C1Nc2ccccc2CC[C@@H]1NC(=O)N1CC[C@H](C2CCCC2)C1	654631108	25	341.21	3.16
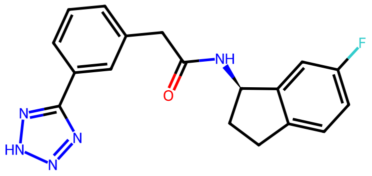	O=C(Cc1cccc(-c2nn[nH]n2)c1)N[C@@H]1CCc2ccc(F)cc21	823092689	25	337.13	2.35
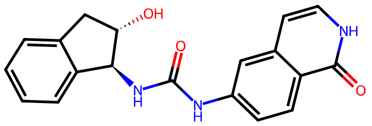	O=C(Nc1ccc2c(=O)[nH]ccc2c1)N[C@H]1c2ccccc2C[C@@H]1O	1219414260	25	335.13	2.31

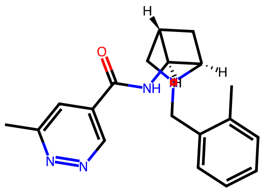	Cc1cc(C(=O)N[C@H]2[C@H]3C[C@H]2N(Cc2ccccc2C)C3)cnn1	1037383355	24	322.18	2.1
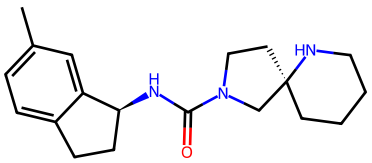	Cc1ccc2c(c1)[C@@H](NC(=O)N1CC[C@]3(CCCCN3)C1)CC2	1553507833	23	313.22	2.91
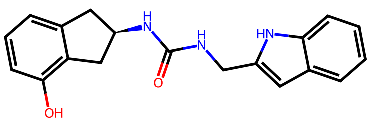	O=C(NCc1cc2ccccc2[nH]1)N[C@@H]1Cc2cccc(O)c2C1	428554698	24	321.15	2.84
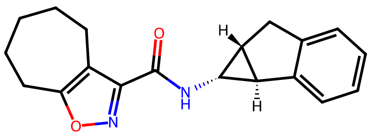	O=C(N[C@H]1[C@@H]2c3ccccc3C[C@H]21)c1noc2c1CCCCC2	156263494	23	308.15	3.01
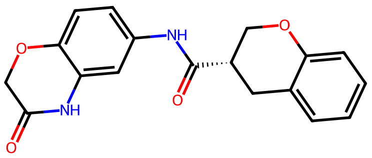	O=C1COc2ccc(NC(=O)[C@@H]3COc4ccccc4C3)cc2N1	248071820	24	324.11	2.21
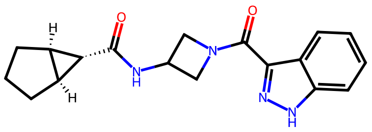	O=C(NC1CN(C(=O)c2n[nH]c3ccccc23)C1)[C@@H]1[C@H]2CCC[C@H]21	990708208	24	324.16	1.55
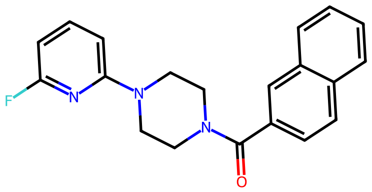	O=C(c1ccc2ccccc2c1)N1CCN(c2cccc(F)n2)CC1	919963570	25	335.14	3.34
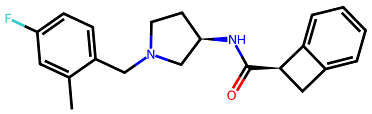	Cc1cc(F)ccc1CN1CC[C@@H](NC(=O)[C@@H]2Cc3ccccc32)C1	1015636267	25	338.18	3.16

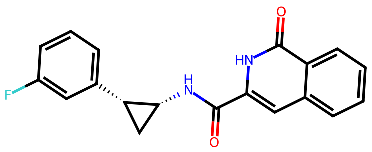	O=C(N[C@@H]1C[C@@H]1c1cccc(F)c1)c1cc2ccccc2c(=O)[nH]1	67333740	24	322.11	2.95
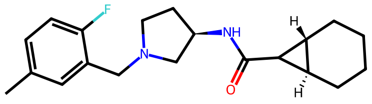	Cc1ccc(F)c(CN2CC[C@@H](NC(=O)C3[C@@H]4CCCC[C@@H]34)C2)c1	1032835044	24	330.21	3.26
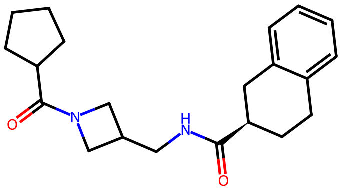	O=C(NCC1CN(C(=O)C2CCCC2)C1)[C@@H]1CCc2ccccc2C1	936099902	25	340.22	2.56
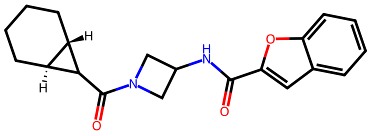	O=C(NC1CN(C(=O)C2[C@H]3CCCC[C@H]23)C1)c1cc2ccccc2o1	1000050510	25	338.16	2.81
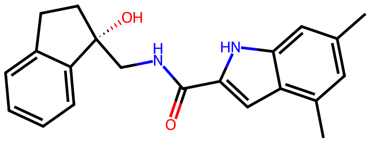	Cc1cc(C)c2cc(C(=O)NC[C@]3(O)CCc4ccccc43)[nH]c2c1	752555134	25	334.17	3.35
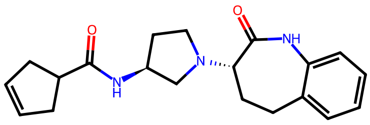	O=C(N[C@H]1CCN([C@H]2CCc3ccccc3NC2=O)C1)C1CC=CC1	1010415977	25	339.19	2.1
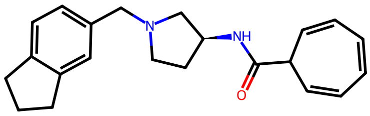	O=C(N[C@H]1CCN(Cc2ccc3c(c2)CCC3)C1)C1C=CC=CC=C1	1014591425	25	334.2	3.16
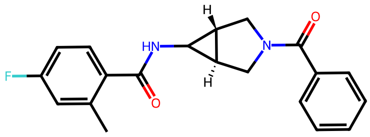	Cc1cc(F)ccc1C(=O)NC1[C@@H]2CN(C(=O)c3ccccc3)C[C@@H]12	1013518260	25	338.14	2.63

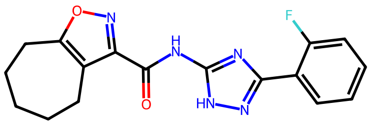	O=C(Nc1nc(-c2ccccc2F)n[nH]1)c1noc2c1CCCCC2	119960389	25	341.13	3.12
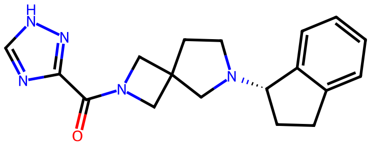	O=C(c1nc[nH]n1)N1CC2(CCN([C@H]3CCc4ccccc43)C2)C1	1073067222	24	323.17	1.64
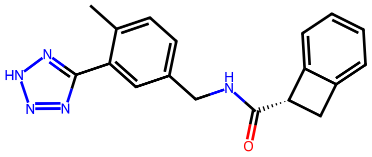	Cc1ccc(CNC(=O)[C@H]2Cc3ccccc32)cc1-c1nn[nH]n1	1581196840	24	319.14	2.13
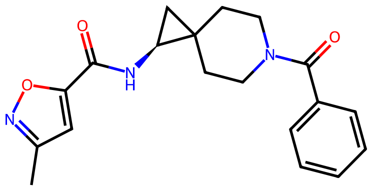	Cc1cc(C(=O)N[C@H]2CC23CCN(C(=O)c2ccccc2)CC3)on1	963028223	25	339.16	2.41
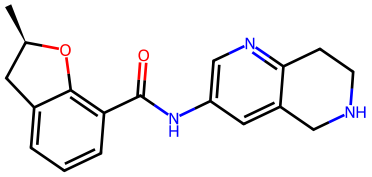	C[C@@H]1Cc2cccc(C(=O)Nc3cnc4c(c3)CNCC4)c2O1	1576866158	23	309.15	2.3
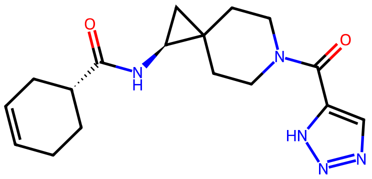	O=C(N[C@H]1CC12CCN(C(=O)c1cnn[nH]1)CC2)[C@H]1CC=CCC1	962970776	24	329.19	1.27
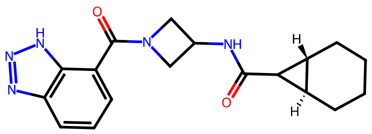	O=C(NC1CN(C(=O)c2cccc3nn[nH]c23)C1)C1[C@@H]2CCCC[C@@H]12	990651879	25	339.17	1.33
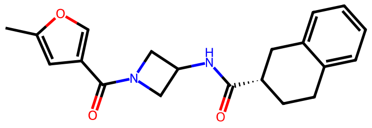	Cc1cc(C(=O)N2CC(NC(=O)[C@H]3CCc4ccccc4C3)C2)co1	1000537666	25	338.16	2.33

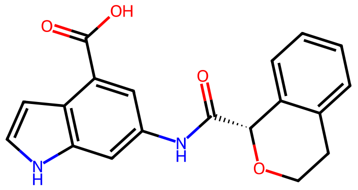	O=C(O)c1cc(NC(=O)[C@H]2OCCc3ccccc32)cc2[nH]ccc12	1602278162	25	336.11	3.12
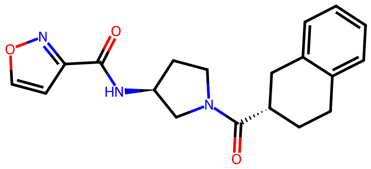	O=C(N[C@H]1CCN(C(=O)[C@H]2CCc3ccccc3C2)C1)c1ccon1	937718553	25	339.16	1.81
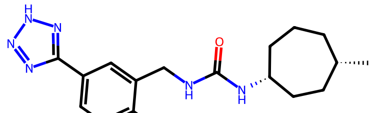	C[C@H]1CCC[C@@H](NC(=O)NCc2cc(-c3nn[nH]n3)ccc2F)CC1	1603888445	25	346.19	2.77
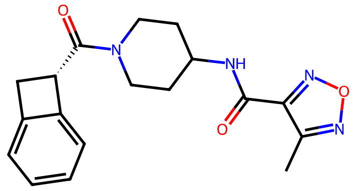	Cc1nonc1C(=O)NC1CCN(C(=O)[C@H]2Cc3ccccc32)CC1	948063519	25	340.15	1.44
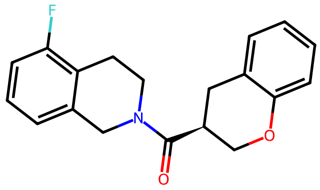	O=C([C@@H]1COc2ccccc2C1)N1CCc2c(F)cccc2C1	270531820	23	311.13	2.96
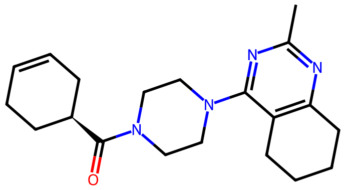	Cc1nc2c(c(N3CCN(C(=O)[C@@H]4CC=CCC4)CC3)n1)CCCC2	1055721288	25	340.23	2.67
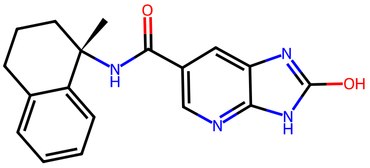	C[C@]1(NC(=O)c2cnc3[nH]c(O)nc3c2)CCCc2ccccc21	564884054	24	322.14	2.65
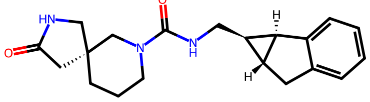	O=C1C[C@]2(CCCN(C(=O)NC[C@@H]3[C@H]4Cc5ccccc5[C@@H]43)C2)CN1	332906351	25	339.19	1.88

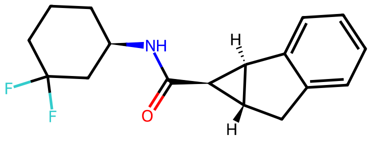	O=C(N[C@@H]1CCCC(F)(F)C1)[C@@H]1[C@H]2Cc3ccccc3[C@@H]21	626188118	21	291.14	3.27
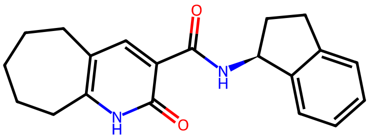	O=C(N[C@H]1CCc2ccccc21)c1cc2c([nH]c1=O)CCCCC2	78051036	24	322.17	3.06
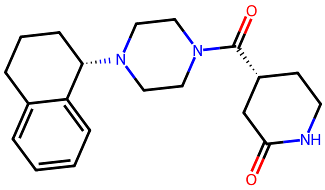	O=C1C[C@H](C(=O)N2CCN([C@H]3CCCc4ccccc43)CC2)CCN1	1381092586	25	341.21	1.73
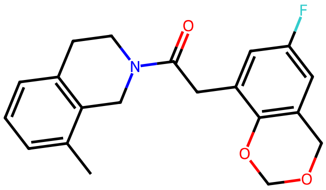	Cc1cccc2c1CN(C(=O)Cc1cc(F)cc3c1OCOC3)CC2	277005261	25	341.14	3.13
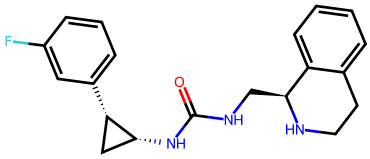	O=C(NC[C@@H]1NCCc2ccccc21)N[C@@H]1C[C@@H]1c1cccc(F)c1	572825140	25	339.17	2.87
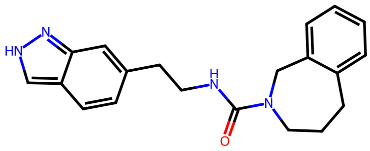	O=C(NCCc1ccc2c[nH]nc2c1)N1CCCc2ccccc2C1	504967859	25	334.18	3.26
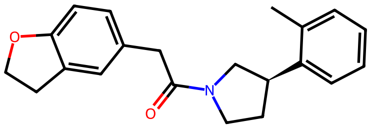	Cc1ccccc1[C@H]1CCN(C(=O)Cc2ccc3c(c2)CCO3)C1	504865777	24	321.17	3.49
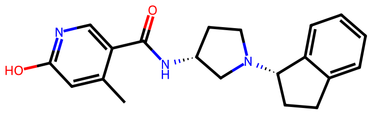	Cc1cc(O)ncc1C(=O)N[C@@H]1CCN([C@H]2CCc3ccccc32)C1	1015680496	25	337.18	2.59

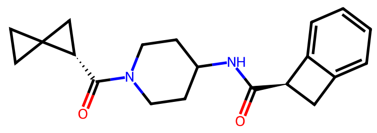	O=C(NC1CCN(C(=O)[C@H]2CC23CC3)CC1)[C@@H]1Cc2ccccc21	948571711	24	324.18	2.23
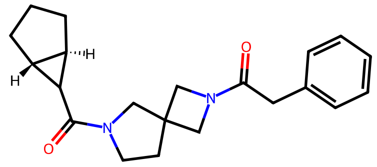	O=C(Cc1ccccc1)N1CC2(CCN(C(=O)C3[C@@H]4CCC[C@@H]34)C2)C1	1019171306	25	338.2	2.34
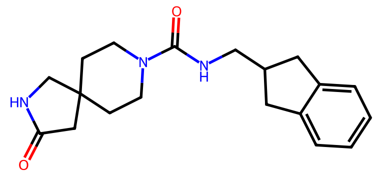	O=C1CC2(CCN(C(=O)NCC3Cc4ccccc4C3)CC2)CN1	531442532	24	327.19	1.71
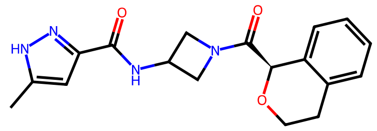	Cc1cc(C(=O)NC2CN(C(=O)[C@@H]3OCCc4ccccc43)C2)n[nH]1	990370873	25	340.15	0.97
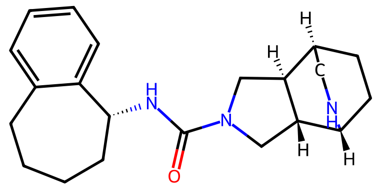	O=C(N[C@@H]1CCCCc2ccccc21)N1C[C@@H]2[C@@H](C1)[C@H]1CC[C@H]2CN1	626961581	25	339.23	3.09
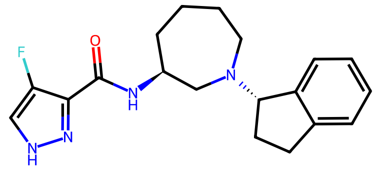	O=C(N[C@H]1CCCCN([C@H]2CCc3ccccc32)C1)c1n[nH]cc1F	1034381098	25	342.19	2.82
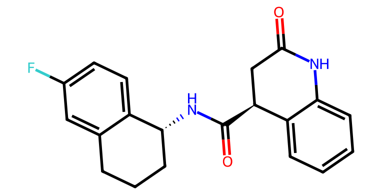	O=C1C[C@H](C(=O)N[C@@H]2CCCc3cc(F)ccc32)c2ccccc2N1	157618617	25	338.14	3.45
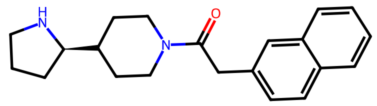	O=C(Cc1ccc2ccccc2c1)N1CCC([C@H]2CCCN2)CC1	261600226	24	322.2	3.37

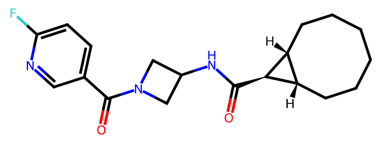	O=C(NC1CN(C(=O)c2ccc(F)nc2)C1)[C@@H]1[C@H]2CCCCCC[C@H]21	1000484318	25	345.19	2.38
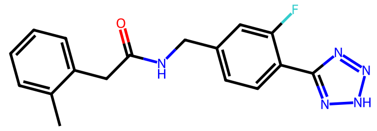	Cc1ccccc1CC(=O)NCc1ccc(-c2nn[nH]n2)c(F)c1	1581258653	24	325.13	2.17
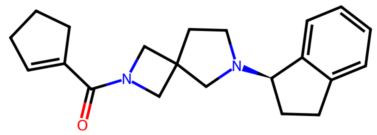	O=C(C1=CCCC1)N1CC2(CCN([C@@H]3CCc4ccccc43)C2)C1	1072615589	24	322.2	3.32
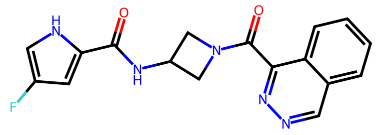	O=C(NC1CN(C(=O)c2nncc3ccccc23)C1)c1cc(F)c[nH]1	996213802	25	339.11	1.35
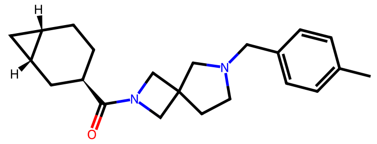	Cc1ccc(CN2CCC3(C2)CN(C(=O)[C@@H]2CC[C@H]4C[C@H]4C2)C3)cc1	1072879511	25	338.24	3.47
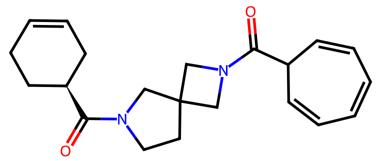	O=C(C1C=CC=CC=C1)N1CC2(CCN(C(=O)[C@@H]3CC=CCC3)C2)C1	1019080587	25	338.2	2.7
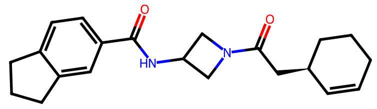	O=C(NC1CN(C(=O)C[C@@H]2C=CCCC2)C1)c1ccc2c(c1)CCC2	991368725	25	338.2	2.86
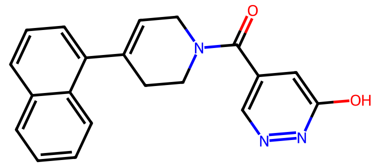	O=C(c1cnnc(O)c1)N1CC=C(c2cccc3ccccc23)CC1	1136453266	25	331.13	3.26

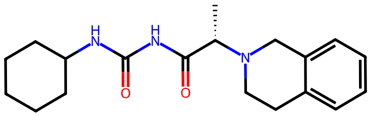	C[C@@H](C(=O)NC(=O)NC1CCCCC1)N1CCc2ccccc2C1	38332293	24	329.21	2.59
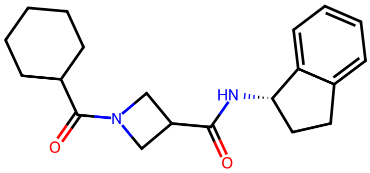	O=C(N[C@H]1CCc2ccccc21)C1CN(C(=O)C2CCCCC2)C1	454415998	24	326.2	2.83
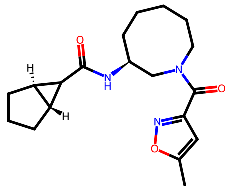	Cc1cc(C(=O)N2CCCCC[C@H](NC(=O)C3[C@@H]4CCC[C@@H]34)C2)no1	986888454	25	345.21	2.53
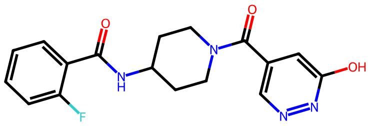	O=C(NC1CCN(C(=O)c2cnnc(O)c2)CC1)c1ccccc1F	947096041	25	344.13	1.36
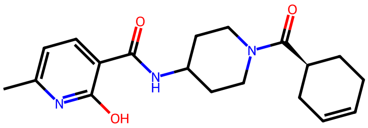	Cc1ccc(C(=O)NC2CCN(C(=O)[C@@H]3CC=CCC3)CC2)c(O)n1	944127722	25	343.19	2.17
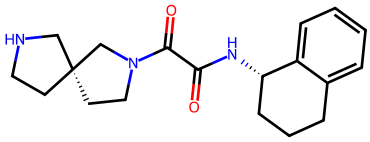	O=C(N[C@H]1CCCc2ccccc21)C(=O)N1CC[C@@]2(CCNC2)C1	514663740	24	327.19	1.39
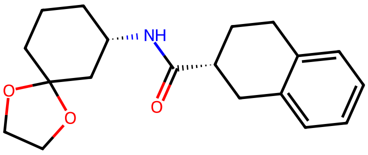	O=C(N[C@H]1CCCC2(C1)OCCO2)[C@@H]1CCc2ccccc2C1	715634485	23	315.18	2.59
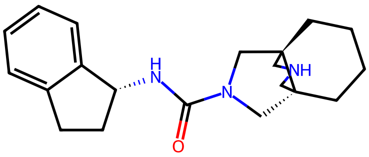	O=C(N[C@@H]1CCc2ccccc21)N1C[C@@]23CCCC[C@@]2(CNC3)C1	1553507654	24	325.22	2.85

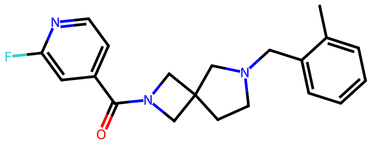	Cc1ccccc1CN1CCC2(C1)CN(C(=O)c1ccnc(F)c1)C2	1072536086	25	339.17	2.88
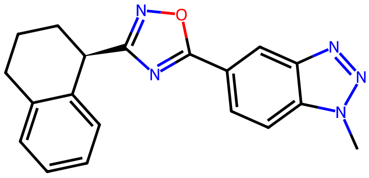	Cn1nnc2cc(-c3nc([C@@H]4CCCc5ccccc54)no3)ccc21	273760911	25	331.14	3.49
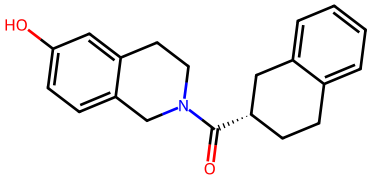	O=C([C@H]1CCc2ccccc2C1)N1CCc2cc(O)ccc2C1	1336312162	23	307.16	3.08
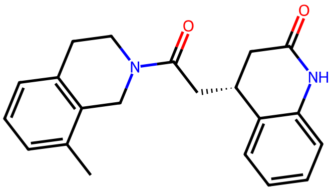	Cc1cccc2c1CN(C(=O)C[C@@H]1CC(=O)Nc3ccccc31)CC2	935646794	25	334.17	3.4
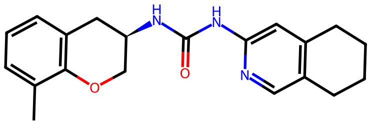	Cc1cccc2c1OC[C@H](NC(=O)Nc1cc3c(cn1)CCCC3)C2	1672342115	25	337.18	3.39
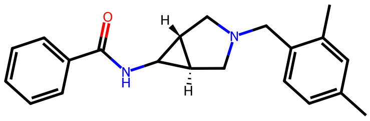	Cc1ccc(CN2C[C@H]3C(NC(=O)c4ccccc4)[C@@H]3C2)c(C)c1	958115687	24	320.19	3.16
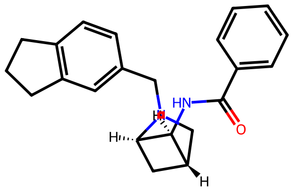	O=C(N[C@H]1[C@H]2C[C@H]1N(Cc1ccc3c(c1)CCC3)C2)c1ccccc1	1037284697	25	332.19	3.18
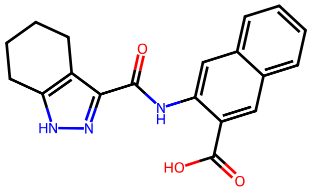	O=C(O)c1cc2ccccc2cc1NC(=O)c1n[nH]c2c1CCCC2	238019169	25	335.13	3.39

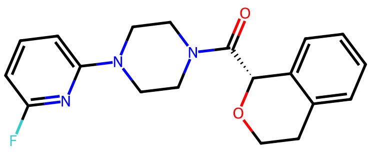	O=C([C@H]1OCCc2ccccc21)N1CCN(c2cccc(F)n2)CC1	1055978677	25	341.15	2.18
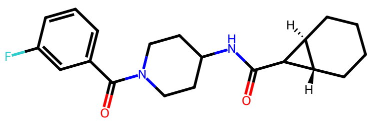	O=C(NC1CCN(C(=O)c2cccc(F)c2)CC1)C1[C@H]2CCCC[C@H]12	943774551	25	344.19	2.98
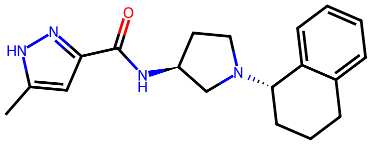	Cc1cc(C(=O)N[C@H]2CCN([C@H]3CCCc4ccccc43)C2)n[nH]1	1010333904	24	324.2	2.6
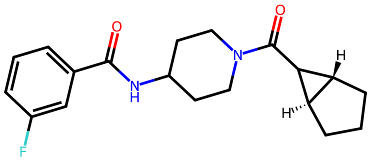	O=C(NC1CCN(C(=O)C2[C@@H]3CCC[C@@H]23)CC1)c1cccc(F)c1	946066010	24	330.17	2.59
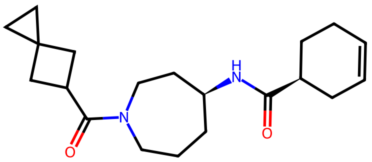	O=C(N[C@H]1CCCN(C(=O)C2CC3(CC3)C2)CC1)[C@@H]1CC=CCC1	964740738	24	330.23	3.03
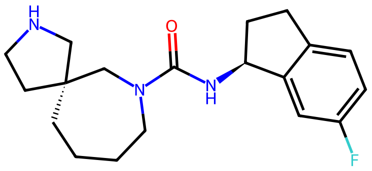	O=C(N[C@H]1CCc2ccc(F)cc21)N1CCCC[C@@]2(CCNC2)C1	891523612	24	331.21	2.99
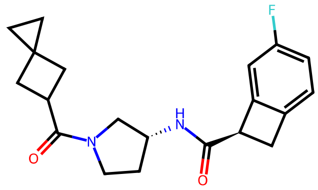	O=C(N[C@@H]1CCN(C(=O)C2CC3(CC3)C2)C1)[C@@H]1Cc2ccc(F)cc21	942285076	25	342.17	2.37
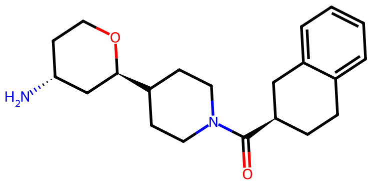	N[C@@H]1CCO[C@@H](C2CCN(C(=O)[C@@H]3CCc4ccccc4C3)CC2)C1	566407982	25	342.23	2.54

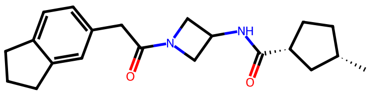	C[C@H]1CC[C@@H](C(=O)NC2CN(C(=O)Cc3ccc4c(c3)CCC4)C2)C1	991428361	25	340.22	2.48
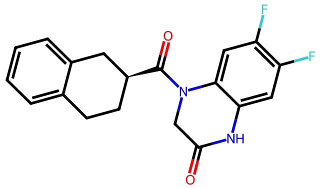	O=C1CN(C(=O)[C@H]2CCc3ccccc3C2)c2cc(F)c(F)cc2N1	1626349867	25	342.12	3.06
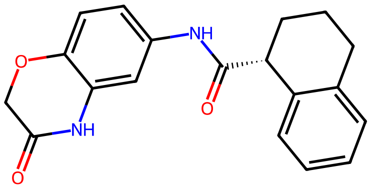	O=C1COc2ccc(NC(=O)[C@@H]3CCCc4ccccc43)cc2N1	44336017	24	322.13	3.08
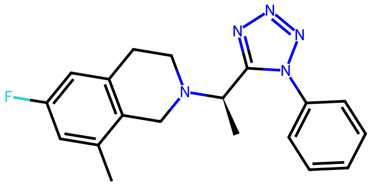	Cc1cc(F)cc2c1CN([C@H](C)c1nnnn1-c1ccccc1)CC2	337383514	25	337.17	3.23
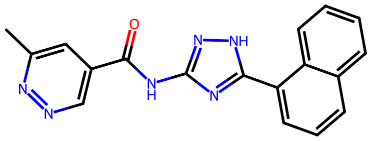	Cc1cc(C(=O)Nc2n[nH]c(-c3cccc4ccccc34)n2)cnn1	348728186	25	330.12	2.98
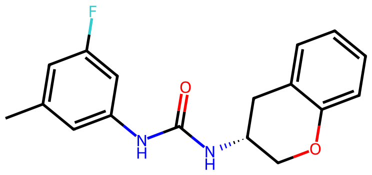	Cc1cc(F)cc(NC(=O)N[C@H]2COc3ccccc3C2)c1	178260005	22	300.13	3.26
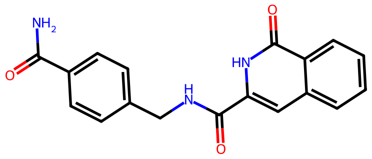	NC(=O)c1ccc(CNC(=O)c2cc3ccccc3c(=O)[nH]2)cc1	56825410	24	321.11	1.56
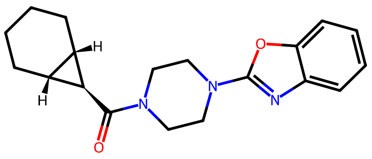	O=C([C@@H]1[C@H]2CCCC[C@H]21)N1CCN(c2nc3ccccc3o2)CC1	245455839	24	325.18	2.91

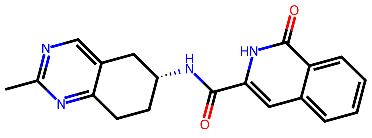	Cc1ncc2c(n1)CC[C@@H](NC(=O)c1cc3ccccc3c(=O)[nH]1)C2	30387010	25	334.14	1.91
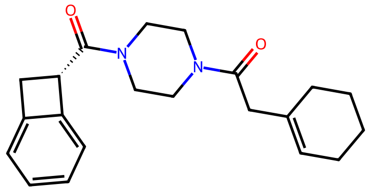	O=C(CC1=CCCCC1)N1CCN(C(=O)[C@H]2Cc3ccccc32)CC1	1010710342	25	338.2	2.89
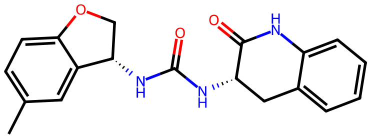	Cc1ccc2c(c1)[C@@H](NC(=O)N[C@H]1Cc3ccccc3NC1=O)CO2	636934829	25	337.14	2.29
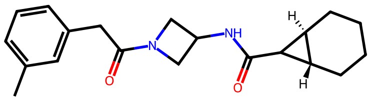	Cc1cccc(CC(=O)N2CC(NC(=O)C3[C@H]4CCCC[C@H]34)C2)c1	990648583	24	326.2	2.3
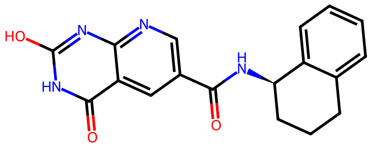	O=C(N[C@@H]1CCCc2ccccc21)c1cnc2nc(O)[nH]c(=O)c2c1	47476959	25	336.12	1.83
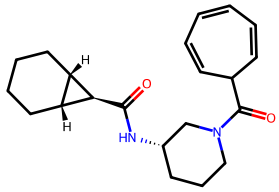	O=C(N[C@H]1CCCN(C(=O)C2C=CC=CC=C2)C1)[C@@H]1[C@H]2CCCC[C@H]21	951022595	25	340.22	2.83
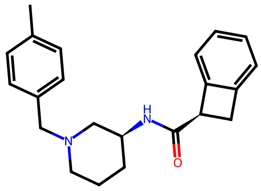	Cc1ccc(CN2CCC[C@H](NC(=O)[C@@H]3Cc4ccccc43)C2)cc1	1008373612	25	334.2	3.42
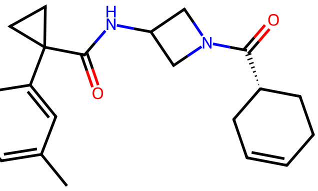	Cc1cccc(C2(C(=O)NC3CN(C(=O)[C@H]4CC=CCC4)C3)CC2)c1	993893399	25	338.2	2.71

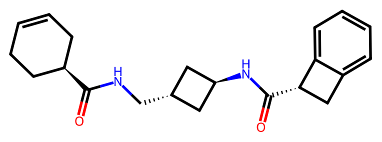	O=C(NC[C@H]1C[C@H](NC(=O)[C@H]2Cc3ccccc32)C1)[C@@H]1CC=CCC1	979475050	25	338.2	2.69
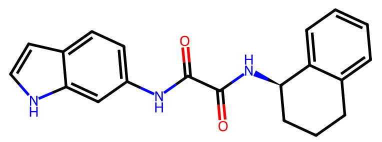	O=C(Nc1ccc2cc[nH]c2c1)C(=O)N[C@@H]1CCCc2ccccc21	192487047	25	333.15	3.3
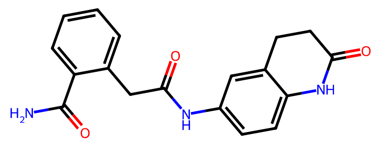	NC(=O)c1ccccc1CC(=O)Nc1ccc2c(c1)CCC(=O)N2	192026979	24	323.13	1.85
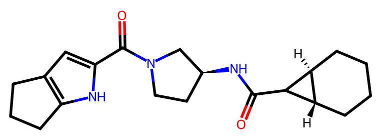	O=C(N[C@H]1CCN(C(=O)c2cc3c([nH]2)CCC3)C1)C1[C@H]2CCCC[C@H]12	937788573	25	341.21	2.27
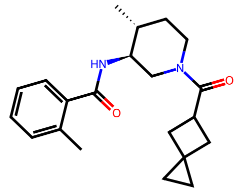	Cc1ccccc1C(=O)N[C@@H]1CN(C(=O)C2CC3(CC3)C2)CC[C@H]1C	1004429300	25	340.22	3.15
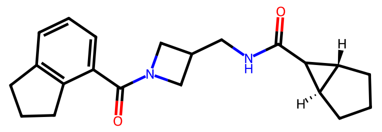	O=C(NCC1CN(C(=O)c2cccc3c2CCC3)C1)C1[C@@H]2CCC[C@@H]12	1001355157	25	338.2	2.41
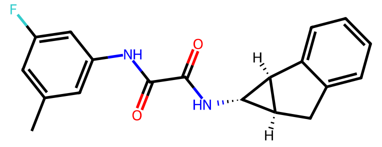	Cc1cc(F)cc(NC(=O)C(=O)N[C@H]2[C@@H]3Cc4ccccc4[C@H]23)c1	153510461	24	324.13	2.53
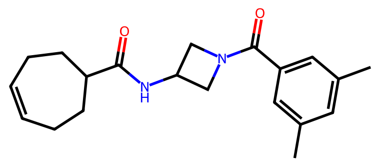	Cc1cc(C)cc(C(=O)N2CC(NC(=O)C3CCC=CCC3)C2)c1	991822685	24	326.2	2.99

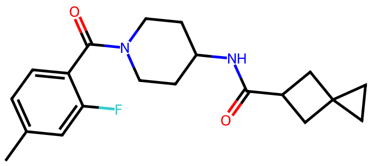	Cc1ccc(C(=O)N2CCC(NC(=O)C3CC4(CC4)C3)CC2)c(F)c1	948559262	25	344.19	3.05
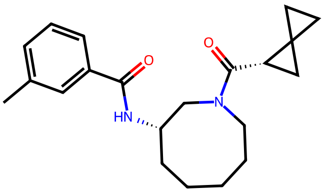	Cc1cccc(C(=O)N[C@H]2CCCCCN(C(=O)[C@H]3CC34CC4)C2)c1	987168606	25	340.22	3.3
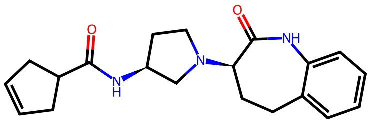	O=C(N[C@H]1CCN([C@@H]2CCc3ccccc3NC2=O)C1)C1CC=CC1	1010415974	25	339.19	2.1
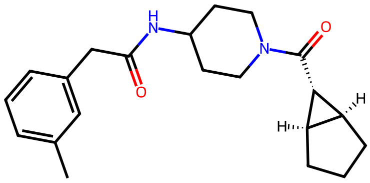	Cc1cccc(CC(=O)NC2CCN(C(=O)[C@@H]3[C@H]4CCC[C@H]43)CC2)c1	946078306	25	340.22	2.69
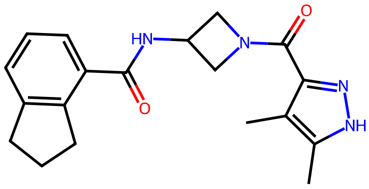	Cc1[nH]nc(C(=O)N2CC(NC(=O)c3cccc4c3CCC4)C2)c1C	994403518	25	338.17	1.77
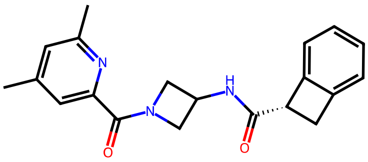	Cc1cc(C)nc(C(=O)N2CC(NC(=O)[C@H]3Cc4ccccc43)C2)c1	998693638	25	335.16	1.98
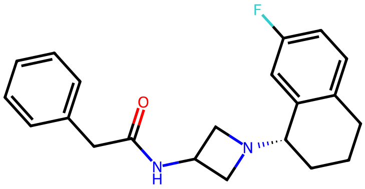	O=C(Cc1ccccc1)NC1CN([C@H]2CCCc3ccc(F)cc32)C1	1030538386	25	338.18	3.25
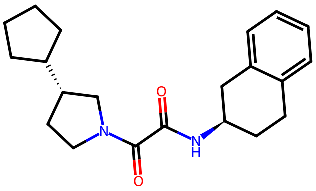	O=C(N[C@@H]1CCc2ccccc2C1)C(=O)N1CC[C@H](C2CCCC2)C1	420892110	25	340.22	2.7

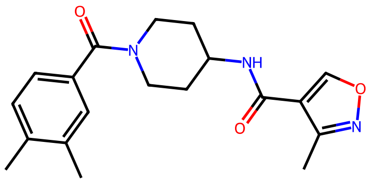	Cc1ccc(C(=O)N2CCC(NC(=O)c3conc3C)CC2)cc1C	948351682	25	341.17	2.63
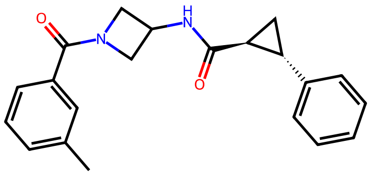	Cc1cccc(C(=O)N2CC(NC(=O)[C@H]3C[C@@H]3c3ccccc3)C2)c1	991639649	25	334.17	2.74
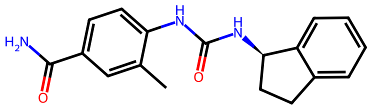	Cc1cc(C(N)=O)ccc1NC(=O)N[C@@H]1CCc2ccccc21	45729887	23	309.15	2.9
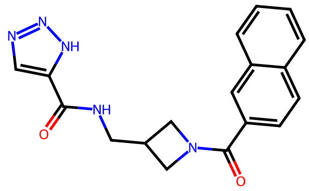	O=C(NCC1CN(C(=O)c2ccc3ccccc3c2)C1)c1cnn[nH]1	1003113505	25	335.14	1.46
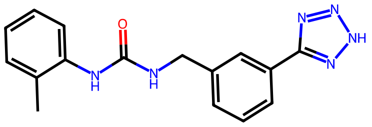	Cc1ccccc1NC(=O)NCc1cccc(-c2nn[nH]n2)c1	1592259522	23	308.14	2.5
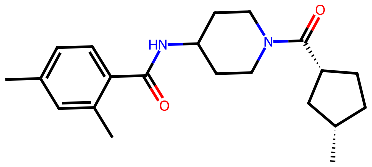	Cc1ccc(C(=O)NC2CCN(C(=O)[C@@H]3CC[C@H](C)C3)CC2)c(C)c1	946734175	25	342.23	3.46
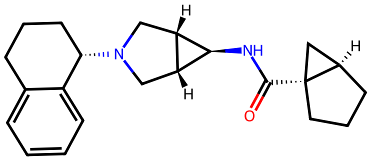	O=C(N[C@H]1[C@@H]2CN([C@H]3CCCc4ccccc43)C[C@@H]21)[C@@]12CCC[C@@H]1C2	1025913222	25	336.22	3.3
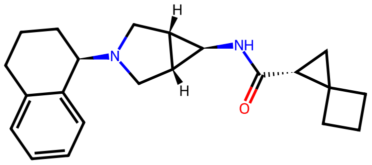	O=C(N[C@H]1[C@@H]2CN([C@@H]3CCCc4ccccc43)C[C@@H]21)[C@@H]1CC12CCC2	1026078138	25	336.22	3.3

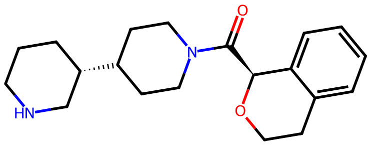	O=C([C@@H]1OCCc2ccccc21)N1CCC([C@H]2CCCNC2)CC1	658957121	24	328.22	2.54
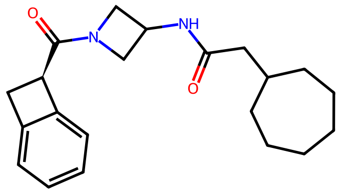	O=C(CC1CCCCCC1)NC1CN(C(=O)[C@@H]2Cc3ccccc32)C1	1000581627	25	340.22	3.01
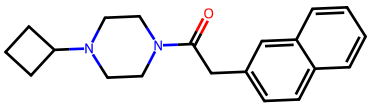	O=C(Cc1ccc2ccccc2c1)N1CCN(C2CCC2)CC1	354357212	23	308.19	3.08
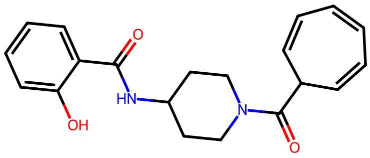	O=C(NC1CCN(C(=O)C2C=CC=CC=C2)CC1)c1ccccc1O	946949067	25	338.16	2.41
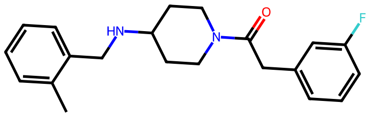	Cc1ccccc1CNC1CCN(C(=O)Cc2cccc(F)c2)CC1	997254233	25	340.2	3.46
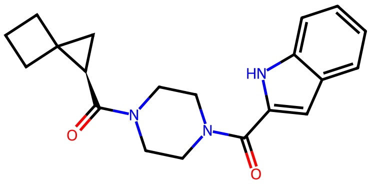	O=C(c1cc2ccccc2[nH]1)N1CCN(C(=O)[C@@H]2CC23CCC3)CC1	675318808	25	337.18	2.64
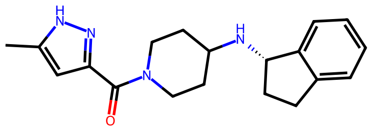	Cc1cc(C(=O)N2CCC(N[C@H]3CCc4ccccc43)CC2)n[nH]1	990086107	24	324.2	2.6
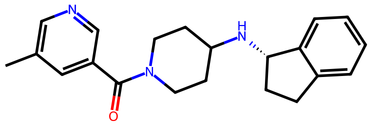	Cc1cncc(C(=O)N2CCC(N[C@H]3CCc4ccccc43)CC2)c1	990594022	25	335.2	3.27

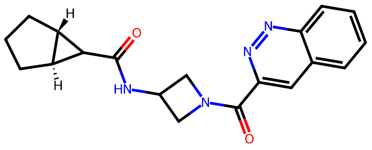	O=C(NC1CN(C(=O)c2cc3ccccc3nn2)C1)C1[C@H]2CCC[C@H]12	990706846	25	336.16	1.62
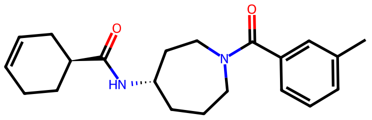	Cc1cccc(C(=O)N2CCC[C@H](NC(=O)[C@@H]3CC=CCC3)CC2)c1	964717488	25	340.22	3.46
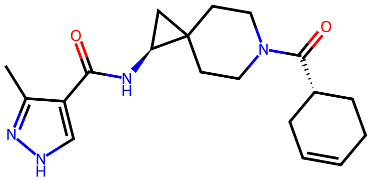	Cc1n[nH]cc1C(=O)N[C@H]1CC12CCN(C(=O)[C@H]1CC=CCC1)CC2	964568814	25	342.21	2.19
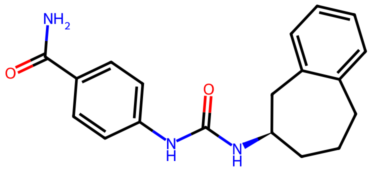	NC(=O)c1ccc(NC(=O)N[C@@H]2CCCc3ccccc3C2)cc1	929401711	24	323.16	2.85
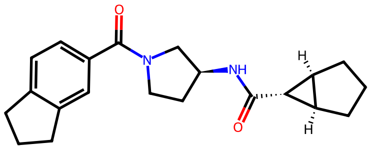	O=C(N[C@H]1CCN(C(=O)c2ccc3c(c2)CCC3)C1)[C@@H]1[C@H]2CCC[C@H]21	937805350	25	338.2	2.55
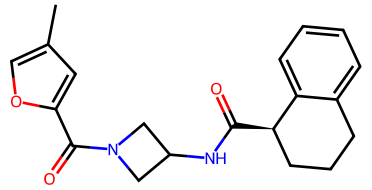	Cc1coc(C(=O)N2CC(NC(=O)[C@@H]3CCCc4ccccc43)C2)c1	995075511	25	338.16	2.65
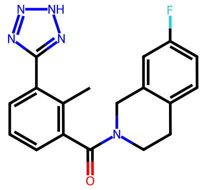	Cc1c(C(=O)N2CCc3ccc(F)cc3C2)cccc1-c1nn[nH]n1	1609380335	25	337.13	2.51
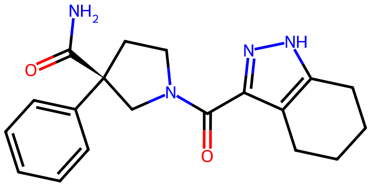	NC(=O)[C@@]1(c2ccccc2)CCN(C(=O)c2n[nH]c3c2CCCC3)C1	665646828	25	338.17	1.56

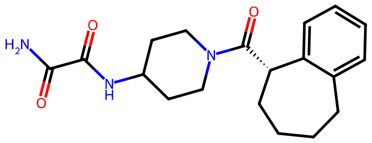	NC(=O)C(=O)NC1CCN(C(=O)[C@H]2CCCCc3ccccc32)CC1	947145653	25	343.19	1.09
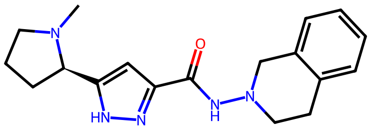	CN1CCC[C@@H]1c1cc(C(=O)NN2CCc3ccccc3C2)n[nH]1	898725426	24	325.19	1.88
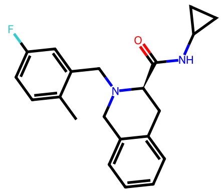	Cc1ccc(F)cc1CN1Cc2ccccc2C[C@@H]1C(=O)NC1CC1	1473618327	25	338.18	3.34
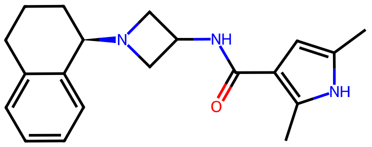	Cc1cc(C(=O)NC2CN([C@@H]3CCCc4ccccc43)C2)c(C)[nH]1	1031019028	24	323.2	3.12
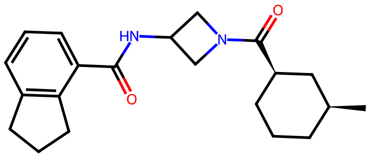	C[C@H]1CCC[C@@H](C(=O)N2CC(NC(=O)c3cccc4c3CCC4)C2)C1	994404452	25	340.22	2.94
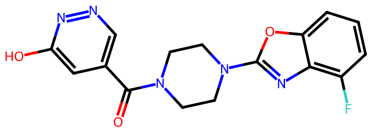	O=C(c1cnnc(O)c1)N1CCN(c2nc3c(F)cccc3o2)CC1	1055931836	25	343.11	1.42
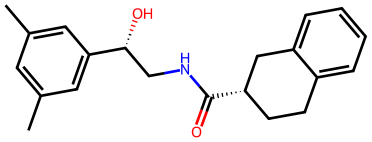	Cc1cc(C)cc([C@H](O)CNC(=O)[C@H]2CCc3ccccc3C2)c1	846092949	24	323.19	3.26
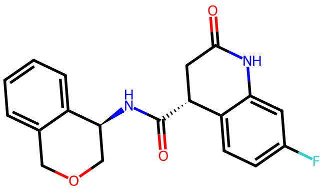	O=C1C[C@@H](C(=O)N[C@H]2COCc3ccccc32)c2ccc(F)cc2N1	577364419	25	340.12	2.64

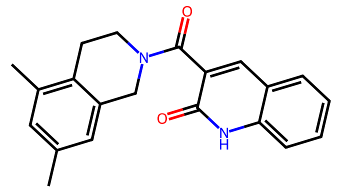	Cc1cc(C)c2c(c1)CN(C(=O)c1cc3ccccc3[nH]c1=O)CC2	187734065	25	332.15	3.34
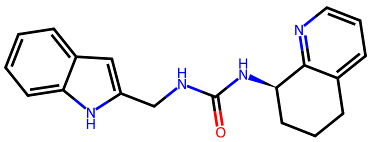	O=C(NCc1cc2ccccc2[nH]1)N[C@@H]1CCCc2cccnc21	915356995	24	320.16	3.44
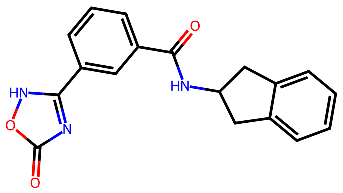	O=C(NC1Cc2ccccc2C1)c1cccc(-c2nc(=O)o[nH]2)c1	622512735	24	321.11	1.93
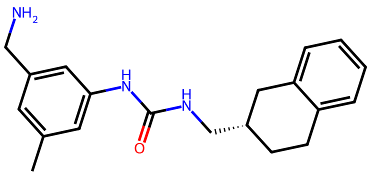	Cc1cc(CN)cc(NC(=O)NC[C@H]2CCc3ccccc3C2)c1	644190614	24	323.2	3.38
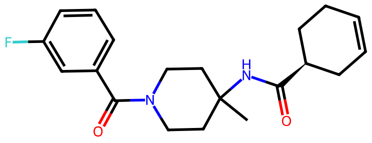	CC1(NC(=O)[C@@H]2CC=CCC2)CCN(C(=O)c2cccc(F)c2)CC1	956288841	25	344.19	3.29
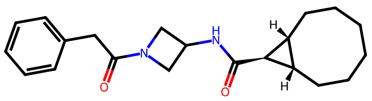	O=C(NC1CN(C(=O)Cc2ccccc2)C1)[C@@H]1[C@H]2CCCCCC[C@H]21	1000507613	25	340.22	2.77
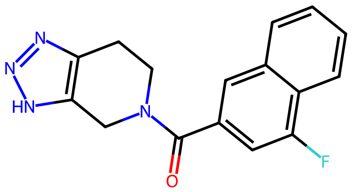	O=C(c1cc(F)c2ccccc2c1)N1CCc2nn[nH]c2C1	340876903	22	296.11	2.3
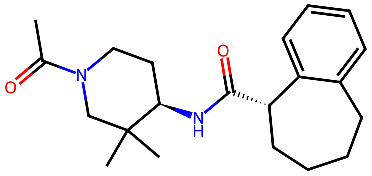	CC(=O)N1CC[C@@H](NC(=O)[C@H]2CCCCc3ccccc32)C(C)(C)C1	967056852	25	342.23	3.26

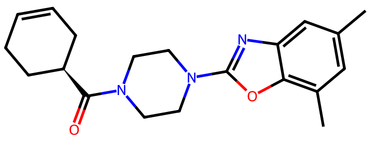	Cc1cc(C)c2oc(N3CCN(C(=O)[C@@H]4CC=CCC4)CC3)nc2c1	1055721271	25	339.19	3.45
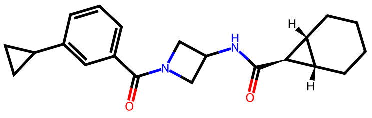	O=C(NC1CN(C(=O)c2cccc(C3CC3)c2)C1)[C@@H]1[C@H]2CCCC[C@H]21	990653947	25	338.2	2.94
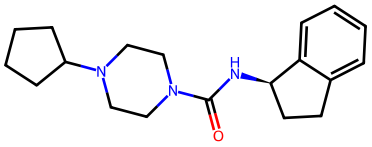	O=C(N[C@@H]1CCc2ccccc21)N1CCN(C2CCCC2)CC1	45304764	23	313.22	2.94
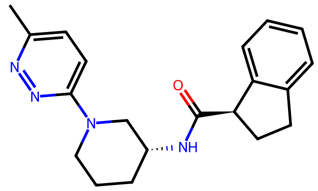	Cc1ccc(N2CCC[C@@H](NC(=O)[C@@H]3CCc4ccccc43)C2)nn1	332873806	25	336.2	2.6
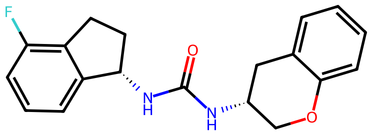	O=C(N[C@H]1CCc2c(F)cccc21)N[C@H]1COc2ccccc2C1	619391773	24	326.14	3.12
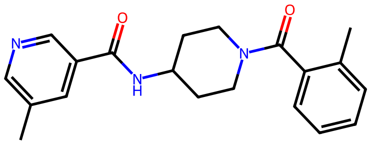	Cc1cncc(C(=O)NC2CCN(C(=O)c3ccccc3C)CC2)c1	943772303	25	337.18	2.73
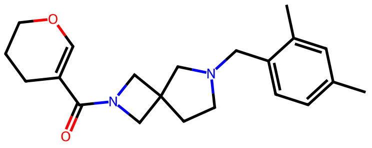	Cc1ccc(CN2CCC3(C2)CN(C(=O)C2=COCCC2)C3)c(C)c1	1072537409	25	340.22	3.03
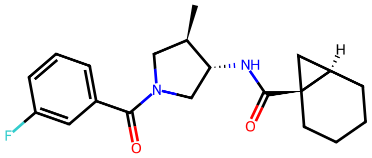	C[C@@H]1CN(C(=O)c2cccc(F)c2)C[C@H]1NC(=O)[C@]12CCCC[C@@H]1C2	939980505	25	344.19	2.98

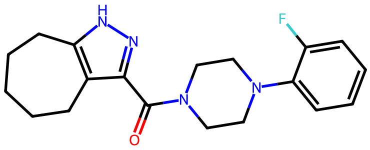	O=C(c1n[nH]c2c1CCCCC2)N1CCN(c2ccccc2F)CC1	190110209	25	342.19	2.78
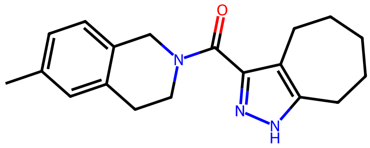	Cc1ccc2c(c1)CCN(C(=O)c1n[nH]c3c1CCCCC3)C2	360226750	23	309.18	3.19
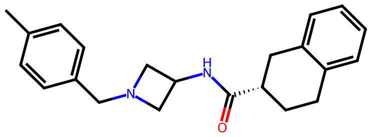	Cc1ccc(CN2CC(NC(=O)[C@H]3CCc4ccccc4C3)C2)cc1	1031013760	25	334.2	3.1
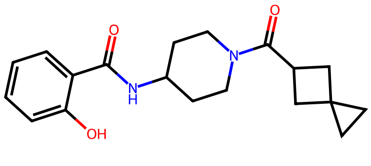	O=C(NC1CCN(C(=O)C2CC3(CC3)C2)CC1)c1ccccc1O	948300144	24	328.18	2.3
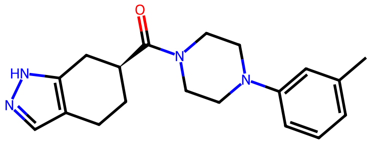	Cc1cccc(N2CCN(C(=O)[C@H]3CCc4cn[nH]c4C3)CC2)c1	79375773	24	324.2	2.17
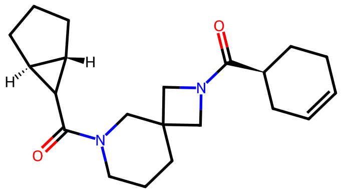	O=C(C1[C@H]2CCC[C@H]12)N1CCCC2(C1)CN(C(=O)[C@@H]1CC=CCC1)C2	1010531132	25	342.23	2.84
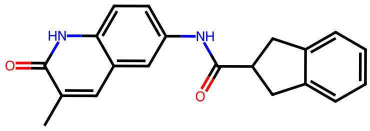	Cc1cc2cc(NC(=O)C3Cc4ccccc4C3)ccc2[nH]c1=O	1468333299	24	318.14	3.19
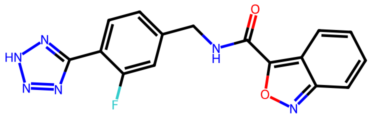	O=C(NCc1ccc(-c2nn[nH]n2)c(F)c1)c1onc2ccccc12	1582546457	25	338.09	2.08

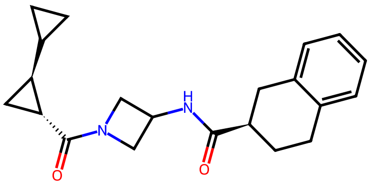	O=C(NC1CN(C(=O)[C@@H]2C[C@H]2C2CC2)C1)[C@@H]1CCc2ccccc2C1	1000544528	25	338.2	2.16
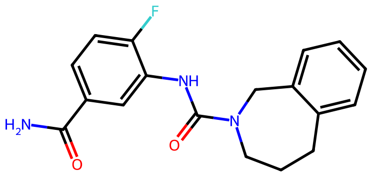	NC(=O)c1ccc(F)c(NC(=O)N2CCCc3ccccc3C2)c1	171636086	24	327.14	2.9
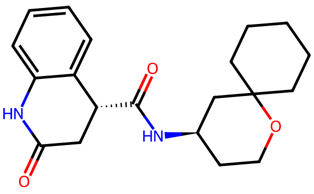	O=C1C[C@@H](C(=O)N[C@@H]2CCOC3(CCCCC3)C2)c2ccccc2N1	162335313	25	342.19	3.11
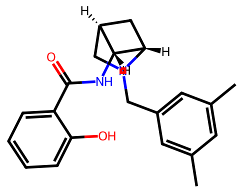	Cc1cc(C)cc(CN2C[C@H]3C[C@H]2[C@@H]3NC(=O)c2ccccc2O)c1	1037311090	25	336.18	3.01
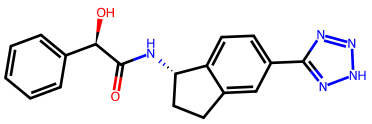	O=C(N[C@H]1CCc2cc(-c3nn[nH]n3)ccc21)[C@H](O)c1ccccc1	1606683634	25	335.14	1.7
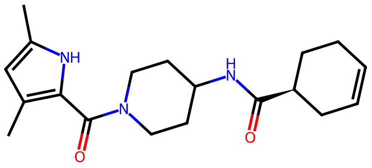	Cc1cc(C)c(C(=O)N2CCC(NC(=O)[C@@H]3CC=CCC3)CC2)[nH]1	947335624	24	329.21	2.71
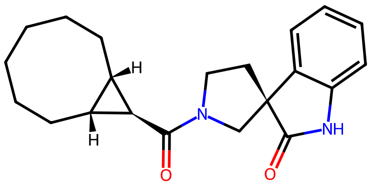	O=C([C@@H]1[C@H]2CCCCCC[C@H]21)N1CC[C@]2(C1)C(=O)Nc1ccccc12	613673817	25	338.2	3.33
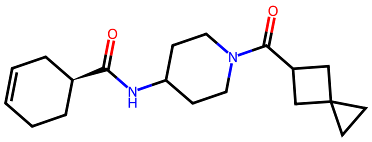	O=C(NC1CCN(C(=O)C2CC3(CC3)C2)CC1)[C@@H]1CC=CCC1	948300001	23	316.22	2.64

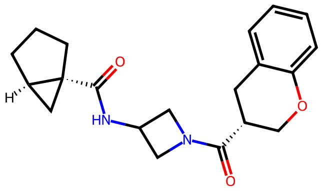	O=C([C@H]1COc2ccccc2C1)N1CC(NC(=O)[C@@]23CCC[C@@H]2C3)C1	997553619	25	340.18	1.75
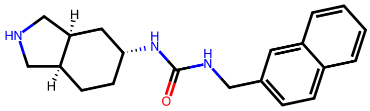	O=C(NCc1ccc2ccccc2c1)N[C@@H]1CC[C@H]2CNC[C@H]2C1	565999466	24	323.2	3.03
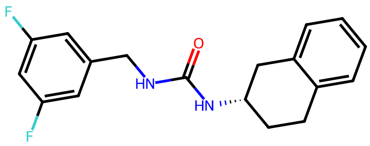	O=C(NCc1cc(F)cc(F)c1)N[C@H]1CCc2ccccc2C1	623845880	23	316.14	3.32
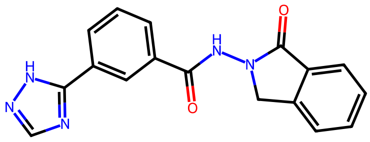	O=C(NN1Cc2ccccc2C1=O)c1cccc(-c2ncn[nH]2)c1	183818211	24	319.11	1.77
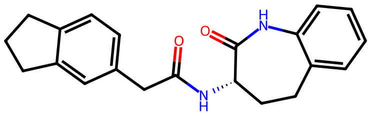	O=C(Cc1ccc2c(c1)CCC2)N[C@H]1CCc2ccccc2NC1=O	654004449	25	334.17	2.79
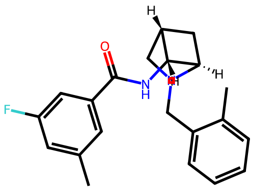	Cc1cc(F)cc(C(=O)N[C@@H]2[C@H]3C[C@H]2N(Cc2ccccc2C)C3)c1	1037588405	25	338.18	3.45
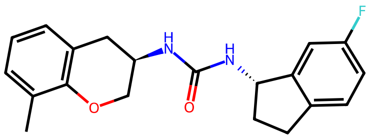	Cc1cccc2c1OC[C@H](NC(=O)N[C@H]1CCc3ccc(F)cc31)C2	573147370	25	340.16	3.42
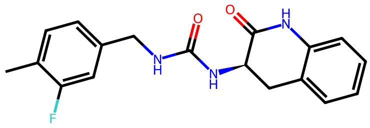	Cc1ccc(CNC(=O)N[C@@H]2Cc3ccccc3NC2=O)cc1F	1413938606	24	327.14	2.5

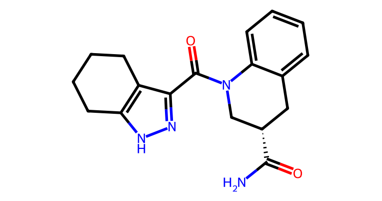	NC(=O)[C@H]1Cc2ccccc2N(C(=O)c2n[nH]c3c2CCCC3)C1	602126763	24	324.16	1.59
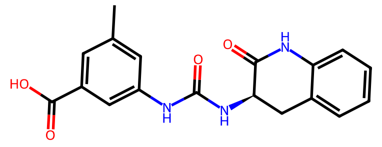	Cc1cc(NC(=O)N[C@@H]2Cc3ccccc3NC2=O)cc(C(=O)O)c1	1600270158	25	339.12	2.38
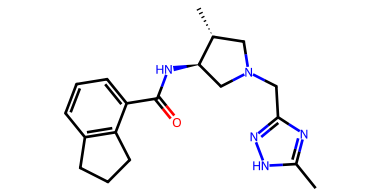	Cc1nc(CN2C[C@@H](NC(=O)c3cccc4c3CCC4)[C@H](C)C2)n[nH]1	1081028400	25	339.21	1.85
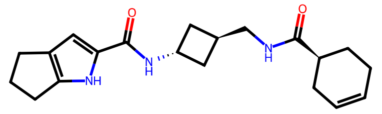	O=C(N[C@H]1C[C@H](CNC(=O)[C@@H]2CC=CCC2)C1)c1cc2c([nH]1)CCC2	979484790	25	341.21	2.48
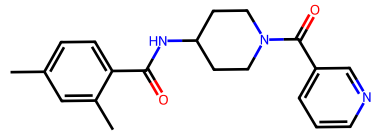	Cc1ccc(C(=O)NC2CCN(C(=O)c3cccnc3)CC2)c(C)c1	943676391	25	337.18	2.73
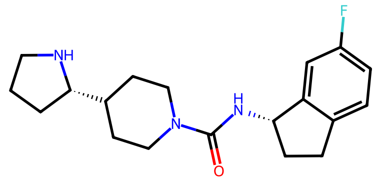	O=C(N[C@H]1CCc2ccc(F)cc21)N1CCC([C@@H]2CCCN2)CC1	518497267	24	331.21	2.99
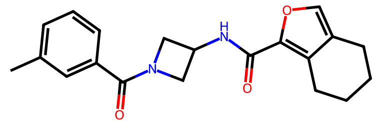	Cc1cccc(C(=O)N2CC(NC(=O)c3occ4c3CCCC4)C2)c1	997218284	25	338.16	2.72
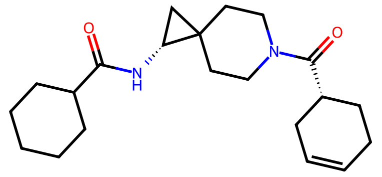	O=C(N[C@@H]1CC12CCN(C(=O)[C@H]1CC=CCC1)CC2)C1CCCCC1	962750264	25	344.25	3.42

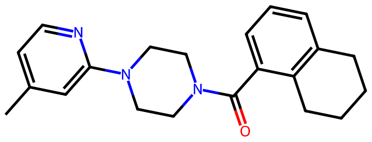	Cc1ccnc(N2CCN(C(=O)c3cccc4c3CCCC4)CC2)c1	450147328	25	335.2	3.23
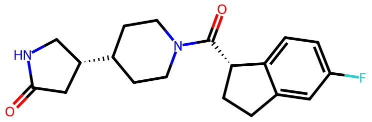	O=C1C[C@@H](C2CCN(C(=O)[C@H]3CCc4cc(F)ccc43)CC2)CN1	1551710035	24	330.17	2.23
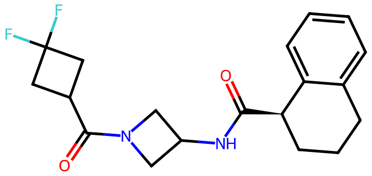	O=C(NC1CN(C(=O)C2CC(F)(F)C2)C1)[C@@H]1CCCc2ccccc21	995068164	25	348.16	2.48
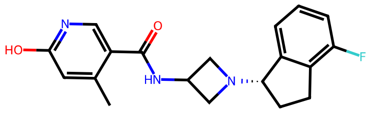	Cc1cc(O)ncc1C(=O)NC1CN([C@H]2CCc3c(F)cccc32)C1	1030933887	25	341.15	2.34
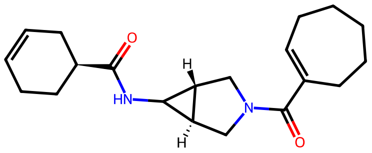	O=C(NC1[C@@H]2CN(C(=O)C3=CCCCCC3)C[C@@H]12)[C@@H]1CC=CCC1	1012758719	24	328.22	2.81
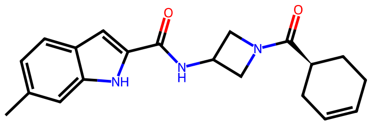	Cc1ccc2cc(C(=O)NC3CN(C(=O)[C@@H]4CC=CCC4)C3)[nH]c2c1	996606203	25	337.18	2.77
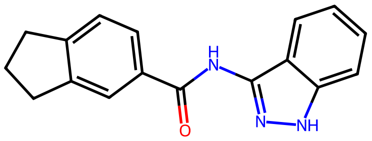	O=C(Nc1n[nH]c2ccccc12)c1ccc2c(c1)CCC2	55351510	21	277.12	3.3
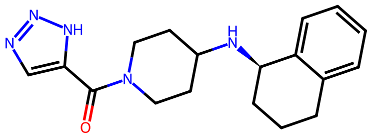	O=C(c1cnn[nH]1)N1CCC(N[C@@H]2CCCc3ccccc32)CC1	996753861	24	325.19	2.08

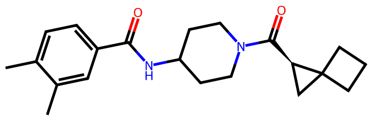	Cc1ccc(C(=O)NC2CCN(C(=O)[C@@H]3CC34CCC4)CC2)cc1C	946755466	25	340.22	3.21
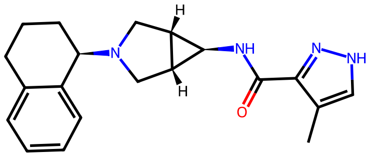	Cc1c[nH]nc1C(=O)N[C@H]1[C@@H]2CN([C@@H]3CCCc4ccccc43)C[C@@H]21	958813487	25	336.2	2.46
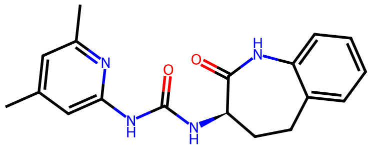	Cc1cc(C)nc(NC(=O)N[C@@H]2CCc3ccccc3NC2=O)c1	654039083	24	324.16	2.77
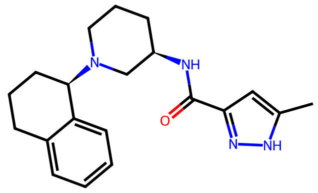	Cc1cc(C(=O)N[C@@H]2CCCN([C@@H]3CCCc4ccccc43)C2)n[nH]1	1006857947	25	338.21	2.99
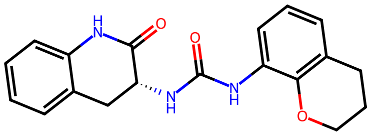	O=C(Nc1cccc2c1OCCC2)N[C@@H]1Cc2ccccc2NC1=O	529022325	25	337.14	2.7
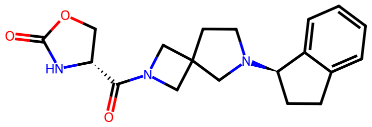	O=C1N[C@@H](C(=O)N2CC3(CCN([C@@H]4CCc5ccccc54)C3)C2)CO1	1072686788	25	341.17	1.32
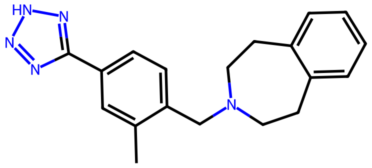	Cc1cc(-c2nn[nH]n2)ccc1CN1CCc2ccccc2CC1	1605542168	24	319.18	2.78
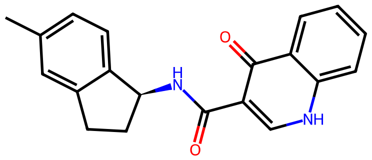	Cc1ccc2c(c1)CC[C@@H]2NC(=O)c1c[nH]c2ccccc2c1=O	55661577	24	318.14	3.25

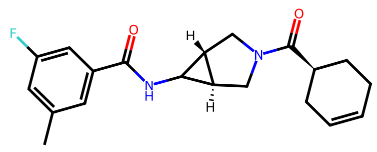	Cc1cc(F)cc(C(=O)NC2[C@@H]3CN(C(=O)[C@@H]4CC=CCC4)C[C@@H]23)c1	1013862334	25	342.17	2.68
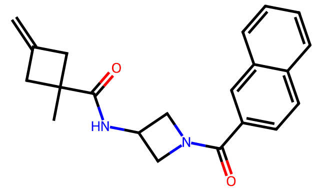	C=C1CC(C)(C(=O)NC2CN(C(=O)c3ccc4ccccc4c3)C2)C1	999284755	25	334.17	3.14
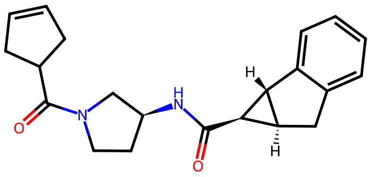	O=C(N[C@H]1CCN(C(=O)C2CC=CC2)C1)[C@@H]1[C@@H]2Cc3ccccc3[C@H]21	937966145	25	336.18	2.26
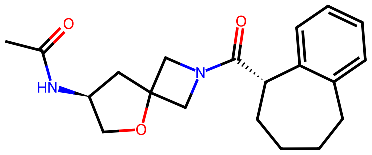	CC(=O)N[C@@H]1COC2(C1)CN(C(=O)[C@H]1CCCCc3ccccc31)C2	965576150	25	342.19	2.0
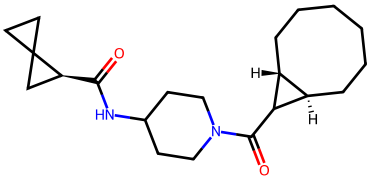	O=C(NC1CCN(C(=O)C2[C@@H]3CCCCCC[C@@H]23)CC1)[C@H]1CC12CC2	948568086	25	344.25	3.11
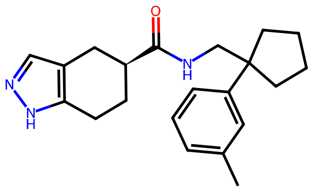	Cc1cccc(C2(CNC(=O)[C@H]3CCc4[nH]ncc4C3)CCCC2)c1	270183607	25	337.22	3.45
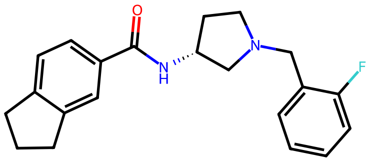	O=C(N[C@@H]1CCN(Cc2ccccc2F)C1)c1ccc2c(c1)CCC2	1016225820	25	338.18	3.32
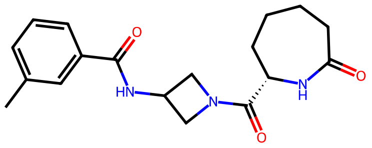	Cc1cccc(C(=O)NC2CN(C(=O)[C@@H]3CCCCC(=O)N3)C2)c1	990144031	24	329.17	0.99

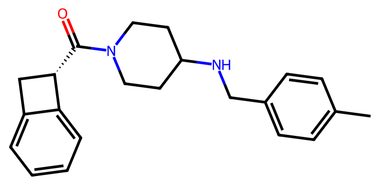	Cc1ccc(CNC2CCN(C(=O)[C@H]3Cc4ccccc43)CC2)cc1	996934956	25	334.2	3.42
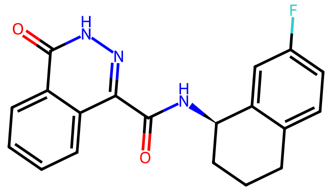	O=C(N[C@@H]1CCCc2ccc(F)cc21)c1n[nH]c(=O)c2ccccc12	93190709	25	337.12	2.87
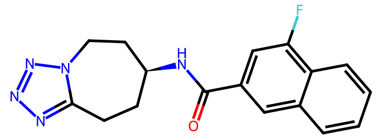	O=C(N[C@H]1CCc2nnnn2CC1)c1cc(F)c2ccccc2c1	580180785	24	325.13	2.1
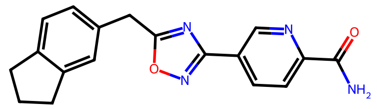	NC(=O)c1ccc(-c2noc(Cc3ccc4c(c3)CCC4)n2)cn1	544593997	24	320.13	2.31
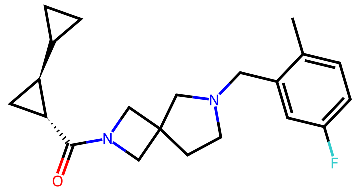	Cc1ccc(F)cc1CN1CCC2(C1)CN(C(=O)[C@@H]1C[C@H]1C1CC1)C2	1073047587	25	342.21	3.21
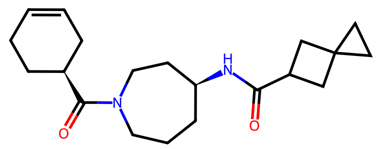	O=C(N[C@H]1CCCN(C(=O)[C@@H]2CC=CCC2)CC1)C1CC2(CC2)C1	965181763	24	330.23	3.03
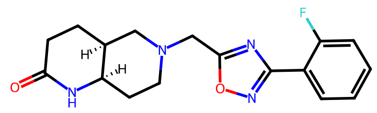	O=C1CC[C@H]2CN(Cc3nc(-c4ccccc4F)no3)CC[C@H]2N1	661599918	24	330.15	1.98
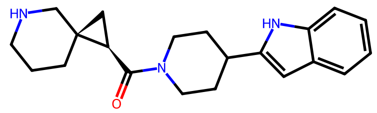	O=C([C@@H]1C[C@]12CCCNC2)N1CCC(c2cc3ccccc3[nH]2)CC1	848397462	25	337.22	3.26

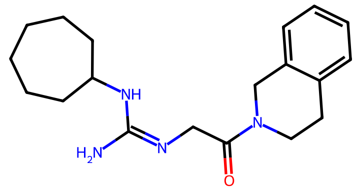	N/C(=N/CC(=O)N1CCc2ccccc2C1)NC1CCCCCC1	92278024	24	328.23	2.2
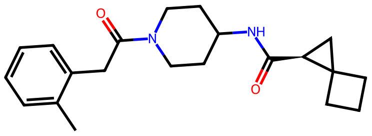	Cc1ccccc1CC(=O)N1CCC(NC(=O)[C@H]2CC23CCC3)CC1	946717922	25	340.22	2.83
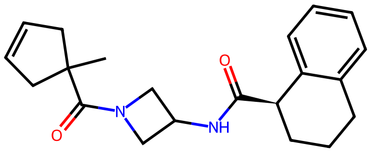	CC1(C(=O)N2CC(NC(=O)[C@@H]3CCCc4ccccc43)C2)CC=CC1	995071042	25	338.2	2.79
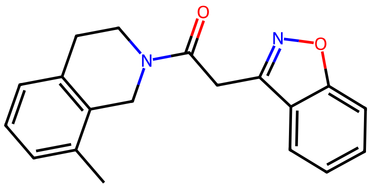	Cc1cccc2c1CN(C(=O)Cc1noc3ccccc13)CC2	346975672	23	306.14	3.26
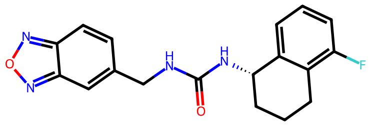	O=C(NCc1ccc2nonc2c1)N[C@H]1CCCc2c(F)cccc21	1229312091	25	340.13	3.24
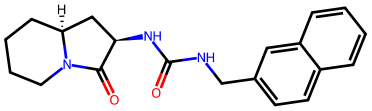	O=C(NCc1ccc2ccccc2c1)N[C@@H]1C[C@@H]2CCCCN2C1=O	556740145	25	337.18	2.79
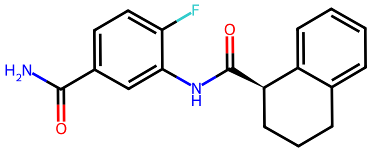	NC(=O)c1ccc(F)c(NC(=O)[C@@H]2CCCc3ccccc32)c1	44630919	23	312.13	2.98
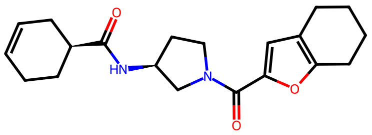	O=C(N[C@H]1CCN(C(=O)c2cc3c(o2)CCCC3)C1)[C@@H]1CC=CCC1	937672100	25	342.19	2.85

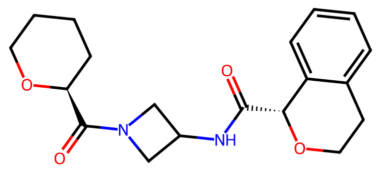	O=C(NC1CN(C(=O)[C@@H]2CCCCO2)C1)[C@H]1OCCc2ccccc21	997321176	25	344.17	1.2
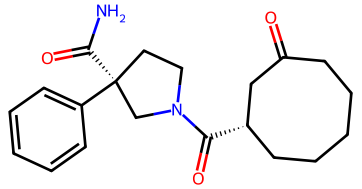	NC(=O)[C@]1(c2ccccc2)CCN(C(=O)[C@H]2CCCCCC(=O)C2)C1	665993667	25	342.19	2.18
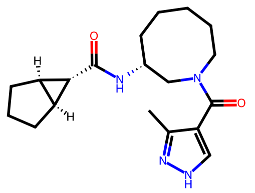	Cc1n[nH]cc1C(=O)N1CCCCC[C@@H](NC(=O)[C@@H]2[C@H]3CCC[C@H]32)C1	987093628	25	344.22	2.27
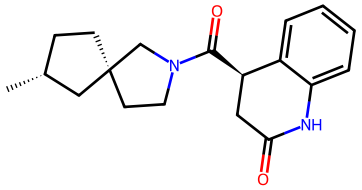	C[C@@H]1CC[C@]2(CCN(C(=O)[C@@H]3CC(=O)Nc4ccccc43)C2)C1	359157415	23	312.18	3.15
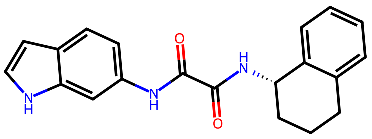	O=C(Nc1ccc2cc[nH]c2c1)C(=O)N[C@H]1CCCc2ccccc21	192487036	25	333.15	3.3
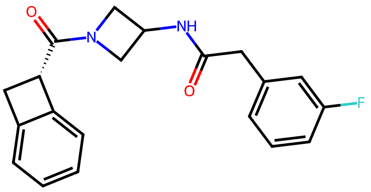	O=C(Cc1cccc(F)c1)NC1CN(C(=O)[C@H]2Cc3ccccc32)C1	1000077005	25	338.14	2.04
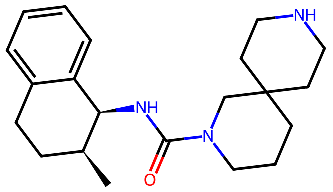	C[C@H]1CCc2ccccc2[C@H]1NC(=O)N1CCCC2(CCNCC2)C1	1325365403	25	341.25	3.49
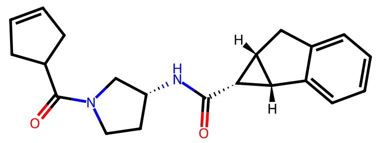	O=C(N[C@@H]1CCN(C(=O)C2CC=CC2)C1)[C@H]1[C@H]2c3ccccc3C[C@H]21	940494201	25	336.18	2.26

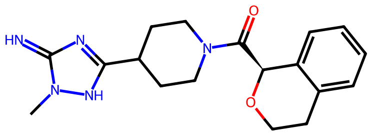	Cn1[nH]c(C2CCN(C(=O)[C@@H]3OCCc4ccccc43)CC2)nc1=N	614296076	25	341.19	1.25
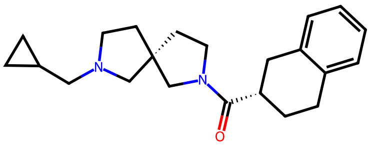	O=C([C@H]1CCc2ccccc2C1)N1CC[C@]2(CCN(CC3CC3)C2)C1	1041708434	25	338.24	3.13
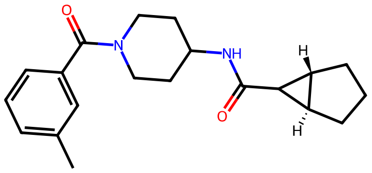	Cc1cccc(C(=O)N2CCC(NC(=O)C3[C@@H]4CCC[C@@H]34)CC2)c1	943804529	24	326.2	2.76
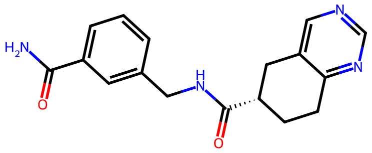	NC(=O)c1cccc(CNC(=O)[C@H]2CCc3ncncc3C2)c1	648237571	23	310.14	1.0
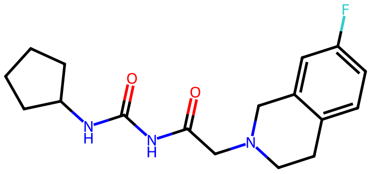	O=C(CN1CCc2ccc(F)cc2C1)NC(=O)NC1CCCC1	179071434	23	319.17	1.95
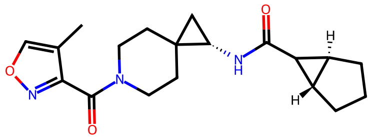	Cc1conc1C(=O)N1CCC2(CC1)C[C@@H]2NC(=O)C1[C@H]2CCC[C@H]12	963487318	25	343.19	2.14
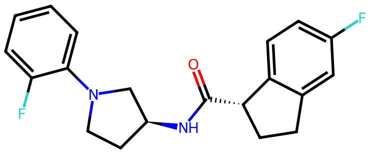	O=C(N[C@H]1CCN(c2ccccc2F)C1)[C@H]1CCc2cc(F)ccc21	615196903	25	342.15	3.39
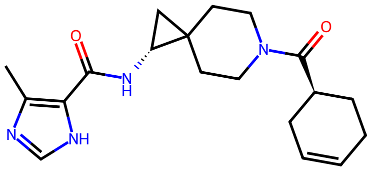	Cc1nc[nH]c1C(=O)N[C@@H]1CC12CCN(C(=O)[C@@H]1CC=CCC1)CC2	964278541	25	342.21	2.19

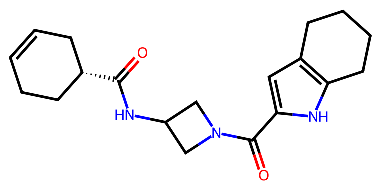	O=C(NC1CN(C(=O)c2cc3c([nH]2)CCCC3)C1)[C@H]1CC=CCC1	990216927	24	327.19	2.19
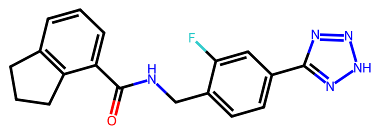	O=C(NCc1ccc(-c2nn[nH]n2)cc1F)c1cccc2c1CCC2	738102602	25	337.13	2.42
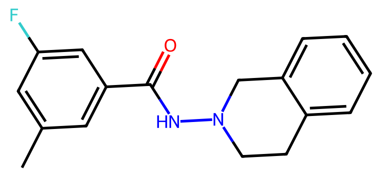	Cc1cc(F)cc(C(=O)NN2CCc3ccccc3C2)c1	1412324154	21	284.13	2.84
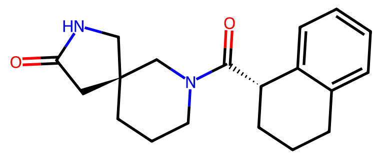	O=C1C[C@@]2(CCCN(C(=O)[C@H]3CCCc4ccccc43)C2)CN1	362206874	23	312.18	2.24
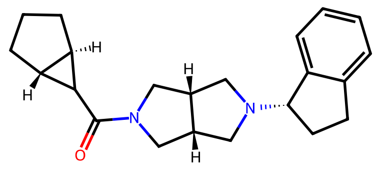	O=C(C1[C@@H]2CCC[C@@H]12)N1C[C@@H]2CN([C@H]3CCc4ccccc43)C[C@@H]2C1	1048746899	25	336.22	3.11
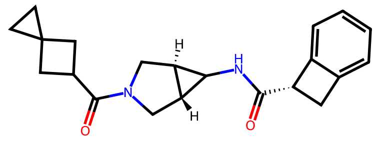	O=C(NC1[C@@H]2CN(C(=O)C3CC4(CC4)C3)C[C@@H]12)[C@H]1Cc2ccccc21	1013686172	25	336.18	2.09
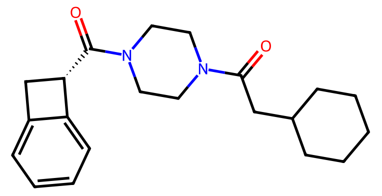	O=C(CC1CCCCC1)N1CCN(C(=O)[C@H]2Cc3ccccc32)CC1	358329157	25	340.22	2.97
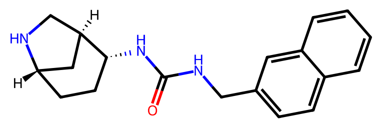	O=C(NCc1ccc2ccccc2c1)N[C@@H]1CC[C@H]2C[C@H]1CN2	1551519511	23	309.18	2.78

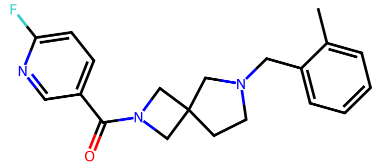	Cc1ccccc1CN1CCC2(C1)CN(C(=O)c1ccc(F)nc1)C2	1072558833	25	339.17	2.88
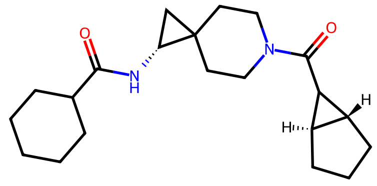	O=C(N[C@@H]1CC12CCN(C(=O)C1[C@@H]3CCC[C@@H]13)CC2)C1CCCCC1	962749742	25	344.25	3.11
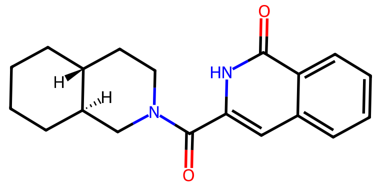	O=C(c1cc2ccccc2c(=O)[nH]1)N1CC[C@H]2CCCC[C@@H]2C1	53251870	23	310.17	3.18
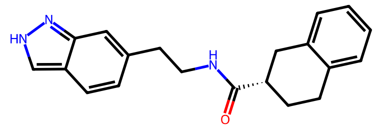	O=C(NCCc1ccc2c[nH]nc2c1)[C@H]1CCc2ccccc2C1	652023478	24	319.17	3.03
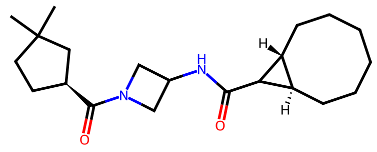	CC1(C)CC[C@H](C(=O)N2CC(NC(=O)C3[C@@H]4CCCCCC[C@@H]34)C2)C1	1000508400	25	346.26	3.36
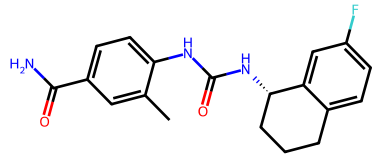	Cc1cc(C(N)=O)ccc1NC(=O)N[C@H]1CCCc2ccc(F)cc21	792864570	25	341.15	3.43
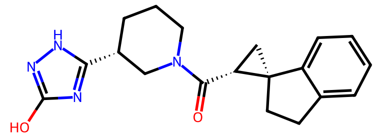	O=C([C@@H]1C[C@]12CCc1ccccc12)N1CCC[C@@H](c2nc(O)n[nH]2)C1	334018336	25	338.17	2.12
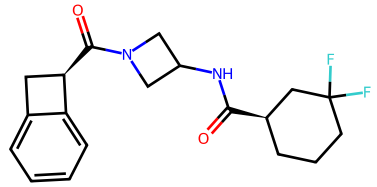	O=C(NC1CN(C(=O)[C@@H]2Cc3ccccc32)C1)[C@@H]1CCCC(F)(F)C1	999754421	25	348.16	2.48

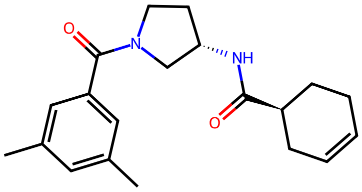	Cc1cc(C)cc(C(=O)N2CC[C@H](NC(=O)[C@@H]3CC=CCC3)C2)c1	937667609	24	326.2	2.99
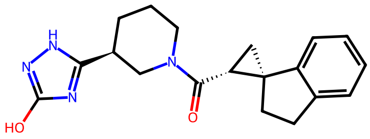	O=C([C@@H]1C[C@]12CCc1ccccc12)N1CCC[C@H](c2nc(O)n[nH]2)C1	334018334	25	338.17	2.12
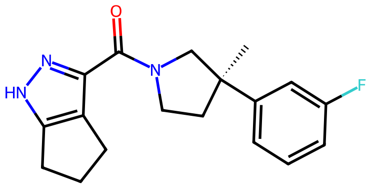	C[C@]1(c2cccc(F)c2)CCN(C(=O)c2n[nH]c3c2CCC3)C1	652618501	23	313.16	2.84
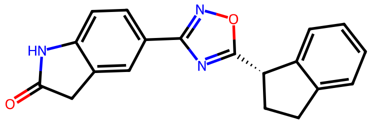	O=C1Cc2cc(-c3noc([C@H]4CCc5ccccc54)n3)ccc2N1	355980760	24	317.12	3.31
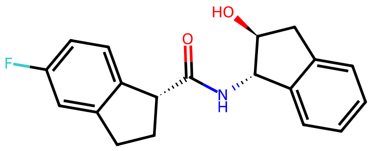	O=C(N[C@H]1c2ccccc2C[C@@H]1O)[C@@H]1CCc2cc(F)ccc21	616705774	23	311.13	2.63
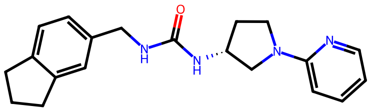	O=C(NCc1ccc2c(c1)CCC2)N[C@@H]1CCN(c2ccccn2)C1	1645627974	25	336.2	2.65
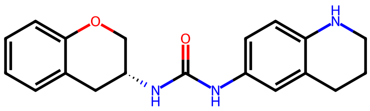	O=C(Nc1ccc2c(c1)CCCN2)N[C@H]1COc2ccccc2C1	415882928	24	323.16	3.17
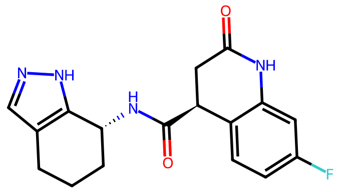	O=C1C[C@H](C(=O)N[C@@H]2CCCc3cn[nH]c32)c2ccc(F)cc2N1	268992688	24	328.13	2.17

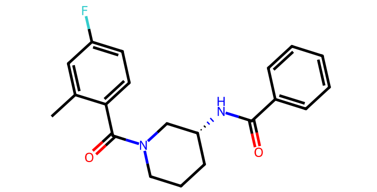	Cc1cc(F)ccc1C(=O)N1CCC[C@@H](NC(=O)c2ccccc2)C1	951502822	25	340.16	3.17
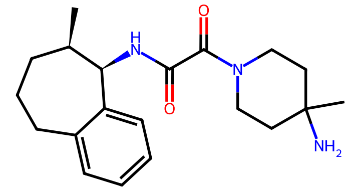	C[C@@H]1CCCc2ccccc2[C@@H]1NC(=O)C(=O)N1CCC(C)(N)CC1	642482410	25	343.23	2.16
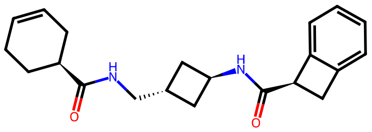	O=C(NC[C@H]1C[C@H](NC(=O)[C@@H]2Cc3ccccc32)C1)[C@@H]1CC=CCC1	979475049	25	338.2	2.69
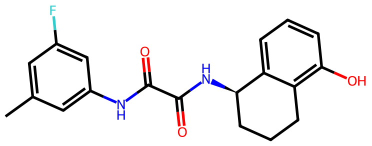	Cc1cc(F)cc(NC(=O)C(=O)N[C@@H]2CCCc3c(O)cccc32)c1	192329426	25	342.14	2.97
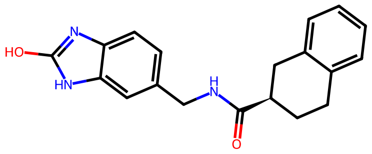	O=C(NCc1ccc2nc(O)[nH]c2c1)[C@@H]1CCc2ccccc2C1	521053327	24	321.15	2.69
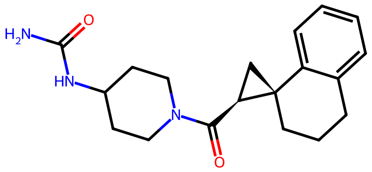	NC(=O)NC1CCN(C(=O)[C@H]2C[C@@]23CCCc2ccccc23)CC1	176063149	24	327.19	1.94
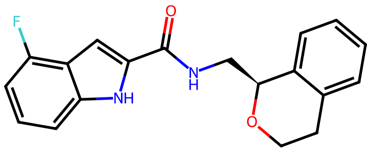	O=C(NC[C@@H]1OCCc2ccccc21)c1cc2c(F)cccc2[nH]1	377653041	24	324.13	3.35
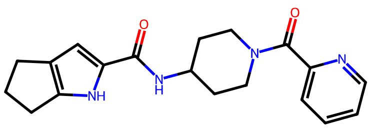	O=C(NC1CCN(C(=O)c2ccccn2)CC1)c1cc2c([nH]1)CCC2	943576304	25	338.17	1.93

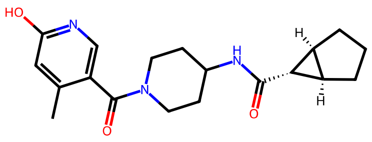	Cc1cc(O)ncc1C(=O)N1CCC(NC(=O)[C@@H]2[C@H]3CCC[C@H]32)CC1	948305872	25	343.19	1.86
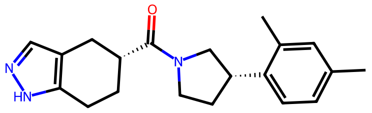	Cc1ccc([C@@H]2CCN(C(=O)[C@@H]3CCc4[nH]ncc4C3)C2)c(C)c1	279625310	24	323.2	3.15
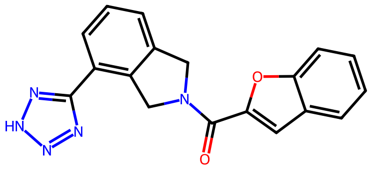	O=C(c1cc2ccccc2o1)N1Cc2cccc(-c3nn[nH]n3)c2C1	1607007730	25	331.11	2.77
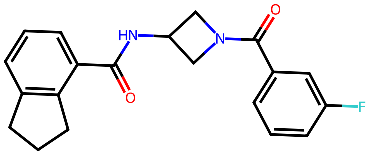	O=C(NC1CN(C(=O)c2cccc(F)c2)C1)c1cccc2c1CCC2	994397881	25	338.14	2.57
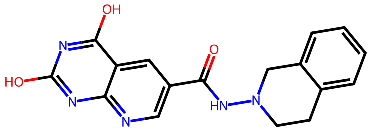	O=C(NN1CCc2ccccc2C1)c1cnc2nc(O)nc(O)c2c1	1165596242	25	337.12	1.14
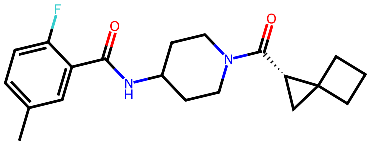	Cc1ccc(F)c(C(=O)NC2CCN(C(=O)[C@H]3CC34CCC4)CC2)c1	946755433	25	344.19	3.05
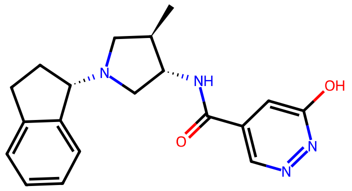	C[C@@H]1CN([C@H]2CCc3ccccc32)C[C@H]1NC(=O)c1cnnc(O)c1	1079911324	25	338.17	1.92
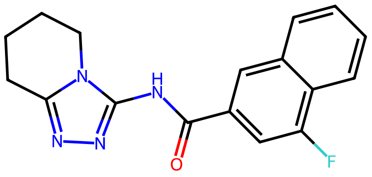	O=C(Nc1nnc2n1CCCC2)c1cc(F)c2ccccc2c1	345992955	23	310.12	3.16

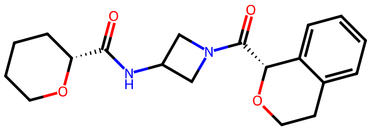	O=C(NC1CN(C(=O)[C@H]2OCCc3ccccc32)C1)[C@H]1CCCCO1	990854099	25	344.17	1.2
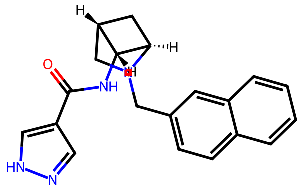	O=C(N[C@@H]1[C@H]2C[C@H]1N(Cc1ccc3ccccc3c1)C2)c1cn[nH]c1	1037322143	25	332.16	2.57
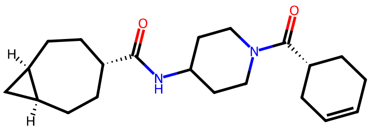	O=C(NC1CCN(C(=O)[C@H]2CC=CCC2)CC1)[C@@H]1CC[C@H]2C[C@H]2CC1	944114449	25	344.25	3.28
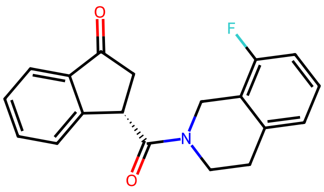	O=C1C[C@H](C(=O)N2CCc3cccc(F)c3C2)c2ccccc21	277143748	23	309.12	3.08
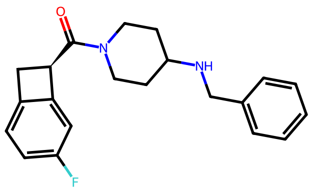	O=C([C@@H]1Cc2ccc(F)cc21)N1CCC(NCc2ccccc2)CC1	996915411	25	338.18	3.25
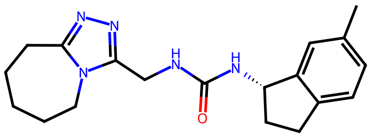	Cc1ccc2c(c1)[C@@H](NC(=O)NCc1nnc3n1CCCCC3)CC2	592477381	25	339.21	2.8
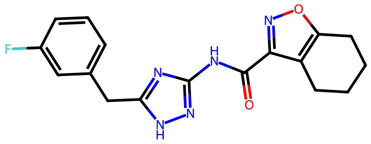	O=C(Nc1n[nH]c(Cc2cccc(F)c2)n1)c1noc2c1CCCC2	580851875	25	341.13	2.65
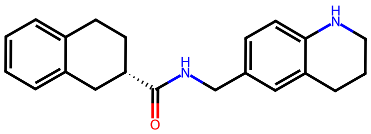	O=C(NCc1ccc2c(c1)CCCN2)[C@H]1CCc2ccccc2C1	381423032	24	320.19	3.47

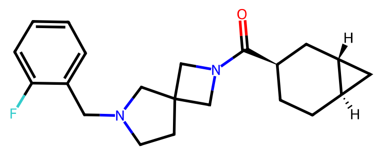	O=C([C@@H]1CC[C@@H]2C[C@H]2C1)N1CC2(CCN(Cc3ccccc3F)C2)C1	1072879374	25	342.21	3.3
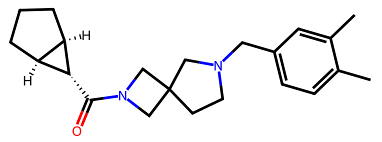	Cc1ccc(CN2CCC3(C2)CN(C(=O)[C@@H]2[C@H]4CCC[C@H]42)C3)cc1C	1072592000	25	338.24	3.38
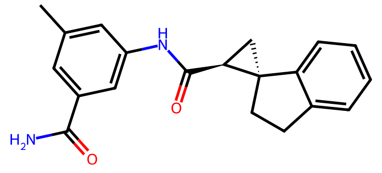	Cc1cc(NC(=O)[C@H]2C[C@]23CCc2ccccc23)cc(C(N)=O)c1	456173205	24	320.15	2.94
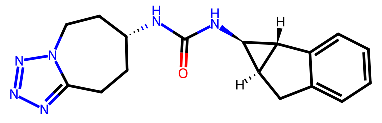	O=C(N[C@@H]1CCc2nnnn2CC1)N[C@H]1[C@@H]2c3ccccc3C[C@H]21	333787068	24	324.17	1.02
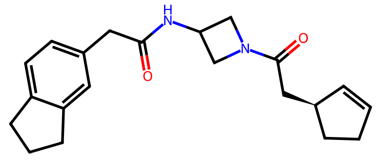	O=C(Cc1ccc2c(c1)CCC2)NC1CN(C(=O)C[C@H]2C=CCC2)C1	991383283	25	338.2	2.4
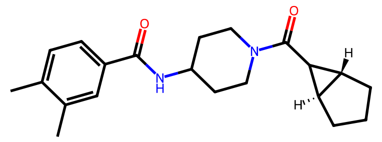	Cc1ccc(C(=O)NC2CCN(C(=O)C3[C@@H]4CCC[C@@H]34)CC2)cc1C	946081168	25	340.22	3.07
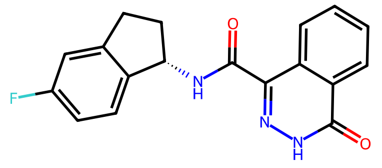	O=C(N[C@H]1CCc2cc(F)ccc21)c1n[nH]c(=O)c2ccccc12	72022052	24	323.11	2.48
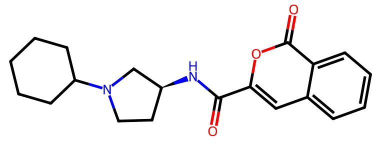	O=C(N[C@H]1CCN(C2CCCCC2)C1)c1cc2ccccc2c(=O)o1	618669338	25	340.18	2.93

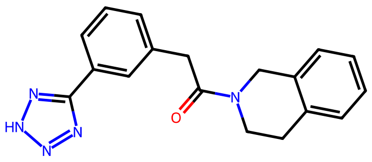	O=C(Cc1cccc(-c2nn[nH]n2)c1)N1CCc2ccccc2C1	823092352	24	319.14	1.99
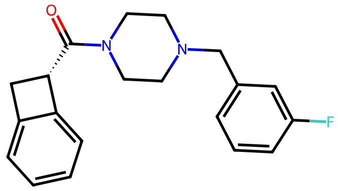	O=C([C@H]1Cc2ccccc21)N1CCN(Cc2cccc(F)c2)CC1	348059513	24	324.16	2.81
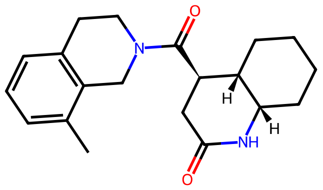	Cc1cccc2c1CN(C(=O)[C@@H]1CC(=O)N[C@H]3CCCC[C@H]31)CC2	614003100	24	326.2	2.57
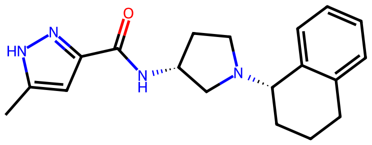	Cc1cc(C(=O)N[C@@H]2CCN([C@H]3CCCc4ccccc43)C2)n[nH]1	1010333902	24	324.2	2.6
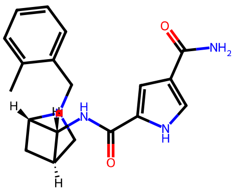	Cc1ccccc1CN1C[C@H]2C[C@H]1[C@@H]2NC(=O)c1cc(C(N)=O)c[nH]1	1037645110	25	338.17	1.42
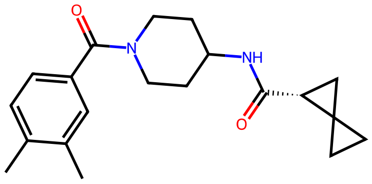	Cc1ccc(C(=O)N2CCC(NC(=O)[C@@H]3CC34CC4)CC2)cc1C	948351648	24	326.2	2.82
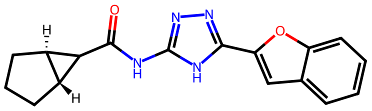	O=C(Nc1nnc(-c2cc3ccccc3o2)[nH]1)C1[C@@H]2CCC[C@@H]12	276777934	23	308.13	3.2
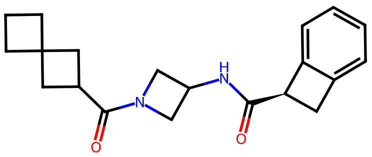	O=C(NC1CN(C(=O)C2CC3(CCC3)C2)C1)[C@@H]1Cc2ccccc21	998688743	24	324.18	2.23

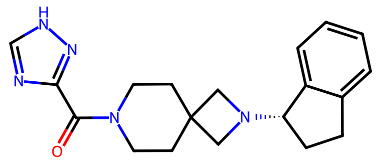	O=C(c1nc[nH]n1)N1CCC2(CC1)CN([C@H]1CCc3ccccc31)C2	1035820223	25	337.19	2.03
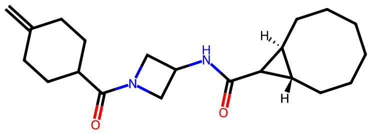	C=C1CCC(C(=O)N2CC(NC(=O)C3[C@H]4CCCCCC[C@H]34)C2)CC1	1000501570	25	344.25	3.28
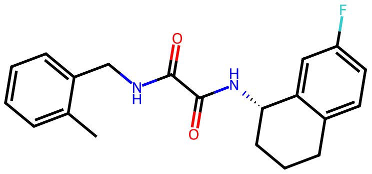	Cc1ccccc1CNC(=O)C(=O)N[C@H]1CCCc2ccc(F)cc21	1346999770	25	340.16	2.94
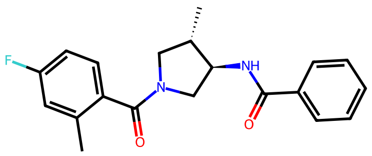	Cc1cc(F)ccc1C(=O)N1C[C@H](C)[C@@H](NC(=O)c2ccccc2)C1	966657092	25	340.16	3.02
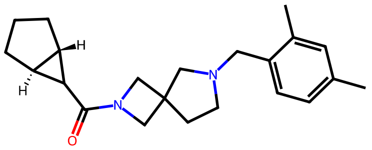	Cc1ccc(CN2CCC3(C2)CN(C(=O)C2[C@H]4CCC[C@H]24)C3)c(C)c1	1072592455	25	338.24	3.38
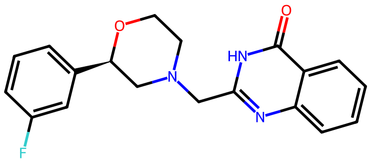	O=c1[nH]c(CN2CCO[C@H](c3cccc(F)c3)C2)nc2ccccc12	66849103	25	339.14	2.64
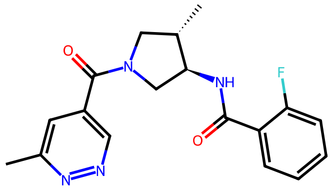	Cc1cc(C(=O)N2C[C@H](C)[C@@H](NC(=O)c3ccccc3F)C2)cnn1	966347174	25	342.15	1.81
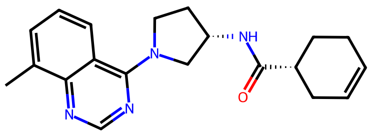	Cc1cccc2c(N3CC[C@H](NC(=O)[C@H]4CC=CCC4)C3)ncnc12	1058297482	25	336.2	2.99

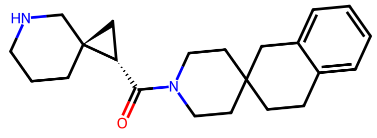	O=C([C@H]1C[C@]12CCCNC2)N1CCC2(CCc3ccccc3C2)CC1	655482564	25	338.24	3.17
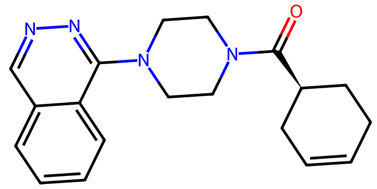	O=C([C@@H]1CC=CCC1)N1CCN(c2nncc3ccccc23)CC1	96418529	24	322.18	2.63
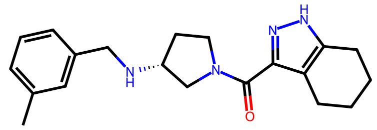	Cc1cccc(CN[C@@H]2CCN(C(=O)c3n[nH]c4c3CCCC4)C2)c1	969182013	25	338.21	2.6
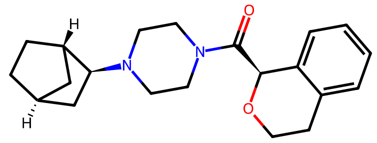	O=C([C@@H]1OCCc2ccccc21)N1CCN([C@H]2C[C@H]3CC[C@H]2C3)CC1	441469646	25	340.22	2.63
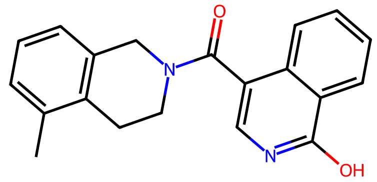	Cc1cccc2c1CCN(C(=O)c1cnc(O)c3ccccc13)C2	786897126	24	318.14	3.45
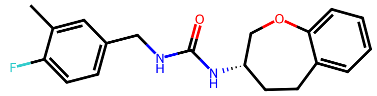	Cc1cc(CNC(=O)N[C@H]2CCc3ccccc3OC2)ccc1F	510825142	24	328.16	3.33
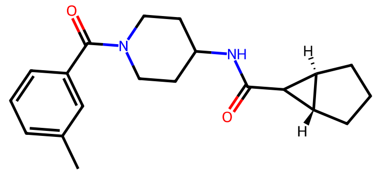	Cc1cccc(C(=O)N2CCC(NC(=O)C3[C@H]4CCC[C@H]34)CC2)c1	943804530	24	326.2	2.76
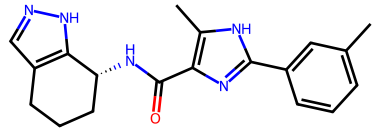	Cc1cccc(-c2nc(C(=O)N[C@@H]3CCCc4cn[nH]c43)c(C)[nH]2)c1	270348182	25	335.17	3.22

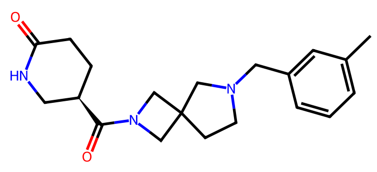	Cc1cccc(CN2CCC3(C2)CN(C(=O)[C@@H]2CCC(=O)NC2)C3)c1	1072886291	25	341.21	1.56
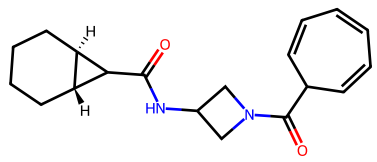	O=C(NC1CN(C(=O)C2C=CC=CC=C2)C1)C1[C@@H]2CCCC[C@@H]12	990669221	23	312.18	2.05
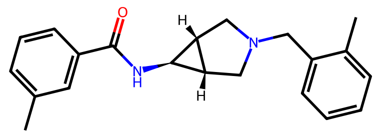	Cc1cccc(C(=O)N[C@H]2[C@@H]3CN(Cc4ccccc4C)C[C@@H]32)c1	958151332	24	320.19	3.16
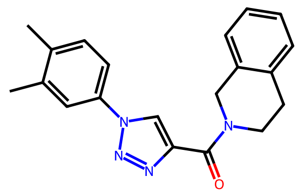	Cc1ccc(-n2cc(C(=O)N3CCc4ccccc4C3)nn2)cc1C	23127101	25	332.16	3.08
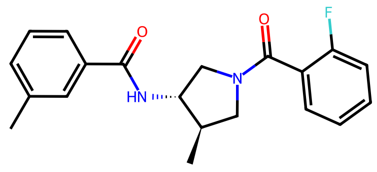	Cc1cccc(C(=O)N[C@@H]2CN(C(=O)c3ccccc3F)C[C@H]2C)c1	947211119	25	340.16	3.02
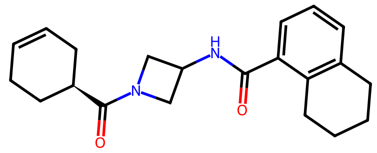	O=C(NC1CN(C(=O)[C@@H]2CC=CC C2)C1)c1cccc2c1CCCC2	991900412	25	338.2	2.86
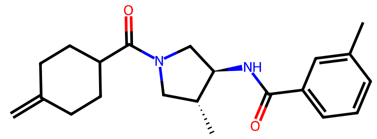	C=C1CCC(C(=O)N2C[C@@H](NC(=O)c3cccc(C)c3)[C@H](C)C2)CC1	958540857	25	340.22	3.32
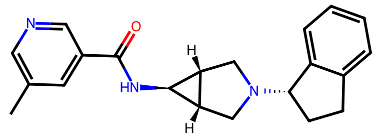	Cc1cncc(C(=O)N[C@H]2[C@@H]3CN([C@H]4CCc5ccccc54)C[C@@H]32)c1	958470708	25	333.18	2.74

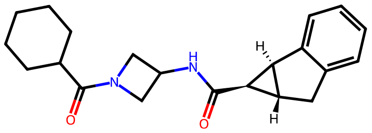	O=C(NC1CN(C(=O)C2CCCCC2)C1)[C@@H]1[C@H]2Cc3ccccc3[C@@H]21	991647245	25	338.2	2.48
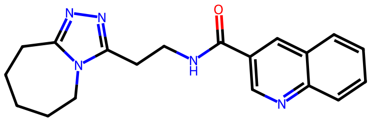	O=C(NCCc1nnc2n1CCCCC2)c1cnc2ccccc2c1	293571448	25	335.17	2.53
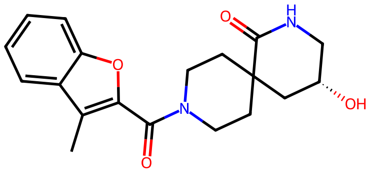	Cc1c(C(=O)N2CCC3(CC2)C[C@@H](O)CNC3=O)oc2ccccc12	885026005	25	342.16	1.84
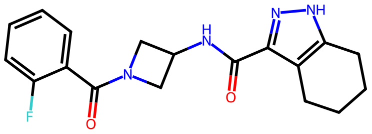	O=C(NC1CN(C(=O)c2ccccc2F)C1)c1n[nH]c2c1CCCC2	995171762	25	342.15	1.68
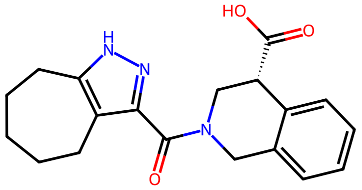	O=C(O)[C@H]1CN(C(=O)c2n[nH]c3c2CCCCC3)Cc2ccccc21	909425708	25	339.16	2.5
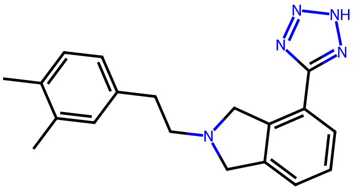	Cc1ccc(CCN2Cc3cccc(-c4nn[nH]n4)c3C2)cc1C	1605844158	24	319.18	3.04
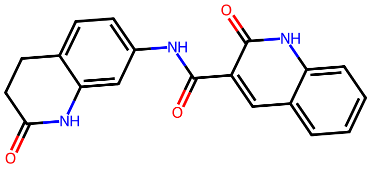	O=C1CCc2ccc(NC(=O)c3cc4ccccc4[nH]c3=O)cc2N1	172659113	25	333.11	2.67
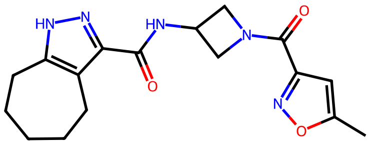	Cc1cc(C(=O)N2CC(NC(=O)c3n[nH]c4c3CCCCC4)C2)no1	991765157	25	343.16	1.23

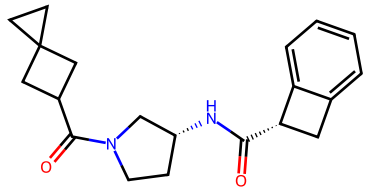	O=C(N[C@@H]1CCN(C(=O)C2CC3(CC3)C2)C1)[C@H]1Cc2ccccc21	942302867	24	324.18	2.23
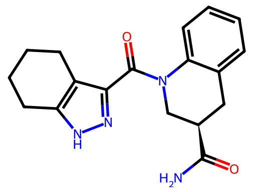	NC(=O)[C@@H]1Cc2ccccc2N(C(=O)c2n[nH]c3c2CCCC3)C1	602126762	24	324.16	1.59
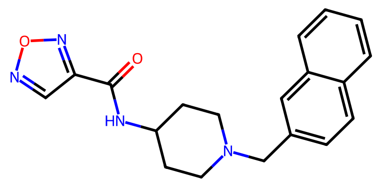	O=C(NC1CCN(Cc2ccc3ccccc3c2)CC1)c1cnon1	1003817498	25	336.16	2.62
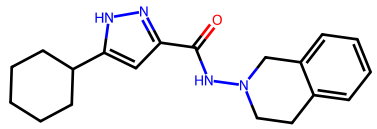	O=C(NN1CCc2ccccc2C1)c1cc(C2CCCCC2)[nH]n1	1469894177	24	324.2	3.16
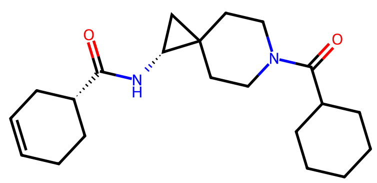	O=C(N[C@@H]1CC12CCN(C(=O)C1CCCCC1)CC2)[C@H]1CC=CCC1	963027286	25	344.25	3.42
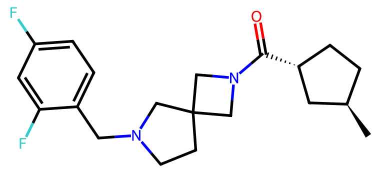	C[C@@H]1CC[C@@H](C(=O)N2CC3(CCN(Cc4ccc(F)cc4F)C3)C2)C1	1073031350	25	348.2	3.44
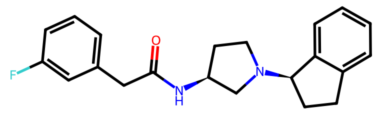	O=C(Cc1cccc(F)c1)N[C@H]1CCN([C@@H]2CCc3ccccc32)C1	1016184462	25	338.18	3.25
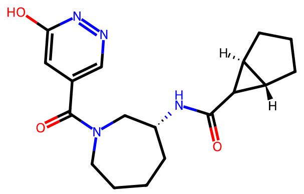	O=C(N[C@@H]1CCCCN(C(=O)c2cnnc(O)c2)C1)C1[C@H]2CCC[C@H]12	943166098	25	344.18	1.34

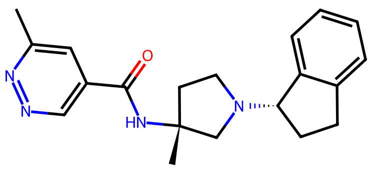	Cc1cc(C(=O)N[C@]2(C)CCN([C@H]3CCc4ccccc43)C2)cnn1	1046269699	25	336.2	2.67
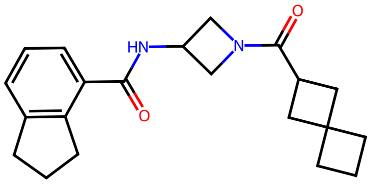	O=C(NC1CN(C(=O)C2CC3(CCC3)C2)C1)c1cccc2c1CCC2	994397320	25	338.2	2.7
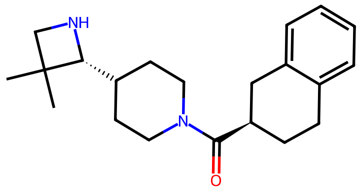	CC1(C)CN[C@@H]1C1CCN(C(=O)[C@@H]2CCc3ccccc3C2)CC1	653164894	24	326.24	3.03
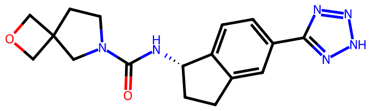	O=C(N[C@H]1CCc2cc(-c3nn[nH]n3)ccc21)N1CCC2(COC2)C1	1606686623	25	340.16	1.29
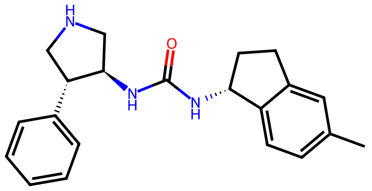	Cc1ccc2c(c1)CC[C@H]2NC(=O)N[C@@H]1CNC[C@H]1c1ccccc1	567595565	25	335.2	3.04
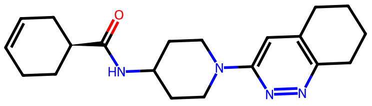	O=C(NC1CCN(c2cc3c(nn2)CCCC3)CC1)[C@@H]1CC=CCC1	225793293	25	340.23	2.8
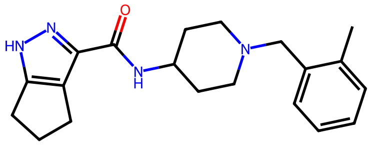	Cc1ccccc1CN1CCC(NC(=O)c2n[nH]c3c2CCC3)CC1	120123828	25	338.21	2.6
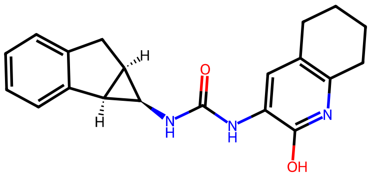	O=C(Nc1cc2c(nc1O)CCCC2)N[C@@H]1[C@@H]2Cc3ccccc3[C@@H]21	650270659	25	335.16	3.13

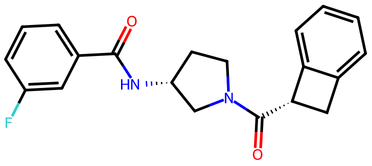	O=C(N[C@@H]1CCN(C(=O)[C@H]2Cc3ccccc32)C1)c1cccc(F)c1	939219755	25	338.14	2.5
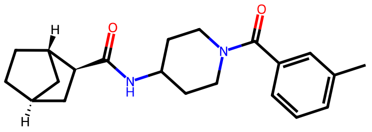	Cc1cccc(C(=O)N2CCC(NC(=O)[C@H]3C[C@H]4CC[C@H]3C4)CC2)c1	943793065	25	340.22	3.15
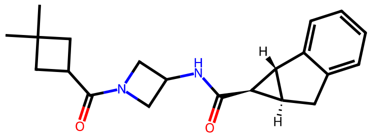	CC1(C)CC(C(=O)N2CC(NC(=O)[C@@H]3[C@@H]4Cc5ccccc5[C@H]43)C2)C1	991644354	25	338.2	2.34
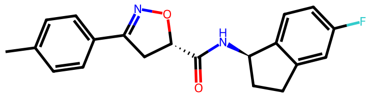	Cc1ccc(C2=NO[C@H](C(=O)N[C@@H]3CCc4cc(F)ccc43)C2)cc1	1526637388	25	338.14	3.43
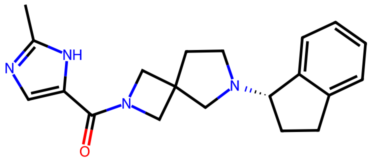	Cc1ncc(C(=O)N2CC3(CCN([C@H]4CCc5ccccc54)C3)C2)[nH]1	1072550336	25	336.2	2.55
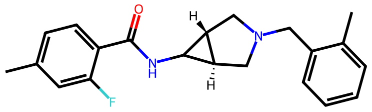	Cc1ccc(C(=O)NC2[C@@H]3CN(Cc4ccccc4C)C[C@@H]23)c(F)c1	961878491	25	338.18	3.3
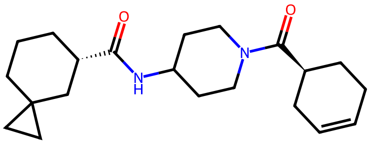	O=C(NC1CCN(C(=O)[C@@H]2CC=CCC2)CC1)[C@H]1CCCC2(CC2)C1	944106680	25	344.25	3.42
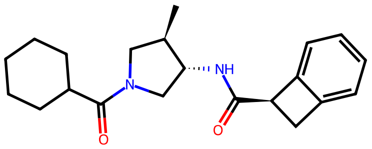	C[C@@H]1CN(C(=O)C2CCCCC2)C[C@H]1NC(=O)[C@@H]1Cc2ccccc21	939656309	25	340.22	2.87

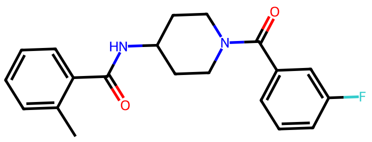	Cc1ccccc1C(=O)NC1CCN(C(=O)c2cccc(F)c2)CC1	32307961	25	340.16	3.17
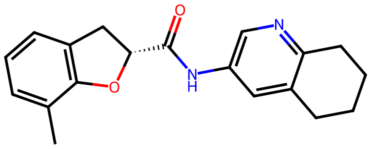	Cc1cccc2c1O[C@@H](C(=O)Nc1cnc3c(c1)CCCC3)C2	333759407	23	308.15	3.21
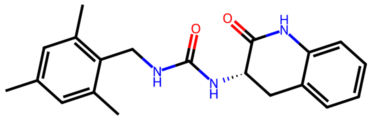	Cc1cc(C)c(CNC(=O)N[C@H]2Cc3ccccc3NC2=O)c(C)c1	1365100495	25	337.18	2.97
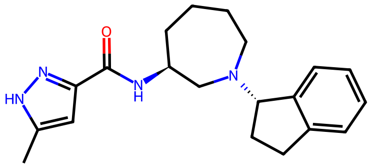	Cc1cc(C(=O)N[C@H]2CCCCN([C@H]3CCc4ccccc43)C2)n[nH]1	1034123584	25	338.21	2.99
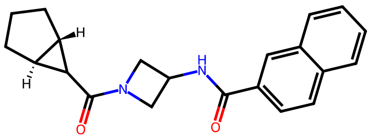	O=C(NC1CN(C(=O)C2[C@H]3CCC[C@H]23)C1)c1ccc2ccccc2c1	999446199	25	334.17	2.83
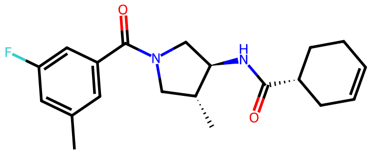	Cc1cc(F)cc(C(=O)N2C[C@@H](NC(=O)[C@H]3CC=CCC3)[C@H](C)C2)c1	959723140	25	344.19	3.07
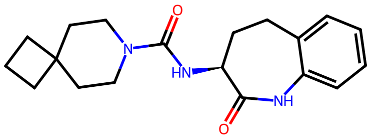	O=C1Nc2ccccc2CC[C@@H]1NC(=O)N1CCC2(CCC2)CC1	654634280	24	327.19	2.92
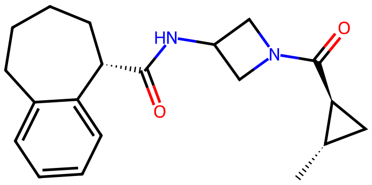	C[C@H]1C[C@@H]1C(=O)N1CC(NC(=O)[C@H]2CCCCc3ccccc32)C1	994323859	24	326.2	2.48

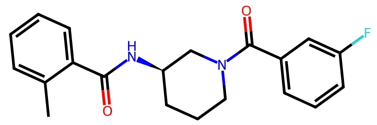	Cc1ccccc1C(=O)N[C@@H]1CCCN(C(=O)c2cccc(F)c2)C1	585989374	25	340.16	3.17
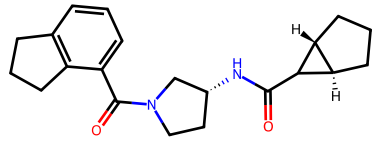	O=C(N[C@@H]1CCN(C(=O)c2cccc3c2CCC3)C1)C1[C@@H]2CCC[C@@H]12	939930783	25	338.2	2.55
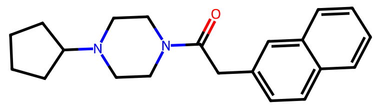	O=C(Cc1ccc2ccccc2c1)N1CCN(C2CCCC2)CC1	84605829	24	322.2	3.47
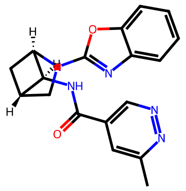	Cc1cc(C(=O)N[C@H]2[C@H]3C[C@H]2N(c2nc4ccccc4o2)C3)cnn1	1064241261	25	335.14	1.93
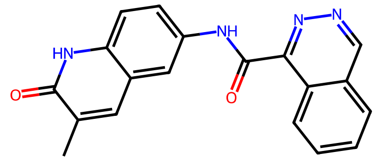	Cc1cc2cc(NC(=O)c3nncc4ccccc34)ccc2[nH]c1=O	1641294614	25	330.11	3.03
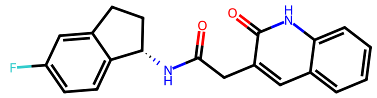	O=C(Cc1cc2ccccc2[nH]c1=O)N[C@H]1CCc2cc(F)ccc21	55658385	25	336.13	3.01
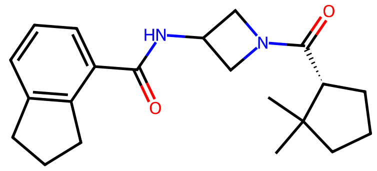	CC1(C)CCC[C@H]1C(=O)N1CC(NC(=O)c2cccc3c2CCC3)C1	994393879	25	340.22	2.94
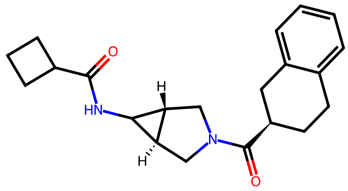	O=C(NC1[C@@H]2CN(C(=O)[C@@H]3CCc4ccccc4C3)C[C@@H]12)C1CCC1	1012656073	25	338.2	2.16

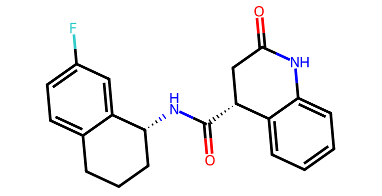	O=C1C[C@@H](C(=O)N[C@@H]2CCCc3ccc(F)cc32)c2ccccc2N1	93062918	25	338.14	3.45
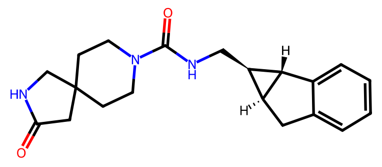	O=C1CC2(CCN(C(=O)NC[C@@H]3[C@@H]4Cc5ccccc5[C@@H]34)CC2)CN1	269043553	25	339.19	1.88
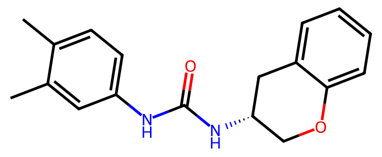	Cc1ccc(NC(=O)N[C@H]2COc3ccccc3C2)cc1C	21101601	22	296.15	3.43
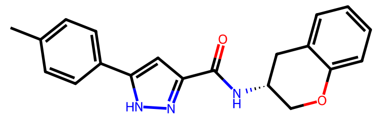	Cc1ccc(-c2cc(C(=O)N[C@H]3COc4ccccc4C3)n[nH]2)cc1	516105195	25	333.15	3.12
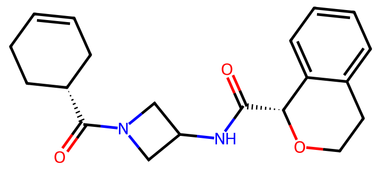	O=C(NC1CN(C(=O)[C@H]2CC=CCC2)C1)[C@H]1OCCc2ccccc21	997322121	25	340.18	1.98
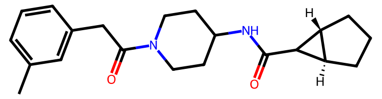	Cc1cccc(CC(=O)N2CCC(NC(=O)C3[C@@H]4CCC[C@@H]34)CC2)c1	948068519	25	340.22	2.69
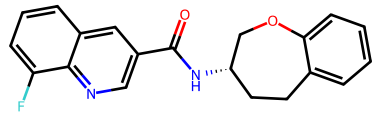	O=C(N[C@H]1CCc2ccccc2OC1)c1cnc2c(F)cccc2c1	353143322	25	336.13	3.5
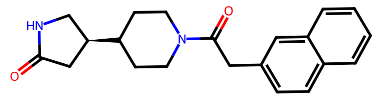	O=C1C[C@H](C2CCN(C(=O)Cc3ccc4ccccc4c3)CC2)CN1	1551713760	25	336.18	2.76

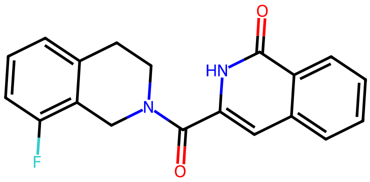	O=C(c1cc2ccccc2c(=O)[nH]1)N1CCc2cccc(F)c2C1	128075008	24	322.11	2.87
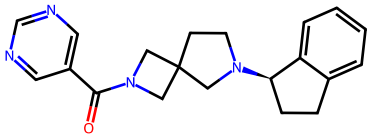	O=C(c1cncnc1)N1CC2(CCN([C@@H]3CCc4ccccc43)C2)C1	1072656226	25	334.18	2.31
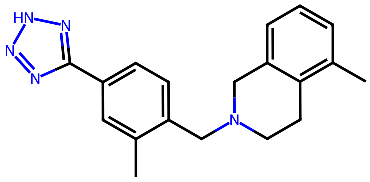	Cc1cc(-c2nn[nH]n2)ccc1CN1CCc2c(C)cccc2C1	1591866122	24	319.18	3.04
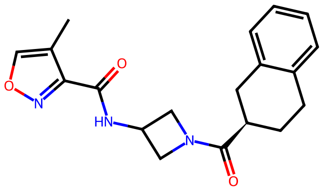	Cc1conc1C(=O)NC1CN(C(=O)[C@@H]2CCc3ccccc3C2)C1	999667491	25	339.16	1.73
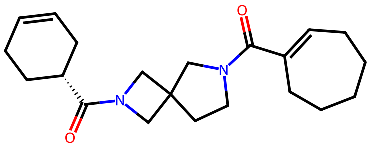	O=C(C1=CCCCCC1)N1CCC2(C1)CN(C(=O)[C@H]1CC=CCC1)C2	1018770074	25	342.23	3.29
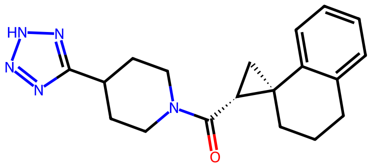	O=C([C@@H]1C[C@]12CCCc1ccccc12)N1CCC(c2nn[nH]n2)CC1	913743893	25	337.19	2.2
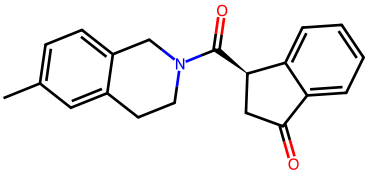	Cc1ccc2c(c1)CCN(C(=O)[C@@H]1CC(=O)c3ccccc31)C2	360219338	23	305.14	3.25
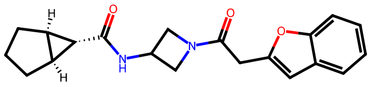	O=C(NC1CN(C(=O)Cc2cc3ccccc3o2)C1)[C@@H]1[C@H]2CCC[C@H]21	990708151	25	338.16	2.35

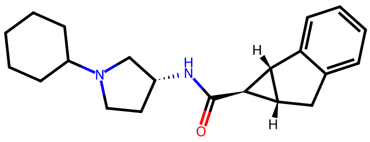	O=C(N[C@@H]1CCN(C2CCCCC2)C1)[C@@H]1[C@H]2Cc3ccccc3[C@H]21	628092779	24	324.22	3.1
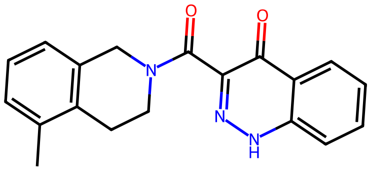	Cc1cccc2c1CCN(C(=O)c1n[nH]c3ccccc3c1=O)C2	171870517	24	319.13	2.43
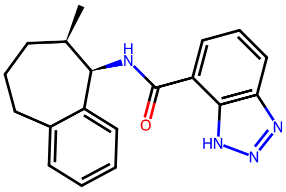	C[C@@H]1CCCc2ccccc2[C@@H]1NC(=O)c1cccc2nn[nH]c12	1138085095	24	320.16	3.4
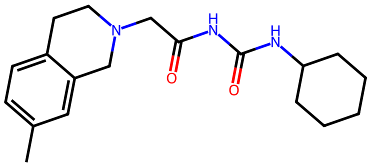	Cc1ccc2c(c1)CN(CC(=O)NC(=O)NC1CCCCC1)CC2	1363801562	24	329.21	2.51
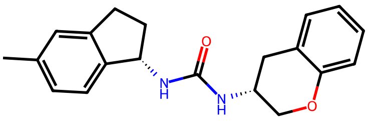	Cc1ccc2c(c1)CC[C@@H]2NC(=O)N[C@H]1COc2ccccc2C1	516689624	24	322.17	3.29
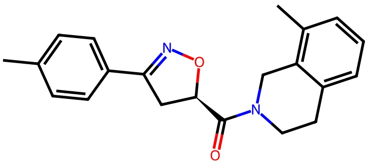	Cc1ccc(C2=NO[C@@H](C(=O)N3CCc4cccc(C)c4C3)C2)cc1	1435877049	25	334.17	3.38
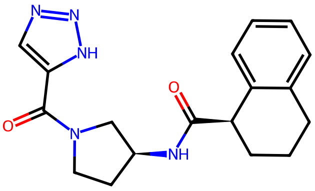	O=C(N[C@H]1CCN(C(=O)c2cnn[nH]2)C1)[C@@H]1CCCc2ccccc21	938222195	25	339.17	1.26
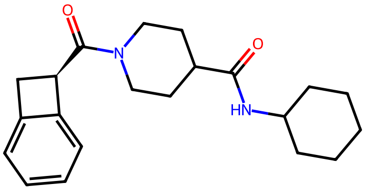	O=C(NC1CCCCC1)C1CCN(C(=O)[C@@H]2Cc3ccccc32)CC1	358191265	25	340.22	3.01

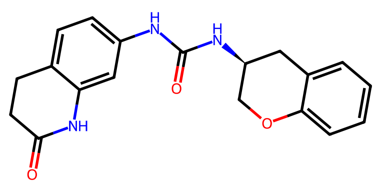	O=C1CCc2ccc(NC(=O)N[C@@H]3COc4ccccc4C3)cc2N1	173739352	25	337.14	2.7
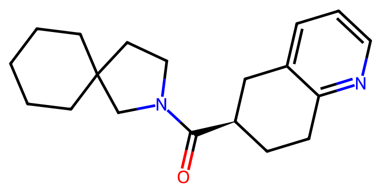	O=C([C@@H]1CCc2ncccc2C1)N1CCC2(CCCCC2)C1	1875307446	22	298.2	3.37
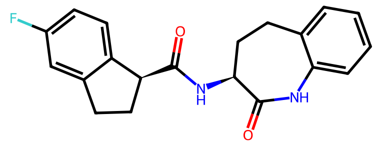	O=C1Nc2ccccc2CC[C@@H]1NC(=O)[C@H]1CCc2cc(F)ccc21	616691831	25	338.14	2.93
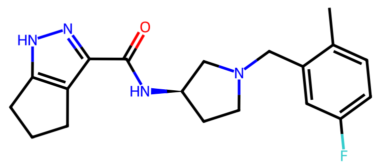	Cc1ccc(F)cc1CN1CC[C@@H](NC(=O)c2n[nH]c3c2CCC3)C1	1015079416	25	342.19	2.35
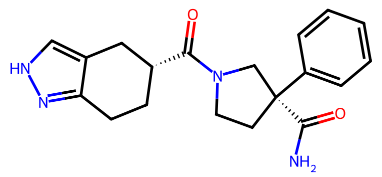	NC(=O)[C@]1(c2ccccc2)CCN(C(=O)[C@@H]2CCc3n[nH]cc3C2)C1	1363444251	25	338.17	1.17
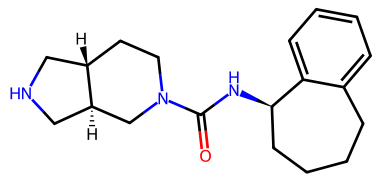	O=C(N[C@@H]1CCCCc2ccccc21)N1CC[C@H]2CNC[C@@H]2C1	578333972	23	313.22	2.71
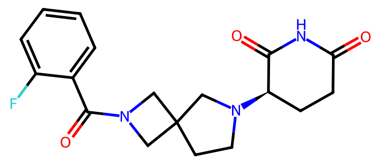	O=C1CC[C@@H](N2CCC3(CN(C(=O)c4ccccc4F)C3)C2)C(=O)N1	1072408669	25	345.15	0.78
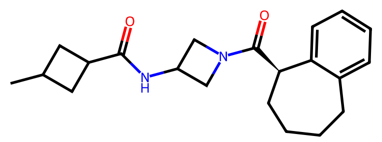	CC1CC(C(=O)NC2CN(C(=O)[C@@H]3CCCCc4ccccc43)C2)C1	990758781	25	340.22	2.87

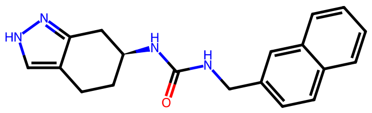	O=C(NCc1ccc2ccccc2c1)N[C@H]1CCc2c[nH]nc2C1	372745930	24	320.16	2.92
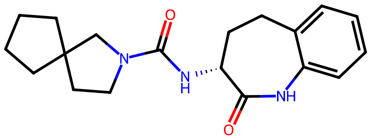	O=C1Nc2ccccc2CC[C@H]1NC(=O)N1CCC2(CCCC2)C1	847396012	24	327.19	2.92
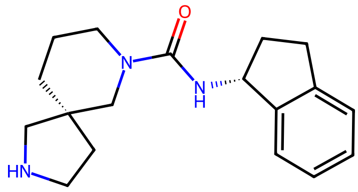	O=C(N[C@@H]1CCc2ccccc21)N1CCC[C@@]2(CCNC2)C1	335612079	22	299.2	2.46
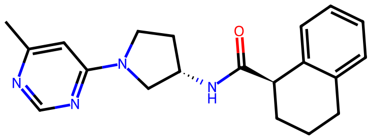	Cc1cc(N2CC[C@H](NC(=O)[C@@H]3CCCc4ccccc43)C2)ncn1	1058592334	25	336.2	2.6
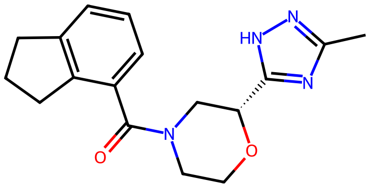	Cc1n[nH]c([C@H]2CN(C(=O)c3cccc4c3CCC4)CCO2)n1	173530013	23	312.16	1.82
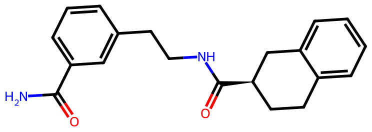	NC(=O)c1cccc(CCNC(=O)[C@@H]2CCc3ccccc3C2)c1	521882937	24	322.17	2.25
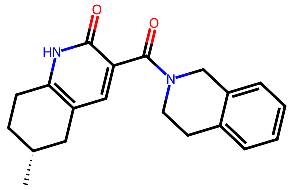	C[C@@H]1CCc2[nH]c(=O)c(C(=O)N3CCc4ccccc4C3)cc2C1	49551329	24	322.17	2.7
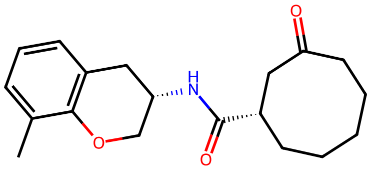	Cc1cccc2c1OC[C@@H](NC(=O)[C@H]1CCCCCC(=O)C1)C2	517228903	23	315.18	2.95

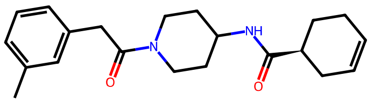	Cc1cccc(CC(=O)N2CCC(NC(=O)[C@@H]3CC=CCC3)CC2)c1	948068618	25	340.22	3.0
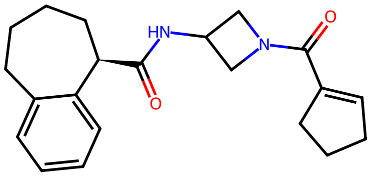	O=C(NC1CN(C(=O)C2=CCCC2)C1)[C@@H]1CCCCc2ccccc21	994304237	25	338.2	2.93
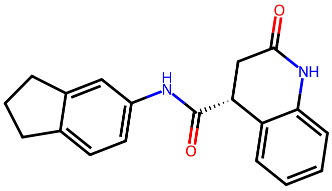	O=C1C[C@@H](C(=O)Nc2ccc3c(c2)CCC3)c2ccccc2N1	10642690	23	306.14	3.24
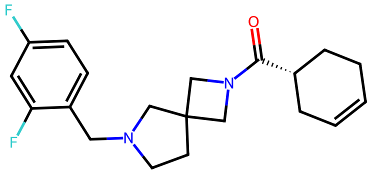	O=C([C@H]1CC=CCC1)N1CC2(CCN(Cc3ccc(F)cc3F)C2)C1	1072457548	25	346.19	3.36
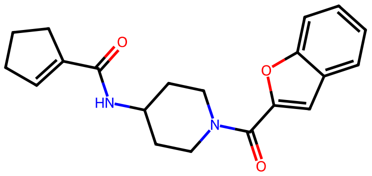	O=C(NC1CCN(C(=O)c2cc3ccccc3o2)CC1)C1=CCCC1	948479304	25	338.16	3.26
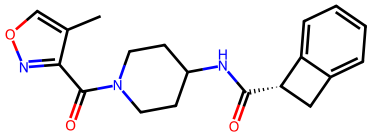	Cc1conc1C(=O)N1CCC(NC(=O)[C@H]2Cc3ccccc32)CC1	948376862	25	339.16	2.04
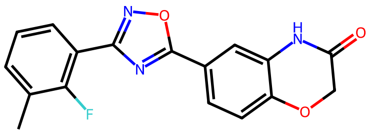	Cc1cccc(-c2noc(-c3ccc4c(c3)NC(=O)CO4)n2)c1F	362591882	24	325.09	3.18
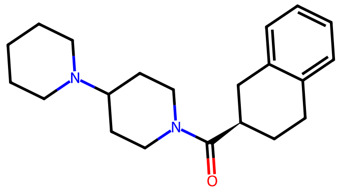	O=C([C@@H]1CCc2ccccc2C1)N1CCC(N2CCCCC2)CC1	566324137	24	326.24	3.27

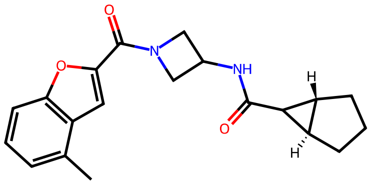	Cc1cccc2oc(C(=O)N3CC(NC(=O)C4[C@@H]5CCC[C@@H]45)C3)cc12	990700293	25	338.16	2.73
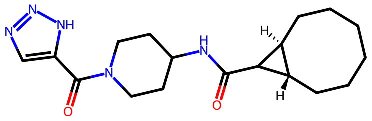	O=C(NC1CCN(C(=O)c2cnn[nH]2)CC1)C1[C@H]2CCCCCC[C@H]12	947373280	25	345.22	1.74
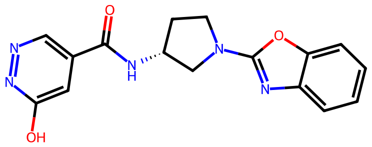	O=C(N[C@@H]1CCN(c2nc3ccccc3o2)C1)c1cnnc(O)c1	1058473187	24	325.12	1.33
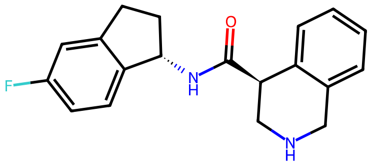	O=C(N[C@H]1CCc2cc(F)ccc21)[C@@H]1CNCc2ccccc21	580069633	23	310.15	2.82
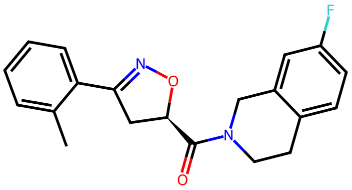	Cc1ccccc1C1=NO[C@@H](C(=O)N2CCc3ccc(F)cc3C2)C1	1357172321	25	338.14	3.21
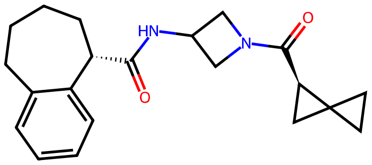	O=C(NC1CN(C(=O)[C@@H]2CC23CC3)C1)[C@H]1CCCCc2ccccc21	994322765	25	338.2	2.62
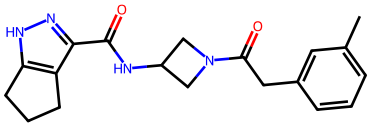	Cc1cccc(CC(=O)N2CC(NC(=O)c3n[nH]c4c3CCC4)C2)c1	995167411	25	338.17	1.39
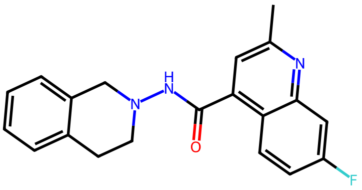	Cc1cc(C(=O)NN2CCc3ccccc3C2)c2ccc(F)cc2n1	1165595873	25	335.14	3.39

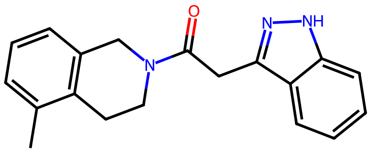	Cc1cccc2c1CCN(C(=O)Cc1n[nH]c3ccccc13)C2	171544027	23	305.15	3.0
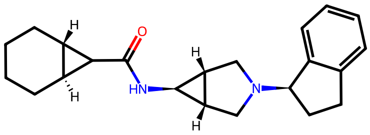	O=C(N[C@H]1[C@@H]2CN([C@@H]3CCc4ccccc43)C[C@@H]21)C1[C@H]2CCCC[C@H]12	1025723608	25	336.22	3.16
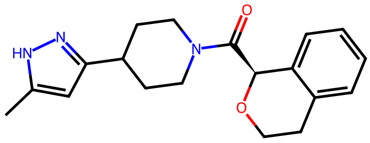	Cc1cc(C2CCN(C(=O)[C@@H]3OCCc4ccccc43)CC2)n[nH]1	329521298	24	325.18	2.74
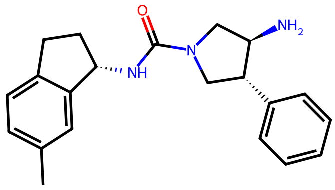	Cc1ccc2c(c1)[C@@H](NC(=O)N1C[C@@H](N)[C@H](c3ccccc3)C1)CC2	841791976	25	335.2	3.12
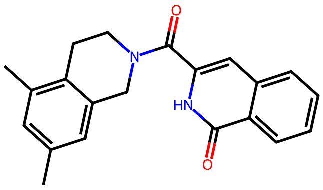	Cc1cc(C)c2c(c1)CN(C(=O)c1cc3ccccc3c(=O)[nH]1)CC2	187760724	25	332.15	3.34
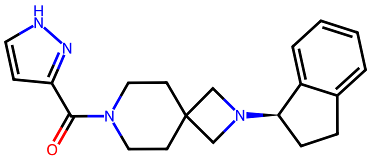	O=C(c1cc[nH]n1)N1CCC2(CC1)CN([C@@H]1CCc3ccccc31)C2	1035657911	25	336.2	2.64
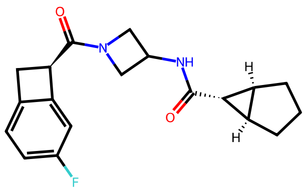	O=C(NC1CN(C(=O)[C@@H]2Cc3ccc(F)cc32)C1)[C@@H]1[C@H]2CCC[C@H]21	990707702	24	328.16	1.84
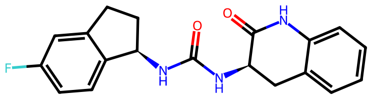	O=C(N[C@@H]1Cc2ccccc2NC1=O)N[C@@H]1CCc2cc(F)ccc21	636635619	25	339.14	2.68

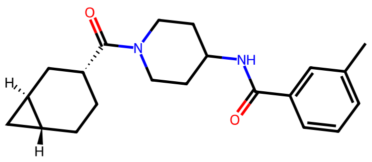	Cc1cccc(C(=O)NC2CCN(C(=O)[C@@H]3CC[C@@H]4C[C@H]4C3)CC2)c1	870837573	25	340.22	3.15
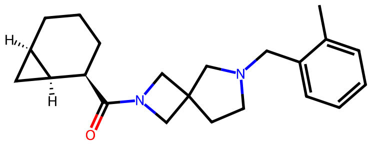	Cc1ccccc1CN1CCC2(C1)CN(C(=O)[C@@H]1CCC[C@@H]3C[C@@H]31)C2	1072874524	25	338.24	3.47
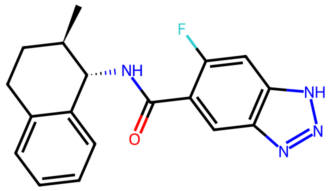	C[C@@H]1CCc2ccccc2[C@H]1NC(=O)c1cc2nn[nH]c2cc1F	781961684	24	324.14	3.15
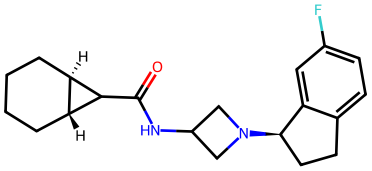	O=C(NC1CN([C@@H]2CCc3ccc(F)cc32)C1)C1[C@@H]2CCCC[C@@H]12	1030185895	24	328.2	3.05
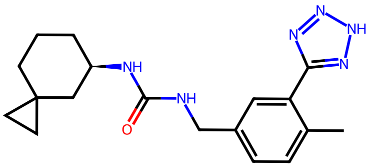	Cc1ccc(CNC(=O)N[C@@H]2CCCC3(CC3)C2)cc1-c1nn[nH]n1	1592159826	25	340.2	2.7
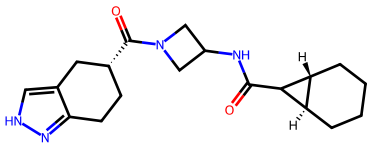	O=C(NC1CN(C(=O)[C@@H]2CCc3n[nH]cc3C2)C1)C1[C@@H]2CCCC[C@@H]12	990649340	25	342.21	1.28
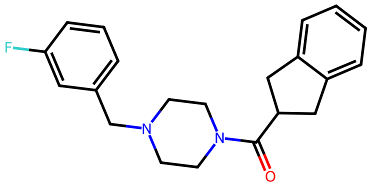	O=C(C1Cc2ccccc2C1)N1CCN(Cc2cccc(F)c2)CC1	346501404	25	338.18	2.88
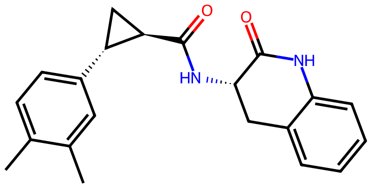	Cc1ccc([C@@H]2C[C@H]2C(=O)N[C@@H]2Cc3ccccc3NC2=O)cc1C	628943216	25	334.17	3.09

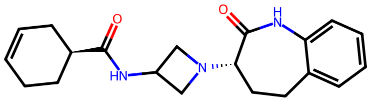	O=C(NC1CN([C@H]2CCc3ccccc3NC2=O)C1)[C@@H]1CC=CCC1	1030098287	25	339.19	2.1
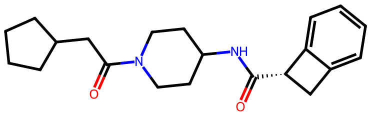	O=C(NC1CCN(C(=O)CC2CCCC2)CC1)[C@H]1Cc2ccccc21	943770177	25	340.22	3.01
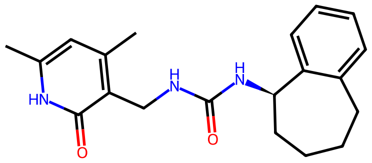	Cc1cc(C)c(CNC(=O)N[C@@H]2CCCCc3ccccc32)c(=O)[nH]1	69657184	25	339.19	3.26
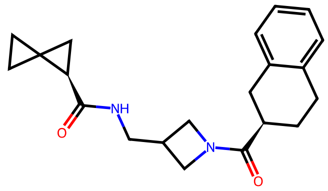	O=C(NCC1CN(C(=O)[C@@H]2CCc3ccccc3C2)C1)[C@@H]1CC12CC2	936091180	25	338.2	2.17
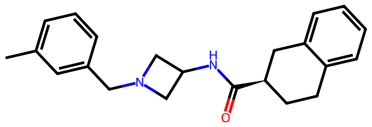	Cc1cccc(CN2CC(NC(=O)[C@@H]3CCc4ccccc4C3)C2)c1	1031012444	25	334.2	3.1
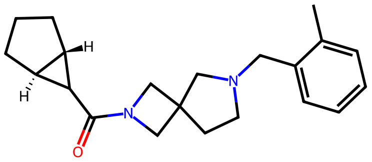	Cc1ccccc1CN1CCC2(C1)CN(C(=O)C1[C@H]3CCC[C@H]13)C2	1072590581	24	324.22	3.08
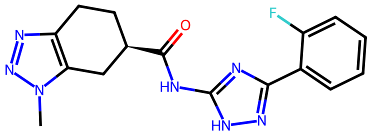	Cn1nnc2c1C[C@H](C(=O)Nc1nc(-c3ccccc3F)n[nH]1)CC2	290367880	25	341.14	1.48
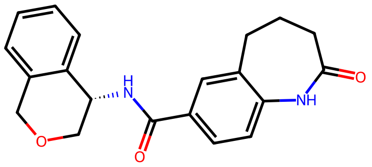	O=C1CCCc2cc(C(=O)N[C@@H]3COCc4ccccc43)ccc2N1	667902075	25	336.15	2.96

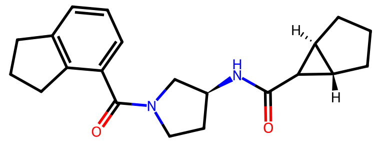	O=C(N[C@H]1CCN(C(=O)c2cccc3c2CCC3)C1)C1[C@H]2CCC[C@H]12	937800261	25	338.2	2.55
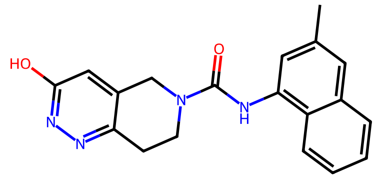	Cc1cc(NC(=O)N2CCc3nnc(O)cc3C2)c2ccccc2c1	1672036976	25	334.14	3.23
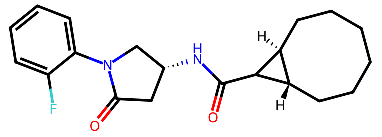	O=C(N[C@@H]1CC(=O)N(c2ccccc2F)C1)C1[C@H]2CCCCCC[C@H]12	1460432393	25	344.19	3.26
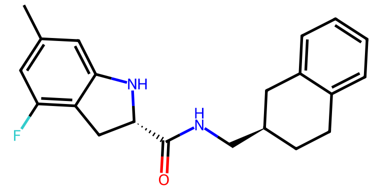	Cc1cc(F)c2c(c1)N[C@H](C(=O)NC[C@@H]1CCc3ccccc3C1)C2	1579610646	25	338.18	3.39
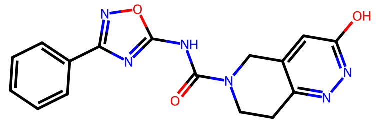	O=C(Nc1nc(-c2ccccc2)no1)N1CCc2nnc(O)cc2C1	1670488436	25	338.11	1.82
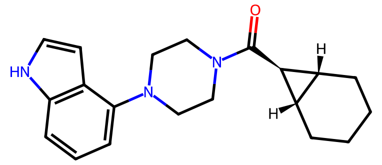	O=C([C@@H]1[C@H]2CCCC[C@H]21)N1CCN(c2cccc3[nH]ccc23)CC1	616751582	24	323.2	3.25
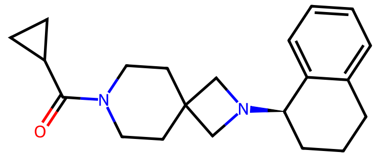	O=C(C1CC1)N1CCC2(CC1)CN([C@@H]1CCCc3ccccc31)C2	1035634714	24	324.22	3.4
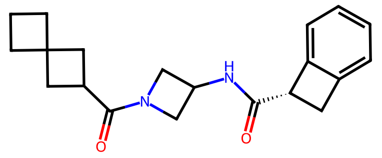	O=C(NC1CN(C(=O)C2CC3(CCC3)C2)C1)[C@H]1Cc2ccccc21	998688740	24	324.18	2.23

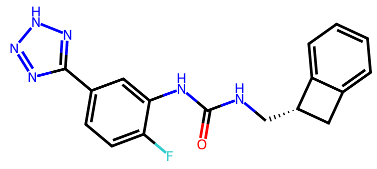	O=C(NC[C@H]1Cc2ccccc21)Nc1cc(-c2nn[nH]n2)ccc1F	1606753411	25	338.13	2.47
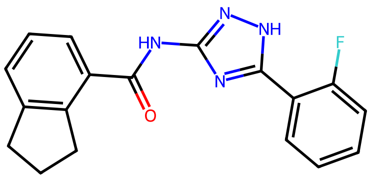	O=C(Nc1n[nH]c(-c2ccccc2F)n1)c1cccc2c1CCC2	354828575	24	322.12	3.35
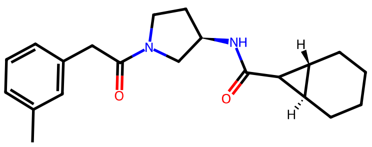	Cc1cccc(CC(=O)N2CC[C@@H](NC(=O)C3[C@@H]4CCCC[C@@H]34)C2)c1	939894995	25	340.22	2.69
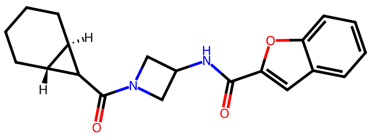	O=C(NC1CN(C(=O)C2[C@@H]3CCCC[C@@H]23)C1)c1cc2ccccc2o1	1000050513	25	338.16	2.81
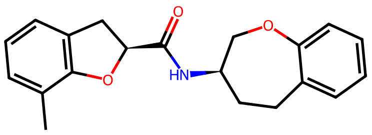	Cc1cccc2c1O[C@H](C(=O)N[C@@H]1CCc3ccccc3OC1)C2	451487665	24	323.15	2.81
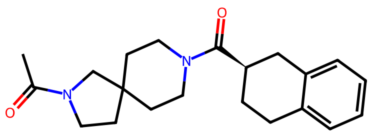	CC(=O)N1CCC2(CCN(C(=O)[C@@H]3CCc4ccccc4C3)CC2)C1	944428796	25	340.22	2.65
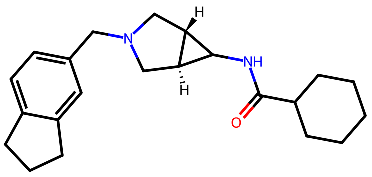	O=C(NC1[C@H]2CN(Cc3ccc4c(c3)CCC4)C[C@H]12)C1CCCCC1	958102800	25	338.24	3.3
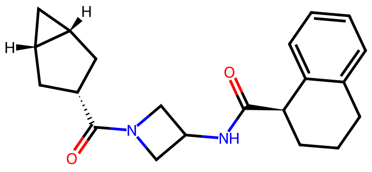	O=C(NC1CN(C(=O)[C@@H]2C[C@@H]3C[C@@H]3C2)C1)[C@@H]1CCCc2ccccc21	995073269	25	338.2	2.48

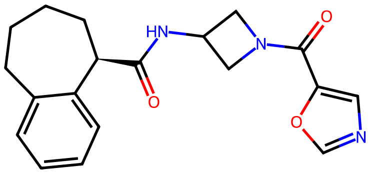	O=C(NC1CN(C(=O)c2cnco2)C1)[C@@H]1CCCCc2ccccc21	994320611	25	339.16	2.13
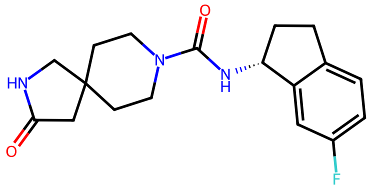	O=C1CC2(CCN(C(=O)N[C@@H]3CCc4ccc(F)cc43)CC2)CN1	74133673	24	331.17	2.12
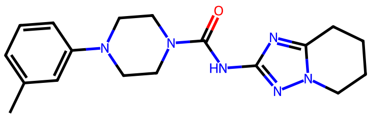	Cc1cccc(N2CCN(C(=O)Nc3nc4n(n3)CCCC4)CC2)c1	333609592	25	340.2	2.28
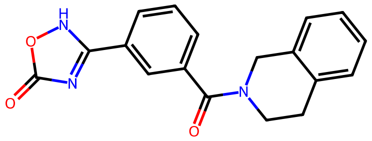	O=C(c1cccc(-c2nc(=O)o[nH]2)c1)N1CCc2ccccc2C1	1618400369	24	321.11	2.23
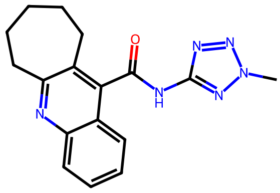	Cn1nnc(NC(=O)c2c3c(nc4ccccc24)CCCCC3)n1	889633823	24	322.15	2.28
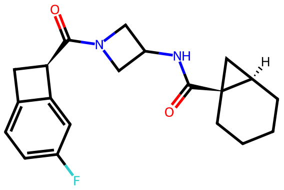	O=C([C@@H]1Cc2ccc(F)cc21)N1CC(NC(=O)[C@]23CCCC[C@@H]2C3)C1	999232963	25	342.17	2.37
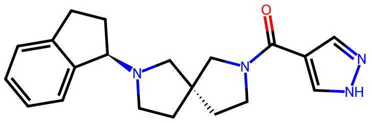	O=C(c1cn[nH]c1)N1CC[C@@]2(CCN([C@@H]3CCc4ccccc43)C2)C1	1041104346	25	336.2	2.64
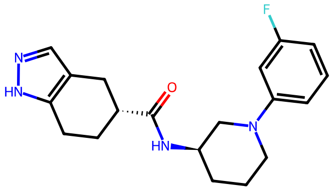	O=C(N[C@@H]1CCCN(c2cccc(F)c2)C1)[C@@H]1CCc2[nH]ncc2C1	270282294	25	342.19	2.44

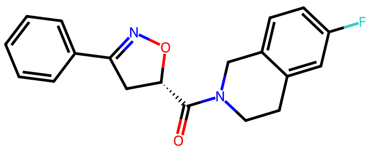	O=C([C@@H]1CC(c2ccccc2)=NO1)N1CCc2cc(F)ccc2C1	121929089	24	324.13	2.9
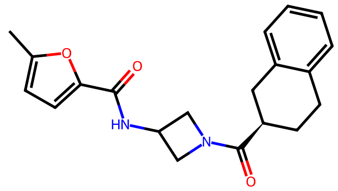	Cc1ccc(C(=O)NC2CN(C(=O)[C@@H]3CCc4ccccc4C3)C2)o1	990163778	25	338.16	2.33
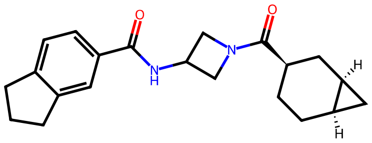	O=C(NC1CN(C(=O)[C@@H]2CC[C@@H]3C[C@@H]3C2)C1)c1ccc2c(c1)CCC2	991373582	25	338.2	2.55
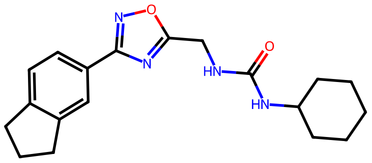	O=C(NCc1nc(-c2ccc3c(c2)CCC3)no1)NC1CCCCC1	570439025	25	340.19	3.36
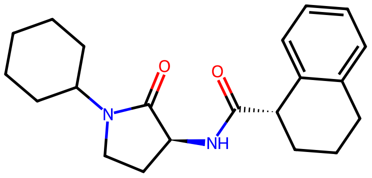	O=C(N[C@H]1CCN(C2CCCCC2)C1=O)[C@H]1CCCc2ccccc21	675913094	25	340.22	3.16
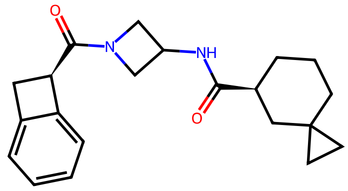	O=C(NC1CN(C(=O)[C@@H]2Cc3ccccc32)C1)[C@H]1CCCC2(CC2)C1	991515471	25	338.2	2.62
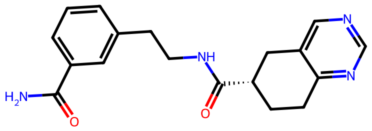	NC(=O)c1cccc(CCNC(=O)[C@H]2CCc3ncncc3C2)c1	648268876	24	324.16	1.04
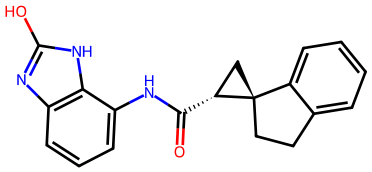	O=C(Nc1cccc2nc(O)[nH]c12)[C@@H]1C[C@@]12CCc1ccccc12	563395846	24	319.13	3.11

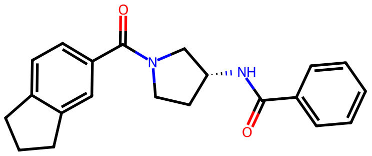	O=C(N[C@@H]1CCN(C(=O)c2ccc3c(c2)CCC3)C1)c1ccccc1	939183035	25	334.17	2.82
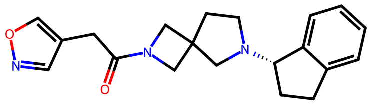	O=C(Cc1cnoc1)N1CC2(CCN([C@H]3CCc4ccccc43)C2)C1	1072909146	25	337.18	2.44
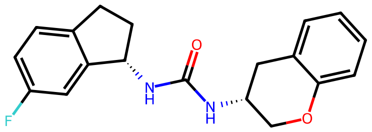	O=C(N[C@H]1CCc2ccc(F)cc21)N[C@H]1COc2ccccc2C1	106536593	24	326.14	3.12
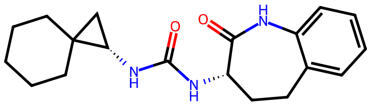	O=C(N[C@H]1CCc2ccccc2NC1=O)N[C@H]1CC12CCCCC2	656111465	24	327.19	2.96
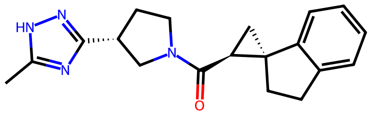	Cc1nc([C@@H]2CCN(C(=O)[C@H]3C[C@]34CCc3ccccc34)C2)n[nH]1	333996519	24	322.18	2.33
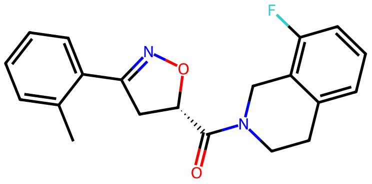	Cc1ccccc1C1=NO[C@H](C(=O)N2CCc3cccc(F)c3C2)C1	1584005262	25	338.14	3.21
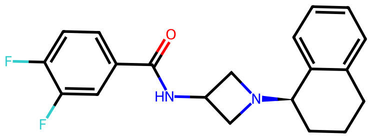	O=C(NC1CN([C@@H]2CCCc3ccccc32)C1)c1ccc(F)c(F)c1	1030446358	25	342.15	3.46
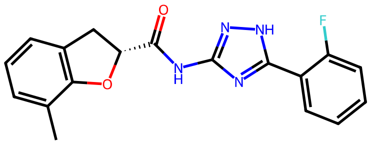	Cc1cccc2c1O[C@@H](C(=O)Nc1n[nH]c(-c3ccccc3F)n1)C2	1558919661	25	338.12	2.86

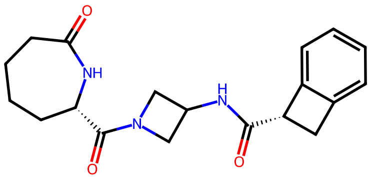	O=C1CCCC[C@@H](C(=O)N2CC(NC(=O)[C@H]3Cc4ccccc43)C2)N1	998690529	25	341.17	0.71
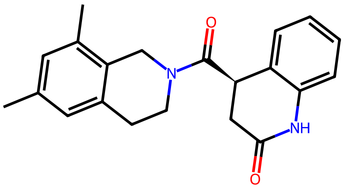	Cc1cc(C)c2c(c1)CCN(C(=O)[C@@H]1CC(=O)Nc3ccccc31)C2	188052677	25	334.17	3.31
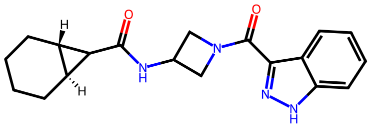	O=C(NC1CN(C(=O)c2n[nH]c3ccccc23)C1)C1[C@H]2CCCC[C@H]12	990671040	25	338.17	1.94
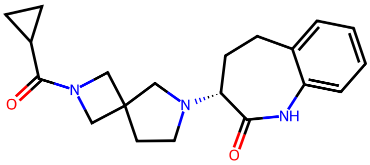	O=C1Nc2ccccc2CC[C@H]1N1CCC2(CN(C(=O)C3CC3)C2)C1	1072378284	25	339.19	1.88
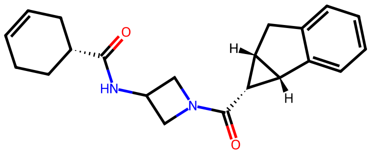	O=C(NC1CN(C(=O)[C@H]2[C@H]3c4ccccc4C[C@H]32)C1)[C@H]1CC=CCC1	990220877	25	336.18	2.26
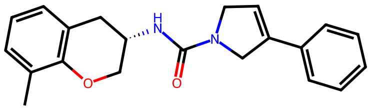	Cc1cccc2c1OC[C@@H](NC(=O)N1CC=C(c3ccccc3)C1)C2	462618829	25	334.17	3.41
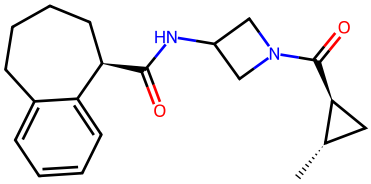	C[C@H]1C[C@@H]1C(=O)N1CC(NC(=O)[C@@H]2CCCCc3ccccc32)C1	994323868	24	326.2	2.48
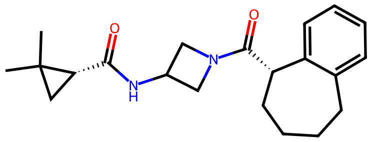	CC1(C)C[C@H]1C(=O)NC1CN(C(=O)[C@H]2CCCCc3ccccc32)C1	990877258	25	340.22	2.87

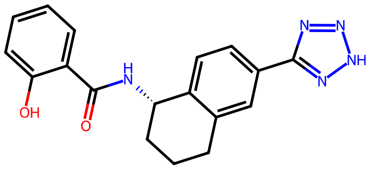	O=C(N[C@H]1CCCc2cc(-c3nn[nH]n3)ccc21)c1ccccc1O	1606671368	25	335.14	2.38
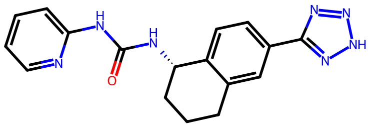	O=C(Nc1ccccn1)N[C@H]1CCCc2cc(-c3nn[nH]n3)ccc21	1593254197	25	335.15	2.46
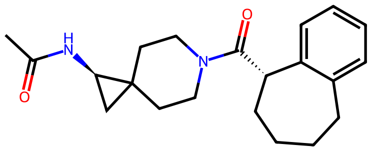	CC(=O)N[C@@H]1CC12CCN(C(=O)[C@H]1CCCCc3ccccc31)CC2	962352477	25	340.22	3.01
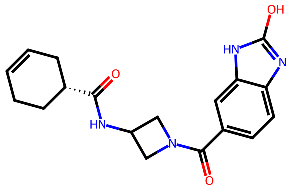	O=C(NC1CN(C(=O)c2ccc3nc(O)[nH]c3c2)C1)[C@H]1CC=CCC1	990222554	25	340.15	1.57
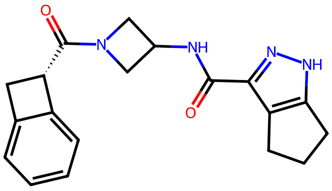	O=C(NC1CN(C(=O)[C@H]2Cc3ccccc32)C1)c1n[nH]c2c1CCC2	995165352	25	336.16	1.18
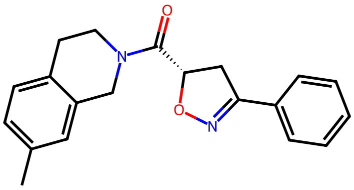	Cc1ccc2c(c1)CN(C(=O)[C@@H]1CC(c3ccccc3)=NO1)CC2	1585658345	24	320.15	3.07
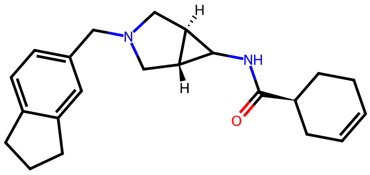	O=C(NC1[C@@H]2CN(Cc3ccc4c(c3)CCC4)C[C@@H]12)[C@@H]1CC=CCC1	958208416	25	336.22	3.08
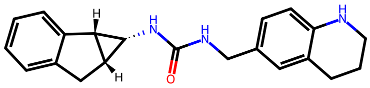	O=C(NCc1ccc2c(c1)CCCN2)N[C@@H]1[C@@H]2Cc3ccccc3[C@@H]21	572264081	25	333.18	3.18

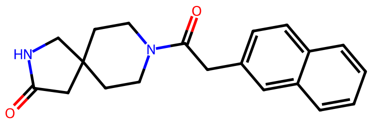	O=C1CC2(CCN(C(=O)Cc3ccc4ccccc4c3)CC2)CN1	267585823	24	322.17	2.51
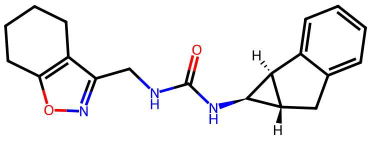	O=C(NCc1noc2c1CCCC2)N[C@@H]1[C@H]2Cc3ccccc3[C@@H]21	288004894	24	323.16	2.69
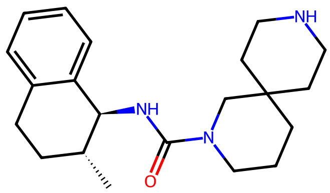	C[C@@H]1CCc2ccccc2[C@H]1NC(=O)N1CCCC2(CCNCC2)C1	1325365401	25	341.25	3.49
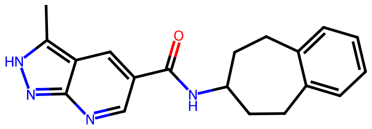	Cc1[nH]nc2ncc(C(=O)NC3CCc4ccccc4CC3)cc12	573445080	24	320.16	2.94
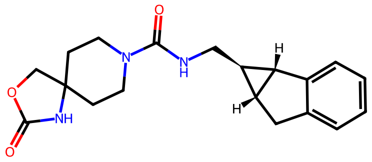	O=C1NC2(CCN(C(=O)NC[C@@H]3[C@H]4Cc5ccccc5[C@H]43)CC2)CO1	495141963	25	341.17	1.86
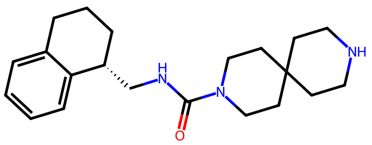	O=C(NC[C@H]1CCCc2ccccc21)N1CCC2(CCNCC2)CC1	417069539	25	341.25	3.28
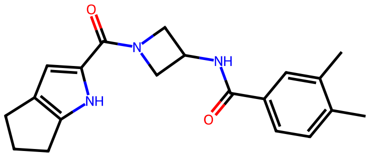	Cc1ccc(C(=O)NC2CN(C(=O)c3cc4c([nH]3)CCC4)C2)cc1C	999541243	25	337.18	2.37
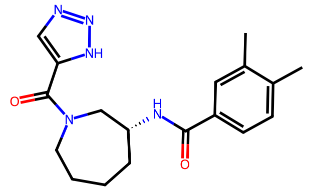	Cc1ccc(C(=O)N[C@@H]2CCCCN(C(=O)c3cnn[nH]3)C2)cc1C	943526689	25	341.19	1.85

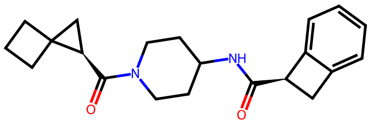	O=C(NC1CCN(C(=O)[C@@H]2CC23CCC3)CC1)[C@@H]1Cc2ccccc21	946759057	25	338.2	2.62
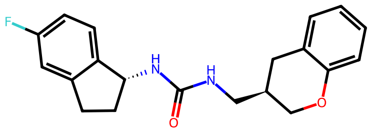	O=C(NC[C@@H]1COc2ccccc2C1)N[C@@H]1CCc2cc(F)ccc21	56839436	25	340.16	3.36
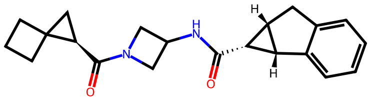	O=C(NC1CN(C(=O)[C@@H]2CC23CCC3)C1)[C@H]1[C@H]2c3ccccc3C[C@H]21	991647427	25	336.18	2.09
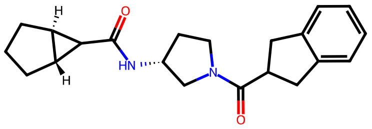	O=C(N[C@@H]1CCN(C(=O)C2Cc3ccccc3C2)C1)C1[C@@H]2CCC[C@@H]12	936250510	25	338.2	2.16
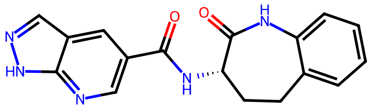	O=C(N[C@H]1CCc2ccccc2NC1=O)c1cnc2[nH]ncc2c1	1640486500	24	321.12	1.64
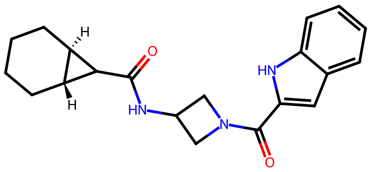	O=C(NC1CN(C(=O)c2cc3ccccc3[nH]2)C1)C1[C@@H]2CCCC[C@@H]12	990663284	25	337.18	2.54
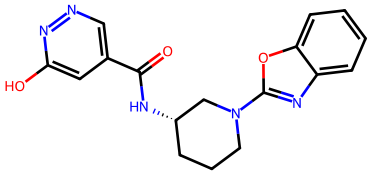	O=C(N[C@H]1CCCN(c2nc3ccccc3o2)C1)c1cnnc(O)c1	1058205919	25	339.13	1.72
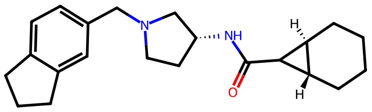	O=C(N[C@@H]1CCN(Cc2ccc3c(c2)CCC3)C1)C1[C@H]2CCCC[C@H]12	1010439987	25	338.24	3.3

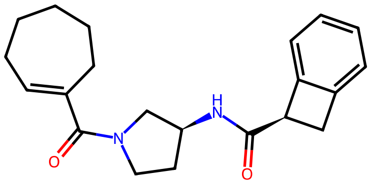	O=C(N[C@H]1CCN(C(=O)C2=CCCCCC2)C1)[C@@H]1Cc2ccccc21	938576867	25	338.2	2.93
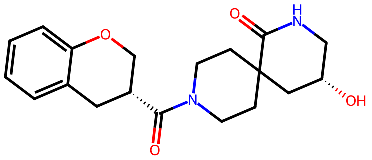	O=C([C@H]1COc2ccccc2C1)N1CCC2(CC1)C[C@@H](O)CNC2=O	885026443	25	344.17	0.73
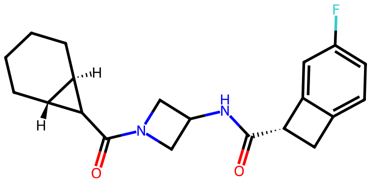	O=C(NC1CN(C(=O)C2[C@@H]3CCCC[C@@H]23)C1)[C@H]1Cc2ccc(F)cc21	998488014	25	342.17	2.23
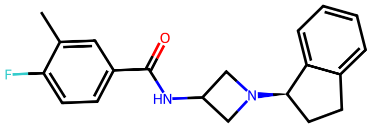	Cc1cc(C(=O)NC2CN([C@@H]3CCc4ccccc43)C2)ccc1F	1030879556	24	324.16	3.24
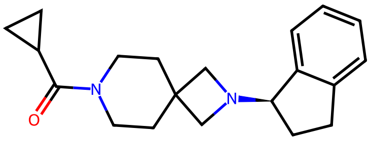	O=C(C1CC1)N1CCC2(CC1)CN([C@@H]1CCc3ccccc31)C2	1035631953	23	310.2	3.01
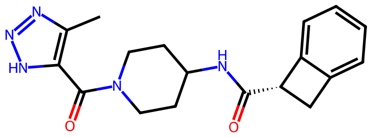	Cc1nn[nH]c1C(=O)N1CCC(NC(=O)[C@H]2Cc3ccccc32)CC1	947680472	25	339.17	1.17
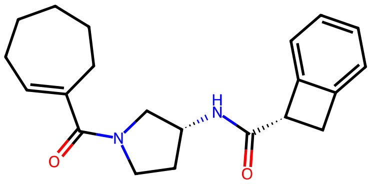	O=C(N[C@@H]1CCN(C(=O)C2=CCCCCC2)C1)[C@H]1Cc2ccccc21	942304983	25	338.2	2.93
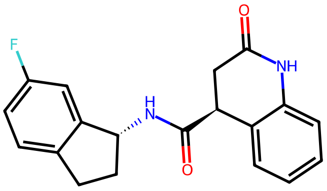	O=C1C[C@H](C(=O)N[C@@H]2CCc3ccc(F)cc32)c2ccccc2N1	106680928	24	324.13	3.06

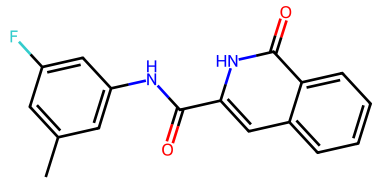	Cc1cc(F)cc(NC(=O)c2cc3ccccc3c(=O)[nH]2)c1	155205911	22	296.1	3.23
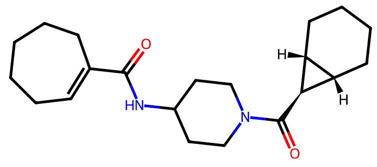	O=C(NC1CCN(C(=O)[C@@H]2[C@H]3CCCC[C@H]32)CC1)C1=CCCCCC1	945957343	25	344.25	3.42
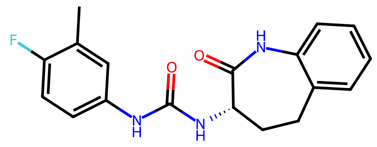	Cc1cc(NC(=O)N[C@H]2CCc3ccccc3NC2=O)ccc1F	1192407370	24	327.14	3.21
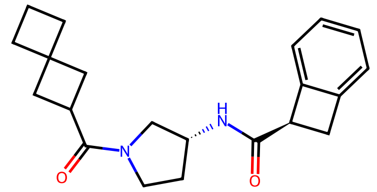	O=C(N[C@@H]1CCN(C(=O)C2CC3(CCC3)C2)C1)[C@@H]1Cc2ccccc21	942304942	25	338.2	2.62
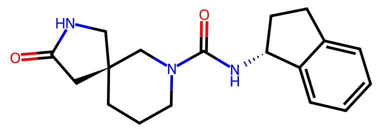	O=C1C[C@@]2(CCCN(C(=O)N[C@@H]3CCc4ccccc43)C2)CN1	340919596	23	313.18	1.99
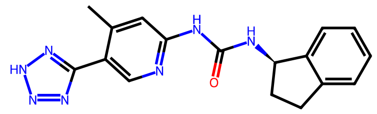	Cc1cc(NC(=O)N[C@@H]2CCc3ccccc32)ncc1-c1nn[nH]n1	1605713127	25	335.15	2.38
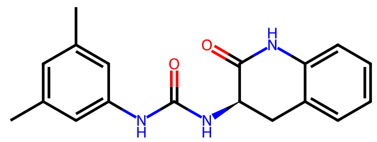	Cc1cc(C)cc(NC(=O)N[C@@H]2Cc3ccccc3NC2=O)c1	1202850234	23	309.15	2.99
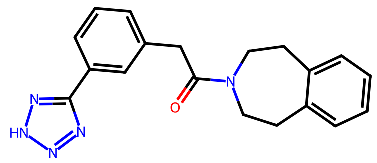	O=C(Cc1cccc(-c2nn[nH]n2)c1)N1CCc2ccccc2CC1	823092195	25	333.16	2.04

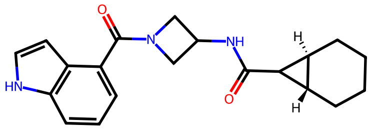	O=C(NC1CN(C(=O)c2cccc3[nH]ccc23)C1)C1[C@H]2CCCC[C@H]12	990659246	25	337.18	2.54
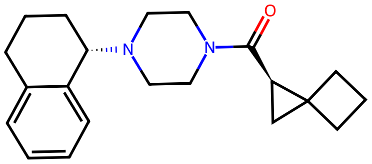	O=C([C@@H]1CC12CCC2)N1CCN([C@H]2CCCc3ccccc32)CC1	950851296	24	324.22	3.4
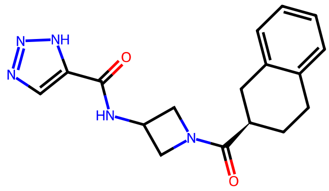	O=C(NC1CN(C(=O)[C@@H]2CCc3ccccc3C2)C1)c1cnn[nH]1	995185854	24	325.15	0.55
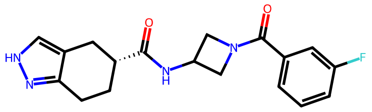	O=C(NC1CN(C(=O)c2cccc(F)c2)C1)[C@@H]1CCc2n[nH]cc2C1	996396452	25	342.15	1.29
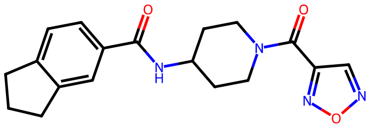	O=C(NC1CCN(C(=O)c2cnon2)CC1)c1ccc2c(c1)CCC2	947961049	25	340.15	1.59
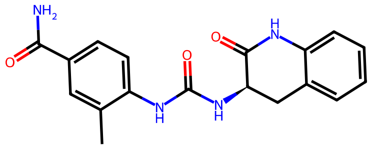	Cc1cc(C(N)=O)ccc1NC(=O)N[C@@H]1Cc2ccccc2NC1=O	1425222388	25	338.14	1.78
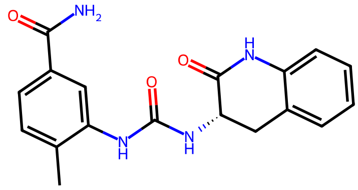	Cc1ccc(C(N)=O)cc1NC(=O)N[C@H]1Cc2ccccc2NC1=O	1364252568	25	338.14	1.78
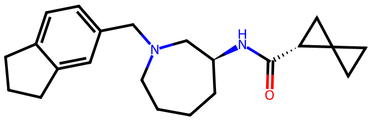	O=C(N[C@H]1CCCCN(Cc2ccc3c(c2)CCC3)C1)[C@@H]1CC12CC2	1034416879	25	338.24	3.45

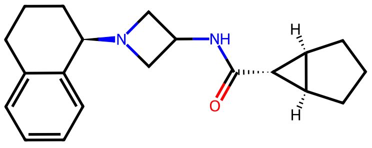	O=C(NC1CN([C@@H]2CCCc3ccccc32)C1)[C@@H]1[C@H]2CCC[C@H]21	1030193406	23	310.2	2.91
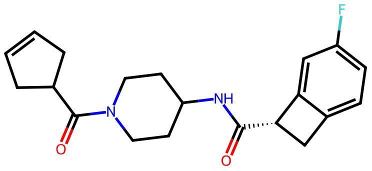	O=C(NC1CCN(C(=O)C2CC=CC2)CC1)[C@H]1Cc2ccc(F)cc21	945689794	25	342.17	2.54
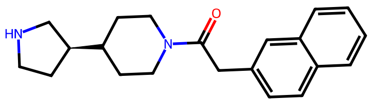	O=C(Cc1ccc2ccccc2c1)N1CCC([C@H]2CCNC2)CC1	289493264	24	322.2	3.23
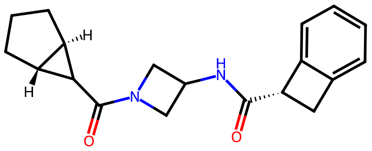	O=C(NC1CN(C(=O)C2[C@@H]3CCC[C@@H]23)C1)[C@H]1Cc2ccccc21	998685007	23	310.17	1.7
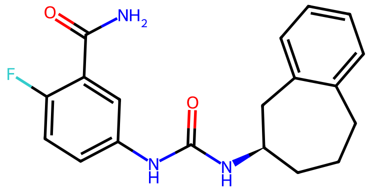	NC(=O)c1cc(NC(=O)N[C@@H]2CCCc3ccccc3C2)ccc1F	929633906	25	341.15	2.99
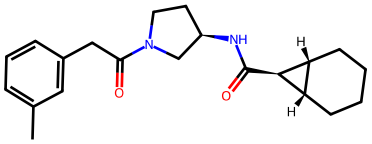	Cc1cccc(CC(=O)N2CC[C@@H](NC(=O)[C@@H]3[C@H]4CCCC[C@H]43)C2)c1	939894997	25	340.22	2.69
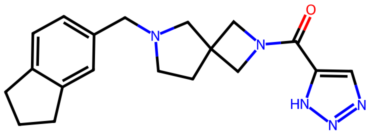	O=C(c1cnn[nH]1)N1CC2(CCN(Cc3ccc4c(c3)CCC4)C2)C1	1072888692	25	337.19	1.64
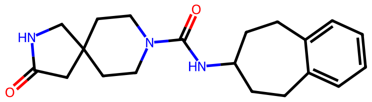	O=C1CC2(CCN(C(=O)NC3CCc4ccccc4CC3)CC2)CN1	564054272	25	341.21	2.25

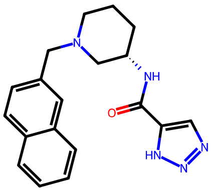	O=C(N[C@H]1CCCN(Cc2ccc3ccccc3c2)C1)c1cnn[nH]1	1007757661	25	335.17	2.35
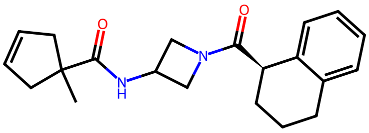	CC1(C(=O)NC2CN(C(=O)[C@@H]3CCCc4ccccc43)C2)CC=CC1	999071985	25	338.2	2.79
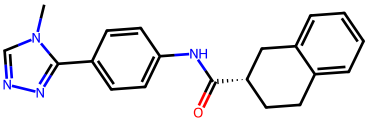	Cn1cnnc1-c1ccc(NC(=O)[C@H]2CCc3ccccc3C2)cc1	565053224	25	332.16	3.23
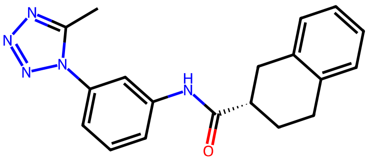	Cc1nnnn1-c1cccc(NC(=O)[C@H]2CCc3ccccc3C2)c1	565541691	25	333.16	2.71
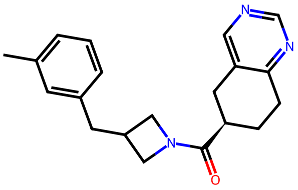	Cc1cccc(CC2CN(C(=O)[C@@H]3CCc4ncncc4C3)C2)c1	648818820	24	321.18	2.59
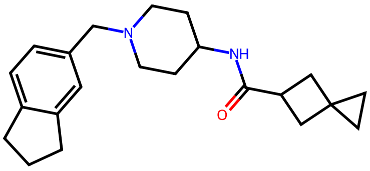	O=C(NC1CCN(Cc2ccc3c(c2)CCC3)CC1)C1CC2(CC2)C1	1003599942	25	338.24	3.45
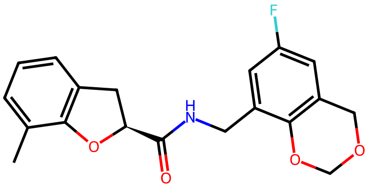	Cc1cccc2c1O[C@H](C(=O)NCc1cc(F)cc3c1OCOC3)C2	449824924	25	343.12	2.62
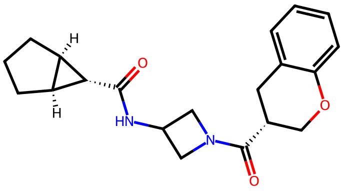	O=C(NC1CN(C(=O)[C@H]2COc3ccccc3C2)C1)[C@@H]1[C@H]2CCC[C@H]21	990708411	25	340.18	1.61

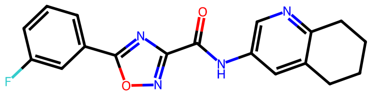	O=C(Nc1cnc2c(c1)CCCC2)c1noc(-c2cccc(F)c2)n1	679914429	25	338.12	3.4
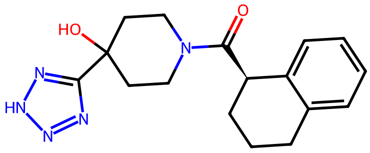	O=C([C@@H]1CCCc2ccccc21)N1CCC(O)(c2nn[nH]n2)CC1	807992308	24	327.17	1.13
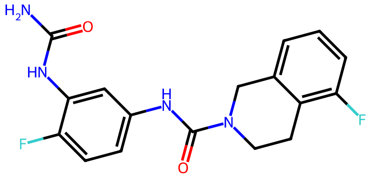	NC(=O)Nc1cc(NC(=O)N2CCc3c(F)cccc3C2)ccc1F	1476634543	25	346.12	3.05
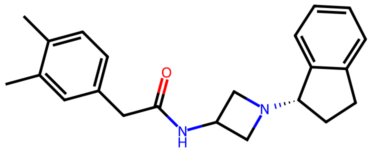	Cc1ccc(CC(=O)NC2CN([C@H]3CCc4ccccc43)C2)cc1C	1031357546	25	334.2	3.33
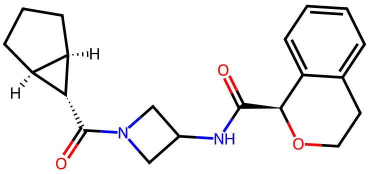	O=C(NC1CN(C(=O)[C@@H]2[C@H]3CCC[C@H]32)C1)[C@@H]1OCCc2ccccc21	997327093	25	340.18	1.67
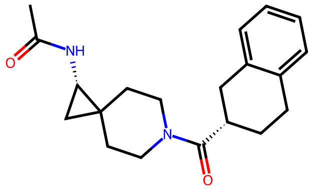	CC(=O)N[C@H]1CC12CCN(C(=O)[C@H]1CCc3ccccc3C1)CC2	962445463	24	326.2	2.31
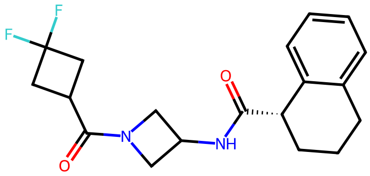	O=C(NC1CN(C(=O)C2CC(F)(F)C2)C1)[C@H]1CCCc2ccccc21	995068161	25	348.16	2.48
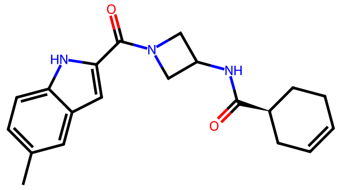	Cc1ccc2[nH]c(C(=O)N3CC(NC(=O)[C@@H]4CC=CCC4)C3)cc2c1	990216945	25	337.18	2.77

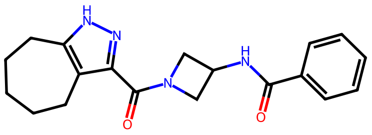	O=C(NC1CN(C(=O)c2n[nH]c3c2CCCCC3)C1)c1ccccc1	990097788	25	338.17	1.93
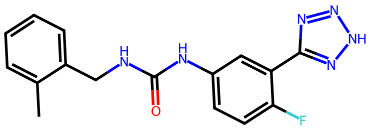	Cc1ccccc1CNC(=O)Nc1ccc(F)c(-c2nn[nH]n2)c1	737427883	24	326.13	2.64
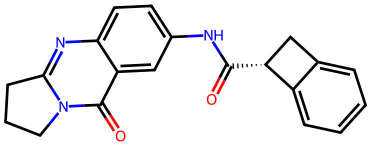	O=C(Nc1ccc2nc3n(c(=O)c2c1)CCC3)[C@@H]1Cc2ccccc21	607790917	25	331.13	2.62
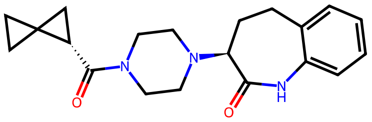	O=C1Nc2ccccc2CC[C@@H]1N1CCN(C(=O)[C@H]2CC23CC3)CC1	950707454	25	339.19	1.88
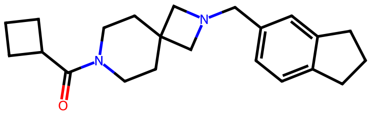	O=C(C1CCC1)N1CCC2(CC1)CN(Cc1ccc3c(c1)CCC3)C2	1035631530	25	338.24	3.4
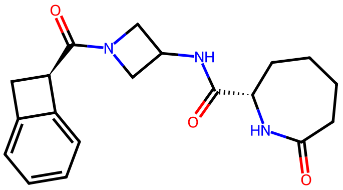	O=C1CCCC[C@@H](C(=O)NC2CN(C(=O)[C@@H]3Cc4ccccc43)C2)N1	999369220	25	341.17	0.71
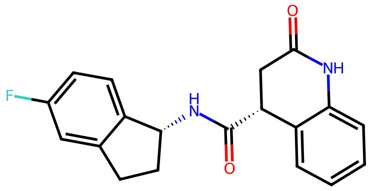	O=C1C[C@@H](C(=O)N[C@@H]2CCc3cc(F)ccc32)c2ccccc2N1	56315390	24	324.13	3.06
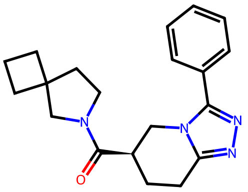	O=C([C@@H]1CCc2nnc(-c3ccccc3)n2C1)N1CCC2(CCC2)C1	564287936	25	336.2	2.91

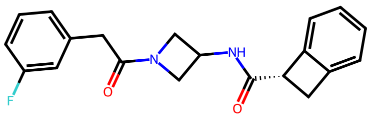	O=C(NC1CN(C(=O)Cc2cccc(F)c2)C1)[C@H]1Cc2ccccc21	998692382	25	338.14	2.04
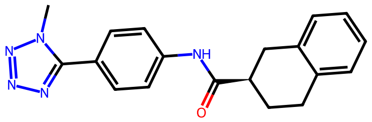	Cn1nnnc1-c1ccc(NC(=O)[C@@H]2CCc3ccccc3C2)cc1	1131225313	25	333.16	2.62
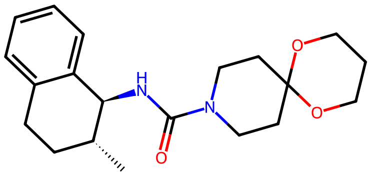	C[C@@H]1CCc2ccccc2[C@H]1NC(=O)N1CCC2(CC1)OCCCO2	838533234	25	344.21	3.25
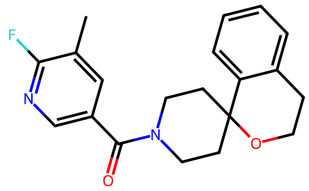	Cc1cc(C(=O)N2CCC3(CC2)OCCc2ccccc23)cnc1F	857628083	25	340.16	3.23
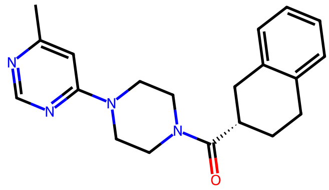	Cc1cc(N2CCN(C(=O)[C@H]3CCc4ccccc4C3)CC2)ncn1	1056033867	25	336.2	2.24
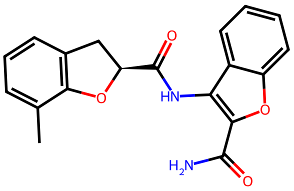	Cc1cccc2c1O[C@H](C(=O)Nc1c(C(N)=O)oc3ccccc13)C2	1352452944	25	336.11	2.78
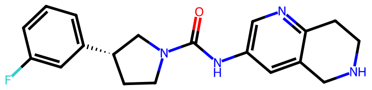	O=C(Nc1cnc2c(c1)CNCC2)N1CC[C@H](c2cccc(F)c2)C1	667795523	25	340.17	2.89
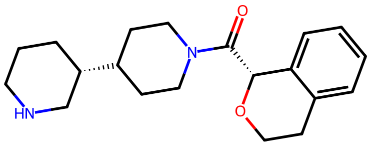	O=C([C@H]1OCCc2ccccc21)N1CCC([C@H]2CCCNC2)CC1	658957118	24	328.22	2.54

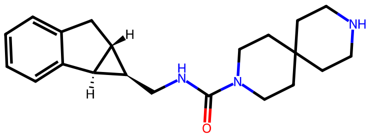	O=C(NC[C@@H]1[C@H]2Cc3ccccc3[C@@H]21)N1CCC2(CCNCC2)CC1	417915134	25	339.23	2.75
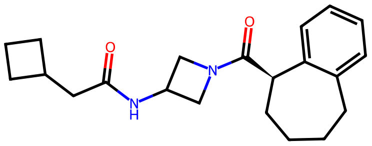	O=C(CC1CCC1)NC1CN(C(=O)[C@@H]2CCCCc3ccccc32)C1	990646078	25	340.22	3.01
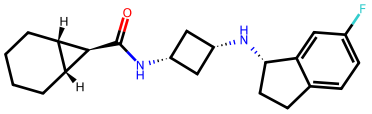	O=C(N[C@H]1C[C@@H](N[C@H]2CCc3ccc(F)cc32)C1)[C@@H]1[C@H]2CCCC[C@H]21	1021931912	25	342.21	3.49
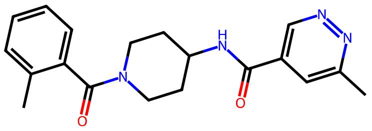	Cc1cc(C(=O)NC2CCN(C(=O)c3ccccc3C)CC2)cnn1	943773245	25	338.17	2.13
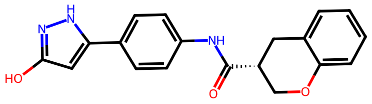	O=C(Nc1ccc(-c2cc(O)n[nH]2)cc1)[C@H]1COc2ccccc2C1	339118674	25	335.13	2.97
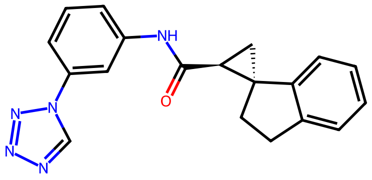	O=C(Nc1cccc(-n2cnnn2)c1)[C@H]1C[C@]12CCc1ccccc12	174630298	25	331.14	2.5
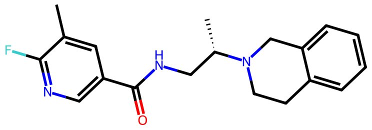	Cc1cc(C(=O)NC[C@H](C)N2CCc3ccccc3C2)cnc1F	857576661	24	327.17	2.71
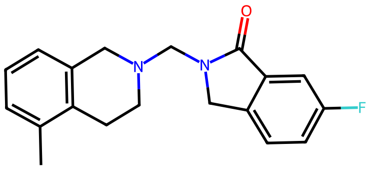	Cc1cccc2c1CCN(CN1Cc3ccc(F)cc3C1=O)C2	771617865	23	310.15	3.11

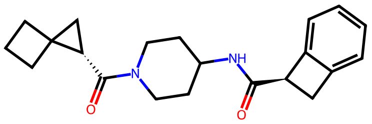	O=C(NC1CCN(C(=O)[C@H]2CC23CCC3)CC1)[C@@H]1Cc2ccccc21	946759058	25	338.2	2.62
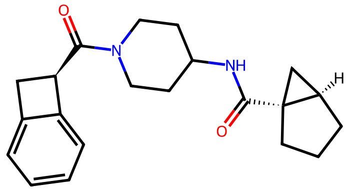	O=C([C@@H]1Cc2ccccc21)N1CCC(NC(=O)[C@@]23CCC[C@@H]2C3)CC1	948059931	25	338.2	2.62
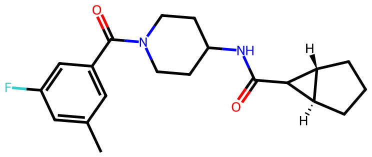	Cc1cc(F)cc(C(=O)N2CCC(NC(=O)C3[C@@H]4CCC[C@@H]34)CC2)c1	948549845	25	344.19	2.9
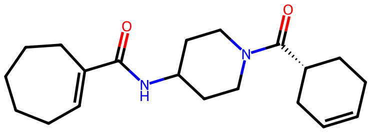	O=C(NC1CCN(C(=O)[C@H]2CC=CCC2)CC1)C1=CCCCCC1	944113723	24	330.23	3.34
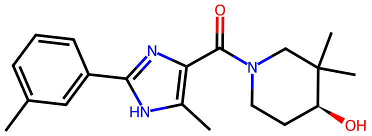	Cc1cccc(-c2nc(C(=O)N3CC[C@H](O)C(C)(C)C3)c(C)[nH]2)c1	275766348	24	327.19	2.93
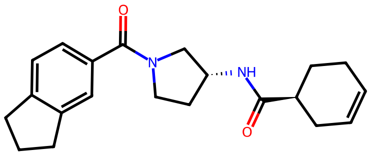	O=C(N[C@@H]1CCN(C(=O)c2ccc3c(c2)CCC3)C1)[C@@H]1CC=CCC1	939379577	25	338.2	2.86
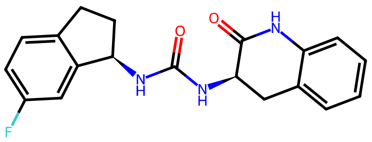	O=C(N[C@@H]1Cc2ccccc2NC1=O)N[C@@H]1CCc2ccc(F)cc21	636743796	25	339.14	2.68
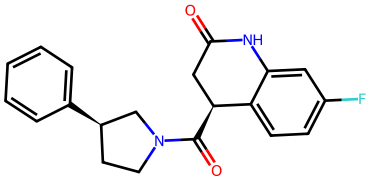	O=C1C[C@H](C(=O)N2CC[C@@H](c3ccccc3)C2)c2ccc(F)cc2N1	121578978	25	338.14	3.27

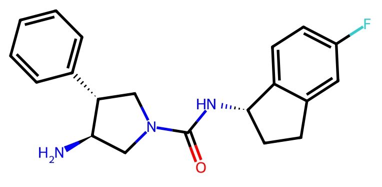	N[C@@H]1CN(C(=O)N[C@H]2CCc3cc(F)ccc32)C[C@H]1c1ccccc1	333949046	25	339.17	2.95
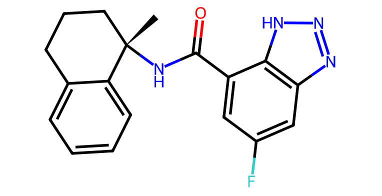	C[C@]1(NC(=O)c2cc(F)cc3nn[nH]c23)CCCc2ccccc21	337449016	24	324.14	3.08
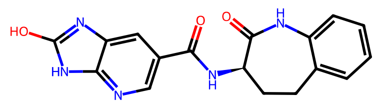	O=C(N[C@@H]1CCc2ccccc2NC1=O)c1cnc2[nH]c(O)nc2c1	1437231495	25	337.12	1.35
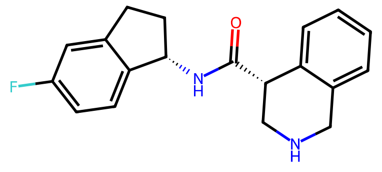	O=C(N[C@H]1CCc2cc(F)ccc21)[C@H]1CNCc2ccccc21	580069635	23	310.15	2.82
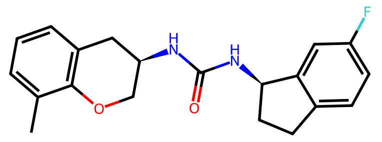	Cc1cccc2c1OC[C@H](NC(=O)N[C@@H]1CCc3ccc(F)cc31)C2	573147372	25	340.16	3.42
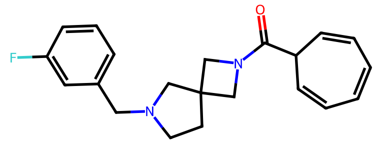	O=C(C1C=CC=CC=C1)N1CC2(CCN(Cc3cccc(F)c3)C2)C1	1072785159	25	338.18	3.16
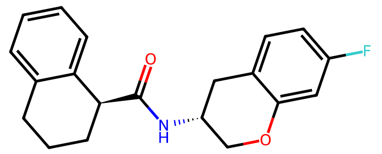	O=C(N[C@H]1COc2cc(F)ccc2C1)[C@H]1CCCc2ccccc21	1618175292	24	325.15	3.37
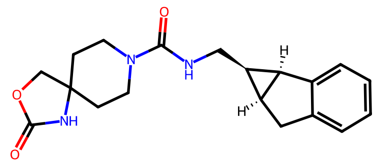	O=C1NC2(CCN(C(=O)NC[C@H]3[C@H]4c5ccccc5C[C@H]43)CC2)CO1	495141965	25	341.17	1.86

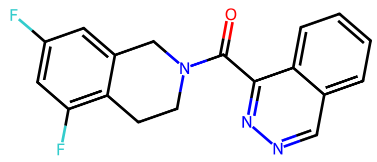	O=C(c1nncc2ccccc12)N1CCc2c(F)cc(F)cc2C1	1519576338	24	325.1	3.11
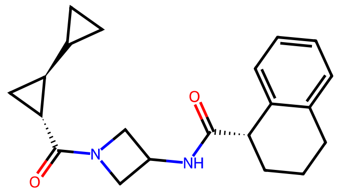	O=C(NC1CN(C(=O)[C@@H]2C[C@H]2C2CC2)C1)[C@H]1CCCc2ccccc21	995075704	25	338.2	2.48
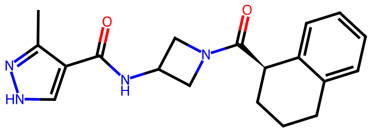	Cc1n[nH]cc1C(=O)NC1CN(C(=O)[C@@H]2CCCc3ccccc32)C1	997295284	25	338.17	1.78
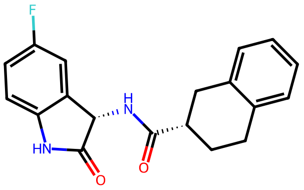	O=C(N[C@@H]1C(=O)Nc2ccc(F)cc21)[C@H]1CCc2ccccc2C1	102324740	24	324.13	2.74
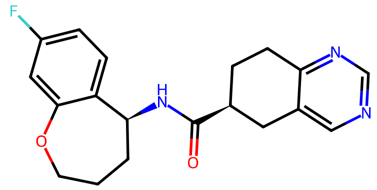	O=C(N[C@H]1CCCOc2cc(F)ccc21)[C@H]1CCc2ncncc2C1	648347053	25	341.15	2.75
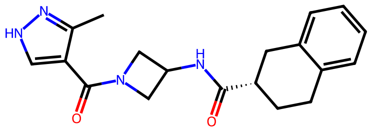	Cc1n[nH]cc1C(=O)N1CC(NC(=O)[C@H]2CCc3ccccc3C2)C1	1000544999	25	338.17	1.46
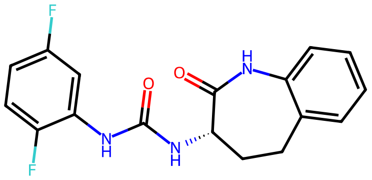	O=C(Nc1cc(F)ccc1F)N[C@H]1CCc2ccccc2NC1=O	1202661714	24	331.11	3.04
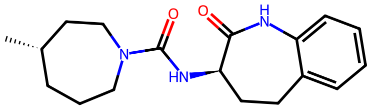	C[C@H]1CCCN(C(=O)N[C@@H]2CCc3ccccc3NC2=O)CC1	654629903	23	315.19	2.77

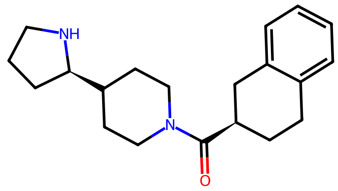	O=C([C@@H]1CCc2ccccc2C1)N1CCC([C@H]2CCCN2)CC1	324106686	23	312.22	2.78
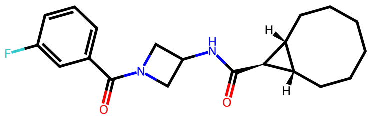	O=C(NC1CN(C(=O)c2cccc(F)c2)C1)[C@@H]1[C@H]2CCCCCC[C@H]21	1000465997	25	344.19	2.98
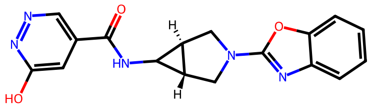	O=C(NC1[C@H]2CN(c3nc4ccccc4o3)C[C@H]12)c1cnnc(O)c1	1056333222	25	337.12	1.19
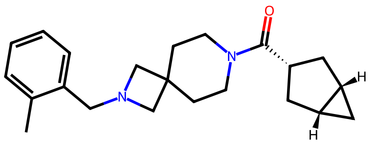	Cc1ccccc1CN1CC2(CCN(C(=O)[C@@H]3C[C@@H]4C[C@@H]4C3)CC2)C1	1035771318	25	338.24	3.47
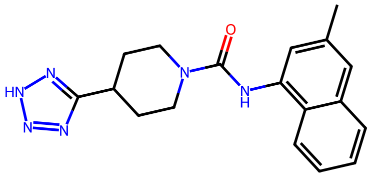	Cc1cc(NC(=O)N2CCC(c3nn[nH]n3)CC2)c2ccccc2c1	1677702865	25	336.17	3.07
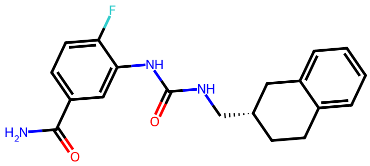	NC(=O)c1ccc(F)c(NC(=O)NC[C@H]2CCc3ccccc3C2)c1	605030402	25	341.15	2.85
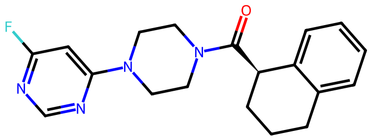	O=C([C@@H]1CCCc2ccccc21)N1CCN(c2cc(F)ncn2)CC1	908912259	25	340.17	2.38
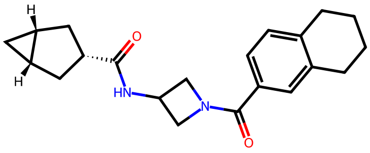	O=C(NC1CN(C(=O)c2ccc3c(c2)CCCC3)C1)[C@@H]1C[C@@H]2C[C@@H]2C1	998004762	25	338.2	2.55

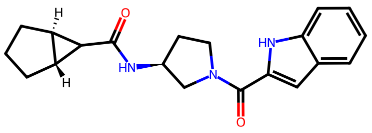	O=C(N[C@H]1CCN(C(=O)c2cc3ccccc3[nH]2)C1)C1[C@@H]2CCC[C@@H]12	937804051	25	337.18	2.54
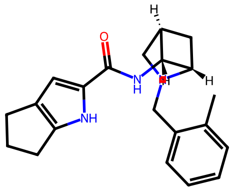	Cc1ccccc1CN1C[C@H]2C[C@H]1[C@@H]2NC(=O)c1cc2c([nH]1)CCC2	1037675227	25	335.2	2.81
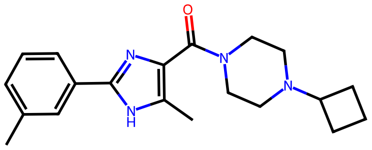	Cc1cccc(-c2nc(C(=O)N3CCN(C4CCC4)CC3)c(C)[nH]2)c1	270275803	25	338.21	3.0
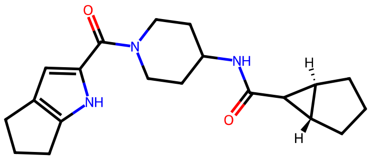	O=C(NC1CCN(C(=O)c2cc3c([nH]2)CCC3)CC1)C1[C@H]2CCC[C@H]12	947445683	25	341.21	2.27
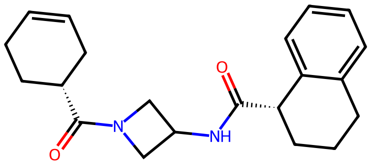	O=C(NC1CN(C(=O)[C@H]2CC=CCC2)C1)[C@H]1CCCc2ccccc21	995071179	25	338.2	2.79
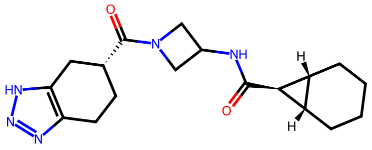	O=C(NC1CN(C(=O)[C@@H]2CCc3nn[nH]c3C2)C1)[C@@H]1[C@H]2CCCC[C@H]21	990645369	25	343.2	0.67
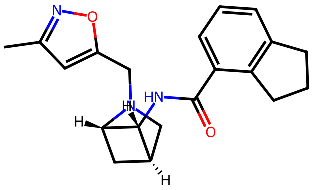	Cc1cc(CN2C[C@H]3C[C@H]2[C@@H]3NC(=O)c2cccc3c2CCC3)on1	1037747787	25	337.18	2.47
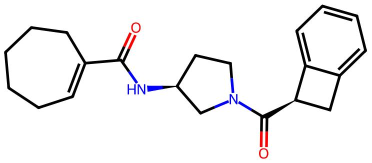	O=C(N[C@H]1CCN(C(=O)[C@@H]2Cc3ccccc32)C1)C1=CCCCCC1	937872719	25	338.2	2.93

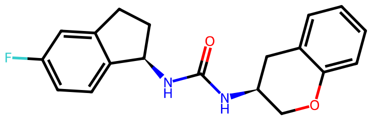	O=C(N[C@@H]1COc2ccccc2C1)N[C@@H]1CCc2cc(F)ccc21	193788731	24	326.14	3.12
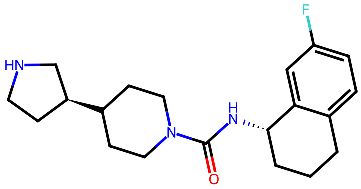	O=C(N[C@H]1CCCc2ccc(F)cc21)N1CCC([C@H]2CCNC2)CC1	418118510	25	345.22	3.23
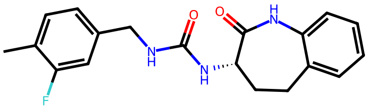	Cc1ccc(CNC(=O)N[C@H]2CCc3ccccc3NC2=O)cc1F	847278771	25	341.15	2.89
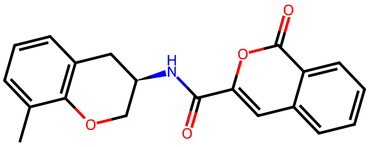	Cc1cccc2c1OC[C@H](NC(=O)c1cc3ccccc3c(=O)o1)C2	365990014	25	335.12	2.83
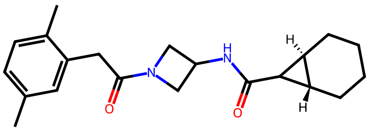	Cc1ccc(C)c(CC(=O)N2CC(NC(=O)C3[C@H]4CCCC[C@H]34)C2)c1	990647988	25	340.22	2.61
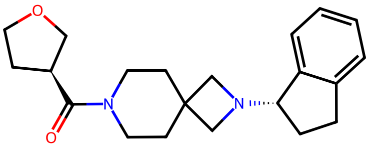	O=C([C@H]1CCOC1)N1CCC2(CC1)CN([C@H]1CCc3ccccc31)C2	1035663236	25	340.22	2.63
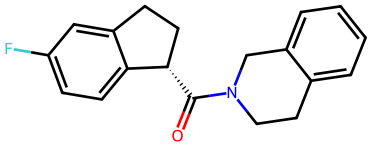	O=C([C@H]1CCc2cc(F)ccc21)N1CCc2ccccc2C1	665059915	22	295.14	3.44
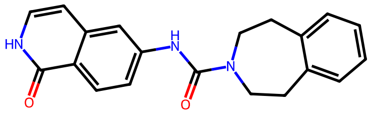	O=C(Nc1ccc2c(=O)[nH]ccc2c1)N1CCc2ccccc2CC1	1219415336	25	333.15	3.16

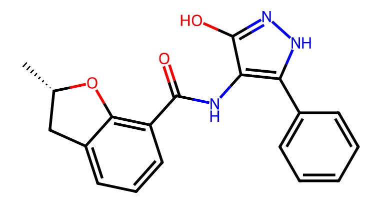	C[C@H]1Cc2cccc(C(=O)Nc3c(O)n[nH]c3-c3ccccc3)c2O1	1298438534	25	335.13	3.36
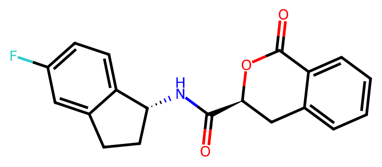	O=C1O[C@H](C(=O)N[C@@H]2CCc3cc(F)ccc32)Cc2ccccc21	55657740	24	325.11	2.71
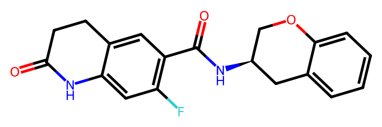	O=C1CCc2cc(C(=O)N[C@H]3COc4ccccc4C3)c(F)cc2N1	178490609	25	340.12	2.44
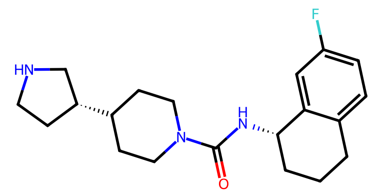	O=C(N[C@H]1CCCc2ccc(F)cc21)N1CCC([C@@H]2CCNC2)CC1	418118499	25	345.22	3.23
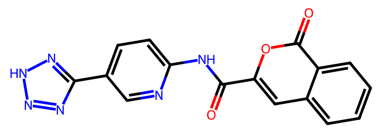	O=C(Nc1ccc(-c2nn[nH]n2)cn1)c1cc2ccccc2c(=O)o1	1593223190	25	334.08	1.62
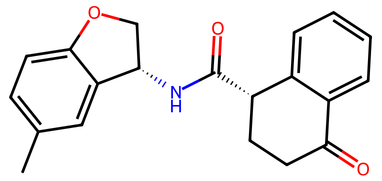	Cc1ccc2c(c1)[C@@H](NC(=O)[C@H]1CCC(=O)c3ccccc31)CO2	375070912	24	321.14	3.31
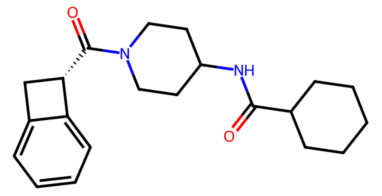	O=C(NC1CCN(C(=O)[C@H]2Cc3ccccc32)CC1)C1CCCCC1	353779967	25	340.22	3.01
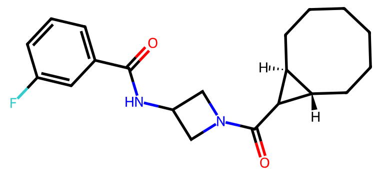	O=C(NC1CN(C(=O)C2[C@H]3CCCCCC[C@H]23)C1)c1cccc(F)c1	990118129	25	344.19	2.98

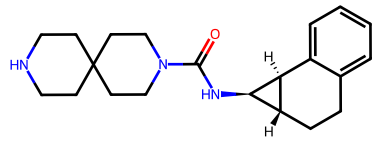	O=C(N[C@@H]1[C@H]2CCc3ccccc3[C@@H]21)N1CCC2(CCNCC2)CC1	417916847	25	339.23	2.89
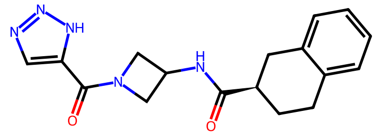	O=C(NC1CN(C(=O)c2cnn[nH]2)C1)[C@@H]1CCc2ccccc2C1	1000545762	24	325.15	0.55
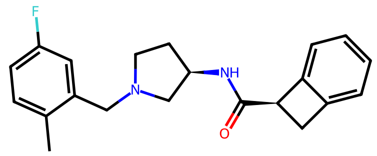	Cc1ccc(F)cc1CN1CC[C@@H](NC(=O)[C@@H]2Cc3ccccc32)C1	1015636087	25	338.18	3.16
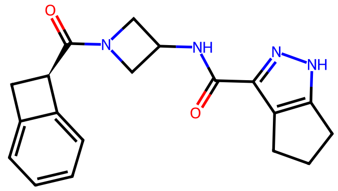	O=C(NC1CN(C(=O)[C@@H]2Cc3ccccc32)C1)c1n[nH]c2c1CCC2	995165356	25	336.16	1.18
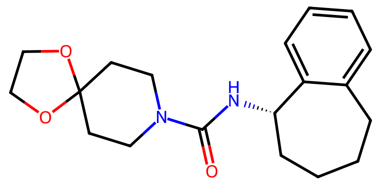	O=C(N[C@H]1CCCCc2ccccc21)N1CCC2(CC1)OCCO2	65594061	24	330.19	3.0
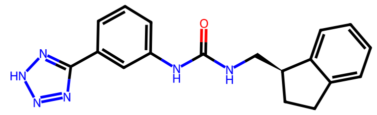	O=C(NC[C@@H]1CCc2ccccc21)Nc1cccc(-c2nn[nH]n2)c1	738051363	25	334.15	2.72
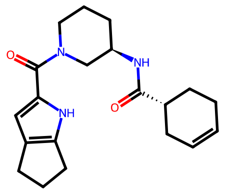	O=C(N[C@@H]1CCCN(C(=O)c2cc3c([nH]2)CCC3)C1)[C@H]1CC=CCC1	951367691	25	341.21	2.58
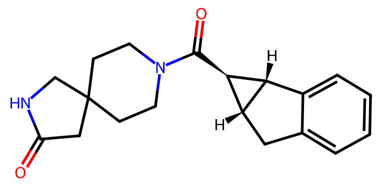	O=C1CC2(CCN(C(=O)[C@@H]3[C@H]4Cc5ccccc5[C@H]43)CC2)CN1	859774220	23	310.17	1.7

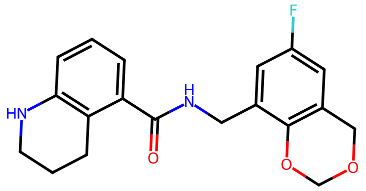	O=C(NCc1cc(F)cc2c1OCOC2)c1cccc2c1CCCN2	190043112	25	342.14	2.98
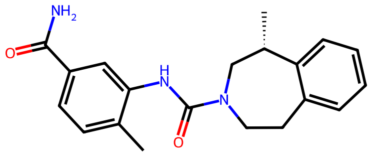	Cc1ccc(C(N)=O)cc1NC(=O)N1CCc2ccccc2[C@@H](C)C1	650014979	25	337.18	3.29
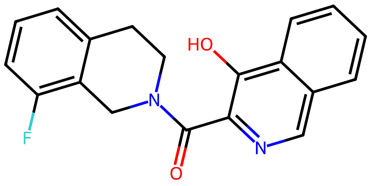	O=C(c1ncc2ccccc2c1O)N1CCc2cccc(F)c2C1	1295921119	24	322.11	3.28
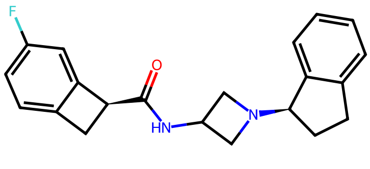	O=C(NC1CN([C@@H]2CCc3ccccc32)C1)[C@H]1Cc2ccc(F)cc21	1030847159	25	336.16	2.95
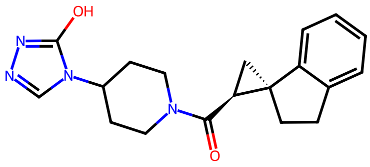	O=C([C@H]1C[C@]12CCc1ccccc12)N1CCC(n2cnnc2O)CC1	517586133	25	338.17	2.05
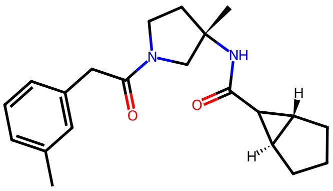	Cc1cccc(CC(=O)N2CC[C@](C)(NC(=O)C3[C@@H]4CCC[C@@H]34)C2)c1	956803742	25	340.22	2.69
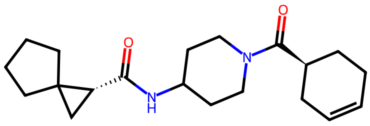	O=C(NC1CCN(C(=O)[C@@H]2CC=CCC2)CC1)[C@@H]1CC12CCCC2	944105939	24	330.23	3.03
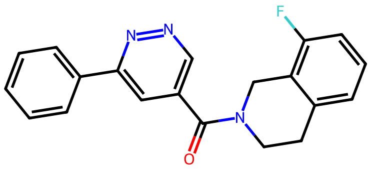	O=C(c1cnnc(-c2ccccc2)c1)N1CCc2ccc(F)c2C1	355125375	25	333.13	3.48

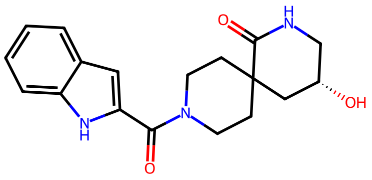	O=C(c1cc2ccccc2[nH]1)N1CCC2(CC1)C[C@@H](O)CNC2=O	885024747	24	327.16	1.27
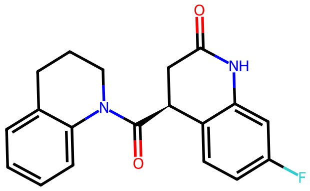	O=C1C[C@H](C(=O)N2CCCc3ccccc32)c2ccc(F)cc2N1	25894705	24	324.13	3.23
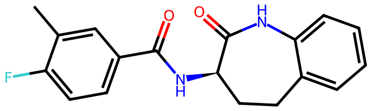	Cc1cc(C(=O)N[C@@H]2CCc3ccccc3NC2=O)ccc1F	1417561931	23	312.13	2.82
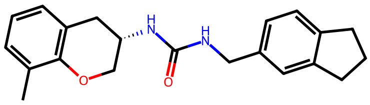	Cc1cccc2c1OC[C@@H](NC(=O)NCc1ccc3c(c1)CCC3)C2	1467860261	25	336.18	3.29
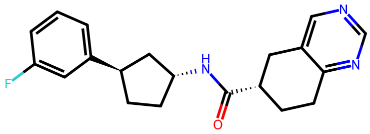	O=C(N[C@@H]1CC[C@@H](c2cccc(F)c2)C1)[C@H]1CCc2ncncc2C1	648378422	25	339.17	3.17
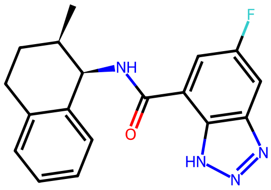	C[C@@H]1CCc2ccccc2[C@@H]1NC(=O)c1cc(F)cc2nn[nH]c12	337463286	24	324.14	3.15
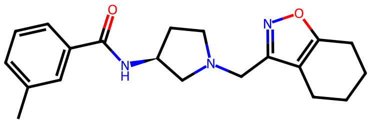	Cc1cccc(C(=O)N[C@H]2CCN(Cc3noc4c3CCCC4)C2)c1	1010019291	25	339.19	2.87
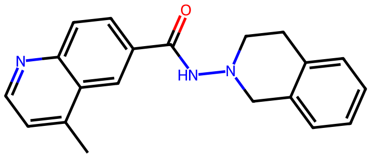	Cc1ccnc2ccc(C(=O)NN3CCc4ccccc4C3)cc12	1452971643	24	317.15	3.25

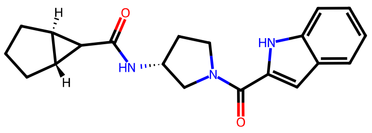	O=C(N[C@@H]1CCN(C(=O)c2cc3ccccc3[nH]2)C1)C1[C@@H]2CCC[C@@H]12	936158055	25	337.18	2.54
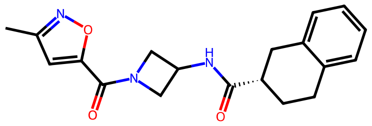	Cc1cc(C(=O)N2CC(NC(=O)[C@H]3CCc4ccccc4C3)C2)on1	1000540032	25	339.16	1.73
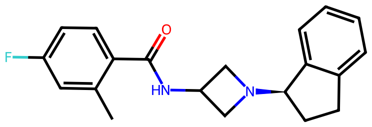	Cc1cc(F)ccc1C(=O)NC1CN([C@@H]2CCc3ccccc32)C1	1031287793	24	324.16	3.24
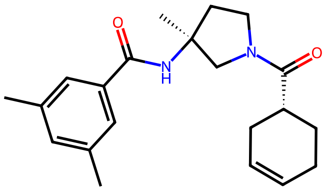	Cc1cc(C)cc(C(=O)N[C@@]2(C)CCN(C(=O)[C@H]3CC=CCC3)C2)c1	957560539	25	340.22	3.38
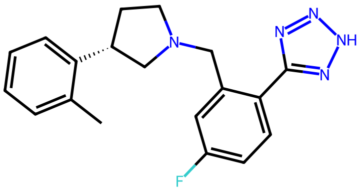	Cc1ccccc1[C@@H]1CCN(Cc2cc(F)ccc2-c2nn[nH]n2)C1	822351182	25	337.17	3.3
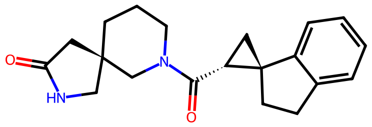	O=C1C[C@]2(CCCN(C(=O)[C@@H]3C[C@@]34CCc3ccccc34)C2)CN1	341499948	24	324.18	2.02
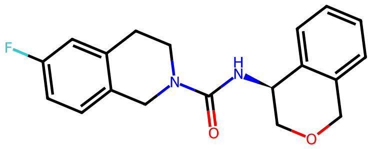	O=C(N[C@@H]1COCc2ccccc21)N1CCc2cc(F)ccc2C1	419565764	24	326.14	3.16
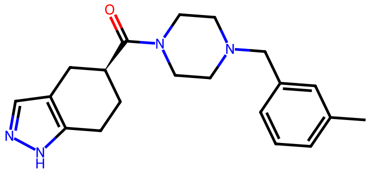	Cc1cccc(CN2CCN(C(=O)[C@H]3CCc4[nH]ncc4C3)CC2)c1	270173612	25	338.21	2.17

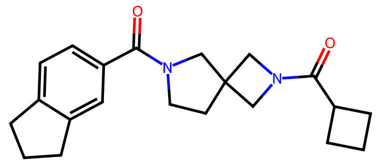	O=C(c1ccc2c(c1)CCC2)N1CCC2(C1)CN(C(=O)C1CCC1)C2	1018669584	25	338.2	2.65
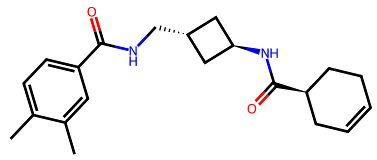	Cc1ccc(C(=O)NC[C@H]2C[C@H](NC(=O)[C@@H]3CC=CCC3)C2)cc1C	980507486	25	340.22	3.28
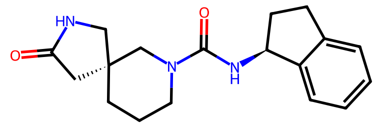	O=C1C[C@]2(CCCN(C(=O)N[C@H]3CCc4ccccc43)C2)CN1	340919592	23	313.18	1.99
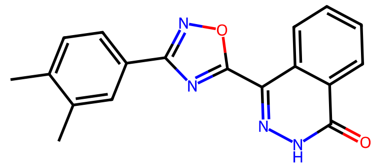	Cc1ccc(-c2noc(-c3n[nH]c(=O)c4ccccc34)n2)cc1C	67172493	24	318.11	3.26
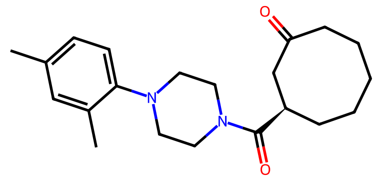	Cc1ccc(N2CCN(C(=O)[C@@H]3CCCCCC(=O)C3)CC2)c(C)c1	1557625164	25	342.23	3.49
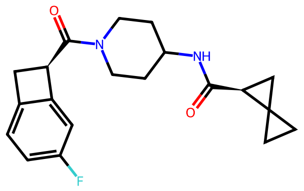	O=C(NC1CCN(C(=O)[C@@H]2Cc3ccc(F)cc32)CC1)[C@H]1CC12CC2	948026947	25	342.17	2.37
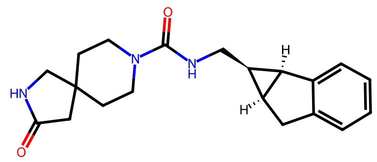	O=C1CC2(CCN(C(=O)NC[C@H]3[C@H]4c5ccccc5C[C@H]43)CC2)CN1	269043550	25	339.19	1.88
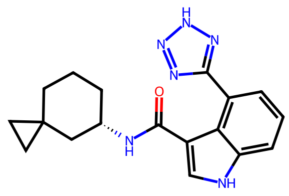	O=C(N[C@H]1CCCC2(CC2)C1)c1c[nH]c2cccc(-c3nn[nH]n3)c12	1606667815	25	336.17	2.8

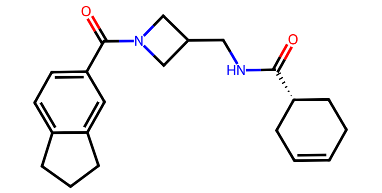	O=C(NCC1CN(C(=O)c2ccc3c(c2)CCC3)C1)[C@H]1CC=CCC1	1000899203	25	338.2	2.72
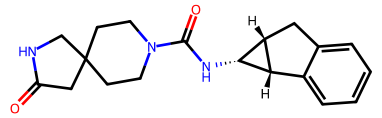	O=C1CC2(CCN(C(=O)N[C@@H]3[C@@H]4Cc5ccccc5[C@@H]43)CC2)CN1	268681929	24	325.18	1.64
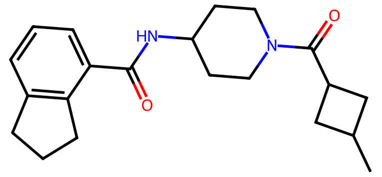	CC1CC(C(=O)N2CCC(NC(=O)c3cccc4c3CCC4)CC2)C1	946353070	25	340.22	2.94
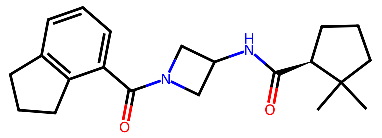	CC1(C)CCC[C@@H]1C(=O)NC1CN(C(=O)c2cccc3c2CCC3)C1	997894714	25	340.22	2.94
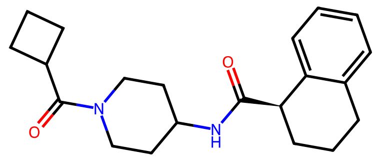	O=C(NC1CCN(C(=O)C2CCC2)CC1)[C@@H]1CCCc2ccccc21	943328057	25	340.22	3.01
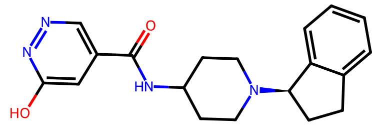	O=C(NC1CCN([C@@H]2CCc3ccccc32)CC1)c1cnnc(O)c1	1003085220	25	338.17	2.06
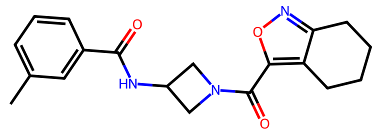	Cc1cccc(C(=O)NC2CN(C(=O)c3onc4c3CCCC4)C2)c1	990139153	25	339.16	2.12
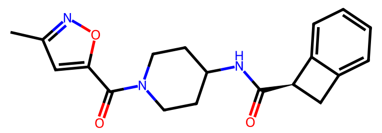	Cc1cc(C(=O)N2CCC(NC(=O)[C@@H]3Cc4ccccc43)CC2)on1	944152633	25	339.16	2.04

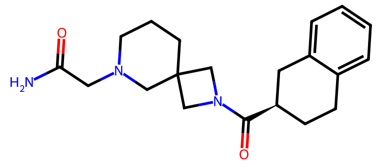	NC(=O)CN1CCCC2(C1)CN(C(=O)[C@@H]1CCc3ccccc3C1)C2	982037134	25	341.21	1.2
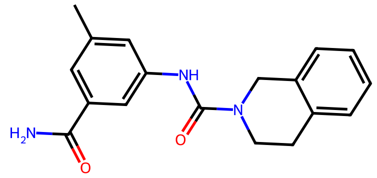	Cc1cc(NC(=O)N2CCc3ccccc3C2)cc(C(N)=O)c1	1476622440	23	309.15	2.68
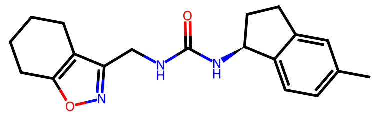	Cc1ccc2c(c1)CC[C@@H]2NC(=O)NCc1noc2c1CCCC2	287000458	24	325.18	3.35
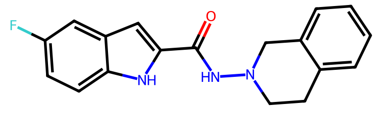	O=C(NN1CCc2ccccc2C1)c1cc2cc(F)ccc2[nH]1	1300426662	23	309.13	3.01
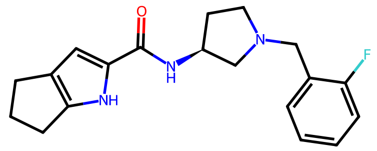	O=C(N[C@H]1CCN(Cc2ccccc2F)C1)c1cc2c([nH]1)CCC2	1015978285	24	327.17	2.65
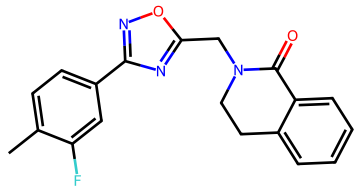	Cc1ccc(-c2noc(CN3CCc4ccccc4C3=O)n2)cc1F	345701286	25	337.12	3.38
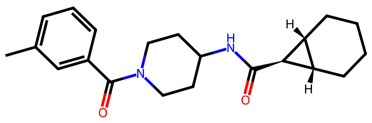	Cc1cccc(C(=O)N2CCC(NC(=O)[C@@H]3[C@H]4CCCC[C@H]43)CC2)c1	943805876	25	340.22	3.15
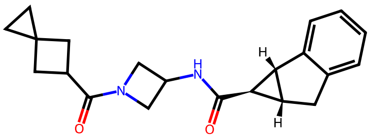	O=C(NC1CN(C(=O)C2CC3(CC3)C2)C1)[C@@H]1[C@H]2Cc3ccccc3[C@H]21	991646008	25	336.18	2.09

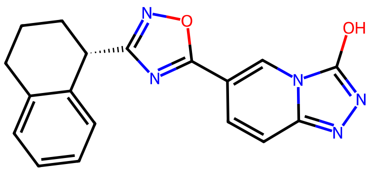	Oc1nnc2ccc(-c3nc([C@H]4CCCc5ccccc54)no3)cn12	355884904	25	333.12	2.95
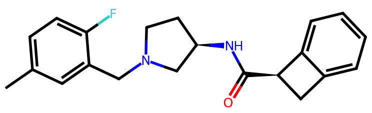	Cc1ccc(F)c(CN2CC[C@@H](NC(=O)[C@@H]3Cc4ccccc43)C2)c1	1015632558	25	338.18	3.16
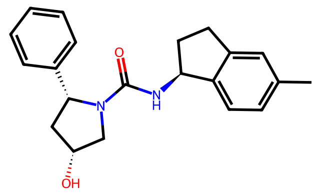	Cc1ccc2c(c1)CC[C@@H]2NC(=O)N1C[C@H](O)C[C@@H]1c1ccccc1	413162453	25	336.18	3.5
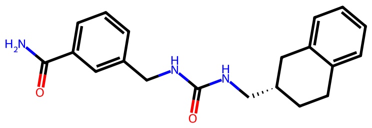	NC(=O)c1cccc(CNC(=O)NC[C@H]2CCc3ccccc3C2)c1	605011864	25	337.18	2.39
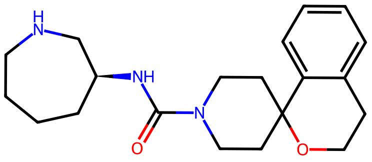	O=C(N[C@H]1CCCCNC1)N1CCC2(CC1)OCCc1ccccc12	561207823	25	343.23	2.4
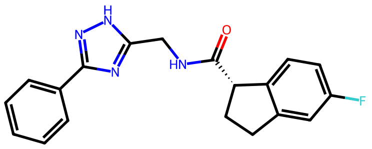	O=C(NCc1nc(-c2ccccc2)n[nH]1)[C@H]1CCc2cc(F)ccc21	1443014445	25	336.14	2.96
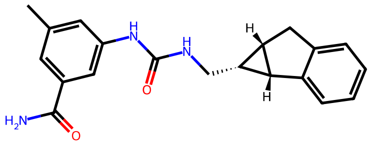	Cc1cc(NC(=O)NC[C@H]2[C@H]3c4ccccc4C[C@H]32)cc(C(N)=O)c1	583608642	25	335.16	2.8
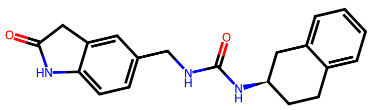	O=C1Cc2cc(CNC(=O)N[C@@H]3CCc4ccccc4C3)ccc2N1	1875253025	25	335.16	2.54

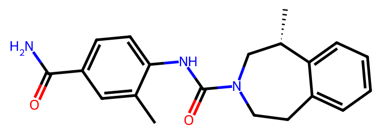	Cc1cc(C(N)=O)ccc1NC(=O)N1CCc2cc ccc2[C@@H](C)C1	844504867	25	337.18	3.29
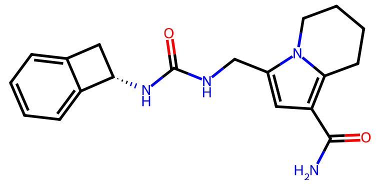	NC(=O)c1cc(CNC(=O)N[C@H]2Cc3cc ccc32)n2c1CCCC2	1551273685	25	338.17	2.02
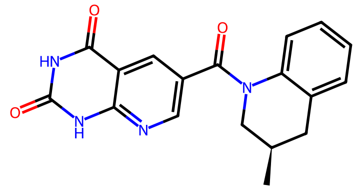	C[C@@H]1Cc2ccccc2N(C(=O)c2cnc3 [nH]c(=O)[nH]c(=O)c3c2)C1	104523322	25	336.12	1.45
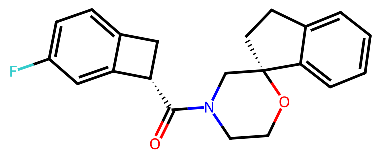	O=C([C@H]1Cc2ccc(F)cc21)N1CCO[C@@]2(CCc3ccccc32)C1	684466781	25	337.15	3.17
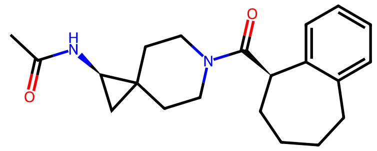	CC(=O)N[C@@H]1CC12CCN(C(=O)[C@@H]1CCCCc3ccccc31)CC2	962352478	25	340.22	3.01
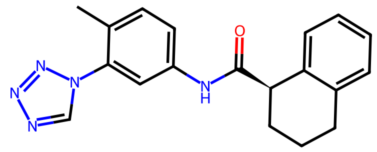	Cc1ccc(NC(=O)[C@@H]2CCCc3cccc c32)cc1-n1cnnn1	46271144	25	333.16	3.03
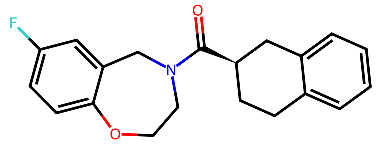	O=C([C@@H]1CCc2ccccc2C1)N1CC Oc2ccc(F)cc2C1	563259815	24	325.15	3.35
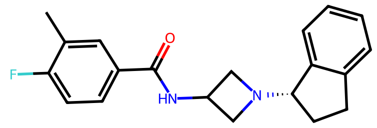	Cc1cc(C(=O)NC2CN([C@H]3CCc4ccc cc43)C2)ccc1F	1030879554	24	324.16	3.24

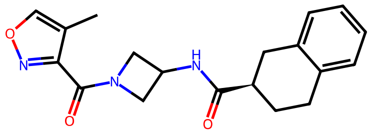	Cc1conc1C(=O)N1CC(NC(=O)[C@@H]2CCc3ccccc3C2)C1	1000538491	25	339.16	1.73
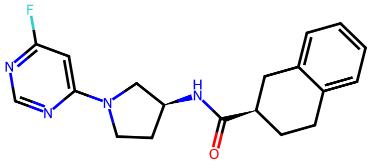	O=C(N[C@H]1CCN(c2cc(F)ncn2)C1)[C@@H]1CCc2ccccc2C1	1058559467	25	340.17	2.12
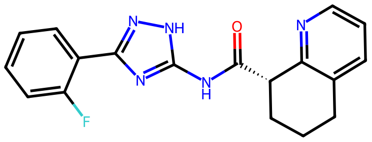	O=C(Nc1nc(-c2ccccc2F)n[nH]1)[C@H]1CCCc2cccnc21	193218007	25	337.13	3.06
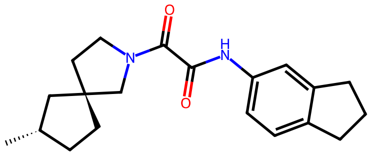	C[C@H]1CC[C@]2(CCN(C(=O)C(=O)Nc3ccc4c(c3)CCC4)C2)C1	359461242	24	326.2	3.15
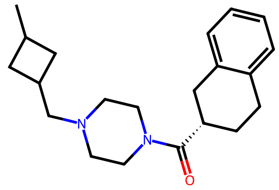	CC1CC(CN2CCN(C(=O)[C@H]3CCc4ccccc4C3)CC2)C1	950721534	24	326.24	2.98
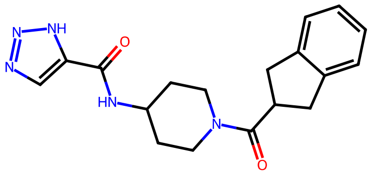	O=C(NC1CCN(C(=O)C2Cc3ccccc3C2)CC1)c1cnn[nH]1	948551689	25	339.17	0.94
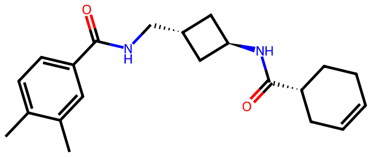	Cc1ccc(C(=O)NC[C@H]2C[C@H](NC(=O)[C@H]3CC=CCC3)C2)cc1C	980507485	25	340.22	3.28
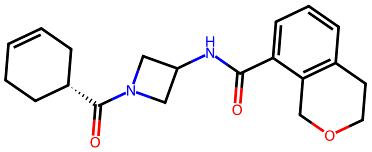	O=C(NC1CN(C(=O)[C@H]2CC=CCC2)C1)c1cccc2c1COCC2	993315347	25	340.18	2.06

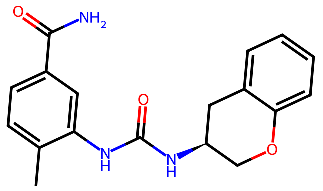	Cc1ccc(C(N)=O)cc1NC(=O)N[C@@H]1COc2ccccc2C1	194578185	24	325.14	2.22
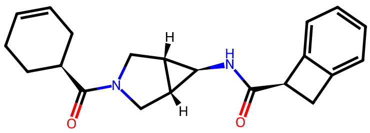	O=C(N[C@H]1[C@@H]2CN(C(=O)[C@@H]3CC=CCC3)C[C@@H]21)[C@@H]1Cc2ccccc21	977081564	25	336.18	2.26
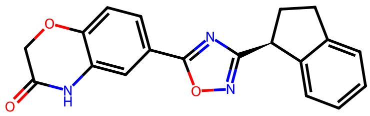	O=C1COc2ccc(-c3nc([C@H]4CCc5ccccc54)no3)cc2N1	356605613	25	333.11	3.15
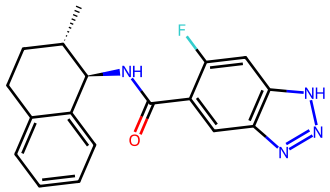	C[C@H]1CCc2ccccc2[C@@H]1NC(=O)c1cc2nn[nH]c2cc1F	781961680	24	324.14	3.15
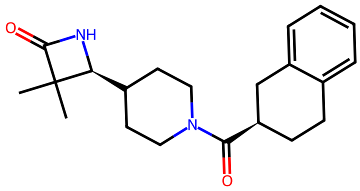	CC1(C)C(=O)N[C@H]1C1CCN(C(=O)[C@@H]2CCc3ccccc3C2)CC1	577184880	25	340.22	2.55
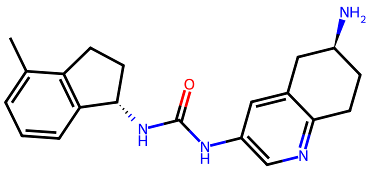	Cc1cccc2c1CC[C@@H]2NC(=O)Nc1cnc2c(c1)C[C@@H](N)CC2	579450949	25	336.2	3.02
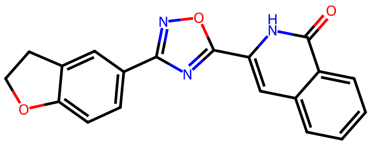	O=c1[nH]c(-c2nc(-c3ccc4c(c3)CCO4)no2)cc2ccccc12	154886715	25	331.1	3.18
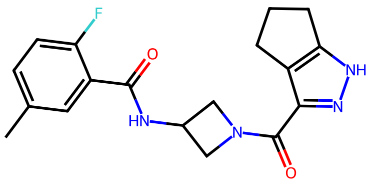	Cc1ccc(F)c(C(=O)NC2CN(C(=O)c3n[nH]c4c3CCC4)C2)c1	1000529544	25	342.15	1.6

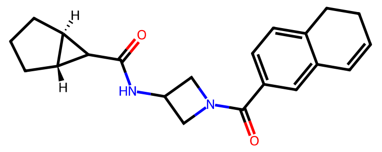	O=C(NC1CN(C(=O)c2ccc3c(c2)C=CCC3)C1)C1[C@@H]2CCC[C@@H]12	990702815	25	336.18	2.63
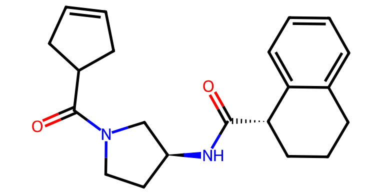	O=C(N[C@H]1CCN(C(=O)C2CC=CC2)C1)[C@H]1CCCc2ccccc21	938221968	25	338.2	2.79
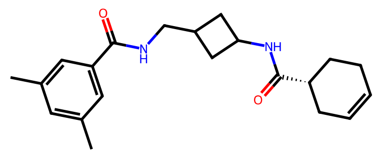	Cc1cc(C)cc(C(=O)NCC2CC(NC(=O)[C@H]3CC=CCC3)C2)c1	992389204	25	340.22	3.28
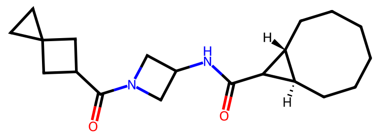	O=C(NC1CN(C(=O)C2CC3(CC3)C2)C1)C1[C@@H]2CCCCCC[C@@H]12	1000510027	24	330.23	2.72
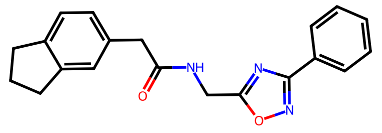	O=C(Cc1ccc2c(c1)CCC2)NCc1nc(-c2ccccc2)no1	44779530	25	333.15	3.08
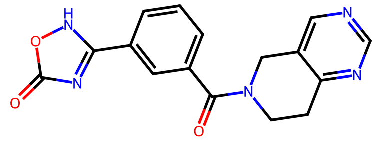	O=C(c1cccc(-c2nc(=O)o[nH]2)c1)N1CCc2ncncc2C1	1153217656	24	323.1	1.02
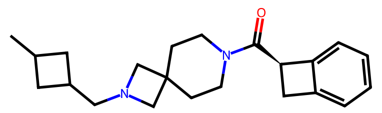	CC1CC(CN2CC3(CCN(C(=O)[C@@H]4Cc5ccccc54)CC3)C2)C1	1035779833	25	338.24	3.3
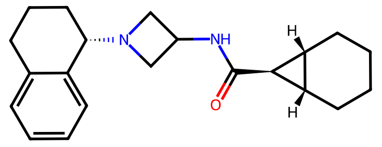	O=C(NC1CN([C@H]2CCCc3ccccc32)C1)[C@@H]1[C@H]2CCCC[C@H]21	1030187237	24	324.22	3.3

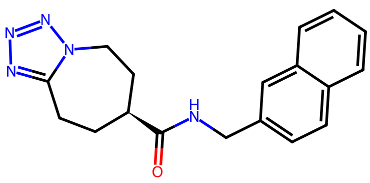	O=C(NCc1ccc2ccccc2c1)[C@H]1CCc2nnnn2CC1	651074465	24	321.16	2.1
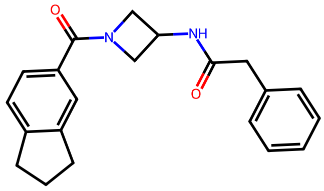	O=C(Cc1ccccc1)NC1CN(C(=O)c2ccc3c(c2)CCC3)C1	995107612	25	334.17	2.36
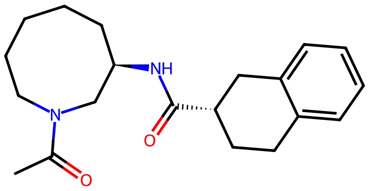	CC(=O)N1CCCCC[C@@H](NC(=O)[C@H]2CCc3ccccc3C2)C1	986791973	24	328.22	2.7
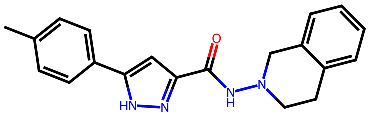	Cc1ccc(-c2cc(C(=O)NN3CCc4ccccc4C3)n[nH]2)cc1	1628726121	25	332.16	3.09
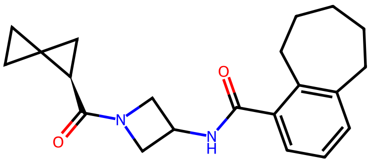	O=C(NC1CN(C(=O)[C@@H]2CC23CC3)C1)c1cccc2c1CCCCC2	994066606	25	338.2	2.7
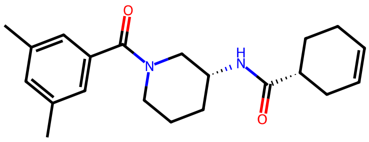	Cc1cc(C)cc(C(=O)N2CCC[C@@H](NC(=O)[C@H]3CC=CCC3)C2)c1	951988426	25	340.22	3.38
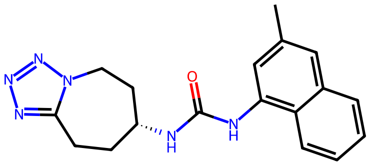	Cc1cc(NC(=O)N[C@@H]2CCc3nnnn3CC2)c2ccccc2c1	574910276	25	336.17	2.66
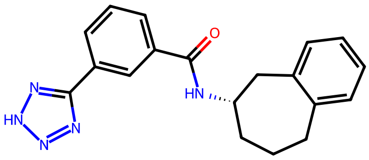	O=C(N[C@H]1CCCc2ccccc2C1)c1cccc(-c2nn[nH]n2)c1	1592985825	25	333.16	2.54

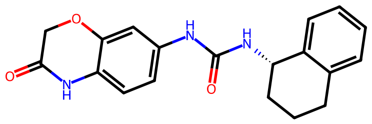	O=C1COc2cc(NC(=O)N[C@H]3CCCc4ccccc43)ccc2N1	126625361	25	337.14	3.22
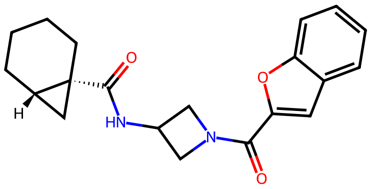	O=C(c1cc2ccccc2o1)N1CC(NC(=O)[C@@]23CCCC[C@H]2C3)C1	999284290	25	338.16	2.95
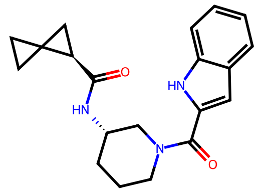	O=C(N[C@H]1CCCN(C(=O)c2cc3ccccc3[nH]2)C1)[C@@H]1CC12CC2	952080981	25	337.18	2.69
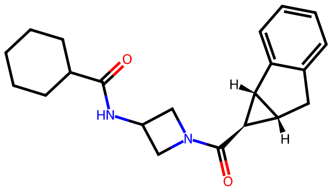	O=C(NC1CN(C(=O)[C@@H]2[C@H]3Cc4ccccc4[C@H]32)C1)C1CCCCC1	990104222	25	338.2	2.48
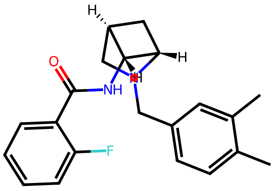	Cc1ccc(CN2C[C@H]3C[C@H]2[C@@H]3NC(=O)c2ccccc2F)cc1C	1037286203	25	338.18	3.45
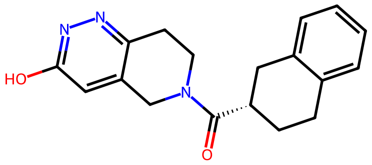	O=C([C@H]1CCc2ccccc2C1)N1CCc2nnc(O)cc2C1	563891972	23	309.15	1.87
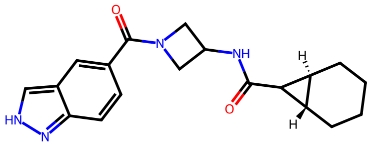	O=C(NC1CN(C(=O)c2ccc3n[nH]cc3c2)C1)C1[C@H]2CCCC[C@H]12	990643513	25	338.17	1.94
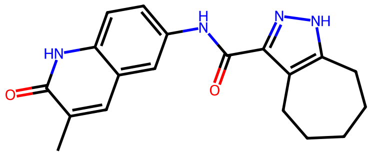	Cc1cc2cc(NC(=O)c3n[nH]c4c3CCCCC4)ccc2[nH]c1=O	908457028	25	336.16	3.08

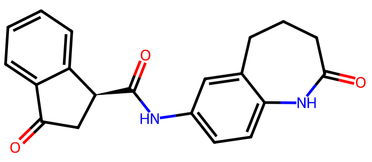	O=C1CCCc2cc(NC(=O)[C@H]3CC(=O)c4ccccc43)ccc2N1	277177335	25	334.13	3.27
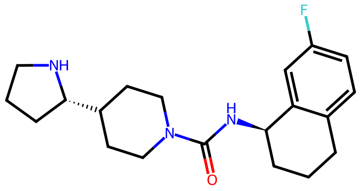	O=C(N[C@@H]1CCCc2ccc(F)cc21)N1CCC([C@@H]2CCCN2)CC1	418114491	25	345.22	3.38
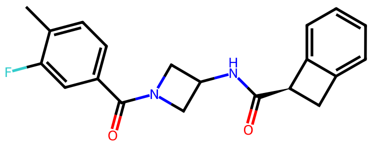	Cc1ccc(C(=O)N2CC(NC(=O)[C@@H]3Cc4ccccc43)C2)cc1F	998686464	25	338.14	2.41
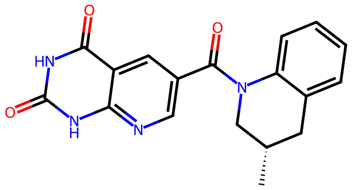	C[C@H]1Cc2ccccc2N(C(=O)c2cnc3[nH]c(=O)[nH]c(=O)c3c2)C1	104523328	25	336.12	1.45
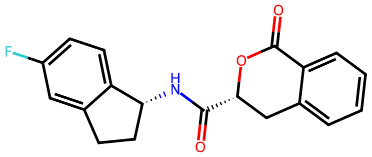	O=C1O[C@@H](C(=O)N[C@@H]2CCc3cc(F)ccc32)Cc2ccccc21	55657739	24	325.11	2.71
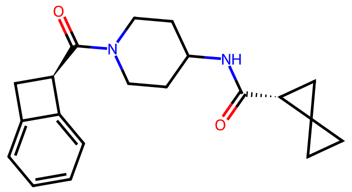	O=C(NC1CCN(C(=O)[C@@H]2Cc3ccccc32)CC1)[C@@H]1CC12CC2	948059408	24	324.18	2.23
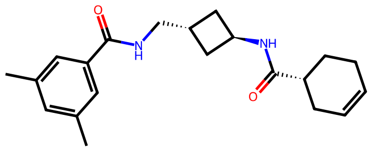	Cc1cc(C)cc(C(=O)NC[C@H]2C[C@H](NC(=O)[C@H]3CC=CCC3)C2)c1	980498374	25	340.22	3.28
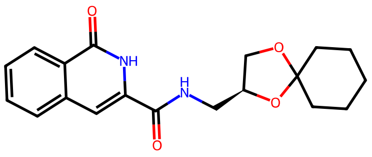	O=C(NC[C@H]1COC2(CCCCC2)O1)c1cc2ccccc2c(=O)[nH]1	181699980	25	342.16	2.33

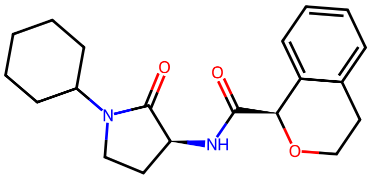	O=C(N[C@H]1CCN(C2CCCCC2)C1=O)[C@@H]1OCCc2ccccc21	124802121	25	342.19	2.35
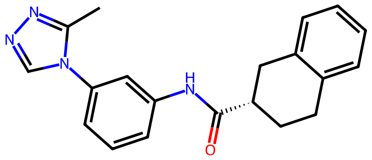	Cc1nncn1-c1cccc(NC(=O)[C@H]2CCc3ccccc3C2)c1	565045840	25	332.16	3.32
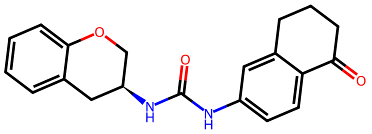	O=C(Nc1ccc2c(c1)CCCC2=O)N[C@@H]1COc2ccccc2C1	575996827	25	336.15	3.33
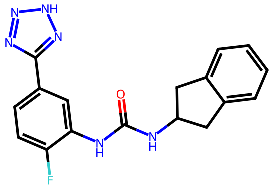	O=C(Nc1cc(-c2nn[nH]n2)ccc1F)NC1Cc2ccccc2C1	1582633860	25	338.13	2.29
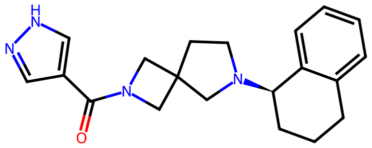	O=C(c1cn[nH]c1)N1CC2(CCN([C@@H]3CCCc4ccccc43)C2)C1	1072511764	25	336.2	2.64
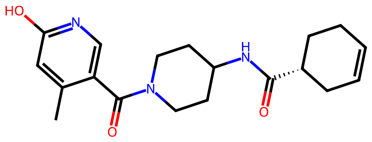	Cc1cc(O)ncc1C(=O)N1CCC(NC(=O)[C@H]2CC=CCC2)CC1	948305539	25	343.19	2.17
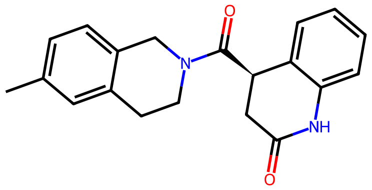	Cc1ccc2c(c1)CCN(C(=O)[C@@H]1CC(=O)Nc3ccccc31)C2	360195824	24	320.15	3.01
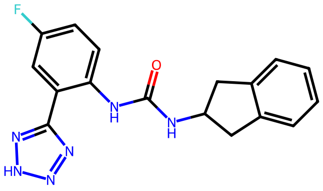	O=C(Nc1ccc(F)cc1-c1nn[nH]n1)NC1Cc2ccccc2C1	1593231245	25	338.13	2.29

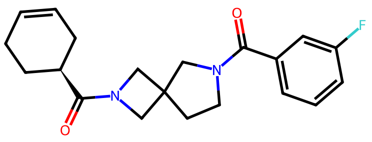	O=C(c1cccc(F)c1)N1CCC2(C1)CN(C(=O)[C@@H]1CC=CCC1)C2	1018769541	25	342.17	2.86
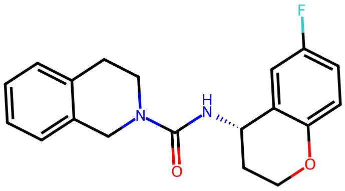	O=C(N[C@H]1CCOc2ccc(F)cc21)N1CCc2ccccc2C1	69461176	24	326.14	3.42
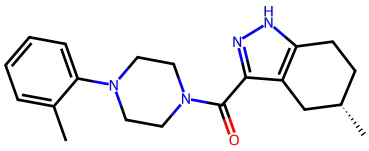	Cc1ccccc1N1CCN(C(=O)c2n[nH]c3c2C[C@@H](C)CC3)CC1	1362163737	25	338.21	2.81
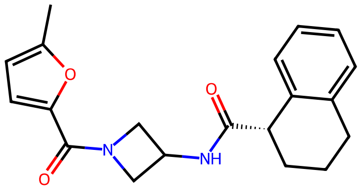	Cc1ccc(C(=O)N2CC(NC(=O)[C@H]3CCCc4ccccc43)C2)o1	995068216	25	338.16	2.65
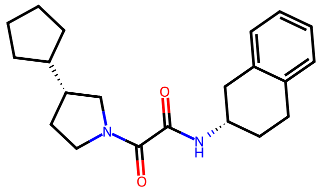	O=C(N[C@H]1CCc2ccccc2C1)C(=O)N1CC[C@H](C2CCCC2)C1	420892106	25	340.22	2.7
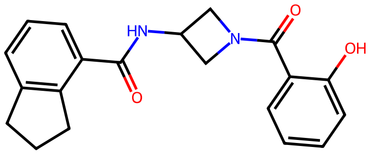	O=C(NC1CN(C(=O)c2ccccc2O)C1)c1cccc2c1CCC2	994404301	25	336.15	2.14
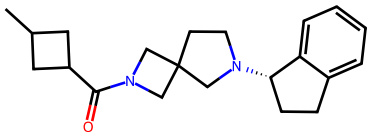	CC1CC(C(=O)N2CC3(CCN([C@H]4CCc5ccccc54)C3)C2)C1	1072606578	24	324.22	3.25
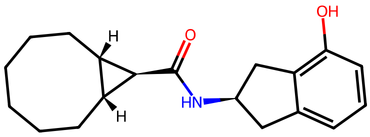	O=C(N[C@@H]1Cc2cccc(O)c2C1)[C@@H]1[C@H]2CCCCCC[C@H]21	613999380	22	299.19	3.19

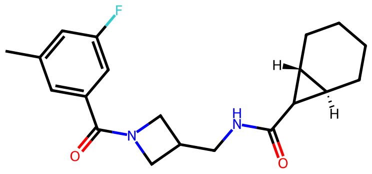	Cc1cc(F)cc(C(=O)N2CC(CNC(=O)C3[C@@H]4CCCC[C@@H]34)C2)c1	1001335770	25	344.19	2.76
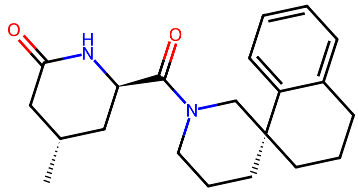	C[C@@H]1CC(=O)N[C@@H](C(=O)N2CCC[C@]3(CCCc4ccccc43)C2)C1	342512941	25	340.22	2.8
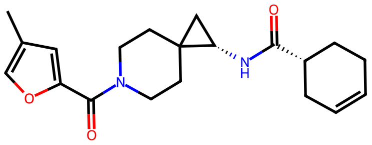	Cc1coc(C(=O)N2CCC3(CC2)C[C@@H]3NC(=O)[C@H]2CC=CCC2)c1	963028186	25	342.19	3.06
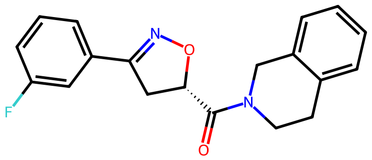	O=C([C@@H]1CC(c2cccc(F)c2)=NO1)N1CCc2ccccc2C1	915045124	24	324.13	2.9
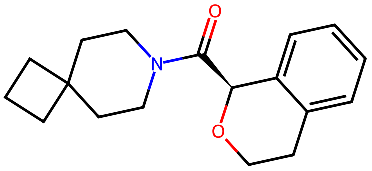	O=C([C@@H]1OCCc2ccccc21)N1CCC2(CCC2)CC1	672254117	21	285.17	3.09
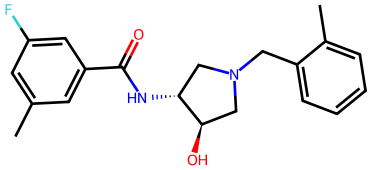	Cc1cc(F)cc(C(=O)N[C@@H]2CN(Cc3ccccc3C)C[C@H]2O)c1	1077722569	25	342.17	2.42
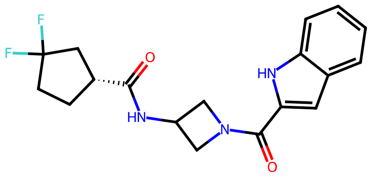	O=C(NC1CN(C(=O)c2cc3ccccc3[nH]2)C1)[C@@H]1CCC(F)(F)C1	994164875	25	347.14	2.54
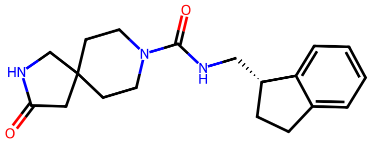	O=C1CC2(CCN(C(=O)NC[C@H]3CCc4ccccc43)CC2)CN1	371839497	24	327.19	2.03

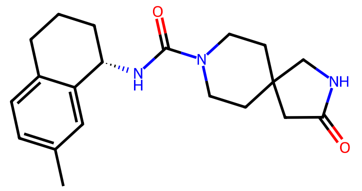	Cc1ccc2c(c1)[C@@H](NC(=O)N1CCC3(CC1)CNC(=O)C3)CCC2	288904529	25	341.21	2.68
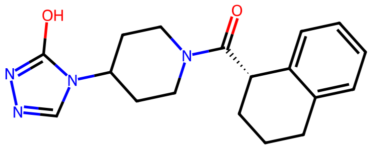	O=C([C@H]1CCCc2ccccc21)N1CCC(n2cnnc2O)CC1	569949193	24	326.17	2.27
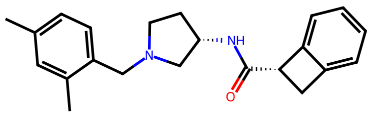	Cc1ccc(CN2CC[C@H](NC(=O)[C@H]3Cc4ccccc43)C2)c(C)c1	1015634759	25	334.2	3.33
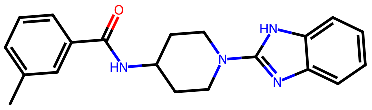	Cc1cccc(C(=O)NC2CCN(c3nc4ccccc4[nH]3)CC2)c1	377540621	25	334.18	3.27
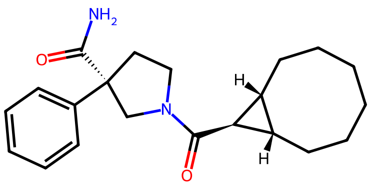	NC(=O)[C@]1(c2ccccc2)CCN(C(=O)[C@@H]2[C@H]3CCCCCC[C@H]32)C1	616272794	25	340.22	2.86
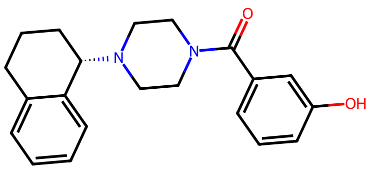	O=C(c1cccc(O)c1)N1CCN([C@H]2CCCc3ccccc32)CC1	81011365	25	336.18	3.23
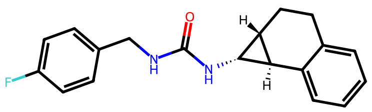	O=C(NCc1ccc(F)cc1)N[C@H]1[C@@H]2c3ccccc3CC[C@H]21	152958672	23	310.15	3.35
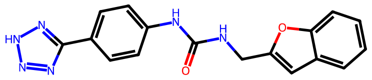	O=C(NCc1cc2ccccc2o1)Nc1ccc(-c2nn[nH]n2)cc1	1609552929	25	334.12	2.93

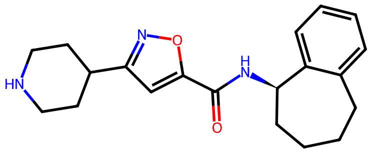	O=C(N[C@@H]1CCCCc2ccccc21)c1cc(C2CCNCC2)no1	662434872	25	339.19	3.34
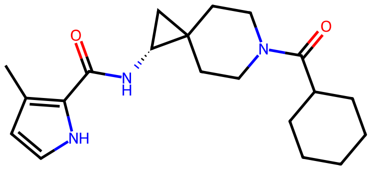	Cc1cc[nH]c1C(=O)N[C@@H]1CC12CCN(C(=O)C1CCCCC1)CC2	963755802	25	343.23	3.01
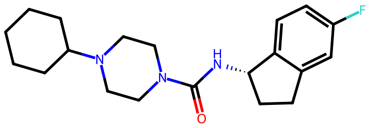	O=C(N[C@H]1CCc2cc(F)ccc21)N1CCN(C2CCCCC2)CC1	485155805	25	345.22	3.47
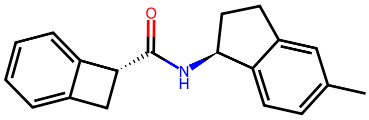	Cc1ccc2c(c1)CC[C@@H]2NC(=O)[C@@H]1Cc2ccccc21	358220723	21	277.15	3.44
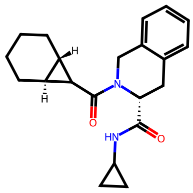	O=C(NC1CC1)[C@H]1Cc2ccccc2CN1C(=O)C1[C@H]2CCCC[C@H]12	650214804	25	338.2	2.65
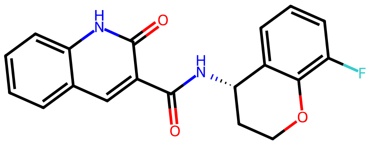	O=C(N[C@H]1CCOc2c(F)cccc21)c1cc2ccccc2[nH]c1=O	189023100	25	338.11	2.92
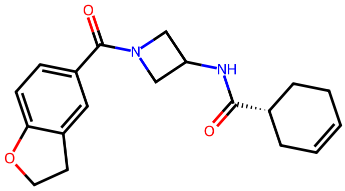	O=C(NC1CN(C(=O)c2ccc3c(c2)CCO3)C1)[C@H]1CC=CCC1	990219835	24	326.16	1.92
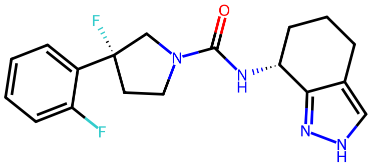	O=C(N[C@@H]1CCCc2c[nH]nc21)N1CC[C@@](F)(c2ccccc2F)C1	555183100	25	346.16	3.21

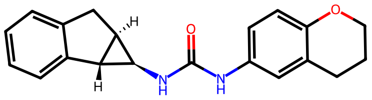	O=C(Nc1ccc2c(c1)CCCO2)N[C@H]1[C@@H]2c3ccccc3C[C@H]21	178217963	24	320.15	3.47
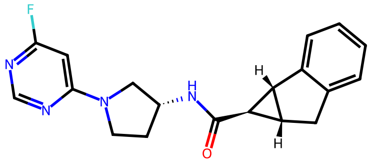	O=C(N[C@@H]1CCN(c2cc(F)ncn2)C1)[C@@H]1[C@H]2Cc3ccccc3[C@H]21	1058566671	25	338.15	1.9
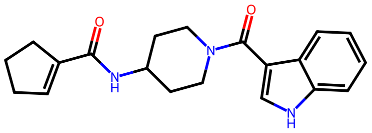	O=C(NC1CCN(C(=O)c2c[nH]c3ccccc23)CC1)C1=CCCC1	947317839	25	337.18	3.0
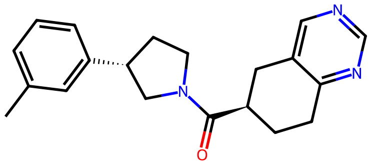	Cc1cccc([C@@H]2CCN(C(=O)[C@@H]3CCc4ncncc4C3)C2)c1	648353007	24	321.18	2.91
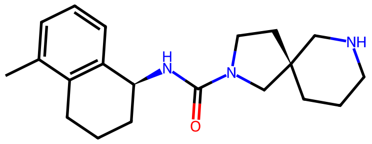	Cc1cccc2c1CCC[C@@H]2NC(=O)N1CC[C@@]2(CCCNC2)C1	851681295	24	327.23	3.16
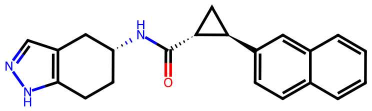	O=C(N[C@@H]1CCc2[nH]ncc2C1)[C@@H]1C[C@H]1c1ccc2ccccc2c1	172212460	25	331.17	3.34
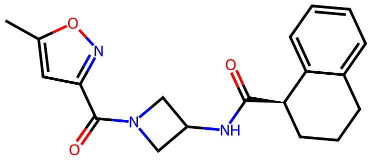	Cc1cc(C(=O)N2CC(NC(=O)[C@@H]3CCCc4ccccc43)C2)no1	995071068	25	339.16	2.04
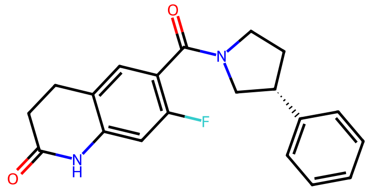	O=C1CCc2cc(C(=O)N3CC[C@H](c4ccccc4)C3)c(F)cc2N1	136103352	25	338.14	3.34

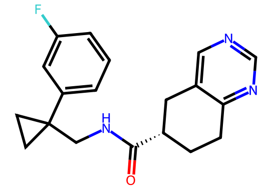	O=C(NCC1(c2cccc(F)c2)CC1)[C@H]1CCc2ncncc2C1	648296660	24	325.16	2.57
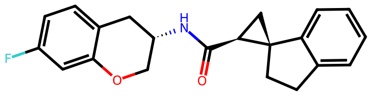	O=C(N[C@@H]1COc2cc(F)ccc2C1)[C@H]1C[C@@]12CCc1ccccc12	618651824	25	337.15	3.15
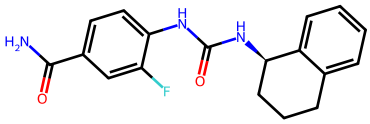	NC(=O)c1ccc(NC(=O)N[C@@H]2CCCc3ccccc32)c(F)c1	80139049	24	327.14	3.12
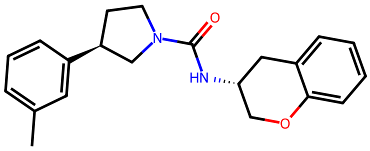	Cc1cccc([C@H]2CCN(C(=O)N[C@H]3COc4ccccc4C3)C2)c1	542426545	25	336.18	3.5
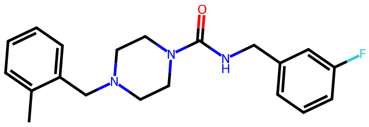	Cc1ccccc1CN1CCN(C(=O)NCc2cccc(F)c2)CC1	410175215	25	341.19	3.16
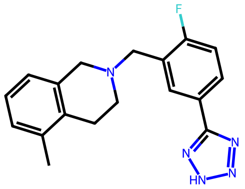	Cc1cccc2c1CCN(Cc1cc(-c3nn[nH]n3)ccc1F)C2	737408082	24	323.15	2.87
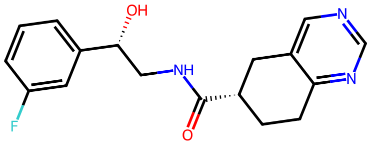	O=C(NC[C@@H](O)c1cccc(F)c1)[C@H]1CCc2ncncc2C1	843824868	23	315.14	1.57
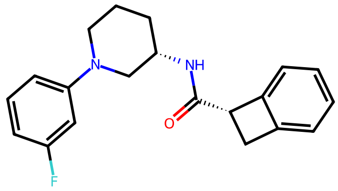	O=C(N[C@H]1CCCN(c2cccc(F)c2)C1)[C@H]1Cc2ccccc21	358496087	24	324.16	3.25

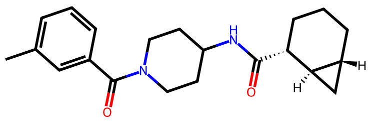	Cc1cccc(C(=O)N2CCC(NC(=O)[C@@H]3CCC[C@@H]4C[C@H]43)CC2)c1	943833610	25	340.22	3.15
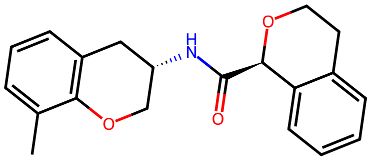	Cc1cccc2c1OC[C@@H](NC(=O)[C@H]1OCCc3ccccc31)C2	291712144	24	323.15	2.73
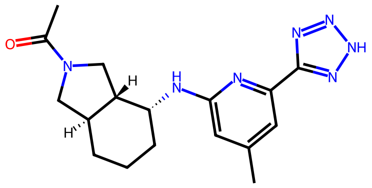	CC(=O)N1C[C@@H]2CCC[C@@H](Nc3cc(C)cc(-c4nn[nH]n4)n3)[C@H]2C1	1588809923	25	341.2	1.63
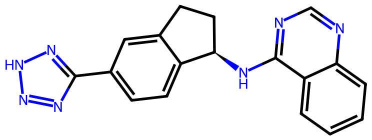	c1ccc2c(N[C@@H]3CCc4cc(-c5nn[nH]n5)ccc43)ncnc2c1	1593580518	25	329.14	2.91
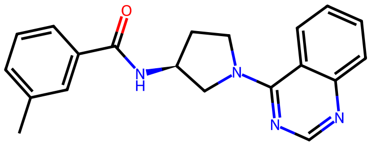	Cc1cccc(C(=O)N[C@H]2CCN(c3ncnc4ccccc34)C2)c1	1058301094	25	332.16	2.95
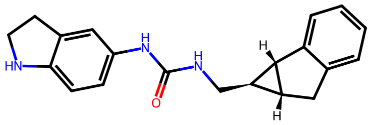	O=C(NC[C@@H]1[C@H]2Cc3ccccc3[C@H]21)Nc1ccc2c(c1)CCN2	576449347	24	319.17	3.36
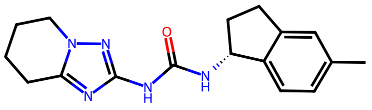	Cc1ccc2c(c1)CC[C@H]2NC(=O)Nc1nc2n(n1)CCCC2	333229709	23	311.17	2.73
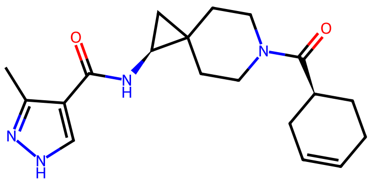	Cc1n[nH]cc1C(=O)N[C@H]1CC12CCN(C(=O)[C@@H]1CC=CCC1)CC2	964568816	25	342.21	2.19

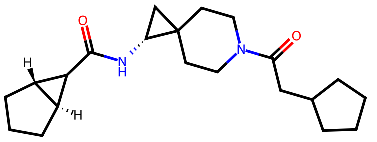	O=C(N[C@@H]1CC12CCN(C(=O)CC1CCCC1)CC2)C1[C@H]2CCC[C@H]12	963470285	25	344.25	3.11
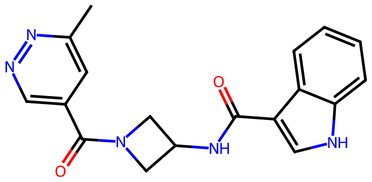	Cc1cc(C(=O)N2CC(NC(=O)c3c[nH]c4ccccc34)C2)cnn1	995032109	25	335.14	1.52
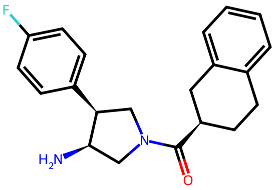	N[C@@H]1CN(C(=O)[C@@H]2CCc3ccccc3C2)C[C@@H]1c1ccc(F)cc1	1549122620	25	338.18	2.88
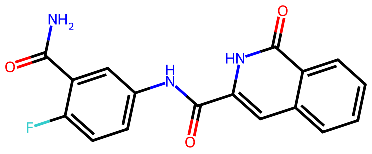	NC(=O)c1cc(NC(=O)c2cc3ccccc3c(=O)[nH]2)ccc1F	33064766	24	325.09	2.02
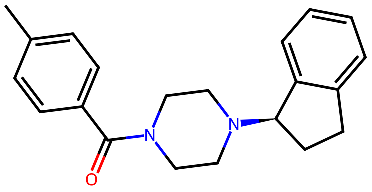	Cc1ccc(C(=O)N2CCN([C@@H]3CCc4ccccc43)CC2)cc1	49719890	24	320.19	3.44
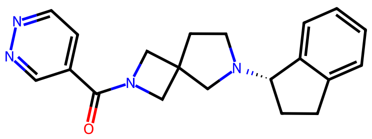	O=C(c1ccnnc1)N1CC2(CCN([C@H]3CCc4ccccc43)C2)C1	1073012182	25	334.18	2.31
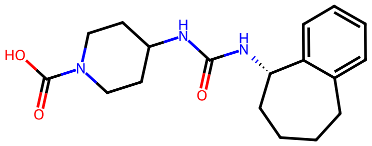	O=C(NC1CCN(C(=O)O)CC1)N[C@H]1CCCCc2ccccc21	738486179	24	331.19	2.9
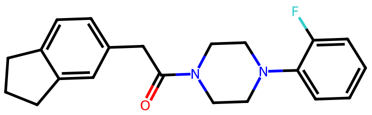	O=C(Cc1ccc2c(c1)CCC2)N1CCN(c2ccccc2F)CC1	514650185	25	338.18	3.21

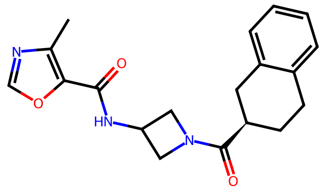	Cc1ncoc1C(=O)NC1CN(C(=O)[C@@H]2CCc3ccccc3C2)C1	990668084	25	339.16	1.73
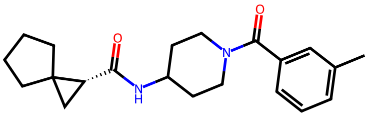	Cc1cccc(C(=O)N2CCC(NC(=O)[C@@H]3CC34CCCC4)CC2)c1	943835547	25	340.22	3.3
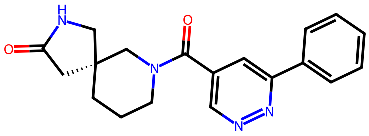	O=C1C[C@]2(CCCN(C(=O)c3cnnc(-c4ccccc4)c3)C2)CN1	580252102	25	336.16	1.89
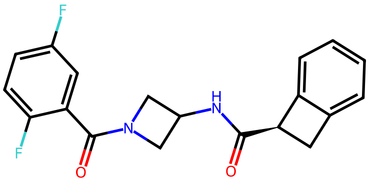	O=C(NC1CN(C(=O)c2cc(F)ccc2F)C1)[C@@H]1Cc2ccccc21	998687196	25	342.12	2.25
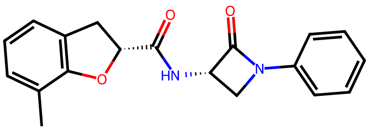	Cc1cccc2c1O[C@@H](C(=O)N[C@H]1CN(c3ccccc3)C1=O)C2	562693062	24	322.13	1.83
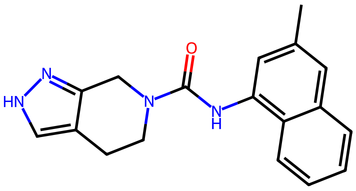	Cc1cc(NC(=O)N2CCc3c[nH]nc3C2)c2ccccc2c1	1677702368	23	306.15	3.46
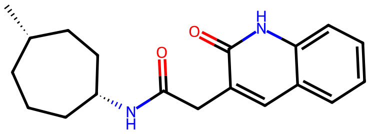	C[C@H]1CCC[C@@H](NC(=O)Cc2cc3ccccc3[nH]c2=O)CC1	188924036	23	312.18	3.16
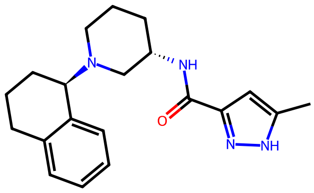	Cc1cc(C(=O)N[C@H]2CCCN([C@@H]3CCCc4ccccc43)C2)n[nH]1	1006857945	25	338.21	2.99

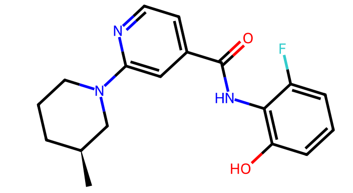	C[C@H]1CCCN(c2cc(C(=O)Nc3c(O)cccc3F)ccn2)C1	643209611	24	329.15	3.41
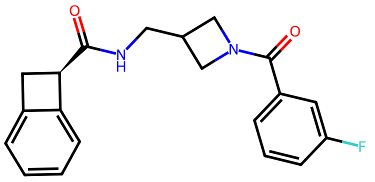	O=C(NCC1CN(C(=O)c2cccc(F)c2)C1)[C@@H]1Cc2ccccc21	1003950801	25	338.14	2.35
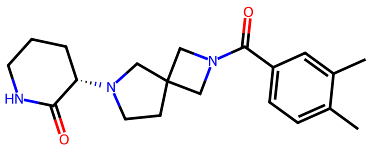	Cc1ccc(C(=O)N2CC3(CCN([C@H]4CCCNC4=O)C3)C2)cc1C	1073402281	25	341.21	1.73
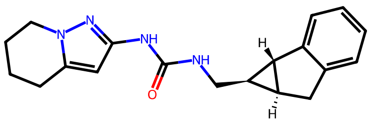	O=C(NC[C@@H]1[C@@H]2Cc3ccccc3[C@@H]12)Nc1cc2n(n1)CCCC2	287410913	24	322.18	2.93
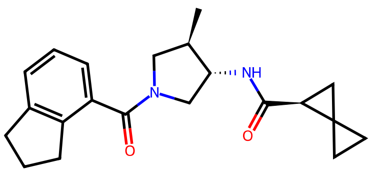	C[C@@H]1CN(C(=O)c2cccc3c2CCC3)C[C@H]1NC(=O)[C@H]1CC12CC2	940668009	25	338.2	2.55
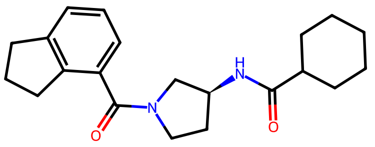	O=C(N[C@H]1CCN(C(=O)c2cccc3c2CCC3)C1)C1CCCCC1	937627153	25	340.22	3.09
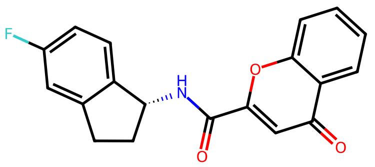	O=C(N[C@@H]1CCc2cc(F)ccc21)c1cc(=O)c2ccccc2o1	55657647	24	323.1	3.35
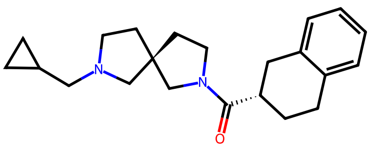	O=C([C@H]1CCc2ccccc2C1)N1CC[C@@]2(CCN(CC3CC3)C2)C1	1041708433	25	338.24	3.13

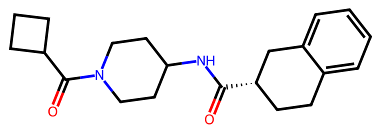	O=C(NC1CCN(C(=O)C2CCC2)CC1)[C@H]1CCc2ccccc2C1	943344873	25	340.22	2.7
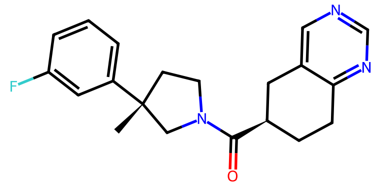	C[C@@]1(c2cccc(F)c2)CCN(C(=O)[C@@H]2CCc3ncncc3C2)C1	650218235	25	339.17	2.91
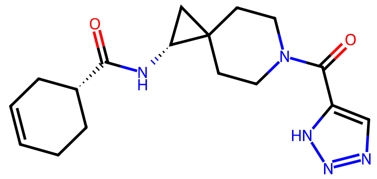	O=C(N[C@@H]1CC12CCN(C(=O)c1cnn[nH]1)CC2)[C@H]1CC=CCC1	962970774	24	329.19	1.27
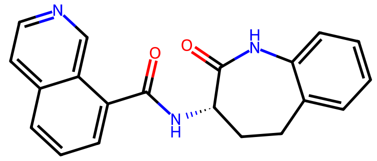	O=C(N[C@H]1CCc2ccccc2NC1=O)c1cccc2ccncc12	1526999710	25	331.13	2.92
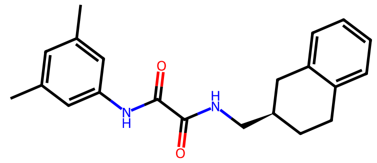	Cc1cc(C)cc(NC(=O)C(=O)NC[C@@H]2CCc3ccccc3C2)c1	117519614	25	336.18	3.16
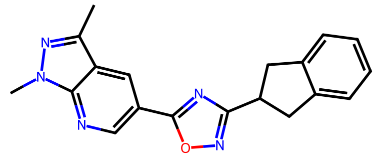	Cc1nn(C)c2ncc(-c3nc(C4Cc5ccccc5C4)no3)cc12	547090208	25	331.14	3.21
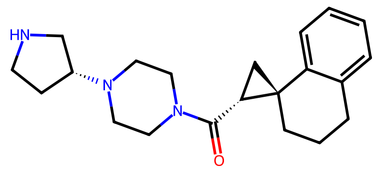	O=C([C@@H]1C[C@@]12CCCc1ccccc12)N1CCN([C@@H]2CCNC2)CC1	343604818	25	339.23	1.79
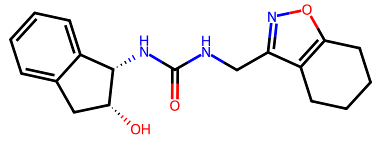	O=C(NCc1noc2c1CCCC2)N[C@H]1c2ccccc2C[C@H]1O	287953119	24	327.16	2.01

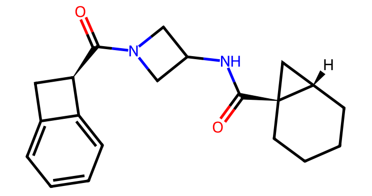	O=C([C@@H]1Cc2ccccc21)N1CC(NC(=O)[C@]23CCCC[C@H]2C3)C1	999286402	24	324.18	2.23
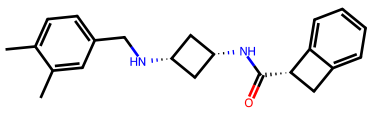	Cc1ccc(CN[C@H]2C[C@@H](NC(=O)[C@H]3Cc4ccccc43)C2)cc1C	1022632032	25	334.2	3.38
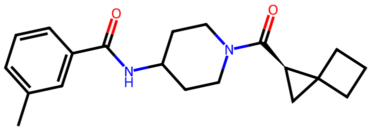	Cc1cccc(C(=O)NC2CCN(C(=O)[C@@H]3CC34CCC4)CC2)c1	870833639	24	326.2	2.91
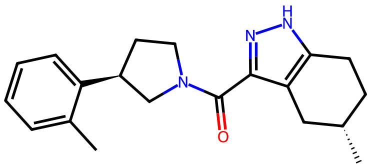	Cc1ccccc1[C@H]1CCN(C(=O)c2n[nH]c3c2C[C@@H](C)CC3)C1	1142810723	24	323.2	3.47
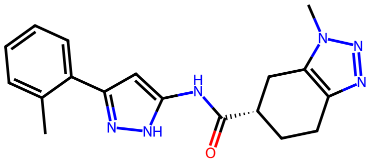	Cc1ccccc1-c1cc(NC(=O)[C@H]2CCc3nnn(C)c3C2)[nH]n1	290323166	25	336.17	2.26
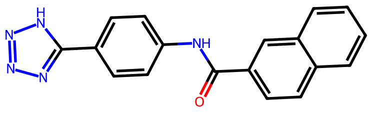	O=C(Nc1ccc(-c2nnn[nH]2)cc1)c1ccc2ccccc2c1	123619243	24	315.11	3.27
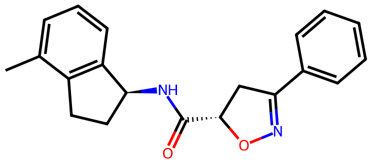	Cc1cccc2c1CC[C@@H]2NC(=O)[C@@H]1CC(c2ccccc2)=NO1	729441365	24	320.15	3.29
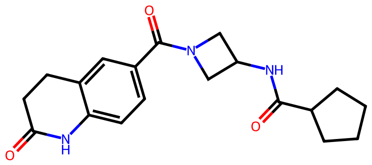	O=C1CCc2cc(C(=O)N3CC(NC(=O)C4CCCC4)C3)ccc2N1	990035351	25	341.17	1.7

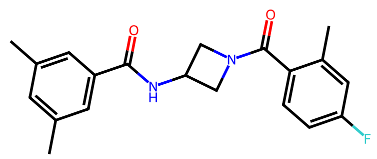	Cc1cc(C)cc(C(=O)NC2CN(C(=O)c3ccc(F)cc3C)C2)c1	999485608	25	340.16	3.01
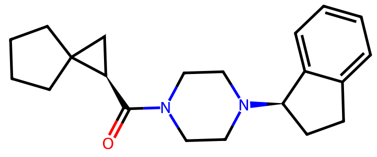	O=C([C@@H]1CC12CCCC2)N1CCN([C@@H]2CCc3ccccc32)CC1	957078630	24	324.22	3.4
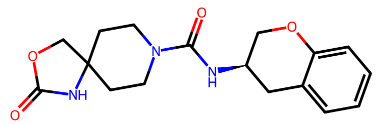	O=C1NC2(CCN(C(=O)N[C@H]3COc4ccccc4C3)CC2)CO1	495267347	24	331.15	1.27
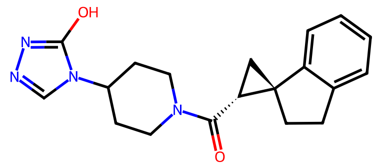	O=C([C@@H]1C[C@@]12CCc1ccccc12)N1CCC(n2cnnc2O)CC1	517586135	25	338.17	2.05
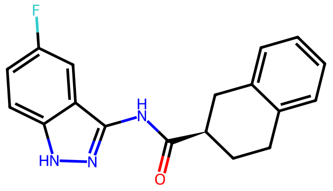	O=C(Nc1n[nH]c2ccc(F)cc12)[C@@H]1CCc2ccccc2C1	1628081700	23	309.13	3.45
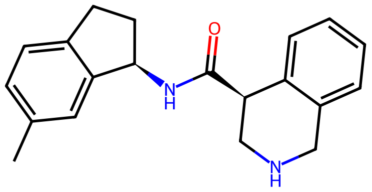	Cc1ccc2c(c1)[C@H](NC(=O)[C@@H]1CNCc3ccccc31)CC2	645946689	23	306.17	2.99
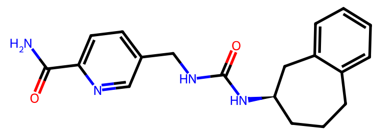	NC(=O)c1ccc(CNC(=O)N[C@@H]2CCc3ccccc3C2)cn1	893536090	25	338.17	1.93
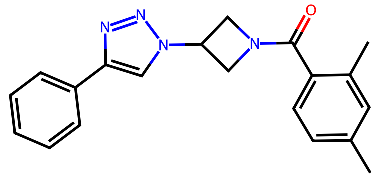	Cc1ccc(C(=O)N2CC(n3cc(-c4ccccc4)nn3)C2)c(C)c1	238836148	25	332.16	3.26

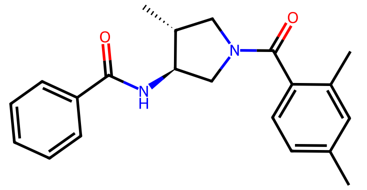	Cc1ccc(C(=O)N2C[C@@H](NC(=O)c3ccccc3)[C@H](C)C2)c(C)c1	959621336	25	336.18	3.19
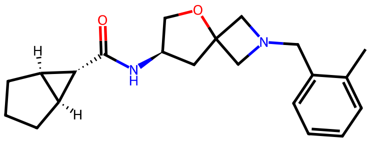	Cc1ccccc1CN1CC2(C[C@@H](NC(=O)[C@@H]3[C@H]4CCC[C@H]43)CO2)C1	1053769415	25	340.22	2.5
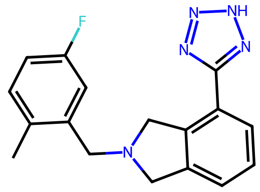	Cc1ccc(F)cc1CN1Cc2cccc(-c3nn[nH]n3)c2C1	1592173241	23	309.14	2.83
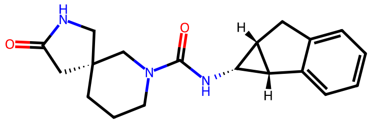	O=C1C[C@]2(CCCN(C(=O)N[C@@H]3[C@@H]4Cc5ccccc5[C@@H]43)C2)CN1	332911912	24	325.18	1.64
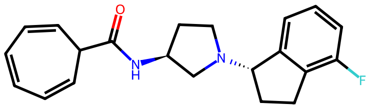	O=C(N[C@H]1CCN([C@H]2CCc3c(F)cccc32)C1)C1C=CC=CC=C1	1014591446	25	338.18	3.3
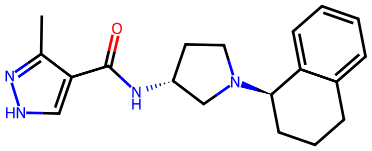	Cc1n[nH]cc1C(=O)N[C@@H]1CCN([C@@H]2CCCc3ccccc32)C1	1015462722	24	324.2	2.6
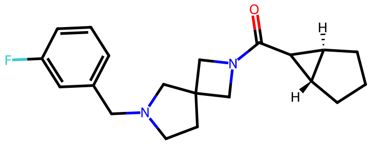	O=C(C1[C@H]2CCC[C@H]12)N1CC2(CCN(Cc3cccc(F)c3)C2)C1	1072591755	24	328.2	2.91
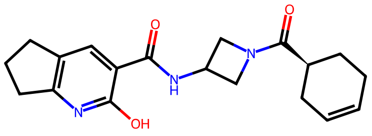	O=C(NC1CN(C(=O)[C@@H]2CC=CCC2)C1)c1cc2c(nc1O)CCC2	991734730	25	341.17	1.57

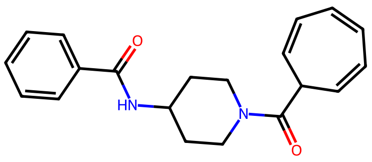	O=C(NC1CCN(C(=O)C2C=CC=CC=C2)CC1)c1ccccc1	367350911	24	322.17	2.71
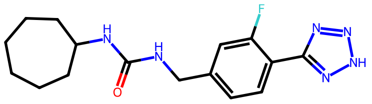	O=C(NCc1ccc(-c2nn[nH]n2)c(F)c1)NC1CCCCCC1	1593126158	24	332.18	2.53
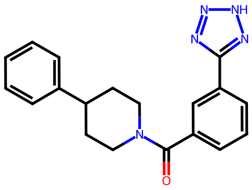	O=C(c1cccc(-c2nn[nH]n2)c1)N1CCC(c2ccccc2)CC1	1609583195	25	333.16	2.89
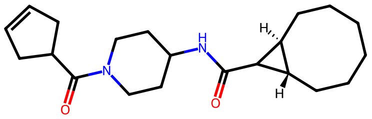	O=C(NC1CCN(C(=O)C2CC=CC2)CC1)C1[C@H]2CCCCCC[C@H]12	945717730	25	344.25	3.28
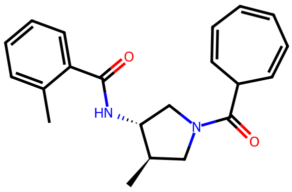	Cc1ccccc1C(=O)N[C@@H]1CN(C(=O)C2C=CC=CC=C2)C[C@H]1C	949636442	25	336.18	2.87
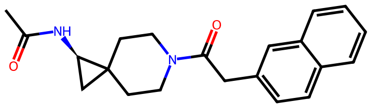	CC(=O)N[C@@H]1CC12CCN(C(=O)Cc1ccc3ccccc3c1)CC2	962444812	25	336.18	2.9
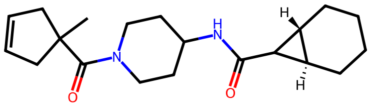	CC1(C(=O)N2CCC(NC(=O)C3[C@@H]4CCCC[C@@H]34)CC2)CC=CC1	948178973	24	330.23	2.89
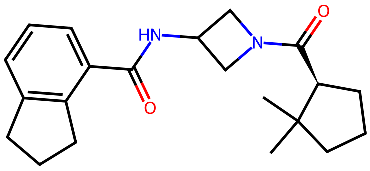	CC1(C)CCC[C@@H]1C(=O)N1CC(NC(=O)c2cccc3c2CCC3)C1	994393874	25	340.22	2.94

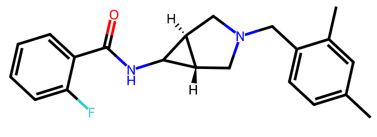	Cc1ccc(CN2C[C@@H]3C(NC(=O)c4ccccc4F)[C@H]3C2)c(C)c1	958107004	25	338.18	3.3
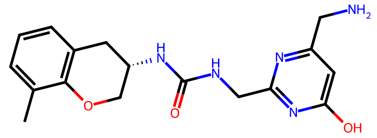	Cc1cccc2c1OC[C@@H](NC(=O)NCc1nc(O)cc(CN)n1)C2	659493996	25	343.16	0.75
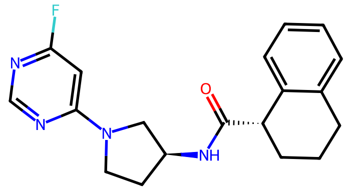	O=C(N[C@H]1CCN(c2cc(F)ncn2)C1)[C@H]1CCCc2ccccc21	1058591965	25	340.17	2.43
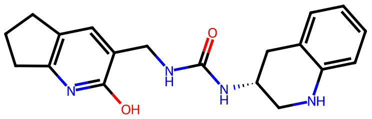	O=C(NCc1cc2c(nc1O)CCC2)N[C@H]1CNc2ccccc2C1	652150829	25	338.17	2.11
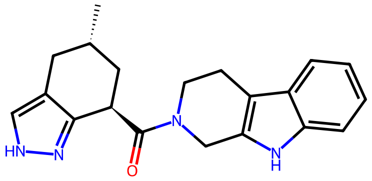	C[C@H]1Cc2c[nH]nc2[C@H](C(=O)N2CCc3c([nH]c4ccccc34)C2)C1	623206516	25	334.18	3.14
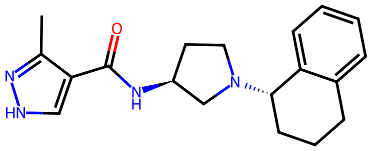	Cc1n[nH]cc1C(=O)N[C@H]1CCN([C@H]2CCCc3ccccc32)C1	1015462725	24	324.2	2.6
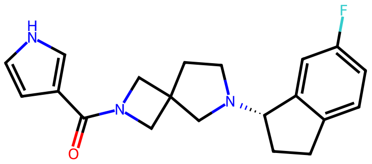	O=C(c1cc[nH]c1)N1CC2(CCN([C@H]3CCc4ccc(F)cc43)C2)C1	1072771102	25	339.17	2.99
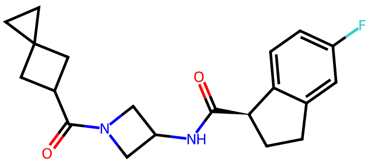	O=C(NC1CN(C(=O)C2CC3(CC3)C2)C1)[C@@H]1CCc2cc(F)ccc21	996096859	25	342.17	2.37

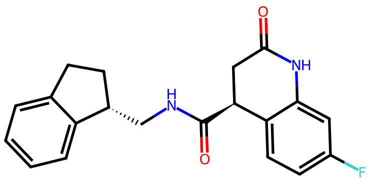	O=C1C[C@H](C(=O)NC[C@H]2CCc3ccccc32)c2ccc(F)cc2N1	110378222	25	338.14	3.1
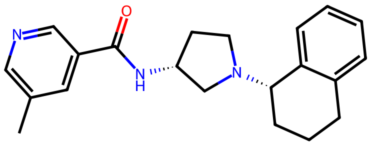	Cc1cncc(C(=O)N[C@@H]2CCN([C@H]3CCCc4ccccc43)C2)c1	1010436913	25	335.2	3.27
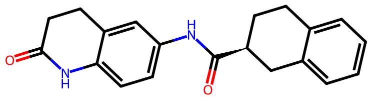	O=C1CCc2cc(NC(=O)[C@H]3CCc4ccccc4C3)ccc2N1	565052922	24	320.15	3.31
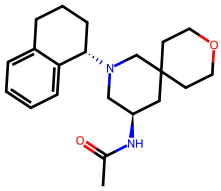	CC(=O)N[C@H]1CN([C@H]2CCCc3ccccc32)CC2(CCOCC2)C1	1089916370	25	342.23	3.07
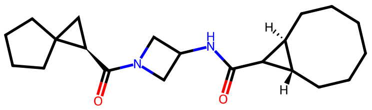	O=C(NC1CN(C(=O)[C@@H]2CC23CCCC3)C1)C1[C@H]2CCCCCC[C@H]12	1000500591	25	344.25	3.11
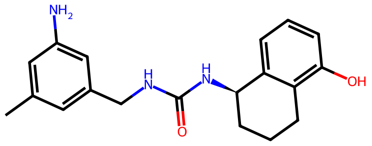	Cc1cc(N)cc(CNC(=O)N[C@@H]2CCCc3c(O)cccc32)c1	418079993	24	325.18	3.16
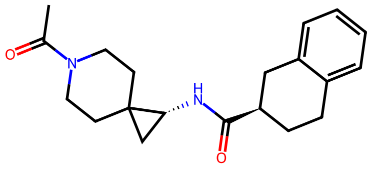	CC(=O)N1CCC2(CC1)C[C@H]2NC(=O)[C@@H]1CCc2ccccc2C1	965695114	24	326.2	2.31
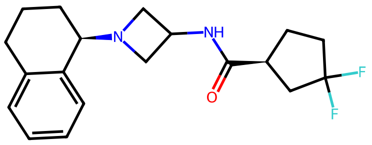	O=C(NC1CN([C@@H]2CCCc3ccccc32)C1)[C@H]1CCC(F)(F)C1	1030412205	24	334.19	3.3

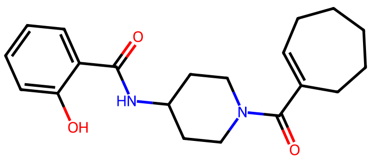	O=C(NC1CCN(C(=O)C2=CCCCCC2)CC1)c1ccccc1O	946548595	25	342.19	3.0
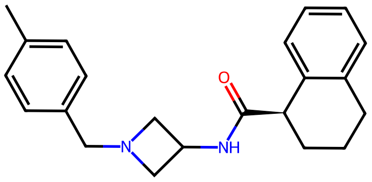	Cc1ccc(CN2CC(NC(=O)[C@@H]3CCCc4ccccc43)C2)cc1	1030530563	25	334.2	3.42
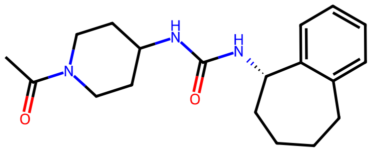	CC(=O)N1CCC(NC(=O)N[C@H]2CCCCc3ccccc32)CC1	65613671	24	329.21	2.76
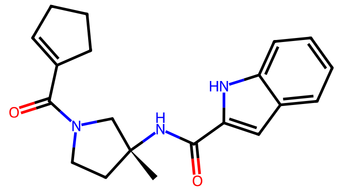	C[C@@]1(NC(=O)c2cc3ccccc3[nH]2)CCN(C(=O)C2=CCCC2)C1	957652172	25	337.18	3.0
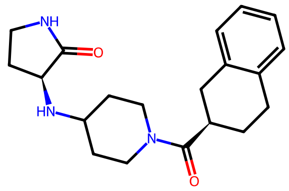	O=C1NCC[C@@H]1NC1CCN(C(=O)[C@@H]2CCc3ccccc3C2)CC1	997036731	25	341.21	1.26
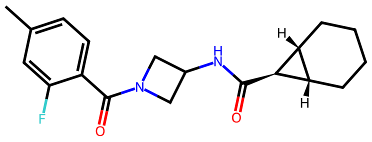	Cc1ccc(C(=O)N2CC(NC(=O)[C@@H]3[C@H]4CCCC[C@H]43)C2)c(F)c1	990659913	24	330.17	2.51
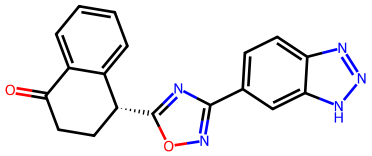	O=C1CC[C@@H](c2nc(-c3ccc4nn[nH]c4c3)no2)c2ccccc21	350488876	25	331.11	3.12
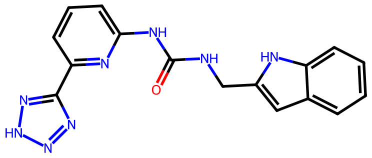	O=C(NCc1cc2ccccc2[nH]1)Nc1cccc(-c2nn[nH]n2)n1	1606817684	25	334.13	2.06

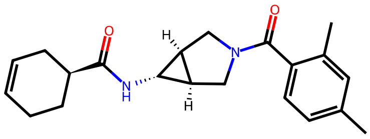	Cc1ccc(C(=O)N2C[C@H]3[C@H](NC(=O)[C@@H]4CC=CCC4)[C@H]3C2)c(C)c1	976182680	25	338.2	2.85
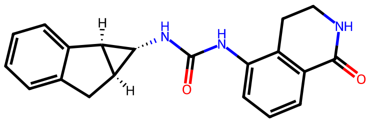	O=C(Nc1cccc2c1CCNC2=O)N[C@@H]1[C@H]2Cc3ccccc3[C@H]21	557142760	25	333.15	2.43
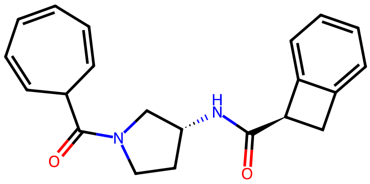	O=C(N[C@@H]1CCN(C(=O)C2C=CC=CC=C2)C1)[C@@H]1Cc2ccccc21	942304989	25	334.17	2.34
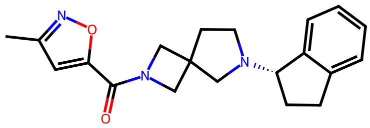	Cc1cc(C(=O)N2CC3(CCN([C@H]4CCc5ccccc54)C3)C2)on1	1072462438	25	337.18	2.82
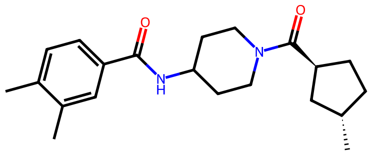	Cc1ccc(C(=O)NC2CCN(C(=O)[C@H]3CC[C@H](C)C3)CC2)cc1C	946734332	25	342.23	3.46
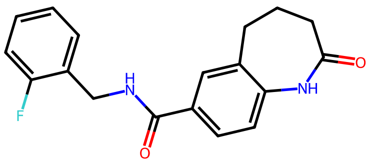	O=C1CCCc2cc(C(=O)NCc3ccccc3F)ccc2N1	170553305	23	312.13	3.03
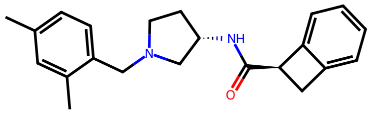	Cc1ccc(CN2CC[C@H](NC(=O)[C@@H]3Cc4ccccc43)C2)c(C)c1	1015634761	25	334.2	3.33
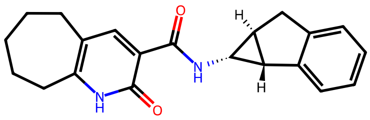	O=C(N[C@@H]1[C@H]2Cc3ccccc3[C@@H]21)c1cc2c([nH]c1=O)CCCCC2	130041656	25	334.17	2.71

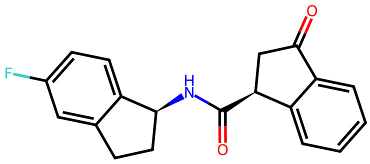	O=C1C[C@H](C(=O)N[C@H]2CCc3cc(F)ccc32)c2ccccc21	277338893	23	309.12	3.3
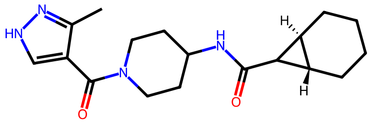	Cc1n[nH]cc1C(=O)N1CCC(NC(=O)C2[C@H]3CCCC[C@H]23)CC1	947668624	24	330.21	1.88
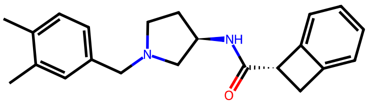	Cc1ccc(CN2CC[C@@H](NC(=O)[C@H]3Cc4ccccc43)C2)cc1C	1015636498	25	334.2	3.33
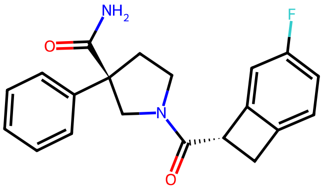	NC(=O)[C@@]1(c2ccccc2)CCN(C(=O)[C@H]2Cc3ccc(F)cc32)C1	665647121	25	338.14	2.12
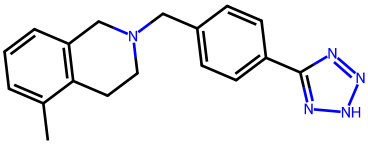	Cc1cccc2c1CCN(Cc1ccc(-c3nn[nH]n3)cc1)C2	737409008	23	305.16	2.73
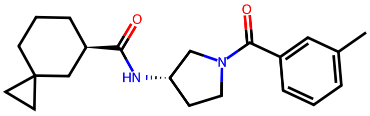	Cc1cccc(C(=O)N2CC[C@H](NC(=O)[C@@H]3CCCC4(CC4)C3)C2)c1	937948925	25	340.22	3.3
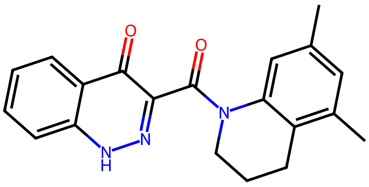	Cc1cc(C)c2c(c1)N(C(=O)c1n[nH]c3ccccc3c1=O)CCC2	118328773	25	333.15	3.13
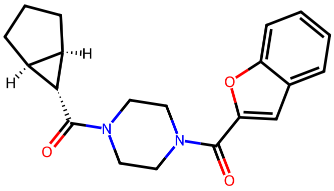	O=C(c1cc2ccccc2o1)N1CCN(C(=O)[C@@H]2[C@H]3CCC[C@H]32)CC1	1010925356	25	338.16	2.76

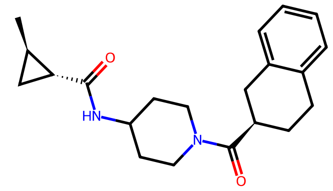	C[C@@H]1C[C@H]1C(=O)NC1CCN(C(=O)[C@@H]2CCc3ccccc3C2)CC1	948572114	25	340.22	2.55
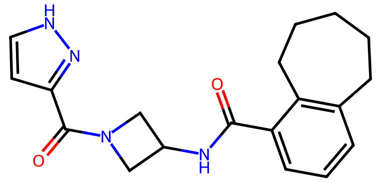	O=C(NC1CN(C(=O)c2cc[nH]n2)C1)c1cccc2c1CCCCC2	994061187	25	338.17	1.93
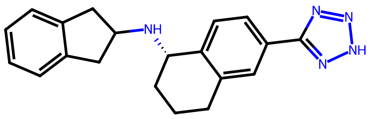	c1ccc2c(c1)CC(N[C@H]1CCCc3cc(-c4nn[nH]n4)ccc31)C2	1593580217	25	331.18	3.0
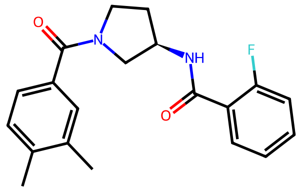	Cc1ccc(C(=O)N2CC[C@@H](NC(=O)c3ccccc3F)C2)cc1C	939176076	25	340.16	3.09
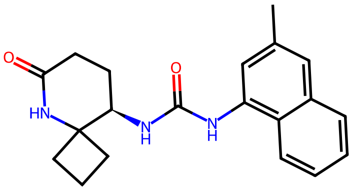	Cc1cc(NC(=O)N[C@@H]2CCC(=O)NC23CCC3)c2ccccc2c1	297524485	25	337.18	3.47
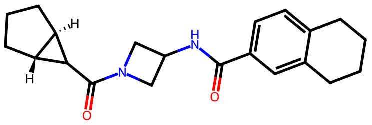	O=C(NC1CN(C(=O)C2[C@@H]3CCC[C@@H]23)C1)c1ccc2c(c1)CCCC2	1000057098	25	338.2	2.55
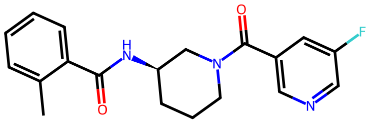	Cc1ccccc1C(=O)N[C@@H]1CCCN(C(=O)c2cncc(F)c2)C1	950391759	25	341.15	2.56
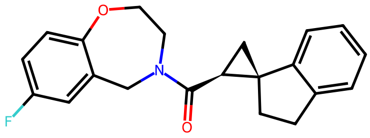	O=C([C@H]1C[C@@]12CCc1ccccc12)N1CCOc2ccc(F)cc2C1	470582328	25	337.15	3.45

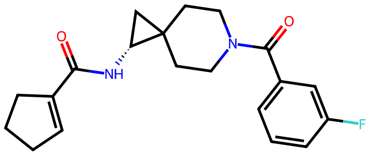	O=C(N[C@@H]1CC12CCN(C(=O)c1cccc(F)c1)CC2)C1=CCCC1	963565945	25	342.17	3.05
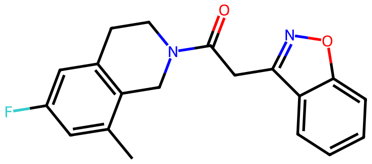	Cc1cc(F)cc2c1CN(C(=O)Cc1noc3ccccc13)CC2	337274694	24	324.13	3.4
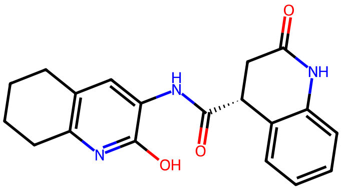	O=C1C[C@@H](C(=O)Nc2cc3c(nc2O)CCCC3)c2ccccc2N1	1461364355	25	337.14	2.73
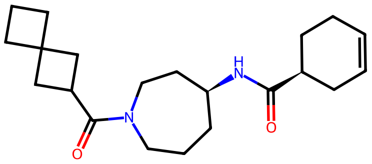	O=C(N[C@H]1CCCN(C(=O)C2CC3(CCC3)C2)CC1)[C@@H]1CC=CCC1	964717425	25	344.25	3.42
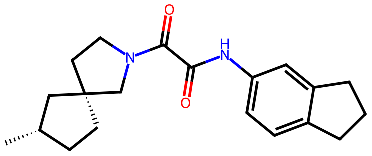	C[C@H]1CC[C@@]2(CCN(C(=O)C(=O)Nc3ccc4c(c3)CCC4)C2)C1	359461239	24	326.2	3.15
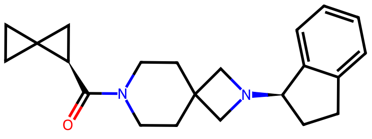	O=C([C@@H]1CC12CC2)N1CCC2(CC1)CN([C@@H]1CCc3ccccc31)C2	1035808194	25	336.22	3.4
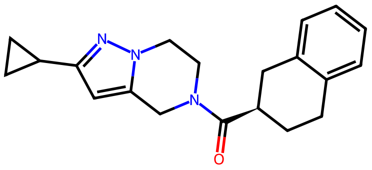	O=C([C@@H]1CCc2ccccc2C1)N1CCn2nc(C3CC3)cc2C1	834434006	24	321.18	2.91
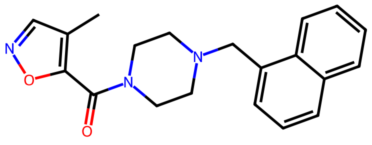	Cc1cnoc1C(=O)N1CCN(Cc2cccc3ccccc23)CC1	73403322	25	335.16	3.09

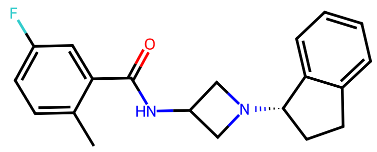	Cc1ccc(F)cc1C(=O)NC1CN([C@H]2CCc3ccccc32)C1	1030758779	24	324.16	3.24
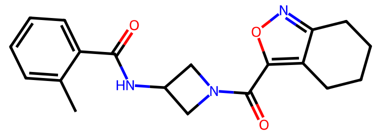	Cc1ccccc1C(=O)NC1CN(C(=O)c2onc3c2CCCC3)C1	990107458	25	339.16	2.12
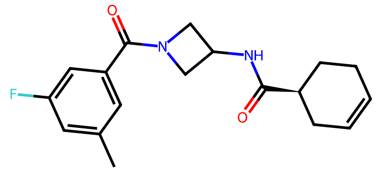	Cc1cc(F)cc(C(=O)N2CC(NC(=O)[C@@H]3CC=CCC3)C2)c1	990222824	23	316.16	2.43
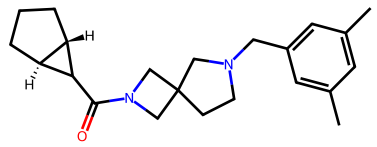	Cc1cc(C)cc(CN2CCC3(C2)CN(C(=O)C2[C@H]4CCC[C@H]24)C3)c1	1072591328	25	338.24	3.38
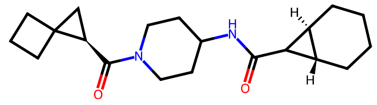	O=C(NC1CCN(C(=O)[C@@H]2CC23CCC3)CC1)C1[C@H]2CCCC[C@H]12	946763516	24	330.23	2.72
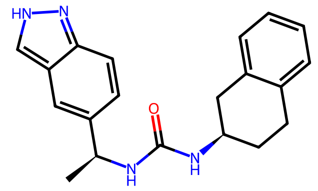	C[C@H](NC(=O)N[C@@H]1CCc2ccccc2C1)c1ccc2n[nH]cc2c1	554534194	25	334.18	3.48
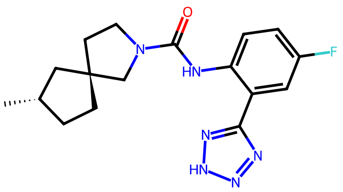	C[C@H]1CC[C@]2(CCN(C(=O)Nc3ccc(F)cc3-c3nn[nH]n3)C2)C1	824914917	25	344.18	3.05
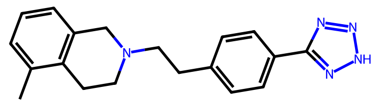	Cc1cccc2c1CCN(CCc1ccc(-c3nn[nH]n3)cc1)C2	1592243749	24	319.18	2.78

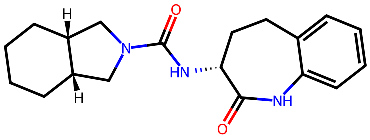	O=C1Nc2ccccc2CC[C@H]1NC(=O)N1C[C@H]2CCCC[C@H]2C1	654634222	24	327.19	2.77
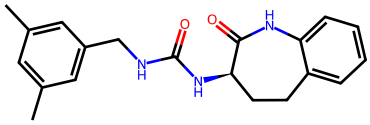	Cc1cc(C)cc(CNC(=O)N[C@@H]2CCc3ccccc3NC2=O)c1	661720340	25	337.18	3.06
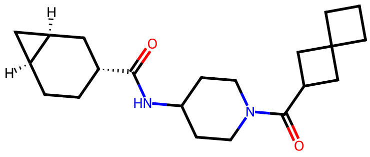	O=C(NC1CCN(C(=O)C2CC3(CCC3)C2)CC1)[C@@H]1CC[C@H]2C[C@H]2C1	947886369	25	344.25	3.11
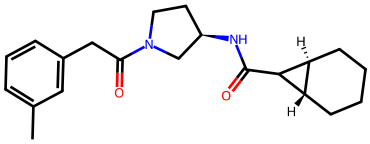	Cc1cccc(CC(=O)N2CC[C@@H](NC(=O)C3[C@H]4CCCC[C@H]34)C2)c1	939894996	25	340.22	2.69
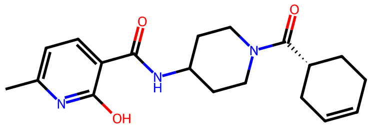	Cc1ccc(C(=O)NC2CCN(C(=O)[C@H]3CC=CCC3)CC2)c(O)n1	944127721	25	343.19	2.17
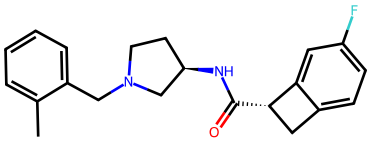	Cc1ccccc1CN1CC[C@@H](NC(=O)[C@H]2Cc3ccc(F)cc32)C1	1015615717	25	338.18	3.16
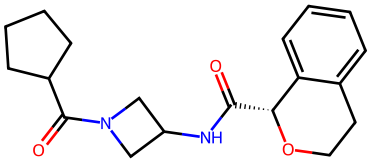	O=C(NC1CN(C(=O)C2CCCC2)C1)[C@H]1OCCc2ccccc21	997326996	24	328.18	1.82
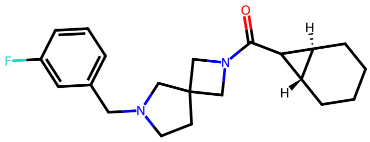	O=C(C1[C@H]2CCCC[C@H]12)N1CC2(CCN(Cc3cccc(F)c3)C2)C1	1072580949	25	342.21	3.3

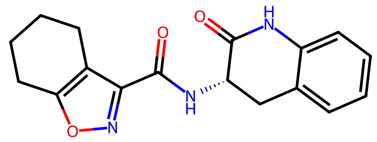	O=C(N[C@H]1Cc2ccccc2NC1=O)c1noc2c1CCCC2	529952315	23	311.13	1.85
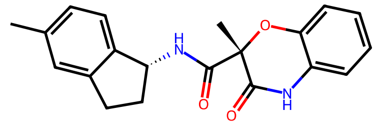	Cc1ccc2c(c1)CC[C@H]2NC(=O)[C@]1(C)Oc2ccccc2NC1=O	630814615	25	336.15	2.89
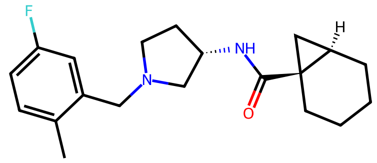	Cc1ccc(F)cc1CN1CC[C@H](NC(=O)[C@]23CCCC[C@@H]2C3)C1	1015672620	24	330.21	3.4
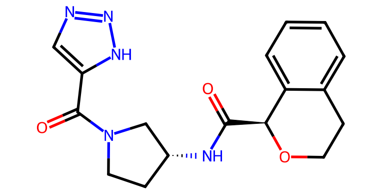	O=C(N[C@@H]1CCN(C(=O)c2cnn[nH]2)C1)[C@@H]1OCCc2ccccc21	941964762	25	341.15	0.45
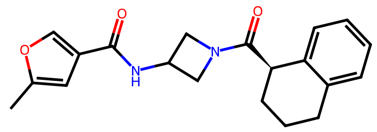	Cc1cc(C(=O)NC2CN(C(=O)[C@@H]3CCCc4ccccc43)C2)co1	991007724	25	338.16	2.65
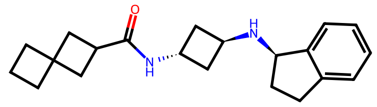	O=C(N[C@H]1C[C@H](N[C@@H]2CCc3ccccc32)C1)C1CC2(CCC2)C1	1020487867	24	324.22	3.49
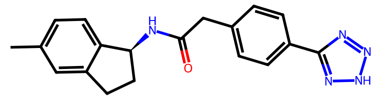	Cc1ccc2c(c1)CC[C@@H]2NC(=O)Cc1ccc(-c2nn[nH]n2)cc1	1592201698	25	333.16	2.52
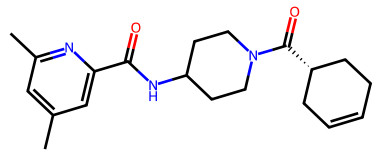	Cc1cc(C)nc(C(=O)NC2CCN(C(=O)[C@H]3CC=CCC3)CC2)c1	944126889	25	341.21	2.78

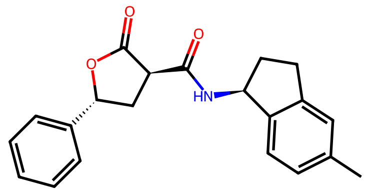	Cc1ccc2c(c1)CC[C@@H]2NC(=O)[C@H]1C[C@H](c2ccccc2)OC1=O	290211514	25	335.15	3.4
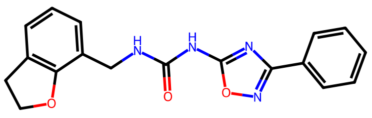	O=C(NCc1cccc2c1OCC2)Nc1nc(-c2ccccc2)no1	1310519045	25	336.12	2.99
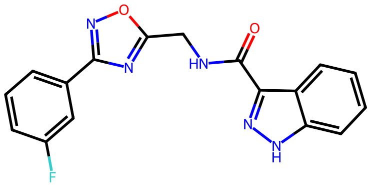	O=C(NCc1nc(-c2cccc(F)c2)no1)c1n[nH]c2ccccc12	366788357	25	337.1	2.68
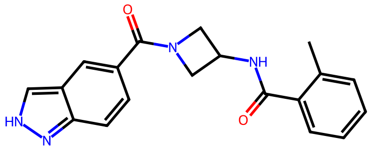	Cc1ccccc1C(=O)NC1CN(C(=O)c2ccc3n[nH]cc3c2)C1	990110770	25	334.14	2.13
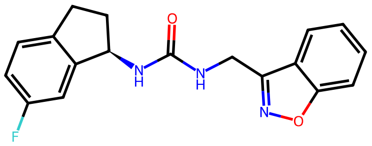	O=C(NCc1noc2ccccc12)N[C@@H]1CCc2ccc(F)cc21	1668676238	24	325.12	3.45
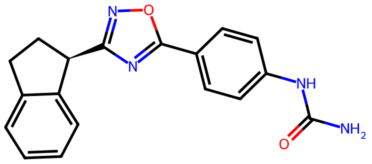	NC(=O)Nc1ccc(-c2nc([C@@H]3CCc4ccccc43)no2)cc1	356583668	24	320.13	3.31
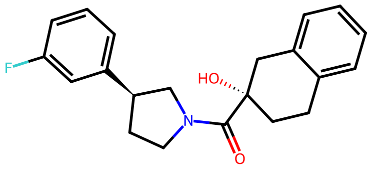	O=C(N1CC[C@@H](c2cccc(F)c2)C1)[C@]1(O)CCc2ccccc2C1	786105134	25	339.16	3.06
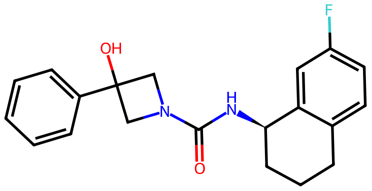	O=C(N[C@@H]1CCCc2ccc(F)cc21)N1CC(O)(c2ccccc2)C1	812207963	25	340.16	3.12

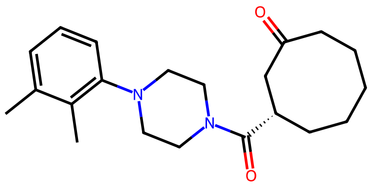	Cc1cccc(N2CCN(C(=O)[C@H]3CCCCCC(=O)C3)CC2)c1C	485433249	25	342.23	3.49
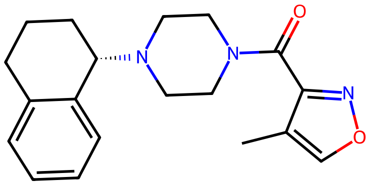	Cc1conc1C(=O)N1CCN([C@H]2CCCc3ccccc32)CC1	957090515	24	325.18	2.82
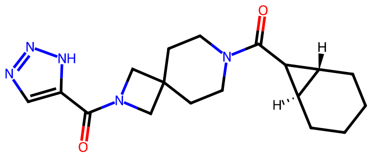	O=C(c1cnn[nH]1)N1CC2(CCN(C(=O)C3[C@@H]4CCCC[C@@H]34)CC2)C1	944898424	25	343.2	1.31
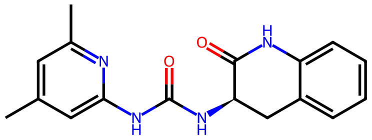	Cc1cc(C)nc(NC(=O)N[C@@H]2Cc3ccccc3NC2=O)c1	1364105975	23	310.14	2.38
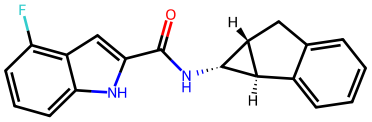	O=C(N[C@H]1[C@@H]2c3ccccc3C[C@H]21)c1cc2c(F)cccc2[nH]1	152294004	23	306.12	3.38
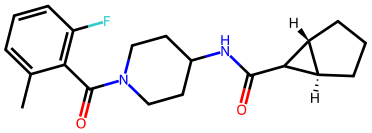	Cc1cccc(F)c1C(=O)N1CCC(NC(=O)C2[C@@H]3CCC[C@@H]23)CC1	947289439	25	344.19	2.9
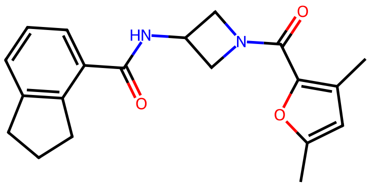	Cc1cc(C)c(C(=O)N2CC(NC(=O)c3cccc4c3CCC4)C2)o1	994403054	25	338.16	2.64
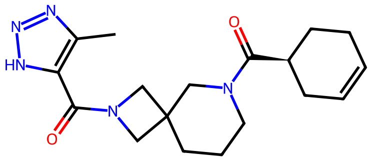	Cc1nn[nH]c1C(=O)N1CC2(CCCN(C(=O)[C@@H]3CC=CCC3)C2)C1	1010766439	25	343.2	1.53

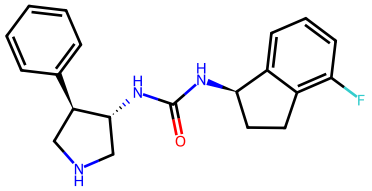	O=C(N[C@@H]1CNC[C@H]1c1ccccc1)N[C@@H]1CCc2c(F)cccc21	568490114	25	339.17	2.87
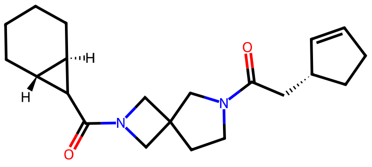	O=C(C[C@@H]1C=CCC1)N1CCC2(C1)CN(C(=O)C1[C@@H]3CCCC[C@@H]13)C2	1018904629	25	342.23	2.84
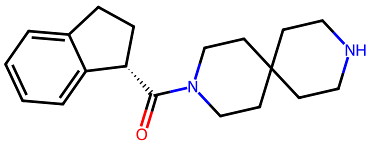	O=C([C@H]1CCc2ccccc21)N1CCC2(CCNCC2)CC1	336209712	22	298.2	2.71
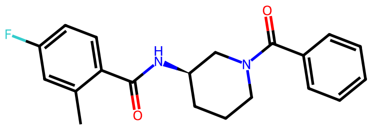	Cc1cc(F)ccc1C(=O)N[C@@H]1CCCN(C(=O)c2ccccc2)C1	949807208	25	340.16	3.17
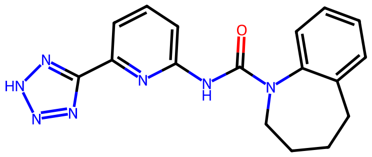	O=C(Nc1cccc(-c2nn[nH]n2)n1)N1CCCCc2ccccc21	1593244096	25	335.15	2.64
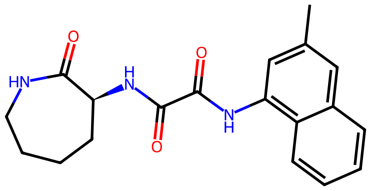	Cc1cc(NC(=O)C(=O)N[C@H]2CCCCNC2=O)c2ccccc2c1	296484594	25	339.16	1.87
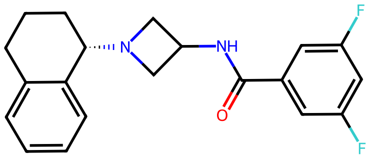	O=C(NC1CN([C@H]2CCCc3ccccc32)C1)c1cc(F)cc(F)c1	1031355770	25	342.15	3.46
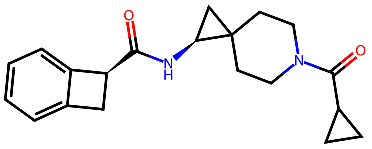	O=C(N[C@H]1CC12CCN(C(=O)C1CC1)CC2)[C@H]1Cc2ccccc21	965007720	24	324.18	2.23

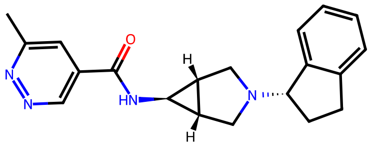	Cc1cc(C(=O)N[C@H]2[C@@H]3CN([C@H]4CCc5ccccc54)C[C@@H]32)cnn1	958675599	25	334.18	2.13
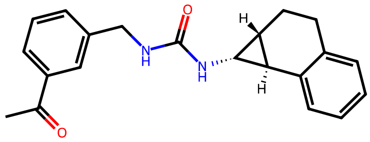	CC(=O)c1cccc(CNC(=O)N[C@H]2[C@@H]3c4ccccc4CC[C@H]32)c1	921557143	25	334.17	3.42
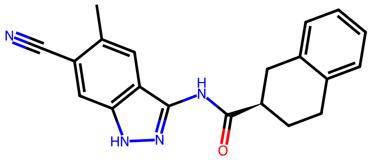	Cc1cc2c(NC(=O)[C@@H]3CCc4ccccc4C3)n[nH]c2cc1C#N	1144854429	25	330.15	3.49
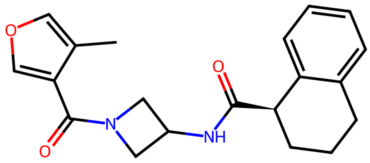	Cc1cocc1C(=O)N1CC(NC(=O)[C@@H]2CCCc3ccccc32)C1	995075079	25	338.16	2.65
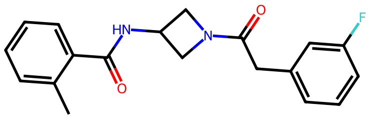	Cc1ccccc1C(=O)NC1CN(C(=O)Cc2cccc(F)c2)C1	990110566	24	326.14	2.32
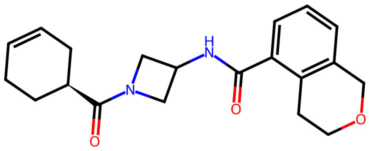	O=C(NC1CN(C(=O)[C@@H]2CC=CCC2)C1)c1cccc2c1CCOC2	996911265	25	340.18	2.06
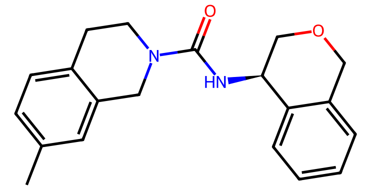	Cc1ccc2c(c1)CN(C(=O)N[C@H]1COCc3ccccc31)CC2	665161701	24	322.17	3.33
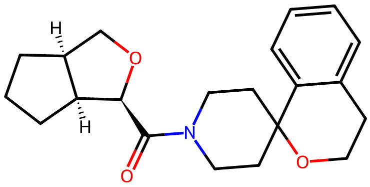	O=C([C@@H]1OC[C@@H]2CCC[C@@H]21)N1CCC2(CC1)OCCc1ccccc12	880252953	25	341.2	2.89

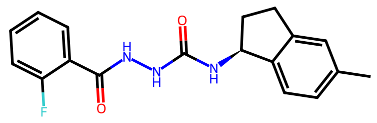	Cc1ccc2c(c1)CC[C@@H]2NC(=O)NNC(=O)c1ccccc1F	56841866	24	327.14	2.77
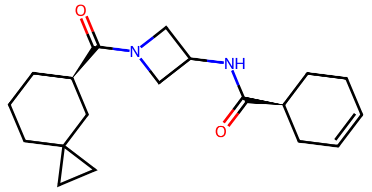	O=C(NC1CN(C(=O)[C@@H]2CCCC3(CC3)C2)C1)[C@@H]1CC=CCC1	990211580	23	316.22	2.64
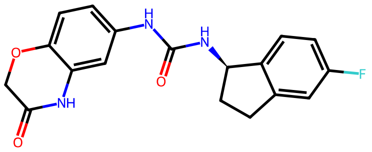	O=C1COc2ccc(NC(=O)N[C@@H]3CCc4cc(F)ccc43)cc2N1	56575963	25	341.12	2.97
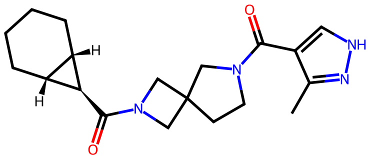	Cc1n[nH]cc1C(=O)N1CCC2(C1)CN(C(=O)[C@@H]1[C@H]3CCCC[C@H]31)C2	1018905920	25	342.21	1.83
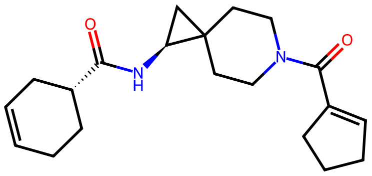	O=C(N[C@H]1CC12CCN(C(=O)C1=CCCC1)CC2)[C@H]1CC=CCC1	963030455	24	328.22	2.95
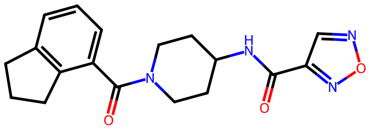	O=C(NC1CCN(C(=O)c2cccc3c2CCC3)CC1)c1cnon1	947160707	25	340.15	1.59
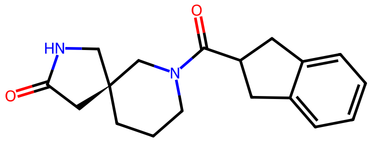	O=C1C[C@@]2(CCCN(C(=O)C3Cc4ccccc4C3)C2)CN1	334709978	22	298.17	1.53
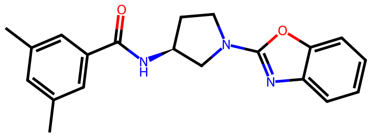	Cc1cc(C)cc(C(=O)N[C@H]2CCN(c3nc4ccccc4o3)C2)c1	1058358163	25	335.16	3.45

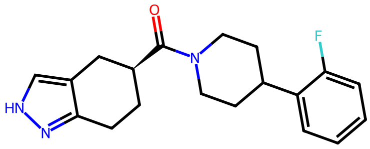	O=C([C@H]1CCc2n[nH]cc2C1)N1CCC(c2ccccc2F)CC1	534553937	24	327.17	3.06
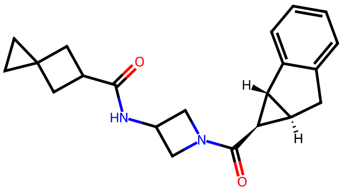	O=C(NC1CN(C(=O)[C@@H]2[C@@H]3Cc4ccccc4[C@H]32)C1)C1CC2(CC2)C1	999346113	25	336.18	2.09
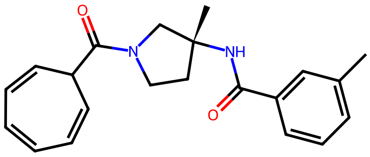	Cc1cccc(C(=O)N[C@]2(C)CCN(C(=O)C3C=CC=CC=C3)C2)c1	956667484	25	336.18	3.01
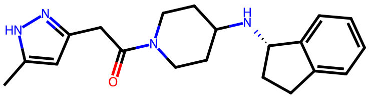	Cc1cc(CC(=O)N2CCC(N[C@H]3CCc4ccccc43)CC2)n[nH]1	990974754	25	338.21	2.53
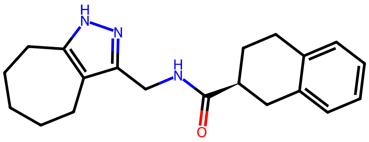	O=C(NCc1n[nH]c2c1CCCCC2)[C@H]1CCc2ccccc2C1	219002510	24	323.2	3.1
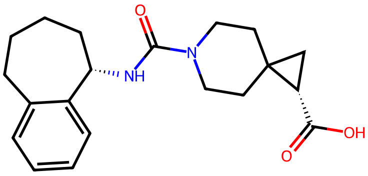	O=C(O)[C@H]1CC12CCN(C(=O)N[C@H]1CCCCc3ccccc31)CC2	739527326	25	342.19	3.35
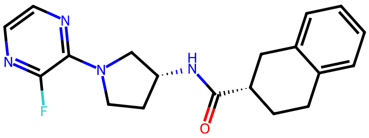	O=C(N[C@@H]1CCN(c2nccnc2F)C1)[C@H]1CCc2ccccc2C1	1058559516	25	340.17	2.12
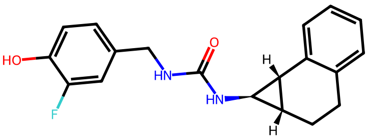	O=C(NCc1ccc(O)c(F)c1)N[C@@H]1[C@H]2CCc3ccccc3[C@H]21	128891470	24	326.14	3.06

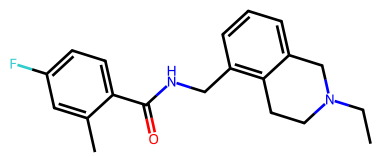	CCN1CCc2c(CNC(=O)c3ccc(F)cc3C)cccc2C1	1051305657	24	326.18	3.44
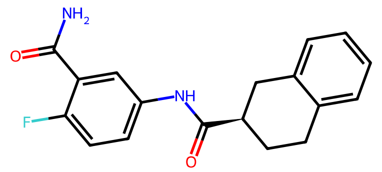	NC(=O)c1cc(NC(=O)[C@@H]2CCc3ccccc3C2)ccc1F	1361333083	23	312.13	2.67
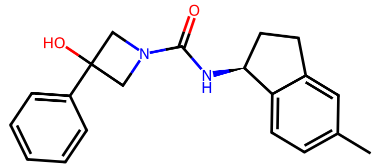	Cc1ccc2c(c1)CC[C@@H]2NC(=O)N1CC(O)(c2ccccc2)C1	812012733	24	322.17	2.9
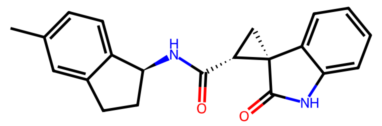	Cc1ccc2c(c1)CC[C@@H]2NC(=O)[C@@H]1C[C@]12C(=O)Nc1ccccc12	930567644	25	332.15	3.01
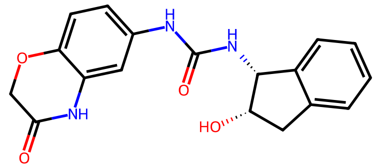	O=C1COc2ccc(NC(=O)N[C@@H]3c4ccccc4C[C@@H]3O)cc2N1	586770903	25	339.12	1.8
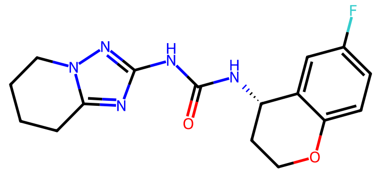	O=C(Nc1nc2n(n1)CCCC2)N[C@H]1CCOc2ccc(F)cc21	333628119	24	331.14	2.4
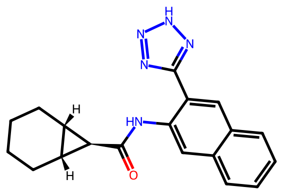	O=C(Nc1cc2ccccc2cc1-c1nn[nH]n1)[C@@H]1[C@H]2CCCC[C@H]21	1593212522	25	333.16	3.39
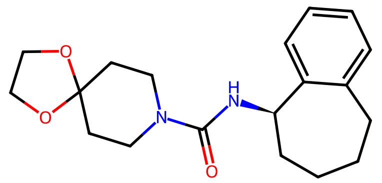	O=C(N[C@@H]1CCCCc2ccccc21)N1CCC2(CC1)OCCO2	65594062	24	330.19	3.0

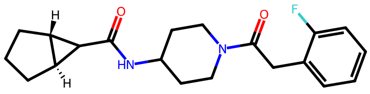	O=C(NC1CCN(C(=O)Cc2ccccc2F)CC1)C1[C@H]2CCC[C@H]12	948348123	25	344.19	2.52
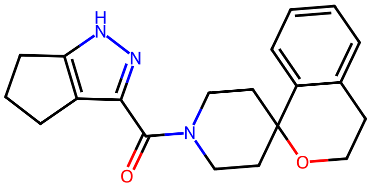	O=C(c1n[nH]c2c1CCC2)N1CCC2(CC1)OCCc1ccccc12	562642425	25	337.18	2.6
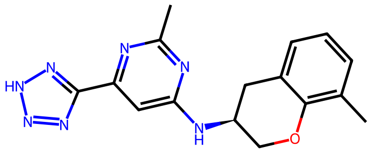	Cc1nc(N[C@@H]2COc3c(C)cccc3C2)cc(-c2nn[nH]n2)n1	1606066133	24	323.15	1.69
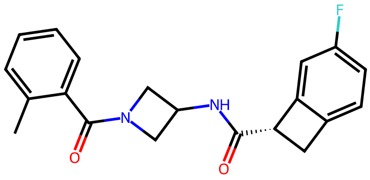	Cc1ccccc1C(=O)N1CC(NC(=O)[C@H]2Cc3ccc(F)cc32)C1	998489078	25	338.14	2.41
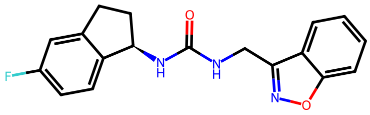	O=C(NCc1noc2ccccc12)N[C@@H]1CCc2cc(F)ccc21	1224628610	24	325.12	3.45
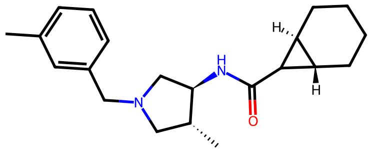	Cc1cccc(CN2C[C@@H](NC(=O)C3[C@H]4CCCC[C@H]34)[C@H](C)C2)c1	1079530626	24	326.24	3.37
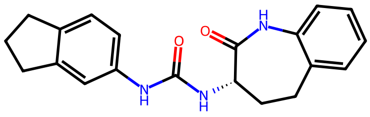	O=C(Nc1ccc2c(c1)CCC2)N[C@H]1CCc2ccccc2NC1=O	653926050	25	335.16	3.25
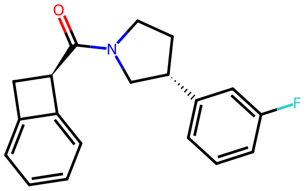	O=C([C@@H]1Cc2ccccc21)N1CC[C@H](c2cccc(F)c2)C1	460157386	22	295.14	3.48

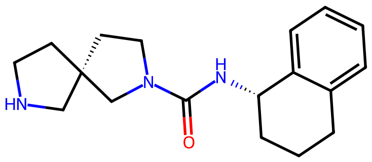	O=C(N[C@H]1CCCc2ccccc21)N1CC[C@]2(CCNC2)C1	335270128	22	299.2	2.46
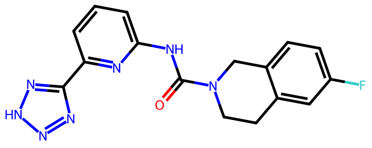	O=C(Nc1cccc(-c2nn[nH]n2)n1)N1CCc2cc(F)ccc2C1	1582644798	25	339.12	1.99
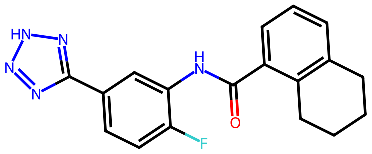	O=C(Nc1cc(-c2nn[nH]n2)ccc1F)c1cccc2c1CCCC2	1582634978	25	337.13	3.14
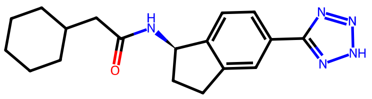	O=C(CC1CCCCC1)N[C@@H]1CCc2cc(-c3nn[nH]n3)ccc21	1592819240	24	325.19	2.94
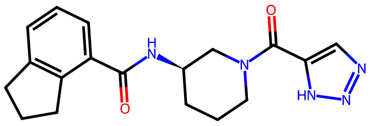	O=C(N[C@@H]1CCCN(C(=O)c2cnn[nH]2)C1)c1cccc2c1CCC2	951314336	25	339.17	1.33
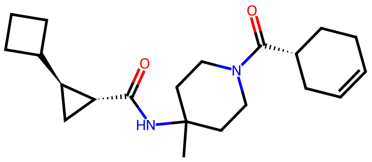	CC1(NC(=O)[C@@H]2C[C@H]2C2CCC2)CCN(C(=O)[C@H]2CC=CCC2)CC1	956382557	25	344.25	3.28
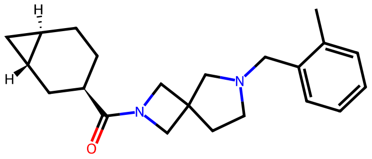	Cc1ccccc1CN1CCC2(C1)CN(C(=O)[C@@H]1CC[C@@H]3C[C@H]3C1)C2	1072879085	25	338.24	3.47
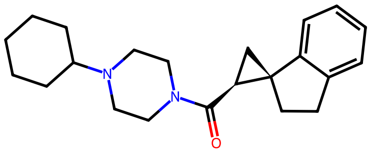	O=C([C@H]1C[C@@]12CCc1ccccc12)N1CCN(C2CCCCC2)CC1	482064720	25	338.24	3.37

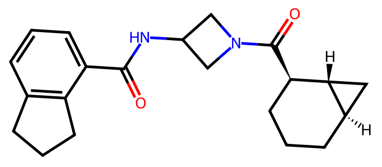	O=C(NC1CN(C(=O)[C@@H]2CCC[C@@H]3C[C@H]32)C1)c1cccc2c1CCC2	994398012	25	338.2	2.55
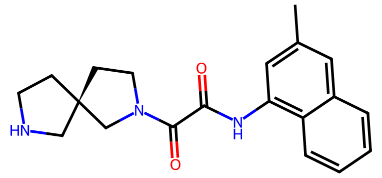	Cc1cc(NC(=O)C(=O)N2CC[C@@]3(CCNC3)C2)c2ccccc2c1	667474034	25	337.18	2.3
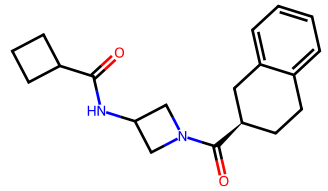	O=C(NC1CN(C(=O)[C@@H]2CCc3ccccc3C2)C1)C1CCC1	517574518	23	312.18	1.92
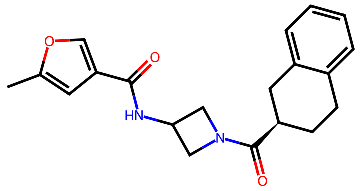	Cc1cc(C(=O)NC2CN(C(=O)[C@@H]3CCc4ccccc4C3)C2)co1	991028494	25	338.16	2.33
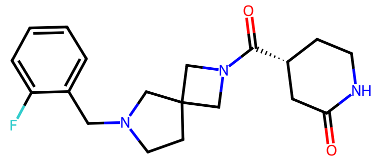	O=C1C[C@H](C(=O)N2CC3(CCN(Cc4ccccc4F)C3)C2)CCN1	1072612545	25	345.19	1.39
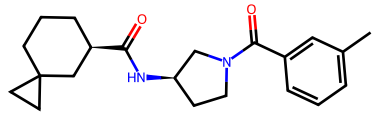	Cc1cccc(C(=O)N2CC[C@@H](NC(=O)[C@@H]3CCCC4(CC4)C3)C2)c1	940429528	25	340.22	3.3
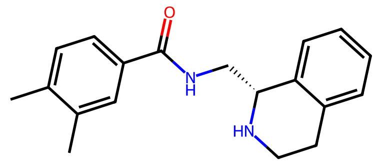	Cc1ccc(C(=O)NC[C@H]2NCCc3ccccc32)cc1C	262562970	22	294.17	2.92
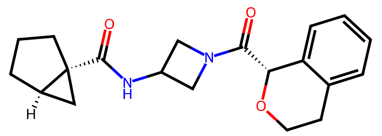	O=C([C@H]1OCCc2ccccc21)N1CC(NC(=O)[C@@]23CCC[C@@H]2C3)C1	997552125	25	340.18	1.82

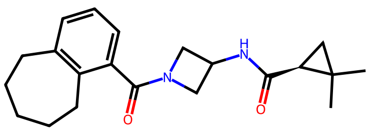	CC1(C)C[C@@H]1C(=O)NC1CN(C(=O)c2cccc3c2CCCCC3)C1	990887461	25	340.22	2.94
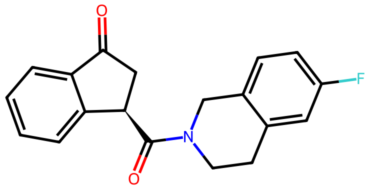	O=C1C[C@@H](C(=O)N2CCc3cc(F)ccc3C2)c2ccccc21	277194102	23	309.12	3.08
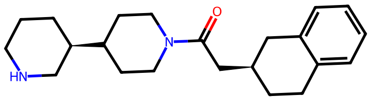	O=C(C[C@@H]1CCc2ccccc2C1)N1CCC([C@@H]2CCCNC2)CC1	658956228	25	340.25	3.42
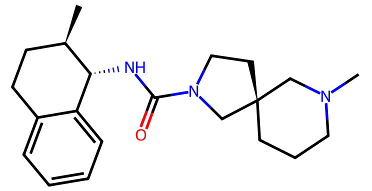	C[C@@H]1CCc2ccccc2[C@H]1NC(=O)N1CC[C@@]2(CCCN(C)C2)C1	458585722	25	341.25	3.44
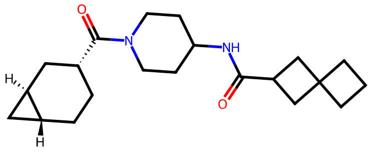	O=C(NC1CCN(C(=O)[C@@H]2CC[C@@H]3C[C@H]3C2)CC1)C1CC2(CC2)C1	947210534	25	344.25	3.11
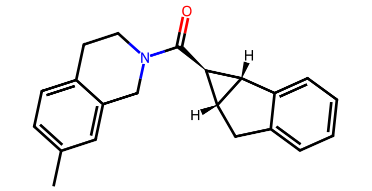	Cc1ccc2c(c1)CN(C(=O)[C@@H]1[C@H]3Cc4ccccc4[C@H]31)CC2	628058151	23	303.16	3.47
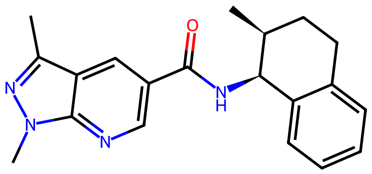	Cc1nn(C)c2ncc(C(=O)N[C@@H]3c4ccccc4CC[C@@H]3C)cc12	179440782	25	334.18	3.33
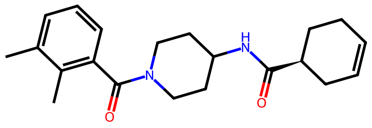	Cc1cccc(C(=O)N2CCC(NC(=O)[C@@H]3CC=CCC3)CC2)c1C	948342621	25	340.22	3.38

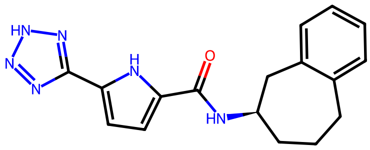	O=C(N[C@@H]1CCCc2ccccc2C1)c1ccc(-c2nn[nH]n2)[nH]1	1606671198	24	322.15	1.87
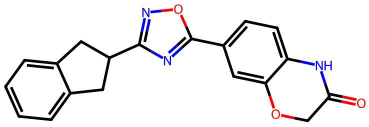	O=C1COc2cc(-c3nc(C4Cc5ccccc5C4)no3)ccc2N1	545672106	25	333.11	2.95
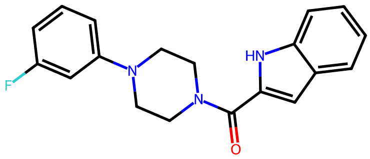	O=C(c1cc2ccccc2[nH]1)N1CCN(c2cccc(F)c2)CC1	618148150	24	323.14	3.27
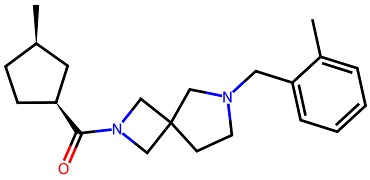	Cc1ccccc1CN1CCC2(C1)CN(C(=O)[C@H]1CC[C@@H](C)C1)C2	1073033270	24	326.24	3.47
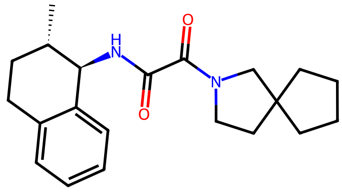	C[C@H]1CCc2ccccc2[C@@H]1NC(=O)C(=O)N1CCC2(CCCC2)C1	807691450	25	340.22	3.22
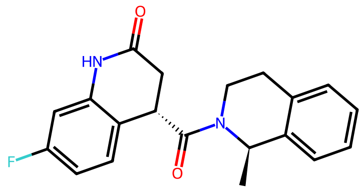	C[C@@H]1c2ccccc2CCN1C(=O)[C@H]1CC(=O)Nc2cc(F)ccc21	171507265	25	338.14	3.4
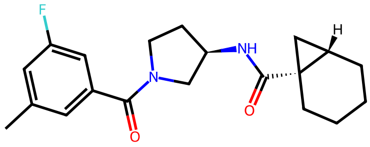	Cc1cc(F)cc(C(=O)N2CC[C@@H](NC(=O)[C@@]34CCCC[C@H]3C4)C2)c1	936243691	25	344.19	3.05
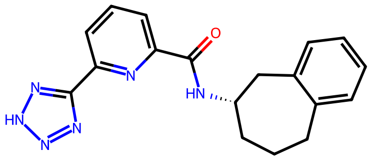	O=C(N[C@H]1CCCc2ccccc2C1)c1cccc(-c2nn[nH]n2)n1	1592985274	25	334.15	1.94

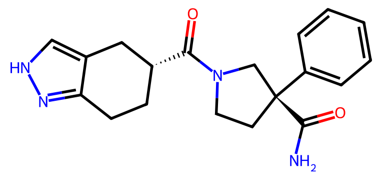	NC(=O)[C@@]1(c2ccccc2)CCN(C(=O)[C@@H]2CCc3n[nH]cc3C2)C1	1363444254	25	338.17	1.17
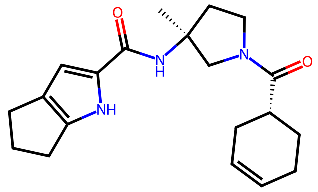	C[C@]1(NC(=O)c2cc3c([nH]2)CCC3)CCN(C(=O)[C@H]2CC=CCC2)C1	957207193	25	341.21	2.58
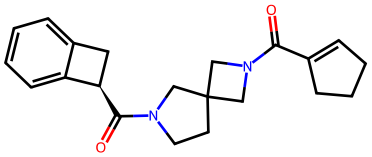	O=C(C1=CCCC1)N1CCC2(CCN(C(=O)[C@@H]3Cc4ccccc43)C2)C1	1018937658	25	336.18	2.5
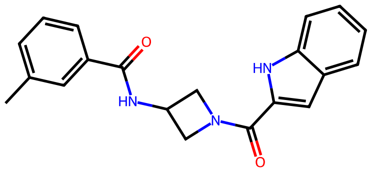	Cc1cccc(C(=O)NC2CN(C(=O)c3cc4ccccc4[nH]3)C2)c1	990143008	25	333.15	2.73
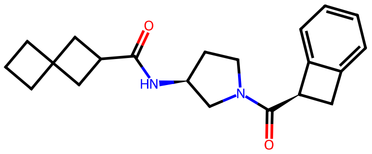	O=C(N[C@H]1CCN(C(=O)[C@@H]2Cc3ccccc32)C1)C1CC2(CCC2)C1	938468950	25	338.2	2.62
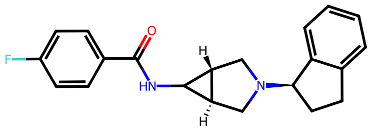	O=C(NC1[C@@H]2CN([C@@H]3CCc4ccccc43)C[C@@H]12)c1ccc(F)cc1	958085099	25	336.16	3.17
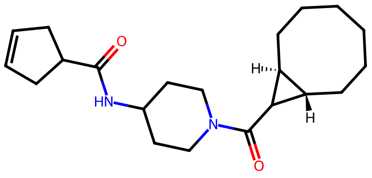	O=C(NC1CCN(C(=O)C2[C@H]3CCCCCC[C@H]23)CC1)C1CC=CC1	948562334	25	344.25	3.28
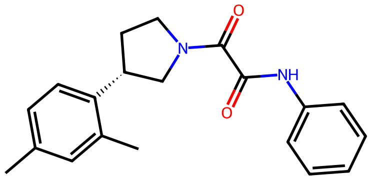	Cc1ccc([C@@H]2CCN(C(=O)C(=O)Nc3ccccc3)C2)c(C)c1	351765207	24	322.17	3.26

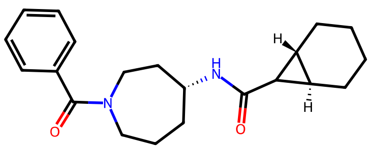	O=C(N[C@@H]1CCCN(C(=O)c2cccc c2)CC1)C1[C@@H]2CCCC[C@@H]1 2	964825995	25	340.22	3.23
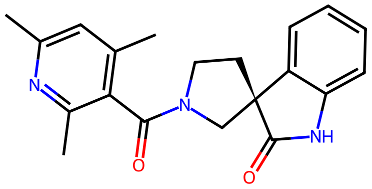	Cc1cc(C)c(C(=O)N2CC[C@]3(C2)C(= O)Nc2ccccc23)c(C)n1	907526234	25	335.16	2.74
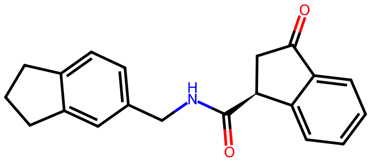	O=C1C[C@H](C(=O)NCc2ccc3c(c2)C CC3)c2ccccc21	1320686685	23	305.14	3.16
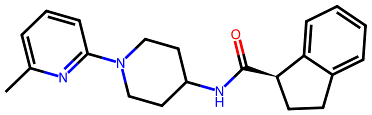	Cc1cccc(N2CCC(NC(=O)[C@@H]3C Cc4ccccc43)CC2)n1	342185504	25	335.2	3.21
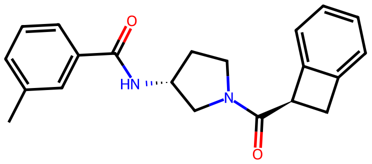	Cc1cccc(C(=O)N[C@@H]2CCN(C(=O)[C@@H]3Cc4ccccc43)C2)c1	939249080	25	334.17	2.67
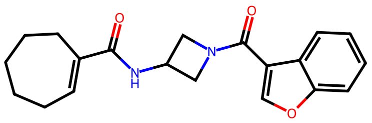	O=C(NC1CN(C(=O)c2coc3ccccc23)C 1)C1=CCCCCC1	991004136	25	338.16	3.26
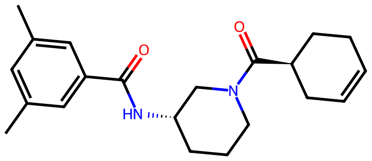	Cc1cc(C)cc(C(=O)N[C@H]2CCCN(C(=O)[C@@H]3CC=CCC3)C2)c1	949985076	25	340.22	3.38
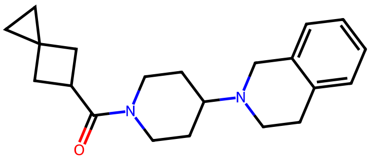	O=C(C1CC2(CC2)C1)N1CCC(N2CCc 3ccccc3C2)CC1	667386871	24	324.22	3.23

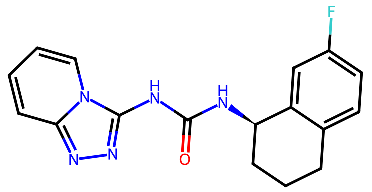	O=C(Nc1nnc2ccccn12)N[C@@H]1CCCc2ccc(F)cc21	93256292	24	325.13	3.07
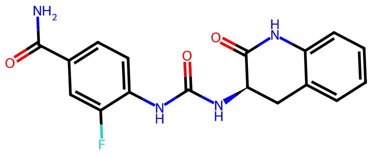	NC(=O)c1ccc(NC(=O)N[C@@H]2Cc3ccccc3NC2=O)c(F)c1	1365041730	25	342.11	1.61
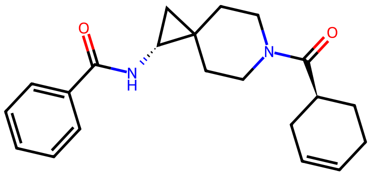	O=C(N[C@@H]1CC12CCN(C(=O)[C@@H]1CC=CCC1)CC2)c1ccccc1	962784349	25	338.2	3.15
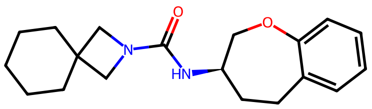	O=C(N[C@@H]1CCc2ccccc2OC1)N1CC2(CCCCC2)C1	802404942	23	314.2	3.36
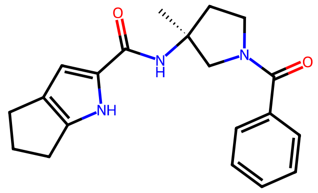	C[C@]1(NC(=O)c2cc3c([nH]2)CCC3)CCN(C(=O)c2ccccc2)C1	957207576	25	337.18	2.54
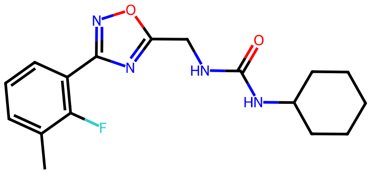	Cc1cccc(-c2noc(CNC(=O)NC3CCCCC3)n2)c1F	485857977	24	332.16	3.32
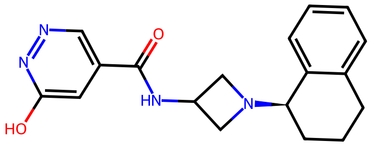	O=C(NC1CN([C@@H]2CCCc3ccccc32)C1)c1cnnc(O)c1	1030485673	24	324.16	1.67
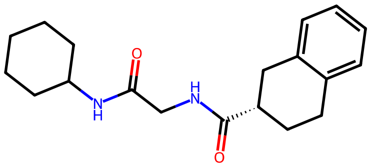	O=C(CNC(=O)[C@H]1CCc2ccccc2C1)NC1CCCCC1	565053071	23	314.2	2.36

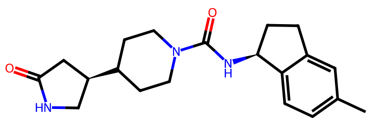	Cc1ccc2c(c1)CC[C@@H]2NC(=O)N1CCC([C@H]2CNC(=O)C2)CC1	1553385643	25	341.21	2.54
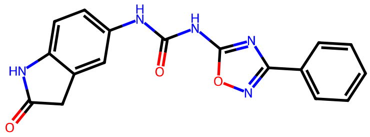	O=C1Cc2cc(NC(=O)Nc3nc(-c4ccccc4)no3)ccc2N1	1660466422	25	335.1	2.88
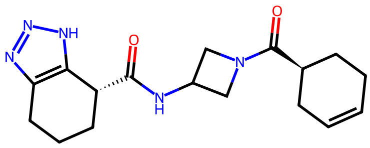	O=C(NC1CN(C(=O)[C@@H]2CC=CC2)C1)[C@@H]1CCCc2nn[nH]c21	998568051	24	329.19	0.91
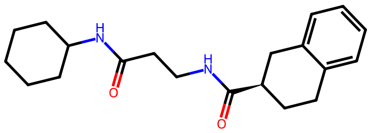	O=C(CCNC(=O)[C@@H]1CCc2ccccc2C1)NC1CCCCC1	649642196	24	328.22	2.75
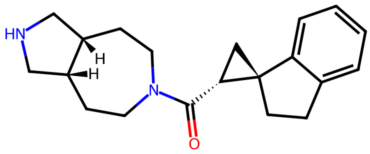	O=C([C@@H]1C[C@@]12CCc1ccccc12)N1CC[C@@H]2CNC[C@@H]2CC1	261680100	23	310.2	2.35
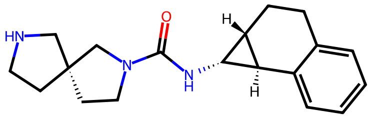	O=C(N[C@H]1[C@@H]2c3ccccc3CC[C@H]21)N1CC[C@@]2(CCNC2)C1	417909170	23	311.2	2.11
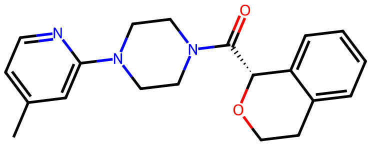	Cc1ccnc(N2CCN(C(=O)[C@H]3OCCc4ccccc43)CC2)c1	124813170	25	337.18	2.35
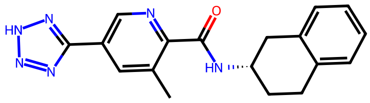	Cc1cc(-c2nn[nH]n2)cnc1C(=O)N[C@H]1CCc2ccccc2C1	1605549018	25	334.15	1.86

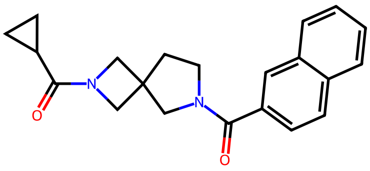	O=C(c1ccc2ccccc2c1)N1CCC2(C1)CN(C(=O)C1CC1)C2	1018666735	25	334.17	2.92
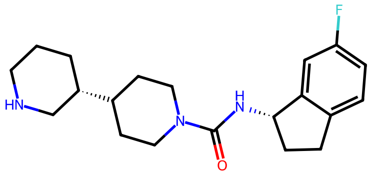	O=C(N[C@H]1CCc2ccc(F)cc21)N1CCC([C@H]2CCCNC2)CC1	659452474	25	345.22	3.23
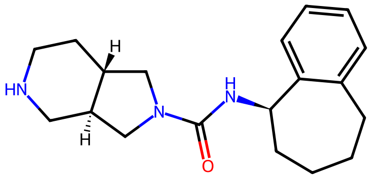	O=C(N[C@@H]1CCCCc2ccccc21)N1C[C@H]2CCNC[C@@H]2C1	583252284	23	313.22	2.71
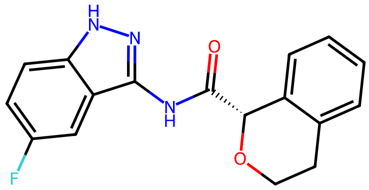	O=C(Nc1n[nH]c2ccc(F)cc12)[C@H]1OCCc2ccccc21	442152245	23	311.11	2.95
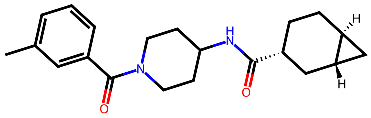	Cc1cccc(C(=O)N2CCC(NC(=O)[C@@H]3CC[C@H]4C[C@@H]4C3)CC2)c1	943836127	25	340.22	3.15
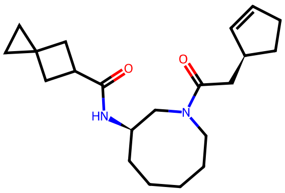	O=C(N[C@@H]1CCCCCN(C(=O)C[C@H]2C=CCC2)C1)C1CC2(CC2)C1	986878687	25	344.25	3.42
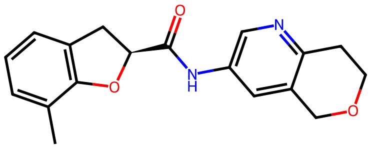	Cc1cccc2c1O[C@H](C(=O)Nc1cnc3c(c1)COCC3)C2	649409278	23	310.13	2.4
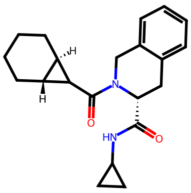	O=C(NC1CC1)[C@H]1Cc2ccccc2CN1C(=O)C1[C@@H]2CCCC[C@@H]12	650214803	25	338.2	2.65

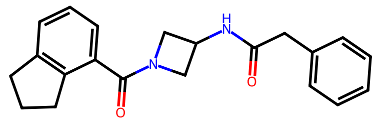	O=C(Cc1ccccc1)NC1CN(C(=O)c2cccc3c2CCC3)C1	995109393	25	334.17	2.36
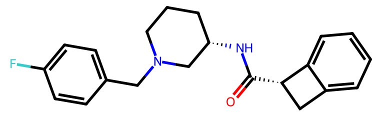	O=C(N[C@H]1CCCN(Cc2ccc(F)cc2)C1)[C@H]1Cc2ccccc21	1008355308	25	338.18	3.25
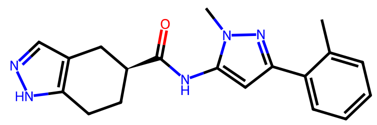	Cc1ccccc1-c1cc(NC(=O)[C@H]2CCc3[nH]ncc3C2)n(C)n1	270161179	25	335.17	2.86
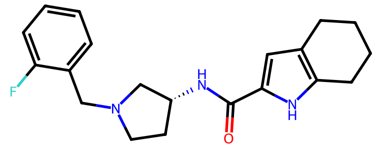	O=C(N[C@@H]1CCN(Cc2ccccc2F)C1)c1cc2c([nH]1)CCCC2	1015953770	25	341.19	3.04
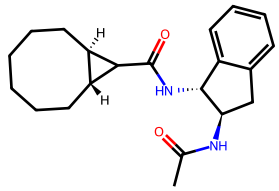	CC(=O)N[C@@H]1Cc2ccccc2[C@H]1NC(=O)C1[C@@H]2CCCCCC[C@@H]12	1001896699	25	340.22	3.12
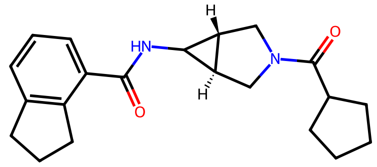	O=C(NC1[C@@H]2CN(C(=O)C3CCCC3)C[C@@H]12)c1cccc2c1CCC2	1013298157	25	338.2	2.55
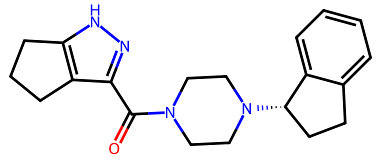	O=C(c1n[nH]c2c1CCC2)N1CCN([C@H]2CCc3ccccc32)CC1	949062968	25	336.2	2.34
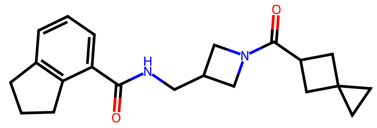	O=C(NCC1CN(C(=O)C2CC3(CC3)C2)C1)c1cccc2c1CCC2	1002703080	25	338.2	2.55

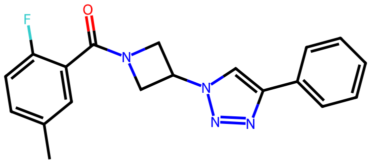	Cc1ccc(F)c(C(=O)N2CC(n3cc(-c4ccccc4)nn3)C2)c1	368602711	25	336.14	3.09
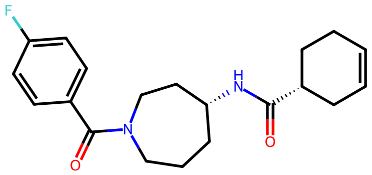	O=C(N[C@@H]1CCCN(C(=O)c2ccc(F)cc2)CC1)[C@H]1CC=CCC1	964747729	25	344.19	3.29
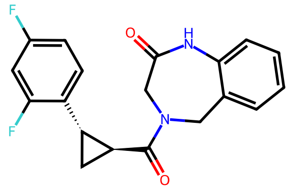	O=C1CN(C(=O)[C@H]2C[C@@H]2c2ccc(F)cc2F)Cc2ccccc2N1	655315435	25	342.12	3.05
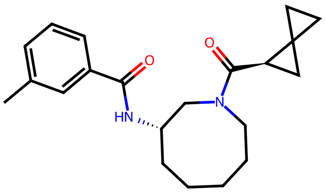	Cc1cccc(C(=O)N[C@H]2CCCCCN(C(=O)[C@@H]3CC34CC4)C2)c1	987168604	25	340.22	3.3
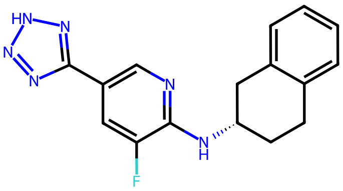	Fc1cc(-c2nn[nH]n2)cnc1N[C@H]1CCc2ccccc2C1	1592639945	23	310.13	2.37
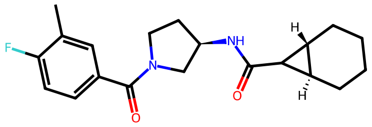	Cc1cc(C(=O)N2CC[C@@H](NC(=O)C3[C@@H]4CCCC[C@@H]34)C2)ccc1F	939895630	25	344.19	2.9
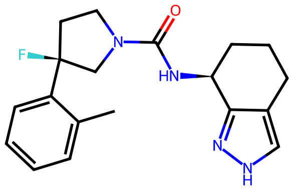	Cc1ccccc1[C@]1(F)CCN(C(=O)N[C@H]2CCCc3c[nH]nc32)C1	554600164	25	342.19	3.38
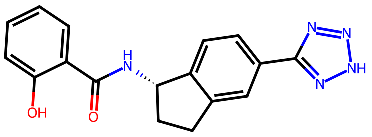	O=C(N[C@H]1CCc2cc(-c3nn[nH]n3)ccc21)c1ccccc1O	1606684166	24	321.12	1.99

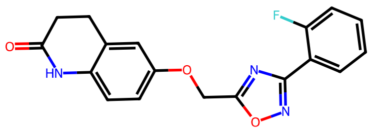	O=C1CCc2cc(OCc3nc(-c4ccccc4F)no3)ccc2N1	355796895	25	339.1	3.34
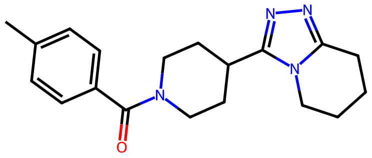	Cc1ccc(C(=O)N2CCC(c3nnc4n3CCCC4)CC2)cc1	274637940	24	324.2	2.94
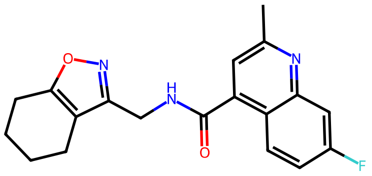	Cc1cc(C(=O)NCc2noc3c2CCCC3)c2cc(F)cc2n1	1327062598	25	339.14	3.48
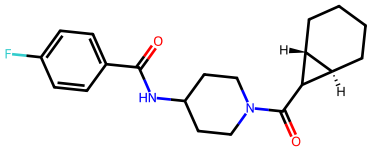	O=C(NC1CCN(C(=O)C2[C@@H]3CCCC[C@@H]23)CC1)c1ccc(F)cc1	468508927	25	344.19	2.98
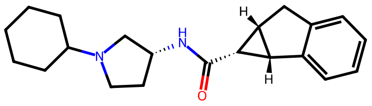	O=C(N[C@@H]1CCN(C2CCCCC2)C1)[C@H]1[C@H]2c3ccccc3C[C@H]21	628092776	24	324.22	3.1
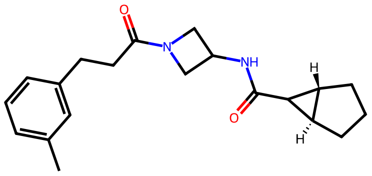	Cc1cccc(CCC(=O)N2CC(NC(=O)C3[C@@H]4CCC[C@@H]34)C2)c1	990707485	24	326.2	2.3
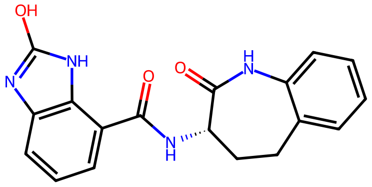	O=C(N[C@H]1CCc2ccccc2NC1=O)c1cccc2nc(O)[nH]c12	1583191152	25	336.12	1.95
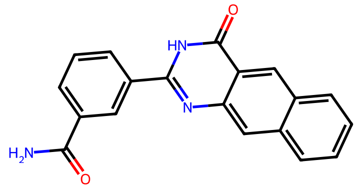	NC(=O)c1cccc(-c2nc3cc4ccccc4cc3c(=O)[nH]2)c1	77256602	24	315.1	2.84

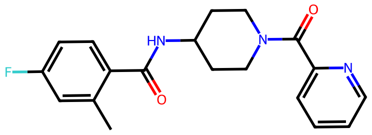	Cc1cc(F)ccc1C(=O)NC1CCN(C(=O)c2ccccn2)CC1	943590363	25	341.15	2.56
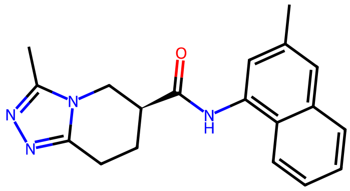	Cc1cc(NC(=O)[C@H]2CCc3nnc(C)n3C2)c2ccccc2c1	651026219	24	320.16	3.25
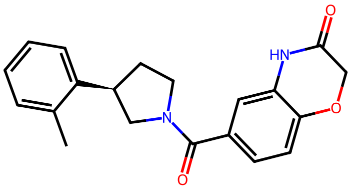	Cc1ccccc1[C@H]1CCN(C(=O)c2ccc3c(c2)NC(=O)CO3)C1	299718693	25	336.15	2.96
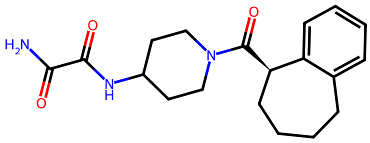	NC(=O)C(=O)NC1CCN(C(=O)[C@@H]2CCCCc3ccccc32)CC1	947145654	25	343.19	1.09
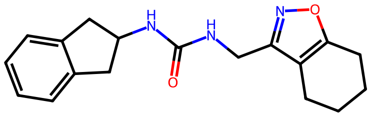	O=C(NCc1noc2c1CCCC2)NC1Cc2ccccc2C1	288089205	23	311.16	2.52
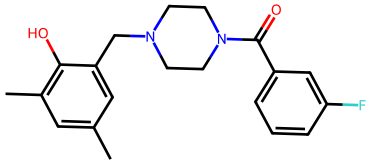	Cc1cc(C)c(O)c(CN2CCN(C(=O)c3cccc(F)c3)CC2)c1	164062820	25	342.17	3.11
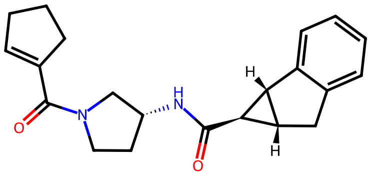	O=C(N[C@@H]1CCN(C(=O)C2=CCCC2)C1)[C@@H]1[C@H]2Cc3ccccc3[C@H]21	940495253	25	336.18	2.4
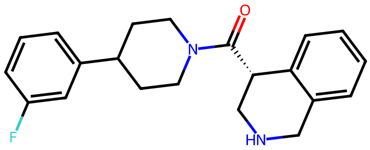	O=C([C@H]1CNCc2ccccc21)N1CCC(c2cccc(F)c2)CC1	1341239464	25	338.18	3.42

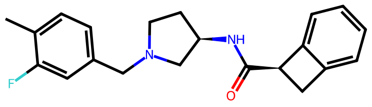	Cc1ccc(CN2CC[C@@H](NC(=O)[C@@H]3Cc4ccccc43)C2)cc1F	1015634520	25	338.18	3.16
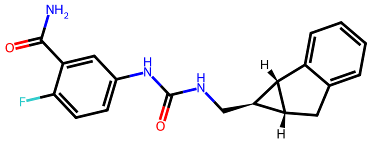	NC(=O)c1cc(NC(=O)NC[C@@H]2[C@H]3Cc4ccccc4[C@H]32)ccc1F	121680102	25	339.14	2.63
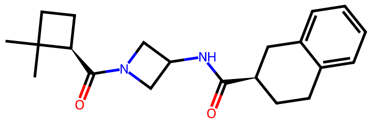	CC1(C)CC[C@H]1C(=O)N1CC(NC(=O)[C@@H]2CCc3ccccc3C2)C1	1000545825	25	340.22	2.55
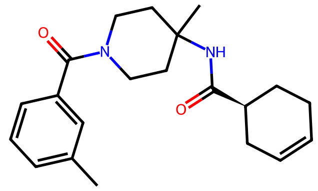	Cc1cccc(C(=O)N2CCC(C)(NC(=O)[C@@H]3CC=CCC3)CC2)c1	956288785	25	340.22	3.46
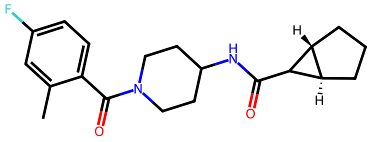	Cc1cc(F)ccc1C(=O)N1CCC(NC(=O)C2[C@@H]3CCC[C@@H]23)CC1	947646361	25	344.19	2.9
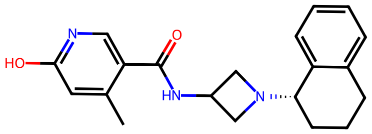	Cc1cc(O)ncc1C(=O)NC1CN([C@H]2CCCc3ccccc32)C1	1030933557	25	337.18	2.59
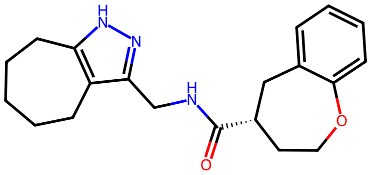	O=C(NCc1n[nH]c2c1CCCCC2)[C@H]1CCOc2ccccc2C1	670467222	25	339.19	2.94
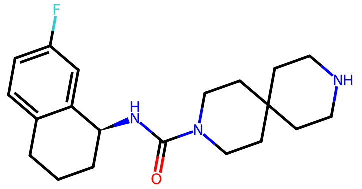	O=C(N[C@H]1CCCc2ccc(F)cc21)N1CCC2(CCNCC2)CC1	418115315	25	345.22	3.38

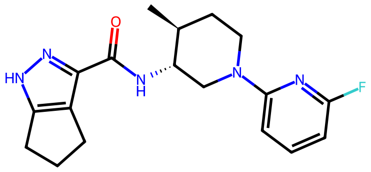	C[C@H]1CCN(c2cccc(F)n2)C[C@@H]1NC(=O)c1n[nH]c2c1CCC2	1068000589	25	343.18	2.08
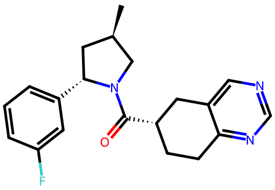	C[C@@H]1C[C@@H](c2cccc(F)c2)N(C(=O)[C@H]2CCc3ncncc3C2)C1	648798079	25	339.17	3.33
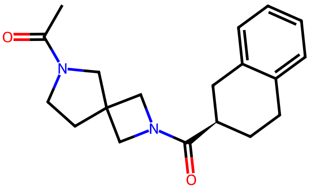	CC(=O)N1CCC2(C1)CN(C(=O)[C@@H]1CCc3ccccc3C1)C2	1019491441	23	312.18	1.87
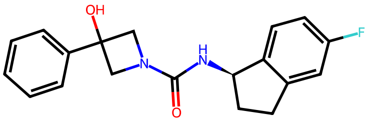	O=C(N[C@@H]1CCc2cc(F)ccc21)N1CC(O)(c2ccccc2)C1	812012556	24	326.14	2.73
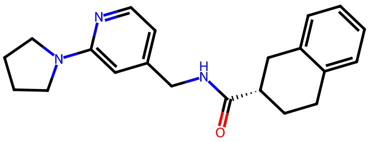	O=C(NCc1ccnc(N2CCCC2)c1)[C@H]1CCc2ccccc2C1	570806118	25	335.2	3.1
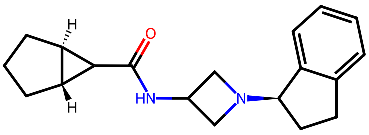	O=C(NC1CN([C@@H]2CCc3ccccc32)C1)C1[C@@H]2CCC[C@@H]12	1030201046	22	296.19	2.52
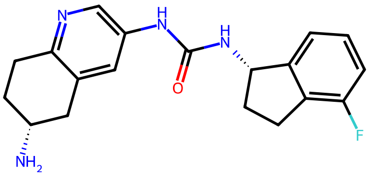	N[C@@H]1CCc2ncc(NC(=O)N[C@H]3CCc4c(F)cccc43)cc2C1	333286795	25	340.17	2.85
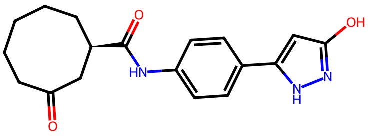	O=C1CCCCC[C@@H](C(=O)Nc2ccc(-c3cc(O)n[nH]3)cc2)C1	565013425	24	327.16	3.26

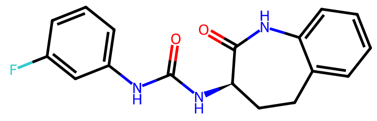	O=C(Nc1cccc(F)c1)N[C@@H]1CCc2ccccc2NC1=O	1202237215	23	313.12	2.9
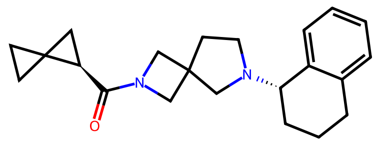	O=C([C@@H]1CC12CC2)N1CC2(CCN([C@H]3CCCc4ccccc43)C2)C1	1073012102	25	336.22	3.4
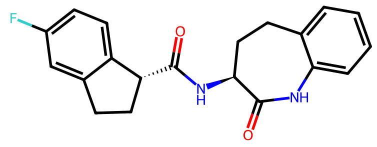	O=C1Nc2ccccc2CC[C@@H]1NC(=O)[C@@H]1CCc2cc(F)ccc21	616691822	25	338.14	2.93
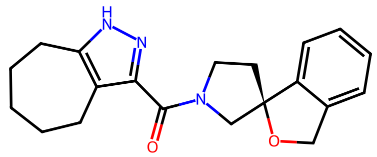	O=C(c1n[nH]c2c1CCCCC2)N1CC[C@]2(C1)OCc1ccccc12	188741558	25	337.18	2.95
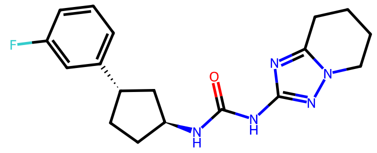	O=C(Nc1nc2n(n1)CCCC2)N[C@H]1CC[C@H](c2cccc(F)c2)C1	580161372	25	343.18	3.21
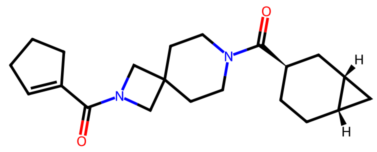	O=C(C1=CCCC1)N1CC2(CCN(C(=O)[C@@H]3CC[C@H]4C[C@H]4C3)CC2)C1	944966799	25	342.23	2.98
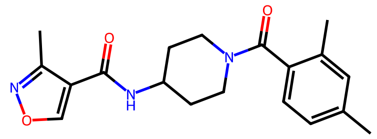	Cc1ccc(C(=O)N2CCC(NC(=O)c3conc3C)CC2)c(C)c1	948346760	25	341.17	2.63
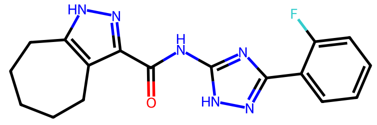	O=C(Nc1nc(-c2ccccc2F)n[nH]1)c1n[nH]c2c1CCCCC2	188142041	25	340.14	2.86

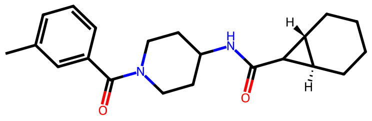	Cc1cccc(C(=O)N2CCC(NC(=O)C3[C@@H]4CCCC[C@@H]34)CC2)c1	943805878	25	340.22	3.15
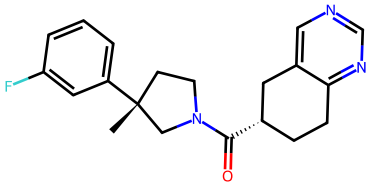	C[C@@]1(c2cccc(F)c2)CCN(C(=O)[C@H]2CCc3ncncc3C2)C1	650218236	25	339.17	2.91
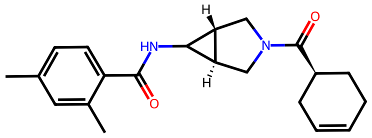	Cc1ccc(C(=O)NC2[C@@H]3CN(C(=O)[C@@H]4CC=CCC4)C[C@@H]23)c(C)c1	1013775083	25	338.2	2.85
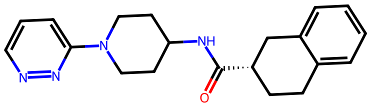	O=C(NC1CCN(c2cccnn2)CC1)[C@H]1CCc2ccccc2C1	565049585	25	336.2	2.37
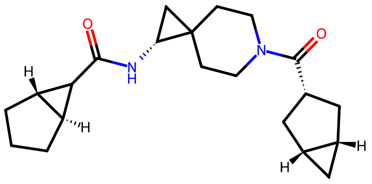	O=C(N[C@@H]1CC12CCN(C(=O)[C@@H]1C[C@@H]3C[C@@H]3C1)CC2)C1[C@H]2CCC[C@H]12	963491145	25	342.23	2.58
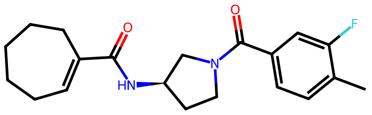	Cc1ccc(C(=O)N2CC[C@@H](NC(=O)C3=CCCCCC3)C2)cc1F	940159512	25	344.19	3.36
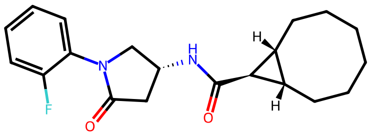	O=C(N[C@@H]1CC(=O)N(c2ccccc2F)C1)[C@@H]1[C@H]2CCCCCC[C@H]21	1460432392	25	344.19	3.26
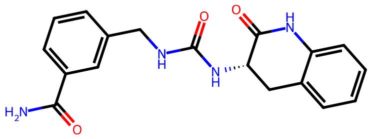	NC(=O)c1cccc(CNC(=O)N[C@H]2Cc3ccccc3NC2=O)c1	1414530159	25	338.14	1.15

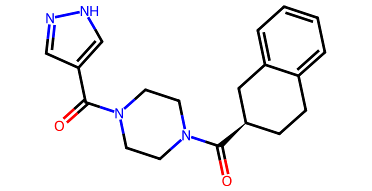	O=C(c1cn[nH]c1)N1CCN(C(=O)[C@@H]2CCc3ccccc3C2)CC1	1010446614	25	338.17	1.5
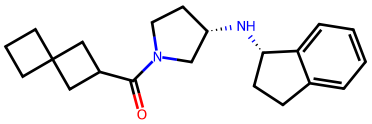	O=C(C1CC2(CCC2)C1)N1CC[C@H](N[C@H]2CCc3ccccc32)C1	1019261482	24	324.22	3.44
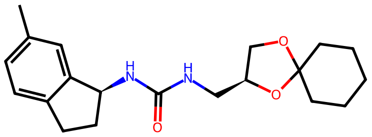	Cc1ccc2c(c1)[C@@H](NC(=O)NC[C@H]1COC3(CCCCC3)O1)CC2	775738032	25	344.21	3.36
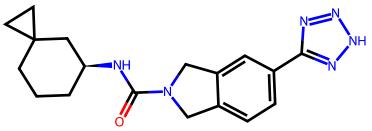	O=C(N[C@H]1CCCC2(CC2)C1)N1Cc2ccc(-c3nn[nH]n3)cc2C1	1592979464	25	338.19	2.61
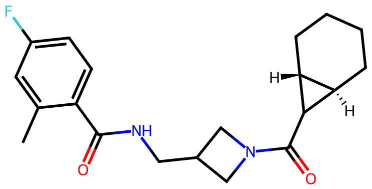	Cc1cc(F)ccc1C(=O)NCC1CN(C(=O)C2[C@@H]3CCCC[C@@H]23)C1	1003487525	25	344.19	2.76
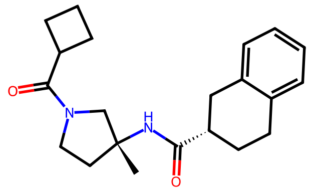	C[C@@]1(NC(=O)[C@H]2CCc3ccccc3C2)CCN(C(=O)C2CCC2)C1	957727570	25	340.22	2.7
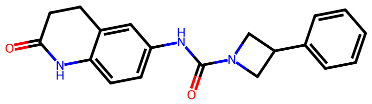	O=C1CCc2cc(NC(=O)N3CC(c4ccccc4)C3)ccc2N1	639506643	24	321.15	3.2
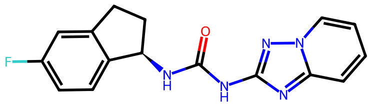	O=C(Nc1nc2ccccn2n1)N[C@@H]1CCc2cc(F)ccc21	183049032	23	311.12	2.68

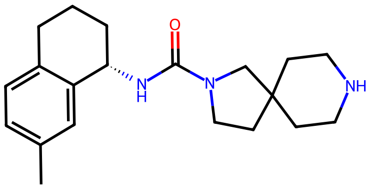	Cc1ccc2c(c1)[C@@H](NC(=O)N1CCC3(CCNCC3)C1)CCC2	418195475	24	327.23	3.16
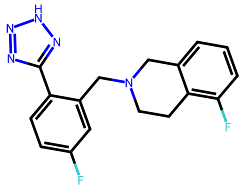	Fc1ccc(-c2nn[nH]n2)c(CN2CCc3c(F)cccc3C2)c1	826373461	24	327.13	2.7
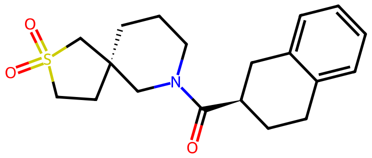	O=C([C@@H]1CCc2ccccc2C1)N1CCC[C@@]2(CCS(=O)(=O)C2)C1	576640526	24	347.16	2.22
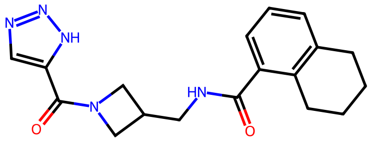	O=C(NCC1CN(C(=O)c2cnn[nH]2)C1)c1cccc2c1CCCC2	1002099123	25	339.17	1.19
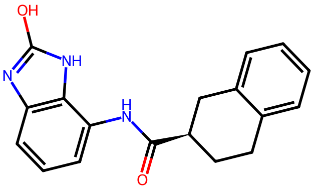	O=C(Nc1cccc2nc(O)[nH]c12)[C@@H]1CCc2ccccc2C1	1641882707	23	307.13	3.01
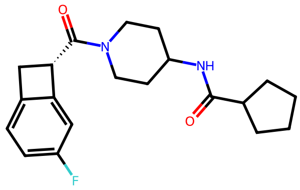	O=C(NC1CCN(C(=O)[C@H]2Cc3ccc(F)cc32)CC1)C1CCCC1	948024320	25	344.19	2.76
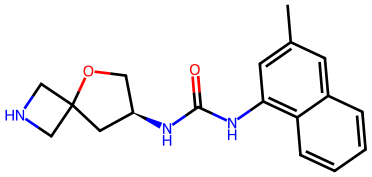	Cc1cc(NC(=O)N[C@@H]2COC3(CNC3)C2)c2ccccc2c1	1579630980	23	311.16	2.4
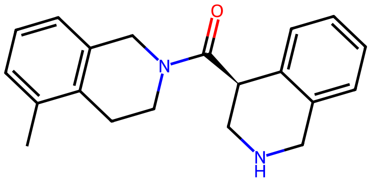	Cc1cccc2c1CCN(C(=O)[C@@H]1CNCc3ccccc31)C2	828739820	23	306.17	2.77

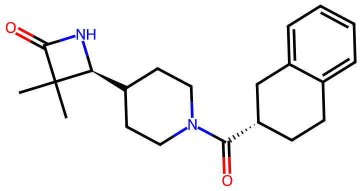	CC1(C)C(=O)N[C@H]1C1CCN(C(=O)[C@H]2CCc3ccccc3C2)CC1	577184882	25	340.22	2.55
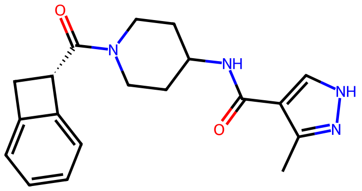	Cc1n[nH]cc1C(=O)NC1CCN(C(=O)[C@H]2Cc3ccccc32)CC1	948062626	25	338.17	1.78
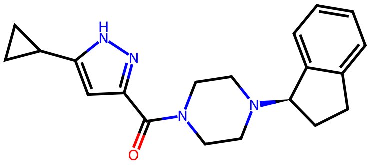	O=C(c1cc(C2CC2)[nH]n1)N1CCN([C@@H]2CCc3ccccc32)CC1	950671886	25	336.2	2.73
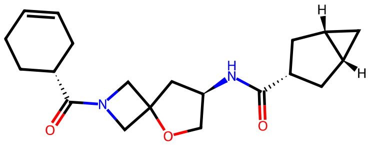	O=C(N[C@H]1COC2(C1)CN(C(=O)[C@H]1CC=CCC1)C2)[C@@H]1C[C@@H]2C[C@@H]2C1	965785058	25	344.21	1.87
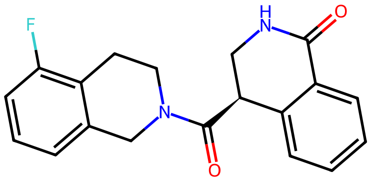	O=C1NC[C@H](C(=O)N2CCc3c(F)cccc3C2)c2ccccc21	860435419	24	324.13	2.24
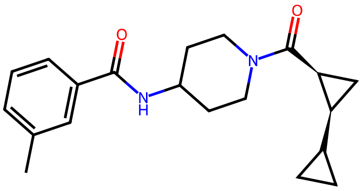	Cc1cccc(C(=O)NC2CCN(C(=O)[C@H]3C[C@H]3C3CC3)CC2)c1	1549880909	24	326.2	2.76
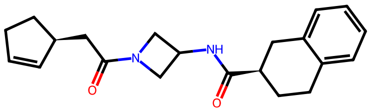	O=C(NC1CN(C(=O)C[C@H]2C=CCC2)C1)[C@@H]1CCc2ccccc2C1	1000541692	25	338.2	2.47
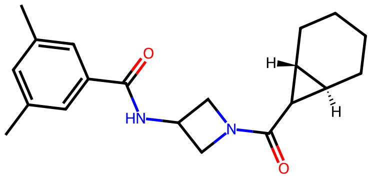	Cc1cc(C)cc(C(=O)NC2CN(C(=O)C3[C@@H]4CCCC[C@@H]34)C2)c1	999490012	24	326.2	2.68

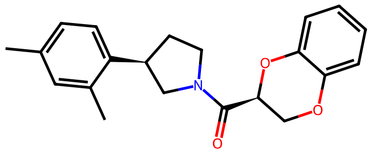	Cc1ccc([C@H]2CCN(C(=O)[C@@H]3COc4ccccc4O3)C2)c(C)c1	348808358	25	337.17	3.46
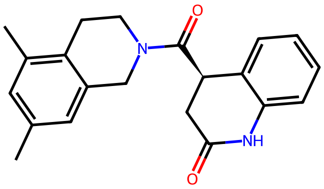	Cc1cc(C)c2c(c1)CN(C(=O)[C@@H]1CC(=O)Nc3ccccc31)CC2	187793684	25	334.17	3.31
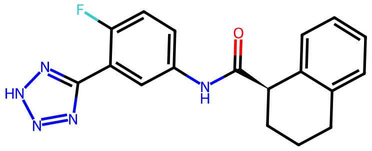	O=C(Nc1ccc(F)c(-c2nn[nH]n2)c1)[C@@H]1CCCc2ccccc21	738162792	25	337.13	3.06
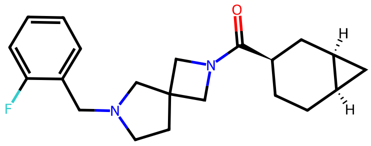	O=C([C@@H]1CC[C@@H]2C[C@@H]2C1)N1CC2(CCN(Cc3ccccc3F)C2)C1	1072879371	25	342.21	3.3
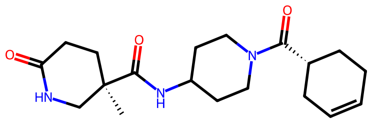	C[C@@]1(C(=O)NC2CCN(C(=O)[C@H]3CC=CCC3)CC2)CCC(=O)NC1	944126595	25	347.22	1.37
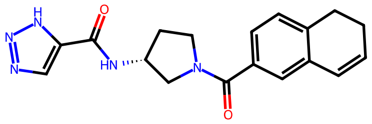	O=C(N[C@@H]1CCN(C(=O)c2ccc3c(c2)C=CCC3)C1)c1cnn[nH]1	941443324	25	337.15	1.41
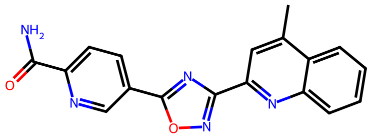	Cc1cc(-c2noc(-c3ccc(C(N)=O)nc3)n2)nc2ccccc12	350778847	25	331.11	2.75
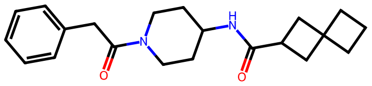	O=C(NC1CCN(C(=O)Cc2ccccc2)CC1)C1CC2(CCC2)C1	947344793	25	340.22	2.92

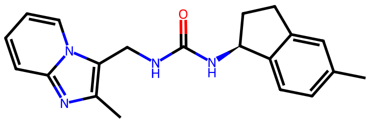	Cc1ccc2c(c1)CC[C@@H]2NC(=O)NCc1c(C)nc2ccccn12	892278033	25	334.18	3.44
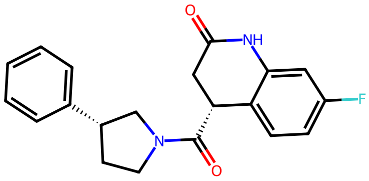	O=C1C[C@@H](C(=O)N2CC[C@H](c3ccccc3)C2)c2ccc(F)cc2N1	121578824	25	338.14	3.27
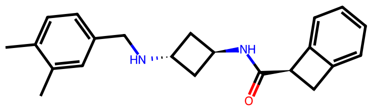	Cc1ccc(CN[C@H]2C[C@H](NC(=O)[C@@H]3Cc4ccccc43)C2)cc1C	1020532292	25	334.2	3.38
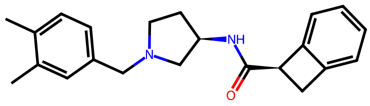	Cc1ccc(CN2CC[C@@H](NC(=O)[C@@H]3Cc4ccccc43)C2)cc1C	1015636501	25	334.2	3.33
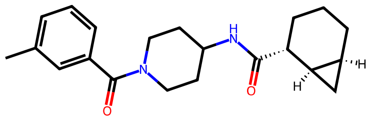	Cc1cccc(C(=O)N2CCC(NC(=O)[C@@H]3CCC[C@H]4C[C@H]43)CC2)c1	943833614	25	340.22	3.15
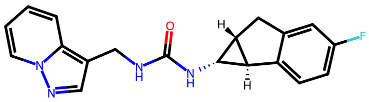	O=C(NCc1cnn2ccccc12)N[C@H]1[C@@H]2c3ccc(F)cc3C[C@H]21	664876623	25	336.14	2.61
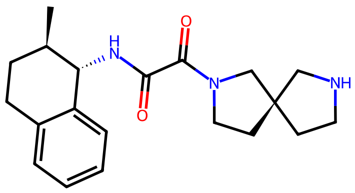	C[C@@H]1CCc2ccccc2[C@H]1NC(=O)C(=O)N1CC[C@@]2(CCNC2)C1	580764513	25	341.21	1.64
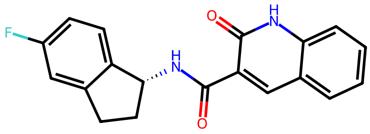	O=C(N[C@@H]1CCc2cc(F)ccc21)c1cc2ccccc2[nH]c1=O	194277761	24	322.11	3.08

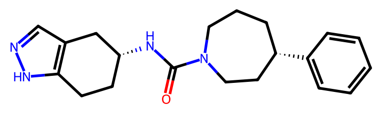	O=C(N[C@@H]1CCc2[nH]ncc2C1)N1CCC[C@H](c2ccccc2)CC1	154092128	25	338.21	3.25
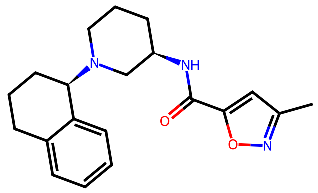	Cc1cc(C(=O)N[C@@H]2CCCN([C@@H]3CCCc4ccccc43)C2)on1	1006742590	25	339.19	3.25
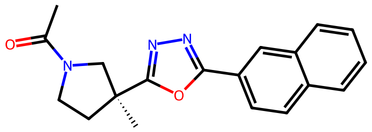	CC(=O)N1CC[C@](C)(c2nnc(-c3ccc4ccccc4c3)o2)C1	1329261361	24	321.15	3.4
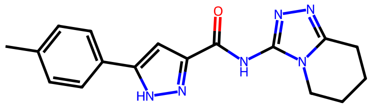	Cc1ccc(-c2cc(C(=O)Nc3nnc4n3CCCC4)n[nH]2)cc1	780791004	24	322.15	2.57
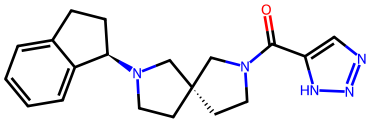	O=C(c1cnn[nH]1)N1CC[C@@]2(CCN([C@@H]3CCc4ccccc43)C2)C1	1041434227	25	337.19	2.03
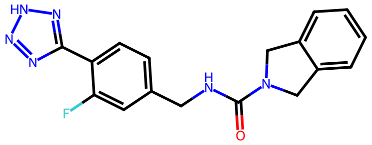	O=C(NCc1ccc(-c2nn[nH]n2)c(F)c1)N1Cc2ccccc2C1	1582545518	25	338.13	2.23
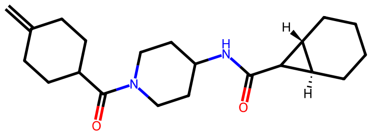	C=C1CCC(C(=O)N2CCC(NC(=O)C3[C@@H]4CCCC[C@@H]34)CC2)CC1	948190106	25	344.25	3.28
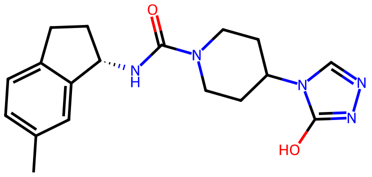	Cc1ccc2c(c1)[C@@H](NC(=O)N1CCC(n3cnnc3O)CC1)CC2	664023891	25	341.19	2.33

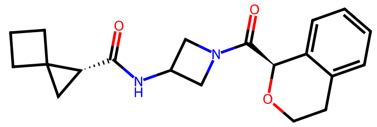	O=C(NC1CN(C(=O)[C@@H]2OCCc3ccccc32)C1)[C@@H]1CC12CCC2	991553818	25	340.18	1.82
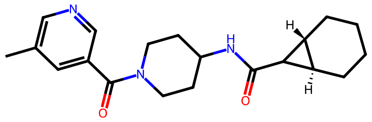	Cc1cncc(C(=O)N2CCC(NC(=O)C3[C@@H]4CCCC[C@@H]34)CC2)c1	945879731	25	341.21	2.55
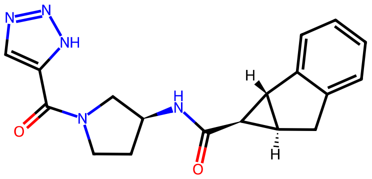	O=C(N[C@H]1CCN(C(=O)c2cnn[nH]2)C1)[C@@H]1[C@@H]2Cc3ccccc3[C@H]21	937965993	25	337.15	0.72
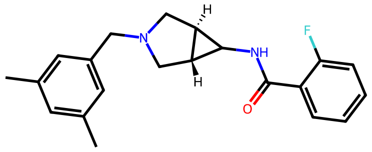	Cc1cc(C)cc(CN2C[C@H]3C(NC(=O)c4ccccc4F)[C@@H]3C2)c1	958088844	25	338.18	3.3
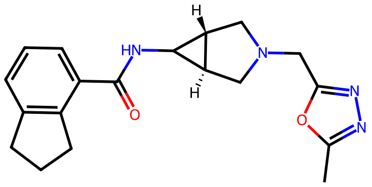	Cc1nnc(CN2C[C@H]3C(NC(=O)c4cccc5c4CCC5)[C@@H]3C2)o1	962977198	25	338.17	1.73
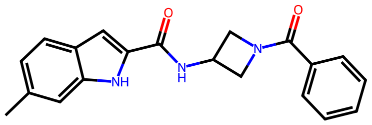	Cc1ccc2cc(C(=O)NC3CN(C(=O)c4ccccc4)C3)[nH]c2c1	996604306	25	333.15	2.73
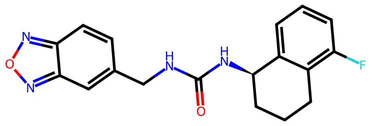	O=C(NCc1ccc2nonc2c1)N[C@@H]1CCCc2c(F)cccc21	1229312092	25	340.13	3.24
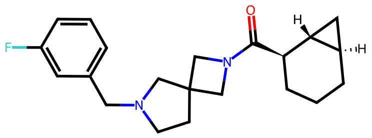	O=C([C@@H]1CCC[C@@H]2C[C@H]21)N1CC2(CCN(Cc3cccc(F)c3)C2)C1	1072875156	25	342.21	3.3

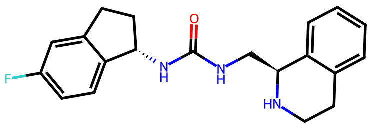	O=C(NC[C@@H]1NCCc2ccccc21)N[C@H]1CCc2cc(F)ccc21	557162127	25	339.17	3.0
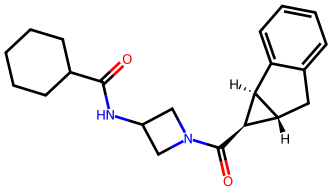	O=C(NC1CN(C(=O)[C@@H]2[C@H]3Cc4ccccc4[C@@H]32)C1)C1CCCCC1	990104215	25	338.2	2.48
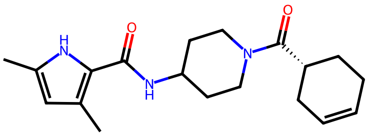	Cc1cc(C)c(C(=O)NC2CCN(C(=O)[C@H]3CC=CCC3)CC2)[nH]1	944126469	24	329.21	2.71
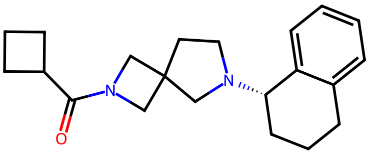	O=C(C1CCC1)N1CC2(CCN([C@H]3CCCc4ccccc43)C2)C1	1072376514	24	324.22	3.4
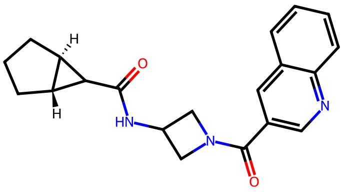	O=C(NC1CN(C(=O)c2cnc3ccccc3c2)C1)C1[C@@H]2CCC[C@@H]12	990713126	25	335.16	2.22
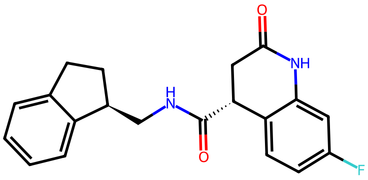	O=C1C[C@@H](C(=O)NC[C@@H]2CCc3ccccc32)c2ccc(F)cc2N1	110378220	25	338.14	3.1
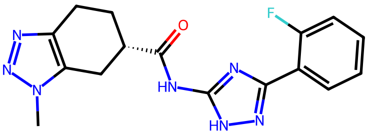	Cn1nnc2c1C[C@@H](C(=O)Nc1nc(-c3ccccc3F)n[nH]1)CC2	290367884	25	341.14	1.48
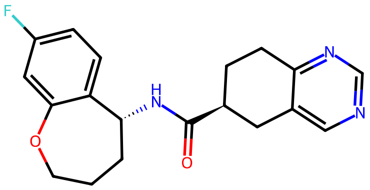	O=C(N[C@@H]1CCCOc2cc(F)ccc21)[C@H]1CCc2ncncc2C1	648347055	25	341.15	2.75

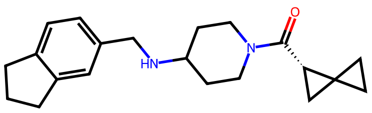	O=C([C@H]1CC12CC2)N1CCC(NCc2ccc3c(c2)CCC3)CC1	997044991	24	324.22	3.06
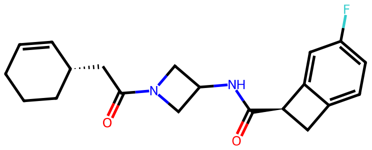	O=C(NC1CN(C(=O)C[C@H]2C=CCCC2)C1)[C@@H]1Cc2ccc(F)cc21	998490095	25	342.17	2.54
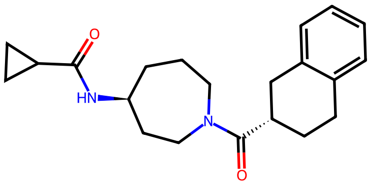	O=C(N[C@H]1CCCN(C(=O)[C@H]2CCc3ccccc3C2)CC1)C1CC1	964676108	25	340.22	2.7
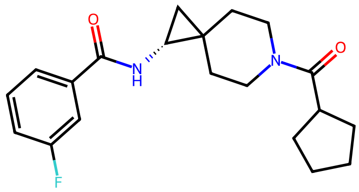	O=C(N[C@@H]1CC12CCN(C(=O)C1CCCC1)CC2)c1cccc(F)c1	962812046	25	344.19	3.13
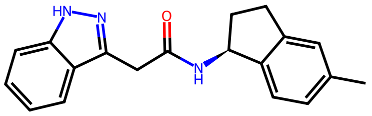	Cc1ccc2c(c1)CC[C@@H]2NC(=O)Cc1n[nH]c2ccccc12	50610421	23	305.15	3.22
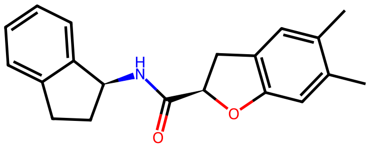	Cc1cc2c(cc1C)O[C@@H](C(=O)N[C@H]1CCc3ccccc31)C2	463350913	23	307.16	3.41
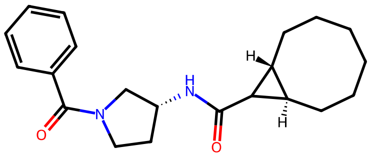	O=C(N[C@@H]1CCN(C(=O)c2ccccc2)C1)C1[C@@H]2CCCCCC[C@@H]12	942504705	25	340.22	3.23
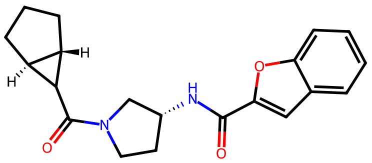	O=C(N[C@@H]1CCN(C(=O)C2[C@H]3CCC[C@H]23)C1)c1cc2ccccc2o1	942453625	25	338.16	2.81

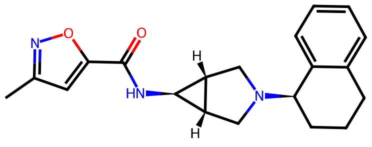	Cc1cc(C(=O)N[C@H]2[C@@H]3CN([C@@H]4CCCc5ccccc54)C[C@@H]32)on1	958216849	25	337.18	2.72
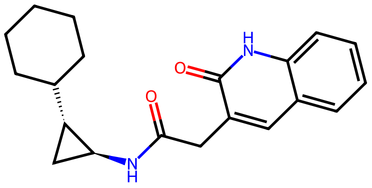	O=C(Cc1cc2ccccc2[nH]c1=O)N[C@H]1C[C@@H]1C1CCCCC1	152678196	24	324.18	3.16
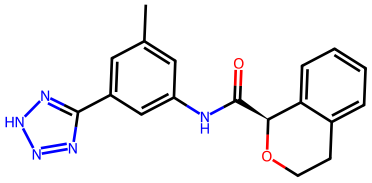	Cc1cc(NC(=O)[C@@H]2OCCc3ccccc32)cc(-c2nn[nH]n2)c1	1580979877	25	335.14	2.43
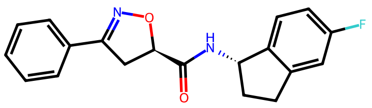	O=C(N[C@H]1CCc2cc(F)ccc21)[C@H]1CC(c2ccccc2)=NO1	56318608	24	324.13	3.12
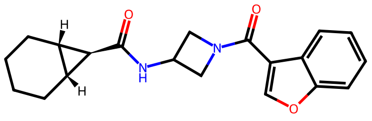	O=C(NC1CN(C(=O)c2coc3ccccc23)C1)[C@@H]1[C@H]2CCCC[C@H]21	990655471	25	338.16	2.81
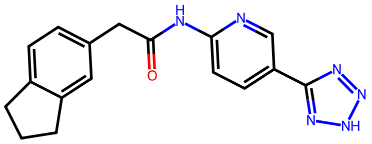	O=C(Cc1ccc2c(c1)CCC2)Nc1ccc(-c2nn[nH]n2)cn1	737965625	24	320.14	1.93
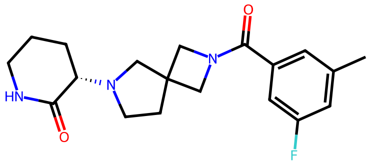	Cc1cc(F)cc(C(=O)N2CC3(CCN([C@H]4CCCNC4=O)C3)C2)c1	1073007308	25	345.19	1.56
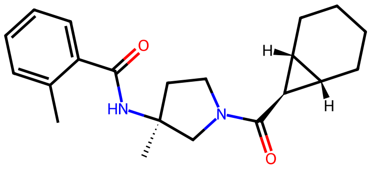	Cc1ccccc1C(=O)N[C@@]1(C)CCN(C(=O)[C@@H]2[C@H]3CCCC[C@H]32)C1	956661609	25	340.22	3.15

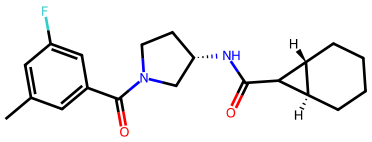	Cc1cc(F)cc(C(=O)N2CC[C@H](NC(=O)C3[C@@H]4CCCC[C@@H]34)C2)c1	937794554	25	344.19	2.9
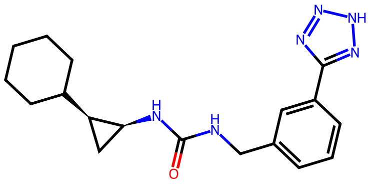	O=C(NCc1cccc(-c2nn[nH]n2)c1)N[C@H]1C[C@H]1C1CCCCC1	823257228	25	340.2	2.63
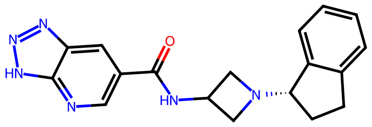	O=C(NC1CN([C@H]2CCc3ccccc32)C1)c1cnc2[nH]nnc2c1	1030450891	25	334.15	1.45
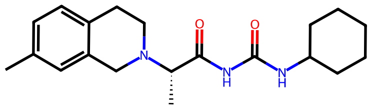	Cc1ccc2c(c1)CN([C@@H](C)C(=O)NC(=O)NC1CCCCC1)CC2	1364756811	25	343.23	2.9
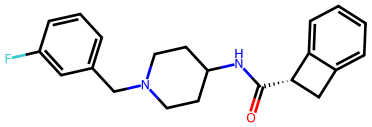	O=C(NC1CCN(Cc2cccc(F)c2)CC1)[C@H]1Cc2ccccc21	1003538381	25	338.18	3.25
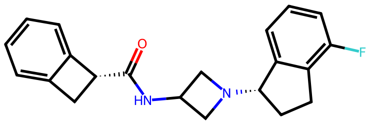	O=C(NC1CN([C@H]2CCc3c(F)cccc32)C1)[C@@H]1Cc2ccccc21	1030881521	25	336.16	2.95
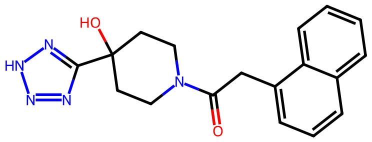	O=C(Cc1cccc2ccccc12)N1CCC(O)(c2nn[nH]n2)CC1	585139781	25	337.15	1.41
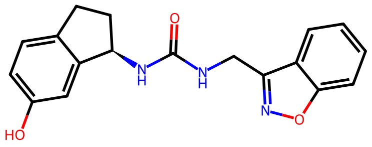	O=C(NCc1noc2ccccc12)N[C@@H]1CCc2ccc(O)cc21	1684163098	24	323.13	3.02

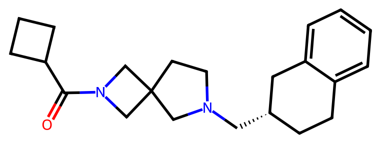	O=C(C1CCC1)N1CC2(CCN(C[C@H]3CCc4ccccc4C3)C2)C1	1072389138	25	338.24	3.13
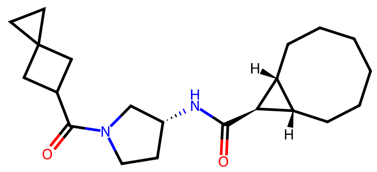	O=C(N[C@@H]1CCN(C(=O)C2CC3(CC3)C2)C1)[C@@H]1[C@H]2CCCCCC[C@H]21	942510228	25	344.25	3.11
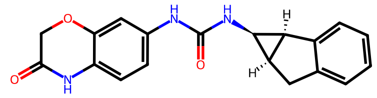	O=C1COc2cc(NC(=O)N[C@@H]3[C@@H]4Cc5ccccc5[C@@H]43)ccc2N1	150784694	25	335.13	2.48
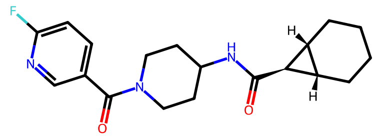	O=C(NC1CCN(C(=O)c2ccc(F)nc2)CC1)[C@@H]1[C@H]2CCCC[C@H]21	945398769	25	345.19	2.38
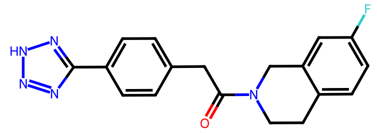	O=C(Cc1ccc(-c2nn[nH]n2)cc1)N1CCc2ccc(F)cc2C1	823078674	25	337.13	2.13
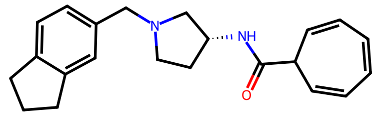	O=C(N[C@@H]1CCN(Cc2ccc3c(c2)CCC3)C1)C1C=CC=CC=C1	1014591430	25	334.2	3.16
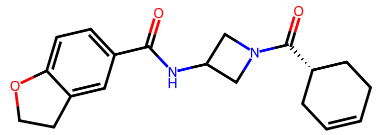	O=C(NC1CN(C(=O)[C@H]2CC=CCC2)C1)c1ccc2c(c1)CCO2	1000427612	24	326.16	1.92
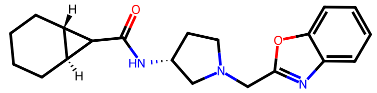	O=C(N[C@@H]1CCN(Cc2nc3ccccc3o2)C1)C1[C@H]2CCCC[C@H]12	1010437583	25	339.19	2.95

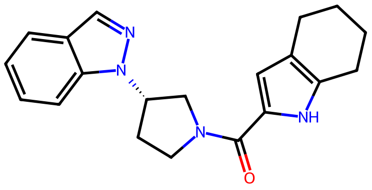	O=C(c1cc2c([nH]1)CCCC2)N1CC[C@H](n2ncc3ccccc32)C1	560208142	25	334.18	3.33
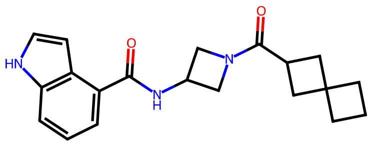	O=C(NC1CN(C(=O)C2CC3(CCC3)C2)C1)c1cccc2[nH]ccc12	1000399251	25	337.18	2.69
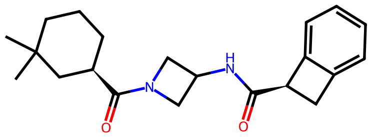	CC1(C)CCC[C@@H](C(=O)N2CC(NC(=O)[C@@H]3Cc4ccccc43)C2)C1	998686752	25	340.22	2.87
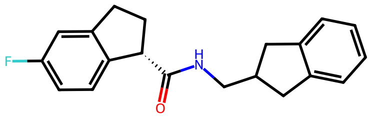	O=C(NCC1Cc2ccccc2C1)[C@H]1CCc2cc(F)ccc21	828058528	23	309.15	3.39
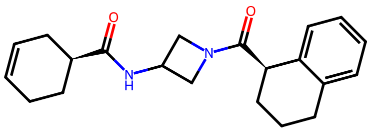	O=C(NC1CN(C(=O)[C@@H]2CCCc3ccccc32)C1)[C@@H]1CC=CCC1	990202683	25	338.2	2.79
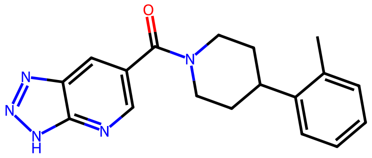	Cc1ccccc1C1CCN(C(=O)c2cnc3[nH]nnc3c2)CC1	329653901	24	321.16	2.68
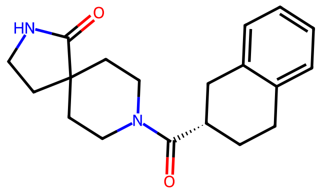	O=C([C@H]1CCc2ccccc2C1)N1CCC2(CCNC2=O)CC1	831767321	23	312.18	1.92
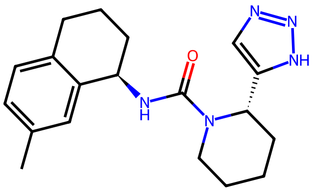	Cc1ccc2c(c1)[C@H](NC(=O)N1CCCC[C@H]1c1cnn[nH]1)CCC2	1577409725	25	339.21	3.43

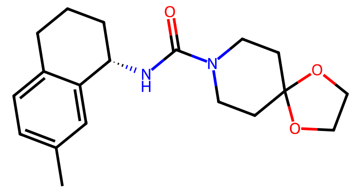	Cc1ccc2c(c1)[C@@H](NC(=O)N1CCC3(CC1)OCCO3)CCC2	179558980	24	330.19	2.92
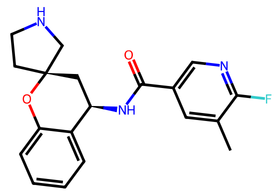	Cc1cc(C(=O)N[C@@H]2C[C@]3(CCNC3)Oc3ccccc32)cnc1F	1263253825	25	341.15	2.51
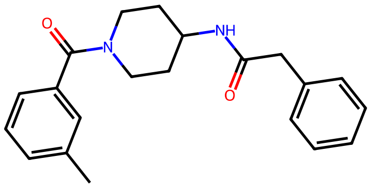	Cc1cccc(C(=O)N2CCC(NC(=O)Cc3ccccc3)CC2)c1	75057906	25	336.18	2.96
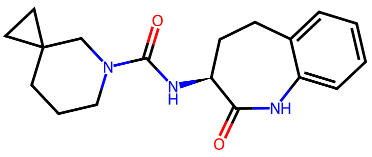	O=C1Nc2ccccc2CC[C@@H]1NC(=O)N1CCCC2(CC2)C1	654632645	23	313.18	2.53
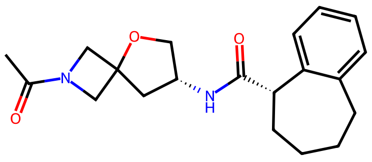	CC(=O)N1CC2(C[C@@H](NC(=O)[C@H]3CCCCc4ccccc43)CO2)C1	965739648	25	342.19	2.0
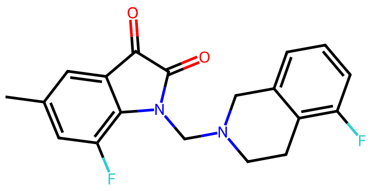	Cc1cc(F)c2c(c1)C(=O)C(=O)N2CN1CCc2c(F)cccc2C1	156960412	25	342.12	2.82
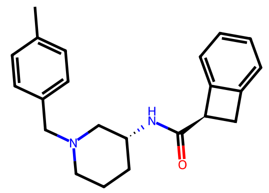	Cc1ccc(CN2CCC[C@@H](NC(=O)[C@@H]3Cc4ccccc43)C2)cc1	1008373616	25	334.2	3.42
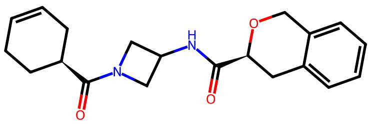	O=C(NC1CN(C(=O)[C@@H]2CC=CCC2)C1)[C@@H]1Cc2ccccc2CO1	997728091	25	340.18	1.81

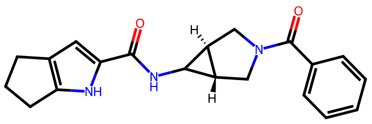	O=C(NC1[C@H]2CN(C(=O)c3ccccc3)C[C@H]12)c1cc2c([nH]1)CCC2	1013426634	25	335.16	2.0
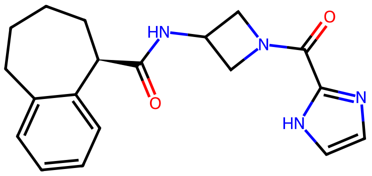	O=C(NC1CN(C(=O)c2ncc[nH]2)C1)[C@@H]1CCCCc2ccccc21	994306549	25	338.17	1.86
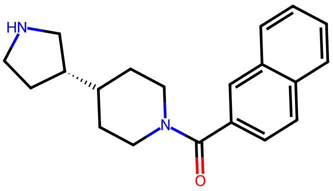	O=C(c1ccc2ccccc2c1)N1CCC([C@@H]2CCNC2)CC1	290009597	23	308.19	3.3
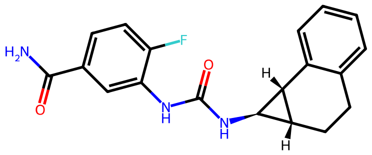	NC(=O)c1ccc(F)c(NC(=O)N[C@@H]2[C@H]3CCc4ccccc4[C@H]32)c1	178923018	25	339.14	2.77
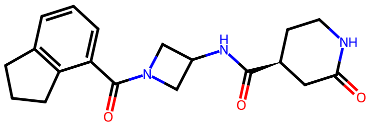	O=C1C[C@@H](C(=O)NC2CN(C(=O)c3cccc4c3CCC4)C2)CCN1	990804003	25	341.17	0.64
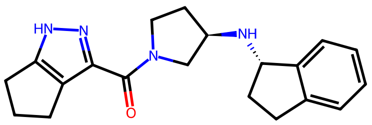	O=C(c1n[nH]c2c1CCC2)N1CC[C@@H](N[C@H]2CCc3ccccc32)C1	969180677	25	336.2	2.39
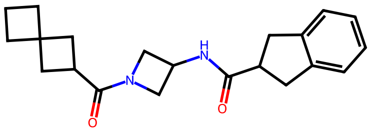	O=C(NC1CN(C(=O)C2CC3(CCC3)C2)C1)C1Cc2ccccc2C1	1000447599	25	338.2	2.31
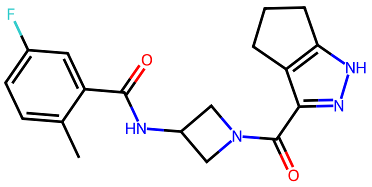	Cc1ccc(F)cc1C(=O)NC1CN(C(=O)c2n[nH]c3c2CCC3)C1	996646306	25	342.15	1.6

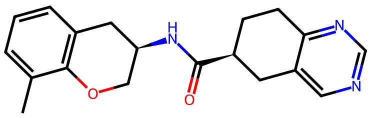	Cc1cccc2c1OC[C@H](NC(=O)[C@H]1CCc3ncncc3C1)C2	648396895	24	323.16	2.01
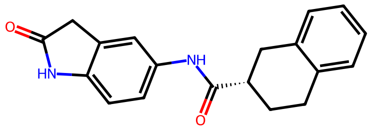	O=C1Cc2cc(NC(=O)[C@H]3CCc4ccccc4C3)ccc2N1	566326842	23	306.14	2.92
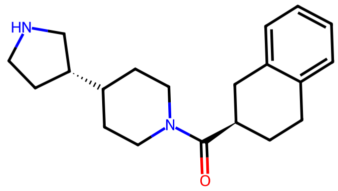	O=C([C@@H]1CCc2ccccc2C1)N1CCC([C@@H]2CCNC2)CC1	582613857	23	312.22	2.64
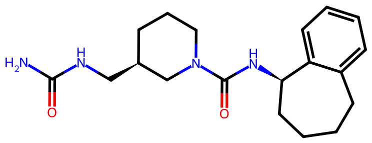	NC(=O)NC[C@H]1CCCN(C(=O)N[C@@H]2CCCCc3ccccc32)C1	72230553	25	344.22	2.54
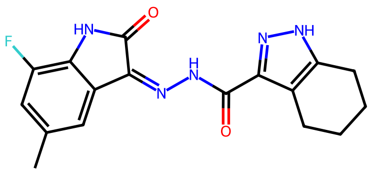	Cc1cc(F)c2c(c1)/C(=N/NC(=O)c1n[nH]c3c1CCCC3)C(=O)N2	46414991	25	341.13	1.82
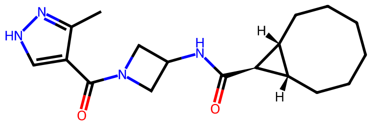	Cc1n[nH]cc1C(=O)N1CC(NC(=O)[C@@H]2[C@H]3CCCCCC[C@H]32)C1	1000510979	24	330.21	1.88
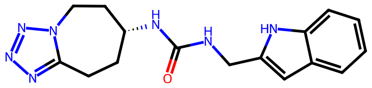	O=C(NCc1cc2ccccc2[nH]1)N[C@@H]1CCc2nnnn2CC1	571394496	24	325.17	1.36
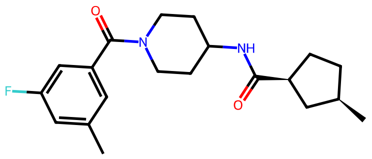	Cc1cc(F)cc(C(=O)N2CCC(NC(=O)[C@H]3CC[C@@H](C)C3)CC2)c1	948549820	25	346.21	3.29

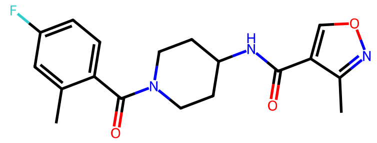	Cc1cc(F)ccc1C(=O)N1CCC(NC(=O)c2conc2C)CC1	947646536	25	345.15	2.47
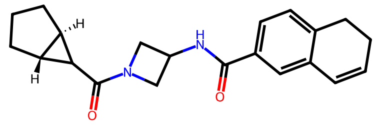	O=C(NC1CN(C(=O)C2[C@@H]3CCC[C@@H]23)C1)c1ccc2c(c1)C=CCC2	995372072	25	336.18	2.63
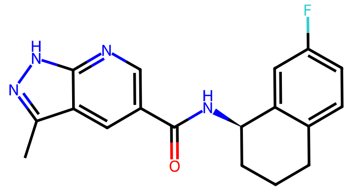	Cc1n[nH]c2ncc(C(=O)N[C@@H]3CCCc4ccc(F)cc43)cc12	93062665	24	324.14	3.21
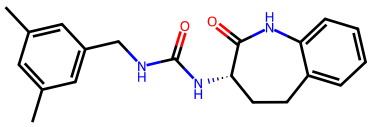	Cc1cc(C)cc(CNC(=O)N[C@H]2CCc3ccccc3NC2=O)c1	661720341	25	337.18	3.06
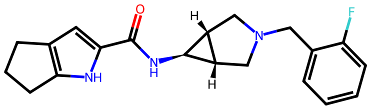	O=C(N[C@H]1[C@@H]2CN(Cc3ccccc3F)C[C@@H]21)c1cc2c([nH]1)CCC2	962270975	25	339.17	2.5
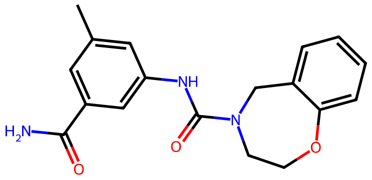	Cc1cc(NC(=O)N2CCOc3ccccc3C2)cc(C(N)=O)c1	1364370130	24	325.14	2.52
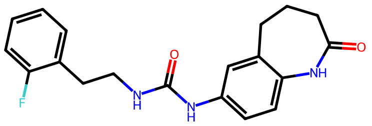	O=C1CCCc2cc(NC(=O)NCCc3ccccc3F)ccc2N1	171527567	25	341.15	3.46
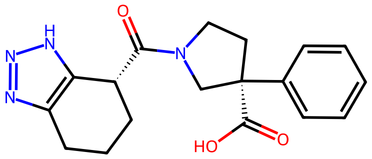	O=C([C@@H]1CCCc2nn[nH]c21)N1CC[C@@](C(=O)O)(c2ccccc2)C1	909857197	25	340.15	1.48

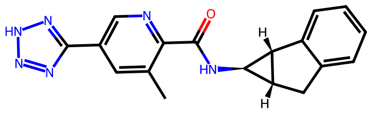	Cc1cc(-c2nn[nH]n2)cnc1C(=O)N[C@@H]1[C@H]2Cc3ccccc3[C@H]21	1591880481	25	332.14	1.64
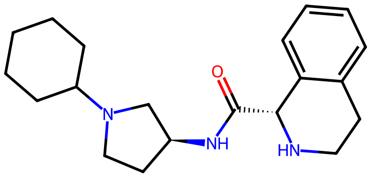	O=C(N[C@H]1CCN(C2CCCCC2)C1)[C@H]1NCCc2ccccc21	662878150	24	327.23	2.4
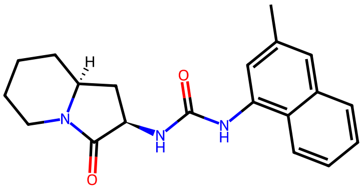	Cc1cc(NC(=O)N[C@@H]2C[C@@H]3CCCCN3C2=O)c2ccccc2c1	576594479	25	337.18	3.42
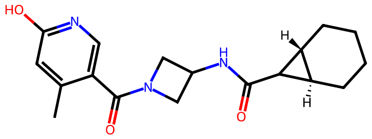	Cc1cc(O)ncc1C(=O)N1CC(NC(=O)C2[C@@H]3CCCC[C@@H]23)C1	990674532	24	329.17	1.47
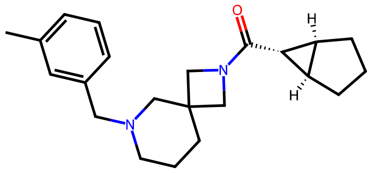	Cc1cccc(CN2CCCC3(C2)CN(C(=O)[C@@H]2[C@H]4CCC[C@H]42)C3)c1	981696299	25	338.24	3.47
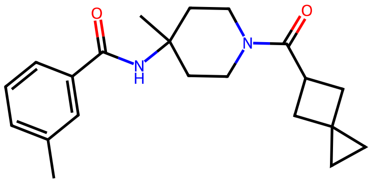	Cc1cccc(C(=O)NC2(C)CCN(C(=O)C3CC4(CC4)C3)CC2)c1	956284943	25	340.22	3.3
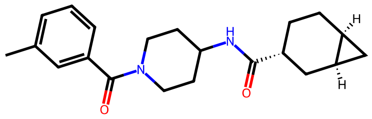	Cc1cccc(C(=O)N2CCC(NC(=O)[C@@H]3CC[C@H]4C[C@H]4C3)CC2)c1	943836126	25	340.22	3.15
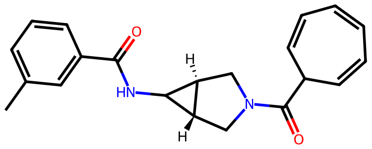	Cc1cccc(C(=O)NC2[C@H]3CN(C(=O)C4C=CC=CC=C4)C[C@H]23)c1	1012721667	25	334.17	2.48

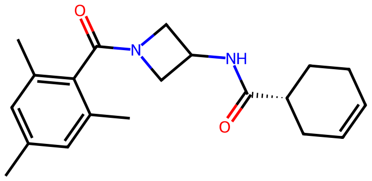	Cc1cc(C)c(C(=O)N2CC(NC(=O)[C@H]3CC=CCC3)C2)c(C)c1	990214461	24	326.2	2.91
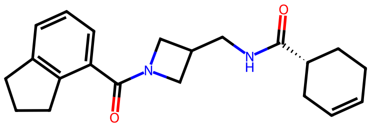	O=C(NCC1CN(C(=O)c2cccc3c2CCC3)C1)[C@H]1CC=CCC1	1000895364	25	338.2	2.72
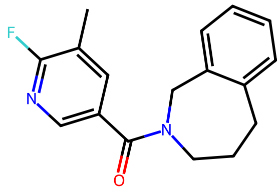	Cc1cc(C(=O)N2CCCc3ccccc3C2)cnc1F	857586663	21	284.13	3.12
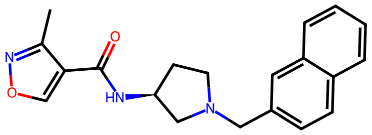	Cc1nocc1C(=O)N[C@H]1CCN(Cc2ccc3ccccc3c2)C1	1015647808	25	335.16	3.14
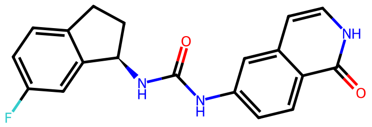	O=C(Nc1ccc2c(=O)[nH]ccc2c1)N[C@@H]1CCc2ccc(F)cc21	1679091269	25	337.12	3.48
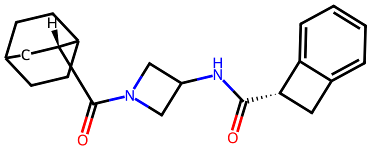	O=C(NC1CN(C(=O)[C@@H]2CC3CCC2CC3)C1)[C@H]1Cc2ccccc21	998687482	25	338.2	2.48
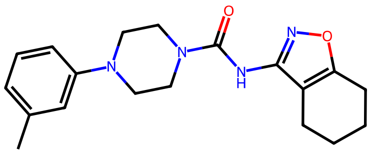	Cc1cccc(N2CCN(C(=O)Nc3noc4c3CCCC4)CC2)c1	934363397	25	340.19	3.22
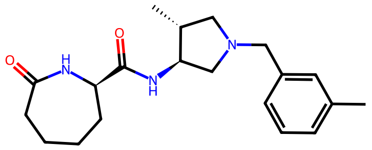	Cc1cccc(CN2C[C@@H](NC(=O)[C@H]3CCCCC(=O)N3)[C@H](C)C2)c1	1080422633	25	343.23	1.99

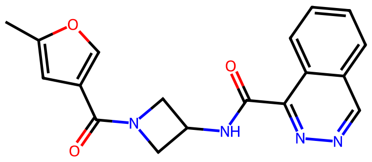	Cc1cc(C(=O)N2CC(NC(=O)c3nncc4ccccc34)C2)co1	996895082	25	336.12	1.79
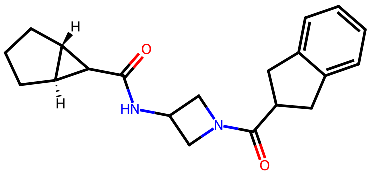	O=C(NC1CN(C(=O)C2Cc3ccccc3C2)C1)C1[C@H]2CCC[C@H]12	990710894	24	324.18	1.77
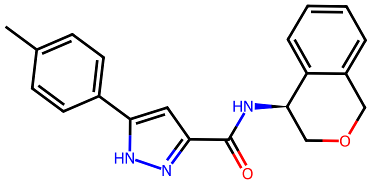	Cc1ccc(-c2cc(C(=O)N[C@@H]3COCc4ccccc43)n[nH]2)cc1	1638320976	25	333.15	3.39
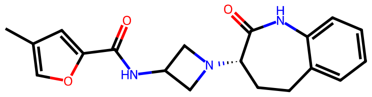	Cc1coc(C(=O)NC2CN([C@H]3CCc4ccccc4NC3=O)C2)c1	1030816130	25	339.16	1.96
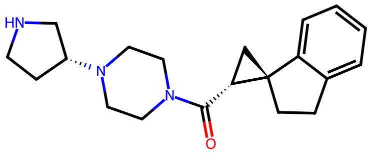	O=C([C@@H]1C[C@@]12CCc1ccccc12)N1CCN([C@@H]2CCNC2)CC1	289308551	24	325.22	1.4
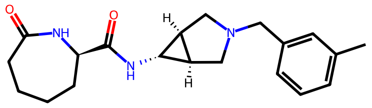	Cc1cccc(CN2C[C@H]3[C@H](NC(=O)[C@H]4CCCCC(=O)N4)[C@H]3C2)c1	961764080	25	341.21	1.6
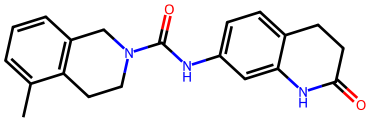	Cc1cccc2c1CCN(C(=O)Nc1ccc3c(c1)NC(=O)CC3)C2	743193571	25	335.16	3.47
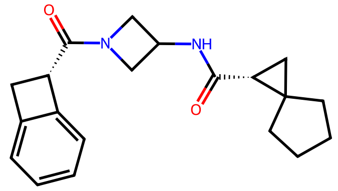	O=C(NC1CN(C(=O)[C@H]2Cc3ccccc32)C1)[C@@H]1CC12CCCC2	999634041	24	324.18	2.23

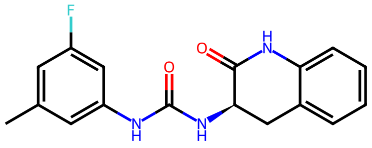	Cc1cc(F)cc(NC(=O)N[C@@H]2Cc3ccccc3NC2=O)c1	1364318174	23	313.12	2.82
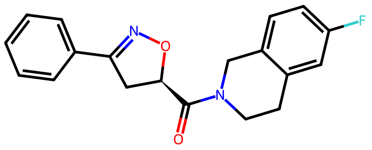	O=C([C@H]1CC(c2ccccc2)=NO1)N1CCc2cc(F)ccc2C1	121929269	24	324.13	2.9
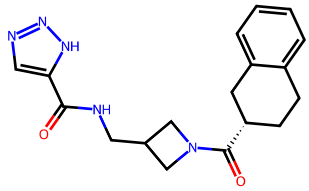	O=C(NCC1CN(C(=O)[C@H]2CCc3ccccc3C2)C1)c1cnn[nH]1	1003127294	25	339.17	0.8
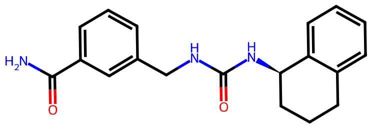	NC(=O)c1cccc(CNC(=O)N[C@@H]2CCCc3ccccc32)c1	71869613	24	323.16	2.66
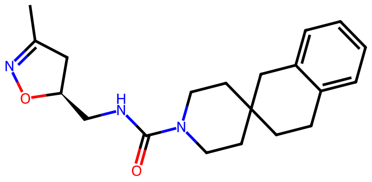	CC1=NO[C@H](CNC(=O)N2CCC3(CCc4ccccc4C3)CC2)C1	1470999612	25	341.21	3.13
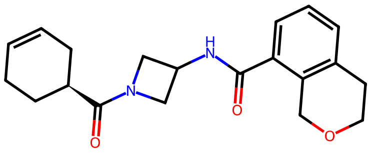	O=C(NC1CN(C(=O)[C@@H]2CC=CCC2)C1)c1cccc2c1COCC2	993315349	25	340.18	2.06
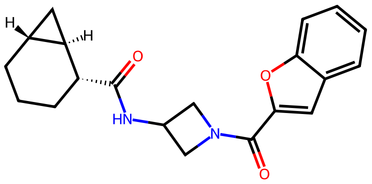	O=C(NC1CN(C(=O)c2cc3ccccc3o2)C1)[C@@H]1CCC[C@@H]2C[C@H]21	994726086	25	338.16	2.81
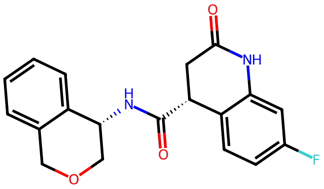	O=C1C[C@@H](C(=O)N[C@@H]2COCc3ccccc32)c2ccc(F)cc2N1	577364417	25	340.12	2.64

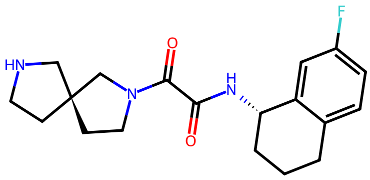	O=C(N[C@H]1CCCc2ccc(F)cc21)C(=O)N1CC[C@]2(CCNC2)C1	517101653	25	345.19	1.53
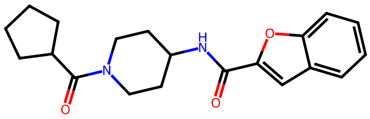	O=C(NC1CCN(C(=O)C2CCCC2)CC1)c1cc2ccccc2o1	943367021	25	340.18	3.34
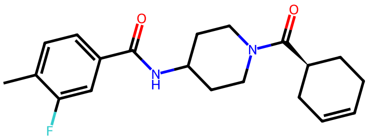	Cc1ccc(C(=O)NC2CCN(C(=O)[C@@H]3CC=CCC3)CC2)cc1F	944127720	25	344.19	3.21
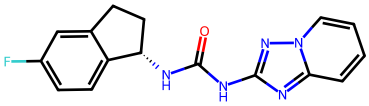	O=C(Nc1nc2ccccn2n1)N[C@H]1CCc2cc(F)ccc21	183049052	23	311.12	2.68
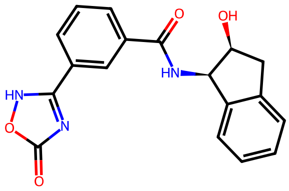	O=C(N[C@@H]1c2ccccc2C[C@@H]1O)c1cccc(-c2nc(=O)o[nH]2)c1	1566325812	25	337.11	1.42
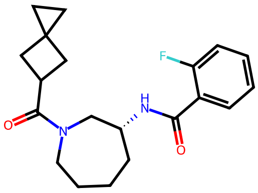	O=C(N[C@@H]1CCCCN(C(=O)C2CC3(CC3)C2)C1)c1ccccc1F	943054915	25	344.19	3.13
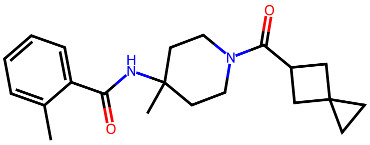	Cc1ccccc1C(=O)NC1(C)CCN(C(=O)C2CC3(CC3)C2)CC1	956274464	25	340.22	3.3
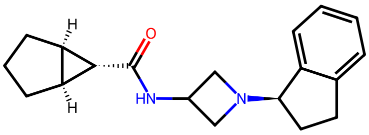	O=C(NC1CN([C@@H]2CCc3ccccc32)C1)[C@@H]1[C@H]2CCC[C@H]21	1030201049	22	296.19	2.52

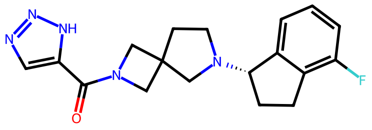	O=C(c1cnn[nH]1)N1CC2(CCN([C@H]3CCc4c(F)cccc43)C2)C1	1072892463	25	341.17	1.78
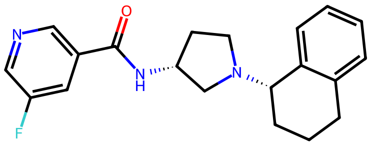	O=C(N[C@@H]1CCN([C@H]2CCCc3ccccc32)C1)c1cncc(F)c1	1010513580	25	339.17	3.1
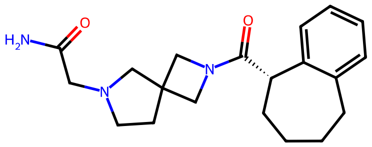	NC(=O)CN1CCC2(C1)CN(C(=O)[C@H]1CCCCc3ccccc31)C2	1073469975	25	341.21	1.52
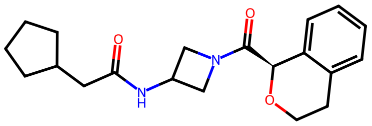	O=C(CC1CCCC1)NC1CN(C(=O)[C@@H]2OCCc3ccccc32)C1	990107250	25	342.19	2.21
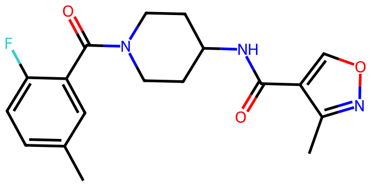	Cc1ccc(F)c(C(=O)N2CCC(NC(=O)c3conc3C)CC2)c1	948575721	25	345.15	2.47
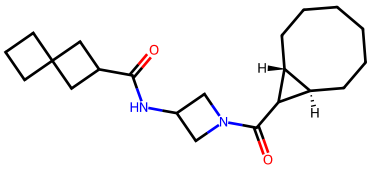	O=C(NC1CN(C(=O)C2[C@@H]3CCCCCC[C@@H]23)C1)C1CC2(CCC2)C1	997934839	25	344.25	3.11
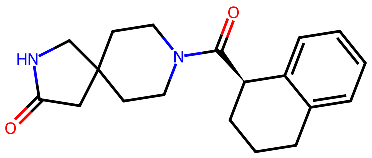	O=C1CC2(CCN(C(=O)[C@@H]3CCCc4ccccc43)CC2)CN1	267751683	23	312.18	2.24
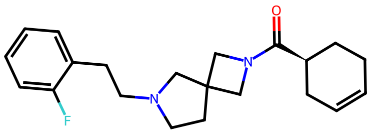	O=C([C@@H]1CC=CCC1)N1CC2(CCN(CCc3ccccc3F)C2)C1	1072457707	25	342.21	3.26

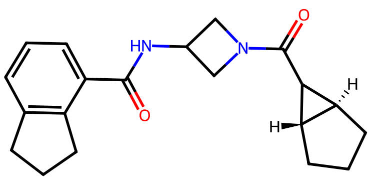	O=C(NC1CN(C(=O)C2[C@H]3CCC[C@H]23)C1)c1cccc2c1CCC2	994404493	24	324.18	2.16
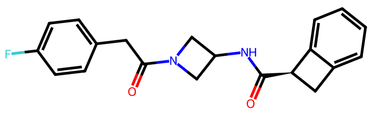	O=C(NC1CN(C(=O)Cc2ccc(F)cc2)C1)[C@@H]1Cc2ccccc21	998686068	25	338.14	2.04
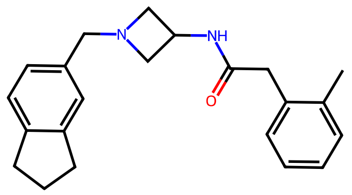	Cc1ccccc1CC(=O)NC1CN(Cc2ccc3c(c2)CCC3)C1	1031371051	25	334.2	3.03
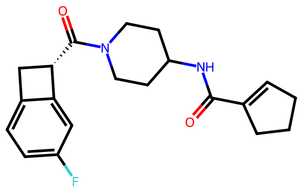	O=C(NC1CCN(C(=O)[C@H]2Cc3ccc(F)cc32)CC1)C1=CCCC1	948024619	25	342.17	2.68
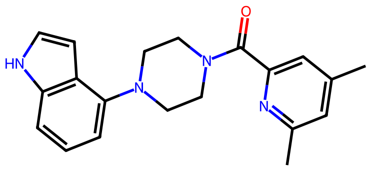	Cc1cc(C)nc(C(=O)N2CCN(c3cccc4[nH]ccc34)CC2)c1	855064930	25	334.18	3.14
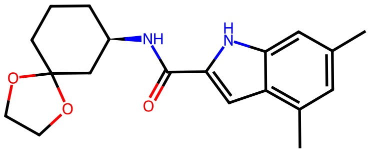	Cc1cc(C)c2cc(C(=O)N[C@@H]3CCCC4(C3)OCCO4)[nH]c2c1	782164398	24	328.18	3.2
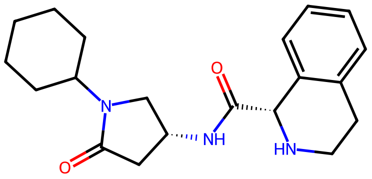	O=C(N[C@@H]1CC(=O)N(C2CCCCC2)C1)[C@H]1NCCc2ccccc21	576228605	25	341.21	1.92
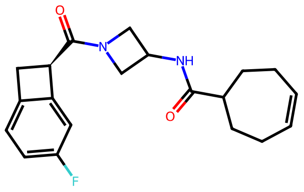	O=C(NC1CN(C(=O)[C@@H]2Cc3ccc(F)cc32)C1)C1CCC=CCC1	991816875	25	342.17	2.54

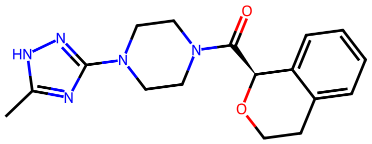	Cc1nc(N2CCN(C(=O)[C@@H]3OCCc4ccccc43)CC2)n[nH]1	898179201	24	327.17	1.08
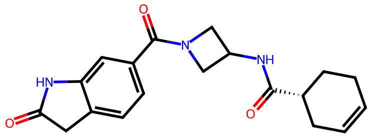	O=C1Cc2ccc(C(=O)N3CC(NC(=O)[C@H]4CC=CCC4)C3)cc2N1	990217327	25	339.16	1.48
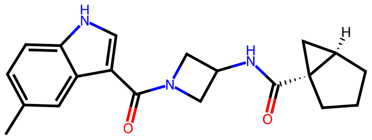	Cc1ccc2[nH]cc(C(=O)N3CC(NC(=O)[C@@]45CCC[C@@H]4C5)C3)c2c1	997554404	25	337.18	2.61
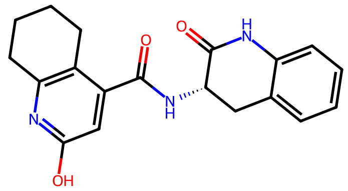	O=C(N[C@H]1Cc2ccccc2NC1=O)c1cc(O)nc2c1CCCC2	827439119	25	337.14	1.96
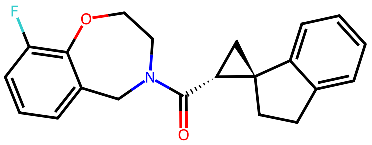	O=C([C@@H]1C[C@@]12CCc1ccccc12)N1CCOc2c(F)cccc2C1	375927258	25	337.15	3.45
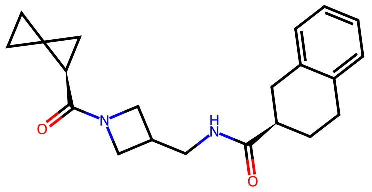	O=C(NCC1CN(C(=O)[C@@H]2CC23CC3)C1)[C@@H]1CCc2ccccc2C1	936100053	25	338.2	2.17
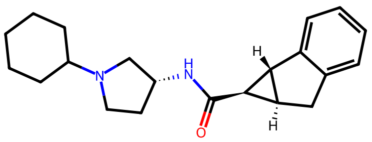	O=C(N[C@@H]1CCN(C2CCCCC2)C1)[C@@H]1[C@@H]2Cc3ccccc3[C@H]21	628092777	24	324.22	3.1
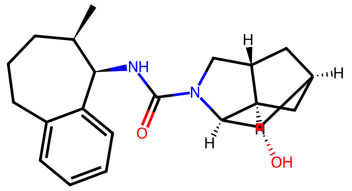	C[C@@H]1CCCc2ccccc2[C@@H]1NC(=O)N1C[C@@H]2C[C@H]3C[C@H]2[C@H]1[C@H]3O	249610379	25	340.22	3.11

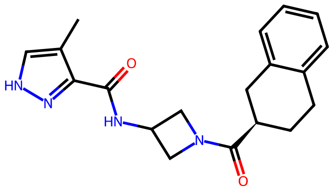	Cc1c[nH]nc1C(=O)NC1CN(C(=O)[C@@H]2CCc3ccccc3C2)C1	991258159	25	338.17	1.46
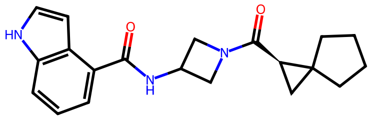	O=C(NC1CN(C(=O)[C@@H]2CC23CCCC3)C1)c1cccc2[nH]ccc12	1000410684	25	337.18	2.69
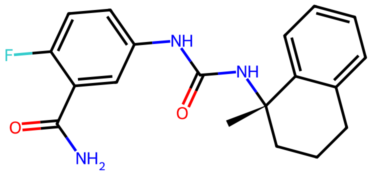	C[C@]1(NC(=O)Nc2ccc(F)c(C(N)=O)c2)CCCc2ccccc21	604092922	25	341.15	3.3
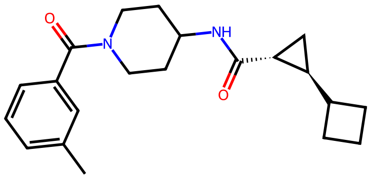	Cc1cccc(C(=O)N2CCC(NC(=O)[C@@H]3C[C@H]3C3CCC3)CC2)c1	943833955	25	340.22	3.15
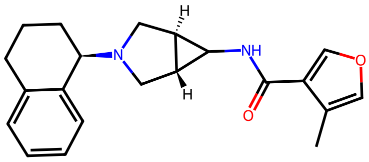	Cc1cocc1C(=O)NC1[C@@H]2CN([C@@H]3CCCc4ccccc43)C[C@@H]12	961521048	25	336.18	3.33
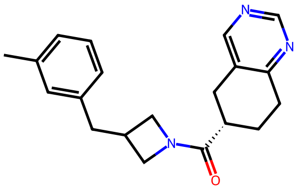	Cc1cccc(CC2CN(C(=O)[C@H]3CCc4ncncc4C3)C2)c1	648818819	24	321.18	2.59
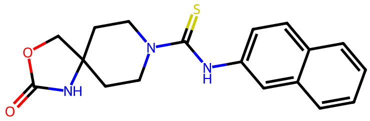	O=C1NC2(CCN(C(=S)Nc3ccc4ccccc4c3)CC2)CO1	495252510	24	341.12	3.11
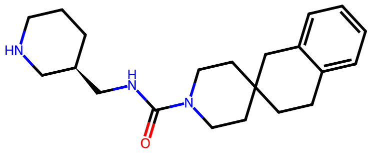	O=C(NC[C@@H]1CCCNC1)N1CCC2(CCc3ccccc3C2)CC1	652202555	25	341.25	2.97

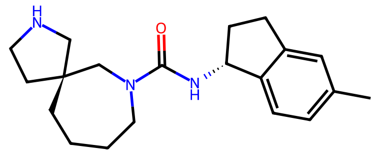	Cc1ccc2c(c1)CC[C@H]2NC(=O)N1CCCC[C@]2(CCNC2)C1	891420225	24	327.23	3.16
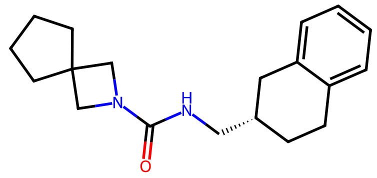	O=C(NC[C@H]1CCc2ccccc2C1)N1CC2(CCCC2)C1	681792088	22	298.2	3.38
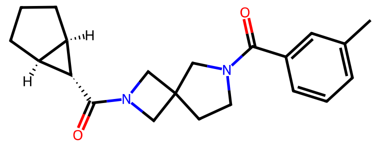	Cc1cccc(C(=O)N2CCC3(C2)CN(C(=O)[C@@H]2[C@H]4CCC[C@H]42)C3)c1	1018911086	25	338.2	2.72
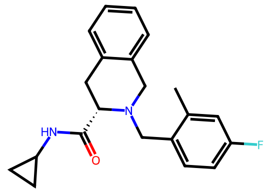	Cc1cc(F)ccc1CN1Cc2ccccc2C[C@H]1C(=O)NC1CC1	1653985162	25	338.18	3.34
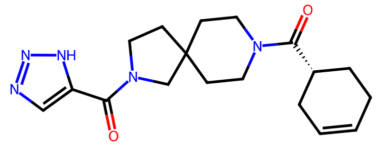	O=C(c1cnn[nH]1)N1CCC2(CCN(C(=O)[C@H]3CC=CCC3)CC2)C1	944355404	25	343.2	1.62
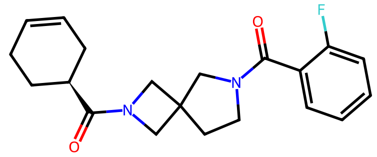	O=C(c1ccccc1F)N1CCC2(C1)CN(C(=O)[C@@H]1CC=CCC1)C2	1018768024	25	342.17	2.86
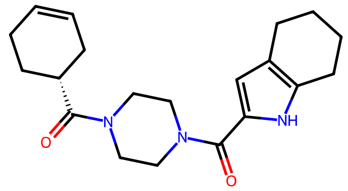	O=C(c1cc2c([nH]1)CCCC2)N1CCN(C(=O)[C@H]2CC=CCC2)CC1	1010302802	25	341.21	2.53
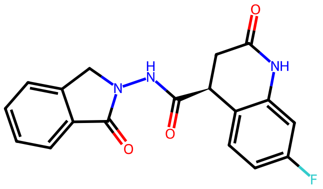	O=C1C[C@H](C(=O)NN2Cc3ccccc3C2=O)c2ccc(F)cc2N1	183855492	25	339.1	1.94

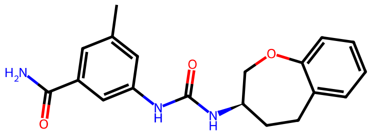	Cc1cc(NC(=O)N[C@@H]2CCc3ccccc3OC2)cc(C(N)=O)c1	455844717	25	339.16	2.61
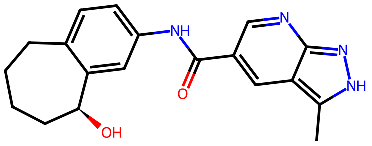	Cc1[nH]nc2ncc(C(=O)Nc3ccc4c(c3)[C@@H](O)CCCC4)cc12	855555213	25	336.16	3.28
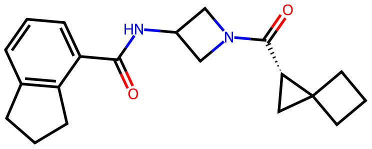	O=C(NC1CN(C(=O)[C@H]2CC23CCC3)C1)c1cccc2c1CCC2	994401782	24	324.18	2.31
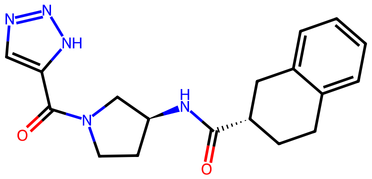	O=C(N[C@H]1CCN(C(=O)c2cnn[nH]2)C1)[C@H]1CCc2ccccc2C1	938982827	25	339.17	0.94
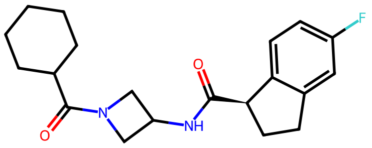	O=C(NC1CN(C(=O)C2CCCCC2)C1)[C@@H]1CCc2cc(F)ccc21	996100648	25	344.19	2.76
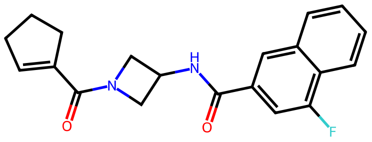	O=C(NC1CN(C(=O)C2=CCCC2)C1)c1cc(F)c2ccccc2c1	994125439	25	338.14	3.03
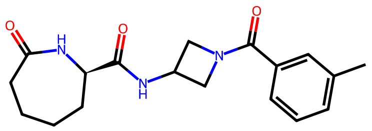	Cc1cccc(C(=O)N2CC(NC(=O)[C@H]3CCCCC(=O)N3)C2)c1	999380455	24	329.17	0.99
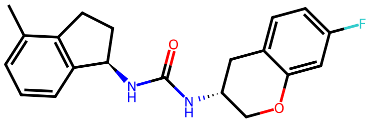	Cc1cccc2c1CC[C@H]2NC(=O)N[C@H]1COc2cc(F)ccc2C1	1677246833	25	340.16	3.42

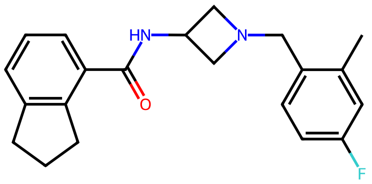	Cc1cc(F)ccc1CN1CC(NC(=O)c2cccc3c2CCC3)C1	1031467545	25	338.18	3.24
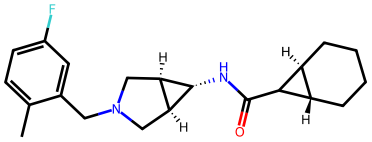	Cc1ccc(F)cc1CN1C[C@H]2[C@H](NC(=O)C3[C@H]4CCCC[C@H]34)[C@H]2C1	1025723258	25	342.21	3.12
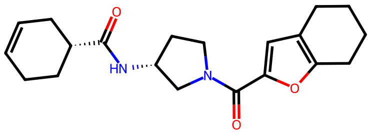	O=C(N[C@@H]1CCN(C(=O)c2cc3c(o2)CCCC3)C1)[C@H]1CC=CCC1	939379940	25	342.19	2.85
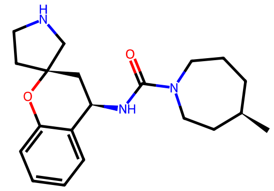	C[C@@H]1CCCN(C(=O)N[C@@H]2C[C@]3(CCNC3)Oc3ccccc32)CC1	1337005305	25	343.23	3.07
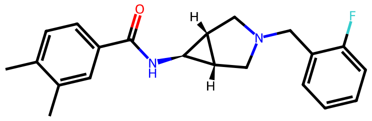	Cc1ccc(C(=O)N[C@H]2[C@@H]3CN(Cc4ccccc4F)C[C@@H]32)cc1C	962461923	25	338.18	3.3
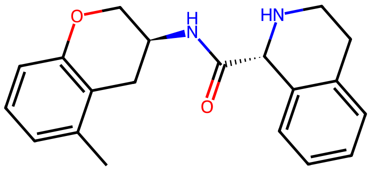	Cc1cccc2c1C[C@H](NC(=O)[C@@H]1NCCc3ccccc31)CO2	638472223	24	322.17	2.3
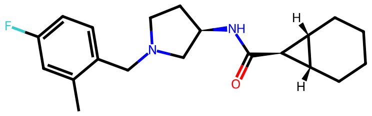	Cc1cc(F)ccc1CN1CC[C@@H](NC(=O)[C@@H]2[C@H]3CCCC[C@H]32)C1	1010439875	24	330.21	3.26
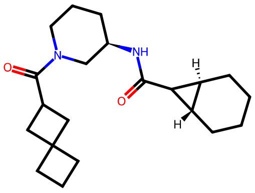	O=C(N[C@@H]1CCCN(C(=O)C2CC3(CCC3)C2)C1)C1[C@H]2CCCC[C@H]12	951683773	25	344.25	3.11

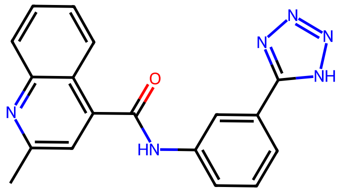	Cc1cc(C(=O)Nc2cccc(-c3nnn[nH]3)c2)c2ccccc2n1	70731430	25	330.12	2.98
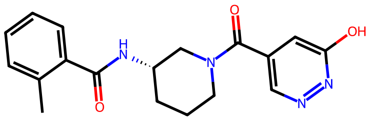	Cc1ccccc1C(=O)N[C@H]1CCCN(C(=O)c2cnnc(O)c2)C1	951118000	25	340.15	1.53
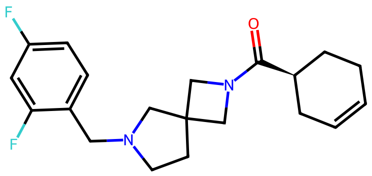	O=C([C@@H]1CC=CCC1)N1CC2(CCN(Cc3ccc(F)cc3F)C2)C1	1072457549	25	346.19	3.36
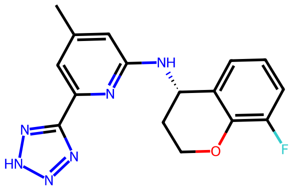	Cc1cc(N[C@H]2CCOc3c(F)cccc32)nc(-c2nn[nH]n2)c1	1592054823	24	326.13	2.64
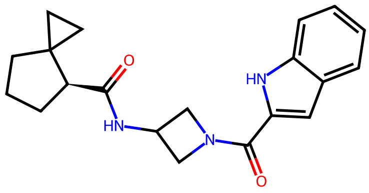	O=C(NC1CN(C(=O)c2cc3ccccc3[nH]2)C1)[C@H]1CCCC12CC2	994866080	25	337.18	2.69
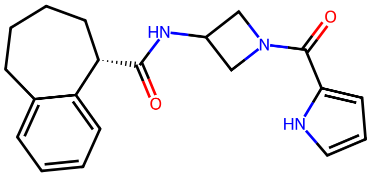	O=C(NC1CN(C(=O)c2ccc[nH]2)C1)[C@H]1CCCCc2ccccc21	994304875	25	337.18	2.47
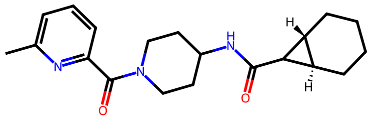	Cc1cccc(C(=O)N2CCC(NC(=O)C3[C@@H]4CCCC[C@@H]34)CC2)n1	944330721	25	341.21	2.55
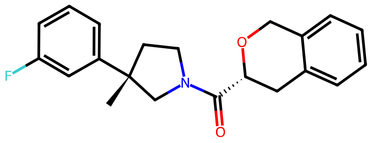	C[C@@]1(c2cccc(F)c2)CCN(C(=O)[C@H]2Cc3ccccc3CO2)C1	855011922	25	339.16	3.46

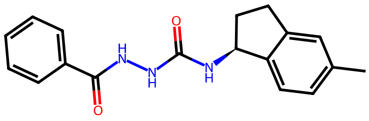	Cc1ccc2c(c1)CC[C@@H]2NC(=O)NNC(=O)c1ccccc1	56841730	23	309.15	2.63
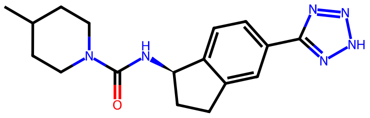	CC1CCN(C(=O)N[C@@H]2CCc3cc(-c4nn[nH]n4)ccc32)CC1	1609166925	24	326.19	2.3
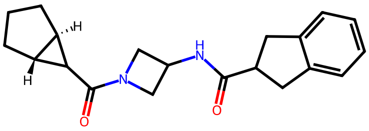	O=C(NC1CN(C(=O)C2[C@@H]3CCC[C@@H]23)C1)C1Cc2ccccc2C1	1000449328	24	324.18	1.77
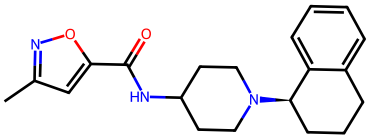	Cc1cc(C(=O)NC2CCN([C@@H]3CCCc4ccccc43)CC2)on1	1002333122	25	339.19	3.25
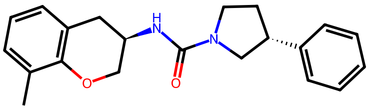	Cc1cccc2c1OC[C@H](NC(=O)N1CC[C@H](c3ccccc3)C1)C2	462665453	25	336.18	3.5
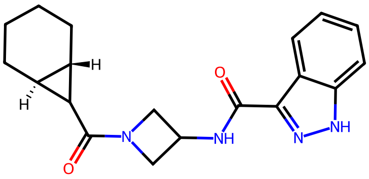	O=C(NC1CN(C(=O)C2[C@H]3CCCC[C@H]23)C1)c1n[nH]c2ccccc12	999527328	25	338.17	1.94
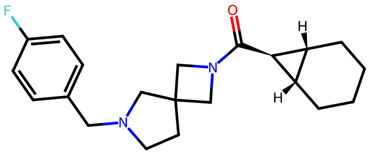	O=C([C@@H]1[C@H]2CCCC[C@H]21)N1CC2(CCN(Cc3ccc(F)cc3)C2)C1	1072580731	25	342.21	3.3
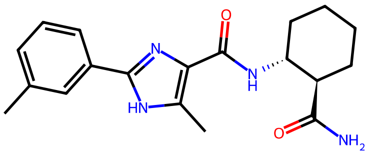	Cc1cccc(-c2nc(C(=O)N[C@@H]3CCCC[C@H]3C(N)=O)c(C)[nH]2)c1	270385603	25	340.19	2.47

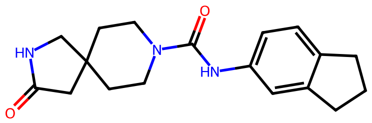	O=C1CC2(CCN(C(=O)Nc3ccc4c(c3)CCC4)CC2)CN1	267676919	23	313.18	2.31
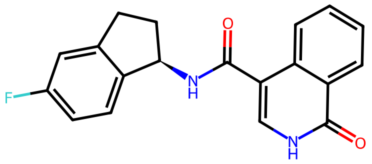	O=C(N[C@@H]1CCc2cc(F)ccc21)c1c[nH]c(=O)c2ccccc12	190286913	24	322.11	3.08
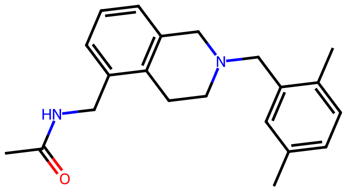	CC(=O)NCc1cccc2c1CCN(Cc1cc(C)ccc1C)C2	1051272349	24	322.2	3.5
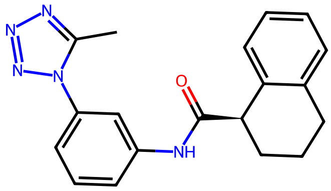	Cc1nnnn1-c1cccc(NC(=O)[C@@H]2CCCc3ccccc32)c1	41985732	25	333.16	3.03
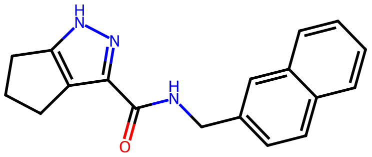	O=C(NCc1ccc2ccccc2c1)c1n[nH]c2c1CCC2	122276914	22	291.14	2.98
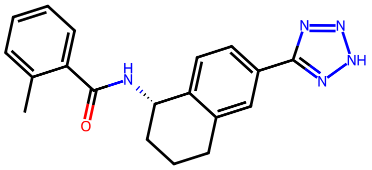	Cc1ccccc1C(=O)N[C@H]1CCCc2cc(-c3nn[nH]n3)ccc21	1605932812	25	333.16	2.98
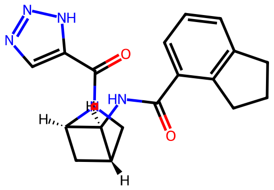	O=C(N[C@H]1[C@H]2C[C@H]1N(C(=O)c1cnn[nH]1)C2)c1cccc2c1CCC2	947377531	25	337.15	0.94
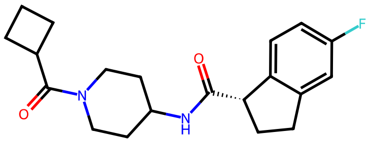	O=C(NC1CCN(C(=O)C2CCC2)CC1)[C@H]1CCc2cc(F)ccc21	943344974	25	344.19	2.76

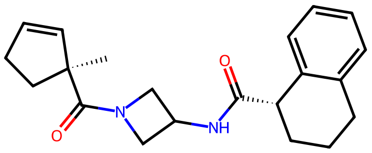	C[C@@]1(C(=O)N2CC(NC(=O)[C@H]3CCCc4ccccc43)C2)C=CCC1	995076433	25	338.2	2.79
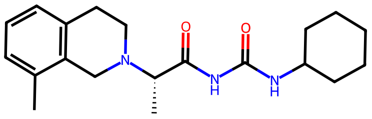	Cc1cccc2c1CN([C@@H](C)C(=O)NC(=O)NC1CCCCC1)CC2	429556448	25	343.23	2.9
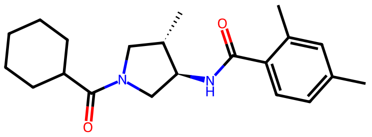	Cc1ccc(C(=O)N[C@H]2CN(C(=O)C3CCCCC3)C[C@@H]2C)c(C)c1	966119199	25	342.23	3.46
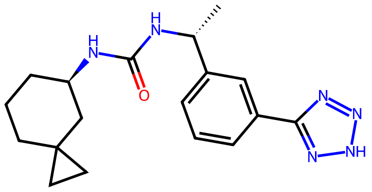	C[C@@H](NC(=O)N[C@@H]1CCCC2(CC2)C1)c1cccc(-c2nn[nH]n2)c1	1603626306	25	340.2	2.95
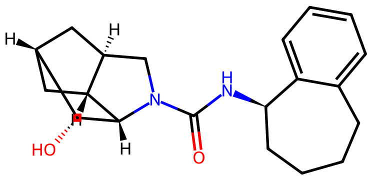	O=C(N[C@@H]1CCCCc2ccccc21)N1C[C@@H]2C[C@H]3C[C@H]2[C@H]1[C@@H]3O	570782955	24	326.2	2.86
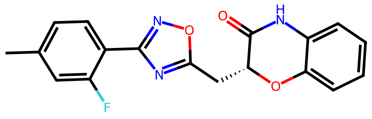	Cc1ccc(-c2noc(C[C@H]3Oc4ccccc4NC3=O)n2)c(F)c1	445383645	25	339.1	3.13
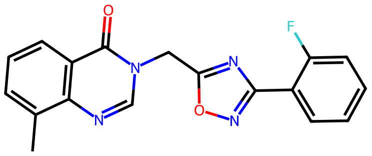	Cc1cccc2c(=O)n(Cc3nc(-c4ccccc4F)no3)cnc12	185180428	25	336.1	2.94
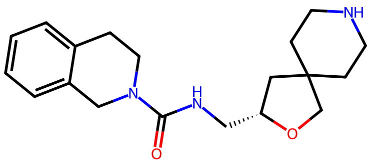	O=C(NC[C@@H]1CC2(CCNCC2)CO1)N1CCc2ccccc2C1	871673821	24	329.21	1.91

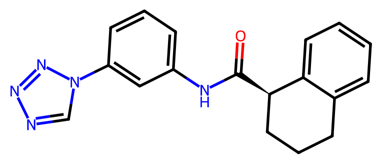	O=C(Nc1cccc(-n2cnnn2)c1)[C@@H]1CCCc2ccccc21	44363286	24	319.14	2.72
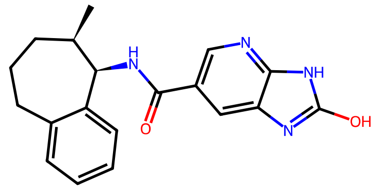	C[C@@H]1CCCc2ccccc2[C@@H]1NC(=O)c1cnc2[nH]c(O)nc2c1	572563543	25	336.16	3.11
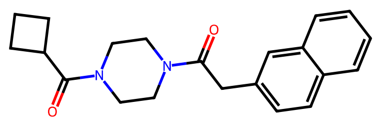	O=C(Cc1ccc2ccccc2c1)N1CCN(C(=O)C2CCC2)CC1	1009719903	25	336.18	2.85
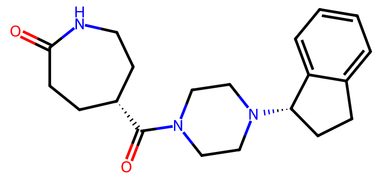	O=C1CC[C@@H](C(=O)N2CCN([C@H]3CCc4ccccc43)CC2)CCN1	949488673	25	341.21	1.73
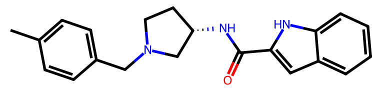	Cc1ccc(CN2CC[C@H](NC(=O)c3cc4ccccc4[nH]3)C2)cc1	1016211993	25	333.18	3.48
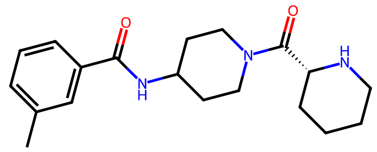	Cc1cccc(C(=O)NC2CCN(C(=O)[C@H]3CCCCN3)CC2)c1	877227050	24	329.21	1.86
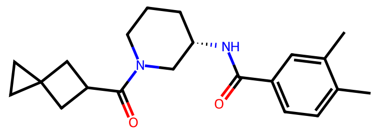	Cc1ccc(C(=O)N[C@H]2CCCN(C(=O)C3CC4(CC4)C3)C2)cc1C	951967144	25	340.22	3.21
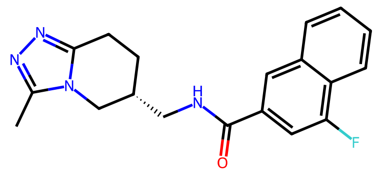	Cc1nnc2n1C[C@@H](CNC(=O)c1cc(F)c3ccccc3c1)CC2	559880753	25	338.15	2.87

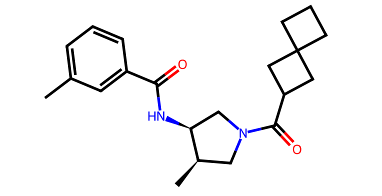	Cc1cccc(C(=O)N[C@H]2CN(C(=O)C3CC4(CCC4)C3)C[C@H]2C)c1	966699087	25	340.22	3.15
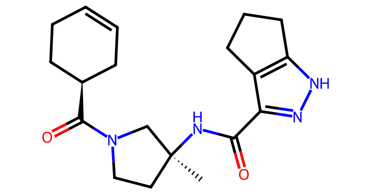	C[C@]1(NC(=O)c2n[nH]c3c2CCC3)CCN(C(=O)[C@@H]2CC=CCC2)C1	957184819	25	342.21	1.98
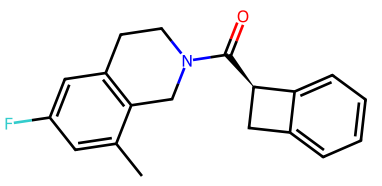	Cc1cc(F)cc2c1CN(C(=O)[C@@H]1Cc3ccccc31)CC2	337328995	22	295.14	3.36
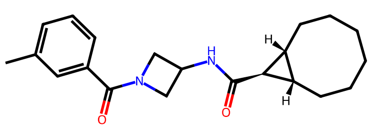	Cc1cccc(C(=O)N2CC(NC(=O)[C@@H]3[C@H]4CCCCCC[C@H]43)C2)c1	1000468302	25	340.22	3.15
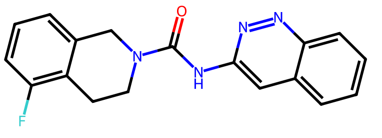	O=C(Nc1cc2ccccc2nn1)N1CCc2c(F)cccc2C1	1662976479	24	322.12	3.36
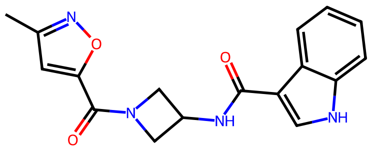	Cc1cc(C(=O)N2CC(NC(=O)c3c[nH]c4ccccc34)C2)on1	995029328	24	324.12	1.72
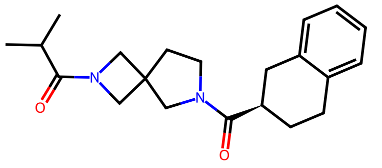	CC(C)C(=O)N1CC2(CCN(C(=O)[C@@H]3CCc4ccccc4C3)C2)C1	1018800770	25	340.22	2.51
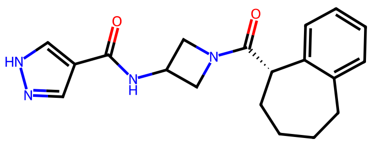	O=C(NC1CN(C(=O)[C@H]2CCCCc3ccccc32)C1)c1cn[nH]c1	990323610	25	338.17	1.86

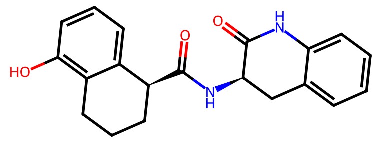	O=C(N[C@@H]1Cc2ccccc2NC1=O)[C@H]1CCCc2c(O)cccc21	529949072	25	336.15	2.49
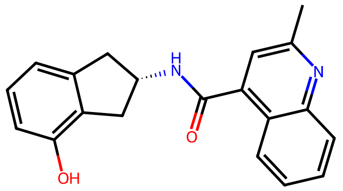	Cc1cc(C(=O)N[C@H]2Cc3cccc(O)c3C2)c2ccccc2n1	430239332	24	318.14	3.15
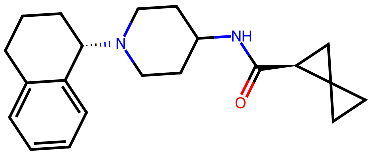	O=C(NC1CCN([C@H]2CCCc3ccccc32)CC1)[C@H]1CC12CC2	1003695566	24	324.22	3.44
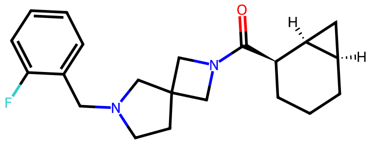	O=C([C@@H]1CCC[C@@H]2C[C@@H]21)N1CC2(CCN(Cc3ccccc3F)C2)C1	1072875762	25	342.21	3.3
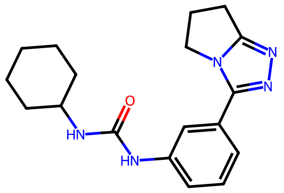	O=C(Nc1cccc(-c2nnc3n2CCC3)c1)NC1CCCCC1	76101612	24	325.19	3.35
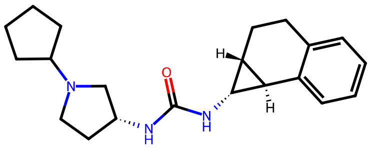	O=C(N[C@@H]1CCN(C2CCCC2)C1)N[C@H]1[C@@H]2c3ccccc3CC[C@H]21	128831205	25	339.23	3.03
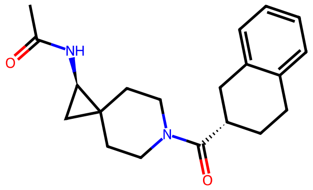	CC(=O)N[C@@H]1CC12CCN(C(=O)[C@H]1CCc3ccccc3C1)CC2	962445466	24	326.2	2.31
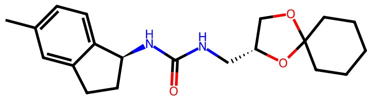	Cc1ccc2c(c1)CC[C@@H]2NC(=O)NC[C@@H]1COC2(CCCCC2)O1	775727362	25	344.21	3.36

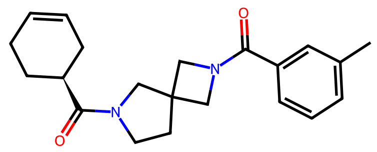	Cc1cccc(C(=O)N2CC3(CCN(C(=O)[C@@H]4CC=CCC4)C3)C2)c1	1018737300	25	338.2	3.03
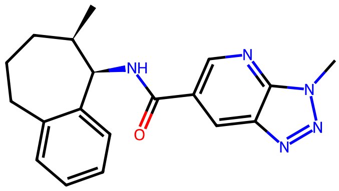	C[C@@H]1CCCc2ccccc2[C@@H]1NC(=O)c1cnc2c(c1)nnn2C	183879426	25	335.17	2.81
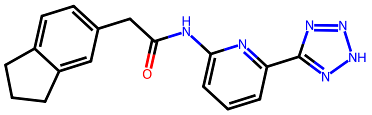	O=C(Cc1ccc2c(c1)CCC2)Nc1cccc(-c2nn[nH]n2)n1	1606599997	24	320.14	1.93
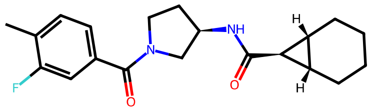	Cc1ccc(C(=O)N2CC[C@@H](NC(=O)[C@@H]3[C@H]4CCCC[C@H]43)C2)cc1F	939903508	25	344.19	2.9
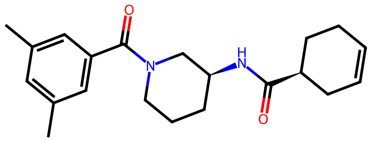	Cc1cc(C)cc(C(=O)N2CCC[C@H](NC(=O)[C@@H]3CC=CCC3)C2)c1	951988425	25	340.22	3.38
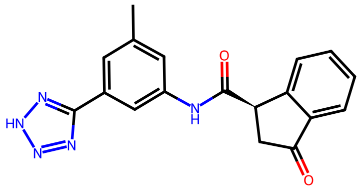	Cc1cc(NC(=O)[C@@H]2CC(=O)c3ccccc32)cc(-c2nn[nH]n2)c1	1592016804	25	333.12	2.48
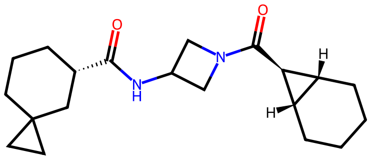	O=C(NC1CN(C(=O)[C@@H]2[C@H]3CCCC[C@H]32)C1)[C@H]1CCCC2(CC2)C1	991507775	24	330.23	2.72
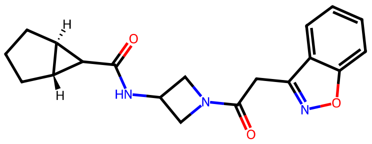	O=C(NC1CN(C(=O)Cc2noc3ccccc23)C1)C1[C@@H]2CCC[C@@H]12	990719804	25	339.16	1.74

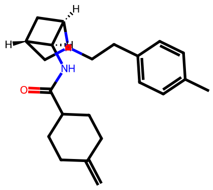	C=C1CCC(C(=O)N[C@H]2[C@H]3C[C@H]2N(CCc2ccc(C)cc2)C3)CC1	1037580742	25	338.24	3.47
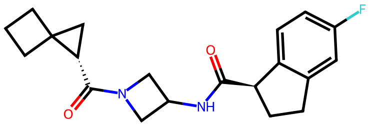	O=C(NC1CN(C(=O)[C@H]2CC23CCC3)C1)[C@@H]1CCc2cc(F)ccc21	996091270	25	342.17	2.37
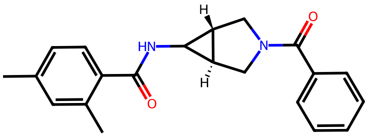	Cc1ccc(C(=O)NC2[C@@H]3CN(C(=O)c4ccccc4)C[C@@H]23)c(C)c1	1013775347	25	334.17	2.8
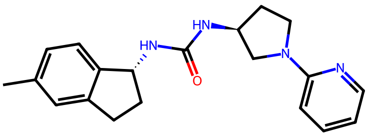	Cc1ccc2c(c1)CC[C@H]2NC(=O)N[C@H]1CCN(c2ccccn2)C1	269903447	25	336.2	2.96
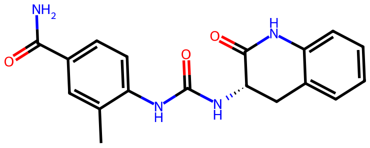	Cc1cc(C(N)=O)ccc1NC(=O)N[C@H]1Cc2ccccc2NC1=O	1425222382	25	338.14	1.78
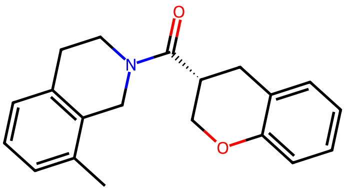	Cc1cccc2c1CN(C(=O)[C@H]1COc3ccccc3C1)CC2	469948526	23	307.16	3.13
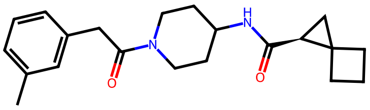	Cc1cccc(CC(=O)N2CCC(NC(=O)[C@H]3CC34CCC4)CC2)c1	948063483	25	340.22	2.83
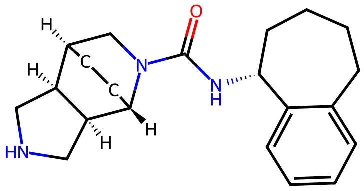	O=C(N[C@@H]1CCCCc2ccccc21)N1C[C@H]2CC[C@H]1[C@H]1CNC[C@@H]21	891473394	25	339.23	3.09

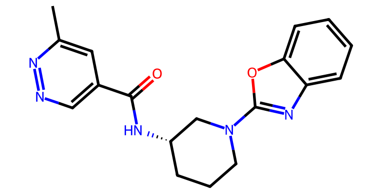	Cc1cc(C(=O)N[C@H]2CCCN(c3nc4ccccc4o3)C2)cnn1	1058149143	25	337.15	2.33
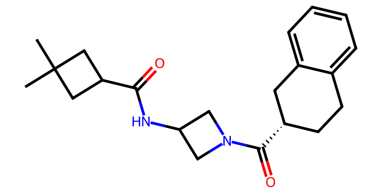	CC1(C)CC(C(=O)NC2CN(C(=O)[C@H]3CCc4ccccc4C3)C2)C1	991151697	25	340.22	2.55
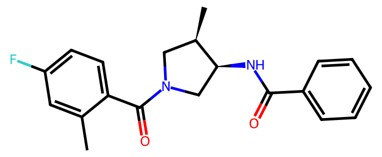	Cc1cc(F)ccc1C(=O)N1C[C@@H](C)[C@@H](NC(=O)c2ccccc2)C1	966657091	25	340.16	3.02
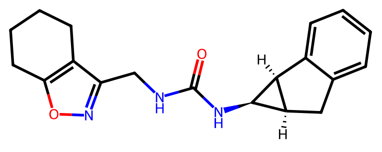	O=C(NCc1noc2c1CCCC2)N[C@@H]1[C@@H]2Cc3ccccc3[C@@H]21	288004890	24	323.16	2.69
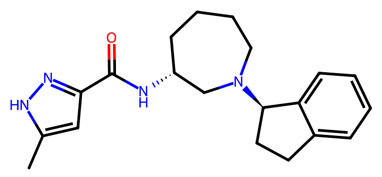	Cc1cc(C(=O)N[C@@H]2CCCCN([C@@H]3CCc4ccccc43)C2)n[nH]1	1034123586	25	338.21	2.99
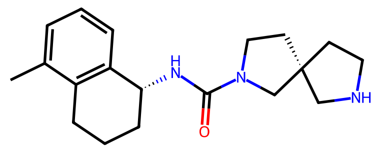	Cc1cccc2c1CCC[C@H]2NC(=O)N1CC[C@@]2(CCNC2)C1	659628757	23	313.22	2.77
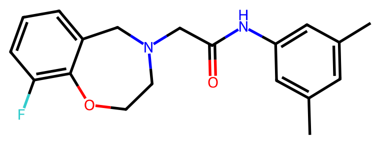	Cc1cc(C)cc(NC(=O)CN2CCOc3c(F)cccc3C2)c1	434624112	24	328.16	3.28
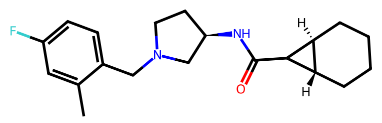	Cc1cc(F)ccc1CN1CC[C@@H](NC(=O)C2[C@H]3CCCC[C@H]23)C1	1032834928	24	330.21	3.26

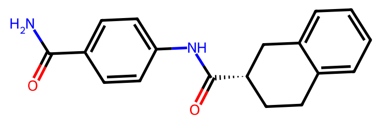	NC(=O)c1ccc(NC(=O)[C@H]2CCc3ccccc3C2)cc1	1361319652	22	294.14	2.53
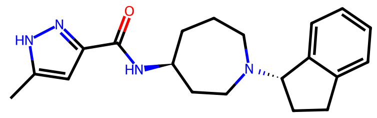	Cc1cc(C(=O)N[C@H]2CCCN([C@H]3CCc4ccccc43)CC2)n[nH]1	1052236597	25	338.21	2.99
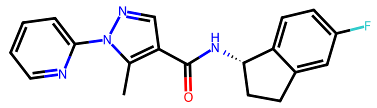	Cc1c(C(=O)N[C@H]2CCc3cc(F)ccc32)cnn1-c1ccccn1	55657477	25	336.14	3.13
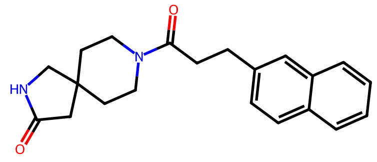	O=C1CC2(CCN(C(=O)CCc3ccc4ccccc4c3)CC2)CN1	73316846	25	336.18	2.9
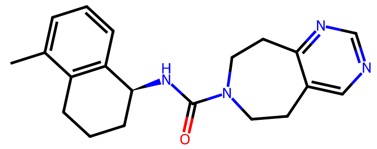	Cc1cccc2c1CCC[C@@H]2NC(=O)N1CCc2cncnc2CC1	1549363331	25	336.2	2.97
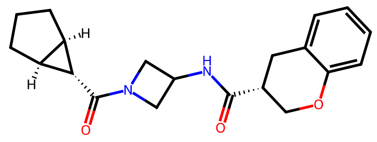	O=C(NC1CN(C(=O)[C@@H]2[C@H]3CCC[C@H]32)C1)[C@H]1COc2ccccc2C1	995466376	25	340.18	1.61
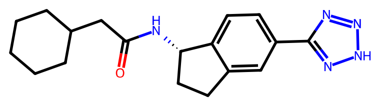	O=C(CC1CCCCC1)N[C@H]1CCc2cc(-c3nn[nH]n3)ccc21	1592819239	24	325.19	2.94
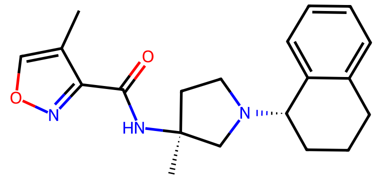	Cc1conc1C(=O)N[C@@]1(C)CCN([C@H]2CCCc3ccccc32)C1	1046752857	25	339.19	3.25

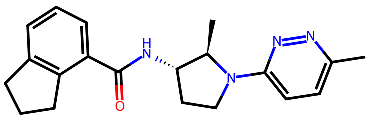	Cc1ccc(N2CC[C@H](NC(=O)c3cccc4 c3CCC4)[C@H]2C)nn1	1050385151	25	336.2	2.67
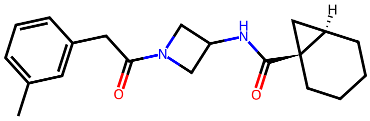	Cc1cccc(CC(=O)N2CC(NC(=O)[C@]3 4CCCC[C@@H]3C4)C2)c1	999285590	24	326.2	2.44
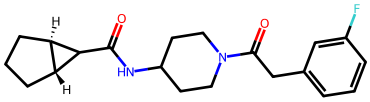	O=C(NC1CCN(C(=O)Cc2cccc(F)c2)C C1)C1[C@@H]2CCC[C@@H]12	948481087	25	344.19	2.52
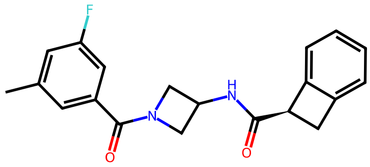	Cc1cc(F)cc(C(=O)N2CC(NC(=O)[C@ @H]3Cc4ccccc43)C2)c1	998694514	25	338.14	2.41
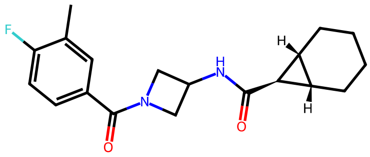	Cc1cc(C(=O)N2CC(NC(=O)[C@@H]3 [C@H]4CCCC[C@H]43)C2)ccc1F	990652019	24	330.17	2.51
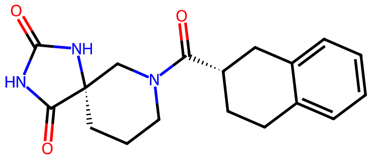	O=C1NC(=O)[C@@]2(CCCN(C(=O)[C@H]3CCc4ccccc4C3)C2)N1	392626371	24	327.16	0.99
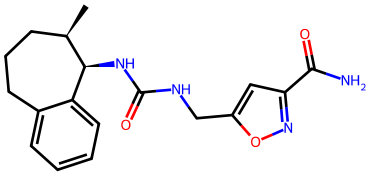	C[C@@H]1CCCc2ccccc2[C@@H]1N C(=O)NCc1cc(C(N)=O)no1	274229070	25	342.17	2.29
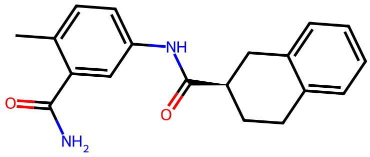	Cc1ccc(NC(=O)[C@@H]2CCc3ccccc3 C2)cc1C(N)=O	575410125	23	308.15	2.84

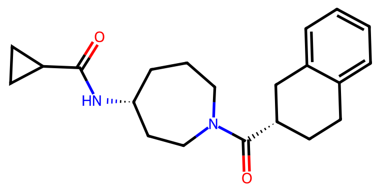	O=C(N[C@@H]1CCCN(C(=O)[C@H]2CCc3ccccc3C2)CC1)C1CC1	964676110	25	340.22	2.7
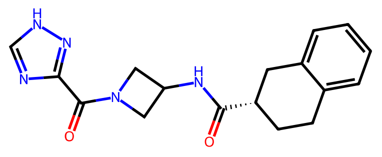	O=C(NC1CN(C(=O)c2nc[nH]n2)C1)[C@H]1CCc2ccccc2C1	1000537334	24	325.15	0.55
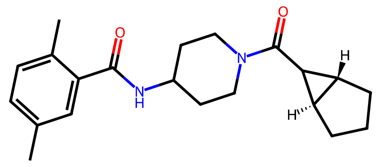	Cc1ccc(C)c(C(=O)NC2CCN(C(=O)C3[C@@H]4CCC[C@@H]34)CC2)c1	946079866	25	340.22	3.07
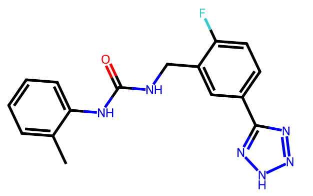	Cc1ccccc1NC(=O)NCc1cc(-c2nn[nH]n2)ccc1F	822362444	24	326.13	2.64
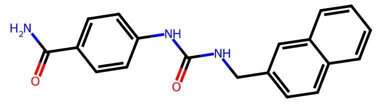	NC(=O)c1ccc(NC(=O)NCc2ccc3ccccc3c2)cc1	69070518	24	319.13	3.26
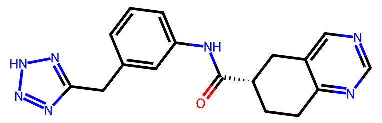	O=C(Nc1cccc(Cc2nn[nH]n2)c1)[C@H]1CCc2ncncc2C1	1637393779	25	335.15	1.32
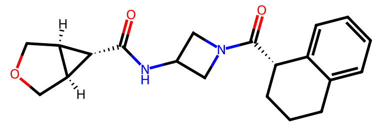	O=C(NC1CN(C(=O)[C@H]2CCCc3ccccc32)C1)[C@@H]1[C@H]2COC[C@H]21	997923271	25	340.18	1.33
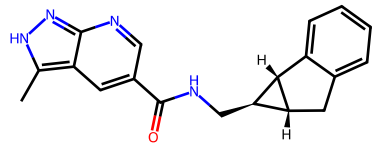	Cc1[nH]nc2ncc(C(=O)NC[C@@H]3[C@H]4Cc5ccccc5[C@H]43)cc12	246236469	24	318.15	2.58

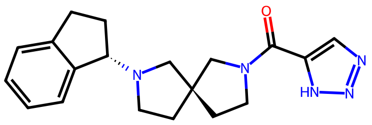	O=C(c1cnn[nH]1)N1CC[C@]2(CCN([C@H]3CCc4ccccc43)C2)C1	1041434226	25	337.19	2.03
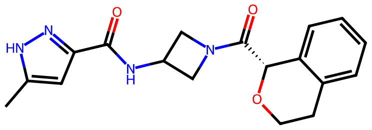	Cc1cc(C(=O)NC2CN(C(=O)[C@H]3OCCc4ccccc43)C2)n[nH]1	990370870	25	340.15	0.97
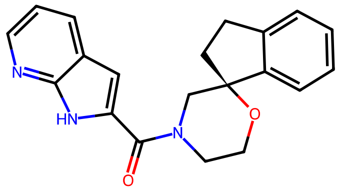	O=C(c1cc2cccnc2[nH]1)N1CCO[C@]2(CCc3ccccc32)C1	665384201	25	333.15	2.88
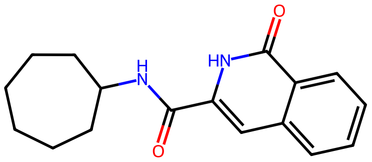	O=C(NC1CCCCCC1)c1cc2ccccc2c(=O)[nH]1	6590068	21	284.15	2.98
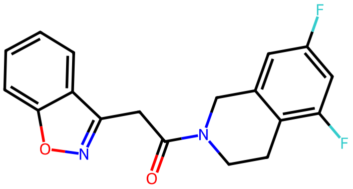	O=C(Cc1noc2ccccc12)N1CCc2c(F)cc(F)cc2C1	122573488	24	328.1	3.23
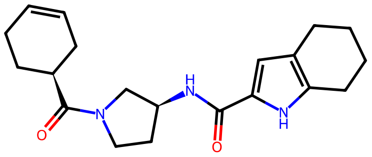	O=C(N[C@H]1CCN(C(=O)[C@@H]2CC=CCC2)C1)c1cc2c([nH]1)CCCC2	938789835	25	341.21	2.58
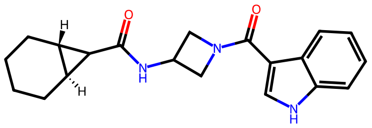	O=C(NC1CN(C(=O)c2c[nH]c3ccccc23)C1)C1[C@H]2CCCC[C@H]12	990665764	25	337.18	2.54
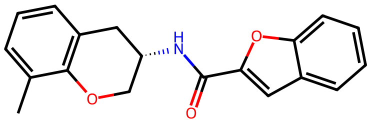	Cc1cccc2c1OC[C@@H](NC(=O)c1cc3ccccc3o1)C2	685822558	23	307.12	3.47

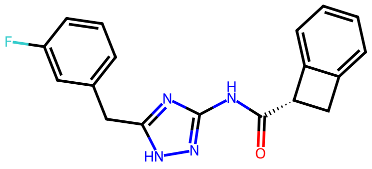	O=C(Nc1n[nH]c(Cc2cccc(F)c2)n1)[C@H]1Cc2ccccc21	358555357	24	322.12	2.81
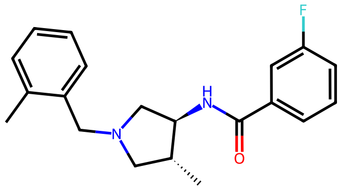	Cc1ccccc1CN1C[C@@H](NC(=O)c2cccc(F)c2)[C@H](C)C1	1079378005	24	326.18	3.38
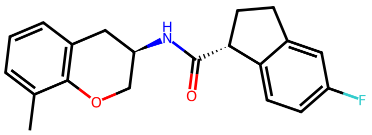	Cc1cccc2c1OC[C@H](NC(=O)[C@@H]1CCc3cc(F)ccc31)C2	615059820	24	325.15	3.28
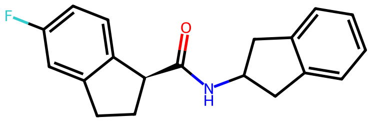	O=C(NC1Cc2ccccc2C1)[C@H]1CCc2cc(F)ccc21	613521223	22	295.14	3.14
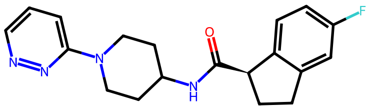	O=C(NC1CCN(c2cccnn2)CC1)[C@@H]1CCc2cc(F)ccc21	614003200	25	340.17	2.43
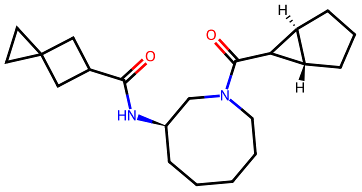	O=C(N[C@@H]1CCCCCN(C(=O)C2[C@H]3CCC[C@H]23)C1)C1CC2(CC2)C1	986974524	25	344.25	3.11
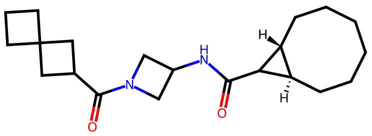	O=C(NC1CN(C(=O)C2CC3(CCC3)C2)C1)C1[C@@H]2CCCCCC[C@@H]12	1000507785	25	344.25	3.11
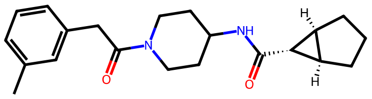	Cc1cccc(CC(=O)N2CCC(NC(=O)[C@@H]3[C@H]4CCC[C@H]43)CC2)c1	948068521	25	340.22	2.69

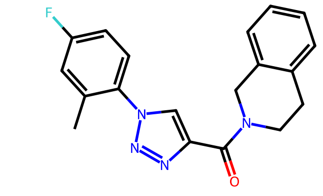	Cc1cc(F)ccc1-n1cc(C(=O)N2CCc3ccccc3C2)nn1	179958421	25	336.14	2.91
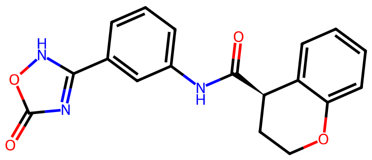	O=C(Nc1cccc(-c2nc(=O)o[nH]2)c1)[C@@H]1CCOc2ccccc21	591188481	25	337.11	2.53
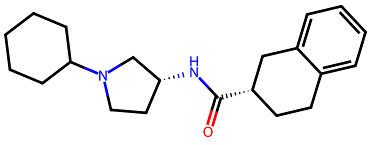	O=C(N[C@@H]1CCN(C2CCCCC2)C1)[C@H]1CCc2ccccc2C1	618656947	24	326.24	3.31
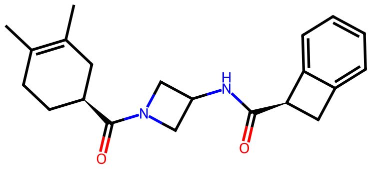	CC1=C(C)C[C@@H](C(=O)N2CC(NC(=O)[C@@H]3Cc4ccccc43)C2)CC1	998686645	25	338.2	2.79
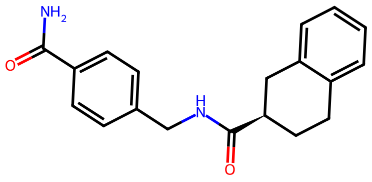	NC(=O)c1ccc(CNC(=O)[C@@H]2CCc3ccccc3C2)cc1	584722441	23	308.15	2.21
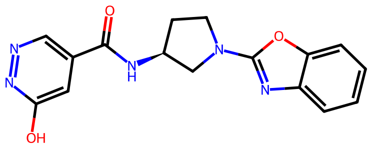	O=C(N[C@H]1CCN(c2nc3ccccc3o2)C1)c1cnnc(O)c1	1058473188	24	325.12	1.33
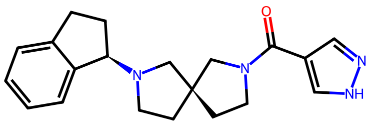	O=C(c1cn[nH]c1)N1CC[C@]2(CCN([C@@H]3CCc4ccccc43)C2)C1	1041104345	25	336.2	2.64
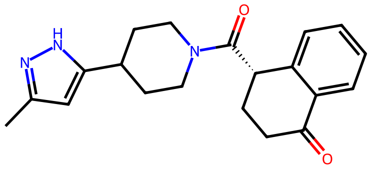	Cc1cc(C2CCN(C(=O)[C@H]3CCC(=O)c4ccccc43)CC2)[nH]n1	285722555	25	337.18	3.18

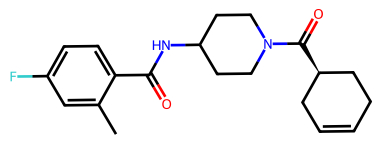	Cc1cc(F)ccc1C(=O)NC1CCN(C(=O)[C@@H]2CC=CCC2)CC1	944113609	25	344.19	3.21
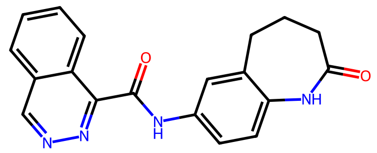	O=C1CCCc2cc(NC(=O)c3nncc4ccccc34)ccc2N1	1125844856	25	332.13	3.16
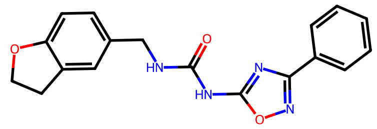	O=C(NCc1ccc2c(c1)CCO2)Nc1nc(-c2ccccc2)no1	1676056104	25	336.12	2.99
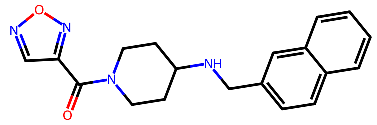	O=C(c1cnon1)N1CCC(NCc2ccc3ccccc3c2)CC1	997099813	25	336.16	2.62
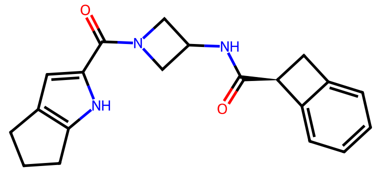	O=C(NC1CN(C(=O)c2cc3c([nH]2)CCC3)C1)[C@H]1Cc2ccccc21	998680745	25	335.16	1.78
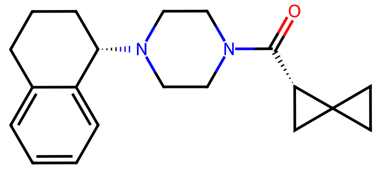	O=C([C@H]1CC12CC2)N1CCN([C@H]2CCCc3ccccc32)CC1	950710786	23	310.2	3.01
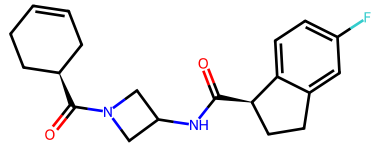	O=C(NC1CN(C(=O)[C@@H]2CC=CCC2)C1)[C@@H]1CCc2cc(F)ccc21	996094472	25	342.17	2.54
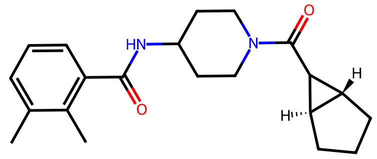	Cc1cccc(C(=O)NC2CCN(C(=O)C3[C@@H]4CCC[C@@H]34)CC2)c1C	946079487	25	340.22	3.07

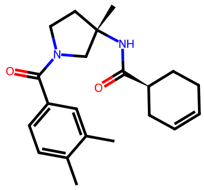	Cc1ccc(C(=O)N2CC[C@](C)(NC(=O)[C@@H]3CC=CCC3)C2)cc1C	956691100	25	340.22	3.38
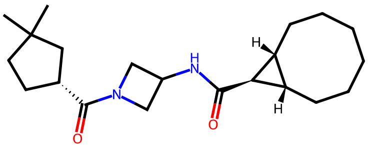	CC1(C)CC[C@@H](C(=O)N2CC(NC(=O)[C@@H]3[C@H]4CCCCCC[C@H]43)C2)C1	1000508396	25	346.26	3.36
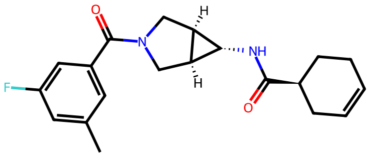	Cc1cc(F)cc(C(=O)N2C[C@H]3[C@H](NC(=O)[C@@H]4CC=CCC4)[C@H]3C2)c1	976175660	25	342.17	2.68
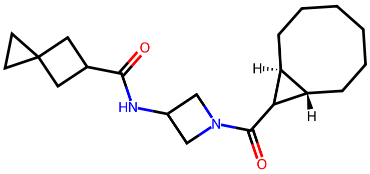	O=C(NC1CN(C(=O)C2[C@H]3CCCCCC[C@H]23)C1)C1CC2(CC2)C1	999347714	24	330.23	2.72
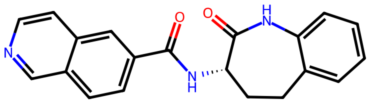	O=C(N[C@H]1CCc2ccccc2NC1=O)c1ccc2cnccc2c1	1537777754	25	331.13	2.92
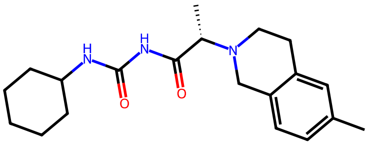	Cc1ccc2c(c1)CCN([C@@H](C)C(=O)NC(=O)NC1CCCCC1)C2	361841068	25	343.23	2.9
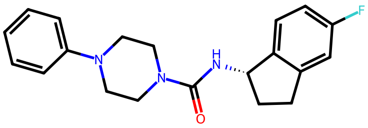	O=C(N[C@H]1CCc2cc(F)ccc21)N1CCN(c2ccccc2)CC1	56838593	25	339.17	3.34
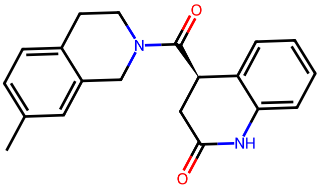	Cc1ccc2c(c1)CN(C(=O)[C@@H]1CC(=O)Nc3ccccc31)CC2	1130662838	24	320.15	3.01

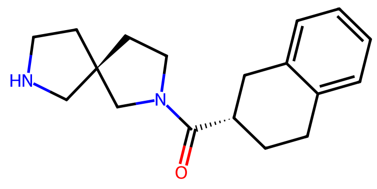	O=C([C@H]1CCc2ccccc2C1)N1CC[C@@]2(CCNC2)C1	314203819	21	284.19	2.0
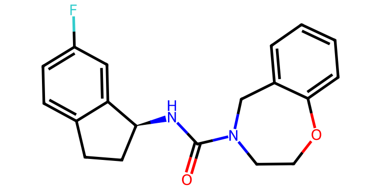	O=C(N[C@H]1CCc2ccc(F)cc21)N1CCOc2ccccc2C1	592546942	24	326.14	3.42
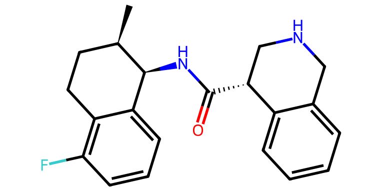	C[C@@H]1CCc2c(F)cccc2[C@@H]1NC(=O)[C@@H]1CNCc2ccccc21	566190195	25	338.18	3.45
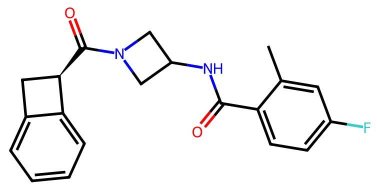	Cc1cc(F)ccc1C(=O)NC1CN(C(=O)[C@@H]2Cc3ccccc32)C1	997181369	25	338.14	2.41
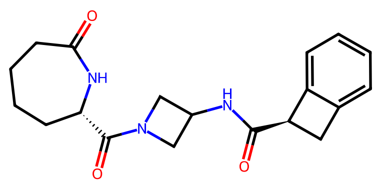	O=C1CCCC[C@@H](C(=O)N2CC(NC(=O)[C@@H]3Cc4ccccc43)C2)N1	998690526	25	341.17	0.71
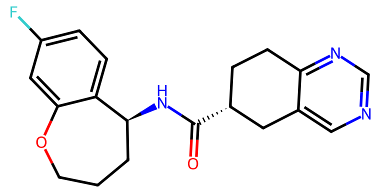	O=C(N[C@H]1CCCOc2cc(F)ccc21)[C@@H]1CCc2ncncc2C1	648347052	25	341.15	2.75
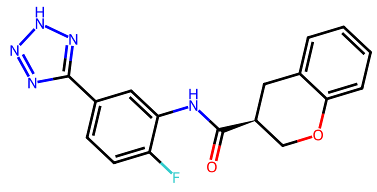	O=C(Nc1cc(-c2nn[nH]n2)ccc1F)[C@@H]1COc2ccccc2C1	1275482840	25	339.11	2.2
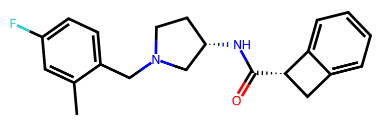	Cc1cc(F)ccc1CN1CC[C@H](NC(=O)[C@H]2Cc3ccccc32)C1	1015636265	25	338.18	3.16

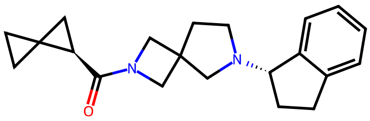	O=C([C@@H]1CC12CC2)N1CC2(CCN([C@H]3CCc4ccccc43)C2)C1	1073013328	24	322.2	3.01
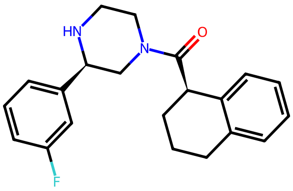	O=C([C@@H]1CCCc2ccccc21)N1CCN[C@H](c2cccc(F)c2)C1	337589466	25	338.18	3.42
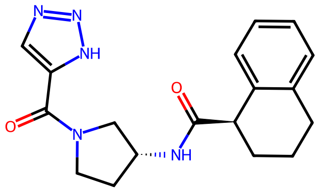	O=C(N[C@@H]1CCN(C(=O)c2cnn[nH]2)C1)[C@@H]1CCCc2ccccc21	941389137	25	339.17	1.26
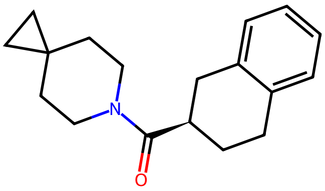	O=C([C@@H]1CCc2ccccc2C1)N1CCC2(CC1)CC2	649715266	20	269.18	3.19
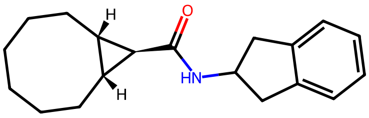	O=C(NC1Cc2ccccc2C1)[C@@H]1[C@H]2CCCCCC[C@H]21	1127809050	21	283.19	3.49
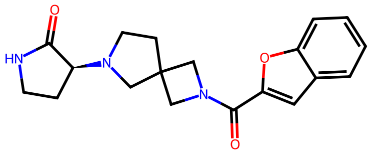	O=C1NCC[C@@H]1N1CCC2(CN(C(=O)c3cc4ccccc4o3)C2)C1	1073403947	25	339.16	1.47
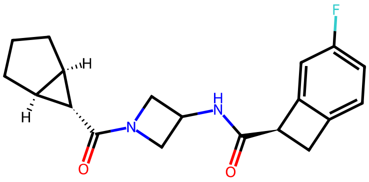	O=C(NC1CN(C(=O)[C@@H]2[C@H]3CCC[C@H]32)C1)[C@@H]1Cc2ccc(F)cc21	998486443	24	328.16	1.84
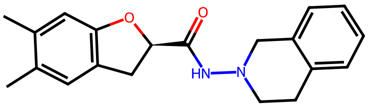	Cc1cc2c(cc1C)O[C@@H](C(=O)NN1CCc3ccccc3C1)C2	1469893173	24	322.17	2.7

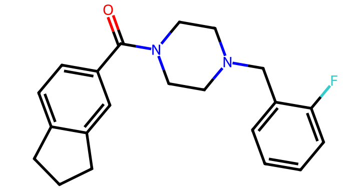	O=C(c1ccc2c(c1)CCC2)N1CCN(Cc2ccccc2F)CC1	23441717	25	338.18	3.27
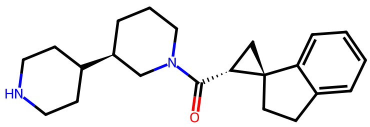	O=C([C@@H]1C[C@@]12CCc1ccccc12)N1CCC[C@H](C2CCNCC2)C1	662596887	25	338.24	3.13
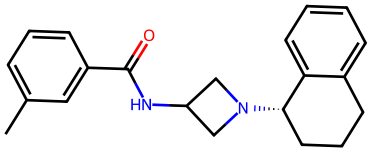	Cc1cccc(C(=O)NC2CN([C@H]3CCCc4ccccc43)C2)c1	1030074877	24	320.19	3.49
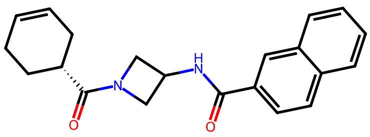	O=C(NC1CN(C(=O)[C@H]2CC=CCC2)C1)c1ccc2ccccc2c1	999448734	25	334.17	3.14
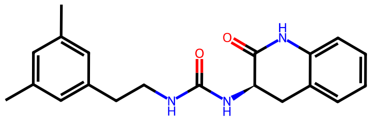	Cc1cc(C)cc(CCNC(=O)N[C@@H]2Cc3ccccc3NC2=O)c1	1365110150	25	337.18	2.71
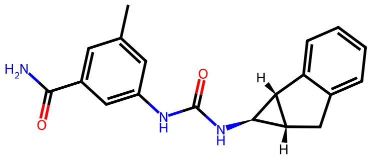	Cc1cc(NC(=O)N[C@@H]2[C@H]3Cc4ccccc4[C@H]32)cc(C(N)=O)c1	576560111	24	321.15	2.55
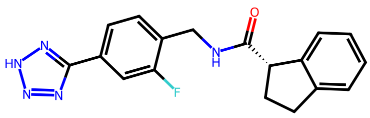	O=C(NCc1ccc(-c2nn[nH]n2)cc1F)[C@H]1CCc2ccccc21	738100553	25	337.13	2.35
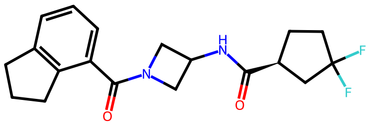	O=C(NC1CN(C(=O)c2cccc3c2CCC3)C1)[C@H]1CCC(F)(F)C1	994170613	25	348.16	2.55

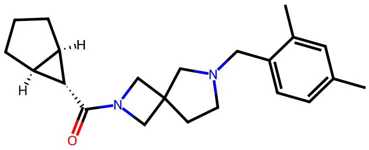	Cc1ccc(CN2CCC3(C2)CN(C(=O)[C@@H]2[C@H]4CCC[C@H]42)C3)c(C)c1	1072592456	25	338.24	3.38
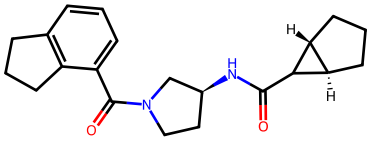	O=C(N[C@H]1CCN(C(=O)c2cccc3c2CCC3)C1)C1[C@@H]2CCC[C@@H]12	937800260	25	338.2	2.55
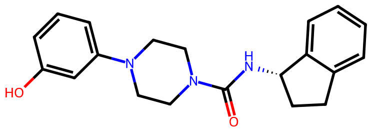	O=C(N[C@H]1CCc2ccccc21)N1CCN(c2cccc(O)c2)CC1	121641480	25	337.18	2.91
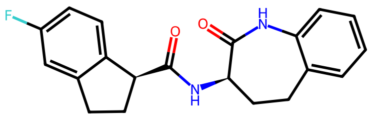	O=C(N[C@@H]1CCc2ccccc2NC1=O)[C@H]1CCc2cc(F)ccc21	616691827	25	338.14	2.93
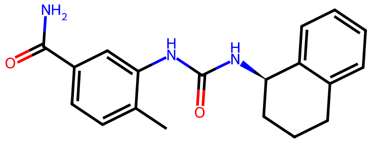	Cc1ccc(C(N)=O)cc1NC(=O)N[C@@H]1CCCc2ccccc21	69605306	24	323.16	3.29
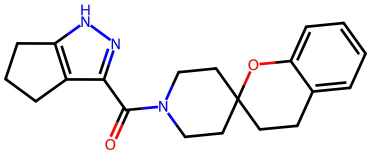	O=C(c1n[nH]c2c1CCC2)N1CCC2(CCc3ccccc3O2)CC1	620231425	25	337.18	2.9
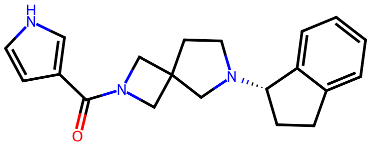	O=C(c1cc[nH]c1)N1CC2(CCN([C@H]3CCc4ccccc43)C2)C1	1072769008	24	321.18	2.85
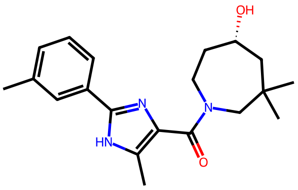	Cc1cccc(-c2nc(C(=O)N3CC[C@H](O)CC(C)(C)C3)c(C)[nH]2)c1	296786926	25	341.21	3.32

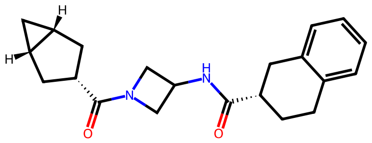	O=C(NC1CN(C(=O)[C@@H]2C[C@@H]3C[C@@H]3C2)C1)[C@H]1CCc2ccccc2C1	1000543127	25	338.2	2.16
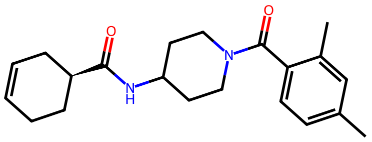	Cc1ccc(C(=O)N2CCC(NC(=O)[C@@H]3CC=CCC3)CC2)c(C)c1	948346323	25	340.22	3.38
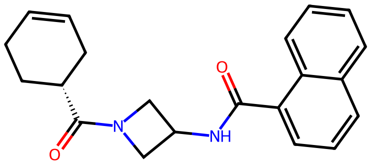	O=C(NC1CN(C(=O)[C@H]2CC=CCC2)C1)c1cccc2ccccc12	999544535	25	334.17	3.14
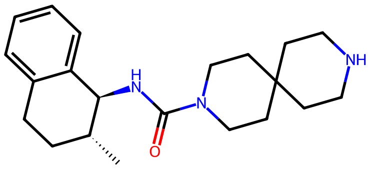	C[C@@H]1CCc2ccccc2[C@H]1NC(=O)N1CCC2(CCNCC2)CC1	417945385	25	341.25	3.49
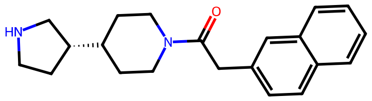	O=C(Cc1ccc2ccccc2c1)N1CCC([C@@H]2CCNC2)CC1	289493265	24	322.2	3.23
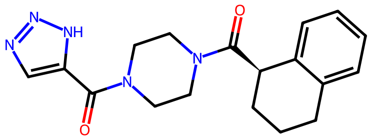	O=C(c1cnn[nH]1)N1CCN(C(=O)[C@@H]2CCCc3ccccc32)CC1	1011403011	25	339.17	1.21
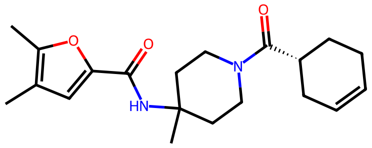	Cc1cc(C(=O)NC2(C)CCN(C(=O)[C@H]3CC=CCC3)CC2)oc1C	956339952	25	344.21	3.36
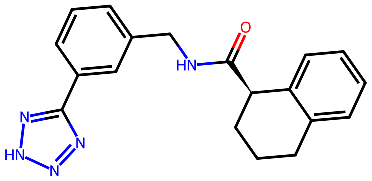	O=C(NCc1cccc(-c2nn[nH]n2)c1)[C@@H]1CCCc2ccccc21	1606835305	25	333.16	2.6

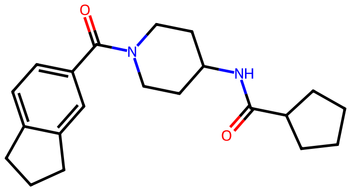	O=C(NC1CCN(C(=O)c2ccc3c(c2)CCC3)CC1)C1CCCC1	946720617	25	340.22	3.09
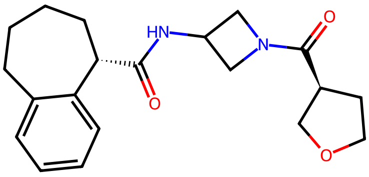	O=C(NC1CN(C(=O)[C@H]2CCOC2)C1)[C@H]1CCCCc2ccccc21	994305240	25	342.19	1.86
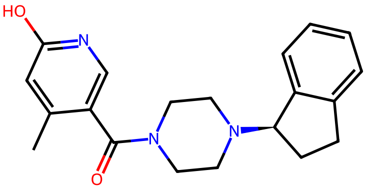	Cc1cc(O)ncc1C(=O)N1CCN([C@@H]2CCc3ccccc32)CC1	950426529	25	337.18	2.54
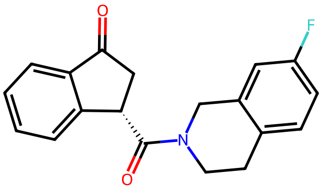	O=C1C[C@H](C(=O)N2CCc3ccc(F)cc3C2)c2ccccc21	277280532	23	309.12	3.08
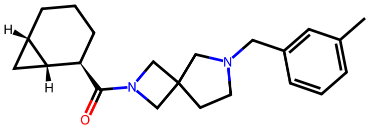	Cc1cccc(CN2CCC3(C2)CN(C(=O)[C@@H]2CCC[C@H]4C[C@H]42)C3)c1	1072875666	25	338.24	3.47
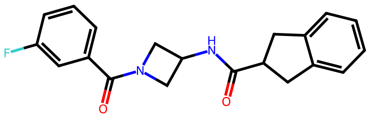	O=C(NC1CN(C(=O)c2cccc(F)c2)C1)C1Cc2ccccc2C1	1000447462	25	338.14	2.18
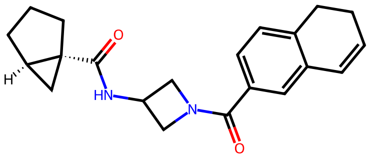	O=C(c1ccc2c(c1)C=CCC2)N1CC(NC(=O)[C@@]23CCC[C@@H]2C3)C1	997537538	25	336.18	2.78
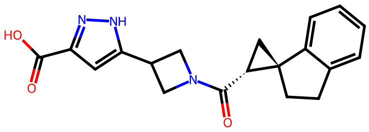	O=C(O)c1cc(C2CN(C(=O)[C@@H]3C[C@@]34CCc3ccccc34)C2)[nH]n1	1602253149	25	337.14	1.94

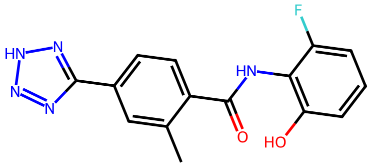	Cc1cc(-c2nn[nH]n2)ccc1C(=O)Nc1c(O)cccc1F	826249423	23	313.1	2.27
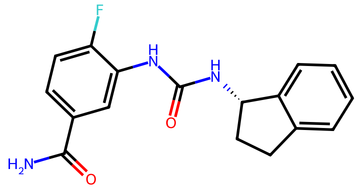	NC(=O)c1ccc(F)c(NC(=O)N[C@H]2CCc3ccccc32)c1	47874814	23	313.12	2.73
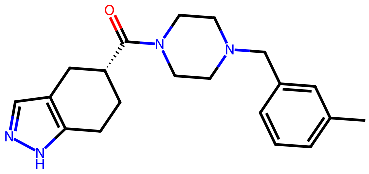	Cc1cccc(CN2CCN(C(=O)[C@@H]3CCc4[nH]ncc4C3)CC2)c1	270173616	25	338.21	2.17
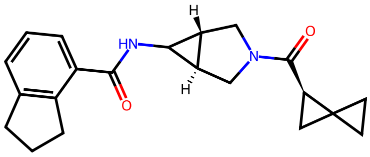	O=C(NC1[C@@H]2CN(C(=O)[C@@H]3CC34CC4)C[C@@H]12)c1cccc2c1CCC2	1013293654	25	336.18	2.16
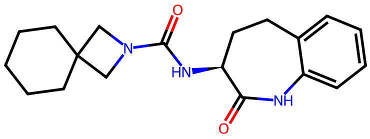	O=C1Nc2ccccc2CC[C@@H]1NC(=O)N1CC2(CCCCC2)C1	654628864	24	327.19	2.92
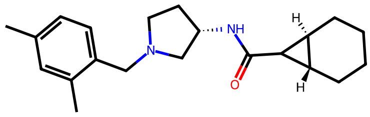	Cc1ccc(CN2CC[C@H](NC(=O)C3[C@H]4CCCC[C@H]34)C2)c(C)c1	1010438647	24	326.24	3.43
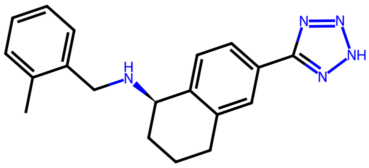	Cc1ccccc1CN[C@@H]1CCCc2cc(-c3nn[nH]n3)ccc21	826307431	24	319.18	3.34
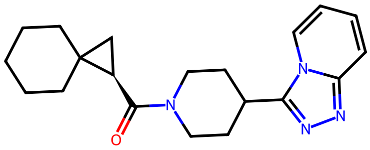	O=C([C@@H]1CC12CCCCC2)N1CCC(c2nnc3ccccn23)CC1	370568828	25	338.21	3.41

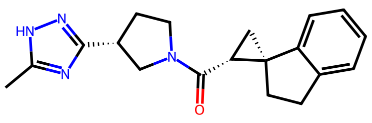	Cc1nc([C@@H]2CCN(C(=O)[C@@H]3C[C@]34CCc3ccccc34)C2)n[nH]1	333996517	24	322.18	2.33
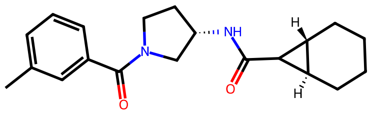	Cc1cccc(C(=O)N2CC[C@H](NC(=O)C3[C@@H]4CCCC[C@@H]34)C2)c1	937787186	24	326.2	2.76
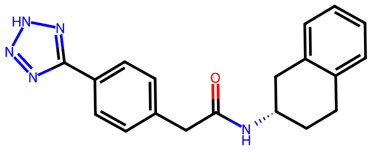	O=C(Cc1ccc(-c2nn[nH]n2)cc1)N[C@H]1CCc2ccccc2C1	1592911607	25	333.16	2.08
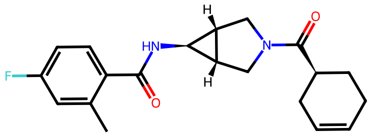	Cc1cc(F)ccc1C(=O)N[C@H]1[C@@H]2CN(C(=O)[C@@H]3CC=CCC3)C[C@@H]21	976919008	25	342.17	2.68
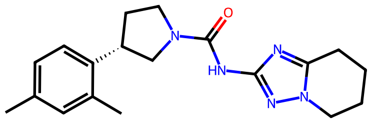	Cc1ccc([C@@H]2CCN(C(=O)Nc3nc4n(n3)CCCC4)C2)c(C)c1	333723935	25	339.21	3.25
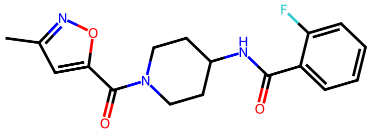	Cc1cc(C(=O)N2CCC(NC(=O)c3ccccc3F)CC2)on1	43065243	24	331.13	2.16
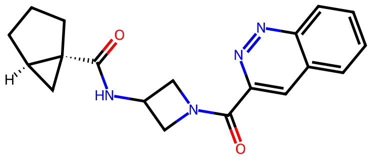	O=C(c1cc2ccccc2nn1)N1CC(NC(=O)[C@@]23CCC[C@@H]2C3)C1	997552814	25	336.16	1.76
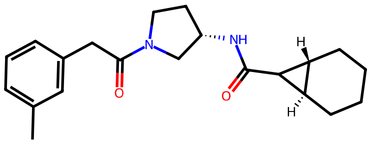	Cc1cccc(CC(=O)N2CC[C@H](NC(=O)C3[C@@H]4CCCC[C@@H]34)C2)c1	937788593	25	340.22	2.69

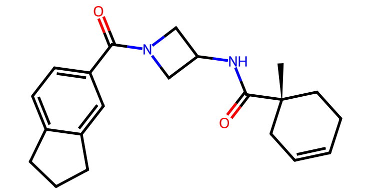	C[C@]1(C(=O)NC2CN(C(=O)c3ccc4c(c3)CCC4)C2)CC=CCC1	991914554	25	338.2	2.86
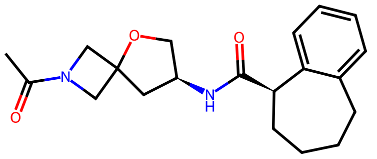	CC(=O)N1CC2(C[C@H](NC(=O)[C@@H]3CCCCc4ccccc43)CO2)C1	965739649	25	342.19	2.0
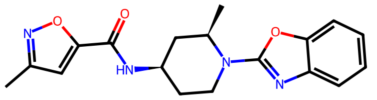	Cc1cc(C(=O)N[C@@H]2CCN(c3nc4ccccc4o3)[C@H](C)C2)on1	1067507716	25	340.15	2.91
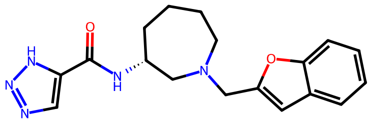	O=C(N[C@@H]1CCCCN(Cc2cc3ccccc3o2)C1)c1cnn[nH]1	1034284664	25	339.17	2.34
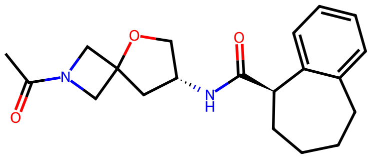	CC(=O)N1CC2(C[C@@H](NC(=O)[C@@H]3CCCCc4ccccc43)CO2)C1	965739650	25	342.19	2.0
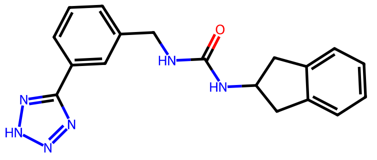	O=C(NCc1cccc(-c2nn[nH]n2)c1)NC1Cc2ccccc2C1	1609556000	25	334.15	1.83
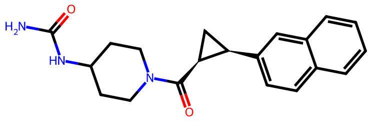	NC(=O)NC1CCN(C(=O)[C@H]2C[C@H]2c2ccc3ccccc3c2)CC1	176057369	25	337.18	2.6
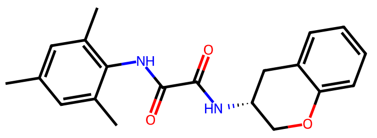	Cc1cc(C)c(NC(=O)C(=O)N[C@H]2COc3ccccc3C2)c(C)c1	42049452	25	338.16	2.67

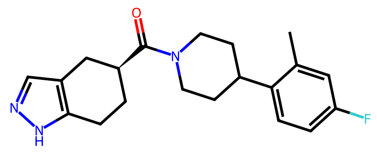	Cc1cc(F)ccc1C1CCN(C(=O)[C@H]2CCc3[nH]ncc3C2)CC1	292954878	25	341.19	3.37
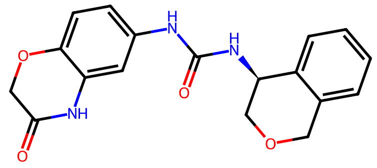	O=C1COc2ccc(NC(=O)N[C@@H]3COCc4ccccc43)cc2N1	1464967628	25	339.12	2.41
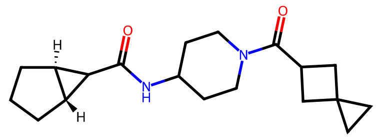	O=C(NC1CCN(C(=O)C2CC3(CC3)C2)CC1)C1[C@@H]2CCC[C@@H]12	948299970	23	316.22	2.33
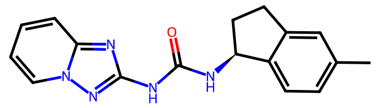	Cc1ccc2c(c1)CC[C@@H]2NC(=O)Nc1nc2ccccn2n1	353914955	23	307.14	2.85
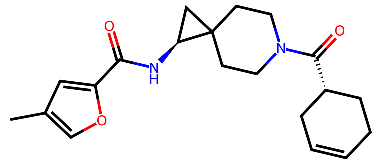	Cc1coc(C(=O)N[C@H]2CC23CCN(C(=O)[C@H]2CC=CCC2)CC3)c1	964714526	25	342.19	3.06
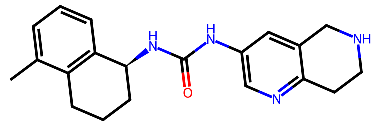	Cc1cccc2c1CCC[C@@H]2NC(=O)Nc1cnc2c(c1)CNCC2	659019476	25	336.2	3.23
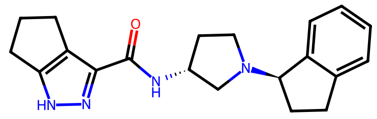	O=C(N[C@@H]1CCN([C@@H]2CCc3ccccc32)C1)c1n[nH]c2c1CCC2	1015070832	25	336.2	2.39
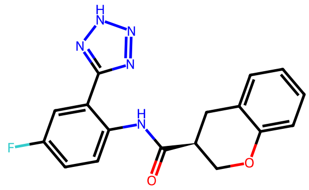	O=C(Nc1ccc(F)cc1-c1nn[nH]n1)[C@@H]1COc2ccccc2C1	823343643	25	339.11	2.2

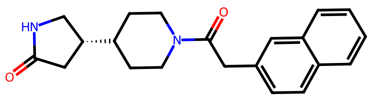	O=C1C[C@@H](C2CCN(C(=O)Cc3ccc4ccccc4c3)CC2)CN1	1551713765	25	336.18	2.76
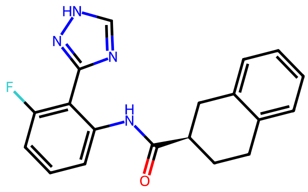	O=C(Nc1cccc(F)c1-c1nc[nH]n1)[C@@H]1CCc2ccccc2C1	1639693334	25	336.14	3.35
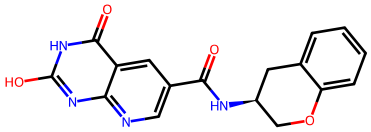	O=C(N[C@@H]1COc2ccccc2C1)c1cnc2nc(O)[nH]c(=O)c2c1	53047092	25	338.1	0.76
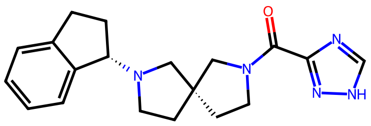	O=C(c1nc[nH]n1)N1CC[C@@]2(CCN([C@H]3CCc4ccccc43)C2)C1	1041748562	25	337.19	2.03
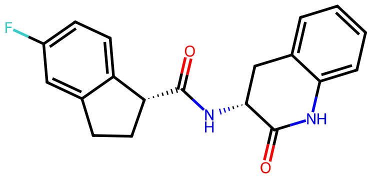	O=C1Nc2ccccc2C[C@H]1NC(=O)[C@@H]1CCc2cc(F)ccc21	529945399	24	324.13	2.54
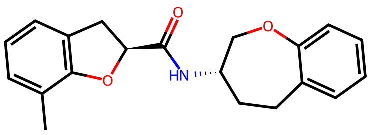	Cc1cccc2c1O[C@H](C(=O)N[C@H]1CCc3ccccc3OC1)C2	451487670	24	323.15	2.81
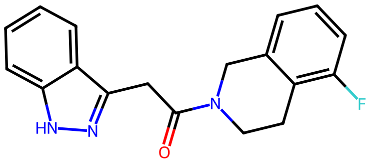	O=C(Cc1n[nH]c2ccccc12)N1CCc2c(F)cccc2C1	162754428	23	309.13	2.83
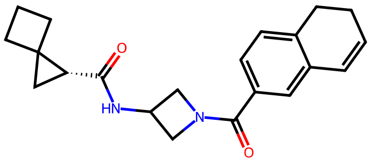	O=C(NC1CN(C(=O)c2ccc3c(c2)C=CCC3)C1)[C@@H]1CC12CCC2	991534286	25	336.18	2.78

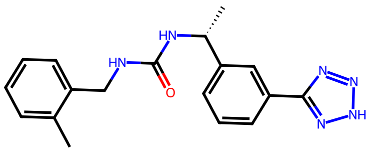	Cc1ccccc1CNC(=O)N[C@H](C)c1cccc(-c2nn[nH]n2)c1	822361881	25	336.17	2.74
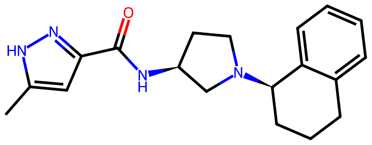	Cc1cc(C(=O)N[C@H]2CCN([C@@H]3CCCc4ccccc43)C2)n[nH]1	1010333900	24	324.2	2.6
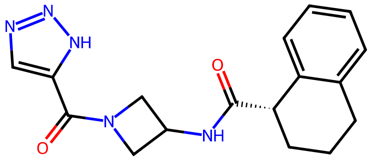	O=C(NC1CN(C(=O)c2cnn[nH]2)C1)[C@H]1CCCc2ccccc21	995078852	24	325.15	0.87
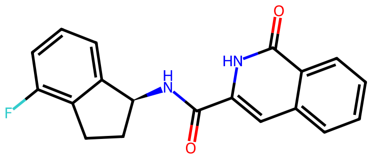	O=C(N[C@H]1CCc2c(F)cccc21)c1cc2ccccc2c(=O)[nH]1	56325506	24	322.11	3.08
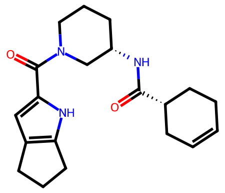	O=C(N[C@H]1CCCN(C(=O)c2cc3c([nH]2)CCC3)C1)[C@H]1CC=CCC1	951367692	25	341.21	2.58
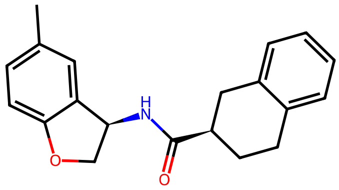	Cc1ccc2c(c1)[C@@H](NC(=O)[C@@H]1CCc3ccccc3C1)CO2	567392392	23	307.16	3.35
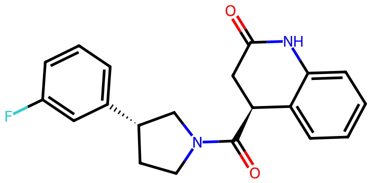	O=C1C[C@H](C(=O)N2CC[C@H](c3cccc(F)c3)C2)c2ccccc2N1	264253026	25	338.14	3.27
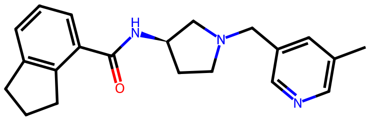	Cc1cncc(CN2CC[C@@H](NC(=O)c3cccc4c3CCC4)C2)c1	1016380444	25	335.2	2.88

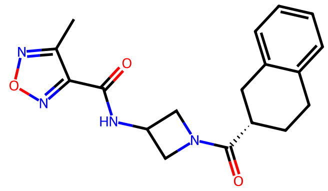	Cc1nonc1C(=O)NC1CN(C(=O)[C@H]2CCc3ccccc3C2)C1	994941986	25	340.15	1.12
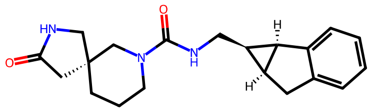	O=C1C[C@]2(CCCN(C(=O)NC[C@H]3[C@H]4c5ccccc5C[C@H]43)C2)CN1	332906349	25	339.19	1.88
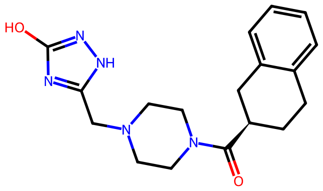	O=C([C@@H]1CCc2ccccc2C1)N1CCN(Cc2nc(O)n[nH]2)CC1	950725781	25	341.19	0.96
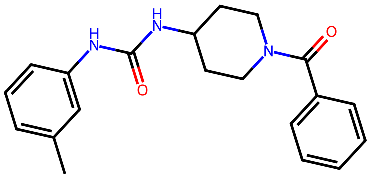	Cc1cccc(NC(=O)NC2CCN(C(=O)c3ccccc3)CC2)c1	18177504	25	337.18	3.42
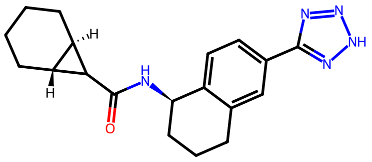	O=C(N[C@@H]1CCCc2cc(-c3nn[nH]n3)ccc21)C1[C@@H]2CCCC[C@@H]12	1592985264	25	337.19	2.8
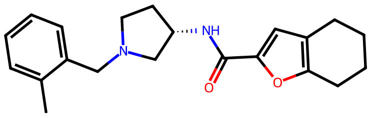	Cc1ccccc1CN1CC[C@H](NC(=O)c2cc3c(o2)CCCC3)C1	1014682870	25	338.2	3.47
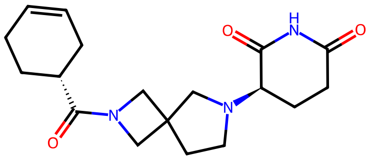	O=C1CC[C@@H](N2CCC3(CN(C(=O)[C@H]4CC=CCC4)C3)C2)C(=O)N1	1072457645	24	331.19	0.68
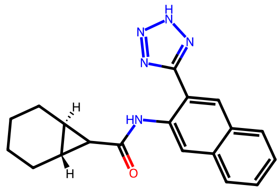	O=C(Nc1cc2ccccc2cc1-c1nn[nH]n1)C1[C@@H]2CCCC[C@@H]12	1593212524	25	333.16	3.39

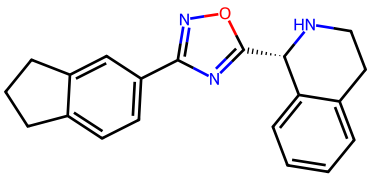	c1ccc2c(c1)CCN[C@H]2c1nc(-c2ccc3c(c2)CCC3)no1	326754341	24	317.15	3.46
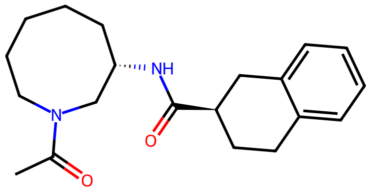	CC(=O)N1CCCCC[C@H](NC(=O)[C@@H]2CCc3ccccc3C2)C1	986791969	24	328.22	2.7
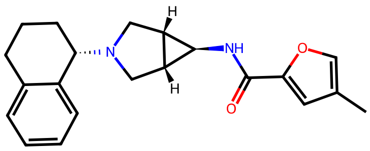	Cc1coc(C(=O)N[C@H]2[C@@H]3CN([C@H]4CCCc5ccccc54)C[C@@H]32)c1	961517320	25	336.18	3.33
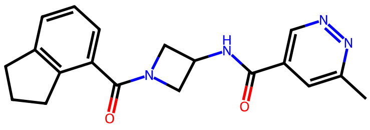	Cc1cc(C(=O)NC2CN(C(=O)c3cccc4c3CCC4)C2)cnn1	991035202	25	336.16	1.53
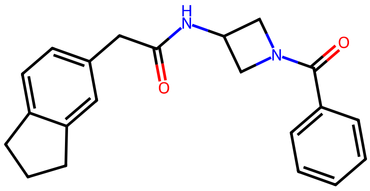	O=C(Cc1ccc2c(c1)CCC2)NC1CN(C(=O)c2ccccc2)C1	991387892	25	334.17	2.36
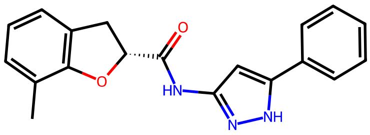	Cc1cccc2c1O[C@@H](C(=O)Nc1cc(-c3ccccc3)[nH]n1)C2	1143768602	24	319.13	3.33
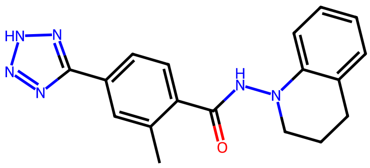	Cc1cc(-c2nn[nH]n2)ccc1C(=O)NN1CCCc2ccccc21	1609387385	25	334.15	2.27
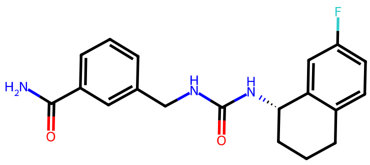	NC(=O)c1cccc(CNC(=O)N[C@H]2CCCc3ccc(F)cc32)c1	93119745	25	341.15	2.8

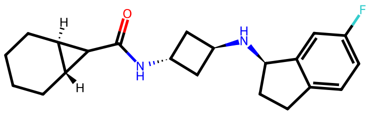	O=C(N[C@H]1C[C@H](N[C@@H]2CCc3ccc(F)cc32)C1)C1[C@@H]2CCCC[C@@H]12	1020047965	25	342.21	3.49
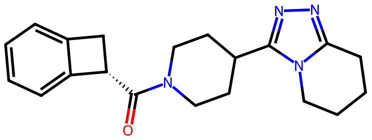	O=C([C@H]1Cc2ccccc21)N1CCC(c2nnc3n2CCCC3)CC1	330011645	25	336.2	2.66
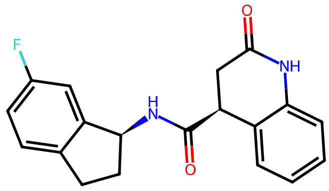	O=C1C[C@H](C(=O)N[C@H]2CCc3cc(F)cc32)c2ccccc2N1	106680932	24	324.13	3.06
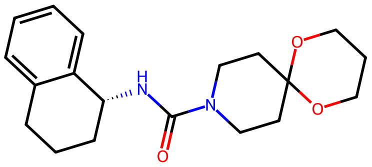	O=C(N[C@@H]1CCCc2ccccc21)N1CCC2(CC1)OCCCO2	297426007	24	330.19	3.0
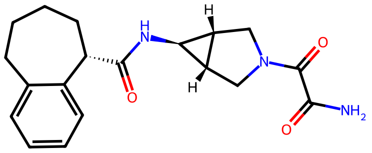	NC(=O)C(=O)N1C[C@H]2[C@H](NC(=O)[C@H]3CCCCc4ccccc43)[C@H]2C1	976704992	25	341.17	0.55
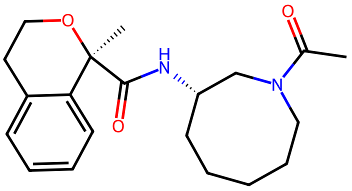	CC(=O)N1CCCCC[C@H](NC(=O)[C@]2(C)OCCc3ccccc32)C1	986795752	25	344.21	2.38
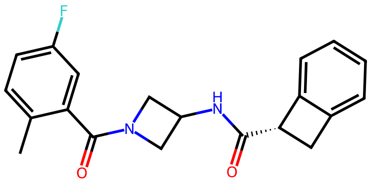	Cc1ccc(F)cc1C(=O)N1CC(NC(=O)[C@H]2Cc3ccccc32)C1	998688191	25	338.14	2.41
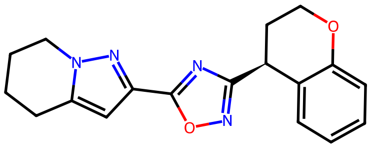	c1ccc2c(c1)OCC[C@@H]2c1noc(-c2cc3n(n2)CCCC3)n1	351090230	24	322.14	3.18

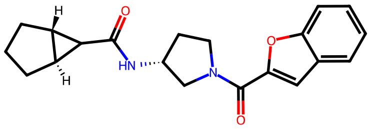	O=C(N[C@@H]1CCN(C(=O)c2cc3ccccc3o2)C1)C1[C@H]2CCC[C@H]12	936129673	25	338.16	2.81
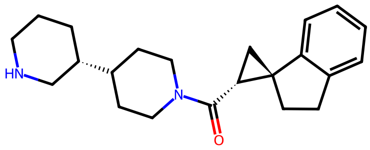	O=C([C@@H]1C[C@@]12CCc1ccccc12)N1CCC([C@H]2CCCNC2)CC1	658956685	25	338.24	3.13
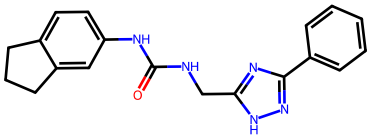	O=C(NCc1nc(-c2ccccc2)n[nH]1)Nc1ccc2c(c1)CCC2	731899823	25	333.16	3.28
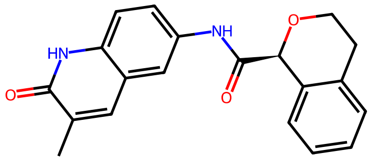	Cc1cc2cc(NC(=O)[C@H]3OCCc4ccccc43)ccc2[nH]c1=O	1569493788	25	334.13	3.09
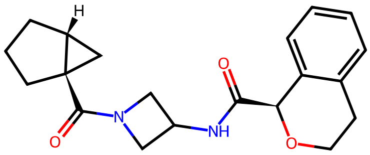	O=C(NC1CN(C(=O)[C@@]23CCC[C@@H]2C3)C1)[C@@H]1OCCc2ccccc21	997321319	25	340.18	1.82
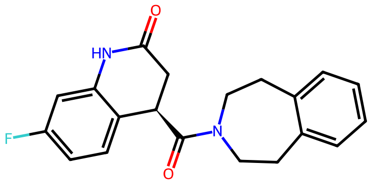	O=C1C[C@@H](C(=O)N2CCc3ccccc3CC2)c2ccc(F)cc2N1	116071684	25	338.14	2.88
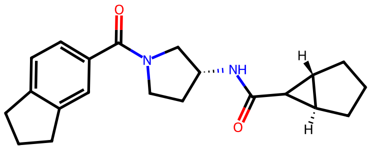	O=C(N[C@@H]1CCN(C(=O)c2ccc3c(c2)CCC3)C1)C1[C@@H]2CCC[C@@H]12	939933090	25	338.2	2.55
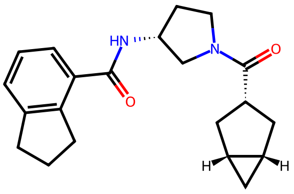	O=C(N[C@@H]1CCN(C(=O)[C@@H]2C[C@@H]3C[C@@H]3C2)C1)c1cccc2c1CCC2	941156998	25	338.2	2.55

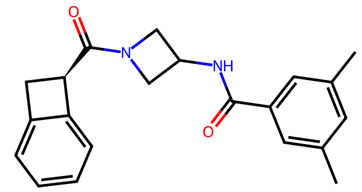	Cc1cc(C)cc(C(=O)NC2CN(C(=O)[C@@H]3Cc4ccccc43)C2)c1	999493666	25	334.17	2.58
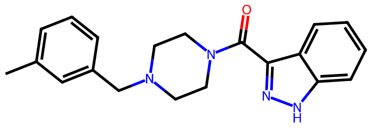	Cc1cccc(CN2CCN(C(=O)c3n[nH]c4ccccc34)CC2)c1	38411394	25	334.18	2.83
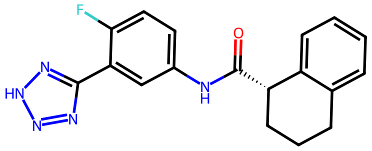	O=C(Nc1ccc(F)c(-c2nn[nH]n2)c1)[C@H]1CCCc2ccccc21	738162791	25	337.13	3.06
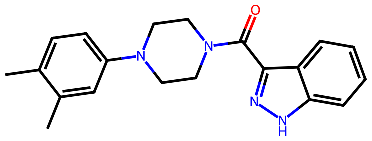	Cc1ccc(N2CCN(C(=O)c3n[nH]c4ccccc34)CC2)cc1C	126485424	25	334.18	3.14
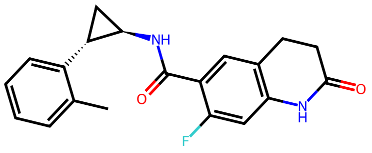	Cc1ccccc1[C@@H]1C[C@H]1NC(=O)c1cc2c(cc1F)NC(=O)CC2	127305198	25	338.14	3.3
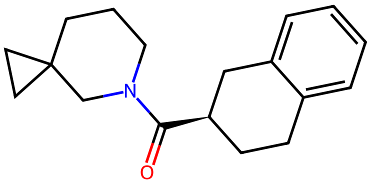	O=C([C@@H]1CCc2ccccc2C1)N1CCCC2(CC2)C1	569369615	20	269.18	3.19
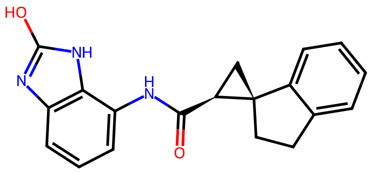	O=C(Nc1cccc2nc(O)[nH]c12)[C@H]1C[C@@]12CCc1ccccc12	563395841	24	319.13	3.11
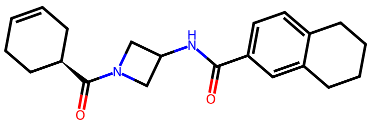	O=C(NC1CN(C(=O)[C@@H]2CC=CCC2)C1)c1ccc2c(c1)CCCC2	1000060305	25	338.2	2.86

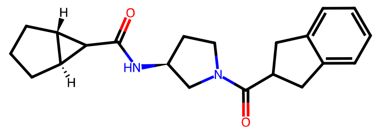	O=C(N[C@H]1CCN(C(=O)C2Cc3cccc c3C2)C1)C1[C@H]2CCC[C@H]12	937802015	25	338.2	2.16
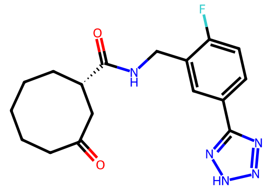	O=C1CCCCC[C@H](C(=O)NCc2cc(-c 3nn[nH]n3)ccc2F)C1	1607045313	25	345.16	2.16
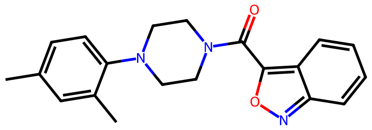	Cc1ccc(N2CCN(C(=O)c3onc4ccccc34)CC2)c(C)c1	1557622842	25	335.16	3.41
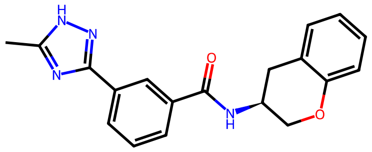	Cc1nc(-c2cccc(C(=O)N[C@@H]3COc 4ccccc4C3)c2)n[nH]1	96446771	25	334.14	2.51
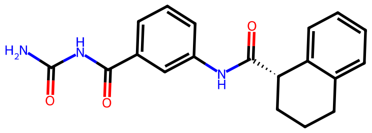	NC(=O)NC(=O)c1cccc(NC(=O)[C@H] 2CCCc3ccccc32)c1	338744769	25	337.14	2.55
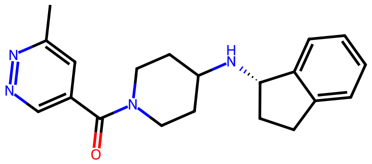	Cc1cc(C(=O)N2CCC(N[C@H]3CCc4c cccc43)CC2)cnn1	991759567	25	336.2	2.67
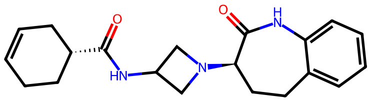	O=C(NC1CN([C@@H]2CCc3ccccc3N C2=O)C1)[C@H]1CC=CCC1	1030098286	25	339.19	2.1
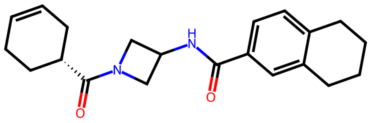	O=C(NC1CN(C(=O)[C@H]2CC=CCC 2)C1)c1ccc2c(c1)CCCC2	1000060298	25	338.2	2.86

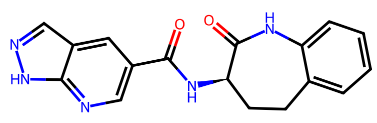	O=C(N[C@@H]1CCc2ccccc2NC1=O)c1cnc2[nH]ncc2c1	1640486501	24	321.12	1.64
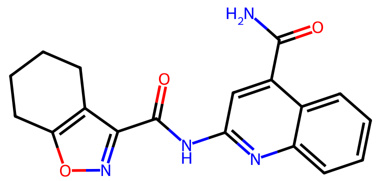	NC(=O)c1cc(NC(=O)c2noc3c2CCCC3)nc2ccccc12	1533764591	25	336.12	2.45
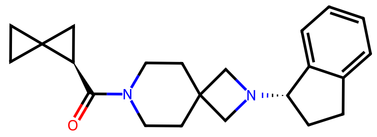	O=C([C@@H]1CC12CC2)N1CCC2(CC1)CN([C@H]1CCc3ccccc31)C2	1035808193	25	336.22	3.4
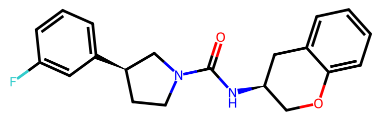	O=C(N[C@@H]1COc2ccccc2C1)N1CC[C@@H](c2cccc(F)c2)C1	552634902	25	340.16	3.33
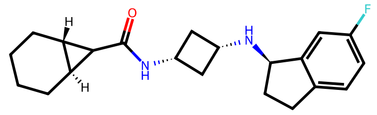	O=C(N[C@H]1C[C@@H](N[C@@H]2CCc3ccc(F)cc32)C1)C1[C@H]2CCCC[C@H]12	1021931911	25	342.21	3.49
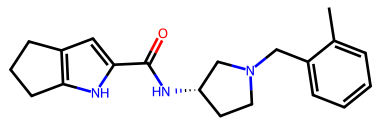	Cc1ccccc1CN1CC[C@H](NC(=O)c2cc3c([nH]2)CCC3)C1	1015979530	24	323.2	2.82
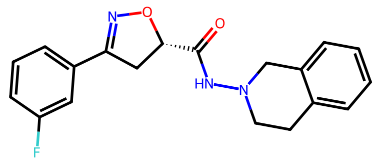	O=C(NN1CCc2ccccc2C1)[C@@H]1CC(c2cccc(F)c2)=NO1	1418366062	25	339.14	2.41
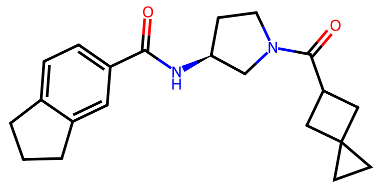	O=C(N[C@H]1CCN(C(=O)C2CC3(CC3)C2)C1)c1ccc2c(c1)CCC2	937934089	25	338.2	2.7

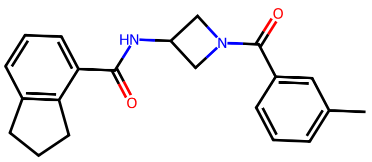	Cc1cccc(C(=O)N2CC(NC(=O)c3cccc4c3CCC4)C2)c1	994397730	25	334.17	2.74
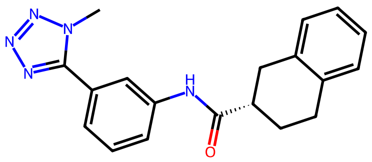	Cn1nnnc1-c1cccc(NC(=O)[C@H]2CCc3ccccc3C2)c1	570283442	25	333.16	2.62
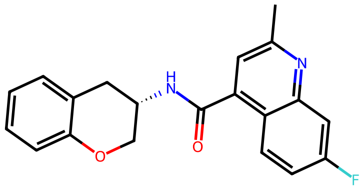	Cc1cc(C(=O)N[C@@H]2COc3ccccc3C2)c2ccc(F)cc2n1	53046050	25	336.13	3.42
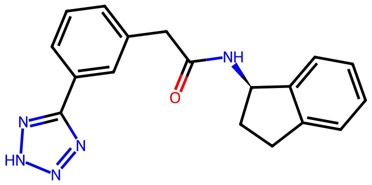	O=C(Cc1cccc(-c2nn[nH]n2)c1)N[C@@H]1CCc2ccccc21	1582146677	24	319.14	2.21
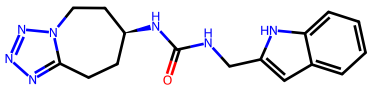	O=C(NCc1cc2ccccc2[nH]1)N[C@H]1CCc2nnnn2CC1	571394498	24	325.17	1.36
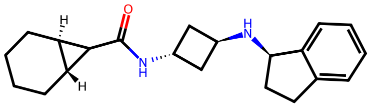	O=C(N[C@H]1C[C@H](N[C@@H]2CCc3ccccc32)C1)C1[C@@H]2CCCC[C@@H]12	1020052290	24	324.22	3.35
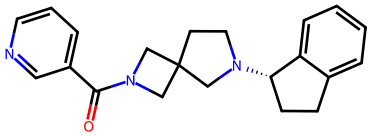	O=C(c1cccnc1)N1CC2(CCN([C@H]3CCc4ccccc43)C2)C1	1072400267	25	333.18	2.92
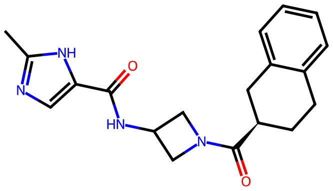	Cc1ncc(C(=O)NC2CN(C(=O)[C@@H]3CCc4ccccc4C3)C2)[nH]1	990503002	25	338.17	1.46

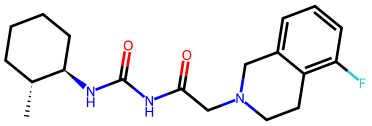	C[C@@H]1CCCC[C@H]1NC(=O)NC(=O)CN1CCc2c(F)cccc2C1	339779910	25	347.2	2.59
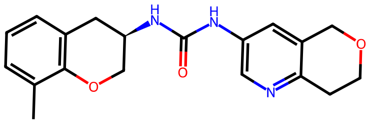	Cc1cccc2c1OC[C@H](NC(=O)Nc1cnc3c(c1)COCC3)C2	685642161	25	339.16	2.59
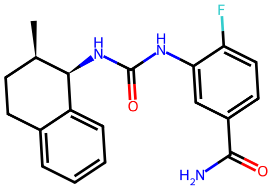	C[C@@H]1CCc2ccccc2[C@@H]1NC(=O)Nc1cc(C(N)=O)ccc1F	179534105	25	341.15	3.37
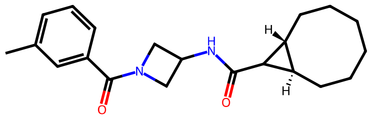	Cc1cccc(C(=O)N2CC(NC(=O)C3[C@@H]4CCCCCC[C@@H]34)C2)c1	1000468303	25	340.22	3.15
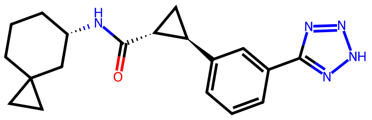	O=C(N[C@H]1CCCC2(CC2)C1)[C@@H]1C[C@H]1c1cccc(-c2nn[nH]n2)c1	1592980094	25	337.19	2.81
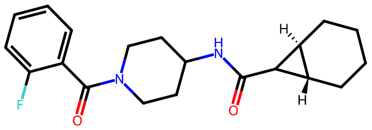	O=C(NC1CCN(C(=O)c2ccccc2F)CC1)C1[C@H]2CCCC[C@H]12	174637595	25	344.19	2.98
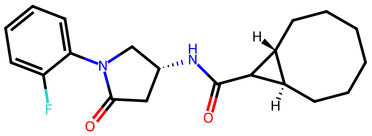	O=C(N[C@@H]1CC(=O)N(c2ccccc2F)C1)C1[C@@H]2CCCCCC[C@@H]12	1460432394	25	344.19	3.26
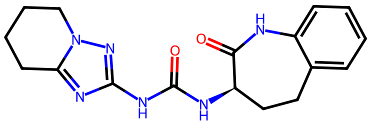	O=C(Nc1nc2n(n1)CCCC2)N[C@@H]1CCc2ccccc2NC1=O	654107356	25	340.16	1.69

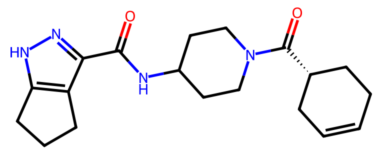	O=C(NC1CCN(C(=O)[C@H]2CC=CC C2)CC1)c1n[nH]c2c1CCC2	944126515	25	342.21	1.98
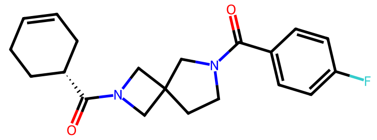	O=C(c1ccc(F)cc1)N1CCC2(C1)CN(C(=O)[C@H]1CC=CCC1)C2	1018769163	25	342.17	2.86
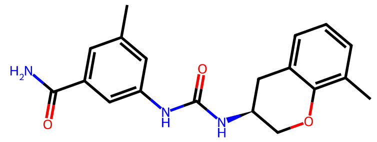	Cc1cc(NC(=O)N[C@@H]2COc3c(C)c ccc3C2)cc(C(N)=O)c1	455824986	25	339.16	2.53
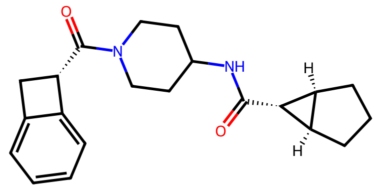	O=C(NC1CCN(C(=O)[C@H]2Cc3cccc c32)CC1)[C@@H]1[C@H]2CCC[C@ H]21	948059460	25	338.2	2.48
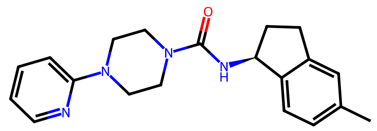	Cc1ccc2c(c1)CC[C@@H]2NC(=O)N1 CCN(c2ccccn2)CC1	56841862	25	336.2	2.91
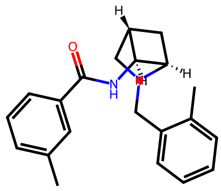	Cc1cccc(C(=O)N[C@H]2[C@H]3C[C @H]2N(Cc2ccccc2C)C3)c1	1037295279	24	320.19	3.31
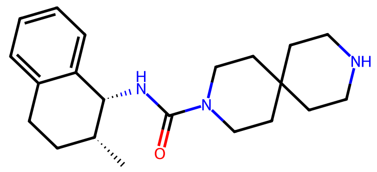	C[C@@H]1CCc2ccccc2[C@@H]1NC(=O)N1CCC2(CCNCC2)CC1	417945402	25	341.25	3.49
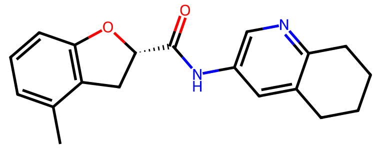	Cc1cccc2c1C[C@@H](C(=O)Nc1cnc3 c(c1)CCCC3)O2	569917854	23	308.15	3.21

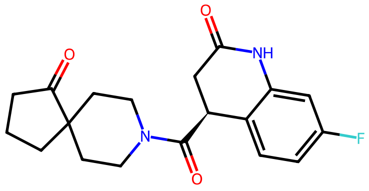	O=C1C[C@H](C(=O)N2CCC3(CCCC3=O)CC2)c2ccc(F)cc2N1	619334237	25	344.15	2.61
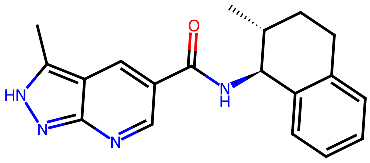	Cc1[nH]nc2ncc(C(=O)N[C@@H]3c4ccccc4CC[C@H]3C)cc12	342885251	24	320.16	3.32
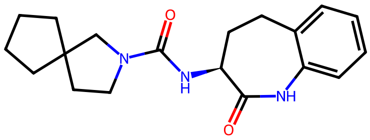	O=C1Nc2ccccc2CC[C@@H]1NC(=O)N1CCC2(CCCC2)C1	847396011	24	327.19	2.92
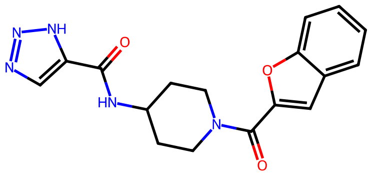	O=C(NC1CCN(C(=O)c2cc3ccccc3o2)CC1)c1cnn[nH]1	948481134	25	339.13	1.59
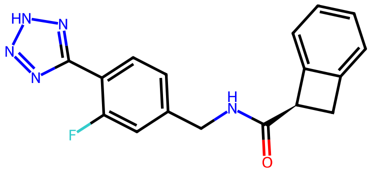	O=C(NCc1ccc(-c2nn[nH]n2)c(F)c1)[C@@H]1Cc2ccccc21	1582545731	24	323.12	1.96
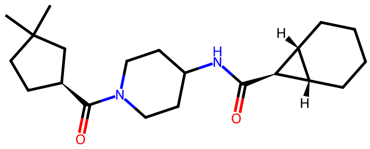	CC1(C)CC[C@H](C(=O)N2CCC(NC(=O)[C@@H]3[C@H]4CCCC[C@H]43)CC2)C1	948254806	25	346.26	3.36
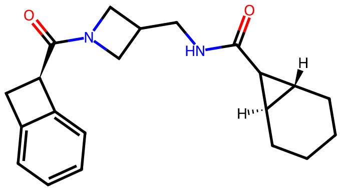	O=C(NCC1CN(C(=O)[C@@H]2Cc3ccccc32)C1)C1[C@@H]2CCCC[C@@H]12	1001321383	25	338.2	2.34
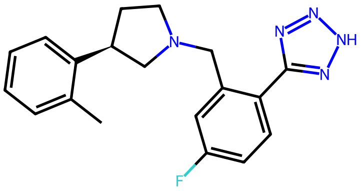	Cc1ccccc1[C@H]1CCN(Cc2cc(F)ccc2-c2nn[nH]n2)C1	822351181	25	337.17	3.3

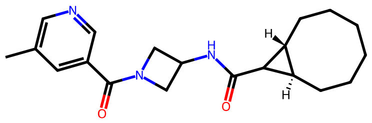	Cc1cncc(C(=O)N2CC(NC(=O)C3[C@@H]4CCCCCC[C@@H]34)C2)c1	1000486569	25	341.21	2.55
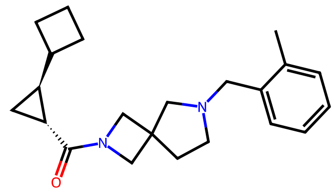	Cc1ccccc1CN1CCC2(C1)CN(C(=O)[C@@H]1C[C@H]1C1CCC1)C2	1073049195	25	338.24	3.47
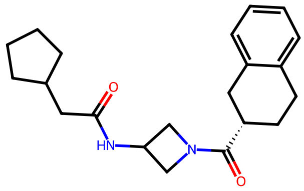	O=C(CC1CCCC1)NC1CN(C(=O)[C@H]2CCc3ccccc3C2)C1	990113150	25	340.22	2.7
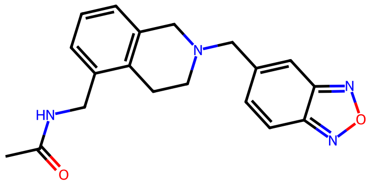	CC(=O)NCc1cccc2c1CCN(Cc1ccc3nonc3c1)C2	1051277728	25	336.16	2.42
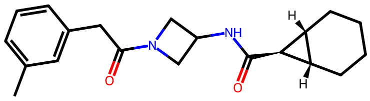	Cc1cccc(CC(=O)N2CC(NC(=O)[C@@H]3[C@H]4CCCC[C@H]43)C2)c1	990648577	24	326.2	2.3
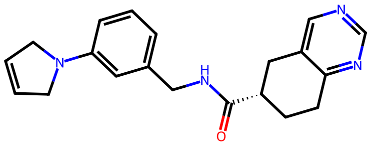	O=C(NCc1cccc(N2CC=CC2)c1)[C@H]1CCc2ncncc2C1	648296092	25	334.18	2.27
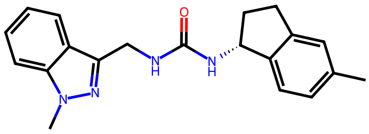	Cc1ccc2c(c1)CC[C@H]2NC(=O)NCc1nn(C)c2ccccc12	680551580	25	334.18	3.37
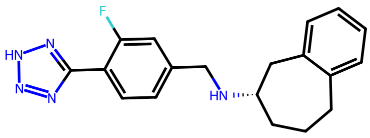	Fc1cc(CN[C@H]2CCCc3ccccc3C2)ccc1-c1nn[nH]n1	1581853085	25	337.17	3.04

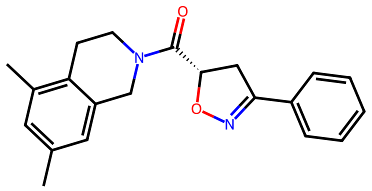	Cc1cc(C)c2c(c1)CN(C(=O)[C@@H]1CC(c3ccccc3)=NO1)CC2	1523545891	25	334.17	3.38
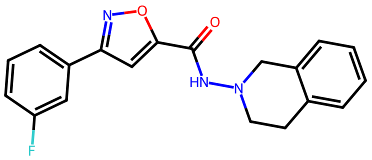	O=C(NN1CCc2ccccc2C1)c1cc(-c2cccc(F)c2)no1	1583867151	25	337.12	3.18
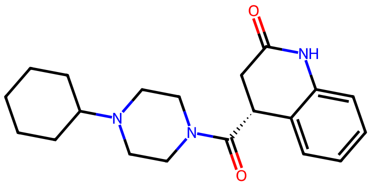	O=C1C[C@@H](C(=O)N2CCN(C3CCCCC3)CC2)c2ccccc2N1	154449370	25	341.21	2.59
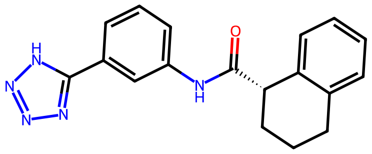	O=C(Nc1cccc(-c2nnn[nH]2)c1)[C@H]1CCCc2ccccc21	74045543	24	319.14	2.93
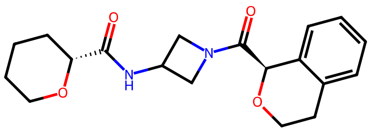	O=C(NC1CN(C(=O)[C@@H]2OCCc3ccccc32)C1)[C@H]1CCCCO1	990854097	25	344.17	1.2
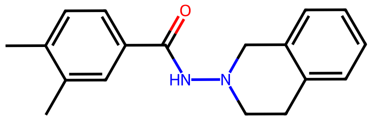	Cc1ccc(C(=O)NN2CCc3ccccc3C2)cc1C	1628725601	21	280.16	3.01
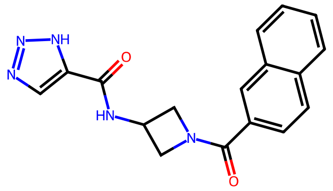	O=C(NC1CN(C(=O)c2ccc3ccccc3c2)C1)c1cnn[nH]1	995177908	24	321.12	1.21
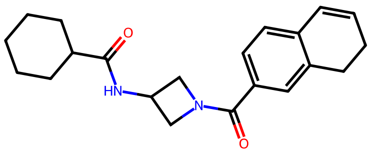	O=C(NC1CN(C(=O)c2ccc3c(c2)CCC=C3)C1)C1CCCCC1	990092743	25	338.2	3.17

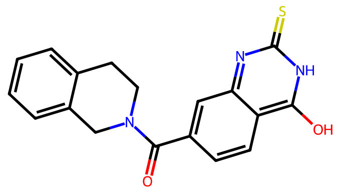	O=C(c1ccc2c(O)[nH]c(=S)nc2c1)N1CCc2ccccc2C1	73843277	24	337.09	3.2
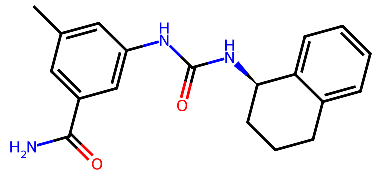	Cc1cc(NC(=O)N[C@@H]2CCCc3ccccc32)cc(C(N)=O)c1	455818932	24	323.16	3.29
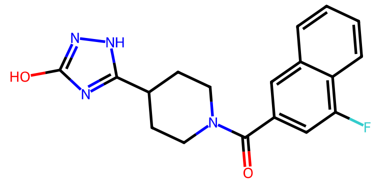	O=C(c1cc(F)c2ccccc2c1)N1CCC(c2nc(O)n[nH]2)CC1	568636829	25	340.13	2.82
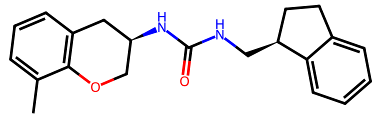	Cc1cccc2c1OC[C@H](NC(=O)NC[C@H]1CCc3ccccc31)C2	1668963683	25	336.18	3.33
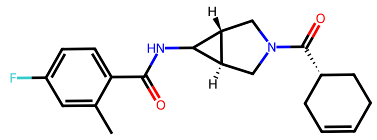	Cc1cc(F)ccc1C(=O)NC1[C@@H]2CN(C(=O)[C@H]3CC=CCC3)C[C@@H]12	1013517352	25	342.17	2.68
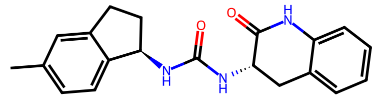	Cc1ccc2c(c1)CC[C@H]2NC(=O)N[C@H]1Cc2ccccc2NC1=O	636635271	25	335.16	2.84
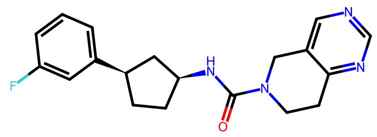	O=C(N[C@H]1CC[C@@H](c2cccc(F)c2)C1)N1CCc2ncncc2C1	883620712	25	340.17	3.02
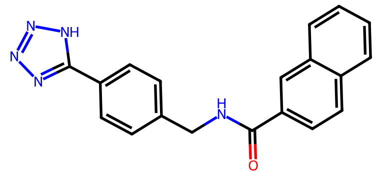	O=C(NCc1ccc(-c2nnn[nH]2)cc1)c1ccc2ccccc2c1	68962413	25	329.13	2.95

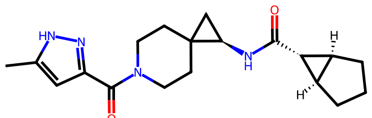	Cc1cc(C(=O)N2CCC3(CC2)C[C@H]3NC(=O)[C@@H]2[C@H]3CCC[C@H]32)n[nH]1	963468254	25	342.21	1.88
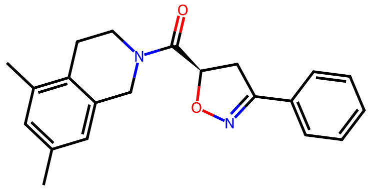	Cc1cc(C)c2c(c1)CN(C(=O)[C@H]1CC(c3ccccc3)=NO1)CC2	1523545896	25	334.17	3.38
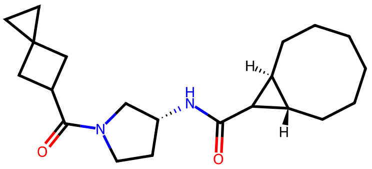	O=C(N[C@@H]1CCN(C(=O)C2CC3(CC3)C2)C1)C1[C@H]2CCCCCC[C@H]12	942510232	25	344.25	3.11
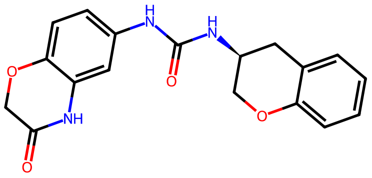	O=C1COc2ccc(NC(=O)N[C@@H]3COc4ccccc4C3)cc2N1	47777239	25	339.12	2.14
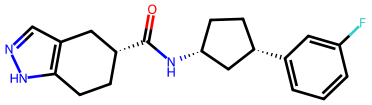	O=C(N[C@@H]1CC[C@H](c2cccc(F)c2)C1)[C@@H]1CCc2[nH]ncc2C1	280364739	24	327.17	3.11
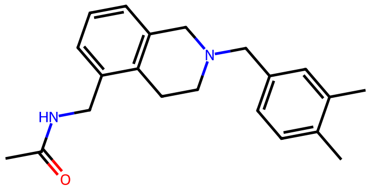	CC(=O)NCc1cccc2c1CCN(Cc1ccc(C)c(C)c1)C2	1051276850	24	322.2	3.5
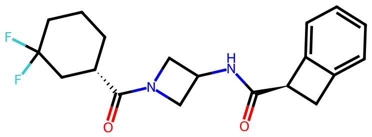	O=C(NC1CN(C(=O)[C@H]2CCCC(F)(F)C2)C1)[C@@H]1Cc2ccccc21	998689504	25	348.16	2.48
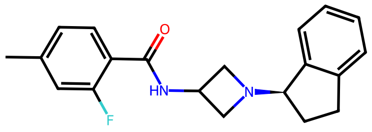	Cc1ccc(C(=O)NC2CN([C@@H]3CCc4ccccc43)C2)c(F)c1	1031005378	24	324.16	3.24

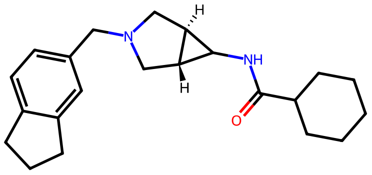	O=C(NC1[C@@H]2CN(Cc3ccc4c(c3)CCC4)C[C@@H]12)C1CCCCC1	958102801	25	338.24	3.3
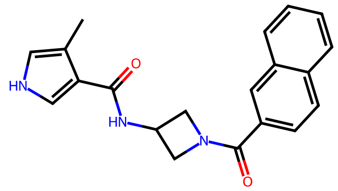	Cc1c[nH]cc1C(=O)NC1CN(C(=O)c2ccc3ccccc3c2)C1	991711188	25	333.15	2.73
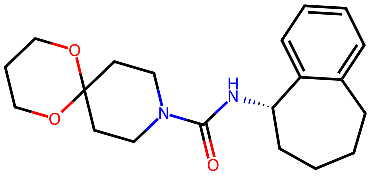	O=C(N[C@H]1CCCCc2ccccc21)N1CCC2(CC1)OCCCO2	836269053	25	344.21	3.39
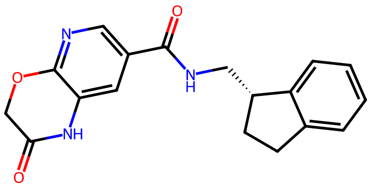	O=C1COc2ncc(C(=O)NC[C@H]3CCc4ccccc43)cc2N1	378432201	24	323.13	1.87
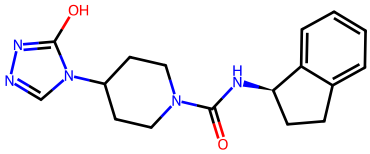	O=C(N[C@@H]1CCc2ccccc21)N1CCC(n2cnnc2O)CC1	666408614	24	327.17	2.02
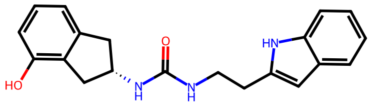	O=C(NCCc1cc2ccccc2[nH]1)N[C@H]1Cc2cccc(O)c2C1	429001431	25	335.16	2.88
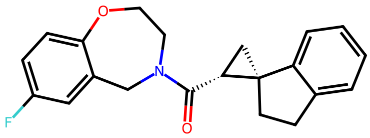	O=C([C@@H]1C[C@]12CCc1ccccc12)N1CCOc2ccc(F)cc2C1	470582327	25	337.15	3.45
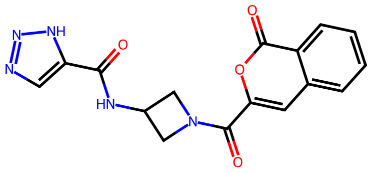	O=C(NC1CN(C(=O)c2cc3ccccc3c(=O)o2)C1)c1cnn[nH]1	995174537	25	339.1	0.17

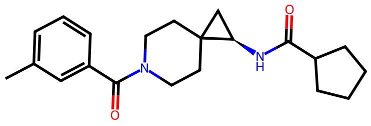	Cc1cccc(C(=O)N2CCC3(CC2)C[C@H]3NC(=O)C2CCCC2)c1	962504721	25	340.22	3.3
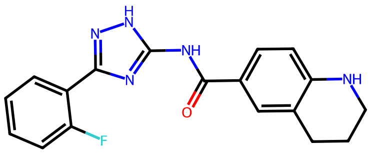	O=C(Nc1nc(-c2ccccc2F)n[nH]1)c1ccc2c(c1)CCCN2	1580793349	25	337.13	3.22
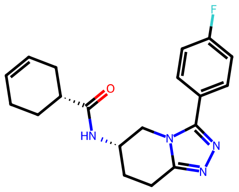	O=C(N[C@H]1CCc2nnc(-c3ccc(F)cc3)n2C1)[C@H]1CC=CCC1	332855339	25	340.17	2.87
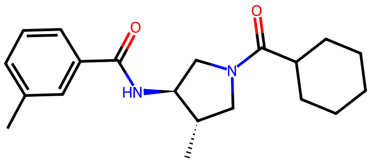	Cc1cccc(C(=O)N[C@H]2CN(C(=O)C3CCCCC3)C[C@@H]2C)c1	966111593	24	328.22	3.15
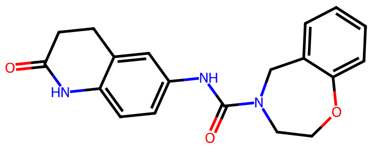	O=C1CCc2cc(NC(=O)N3CCOc4ccccc4C3)ccc2N1	79519362	25	337.14	3.0
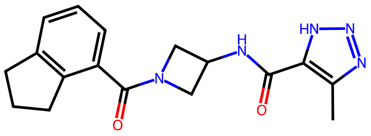	Cc1nn[nH]c1C(=O)NC1CN(C(=O)c2cccc3c2CCC3)C1	997347065	24	325.15	0.86
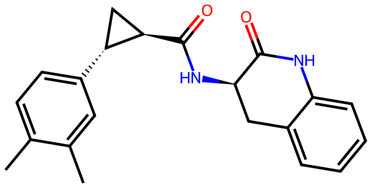	Cc1ccc([C@@H]2C[C@H]2C(=O)N[C@@H]2Cc3ccccc3NC2=O)cc1C	628943217	25	334.17	3.09
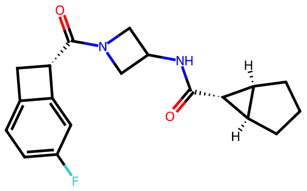	O=C(NC1CN(C(=O)[C@H]2Cc3ccc(F)cc32)C1)[C@@H]1[C@H]2CCC[C@H]21	990707693	24	328.16	1.84

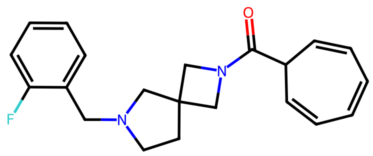	O=C(C1C=CC=CC=C1)N1CC2(CCN(Cc3ccccc3F)C2)C1	1072785334	25	338.18	3.16
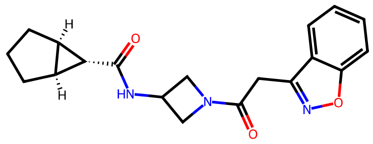	O=C(NC1CN(C(=O)Cc2noc3ccccc23)C1)[C@@H]1[C@H]2CCC[C@H]21	990719810	25	339.16	1.74
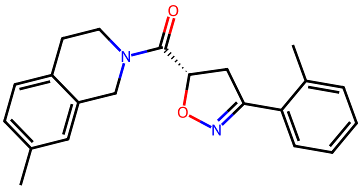	Cc1ccc2c(c1)CN(C(=O)[C@@H]1CC(c3ccccc3C)=NO1)CC2	1531334318	25	334.17	3.38
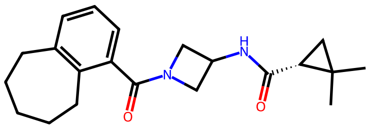	CC1(C)C[C@H]1C(=O)NC1CN(C(=O)c2cccc3c2CCCCC3)C1	990887466	25	340.22	2.94
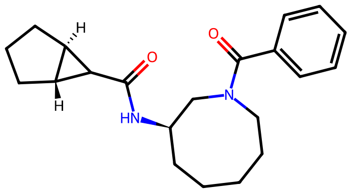	O=C(N[C@@H]1CCCCCN(C(=O)c2ccccc2)C1)C1[C@@H]2CCC[C@@H]12	986852254	25	340.22	3.23
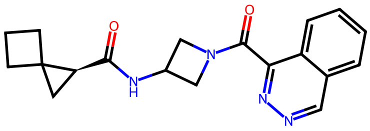	O=C(NC1CN(C(=O)c2nncc3ccccc23)C1)[C@H]1CC12CCC2	991541858	25	336.16	1.76
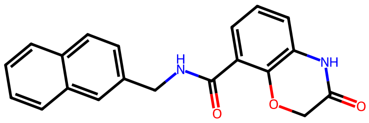	O=C1COc2c(cccc2C(=O)NCc2ccc3ccccc3c2)N1	104241314	25	332.12	3.1
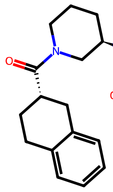	C[C@@H]1C[C@H]1C(=O)N[C@@H]1CCCN(C(=O)[C@H]2CCc3ccccc3C2)C1	966006951	25	340.22	2.55

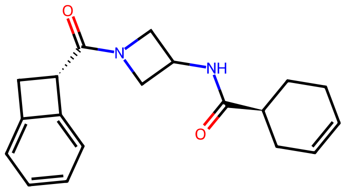	O=C(NC1CN(C(=O)[C@H]2Cc3ccccc32)C1)[C@@H]1CC=CCC1	990214629	23	310.17	2.01
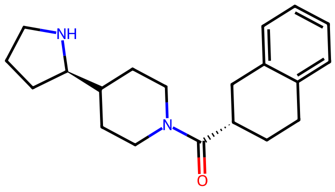	O=C([C@H]1CCc2ccccc2C1)N1CCC([C@H]2CCCN2)CC1	324106684	23	312.22	2.78
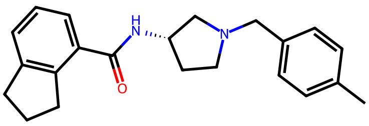	Cc1ccc(CN2CC[C@H](NC(=O)c3cccc4c3CCC4)C2)cc1	1016380141	25	334.2	3.49
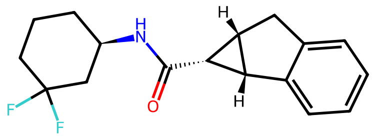	O=C(N[C@@H]1CCCC(F)(F)C1)[C@H]1[C@H]2c3ccccc3C[C@H]21	626188117	21	291.14	3.27
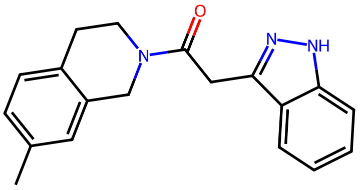	Cc1ccc2c(c1)CN(C(=O)Cc1n[nH]c3ccccc13)CC2	617602852	23	305.15	3.0
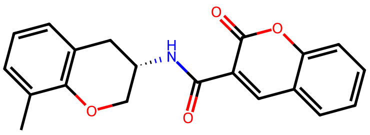	Cc1cccc2c1OC[C@@H](NC(=O)c1cc3ccccc3oc1=O)C2	365645625	25	335.12	2.83
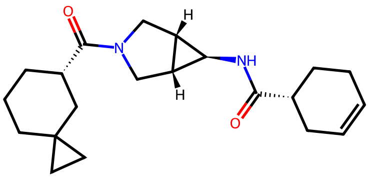	O=C(N[C@H]1[C@@H]2CN(C(=O)[C@H]3CCCC4(CC4)C3)C[C@@H]21)[C@H]1CC=CCC1	976182626	25	342.23	2.89
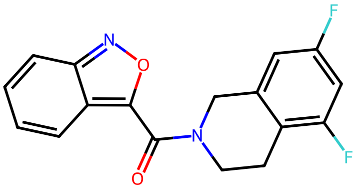	O=C(c1onc2ccccc12)N1CCc2c(F)cc(F)cc2C1	1597020970	23	314.09	3.3

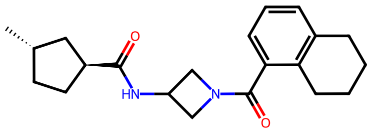	C[C@H]1CC[C@H](C(=O)NC2CN(C(=O)c3cccc4c3CCCC4)C2)C1	991435437	25	340.22	2.94
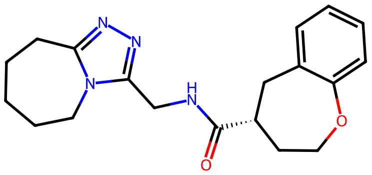	O=C(NCc1nnc2n1CCCCC2)[C@H]1CCOc2ccccc2C1	95530811	25	340.19	2.26
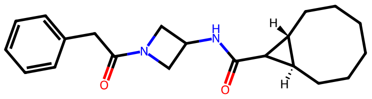	O=C(NC1CN(C(=O)Cc2ccccc2)C1)C1[C@@H]2CCCCCC[C@@H]12	1000507610	25	340.22	2.77
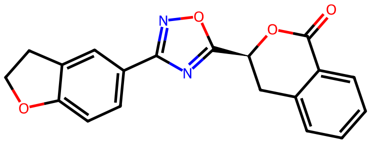	O=C1O[C@H](c2nc(-c3ccc4c(c3)CCO4)no2)Cc2ccccc21	154824740	25	334.1	3.13
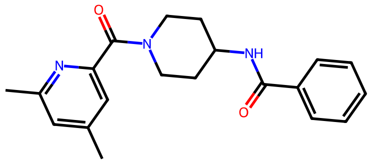	Cc1cc(C)nc(C(=O)N2CCC(NC(=O)c3ccccc3)CC2)c1	800820734	25	337.18	2.73
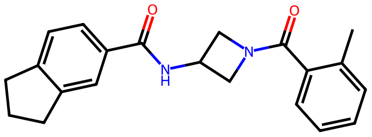	Cc1ccccc1C(=O)N1CC(NC(=O)c2ccc3c(c2)CCC3)C1	991356387	25	334.17	2.74
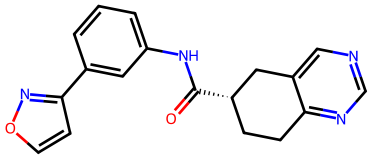	O=C(Nc1cccc(-c2ccon2)c1)[C@H]1CCc2ncncc2C1	1536209961	24	320.13	2.88
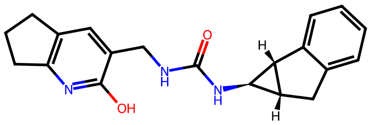	O=C(NCc1cc2c(nc1O)CCC2)N[C@@H]1[C@H]2Cc3ccccc3[C@H]21	675712912	25	335.16	2.41

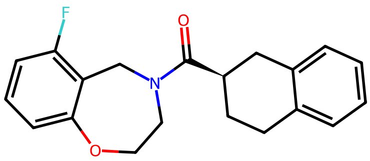	O=C([C@@H]1CCc2ccccc2C1)N1CCOc2cccc(F)c2C1	652001190	24	325.15	3.35
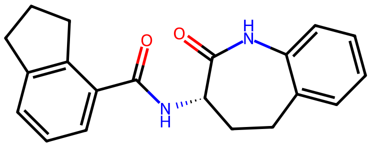	O=C(N[C@H]1CCc2ccccc2NC1=O)c1cccc2c1CCC2	654005150	24	320.15	2.86
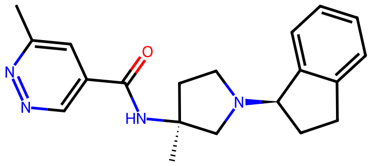	Cc1cc(C(=O)N[C@@]2(C)CCN([C@@H]3CCc4ccccc43)C2)cnn1	1046269701	25	336.2	2.67
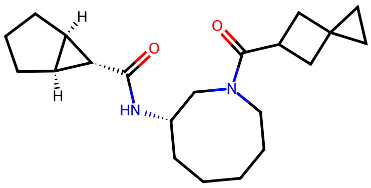	O=C(N[C@H]1CCCCCN(C(=O)C2CC3(CC3)C2)C1)[C@@H]1[C@H]2CCC[C@H]21	987150770	25	344.25	3.11
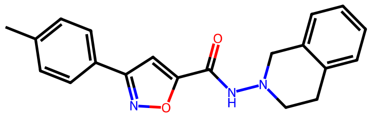	Cc1ccc(-c2cc(C(=O)NN3CCc4ccccc4C3)on2)cc1	1300427038	25	333.15	3.35
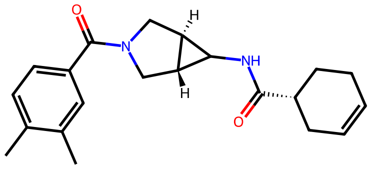	Cc1ccc(C(=O)N2C[C@H]3C(NC(=O)[C@H]4CC=CCC4)[C@@H]3C2)cc1C	1012765472	25	338.2	2.85
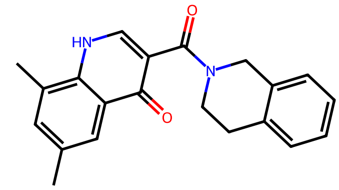	Cc1cc(C)c2[nH]cc(C(=O)N3CCc4ccccc4C3)c(=O)c2c1	1182174239	25	332.15	3.34
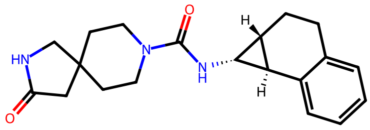	O=C1CC2(CCN(C(=O)N[C@H]3[C@@H]4c5ccccc5CC[C@H]43)CC2)CN1	269450133	25	339.19	2.03

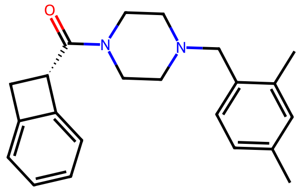	Cc1ccc(CN2CCN(C(=O)[C@H]3Cc4ccccc43)CC2)c(C)c1	949958619	25	334.2	3.29
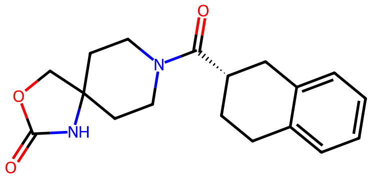	O=C1NC2(CCN(C(=O)[C@H]3CCc4ccccc4C3)CC2)CO1	496739948	23	314.16	1.89
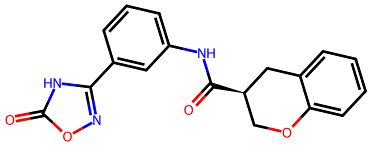	O=C(Nc1cccc(-c2noc(=O)[nH]2)c1)[C@@H]1COc2ccccc2C1	69808716	25	337.11	2.22
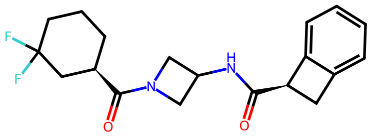	O=C(NC1CN(C(=O)[C@@H]2CCCC(F)(F)C2)C1)[C@@H]1Cc2ccccc21	998689503	25	348.16	2.48
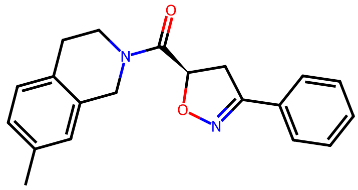	Cc1ccc2c(c1)CN(C(=O)[C@H]1CC(c3ccccc3)=NO1)CC2	1585658346	24	320.15	3.07
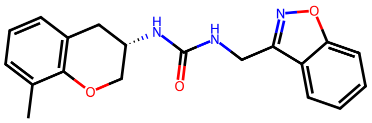	Cc1cccc2c1OC[C@@H](NC(=O)NCc1noc3ccccc13)C2	654568389	25	337.14	2.94
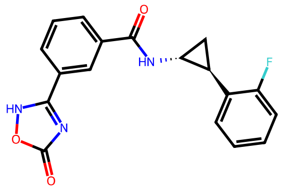	O=C(N[C@@H]1C[C@H]1c1ccccc1F)c1cccc(-c2nc(=O)o[nH]2)c1	1141961862	25	339.1	2.45
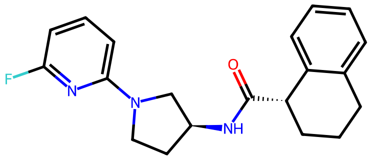	O=C(N[C@H]1CCN(c2cccc(F)n2)C1)[C@H]1CCCc2ccccc21	1058592033	25	339.17	3.04

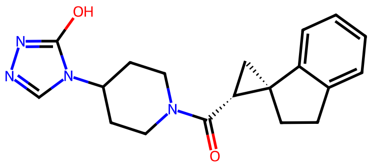	O=C([C@@H]1C[C@]12CCc1ccccc12)N1CCC(n2cnnc2O)CC1	517586134	25	338.17	2.05
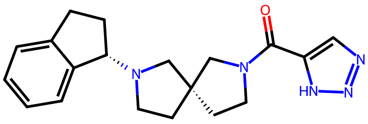	O=C(c1cnn[nH]1)N1CC[C@@]2(CCN([C@H]3CCc4ccccc43)C2)C1	1041434229	25	337.19	2.03
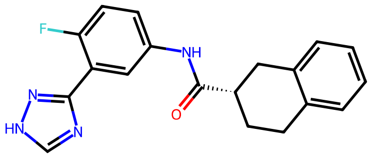	O=C(Nc1ccc(F)c(-c2nc[nH]n2)c1)[C@H]1CCc2ccccc2C1	1128485512	25	336.14	3.35
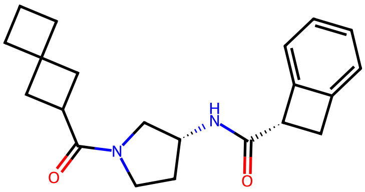	O=C(N[C@@H]1CCN(C(=O)C2CC3(CCC3)C2)C1)[C@H]1Cc2ccccc21	942304939	25	338.2	2.62
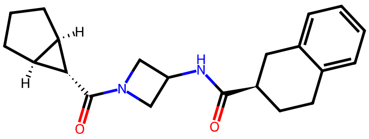	O=C(NC1CN(C(=O)[C@@H]2[C@H]3CCC[C@H]32)C1)[C@@H]1CCc2ccccc2C1	1000537292	25	338.2	2.16
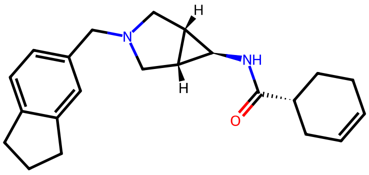	O=C(N[C@H]1[C@@H]2CN(Cc3ccc4c(c3)CCC4)C[C@@H]21)[C@H]1CC=CCC1	958208417	25	336.22	3.08
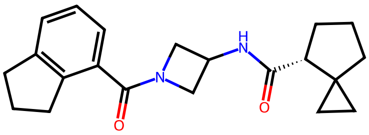	O=C(NC1CN(C(=O)c2cccc3c2CCC3)C1)[C@@H]1CCCC12CC2	994862723	25	338.2	2.7
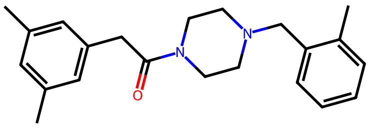	Cc1cc(C)cc(CC(=O)N2CCN(Cc3ccccc3C)CC2)c1	949999039	25	336.22	3.5

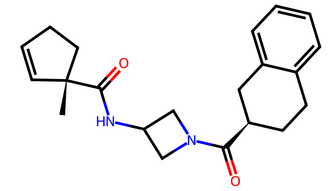	C[C@@]1(C(=O)NC2CN(C(=O)[C@@H]3CCc4ccccc4C3)C2)C=CCC1	997849491	25	338.2	2.47
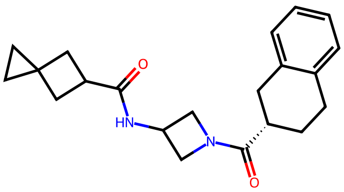	O=C(NC1CN(C(=O)[C@H]2CCc3ccccc3C2)C1)C1CC2(CC2)C1	999346677	25	338.2	2.31
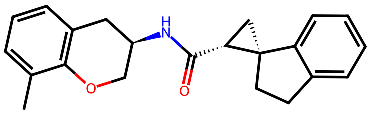	Cc1cccc2c1OC[C@H](NC(=O)[C@@H]1C[C@]13CCc1ccccc13)C2	564516632	25	333.17	3.32
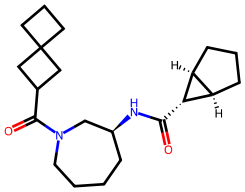	O=C(N[C@H]1CCCCN(C(=O)C2CC3(CCC3)C2)C1)[C@@H]1[C@H]2CCC[C@H]21	943169093	25	344.25	3.11
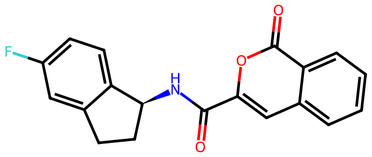	O=C(N[C@H]1CCc2cc(F)ccc21)c1cc2ccccc2c(=O)o1	352463794	24	323.1	3.35
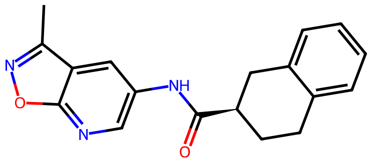	Cc1noc2ncc(NC(=O)[C@@H]3CCc4ccccc4C3)cc12	581887538	23	307.13	3.27
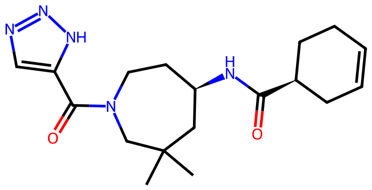	CC1(C)C[C@H](NC(=O)[C@@H]2CC=CCC2)CCN(C(=O)c2cnn[nH]2)C1	990660101	25	345.22	1.91
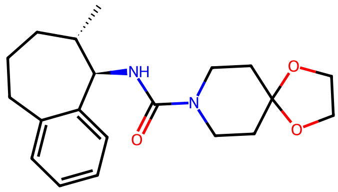	C[C@H]1CCCc2ccccc2[C@@H]1NC(=O)N1CCC2(CC1)OCCO2	185616362	25	344.21	3.25

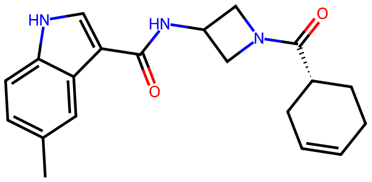	Cc1ccc2[nH]cc(C(=O)NC3CN(C(=O)[C@H]4CC=CCC4)C3)c2c1	994274189	25	337.18	2.77
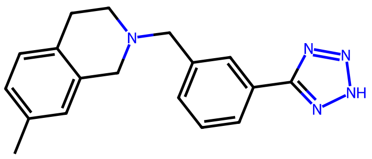	Cc1ccc2c(c1)CN(Cc1cccc(-c3nn[nH]n3)c1)CC2	1139850646	23	305.16	2.73
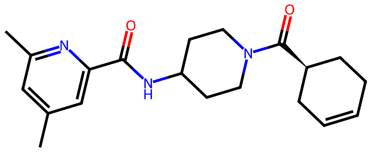	Cc1cc(C)nc(C(=O)NC2CCN(C(=O)[C@@H]3CC=CCC3)CC2)c1	944126890	25	341.21	2.78
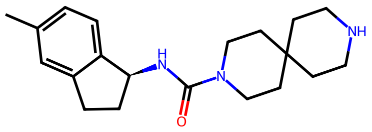	Cc1ccc2c(c1)CC[C@@H]2NC(=O)N1CCC2(CCNCC2)CC1	417263227	24	327.23	3.16
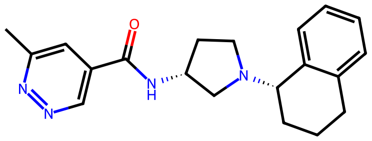	Cc1cc(C(=O)N[C@@H]2CCN([C@H]3CCCc4ccccc43)C2)cnn1	1010758324	25	336.2	2.67
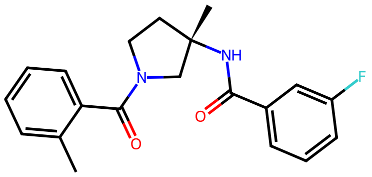	Cc1ccccc1C(=O)N1CC[C@](C)(NC(=O)c2cccc(F)c2)C1	956654801	25	340.16	3.17
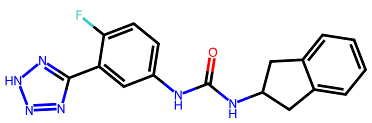	O=C(Nc1ccc(F)c(-c2nn[nH]n2)c1)NC1Cc2ccccc2C1	1593232812	25	338.13	2.29
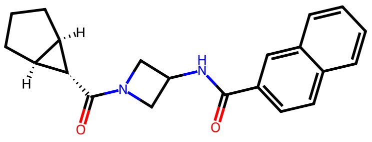	O=C(NC1CN(C(=O)[C@@H]2[C@H]3CCC[C@H]32)C1)c1ccc2ccccc2c1	999446195	25	334.17	2.83

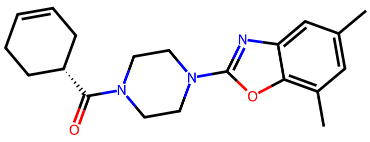	Cc1cc(C)c2oc(N3CCN(C(=O)[C@H]4CC=CCC4)CC3)nc2c1	1055721270	25	339.19	3.45
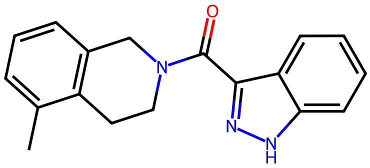	Cc1cccc2c1CCN(C(=O)c1n[nH]c3ccccc13)C2	171536685	22	291.14	3.07
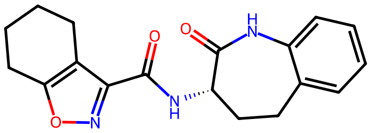	O=C(N[C@H]1CCc2ccccc2NC1=O)c1noc2c1CCCC2	654005096	24	325.14	2.24
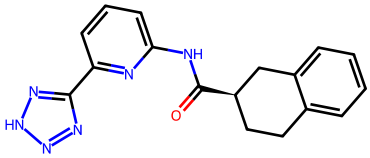	O=C(Nc1cccc(-c2nn[nH]n2)n1)[C@@H]1CCc2ccccc2C1	1582645298	24	320.14	2.01
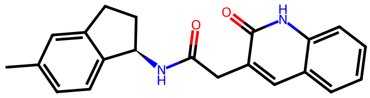	Cc1ccc2c(c1)CC[C@H]2NC(=O)Cc1cc2ccccc2[nH]c1=O	55661730	25	332.15	3.18
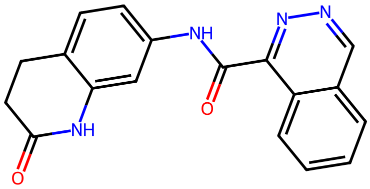	O=C1CCc2ccc(NC(=O)c3nncc4ccccc34)cc2N1	1538042850	24	318.11	2.77
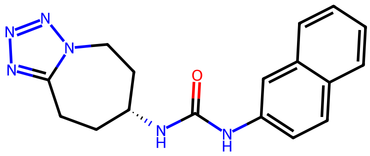	O=C(Nc1ccc2ccccc2c1)N[C@@H]1CCc2nnnn2CC1	817296032	24	322.15	2.35
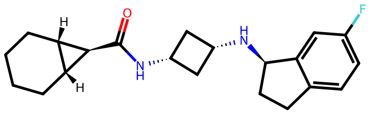	O=C(N[C@H]1C[C@@H](N[C@@H]2CCc3ccc(F)cc32)C1)[C@@H]1[C@H]2CCCC[C@H]21	1021931914	25	342.21	3.49

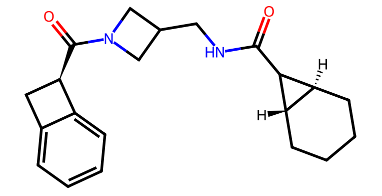	O=C(NCC1CN(C(=O)[C@@H]2Cc3ccccc32)C1)C1[C@H]2CCCC[C@H]12	1001321384	25	338.2	2.34
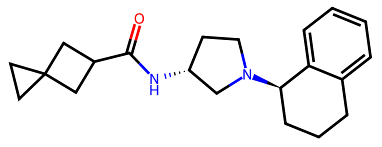	O=C(N[C@@H]1CCN([C@@H]2CCCc3ccccc32)C1)C1CC2(CC2)C1	1015681201	24	324.22	3.44
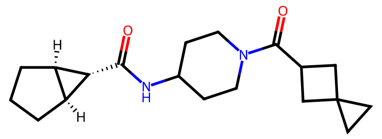	O=C(NC1CCN(C(=O)C2CC3(CC3)C2)CC1)[C@@H]1[C@H]2CCC[C@H]21	948299971	23	316.22	2.33
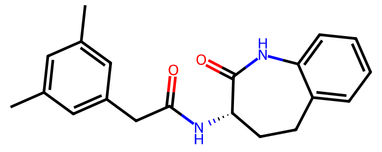	Cc1cc(C)cc(CC(=O)N[C@H]2CCc3ccccc3NC2=O)c1	616692074	24	322.17	2.92
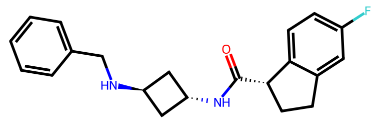	O=C(N[C@H]1C[C@H](NCc2ccccc2)C1)[C@H]1CCc2cc(F)ccc21	1020399748	25	338.18	3.29
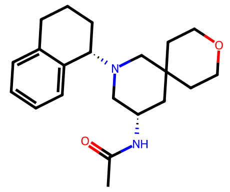	CC(=O)N[C@@H]1CN([C@H]2CCCc3ccccc32)CC2(CCOCC2)C1	1089916369	25	342.23	3.07
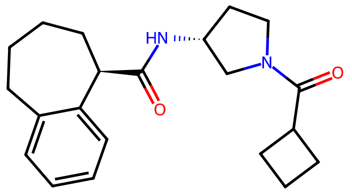	O=C(N[C@@H]1CCN(C(=O)C2CCC2)C1)[C@@H]1CCCCc2ccccc21	941119888	25	340.22	3.01
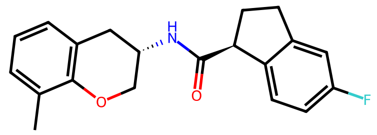	Cc1cccc2c1OC[C@@H](NC(=O)[C@H]1CCc3cc(F)ccc31)C2	615059821	24	325.15	3.28

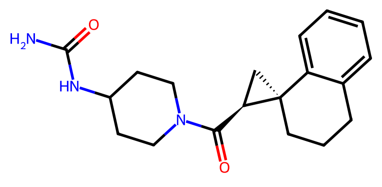	NC(=O)NC1CCN(C(=O)[C@H]2C[C@]23CCCc2ccccc23)CC1	176063166	24	327.19	1.94
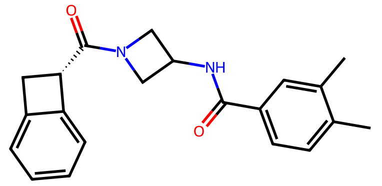	Cc1ccc(C(=O)NC2CN(C(=O)[C@H]3Cc4ccccc43)C2)cc1C	999548888	25	334.17	2.58
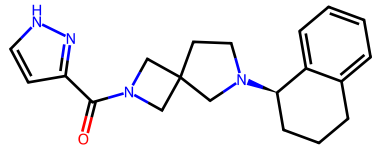	O=C(c1cc[nH]n1)N1CC2(CCN([C@@H]3CCCc4ccccc43)C2)C1	1072500211	25	336.2	2.64
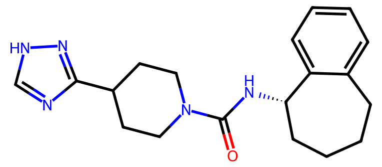	O=C(N[C@H]1CCCCc2ccccc21)N1CCC(c2nc[nH]n2)CC1	83717102	25	339.21	3.16
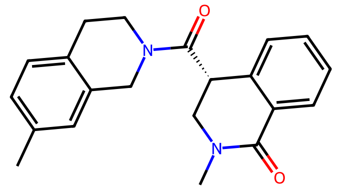	Cc1ccc2c(c1)CN(C(=O)[C@H]1CN(C)C(=O)c3ccccc31)CC2	617605087	25	334.17	2.75
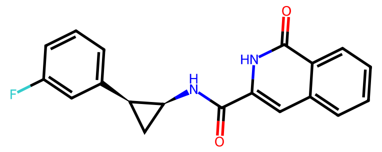	O=C(N[C@H]1C[C@H]1c1cccc(F)c1)c1cc2ccccc2c(=O)[nH]1	67333745	24	322.11	2.95
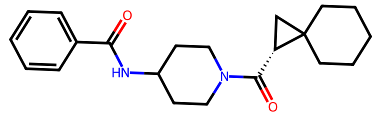	O=C(NC1CCN(C(=O)[C@@H]2CC23CCCCC3)CC1)c1ccccc1	370570937	25	340.22	3.38
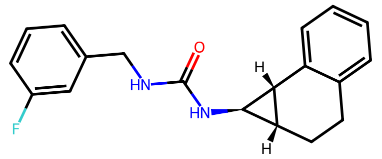	O=C(NCc1cccc(F)c1)N[C@@H]1[C@H]2CCc3ccccc3[C@H]21	126057433	23	310.15	3.35

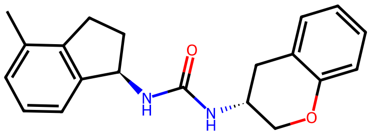	Cc1cccc2c1CC[C@H]2NC(=O)N[C@H]1COc2ccccc2C1	517916000	24	322.17	3.29
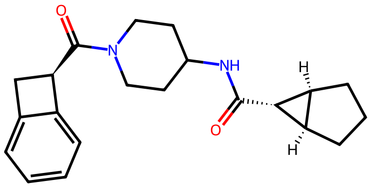	O=C(NC1CCN(C(=O)[C@@H]2Cc3ccccc32)CC1)[C@@H]1[C@H]2CCC[C@H]21	948059457	25	338.2	2.48
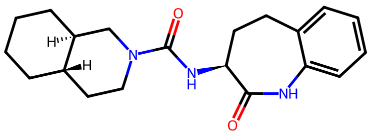	O=C1Nc2ccccc2CC[C@@H]1NC(=O)N1CC[C@@H]2CCCC[C@H]2C1	1349786681	25	341.21	3.16
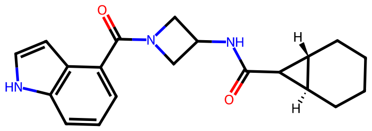	O=C(NC1CN(C(=O)c2cccc3[nH]ccc23)C1)C1[C@@H]2CCCC[C@@H]12	990659248	25	337.18	2.54
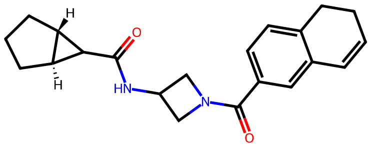	O=C(NC1CN(C(=O)c2ccc3c(c2)C=CCC3)C1)C1[C@H]2CCC[C@H]12	990702816	25	336.18	2.63
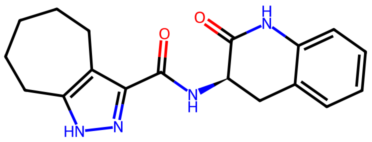	O=C(N[C@@H]1Cc2ccccc2NC1=O)c1n[nH]c2c1CCCCC2	529949006	24	324.16	1.97
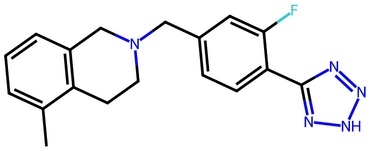	Cc1cccc2c1CCN(Cc1ccc(-c3nn[nH]n3)c(F)c1)C2	1592244522	24	323.15	2.87
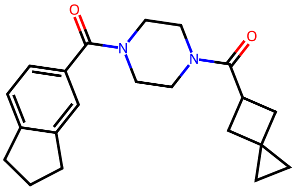	O=C(c1ccc2c(c1)CCC2)N1CCN(C(=O)C2CC3(CC3)C2)CC1	1011121766	25	338.2	2.65

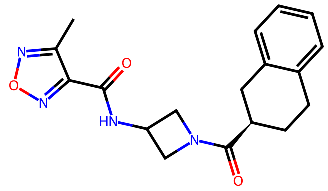	Cc1nonc1C(=O)NC1CN(C(=O)[C@@H]2CCc3ccccc3C2)C1	994941990	25	340.15	1.12
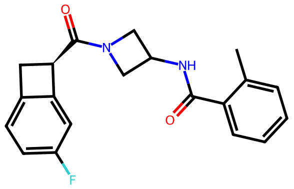	Cc1ccccc1C(=O)NC1CN(C(=O)[C@@H]2Cc3ccc(F)cc32)C1	990110210	25	338.14	2.41
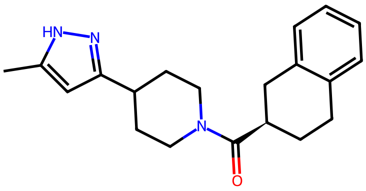	Cc1cc(C2CCN(C(=O)[C@@H]3CCc4ccccc4C3)CC2)n[nH]1	579720007	24	323.2	3.23
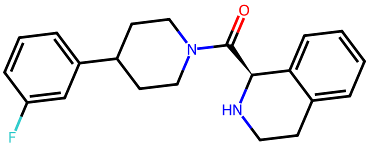	O=C([C@@H]1NCCc2ccccc21)N1CCC(c2cccc(F)c2)CC1	1340470615	25	338.18	3.42
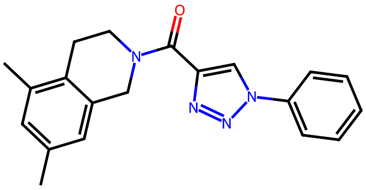	Cc1cc(C)c2c(c1)CN(C(=O)c1cn(-c3ccccc3)nn1)CC2	362463865	25	332.16	3.08
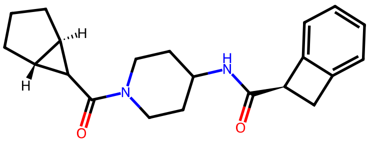	O=C(NC1CCN(C(=O)C2[C@@H]3CCC[C@@H]23)CC1)[C@@H]1Cc2ccccc21	946079927	25	338.2	2.48
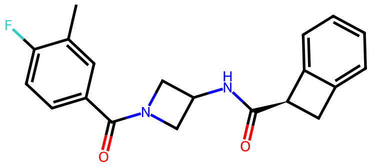	Cc1cc(C(=O)N2CC(NC(=O)[C@@H]3Cc4ccccc43)C2)ccc1F	998692424	25	338.14	2.41
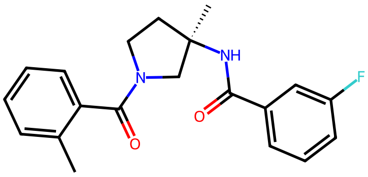	Cc1ccccc1C(=O)N1CC[C@@](C)(NC(=O)c2cccc(F)c2)C1	956654803	25	340.16	3.17

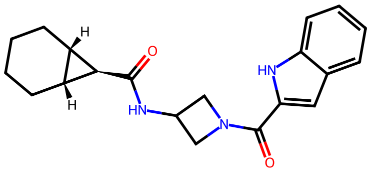	O=C(NC1CN(C(=O)c2cc3ccccc3[nH]2)C1)[C@@H]1[C@H]2CCCC[C@H]21	990663286	25	337.18	2.54
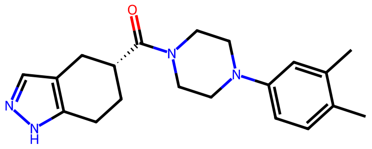	Cc1ccc(N2CCN(C(=O)[C@@H]3CCc4[nH]ncc4C3)CC2)cc1C	270480712	25	338.21	2.48
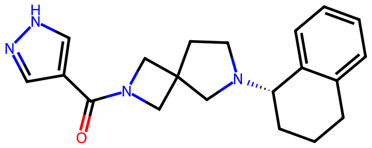	O=C(c1cn[nH]c1)N1CC2(CCN([C@H]3CCCc4ccccc43)C2)C1	1072511765	25	336.2	2.64
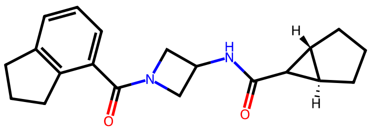	O=C(NC1CN(C(=O)c2cccc3c2CCC3)C1)C1[C@@H]2CCC[C@@H]12	990710297	24	324.18	2.16
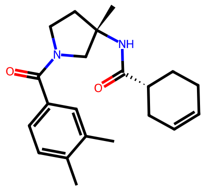	Cc1ccc(C(=O)N2CC[C@](C)(NC(=O)[C@H]3CC=CCC3)C2)cc1C	956691101	25	340.22	3.38
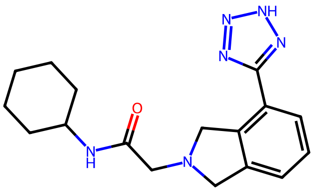	O=C(CN1Cc2cccc(-c3nn[nH]n3)c2C1)NC1CCCCC1	1592869588	24	326.19	1.63
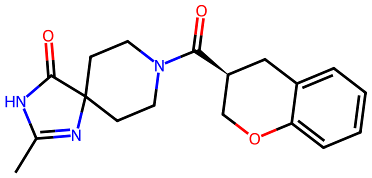	CC1=NC2(CCN(C(=O)[C@@H]3COc4ccccc4C3)CC2)C(=O)N1	244788041	24	327.16	1.15
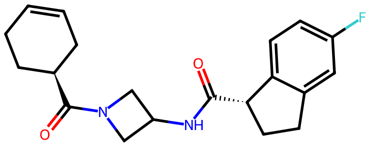	O=C(NC1CN(C(=O)[C@@H]2CC=CCC2)C1)[C@H]1CCc2cc(F)ccc21	996094466	25	342.17	2.54

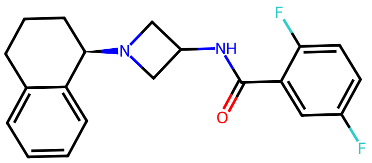	O=C(NC1CN([C@@H]2CCCc3ccccc3 2)C1)c1cc(F)ccc1F	1030447217	25	342.15	3.46
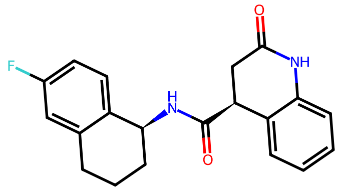	O=C1C[C@H](C(=O)N[C@H]2CCCc3 cc(F)ccc32)c2ccccc2N1	157618904	25	338.14	3.45
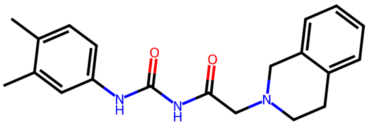	Cc1ccc(NC(=O)NC(=O)CN2CCc3cccc c3C2)cc1C	38533435	25	337.18	3.01
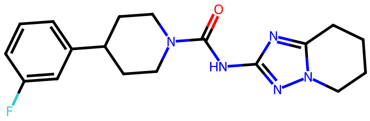	O=C(Nc1nc2n(n1)CCCC2)N1CCC(c2 cccc(F)c2)CC1	836776111	25	343.18	3.17
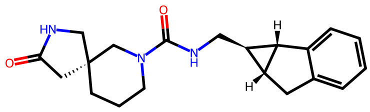	O=C1C[C@]2(CCCN(C(=O)NC[C@@ H]3[C@H]4Cc5ccccc5[C@H]43)C2)C N1	332906347	25	339.19	1.88
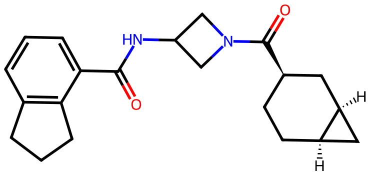	O=C(NC1CN(C(=O)[C@@H]2CC[C@ @H]3C[C@@H]3C2)C1)c1cccc2c1CC C2	994400581	25	338.2	2.55
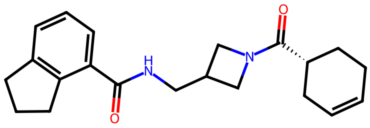	O=C(NCC1CN(C(=O)[C@H]2CC=CC C2)C1)c1cccc2c1CCC2	1002713137	25	338.2	2.72
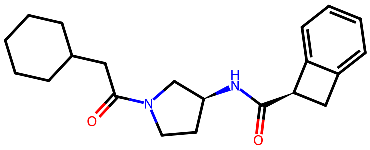	O=C(N[C@H]1CCN(C(=O)CC2CCCC C2)C1)[C@@H]1Cc2ccccc21	938574996	25	340.22	3.01

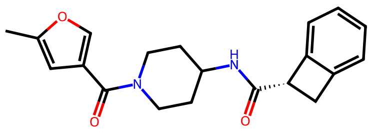	Cc1cc(C(=O)N2CCC(NC(=O)[C@H]3Cc4ccccc43)CC2)co1	946557089	25	338.16	2.65
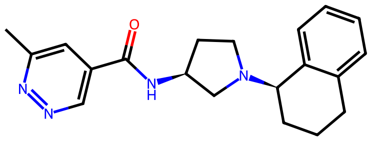	Cc1cc(C(=O)N[C@H]2CCN([C@@H]3CCCc4ccccc43)C2)cnn1	1010758326	25	336.2	2.67
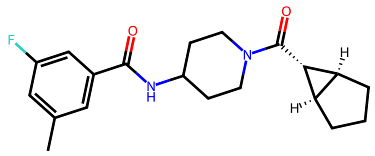	Cc1cc(F)cc(C(=O)NC2CCN(C(=O)[C@@H]3[C@H]4CCC[C@H]43)CC2)c1	946094227	25	344.19	2.9
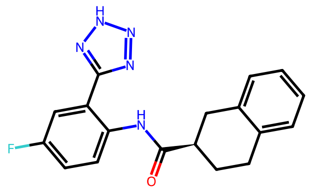	O=C(Nc1ccc(F)cc1-c1nn[nH]n1)[C@@H]1CCc2ccccc2C1	1606922159	25	337.13	2.75
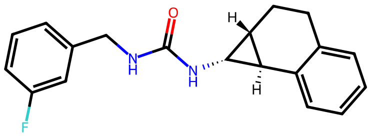	O=C(NCc1cccc(F)c1)N[C@H]1[C@@H]2c3ccccc3CC[C@H]21	126057217	23	310.15	3.35
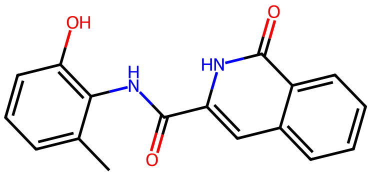	Cc1cccc(O)c1NC(=O)c1cc2ccccc2c(=O)[nH]1	179564404	22	294.1	2.79
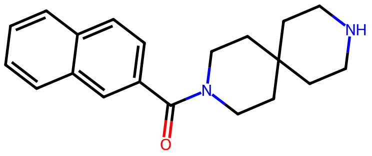	O=C(c1ccc2ccccc2c1)N1CCC2(CCNCC2)CC1	261854667	23	308.19	3.45
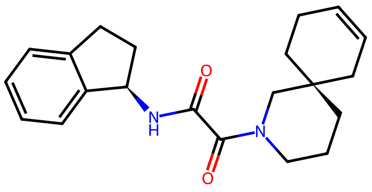	O=C(N[C@@H]1CCc2ccccc21)C(=O)N1CCC[C@@]2(CC=CCC2)C1	339872977	25	338.2	3.14

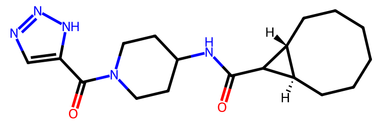	O=C(NC1CCN(C(=O)c2cnn[nH]2)CC1)C1[C@@H]2CCCCCC[C@@H]12	947373281	25	345.22	1.74
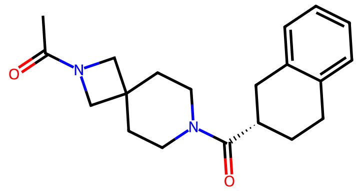	CC(=O)N1CC2(CCN(C(=O)[C@H]3CCc4ccccc4C3)CC2)C1	945071780	24	326.2	2.26
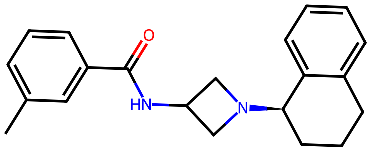	Cc1cccc(C(=O)NC2CN([C@@H]3CCCc4ccccc43)C2)c1	1030074878	24	320.19	3.49
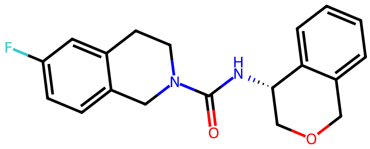	O=C(N[C@H]1COCc2ccccc21)N1CCc2cc(F)ccc2C1	419565765	24	326.14	3.16
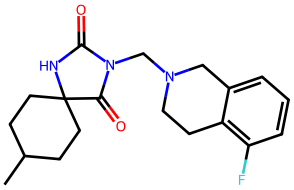	CC1CCC2(CC1)NC(=O)N(CN1CCc3c(F)cccc3C1)C2=O	156979067	25	345.19	2.64
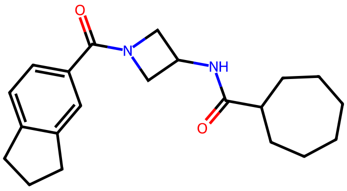	O=C(NC1CN(C(=O)c2ccc3c(c2)CCC3)C1)C1CCCCCC1	990456154	25	340.22	3.09
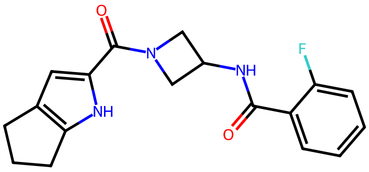	O=C(NC1CN(C(=O)c2cc3c([nH]2)CCC3)C1)c1ccccc1F	990089358	24	327.14	1.9
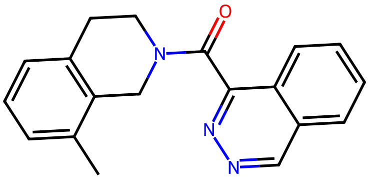	Cc1cccc2c1CN(C(=O)c1nncc3ccccc13)CC2	1537083124	23	303.14	3.14

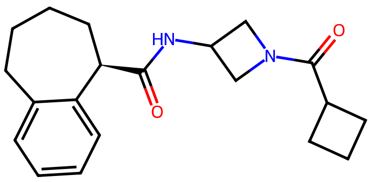	O=C(NC1CN(C(=O)C2CCC2)C1)[C@@H]1CCCCc2ccccc21	994322803	24	326.2	2.62
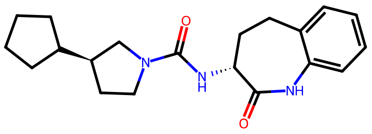	O=C1Nc2ccccc2CC[C@H]1NC(=O)N1CC[C@@H](C2CCCC2)C1	654631106	25	341.21	3.16
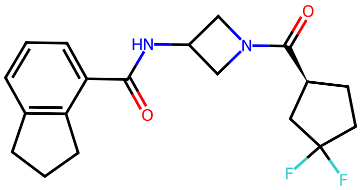	O=C(NC1CN(C(=O)[C@H]2CCC(F)(F)C2)C1)c1cccc2c1CCC2	994395329	25	348.16	2.55
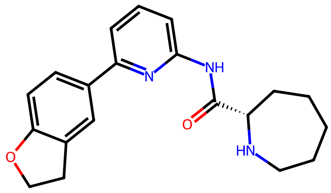	O=C(Nc1cccc(-c2ccc3c(c2)CCO3)n1)[C@@H]1CCCCCN1	571926650	25	337.18	3.15
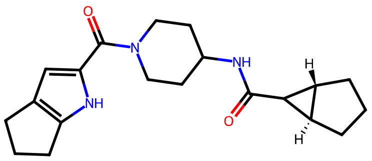	O=C(NC1CCN(C(=O)c2cc3c([nH]2)CCC3)CC1)C1[C@@H]2CCC[C@@H]12	947445685	25	341.21	2.27
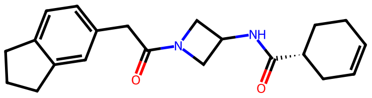	O=C(NC1CN(C(=O)Cc2ccc3c(c2)CCC3)C1)[C@H]1CC=CCC1	990222331	25	338.2	2.4
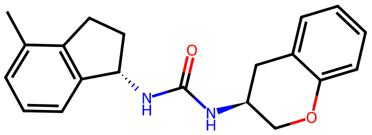	Cc1cccc2c1CC[C@@H]2NC(=O)N[C@@H]1COc2ccccc2C1	517916008	24	322.17	3.29
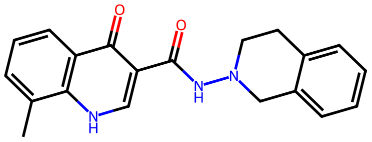	Cc1cccc2c(=O)c(C(=O)NN3CCc4ccccc4C3)c[nH]c12	1161618516	25	333.15	2.54

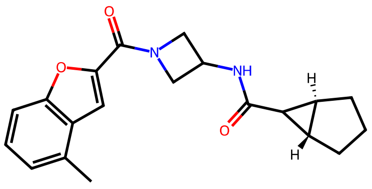	Cc1cccc2oc(C(=O)N3CC(NC(=O)C4[C@H]5CCC[C@H]45)C3)cc12	990700289	25	338.16	2.73
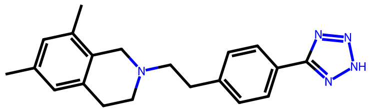	Cc1cc(C)c2c(c1)CCN(CCc1ccc(-c3nn[nH]n3)cc1)C2	1591950562	25	333.2	3.08
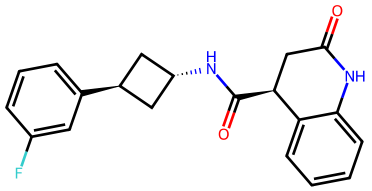	O=C1C[C@H](C(=O)N[C@H]2C[C@H](c3cccc(F)c3)C2)c2ccccc2N1	280609381	25	338.14	3.31
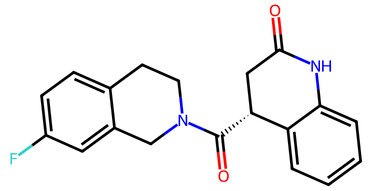	O=C1C[C@@H](C(=O)N2CCc3ccc(F)cc3C2)c2ccccc2N1	151527281	24	324.13	2.84
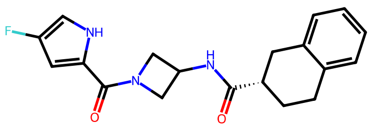	O=C(NC1CN(C(=O)c2cc(F)c[nH]2)C1)[C@H]1CCc2ccccc2C1	1000543715	25	341.15	1.9
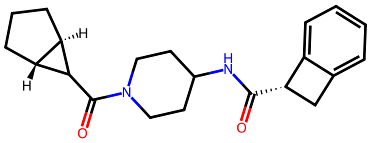	O=C(NC1CCN(C(=O)C2[C@@H]3CCC[C@@H]23)CC1)[C@H]1Cc2ccccc21	946079926	25	338.2	2.48
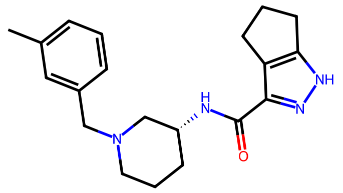	Cc1cccc(CN2CCC[C@@H](NC(=O)c3n[nH]c4c3CCC4)C2)c1	1007748362	25	338.21	2.6
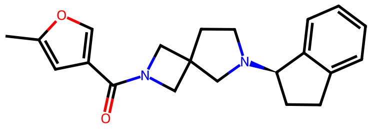	Cc1cc(C(=O)N2CC3(CCN([C@@H]4CCc5ccccc54)C3)C2)co1	1072661189	25	336.18	3.42

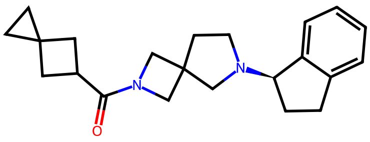	O=C(C1CC2(CC2)C1)N1CC2(CCN([C@@H]3CCc4ccccc43)C2)C1	1072972245	25	336.22	3.4
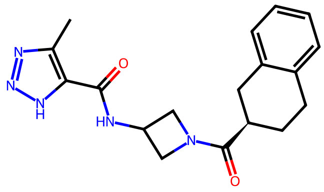	Cc1nn[nH]c1C(=O)NC1CN(C(=O)[C@@H]2CCc3ccccc3C2)C1	997352832	25	339.17	0.86
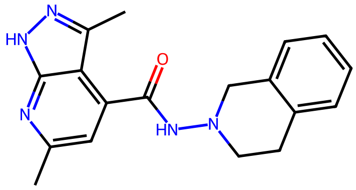	Cc1cc(C(=O)NN2CCc3ccccc3C2)c2c(C)n[nH]c2n1	855710086	24	321.16	2.28
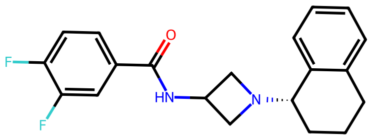	O=C(NC1CN([C@H]2CCCc3ccccc32)C1)c1ccc(F)c(F)c1	1030446356	25	342.15	3.46
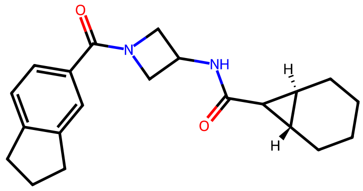	O=C(NC1CN(C(=O)c2ccc3c(c2)CCC3)C1)C1[C@H]2CCCC[C@H]12	990665623	25	338.2	2.55
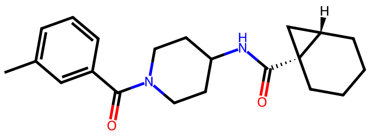	Cc1cccc(C(=O)N2CCC(NC(=O)[C@@]34CCCC[C@H]3C4)CC2)c1	943834391	25	340.22	3.3
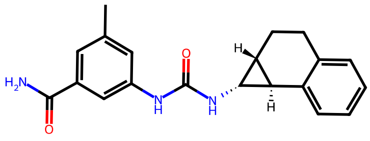	Cc1cc(NC(=O)N[C@H]2[C@@H]3c4ccccc4CC[C@H]32)cc(C(N)=O)c1	565994177	25	335.16	2.94
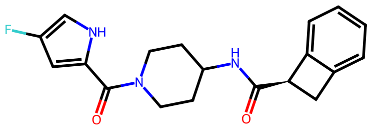	O=C(NC1CCN(C(=O)c2cc(F)c[nH]2)CC1)[C@@H]1Cc2ccccc21	947461562	25	341.15	2.21

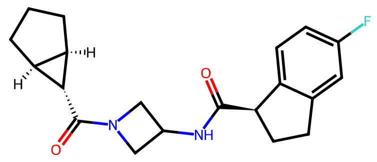	O=C(NC1CN(C(=O)[C@@H]2[C@H]3CCC[C@H]32)C1)[C@@H]1CCc2cc(F)ccc21	996098996	25	342.17	2.23
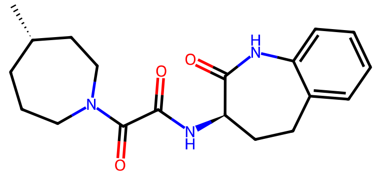	C[C@H]1CCCN(C(=O)C(=O)N[C@@H]2CCc3ccccc3NC2=O)CC1	1327237245	25	343.19	1.7
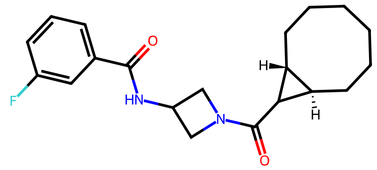	O=C(NC1CN(C(=O)C2[C@@H]3CCCCC[C@@H]23)C1)c1cccc(F)c1	990118128	25	344.19	2.98
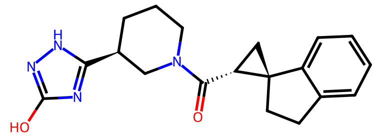	O=C([C@@H]1C[C@@]12CCc1ccccc12)N1CCC[C@H](c2nc(O)n[nH]2)C1	334018338	25	338.17	2.12
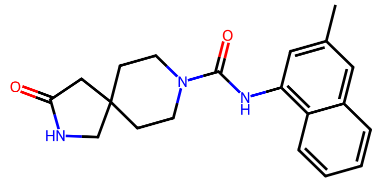	Cc1cc(NC(=O)N2CCC3(CC2)CNC(=O)C3)c2ccccc2c1	296720456	25	337.18	3.28
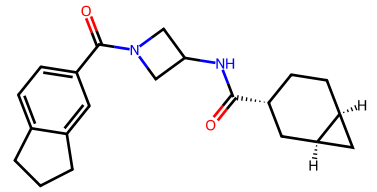	O=C(NC1CN(C(=O)c2ccc3c(c2)CCC3)C1)[C@@H]1CC[C@H]2C[C@H]2C1	994697268	25	338.2	2.55
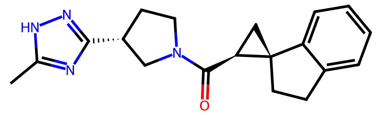	Cc1nc([C@@H]2CCN(C(=O)[C@H]3C[C@@]34CCc3ccccc34)C2)n[nH]1	333996518	24	322.18	2.33
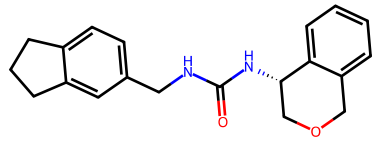	O=C(NCc1ccc2c(c1)CCC2)N[C@H]1COCc2ccccc21	1325350866	24	322.17	3.25

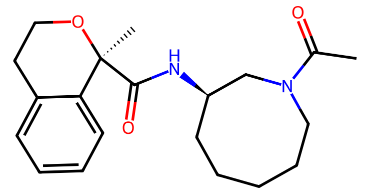	CC(=O)N1CCCCC[C@@H](NC(=O)[C@]2(C)OCCc3ccccc32)C1	986795755	25	344.21	2.38
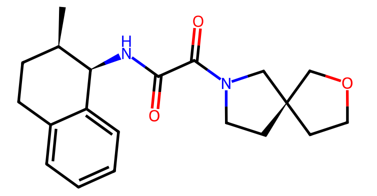	C[C@@H]1CCc2ccccc2[C@@H]1NC(=O)C(=O)N1CC[C@@]2(CCOC2)C1	673299622	25	342.19	2.07
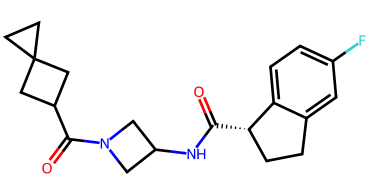	O=C(NC1CN(C(=O)C2CC3(CC3)C2)C1)[C@H]1CCc2cc(F)ccc21	996096856	25	342.17	2.37
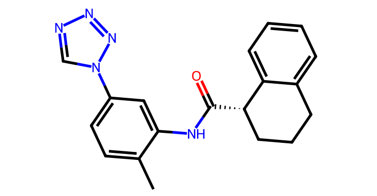	Cc1ccc(-n2cnnn2)cc1NC(=O)[C@H]1CCCc2ccccc21	44601204	25	333.16	3.03
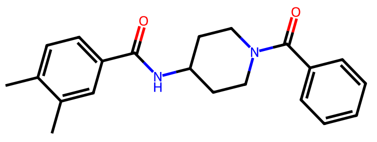	Cc1ccc(C(=O)NC2CCN(C(=O)c3ccccc3)CC2)cc1C	53392747	25	336.18	3.34
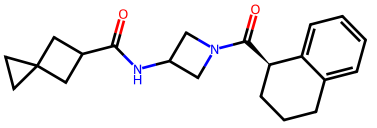	O=C(NC1CN(C(=O)[C@@H]2CCCc3ccccc32)C1)C1CC2(CC2)C1	999341008	25	338.2	2.62
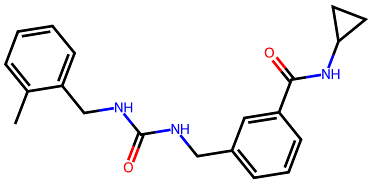	Cc1ccccc1CNC(=O)NCc1cccc(C(=O)NC2CC2)c1	48018905	25	337.18	2.89
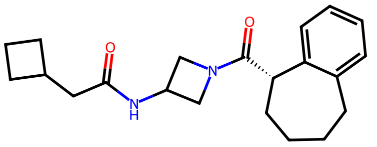	O=C(CC1CCC1)NC1CN(C(=O)[C@H]2CCCCc3ccccc32)C1	990646076	25	340.22	3.01

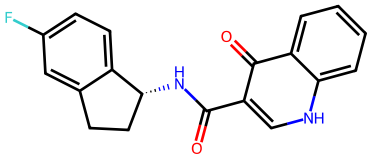	O=C(N[C@@H]1CCc2cc(F)ccc21)c1c[nH]c2ccccc2c1=O	55658184	24	322.11	3.08
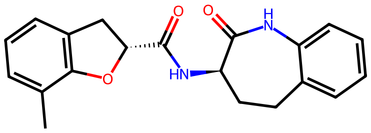	Cc1cccc2c1O[C@@H](C(=O)N[C@@H]1CCc3ccccc3NC1=O)C2	653996570	25	336.15	2.37
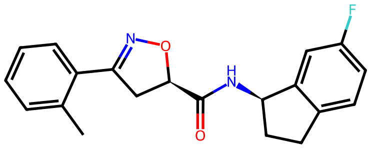	Cc1ccccc1C1=NO[C@@H](C(=O)N[C@@H]2CCc3ccc(F)cc32)C1	1360016917	25	338.14	3.43
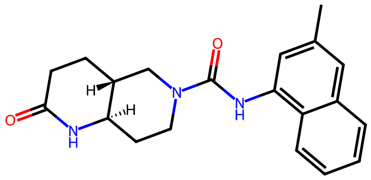	Cc1cc(NC(=O)N2CC[C@H]3NC(=O)CC[C@@H]3C2)c2ccccc2c1	667170964	25	337.18	3.28
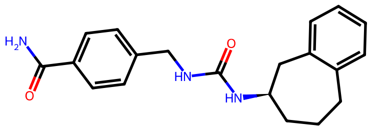	NC(=O)c1ccc(CNC(=O)N[C@@H]2CCCc3ccccc3C2)cc1	891954652	25	337.18	2.53
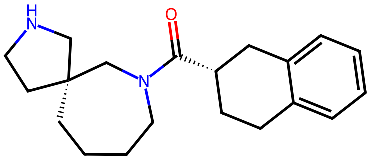	O=C([C@H]1CCc2ccccc2C1)N1CCCC[C@@]2(CCNC2)C1	1550236244	23	312.22	2.78
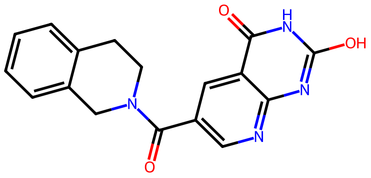	O=C(c1cnc2nc(O)[nH]c(=O)c2c1)N1CCc2ccccc2C1	47402919	24	322.11	1.22
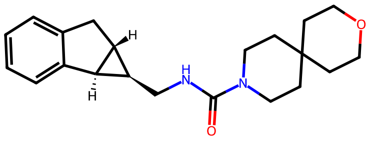	O=C(NC[C@@H]1[C@H]2Cc3ccccc3[C@@H]21)N1CCC2(CCOCC2)CC1	285281121	25	340.22	3.17

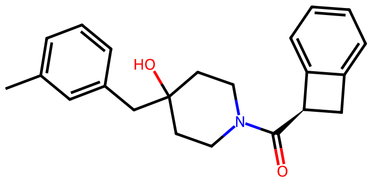	Cc1cccc(CC2(O)CCN(C(=O)[C@@H]3Cc4ccccc43)CC2)c1	357667451	25	335.19	3.23
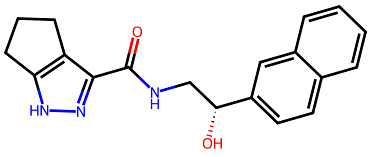	O=C(NC[C@@H](O)c1ccc2ccccc2c1)c1n[nH]c2c1CCC2	109690455	24	321.15	2.52
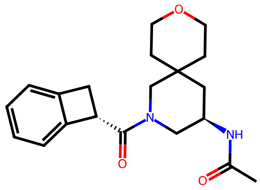	CC(=O)N[C@H]1CN(C(=O)[C@H]2Cc3ccccc32)CC2(CCOCC2)C1	991113933	25	342.19	1.86
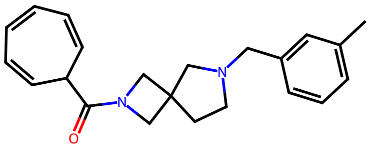	Cc1cccc(CN2CCC3(C2)CN(C(=O)C2C=CC=CC=C2)C3)c1	1072784942	25	334.2	3.33
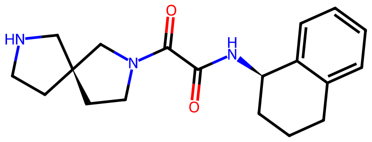	O=C(N[C@@H]1CCCc2ccccc21)C(=O)N1CC[C@]2(CCNC2)C1	514663735	24	327.19	1.39
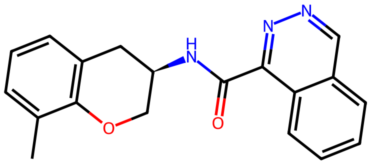	Cc1cccc2c1OC[C@H](NC(=O)c1nncc3ccccc13)C2	1584227573	24	319.13	2.67
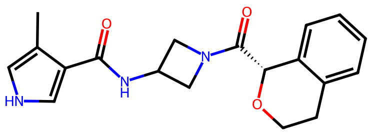	Cc1c[nH]cc1C(=O)NC1CN(C(=O)[C@H]2OCCc3ccccc32)C1	991710100	25	339.16	1.58
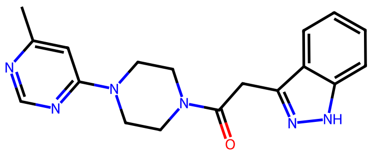	Cc1cc(N2CCN(C(=O)Cc3n[nH]c4cccc c34)CC2)ncn1	1055829722	25	336.17	1.55

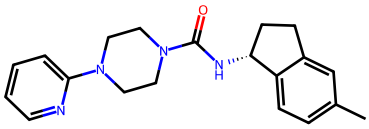	Cc1ccc2c(c1)CC[C@H]2NC(=O)N1CCN(c2ccccn2)CC1	56841863	25	336.2	2.91
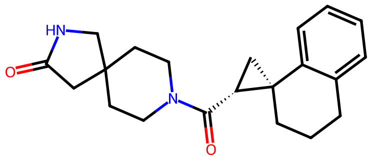	O=C1CC2(CCN(C(=O)[C@@H]3C[C@]34CCCc3ccccc34)CC2)CN1	374098582	25	338.2	2.41
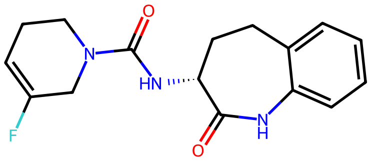	O=C1Nc2ccccc2CC[C@H]1NC(=O)N1CCC=C(F)C1	654629377	22	303.14	2.21
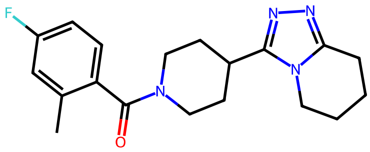	Cc1cc(F)ccc1C(=O)N1CCC(c2nnc3n2CCCC3)CC1	274687841	25	342.19	3.08
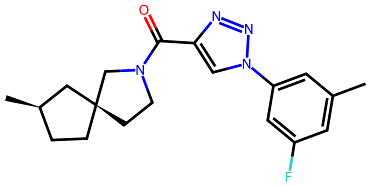	Cc1cc(F)cc(-n2cc(C(=O)N3CC[C@]4(CC[C@@H](C)C4)C3)nn2)c1	329866402	25	342.19	3.37
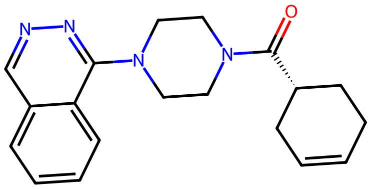	O=C([C@H]1CC=CCC1)N1CCN(c2nncc3ccccc23)CC1	96418528	24	322.18	2.63
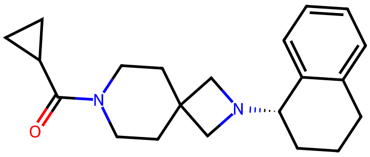	O=C(C1CC1)N1CCC2(CC1)CN([C@H]1CCCc3ccccc31)C2	1035634713	24	324.22	3.4
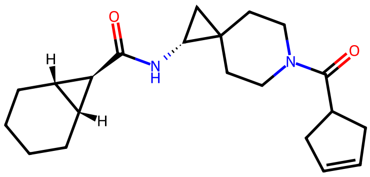	O=C(N[C@@H]1CC12CCN(C(=O)C1CC=CC1)CC2)[C@@H]1[C@H]2CCCC[C@H]21	963446743	25	342.23	2.89

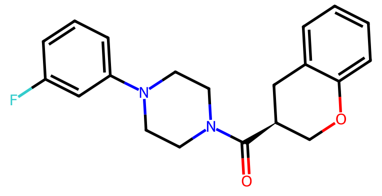	O=C([C@@H]1COc2ccccc2C1)N1CCN(c2cccc(F)c2)CC1	618147879	25	340.16	2.73
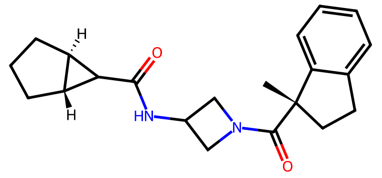	C[C@]1(C(=O)N2CC(NC(=O)C3[C@@H]4CCC[C@@H]34)C2)CCc2ccccc21	990713344	25	338.2	2.26
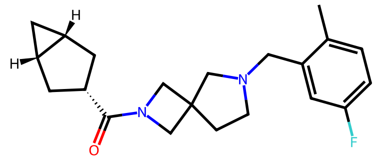	Cc1ccc(F)cc1CN1CCC2(C1)CN(C(=O)[C@@H]1C[C@@H]3C[C@@H]3C1)C2	1072906094	25	342.21	3.21
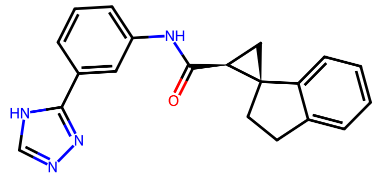	O=C(Nc1cccc(-c2nnc[nH]2)c1)[C@H]1C[C@@]12CCc1ccccc12	266489979	25	330.15	3.31
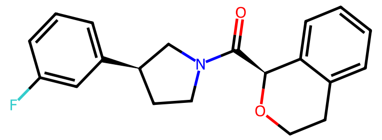	O=C([C@@H]1OCCc2ccccc21)N1CC[C@@H](c2cccc(F)c2)C1	490666559	24	325.15	3.46
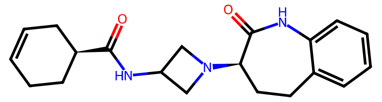	O=C(NC1CN([C@@H]2CCc3ccccc3NC2=O)C1)[C@@H]1CC=CCC1	1030098285	25	339.19	2.1
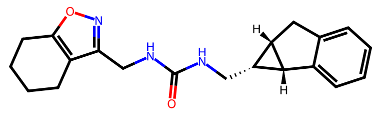	O=C(NCc1noc2c1CCCC2)NC[C@H]1[C@H]2c3ccccc3C[C@H]21	289331211	25	337.18	2.94
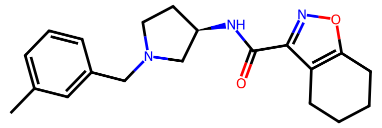	Cc1cccc(CN2CC[C@@H](NC(=O)c3noc4c3CCCC4)C2)c1	634270822	25	339.19	2.87

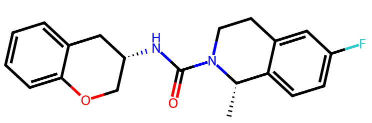	C[C@H]1c2ccc(F)cc2CCN1C(=O)N[C@@H]1COc2ccccc2C1	606644261	25	340.16	3.46
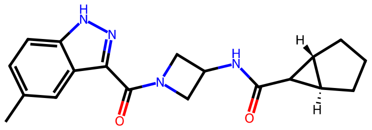	Cc1ccc2[nH]nc(C(=O)N3CC(NC(=O)C4[C@@H]5CCC[C@@H]45)C3)c2c1	990705744	25	338.17	1.86
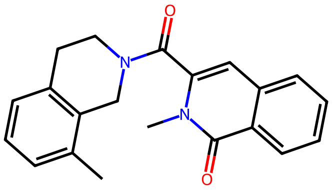	Cc1cccc2c1CN(C(=O)c1cc3ccccc3c(=O)n1C)CC2	355349390	25	332.15	3.05
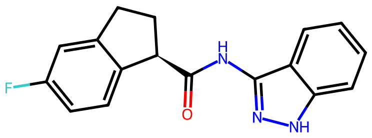	O=C(Nc1n[nH]c2ccccc12)[C@@H]1CCc2cc(F)ccc21	1135394963	22	295.11	3.37
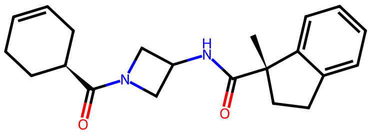	C[C@]1(C(=O)NC2CN(C(=O)[C@@H]3CC=CCC3)C2)CCc2ccccc21	994376602	25	338.2	2.57
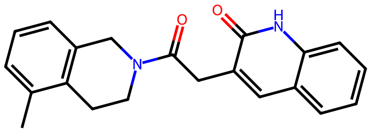	Cc1cccc2c1CCN(C(=O)Cc1cc3ccccc3[nH]c1=O)C2	171542846	25	332.15	2.96
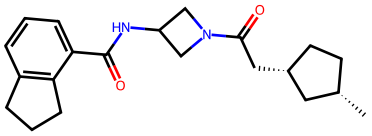	C[C@H]1CC[C@@H](CC(=O)N2CC(NC(=O)c3cccc4c3CCC4)C2)C1	994395386	25	340.22	2.94
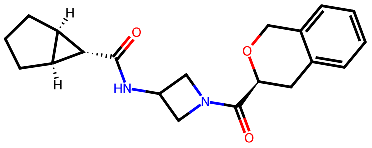	O=C(NC1CN(C(=O)[C@@H]2Cc3ccccc3CO2)C1)[C@@H]1[C@H]2CCC[C@@H]21	990698941	25	340.18	1.5

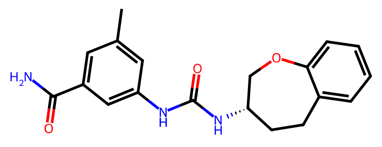	Cc1cc(NC(=O)N[C@H]2CCc3ccccc3OC2)cc(C(N)=O)c1	455844716	25	339.16	2.61
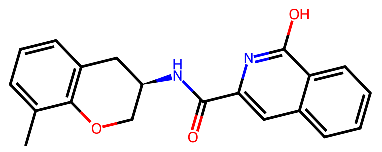	Cc1cccc2c1OC[C@H](NC(=O)c1cc3ccccc3c(O)n1)C2	367943911	25	334.13	2.98
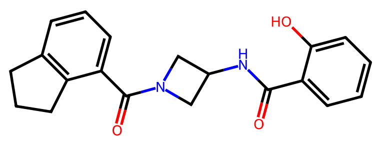	O=C(NC1CN(C(=O)c2cccc3c2CCC3)C1)c1ccccc1O	990276911	25	336.15	2.14
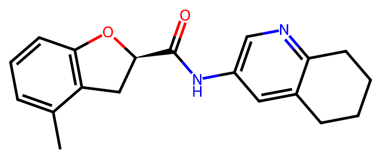	Cc1cccc2c1C[C@H](C(=O)Nc1cnc3c(c1)CCCC3)O2	569917853	23	308.15	3.21
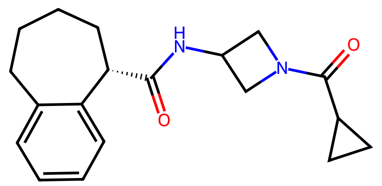	O=C(NC1CN(C(=O)C2CC2)C1)[C@H]1CCCCc2ccccc21	994325471	23	312.18	2.23
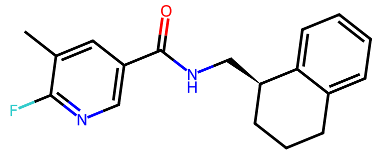	Cc1cc(C(=O)NC[C@@H]2CCCc3ccccc32)cnc1F	857574267	22	298.15	3.38
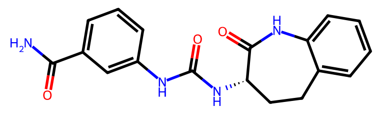	NC(=O)c1cccc(NC(=O)N[C@H]2CCc3ccccc3NC2=O)c1	1425082169	25	338.14	1.86
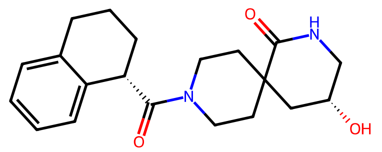	O=C([C@H]1CCCc2ccccc21)N1CCC2(CC1)C[C@@H](O)CNC2=O	885024780	25	342.19	1.6

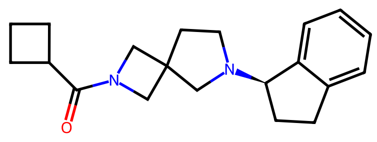	O=C(C1CCC1)N1CC2(CCN([C@@H]3CCc4ccccc43)C2)C1	1072375565	23	310.2	3.01
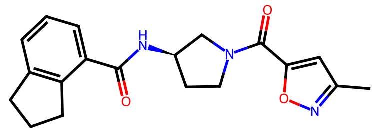	Cc1cc(C(=O)N2CC[C@@H](NC(=O)c3cccc4c3CCC4)C2)on1	941156702	25	339.16	2.12
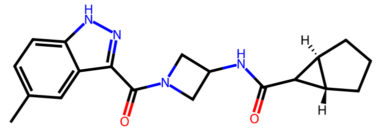	Cc1ccc2[nH]nc(C(=O)N3CC(NC(=O)C4[C@H]5CCC[C@H]45)C3)c2c1	990705759	25	338.17	1.86
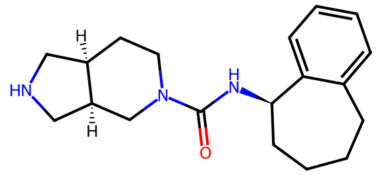	O=C(N[C@@H]1CCCCc2ccccc21)N1CC[C@@H]2CNC[C@@H]2C1	578333971	23	313.22	2.71
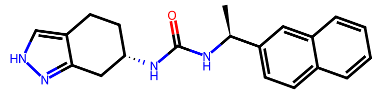	C[C@H](NC(=O)N[C@H]1CCc2c[nH]nc2C1)c1ccc2ccccc2c1	363938597	25	334.18	3.48
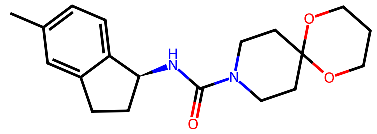	Cc1ccc2c(c1)CC[C@@H]2NC(=O)N1CCC2(CC1)OCCCO2	836252117	24	330.19	2.92
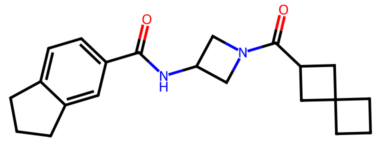	O=C(NC1CN(C(=O)C2CC3(CCC3)C2)C1)c1ccc2c(c1)CCC2	991373929	25	338.2	2.7
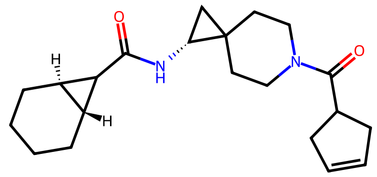	O=C(N[C@@H]1CC12CCN(C(=O)C1CC=CC1)CC2)C1[C@@H]2CCCC[C@@H]12	963446742	25	342.23	2.89

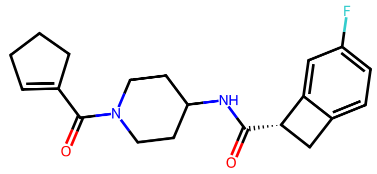	O=C(NC1CCN(C(=O)C2=CCCC2)CC1)[C@H]1Cc2ccc(F)cc21	946449650	25	342.17	2.68
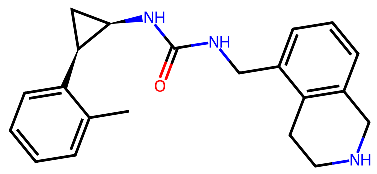	Cc1ccccc1[C@H]1C[C@H]1NC(=O)NCc1cccc2c1CCNC2	1550270210	25	335.2	3.0
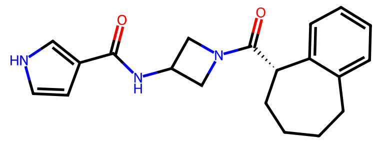	O=C(NC1CN(C(=O)[C@H]2CCCCc3ccccc32)C1)c1cc[nH]c1	993723644	25	337.18	2.47
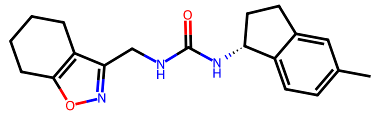	Cc1ccc2c(c1)CC[C@H]2NC(=O)NCc1noc2c1CCCC2	287000460	24	325.18	3.35
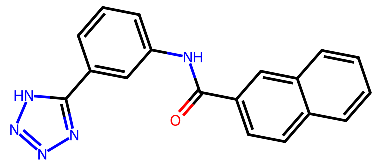	O=C(Nc1cccc(-c2nnn[nH]2)c1)c1ccc2ccccc2c1	20501507	24	315.11	3.27
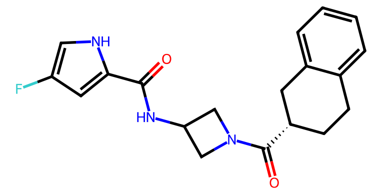	O=C(NC1CN(C(=O)[C@H]2CCc3ccccc3C2)C1)c1cc(F)c[nH]1	996235883	25	341.15	1.9
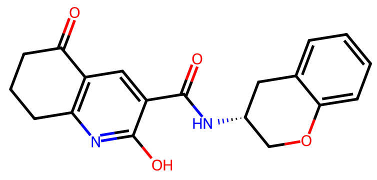	O=C(N[C@H]1COc2ccccc2C1)c1cc2c(nc1O)CCCC2=O	299023614	25	338.13	2.04
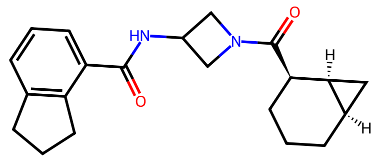	O=C(NC1CN(C(=O)[C@@H]2CCC[C@@H]3C[C@@H]32)C1)c1cccc2c1CCC2	994398022	25	338.2	2.55

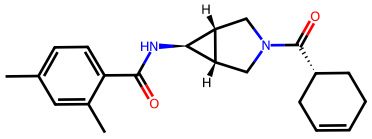	Cc1ccc(C(=O)N[C@H]2[C@@H]3CN(C(=O)[C@H]4CC=CCC4)C[C@@H]32)c(C)c1	977175941	25	338.2	2.85
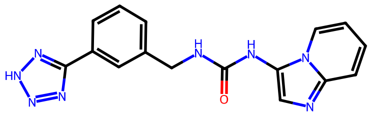	O=C(NCc1cccc(-c2nn[nH]n2)c1)Nc1cnc2ccccn12	1606836940	25	334.13	1.84
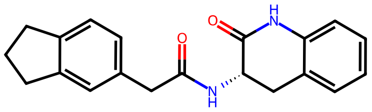	O=C(Cc1ccc2c(c1)CCC2)N[C@H]1Cc2ccccc2NC1=O	529950642	24	320.15	2.4
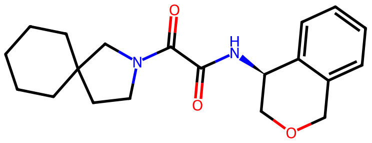	O=C(N[C@@H]1COCc2ccccc21)C(=O)N1CCC2(CCCCC2)C1	1329006203	25	342.19	2.56
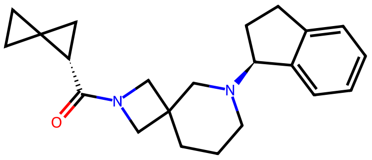	O=C([C@H]1CC12CC2)N1CC2(CCCN([C@H]3CCc4ccccc43)C2)C1	982038870	25	336.22	3.4
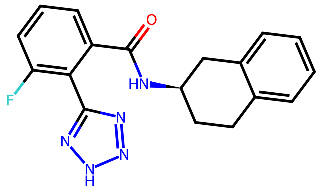	O=C(N[C@@H]1CCc2ccccc2C1)c1cccc(F)c1-c1nn[nH]n1	1592995904	25	337.13	2.29
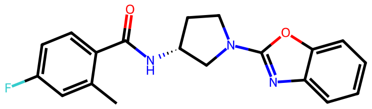	Cc1cc(F)ccc1C(=O)N[C@@H]1CCN(c2nc3ccccc3o2)C1	1058411350	25	339.14	3.28
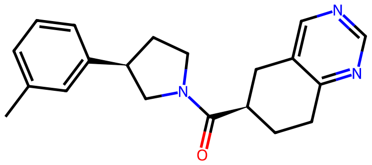	Cc1cccc([C@H]2CCN(C(=O)[C@@H]3CCc4ncncc4C3)C2)c1	648353009	24	321.18	2.91

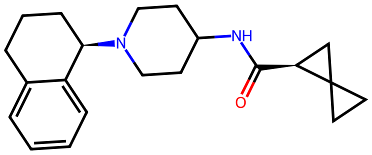	O=C(NC1CCN([C@@H]2CCCc3ccccc32)CC1)[C@H]1CC12CC2	1003695574	24	324.22	3.44
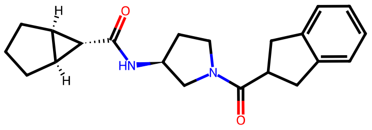	O=C(N[C@H]1CCN(C(=O)C2Cc3ccccc3C2)C1)[C@@H]1[C@H]2CCC[C@H]21	937802016	25	338.2	2.16
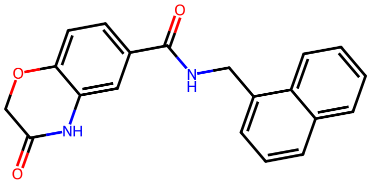	O=C1COc2ccc(C(=O)NCc3cccc4ccccc34)cc2N1	79142973	25	332.12	3.1
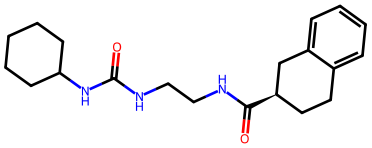	O=C(NCCNC(=O)[C@@H]1CCc2ccccc2C1)NC1CCCCC1	566330203	25	343.23	2.54
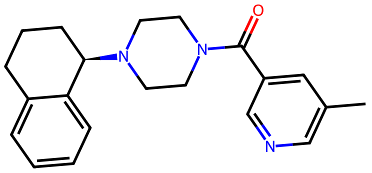	Cc1cncc(C(=O)N2CCN([C@@H]3CCCc4ccccc43)CC2)c1	1681646832	25	335.2	3.23
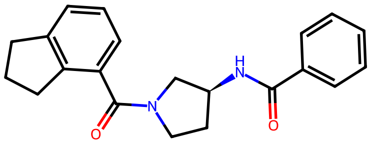	O=C(N[C@H]1CCN(C(=O)c2cccc3c2CCC3)C1)c1ccccc1	937625892	25	334.17	2.82
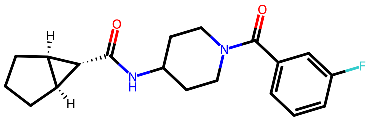	O=C(NC1CCN(C(=O)c2cccc(F)c2)CC1)[C@@H]1[C@H]2CCC[C@H]21	943774783	24	330.17	2.59
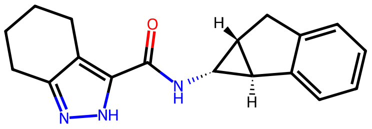	O=C(N[C@H]1[C@@H]2c3ccccc3C[C@H]21)c1[nH]nc2c1CCCC2	130590393	22	293.15	2.36

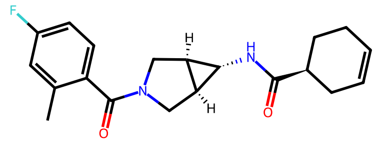	Cc1cc(F)ccc1C(=O)N1C[C@H]2[C@H](NC(=O)[C@@H]3CC=CCC3)[C@H]2C1	976178767	25	342.17	2.68
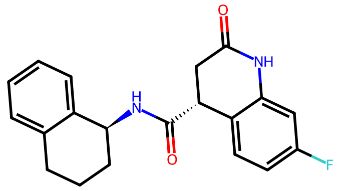	O=C1C[C@@H](C(=O)N[C@H]2CCCc3ccccc32)c2ccc(F)cc2N1	120390969	25	338.14	3.45
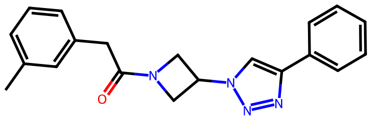	Cc1cccc(CC(=O)N2CC(n3cc(-c4ccccc4)nn3)C2)c1	238917606	25	332.16	2.88
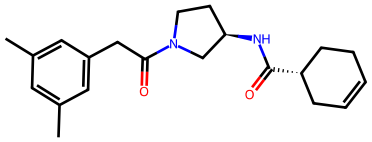	Cc1cc(C)cc(CC(=O)N2CC[C@@H](NC(=O)[C@H]3CC=CCC3)C2)c1	939380878	25	340.22	2.92
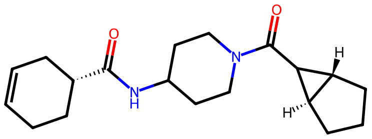	O=C(NC1CCN(C(=O)C2[C@@H]3CCC[C@@H]23)CC1)[C@H]1CC=CCC1	946064269	23	316.22	2.5
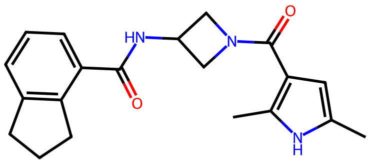	Cc1cc(C(=O)N2CC(NC(=O)c3cccc4c3CCC4)C2)c(C)[nH]1	994399833	25	337.18	2.37
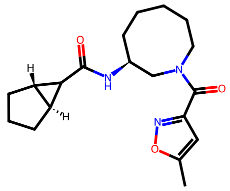	Cc1cc(C(=O)N2CCCCC[C@H](NC(=O)C3[C@H]4CCC[C@H]34)C2)no1	986888450	25	345.21	2.53
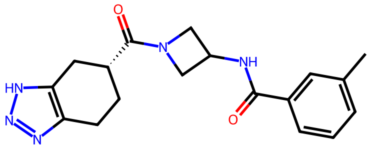	Cc1cccc(C(=O)NC2CN(C(=O)[C@@H]3CCc4nn[nH]c4C3)C2)c1	990129376	25	339.17	0.86

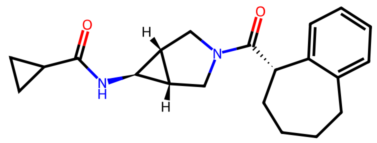	O=C(N[C@H]1[C@@H]2CN(C(=O)[C@H]3CCCCc4ccccc43)C[C@@H]21)C1CC1	976031441	25	338.2	2.48
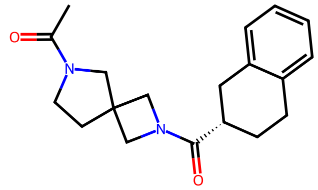	CC(=O)N1CCC2(C1)CN(C(=O)[C@H]1CCc3ccccc3C1)C2	1019491440	23	312.18	1.87
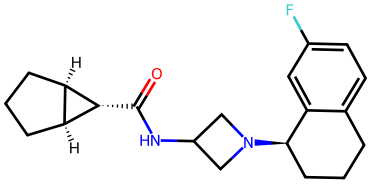	O=C(NC1CN([C@@H]2CCCc3ccc(F)cc32)C1)[C@@H]1[C@H]2CCC[C@H]21	1030201940	24	328.2	3.05
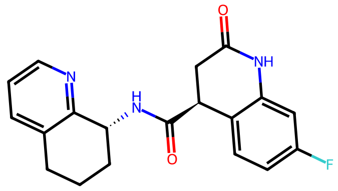	O=C1C[C@H](C(=O)N[C@@H]2CCCc3cccnc32)c2ccc(F)cc2N1	885373633	25	339.14	2.84
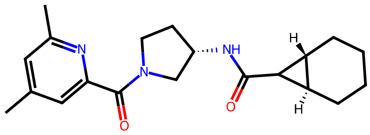	Cc1cc(C)nc(C(=O)N2CC[C@H](NC(=O)C3[C@@H]4CCCC[C@@H]34)C2)c1	937790181	25	341.21	2.47
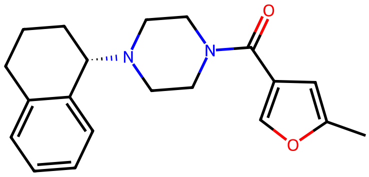	Cc1cc(C(=O)N2CCN([C@H]3CCCc4ccccc43)CC2)co1	1565501957	24	324.18	3.42
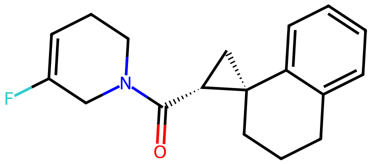	O=C([C@@H]1C[C@]12CCCc1ccccc12)N1CCC=C(F)C1	376854287	21	285.15	3.37
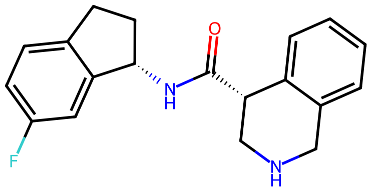	O=C(N[C@H]1CCc2ccc(F)cc21)[C@H]1CNCc2ccccc21	563075020	23	310.15	2.82

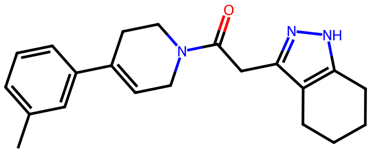	Cc1cccc(C2=CCN(C(=O)Cc3n[nH]c4c3CCCC4)CC2)c1	188739167	25	335.2	3.46
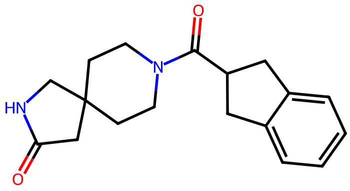	O=C1CC2(CCN(C(=O)C3Cc4ccccc4C3)CC2)CN1	277640350	22	298.17	1.53
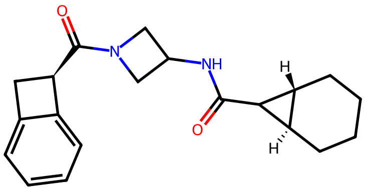	O=C(NC1CN(C(=O)[C@@H]2Cc3ccccc32)C1)C1[C@@H]2CCCC[C@@H]12	990649259	24	324.18	2.09
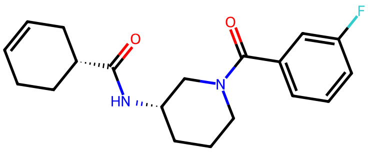	O=C(N[C@H]1CCCN(C(=O)c2cccc(F)c2)C1)[C@H]1CC=CCC1	949850584	24	330.17	2.9
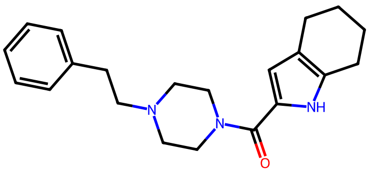	O=C(c1cc2c([nH]1)CCCC2)N1CCN(CCc2ccccc2)CC1	448625155	25	337.22	2.89
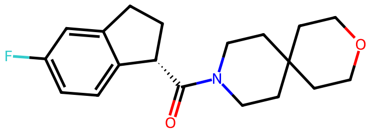	O=C([C@H]1CCc2cc(F)ccc21)N1CCC2(CCOCC2)CC1	614600701	23	317.18	3.27
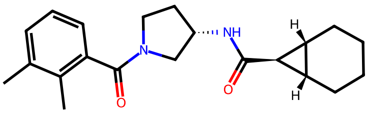	Cc1cccc(C(=O)N2CC[C@H](NC(=O)[C@@H]3[C@H]4CCCC[C@H]43)C2)c1C	937790185	25	340.22	3.07
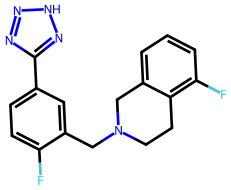	Fc1ccc(-c2nn[nH]n2)cc1CN1CCc2c(F)cccc2C1	826373468	24	327.13	2.7

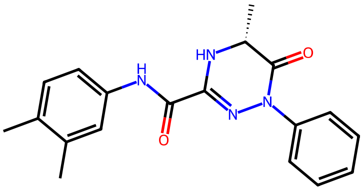	Cc1ccc(NC(=O)C2=NN(c3ccccc3)C(=O)[C@@H](C)N2)cc1C	32643611	25	336.16	2.58
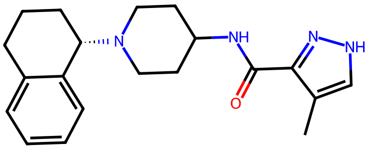	Cc1c[nH]nc1C(=O)NC1CCN([C@H]2CCCc3ccccc32)CC1	1002773738	25	338.21	2.99
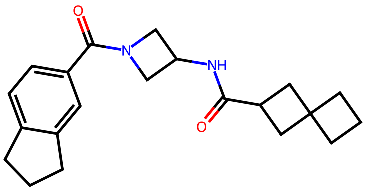	O=C(NC1CN(C(=O)c2ccc3c(c2)CCC3)C1)C1CC2(CCC2)C1	997933454	25	338.2	2.7
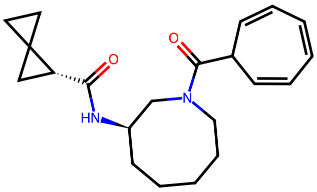	O=C(N[C@@H]1CCCCCN(C(=O)C2C=CC=CC=C2)C1)[C@@H]1CC12CC2	987047822	25	340.22	2.97
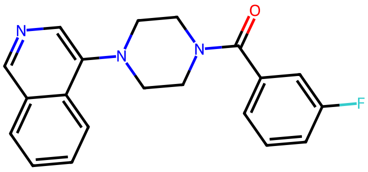	O=C(c1cccc(F)c1)N1CCN(c2cncc3ccccc23)CC1	190387990	25	335.14	3.34
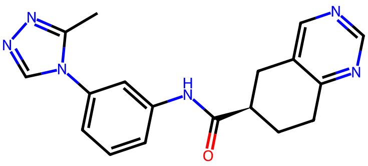	Cc1nncn1-c1cccc(NC(=O)[C@@H]2CCc3ncncc3C2)c1	648339046	25	334.15	2.11
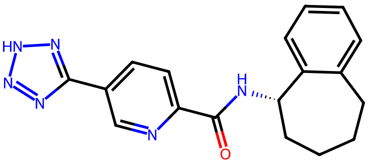	O=C(N[C@H]1CCCCc2ccccc21)c1ccc(-c2nn[nH]n2)cn1	738026726	25	334.15	2.46
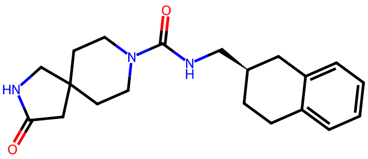	O=C1CC2(CCN(C(=O)NC[C@@H]3CCc4ccccc4C3)CC2)CN1	752015749	25	341.21	2.1

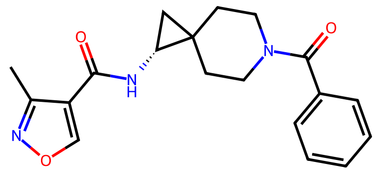	Cc1nocc1C(=O)N[C@@H]1CC12CCN(C(=O)c1ccccc1)CC2	965008928	25	339.16	2.41
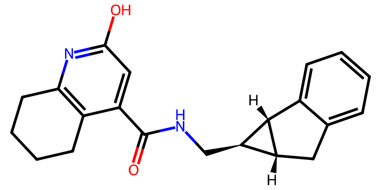	O=C(NC[C@@H]1[C@H]2Cc3ccccc3[C@H]21)c1cc(O)nc2c1CCCC2	934913629	25	334.17	2.98
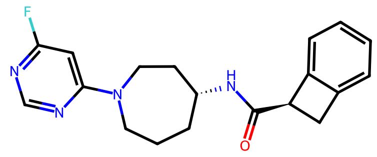	O=C(N[C@@H]1CCCN(c2cc(F)ncn2)CC1)[C@@H]1Cc2ccccc21	1066778779	25	340.17	2.43
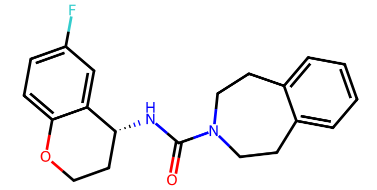	O=C(N[C@@H]1CCOc2ccc(F)cc21)N1CCc2ccccc2CC1	548042081	25	340.16	3.46
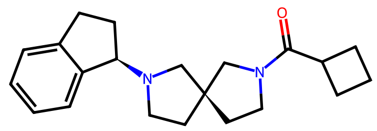	O=C(C1CCC1)N1CC[C@]2(CCN([C@@H]3CCc4ccccc43)C2)C1	1040990572	24	324.22	3.4
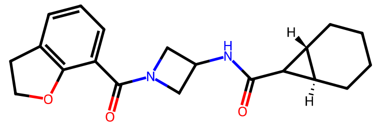	O=C(NC1CN(C(=O)c2cccc3c2OCC3)C1)C1[C@@H]2CCCC[C@@H]12	990662706	25	340.18	2.0
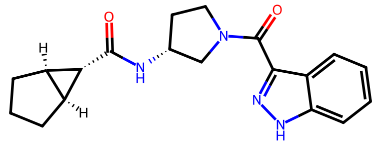	O=C(N[C@@H]1CCN(C(=O)c2n[nH]c3ccccc23)C1)[C@@H]1[C@H]2CCC[C@H]21	939933030	25	338.17	1.94
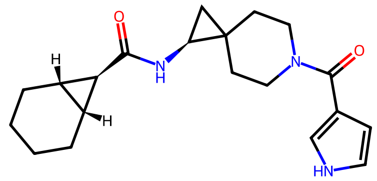	O=C(N[C@H]1CC12CCN(C(=O)c1cc[nH]c1)CC2)[C@@H]1[C@H]2CCCC[C@H]21	963443630	25	341.21	2.56

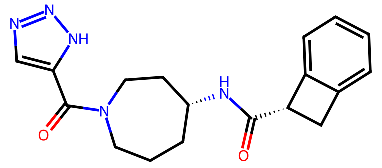	O=C(N[C@@H]1CCCN(C(=O)c2cnn[nH]2)CC1)[C@H]1Cc2ccccc21	965142452	25	339.17	1.26
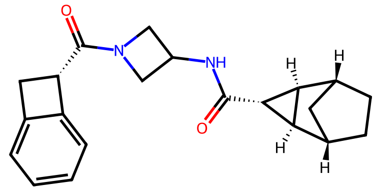	O=C(NC1CN(C(=O)[C@H]2Cc3ccccc32)C1)[C@@H]1[C@H]2[C@@H]3CC[C@@H](C3)[C@H]21	994362265	25	336.18	1.95
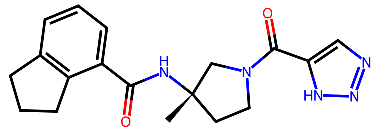	C[C@]1(NC(=O)c2cccc3c2CCC3)CCN(C(=O)c2cnn[nH]2)C1	957143927	25	339.17	1.33
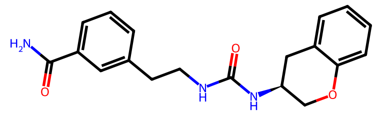	NC(=O)c1cccc(CCNC(=O)N[C@@H]2COc3ccccc3C2)c1	171083721	25	339.16	1.63
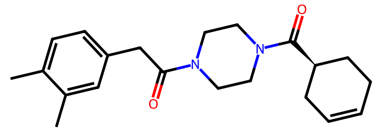	Cc1ccc(CC(=O)N2CCN(C(=O)[C@@H]3CC=CCC3)CC2)cc1C	1010298186	25	340.22	2.87
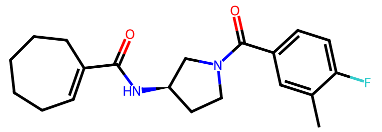	Cc1cc(C(=O)N2CC[C@@H](NC(=O)C3=CCCCCC3)C2)ccc1F	940158824	25	344.19	3.36
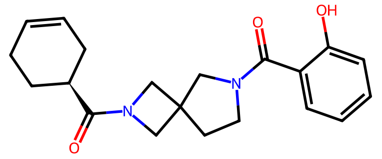	O=C(c1ccccc1O)N1CCC2(C1)CN(C(=O)[C@@H]1CC=CCC1)C2	1018770288	25	340.18	2.42
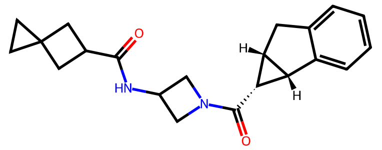	O=C(NC1CN(C(=O)[C@H]2[C@H]3c4ccccc4C[C@H]32)C1)C1CC2(CC2)C1	999346109	25	336.18	2.09

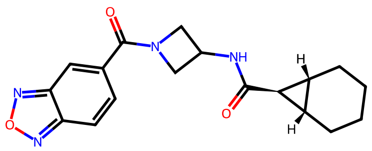	O=C(NC1CN(C(=O)c2ccc3nonc3c2)C1)[C@@H]1[C@H]2CCCC[C@H]21	990651247	25	340.15	1.6
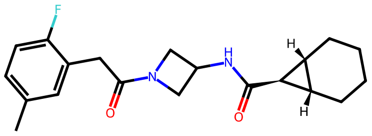	Cc1ccc(F)c(CC(=O)N2CC(NC(=O)[C@@H]3[C@H]4CCCC[C@H]43)C2)c1	990643902	25	344.19	2.44
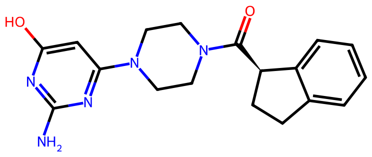	Nc1nc(O)cc(N2CCN(C(=O)[C@@H]3CCc4ccccc43)CC2)n1	426501929	25	339.17	1.14
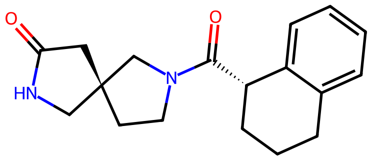	O=C1C[C@@]2(CCN(C(=O)[C@H]3CCCc4ccccc43)C2)CN1	279456502	22	298.17	1.85
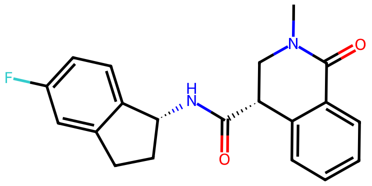	CN1C[C@@H](C(=O)N[C@@H]2CCc3cc(F)ccc32)c2ccccc2C1=O	345833156	25	338.14	2.8
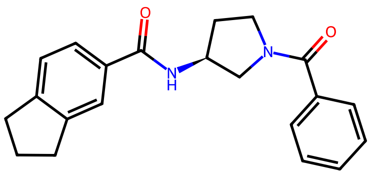	O=C(N[C@H]1CCN(C(=O)c2ccccc2)C1)c1ccc2c(c1)CCC2	937934305	25	334.17	2.82
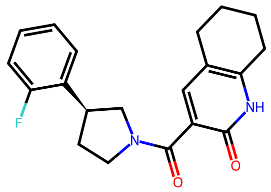	O=C(c1cc2c([nH]c1=O)CCCC2)N1CC[C@@H](c2ccccc2F)C1	68107606	25	340.16	3.02
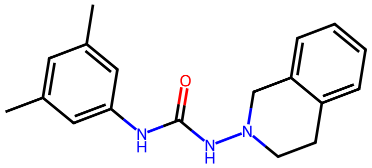	Cc1cc(C)cc(NC(=O)NN2CCc3ccccc3C2)c1	1666856440	22	295.17	3.4

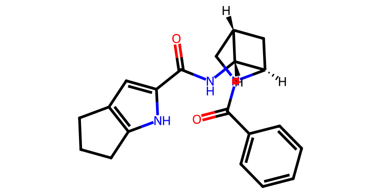	O=C(N[C@@H]1[C@H]2C[C@H]1N(C(=O)c1ccccc1)C2)c1cc2c([nH]1)CCC2	947551921	25	335.16	2.15
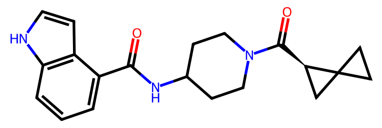	O=C(NC1CCN(C(=O)[C@@H]2CC23CC3)CC1)c1cccc2[nH]ccc12	948557400	25	337.18	2.69
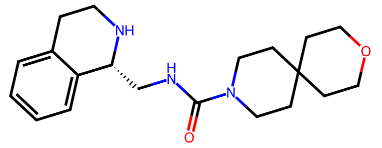	O=C(NC[C@H]1NCCc2ccccc21)N1CCC2(CCOCC2)CC1	841992784	25	343.23	2.48
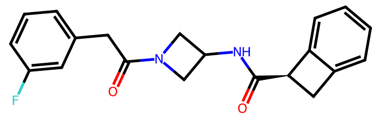	O=C(NC1CN(C(=O)Cc2cccc(F)c2)C1)[C@@H]1Cc2ccccc21	998692381	25	338.14	2.04
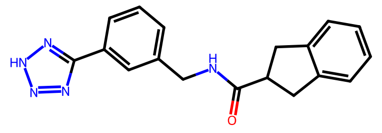	O=C(NCc1cccc(-c2nn[nH]n2)c1)C1Cc2ccccc2C1	1178545032	24	319.14	1.9
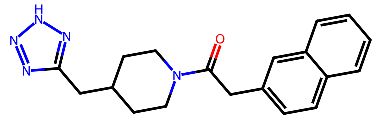	O=C(Cc1ccc2ccccc2c1)N1CCC(Cc2nn[nH]n2)CC1	1123983637	25	335.17	2.38
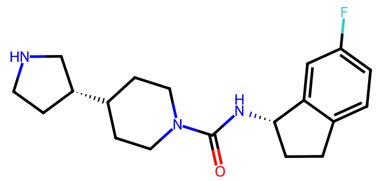	O=C(N[C@H]1CCc2ccc(F)cc21)N1CCC([C@@H]2CCNC2)CC1	580763649	24	331.21	2.84
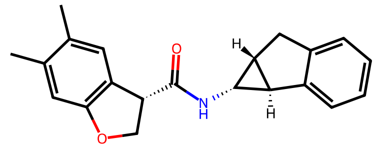	Cc1cc2c(cc1C)[C@H](C(=O)N[C@H]1[C@@H]3c4ccccc4C[C@H]31)CO2	248634217	24	319.16	3.23

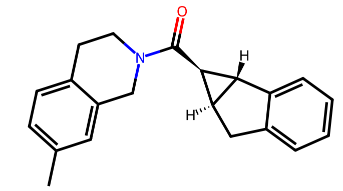	Cc1ccc2c(c1)CN(C(=O)[C@@H]1[C@@H]3Cc4ccccc4[C@H]31)CC2	628058150	23	303.16	3.47
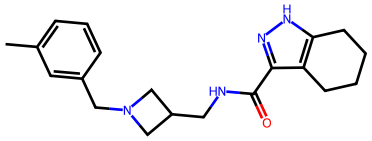	Cc1cccc(CN2CC(CNC(=O)c3n[nH]c4c3CCCC4)C2)c1	1031808399	25	338.21	2.46
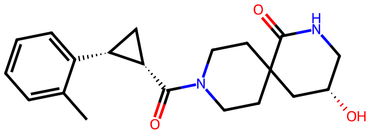	Cc1ccccc1[C@@H]1C[C@@H]1C(=O)N1CCC2(CC1)C[C@@H](O)CNC2=O	1551049841	25	342.19	1.59
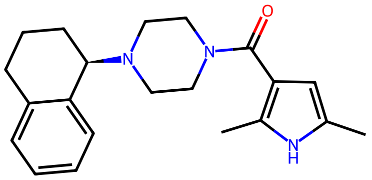	Cc1cc(C(=O)N2CCN([C@@H]3CCCc4ccccc43)CC2)c(C)[nH]1	950851759	25	337.22	3.47
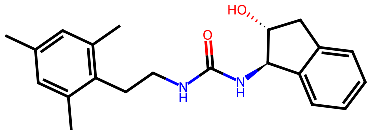	Cc1cc(C)c(CCNC(=O)N[C@@H]2c3ccccc3C[C@H]2O)c(C)c1	506682099	25	338.2	3.11
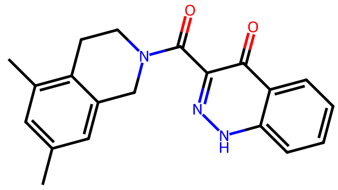	Cc1cc(C)c2c(c1)CN(C(=O)c1n[nH]c3ccccc3c1=O)CC2	188650885	25	333.15	2.74
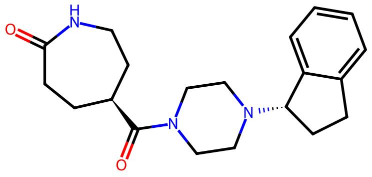	O=C1CC[C@H](C(=O)N2CCN([C@H]3CCc4ccccc43)CC2)CCN1	949488676	25	341.21	1.73
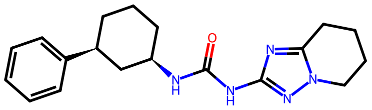	O=C(Nc1nc2n(n1)CCCC2)N[C@@H]1CCC[C@H](c2ccccc2)C1	333601785	25	339.21	3.46

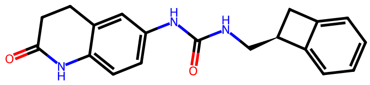	O=C1CCc2cc(NC(=O)NC[C@H]3Cc4ccccc43)ccc2N1	193485098	24	321.15	3.03
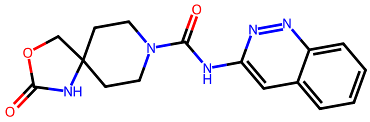	O=C1NC2(CCN(C(=O)Nc3cc4ccccc4nn3)CC2)CO1	888612111	24	327.13	1.74
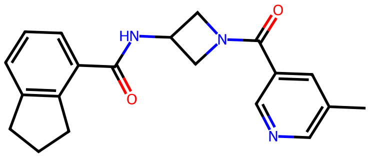	Cc1cncc(C(=O)N2CC(NC(=O)c3cccc4c3CCC4)C2)c1	994406531	25	335.16	2.13
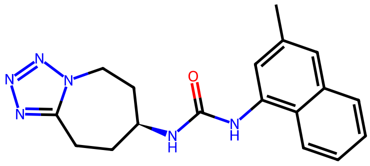	Cc1cc(NC(=O)N[C@H]2CCc3nnnn3CC2)c2ccccc2c1	574910275	25	336.17	2.66
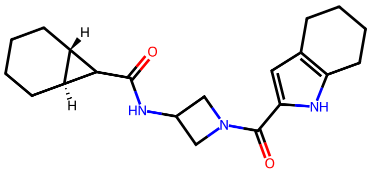	O=C(NC1CN(C(=O)c2cc3c([nH]2)CCCC3)C1)C1[C@H]2CCCC[C@H]12	990649635	25	341.21	2.27
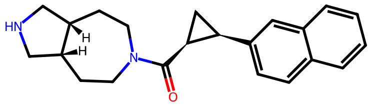	O=C([C@H]1C[C@H]1c1ccc2ccccc2c1)N1CC[C@@H]2CNC[C@@H]2CC1	261879360	25	334.2	3.4
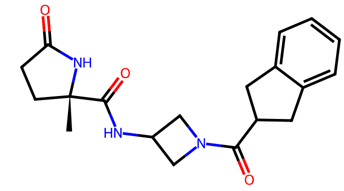	C[C@@]1(C(=O)NC2CN(C(=O)C3Cc4ccccc4C3)C2)CCC(=O)N1	999185984	25	341.17	0.4
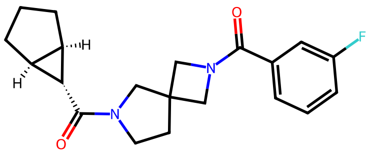	O=C(c1cccc(F)c1)N1CC2(CCN(C(=O)[C@@H]3[C@H]4CCC[C@H]43)C2)C1	1018726469	25	342.17	2.55

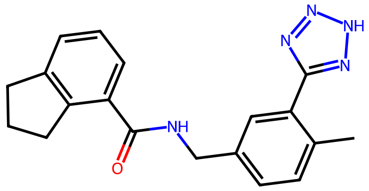	Cc1ccc(CNC(=O)c2cccc3c2CCC3)cc1-c1nn[nH]n1	1605854668	25	333.16	2.59
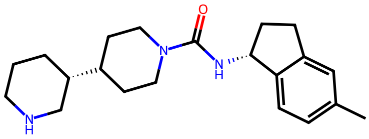	Cc1ccc2c(c1)CC[C@H]2NC(=O)N1CCC([C@H]2CCCNC2)CC1	659441202	25	341.25	3.4
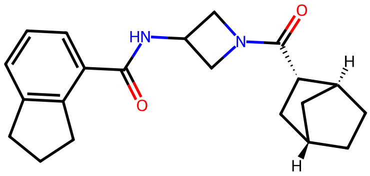	O=C(NC1CN(C(=O)[C@H]2C[C@H]3CC[C@H]2C3)C1)c1cccc2c1CCC2	994399598	25	338.2	2.55
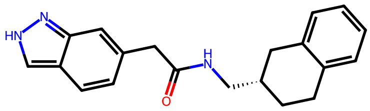	O=C(Cc1ccc2c[nH]nc2c1)NC[C@H]1CCc2ccccc2C1	684445154	24	319.17	3.03
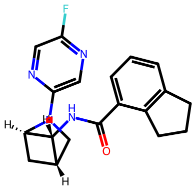	O=C(N[C@@H]1[C@H]2C[C@H]1N(c1cnc(F)cn1)C2)c1cccc2c1CCC2	1064267396	25	338.15	2.11
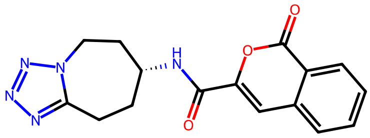	O=C(N[C@@H]1CCc2nnnn2CC1)c1cc2ccccc2c(=O)o1	422074175	24	325.12	0.91
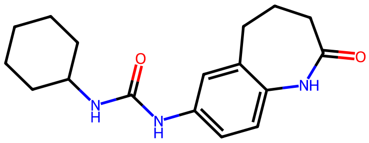	O=C1CCCc2cc(NC(=O)NC3CCCCC3)ccc2N1	71799369	22	301.18	3.42
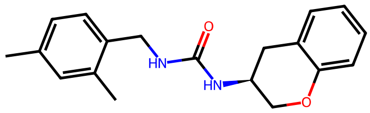	Cc1ccc(CNC(=O)N[C@@H]2COc3ccccc3C2)c(C)c1	616104744	23	310.17	3.11

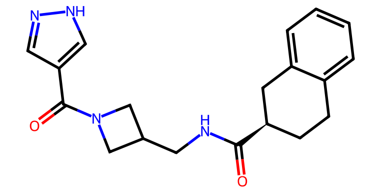	O=C(NCC1CN(C(=O)c2cn[nH]c2)C1)[C@@H]1CCc2ccccc2C1	936099917	25	338.17	1.4
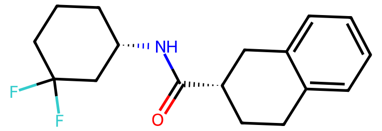	O=C(N[C@H]1CCCC(F)(F)C1)[C@H]1CCc2ccccc2C1	649847877	21	293.16	3.49
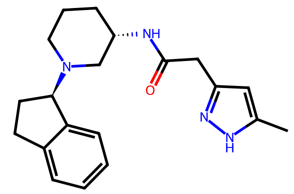	Cc1cc(CC(=O)N[C@H]2CCCN([C@@H]3CCc4ccccc43)C2)n[nH]1	1007063918	25	338.21	2.53
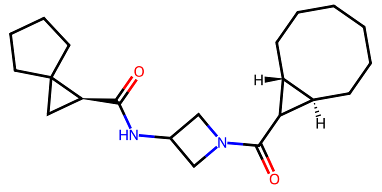	O=C(NC1CN(C(=O)C2[C@@H]3CCCCCC[C@@H]23)C1)[C@H]1CC12CCCC2	999633170	25	344.25	3.11
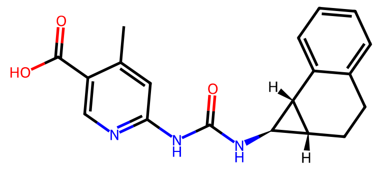	Cc1cc(NC(=O)N[C@@H]2[C@H]3CCc4ccccc4[C@H]32)ncc1C(=O)O	1600266459	25	337.14	2.94
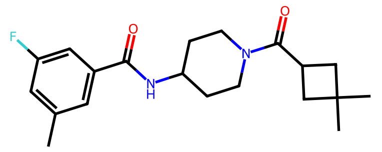	Cc1cc(F)cc(C(=O)NC2CCN(C(=O)C3CC(C)(C)C3)CC2)c1	946628395	25	346.21	3.29
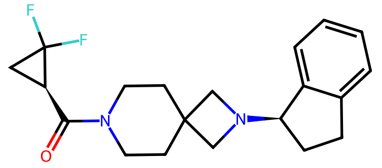	O=C([C@H]1CC1(F)F)N1CCC2(CC1)CN([C@@H]1CCc3ccccc31)C2	1035700080	25	346.19	3.25
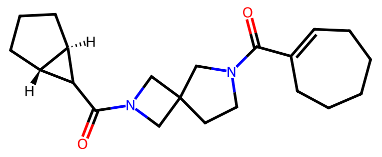	O=C(C1=CCCCCC1)N1CCC2(C1)CN(C(=O)C1[C@@H]3CCC[C@@H]13)C2	1018912677	25	342.23	2.98

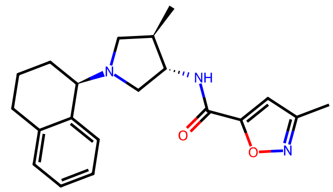	Cc1cc(C(=O)N[C@@H]2CN([C@@H]3CCCc4ccccc43)C[C@H]2C)on1	1079416934	25	339.19	3.11
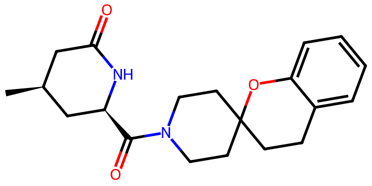	C[C@H]1CC(=O)N[C@@H](C(=O)N2CCC3(CCc4ccccc4O3)CC2)C1	869292011	25	342.19	2.29
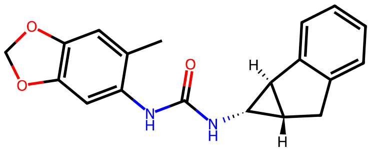	Cc1cc2c(cc1NC(=O)N[C@H]1[C@H]3Cc4ccccc4[C@@H]31)OCO2	183773297	24	322.13	3.18
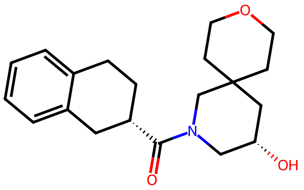	O=C([C@H]1CCc2ccccc2C1)N1C[C@@H](O)CC2(CCOCC2)C1	882954971	24	329.2	2.18
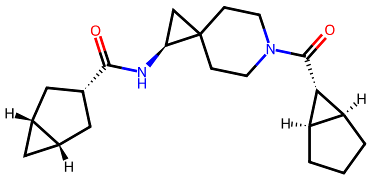	O=C(N[C@H]1CC12CCN(C(=O)[C@@H]1[C@H]3CCC[C@H]31)CC2)[C@@H]1C[C@@H]2C[C@@H]2C1	964766570	25	342.23	2.58
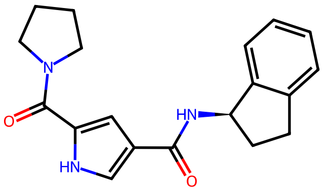	O=C(N[C@@H]1CCc2ccccc21)c1c[nH]c(C(=O)N2CCCC2)c1	51184331	24	323.16	2.67
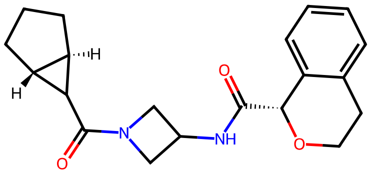	O=C(NC1CN(C(=O)C2[C@@H]3CCC[C@@H]23)C1)[C@H]1OCCc2ccccc21	997327092	25	340.18	1.67
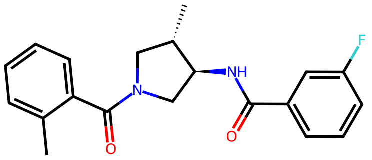	Cc1ccccc1C(=O)N1C[C@H](C)[C@@H](NC(=O)c2cccc(F)c2)C1	966095833	25	340.16	3.02

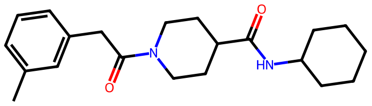	Cc1cccc(CC(=O)N2CCC(C(=O)NC3CCCCC3)CC2)c1	242687795	25	342.23	3.23
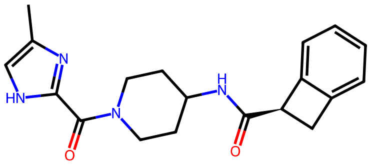	Cc1c[nH]c(C(=O)N2CCC(NC(=O)[C@@H]3Cc4ccccc43)CC2)n1	946870433	25	338.17	1.78
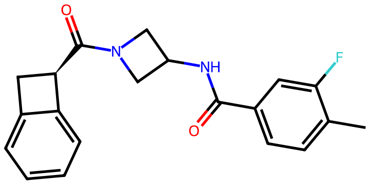	Cc1ccc(C(=O)NC2CN(C(=O)[C@@H]3Cc4ccccc43)C2)cc1F	995442881	25	338.14	2.41
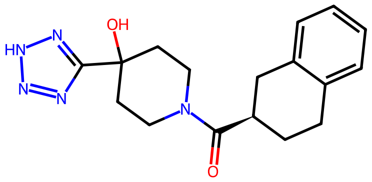	O=C([C@@H]1CCc2ccccc2C1)N1CCC(O)(c2nn[nH]n2)CC1	827763588	24	327.17	0.81
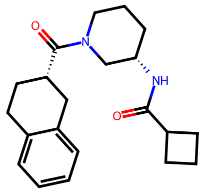	O=C(N[C@H]1CCCN(C(=O)[C@H]2CCc3ccccc3C2)C1)C1CCC1	952147403	25	340.22	2.7
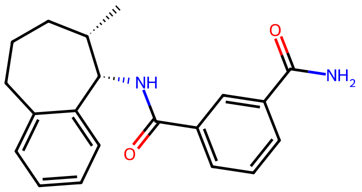	C[C@H]1CCCc2ccccc2[C@H]1NC(=O)c1cccc(C(N)=O)c1	185029410	24	322.17	3.23
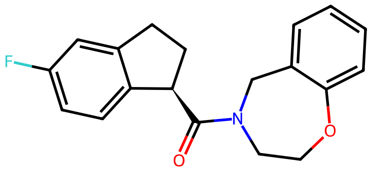	O=C([C@@H]1CCc2cc(F)ccc21)N1CCOc2ccccc2C1	666868276	23	311.13	3.28
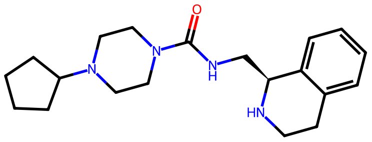	O=C(NC[C@@H]1NCCc2ccccc21)N1CCN(C2CCCC2)CC1	418648731	25	342.24	2.14

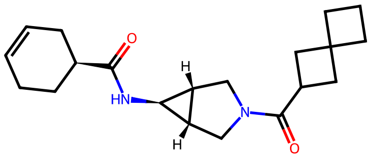	O=C(N[C@H]1[C@@H]2CN(C(=O)C3CC4(CCC4)C3)C[C@@H]21)[C@@H]1CC=CCC1	976170205	24	328.22	2.5
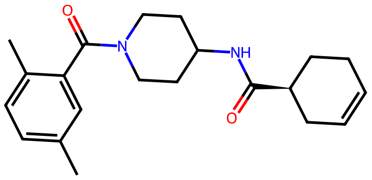	Cc1ccc(C)c(C(=O)N2CCC(NC(=O)[C@@H]3CC=CCC3)CC2)c1	948336350	25	340.22	3.38
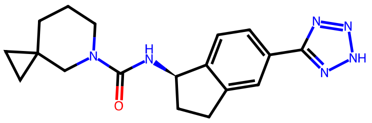	O=C(N[C@@H]1CCc2cc(-c3nn[nH]n3)ccc21)N1CCCC2(CC2)C1	1593000532	25	338.19	2.44
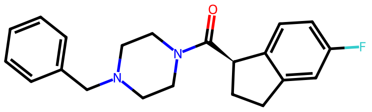	O=C([C@@H]1CCc2cc(F)ccc21)N1CCN(Cc2ccccc2)CC1	949321875	25	338.18	3.2
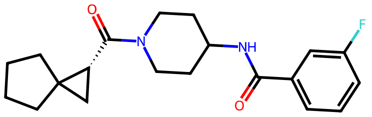	O=C(NC1CCN(C(=O)[C@@H]2CC23CCCC3)CC1)c1cccc(F)c1	948360610	25	344.19	3.13
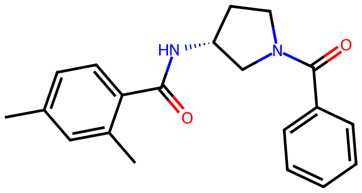	Cc1ccc(C(=O)N[C@@H]2CCN(C(=O)c3ccccc3)C2)c(C)c1	942397071	24	322.17	2.95
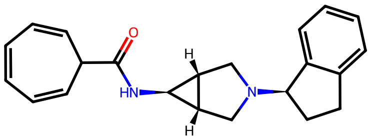	O=C(N[C@H]1[C@@H]2CN([C@@H]3CCc4ccccc43)C[C@@H]21)C1C=CC=CC=C1	960116812	25	332.19	3.02
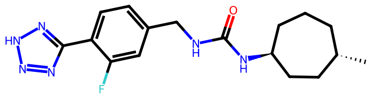	C[C@H]1CCC[C@H](NC(=O)NCc2ccc(-c3nn[nH]n3)c(F)c2)CC1	1609162744	25	346.19	2.77

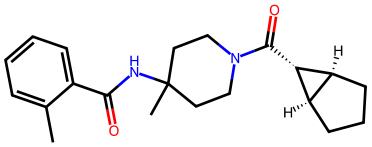	Cc1ccccc1C(=O)NC1(C)CCN(C(=O)[C@@H]2[C@H]3CCC[C@H]32)CC1	956273160	25	340.22	3.15
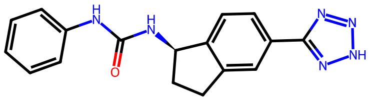	O=C(Nc1ccccc1)N[C@@H]1CCc2cc(-c3nn[nH]n3)ccc21	1593250468	24	320.14	2.68
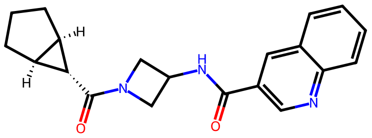	O=C(NC1CN(C(=O)[C@@H]2[C@H]3CCC[C@H]32)C1)c1cnc2ccccc2c1	1000546976	25	335.16	2.22
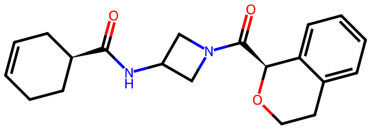	O=C(NC1CN(C(=O)[C@@H]2OCCc3ccccc32)C1)[C@@H]1CC=CCC1	990218187	25	340.18	1.98
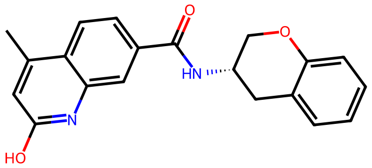	Cc1cc(O)nc2cc(C(=O)N[C@@H]3COc4ccccc4C3)ccc12	367969654	25	334.13	2.98
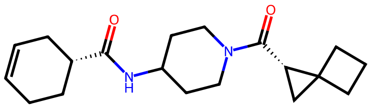	O=C(NC1CCN(C(=O)[C@H]2CC23CCC3)CC1)[C@H]1CC=CCC1	946763927	23	316.22	2.64
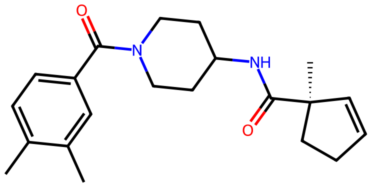	Cc1ccc(C(=O)N2CCC(NC(=O)[C@@]3(C)C=CCC3)CC2)cc1C	948352133	25	340.22	3.38
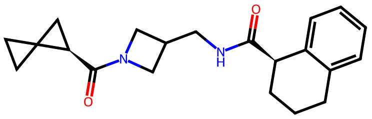	O=C(NCC1CN(C(=O)[C@@H]2CC23CC3)C1)[C@@H]1CCCc2ccccc21	1003035022	25	338.2	2.48

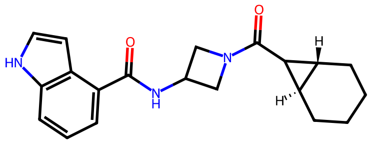	O=C(NC1CN(C(=O)C2[C@@H]3CCCC[C@@H]23)C1)c1cccc2[nH]ccc12	1000403524	25	337.18	2.54
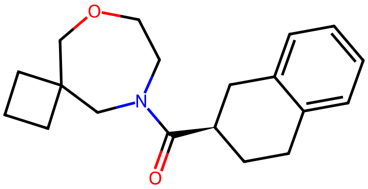	O=C([C@@H]1CCc2ccccc2C1)N1CCOCC2(CCC2)C1	569726898	22	299.19	2.82
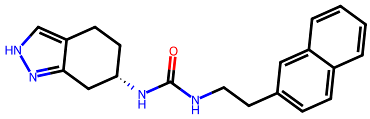	O=C(NCCc1ccc2ccccc2c1)N[C@H]1CCc2c[nH]nc2C1	468039213	25	334.18	2.96
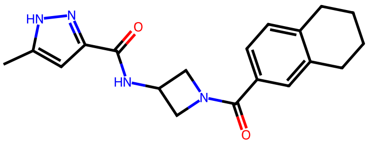	Cc1cc(C(=O)NC2CN(C(=O)c3ccc4c(c3)CCCC4)C2)n[nH]1	990379392	25	338.17	1.85
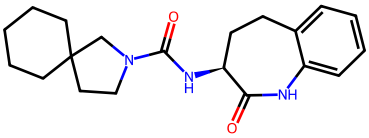	O=C1Nc2ccccc2CC[C@@H]1NC(=O)N1CCC2(CCCCC2)C1	654628051	25	341.21	3.31
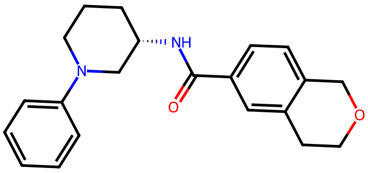	O=C(N[C@H]1CCCN(c2ccccc2)C1)c1ccc2c(c1)CCOC2	668797135	25	336.18	3.16
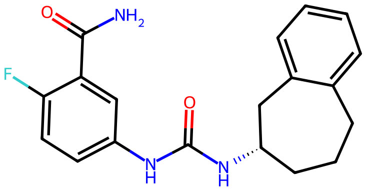	NC(=O)c1cc(NC(=O)N[C@H]2CCCc3ccccc3C2)ccc1F	929633905	25	341.15	2.99
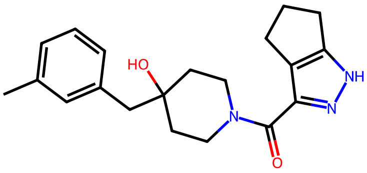	Cc1cccc(CC2(O)CCN(C(=O)c3n[nH]c4c3CCC4)CC2)c1	280297718	25	339.19	2.42

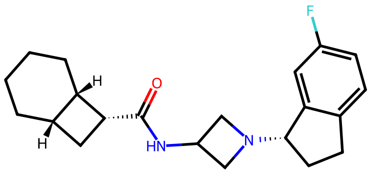	O=C(NC1CN([C@H]2CCc3ccc(F)cc32)C1)[C@@H]1C[C@@H]2CCCC[C@@H]21	1030723340	25	342.21	3.44
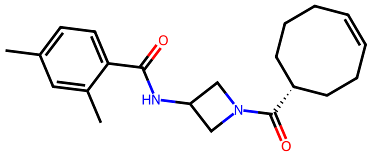	Cc1ccc(C(=O)NC2CN(C(=O)[C@H]3CCC=CCCC3)C2)c(C)c1	999532356	25	340.22	3.38
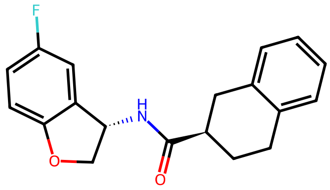	O=C(N[C@@H]1COc2ccc(F)cc21)[C@@H]1CCc2ccccc2C1	573807409	23	311.13	3.18
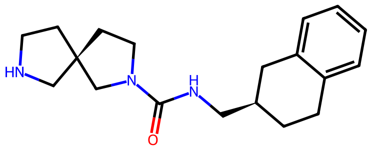	O=C(NC[C@@H]1CCc2ccccc2C1)N1CC[C@@]2(CCNC2)C1	669551282	23	313.22	2.19
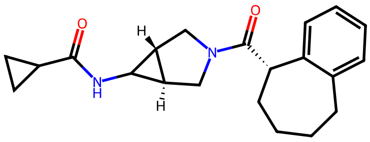	O=C(NC1[C@@H]2CN(C(=O)[C@H]3CCCCc4ccccc43)C[C@@H]12)C1CC1	1012651033	25	338.2	2.48
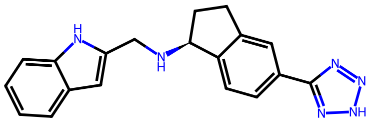	c1ccc2[nH]c(CN[C@H]3CCc4cc(-c5nn[nH]n5)ccc43)cc2c1	1593577874	25	330.16	3.13
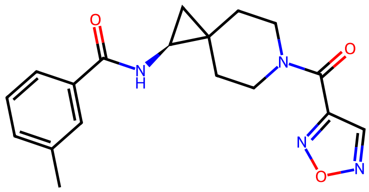	Cc1cccc(C(=O)N[C@H]2CC23CCN(C(=O)c2cnon2)CC3)c1	962878963	25	340.15	1.8
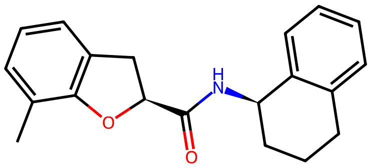	Cc1cccc2c1O[C@H](C(=O)N[C@@H]1CCCc3ccccc31)C2	1616543288	23	307.16	3.49

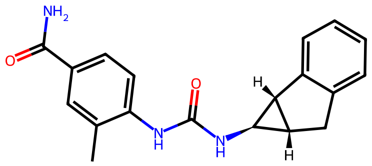	Cc1cc(C(N)=O)ccc1NC(=O)N[C@@H]1[C@H]2Cc3ccccc3[C@H]21	914210265	24	321.15	2.55
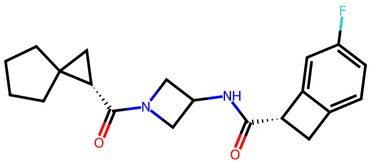	O=C(NC1CN(C(=O)[C@H]2CC23CCCC3)C1)[C@H]1Cc2ccc(F)cc21	998486415	25	342.17	2.37
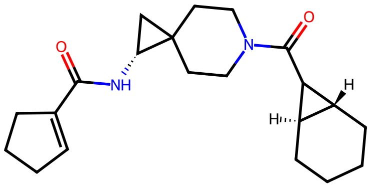	O=C(N[C@@H]1CC12CCN(C(=O)C1[C@@H]3CCCC[C@@H]13)CC2)C1=CCCC1	963562469	25	342.23	3.03
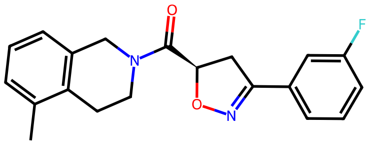	Cc1cccc2c1CCN(C(=O)[C@H]1CC(c3cccc(F)c3)=NO1)C2	729414765	25	338.14	3.21
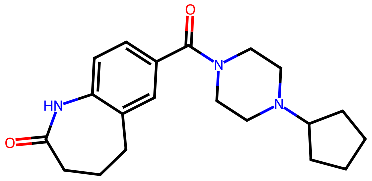	O=C1CCCc2cc(C(=O)N3CCN(C4CCCC4)CC3)ccc2N1	642260586	25	341.21	2.66
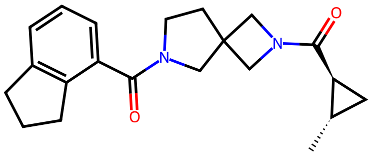	C[C@H]1C[C@@H]1C(=O)N1CC2(CCN(C(=O)c3cccc4c3CCC4)C2)C1	1018732218	25	338.2	2.51
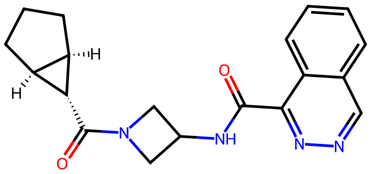	O=C(NC1CN(C(=O)[C@@H]2[C@H]3CCC[C@H]32)C1)c1nncc2ccccc12	996893140	25	336.16	1.62
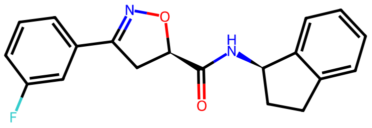	O=C(N[C@@H]1CCc2ccccc21)[C@H]1CC(c2cccc(F)c2)=NO1	47904462	24	324.13	3.12

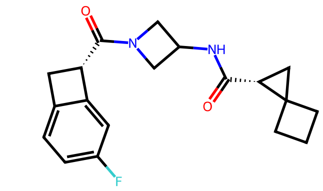	O=C(NC1CN(C(=O)[C@H]2Cc3ccc(F)cc32)C1)[C@@H]1CC12CCC2	991553902	24	328.16	1.98
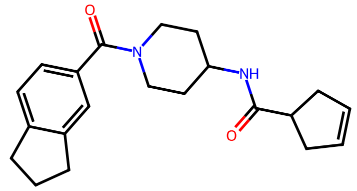	O=C(NC1CCN(C(=O)c2ccc3c(c2)CCC3)CC1)C1CC=CC1	946720436	25	338.2	2.86
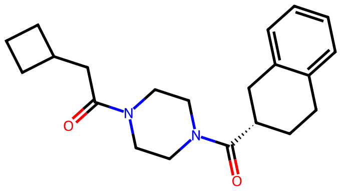	O=C(CC1CCC1)N1CCN(C(=O)[C@H]2CCc3ccccc3C2)CC1	1010896474	25	340.22	2.65
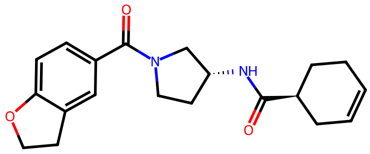	O=C(N[C@@H]1CCN(C(=O)c2ccc3c(c2)CCO3)C1)[C@@H]1CC=CCC1	936237811	25	340.18	2.31
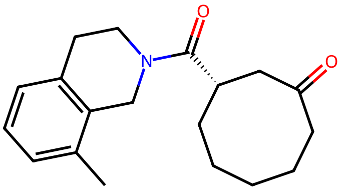	Cc1cccc2c1CN(C(=O)[C@H]1CCCCCC(=O)C1)CC2	487632242	22	299.19	3.42
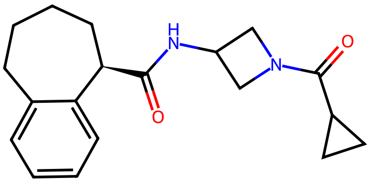	O=C(NC1CN(C(=O)C2CC2)C1)[C@@H]1CCCCc2ccccc21	994325478	23	312.18	2.23
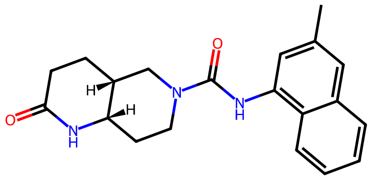	Cc1cc(NC(=O)N2CC[C@@H]3NC(=O)CC[C@@H]3C2)c2ccccc2c1	667170966	25	337.18	3.28
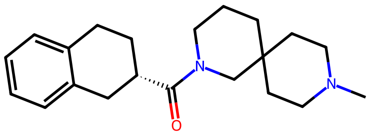	CN1CCC2(CCCN(C(=O)[C@H]3CCc4ccccc4C3)C2)CC1	846064650	24	326.24	3.13

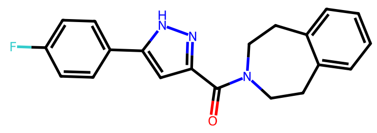	O=C(c1cc(-c2ccc(F)cc2)[nH]n1)N1CCc2ccccc2CC1	116217709	25	335.14	3.46
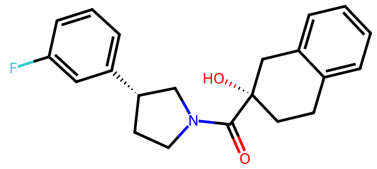	O=C(N1CC[C@H](c2cccc(F)c2)C1)[C@]1(O)CCc2ccccc2C1	786105136	25	339.16	3.06
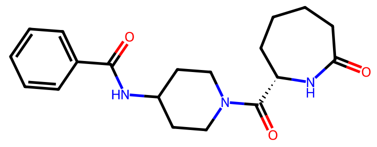	O=C1CCCC[C@@H](C(=O)N2CCC(NC(=O)c3ccccc3)CC2)N1	193732943	25	343.19	1.47
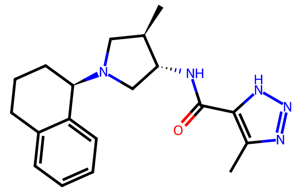	Cc1nn[nH]c1C(=O)N[C@@H]1CN([C@@H]2CCCc3ccccc32)C[C@H]1C	1080246085	25	339.21	2.24
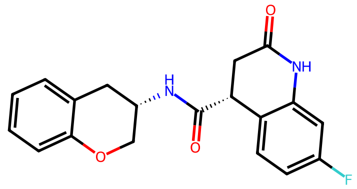	O=C1C[C@@H](C(=O)N[C@@H]2COc3ccccc3C2)c2ccc(F)cc2N1	53045171	25	340.12	2.37
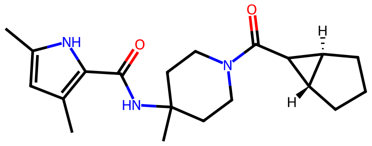	Cc1cc(C)c(C(=O)NC2(C)CCN(C(=O)C3[C@H]4CCC[C@H]34)CC2)[nH]1	956421105	25	343.23	2.79
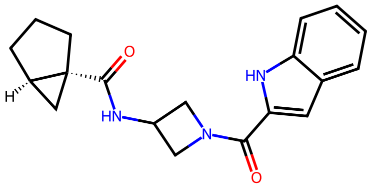	O=C(c1cc2ccccc2[nH]1)N1CC(NC(=O)[C@@]23CCC[C@@H]2C3)C1	997551729	24	323.16	2.3
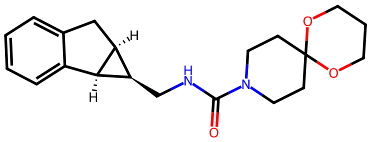	O=C(NC[C@H]1[C@H]2c3ccccc3C[C@H]21)N1CCC2(CC1)OCCCO2	296543122	25	342.19	2.51

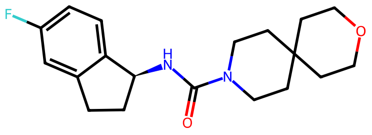	O=C(N[C@H]1CCc2cc(F)ccc21)N1CCC2(CCOCC2)CC1	285265987	24	332.19	3.42
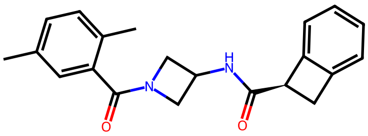	Cc1ccc(C)c(C(=O)N2CC(NC(=O)[C@@H]3Cc4ccccc43)C2)c1	998691937	25	334.17	2.58
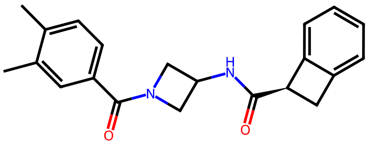	Cc1ccc(C(=O)N2CC(NC(=O)[C@@H]3Cc4ccccc43)C2)cc1C	998692380	25	334.17	2.58
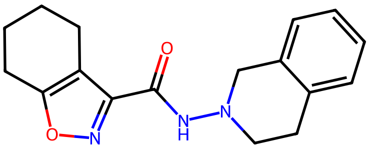	O=C(NN1CCc2ccccc2C1)c1noc2c1CCCC2	855711748	22	297.15	2.26
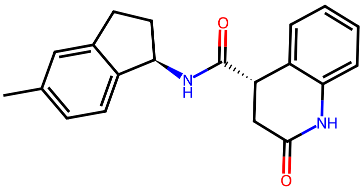	Cc1ccc2c(c1)CC[C@H]2NC(=O)[C@H]1CC(=O)Nc2ccccc21	56316916	24	320.15	3.22
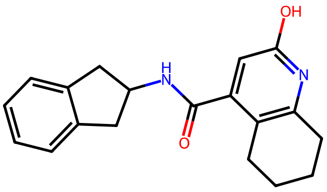	O=C(NC1Cc2ccccc2C1)c1cc(O)nc2c1CCCC2	935397877	23	308.15	2.56
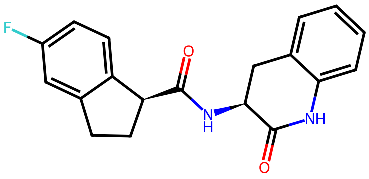	O=C1Nc2ccccc2C[C@@H]1NC(=O)[C@H]1CCc2cc(F)ccc21	529945400	24	324.13	2.54
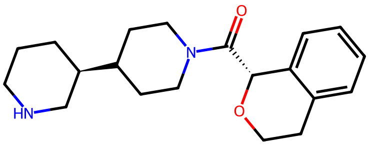	O=C([C@H]1OCCc2ccccc21)N1CCC([C@@H]2CCCNC2)CC1	658957120	24	328.22	2.54

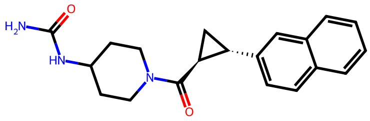	NC(=O)NC1CCN(C(=O)[C@H]2C[C@@H]2c2ccc3ccccc3c2)CC1	176057386	25	337.18	2.6
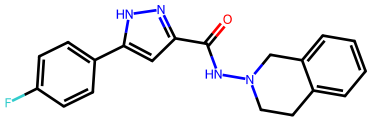	O=C(NN1CCc2ccccc2C1)c1cc(-c2ccc(F)cc2)[nH]n1	1644634994	25	336.14	2.92
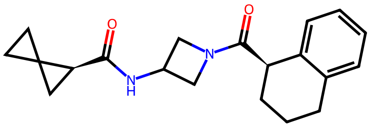	O=C(NC1CN(C(=O)[C@@H]2CCCc3ccccc32)C1)[C@H]1CC12CC2	1000518192	24	324.18	2.23
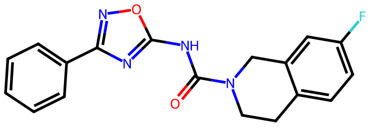	O=C(Nc1nc(-c2ccccc2)no1)N1CCc2ccc(F)cc2C1	1669975238	25	338.12	3.47
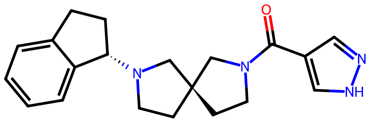	O=C(c1cn[nH]c1)N1CC[C@]2(CCN([C@H]3CCc4ccccc43)C2)C1	1041104343	25	336.2	2.64
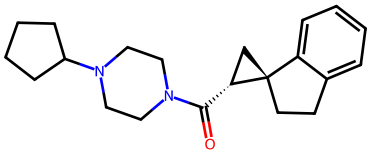	O=C([C@@H]1C[C@@]12CCc1ccccc12)N1CCN(C2CCCC2)CC1	175260533	24	324.22	2.98
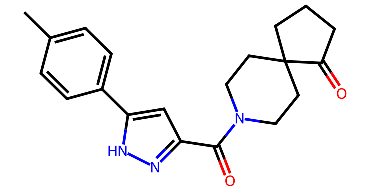	Cc1ccc(-c2cc(C(=O)N3CCC4(CCCC4=O)CC3)n[nH]2)cc1	619333389	25	337.18	3.36
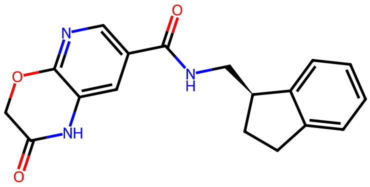	O=C1COc2ncc(C(=O)NC[C@@H]3CCc4ccccc43)cc2N1	378432202	24	323.13	1.87

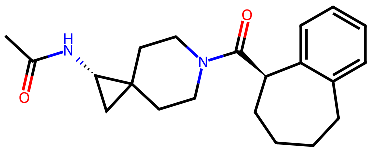	CC(=O)N[C@H]1CC12CCN(C(=O)[C@@H]1CCCCc3ccccc31)CC2	962352474	25	340.22	3.01
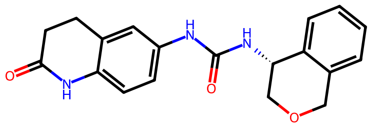	O=C1CCc2cc(NC(=O)N[C@H]3COCc4ccccc43)ccc2N1	562787927	25	337.14	2.96
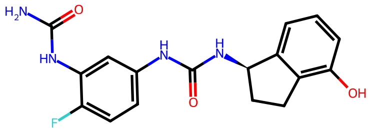	NC(=O)Nc1cc(NC(=O)N[C@@H]2CCc3c(O)cccc32)ccc1F	1434726822	25	344.13	2.83
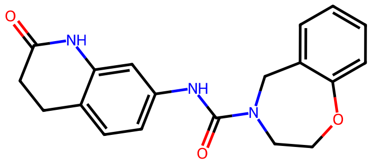	O=C1CCc2ccc(NC(=O)N3CCOc4ccccc4C3)cc2N1	608326798	25	337.14	3.0
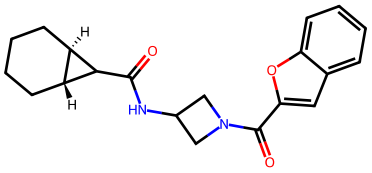	O=C(NC1CN(C(=O)c2cc3ccccc3o2)C1)C1[C@@H]2CCCC[C@@H]12	990674361	25	338.16	2.81
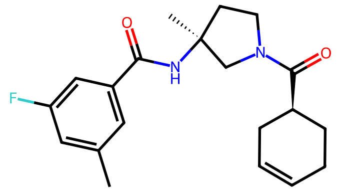	Cc1cc(F)cc(C(=O)N[C@@]2(C)CCN(C(=O)[C@@H]3CC=CCC3)C2)c1	957700734	25	344.19	3.21
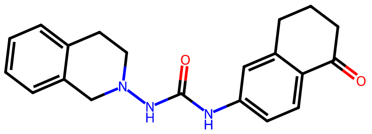	O=C(Nc1ccc2c(c1)CCCC2=O)NN1CCc2ccccc2C1	823631388	25	335.16	3.3
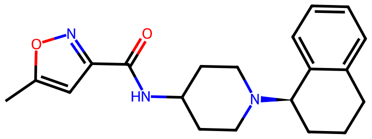	Cc1cc(C(=O)NC2CCN([C@@H]3CCCc4ccccc43)CC2)no1	364019828	25	339.19	3.25

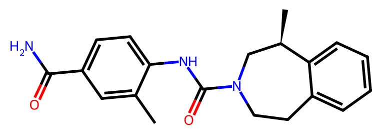	Cc1cc(C(N)=O)ccc1NC(=O)N1CCc2ccccc2[C@H](C)C1	844504868	25	337.18	3.29
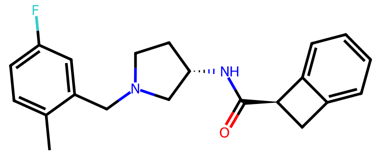	Cc1ccc(F)cc1CN1CC[C@H](NC(=O)[C@@H]2Cc3ccccc32)C1	1015636084	25	338.18	3.16
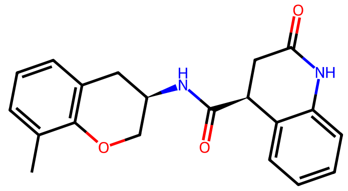	Cc1cccc2c1OC[C@H](NC(=O)[C@H]1CC(=O)Nc3ccccc31)C2	291627697	25	336.15	2.54
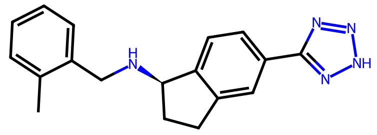	Cc1ccccc1CN[C@@H]1CCc2cc(-c3nn[nH]n3)ccc21	1605940037	23	305.16	2.95
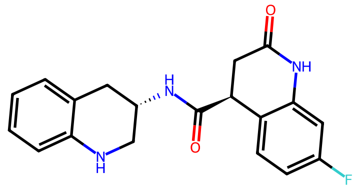	O=C1C[C@H](C(=O)N[C@@H]2CNc3ccccc3C2)c2ccc(F)cc2N1	653205579	25	339.14	2.4
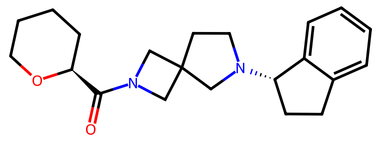	O=C([C@@H]1CCCCO1)N1CC2(CCN([C@H]3CCc4ccccc43)C2)C1	1072628538	25	340.22	2.78
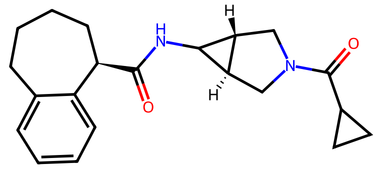	O=C(NC1[C@@H]2CN(C(=O)C3CC3)C[C@@H]12)[C@@H]1CCCCc2ccccc21	1013288179	25	338.2	2.48
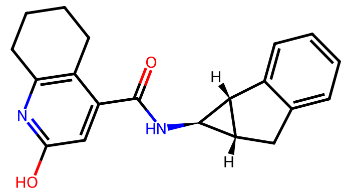	O=C(N[C@@H]1[C@H]2Cc3ccccc3[C@H]21)c1cc(O)nc2c1CCCC2	829905746	24	320.15	2.73

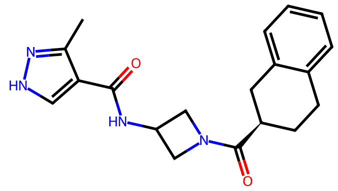	Cc1n[nH]cc1C(=O)NC1CN(C(=O)[C@@H]2CCc3ccccc3C2)C1	997287161	25	338.17	1.46
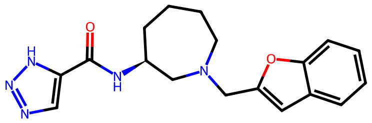	O=C(N[C@H]1CCCCN(Cc2cc3ccccc3o2)C1)c1cnn[nH]1	1034284663	25	339.17	2.34
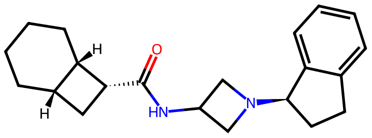	O=C(NC1CN([C@@H]2CCc3ccccc32)C1)[C@@H]1C[C@@H]2CCCC[C@@H]21	1030724046	24	324.22	3.3
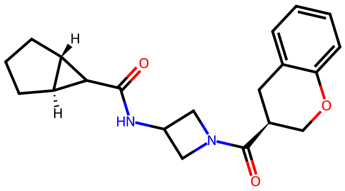	O=C(NC1CN(C(=O)[C@@H]2COc3ccccc3C2)C1)C1[C@H]2CCC[C@H]12	990708409	25	340.18	1.61
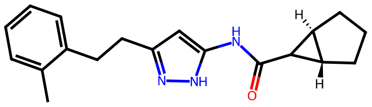	Cc1ccccc1CCc1cc(NC(=O)C2[C@H]3CCC[C@H]23)[nH]n1	182888168	23	309.18	3.49
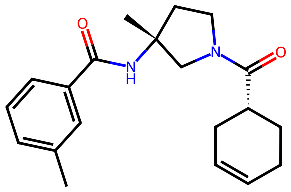	Cc1cccc(C(=O)N[C@]2(C)CCN(C(=O)[C@H]3CC=CCC3)C2)c1	956663618	24	326.2	3.07
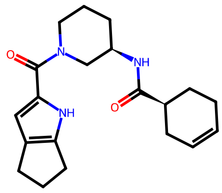	O=C(N[C@@H]1CCCN(C(=O)c2cc3c([nH]2)CCC3)C1)[C@@H]1CC=CCC1	951367690	25	341.21	2.58
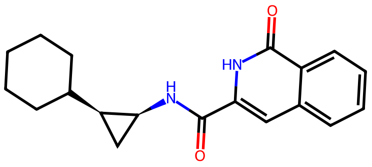	O=C(N[C@H]1C[C@H]1C1CCCCC1)c1cc2ccccc2c(=O)[nH]1	152715069	23	310.17	3.23

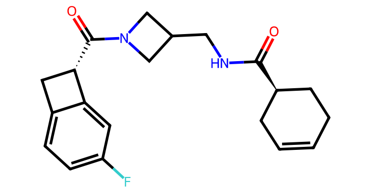	O=C(NCC1CN(C(=O)[C@H]2Cc3ccc(F)cc32)C1)[C@@H]1CC=CCC1	1000868603	25	342.17	2.4
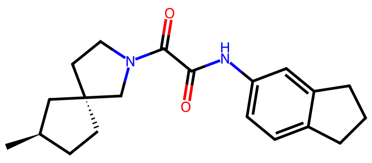	C[C@@H]1CC[C@@]2(CCN(C(=O)C(=O)Nc3ccc4c(c3)CCC4)C2)C1	359461237	24	326.2	3.15
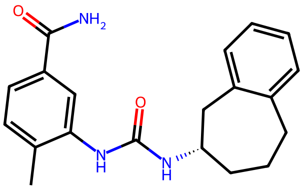	Cc1ccc(C(N)=O)cc1NC(=O)N[C@H]1CCCc2ccccc2C1	930016464	25	337.18	3.16
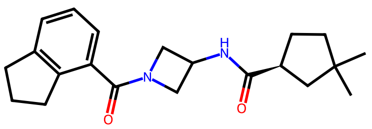	CC1(C)CC[C@H](C(=O)NC2CN(C(=O)c3cccc4c3CCC4)C2)C1	999248585	25	340.22	2.94
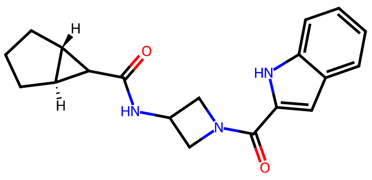	O=C(NC1CN(C(=O)c2cc3ccccc3[nH]2)C1)C1[C@H]2CCC[C@H]12	990707906	24	323.16	2.15
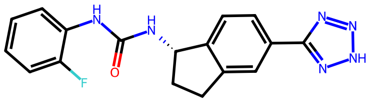	O=C(Nc1ccccc1F)N[C@H]1CCc2cc(-c3nn[nH]n3)ccc21	1606940106	25	338.13	2.81
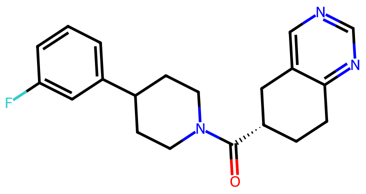	O=C([C@H]1CCc2ncncc2C1)N1CCC(c2cccc(F)c2)CC1	843561291	25	339.17	3.13
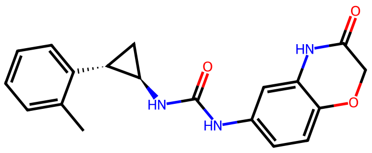	Cc1ccccc1[C@@H]1C[C@H]1NC(=O)Nc1ccc2c(c1)NC(=O)CO2	133701801	25	337.14	3.0

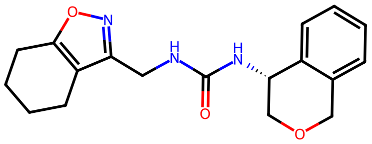	O=C(NCc1noc2c1CCCC2)N[C@H]1COCc2ccccc21	569558258	24	327.16	2.62
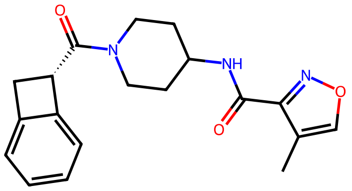	Cc1conc1C(=O)NC1CCN(C(=O)[C@H]2Cc3ccccc32)CC1	948060014	25	339.16	2.04
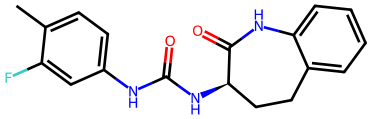	Cc1ccc(NC(=O)N[C@@H]2CCc3ccccc3NC2=O)cc1F	1188002300	24	327.14	3.21
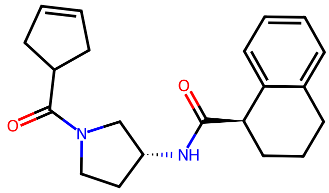	O=C(N[C@@H]1CCN(C(=O)C2CC=CC2)C1)[C@@H]1CCCc2ccccc21	941386877	25	338.2	2.79
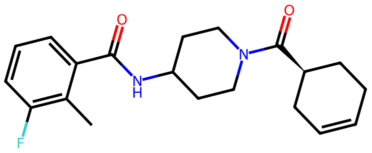	Cc1c(F)cccc1C(=O)NC1CCN(C(=O)[C@@H]2CC=CCC2)CC1	944127518	25	344.19	3.21
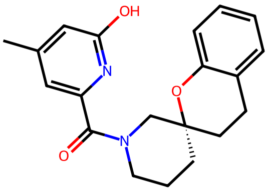	Cc1cc(O)nc(C(=O)N2CCC[C@]3(CCc4ccccc4O3)C2)c1	529905948	25	338.16	3.1
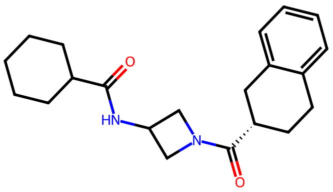	O=C(NC1CN(C(=O)[C@H]2CCc3ccccc3C2)C1)C1CCCCC1	990100029	25	340.22	2.7
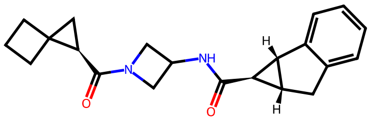	O=C(NC1CN(C(=O)[C@@H]2CC23CCC3)C1)[C@@H]1[C@H]2Cc3ccccc3[C@H]21	991647431	25	336.18	2.09

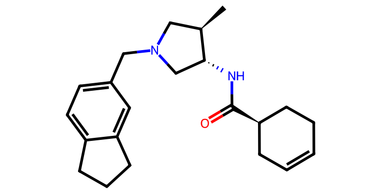	C[C@@H]1CN(Cc2ccc3c(c2)CCC3)C[C@H]1NC(=O)[C@@H]1CC=CCC1	1079399991	25	338.24	3.47
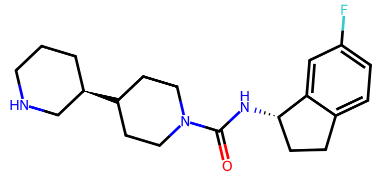	O=C(N[C@H]1CCc2ccc(F)cc21)N1CCC([C@@H]2CCCNC2)CC1	659452471	25	345.22	3.23
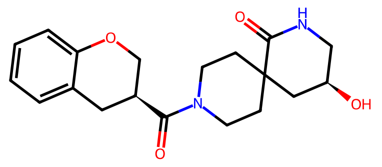	O=C([C@@H]1COc2ccccc2C1)N1CCC2(CC1)C[C@H](O)CNC2=O	885026441	25	344.17	0.73
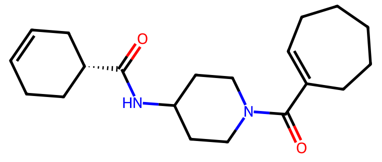	O=C(NC1CCN(C(=O)C2=CCCCCC2)CC1)[C@H]1CC=CCC1	946549008	24	330.23	3.34
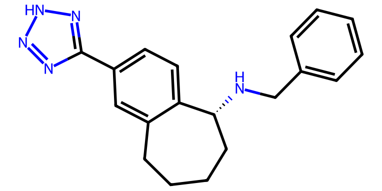	c1ccc(CN[C@@H]2CCCCc3cc(-c4nn[nH]n4)ccc32)cc1	1609617304	24	319.18	3.42
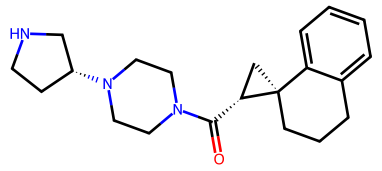	O=C([C@@H]1C[C@]12CCCc1ccccc12)N1CCN([C@@H]2CCNC2)CC1	343604816	25	339.23	1.79
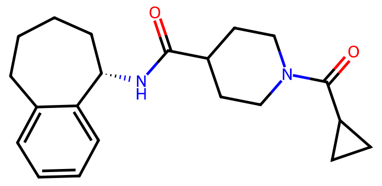	O=C(N[C@H]1CCCCc2ccccc21)C1CCN(C(=O)C2CC2)CC1	58393025	25	340.22	3.22
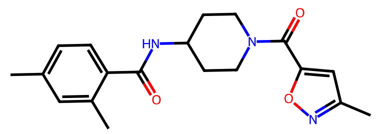	Cc1ccc(C(=O)NC2CCN(C(=O)c3cc(C)no3)CC2)c(C)c1	944153738	25	341.17	2.63

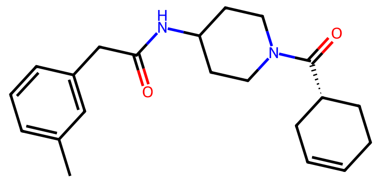	Cc1cccc(CC(=O)NC2CCN(C(=O)[C@H]3CC=CCC3)CC2)c1	944105670	25	340.22	3.0
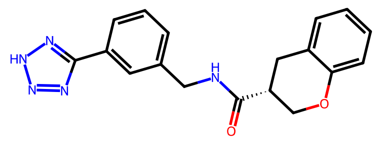	O=C(NCc1cccc(-c2nn[nH]n2)c1)[C@H]1COc2ccccc2C1	1606836702	25	335.14	1.73
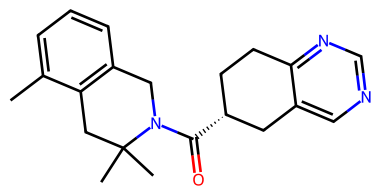	Cc1cccc2c1CC(C)(C)N(C(=O)[C@@H]1CCc3ncncc3C1)C2	648406347	25	335.2	3.25
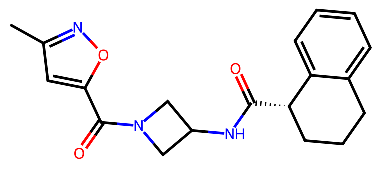	Cc1cc(C(=O)N2CC(NC(=O)[C@H]3CCCc4ccccc43)C2)on1	995071666	25	339.16	2.04
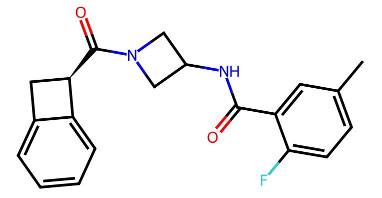	Cc1ccc(F)c(C(=O)NC2CN(C(=O)[C@@H]3Cc4ccccc43)C2)c1	1000538488	25	338.14	2.41
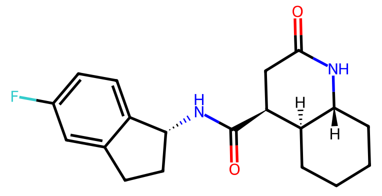	O=C1C[C@H](C(=O)N[C@@H]2CCc3cc(F)ccc32)[C@@H]2CCCC[C@H]2N1	667094753	24	330.17	2.62
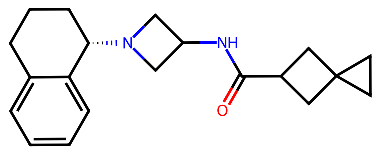	O=C(NC1CN([C@H]2CCCc3ccccc32)C1)C1CC2(CC2)C1	1030932353	23	310.2	3.05
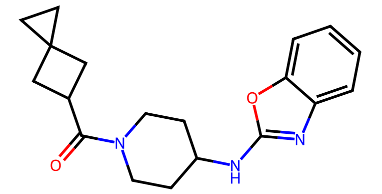	O=C(C1CC2(CC2)C1)N1CCC(Nc2nc3ccccc3o2)CC1	1057424005	24	325.18	3.42

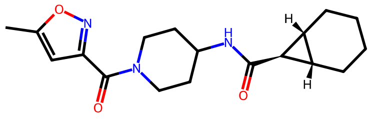	Cc1cc(C(=O)N2CCC(NC(=O)[C@@H]3[C@H]4CCCC[C@H]43)CC2)no1	944001915	24	331.19	2.14
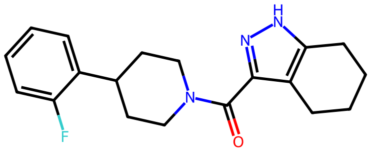	O=C(c1n[nH]c2c1CCCC2)N1CCC(c2ccccc2F)CC1	605560862	24	327.17	3.45
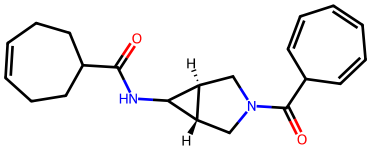	O=C(NC1[C@H]2CN(C(=O)C3C=CC=CC=C3)C[C@H]12)C1CCC=CCC1	1013133103	25	338.2	2.6
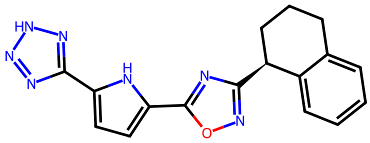	c1ccc2c(c1)CCC[C@@H]2c1noc(-c2cc(-c3nn[nH]n3)[nH]2)n1	1593580818	25	333.13	2.71
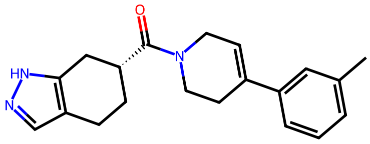	Cc1cccc(C2=CCN(C(=O)[C@@H]3CCc4cn[nH]c4C3)CC2)c1	92696475	24	321.18	3.14
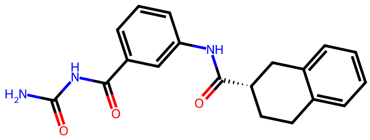	NC(=O)NC(=O)c1cccc(NC(=O)[C@H]2CCc3ccccc3C2)c1	1412344531	25	337.14	2.24
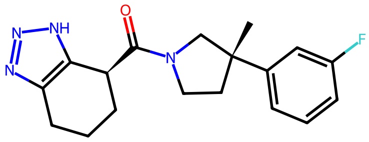	C[C@@]1(c2cccc(F)c2)CCN(C(=O)[C@H]2CCCc3nn[nH]c32)C1	616516309	24	328.17	2.55
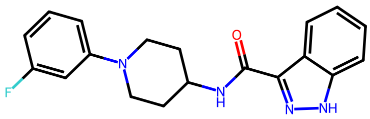	O=C(NC1CCN(c2cccc(F)c2)CC1)c1n[nH]c2ccccc12	363620432	25	338.15	3.1

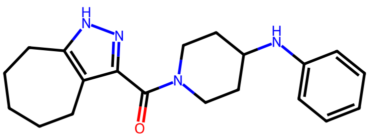	O=C(c1n[nH]c2c1CCCCC2)N1CCC(Nc2ccccc2)CC1	1321813275	25	338.21	3.4
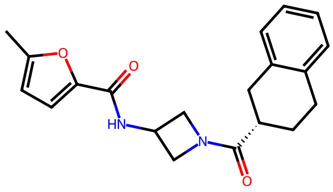	Cc1ccc(C(=O)NC2CN(C(=O)[C@H]3CCc4ccccc4C3)C2)o1	990163774	25	338.16	2.33
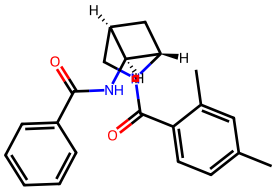	Cc1ccc(C(=O)N2C[C@H]3C[C@H]2[C@H]3NC(=O)c2ccccc2)c(C)c1	946597109	25	334.17	2.95
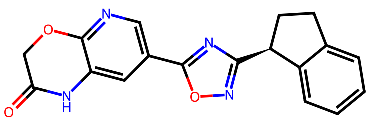	O=C1COc2ncc(-c3nc([C@H]4CCc5ccccc54)no3)cc2N1	356608042	25	334.11	2.54
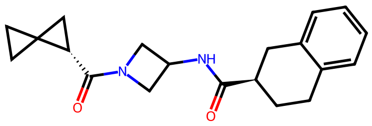	O=C(NC1CN(C(=O)[C@H]2CC23CC3)C1)[C@@H]1CCc2ccccc2C1	1000545203	24	324.18	1.92
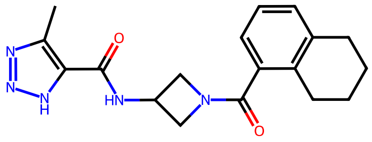	Cc1nn[nH]c1C(=O)NC1CN(C(=O)c2cccc3c2CCCC3)C1	997342124	25	339.17	1.25
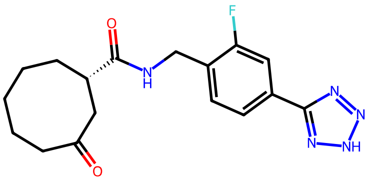	O=C1CCCCC[C@H](C(=O)NCc2ccc(-c3nn[nH]n3)cc2F)C1	1593392983	25	345.16	2.16
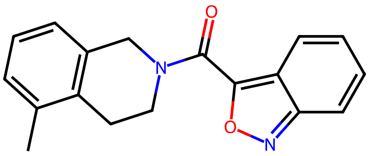	Cc1cccc2c1CCN(C(=O)c1onc3ccccc13)C2	1585519258	22	292.12	3.33

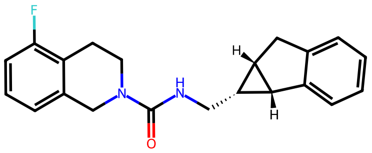	O=C(NC[C@H]1[C@H]2c3ccccc3C[C@H]21)N1CCc2c(F)cccc2C1	125953354	25	336.16	3.48
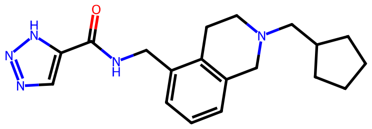	O=C(NCc1cccc2c1CCN(CC1CCCC1)C2)c1cnn[nH]1	1051291943	25	339.21	2.28
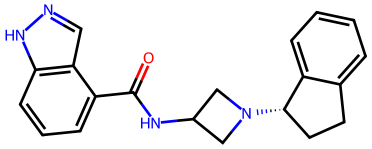	O=C(NC1CN([C@H]2CCc3ccccc32)C1)c1cccc2[nH]ncc12	1030773334	25	332.16	2.66
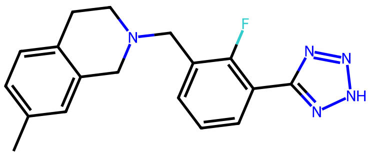	Cc1ccc2c(c1)CN(Cc1cccc(-c3nn[nH]n3)c1F)CC2	1581214928	24	323.15	2.87
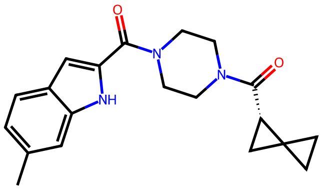	Cc1ccc2cc(C(=O)N3CCN(C(=O)[C@H]4CC45CC5)CC3)[nH]c2c1	1011712396	25	337.18	2.56
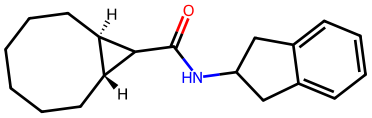	O=C(NC1Cc2ccccc2C1)C1[C@@H]2CCCCCC[C@@H]12	1127808947	21	283.19	3.49
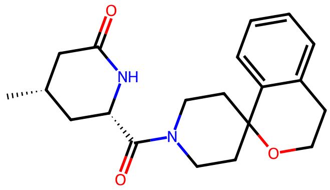	C[C@@H]1CC(=O)N[C@H](C(=O)N2CCC3(CC2)OCCc2ccccc23)C1	564708037	25	342.19	1.99
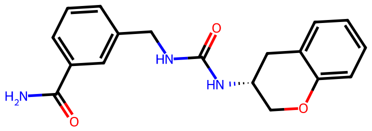	NC(=O)c1cccc(CNC(=O)N[C@H]2COc3ccccc3C2)c1	193683953	24	325.14	1.59

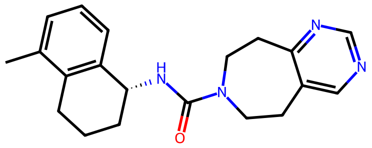	Cc1cccc2c1CCC[C@H]2NC(=O)N1CCc2cncnc2CC1	1549363333	25	336.2	2.97
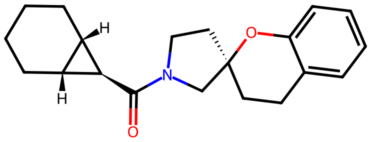	O=C([C@@H]1[C@H]2CCCC[C@H]21)N1CC[C@]2(CCc3ccccc3O2)C1	377960989	23	311.19	3.42
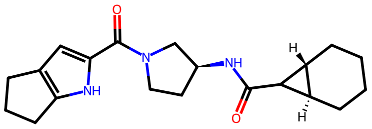	O=C(N[C@H]1CCN(C(=O)c2cc3c([nH]2)CCC3)C1)C1[C@@H]2CCCC[C@@H]12	937788575	25	341.21	2.27
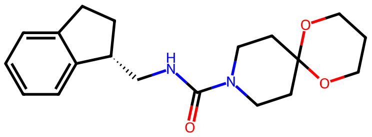	O=C(NC[C@H]1CCc2ccccc21)N1CCC2(CC1)OCCCO2	838428824	24	330.19	2.66
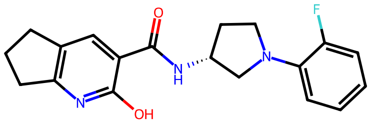	O=C(N[C@@H]1CCN(c2ccccc2F)C1)c1cc2c(nc1O)CCC2	817028078	25	341.15	2.42
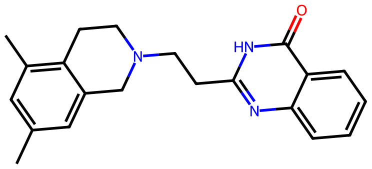	Cc1cc(C)c2c(c1)CN(CCc1nc3ccccc3c(=O)[nH]1)CC2	186720214	25	333.18	3.14
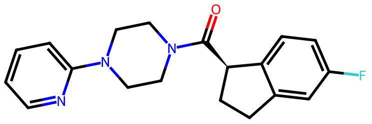	O=C([C@@H]1CCc2cc(F)ccc21)N1CCN(c2ccccn2)CC1	665153897	24	325.16	2.6
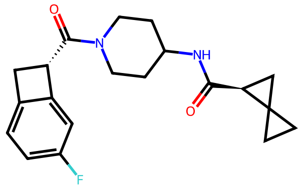	O=C(NC1CCN(C(=O)[C@H]2Cc3ccc(F)cc32)CC1)[C@H]1CC12CC2	948026946	25	342.17	2.37

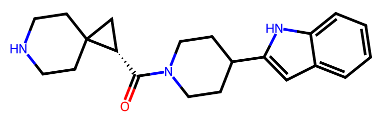	O=C([C@H]1CC12CCNCC2)N1CCC(c2cc3ccccc3[nH]2)CC1	827851355	25	337.22	3.26
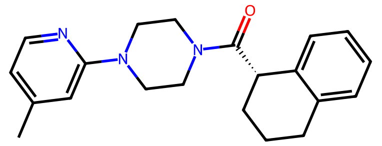	Cc1ccnc(N2CCN(C(=O)[C@H]3CCCc4ccccc43)CC2)c1	361928736	25	335.2	3.16
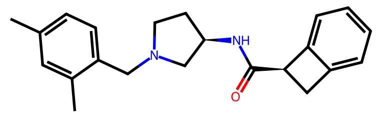	Cc1ccc(CN2CC[C@@H](NC(=O)[C@@H]3Cc4ccccc43)C2)c(C)c1	1015634762	25	334.2	3.33
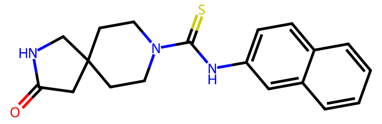	O=C1CC2(CCN(C(=S)Nc3ccc4ccccc4c3)CC2)CN1	751899779	24	339.14	3.14
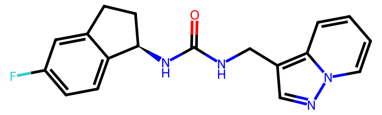	O=C(NCc1cnn2ccccc12)N[C@@H]1CCc2cc(F)ccc21	344430382	24	324.14	2.96
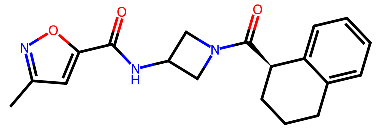	Cc1cc(C(=O)NC2CN(C(=O)[C@@H]3CCCc4ccccc43)C2)on1	990222964	25	339.16	2.04
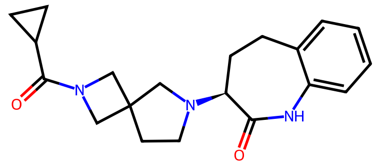	O=C1Nc2ccccc2CC[C@@H]1N1CCC2(CN(C(=O)C3CC3)C2)C1	1072378283	25	339.19	1.88
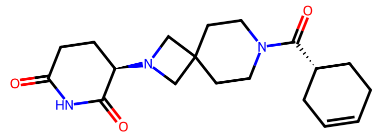	O=C1CC[C@@H](N2CC3(CCN(C(=O)[C@H]4CC=CCC4)CC3)C2)C(=O)N1	1035648626	25	345.21	1.07

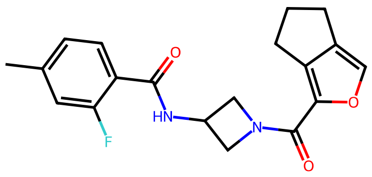	Cc1ccc(C(=O)NC2CN(C(=O)c3occ4c3CCC4)C2)c(F)c1	1000490519	25	342.14	2.47
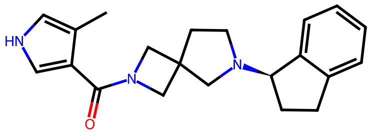	Cc1c[nH]cc1C(=O)N1CC2(CCN([C@@H]3CCc4ccccc43)C2)C1	1073428025	25	335.2	3.16
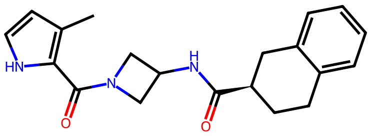	Cc1cc[nH]c1C(=O)N1CC(NC(=O)[C@@H]2CCc3ccccc3C2)C1	1000536797	25	337.18	2.07
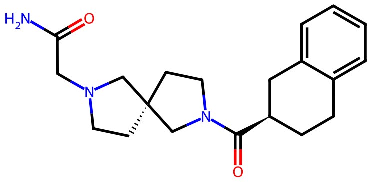	NC(=O)CN1CC[C@@]2(CCN(C(=O)[C@@H]3CCc4ccccc4C3)C2)C1	1041710113	25	341.21	1.2
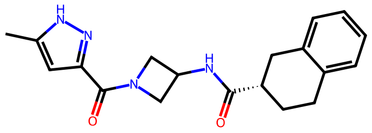	Cc1cc(C(=O)N2CC(NC(=O)[C@H]3CCc4ccccc4C3)C2)n[nH]1	1000537641	25	338.17	1.46
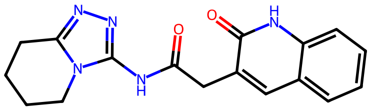	O=C(Cc1cc2ccccc2[nH]c1=O)Nc1nnc2n1CCCC2	180897474	24	323.14	1.64
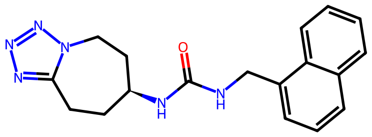	O=C(NCc1cccc2ccccc12)N[C@H]1CCc2nnnn2CC1	643377778	25	336.17	2.03
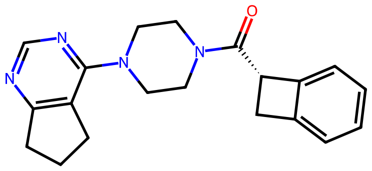	O=C([C@H]1Cc2ccccc21)N1CCN(c2ncnc3c2CCC3)CC1	1055998638	25	334.18	1.95

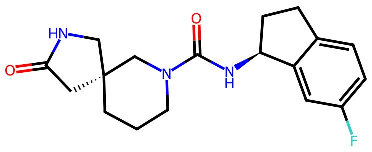	O=C1C[C@]2(CCCN(C(=O)N[C@H]3CCc4ccc(F)cc43)C2)CN1	608426947	24	331.17	2.12
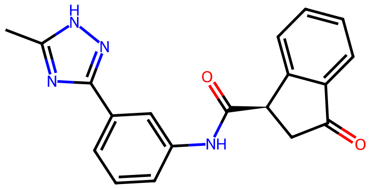	Cc1nc(-c2cccc(NC(=O)[C@@H]3CC(=O)c4ccccc43)c2)n[nH]1	365155424	25	332.13	3.09
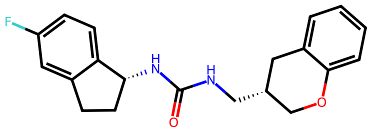	O=C(NC[C@H]1COc2ccccc2C1)N[C@@H]1CCc2cc(F)ccc21	56839437	25	340.16	3.36
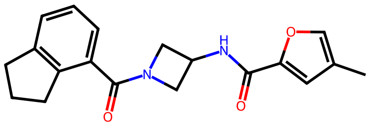	Cc1coc(C(=O)NC2CN(C(=O)c3cccc4c3CCC4)C2)c1	997979411	24	324.15	2.33
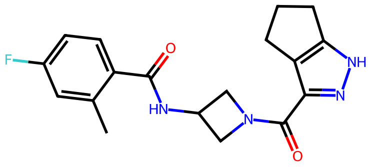	Cc1cc(F)ccc1C(=O)NC1CN(C(=O)c2n[nH]c3c2CCC3)C1	997190240	25	342.15	1.6
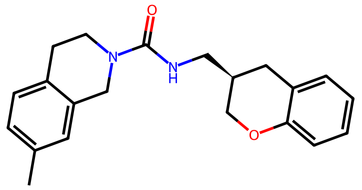	Cc1ccc2c(c1)CN(C(=O)NC[C@@H]1COc3ccccc3C1)CC2	851972741	25	336.18	3.31
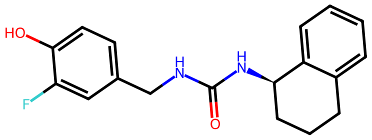	O=C(NCc1ccc(O)c(F)c1)N[C@@H]1CCCc2ccccc21	155031080	23	314.14	3.41
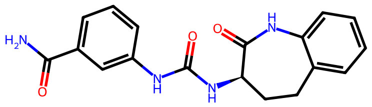	NC(=O)c1cccc(NC(=O)N[C@@H]2CCc3ccccc3NC2=O)c1	1425082170	25	338.14	1.86

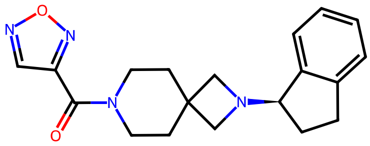	O=C(c1cnon1)N1CCC2(CC1)CN([C@@H]1CCc3ccccc31)C2	1035821214	25	338.17	2.3
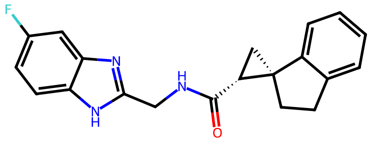	O=C(NCc1nc2cc(F)ccc2[nH]1)[C@@H]1C[C@]12CCc1ccccc12	176114677	25	335.14	3.22
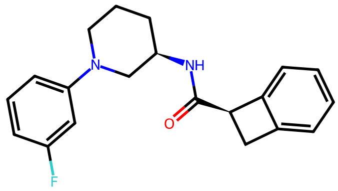	O=C(N[C@@H]1CCCN(c2cccc(F)c2)C1)[C@@H]1Cc2ccccc21	358496091	24	324.16	3.25
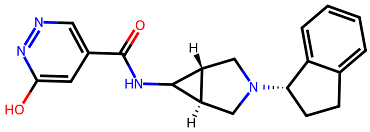	O=C(NC1[C@@H]2CN([C@H]3CCc4ccccc43)C[C@@H]12)c1cnnc(O)c1	960411883	25	336.16	1.53
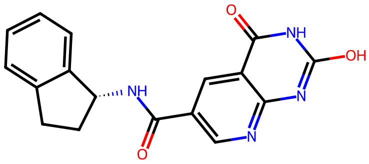	O=C(N[C@@H]1CCc2ccccc21)c1cnc2nc(O)[nH]c(=O)c2c1	51184219	24	322.11	1.44
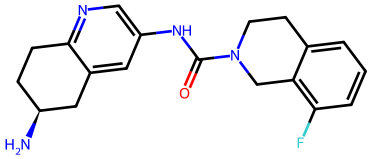	N[C@H]1CCc2ncc(NC(=O)N3CCc4cccc(F)c4C3)cc2C1	333349659	25	340.17	2.63
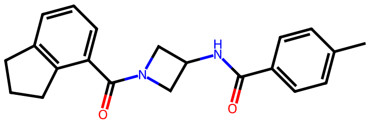	Cc1ccc(C(=O)NC2CN(C(=O)c3cccc4c3CCC4)C2)cc1	999439352	25	334.17	2.74
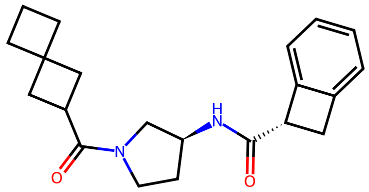	O=C(N[C@H]1CCN(C(=O)C2CC3(CCC3)C2)C1)[C@H]1Cc2ccccc21	938570785	25	338.2	2.62

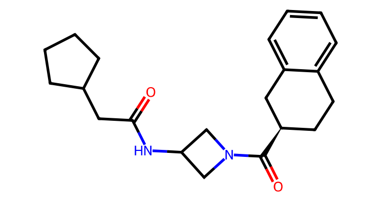	O=C(CC1CCCC1)NC1CN(C(=O)[C@@H]2CCc3ccccc3C2)C1	990113149	25	340.22	2.7
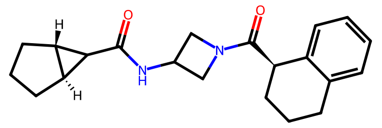	O=C(NC1CN(C(=O)[C@@H]2CCCc3ccccc32)C1)C1[C@H]2CCC[C@H]12	990708392	25	338.2	2.48
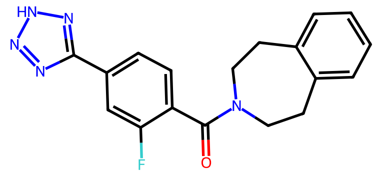	O=C(c1ccc(-c2nn[nH]n2)cc1F)N1CCc2ccccc2CC1	1582700716	25	337.13	2.25
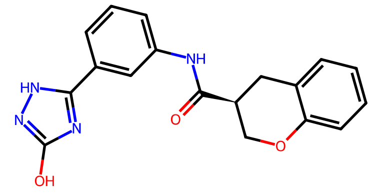	O=C(Nc1cccc(-c2nc(O)n[nH]2)c1)[C@@H]1COc2ccccc2C1	377134939	25	336.12	2.37
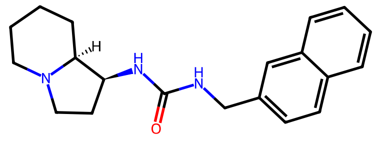	O=C(NCc1ccc2ccccc2c1)N[C@H]1CCN2CCCC[C@@H]12	356229343	24	323.2	3.27
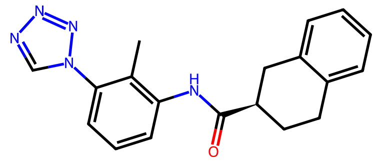	Cc1c(NC(=O)[C@@H]2CCc3ccccc3C2)cccc1-n1cnnn1	566299267	25	333.16	2.71
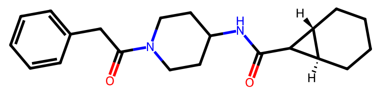	O=C(NC1CCN(C(=O)Cc2ccccc2)CC1)C1[C@@H]2CCCC[C@@H]12	947344906	25	340.22	2.77
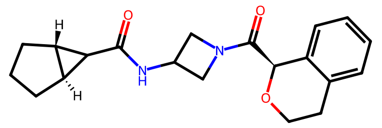	O=C(NC1CN(C(=O)[C@@H]2OCCc3ccccc32)C1)C1[C@H]2CCC[C@H]12	990713009	25	340.18	1.67

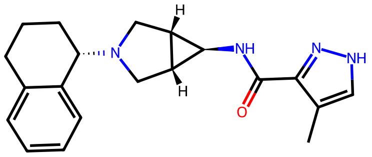	Cc1c[nH]nc1C(=O)N[C@H]1[C@@H]2CN([C@H]3CCCc4ccccc43)C[C@@H]21	958813486	25	336.2	2.46
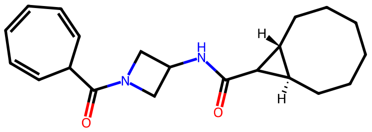	O=C(NC1CN(C(=O)C2C=CC=CC=C2)C1)C1[C@@H]2CCCCCC[C@@H]12	1000507828	25	340.22	2.83
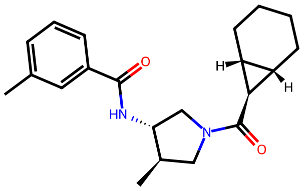	Cc1cccc(C(=O)N[C@@H]2CN(C(=O)[C@@H]3[C@H]4CCCC[C@H]43)C[C@H]2C)c1	948157459	25	340.22	3.01
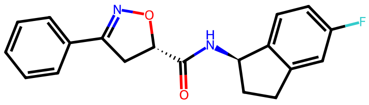	O=C(N[C@@H]1CCc2cc(F)ccc21)[C@@H]1CC(c2ccccc2)=NO1	56318601	24	324.13	3.12
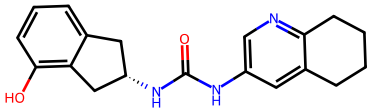	O=C(Nc1cnc2c(c1)CCCC2)N[C@H]1Cc2cccc(O)c2C1	562383493	24	323.16	2.95
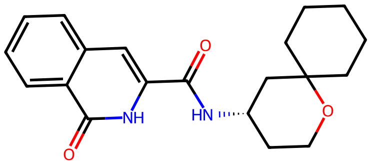	O=C(N[C@H]1CCOC2(CCCCC2)C1)c1cc2ccccc2c(=O)[nH]1	161530720	25	340.18	3.14
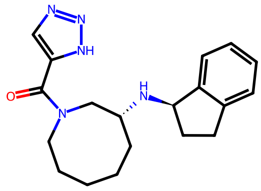	O=C(c1cnn[nH]1)N1CCCCC[C@@H](N[C@@H]2CCc3ccccc32)C1	1088340448	25	339.21	2.47
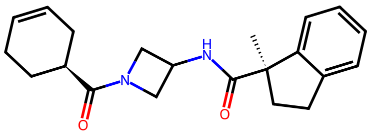	C[C@@]1(C(=O)NC2CN(C(=O)[C@@H]3CC=CCC3)C2)CCc2ccccc21	994376603	25	338.2	2.57

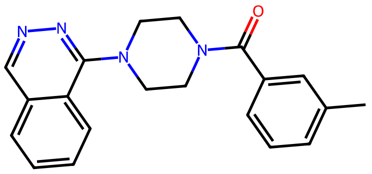	Cc1cccc(C(=O)N2CCN(c3nncc4ccccc34)CC2)c1	74795478	25	332.16	2.9
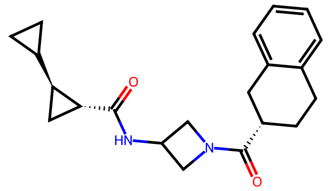	O=C(NC1CN(C(=O)[C@H]2CCc3ccccc3C2)C1)[C@@H]1C[C@H]1C1CC1	992004871	25	338.2	2.16
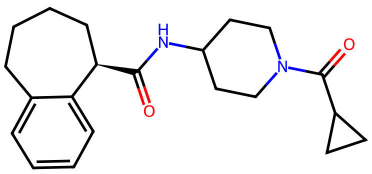	O=C(NC1CCN(C(=O)C2CC2)CC1)[C@@H]1CCCCc2ccccc21	943234841	25	340.22	3.01
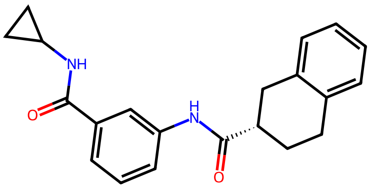	O=C(NC1CC1)c1cccc(NC(=O)[C@H]2CCc3ccccc3C2)c1	576163156	25	334.17	3.32
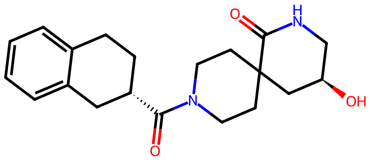	O=C([C@H]1CCc2ccccc2C1)N1CCC2(CC1)C[C@H](O)CNC2=O	885016106	25	342.19	1.28
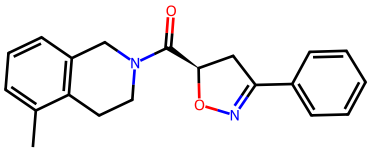	Cc1cccc2c1CCN(C(=O)[C@H]1CC(c3ccccc3)=NO1)C2	729415260	24	320.15	3.07
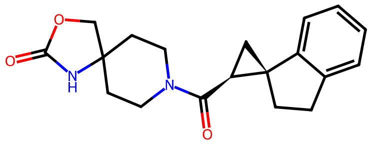	O=C1NC2(CCN(C(=O)[C@H]3C[C@@]34CCc3ccccc34)CC2)CO1	495157008	24	326.16	1.99
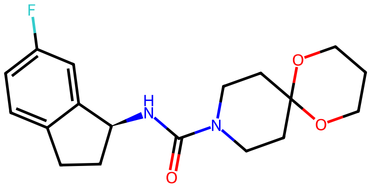	O=C(N[C@H]1CCc2ccc(F)cc21)N1CCC2(CC1)OCCCO2	836288454	24	334.17	2.75

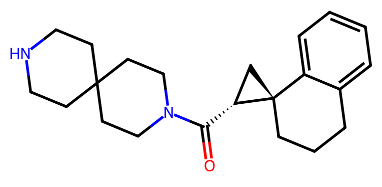	O=C([C@@H]1C[C@@]12CCCc1ccc cc12)N1CCC2(CCNCC2)CC1	357887481	25	338.24	3.27
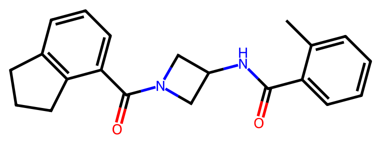	Cc1ccccc1C(=O)NC1CN(C(=O)c2cccc 3c2CCC3)C1	990110024	25	334.17	2.74
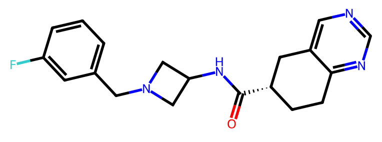	O=C(NC1CN(Cc2cccc(F)c2)C1)[C@H]1CCc2ncncc2C1	1030428228	25	340.17	1.72
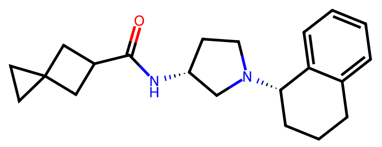	O=C(N[C@@H]1CCN([C@H]2CCCc3 ccccc32)C1)C1CC2(CC2)C1	1015681202	24	324.22	3.44
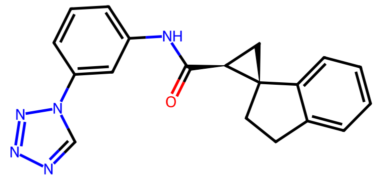	O=C(Nc1cccc(-n2cnnn2)c1)[C@H]1C[C@@]12CCc1ccccc12	174630286	25	331.14	2.5
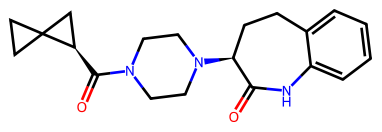	O=C1Nc2ccccc2CC[C@@H]1N1CCN (C(=O)[C@@H]2CC23CC3)CC1	950707453	25	339.19	1.88
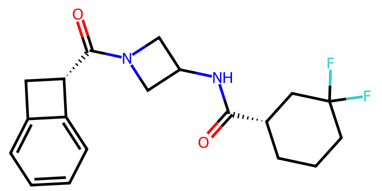	O=C(NC1CN(C(=O)[C@H]2Cc3ccccc 32)C1)[C@H]1CCCC(F)(F)C1	999754410	25	348.16	2.48
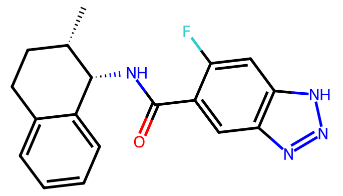	C[C@H]1CCc2ccccc2[C@H]1NC(=O) c1cc2nn[nH]c2cc1F	781961679	24	324.14	3.15

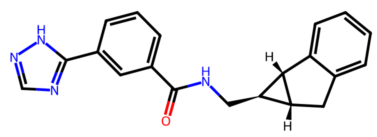	O=C(NC[C@@H]1[C@H]2Cc3ccccc3[C@H]21)c1cccc(-c2ncn[nH]2)c1	157757632	25	330.15	2.79
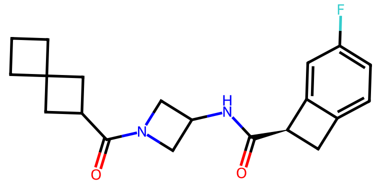	O=C(NC1CN(C(=O)C2CC3(CCC3)C2)C1)[C@@H]1Cc2ccc(F)cc21	998490204	25	342.17	2.37
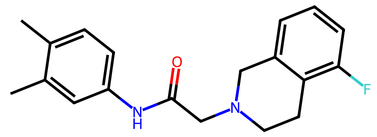	Cc1ccc(NC(=O)CN2CCc3c(F)cccc3C2)cc1C	757149676	23	312.16	3.44
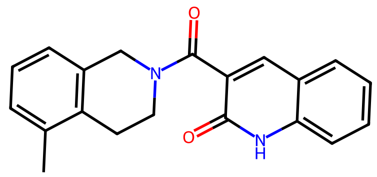	Cc1cccc2c1CCN(C(=O)c1cc3ccccc3[nH]c1=O)C2	171540512	24	318.14	3.04
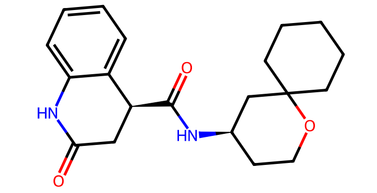	O=C1C[C@H](C(=O)N[C@@H]2CCOC3(CCCCC3)C2)c2ccccc2N1	162335414	25	342.19	3.11
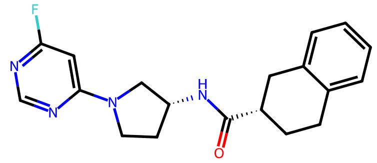	O=C(N[C@@H]1CCN(c2cc(F)ncn2)C1)[C@H]1CCc2ccccc2C1	1058559470	25	340.17	2.12
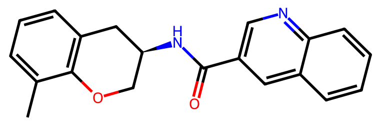	Cc1cccc2c1OC[C@H](NC(=O)c1cnc3ccccc3c1)C2	1527464546	24	318.14	3.28
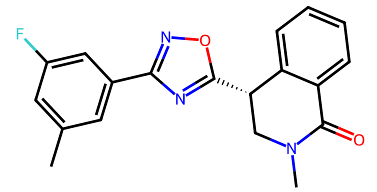	Cc1cc(F)cc(-c2noc([C@H]3CN(C)C(=O)c4ccccc43)n2)c1	377438648	25	337.12	3.4

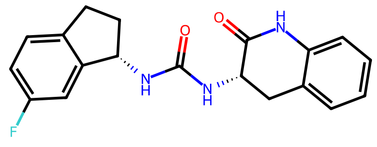	O=C(N[C@H]1Cc2ccccc2NC1=O)N[C@H]1CCc2ccc(F)cc21	636743797	25	339.14	2.68
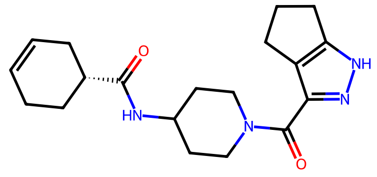	O=C(NC1CCN(C(=O)c2n[nH]c3c2CCC3)CC1)[C@H]1CC=CCC1	947366676	25	342.21	1.98
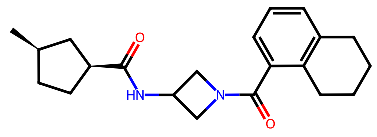	C[C@@H]1CC[C@H](C(=O)NC2CN(C(=O)c3cccc4c3CCCC4)C2)C1	991435439	25	340.22	2.94
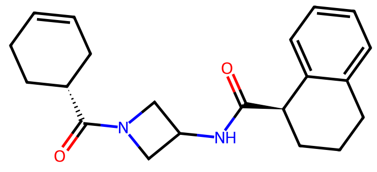	O=C(NC1CN(C(=O)[C@H]2CC=CCC2)C1)[C@@H]1CCCc2ccccc21	995071181	25	338.2	2.79
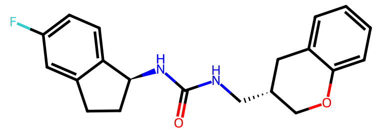	O=C(NC[C@H]1COc2ccccc2C1)N[C@H]1CCc2cc(F)ccc21	56839439	25	340.16	3.36
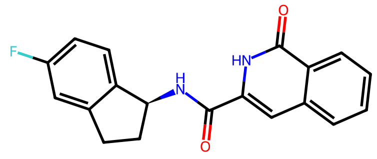	O=C(N[C@H]1CCc2cc(F)ccc21)c1cc2ccccc2c(=O)[nH]1	56314793	24	322.11	3.08
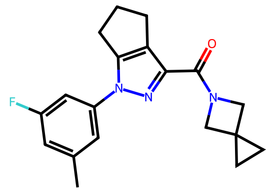	Cc1cc(F)cc(-n2nc(C(=O)N3CC4(CC4)C3)c3c2CCC3)c1	424854514	24	325.16	3.04
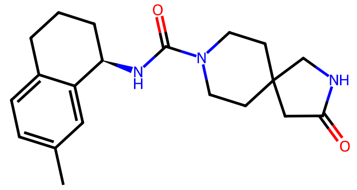	Cc1ccc2c(c1)[C@H](NC(=O)N1CCC3(CC1)CNC(=O)C3)CCC2	293530208	25	341.21	2.68

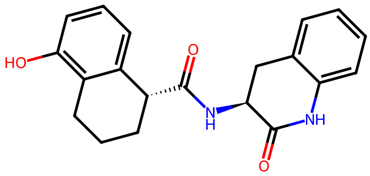	O=C1Nc2ccccc2C[C@@H]1NC(=O)[C@@H]1CCCc2c(O)cccc21	529949078	25	336.15	2.49
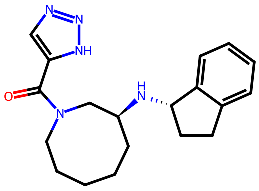	O=C(c1cnn[nH]1)N1CCCCC[C@H](N[C@H]2CCc3ccccc32)C1	1088340449	25	339.21	2.47
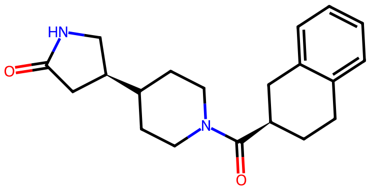	O=C1C[C@H](C2CCN(C(=O)[C@@H]3CCc4ccccc4C3)CC2)CN1	1551708573	24	326.2	2.17
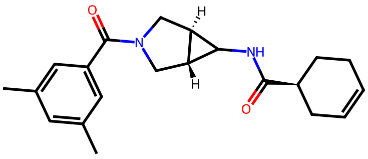	Cc1cc(C)cc(C(=O)N2C[C@H]3C(NC(=O)[C@@H]4CC=CCC4)[C@@H]3C2)c1	1012767543	25	338.2	2.85
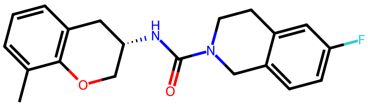	Cc1cccc2c1OC[C@@H](NC(=O)N1CCc3cc(F)ccc3C1)C2	462615353	25	340.16	3.21
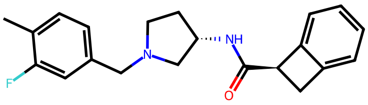	Cc1ccc(CN2CC[C@H](NC(=O)[C@@H]3Cc4ccccc43)C2)cc1F	1015634523	25	338.18	3.16
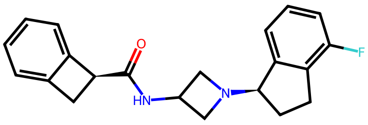	O=C(NC1CN([C@@H]2CCc3c(F)cccc32)C1)[C@H]1Cc2ccccc21	1030881519	25	336.16	2.95
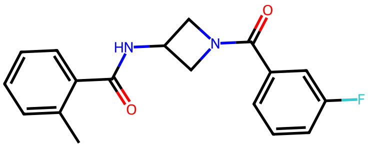	Cc1ccccc1C(=O)NC1CN(C(=O)c2cccc(F)c2)C1	990106438	23	312.13	2.39

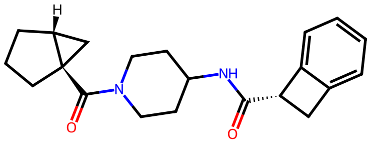	O=C(NC1CCN(C(=O)[C@@]23CCC[C@@H]2C3)CC1)[C@H]1Cc2ccccc21	947751926	25	338.2	2.62
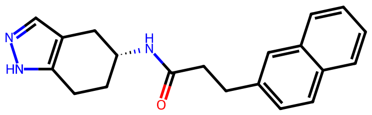	O=C(CCc1ccc2ccccc2c1)N[C@@H]1CCc2[nH]ncc2C1	172037300	24	319.17	3.17
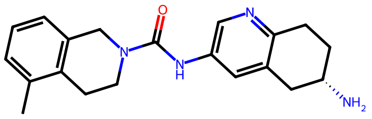	Cc1cccc2c1CCN(C(=O)Nc1cnc3c(c1)C[C@@H](N)CC3)C2	841323386	25	336.2	2.8
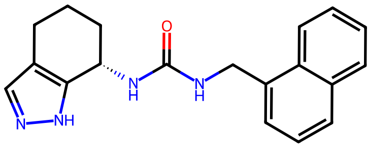	O=C(NCc1cccc2ccccc12)N[C@H]1CCCc2cn[nH]c21	268940219	24	320.16	3.44
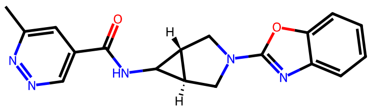	Cc1cc(C(=O)NC2[C@@H]3CN(c4nc5ccccc5o4)C[C@@H]23)cnn1	1056166504	25	335.14	1.79
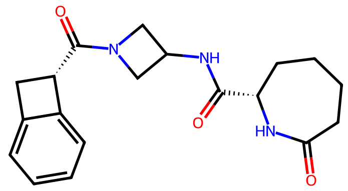	O=C1CCCC[C@@H](C(=O)NC2CN(C(=O)[C@H]3Cc4ccccc43)C2)N1	999369227	25	341.17	0.71
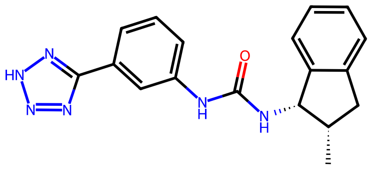	C[C@H]1Cc2ccccc2[C@H]1NC(=O)Nc1cccc(-c2nn[nH]n2)c1	736299648	25	334.15	2.92
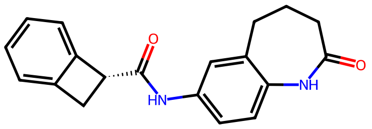	O=C1CCCc2cc(NC(=O)[C@@H]3Cc4ccccc43)ccc2N1	607823482	23	306.14	3.24

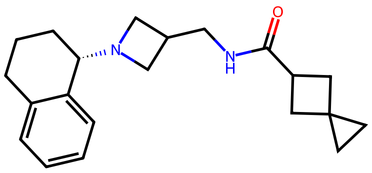	O=C(NCC1CN([C@H]2CCCc3ccccc3 2)C1)C1CC2(CC2)C1	1031967789	24	324.22	3.3
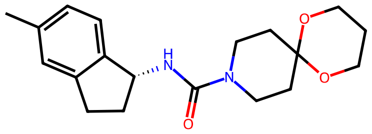	Cc1ccc2c(c1)CC[C@H]2NC(=O)N1C CC2(CC1)OCCCO2	836252118	24	330.19	2.92
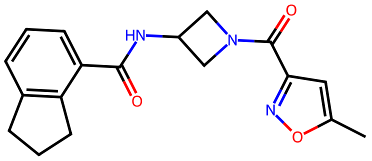	Cc1cc(C(=O)N2CC(NC(=O)c3cccc4c3 CCC4)C2)no1	994394172	24	325.14	1.73
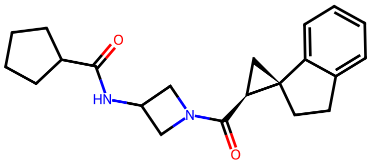	O=C(NC1CN(C(=O)[C@H]2C[C@@]2 3CCc2ccccc23)C1)C1CCCC1	990036736	25	338.2	2.41
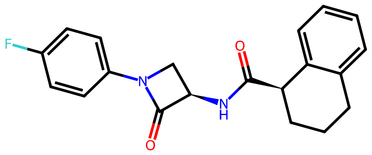	O=C(N[C@@H]1CN(c2ccc(F)cc2)C1= O)[C@@H]1CCCc2ccccc21	360848255	25	338.14	2.78
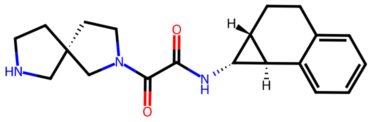	O=C(N[C@H]1[C@@H]2c3ccccc3CC[C@H]21)C(=O)N1CC[C@]2(CCNC2) C1	1328748179	25	339.19	1.04
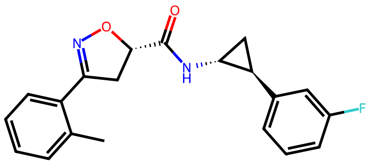	Cc1ccccc1C1=NO[C@H](C(=O)N[C@ @H]2C[C@H]2c2cccc(F)c2)C1	1128622826	25	338.14	3.3
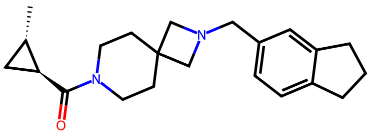	C[C@H]1C[C@@H]1C(=O)N1CCC2(CC1)CN(Cc1ccc3c(c1)CCC3)C2	1035641731	25	338.24	3.26

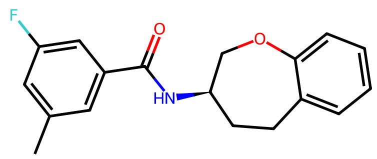	Cc1cc(F)cc(C(=O)N[C@@H]2CCc3ccccc3OC2)c1	511610968	22	299.13	3.26
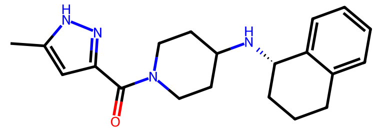	Cc1cc(C(=O)N2CCC(N[C@H]3CCCc4ccccc43)CC2)n[nH]1	990066342	25	338.21	2.99
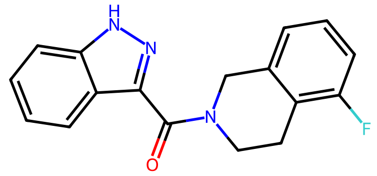	O=C(c1n[nH]c2ccccc12)N1CCc2c(F)cccc2C1	160918827	22	295.11	2.9
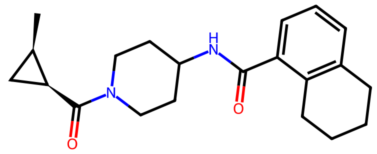	C[C@@H]1C[C@@H]1C(=O)N1CCC(NC(=O)c2cccc3c2CCCC3)CC1	943825824	25	340.22	2.94
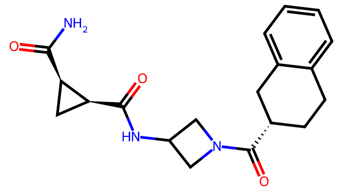	NC(=O)[C@@H]1C[C@@H]1C(=O)NC1CN(C(=O)[C@H]2CCc3ccccc3C2)C1	999929948	25	341.17	0.24
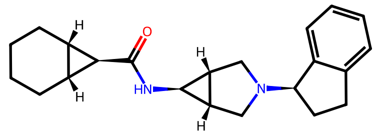	O=C(N[C@H]1[C@@H]2CN([C@@H]3CCc4ccccc43)C[C@@H]21)[C@@H]1[C@H]2CCCC[C@H]21	1025723607	25	336.22	3.16
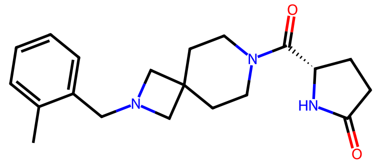	Cc1ccccc1CN1CC2(CCN(C(=O)[C@@H]3CCC(=O)N3)CC2)C1	1035839897	25	341.21	1.7
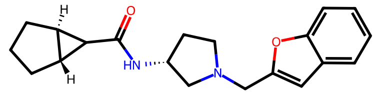	O=C(N[C@@H]1CCN(Cc2cc3ccccc3o2)C1)C1[C@@H]2CCC[C@@H]12	1010475718	24	324.18	3.17

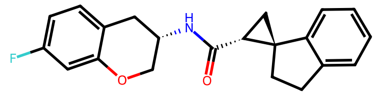	O=C(N[C@@H]1COc2cc(F)ccc2C1)[C@@H]1C[C@@]12CCc1ccccc12	618651819	25	337.15	3.15
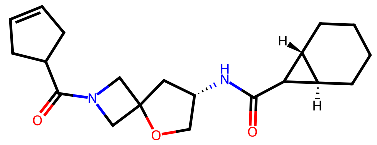	O=C(N[C@@H]1COC2(C1)CN(C(=O)C1CC=CC1)C2)C1[C@@H]2CCCC[C@@H]12	965651918	25	344.21	1.87
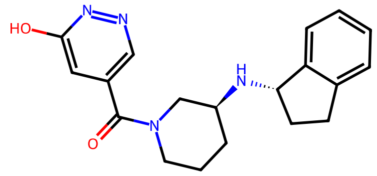	O=C(c1cnnc(O)c1)N1CCC[C@H](N[C@H]2CCc3ccccc32)C1	999440208	25	338.17	2.06
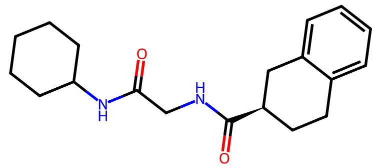	O=C(CNC(=O)[C@@H]1CCc2ccccc2C1)NC1CCCCC1	565053072	23	314.2	2.36
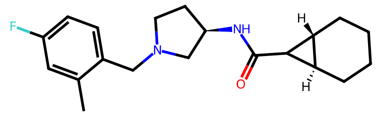	Cc1cc(F)ccc1CN1CC[C@@H](NC(=O)C2[C@@H]3CCCC[C@@H]23)C1	1032834927	24	330.21	3.26
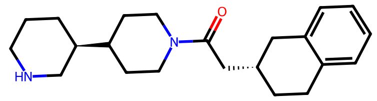	O=C(C[C@H]1CCc2ccccc2C1)N1CCC([C@@H]2CCCNC2)CC1	658956229	25	340.25	3.42
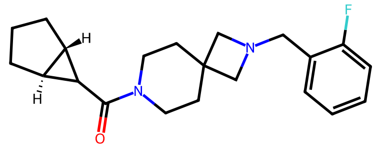	O=C(C1[C@H]2CCC[C@H]12)N1CCC2(CC1)CN(Cc1ccccc1F)C2	1035679280	25	342.21	3.3
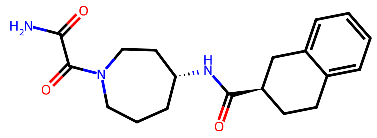	NC(=O)C(=O)N1CCC[C@@H](NC(=O)[C@@H]2CCc3ccccc3C2)CC1	965222099	25	343.19	0.77

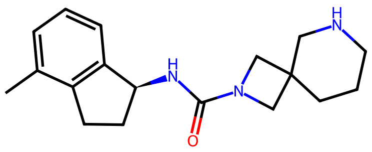	Cc1cccc2c1CC[C@@H]2NC(=O)N1CC2(CCCNC2)C1	1579777260	22	299.2	2.38
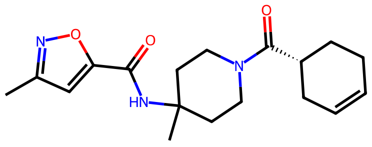	Cc1cc(C(=O)NC2(C)CCN(C(=O)[C@H]3CC=CCC3)CC2)on1	956288676	24	331.19	2.45
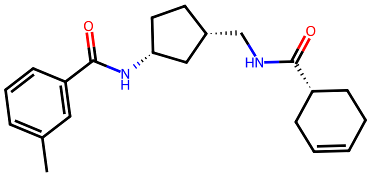	Cc1cccc(C(=O)N[C@@H]2CC[C@H](CNC(=O)[C@H]3CC=CCC3)C2)c1	980895461	25	340.22	3.37
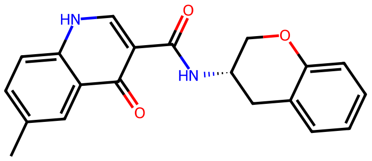	Cc1ccc2[nH]cc(C(=O)N[C@@H]3COc4ccccc4C3)c(=O)c2c1	1641160323	25	334.13	2.57
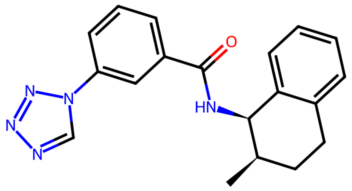	C[C@@H]1CCc2ccccc2[C@@H]1NC(=O)c1cccc(-n2cnnn2)c1	122723954	25	333.16	2.72
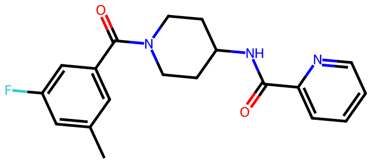	Cc1cc(F)cc(C(=O)N2CCC(NC(=O)c3ccccn3)CC2)c1	948546204	25	341.15	2.56
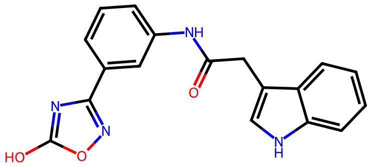	O=C(Cc1c[nH]c2ccccc12)Nc1cccc(-c2noc(O)n2)c1	57753527	25	334.11	3.1
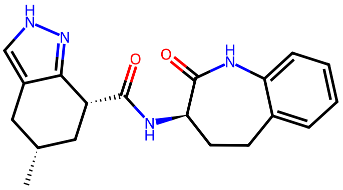	C[C@@H]1Cc2c[nH]nc2[C@H](C(=O)N[C@@H]2CCc3ccccc3NC2=O)C1	623225355	25	338.17	2.15

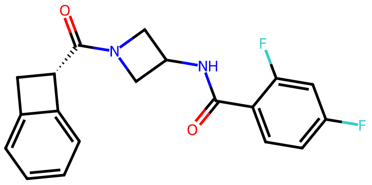	O=C(NC1CN(C(=O)[C@H]2Cc3ccccc32)C1)c1ccc(F)cc1F	999515005	25	342.12	2.25
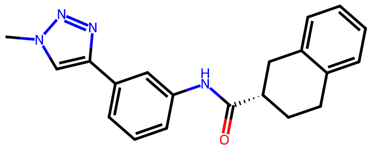	Cn1cc(-c2cccc(NC(=O)[C@H]3CCc4ccccc4C3)c2)nn1	620270389	25	332.16	3.23
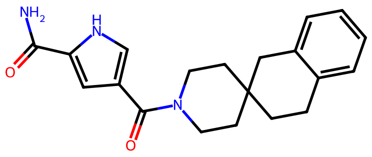	NC(=O)c1cc(C(=O)N2CCC3(CCc4ccccc4C3)CC2)c[nH]1	652526482	25	337.18	2.52
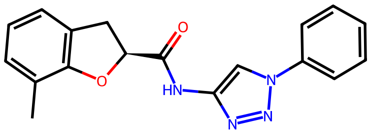	Cc1cccc2c1O[C@H](C(=O)Nc1cn(-c3ccccc3)nn1)C2	1540300366	24	320.13	2.52
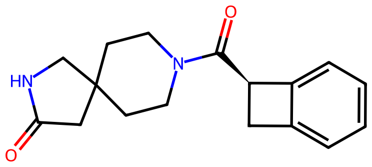	O=C1CC2(CCN(C(=O)[C@@H]3Cc4ccccc43)CC2)CN1	90985319	21	284.15	1.45
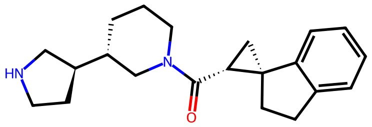	O=C([C@@H]1C[C@]12CCc1ccccc12)N1CCC[C@@H]([C@H]2CCNC2)C1	449212220	24	324.22	2.74
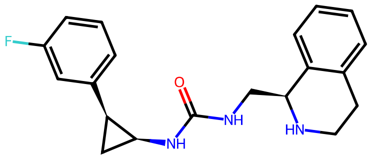	O=C(NC[C@@H]1NCCc2ccccc21)N[C@H]1C[C@H]1c1cccc(F)c1	572825139	25	339.17	2.87
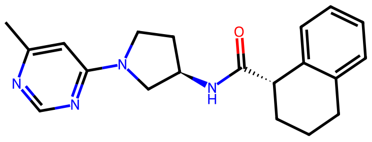	Cc1cc(N2CC[C@@H](NC(=O)[C@H]3CCCc4ccccc43)C2)ncn1	1058592333	25	336.2	2.6

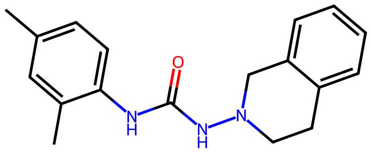	Cc1ccc(NC(=O)NN2CCc3ccccc3C2)c(C)c1	1657995126	22	295.17	3.4
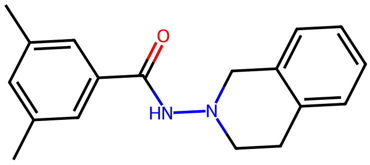	Cc1cc(C)cc(C(=O)NN2CCc3ccccc3C2)c1	1146331167	21	280.16	3.01
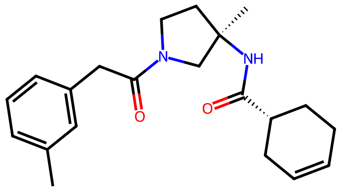	Cc1cccc(CC(=O)N2CC[C@@](C)(NC(=O)[C@H]3CC=CCC3)C2)c1	956691327	25	340.22	3.0
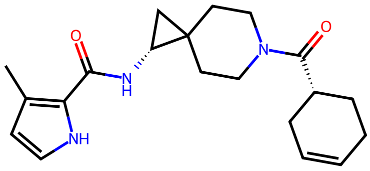	Cc1cc[nH]c1C(=O)N[C@@H]1CC12CCN(C(=O)[C@H]1CC=CCC1)CC2	963754049	25	341.21	2.79
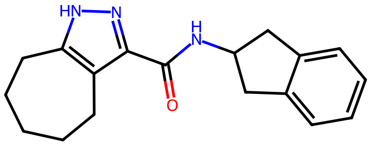	O=C(NC1Cc2ccccc2C1)c1n[nH]c2c1CCCCC2	191027908	22	295.17	2.58
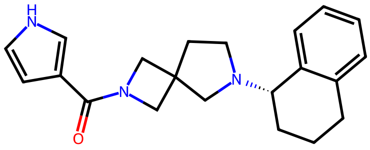	O=C(c1cc[nH]c1)N1CC2(CCN([C@H]3CCCc4ccccc43)C2)C1	1072771015	25	335.2	3.24
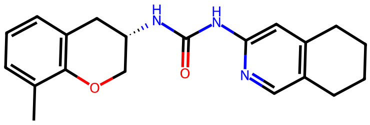	Cc1cccc2c1OC[C@@H](NC(=O)Nc1cc3c(cn1)CCCC3)C2	1672342114	25	337.18	3.39
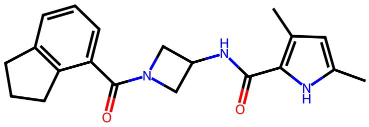	Cc1cc(C)c(C(=O)NC2CN(C(=O)c3cccc4c3CCC4)C2)[nH]1	995092185	25	337.18	2.37

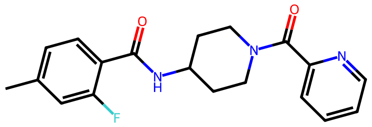	Cc1ccc(C(=O)NC2CCN(C(=O)c3ccccn3)CC2)c(F)c1	943598246	25	341.15	2.56
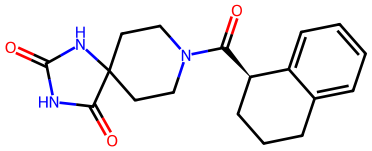	O=C1NC(=O)C2(CCN(C(=O)[C@@H]3CCCc4ccccc43)CC2)N1	44385259	24	327.16	1.31
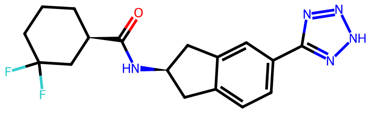	O=C(N[C@@H]1Cc2ccc(-c3nn[nH]n3)cc2C1)[C@@H]1CCCC(F)(F)C1	1593001683	25	347.16	2.28
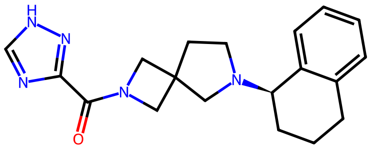	O=C(c1nc[nH]n1)N1CC2(CCN([C@@H]3CCCc4ccccc43)C2)C1	1073067074	25	337.19	2.03
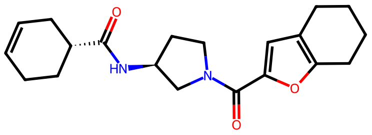	O=C(N[C@H]1CCN(C(=O)c2cc3c(o2)CCCC3)C1)[C@H]1CC=CCC1	937672099	25	342.19	2.85
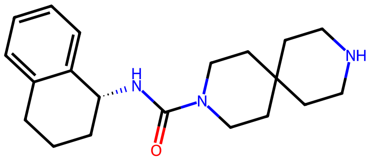	O=C(N[C@@H]1CCCc2ccccc21)N1CCC2(CCNCC2)CC1	416800385	24	327.23	3.24
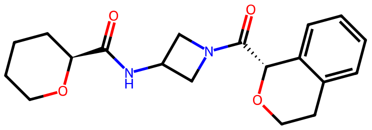	O=C(NC1CN(C(=O)[C@H]2OCCc3ccccc32)C1)[C@@H]1CCCCO1	990854098	25	344.17	1.2
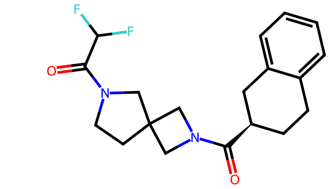	O=C(C(F)F)N1CCC2(C1)CN(C(=O)[C@@H]1CCc3ccccc3C1)C2	1019491872	25	348.16	2.12

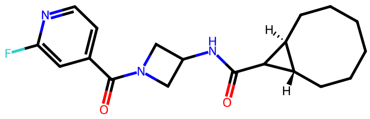	O=C(NC1CN(C(=O)c2ccnc(F)c2)C1)C1[C@H]2CCCCCC[C@H]12	1000484297	25	345.19	2.38
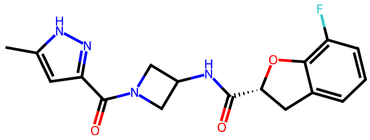	Cc1cc(C(=O)N2CC(NC(=O)[C@H]3Cc4cccc(F)c4O3)C2)n[nH]1	992546127	25	344.13	0.8
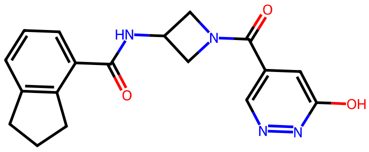	O=C(NC1CN(C(=O)c2cnnc(O)c2)C1)c1cccc2c1CCC2	994396922	25	338.14	0.93
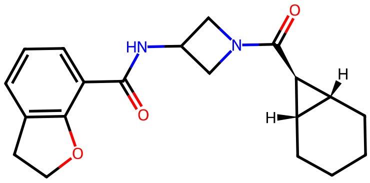	O=C(NC1CN(C(=O)[C@@H]2[C@H]3CCCC[C@H]32)C1)c1cccc2c1OCC2	1000416625	25	340.18	2.0
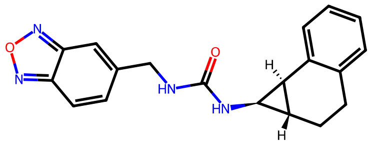	O=C(NCc1ccc2nonc2c1)N[C@@H]1[C@H]2CCc3ccccc3[C@@H]21	893766475	25	334.14	2.75
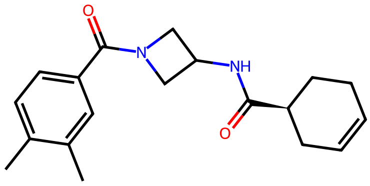	Cc1ccc(C(=O)N2CC(NC(=O)[C@@H]3CC=CCC3)C2)cc1C	990215417	23	312.18	2.6
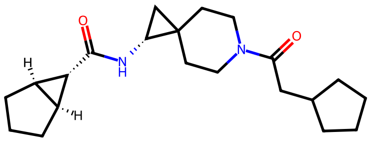	O=C(N[C@@H]1CC12CCN(C(=O)CC1CCCC1)CC2)[C@@H]1[C@H]2CCC[C@H]21	963470283	25	344.25	3.11
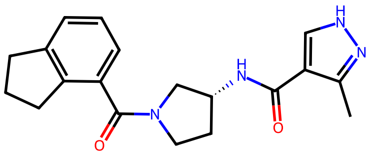	Cc1n[nH]cc1C(=O)N[C@@H]1CCN(C(=O)c2cccc3c2CCC3)C1	941952952	25	338.17	1.85

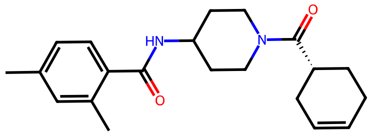	Cc1ccc(C(=O)NC2CCN(C(=O)[C@H]3CC=CCC3)CC2)c(C)c1	944107484	25	340.22	3.38
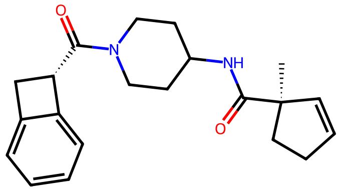	C[C@]1(C(=O)NC2CCN(C(=O)[C@H]3Cc4ccccc43)CC2)C=CCC1	948062445	25	338.2	2.79
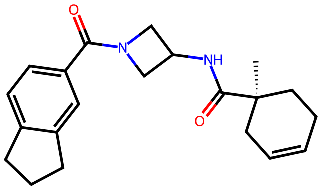	C[C@@]1(C(=O)NC2CN(C(=O)c3ccc4c(c3)CCC4)C2)CC=CCC1	991914556	25	338.2	2.86
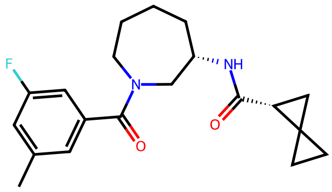	Cc1cc(F)cc(C(=O)N2CCCC[C@H](NC(=O)[C@@H]3CC34CC4)C2)c1	943574024	25	344.19	3.05
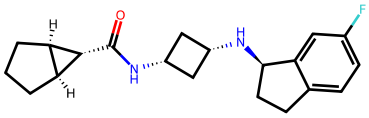	O=C(N[C@H]1C[C@@H](N[C@@H]2CCc3ccc(F)cc32)C1)[C@@H]1[C@H]2CCC[C@H]21	1021941876	24	328.2	3.1
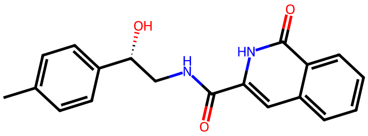	Cc1ccc([C@H](O)CNC(=O)c2cc3ccccc3c(=O)[nH]2)cc1	133664046	24	322.13	2.3
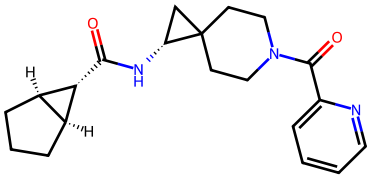	O=C(N[C@@H]1CC12CCN(C(=O)c1ccccn1)CC2)[C@@H]1[C@H]2CCC[C@H]21	963471477	25	339.19	2.24
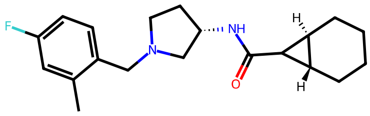	Cc1cc(F)ccc1CN1CC[C@H](NC(=O)C2[C@H]3CCCC[C@H]23)C1	1010439872	24	330.21	3.26

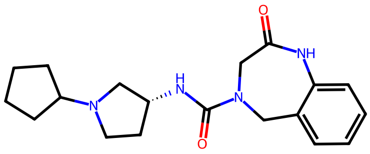	O=C1CN(C(=O)N[C@@H]2CCN(C3CCCC3)C2)Cc2ccccc2N1	582368604	25	342.21	2.17
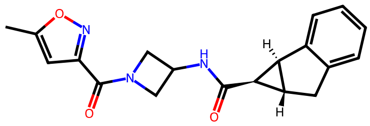	Cc1cc(C(=O)N2CC(NC(=O)[C@@H]3[C@H]4Cc5ccccc5[C@@H]43)C2)no1	991655535	25	337.14	1.51
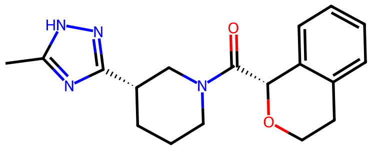	Cc1nc([C@H]2CCCN(C(=O)[C@H]3OCCc4ccccc43)C2)n[nH]1	269737417	24	326.17	2.13
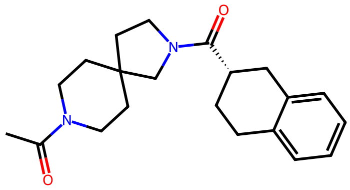	CC(=O)N1CCC2(CC1)CCN(C(=O)[C@H]1CCc3ccccc3C1)C2	944337433	25	340.22	2.65
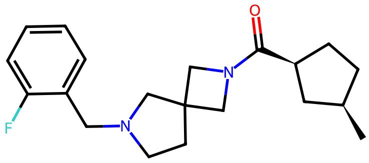	C[C@@H]1CC[C@H](C(=O)N2CC3(CCN(Cc4ccccc4F)C3)C2)C1	1073031873	24	330.21	3.3
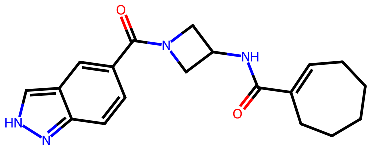	O=C(NC1CN(C(=O)c2ccc3n[nH]cc3c2)C1)C1=CCCCCC1	991009509	25	338.17	2.39
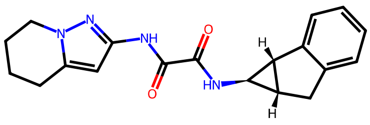	O=C(Nc1cc2n(n1)CCCC2)C(=O)N[C@@H]1[C@H]2Cc3ccccc3[C@H]21	288866889	25	336.16	1.61
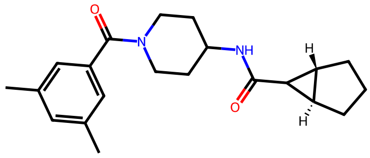	Cc1cc(C)cc(C(=O)N2CCC(NC(=O)C3[C@@H]4CCC[C@@H]34)CC2)c1	948338796	25	340.22	3.07

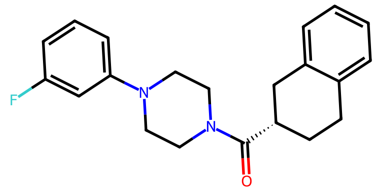	O=C([C@H]1CCc2ccccc2C1)N1CCN(c2cccc(F)c2)CC1	1125059844	25	338.18	3.28
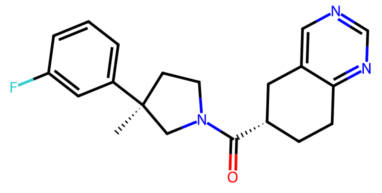	C[C@]1(c2cccc(F)c2)CCN(C(=O)[C@H]2CCc3ncncc3C2)C1	650218238	25	339.17	2.91
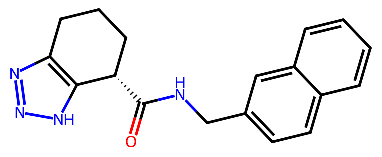	O=C(NCc1ccc2ccccc2c1)[C@H]1CCCc2nn[nH]c21	821045032	23	306.15	2.69
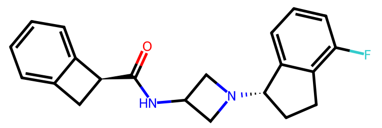	O=C(NC1CN([C@H]2CCc3c(F)cccc32)C1)[C@H]1Cc2ccccc21	1030881520	25	336.16	2.95
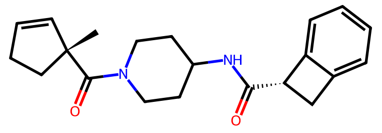	C[C@]1(C(=O)N2CCC(NC(=O)[C@H]3Cc4ccccc43)CC2)C=CCC1	947852259	25	338.2	2.79
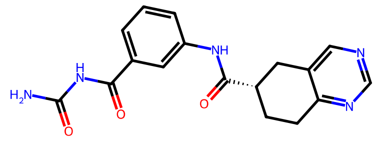	NC(=O)NC(=O)c1cccc(NC(=O)[C@H]2CCc3ncncc3C2)c1	1463414760	25	339.13	1.03
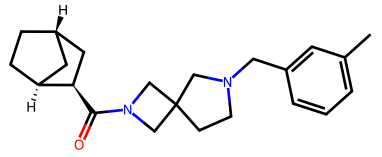	Cc1cccc(CN2CCC3(C2)CN(C(=O)[C@@H]2C[C@H]4CC[C@H]2C4)C3)c1	1072470540	25	338.24	3.47
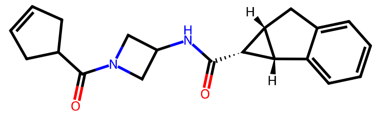	O=C(NC1CN(C(=O)C2CC=CC2)C1)[C@H]1[C@H]2c3ccccc3C[C@H]21	991654325	24	322.17	1.87

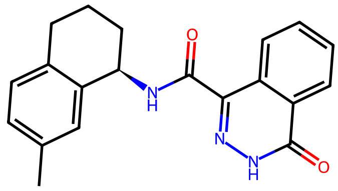	Cc1ccc2c(c1)[C@H](NC(=O)c1n[nH]c(=O)c3ccccc13)CCC2	179472994	25	333.15	3.04
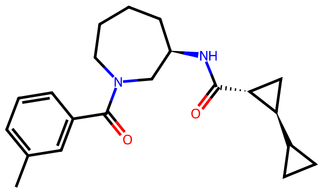	Cc1cccc(C(=O)N2CCCC[C@@H](NC(=O)[C@@H]3C[C@H]3C3CC3)C2)c1	943281276	25	340.22	3.15
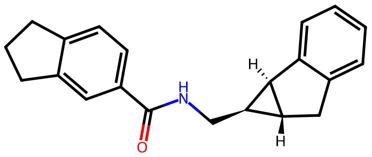	O=C(NC[C@@H]1[C@H]2Cc3ccccc3[C@@H]21)c1ccc2c(c1)CCC2	488689589	23	303.16	3.49
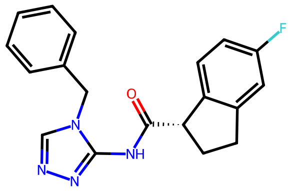	O=C(Nc1nncn1Cc1ccccc1)[C@H]1CCc2cc(F)ccc21	1618159646	25	336.14	3.13
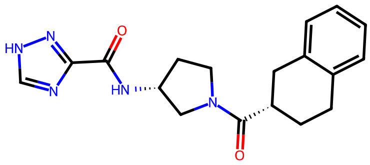	O=C(N[C@@H]1CCN(C(=O)[C@H]2CCc3ccccc3C2)C1)c1nc[nH]n1	936274029	25	339.17	0.94
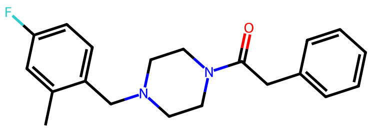	Cc1cc(F)ccc1CN1CCN(C(=O)Cc2ccccc2)CC1	349830856	24	326.18	3.02
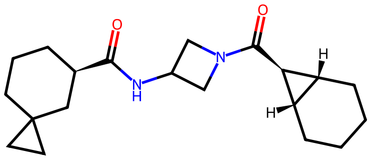	O=C(NC1CN(C(=O)[C@@H]2[C@H]3CCCC[C@H]32)C1)[C@@H]1CCCC2(CC2)C1	991507779	24	330.23	2.72
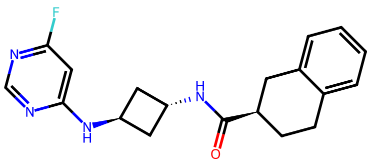	O=C(N[C@H]1C[C@H](Nc2cc(F)ncn2)C1)[C@@H]1CCc2ccccc2C1	1059425510	25	340.17	2.48

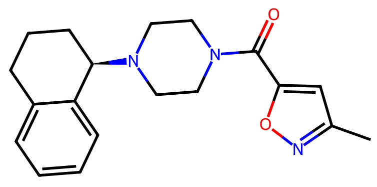	Cc1cc(C(=O)N2CCN([C@@H]3CCCc4ccccc43)CC2)on1	65514817	24	325.18	2.82
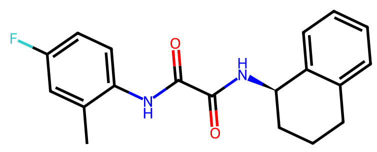	Cc1cc(F)ccc1NC(=O)C(=O)N[C@@H]1CCCc2ccccc21	48648590	24	326.14	3.27
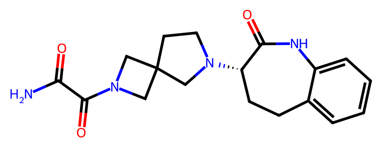	NC(=O)C(=O)N1CC2(CCN([C@H]3CCc4ccccc4NC3=O)C2)C1	1072935839	25	342.17	-0.04
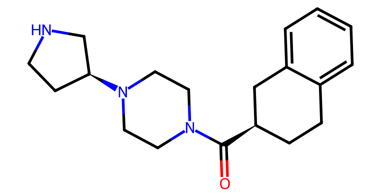	O=C([C@@H]1CCc2ccccc2C1)N1CCN([C@H]2CCNC2)CC1	392053485	23	313.22	1.3
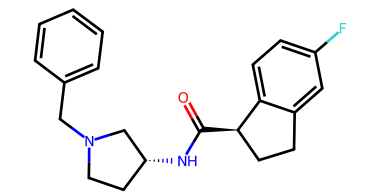	O=C(N[C@@H]1CCN(Cc2ccccc2)C1)[C@@H]1CCc2cc(F)ccc21	665576580	25	338.18	3.25
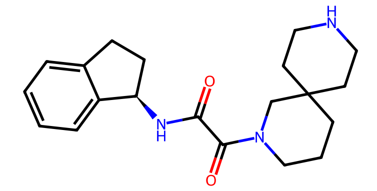	O=C(N[C@@H]1CCc2ccccc21)C(=O)N1CCCC2(CCNCC2)C1	1330533310	25	341.21	1.78
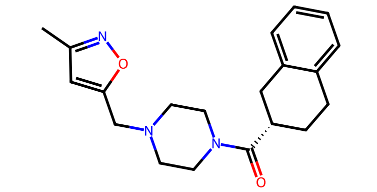	Cc1cc(CN2CCN(C(=O)[C@H]3CCc4ccccc4C3)CC2)on1	569814637	25	339.19	2.43
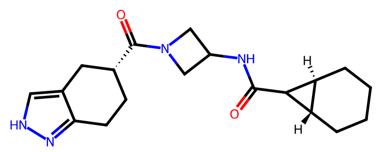	O=C(NC1CN(C(=O)[C@@H]2CCc3[nH]cc3C2)C1)C1[C@H]2CCCC[C@H]12	990649349	25	342.21	1.28

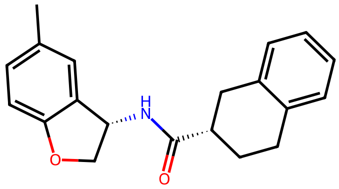	Cc1ccc2c(c1)[C@H](NC(=O)[C@H]1CCc3ccccc3C1)CO2	567392393	23	307.16	3.35
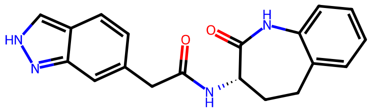	O=C(Cc1ccc2c[nH]nc2c1)N[C@H]1CCc2ccccc2NC1=O	1469664455	25	334.14	2.18
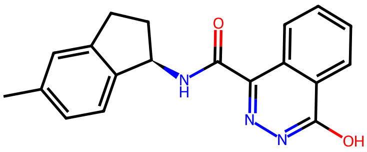	Cc1ccc2c(c1)CC[C@H]2NC(=O)c1nnc(O)c2ccccc12	351393631	24	319.13	3.06
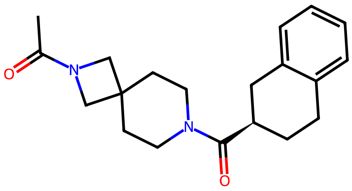	CC(=O)N1CC2(CCN(C(=O)[C@@H]3CCc4ccccc4C3)CC2)C1	945071779	24	326.2	2.26
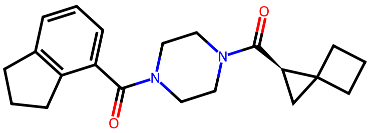	O=C(c1cccc2c1CCC2)N1CCN(C(=O)[C@@H]2CC23CCC3)CC1	1011144436	25	338.2	2.65
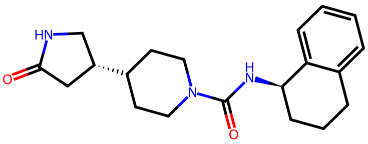	O=C1C[C@@H](C2CCN(C(=O)N[C@@H]3CCCc4ccccc43)CC2)CN1	1553351032	25	341.21	2.62
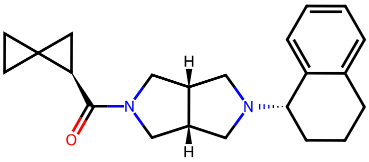	O=C([C@@H]1CC12CC2)N1C[C@@H]2CN([C@H]3CCCc4ccccc43)C[C@@H]2C1	1049082781	25	336.22	3.25
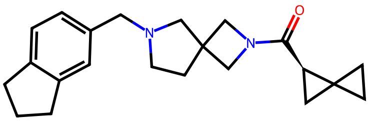	O=C([C@@H]1CC12CC2)N1CC2(CCN(Cc3ccc4c(c3)CCC4)C2)C1	1073012271	25	336.22	3.01

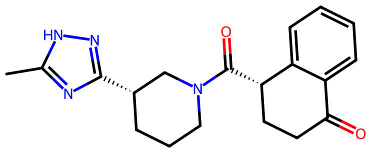	Cc1nc([C@H]2CCCN(C(=O)[C@H]3CCC(=O)c4ccccc43)C2)n[nH]1	285396555	25	338.17	2.58
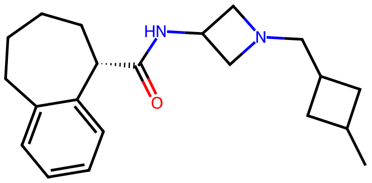	CC1CC(CN2CC(NC(=O)[C@H]3CCCCc4ccccc43)C2)C1	1031451498	24	326.24	3.34
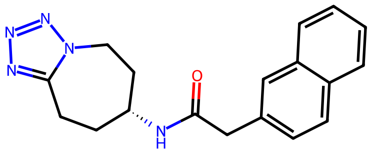	O=C(Cc1ccc2ccccc2c1)N[C@@H]1CCc2nnnn2CC1	564815994	24	321.16	1.89
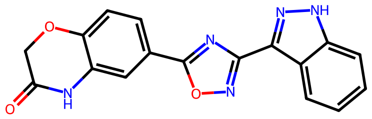	O=C1COc2ccc(-c3nc(-c4n[nH]c5ccccc45)no3)cc2N1	356760675	25	333.09	2.61
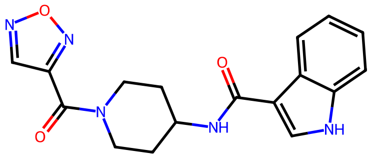	O=C(NC1CCN(C(=O)c2cnon2)CC1)c1c[nH]c2ccccc12	947963974	25	339.13	1.59
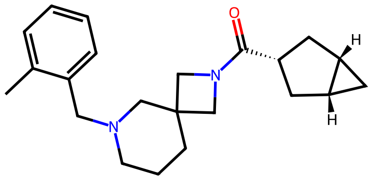	Cc1ccccc1CN1CCCC2(C1)CN(C(=O)[C@@H]1C[C@@H]3C[C@@H]3C1)C2	981954537	25	338.24	3.47
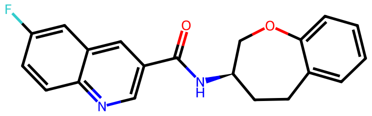	O=C(N[C@@H]1CCc2ccccc2OC1)c1cnc2ccc(F)cc2c1	338058790	25	336.13	3.5
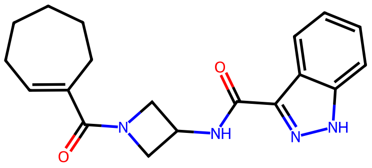	O=C(NC1CN(C(=O)C2=CCCCCC2)C1)c1n[nH]c2ccccc12	999525585	25	338.17	2.39

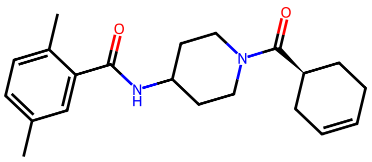	Cc1ccc(C)c(C(=O)NC2CCN(C(=O)[C@@H]3CC=CCC3)CC2)c1	944106197	25	340.22	3.38
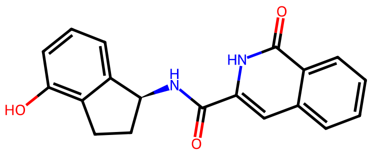	O=C(N[C@H]1CCc2c(O)cccc21)c1cc2ccccc2c(=O)[nH]1	74141578	24	320.12	2.65
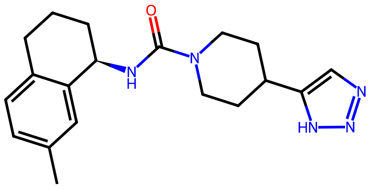	Cc1ccc2c(c1)[C@H](NC(=O)N1CCC(c3cnn[nH]3)CC1)CCC2	1577409771	25	339.21	3.08
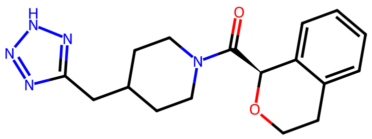	O=C([C@@H]1OCCc2ccccc21)N1CCC(Cc2nn[nH]n2)CC1	1548804638	24	327.17	1.29
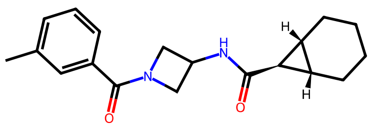	Cc1cccc(C(=O)N2CC(NC(=O)[C@@H]3[C@H]4CCCC[C@H]43)C2)c1	990641535	23	312.18	2.37
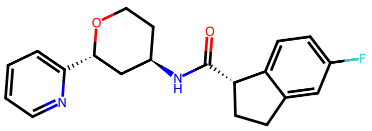	O=C(N[C@@H]1CCO[C@@H](c2ccccn2)C1)[C@H]1CCc2cc(F)ccc21	615979990	25	340.16	3.29
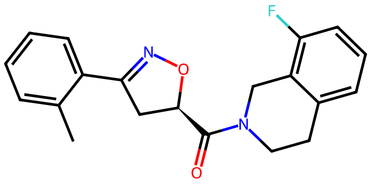	Cc1ccccc1C1=NO[C@@H](C(=O)N2CCc3cccc(F)c3C2)C1	1584005263	25	338.14	3.21
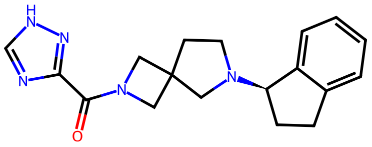	O=C(c1nc[nH]n1)N1CC2(CCN([C@@H]3CCc4ccccc43)C2)C1	1073067223	24	323.17	1.64

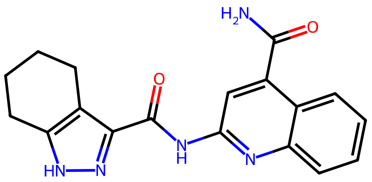	NC(=O)c1cc(NC(=O)c2n[nH]c3c2CCCC3)nc2ccccc12	1457952165	25	335.14	2.19
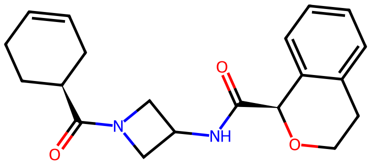	O=C(NC1CN(C(=O)[C@@H]2CC=CCC2)C1)[C@@H]1OCCc2ccccc21	997322097	25	340.18	1.98
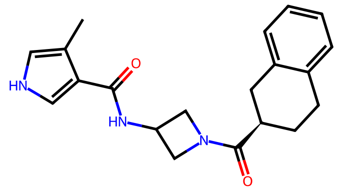	Cc1c[nH]cc1C(=O)NC1CN(C(=O)[C@@H]2CCc3ccccc3C2)C1	991708284	25	337.18	2.07
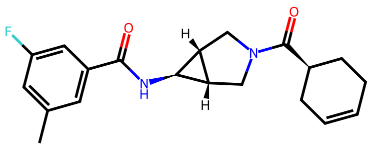	Cc1cc(F)cc(C(=O)N[C@H]2[C@@H]3CN(C(=O)[C@@H]4CC=CCC4)C[C@@H]32)c1	977339011	25	342.17	2.68
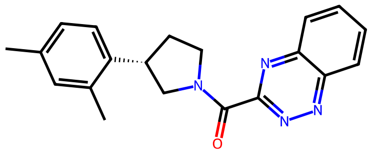	Cc1ccc([C@@H]2CCN(C(=O)c3nnc4ccccc4n3)C2)c(C)c1	290775137	25	332.16	3.27
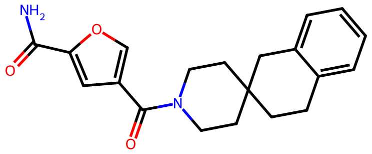	NC(=O)c1cc(C(=O)N2CCC3(CCc4ccccc4C3)CC2)co1	652525273	25	338.16	2.79
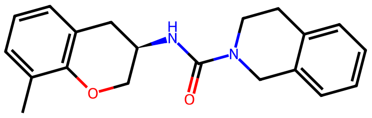	Cc1cccc2c1OC[C@H](NC(=O)N1CCc3ccccc3C1)C2	571459172	24	322.17	3.07
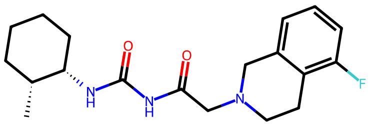	C[C@@H]1CCCC[C@@H]1NC(=O)NC(=O)CN1CCc2c(F)cccc2C1	339779912	25	347.2	2.59

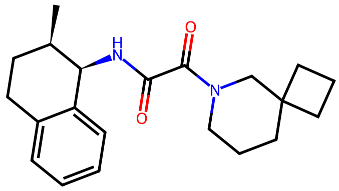	C[C@@H]1CCc2ccccc2[C@@H]1NC(=O)C(=O)N1CCCC2(CCC2)C1	359648123	25	340.22	3.22
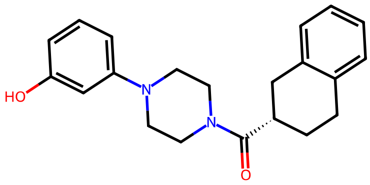	O=C([C@H]1CCc2ccccc2C1)N1CCN(c2cccc(O)c2)CC1	565065055	25	336.18	2.85
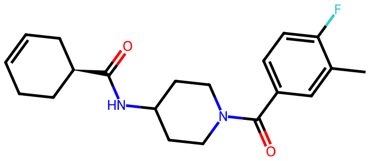	Cc1cc(C(=O)N2CCC(NC(=O)[C@@H]3CC=CCC3)CC2)ccc1F	948053325	25	344.19	3.21
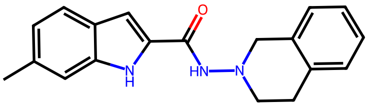	Cc1ccc2cc(C(=O)NN3CCc4ccccc4C3)[nH]c2c1	1452972057	23	305.15	3.18
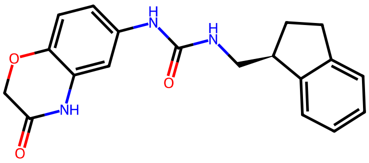	O=C1COc2ccc(NC(=O)NC[C@H]3CCc4ccccc43)cc2N1	111714336	25	337.14	2.87
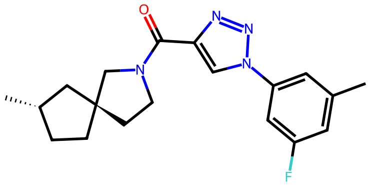	Cc1cc(F)cc(-n2cc(C(=O)N3CC[C@]4(CC[C@H](C)C4)C3)nn2)c1	329866400	25	342.19	3.37
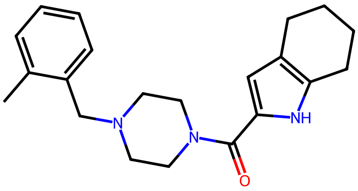	Cc1ccccc1CN1CCN(C(=O)c2cc3c([nH]2)CCCC3)CC1	957089023	25	337.22	3.16
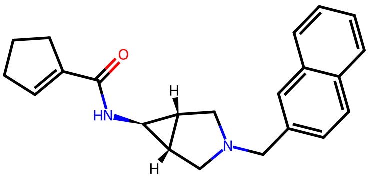	O=C(N[C@H]1[C@@H]2CN(Cc3ccc4ccccc4c3)C[C@@H]21)C1=CCCC1	958559996	25	332.19	3.5

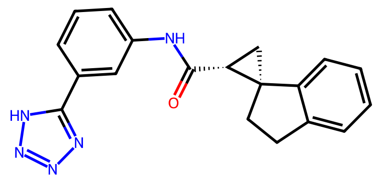	O=C(Nc1cccc(-c2nnn[nH]2)c1)[C@@H]1C[C@]12CCc1ccccc12	266615507	25	331.14	2.71
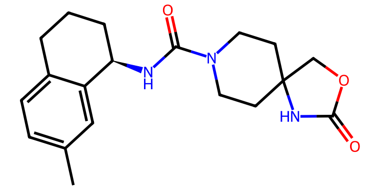	Cc1ccc2c(c1)[C@H](NC(=O)N1CCC3(CC1)COC(=O)N3)CCC2	495850318	25	343.19	2.66
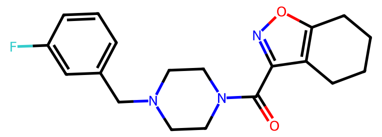	O=C(c1noc2c1CCCC2)N1CCN(Cc2ccc(F)c2)CC1	20614119	25	343.17	2.65
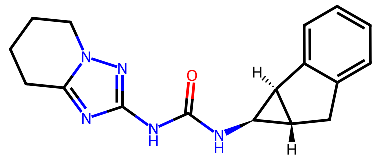	O=C(Nc1nc2n(n1)CCCC2)N[C@@H]1[C@H]2Cc3ccccc3[C@@H]21	653706405	23	309.16	2.07
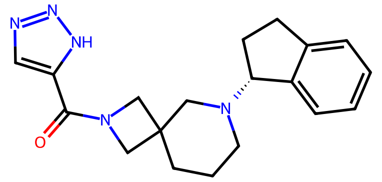	O=C(c1cnn[nH]1)N1CC2(CCCN([C@@H]3CCc4ccccc43)C2)C1	981867502	25	337.19	2.03
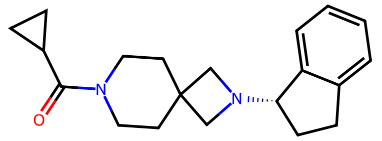	O=C(C1CC1)N1CCC2(CC1)CN([C@H]1CCc3ccccc31)C2	1035631955	23	310.2	3.01
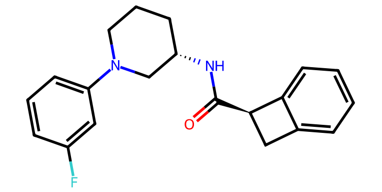	O=C(N[C@H]1CCCN(c2cccc(F)c2)C1)[C@@H]1Cc2ccccc21	358496089	24	324.16	3.25
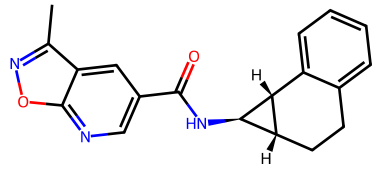	Cc1noc2ncc(C(=O)N[C@@H]3[C@H]4CCc5ccccc5[C@H]43)cc12	246203496	24	319.13	2.99

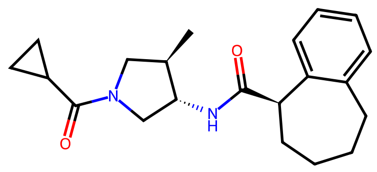	C[C@@H]1CN(C(=O)C2CC2)C[C@H]1NC(=O)[C@@H]1CCCCc2ccccc21	947037174	25	340.22	2.87
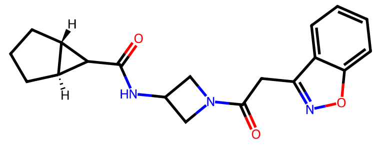	O=C(NC1CN(C(=O)Cc2noc3ccccc23)C1)C1[C@H]2CCC[C@H]12	990719815	25	339.16	1.74
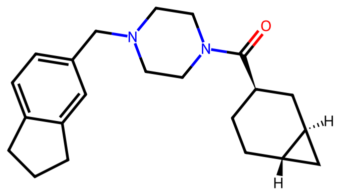	O=C([C@@H]1CC[C@H]2C[C@@H]2C1)N1CCN(Cc2ccc3c(c2)CCC3)CC1	948952251	25	338.24	3.26
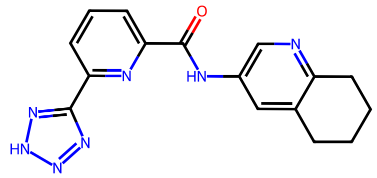	O=C(Nc1cnc2c(c1)CCCC2)c1cccc(-c2nn[nH]n2)n1	1593258502	24	321.13	1.79
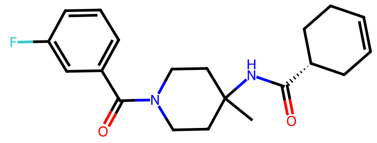	CC1(NC(=O)[C@H]2CC=CCC2)CCN(C(=O)c2cccc(F)c2)CC1	956288840	25	344.19	3.29
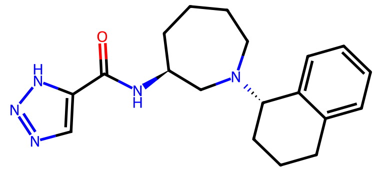	O=C(N[C@H]1CCCCN([C@H]2CCCc3ccccc32)C1)c1cnn[nH]1	1034291874	25	339.21	2.47
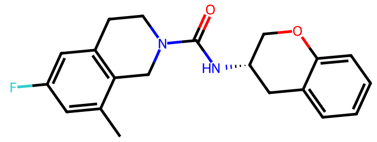	Cc1cc(F)cc2c1CN(C(=O)N[C@@H]1COc3ccccc3C1)CC2	507260217	25	340.16	3.21
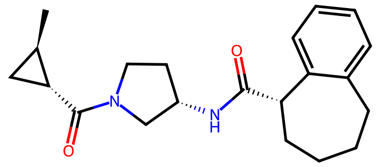	C[C@@H]1C[C@H]1C(=O)N1CC[C@H](NC(=O)[C@H]2CCCCc3ccccc32)C1	938139000	25	340.22	2.87

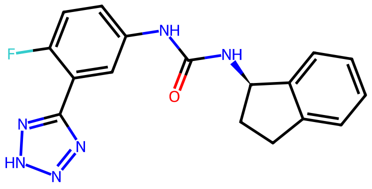	O=C(Nc1ccc(F)c(-c2nn[nH]n2)c1)N[C@@H]1CCc2ccccc21	1606921130	25	338.13	2.81
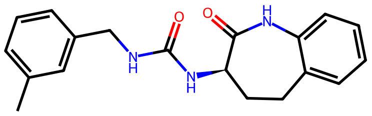	Cc1cccc(CNC(=O)N[C@@H]2CCc3ccccc3NC2=O)c1	654366413	24	323.16	2.75
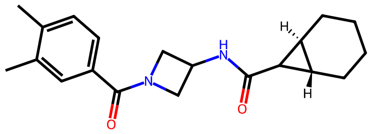	Cc1ccc(C(=O)N2CC(NC(=O)C3[C@H]4CCCC[C@H]34)C2)cc1C	990666518	24	326.2	2.68
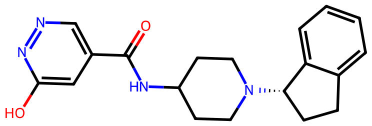	O=C(NC1CCN([C@H]2CCc3ccccc32)CC1)c1cnnc(O)c1	1003085212	25	338.17	2.06
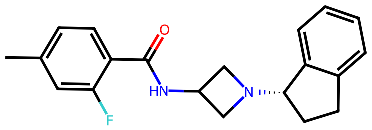	Cc1ccc(C(=O)NC2CN([C@H]3CCc4ccccc43)C2)c(F)c1	1031005377	24	324.16	3.24
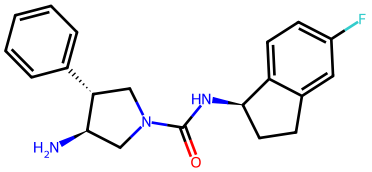	N[C@@H]1CN(C(=O)N[C@@H]2CCc3cc(F)ccc32)C[C@H]1c1ccccc1	333949045	25	339.17	2.95
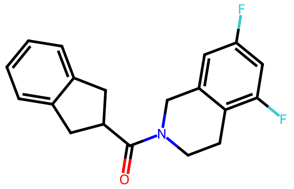	O=C(C1Cc2ccccc2C1)N1CCc2c(F)cc(F)cc2C1	346295313	23	313.13	3.26
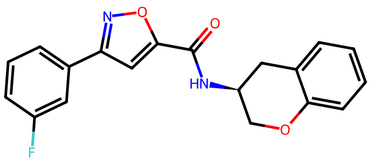	O=C(N[C@@H]1COc2ccccc2C1)c1cc(-c2cccc(F)c2)no1	53046836	25	338.11	3.21

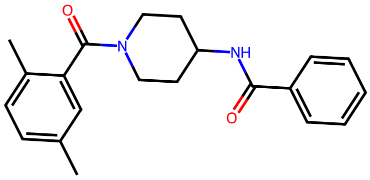	Cc1ccc(C)c(C(=O)N2CCC(NC(=O)c3ccccc3)CC2)c1	10779583	25	336.18	3.34
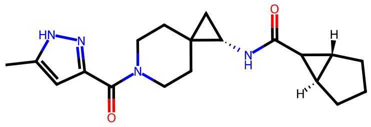	Cc1cc(C(=O)N2CCC3(CC2)C[C@@H]3NC(=O)C2[C@@H]3CCC[C@@H]23)n[nH]1	963468258	25	342.21	1.88
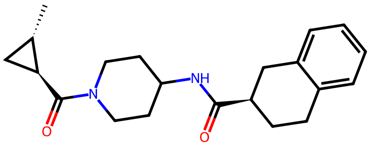	C[C@H]1C[C@@H]1C(=O)N1CCC(NC(=O)[C@@H]2CCc3ccccc3C2)CC1	947839559	25	340.22	2.55
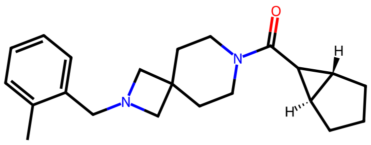	Cc1ccccc1CN1CC2(CCN(C(=O)C3[C@@H]4CCC[C@@H]34)CC2)C1	1035680957	25	338.24	3.47
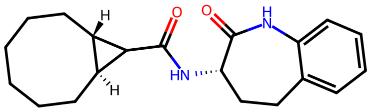	O=C(N[C@H]1CCc2ccccc2NC1=O)C1[C@H]2CCCCCC[C@H]12	616693214	24	326.2	3.27
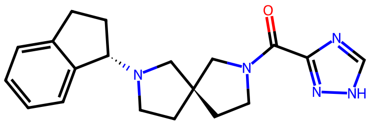	O=C(c1nc[nH]n1)N1CC[C@]2(CCN([C@H]3CCc4ccccc43)C2)C1	1041748553	25	337.19	2.03
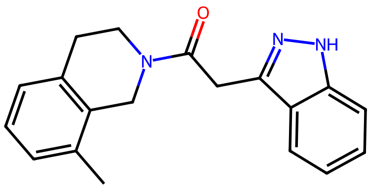	Cc1cccc2c1CN(C(=O)Cc1n[nH]c3ccccc13)CC2	277075268	23	305.15	3.0
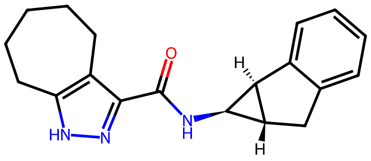	O=C(N[C@@H]1[C@H]2Cc3ccccc3[C@@H]21)c1n[nH]c2c1CCCCC2	190676940	23	307.17	2.75

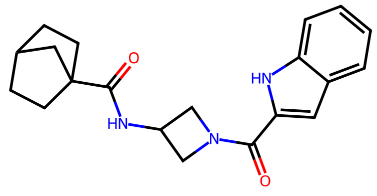	O=C(c1cc2ccccc2[nH]1)N1CC(NC(=O)C23CCC(CC2)C3)C1	995094902	25	337.18	2.69
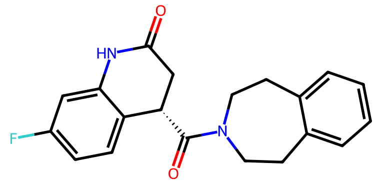	O=C1C[C@H](C(=O)N2CCc3ccccc3CC2)c2ccc(F)cc2N1	116071686	25	338.14	2.88
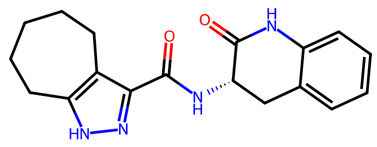	O=C(N[C@H]1Cc2ccccc2NC1=O)c1n[nH]c2c1CCCCC2	529949005	24	324.16	1.97
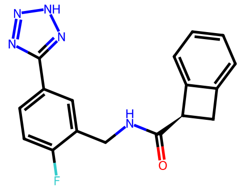	O=C(NCc1cc(-c2nn[nH]n2)ccc1F)[C@@H]1Cc2ccccc21	823234815	24	323.12	1.96
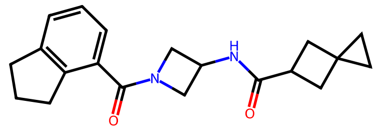	O=C(NC1CN(C(=O)c2cccc3c2CCC3)C1)C1CC2(CC2)C1	999336048	24	324.18	2.31
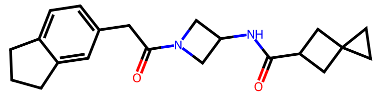	O=C(NC1CN(C(=O)Cc2ccc3c(c2)CCC3)C1)C1CC2(CC2)C1	999346252	25	338.2	2.23
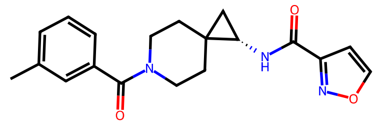	Cc1cccc(C(=O)N2CCC3(CC2)C[C@@H]3NC(=O)c2ccon2)c1	963276048	25	339.16	2.41
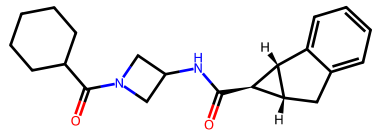	O=C(NC1CN(C(=O)C2CCCCC2)C1)[C@@H]1[C@H]2Cc3ccccc3[C@H]21	991647254	25	338.2	2.48

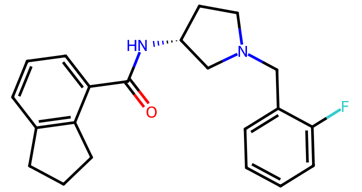	O=C(N[C@@H]1CCN(Cc2ccccc2F)C1)c1cccc2c1CCC2	1016382194	25	338.18	3.32
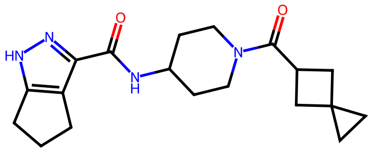	O=C(NC1CCN(C(=O)C2CC3(CC3)C2)CC1)c1n[nH]c2c1CCC2	948305950	25	342.21	1.81
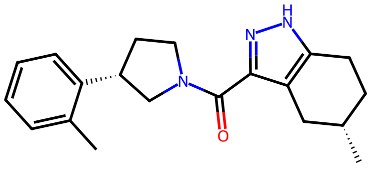	Cc1ccccc1[C@@H]1CCN(C(=O)c2n[nH]c3c2C[C@@H](C)CC3)C1	1142810720	24	323.2	3.47
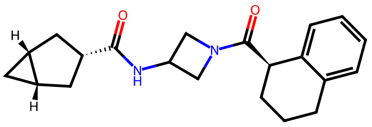	O=C(NC1CN(C(=O)[C@@H]2CCCc3ccccc32)C1)[C@@H]1C[C@@H]2C[C@@H]2C1	998005793	25	338.2	2.48
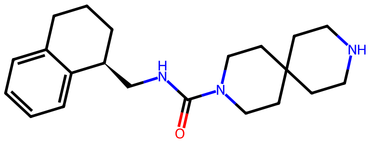	O=C(NC[C@@H]1CCCc2ccccc21)N1CCC2(CCNCC2)CC1	417069540	25	341.25	3.28
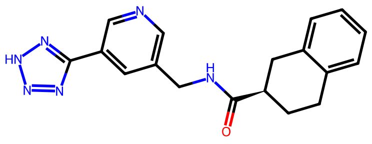	O=C(NCc1cncc(-c2nn[nH]n2)c1)[C@@H]1CCc2ccccc2C1	1582557448	25	334.15	1.68
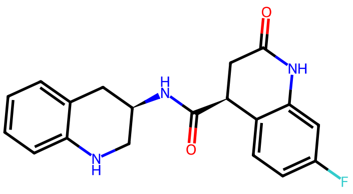	O=C1C[C@H](C(=O)N[C@H]2CNc3ccccc3C2)c2ccc(F)cc2N1	653205577	25	339.14	2.4
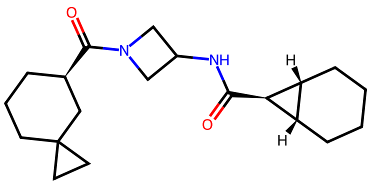	O=C(NC1CN(C(=O)[C@@H]2CCCC3(CC3)C2)C1)[C@@H]1[C@H]2CCCC[C@H]21	990651874	24	330.23	2.72

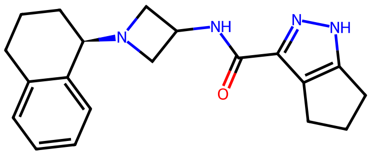	O=C(NC1CN([C@@H]2CCCc3ccccc32)C1)c1n[nH]c2c1CCC2	1030597966	25	336.2	2.39
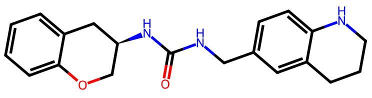	O=C(NCc1ccc2c(c1)CCCN2)N[C@H]1COc2ccccc2C1	579452371	25	337.18	2.85
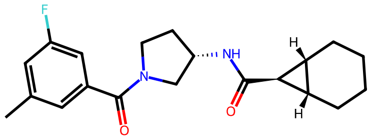	Cc1cc(F)cc(C(=O)N2CC[C@H](NC(=O)[C@@H]3[C@H]4CCCC[C@H]43)C2)c1	937794556	25	344.19	2.9
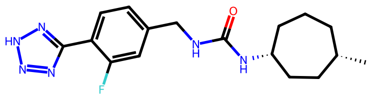	C[C@H]1CCC[C@@H](NC(=O)NCc2ccc(-c3nn[nH]n3)c(F)c2)CC1	1609162746	25	346.19	2.77
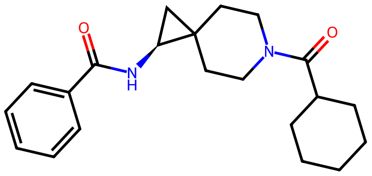	O=C(N[C@H]1CC12CCN(C(=O)C1CCCCC1)CC2)c1ccccc1	962786785	25	340.22	3.38
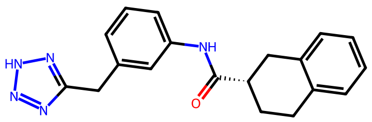	O=C(Nc1cccc(Cc2nn[nH]n2)c1)[C@H]1CCc2ccccc2C1	910048433	25	333.16	2.53
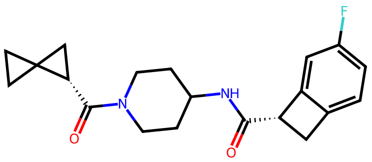	O=C(NC1CCN(C(=O)[C@H]2CC23CC3)CC1)[C@H]1Cc2ccc(F)cc21	948568395	25	342.17	2.37
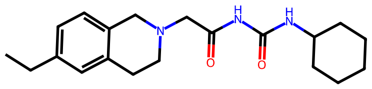	CCc1ccc2c(c1)CCN(CC(=O)NC(=O)NC1CCCCC1)C2	1434494560	25	343.23	2.77

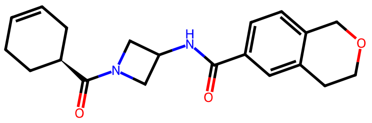	O=C(NC1CN(C(=O)[C@@H]2CC=CCC2)C1)c1ccc2c(c1)CCOC2	996918766	25	340.18	2.06
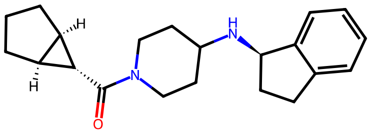	O=C([C@@H]1[C@H]2CCC[C@H]21)N1CCC(N[C@@H]2CCc3ccccc32)CC1	990770127	24	324.22	3.3
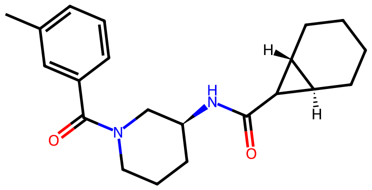	Cc1cccc(C(=O)N2CCC[C@H](NC(=O)C3[C@@H]4CCCC[C@@H]34)C2)c1	949869537	25	340.22	3.15
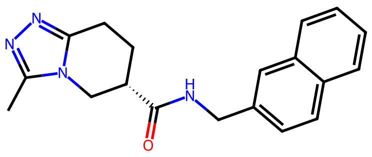	Cc1nnc2n1C[C@@H](C(=O)NCc1ccc3ccccc3c1)CC2	750720034	24	320.16	2.62
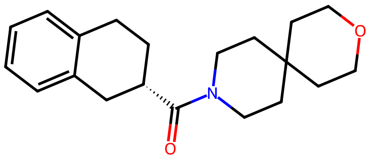	O=C([C@H]1CCc2ccccc2C1)N1CCC2(CCOCC2)CC1	650011911	23	313.2	3.21
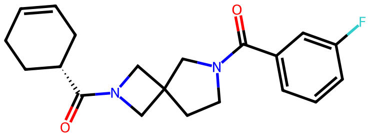	O=C(c1cccc(F)c1)N1CCC2(C1)CN(C(=O)[C@H]1CC=CCC1)C2	1018769540	25	342.17	2.86
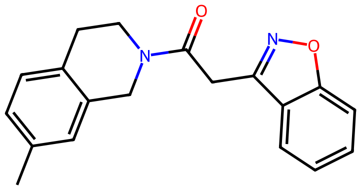	Cc1ccc2c(c1)CN(C(=O)Cc1noc3ccccc13)CC2	617602501	23	306.14	3.26
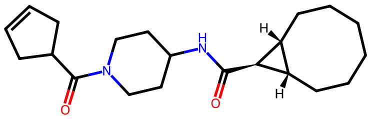	O=C(NC1CCN(C(=O)C2CC=CC2)CC1)[C@@H]1[C@H]2CCCCCC[C@H]21	945717726	25	344.25	3.28

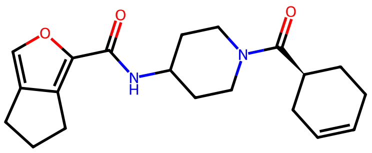	O=C(NC1CCN(C(=O)[C@@H]2CC=CCC2)CC1)c1occ2c1CCC2	944127312	25	342.19	2.85
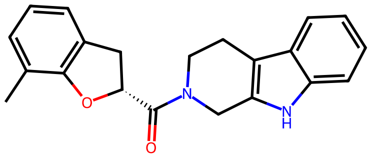	Cc1cccc2c1O[C@@H](C(=O)N1CCc3c([nH]c4ccccc34)C1)C2	450276639	25	332.15	3.36
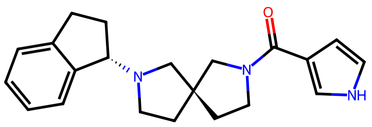	O=C(c1cc[nH]c1)N1CC[C@]2(CCN([C@H]3CCc4ccccc43)C2)C1	1041312084	25	335.2	3.24
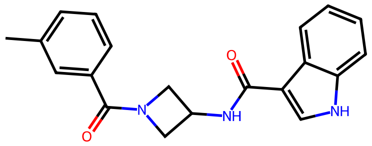	Cc1cccc(C(=O)N2CC(NC(=O)c3c[nH]c4ccccc34)C2)c1	995031373	25	333.15	2.73
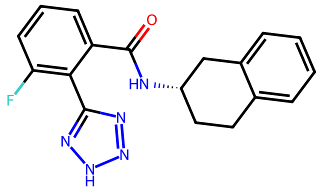	O=C(N[C@H]1CCc2ccccc2C1)c1cccc(F)c1-c1nn[nH]n1	1592995906	25	337.13	2.29
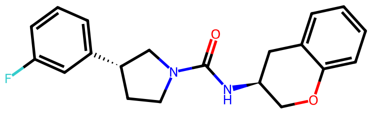	O=C(N[C@@H]1COc2ccccc2C1)N1CC[C@H](c2cccc(F)c2)C1	552634905	25	340.16	3.33
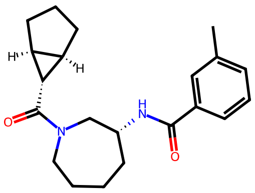	Cc1cccc(C(=O)N[C@@H]2CCCCN(C(=O)[C@@H]3[C@H]4CCC[C@H]43)C2)c1	943063360	25	340.22	3.15
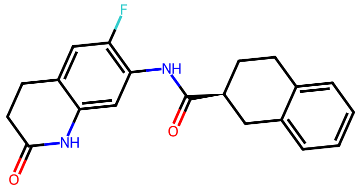	O=C1CCc2cc(F)c(NC(=O)[C@H]3CCc4ccccc4C3)cc2N1	569582395	25	338.14	3.45

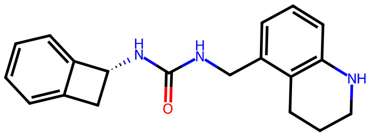	O=C(NCc1cccc2c1CCCN2)N[C@@H]1Cc2ccccc21	1548923833	23	307.17	3.14
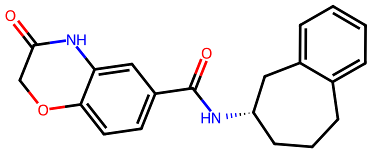	O=C1COc2ccc(C(=O)N[C@H]3CCCc4ccccc4C3)cc2N1	888254504	25	336.15	2.69
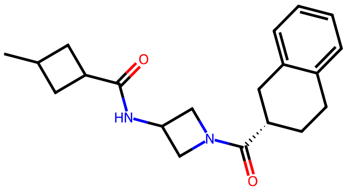	CC1CC(C(=O)NC2CN(C(=O)[C@H]3CCc4ccccc4C3)C2)C1	990755015	24	326.2	2.16
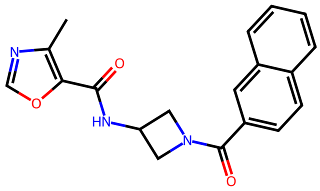	Cc1ncoc1C(=O)NC1CN(C(=O)c2ccc3ccccc3c2)C1	990659287	25	335.13	2.39
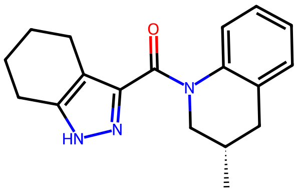	C[C@H]1Cc2ccccc2N(C(=O)c2n[nH]c3c2CCCC3)C1	62222684	22	295.17	3.13
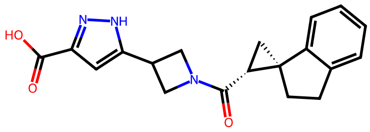	O=C(O)c1cc(C2CN(C(=O)[C@@H]3C[C@]34CCc3ccccc34)C2)[nH]n1	1602253152	25	337.14	1.94
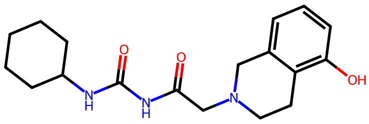	O=C(CN1CCc2c(O)cccc2C1)NC(=O)NC1CCCCC1	599645963	24	331.19	1.91
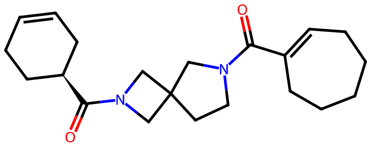	O=C(C1=CCCCCC1)N1CCC2(C1)CN(C(=O)[C@@H]1CC=CCC1)C2	1018770073	25	342.23	3.29

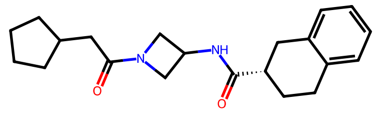	O=C(NC1CN(C(=O)CC2CCCC2)C1)[C@H]1CCc2ccccc2C1	1000540141	25	340.22	2.7
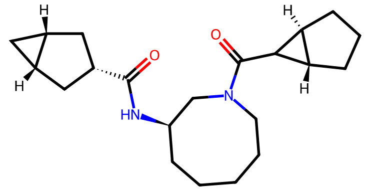	O=C(N[C@@H]1CCCCCN(C(=O)C2[C@H]3CCC[C@H]23)C1)[C@@H]1C[C@@H]2C[C@@H]2C1	986984636	25	344.25	2.97
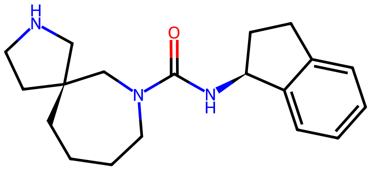	O=C(N[C@H]1CCc2ccccc21)N1CCCC[C@]2(CCNC2)C1	1552956350	23	313.22	2.85
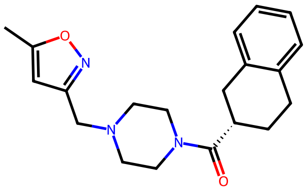	Cc1cc(CN2CCN(C(=O)[C@H]3CCc4ccccc4C3)CC2)no1	583938293	25	339.19	2.43
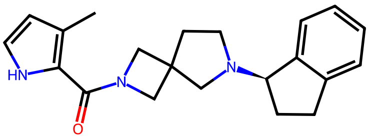	Cc1cc[nH]c1C(=O)N1CC2(CCN([C@@H]3CCc4ccccc43)C2)C1	1072683702	25	335.2	3.16
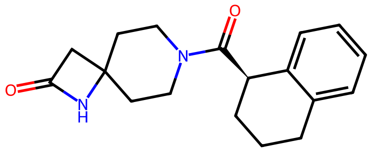	O=C1CC2(CCN(C(=O)[C@@H]3CCCc4ccccc43)CC2)N1	1325280061	22	298.17	1.99
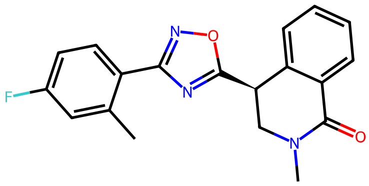	Cc1cc(F)ccc1-c1noc([C@@H]2CN(C)C(=O)c3ccccc32)n1	377514950	25	337.12	3.4
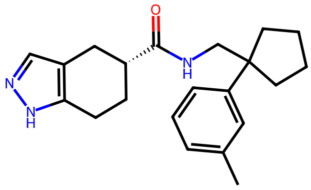	Cc1ccc(C2(CNC(=O)[C@@H]3CCc4[nH]ncc4C3)CCCC2)c1	270183609	25	337.22	3.45

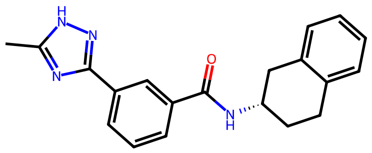	Cc1nc(-c2cccc(C(=O)N[C@H]3CCc4ccccc4C3)c2)n[nH]1	81112572	25	332.16	3.07
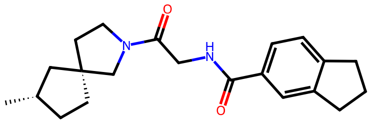	C[C@H]1CC[C@@]2(CCN(C(=O)CNC(=O)c3ccc4c(c3)CCC4)C2)C1	120445254	25	340.22	2.94
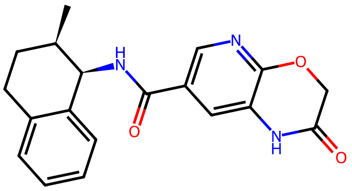	C[C@@H]1CCc2ccccc2[C@@H]1NC(=O)c1cnc2c(c1)NC(=O)CO2	271422755	25	337.14	2.47
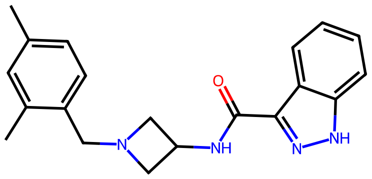	Cc1ccc(CN2CC(NC(=O)c3n[nH]c4ccccc34)C2)c(C)c1	1031341877	25	334.18	2.79
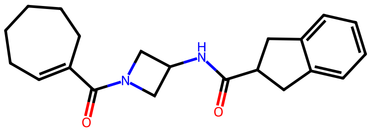	O=C(NC1CN(C(=O)C2=CCCCCC2)C1)C1Cc2ccccc2C1	1000454143	25	338.2	2.62
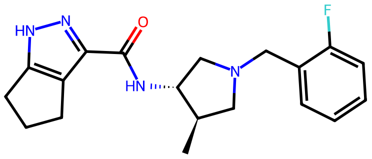	C[C@@H]1CN(Cc2ccccc2F)C[C@H]1NC(=O)c1n[nH]c2c1CCC2	1080026418	25	342.19	2.29
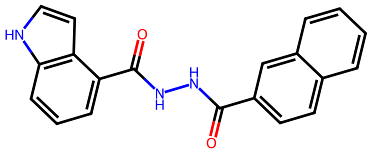	O=C(NNC(=O)c1cccc2[nH]ccc12)c1ccc2ccccc2c1	81555818	25	329.12	3.4
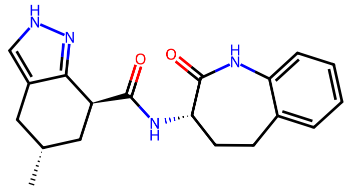	C[C@@H]1Cc2c[nH]nc2[C@@H](C(=O)N[C@H]2CCc3ccccc3NC2=O)C1	623225356	25	338.17	2.15

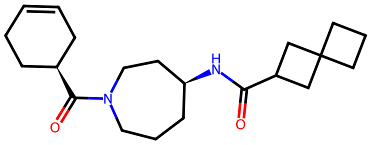	O=C(N[C@H]1CCCN(C(=O)[C@@H]2CC=CCC2)CC1)C1CC2(CCC2)C1	965098553	25	344.25	3.42
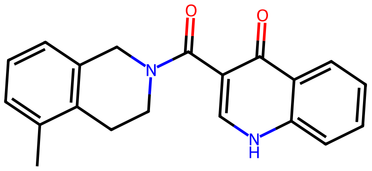	Cc1cccc2c1CCN(C(=O)c1c[nH]c3cccc c3c1=O)C2	171542044	24	318.14	3.04
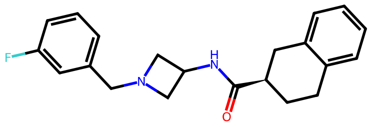	O=C(NC1CN(Cc2cccc(F)c2)C1)[C@@H]1CCc2ccccc2C1	1031012236	25	338.18	2.93
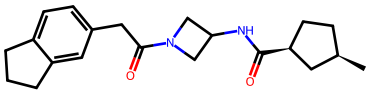	C[C@@H]1CC[C@H](C(=O)NC2CN(C(=O)Cc3ccc4c(c3)CCC4)C2)C1	991428365	25	340.22	2.48
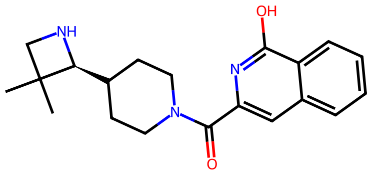	CC1(C)CN[C@H]1C1CCN(C(=O)c2cc3ccccc3c(O)n2)CC1	424048418	25	339.19	2.79
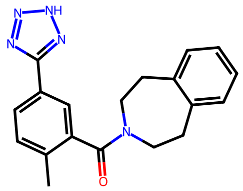	Cc1ccc(-c2nn[nH]n2)cc1C(=O)N1CCc2ccccc2CC1	1592096476	25	333.16	2.42
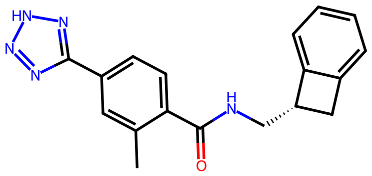	Cc1cc(-c2nn[nH]n2)ccc1C(=O)NC[C@H]1Cc2ccccc21	826248273	24	319.14	2.24
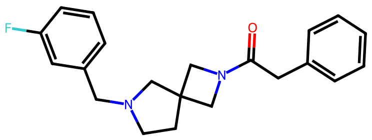	O=C(Cc1ccccc1)N1CC2(CCN(Cc3cccc(F)c3)C2)C1	1072862318	25	338.18	3.1

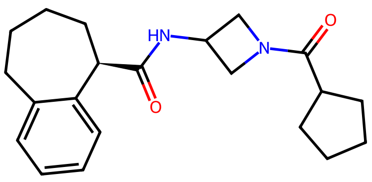	O=C(NC1CN(C(=O)C2CCCC2)C1)[C@@H]1CCCCc2ccccc21	994322329	25	340.22	3.01
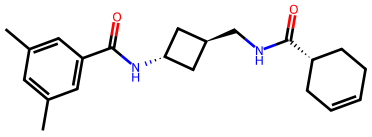	Cc1cc(C)cc(C(=O)N[C@H]2C[C@H](CNC(=O)[C@H]3CC=CCC3)C2)c1	979485721	25	340.22	3.28
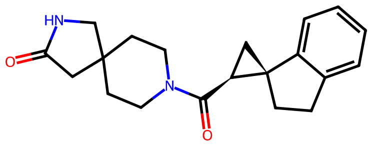	O=C1CC2(CCN(C(=O)[C@H]3C[C@@]34CCc3ccccc34)CC2)CN1	267619871	24	324.18	2.02
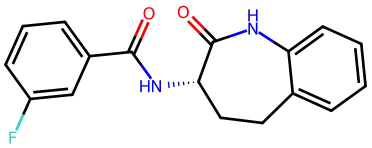	O=C(N[C@H]1CCc2ccccc2NC1=O)c1cccc(F)c1	1361647443	22	298.11	2.51
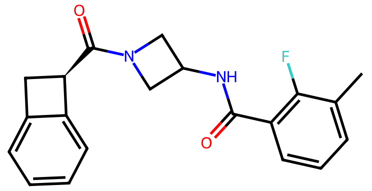	Cc1cccc(C(=O)NC2CN(C(=O)[C@@H]3Cc4ccccc43)C2)c1F	993678311	25	338.14	2.41
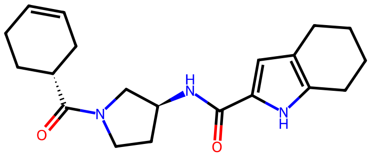	O=C(N[C@H]1CCN(C(=O)[C@H]2CC=CCC2)C1)c1cc2c([nH]1)CCCC2	938789834	25	341.21	2.58
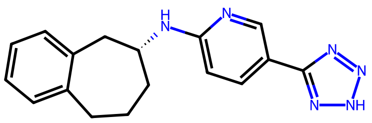	c1ccc2c(c1)CCC[C@@H](Nc1ccc(-c3nn[nH]n3)cn1)C2	1593581948	23	306.16	2.62
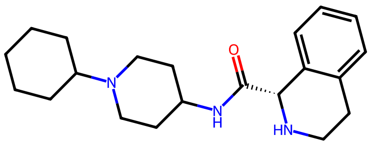	O=C(NC1CCN(C2CCCCC2)CC1)[C@H]1NCCc2ccccc21	237990454	25	341.25	2.79

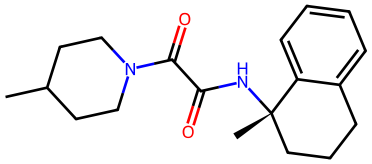	CC1CCN(C(=O)C(=O)N[C@@]2(C)CCCc3ccccc32)CC1	444144270	23	314.2	2.61
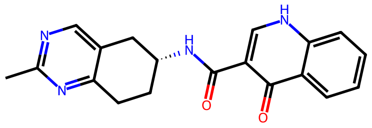	Cc1ncc2c(n1)CC[C@@H](NC(=O)c1c[nH]c3ccccc3c1=O)C2	360568532	25	334.14	1.91
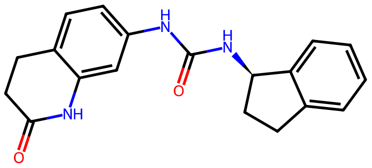	O=C1CCc2ccc(NC(=O)N[C@@H]3CCc4ccccc43)cc2N1	266378615	24	321.15	3.38
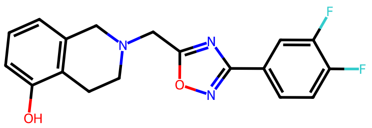	Oc1cccc2c1CCN(Cc1nc(-c3ccc(F)c(F)c3)no1)C2	1659688131	25	343.11	3.28
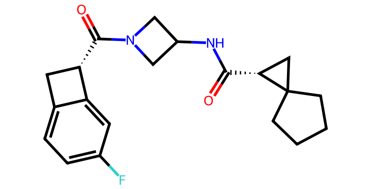	O=C(NC1CN(C(=O)[C@H]2Cc3ccc(F)cc32)C1)[C@@H]1CC12CCCC2	999626536	25	342.17	2.37
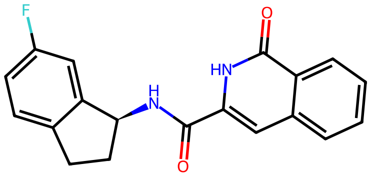	O=C(N[C@H]1CCc2ccc(F)cc21)c1cc2ccccc2c(=O)[nH]1	106797655	24	322.11	3.08
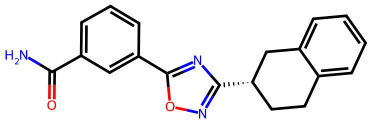	NC(=O)c1cccc(-c2nc([C@H]3CCc4ccccc4C3)no2)c1	680773703	24	319.13	3.11
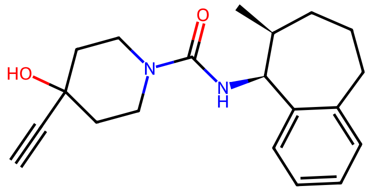	C#CC1(O)CCN(C(=O)N[C@@H]2c3ccccc3CCC[C@@H]2C)CC1	1345843217	24	326.2	2.87

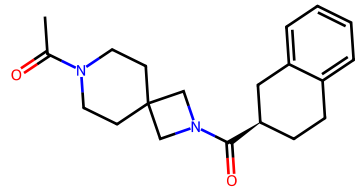	CC(=O)N1CCC2(CC1)CN(C(=O)[C@@H]1CCc3ccccc3C1)C2	944809116	24	326.2	2.26
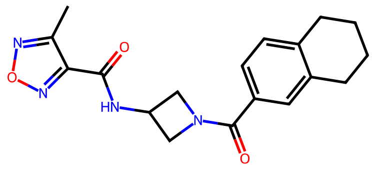	Cc1nonc1C(=O)NC1CN(C(=O)c2ccc3c(c2)CCCC3)C1	994942364	25	340.15	1.51
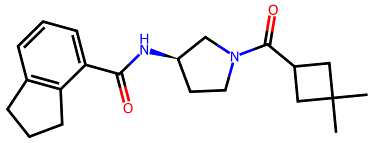	CC1(C)CC(C(=O)N2CC[C@@H](NC(=O)c3cccc4c3CCC4)C2)C1	941157595	25	340.22	2.94
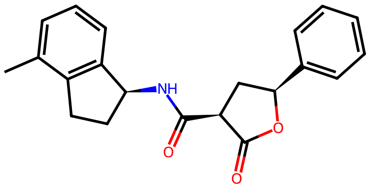	Cc1cccc2c1CC[C@@H]2NC(=O)[C@H]1C[C@@H](c2ccccc2)OC1=O	809666561	25	335.15	3.4
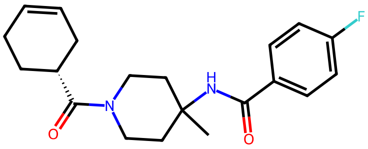	CC1(NC(=O)c2ccc(F)cc2)CCN(C(=O)[C@H]2CC=CCC2)CC1	956270903	25	344.19	3.29
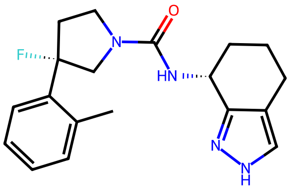	Cc1ccccc1[C@@]1(F)CCN(C(=O)N[C@@H]2CCCc3c[nH]nc32)C1	554600166	25	342.19	3.38
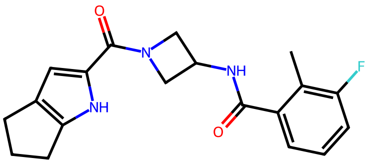	Cc1c(F)cccc1C(=O)NC1CN(C(=O)c2cc3c([nH]2)CCC3)C1	993590034	25	341.15	2.21
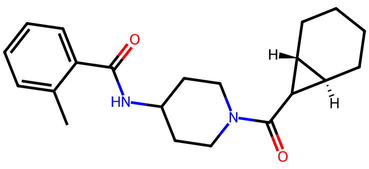	Cc1ccccc1C(=O)NC1CCN(C(=O)C2[C@@H]3CCCC[C@@H]23)CC1	174637970	25	340.22	3.15

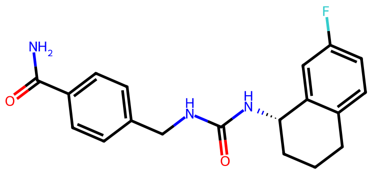	NC(=O)c1ccc(CNC(=O)N[C@H]2CCCc3ccc(F)cc32)cc1	93355069	25	341.15	2.8
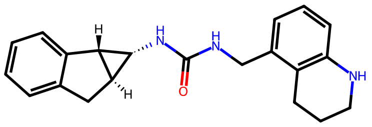	O=C(NCc1cccc2c1CCCN2)N[C@@H]1[C@H]2Cc3ccccc3[C@@H]21	573701145	25	333.18	3.18
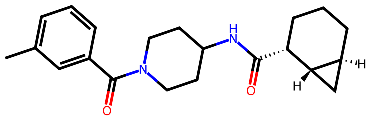	Cc1cccc(C(=O)N2CCC(NC(=O)[C@@H]3CCC[C@H]4C[C@@H]43)CC2)c1	943833612	25	340.22	3.15
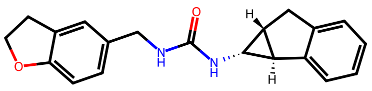	O=C(NCc1ccc2c(c1)CCO2)N[C@H]1[C@@H]2c3ccccc3C[C@H]21	532173591	24	320.15	2.76
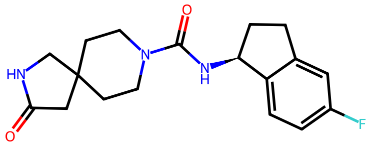	O=C1CC2(CCN(C(=O)N[C@H]3CCc4cc(F)ccc43)CC2)CN1	73834868	24	331.17	2.12
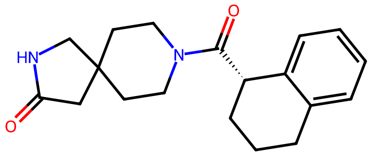	O=C1CC2(CCN(C(=O)[C@H]3CCCc4ccccc43)CC2)CN1	267751681	23	312.18	2.24
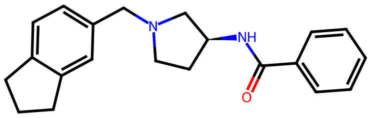	O=C(N[C@H]1CCN(Cc2ccc3c(c2)CCC3)C1)c1ccccc1	1009949227	24	320.19	3.18
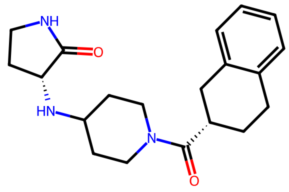	O=C1NCC[C@H]1NC1CCN(C(=O)[C@H]2CCc3ccccc3C2)CC1	997036732	25	341.21	1.26

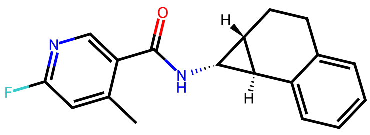	Cc1cc(F)ncc1C(=O)N[C@H]1[C@@H]2c3ccccc3CC[C@H]21	866831348	22	296.13	2.99
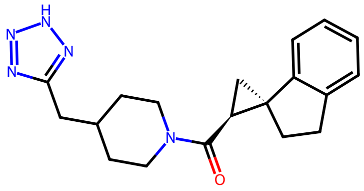	O=C([C@H]1C[C@]12CCc1ccccc12)N1CCC(Cc2nn[nH]n2)CC1	1548804791	25	337.19	1.88
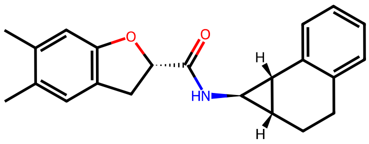	Cc1cc2c(cc1C)O[C@H](C(=O)N[C@@H]1[C@H]3CCc4ccccc4[C@H]31)C2	435966334	25	333.17	3.45
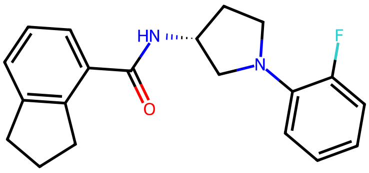	O=C(N[C@@H]1CCN(c2ccccc2F)C1)c1cccc2c1CCC2	345247247	24	324.16	3.32
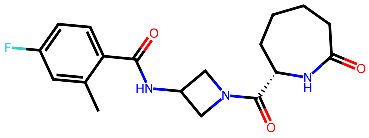	Cc1cc(F)ccc1C(=O)NC1CN(C(=O)[C@@H]2CCCCC(=O)N2)C1	997178481	25	347.16	1.13
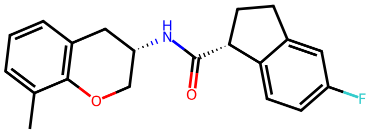	Cc1cccc2c1OC[C@@H](NC(=O)[C@@H]1CCc3cc(F)ccc31)C2	615059819	24	325.15	3.28
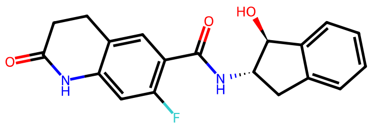	O=C1CCc2cc(C(=O)N[C@H]3Cc4ccccc4[C@@H]3O)c(F)cc2N1	178695304	25	340.12	2.1
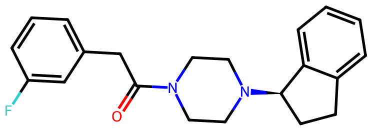	O=C(Cc1cccc(F)c1)N1CCN([C@@H]2CCc3ccccc32)CC1	957558376	25	338.18	3.2

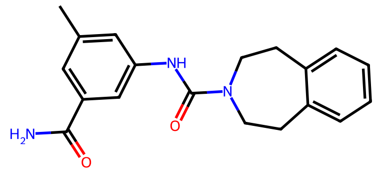	Cc1cc(NC(=O)N2CCc3ccccc3CC2)cc(C(N)=O)c1	581472369	24	323.16	2.73
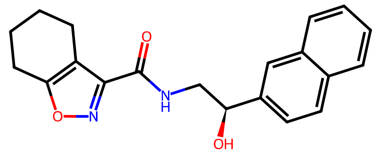	O=C(NC[C@H](O)c1ccc2ccccc2c1)c1noc2c1CCCC2	109691396	25	336.15	3.17
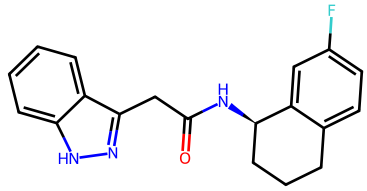	O=C(Cc1n[nH]c2ccccc12)N[C@@H]1CCCc2ccc(F)cc21	89392166	24	323.14	3.44
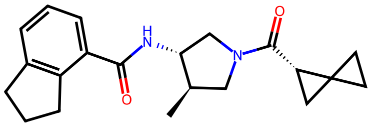	C[C@@H]1CN(C(=O)[C@H]2CC23CC3)C[C@H]1NC(=O)c1cccc2c1CCC2	959763893	25	338.2	2.55
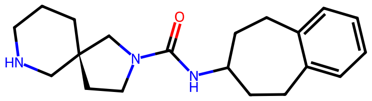	O=C(NC1CCc2ccccc2CC1)N1CC[C@@]2(CCCNC2)C1	514850129	24	327.23	2.72
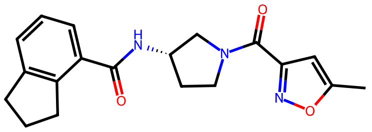	Cc1cc(C(=O)N2CC[C@H](NC(=O)c3cccc4c3CCC4)C2)no1	938146884	25	339.16	2.12
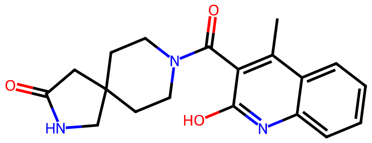	Cc1c(C(=O)N2CCC3(CC2)CNC(=O)C3)c(O)nc2ccccc12	267603462	25	339.16	1.99
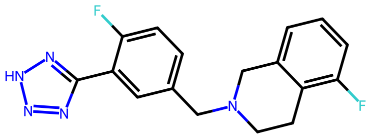	Fc1ccc(CN2CCc3c(F)cccc3C2)cc1-c1nn[nH]n1	826374802	24	327.13	2.7

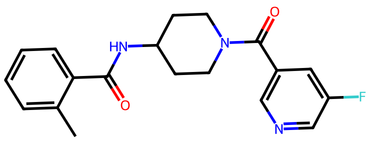	Cc1ccccc1C(=O)NC1CCN(C(=O)c2cncc(F)c2)CC1	121173846	25	341.15	2.56
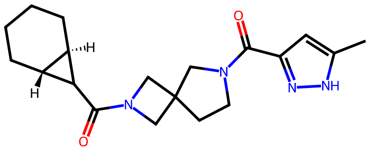	Cc1cc(C(=O)N2CCC3(C2)CN(C(=O)C2[C@@H]4CCCC[C@@H]24)C3)n[nH]1	1018904672	25	342.21	1.83
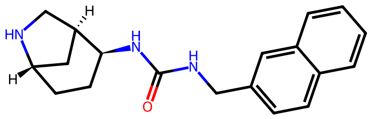	O=C(NCc1ccc2ccccc2c1)N[C@H]1CC[C@H]2C[C@H]1CN2	1551519508	23	309.18	2.78
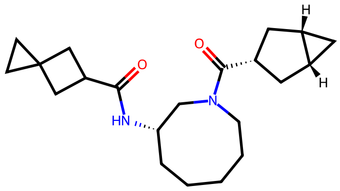	O=C(N[C@H]1CCCCCN(C(=O)[C@@H]2C[C@@H]3C[C@@H]3C2)C1)C1CC2(CC2)C1	987118565	25	344.25	3.11
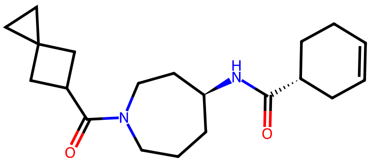	O=C(N[C@H]1CCCN(C(=O)C2CC3(CC3)C2)CC1)[C@H]1CC=CCC1	964740737	24	330.23	3.03
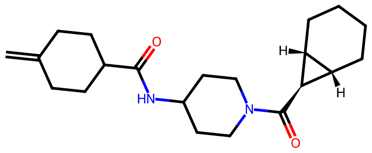	C=C1CCC(C(=O)NC2CCN(C(=O)[C@@H]3[C@H]4CCCC[C@H]43)CC2)CC1	945958148	25	344.25	3.28
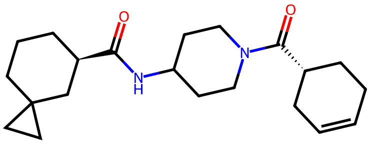	O=C(NC1CCN(C(=O)[C@H]2CC=CCC2)CC1)[C@@H]1CCCC2(CC2)C1	944106677	25	344.25	3.42
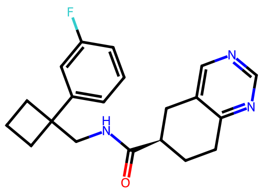	O=C(NCC1(c2cccc(F)c2)CCC1)[C@@H]1CCc2ncncc2C1	830870519	25	339.17	2.96

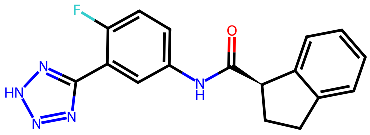	O=C(Nc1ccc(F)c(-c2nn[nH]n2)c1)[C@@H]1CCc2ccccc21	738160832	24	323.12	2.67
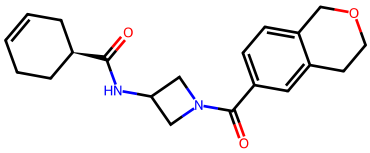	O=C(NC1CN(C(=O)c2ccc3c(c2)CCOC3)C1)[C@@H]1CC=CCC1	990213184	25	340.18	2.06
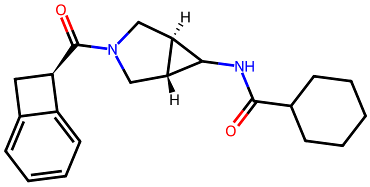	O=C(NC1[C@@H]2CN(C(=O)[C@@H]3Cc4ccccc43)C[C@@H]12)C1CCCCC1	1012708695	25	338.2	2.48
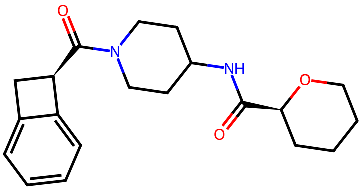	O=C(NC1CCN(C(=O)[C@@H]2Cc3ccccc32)CC1)[C@@H]1CCCCO1	948059461	25	342.19	2.0
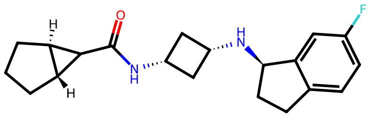	O=C(N[C@H]1C[C@@H](N[C@@H]2CCc3cc(F)cc32)C1)C1[C@@H]2CCC[C@@H]12	1021941881	24	328.2	3.1
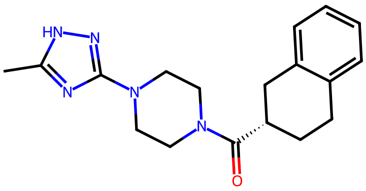	Cc1nc(N2CCN(C(=O)[C@H]3CCc4ccccc4C3)CC2)n[nH]1	898184622	24	325.19	1.57
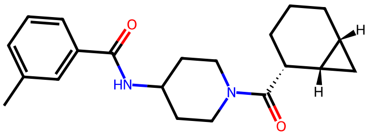	Cc1cccc(C(=O)NC2CCN(C(=O)[C@@H]3CCC[C@@H]4C[C@@H]43)CC2)c1	870838810	25	340.22	3.15
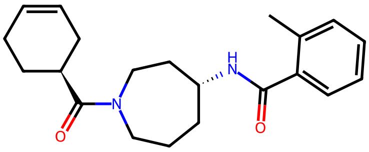	Cc1ccccc1C(=O)N[C@@H]1CCCN(C(=O)[C@@H]2CC=CCC2)CC1	964705516	25	340.22	3.46

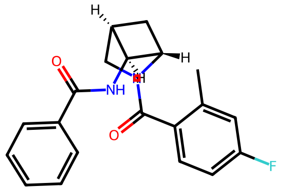	Cc1cc(F)ccc1C(=O)N1C[C@H]2C[C@H]1[C@H]2NC(=O)c1ccccc1	946597204	25	338.14	2.78
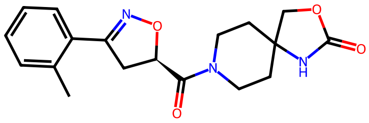	Cc1ccccc1C1=NO[C@@H](C(=O)N2CCC3(CC2)COC(=O)N3)C1	907337711	25	343.15	1.59
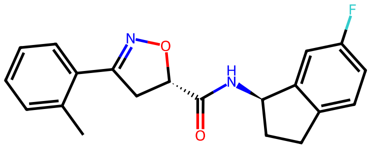	Cc1ccccc1C1=NO[C@H](C(=O)N[C@@H]2CCc3ccc(F)cc32)C1	1360016918	25	338.14	3.43
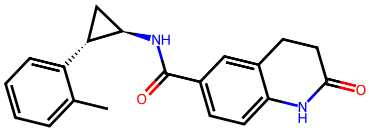	Cc1ccccc1[C@@H]1C[C@H]1NC(=O)c1ccc2c(c1)CCC(=O)N2	154575931	24	320.15	3.17
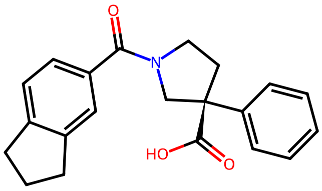	O=C(c1ccc2c(c1)CCC2)N1CC[C@](C(=O)O)(c2ccccc2)C1	569728926	25	335.15	3.04
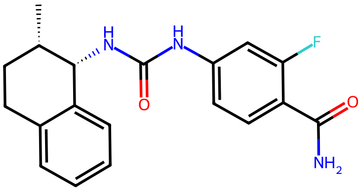	C[C@H]1CCc2ccccc2[C@H]1NC(=O)Nc1ccc(C(N)=O)c(F)c1	179538979	25	341.15	3.37
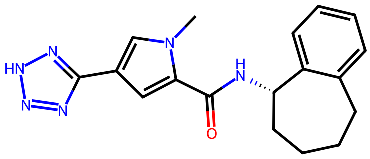	Cn1cc(-c2nn[nH]n2)cc1C(=O)N[C@H]1CCCCc2ccccc21	822632099	25	336.17	2.4
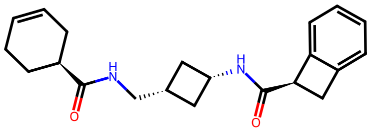	O=C(NC[C@H]1C[C@@H](NC(=O)[C@@H]2Cc3ccccc32)C1)[C@@H]1CC=CCC1	978582709	25	338.2	2.69

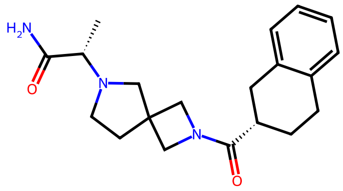	C[C@@H](C(N)=O)N1CCC2(CN(C(=O)[C@H]3CCc4ccccc4C3)C2)C1	1073013594	25	341.21	1.2
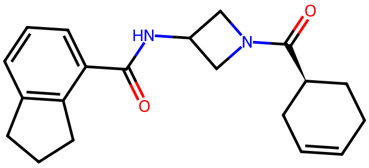	O=C(NC1CN(C(=O)[C@@H]2CC=CCC2)C1)c1cccc2c1CCC2	994397754	24	324.18	2.47
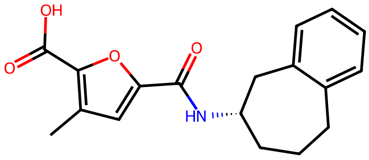	Cc1cc(C(=O)N[C@H]2CCCc3ccccc3C2)oc1C(=O)O	1607577641	23	313.13	2.96
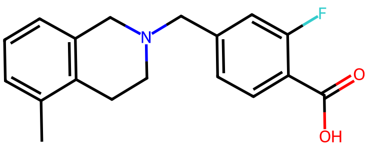	Cc1cccc2c1CCN(Cc1ccc(C(=O)O)c(F)c1)C2	832973462	22	299.13	3.39
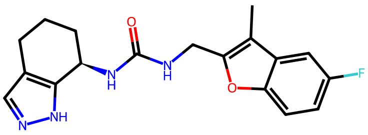	Cc1c(CNC(=O)N[C@@H]2CCCc3cn[nH]c32)oc2ccc(F)cc12	268868688	25	342.15	3.48
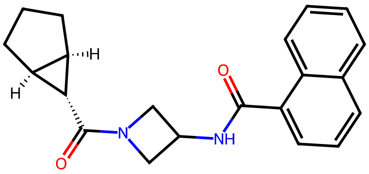	O=C(NC1CN(C(=O)[C@@H]2[C@H]3CCC[C@H]32)C1)c1cccc2ccccc12	999540248	25	334.17	2.83
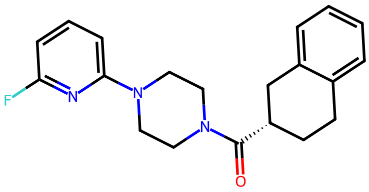	O=C([C@H]1CCc2ccccc2C1)N1CCN(c2cccc(F)n2)CC1	844560827	25	339.17	2.67
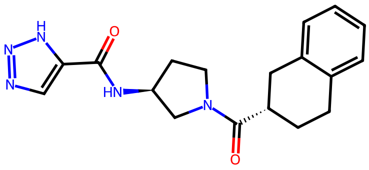	O=C(N[C@H]1CCN(C(=O)[C@H]2CCc3ccccc3C2)C1)c1cnn[nH]1	938241446	25	339.17	0.94

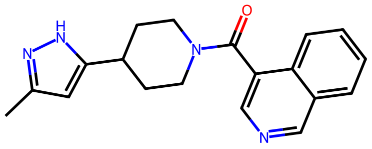	Cc1cc(C2CCN(C(=O)c3cncc4ccccc34)CC2)[nH]n1	273586237	24	320.16	3.29
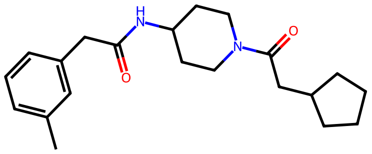	Cc1cccc(CC(=O)NC2CCN(C(=O)CC3CCCC3)CC2)c1	943769741	25	342.23	3.23
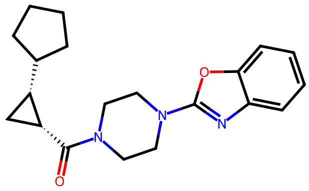	O=C([C@@H]1C[C@@H]1C1CCCC1)N1CCN(c2nc3ccccc3o2)CC1	651361196	25	339.19	3.3
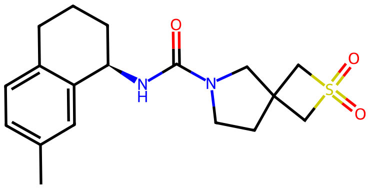	Cc1ccc2c(c1)[C@H](NC(=O)N1CCC3(C1)CS(=O)(=O)C3)CCC2	1550012887	24	348.15	2.2
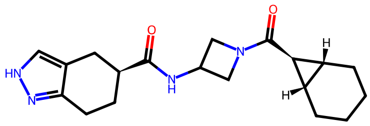	O=C(NC1CN(C(=O)[C@@H]2[C@H]3CCCC[C@H]32)C1)[C@H]1CCc2n[nH]cc2C1	996397685	25	342.21	1.28
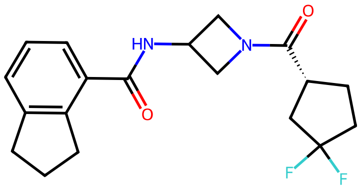	O=C(NC1CN(C(=O)[C@@H]2CCC(F)(F)C2)C1)c1cccc2c1CCC2	994395327	25	348.16	2.55
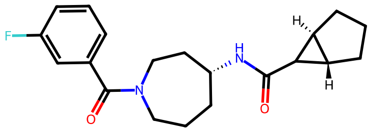	O=C(N[C@@H]1CCCN(C(=O)c2cccc(F)c2)CC1)C1[C@H]2CCC[C@H]12	964819277	25	344.19	2.98
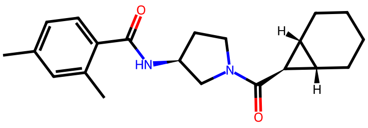	Cc1ccc(C(=O)N[C@H]2CCN(C(=O)[C@@H]3[C@H]4CCCC[C@H]43)C2)c(C)c1	938757798	25	340.22	3.07

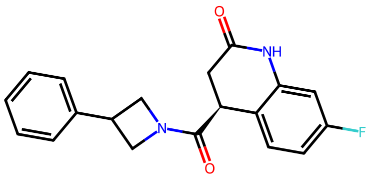	O=C1C[C@H](C(=O)N2CC(c3ccccc3)C2)c2ccc(F)cc2N1	633806366	24	324.13	2.88
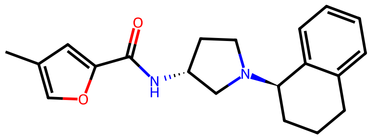	Cc1coc(C(=O)N[C@@H]2CCN([C@@H]3CCCc4ccccc43)C2)c1	1015573794	24	324.18	3.47
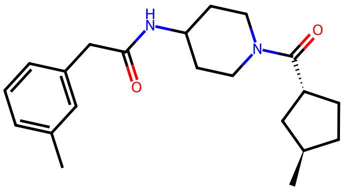	Cc1cccc(CC(=O)NC2CCN(C(=O)[C@@H]3CC[C@@H](C)C3)CC2)c1	946734662	25	342.23	3.08
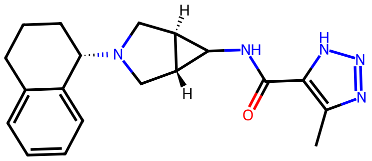	Cc1nn[nH]c1C(=O)NC1[C@@H]2CN([C@H]3CCCc4ccccc43)C[C@@H]12	961448321	25	337.19	1.85
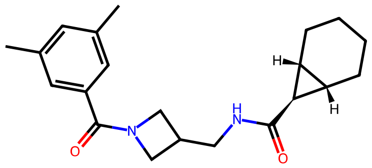	Cc1cc(C)cc(C(=O)N2CC(CNC(=O)[C@@H]3[C@H]4CCCC[C@H]43)C2)c1	1001322779	25	340.22	2.93
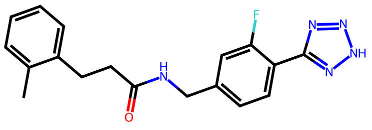	Cc1ccccc1CCC(=O)NCc1ccc(-c2nn[nH]n2)c(F)c1	1592255686	25	339.15	2.56
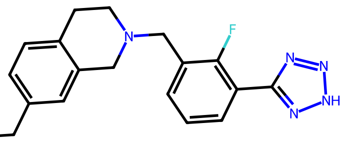	CCc1ccc2c(c1)CN(Cc1cccc(-c3nn[nH]n3)c1F)CC2	1604528731	25	337.17	3.13
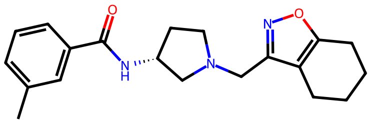	Cc1cccc(C(=O)N[C@@H]2CCN(Cc3noc4c3CCCC4)C2)c1	1010019306	25	339.19	2.87

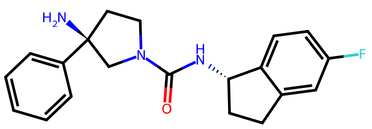	N[C@@]1(c2ccccc2)CCN(C(=O)N[C@H]2CCc3cc(F)ccc32)C1	1551528922	25	339.17	3.08
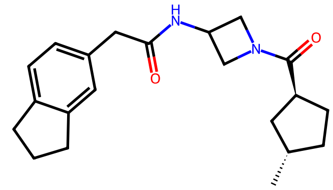	C[C@H]1CC[C@H](C(=O)N2CC(NC(=O)Cc3ccc4c(c3)CCC4)C2)C1	991384200	25	340.22	2.48
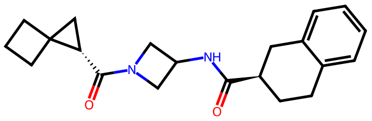	O=C(NC1CN(C(=O)[C@H]2CC23CCC3)C1)[C@@H]1CCc2ccccc2C1	1000545881	25	338.2	2.31
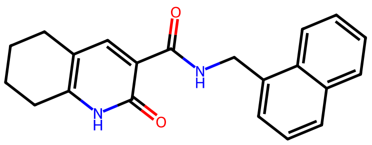	O=C(NCc1cccc2ccccc12)c1cc2c([nH]c1=O)CCCC2	68088274	25	332.15	3.34
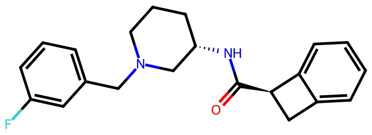	O=C(N[C@H]1CCCN(Cc2cccc(F)c2)C1)[C@@H]1Cc2ccccc21	1008359797	25	338.18	3.25
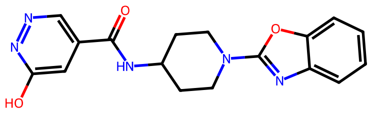	O=C(NC1CCN(c2nc3ccccc3o2)CC1)c1cnnc(O)c1	1057908708	25	339.13	1.72
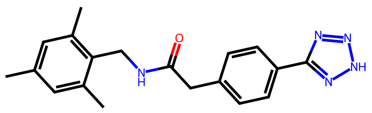	Cc1cc(C)c(CNC(=O)Cc2ccc(-c3nn[nH]n3)cc2)c(C)c1	1591946349	25	335.17	2.65
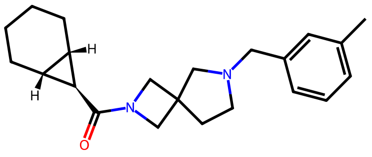	Cc1cccc(CN2CCC3(C2)CN(C(=O)[C@@H]2[C@H]4CCCC[C@H]42)C3)c1	1072580644	25	338.24	3.47

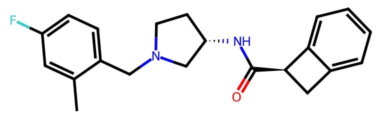	Cc1cc(F)ccc1CN1CC[C@H](NC(=O)[C@@H]2Cc3ccccc32)C1	1015636264	25	338.18	3.16
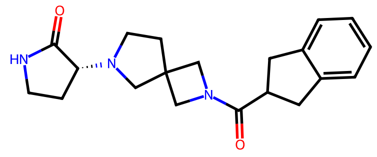	O=C1NCC[C@H]1N1CCC2(CN(C(=O)C3Cc4ccccc4C3)C2)C1	1073009956	25	339.19	0.82
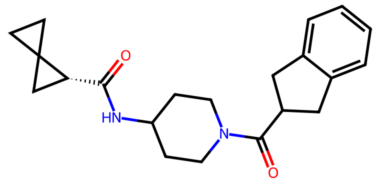	O=C(NC1CCN(C(=O)C2Cc3ccccc3C2)CC1)[C@@H]1CC12CC2	948551343	25	338.2	2.31
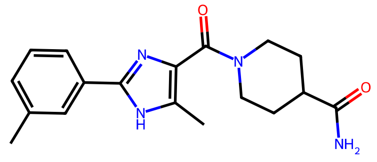	Cc1cccc(-c2nc(C(=O)N3CCC(C(N)=O)CC3)c(C)[nH]2)c1	270150666	24	326.17	2.03
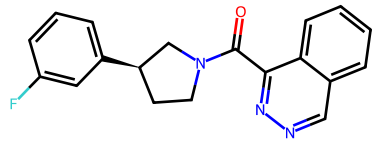	O=C(c1nncc2ccccc12)N1CC[C@@H](c2cccc(F)c2)C1	1519843546	24	321.13	3.4
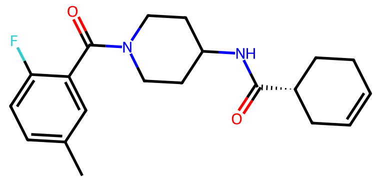	Cc1ccc(F)c(C(=O)N2CCC(NC(=O)[C@H]3CC=CCC3)CC2)c1	948572042	25	344.19	3.21
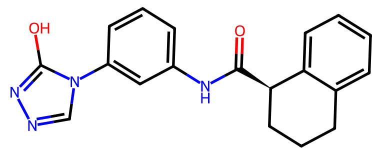	O=C(Nc1cccc(-n2cnnc2O)c1)[C@@H]1CCCc2ccccc21	364279208	25	334.14	3.03
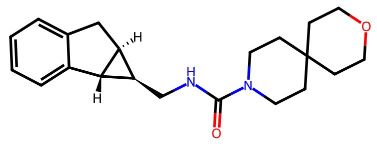	O=C(NC[C@@H]1[C@@H]2Cc3ccccc3[C@@H]12)N1CCC2(CCOCC2)CC1	285281114	25	340.22	3.17

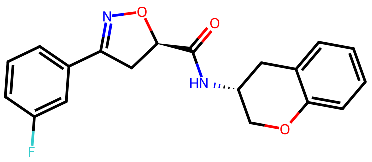	O=C(N[C@H]1COc2ccccc2C1)[C@H]1CC(c2cccc(F)c2)=NO1	194461073	25	340.12	2.44
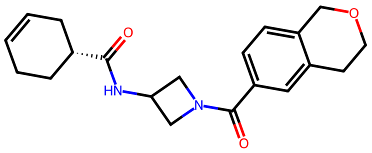	O=C(NC1CN(C(=O)c2ccc3c(c2)CCOC3)C1)[C@H]1CC=CCC1	990213182	25	340.18	2.06
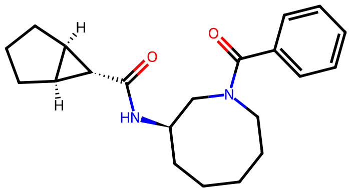	O=C(N[C@@H]1CCCCCN(C(=O)c2ccccc2)C1)[C@@H]1[C@H]2CCC[C@H]21	986852256	25	340.22	3.23
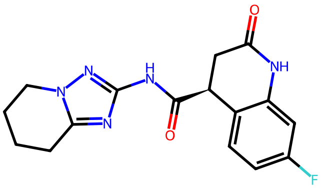	O=C1C[C@H](C(=O)Nc2nc3n(n2)CCCC3)c2ccc(F)cc2N1	333579239	24	329.13	1.82
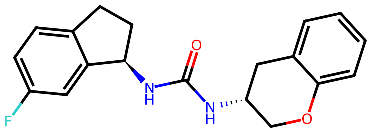	O=C(N[C@H]1COc2ccccc2C1)N[C@@H]1CCc2ccc(F)cc21	106536591	24	326.14	3.12
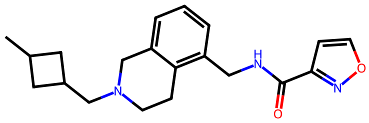	CC1CC(CN2CCc3c(CNC(=O)c4ccon4)cccc3C2)C1	1051282156	25	339.19	3.01
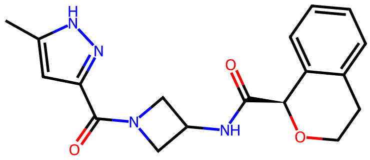	Cc1cc(C(=O)N2CC(NC(=O)[C@@H]3OCCc4ccccc43)C2)n[nH]1	997325485	25	340.15	0.97
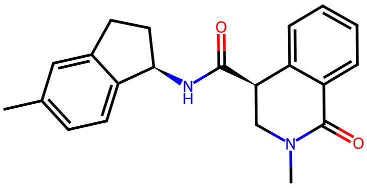	Cc1ccc2c(c1)CC[C@H]2NC(=O)[C@@H]1CN(C)C(=O)c2ccccc21	345861740	25	334.17	2.97

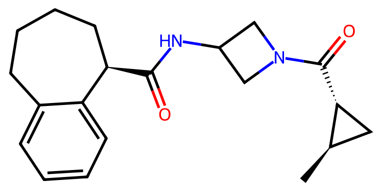	C[C@@H]1C[C@H]1C(=O)N1CC(NC(=O)[C@@H]2CCCCc3ccccc32)C1	994323839	24	326.2	2.48
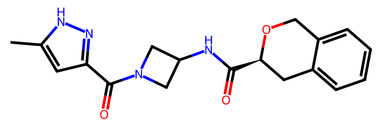	Cc1cc(C(=O)N2CC(NC(=O)[C@@H]3Cc4ccccc4CO3)C2)n[nH]1	997728484	25	340.15	0.8
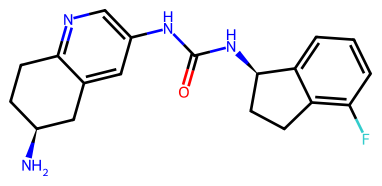	N[C@H]1CCc2ncc(NC(=O)N[C@@H]3CCc4c(F)cccc43)cc2C1	333286786	25	340.17	2.85
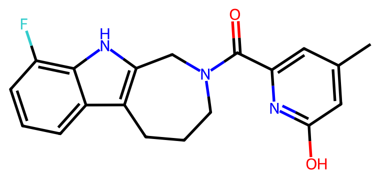	Cc1cc(O)nc(C(=O)N2CCCc3c([nH]c4c(F)cccc34)C2)c1	560491534	25	339.14	3.3
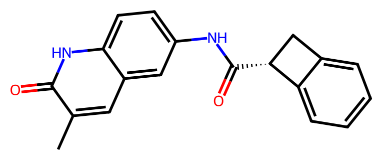	Cc1cc2cc(NC(=O)[C@@H]3Cc4ccccc43)ccc2[nH]c1=O	1569494293	23	304.12	3.11
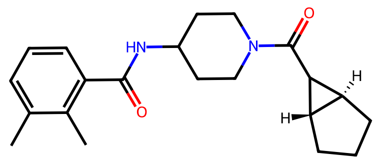	Cc1cccc(C(=O)NC2CCN(C(=O)C3[C@H]4CCC[C@H]34)CC2)c1C	946079489	25	340.22	3.07
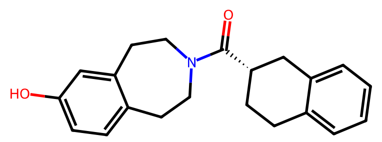	O=C([C@H]1CCc2ccccc2C1)N1CCc2ccc(O)cc2CC1	653018830	24	321.17	3.12
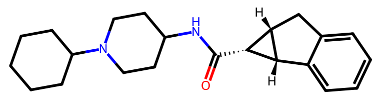	O=C(NC1CCN(C2CCCCC2)CC1)[C@H]1[C@H]2c3ccccc3C[C@H]21	625970888	25	338.24	3.49

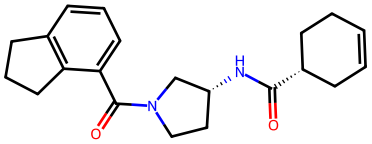	O=C(N[C@@H]1CCN(C(=O)c2cccc3c2CCC3)C1)[C@H]1CC=CCC1	939378581	25	338.2	2.86
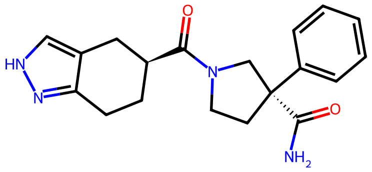	NC(=O)[C@]1(c2ccccc2)CCN(C(=O)[C@H]2CCc3n[nH]cc3C2)C1	1363444253	25	338.17	1.17
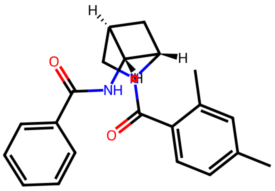	Cc1ccc(C(=O)N2C[C@H]3C[C@H]2[C@@H]3NC(=O)c2ccccc2)c(C)c1	946597110	25	334.17	2.95
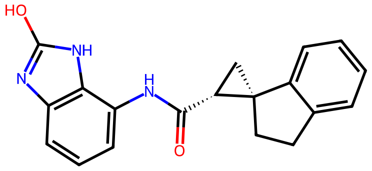	O=C(Nc1cccc2nc(O)[nH]c12)[C@@H]1C[C@]12CCc1ccccc12	563395843	24	319.13	3.11
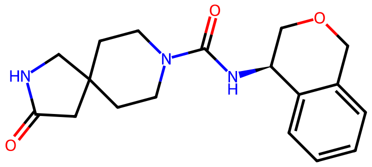	O=C1CC2(CCN(C(=O)N[C@H]3COCc4ccccc43)CC2)CN1	1365204069	24	329.17	1.57
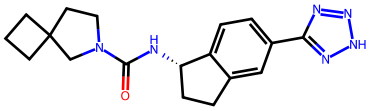	O=C(N[C@H]1CCc2cc(-c3nn[nH]n3)ccc21)N1CCC2(CCC2)C1	1606684005	25	338.19	2.44
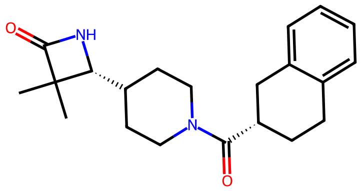	CC1(C)C(=O)N[C@@H]1C1CCN(C(=O)[C@H]2CCc3ccccc3C2)CC1	577184883	25	340.22	2.55
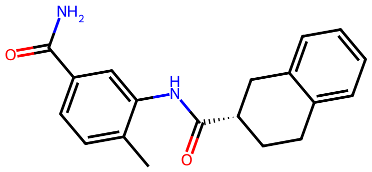	Cc1ccc(C(N)=O)cc1NC(=O)[C@H]1CCc2ccccc2C1	566309154	23	308.15	2.84

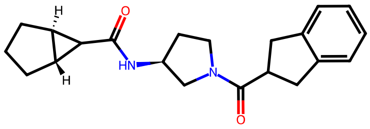	O=C(N[C@H]1CCN(C(=O)C2Cc3cccc c3C2)C1)C1[C@@H]2CCC[C@@H]1 2	937802017	25	338.2	2.16
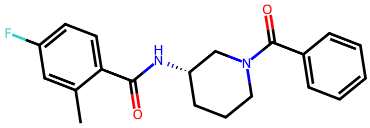	Cc1cc(F)ccc1C(=O)N[C@H]1CCCN(C (=O)c2ccccc2)C1	949807207	25	340.16	3.17
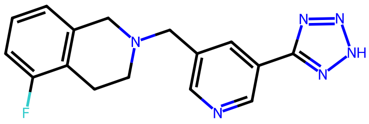	Fc1cccc2c1CCN(Cc1cncc(-c3nn[nH]n 3)c1)C2	1592658234	23	310.13	1.96
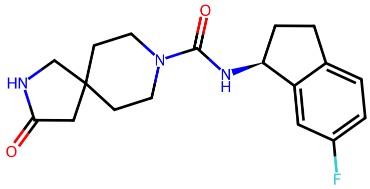	O=C1CC2(CCN(C(=O)N[C@H]3CCc4 ccc(F)cc43)CC2)CN1	74133674	24	331.17	2.12
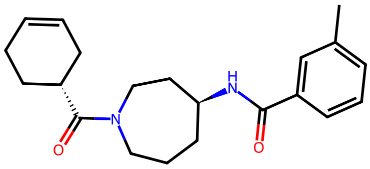	Cc1cccc(C(=O)N[C@H]2CCCN(C(=O) [C@H]3CC=CCC3)CC2)c1	964699321	25	340.22	3.46
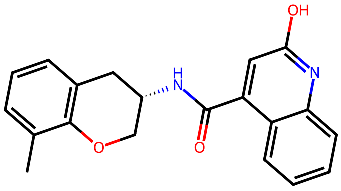	Cc1cccc2c1OC[C@@H](NC(=O)c1cc(O)nc3ccccc13)C2	484928706	25	334.13	2.98
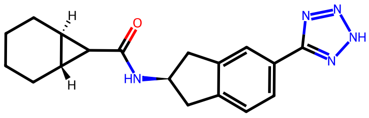	O=C(N[C@@H]1Cc2ccc(-c3nn[nH]n3) cc2C1)C1[C@@H]2CCCC[C@@H]12	1593001820	24	323.17	1.89
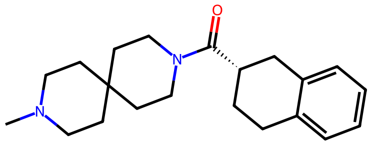	CN1CCC2(CC1)CCN(C(=O)[C@H]1C Cc3ccccc3C1)CC2	1118988300	24	326.24	3.13

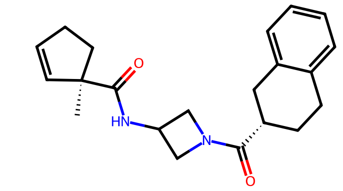	C[C@]1(C(=O)NC2CN(C(=O)[C@H]3CCc4ccccc4C3)C2)C=CCC1	997849496	25	338.2	2.47
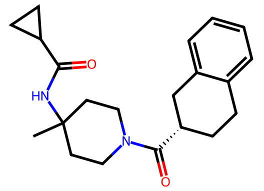	CC1(NC(=O)C2CC2)CCN(C(=O)[C@H]2CCc3ccccc3C2)CC1	956263577	25	340.22	2.7
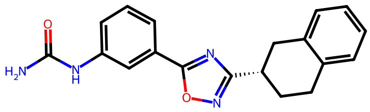	NC(=O)Nc1cccc(-c2nc([C@H]3CCc4cccc4C3)no2)c1	680770252	25	334.14	3.5
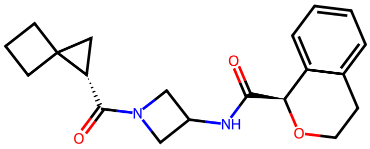	O=C(NC1CN(C(=O)[C@H]2CC23CCC3)C1)[C@@H]1OCCc2ccccc21	997326404	25	340.18	1.82
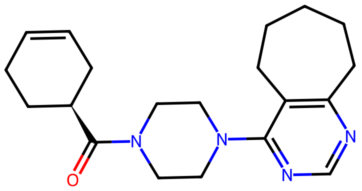	O=C([C@@H]1CC=CCC1)N1CCN(c2ncnc3c2CCCCC3)CC1	1055720898	25	340.23	2.75
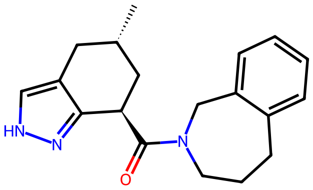	C[C@H]1Cc2c[nH]nc2[C@H](C(=O)N2CCCc3ccccc3C2)C1	623191806	23	309.18	3.05
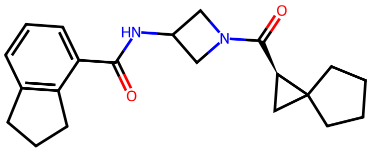	O=C(NC1CN(C(=O)[C@@H]2CC23CCCC3)C1)c1cccc2c1CCC2	994404394	25	338.2	2.7
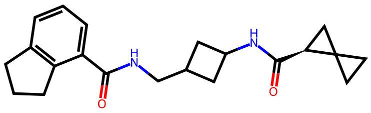	O=C(NCC1CC(NC(=O)[C@H]2CC23CC3)C1)c1cccc2c1CCC2	992200886	25	338.2	2.6

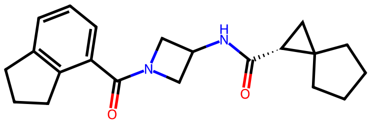	O=C(NC1CN(C(=O)c2cccc3c2CCC3)C1)[C@@H]1CC12CCCC2	999624801	25	338.2	2.7
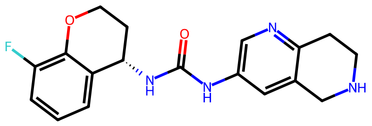	O=C(Nc1cnc2c(c1)CNCC2)N[C@H]1CCOc2c(F)cccc21	667803918	25	342.15	2.51
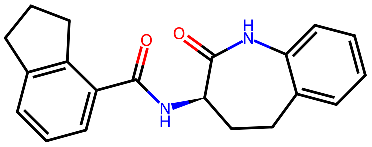	O=C(N[C@@H]1CCc2ccccc2NC1=O)c1cccc2c1CCC2	654005149	24	320.15	2.86
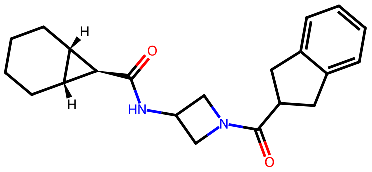	O=C(NC1CN(C(=O)C2Cc3ccccc3C2)C1)[C@@H]1[C@H]2CCCC[C@H]21	990659116	25	338.2	2.16
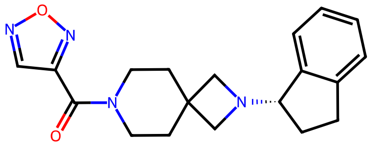	O=C(c1cnon1)N1CCC2(CC1)CN([C@H]1CCc3ccccc31)C2	1035821213	25	338.17	2.3
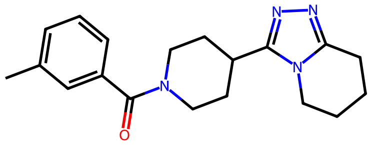	Cc1cccc(C(=O)N2CCC(c3nnc4n3CCCC4)CC2)c1	274666208	24	324.2	2.94
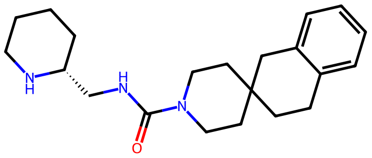	O=C(NC[C@H]1CCCCN1)N1CCC2(CCc3ccccc3C2)CC1	1580647919	25	341.25	3.11
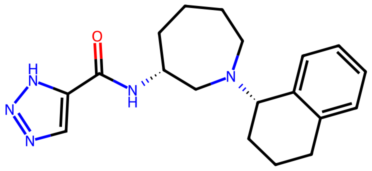	O=C(N[C@@H]1CCCCN([C@H]2CCCc3ccccc32)C1)c1cnn[nH]1	1034291873	25	339.21	2.47

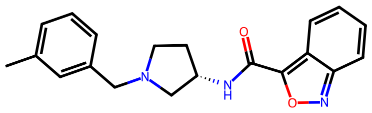	Cc1cccc(CN2CC[C@H](NC(=O)c3onc4ccccc34)C2)c1	1301783411	25	335.16	3.14
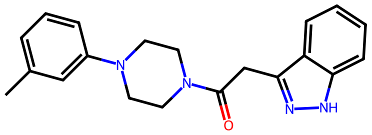	Cc1cccc(N2CCN(C(=O)Cc3n[nH]c4ccccc34)CC2)c1	58415603	25	334.18	2.76
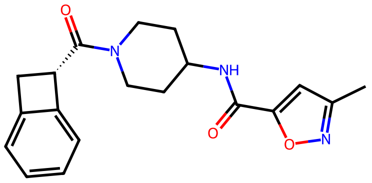	Cc1cc(C(=O)NC2CCN(C(=O)[C@H]3Cc4ccccc43)CC2)on1	948059503	25	339.16	2.04
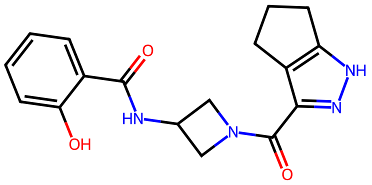	O=C(NC1CN(C(=O)c2n[nH]c3c2CCC3)C1)c1ccccc1O	990276206	24	326.14	0.86
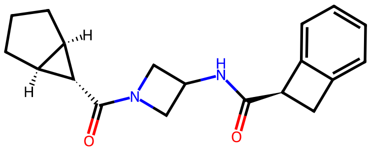	O=C(NC1CN(C(=O)[C@@H]2[C@H]3CCC[C@H]32)C1)[C@@H]1Cc2ccccc21	998685009	23	310.17	1.7
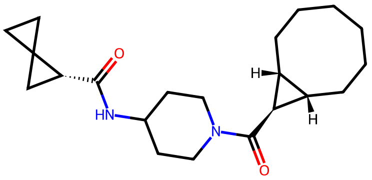	O=C(NC1CCN(C(=O)[C@@H]2[C@H]3CCCCCC[C@H]32)CC1)[C@@H]1CC12CC2	948568088	25	344.25	3.11
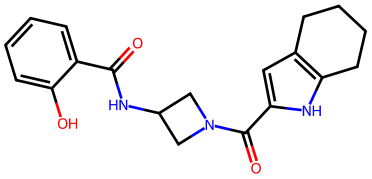	O=C(NC1CN(C(=O)c2cc3c([nH]2)CCCC3)C1)c1ccccc1O	990280469	25	339.16	1.85
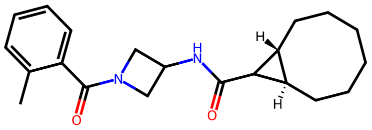	Cc1ccccc1C(=O)N1CC(NC(=O)C2[C@@H]3CCCCCC[C@@H]23)C1	1000466635	25	340.22	3.15

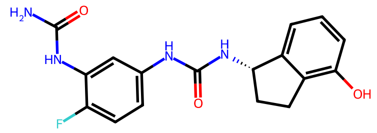	NC(=O)Nc1cc(NC(=O)N[C@H]2CCc3c(O)cccc32)ccc1F	1434726825	25	344.13	2.83
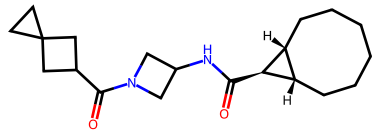	O=C(NC1CN(C(=O)C2CC3(CC3)C2)C1)[C@@H]1[C@H]2CCCCCC[C@H]21	1000510025	24	330.23	2.72
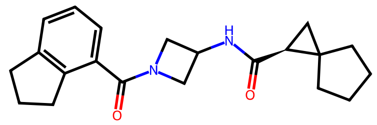	O=C(NC1CN(C(=O)c2cccc3c2CCC3)C1)[C@H]1CC12CCCC2	999624792	25	338.2	2.7
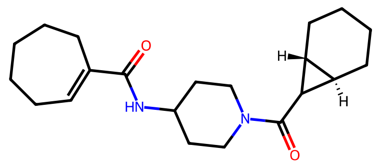	O=C(NC1CCN(C(=O)C2[C@@H]3CCCC[C@@H]23)CC1)C1=CCCCCC1	945957344	25	344.25	3.42
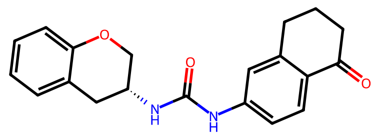	O=C(Nc1ccc2c(c1)CCCC2=O)N[C@H]1COc2ccccc2C1	575996828	25	336.15	3.33
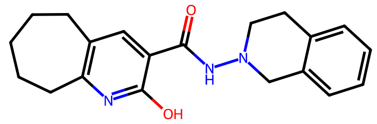	O=C(NN1CCc2ccccc2C1)c1cc2c(nc1O)CCCCC2	855710734	25	337.18	2.76
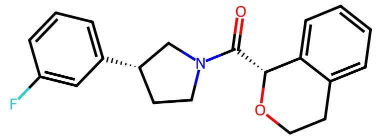	O=C([C@H]1OCCc2ccccc21)N1CC[C@H](c2cccc(F)c2)C1	490666384	24	325.15	3.46
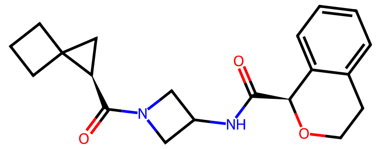	O=C(NC1CN(C(=O)[C@@H]2CC23CCC3)C1)[C@@H]1OCCc2ccccc21	997326402	25	340.18	1.82

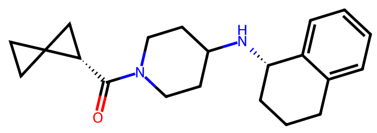	O=C([C@H]1CC12CC2)N1CCC(N[C@H]2CCCc3ccccc32)CC1	997040902	24	324.22	3.44
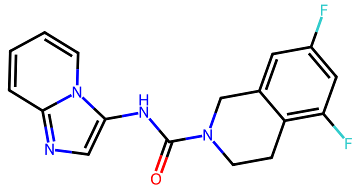	O=C(Nc1cnc2ccccn12)N1CCc2c(F)cc(F)cc2C1	1563040913	24	328.11	3.2
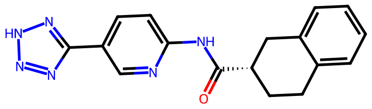	O=C(Nc1ccc(-c2nn[nH]n2)cn1)[C@H]1CCc2ccccc2C1	1582638388	24	320.14	2.01
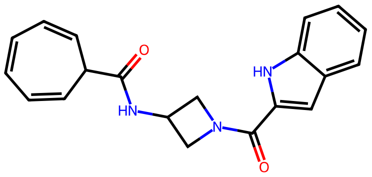	O=C(NC1CN(C(=O)c2cc3ccccc3[nH]2)C1)C1C=CC=CC=C1	992876791	25	333.15	2.41
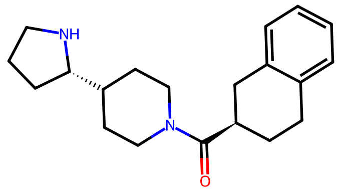	O=C([C@@H]1CCc2ccccc2C1)N1CCC([C@@H]2CCCN2)CC1	324106681	23	312.22	2.78
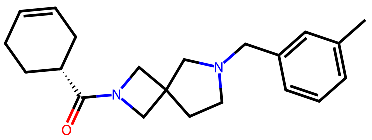	Cc1cccc(CN2CCC3(C2)CN(C(=O)[C@H]2CC=CCC2)C3)c1	1072458053	24	324.22	3.39
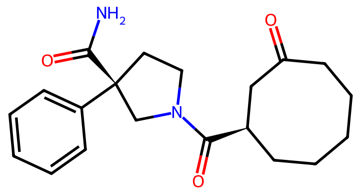	NC(=O)[C@@]1(c2ccccc2)CCN(C(=O)[C@@H]2CCCCCC(=O)C2)C1	665993668	25	342.19	2.18
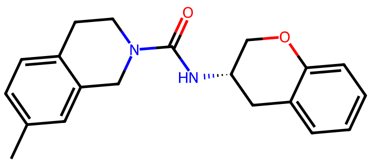	Cc1ccc2c(c1)CN(C(=O)N[C@@H]1COc3ccccc3C1)CC2	1220856150	24	322.17	3.07

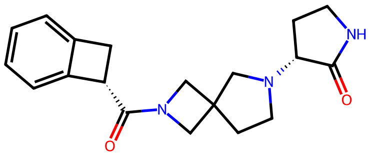	O=C1NCC[C@H]1N1CCC2(CN(C(=O)[C@H]3Cc4ccccc43)C2)C1	1072931219	24	325.18	0.75
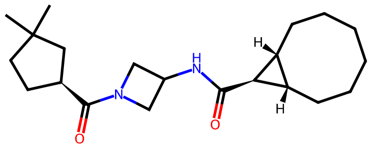	CC1(C)CC[C@H](C(=O)N2CC(NC(=O)[C@@H]3[C@H]4CCCCCC[C@H]43)C2)C1	1000508398	25	346.26	3.36
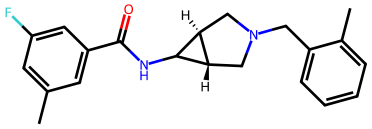	Cc1cc(F)cc(C(=O)NC2[C@H]3CN(Cc4ccccc4C)C[C@H]23)c1	961852750	25	338.18	3.3
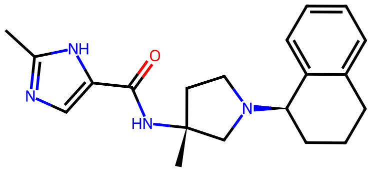	Cc1ncc(C(=O)N[C@]2(C)CCN([C@@H]3CCCc4ccccc43)C2)[nH]1	1046199614	25	338.21	2.99
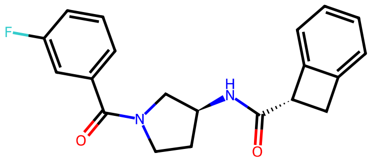	O=C(N[C@H]1CCN(C(=O)c2cccc(F)c2)C1)[C@H]1Cc2ccccc21	938574408	25	338.14	2.5
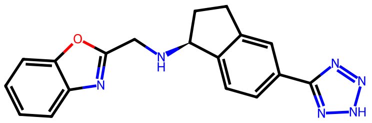	c1ccc2oc(CN[C@H]3CCc4cc(-c5nn[nH]n5)ccc43)nc2c1	1593581735	25	332.14	2.79
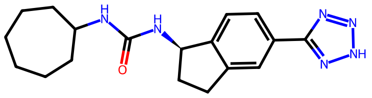	O=C(NC1CCCCCC1)N[C@@H]1CCc2cc(-c3nn[nH]n3)ccc21	1592976560	25	340.2	2.88
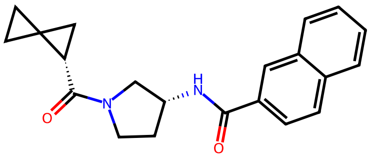	O=C(N[C@@H]1CCN(C(=O)[C@H]2CC23CC3)C1)c1ccc2ccccc2c1	936266687	25	334.17	2.97

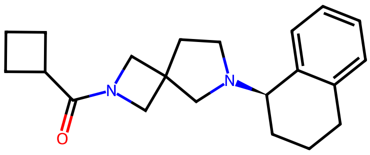	O=C(C1CCC1)N1CC2(CCN([C@@H]3CCCc4ccccc43)C2)C1	1072376515	24	324.22	3.4
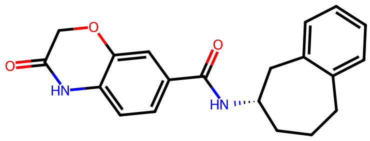	O=C1COc2cc(C(=O)N[C@H]3CCCc4ccccc4C3)ccc2N1	888253538	25	336.15	2.69
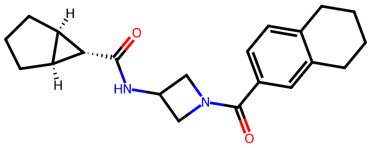	O=C(NC1CN(C(=O)c2ccc3c(c2)CCCC3)C1)[C@@H]1[C@H]2CCC[C@H]21	990711838	25	338.2	2.55
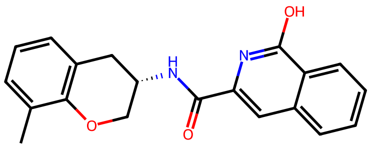	Cc1cccc2c1OC[C@@H](NC(=O)c1cc3ccccc3c(O)n1)C2	367943910	25	334.13	2.98
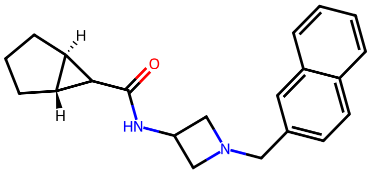	O=C(NC1CN(Cc2ccc3ccccc3c2)C1)C1[C@@H]2CCC[C@@H]12	1030192604	24	320.19	3.19
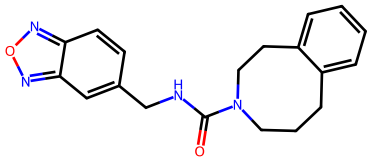	O=C(NCc1ccc2nonc2c1)N1CCCc2ccccc2CC1	1638902669	25	336.16	2.92
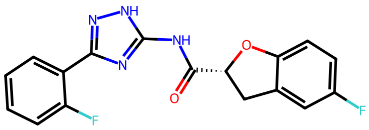	O=C(Nc1nc(-c2ccccc2F)n[nH]1)[C@H]1Cc2cc(F)ccc2O1	175971571	25	342.09	2.69
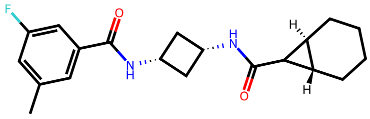	Cc1cc(F)cc(C(=O)N[C@H]2C[C@@H](NC(=O)C3[C@H]4CCCC[C@H]34)C2)c1	973991891	25	344.19	2.95

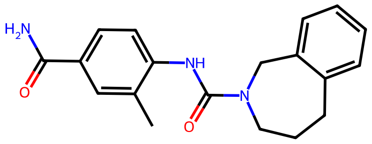	Cc1cc(C(N)=O)ccc1NC(=O)N1CCCc2ccccc2C1	730119661	24	323.16	3.07
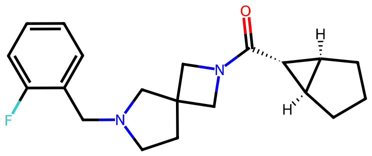	O=C([C@@H]1[C@H]2CCC[C@H]21)N1CC2(CCN(Cc3ccccc3F)C2)C1	1072590900	24	328.2	2.91
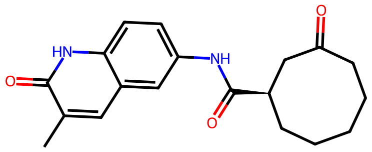	Cc1cc2cc(NC(=O)[C@@H]3CCCCCC(=O)C3)ccc2[nH]c1=O	908456671	24	326.16	3.31
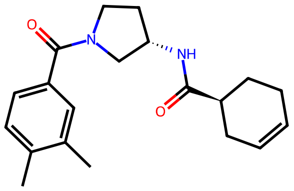	Cc1ccc(C(=O)N2CC[C@H](NC(=O)[C@@H]3CC=CCC3)C2)cc1C	937671977	24	326.2	2.99
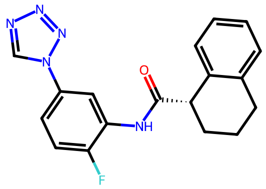	O=C(Nc1cc(-n2cnnn2)ccc1F)[C@H]1CCCc2ccccc21	47180237	25	337.13	2.86
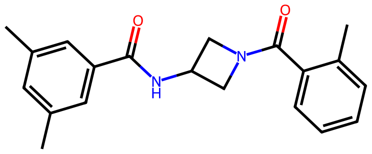	Cc1cc(C)cc(C(=O)NC2CN(C(=O)c3ccccc3C)C2)c1	999489393	24	322.17	2.87
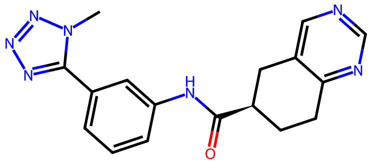	Cn1nnnc1-c1cccc(NC(=O)[C@@H]2CCc3ncncc3C2)c1	648228641	25	335.15	1.41
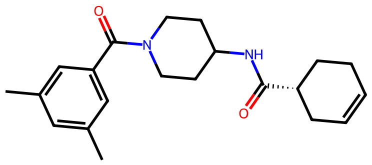	Cc1cc(C)cc(C(=O)N2CCC(NC(=O)[C@H]3CC=CCC3)CC2)c1	948338268	25	340.22	3.38

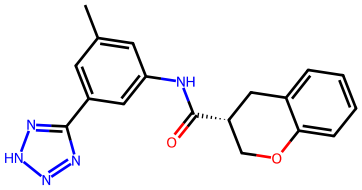	Cc1cc(NC(=O)[C@H]2COc3ccccc3C2)cc(-c2nn[nH]n2)c1	1592017220	25	335.14	2.36
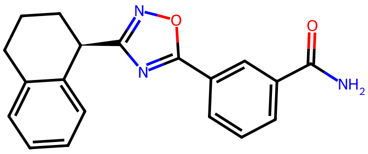	NC(=O)c1cccc(-c2nc([C@@H]3CCCc4ccccc43)no2)c1	355883179	24	319.13	3.3
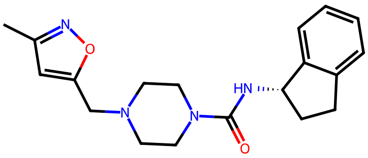	Cc1cc(CN2CCN(C(=O)N[C@H]3CCc4ccccc43)CC2)on1	45596364	25	340.19	2.5
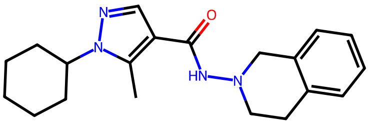	Cc1c(C(=O)NN2CCc3ccccc3C2)cnn1C1CCCCC1	855709998	25	338.21	3.4
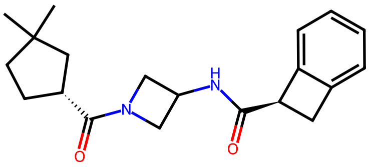	CC1(C)CC[C@@H](C(=O)N2CC(NC(=O)[C@@H]3Cc4ccccc43)C2)C1	998691792	24	326.2	2.48
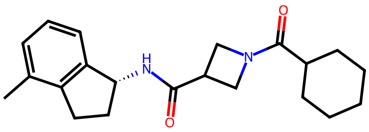	Cc1cccc2c1CC[C@H]2NC(=O)C1CN(C(=O)C2CCCCC2)C1	1360165243	25	340.22	3.14
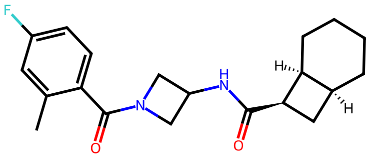	Cc1cc(F)ccc1C(=O)N1CC(NC(=O)[C@@H]2C[C@@H]3CCCC[C@@H]32)C1	997652232	25	344.19	2.9
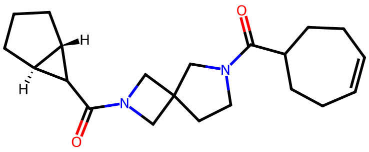	O=C(C1CCC=CCC1)N1CCC2(C1)CN(C(=O)C1[C@H]3CCC[C@H]13)C2	1018914021	25	342.23	2.84

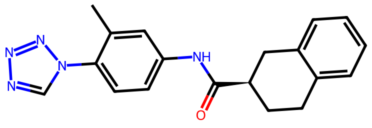	Cc1cc(NC(=O)[C@@H]2CCc3ccccc3C2)ccc1-n1cnnn1	571332534	25	333.16	2.71
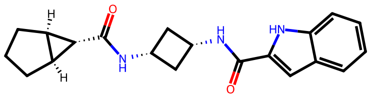	O=C(N[C@H]1C[C@@H](NC(=O)[C@@H]2[C@H]3CCC[C@H]32)C1)c1cc2ccccc2[nH]1	973995846	25	337.18	2.59
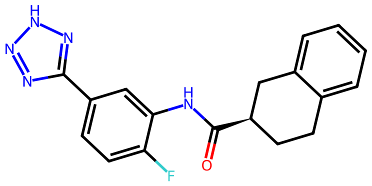	O=C(Nc1cc(-c2nn[nH]n2)ccc1F)[C@@H]1CCc2ccccc2C1	1525064649	25	337.13	2.75
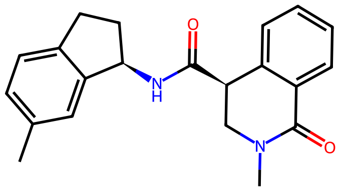	Cc1ccc2c(c1)[C@H](NC(=O)[C@@H]1CN(C)C(=O)c3ccccc31)CC2	683741568	25	334.17	2.97
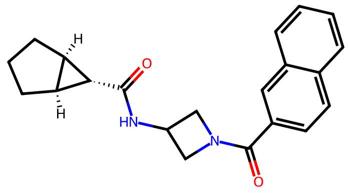	O=C(NC1CN(C(=O)c2ccc3ccccc3c2)C1)[C@@H]1[C@H]2CCC[C@H]21	990691217	25	334.17	2.83
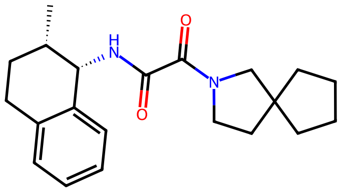	C[C@H]1CCc2ccccc2[C@H]1NC(=O)C(=O)N1CCC2(CCCC2)C1	807691452	25	340.22	3.22
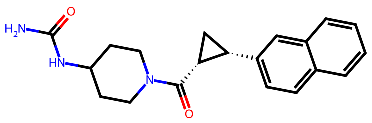	NC(=O)NC1CCN(C(=O)[C@@H]2C[C@@H]2c2ccc3ccccc3c2)CC1	176057418	25	337.18	2.6

Supplementary Table 2. Final Selection of 34 Lead Compounds with Favorable ADMET Profiles

zinc_id	score	Query	SMILES	DILI	Cyto-toxicity	HLM	Cyp1A2 Inhibitor	Cyp3A4 Inhibitor	Cyp2D6 Inhibitor	Cyp2C9 Inhibitor	Cyp2C19 Inhibitor	BBB	P-gp Inhibitor	P-gp Substrate	hERG Blocker	MMP	AMES	MRTD (mg/day)
1000500594	-7.509	1000500594	O=C(NC1CN(C(=O)[C@@H]2CC23CCCC3)C1)[C@@H]1[C@H]2CCCCCC[C@H]21	No	No	Yes	No	No	No	No	No	No	No	No	No	No	No	119
1000507824	-8.323	1000507824	O=C(NC1CN(C(=O)C2C=CC=CC=C2)C1)[C@@H]1[C@H]2CCCCCC[C@H]21	No	No	Yes	No	No	No	No	No	No	No	No	No	No	No	128
1000507789	-8.297	1000507789	O=C(NC1CN(C(=O)C2CC3(CCC3)C2)C1)C1[C@H]2CCCCCC[C@H]12	No	No	Yes	No	No	No	No	No	No	No	No	No	No	No	115
1000507828	-8.123	1000507828	O=C(NC1CN(C(=O)C2C=CC=CC=C2)C1)C1[C@@H]2CCCCCC[C@@H]12	No	No	Yes	No	No	No	No	No	No	No	No	No	No	No	128
1000510027	-7.422	1000510027	O=C(NC1CN(C(=O)C2CC3(CC3)C2)C1)C1[C@@H]2CCCCCC[C@@H]12	No	No	Yes	No	No	No	No	No	No	No	No	No	No	No	111
999347714	-7.644	999347714	O=C(NC1CN(C(=O)C2[C@H]3CCCCCC[C@H]23)C1)C1CC2(CC2)C1	No	No	Yes	No	No	No	No	No	No	No	No	No	No	No	113
999347713	-7.838	999347713	O=C(NC1CN(C(=O)[C@@H]2[C@H]3CCCCCC[C@H]32)C1)C1CC2(CC2)C1	No	No	Yes	No	No	No	No	No	No	No	No	No	No	No	113
1000507785	-8.27	1000507785	O=C(NC1CN(C(=O)C2CC3(CCC3)C2)C1)C1[C@@H]2CCCCCC[C@@H]12	No	No	Yes	No	No	No	No	No	No	No	No	No	No	No	115
1000507794	-8.03	1000507794	O=C(NC1CN(C(=O)C2CC3(CCC3)C2)C1)[C@@H]1[C@H]2CCCCCC[C@H]21	No	No	Yes	No	No	No	No	No	No	No	No	No	No	No	115
997934840	-8.008	997934840	O=C(NC1CN(C(=O)C2[C@H]3CCCCCC[C@H]23)C1)C1CC2(CCC2)C1	No	No	Yes	No	No	No	No	No	No	No	No	No	No	No	117
1000500592	-7.509	1000500592	O=C(NC1CN(C(=O)[C@H]2CC23CCCC3)C1)[C@@H]1[C@H]2CCCCCC[C@H]21	No	No	Yes	No	No	No	No	No	No	No	No	No	No	No	119
997934841	-7.706	997934841	O=C(NC1CN(C(=O)[C@@H]2[C@H]3CCCCCC[C@H]32)C1)C1CC2(CCC2)C1	No	No	Yes	No	No	No	No	No	No	No	No	No	No	No	117
1000500595	-7.812	1000500595	O=C(NC1CN(C(=O)[C@@H]2CC23CCCC3)C1)C1[C@@H]2CCCCCC[C@@H]12	No	No	Yes	No	No	No	No	No	No	No	No	No	No	No	119
997934839	-8.261	997934839	O=C(NC1CN(C(=O)C2[C@@H]3CCCCCC[C@@H]23)C1)C1CC2(CCC2)C1	No	No	Yes	No	No	No	No	No	No	No	No	No	No	No	117
999347715	-7.763	999347715	O=C(NC1CN(C(=O)C2[C@@H]3CCCCCC[C@@H]23)C1)C1CC2(CC2)C1	No	No	Yes	No	No	No	No	No	No	No	No	No	No	No	113
1000510026	-7.658	1000510026	O=C(NC1CN(C(=O)C2CC3(CC3)C2)C1)C1[C@H]2CCCCCC[C@H]12	No	No	Yes	No	No	No	No	No	No	No	No	No	No	No	111
987150770	-8.229	987150770	O=C(N[C@H]1CCCCCN(C(=O)C2CC3(CC3)C2)C1)[C@@H]1[C@H]2CCC[C@H]21	No	No	Yes	No	No	No	No	No	No	No	No	No	No	No	114
1000500591	-8.321	1000500591	O=C(NC1CN(C(=O)[C@@H]2CC23CCCC3)C1)C1[C@H]2CCCCCC[C@H]12	No	No	Yes	No	No	No	No	No	No	No	No	No	No	No	119
987150764	-8.713	987150764	O=C(N[C@@H]1CCCCCN(C(=O)C2CC3(CC3)C2)C1)[C@@H]1[C@H]2CCC[C@H]21	No	No	Yes	No	No	No	No	No	No	No	No	No	No	No	114
1000507831	-8.196	1000507831	O=C(NC1CN(C(=O)C2C=CC=CC=C2)C1)C1[C@H]2CCCCCC[C@H]12	No	No	Yes	No	No	No	No	No	No	No	No	No	No	No	128
1000510025	-7.9	1000510025	O=C(NC1CN(C(=O)C2CC3(CC3)C2)C1)[C@@H]1[C@H]2CCCCCC[C@H]21	No	No	Yes	No	No	No	No	No	No	No	No	No	No	No	111
946492296	-7.723	946492296	CC1(C)C[C@H]1C(=O)N1CCC(NC(=O)[C@@H]2[C@H]3CCCCCC[C@H]32)CC1	No	No	Yes	No	No	No	No	No	No	No	No	No	No	No	122
992875268	-8.259	992875268	O=C(NC1CN(C(=O)C2[C@@H]3CCCCCC[C@@H]23)C1)C1C=CC=CC=C1	No	No	Yes	No	No	No	No	No	No	No	No	No	No	No	134
327955030	-8.88	327955030	O=C(O)c1cccc2c1CCN(C(=O)[C@@H]1CCc3ccccc3C1)C2	No	No	Yes	No	No	No	No	No	No	No	No	No	No	No	140
1000511861	-7.543	1000511861	O=C(NC1CN(C(=O)[C@H]2CC23CC3)C1)[C@@H]1[C@H]2CCCCCC[C@H]21	No	No	Yes	No	No	No	No	No	No	No	No	No	No	No	108
1000511927	-7.625	1000511927	O=C(NC1CN(C(=O)[C@@H]2CC23CCC3)C1)C1[C@@H]2CCCCCC[C@@H]12	No	No	Yes	No	No	No	No	No	No	No	No	No	No	No	113
1000507941	-8.337	1000507941	O=C(NC1CN(C(=O)[C@@H]2C[C@@H]3C[C@@H]3C2)C1)C1[C@H]2CCCCCC[C@H]12	No	No	Yes	No	No	No	No	No	No	No	No	No	No	No	121
944966799	-7.54	944966799	O=C(C1=CCCC1)N1CC2(CCN(C(=O)[C@@H]3CC[C@H]4C[C@H]4C3)CC2)C1	No	No	Yes	No	No	No	No	No	No	No	No	No	No	No	98
999633190	-8.283	999633190	O=C(NC1CN(C(=O)C2[C@@H]3CCCCCC[C@@H]23)C1)[C@@H]1CC12CCCC2	No	No	Yes	No	No	No	No	No	No	No	No	No	No	No	123
1000511860	-7.841	1000511860	O=C(NC1CN(C(=O)[C@@H]2CC23CC3)C1)C1[C@H]2CCCCCC[C@H]12	No	No	Yes	No	No	No	No	No	No	No	No	No	No	No	108
943770141	-8.453	943770141	C=C1CCC(C(=O)NC2CCN(C(=O)c3ccccc3C)CC2)CC1	No	No	Yes	No	No	No	No	No	No	No	No	No	No	No	144
999633170	-7.998	999633170	O=C(NC1CN(C(=O)C2[C@@H]3CCCCCC[C@@H]23)C1)[C@H]1CC12CCCC2	No	No	Yes	No	No	No	No	No	No	No	No	No	No	No	123
990645299	-8.242	990645299	O=C(NC1CN(C(=O)[C@@H]2CCCc3nn[nH]c32)C1)C1[C@H]2CCCC[C@H]12	No	No	Yes	No	No	No	No	No	No	No	No	No	No	No	122
956332063	-8.13	956332063	CC1(NC(=O)[C@@H]2[C@H]3CCC[C@H]32)CCN(C(=O)C2C=CC=CC=C2)CC1	No	No	Yes	No	No	No	No	No	No	No	No	No	No	No	107

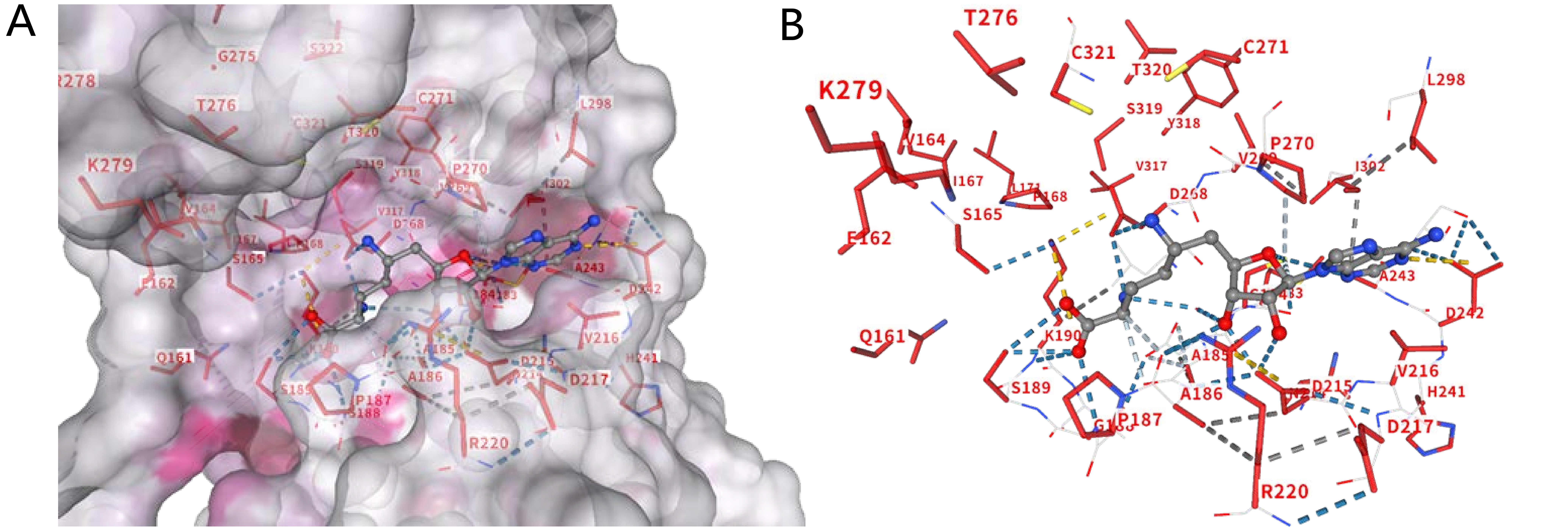

Supplementary Figure 1. Docking results of sinefungin with the NSUN2 AlphaFold-predicted structure using the CB-DOCK public platform (CB-Dock2: An accurate protein-ligand blind docking tool). A shows Sinafincon bound within the protein pocket of NSUN2, with a Vina Score of -3.3 kcal/mol. The figure illustrates that despite the relatively high binding energy, Sinafincon still forms a considerable number of hydrogen bonds interacting with the protein pocket. Supplementary Fig. 1B presents the docking model of the protein pocket with Sinafincon, displayed using a ball-and-stick model for the small molecule and a stick model for the interacting residues. (The dashed lines in the figure represent different interaction types: blue for hydrogen bonds, light blue for weak hydrogen bonds, yellow for ionic interactions, and gray for hydrophobic interactions.)

Supplementary Data

The Supplementary Data 1 has been uploaded in https://www.jianguoyun.com/p/DfbjtOgQ887bChj3sJQGIAA, Supplementary Data 2 in https://www.jianguoyun.com/p/DfK-aqcQ887bChj-i58GIAA.

Supplementary Data 1. 101,070,481 Lead Compounds

Supplementary Data 2. Top 12,000 Lead Compounds Identified by Virtual Screening
